# Succinate supplementation ameliorates musculoskeletal defects caused by *PLOD3* mutations in a BCARD syndrome model

**DOI:** 10.1186/s13073-026-01608-y

**Published:** 2026-03-13

**Authors:** Dharmendra Choudhary, Gokhan Unlu, Taylor H. Nagai, David B. Melville, Alexandra Scalici, Mais O. Hashem, Dylan J. Ritter, Georg Schmidt, Cory L. Guthrie, Eric R. Gamazon, Fowzan S. Alkuraya, Nancy J. Cox, Ela W. Knapik

**Affiliations:** 1 Department of Medicine, Division of Genetic Medicine, Vanderbilt University Medical Center, 1165 Light Hall, 2215 Garland Ave., Nashville, TN 37232 USA; 2 Vanderbilt Genetic Institute, Vanderbilt University Medical Center, Nashville, TN 37232 USA; 3 Department of Cell and Developmental Biology, Vanderbilt University, Nashville, TN 37232 USA; 4 Genomic Medicine Center of Excellence, King Faisal Specialist Hospital and Research Center, Riyadh, Saudi Arabia; 5 Developmental Biology, Institute Biology I, University of Freiburg, Hauptstrasse 1, Freiburg, 79104 Germany; 6 Clare Hall, University of Cambridge, Cambridge, CB3 9AL UK; 7 College of Medicine, Alfaisal University, Riyadh, Saudi Arabia; 8 Present address: Laboratory of Metabolic Regulation and Genetics, The Rockefeller University, New York, NY 10065 USA; 9 Present address: Department of Psychiatry, Yale University School of Medicine, New Haven, 06510 CT USA

**Keywords:** BCARD, PLOD3, ECM, Collagen, Succinate, Variant fibroblasts, Zebrafish.

## Abstract

**Background:**

BCARD syndrome is a rare complex connective tissue disorder associated with variants in the *PLOD3* gene, presenting with musculoskeletal, vascular, and sensory deficits. The role of PLOD3 in post-translational modifications of collagens has been established. However, limited treatment options exist to correct connective tissue deficits linked to PLOD3, largely due to sparse knowledge of cellular and molecular mechanisms driving phenotypic changes.

**Methods:**

To explain the mechanisms of *PLOD3* genotype-phenotype associations, we have used clinical data, molecular assays in patient-derived fibroblasts, perturbation experiments in zebrafish models, cellular and molecular experiments, and unbiased genome- and transcriptome-wide approaches.

**Results:**

We show that wild-type human *PLOD3* mRNA partially rescued musculoskeletal, vascular, and brain phenotypes in zebrafish *plod3* mutants, while clinically identified variants had only a limited effect, validating the pathogenicity of the variants and the high conservation of PLOD3 function across vertebrates. We found that, at the molecular level, organ systems selectively upregulated the PERK pathway of the Unfolded Protein Response and subsequently activated autophagy as an adaptive response to an extracellular matrix (ECM) protein backlog; however, autophagy inhibitors did not rescue the *plod3* mutant phenotypes. Bulk RNA-seq analysis of *plod3* mutants revealed downregulation of genes in metabolic pathways, including the electron transport chain and the tricarboxylic acid (TCA) cycle, consistent with structural defects in electron micrographs of mitochondria. Search of Drug Repurposing Data Portals identified a dietary supplement, succinate, to be associated with *PLOD3* and 25 additional genes, involved in the TCA cycle and collagen synthetic pathways. We showed that treatment with succinate ameliorated BCARD features, i.e., musculoskeletal defects, and restored reduced expression of TCA cycle genes in the zebrafish model.

**Conclusions:**

Our data indicate that the interaction between ECM synthesis and mitochondrial energy metabolism offers an entry point for novel therapies to prevent complex connective tissue decline in BCARD and, potentially, in other rare and common musculoskeletal disorders and conditions such as aging, cancer, or injury. Moreover, the genetic models developed here, and succinate, should be valuable tools in future studies of the underlying mechanisms of the BCARD extensive medical phenome.

**Supplementary Information:**

The online version contains supplementary material available at 10.1186/s13073-026-01608-y.

## Background

Recessive deleterious mutations in the *PLOD3* gene are associated with the BCARD syndrome (Bone Abnormalities, Cataracts, Arterial Rupture, and Deafness; OMIM# 612394), a complex connective tissue disorder characterized by the core phenotypes highlighted in the syndrome’s acronym and a spectrum of manifestations observed in published cases, including bone fragility, scoliosis, muscle contractures, aneurysms, and craniofacial dysmorphism [1–8]. The overall severity of the clinical presentation of BCARD syndrome ranges from cases of in utero demise [1] to less affected adult individuals [2]. Although clinical cases identified variants in all functional domains of PLOD3, detailed mechanistic studies are lacking. Furthermore, while BCARD is a rare syndrome, connective tissue deficiencies are prevalent within the general population.

Zebrafish has been an effective genetic tool for identifying causative genes of human syndromes associated with ECM dysfunction [9, 10]. For example, studies of genetic zebrafish mutations in genes of the secretory machinery, such as *sec23A*, *sec24D*, *sec24C*, and *ric1*, led to the identification of genes and underlying mechanisms responsible for rare diseases such as Craniolenticulosutural Dysplasia (CLSD), Osteogenesis Imperfecta, SEC24C-associated complex connective tissues disorder, and CATIFA, respectively [11–14].

The architecture of complex connective tissues is defined by extracellular matrix (ECM) scaffolds constructed of structural proteins, such as collagens, which are the main components of the ECM [15]. Matrices need to be established and maintained during embryonic development and throughout adult stages and aging. Failure to produce, renew, or maintain the ECM could result from mutations in the structural proteins themselves, their trafficking and assembly apparatus, or the enzymes that catalyze their post-translational modifications. Despite the importance of the ECM in ontogeny and age-related human diseases, there are few pharmacological options available to support matrix maintenance and repair.

ECM crosslinking and stability largely depend on collagen post-translational modifications. Collagen’s lysine residues first undergo hydroxylation conducted by one of the PLOD enzymes. PLOD3 [procollagen-lysine, 2-oxoglutarate 5-dioxygenase 3, also called lysyl hydroxylase 3 (LH3)] [16] catalyzes the first step in the conversion of 2-oxoglutarate in the presence of vitamin C and molecular oxygen, producing hydroxylated lysine residues within collagen, and succinate and CO_2_ as reaction byproducts. Subsequently, the homodimeric GLT25D1/COLGALT1, in complex with PLOD3, executes the attachment of a galactosyl group to the hydroxylated lysine, followed by the final step conducted by PLOD3 to attach a glucosyl group in the presence of Mn2 + ion [17]. This process generates a well-conserved collagen modification resulting in α-(1,2)-glucosyl-β-(1,O)-galactosyl-5-hydroxylysine (Glc-Gal-Hyl) [17–21]. Interestingly, variants in *PLOD3* were shown to disrupt all three steps of the Glc-Gal-Hyl modification including the galactosyltransferase activity performed by GLT25D1/COLGALT1 [17, 19, 21].

Using a BCARD patient’s skin fibroblasts and zebrafish models carrying *PLOD3* variants, we show that cells respond to *PLOD3* deficiency by activating ER stress and autophagy pathways to counter collagen accumulation and ECM dysfunction. Using omics approaches and pharmacological databases, we uncovered a novel connection between ECM synthesis and metabolic pathways in mitochondria. We show that treatment with succinate, a tricarboxylic acid (TCA) cycle intermediate and dietary supplement, reverses transcriptional trends of mitochondrial enzymes observed in *plod3* mutants and improves overall larval development and musculoskeletal architecture.

## Methods

### Fish husbandry and breeding

Zebrafish were raised and maintained under standard laboratory conditions as previously described [22]. All experiments were conducted under the guidelines established by the Institutional Animal Care and Use Committee (IACUC) at Vanderbilt University Medical Center, following approved animal protocol (M1700020). Zebrafish lines were raised at Vanderbilt University Medical Center fish facility at 28.5 °C with a constant photoperiod (14 h light: 10 h dark) under standard aquaculture conditions. Fish were fed with tropical flake food (Tetramin, Tetra) and hatched brine shrimp (Bio-Marine). Fish were staged and imaged at indicated developmental stages, i.e., embryos till hatching at 72 h post-fertilization (hpf), and larvae post hatching, days post-fertilization (dpf), as previously described [23]. At the end of study fish were euthanized by overdose of MS-222 / tricaine (Syncaine, Syndel), or by rapid cooling. *Maggot (mgt)* alleles *m350*, *m503*, and *m635* were isolated in a large-scale ENU mutagenesis screen at the Massachusetts General Hospital and previously described [24]; fish were kept in the wild-type, AB genetic background for functional phenotypic analysis [25].

### Genetic manipulations in zebrafish

#### mRNA overexpression constructs

A human *PLOD3* (*hPLOD3*) cDNA clone was obtained from Harvard Medical School (Clone ID HsCD00330495). ClaI and XhoI sites were added, flanking the coding sequence for directional insertion into the pCS2 + plasmid using primers shown in Additional file 1: Table S1. Zebrafish *plod3* (*zplod3*) cDNA was generated from reverse-transcribed mRNA. Total RNA was isolated from 1 dpf embryos and reverse transcribed to cDNA using M-MLV Reverse Transcriptase (Promega, M1701). The *zplod3* cDNA was amplified with primers containing ClaI and XhoI restriction sites (Additional file 1: Table S1). All final products were sequence verified. The following PLOD3 nomenclature is used throughout the paper: human gene and mRNA are referred as *PLOD3*, protein as PLOD3; the zebrafish gene and mRNA are denoted *plod3* and protein Plod3. When the text refers to human and zebrafish, the human nomenclature is used.

#### Overexpression and rescue experiments

Previously identified human variants in *PLOD3* [1–3] were introduced into the wild-type *PLOD3* construct using Q5^®^ site-directed mutagenesis (New England Biolabs) with primers listed in Additional file 1: Table S2. These plasmids were linearized and used for in vitro transcription of capped mRNA with the mMESSAGE mMACHINE SP6 transcription kit (Thermo Fisher Scientific). The concentration of the mRNAs was titered to establish a minimal effective dose of 300 pg, which was micro-injected into 1-cell stage zebrafish embryos [26]. Embryos were raised to 4 dpf and scored for craniofacial morphology (by live imaging) and cartilage shape (by Alcian blue staining). Whole body length and eye diameter were measured and compared to buffer-injected siblings as a control. All larvae were imaged by an AxioImager.Z1 equipped with a 20X/0.80 Plan Apochromat objective and HRc camera (Zeiss).

### Tissue staining and transmission electron microscopy (TEM)

#### Cartilage staining (Alcian Blue)

Zebrafish larvae were fixed at 4 dpf in 4% phosphate-buffered paraformaldehyde (PFA) overnight at 4 °C. After fixation, larvae were washed in PBT (PBS and 0.1% TritonX-100) twice for 5 min (min) each, incubated in 0.02% alcian blue and 60 mM MgCl_2_ in 70% ethanol overnight at room temperature (RT) on a shaker. Afterwards, samples were washed in PBT and bleached with 1.5% H_2_O_2_ and 1% KOH for 1 h (hr). The bleach was removed, and the larvae were rinsed with 0.25% KOH in 20% glycerol and stored in 0.1% KOH in 50% glycerol for further imaging [26]. Stained larvae were imaged with a Stemi 2000-C stereomicroscope (Zeiss) with an Axiocam HRc camera under transmitted light illumination.

#### Calcified tissue staining (Alizarin Red)

Zebrafish larvae were fixed at 5 dpf in 2% PFA for 2 h at RT, rinsed in PBT (PBS-0.1% tween), and dehydrated in 50% ethanol-PBT for 10 min. Subsequently, larvae were stained with 0.05% alizarin red (MP, USA) and 10 mM MgCl_2_ in 50% ethanol overnight at RT on a shaker. Afterwards, samples were washed in water and bleached with 1.5% H_2_O_2_ and 1% KOH for 20 min; following washes, samples were stored in 0.1% KOH in 50% glycerol [14]. Stained larvae were imaged with a Stemi 2000-C stereomicroscope (Zeiss) with an Axiocam HRc camera under transmitted light illumination.

#### TEM

Zebrafish WT and *mgt* embryos were fixed at 59, 72, and 96 hpf in 2% glutaraldehyde in 0.1 M sodium cacodylate and incubated at RT for 1 h first, then overnight at 4 °C. The next day, samples underwent TEM sample processing as previously described [12]. 70-nm sections were collected on a Leica Ultracut Microtome and analyzed on a Phillips CM-12 Transmission Electron Microscope (TEM) provided by the Vanderbilt Cell Imaging Shared Resource (CISR).

### Live imaging and immunofluorescence

#### Live imaging of zebrafish larvae and morphometric analysis

Fish were staged and imaged at the indicated developmental stages, as previously described [23]. Larvae were grown in embryo medium and, for subsequent live imaging, were supplemented with 0.003% PTU (1-phenyl-2-thiourea, Sigma-Aldrich, P7629) to prevent pigmentation. For imaging and quantification of body length parameters, 4 dpf larvae were anesthetized with tricaine, mounted in 3% methylcellulose (Sigma), and imaged with a Stemi 2000-C stereomicroscope (Zeiss) with an Axiocam HRc camera under transmitted light illumination. Using dorsal views in ImageJ, body lengths were measured from jaw protrusion to the caudal fin fold; lateral views were used for measuring eye diameter.

#### Transgenic zebrafish

Transgenic lines eGFP-GABARAP [27] and flk1-eGFP [28] were maintained by in-crosses. Zebrafish *gabarap* is 94% identical to mammalian GABARAP, which is homologous to yeast Atg8. The Zebrafish eGFP-GABARAP line was genome-edited with CRISPR/Cas9 in the transient and stable *mgt*^*m635*^ background and used for in-vivo autophagy studies. *flk1* is an endothelial-specific receptor of vascular endothelial growth factor (VEGF), known as *KDR* and *VEGFR2* [29, 30]. The *flk1* gene function is conserved from fish to mammals. Stable *mgt*^*m635*^ in the Tg (flk1:eGFP) background was used for in-vivo blood vessel-related studies.

#### Microangiography

Carboxylate-modified microspheres of 0.02 μm size (FluoSpheres^®^, ThermoFisher Scientific F8786), emitting red fluorescence (580/605), were diluted 1:5 in 0.3X Danieau buffer to 0.4% final concentration. Nanobeads were resuspended by a heat systems sonicator and then injected into the common cardinal vein of 3 dpf Tg(flk1:eGFP) in *mgt*^*m635*^ genetic background zebrafish larvae to mark blood plasma in red [31]. Injected live larvae were lightly anesthetized in tricaine and mounted sagittally in 3% methylcellulose (Sigma, M0387) for imaging. Multichannel images were acquired with an AxioImager Z1 Fluorescent microscope equipped with an Apotome (Zeiss) and an EC Plan Neofluar 20x.80 plan.

#### Immunofluorescence whole mount staining

Zebrafish larvae at the specified stages were fixed in Prefer fixative (Anatech, NC9053360) for 48 h, rinsed in PBT, post-fixed in 4% PFA for 10 min, and then permeabilized in 0.5% Triton X-100 in PBS. Next, larvae were bleached with 1.5% H_2_O_2_ / 1% KOH (Fisher Scientific, SP208 (Potassium Hydroxide), H325 (Hydrogen Peroxide), rinsed, blocked in 10% goat serum (Sigma, G9023), 1% DMSO (Sigma, D8418), and 1% BSA (Sigma, A8022), and incubated in primary antibody overnight at 4 °C. Secondary antibody incubation with fluorophore-conjugated antibodies followed at 4 °C overnight (Additional file 1: Table 3). After rinsing in PBT, DAPI (4’,6-diamidino-2-phenylindole, Invitrogen, D1306) staining was performed, and larvae were post-fixed in 4% PFA for 20 min, followed by clearing and storage in 90% glycerol. Stained larvae were imaged on the Nikon spinning disk confocal microscope. Images were presented as maximum-intensity z-projections generated by Nikon Elements software, as previously described [32].

#### Immunohistochemistry on sections

4 dpf larvae were fixed in 4% PFA at 4 °C overnight. After fixation, larvae were washed in PBT twice at 5 min each and cleared in 30% sucrose at 4 °C overnight. Larvae were mounted in Optimal Cutting Temperature (OCT) media (Sakura, Fine Technical Co, 4583) and cooled at -80 °C for at least 30 min. 14-µm thick cryo-sections of zebrafish tissues were obtained using a CM 3050 cryostat (Leica) at -20 °C and transferred onto *Superfrost* slides (Fisher Scientific). Sections were washed in PBT, blocked in 2 mg/ml BSA, 2% goat serum, and 2% DMSO in PBT, and incubated with primary antibody at 4 °C overnight, followed by fluorescent conjugated secondary antibodies (Additional file 1: Table S3). DAPI was used for nuclear counterstaining [14].

#### Immunoblotting

For Western Blot (WB) analysis, proteins were isolated by homogenizing 60 WT and 60 *mgt* larvae at 4 dpf in tissue cell lysis buffer (GoldBio-181) containing protease and phosphatase inhibitors (GoldBio, GB451 & GB331). Protein was quantified using the bicinchoninic acid (BCA) assay (ThermoFisher Scientific, 23227). Proteins were separated by sodium dodecyl sulfate-polyacrylamide gel electrophoresis (SDS-PAGE) on 4–20% Mini-Protean TGX Precast Gel (Bio-Rad, 4561094). Proteins were transferred to a polyvinylidene fluoride (PVDF) membrane (Bio-Rad,1620174) for immunoblotting using an electrophoretic transfer apparatus (Bio-Rad). The membrane was blocked with 5% non-fat milk (Bio-Rad 1706404) and incubated with the primary antibody against the tested protein (Additional file 1: Table S3). To assess the amount of loaded proteins, membranes were stripped in stripping buffer (ThermoFisher Scientific, 21059) and probed with HRP-conjugated secondary antibodies with anti-alpha tubulin antibody [DM1A] as a loading control [33]. Signal detection was performed using Western Lightning Plus-ECL, Enhanced Chemiluminescence Substrate (ThermoFisher Scientific, 34580).

### Human cell culture, TEM, and immunofluorescence analysis

Human subjects 

Dermal fibroblasts obtained from a single patient carrying the *PLOD3* variant were isolated from a skin biopsy. The sample was collected only after signing a written informed consent form by the legal guardians. The research was approved by the local IRB (KFSHRC RAC# 2080006).

#### Culture conditions

Dermal fibroblasts carrying the *PLOD3* variant c.1354 C > T transition in the AC domain were cultured in DMEM medium (GIBCO, 11995065) supplied with 10% bovine serum (Gibco A3160501), 100 ug/ml streptomycin, 100 units/ml penicillin (Gibco, 15140122), at 37 °C with 5% CO_2_. BJ skin fibroblasts (ATCC CRL-2522) controlled all cell culture-related experiments. Control and variant fibroblasts were cultured in dishes (Nunc) and grown to approximately 80–90% confluency at 37 °C in a 5% CO_2_ incubator. Before harvesting, cells were treated with 1x ascorbate (25 mM of L-ascorbic acid (Sigma, 255564) and 100 mM of 2-phospho-L-ascorbic acid trisodium salt, (Sigma, 49752) for 1 h at 37 °C with 5% CO_2_. After ascorbate treatment, cells were washed twice with PBS and collected in a cell lysis buffer containing protease and phosphatase inhibitors (Goldbio, GB331 and GB451). Collected proteins were separated by sodium dodecyl sulfate-polyacrylamide gel electrophoresis (SDS-PAGE) on 10% Mini-Protean TGX Precast Gel (Bio-Rad, 4561035).

*Western blotting* was performed, as described for the zebrafish samples in the above section. Band density in the blots was calculated using FIJI software [34].

#### Immunohistochemistry

Control and *PLOD3* variant fibroblasts were cultured in 4-well chamber slides (Millicell EZslides, PEZGS0416). 1500 cells per well were cultured and grown until approximately 70% confluency. Before staining, cells were treated with 1x ascorbate for 1 h at 37 °C with 5% CO_2_. Cells were washed and fixed in 4% PFA at RT for 20 min. Further, cells were permeabilized PBT and blocked in 1% BSA. Cells were incubated with the primary antibody overnight at 4 °C, followed by incubation with a fluorophore-conjugated secondary antibody overnight at 4 °C (Additional file 1: Table S3). WT and variant cells were incubated with Opti-MEM (Gibco 11058) and LysoTracker for 1 h, washed with Dulbecco’s phosphate-buffered saline (Gibco, 14190144), and fixed in 4% PFA. DAPI was used as the nuclear counterstain. Images were acquired with an AxioImager.Z1 Fluorescent microscope equipped with an Apotome (Zeiss) and an EC Plan Neofluar 100x/1.30 oil objective under the same settings for both control and patient fibroblasts [14].

#### Image analysis of cultured cells

Co-localization analysis of COL1 /ER, and LC3B/Lysosome, were performed using FIJI’s Coloc2 function. ImageJ’s ' Measure ' tool measured the fluorescence intensity of different protein signals per cell and total cellular area. Pearson’s *r* values were recorded from multiple WT and variant cells and compared using the Student’s t-test, as indicated in individual figures. Saturated pixels (255 values in the dynamic range spectrum) and background-level low pixels (1–10 values) were eliminated from the intensity datasets.

#### TEM

Patient-derived skin fibroblasts and WT controls were grown in 10-cm^2^ dishes up to 70% confluency. Cells were fixed in 2.5% glutaraldehyde and 0.1 M sodium cacodylate at RT for 1 h, then overnight at 4 °C [14, 35]. Samples were then processed for TEM imaging by CISR core at Vanderbilt University.

### RNA-seq and gene expression analysis

*Quantitative real-time PCR (qRT-PCR)* was performed using TRIzol (Invitrogen,15596018) reagent [36, 37]. Total RNA was isolated from three batches of 30 larvae for vehicle-treated wild type, *mgt*^*−/−*^, and SA-treated *mgt*^*−/−*^ samples. RNA (500 ng) was used to synthesize cDNA. Samples were mixed with an 18-nucleotide poly T primer and allowed to anneal before the reverse transcription reaction was initiated by adding dNTPs and M-MLV Reverse Transcriptase. Each qRT-PCR reaction was performed with 20 ng of total cDNA, SYBR Green Real-Time PCR master mix (ThermoFisher Scientific 4309155), and 2 µM of each primer. Primers used are listed in Additional file 1: Table S4. qRT-PCR reactions were run on a CFX96 (Bio-Rad) system. Data were analyzed using the -ΔΔCt method, and the Student’s t-test (CI = 95%). Each sample was independently obtained from three independent zebrafish clutches.

#### RNA isolation & cDNA library preparation

Total RNA was isolated from 4 dpf whole zebrafish larvae by TRIzol/Chloroform extraction. Total RNA, retained in the aqueous layer, was precipitated through a series of isopropanol and ethanol washes. Total RNA was isolated from three batches of 60 larvae each for wild-type (AB) and *mgt*^*−/−*^ samples [36]. Total RNA was submitted to the Vanderbilt Technologies for Advanced Genomics (*VANTAGE*) core for RNA quality assessment (RIM value) and cDNA libraries construction. RNA samples with an RNA integrity number (RIN)$\:\ge\:$ 8.5 were selected for RNA-Seq analysis. Paired-end sequencing reads were obtained from the Illumina HiSeq3000 platform at VANTAGE.

#### RNA-seq data processing and differential expression analysis

The sequencing reads obtained from VANTAGE were quality-assessed using FASTQC to ensure the accuracy of base calls (Phred $\:\ge\:$ 30), assess missing base calls (N content), and identify overrepresented sequences, such as sequencing adaptors [38]. Adaptor sequences were removed using CutAdapt [39] and sequences were aligned to the zebrafish reference genome (GRCz11) using STAR [40]. Read counts for each gene were obtained using HT-seq [41]. Differential expression analysis was conducted using DeSeq2 [42]. Statistical significance was determined using a log_2_(FC)>1.25 and a false discovery rate (FDR)< 0.05.

#### Gene set enrichment analysis (GSEA)

Statistically significant differentially expressed genes (FDR < 0.05) and their log2fold change values as weights were input to WebGestalt, mapped to human orthologues, and enrichment analysis was performed among pathways in the KEGG database [43, 44]. Normalized enrichment scores were plotted for the top up and downregulated significant pathways (FDR < 0.05).

#### Enrichr analysis

Zebrafish genes identified in the RNA-seq analysis that showed nominally significant (*p* < 0.05) expression change between WT and *mgt*^*−/−*^ samples were converted to their human orthologues using the HUGO Gene Nomenclature Committee (HGNC) HCOP tool and the ZFIN database [45]. The genes were then entered into Enrichr [46] and analyzed using Reactome and Gene Ontology cellular component pathways.

#### BROAD drug repurposing hub

The BROAD Drug Repurposing Hub [47] is a database of curated FDA-approved drugs, clinical trial drugs, and pre-clinical compounds whose identities have been experimentally confirmed and annotated with literature-reported targets. Our study used this database to identify existing therapeutic compounds for the *PLOD3* gene (Additional file 1: Table S5).

#### STRING database

STRING (Search Tool for the Retrieval of Interacting Genes/Proteins) is a database of tested and predicted protein-protein interactions based on scientific literature [48]. We used STRING to identify functional interactions between succinic acid targets obtained from the BROAD Drug Repurposing Hub and to generate a protein-protein interaction network.

### Chemical treatments

#### Succinic acid

Succinic acid (Sigma 398055) was dissolved in egg water to a 100 mM stock solution, and the pH was adjusted to 7.2. Fish were treated starting after gastrulation (10 hpf) to the 4 dpf stage. Dose response was tested in serial dilutions ranging from 100 µM − 800 µM concentration in 30 ml egg water in a 10 cm dish. Wild-type and *mgt* fish were exposed to egg water as a control to drug treatment. Following the treatment regime, images were taken, and two scorers blindly evaluated the results to determine changes in phenotype. Larvae were measured for length and further stained with TSP4 antibody to quantify angles of intersomitic boundaries to the horizontal midline. TSP4 staining was imaged using a Nikon confocal spinning-disk microscope.

### Statistical analysis

All statistical analysis was performed in GraphPad Prism 9.3.1 and R 4.3.3. All *zplod3* and *hPLOD3* variant rescue experiments were graphed with bars representing mean ± SEM. Data for each rescue experiment were analyzed by one-way ANOVA with Tukey’s multiple comparisons test. All Western blot fold changes were measured in ImageJ, normalized to alpha-tubulin expression, and compared to the wild-type expression. Bars represented mean ± SEM and were analyzed by Student’s t-test. Immunofluorescence analysis bars are graphed by mean ± SEM and were assessed by the Mann-Whitney U test for significance. qPCR expression differences were graphed by mean ± SEM and tested by Student’s t-test. Significance is represented by *p < 0.05; **p < 0.01; ***p < 0.001; ****p < 0.0001; ns = not significant.

## Results

### Zebrafish mutants recapitulate core BCARD features resulting from PLOD3 genetic variation

**Fig. 1:**
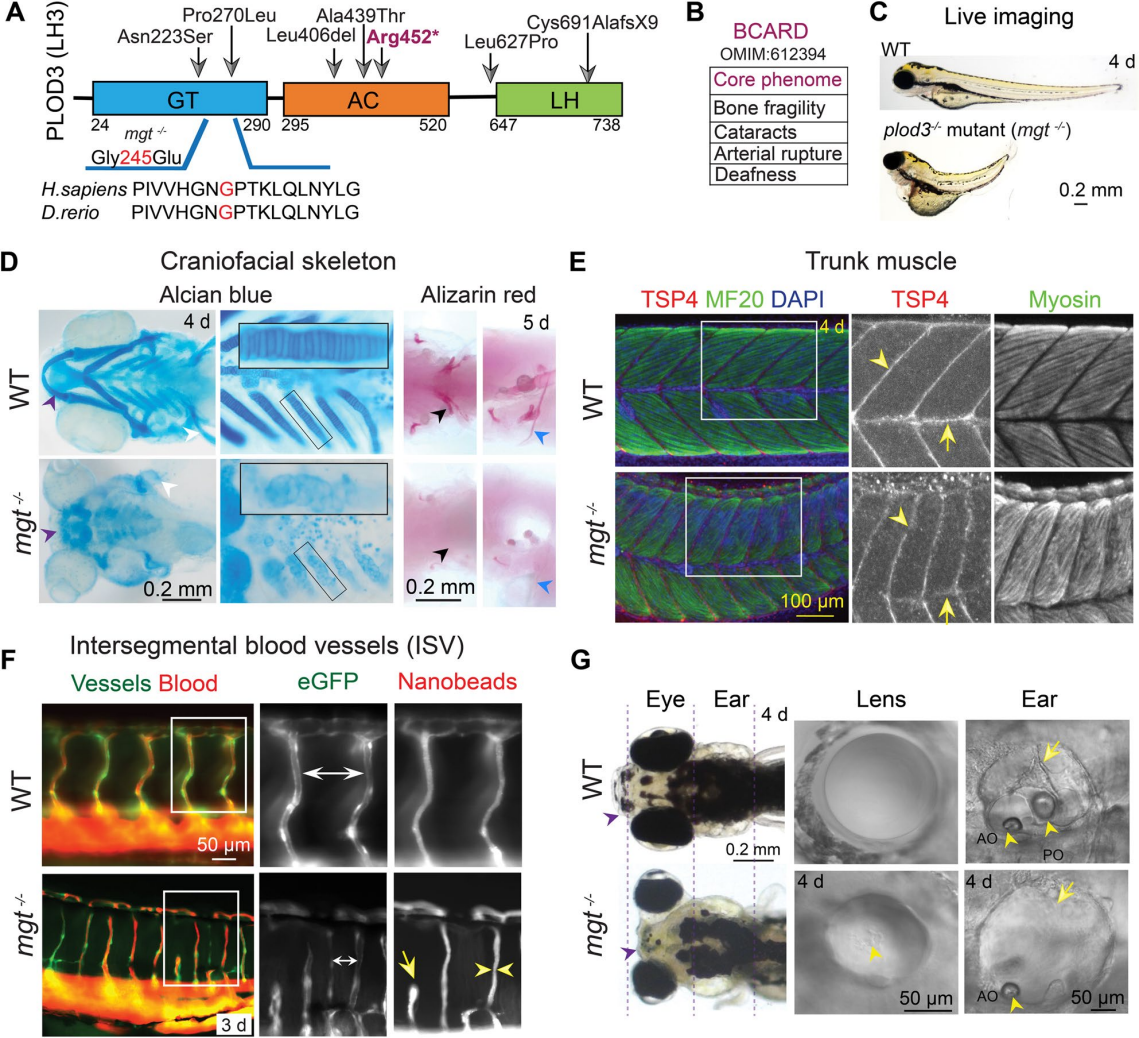
Genetic variants in the *PLOD3* gene lead to similar phenotypes in humans and zebrafish. **A** Schematic diagram of human PLOD3/LH3 depicts the highly conserved glycosyltransferase domain (GT), accessory domain (AC), and lysyl hydroxylase domain (LH); positions numbered as listed in the UniProt database. Arrows indicate clinically identified variants and resulting substitutions. The zebrafish *mgt*^*m635*^ mutant line carries a missense mutation, G245E, in the GT domain of *plod3*. **B** Core phenome of the BCARD syndrome. **C** Live image of the zebrafish *mgt*^*m635*^ mutant and WT at 4 dpf. Lateral views show a smaller head, a shorter body length with scoliosis, reduced eye size, an absence of a protruding jaw, and heart defects with occasional blood cell extravasations. **D** Zebrafish craniofacial cartilage skeleton stained with Alcian blue shows short and malformed Meckel’s (purple arrowheads) and kinked pectoral fins (white arrowheads) in WT and *mgt* ^*−/−*^ mutants (ventral view, 4 dpf, *n* = 24 per each group). Flat-mounted pharyngeal skeleton (inset, 3rd ceratobranchial) shows stacked chondrocytes in WT and clusters of rounded, smaller cells in *mgt* ^*−/−*^ larvae with reduced Alcian blue staining of the ECM compared with WT cartilage. Alizarin red staining of *mgt* ^*−/−*^ larvae shows largely absent ossifications in the pharyngeal teeth (black arrowheads) and the pectoral girdle, cleithrum bone (blue arrowheads), ventral (left) and lateral (right) views at 5 dpf; *n* = 12. **E** Whole-mount immunostaining of WT and *mgt* ^*−/−*^ larvae for myosin (MF20, muscle) and TSP4 (tendons). Maximum intensity projections of confocal z-stacks in all groups are shown. Intersomitic boundaries (arrowheads) and horizontal myoseptum/notochord (arrows) are marked. DAPI staining (blue) in merged images marks nuclei. Lateral views of trunk muscles; *n* = 8 animals per group. **F** Microangiography depicts maximum-intensity projections of trunk intersegmental vessels (ISVs) in Tg(flk1:eGFP) background (green). The 3 dpf WT and *mgt* ^*−/−*^ larvae were injected with fluorescent nanobeads (red) to mark plasma. Magnified views of boxed areas show narrowly spaced ISVs (double-sided arrows), variable width of the vessels (arrowheads), and abnormal sprouting and patency in the mutant compared to WT (arrow; *n* = 8). **G** Live images of WT and *mgt* ^*−/−*^ larvae showing small head, micrognathia (arrowhead), small eyes with prominent proptosis (dashed lines), distended ears (distal dashed lines), and kinked pectoral fins in mutants (*n* = 12). Lens images under DIC illumination in live *mgt* ^*−/−*^ zebrafish show smaller, proptotic lenses, with numerous artifacts consistent with cataract appearance (arrowhead). The ear panels show semicircular canals (arrows) and otoliths (arrowheads) in WT and *mgt* ^*−/−*^ larvae. Mutants lack one or both otoliths and have stunted semicircular canals (*n* = 6 per group)

Pathogenic variants in the human *PLOD3* gene have been reported and are associated with BCARD syndrome (Fig. 1A) [2] Overall, besides the core symptoms used in the acronym (bone fragility, cataracts, arterial rupture, and deafness), the shared features reported in clinical cases are: joint and bone deformities, including scoliosis and craniofacial dysmorphism, muscle weakness with contractures, connective tissue dysfunction, including skin blistering, cardiovascular anomalies, vessel fragility with hemorrhage, and aneurysms (Fig. 1B, Additional file 1: Table S6) [1, 3–8].

Although the core BCARD symptoms have been reported, the cellular and molecular mechanisms of disease phenotypes have been sparsely characterized. To complement our human cell culture model and to examine deficits across multiple tissues, we have developed zebrafish in vivo models of BCARD syndrome based on the ENU-induced zebrafish mutation called *maggot (mgt*^*m635*^*)*. Since chemically induced variants are not marked in any way, we used genetic linkage analysis and a positional cloning strategy to identify a 160-kbp critical region containing four candidate genes on zebrafish chromosome 23 (Additional file 2: Fig. S1A) [49, 50]. Sanger sequencing revealed a G > A transition at base pair 843 of the *plod3* gene in *mgt*^*m635*^ mutants, predicted to cause a G245E missense mutation in a highly conserved region of the GT glycosyltransferase domain (Fig. 1A; Additional file 2: Fig. S1B).

The *mgt*^*m635*^ mutation was isolated based on a complex skeletal phenotype with craniofacial dysmorphology (Fig. 1C; Additional file 2: Fig. S1C) [24]. Alcian blue and alizarin red staining revealed a smaller and misshapen craniofacial cartilage skeleton that failed to calcify in mutants as compared to wild-type (WT) siblings (Fig. 1D). Zebrafish and human *PLOD3* genes are highly conserved (homology 79% and 65% identical, Additional file 2: Fig. S2A). To test whether *mgt* mutants recapitulate other BCARD phenotypes such as vascular anomalies (Additional file 1: Table S6), we examined trunk muscle and intersegmental vessels (ISV) (Fig. 1E). In a transgenic Tg (flk1:eGFP) background, we found deficits in sprouting of individual vessels, as well as variable vessel diameter and connectivity to the collecting dorsal vessel (Fig. 1F). To test whether the newly formed vessels are patent, we injected fluorescently-labeled nanobeads (0.02 μm) into the common cardinal vein of 3 dpf larvae [28, 51]. The angiograms revealed that some vessels failed to tubulate and were not patent, indicating poor vessel formation, which was associated with vessel rupture and nanobeads extravasation in a subset of animals, in line with BCARD vascular and aneurysm phenotypes.

Sensory hearing loss and cataracts are among the core phenotypes in individuals with PLOD3 deficiency. Live imaging of zebrafish mutants showed small eyes, prominent lens proptosis, with extensive lens damage typical of cataracts [13], while larval ears were distended and largely lacking the semicircular canals, with malformed cristae and absent inner ear otolith(s) (Fig. 1G), consistent with clinically observed sensory hearing loss in BCARD.

Since *mgt*^*m635*^ carries a missense allele, we assessed the severity of the mutant phenotype as compared to the corresponding complete loss-of-function model. We generated a zebrafish *plod3* knockout using CRISPR/Cas9 gene editing by targeting exon 4 (Additional file 2: Fig. S2 B-E). We obtained mosaic deletions and substitutions leading to a putative early stop codon predicted to result in frameshifts and a truncated protein, and found that edited larvae exhibited similar phenotypes to *mgt*^*m635*^ mutants, with shorter, twisted body and malformed heads, providing an independent confirmation that the *mgt*^*m635*^ missense allele is likely a knockout or a severe loss-of-function allele.

### PLOD3 c.1354 C > T variant is pathogenic

**Fig. 2:**
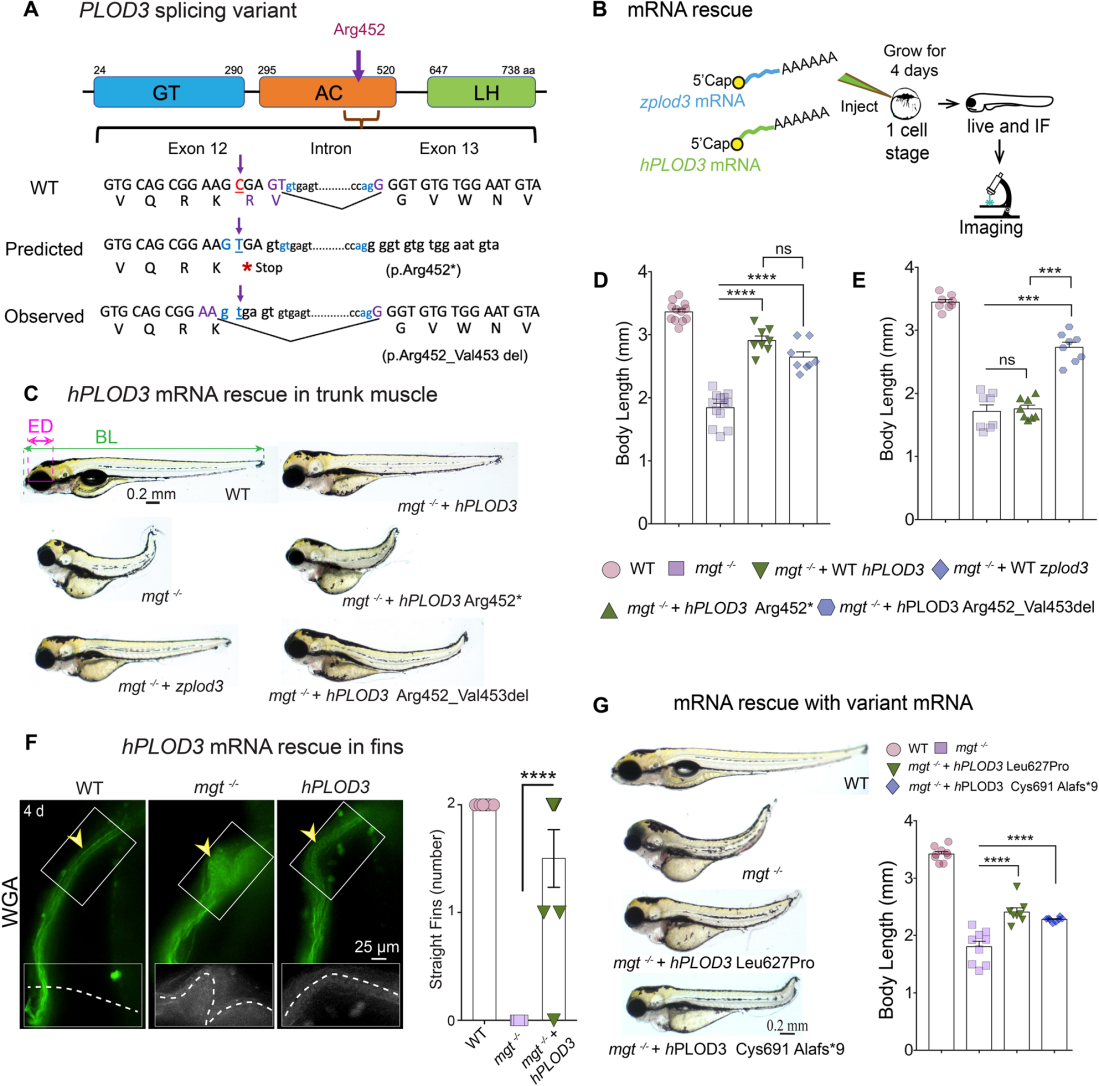
Genetic variants in the *PLOD3* gene lead to similar phenotypes in zebrafish. **A** Schematic depiction of the human *PLOD3* gene carrying the c.1354 C > T transition in the AC domain (red) that was expected to generate Arg452*, a predicted early stop codon, thereby truncating the peptide. However, sequencing of the cDNA isolated from cultured patient fibroblasts revealed a premature splice site in exon 12, resulting in the deletion of p.Arg452_Val453 and correct splicing to exon 13. **B** Experimental design of zebrafish (z) and human (h) *PLOD3* mRNA overexpression (OE) for rescue experiments. **C** Rescue experiments with zebrafish (z) and human (h) *PLOD3* mRNA OE. Live imaging of larvae at 4 dpf WT, *mgt* ^*−/−*^, *mgt* ^*−/−*^ + *zplod3* mRNA, and *mgt* ^*−/−*^ + *hPLOD3* mRNA and two variants (predicted *PLOD3* Arg452* and observed Arg452_Val453del). ED = Eye diameter, BL= Body length; lateral views. **D**,** E** quantification of the rescue experiments in C. D. Quantification of overexpression (OE) experiments for body length after mRNA microinjection with wild-type zebrafish *plod3* and human *PLOD3* mRNAs. The *n* numbers for body length = WT (14) *mgt* ^*−/−*^ (14), *mgt* ^*−/−*^ + z-*plod3* (8), and *mgt* ^*−/−*^ + h-*PLOD3* (8). E. *n* = 8 for each group. One-way ANOVA with Tukey’s multiple comparisons test with 95% CL of diff was conducted for analysis, ****p < 0.0001, ***p < 0.001, **p < 0.01, and ns = non-significant. **F** Whole-mount staining of WT, *mgt* ^*−/−*^ and *mgt* ^*−/−*^ + *h-PLOD3* mRNA injected larvae with fluorescently-conjugated WGA lectin (green) to mark N-glycosylated proteins in the pectoral fins. Panels of magnified views of boxed areas show kinked pectoral fins (yellow arrowhead) in *mgt* ^*−/−*^ fish that were partially rescued with *hPLOD3* mRNA. Quantification, WT *n* = 8, *mgt*^*−/−*^ (*n* = 9), and *mgt*^*−/−*^ + *hPLOD3* mRNA (*n* = 8). One-way ANOVA with Tukey’s multiple comparisons test with 95% CL of diff was conducted to compare fins between *mgt* ^*−/−*^ and *mgt* ^*−/−*^ injected with an mRNA rescue group. ****p < 0.0001. **G** Rescue experiments of the body length using variants Leu627Pro and Cys690*. The one-way ANOVA with Tukey’s multiple comparisons test with 95% CL of diff was conducted for comparison between *mgt* ^*−/−*^ and *mgt* ^*−/−*^ injected with *hPLOD3* variants mRNA; ****p < 0.0001. Error bars are the standard error of the mean

We have previously identified a *PLOD3* c.1354 C > T variant in individuals presenting with developmental delay, microcephaly, midfacial hypoplasia, depressed nasal bridge, micrognathia, truncal hypotonia, and distal arthrogryposis (joint contractures) [2]. We established cells in culture from biopsied dermal fibroblasts and confirmed by cDNA sequencing the c.1354 C > T variant in exon 12 adjacent to a splice site, with the mutation predicted to result in p.Arg452* (Fig. 2A). However, cDNA sequencing revealed that rather than resulting in a stop codon, the c.1354 C > T transition created a premature GT splice donor site leading to a 6-bp in frame deletion (Fig. 2A). This created a two-amino acid deletion, p.Arg452_Val453, followed by a normal sequence of exon 13 in the tested sample.

We set out to assess the specificity of the phenotypes and to test whether the human c.1354 C > T variant is pathogenic. In a series of rescue experiments, the in vitro transcribed full-length zebrafish *plod3* (*zplod3*) and human (*hPLOD3*) mRNAs were injected into one-cell stage embryos obtained from crosses of *mgt* ^*m635+/−*^ carriers (Fig. 2B). Embryos were injected with 300 pg of mRNAs (minimal effective dose, Additional file 2: Fig. S3A) or with the dilution medium as a control, and their genotype was verified by sequencing. We grew larvae to 4 dpf and measured eye diameter (ED) and body length (BL) in a double-blinded assessment (Fig. 2C-E; Additional file 2: Fig. S3B-C). We found that both zebrafish and human *PLOD3* mRNAs effectively improved tested phenotypes in *mgt*^*m635*^ mutants, while having no effect on WT larvae.

The zebrafish mutation is in the catalytic GT domain and likely represents a null or severe hypomorphic allele, lethal by 5 dpf. The human c.1354 C > T *PLOD3* variant is in the AC domain and appears to result in a less severe clinical phenotype. To test the pathogenicity of this variant, we overexpressed *hPLOD3* mRNA truncated at 1354 C and a second mRNA in which site-directed mutagenesis removed the 6 nucleotides that were missing in the patient’s cDNA. As expected, the truncated mRNA failed to rescue the *mgt*^*m635*^ mutants, while the 6 bp deletion partially restored the body length and eye diameter, establishing this variant as a partial loss-of-function allele (Fig. 2C, D, E; Additional file 2: Fig. S3B-C). Furthermore, the morphology of the pectoral fins (forelimbs), revealed by a lectin staining for glycans, showed consistent improvement in *maggot* mutants supplemented with WT *hPLOD3* (Fig. 2F). We have also tested two additional variants identified in clinical cases, p. Leu627Pro and p.Cys691Ala fs*9, in the LH domain of PLOD3 (Fig. 2G), and found that they are both hypomorphic alleles and partially rescued the axial phenotypes. Our results are further supported by in silico variant modeling using the AlphaMissense algorithm [52], (Additional file 3: Table S7), which showed that the human G256 amino acid, homologous to the *mgt* G245E, cannot be substituted for any other amino acid (score 0.989/1), rendering it a null allele (Additional file 2: Fig. S3D). Similarly, the amino acids Arg452 and Leu627 are not tolerant to the majority of substitutions.

The PLOD3 deficiency also affects muscle and skeletal tissues of the extremities. The zebrafish mutants consistently displayed short, kinked pectoral fins, and their mobility was impaired. We stained trunk muscles with antibodies against α-myosin (MF20) and thrombospondin 4 (TSP4), which is associated with tendons and the ECM, to evaluate muscle structure (Additional file 2: Fig. S4A). The *hPLOD3* mRNA overexpression (OE) partially rescued the architecture of the trunk muscles, intersomitic connective tissue, kinked pectoral fins, and intersomitic vasculature (Fig. 2F; Additional file 2: Fig. S4B). Furthermore, injected embryos partially regained mobility and could swim around the dish. To test for potential neural deficits in *mgt*^*m635*^, we labeled the brain and motor tracts traveling through the cerebellum with acetylated tubulin, which marks axonal tracts, and wheat germ agglutinin (WGA), which binds N-glycosylated proteins. Smaller cerebellum and optic tecta (to which retinal tracts project) phenotypes were restored to almost WT levels by *hPLOD3* mRNA OE in *mgt*^*m635*^ ( Additional file 2: Fig. S4C-F). Together, these data underscore the close conservation of PLOD3 function from human to zebrafish, validate the specificity of the PLOD3 phenotypes, and confirm the pathogenicity of the human variants.

### PLOD3 mutations lead to intracellular accumulation of procollagens

**Fig. 3:**
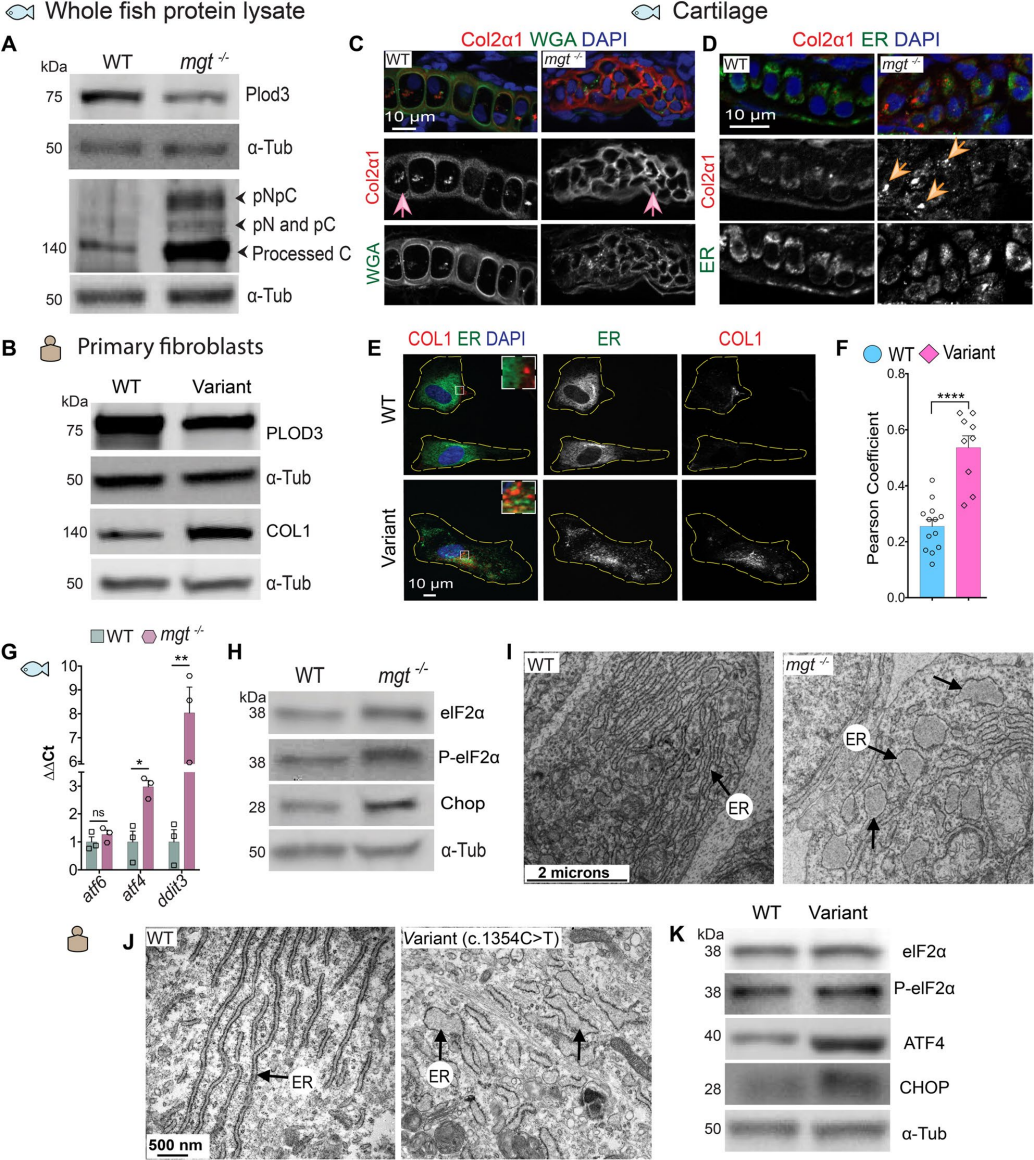
Reduced expression of PLOD3 disrupts procollagen trafficking and induces ER stress in *mgt* ^*−/−*^ embryos and human BCARD variant fibroblasts. **A** Western blot (WB) of whole zebrafish protein lysates for Plod3 and type II collagen to assess unprocessed (pNpC), processing intermediates (pN or pC), and processed, mature collagen. α-Tub staining served as a loading control. **B** WB of protein lysates from human fibroblasts stained with PLOD3 (*n* = 3 blots) and COL1 (*n* = 4 blots) antibodies from WT (BJ fibroblasts) and *PLOD3* (c.1353 C > T) variant dermal fibroblasts. α-Tub staining served as a loading control. **C** Cryosections of zebrafish craniofacial cartilage stained with Col2 recognizing antibody (red) and WGA (cell membranes, green) treated with proteinase K in WT and *mgt* ^*−/−*^ at 4 dpf. In WT, Col2 signal localizes to ECM and marks stacked chondrocyte boundaries, whereas in *mgt* ^*−/−*^, cartilage elements and cells are misshapen (ECM pink arrow, *n* = 5 animals per group). DAPI marks nuclei in blue. **D** Cryosections that were not treated with proteinase K and stained with Col2 (red) and PDI (ER marker, green) antibodies revealed cytoplasmic accumulations and large intracellular deposits of Col2 in *mgt* ^*−/−*^ cartilage that co-localize with PDI (orange arrows, *n* = 3 animals per group). DAPI marks nuclei in blue. **E** IF images of WT and *PLOD3* (c.1353 C > T) variant dermal fibroblasts show COL1 (red) staining partially co-localized with ERp57 (ER marker, green). Higher-magnification insets of the boxed areas show partial co-localization. DAPI marks nuclei in blue. **F** Pearson coefficient analysis of the co-localization of COL1 and ERp57; WT *n* = 13, variant *n* = 9. Data were analyzed with a two-tailed Student’s t-test, CI = 95%. Mean and SEM values are indicated with bars; ****p < 0.0001. **G** Expression analysis by qPCR of ER stress pathways-associated genes *atf6*, *atf4*, *ddit3* (Chop) normalized to *β-actin* in 4 dpf zebrafish. Data were analyzed with a two-tailed Student’s t-test, CI = 95%. Mean and SEM values are indicated with bars **p < 0.01, *p < 0.05, and ns = non-significant. **H** WB analysis in whole zebra-fish lysates at 4 dpf of the ER stress markers elF2α, its phosphorylated form P-elF2α, Chop, and α-Tub (as loading control). **I** TEM images of 3 dpf zebrafish chondrocytes showing distended rough ER in *mgt* ^*−/−*^ mutants compared to WT (black arrows). **J** TEM images of control BJ fibroblasts show tightly packed ER (arrow) and distended rough ER (arrows) in variant *PLOD3* (c.1353 C > T) dermal fibroblasts. **K** WB of protein lysates from human WT and variant *PLOD3* (c.1353 C > T) dermal fibroblasts stained with elF2α, P-elF2α, ATF4, CHOP, and α-Tub (as loading control)

PLOD3 catalyzes post-translational modifications essential for collagen cross-linking [53, 54]. To test the effect of variants on PLOD3 function, we analyzed PLOD3 protein levels in *mgt*^*m635*^ mutants (Fig. 3A; Additional file 2: Fig. S5A) and patient skin fibroblasts by Western immunoblotting (Fig. 3B; Additional file 2: Fig. S5B). We found significantly reduced PLOD3 levels in both cases compared to the corresponding controls. Western blotting (WB) of zebrafish protein lysates for type II collagen revealed that the lack of Plod3 in *mgt*^*m635*^ correlated with a high proportion of unprocessed procollagen N- and C-peptides, and significantly increased amounts of the fully processed form (Fig. 3A; Additional file 2: Fig. S5A). In the patient’s skin fibroblasts, type I collagen was also more abundant than in controls (Fig. 3B; Additional file 2: Fig. S5B). Consistent with the WB data, immunostaining of zebrafish craniofacial cartilage treated with proteinase K showed misshapen and disorganized chondrocytes (counterstained with WGA), and type II collagen present in extracellular space (Fig. 3C), whereas without proteinase K treatment, it showed intracellular accumulation of large amounts of type II collagen that were partially co-localized with the ER marker in *mgt*^*m635*^ mutants (Fig. 3D). Similarly, type I collagen accumulated in patient-derived fibroblasts and partially co-localized with ERp57 (Fig. 3E, F). We also tested collagen type II distribution in the notochord, the hydrostatic axial skeleton of the larvae that contributes to body length extension. Unlike in WT siblings, the notochord sheath in *mgt*^*m635*^ mutants was not fully stretched, and the notochord appeared compressed with irregular distribution of collagen-positive thickenings and interruptions of the basement membrane ( Additional file 2: Fig. S5C).

Transmission electron microscopy (TEM) revealed ECM deficits in craniofacial cartilage (Fig. S5D; Additional file 2: Fig. S5D) and poorly stacked chondrocytes ( Additional file 2: Fig. S5E). Chondrocytes, as they mature and secrete large amounts of ECM, lose their cell-cell contacts and become solitary, stacked cells suspended in dense ECM [55]. This process was disrupted in *mgt*^*m635*^ cartilage, and cells remained connected to each other (Fig. S5E), surrounded by sparse extracellular matrix lacking collagen fibrils ( Additional file 2: Fig. S5D). Together, these data indicate that the PLOD3 *deficiency* impairs fibrillar collagen presence in the ECM and compromises the integrity of skeletal matrices.

### PLOD3 mutations specifically activate the PERK stress pathway

Intracellular accumulation of misfolded proteins typically activates ER stress response pathways [56]. The quantitative PCR analysis of the canonical ER stress response pathways, IRE1 and ATF6, did not show significant changes at the levels of *xbp1(data not shown)* and *atf6* genes, respectively. However, the PERK pathway was upregulated as shown by increased levels of its targets *atf4* and *ddit3* (Chop) in 4 dpf *mgt*^*m635*^ zebrafish (Fig. 3G). The transcriptional changes corresponded to upregulated phosphorylated P-elF2a and Chop on immunoblots of *mgt*^*m635*^ zebrafish lysates compared to WT (Fig. 3H; Additional file 2: Fig. S5F). The molecular data were consistent with TEM analysis, which showed distended ER in zebrafish chondrocytes (Fig. 3I). Comparative TEM and immunoblot analyses in the patient’s fibroblasts confirmed these findings (Fig. 3J, K; Additional file 2: Fig. S5G). Both protein backlog and the PERK pathway are known activators of autophagy [57–61], driving the return of macromolecular resources to cellular metabolism.

### PLOD3-deficient human fibroblasts and zebrafish mutants activate autophagy

**Fig. 4:**
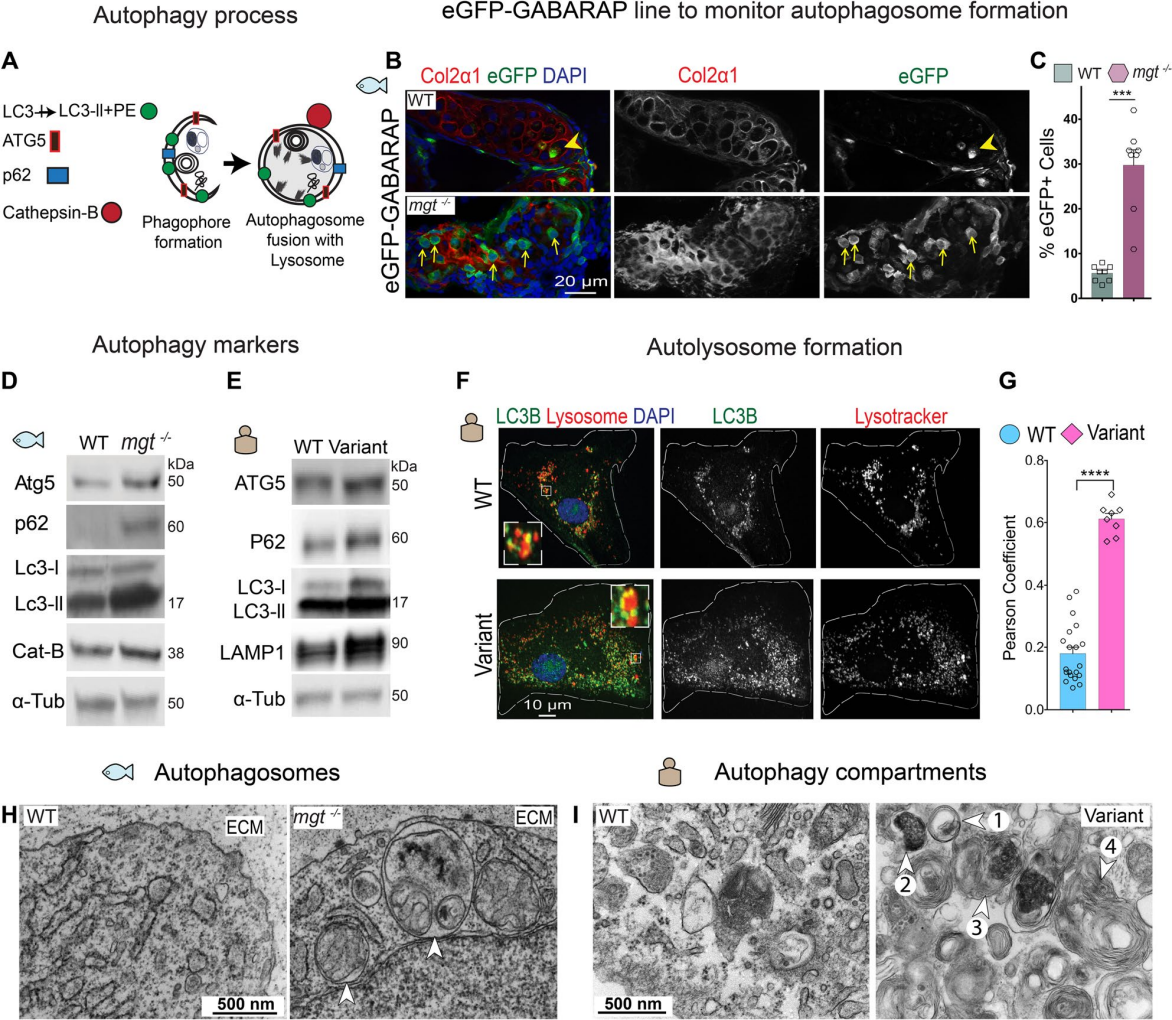
PLOD3 deficiency leads to increased autophagy. **A** Schematic diagram of autophagosome formation. **B** Cryosections of the craniofacial cartilage chondrocytes stained with Col2 antibodies (red) in Tg (eGFP-GABARAP) genetic background (green), with protease K treatment from WT and *mgt* ^*−/−*^ zebrafish at 4 dpf. The arrowhead marks an eGFP^+^ chondrocyte in WT, and the yellow arrows in *mgt* ^*−/−*^; nuclei stained with DAPI (blue). **C** Quantification of eGFP-GABARAP^+^ chondrocytes as a percentage of the total number of chondrocytes; WT, *n* = 288 cells (*N* = 6 WT larvae); mutant, *n* = 250 cells (*N* = 8 *mgt* ^*−/−*^ larvae). Data were analyzed with a two-tailed Student’s t-test, CI = 95%. Mean and SEM values are indicated with bars. ****p < 0.0001. **D** WB of whole zebrafish protein lysates in WT and *mgt*^*−/−*^ at 4dpf labeled for Atg5, p62, Lc3l, Lc3ll, and Cathepsin B. α-Tub served as a loading control. **E** WB of protein lysates from human fibroblasts, WT and variant (c.1353 C > T), stained for ATG5, p62, LC3, LC3ll, and LAMP1, α-Tub served as loading control. **F** IF images of co-immunostaining for LC3B (green) and Lysosomes (Red, Lysotracker) in control fibroblasts (WT) and *PLOD3* (c.1353 C > T) variant cells, nuclei stained with DAPI (blue). Inset of the boxed area highlights co-localization of the two markers. **G** Pearson coefficient analysis of the co-localization of LC3B and Lysotracker, WT *n* = 20 and variant *n* = 8. Data were analyzed with a two-tailed Student’s t-test, CI = 95%. Mean and SEM values are indicated with bars; ****p < 0.0001. **H** TEM image of 3 dpf WT zebrafish shows a healthy cytoplasmic compartment in a chondrocyte immersed in dense ECM, while *mgt* ^*−/−*^ chondrocyte contains large autophagosomes (arrowheads) in the cytoplasm. **I** TEM image of human WT fibroblast shows normal ER, Golgi, and other secretory compartments, while the human *PLOD3* variant (c.1353 C > T) fibroblast shows degradative compartments: autophagosome (arrowhead 1), lysosome (arrowhead 2), autolysosome (arrowhead 3), and multilamellar bodies (arrowhead 4)

Canonical degradative autophagy is initiated to relieve cellular burden by generating membrane-bound compartments encapsulating organelles and/or misfolded cargos for hydrolysis [62]. The autophagy process begins with phagophore initiation and autophagosome formation, followed by fusion with lysosomes and hydrolysis (Fig. 4A) [63]. A core set of autophagy related genes (ATG) and membrane nucleating factors, including LC3-II, Atg5, and p62, drive the autophagy process [58, 60, 64–66].

To assess whether autophagy is activated in response to *plod3* depletion and intracellular procollagen backlog, we CRISPR-edited *plod3* in the zebrafish reporter line Tg(CMV: eGFP-GABARAP). GABARAP is a vertebrate homolog of the yeast Atg8 protein involved in autophagosome formation [27, 67]. We found a significantly increased number of eGFP^+^ chondrocytes in edited larvae, which was replicated in *mgt*^*m635*^ mutants in the same transgenic background (Fig. 4B, C; Additional file 2: Fig. S6A-C). We verified cellular changes in *plod3* mutants by immunoblot analyses of proteins harvested at 4 dpf that revealed accumulation of autophagy-related protein 5 (ATG5), p62 (a cargo adaptor interacting with LC3-II that is subsequently degraded in an autolysosome), LC3-II (autophagosome membrane marker), and lysosomal enzyme cathepsin B (Fig. 4D; Additional file 2: Fig. S6D). Comparative immunoblots of lysates from primary, patient-derived fibroblasts cultured under basal conditions showed a highly significant increase in ATG5, P62, LC3-I and LC3-II, and LAMP1 (Lysosomal-associated membrane protein 1, a lysosomal marker) (Fig. 4E; Additional file 2: Fig. S6E). These findings were consistent with increased immunofluorescence signal in fibroblasts co-stained with LC3B and Lysotracker that showed co-localization of the examined markers in large punctae (Fig. 4F-G). TEM analysis revealed a high density of autophagosomes and autolysosomes in the cytoplasm (Fig. 4H, I; Additional file 2: Fig. S6F), suggesting that autophagy is potentially an underlying cause of the observed phenotypes and might explain the early demise of *plod3*-deficient zebrafish larvae.

### Inhibition of autophagy does not rescue the maggot phenotype

To discern whether the ER stress or upregulated autophagy are the underlying cause of the *mgt* phenotypes, we treated zebrafish mutants with 11 ER stress inhibitors (Additional file 1: Table S8), beginning after gastrulation up to 4 dpf, and evaluated live phenotypes. There was no phenotype improvement compared to vehicle treated controls (data not shown).

Next, we treated mutants with autophagy inhibitors [68] that block consecutive autophagy steps: autophagosome formation with 3-Methyladenine (3-MA), Chloroquine (Chloq) that targets autophagosome-lysosome fusion, and Bafilomycin A1 (BafA1), a specific vacuolar H^+^ ATPase (V-ATPase) inhibitor ( Additional file 2: Fig. S7A, B). In addition to live phenotypes, we evaluated levels of collagen type II and LC3-II expression on immunoblots. We did not find statistically significant improvements of either readout after treatment; instead, larvae were dying slightly sooner compared to vehicle treated controls ( Additional file 2: Fig. S7C-E).

Taken together, deficits in procollagen processing and impaired ECM integrity in *plod3* mutants led to selective upregulation of the PERK branch of unfolded protein response (UPR), and to autophagy. The molecular changes accompanying cellular remodeling in fibroblasts, muscle cells, and chondrocytes included distended ER, autophagosome formation, and impaired integrity of mitochondrial membranes. However, neither ER stress nor inhibition of autophagy improved the phenotypes. Thus, they do not appear to be the underlying cause of the *plod3-*deficiency phenotypes but instead seem to be an adaptive response to the protein backlog.

### Metabolic genes are dysregulated in PLOD3-deficiency

In search of molecular mechanisms driving BCARD phenotypes, we turned to transcriptomics using unbiased bulk RNA-seq of zebrafish mutants (Additional file 4 and 5, Tables S9 and S10). We conducted paired-end sequencing with 50 million reads per sample using the Illumina HiSeq3000 platform and compared *mgt* mutants to WT animals. Following quality control processing, reads were aligned to the zebrafish reference genome (GRCz11) using STAR [40], and these were further converted to their human orthologs using the HUGO Gene Nomenclature Committee (HGNC) HCOP tool and the ZFIN database [45, 46].

**Fig. 5:**
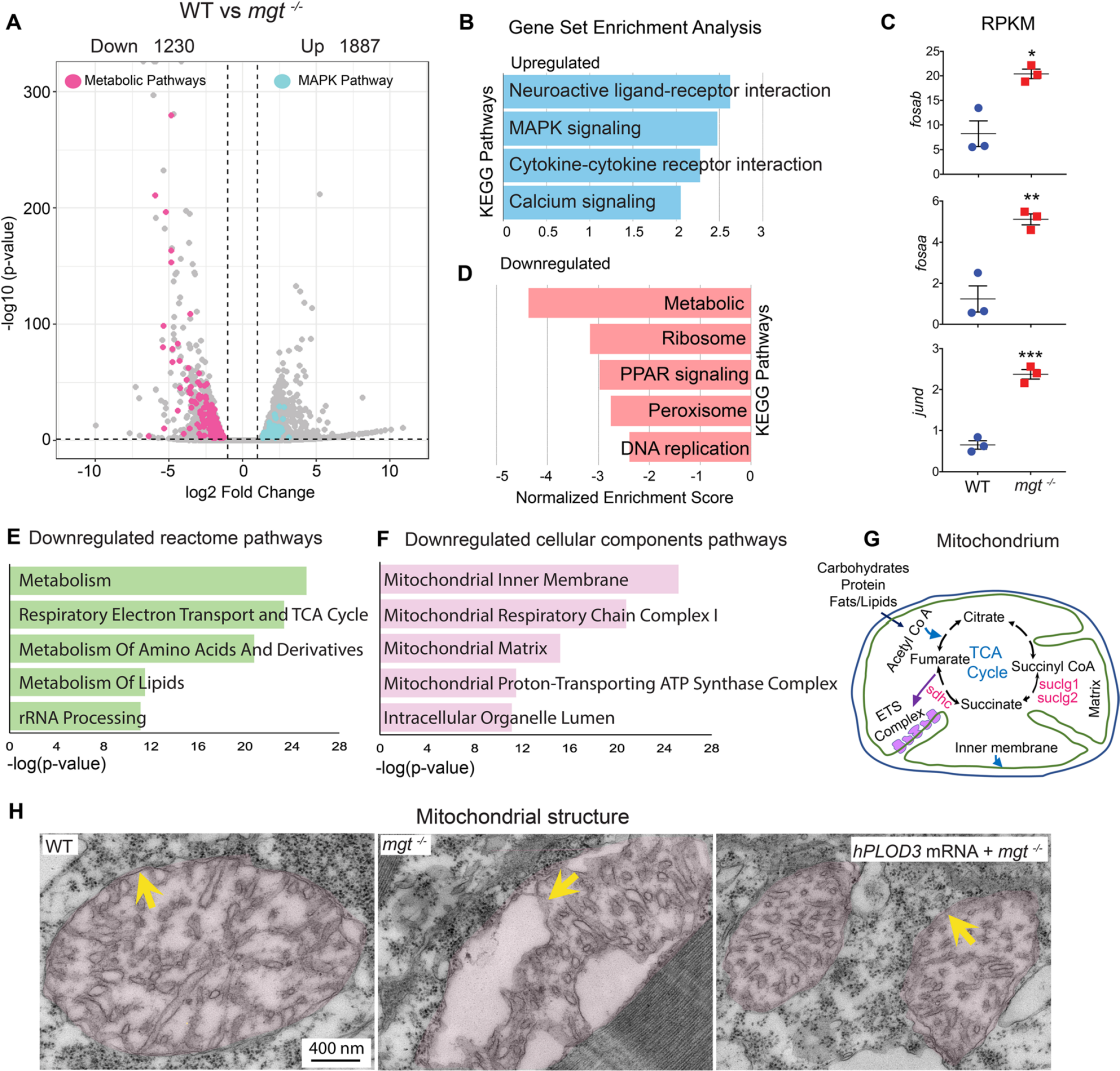
Gene expression and pathway changes in *plod3* deficiency. **A** RNA-seq data analysis presented as a volcano plot of differentially expressed genes between 4 dpf WT and *mgt*^*−/−*^ zebrafish. Significant genes (FC > 1.25 and adjusted *p* < 0.05) belonging to the KEGG metabolic (pink) and MAPK pathways (blue) are highlighted. Each RNA-seq technical replicate sample contains RNA from 60 embryos, with 3 independent technical replicates for each WT and *mgt*^*−/−*^ groups. **B** Gene set enrichment analysis (GSEA) of upregulated KEGG pathways that reached significance (*FDR* ≤ 0.05). **C** Expression changes in MAPK pathway-associated genes *fosaa*,* fosab*, and *jund* in WT and *mgt*^*−/−*^ zebrafish RNA-seq samples (*n* = 3) represented by RPKM values. Bars represent mean ± SEM, data were analyzed by Student’s t-test; ***p < 0.001, **p < 0.01, *p < 0.05. **D** GSEA of downregulated KEGG pathways that reached significance (*FDR* ≤ 0.05). **E** Human orthologue *Enrichr* analysis of the top 5 downregulated Reactome pathways by p-value. **F** Human orthologue *Enrichr* analysis of the top 5 downregulated Gene Ontology cellular components by p-value. **G** Graphic highlighting downregulated pathways and processes in the mitochondria. **H** TEM of trunk muscle shows mitochondria structure (pink shaded) in WT, *mgt* ^*−/−*^, and *mgt* ^*−/−*^ + *hPLOD3* mRNA at 4 dpf. WT mitochondrion with enclosing double membrane (yellow arrow) and properly arranged cristae, *mgt* ^*−/−*^ with interrupted double membranes, and *mgt* ^*−/−*^ injected with 300 pg *hPLOD3* mRNA showing mitochondria structure similar to the WT

Differential expression analysis identified 3,117 differentially regulated genes in *mgt* mutants with at least 1.25-fold change and *p* < 0.05 (Fig. 5A; Additional file 6 and 7, Tables S11 to S12). Prominently among the 1,887 upregulated transcripts, there was significant enrichment of genes encoding components of the MAPK pathway, including *jund*, *fosaa*, and *fosab* (Fig. 5B, C), which we further confirmed at the protein level in zebrafish mutant larvae and patient’s fibroblasts ( Additional file 2: Fig. S7F-I). MAPK signaling plays an important role in the regulation of autophagy, thus corroborating the findings described above (Fig. 4).

Interestingly, metabolic and synthetic pathways were downregulated in gene set enrichment analyses (GSEA) (Fig. 5D). To further explore the nature of differentially expressed genes, we mapped the zebrafish genes to their human orthologs applying the HCOP tool (HUGO Gene Nomenclature Committee, HGNC) and the ZFIN database [45, 69]. Then, we used the Enrichr [46] tool and examined the Reactome and Gene Ontology Cellular Components pathways. We found Respiratory Electron Transport and TCA Cycle, as well as Metabolism of Lipids and Amino Acid Derivatives, to be affected. Consistent with the subcellular localization of these processes, mitochondrial functions were prominently represented in Cellular Components pathways (Fig. 5E-G).

TEM examination of muscle cells revealed that mitochondrial outer and inner membrane integrity was disrupted in the *plod3* KO in zebrafish, and it could be restored by overexpression of *hPLOD3* mRNA. (Fig. 5H), suggesting that mitochondrial structural defects are an integral *plod3*-deficiency phenotype.

In conclusion, the RNA-seq data corroborated our results in *plod3* knockout and showed transcriptional upregulation of UPR and autophagy signaling pathways. They also revealed downregulation of transcripts associated with mitochondrial lipid and amino acid metabolism and energy cycles, both being consistent with ultrastructural defects in mitochondrial membranes revealed by TEM.

### Succinate restores musculoskeletal architecture in PLOD3 deficiency

The RNA-seq findings pointed to energy metabolism and mitochondrial integrity as key features of the *PLOD3* deficiency phenotypes. ECM production is an energy demanding process, and collagen has a high content of glycine and proline, whose synthesis involves steps that take place in mitochondria [70–72].

**Fig. 6:**
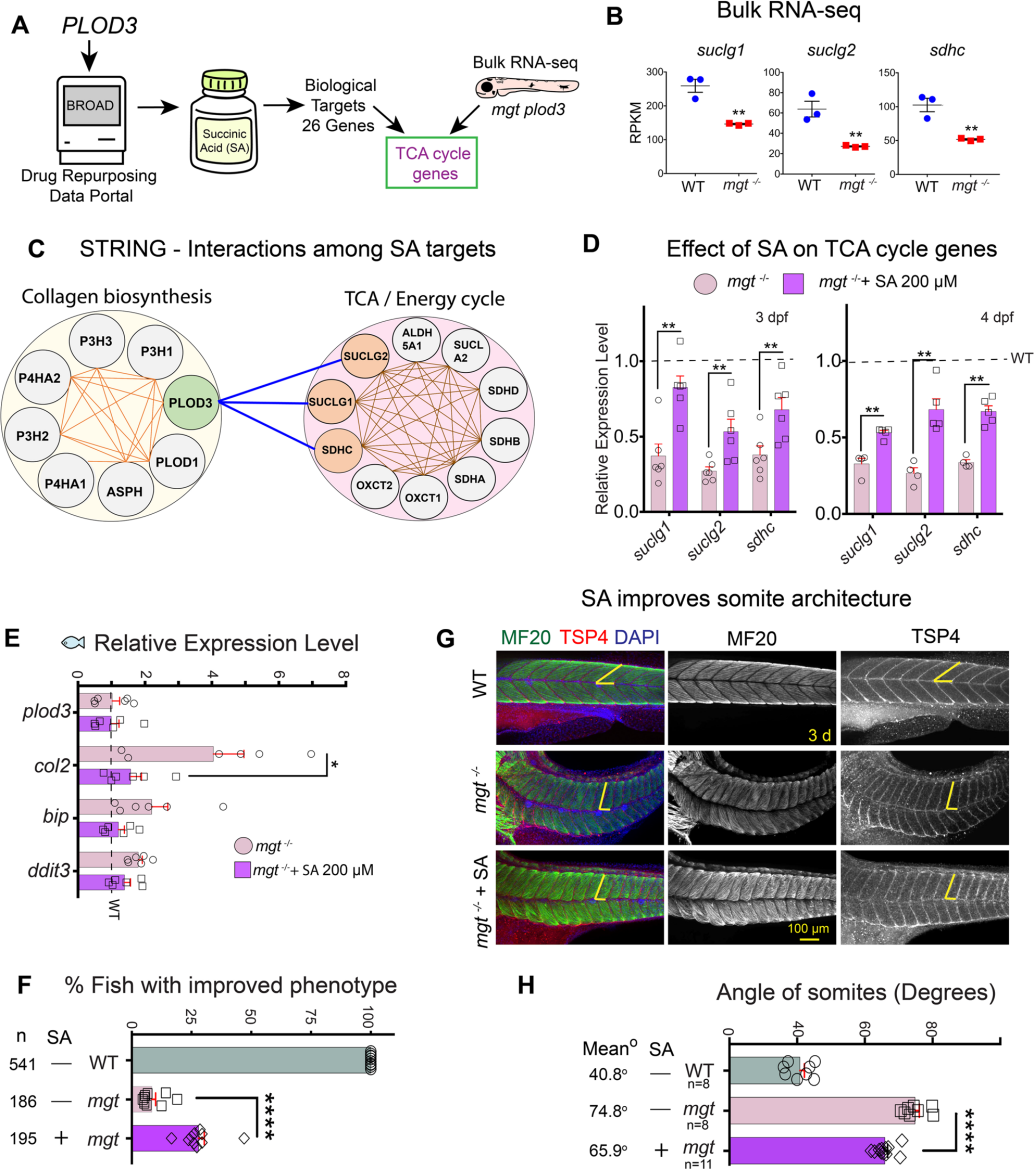
Succinate supplementation rescues musculoskeletal phenotypes. **A** Search strategy for PLOD3 targeting drugs and affected pathways. **B** RNA-seq results (RPKM values) of TCA cycle genes *suclg1*,* suclg2*,* and sdhc*; *n* = 3 biological replicates. **C** STRING (Search Tool for the Retrieval of Interacting Genes/Proteins) web tool analyses of protein-protein interactions among the 26 Succinic Acid (SA) targets. Identified novel interactions between *PLOD3* and TCA cycle genes are indicated by blue lines. Orange lines mark previously reported interactions. **D** Treatment with succinate increases expression of TCA cycle *suclg1*,* suclg2*, and *sdhc* genes in *mgt* ^*−/−*^. Relative expression levels in a qPCR assay normalized to *β-actin* transcript levels with WT expression set as 1 (dashed line). SA-treated larvae were assayed at 3 dpf (*n* = 6 replicates) and at 4 dpf (*n* = 4 replicates), comparing *mgt* ^*−/−*^ to *mgt* ^*−/−*^ treated with SA. Each replicate used an RNA sample from 30 zebrafish larvae. **E** Relative expression by qPCR of *plod3*,* col2*,* bip*, and *ddit3* normalized to *β-actin* transcript levels at 4 dpf larvae (*n* = 6 replicates), comparing *mgt* ^*−/−*^ to *mgt* ^*−/−*^ treated with SA. Each replicate used RNA from 30 zebrafish larvae. WT is set to 1 and marked on the graph with a dashed line. Data were analyzed with a two-tailed Student’s t-test, CI = 95%. Mean and SEM values are indicated with bars. Significance *p < 0.05. **F** Quantification of the % larvae with rescued live phenotypes at 3 dpf after SA treatment. Larvae with straighter, longer trunks were considered rescued/improved phenotype (n= number of examined animals, *N* = 10 experimental replicates). **G** Analysis of somite architecture by whole-mount immunostaining. WT, *mgt* ^*−/−*^, and *mgt* ^*−/−*^ +SA were stained for myosin (MF20, muscle) and TSP4 (tendons) and are shown as maximum intensity projections of confocal images (lateral views of trunk muscles). Yellow lines depict somite angle. **H** Quantification of somite angle was measured in the three experimental groups. Measurements for each animal were taken in three somites immediately anterior to the anal opening (as a landmark point), and the average angle in degrees was plotted for each animal. n=number of tested animals, Mean = mean angle in degrees for each group. Data in **b** and **d** were analyzed with a two-tailed Student’s t-test, CI = 95%. Mean and SEM values are indicated with bars. **p < 0.01. Data in **f** and **h** were analyzed by one-way ANOVA with Tukey’s multiple comparisons test. Mean and SEM values are indicated with bars. ****p < 0.0001

To identify molecules that could alleviate deficits in cellular pathways affected by PLOD3 deficiency, we searched the Drug Repurposing Data Portal (Broad) for known compounds that were laboratory tested and literature annotated to target individual genes [47]. We found the dietary supplement and TCA cycle intermediate –succinic acid (SA), which is also a product of PLOD3-catalyzed lysine hydroxylation of procollagen [19], to be the only compound in the Drug Repurposing Data Portal associated with *PLOD3*. To follow up, we conducted a reverse search for all targets of succinate reported in the Broad Portal and identified a total of 26 genes (Fig. 6A; Additional file 1: Table S5). Interestingly, many SA targets were downregulated in RNA-seq zebrafish data (Fig. 6B). To examine potential gene-gene interactions among SA targets, we used Search Tool for the Retrieval of Interacting Genes/Proteins (STRING) [48] and found that they fall into two main categories: collagen biosynthesis (8 genes), and TCA cycle and energy metabolism (10 genes) (Fig. 6C). For the remaining 8 genes STRING did not offer known associations; however, these genes were mitochondrial transporters or enzymes known to work in energy metabolism pathways (Additional file 1: Table S5). Taken together, the RNA-seq data revealed a connection between collagen biosynthesis genes and SA processing enzymes (*suclg1*, *suclg2*, and *sdhc*) that were substantially downregulated in *mgt* mutants (Fig. 6B, C).

We reasoned that if SA levels are affected by *plod3* deficiency, supplementation might reverse phenotypes associated with BCARD and restore normal transcriptional dynamics in *plod3* mutants. We established a minimum effective dose of SA to rescue notochord extension and body length to be 200 µM. We evaluated the live phenotype of SA-treated larvae in multiple independent experiments (following procedures established for mRNA rescue experiments) and found significant improvement in body length and patterning. Furthermore, evaluation of treated animals by qPCR showed that transcripts of SA processing enzymes (*suclg1*, *suclg2*, and *sdhc*) rebounded towards WT levels after SA treatment (Fig. 6D). Expression analysis of SA-treated animals by qPCR found no significant changes in transcript levels of *plod3*, and the UPR genes *bip and ddit3;* however, both trended towards WT levels. Whereas, collagen type II was significantly downregulated, almost reaching WT levels (Fig. 6E).

We examined trunk muscle architecture and notochord extension because these two phenotypes are the hallmarks of zebrafish *mgt* mutants, and muscle dysfunction is highly prevalent in BCARD individuals. We examined zebrafish trunk muscles by measuring the angle of intersomitic boundaries to the horizontal myoseptum (averaging 3 measurements per embryo at the somites before anal opening) in larvae stained with MF20 and Tsp4. We found that SA treatment significantly reduced the somite angle in treated animals by approximately 12%, trending towards WT levels (Fig. 6F-H).

Overall, treatment with SA reversed reduced expression of *suclg1*, *suclg2* and *sdhc* enzymes trending towards WT levels, and improved the architecture of the trunk muscles in *plod3*-mutant zebrafish.

## Discussion

We have investigated clinically relevant BCARD phenotypes associated with recessive deleterious mutations in the *PLOD3* gene in zebrafish models of PLOD3 deficiency, matched with molecular and cellular studies in patient’s skin fibroblasts. We show that the core clinical features in BCARD syndrome are recapitulated in the zebrafish loss-of-function models. These include cartilage, bone, ocular, ear, and vascular defects [2]. Besides the core features, contributing to the BCARD syndrome acronym, individuals carrying ten distinct *PLOD3* variants also presented with developmental dysmorphology within craniofacial structures, axial skeleton and limb defects, muscle dysfunction, skin abnormalities, and brain anomalies and dysfunction [3–8]. In many subjects, sudden arterial rupture and intracranial hemorrhage are the catastrophic outcomes of the disease. These additional phenotypes also manifested in zebrafish *plod3* mutants, indicating that zebrafish offer a robust in vivo system to examine the wide spectrum of cellular and molecular mechanisms underlying PLOD3 deficiency symptoms. This is particularly important because prior studies in animal models focused on neuronal and eye phenotypes [73, 74], while the musculoskeletal deficits have not been examined and remain poorly understood.

The zebrafish and human enzymes exhibit high sequence homology (~ 80%) and are functionally conserved across vertebrates, as shown by our rescue experiments with *hPLOD3* mRNA in zebrafish knockout animals. We observed broad improvements across multiple organ systems, including skeletal muscle of the trunk and the structure-function of the forelimbs, as well as the architecture of the axial vasculature. However, some organ systems, e.g., craniofacial skeleton, were restored to a lesser degree, possibly because development of the craniofacial structures is tightly regulated in time and space, and the rescue experiments, by mRNA injection into a 1-cell stage embryo, result in broad overexpression that does not recapitulate the native spatial and temporal dynamics of Plod3 protein function. Interestingly, the relatively simpler structure of pectoral fins was sufficiently improved to restore their function in larval swimming behavior.

The *maggot* G245E substitution corresponds to the human G256 amino acid in PLOD3, a critical residue within the catalytic site of glycosyltransferase in the GT domain [17, 21]. Experiments with purified PLOD3 in complex with GLT24D1 measuring galactosyltransferase and glucosyltransferase activity in vitro revealed that a *PLOD3* G256A mutation results in a highly significant loss of glucosyltransferase activity and, surprisingly, markedly reduced galactosyltransferase activity of GLT24D1 [17]. These data indicate that the *mgt* missense mutation disrupts not only the essential glucosyltransferase activity of PLOD3, but also the overall complex function, likely rendering it a null allele. This is consistent with the early developmental lethal phenotype of the zebrafish G245E mutation and further underscores the highly conserved function of the human and zebrafish enzymes.

The patient variant c.1354 C > T corresponding to position R452 was originally thought to represent a null allele due to predicted formation of a premature stop codon [2]; however, our detailed molecular analysis in biopsied skin fibroblast revealed that the cDNA carries a deletion removing R452 and V453, while the remaining part of the peptide is complete. This finding is consistent with partial rescue of the null *maggot* allele by the mutated mRNA lacking R452 and V453 residues, but no rescue with the R452* truncated mRNA. Similarly, the patient’s clinical manifestations are moderately severe at 6 years of age, suggesting that the individual carries the two-amino-acid deletion in most cells.

Recent structural analysis revealed that PLOD3 R452 contributes to the interaction surface with the GLT24D1 enzyme in the complex. Consistent with the structural data, 293T cells transfected with a construct with an alanine substitution in this position resulted in a decrease or complete loss of complex formation in pull-down experiments using Strep-tagged GLT24D1 [17]. Our AlphaMissense prediction analysis further confirmed that deletion/substitution of R452 would be deleterious to protein function [52]. We have also tested two additional variants in the LH domain active site, C691* and L627P ( Additional file 2: Fig. S8A). Both have partially rescued the notochord extension in the *mgt* mutant, indicating that the two alleles are strong hypomorphs [1, 3, 21]. Together, our results have provided strong evidence supporting the pathogenicity of human variants and zebrafish mutations using in vivo replacement experiments. Cross-referencing of zebrafish and human phenotypes highlighted that ECM processing mechanisms are evolutionarily conserved and that zebrafish can serve as an informative preclinical model.

In cells producing large quantities of ECM proteins, such as collagens, activation of the basal levels of ER stress and unfolded protein response (UPR) pathways is necessary to sustain physiological cellular functions, as shown for chondrocytes [75, 76]. ECM deficits, resulting from a range of genetic mutations affecting the structural proteins themselves, their trafficking and assembly apparatus, as well as enzymes catalyzing their post-translational modifications, further activate ER stress and UPR pathways [32]. In this respect, PLOD3/BCARD shares the cellular phenotypes of protein backlog in vesicular compartments of the anterograde secretory pathway and ER stress responses as observed in mutations in SEC24D/Osteogenesis Imperfecta and RIC1/CATIFA syndrome, which disrupt pre- and post-Golgi collagen traffic, respectively [12, 14]. However, our drug treatment experiments with UPR and autophagy inhibitors failed to rescue Plod3 deficiency phenotypes in zebrafish, suggesting that the observed upregulation of the PERK pathway of the UPR [56, 77] in *plod3* mutants and phosphorylation of MAP kinase pathway components [57, 78, 79], leading to activation of autophagy [80, 81], are likely adaptive mechanisms intended to clear a backlog of unprocessed collagens and not the underlying cause of the observed phenotypes.

The PLOD3 deficiency, as revealed by TEM imaging, is characterized by mitochondrial dysmorphology and autophagy, and correlates with deficits in mitochondrial biosynthetic pathways, as shown by expression analyses in zebrafish. Importantly, BCARD tissue-level phenotypes of muscle weakness and neuronal phenotypes are shared with mitochondrial cytopathies that could be explained by deficits in mitochondrial biosynthetic pathways and energy metabolism [82].

The link to energy metabolism is underscored by the fact that succinate is one of the main products of the PLOD3 enzymatic activity during lysyl hydroxylation of collagen. Succinate is also a TCA cycle intermediate and thereby a key metabolite in mitochondrial energy generation pathways [83, 84]. This association raises the possibility that perturbations in succinate metabolism due to PLOD3 deficiency might be responsible for the mitochondrial dysmorphology detected by TEM in muscle cells and the zebrafish RNA-seq data that showed downregulation of TCA cycle genes, including *suclg1*, *suclg2*, and *sdhc* [85–87]. Furthermore, the 26 target genes of succinate identified using the STRING database fall into two main groups, the collagen biosynthesis network, and the TCA cycle and energy metabolism (Fig. 5). Defective mitochondrial structure and function may further compromise ECM integrity since collagen production is energetically demanding [72, 88], and glycine and proline, the two main amino acids needed for collagen synthesis can be synthesized in mitochondria [70, 71].

The electron chain complex II on the inner mitochondrial membrane includes the SDHC [86] enzyme that could be directly responding to succinate, bypassing the need for upstream factors [89]. Consistent with this possibility, treatment of zebrafish *plod3* mutants with succinic acid led to significant recovery of transcript levels of the three TCA cycle genes, trending towards wild-type levels. This evidence, together with the in vivo improvement in trunk muscle architecture and notochord extension, indicates that future treatment options based on this observation could be developed for individuals with ECM deficits caused either by rare genetic diseases or other conditions such as aging, cancer, or injury. Moreover, the use of succinate in PLOD3 deficiency models coupled with high-throughput readouts may offer additional insights into molecular mechanisms contributing to the phenotypic manifestations of BCARD syndrome.

Our findings suggest that mitochondrial energy metabolism processes might be, in part, the underlying biological mechanism of PLOD3 deficiency, as we found an overall improvement of the musculoskeletal phenotypes in a highly significant number of animals treated with succinic acid, which is currently readily available as a nutritional supplement. The structure-function improvements were particularly impressive in comparison to treatments with ER stress blockers and autophagy inhibitors, where phenotypes did not improve and, in fact, further worsened. Of particular interest was the fact that the improvements were most obvious in the architecture of skeletal trunk muscle and notochord length, two tissues that were almost completely rescued by mRNA overexpression in *mgt* mutants. It would be of interest to test whether other ‘drug probes’ would be more effective in the rescue of other deficits, e.g., vascular or ear phenotypes. Future screens of chemical libraries would be needed to identify novel therapeutic leads, and zebrafish models will be helpful in this endeavor.

Taken together, our findings have enhanced understanding of BCARD disease mechanisms at the molecular, cellular, and tissue levels, and will aid in clinical differential diagnosis, as well as development of treatment options for a range of genetic disorders affecting ECM synthesis, processing, and maintenance.

## Conclusions

We describe novel animal models and functional validation of rare disease variants in the extracellular matrix-modifying enzyme PLOD3 that are associated with BCARD syndrome. Our data clearly demonstrate a previously unknown mechanistic connection between energy metabolism and BCARD disease phenotypes caused by the PLOD3 deficiency. We propose a path forward to alleviate symptoms of musculoskeletal deficits in BCARD by strengthening mitochondrial energy metabolism with a dietary supplement, succinic acid. We set an example of how multi-omics approaches, animal models, and drug repurposing portals could bolster treatment options for rare diseases. Future studies on succinate requirement for specific metabolic processes will further help understand the physiologically relevant role of the PLOD3-dependent succinate production during collagen processing.

## Supplementary Information

Additional file 1: Table S1. Sequencing primers. Table S2: Q5® site-directed mutagenesis. Table S3: Primary and secondary Antibodies. Table S4: Primers for qPCR. Table S5: Succinic-acid targets. Table S6: BCARD Phenotype. Table S8: ER Stress Inhibitors.

Additional file 2. Additional methods: Genetic Mapping and Positional Cloning of Zebrafish Mutations, Genetic Manipulations in Zebrafish, CRISPR/Cas9 genome editing, Brain anatomy measurements, Data Portals, and Chemical treatments. Fig. S1: Genetic analysis of the zebrafish *plod3* mutations. Fig. S2: *plod3* CRISPR edited gene knockout phenotypes. Fig. S3: Clinical variants in *PLOD3* are pathogenic. Fig. S4: Rescue of zebrafish muscle, vessel, and brain phenotypes with human *PLOD3* RNA. Fig. S5: Reduced expression of *PLOD3* disrupts procollagen trafficking and induces ER stress in zebrafish *mgt* −/− mutants and human fibroblasts. Fig. S6: Increased autophagy in *PLOD3* deficiency. Fig. S7: Autophagy inhibitors do not improve *PLOD3* deficiency outcomes. Fig. S8: Human *PLOD3* variants and functional domains.

Additional file 3: Table S7. AlphaMissense algorithm data.

Additional file 4: Table S9. DE_results WT vs *mgt*.

Additional file 5: Table S10. RPKM values.

Additional file 6: Table S11. Enrichr Reactome downregulated.

Additional file 7: Table S12. Enrichr Cellular components downregulated.

## Data Availability

The RNA-seq datasets generated and analyzed during the current study are available in the Gene Expression Omnibus (GEO) repository, accession number [GSE318257] (https://www.ncbi.nlm.nih.gov/geo/query/acc.cgi?acc=GSE318257) [90]. All analyzed data are provided in additional files.

## Declarations

All animal studies were conducted under the guidelines established by the Institutional Animal Care and Use Committee (IACUC) at Vanderbilt University Medical Center, following approved animal protocols (Protocol number M1700020).

## Abbreviations

ECM: Extracellular Matrix
PLOD3: procollagen-lysine, 2-oxoglutarate 5-dioxygenase 3
BCARD: Bone Abnormalities, Cataracts, Arterial Rupture, and Deafness
ER: Endoplasmic Reticulum
N: Nucleus
TCA: Tricarboxylic Acid
WT: Wild Type
KO: Knock Out
mgt: maggot (plod3 KO zebrafish line)
mRNA: messenger Ribonucleic Acid
CRISPR: Clustered Regularly Interspaced Short Palindromic Repeats
CAS9: CRISPR-Associated protein 9
gRNA: guide Ribonucleic Acid
COL2α1: Collagen type II alpha 1 chain
eGFP: Enhanced Green Fluorescent Protein
h: Human
z: Zebrafish
pg: picogram
ED: Eye Diameter
BL: Body Length
MF20: Muscle marker, α-myosin
TSP4: Thrombospondin 4
DAPI: 4′, 6-Diamidino-2-Phenylindole
Ace Tub: Acetylated Tubulin
WGA: Wheat Germ Agglutinin
TEM: Transmission Electron Microscopy
PERK: Protein kinase R like Endoplasmic Reticulum (ER) Kinase pathway
ATF6: Activating Transcription Factor 6
Atf4: Activating Transcription Factor 4
Ddit3: DNA damage-inducible transcript 3
LC3: Microtubule-associated protein 1 light chain 3
Atg5: Autophagy-related protein 5
p62: ubiquitin-binding protein p62/Sequestrosome-1
LAMP1: Lysosomal-associated membrane protein 1
GABARAP: Gamma-AminoButyric Acid Receptor-Associated Protein
GSEA: Gene set enrichment analysis
ZFIN: Zebrafish Information Network
STRING: Search Tool for the Retrieval of Interacting Genes/Proteins
RNA-seq: RNA sequencing)
SA: Succinic Acid
SUCLG1: Succinate-CoA ligase GDP/ADP-forming subunit alpha
SUCLG2: Succinate-CoA ligase, GDP-forming, subunit beta
SDHC: Succinate dehydrogenase complex subunit C
