# Supplementary material for: Succinate supplementation ameliorates musculoskeletal defects caused by PLOD3 mutations in a BCARD syndrome model
Source: Genome Med. 2026 Mar 13;18:29. doi: 10.1186/s13073-026-01608-y (PMC12994257; doi:10.1186/s13073-026-01608-y)
Supplement: Supplementary file 1 — Additional file 1: Table S1. Sequencing primers. Table S2: Q5® site-directed mutagenesis. Table S3: Primary and secondary Antibodies. Table S4: Primers for qPCR. Table S5: Succinic-acid targets. Table S6: BCARD Phenotype. Table S8: ER Stress Inhibitors. [file 13073_2026_1608_MOESM1_ESM.pdf]

Table S1: Sequencing primers

	Details	FWD primer	REV Primer
1	*plod3* cDNA amplification	5’-TAGCGACGGGAAAACTGAAC-3'	5’-CAAATCTGATGGAGGGAGGA-3'
2	*mgt*^{m635} cDNA mutation	5’-CAGCTGTTCTACACACGCATC-3'	5’-CAACGTAGCGAGTCTCTCCAG-3'
3	*plod3* CRISPR mutation	5’-GCCTTTATAGTGTGCGTGTTTG-3'	5’-GAAATTAAGTCGCTGCAgtgtg-3'
4	hPLOD3 cDNA	ClaI (5’atcgatATGACCTCCTCGGGGCCT-3')	XhoI (5’ctcgagTCAGGGGTCGACAAAGGA-3’)
5	zebrafish *PLOD3* (zPLOD3) cDNA	ClaI 5’-actcATCGATTAGCGACGGGAAAACTGAAC-3’	XhoI 5’-actcCTCGAGCAAATCTGATGGAGGGAGGA-3’

Table S2: Q5® site-directed mutagenesis

	Mutation	FWD primer	REV Primer
1	1354C>T (Arg452 to stop) Truncating Mutation	5'GCAGCGGAAGtGAGTGG GTGT-3'	5'ACCAGCTCCACGTAGT CC-3'
2	1354C>T (Arg452_Val453del) 2 AA Deletion	5'GGTGTGTGGAATGTACC ATACATC-3'	5'CTTCCGCTGCACCAG CTC-3'
3	1880T>C (Leu627Pro) Missense mutation	5'- CTGCAGCTGCcGCGGACG TAT-3'	5'- GCCACCTCCCTCATAGT CCAG-3'
4	2071delT (Cys690 to frameshift)	5'- GCCGCTTCCTGCGCTACG- 3'	5'- GCCACCTCCCTCATAGT CCAG-3'

Table S3: Primary and secondary Antibodies

	Antibody Name	Catalog Number	Manufacture	Conc
1.	PLOD3 Antibody	11027-1-AP	Proteintech	1:500
2.	Collagen Type II Antibody WB	600-401-104-0.1	Rockland	1:500
3.	Collagen Type II Antibody IF	II-II6B3	DSHB	1:500
4.	Anti-Collagen I antibody [EPR7785] WB	ab138492	abcam	1:500 WB,
5.	Anti-Collagen I antibody IF	ab34710	abcam	1:200 IF
6.	eIF2α Antibody	#9722	CST	1:500
7.	Phospho-eIF2α (Ser51) Antibody	#9721	CST	1:500
8.	ATG5 antibody	ab108327	abcam	1:500
9.	Anti-SQSTM1 / p62 antibody	ab155686	abcam	1:500
10.	LC3A/B Antibody	PA1-16931	Thermo	1:500, 1:200 IF
11.	Cathepsin B Antibody	M1506-1	Huabio	1:500
12.	ATF4	PA5-72620	Thermo	1:500
13.	LAMP1	Ab24170	abcam	1:500 WB, 1:200 IF
14.	CHOP	2895	CST	1:500
15.	ERp57	**MA5-31366**	Thermo	1:200
16.	GM130	610822	BD-Biosciences	1;200
17.	PDI	P7496	Sigma	1:200
18.	P44/42 MAPK	4696	CST	1:500
19.	Phospho P44/42 MAPK	9106	CST	1:500
Secondary				
20.	Anti-alpha Tubulin Antibody	ab40742	abcam	1:3000
22.	Anti-Rabbit IgG (H+L), HRP Conjugate	W4011	Promega	1:3000
23.	Anti-Mouse IgG (H+L), HRP Conjugate	W4021	Promega	1:3000
21.	GFP (Chicken IgY)	A10262	Vanderbilt Molecular and Cell Biology Core	1:500
22.	Chicken IgY-Alexa488	A11039	Invitrogen	1:500
23.	WGA-488	W11261	Invitrogen	1:500
22.	Anti-mouse GFP-488	A21121	Invitrogen	1:500

23.	Anti-mouse GFP-555	A21127	Invitrogen	1:500
24.	Anti-rabbit GFP-488	A11034	Invitrogen	1:500
25.	Anti-rabbit GFP-555	A21428	Invitrogen	1:500
Dyes				
26.	DAPI	D1306	Invitrogen	1:5000
27.	LysoTracker	L7528	Thermo	50 nM

Table S4: Primers

Name	Forward	Reverse
β-actin 1	CCATGGATGAGGAAATCGCTGC	GTCACACCATCACCAGAGTCC
zAtf4	TTAGCCATTGCTCCGATAGC	GCTGCGGTTTTATTCTGCTC
zAtf6	CTGTGGTGAAACCTCCACCT	CATGGTGACCACAGGAGATG
zDdit3	AAGGAAAGTGCAGGAGCTGA	TCACGCTCTCCACAAGAAGA
Zplod3	CGACGGGAAAACTGAACTGAC	GCCATCGAACCTTCTGACCT
Col2	TTACAAGAAGCAGACTGCGC	ACCTGAAGAAGGCCATTCTG
β-actin 2	ACTGTATTGTCTGGTGGTAC	TACTCCTGCTTGCTAATCC
Bip	AAGAGGCCGAAGAGAAGGAC	AGCAGCAGAGCCTCGAAATA
Suclg1	CAGCAAGCAGGAGTGAGACA	TCACGGAGTTGAAGACTGGC
sdhc	CCTCCGAACACTCAAGCTCA	ACTCTCCGGTAACACCAACG
suclg2	CAAACTTCTTGGACCTGGGC	TTCCTTCGAGTCTGACCACC

Table S5: Succinic-acid targets

N	Succinic-acid targets (Broad Institute data portal)	
1	PLOD1	procollagen-lysine,2-oxoglutarate 5-dioxygenase 1
2	PLOD3	procollagen-lysine,2-oxoglutarate 5-dioxygenase 3
3	SDHA	succinate dehydrogenase complex flavoprotein subunit A
4	SDHB	succinate dehydrogenase complex iron sulfur subunit B
5	SDHC	succinate Dehydrogenase Complex Subunit C
6	SDHD	succinate dehydrogenase complex, subunit D
7	ALDH5A1	aldehyde dehydrogenase 5 family member A1
8	ASPH	aspartyl/asparaginyl beta-hydroxylase
9	BBOX1	gamma-butyrobetaine hydroxylase 1
10	HSD17B6	hydroxysteroid 17-beta dehydrogenase 6
11	OXCT1	3-oxoacid CoA-transferase 1
12	OXCT2	3-Oxoacid CoA-Transferase 2
13	P3H1	prolyl 3-hydroxylase 1
14	P3H2	prolyl 3-hydroxylase 2
15	P3H3	prolyl 3-hydroxylase 3
16	P4HA1	prolyl 4-hydroxylase subunit alpha 1
17	P4HA2	prolyl 4-hydroxylase subunit alpha 2
18	SLC13A1	solute carrier family 13 member 1
19	SLC13A2	solute carrier family 13 member 2
20	SLC13A3	solute carrier family 13 member 3
21	SLC25A10	solute carrier family 25, member 10
22	SUCLA2	succinate--CoA ligase [ADP-forming] subunit beta
23	SUCLG1	succinate-CoA ligase GDP/ADP-forming subunit alpha
24	SUCLG2	succinate-CoA ligase [GDP-forming] subunit beta
25	SUCNR1	succinate receptor 1
26	TMLHE	trimethyllysine hydroxylase, epsilon

Table S6:

BCARD Phenotype	
Organ	Phenotype
Muscle	Reduced muscle mass,<br>Muscle atrophy,<br>Joint contractures (elbows, fingers),<br>Club foot (TEV),<br>Diaphragmatic Eventration
Skeleton	Osteopenia, Low Bone Mineral Density,<br>Fractures,<br>Scoliosis, Kyphoscoliosis,<br>Flat Vertebrae (Platyspondyly),<br>Small capital femoral epiphyses,<br>Prominent knees,<br>Midfacial hypoplasia, Shallow orbits,<br>J-shaped sella turcica,<br>Teeth abnormalities
Dysmorphic features	Low set, simple ears,<br>Long philtrum, Short nose, anteverted nares,<br>Downturned corners of the mouth,<br>Decreased palmar creases,<br>Dysplastic nails and tapered fingers,<br>Coarse hair
Ear	Sensorineural hearing loss
Eye	Cataracts, Retinal thinning,<br>Optically empty vitreous<br>Myopia
Neurologic	Developmental delay,<br>Speech deficits,<br>Ptosis
CV	Aneurysms (hemorrhage),<br>Bruisability and blistering of skin

Table S8: ER Stress Inhibitors

No	Catalog no	Name
1	HY-15654	Sodium phenylbutyrate
2	HY-19696A	Tauroursodeoxycholate Sodium
3	HY-12837	EN460
4	HY-101455	CDN1163
5	HY-15486	Salubrinal
6	HY-17537	APY29
7	HY-13820	GSK2656157
8	HY-15845	STF-083010
9	HY-19710	MKC3946
10	HY-12825	BHPI
11	HY-100545	BAPTA-AM
