# Supplementary material for: Succinate supplementation ameliorates musculoskeletal defects caused by PLOD3 mutations in a BCARD syndrome model
Source: Genome Med. 2026 Mar 13;18:29. doi: 10.1186/s13073-026-01608-y (PMC12994257; doi:10.1186/s13073-026-01608-y)
Supplement: Supplementary file 2 — Additional file 2. Additional methods: Genetic Mapping and Positional Cloning of Zebrafish Mutations, Genetic Manipulations in Zebrafish, CRISPR/Cas9 genome editing, Brain anatomy measurements, Data Portals, and Chemical treatments. Fig. S1: Genetic analysis of the zebrafish plod3 mutations. Fig. S2: plod3 CRISPR edited gene knockout phenotypes. Fig. S3: Clinical variants in PLOD3 are pathogenic. Fig. S4: Rescue of zebrafish muscle, vessel, and brain phenotypes with human PLOD3 RNA. Fig. S5: Reduced expression of PLOD3 disrupts procollagen trafficking and induces ER stress in zebrafish mgt −/− mutants and human fibroblasts. Fig. S6: Increased autophagy in PLOD3 deficiency. Fig. S7: Autophagy inhibitors do not improve PLOD3 deficiency outcomes. Fig. S8: Human PLOD3 variants and functional domains. [file 13073_2026_1608_MOESM2_ESM.pdf]

Additional information

Succinate supplementation ameliorates musculoskeletal defects caused by *PLOD3* mutations in a BCARD syndrome model

Dharmendra Choudhary [1,2], Gokhan Unlu[1,2,3,&], Taylor Nagai[1,2], David B. Melville[1,3], Alexandra Scalici[1,2,$], Mais O. Hashem[4], Dylan J. Ritter[1,2,3], Georg Schmidt[5], Cory L. Guthrie[1,] Eric R. Gamazon[1,2,6], Fowzan S. Alkuraya[4,7], Nancy J. Cox[1,2], Ela W. Knapik[1,2,3,5]*

[1]Department of Medicine, Division of Genetic Medicine, [2]Vanderbilt Genetic Institute, Vanderbilt University Medical Center, TN 37232, U.S.A.; [3]Department of Cell and Developmental Biology, Vanderbilt University, TN 37232, U.S.A.; [4]Genomic Medicine Center of Excellence, King Faisal Specialist Hospital and Research Center, Riyadh, Saudi Arabia; [5]Developmental Biology, Institute Biology I, University of Freiburg, Hauptstrasse 1, D-79104 Freiburg, Germany; [6]Clare Hall, University of Cambridge, Cambridge, CB3 9AL United Kingdom, [7]College of Medicine, Alfaisal University, Riyadh, Saudi Arabia;

[&] Current address: Laboratory of Metabolic Regulation and Genetics, The Rockefeller University, New York, NY 10065, U.S.A.

[$] Current address: Department of Psychiatry, Yale University School of Medicine, New Haven, CT 06510, U.S.A.

* Correspondence:

Ela W. Knapik

1165 Light Hall, 2215 Garland Ave., Nashville, TN 37232, U.S.A.

Email: ela.knapik@vumc.org

Phone: +1 (615) 322-7569

Supplementary Materials and Methods

Genetic Mapping and Positional Cloning of Zebrafish Mutations

The *maggot* locus was mapped to the distal arm of chromosome 23 using the zebrafish genetic linkage map in an F2 intercross by bulked segregant analysis [1]. Mutation-carrying AB fish and India line were used for the cross [2]. We constructed a physical map of chromosome 23 using BAC clone sequences in the Zv9 assembly (NCBI) and restricted the critical interval to a 0.33 cM region containing four candidate genes, including *plod3*. Direct Sanger cDNA sequencing identified *plod3* as the affected gene. For genotyping of the *maggot (mgt*^{m635}*)* mutation, *plod3* cDNA was amplified using (5'-TAGCGACGGGAAAACTGAAC-3') and (5'-CAAATCTGATGGAGGGAGGA-3'); and the mutation was identified using 5'-CAGCTGTTCTACACACGCATC-3' and 5'-CAACGTAGCGAGTCTCTCCAG-3' primers and Sanger sequencing. The sequence homology analysis was conducted with Clustal Omega (EMBL-EBI) using zebrafish *Plod3* (Zv11 assembly) and human *PLOD3* primary sequences (Uniprot, no.060568).

Genetic Manipulations in Zebrafish and Brain Imaging

CRISPR/Cas9 genome editing: CRISPR/Cas9 target sites within the zebrafish *plod3* gene (GRCz11 assembly, ENSDARG00000076317) were identified using the CHOPCHOP web tool [3]. The following site in exon 4 of the *plod3* gene was selected for this study: 5'-GGTGATGTGTCGGACTGTCGG-3'. As previously described, a cloning-free method to generate sgRNA (single guide RNA) templates was performed [4]. Guide RNAs were synthesized with a MEGAshortscript™ T7 transcription kit (ThermoFisher Scientific, AM1354). To generate mutations with the CRISPR/Cas9 system, a mixture of 500 pg purified Cas9 protein (PNA Bio Inc, # CP01) and 300 pg gRNA was injected into one-cell stage embryos. Injected embryos were grown to 4 dpf for phenotypic analysis. Mutations generated in injected embryos were detected via direct Sanger sequencing of the region flanking the target site using primers (Additional Table 1). PCR-amplified products were cloned into the pGEM-T Easy vector (Promega, A1360) and sequenced using SP6 primers to detect mutations created by the CRISPR/Cas9 editing.

Brain anatomy measurements: Whole-mount zebrafish larvae stained with Acetylated Tubulin and WGA were imaged on a Nikon Spinning Disk Confocal Microscope with a Plan Apo Lambda ×10/0.45 NA objective

[5,6]. Maximum-intensity z-projections (150–200 µm depth) were created in Nikon Elements software and analyzed in ImageJ using Freehand selection and measurement tools. Measurements of the cerebellar area and optic tecta were performed using ImageJ on whole-mount immunostained images. Arbitrary units (a.u.) of areas were plotted.

Data Portals

Protein structure: The predicted protein structure of zebrafish Plod3 (zPlod3) was obtained using I-TASSER [7,8]. The predicted zPlod3 structure was compared to a published human PLOD3 (hPLOD3) structure (PDB #6FXK). The image was generated to show structural changes between hPLOD3 and zPlod3. For 3D structure analysis, we used data portals: RCSB (Research Collaboratory for Structural Bioinformatics Protein Data Bank) [9], PBM (protein binding microarrays) [10], and *UCSF Chimera* [11]. Homology between human and zebrafish proteins was calculated through Clustal Omega [12].

AlphaFold protein structure platform: AlphaFold was used to predict the 3D structure of variants of PLOD3 based on primary amino acid sequences using machine-learning models[13]. We used AlphaMissense [13] to predict the pathogenicity of PLOD3 amino acid variants associated with human and zebrafish models.

In-silico translation of *Plod3*: ExPASy (Expert Protein Analysis System) (https://web.expasy.org/translate/) is part of the Swiss Institute of Bioinformatics (SIB). It was used for the *in-silico* translation of DNA to protein [14].

Gene Expression

Immunohistochemistry: Notochord Col2 staining procedures were performed as prescribed in the main Methods section.

Western blotting (WB): As described in the main Methods section, WB of MAPK and its phosphorylated form was performed in human variant fibroblasts and zebrafish samples.

Chemical treatments

ER Stress inhibitors: As listed in Table S8, ER stress inhibitors were dissolved according to the manufacturer's instructions (MedCemExpress). Larvae were kept with inhibitors at final

concentrations of 10 μM, 1 μM, and 0.1 μM from 1 dpf to 4 dpf, and the egg water was changed daily. The larvae were evaluated for survival, locomotion, length, trunk angles, vascular defects, and cardiac edema.

Autophagy inhibitors: The following inhibitors of autophagic flux were used: Chloroquine (tlrl-chq-4, InvivoGen) targets autophagosome-lysosome fusion; 3-Methyladenine (3-MA, inh-3ma-2, InvivoGen) inhibits lysosome maturation and the double-membrane-bound autophagosome formation process; Bafilomycin A1 (BafA1, tlrl-baf1, InvivoGen), a specific vacuolar H^+ ATPase (V-ATPase) inhibitor. BafA1 inhibits H^+ translocation in acidic intracellular compartments (i.e., endosomes, lysosomes, and vesicles) and their resident hydrolase activity. Chloroquine (Chloq, 5 μM), 3-methyladenine (10 mM), and Bafilomycin A1 (25 nM) treatment were given at 24 hpf, and protein was collected at 72 hpf [15]. Vehicle-treated WT and *mgt*$^{-/-}$ were used as controls. After treatment**,** Lamp1 and Col2 protein expression were analyzed by WB.

ADDITIONAL DATA FIGURES

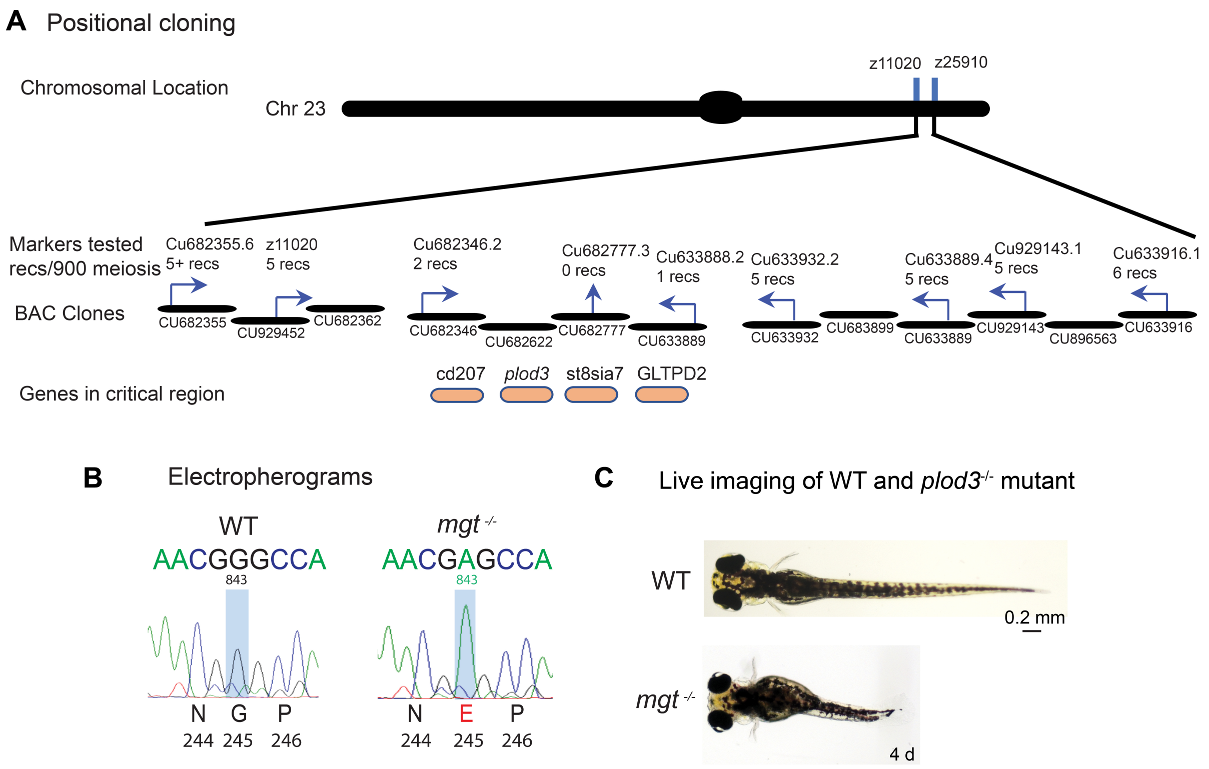

Fig. S1: Genetic mapping of the zebrafish *plod3* mutation.
A, Genetic linkage analysis, physical mapping, and positional cloning localized the *mgt* mutation to zebrafish chromosome 23. Genetic markers and the number of recombination events (recs in 900 meioses) in a critical region (BAC derived) are depicted. The 4 protein-coding genes (orange) were genotyped for segregating SNPs.
B, Electropherograms of wild-type (WT) and *mgt* $^{-/-}$ larvae show the 843G>A point mutation in *plod3* predicted to translate to Gly245Glu (G245E).
C, Live images of WT and *mgt* $^{-/-}$ mutant larvae at 4 days show a smaller head, shorter body length with scoliosis, reduced eye size, and absence of protruding jaw in *mgt* $^{-/-}$ larvae (*n* = 8, dorsal views).

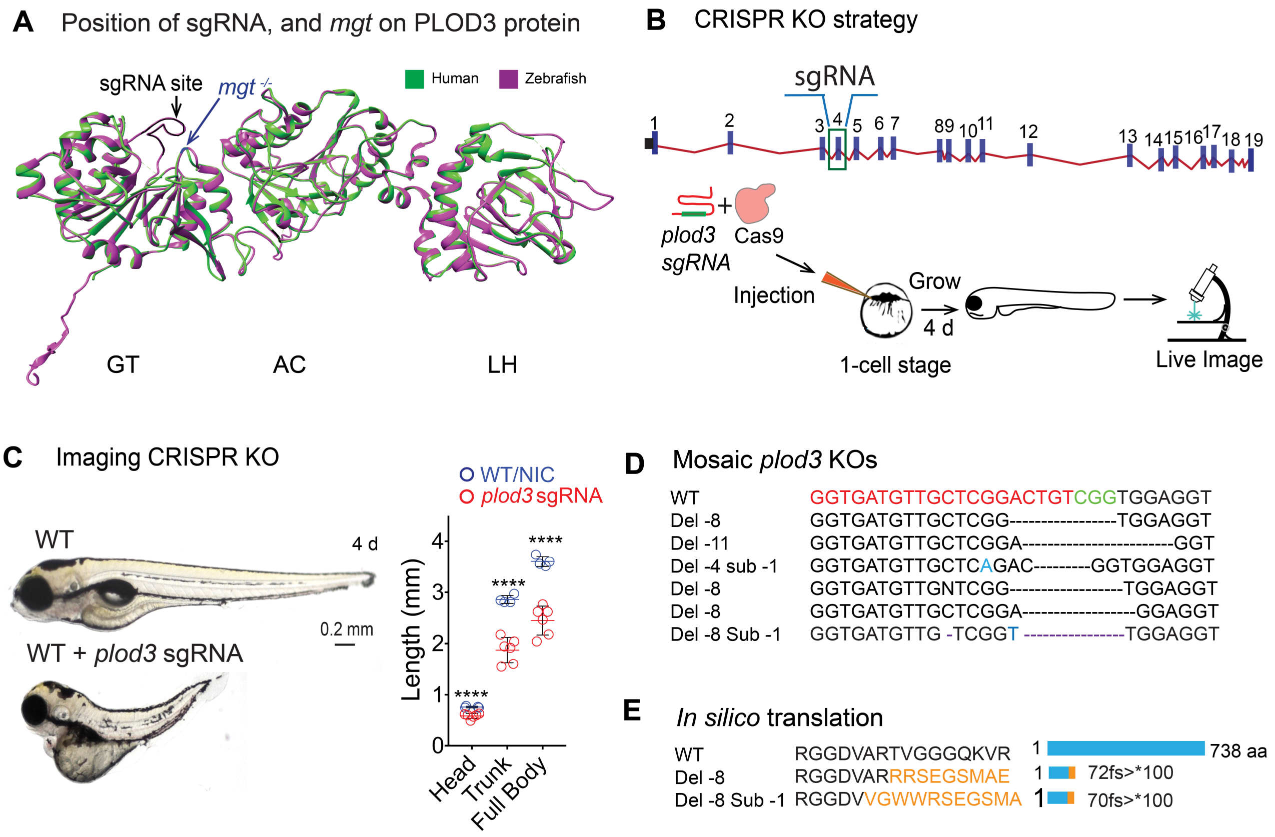

Fig. S2: *plod3* CRISPR-edited gene knockout phenotypes.

A, Predicted 3D-protein structure of zebrafish Plod3 protein and its alignment with human PLOD3 (PDB 6FXK, RCSB Protein Data Bank). Positions of zebrafish *mgt* $^{-/-}$ mutant and CRISPR site is marked.

B, Experimental design to generate CRISPR-edited gene knockout (KO) using sgRNA (single guide RNA) and Cas9 protein injections into 1-cell stage embryos targeting exon 4 of the *plod3* gene.

C, Assessment of KO animals after CRISPR editing in live larvae at 4 dpf. Quantification of the head, trunk, and full-body length; the head-trunk boundary was defined at the pectoral fin attachment site. WT/NIC (not injected controls), *n* = 4 larvae; in mutants, *n* = 6 larvae per group. Statistical analysis of body length measured by FIJI and calculated using Prism. Two-tailed Student's t-test, CI = 95% was conducted. ****p<0.0001. Error bars are the standard error of the mean.

D, Representative mutations detected in *plod3* ^{sgRNA} mosaic mutants by direct sequencing. Sequence analysis of the representative edited individual animals. The guide oligonucleotide (red text), the PAM (Protospacer Adjacent Motif, green), insertions (blue), and dashes mark deleted sequences as compared to the WT.

E, In-silico translation for predicted Plod3 peptide lengths in CRISPR-edited animals.

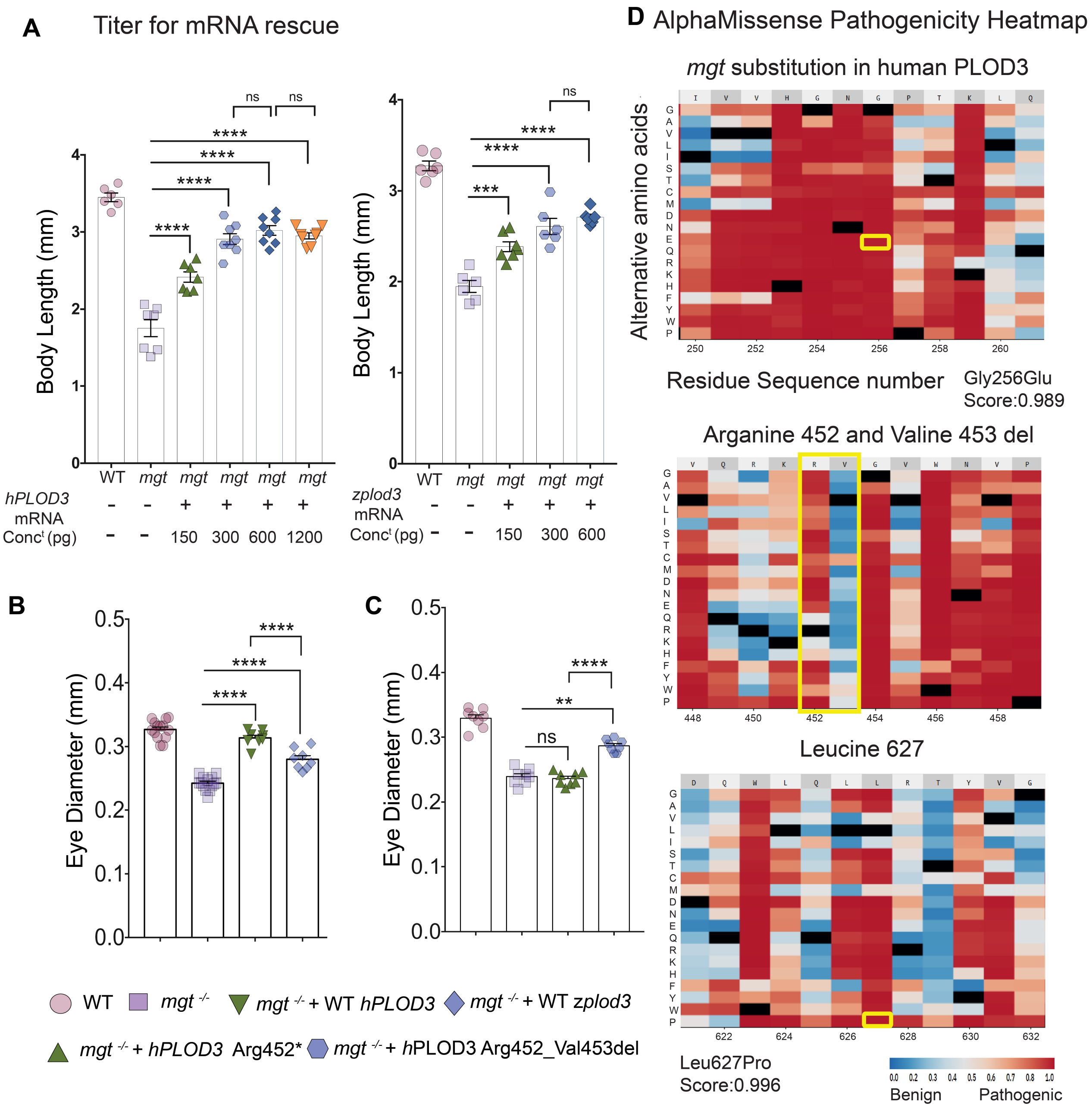

Fig. S3: Clinical variants in *PLOD3* are pathogenic.
A, Titer of human *PLOD3* and zebrafish *plod3* mRNAs to determine a minimal effective dose.
B, Quantification of overexpression (OE) experiments for eye diameter after mRNA microinjection with wild-type zebrafish *plod3* and human *PLOD3* mRNAs. The *n* numbers for eye diameter = WT (12) *mgt* $^{-/-}$ (13), *mgt* $^{-/-}$ + *z-plod3* (8), *mgt* $^{-/-}$ + h-*PLOD3* (8) ****p<0.0001, ns=non-significant.
C, Quantification of OE experiments with human variant *PLOD3* mRNAs (truncated at Arg452, and Arg452_Val453 deletion). Graph showing eye diameter measurements at 4 dpf, *n* = 8 for each group. One-way ANOVA with Tukey's multiple comparisons test with 95% CL of diff was conducted for analysis, ****p<0.0001, ***p<0.001, **p<0.01, and ns=non-significant.
D, Predicted variant pathogenicity index by AlphaMissense algorithm for PLOD3. The algorithm identified a homologous to zebrafish *mgt* mutation, a G256E substitution in human PLOD3 as likely pathogenic with a score of 0.989. The Arg452_Val453 deletion is marked as potentially highly pathogenic for Arg452 and benign for Val453. The Lue627Pro is also predicted to be highly pathogenic, with a score of 0.996.

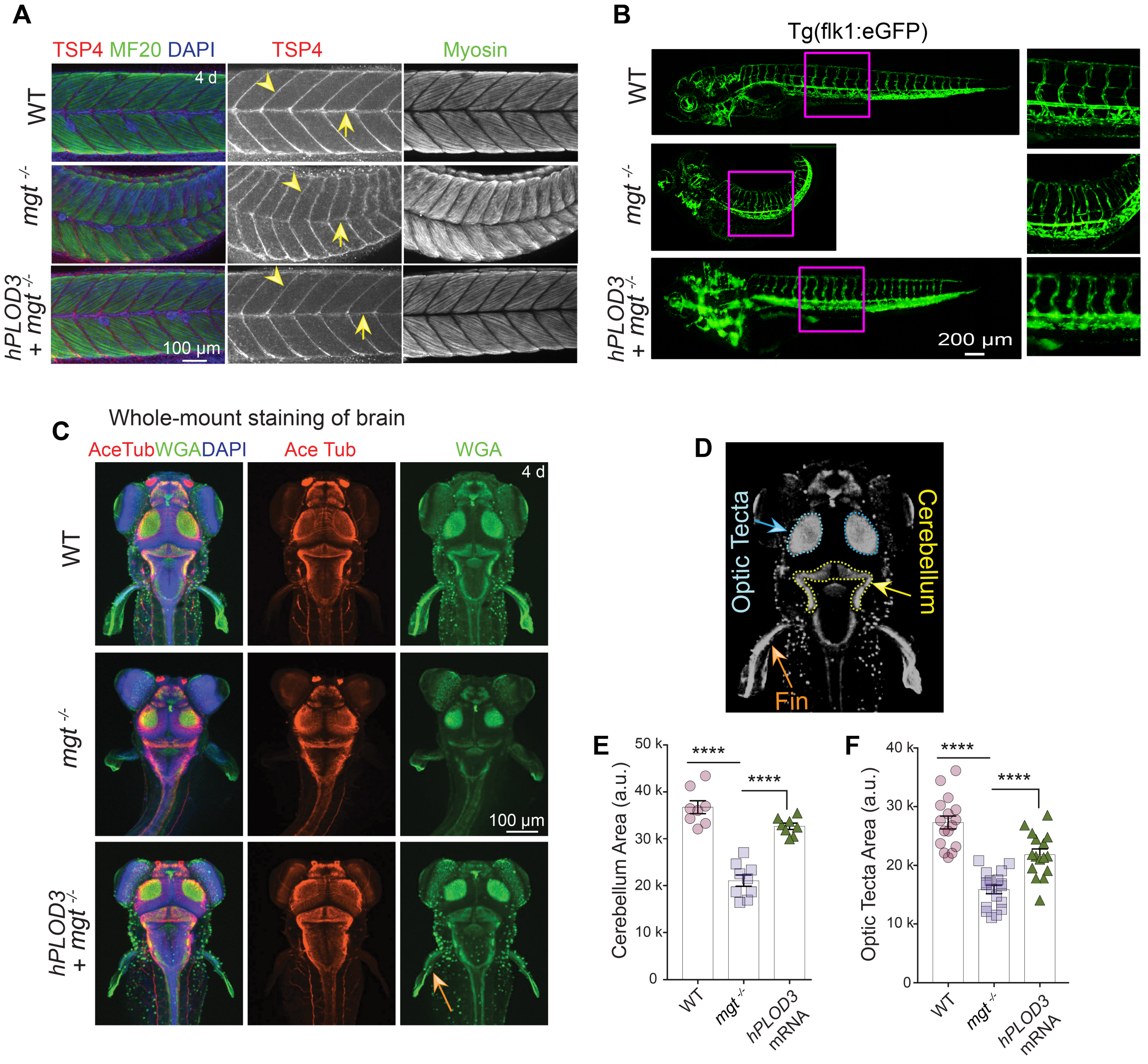

Fig. S4: Rescue of zebrafish muscle, vascular, and brain phenotypes with human *PLOD3* mRNA.
A, Whole-mount immunostaining of WT, *mgt* $^{-/-}$ and *mgt* $^{-/-}$ + *h-PLOD3* mRNA-injected larvae for myosin (MF20, muscle) and TSP4 (tendons). Maximum intensity projections of confocal z-stacks in all groups are shown. Intersomitic boundaries (arrowheads) and horizontal myoseptum/notochord (arrows) are marked. DAPI staining (blue) in merged images marks nuclei. Lateral views of trunk muscles; *n* = 8 animals per group.
B, Maximum-intensity projections of WT, *mgt* $^{-/-}$ mutant, and *hPLOD3*-injected *mgt* $^{-/-}$ whole larvae at 3 dpf in Tg(flk1:eGFP) background (green). The boxed areas, magnified in the right panels, show normal vasculature in rescued animals.
C, Whole-mount immunostaining images of 4 dpf zebrafish larvae with acetylated tubulin (Ace Tub, red), labeling axonal projections, and WGA (green), marking N-glycosylated proteins in neural plasma membranes and ECM. Visualization using maximum intensity projections of confocal z-stacks. DAPI staining marks nuclei blue. The arrow points to the straight pectoral fin in the rescue experiment.
D, Example of WT image to demarcate measured structures (dashed lines); paired fins (orange arrow), cerebellum (yellow arrow), and paired optic tecta (blue arrow); animals numbers for respective groups: WT (*n* = 8), *mgt* $^{-/-}$ (n=9), and *mgt* $^{-/-}$ + *hPLOD3* mRNA (n=8).
E, F, Quantification of the rescue experiments: **E**, cerebellum area (a.u., arbitrary units), **F**, optic tectal area (in arbitrary units). One-way ANOVA with Tukey's multiple comparisons test with 95% CL of diff was conducted to compare *mgt* $^{-/-}$ and *mgt* $^{-/-}$ injected with *hPLOD3* mRNA. ****p<0.0001.

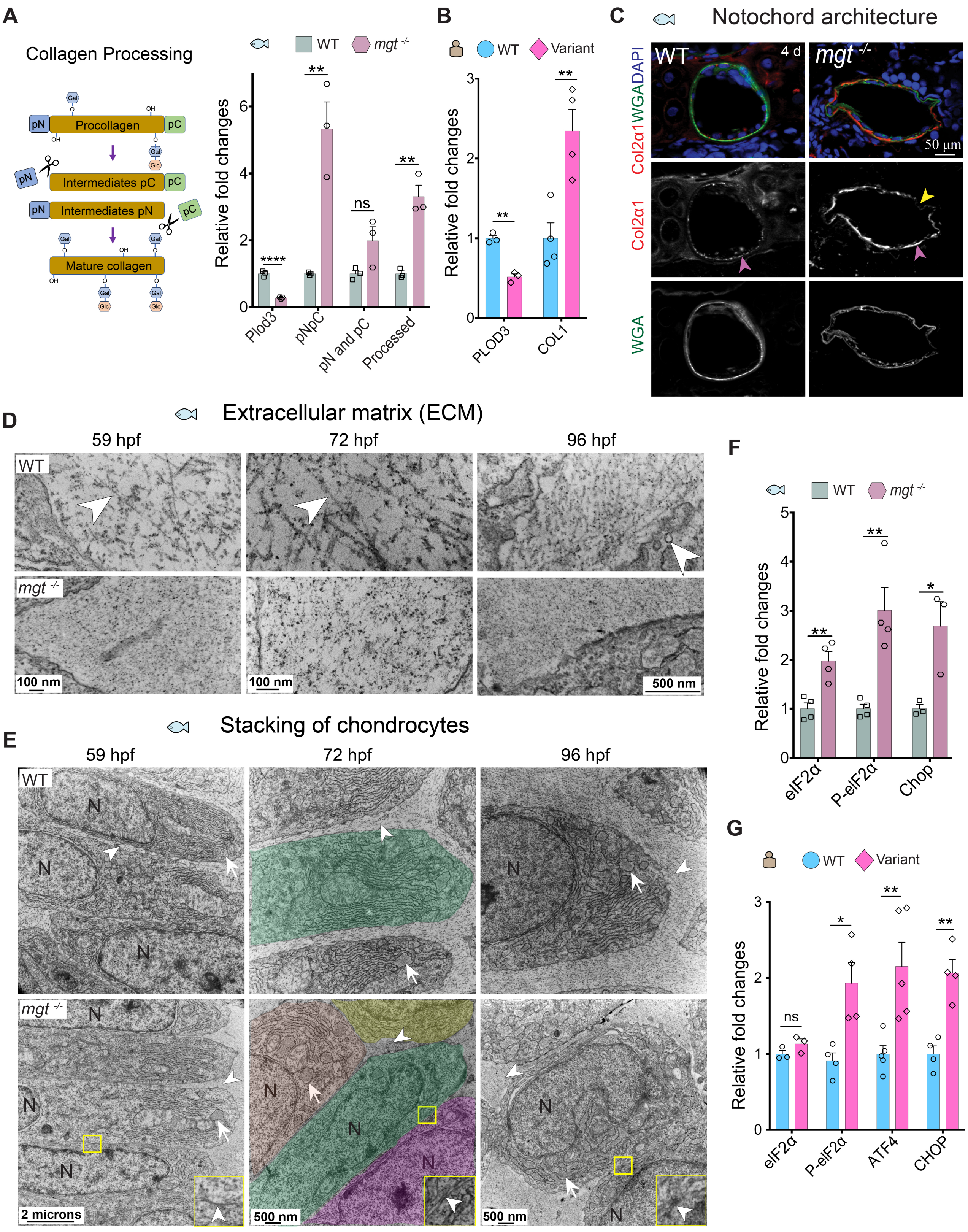
A
Collagen Processing
Procollagen
Intermediates pC
Intermediates pN
Mature collagen
pN
pC
WT
mgt -/-
Relative fold changes
Plod3
pNpC
pN and pC
Processed
B
WT
Variant
PLOD3
COL1
C
Notochord architecture
WT
4 d
mgt -/-
50 µm
Col2α1WGADAPI
Col2α1
WGA
D
Extracellular matrix (ECM)
59 hpf
72 hpf
96 hpf
100 nm
500 nm
E
Stacking of chondrocytes
2 microns
500 nm
F
eIF2α
P-eIF2α
Chop
G
ATF4
CHOP

Fig. S5: Reduced expression of PLOD3 disrupts procollagen processing and induces ER stress in zebrafish *mgt* $^{-/-}$ mutants and human fibroblasts.

A, Graphic represents steps in collagen processing, with proteases cleaving N-terminal and C-terminal peptides. The Plod3 complex is active in the ER, catalyzing lysine hydroxylation and addition of alpha-1,2-glucose to galactosyl hydroxylysines (Glc-T, GT), and Plod3 is secreted to the extracellular matrix to further process collagens. Western blotting (WB) quantification of the relative expression fold change of Plod3 (n=3 blots) and Col2 forms (n=3 blots) after normalization to α-Tubulin (α-Tub) in whole zebrafish protein lysates.

B, WB quantification of the relative fold change expression of PLOD3 (n=3 blots) and COL1 (n=4 blots) after normalization to α-Tub in control BJ fibroblasts (WT) and *PLOD3* (c.1353C>T) dermal fibroblasts.

C, Cryosections (perpendicular to the long body axis) of zebrafish larvae at 4 dpf labeled with Col2 antibody (red) and WGA (green) in WT and *mgt* $^{-/-}$. Nuclei stained blue with DAPI. In WT, Col2 and WGA mark an uninterrupted, round notochord sheath. Whereas in *mgt* $^{-/-}$, notochord appears flatten with interruption in continuity of the membrane and uneven staining of WGA and Col2, including regions of Col2 accumulation (pink arrowhead) and absent staining (yellow arrowhead), n=5 animals per group**.**

D, Transmission electron microscopy (TEM) analysis of ECM in craniofacial cartilage at 59 hpf, 72 hpf, and 96 hpf. WT cartilage at 59 hpf shows crosslinked ECM (arrowheads) and progressive crosslinking at 72 hpf and 96 hpf. Whereas in *mgt* $^{-/-}$, ECM has fewer collagen fibrils, and ECM crosslinking is progressively diminished.

E, Ultra-structure of craniofacial chondrocytes by TEM at 59 hpf, 72 hpf and 96 hpf. At 59 hpf, WT chondrocytes begin separating from each other, start losing cell-cell junctions, and begin secreting ECM. At 72 hpf, WT chondrocytes are stacked (pseudo-colored cells) and separated from each other by ECM (arrowheads), processes that continue at 96 hpf. Normal levels of lightly distended ER are typical of highly secretory chondrocytes (arrows). Whereas, *mgt* $^{-/-}$ chondrocytes at these stages are irregularly shaped, fail to stack (pseudo-colored cells at 72 hpf), and remain attached to neighboring cells by tight junctions (arrowheads in higher magnification insets). Cells contain distended rough ER (arrows). The ECM is limited in occupied space and density (arrowheads).

F, WB quantification of the relative fold change of eIF2α (n=4 of WB replicates), P-eIF2α (n=4 of WB replicates), and Chop (n=3 of WB replicates) after normalization to α-Tub in whole zebrafish protein lysates.

G, WB quantification of the relative fold change of eIF2α (n=3 of WB replicates), P-eIF2α (n=4 of WB replicates), ATF4 (n=5 of WB replicates), and CHOP (n=4 of WB replicates) after normalization to α-Tub in control BJ fibroblasts (WT) and patient's *PLOD3* (c.1353C>T) dermal fibroblast lysates.

Data in **A, B, F, G** were analyzed with a two-tailed Student's t-test, CI= 95%. Mean and SEM values are indicated with bars; significance ****p<0.0001, **p<0.01, *p<0.05, and ns=non-significant.

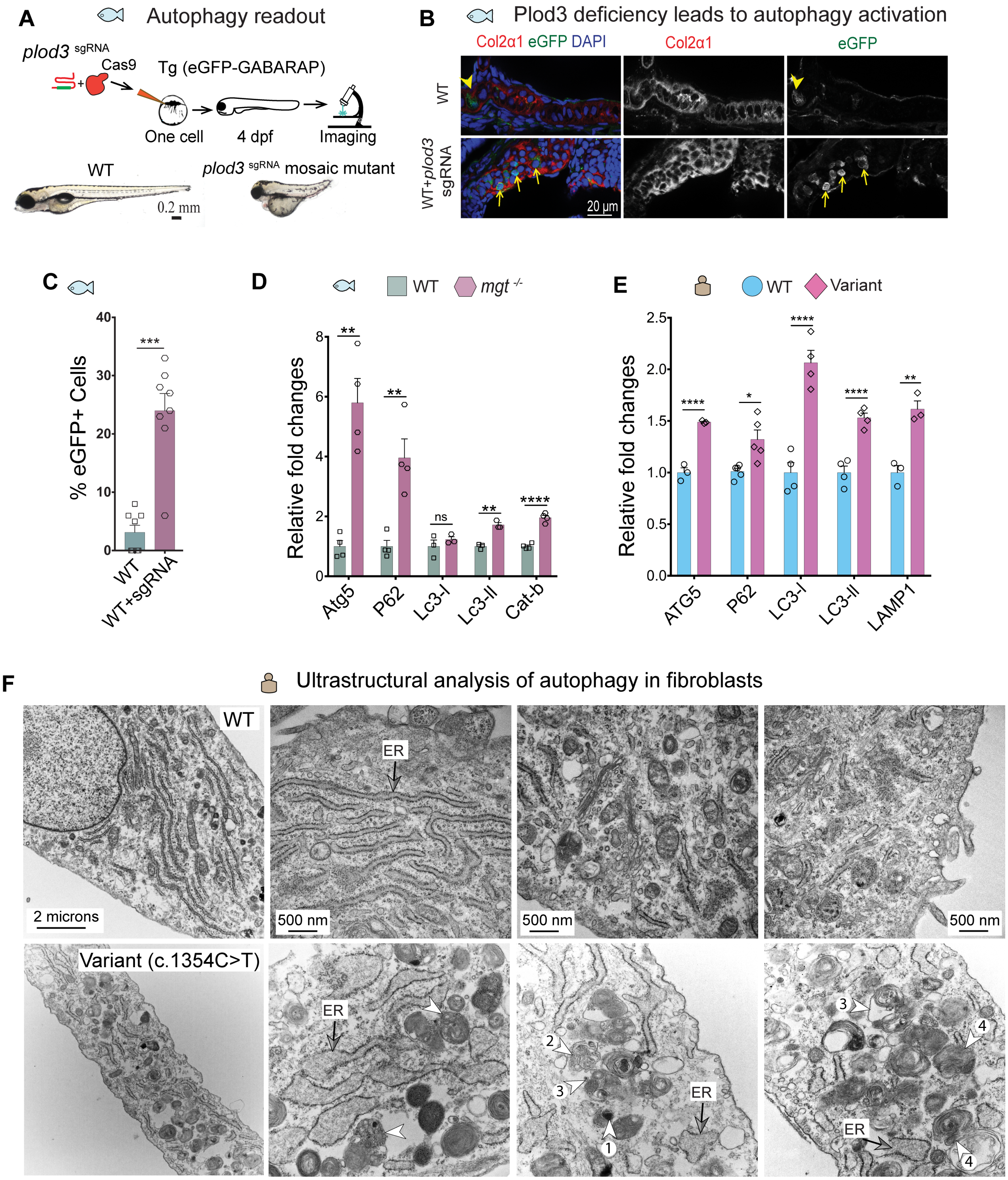

Fig. S6: Increased autophagy in PLOD3 deficiency.

A, Experimental design to generate CRISPR-edited *plod3* gene knockout in Tg(eGFP-GABARAP) autophagy reporter line using sgRNA (single guide) and CAS9 protein injections into 1-cell stage zebrafish embryos.

B, Cryosections of craniofacial cartilage stained with Col2 antibody (red) in Tg(eGFP-GABARAP) background (green, treated with proteinase K) of WT and *mgt* $^{-/-}$ zebrafish. Yellow arrows mark eGFP^{+} chondrocytes in WT and *mgt* $^{-/-}$ samples. Nuclei stained with DAPI in blue.

C, Quantification of eGFP-GABARAP^{+} chondrocytes (undergoing autophagy) in panel **B**, calculated as a percentage of total chondrocytes on analyzed sections. For WT, n=106 cells (N=6 WT larvae), for *mgt* $^{-/-}$ mutants, n=243 cells (N=8 larvae).

D, Quantification of the relative fold change of Atg5 (n=4 of WB replicates), p62 (n=4 of WB replicates), Lc3 I and II (n=3 of WB replicates), and Cathepsin B (n=4 of WB replicates) after normalization to α-Tub in whole zebrafish protein lysates.
E, Quantification of the relative fold change of ATG5 (n=3 of WB replicates), p62 (n=5 of WB replicates), LC3-I and LC3-II (n=4 of WB replicates), and LAMP1 (n=3 of WB replicates) after normalization to α-Tub in control BJ fibroblasts (WT) and patient's *PLOD3* (c.1353C>T) dermal fibroblasts.
F, TEM images of WT fibroblasts and *PLOD3* (c.1353C>T) variant patient's fibroblasts. WT cells in culture show moderately distended rough ER (arrow) and the typical appearance of other organelles. The *PLOD3* variant (c.1353C>T) patient's cells show large, distended ER (arrow), and accumulation of vesicular structures consistent with increased autophagy, including lysosomes (arrowhead 1), autophagosomes (arrowhead 2), autolysosomes (arrowhead 3), and multilamellar bodies (arrowhead 4).
Data in **C-E** were analyzed with a two-tailed Student's t-test, CI= 95%. Mean and SEM values are indicated with bars; significance ****p<0.0001, ***p<0.001, **p<0.01, *p<0.05, and ns =non-significant.

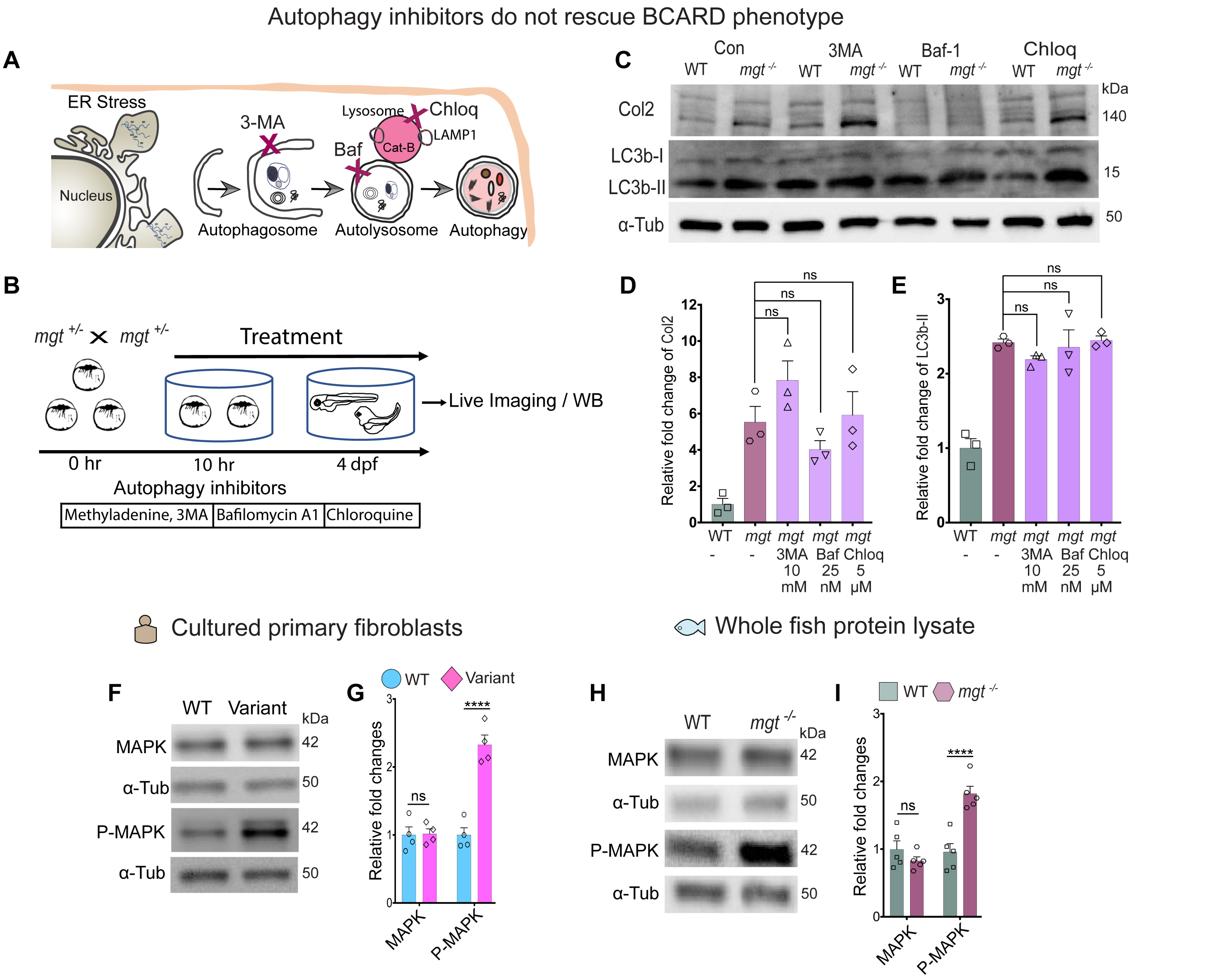

Fig. S7: Autophagy inhibitors do not improve PLOD3 deficiency outcomes.

A, Schematic diagram of autophagy inhibitors marking distinct steps blocked by 3-methyladenine (3MA), Bafilomycin A1 (Baf), and Chloroquine (Chloq).

B, Experimental design of the *in-vivo* zebrafish WT and mutant treatment strategy and phenotypic evaluation.

C, WB of whole zebrafish protein lysates with Col2 and LC3b-II antibodies in WT and *mgt* $^{-/-}$ zebrafish treated with autophagy inhibitors 3-methyladenine (3MA, 10 mM), Bafilomycin A1 (Baf, 25 nM), and Chloroquine (Chloq, 5 µM). Treatments were initiated after gastrulation at 10 hpf and continued with daily solution changes until protein collection at 4 dpf. Vehicle-treated WT and *mgt* $^{-/-}$ acted as control (Con). Results were normalized to α-Tub.

D,**E,** Quantification of the relative fold change of Col2 and LC3b-II protein expression compared to controls (n=3 replicates) after normalization to α-Tub. Data in **D** and **E** were analyzed with one-way ANOVA with Tukey's multiple comparisons test with 95% CL of diff, which was conducted for comparison between *mgt* $^{-/-}$ and *mgt* $^{-/-}$ drug-treated groups. Significance ns=non-significant.

F, MAPK expression in WT and *PLOD3* (c.1353C>T) patient's fibroblasts in whole protein lysates on WB stained MAPK and P-MAPK.

G, Quantification of the relative fold change of MAPK (n=4 replicates) and P-MAPK (n=4 replicates) after normalization to α-Tub.

H, WB of whole zebrafish protein lysates with MAPK and P-MAPK antibodies.

I, Quantification of the relative fold change of MAPK (n=5 replicates) and P-MAPK (n=5 replicates) after normalization to α-Tub.

Data in **G,** and **I,** were analyzed with two-tailed Student's t-test, CI= 95%. Mean and SEM values are indicated with bars. ****p<0.0001, ns=non-significant

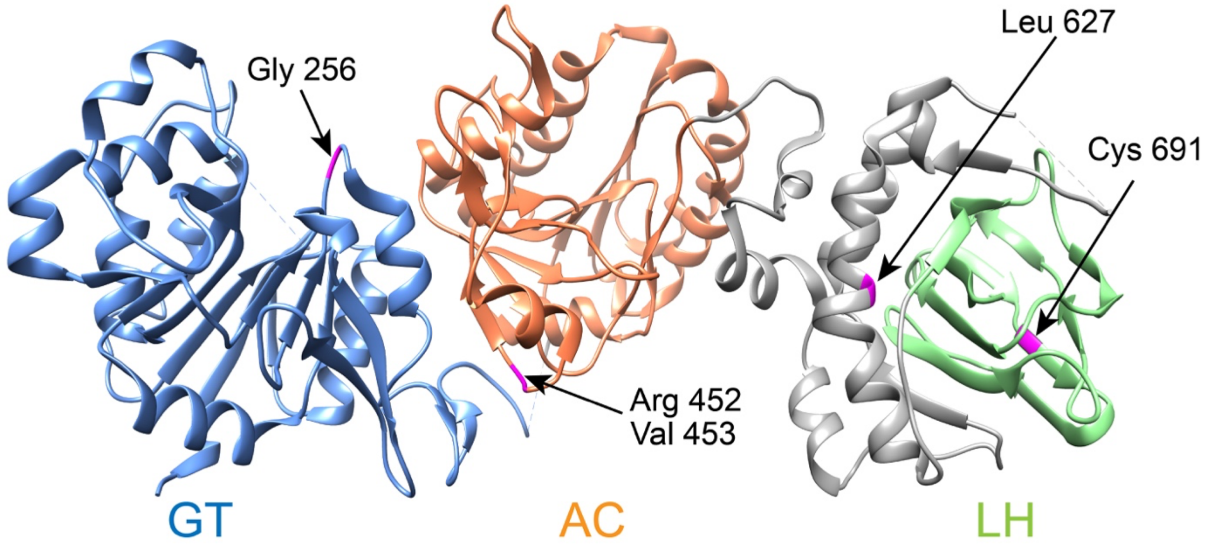

Fig. S8: A: Human PLOD3 variants and functional domains: GT, glycosyltransferase domain, AC, accessory domain, LH Lysyl hydroxylase domain. Position of variants tested in this study: Gly256 is the homologous position to the zebrafish *mgt* mutation Gly245Glu, amino acids affected in our clinical case Arg452, Val453, and variants in Leu627 and Cys691 that were tested in PLOD3 replacement experiments in zebrafish.

Additional References:

1. Michelmore RW, Paran I, Kesseli RV. Identification of markers linked to disease-resistance genes by bulked segregant analysis: a rapid method to detect markers in specific genomic regions by using segregating populations. Proc Natl Acad Sci U S A. 1991;88:9828–32. https://doi.org/10.1073/pnas.88.21.9828

2. Knapik EW, Goodman A, Atkinson OS, Roberts CT, Shiozawa M, Sim CU, et al. A reference cross DNA panel for zebrafish (Danio rerio) anchored with simple sequence length polymorphisms. Dev Camb Engl. 1996;123:451–60. https://doi.org/10.1242/dev.123.1.451

3. Labun K, Montague TG, Krause M, Torres Cleuren YN, Tjeldnes H, Valen E. CHOPCHOP v3: expanding the CRISPR web toolbox beyond genome editing. Nucleic Acids Res. 2019;47:W171–4. https://doi.org/10.1093/nar/gkz365

4. Jao L-E, Wente SR, Chen W. Efficient multiplex biallelic zebrafish genome editing using a CRISPR nuclease system. Proc Natl Acad Sci U S A. 2013;110:13904–9. https://doi.org/10.1073/pnas.1308335110

5. Unlu G, Qi X, Gamazon ER, Melville DB, Patel N, Rushing AR, et al. Phenome-based approach identifies RIC1-linked Mendelian syndrome through zebrafish models, biobank associations, and clinical studies. Nat Med. 2020;26:98–109. https://doi.org/10.1038/s41591-019-0705-y

6. Luderman LN, Michaels MT, Levic DS, Knapik EW. Zebrafish Erc1b mediates motor innervation and organization of craniofacial muscles in control of jaw movement. Dev Dyn. 2022;dvdy.511. https://doi.org/10.1002/dvdy.511

7. Yang J, Zhang Y. I-TASSER server: new development for protein structure and function predictions. Nucleic Acids Res. 2015;43:W174–81. https://doi.org/10.1093/nar/gkv342

8. Zheng W, Zhang C, Li Y, Pearce R, Bell EW, Zhang Y. Folding non-homologous proteins by coupling deep-learning contact maps with I-TASSER assembly simulations. Cell Rep Methods. 2021;1:100014. https://doi.org/10.1016/j.crmeth.2021.100014

9. Burley SK, Bhikadiya C, Bi C, Bittrich S, Chao H, Chen L, et al. RCSB Protein Data Bank (RCSB.org): delivery of experimentally-determined PDB structures alongside one million computed structure models of proteins from artificial intelligence/machine learning. Nucleic Acids Res. 2023;51:D488–508. https://doi.org/10.1093/nar/gkac1077

10. Berger MF, Bulyk ML. Protein binding microarrays (PBMs) for rapid, high-throughput characterization of the sequence specificities of DNA binding proteins. Methods Mol Biol Clifton NJ. 2006;338:245–60. https://doi.org/10.1385/1-59745-097-9:245

11. Pettersen EF, Goddard TD, Huang CC, Couch GS, Greenblatt DM, Meng EC, et al. UCSF Chimera--a visualization system for exploratory research and analysis. J Comput Chem. 2004;25:1605–12. https://doi.org/10.1002/jcc.20084

12. Madeira F, Pearce M, Tivey ARN, Basutkar P, Lee J, Edbali O, et al. Search and sequence analysis tools services from EMBL-EBI in 2022. Nucleic Acids Res. 2022;50:W276–9. https://doi.org/10.1093/nar/gkac240

13. Cheng J, Novati G, Pan J, Bycroft C, Žemgulytė A, Applebaum T, et al. Accurate proteome-wide missense variant effect prediction with AlphaMissense. Science. 2023;381:eadg7492. https://doi.org/10.1126/science.adg7492

14. Gasteiger E, Gattiker A, Hoogland C, Ivanyi I, Appel RD, Bairoch A. ExPASy: The proteomics server for in-depth protein knowledge and analysis. Nucleic Acids Res. 2003;31:3784–8. https://doi.org/10.1093/nar/gkg563

15. Jia X-E, Ma K, Xu T, Gao L, Wu S, Fu C, et al. Mutation of kri1l causes definitive hematopoiesis failure via PERK-dependent excessive autophagy induction. Cell Res. 2015;25:946–62. https://doi.org/10.1038/cr.2015.81
