# Supplementary material for: Succinate supplementation ameliorates musculoskeletal defects caused by PLOD3 mutations in a BCARD syndrome model
Source: Genome Med. 2026 Mar 13;18:29. doi: 10.1186/s13073-026-01608-y (PMC12994257; doi:10.1186/s13073-026-01608-y)
Supplement: Supplementary file 7 — Additional file 7: Table S12. Enrichr Cellular components downregulated. [file 13073_2026_1608_MOESM7_ESM.pdf]

Table S12: Enrichr Cellular components down

Term	Overlap	P-value	Adjusted P-value	Old P-value	Old Adjusted P-value	Odds Ratio	Combined Score	Genes
Mitochondrial Inner Membrane (GO:0005743)	72/409	1.42E-29	3.31E-27	0	0	6.225756677	413.5342099	MRPS17;NDUFA11;NDUFA12;AQP8;NDUFA10;MRPL36;MRPL34;COX6A1;COX7C;COX6A2;MRPL4;MRPL42;MPC2;CHCHD1;COA5;MRPS24;NDUFC2;SDHC;MRPL48;COX6B2;HADHB;SMDT1;NDUFS8;PLSCR3;NDUFS7;
Mitochondrial Membrane (GO:0031966)	83/595	1.13E-26	1.31E-24	0	0	4.760789564	284.4506569	MRPS17;NDUFA11;NDUFA12;AQP8;NDUFA10;MRPL36;MRPL34;COX6A1;COX7C;COX6A2;MRPL4;MRPL42;AIFM2;MPC2;ACADM;CHCHD1;TMEM14A;TMEM14B;COA5;TMEM14C;MRPS24;NDUFC2;SDHC;MRPL48;
Mitochondrial Matrix (GO:0005759)	50/407	3.49E-14	2.71E-12	0	0	3.943452381	122.1982449	ACAA2;ECI1;ETFB;MRPL34;GCSH;ACADL;ALDH2;C1QBP;ACADM;HADH;ACADS;MCCC2;GSTK1;GPX1;TSTD1;AMT;HOGA1;MRPL48;NIPSNAP1;SMDT1;NDUFS8;NDUFS7;BOLA3;SUCLG2;SUCLG1;CASQ1;ALDH7A1;
Intracellular Organelle Lumen (GO:0070013)	81/912	5.41E-14	3.15E-12	0	0	2.805121736	85.69085057	SERPINA1;ACAA2;ECI1;DBI;ETFB;PCSK5;GCSH;ACADL;ALDH2;AIFM2;FTH1;C1QBP;ACADM;HADH;GYG1;ACADS;MCCC2;GSTK1;GPX1;IGFBP5;TSTD1;AHSG;TNNC1;OMD;TMT1A;AMT;NME2;APOA1;APOA4;HOGA1;
Brush Border Membrane (GO:0031526)	11/40	1.12E-07	3.87E-06	0	0	10.26912071	164.3809208	SLC34A2;ACE2;SLC6A19;SLC22A5;AQP8;SLC34A1;CA4;SLC5A1;CD36;PDZK1;SLC28A1
Cytosolic Small Ribosomal Subunit (GO:0022627)	11/40	1.12E-07	3.87E-06	0	0	10.26912071	164.3809208	RPS14;RPS25;RPS17;RPS9;RPS8;RPS3A;RPS20;FAU;RPS11;RPS27A;RPS24
Small Ribosomal Subunit (GO:0015935)	11/41	1.48E-07	3.87E-06	0	0	9.926300985	156.1195855	RPS14;RPS25;RPS17;RPS9;RPS8;RPS3A;RPS20;FAU;RPS11;RPS27A;RPS24
Microbody Lumen (GO:0031907)	12/50	1.54E-07	3.87E-06	0	0	8.557449963	134.2391235	FABP1;GSTK1;GRHPR;NUDT7;AMACR;DAO;EHHADH;IDH1;ECI2;PIPOX;ACAA1;DHRS4
Peroxisomal Matrix (GO:0005782)	12/50	1.54E-07	3.87E-06	0	0	8.557449963	134.2391235	FABP1;GSTK1;GRHPR;NUDT7;AMACR;DAO;EHHADH;IDH1;ECI2;PIPOX;ACAA1;DHRS4
CMG Complex (GO:0071162)	6/9	1.66E-07	3.87E-06	0	0	53.84078212	840.5419784	GINS1;GINS2;GINS3;GINS4;MCM3;MCM5
Peroxisome (GO:0005777)	18/134	1.6E-06	3.11E-05	0	0	4.223599138	56.3651206	GSTK1;AOC1;ABCD3;IDH1;ECI2;MGST1;PIPOX;DHRS4;SOD1;FABP1;GRHPR;NUDT7;AMACR;DAO;EHHADH;MVD;ACAA1;SLC27A2
Cytosolic Large Ribosomal Subunit (GO:0022625)	11/51	1.6E-06	3.11E-05	0	0	7.440857947	99.29019903	RPL32;RPL31;RPLP1;RPL7L1;RPL22;RPL11;RPL36;RPL36A;RPL13A;RPL35A;RPL7
Large Ribosomal Subunit (GO:0015934)	11/52	1.97E-06	3.53E-05	0	0	7.258996261	95.37672441	RPL32;RPL31;RPLP1;RPL7L1;RPL22;RPL11;RPL36;RPL36A;RPL13A;RPL35A;RPL7
Endoplasmic Reticulum Membrane (GO:0005789)	54/872	8.22E-05	0.001367271	0	0	1.824297615	17.1610205	MOGAT3;MOGAT2;MSMO1;CYP46A1;FTCD;FADS2;SDR16C5;NSDHL;CYP4V2;CYP2R1;LRAT;TMEM14A;PCYT1B;DGAT2;DGAT1;ELOVL2;ACSL5;SLC51A;CASQ1;HPD;SLC27A2;PON3;SLC35D1;FAXDC2;MGST3;
Chylomicron (GO:0042627)	4/9	1.84E-04	0.002760096	0	0	21.47409471	184.7417597	APOC2;APOC1;APOA4;APOB
Preribosome, Large Subunit Precursor (GO:0030687)	5/16	1.9E-04	0.002760096	0	0	12.21440345	104.6889129	NIP7;MRTO4;RRS1;NOC2L;RRP15
Intracellular Membraneless Organelle (GO:0043232)	72/1310	2.38E-04	0.003244796	0	0	1.614117062	13.46497033	POP5;FEN1;PINX1;HMGB2;GPATCH4;NOC2L;PWP1;RRP8;RPL7;GYS2;RPS14;FBL;SDR16C5;NSDHL;RPS17;HINT1;AIFM2;RPL36;BUD23;RPS11;RPS9;DGAT2;ANXA2;TMOD4;TMT1A;FAAH2;RPL13A;ACSL5;CDPF1;
Small-Subunit Processome (GO:0032040)	10/72	2.51E-04	0.003244796	0	0	4.353026459	36.09255102	FBL;RPS14;RPS17;RPS9;RPS8;RPS3A;SNU13;RPS11;RPS27A;RPS24
Cytoplasmic Vesicle Lumen (GO:0060205)	13/117	3.11E-04	0.003819187	0	0	3.380465444	27.29495061	SERPINB1;GSN;UCMA;AHSG;IDH1;GSTP1;NME2;APOA1;TF;GNPDA1;BIN2;GYG1;S100A11
Nucleolus (GO:0005730)	48/805	4.76E-04	0.00554846	0	0	1.742408147	13.32862268	POP5;FEN1;DDX27;NIP7;RPL11;PINX1;HMGB2;GPATCH4;SNU13;NOC2L;PWP1;RRP8;RPL7;SELENBP1;RPS14;FBL;EXOSC5;RPL36;RRS1;POLR2E;LLPH;BUD23;RPS27A;RPS11;EXOSC2;LYAR;RPS9;RPL13A;ACSL5;
Nuclear Lumen (GO:0031981)	47/814	0.00107103	0.011883333	0	0	1.680461635	11.49290312	POP5;FEN1;DDX27;NIP7;RPL11;PINX1;HMGB2;GPATCH4;SNU13;NOC2L;PWP1;RRP8;RPL7;SELENBP1;RPS14;FBL;EXOSC5;RPL36;RRS1;LLPH;BUD23;RPS27A;RPS11;EXOSC2;LYAR;RPS9;RPL13A;ACSL5;CDPF1;
Secretory Granule Lumen (GO:0034774)	23/316	0.001203407	0.012227907	0	0	2.13203162	14.33279225	SERPINB1;AOC1;SERPINA1;GSN;ANXA2;UCMA;AHSG;GSTP1;IDH1;SERPINF2;NME2;APOA1;PLAC8;TF;SCCPDH;TTR;GNPDA1;BIN2;AIFM2;ACAA1;GYG1;NIT2;S100A11
Ribosome (GO:0005840)	7/46	0.001218052	0.012227907	0	0	4.829585799	32.40894613	PSMA6;RPS25;RPS17;RPS9;RPL36A;RPL13A;RPS11
Lipid Droplet (GO:0005811)	10/88	0.001259527	0.012227907	0	0	3.45721694	23.08390363	SDR16C5;HSD3B7;NSDHL;DGAT2;SCCPDH;ANXA2;AIFM2;FAAH2;TMT1A;CIDEB
Nuclear Chromosome (GO:0000228)	10/90	0.001496939	0.013951469	0	0	3.370435393	21.92243455	GINS1;GINS2;CDC45;TTR;GINS3;PINX1;GINS4;MCM3;RRS1;MCM5
Triglyceride-Rich Plasma Lipoprotein Particle (GO:0034385)	4/15	0.001672193	0.013965719	0	0	9.757913396	62.38838518	APOC2;APOC1;APOA4;APOB
Very-Low-Density Lipoprotein Particle (GO:0034361)	4/15	0.001672193	0.013965719	0	0	9.757913396	62.38838518	APOC2;APOC1;APOA4;APOB
Cell Projection Membrane (GO:0031253)	11/107	0.001678284	0.013965719	0	0	3.09133263	19.75356466	SLC34A2;ACE2;SLC6A19;SLC22A5;AQP8;SLC34A1;CA4;PDZK1;SLC5A1;CD36;SLC28A1
Low-Density Lipoprotein Particle (GO:0034362)	3/9	0.003343088	0.026859985	0	0	13.40194715	76.40262827	APOC2;APOA4;APOB
Lysosomal Lumen (GO:0043202)	9/86	0.003823089	0.02969266	0	0	3.147647584	17.52199875	CD74;TCN2;HEXA;OMD;IFI30;APOB;GC;GYG1;SPAM1
Mitochondrial Intermembrane Space (GO:0005758)	8/71	0.003976594	0.029888592	0	0	3.417366947	18.88891363	TIMM8B;NDUFA8;GATM;NDUFB7;CMC4;CHCHD10;COX5A;SOD1
High-Density Lipoprotein Particle (GO:0034364)	4/19	0.004233523	0.030825342	0	0	7.154317549	39.09634709	APOC2;PON1;APOC1;APOA4
Ficolin-1-Rich Granule Lumen (GO:1904813)	11/123	0.004982482	0.035179344	0	0	2.647503516	14.03660586	SERPINA1;GSN;GNPDA1;BIN2;FTH1;IDH1;GSTP1;FGL2;NME2;LTA4H;GYG1
Vesicle Membrane (GO:0012506)	8/77	0.006518208	0.044668894	0	0	3.119230301	15.69957215	BAIAP2L2;ZNRF2;ANXA1;ANXA2;IYD;ANXA4;ANXA5;AP1S3
Focal Adhesion (GO:0005925)	24/387	0.007394775	0.049228074	0	0	1.79165976	8.791641481	RPS9;ANXA1;GSN;RPL31;RPS8;RPLP1;TNNC1;RPL22;ANXA5;NME2;RPL13A;RPS3A;RPL7;DPP4;RPS14;CNN1;RPS17;CSRP1;PLAU;FLNB;RPS11;PFN1;MAPRE1;TGM2
Cell-Substrate Junction (GO:0030055)	24/395	0.009387539	0.06075824	0	0	1.752284154	8.180314435	RPS9;ANXA1;GSN;RPL31;RPS8;RPLP1;TNNC1;RPL22;ANXA5;NME2;RPL13A;RPS3A;RPL7;DPP4;RPS14;CNN1;RPS17;CSRP1;PLAU;FLNB;RPS11;PFN1;MAPRE1;TGM2
ATP-binding Cassette (ABC) Transporter Complex (GO:0043190)	2/5	0.012102455	0.076212758	0	0	17.84722222	78.78383078	ABCG8;ABCG5
Vacuolar Lumen (GO:0005775)	12/163	0.015346707	0.094099549	0	0	2.14088238	8.942153827	PLAC8;CD74;TTR;TCN2;ANXA2;OMD;HEXA;IFI30;APOB;GC;GYG1;SPAM1
Specific Granule Membrane (GO:0035579)	8/90	0.016020676	0.095713267	0	0	2.62294186	10.8429142	CEACAM1;PGRMC1;DGAT1;PLAU;MOSPD2;CD36;SLC2A5;SLC27A2
ATPase Dependent Transmembrane Transport Complex (GO:0098533)	2/6	0.017721966	0.101521901	0	0	13.38472222	53.97992105	ABCG8;ABCG5
Microvillus (GO:0005902)	6/58	0.017864369	0.101521901	0	0	3.098302535	12.47050386	VIL1;MYO7B;USH1C;ANKS4B;CALML4;ENPP7
Myofibril (GO:0030016)	4/29	0.019525262	0.108318718	0	0	4.290362117	16.88706326	PSMA6;DES;TMOD4;MYL2
Vesicle (GO:0031982)	15/230	0.020195792	0.109433011	0	0	1.881155225	7.340796347	SLC34A2;CBR1;ANXA1;ANXA2;AQP8;SLC34A1;ANXA4;GSTP1;ANXA5;APOA1;APOA4;SLC5A1;BAIAP2L2;TF;RPS27A
Collagen-Containing Extracellular Matrix (GO:0062023)	22/383	0.022294605	0.118060069	0	0	1.646909379	6.263872496	APCS;SERPINB1;SERPINA1;ANXA1;ANXA2;AHSG;ANXA4;TNNC1;SERPINF2;ANXA5;ADIPOQ;FGL2;DPT;APOA1;APOA4;MFAP5;VTN;GH1;AIFM2;ANGPTL3;TGM2;CPA4
Spherical High-Density Lipoprotein Particle (GO:0034366)	2/7	0.024222548	0.125418972	0	0	10.70722222	39.83591338	APOC2;PON1
Smooth Endoplasmic Reticulum (GO:0005790)	3/19	0.02950077	0.146640626	0	0	5.023122392	17.69816267	AQP8;CASQ1;FTCD
Specific Granule (GO:0042581)	11/159	0.029579869	0.146640626	0	0	1.999752917	7.040452617	CEACAM1;PGRMC1;AOC1;DGAT1;PLAU;AIFM2;MOSPD2;CD36;SLC2A5;SLC27A2;NIT2
Endoplasmic Reticulum Lumen (GO:0005788)	17/289	0.033845876	0.164293522	0	0	1.684929078	5.705065604	SERPINA1;SERPIND1;IGFBP5;AHSG;TNNC1;EBI3;MTTP;APOA1;DBI;APOA4;TF;ESD;MELTF;CASQ1;APOB;SLC27A2;CES2
Mitochondrial Ribosome (GO:0005761)	3/21	0.038403474	0.182612438	0	0	4.464534075	14.5526281	MRPL18;MRPL48;MRPL34
Nucleolar Exosome (RNase Complex) (GO:0101019)	2/9	0.03958785	0.184479383	0	0	7.647222222	24.69466248	EXOSC5;EXOSC2
Tertiary Granule Lumen (GO:1904724)	5/54	0.044747511	0.204434705	0	0	2.736600917	8.501851341	FTH1;IDH1;TMT1A;LTA4H;NIT2
Secretory Granule Membrane (GO:0030667)	16/279	0.046849772	0.209923017	0	0	1.638535529	5.015244515	SLC30A8;DGAT1;MGST1;MOSPD2;NDUFC2;SLC2A5;CUZD1;CYB5R1;CEACAM1;PGRMC1;PLAU;ANPEP;CA4;TEKT3;CD36;SLC27A2
RNA Polymerase I Complex (GO:0005736)	2/11	0.057678541	0.248872221	0	0	5.947222222	16.96665238	POLR2E;POLR1E
Intracellular Vesicle (GO:0097708)	2/11	0.057678541	0.248872221	0	0	5.947222222	16.96665238	AQP8;SLC5A1
Endosome Lumen (GO:0031904)	3/25	0.059779935	0.253249542	0	0	3.652041977	10.28811346	GH1;AP4S1;APOB
Cornified Envelope (GO:0001533)	4/42	0.06399097	0.266248143	0	0	2.820700777	7.754143952	SCEL;CNFN;EVPL;PPL
Serine-Type Endopeptidase Complex (GO:1905370)	2/12	0.067599264	0.271562559	0	0	5.352222222	14.41973333	PLAU;MBL2
Serine-Type Peptidase Complex (GO:1905286)	2/12	0.067599264	0.271562559	0	0	5.352222222	14.41973333	PLAU;MBL2
Ficolin-1-Rich Granule (GO:0101002)	11/184	0.070467132	0.278285454	0	0	1.708535564	4.532076625	SERPINA1;GSN;GNPDA1;BIN2;FTH1;IDH1;GSTP1;FGL2;NME2;LTA4H;GYG1
Endopeptidase Complex (GO:1905369)	2/14	0.088927043	0.345333351	0	0	4.459722222	10.79225567	PLAU;MBL2
Actin-Based Cell Projection (GO:0098858)	6/88	0.098530041	0.376352451	0	0	1.961711405	4.546057834	VIL1;MYO7B;USH1C;ANKS4B;CALML4;ENPP7
NADPH Oxidase Complex (GO:0043020)	2/15	0.100237454	0.376698818	0	0	4.116452991	9.468720209	DUOX1;DUOX2
Platelet Alpha Granule (GO:0031091)	6/90	0.106803998	0.394192298	0	0	1.914804469	4.282957887	CYB5R1;SERPINA1;SCCPDH;AHSG;SERPINF2;CD36
Basolateral Plasma Membrane (GO:0016323)	11/200	0.109798971	0.394192298	0	0	1.562587904	3.451919374	SLC26A1;ANXA2;AQP8;SLC23A1;AQP10;MTTP;CA4;SLC51A;ABCB11;RHBG;CLCN2
RNA Polymerase III Complex (GO:0005666)	2/16	0.111919166	0.394192298	0	0	3.822222222	8.370584097	POLR3GL;POLR2E
Platelet Alpha Granule Membrane (GO:0031092)	2/16	0.111919166	0.394192298	0	0	3.822222222	8.370584097	CYB5R1;CD36
Intermediate Filament (GO:0005882)	5/71	0.113351433	0.394192298	0	0	2.029922658	4.419673986	DES;KRT18;KRT5;EVPL;PPL
Microbody Membrane (GO:0031903)	4/53	0.123833736	0.418163195	0	0	2.186231596	4.566634341	ABCD3;MGST1;SLC27A2;DHRS4
Peroxisomal Membrane (GO:0005778)	4/53	0.123833736	0.418163195	0	0	2.186231596	4.566634341	ABCD3;MGST1;SLC27A2;DHRS4
Intermediate Filament Cytoskeleton (GO:0045111)	5/78	0.150959108	0.483101429	0	0	1.834603848	3.468770412	DES;KRT18;KRT5;EVPL;PPL
Actin Cytoskeleton (GO:0015629)	17/361	0.160857072	0.483101429	0	0	1.327222497	2.42515279	CLIC5;ANXA1;GSN;TPM2;MYO7B;NPM3;GYS2;VIL1;CNN1;BIN2;MYL2;FLNB;FILIP1;MYZAP;MYOZ1;MYOZ2;PLS1
Podosome (GO:0002102)	2/20	0.161566681	0.483101429	0	0	2.972222222	5.417877634	GSN;BIN2
Mitochondrial Outer Membrane (GO:0005741)	9/171	0.166568228	0.483101429	0	0	1.489481066	2.6696718	HADHB;CYB5A;PGRMC1;DAO;IFI27;AIFM2;MGST3;COX14;RPS27A
H3 Histone Acetyltransferase Complex (GO:0070775)	1/5	0.167945132	0.483101429	0	0	6.683079057	11.92340129	ING5
MOZ/MORF Histone Acetyltransferase Complex (GO:0070776)	1/5	0.167945132	0.483101429	0	0	6.683079057	11.92340129	ING5
Astrocyte End-Foot (GO:0097450)	1/5	0.167945132	0.483101429	0	0	6.683079057	11.92340129	CLCN2
Box C/D RNP Complex (GO:0170049)	1/5	0.167945132	0.483101429	0	0	6.683079057	11.92340129	SNU13
Box C/D Methylation Guide snoRNP Complex (GO:0031428)	1/5	0.167945132	0.483101429	0	0	6.683079057	11.92340129	SNU13
External Side of Apical Plasma Membrane (GO:0098591)	1/5	0.167945132	0.483101429	0	0	6.683079057	11.92340129	LCT
Serine Protease Inhibitor Complex (GO:0097180)	1/5	0.167945132	0.483101429	0	0	6.683079057	11.92340129	PLAU
Uniplex Complex (GO:1990246)	1/5	0.167945132	0.483101429	0	0	6.683079057	11.92340129	SMDT1
Cortical Cytoskeleton (GO:0030863)	4/63	0.192460741	0.546870155	0	0	1.814739625	2.990442442	GYS2;GSN;MYZAP;MAPRE1
U7 snRNP (GO:0005683)	1/6	0.197989782	0.550716266	0	0	5.346185853	8.658361054	SNRPE
Chromosome (GO:0005694)	9/179	0.199709856	0.550716266	0	0	1.418793829	2.285520348	RSL1D1;DDX27;PINX1;HMGB2;CCDC86;LLPH;NOC2L;GNL3;BYSL
U2 snRNP (GO:0005686)	2/23	0.200905076	0.550716266	0	0	2.547222222	4.088094876	SF3B5;SNRPE
Polymeric Cytoskeletal Fiber (GO:0099513)	13/280	0.214055241	0.562886891	0	0	1.305541909	2.012520483	ANXA1;TRIM63;TPM2;KRT5;EVPL;PPL;TPPP3;KRT18;DES;KIF16B;TPPP;MAPRE1;PLS1
U12-type Spliceosomal Complex (GO:0005689)	2/24	0.21425974	0.562886891	0	0	2.431313131	3.745598979	SF3B5;SNRPE
Lysosome (GO:0005764)	23/532	0.214645897	0.562886891	0	0	1.213316245	1.8670093	CD74;ENPEP;ANXA2;HEXA;OMD;TM4SF5;TMEM97;IFI30;DPP4;NEU3;P2RX4;TCN2;ANPEP;SLC17A5;CHMP4C;AP1S3;OCIAD2;GC;APOB;RNF152;GYG1;ADA;SPAM1
Platelet Alpha Granule Lumen (GO:0031093)	4/66	0.215008297	0.562886891	0	0	1.726660077	2.654012356	SERPINA1;SCCPDH;AHSG;SERPINF2
HFE-transferrin Receptor Complex (GO:1990712)	1/7	0.226951	0.570456078	0	0	4.454923717	6.606746076	TF
Ribonuclease MRP Complex (GO:0000172)	1/7	0.226951	0.570456078	0	0	4.454923717	6.606746076	POP5
Spectrin-Associated Cytoskeleton (GO:0014731)	1/7	0.226951	0.570456078	0	0	4.454923717	6.606746076	RHBG
Rough Endoplasmic Reticulum (GO:0005791)	2/25	0.227692769	0.570456078	0	0	2.325483092	3.441152354	LRAT;TMEM97
Tertiary Granule (GO:0070820)	8/163	0.236798909	0.586958999	0	0	1.382343905	1.991327193	CEACAM1;PLAU;AIFM2;FTH1;IDH1;TMT1A;LTA4H;NIT2
U2-type Precatalytic Spliceosome (GO:0071005)	3/48	0.250009685	0.593460595	0	0	1.783310153	2.472123725	SF3B5;SNU13;SNRPE
MICOS Complex (GO:0061617)	1/8	0.2548678	0.593460595	0	0	3.818307906	5.219666233	CHCHD10
U4 snRNP (GO:0005687)	1/8	0.2548678	0.593460595	0	0	3.818307906	5.219666233	SNRPE
Dendrite Cytoplasm (GO:0032839)	1/8	0.2548678	0.593460595	0	0	3.818307906	5.219666233	SOD1
Transferase Complex, Transferring Phosphorus-Containing Groups (G	1/8	0.2548678	0.593460595	0	0	3.818307906	5.219666233	PKLR
Cortical Actin Cytoskeleton (GO:0030864)	3/49	0.2597982	0.593460595	0	0	1.744451835	2.351259583	GYS2;GSN;MYZAP
Multivesicular Body (GO:0005771)	3/49	0.2597982	0.593460595	0	0	1.744451835	2.351259583	CHMP4C;LRAT;MBL2
Precatalytic Spliceosome (GO:0071011)	3/49	0.2597982	0.593460595	0	0	1.744451835	2.351259583	SF3B5;SNRPE;SNU13
U2-type Catalytic Step 2 Spliceosome (GO:0071007)	2/28	0.268239505	0.600959659	0	0	2.056837607	2.706541234	PPIL1;SNRPE
Condensed Nuclear Chromosome (GO:0000794)	2/28	0.268239505	0.600959659	0	0	2.056837607	2.706541234	TTR;RRS1
90S Preribosome (GO:0030686)	1/9	0.281777798	0.602332356	0	0	3.340846047	4.231637445	NOC2L
Amphisome Membrane (GO:1904930)	1/9	0.281777798	0.602332356	0	0	3.340846047	4.231637445	CHMP4C
Autolysosome (GO:0044754)	1/9	0.281777798	0.602332356	0	0	3.340846047	4.231637445	FTH1
Multimeric Ribonuclease P Complex (GO:0030681)	1/9	0.281777798	0.602332356	0	0	3.340846047	4.231637445	POP5
Perisynaptic Extracellular Matrix (GO:0098966)	1/9	0.281777798	0.602332356	0	0	3.340846047	4.231637445	TNNC1
Sarcolemma (GO:0042383)	3/52	0.289423813	0.613052259	0	0	1.637393205	2.030143551	DES;ANXA5;SLC2A5
COPII-coated ER to Golgi Transport Vesicle (GO:0030134)	4/77	0.302501423	0.621290631	0	0	1.465638951	1.752419498	CD74;SEC16B;SERPINA1;TMED6
Adherens Junction (GO:0005912)	7/151	0.304332301	0.621290631	0	0	1.300874126	1.547565493	CEACAM1;ANXA1;PDLIM2;KRT18;ANXA2;PARK7;S100A11
Astrocyte Projection (GO:0097449)	1/10	0.307717253	0.621290631	0	0	2.969486824	3.499759747	CLCN2
Neuron Projection Cytoplasm (GO:0120111)	1/10	0.307717253	0.621290631	0	0	2.969486824	3.499759747	SOD1
Sarcoplasmic Reticulum Membrane (GO:0033017)	2/31	0.3087517	0.621290631	0	0	1.843773946	2.166836117	DHRS7C;CASQ1
ER to Golgi Transport Vesicle Membrane (GO:0012507)	3/54	0.309312074	0.621290631	0	0	1.573018081	1.845786594	CD74;SEC16B;CIDEB
Spliceosomal snRNP Complex (GO:0097525)	3/56	0.329241498	0.640510573	0	0	1.513501456	1.68144527	SF3B5;SNRPE;SNU13
AP-1 Adaptor Complex (GO:0030121)	1/11	0.332721123	0.640510573	0	0	2.672399445	2.940843591	AP1S3
Amphisome (GO:0044753)	1/11	0.332721123	0.640510573	0	0	2.672399445	2.940843591	CHMP4C
Microfibril (GO:0001527)	1/11	0.332721123	0.640510573	0	0	2.672399445	2.940843591	MFAP5
Primary Lysosome (GO:0005766)	1/11	0.332721123	0.640510573	0	0	2.672399445	2.940843591	HEXA
Bicellular Tight Junction (GO:0005923)	4/81	0.335374635	0.640510573	0	0	1.389212459	1.517724418	AOC1;CLDN15;CLDN34;F11R
Apical Junction Complex (GO:0043296)	5/107	0.343747434	0.650829116	0	0	1.311018131	1.399968213	AOC1;CLDN15;NHERF4;CLDN34;F11R
Lytic Vacuole (GO:0000323)	10/236	0.347989554	0.650829116	0	0	1.184001193	1.249811315	NEU3;CD74;HEXA;SLC17A5;TMEM97;OCIAD2;IFI30;RNF152;ADA;SPAM1
Transport Vesicle Membrane (GO:0030658)	3/58	0.349157251	0.650829116	0	0	1.458313314	1.534485222	CD74;SEC16B;CA4
U4/U6 X U5 tri-snRNP Complex (GO:0046540)	2/35	0.361976535	0.664098683	0	0	1.619949495	1.646153619	SNU13;SNRPE
Spliceosomal tri-snRNP Complex (GO:0097526)	2/35	0.361976535	0.664098683	0	0	1.619949495	1.646153619	SNU13;SNRPE
Specific Granule Lumen (GO:0035580)	3/61	0.378894987	0.681176744	0	0	1.382667498	1.341873542	AOC1;AIFM2;NIT2
Cul4A-RING E3 Ubiquitin Ligase Complex (GO:0031464)	1/13	0.380055694	0.681176744	0	0	2.226768377	2.154259172	DDB2
Clathrin Coat of trans-Golgi Network Vesicle (GO:0030130)	1/13	0.380055694	0.681176744	0	0	2.226768377	2.154259172	AP1S3
Condensed Chromosome (GO:0000793)	3/62	0.388748079	0.691437422	0	0	1.359161736	1.284168299	TTR;HMGB2;RRS1
MHC Class II Protein Complex (GO:0042613)	1/14	0.402450201	0.710385582	0	0	2.055371813	1.870766363	CD74
Keratin Filament (GO:0045095)	2/39	0.41359268	0.721172109	0	0	1.44451952	1.275328228	KRT18;KRT5
U2-type Spliceosomal Complex (GO:0005684)	4/91	0.417641581	0.721172109	0	0	1.228892518	1.072984983	SF3B5;PPIL1;SNRPE;SNU13
T Cell Receptor Complex (GO:0042101)	1/15	0.424036819	0.721172109	0	0	1.908460472	1.637335015	CEACAM1
Mitotic Spindle Microtubule (GO:1990498)	1/15	0.424036819	0.721172109	0	0	1.908460472	1.637335015	MAPRE1
Secondary Lysosome (GO:0005767)	1/15	0.424036819	0.721172109	0	0	1.908460472	1.637335015	FTH1
Clathrin-Coated Endocytic Vesicle Membrane (GO:0030669)	3/66	0.427723968	0.722171627	0	0	1.272600834	1.080790907	CD74;TF;APOB
Endocytic Vesicle (GO:0030139)	8/201	0.440241579	0.72992116	0	0	1.107966503	0.909010796	DPP4;ACE2;CD74;TF;APOA1;CD36;APOB;RPS27A
RNA Polymerase II, Core Complex (GO:0005665)	1/16	0.444844656	0.72992116	0	0	1.781137309	1.442774915	POLR2E
U1 snRNP (GO:0005685)	1/16	0.444844656	0.72992116	0	0	1.781137309	1.442774915	SNRPE
Acrosomal Membrane (GO:0002080)	1/16	0.444844656	0.72992116	0	0	1.781137309	1.442774915	TEKT3
Tight Junction (GO:0070160)	4/96	0.458003672	0.746257731	0	0	1.161802107	0.907225796	AOC1;CLDN15;CLDN34;F11R
Lytic Vacuole Membrane (GO:0098852)	12/314	0.461715269	0.747049049	0	0	1.061990486	0.820713553	DPP4;NEU3;CD74;ENPEP;P2RX4;ANXA2;ANPEP;SLC17A5;CHMP4C;TM4SF5;AP1S3;RNF152
U5 snRNP (GO:0005682)	1/17	0.464901769	0.747049049	0	0	1.669729542	1.278894522	SNRPE
Cell-Cell Junction (GO:0005911)	12/316	0.470509352	0.750516831	0	0	1.054892513	0.795325074	BAIAP2L2;CEACAM1;ANXA1;KRT18;PDLIM2;DES;ANXA2;CLDN15;NHERF4;PARK7;F11R;S100A11
Spindle Microtubule (GO:0005876)	3/71	0.475126635	0.750516831	0	0	1.178720445	0.877173003	CHMP4C;RMDN1;MAPRE1
Dense Core Granule (GO:0031045)	1/18	0.484235207	0.750516831	0	0	1.571428571	1.139575683	ECRG4
Endoribonuclease Complex (GO:1902555)	1/18	0.484235207	0.750516831	0	0	1.571428571	1.139575683	POP5
Kinetochore Microtubule (GO:0005828)	1/18	0.484235207	0.750516831	0	0	1.571428571	1.139575683	CHMP4C
Early Endosome (GO:0005769)	12/320	0.488024732	0.750516831	0	0	1.040973111	0.746782861	PRDX3;NEU3;TF;ANXA1;KIF16B;APOC2;APOA1;APOA4;AP1S3;SLC5A1;MELTF;APOB
Azurophil Granule (GO:0042582)	6/155	0.489607546	0.750516831	0	0	1.075831427	0.768306235	PLAC8;TTR;ANXA2;HEXA;MGST1;NDUFC2
Multivesicular Body Membrane (GO:0032585)	1/19	0.502871047	0.765810156	0	0	1.484049931	1.020167844	CHMP4C
Lysosomal Membrane (GO:0005765)	14/383	0.519497965	0.76869433	0	0	1.013297506	0.663600821	CD74;ENPEP;ANXA2;MGST1;NDUFC2;TM4SF5;DPP4;NEU3;P2RX4;ANPEP;SLC17A5;CHMP4C;AP1S3;RNF152
MHC Protein Complex (GO:0042611)	1/20	0.520834425	0.76869433	0	0	1.405869042	0.917080837	CD74
Clathrin Adaptor Complex (GO:0030131)	1/20	0.520834425	0.76869433	0	0	1.405869042	0.917080837	AP1S3
Cell-Cell Contact Zone (GO:0044291)	2/48	0.521260533	0.76869433	0	0	1.161352657	0.75662741	BAIAP2L2;DES
Sarcoplasmic Reticulum (GO:0016529)	2/48	0.521260533	0.76869433	0	0	1.161352657	0.75662741	DHRS7C;CASQ1
Endocytic Vesicle Lumen (GO:0071682)	1/21	0.538149575	0.778933947	0	0	1.335506241	0.827504691	APOB
Pericentric Heterochromatin (GO:0005721)	1/21	0.538149575	0.778933947	0	0	1.335506241	0.827504691	HELLS
Actin Filament (GO:0005884)	3/78	0.538233328	0.778933947	0	0	1.068317107	0.661783046	ANXA1;TPM2;PLS1
Clathrin-Coated Endocytic Vesicle (GO:0045334)	3/82	0.572283106	0.823098541	0	0	1.014013838	0.565942893	CD74;TF;APOB
Coated Vesicle Membrane (GO:0030662)	2/54	0.585317428	0.836680741	0	0	1.027029915	0.550078215	CD74;SEC16B
Motile Cilium (GO:0031514)	3/85	0.596772518	0.847853638	0	0	0.97676312	0.504223955	ANXA1;SORD;SPA17
SAGA Complex (GO:0000124)	1/25	0.601382672	0.849225228	0	0	1.112690707	0.56582973	SF3B5
Clathrin-Coated Vesicle Membrane (GO:0030665)	3/88	0.620328498	0.865780069	0	0	0.942141864	0.449878492	CD74;TF;APOB
SCF Ubiquitin Ligase Complex (GO:0019005)	2/58	0.624376196	0.865780069	0	0	0.953472222	0.449087528	FBXO2;NCCRP1
Azurophil Granule Membrane (GO:0035577)	2/58	0.624376196	0.865780069	0	0	0.953472222	0.449087528	MGST1;NDUFC2
Azurophil Granule Lumen (GO:0035578)	3/89	0.627969235	0.865780069	0	0	0.931138209	0.433225184	PLAC8;TTR;ANXA2
Extracellular Membrane-Bounded Organelle (GO:0065010)	2/59	0.633684337	0.868520298	0	0	0.936695906	0.427324737	CBR1;APOA4
Exocytic Vesicle (GO:0070382)	1/29	0.65596867	0.884495256	0	0	0.953536755	0.402051383	SYT15
Nuclear Inner Membrane (GO:0005637)	1/29	0.65596867	0.884495256	0	0	0.953536755	0.402051383	IFI27
Caveola (GO:0005901)	2/62	0.660524354	0.884495256	0	0	0.889722222	0.368986742	ASAH2;CD36
Extracellular Vesicle (GO:1903561)	2/62	0.660524354	0.884495256	0	0	0.889722222	0.368986742	CBR1;APOA4
Secretory Vesicle (GO:0099503)	1/31	0.680394659	0.905896888	0	0	0.889875173	0.342675151	SYT15
Intercalated Disc (GO:0014704)	1/32	0.691950274	0.908328277	0	0	0.861124782	0.31710161	DES
Lumenal Side of Endoplasmic Reticulum Membrane (GO:0098553)	1/32	0.691950274	0.908328277	0	0	0.861124782	0.31710161	CD74
Cytoskeleton (GO:0005856)	23/694	0.693916023	0.908328277	0	0	0.912440382	0.333409667	CLIC5;GSN;TMOD4;TPM2;MYO7B;NPM3;EVPL;PPL;HYPK;GYS2;VIL1;CNN1;HINT1;KRT18;DES;MYL2;KRT15;TEKT3;FLNB;FILIP1;MYOZ1;RHBG;MYOZ2
Microtubule (GO:0005874)	6/194	0.705666695	0.916586034	0	0	0.85091525	0.296639485	TPPP3;TRIM63;KIF16B;RMDN1;TPPP;MAPRE1
Heterotrimeric G-protein Complex (GO:0005834)	1/34	0.713824805	0.916586034	0	0	0.808851343	0.272678119	GNG13
Endocytic Vesicle Membrane (GO:0030666)	5/165	0.715055591	0.916586034	0	0	0.833246165	0.279466589	CD74;TF;CD36;APOB;RPS27A
Spindle (GO:0005819)	7/227	0.715959907	0.916586034	0	0	0.848099174	0.283376318	KIF16B;SLC34A1;PINX1;MYF6;RMDN1;TPPP;MAPRE1
Recycling Endosome Membrane (GO:0055038)	2/70	0.724383496	0.916913596	0	0	0.784722222	0.253021389	NEU3;SLC39A4
Cul4-RING E3 Ubiquitin Ligase Complex (GO:0080008)	1/36	0.73414802	0.916913596	0	0	0.762552011	0.235662588	DDB2
Autophagosome Membrane (GO:0000421)	1/36	0.73414802	0.916913596	0	0	0.762552011	0.235662588	CHMP4C
Acrosomal Vesicle (GO:0001669)	2/72	0.738671614	0.916913596	0	0	0.762222222	0.2308785	TEKT3;SPAM1
Tertiary Granule Membrane (GO:0070821)	2/72	0.738671614	0.916913596	0	0	0.762222222	0.2308785	CEACAM1;PLAU
Supramolecular Fiber (GO:0099512)	1/37	0.743762531	0.916913596	0	0	0.741331484	0.219458935	MFAP5
Vesicle Coat (GO:0030120)	1/37	0.743762531	0.916913596	0	0	0.741331484	0.219458935	CIDEB
trans-Golgi Network Membrane (GO:0032588)	3/108	0.752836236	0.923214963	0	0	0.761891516	0.216306759	CD74;AP4S1;AP1S3
SAGA-type Complex (GO:0070461)	1/39	0.761962338	0.929514266	0	0	0.702241039	0.19090995	SF3B5
Cytoplasmic Side of Plasma Membrane (GO:0009898)	3/113	0.779468433	0.945917421	0	0	0.727070426	0.181144571	NDUFAB1;CHMP4C;MYZAP
Plasma Membrane Raft (GO:0044853)	2/83	0.806371663	0.953716556	0	0	0.658333333	0.14168026	ASAH2;CD36
Voltage-Gated Potassium Channel Complex (GO:0008076)	2/83	0.806371663	0.953716556	0	0	0.658333333	0.14168026	KCNA7;STX1A
Coated Vesicle (GO:0030135)	2/84	0.811680431	0.953716556	0	0	0.650271003	0.135678117	SERPINA1;TMED6
Mitotic Spindle (GO:0072686)	4/155	0.815123058	0.953716556	0	0	0.705676179	0.144251633	SLC34A1;MYF6;TPPP;MAPRE1
Recycling Endosome (GO:0055037)	4/155	0.815123058	0.953716556	0	0	0.705676179	0.144251633	NEU3;TF;MELTF;SLC39A4
Extrinsic Component of Cytoplasmic Side of Plasma Membrane (GO:0	1/46	0.816079238	0.953716556	0	0	0.592787795	0.120480458	GNG13
Late Endosome (GO:0005770)	6/222	0.816608319	0.953716556	0	0	0.73952514	0.149824623	CD74;TF;ANXA2;CHMP4C;LRAT;MBL2
Cytoplasmic Vesicle Membrane (GO:0030659)	12/415	0.821640579	0.953716556	0	0	0.791598225	0.155511238	ACE2;CD74;ZNRF2;TF;SLC30A8;IYD;ANPEP;CA4;AP1S3;CD36;RPS27A;APOB
Cyclin-Dependent Protein Kinase Holoenzyme Complex (GO:0000307)	1/47	0.822734025	0.953716556	0	0	0.579870952	0.113145758	CDPF1
Potassium Channel Complex (GO:0034705)	2/90	0.84088345	0.965618526	0	0	0.605744949	0.10497694	KCNA7;STX1A
Basement Membrane (GO:0005604)	1/50	0.84128996	0.965618526	0	0	0.544283733	0.094062515	TNNC1
Calcium Channel Complex (GO:0034704)	1/51	0.847033691	0.967445344	0	0	0.533370319	0.088547371	SMDT1
Endoplasmic reticulum-Golgi Intermediate Compartment Membrane (G	1/52	0.852569831	0.968630415	0	0	0.522884882	0.083400222	SERPINA1
Asymmetric Synapse (GO:0032279)	3/131	0.856385689	0.968630415	0	0	0.624239395	0.096778601	RPS14;RPS25;RPL7
Membrane Raft (GO:0045121)	4/171	0.869409043	0.968717412	0	0	0.637532734	0.089217325	ACE2;ASAH2;ANXA2;CD36
Vacuolar Membrane (GO:0005774)	1/56	0.872784337	0.968717412	0	0	0.484756021	0.065959196	CHMP4C
Golgi Lumen (GO:0005796)	2/99	0.877037895	0.968717412	0	0	0.549284078	0.07206886	OMD;PCSK5
Late Endosome Membrane (GO:0031902)	2/99	0.877037895	0.968717412	0	0	0.549284078	0.07206886	ANXA2;CHMP4C
Filopodium (GO:0030175)	1/57	0.877389653	0.968717412	0	0	0.476074896	0.06227254	VIL1
Golgi Membrane (GO:0000139)	13/476	0.881408118	0.968717412	0	0	0.745109926	0.094058591	ARF4;CD74;SLC30A8;MOGAT3;MOGAT2;PCSK5;FTCD;APOO;B3GNT5;GCNT4;CHPT1;AP1S3;HPD
Glutamatergic Synapse (GO:0098978)	2/102	0.887298222	0.970612609	0	0	0.532722222	0.063699801	SH3GL3;SH3GL1
Serine/Threonine Protein Kinase Complex (GO:1902554)	1/72	0.929493602	0.999754895	0	0	0.375202672	0.027433077	CDPF1
Postsynaptic Density (GO:0014069)	3/164	0.938449847	0.999754895	0	0	0.49543448	0.031472903	RPS14;RPS25;RPL7
Early Endosome Membrane (GO:0031901)	2/124	0.941435791	0.999754895	0	0	0.436156648	0.026321675	NEU3;ANXA1
Autophagosome (GO:0005776)	1/81	0.949421849	0.999754895	0	0	0.332836338	0.017274892	CHMP4C
trans-Golgi Network (GO:0005802)	5/251	0.951040081	0.999754895	0	0	0.539510834	0.027082943	CD74;CA4;AP4S1;AP1S3;PCSK5
P-body (GO:0000932)	1/83	0.953021926	0.999754895	0	0	0.324684551	0.015622966	PSMA6
Bounding Membrane of Organelle (GO:0098588)	23/881	0.962392817	0.999754895	0	0	0.706403754	0.027078277	ARF4;CD74;SERPINA1;SLC30A8;MOGAT3;MOGAT2;PCSK5;FTCD;ACE2;APOO;TF;ANPEP;B3GNT5;CA4;DHRS7C;GCNT4;CHPT1;AP1S3;CD36;CASQ1;RPS27A;APOB;HPD
Endosome Membrane (GO:0010008)	8/392	0.974049056	0.999754895	0	0	0.551295518	0.01449555	NEU3;TF;ANXA1;ANXA2;ABCB11;RPS27A;APOB;SLC39A4
Microtubule Cytoskeleton (GO:0015630)	8/393	0.97458594	0.999754895	0	0	0.549834479	0.014154155	TPPP3;TRIM63;KIF16B;PINX1;TEKT3;TPPP;MAPRE1;HYPK
Phagocytic Vesicle (GO:0045335)	1/100	0.974927759	0.999754895	0	0	0.268692473	0.006822614	CD36
Nuclear Membrane (GO:0031965)	4/239	0.974988835	0.999754895	0	0	0.451443134	0.01143472	IFI27;ANXA4;TMEM97;SLC5A1
cullin-RING Ubiquitin Ligase Complex (GO:0031461)	3/219	0.986755838	0.999754895	0	0	0.36821975	0.004909344	FBXO2;NCCRP1;DDB2
Sperm Flagellum (GO:0036126)	1/126	0.990412579	0.999754895	0	0	0.21251595	0.00204731	TEKT3
9+2 Motile Cilium (GO:0097729)	1/140	0.994289	0.999754895	0	0	0.190971772	0.001093766	TEKT3
Cilium (GO:0005929)	6/402	0.99676285	0.999754895	0	0	0.399568309	0.001295561	PSMA6;RSL1D1;ANXA1;SORD;USH1C;SPA17
Dendrite (GO:0030425)	3/281	0.997927322	0.999754895	0	0	0.285168249	5.92E-04	APOD;CYP46A1;SOD1
Intracellular Membrane-Bounded Organelle (GO:0043231)	169/5597	0.997990555	0.999754895	0	0	0.779778951	0.001568499	TP53RK;FOXI3;AQP8;ECI2;BCCIP;THYN1;AHR;PARK7;NOC2L;IFI30;PWP1;RPL7;FBL;MPC2;FTH1;TEKT3;AP1S3;TGM2;RPS9;DGAT2;SLC30A8;RPS8;DAPK2;RPL22;ACOT13;HSD11B1L;CDPF1;BHLHA15;KRT5;DKC1;
Neuron Projection (GO:0043005)	8/560	0.999496035	0.999754895	0	0	0.380099866	1.92E-04	PGRMC1;UCMA;STMN1;APOD;PARK7;ACADM;CYP46A1;STX1A
Axon (GO:0030424)	1/217	0.999669101	0.999754895	0	0	0.122399445	4.05E-05	PARK7
Nucleus (GO:0005634)	138/4879	0.999754895	0.999754895	0	0	0.724554527	1.78E-04	TP53RK;FOXI3;BCCIP;THYN1;AHR;PARK7;NOC2L;PWP1;RPL7;FBL;MPC2;FTH1;TEKT3;TGM2;RPS9;RPS8;DAPK2;RPL22;ACOT13;CDPF1;BHLHA15;KRT5;DKC1;CCDC86;SNRPE;NUPR1;PFN1;GCM2;CDCA7;SNU13;
