# Supplementary material for: Succinate supplementation ameliorates musculoskeletal defects caused by PLOD3 mutations in a BCARD syndrome model
Source: Genome Med. 2026 Mar 13;18:29. doi: 10.1186/s13073-026-01608-y (PMC12994257; doi:10.1186/s13073-026-01608-y)
Supplement: Supplementary file 3 — Additional file 3: Table S7. AlphaMissense algorithm data. [file 13073_2026_1608_MOESM3_ESM.pdf]

Table 7	Plod3_alphamissense_data	
**protein_variant**	am_pathogenicity	am_class
**M1A**	0.1906	LBen
**M1C**	0.2039	LBen
**M1D**	0.5134	Amb
**M1E**	0.3586	Amb
**M1F**	0.1506	LBen
**M1G**	0.2885	LBen
**M1H**	0.3238	LBen
**M1I**	0.2822	LBen
**M1K**	0.2415	LBen
**M1L**	0.1198	LBen
**M1N**	0.3644	Amb
**M1P**	0.283	LBen
**M1Q**	0.1506	LBen
**M1R**	0.1792	LBen
**M1S**	0.2382	LBen
**M1T**	0.2356	LBen
**M1V**	0.0991	LBen
**M1W**	0.2667	LBen
**M1Y**	0.3135	LBen
**T2A**	0.0514	LBen
**T2C**	0.2253	LBen
**T2D**	0.1448	LBen
**T2E**	0.1453	LBen
**T2F**	0.112	LBen
**T2G**	0.0957	LBen
**T2H**	0.1203	LBen
**T2I**	0.1115	LBen
**T2K**	0.1349	LBen
**T2L**	0.1036	LBen
**T2M**	0.1676	LBen
**T2N**	0.0921	LBen
**T2P**	0.0538	LBen
**T2Q**	0.1328	LBen
**T2R**	0.095	LBen
**T2S**	0.0739	LBen
**T2V**	0.1121	LBen

**T2W**	0.2803	LBen
**T2Y**	0.1401	LBen
**S3A**	0.0504	LBen
**S3C**	0.0925	LBen
**S3D**	0.1292	LBen
**S3E**	0.1385	LBen
**S3F**	0.0876	LBen
**S3G**	0.0686	LBen
**S3H**	0.1425	LBen
**S3I**	0.1144	LBen
**S3K**	0.2315	LBen
**S3L**	0.1175	LBen
**S3M**	0.2435	LBen
**S3N**	0.1252	LBen
**S3P**	0.0643	LBen
**S3Q**	0.1832	LBen
**S3R**	0.1455	LBen
**S3T**	0.0687	LBen
**S3V**	0.096	LBen
**S3W**	0.1283	LBen
**S3Y**	0.1032	LBen
**S4A**	0.0538	LBen
**S4C**	0.0797	LBen
**S4D**	0.1473	LBen
**S4E**	0.1381	LBen
**S4F**	0.0954	LBen
**S4G**	0.061	LBen
**S4H**	0.1363	LBen
**S4I**	0.119	LBen
**S4K**	0.219	LBen
**S4L**	0.0993	LBen
**S4M**	0.2169	LBen
**S4N**	0.1333	LBen
**S4P**	0.0606	LBen
**S4Q**	0.1659	LBen
**S4R**	0.1381	LBen
**S4T**	0.068	LBen
**S4V**	0.1023	LBen
**S4W**	0.0874	LBen
**S4Y**	0.0988	LBen
**G5A**	0.0674	LBen

**G5C**	0.1259	LBen
**G5D**	0.099	LBen
**G5E**	0.0991	LBen
**G5F**	0.137	LBen
**G5H**	0.1678	LBen
**G5I**	0.104	LBen
**G5K**	0.2246	LBen
**G5L**	0.1111	LBen
**G5M**	0.2465	LBen
**G5N**	0.1794	LBen
**G5P**	0.1479	LBen
**G5Q**	0.1613	LBen
**G5R**	0.147	LBen
**G5S**	0.0877	LBen
**G5T**	0.0889	LBen
**G5V**	0.0778	LBen
**G5W**	0.1363	LBen
**G5Y**	0.1564	LBen
**P6A**	0.0486	LBen
**P6C**	0.1599	LBen
**P6D**	0.1351	LBen
**P6E**	0.093	LBen
**P6F**	0.116	LBen
**P6G**	0.0836	LBen
**P6H**	0.0882	LBen
**P6I**	0.1205	LBen
**P6K**	0.1068	LBen
**P6L**	0.0825	LBen
**P6M**	0.1835	LBen
**P6N**	0.1372	LBen
**P6Q**	0.076	LBen
**P6R**	0.0599	LBen
**P6S**	0.0688	LBen
**P6T**	0.0641	LBen
**P6V**	0.0932	LBen
**P6W**	0.1408	LBen
**P6Y**	0.143	LBen
**G7A**	0.0672	LBen
**G7C**	0.1292	LBen
**G7D**	0.1294	LBen
**G7E**	0.0967	LBen
**G7F**	0.1434	LBen

**G7H**	0.1891	LBen
**G7I**	0.1134	LBen
**G7K**	0.2123	LBen
**G7L**	0.1141	LBen
**G7M**	0.2413	LBen
**G7N**	0.2082	LBen
**G7P**	0.1483	LBen
**G7Q**	0.1559	LBen
**G7R**	0.1448	LBen
**G7S**	0.1011	LBen
**G7T**	0.0977	LBen
**G7V**	0.0814	LBen
**G7W**	0.1271	LBen
**G7Y**	0.1718	LBen
**P8A**	0.0584	LBen
**P8C**	0.1436	LBen
**P8D**	0.1535	LBen
**P8E**	0.1008	LBen
**P8F**	0.1087	LBen
**P8G**	0.0883	LBen
**P8H**	0.0862	LBen
**P8I**	0.1095	LBen
**P8K**	0.1135	LBen
**P8L**	0.0712	LBen
**P8M**	0.1776	LBen
**P8N**	0.1424	LBen
**P8Q**	0.0743	LBen
**P8R**	0.0626	LBen
**P8S**	0.0713	LBen
**P8T**	0.0746	LBen
**P8V**	0.0916	LBen
**P8W**	0.1245	LBen
**P8Y**	0.1362	LBen
**R9A**	0.0751	LBen
**R9C**	0.1901	LBen
**R9D**	0.2397	LBen
**R9E**	0.186	LBen
**R9F**	0.1497	LBen
**R9G**	0.0733	LBen
**R9H**	0.1665	LBen
**R9I**	0.1127	LBen
**R9K**	0.1393	LBen

**R9L**	0.0845	LBen
**R9M**	0.2778	LBen
**R9N**	0.2579	LBen
**R9P**	0.0835	LBen
**R9Q**	0.1447	LBen
**R9S**	0.1311	LBen
**R9T**	0.1173	LBen
**R9V**	0.1115	LBen
**R9W**	0.1705	LBen
**R9Y**	0.2132	LBen
**F10A**	0.0995	LBen
**F10C**	0.1068	LBen
**F10D**	0.3008	LBen
**F10E**	0.3069	LBen
**F10G**	0.1529	LBen
**F10H**	0.2243	LBen
**F10I**	0.0801	LBen
**F10K**	0.3158	LBen
**F10L**	0.1511	LBen
**F10M**	0.221	LBen
**F10N**	0.2221	LBen
**F10P**	0.1588	LBen
**F10Q**	0.2457	LBen
**F10R**	0.1747	LBen
**F10S**	0.122	LBen
**F10T**	0.1546	LBen
**F10V**	0.0782	LBen
**F10W**	0.123	LBen
**F10Y**	0.136	LBen
**L11A**	0.1012	LBen
**L11C**	0.1661	LBen
**L11D**	0.4631	Amb
**L11E**	0.317	LBen
**L11F**	0.064	LBen
**L11G**	0.1542	LBen
**L11H**	0.1823	LBen
**L11I**	0.0907	LBen
**L11K**	0.2908	LBen
**L11M**	0.1716	LBen
**L11N**	0.3079	LBen
**L11P**	0.1051	LBen
**L11Q**	0.1626	LBen

**L11R**	0.1398	LBen
**L11S**	0.1394	LBen
**L11T**	0.1427	LBen
**L11V**	0.0847	LBen
**L11W**	0.1024	LBen
**L11Y**	0.2108	LBen
**L12A**	0.0999	LBen
**L12C**	0.1778	LBen
**L12D**	0.5147	Amb
**L12E**	0.3376	LBen
**L12F**	0.0677	LBen
**L12G**	0.1576	LBen
**L12H**	0.2136	LBen
**L12I**	0.0974	LBen
**L12K**	0.2998	LBen
**L12M**	0.1699	LBen
**L12N**	0.3279	LBen
**L12P**	0.1185	LBen
**L12Q**	0.1768	LBen
**L12R**	0.163	LBen
**L12S**	0.1624	LBen
**L12T**	0.1525	LBen
**L12V**	0.0847	LBen
**L12W**	0.1214	LBen
**L12Y**	0.2278	LBen
**L13A**	0.0816	LBen
**L13C**	0.1581	LBen
**L13D**	0.4773	Amb
**L13E**	0.33	LBen
**L13F**	0.0686	LBen
**L13G**	0.1137	LBen
**L13H**	0.2224	LBen
**L13I**	0.0917	LBen
**L13K**	0.3221	LBen
**L13M**	0.161	LBen
**L13N**	0.3033	LBen
**L13P**	0.1028	LBen
**L13Q**	0.1831	LBen
**L13R**	0.1856	LBen
**L13S**	0.1475	LBen
**L13T**	0.1361	LBen
**L13V**	0.0762	LBen

**L13W**	0.1229	LBen
**L13Y**	0.2247	LBen
**L14A**	0.1128	LBen
**L14C**	0.2251	LBen
**L14D**	0.5815	LPath
**L14E**	0.3635	Amb
**L14F**	0.0733	LBen
**L14G**	0.1824	LBen
**L14H**	0.2583	LBen
**L14I**	0.0925	LBen
**L14K**	0.3667	Amb
**L14M**	0.1457	LBen
**L14N**	0.3668	Amb
**L14P**	0.1389	LBen
**L14Q**	0.203	LBen
**L14R**	0.235	LBen
**L14S**	0.1699	LBen
**L14T**	0.1685	LBen
**L14V**	0.0896	LBen
**L14W**	0.1458	LBen
**L14Y**	0.2582	LBen
**P15A**	0.062	LBen
**P15C**	0.1387	LBen
**P15D**	0.3559	Amb
**P15E**	0.2682	LBen
**P15F**	0.125	LBen
**P15G**	0.1026	LBen
**P15H**	0.1418	LBen
**P15I**	0.1332	LBen
**P15K**	0.304	LBen
**P15L**	0.0744	LBen
**P15M**	0.2207	LBen
**P15N**	0.229	LBen
**P15Q**	0.14	LBen
**P15R**	0.1584	LBen
**P15S**	0.0855	LBen
**P15T**	0.097	LBen
**P15V**	0.0999	LBen
**P15W**	0.1794	LBen
**P15Y**	0.186	LBen
**L16A**	0.0922	LBen
**L16C**	0.1855	LBen

**L16D**	0.4541	Amb
**L16E**	0.2872	LBen
**L16F**	0.0657	LBen
**L16G**	0.1495	LBen
**L16H**	0.2132	LBen
**L16I**	0.0962	LBen
**L16K**	0.3186	LBen
**L16M**	0.1468	LBen
**L16N**	0.2931	LBen
**L16P**	0.114	LBen
**L16Q**	0.1685	LBen
**L16R**	0.1733	LBen
**L16S**	0.1392	LBen
**L16T**	0.1547	LBen
**L16V**	0.0801	LBen
**L16W**	0.1297	LBen
**L16Y**	0.2172	LBen
**L17A**	0.081	LBen
**L17C**	0.1589	LBen
**L17D**	0.3963	Amb
**L17E**	0.2468	LBen
**L17F**	0.0608	LBen
**L17G**	0.1428	LBen
**L17H**	0.1596	LBen
**L17I**	0.079	LBen
**L17K**	0.2431	LBen
**L17M**	0.1348	LBen
**L17N**	0.2327	LBen
**L17P**	0.0849	LBen
**L17Q**	0.1291	LBen
**L17R**	0.1261	LBen
**L17S**	0.1188	LBen
**L17T**	0.1183	LBen
**L17V**	0.071	LBen
**L17W**	0.1086	LBen
**L17Y**	0.1878	LBen
**L18A**	0.0857	LBen
**L18C**	0.1777	LBen
**L18D**	0.3132	LBen
**L18E**	0.1858	LBen
**L18F**	0.0683	LBen
**L18G**	0.1438	LBen

**L18H**	0.1318	LBen
**L18I**	0.0871	LBen
**L18K**	0.1888	LBen
**L18M**	0.1326	LBen
**L18N**	0.2118	LBen
**L18P**	0.0654	LBen
**L18Q**	0.1006	LBen
**L18R**	0.0995	LBen
**L18S**	0.115	LBen
**L18T**	0.1205	LBen
**L18V**	0.0768	LBen
**L18W**	0.0991	LBen
**L18Y**	0.1803	LBen
**P19A**	0.062	LBen
**P19C**	0.167	LBen
**P19D**	0.218	LBen
**P19E**	0.1517	LBen
**P19F**	0.1358	LBen
**P19G**	0.1027	LBen
**P19H**	0.0961	LBen
**P19I**	0.1408	LBen
**P19K**	0.1843	LBen
**P19L**	0.0853	LBen
**P19M**	0.2225	LBen
**P19N**	0.1877	LBen
**P19Q**	0.0865	LBen
**P19R**	0.0901	LBen
**P19S**	0.0738	LBen
**P19T**	0.08	LBen
**P19V**	0.1049	LBen
**P19W**	0.1768	LBen
**P19Y**	0.1595	LBen
**P20A**	0.0486	LBen
**P20C**	0.1773	LBen
**P20D**	0.1819	LBen
**P20E**	0.1221	LBen
**P20F**	0.167	LBen
**P20G**	0.093	LBen
**P20H**	0.1015	LBen
**P20I**	0.1676	LBen
**P20K**	0.1614	LBen
**P20L**	0.0964	LBen

**P20M**	0.222	LBen
**P20N**	0.186	LBen
**P20Q**	0.0878	LBen
**P20R**	0.077	LBen
**P20S**	0.0725	LBen
**P20T**	0.0718	LBen
**P20V**	0.106	LBen
**P20W**	0.1922	LBen
**P20Y**	0.1874	LBen
**A21C**	0.2306	LBen
**A21D**	0.1437	LBen
**A21E**	0.1154	LBen
**A21F**	0.1582	LBen
**A21G**	0.0744	LBen
**A21H**	0.2195	LBen
**A21I**	0.1689	LBen
**A21K**	0.2225	LBen
**A21L**	0.1148	LBen
**A21M**	0.2191	LBen
**A21N**	0.2013	LBen
**A21P**	0.0786	LBen
**A21Q**	0.1539	LBen
**A21R**	0.1475	LBen
**A21S**	0.0927	LBen
**A21T**	0.094	LBen
**A21V**	0.098	LBen
**A21W**	0.2399	LBen
**A21Y**	0.2228	LBen
**A22C**	0.2336	LBen
**A22D**	0.1365	LBen
**A22E**	0.1388	LBen
**A22F**	0.1633	LBen
**A22G**	0.0787	LBen
**A22H**	0.2264	LBen
**A22I**	0.1733	LBen
**A22K**	0.2563	LBen
**A22L**	0.1238	LBen
**A22M**	0.2599	LBen
**A22N**	0.1768	LBen
**A22P**	0.0799	LBen
**A22Q**	0.1797	LBen
**A22R**	0.1548	LBen

**A22S**	0.0863	LBen
**A22T**	0.0916	LBen
**A22V**	0.1047	LBen
**A22W**	0.2805	LBen
**A22Y**	0.2308	LBen
**S23A**	0.0493	LBen
**S23C**	0.0841	LBen
**S23D**	0.1547	LBen
**S23E**	0.1539	LBen
**S23F**	0.0995	LBen
**S23G**	0.0632	LBen
**S23H**	0.1512	LBen
**S23I**	0.1263	LBen
**S23K**	0.2604	LBen
**S23L**	0.098	LBen
**S23M**	0.193	LBen
**S23N**	0.138	LBen
**S23P**	0.0538	LBen
**S23Q**	0.1779	LBen
**S23R**	0.1585	LBen
**S23T**	0.072	LBen
**S23V**	0.0988	LBen
**S23W**	0.1155	LBen
**S23Y**	0.1171	LBen
**A24C**	0.2353	LBen
**A24D**	0.119	LBen
**A24E**	0.1417	LBen
**A24F**	0.1688	LBen
**A24G**	0.0793	LBen
**A24H**	0.2151	LBen
**A24I**	0.1876	LBen
**A24K**	0.2625	LBen
**A24L**	0.1313	LBen
**A24M**	0.289	LBen
**A24N**	0.1745	LBen
**A24P**	0.077	LBen
**A24Q**	0.1839	LBen
**A24R**	0.1513	LBen
**A24S**	0.0827	LBen
**A24T**	0.0944	LBen
**A24V**	0.1121	LBen
**A24W**	0.3215	LBen

**A24Y**	0.2321	LBen
**S25A**	0.0538	LBen
**S25C**	0.1016	LBen
**S25D**	0.1496	LBen
**S25E**	0.1507	LBen
**S25F**	0.1203	LBen
**S25G**	0.0685	LBen
**S25H**	0.1839	LBen
**S25I**	0.1273	LBen
**S25K**	0.2766	LBen
**S25L**	0.0937	LBen
**S25M**	0.1866	LBen
**S25N**	0.1338	LBen
**S25P**	0.0653	LBen
**S25Q**	0.2081	LBen
**S25R**	0.1944	LBen
**S25T**	0.0714	LBen
**S25V**	0.1072	LBen
**S25W**	0.1369	LBen
**S25Y**	0.1363	LBen
**D26A**	0.0777	LBen
**D26C**	0.2637	LBen
**D26E**	0.0857	LBen
**D26F**	0.2373	LBen
**D26G**	0.0737	LBen
**D26H**	0.1156	LBen
**D26I**	0.1711	LBen
**D26K**	0.1784	LBen
**D26L**	0.1538	LBen
**D26M**	0.3817	Amb
**D26N**	0.0956	LBen
**D26P**	0.1528	LBen
**D26Q**	0.1303	LBen
**D26R**	0.1269	LBen
**D26S**	0.077	LBen
**D26T**	0.1193	LBen
**D26V**	0.1049	LBen
**D26W**	0.4343	Amb
**D26Y**	0.1103	LBen
**R27A**	0.0977	LBen
**R27C**	0.1325	LBen
**R27D**	0.1458	LBen

**R27E**	0.107	LBen
**R27F**	0.1822	LBen
**R27G**	0.0687	LBen
**R27H**	0.0825	LBen
**R27I**	0.1171	LBen
**R27K**	0.0901	LBen
**R27L**	0.0889	LBen
**R27M**	0.1656	LBen
**R27N**	0.1741	LBen
**R27P**	0.09	LBen
**R27Q**	0.0736	LBen
**R27S**	0.117	LBen
**R27T**	0.0919	LBen
**R27V**	0.1211	LBen
**R27W**	0.1105	LBen
**R27Y**	0.1599	LBen
**P28A**	0.0698	LBen
**P28C**	0.2491	LBen
**P28D**	0.1492	LBen
**P28E**	0.1038	LBen
**P28F**	0.1883	LBen
**P28G**	0.1208	LBen
**P28H**	0.1007	LBen
**P28I**	0.1569	LBen
**P28K**	0.1347	LBen
**P28L**	0.0858	LBen
**P28M**	0.2129	LBen
**P28N**	0.1562	LBen
**P28Q**	0.0819	LBen
**P28R**	0.0785	LBen
**P28S**	0.077	LBen
**P28T**	0.0782	LBen
**P28V**	0.117	LBen
**P28W**	0.2229	LBen
**P28Y**	0.1926	LBen
**R29A**	0.0905	LBen
**R29C**	0.1588	LBen
**R29D**	0.1605	LBen
**R29E**	0.1078	LBen
**R29F**	0.2163	LBen
**R29G**	0.064	LBen
**R29H**	0.0962	LBen

**R29I**	0.1434	LBen
**R29K**	0.0899	LBen
**R29L**	0.096	LBen
**R29M**	0.1699	LBen
**R29N**	0.1969	LBen
**R29P**	0.0839	LBen
**R29Q**	0.0784	LBen
**R29S**	0.1246	LBen
**R29T**	0.0955	LBen
**R29V**	0.1351	LBen
**R29W**	0.1094	LBen
**R29Y**	0.2058	LBen
**G30A**	0.0801	LBen
**G30C**	0.1223	LBen
**G30D**	0.0726	LBen
**G30E**	0.0716	LBen
**G30F**	0.1873	LBen
**G30H**	0.1235	LBen
**G30I**	0.1112	LBen
**G30K**	0.127	LBen
**G30L**	0.1388	LBen
**G30M**	0.2049	LBen
**G30N**	0.1128	LBen
**G30P**	0.16	LBen
**G30Q**	0.1093	LBen
**G30R**	0.0958	LBen
**G30S**	0.0782	LBen
**G30T**	0.0882	LBen
**G30V**	0.0814	LBen
**G30W**	0.1234	LBen
**G30Y**	0.1375	LBen
**R31A**	0.0805	LBen
**R31C**	0.1266	LBen
**R31D**	0.1131	LBen
**R31E**	0.0972	LBen
**R31F**	0.1879	LBen
**R31G**	0.0584	LBen
**R31H**	0.0825	LBen
**R31I**	0.105	LBen
**R31K**	0.0752	LBen
**R31L**	0.0941	LBen
**R31M**	0.1266	LBen

**R31N**	0.1236	LBen
**R31P**	0.0887	LBen
**R31Q**	0.0728	LBen
**R31S**	0.0851	LBen
**R31T**	0.0744	LBen
**R31V**	0.1089	LBen
**R31W**	0.1072	LBen
**R31Y**	0.1548	LBen
**D32A**	0.0759	LBen
**D32C**	0.248	LBen
**D32E**	0.0802	LBen
**D32F**	0.1763	LBen
**D32G**	0.0787	LBen
**D32H**	0.0887	LBen
**D32I**	0.1242	LBen
**D32K**	0.1138	LBen
**D32L**	0.1135	LBen
**D32M**	0.2542	LBen
**D32N**	0.0765	LBen
**D32P**	0.1283	LBen
**D32Q**	0.1033	LBen
**D32R**	0.1012	LBen
**D32S**	0.0736	LBen
**D32T**	0.104	LBen
**D32V**	0.0852	LBen
**D32W**	0.3526	Amb
**D32Y**	0.0849	LBen
**P33A**	0.0582	LBen
**P33C**	0.2614	LBen
**P33D**	0.1424	LBen
**P33E**	0.1135	LBen
**P33F**	0.1837	LBen
**P33G**	0.1269	LBen
**P33H**	0.0944	LBen
**P33I**	0.1357	LBen
**P33K**	0.1253	LBen
**P33L**	0.0831	LBen
**P33M**	0.1703	LBen
**P33N**	0.1518	LBen
**P33Q**	0.0902	LBen
**P33R**	0.0725	LBen
**P33S**	0.0776	LBen

**P33T**	0.065	LBen
**P33V**	0.1019	LBen
**P33W**	0.2229	LBen
**P33Y**	0.1818	LBen
**V34A**	0.109	LBen
**V34C**	0.5679	LPath
**V34D**	0.1706	LBen
**V34E**	0.141	LBen
**V34F**	0.1056	LBen
**V34G**	0.099	LBen
**V34H**	0.3323	LBen
**V34I**	0.0792	LBen
**V34K**	0.1875	LBen
**V34L**	0.0993	LBen
**V34M**	0.1287	LBen
**V34N**	0.192	LBen
**V34P**	0.1823	LBen
**V34Q**	0.1791	LBen
**V34R**	0.1485	LBen
**V34S**	0.1409	LBen
**V34T**	0.1553	LBen
**V34W**	0.4435	Amb
**V34Y**	0.318	LBen
**N35A**	0.1371	LBen
**N35C**	0.247	LBen
**N35D**	0.072	LBen
**N35E**	0.1683	LBen
**N35F**	0.2989	LBen
**N35G**	0.227	LBen
**N35H**	0.0774	LBen
**N35I**	0.1189	LBen
**N35K**	0.1347	LBen
**N35L**	0.1341	LBen
**N35M**	0.2271	LBen
**N35P**	0.1666	LBen
**N35Q**	0.1681	LBen
**N35R**	0.1454	LBen
**N35S**	0.0783	LBen
**N35T**	0.1115	LBen
**N35V**	0.1347	LBen
**N35W**	0.4811	Amb
**N35Y**	0.1044	LBen

**P36A**	0.075	LBen
**P36C**	0.4916	Amb
**P36D**	0.2937	LBen
**P36E**	0.1615	LBen
**P36F**	0.429	Amb
**P36G**	0.1938	LBen
**P36H**	0.1717	LBen
**P36I**	0.2643	LBen
**P36K**	0.1798	LBen
**P36L**	0.1163	LBen
**P36M**	0.2695	LBen
**P36N**	0.2779	LBen
**P36Q**	0.1315	LBen
**P36R**	0.1019	LBen
**P36S**	0.1075	LBen
**P36T**	0.0829	LBen
**P36V**	0.1651	LBen
**P36W**	0.418	Amb
**P36Y**	0.3655	Amb
**E37A**	0.1415	LBen
**E37C**	0.7656	LPath
**E37D**	0.1024	LBen
**E37F**	0.6842	LPath
**E37G**	0.0779	LBen
**E37H**	0.302	LBen
**E37I**	0.4848	Amb
**E37K**	0.1052	LBen
**E37L**	0.3587	Amb
**E37M**	0.4831	Amb
**E37N**	0.1968	LBen
**E37P**	0.4522	Amb
**E37Q**	0.101	LBen
**E37R**	0.1303	LBen
**E37S**	0.1458	LBen
**E37T**	0.2325	LBen
**E37V**	0.2891	LBen
**E37W**	0.6996	LPath
**E37Y**	0.4962	Amb
**K38A**	0.2928	LBen
**K38C**	0.7431	LPath
**K38D**	0.2469	LBen
**K38E**	0.1132	LBen

**K38F**	0.7236	LPath
**K38G**	0.2872	LBen
**K38H**	0.2123	LBen
**K38I**	0.4562	Amb
**K38L**	0.354	Amb
**K38M**	0.2233	LBen
**K38N**	0.1423	LBen
**K38P**	0.6632	LPath
**K38Q**	0.1103	LBen
**K38R**	0.0893	LBen
**K38S**	0.2406	LBen
**K38T**	0.1688	LBen
**K38V**	0.4029	Amb
**K38W**	0.7172	LPath
**K38Y**	0.4402	Amb
**L39A**	0.8843	LPath
**L39C**	0.8764	LPath
**L39D**	0.9951	LPath
**L39E**	0.9807	LPath
**L39F**	0.3735	Amb
**L39G**	0.9652	LPath
**L39H**	0.9492	LPath
**L39I**	0.1876	LBen
**L39K**	0.9724	LPath
**L39M**	0.2626	LBen
**L39N**	0.9781	LPath
**L39P**	0.9631	LPath
**L39Q**	0.9325	LPath
**L39R**	0.9402	LPath
**L39S**	0.9715	LPath
**L39T**	0.8971	LPath
**L39V**	0.2063	LBen
**L39W**	0.8271	LPath
**L39Y**	0.8789	LPath
**L40A**	0.403	Amb
**L40C**	0.6719	LPath
**L40D**	0.8727	LPath
**L40E**	0.4392	Amb
**L40F**	0.2094	LBen
**L40G**	0.8208	LPath
**L40H**	0.2662	LBen
**L40I**	0.1211	LBen

**L40K**	0.3729	Amb
**L40M**	0.1407	LBen
**L40N**	0.749	LPath
**L40P**	0.9568	LPath
**L40Q**	0.1968	LBen
**L40R**	0.2295	LBen
**L40S**	0.5225	Amb
**L40T**	0.3313	LBen
**L40V**	0.1108	LBen
**L40W**	0.3089	LBen
**L40Y**	0.4723	Amb
**V41A**	0.6374	LPath
**V41C**	0.9409	LPath
**V41D**	0.9876	LPath
**V41E**	0.9769	LPath
**V41F**	0.6501	LPath
**V41G**	0.7441	LPath
**V41H**	0.9904	LPath
**V41I**	0.1059	LBen
**V41K**	0.9852	LPath
**V41L**	0.2561	LBen
**V41M**	0.4756	Amb
**V41N**	0.9582	LPath
**V41P**	0.9736	LPath
**V41Q**	0.9719	LPath
**V41R**	0.9743	LPath
**V41S**	0.8713	LPath
**V41T**	0.7739	LPath
**V41W**	0.9909	LPath
**V41Y**	0.9751	LPath
**I42A**	0.8077	LPath
**I42C**	0.8633	LPath
**I42D**	0.9924	LPath
**I42E**	0.9783	LPath
**I42F**	0.2169	LBen
**I42G**	0.9631	LPath
**I42H**	0.9466	LPath
**I42K**	0.9477	LPath
**I42L**	0.0874	LBen
**I42M**	0.1228	LBen
**I42N**	0.9227	LPath
**I42P**	0.9859	LPath

**I42Q**	0.952	LPath
**I42R**	0.9222	LPath
**I42S**	0.8813	LPath
**I42T**	0.8134	LPath
**I42V**	0.1011	LBen
**I42W**	0.9092	LPath
**I42Y**	0.7587	LPath
**T43A**	0.7604	LPath
**T43C**	0.9604	LPath
**T43D**	0.9924	LPath
**T43E**	0.9934	LPath
**T43F**	0.9937	LPath
**T43G**	0.8736	LPath
**T43H**	0.9828	LPath
**T43I**	0.987	LPath
**T43K**	0.9897	LPath
**T43L**	0.9478	LPath
**T43M**	0.8516	LPath
**T43N**	0.9541	LPath
**T43P**	0.9486	LPath
**T43Q**	0.9839	LPath
**T43R**	0.9837	LPath
**T43S**	0.6385	LPath
**T43V**	0.9466	LPath
**T43W**	0.9984	LPath
**T43Y**	0.9958	LPath
**V44A**	0.2376	LBen
**V44C**	0.943	LPath
**V44D**	0.9774	LPath
**V44E**	0.9567	LPath
**V44F**	0.9268	LPath
**V44G**	0.6944	LPath
**V44H**	0.9931	LPath
**V44I**	0.2241	LBen
**V44K**	0.9812	LPath
**V44L**	0.8799	LPath
**V44M**	0.8092	LPath
**V44N**	0.9373	LPath
**V44P**	0.9416	LPath
**V44Q**	0.9633	LPath
**V44R**	0.9556	LPath
**V44S**	0.6036	LPath

**V44T**	0.5327	Amb
**V44W**	0.9986	LPath
**V44Y**	0.9914	LPath
**A45C**	0.9615	LPath
**A45D**	0.9911	LPath
**A45E**	0.9896	LPath
**A45F**	0.985	LPath
**A45G**	0.5193	Amb
**A45H**	0.9928	LPath
**A45I**	0.9895	LPath
**A45K**	0.9955	LPath
**A45L**	0.9397	LPath
**A45M**	0.9744	LPath
**A45N**	0.9868	LPath
**A45P**	0.9865	LPath
**A45Q**	0.9801	LPath
**A45R**	0.9775	LPath
**A45S**	0.4934	Amb
**A45T**	0.9219	LPath
**A45V**	0.9572	LPath
**A45W**	0.9983	LPath
**A45Y**	0.9938	LPath
**T46A**	0.6508	LPath
**T46C**	0.9844	LPath
**T46D**	0.9588	LPath
**T46E**	0.947	LPath
**T46F**	0.9744	LPath
**T46G**	0.8632	LPath
**T46H**	0.9516	LPath
**T46I**	0.9587	LPath
**T46K**	0.9057	LPath
**T46L**	0.8208	LPath
**T46M**	0.6607	LPath
**T46N**	0.8789	LPath
**T46P**	0.7655	LPath
**T46Q**	0.9199	LPath
**T46R**	0.8866	LPath
**T46S**	0.5976	LPath
**T46V**	0.854	LPath
**T46W**	0.9916	LPath
**T46Y**	0.9824	LPath
**A47C**	0.5666	LPath

**A47D**	0.1465	LBen
**A47E**	0.0956	LBen
**A47F**	0.3173	LBen
**A47G**	0.1283	LBen
**A47H**	0.2897	LBen
**A47I**	0.2593	LBen
**A47K**	0.1646	LBen
**A47L**	0.1704	LBen
**A47M**	0.1953	LBen
**A47N**	0.1659	LBen
**A47P**	0.7302	LPath
**A47Q**	0.1502	LBen
**A47R**	0.159	LBen
**A47S**	0.0773	LBen
**A47T**	0.0735	LBen
**A47V**	0.1281	LBen
**A47W**	0.5992	LPath
**A47Y**	0.3808	Amb
**E48A**	0.1394	LBen
**E48C**	0.8624	LPath
**E48D**	0.208	LBen
**E48F**	0.688	LPath
**E48G**	0.1762	LBen
**E48H**	0.3477	Amb
**E48I**	0.3287	LBen
**E48K**	0.0663	LBen
**E48L**	0.3233	LBen
**E48M**	0.3685	Amb
**E48N**	0.2688	LBen
**E48P**	0.256	LBen
**E48Q**	0.0868	LBen
**E48R**	0.0979	LBen
**E48S**	0.2046	LBen
**E48T**	0.2019	LBen
**E48V**	0.1905	LBen
**E48W**	0.8064	LPath
**E48Y**	0.5595	Amb
**T49A**	0.4914	Amb
**T49C**	0.9456	LPath
**T49D**	0.5145	Amb
**T49E**	0.657	LPath
**T49F**	0.886	LPath

**T49G**	0.528	Amb
**T49H**	0.6076	LPath
**T49I**	0.8669	LPath
**T49K**	0.5614	Amb
**T49L**	0.6243	LPath
**T49M**	0.3502	Amb
**T49N**	0.1223	LBen
**T49P**	0.5922	LPath
**T49Q**	0.5867	LPath
**T49R**	0.5231	Amb
**T49S**	0.2611	LBen
**T49V**	0.7372	LPath
**T49W**	0.9284	LPath
**T49Y**	0.8189	LPath
**E50A**	0.7121	LPath
**E50C**	0.9922	LPath
**E50D**	0.2844	LBen
**E50F**	0.9969	LPath
**E50G**	0.6692	LPath
**E50H**	0.9482	LPath
**E50I**	0.9871	LPath
**E50K**	0.8582	LPath
**E50L**	0.9824	LPath
**E50M**	0.978	LPath
**E50N**	0.8301	LPath
**E50P**	0.9267	LPath
**E50Q**	0.647	LPath
**E50R**	0.8998	LPath
**E50S**	0.7151	LPath
**E50T**	0.907	LPath
**E50V**	0.9432	LPath
**E50W**	0.9978	LPath
**E50Y**	0.991	LPath
**G51A**	0.636	LPath
**G51C**	0.9684	LPath
**G51D**	0.9834	LPath
**G51E**	0.9702	LPath
**G51F**	0.9954	LPath
**G51H**	0.9962	LPath
**G51I**	0.9827	LPath
**G51K**	0.9958	LPath
**G51L**	0.9921	LPath

**G51M**	0.9933	LPath
**G51N**	0.9799	LPath
**G51P**	0.9803	LPath
**G51Q**	0.9894	LPath
**G51R**	0.9886	LPath
**G51S**	0.7416	LPath
**G51T**	0.9449	LPath
**G51V**	0.9615	LPath
**G51W**	0.9918	LPath
**G51Y**	0.9931	LPath
**Y52A**	0.9737	LPath
**Y52C**	0.72	LPath
**Y52D**	0.977	LPath
**Y52E**	0.9928	LPath
**Y52F**	0.1703	LBen
**Y52G**	0.947	LPath
**Y52H**	0.8234	LPath
**Y52I**	0.9439	LPath
**Y52K**	0.987	LPath
**Y52L**	0.7316	LPath
**Y52M**	0.9402	LPath
**Y52N**	0.8884	LPath
**Y52P**	0.9961	LPath
**Y52Q**	0.9857	LPath
**Y52R**	0.9614	LPath
**Y52S**	0.9298	LPath
**Y52T**	0.9804	LPath
**Y52V**	0.8948	LPath
**Y52W**	0.761	LPath
**L53A**	0.1156	LBen
**L53C**	0.2195	LBen
**L53D**	0.2741	LBen
**L53E**	0.1046	LBen
**L53F**	0.1187	LBen
**L53G**	0.1532	LBen
**L53H**	0.0651	LBen
**L53I**	0.0958	LBen
**L53K**	0.0619	LBen
**L53M**	0.1089	LBen
**L53N**	0.1511	LBen
**L53P**	0.65	LPath
**L53Q**	0.0453	LBen

**L53R**	0.037	LBen
**L53S**	0.1283	LBen
**L53T**	0.1116	LBen
**L53V**	0.0887	LBen
**L53W**	0.1018	LBen
**L53Y**	0.1594	LBen
**R54A**	0.9818	LPath
**R54C**	0.829	LPath
**R54D**	0.9944	LPath
**R54E**	0.9632	LPath
**R54F**	0.9936	LPath
**R54G**	0.968	LPath
**R54H**	0.7571	LPath
**R54I**	0.9766	LPath
**R54K**	0.9109	LPath
**R54L**	0.9267	LPath
**R54M**	0.9879	LPath
**R54N**	0.9823	LPath
**R54P**	0.9918	LPath
**R54Q**	0.7107	LPath
**R54S**	0.9799	LPath
**R54T**	0.9796	LPath
**R54V**	0.9792	LPath
**R54W**	0.8528	LPath
**R54Y**	0.9706	LPath
**F55A**	0.9923	LPath
**F55C**	0.9535	LPath
**F55D**	0.9986	LPath
**F55E**	0.9988	LPath
**F55G**	0.991	LPath
**F55H**	0.9847	LPath
**F55I**	0.9632	LPath
**F55K**	0.9985	LPath
**F55L**	0.9865	LPath
**F55M**	0.963	LPath
**F55N**	0.9925	LPath
**F55P**	0.9998	LPath
**F55Q**	0.997	LPath
**F55R**	0.9952	LPath
**F55S**	0.9879	LPath
**F55T**	0.9896	LPath
**F55V**	0.9411	LPath

**F55W**	0.9242	LPath
**F55Y**	0.5376	Amb
**L56A**	0.3049	LBen
**L56C**	0.4509	Amb
**L56D**	0.855	LPath
**L56E**	0.3409	Amb
**L56F**	0.2167	LBen
**L56G**	0.6133	LPath
**L56H**	0.2136	LBen
**L56I**	0.1037	LBen
**L56K**	0.1506	LBen
**L56M**	0.1065	LBen
**L56N**	0.5223	Amb
**L56P**	0.9504	LPath
**L56Q**	0.0865	LBen
**L56R**	0.0949	LBen
**L56S**	0.3201	LBen
**L56T**	0.254	LBen
**L56V**	0.1016	LBen
**L56W**	0.2409	LBen
**L56Y**	0.4424	Amb
**R57A**	0.2543	LBen
**R57C**	0.1738	LBen
**R57D**	0.3439	Amb
**R57E**	0.172	LBen
**R57F**	0.4117	Amb
**R57G**	0.1532	LBen
**R57H**	0.0683	LBen
**R57I**	0.345	Amb
**R57K**	0.1012	LBen
**R57L**	0.1839	LBen
**R57M**	0.3111	LBen
**R57N**	0.2817	LBen
**R57P**	0.7917	LPath
**R57Q**	0.0661	LBen
**R57S**	0.2296	LBen
**R57T**	0.2025	LBen
**R57V**	0.3643	Amb
**R57W**	0.1261	LBen
**R57Y**	0.2283	LBen
**S58A**	0.3874	Amb
**S58C**	0.5336	Amb

**S58D**	0.9502	LPath
**S58E**	0.976	LPath
**S58F**	0.9409	LPath
**S58G**	0.3331	LBen
**S58H**	0.9289	LPath
**S58I**	0.9175	LPath
**S58K**	0.9941	LPath
**S58L**	0.6994	LPath
**S58M**	0.7193	LPath
**S58N**	0.7437	LPath
**S58P**	0.9714	LPath
**S58Q**	0.9659	LPath
**S58R**	0.9912	LPath
**S58T**	0.0961	LBen
**S58V**	0.8241	LPath
**S58W**	0.9456	LPath
**S58Y**	0.8869	LPath
**A59C**	0.6174	LPath
**A59D**	0.9818	LPath
**A59E**	0.936	LPath
**A59F**	0.9109	LPath
**A59G**	0.322	LBen
**A59H**	0.9723	LPath
**A59I**	0.4778	Amb
**A59K**	0.9613	LPath
**A59L**	0.4641	Amb
**A59M**	0.4657	Amb
**A59N**	0.9295	LPath
**A59P**	0.9762	LPath
**A59Q**	0.8889	LPath
**A59R**	0.9249	LPath
**A59S**	0.1741	LBen
**A59T**	0.1659	LBen
**A59V**	0.1999	LBen
**A59W**	0.983	LPath
**A59Y**	0.9668	LPath
**E60A**	0.0958	LBen
**E60C**	0.7275	LPath
**E60D**	0.1286	LBen
**E60F**	0.5747	LPath
**E60G**	0.1	LBen
**E60H**	0.195	LBen

**E60I**	0.3175	LBen
**E60K**	0.0709	LBen
**E60L**	0.2731	LBen
**E60M**	0.3565	Amb
**E60N**	0.1607	LBen
**E60P**	0.7369	LPath
**E60Q**	0.0747	LBen
**E60R**	0.0843	LBen
**E60S**	0.1174	LBen
**E60T**	0.1467	LBen
**E60V**	0.1967	LBen
**E60W**	0.6806	LPath
**E60Y**	0.3895	Amb
**F61A**	0.4603	Amb
**F61C**	0.2811	LBen
**F61D**	0.6661	LPath
**F61E**	0.6571	LPath
**F61G**	0.7446	LPath
**F61H**	0.3118	LBen
**F61I**	0.2006	LBen
**F61K**	0.6513	LPath
**F61L**	0.8111	LPath
**F61M**	0.4996	Amb
**F61N**	0.4655	Amb
**F61P**	0.9849	LPath
**F61Q**	0.5814	LPath
**F61R**	0.5053	Amb
**F61S**	0.3729	Amb
**F61T**	0.4239	Amb
**F61V**	0.1993	LBen
**F61W**	0.4009	Amb
**F61Y**	0.0624	LBen
**F62A**	0.9276	LPath
**F62C**	0.6693	LPath
**F62D**	0.9764	LPath
**F62E**	0.9714	LPath
**F62G**	0.9229	LPath
**F62H**	0.6522	LPath
**F62I**	0.9093	LPath
**F62K**	0.9795	LPath
**F62L**	0.9237	LPath
**F62M**	0.8863	LPath

**F62N**	0.8227	LPath
**F62P**	0.9972	LPath
**F62Q**	0.9027	LPath
**F62R**	0.9072	LPath
**F62S**	0.7771	LPath
**F62T**	0.9456	LPath
**F62V**	0.8537	LPath
**F62W**	0.6455	LPath
**F62Y**	0.2874	LBen
**N63A**	0.3842	Amb
**N63C**	0.6455	LPath
**N63D**	0.1639	LBen
**N63E**	0.4233	Amb
**N63F**	0.8454	LPath
**N63G**	0.2331	LBen
**N63H**	0.1367	LBen
**N63I**	0.7537	LPath
**N63K**	0.3441	Amb
**N63L**	0.5377	Amb
**N63M**	0.7764	LPath
**N63P**	0.7717	LPath
**N63Q**	0.3835	Amb
**N63R**	0.3052	LBen
**N63S**	0.1024	LBen
**N63T**	0.3815	Amb
**N63V**	0.7108	LPath
**N63W**	0.8955	LPath
**N63Y**	0.4148	Amb
**Y64A**	0.9728	LPath
**Y64C**	0.6688	LPath
**Y64D**	0.9723	LPath
**Y64E**	0.9942	LPath
**Y64F**	0.118	LBen
**Y64G**	0.9676	LPath
**Y64H**	0.9191	LPath
**Y64I**	0.8065	LPath
**Y64K**	0.9944	LPath
**Y64L**	0.7246	LPath
**Y64M**	0.8914	LPath
**Y64N**	0.9299	LPath
**Y64P**	0.9966	LPath
**Y64Q**	0.9913	LPath

**Y64R**	0.9856	LPath
**Y64S**	0.9434	LPath
**Y64T**	0.9689	LPath
**Y64V**	0.7336	LPath
**Y64W**	0.8137	LPath
**T65A**	0.1397	LBen
**T65C**	0.7412	LPath
**T65D**	0.336	LBen
**T65E**	0.282	LBen
**T65F**	0.5251	Amb
**T65G**	0.379	Amb
**T65H**	0.242	LBen
**T65I**	0.4785	Amb
**T65K**	0.1704	LBen
**T65L**	0.2744	LBen
**T65M**	0.1855	LBen
**T65N**	0.183	LBen
**T65P**	0.2481	LBen
**T65Q**	0.2309	LBen
**T65R**	0.1321	LBen
**T65S**	0.1191	LBen
**T65V**	0.3672	Amb
**T65W**	0.7289	LPath
**T65Y**	0.49	Amb
**V66A**	0.6065	LPath
**V66C**	0.9209	LPath
**V66D**	0.967	LPath
**V66E**	0.9176	LPath
**V66F**	0.433	Amb
**V66G**	0.6948	LPath
**V66H**	0.9537	LPath
**V66I**	0.0944	LBen
**V66K**	0.9007	LPath
**V66L**	0.3696	Amb
**V66M**	0.3878	Amb
**V66N**	0.9157	LPath
**V66P**	0.9102	LPath
**V66Q**	0.9004	LPath
**V66R**	0.8582	LPath
**V66S**	0.7762	LPath
**V66T**	0.6009	LPath
**V66W**	0.9575	LPath

**V66Y**	0.8813	LPath
**R67A**	0.403	Amb
**R67C**	0.2005	LBen
**R67D**	0.6758	LPath
**R67E**	0.2245	LBen
**R67F**	0.5546	Amb
**R67G**	0.3385	LBen
**R67H**	0.0949	LBen
**R67I**	0.2668	LBen
**R67K**	0.079	LBen
**R67L**	0.2147	LBen
**R67M**	0.2634	LBen
**R67N**	0.5262	Amb
**R67P**	0.9018	LPath
**R67Q**	0.0783	LBen
**R67S**	0.4302	Amb
**R67T**	0.2051	LBen
**R67V**	0.3145	LBen
**R67W**	0.1972	LBen
**R67Y**	0.4011	Amb
**T68A**	0.2397	LBen
**T68C**	0.7022	LPath
**T68D**	0.9059	LPath
**T68E**	0.8462	LPath
**T68F**	0.8563	LPath
**T68G**	0.7292	LPath
**T68H**	0.7473	LPath
**T68I**	0.4427	Amb
**T68K**	0.7742	LPath
**T68L**	0.4823	Amb
**T68M**	0.3287	LBen
**T68N**	0.5605	Amb
**T68P**	0.2609	LBen
**T68Q**	0.7508	LPath
**T68R**	0.7421	LPath
**T68S**	0.3682	Amb
**T68V**	0.262	LBen
**T68W**	0.9684	LPath
**T68Y**	0.8668	LPath
**L69A**	0.7237	LPath
**L69C**	0.8788	LPath
**L69D**	0.9926	LPath

**L69E**	0.9488	LPath
**L69F**	0.4571	Amb
**L69G**	0.9602	LPath
**L69H**	0.8911	LPath
**L69I**	0.196	LBen
**L69K**	0.9138	LPath
**L69M**	0.2989	LBen
**L69N**	0.9721	LPath
**L69P**	0.9503	LPath
**L69Q**	0.8453	LPath
**L69R**	0.7969	LPath
**L69S**	0.9313	LPath
**L69T**	0.7851	LPath
**L69V**	0.1996	LBen
**L69W**	0.7847	LPath
**L69Y**	0.8919	LPath
**G70A**	0.856	LPath
**G70C**	0.9653	LPath
**G70D**	0.9799	LPath
**G70E**	0.9836	LPath
**G70F**	0.9876	LPath
**G70H**	0.9913	LPath
**G70I**	0.9827	LPath
**G70K**	0.9928	LPath
**G70L**	0.9805	LPath
**G70M**	0.9933	LPath
**G70N**	0.9903	LPath
**G70P**	0.9934	LPath
**G70Q**	0.9884	LPath
**G70R**	0.9806	LPath
**G70S**	0.9029	LPath
**G70T**	0.9795	LPath
**G70V**	0.9687	LPath
**G70W**	0.9821	LPath
**G70Y**	0.9863	LPath
**L71A**	0.3555	Amb
**L71C**	0.7132	LPath
**L71D**	0.7912	LPath
**L71E**	0.3703	Amb
**L71F**	0.1589	LBen
**L71G**	0.6538	LPath
**L71H**	0.2435	LBen

**L71I**	0.1551	LBen
**L71K**	0.1902	LBen
**L71M**	0.1238	LBen
**L71N**	0.5734	LPath
**L71P**	0.589	LPath
**L71Q**	0.1499	LBen
**L71R**	0.121	LBen
**L71S**	0.4237	Amb
**L71T**	0.3527	Amb
**L71V**	0.1613	LBen
**L71W**	0.2249	LBen
**L71Y**	0.4009	Amb
**G72A**	0.3995	Amb
**G72C**	0.6489	LPath
**G72D**	0.2267	LBen
**G72E**	0.3248	LBen
**G72F**	0.8556	LPath
**G72H**	0.4451	Amb
**G72I**	0.8831	LPath
**G72K**	0.4537	Amb
**G72L**	0.791	LPath
**G72M**	0.8307	LPath
**G72N**	0.3456	Amb
**G72P**	0.9485	LPath
**G72Q**	0.4043	Amb
**G72R**	0.3528	Amb
**G72S**	0.2152	LBen
**G72T**	0.5064	Amb
**G72V**	0.7746	LPath
**G72W**	0.646	LPath
**G72Y**	0.707	LPath
**E73A**	0.2498	LBen
**E73C**	0.9175	LPath
**E73D**	0.2596	LBen
**E73F**	0.8241	LPath
**E73G**	0.2526	LBen
**E73H**	0.4898	Amb
**E73I**	0.5237	Amb
**E73K**	0.1093	LBen
**E73L**	0.4751	Amb
**E73M**	0.4793	Amb
**E73N**	0.4021	Amb

**E73P**	0.9133	LPath
**E73Q**	0.1064	LBen
**E73R**	0.1964	LBen
**E73S**	0.2892	LBen
**E73T**	0.3	LBen
**E73V**	0.3126	LBen
**E73W**	0.9043	LPath
**E73Y**	0.7296	LPath
**E74A**	0.0963	LBen
**E74C**	0.7457	LPath
**E74D**	0.1016	LBen
**E74F**	0.5754	LPath
**E74G**	0.1291	LBen
**E74H**	0.2953	LBen
**E74I**	0.2316	LBen
**E74K**	0.0792	LBen
**E74L**	0.2536	LBen
**E74M**	0.3094	LBen
**E74N**	0.1699	LBen
**E74P**	0.2254	LBen
**E74Q**	0.096	LBen
**E74R**	0.1329	LBen
**E74S**	0.1398	LBen
**E74T**	0.1385	LBen
**E74V**	0.1353	LBen
**E74W**	0.7392	LPath
**E74Y**	0.4262	Amb
**W75A**	0.9925	LPath
**W75C**	0.9978	LPath
**W75D**	0.9956	LPath
**W75E**	0.996	LPath
**W75F**	0.9023	LPath
**W75G**	0.9589	LPath
**W75H**	0.9934	LPath
**W75I**	0.9909	LPath
**W75K**	0.9983	LPath
**W75L**	0.9837	LPath
**W75M**	0.9941	LPath
**W75N**	0.996	LPath
**W75P**	0.9969	LPath
**W75Q**	0.9984	LPath
**W75R**	0.9976	LPath

**W75S**	0.9871	LPath
**W75T**	0.9927	LPath
**W75V**	0.9919	LPath
**W75Y**	0.9569	LPath
**R76A**	0.2409	LBen
**R76C**	0.1745	LBen
**R76D**	0.5455	Amb
**R76E**	0.215	LBen
**R76F**	0.4132	Amb
**R76G**	0.179	LBen
**R76H**	0.084	LBen
**R76I**	0.2032	LBen
**R76K**	0.0708	LBen
**R76L**	0.1327	LBen
**R76M**	0.1733	LBen
**R76N**	0.367	Amb
**R76P**	0.7506	LPath
**R76Q**	0.0792	LBen
**R76S**	0.3089	LBen
**R76T**	0.1454	LBen
**R76V**	0.2459	LBen
**R76W**	0.1575	LBen
**R76Y**	0.315	LBen
**G77A**	0.8789	LPath
**G77C**	0.9738	LPath
**G77D**	0.9774	LPath
**G77E**	0.984	LPath
**G77F**	0.996	LPath
**G77H**	0.9961	LPath
**G77I**	0.9896	LPath
**G77K**	0.9938	LPath
**G77L**	0.9933	LPath
**G77M**	0.9955	LPath
**G77N**	0.9913	LPath
**G77P**	0.998	LPath
**G77Q**	0.9929	LPath
**G77R**	0.9853	LPath
**G77S**	0.8579	LPath
**G77T**	0.9716	LPath
**G77V**	0.9767	LPath
**G77W**	0.9855	LPath
**G77Y**	0.9936	LPath

**G78A**	0.8557	LPath
**G78C**	0.9493	LPath
**G78D**	0.9351	LPath
**G78E**	0.9459	LPath
**G78F**	0.9885	LPath
**G78H**	0.9772	LPath
**G78I**	0.9793	LPath
**G78K**	0.9568	LPath
**G78L**	0.983	LPath
**G78M**	0.9863	LPath
**G78N**	0.943	LPath
**G78P**	0.9973	LPath
**G78Q**	0.9527	LPath
**G78R**	0.9035	LPath
**G78S**	0.7058	LPath
**G78T**	0.9301	LPath
**G78V**	0.9631	LPath
**G78W**	0.9615	LPath
**G78Y**	0.9836	LPath
**D79A**	0.7261	LPath
**D79C**	0.9631	LPath
**D79E**	0.7141	LPath
**D79F**	0.9386	LPath
**D79G**	0.816	LPath
**D79H**	0.7774	LPath
**D79I**	0.8727	LPath
**D79K**	0.915	LPath
**D79L**	0.8812	LPath
**D79M**	0.9448	LPath
**D79N**	0.1928	LBen
**D79P**	0.9723	LPath
**D79Q**	0.8849	LPath
**D79R**	0.8956	LPath
**D79S**	0.4906	Amb
**D79T**	0.8386	LPath
**D79V**	0.7182	LPath
**D79W**	0.9855	LPath
**D79Y**	0.6804	LPath
**V80A**	0.8467	LPath
**V80C**	0.9876	LPath
**V80D**	0.9691	LPath
**V80E**	0.9467	LPath

**V80F**	0.8304	LPath
**V80G**	0.8652	LPath
**V80H**	0.9878	LPath
**V80I**	0.1841	LBen
**V80K**	0.9609	LPath
**V80L**	0.8242	LPath
**V80M**	0.5519	Amb
**V80N**	0.938	LPath
**V80P**	0.9574	LPath
**V80Q**	0.9554	LPath
**V80R**	0.9315	LPath
**V80S**	0.8843	LPath
**V80T**	0.7208	LPath
**V80W**	0.9957	LPath
**V80Y**	0.9796	LPath
**A81C**	0.8273	LPath
**A81D**	0.7886	LPath
**A81E**	0.5014	Amb
**A81F**	0.5753	LPath
**A81G**	0.2331	LBen
**A81H**	0.7141	LPath
**A81I**	0.5783	LPath
**A81K**	0.6034	LPath
**A81L**	0.2872	LBen
**A81M**	0.4332	Amb
**A81N**	0.626	LPath
**A81P**	0.7645	LPath
**A81Q**	0.3966	Amb
**A81R**	0.4202	Amb
**A81S**	0.1353	LBen
**A81T**	0.2052	LBen
**A81V**	0.3187	LBen
**A81W**	0.8844	LPath
**A81Y**	0.7497	LPath
**R82A**	0.5433	Amb
**R82C**	0.2845	LBen
**R82D**	0.799	LPath
**R82E**	0.5029	Amb
**R82F**	0.672	LPath
**R82G**	0.373	Amb
**R82H**	0.1169	LBen
**R82I**	0.5009	Amb

**R82K**	0.1441	LBen
**R82L**	0.3506	Amb
**R82M**	0.4477	Amb
**R82N**	0.6388	LPath
**R82P**	0.9577	LPath
**R82Q**	0.1213	LBen
**R82S**	0.6055	LPath
**R82T**	0.3987	Amb
**R82V**	0.5112	Amb
**R82W**	0.237	LBen
**R82Y**	0.4915	Amb
**T83A**	0.2693	LBen
**T83C**	0.8206	LPath
**T83D**	0.7478	LPath
**T83E**	0.628	LPath
**T83F**	0.5066	Amb
**T83G**	0.3493	Amb
**T83H**	0.4148	Amb
**T83I**	0.7895	LPath
**T83K**	0.4128	Amb
**T83L**	0.3623	Amb
**T83M**	0.1813	LBen
**T83N**	0.3542	Amb
**T83P**	0.7455	LPath
**T83Q**	0.4297	Amb
**T83R**	0.3144	LBen
**T83S**	0.2088	LBen
**T83V**	0.6043	LPath
**T83W**	0.7106	LPath
**T83Y**	0.4732	Amb
**V84A**	0.2631	LBen
**V84C**	0.9332	LPath
**V84D**	0.8751	LPath
**V84E**	0.6479	LPath
**V84F**	0.4873	Amb
**V84G**	0.5372	Amb
**V84H**	0.9003	LPath
**V84I**	0.1081	LBen
**V84K**	0.6446	LPath
**V84L**	0.4951	Amb
**V84M**	0.317	LBen
**V84N**	0.8046	LPath

**V84P**	0.6092	LPath
**V84Q**	0.61	LPath
**V84R**	0.5134	Amb
**V84S**	0.5074	Amb
**V84T**	0.2872	LBen
**V84W**	0.9516	LPath
**V84Y**	0.9074	LPath
**G85A**	0.925	LPath
**G85C**	0.98	LPath
**G85D**	0.9904	LPath
**G85E**	0.9917	LPath
**G85F**	0.9961	LPath
**G85H**	0.9971	LPath
**G85I**	0.9939	LPath
**G85K**	0.9955	LPath
**G85L**	0.9935	LPath
**G85M**	0.9975	LPath
**G85N**	0.9955	LPath
**G85P**	0.9978	LPath
**G85Q**	0.995	LPath
**G85R**	0.9828	LPath
**G85S**	0.926	LPath
**G85T**	0.9839	LPath
**G85V**	0.9859	LPath
**G85W**	0.986	LPath
**G85Y**	0.995	LPath
**G86A**	0.9311	LPath
**G86C**	0.9708	LPath
**G86D**	0.9879	LPath
**G86E**	0.9891	LPath
**G86F**	0.9926	LPath
**G86H**	0.9972	LPath
**G86I**	0.989	LPath
**G86K**	0.9939	LPath
**G86L**	0.9926	LPath
**G86M**	0.9954	LPath
**G86N**	0.994	LPath
**G86P**	0.9986	LPath
**G86Q**	0.9944	LPath
**G86R**	0.9782	LPath
**G86S**	0.9208	LPath
**G86T**	0.9837	LPath

**G86V**	0.9798	LPath
**G86W**	0.9782	LPath
**G86Y**	0.9928	LPath
**G87A**	0.8345	LPath
**G87C**	0.9724	LPath
**G87D**	0.9963	LPath
**G87E**	0.9943	LPath
**G87F**	0.997	LPath
**G87H**	0.9984	LPath
**G87I**	0.9939	LPath
**G87K**	0.997	LPath
**G87L**	0.9947	LPath
**G87M**	0.9972	LPath
**G87N**	0.997	LPath
**G87P**	0.9984	LPath
**G87Q**	0.9959	LPath
**G87R**	0.9853	LPath
**G87S**	0.897	LPath
**G87T**	0.9886	LPath
**G87V**	0.9863	LPath
**G87W**	0.9916	LPath
**G87Y**	0.9965	LPath
**Q88A**	0.8989	LPath
**Q88C**	0.9927	LPath
**Q88D**	0.9847	LPath
**Q88E**	0.4687	Amb
**Q88F**	0.9809	LPath
**Q88G**	0.9069	LPath
**Q88H**	0.9196	LPath
**Q88I**	0.9718	LPath
**Q88K**	0.8227	LPath
**Q88L**	0.8406	LPath
**Q88M**	0.8248	LPath
**Q88N**	0.9325	LPath
**Q88P**	0.9545	LPath
**Q88R**	0.8416	LPath
**Q88S**	0.9274	LPath
**Q88T**	0.953	LPath
**Q88V**	0.9532	LPath
**Q88W**	0.9801	LPath
**Q88Y**	0.9847	LPath
**K89A**	0.9946	LPath

**K89C**	0.9993	LPath
**K89D**	0.9976	LPath
**K89E**	0.9915	LPath
**K89F**	0.9996	LPath
**K89G**	0.9951	LPath
**K89H**	0.9912	LPath
**K89I**	0.9957	LPath
**K89L**	0.9933	LPath
**K89M**	0.9806	LPath
**K89N**	0.997	LPath
**K89P**	0.9979	LPath
**K89Q**	0.9927	LPath
**K89R**	0.8642	LPath
**K89S**	0.998	LPath
**K89T**	0.9857	LPath
**K89V**	0.9952	LPath
**K89W**	0.9993	LPath
**K89Y**	0.9979	LPath
**V90A**	0.9369	LPath
**V90C**	0.9899	LPath
**V90D**	0.991	LPath
**V90E**	0.9782	LPath
**V90F**	0.9518	LPath
**V90G**	0.8682	LPath
**V90H**	0.9967	LPath
**V90I**	0.1715	LBen
**V90K**	0.9882	LPath
**V90L**	0.8668	LPath
**V90M**	0.9044	LPath
**V90N**	0.9713	LPath
**V90P**	0.9871	LPath
**V90Q**	0.9851	LPath
**V90R**	0.9794	LPath
**V90S**	0.9642	LPath
**V90T**	0.9359	LPath
**V90W**	0.9983	LPath
**V90Y**	0.9912	LPath
**R91A**	0.8018	LPath
**R91C**	0.284	LBen
**R91D**	0.9426	LPath
**R91E**	0.7026	LPath
**R91F**	0.8686	LPath

**R91G**	0.6784	LPath
**R91H**	0.1863	LBen
**R91I**	0.5431	Amb
**R91K**	0.2833	LBen
**R91L**	0.4498	Amb
**R91M**	0.6487	LPath
**R91N**	0.7848	LPath
**R91P**	0.9841	LPath
**R91Q**	0.1616	LBen
**R91S**	0.8084	LPath
**R91T**	0.6625	LPath
**R91V**	0.6417	LPath
**R91W**	0.3376	LBen
**R91Y**	0.6987	LPath
**W92A**	0.7589	LPath
**W92C**	0.8372	LPath
**W92D**	0.9562	LPath
**W92E**	0.9311	LPath
**W92F**	0.306	LBen
**W92G**	0.6413	LPath
**W92H**	0.881	LPath
**W92I**	0.5743	LPath
**W92K**	0.958	LPath
**W92L**	0.2285	LBen
**W92M**	0.5652	LPath
**W92N**	0.9337	LPath
**W92P**	0.9956	LPath
**W92Q**	0.9232	LPath
**W92R**	0.9332	LPath
**W92S**	0.7702	LPath
**W92T**	0.8536	LPath
**W92V**	0.6586	LPath
**W92Y**	0.5619	Amb
**L93A**	0.9509	LPath
**L93C**	0.9536	LPath
**L93D**	0.9993	LPath
**L93E**	0.9924	LPath
**L93F**	0.6716	LPath
**L93G**	0.9856	LPath
**L93H**	0.9838	LPath
**L93I**	0.4962	Amb
**L93K**	0.9895	LPath

**L93M**	0.3308	LBen
**L93N**	0.9951	LPath
**L93P**	0.9969	LPath
**L93Q**	0.9624	LPath
**L93R**	0.9756	LPath
**L93S**	0.992	LPath
**L93T**	0.9675	LPath
**L93V**	0.522	Amb
**L93W**	0.9239	LPath
**L93Y**	0.9707	LPath
**K94A**	0.6643	LPath
**K94C**	0.8447	LPath
**K94D**	0.9516	LPath
**K94E**	0.398	Amb
**K94F**	0.9568	LPath
**K94G**	0.7154	LPath
**K94H**	0.5703	LPath
**K94I**	0.7667	LPath
**K94L**	0.6151	LPath
**K94M**	0.4748	Amb
**K94N**	0.8512	LPath
**K94P**	0.983	LPath
**K94Q**	0.1425	LBen
**K94R**	0.0878	LBen
**K94S**	0.7507	LPath
**K94T**	0.5856	LPath
**K94V**	0.7114	LPath
**K94W**	0.8894	LPath
**K94Y**	0.8736	LPath
**K95A**	0.156	LBen
**K95C**	0.6234	LPath
**K95D**	0.2282	LBen
**K95E**	0.0747	LBen
**K95F**	0.6341	LPath
**K95G**	0.2111	LBen
**K95H**	0.2218	LBen
**K95I**	0.2937	LBen
**K95L**	0.2155	LBen
**K95M**	0.1503	LBen
**K95N**	0.169	LBen
**K95P**	0.6683	LPath
**K95Q**	0.0877	LBen

**K95R**	0.0764	LBen
**K95S**	0.1878	LBen
**K95T**	0.1024	LBen
**K95V**	0.2459	LBen
**K95W**	0.5651	LPath
**K95Y**	0.4606	Amb
**E96A**	0.2425	LBen
**E96C**	0.9422	LPath
**E96D**	0.605	LPath
**E96F**	0.8904	LPath
**E96G**	0.3914	Amb
**E96H**	0.7937	LPath
**E96I**	0.7049	LPath
**E96K**	0.5753	LPath
**E96L**	0.7894	LPath
**E96M**	0.7459	LPath
**E96N**	0.6696	LPath
**E96P**	0.9843	LPath
**E96Q**	0.299	LBen
**E96R**	0.6871	LPath
**E96S**	0.5174	Amb
**E96T**	0.5806	LPath
**E96V**	0.4322	Amb
**E96W**	0.9716	LPath
**E96Y**	0.8128	LPath
**M97A**	0.6995	LPath
**M97C**	0.946	LPath
**M97D**	0.9975	LPath
**M97E**	0.9786	LPath
**M97F**	0.9056	LPath
**M97G**	0.944	LPath
**M97H**	0.9954	LPath
**M97I**	0.6025	LPath
**M97K**	0.9709	LPath
**M97L**	0.1815	LBen
**M97N**	0.9762	LPath
**M97P**	0.9968	LPath
**M97Q**	0.9536	LPath
**M97R**	0.972	LPath
**M97S**	0.8905	LPath
**M97T**	0.4564	Amb
**M97V**	0.1613	LBen

**M97W**	0.9926	LPath
**M97Y**	0.9922	LPath
**E98A**	0.1128	LBen
**E98C**	0.8183	LPath
**E98D**	0.1855	LBen
**E98F**	0.7311	LPath
**E98G**	0.0877	LBen
**E98H**	0.4281	Amb
**E98I**	0.3409	Amb
**E98K**	0.1003	LBen
**E98L**	0.2845	LBen
**E98M**	0.3572	Amb
**E98N**	0.2876	LBen
**E98P**	0.7358	LPath
**E98Q**	0.0992	LBen
**E98R**	0.1592	LBen
**E98S**	0.1908	LBen
**E98T**	0.212	LBen
**E98V**	0.1884	LBen
**E98W**	0.7934	LPath
**E98Y**	0.5959	LPath
**K99A**	0.2121	LBen
**K99C**	0.6453	LPath
**K99D**	0.3117	LBen
**K99E**	0.1191	LBen
**K99F**	0.6067	LPath
**K99G**	0.2661	LBen
**K99H**	0.246	LBen
**K99I**	0.2235	LBen
**K99L**	0.2145	LBen
**K99M**	0.1512	LBen
**K99N**	0.1857	LBen
**K99P**	0.3647	Amb
**K99Q**	0.1181	LBen
**K99R**	0.0857	LBen
**K99S**	0.2547	LBen
**K99T**	0.1152	LBen
**K99V**	0.2252	LBen
**K99W**	0.6516	LPath
**K99Y**	0.4615	Amb
**Y100A**	0.5149	Amb
**Y100C**	0.2183	LBen

**Y100D**	0.3681	Amb
**Y100E**	0.5117	Amb
**Y100F**	0.1085	LBen
**Y100G**	0.5919	LPath
**Y100H**	0.1104	LBen
**Y100I**	0.4122	Amb
**Y100K**	0.628	LPath
**Y100L**	0.3485	Amb
**Y100M**	0.5341	Amb
**Y100N**	0.2323	LBen
**Y100P**	0.8655	LPath
**Y100Q**	0.4263	Amb
**Y100R**	0.4628	Amb
**Y100S**	0.3459	Amb
**Y100T**	0.5008	Amb
**Y100V**	0.3404	Amb
**Y100W**	0.3077	LBen
**A101C**	0.3883	Amb
**A101D**	0.1754	LBen
**A101E**	0.1309	LBen
**A101F**	0.2614	LBen
**A101G**	0.1012	LBen
**A101H**	0.2506	LBen
**A101I**	0.1707	LBen
**A101K**	0.1501	LBen
**A101L**	0.1474	LBen
**A101M**	0.1742	LBen
**A101N**	0.2041	LBen
**A101P**	0.2244	LBen
**A101Q**	0.1719	LBen
**A101R**	0.1329	LBen
**A101S**	0.0817	LBen
**A101T**	0.0779	LBen
**A101V**	0.0986	LBen
**A101W**	0.4746	Amb
**A101Y**	0.3295	LBen
**D102A**	0.0962	LBen
**D102C**	0.3724	Amb
**D102E**	0.091	LBen
**D102F**	0.352	Amb
**D102G**	0.0729	LBen
**D102H**	0.1206	LBen

**D102I**	0.2228	LBen
**D102K**	0.1415	LBen
**D102L**	0.2049	LBen
**D102M**	0.3915	Amb
**D102N**	0.0771	LBen
**D102P**	0.3592	Amb
**D102Q**	0.1438	LBen
**D102R**	0.1465	LBen
**D102S**	0.0754	LBen
**D102T**	0.1377	LBen
**D102V**	0.1356	LBen
**D102W**	0.6125	LPath
**D102Y**	0.1461	LBen
**R103A**	0.1383	LBen
**R103C**	0.1418	LBen
**R103D**	0.1856	LBen
**R103E**	0.1035	LBen
**R103F**	0.3546	Amb
**R103G**	0.0918	LBen
**R103H**	0.0792	LBen
**R103I**	0.1855	LBen
**R103K**	0.0778	LBen
**R103L**	0.124	LBen
**R103M**	0.1507	LBen
**R103N**	0.209	LBen
**R103P**	0.4208	Amb
**R103Q**	0.0572	LBen
**R103S**	0.1483	LBen
**R103T**	0.0939	LBen
**R103V**	0.1887	LBen
**R103W**	0.1159	LBen
**R103Y**	0.2376	LBen
**E104A**	0.1153	LBen
**E104C**	0.7642	LPath
**E104D**	0.09	LBen
**E104F**	0.6981	LPath
**E104G**	0.0877	LBen
**E104H**	0.2689	LBen
**E104I**	0.3972	Amb
**E104K**	0.0707	LBen
**E104L**	0.3325	LBen
**E104M**	0.3949	Amb

**E104N**	0.1615	LBen
**E104P**	0.2051	LBen
**E104Q**	0.0962	LBen
**E104R**	0.1241	LBen
**E104S**	0.1328	LBen
**E104T**	0.1623	LBen
**E104V**	0.23	LBen
**E104W**	0.7224	LPath
**E104Y**	0.4992	Amb
**D105A**	0.2781	LBen
**D105C**	0.7798	LPath
**D105E**	0.1871	LBen
**D105F**	0.7428	LPath
**D105G**	0.1378	LBen
**D105H**	0.3136	LBen
**D105I**	0.6397	LPath
**D105K**	0.4041	Amb
**D105L**	0.6125	LPath
**D105M**	0.7486	LPath
**D105N**	0.1049	LBen
**D105P**	0.7565	LPath
**D105Q**	0.3801	Amb
**D105R**	0.4482	Amb
**D105S**	0.1797	LBen
**D105T**	0.341	Amb
**D105V**	0.4365	Amb
**D105W**	0.8916	LPath
**D105Y**	0.3181	LBen
**M106A**	0.25	LBen
**M106C**	0.6708	LPath
**M106D**	0.6037	LPath
**M106E**	0.2905	LBen
**M106F**	0.3931	Amb
**M106G**	0.5016	Amb
**M106H**	0.4328	Amb
**M106I**	0.232	LBen
**M106K**	0.1439	LBen
**M106L**	0.0801	LBen
**M106N**	0.4172	Amb
**M106P**	0.8714	LPath
**M106Q**	0.2169	LBen
**M106R**	0.1552	LBen

**M106S**	0.3091	LBen
**M106T**	0.0972	LBen
**M106V**	0.0781	LBen
**M106W**	0.5676	LPath
**M106Y**	0.5864	LPath
**I107A**	0.5232	Amb
**I107C**	0.785	LPath
**I107D**	0.9125	LPath
**I107E**	0.8541	LPath
**I107F**	0.2815	LBen
**I107G**	0.7816	LPath
**I107H**	0.7967	LPath
**I107K**	0.6875	LPath
**I107L**	0.1209	LBen
**I107M**	0.1518	LBen
**I107N**	0.5742	LPath
**I107P**	0.8363	LPath
**I107Q**	0.7776	LPath
**I107R**	0.6107	LPath
**I107S**	0.6094	LPath
**I107T**	0.4541	Amb
**I107V**	0.0628	LBen
**I107W**	0.8616	LPath
**I107Y**	0.6528	LPath
**I108A**	0.8306	LPath
**I108C**	0.9074	LPath
**I108D**	0.9971	LPath
**I108E**	0.9926	LPath
**I108F**	0.3722	Amb
**I108G**	0.9704	LPath
**I108H**	0.9846	LPath
**I108K**	0.9869	LPath
**I108L**	0.1442	LBen
**I108M**	0.2266	LBen
**I108N**	0.9659	LPath
**I108P**	0.9838	LPath
**I108Q**	0.9864	LPath
**I108R**	0.9769	LPath
**I108S**	0.9202	LPath
**I108T**	0.8083	LPath
**I108V**	0.0778	LBen
**I108W**	0.9764	LPath

**I108Y**	0.9231	LPath
**M109A**	0.7573	LPath
**M109C**	0.8939	LPath
**M109D**	0.9982	LPath
**M109E**	0.9884	LPath
**M109F**	0.6966	LPath
**M109G**	0.9462	LPath
**M109H**	0.9864	LPath
**M109I**	0.5994	LPath
**M109K**	0.9686	LPath
**M109L**	0.0881	LBen
**M109N**	0.9811	LPath
**M109P**	0.9945	LPath
**M109Q**	0.9451	LPath
**M109R**	0.9729	LPath
**M109S**	0.8989	LPath
**M109T**	0.7033	LPath
**M109V**	0.184	LBen
**M109W**	0.9823	LPath
**M109Y**	0.9754	LPath
**F110A**	0.9132	LPath
**F110C**	0.8374	LPath
**F110D**	0.9953	LPath
**F110E**	0.9947	LPath
**F110G**	0.9764	LPath
**F110H**	0.9631	LPath
**F110I**	0.7556	LPath
**F110K**	0.9966	LPath
**F110L**	0.9842	LPath
**F110M**	0.8378	LPath
**F110N**	0.9697	LPath
**F110P**	0.9995	LPath
**F110Q**	0.9904	LPath
**F110R**	0.9921	LPath
**F110S**	0.8927	LPath
**F110T**	0.8947	LPath
**F110V**	0.5884	LPath
**F110W**	0.9042	LPath
**F110Y**	0.5797	LPath
**V111A**	0.4861	Amb
**V111C**	0.928	LPath
**V111D**	0.9847	LPath

**V111E**	0.9627	LPath
**V111F**	0.8022	LPath
**V111G**	0.7745	LPath
**V111H**	0.9906	LPath
**V111I**	0.1112	LBen
**V111K**	0.9843	LPath
**V111L**	0.4474	Amb
**V111M**	0.4309	Amb
**V111N**	0.9428	LPath
**V111P**	0.9912	LPath
**V111Q**	0.9625	LPath
**V111R**	0.9745	LPath
**V111S**	0.6497	LPath
**V111T**	0.2663	LBen
**V111W**	0.9957	LPath
**V111Y**	0.9901	LPath
**D112A**	0.9885	LPath
**D112C**	0.9973	LPath
**D112E**	0.976	LPath
**D112F**	0.997	LPath
**D112G**	0.9824	LPath
**D112H**	0.9936	LPath
**D112I**	0.998	LPath
**D112K**	0.9972	LPath
**D112L**	0.9942	LPath
**D112M**	0.9992	LPath
**D112N**	0.9675	LPath
**D112P**	0.996	LPath
**D112Q**	0.9961	LPath
**D112R**	0.9951	LPath
**D112S**	0.9712	LPath
**D112T**	0.9967	LPath
**D112V**	0.9937	LPath
**D112W**	0.9984	LPath
**D112Y**	0.9809	LPath
**S113A**	0.2852	LBen
**S113C**	0.4844	Amb
**S113D**	0.9885	LPath
**S113E**	0.9923	LPath
**S113F**	0.9904	LPath
**S113G**	0.3103	LBen
**S113H**	0.9848	LPath

**S113I**	0.9694	LPath
**S113K**	0.9981	LPath
**S113L**	0.9338	LPath
**S113M**	0.963	LPath
**S113N**	0.9518	LPath
**S113P**	0.9873	LPath
**S113Q**	0.987	LPath
**S113R**	0.9916	LPath
**S113T**	0.7461	LPath
**S113V**	0.9574	LPath
**S113W**	0.9927	LPath
**S113Y**	0.9839	LPath
**Y114A**	0.9668	LPath
**Y114C**	0.8315	LPath
**Y114D**	0.9184	LPath
**Y114E**	0.9867	LPath
**Y114F**	0.2609	LBen
**Y114G**	0.9326	LPath
**Y114H**	0.9276	LPath
**Y114I**	0.9771	LPath
**Y114K**	0.9903	LPath
**Y114L**	0.921	LPath
**Y114M**	0.9597	LPath
**Y114N**	0.8189	LPath
**Y114P**	0.989	LPath
**Y114Q**	0.983	LPath
**Y114R**	0.97	LPath
**Y114S**	0.8349	LPath
**Y114T**	0.9621	LPath
**Y114V**	0.9354	LPath
**Y114W**	0.8073	LPath
**D115A**	0.9928	LPath
**D115C**	0.999	LPath
**D115E**	0.9935	LPath
**D115F**	0.9988	LPath
**D115G**	0.9905	LPath
**D115H**	0.9923	LPath
**D115I**	0.9987	LPath
**D115K**	0.997	LPath
**D115L**	0.9964	LPath
**D115M**	0.9996	LPath
**D115N**	0.9595	LPath

**D115P**	0.9991	LPath
**D115Q**	0.9973	LPath
**D115R**	0.9934	LPath
**D115S**	0.9786	LPath
**D115T**	0.9971	LPath
**D115V**	0.9953	LPath
**D115W**	0.9996	LPath
**D115Y**	0.9839	LPath
**V116A**	0.5402	Amb
**V116C**	0.9446	LPath
**V116D**	0.9683	LPath
**V116E**	0.9559	LPath
**V116F**	0.9228	LPath
**V116G**	0.702	LPath
**V116H**	0.9939	LPath
**V116I**	0.1536	LBen
**V116K**	0.9874	LPath
**V116L**	0.5982	LPath
**V116M**	0.6415	LPath
**V116N**	0.8849	LPath
**V116P**	0.9978	LPath
**V116Q**	0.9697	LPath
**V116R**	0.9761	LPath
**V116S**	0.7055	LPath
**V116T**	0.5176	Amb
**V116W**	0.999	LPath
**V116Y**	0.9928	LPath
**I117A**	0.7657	LPath
**I117C**	0.875	LPath
**I117D**	0.9854	LPath
**I117E**	0.9632	LPath
**I117F**	0.3541	Amb
**I117G**	0.9442	LPath
**I117H**	0.9302	LPath
**I117K**	0.9418	LPath
**I117L**	0.0807	LBen
**I117M**	0.1348	LBen
**I117N**	0.8414	LPath
**I117P**	0.9821	LPath
**I117Q**	0.9036	LPath
**I117R**	0.9139	LPath
**I117S**	0.8083	LPath

**I117T**	0.707	LPath
**I117V**	0.0842	LBen
**I117W**	0.9436	LPath
**I117Y**	0.852	LPath
**L118A**	0.8593	LPath
**L118C**	0.8612	LPath
**L118D**	0.9971	LPath
**L118E**	0.9846	LPath
**L118F**	0.1056	LBen
**L118G**	0.9735	LPath
**L118H**	0.9227	LPath
**L118I**	0.1282	LBen
**L118K**	0.9815	LPath
**L118M**	0.1885	LBen
**L118N**	0.986	LPath
**L118P**	0.8475	LPath
**L118Q**	0.94	LPath
**L118R**	0.9526	LPath
**L118S**	0.9592	LPath
**L118T**	0.8917	LPath
**L118V**	0.1851	LBen
**L118W**	0.5928	LPath
**L118Y**	0.7663	LPath
**A119C**	0.6746	LPath
**A119D**	0.9081	LPath
**A119E**	0.8828	LPath
**A119F**	0.869	LPath
**A119G**	0.2643	LBen
**A119H**	0.9355	LPath
**A119I**	0.6757	LPath
**A119K**	0.9789	LPath
**A119L**	0.4224	Amb
**A119M**	0.5678	LPath
**A119N**	0.7346	LPath
**A119P**	0.9521	LPath
**A119Q**	0.8642	LPath
**A119R**	0.9464	LPath
**A119S**	0.1229	LBen
**A119T**	0.186	LBen
**A119V**	0.349	Amb
**A119W**	0.9765	LPath
**A119Y**	0.9369	LPath

**G120A**	0.1458	LBen
**G120C**	0.5514	Amb
**G120D**	0.6017	LPath
**G120E**	0.7226	LPath
**G120F**	0.9502	LPath
**G120H**	0.8608	LPath
**G120I**	0.8692	LPath
**G120K**	0.9224	LPath
**G120L**	0.8634	LPath
**G120M**	0.8913	LPath
**G120N**	0.6555	LPath
**G120P**	0.9918	LPath
**G120Q**	0.7798	LPath
**G120R**	0.8032	LPath
**G120S**	0.1087	LBen
**G120T**	0.4283	Amb
**G120V**	0.7169	LPath
**G120W**	0.9053	LPath
**G120Y**	0.8926	LPath
**S121A**	0.1424	LBen
**S121C**	0.22	LBen
**S121D**	0.2798	LBen
**S121E**	0.4812	Amb
**S121F**	0.3593	Amb
**S121G**	0.0752	LBen
**S121H**	0.3318	LBen
**S121I**	0.3819	Amb
**S121K**	0.5795	LPath
**S121L**	0.1901	LBen
**S121M**	0.3409	Amb
**S121N**	0.1337	LBen
**S121P**	0.156	LBen
**S121Q**	0.4941	Amb
**S121R**	0.5252	Amb
**S121T**	0.1054	LBen
**S121V**	0.384	Amb
**S121W**	0.4607	Amb
**S121Y**	0.276	LBen
**P122A**	0.1925	LBen
**P122C**	0.8949	LPath
**P122D**	0.9362	LPath
**P122E**	0.7434	LPath

**P122F**	0.9638	LPath
**P122G**	0.6909	LPath
**P122H**	0.68	LPath
**P122I**	0.876	LPath
**P122K**	0.7425	LPath
**P122L**	0.6552	LPath
**P122M**	0.8742	LPath
**P122N**	0.8983	LPath
**P122Q**	0.5126	Amb
**P122R**	0.5108	Amb
**P122S**	0.3329	LBen
**P122T**	0.3676	Amb
**P122V**	0.693	LPath
**P122W**	0.9679	LPath
**P122Y**	0.9428	LPath
**T123A**	0.0572	LBen
**T123C**	0.2754	LBen
**T123D**	0.1241	LBen
**T123E**	0.1011	LBen
**T123F**	0.1209	LBen
**T123G**	0.0953	LBen
**T123H**	0.1191	LBen
**T123I**	0.0909	LBen
**T123K**	0.092	LBen
**T123L**	0.0681	LBen
**T123M**	0.0817	LBen
**T123N**	0.0763	LBen
**T123P**	0.0754	LBen
**T123Q**	0.1038	LBen
**T123R**	0.0729	LBen
**T123S**	0.0667	LBen
**T123V**	0.0847	LBen
**T123W**	0.2292	LBen
**T123Y**	0.1289	LBen
**E124A**	0.5519	Amb
**E124C**	0.9833	LPath
**E124D**	0.418	Amb
**E124F**	0.9588	LPath
**E124G**	0.6329	LPath
**E124H**	0.8688	LPath
**E124I**	0.8317	LPath
**E124K**	0.5906	LPath

**E124L**	0.8351	LPath
**E124M**	0.8787	LPath
**E124N**	0.8009	LPath
**E124P**	0.9779	LPath
**E124Q**	0.4163	Amb
**E124R**	0.6652	LPath
**E124S**	0.683	LPath
**E124T**	0.7406	LPath
**E124V**	0.6513	LPath
**E124W**	0.9839	LPath
**E124Y**	0.9161	LPath
**L125A**	0.7322	LPath
**L125C**	0.8413	LPath
**L125D**	0.9974	LPath
**L125E**	0.9855	LPath
**L125F**	0.4965	Amb
**L125G**	0.9473	LPath
**L125H**	0.9611	LPath
**L125I**	0.1265	LBen
**L125K**	0.9869	LPath
**L125M**	0.2365	LBen
**L125N**	0.9821	LPath
**L125P**	0.9852	LPath
**L125Q**	0.9379	LPath
**L125R**	0.9671	LPath
**L125S**	0.9427	LPath
**L125T**	0.7808	LPath
**L125V**	0.1196	LBen
**L125W**	0.8994	LPath
**L125Y**	0.9338	LPath
**L126A**	0.5987	LPath
**L126C**	0.7262	LPath
**L126D**	0.9786	LPath
**L126E**	0.7854	LPath
**L126F**	0.3702	Amb
**L126G**	0.8421	LPath
**L126H**	0.5985	LPath
**L126I**	0.1568	LBen
**L126K**	0.6057	LPath
**L126M**	0.2043	LBen
**L126N**	0.8831	LPath
**L126P**	0.959	LPath

**L126Q**	0.3609	Amb
**L126R**	0.3934	Amb
**L126S**	0.7227	LPath
**L126T**	0.6054	LPath
**L126V**	0.1573	LBen
**L126W**	0.45	Amb
**L126Y**	0.6884	LPath
**K127A**	0.305	LBen
**K127C**	0.6587	LPath
**K127D**	0.4347	Amb
**K127E**	0.1222	LBen
**K127F**	0.6929	LPath
**K127G**	0.2677	LBen
**K127H**	0.2035	LBen
**K127I**	0.4735	Amb
**K127L**	0.3017	LBen
**K127M**	0.2431	LBen
**K127N**	0.3441	Amb
**K127P**	0.8155	LPath
**K127Q**	0.089	LBen
**K127R**	0.0662	LBen
**K127S**	0.3096	LBen
**K127T**	0.1971	LBen
**K127V**	0.4192	Amb
**K127W**	0.4324	Amb
**K127Y**	0.468	Amb
**K128A**	0.7182	LPath
**K128C**	0.8829	LPath
**K128D**	0.9179	LPath
**K128E**	0.6033	LPath
**K128F**	0.9398	LPath
**K128G**	0.8329	LPath
**K128H**	0.4791	Amb
**K128I**	0.7164	LPath
**K128L**	0.6383	LPath
**K128M**	0.5083	Amb
**K128N**	0.7501	LPath
**K128P**	0.9848	LPath
**K128Q**	0.3312	LBen
**K128R**	0.1181	LBen
**K128S**	0.8037	LPath
**K128T**	0.5124	Amb

**K128V**	0.6937	LPath
**K128W**	0.9256	LPath
**K128Y**	0.7909	LPath
**F129A**	0.9569	LPath
**F129C**	0.9473	LPath
**F129D**	0.9979	LPath
**F129E**	0.997	LPath
**F129G**	0.9877	LPath
**F129H**	0.9664	LPath
**F129I**	0.8273	LPath
**F129K**	0.9966	LPath
**F129L**	0.9756	LPath
**F129M**	0.889	LPath
**F129N**	0.9904	LPath
**F129P**	0.9997	LPath
**F129Q**	0.9928	LPath
**F129R**	0.9894	LPath
**F129S**	0.967	LPath
**F129T**	0.9738	LPath
**F129V**	0.8097	LPath
**F129W**	0.7273	LPath
**F129Y**	0.3494	Amb
**V130A**	0.0856	LBen
**V130C**	0.4293	Amb
**V130D**	0.1666	LBen
**V130E**	0.1161	LBen
**V130F**	0.0733	LBen
**V130G**	0.0969	LBen
**V130H**	0.1735	LBen
**V130I**	0.0643	LBen
**V130K**	0.1175	LBen
**V130L**	0.0712	LBen
**V130M**	0.0834	LBen
**V130N**	0.1194	LBen
**V130P**	0.5599	Amb
**V130Q**	0.1029	LBen
**V130R**	0.0858	LBen
**V130S**	0.0849	LBen
**V130T**	0.0953	LBen
**V130W**	0.3507	Amb
**V130Y**	0.2259	LBen
**Q131A**	0.1612	LBen

**Q131C**	0.5902	LPath
**Q131D**	0.2399	LBen
**Q131E**	0.0713	LBen
**Q131F**	0.5683	LPath
**Q131G**	0.1874	LBen
**Q131H**	0.1729	LBen
**Q131I**	0.359	Amb
**Q131K**	0.1037	LBen
**Q131L**	0.1129	LBen
**Q131M**	0.3338	LBen
**Q131N**	0.2601	LBen
**Q131P**	0.3102	LBen
**Q131R**	0.0858	LBen
**Q131S**	0.2038	LBen
**Q131T**	0.1994	LBen
**Q131V**	0.2436	LBen
**Q131W**	0.356	Amb
**Q131Y**	0.3488	Amb
**S132A**	0.0865	LBen
**S132C**	0.1158	LBen
**S132D**	0.4507	Amb
**S132E**	0.538	Amb
**S132F**	0.2233	LBen
**S132G**	0.0997	LBen
**S132H**	0.3715	Amb
**S132I**	0.2738	LBen
**S132K**	0.7533	LPath
**S132L**	0.1353	LBen
**S132M**	0.21	LBen
**S132N**	0.1906	LBen
**S132P**	0.6673	LPath
**S132Q**	0.5292	Amb
**S132R**	0.6567	LPath
**S132T**	0.0802	LBen
**S132V**	0.2456	LBen
**S132W**	0.2881	LBen
**S132Y**	0.1875	LBen
**G133A**	0.1893	LBen
**G133C**	0.3482	Amb
**G133D**	0.1031	LBen
**G133E**	0.1231	LBen
**G133F**	0.7566	LPath

**G133H**	0.2048	LBen
**G133I**	0.6361	LPath
**G133K**	0.1753	LBen
**G133L**	0.6493	LPath
**G133M**	0.6862	LPath
**G133N**	0.1599	LBen
**G133P**	0.5865	LPath
**G133Q**	0.1511	LBen
**G133R**	0.1168	LBen
**G133S**	0.0955	LBen
**G133T**	0.2356	LBen
**G133V**	0.4581	Amb
**G133W**	0.5071	Amb
**G133Y**	0.4894	Amb
**S134A**	0.1075	LBen
**S134C**	0.0899	LBen
**S134D**	0.6027	LPath
**S134E**	0.7069	LPath
**S134F**	0.3945	Amb
**S134G**	0.1005	LBen
**S134H**	0.3423	Amb
**S134I**	0.3677	Amb
**S134K**	0.7734	LPath
**S134L**	0.2985	LBen
**S134M**	0.4467	Amb
**S134N**	0.2123	LBen
**S134P**	0.5696	LPath
**S134Q**	0.5618	Amb
**S134R**	0.6089	LPath
**S134T**	0.1446	LBen
**S134V**	0.3287	LBen
**S134W**	0.4932	Amb
**S134Y**	0.2996	LBen
**R135A**	0.1913	LBen
**R135C**	0.1056	LBen
**R135D**	0.2366	LBen
**R135E**	0.1968	LBen
**R135F**	0.348	Amb
**R135G**	0.1066	LBen
**R135H**	0.0587	LBen
**R135I**	0.2861	LBen
**R135K**	0.101	LBen

**R135L**	0.1568	LBen
**R135M**	0.286	LBen
**R135N**	0.1919	LBen
**R135P**	0.2283	LBen
**R135Q**	0.0722	LBen
**R135S**	0.1582	LBen
**R135T**	0.1502	LBen
**R135V**	0.2822	LBen
**R135W**	0.1517	LBen
**R135Y**	0.187	LBen
**L136A**	0.6088	LPath
**L136C**	0.692	LPath
**L136D**	0.9948	LPath
**L136E**	0.9723	LPath
**L136F**	0.4036	Amb
**L136G**	0.9012	LPath
**L136H**	0.9329	LPath
**L136I**	0.1025	LBen
**L136K**	0.9748	LPath
**L136M**	0.1807	LBen
**L136N**	0.9671	LPath
**L136P**	0.9497	LPath
**L136Q**	0.8991	LPath
**L136R**	0.9394	LPath
**L136S**	0.8815	LPath
**L136T**	0.6997	LPath
**L136V**	0.0791	LBen
**L136W**	0.8757	LPath
**L136Y**	0.8934	LPath
**L137A**	0.706	LPath
**L137C**	0.7563	LPath
**L137D**	0.9896	LPath
**L137E**	0.9249	LPath
**L137F**	0.4299	Amb
**L137G**	0.9384	LPath
**L137H**	0.7577	LPath
**L137I**	0.1037	LBen
**L137K**	0.9075	LPath
**L137M**	0.2318	LBen
**L137N**	0.9174	LPath
**L137P**	0.9766	LPath
**L137Q**	0.6568	LPath

**L137R**	0.7841	LPath
**L137S**	0.7936	LPath
**L137T**	0.6505	LPath
**L137V**	0.0973	LBen
**L137W**	0.7445	LPath
**L137Y**	0.7971	LPath
**F138A**	0.9858	LPath
**F138C**	0.9308	LPath
**F138D**	0.9994	LPath
**F138E**	0.9994	LPath
**F138G**	0.9963	LPath
**F138H**	0.9934	LPath
**F138I**	0.6217	LPath
**F138K**	0.9993	LPath
**F138L**	0.9639	LPath
**F138M**	0.927	LPath
**F138N**	0.9978	LPath
**F138P**	0.9987	LPath
**F138Q**	0.9981	LPath
**F138R**	0.9973	LPath
**F138S**	0.9917	LPath
**F138T**	0.9934	LPath
**F138V**	0.6871	LPath
**F138W**	0.95	LPath
**F138Y**	0.8289	LPath
**S139A**	0.1172	LBen
**S139C**	0.5442	Amb
**S139D**	0.9946	LPath
**S139E**	0.996	LPath
**S139F**	0.9858	LPath
**S139G**	0.1665	LBen
**S139H**	0.9897	LPath
**S139I**	0.9894	LPath
**S139K**	0.9994	LPath
**S139L**	0.8625	LPath
**S139M**	0.9505	LPath
**S139N**	0.9715	LPath
**S139P**	0.9443	LPath
**S139Q**	0.9896	LPath
**S139R**	0.998	LPath
**S139T**	0.5972	LPath
**S139V**	0.9658	LPath

**S139W**	0.9939	LPath
**S139Y**	0.9876	LPath
**A140C**	0.9415	LPath
**A140D**	0.9965	LPath
**A140E**	0.9926	LPath
**A140F**	0.9976	LPath
**A140G**	0.5859	LPath
**A140H**	0.9967	LPath
**A140I**	0.9941	LPath
**A140K**	0.9983	LPath
**A140L**	0.9718	LPath
**A140M**	0.9743	LPath
**A140N**	0.9877	LPath
**A140P**	0.9927	LPath
**A140Q**	0.9841	LPath
**A140R**	0.9891	LPath
**A140S**	0.2057	LBen
**A140T**	0.5472	Amb
**A140V**	0.9254	LPath
**A140W**	0.9995	LPath
**A140Y**	0.9987	LPath
**E141A**	0.9746	LPath
**E141C**	0.9971	LPath
**E141D**	0.9307	LPath
**E141F**	0.9997	LPath
**E141G**	0.9473	LPath
**E141H**	0.9978	LPath
**E141I**	0.9991	LPath
**E141K**	0.9831	LPath
**E141L**	0.9986	LPath
**E141M**	0.9986	LPath
**E141N**	0.993	LPath
**E141P**	0.9989	LPath
**E141Q**	0.9698	LPath
**E141R**	0.9861	LPath
**E141S**	0.9745	LPath
**E141T**	0.9935	LPath
**E141V**	0.9954	LPath
**E141W**	0.9998	LPath
**E141Y**	0.9992	LPath
**S142A**	0.0875	LBen
**S142C**	0.2128	LBen

**S142D**	0.3651	Amb
**S142E**	0.4439	Amb
**S142F**	0.4968	Amb
**S142G**	0.0508	LBen
**S142H**	0.4433	Amb
**S142I**	0.2035	LBen
**S142K**	0.6664	LPath
**S142L**	0.2402	LBen
**S142M**	0.2991	LBen
**S142N**	0.1333	LBen
**S142P**	0.2593	LBen
**S142Q**	0.4759	Amb
**S142R**	0.5987	LPath
**S142T**	0.1046	LBen
**S142V**	0.202	LBen
**S142W**	0.5971	LPath
**S142Y**	0.3972	Amb
**F143A**	0.9281	LPath
**F143C**	0.8805	LPath
**F143D**	0.9804	LPath
**F143E**	0.9852	LPath
**F143G**	0.9693	LPath
**F143H**	0.8698	LPath
**F143I**	0.7062	LPath
**F143K**	0.9825	LPath
**F143L**	0.964	LPath
**F143M**	0.8951	LPath
**F143N**	0.9273	LPath
**F143P**	0.9984	LPath
**F143Q**	0.962	LPath
**F143R**	0.9418	LPath
**F143S**	0.8413	LPath
**F143T**	0.9098	LPath
**F143V**	0.6592	LPath
**F143W**	0.8388	LPath
**F143Y**	0.3081	LBen
**C144A**	0.8188	LPath
**C144D**	0.998	LPath
**C144E**	0.9979	LPath
**C144F**	0.9452	LPath
**C144G**	0.8403	LPath
**C144H**	0.9914	LPath

**C144I**	0.6744	LPath
**C144K**	0.9984	LPath
**C144L**	0.5985	LPath
**C144M**	0.9555	LPath
**C144N**	0.9832	LPath
**C144P**	0.9456	LPath
**C144Q**	0.9904	LPath
**C144R**	0.9732	LPath
**C144S**	0.8726	LPath
**C144T**	0.9307	LPath
**C144V**	0.6942	LPath
**C144W**	0.989	LPath
**C144Y**	0.9797	LPath
**W145A**	0.9897	LPath
**W145C**	0.9957	LPath
**W145D**	0.9979	LPath
**W145E**	0.9976	LPath
**W145F**	0.762	LPath
**W145G**	0.9713	LPath
**W145H**	0.9849	LPath
**W145I**	0.9862	LPath
**W145K**	0.9986	LPath
**W145L**	0.956	LPath
**W145M**	0.9948	LPath
**W145N**	0.9968	LPath
**W145P**	0.9957	LPath
**W145Q**	0.9975	LPath
**W145R**	0.9953	LPath
**W145S**	0.9611	LPath
**W145T**	0.9915	LPath
**W145V**	0.9843	LPath
**W145Y**	0.9117	LPath
**P146A**	0.9735	LPath
**P146C**	0.9983	LPath
**P146D**	0.9944	LPath
**P146E**	0.9885	LPath
**P146F**	0.9992	LPath
**P146G**	0.9825	LPath
**P146H**	0.9927	LPath
**P146I**	0.9955	LPath
**P146K**	0.9893	LPath
**P146L**	0.9697	LPath

**P146M**	0.9961	LPath
**P146N**	0.996	LPath
**P146Q**	0.9895	LPath
**P146R**	0.9718	LPath
**P146S**	0.9926	LPath
**P146T**	0.9846	LPath
**P146V**	0.9896	LPath
**P146W**	0.9985	LPath
**P146Y**	0.9979	LPath
**E147A**	0.1757	LBen
**E147C**	0.8711	LPath
**E147D**	0.1154	LBen
**E147F**	0.8254	LPath
**E147G**	0.3202	LBen
**E147H**	0.4674	Amb
**E147I**	0.4743	Amb
**E147K**	0.2121	LBen
**E147L**	0.4439	Amb
**E147M**	0.5527	Amb
**E147N**	0.2403	LBen
**E147P**	0.8485	LPath
**E147Q**	0.1401	LBen
**E147R**	0.2772	LBen
**E147S**	0.2067	LBen
**E147T**	0.3493	Amb
**E147V**	0.2511	LBen
**E147W**	0.9187	LPath
**E147Y**	0.6945	LPath
**W148A**	0.3514	Amb
**W148C**	0.7877	LPath
**W148D**	0.6615	LPath
**W148E**	0.5812	LPath
**W148F**	0.3775	Amb
**W148G**	0.2699	LBen
**W148H**	0.6542	LPath
**W148I**	0.567	LPath
**W148K**	0.6419	LPath
**W148L**	0.3762	Amb
**W148M**	0.5438	Amb
**W148N**	0.6975	LPath
**W148P**	0.5715	LPath
**W148Q**	0.6277	LPath

**W148R**	0.5677	LPath
**W148S**	0.36	Amb
**W148T**	0.4458	Amb
**W148V**	0.4526	Amb
**W148Y**	0.4875	Amb
**G149A**	0.0836	LBen
**G149C**	0.2077	LBen
**G149D**	0.1436	LBen
**G149E**	0.1132	LBen
**G149F**	0.4108	Amb
**G149H**	0.2023	LBen
**G149I**	0.1912	LBen
**G149K**	0.2193	LBen
**G149L**	0.2698	LBen
**G149M**	0.3124	LBen
**G149N**	0.1648	LBen
**G149P**	0.6821	LPath
**G149Q**	0.1753	LBen
**G149R**	0.1574	LBen
**G149S**	0.0718	LBen
**G149T**	0.1099	LBen
**G149V**	0.1288	LBen
**G149W**	0.2781	LBen
**G149Y**	0.2726	LBen
**L150A**	0.694	LPath
**L150C**	0.9146	LPath
**L150D**	0.9618	LPath
**L150E**	0.7975	LPath
**L150F**	0.4054	Amb
**L150G**	0.919	LPath
**L150H**	0.718	LPath
**L150I**	0.2743	LBen
**L150K**	0.6349	LPath
**L150M**	0.2361	LBen
**L150N**	0.8943	LPath
**L150P**	0.9365	LPath
**L150Q**	0.5024	Amb
**L150R**	0.5209	Amb
**L150S**	0.813	LPath
**L150T**	0.7382	LPath
**L150V**	0.3049	LBen
**L150W**	0.5784	LPath

**L150Y**	0.745	LPath
**A151C**	0.674	LPath
**A151D**	0.6926	LPath
**A151E**	0.3299	LBen
**A151F**	0.5898	LPath
**A151G**	0.2415	LBen
**A151H**	0.6935	LPath
**A151I**	0.4063	Amb
**A151K**	0.5214	Amb
**A151L**	0.3081	LBen
**A151M**	0.2892	LBen
**A151N**	0.588	LPath
**A151P**	0.7851	LPath
**A151Q**	0.3511	Amb
**A151R**	0.4194	Amb
**A151S**	0.1188	LBen
**A151T**	0.1434	LBen
**A151V**	0.1809	LBen
**A151W**	0.866	LPath
**A151Y**	0.7257	LPath
**E152A**	0.1282	LBen
**E152C**	0.8115	LPath
**E152D**	0.1007	LBen
**E152F**	0.7278	LPath
**E152G**	0.0955	LBen
**E152H**	0.3434	Amb
**E152I**	0.4184	Amb
**E152K**	0.104	LBen
**E152L**	0.3456	Amb
**E152M**	0.4678	Amb
**E152N**	0.2136	LBen
**E152P**	0.3147	LBen
**E152Q**	0.109	LBen
**E152R**	0.1612	LBen
**E152S**	0.1379	LBen
**E152T**	0.2157	LBen
**E152V**	0.2386	LBen
**E152W**	0.8253	LPath
**E152Y**	0.5812	LPath
**Q153A**	0.1793	LBen
**Q153C**	0.542	Amb
**Q153D**	0.2607	LBen

**Q153E**	0.0866	LBen
**Q153F**	0.554	Amb
**Q153G**	0.2059	LBen
**Q153H**	0.1519	LBen
**Q153I**	0.3357	LBen
**Q153K**	0.066	LBen
**Q153L**	0.1107	LBen
**Q153M**	0.2871	LBen
**Q153N**	0.2251	LBen
**Q153P**	0.3449	Amb
**Q153R**	0.0723	LBen
**Q153S**	0.2091	LBen
**Q153T**	0.1864	LBen
**Q153V**	0.2496	LBen
**Q153W**	0.3666	Amb
**Q153Y**	0.31	LBen
**Y154A**	0.9951	LPath
**Y154C**	0.9727	LPath
**Y154D**	0.9916	LPath
**Y154E**	0.9971	LPath
**Y154F**	0.4712	Amb
**Y154G**	0.9835	LPath
**Y154H**	0.9531	LPath
**Y154I**	0.9749	LPath
**Y154K**	0.9977	LPath
**Y154L**	0.9438	LPath
**Y154M**	0.984	LPath
**Y154N**	0.957	LPath
**Y154P**	0.9952	LPath
**Y154Q**	0.9971	LPath
**Y154R**	0.9951	LPath
**Y154S**	0.9806	LPath
**Y154T**	0.9947	LPath
**Y154V**	0.9685	LPath
**Y154W**	0.9259	LPath
**P155A**	0.5923	LPath
**P155C**	0.9724	LPath
**P155D**	0.959	LPath
**P155E**	0.8517	LPath
**P155F**	0.981	LPath
**P155G**	0.8944	LPath
**P155H**	0.8549	LPath

**P155I**	0.9373	LPath
**P155K**	0.886	LPath
**P155L**	0.6824	LPath
**P155M**	0.9365	LPath
**P155N**	0.9688	LPath
**P155Q**	0.7781	LPath
**P155R**	0.7156	LPath
**P155S**	0.7641	LPath
**P155T**	0.6931	LPath
**P155V**	0.857	LPath
**P155W**	0.9788	LPath
**P155Y**	0.978	LPath
**E156A**	0.0906	LBen
**E156C**	0.7481	LPath
**E156D**	0.1072	LBen
**E156F**	0.5619	Amb
**E156G**	0.1444	LBen
**E156H**	0.264	LBen
**E156I**	0.2295	LBen
**E156K**	0.0903	LBen
**E156L**	0.2198	LBen
**E156M**	0.3016	LBen
**E156N**	0.1869	LBen
**E156P**	0.1821	LBen
**E156Q**	0.0926	LBen
**E156R**	0.1287	LBen
**E156S**	0.1321	LBen
**E156T**	0.1523	LBen
**E156V**	0.1278	LBen
**E156W**	0.7264	LPath
**E156Y**	0.4308	Amb
**V157A**	0.1461	LBen
**V157C**	0.8951	LPath
**V157D**	0.6051	LPath
**V157E**	0.4696	Amb
**V157F**	0.4108	Amb
**V157G**	0.26	LBen
**V157H**	0.8244	LPath
**V157I**	0.1129	LBen
**V157K**	0.6319	LPath
**V157L**	0.4366	Amb
**V157M**	0.3078	LBen

**V157N**	0.4975	Amb
**V157P**	0.4668	Amb
**V157Q**	0.5946	LPath
**V157R**	0.5709	LPath
**V157S**	0.2824	LBen
**V157T**	0.1643	LBen
**V157W**	0.926	LPath
**V157Y**	0.8248	LPath
**G158A**	0.0939	LBen
**G158C**	0.2684	LBen
**G158D**	0.1436	LBen
**G158E**	0.1268	LBen
**G158F**	0.4171	Amb
**G158H**	0.2163	LBen
**G158I**	0.2009	LBen
**G158K**	0.2138	LBen
**G158L**	0.2661	LBen
**G158M**	0.3386	LBen
**G158N**	0.1978	LBen
**G158P**	0.3539	Amb
**G158Q**	0.1777	LBen
**G158R**	0.1342	LBen
**G158S**	0.0792	LBen
**G158T**	0.1143	LBen
**G158V**	0.1331	LBen
**G158W**	0.3003	LBen
**G158Y**	0.3022	LBen
**T159A**	0.0623	LBen
**T159C**	0.311	LBen
**T159D**	0.1824	LBen
**T159E**	0.1477	LBen
**T159F**	0.129	LBen
**T159G**	0.1226	LBen
**T159H**	0.131	LBen
**T159I**	0.1064	LBen
**T159K**	0.1139	LBen
**T159L**	0.0723	LBen
**T159M**	0.0758	LBen
**T159N**	0.0763	LBen
**T159P**	0.0763	LBen
**T159Q**	0.1213	LBen
**T159R**	0.0876	LBen

**T159S**	0.0699	LBen
**T159V**	0.0944	LBen
**T159W**	0.2819	LBen
**T159Y**	0.1608	LBen
**G160A**	0.6933	LPath
**G160C**	0.9412	LPath
**G160D**	0.9567	LPath
**G160E**	0.9415	LPath
**G160F**	0.9913	LPath
**G160H**	0.9862	LPath
**G160I**	0.981	LPath
**G160K**	0.9837	LPath
**G160L**	0.9828	LPath
**G160M**	0.9881	LPath
**G160N**	0.962	LPath
**G160P**	0.9869	LPath
**G160Q**	0.9583	LPath
**G160R**	0.946	LPath
**G160S**	0.5958	LPath
**G160T**	0.9137	LPath
**G160V**	0.951	LPath
**G160W**	0.9715	LPath
**G160Y**	0.9849	LPath
**K161A**	0.8878	LPath
**K161C**	0.8829	LPath
**K161D**	0.9595	LPath
**K161E**	0.749	LPath
**K161F**	0.9527	LPath
**K161G**	0.874	LPath
**K161H**	0.5454	Amb
**K161I**	0.916	LPath
**K161L**	0.7912	LPath
**K161M**	0.6968	LPath
**K161N**	0.7606	LPath
**K161P**	0.9647	LPath
**K161Q**	0.4386	Amb
**K161R**	0.1369	LBen
**K161S**	0.8468	LPath
**K161T**	0.8143	LPath
**K161V**	0.8841	LPath
**K161W**	0.9429	LPath
**K161Y**	0.7921	LPath

**R162A**	0.8437	LPath
**R162C**	0.5087	Amb
**R162D**	0.9787	LPath
**R162E**	0.8588	LPath
**R162F**	0.9734	LPath
**R162G**	0.7942	LPath
**R162H**	0.3923	Amb
**R162I**	0.8669	LPath
**R162K**	0.2615	LBen
**R162L**	0.7818	LPath
**R162M**	0.8769	LPath
**R162N**	0.9496	LPath
**R162P**	0.5013	Amb
**R162Q**	0.269	LBen
**R162S**	0.9288	LPath
**R162T**	0.8645	LPath
**R162V**	0.8714	LPath
**R162W**	0.7248	LPath
**R162Y**	0.8866	LPath
**F163A**	0.9971	LPath
**F163C**	0.972	LPath
**F163D**	0.9986	LPath
**F163E**	0.9991	LPath
**F163G**	0.9973	LPath
**F163H**	0.9664	LPath
**F163I**	0.9806	LPath
**F163K**	0.9982	LPath
**F163L**	0.9976	LPath
**F163M**	0.99	LPath
**F163N**	0.9886	LPath
**F163P**	0.9996	LPath
**F163Q**	0.9972	LPath
**F163R**	0.9924	LPath
**F163S**	0.9908	LPath
**F163T**	0.9976	LPath
**F163V**	0.9764	LPath
**F163W**	0.8781	LPath
**F163Y**	0.1723	LBen
**L164A**	0.9829	LPath
**L164C**	0.9784	LPath
**L164D**	0.9999	LPath
**L164E**	0.9989	LPath

**L164F**	0.9747	LPath
**L164G**	0.9972	LPath
**L164H**	0.9973	LPath
**L164I**	0.6353	LPath
**L164K**	0.998	LPath
**L164M**	0.8525	LPath
**L164N**	0.9989	LPath
**L164P**	0.9853	LPath
**L164Q**	0.9951	LPath
**L164R**	0.9914	LPath
**L164S**	0.9977	LPath
**L164T**	0.9924	LPath
**L164V**	0.7352	LPath
**L164W**	0.9948	LPath
**L164Y**	0.9975	LPath
**N165A**	0.9171	LPath
**N165C**	0.4268	Amb
**N165D**	0.9669	LPath
**N165E**	0.9957	LPath
**N165F**	0.9948	LPath
**N165G**	0.8478	LPath
**N165H**	0.9086	LPath
**N165I**	0.9603	LPath
**N165K**	0.9961	LPath
**N165L**	0.8942	LPath
**N165M**	0.9606	LPath
**N165P**	0.9766	LPath
**N165Q**	0.9851	LPath
**N165R**	0.992	LPath
**N165S**	0.438	Amb
**N165T**	0.9084	LPath
**N165V**	0.9383	LPath
**N165W**	0.9989	LPath
**N165Y**	0.9633	LPath
**S166A**	0.5001	Amb
**S166C**	0.8916	LPath
**S166D**	0.9951	LPath
**S166E**	0.9952	LPath
**S166F**	0.9908	LPath
**S166G**	0.847	LPath
**S166H**	0.9865	LPath
**S166I**	0.9905	LPath

**S166K**	0.9986	LPath
**S166L**	0.9566	LPath
**S166M**	0.9767	LPath
**S166N**	0.9885	LPath
**S166P**	0.9917	LPath
**S166Q**	0.9905	LPath
**S166R**	0.9969	LPath
**S166T**	0.8533	LPath
**S166V**	0.9848	LPath
**S166W**	0.9899	LPath
**S166Y**	0.9867	LPath
**G167A**	0.8699	LPath
**G167C**	0.9783	LPath
**G167D**	0.9912	LPath
**G167E**	0.9893	LPath
**G167F**	0.9969	LPath
**G167H**	0.9977	LPath
**G167I**	0.9938	LPath
**G167K**	0.9926	LPath
**G167L**	0.9936	LPath
**G167M**	0.9965	LPath
**G167N**	0.9961	LPath
**G167P**	0.9978	LPath
**G167Q**	0.992	LPath
**G167R**	0.9714	LPath
**G167S**	0.899	LPath
**G167T**	0.9795	LPath
**G167V**	0.9823	LPath
**G167W**	0.9925	LPath
**G167Y**	0.9971	LPath
**G168A**	0.2658	LBen
**G168C**	0.6525	LPath
**G168D**	0.991	LPath
**G168E**	0.9851	LPath
**G168F**	0.9848	LPath
**G168H**	0.994	LPath
**G168I**	0.8707	LPath
**G168K**	0.9971	LPath
**G168L**	0.8698	LPath
**G168M**	0.8894	LPath
**G168N**	0.9625	LPath
**G168P**	0.999	LPath

**G168Q**	0.9809	LPath
**G168R**	0.9901	LPath
**G168S**	0.3304	LBen
**G168T**	0.7694	LPath
**G168V**	0.8095	LPath
**G168W**	0.9899	LPath
**G168Y**	0.9873	LPath
**F169A**	0.9753	LPath
**F169C**	0.8748	LPath
**F169D**	0.9989	LPath
**F169E**	0.9984	LPath
**F169G**	0.9913	LPath
**F169H**	0.9887	LPath
**F169I**	0.441	Amb
**F169K**	0.9982	LPath
**F169L**	0.9454	LPath
**F169M**	0.7424	LPath
**F169N**	0.9933	LPath
**F169P**	0.9997	LPath
**F169Q**	0.9964	LPath
**F169R**	0.9953	LPath
**F169S**	0.9834	LPath
**F169T**	0.986	LPath
**F169V**	0.5904	LPath
**F169W**	0.9122	LPath
**F169Y**	0.5514	Amb
**I170A**	0.4005	Amb
**I170C**	0.9038	LPath
**I170D**	0.998	LPath
**I170E**	0.9923	LPath
**I170F**	0.5139	Amb
**I170G**	0.9512	LPath
**I170H**	0.9924	LPath
**I170K**	0.9899	LPath
**I170L**	0.1354	LBen
**I170M**	0.0889	LBen
**I170N**	0.9815	LPath
**I170P**	0.9899	LPath
**I170Q**	0.9779	LPath
**I170R**	0.974	LPath
**I170S**	0.7639	LPath
**I170T**	0.3813	Amb

**I170V**	0.0871	LBen
**I170W**	0.9845	LPath
**I170Y**	0.9724	LPath
**G171A**	0.7325	LPath
**G171C**	0.9654	LPath
**G171D**	0.9962	LPath
**G171E**	0.9986	LPath
**G171F**	0.9983	LPath
**G171H**	0.999	LPath
**G171I**	0.9955	LPath
**G171K**	0.9993	LPath
**G171L**	0.9949	LPath
**G171M**	0.9985	LPath
**G171N**	0.9978	LPath
**G171P**	0.9993	LPath
**G171Q**	0.9982	LPath
**G171R**	0.9952	LPath
**G171S**	0.8021	LPath
**G171T**	0.9801	LPath
**G171V**	0.9876	LPath
**G171W**	0.9978	LPath
**G171Y**	0.998	LPath
**F172A**	0.6189	LPath
**F172C**	0.4955	Amb
**F172D**	0.7157	LPath
**F172E**	0.7848	LPath
**F172G**	0.8082	LPath
**F172H**	0.4379	Amb
**F172I**	0.3729	Amb
**F172K**	0.7579	LPath
**F172L**	0.8607	LPath
**F172M**	0.6152	LPath
**F172N**	0.5889	LPath
**F172P**	0.8747	LPath
**F172Q**	0.7254	LPath
**F172R**	0.6147	LPath
**F172S**	0.4191	Amb
**F172T**	0.5526	Amb
**F172V**	0.3343	LBen
**F172W**	0.3903	Amb
**F172Y**	0.1192	LBen
**A173C**	0.7079	LPath

**A173D**	0.9627	LPath
**A173E**	0.9494	LPath
**A173F**	0.894	LPath
**A173G**	0.2035	LBen
**A173H**	0.9551	LPath
**A173I**	0.6883	LPath
**A173K**	0.977	LPath
**A173L**	0.6273	LPath
**A173M**	0.7381	LPath
**A173N**	0.8994	LPath
**A173P**	0.7571	LPath
**A173Q**	0.9135	LPath
**A173R**	0.9432	LPath
**A173S**	0.1309	LBen
**A173T**	0.2696	LBen
**A173V**	0.3664	Amb
**A173W**	0.9833	LPath
**A173Y**	0.9512	LPath
**T174A**	0.0552	LBen
**T174C**	0.2591	LBen
**T174D**	0.1441	LBen
**T174E**	0.1255	LBen
**T174F**	0.2051	LBen
**T174G**	0.0861	LBen
**T174H**	0.0957	LBen
**T174I**	0.1727	LBen
**T174K**	0.0801	LBen
**T174L**	0.0891	LBen
**T174M**	0.0959	LBen
**T174N**	0.072	LBen
**T174P**	0.0369	LBen
**T174Q**	0.0923	LBen
**T174R**	0.0671	LBen
**T174S**	0.0643	LBen
**T174V**	0.124	LBen
**T174W**	0.3339	LBen
**T174Y**	0.1778	LBen
**T175A**	0.0703	LBen
**T175C**	0.4103	Amb
**T175D**	0.2344	LBen
**T175E**	0.2136	LBen
**T175F**	0.2954	LBen

**T175G**	0.2397	LBen
**T175H**	0.2263	LBen
**T175I**	0.1592	LBen
**T175K**	0.2374	LBen
**T175L**	0.1314	LBen
**T175M**	0.1188	LBen
**T175N**	0.1197	LBen
**T175P**	0.1856	LBen
**T175Q**	0.2182	LBen
**T175R**	0.1779	LBen
**T175S**	0.0991	LBen
**T175V**	0.1123	LBen
**T175W**	0.5954	LPath
**T175Y**	0.2631	LBen
**I176A**	0.7053	LPath
**I176C**	0.8959	LPath
**I176D**	0.9911	LPath
**I176E**	0.98	LPath
**I176F**	0.4049	Amb
**I176G**	0.939	LPath
**I176H**	0.9718	LPath
**I176K**	0.9689	LPath
**I176L**	0.1423	LBen
**I176M**	0.1735	LBen
**I176N**	0.9119	LPath
**I176P**	0.9763	LPath
**I176Q**	0.9606	LPath
**I176R**	0.9527	LPath
**I176S**	0.8446	LPath
**I176T**	0.6927	LPath
**I176V**	0.0678	LBen
**I176W**	0.9737	LPath
**I176Y**	0.9181	LPath
**H177A**	0.2503	LBen
**H177C**	0.1823	LBen
**H177D**	0.2014	LBen
**H177E**	0.2737	LBen
**H177F**	0.2786	LBen
**H177G**	0.2324	LBen
**H177I**	0.3937	Amb
**H177K**	0.2846	LBen
**H177L**	0.1463	LBen

**H177M**	0.5632	Amb
**H177N**	0.0864	LBen
**H177P**	0.4299	Amb
**H177Q**	0.1601	LBen
**H177R**	0.1007	LBen
**H177S**	0.1521	LBen
**H177T**	0.2755	LBen
**H177V**	0.3159	LBen
**H177W**	0.321	LBen
**H177Y**	0.0715	LBen
**Q178A**	0.1605	LBen
**Q178C**	0.4216	Amb
**Q178D**	0.1569	LBen
**Q178E**	0.0613	LBen
**Q178F**	0.5678	LPath
**Q178G**	0.1325	LBen
**Q178H**	0.0988	LBen
**Q178I**	0.4395	Amb
**Q178K**	0.0664	LBen
**Q178L**	0.1553	LBen
**Q178M**	0.359	Amb
**Q178N**	0.156	LBen
**Q178P**	0.2831	LBen
**Q178R**	0.0649	LBen
**Q178S**	0.1555	LBen
**Q178T**	0.1483	LBen
**Q178V**	0.3005	LBen
**Q178W**	0.2817	LBen
**Q178Y**	0.2759	LBen
**I179A**	0.2415	LBen
**I179C**	0.7336	LPath
**I179D**	0.9422	LPath
**I179E**	0.8876	LPath
**I179F**	0.2226	LBen
**I179G**	0.7298	LPath
**I179H**	0.8434	LPath
**I179K**	0.8425	LPath
**I179L**	0.0921	LBen
**I179M**	0.0778	LBen
**I179N**	0.6739	LPath
**I179P**	0.9316	LPath
**I179Q**	0.8041	LPath

**I179R**	0.784	LPath
**I179S**	0.4575	Amb
**I179T**	0.2113	LBen
**I179V**	0.0599	LBen
**I179W**	0.875	LPath
**I179Y**	0.7491	LPath
**V180A**	0.5856	LPath
**V180C**	0.9318	LPath
**V180D**	0.9858	LPath
**V180E**	0.959	LPath
**V180F**	0.7459	LPath
**V180G**	0.74	LPath
**V180H**	0.9844	LPath
**V180I**	0.1126	LBen
**V180K**	0.9729	LPath
**V180L**	0.4564	Amb
**V180M**	0.5586	Amb
**V180N**	0.9459	LPath
**V180P**	0.9761	LPath
**V180Q**	0.9446	LPath
**V180R**	0.9487	LPath
**V180S**	0.7449	LPath
**V180T**	0.5199	Amb
**V180W**	0.9936	LPath
**V180Y**	0.9829	LPath
**R181A**	0.1766	LBen
**R181C**	0.1049	LBen
**R181D**	0.3119	LBen
**R181E**	0.1604	LBen
**R181F**	0.3798	Amb
**R181G**	0.0901	LBen
**R181H**	0.0706	LBen
**R181I**	0.2378	LBen
**R181K**	0.0842	LBen
**R181L**	0.1362	LBen
**R181M**	0.1856	LBen
**R181N**	0.2569	LBen
**R181P**	0.6568	LPath
**R181Q**	0.0608	LBen
**R181S**	0.1642	LBen
**R181T**	0.1152	LBen
**R181V**	0.2583	LBen

**R181W**	0.122	LBen
**R181Y**	0.2499	LBen
**Q182A**	0.2648	LBen
**Q182C**	0.6356	LPath
**Q182D**	0.3438	Amb
**Q182E**	0.0874	LBen
**Q182F**	0.6596	LPath
**Q182G**	0.2393	LBen
**Q182H**	0.2052	LBen
**Q182I**	0.4377	Amb
**Q182K**	0.092	LBen
**Q182L**	0.1288	LBen
**Q182M**	0.3726	Amb
**Q182N**	0.3012	LBen
**Q182P**	0.2235	LBen
**Q182R**	0.0919	LBen
**Q182S**	0.2858	LBen
**Q182T**	0.2563	LBen
**Q182V**	0.3207	LBen
**Q182W**	0.417	Amb
**Q182Y**	0.4137	Amb
**W183A**	0.9667	LPath
**W183C**	0.9838	LPath
**W183D**	0.9945	LPath
**W183E**	0.9887	LPath
**W183F**	0.8341	LPath
**W183G**	0.8381	LPath
**W183H**	0.9813	LPath
**W183I**	0.9842	LPath
**W183K**	0.9947	LPath
**W183L**	0.9492	LPath
**W183M**	0.9779	LPath
**W183N**	0.9916	LPath
**W183P**	0.9969	LPath
**W183Q**	0.99	LPath
**W183R**	0.9895	LPath
**W183S**	0.9439	LPath
**W183T**	0.9819	LPath
**W183V**	0.9735	LPath
**W183Y**	0.8859	LPath
**K184A**	0.2785	LBen
**K184C**	0.71	LPath

**K184D**	0.2931	LBen
**K184E**	0.1144	LBen
**K184F**	0.7382	LPath
**K184G**	0.3543	Amb
**K184H**	0.2337	LBen
**K184I**	0.3401	Amb
**K184L**	0.3405	Amb
**K184M**	0.2296	LBen
**K184N**	0.2067	LBen
**K184P**	0.4926	Amb
**K184Q**	0.1088	LBen
**K184R**	0.0854	LBen
**K184S**	0.2657	LBen
**K184T**	0.1308	LBen
**K184V**	0.3203	LBen
**K184W**	0.713	LPath
**K184Y**	0.5408	Amb
**Y185A**	0.5057	Amb
**Y185C**	0.1579	LBen
**Y185D**	0.2664	LBen
**Y185E**	0.5516	Amb
**Y185F**	0.1252	LBen
**Y185G**	0.2883	LBen
**Y185H**	0.1968	LBen
**Y185I**	0.6247	LPath
**Y185K**	0.5438	Amb
**Y185L**	0.411	Amb
**Y185M**	0.6021	LPath
**Y185N**	0.1772	LBen
**Y185P**	0.8379	LPath
**Y185Q**	0.4642	Amb
**Y185R**	0.4425	Amb
**Y185S**	0.2754	LBen
**Y185T**	0.5462	Amb
**Y185V**	0.5088	Amb
**Y185W**	0.4403	Amb
**K186A**	0.2758	LBen
**K186C**	0.7066	LPath
**K186D**	0.4301	Amb
**K186E**	0.1154	LBen
**K186F**	0.828	LPath
**K186G**	0.3144	LBen

**K186H**	0.2937	LBen
**K186I**	0.5212	Amb
**K186L**	0.3794	Amb
**K186M**	0.2939	LBen
**K186N**	0.3745	Amb
**K186P**	0.6564	LPath
**K186Q**	0.1058	LBen
**K186R**	0.0764	LBen
**K186S**	0.3214	LBen
**K186T**	0.1937	LBen
**K186V**	0.4265	Amb
**K186W**	0.7587	LPath
**K186Y**	0.7053	LPath
**D187A**	0.8592	LPath
**D187C**	0.99	LPath
**D187E**	0.7344	LPath
**D187F**	0.9888	LPath
**D187G**	0.8263	LPath
**D187H**	0.9407	LPath
**D187I**	0.9724	LPath
**D187K**	0.9718	LPath
**D187L**	0.9669	LPath
**D187M**	0.9891	LPath
**D187N**	0.4279	Amb
**D187P**	0.9654	LPath
**D187Q**	0.9706	LPath
**D187R**	0.9714	LPath
**D187S**	0.7538	LPath
**D187T**	0.9314	LPath
**D187V**	0.9102	LPath
**D187W**	0.9953	LPath
**D187Y**	0.8793	LPath
**D188A**	0.1584	LBen
**D188C**	0.6205	LPath
**D188E**	0.1787	LBen
**D188F**	0.5904	LPath
**D188G**	0.1513	LBen
**D188H**	0.196	LBen
**D188I**	0.4131	Amb
**D188K**	0.3208	LBen
**D188L**	0.4375	Amb
**D188M**	0.6067	LPath

**D188N**	0.0813	LBen
**D188P**	0.8188	LPath
**D188Q**	0.3015	LBen
**D188R**	0.2942	LBen
**D188S**	0.0968	LBen
**D188T**	0.1933	LBen
**D188V**	0.2735	LBen
**D188W**	0.8396	LPath
**D188Y**	0.2302	LBen
**D189A**	0.4401	Amb
**D189C**	0.896	LPath
**D189E**	0.526	Amb
**D189F**	0.8905	LPath
**D189G**	0.4704	Amb
**D189H**	0.5987	LPath
**D189I**	0.8757	LPath
**D189K**	0.8012	LPath
**D189L**	0.8708	LPath
**D189M**	0.901	LPath
**D189N**	0.2761	LBen
**D189P**	0.9883	LPath
**D189Q**	0.7021	LPath
**D189R**	0.7912	LPath
**D189S**	0.2886	LBen
**D189T**	0.6664	LPath
**D189V**	0.7254	LPath
**D189W**	0.9723	LPath
**D189Y**	0.5853	LPath
**D190A**	0.981	LPath
**D190C**	0.9965	LPath
**D190E**	0.9909	LPath
**D190F**	0.9988	LPath
**D190G**	0.9759	LPath
**D190H**	0.9908	LPath
**D190I**	0.9978	LPath
**D190K**	0.9959	LPath
**D190L**	0.9957	LPath
**D190M**	0.9992	LPath
**D190N**	0.908	LPath
**D190P**	0.9983	LPath
**D190Q**	0.9974	LPath
**D190R**	0.993	LPath

**D190S**	0.9095	LPath
**D190T**	0.9883	LPath
**D190V**	0.9924	LPath
**D190W**	0.9996	LPath
**D190Y**	0.9842	LPath
**D191A**	0.9917	LPath
**D191C**	0.9986	LPath
**D191E**	0.986	LPath
**D191F**	0.9987	LPath
**D191G**	0.992	LPath
**D191H**	0.9934	LPath
**D191I**	0.9959	LPath
**D191K**	0.9945	LPath
**D191L**	0.9943	LPath
**D191M**	0.9994	LPath
**D191N**	0.9635	LPath
**D191P**	0.9974	LPath
**D191Q**	0.9972	LPath
**D191R**	0.9921	LPath
**D191S**	0.9791	LPath
**D191T**	0.9949	LPath
**D191V**	0.991	LPath
**D191W**	0.9995	LPath
**D191Y**	0.9847	LPath
**Q192A**	0.9241	LPath
**Q192C**	0.9991	LPath
**Q192D**	0.9942	LPath
**Q192E**	0.8021	LPath
**Q192F**	0.9979	LPath
**Q192G**	0.9609	LPath
**Q192H**	0.9948	LPath
**Q192I**	0.9766	LPath
**Q192K**	0.9556	LPath
**Q192L**	0.9465	LPath
**Q192M**	0.9571	LPath
**Q192N**	0.9698	LPath
**Q192P**	0.9746	LPath
**Q192R**	0.966	LPath
**Q192S**	0.9346	LPath
**Q192T**	0.9479	LPath
**Q192V**	0.9683	LPath
**Q192W**	0.999	LPath

**Q192Y**	0.9985	LPath
**L193A**	0.7822	LPath
**L193C**	0.8763	LPath
**L193D**	0.9659	LPath
**L193E**	0.8068	LPath
**L193F**	0.5196	Amb
**L193G**	0.8983	LPath
**L193H**	0.585	LPath
**L193I**	0.4509	Amb
**L193K**	0.5161	Amb
**L193M**	0.4175	Amb
**L193N**	0.855	LPath
**L193P**	0.9858	LPath
**L193Q**	0.4582	Amb
**L193R**	0.2599	LBen
**L193S**	0.8737	LPath
**L193T**	0.8486	LPath
**L193V**	0.4438	Amb
**L193W**	0.6301	LPath
**L193Y**	0.6446	LPath
**F194A**	0.9813	LPath
**F194C**	0.9441	LPath
**F194D**	0.9965	LPath
**F194E**	0.9961	LPath
**F194G**	0.9912	LPath
**F194H**	0.9201	LPath
**F194I**	0.9319	LPath
**F194K**	0.9954	LPath
**F194L**	0.9868	LPath
**F194M**	0.9707	LPath
**F194N**	0.9861	LPath
**F194P**	0.9987	LPath
**F194Q**	0.9903	LPath
**F194R**	0.9809	LPath
**F194S**	0.9763	LPath
**F194T**	0.9891	LPath
**F194V**	0.9142	LPath
**F194W**	0.8205	LPath
**F194Y**	0.2923	LBen
**Y195A**	0.9851	LPath
**Y195C**	0.7344	LPath
**Y195D**	0.9924	LPath

**Y195E**	0.998	LPath
**Y195F**	0.1424	LBen
**Y195G**	0.9763	LPath
**Y195H**	0.947	LPath
**Y195I**	0.9487	LPath
**Y195K**	0.9982	LPath
**Y195L**	0.7626	LPath
**Y195M**	0.9435	LPath
**Y195N**	0.9664	LPath
**Y195P**	0.9958	LPath
**Y195Q**	0.9956	LPath
**Y195R**	0.9932	LPath
**Y195S**	0.9517	LPath
**Y195T**	0.9857	LPath
**Y195V**	0.9134	LPath
**Y195W**	0.7903	LPath
**T196A**	0.6966	LPath
**T196C**	0.9286	LPath
**T196D**	0.9839	LPath
**T196E**	0.9738	LPath
**T196F**	0.9877	LPath
**T196G**	0.8271	LPath
**T196H**	0.9192	LPath
**T196I**	0.9657	LPath
**T196K**	0.9659	LPath
**T196L**	0.8624	LPath
**T196M**	0.7408	LPath
**T196N**	0.8614	LPath
**T196P**	0.9639	LPath
**T196Q**	0.8973	LPath
**T196R**	0.9454	LPath
**T196S**	0.3778	Amb
**T196V**	0.8882	LPath
**T196W**	0.9937	LPath
**T196Y**	0.9841	LPath
**R197A**	0.3313	LBen
**R197C**	0.1535	LBen
**R197D**	0.5167	Amb
**R197E**	0.2557	LBen
**R197F**	0.4166	Amb
**R197G**	0.1714	LBen
**R197H**	0.068	LBen

**R197I**	0.309	LBen
**R197K**	0.0859	LBen
**R197L**	0.1754	LBen
**R197M**	0.2556	LBen
**R197N**	0.3144	LBen
**R197P**	0.9095	LPath
**R197Q**	0.0765	LBen
**R197S**	0.2988	LBen
**R197T**	0.1933	LBen
**R197V**	0.3532	Amb
**R197W**	0.141	LBen
**R197Y**	0.2568	LBen
**L198A**	0.3285	LBen
**L198C**	0.4665	Amb
**L198D**	0.9196	LPath
**L198E**	0.5827	LPath
**L198F**	0.2554	LBen
**L198G**	0.7177	LPath
**L198H**	0.3503	Amb
**L198I**	0.081	LBen
**L198K**	0.4143	Amb
**L198M**	0.1214	LBen
**L198N**	0.6202	LPath
**L198P**	0.9171	LPath
**L198Q**	0.2123	LBen
**L198R**	0.2594	LBen
**L198S**	0.4634	Amb
**L198T**	0.3279	LBen
**L198V**	0.0902	LBen
**L198W**	0.363	Amb
**L198Y**	0.5124	Amb
**Y199A**	0.9855	LPath
**Y199C**	0.823	LPath
**Y199D**	0.993	LPath
**Y199E**	0.9978	LPath
**Y199F**	0.107	LBen
**Y199G**	0.9849	LPath
**Y199H**	0.8909	LPath
**Y199I**	0.9424	LPath
**Y199K**	0.9971	LPath
**Y199L**	0.8737	LPath
**Y199M**	0.96	LPath

**Y199N**	0.9715	LPath
**Y199P**	0.9966	LPath
**Y199Q**	0.993	LPath
**Y199R**	0.9889	LPath
**Y199S**	0.9556	LPath
**Y199T**	0.9883	LPath
**Y199V**	0.9101	LPath
**Y199W**	0.7588	LPath
**L200A**	0.7213	LPath
**L200C**	0.817	LPath
**L200D**	0.9941	LPath
**L200E**	0.9357	LPath
**L200F**	0.8226	LPath
**L200G**	0.9386	LPath
**L200H**	0.9032	LPath
**L200I**	0.1216	LBen
**L200K**	0.8691	LPath
**L200M**	0.3197	LBen
**L200N**	0.9394	LPath
**L200P**	0.9876	LPath
**L200Q**	0.7387	LPath
**L200R**	0.6838	LPath
**L200S**	0.8752	LPath
**L200T**	0.4816	Amb
**L200V**	0.1256	LBen
**L200W**	0.9278	LPath
**L200Y**	0.9682	LPath
**D201A**	0.2751	LBen
**D201C**	0.8194	LPath
**D201E**	0.2735	LBen
**D201F**	0.7517	LPath
**D201G**	0.6019	LPath
**D201H**	0.2616	LBen
**D201I**	0.5352	Amb
**D201K**	0.5396	Amb
**D201L**	0.5429	Amb
**D201M**	0.7492	LPath
**D201N**	0.1156	LBen
**D201P**	0.9243	LPath
**D201Q**	0.3678	Amb
**D201R**	0.5075	Amb
**D201S**	0.1797	LBen

**D201T**	0.459	Amb
**D201V**	0.3289	LBen
**D201W**	0.9291	LPath
**D201Y**	0.2762	LBen
**P202A**	0.0631	LBen
**P202C**	0.4007	Amb
**P202D**	0.2437	LBen
**P202E**	0.131	LBen
**P202F**	0.3613	Amb
**P202G**	0.1873	LBen
**P202H**	0.1075	LBen
**P202I**	0.2465	LBen
**P202K**	0.1311	LBen
**P202L**	0.1062	LBen
**P202M**	0.2409	LBen
**P202N**	0.2191	LBen
**P202Q**	0.0893	LBen
**P202R**	0.0754	LBen
**P202S**	0.0861	LBen
**P202T**	0.0768	LBen
**P202V**	0.1607	LBen
**P202W**	0.3848	Amb
**P202Y**	0.3233	LBen
**G203A**	0.0724	LBen
**G203C**	0.1607	LBen
**G203D**	0.1016	LBen
**G203E**	0.085	LBen
**G203F**	0.2724	LBen
**G203H**	0.131	LBen
**G203I**	0.1162	LBen
**G203K**	0.124	LBen
**G203L**	0.158	LBen
**G203M**	0.2293	LBen
**G203N**	0.149	LBen
**G203P**	0.4447	Amb
**G203Q**	0.1056	LBen
**G203R**	0.0898	LBen
**G203S**	0.0723	LBen
**G203T**	0.0866	LBen
**G203V**	0.0784	LBen
**G203W**	0.192	LBen
**G203Y**	0.1869	LBen

**L204A**	0.2093	LBen
**L204C**	0.5168	Amb
**L204D**	0.6068	LPath
**L204E**	0.2382	LBen
**L204F**	0.1228	LBen
**L204G**	0.4506	Amb
**L204H**	0.1762	LBen
**L204I**	0.0848	LBen
**L204K**	0.1428	LBen
**L204M**	0.1085	LBen
**L204N**	0.3641	Amb
**L204P**	0.5471	Amb
**L204Q**	0.0932	LBen
**L204R**	0.1055	LBen
**L204S**	0.255	LBen
**L204T**	0.183	LBen
**L204V**	0.0816	LBen
**L204W**	0.1708	LBen
**L204Y**	0.2941	LBen
**R205A**	0.9217	LPath
**R205C**	0.6939	LPath
**R205D**	0.9901	LPath
**R205E**	0.8248	LPath
**R205F**	0.9871	LPath
**R205G**	0.879	LPath
**R205H**	0.6269	LPath
**R205I**	0.9294	LPath
**R205K**	0.4125	Amb
**R205L**	0.8298	LPath
**R205M**	0.9243	LPath
**R205N**	0.9848	LPath
**R205P**	0.9724	LPath
**R205Q**	0.2953	LBen
**R205S**	0.9706	LPath
**R205T**	0.9345	LPath
**R205V**	0.9257	LPath
**R205W**	0.7688	LPath
**R205Y**	0.9578	LPath
**E206A**	0.1643	LBen
**E206C**	0.8433	LPath
**E206D**	0.1541	LBen
**E206F**	0.7863	LPath

E206G	0.1043	LBen
E206H	0.3482	Amb
E206I	0.4973	Amb
E206K	0.0963	LBen
E206L	0.4015	Amb
E206M	0.4788	Amb
E206N	0.2533	LBen
E206P	0.7834	LPath
E206Q	0.092	LBen
E206R	0.142	LBen
E206S	0.198	LBen
E206T	0.2419	LBen
E206V	0.2977	LBen
E206W	0.7962	LPath
E206Y	0.6069	LPath
K207A	0.2366	LBen
K207C	0.6956	LPath
K207D	0.3187	LBen
K207E	0.1122	LBen
K207F	0.6683	LPath
K207G	0.2866	LBen
K207H	0.2268	LBen
K207I	0.3012	LBen
K207L	0.2803	LBen
K207M	0.1631	LBen
K207N	0.215	LBen
K207P	0.7311	LPath
K207Q	0.096	LBen
K207R	0.0832	LBen
K207S	0.2391	LBen
K207T	0.1177	LBen
K207V	0.2963	LBen
K207W	0.6681	LPath
K207Y	0.4802	Amb
L208A	0.3023	LBen
L208C	0.3673	Amb
L208D	0.6157	LPath
L208E	0.3586	Amb
L208F	0.0615	LBen
L208G	0.5011	Amb
L208H	0.1083	LBen
L208I	0.1172	LBen

**L208K**	0.2974	LBen
**L208M**	0.1163	LBen
**L208N**	0.3092	LBen
**L208P**	0.7594	LPath
**L208Q**	0.1397	LBen
**L208R**	0.1708	LBen
**L208S**	0.3128	LBen
**L208T**	0.2993	LBen
**L208V**	0.1178	LBen
**L208W**	0.0931	LBen
**L208Y**	0.125	LBen
**S209A**	0.082	LBen
**S209C**	0.0912	LBen
**S209D**	0.1229	LBen
**S209E**	0.1767	LBen
**S209F**	0.2066	LBen
**S209G**	0.0529	LBen
**S209H**	0.1058	LBen
**S209I**	0.174	LBen
**S209K**	0.1682	LBen
**S209L**	0.1296	LBen
**S209M**	0.1908	LBen
**S209N**	0.0752	LBen
**S209P**	0.33	LBen
**S209Q**	0.1487	LBen
**S209R**	0.1369	LBen
**S209T**	0.1037	LBen
**S209V**	0.1856	LBen
**S209W**	0.2209	LBen
**S209Y**	0.1328	LBen
**L210A**	0.6505	LPath
**L210C**	0.7055	LPath
**L210D**	0.9934	LPath
**L210E**	0.9569	LPath
**L210F**	0.2506	LBen
**L210G**	0.917	LPath
**L210H**	0.8883	LPath
**L210I**	0.1049	LBen
**L210K**	0.9455	LPath
**L210M**	0.1139	LBen
**L210N**	0.9637	LPath
**L210P**	0.9474	LPath

**L210Q**	0.8077	LPath
**L210R**	0.8846	LPath
**L210S**	0.8689	LPath
**L210T**	0.7643	LPath
**L210V**	0.1177	LBen
**L210W**	0.7118	LPath
**L210Y**	0.8191	LPath
**N211A**	0.1098	LBen
**N211C**	0.1594	LBen
**N211D**	0.0962	LBen
**N211E**	0.212	LBen
**N211F**	0.3864	Amb
**N211G**	0.1208	LBen
**N211H**	0.0604	LBen
**N211I**	0.1943	LBen
**N211K**	0.1445	LBen
**N211L**	0.1902	LBen
**N211M**	0.2634	LBen
**N211P**	0.7216	LPath
**N211Q**	0.1391	LBen
**N211R**	0.14	LBen
**N211S**	0.0542	LBen
**N211T**	0.0825	LBen
**N211V**	0.1794	LBen
**N211W**	0.5491	Amb
**N211Y**	0.1208	LBen
**L212A**	0.9445	LPath
**L212C**	0.9637	LPath
**L212D**	0.9994	LPath
**L212E**	0.994	LPath
**L212F**	0.9631	LPath
**L212G**	0.9906	LPath
**L212H**	0.9902	LPath
**L212I**	0.363	Amb
**L212K**	0.9893	LPath
**L212M**	0.6741	LPath
**L212N**	0.9948	LPath
**L212P**	0.9892	LPath
**L212Q**	0.9789	LPath
**L212R**	0.963	LPath
**L212S**	0.9918	LPath
**L212T**	0.9206	LPath

**L212V**	0.3559	Amb
**L212W**	0.9921	LPath
**L212Y**	0.9943	LPath
**D213A**	0.9896	LPath
**D213C**	0.9937	LPath
**D213E**	0.985	LPath
**D213F**	0.9991	LPath
**D213G**	0.9872	LPath
**D213H**	0.9948	LPath
**D213I**	0.9983	LPath
**D213K**	0.9982	LPath
**D213L**	0.9954	LPath
**D213M**	0.9995	LPath
**D213N**	0.9774	LPath
**D213P**	0.9977	LPath
**D213Q**	0.9975	LPath
**D213R**	0.996	LPath
**D213S**	0.9776	LPath
**D213T**	0.9968	LPath
**D213V**	0.9919	LPath
**D213W**	0.9996	LPath
**D213Y**	0.9903	LPath
**H214A**	0.7697	LPath
**H214C**	0.5629	Amb
**H214D**	0.6757	LPath
**H214E**	0.8045	LPath
**H214F**	0.8159	LPath
**H214G**	0.6851	LPath
**H214I**	0.8572	LPath
**H214K**	0.8185	LPath
**H214L**	0.6059	LPath
**H214M**	0.8783	LPath
**H214N**	0.2652	LBen
**H214P**	0.5974	LPath
**H214Q**	0.6036	LPath
**H214R**	0.5779	LPath
**H214S**	0.4315	Amb
**H214T**	0.5733	LPath
**H214V**	0.7948	LPath
**H214W**	0.783	LPath
**H214Y**	0.4175	Amb
**K215A**	0.6097	LPath

**K215C**	0.846	LPath
**K215D**	0.8444	LPath
**K215E**	0.3376	LBen
**K215F**	0.8842	LPath
**K215G**	0.6812	LPath
**K215H**	0.3709	Amb
**K215I**	0.6911	LPath
**K215L**	0.5865	LPath
**K215M**	0.4474	Amb
**K215N**	0.7578	LPath
**K215P**	0.9104	LPath
**K215Q**	0.1616	LBen
**K215R**	0.0801	LBen
**K215S**	0.6607	LPath
**K215T**	0.2811	LBen
**K215V**	0.6089	LPath
**K215W**	0.84	LPath
**K215Y**	0.7607	LPath
**S216A**	0.1335	LBen
**S216C**	0.0952	LBen
**S216D**	0.9705	LPath
**S216E**	0.9782	LPath
**S216F**	0.9594	LPath
**S216G**	0.1935	LBen
**S216H**	0.9552	LPath
**S216I**	0.9481	LPath
**S216K**	0.9971	LPath
**S216L**	0.7262	LPath
**S216M**	0.8305	LPath
**S216N**	0.6984	LPath
**S216P**	0.9851	LPath
**S216Q**	0.9548	LPath
**S216R**	0.9898	LPath
**S216T**	0.5284	Amb
**S216V**	0.8631	LPath
**S216W**	0.9727	LPath
**S216Y**	0.9191	LPath
**R217A**	0.268	LBen
**R217C**	0.1837	LBen
**R217D**	0.5063	Amb
**R217E**	0.2364	LBen
**R217F**	0.4156	Amb

**R217G**	0.2001	LBen
**R217H**	0.0846	LBen
**R217I**	0.2486	LBen
**R217K**	0.1081	LBen
**R217L**	0.1894	LBen
**R217M**	0.2434	LBen
**R217N**	0.4094	Amb
**R217P**	0.8238	LPath
**R217Q**	0.0744	LBen
**R217S**	0.2889	LBen
**R217T**	0.1676	LBen
**R217V**	0.2534	LBen
**R217W**	0.1341	LBen
**R217Y**	0.3007	LBen
**I218A**	0.9554	LPath
**I218C**	0.9781	LPath
**I218D**	0.9984	LPath
**I218E**	0.9975	LPath
**I218F**	0.5775	LPath
**I218G**	0.9932	LPath
**I218H**	0.9919	LPath
**I218K**	0.9937	LPath
**I218L**	0.1406	LBen
**I218M**	0.2914	LBen
**I218N**	0.9732	LPath
**I218P**	0.9923	LPath
**I218Q**	0.9937	LPath
**I218R**	0.9895	LPath
**I218S**	0.972	LPath
**I218T**	0.9649	LPath
**I218V**	0.2001	LBen
**I218W**	0.9874	LPath
**I218Y**	0.941	LPath
**F219A**	0.9963	LPath
**F219C**	0.9831	LPath
**F219D**	0.9997	LPath
**F219E**	0.9997	LPath
**F219G**	0.9982	LPath
**F219H**	0.9924	LPath
**F219I**	0.9421	LPath
**F219K**	0.9996	LPath
**F219L**	0.9916	LPath

**F219M**	0.974	LPath
**F219N**	0.9982	LPath
**F219P**	0.9999	LPath
**F219Q**	0.9987	LPath
**F219R**	0.9982	LPath
**F219S**	0.9929	LPath
**F219T**	0.9952	LPath
**F219V**	0.9454	LPath
**F219W**	0.948	LPath
**F219Y**	0.8763	LPath
**Q220A**	0.9087	LPath
**Q220C**	0.9747	LPath
**Q220D**	0.9961	LPath
**Q220E**	0.7375	LPath
**Q220F**	0.9805	LPath
**Q220G**	0.9462	LPath
**Q220H**	0.8905	LPath
**Q220I**	0.9681	LPath
**Q220K**	0.9579	LPath
**Q220L**	0.7948	LPath
**Q220M**	0.7995	LPath
**Q220N**	0.9535	LPath
**Q220P**	0.9947	LPath
**Q220R**	0.9548	LPath
**Q220S**	0.8779	LPath
**Q220T**	0.9433	LPath
**Q220V**	0.9431	LPath
**Q220W**	0.9926	LPath
**Q220Y**	0.9912	LPath
**N221A**	0.8604	LPath
**N221C**	0.686	LPath
**N221D**	0.966	LPath
**N221E**	0.9948	LPath
**N221F**	0.9944	LPath
**N221G**	0.9126	LPath
**N221H**	0.9214	LPath

**N221I**	0.9204	LPath
**N221K**	0.9965	LPath
**N221L**	0.8997	LPath
**N221M**	0.9236	LPath
**N221P**	0.9575	LPath
**N221Q**	0.9882	LPath
**N221R**	0.9961	LPath
**N221S**	0.4218	Amb
**N221T**	0.6826	LPath
**N221V**	0.8548	LPath
**N221W**	0.9992	LPath
**N221Y**	0.9629	LPath
**L222A**	0.9689	LPath
**L222C**	0.974	LPath
**L222D**	0.9997	LPath
**L222E**	0.997	LPath
**L222F**	0.9005	LPath
**L222G**	0.9955	LPath
**L222H**	0.9963	LPath
**L222I**	0.4015	Amb
**L222K**	0.997	LPath
**L222M**	0.4718	Amb
**L222N**	0.9973	LPath
**L222P**	0.9982	LPath
**L222Q**	0.9879	LPath
**L222R**	0.9919	LPath
**L222S**	0.9944	LPath
**L222T**	0.9654	LPath
**L222V**	0.4938	Amb
**L222W**	0.9897	LPath
**L222Y**	0.9931	LPath
**N223A**	0.8831	LPath
**N223C**	0.7988	LPath
**N223D**	0.846	LPath
**N223E**	0.9687	LPath
**N223F**	0.7703	LPath
**N223G**	0.8609	LPath
**N223H**	0.2118	LBen
**N223I**	0.9377	LPath
**N223K**	0.9716	LPath
**N223L**	0.8137	LPath
**N223M**	0.9054	LPath

**N223P**	0.9708	LPath
**N223Q**	0.9005	LPath
**N223R**	0.9159	LPath
**N223S**	0.3383	LBen
**N223T**	0.8667	LPath
**N223V**	0.926	LPath
**N223W**	0.8914	LPath
**N223Y**	0.3286	LBen
**G224A**	0.7923	LPath
**G224C**	0.9483	LPath
**G224D**	0.9685	LPath
**G224E**	0.9722	LPath
**G224F**	0.9733	LPath
**G224H**	0.9773	LPath
**G224I**	0.9749	LPath
**G224K**	0.9858	LPath
**G224L**	0.9722	LPath
**G224M**	0.9802	LPath
**G224N**	0.9564	LPath
**G224P**	0.9988	LPath
**G224Q**	0.9673	LPath
**G224R**	0.9537	LPath
**G224S**	0.7069	LPath
**G224T**	0.9348	LPath
**G224V**	0.9522	LPath
**G224W**	0.9694	LPath
**G224Y**	0.9697	LPath
**A225C**	0.7815	LPath
**A225D**	0.9225	LPath
**A225E**	0.8954	LPath
**A225F**	0.8742	LPath
**A225G**	0.565	LPath
**A225H**	0.9197	LPath
**A225I**	0.8393	LPath
**A225K**	0.9788	LPath
**A225L**	0.7832	LPath
**A225M**	0.7855	LPath
**A225N**	0.7928	LPath
**A225P**	0.9917	LPath
**A225Q**	0.8248	LPath
**A225R**	0.9214	LPath
**A225S**	0.1575	LBen

**A225T**	0.5327	Amb
**A225V**	0.4918	Amb
**A225W**	0.9861	LPath
**A225Y**	0.9495	LPath
**L226A**	0.3311	LBen
**L226C**	0.8425	LPath
**L226D**	0.8987	LPath
**L226E**	0.5605	Amb
**L226F**	0.3027	LBen
**L226G**	0.6976	LPath
**L226H**	0.5476	Amb
**L226I**	0.0968	LBen
**L226K**	0.5007	Amb
**L226M**	0.1406	LBen
**L226N**	0.6654	LPath
**L226P**	0.6854	LPath
**L226Q**	0.2992	LBen
**L226R**	0.3794	Amb
**L226S**	0.4515	Amb
**L226T**	0.2788	LBen
**L226V**	0.0993	LBen
**L226W**	0.4914	Amb
**L226Y**	0.6748	LPath
**D227A**	0.5243	Amb
**D227C**	0.952	LPath
**D227E**	0.2541	LBen
**D227F**	0.9312	LPath
**D227G**	0.3717	Amb
**D227H**	0.7322	LPath
**D227I**	0.9155	LPath
**D227K**	0.8385	LPath
**D227L**	0.8252	LPath
**D227M**	0.9364	LPath
**D227N**	0.4006	Amb
**D227P**	0.9216	LPath
**D227Q**	0.6841	LPath
**D227R**	0.8071	LPath
**D227S**	0.4004	Amb
**D227T**	0.6981	LPath
**D227V**	0.7406	LPath

**D227W**	0.9688	LPath
**D227Y**	0.7165	LPath
**E228A**	0.9186	LPath
**E228C**	0.9976	LPath
**E228D**	0.4479	Amb
**E228F**	0.9989	LPath
**E228G**	0.9195	LPath
**E228H**	0.986	LPath
**E228I**	0.9945	LPath
**E228K**	0.9344	LPath
**E228L**	0.9928	LPath
**E228M**	0.989	LPath
**E228N**	0.932	LPath
**E228P**	0.9987	LPath
**E228Q**	0.8278	LPath
**E228R**	0.9601	LPath
**E228S**	0.9165	LPath
**E228T**	0.9704	LPath
**E228V**	0.9778	LPath
**E228W**	0.9994	LPath
**E228Y**	0.9964	LPath
**V229A**	0.5582	Amb
**V229C**	0.9726	LPath
**V229D**	0.9713	LPath
**V229E**	0.9375	LPath
**V229F**	0.7269	LPath
**V229G**	0.7642	LPath
**V229H**	0.9875	LPath
**V229I**	0.0965	LBen
**V229K**	0.973	LPath
**V229L**	0.4898	Amb
**V229M**	0.3944	Amb
**V229N**	0.9205	LPath
**V229P**	0.9866	LPath
**V229Q**	0.9549	LPath
**V229R**	0.9667	LPath
**V229S**	0.8085	LPath
**V229T**	0.5163	Amb
**V229W**	0.9891	LPath
**V229Y**	0.9683	LPath
**V230A**	0.3161	LBen
**V230C**	0.9199	LPath

**V230D**	0.6842	LPath
**V230E**	0.5178	Amb
**V230F**	0.2522	LBen
**V230G**	0.4202	Amb
**V230H**	0.765	LPath
**V230I**	0.0946	LBen
**V230K**	0.6454	LPath
**V230L**	0.291	LBen
**V230M**	0.249	LBen
**V230N**	0.5456	Amb
**V230P**	0.9576	LPath
**V230Q**	0.5127	Amb
**V230R**	0.5385	Amb
**V230S**	0.3699	Amb
**V230T**	0.3488	Amb
**V230W**	0.9092	LPath
**V230Y**	0.7224	LPath
**L231A**	0.919	LPath
**L231C**	0.9429	LPath
**L231D**	0.9979	LPath
**L231E**	0.978	LPath
**L231F**	0.6229	LPath
**L231G**	0.981	LPath
**L231H**	0.962	LPath
**L231I**	0.215	LBen
**L231K**	0.9633	LPath
**L231M**	0.2491	LBen
**L231N**	0.9842	LPath
**L231P**	0.9283	LPath
**L231Q**	0.9031	LPath
**L231R**	0.9323	LPath
**L231S**	0.9725	LPath
**L231T**	0.9125	LPath
**L231V**	0.2627	LBen
**L231W**	0.8872	LPath
**L231Y**	0.9488	LPath
**K232A**	0.8877	LPath
**K232C**	0.9573	LPath
**K232D**	0.9613	LPath
**K232E**	0.8574	LPath
**K232F**	0.9692	LPath
**K232G**	0.9434	LPath

K232H	0.5557	Amb
K232I	0.8511	LPath
K232L	0.8112	LPath
K232M	0.7514	LPath
K232N	0.9238	LPath
K232P	0.9872	LPath
K232Q	0.4882	Amb
K232R	0.1608	LBen
K232S	0.9096	LPath
K232T	0.7321	LPath
K232V	0.794	LPath
K232W	0.954	LPath
K232Y	0.8977	LPath
F233A	0.9168	LPath
F233C	0.9455	LPath
F233D	0.967	LPath
F233E	0.9601	LPath
F233G	0.9519	LPath
F233H	0.915	LPath
F233I	0.7935	LPath
F233K	0.9762	LPath
F233L	0.978	LPath
F233M	0.8237	LPath
F233N	0.9236	LPath
F233P	0.9932	LPath
F233Q	0.9474	LPath
F233R	0.9452	LPath
F233S	0.8903	LPath
F233T	0.8897	LPath
F233V	0.7799	LPath
F233W	0.6965	LPath
F233Y	0.4367	Amb
D234A	0.23	LBen
D234C	0.7975	LPath
D234E	0.1502	LBen
D234F	0.7145	LPath
D234G	0.173	LBen
D234H	0.327	LBen
D234I	0.5545	Amb
D234K	0.5313	Amb

**D234L**	0.5076	Amb
**D234M**	0.757	LPath
**D234N**	0.1644	LBen
**D234P**	0.7967	LPath
**D234Q**	0.3221	LBen
**D234R**	0.4699	Amb
**D234S**	0.1375	LBen
**D234T**	0.329	LBen
**D234V**	0.372	Amb
**D234W**	0.8736	LPath
**D234Y**	0.3952	Amb
**R235A**	0.1782	LBen
**R235C**	0.2727	LBen
**R235D**	0.2758	LBen
**R235E**	0.1545	LBen
**R235F**	0.4358	Amb
**R235G**	0.0948	LBen
**R235H**	0.0865	LBen
**R235I**	0.2619	LBen
**R235K**	0.0796	LBen
**R235L**	0.1791	LBen
**R235M**	0.2398	LBen
**R235N**	0.2564	LBen
**R235P**	0.169	LBen
**R235Q**	0.0718	LBen
**R235S**	0.1973	LBen
**R235T**	0.1349	LBen
**R235V**	0.278	LBen
**R235W**	0.1506	LBen
**R235Y**	0.295	LBen
**N236A**	0.1901	LBen
**N236C**	0.4067	Amb
**N236D**	0.0836	LBen
**N236E**	0.1788	LBen
**N236F**	0.4374	Amb
**N236G**	0.1725	LBen
**N236H**	0.0849	LBen
**N236I**	0.2981	LBen
**N236K**	0.1524	LBen
**N236L**	0.2517	LBen
**N236M**	0.3397	LBen
**N236P**	0.4572	Amb

**N236Q**	0.177	LBen
**N236R**	0.1728	LBen
**N236S**	0.0792	LBen
**N236T**	0.128	LBen
**N236V**	0.2908	LBen
**N236W**	0.5922	LPath
**N236Y**	0.142	LBen
**R237A**	0.2451	LBen
**R237C**	0.2298	LBen
**R237D**	0.4702	Amb
**R237E**	0.2414	LBen
**R237F**	0.4347	Amb
**R237G**	0.1614	LBen
**R237H**	0.0889	LBen
**R237I**	0.2435	LBen
**R237K**	0.0995	LBen
**R237L**	0.191	LBen
**R237M**	0.2275	LBen
**R237N**	0.38	Amb
**R237P**	0.6967	LPath
**R237Q**	0.0823	LBen
**R237S**	0.285	LBen
**R237T**	0.1495	LBen
**R237V**	0.2703	LBen
**R237W**	0.1465	LBen
**R237Y**	0.3173	LBen
**V238A**	0.2952	LBen
**V238C**	0.9093	LPath
**V238D**	0.9405	LPath
**V238E**	0.8844	LPath
**V238F**	0.6121	LPath
**V238G**	0.4047	Amb
**V238H**	0.96	LPath
**V238I**	0.1947	LBen
**V238K**	0.9438	LPath
**V238L**	0.7417	LPath
**V238M**	0.5646	LPath
**V238N**	0.8362	LPath
**V238P**	0.785	LPath
**V238Q**	0.879	LPath
**V238R**	0.8994	LPath
**V238S**	0.4504	Amb

**V238T**	0.4535	Amb
**V238W**	0.9803	LPath
**V238Y**	0.9136	LPath
**R239A**	0.9093	LPath
**R239C**	0.6079	LPath
**R239D**	0.9726	LPath
**R239E**	0.8186	LPath
**R239F**	0.872	LPath
**R239G**	0.7956	LPath
**R239H**	0.2722	LBen
**R239I**	0.8072	LPath
**R239K**	0.419	Amb
**R239L**	0.7155	LPath
**R239M**	0.8049	LPath
**R239N**	0.9441	LPath
**R239P**	0.9893	LPath
**R239Q**	0.2448	LBen
**R239S**	0.8946	LPath
**R239T**	0.8054	LPath
**R239V**	0.8315	LPath
**R239W**	0.337	LBen
**R239Y**	0.734	LPath
**I240A**	0.6557	LPath
**I240C**	0.8917	LPath
**I240D**	0.9967	LPath
**I240E**	0.991	LPath
**I240F**	0.4976	Amb
**I240G**	0.9496	LPath
**I240H**	0.9886	LPath
**I240K**	0.9906	LPath
**I240L**	0.1392	LBen
**I240M**	0.1909	LBen
**I240N**	0.9582	LPath
**I240P**	0.9337	LPath
**I240Q**	0.9821	LPath
**I240R**	0.9833	LPath
**I240S**	0.8675	LPath
**I240T**	0.822	LPath
**I240V**	0.0929	LBen
**I240W**	0.9816	LPath
**I240Y**	0.9435	LPath
**R241A**	0.9564	LPath

**R241C**	0.7072	LPath
**R241D**	0.9946	LPath
**R241E**	0.8981	LPath
**R241F**	0.9553	LPath
**R241G**	0.9145	LPath
**R241H**	0.3939	Amb
**R241I**	0.9085	LPath
**R241K**	0.4835	Amb
**R241L**	0.8353	LPath
**R241M**	0.9125	LPath
**R241N**	0.9837	LPath
**R241P**	0.9965	LPath
**R241Q**	0.2992	LBen
**R241S**	0.9593	LPath
**R241T**	0.9299	LPath
**R241V**	0.9187	LPath
**R241W**	0.4733	Amb
**R241Y**	0.8738	LPath
**N242A**	0.9939	LPath
**N242C**	0.9784	LPath
**N242D**	0.9847	LPath
**N242E**	0.9971	LPath
**N242F**	0.9989	LPath
**N242G**	0.9905	LPath
**N242H**	0.9581	LPath
**N242I**	0.9907	LPath
**N242K**	0.9984	LPath
**N242L**	0.9823	LPath
**N242M**	0.991	LPath
**N242P**	0.9981	LPath
**N242Q**	0.9971	LPath
**N242R**	0.9977	LPath
**N242S**	0.8313	LPath
**N242T**	0.9771	LPath
**N242V**	0.9873	LPath
**N242W**	0.9994	LPath
**N242Y**	0.9839	LPath
**V243A**	0.3438	Amb
**V243C**	0.9674	LPath
**V243D**	0.7925	LPath
**V243E**	0.5862	LPath
**V243F**	0.3802	Amb

**V243G**	0.4834	Amb
**V243H**	0.855	LPath
**V243I**	0.0945	LBen
**V243K**	0.6981	LPath
**V243L**	0.3162	LBen
**V243M**	0.2039	LBen
**V243N**	0.63	LPath
**V243P**	0.9211	LPath
**V243Q**	0.6061	LPath
**V243R**	0.6186	LPath
**V243S**	0.4362	Amb
**V243T**	0.2532	LBen
**V243W**	0.9439	LPath
**V243Y**	0.8466	LPath
**A244C**	0.8153	LPath
**A244D**	0.687	LPath
**A244E**	0.5027	Amb
**A244F**	0.4561	Amb
**A244G**	0.3207	LBen
**A244H**	0.7004	LPath
**A244I**	0.3089	LBen
**A244K**	0.7375	LPath
**A244L**	0.2191	LBen
**A244M**	0.315	LBen
**A244N**	0.5179	Amb
**A244P**	0.785	LPath
**A244Q**	0.476	Amb
**A244R**	0.6078	LPath
**A244S**	0.1569	LBen
**A244T**	0.1625	LBen
**A244V**	0.1566	LBen
**A244W**	0.8994	LPath
**A244Y**	0.7044	LPath
**Y245A**	0.7828	LPath
**Y245C**	0.3769	Amb
**Y245D**	0.6903	LPath

**Y245E**	0.884	LPath
**Y245F**	0.0954	LBen
**Y245G**	0.6526	LPath
**Y245H**	0.2919	LBen
**Y245I**	0.9267	LPath
**Y245K**	0.9199	LPath
**Y245L**	0.7231	LPath
**Y245M**	0.7896	LPath
**Y245N**	0.3489	Amb
**Y245P**	0.9879	LPath
**Y245Q**	0.7818	LPath
**Y245R**	0.7889	LPath
**Y245S**	0.3934	Amb
**Y245T**	0.5182	Amb
**Y245V**	0.787	LPath
**Y245W**	0.397	Amb
**D246A**	0.7587	LPath
**D246C**	0.9837	LPath
**D246E**	0.6689	LPath
**D246F**	0.9787	LPath
**D246G**	0.5842	LPath
**D246H**	0.811	LPath
**D246I**	0.9779	LPath
**D246K**	0.9286	LPath
**D246L**	0.9455	LPath
**D246M**	0.9835	LPath
**D246N**	0.2698	LBen
**D246P**	0.9895	LPath
**D246Q**	0.8351	LPath
**D246R**	0.8724	LPath
**D246S**	0.4636	Amb
**D246T**	0.8771	LPath
**D246V**	0.9263	LPath
**D246W**	0.9948	LPath
**D246Y**	0.8411	LPath
**T247A**	0.4328	Amb
**T247C**	0.8535	LPath
**T247D**	0.9209	LPath
**T247E**	0.8675	LPath
**T247F**	0.913	LPath
**T247G**	0.6927	LPath
**T247H**	0.7779	LPath

**T247I**	0.9102	LPath
**T247K**	0.9	LPath
**T247L**	0.6755	LPath
**T247M**	0.4834	Amb
**T247N**	0.5977	LPath
**T247P**	0.9626	LPath
**T247Q**	0.7508	LPath
**T247R**	0.8086	LPath
**T247S**	0.1892	LBen
**T247V**	0.7683	LPath
**T247W**	0.9761	LPath
**T247Y**	0.9183	LPath
**L248A**	0.5042	Amb
**L248C**	0.8023	LPath
**L248D**	0.895	LPath
**L248E**	0.5595	Amb
**L248F**	0.1417	LBen
**L248G**	0.8709	LPath
**L248H**	0.3705	Amb
**L248I**	0.1207	LBen
**L248K**	0.5719	LPath
**L248M**	0.1465	LBen
**L248N**	0.6451	LPath
**L248P**	0.8727	LPath
**L248Q**	0.2871	LBen
**L248R**	0.3799	Amb
**L248S**	0.4901	Amb
**L248T**	0.4066	Amb
**L248V**	0.128	LBen
**L248W**	0.3123	LBen
**L248Y**	0.389	Amb
**P249A**	0.866	LPath
**P249C**	0.994	LPath
**P249D**	0.999	LPath
**P249E**	0.9982	LPath
**P249F**	0.9996	LPath
**P249G**	0.9852	LPath
**P249H**	0.9973	LPath
**P249I**	0.9977	LPath
**P249K**	0.9993	LPath
**P249L**	0.9773	LPath
**P249M**	0.9981	LPath

**P249N**	0.9974	LPath
**P249Q**	0.9956	LPath
**P249R**	0.9961	LPath
**P249S**	0.963	LPath
**P249T**	0.9784	LPath
**P249V**	0.9883	LPath
**P249W**	0.9999	LPath
**P249Y**	0.9997	LPath
**I250A**	0.1889	LBen
**I250C**	0.6935	LPath
**I250D**	0.8841	LPath
**I250E**	0.8176	LPath
**I250F**	0.4566	Amb
**I250G**	0.6232	LPath
**I250H**	0.8873	LPath
**I250K**	0.8836	LPath
**I250L**	0.1494	LBen
**I250M**	0.1247	LBen
**I250N**	0.5112	Amb
**I250P**	0.7634	LPath
**I250Q**	0.7798	LPath
**I250R**	0.7995	LPath
**I250S**	0.283	LBen
**I250T**	0.1576	LBen
**I250V**	0.0462	LBen
**I250W**	0.949	LPath
**I250Y**	0.8715	LPath
**V251A**	0.476	Amb
**V251C**	0.9456	LPath
**V251D**	0.9934	LPath
**V251E**	0.9812	LPath
**V251F**	0.6576	LPath
**V251G**	0.8494	LPath
**V251H**	0.9922	LPath
**V251I**	0.0799	LBen
**V251K**	0.9913	LPath
**V251L**	0.4086	Amb
**V251M**	0.4249	Amb
**V251N**	0.975	LPath
**V251P**	0.9892	LPath
**V251Q**	0.9777	LPath
**V251R**	0.9829	LPath

**V251S**	0.832	LPath
**V251T**	0.6001	LPath
**V251W**	0.991	LPath
**V251Y**	0.9769	LPath
**V252A**	0.6376	LPath
**V252C**	0.923	LPath
**V252D**	0.9957	LPath
**V252E**	0.9881	LPath
**V252F**	0.5216	Amb
**V252G**	0.838	LPath
**V252H**	0.9937	LPath
**V252I**	0.0676	LBen
**V252K**	0.9939	LPath
**V252L**	0.257	LBen
**V252M**	0.3858	Amb
**V252N**	0.9779	LPath
**V252P**	0.9885	LPath
**V252Q**	0.9843	LPath
**V252R**	0.989	LPath
**V252S**	0.8886	LPath
**V252T**	0.8139	LPath
**V252W**	0.9885	LPath
**V252Y**	0.9633	LPath
**H253A**	0.9953	LPath
**H253C**	0.9549	LPath
**H253D**	0.9961	LPath
**H253E**	0.9962	LPath
**H253F**	0.9811	LPath
**H253G**	0.9959	LPath
**H253I**	0.995	LPath
**H253K**	0.9945	LPath
**H253L**	0.9597	LPath
**H253M**	0.9944	LPath
**H253N**	0.9772	LPath
**H253P**	0.9843	LPath
**H253Q**	0.9886	LPath
**H253R**	0.9811	LPath
**H253S**	0.9887	LPath
**H253T**	0.9953	LPath
**H253V**	0.9956	LPath
**H253W**	0.9555	LPath
**H253Y**	0.9681	LPath

**G254A**	0.7124	LPath
**G254C**	0.9732	LPath
**G254D**	0.9973	LPath
**G254E**	0.997	LPath
**G254F**	0.9984	LPath
**G254H**	0.9991	LPath
**G254I**	0.9964	LPath
**G254K**	0.999	LPath
**G254L**	0.996	LPath
**G254M**	0.9984	LPath
**G254N**	0.9979	LPath
**G254P**	0.9996	LPath
**G254Q**	0.9968	LPath
**G254R**	0.993	LPath
**G254S**	0.8472	LPath
**G254T**	0.9868	LPath
**G254V**	0.9901	LPath
**G254W**	0.9978	LPath
**G254Y**	0.9988	LPath
**N255A**	0.9916	LPath
**N255C**	0.9809	LPath
**N255D**	0.9809	LPath
**N255E**	0.9974	LPath
**N255F**	0.9966	LPath
**N255G**	0.9832	LPath
**N255H**	0.9787	LPath
**N255I**	0.9914	LPath
**N255K**	0.9979	LPath
**N255L**	0.9835	LPath
**N255M**	0.9911	LPath
**N255P**	0.9936	LPath
**N255Q**	0.997	LPath
**N255R**	0.9958	LPath
**N255S**	0.7963	LPath
**N255T**	0.9809	LPath
**N255V**	0.9915	LPath
**N255W**	0.9985	LPath
**N255Y**	0.9733	LPath
**G256A**	0.9284	LPath
**G256C**	0.9866	LPath
**G256D**	0.9926	LPath
**G256E**	0.989	LPath

**G256F**	0.9867	LPath
**G256H**	0.9957	LPath
**G256I**	0.9596	LPath
**G256K**	0.9926	LPath
**G256L**	0.9824	LPath
**G256M**	0.9928	LPath
**G256N**	0.9919	LPath
**G256P**	0.9977	LPath
**G256Q**	0.9902	LPath
**G256R**	0.9777	LPath
**G256S**	0.877	LPath
**G256T**	0.9747	LPath
**G256V**	0.9514	LPath
**G256W**	0.9886	LPath
**G256Y**	0.9899	LPath
**P257A**	0.2719	LBen
**P257C**	0.9266	LPath
**P257D**	0.9197	LPath
**P257E**	0.7788	LPath
**P257F**	0.8085	LPath
**P257G**	0.7764	LPath
**P257H**	0.5477	Amb
**P257I**	0.7028	LPath
**P257K**	0.712	LPath
**P257L**	0.3368	LBen
**P257M**	0.703	LPath
**P257N**	0.8089	LPath
**P257Q**	0.5204	Amb
**P257R**	0.4608	Amb
**P257S**	0.5234	Amb
**P257T**	0.4914	Amb
**P257V**	0.5738	LPath
**P257W**	0.8956	LPath
**P257Y**	0.8133	LPath
**T258A**	0.2807	LBen
**T258C**	0.9141	LPath
**T258D**	0.9764	LPath
**T258E**	0.9412	LPath
**T258F**	0.9376	LPath
**T258G**	0.6621	LPath
**T258H**	0.9219	LPath
**T258I**	0.8725	LPath

**T258K**	0.9408	LPath
**T258L**	0.5975	LPath
**T258M**	0.4633	Amb
**T258N**	0.7625	LPath
**T258P**	0.8248	LPath
**T258Q**	0.861	LPath
**T258R**	0.8814	LPath
**T258S**	0.3091	LBen
**T258V**	0.6665	LPath
**T258W**	0.9829	LPath
**T258Y**	0.9558	LPath
**K259A**	0.9841	LPath
**K259C**	0.9962	LPath
**K259D**	0.9947	LPath
**K259E**	0.979	LPath
**K259F**	0.9985	LPath
**K259G**	0.9794	LPath
**K259H**	0.9619	LPath
**K259I**	0.993	LPath
**K259L**	0.9844	LPath
**K259M**	0.9795	LPath
**K259N**	0.9941	LPath
**K259P**	0.9917	LPath
**K259Q**	0.9313	LPath
**K259R**	0.4251	Amb
**K259S**	0.9853	LPath
**K259T**	0.9693	LPath
**K259V**	0.9851	LPath
**K259W**	0.9968	LPath
**K259Y**	0.9941	LPath
**L260A**	0.5823	LPath
**L260C**	0.8639	LPath
**L260D**	0.8902	LPath
**L260E**	0.6104	LPath
**L260F**	0.3674	Amb
**L260G**	0.7617	LPath
**L260H**	0.4889	Amb
**L260I**	0.1463	LBen
**L260K**	0.4919	Amb
**L260M**	0.2043	LBen
**L260N**	0.6251	LPath
**L260P**	0.6802	LPath

**L260Q**	0.2914	LBen
**L260R**	0.3362	LBen
**L260S**	0.668	LPath
**L260T**	0.4754	Amb
**L260V**	0.1556	LBen
**L260W**	0.4494	Amb
**L260Y**	0.5923	LPath
**Q261A**	0.3081	LBen
**Q261C**	0.9139	LPath
**Q261D**	0.7374	LPath
**Q261E**	0.1819	LBen
**Q261F**	0.7716	LPath
**Q261G**	0.4959	Amb
**Q261H**	0.3411	Amb
**Q261I**	0.5927	LPath
**Q261K**	0.2897	LBen
**Q261L**	0.2171	LBen
**Q261M**	0.5143	Amb
**Q261N**	0.4929	Amb
**Q261P**	0.2283	LBen
**Q261R**	0.2691	LBen
**Q261S**	0.3528	Amb
**Q261T**	0.38	Amb
**Q261V**	0.4212	Amb
**Q261W**	0.7612	LPath
**Q261Y**	0.5643	LPath
**L262A**	0.9515	LPath
**L262C**	0.9573	LPath
**L262D**	0.9989	LPath
**L262E**	0.9914	LPath
**L262F**	0.7375	LPath
**L262G**	0.9852	LPath
**L262H**	0.982	LPath
**L262I**	0.3605	Amb
**L262K**	0.9898	LPath
**L262M**	0.3386	LBen
**L262N**	0.9877	LPath
**L262P**	0.9963	LPath
**L262Q**	0.9622	LPath
**L262R**	0.9783	LPath
**L262S**	0.988	LPath
**L262T**	0.9534	LPath

**L262V**	0.4173	Amb
**L262W**	0.956	LPath
**L262Y**	0.9678	LPath
**N263A**	0.8544	LPath
**N263C**	0.89	LPath
**N263D**	0.8544	LPath
**N263E**	0.976	LPath
**N263F**	0.9771	LPath
**N263G**	0.828	LPath
**N263H**	0.7601	LPath
**N263I**	0.9089	LPath
**N263K**	0.9865	LPath
**N263L**	0.8267	LPath
**N263M**	0.925	LPath
**N263P**	0.976	LPath
**N263Q**	0.9503	LPath
**N263R**	0.9693	LPath
**N263S**	0.3079	LBen
**N263T**	0.7482	LPath
**N263V**	0.8876	LPath
**N263W**	0.9915	LPath
**N263Y**	0.8409	LPath
**Y264A**	0.6854	LPath
**Y264C**	0.5664	LPath
**Y264D**	0.745	LPath
**Y264E**	0.9185	LPath
**Y264F**	0.2096	LBen
**Y264G**	0.6236	LPath
**Y264H**	0.4142	Amb
**Y264I**	0.8261	LPath
**Y264K**	0.909	LPath
**Y264L**	0.6921	LPath
**Y264M**	0.8257	LPath
**Y264N**	0.3999	Amb
**Y264P**	0.9867	LPath
**Y264Q**	0.7749	LPath

**Y264R**	0.726	LPath
**Y264S**	0.2838	LBen
**Y264T**	0.6223	LPath
**Y264V**	0.6701	LPath
**Y264W**	0.7642	LPath
**L265A**	0.8688	LPath
**L265C**	0.9491	LPath
**L265D**	0.9978	LPath
**L265E**	0.9843	LPath
**L265F**	0.2461	LBen
**L265G**	0.9835	LPath
**L265H**	0.9278	LPath
**L265I**	0.2426	LBen
**L265K**	0.9783	LPath
**L265M**	0.2448	LBen
**L265N**	0.9894	LPath
**L265P**	0.9911	LPath
**L265Q**	0.9231	LPath
**L265R**	0.9463	LPath
**L265S**	0.9625	LPath
**L265T**	0.8753	LPath
**L265V**	0.2913	LBen
**L265W**	0.6532	LPath
**L265Y**	0.8006	LPath
**G266A**	0.1978	LBen
**G266C**	0.6653	LPath
**G266D**	0.9081	LPath
**G266E**	0.8858	LPath
**G266F**	0.9755	LPath
**G266H**	0.9666	LPath
**G266I**	0.9418	LPath
**G266K**	0.9855	LPath
**G266L**	0.9225	LPath
**G266M**	0.9371	LPath
**G266N**	0.884	LPath
**G266P**	0.9965	LPath
**G266Q**	0.9173	LPath
**G266R**	0.9551	LPath
**G266S**	0.1707	LBen
**G266T**	0.5599	Amb
**G266V**	0.8385	LPath

**G266W**	0.9552	LPath
**G266Y**	0.9662	LPath
**N267A**	0.9216	LPath
**N267C**	0.8796	LPath
**N267D**	0.9381	LPath
**N267E**	0.9906	LPath
**N267F**	0.9931	LPath
**N267G**	0.8913	LPath
**N267H**	0.8748	LPath
**N267I**	0.9627	LPath
**N267K**	0.9942	LPath
**N267L**	0.9379	LPath
**N267M**	0.9676	LPath
**N267P**	0.9893	LPath
**N267Q**	0.9777	LPath
**N267R**	0.9879	LPath
**N267S**	0.3522	Amb
**N267T**	0.8701	LPath
**N267V**	0.952	LPath
**N267W**	0.9975	LPath
**N267Y**	0.9383	LPath
**Y268A**	0.9917	LPath
**Y268C**	0.9772	LPath
**Y268D**	0.9869	LPath
**Y268E**	0.9971	LPath
**Y268F**	0.5802	LPath
**Y268G**	0.9804	LPath
**Y268H**	0.9789	LPath
**Y268I**	0.9752	LPath
**Y268K**	0.9976	LPath
**Y268L**	0.9269	LPath
**Y268M**	0.9872	LPath
**Y268N**	0.9644	LPath
**Y268P**	0.9963	LPath
**Y268Q**	0.9971	LPath
**Y268R**	0.9879	LPath
**Y268S**	0.9654	LPath
**Y268T**	0.9878	LPath
**Y268V**	0.9435	LPath
**Y268W**	0.9374	LPath

**V269A**	0.7121	LPath
**V269C**	0.9453	LPath
**V269D**	0.9915	LPath
**V269E**	0.9733	LPath
**V269F**	0.6049	LPath
**V269G**	0.8723	LPath
**V269H**	0.9867	LPath
**V269I**	0.0728	LBen
**V269K**	0.9858	LPath
**V269L**	0.3811	Amb
**V269M**	0.452	Amb
**V269N**	0.9667	LPath
**V269P**	0.9896	LPath
**V269Q**	0.9659	LPath
**V269R**	0.9743	LPath
**V269S**	0.8479	LPath
**V269T**	0.7488	LPath
**V269W**	0.9907	LPath
**V269Y**	0.9621	LPath
**P270A**	0.4216	Amb
**P270C**	0.9427	LPath
**P270D**	0.9861	LPath
**P270E**	0.9707	LPath
**P270F**	0.9795	LPath
**P270G**	0.8186	LPath
**P270H**	0.9394	LPath
**P270I**	0.9504	LPath
**P270K**	0.9811	LPath
**P270L**	0.7823	LPath
**P270M**	0.9395	LPath
**P270N**	0.969	LPath
**P270Q**	0.9106	LPath
**P270R**	0.9127	LPath
**P270S**	0.7337	LPath
**P270T**	0.7503	LPath
**P270V**	0.865	LPath
**P270W**	0.9896	LPath
**P270Y**	0.9817	LPath
**N271A**	0.1739	LBen

**N271C**	0.3746	Amb
**N271D**	0.1008	LBen
**N271E**	0.2618	LBen
**N271F**	0.4355	Amb
**N271G**	0.1681	LBen
**N271H**	0.1001	LBen
**N271I**	0.3646	Amb
**N271K**	0.211	LBen
**N271L**	0.2387	LBen
**N271M**	0.3336	LBen
**N271P**	0.6633	LPath
**N271Q**	0.2183	LBen
**N271R**	0.1881	LBen
**N271S**	0.0772	LBen
**N271T**	0.1691	LBen
**N271V**	0.2936	LBen
**N271W**	0.6011	LPath
**N271Y**	0.141	LBen
**G272A**	0.0956	LBen
**G272C**	0.2361	LBen
**G272D**	0.5147	Amb
**G272E**	0.3919	Amb
**G272F**	0.5189	Amb
**G272H**	0.4634	Amb
**G272I**	0.2611	LBen
**G272K**	0.5913	LPath
**G272L**	0.3668	Amb
**G272M**	0.4177	Amb
**G272N**	0.3759	Amb
**G272P**	0.9525	LPath
**G272Q**	0.3849	Amb
**G272R**	0.3676	Amb
**G272S**	0.0921	LBen
**G272T**	0.1524	LBen
**G272V**	0.1883	LBen
**G272W**	0.4955	Amb
**G272Y**	0.4523	Amb
**W273A**	0.9769	LPath
**W273C**	0.9911	LPath
**W273D**	0.9968	LPath
**W273E**	0.9946	LPath

**W273F**	0.4858	Amb
**W273G**	0.9251	LPath
**W273H**	0.974	LPath
**W273I**	0.9699	LPath
**W273K**	0.9965	LPath
**W273L**	0.9359	LPath
**W273M**	0.9749	LPath
**W273N**	0.9919	LPath
**W273P**	0.9976	LPath
**W273Q**	0.9911	LPath
**W273R**	0.9902	LPath
**W273S**	0.9543	LPath
**W273T**	0.9815	LPath
**W273V**	0.9606	LPath
**W273Y**	0.7834	LPath
**T274A**	0.1643	LBen
**T274C**	0.6857	LPath
**T274D**	0.4809	Amb
**T274E**	0.4778	Amb
**T274F**	0.3434	Amb
**T274G**	0.2744	LBen
**T274H**	0.2716	LBen
**T274I**	0.3303	LBen
**T274K**	0.2712	LBen
**T274L**	0.1368	LBen
**T274M**	0.121	LBen
**T274N**	0.1165	LBen
**T274P**	0.5459	Amb
**T274Q**	0.2982	LBen
**T274R**	0.2298	LBen
**T274S**	0.1259	LBen
**T274V**	0.2765	LBen
**T274W**	0.6859	LPath
**T274Y**	0.4	Amb
**P275A**	0.0835	LBen
**P275C**	0.646	LPath
**P275D**	0.4485	Amb
**P275E**	0.2386	LBen
**P275F**	0.2798	LBen

**P275G**	0.2676	LBen
**P275H**	0.1578	LBen
**P275I**	0.2422	LBen
**P275K**	0.2557	LBen
**P275L**	0.1014	LBen
**P275M**	0.2525	LBen
**P275N**	0.3046	LBen
**P275Q**	0.1394	LBen
**P275R**	0.1324	LBen
**P275S**	0.1175	LBen
**P275T**	0.1096	LBen
**P275V**	0.1679	LBen
**P275W**	0.3831	Amb
**P275Y**	0.2698	LBen
**E276A**	0.0897	LBen
**E276C**	0.7647	LPath
**E276D**	0.1103	LBen
**E276F**	0.4775	Amb
**E276G**	0.099	LBen
**E276H**	0.2163	LBen
**E276I**	0.2246	LBen
**E276K**	0.0911	LBen
**E276L**	0.1948	LBen
**E276M**	0.2908	LBen
**E276N**	0.1657	LBen
**E276P**	0.3136	LBen
**E276Q**	0.0727	LBen
**E276R**	0.1092	LBen
**E276S**	0.107	LBen
**E276T**	0.124	LBen
**E276V**	0.143	LBen
**E276W**	0.5916	LPath
**E276Y**	0.3697	Amb
**G277A**	0.0972	LBen
**G277C**	0.3765	Amb
**G277D**	0.126	LBen
**G277E**	0.1055	LBen
**G277F**	0.3452	Amb
**G277H**	0.2234	LBen
**G277I**	0.2003	LBen
**G277K**	0.1959	LBen

**G277L**	0.2557	LBen
**G277M**	0.2981	LBen
**G277N**	0.1689	LBen
**G277P**	0.7417	LPath
**G277Q**	0.1843	LBen
**G277R**	0.1517	LBen
**G277S**	0.0959	LBen
**G277T**	0.1043	LBen
**G277V**	0.1341	LBen
**G277W**	0.2664	LBen
**G277Y**	0.2513	LBen
**G278A**	0.2625	LBen
**G278C**	0.5886	LPath
**G278D**	0.3715	Amb
**G278E**	0.3485	Amb
**G278F**	0.6596	LPath
**G278H**	0.4835	Amb
**G278I**	0.4633	Amb
**G278K**	0.4564	Amb
**G278L**	0.5839	LPath
**G278M**	0.6333	LPath
**G278N**	0.3697	Amb
**G278P**	0.8106	LPath
**G278Q**	0.3808	Amb
**G278R**	0.3375	LBen
**G278S**	0.1452	LBen
**G278T**	0.2756	LBen
**G278V**	0.3981	Amb
**G278W**	0.6164	LPath
**G278Y**	0.5917	LPath
**C279A**	0.9785	LPath
**C279D**	0.9907	LPath
**C279E**	0.9939	LPath
**C279F**	0.9477	LPath
**C279G**	0.9065	LPath
**C279H**	0.9881	LPath
**C279I**	0.9837	LPath
**C279K**	0.9961	LPath
**C279L**	0.9624	LPath
**C279M**	0.9878	LPath

**C279N**	0.9888	LPath
**C279P**	0.9867	LPath
**C279Q**	0.9935	LPath
**C279R**	0.9691	LPath
**C279S**	0.9797	LPath
**C279T**	0.988	LPath
**C279V**	0.9774	LPath
**C279W**	0.9632	LPath
**C279Y**	0.9649	LPath
**G280A**	0.0931	LBen
**G280C**	0.2505	LBen
**G280D**	0.1759	LBen
**G280E**	0.1411	LBen
**G280F**	0.3387	LBen
**G280H**	0.2205	LBen
**G280I**	0.1508	LBen
**G280K**	0.2473	LBen
**G280L**	0.2128	LBen
**G280M**	0.3285	LBen
**G280N**	0.206	LBen
**G280P**	0.5261	Amb
**G280Q**	0.1848	LBen
**G280R**	0.1352	LBen
**G280S**	0.0769	LBen
**G280T**	0.1039	LBen
**G280V**	0.1148	LBen
**G280W**	0.2662	LBen
**G280Y**	0.2813	LBen
**F281A**	0.2302	LBen
**F281C**	0.341	Amb
**F281D**	0.2765	LBen
**F281E**	0.4428	Amb
**F281G**	0.375	Amb
**F281H**	0.3038	LBen
**F281I**	0.1095	LBen
**F281K**	0.4824	Amb
**F281L**	0.5177	Amb
**F281M**	0.3513	Amb
**F281N**	0.2338	LBen

**F281P**	0.6091	LPath
**F281Q**	0.4281	Amb
**F281R**	0.3519	Amb
**F281S**	0.1404	LBen
**F281T**	0.2136	LBen
**F281V**	0.1238	LBen
**F281W**	0.3149	LBen
**F281Y**	0.122	LBen
**C282A**	0.9566	LPath
**C282D**	0.9809	LPath
**C282E**	0.9837	LPath
**C282F**	0.9221	LPath
**C282G**	0.7815	LPath
**C282H**	0.9618	LPath
**C282I**	0.9662	LPath
**C282K**	0.9896	LPath
**C282L**	0.9342	LPath
**C282M**	0.983	LPath
**C282N**	0.9807	LPath
**C282P**	0.9727	LPath
**C282Q**	0.9707	LPath
**C282R**	0.8995	LPath
**C282S**	0.9461	LPath
**C282T**	0.9723	LPath
**C282V**	0.9538	LPath
**C282W**	0.9284	LPath
**C282Y**	0.9562	LPath
**N283A**	0.1025	LBen
**N283C**	0.2426	LBen
**N283D**	0.0607	LBen
**N283E**	0.1061	LBen
**N283F**	0.2294	LBen
**N283G**	0.1052	LBen
**N283H**	0.0613	LBen
**N283I**	0.1234	LBen
**N283K**	0.0997	LBen
**N283L**	0.1079	LBen
**N283M**	0.2216	LBen
**N283P**	0.1669	LBen
**N283Q**	0.1066	LBen
**N283R**	0.0928	LBen

**N283S**	0.0584	LBen
**N283T**	0.0912	LBen
**N283V**	0.121	LBen
**N283W**	0.3301	LBen
**N283Y**	0.0924	LBen
**Q284A**	0.1224	LBen
**Q284C**	0.4119	Amb
**Q284D**	0.1656	LBen
**Q284E**	0.0695	LBen
**Q284F**	0.3346	LBen
**Q284G**	0.113	LBen
**Q284H**	0.1016	LBen
**Q284I**	0.1934	LBen
**Q284K**	0.0722	LBen
**Q284L**	0.0918	LBen
**Q284M**	0.2299	LBen
**Q284N**	0.1754	LBen
**Q284P**	0.1062	LBen
**Q284R**	0.0577	LBen
**Q284S**	0.125	LBen
**Q284T**	0.1049	LBen
**Q284V**	0.1428	LBen
**Q284W**	0.1678	LBen
**Q284Y**	0.2261	LBen
**D285A**	0.1231	LBen
**D285C**	0.5747	LPath
**D285E**	0.1256	LBen
**D285F**	0.3143	LBen
**D285G**	0.0703	LBen
**D285H**	0.1485	LBen
**D285I**	0.2441	LBen
**D285K**	0.1886	LBen
**D285L**	0.2214	LBen
**D285M**	0.4363	Amb
**D285N**	0.0758	LBen
**D285P**	0.3034	LBen
**D285Q**	0.1711	LBen
**D285R**	0.1772	LBen
**D285S**	0.0997	LBen

**D285T**	0.1767	LBen
**D285V**	0.1549	LBen
**D285W**	0.5996	LPath
**D285Y**	0.1218	LBen
**R286A**	0.211	LBen
**R286C**	0.2165	LBen
**R286D**	0.393	Amb
**R286E**	0.1918	LBen
**R286F**	0.3049	LBen
**R286G**	0.1235	LBen
**R286H**	0.0917	LBen
**R286I**	0.1635	LBen
**R286K**	0.0935	LBen
**R286L**	0.1188	LBen
**R286M**	0.206	LBen
**R286N**	0.3483	Amb
**R286P**	0.2397	LBen
**R286Q**	0.0814	LBen
**R286S**	0.2331	LBen
**R286T**	0.137	LBen
**R286V**	0.2064	LBen
**R286W**	0.1181	LBen
**R286Y**	0.2642	LBen
**R287A**	0.2339	LBen
**R287C**	0.2227	LBen
**R287D**	0.3845	Amb
**R287E**	0.1834	LBen
**R287F**	0.2797	LBen
**R287G**	0.1589	LBen
**R287H**	0.0878	LBen
**R287I**	0.14	LBen
**R287K**	0.0999	LBen
**R287L**	0.1163	LBen
**R287M**	0.194	LBen
**R287N**	0.357	Amb
**R287P**	0.3285	LBen
**R287Q**	0.0776	LBen
**R287S**	0.282	LBen
**R287T**	0.1599	LBen
**R287V**	0.1872	LBen
**R287W**	0.1121	LBen

**R287Y**	0.2251	LBen
**T288A**	0.0628	LBen
**T288C**	0.3583	Amb
**T288D**	0.1208	LBen
**T288E**	0.1081	LBen
**T288F**	0.098	LBen
**T288G**	0.1335	LBen
**T288H**	0.0976	LBen
**T288I**	0.0687	LBen
**T288K**	0.087	LBen
**T288L**	0.0604	LBen
**T288M**	0.0773	LBen
**T288N**	0.065	LBen
**T288P**	0.0517	LBen
**T288Q**	0.0949	LBen
**T288R**	0.0734	LBen
**T288S**	0.0702	LBen
**T288V**	0.0749	LBen
**T288W**	0.264	LBen
**T288Y**	0.1267	LBen
**L289A**	0.1871	LBen
**L289C**	0.6418	LPath
**L289D**	0.4807	Amb
**L289E**	0.2507	LBen
**L289F**	0.0952	LBen
**L289G**	0.3386	LBen
**L289H**	0.2272	LBen
**L289I**	0.0876	LBen
**L289K**	0.2814	LBen
**L289M**	0.1389	LBen
**L289N**	0.3252	LBen
**L289P**	0.135	LBen
**L289Q**	0.1634	LBen
**L289R**	0.1772	LBen
**L289S**	0.201	LBen
**L289T**	0.2072	LBen
**L289V**	0.0909	LBen
**L289W**	0.1686	LBen
**L289Y**	0.2852	LBen
**P290A**	0.0651	LBen

**P290C**	0.4034	Amb
**P290D**	0.1778	LBen
**P290E**	0.1191	LBen
**P290F**	0.242	LBen
**P290G**	0.1527	LBen
**P290H**	0.104	LBen
**P290I**	0.1978	LBen
**P290K**	0.1481	LBen
**P290L**	0.0822	LBen
**P290M**	0.2149	LBen
**P290N**	0.1848	LBen
**P290Q**	0.0877	LBen
**P290R**	0.0879	LBen
**P290S**	0.076	LBen
**P290T**	0.0713	LBen
**P290V**	0.1323	LBen
**P290W**	0.2662	LBen
**P290Y**	0.2458	LBen
**G291A**	0.0688	LBen
**G291C**	0.2115	LBen
**G291D**	0.0691	LBen
**G291E**	0.0682	LBen
**G291F**	0.2395	LBen
**G291H**	0.1348	LBen
**G291I**	0.1286	LBen
**G291K**	0.1212	LBen
**G291L**	0.1543	LBen
**G291M**	0.2128	LBen
**G291N**	0.13	LBen
**G291P**	0.1708	LBen
**G291Q**	0.1069	LBen
**G291R**	0.0897	LBen
**G291S**	0.0714	LBen
**G291T**	0.081	LBen
**G291V**	0.0851	LBen
**G291W**	0.1623	LBen
**G291Y**	0.1911	LBen
**G292A**	0.0906	LBen
**G292C**	0.1976	LBen

**G292D**	0.0662	LBen
**G292E**	0.0702	LBen
**G292F**	0.2044	LBen
**G292H**	0.131	LBen
**G292I**	0.1092	LBen
**G292K**	0.1238	LBen
**G292L**	0.1588	LBen
**G292M**	0.2131	LBen
**G292N**	0.1112	LBen
**G292P**	0.2184	LBen
**G292Q**	0.1056	LBen
**G292R**	0.097	LBen
**G292S**	0.0701	LBen
**G292T**	0.0888	LBen
**G292V**	0.0881	LBen
**G292W**	0.1586	LBen
**G292Y**	0.1637	LBen
**Q293A**	0.1217	LBen
**Q293C**	0.4539	Amb
**Q293D**	0.1511	LBen
**Q293E**	0.0737	LBen
**Q293F**	0.3054	LBen
**Q293G**	0.142	LBen
**Q293H**	0.1171	LBen
**Q293I**	0.1645	LBen
**Q293K**	0.0765	LBen
**Q293L**	0.0885	LBen
**Q293M**	0.2241	LBen
**Q293N**	0.148	LBen
**Q293P**	0.09	LBen
**Q293R**	0.0802	LBen
**Q293S**	0.1086	LBen
**Q293T**	0.1113	LBen
**Q293V**	0.1275	LBen
**Q293W**	0.2471	LBen
**Q293Y**	0.227	LBen
**P294A**	0.0586	LBen
**P294C**	0.3721	Amb
**P294D**	0.1408	LBen
**P294E**	0.0978	LBen
**P294F**	0.1865	LBen

**P294G**	0.1667	LBen
**P294H**	0.0951	LBen
**P294I**	0.1245	LBen
**P294K**	0.1127	LBen
**P294L**	0.0708	LBen
**P294M**	0.1583	LBen
**P294N**	0.1385	LBen
**P294Q**	0.0725	LBen
**P294R**	0.0833	LBen
**P294S**	0.0672	LBen
**P294T**	0.0673	LBen
**P294V**	0.0928	LBen
**P294W**	0.2964	LBen
**P294Y**	0.1973	LBen
**P295A**	0.0631	LBen
**P295C**	0.413	Amb
**P295D**	0.2938	LBen
**P295E**	0.1585	LBen
**P295F**	0.1701	LBen
**P295G**	0.2076	LBen
**P295H**	0.126	LBen
**P295I**	0.1459	LBen
**P295K**	0.2154	LBen
**P295L**	0.0587	LBen
**P295M**	0.1833	LBen
**P295N**	0.2702	LBen
**P295Q**	0.1064	LBen
**P295R**	0.1191	LBen
**P295S**	0.0853	LBen
**P295T**	0.0877	LBen
**P295V**	0.1064	LBen
**P295W**	0.2523	LBen
**P295Y**	0.1841	LBen
**P296A**	0.1258	LBen
**P296C**	0.7697	LPath
**P296D**	0.6763	LPath
**P296E**	0.3971	Amb
**P296F**	0.7478	LPath
**P296G**	0.4881	Amb

**P296H**	0.3769	Amb
**P296I**	0.5709	LPath
**P296K**	0.4413	Amb
**P296L**	0.2256	LBen
**P296M**	0.6187	LPath
**P296N**	0.6809	LPath
**P296Q**	0.2489	LBen
**P296R**	0.2335	LBen
**P296S**	0.2117	LBen
**P296T**	0.2332	LBen
**P296V**	0.3644	Amb
**P296W**	0.8205	LPath
**P296Y**	0.7031	LPath
**R297A**	0.1999	LBen
**R297C**	0.2765	LBen
**R297D**	0.4051	Amb
**R297E**	0.2236	LBen
**R297F**	0.4403	Amb
**R297G**	0.1431	LBen
**R297H**	0.0971	LBen
**R297I**	0.2526	LBen
**R297K**	0.0926	LBen
**R297L**	0.148	LBen
**R297M**	0.2461	LBen
**R297N**	0.3639	Amb
**R297P**	0.213	LBen
**R297Q**	0.0878	LBen
**R297S**	0.2376	LBen
**R297T**	0.1389	LBen
**R297V**	0.2432	LBen
**R297W**	0.182	LBen
**R297Y**	0.3518	Amb
**V298A**	0.5095	Amb
**V298C**	0.9347	LPath
**V298D**	0.9711	LPath
**V298E**	0.9456	LPath
**V298F**	0.6384	LPath
**V298G**	0.5894	LPath
**V298H**	0.982	LPath
**V298I**	0.1022	LBen
**V298K**	0.9695	LPath

**V298L**	0.3377	LBen
**V298M**	0.5157	Amb
**V298N**	0.9311	LPath
**V298P**	0.9162	LPath
**V298Q**	0.9449	LPath
**V298R**	0.937	LPath
**V298S**	0.7791	LPath
**V298T**	0.649	LPath
**V298W**	0.9854	LPath
**V298Y**	0.9497	LPath
**F299A**	0.304	LBen
**F299C**	0.2096	LBen
**F299D**	0.7897	LPath
**F299E**	0.6905	LPath
**F299G**	0.5209	Amb
**F299H**	0.278	LBen
**F299I**	0.1337	LBen
**F299K**	0.5909	LPath
**F299L**	0.2086	LBen
**F299M**	0.2039	LBen
**F299N**	0.4773	Amb
**F299P**	0.953	LPath
**F299Q**	0.3495	Amb
**F299R**	0.353	Amb
**F299S**	0.2463	LBen
**F299T**	0.2734	LBen
**F299V**	0.1041	LBen
**F299W**	0.2661	LBen
**F299Y**	0.131	LBen
**L300A**	0.5728	LPath
**L300C**	0.8148	LPath
**L300D**	0.9947	LPath
**L300E**	0.962	LPath
**L300F**	0.2531	LBen
**L300G**	0.9202	LPath
**L300H**	0.8987	LPath
**L300I**	0.1031	LBen
**L300K**	0.956	LPath
**L300M**	0.1536	LBen
**L300N**	0.9734	LPath

**L300P**	0.9305	LPath
**L300Q**	0.8336	LPath
**L300R**	0.8963	LPath
**L300S**	0.8388	LPath
**L300T**	0.6008	LPath
**L300V**	0.0861	LBen
**L300W**	0.7073	LPath
**L300Y**	0.857	LPath
**A301C**	0.8946	LPath
**A301D**	0.9792	LPath
**A301E**	0.9861	LPath
**A301F**	0.9773	LPath
**A301G**	0.098	LBen
**A301H**	0.9889	LPath
**A301I**	0.9634	LPath
**A301K**	0.9969	LPath
**A301L**	0.8943	LPath
**A301M**	0.9242	LPath
**A301N**	0.959	LPath
**A301P**	0.9691	LPath
**A301Q**	0.9755	LPath
**A301R**	0.9899	LPath
**A301S**	0.1808	LBen
**A301T**	0.5695	LPath
**A301V**	0.7956	LPath
**A301W**	0.9974	LPath
**A301Y**	0.9921	LPath
**V302A**	0.3505	Amb
**V302C**	0.9034	LPath
**V302D**	0.9868	LPath
**V302E**	0.9721	LPath
**V302F**	0.6105	LPath
**V302G**	0.6499	LPath
**V302H**	0.9894	LPath
**V302I**	0.0938	LBen
**V302K**	0.9876	LPath
**V302L**	0.262	LBen
**V302M**	0.4031	Amb
**V302N**	0.9549	LPath

**V302P**	0.967	LPath
**V302Q**	0.9649	LPath
**V302R**	0.9677	LPath
**V302S**	0.7396	LPath
**V302T**	0.6132	LPath
**V302W**	0.9906	LPath
**V302Y**	0.9652	LPath
**F303A**	0.9779	LPath
**F303C**	0.9699	LPath
**F303D**	0.997	LPath
**F303E**	0.997	LPath
**F303G**	0.9912	LPath
**F303H**	0.9543	LPath
**F303I**	0.9128	LPath
**F303K**	0.9978	LPath
**F303L**	0.9867	LPath
**F303M**	0.9231	LPath
**F303N**	0.9823	LPath
**F303P**	0.9995	LPath
**F303Q**	0.9905	LPath
**F303R**	0.9926	LPath
**F303S**	0.9573	LPath
**F303T**	0.9758	LPath
**F303V**	0.9	LPath
**F303W**	0.8892	LPath
**F303Y**	0.5991	LPath
**V304A**	0.5658	LPath
**V304C**	0.9496	LPath
**V304D**	0.9833	LPath
**V304E**	0.9546	LPath
**V304F**	0.7043	LPath
**V304G**	0.8309	LPath
**V304H**	0.9879	LPath
**V304I**	0.0864	LBen
**V304K**	0.9798	LPath
**V304L**	0.3497	Amb
**V304M**	0.4963	Amb
**V304N**	0.9628	LPath
**V304P**	0.9581	LPath
**V304Q**	0.9496	LPath
**V304R**	0.9529	LPath

**V304S**	0.8073	LPath
**V304T**	0.6308	LPath
**V304W**	0.9851	LPath
**V304Y**	0.9758	LPath
**E305A**	0.6055	LPath
**E305C**	0.9683	LPath
**E305D**	0.7862	LPath
**E305F**	0.9871	LPath
**E305G**	0.5842	LPath
**E305H**	0.9155	LPath
**E305I**	0.9367	LPath
**E305K**	0.8559	LPath
**E305L**	0.895	LPath
**E305M**	0.9309	LPath
**E305N**	0.8763	LPath
**E305P**	0.8599	LPath
**E305Q**	0.4361	Amb
**E305R**	0.8425	LPath
**E305S**	0.6502	LPath
**E305T**	0.7608	LPath
**E305V**	0.8107	LPath
**E305W**	0.993	LPath
**E305Y**	0.9733	LPath
**Q306A**	0.3114	LBen
**Q306C**	0.8925	LPath
**Q306D**	0.6755	LPath
**Q306E**	0.1638	LBen
**Q306F**	0.8043	LPath
**Q306G**	0.3767	Amb
**Q306H**	0.3595	Amb
**Q306I**	0.5553	Amb
**Q306K**	0.1296	LBen
**Q306L**	0.2299	LBen
**Q306M**	0.4735	Amb
**Q306N**	0.4364	Amb
**Q306P**	0.5747	LPath
**Q306R**	0.157	LBen
**Q306S**	0.3336	LBen
**Q306T**	0.2757	LBen

**Q306V**	0.3597	Amb
**Q306W**	0.7743	LPath
**Q306Y**	0.6479	LPath
**P307A**	0.107	LBen
**P307C**	0.8775	LPath
**P307D**	0.8457	LPath
**P307E**	0.6236	LPath
**P307F**	0.9565	LPath
**P307G**	0.5518	Amb
**P307H**	0.6406	LPath
**P307I**	0.8411	LPath
**P307K**	0.6517	LPath
**P307L**	0.5012	Amb
**P307M**	0.7921	LPath
**P307N**	0.7421	LPath
**P307Q**	0.4264	Amb
**P307R**	0.4324	Amb
**P307S**	0.3086	LBen
**P307T**	0.356	Amb
**P307V**	0.6143	LPath
**P307W**	0.9654	LPath
**P307Y**	0.9199	LPath
**T308A**	0.2467	LBen
**T308C**	0.8113	LPath
**T308D**	0.9774	LPath
**T308E**	0.9205	LPath
**T308F**	0.8481	LPath
**T308G**	0.7333	LPath
**T308H**	0.8679	LPath
**T308I**	0.4231	Amb
**T308K**	0.9026	LPath
**T308L**	0.2821	LBen
**T308M**	0.2039	LBen
**T308N**	0.7067	LPath
**T308P**	0.6942	LPath
**T308Q**	0.7802	LPath
**T308R**	0.876	LPath
**T308S**	0.3232	LBen
**T308V**	0.2424	LBen
**T308W**	0.9709	LPath
**T308Y**	0.9216	LPath

**P309A**	0.411	Amb
**P309C**	0.9723	LPath
**P309D**	0.9962	LPath
**P309E**	0.9872	LPath
**P309F**	0.9958	LPath
**P309G**	0.937	LPath
**P309H**	0.974	LPath
**P309I**	0.9729	LPath
**P309K**	0.9946	LPath
**P309L**	0.8785	LPath
**P309M**	0.9846	LPath
**P309N**	0.9916	LPath
**P309Q**	0.9703	LPath
**P309R**	0.9641	LPath
**P309S**	0.8384	LPath
**P309T**	0.8787	LPath
**P309V**	0.8942	LPath
**P309W**	0.9979	LPath
**P309Y**	0.9944	LPath
**F310A**	0.9903	LPath
**F310C**	0.9843	LPath
**F310D**	0.9975	LPath
**F310E**	0.9985	LPath
**F310G**	0.9917	LPath
**F310H**	0.9767	LPath
**F310I**	0.9891	LPath
**F310K**	0.9991	LPath
**F310L**	0.995	LPath
**F310M**	0.9827	LPath
**F310N**	0.993	LPath
**F310P**	0.9995	LPath
**F310Q**	0.9953	LPath
**F310R**	0.9953	LPath
**F310S**	0.9825	LPath
**F310T**	0.9943	LPath
**F310V**	0.9773	LPath
**F310W**	0.9248	LPath
**F310Y**	0.8057	LPath
**L311A**	0.8595	LPath

**L311C**	0.927	LPath
**L311D**	0.9974	LPath
**L311E**	0.9799	LPath
**L311F**	0.1938	LBen
**L311G**	0.9537	LPath
**L311H**	0.9353	LPath
**L311I**	0.2092	LBen
**L311K**	0.9831	LPath
**L311M**	0.2798	LBen
**L311N**	0.9816	LPath
**L311P**	0.9586	LPath
**L311Q**	0.8712	LPath
**L311R**	0.9471	LPath
**L311S**	0.9324	LPath
**L311T**	0.7808	LPath
**L311V**	0.225	LBen
**L311W**	0.7687	LPath
**L311Y**	0.8728	LPath
**P312A**	0.1276	LBen
**P312C**	0.7503	LPath
**P312D**	0.4784	Amb
**P312E**	0.2919	LBen
**P312F**	0.6974	LPath
**P312G**	0.3044	LBen
**P312H**	0.2205	LBen
**P312I**	0.5972	LPath
**P312K**	0.3326	LBen
**P312L**	0.2248	LBen
**P312M**	0.5797	LPath
**P312N**	0.4221	Amb
**P312Q**	0.1631	LBen
**P312R**	0.1721	LBen
**P312S**	0.1435	LBen
**P312T**	0.1632	LBen
**P312V**	0.383	Amb
**P312W**	0.6981	LPath
**P312Y**	0.5635	Amb
**R313A**	0.2243	LBen
**R313C**	0.2095	LBen
**R313D**	0.4895	Amb

**R313E**	0.2949	LBen
**R313F**	0.4807	Amb
**R313G**	0.1497	LBen
**R313H**	0.1044	LBen
**R313I**	0.2604	LBen
**R313K**	0.1381	LBen
**R313L**	0.142	LBen
**R313M**	0.3298	LBen
**R313N**	0.4461	Amb
**R313P**	0.3179	LBen
**R313Q**	0.0926	LBen
**R313S**	0.2824	LBen
**R313T**	0.1954	LBen
**R313V**	0.2588	LBen
**R313W**	0.1991	LBen
**R313Y**	0.3529	Amb
**F314A**	0.9359	LPath
**F314C**	0.8993	LPath
**F314D**	0.9952	LPath
**F314E**	0.9947	LPath
**F314G**	0.9751	LPath
**F314H**	0.9473	LPath
**F314I**	0.8109	LPath
**F314K**	0.9958	LPath
**F314L**	0.967	LPath
**F314M**	0.8707	LPath
**F314N**	0.976	LPath
**F314P**	0.9989	LPath
**F314Q**	0.9791	LPath
**F314R**	0.9879	LPath
**F314S**	0.9386	LPath
**F314T**	0.9516	LPath
**F314V**	0.7615	LPath
**F314W**	0.8344	LPath
**F314Y**	0.5315	Amb
**L315A**	0.8569	LPath
**L315C**	0.8966	LPath
**L315D**	0.996	LPath
**L315E**	0.9678	LPath
**L315F**	0.1613	LBen
**L315G**	0.9516	LPath

**L315H**	0.9006	LPath
**L315I**	0.2164	LBen
**L315K**	0.9655	LPath
**L315M**	0.2506	LBen
**L315N**	0.9792	LPath
**L315P**	0.9693	LPath
**L315Q**	0.8169	LPath
**L315R**	0.8851	LPath
**L315S**	0.9288	LPath
**L315T**	0.8112	LPath
**L315V**	0.2747	LBen
**L315W**	0.5424	Amb
**L315Y**	0.8319	LPath
**Q316A**	0.2255	LBen
**Q316C**	0.6941	LPath
**Q316D**	0.2349	LBen
**Q316E**	0.0825	LBen
**Q316F**	0.7425	LPath
**Q316G**	0.1652	LBen
**Q316H**	0.1522	LBen
**Q316I**	0.6665	LPath
**Q316K**	0.0971	LBen
**Q316L**	0.2188	LBen
**Q316M**	0.5469	Amb
**Q316N**	0.2423	LBen
**Q316P**	0.326	LBen
**Q316R**	0.0755	LBen
**Q316S**	0.1992	LBen
**Q316T**	0.2711	LBen
**Q316V**	0.4841	Amb
**Q316W**	0.419	Amb
**Q316Y**	0.4448	Amb
**R317A**	0.6688	LPath
**R317C**	0.451	Amb
**R317D**	0.759	LPath
**R317E**	0.5449	Amb
**R317F**	0.8752	LPath
**R317G**	0.3947	Amb
**R317H**	0.1562	LBen

**R317I**	0.698	LPath
**R317K**	0.2788	LBen
**R317L**	0.5062	Amb
**R317M**	0.6878	LPath
**R317N**	0.6586	LPath
**R317P**	0.9449	LPath
**R317Q**	0.1384	LBen
**R317S**	0.6313	LPath
**R317T**	0.55	Amb
**R317V**	0.7373	LPath
**R317W**	0.4136	Amb
**R317Y**	0.6164	LPath
**L318A**	0.7613	LPath
**L318C**	0.8772	LPath
**L318D**	0.9962	LPath
**L318E**	0.9742	LPath
**L318F**	0.4197	Amb
**L318G**	0.9597	LPath
**L318H**	0.9353	LPath
**L318I**	0.1366	LBen
**L318K**	0.9732	LPath
**L318M**	0.2255	LBen
**L318N**	0.9722	LPath
**L318P**	0.9574	LPath
**L318Q**	0.8729	LPath
**L318R**	0.9335	LPath
**L318S**	0.9187	LPath
**L318T**	0.7564	LPath
**L318V**	0.1527	LBen
**L318W**	0.7979	LPath
**L318Y**	0.9011	LPath
**L319A**	0.16	LBen
**L319C**	0.4453	Amb
**L319D**	0.5439	Amb
**L319E**	0.2322	LBen
**L319F**	0.1275	LBen
**L319G**	0.2742	LBen
**L319H**	0.1532	LBen
**L319I**	0.1496	LBen
**L319K**	0.1604	LBen
**L319M**	0.1255	LBen

**L319N**	0.3049	LBen
**L319P**	0.2969	LBen
**L319Q**	0.0834	LBen
**L319R**	0.0899	LBen
**L319S**	0.1729	LBen
**L319T**	0.1625	LBen
**L319V**	0.1124	LBen
**L319W**	0.1827	LBen
**L319Y**	0.2748	LBen
**L320A**	0.0889	LBen
**L320C**	0.3936	Amb
**L320D**	0.231	LBen
**L320E**	0.1036	LBen
**L320F**	0.1077	LBen
**L320G**	0.1665	LBen
**L320H**	0.0887	LBen
**L320I**	0.1269	LBen
**L320K**	0.1075	LBen
**L320M**	0.1081	LBen
**L320N**	0.1535	LBen
**L320P**	0.3325	LBen
**L320Q**	0.051	LBen
**L320R**	0.0614	LBen
**L320S**	0.0941	LBen
**L320T**	0.099	LBen
**L320V**	0.0953	LBen
**L320W**	0.1553	LBen
**L320Y**	0.2155	LBen
**L321A**	0.395	Amb
**L321C**	0.8373	LPath
**L321D**	0.9243	LPath
**L321E**	0.6746	LPath
**L321F**	0.1636	LBen
**L321G**	0.7351	LPath
**L321H**	0.564	LPath
**L321I**	0.1426	LBen
**L321K**	0.7612	LPath
**L321M**	0.1199	LBen
**L321N**	0.7556	LPath

**L321P**	0.6759	LPath
**L321Q**	0.2912	LBen
**L321R**	0.6219	LPath
**L321S**	0.5534	Amb
**L321T**	0.3298	LBen
**L321V**	0.1652	LBen
**L321W**	0.3126	LBen
**L321Y**	0.6263	LPath
**D322A**	0.2043	LBen
**D322C**	0.8019	LPath
**D322E**	0.195	LBen
**D322F**	0.7895	LPath
**D322G**	0.1833	LBen
**D322H**	0.2593	LBen
**D322I**	0.6304	LPath
**D322K**	0.4069	Amb
**D322L**	0.5737	LPath
**D322M**	0.8224	LPath
**D322N**	0.1217	LBen
**D322P**	0.5785	LPath
**D322Q**	0.292	LBen
**D322R**	0.3325	LBen
**D322S**	0.1394	LBen
**D322T**	0.3474	Amb
**D322V**	0.4087	Amb
**D322W**	0.9085	LPath
**D322Y**	0.3768	Amb
**Y323A**	0.9292	LPath
**Y323C**	0.8052	LPath
**Y323D**	0.9147	LPath
**Y323E**	0.9776	LPath
**Y323F**	0.1603	LBen
**Y323G**	0.9155	LPath
**Y323H**	0.7924	LPath
**Y323I**	0.7589	LPath
**Y323K**	0.9839	LPath
**Y323L**	0.6264	LPath
**Y323M**	0.9028	LPath
**Y323N**	0.8175	LPath
**Y323P**	0.9675	LPath

**Y323Q**	0.9682	LPath
**Y323R**	0.9594	LPath
**Y323S**	0.7632	LPath
**Y323T**	0.8852	LPath
**Y323V**	0.687	LPath
**Y323W**	0.7027	LPath
**P324A**	0.095	LBen
**P324C**	0.6196	LPath
**P324D**	0.3251	LBen
**P324E**	0.246	LBen
**P324F**	0.6076	LPath
**P324G**	0.3907	Amb
**P324H**	0.1705	LBen
**P324I**	0.4763	Amb
**P324K**	0.344	Amb
**P324L**	0.1728	LBen
**P324M**	0.5003	Amb
**P324N**	0.3836	Amb
**P324Q**	0.1587	LBen
**P324R**	0.1621	LBen
**P324S**	0.1082	LBen
**P324T**	0.143	LBen
**P324V**	0.3099	LBen
**P324W**	0.6989	LPath
**P324Y**	0.5366	Amb
**P325A**	0.0601	LBen
**P325C**	0.4239	Amb
**P325D**	0.2924	LBen
**P325E**	0.1628	LBen
**P325F**	0.304	LBen
**P325G**	0.1694	LBen
**P325H**	0.1061	LBen
**P325I**	0.2364	LBen
**P325K**	0.121	LBen
**P325L**	0.0919	LBen
**P325M**	0.2432	LBen
**P325N**	0.2359	LBen
**P325Q**	0.0896	LBen
**P325R**	0.0679	LBen
**P325S**	0.0836	LBen
**P325T**	0.0832	LBen

**P325V**	0.1547	LBen
**P325W**	0.3157	LBen
**P325Y**	0.2553	LBen
**D326A**	0.1002	LBen
**D326C**	0.5151	Amb
**D326E**	0.0924	LBen
**D326F**	0.434	Amb
**D326G**	0.0885	LBen
**D326H**	0.1153	LBen
**D326I**	0.3168	LBen
**D326K**	0.1648	LBen
**D326L**	0.2378	LBen
**D326M**	0.4738	Amb
**D326N**	0.0818	LBen
**D326P**	0.4028	Amb
**D326Q**	0.1323	LBen
**D326R**	0.1533	LBen
**D326S**	0.0752	LBen
**D326T**	0.1617	LBen
**D326V**	0.1827	LBen
**D326W**	0.6851	LPath
**D326Y**	0.176	LBen
**R327A**	0.2286	LBen
**R327C**	0.1756	LBen
**R327D**	0.5412	Amb
**R327E**	0.2685	LBen
**R327F**	0.5117	Amb
**R327G**	0.1804	LBen
**R327H**	0.0913	LBen
**R327I**	0.3151	LBen
**R327K**	0.1192	LBen
**R327L**	0.179	LBen
**R327M**	0.2618	LBen
**R327N**	0.4529	Amb
**R327P**	0.7747	LPath
**R327Q**	0.0749	LBen
**R327S**	0.2903	LBen
**R327T**	0.1805	LBen
**R327V**	0.3105	LBen

**R327W**	0.2039	LBen
**R327Y**	0.3688	Amb
**V328A**	0.3535	Amb
**V328C**	0.84	LPath
**V328D**	0.8869	LPath
**V328E**	0.7778	LPath
**V328F**	0.2207	LBen
**V328G**	0.4888	Amb
**V328H**	0.8694	LPath
**V328I**	0.0697	LBen
**V328K**	0.7872	LPath
**V328L**	0.0932	LBen
**V328M**	0.1255	LBen
**V328N**	0.7398	LPath
**V328P**	0.9065	LPath
**V328Q**	0.7194	LPath
**V328R**	0.706	LPath
**V328S**	0.5548	Amb
**V328T**	0.3938	Amb
**V328W**	0.8573	LPath
**V328Y**	0.7356	LPath
**T329A**	0.0755	LBen
**T329C**	0.4101	Amb
**T329D**	0.2441	LBen
**T329E**	0.2325	LBen
**T329F**	0.1938	LBen
**T329G**	0.1808	LBen
**T329H**	0.1412	LBen
**T329I**	0.1554	LBen
**T329K**	0.1444	LBen
**T329L**	0.1032	LBen
**T329M**	0.1081	LBen
**T329N**	0.1022	LBen
**T329P**	0.2948	LBen
**T329Q**	0.1515	LBen
**T329R**	0.1111	LBen
**T329S**	0.0772	LBen
**T329V**	0.1423	LBen
**T329W**	0.4397	Amb
**T329Y**	0.2047	LBen
**L330A**	0.7444	LPath

**L330C**	0.83	LPath
**L330D**	0.9984	LPath
**L330E**	0.9921	LPath
**L330F**	0.323	LBen
**L330G**	0.9504	LPath
**L330H**	0.9708	LPath
**L330I**	0.1342	LBen
**L330K**	0.9894	LPath
**L330M**	0.26	LBen
**L330N**	0.9907	LPath
**L330P**	0.968	LPath
**L330Q**	0.9586	LPath
**L330R**	0.9569	LPath
**L330S**	0.9574	LPath
**L330T**	0.8797	LPath
**L330V**	0.1079	LBen
**L330W**	0.8556	LPath
**L330Y**	0.9054	LPath
**F331A**	0.9718	LPath
**F331C**	0.9215	LPath
**F331D**	0.9973	LPath
**F331E**	0.9965	LPath
**F331G**	0.9883	LPath
**F331H**	0.927	LPath
**F331I**	0.6988	LPath
**F331K**	0.9961	LPath
**F331L**	0.9296	LPath
**F331M**	0.8942	LPath
**F331N**	0.9891	LPath
**F331P**	0.9996	LPath
**F331Q**	0.9873	LPath
**F331R**	0.9828	LPath
**F331S**	0.9573	LPath
**F331T**	0.9747	LPath
**F331V**	0.7054	LPath
**F331W**	0.7898	LPath
**F331Y**	0.3651	Amb
**L332A**	0.8164	LPath
**L332C**	0.835	LPath

**L332D**	0.9978	LPath
**L332E**	0.9842	LPath
**L332F**	0.2394	LBen
**L332G**	0.9689	LPath
**L332H**	0.9422	LPath
**L332I**	0.0844	LBen
**L332K**	0.9857	LPath
**L332M**	0.2249	LBen
**L332N**	0.9868	LPath
**L332P**	0.9743	LPath
**L332Q**	0.924	LPath
**L332R**	0.9511	LPath
**L332S**	0.9432	LPath
**L332T**	0.8602	LPath
**L332V**	0.1052	LBen
**L332W**	0.7324	LPath
**L332Y**	0.8647	LPath
**H333A**	0.9675	LPath
**H333C**	0.7255	LPath
**H333D**	0.9831	LPath
**H333E**	0.9798	LPath
**H333F**	0.8699	LPath
**H333G**	0.9697	LPath
**H333I**	0.9815	LPath
**H333K**	0.9171	LPath
**H333L**	0.8513	LPath
**H333M**	0.9848	LPath
**H333N**	0.8724	LPath
**H333P**	0.9718	LPath
**H333Q**	0.8746	LPath
**H333R**	0.4802	Amb
**H333S**	0.9211	LPath
**H333T**	0.9782	LPath
**H333V**	0.9582	LPath
**H333W**	0.7612	LPath
**H333Y**	0.4311	Amb
**N334A**	0.8223	LPath
**N334C**	0.5038	Amb
**N334D**	0.7836	LPath
**N334E**	0.982	LPath

**N334F**	0.9903	LPath
**N334G**	0.8339	LPath
**N334H**	0.693	LPath
**N334I**	0.8706	LPath
**N334K**	0.9849	LPath
**N334L**	0.8308	LPath
**N334M**	0.9309	LPath
**N334P**	0.975	LPath
**N334Q**	0.9453	LPath
**N334R**	0.9706	LPath
**N334S**	0.1688	LBen
**N334T**	0.5799	LPath
**N334V**	0.7926	LPath
**N334W**	0.9942	LPath
**N334Y**	0.9037	LPath
**N335A**	0.1286	LBen
**N335C**	0.2771	LBen
**N335D**	0.1518	LBen
**N335E**	0.3087	LBen
**N335F**	0.543	Amb
**N335G**	0.1412	LBen
**N335H**	0.0765	LBen
**N335I**	0.3154	LBen
**N335K**	0.1927	LBen
**N335L**	0.2424	LBen
**N335M**	0.3032	LBen
**N335P**	0.684	LPath
**N335Q**	0.1726	LBen
**N335R**	0.165	LBen
**N335S**	0.0483	LBen
**N335T**	0.1034	LBen
**N335V**	0.2742	LBen
**N335W**	0.6394	LPath
**N335Y**	0.1848	LBen
**E336A**	0.3104	LBen
**E336C**	0.9509	LPath
**E336D**	0.7826	LPath
**E336F**	0.9711	LPath
**E336G**	0.7506	LPath
**E336H**	0.9	LPath
**E336I**	0.5666	LPath

**E336K**	0.6471	LPath
**E336L**	0.8194	LPath
**E336M**	0.7431	LPath
**E336N**	0.7822	LPath
**E336P**	0.9813	LPath
**E336Q**	0.3092	LBen
**E336R**	0.7033	LPath
**E336S**	0.5756	LPath
**E336T**	0.5487	Amb
**E336V**	0.2104	LBen
**E336W**	0.9943	LPath
**E336Y**	0.9575	LPath
**V337A**	0.3288	LBen
**V337C**	0.9516	LPath
**V337D**	0.4088	Amb
**V337E**	0.3363	LBen
**V337F**	0.4071	Amb
**V337G**	0.3127	LBen
**V337H**	0.7807	LPath
**V337I**	0.1405	LBen
**V337K**	0.55	Amb
**V337L**	0.4867	Amb
**V337M**	0.3942	Amb
**V337N**	0.4409	Amb
**V337P**	0.4042	Amb
**V337Q**	0.5117	Amb
**V337R**	0.4644	Amb
**V337S**	0.4043	Amb
**V337T**	0.444	Amb
**V337W**	0.9382	LPath
**V337Y**	0.715	LPath
**F338A**	0.5932	LPath
**F338C**	0.4298	Amb
**F338D**	0.7114	LPath
**F338E**	0.7895	LPath
**F338G**	0.7246	LPath
**F338H**	0.368	Amb
**F338I**	0.3377	LBen
**F338K**	0.8041	LPath

**F338L**	0.7658	LPath
**F338M**	0.6393	LPath
**F338N**	0.5546	Amb
**F338P**	0.8873	LPath
**F338Q**	0.6677	LPath
**F338R**	0.6372	LPath
**F338S**	0.4211	Amb
**F338T**	0.5975	LPath
**F338V**	0.3299	LBen
**F338W**	0.3684	Amb
**F338Y**	0.085	LBen
**H339A**	0.9785	LPath
**H339C**	0.8761	LPath
**H339D**	0.9748	LPath
**H339E**	0.9799	LPath
**H339F**	0.9623	LPath
**H339G**	0.9778	LPath
**H339I**	0.9887	LPath
**H339K**	0.9908	LPath
**H339L**	0.8171	LPath
**H339M**	0.9808	LPath
**H339N**	0.8514	LPath
**H339P**	0.9481	LPath
**H339Q**	0.9364	LPath
**H339R**	0.9528	LPath
**H339S**	0.9202	LPath
**H339T**	0.9826	LPath
**H339V**	0.9826	LPath
**H339W**	0.9266	LPath
**H339Y**	0.8602	LPath
**E340A**	0.5175	Amb
**E340C**	0.9896	LPath
**E340D**	0.7639	LPath
**E340F**	0.9734	LPath
**E340G**	0.5326	Amb
**E340H**	0.8913	LPath
**E340I**	0.852	LPath
**E340K**	0.4874	Amb
**E340L**	0.8021	LPath
**E340M**	0.8812	LPath
**E340N**	0.8838	LPath

**E340P**	0.989	LPath
**E340Q**	0.3208	LBen
**E340R**	0.6061	LPath
**E340S**	0.6854	LPath
**E340T**	0.7649	LPath
**E340V**	0.7201	LPath
**E340W**	0.9925	LPath
**E340Y**	0.9477	LPath
**P341A**	0.0683	LBen
**P341C**	0.5673	LPath
**P341D**	0.2596	LBen
**P341E**	0.1467	LBen
**P341F**	0.4614	Amb
**P341G**	0.2037	LBen
**P341H**	0.1309	LBen
**P341I**	0.2526	LBen
**P341K**	0.1329	LBen
**P341L**	0.1085	LBen
**P341M**	0.2471	LBen
**P341N**	0.2328	LBen
**P341Q**	0.0828	LBen
**P341R**	0.088	LBen
**P341S**	0.0989	LBen
**P341T**	0.0799	LBen
**P341V**	0.1632	LBen
**P341W**	0.4824	Amb
**P341Y**	0.3889	Amb
**H342A**	0.3425	Amb
**H342C**	0.5526	Amb
**H342D**	0.2026	LBen
**H342E**	0.3626	Amb
**H342F**	0.4673	Amb
**H342G**	0.4744	Amb
**H342I**	0.4424	Amb
**H342K**	0.522	Amb
**H342L**	0.1502	LBen
**H342M**	0.5581	Amb
**H342N**	0.1797	LBen
**H342P**	0.4666	Amb

**H342Q**	0.2336	LBen
**H342R**	0.2533	LBen
**H342S**	0.3	LBen
**H342T**	0.4449	Amb
**H342V**	0.3586	Amb
**H342W**	0.5907	LPath
**H342Y**	0.1796	LBen
**I343A**	0.6672	LPath
**I343C**	0.9157	LPath
**I343D**	0.9889	LPath
**I343E**	0.967	LPath
**I343F**	0.3755	Amb
**I343G**	0.9469	LPath
**I343H**	0.9545	LPath
**I343K**	0.9564	LPath
**I343L**	0.1413	LBen
**I343M**	0.1795	LBen
**I343N**	0.9198	LPath
**I343P**	0.9637	LPath
**I343Q**	0.9439	LPath
**I343R**	0.933	LPath
**I343S**	0.8293	LPath
**I343T**	0.5297	Amb
**I343V**	0.0748	LBen
**I343W**	0.9571	LPath
**I343Y**	0.9046	LPath
**A344C**	0.492	Amb
**A344D**	0.1246	LBen
**A344E**	0.1146	LBen
**A344F**	0.2524	LBen
**A344G**	0.1226	LBen
**A344H**	0.2323	LBen
**A344I**	0.1841	LBen
**A344K**	0.2101	LBen
**A344L**	0.1275	LBen
**A344M**	0.1641	LBen
**A344N**	0.1513	LBen
**A344P**	0.5231	Amb
**A344Q**	0.1615	LBen
**A344R**	0.1592	LBen

**A344S**	0.0788	LBen
**A344T**	0.0733	LBen
**A344V**	0.1028	LBen
**A344W**	0.4788	Amb
**A344Y**	0.3078	LBen
**D345A**	0.0644	LBen
**D345C**	0.4169	Amb
**D345E**	0.0811	LBen
**D345F**	0.3198	LBen
**D345G**	0.0799	LBen
**D345H**	0.1065	LBen
**D345I**	0.1817	LBen
**D345K**	0.1201	LBen
**D345L**	0.1588	LBen
**D345M**	0.3271	LBen
**D345N**	0.072	LBen
**D345P**	0.4227	Amb
**D345Q**	0.1035	LBen
**D345R**	0.1039	LBen
**D345S**	0.064	LBen
**D345T**	0.1063	LBen
**D345V**	0.1148	LBen
**D345W**	0.5921	LPath
**D345Y**	0.1416	LBen
**S346A**	0.059	LBen
**S346C**	0.0943	LBen
**S346D**	0.2364	LBen
**S346E**	0.2768	LBen
**S346F**	0.1086	LBen
**S346G**	0.0897	LBen
**S346H**	0.1476	LBen
**S346I**	0.1116	LBen
**S346K**	0.3902	Amb
**S346L**	0.0928	LBen
**S346M**	0.1776	LBen
**S346N**	0.1252	LBen
**S346P**	0.4941	Amb
**S346Q**	0.2427	LBen
**S346R**	0.2532	LBen
**S346T**	0.0796	LBen

**S346V**	0.1135	LBen
**S346W**	0.1632	LBen
**S346Y**	0.0964	LBen
**W347A**	0.6712	LPath
**W347C**	0.9098	LPath
**W347D**	0.9509	LPath
**W347E**	0.8903	LPath
**W347F**	0.2619	LBen
**W347G**	0.4728	Amb
**W347H**	0.8388	LPath
**W347I**	0.5543	Amb
**W347K**	0.896	LPath
**W347L**	0.3307	LBen
**W347M**	0.6618	LPath
**W347N**	0.9351	LPath
**W347P**	0.9799	LPath
**W347Q**	0.8536	LPath
**W347R**	0.8486	LPath
**W347S**	0.5977	LPath
**W347T**	0.7177	LPath
**W347V**	0.5474	Amb
**W347Y**	0.5348	Amb
**P348A**	0.0755	LBen
**P348C**	0.4239	Amb
**P348D**	0.1968	LBen
**P348E**	0.1203	LBen
**P348F**	0.2842	LBen
**P348G**	0.1583	LBen
**P348H**	0.0948	LBen
**P348I**	0.1916	LBen
**P348K**	0.1354	LBen
**P348L**	0.0848	LBen
**P348M**	0.2121	LBen
**P348N**	0.1731	LBen
**P348Q**	0.0847	LBen
**P348R**	0.0863	LBen
**P348S**	0.0811	LBen
**P348T**	0.0802	LBen
**P348V**	0.1394	LBen
**P348W**	0.3311	LBen
**P348Y**	0.2524	LBen

**Q349A**	0.136	LBen
**Q349C**	0.5764	LPath
**Q349D**	0.2189	LBen
**Q349E**	0.0808	LBen
**Q349F**	0.4154	Amb
**Q349G**	0.1596	LBen
**Q349H**	0.1233	LBen
**Q349I**	0.2422	LBen
**Q349K**	0.0822	LBen
**Q349L**	0.0867	LBen
**Q349M**	0.2843	LBen
**Q349N**	0.223	LBen
**Q349P**	0.1216	LBen
**Q349R**	0.0699	LBen
**Q349S**	0.1473	LBen
**Q349T**	0.1502	LBen
**Q349V**	0.1701	LBen
**Q349W**	0.2691	LBen
**Q349Y**	0.2808	LBen
**L350A**	0.7382	LPath
**L350C**	0.8981	LPath
**L350D**	0.9779	LPath
**L350E**	0.8758	LPath
**L350F**	0.2096	LBen
**L350G**	0.9062	LPath
**L350H**	0.7135	LPath
**L350I**	0.1455	LBen
**L350K**	0.8562	LPath
**L350M**	0.1803	LBen
**L350N**	0.9074	LPath
**L350P**	0.9098	LPath
**L350Q**	0.6539	LPath
**L350R**	0.719	LPath
**L350S**	0.8711	LPath
**L350T**	0.7685	LPath
**L350V**	0.1872	LBen
**L350W**	0.3823	Amb
**L350Y**	0.5935	LPath
**Q351A**	0.2037	LBen
**Q351C**	0.6278	LPath

**Q351D**	0.4268	Amb
**Q351E**	0.0847	LBen
**Q351F**	0.7349	LPath
**Q351G**	0.2024	LBen
**Q351H**	0.1814	LBen
**Q351I**	0.4542	Amb
**Q351K**	0.0772	LBen
**Q351L**	0.1504	LBen
**Q351M**	0.4181	Amb
**Q351N**	0.3676	Amb
**Q351P**	0.276	LBen
**Q351R**	0.0649	LBen
**Q351S**	0.2479	LBen
**Q351T**	0.2202	LBen
**Q351V**	0.299	LBen
**Q351W**	0.4178	Amb
**Q351Y**	0.4612	Amb
**D352A**	0.0883	LBen
**D352C**	0.4443	Amb
**D352E**	0.0972	LBen
**D352F**	0.3823	Amb
**D352G**	0.0702	LBen
**D352H**	0.1171	LBen
**D352I**	0.2494	LBen
**D352K**	0.1301	LBen
**D352L**	0.2093	LBen
**D352M**	0.387	Amb
**D352N**	0.0803	LBen
**D352P**	0.3009	LBen
**D352Q**	0.124	LBen
**D352R**	0.1228	LBen
**D352S**	0.0789	LBen
**D352T**	0.1377	LBen
**D352V**	0.1471	LBen
**D352W**	0.5797	LPath
**D352Y**	0.1464	LBen
**H353A**	0.1666	LBen
**H353C**	0.2037	LBen
**H353D**	0.1811	LBen

**H353E**	0.2398	LBen
**H353F**	0.2548	LBen
**H353G**	0.212	LBen
**H353I**	0.2314	LBen
**H353K**	0.1864	LBen
**H353L**	0.1188	LBen
**H353M**	0.395	Amb
**H353N**	0.1093	LBen
**H353P**	0.3215	LBen
**H353Q**	0.139	LBen
**H353R**	0.0739	LBen
**H353S**	0.1344	LBen
**H353T**	0.1591	LBen
**H353V**	0.2031	LBen
**H353W**	0.3104	LBen
**H353Y**	0.0898	LBen
**F354A**	0.9546	LPath
**F354C**	0.8883	LPath
**F354D**	0.9933	LPath
**F354E**	0.9955	LPath
**F354G**	0.9726	LPath
**F354H**	0.9337	LPath
**F354I**	0.6454	LPath
**F354K**	0.9937	LPath
**F354L**	0.9343	LPath
**F354M**	0.9112	LPath
**F354N**	0.9829	LPath
**F354P**	0.9948	LPath
**F354Q**	0.9883	LPath
**F354R**	0.9758	LPath
**F354S**	0.9459	LPath
**F354T**	0.9629	LPath
**F354V**	0.6166	LPath
**F354W**	0.8156	LPath
**F354Y**	0.3977	Amb
**S355A**	0.0659	LBen
**S355C**	0.1842	LBen
**S355D**	0.2016	LBen
**S355E**	0.2433	LBen
**S355F**	0.1544	LBen

**S355G**	0.0817	LBen
**S355H**	0.1634	LBen
**S355I**	0.1506	LBen
**S355K**	0.2888	LBen
**S355L**	0.0914	LBen
**S355M**	0.1953	LBen
**S355N**	0.1223	LBen
**S355P**	0.087	LBen
**S355Q**	0.2279	LBen
**S355R**	0.1998	LBen
**S355T**	0.0828	LBen
**S355V**	0.1452	LBen
**S355W**	0.217	LBen
**S355Y**	0.1455	LBen
**A356C**	0.4543	Amb
**A356D**	0.1113	LBen
**A356E**	0.1282	LBen
**A356F**	0.2325	LBen
**A356G**	0.1077	LBen
**A356H**	0.1974	LBen
**A356I**	0.2048	LBen
**A356K**	0.1777	LBen
**A356L**	0.1308	LBen
**A356M**	0.1597	LBen
**A356N**	0.1341	LBen
**A356P**	0.3248	LBen
**A356Q**	0.163	LBen
**A356R**	0.1491	LBen
**A356S**	0.0677	LBen
**A356T**	0.0659	LBen
**A356V**	0.1077	LBen
**A356W**	0.4593	Amb
**A356Y**	0.284	LBen
**V357A**	0.1374	LBen
**V357C**	0.7588	LPath
**V357D**	0.6149	LPath
**V357E**	0.5027	Amb
**V357F**	0.2247	LBen
**V357G**	0.198	LBen
**V357H**	0.7112	LPath

**V357I**	0.0846	LBen
**V357K**	0.499	Amb
**V357L**	0.1933	LBen
**V357M**	0.1622	LBen
**V357N**	0.45	Amb
**V357P**	0.6252	LPath
**V357Q**	0.4598	Amb
**V357R**	0.414	Amb
**V357S**	0.2317	LBen
**V357T**	0.2067	LBen
**V357W**	0.8085	LPath
**V357Y**	0.6493	LPath
**K358A**	0.6065	LPath
**K358C**	0.8341	LPath
**K358D**	0.7833	LPath
**K358E**	0.3307	LBen
**K358F**	0.8607	LPath
**K358G**	0.7276	LPath
**K358H**	0.2714	LBen
**K358I**	0.5971	LPath
**K358L**	0.4747	Amb
**K358M**	0.3815	Amb
**K358N**	0.6449	LPath
**K358P**	0.9436	LPath
**K358Q**	0.1542	LBen
**K358R**	0.0913	LBen
**K358S**	0.5888	LPath
**K358T**	0.2533	LBen
**K358V**	0.5355	Amb
**K358W**	0.798	LPath
**K358Y**	0.7169	LPath
**L359A**	0.3526	Amb
**L359C**	0.7069	LPath
**L359D**	0.8346	LPath
**L359E**	0.4623	Amb
**L359F**	0.1897	LBen
**L359G**	0.7233	LPath
**L359H**	0.3556	Amb
**L359I**	0.1055	LBen
**L359K**	0.3896	Amb
**L359M**	0.1329	LBen

**L359N**	0.6352	LPath
**L359P**	0.7581	LPath
**L359Q**	0.2315	LBen
**L359R**	0.2288	LBen
**L359S**	0.5292	Amb
**L359T**	0.3073	LBen
**L359V**	0.0977	LBen
**L359W**	0.2447	LBen
**L359Y**	0.4245	Amb
**V360A**	0.3279	LBen
**V360C**	0.9401	LPath
**V360D**	0.8936	LPath
**V360E**	0.7552	LPath
**V360F**	0.402	Amb
**V360G**	0.628	LPath
**V360H**	0.86	LPath
**V360I**	0.1032	LBen
**V360K**	0.7936	LPath
**V360L**	0.3289	LBen
**V360M**	0.3743	Amb
**V360N**	0.8675	LPath
**V360P**	0.9326	LPath
**V360Q**	0.7139	LPath
**V360R**	0.6301	LPath
**V360S**	0.5878	LPath
**V360T**	0.3585	Amb
**V360W**	0.9127	LPath
**V360Y**	0.8456	LPath
**G361A**	0.5545	Amb
**G361C**	0.8313	LPath
**G361D**	0.8932	LPath
**G361E**	0.8826	LPath
**G361F**	0.9176	LPath
**G361H**	0.9296	LPath
**G361I**	0.8785	LPath
**G361K**	0.9413	LPath
**G361L**	0.8401	LPath
**G361M**	0.9131	LPath
**G361N**	0.9072	LPath
**G361P**	0.9582	LPath

**G361Q**	0.9006	LPath
**G361R**	0.8743	LPath
**G361S**	0.445	Amb
**G361T**	0.7295	LPath
**G361V**	0.8042	LPath
**G361W**	0.8689	LPath
**G361Y**	0.9102	LPath
**P362A**	0.393	Amb
**P362C**	0.9671	LPath
**P362D**	0.9692	LPath
**P362E**	0.8893	LPath
**P362F**	0.9661	LPath
**P362G**	0.8597	LPath
**P362H**	0.812	LPath
**P362I**	0.9158	LPath
**P362K**	0.9055	LPath
**P362L**	0.6529	LPath
**P362M**	0.8964	LPath
**P362N**	0.9442	LPath
**P362Q**	0.7941	LPath
**P362R**	0.7382	LPath
**P362S**	0.6481	LPath
**P362T**	0.6358	LPath
**P362V**	0.8054	LPath
**P362W**	0.9693	LPath
**P362Y**	0.9637	LPath
**E363A**	0.6642	LPath
**E363C**	0.9946	LPath
**E363D**	0.7429	LPath
**E363F**	0.9935	LPath
**E363G**	0.6179	LPath
**E363H**	0.9469	LPath
**E363I**	0.9613	LPath
**E363K**	0.622	LPath
**E363L**	0.9384	LPath
**E363M**	0.9544	LPath
**E363N**	0.9316	LPath
**E363P**	0.9656	LPath
**E363Q**	0.5423	Amb

**E363R**	0.6932	LPath
**E363S**	0.789	LPath
**E363T**	0.8609	LPath
**E363V**	0.8694	LPath
**E363W**	0.9944	LPath
**E363Y**	0.984	LPath
**E364A**	0.5524	Amb
**E364C**	0.9931	LPath
**E364D**	0.6013	LPath
**E364F**	0.9836	LPath
**E364G**	0.6073	LPath
**E364H**	0.9505	LPath
**E364I**	0.9082	LPath
**E364K**	0.6995	LPath
**E364L**	0.902	LPath
**E364M**	0.9057	LPath
**E364N**	0.8603	LPath
**E364P**	0.9798	LPath
**E364Q**	0.6057	LPath
**E364R**	0.8076	LPath
**E364S**	0.795	LPath
**E364T**	0.8039	LPath
**E364V**	0.7145	LPath
**E364W**	0.9951	LPath
**E364Y**	0.9634	LPath
**A365C**	0.62	LPath
**A365D**	0.0959	LBen
**A365E**	0.114	LBen
**A365F**	0.2354	LBen
**A365G**	0.091	LBen
**A365H**	0.2928	LBen
**A365I**	0.2393	LBen
**A365K**	0.2659	LBen
**A365L**	0.1515	LBen
**A365M**	0.1911	LBen
**A365N**	0.1112	LBen
**A365P**	0.2759	LBen
**A365Q**	0.1944	LBen
**A365R**	0.2322	LBen
**A365S**	0.0717	LBen

**A365T**	0.0837	LBen
**A365V**	0.1195	LBen
**A365W**	0.6147	LPath
**A365Y**	0.3224	LBen
**L366A**	0.5537	Amb
**L366C**	0.8434	LPath
**L366D**	0.9268	LPath
**L366E**	0.7231	LPath
**L366F**	0.2718	LBen
**L366G**	0.8038	LPath
**L366H**	0.5875	LPath
**L366I**	0.1646	LBen
**L366K**	0.585	LPath
**L366M**	0.2004	LBen
**L366N**	0.8061	LPath
**L366P**	0.7891	LPath
**L366Q**	0.391	Amb
**L366R**	0.3707	Amb
**L366S**	0.6711	LPath
**L366T**	0.5602	Amb
**L366V**	0.1505	LBen
**L366W**	0.5366	Amb
**L366Y**	0.6557	LPath
**S367A**	0.1179	LBen
**S367C**	0.3537	Amb
**S367D**	0.3183	LBen
**S367E**	0.4445	Amb
**S367F**	0.314	LBen
**S367G**	0.0885	LBen
**S367H**	0.3219	LBen
**S367I**	0.3132	LBen
**S367K**	0.5053	Amb
**S367L**	0.1048	LBen
**S367M**	0.2191	LBen
**S367N**	0.1241	LBen
**S367P**	0.1602	LBen
**S367Q**	0.4269	Amb
**S367R**	0.4698	Amb
**S367T**	0.0737	LBen
**S367V**	0.2964	LBen

**S367W**	0.4237	Amb
**S367Y**	0.2701	LBen
**P368A**	0.0539	LBen
**P368C**	0.5296	Amb
**P368D**	0.3485	Amb
**P368E**	0.1802	LBen
**P368F**	0.3606	Amb
**P368G**	0.1686	LBen
**P368H**	0.1517	LBen
**P368I**	0.2368	LBen
**P368K**	0.2705	LBen
**P368L**	0.0996	LBen
**P368M**	0.2245	LBen
**P368N**	0.2335	LBen
**P368Q**	0.1094	LBen
**P368R**	0.1617	LBen
**P368S**	0.0733	LBen
**P368T**	0.0731	LBen
**P368V**	0.1477	LBen
**P368W**	0.4807	Amb
**P368Y**	0.3762	Amb
**G369A**	0.1145	LBen
**G369C**	0.6044	LPath
**G369D**	0.6484	LPath
**G369E**	0.4725	Amb
**G369F**	0.8126	LPath
**G369H**	0.6811	LPath
**G369I**	0.5914	LPath
**G369K**	0.7122	LPath
**G369L**	0.6892	LPath
**G369M**	0.7122	LPath
**G369N**	0.6455	LPath
**G369P**	0.7697	LPath
**G369Q**	0.5214	Amb
**G369R**	0.4903	Amb
**G369S**	0.1282	LBen
**G369T**	0.2695	LBen
**G369V**	0.3697	Amb
**G369W**	0.6046	LPath
**G369Y**	0.7306	LPath
**E370A**	0.2056	LBen

**E370C**	0.9544	LPath
**E370D**	0.2338	LBen
**E370F**	0.8722	LPath
**E370G**	0.3041	LBen
**E370H**	0.5976	LPath
**E370I**	0.5786	LPath
**E370K**	0.1743	LBen
**E370L**	0.5611	Amb
**E370M**	0.5701	LPath
**E370N**	0.4543	Amb
**E370P**	0.8795	LPath
**E370Q**	0.1458	LBen
**E370R**	0.2777	LBen
**E370S**	0.3414	Amb
**E370T**	0.39	Amb
**E370V**	0.355	Amb
**E370W**	0.9491	LPath
**E370Y**	0.7904	LPath
**A371C**	0.9566	LPath
**A371D**	0.9767	LPath
**A371E**	0.9713	LPath
**A371F**	0.9746	LPath
**A371G**	0.1783	LBen
**A371H**	0.9891	LPath
**A371I**	0.955	LPath
**A371K**	0.9928	LPath
**A371L**	0.8721	LPath
**A371M**	0.9065	LPath
**A371N**	0.9659	LPath
**A371P**	0.9652	LPath
**A371Q**	0.9651	LPath
**A371R**	0.9775	LPath
**A371S**	0.2616	LBen
**A371T**	0.7356	LPath
**A371V**	0.7978	LPath
**A371W**	0.9964	LPath
**A371Y**	0.9861	LPath
**R372A**	0.9859	LPath
**R372C**	0.9042	LPath
**R372D**	0.9975	LPath

**R372E**	0.9642	LPath
**R372F**	0.99	LPath
**R372G**	0.9698	LPath
**R372H**	0.6378	LPath
**R372I**	0.9874	LPath
**R372K**	0.6176	LPath
**R372L**	0.95	LPath
**R372M**	0.9823	LPath
**R372N**	0.996	LPath
**R372P**	0.9963	LPath
**R372Q**	0.6389	LPath
**R372S**	0.9909	LPath
**R372T**	0.9893	LPath
**R372V**	0.9841	LPath
**R372W**	0.7834	LPath
**R372Y**	0.9591	LPath
**D373A**	0.7426	LPath
**D373C**	0.9759	LPath
**D373E**	0.7507	LPath
**D373F**	0.9816	LPath
**D373G**	0.5707	LPath
**D373H**	0.8639	LPath
**D373I**	0.9543	LPath
**D373K**	0.9504	LPath
**D373L**	0.9362	LPath
**D373M**	0.9708	LPath
**D373N**	0.1739	LBen
**D373P**	0.9942	LPath
**D373Q**	0.9055	LPath
**D373R**	0.9437	LPath
**D373S**	0.4451	Amb
**D373T**	0.7314	LPath
**D373V**	0.8556	LPath
**D373W**	0.9954	LPath
**D373Y**	0.8647	LPath
**M374A**	0.6013	LPath
**M374C**	0.9387	LPath
**M374D**	0.9092	LPath
**M374E**	0.6317	LPath

**M374F**	0.4875	Amb
**M374G**	0.8149	LPath
**M374H**	0.7017	LPath
**M374I**	0.6576	LPath
**M374K**	0.3281	LBen
**M374L**	0.1835	LBen
**M374N**	0.7904	LPath
**M374P**	0.9916	LPath
**M374Q**	0.4113	Amb
**M374R**	0.3245	LBen
**M374S**	0.6589	LPath
**M374T**	0.3989	Amb
**M374V**	0.2015	LBen
**M374W**	0.7599	LPath
**M374Y**	0.7473	LPath
**A375C**	0.9467	LPath
**A375D**	0.9806	LPath
**A375E**	0.9799	LPath
**A375F**	0.9762	LPath
**A375G**	0.1088	LBen
**A375H**	0.9916	LPath
**A375I**	0.9449	LPath
**A375K**	0.9968	LPath
**A375L**	0.8549	LPath
**A375M**	0.9099	LPath
**A375N**	0.9633	LPath
**A375P**	0.973	LPath
**A375Q**	0.9747	LPath
**A375R**	0.9904	LPath
**A375S**	0.2328	LBen
**A375T**	0.6347	LPath
**A375V**	0.7338	LPath
**A375W**	0.9979	LPath
**A375Y**	0.992	LPath
**M376A**	0.4936	Amb
**M376C**	0.9427	LPath
**M376D**	0.9901	LPath
**M376E**	0.893	LPath
**M376F**	0.5884	LPath
**M376G**	0.8392	LPath

**M376H**	0.9508	LPath
**M376I**	0.5576	Amb
**M376K**	0.7609	LPath
**M376L**	0.1604	LBen
**M376N**	0.9378	LPath
**M376P**	0.9909	LPath
**M376Q**	0.7103	LPath
**M376R**	0.7273	LPath
**M376S**	0.7298	LPath
**M376T**	0.3834	Amb
**M376V**	0.1047	LBen
**M376W**	0.9204	LPath
**M376Y**	0.9416	LPath
**D377A**	0.3357	LBen
**D377C**	0.925	LPath
**D377E**	0.2957	LBen
**D377F**	0.7722	LPath
**D377G**	0.3139	LBen
**D377H**	0.4259	Amb
**D377I**	0.7698	LPath
**D377K**	0.7319	LPath
**D377L**	0.6674	LPath
**D377M**	0.8397	LPath
**D377N**	0.1701	LBen
**D377P**	0.9685	LPath
**D377Q**	0.538	Amb
**D377R**	0.6816	LPath
**D377S**	0.2577	LBen
**D377T**	0.5008	Amb
**D377V**	0.5193	Amb
**D377W**	0.9382	LPath
**D377Y**	0.3716	Amb
**L378A**	0.0784	LBen
**L378C**	0.3372	LBen
**L378D**	0.2318	LBen
**L378E**	0.1502	LBen
**L378F**	0.0986	LBen
**L378G**	0.1825	LBen
**L378H**	0.1076	LBen
**L378I**	0.0742	LBen

**L378K**	0.1237	LBen
**L378M**	0.0857	LBen
**L378N**	0.1261	LBen
**L378P**	0.7035	LPath
**L378Q**	0.0845	LBen
**L378R**	0.0794	LBen
**L378S**	0.0895	LBen
**L378T**	0.0741	LBen
**L378V**	0.0709	LBen
**L378W**	0.1548	LBen
**L378Y**	0.1853	LBen
**C379A**	0.9752	LPath
**C379D**	0.9982	LPath
**C379E**	0.9986	LPath
**C379F**	0.9559	LPath
**C379G**	0.9243	LPath
**C379H**	0.9945	LPath
**C379I**	0.9876	LPath
**C379K**	0.9991	LPath
**C379L**	0.9446	LPath
**C379M**	0.9908	LPath
**C379N**	0.9966	LPath
**C379P**	0.9948	LPath
**C379Q**	0.9969	LPath
**C379R**	0.9888	LPath
**C379S**	0.9818	LPath
**C379T**	0.9917	LPath
**C379V**	0.9744	LPath
**C379W**	0.9907	LPath
**C379Y**	0.9871	LPath
**R380A**	0.8733	LPath
**R380C**	0.508	Amb
**R380D**	0.9846	LPath
**R380E**	0.8396	LPath
**R380F**	0.9104	LPath
**R380G**	0.7423	LPath
**R380H**	0.3242	LBen
**R380I**	0.7544	LPath
**R380K**	0.4161	Amb
**R380L**	0.5732	LPath
**R380M**	0.8403	LPath

**R380N**	0.9643	LPath
**R380P**	0.9823	LPath
**R380Q**	0.1978	LBen
**R380S**	0.8847	LPath
**R380T**	0.859	LPath
**R380V**	0.8241	LPath
**R380W**	0.4707	Amb
**R380Y**	0.8236	LPath
**Q381A**	0.2528	LBen
**Q381C**	0.8038	LPath
**Q381D**	0.3944	Amb
**Q381E**	0.0949	LBen
**Q381F**	0.8051	LPath
**Q381G**	0.2444	LBen
**Q381H**	0.2641	LBen
**Q381I**	0.5346	Amb
**Q381K**	0.0863	LBen
**Q381L**	0.1748	LBen
**Q381M**	0.4575	Amb
**Q381N**	0.3567	Amb
**Q381P**	0.5945	LPath
**Q381R**	0.0842	LBen
**Q381S**	0.2946	LBen
**Q381T**	0.2535	LBen
**Q381V**	0.3569	Amb
**Q381W**	0.5866	LPath
**Q381Y**	0.5945	LPath
**D382A**	0.2549	LBen
**D382C**	0.8353	LPath
**D382E**	0.2752	LBen
**D382F**	0.6335	LPath
**D382G**	0.3844	Amb
**D382H**	0.264	LBen
**D382I**	0.4335	Amb
**D382K**	0.4633	Amb
**D382L**	0.4274	Amb
**D382M**	0.6453	LPath
**D382N**	0.1072	LBen
**D382P**	0.885	LPath
**D382Q**	0.3758	Amb

**D382R**	0.4625	Amb
**D382S**	0.1759	LBen
**D382T**	0.3797	Amb
**D382V**	0.2648	LBen
**D382W**	0.8758	LPath
**D382Y**	0.2244	LBen
**P383A**	0.0699	LBen
**P383C**	0.5386	Amb
**P383D**	0.2188	LBen
**P383E**	0.1341	LBen
**P383F**	0.359	Amb
**P383G**	0.1875	LBen
**P383H**	0.1365	LBen
**P383I**	0.2209	LBen
**P383K**	0.1308	LBen
**P383L**	0.0935	LBen
**P383M**	0.219	LBen
**P383N**	0.2264	LBen
**P383Q**	0.1016	LBen
**P383R**	0.0863	LBen
**P383S**	0.0909	LBen
**P383T**	0.0801	LBen
**P383V**	0.1497	LBen
**P383W**	0.4065	Amb
**P383Y**	0.3704	Amb
**E384A**	0.0658	LBen
**E384C**	0.6406	LPath
**E384D**	0.0773	LBen
**E384F**	0.4074	Amb
**E384G**	0.0728	LBen
**E384H**	0.1614	LBen
**E384I**	0.219	LBen
**E384K**	0.0732	LBen
**E384L**	0.1578	LBen
**E384M**	0.2497	LBen
**E384N**	0.1101	LBen
**E384P**	0.4358	Amb
**E384Q**	0.072	LBen
**E384R**	0.0943	LBen

**E384S**	0.0773	LBen
**E384T**	0.0948	LBen
**E384V**	0.1327	LBen
**E384W**	0.559	Amb
**E384Y**	0.3093	LBen
**C385A**	0.8928	LPath
**C385D**	0.974	LPath
**C385E**	0.9872	LPath
**C385F**	0.9332	LPath
**C385G**	0.7009	LPath
**C385H**	0.9775	LPath
**C385I**	0.9486	LPath
**C385K**	0.9935	LPath
**C385L**	0.9173	LPath
**C385M**	0.9539	LPath
**C385N**	0.9444	LPath
**C385P**	0.9881	LPath
**C385Q**	0.9824	LPath
**C385R**	0.9552	LPath
**C385S**	0.8885	LPath
**C385T**	0.9315	LPath
**C385V**	0.888	LPath
**C385W**	0.9647	LPath
**C385Y**	0.9514	LPath
**E386A**	0.1512	LBen
**E386C**	0.8855	LPath
**E386D**	0.0796	LBen
**E386F**	0.8451	LPath
**E386G**	0.1578	LBen
**E386H**	0.4003	Amb
**E386I**	0.596	LPath
**E386K**	0.211	LBen
**E386L**	0.537	Amb
**E386M**	0.6285	LPath
**E386N**	0.2176	LBen
**E386P**	0.4932	Amb
**E386Q**	0.1413	LBen
**E386R**	0.2701	LBen
**E386S**	0.1544	LBen
**E386T**	0.2437	LBen

**E386V**	0.366	Amb
**E386W**	0.9194	LPath
**E386Y**	0.7033	LPath
**F387A**	0.8625	LPath
**F387C**	0.7735	LPath
**F387D**	0.9604	LPath
**F387E**	0.9733	LPath
**F387G**	0.925	LPath
**F387H**	0.8288	LPath
**F387I**	0.5216	Amb
**F387K**	0.9853	LPath
**F387L**	0.9487	LPath
**F387M**	0.8186	LPath
**F387N**	0.8872	LPath
**F387P**	0.9985	LPath
**F387Q**	0.9648	LPath
**F387R**	0.954	LPath
**F387S**	0.7784	LPath
**F387T**	0.8814	LPath
**F387V**	0.5032	Amb
**F387W**	0.5618	Amb
**F387Y**	0.1225	LBen
**Y388A**	0.8708	LPath
**Y388C**	0.4635	Amb
**Y388D**	0.9792	LPath
**Y388E**	0.9925	LPath
**Y388F**	0.0889	LBen
**Y388G**	0.9047	LPath
**Y388H**	0.7839	LPath
**Y388I**	0.6935	LPath
**Y388K**	0.9944	LPath
**Y388L**	0.3898	Amb
**Y388M**	0.8245	LPath
**Y388N**	0.8933	LPath
**Y388P**	0.9911	LPath
**Y388Q**	0.9708	LPath
**Y388R**	0.9729	LPath
**Y388S**	0.7333	LPath
**Y388T**	0.8958	LPath
**Y388V**	0.5626	Amb

**Y388W**	0.727	LPath
**F389A**	0.9211	LPath
**F389C**	0.7081	LPath
**F389D**	0.9989	LPath
**F389E**	0.9989	LPath
**F389G**	0.9708	LPath
**F389H**	0.9865	LPath
**F389I**	0.45	Amb
**F389K**	0.9992	LPath
**F389L**	0.8079	LPath
**F389M**	0.7663	LPath
**F389N**	0.9959	LPath
**F389P**	0.9995	LPath
**F389Q**	0.9959	LPath
**F389R**	0.9958	LPath
**F389S**	0.9617	LPath
**F389T**	0.9695	LPath
**F389V**	0.3977	Amb
**F389W**	0.8969	LPath
**F389Y**	0.6443	LPath
**S390A**	0.3325	LBen
**S390C**	0.3666	Amb
**S390D**	0.9766	LPath
**S390E**	0.9892	LPath
**S390F**	0.8462	LPath
**S390G**	0.443	Amb
**S390H**	0.8692	LPath
**S390I**	0.7684	LPath
**S390K**	0.9987	LPath
**S390L**	0.6013	LPath
**S390M**	0.7607	LPath
**S390N**	0.536	Amb
**S390P**	0.9829	LPath
**S390Q**	0.959	LPath
**S390R**	0.9951	LPath
**S390T**	0.3755	Amb
**S390V**	0.676	LPath
**S390W**	0.9322	LPath
**S390Y**	0.8511	LPath
**L391A**	0.6354	LPath
**L391C**	0.8627	LPath

**L391D**	0.9973	LPath
**L391E**	0.9706	LPath
**L391F**	0.4153	Amb
**L391G**	0.955	LPath
**L391H**	0.9582	LPath
**L391I**	0.0972	LBen
**L391K**	0.9775	LPath
**L391M**	0.1447	LBen
**L391N**	0.9825	LPath
**L391P**	0.9889	LPath
**L391Q**	0.8826	LPath
**L391R**	0.9462	LPath
**L391S**	0.9025	LPath
**L391T**	0.6482	LPath
**L391V**	0.0775	LBen
**L391W**	0.8495	LPath
**L391Y**	0.9318	LPath
**D392A**	0.9504	LPath
**D392C**	0.9957	LPath
**D392E**	0.8799	LPath
**D392F**	0.9948	LPath
**D392G**	0.9322	LPath
**D392H**	0.9728	LPath
**D392I**	0.9952	LPath
**D392K**	0.993	LPath
**D392L**	0.9843	LPath
**D392M**	0.9973	LPath
**D392N**	0.8196	LPath
**D392P**	0.9959	LPath
**D392Q**	0.9806	LPath
**D392R**	0.9859	LPath
**D392S**	0.8726	LPath
**D392T**	0.9758	LPath
**D392V**	0.9795	LPath
**D392W**	0.9984	LPath
**D392Y**	0.9635	LPath
**A393C**	0.7808	LPath
**A393D**	0.9528	LPath
**A393E**	0.9425	LPath
**A393F**	0.9436	LPath

**A393G**	0.2111	LBen
**A393H**	0.9732	LPath
**A393I**	0.6755	LPath
**A393K**	0.9886	LPath
**A393L**	0.7043	LPath
**A393M**	0.7007	LPath
**A393N**	0.8821	LPath
**A393P**	0.8346	LPath
**A393Q**	0.9328	LPath
**A393R**	0.9709	LPath
**A393S**	0.1255	LBen
**A393T**	0.2024	LBen
**A393V**	0.3496	Amb
**A393W**	0.9917	LPath
**A393Y**	0.9799	LPath
**D394A**	0.5554	Amb
**D394C**	0.9626	LPath
**D394E**	0.4015	Amb
**D394F**	0.9455	LPath
**D394G**	0.5828	LPath
**D394H**	0.8302	LPath
**D394I**	0.8829	LPath
**D394K**	0.9045	LPath
**D394L**	0.8345	LPath
**D394M**	0.9366	LPath
**D394N**	0.3224	LBen
**D394P**	0.94	LPath
**D394Q**	0.7869	LPath
**D394R**	0.8937	LPath
**D394S**	0.3687	Amb
**D394T**	0.6545	LPath
**D394V**	0.6839	LPath
**D394W**	0.9906	LPath
**D394Y**	0.7996	LPath
**A395C**	0.5914	LPath
**A395D**	0.9526	LPath
**A395E**	0.9396	LPath
**A395F**	0.911	LPath
**A395G**	0.3172	LBen

**A395H**	0.9611	LPath
**A395I**	0.3933	Amb
**A395K**	0.9823	LPath
**A395L**	0.5254	Amb
**A395M**	0.5008	Amb
**A395N**	0.8047	LPath
**A395P**	0.9595	LPath
**A395Q**	0.922	LPath
**A395R**	0.9569	LPath
**A395S**	0.1446	LBen
**A395T**	0.0957	LBen
**A395V**	0.1309	LBen
**A395W**	0.9903	LPath
**A395Y**	0.9674	LPath
**V396A**	0.227	LBen
**V396C**	0.8763	LPath
**V396D**	0.7912	LPath
**V396E**	0.5949	LPath
**V396F**	0.3526	Amb
**V396G**	0.4353	Amb
**V396H**	0.831	LPath
**V396I**	0.0862	LBen
**V396K**	0.8207	LPath
**V396L**	0.2402	LBen
**V396M**	0.1968	LBen
**V396N**	0.531	Amb
**V396P**	0.9108	LPath
**V396Q**	0.5505	Amb
**V396R**	0.7226	LPath
**V396S**	0.3155	LBen
**V396T**	0.3236	LBen
**V396W**	0.9533	LPath
**V396Y**	0.8177	LPath
**L397A**	0.7498	LPath
**L397C**	0.8543	LPath
**L397D**	0.9905	LPath
**L397E**	0.9617	LPath
**L397F**	0.2331	LBen
**L397G**	0.945	LPath
**L397H**	0.8999	LPath

**L397I**	0.0832	LBen
**L397K**	0.9598	LPath
**L397M**	0.1884	LBen
**L397N**	0.9495	LPath
**L397P**	0.6245	LPath
**L397Q**	0.8802	LPath
**L397R**	0.9246	LPath
**L397S**	0.916	LPath
**L397T**	0.7974	LPath
**L397V**	0.1139	LBen
**L397W**	0.736	LPath
**L397Y**	0.8321	LPath
**T398A**	0.1263	LBen
**T398C**	0.5744	LPath
**T398D**	0.3493	Amb
**T398E**	0.2843	LBen
**T398F**	0.4171	Amb
**T398G**	0.2323	LBen
**T398H**	0.2904	LBen
**T398I**	0.3006	LBen
**T398K**	0.2394	LBen
**T398L**	0.1521	LBen
**T398M**	0.1526	LBen
**T398N**	0.1519	LBen
**T398P**	0.205	LBen
**T398Q**	0.2245	LBen
**T398R**	0.1919	LBen
**T398S**	0.1251	LBen
**T398V**	0.2182	LBen
**T398W**	0.6885	LPath
**T398Y**	0.4419	Amb
**N399A**	0.6047	LPath
**N399C**	0.6826	LPath
**N399D**	0.18	LBen
**N399E**	0.4702	Amb
**N399F**	0.9187	LPath
**N399G**	0.6963	LPath
**N399H**	0.1786	LBen
**N399I**	0.7715	LPath
**N399K**	0.676	LPath

**N399L**	0.7389	LPath
**N399M**	0.8322	LPath
**N399P**	0.8802	LPath
**N399Q**	0.5545	Amb
**N399R**	0.5808	LPath
**N399S**	0.1395	LBen
**N399T**	0.4731	Amb
**N399V**	0.6947	LPath
**N399W**	0.9394	LPath
**N399Y**	0.5107	Amb
**L400A**	0.0634	LBen
**L400C**	0.3104	LBen
**L400D**	0.1955	LBen
**L400E**	0.0811	LBen
**L400F**	0.1184	LBen
**L400G**	0.1289	LBen
**L400H**	0.0733	LBen
**L400I**	0.0884	LBen
**L400K**	0.0732	LBen
**L400M**	0.1007	LBen
**L400N**	0.1294	LBen
**L400P**	0.0394	LBen
**L400Q**	0.0449	LBen
**L400R**	0.0422	LBen
**L400S**	0.0712	LBen
**L400T**	0.0631	LBen
**L400V**	0.0748	LBen
**L400W**	0.1365	LBen
**L400Y**	0.1899	LBen
**Q401A**	0.1998	LBen
**Q401C**	0.6784	LPath
**Q401D**	0.1658	LBen
**Q401E**	0.0655	LBen
**Q401F**	0.688	LPath
**Q401G**	0.1672	LBen
**Q401H**	0.1515	LBen
**Q401I**	0.6256	LPath
**Q401K**	0.0811	LBen
**Q401L**	0.1861	LBen
**Q401M**	0.468	Amb
**Q401N**	0.1965	LBen

**Q401P**	0.1807	LBen
**Q401R**	0.0728	LBen
**Q401S**	0.1912	LBen
**Q401T**	0.232	LBen
**Q401V**	0.4159	Amb
**Q401W**	0.4074	Amb
**Q401Y**	0.3977	Amb
**T402A**	0.0839	LBen
**T402C**	0.5746	LPath
**T402D**	0.8931	LPath
**T402E**	0.8776	LPath
**T402F**	0.6911	LPath
**T402G**	0.3732	Amb
**T402H**	0.8223	LPath
**T402I**	0.2254	LBen
**T402K**	0.8987	LPath
**T402L**	0.2135	LBen
**T402M**	0.1534	LBen
**T402N**	0.4604	Amb
**T402P**	0.5825	LPath
**T402Q**	0.8061	LPath
**T402R**	0.865	LPath
**T402S**	0.1593	LBen
**T402V**	0.1436	LBen
**T402W**	0.9486	LPath
**T402Y**	0.845	LPath
**L403A**	0.8236	LPath
**L403C**	0.9553	LPath
**L403D**	0.9973	LPath
**L403E**	0.981	LPath
**L403F**	0.4623	Amb
**L403G**	0.9702	LPath
**L403H**	0.9695	LPath
**L403I**	0.1673	LBen
**L403K**	0.9777	LPath
**L403M**	0.2922	LBen
**L403N**	0.9884	LPath
**L403P**	0.8295	LPath
**L403Q**	0.9322	LPath
**L403R**	0.9522	LPath

**L403S**	0.9586	LPath
**L403T**	0.8641	LPath
**L403V**	0.1865	LBen
**L403W**	0.8449	LPath
**L403Y**	0.9464	LPath
**R404A**	0.2895	LBen
**R404C**	0.2119	LBen
**R404D**	0.4332	Amb
**R404E**	0.2542	LBen
**R404F**	0.4839	Amb
**R404G**	0.1548	LBen
**R404H**	0.0794	LBen
**R404I**	0.3458	Amb
**R404K**	0.1168	LBen
**R404L**	0.218	LBen
**R404M**	0.356	Amb
**R404N**	0.365	Amb
**R404P**	0.3991	Amb
**R404Q**	0.0776	LBen
**R404S**	0.2866	LBen
**R404T**	0.2076	LBen
**R404V**	0.3678	Amb
**R404W**	0.1547	LBen
**R404Y**	0.301	LBen
**I405A**	0.31	LBen
**I405C**	0.6524	LPath
**I405D**	0.4917	Amb
**I405E**	0.4373	Amb
**I405F**	0.1329	LBen
**I405G**	0.4906	Amb
**I405H**	0.2934	LBen
**I405K**	0.3007	LBen
**I405L**	0.0747	LBen
**I405M**	0.1011	LBen
**I405N**	0.1845	LBen
**I405P**	0.7856	LPath
**I405Q**	0.3266	LBen
**I405R**	0.2092	LBen
**I405S**	0.2219	LBen

**I405T**	0.2567	LBen
**I405V**	0.1072	LBen
**I405W**	0.5446	Amb
**I405Y**	0.299	LBen
**L406A**	0.8056	LPath
**L406C**	0.9261	LPath
**L406D**	0.9981	LPath
**L406E**	0.9863	LPath
**L406F**	0.6999	LPath
**L406G**	0.9622	LPath
**L406H**	0.9697	LPath
**L406I**	0.3418	Amb
**L406K**	0.9895	LPath
**L406M**	0.3405	Amb
**L406N**	0.9846	LPath
**L406P**	0.9823	LPath
**L406Q**	0.9237	LPath
**L406R**	0.9683	LPath
**L406S**	0.9462	LPath
**L406T**	0.8813	LPath
**L406V**	0.3525	Amb
**L406W**	0.9522	LPath
**L406Y**	0.9758	LPath
**I407A**	0.3907	Amb
**I407C**	0.9275	LPath
**I407D**	0.9854	LPath
**I407E**	0.9082	LPath
**I407F**	0.4284	Amb
**I407G**	0.8859	LPath
**I407H**	0.9395	LPath
**I407K**	0.8727	LPath
**I407L**	0.1223	LBen
**I407M**	0.0741	LBen
**I407N**	0.8842	LPath
**I407P**	0.9597	LPath
**I407Q**	0.8027	LPath
**I407R**	0.7877	LPath
**I407S**	0.6366	LPath
**I407T**	0.2743	LBen
**I407V**	0.1059	LBen

**I407W**	0.9356	LPath
**I407Y**	0.9063	LPath
**E408A**	0.1698	LBen
**E408C**	0.9374	LPath
**E408D**	0.4	Amb
**E408F**	0.9049	LPath
**E408G**	0.1829	LBen
**E408H**	0.5938	LPath
**E408I**	0.7098	LPath
**E408K**	0.1747	LBen
**E408L**	0.5699	LPath
**E408M**	0.6044	LPath
**E408N**	0.5283	Amb
**E408P**	0.9567	LPath
**E408Q**	0.1092	LBen
**E408R**	0.2123	LBen
**E408S**	0.2667	LBen
**E408T**	0.3141	LBen
**E408V**	0.4773	Amb
**E408W**	0.9254	LPath
**E408Y**	0.8363	LPath
**E409A**	0.1872	LBen
**E409C**	0.9255	LPath
**E409D**	0.4342	Amb
**E409F**	0.8787	LPath
**E409G**	0.2095	LBen
**E409H**	0.5952	LPath
**E409I**	0.7285	LPath
**E409K**	0.285	LBen
**E409L**	0.5138	Amb
**E409M**	0.5712	LPath
**E409N**	0.4538	Amb
**E409P**	0.9711	LPath
**E409Q**	0.111	LBen
**E409R**	0.3986	Amb
**E409S**	0.3096	LBen
**E409T**	0.3962	Amb
**E409V**	0.505	Amb
**E409W**	0.924	LPath
**E409Y**	0.7698	LPath

**N410A**	0.858	LPath
**N410C**	0.9409	LPath
**N410D**	0.2081	LBen
**N410E**	0.6366	LPath
**N410F**	0.9785	LPath
**N410G**	0.5991	LPath
**N410H**	0.4473	Amb
**N410I**	0.9447	LPath
**N410K**	0.581	LPath
**N410L**	0.9412	LPath
**N410M**	0.929	LPath
**N410P**	0.9616	LPath
**N410Q**	0.643	LPath
**N410R**	0.6551	LPath
**N410S**	0.3587	Amb
**N410T**	0.7593	LPath
**N410V**	0.9375	LPath
**N410W**	0.9764	LPath
**N410Y**	0.6987	LPath
**R411A**	0.7035	LPath
**R411C**	0.285	LBen
**R411D**	0.9228	LPath
**R411E**	0.5385	Amb
**R411F**	0.7917	LPath
**R411G**	0.5149	Amb
**R411H**	0.1584	LBen
**R411I**	0.5889	LPath
**R411K**	0.1222	LBen
**R411L**	0.3812	Amb
**R411M**	0.543	Amb
**R411N**	0.8693	LPath
**R411P**	0.9334	LPath
**R411Q**	0.1124	LBen
**R411S**	0.7814	LPath
**R411T**	0.5825	LPath
**R411V**	0.6266	LPath
**R411W**	0.238	LBen
**R411Y**	0.5941	LPath
**K412A**	0.5269	Amb
**K412C**	0.9205	LPath
**K412D**	0.6407	LPath

**K412E**	0.3325	LBen
**K412F**	0.8958	LPath
**K412G**	0.5657	LPath
**K412H**	0.4334	Amb
**K412I**	0.7913	LPath
**K412L**	0.5667	LPath
**K412M**	0.4424	Amb
**K412N**	0.5437	Amb
**K412P**	0.5852	LPath
**K412Q**	0.2288	LBen
**K412R**	0.1179	LBen
**K412S**	0.5464	Amb
**K412T**	0.3493	Amb
**K412V**	0.6996	LPath
**K412W**	0.8653	LPath
**K412Y**	0.7383	LPath
**V413A**	0.4905	Amb
**V413C**	0.9525	LPath
**V413D**	0.9869	LPath
**V413E**	0.9739	LPath
**V413F**	0.6744	LPath
**V413G**	0.5657	LPath
**V413H**	0.9927	LPath
**V413I**	0.1113	LBen
**V413K**	0.9875	LPath
**V413L**	0.4899	Amb
**V413M**	0.5234	Amb
**V413N**	0.9612	LPath
**V413P**	0.9721	LPath
**V413Q**	0.9758	LPath
**V413R**	0.9767	LPath
**V413S**	0.8531	LPath
**V413T**	0.7909	LPath
**V413W**	0.992	LPath
**V413Y**	0.9675	LPath
**I414A**	0.5401	Amb
**I414C**	0.9419	LPath
**I414D**	0.9938	LPath
**I414E**	0.977	LPath
**I414F**	0.6117	LPath

**I414G**	0.9477	LPath
**I414H**	0.9719	LPath
**I414K**	0.9521	LPath
**I414L**	0.1808	LBen
**I414M**	0.1975	LBen
**I414N**	0.9238	LPath
**I414P**	0.9819	LPath
**I414Q**	0.9402	LPath
**I414R**	0.9129	LPath
**I414S**	0.7366	LPath
**I414T**	0.4137	Amb
**I414V**	0.1199	LBen
**I414W**	0.9759	LPath
**I414Y**	0.9529	LPath
**A415C**	0.8755	LPath
**A415D**	0.9968	LPath
**A415E**	0.9949	LPath
**A415F**	0.9713	LPath
**A415G**	0.247	LBen
**A415H**	0.9941	LPath
**A415I**	0.9261	LPath
**A415K**	0.9988	LPath
**A415L**	0.8699	LPath
**A415M**	0.8945	LPath
**A415N**	0.9826	LPath
**A415P**	0.9927	LPath
**A415Q**	0.9832	LPath
**A415R**	0.9949	LPath
**A415S**	0.1646	LBen
**A415T**	0.3962	Amb
**A415V**	0.7172	LPath
**A415W**	0.9986	LPath
**A415Y**	0.9944	LPath
**P416A**	0.8042	LPath
**P416C**	0.9935	LPath
**P416D**	0.9991	LPath
**P416E**	0.9981	LPath
**P416F**	0.9986	LPath
**P416G**	0.9691	LPath

**P416H**	0.9966	LPath
**P416I**	0.9937	LPath
**P416K**	0.9987	LPath
**P416L**	0.9343	LPath
**P416M**	0.994	LPath
**P416N**	0.9984	LPath
**P416Q**	0.9944	LPath
**P416R**	0.9942	LPath
**P416S**	0.9608	LPath
**P416T**	0.9544	LPath
**P416V**	0.9703	LPath
**P416W**	0.9996	LPath
**P416Y**	0.9993	LPath
**M417A**	0.8391	LPath
**M417C**	0.9702	LPath
**M417D**	0.9978	LPath
**M417E**	0.9731	LPath
**M417F**	0.8187	LPath
**M417G**	0.948	LPath
**M417H**	0.9765	LPath
**M417I**	0.8783	LPath
**M417K**	0.9102	LPath
**M417L**	0.1714	LBen
**M417N**	0.9703	LPath
**M417P**	0.9903	LPath
**M417Q**	0.7947	LPath
**M417R**	0.9172	LPath
**M417S**	0.9029	LPath
**M417T**	0.8562	LPath
**M417V**	0.2867	LBen
**M417W**	0.9875	LPath
**M417Y**	0.9749	LPath
**L418A**	0.9322	LPath
**L418C**	0.958	LPath
**L418D**	0.999	LPath
**L418E**	0.9915	LPath
**L418F**	0.6157	LPath
**L418G**	0.9864	LPath
**L418H**	0.9843	LPath
**L418I**	0.2426	LBen

**L418K**	0.992	LPath
**L418M**	0.266	LBen
**L418N**	0.9904	LPath
**L418P**	0.9938	LPath
**L418Q**	0.9499	LPath
**L418R**	0.9792	LPath
**L418S**	0.9845	LPath
**L418T**	0.9381	LPath
**L418V**	0.237	LBen
**L418W**	0.9646	LPath
**L418Y**	0.9663	LPath
**S419A**	0.2231	LBen
**S419C**	0.476	Amb
**S419D**	0.9619	LPath
**S419E**	0.9519	LPath
**S419F**	0.8053	LPath
**S419G**	0.2637	LBen
**S419H**	0.8797	LPath
**S419I**	0.676	LPath
**S419K**	0.9836	LPath
**S419L**	0.4316	Amb
**S419M**	0.6083	LPath
**S419N**	0.7069	LPath
**S419P**	0.9447	LPath
**S419Q**	0.8845	LPath
**S419R**	0.9605	LPath
**S419T**	0.1824	LBen
**S419V**	0.5982	LPath
**S419W**	0.8645	LPath
**S419Y**	0.789	LPath
**R420A**	0.9661	LPath
**R420C**	0.8064	LPath
**R420D**	0.9954	LPath
**R420E**	0.957	LPath
**R420F**	0.9803	LPath
**R420G**	0.9488	LPath
**R420H**	0.5977	LPath
**R420I**	0.9019	LPath
**R420K**	0.3733	Amb
**R420L**	0.8259	LPath

**R420M**	0.9438	LPath
**R420N**	0.9905	LPath
**R420P**	0.9886	LPath
**R420Q**	0.4434	Amb
**R420S**	0.9776	LPath
**R420T**	0.9578	LPath
**R420V**	0.9184	LPath
**R420W**	0.7691	LPath
**R420Y**	0.9415	LPath
**H421A**	0.3807	Amb
**H421C**	0.5529	Amb
**H421D**	0.3646	Amb
**H421E**	0.3721	Amb
**H421F**	0.4136	Amb
**H421G**	0.5279	Amb
**H421I**	0.5224	Amb
**H421K**	0.4616	Amb
**H421L**	0.2663	LBen
**H421M**	0.6594	LPath
**H421N**	0.2042	LBen
**H421P**	0.1246	LBen
**H421Q**	0.26	LBen
**H421R**	0.2081	LBen
**H421S**	0.2953	LBen
**H421T**	0.4168	Amb
**H421V**	0.4168	Amb
**H421W**	0.4419	Amb
**H421Y**	0.1495	LBen
**G422A**	0.4491	Amb
**G422C**	0.775	LPath
**G422D**	0.4712	Amb
**G422E**	0.4727	Amb
**G422F**	0.9231	LPath
**G422H**	0.578	LPath
**G422I**	0.916	LPath
**G422K**	0.654	LPath
**G422L**	0.8513	LPath
**G422M**	0.8904	LPath
**G422N**	0.4185	Amb
**G422P**	0.9859	LPath
**G422Q**	0.4333	Amb

**G422R**	0.4492	Amb
**G422S**	0.1656	LBen
**G422T**	0.5522	Amb
**G422V**	0.8441	LPath
**G422W**	0.8177	LPath
**G422Y**	0.836	LPath
**K423A**	0.9607	LPath
**K423C**	0.9806	LPath
**K423D**	0.9874	LPath
**K423E**	0.9434	LPath
**K423F**	0.9848	LPath
**K423G**	0.9628	LPath
**K423H**	0.7786	LPath
**K423I**	0.9653	LPath
**K423L**	0.9164	LPath
**K423M**	0.8881	LPath
**K423N**	0.9701	LPath
**K423P**	0.9914	LPath
**K423Q**	0.6366	LPath
**K423R**	0.1467	LBen
**K423S**	0.9651	LPath
**K423T**	0.9248	LPath
**K423V**	0.9452	LPath
**K423W**	0.9762	LPath
**K423Y**	0.9513	LPath
**L424A**	0.946	LPath
**L424C**	0.9939	LPath
**L424D**	0.9978	LPath
**L424E**	0.9943	LPath
**L424F**	0.8404	LPath
**L424G**	0.9787	LPath
**L424H**	0.9806	LPath
**L424I**	0.8046	LPath
**L424K**	0.9853	LPath
**L424M**	0.7307	LPath
**L424N**	0.9815	LPath
**L424P**	0.9824	LPath
**L424Q**	0.974	LPath
**L424R**	0.9445	LPath
**L424S**	0.9866	LPath

**L424T**	0.9782	LPath
**L424V**	0.8323	LPath
**L424W**	0.9349	LPath
**L424Y**	0.924	LPath
**W425A**	0.9913	LPath
**W425C**	0.9984	LPath
**W425D**	0.9988	LPath
**W425E**	0.9988	LPath
**W425F**	0.7314	LPath
**W425G**	0.9792	LPath
**W425H**	0.9917	LPath
**W425I**	0.995	LPath
**W425K**	0.9996	LPath
**W425L**	0.9821	LPath
**W425M**	0.9969	LPath
**W425N**	0.9986	LPath
**W425P**	0.9977	LPath
**W425Q**	0.9987	LPath
**W425R**	0.998	LPath
**W425S**	0.9818	LPath
**W425T**	0.9954	LPath
**W425V**	0.9928	LPath
**W425Y**	0.9509	LPath
**S426A**	0.8591	LPath
**S426C**	0.972	LPath
**S426D**	0.9948	LPath
**S426E**	0.9967	LPath
**S426F**	0.9989	LPath
**S426G**	0.8914	LPath
**S426H**	0.9942	LPath
**S426I**	0.9974	LPath
**S426K**	0.9993	LPath
**S426L**	0.9899	LPath
**S426M**	0.9901	LPath
**S426N**	0.9859	LPath
**S426P**	0.9983	LPath
**S426Q**	0.9953	LPath
**S426R**	0.9991	LPath
**S426T**	0.5281	Amb

**S426V**	0.9935	LPath
**S426W**	0.9971	LPath
**S426Y**	0.9969	LPath
**N427A**	0.9856	LPath
**N427C**	0.9842	LPath
**N427D**	0.9737	LPath
**N427E**	0.9987	LPath
**N427F**	0.9992	LPath
**N427G**	0.9742	LPath
**N427H**	0.9741	LPath
**N427I**	0.9928	LPath
**N427K**	0.9993	LPath
**N427L**	0.9823	LPath
**N427M**	0.9945	LPath
**N427P**	0.9825	LPath
**N427Q**	0.997	LPath
**N427R**	0.9979	LPath
**N427S**	0.5094	Amb
**N427T**	0.8435	LPath
**N427V**	0.9859	LPath
**N427W**	0.9997	LPath
**N427Y**	0.9926	LPath
**F428A**	0.997	LPath
**F428C**	0.9924	LPath
**F428D**	0.9997	LPath
**F428E**	0.9998	LPath
**F428G**	0.9977	LPath
**F428H**	0.9931	LPath
**F428I**	0.99	LPath
**F428K**	0.9998	LPath
**F428L**	0.999	LPath
**F428M**	0.9958	LPath
**F428N**	0.9987	LPath
**F428P**	0.9999	LPath
**F428Q**	0.9993	LPath
**F428R**	0.9987	LPath
**F428S**	0.9962	LPath
**F428T**	0.998	LPath
**F428V**	0.9819	LPath
**F428W**	0.9731	LPath

**F428Y**	0.7565	LPath
**W429A**	0.9967	LPath
**W429C**	0.9995	LPath
**W429D**	0.9999	LPath
**W429E**	0.9998	LPath
**W429F**	0.9589	LPath
**W429G**	0.9928	LPath
**W429H**	0.9983	LPath
**W429I**	0.9986	LPath
**W429K**	0.9999	LPath
**W429L**	0.9921	LPath
**W429M**	0.9994	LPath
**W429N**	0.9999	LPath
**W429P**	0.9993	LPath
**W429Q**	0.9998	LPath
**W429R**	0.9995	LPath
**W429S**	0.9945	LPath
**W429T**	0.9981	LPath
**W429V**	0.9985	LPath
**W429Y**	0.9923	LPath
**G430A**	0.8885	LPath
**G430C**	0.9852	LPath
**G430D**	0.9947	LPath
**G430E**	0.996	LPath
**G430F**	0.9978	LPath
**G430H**	0.9989	LPath
**G430I**	0.9959	LPath
**G430K**	0.999	LPath
**G430L**	0.9941	LPath
**G430M**	0.9965	LPath
**G430N**	0.9961	LPath
**G430P**	0.999	LPath
**G430Q**	0.998	LPath
**G430R**	0.9974	LPath
**G430S**	0.9298	LPath
**G430T**	0.9867	LPath
**G430V**	0.9884	LPath
**G430W**	0.9975	LPath
**G430Y**	0.9975	LPath
**A431C**	0.9362	LPath

**A431D**	0.6358	LPath
**A431E**	0.8312	LPath
**A431F**	0.8574	LPath
**A431G**	0.4152	Amb
**A431H**	0.8832	LPath
**A431I**	0.9503	LPath
**A431K**	0.9537	LPath
**A431L**	0.7881	LPath
**A431M**	0.7698	LPath
**A431N**	0.6966	LPath
**A431P**	0.9963	LPath
**A431Q**	0.7579	LPath
**A431R**	0.8728	LPath
**A431S**	0.1356	LBen
**A431T**	0.518	Amb
**A431V**	0.8076	LPath
**A431W**	0.9733	LPath
**A431Y**	0.9092	LPath
**L432A**	0.9168	LPath
**L432C**	0.9657	LPath
**L432D**	0.9979	LPath
**L432E**	0.9743	LPath
**L432F**	0.8616	LPath
**L432G**	0.9879	LPath
**L432H**	0.9712	LPath
**L432I**	0.1885	LBen
**L432K**	0.9423	LPath
**L432M**	0.4423	Amb
**L432N**	0.9827	LPath
**L432P**	0.9906	LPath
**L432Q**	0.8961	LPath
**L432R**	0.8714	LPath
**L432S**	0.9705	LPath
**L432T**	0.8537	LPath
**L432V**	0.2064	LBen
**L432W**	0.9649	LPath
**L432Y**	0.9797	LPath
**S433A**	0.3478	Amb
**S433C**	0.7326	LPath
**S433D**	0.9009	LPath
**S433E**	0.9684	LPath

**S433F**	0.8761	LPath
**S433G**	0.2558	LBen
**S433H**	0.8356	LPath
**S433I**	0.8633	LPath
**S433K**	0.9886	LPath
**S433L**	0.4607	Amb
**S433M**	0.6865	LPath
**S433N**	0.4893	Amb
**S433P**	0.9755	LPath
**S433Q**	0.9244	LPath
**S433R**	0.9812	LPath
**S433T**	0.3486	Amb
**S433V**	0.8607	LPath
**S433W**	0.9353	LPath
**S433Y**	0.8379	LPath
**P434A**	0.0946	LBen
**P434C**	0.8073	LPath
**P434D**	0.5397	Amb
**P434E**	0.3159	LBen
**P434F**	0.6769	LPath
**P434G**	0.3496	Amb
**P434H**	0.2556	LBen
**P434I**	0.4662	Amb
**P434K**	0.3417	Amb
**P434L**	0.1588	LBen
**P434M**	0.399	Amb
**P434N**	0.4296	Amb
**P434Q**	0.1768	LBen
**P434R**	0.1804	LBen
**P434S**	0.1463	LBen
**P434T**	0.1373	LBen
**P434V**	0.2986	LBen
**P434W**	0.728	LPath
**P434Y**	0.638	LPath
**D435A**	0.3202	LBen
**D435C**	0.9034	LPath
**D435E**	0.1495	LBen
**D435F**	0.8287	LPath
**D435G**	0.2371	LBen
**D435H**	0.5223	Amb

**D435I**	0.7798	LPath
**D435K**	0.6069	LPath
**D435L**	0.6443	LPath
**D435M**	0.8072	LPath
**D435N**	0.1487	LBen
**D435P**	0.9191	LPath
**D435Q**	0.4165	Amb
**D435R**	0.613	LPath
**D435S**	0.1856	LBen
**D435T**	0.4314	Amb
**D435V**	0.5353	Amb
**D435W**	0.9429	LPath
**D435Y**	0.5294	Amb
**E436A**	0.162	LBen
**E436C**	0.9001	LPath
**E436D**	0.1531	LBen
**E436F**	0.8391	LPath
**E436G**	0.0966	LBen
**E436H**	0.4679	Amb
**E436I**	0.7267	LPath
**E436K**	0.2137	LBen
**E436L**	0.5173	Amb
**E436M**	0.6183	LPath
**E436N**	0.2431	LBen
**E436P**	0.8749	LPath
**E436Q**	0.1302	LBen
**E436R**	0.2815	LBen
**E436S**	0.1646	LBen
**E436T**	0.3239	LBen
**E436V**	0.4961	Amb
**E436W**	0.9483	LPath
**E436Y**	0.7207	LPath
**Y437A**	0.9805	LPath
**Y437C**	0.9753	LPath
**Y437D**	0.9617	LPath
**Y437E**	0.9941	LPath
**Y437F**	0.1721	LBen
**Y437G**	0.9476	LPath
**Y437H**	0.9525	LPath

**Y437I**	0.9649	LPath
**Y437K**	0.9926	LPath
**Y437L**	0.9517	LPath
**Y437M**	0.97	LPath
**Y437N**	0.9091	LPath
**Y437P**	0.9976	LPath
**Y437Q**	0.9915	LPath
**Y437R**	0.982	LPath
**Y437S**	0.9516	LPath
**Y437T**	0.982	LPath
**Y437V**	0.9313	LPath
**Y437W**	0.8717	LPath
**Y438A**	0.9959	LPath
**Y438C**	0.9979	LPath
**Y438D**	0.9977	LPath
**Y438E**	0.9993	LPath
**Y438F**	0.7379	LPath
**Y438G**	0.9887	LPath
**Y438H**	0.9982	LPath
**Y438I**	0.9918	LPath
**Y438K**	0.9994	LPath
**Y438L**	0.954	LPath
**Y438M**	0.9961	LPath
**Y438N**	0.9948	LPath
**Y438P**	0.9837	LPath
**Y438Q**	0.9994	LPath
**Y438R**	0.9971	LPath
**Y438S**	0.9866	LPath
**Y438T**	0.9969	LPath
**Y438V**	0.9813	LPath
**Y438W**	0.9785	LPath
**A439C**	0.9334	LPath
**A439D**	0.9613	LPath
**A439E**	0.8091	LPath
**A439F**	0.8503	LPath
**A439G**	0.5735	LPath
**A439H**	0.9128	LPath
**A439I**	0.8602	LPath
**A439K**	0.9317	LPath
**A439L**	0.5813	LPath

**A439M**	0.656	LPath
**A439N**	0.8913	LPath
**A439P**	0.9977	LPath
**A439Q**	0.6276	LPath
**A439R**	0.7909	LPath
**A439S**	0.1781	LBen
**A439T**	0.4696	Amb
**A439V**	0.6374	LPath
**A439W**	0.9747	LPath
**A439Y**	0.9251	LPath
**R440A**	0.9953	LPath
**R440C**	0.9919	LPath
**R440D**	0.9981	LPath
**R440E**	0.9892	LPath
**R440F**	0.9968	LPath
**R440G**	0.9948	LPath
**R440H**	0.9573	LPath
**R440I**	0.9934	LPath
**R440K**	0.9613	LPath
**R440L**	0.9831	LPath
**R440M**	0.9977	LPath
**R440N**	0.9983	LPath
**R440P**	0.9896	LPath
**R440Q**	0.9557	LPath
**R440S**	0.9967	LPath
**R440T**	0.9974	LPath
**R440V**	0.9929	LPath
**R440W**	0.9556	LPath
**R440Y**	0.9922	LPath
**S441A**	0.3242	LBen
**S441C**	0.8415	LPath
**S441D**	0.9904	LPath
**S441E**	0.9928	LPath
**S441F**	0.9929	LPath
**S441G**	0.6869	LPath
**S441H**	0.9843	LPath
**S441I**	0.9947	LPath
**S441K**	0.9989	LPath
**S441L**	0.939	LPath
**S441M**	0.9603	LPath

**S441N**	0.9705	LPath
**S441P**	0.9952	LPath
**S441Q**	0.9857	LPath
**S441R**	0.9976	LPath
**S441T**	0.7941	LPath
**S441V**	0.982	LPath
**S441W**	0.9896	LPath
**S441Y**	0.9882	LPath
**E442A**	0.7209	LPath
**E442C**	0.9966	LPath
**E442D**	0.6664	LPath
**E442F**	0.9921	LPath
**E442G**	0.776	LPath
**E442H**	0.9588	LPath
**E442I**	0.9531	LPath
**E442K**	0.753	LPath
**E442L**	0.9614	LPath
**E442M**	0.9543	LPath
**E442N**	0.9145	LPath
**E442P**	0.9254	LPath
**E442Q**	0.6466	LPath
**E442R**	0.836	LPath
**E442S**	0.7991	LPath
**E442T**	0.8789	LPath
**E442V**	0.8716	LPath
**E442W**	0.9967	LPath
**E442Y**	0.987	LPath
**D443A**	0.9892	LPath
**D443C**	0.9988	LPath
**D443E**	0.9812	LPath
**D443F**	0.998	LPath
**D443G**	0.9796	LPath
**D443H**	0.996	LPath
**D443I**	0.9969	LPath
**D443K**	0.9988	LPath
**D443L**	0.9955	LPath
**D443M**	0.9992	LPath
**D443N**	0.9192	LPath
**D443P**	0.9989	LPath
**D443Q**	0.9981	LPath

**D443R**	0.9982	LPath
**D443S**	0.9821	LPath
**D443T**	0.9933	LPath
**D443V**	0.9908	LPath
**D443W**	0.9995	LPath
**D443Y**	0.9831	LPath
**Y444A**	0.9927	LPath
**Y444C**	0.9941	LPath
**Y444D**	0.9861	LPath
**Y444E**	0.9977	LPath
**Y444F**	0.7737	LPath
**Y444G**	0.9812	LPath
**Y444H**	0.996	LPath
**Y444I**	0.9873	LPath
**Y444K**	0.9983	LPath
**Y444L**	0.9335	LPath
**Y444M**	0.9896	LPath
**Y444N**	0.9785	LPath
**Y444P**	0.9951	LPath
**Y444Q**	0.9988	LPath
**Y444R**	0.9954	LPath
**Y444S**	0.9668	LPath
**Y444T**	0.988	LPath
**Y444V**	0.9633	LPath
**Y444W**	0.9793	LPath
**V445A**	0.7728	LPath
**V445C**	0.9892	LPath
**V445D**	0.9773	LPath
**V445E**	0.916	LPath
**V445F**	0.6814	LPath
**V445G**	0.782	LPath
**V445H**	0.9787	LPath
**V445I**	0.1293	LBen
**V445K**	0.9127	LPath
**V445L**	0.5773	LPath
**V445M**	0.4537	Amb
**V445N**	0.9478	LPath
**V445P**	0.9541	LPath
**V445Q**	0.8788	LPath
**V445R**	0.8495	LPath

**V445S**	0.8646	LPath
**V445T**	0.7239	LPath
**V445W**	0.9836	LPath
**V445Y**	0.958	LPath
**E446A**	0.4199	Amb
**E446C**	0.9882	LPath
**E446D**	0.174	LBen
**E446F**	0.9791	LPath
**E446G**	0.5536	Amb
**E446H**	0.9101	LPath
**E446I**	0.9539	LPath
**E446K**	0.8468	LPath
**E446L**	0.8668	LPath
**E446M**	0.9229	LPath
**E446N**	0.7113	LPath
**E446P**	0.9279	LPath
**E446Q**	0.4985	Amb
**E446R**	0.8728	LPath
**E446S**	0.5234	Amb
**E446T**	0.803	LPath
**E446V**	0.8358	LPath
**E446W**	0.9923	LPath
**E446Y**	0.9554	LPath
**L447A**	0.8945	LPath
**L447C**	0.9697	LPath
**L447D**	0.9974	LPath
**L447E**	0.9828	LPath
**L447F**	0.8559	LPath
**L447G**	0.9853	LPath
**L447H**	0.9825	LPath
**L447I**	0.1137	LBen
**L447K**	0.9833	LPath
**L447M**	0.297	LBen
**L447N**	0.9795	LPath
**L447P**	0.9915	LPath
**L447Q**	0.9612	LPath
**L447R**	0.9717	LPath
**L447S**	0.9799	LPath
**L447T**	0.8626	LPath
**L447V**	0.2061	LBen

**L447W**	0.9654	LPath
**L447Y**	0.9764	LPath
**V448A**	0.8419	LPath
**V448C**	0.9833	LPath
**V448D**	0.996	LPath
**V448E**	0.9772	LPath
**V448F**	0.8976	LPath
**V448G**	0.8983	LPath
**V448H**	0.9916	LPath
**V448I**	0.1846	LBen
**V448K**	0.9842	LPath
**V448L**	0.754	LPath
**V448M**	0.8662	LPath
**V448N**	0.9806	LPath
**V448P**	0.9907	LPath
**V448Q**	0.9533	LPath
**V448R**	0.9476	LPath
**V448S**	0.9073	LPath
**V448T**	0.8774	LPath
**V448W**	0.9959	LPath
**V448Y**	0.9839	LPath
**Q449A**	0.5438	Amb
**Q449C**	0.9677	LPath
**Q449D**	0.8004	LPath
**Q449E**	0.1628	LBen
**Q449F**	0.9432	LPath
**Q449G**	0.4463	Amb
**Q449H**	0.6137	LPath
**Q449I**	0.8888	LPath
**Q449K**	0.2876	LBen
**Q449L**	0.3125	LBen
**Q449M**	0.6876	LPath
**Q449N**	0.5916	LPath
**Q449P**	0.9396	LPath
**Q449R**	0.2526	LBen
**Q449S**	0.5064	Amb
**Q449T**	0.5627	Amb
**Q449V**	0.7558	LPath
**Q449W**	0.8927	LPath
**Q449Y**	0.8659	LPath

**R450A**	0.4695	Amb
**R450C**	0.3098	LBen
**R450D**	0.6046	LPath
**R450E**	0.3172	LBen
**R450F**	0.6083	LPath
**R450G**	0.0937	LBen
**R450H**	0.0962	LBen
**R450I**	0.6922	LPath
**R450K**	0.1259	LBen
**R450L**	0.3192	LBen
**R450M**	0.3978	Amb
**R450N**	0.4538	Amb
**R450P**	0.9107	LPath
**R450Q**	0.0858	LBen
**R450S**	0.4392	Amb
**R450T**	0.465	Amb
**R450V**	0.6925	LPath
**R450W**	0.1719	LBen
**R450Y**	0.347	Amb
**K451A**	0.6732	LPath
**K451C**	0.9092	LPath
**K451D**	0.7374	LPath
**K451E**	0.3032	LBen
**K451F**	0.9371	LPath
**K451G**	0.6909	LPath
**K451H**	0.3801	Amb
**K451I**	0.7011	LPath
**K451L**	0.633	LPath
**K451M**	0.5135	Amb
**K451N**	0.5349	Amb
**K451P**	0.9814	LPath
**K451Q**	0.1636	LBen
**K451R**	0.0934	LBen
**K451S**	0.6228	LPath
**K451T**	0.3793	Amb
**K451V**	0.656	LPath
**K451W**	0.8915	LPath
**K451Y**	0.8339	LPath
**R452A**	0.9643	LPath
**R452C**	0.8529	LPath

**R452D**	0.9928	LPath
**R452E**	0.911	LPath
**R452F**	0.9739	LPath
**R452G**	0.9578	LPath
**R452H**	0.5941	LPath
**R452I**	0.8491	LPath
**R452K**	0.4395	Amb
**R452L**	0.8055	LPath
**R452M**	0.9066	LPath
**R452N**	0.9853	LPath
**R452P**	0.9933	LPath
**R452Q**	0.4547	Amb
**R452S**	0.9871	LPath
**R452T**	0.9604	LPath
**R452V**	0.8977	LPath
**R452W**	0.738	LPath
**R452Y**	0.9265	LPath
**V453A**	0.1063	LBen
**V453C**	0.7098	LPath
**V453D**	0.2882	LBen
**V453E**	0.1258	LBen
**V453F**	0.1446	LBen
**V453G**	0.1718	LBen
**V453H**	0.3697	Amb
**V453I**	0.072	LBen
**V453K**	0.1321	LBen
**V453L**	0.1054	LBen
**V453M**	0.09	LBen
**V453N**	0.2328	LBen
**V453P**	0.4271	Amb
**V453Q**	0.1423	LBen
**V453R**	0.1099	LBen
**V453S**	0.1472	LBen
**V453T**	0.0986	LBen
**V453W**	0.5416	Amb
**V453Y**	0.432	Amb
**G454A**	0.8435	LPath
**G454C**	0.9712	LPath
**G454D**	0.9861	LPath
**G454E**	0.9905	LPath

**G454F**	0.9963	LPath
**G454H**	0.9969	LPath
**G454I**	0.9944	LPath
**G454K**	0.9971	LPath
**G454L**	0.9932	LPath
**G454M**	0.9956	LPath
**G454N**	0.9917	LPath
**G454P**	0.9986	LPath
**G454Q**	0.9942	LPath
**G454R**	0.9908	LPath
**G454S**	0.8466	LPath
**G454T**	0.9734	LPath
**G454V**	0.9844	LPath
**G454W**	0.9928	LPath
**G454Y**	0.9957	LPath
**V455A**	0.4344	Amb
**V455C**	0.8591	LPath
**V455D**	0.7866	LPath
**V455E**	0.6234	LPath
**V455F**	0.266	LBen
**V455G**	0.5146	Amb
**V455H**	0.8041	LPath
**V455I**	0.0686	LBen
**V455K**	0.661	LPath
**V455L**	0.1486	LBen
**V455M**	0.1883	LBen
**V455N**	0.5929	LPath
**V455P**	0.9386	LPath
**V455Q**	0.6237	LPath
**V455R**	0.5598	Amb
**V455S**	0.5187	Amb
**V455T**	0.3833	Amb
**V455W**	0.8596	LPath
**V455Y**	0.7134	LPath
**W456A**	0.9969	LPath
**W456C**	0.9976	LPath
**W456D**	0.9996	LPath
**W456E**	0.9993	LPath
**W456F**	0.7282	LPath
**W456G**	0.9877	LPath

**W456H**	0.9933	LPath
**W456I**	0.9951	LPath
**W456K**	0.9996	LPath
**W456L**	0.9882	LPath
**W456M**	0.9967	LPath
**W456N**	0.9993	LPath
**W456P**	0.9996	LPath
**W456Q**	0.9992	LPath
**W456R**	0.9987	LPath
**W456S**	0.9943	LPath
**W456T**	0.9976	LPath
**W456V**	0.9946	LPath
**W456Y**	0.9541	LPath
**N457A**	0.885	LPath
**N457C**	0.9019	LPath
**N457D**	0.936	LPath
**N457E**	0.982	LPath
**N457F**	0.9868	LPath
**N457G**	0.9454	LPath
**N457H**	0.81	LPath
**N457I**	0.803	LPath
**N457K**	0.9839	LPath
**N457L**	0.8696	LPath
**N457M**	0.9051	LPath
**N457P**	0.9751	LPath
**N457Q**	0.962	LPath
**N457R**	0.9741	LPath
**N457S**	0.4826	Amb
**N457T**	0.8053	LPath
**N457V**	0.7661	LPath
**N457W**	0.9954	LPath
**N457Y**	0.9089	LPath
**V458A**	0.7742	LPath
**V458C**	0.967	LPath
**V458D**	0.9946	LPath
**V458E**	0.9821	LPath
**V458F**	0.9368	LPath
**V458G**	0.8709	LPath
**V458H**	0.9951	LPath
**V458I**	0.1483	LBen

**V458K**	0.9916	LPath
**V458L**	0.8289	LPath
**V458M**	0.7824	LPath
**V458N**	0.9658	LPath
**V458P**	0.9918	LPath
**V458Q**	0.9742	LPath
**V458R**	0.9824	LPath
**V458S**	0.7709	LPath
**V458T**	0.7105	LPath
**V458W**	0.9992	LPath
**V458Y**	0.995	LPath
**P459A**	0.7644	LPath
**P459C**	0.9926	LPath
**P459D**	0.9976	LPath
**P459E**	0.9953	LPath
**P459F**	0.9984	LPath
**P459G**	0.9718	LPath
**P459H**	0.9903	LPath
**P459I**	0.9938	LPath
**P459K**	0.9976	LPath
**P459L**	0.9445	LPath
**P459M**	0.9954	LPath
**P459N**	0.995	LPath
**P459Q**	0.986	LPath
**P459R**	0.9844	LPath
**P459S**	0.929	LPath
**P459T**	0.955	LPath
**P459V**	0.9804	LPath
**P459W**	0.999	LPath
**P459Y**	0.998	LPath
**Y460A**	0.9844	LPath
**Y460C**	0.7854	LPath
**Y460D**	0.9931	LPath
**Y460E**	0.9978	LPath
**Y460F**	0.1391	LBen
**Y460G**	0.9792	LPath
**Y460H**	0.8318	LPath
**Y460I**	0.9765	LPath
**Y460K**	0.9977	LPath
**Y460L**	0.9117	LPath

**Y460M**	0.9664	LPath
**Y460N**	0.9412	LPath
**Y460P**	0.9987	LPath
**Y460Q**	0.9928	LPath
**Y460R**	0.9888	LPath
**Y460S**	0.9366	LPath
**Y460T**	0.9888	LPath
**Y460V**	0.9335	LPath
**Y460W**	0.7357	LPath
**I461A**	0.8655	LPath
**I461C**	0.9431	LPath
**I461D**	0.9979	LPath
**I461E**	0.9951	LPath
**I461F**	0.6094	LPath
**I461G**	0.9809	LPath
**I461H**	0.9915	LPath
**I461K**	0.9934	LPath
**I461L**	0.1686	LBen
**I461M**	0.3041	LBen
**I461N**	0.9722	LPath
**I461P**	0.9892	LPath
**I461Q**	0.9874	LPath
**I461R**	0.9862	LPath
**I461S**	0.9225	LPath
**I461T**	0.8606	LPath
**I461V**	0.0908	LBen
**I461W**	0.9928	LPath
**I461Y**	0.9687	LPath
**S462A**	0.1053	LBen
**S462C**	0.3264	LBen
**S462D**	0.9573	LPath
**S462E**	0.9665	LPath
**S462F**	0.7468	LPath
**S462G**	0.173	LBen
**S462H**	0.8494	LPath
**S462I**	0.8288	LPath
**S462K**	0.9869	LPath
**S462L**	0.4318	Amb
**S462M**	0.5948	LPath
**S462N**	0.5836	LPath

**S462P**	0.9636	LPath
**S462Q**	0.8858	LPath
**S462R**	0.9661	LPath
**S462T**	0.1752	LBen
**S462V**	0.6679	LPath
**S462W**	0.8653	LPath
**S462Y**	0.7295	LPath
**Q463A**	0.3306	LBen
**Q463C**	0.9009	LPath
**Q463D**	0.7722	LPath
**Q463E**	0.2098	LBen
**Q463F**	0.9083	LPath
**Q463G**	0.2923	LBen
**Q463H**	0.383	Amb
**Q463I**	0.8509	LPath
**Q463K**	0.3563	Amb
**Q463L**	0.3228	LBen
**Q463M**	0.5858	LPath
**Q463N**	0.4126	Amb
**Q463P**	0.7738	LPath
**Q463R**	0.3269	LBen
**Q463S**	0.2552	LBen
**Q463T**	0.3791	Amb
**Q463V**	0.6525	LPath
**Q463W**	0.8923	LPath
**Q463Y**	0.8111	LPath
**A464C**	0.5942	LPath
**A464D**	0.9702	LPath
**A464E**	0.9731	LPath
**A464F**	0.8957	LPath
**A464G**	0.2883	LBen
**A464H**	0.9837	LPath
**A464I**	0.4175	Amb
**A464K**	0.9948	LPath
**A464L**	0.4869	Amb
**A464M**	0.6082	LPath
**A464N**	0.8983	LPath
**A464P**	0.9359	LPath
**A464Q**	0.9677	LPath
**A464R**	0.9854	LPath

**A464S**	0.1968	LBen
**A464T**	0.1797	LBen
**A464V**	0.1605	LBen
**A464W**	0.9928	LPath
**A464Y**	0.9713	LPath
**Y465A**	0.9067	LPath
**Y465C**	0.5165	Amb
**Y465D**	0.988	LPath
**Y465E**	0.9945	LPath
**Y465F**	0.111	LBen
**Y465G**	0.9397	LPath
**Y465H**	0.8627	LPath
**Y465I**	0.7122	LPath
**Y465K**	0.9944	LPath
**Y465L**	0.5803	LPath
**Y465M**	0.7626	LPath
**Y465N**	0.9142	LPath
**Y465P**	0.9948	LPath
**Y465Q**	0.9817	LPath
**Y465R**	0.9791	LPath
**Y465S**	0.8041	LPath
**Y465T**	0.8672	LPath
**Y465V**	0.5058	Amb
**Y465W**	0.5894	LPath
**V466A**	0.2406	LBen
**V466C**	0.7578	LPath
**V466D**	0.9706	LPath
**V466E**	0.9231	LPath
**V466F**	0.329	LBen
**V466G**	0.4454	Amb
**V466H**	0.9596	LPath
**V466I**	0.0807	LBen
**V466K**	0.9522	LPath
**V466L**	0.0636	LBen
**V466M**	0.1048	LBen
**V466N**	0.8893	LPath
**V466P**	0.9722	LPath
**V466Q**	0.8691	LPath
**V466R**	0.9123	LPath
**V466S**	0.5778	LPath

**V466T**	0.4573	Amb
**V466W**	0.9567	LPath
**V466Y**	0.9037	LPath
**I467A**	0.8157	LPath
**I467C**	0.9289	LPath
**I467D**	0.9975	LPath
**I467E**	0.9911	LPath
**I467F**	0.3766	Amb
**I467G**	0.9714	LPath
**I467H**	0.9808	LPath
**I467K**	0.9846	LPath
**I467L**	0.136	LBen
**I467M**	0.1769	LBen
**I467N**	0.9671	LPath
**I467P**	0.9919	LPath
**I467Q**	0.9717	LPath
**I467R**	0.9703	LPath
**I467S**	0.9048	LPath
**I467T**	0.8732	LPath
**I467V**	0.1221	LBen
**I467W**	0.9694	LPath
**I467Y**	0.9334	LPath
**R468A**	0.3138	LBen
**R468C**	0.2465	LBen
**R468D**	0.6286	LPath
**R468E**	0.3368	LBen
**R468F**	0.6039	LPath
**R468G**	0.2208	LBen
**R468H**	0.0976	LBen
**R468I**	0.4779	Amb
**R468K**	0.116	LBen
**R468L**	0.2106	LBen
**R468M**	0.3609	Amb
**R468N**	0.5352	Amb
**R468P**	0.5185	Amb
**R468Q**	0.0865	LBen
**R468S**	0.3431	Amb
**R468T**	0.2447	LBen
**R468V**	0.4877	Amb
**R468W**	0.1862	LBen

**R468Y**	0.4294	Amb
**G469A**	0.1715	LBen
**G469C**	0.6648	LPath
**G469D**	0.8781	LPath
**G469E**	0.8009	LPath
**G469F**	0.968	LPath
**G469H**	0.8982	LPath
**G469I**	0.8614	LPath
**G469K**	0.8483	LPath
**G469L**	0.8845	LPath
**G469M**	0.8836	LPath
**G469N**	0.8458	LPath
**G469P**	0.9736	LPath
**G469Q**	0.8025	LPath
**G469R**	0.6946	LPath
**G469S**	0.2038	LBen
**G469T**	0.5543	Amb
**G469V**	0.6937	LPath
**G469W**	0.8927	LPath
**G469Y**	0.9428	LPath
**D470A**	0.1237	LBen
**D470C**	0.5424	Amb
**D470E**	0.0856	LBen
**D470F**	0.4794	Amb
**D470G**	0.1215	LBen
**D470H**	0.1535	LBen
**D470I**	0.3009	LBen
**D470K**	0.1493	LBen
**D470L**	0.2885	LBen
**D470M**	0.4633	Amb
**D470N**	0.0882	LBen
**D470P**	0.4027	Amb
**D470Q**	0.1443	LBen
**D470R**	0.1578	LBen
**D470S**	0.0894	LBen
**D470T**	0.1802	LBen
**D470V**	0.1866	LBen
**D470W**	0.7159	LPath
**D470Y**	0.201	LBen
**T471A**	0.0931	LBen

**T471C**	0.5429	Amb
**T471D**	0.6611	LPath
**T471E**	0.6239	LPath
**T471F**	0.3527	Amb
**T471G**	0.4563	Amb
**T471H**	0.4692	Amb
**T471I**	0.1229	LBen
**T471K**	0.5154	Amb
**T471L**	0.096	LBen
**T471M**	0.1171	LBen
**T471N**	0.2955	LBen
**T471P**	0.4887	Amb
**T471Q**	0.511	Amb
**T471R**	0.383	Amb
**T471S**	0.1816	LBen
**T471V**	0.1052	LBen
**T471W**	0.7527	LPath
**T471Y**	0.4677	Amb
**L472A**	0.8816	LPath
**L472C**	0.9735	LPath
**L472D**	0.9983	LPath
**L472E**	0.9866	LPath
**L472F**	0.6139	LPath
**L472G**	0.9804	LPath
**L472H**	0.9779	LPath
**L472I**	0.2576	LBen
**L472K**	0.9848	LPath
**L472M**	0.3785	Amb
**L472N**	0.9907	LPath
**L472P**	0.9541	LPath
**L472Q**	0.941	LPath
**L472R**	0.952	LPath
**L472S**	0.9721	LPath
**L472T**	0.9297	LPath
**L472V**	0.28	LBen
**L472W**	0.8968	LPath
**L472Y**	0.9603	LPath
**R473A**	0.7564	LPath
**R473C**	0.4592	Amb
**R473D**	0.9255	LPath

**R473E**	0.6818	LPath
**R473F**	0.8596	LPath
**R473G**	0.5992	LPath
**R473H**	0.206	LBen
**R473I**	0.7094	LPath
**R473K**	0.2861	LBen
**R473L**	0.5023	Amb
**R473M**	0.7407	LPath
**R473N**	0.8712	LPath
**R473P**	0.9116	LPath
**R473Q**	0.1467	LBen
**R473S**	0.808	LPath
**R473T**	0.6828	LPath
**R473V**	0.7466	LPath
**R473W**	0.3557	Amb
**R473Y**	0.7266	LPath
**M474A**	0.0797	LBen
**M474C**	0.4881	Amb
**M474D**	0.2191	LBen
**M474E**	0.105	LBen
**M474F**	0.234	LBen
**M474G**	0.1416	LBen
**M474H**	0.1337	LBen
**M474I**	0.17	LBen
**M474K**	0.0509	LBen
**M474L**	0.0913	LBen
**M474N**	0.1009	LBen
**M474P**	0.4814	Amb
**M474Q**	0.071	LBen
**M474R**	0.0475	LBen
**M474S**	0.0763	LBen
**M474T**	0.0459	LBen
**M474V**	0.0829	LBen
**M474W**	0.2926	LBen
**M474Y**	0.2829	LBen
**E475A**	0.1578	LBen
**E475C**	0.8866	LPath
**E475D**	0.1803	LBen
**E475F**	0.7024	LPath
**E475G**	0.219	LBen

**E475H**	0.4621	Amb
**E475I**	0.4009	Amb
**E475K**	0.2838	LBen
**E475L**	0.3712	Amb
**E475M**	0.5059	Amb
**E475N**	0.3436	Amb
**E475P**	0.753	LPath
**E475Q**	0.1505	LBen
**E475R**	0.3356	LBen
**E475S**	0.2507	LBen
**E475T**	0.283	LBen
**E475V**	0.2247	LBen
**E475W**	0.8955	LPath
**E475Y**	0.6113	LPath
**L476A**	0.4774	Amb
**L476C**	0.7215	LPath
**L476D**	0.8643	LPath
**L476E**	0.6441	LPath
**L476F**	0.1588	LBen
**L476G**	0.7046	LPath
**L476H**	0.4018	Amb
**L476I**	0.1183	LBen
**L476K**	0.5364	Amb
**L476M**	0.1463	LBen
**L476N**	0.601	LPath
**L476P**	0.7773	LPath
**L476Q**	0.3415	Amb
**L476R**	0.3795	Amb
**L476S**	0.5748	LPath
**L476T**	0.5041	Amb
**L476V**	0.1322	LBen
**L476W**	0.29	LBen
**L476Y**	0.4284	Amb
**P477A**	0.0803	LBen
**P477C**	0.4466	Amb
**P477D**	0.2613	LBen
**P477E**	0.175	LBen
**P477F**	0.3081	LBen
**P477G**	0.17	LBen
**P477H**	0.118	LBen

**P477I**	0.254	LBen
**P477K**	0.1568	LBen
**P477L**	0.0917	LBen
**P477M**	0.2568	LBen
**P477N**	0.2146	LBen
**P477Q**	0.0958	LBen
**P477R**	0.0914	LBen
**P477S**	0.0849	LBen
**P477T**	0.0884	LBen
**P477V**	0.1723	LBen
**P477W**	0.3576	Amb
**P477Y**	0.2921	LBen
**Q478A**	0.146	LBen
**Q478C**	0.533	Amb
**Q478D**	0.1954	LBen
**Q478E**	0.0737	LBen
**Q478F**	0.4631	Amb
**Q478G**	0.1295	LBen
**Q478H**	0.1353	LBen
**Q478I**	0.3193	LBen
**Q478K**	0.0884	LBen
**Q478L**	0.1135	LBen
**Q478M**	0.3211	LBen
**Q478N**	0.2032	LBen
**Q478P**	0.1024	LBen
**Q478R**	0.0744	LBen
**Q478S**	0.1402	LBen
**Q478T**	0.1582	LBen
**Q478V**	0.1988	LBen
**Q478W**	0.2849	LBen
**Q478Y**	0.3071	LBen
**R479A**	0.2188	LBen
**R479C**	0.3432	Amb
**R479D**	0.4069	Amb
**R479E**	0.1937	LBen
**R479F**	0.4894	Amb
**R479G**	0.117	LBen
**R479H**	0.1079	LBen
**R479I**	0.2375	LBen
**R479K**	0.072	LBen

**R479L**	0.1849	LBen
**R479M**	0.2017	LBen
**R479N**	0.3781	Amb
**R479P**	0.21	LBen
**R479Q**	0.0846	LBen
**R479S**	0.2773	LBen
**R479T**	0.145	LBen
**R479V**	0.2679	LBen
**R479W**	0.1609	LBen
**R479Y**	0.3578	Amb
**D480A**	0.0942	LBen
**D480C**	0.5141	Amb
**D480E**	0.0796	LBen
**D480F**	0.4131	Amb
**D480G**	0.0901	LBen
**D480H**	0.1396	LBen
**D480I**	0.2683	LBen
**D480K**	0.133	LBen
**D480L**	0.2282	LBen
**D480M**	0.3646	Amb
**D480N**	0.0728	LBen
**D480P**	0.2793	LBen
**D480Q**	0.1285	LBen
**D480R**	0.1421	LBen
**D480S**	0.0862	LBen
**D480T**	0.1409	LBen
**D480V**	0.1497	LBen
**D480W**	0.6253	LPath
**D480Y**	0.1611	LBen
**V481A**	0.2661	LBen
**V481C**	0.8408	LPath
**V481D**	0.6595	LPath
**V481E**	0.6257	LPath
**V481F**	0.2016	LBen
**V481G**	0.3389	LBen
**V481H**	0.7671	LPath
**V481I**	0.0778	LBen
**V481K**	0.7213	LPath
**V481L**	0.1733	LBen
**V481M**	0.1931	LBen

**V481N**	0.502	Amb
**V481P**	0.5594	Amb
**V481Q**	0.6589	LPath
**V481R**	0.5995	LPath
**V481S**	0.3628	Amb
**V481T**	0.3064	LBen
**V481W**	0.84	LPath
**V481Y**	0.6282	LPath
**F482A**	0.9842	LPath
**F482C**	0.9753	LPath
**F482D**	0.9963	LPath
**F482E**	0.9967	LPath
**F482G**	0.9897	LPath
**F482H**	0.9619	LPath
**F482I**	0.9473	LPath
**F482K**	0.9969	LPath
**F482L**	0.992	LPath
**F482M**	0.9767	LPath
**F482N**	0.9875	LPath
**F482P**	0.9977	LPath
**F482Q**	0.9925	LPath
**F482R**	0.9881	LPath
**F482S**	0.973	LPath
**F482T**	0.9882	LPath
**F482V**	0.9306	LPath
**F482W**	0.8687	LPath
**F482Y**	0.5077	Amb
**S483A**	0.0831	LBen
**S483C**	0.2016	LBen
**S483D**	0.4361	Amb
**S483E**	0.4641	Amb
**S483F**	0.2113	LBen
**S483G**	0.0915	LBen
**S483H**	0.2669	LBen
**S483I**	0.1899	LBen
**S483K**	0.5046	Amb
**S483L**	0.1069	LBen
**S483M**	0.2369	LBen
**S483N**	0.175	LBen
**S483P**	0.4353	Amb

**S483Q**	0.3956	Amb
**S483R**	0.331	LBen
**S483T**	0.08	LBen
**S483V**	0.1933	LBen
**S483W**	0.3149	LBen
**S483Y**	0.2013	LBen
**G484A**	0.076	LBen
**G484C**	0.1635	LBen
**G484D**	0.0957	LBen
**G484E**	0.1004	LBen
**G484F**	0.2375	LBen
**G484H**	0.1197	LBen
**G484I**	0.1149	LBen
**G484K**	0.1481	LBen
**G484L**	0.1509	LBen
**G484M**	0.2205	LBen
**G484N**	0.1299	LBen
**G484P**	0.3575	Amb
**G484Q**	0.1178	LBen
**G484R**	0.0928	LBen
**G484S**	0.0657	LBen
**G484T**	0.0831	LBen
**G484V**	0.0852	LBen
**G484W**	0.16	LBen
**G484Y**	0.1789	LBen
**S485A**	0.0749	LBen
**S485C**	0.1956	LBen
**S485D**	0.1722	LBen
**S485E**	0.2105	LBen
**S485F**	0.1448	LBen
**S485G**	0.0665	LBen
**S485H**	0.1822	LBen
**S485I**	0.1257	LBen
**S485K**	0.2715	LBen
**S485L**	0.0907	LBen
**S485M**	0.1539	LBen
**S485N**	0.091	LBen
**S485P**	0.0966	LBen
**S485Q**	0.2206	LBen
**S485R**	0.217	LBen

**S485T**	0.0753	LBen
**S485V**	0.1295	LBen
**S485W**	0.2105	LBen
**S485Y**	0.1496	LBen
**D486A**	0.1205	LBen
**D486C**	0.6291	LPath
**D486E**	0.1061	LBen
**D486F**	0.4294	Amb
**D486G**	0.1012	LBen
**D486H**	0.1842	LBen
**D486I**	0.2675	LBen
**D486K**	0.2235	LBen
**D486L**	0.2657	LBen
**D486M**	0.4562	Amb
**D486N**	0.0859	LBen
**D486P**	0.4231	Amb
**D486Q**	0.196	LBen
**D486R**	0.2249	LBen
**D486S**	0.0899	LBen
**D486T**	0.1595	LBen
**D486V**	0.1668	LBen
**D486W**	0.7726	LPath
**D486Y**	0.2099	LBen
**T487A**	0.0643	LBen
**T487C**	0.4866	Amb
**T487D**	0.2556	LBen
**T487E**	0.2252	LBen
**T487F**	0.1961	LBen
**T487G**	0.1461	LBen
**T487H**	0.1999	LBen
**T487I**	0.1187	LBen
**T487K**	0.1631	LBen
**T487L**	0.0745	LBen
**T487M**	0.0803	LBen
**T487N**	0.1043	LBen
**T487P**	0.1586	LBen
**T487Q**	0.1751	LBen
**T487R**	0.1238	LBen
**T487S**	0.0813	LBen
**T487V**	0.1012	LBen

**T487W**	0.3914	Amb
**T487Y**	0.2316	LBen
**D488A**	0.9161	LPath
**D488C**	0.9926	LPath
**D488E**	0.8963	LPath
**D488F**	0.9823	LPath
**D488G**	0.9558	LPath
**D488H**	0.9697	LPath
**D488I**	0.9751	LPath
**D488K**	0.9883	LPath
**D488L**	0.9566	LPath
**D488M**	0.992	LPath
**D488N**	0.8776	LPath
**D488P**	0.9798	LPath
**D488Q**	0.9816	LPath
**D488R**	0.9815	LPath
**D488S**	0.8659	LPath
**D488T**	0.971	LPath
**D488V**	0.9382	LPath
**D488W**	0.997	LPath
**D488Y**	0.919	LPath
**P489A**	0.0894	LBen
**P489C**	0.7701	LPath
**P489D**	0.7714	LPath
**P489E**	0.558	Amb
**P489F**	0.7083	LPath
**P489G**	0.4384	Amb
**P489H**	0.4236	Amb
**P489I**	0.4565	Amb
**P489K**	0.7008	LPath
**P489L**	0.2323	LBen
**P489M**	0.4855	Amb
**P489N**	0.5914	LPath
**P489Q**	0.3595	Amb
**P489R**	0.4408	Amb
**P489S**	0.1509	LBen
**P489T**	0.1445	LBen
**P489V**	0.2923	LBen
**P489W**	0.7985	LPath
**P489Y**	0.7238	LPath

**D490A**	0.9347	LPath
**D490C**	0.9962	LPath
**D490E**	0.873	LPath
**D490F**	0.9948	LPath
**D490G**	0.9378	LPath
**D490H**	0.9803	LPath
**D490I**	0.9894	LPath
**D490K**	0.9909	LPath
**D490L**	0.9786	LPath
**D490M**	0.9966	LPath
**D490N**	0.8474	LPath
**D490P**	0.9929	LPath
**D490Q**	0.9816	LPath
**D490R**	0.985	LPath
**D490S**	0.8705	LPath
**D490T**	0.9714	LPath
**D490V**	0.9577	LPath
**D490W**	0.999	LPath
**D490Y**	0.9662	LPath
**M491A**	0.9845	LPath
**M491C**	0.9965	LPath
**M491D**	0.9982	LPath
**M491E**	0.9858	LPath
**M491F**	0.9364	LPath
**M491G**	0.9932	LPath
**M491H**	0.9899	LPath
**M491I**	0.953	LPath
**M491K**	0.9527	LPath
**M491L**	0.7041	LPath
**M491N**	0.9859	LPath
**M491P**	0.9984	LPath
**M491Q**	0.9382	LPath
**M491R**	0.9539	LPath
**M491S**	0.9825	LPath
**M491T**	0.971	LPath
**M491V**	0.5957	LPath
**M491W**	0.9929	LPath
**M491Y**	0.9888	LPath
**A492C**	0.765	LPath
**A492D**	0.5008	Amb

**A492E**	0.4123	Amb
**A492F**	0.5007	Amb
**A492G**	0.2003	LBen
**A492H**	0.5826	LPath
**A492I**	0.3501	Amb
**A492K**	0.6825	LPath
**A492L**	0.2609	LBen
**A492M**	0.3237	LBen
**A492N**	0.3524	Amb
**A492P**	0.9359	LPath
**A492Q**	0.4194	Amb
**A492R**	0.4878	Amb
**A492S**	0.0927	LBen
**A492T**	0.1207	LBen
**A492V**	0.1674	LBen
**A492W**	0.8684	LPath
**A492Y**	0.636	LPath
**F493A**	0.8636	LPath
**F493C**	0.7673	LPath
**F493D**	0.9976	LPath
**F493E**	0.9976	LPath
**F493G**	0.9724	LPath
**F493H**	0.976	LPath
**F493I**	0.5401	Amb
**F493K**	0.9973	LPath
**F493L**	0.8465	LPath
**F493M**	0.6863	LPath
**F493N**	0.9877	LPath
**F493P**	0.9986	LPath
**F493Q**	0.9907	LPath
**F493R**	0.9891	LPath
**F493S**	0.9343	LPath
**F493T**	0.9462	LPath
**F493V**	0.4585	Amb
**F493W**	0.8213	LPath
**F493Y**	0.5291	Amb
**C494A**	0.842	LPath
**C494D**	0.9988	LPath
**C494E**	0.9992	LPath
**C494F**	0.988	LPath

**C494G**	0.7788	LPath
**C494H**	0.9958	LPath
**C494I**	0.9939	LPath
**C494K**	0.9998	LPath
**C494L**	0.9757	LPath
**C494M**	0.9901	LPath
**C494N**	0.9882	LPath
**C494P**	0.9936	LPath
**C494Q**	0.9948	LPath
**C494R**	0.9944	LPath
**C494S**	0.7429	LPath
**C494T**	0.9449	LPath
**C494V**	0.9729	LPath
**C494W**	0.9975	LPath
**C494Y**	0.9969	LPath
**K495A**	0.5528	Amb
**K495C**	0.8571	LPath
**K495D**	0.8855	LPath
**K495E**	0.4039	Amb
**K495F**	0.871	LPath
**K495G**	0.6169	LPath
**K495H**	0.362	Amb
**K495I**	0.7091	LPath
**K495L**	0.5551	Amb
**K495M**	0.4282	Amb
**K495N**	0.7506	LPath
**K495P**	0.9731	LPath
**K495Q**	0.1484	LBen
**K495R**	0.0766	LBen
**K495S**	0.5981	LPath
**K495T**	0.4311	Amb
**K495V**	0.6211	LPath
**K495W**	0.8112	LPath
**K495Y**	0.7838	LPath
**S496A**	0.0892	LBen
**S496C**	0.1826	LBen
**S496D**	0.2397	LBen
**S496E**	0.3428	Amb
**S496F**	0.2018	LBen
**S496G**	0.0766	LBen

**S496H**	0.164	LBen
**S496I**	0.1341	LBen
**S496K**	0.3528	Amb
**S496L**	0.1381	LBen
**S496M**	0.1934	LBen
**S496N**	0.0773	LBen
**S496P**	0.7711	LPath
**S496Q**	0.2753	LBen
**S496R**	0.2401	LBen
**S496T**	0.0788	LBen
**S496V**	0.1694	LBen
**S496W**	0.2708	LBen
**S496Y**	0.1285	LBen
**F497A**	0.3025	LBen
**F497C**	0.1593	LBen
**F497D**	0.967	LPath
**F497E**	0.9366	LPath
**F497G**	0.693	LPath
**F497H**	0.7503	LPath
**F497I**	0.0671	LBen
**F497K**	0.9528	LPath
**F497L**	0.1512	LBen
**F497M**	0.1497	LBen
**F497N**	0.7951	LPath
**F497P**	0.9556	LPath
**F497Q**	0.7842	LPath
**F497R**	0.852	LPath
**F497S**	0.4521	Amb
**F497T**	0.418	Amb
**F497V**	0.0601	LBen
**F497W**	0.4523	Amb
**F497Y**	0.3207	LBen
**R498A**	0.9928	LPath
**R498C**	0.9345	LPath
**R498D**	0.9975	LPath
**R498E**	0.9727	LPath
**R498F**	0.994	LPath
**R498G**	0.9799	LPath
**R498H**	0.7486	LPath
**R498I**	0.9777	LPath

**R498K**	0.8389	LPath
**R498L**	0.9469	LPath
**R498M**	0.9897	LPath
**R498N**	0.9958	LPath
**R498P**	0.996	LPath
**R498Q**	0.6783	LPath
**R498S**	0.993	LPath
**R498T**	0.9892	LPath
**R498V**	0.983	LPath
**R498W**	0.7945	LPath
**R498Y**	0.9756	LPath
**D499A**	0.1536	LBen
**D499C**	0.6786	LPath
**D499E**	0.0857	LBen
**D499F**	0.6107	LPath
**D499G**	0.144	LBen
**D499H**	0.2531	LBen
**D499I**	0.4139	Amb
**D499K**	0.2571	LBen
**D499L**	0.3382	LBen
**D499M**	0.5213	Amb
**D499N**	0.0922	LBen
**D499P**	0.908	LPath
**D499Q**	0.1811	LBen
**D499R**	0.276	LBen
**D499S**	0.1139	LBen
**D499T**	0.2338	LBen
**D499V**	0.2379	LBen
**D499W**	0.8231	LPath
**D499Y**	0.2639	LBen
**K500A**	0.3604	Amb
**K500C**	0.7953	LPath
**K500D**	0.5602	Amb
**K500E**	0.1421	LBen
**K500F**	0.8211	LPath
**K500G**	0.4083	Amb
**K500H**	0.2908	LBen
**K500I**	0.5646	LPath
**K500L**	0.3592	Amb
**K500M**	0.2691	LBen

**K500N**	0.4395	Amb
**K500P**	0.9253	LPath
**K500Q**	0.1004	LBen
**K500R**	0.0747	LBen
**K500S**	0.3867	Amb
**K500T**	0.2658	LBen
**K500V**	0.4881	Amb
**K500W**	0.7437	LPath
**K500Y**	0.6678	LPath
**G501A**	0.4326	Amb
**G501C**	0.7304	LPath
**G501D**	0.3765	Amb
**G501E**	0.4696	Amb
**G501F**	0.9371	LPath
**G501H**	0.8025	LPath
**G501I**	0.8882	LPath
**G501K**	0.8617	LPath
**G501L**	0.8929	LPath
**G501M**	0.8846	LPath
**G501N**	0.4371	Amb
**G501P**	0.9886	LPath
**G501Q**	0.6789	LPath
**G501R**	0.7876	LPath
**G501S**	0.2351	LBen
**G501T**	0.5934	LPath
**G501V**	0.7615	LPath
**G501W**	0.869	LPath
**G501Y**	0.8368	LPath
**I502A**	0.6591	LPath
**I502C**	0.7969	LPath
**I502D**	0.9063	LPath
**I502E**	0.887	LPath
**I502F**	0.2217	LBen
**I502G**	0.8494	LPath
**I502H**	0.7069	LPath
**I502K**	0.7479	LPath
**I502L**	0.1407	LBen
**I502M**	0.2	LBen
**I502N**	0.5434	Amb
**I502P**	0.9406	LPath

**I502Q**	0.7892	LPath
**I502R**	0.6326	LPath
**I502S**	0.6057	LPath
**I502T**	0.5729	LPath
**I502V**	0.0913	LBen
**I502W**	0.8345	LPath
**I502Y**	0.5535	Amb
**F503A**	0.9496	LPath
**F503C**	0.9074	LPath
**F503D**	0.9861	LPath
**F503E**	0.9913	LPath
**F503G**	0.9731	LPath
**F503H**	0.8187	LPath
**F503I**	0.8965	LPath
**F503K**	0.9922	LPath
**F503L**	0.9679	LPath
**F503M**	0.9134	LPath
**F503N**	0.9399	LPath
**F503P**	0.99	LPath
**F503Q**	0.969	LPath
**F503R**	0.9722	LPath
**F503S**	0.8826	LPath
**F503T**	0.9671	LPath
**F503V**	0.8717	LPath
**F503W**	0.8668	LPath
**F503Y**	0.4599	Amb
**L504A**	0.8588	LPath
**L504C**	0.8955	LPath
**L504D**	0.9981	LPath
**L504E**	0.9843	LPath
**L504F**	0.432	Amb
**L504G**	0.9668	LPath
**L504H**	0.9444	LPath
**L504I**	0.2691	LBen
**L504K**	0.9842	LPath
**L504M**	0.1691	LBen
**L504N**	0.9767	LPath
**L504P**	0.9107	LPath
**L504Q**	0.8387	LPath
**L504R**	0.9603	LPath

**L504S**	0.9241	LPath
**L504T**	0.8894	LPath
**L504V**	0.2944	LBen
**L504W**	0.9188	LPath
**L504Y**	0.9411	LPath
**H505A**	0.8961	LPath
**H505C**	0.6456	LPath
**H505D**	0.8854	LPath
**H505E**	0.8978	LPath
**H505F**	0.3894	Amb
**H505G**	0.9155	LPath
**H505I**	0.8307	LPath
**H505K**	0.9267	LPath
**H505L**	0.4734	Amb
**H505M**	0.8777	LPath
**H505N**	0.5455	Amb
**H505P**	0.8358	LPath
**H505Q**	0.7172	LPath
**H505R**	0.6808	LPath
**H505S**	0.7598	LPath
**H505T**	0.8692	LPath
**H505V**	0.7899	LPath
**H505W**	0.5169	Amb
**H505Y**	0.1409	LBen
**L506A**	0.7364	LPath
**L506C**	0.8382	LPath
**L506D**	0.9975	LPath
**L506E**	0.9843	LPath
**L506F**	0.7611	LPath
**L506G**	0.9648	LPath
**L506H**	0.9742	LPath
**L506I**	0.0808	LBen
**L506K**	0.9897	LPath
**L506M**	0.3182	LBen
**L506N**	0.9764	LPath
**L506P**	0.9869	LPath
**L506Q**	0.9381	LPath
**L506R**	0.9681	LPath
**L506S**	0.9226	LPath
**L506T**	0.7662	LPath

**L506V**	0.0803	LBen
**L506W**	0.9785	LPath
**L506Y**	0.9775	LPath
**S507A**	0.2353	LBen
**S507C**	0.2666	LBen
**S507D**	0.6464	LPath
**S507E**	0.8887	LPath
**S507F**	0.8441	LPath
**S507G**	0.2577	LBen
**S507H**	0.7253	LPath
**S507I**	0.5044	Amb
**S507K**	0.9576	LPath
**S507L**	0.365	Amb
**S507M**	0.5736	LPath
**S507N**	0.3154	LBen
**S507P**	0.8989	LPath
**S507Q**	0.8647	LPath
**S507R**	0.9097	LPath
**S507T**	0.1074	LBen
**S507V**	0.4644	Amb
**S507W**	0.8072	LPath
**S507Y**	0.7466	LPath
**N508A**	0.9635	LPath
**N508C**	0.9402	LPath
**N508D**	0.9506	LPath
**N508E**	0.9932	LPath
**N508F**	0.9965	LPath
**N508G**	0.9392	LPath
**N508H**	0.9051	LPath
**N508I**	0.9733	LPath
**N508K**	0.9948	LPath
**N508L**	0.9306	LPath
**N508M**	0.9815	LPath
**N508P**	0.9715	LPath
**N508Q**	0.9851	LPath
**N508R**	0.987	LPath
**N508S**	0.4437	Amb
**N508T**	0.877	LPath
**N508V**	0.9593	LPath
**N508W**	0.9982	LPath

**N508Y**	0.9644	LPath
**Q509A**	0.305	LBen
**Q509C**	0.5999	LPath
**Q509D**	0.5883	LPath
**Q509E**	0.0976	LBen
**Q509F**	0.6568	LPath
**Q509G**	0.3095	LBen
**Q509H**	0.2491	LBen
**Q509I**	0.4167	Amb
**Q509K**	0.102	LBen
**Q509L**	0.125	LBen
**Q509M**	0.388	Amb
**Q509N**	0.4806	Amb
**Q509P**	0.5482	Amb
**Q509R**	0.0661	LBen
**Q509S**	0.3586	Amb
**Q509T**	0.2454	LBen
**Q509V**	0.2862	LBen
**Q509W**	0.4098	Amb
**Q509Y**	0.4707	Amb
**H510A**	0.217	LBen
**H510C**	0.3386	LBen
**H510D**	0.1201	LBen
**H510E**	0.1173	LBen
**H510F**	0.3214	LBen
**H510G**	0.2616	LBen
**H510I**	0.3114	LBen
**H510K**	0.1434	LBen
**H510L**	0.133	LBen
**H510M**	0.3765	Amb
**H510N**	0.1077	LBen
**H510P**	0.4197	Amb
**H510Q**	0.0967	LBen
**H510R**	0.0788	LBen
**H510S**	0.191	LBen
**H510T**	0.2266	LBen
**H510V**	0.2123	LBen
**H510W**	0.3182	LBen
**H510Y**	0.1153	LBen
**E511A**	0.1697	LBen

**E511C**	0.8782	LPath
**E511D**	0.091	LBen
**E511F**	0.6689	LPath
**E511G**	0.2365	LBen
**E511H**	0.3658	Amb
**E511I**	0.3557	Amb
**E511K**	0.1998	LBen
**E511L**	0.4019	Amb
**E511M**	0.5187	Amb
**E511N**	0.2269	LBen
**E511P**	0.3499	Amb
**E511Q**	0.1517	LBen
**E511R**	0.2538	LBen
**E511S**	0.1779	LBen
**E511T**	0.2041	LBen
**E511V**	0.222	LBen
**E511W**	0.8399	LPath
**E511Y**	0.4917	Amb
**F512A**	0.937	LPath
**F512C**	0.9372	LPath
**F512D**	0.9792	LPath
**F512E**	0.9875	LPath
**F512G**	0.963	LPath
**F512H**	0.9108	LPath
**F512I**	0.8956	LPath
**F512K**	0.9936	LPath
**F512L**	0.9799	LPath
**F512M**	0.9052	LPath
**F512N**	0.9483	LPath
**F512P**	0.9935	LPath
**F512Q**	0.9829	LPath
**F512R**	0.9841	LPath
**F512S**	0.9137	LPath
**F512T**	0.9513	LPath
**F512V**	0.8724	LPath
**F512W**	0.587	LPath
**F512Y**	0.2216	LBen
**G513A**	0.9156	LPath
**G513C**	0.9822	LPath
**G513D**	0.9913	LPath

**G513E**	0.9938	LPath
**G513F**	0.9974	LPath
**G513H**	0.9977	LPath
**G513I**	0.9957	LPath
**G513K**	0.998	LPath
**G513L**	0.9938	LPath
**G513M**	0.9985	LPath
**G513N**	0.9967	LPath
**G513P**	0.9973	LPath
**G513Q**	0.9945	LPath
**G513R**	0.9899	LPath
**G513S**	0.8969	LPath
**G513T**	0.9875	LPath
**G513V**	0.9901	LPath
**G513W**	0.9925	LPath
**G513Y**	0.9972	LPath
**R514A**	0.9635	LPath
**R514C**	0.571	LPath
**R514D**	0.9796	LPath
**R514E**	0.8911	LPath
**R514F**	0.8476	LPath
**R514G**	0.8907	LPath
**R514H**	0.1082	LBen
**R514I**	0.9169	LPath
**R514K**	0.5463	Amb
**R514L**	0.7981	LPath
**R514M**	0.9244	LPath
**R514N**	0.9368	LPath
**R514P**	0.9922	LPath
**R514Q**	0.337	LBen
**R514S**	0.9411	LPath
**R514T**	0.9361	LPath
**R514V**	0.9221	LPath
**R514W**	0.2907	LBen
**R514Y**	0.5092	Amb
**L515A**	0.9466	LPath
**L515C**	0.9772	LPath
**L515D**	0.9977	LPath
**L515E**	0.9787	LPath
**L515F**	0.8591	LPath

**L515G**	0.9865	LPath
**L515H**	0.9632	LPath
**L515I**	0.2872	LBen
**L515K**	0.9706	LPath
**L515M**	0.4655	Amb
**L515N**	0.9722	LPath
**L515P**	0.9931	LPath
**L515Q**	0.8932	LPath
**L515R**	0.9374	LPath
**L515S**	0.9766	LPath
**L515T**	0.9219	LPath
**L515V**	0.4208	Amb
**L515W**	0.9499	LPath
**L515Y**	0.9583	LPath
**L516A**	0.6889	LPath
**L516C**	0.8947	LPath
**L516D**	0.9925	LPath
**L516E**	0.9314	LPath
**L516F**	0.7654	LPath
**L516G**	0.9401	LPath
**L516H**	0.8887	LPath
**L516I**	0.134	LBen
**L516K**	0.8701	LPath
**L516M**	0.3224	LBen
**L516N**	0.9387	LPath
**L516P**	0.9061	LPath
**L516Q**	0.709	LPath
**L516R**	0.7743	LPath
**L516S**	0.871	LPath
**L516T**	0.5714	LPath
**L516V**	0.127	LBen
**L516W**	0.8898	LPath
**L516Y**	0.9471	LPath
**A517C**	0.7542	LPath
**A517D**	0.5439	Amb
**A517E**	0.6016	LPath
**A517F**	0.5578	Amb
**A517G**	0.2312	LBen
**A517H**	0.6565	LPath
**A517I**	0.5729	LPath

**A517K**	0.8761	LPath
**A517L**	0.3213	LBen
**A517M**	0.4545	Amb
**A517N**	0.3479	Amb
**A517P**	0.7159	LPath
**A517Q**	0.5483	Amb
**A517R**	0.8021	LPath
**A517S**	0.0897	LBen
**A517T**	0.1461	LBen
**A517V**	0.2638	LBen
**A517W**	0.9022	LPath
**A517Y**	0.7226	LPath
**T518A**	0.1083	LBen
**T518C**	0.6655	LPath
**T518D**	0.8041	LPath
**T518E**	0.7846	LPath
**T518F**	0.6403	LPath
**T518G**	0.3047	LBen
**T518H**	0.6099	LPath
**T518I**	0.3692	Amb
**T518K**	0.6226	LPath
**T518L**	0.153	LBen
**T518M**	0.1738	LBen
**T518N**	0.3019	LBen
**T518P**	0.1525	LBen
**T518Q**	0.5597	Amb
**T518R**	0.5351	Amb
**T518S**	0.1666	LBen
**T518V**	0.2203	LBen
**T518W**	0.9056	LPath
**T518Y**	0.7176	LPath
**S519A**	0.0828	LBen
**S519C**	0.4044	Amb
**S519D**	0.6082	LPath
**S519E**	0.6674	LPath
**S519F**	0.4504	Amb
**S519G**	0.0952	LBen
**S519H**	0.5346	Amb
**S519I**	0.4132	Amb
**S519K**	0.8699	LPath

**S519L**	0.2082	LBen
**S519M**	0.338	LBen
**S519N**	0.1956	LBen
**S519P**	0.3885	Amb
**S519Q**	0.582	LPath
**S519R**	0.819	LPath
**S519T**	0.1101	LBen
**S519V**	0.315	LBen
**S519W**	0.6335	LPath
**S519Y**	0.4519	Amb
**R520A**	0.3355	LBen
**R520C**	0.3092	LBen
**R520D**	0.5007	Amb
**R520E**	0.4104	Amb
**R520F**	0.6443	LPath
**R520G**	0.1617	LBen
**R520H**	0.0949	LBen
**R520I**	0.5034	Amb
**R520K**	0.1435	LBen
**R520L**	0.295	LBen
**R520M**	0.5031	Amb
**R520N**	0.4095	Amb
**R520P**	0.5947	LPath
**R520Q**	0.1114	LBen
**R520S**	0.3091	LBen
**R520T**	0.2691	LBen
**R520V**	0.4957	Amb
**R520W**	0.2933	LBen
**R520Y**	0.4691	Amb
**Y521A**	0.9367	LPath
**Y521C**	0.6208	LPath
**Y521D**	0.9479	LPath
**Y521E**	0.9802	LPath
**Y521F**	0.1275	LBen
**Y521G**	0.8971	LPath
**Y521H**	0.8303	LPath
**Y521I**	0.8652	LPath
**Y521K**	0.9843	LPath
**Y521L**	0.8288	LPath
**Y521M**	0.8677	LPath

**Y521N**	0.8476	LPath
**Y521P**	0.9777	LPath
**Y521Q**	0.9615	LPath
**Y521R**	0.9535	LPath
**Y521S**	0.8481	LPath
**Y521T**	0.9026	LPath
**Y521V**	0.7188	LPath
**Y521W**	0.7091	LPath
**D522A**	0.1697	LBen
**D522C**	0.73	LPath
**D522E**	0.1712	LBen
**D522F**	0.6928	LPath
**D522G**	0.2388	LBen
**D522H**	0.2218	LBen
**D522I**	0.411	Amb
**D522K**	0.3394	LBen
**D522L**	0.4258	Amb
**D522M**	0.6552	LPath
**D522N**	0.0812	LBen
**D522P**	0.6928	LPath
**D522Q**	0.2363	LBen
**D522R**	0.2805	LBen
**D522S**	0.1064	LBen
**D522T**	0.2431	LBen
**D522V**	0.2419	LBen
**D522W**	0.9104	LPath
**D522Y**	0.32	LBen
**T523A**	0.0988	LBen
**T523C**	0.6057	LPath
**T523D**	0.4893	Amb
**T523E**	0.3382	LBen
**T523F**	0.3971	Amb
**T523G**	0.2587	LBen
**T523H**	0.3562	Amb
**T523I**	0.1437	LBen
**T523K**	0.1966	LBen
**T523L**	0.0845	LBen
**T523M**	0.0882	LBen
**T523N**	0.211	LBen
**T523P**	0.0797	LBen

**T523Q**	0.2647	LBen
**T523R**	0.1728	LBen
**T523S**	0.141	LBen
**T523V**	0.1259	LBen
**T523W**	0.7035	LPath
**T523Y**	0.4776	Amb
**E524A**	0.083	LBen
**E524C**	0.651	LPath
**E524D**	0.0664	LBen
**E524F**	0.5776	LPath
**E524G**	0.0773	LBen
**E524H**	0.18	LBen
**E524I**	0.331	LBen
**E524K**	0.0889	LBen
**E524L**	0.2411	LBen
**E524M**	0.3572	Amb
**E524N**	0.1034	LBen
**E524P**	0.4687	Amb
**E524Q**	0.0774	LBen
**E524R**	0.1122	LBen
**E524S**	0.0748	LBen
**E524T**	0.1055	LBen
**E524V**	0.1871	LBen
**E524W**	0.704	LPath
**E524Y**	0.3901	Amb
**H525A**	0.6212	LPath
**H525C**	0.4327	Amb
**H525D**	0.6972	LPath
**H525E**	0.6365	LPath
**H525F**	0.5899	LPath
**H525G**	0.616	LPath
**H525I**	0.7018	LPath
**H525K**	0.2802	LBen
**H525L**	0.3397	LBen
**H525M**	0.7843	LPath
**H525N**	0.35	Amb
**H525P**	0.4386	Amb
**H525Q**	0.3271	LBen
**H525R**	0.0939	LBen
**H525S**	0.6257	LPath

**H525T**	0.6513	LPath
**H525V**	0.5628	Amb
**H525W**	0.5643	LPath
**H525Y**	0.2502	LBen
**L526A**	0.2566	LBen
**L526C**	0.5977	LPath
**L526D**	0.7466	LPath
**L526E**	0.3517	Amb
**L526F**	0.2002	LBen
**L526G**	0.5865	LPath
**L526H**	0.2523	LBen
**L526I**	0.1556	LBen
**L526K**	0.2369	LBen
**L526M**	0.2105	LBen
**L526N**	0.5622	Amb
**L526P**	0.3152	LBen
**L526Q**	0.1495	LBen
**L526R**	0.1182	LBen
**L526S**	0.3681	Amb
**L526T**	0.2723	LBen
**L526V**	0.1208	LBen
**L526W**	0.2768	LBen
**L526Y**	0.3701	Amb
**H527A**	0.7571	LPath
**H527C**	0.4706	Amb
**H527D**	0.8044	LPath
**H527E**	0.7553	LPath
**H527F**	0.3896	Amb
**H527G**	0.8509	LPath
**H527I**	0.8303	LPath
**H527K**	0.6895	LPath
**H527L**	0.5157	Amb
**H527M**	0.8695	LPath
**H527N**	0.2385	LBen
**H527P**	0.8278	LPath
**H527Q**	0.4674	Amb
**H527R**	0.2829	LBen
**H527S**	0.5366	Amb
**H527T**	0.7206	LPath
**H527V**	0.7467	LPath

**H527W**	0.5299	Amb
**H527Y**	0.1287	LBen
**P528A**	0.1631	LBen
**P528C**	0.7619	LPath
**P528D**	0.8564	LPath
**P528E**	0.8305	LPath
**P528F**	0.9812	LPath
**P528G**	0.409	Amb
**P528H**	0.6374	LPath
**P528I**	0.9544	LPath
**P528K**	0.9227	LPath
**P528L**	0.7373	LPath
**P528M**	0.918	LPath
**P528N**	0.4019	Amb
**P528Q**	0.642	LPath
**P528R**	0.7184	LPath
**P528S**	0.1804	LBen
**P528T**	0.4772	Amb
**P528V**	0.8433	LPath
**P528W**	0.9742	LPath
**P528Y**	0.9517	LPath
**D529A**	0.8909	LPath
**D529C**	0.9903	LPath
**D529E**	0.5795	LPath
**D529F**	0.9964	LPath
**D529G**	0.9262	LPath
**D529H**	0.9498	LPath
**D529I**	0.9926	LPath
**D529K**	0.9837	LPath
**D529L**	0.9823	LPath
**D529M**	0.9962	LPath
**D529N**	0.7679	LPath
**D529P**	0.9871	LPath
**D529Q**	0.9534	LPath
**D529R**	0.9794	LPath
**D529S**	0.8185	LPath
**D529T**	0.9723	LPath
**D529V**	0.9712	LPath
**D529W**	0.9991	LPath
**D529Y**	0.9736	LPath

**L530A**	0.8891	LPath
**L530C**	0.9496	LPath
**L530D**	0.9977	LPath
**L530E**	0.9857	LPath
**L530F**	0.5454	Amb
**L530G**	0.9817	LPath
**L530H**	0.9566	LPath
**L530I**	0.2693	LBen
**L530K**	0.9711	LPath
**L530M**	0.2207	LBen
**L530N**	0.9847	LPath
**L530P**	0.982	LPath
**L530Q**	0.908	LPath
**L530R**	0.9355	LPath
**L530S**	0.9687	LPath
**L530T**	0.908	LPath
**L530V**	0.3148	LBen
**L530W**	0.8947	LPath
**L530Y**	0.9406	LPath
**W531A**	0.9649	LPath
**W531C**	0.9851	LPath
**W531D**	0.993	LPath
**W531E**	0.9905	LPath
**W531F**	0.6217	LPath
**W531G**	0.8551	LPath
**W531H**	0.9199	LPath
**W531I**	0.9918	LPath
**W531K**	0.988	LPath
**W531L**	0.9258	LPath
**W531M**	0.9852	LPath
**W531N**	0.9867	LPath
**W531P**	0.9946	LPath
**W531Q**	0.9785	LPath
**W531R**	0.9538	LPath
**W531S**	0.9138	LPath
**W531T**	0.9777	LPath
**W531V**	0.9828	LPath
**W531Y**	0.6496	LPath
**Q532A**	0.7286	LPath
**Q532C**	0.9741	LPath

**Q532D**	0.9536	LPath
**Q532E**	0.1656	LBen
**Q532F**	0.9932	LPath
**Q532G**	0.7983	LPath
**Q532H**	0.8871	LPath
**Q532I**	0.9768	LPath
**Q532K**	0.908	LPath
**Q532L**	0.7488	LPath
**Q532M**	0.7361	LPath
**Q532N**	0.6533	LPath
**Q532P**	0.9859	LPath
**Q532R**	0.911	LPath
**Q532S**	0.7363	LPath
**Q532T**	0.8229	LPath
**Q532V**	0.9148	LPath
**Q532W**	0.996	LPath
**Q532Y**	0.989	LPath
**I533A**	0.829	LPath
**I533C**	0.9254	LPath
**I533D**	0.9895	LPath
**I533E**	0.9825	LPath
**I533F**	0.5321	Amb
**I533G**	0.9658	LPath
**I533H**	0.9576	LPath
**I533K**	0.969	LPath
**I533L**	0.1562	LBen
**I533M**	0.2123	LBen
**I533N**	0.8998	LPath
**I533P**	0.9305	LPath
**I533Q**	0.9471	LPath
**I533R**	0.9299	LPath
**I533S**	0.881	LPath
**I533T**	0.9468	LPath
**I533V**	0.1729	LBen
**I533W**	0.9618	LPath
**I533Y**	0.8454	LPath
**F534A**	0.5097	Amb
**F534C**	0.7619	LPath
**F534D**	0.8193	LPath
**F534E**	0.8325	LPath

**F534G**	0.7347	LPath
**F534H**	0.6096	LPath
**F534I**	0.569	LPath
**F534K**	0.8563	LPath
**F534L**	0.8981	LPath
**F534M**	0.6447	LPath
**F534N**	0.6695	LPath
**F534P**	0.9492	LPath
**F534Q**	0.7109	LPath
**F534R**	0.7171	LPath
**F534S**	0.3999	Amb
**F534T**	0.4915	Amb
**F534V**	0.4506	Amb
**F534W**	0.666	LPath
**F534Y**	0.3364	LBen
**D535A**	0.373	Amb
**D535C**	0.8718	LPath
**D535E**	0.2187	LBen
**D535F**	0.8413	LPath
**D535G**	0.4439	Amb
**D535H**	0.4083	Amb
**D535I**	0.7219	LPath
**D535K**	0.7794	LPath
**D535L**	0.6484	LPath
**D535M**	0.8841	LPath
**D535N**	0.2139	LBen
**D535P**	0.9304	LPath
**D535Q**	0.5016	Amb
**D535R**	0.6958	LPath
**D535S**	0.2172	LBen
**D535T**	0.4736	Amb
**D535V**	0.5252	Amb
**D535W**	0.9511	LPath
**D535Y**	0.509	Amb
**N536A**	0.9835	LPath
**N536C**	0.9499	LPath
**N536D**	0.9768	LPath
**N536E**	0.9941	LPath
**N536F**	0.9973	LPath
**N536G**	0.9922	LPath

**N536H**	0.9078	LPath
**N536I**	0.9745	LPath
**N536K**	0.9981	LPath
**N536L**	0.9647	LPath
**N536M**	0.9866	LPath
**N536P**	0.9928	LPath
**N536Q**	0.9912	LPath
**N536R**	0.996	LPath
**N536S**	0.691	LPath
**N536T**	0.9409	LPath
**N536V**	0.9752	LPath
**N536W**	0.9993	LPath
**N536Y**	0.9732	LPath
**P537A**	0.1683	LBen
**P537C**	0.902	LPath
**P537D**	0.8077	LPath
**P537E**	0.4447	Amb
**P537F**	0.8808	LPath
**P537G**	0.6598	LPath
**P537H**	0.4899	Amb
**P537I**	0.6253	LPath
**P537K**	0.5753	LPath
**P537L**	0.325	LBen
**P537M**	0.6159	LPath
**P537N**	0.7666	LPath
**P537Q**	0.2977	LBen
**P537R**	0.3384	LBen
**P537S**	0.3136	LBen
**P537T**	0.1966	LBen
**P537V**	0.4174	Amb
**P537W**	0.9141	LPath
**P537Y**	0.8574	LPath
**V538A**	0.1091	LBen
**V538C**	0.7552	LPath
**V538D**	0.2382	LBen
**V538E**	0.1254	LBen
**V538F**	0.1879	LBen
**V538G**	0.1803	LBen
**V538H**	0.4127	Amb
**V538I**	0.0812	LBen

**V538K**	0.223	LBen
**V538L**	0.0731	LBen
**V538M**	0.0971	LBen
**V538N**	0.2422	LBen
**V538P**	0.4103	Amb
**V538Q**	0.1535	LBen
**V538R**	0.1852	LBen
**V538S**	0.1752	LBen
**V538T**	0.1327	LBen
**V538W**	0.5688	LPath
**V538Y**	0.4799	Amb
**D539A**	0.8883	LPath
**D539C**	0.9876	LPath
**D539E**	0.6621	LPath
**D539F**	0.9827	LPath
**D539G**	0.9192	LPath
**D539H**	0.9755	LPath
**D539I**	0.9693	LPath
**D539K**	0.9909	LPath
**D539L**	0.9445	LPath
**D539M**	0.9922	LPath
**D539N**	0.93	LPath
**D539P**	0.9889	LPath
**D539Q**	0.9652	LPath
**D539R**	0.9824	LPath
**D539S**	0.8989	LPath
**D539T**	0.9661	LPath
**D539V**	0.9101	LPath
**D539W**	0.9948	LPath
**D539Y**	0.9296	LPath
**W540A**	0.9936	LPath
**W540C**	0.9976	LPath
**W540D**	0.9995	LPath
**W540E**	0.9993	LPath
**W540F**	0.7308	LPath
**W540G**	0.9753	LPath
**W540H**	0.9963	LPath
**W540I**	0.9936	LPath
**W540K**	0.9997	LPath
**W540L**	0.9739	LPath

**W540M**	0.9942	LPath
**W540N**	0.9995	LPath
**W540P**	0.9995	LPath
**W540Q**	0.9992	LPath
**W540R**	0.9983	LPath
**W540S**	0.9933	LPath
**W540T**	0.9969	LPath
**W540V**	0.9901	LPath
**W540Y**	0.9515	LPath
**K541A**	0.2879	LBen
**K541C**	0.7068	LPath
**K541D**	0.6723	LPath
**K541E**	0.1045	LBen
**K541F**	0.8635	LPath
**K541G**	0.4494	Amb
**K541H**	0.307	LBen
**K541I**	0.5094	Amb
**K541L**	0.3845	Amb
**K541M**	0.2636	LBen
**K541N**	0.5553	Amb
**K541P**	0.9011	LPath
**K541Q**	0.0714	LBen
**K541R**	0.0702	LBen
**K541S**	0.4347	Amb
**K541T**	0.2063	LBen
**K541V**	0.4137	Amb
**K541W**	0.704	LPath
**K541Y**	0.711	LPath
**E542A**	0.3367	LBen
**E542C**	0.9728	LPath
**E542D**	0.2992	LBen
**E542F**	0.9512	LPath
**E542G**	0.451	Amb
**E542H**	0.8125	LPath
**E542I**	0.8057	LPath
**E542K**	0.6012	LPath
**E542L**	0.7125	LPath
**E542M**	0.7763	LPath
**E542N**	0.6527	LPath
**E542P**	0.9845	LPath

**E542Q**	0.2871	LBen
**E542R**	0.6578	LPath
**E542S**	0.4972	Amb
**E542T**	0.5937	LPath
**E542V**	0.6177	LPath
**E542W**	0.9718	LPath
**E542Y**	0.8963	LPath
**Q543A**	0.193	LBen
**Q543C**	0.6597	LPath
**Q543D**	0.5415	Amb
**Q543E**	0.1183	LBen
**Q543F**	0.7166	LPath
**Q543G**	0.3867	Amb
**Q543H**	0.2357	LBen
**Q543I**	0.3732	Amb
**Q543K**	0.1096	LBen
**Q543L**	0.1177	LBen
**Q543M**	0.3462	Amb
**Q543N**	0.3447	Amb
**Q543P**	0.6701	LPath
**Q543R**	0.1075	LBen
**Q543S**	0.3188	LBen
**Q543T**	0.1816	LBen
**Q543V**	0.256	LBen
**Q543W**	0.6185	LPath
**Q543Y**	0.4893	Amb
**Y544A**	0.991	LPath
**Y544C**	0.9477	LPath
**Y544D**	0.9914	LPath
**Y544E**	0.9982	LPath
**Y544F**	0.3903	Amb
**Y544G**	0.9697	LPath
**Y544H**	0.9446	LPath
**Y544I**	0.9938	LPath
**Y544K**	0.9972	LPath
**Y544L**	0.9665	LPath
**Y544M**	0.9895	LPath
**Y544N**	0.9404	LPath
**Y544P**	0.9993	LPath
**Y544Q**	0.9961	LPath

**Y544R**	0.9887	LPath
**Y544S**	0.9785	LPath
**Y544T**	0.9948	LPath
**Y544V**	0.9784	LPath
**Y544W**	0.8814	LPath
**I545A**	0.93	LPath
**I545C**	0.9576	LPath
**I545D**	0.9988	LPath
**I545E**	0.9953	LPath
**I545F**	0.6312	LPath
**I545G**	0.9893	LPath
**I545H**	0.9905	LPath
**I545K**	0.9887	LPath
**I545L**	0.1151	LBen
**I545M**	0.3401	Amb
**I545N**	0.9859	LPath
**I545P**	0.9902	LPath
**I545Q**	0.9868	LPath
**I545R**	0.9735	LPath
**I545S**	0.9635	LPath
**I545T**	0.9081	LPath
**I545V**	0.126	LBen
**I545W**	0.9808	LPath
**I545Y**	0.964	LPath
**H546A**	0.7343	LPath
**H546C**	0.6846	LPath
**H546D**	0.6019	LPath
**H546E**	0.904	LPath
**H546F**	0.9317	LPath
**H546G**	0.8445	LPath
**H546I**	0.9612	LPath
**H546K**	0.9627	LPath
**H546L**	0.7573	LPath
**H546M**	0.9387	LPath
**H546N**	0.2555	LBen
**H546P**	0.5982	LPath
**H546Q**	0.8018	LPath
**H546R**	0.8761	LPath
**H546S**	0.3732	Amb
**H546T**	0.8567	LPath

**H546V**	0.9358	LPath
**H546W**	0.9051	LPath
**H546Y**	0.6821	LPath
**E547A**	0.1047	LBen
**E547C**	0.7312	LPath
**E547D**	0.1348	LBen
**E547F**	0.681	LPath
**E547G**	0.1174	LBen
**E547H**	0.2434	LBen
**E547I**	0.329	LBen
**E547K**	0.0735	LBen
**E547L**	0.2991	LBen
**E547M**	0.3828	Amb
**E547N**	0.2522	LBen
**E547P**	0.139	LBen
**E547Q**	0.083	LBen
**E547R**	0.1043	LBen
**E547S**	0.1556	LBen
**E547T**	0.1725	LBen
**E547V**	0.1834	LBen
**E547W**	0.702	LPath
**E547Y**	0.5029	Amb
**N548A**	0.5388	Amb
**N548C**	0.9081	LPath
**N548D**	0.2205	LBen
**N548E**	0.4686	Amb
**N548F**	0.87	LPath
**N548G**	0.5309	Amb
**N548H**	0.2907	LBen
**N548I**	0.7826	LPath
**N548K**	0.573	LPath
**N548L**	0.6373	LPath
**N548M**	0.794	LPath
**N548P**	0.93	LPath
**N548Q**	0.5992	LPath
**N548R**	0.6002	LPath
**N548S**	0.2794	LBen
**N548T**	0.491	Amb
**N548V**	0.7485	LPath
**N548W**	0.9247	LPath

**N548Y**	0.389	Amb
**Y549A**	0.9719	LPath
**Y549C**	0.8717	LPath
**Y549D**	0.9788	LPath
**Y549E**	0.9936	LPath
**Y549F**	0.1652	LBen
**Y549G**	0.9607	LPath
**Y549H**	0.7188	LPath
**Y549I**	0.9461	LPath
**Y549K**	0.9944	LPath
**Y549L**	0.8817	LPath
**Y549M**	0.9631	LPath
**Y549N**	0.9348	LPath
**Y549P**	0.9944	LPath
**Y549Q**	0.9839	LPath
**Y549R**	0.9729	LPath
**Y549S**	0.9115	LPath
**Y549T**	0.9728	LPath
**Y549V**	0.9086	LPath
**Y549W**	0.5432	Amb
**S550A**	0.144	LBen
**S550C**	0.3982	Amb
**S550D**	0.3423	Amb
**S550E**	0.462	Amb
**S550F**	0.3247	LBen
**S550G**	0.1163	LBen
**S550H**	0.3458	Amb
**S550I**	0.4002	Amb
**S550K**	0.6252	LPath
**S550L**	0.2087	LBen
**S550M**	0.3553	Amb
**S550N**	0.2465	LBen
**S550P**	0.2357	LBen
**S550Q**	0.4926	Amb
**S550R**	0.4802	Amb
**S550T**	0.0759	LBen
**S550V**	0.3613	Amb
**S550W**	0.3376	LBen
**S550Y**	0.3103	LBen
**R551A**	0.1239	LBen

**R551C**	0.1492	LBen
**R551D**	0.3112	LBen
**R551E**	0.1463	LBen
**R551F**	0.245	LBen
**R551G**	0.1179	LBen
**R551H**	0.0754	LBen
**R551I**	0.1467	LBen
**R551K**	0.0807	LBen
**R551L**	0.0752	LBen
**R551M**	0.1384	LBen
**R551N**	0.293	LBen
**R551P**	0.4259	Amb
**R551Q**	0.0682	LBen
**R551S**	0.179	LBen
**R551T**	0.1127	LBen
**R551V**	0.1444	LBen
**R551W**	0.0923	LBen
**R551Y**	0.1963	LBen
**A552C**	0.6577	LPath
**A552D**	0.8323	LPath
**A552E**	0.7351	LPath
**A552F**	0.4347	Amb
**A552G**	0.2529	LBen
**A552H**	0.7674	LPath
**A552I**	0.2482	LBen
**A552K**	0.9021	LPath
**A552L**	0.2554	LBen
**A552M**	0.3362	LBen
**A552N**	0.6353	LPath
**A552P**	0.8142	LPath
**A552Q**	0.705	LPath
**A552R**	0.8115	LPath
**A552S**	0.1371	LBen
**A552T**	0.165	LBen
**A552V**	0.1212	LBen
**A552W**	0.8456	LPath
**A552Y**	0.6716	LPath
**L553A**	0.2233	LBen
**L553C**	0.7173	LPath
**L553D**	0.8333	LPath

**L553E**	0.3248	LBen
**L553F**	0.2028	LBen
**L553G**	0.5253	Amb
**L553H**	0.3555	Amb
**L553I**	0.1283	LBen
**L553K**	0.1955	LBen
**L553M**	0.1137	LBen
**L553N**	0.5967	LPath
**L553P**	0.7427	LPath
**L553Q**	0.1213	LBen
**L553R**	0.1275	LBen
**L553S**	0.3398	LBen
**L553T**	0.2323	LBen
**L553V**	0.1034	LBen
**L553W**	0.2534	LBen
**L553Y**	0.5215	Amb
**E554A**	0.0713	LBen
**E554C**	0.5371	Amb
**E554D**	0.0927	LBen
**E554F**	0.4361	Amb
**E554G**	0.0883	LBen
**E554H**	0.1464	LBen
**E554I**	0.2357	LBen
**E554K**	0.0809	LBen
**E554L**	0.1605	LBen
**E554M**	0.2537	LBen
**E554N**	0.1387	LBen
**E554P**	0.3824	Amb
**E554Q**	0.0683	LBen
**E554R**	0.0923	LBen
**E554S**	0.0955	LBen
**E554T**	0.1182	LBen
**E554V**	0.1419	LBen
**E554W**	0.494	Amb
**E554Y**	0.3085	LBen
**G555A**	0.1102	LBen
**G555C**	0.3061	LBen
**G555D**	0.1079	LBen
**G555E**	0.0968	LBen
**G555F**	0.4675	Amb

**G555H**	0.2121	LBen
**G555I**	0.2579	LBen
**G555K**	0.1553	LBen
**G555L**	0.2777	LBen
**G555M**	0.31	LBen
**G555N**	0.1687	LBen
**G555P**	0.4481	Amb
**G555Q**	0.1582	LBen
**G555R**	0.1275	LBen
**G555S**	0.0979	LBen
**G555T**	0.1272	LBen
**G555V**	0.1623	LBen
**G555W**	0.2496	LBen
**G555Y**	0.3044	LBen
**E556A**	0.0884	LBen
**E556C**	0.5181	Amb
**E556D**	0.0797	LBen
**E556F**	0.4015	Amb
**E556G**	0.08	LBen
**E556H**	0.1521	LBen
**E556I**	0.2129	LBen
**E556K**	0.0638	LBen
**E556L**	0.1758	LBen
**E556M**	0.2335	LBen
**E556N**	0.1243	LBen
**E556P**	0.1626	LBen
**E556Q**	0.0738	LBen
**E556R**	0.0882	LBen
**E556S**	0.0926	LBen
**E556T**	0.1118	LBen
**E556V**	0.1357	LBen
**E556W**	0.4633	Amb
**E556Y**	0.2751	LBen
**G557A**	0.0949	LBen
**G557C**	0.2356	LBen
**G557D**	0.0808	LBen
**G557E**	0.09	LBen
**G557F**	0.3409	Amb
**G557H**	0.1691	LBen
**G557I**	0.1727	LBen

**G557K**	0.1686	LBen
**G557L**	0.2364	LBen
**G557M**	0.264	LBen
**G557N**	0.1305	LBen
**G557P**	0.3418	Amb
**G557Q**	0.1555	LBen
**G557R**	0.1353	LBen
**G557S**	0.0858	LBen
**G557T**	0.1047	LBen
**G557V**	0.1128	LBen
**G557W**	0.2089	LBen
**G557Y**	0.2051	LBen
**I558A**	0.1137	LBen
**I558C**	0.4369	Amb
**I558D**	0.2404	LBen
**I558E**	0.1711	LBen
**I558F**	0.0644	LBen
**I558G**	0.2317	LBen
**I558H**	0.1771	LBen
**I558K**	0.1373	LBen
**I558L**	0.0537	LBen
**I558M**	0.0612	LBen
**I558N**	0.1394	LBen
**I558P**	0.1813	LBen
**I558Q**	0.1356	LBen
**I558R**	0.098	LBen
**I558S**	0.1205	LBen
**I558T**	0.0968	LBen
**I558V**	0.0677	LBen
**I558W**	0.2128	LBen
**I558Y**	0.1935	LBen
**V559A**	0.1947	LBen
**V559C**	0.8788	LPath
**V559D**	0.7108	LPath
**V559E**	0.4606	Amb
**V559F**	0.2143	LBen
**V559G**	0.3213	LBen
**V559H**	0.737	LPath
**V559I**	0.0855	LBen
**V559K**	0.466	Amb

**V559L**	0.2048	LBen
**V559M**	0.1629	LBen
**V559N**	0.6249	LPath
**V559P**	0.4297	Amb
**V559Q**	0.4274	Amb
**V559R**	0.3706	Amb
**V559S**	0.3355	LBen
**V559T**	0.1918	LBen
**V559W**	0.7359	LPath
**V559Y**	0.6522	LPath
**E560A**	0.2633	LBen
**E560C**	0.9375	LPath
**E560D**	0.4028	Amb
**E560F**	0.8875	LPath
**E560G**	0.4217	Amb
**E560H**	0.684	LPath
**E560I**	0.5721	LPath
**E560K**	0.3413	Amb
**E560L**	0.4505	Amb
**E560M**	0.6323	LPath
**E560N**	0.6739	LPath
**E560P**	0.7945	LPath
**E560Q**	0.1705	LBen
**E560R**	0.3976	Amb
**E560S**	0.3846	Amb
**E560T**	0.429	Amb
**E560V**	0.365	Amb
**E560W**	0.9467	LPath
**E560Y**	0.8433	LPath
**Q561A**	0.5047	Amb
**Q561C**	0.9358	LPath
**Q561D**	0.8166	LPath
**Q561E**	0.1078	LBen
**Q561F**	0.9201	LPath
**Q561G**	0.6675	LPath
**Q561H**	0.769	LPath
**Q561I**	0.765	LPath
**Q561K**	0.4068	Amb
**Q561L**	0.371	Amb
**Q561M**	0.3348	LBen

**Q561N**	0.6431	LPath
**Q561P**	0.7852	LPath
**Q561R**	0.4671	Amb
**Q561S**	0.492	Amb
**Q561T**	0.3743	Amb
**Q561V**	0.5101	Amb
**Q561W**	0.9448	LPath
**Q561Y**	0.8829	LPath
**P562A**	0.6225	LPath
**P562C**	0.9836	LPath
**P562D**	0.9841	LPath
**P562E**	0.9675	LPath
**P562F**	0.9832	LPath
**P562G**	0.9484	LPath
**P562H**	0.9487	LPath
**P562I**	0.8578	LPath
**P562K**	0.9701	LPath
**P562L**	0.7235	LPath
**P562M**	0.9466	LPath
**P562N**	0.9831	LPath
**P562Q**	0.9417	LPath
**P562R**	0.9128	LPath
**P562S**	0.9106	LPath
**P562T**	0.8383	LPath
**P562V**	0.7414	LPath
**P562W**	0.991	LPath
**P562Y**	0.98	LPath
**C563A**	0.9959	LPath
**C563D**	0.9997	LPath
**C563E**	0.9998	LPath
**C563F**	0.9922	LPath
**C563G**	0.9867	LPath
**C563H**	0.9994	LPath
**C563I**	0.9974	LPath
**C563K**	0.9999	LPath
**C563L**	0.988	LPath
**C563M**	0.9983	LPath
**C563N**	0.9996	LPath
**C563P**	0.9991	LPath
**C563Q**	0.9998	LPath

**C563R**	0.9982	LPath
**C563S**	0.9969	LPath
**C563T**	0.9991	LPath
**C563V**	0.995	LPath
**C563W**	0.9974	LPath
**C563Y**	0.9976	LPath
**P564A**	0.2845	LBen
**P564C**	0.9418	LPath
**P564D**	0.8583	LPath
**P564E**	0.6136	LPath
**P564F**	0.9402	LPath
**P564G**	0.7576	LPath
**P564H**	0.6014	LPath
**P564I**	0.7882	LPath
**P564K**	0.6781	LPath
**P564L**	0.361	Amb
**P564M**	0.7076	LPath
**P564N**	0.7383	LPath
**P564Q**	0.3772	Amb
**P564R**	0.4371	Amb
**P564S**	0.3731	Amb
**P564T**	0.2324	LBen
**P564V**	0.6169	LPath
**P564W**	0.9413	LPath
**P564Y**	0.9312	LPath
**D565A**	0.98	LPath
**D565C**	0.9985	LPath
**D565E**	0.9759	LPath
**D565F**	0.998	LPath
**D565G**	0.9754	LPath
**D565H**	0.9932	LPath
**D565I**	0.9982	LPath
**D565K**	0.9977	LPath
**D565L**	0.9935	LPath
**D565M**	0.9991	LPath
**D565N**	0.9564	LPath
**D565P**	0.9983	LPath
**D565Q**	0.9958	LPath
**D565R**	0.9952	LPath
**D565S**	0.9608	LPath

**D565T**	0.9928	LPath
**D565V**	0.992	LPath
**D565W**	0.9994	LPath
**D565Y**	0.9834	LPath
**V566A**	0.9126	LPath
**V566C**	0.9844	LPath
**V566D**	0.9969	LPath
**V566E**	0.9933	LPath
**V566F**	0.9889	LPath
**V566G**	0.9457	LPath
**V566H**	0.9986	LPath
**V566I**	0.2761	LBen
**V566K**	0.9973	LPath
**V566L**	0.9601	LPath
**V566M**	0.9628	LPath
**V566N**	0.9853	LPath
**V566P**	0.9986	LPath
**V566Q**	0.9937	LPath
**V566R**	0.9892	LPath
**V566S**	0.9348	LPath
**V566T**	0.843	LPath
**V566W**	0.9998	LPath
**V566Y**	0.9983	LPath
**Y567A**	0.9745	LPath
**Y567C**	0.8159	LPath
**Y567D**	0.9883	LPath
**Y567E**	0.9965	LPath
**Y567F**	0.1134	LBen
**Y567G**	0.9713	LPath
**Y567H**	0.8259	LPath
**Y567I**	0.941	LPath
**Y567K**	0.9942	LPath
**Y567L**	0.8949	LPath
**Y567M**	0.959	LPath
**Y567N**	0.946	LPath
**Y567P**	0.9975	LPath
**Y567Q**	0.9852	LPath
**Y567R**	0.9718	LPath
**Y567S**	0.906	LPath
**Y567T**	0.976	LPath

**Y567V**	0.8877	LPath
**Y567W**	0.6763	LPath
**W568A**	0.7985	LPath
**W568C**	0.9294	LPath
**W568D**	0.9867	LPath
**W568E**	0.9818	LPath
**W568F**	0.5963	LPath
**W568G**	0.7103	LPath
**W568H**	0.8999	LPath
**W568I**	0.9011	LPath
**W568K**	0.9939	LPath
**W568L**	0.7183	LPath
**W568M**	0.9198	LPath
**W568N**	0.9699	LPath
**W568P**	0.9962	LPath
**W568Q**	0.9578	LPath
**W568R**	0.9704	LPath
**W568S**	0.6234	LPath
**W568T**	0.8478	LPath
**W568V**	0.8415	LPath
**W568Y**	0.7468	LPath
**F569A**	0.9929	LPath
**F569C**	0.9775	LPath
**F569D**	0.9996	LPath
**F569E**	0.9994	LPath
**F569G**	0.9956	LPath
**F569H**	0.9918	LPath
**F569I**	0.8581	LPath
**F569K**	0.9992	LPath
**F569L**	0.9807	LPath
**F569M**	0.9353	LPath
**F569N**	0.9974	LPath
**F569P**	0.9999	LPath
**F569Q**	0.9974	LPath
**F569R**	0.998	LPath
**F569S**	0.9931	LPath
**F569T**	0.9942	LPath
**F569V**	0.8447	LPath
**F569W**	0.9318	LPath
**F569Y**	0.6845	LPath

**P570A**	0.6395	LPath
**P570C**	0.981	LPath
**P570D**	0.9966	LPath
**P570E**	0.9803	LPath
**P570F**	0.9946	LPath
**P570G**	0.9589	LPath
**P570H**	0.9556	LPath
**P570I**	0.9884	LPath
**P570K**	0.9897	LPath
**P570L**	0.8881	LPath
**P570M**	0.9884	LPath
**P570N**	0.9921	LPath
**P570Q**	0.9444	LPath
**P570R**	0.9451	LPath
**P570S**	0.8477	LPath
**P570T**	0.9323	LPath
**P570V**	0.9571	LPath
**P570W**	0.9952	LPath
**P570Y**	0.9906	LPath
**L571A**	0.8758	LPath
**L571C**	0.9384	LPath
**L571D**	0.9991	LPath
**L571E**	0.9947	LPath
**L571F**	0.2976	LBen
**L571G**	0.9818	LPath
**L571H**	0.9871	LPath
**L571I**	0.1509	LBen
**L571K**	0.9958	LPath
**L571M**	0.2249	LBen
**L571N**	0.995	LPath
**L571P**	0.9864	LPath
**L571Q**	0.976	LPath
**L571R**	0.9879	LPath
**L571S**	0.9755	LPath
**L571T**	0.9189	LPath
**L571V**	0.1485	LBen
**L571W**	0.9016	LPath
**L571Y**	0.9525	LPath
**L572A**	0.769	LPath
**L572C**	0.7878	LPath

L572D	0.997	LPath
L572E	0.9886	LPath
L572F	0.116	LBen
L572G	0.8978	LPath
L572H	0.9451	LPath
L572I	0.2607	LBen
L572K	0.9887	LPath
L572M	0.203	LBen
L572N	0.9838	LPath
L572P	0.9758	LPath
L572Q	0.9246	LPath
L572R	0.9612	LPath
L572S	0.9357	LPath
L572T	0.8237	LPath
L572V	0.1998	LBen
L572W	0.7207	LPath
L572Y	0.8542	LPath
S573A	0.2929	LBen
S573C	0.3962	Amb
S573D	0.7068	LPath
S573E	0.8691	LPath
S573F	0.9166	LPath
S573G	0.1629	LBen
S573H	0.6947	LPath
S573I	0.9107	LPath
S573K	0.845	LPath
S573L	0.4634	Amb
S573M	0.6591	LPath
S573N	0.2838	LBen
S573P	0.8997	LPath
S573Q	0.7786	LPath
S573R	0.7882	LPath
S573T	0.1015	LBen
S573V	0.8237	LPath
S573W	0.8608	LPath
S573Y	0.7883	LPath
E574A	0.1281	LBen
E574C	0.7868	LPath
E574D	0.0706	LBen
E574F	0.6708	LPath

E574G	0.1302	LBen
E574H	0.2748	LBen
E574I	0.3595	Amb
E574K	0.1139	LBen
E574L	0.3081	LBen
E574M	0.4329	Amb
E574N	0.1472	LBen
E574P	0.1913	LBen
E574Q	0.1054	LBen
E574R	0.153	LBen
E574S	0.1195	LBen
E574T	0.1763	LBen
E574V	0.2084	LBen
E574W	0.764	LPath
E574Y	0.4835	Amb
Q575A	0.1386	LBen
Q575C	0.6157	LPath
Q575D	0.3297	LBen
Q575E	0.0827	LBen
Q575F	0.6444	LPath
Q575G	0.2089	LBen
Q575H	0.2064	LBen
Q575I	0.3	LBen
Q575K	0.0794	LBen
Q575L	0.1114	LBen
Q575M	0.3063	LBen
Q575N	0.3163	LBen
Q575P	0.1752	LBen
Q575R	0.0727	LBen
Q575S	0.2228	LBen
Q575T	0.1265	LBen
Q575V	0.1868	LBen
Q575W	0.4485	Amb
Q575Y	0.4843	Amb
M576A	0.4113	Amb
M576C	0.8301	LPath
M576D	0.9904	LPath
M576E	0.9681	LPath
M576F	0.3045	LBen
M576G	0.5358	Amb

**M576H**	0.9209	LPath
**M576I**	0.8926	LPath
**M576K**	0.9151	LPath
**M576L**	0.2546	LBen
**M576N**	0.9096	LPath
**M576P**	0.9784	LPath
**M576Q**	0.7802	LPath
**M576R**	0.9023	LPath
**M576S**	0.657	LPath
**M576T**	0.5205	Amb
**M576V**	0.3519	Amb
**M576W**	0.9053	LPath
**M576Y**	0.7924	LPath
**C577A**	0.5808	LPath
**C577D**	0.9954	LPath
**C577E**	0.9966	LPath
**C577F**	0.9848	LPath
**C577G**	0.4619	Amb
**C577H**	0.9948	LPath
**C577I**	0.9818	LPath
**C577K**	0.9984	LPath
**C577L**	0.9279	LPath
**C577M**	0.9725	LPath
**C577N**	0.978	LPath
**C577P**	0.9915	LPath
**C577Q**	0.9892	LPath
**C577R**	0.981	LPath
**C577S**	0.7529	LPath
**C577T**	0.8337	LPath
**C577V**	0.9196	LPath
**C577W**	0.9946	LPath
**C577Y**	0.9926	LPath
**D578A**	0.4943	Amb
**D578C**	0.9404	LPath
**D578E**	0.3446	Amb
**D578F**	0.937	LPath
**D578G**	0.4457	Amb
**D578H**	0.5153	Amb
**D578I**	0.8594	LPath
**D578K**	0.5903	LPath

**D578L**	0.8008	LPath
**D578M**	0.9014	LPath
**D578N**	0.1203	LBen
**D578P**	0.9873	LPath
**D578Q**	0.53	Amb
**D578R**	0.5418	Amb
**D578S**	0.2187	LBen
**D578T**	0.4763	Amb
**D578V**	0.6805	LPath
**D578W**	0.9743	LPath
**D578Y**	0.6313	LPath
**E579A**	0.3689	Amb
**E579C**	0.9579	LPath
**E579D**	0.1477	LBen
**E579F**	0.9679	LPath
**E579G**	0.5365	Amb
**E579H**	0.464	Amb
**E579I**	0.9351	LPath
**E579K**	0.5431	Amb
**E579L**	0.8686	LPath
**E579M**	0.8698	LPath
**E579N**	0.4872	Amb
**E579P**	0.9935	LPath
**E579Q**	0.2338	LBen
**E579R**	0.6675	LPath
**E579S**	0.4178	Amb
**E579T**	0.7348	LPath
**E579V**	0.8315	LPath
**E579W**	0.9859	LPath
**E579Y**	0.9238	LPath
**L580A**	0.8303	LPath
**L580C**	0.9583	LPath
**L580D**	0.9976	LPath
**L580E**	0.9841	LPath
**L580F**	0.26	LBen
**L580G**	0.9791	LPath
**L580H**	0.9701	LPath
**L580I**	0.1582	LBen
**L580K**	0.9811	LPath
**L580M**	0.1344	LBen

**L580N**	0.9879	LPath
**L580P**	0.9962	LPath
**L580Q**	0.9363	LPath
**L580R**	0.9672	LPath
**L580S**	0.9706	LPath
**L580T**	0.8169	LPath
**L580V**	0.2026	LBen
**L580W**	0.6362	LPath
**L580Y**	0.8766	LPath
**V581A**	0.4738	Amb
**V581C**	0.9792	LPath
**V581D**	0.9765	LPath
**V581E**	0.8328	LPath
**V581F**	0.8813	LPath
**V581G**	0.7435	LPath
**V581H**	0.9713	LPath
**V581I**	0.1207	LBen
**V581K**	0.7724	LPath
**V581L**	0.6738	LPath
**V581M**	0.6586	LPath
**V581N**	0.953	LPath
**V581P**	0.9718	LPath
**V581Q**	0.7756	LPath
**V581R**	0.6226	LPath
**V581S**	0.7909	LPath
**V581T**	0.529	Amb
**V581W**	0.9936	LPath
**V581Y**	0.9843	LPath
**A582C**	0.5738	LPath
**A582D**	0.135	LBen
**A582E**	0.0597	LBen
**A582F**	0.4746	Amb
**A582G**	0.1072	LBen
**A582H**	0.3415	Amb
**A582I**	0.3581	Amb
**A582K**	0.1985	LBen
**A582L**	0.1758	LBen
**A582M**	0.1866	LBen
**A582N**	0.2171	LBen
**A582P**	0.9125	LPath

**A582Q**	0.1106	LBen
**A582R**	0.1909	LBen
**A582S**	0.0848	LBen
**A582T**	0.0833	LBen
**A582V**	0.1594	LBen
**A582W**	0.645	LPath
**A582Y**	0.514	Amb
**E583A**	0.5818	LPath
**E583C**	0.9749	LPath
**E583D**	0.9381	LPath
**E583F**	0.9948	LPath
**E583G**	0.8377	LPath
**E583H**	0.9693	LPath
**E583I**	0.7636	LPath
**E583K**	0.8494	LPath
**E583L**	0.888	LPath
**E583M**	0.8074	LPath
**E583N**	0.9326	LPath
**E583P**	0.9974	LPath
**E583Q**	0.5396	Amb
**E583R**	0.9145	LPath
**E583S**	0.7425	LPath
**E583T**	0.4024	Amb
**E583V**	0.4878	Amb
**E583W**	0.9989	LPath
**E583Y**	0.9935	LPath
**M584A**	0.7051	LPath
**M584C**	0.8922	LPath
**M584D**	0.9983	LPath
**M584E**	0.9836	LPath
**M584F**	0.8995	LPath
**M584G**	0.9481	LPath
**M584H**	0.9932	LPath
**M584I**	0.9078	LPath
**M584K**	0.9786	LPath
**M584L**	0.4526	Amb
**M584N**	0.9722	LPath
**M584P**	0.9989	LPath
**M584Q**	0.9281	LPath
**M584R**	0.9748	LPath

**M584S**	0.8883	LPath
**M584T**	0.5975	LPath
**M584V**	0.223	LBen
**M584W**	0.9957	LPath
**M584Y**	0.9913	LPath
**E585A**	0.864	LPath
**E585C**	0.9951	LPath
**E585D**	0.8964	LPath
**E585F**	0.9984	LPath
**E585G**	0.8868	LPath
**E585H**	0.9797	LPath
**E585I**	0.9872	LPath
**E585K**	0.9441	LPath
**E585L**	0.9731	LPath
**E585M**	0.983	LPath
**E585N**	0.9149	LPath
**E585P**	0.9977	LPath
**E585Q**	0.7907	LPath
**E585R**	0.9389	LPath
**E585S**	0.9352	LPath
**E585T**	0.9571	LPath
**E585V**	0.95	LPath
**E585W**	0.999	LPath
**E585Y**	0.9941	LPath
**H586A**	0.2026	LBen
**H586C**	0.3587	Amb
**H586D**	0.1866	LBen
**H586E**	0.2612	LBen
**H586F**	0.2849	LBen
**H586G**	0.2327	LBen
**H586I**	0.4009	Amb
**H586K**	0.2707	LBen
**H586L**	0.1536	LBen
**H586M**	0.4686	Amb
**H586N**	0.0626	LBen
**H586P**	0.8255	LPath
**H586Q**	0.1605	LBen
**H586R**	0.1096	LBen
**H586S**	0.1482	LBen
**H586T**	0.2031	LBen

**H586V**	0.3232	LBen
**H586W**	0.3984	Amb
**H586Y**	0.1041	LBen
**Y587A**	0.4846	Amb
**Y587C**	0.1846	LBen
**Y587D**	0.3788	Amb
**Y587E**	0.6106	LPath
**Y587F**	0.0648	LBen
**Y587G**	0.4839	Amb
**Y587H**	0.1032	LBen
**Y587I**	0.6568	LPath
**Y587K**	0.6366	LPath
**Y587L**	0.523	Amb
**Y587M**	0.661	LPath
**Y587N**	0.2055	LBen
**Y587P**	0.9507	LPath
**Y587Q**	0.4325	Amb
**Y587R**	0.3902	Amb
**Y587S**	0.2579	LBen
**Y587T**	0.5538	Amb
**Y587V**	0.5219	Amb
**Y587W**	0.3332	LBen
**G588A**	0.5861	LPath
**G588C**	0.863	LPath
**G588D**	0.63	LPath
**G588E**	0.6911	LPath
**G588F**	0.982	LPath
**G588H**	0.7111	LPath
**G588I**	0.9687	LPath
**G588K**	0.7511	LPath
**G588L**	0.9335	LPath
**G588M**	0.9638	LPath
**G588N**	0.5584	Amb
**G588P**	0.9829	LPath
**G588Q**	0.5869	LPath
**G588R**	0.5639	Amb
**G588S**	0.2367	LBen
**G588T**	0.8008	LPath
**G588V**	0.9355	LPath
**G588W**	0.939	LPath

**G588Y**	0.9451	LPath
**Q589A**	0.1921	LBen
**Q589C**	0.6213	LPath
**Q589D**	0.3615	Amb
**Q589E**	0.0866	LBen
**Q589F**	0.5661	LPath
**Q589G**	0.1922	LBen
**Q589H**	0.1852	LBen
**Q589I**	0.352	Amb
**Q589K**	0.0789	LBen
**Q589L**	0.1115	LBen
**Q589M**	0.3192	LBen
**Q589N**	0.2934	LBen
**Q589P**	0.4086	Amb
**Q589R**	0.0775	LBen
**Q589S**	0.2144	LBen
**Q589T**	0.2069	LBen
**Q589V**	0.2211	LBen
**Q589W**	0.493	Amb
**Q589Y**	0.4157	Amb
**W590A**	0.9972	LPath
**W590C**	0.9995	LPath
**W590D**	0.999	LPath
**W590E**	0.9993	LPath
**W590F**	0.8769	LPath
**W590G**	0.9866	LPath
**W590H**	0.9977	LPath
**W590I**	0.9975	LPath
**W590K**	0.9997	LPath
**W590L**	0.9927	LPath
**W590M**	0.9984	LPath
**W590N**	0.9994	LPath
**W590P**	0.997	LPath
**W590Q**	0.9997	LPath
**W590R**	0.9994	LPath
**W590S**	0.996	LPath
**W590T**	0.9982	LPath
**W590V**	0.9974	LPath
**W590Y**	0.9707	LPath
**S591A**	0.7401	LPath

**S591C**	0.9806	LPath
**S591D**	0.9907	LPath
**S591E**	0.9871	LPath
**S591F**	0.9949	LPath
**S591G**	0.8724	LPath
**S591H**	0.9857	LPath
**S591I**	0.9926	LPath
**S591K**	0.9982	LPath
**S591L**	0.9785	LPath
**S591M**	0.9865	LPath
**S591N**	0.9845	LPath
**S591P**	0.9939	LPath
**S591Q**	0.9894	LPath
**S591R**	0.9968	LPath
**S591T**	0.9076	LPath
**S591V**	0.9913	LPath
**S591W**	0.9828	LPath
**S591Y**	0.9872	LPath
**G592A**	0.2342	LBen
**G592C**	0.6946	LPath
**G592D**	0.4058	Amb
**G592E**	0.4793	Amb
**G592F**	0.8554	LPath
**G592H**	0.5761	LPath
**G592I**	0.7778	LPath
**G592K**	0.5838	LPath
**G592L**	0.6991	LPath
**G592M**	0.7592	LPath
**G592N**	0.41	Amb
**G592P**	0.9338	LPath
**G592Q**	0.4869	Amb
**G592R**	0.4193	Amb
**G592S**	0.1154	LBen
**G592T**	0.318	LBen
**G592V**	0.6108	LPath
**G592W**	0.7347	LPath
**G592Y**	0.7821	LPath
**G593A**	0.8419	LPath
**G593C**	0.9725	LPath
**G593D**	0.9754	LPath

**G593E**	0.9781	LPath
**G593F**	0.9927	LPath
**G593H**	0.9892	LPath
**G593I**	0.989	LPath
**G593K**	0.9884	LPath
**G593L**	0.9892	LPath
**G593M**	0.9937	LPath
**G593N**	0.9684	LPath
**G593P**	0.9957	LPath
**G593Q**	0.9791	LPath
**G593R**	0.9641	LPath
**G593S**	0.6902	LPath
**G593T**	0.9628	LPath
**G593V**	0.9814	LPath
**G593W**	0.981	LPath
**G593Y**	0.9893	LPath
**R594A**	0.2247	LBen
**R594C**	0.2419	LBen
**R594D**	0.4855	Amb
**R594E**	0.2606	LBen
**R594F**	0.4824	Amb
**R594G**	0.0983	LBen
**R594H**	0.0833	LBen
**R594I**	0.3536	Amb
**R594K**	0.0811	LBen
**R594L**	0.1628	LBen
**R594M**	0.2591	LBen
**R594N**	0.3679	Amb
**R594P**	0.8215	LPath
**R594Q**	0.0766	LBen
**R594S**	0.2125	LBen
**R594T**	0.1573	LBen
**R594V**	0.3353	LBen
**R594W**	0.213	LBen
**R594Y**	0.3765	Amb
**H595A**	0.8509	LPath
**H595C**	0.7955	LPath
**H595D**	0.7328	LPath
**H595E**	0.8626	LPath
**H595F**	0.7227	LPath

**H595G**	0.8192	LPath
**H595I**	0.9233	LPath
**H595K**	0.8189	LPath
**H595L**	0.5704	LPath
**H595M**	0.8935	LPath
**H595N**	0.1696	LBen
**H595P**	0.5597	Amb
**H595Q**	0.7066	LPath
**H595R**	0.5968	LPath
**H595S**	0.6379	LPath
**H595T**	0.8447	LPath
**H595V**	0.8953	LPath
**H595W**	0.6664	LPath
**H595Y**	0.3345	LBen
**E596A**	0.233	LBen
**E596C**	0.9361	LPath
**E596D**	0.2831	LBen
**E596F**	0.7967	LPath
**E596G**	0.3463	Amb
**E596H**	0.3883	Amb
**E596I**	0.4694	Amb
**E596K**	0.1548	LBen
**E596L**	0.5067	Amb
**E596M**	0.5292	Amb
**E596N**	0.4257	Amb
**E596P**	0.9095	LPath
**E596Q**	0.11	LBen
**E596R**	0.2002	LBen
**E596S**	0.2652	LBen
**E596T**	0.2135	LBen
**E596V**	0.2661	LBen
**E596W**	0.8988	LPath
**E596Y**	0.7114	LPath
**D597A**	0.9965	LPath
**D597C**	0.9996	LPath
**D597E**	0.9955	LPath
**D597F**	0.9995	LPath
**D597G**	0.9919	LPath
**D597H**	0.9985	LPath
**D597I**	0.9992	LPath

D597K	0.9984	LPath
D597L	0.9986	LPath
D597M	0.9998	LPath
D597N	0.9891	LPath
D597P	0.9992	LPath
D597Q	0.9992	LPath
D597R	0.9984	LPath
D597S	0.9952	LPath
D597T	0.9981	LPath
D597V	0.9938	LPath
D597W	0.9999	LPath
D597Y	0.9923	LPath
S598A	0.0727	LBen
S598C	0.262	LBen
S598D	0.1992	LBen
S598E	0.2093	LBen
S598F	0.1969	LBen
S598G	0.0814	LBen
S598H	0.1697	LBen
S598I	0.1505	LBen
S598K	0.2369	LBen
S598L	0.1057	LBen
S598M	0.1933	LBen
S598N	0.0976	LBen
S598P	0.1057	LBen
S598Q	0.2031	LBen
S598R	0.1878	LBen
S598T	0.0759	LBen
S598V	0.1531	LBen
S598W	0.273	LBen
S598Y	0.1941	LBen
R599A	0.996	LPath
R599C	0.9847	LPath
R599D	0.9969	LPath
R599E	0.9861	LPath
R599F	0.9983	LPath
R599G	0.9924	LPath
R599H	0.9422	LPath
R599I	0.994	LPath
R599K	0.9858	LPath

**R599L**	0.9793	LPath
**R599M**	0.9989	LPath
**R599N**	0.9965	LPath
**R599P**	0.9947	LPath
**R599Q**	0.9635	LPath
**R599S**	0.9952	LPath
**R599T**	0.9959	LPath
**R599V**	0.9944	LPath
**R599W**	0.9667	LPath
**R599Y**	0.9916	LPath
**L600A**	0.9811	LPath
**L600C**	0.9985	LPath
**L600D**	0.9994	LPath
**L600E**	0.9955	LPath
**L600F**	0.98	LPath
**L600G**	0.9967	LPath
**L600H**	0.9952	LPath
**L600I**	0.4163	Amb
**L600K**	0.9889	LPath
**L600M**	0.8273	LPath
**L600N**	0.9954	LPath
**L600P**	0.9915	LPath
**L600Q**	0.9905	LPath
**L600R**	0.9755	LPath
**L600S**	0.997	LPath
**L600T**	0.981	LPath
**L600V**	0.7662	LPath
**L600W**	0.991	LPath
**L600Y**	0.9962	LPath
**A601C**	0.7686	LPath
**A601D**	0.4097	Amb
**A601E**	0.3247	LBen
**A601F**	0.5167	Amb
**A601G**	0.2156	LBen
**A601H**	0.5853	LPath
**A601I**	0.3724	Amb
**A601K**	0.673	LPath
**A601L**	0.2085	LBen
**A601M**	0.3049	LBen
**A601N**	0.3248	LBen

**A601P**	0.3884	Amb
**A601Q**	0.3372	LBen
**A601R**	0.5364	Amb
**A601S**	0.0832	LBen
**A601T**	0.1052	LBen
**A601V**	0.1621	LBen
**A601W**	0.8174	LPath
**A601Y**	0.6751	LPath
**G602A**	0.6117	LPath
**G602C**	0.9277	LPath
**G602D**	0.8362	LPath
**G602E**	0.9127	LPath
**G602F**	0.9698	LPath
**G602H**	0.9448	LPath
**G602I**	0.9564	LPath
**G602K**	0.9719	LPath
**G602L**	0.9438	LPath
**G602M**	0.9519	LPath
**G602N**	0.8321	LPath
**G602P**	0.9917	LPath
**G602Q**	0.9268	LPath
**G602R**	0.927	LPath
**G602S**	0.292	LBen
**G602T**	0.5911	LPath
**G602V**	0.8922	LPath
**G602W**	0.9359	LPath
**G602Y**	0.9637	LPath
**G603A**	0.835	LPath
**G603C**	0.9545	LPath
**G603D**	0.9536	LPath
**G603E**	0.972	LPath
**G603F**	0.9811	LPath
**G603H**	0.9673	LPath
**G603I**	0.9675	LPath
**G603K**	0.9782	LPath
**G603L**	0.9778	LPath
**G603M**	0.9852	LPath
**G603N**	0.9207	LPath
**G603P**	0.9947	LPath
**G603Q**	0.9604	LPath

**G603R**	0.9402	LPath
**G603S**	0.6058	LPath
**G603T**	0.9004	LPath
**G603V**	0.9562	LPath
**G603W**	0.9745	LPath
**G603Y**	0.9734	LPath
**Y604A**	0.9904	LPath
**Y604C**	0.9755	LPath
**Y604D**	0.9814	LPath
**Y604E**	0.9918	LPath
**Y604F**	0.62	LPath
**Y604G**	0.9675	LPath
**Y604H**	0.9749	LPath
**Y604I**	0.9806	LPath
**Y604K**	0.9912	LPath
**Y604L**	0.9488	LPath
**Y604M**	0.9847	LPath
**Y604N**	0.9535	LPath
**Y604P**	0.9897	LPath
**Y604Q**	0.9913	LPath
**Y604R**	0.9621	LPath
**Y604S**	0.9466	LPath
**Y604T**	0.9837	LPath
**Y604V**	0.9474	LPath
**Y604W**	0.8696	LPath
**E605A**	0.9943	LPath
**E605C**	0.999	LPath
**E605D**	0.9974	LPath
**E605F**	0.9999	LPath
**E605G**	0.9897	LPath
**E605H**	0.9992	LPath
**E605I**	0.9994	LPath
**E605K**	0.9929	LPath
**E605L**	0.9992	LPath
**E605M**	0.9996	LPath
**E605N**	0.9994	LPath
**E605P**	0.999	LPath
**E605Q**	0.9857	LPath
**E605R**	0.9911	LPath
**E605S**	0.9961	LPath

**E605T**	0.9986	LPath
**E605V**	0.9985	LPath
**E605W**	0.9999	LPath
**E605Y**	0.9997	LPath
**N606A**	0.7432	LPath
**N606C**	0.9778	LPath
**N606D**	0.9094	LPath
**N606E**	0.9842	LPath
**N606F**	0.983	LPath
**N606G**	0.8997	LPath
**N606H**	0.8148	LPath
**N606I**	0.9503	LPath
**N606K**	0.9856	LPath
**N606L**	0.8948	LPath
**N606M**	0.9124	LPath
**N606P**	0.944	LPath
**N606Q**	0.9636	LPath
**N606R**	0.9778	LPath
**N606S**	0.3524	Amb
**N606T**	0.7304	LPath
**N606V**	0.9197	LPath
**N606W**	0.9954	LPath
**N606Y**	0.8715	LPath
**V607A**	0.9846	LPath
**V607C**	0.9957	LPath
**V607D**	0.9971	LPath
**V607E**	0.9835	LPath
**V607F**	0.9425	LPath
**V607G**	0.9872	LPath
**V607H**	0.9931	LPath
**V607I**	0.3832	Amb
**V607K**	0.9934	LPath
**V607L**	0.9768	LPath
**V607M**	0.9786	LPath
**V607N**	0.988	LPath
**V607P**	0.9951	LPath
**V607Q**	0.9819	LPath
**V607R**	0.9672	LPath
**V607S**	0.9733	LPath
**V607T**	0.9631	LPath

**V607W**	0.9989	LPath
**V607Y**	0.9918	LPath
**P608A**	0.9666	LPath
**P608C**	0.9991	LPath
**P608D**	0.9993	LPath
**P608E**	0.998	LPath
**P608F**	0.9997	LPath
**P608G**	0.9967	LPath
**P608H**	0.9977	LPath
**P608I**	0.9993	LPath
**P608K**	0.9988	LPath
**P608L**	0.9889	LPath
**P608M**	0.9993	LPath
**P608N**	0.9994	LPath
**P608Q**	0.9974	LPath
**P608R**	0.9927	LPath
**P608S**	0.9932	LPath
**P608T**	0.996	LPath
**P608V**	0.9974	LPath
**P608W**	0.9996	LPath
**P608Y**	0.9995	LPath
**T609A**	0.9481	LPath
**T609C**	0.9977	LPath
**T609D**	0.999	LPath
**T609E**	0.997	LPath
**T609F**	0.999	LPath
**T609G**	0.9915	LPath
**T609H**	0.9975	LPath
**T609I**	0.9965	LPath
**T609K**	0.9965	LPath
**T609L**	0.9718	LPath
**T609M**	0.9704	LPath
**T609N**	0.9964	LPath
**T609P**	0.9747	LPath
**T609Q**	0.9965	LPath
**T609R**	0.9944	LPath
**T609S**	0.9579	LPath
**T609V**	0.9771	LPath
**T609W**	0.9995	LPath
**T609Y**	0.9992	LPath

**V610A**	0.788	LPath
**V610C**	0.991	LPath
**V610D**	0.7572	LPath
**V610E**	0.7607	LPath
**V610F**	0.9124	LPath
**V610G**	0.9141	LPath
**V610H**	0.9647	LPath
**V610I**	0.1279	LBen
**V610K**	0.8137	LPath
**V610L**	0.8265	LPath
**V610M**	0.6875	LPath
**V610N**	0.8072	LPath
**V610P**	0.9927	LPath
**V610Q**	0.7389	LPath
**V610R**	0.5534	Amb
**V610S**	0.8134	LPath
**V610T**	0.7081	LPath
**V610W**	0.993	LPath
**V610Y**	0.9819	LPath
**D611A**	0.9988	LPath
**D611C**	0.9998	LPath
**D611E**	0.9986	LPath
**D611F**	0.9997	LPath
**D611G**	0.9976	LPath
**D611H**	0.9987	LPath
**D611I**	0.9998	LPath
**D611K**	0.9996	LPath
**D611L**	0.9991	LPath
**D611M**	0.9999	LPath
**D611N**	0.995	LPath
**D611P**	0.9997	LPath
**D611Q**	0.9996	LPath
**D611R**	0.9992	LPath
**D611S**	0.9968	LPath
**D611T**	0.9996	LPath
**D611V**	0.9992	LPath
**D611W**	0.9999	LPath
**D611Y**	0.9962	LPath
**I612A**	0.9832	LPath
**I612C**	0.996	LPath

**I612D**	0.9996	LPath
**I612E**	0.9991	LPath
**I612F**	0.9887	LPath
**I612G**	0.9984	LPath
**I612H**	0.9991	LPath
**I612K**	0.9981	LPath
**I612L**	0.8094	LPath
**I612M**	0.8315	LPath
**I612N**	0.9897	LPath
**I612P**	0.9992	LPath
**I612Q**	0.998	LPath
**I612R**	0.9955	LPath
**I612S**	0.9764	LPath
**I612T**	0.9041	LPath
**I612V**	0.4153	Amb
**I612W**	0.9996	LPath
**I612Y**	0.9981	LPath
**H613A**	0.9981	LPath
**H613C**	0.9876	LPath
**H613D**	0.9987	LPath
**H613E**	0.9985	LPath
**H613F**	0.9925	LPath
**H613G**	0.9989	LPath
**H613I**	0.9992	LPath
**H613K**	0.9988	LPath
**H613L**	0.9772	LPath
**H613M**	0.9984	LPath
**H613N**	0.9947	LPath
**H613P**	0.9656	LPath
**H613Q**	0.9959	LPath
**H613R**	0.9952	LPath
**H613S**	0.9956	LPath
**H613T**	0.9991	LPath
**H613V**	0.999	LPath
**H613W**	0.9822	LPath
**H613Y**	0.9897	LPath
**M614A**	0.9169	LPath
**M614C**	0.946	LPath
**M614D**	0.9992	LPath
**M614E**	0.9931	LPath

**M614F**	0.7934	LPath
**M614G**	0.9505	LPath
**M614H**	0.9923	LPath
**M614I**	0.9844	LPath
**M614K**	0.9876	LPath
**M614L**	0.3035	LBen
**M614N**	0.9876	LPath
**M614P**	0.9958	LPath
**M614Q**	0.9417	LPath
**M614R**	0.9825	LPath
**M614S**	0.9427	LPath
**M614T**	0.6823	LPath
**M614V**	0.6799	LPath
**M614W**	0.9913	LPath
**M614Y**	0.9909	LPath
**K615A**	0.6138	LPath
**K615C**	0.8537	LPath
**K615D**	0.8051	LPath
**K615E**	0.5033	Amb
**K615F**	0.9281	LPath
**K615G**	0.5756	LPath
**K615H**	0.3348	LBen
**K615I**	0.6916	LPath
**K615L**	0.5673	LPath
**K615M**	0.4762	Amb
**K615N**	0.3528	Amb
**K615P**	0.9386	LPath
**K615Q**	0.1848	LBen
**K615R**	0.0756	LBen
**K615S**	0.5613	Amb
**K615T**	0.2797	LBen
**K615V**	0.5928	LPath
**K615W**	0.8708	LPath
**K615Y**	0.7794	LPath
**Q616A**	0.8543	LPath
**Q616C**	0.9993	LPath
**Q616D**	0.9256	LPath
**Q616E**	0.3944	Amb
**Q616F**	0.9981	LPath
**Q616G**	0.9237	LPath

**Q616H**	0.985	LPath
**Q616I**	0.9859	LPath
**Q616K**	0.9493	LPath
**Q616L**	0.9305	LPath
**Q616M**	0.8986	LPath
**Q616N**	0.9291	LPath
**Q616P**	0.9812	LPath
**Q616R**	0.9626	LPath
**Q616S**	0.8721	LPath
**Q616T**	0.908	LPath
**Q616V**	0.9683	LPath
**Q616W**	0.9989	LPath
**Q616Y**	0.9976	LPath
**V617A**	0.6047	LPath
**V617C**	0.9768	LPath
**V617D**	0.9864	LPath
**V617E**	0.961	LPath
**V617F**	0.7977	LPath
**V617G**	0.827	LPath
**V617H**	0.9913	LPath
**V617I**	0.0873	LBen
**V617K**	0.9766	LPath
**V617L**	0.4961	Amb
**V617M**	0.5541	Amb
**V617N**	0.9683	LPath
**V617P**	0.9759	LPath
**V617Q**	0.9672	LPath
**V617R**	0.9603	LPath
**V617S**	0.8662	LPath
**V617T**	0.6911	LPath
**V617W**	0.992	LPath
**V617Y**	0.9811	LPath
**G618A**	0.2165	LBen
**G618C**	0.4597	Amb
**G618D**	0.1456	LBen
**G618E**	0.2966	LBen
**G618F**	0.8494	LPath
**G618H**	0.346	Amb
**G618I**	0.8164	LPath
**G618K**	0.4992	Amb

**G618L**	0.7155	LPath
**G618M**	0.7727	LPath
**G618N**	0.1967	LBen
**G618P**	0.9759	LPath
**G618Q**	0.2943	LBen
**G618R**	0.3211	LBen
**G618S**	0.1096	LBen
**G618T**	0.4238	Amb
**G618V**	0.6714	LPath
**G618W**	0.7186	LPath
**G618Y**	0.6709	LPath
**Y619A**	0.9489	LPath
**Y619C**	0.5783	LPath
**Y619D**	0.9654	LPath
**Y619E**	0.9919	LPath
**Y619F**	0.0573	LBen
**Y619G**	0.9046	LPath
**Y619H**	0.9012	LPath
**Y619I**	0.8938	LPath
**Y619K**	0.991	LPath
**Y619L**	0.4486	Amb
**Y619M**	0.674	LPath
**Y619N**	0.9146	LPath
**Y619P**	0.9962	LPath
**Y619Q**	0.9825	LPath
**Y619R**	0.983	LPath
**Y619S**	0.9236	LPath
**Y619T**	0.9757	LPath
**Y619V**	0.8309	LPath
**Y619W**	0.4891	Amb
**E620A**	0.7394	LPath
**E620C**	0.9921	LPath
**E620D**	0.2861	LBen
**E620F**	0.9913	LPath
**E620G**	0.6457	LPath
**E620H**	0.8171	LPath
**E620I**	0.9812	LPath
**E620K**	0.7899	LPath
**E620L**	0.9757	LPath
**E620M**	0.9727	LPath

**E620N**	0.7342	LPath
**E620P**	0.9804	LPath
**E620Q**	0.464	Amb
**E620R**	0.8094	LPath
**E620S**	0.6728	LPath
**E620T**	0.8792	LPath
**E620V**	0.9431	LPath
**E620W**	0.9948	LPath
**E620Y**	0.9766	LPath
**D621A**	0.163	LBen
**D621C**	0.8308	LPath
**D621E**	0.0843	LBen
**D621F**	0.6901	LPath
**D621G**	0.1809	LBen
**D621H**	0.2912	LBen
**D621I**	0.4595	Amb
**D621K**	0.3059	LBen
**D621L**	0.3895	Amb
**D621M**	0.5881	LPath
**D621N**	0.1309	LBen
**D621P**	0.4665	Amb
**D621Q**	0.213	LBen
**D621R**	0.3262	LBen
**D621S**	0.1377	LBen
**D621T**	0.2499	LBen
**D621V**	0.2553	LBen
**D621W**	0.8896	LPath
**D621Y**	0.334	LBen
**Q622A**	0.2532	LBen
**Q622C**	0.767	LPath
**Q622D**	0.8509	LPath
**Q622E**	0.0737	LBen
**Q622F**	0.9202	LPath
**Q622G**	0.6274	LPath
**Q622H**	0.2734	LBen
**Q622I**	0.6896	LPath
**Q622K**	0.4142	Amb
**Q622L**	0.539	Amb
**Q622M**	0.6741	LPath
**Q622N**	0.5367	Amb

**Q622P**	0.26	LBen
**Q622R**	0.3603	Amb
**Q622S**	0.3645	Amb
**Q622T**	0.5097	Amb
**Q622V**	0.4468	Amb
**Q622W**	0.9157	LPath
**Q622Y**	0.8139	LPath
**W623A**	0.9792	LPath
**W623C**	0.992	LPath
**W623D**	0.9989	LPath
**W623E**	0.9981	LPath
**W623F**	0.5073	Amb
**W623G**	0.9245	LPath
**W623H**	0.9885	LPath
**W623I**	0.9777	LPath
**W623K**	0.9993	LPath
**W623L**	0.8953	LPath
**W623M**	0.9776	LPath
**W623N**	0.9973	LPath
**W623P**	0.9995	LPath
**W623Q**	0.9983	LPath
**W623R**	0.9983	LPath
**W623S**	0.9727	LPath
**W623T**	0.9849	LPath
**W623V**	0.956	LPath
**W623Y**	0.7391	LPath
**L624A**	0.8437	LPath
**L624C**	0.9174	LPath
**L624D**	0.9773	LPath
**L624E**	0.7891	LPath
**L624F**	0.6929	LPath
**L624G**	0.9022	LPath
**L624H**	0.6421	LPath
**L624I**	0.3447	Amb
**L624K**	0.6666	LPath
**L624M**	0.2915	LBen
**L624N**	0.7801	LPath
**L624P**	0.9909	LPath
**L624Q**	0.3411	Amb
**L624R**	0.4731	Amb

**L624S**	0.8641	LPath
**L624T**	0.7781	LPath
**L624V**	0.3826	Amb
**L624W**	0.7633	LPath
**L624Y**	0.842	LPath
**Q625A**	0.288	LBen
**Q625C**	0.7421	LPath
**Q625D**	0.6052	LPath
**Q625E**	0.1147	LBen
**Q625F**	0.5802	LPath
**Q625G**	0.407	Amb
**Q625H**	0.127	LBen
**Q625I**	0.6676	LPath
**Q625K**	0.167	LBen
**Q625L**	0.1385	LBen
**Q625M**	0.4482	Amb
**Q625N**	0.3858	Amb
**Q625P**	0.8195	LPath
**Q625R**	0.1257	LBen
**Q625S**	0.3027	LBen
**Q625T**	0.3374	LBen
**Q625V**	0.4551	Amb
**Q625W**	0.5412	Amb
**Q625Y**	0.3096	LBen
**L626A**	0.8316	LPath
**L626C**	0.8595	LPath
**L626D**	0.9978	LPath
**L626E**	0.9874	LPath
**L626F**	0.1576	LBen
**L626G**	0.9695	LPath
**L626H**	0.9464	LPath
**L626I**	0.1087	LBen
**L626K**	0.988	LPath
**L626M**	0.158	LBen
**L626N**	0.9833	LPath
**L626P**	0.9908	LPath
**L626Q**	0.9356	LPath
**L626R**	0.9683	LPath
**L626S**	0.9632	LPath
**L626T**	0.8468	LPath

**L626V**	0.1083	LBen
**L626W**	0.7303	LPath
**L626Y**	0.8132	LPath
**L627A**	0.9674	LPath
**L627C**	0.9745	LPath
**L627D**	0.9997	LPath
**L627E**	0.9948	LPath
**L627F**	0.9415	LPath
**L627G**	0.9946	LPath
**L627H**	0.9929	LPath
**L627I**	0.3766	Amb
**L627K**	0.9943	LPath
**L627M**	0.5181	Amb
**L627N**	0.9958	LPath
**L627P**	0.9964	LPath
**L627Q**	0.9653	LPath
**L627R**	0.986	LPath
**L627S**	0.9921	LPath
**L627T**	0.959	LPath
**L627V**	0.4796	Amb
**L627W**	0.9857	LPath
**L627Y**	0.9936	LPath
**R628A**	0.4539	Amb
**R628C**	0.3227	LBen
**R628D**	0.7057	LPath
**R628E**	0.4049	Amb
**R628F**	0.7455	LPath
**R628G**	0.2903	LBen
**R628H**	0.0989	LBen
**R628I**	0.5448	Amb
**R628K**	0.1265	LBen
**R628L**	0.3063	LBen
**R628M**	0.4965	Amb
**R628N**	0.6118	LPath
**R628P**	0.9519	LPath
**R628Q**	0.0888	LBen
**R628S**	0.4747	Amb
**R628T**	0.3811	Amb
**R628V**	0.5361	Amb
**R628W**	0.2877	LBen

**R628Y**	0.5025	Amb
**T629A**	0.0767	LBen
**T629C**	0.4475	Amb
**T629D**	0.1595	LBen
**T629E**	0.1441	LBen
**T629F**	0.2338	LBen
**T629G**	0.2099	LBen
**T629H**	0.1291	LBen
**T629I**	0.1739	LBen
**T629K**	0.1518	LBen
**T629L**	0.0953	LBen
**T629M**	0.1029	LBen
**T629N**	0.0671	LBen
**T629P**	0.7149	LPath
**T629Q**	0.1208	LBen
**T629R**	0.1187	LBen
**T629S**	0.0697	LBen
**T629V**	0.1491	LBen
**T629W**	0.5085	Amb
**T629Y**	0.2214	LBen
**Y630A**	0.8665	LPath
**Y630C**	0.484	Amb
**Y630D**	0.9349	LPath
**Y630E**	0.9683	LPath
**Y630F**	0.068	LBen
**Y630G**	0.9331	LPath
**Y630H**	0.525	Amb
**Y630I**	0.7433	LPath
**Y630K**	0.9812	LPath
**Y630L**	0.7977	LPath
**Y630M**	0.8381	LPath
**Y630N**	0.8295	LPath
**Y630P**	0.9945	LPath
**Y630Q**	0.9386	LPath
**Y630R**	0.9368	LPath
**Y630S**	0.7061	LPath
**Y630T**	0.8324	LPath
**Y630V**	0.5752	LPath
**Y630W**	0.3789	Amb
**V631A**	0.2725	LBen

**V631C**	0.9385	LPath
**V631D**	0.9914	LPath
**V631E**	0.9725	LPath
**V631F**	0.7116	LPath
**V631G**	0.699	LPath
**V631H**	0.9918	LPath
**V631I**	0.1003	LBen
**V631K**	0.9849	LPath
**V631L**	0.4693	Amb
**V631M**	0.4405	Amb
**V631N**	0.9776	LPath
**V631P**	0.9539	LPath
**V631Q**	0.9627	LPath
**V631R**	0.9692	LPath
**V631S**	0.711	LPath
**V631T**	0.4061	Amb
**V631W**	0.9928	LPath
**V631Y**	0.9856	LPath
**G632A**	0.0715	LBen
**G632C**	0.2788	LBen
**G632D**	0.712	LPath
**G632E**	0.4313	Amb
**G632F**	0.7219	LPath
**G632H**	0.6195	LPath
**G632I**	0.259	LBen
**G632K**	0.6801	LPath
**G632L**	0.4163	Amb
**G632M**	0.4616	Amb
**G632N**	0.5254	Amb
**G632P**	0.9401	LPath
**G632Q**	0.4343	Amb
**G632R**	0.5265	Amb
**G632S**	0.0835	LBen
**G632T**	0.1386	LBen
**G632V**	0.1521	LBen
**G632W**	0.6585	LPath
**G632Y**	0.6529	LPath
**P633A**	0.2754	LBen
**P633C**	0.905	LPath
**P633D**	0.8315	LPath

**P633E**	0.5819	LPath
**P633F**	0.8807	LPath
**P633G**	0.672	LPath
**P633H**	0.4831	Amb
**P633I**	0.6906	LPath
**P633K**	0.4778	Amb
**P633L**	0.3958	Amb
**P633M**	0.7374	LPath
**P633N**	0.7646	LPath
**P633Q**	0.3635	Amb
**P633R**	0.3121	LBen
**P633S**	0.4386	Amb
**P633T**	0.3238	LBen
**P633V**	0.5073	Amb
**P633W**	0.9249	LPath
**P633Y**	0.8583	LPath
**M634A**	0.6951	LPath
**M634C**	0.9622	LPath
**M634D**	0.9978	LPath
**M634E**	0.9695	LPath
**M634F**	0.6737	LPath
**M634G**	0.9618	LPath
**M634H**	0.981	LPath
**M634I**	0.3183	LBen
**M634K**	0.8755	LPath
**M634L**	0.1315	LBen
**M634N**	0.9823	LPath
**M634P**	0.9595	LPath
**M634Q**	0.8677	LPath
**M634R**	0.8928	LPath
**M634S**	0.9202	LPath
**M634T**	0.7396	LPath
**M634V**	0.0737	LBen
**M634W**	0.9721	LPath
**M634Y**	0.9666	LPath
**T635A**	0.1531	LBen
**T635C**	0.7801	LPath
**T635D**	0.9813	LPath
**T635E**	0.9269	LPath
**T635F**	0.9422	LPath

**T635G**	0.6248	LPath
**T635H**	0.9373	LPath
**T635I**	0.5701	LPath
**T635K**	0.9384	LPath
**T635L**	0.3704	Amb
**T635M**	0.1902	LBen
**T635N**	0.696	LPath
**T635P**	0.8709	LPath
**T635Q**	0.7873	LPath
**T635R**	0.9081	LPath
**T635S**	0.3791	Amb
**T635V**	0.2886	LBen
**T635W**	0.9875	LPath
**T635Y**	0.9631	LPath
**E636A**	0.2243	LBen
**E636C**	0.9662	LPath
**E636D**	0.4418	Amb
**E636F**	0.9528	LPath
**E636G**	0.3349	LBen
**E636H**	0.7297	LPath
**E636I**	0.7152	LPath
**E636K**	0.2816	LBen
**E636L**	0.6582	LPath
**E636M**	0.7156	LPath
**E636N**	0.6028	LPath
**E636P**	0.9804	LPath
**E636Q**	0.1679	LBen
**E636R**	0.3423	Amb
**E636S**	0.3648	Amb
**E636T**	0.4536	Amb
**E636V**	0.461	Amb
**E636W**	0.9821	LPath
**E636Y**	0.9041	LPath
**S637A**	0.077	LBen
**S637C**	0.1267	LBen
**S637D**	0.1958	LBen
**S637E**	0.2071	LBen
**S637F**	0.1233	LBen
**S637G**	0.0835	LBen
**S637H**	0.06	LBen

**S637I**	0.1007	LBen
**S637K**	0.1211	LBen
**S637L**	0.0989	LBen
**S637M**	0.1414	LBen
**S637N**	0.0719	LBen
**S637P**	0.6014	LPath
**S637Q**	0.1146	LBen
**S637R**	0.0885	LBen
**S637T**	0.0712	LBen
**S637V**	0.1229	LBen
**S637W**	0.1665	LBen
**S637Y**	0.0759	LBen
**L638A**	0.7494	LPath
**L638C**	0.8347	LPath
**L638D**	0.9894	LPath
**L638E**	0.921	LPath
**L638F**	0.3312	LBen
**L638G**	0.9553	LPath
**L638H**	0.7283	LPath
**L638I**	0.1791	LBen
**L638K**	0.8408	LPath
**L638M**	0.2331	LBen
**L638N**	0.9316	LPath
**L638P**	0.9815	LPath
**L638Q**	0.6278	LPath
**L638R**	0.5957	LPath
**L638S**	0.8759	LPath
**L638T**	0.7687	LPath
**L638V**	0.1641	LBen
**L638W**	0.6715	LPath
**L638Y**	0.7359	LPath
**F639A**	0.9982	LPath
**F639C**	0.9954	LPath
**F639D**	0.9991	LPath
**F639E**	0.9994	LPath
**F639G**	0.9985	LPath
**F639H**	0.9829	LPath
**F639I**	0.9876	LPath
**F639K**	0.9993	LPath
**F639L**	0.9956	LPath

**F639M**	0.9913	LPath
**F639N**	0.9968	LPath
**F639P**	0.9997	LPath
**F639Q**	0.9979	LPath
**F639R**	0.9975	LPath
**F639S**	0.995	LPath
**F639T**	0.9986	LPath
**F639V**	0.9884	LPath
**F639W**	0.8517	LPath
**F639Y**	0.3081	LBen
**P640A**	0.2151	LBen
**P640C**	0.8759	LPath
**P640D**	0.8849	LPath
**P640E**	0.607	LPath
**P640F**	0.8452	LPath
**P640G**	0.7293	LPath
**P640H**	0.5026	Amb
**P640I**	0.6065	LPath
**P640K**	0.6264	LPath
**P640L**	0.2329	LBen
**P640M**	0.646	LPath
**P640N**	0.8446	LPath
**P640Q**	0.3903	Amb
**P640R**	0.3529	Amb
**P640S**	0.3659	Amb
**P640T**	0.2673	LBen
**P640V**	0.4507	Amb
**P640W**	0.8669	LPath
**P640Y**	0.8252	LPath
**G641A**	0.8852	LPath
**G641C**	0.9809	LPath
**G641D**	0.9875	LPath
**G641E**	0.9924	LPath
**G641F**	0.9924	LPath
**G641H**	0.9934	LPath
**G641I**	0.9905	LPath
**G641K**	0.9975	LPath
**G641L**	0.9869	LPath
**G641M**	0.9957	LPath
**G641N**	0.9869	LPath

**G641P**	0.9968	LPath
**G641Q**	0.9916	LPath
**G641R**	0.9885	LPath
**G641S**	0.8234	LPath
**G641T**	0.9683	LPath
**G641V**	0.9801	LPath
**G641W**	0.9894	LPath
**G641Y**	0.9919	LPath
**Y642A**	0.9961	LPath
**Y642C**	0.9721	LPath
**Y642D**	0.9962	LPath
**Y642E**	0.9985	LPath
**Y642F**	0.5305	Amb
**Y642G**	0.9877	LPath
**Y642H**	0.9877	LPath
**Y642I**	0.9795	LPath
**Y642K**	0.9984	LPath
**Y642L**	0.9403	LPath
**Y642M**	0.9881	LPath
**Y642N**	0.9807	LPath
**Y642P**	0.9971	LPath
**Y642Q**	0.9978	LPath
**Y642R**	0.9942	LPath
**Y642S**	0.9774	LPath
**Y642T**	0.994	LPath
**Y642V**	0.9641	LPath
**Y642W**	0.9333	LPath
**H643A**	0.4408	Amb
**H643C**	0.5829	LPath
**H643D**	0.4655	Amb
**H643E**	0.4962	Amb
**H643F**	0.2584	LBen
**H643G**	0.5161	Amb
**H643I**	0.518	Amb
**H643K**	0.4796	Amb
**H643L**	0.2128	LBen
**H643M**	0.6543	LPath
**H643N**	0.2229	LBen
**H643P**	0.404	Amb
**H643Q**	0.2701	LBen

**H643R**	0.1709	LBen
**H643S**	0.3332	LBen
**H643T**	0.4041	Amb
**H643V**	0.3792	Amb
**H643W**	0.4187	Amb
**H643Y**	0.092	LBen
**T644A**	0.2073	LBen
**T644C**	0.6022	LPath
**T644D**	0.9149	LPath
**T644E**	0.914	LPath
**T644F**	0.8473	LPath
**T644G**	0.4273	Amb
**T644H**	0.7541	LPath
**T644I**	0.7978	LPath
**T644K**	0.8999	LPath
**T644L**	0.3987	Amb
**T644M**	0.3737	Amb
**T644N**	0.5575	Amb
**T644P**	0.1945	LBen
**T644Q**	0.7703	LPath
**T644R**	0.8213	LPath
**T644S**	0.2247	LBen
**T644V**	0.5545	Amb
**T644W**	0.9505	LPath
**T644Y**	0.8985	LPath
**K645A**	0.5566	Amb
**K645C**	0.9346	LPath
**K645D**	0.7806	LPath
**K645E**	0.3694	Amb
**K645F**	0.9562	LPath
**K645G**	0.642	LPath
**K645H**	0.49	Amb
**K645I**	0.8314	LPath
**K645L**	0.7143	LPath
**K645M**	0.6565	LPath
**K645N**	0.7921	LPath
**K645P**	0.7201	LPath
**K645Q**	0.1982	LBen
**K645R**	0.0859	LBen
**K645S**	0.6646	LPath

**K645T**	0.3894	Amb
**K645V**	0.6607	LPath
**K645W**	0.9126	LPath
**K645Y**	0.8906	LPath
**A646C**	0.355	Amb
**A646D**	0.817	LPath
**A646E**	0.7588	LPath
**A646F**	0.7105	LPath
**A646G**	0.1448	LBen
**A646H**	0.8047	LPath
**A646I**	0.5125	Amb
**A646K**	0.9006	LPath
**A646L**	0.2896	LBen
**A646M**	0.2905	LBen
**A646N**	0.453	Amb
**A646P**	0.5261	Amb
**A646Q**	0.6879	LPath
**A646R**	0.8257	LPath
**A646S**	0.1223	LBen
**A646T**	0.074	LBen
**A646V**	0.2191	LBen
**A646W**	0.9194	LPath
**A646Y**	0.7937	LPath
**R647A**	0.4866	Amb
**R647C**	0.4123	Amb
**R647D**	0.7376	LPath
**R647E**	0.4244	Amb
**R647F**	0.6146	LPath
**R647G**	0.338	LBen
**R647H**	0.1065	LBen
**R647I**	0.5013	Amb
**R647K**	0.1258	LBen
**R647L**	0.3224	LBen
**R647M**	0.433	Amb
**R647N**	0.647	LPath
**R647P**	0.8262	LPath
**R647Q**	0.0991	LBen
**R647S**	0.5166	Amb
**R647T**	0.3375	LBen
**R647V**	0.4698	Amb

**R647W**	0.181	LBen
**R647Y**	0.4394	Amb
**A648C**	0.9231	LPath
**A648D**	0.9922	LPath
**A648E**	0.9891	LPath
**A648F**	0.9818	LPath
**A648G**	0.5968	LPath
**A648H**	0.981	LPath
**A648I**	0.9897	LPath
**A648K**	0.9979	LPath
**A648L**	0.9596	LPath
**A648M**	0.9645	LPath
**A648N**	0.9741	LPath
**A648P**	0.9812	LPath
**A648Q**	0.973	LPath
**A648R**	0.9892	LPath
**A648S**	0.1768	LBen
**A648T**	0.7478	LPath
**A648V**	0.919	LPath
**A648W**	0.9975	LPath
**A648Y**	0.9923	LPath
**V649A**	0.3437	Amb
**V649C**	0.927	LPath
**V649D**	0.63	LPath
**V649E**	0.5288	Amb
**V649F**	0.1801	LBen
**V649G**	0.3771	Amb
**V649H**	0.714	LPath
**V649I**	0.0623	LBen
**V649K**	0.6674	LPath
**V649L**	0.127	LBen
**V649M**	0.1519	LBen
**V649N**	0.4061	Amb
**V649P**	0.8133	LPath
**V649Q**	0.4968	Amb
**V649R**	0.5549	Amb
**V649S**	0.36	Amb
**V649T**	0.3577	Amb
**V649W**	0.8429	LPath
**V649Y**	0.6249	LPath

**M650A**	0.9881	LPath
**M650C**	0.9884	LPath
**M650D**	0.9988	LPath
**M650E**	0.994	LPath
**M650F**	0.919	LPath
**M650G**	0.9922	LPath
**M650H**	0.9928	LPath
**M650I**	0.9872	LPath
**M650K**	0.9782	LPath
**M650L**	0.3538	Amb
**M650N**	0.9916	LPath
**M650P**	0.998	LPath
**M650Q**	0.9613	LPath
**M650R**	0.9783	LPath
**M650S**	0.9875	LPath
**M650T**	0.9864	LPath
**M650V**	0.8731	LPath
**M650W**	0.992	LPath
**M650Y**	0.9892	LPath
**N651A**	0.8584	LPath
**N651C**	0.8808	LPath
**N651D**	0.9017	LPath
**N651E**	0.9936	LPath
**N651F**	0.9957	LPath
**N651G**	0.9399	LPath
**N651H**	0.8977	LPath
**N651I**	0.9578	LPath
**N651K**	0.9976	LPath
**N651L**	0.9251	LPath
**N651M**	0.9021	LPath
**N651P**	0.9928	LPath
**N651Q**	0.9785	LPath
**N651R**	0.9971	LPath
**N651S**	0.3377	LBen
**N651T**	0.8565	LPath
**N651V**	0.9392	LPath
**N651W**	0.9993	LPath
**N651Y**	0.9638	LPath
**F652A**	0.9989	LPath
**F652C**	0.9989	LPath

**F652D**	0.9997	LPath
**F652E**	0.9995	LPath
**F652G**	0.9995	LPath
**F652H**	0.9978	LPath
**F652I**	0.9956	LPath
**F652K**	0.9996	LPath
**F652L**	0.9998	LPath
**F652M**	0.997	LPath
**F652N**	0.9994	LPath
**F652P**	1	LPath
**F652Q**	0.9993	LPath
**F652R**	0.9981	LPath
**F652S**	0.9993	LPath
**F652T**	0.999	LPath
**F652V**	0.996	LPath
**F652W**	0.991	LPath
**F652Y**	0.9732	LPath
**V653A**	0.8135	LPath
**V653C**	0.9833	LPath
**V653D**	0.9986	LPath
**V653E**	0.9933	LPath
**V653F**	0.8776	LPath
**V653G**	0.954	LPath
**V653H**	0.9983	LPath
**V653I**	0.1333	LBen
**V653K**	0.9966	LPath
**V653L**	0.6385	LPath
**V653M**	0.4555	Amb
**V653N**	0.9926	LPath
**V653P**	0.9973	LPath
**V653Q**	0.9876	LPath
**V653R**	0.9929	LPath
**V653S**	0.9344	LPath
**V653T**	0.798	LPath
**V653W**	0.9988	LPath
**V653Y**	0.9969	LPath
**V654A**	0.9631	LPath
**V654C**	0.9908	LPath
**V654D**	0.9977	LPath
**V654E**	0.9936	LPath

V654F	0.9882	LPath
V654G	0.951	LPath
V654H	0.9989	LPath
V654I	0.5264	Amb
V654K	0.9963	LPath
V654L	0.9682	LPath
V654M	0.9842	LPath
V654N	0.9895	LPath
V654P	0.9963	LPath
V654Q	0.9912	LPath
V654R	0.9823	LPath
V654S	0.9549	LPath
V654T	0.9535	LPath
V654W	0.9997	LPath
V654Y	0.9982	LPath
R655A	0.9945	LPath
R655C	0.9207	LPath
R655D	0.9994	LPath
R655E	0.993	LPath
R655F	0.9989	LPath
R655G	0.9933	LPath
R655H	0.8836	LPath
R655I	0.9951	LPath
R655K	0.849	LPath
R655L	0.9759	LPath
R655M	0.9974	LPath
R655N	0.9985	LPath
R655P	0.9985	LPath
R655Q	0.8617	LPath
R655S	0.9966	LPath
R655T	0.9973	LPath
R655V	0.9944	LPath
R655W	0.9636	LPath
R655Y	0.9914	LPath
Y656A	0.999	LPath
Y656C	0.9928	LPath
Y656D	0.9984	LPath
Y656E	0.9996	LPath
Y656F	0.8947	LPath
Y656G	0.9924	LPath

**Y656H**	0.9989	LPath
**Y656I**	0.9957	LPath
**Y656K**	0.9995	LPath
**Y656L**	0.9598	LPath
**Y656M**	0.9974	LPath
**Y656N**	0.9944	LPath
**Y656P**	0.9989	LPath
**Y656Q**	0.9996	LPath
**Y656R**	0.9985	LPath
**Y656S**	0.9932	LPath
**Y656T**	0.9985	LPath
**Y656V**	0.9907	LPath
**Y656W**	0.9837	LPath
**R657A**	0.8725	LPath
**R657C**	0.51	Amb
**R657D**	0.9668	LPath
**R657E**	0.837	LPath
**R657F**	0.8931	LPath
**R657G**	0.8106	LPath
**R657H**	0.1327	LBen
**R657I**	0.8565	LPath
**R657K**	0.1928	LBen
**R657L**	0.5655	LPath
**R657M**	0.8106	LPath
**R657N**	0.9336	LPath
**R657P**	0.993	LPath
**R657Q**	0.1868	LBen
**R657S**	0.8628	LPath
**R657T**	0.7171	LPath
**R657V**	0.8505	LPath
**R657W**	0.4875	Amb
**R657Y**	0.7568	LPath
**P658A**	0.5345	Amb
**P658C**	0.9739	LPath
**P658D**	0.9739	LPath
**P658E**	0.8695	LPath
**P658F**	0.9783	LPath
**P658G**	0.9198	LPath
**P658H**	0.8439	LPath
**P658I**	0.9295	LPath

**P658K**	0.8475	LPath
**P658L**	0.6653	LPath
**P658M**	0.8847	LPath
**P658N**	0.956	LPath
**P658Q**	0.7265	LPath
**P658R**	0.6485	LPath
**P658S**	0.7591	LPath
**P658T**	0.6441	LPath
**P658V**	0.8247	LPath
**P658W**	0.9675	LPath
**P658Y**	0.9706	LPath
**D659A**	0.3007	LBen
**D659C**	0.9004	LPath
**D659E**	0.2508	LBen
**D659F**	0.8313	LPath
**D659G**	0.2057	LBen
**D659H**	0.4376	Amb
**D659I**	0.7508	LPath
**D659K**	0.5715	LPath
**D659L**	0.6507	LPath
**D659M**	0.8047	LPath
**D659N**	0.1326	LBen
**D659P**	0.8743	LPath
**D659Q**	0.4198	Amb
**D659R**	0.5707	LPath
**D659S**	0.1598	LBen
**D659T**	0.3393	LBen
**D659V**	0.5507	Amb
**D659W**	0.9298	LPath
**D659Y**	0.5346	Amb
**E660A**	0.8018	LPath
**E660C**	0.9915	LPath
**E660D**	0.9115	LPath
**E660F**	0.996	LPath
**E660G**	0.7277	LPath
**E660H**	0.9725	LPath
**E660I**	0.9682	LPath
**E660K**	0.8174	LPath
**E660L**	0.9673	LPath
**E660M**	0.9686	LPath

**E660N**	0.9693	LPath
**E660P**	0.9955	LPath
**E660Q**	0.5727	LPath
**E660R**	0.8386	LPath
**E660S**	0.8459	LPath
**E660T**	0.9022	LPath
**E660V**	0.9171	LPath
**E660W**	0.9984	LPath
**E660Y**	0.9922	LPath
**Q661A**	0.9059	LPath
**Q661C**	0.9994	LPath
**Q661D**	0.9921	LPath
**Q661E**	0.8274	LPath
**Q661F**	0.9842	LPath
**Q661G**	0.946	LPath
**Q661H**	0.9942	LPath
**Q661I**	0.9541	LPath
**Q661K**	0.9512	LPath
**Q661L**	0.8837	LPath
**Q661M**	0.8744	LPath
**Q661N**	0.9295	LPath
**Q661P**	0.9579	LPath
**Q661R**	0.9735	LPath
**Q661S**	0.9065	LPath
**Q661T**	0.9148	LPath
**Q661V**	0.9542	LPath
**Q661W**	0.9975	LPath
**Q661Y**	0.9951	LPath
**P662A**	0.1464	LBen
**P662C**	0.8854	LPath
**P662D**	0.789	LPath
**P662E**	0.7105	LPath
**P662F**	0.7664	LPath
**P662G**	0.5737	LPath
**P662H**	0.3637	Amb
**P662I**	0.8904	LPath
**P662K**	0.7124	LPath
**P662L**	0.497	Amb
**P662M**	0.8426	LPath
**P662N**	0.7312	LPath

**P662Q**	0.4809	Amb
**P662R**	0.3414	Amb
**P662S**	0.2294	LBen
**P662T**	0.4956	Amb
**P662V**	0.7323	LPath
**P662W**	0.8028	LPath
**P662Y**	0.6564	LPath
**S663A**	0.0694	LBen
**S663C**	0.2573	LBen
**S663D**	0.6032	LPath
**S663E**	0.5473	Amb
**S663F**	0.1032	LBen
**S663G**	0.1869	LBen
**S663H**	0.3313	LBen
**S663I**	0.4217	Amb
**S663K**	0.7419	LPath
**S663L**	0.1296	LBen
**S663M**	0.268	LBen
**S663N**	0.3592	Amb
**S663P**	0.9318	LPath
**S663Q**	0.442	Amb
**S663R**	0.5716	LPath
**S663T**	0.1947	LBen
**S663V**	0.2915	LBen
**S663W**	0.2187	LBen
**S663Y**	0.1187	LBen
**L664A**	0.9927	LPath
**L664C**	0.9987	LPath
**L664D**	0.9998	LPath
**L664E**	0.9982	LPath
**L664F**	0.9943	LPath
**L664G**	0.9965	LPath
**L664H**	0.9987	LPath
**L664I**	0.9538	LPath
**L664K**	0.997	LPath
**L664M**	0.978	LPath
**L664N**	0.9988	LPath
**L664P**	0.998	LPath
**L664Q**	0.9974	LPath
**L664R**	0.989	LPath

**L664S**	0.9984	LPath
**L664T**	0.9957	LPath
**L664V**	0.9708	LPath
**L664W**	0.9976	LPath
**L664Y**	0.9985	LPath
**R665A**	0.7687	LPath
**R665C**	0.6155	LPath
**R665D**	0.9609	LPath
**R665E**	0.7075	LPath
**R665F**	0.8375	LPath
**R665G**	0.7395	LPath
**R665H**	0.2632	LBen
**R665I**	0.5767	LPath
**R665K**	0.2109	LBen
**R665L**	0.4363	Amb
**R665M**	0.6288	LPath
**R665N**	0.9299	LPath
**R665P**	0.8046	LPath
**R665Q**	0.1555	LBen
**R665S**	0.8465	LPath
**R665T**	0.6997	LPath
**R665V**	0.5704	LPath
**R665W**	0.4377	Amb
**R665Y**	0.7453	LPath
**P666A**	0.5113	Amb
**P666C**	0.9848	LPath
**P666D**	0.9909	LPath
**P666E**	0.961	LPath
**P666F**	0.9895	LPath
**P666G**	0.9434	LPath
**P666H**	0.9375	LPath
**P666I**	0.9769	LPath
**P666K**	0.9736	LPath
**P666L**	0.8098	LPath
**P666M**	0.972	LPath
**P666N**	0.9843	LPath
**P666Q**	0.9135	LPath
**P666R**	0.8888	LPath
**P666S**	0.8463	LPath
**P666T**	0.9115	LPath

**P666V**	0.9372	LPath
**P666W**	0.99	LPath
**P666Y**	0.9825	LPath
**H667A**	0.9983	LPath
**H667C**	0.9823	LPath
**H667D**	0.9995	LPath
**H667E**	0.9991	LPath
**H667F**	0.9952	LPath
**H667G**	0.9988	LPath
**H667I**	0.999	LPath
**H667K**	0.9981	LPath
**H667L**	0.9863	LPath
**H667M**	0.9992	LPath
**H667N**	0.9979	LPath
**H667P**	0.9905	LPath
**H667Q**	0.9982	LPath
**H667R**	0.9911	LPath
**H667S**	0.9968	LPath
**H667T**	0.9991	LPath
**H667V**	0.9984	LPath
**H667W**	0.9881	LPath
**H667Y**	0.9919	LPath
**H668A**	0.9948	LPath
**H668C**	0.9765	LPath
**H668D**	0.9969	LPath
**H668E**	0.9979	LPath
**H668F**	0.9928	LPath
**H668G**	0.997	LPath
**H668I**	0.9992	LPath
**H668K**	0.9987	LPath
**H668L**	0.9896	LPath
**H668M**	0.9978	LPath
**H668N**	0.9825	LPath
**H668P**	0.9897	LPath
**H668Q**	0.9937	LPath
**H668R**	0.9953	LPath
**H668S**	0.9794	LPath
**H668T**	0.9971	LPath
**H668V**	0.9989	LPath
**H668W**	0.98	LPath

**H668Y**	0.9884	LPath
**D669A**	0.9996	LPath
**D669C**	0.9999	LPath
**D669E**	0.9991	LPath
**D669F**	0.9999	LPath
**D669G**	0.9994	LPath
**D669H**	0.9993	LPath
**D669I**	0.9999	LPath
**D669K**	0.9997	LPath
**D669L**	0.9997	LPath
**D669M**	1	LPath
**D669N**	0.9965	LPath
**D669P**	0.9997	LPath
**D669Q**	0.9998	LPath
**D669R**	0.9992	LPath
**D669S**	0.9979	LPath
**D669T**	0.9998	LPath
**D669V**	0.9998	LPath
**D669W**	1	LPath
**D669Y**	0.9983	LPath
**S670A**	0.2228	LBen
**S670C**	0.8377	LPath
**S670D**	0.9914	LPath
**S670E**	0.9901	LPath
**S670F**	0.9249	LPath
**S670G**	0.6886	LPath
**S670H**	0.9389	LPath
**S670I**	0.9398	LPath
**S670K**	0.9982	LPath
**S670L**	0.7626	LPath
**S670M**	0.874	LPath
**S670N**	0.9345	LPath
**S670P**	0.98	LPath
**S670Q**	0.9684	LPath
**S670R**	0.9927	LPath
**S670T**	0.5027	Amb
**S670V**	0.9104	LPath
**S670W**	0.9409	LPath
**S670Y**	0.9245	LPath
**S671A**	0.663	LPath

**S671C**	0.9552	LPath
**S671D**	0.9991	LPath
**S671E**	0.9987	LPath
**S671F**	0.9993	LPath
**S671G**	0.9388	LPath
**S671H**	0.9979	LPath
**S671I**	0.9991	LPath
**S671K**	0.9999	LPath
**S671L**	0.9943	LPath
**S671M**	0.9971	LPath
**S671N**	0.9979	LPath
**S671P**	0.9987	LPath
**S671Q**	0.9976	LPath
**S671R**	0.9994	LPath
**S671T**	0.9654	LPath
**S671V**	0.9967	LPath
**S671W**	0.9991	LPath
**S671Y**	0.9989	LPath
**T672A**	0.916	LPath
**T672C**	0.9933	LPath
**T672D**	0.9954	LPath
**T672E**	0.9971	LPath
**T672F**	0.9963	LPath
**T672G**	0.9724	LPath
**T672H**	0.9898	LPath
**T672I**	0.9921	LPath
**T672K**	0.9971	LPath
**T672L**	0.9476	LPath
**T672M**	0.9668	LPath
**T672N**	0.9697	LPath
**T672P**	0.9642	LPath
**T672Q**	0.9915	LPath
**T672R**	0.991	LPath
**T672S**	0.7514	LPath
**T672V**	0.9648	LPath
**T672W**	0.9986	LPath
**T672Y**	0.9965	LPath
**F673A**	0.9968	LPath
**F673C**	0.9854	LPath
**F673D**	0.9998	LPath

**F673E**	0.9998	LPath
**F673G**	0.999	LPath
**F673H**	0.9936	LPath
**F673I**	0.943	LPath
**F673K**	0.9998	LPath
**F673L**	0.9958	LPath
**F673M**	0.9808	LPath
**F673N**	0.9988	LPath
**F673P**	1	LPath
**F673Q**	0.9992	LPath
**F673R**	0.9991	LPath
**F673S**	0.996	LPath
**F673T**	0.9976	LPath
**F673V**	0.9113	LPath
**F673W**	0.9244	LPath
**F673Y**	0.5482	Amb
**T674A**	0.988	LPath
**T674C**	0.9952	LPath
**T674D**	0.9992	LPath
**T674E**	0.9986	LPath
**T674F**	0.9981	LPath
**T674G**	0.995	LPath
**T674H**	0.992	LPath
**T674I**	0.997	LPath
**T674K**	0.9983	LPath
**T674L**	0.9859	LPath
**T674M**	0.9875	LPath
**T674N**	0.9959	LPath
**T674P**	0.9915	LPath
**T674Q**	0.9957	LPath
**T674R**	0.9971	LPath
**T674S**	0.902	LPath
**T674V**	0.9895	LPath
**T674W**	0.9994	LPath
**T674Y**	0.9985	LPath
**L675A**	0.8598	LPath
**L675C**	0.9023	LPath
**L675D**	0.999	LPath
**L675E**	0.9926	LPath
**L675F**	0.6731	LPath

**L675G**	0.9849	LPath
**L675H**	0.9874	LPath
**L675I**	0.0958	LBen
**L675K**	0.9943	LPath
**L675M**	0.2736	LBen
**L675N**	0.992	LPath
**L675P**	0.9943	LPath
**L675Q**	0.9708	LPath
**L675R**	0.9848	LPath
**L675S**	0.9733	LPath
**L675T**	0.8935	LPath
**L675V**	0.0858	LBen
**L675W**	0.9714	LPath
**L675Y**	0.9781	LPath
**N676A**	0.9468	LPath
**N676C**	0.9281	LPath
**N676D**	0.9323	LPath
**N676E**	0.9952	LPath
**N676F**	0.9979	LPath
**N676G**	0.9611	LPath
**N676H**	0.9357	LPath
**N676I**	0.9647	LPath
**N676K**	0.9976	LPath
**N676L**	0.9087	LPath
**N676M**	0.9673	LPath
**N676P**	0.9885	LPath
**N676Q**	0.9907	LPath
**N676R**	0.9973	LPath
**N676S**	0.5405	Amb
**N676T**	0.8474	LPath
**N676V**	0.9315	LPath
**N676W**	0.9992	LPath
**N676Y**	0.9828	LPath
**V677A**	0.7474	LPath
**V677C**	0.9716	LPath
**V677D**	0.9966	LPath
**V677E**	0.9861	LPath
**V677F**	0.7361	LPath
**V677G**	0.9185	LPath
**V677H**	0.9962	LPath

**V677I**	0.0793	LBen
**V677K**	0.9912	LPath
**V677L**	0.3924	Amb
**V677M**	0.4172	Amb
**V677N**	0.9872	LPath
**V677P**	0.9956	LPath
**V677Q**	0.9831	LPath
**V677R**	0.9852	LPath
**V677S**	0.9368	LPath
**V677T**	0.7621	LPath
**V677W**	0.9952	LPath
**V677Y**	0.9861	LPath
**A678C**	0.9571	LPath
**A678D**	0.9949	LPath
**A678E**	0.9937	LPath
**A678F**	0.9727	LPath
**A678G**	0.5292	Amb
**A678H**	0.9917	LPath
**A678I**	0.9894	LPath
**A678K**	0.9983	LPath
**A678L**	0.9033	LPath
**A678M**	0.9651	LPath
**A678N**	0.9782	LPath
**A678P**	0.8245	LPath
**A678Q**	0.9783	LPath
**A678R**	0.9866	LPath
**A678S**	0.3964	Amb
**A678T**	0.9163	LPath
**A678V**	0.9534	LPath
**A678W**	0.9978	LPath
**A678Y**	0.9926	LPath
**L679A**	0.9815	LPath
**L679C**	0.9856	LPath
**L679D**	0.9999	LPath
**L679E**	0.9992	LPath
**L679F**	0.9589	LPath
**L679G**	0.9952	LPath
**L679H**	0.9988	LPath
**L679I**	0.8392	LPath
**L679K**	0.9987	LPath

**L679M**	0.6971	LPath
**L679N**	0.9991	LPath
**L679P**	0.9959	LPath
**L679Q**	0.9959	LPath
**L679R**	0.9952	LPath
**L679S**	0.9981	LPath
**L679T**	0.9921	LPath
**L679V**	0.8003	LPath
**L679W**	0.9959	LPath
**L679Y**	0.9978	LPath
**N680A**	0.9899	LPath
**N680C**	0.9747	LPath
**N680D**	0.9839	LPath
**N680E**	0.9978	LPath
**N680F**	0.9987	LPath
**N680G**	0.9737	LPath
**N680H**	0.9604	LPath
**N680I**	0.9935	LPath
**N680K**	0.9986	LPath
**N680L**	0.9745	LPath
**N680M**	0.9923	LPath
**N680P**	0.9939	LPath
**N680Q**	0.9937	LPath
**N680R**	0.9958	LPath
**N680S**	0.654	LPath
**N680T**	0.895	LPath
**N680V**	0.9906	LPath
**N680W**	0.9995	LPath
**N680Y**	0.989	LPath
**H681A**	0.264	LBen
**H681C**	0.262	LBen
**H681D**	0.2112	LBen
**H681E**	0.2213	LBen
**H681F**	0.4575	Amb
**H681G**	0.2812	LBen
**H681I**	0.5086	Amb
**H681K**	0.2009	LBen
**H681L**	0.2001	LBen
**H681M**	0.5536	Amb
**H681N**	0.114	LBen

**H681P**	0.6155	LPath
**H681Q**	0.1085	LBen
**H681R**	0.0635	LBen
**H681S**	0.1417	LBen
**H681T**	0.1926	LBen
**H681V**	0.3773	Amb
**H681W**	0.3328	LBen
**H681Y**	0.1391	LBen
**K682A**	0.5676	LPath
**K682C**	0.8582	LPath
**K682D**	0.9304	LPath
**K682E**	0.4354	Amb
**K682F**	0.9522	LPath
**K682G**	0.8038	LPath
**K682H**	0.579	LPath
**K682I**	0.6532	LPath
**K682L**	0.6712	LPath
**K682M**	0.5654	LPath
**K682N**	0.8955	LPath
**K682P**	0.6574	LPath
**K682Q**	0.2105	LBen
**K682R**	0.0848	LBen
**K682S**	0.7784	LPath
**K682T**	0.5034	Amb
**K682V**	0.5033	Amb
**K682W**	0.9188	LPath
**K682Y**	0.887	LPath
**G683A**	0.2872	LBen
**G683C**	0.5697	LPath
**G683D**	0.1259	LBen
**G683E**	0.2337	LBen
**G683F**	0.7075	LPath
**G683H**	0.3857	Amb
**G683I**	0.6636	LPath
**G683K**	0.5742	LPath
**G683L**	0.6845	LPath
**G683M**	0.7286	LPath
**G683N**	0.2244	LBen
**G683P**	0.9451	LPath
**G683Q**	0.4076	Amb

**G683R**	0.4729	Amb
**G683S**	0.1414	LBen
**G683T**	0.3525	Amb
**G683V**	0.4935	Amb
**G683W**	0.5603	Amb
**G683Y**	0.4885	Amb
**L684A**	0.1269	LBen
**L684C**	0.4271	Amb
**L684D**	0.3303	LBen
**L684E**	0.1136	LBen
**L684F**	0.1168	LBen
**L684G**	0.2985	LBen
**L684H**	0.12	LBen
**L684I**	0.0734	LBen
**L684K**	0.0949	LBen
**L684M**	0.0975	LBen
**L684N**	0.225	LBen
**L684P**	0.2889	LBen
**L684Q**	0.0672	LBen
**L684R**	0.0635	LBen
**L684S**	0.1503	LBen
**L684T**	0.1098	LBen
**L684V**	0.0653	LBen
**L684W**	0.1313	LBen
**L684Y**	0.2393	LBen
**D685A**	0.9072	LPath
**D685C**	0.9917	LPath
**D685E**	0.8981	LPath
**D685F**	0.9802	LPath
**D685G**	0.888	LPath
**D685H**	0.8982	LPath
**D685I**	0.9793	LPath
**D685K**	0.9831	LPath
**D685L**	0.9457	LPath
**D685M**	0.9928	LPath
**D685N**	0.7405	LPath
**D685P**	0.9776	LPath
**D685Q**	0.9485	LPath
**D685R**	0.9697	LPath
**D685S**	0.8208	LPath

**D685T**	0.9463	LPath
**D685V**	0.9418	LPath
**D685W**	0.9955	LPath
**D685Y**	0.8954	LPath
**Y686A**	0.9923	LPath
**Y686C**	0.9505	LPath
**Y686D**	0.9895	LPath
**Y686E**	0.9973	LPath
**Y686F**	0.1202	LBen
**Y686G**	0.9758	LPath
**Y686H**	0.9342	LPath
**Y686I**	0.9903	LPath
**Y686K**	0.9976	LPath
**Y686L**	0.9776	LPath
**Y686M**	0.9834	LPath
**Y686N**	0.9771	LPath
**Y686P**	0.998	LPath
**Y686Q**	0.9955	LPath
**Y686R**	0.9888	LPath
**Y686S**	0.9697	LPath
**Y686T**	0.9911	LPath
**Y686V**	0.973	LPath
**Y686W**	0.7897	LPath
**E687A**	0.2019	LBen
**E687C**	0.9445	LPath
**E687D**	0.4811	Amb
**E687F**	0.8915	LPath
**E687G**	0.2505	LBen
**E687H**	0.5233	Amb
**E687I**	0.6326	LPath
**E687K**	0.1255	LBen
**E687L**	0.576	LPath
**E687M**	0.5705	LPath
**E687N**	0.6102	LPath
**E687P**	0.9601	LPath
**E687Q**	0.0738	LBen
**E687R**	0.175	LBen
**E687S**	0.2889	LBen
**E687T**	0.3074	LBen
**E687V**	0.3999	Amb

**E687W**	0.9426	LPath
**E687Y**	0.8098	LPath
**G688A**	0.9161	LPath
**G688C**	0.9899	LPath
**G688D**	0.9259	LPath
**G688E**	0.9714	LPath
**G688F**	0.9953	LPath
**G688H**	0.9959	LPath
**G688I**	0.9925	LPath
**G688K**	0.9926	LPath
**G688L**	0.9932	LPath
**G688M**	0.9947	LPath
**G688N**	0.975	LPath
**G688P**	0.9983	LPath
**G688Q**	0.9911	LPath
**G688R**	0.9883	LPath
**G688S**	0.902	LPath
**G688T**	0.9791	LPath
**G688V**	0.982	LPath
**G688W**	0.9927	LPath
**G688Y**	0.9907	LPath
**G689A**	0.9199	LPath
**G689C**	0.988	LPath
**G689D**	0.9929	LPath
**G689E**	0.9937	LPath
**G689F**	0.9966	LPath
**G689H**	0.9982	LPath
**G689I**	0.9949	LPath
**G689K**	0.9967	LPath
**G689L**	0.9959	LPath
**G689M**	0.9979	LPath
**G689N**	0.9963	LPath
**G689P**	0.9983	LPath
**G689Q**	0.9957	LPath
**G689R**	0.9888	LPath
**G689S**	0.9276	LPath
**G689T**	0.987	LPath
**G689V**	0.9914	LPath
**G689W**	0.9932	LPath
**G689Y**	0.9964	LPath

G690A	0.9206	LPath
G690C	0.9797	LPath
G690D	0.9901	LPath
G690E	0.9922	LPath
G690F	0.9953	LPath
G690H	0.9971	LPath
G690I	0.9907	LPath
G690K	0.9973	LPath
G690L	0.9941	LPath
G690M	0.9974	LPath
G690N	0.9933	LPath
G690P	0.9986	LPath
G690Q	0.9941	LPath
G690R	0.9884	LPath
G690S	0.8923	LPath
G690T	0.9779	LPath
G690V	0.9854	LPath
G690W	0.9934	LPath
G690Y	0.9946	LPath
C691A	0.8438	LPath
C691D	0.9972	LPath
C691E	0.9982	LPath
C691F	0.9858	LPath
C691G	0.8606	LPath
C691H	0.9969	LPath
C691I	0.942	LPath
C691K	0.9994	LPath
C691L	0.9484	LPath
C691M	0.9751	LPath
C691N	0.9826	LPath
C691P	0.9978	LPath
C691Q	0.9952	LPath
C691R	0.9946	LPath
C691S	0.7377	LPath
C691T	0.8809	LPath
C691V	0.7867	LPath
C691W	0.9962	LPath
C691Y	0.9954	LPath
R692A	0.7855	LPath
R692C	0.376	Amb
R692D	0.9325	LPath
R692E	0.7382	LPath

**R692F**	0.7652	LPath
**R692G**	0.6943	LPath
**R692H**	0.0927	LBen
**R692I**	0.7186	LPath
**R692K**	0.2578	LBen
**R692L**	0.4847	Amb
**R692M**	0.7854	LPath
**R692N**	0.7797	LPath
**R692P**	0.9881	LPath
**R692Q**	0.1726	LBen
**R692S**	0.7174	LPath
**R692T**	0.7208	LPath
**R692V**	0.7386	LPath
**R692W**	0.3548	Amb
**R692Y**	0.5062	Amb
**F693A**	0.9993	LPath
**F693C**	0.9984	LPath
**F693D**	0.9996	LPath
**F693E**	0.9996	LPath
**F693G**	0.9992	LPath
**F693H**	0.9959	LPath
**F693I**	0.9945	LPath
**F693K**	0.9996	LPath
**F693L**	0.9982	LPath
**F693M**	0.9914	LPath
**F693N**	0.9988	LPath
**F693P**	0.9999	LPath
**F693Q**	0.9989	LPath
**F693R**	0.9986	LPath
**F693S**	0.9988	LPath
**F693T**	0.9995	LPath
**F693V**	0.9953	LPath
**F693W**	0.9737	LPath
**F693Y**	0.8499	LPath
**L694A**	0.7833	LPath
**L694C**	0.9116	LPath
**L694D**	0.9813	LPath
**L694E**	0.8522	LPath

**L694F**	0.6241	LPath
**L694G**	0.9313	LPath
**L694H**	0.6351	LPath
**L694I**	0.1139	LBen
**L694K**	0.7327	LPath
**L694M**	0.3767	Amb
**L694N**	0.8694	LPath
**L694P**	0.7008	LPath
**L694Q**	0.5383	Amb
**L694R**	0.5251	Amb
**L694S**	0.8206	LPath
**L694T**	0.6328	LPath
**L694V**	0.1632	LBen
**L694W**	0.7565	LPath
**L694Y**	0.8796	LPath
**R695A**	0.9936	LPath
**R695C**	0.9625	LPath
**R695D**	0.996	LPath
**R695E**	0.9861	LPath
**R695F**	0.9974	LPath
**R695G**	0.9849	LPath
**R695H**	0.9144	LPath
**R695I**	0.9939	LPath
**R695K**	0.9679	LPath
**R695L**	0.9747	LPath
**R695M**	0.9985	LPath
**R695N**	0.9967	LPath
**R695P**	0.9864	LPath
**R695Q**	0.9277	LPath
**R695S**	0.9943	LPath
**R695T**	0.9965	LPath
**R695V**	0.9939	LPath
**R695W**	0.9443	LPath
**R695Y**	0.9855	LPath
**Y696A**	0.9178	LPath
**Y696C**	0.9264	LPath
**Y696D**	0.8822	LPath
**Y696E**	0.9433	LPath
**Y696F**	0.601	LPath
**Y696G**	0.8073	LPath

**Y696H**	0.9388	LPath
**Y696I**	0.975	LPath
**Y696K**	0.9744	LPath
**Y696L**	0.8242	LPath
**Y696M**	0.9056	LPath
**Y696N**	0.6584	LPath
**Y696P**	0.9913	LPath
**Y696Q**	0.7745	LPath
**Y696R**	0.9408	LPath
**Y696S**	0.6671	LPath
**Y696T**	0.9054	LPath
**Y696V**	0.915	LPath
**Y696W**	0.921	LPath
**D697A**	0.3115	LBen
**D697C**	0.8815	LPath
**D697E**	0.2491	LBen
**D697F**	0.9183	LPath
**D697G**	0.1612	LBen
**D697H**	0.2912	LBen
**D697I**	0.8583	LPath
**D697K**	0.4222	Amb
**D697L**	0.7975	LPath
**D697M**	0.9152	LPath
**D697N**	0.0783	LBen
**D697P**	0.7903	LPath
**D697Q**	0.3225	LBen
**D697R**	0.3508	Amb
**D697S**	0.1387	LBen
**D697T**	0.4915	Amb
**D697V**	0.73	LPath
**D697W**	0.9658	LPath
**D697Y**	0.5673	LPath
**C698A**	0.9938	LPath
**C698D**	0.9991	LPath
**C698E**	0.9994	LPath
**C698F**	0.9885	LPath
**C698G**	0.9674	LPath
**C698H**	0.9974	LPath
**C698I**	0.9974	LPath
**C698K**	0.9995	LPath

**C698L**	0.9929	LPath
**C698M**	0.9968	LPath
**C698N**	0.9985	LPath
**C698P**	0.9995	LPath
**C698Q**	0.9989	LPath
**C698R**	0.9942	LPath
**C698S**	0.9949	LPath
**C698T**	0.9954	LPath
**C698V**	0.9946	LPath
**C698W**	0.9958	LPath
**C698Y**	0.994	LPath
**V699A**	0.1091	LBen
**V699C**	0.6364	LPath
**V699D**	0.1952	LBen
**V699E**	0.1135	LBen
**V699F**	0.1022	LBen
**V699G**	0.1637	LBen
**V699H**	0.224	LBen
**V699I**	0.0648	LBen
**V699K**	0.1321	LBen
**V699L**	0.0942	LBen
**V699M**	0.0956	LBen
**V699N**	0.1225	LBen
**V699P**	0.5034	Amb
**V699Q**	0.1086	LBen
**V699R**	0.0936	LBen
**V699S**	0.0973	LBen
**V699T**	0.0966	LBen
**V699W**	0.5805	LPath
**V699Y**	0.3424	Amb
**I700A**	0.8769	LPath
**I700C**	0.9428	LPath
**I700D**	0.9916	LPath
**I700E**	0.9821	LPath
**I700F**	0.623	LPath
**I700G**	0.9789	LPath
**I700H**	0.9758	LPath
**I700K**	0.9816	LPath
**I700L**	0.2169	LBen
**I700M**	0.444	Amb

**I700N**	0.9323	LPath
**I700P**	0.9617	LPath
**I700Q**	0.9712	LPath
**I700R**	0.9629	LPath
**I700S**	0.9048	LPath
**I700T**	0.8701	LPath
**I700V**	0.0858	LBen
**I700W**	0.9834	LPath
**I700Y**	0.9436	LPath
**S701A**	0.0718	LBen
**S701C**	0.204	LBen
**S701D**	0.1913	LBen
**S701E**	0.2199	LBen
**S701F**	0.213	LBen
**S701G**	0.0894	LBen
**S701H**	0.1902	LBen
**S701I**	0.2088	LBen
**S701K**	0.3634	Amb
**S701L**	0.1315	LBen
**S701M**	0.2775	LBen
**S701N**	0.1227	LBen
**S701P**	0.0844	LBen
**S701Q**	0.2291	LBen
**S701R**	0.2323	LBen
**S701T**	0.088	LBen
**S701V**	0.182	LBen
**S701W**	0.2664	LBen
**S701Y**	0.1777	LBen
**S702A**	0.0797	LBen
**S702C**	0.3346	LBen
**S702D**	0.6456	LPath
**S702E**	0.6423	LPath
**S702F**	0.423	Amb
**S702G**	0.2144	LBen
**S702H**	0.5647	LPath
**S702I**	0.5524	Amb
**S702K**	0.8837	LPath
**S702L**	0.2251	LBen
**S702M**	0.4519	Amb
**S702N**	0.4313	Amb
**S702P**	0.6494	LPath
**S702Q**	0.6213	LPath

**S702R**	0.7767	LPath
**S702T**	0.1667	LBen
**S702V**	0.3774	Amb
**S702W**	0.6262	LPath
**S702Y**	0.4118	Amb
**P703A**	0.3926	Amb
**P703C**	0.9457	LPath
**P703D**	0.9706	LPath
**P703E**	0.9191	LPath
**P703F**	0.9689	LPath
**P703G**	0.8379	LPath
**P703H**	0.8873	LPath
**P703I**	0.8574	LPath
**P703K**	0.9701	LPath
**P703L**	0.4806	Amb
**P703M**	0.8523	LPath
**P703N**	0.9393	LPath
**P703Q**	0.7848	LPath
**P703R**	0.9046	LPath
**P703S**	0.5381	Amb
**P703T**	0.3543	Amb
**P703V**	0.679	LPath
**P703W**	0.9854	LPath
**P703Y**	0.9786	LPath
**R704A**	0.8665	LPath
**R704C**	0.6799	LPath
**R704D**	0.9736	LPath
**R704E**	0.743	LPath
**R704F**	0.9557	LPath
**R704G**	0.8299	LPath
**R704H**	0.4057	Amb
**R704I**	0.8505	LPath
**R704K**	0.2947	LBen
**R704L**	0.6812	LPath
**R704M**	0.8762	LPath
**R704N**	0.9681	LPath
**R704P**	0.7252	LPath
**R704Q**	0.2083	LBen

**R704S**	0.9346	LPath
**R704T**	0.8886	LPath
**R704V**	0.8283	LPath
**R704W**	0.6191	LPath
**R704Y**	0.8844	LPath
**K705A**	0.9385	LPath
**K705C**	0.9837	LPath
**K705D**	0.9949	LPath
**K705E**	0.9161	LPath
**K705F**	0.9984	LPath
**K705G**	0.9813	LPath
**K705H**	0.9578	LPath
**K705I**	0.932	LPath
**K705L**	0.9114	LPath
**K705M**	0.8623	LPath
**K705N**	0.9891	LPath
**K705P**	0.8759	LPath
**K705Q**	0.7404	LPath
**K705R**	0.2147	LBen
**K705S**	0.979	LPath
**K705T**	0.9025	LPath
**K705V**	0.8676	LPath
**K705W**	0.9964	LPath
**K705Y**	0.9918	LPath
**G706A**	0.9559	LPath
**G706C**	0.9881	LPath
**G706D**	0.9949	LPath
**G706E**	0.9965	LPath
**G706F**	0.9987	LPath
**G706H**	0.9989	LPath
**G706I**	0.9979	LPath
**G706K**	0.9987	LPath
**G706L**	0.9976	LPath
**G706M**	0.9989	LPath
**G706N**	0.9962	LPath
**G706P**	0.9991	LPath
**G706Q**	0.9979	LPath
**G706R**	0.9961	LPath
**G706S**	0.9615	LPath
**G706T**	0.9926	LPath

**G706V**	0.9947	LPath
**G706W**	0.9975	LPath
**G706Y**	0.9976	LPath
**W707A**	0.979	LPath
**W707C**	0.9909	LPath
**W707D**	0.9973	LPath
**W707E**	0.9959	LPath
**W707F**	0.6911	LPath
**W707G**	0.9509	LPath
**W707H**	0.9595	LPath
**W707I**	0.994	LPath
**W707K**	0.9982	LPath
**W707L**	0.9712	LPath
**W707M**	0.9859	LPath
**W707N**	0.9938	LPath
**W707P**	0.9995	LPath
**W707Q**	0.9949	LPath
**W707R**	0.9941	LPath
**W707S**	0.9414	LPath
**W707T**	0.9842	LPath
**W707V**	0.9853	LPath
**W707Y**	0.839	LPath
**A708C**	0.6275	LPath
**A708D**	0.9798	LPath
**A708E**	0.9768	LPath
**A708F**	0.9293	LPath
**A708G**	0.0753	LBen
**A708H**	0.9868	LPath
**A708I**	0.7596	LPath
**A708K**	0.9956	LPath
**A708L**	0.5391	Amb
**A708M**	0.6688	LPath
**A708N**	0.9165	LPath
**A708P**	0.9691	LPath
**A708Q**	0.9609	LPath
**A708R**	0.9882	LPath
**A708S**	0.1347	LBen
**A708T**	0.2012	LBen
**A708V**	0.4169	Amb
**A708W**	0.992	LPath

**A708Y**	0.9839	LPath
**L709A**	0.8769	LPath
**L709C**	0.9338	LPath
**L709D**	0.9984	LPath
**L709E**	0.9902	LPath
**L709F**	0.2607	LBen
**L709G**	0.9777	LPath
**L709H**	0.9605	LPath
**L709I**	0.2014	LBen
**L709K**	0.987	LPath
**L709M**	0.2829	LBen
**L709N**	0.9833	LPath
**L709P**	0.9917	LPath
**L709Q**	0.941	LPath
**L709R**	0.9637	LPath
**L709S**	0.9584	LPath
**L709T**	0.8999	LPath
**L709V**	0.2612	LBen
**L709W**	0.8508	LPath
**L709Y**	0.8833	LPath
**L710A**	0.8784	LPath
**L710C**	0.9404	LPath
**L710D**	0.9994	LPath
**L710E**	0.9938	LPath
**L710F**	0.6538	LPath
**L710G**	0.9853	LPath
**L710H**	0.9851	LPath
**L710I**	0.209	LBen
**L710K**	0.9933	LPath
**L710M**	0.1711	LBen
**L710N**	0.9948	LPath
**L710P**	0.9951	LPath
**L710Q**	0.9413	LPath
**L710R**	0.9822	LPath
**L710S**	0.9718	LPath
**L710T**	0.9357	LPath
**L710V**	0.2256	LBen
**L710W**	0.9581	LPath
**L710Y**	0.9735	LPath
**H711A**	0.9968	LPath

**H711C**	0.9539	LPath
**H711D**	0.9991	LPath
**H711E**	0.9984	LPath
**H711F**	0.9038	LPath
**H711G**	0.9975	LPath
**H711I**	0.9971	LPath
**H711K**	0.9984	LPath
**H711L**	0.9735	LPath
**H711M**	0.9921	LPath
**H711N**	0.9941	LPath
**H711P**	0.9904	LPath
**H711Q**	0.9932	LPath
**H711R**	0.9968	LPath
**H711S**	0.9884	LPath
**H711T**	0.9981	LPath
**H711V**	0.9976	LPath
**H711W**	0.9669	LPath
**H711Y**	0.9639	LPath
**P712A**	0.9215	LPath
**P712C**	0.9966	LPath
**P712D**	0.9991	LPath
**P712E**	0.9977	LPath
**P712F**	0.9994	LPath
**P712G**	0.9894	LPath
**P712H**	0.9961	LPath
**P712I**	0.9979	LPath
**P712K**	0.9985	LPath
**P712L**	0.983	LPath
**P712M**	0.998	LPath
**P712N**	0.9989	LPath
**P712Q**	0.9955	LPath
**P712R**	0.9893	LPath
**P712S**	0.9838	LPath
**P712T**	0.9852	LPath
**P712V**	0.9915	LPath
**P712W**	0.9995	LPath
**P712Y**	0.9992	LPath
**G713A**	0.9763	LPath
**G713C**	0.9964	LPath
**G713D**	0.9988	LPath
**G713E**	0.9986	LPath
**G713F**	0.9996	LPath

**G713H**	0.9998	LPath
**G713I**	0.9994	LPath
**G713K**	0.9996	LPath
**G713L**	0.9987	LPath
**G713M**	0.9997	LPath
**G713N**	0.9995	LPath
**G713P**	0.9994	LPath
**G713Q**	0.9994	LPath
**G713R**	0.9982	LPath
**G713S**	0.9791	LPath
**G713T**	0.9979	LPath
**G713V**	0.9978	LPath
**G713W**	0.9992	LPath
**G713Y**	0.9996	LPath
**R714A**	0.993	LPath
**R714C**	0.9647	LPath
**R714D**	0.9981	LPath
**R714E**	0.9895	LPath
**R714F**	0.9942	LPath
**R714G**	0.9869	LPath
**R714H**	0.8952	LPath
**R714I**	0.9874	LPath
**R714K**	0.8951	LPath
**R714L**	0.97	LPath
**R714M**	0.9942	LPath
**R714N**	0.9962	LPath
**R714P**	0.9971	LPath
**R714Q**	0.898	LPath
**R714S**	0.994	LPath
**R714T**	0.9933	LPath
**R714V**	0.9889	LPath
**R714W**	0.9531	LPath
**R714Y**	0.9839	LPath
**L715A**	0.9872	LPath
**L715C**	0.994	LPath
**L715D**	0.9997	LPath
**L715E**	0.998	LPath
**L715F**	0.9902	LPath

**L715G**	0.9958	LPath
**L715H**	0.9978	LPath
**L715I**	0.8041	LPath
**L715K**	0.9955	LPath
**L715M**	0.9051	LPath
**L715N**	0.9973	LPath
**L715P**	0.9981	LPath
**L715Q**	0.9947	LPath
**L715R**	0.9879	LPath
**L715S**	0.9981	LPath
**L715T**	0.9908	LPath
**L715V**	0.7951	LPath
**L715W**	0.9971	LPath
**L715Y**	0.9973	LPath
**T716A**	0.9798	LPath
**T716C**	0.9972	LPath
**T716D**	0.9977	LPath
**T716E**	0.9969	LPath
**T716F**	0.9955	LPath
**T716G**	0.981	LPath
**T716H**	0.9906	LPath
**T716I**	0.9955	LPath
**T716K**	0.9961	LPath
**T716L**	0.9635	LPath
**T716M**	0.9762	LPath
**T716N**	0.9865	LPath
**T716P**	0.9721	LPath
**T716Q**	0.9912	LPath
**T716R**	0.9925	LPath
**T716S**	0.8862	LPath
**T716V**	0.9846	LPath
**T716W**	0.9977	LPath
**T716Y**	0.9962	LPath
**H717A**	0.9969	LPath
**H717C**	0.9693	LPath
**H717D**	0.9974	LPath
**H717E**	0.9988	LPath
**H717F**	0.9943	LPath
**H717G**	0.9981	LPath
**H717I**	0.9995	LPath

**H717K**	0.999	LPath
**H717L**	0.9878	LPath
**H717M**	0.999	LPath
**H717N**	0.9929	LPath
**H717P**	0.9871	LPath
**H717Q**	0.9975	LPath
**H717R**	0.9951	LPath
**H717S**	0.9927	LPath
**H717T**	0.999	LPath
**H717V**	0.999	LPath
**H717W**	0.991	LPath
**H717Y**	0.9905	LPath
**Y718A**	0.9447	LPath
**Y718C**	0.8488	LPath
**Y718D**	0.9722	LPath
**Y718E**	0.9867	LPath
**Y718F**	0.1312	LBen
**Y718G**	0.9465	LPath
**Y718H**	0.8693	LPath
**Y718I**	0.8974	LPath
**Y718K**	0.9842	LPath
**Y718L**	0.4996	Amb
**Y718M**	0.851	LPath
**Y718N**	0.8997	LPath
**Y718P**	0.9927	LPath
**Y718Q**	0.9507	LPath
**Y718R**	0.9383	LPath
**Y718S**	0.8626	LPath
**Y718T**	0.9544	LPath
**Y718V**	0.8324	LPath
**Y718W**	0.7992	LPath
**H719A**	0.9995	LPath
**H719C**	0.9883	LPath
**H719D**	0.9995	LPath
**H719E**	0.9989	LPath
**H719F**	0.9986	LPath
**H719G**	0.9993	LPath
**H719I**	0.9997	LPath
**H719K**	0.9981	LPath
**H719L**	0.9948	LPath

**H719M**	0.9994	LPath
**H719N**	0.9988	LPath
**H719P**	0.9977	LPath
**H719Q**	0.9976	LPath
**H719R**	0.9944	LPath
**H719S**	0.9986	LPath
**H719T**	0.9995	LPath
**H719V**	0.9996	LPath
**H719W**	0.9928	LPath
**H719Y**	0.9967	LPath
**E720A**	0.7782	LPath
**E720C**	0.9934	LPath
**E720D**	0.9786	LPath
**E720F**	0.9962	LPath
**E720G**	0.9268	LPath
**E720H**	0.9822	LPath
**E720I**	0.971	LPath
**E720K**	0.8557	LPath
**E720L**	0.978	LPath
**E720M**	0.9611	LPath
**E720N**	0.9896	LPath
**E720P**	0.9998	LPath
**E720Q**	0.5331	Amb
**E720R**	0.8752	LPath
**E720S**	0.8856	LPath
**E720T**	0.9377	LPath
**E720V**	0.9044	LPath
**E720W**	0.9989	LPath
**E720Y**	0.9948	LPath
**G721A**	0.9326	LPath
**G721C**	0.9922	LPath
**G721D**	0.9939	LPath
**G721E**	0.9946	LPath
**G721F**	0.9986	LPath
**G721H**	0.9996	LPath
**G721I**	0.9946	LPath
**G721K**	0.9993	LPath
**G721L**	0.9971	LPath
**G721M**	0.9987	LPath
**G721N**	0.9978	LPath

**G721P**	0.9994	LPath
**G721Q**	0.9989	LPath
**G721R**	0.9981	LPath
**G721S**	0.9709	LPath
**G721T**	0.9934	LPath
**G721V**	0.9887	LPath
**G721W**	0.9984	LPath
**G721Y**	0.998	LPath
**L722A**	0.9151	LPath
**L722C**	0.9728	LPath
**L722D**	0.9946	LPath
**L722E**	0.923	LPath
**L722F**	0.7358	LPath
**L722G**	0.9699	LPath
**L722H**	0.8887	LPath
**L722I**	0.5532	Amb
**L722K**	0.7325	LPath
**L722M**	0.4526	Amb
**L722N**	0.9655	LPath
**L722P**	0.9939	LPath
**L722Q**	0.6475	LPath
**L722R**	0.5863	LPath
**L722S**	0.9615	LPath
**L722T**	0.934	LPath
**L722V**	0.6013	LPath
**L722W**	0.8633	LPath
**L722Y**	0.8848	LPath
**P723A**	0.1393	LBen
**P723C**	0.8438	LPath
**P723D**	0.7644	LPath
**P723E**	0.3136	LBen
**P723F**	0.8053	LPath
**P723G**	0.6523	LPath
**P723H**	0.3737	Amb
**P723I**	0.5764	LPath
**P723K**	0.3776	Amb
**P723L**	0.1966	LBen
**P723M**	0.606	LPath
**P723N**	0.7271	LPath
**P723Q**	0.2211	LBen
**P723R**	0.1476	LBen
**P723S**	0.299	LBen

**P723T**	0.2528	LBen
**P723V**	0.3751	Amb
**P723W**	0.8187	LPath
**P723Y**	0.7326	LPath
**T724A**	0.4828	Amb
**T724C**	0.8819	LPath
**T724D**	0.9952	LPath
**T724E**	0.984	LPath
**T724F**	0.9887	LPath
**T724G**	0.9396	LPath
**T724H**	0.9881	LPath
**T724I**	0.3819	Amb
**T724K**	0.9851	LPath
**T724L**	0.5665	LPath
**T724M**	0.6594	LPath
**T724N**	0.9337	LPath
**T724P**	0.7609	LPath
**T724Q**	0.9733	LPath
**T724R**	0.9702	LPath
**T724S**	0.7178	LPath
**T724V**	0.1238	LBen
**T724W**	0.9985	LPath
**T724Y**	0.9938	LPath
**T725A**	0.1554	LBen
**T725C**	0.7902	LPath
**T725D**	0.565	LPath
**T725E**	0.3754	Amb
**T725F**	0.4616	Amb
**T725G**	0.4389	Amb
**T725H**	0.3752	Amb
**T725I**	0.2035	LBen
**T725K**	0.2294	LBen
**T725L**	0.1188	LBen
**T725M**	0.1708	LBen
**T725N**	0.2582	LBen
**T725P**	0.4847	Amb
**T725Q**	0.2802	LBen
**T725R**	0.1751	LBen

**T725S**	0.1546	LBen
**T725V**	0.1585	LBen
**T725W**	0.7405	LPath
**T725Y**	0.5534	Amb
**W726A**	0.071	LBen
**W726C**	0.3189	LBen
**W726D**	0.2162	LBen
**W726E**	0.0963	LBen
**W726F**	0.1457	LBen
**W726G**	0.0593	LBen
**W726H**	0.0767	LBen
**W726I**	0.3042	LBen
**W726K**	0.072	LBen
**W726L**	0.1228	LBen
**W726M**	0.1934	LBen
**W726N**	0.1729	LBen
**W726P**	0.9091	LPath
**W726Q**	0.0571	LBen
**W726R**	0.0526	LBen
**W726S**	0.0597	LBen
**W726T**	0.0963	LBen
**W726V**	0.2079	LBen
**W726Y**	0.1892	LBen
**G727A**	0.9686	LPath
**G727C**	0.9951	LPath
**G727D**	0.9895	LPath
**G727E**	0.9931	LPath
**G727F**	0.9986	LPath
**G727H**	0.998	LPath
**G727I**	0.9982	LPath
**G727K**	0.9958	LPath
**G727L**	0.9971	LPath
**G727M**	0.9988	LPath
**G727N**	0.9933	LPath
**G727P**	0.9987	LPath
**G727Q**	0.996	LPath
**G727R**	0.9921	LPath
**G727S**	0.9665	LPath
**G727T**	0.9944	LPath
**G727V**	0.996	LPath

**G727W**	0.9974	LPath
**G727Y**	0.9971	LPath
**T728A**	0.5294	Amb
**T728C**	0.9397	LPath
**T728D**	0.9463	LPath
**T728E**	0.8933	LPath
**T728F**	0.9213	LPath
**T728G**	0.9354	LPath
**T728H**	0.8991	LPath
**T728I**	0.37	Amb
**T728K**	0.852	LPath
**T728L**	0.4868	Amb
**T728M**	0.3994	Amb
**T728N**	0.7716	LPath
**T728P**	0.7503	LPath
**T728Q**	0.8475	LPath
**T728R**	0.8126	LPath
**T728S**	0.5783	LPath
**T728V**	0.2589	LBen
**T728W**	0.9584	LPath
**T728Y**	0.9368	LPath
**R729A**	0.9974	LPath
**R729C**	0.9717	LPath
**R729D**	0.9991	LPath
**R729E**	0.9925	LPath
**R729F**	0.9994	LPath
**R729G**	0.9945	LPath
**R729H**	0.9701	LPath
**R729I**	0.9978	LPath
**R729K**	0.9872	LPath
**R729L**	0.9914	LPath
**R729M**	0.9995	LPath
**R729N**	0.9988	LPath
**R729P**	0.9981	LPath
**R729Q**	0.9734	LPath
**R729S**	0.9974	LPath
**R729T**	0.9985	LPath
**R729V**	0.9971	LPath
**R729W**	0.9813	LPath
**R729Y**	0.9951	LPath

**Y730A**	0.9987	LPath
**Y730C**	0.991	LPath
**Y730D**	0.9979	LPath
**Y730E**	0.9995	LPath
**Y730F**	0.6655	LPath
**Y730G**	0.9957	LPath
**Y730H**	0.9903	LPath
**Y730I**	0.9963	LPath
**Y730K**	0.9994	LPath
**Y730L**	0.9794	LPath
**Y730M**	0.9972	LPath
**Y730N**	0.9898	LPath
**Y730P**	0.9991	LPath
**Y730Q**	0.9994	LPath
**Y730R**	0.9973	LPath
**Y730S**	0.9926	LPath
**Y730T**	0.9984	LPath
**Y730V**	0.9904	LPath
**Y730W**	0.9382	LPath
**I731A**	0.9952	LPath
**I731C**	0.999	LPath
**I731D**	0.9997	LPath
**I731E**	0.9989	LPath
**I731F**	0.9804	LPath
**I731G**	0.9991	LPath
**I731H**	0.9992	LPath
**I731K**	0.9989	LPath
**I731L**	0.8301	LPath
**I731M**	0.8892	LPath
**I731N**	0.9964	LPath
**I731P**	0.9993	LPath
**I731Q**	0.9973	LPath
**I731R**	0.9966	LPath
**I731S**	0.994	LPath
**I731T**	0.9938	LPath
**I731V**	0.7341	LPath
**I731W**	0.9995	LPath
**I731Y**	0.9979	LPath
**M732A**	0.5709	LPath
**M732C**	0.9287	LPath

**M732D**	0.9987	LPath
**M732E**	0.9909	LPath
**M732F**	0.777	LPath
**M732G**	0.9567	LPath
**M732H**	0.9921	LPath
**M732I**	0.8664	LPath
**M732K**	0.9879	LPath
**M732L**	0.2957	LBen
**M732N**	0.9839	LPath
**M732P**	0.9986	LPath
**M732Q**	0.9357	LPath
**M732R**	0.9845	LPath
**M732S**	0.8237	LPath
**M732T**	0.8042	LPath
**M732V**	0.2961	LBen
**M732W**	0.9926	LPath
**M732Y**	0.99	LPath
**V733A**	0.9768	LPath
**V733C**	0.9972	LPath
**V733D**	0.9942	LPath
**V733E**	0.9907	LPath
**V733F**	0.9792	LPath
**V733G**	0.9629	LPath
**V733H**	0.9989	LPath
**V733I**	0.1682	LBen
**V733K**	0.996	LPath
**V733L**	0.9398	LPath
**V733M**	0.9726	LPath
**V733N**	0.9838	LPath
**V733P**	0.9928	LPath
**V733Q**	0.9942	LPath
**V733R**	0.9906	LPath
**V733S**	0.9789	LPath
**V733T**	0.9615	LPath
**V733W**	0.9997	LPath
**V733Y**	0.9969	LPath
**S734A**	0.4587	Amb
**S734C**	0.6728	LPath
**S734D**	0.9967	LPath
**S734E**	0.9988	LPath
**S734F**	0.9972	LPath
**S734G**	0.7312	LPath

**S734H**	0.9961	LPath
**S734I**	0.9924	LPath
**S734K**	0.9999	LPath
**S734L**	0.9658	LPath
**S734M**	0.98	LPath
**S734N**	0.9759	LPath
**S734P**	0.9982	LPath
**S734Q**	0.9967	LPath
**S734R**	0.9997	LPath
**S734T**	0.4127	Amb
**S734V**	0.9713	LPath
**S734W**	0.998	LPath
**S734Y**	0.9975	LPath
**F735A**	0.9978	LPath
**F735C**	0.999	LPath
**F735D**	0.9994	LPath
**F735E**	0.9993	LPath
**F735G**	0.9992	LPath
**F735H**	0.9972	LPath
**F735I**	0.9973	LPath
**F735K**	0.9996	LPath
**F735L**	0.9997	LPath
**F735M**	0.9973	LPath
**F735N**	0.9992	LPath
**F735P**	0.9999	LPath
**F735Q**	0.9989	LPath
**F735R**	0.9976	LPath
**F735S**	0.998	LPath
**F735T**	0.9988	LPath
**F735V**	0.9967	LPath
**F735W**	0.9863	LPath
**F735Y**	0.9818	LPath
**V736A**	0.8449	LPath
**V736C**	0.9697	LPath
**V736D**	0.9941	LPath
**V736E**	0.9817	LPath
**V736F**	0.9123	LPath
**V736G**	0.9322	LPath

**V736H**	0.9949	LPath
**V736I**	0.0948	LBen
**V736K**	0.993	LPath
**V736L**	0.4774	Amb
**V736M**	0.7445	LPath
**V736N**	0.9603	LPath
**V736P**	0.9804	LPath
**V736Q**	0.9775	LPath
**V736R**	0.9805	LPath
**V736S**	0.9208	LPath
**V736T**	0.8137	LPath
**V736W**	0.9971	LPath
**V736Y**	0.9929	LPath
**D737A**	0.9839	LPath
**D737C**	0.9982	LPath
**D737E**	0.9821	LPath
**D737F**	0.9979	LPath
**D737G**	0.9912	LPath
**D737H**	0.9926	LPath
**D737I**	0.9978	LPath
**D737K**	0.9985	LPath
**D737L**	0.9944	LPath
**D737M**	0.9995	LPath
**D737N**	0.9556	LPath
**D737P**	0.9978	LPath
**D737Q**	0.9963	LPath
**D737R**	0.9963	LPath
**D737S**	0.9545	LPath
**D737T**	0.9944	LPath
**D737V**	0.9932	LPath
**D737W**	0.9994	LPath
**D737Y**	0.9831	LPath
**P738A**	0.8949	LPath
**P738C**	0.9929	LPath
**P738D**	0.9889	LPath
**P738E**	0.983	LPath
**P738F**	0.9967	LPath
**P738G**	0.9643	LPath
**P738H**	0.9837	LPath
**P738I**	0.9887	LPath

**P738K**	0.9918	LPath
**P738L**	0.9675	LPath
**P738M**	0.9923	LPath
**P738N**	0.9909	LPath
**P738Q**	0.9781	LPath
**P738R**	0.9627	LPath
**P738S**	0.9523	LPath
**P738T**	0.9299	LPath
**P738V**	0.9652	LPath
**P738W**	0.9975	LPath
**P738Y**	0.9944	LPath
