# Supplementary material for: Succinate supplementation ameliorates musculoskeletal defects caused by PLOD3 mutations in a BCARD syndrome model
Source: Genome Med. 2026 Mar 13;18:29. doi: 10.1186/s13073-026-01608-y (PMC12994257; doi:10.1186/s13073-026-01608-y)
Supplement: Supplementary file 4 — Additional file 4: Table S9. DE_results WT vs mgt. [file 13073_2026_1608_MOESM4_ESM.pdf]

Table S9: DE_results WT vs mgt

ensembl	baseMean	log2FoldChange	lfcSE	stat	pvalue	padj	external_gene_name	description	chromosome_name	start_position	end_position	hgnc_symbol
ENSDARG00000000069	2852.166733	-1.266754415	0.089274517	-2.988024163	0.002807874	0.024566202	dap	death-associated protein [Source:ZFIN;Acc:ZDB-GENE-000511-3]	24	22070290	22103585	
ENSDARG00000000086	3050.476389	1.2769784	0.094586627	2.928304036	0.003408166	0.029238594	itsn1	intersectin 1 (SH3 domain protein) [Source:ZFIN;Acc:ZDB-GENE-030616-226]	1	46999441	47089818	
ENSDARG00000000183	1195.163633	1.33827991	0.108828634	3.108372277	0.001881209	0.016991809	ptpn4b	protein tyrosine phosphatase non-receptor type 4b [Source:ZFIN;Acc:ZDB-GENE-030131-6259]	22	11778053	11833334	
ENSDARG00000000212	22206.90989	-2.139982958	0.098106415	-11.61986149	3.27E-31	3.37E-29	krt97	keratin 97 [Source:ZFIN;Acc:ZDB-GENE-040718-78]	22	11756040	11759106	
ENSDARG00000000369	287.8187692	-1.652250246	0.160543719	-4.062757793	4.85E-05	0.000594388	prkag3b	protein kinase, AMP-activated, gamma 3b non-catalytic subunit [Source:ZFIN;Acc:ZDB-GENE-060421-6938]	9	14090329	14108896	
ENSDARG00000000472	1156.549911	2.172429503	0.173749791	6.747803833	1.5E-11	4.2E-10	cntn2	contactin 2 [Source:ZFIN;Acc:ZDB-GENE-990630-12]	11	23933016	23987682	
ENSDARG00000000503	15129.65139	2.273535809	0.088574153	14.37818779	7.09E-47	1.42E-44	stx1b	syntaxin 1B [Source:ZFIN;Acc:ZDB-GENE-000330-4]	19	5158306	5254699	
ENSDARG00000001057	3250.499576	-1.328489373	0.093660091	-3.507250204	0.000452763	0.00466153	bysl	bystin-like [Source:ZFIN;Acc:ZDB-GENE-040426-1287]	22	550828	557965	
ENSDARG00000001127	1493.681912	1.856687413	0.146755384	5.837519486	5.3E-09	1.13E-07	slc17a6a	solute carrier family 17 member 6a [Source:NCBI gene;Acc:494492]	25	35250976	35271284	
ENSDARG00000001154	716.2404743	1.769557159	0.187441457	4.10558673	4.03E-05	0.00050089	rimbp2	RIMS binding protein 2 [Source:ZFIN;Acc:ZDB-GENE-040724-96]	8	43941313	44015210	
ENSDARG00000001354	430.8924442	-1.557287058	0.139958299	-3.981807886	6.84E-05	0.000818272	slc6a22.2	solute carrier family 6 member 22, tandem duplicate 2 [Source:ZFIN;Acc:ZDB-GENE-030616-628]	6	41144155	41161017	
ENSDARG00000001463	818.7446146	2.402637487	0.15547225	9.021786763	1.85E-19	9.57E-18	tdh2	L-threonine dehydrogenase 2 [Source:ZFIN;Acc:ZDB-GENE-070620-14]	17	25414033	25425321	
ENSDARG00000001559	1035.550048	2.298428039	0.198466518	6.542302703	6.06E-11	1.59E-09	csmd2	CUB and Sushi multiple domains 2 [Source:ZFIN;Acc:ZDB-GENE-030616-39]	19	36114846	36675023	
ENSDARG00000001686	96.1244352	1.978479499	0.323967056	3.02030556	0.002525198	0.022302953	uckl1a	uridine-cytidine kinase 1-like 1a [Source:ZFIN;Acc:ZDB-GENE-040724-238]	8	23009662	23028536	
ENSDARG00000001754	1861.140289	-1.528852642	0.100535048	-5.260380836	1.44E-07	2.55E-06	noc2l	NOC2-like nucleolar associated transcriptional repressor [Source:ZFIN;Acc:ZDB-GENE-000607-75]	23	23126130	23179417	
ENSDARG00000001788	15128.25204	-1.302557682	0.089583037	-3.377399249	0.000731748	0.007205804	atp5po	ATP synthase peripheral stalk subunit OSCP [Source:ZFIN;Acc:ZDB-GENE-050522-147]	9	33145522	33148873	
ENSDARG00000001803	274.9513878	2.152615588	0.187941953	6.132827548	8.63E-10	2E-08	kcnh6a	potassium voltage-gated channel, subfamily H (eag-related), member 6a [Source:ZFIN;Acc:ZDB-GENE-040702-5]	3	31728871	31784082	
ENSDARG00000001804	249.7208732	-1.556731631	0.168550561	-3.303054151	0.000956379	0.009183655	zgc:112148	zgc:112148 [Source:ZFIN;Acc:ZDB-GENE-050417-235]	3	56861059	56871984	
ENSDARG00000001835	1141.750236	1.842397526	0.141330067	5.960497614	2.51E-09	5.57E-08	nalcn	sodium leak channel, non-selective [Source:ZFIN;Acc:ZDB-GENE-050410-12]	9	31589425	31747106	
ENSDARG00000001870	11933.41408	-1.445389931	0.116107087	-3.83602709	0.000125041	0.001427955	atp1a1a.4	ATPase Na+/K+ transporting subunit alpha 1a, tandem duplicate 4 [Source:ZFIN;Acc:ZDB-GENE-001212-4]	1	1689775	1706159	
ENSDARG00000001937	136.2587469	3.934266278	0.343041019	8.553689252	1.19E-17	5.42E-16	LO018422.1	solute carrier family 2, facilitated glucose transporter member 1-like [Source:NCBI gene;Acc:100334818]	17	48433817	48458789	
ENSDARG00000001953	2074.743147	2.542100071	0.117104085	13.16862743	1.33E-39	2E-37	pfkfb3	6-phosphofructo-2-kinase/fructose-2,6-biphosphatase 3 [Source:ZFIN;Acc:ZDB-GENE-040426-2724]	4	25472505	25485404	
ENSDARG00000001993	12205.79104	1.404063974	0.098517767	4.101432531	4.11E-05	0.000508711	myhb	myosin, heavy chain b [Source:ZFIN;Acc:ZDB-GENE-081124-1]	6	40745437	40756138	
ENSDARG00000001994	10820.39125	2.060773739	0.087171105	12.16886875	4.55E-34	5.17E-32	stxbp1a	syntaxin binding protein 1a [Source:ZFIN;Acc:ZDB-GENE-050626-106]	21	13861589	13921022	
ENSDARG00000002026	740.194285	1.443051459	0.158789122	2.790187729	0.005267749	0.043253439	qkib	QKI, KH domain containing, RNA binding b [Source:ZFIN;Acc:ZDB-GENE-081028-54]	13	3667230	3843692	
ENSDARG00000002165	314.9550502	-1.669919037	0.189540353	-3.534440169	0.00040864	0.004231398	psme1	proteasome activator subunit 1 [Source:ZFIN;Acc:ZDB-GENE-991110-17]	24	12945803	12954482	
ENSDARG00000002255	1567.555872	1.774987085	0.122525763	6.325094954	2.53E-10	6.23E-09	epb41l3a	erythrocyte membrane protein band 4.1-like 3a [Source:ZFIN;Acc:ZDB-GENE-040822-8]	24	41931585	41986895	
ENSDARG00000002259	31.65856104	2.645201967	0.523574267	3.142251387	0.00167654	0.015297287	ca15c	carbonic anhydrase XV c [Source:ZFIN;Acc:ZDB-GENE-061013-737]	12	4338298	4346085	
ENSDARG00000002293	61.92963071	-2.129954915	0.324927634	-3.477558685	0.000506002	0.005150512	si:ch211-197g15.6	si:ch211-197g15.6 [Source:ZFIN;Acc:ZDB-GENE-050208-457]	22	17460871	17463643	
ENSDARG00000002295	101.8969681	4.331268811	0.430096367	7.745400948	9.53E-15	3.45E-13	si:dkey-21p1.3	si:dkey-21p1.3 [Source:ZFIN;Acc:ZDB-GENE-081105-162]	8	14855471	14883692	
ENSDARG00000002304	193.1503819	-1.849629583	0.190279529	-4.465165476	8E-06	0.000113205	gins2	GINS complex subunit 2 [Source:ZFIN;Acc:ZDB-GENE-050419-19]	18	30481030	30499489	
ENSDARG00000002391	546.1627322	-1.550725394	0.139606135	-3.944850941	7.98E-05	0.000946767	tlcd1	TLC domain containing 1 [Source:ZFIN;Acc:ZDB-GENE-041010-173]	21	26080215	26089964	
ENSDARG00000002394	331.2060146	-2.126888315	0.203666348	-5.533011845	3.15E-08	6.09E-07	ugt5d1	UDP glucuronosyltransferase 5 family, polypeptide D1 [Source:ZFIN;Acc:ZDB-GENE-091118-36]	10	25726694	25728744	
ENSDARG00000002396	555.600417	-1.659316461	0.134126737	-4.91562292	8.85E-07	1.43E-05	cry-dash	cryptochrome DASH [Source:NCBI gene;Acc:402986]	24	20194022	20208474	
ENSDARG00000002487	1095.170068	-1.357992915	0.122690716	-2.917848457	0.003524556	0.030163614	noc3l	NOC3-like DNA replication regulator [Source:NCBI gene;Acc:321289]	12	5399680	5418340	
ENSDARG00000002576	83.72969688	1.979269244	0.329582935	2.971237707	0.002966021	0.025850728	cabp5a	calcium binding protein 5a [Source:ZFIN;Acc:ZDB-GENE-040426-1655]	12	3848109	3861759	
ENSDARG00000002600	1188.505641	2.492598696	0.154714025	9.647468569	5.04E-22	3.05E-20	pcsk1	proprotein convertase subtilisin/kexin type 1 [Source:ZFIN;Acc:ZDB-GENE-071009-1]	21	11684830	11722343	
ENSDARG00000002607	544.9582216	1.487691924	0.150387815	3.242895215	0.001183217	0.011136083	unm_sa1614	un-named sa1614 [Source:ZFIN;Acc:ZDB-GENE-120411-1]	11	26476153	26562346	
ENSDARG00000002609	742.3541173	1.663178527	0.146232386	4.535100241	5.76E-06	8.36E-05	rnf145a	ring finger protein 145a [Source:ZFIN;Acc:ZDB-GENE-111012-3]	14	34966598	35012162	
ENSDARG00000002613	365.4934509	-1.634487815	0.154055016	-4.118579407	3.81E-05	0.000476064	rpa3	replication protein A3 [Source:ZFIN;Acc:ZDB-GENE-040426-977]	19	25465025	25467185	
ENSDARG00000002656	1156.691958	2.219542369	0.129200599	9.439138662	3.76E-21	2.16E-19	stxbp5a	syntaxin binding protein 5a (tomosyn) [Source:ZFIN;Acc:ZDB-GENE-041001-161]	20	31745949	31905968	
ENSDARG00000002732	788.812286	-1.427439315	0.125135387	-3.415814872	0.000635915	0.006324126	spon2b	spondin 2b, extracellular matrix protein [Source:ZFIN;Acc:ZDB-GENE-990415-161]	14	17376940	17382534	
ENSDARG00000002754	206.0667131	-1.692767936	0.178594147	-3.87900694	0.000104884	0.001212103	pdcd2l	programmed cell death 2-like [Source:ZFIN;Acc:ZDB-GENE-040426-967]	25	30442389	30454772	
ENSDARG00000002771	392.2232883	3.42447231	0.190891921	12.70075916	5.86E-37	7.82E-35	SLC4A5	si:ch211-48m9.1 [Source:ZFIN;Acc:ZDB-GENE-060531-64]	5	28045764	28109416	SLC4A5
ENSDARG00000002816	207.4006213	2.246571534	0.211328757	5.898731217	3.66E-09	7.95E-08	rasgrf2b	Ras protein-specific guanine nucleotide-releasing factor 2b [Source:NCBI gene;Acc:553520]	5	51443009	51581775	
ENSDARG00000002840	2209.903313	1.782893237	0.096117336	8.145182457	3.79E-16	1.51E-14	si:dkey-28b4.8	si:dkey-28b4.8 [Source:ZFIN;Acc:ZDB-GENE-090501-2]	1	44651727	44704261	
ENSDARG00000002933	523.5754076	1.652900144	0.15280519	4.272761558	1.93E-05	0.000254793	gbx2	gastrulation brain homeobox 2 [Source:ZFIN;Acc:ZDB-GENE-020509-2]	6	16031189	16034286	
ENSDARG00000002945	1321.059123	1.367497303	0.102044325	3.601349731	0.000316569	0.00336134	bgnb	biglycan b [Source:ZFIN;Acc:ZDB-GENE-040426-21]	23	20014030	20051369	
ENSDARG00000002986	574.8899675	-3.518157547	0.187993086	-13.39494764	6.47E-41	1.03E-38	gda	guanine deaminase [Source:ZFIN;Acc:ZDB-GENE-050522-434]	5	25708369	25723079	
ENSDARG00000003020	341.6214808	1.523407288	0.182812011	2.863090253	0.00419531	0.035243135	lrrc4.2	leucine rich repeat containing 4.2 [Source:ZFIN;Acc:ZDB-GENE-030131-7997]	18	10296228	10298162	
ENSDARG00000003041	477.4822144	1.504190425	0.161298772	3.125816882	0.001773119	0.01610824	CABZ01111496.1	E3 ubiquitin-protein ligase pellino homolog 2-like [Source:NCBI gene;Acc:100334245]	20	54174978	54192130	
ENSDARG00000003046	569.856201	1.901387287	0.215496564	4.182838331	2.88E-05	0.000366635	sorbs2a	sorbin and SH3 domain containing 2a [Source:ZFIN;Acc:ZDB-GENE-070308-2]	1	17747656	17803614	
ENSDARG00000003077	67.32936383	2.349039067	0.3806112	3.544401914	0.000393505	0.004091701	gpx9	glutathione peroxidase 9 [Source:ZFIN;Acc:ZDB-GENE-130603-13]	12	45676667	45688953	
ENSDARG00000003113	8970.170454	-2.94113706	0.120444508	-16.11644311	1.96E-58	5.91E-56	ada	adenosine deaminase [Source:NCBI gene;Acc:436919]	23	25946448	25976656	
ENSDARG00000003169	3915.441612	1.265540057	0.092186225	2.880474353	0.003970773	0.033626978	magi1b	membrane associated guanylate kinase, WW and PDZ domain containing 1b [Source:ZFIN;Acc:ZDB-GENE-030131-5110]	11	18873113	19091767	
ENSDARG00000003210	392.8689647	1.772068949	0.158897707	4.85890553	1.18E-06	1.88E-05	ano2a	anoctamin 2a [Source:ZFIN;Acc:ZDB-GENE-091204-400]	18	7283283	7304547	
ENSDARG00000003219	2165.186187	-1.806270145	0.121205375	-6.652098928	2.89E-11	7.78E-10	bin2a	bridging integrator 2a [Source:ZFIN;Acc:ZDB-GENE-041114-126]	23	33739215	33753361	
ENSDARG00000003269	2520.567145	1.283371994	0.096547041	2.935066584	0.003334761	0.028676205	clcn3	chloride channel 3 [Source:ZFIN;Acc:ZDB-GENE-030131-1699]	7	7511914	7643941	
ENSDARG00000003290	269.0092657	2.484574555	0.204289823	7.267002	3.68E-13	1.16E-11	dab1b	DAB adaptor protein 1b [Source:ZFIN;Acc:ZDB-GENE-060421-7958]	2	1522721	1569250	
ENSDARG00000003293	2207.060003	1.55203546	0.102941229	5.362627456	8.2E-08	1.5E-06	sox9a	SRY-box transcription factor 9a [Source:ZFIN;Acc:ZDB-GENE-001103-1]	12	1947593	1951284	
ENSDARG00000003320	944.3097129	2.167593246	0.123526501	9.452168014	3.32E-21	1.92E-19	fam91a1	family with sequence similarity 91 member A1 [Source:NCBI gene;Acc:393413]	19	33093395	33137720	
ENSDARG00000003403	1805.012621	1.816441876	0.116021782	7.036970682	1.96E-12	5.91E-11	tenm1	teneurin transmembrane protein 1 [Source:ZFIN;Acc:ZDB-GENE-060531-26]	5	21295747	21524251	
ENSDARG00000003449	667.4545409	1.873280195	0.158700173	5.502704745	3.74E-08	7.19E-07	pde10a	phosphodiesterase 10A [Source:ZFIN;Acc:ZDB-GENE-040426-1115]	13	4505079	4620715	
ENSDARG00000003469	1598.814171	2.082385354	0.112361061	9.633100127	5.8E-22	3.51E-20	neurod4	neuronal differentiation 4 [Source:ZFIN;Acc:ZDB-GENE-030730-1]	23	28343988	28347039	
ENSDARG00000003495	3642.809832	1.749841487	0.110326579	6.796562492	1.07E-11	3.04E-10	madd	MAP-kinase activating death domain [Source:ZFIN;Acc:ZDB-GENE-030131-8076]	7	34317401	34413398	
ENSDARG00000003523	281.1093261	-3.488769915	0.236650408	-10.5166517	7.24E-26	5.39E-24	itln3	intelectin 3 [Source:ZFIN;Acc:ZDB-GENE-090527-4]	14	16345003	16352209	
ENSDARG00000003544	396.8840444	1.416922251	0.14991356	2.781084328	0.005417766	0.044340186	adarb1b	adenosine deaminase RNA specific B1b [Source:ZFIN;Acc:ZDB-GENE-030219-15]	9	45227028	45541367	
ENSDARG00000003615	734.6591237	-3.79845504	0.183741712	-15.23037427	2.22E-52	5.87E-50	slc26a3.2	solute carrier family 26 member 3, tandem duplicate 2 [Source:ZFIN;Acc:ZDB-GENE-041210-179]	4	18804317	18823032	
ENSDARG00000003635	713.3182189	-1.549148132	0.125842622	-4.363768989	1.28E-05	0.000174683	mogat3b	monoacylglycerol O-acyltransferase 3b [Source:ZFIN;Acc:ZDB-GENE-110302-2]	5	38596630	38604470	
ENSDARG00000003675	810.1649641	-1.360457593	0.118457418	-3.04292969	0.002342871	0.020787698	cryl1	crystallin, lambda 1 [Source:ZFIN;Acc:ZDB-GENE-060810-7]	9	21383620	21401302	
ENSDARG00000003680	2865.924944	1.969955428	0.096251484	10.07730361	6.96E-24	4.77E-22	runx1t1	RUNX1 partner transcriptional co-repressor 1 [Source:ZFIN;Acc:ZDB-GENE-060929-164]	19	33553586	33785914	
ENSDARG00000003706	199.7067212	1.631230609	0.219707951	2.873043998	0.004065375	0.034300832	ryr2b	ryanodine receptor 2b (cardiac) [Source:ZFIN;Acc:ZDB-GENE-061226-3]	17	20009132	20118244	
ENSDARG00000003779	1431.500645	2.108859199	0.104569319	10.60405875	2.85E-26	2.2E-24	ctnnd2b	catenin (cadherin-associated protein), delta 2b [Source:ZFIN;Acc:ZDB-GENE-070912-532]	2	30554256	30693742	
ENSDARG00000003803	227.2531387	2.653164665	0.257510789	6.419787964	1.36E-10	3.5E-09	LHX3	LIM homeobox 3 [Source:HGNC Symbol;Acc:HGNC:6595]	5	71802014	71819807	LHX3
ENSDARG00000003820	4003.387543	2.09750139	0.18099643	6.063663189	1.33E-09	3E-08	nr1d2a	nuclear receptor subfamily 1, group D, member 2a [Source:ZFIN;Acc:ZDB-GENE-040504-1]	16	50089417	50111057	
ENSDARG00000003909	94.15591905	-3.186000929	0.311588309	-7.015670577	2.29E-12	6.86E-11	ftr01	finTRIM family, member 1 [Source:ZFIN;Acc:ZDB-GENE-070705-491]	2	11670270	11677705	
ENSDARG00000003931	4259.571671	-2.097846427	0.114485403	-9.589400901	8.86E-22	5.31E-20	cndp2	carnosine dipeptidase 2 [Source:ZFIN;Acc:ZDB-GENE-030131-5499]	19	20801060	20823099	
ENSDARG00000003941	1163.944202	-1.477984856	0.101843188	-4.69334145	2.69E-06	4.09E-05	rrs1	ribosome biogenesis regulator 1 homolog [Source:ZFIN;Acc:ZDB-GENE-030131-9345]	24	23837519	23839647	
ENSDARG00000003977	419.7462775	-3.272680215	0.186753303	-12.16942448	4.52E-34	5.16E-32	zgc:153896	zgc:153896 [Source:ZFIN;Acc:ZDB-GENE-060825-119]	20	25879826	25887231	
ENSDARG00000003989	291.3305319	2.255930251	0.194399457	6.4605646	1.04E-10	2.69E-09	crhr1	corticotropin releasing hormone receptor 1 [Source:ZFIN;Acc:ZDB-GENE-081231-1]	3	21669545	21889616	
ENSDARG00000003994	482.9831213	1.595984655	0.207148791	2.877084882	0.004013676	0.03393307	syt9a	synaptotagmin IXa [Source:ZFIN;Acc:ZDB-GENE-031002-18]	18	28988373	29060842	
ENSDARG00000004023	2087.352292	1.704828646	0.11998439	5.874336201	4.25E-09	9.15E-08	isl1	ISL LIM homeobox 1 [Source:NCBI gene;Acc:30147]	5	40728613	40734045	
ENSDARG00000004074	904.7467861	1.876942456	0.138705573	6.322330343	2.58E-10	6.33E-09	bach2b	BTB and CNC homology 1, basic leucine zipper transcription factor 2b [Source:ZFIN;Acc:ZDB-GENE-041001-139]	20	23972948	24122881	
ENSDARG00000004141	853.980657	-2.774544137	0.126117131	-14.07060347	5.76E-45	1.08E-42		zgc:92630 [Source:ZFIN;Acc:ZDB-GENE-040718-449]	15	15415623	15437530	
ENSDARG00000004169	1482.090632	-1.667520178	0.119928997	-5.565961508	2.61E-08	5.11E-07	stmn1a	stathmin 1a [Source:ZFIN;Acc:ZDB-GENE-031006-14]	19	17205143	17210928	
ENSDARG00000004187	502.8733914	1.622386533	0.180609348	3.446037209	0.000568872	0.005720856	zgc:122979	zgc:122979 [Source:ZFIN;Acc:ZDB-GENE-051127-45]	21	5169154	5174054	
ENSDARG00000004199	438.2745646	-2.079740773	0.163655753	-6.597634083	4.18E-11	1.11E-09	spam1	sperm adhesion molecule 1 [Source:ZFIN;Acc:ZDB-GENE-050506-63]	25	27531874	27541621	
ENSDARG00000004218	680.4096139	1.503763729	0.128076277	3.933310221	8.38E-05	0.000989211	rnd1b	Rho family GTPase 1b [Source:ZFIN;Acc:ZDB-GENE-060315-7]	23	26979174	27006062	
ENSDARG00000004227	1010.482525	1.80274078	0.113196187	7.091588532	1.33E-12	4.05E-11	pde3a	phosphodiesterase 3A, cGMP-inhibited [Source:ZFIN;Acc:ZDB-GENE-041210-23]	4	2752852	2868112	
ENSDARG00000004251	489.6496889	-1.620130883	0.149092662	-4.159365556	3.19E-05	0.000402945	dhfr	dihydrofolate reductase [Source:ZFIN;Acc:ZDB-GENE-010406-5]	5	26138313	26146488	
ENSDARG00000004274	162.2235754	-1.690919139	0.201005914	-3.437307513	0.000587528	0.005901378	zgc:112496	zgc:112496 [Source:ZFIN;Acc:ZDB-GENE-050320-25]	24	37631632	37640705	
ENSDARG00000004291	915.7939742	1.99067237	0.1466383	6.75589097	1.42E-11	3.98E-10	hecw1b	HECT, C2 and WW domain containing E3 ubiquitin protein ligase 1b [Source:ZFIN;Acc:ZDB-GENE-060503-2]	2	49884696	49978227	
ENSDARG00000004297	3777.556755	1.597077703	0.087931276	6.790276825	1.12E-11	3.17E-10	irf2bpl	interferon regulatory factor 2 binding protein-like [Source:ZFIN;Acc:ZDB-GENE-040426-1968]	17	11327164	11329959	
ENSDARG00000004302	551.5796222	1.642793009	0.141832299	4.532063679	5.84E-06	8.46E-05	slc45a1	solute carrier family 45 member 1 [Source:ZFIN;Acc:ZDB-GENE-070112-2152]	11	40649412	40674070	
ENSDARG00000004305	608.7349599	1.370254185	0.126240141	2.932935461	0.003357737	0.028844131	vangl1	VANGL planar cell polarity protein 1 [Source:ZFIN;Acc:ZDB-GENE-040621-2]	9	33009284	33055440	
ENSDARG00000004358	2139.993211	2.776558546	0.118401606	15.00451388	6.86E-51	1.66E-48	gnb3a	guanine nucleotide binding protein (G protein), beta polypeptide 3a [Source:ZFIN;Acc:ZDB-GENE-040718-134]	16	12022543	12049762	
ENSDARG00000004386	2001.381934	1.646068799	0.150166667	4.30234494	1.69E-05	0.000225642	ELAPOR2	si:dkey-153k10.9 [Source:ZFIN;Acc:ZDB-GENE-030131-3893]	4	9669717	9690419	ELAPOR2
ENSDARG00000004396	190.6785421	-1.52090117	0.185894656	-2.802130954	0.005076626	0.041848405	b3gnt5b	UDP-GlcNAc:betaGal beta-1,3-N-acetylglucosaminyltransferase 5b [Source:NCBI gene;Acc:791470]	2	7749394	7757273	
ENSDARG00000004415	2327.790387	1.745293881	0.094802682	7.861527363	3.79E-15	1.42E-13	tcf7l2	transcription factor 7 like 2 [Source:ZFIN;Acc:ZDB-GENE-991110-8]	12	30977736	31103906	
ENSDARG00000004445	387.493942	1.573166847	0.167924265	3.413246124	0.000641939	0.006376462	grm5a	glutamate receptor, metabotropic 5a [Source:ZFIN;Acc:ZDB-GENE-090821-9]	10	25219728	25272236	
ENSDARG00000004447	241.6726638	1.86139042	0.223095581	3.861082389	0.000112886	0.00129504	aplnr2	apelin receptor 2 [Source:ZFIN;Acc:ZDB-GENE-030131-4347]	7	38685545	38689562	
ENSDARG00000004502	416.7079094	1.884782944	0.153713002	5.756070938	8.61E-09	1.79E-07	rcor3	REST corepressor 3 [Source:ZFIN;Acc:ZDB-GENE-050208-371]	22	38276024	38294242	
ENSDARG00000004527	729.3876791	-1.430342706	0.137959688	-3.119336616	0.001812588	0.016425184	pin4	protein (peptidylprolyl cis/trans isomerase) NIMA-interacting, 4 (parvulin) [Source:NCBI gene;Acc:553574]	14	28432737	28437426	
ENSDARG00000004537	3789.748904	1.450090928	0.105723665	4.257239177	2.07E-05	0.000270859	kmt2a	lysine (K)-specific methyltransferase 2A [Source:ZFIN;Acc:ZDB-GENE-080521-3]	15	23382199	23420170	
ENSDARG00000004588	8234.447854	1.499349796	0.134468313	3.713512772	0.000204402	0.002241458	sox4a	SRY-box transcription factor 4a [Source:ZFIN;Acc:ZDB-GENE-030131-8290]	19	28786149	28789409	
ENSDARG00000004597	403.1944634	1.81063079	0.156178115	5.190424977	2.1E-07	3.64E-06	lrrc4ba	leucine rich repeat containing 4Ba [Source:ZFIN;Acc:ZDB-GENE-080327-25]	3	30369765	30434016	
ENSDARG00000004643	591.3280904	1.819920805	0.147119955	5.573144753	2.5E-08	4.91E-07	cdhr1a	cadherin-related family member 1a [Source:NCBI gene;Acc:449008]	13	29480010	29505946	
ENSDARG00000004687	1468.793161	-1.72336214	0.108281317	-6.680396567	2.38E-11	6.49E-10	acaa1	acetyl-CoA acyltransferase 1 [Source:ZFIN;Acc:ZDB-GENE-040704-48]	24	20325312	20332460	
ENSDARG00000004782	3165.308836	1.855177192	0.111162848	7.693012619	1.44E-14	5.13E-13	fgfr3	fibroblast growth factor receptor 3 [Source:NCBI gene;Acc:58129]	13	13213567	13294872	

ENSDARG00000004789	419.4594316	1.500697422	0.145010104	3.452845068	0.000554707	0.005591304	lrp1ba	low density lipoprotein receptor-related protein 1Ba [Source:ZFIN;Acc:ZDB-GENE-050208-608]	22	14445713	14739491	
ENSDARG00000004836	2577.698266	1.533260009	0.098615816	5.407449124	6.39E-08	1.19E-06	dnajc5ab	DnaJ (Hsp40) homolog, subfamily C, member 5ab [Source:ZFIN;Acc:ZDB-GENE-081021-2]	23	17472702	17509656	
ENSDARG00000004861	967.2755287	1.817198017	0.123777945	6.602129446	4.05E-11	1.08E-09	esrrga	estrogen-related receptor gamma a [Source:ZFIN;Acc:ZDB-GENE-030821-2]	17	29797876	29902187	
ENSDARG00000004904	2019.882333	-2.424186548	0.096626797	-14.7390433	3.62E-49	8.18E-47	gys2	glycogen synthase 2 [Source:ZFIN;Acc:ZDB-GENE-030826-31]	4	16783972	16824556	
ENSDARG00000004948	3879.732889	-1.609365485	0.104373444	-5.838319232	5.27E-09	1.12E-07	bccip	BRCA2 and CDKN1A interacting protein [Source:NCBI gene;Acc:541348]	12	45186533	45197038	
ENSDARG00000004964	320.6954996	-1.983371241	0.202651407	-4.852526098	1.22E-06	1.94E-05	cyp4t8	cytochrome P450, family 4, subfamily T, polypeptide 8 [Source:ZFIN;Acc:ZDB-GENE-031219-3]	16	4615661	4640578	
ENSDARG00000004988	2213.109917	1.732499548	0.176132247	4.1588043	3.2E-05	0.000403733	ppp3ca	protein phosphatase 3, catalytic subunit, alpha isozyme [Source:ZFIN;Acc:ZDB-GENE-091113-24]	21	28958471	29075815	
ENSDARG00000005026	2274.360102	1.367930508	0.098591639	3.731863195	0.000190069	0.002098028	tshz1	teashirt zinc finger homeobox 1 [Source:NCBI gene;Acc:369196]	19	21266008	21416407	
ENSDARG00000005128	478.7863659	-1.467380735	0.138381221	-3.377486725	0.000731515	0.007205804	rnaseh2b	ribonuclease H2, subunit B [Source:ZFIN;Acc:ZDB-GENE-040426-1090]	9	29671553	29679172	
ENSDARG00000005141	652.1851267	1.524119899	0.163928194	3.197252926	0.001387432	0.012888947	camkvb	CaM kinase-like vesicle-associated b [Source:NCBI gene;Acc:393422]	11	35364445	35441348	
ENSDARG00000005221	1054.286762	-2.077099743	0.116588948	-9.238437762	2.5E-20	1.38E-18	desmb	desmin b [Source:ZFIN;Acc:ZDB-GENE-061027-102]	6	13871641	13891727	
ENSDARG00000005251	2173.901821	-1.395030205	0.096789538	-4.081331663	4.48E-05	0.000552326	rpe	ribulose-5-phosphate-3-epimerase [Source:ZFIN;Acc:ZDB-GENE-030131-6837]	9	24025444	24031461	
ENSDARG00000005320	419.1349162	-1.703555614	0.164956414	-4.265100073	2E-05	0.000262317	nipsnap1	nipsnap homolog 1 (C. elegans) [Source:NCBI gene;Acc:30231]	5	12032802	12063381	
ENSDARG00000005332	70.28215905	2.499809583	0.370150754	4.051888495	5.08E-05	0.000621481	lipib	lipase, member Ib [Source:ZFIN;Acc:ZDB-GENE-091118-47]	24	24086249	24101132	
ENSDARG00000005371	83.63458147	-2.081767554	0.289963706	-3.730699855	0.000190949	0.002105888	gprc6a	G protein-coupled receptor, class C, group 6, member A [Source:NCBI gene;Acc:494131]	16	32152612	32159033	
ENSDARG00000005453	1428.079726	1.824030787	0.106837263	7.712952989	1.23E-14	4.43E-13	foxp2	forkhead box P2 [Source:ZFIN;Acc:ZDB-GENE-041203-2]	4	6423041	6623645	
ENSDARG00000005479	5207.516915	1.34138119	0.083137922	4.106203083	4.02E-05	0.000500099	tenm3	Teneurin-3 [Source:UniProtKB/Swiss-Prot;Acc:Q9W7R4]	1	39387876	39779913	
ENSDARG00000005482	3585.931664	1.46859118	0.091619321	5.114545422	3.14E-07	5.37E-06	rapgef2	Rap guanine nucleotide exchange factor (GEF) 2 [Source:ZFIN;Acc:ZDB-GENE-030131-6959]	14	47779846	48033073	
ENSDARG00000005525	834.1208416	-1.738851679	0.123774116	-5.969355327	2.38E-09	5.29E-08	acy3.2	aspartoacylase (aminocyclase) 3, tandem duplicate 2 [Source:NCBI gene;Acc:436619]	1	52489962	52498146	
ENSDARG00000005526	1929.529667	-1.531220043	0.190062091	-2.794981576	0.005190267	0.042659103	igfn1.1	immunoglobulin like and fibronectin type III domain containing 1, tandem duplicate 1 [Source:ZFIN;Acc:ZDB-GENE-050327	6	46878889	46941245	
ENSDARG00000005559	695.573004	1.452468994	0.132031027	3.426989886	0.000610312	0.006100931	pou4f1	POU class 4 homeobox 1 [Source:ZFIN;Acc:ZDB-GENE-000523-2]	6	4539953	4543005	
ENSDARG00000005565	396.6501316	-1.779732402	0.162450549	-4.799813909	1.59E-06	2.5E-05	entpd8	ectonucleoside triphosphate diphosphohydrolase 8 [Source:ZFIN;Acc:ZDB-GENE-040724-142]	6	41038757	41060423	
ENSDARG00000005586	156.8331438	2.125618765	0.252097897	4.465006563	8.01E-06	0.000113225	zbtb20	zinc finger and BTB domain containing 20 [Source:ZFIN;Acc:ZDB-GENE-070112-1992]	5	67533180	67661102	
ENSDARG00000005629	2992.627412	-1.295897211	0.100103468	-2.955913689	0.003117443	0.026992391	smyd2b	SET and MYND domain containing 2b [Source:NCBI gene;Acc:568616]	20	572037	592746	
ENSDARG00000005670	570.9715415	1.979090644	0.143779795	6.80965392	9.78E-12	2.78E-10	ttbk2a	tau tubulin kinase 2a [Source:ZFIN;Acc:ZDB-GENE-120928-3]	17	45216084	45254585	
ENSDARG00000005739	1902.853756	1.600333139	0.196108458	3.061230228	0.002204295	0.019690397	gpm6ba	glycoprotein M6Ba [Source:ZFIN;Acc:ZDB-GENE-030710-9]	1	28961653	29045426	
ENSDARG00000005783	1114.514316	1.852470627	0.139793273	6.098080447	1.07E-09	2.46E-08	ncanb	neurocan b [Source:ZFIN;Acc:ZDB-GENE-050208-586]	22	17864872	18116635	
ENSDARG00000005883	652.6685956	1.631910481	0.227626644	2.776083104	0.005501814	0.044954766	rfx1a	regulatory factor X, 1a (influences HLA class II expression) [Source:ZFIN;Acc:ZDB-GENE-070424-94]	3	19152222	19200651	
ENSDARG00000005943	419.4629084	-3.058177876	0.179087581	-11.49257735	1.44E-30	1.44E-28	htra4	HtrA serine peptidase 4 [Source:ZFIN;Acc:ZDB-GENE-131120-192]	5	26930062	26950374	
ENSDARG00000006029	4129.549464	-2.176104864	0.119366885	-9.852857148	6.66E-23	4.22E-21	lta4h	leukotriene A4 hydrolase [Source:ZFIN;Acc:ZDB-GENE-040426-2477]	4	7653607	7673165	
ENSDARG00000006128	1437.476927	1.363434223	0.111737664	3.252566872	0.001143677	0.010808521	cep170aa	centrosomal protein 170Aa [Source:NCBI gene;Acc:557463]	13	10980755	11072964	
ENSDARG00000006219	2730.931677	-1.416994864	0.088850898	-4.693198078	2.69E-06	4.09E-05	gnl3	guanine nucleotide binding protein-like 3 (nucleolar) [Source:NCBI gene;Acc:321897]	11	3959495	3971035	
ENSDARG00000006220	5076.502922	-2.5646054	0.140155082	-11.16338687	6.16E-29	5.77E-27	ugt1a1	UDP glucuronosyltransferase 1 family, polypeptide A1 [Source:ZFIN;Acc:ZDB-GENE-071004-4]	9	9969987	10003782	
ENSDARG00000006257	2812.396353	1.680548949	0.103657892	6.565336545	5.19E-11	1.37E-09	vldlr	very low density lipoprotein receptor [Source:ZFIN;Acc:ZDB-GENE-040426-803]	10	15255012	15305767	
ENSDARG00000006383	1013.714124	1.858415592	0.193277784	4.441356762	8.94E-06	0.000125006	stxbp5l	syntaxin binding protein 5-like [Source:NCBI gene;Acc:567605]	9	27415754	27649406	
ENSDARG00000006396	1014.068507	1.88345926	0.212294935	4.161471205	3.16E-05	0.000400052	nrcama	neuronal cell adhesion molecule a [Source:ZFIN;Acc:ZDB-GENE-041210-235]	4	13696537	13728375	
ENSDARG00000006420	2496.775636	1.509596541	0.107072652	4.759352956	1.94E-06	3.02E-05	atrnl1b	attractin-like 1b [Source:ZFIN;Acc:ZDB-GENE-030131-8092]	12	29842836	30032188	
ENSDARG00000006427	24621.63736	-4.656547896	0.14664163	-24.93526502	3.09E-137	2.98E-134	fabp2	fatty acid binding protein 2, intestinal [Source:ZFIN;Acc:ZDB-GENE-991019-5]	1	25848231	25851575	
ENSDARG00000006434	675.0878598	-1.538446962	0.1174551	-4.584279121	4.56E-06	6.7E-05	polr1e	RNA polymerase I subunit E [Source:ZFIN;Acc:ZDB-GENE-050522-251]	1	19637312	19648227	
ENSDARG00000006447	51.50650315	-2.545261346	0.372298113	-4.150602145	3.32E-05	0.000418268	slc19a3a	solute carrier family 19 member 3a [Source:ZFIN;Acc:ZDB-GENE-110411-41]	15	35139457	35156791	
ENSDARG00000006467	625.3235369	2.861475906	0.165677164	11.23556116	2.73E-29	2.6E-27	pcdh8	protocadherin 8 [Source:ZFIN;Acc:ZDB-GENE-980526-242]	9	53654866	53666031	
ENSDARG00000006491	861.6699621	-1.461631484	0.122682644	-3.762810034	0.000168015	0.001877712	agpat9l	1-acylglycerol-3-phosphate O-acyltransferase 9, like [Source:NCBI gene;Acc:567414]	5	8919698	8957068	
ENSDARG00000006566	2293.856887	1.394301042	0.120602175	3.269435588	0.001077623	0.010238231	nlk1	nemo-like kinase, type 1 [Source:ZFIN;Acc:ZDB-GENE-040701-1]	3	21066315	21094525	
ENSDARG00000006598	1152.688952	1.455972361	0.11629738	3.920744921	8.83E-05	0.001035424	sgk2b	serum/glucocorticoid regulated kinase 2b [Source:ZFIN;Acc:ZDB-GENE-060929-44]	16	26530426	26537114	
ENSDARG00000006607	547.4635571	1.817948264	0.169502469	4.825583194	1.4E-06	2.21E-05	gpr27	G protein-coupled receptor 27 [Source:ZFIN;Acc:ZDB-GENE-071022-3]	6	43903209	43907321	
ENSDARG00000006747	2085.444763	1.875357149	0.125905315	6.952503525	3.59E-12	1.06E-10	tmem178b	transmembrane protein 178B [Source:NCBI gene;Acc:436669]	4	12111154	12239160	
ENSDARG00000006811	1617.059764	-3.764870353	0.198101321	-13.95684969	2.86E-44	5.2E-42	sult1st6	sulfotransferase family 1, cytosolic sulfotransferase 6 [Source:ZFIN;Acc:ZDB-GENE-040718-343]	12	15197857	15205087	
ENSDARG00000006868	346.3306195	1.746802751	0.184771279	4.041768593	5.3E-05	0.00064672	trh	thyrotropin-releasing hormone [Source:NCBI gene;Acc:266721]	8	54220551	54223316	
ENSDARG00000006923	1427.269797	2.468111366	0.205923094	7.129415823	1.01E-12	3.12E-11	cacna1ab	calcium channel, voltage-dependent, P/Q type, alpha 1A subunit, b [Source:ZFIN;Acc:ZDB-GENE-090514-3]	11	30817943	31026335	
ENSDARG00000007024	2671.701369	-1.598152109	0.133054177	-4.495553031	6.94E-06	9.91E-05	uox	urate oxidase [Source:NCBI gene;Acc:436604]	11	8160894	8168011	
ENSDARG00000007130	1218.564025	-1.542994553	0.126171863	-4.303610483	1.68E-05	0.000224476	mrto4	MRT4 homolog, ribosome maturation factor [Source:ZFIN;Acc:ZDB-GENE-050417-142]	11	41846059	41853874	
ENSDARG00000007149	2351.103904	1.910081989	0.123335768	7.378897515	1.6E-13	5.25E-12	l1cama	L1 cell adhesion molecule, paralog a [Source:NCBI gene;Acc:30634]	23	28582865	28681775	
ENSDARG00000007184	1316.347381	2.091106967	0.112123556	9.731291156	2.22E-22	1.37E-20	zbtb16a	zinc finger and BTB domain containing 16a [Source:NCBI gene;Acc:323269]	21	23159385	23331619	
ENSDARG00000007220	1250.105461	1.814205266	0.107321825	7.586576753	3.28E-14	1.14E-12	ncam1b	neural cell adhesion molecule 1b [Source:ZFIN;Acc:ZDB-GENE-010822-2]	15	18368809	18574716	
ENSDARG00000007276	6076.516747	-4.80009353	0.138487113	-27.44005169	9.13E-166	1.35E-162	ela3l	elastase 3 like [Source:ZFIN;Acc:ZDB-GENE-060710-2]	5	26111596	26118855	
ENSDARG00000007277	134.6372817	-1.620506954	0.22092368	-2.808693725	0.004974294	0.041139956	myf5	myogenic factor 5 [Source:ZFIN;Acc:ZDB-GENE-000616-6]	4	21741228	21745107	
ENSDARG00000007302	656.2948729	-1.408115397	0.144650037	-2.821398502	0.004781477	0.039689101	sh3gl3b	SH3-domain GRB2-like 3b [Source:ZFIN;Acc:ZDB-GENE-040121-4]	25	19522954	19537323	
ENSDARG00000007320	75351.98857	-1.32974111	0.094537583	-3.487936753	0.000486763	0.004974855	rpl7	ribosomal protein L7 [Source:ZFIN;Acc:ZDB-GENE-030131-8654]	2	30177476	30182353	
ENSDARG00000007344	1059.34189	1.487444629	0.133182037	3.659987792	0.000252227	0.002727771	tcap	titin-cap (telethonin) [Source:ZFIN;Acc:ZDB-GENE-070501-5]	3	21240594	21242460	
ENSDARG00000007366	637.3966995	-1.982014408	0.14590224	-6.730632851	1.69E-11	4.7E-10	AMDHD1	si:ch73-71d17.1 [Source:NCBI gene;Acc:402826]	7	19389304	19401501	AMDHD1
ENSDARG00000007371	11.85986851	-4.146279393	0.910912089	-3.453987966	0.000552362	0.005570446	slc26a3.1	solute carrier family 26 member 3 [Source:ZFIN;Acc:ZDB-GENE-041210-180]	4	18824959	18835544	
ENSDARG00000007382	329.6214224	1.473093238	0.167234742	2.828917204	0.004670578	0.038807077	ubtd1a	ubiquitin domain containing 1a [Source:ZFIN;Acc:ZDB-GENE-070424-93]	13	31371536	31378683	
ENSDARG00000007406	702.5624141	1.887979145	0.134630134	6.595693818	4.23E-11	1.12E-09	phox2a	paired like homeobox 2A [Source:ZFIN;Acc:ZDB-GENE-000223-14]	15	47440477	47449248	
ENSDARG00000007418	309.9861717	-1.742210217	0.180064271	-4.121918307	3.76E-05	0.000469682	chmp4c	charged multivesicular body protein 4C [Source:ZFIN;Acc:ZDB-GENE-050417-389]	24	11070110	11076400	
ENSDARG00000007421	4016.687104	-1.806347427	0.091003243	-8.860644931	7.96E-19	3.92E-17	ftcd	formimidoyltransferase cyclodeaminase [Source:ZFIN;Acc:ZDB-GENE-030131-9643]	22	12770877	12786532	
ENSDARG00000007480	1382.591561	2.191551783	0.121607179	9.798367126	1.14E-22	7.18E-21	rpe65a	retinoid isomerohydrolase RPE65 a [Source:NCBI gene;Acc:393724]	2	1428696	1443692	
ENSDARG00000007523	3196.089251	1.444963636	0.100900461	4.409926677	1.03E-05	0.000142929	kmt2e	lysine (K)-specific methyltransferase 2E [Source:ZFIN;Acc:ZDB-GENE-030131-4120]	4	22424451	22472653	
ENSDARG00000007553	586.4777921	2.367680717	0.148967709	9.181054912	4.27E-20	2.3E-18	opn4.1	opsin 4.1 [Source:NCBI gene;Acc:352918]	2	32843133	32846324	
ENSDARG00000007561	219.6838817	1.625015436	0.190941965	3.273326714	0.001062895	0.010113623	cdh23	cadherin-related 23 [Source:ZFIN;Acc:ZDB-GENE-040513-7]	13	43050562	43213012	
ENSDARG00000007576	6626.719893	2.972165724	0.179949361	10.95955945	5.98E-28	5.1E-26	crybb1l1	crystallin, beta B1, like 1 [Source:ZFIN;Acc:ZDB-GENE-100112-3]	14	46301973	46310549	
ENSDARG00000007624	262.0370539	-1.975257116	0.190589005	-5.11706915	3.1E-07	5.3E-06	gins1	GINS complex subunit 1 (Psf1 homolog) [Source:ZFIN;Acc:ZDB-GENE-041010-184]	13	7164345	7170351	
ENSDARG00000007663	3672.632181	1.509416168	0.154657535	3.293833492	0.00098831	0.009457711	amph	amphiphysin [Source:ZFIN;Acc:ZDB-GENE-040426-1711]	2	31891335	31937005	
ENSDARG00000007678	5553.105863	1.902646115	0.108942263	8.285545866	1.18E-16	4.92E-15	ttyh3b	tweety family member 3b [Source:NCBI gene;Acc:798421]	1	11781544	11850353	
ENSDARG00000007709	906.4027874	1.882585046	0.134198266	6.576724676	4.81E-11	1.27E-09	adamts8a	ADAM metallopeptidase with thrombospondin type 1 motif, 8a [Source:ZFIN;Acc:ZDB-GENE-060526-202]	5	30155834	30176970	
ENSDARG00000007744	3206.408749	-1.320996664	0.086881912	-3.694631657	0.000220206	0.002395919	tsr1	TSR1 ribosome maturation factor [Source:ZFIN;Acc:ZDB-GENE-030131-3762]	15	25635326	25656331	
ENSDARG00000007788	2302.514232	1.72639958	0.121001851	6.003210493	1.93E-09	4.3E-08	atp2b1b	ATPase plasma membrane Ca2+ transporting 1b [Source:ZFIN;Acc:ZDB-GENE-080409-1]	25	19609713	19666107	
ENSDARG00000007823	777.5021179	2.424730675	0.14074722	10.12262033	4.39E-24	3.07E-22	atf3	activating transcription factor 3 [Source:ZFIN;Acc:ZDB-GENE-040426-728]	20	37794633	37800737	
ENSDARG00000007824	3878.391142	2.383773146	0.167068558	8.282666498	1.2E-16	5.02E-15	camta1b	calmodulin binding transcription activator 1b [Source:ZFIN;Acc:ZDB-GENE-080229-1]	11	39672874	39926872	
ENSDARG00000007906	175.5479757	2.88789979	0.269535055	7.004282963	2.48E-12	7.42E-11	lrp2b	low density lipoprotein receptor-related protein 2b [Source:ZFIN;Acc:ZDB-GENE-111115-1]	12	16132612	16229402	
ENSDARG00000007944	193.4143525	1.611734965	0.223123341	2.741689693	0.006112406	0.049333599	lhx1b	LIM homeobox 1b [Source:ZFIN;Acc:ZDB-GENE-980526-116]	5	56268436	56274174	
ENSDARG00000007982	373.7612155	1.840085987	0.161847832	5.190591544	2.1E-07	3.64E-06	onecut1	one cut homeobox 1 [Source:ZFIN;Acc:ZDB-GENE-040426-1469]	18	38755023	38760271	
ENSDARG00000007996	1119.690576	1.480964397	0.121861535	3.946810603	7.92E-05	0.000939863	pou2f1b	POU class 2 homeobox 1b [Source:ZFIN;Acc:ZDB-GENE-030131-2422]	9	34319333	34378674	
ENSDARG00000008029	705.6321696	-5.449061546	0.225290317	-19.74812594	8.33E-87	4.75E-84	mep1a.2	meprin A, alpha (PABA peptide hydrolase), tandem duplicate 2 [Source:ZFIN;Acc:ZDB-GENE-041001-208]	20	35484070	35496459	
ENSDARG00000008127	500.5981322	2.061596914	0.141855465	7.48365183	7.23E-14	2.44E-12	pcdh15b	protocadherin-related 15b [Source:ZFIN;Acc:ZDB-GENE-050214-1]	12	6566233	6905375	
ENSDARG00000008131	481.1531021	1.534437925	0.180540481	2.960211047	0.003074284	0.026664683	sox1b	SRY-box transcription factor 1b [Source:NCBI gene;Acc:562710]	1	46194333	46196107	
ENSDARG00000008209	2226.908042	2.246140582	0.12585505	9.901395189	4.11E-23	2.64E-21	myt1la	myelin transcription factor 1-like, a [Source:ZFIN;Acc:ZDB-GENE-041001-172]	20	30445971	30574029	
ENSDARG00000008278	996.5068827	1.39480918	0.126752944	3.114792967	0.001840741	0.016650245	rcor2	REST corepressor 2 [Source:NCBI gene;Acc:402934]	7	24979833	24995640	
ENSDARG00000008329	530.2566803	1.877619987	0.195118643	4.497878686	6.86E-06	9.82E-05	asic1a	acid-sensing (proton-gated) ion channel 1a [Source:NCBI gene;Acc:791696]	8	6967108	7023196	
ENSDARG00000008363	5358.097986	1.447122026	0.08897516	5.025245518	5.03E-07	8.41E-06	mcl1b	MCL1 apoptosis regulator, BCL2 family member b [Source:ZFIN;Acc:ZDB-GENE-030825-1]	16	29586004	29587969	
ENSDARG00000008398	1122.973861	1.903876529	0.154760196	5.840497458	5.2E-09	1.11E-07	cacna1c	calcium channel, voltage-dependent, L type, alpha 1C subunit [Source:ZFIN;Acc:ZDB-GENE-020129-1]	4	20318127	20474972	
ENSDARG00000008454	27.70955498	2.955849886	0.595505097	3.28435457	0.001022162	0.009744511	cpeb1b	cytoplasmic polyadenylation element binding protein 1b [Source:NCBI gene;Acc:30702]	7	48240707	48251235	
ENSDARG00000008457	686.2690177	-1.504378419	0.176195856	-2.86260091	0.004201794	0.035273994	faah2a	fatty acid amide hydrolase 2a [Source:NCBI gene;Acc:436973]	1	40237276	40258430	
ENSDARG00000008485	457.1962954	1.675012296	0.149647904	4.510669897	6.46E-06	9.27E-05	lrp3	low density lipoprotein receptor-related protein 3 [Source:ZFIN;Acc:ZDB-GENE-080116-3]	7	38278860	38310177	
ENSDARG00000008491	2170.261806	-2.148321568	0.130011981	-8.832428795	1.02E-18	4.99E-17	si:ch211-107o10.3	si:ch211-107o10.3 [Source:ZFIN;Acc:ZDB-GENE-030131-4716]	7	30178253	30191357	
ENSDARG00000008553	578.1148181	-1.788845897	0.135998737	-5.80039136	6.62E-09	1.39E-07	zgc:136872	zgc:136872 [Source:ZFIN;Acc:ZDB-GENE-060421-5204]	17	3124129	3166898	
ENSDARG00000008637	3741.604953	-1.629256577	0.147974219	-4.25247439	2.11E-05	0.000275683	mttp	microsomal triglyceride transfer protein [Source:NCBI gene;Acc:406207]	1	12105901	12126625	
ENSDARG00000008662	267.5406889	1.539055217	0.194941281	2.765218401	0.00568847	0.046254057	fign	fidgetin [Source:NCBI gene;Acc:553599]	9	50599362	50755669	
ENSDARG00000008946	252.6198247	-1.522756356	0.187817828	-2.783315953	0.005380638	0.044079437	ccdc127b	coiled-coil domain containing 127b [Source:ZFIN;Acc:ZDB-GENE-060503-656]	19	7292445	7295926	
ENSDARG00000009018	14010.97687	-1.402238901	0.141213307	-2.848448984	0.00439329	0.036721932	rhbg	Rh family, B glycoprotein (gene/pseudogene) [Source:NCBI gene;Acc:337596]	16	29265796	29277164	
ENSDARG00000009026	2692.146559	1.533110474	0.145061571	3.675063426	0.00023779	0.002577191	ank2a	ankyrin 2a, neuronal [Source:ZFIN;Acc:ZDB-GENE-111215-3]	1	14073891	14158563	
ENSDARG00000009153	582.6518568	-5.396592737	0.230773515	-19.0515482	6.38E-81	3.2E-78	pla2g1b	phospholipase A2, group IB (pancreas) [Source:ZFIN;Acc:ZDB-GENE-070117-1]	8	39724138	39733667	
ENSDARG00000009250	7655.7219	-1.436507722	0.079664758	-5.479307727	4.27E-08	8.13E-07	etfb	electron transfer flavoprotein subunit beta [Source:ZFIN;Acc:ZDB-GENE-040426-1931]	16	50214819	50229274	
ENSDARG00000009267	488.0583136	-1.727391261	0.157880227	-4.607234709	4.08E-06	6.04E-05	zgc:136439	zgc:136439 [Source:ZFIN;Acc:ZDB-GENE-060421-2858]	9	41080029	41083181	
ENSDARG00000009281	1839.125852	1.998845638	0.159175616	6.275117161	3.49E-10	8.47E-09	dnm1b	dynamin 1b [Source:ZFIN;Acc:ZDB-GENE-100920-3]	5	63668735	63737734	
ENSDARG00000009299	1961.328479	1.831419017	0.101697603	8.175404268	2.95E-16	1.19E-14	dusp8a	dual specificity phosphatase 8a [Source:ZFIN;Acc:ZDB-GENE-040426-1842]	25	22979743	23052707	
ENSDARG00000009341	2790.227536	-1.990311648	0.108588663	-9.119843856	7.52E-20	3.98E-18	nrap	nebulin-related anchoring protein [Source:ZFIN;Acc:ZDB-GENE-060616-224]	12	30586599	30618440	
ENSDARG00000009386	37.95706307	2.920412836	0.5505608	3.488103104	0.00048646	0.004973786	mpp4l	membrane protein, palmitoylated 4-like [Source:ZFIN;Acc:ZDB-GENE-091118-7]	21	18976623	18993110	
ENSDARG00000009387	919.9372206	1.959421625	0.123437371	7.772537746	7.69E-15	2.81E-13	robo4	roundabout, axon guidance receptor, homolog 4 (Drosophila) [Source:ZFIN;Acc:ZDB-GENE-020809-1]	10	31244619	31284790	
ENSDARG00000009443	3306.742506	-5.190184038	0.139907578	-29.94965741	4.45E-197	9.3E-194	zgc:92137	zgc:92137 [Source:ZFIN;Acc:ZDB-GENE-040801-179]	17	42983983	42988356	
ENSDARG00000009447	31377.81395	-1.395007588	0.121479515	-3.251639486	0.001147415	0.010831611	atp5mc3b	ATP synthase membrane subunit c locus 3b [Source:ZFIN;Acc:ZDB-GENE-020814-1]	6	10830844	10835922	
ENSDARG00000009477	223.1425888	1.940552907	0.202921227	4.635064157	3.57E-06	5.33E-05	prkar2ab	protein kinase, cAMP-dependent, regulatory, type II, alpha, B [Source:ZFIN;Acc:ZDB-GENE-040426-2427]	22	110158	130695	
ENSDARG00000009493	367.1224565	2.217736525	0.166646133	7.307319433	2.73E-13	8.76E-12	MAP3K13	si:ch211-45c16.2 [Source:ZFIN;Acc:ZDB-GENE-081105-15]	9	52694971	52814314	MAP3K13
ENSDARG00000009550	662.7649379	-1.357427484	0.12964503	-2.756970194	0.005833967	0.047299226	foxi3b	forkhead box I3b [Source:ZFIN;Acc:ZDB-GENE-031126-4]	14	34514336	34516587	
ENSDARG00000009612	210.2410076	-4.400423722	0.320797333	-10.59991269	2.98E-26	2.29E-24	chia.3	chitinase, acidic.3 [Source:ZFIN;Acc:ZDB-GENE-040426-2891]	11	24013769	24016813	
ENSDARG00000009637	5175.371068	1.715271673	0.132445718	5.400489245	6.65E-08	1.23E-06	rcvrn3	recoverin 3 [Source:ZFIN;Acc:ZDB-GENE-040426-1661]	19	10339538	10352579	

ENSDARG00000009806	481.5492191	-1.398220215	0.133159773	-2.990544404	0.002784806	0.024372882	smfn	small fragment nuclease [Source:ZFIN;Acc:ZDB-GENE-040426-1709]	10	39893439	39905253	
ENSDARG00000009844	145.9220477	-1.647003707	0.222871141	-2.903039422	0.003695601	0.031473159	dusp23a	dual specificity phosphatase 23a [Source:ZFIN;Acc:ZDB-GENE-030131-2554]	20	43113465	43118411	
ENSDARG00000009901	1471.884566	2.137953312	0.144508359	7.874653902	3.42E-15	1.28E-13	slc38a5a	solute carrier family 38 member 5a [Source:ZFIN;Acc:ZDB-GENE-081105-92]	8	25615781	25625800	
ENSDARG00000009922	1159.66212	2.201965438	0.187255907	6.418838581	1.37E-10	3.51E-09	dmbx1a	diencephalon/mesencephalon homeobox 1a [Source:NCBI gene;Acc:142987]	2	10183640	10192487	
ENSDARG00000009941	1387.571031	1.53452419	0.185020083	2.889006331	0.003864613	0.032794337	erc1a	ELKS/RAB6-interacting/CAST family member 1a [Source:ZFIN;Acc:ZDB-GENE-091214-5]	25	21176937	21253162	
ENSDARG00000009978	11453.15401	-2.02272763	0.08254085	-12.39056333	2.94E-35	3.65E-33	icn	ictacalcin [Source:ZFIN;Acc:ZDB-GENE-030131-8599]	16	23431189	23432552	
ENSDARG00000010023	1091.700497	1.557814435	0.149490627	3.731434167	0.000190393	0.002100681	CU856539.1	hippocampus abundant transcript 1 protein-like [Source:NCBI gene;Acc:557306]	10	2499383	2527342	
ENSDARG00000010042	1808.100911	2.642170765	0.155210134	10.58030634	3.68E-26	2.81E-24	dnm1a	dynamin 1a [Source:NCBI gene;Acc:100307098]	5	1278092	1421927	
ENSDARG00000010047	315.1051863	2.498769308	0.216855738	6.911365705	4.8E-12	1.4E-10	neu3.2	sialidase 3 (membrane sialidase), tandem duplicate 2 [Source:ZFIN;Acc:ZDB-GENE-040801-262]	21	21796663	21799292	
ENSDARG00000010083	2056.299308	2.318491511	0.123038701	10.71607147	8.56E-27	6.88E-25	rbfox3a	RNA binding fox-1 homolog 3a [Source:ZFIN;Acc:ZDB-GENE-111213-1]	12	32729470	33251465	
ENSDARG00000010085	2499.83727	1.86177217	0.101051045	8.528087677	1.49E-17	6.71E-16	p4ha2	procollagen-proline, 2-oxoglutarate 4-dioxygenase (proline 4-hydroxylase), alpha polypeptide 2 [Source:ZFIN;Acc:ZDB-GEI	21	43097798	43131752	
ENSDARG00000010113	6485.902548	-1.329454826	0.102401923	-3.21727187	0.001294159	0.012112112	ndufb8	NADH:ubiquinone oxidoreductase subunit B8 [Source:ZFIN;Acc:ZDB-GENE-040426-1858]	12	48469755	48477031	
ENSDARG00000010130	878.0028138	-2.233285703	0.141833359	-8.695314749	3.46E-18	1.63E-16	smpdl3b	sphingomyelin phosphodiesterase acid like 3B [Source:ZFIN;Acc:ZDB-GENE-041008-132]	16	4710977	4721629	
ENSDARG00000010144	1235.018921	1.868592774	0.149219094	5.820922452	5.85E-09	1.24E-07	plppr3a	phospholipid phosphatase related 3a [Source:ZFIN;Acc:ZDB-GENE-070912-555]	2	26179096	26209425	
ENSDARG00000010186	507.1422616	1.515874081	0.138328314	3.729345532	0.000191978	0.002115379	myo3a	myosin IIIA [Source:ZFIN;Acc:ZDB-GENE-041026-4]	24	6161037	6260333	
ENSDARG00000010250	3733.486809	-1.603699636	0.095689981	-6.30891166	2.81E-10	6.87E-09	nqo1	NAD(P)H dehydrogenase, quinone 1 [Source:ZFIN;Acc:ZDB-GENE-030131-1226]	7	56703254	56722320	
ENSDARG00000010270	148.1849962	2.009994336	0.244473907	4.131297077	3.61E-05	0.000452951	fezf1	FEZ family zinc finger 1 [Source:NCBI gene;Acc:558801]	25	28272908	28276091	
ENSDARG00000010301	1146.689328	1.571911856	0.110937201	5.155275712	2.53E-07	4.35E-06	b4galt6	UDP-Gal:betaGlcNAc beta 1,4- galactosyltransferase, polypeptide 6 [Source:ZFIN;Acc:ZDB-GENE-040426-789]	20	6163932	6196989	
ENSDARG00000010385	348.2228556	1.82162085	0.161954469	5.073159492	3.91E-07	6.62E-06	sept4a	septin 4a [Source:ZFIN;Acc:ZDB-GENE-051120-87]	21	39512437	39546737	
ENSDARG00000010420	1672.867127	2.485666529	0.145569289	10.20590636	1.87E-24	1.32E-22	ndrg1b	N-myc downstream regulated 1b [Source:ZFIN;Acc:ZDB-GENE-040426-1648]	16	31511739	31517227	
ENSDARG00000010658	2352.497766	1.350230683	0.108143593	3.238570798	0.001201302	0.011293589	insig1	insulin induced gene 1 [Source:NCBI gene;Acc:334189]	7	41007773	41014773	
ENSDARG00000010717	1793.78916	-1.428167981	0.096144533	-4.453378363	8.45E-06	0.000118734	chchd10	coiled-coil-helix-coiled-coil-helix domain containing 10 [Source:ZFIN;Acc:ZDB-GENE-040426-1753]	21	15704556	15708626	
ENSDARG00000010816	1475.925458	2.448981241	0.108921325	13.30300785	2.22E-40	3.47E-38	slc7a14a	solute carrier family 7 member 14a [Source:NCBI gene;Acc:566270]	2	25860344	25990784	
ENSDARG00000010862	1508.936018	-1.817313773	0.130849769	-6.24619958	4.21E-10	1.01E-08	amt	aminomethyltransferase [Source:ZFIN;Acc:ZDB-GENE-041010-114]	8	26396552	26408670	
ENSDARG00000010933	261.5293704	1.973338191	0.181102638	5.37451139	7.68E-08	1.41E-06	cacna1fa	calcium channel, voltage-dependent, L type, alpha 1F subunit a [Source:ZFIN;Acc:ZDB-GENE-091204-7]	8	44850710	44868020	
ENSDARG00000010936	1974.074615	-2.10058204	0.17223966	-6.389829399	1.66E-10	4.19E-09	abcb4	ATP-binding cassette, sub-family B (MDR/TAP), member 4 [Source:ZFIN;Acc:ZDB-GENE-080204-52]	16	28629242	28658341	
ENSDARG00000010965	6584.453531	-1.395586206	0.0884268	-4.473600816	7.69E-06	0.000109135	psma8	proteasome 20S subunit alpha 8 [Source:ZFIN;Acc:ZDB-GENE-040426-2194]	20	18306122	18313864	
ENSDARG00000011066	391.721819	1.83424245	0.163875433	5.090710889	3.57E-07	6.07E-06	glra3	glycine receptor, alpha 3 [Source:ZFIN;Acc:ZDB-GENE-020402-1]	1	38416106	38538284	
ENSDARG00000011113	78.762071	2.929716655	0.360525588	5.352509555	8.67E-08	1.59E-06	chrna10a	cholinergic receptor, nicotinic, alpha 10a [Source:ZFIN;Acc:ZDB-GENE-060503-725]	15	38179904	38193383	
ENSDARG00000011171	1741.183036	2.260285678	0.150042063	8.399549109	4.48E-17	1.94E-15	TENM2	si:ch211-12m10.1 [Source:ZFIN;Acc:ZDB-GENE-060503-100]	21	31434251	32008843	TENM2
ENSDARG00000011259	388.0905884	3.428176002	0.198222166	12.24977028	1.68E-34	1.99E-32	trpm1a	transient receptor potential cation channel, subfamily M, member 1a [Source:ZFIN;Acc:ZDB-GENE-070112-1372]	7	29312376	29341364	
ENSDARG00000011376	523.982578	-1.405502508	0.148118678	-2.737686515	0.006187302	0.049841937	dnase1l4.2	deoxyribonuclease 1 like 4, tandem duplicate 2 [Source:ZFIN;Acc:ZDB-GENE-040426-1884]	22	5682635	5700482	
ENSDARG00000011404	1245.544662	-1.623592574	0.108989531	-5.72158234	1.06E-08	2.17E-07	fen1	flap structure-specific endonuclease 1 [Source:NCBI gene;Acc:386707]	24	11908480	11921184	
ENSDARG00000011405	71839.44437	-1.576213456	0.114132059	-5.048655587	4.45E-07	7.49E-06	rps9	ribosomal protein S9 [Source:ZFIN;Acc:ZDB-GENE-010724-15]	16	31921812	31927522	
ENSDARG00000011553	23627.53164	-1.329038525	0.085345579	-3.855366959	0.000115556	0.001324464	atp5pb	ATP synthase peripheral stalk-membrane subunit b [Source:ZFIN;Acc:ZDB-GENE-041010-33]	8	25329032	25338709	
ENSDARG00000011581	400.5984733	-2.133250133	0.205445537	-5.516061102	3.47E-08	6.67E-07	si:dkey-23k10.5	si:dkey-23k10.5 [Source:ZFIN;Acc:ZDB-GENE-030131-4787]	15	36349009	36357889	
ENSDARG00000011582	2646.602446	1.414114728	0.097276039	4.25710929	2.07E-05	0.000270875	sox5	SRY-box transcription factor 5 [Source:ZFIN;Acc:ZDB-GENE-000607-13]	4	16982249	17055950	
ENSDARG00000011600	1390.843195	1.88276661	0.139441879	6.330713696	2.44E-10	6.04E-09	epha4b	eph receptor A4b [Source:ZFIN;Acc:ZDB-GENE-030826-6]	2	40294313	40514151	
ENSDARG00000011640	4760.871762	1.707496667	0.109866474	6.439604719	1.2E-10	3.08E-09	syt5b	synaptotagmin Vb [Source:ZFIN;Acc:ZDB-GENE-050522-134]	24	7686464	7699356	
ENSDARG00000011671	712.6082961	1.520883227	0.133351963	3.90607843	9.38E-05	0.001095685	pde6b	phosphodiesterase 6B, cGMP-specific, rod, beta [Source:ZFIN;Acc:ZDB-GENE-090421-2]	21	20356458	20376607	
ENSDARG00000011693	742.4603878	-2.114674906	0.134809366	-8.268527185	1.36E-16	5.63E-15	ttc36	tetratricopeptide repeat domain 36 [Source:NCBI gene;Acc:492516]	18	42781622	42830563	
ENSDARG00000011785	99.47674354	2.41572286	0.343447237	4.122097099	3.75E-05	0.000469551	tbx6	T-box transcription factor 6 [Source:NCBI gene;Acc:192294]	12	3912544	3923070	
ENSDARG00000011884	179.6681674	2.713643726	0.256993637	6.668039502	2.59E-11	7.02E-10	MFN1	mitofusin 1a [Source:NCBI gene;Acc:100329462]	2	5338759	5356686	MFN1
ENSDARG00000011941	1564.893659	1.532375166	0.105208792	5.060177558	4.19E-07	7.08E-06	bmpr2a	bone morphogenetic protein receptor, type II a (serine/threonine kinase) [Source:ZFIN;Acc:ZDB-GENE-070618-1]	6	12797648	12868401	
ENSDARG00000011983	1720.580872	-1.540108458	0.11913007	-4.533771015	5.79E-06	8.4E-05	zgc:136908	zgc:136908 [Source:ZFIN;Acc:ZDB-GENE-060312-22]	1	55786986	55810730	
ENSDARG00000011989	3456.057629	2.628559769	0.143796686	11.32543326	9.82E-30	9.48E-28	crx	cone-rod homeobox [Source:ZFIN;Acc:ZDB-GENE-010403-1]	5	36932718	36936443	
ENSDARG00000012019	1899.571882	1.687550837	0.157414616	4.367769998	1.26E-05	0.000171608	glra1	glycine receptor, alpha 1 [Source:NCBI gene;Acc:30676]	14	25816874	25911567	
ENSDARG00000012044	341.3919201	-1.631544882	0.180994089	-3.489312205	0.000484265	0.004955377	polr3gla	polymerase (RNA) III (DNA directed) polypeptide G like a [Source:ZFIN;Acc:ZDB-GENE-040912-67]	19	24545731	24555949	
ENSDARG00000012137	216.182314	-2.3912792	0.217795133	-6.388017848	1.68E-10	4.24E-09	cyp46a1.1	cytochrome P450, family 46, subfamily A, polypeptide 1, tandem duplicate 1 [Source:ZFIN;Acc:ZDB-GENE-050522-464]	20	5106568	5120937	
ENSDARG00000012194	6382.363749	-2.108710276	0.110958131	-9.992149902	1.65E-23	1.1E-21	scp2a	sterol carrier protein 2a [Source:ZFIN;Acc:ZDB-GENE-040426-1846]	2	6005199	6039757	
ENSDARG00000012297	211.526823	2.077817352	0.203338361	5.300610012	1.15E-07	2.08E-06	cnga3b	cyclic nucleotide gated channel subunit alpha 3b [Source:ZFIN;Acc:ZDB-GENE-090312-121]	1	10771457	10776547	
ENSDARG00000012306	1083.642789	1.715452478	0.161195687	4.438409559	9.06E-06	0.000126308	syt13	synaptotagmin XIII [Source:ZFIN;Acc:ZDB-GENE-050417-135]	7	38685654	38737718	
ENSDARG00000012311	705.0075791	-1.628612457	0.115275286	-5.453141611	4.95E-08	9.35E-07	myoz2a	myozenin 2a [Source:ZFIN;Acc:ZDB-GENE-040426-1880]	7	69829442	69857692	
ENSDARG00000012341	1152.039346	-1.600562021	0.130665898	-4.596164943	4.3E-06	6.35E-05	capn9	calpain 9 [Source:ZFIN;Acc:ZDB-GENE-010724-2]	1	53881844	53907092	
ENSDARG00000012367	498.2900693	1.773059364	0.205867637	3.755128172	0.000173253	0.00193195	trim46a	tripartite motif containing 46a [Source:ZFIN;Acc:ZDB-GENE-110825-1]	16	23353541	23379464	
ENSDARG00000012381	7465.615002	-1.877312789	0.090419923	-9.702649129	2.94E-22	1.81E-20	hsc70	heat shock cognate 70 [Source:ZFIN;Acc:ZDB-GENE-040426-1221]	15	37834594	37850969	
ENSDARG00000012422	23799.15253	1.797003868	0.183620979	4.340483712	1.42E-05	0.000192897	col11a2	collagen, type XI, alpha 2 [Source:ZFIN;Acc:ZDB-GENE-000208-23]	19	7179740	7225206	
ENSDARG00000012535	44.70924845	-2.018413273	0.368116804	-2.766549263	0.005665302	0.046110492	epx	eosinophil peroxidase [Source:ZFIN;Acc:ZDB-GENE-130425-2]	25	3119810	3139805	
ENSDARG00000012581	370.8490541	1.732660825	0.162367882	4.512350703	6.41E-06	9.21E-05	AL590149.1		17	33206780	33231714	
ENSDARG00000012610	2217.847764	1.390246291	0.115922411	3.366443869	0.000761441	0.007457247	saga	S-antigen; retina and pineal gland (arrestin) a [Source:ZFIN;Acc:ZDB-GENE-040426-1538]	2	5480125	5505094	
ENSDARG00000012640	871.1146983	-2.116542439	0.133555946	-8.360110277	6.27E-17	2.66E-15	cideb	cell death inducing DFFA like effector b [Source:ZFIN;Acc:ZDB-GENE-080514-2]	7	24015507	24022340	
ENSDARG00000012667	1907.849409	1.406774331	0.135334493	3.005695908	0.002649738	0.023320857	tfap2b	transcription factor AP-2 beta [Source:ZFIN;Acc:ZDB-GENE-050417-394]	20	47686629	47704973	
ENSDARG00000012796	553.3678031	-2.40808894	0.164628787	-8.553114933	1.2E-17	5.44E-16	hnmt	histamine N-methyltransferase [Source:NCBI gene;Acc:445242]	9	10138196	10145880	
ENSDARG00000012801	1540.059333	1.837956834	0.103815134	8.071625067	6.94E-16	2.74E-14	pfkpb	phosphofructokinase, platelet b [Source:ZFIN;Acc:ZDB-GENE-070912-634]	2	48119494	48171441	
ENSDARG00000012823	422.3357215	1.531318265	0.147380593	3.605076191	0.000312061	0.00331628	dlgap4b	discs, large (Drosophila) homolog-associated protein 4b [Source:ZFIN;Acc:ZDB-GENE-101215-2]	6	1831111	1874664	
ENSDARG00000012829	979.669	-4.273438907	0.186552281	-17.54703238	6.27E-69	2.46E-66	asah2	N-acylsphingosine amidohydrolase 2 [Source:NCBI gene;Acc:493602]	12	6117682	6142673	
ENSDARG00000012896	1821.421447	1.475612639	0.101383413	4.691227332	2.72E-06	4.12E-05	anos1a	anosmin 1a [Source:ZFIN;Acc:ZDB-GENE-000201-9]	1	31942961	31979510	
ENSDARG00000012903	742.4281108	-4.185680793	0.176561921	-18.04285303	8.98E-73	3.69E-70	slc34a2a	solute carrier family 34 member 2a [Source:ZFIN;Acc:ZDB-GENE-000524-1]	1	14432434	14442902	
ENSDARG00000012915	1345.911171	-1.732811382	0.109485271	-6.693241712	2.18E-11	5.97E-10	ech1	enoyl CoA hydratase 1, peroxisomal [Source:ZFIN;Acc:ZDB-GENE-041010-170]	15	6926987	6946286	
ENSDARG00000012972	16319.54638	-1.432047532	0.083597979	-5.168157604	2.36E-07	4.08E-06	cfl1l	cofilin 1 (non-muscle), like [Source:ZFIN;Acc:ZDB-GENE-040426-2770]	13	33606739	33609427	
ENSDARG00000013000	1223.235243	2.008072107	0.176091938	5.724691984	1.04E-08	2.14E-07	ppfia2	PTPRF interacting protein alpha 2 [Source:ZFIN;Acc:ZDB-GENE-030131-7905]	4	21962316	22207873	
ENSDARG00000013020	675.7844337	1.676689036	0.179858521	3.762340703	0.000168331	0.001880001	dtnbb	dystrobrevin, beta b [Source:ZFIN;Acc:ZDB-GENE-070117-3]	17	37070096	37156520	
ENSDARG00000013022	13.22630235	5.302804468	1.570466024	2.739826525	0.006147162	0.049566307	si:ch211-59h6.1	si:ch211-59h6.1 [Source:ZFIN;Acc:ZDB-GENE-091204-418]	22	7675388	7702958	
ENSDARG00000013044	4606.394539	-1.323172478	0.105724765	-3.05673395	0.002237628	0.019959736	ndufv2	NADH:ubiquinone oxidoreductase core subunit V2 [Source:ZFIN;Acc:ZDB-GENE-040426-1713]	2	54641644	54690184	
ENSDARG00000013056	1324.189	-1.900907365	0.142418006	-6.325796782	2.52E-10	6.22E-09	itpk1a	inositol-tetrakisphosphate 1-kinase a [Source:NCBI gene;Acc:406290]	20	27049523	27086143	
ENSDARG00000013134	186.9902237	2.085440294	0.230679981	4.705394413	2.53E-06	3.87E-05	prph2la	peripherin 2-like a [Source:ZFIN;Acc:ZDB-GENE-130530-548]	17	39873584	39886628	
ENSDARG00000013295	4477.143202	1.29952454	0.10698463	2.799696935	0.00511506	0.042123731	slc2a3a	solute carrier family 2 member 3a [Source:ZFIN;Acc:ZDB-GENE-040718-390]	19	9685165	9712530	
ENSDARG00000013317	6242.404795	1.879920386	0.105038859	8.377093905	5.43E-17	2.32E-15	pygmb	phosphorylase, glycogen, muscle b [Source:ZFIN;Acc:ZDB-GENE-040426-1206]	7	22823889	22852189	
ENSDARG00000013333	7291.81262	-1.280605378	0.080290986	-3.49485529	0.000474319	0.004859542	ndufa10	NADH:ubiquinone oxidoreductase subunit A10 [Source:ZFIN;Acc:ZDB-GENE-030131-670]	9	23740506	23747409	
ENSDARG00000013343	2276.425694	1.500028824	0.114857288	4.353479286	1.34E-05	0.000182297	kif26ba	kinesin family member 26Ba [Source:ZFIN;Acc:ZDB-GENE-070521-4]	17	11507131	11718491	
ENSDARG00000013371	1078.282876	-1.462011331	0.109786555	-4.208268763	2.57E-05	0.000330233	isoc2	isochorismatase domain containing 2 [Source:NCBI gene;Acc:492617]	16	12793911	12809886	
ENSDARG00000013389	97.90003	2.044358478	0.295601748	3.532991553	0.000410886	0.004251294	gabra4	gamma-aminobutyric acid (GABA) A receptor, subunit alpha 4 [Source:ZFIN;Acc:ZDB-GENE-050417-360]	14	4610439	4682114	
ENSDARG00000013478	759.46984	-1.369512655	0.118370444	-3.121663173	0.001798326	0.01630914	rrp15	ribosomal RNA processing 15 homolog [Source:NCBI gene;Acc:327053]	16	4196932	4203022	
ENSDARG00000013491	207.7243852	1.993345596	0.211453532	4.697701611	2.63E-06	4.01E-05	map3k9	mitogen-activated protein kinase kinase kinase 9 [Source:ZFIN;Acc:ZDB-GENE-081113-5]	13	36164639	36184701	
ENSDARG00000013500	1473.044832	1.586820555	0.102563303	5.721545009	1.06E-08	2.17E-07	abca2	ATP-binding cassette, sub-family A (ABC1), member 2 [Source:ZFIN;Acc:ZDB-GENE-050517-1]	5	25088622	25236340	
ENSDARG00000013615	4190.237551	1.555297204	0.133453505	4.160978797	3.17E-05	0.000400714	pbx3b	pre-B-cell leukemia homeobox 3b [Source:ZFIN;Acc:ZDB-GENE-000405-3]	8	33895773	34065573	
ENSDARG00000013669	2544.999271	2.088287617	0.135960147	8.004460417	1.2E-15	4.68E-14	napba	N-ethylmaleimide-sensitive factor attachment protein, beta a [Source:ZFIN;Acc:ZDB-GENE-050522-230]	17	4297076	4318428	
ENSDARG00000013749	1390.939575	-2.26344611	0.140045297	-9.021696087	1.85E-19	9.57E-18	slc25a55b	solute carrier family 25 member 55b [Source:ZFIN;Acc:ZDB-GENE-040801-184]	23	409578	419441	
ENSDARG00000013838	4922.797401	1.657095462	0.089262894	7.361350653	1.82E-13	5.97E-12	sulf2b	sulfatase 2b [Source:ZFIN;Acc:ZDB-GENE-030131-775]	23	15203822	15330230	
ENSDARG00000013855	386.4302822	3.843290123	0.234066733	12.14734828	5.93E-34	6.7E-32	slc12a3	solute carrier family 12 member 3 [Source:ZFIN;Acc:ZDB-GENE-030131-9505]	18	17583479	17598755	
ENSDARG00000013856	4480.809762	-5.916092048	0.158419029	-31.03220672	1.98E-211	4.98E-208	amy2a	amylase alpha 2A [Source:ZFIN;Acc:ZDB-GENE-040426-2393]	2	15612755	15626972	
ENSDARG00000013871	704.0593447	-1.618477931	0.173843621	-3.557668239	0.000374161	0.003911631	slc5a1	solute carrier family 5 member 1 [Source:ZFIN;Acc:ZDB-GENE-040426-1524]	10	17135631	17159761	
ENSDARG00000013926	694.9534282	2.375863323	0.133308334	10.32091001	5.67E-25	4.1E-23	slc16a9a	solute carrier family 16 member 9a [Source:ZFIN;Acc:ZDB-GENE-040426-1364]	17	20653773	20666616	
ENSDARG00000013931	12029.31425	-1.305004503	0.108708792	-2.805702255	0.005020706	0.041464777	eif3m	eukaryotic translation initiation factor 3, subunit M [Source:NCBI gene;Acc:334626]	18	45645357	45658076	
ENSDARG00000013963	1039.111022	4.301955255	0.209446604	15.76514104	5.41E-56	1.56E-53	mipb	major intrinsic protein of lens fiber b [Source:ZFIN;Acc:ZDB-GENE-050706-86]	23	6637611	6641223	
ENSDARG00000013966	2150.400145	-1.301696774	0.104536756	-2.886035353	0.003901283	0.033083146	psma6b	proteasome 20S subunit alpha 6b [Source:ZFIN;Acc:ZDB-GENE-040718-329]	20	16884386	16906767	
ENSDARG00000014031	4331.998371	-1.928649631	0.143710773	-6.461934694	1.03E-10	2.67E-09	abcc2	ATP-binding cassette, sub-family C (CFTR/MRP), member 2 [Source:ZFIN;Acc:ZDB-GENE-040426-1523]	13	681628	711576	
ENSDARG00000014179	6394.910887	-1.376193287	0.105191218	-3.576280371	0.000348518	0.003666449	pfkma	phosphofructokinase, muscle a [Source:ZFIN;Acc:ZDB-GENE-040912-135]	23	27553786	27571667	
ENSDARG00000014201	1601.378971	3.104376988	0.210714995	9.986840239	1.74E-23	1.16E-21	otpa	orthopedia homeobox a [Source:ZFIN;Acc:ZDB-GENE-070216-1]	21	7582036	7586992	
ENSDARG00000014232	337.6808288	-2.209840829	0.183803639	-6.582246341	4.63E-11	1.23E-09	prkg3	protein kinase cGMP-dependent 3 [Source:ZFIN;Acc:ZDB-GENE-030131-4745]	20	616114	641511	
ENSDARG00000014274	602.3433771	-1.403372491	0.143766782	-2.805741957	0.005020088	0.041464777	rfc2	replication factor C (activator 1) 2 [Source:ZFIN;Acc:ZDB-GENE-050306-29]	21	25206289	25213616	
ENSDARG00000014280	151.8742347	2.092033205	0.281507199	3.87923722	0.000104785	0.001211514	dlgap1a	discs, large (Drosophila) homolog-associated protein 1a [Source:NCBI gene;Acc:569898]	2	29491314	29692825	
ENSDARG00000014313	6002.281554	-1.297917621	0.081814197	-3.6413927	0.000271167	0.00292002	atp5pf	ATP synthase peripheral stalk subunit F6 [Source:ZFIN;Acc:ZDB-GENE-040426-2534]	1	610082	614638	
ENSDARG00000014420	10906.06972	2.152341211	0.131001083	8.796425079	1.41E-18	6.83E-17	elavl3	ELAV like neuron-specific RNA binding protein 3 [Source:ZFIN;Acc:ZDB-GENE-980526-76]	3	46800190	46818001	
ENSDARG00000014496	1466.055323	-2.146987252	0.111075752	-10.32617134	5.37E-25	3.89E-23	trpv6	transient receptor potential cation channel, subfamily V, member 6 [Source:ZFIN;Acc:ZDB-GENE-040624-12]	16	12294284	12319822	
ENSDARG00000014499	1015.253463	-1.506192787	0.106449386	-4.755243833	1.98E-06	3.08E-05	nutf2l	nuclear transport factor 2, like [Source:ZFIN;Acc:ZDB-GENE-020416-1]	19	20778011	20783758	
ENSDARG00000014506	844.0015737	-1.450337637	0.124497384	-3.617245787	0.000297755	0.003171936	tmem126a	transmembrane protein 126A [Source:ZFIN;Acc:ZDB-GENE-040426-1770]	10	29269998	29272757	
ENSDARG00000014550	746.7952279	1.839745899	0.127130103	6.605405633	3.96E-11	1.06E-09	rfx3	regulatory factor X, 3 (influences HLA class II expression) [Source:NCBI gene;Acc:570989]	10	600164	641609	
ENSDARG00000014572	4977.161967	-1.541551151	0.125760649	-4.306205126	1.66E-05	0.000222215	chpt1	choline phosphotransferase 1 [Source:NCBI gene;Acc:322605]	4	17707008	17725008	
ENSDARG00000014746	4946.914295	1.33276181	0.086143554	3.862875355	0.00011206	0.001287923	rbfox1	RNA binding fox-1 homolog 1 [Source:NCBI gene;Acc:449554]	3	27982269	28428198	
ENSDARG00000014792	971.5268895	1.781492775	0.151560306	5.156315641	2.52E-07	4.33E-06	lrrc4bb	leucine rich repeat containing 4Bb [Source:ZFIN;Acc:ZDB-GENE-091116-44]	24	38369351	38384432	
ENSDARG00000014803	229.47186	2.952877751	0.265457328	7.356654145	1.89E-13	6.18E-12	cryba1l2	crystallin, beta A1, like 2 [Source:ZFIN;Acc:ZDB-GENE-041010-40]	1	44421920	44434707	
ENSDARG00000014805	309.7071198	-3.105779282	0.172294629	-12.22196707	2.37E-34	2.78E-32	fhl5	four and a half LIM domains 5 [Source:ZFIN;Acc:ZDB-GENE-040822-3]	17	15605572	15611744	
ENSDARG00000014817	4962.856773	-1.301141029	0.086628918	-3.476218309	0.000508538	0.005167942	ranbp1	RAN binding protein 1 [Source:ZFIN;Acc:ZDB-GENE-040426-2548]	5	19006701	19014589	

ENSDARG00000014828	344.4773467	1.631704325	0.193495436	3.264698835	0.001095805	0.010391313	l3mbtl1a	L3MBTL histone methyl-lysine binding protein 1a [Source:ZFIN;Acc:ZDB-GENE-090312-120]	23	4299887	4318418	
ENSDARG00000014840	3098.864129	1.67192304	0.195197914	3.442265467	0.000576864	0.005796581	prph2b	peripherin 2b (retinal degeneration, slow) [Source:ZFIN;Acc:ZDB-GENE-000616-9]	13	3252950	3258772	
ENSDARG00000014849	48.64604474	2.544090425	0.467636381	3.301903973	0.000960309	0.009210824	pih1d2	PIH1 domain containing 2 [Source:NCBI gene;Acc:494086]	15	18157665	18162647	
ENSDARG00000014891	2658.993912	2.316352261	0.099582837	13.21866606	6.85E-40	1.05E-37	robo2	roundabout, axon guidance receptor, homolog 2 (Drosophila) [Source:ZFIN;Acc:ZDB-GENE-001019-1]	15	39096736	39228226	
ENSDARG00000014927	279.7683982	2.212285759	0.208364172	5.818110412	5.95E-09	1.26E-07	uts1	urotensin 1 [Source:ZFIN;Acc:ZDB-GENE-041014-348]	20	38724575	38727396	
ENSDARG00000014941	350.2434127	-1.416012741	0.15040006	-2.766041061	0.005674139	0.046152483	tprkb	Tp53rk binding protein [Source:NCBI gene;Acc:492501]	4	28371732	28375127	
ENSDARG00000014973	446.8178783	2.110974996	0.152581273	7.281201499	3.31E-13	1.05E-11	ntng1a	netrin g1a [Source:ZFIN;Acc:ZDB-GENE-091118-13]	24	29381946	29529252	
ENSDARG00000015002	943.5115556	1.735702365	0.144281212	5.099086384	3.41E-07	5.82E-06	cdh4	cadherin 4, type 1, R-cadherin (retinal) [Source:ZFIN;Acc:ZDB-GENE-991207-1]	11	20371179	20826753	
ENSDARG00000015003	1274.18816	1.425605266	0.143737944	2.960980612	0.003066612	0.026607341	arhgap4b	Rho GTPase activating protein 4b [Source:ZFIN;Acc:ZDB-GENE-090311-38]	23	18615992	18668836	
ENSDARG00000015025	2070.389733	2.689375048	0.152964419	11.04423536	2.34E-28	2.07E-26	l1camb	L1 cell adhesion molecule, paralog b [Source:NCBI gene;Acc:30656]	23	597082	637347	
ENSDARG00000015033	467.7341374	-1.615161934	0.181322498	-3.392639852	0.000692226	0.006846163	slc23a1	solute carrier family 23 member 1 [Source:ZFIN;Acc:ZDB-GENE-080804-2]	21	30091417	30111134	
ENSDARG00000015053	1387.832143	1.498868766	0.116627417	4.27745703	1.89E-05	0.000250269	grip1	glutamate receptor interacting protein 1 [Source:ZFIN;Acc:ZDB-GENE-041210-125]	4	13035870	13255813	
ENSDARG00000015076	218.1260896	4.205747553	0.297446553	10.77755825	4.39E-27	3.6E-25	gja8b	gap junction protein alpha 8 paralog b [Source:ZFIN;Acc:ZDB-GENE-010607-3]	1	47446032	47448850	
ENSDARG00000015123	1585.128267	-1.630139142	0.104004669	-6.058758215	1.37E-09	3.09E-08	dnase1l4.1	deoxyribonuclease 1 like 4, tandem duplicate 1 [Source:ZFIN;Acc:ZDB-GENE-040808-35]	22	5667371	5682494	
ENSDARG00000015134	449.5566436	1.947859122	0.230602037	4.110367517	3.95E-05	0.000492092	camkk1b	calcium/calmodulin-dependent protein kinase kinase 1, alpha b [Source:ZFIN;Acc:ZDB-GENE-080722-13]	15	15314189	15331501	
ENSDARG00000015164	11734.93248	2.208113854	0.121038222	9.981259099	1.84E-23	1.22E-21	mknk2b	MAPK interacting serine/threonine kinase 2b [Source:ZFIN;Acc:ZDB-GENE-030829-2]	8	20438884	20450375	
ENSDARG00000015174	4278.77242	1.47027989	0.164030418	2.867028539	0.004143456	0.034830844	atp6v0a1b	ATPase H+ transporting V0 subunit a1b [Source:ZFIN;Acc:ZDB-GENE-050522-215]	24	7596009	7632187	
ENSDARG00000015228	563.486504	1.747424707	0.16695876	4.476702555	7.58E-06	0.000107684	ero1a	endoplasmic reticulum oxidoreductase alpha [Source:NCBI gene;Acc:393321]	17	15018219	15029639	
ENSDARG00000015240	823.2032229	1.966088667	0.139625686	6.919132826	4.54E-12	1.34E-10	cdkl5	cyclin-dependent kinase-like 5 [Source:ZFIN;Acc:ZDB-GENE-081022-110]	11	30161168	30232133	
ENSDARG00000015263	1199.254723	1.429149513	0.130786704	3.281293132	0.001033323	0.009847167	adma	adrenomedullin a [Source:ZFIN;Acc:ZDB-GENE-120221-5]	7	66874770	66877058	
ENSDARG00000015355	399.8605377	2.967817099	0.178087612	11.04971352	2.2E-28	1.95E-26	fosl1a	FOS-like antigen 1a [Source:ZFIN;Acc:ZDB-GENE-061207-7]	14	30739476	30747686	
ENSDARG00000015422	1005.486321	-1.404854994	0.121276715	-3.338274736	0.000843004	0.00816682	ppil1	peptidylprolyl isomerase (cyclophilin)-like 1 [Source:ZFIN;Acc:ZDB-GENE-051009-1]	6	40647788	40657672	
ENSDARG00000015445	979.9636425	2.043652651	0.205212494	5.085716904	3.66E-07	6.22E-06	lim2.4	lens intrinsic membrane protein 2.4 [Source:ZFIN;Acc:ZDB-GENE-040718-104]	19	10537443	10554352	
ENSDARG00000015537	6122.784388	1.994323321	0.323635746	3.072353199	0.002123783	0.019005016	gad2	glutamate decarboxylase 2 [Source:ZFIN;Acc:ZDB-GENE-030909-9]	24	6133147	6158933	
ENSDARG00000015551	46159.27667	-1.311494517	0.086051993	-3.619840824	0.000294784	0.003141988	fth1a	ferritin, heavy polypeptide 1a [Source:ZFIN;Acc:ZDB-GENE-000831-2]	7	12927944	12936708	
ENSDARG00000015654	1263.20198	-1.421174497	0.111290418	-3.784463259	0.000154041	0.001732336	ca15a	carbonic anhydrase XVa [Source:ZFIN;Acc:ZDB-GENE-070424-7]	12	4320041	4331117	
ENSDARG00000015662	10916.10894	-1.520655781	0.091265105	-5.704872403	1.16E-08	2.37E-07	pla2g12b	phospholipase A2, group XIIB [Source:ZFIN;Acc:ZDB-GENE-040426-2771]	13	4671698	4683595	
ENSDARG00000015678	22.11498168	7.518956208	1.507548384	4.324210274	1.53E-05	0.000206361	si:dkeyp-75b4.9	si:dkeyp-75b4.9 [Source:ZFIN;Acc:ZDB-GENE-090313-396]	1	9986675	9988840	
ENSDARG00000015866	184124.8726	-1.566006255	0.098923534	-5.72165419	1.05E-08	2.17E-07	apoa2	apolipoprotein A-II [Source:ZFIN;Acc:ZDB-GENE-030131-1046]	16	23978978	23980354	
ENSDARG00000015869	1065.714247	-2.31963805	0.124456768	-10.60318431	2.88E-26	2.22E-24	slc22a16	solute carrier family 22 member 16 [Source:ZFIN;Acc:ZDB-GENE-041001-166]	20	31287356	31305503	
ENSDARG00000015876	435.5701577	1.881619024	0.158549567	5.56052622	2.69E-08	5.26E-07	npas1	neuronal PAS domain protein 1 [Source:ZFIN;Acc:ZDB-GENE-060503-934]	15	8424711	8517555	
ENSDARG00000015921	1404.038707	-1.550305151	0.103194651	-5.332690671	9.68E-08	1.76E-06	pwp1	PWP1 homolog, endonuclein [Source:ZFIN;Acc:ZDB-GENE-040426-1049]	4	1825839	1839352	
ENSDARG00000015931	4458.864616	1.848400917	0.104311534	8.133337546	4.18E-16	1.66E-14	snap91b	synaptosome associated protein 91b [Source:ZFIN;Acc:ZDB-GENE-041210-81]	4	5260941	5302866	
ENSDARG00000016044	232.1831761	-1.507600739	0.172919788	-2.935469358	0.003330435	0.028648818	gins4	GINS complex subunit 4 (Sld5 homolog) [Source:ZFIN;Acc:ZDB-GENE-040801-59]	8	52415603	52431522	
ENSDARG00000016074	1060.433667	-1.519594024	0.107626547	-4.82774962	1.38E-06	2.19E-05	cryz	crystallin, zeta (quinone reductase) [Source:ZFIN;Acc:ZDB-GENE-050306-24]	2	11100613	11120220	
ENSDARG00000016081	3195.26636	-2.655101166	0.116332414	-14.22734307	6.2E-46	1.21E-43	cldn15la	claudin 15-like a [Source:ZFIN;Acc:ZDB-GENE-010328-12]	2	5508542	5517438	
ENSDARG00000016085	2157.711199	1.368706956	0.105166063	3.505949976	0.000454981	0.004678603	mbnl3	muscleblind-like splicing regulator 3 [Source:ZFIN;Acc:ZDB-GENE-081002-3]	14	30986754	31080183	
ENSDARG00000016188	2033.511264	1.906710064	0.120812667	7.505091049	6.14E-14	2.09E-12	SBNO2	si:ch73-63e15.2 [Source:ZFIN;Acc:ZDB-GENE-091204-463]	22	17691381	17781213	SBNO2
ENSDARG00000016200	1161.292616	1.336494897	0.120117531	2.801380392	0.00508845	0.041932102	trib3	tribbles pseudokinase 3 [Source:ZFIN;Acc:ZDB-GENE-040426-2609]	11	24058530	24063196	
ENSDARG00000016290	121.5118341	-5.187746237	0.378256528	-11.07118033	1.73E-28	1.55E-26	clca1	chloride channel accessory 1 [Source:ZFIN;Acc:ZDB-GENE-030131-6221]	15	14109652	14120431	
ENSDARG00000016337	151.6569098	2.172358547	0.349302715	3.356282383	0.000789979	0.007712662	lctlb	lactase-like b [Source:ZFIN;Acc:ZDB-GENE-060503-93]	18	19463425	19484919	
ENSDARG00000016348	1293.802071	1.89531991	0.144468399	6.197340828	5.74E-10	1.36E-08	dip2bb	disco-interacting protein 2 homolog Bb [Source:ZFIN;Acc:ZDB-GENE-100729-4]	6	39531135	39605734	
ENSDARG00000016357	1072.564978	-1.391234351	0.108651951	-3.600803733	0.000317235	0.003366983	fmo5	flavin containing dimethylaniline monoxygenase 5 [Source:ZFIN;Acc:ZDB-GENE-030131-6606]	20	15093609	15104709	
ENSDARG00000016368	647.5572083	-1.62014997	0.135073322	-4.591209885	4.41E-06	6.5E-05	zbtb8os	zinc finger and BTB domain containing 8 opposite strand [Source:NCBI gene;Acc:550382]	13	44857087	44888950	
ENSDARG00000016404	2151.603577	1.420005733	0.10704739	3.923549493	8.73E-05	0.001025835	ptch1	patched 1 [Source:ZFIN;Acc:ZDB-GENE-980526-196]	8	29962635	30068277	
ENSDARG00000016480	762.171605	1.551466243	0.123885075	4.451434053	8.53E-06	0.000119614	slc17a7a	solute carrier family 17 member 7a [Source:ZFIN;Acc:ZDB-GENE-050105-5]	3	16612574	16654088	
ENSDARG00000016483	233.1408616	-1.638630402	0.223827428	-2.853226738	0.004327773	0.036234634	baiap2l2a	BAR/IMD domain containing adaptor protein 2 like 2a [Source:ZFIN;Acc:ZDB-GENE-040108-10]	3	24437363	24458281	
ENSDARG00000016484	3104.592197	-1.366825624	0.090909535	-4.035062134	5.46E-05	0.000664183	dkc1	dyskeratosis congenita 1, dyskerin [Source:ZFIN;Acc:ZDB-GENE-031118-120]	21	43702010	43716471	
ENSDARG00000016551	2114.353348	1.407949604	0.103597199	3.937843955	8.22E-05	0.00097254	iqsec1b	IQ motif and Sec7 domain ArfGEF 1b [Source:ZFIN;Acc:ZDB-GENE-120813-1]	11	26704181	27057572	
ENSDARG00000016649	1310.749802	-1.584250515	0.099847909	-5.851404602	4.87E-09	1.05E-07	akr7a3	aldo-keto reductase family 7, member A3 (aflatoxin aldehyde reductase) [Source:ZFIN;Acc:ZDB-GENE-040718-62]	11	41838036	41850086	
ENSDARG00000016667	1393.687244	1.61841602	0.185068762	3.341547288	0.000833128	0.00808363	gabbr1b	gamma-aminobutyric acid (GABA) B receptor, 1b [Source:ZFIN;Acc:ZDB-GENE-060503-5]	19	27040533	27261102	
ENSDARG00000016676	4652.289541	1.831017605	0.104303292	7.967319054	1.62E-15	6.23E-14	gnao1a	guanine nucleotide binding protein (G protein), alpha activating activity polypeptide O, a [Source:ZFIN;Acc:ZDB-GENE-040	18	22793465	22975655	
ENSDARG00000016704	600.8448395	-2.767393827	0.167480213	-10.55285158	4.93E-26	3.74E-24	pbld	phenazine biosynthesis like protein domain containing [Source:ZFIN;Acc:ZDB-GENE-050522-217]	13	22839821	22843805	
ENSDARG00000016739	439.3504924	1.897199026	0.200912475	4.465621291	7.98E-06	0.000113092	lrrc4cb	leucine rich repeat containing 4C, genome duplicate b [Source:ZFIN;Acc:ZDB-GENE-080327-5]	7	50053339	50113581	
ENSDARG00000016754	2128.071806	-1.307859194	0.102097998	-3.01533037	0.002566995	0.022656166	tbca	tubulin cofactor a [Source:ZFIN;Acc:ZDB-GENE-040426-962]	21	7545062	7570765	
ENSDARG00000016771	78022.78182	-2.135056435	0.087737829	-12.93691047	2.79E-38	3.95E-36	tfa	transferrin-a [Source:ZFIN;Acc:ZDB-GENE-980526-35]	2	16780643	16794710	
ENSDARG00000016825	15.11695296	5.499083632	1.56418294	2.876315498	0.004023473	0.034004446	vtg6	vitellogenin 6 [Source:ZFIN;Acc:ZDB-GENE-001201-5]	22	24811396	24818066	
ENSDARG00000016830	9119.061511	1.722013405	0.087513545	8.250304604	1.58E-16	6.51E-15	rimkla	ribosomal modification protein rimK-like family member A [Source:NCBI gene;Acc:447815]	8	26868105	26887308	
ENSDARG00000016855	1060.334652	-1.46376413	0.11771195	-3.939822	8.15E-05	0.000965011	sf3b5	splicing factor 3b, subunit 5 [Source:ZFIN;Acc:ZDB-GENE-040718-181]	13	23897975	23901732	
ENSDARG00000016918	5902.232619	-3.247178383	0.147486218	-15.23653139	2.02E-52	5.4E-50	ace2	angiotensin I converting enzyme 2 [Source:ZFIN;Acc:ZDB-GENE-041114-6]	11	29981678	29996357	
ENSDARG00000016923	54.72547728	2.648238876	0.43882795	3.756002495	0.000172649	0.001926072	si:dkey-94f20.4	si:dkey-94f20.4 [Source:ZFIN;Acc:ZDB-GENE-121214-276]	12	4573696	4576816	
ENSDARG00000016934	267.1248101	1.546095114	0.174673624	3.126374211	0.001769762	0.016095216	ttyh2	tweety family member 2 [Source:ZFIN;Acc:ZDB-GENE-040718-193]	12	38563073	38687993	
ENSDARG00000016999	210.4555853	1.772855155	0.196888284	3.925348619	8.66E-05	0.001018675	lin28a	lin-28 homolog A (C. elegans) [Source:NCBI gene;Acc:394066]	19	15440841	15473157	
ENSDARG00000017007	763.9260129	2.786815125	0.180436342	9.902745244	4.05E-23	2.61E-21	nr4a2a	nuclear receptor subfamily 4, group A, member 2a [Source:ZFIN;Acc:ZDB-GENE-990415-184]	9	5040985	5046315	
ENSDARG00000017162	1059.241224	1.381254265	0.119310323	3.195484316	0.001395964	0.012958611	kif3ca	kinesin family member 3Ca [Source:ZFIN;Acc:ZDB-GENE-111214-2]	20	47383841	47422469	
ENSDARG00000017193	49.31852814	-3.737648024	0.560972323	-4.880183766	1.06E-06	1.7E-05	cnn1a	calponin 1, basic, smooth muscle, a [Source:ZFIN;Acc:ZDB-GENE-090312-141]	1	52214895	52222989	
ENSDARG00000017314	36602.07497	-3.522437688	0.132670203	-19.01284258	1.34E-80	6.57E-78	cela1.6	chymotrypsin like elastase family member 1, tandem duplicate 6 [Source:ZFIN;Acc:ZDB-GENE-040808-55]	22	7439186	7447391	
ENSDARG00000017338	11061.22255	1.813033474	0.098379748	8.264236183	1.41E-16	5.81E-15	kidins220b	kinase D-interacting substrate 220b [Source:NCBI gene;Acc:335881]	20	29743904	29783644	
ENSDARG00000017388	1280.63074	-1.695844459	0.110606341	-6.291180517	3.15E-10	7.68E-09	gstt1b	glutathione S-transferase theta 1b [Source:ZFIN;Acc:ZDB-GENE-040426-1491]	21	15713097	15720534	
ENSDARG00000017391	488.7466539	2.080010507	0.16597286	6.507151286	7.66E-11	1.99E-09	unc13ba	unc-13 homolog Ba (C. elegans) [Source:ZFIN;Acc:ZDB-GENE-100921-10]	5	19094153	19237700	
ENSDARG00000017398	81.20154426	1.83241392	0.302673359	2.750205447	0.005955791	0.048162471	slc44a5a	solute carrier family 44 member 5a [Source:ZFIN;Acc:ZDB-GENE-070705-333]	2	11216814	11468654	
ENSDARG00000017429	846.6775848	2.087874619	0.121690631	8.939674385	3.9E-19	1.98E-17	sphkap	SPHK1 interactor, AKAP domain containing [Source:NCBI gene;Acc:100034387]	18	46395909	46464501	
ENSDARG00000017489	171.8824823	-2.176686805	0.199560204	-5.896400097	3.72E-09	8.06E-08	ifi45	interferon alpha inducible protein 45 [Source:ZFIN;Acc:ZDB-GENE-051030-98]	13	37605896	37608441	
ENSDARG00000017490	4435.225795	-4.829540818	0.107055117	-35.77167467	3.05E-280	1.09E-276	cel.1	carboxyl ester lipase, tandem duplicate 1 [Source:ZFIN;Acc:ZDB-GENE-030131-1201]	21	11401247	11411365	
ENSDARG00000017571	4740.824785	-1.244285339	0.08109395	-3.012374401	0.002592127	0.022845848	mccc2	methylcrotonoyl-CoA carboxylase 2 (beta) [Source:ZFIN;Acc:ZDB-GENE-040426-2493]	5	43808838	43819663	
ENSDARG00000017590	846.3085294	-1.505364943	0.169812211	-2.97602239	0.002920135	0.025477344	nit2	nitrilase family, member 2 [Source:NCBI gene;Acc:402904]	6	37431169	37441520	
ENSDARG00000017624	390031.3808	-1.964975737	0.105047868	-9.186057358	4.08E-20	2.2E-18	krt4	keratin 4 [Source:ZFIN;Acc:ZDB-GENE-000607-83]	6	39308498	39313027	
ENSDARG00000017661	957.70685	1.377055887	0.130348721	2.892670401	0.003819819	0.032447134	braf	B-Raf proto-oncogene, serine/threonine kinase [Source:ZFIN;Acc:ZDB-GENE-040805-1]	4	12074776	12102036	
ENSDARG00000017673	12540.83056	2.425649914	0.105075187	13.56790272	6.21E-42	1.04E-39	nova2	NOVA alternative splicing regulator 2 [Source:ZFIN;Acc:ZDB-GENE-080211-1]	15	28685625	28766824	
ENSDARG00000017720	263.9290874	2.929077484	0.584216644	3.301989946	0.000960015	0.009210824	si:ch211-106h11.1	si:ch211-106h11.1 [Source:ZFIN;Acc:ZDB-GENE-061009-2]	3	5647772	5664123	
ENSDARG00000017742	121.8554103	2.583729723	0.282556992	5.604992152	2.08E-08	4.13E-07	grm6a	glutamate receptor, metabotropic 6a [Source:ZFIN;Acc:ZDB-GENE-060208-1]	8	8532407	8624008	
ENSDARG00000017772	26685.90283	-1.319287379	0.088513598	-3.607212765	0.000309504	0.003290494	mdh1aa	malate dehydrogenase 1Aa, NAD (soluble) [Source:ZFIN;Acc:ZDB-GENE-040204-1]	1	50968908	50990889	
ENSDARG00000017781	2567.782533	-1.331147065	0.098979488	-3.345613022	0.000821009	0.007975288	hsd17b10	hydroxysteroid (17-beta) dehydrogenase 10 [Source:ZFIN;Acc:ZDB-GENE-041010-201]	23	18372048	18381361	
ENSDARG00000017790	1123.840001	1.792369292	0.117456609	6.746059684	1.52E-11	4.25E-10	chrnb2	cholinergic receptor, nicotinic, beta 2 [Source:ZFIN;Acc:ZDB-GENE-070821-3]	16	22618620	22678294	
ENSDARG00000017794	3656.226588	-1.951157264	0.093475704	-10.1754491	2.55E-24	1.8E-22	sdr16c5b	short chain dehydrogenase/reductase family 16C, member 5b [Source:ZFIN;Acc:ZDB-GENE-040426-2861]	7	58699718	58718472	
ENSDARG00000017798	4237.230487	1.625031713	0.104869513	5.96008977	2.52E-09	5.58E-08	bcor	BCL6 corepressor [Source:ZFIN;Acc:ZDB-GENE-040408-1]	22	11144153	11200846	
ENSDARG00000017811	692.7020517	-1.376473957	0.124176291	-3.03177002	0.002431244	0.021503399	si:ch211-210c8.6	si:ch211-210c8.6 [Source:ZFIN;Acc:ZDB-GENE-030131-3630]	23	33752275	33757742	
ENSDARG00000017882	1483.381272	-2.075144516	0.101818445	-10.55942776	4.59E-26	3.49E-24	rdh1	retinol dehydrogenase 1 [Source:ZFIN;Acc:ZDB-GENE-030912-15]	9	48673093	48701035	
ENSDARG00000017985	838.8380212	-2.322668404	0.122158486	-10.82747871	2.55E-27	2.13E-25	C1H4orf33	zgc:77739 [Source:NCBI gene;Acc:393870]	1	12394205	12400783	C1H4orf33
ENSDARG00000018000	778.9461939	1.618609954	0.140771463	4.39442726	1.11E-05	0.00015301	ubp1	upstream binding protein 1 [Source:ZFIN;Acc:ZDB-GENE-060503-870]	19	42806812	42841889	
ENSDARG00000018002	1354.828105	-1.674748465	0.110973276	-6.080278853	1.2E-09	2.73E-08	eci1	enoyl-CoA delta isomerase 1 [Source:ZFIN;Acc:ZDB-GENE-030131-6033]	3	27770110	27777671	
ENSDARG00000018032	1430.297298	1.564852243	0.118210561	4.778356858	1.77E-06	2.76E-05	scn8ab	sodium channel, voltage gated, type VIII, alpha subunit b [Source:ZFIN;Acc:ZDB-GENE-060906-2]	6	39392442	39489190	
ENSDARG00000018048	44.43583821	2.195244407	0.416673293	2.868540956	0.004123698	0.034710391	zgc:171704	zgc:171704 [Source:ZFIN;Acc:ZDB-GENE-070820-16]	16	28792158	28796865	
ENSDARG00000018066	264.785137	1.851325245	0.21935088	3.881111595	0.00010398	0.001203877	ptchd1	patched domain containing 1 [Source:ZFIN;Acc:ZDB-GENE-061220-1]	24	25347503	25364775	
ENSDARG00000018111	995.341368	1.744998543	0.118134569	6.306355089	2.86E-10	6.98E-09	kdm7aa	lysine (K)-specific demethylase 7Aa [Source:NCBI gene;Acc:321248]	4	22484830	22519525	
ENSDARG00000018146	1244.48273	-1.414809507	0.135805553	-3.054437017	0.002254834	0.020084662	gpx1a	glutathione peroxidase 1a [Source:ZFIN;Acc:ZDB-GENE-030410-1]	11	36048579	36156971	
ENSDARG00000018181	281.5603402	-1.445300276	0.15600457	-2.854405333	0.004311748	0.036112508	dnajc28	DnaJ (Hsp40) homolog, subfamily C, member 28 [Source:ZFIN;Acc:ZDB-GENE-050417-130]	9	35872698	35875051	
ENSDARG00000018259	12053.09341	2.444343727	0.087682198	16.47248532	5.78E-61	1.94E-58	atp1a3a	ATPase Na+/K+ transporting subunit alpha 3a [Source:ZFIN;Acc:ZDB-GENE-001212-7]	19	6328234	6385594	
ENSDARG00000018263	5929.584316	-3.541798377	0.134531108	-18.89375939	1.28E-79	5.97E-77	pdia2	protein disulfide isomerase family A, member 2 [Source:ZFIN;Acc:ZDB-GENE-040426-705]	1	9954489	9966195	
ENSDARG00000018264	1058.907014	-1.903962674	0.119558005	-7.560871187	4E-14	1.37E-12	trim101	tripartite motif containing 101 [Source:ZFIN;Acc:ZDB-GENE-040801-100]	23	17454691	17470146	
ENSDARG00000018266	3627.24073	-1.385286303	0.095163267	-4.04868722	5.15E-05	0.000629428	mthfd1a	methylenetetrahydrofolate dehydrogenase (NADP+ dependent) 1a, methenyltetrahydrofolate cyclohydrolase, formyltetrahyc	20	27646772	27687457	
ENSDARG00000018342	320.0975776	-1.791507187	0.194841922	-4.062304344	4.86E-05	0.000595253	ggt1b	gamma-glutamyltransferase 1b [Source:ZFIN;Acc:ZDB-GENE-110408-13]	10	41624711	41664427	
ENSDARG00000018361	342.1477804	-2.545370904	0.192698776	-8.019619725	1.06E-15	4.14E-14	sult1st3	sulfotransferase family 1, cytosolic sulfotransferase 3 [Source:NCBI gene;Acc:368270]	8	46559332	46581448	
ENSDARG00000018426	8718.120889	-1.435903547	0.078174066	-5.576063415	2.46E-08	4.84E-07	aldh7a1	aldehyde dehydrogenase 7 family, member A1 [Source:ZFIN;Acc:ZDB-GENE-030131-6129]	10	16006905	16028082	
ENSDARG00000018461	2341.079419	-1.330561134	0.091092668	-3.628844583	0.000284693	0.003048664	zgc:56095	zgc:56095 [Source:ZFIN;Acc:ZDB-GENE-040426-1948]	24	7741521	7746478	
ENSDARG00000018524	3370.283715	1.293600294	0.105637214	2.779326358	0.005447177	0.044551836	midn	midnolin [Source:NCBI gene;Acc:323713]	22	18725101	18749313	
ENSDARG00000018693	7073.5872	1.306785821	0.106587301	2.878258651	0.003998771	0.03381845	cdh2	cadherin 2, type 1, N-cadherin (neuronal) [Source:NCBI gene;Acc:30291]	20	17581881	17819238	
ENSDARG00000018743	1507.520772	1.548739232	0.122416174	4.482571331	7.37E-06	0.000104942	scamp5a	secretory carrier membrane protein 5a [Source:NCBI gene;Acc:324467]	25	31837228	31863374	
ENSDARG00000018846	3083.900254	-1.406524864	0.111483566	-3.64650036	0.000265836	0.002865072	dgat2	diacylglycerol O-acyltransferase 2 [Source:NCBI gene;Acc:565316]	10	32501961	32524771	
ENSDARG00000018856	921.3319817	1.547032585	0.114547068	4.775614012	1.79E-06	2.8E-05	dclk1a	doublecortin-like kinase 1a [Source:ZFIN;Acc:ZDB-GENE-061013-124]	10	34737322	34871737	
ENSDARG00000018881	1285.057442	-1.574670774	0.122632007	-4.686140163	2.78E-06	4.22E-05	apobec2a	apolipoprotein B mRNA editing enzyme, catalytic polypeptide-like 2a [Source:ZFIN;Acc:ZDB-GENE-050306-15]	22	641342	651719	
ENSDARG00000018896	1499.295011	2.118816937	0.133231009	8.397571586	4.56E-17	1.96E-15	mmp24	matrix metallopeptidase 24 [Source:ZFIN;Acc:ZDB-GENE-040724-262]	6	52362395	52428826	
ENSDARG00000018944	1108.964227	-1.887587708	0.119976567	-7.398008848	1.38E-13	4.58E-12	hoga1	4-hydroxy-2-oxoglutarate aldolase 1 [Source:NCBI gene;Acc:405806]	22	34710036	34732882	
ENSDARG00000018966	1142.469754	-1.45619681	0.106320601	-4.290765903	1.78E-05	0.000236603	cyb5r1	cytochrome b5 reductase 1 [Source:ZFIN;Acc:ZDB-GENE-030131-8497]	11	24786378	24799675	

ENSDARG00000018967	83.17244839	2.195353255	0.344273188	3.472106736	0.000516391	0.005235028	gabbr1a	gamma-aminobutyric acid (GABA) B receptor, 1a [Source:ZFIN;Acc:ZDB-GENE-030904-5]	15	34717514	34845414	
ENSDARG00000019063	1225.863464	1.63693277	0.109410705	5.8214849	5.83E-09	1.24E-07	fat1b	FAT atypical cadherin 1b [Source:ZFIN;Acc:ZDB-GENE-130530-563]	14	30156449	30226882	
ENSDARG00000019081	1901.291803	-1.605476501	0.14159663	-4.276065762	1.9E-05	0.00025144	DHDH	zgc:101723 [Source:NCBI gene;Acc:449558]	18	951092	968706	DHDH
ENSDARG00000019179	428.9469042	3.235694532	0.180251659	12.40318422	2.51E-35	3.15E-33	lrit1a	leucine-rich repeat, immunoglobulin-like and transmembrane domains 1a [Source:ZFIN;Acc:ZDB-GENE-040924-5]	13	29462249	29467558	
ENSDARG00000019208	1150.190023	2.042756001	0.124232192	8.393605442	4.71E-17	2.03E-15	camsap1a	calmodulin regulated spectrin-associated protein 1a [Source:ZFIN;Acc:ZDB-GENE-060518-4]	21	4712134	4764124	
ENSDARG00000019222	1104.476275	-1.363113852	0.101887998	-3.563853052	0.000365451	0.003828537	mrpl24	mitochondrial ribosomal protein L24 [Source:NCBI gene;Acc:436674]	19	7495791	7506134	
ENSDARG00000019228	876.3508329	-2.574514284	0.158181492	-9.953846449	2.43E-23	1.59E-21	mogat2	monoacylglycerol O-acyltransferase 2 [Source:ZFIN;Acc:ZDB-GENE-041010-186]	10	32534814	32610776	
ENSDARG00000019236	1472.897498	-1.56794079	0.117693242	-4.825602411	1.4E-06	2.21E-05	gsr	glutathione reductase [Source:ZFIN;Acc:ZDB-GENE-050522-116]	14	38852969	38873145	
ENSDARG00000019260	1251.099249	-2.67522288	0.152631499	-10.97560389	5.01E-28	4.32E-26	dhrs9	dehydrogenase/reductase (SDR family) member 9 [Source:ZFIN;Acc:ZDB-GENE-030131-1249]	9	48706038	48736388	
ENSDARG00000019328	3710.485713	1.746725605	0.088902806	8.399347992	4.49E-17	1.94E-15	plxna4	plexin A4 [Source:NCBI gene;Acc:325938]	4	15217929	15420452	
ENSDARG00000019365	265.614052	-3.393798722	0.189089233	-12.65962468	9.9E-37	1.3E-34	krt99	keratin 99 [Source:ZFIN;Acc:ZDB-GENE-050417-50]	11	11565996	11575070	
ENSDARG00000019426	2927.445644	1.670436963	0.099923204	6.70952231	1.95E-11	5.39E-10	trioa	trio Rho guanine nucleotide exchange factor a [Source:NCBI gene;Acc:557667]	19	41841147	42045372	
ENSDARG00000019451	653.9111452	2.637833377	0.158781177	10.31503486	6.03E-25	4.35E-23	pcsk2	proprotein convertase subtilisin/kexin type 2 [Source:ZFIN;Acc:ZDB-GENE-090608-1]	13	912123	977652	
ENSDARG00000019492	1384.944833	-1.521019737	0.130061552	-4.005947398	6.18E-05	0.000744704	shbg	sex hormone-binding globulin [Source:ZFIN;Acc:ZDB-GENE-030131-1324]	12	22511015	22524388	
ENSDARG00000019496	2890.28526	-1.83997966	0.089460826	-9.389357345	6.04E-21	3.45E-19	esd	esterase D/formylglutathione hydrolase [Source:ZFIN;Acc:ZDB-GENE-050417-328]	9	25171662	25181697	
ENSDARG00000019507	3170.817581	-1.398354777	0.094340877	-4.222504509	2.42E-05	0.000311477	mcm5	minichromosome maintenance complex component 5 [Source:ZFIN;Acc:ZDB-GENE-021209-1]	3	25999477	26011440	
ENSDARG00000019532	664.1055086	-2.76028378	0.135141848	-13.02545293	8.77E-39	1.29E-36	fads2	fatty acid desaturase 2 [Source:NCBI gene;Acc:140615]	25	4133488	4148719	
ENSDARG00000019658	1049.38524	2.479328021	0.119260816	12.40414133	2.48E-35	3.13E-33	pou2f2a	POU class 2 homeobox 2a [Source:ZFIN;Acc:ZDB-GENE-060310-2]	16	10918252	10991240	
ENSDARG00000019686	262.3092949	-2.819610207	0.221003569	-8.233397375	1.82E-16	7.46E-15	fgl2b	fibrinogen-like 2b [Source:ZFIN;Acc:ZDB-GENE-120709-53]	25	21267579	21273875	
ENSDARG00000019726	1449.515207	1.741326615	0.131819349	5.623807279	1.87E-08	3.73E-07	celsr2	cadherin, EGF LAG seven-pass G-type receptor 2 [Source:ZFIN;Acc:ZDB-GENE-070122-2]	22	413349	465663	
ENSDARG00000019782	265.1030673	2.00183822	0.211893929	4.728017573	2.27E-06	3.5E-05	impg2a	interphotoreceptor matrix proteoglycan 2a [Source:ZFIN;Acc:ZDB-GENE-140106-217]	9	30188764	30190513	
ENSDARG00000019808	2791.287594	-1.618227011	0.141710267	-4.362612686	1.29E-05	0.000175323	evpla	envoplakin a [Source:ZFIN;Acc:ZDB-GENE-030829-19]	6	23057311	23082678	
ENSDARG00000019877	327.7973602	-1.939744582	0.176000134	-5.339453772	9.32E-08	1.69E-06	rbks	ribokinase [Source:ZFIN;Acc:ZDB-GENE-040625-112]	17	40999835	41081806	
ENSDARG00000019907	244.7864128	-1.548163392	0.17622368	-3.110611431	0.001867004	0.016869574	dscc1	DNA replication and sister chromatid cohesion 1 [Source:NCBI gene;Acc:447872]	16	14572008	14587332	
ENSDARG00000019945	1761.616604	2.227840932	0.162243668	7.567881968	3.79E-14	1.31E-12	ptprdb	protein tyrosine phosphatase receptor type Db [Source:ZFIN;Acc:ZDB-GENE-070615-38]	1	19122920	19237612	
ENSDARG00000019986	11887.93156	-1.379355263	0.093703514	-4.048463571	5.16E-05	0.000629723	grhprb	glyoxylate reductase/hydroxypyruvate reductase b [Source:ZFIN;Acc:ZDB-GENE-040426-1847]	1	19835803	19845539	
ENSDARG00000020028	112.7829909	4.281756902	0.465703649	7.04687822	1.83E-12	5.53E-11	cps1	carbamoyl-phosphate synthase 1, mitochondrial [Source:ZFIN;Acc:ZDB-GENE-081105-17]	9	39026311	39139722	
ENSDARG00000020046	61.07951222	-2.644012328	0.36320122	-4.526450456	6E-06	8.67E-05	ahr1a	aryl hydrocarbon receptor 1a [Source:ZFIN;Acc:ZDB-GENE-020531-2]	16	20012190	20038263	
ENSDARG00000020084	304.3342531	2.686741686	0.189212733	8.914525265	4.9E-19	2.45E-17	tg	thyroglobulin [Source:ZFIN;Acc:ZDB-GENE-030519-1]	16	31530762	31598771	
ENSDARG00000020122	141.5589743	1.763903552	0.252283648	3.027955077	0.002462147	0.021769055	endou2	endonuclease, polyU-specific 2 [Source:NCBI gene;Acc:553584]	21	41863540	41873584	
ENSDARG00000020143	4761.600125	-2.076607869	0.092014021	-11.70047633	1.27E-31	1.34E-29	pah	phenylalanine hydroxylase [Source:ZFIN;Acc:ZDB-GENE-031006-2]	4	17391680	17409533	
ENSDARG00000020178	2593.025701	1.66745782	0.178005994	3.749636763	0.000177091	0.001971245	nova1	NOVA alternative splicing regulator 1 [Source:ZFIN;Acc:ZDB-GENE-060526-113]	5	36611128	36626646	
ENSDARG00000020232	2403.472732	-1.364057625	0.09761104	-3.729676725	0.000191726	0.002113528	eif6	eukaryotic translation initiation factor 6 [Source:NCBI gene;Acc:386850]	6	52339548	52348651	
ENSDARG00000020326	790.5031682	1.345894005	0.117044964	2.955223294	0.003124428	0.027034221	tyk2	tyrosine kinase 2 [Source:ZFIN;Acc:ZDB-GENE-071116-4]	3	46479705	46555542	
ENSDARG00000020345	6455.880124	1.281656182	0.085642745	3.288733698	0.001006392	0.009610634	clasp2	cytoplasmic linker associated protein 2 [Source:NCBI gene;Acc:404604]	19	42693855	42802298	
ENSDARG00000020387	756.0409489	-2.169212704	0.12490196	-9.361043693	7.9E-21	4.5E-19	nipsnap3a	nipsnap homolog 3A (C. elegans) [Source:ZFIN;Acc:ZDB-GENE-040426-1037]	14	6520310	6527010	
ENSDARG00000020450	512.5926606	1.584658535	0.182408923	3.205207974	0.001349649	0.012575184	cacng8a	calcium channel, voltage-dependent, gamma subunit 8a [Source:ZFIN;Acc:ZDB-GENE-050417-239]	19	9813287	9829965	
ENSDARG00000020618	1191.68861	-1.344428552	0.114966948	-2.99589194	0.002736434	0.024024913	gatd3a	glutamine amidotransferase like class 1 domain containing 3A [Source:NCBI gene;Acc:550355]	9	32797816	32804953	
ENSDARG00000020645	3040.414031	1.622132896	0.104965228	5.927038016	3.08E-09	6.76E-08	slc7a3a	solute carrier family 7 member 3a [Source:ZFIN;Acc:ZDB-GENE-041114-206]	21	30502002	30537411	
ENSDARG00000020711	295.3710788	-1.505702641	0.166087138	-3.04480316	0.002328326	0.020665955	rrm2	ribonucleotide reductase regulatory subunit M2 [Source:NCBI gene;Acc:100330864]	20	29492469	29499426	
ENSDARG00000020771	1430.545376	2.318673283	0.120029435	10.98624915	4.45E-28	3.87E-26	tnr	tenascin R (restrictin, janusin) [Source:ZFIN;Acc:ZDB-GENE-030804-1]	2	35240485	35474115	
ENSDARG00000020866	7608.001056	-2.32058625	0.091615838	-14.41438813	4.2E-47	8.57E-45	apoa4b.2	apolipoprotein A-IV b, tandem duplicate 2 [Source:ZFIN;Acc:ZDB-GENE-030131-3143]	16	23947196	23948538	
ENSDARG00000020890	7358.54141	-1.934080031	0.100526319	-9.291895347	1.52E-20	8.57E-19	tmod4	tropomodulin 4 (muscle) [Source:ZFIN;Acc:ZDB-GENE-991019-9]	16	29650698	29684858	
ENSDARG00000020982	635.6153016	1.989863236	0.13323677	7.429354816	1.09E-13	3.64E-12	prickle2a	prickle homolog 2a [Source:ZFIN;Acc:ZDB-GENE-061110-112]	8	54081819	54105734	
ENSDARG00000021135	1216.39266	-1.436359713	0.115449271	-3.779666254	0.000157039	0.001763682	dhrs4	dehydrogenase/reductase (SDR family) member 4 [Source:ZFIN;Acc:ZDB-GENE-040426-1498]	7	24039464	24047316	
ENSDARG00000021137	1775.087828	1.665310186	0.096683072	6.881351349	5.93E-12	1.71E-10	adgrv1	adhesion G protein-coupled receptor V1 [Source:NCBI gene;Acc:415105]	5	48683447	48940127	
ENSDARG00000021149	3223.546051	-2.470387244	0.116555142	-12.61537865	1.74E-36	2.27E-34	cbr1l	carbonyl reductase 1-like [Source:ZFIN;Acc:ZDB-GENE-030131-9642]	21	21633213	21641194	
ENSDARG00000021163	1480.760657	2.007690723	0.197842945	5.093387196	3.52E-07	5.99E-06	thrb	thyroid hormone receptor beta [Source:NCBI gene;Acc:30607]	19	17514631	17658253	
ENSDARG00000021172	1168.190603	-3.083440401	0.156526329	-13.3104789	2.01E-40	3.16E-38	cyp2ad2	cytochrome P450, family 2, subfamily AD, polypeptide 2 [Source:ZFIN;Acc:ZDB-GENE-020812-2]	20	25534669	25542183	
ENSDARG00000021208	621.3736451	-1.680705688	0.167047197	-4.07493033	4.6E-05	0.000566349	serpind1	serpin peptidase inhibitor, clade D (heparin cofactor), member 1 [Source:ZFIN;Acc:ZDB-GENE-030711-2]	8	1766206	1781733	
ENSDARG00000021241	172.594905	2.136447841	0.225296188	5.044239097	4.55E-07	7.66E-06	zgc:165604	zgc:165604 [Source:ZFIN;Acc:ZDB-GENE-070615-34]	21	27558381	27570216	
ENSDARG00000021305	138.8150626	2.952589843	0.321170991	6.079595902	1.2E-09	2.73E-08	slc30a2	solute carrier family 30 member 2 [Source:ZFIN;Acc:ZDB-GENE-041014-347]	20	38595935	38617766	
ENSDARG00000021339	23518.14515	-3.836172202	0.094433075	-30.03367413	3.57E-198	8.14E-195	cpa5	carboxypeptidase A5 [Source:ZFIN;Acc:ZDB-GENE-020514-1]	25	18459924	18470695	
ENSDARG00000021343	7101.624452	1.30368369	0.097414075	3.117451882	0.001824217	0.016512685	wsb1	WD repeat and SOCS box containing 1 [Source:NCBI gene;Acc:323226]	15	31161783	31177324	
ENSDARG00000021352	1379.558715	1.794557163	0.20867692	3.807594833	0.000140325	0.001591639	gria1a	glutamate receptor, ionotropic, AMPA 1a [Source:ZFIN;Acc:ZDB-GENE-020125-1]	14	34125855	34276574	
ENSDARG00000021366	1678.769177	-1.378348502	0.100563547	-3.762282789	0.00016837	0.001880001	fbp1a	fructose-1,6-bisphosphatase 1a [Source:ZFIN;Acc:ZDB-GENE-030131-7171]	5	44833313	44843738	
ENSDARG00000021433	1797.436029	-1.465316372	0.097093182	-4.792472154	1.65E-06	2.59E-05	asmtl	acetylserotonin O-methyltransferase-like [Source:ZFIN;Acc:ZDB-GENE-030131-2854]	9	34872733	34882516	
ENSDARG00000021539	1747.677345	1.467423563	0.098958887	4.723411674	2.32E-06	3.57E-05	ephb2b	eph receptor B2b [Source:ZFIN;Acc:ZDB-GENE-050522-415]	11	28166165	28424186	
ENSDARG00000021556	1769.790473	1.545955037	0.110667591	4.933287465	8.09E-07	1.31E-05	ubl3b	ubiquitin-like 3b [Source:ZFIN;Acc:ZDB-GENE-040630-4]	14	30854553	30876708	
ENSDARG00000021573	130.5933154	-2.321153135	0.271439163	-4.867216355	1.13E-06	1.81E-05	slc16a7	solute carrier family 16 member 7 [Source:ZFIN;Acc:ZDB-GENE-051113-248]	23	9867483	9889330	
ENSDARG00000021584	587.669132	1.909480151	0.139998818	6.496341654	8.23E-11	2.14E-09	cntn5	contactin 5 [Source:NCBI gene;Acc:327429]	18	42150947	42395161	
ENSDARG00000021664	1487.247252	1.705284881	0.111494959	6.32571096	2.52E-10	6.22E-09	fzd3a	frizzled class receptor 3a [Source:ZFIN;Acc:ZDB-GENE-990415-225]	20	35208855	35246150	
ENSDARG00000021787	2529.792755	-1.860183554	0.199791342	-4.305409564	1.67E-05	0.000222896	abcb5	ATP-binding cassette, sub-family B (MDR/TAP), member 5 [Source:ZFIN;Acc:ZDB-GENE-030131-6414]	16	19537038	19568795	
ENSDARG00000021805	701.5213111	1.371967896	0.126308761	2.944909707	0.003230489	0.02783677	tle5	TLE family member 5, transcriptional modulator [Source:ZFIN;Acc:ZDB-GENE-040426-1409]	22	21794037	21845685	
ENSDARG00000021864	75116.85869	-1.396681965	0.10128499	-3.916493096	8.98E-05	0.001052249	rplp1	ribosomal protein, large, P1 [Source:ZFIN;Acc:ZDB-GENE-030131-8663]	19	1852078	1855368	
ENSDARG00000021916	265.0677393	2.139285695	0.212041804	5.372929653	7.75E-08	1.42E-06	vax1	ventral anterior homeobox 1 [Source:NCBI gene;Acc:373870]	17	21432693	21441464	
ENSDARG00000021924	885.3330549	1.876242463	0.231516377	3.78479689	0.000153834	0.001730789	hsp70.3	heat shock cognate 70-kd protein, tandem duplicate 3 [Source:ZFIN;Acc:ZDB-GENE-110713-1]	3	26123736	26127091	
ENSDARG00000022165	2459.508282	-1.710640183	0.096972706	-7.328249476	2.33E-13	7.58E-12	mgst1.2	microsomal glutathione S-transferase 1.2 [Source:ZFIN;Acc:ZDB-GENE-040704-59]	4	12718965	12725513	
ENSDARG00000022176	678.7908795	1.993934539	0.204752219	4.854328543	1.21E-06	1.93E-05	IGLON5	zgc:110372 [Source:ZFIN;Acc:ZDB-GENE-050417-297]	16	50434668	50824469	IGLON5
ENSDARG00000022183	352.5487782	-3.153472517	0.181710773	-11.85109988	2.12E-32	2.3E-30	gsto1	glutathione S-transferase omega 1 [Source:ZFIN;Acc:ZDB-GENE-040718-365]	13	25380432	25389051	
ENSDARG00000022261	3158.324635	-2.416571913	0.168871266	-8.388472171	4.93E-17	2.11E-15	pdzk1	PDZ domain containing 1 [Source:ZFIN;Acc:ZDB-GENE-031222-1]	9	5862040	5892075	
ENSDARG00000022438	11168.15225	-1.352605117	0.095581345	-3.689057907	0.000225086	0.002445837	cox6a1	cytochrome c oxidase subunit 6A1 [Source:ZFIN;Acc:ZDB-GENE-030131-7715]	8	39760258	39765996	
ENSDARG00000022503	280.5116316	1.895305351	0.204655532	4.374694113	1.22E-05	0.000166795	pkd2l1	polycystic kidney disease 2-like 1 [Source:ZFIN;Acc:ZDB-GENE-030616-558]	13	25455319	25471376	
ENSDARG00000022531	1509.233819	2.224247222	0.161598877	7.575839909	3.57E-14	1.23E-12	ntn1b	netrin 1b [Source:ZFIN;Acc:ZDB-GENE-990415-168]	3	25708549	25814097	
ENSDARG00000022599	1708.382219	-1.525773785	0.09675163	-5.434262839	5.5E-08	1.03E-06	dnpep	aspartyl aminopeptidase [Source:ZFIN;Acc:ZDB-GENE-040426-852]	6	13896264	13920394	
ENSDARG00000022614	232.4434043	-2.295971096	0.231837561	-5.589996242	2.27E-08	4.48E-07	ddx43	DEAD (Asp-Glu-Ala-Asp) box polypeptide 43 [Source:ZFIN;Acc:ZDB-GENE-080204-77]	13	27332656	27354033	
ENSDARG00000022631	1361.525131	-2.175475076	0.166995192	-7.038975564	1.94E-12	5.84E-11	cyp2p9	cytochrome P450, family 2, subfamily P, polypeptide 9 [Source:ZFIN;Acc:ZDB-GENE-040120-2]	20	25581627	25584869	
ENSDARG00000022650	3141.335089	-1.438578363	0.089862105	-4.880570769	1.06E-06	1.7E-05	cyp2ad3	cytochrome P450, family 2, subfamily AD, polypeptide 3 [Source:ZFIN;Acc:ZDB-GENE-041001-156]	20	25548425	25551676	
ENSDARG00000022689	1119.098425	-1.783313043	0.141504456	-5.53560688	3.1E-08	6.01E-07	itgb1b.2	integrin, beta 1b.2 [Source:ZFIN;Acc:ZDB-GENE-040426-2598]	2	43638983	43653328	
ENSDARG00000022698	424.5266314	-1.71359421	0.162759265	-4.38435385	1.16E-05	0.000159826	lad1	ladinin [Source:ZFIN;Acc:ZDB-GENE-030616-268]	23	5687972	5719453	
ENSDARG00000022712	2371.646968	1.315448066	0.100076901	3.152056714	0.001621248	0.014830518	stat3	signal transducer and activator of transcription 3 (acute-phase response factor) [Source:ZFIN;Acc:ZDB-GENE-980526-68]	3	17030665	17107445	
ENSDARG00000023058	4762.022679	1.27374487	0.089429905	3.060999217	0.002205997	0.019698585	foxo3a	forkhead box O3A [Source:ZFIN;Acc:ZDB-GENE-050116-1]	17	6793001	6810785	
ENSDARG00000023062	2235.469562	1.28457999	0.097672324	2.913619533	0.00357265	0.03054351	ccn1	cellular communication network factor 1 [Source:ZFIN;Acc:ZDB-GENE-040426-3]	23	45456490	45465320	
ENSDARG00000023082	5825.000445	-2.294626836	0.117599268	-11.00880016	3.47E-28	3.03E-26	krt1-19d	keratin, type 1, gene 19d [Source:ZFIN;Acc:ZDB-GENE-060316-1]	19	5324653	5332784	
ENSDARG00000023176	316.2484048	-2.682413889	0.193579537	-8.691073025	3.59E-18	1.69E-16	tdo2b	tryptophan 2,3-dioxygenase b [Source:NCBI gene;Acc:334082]	11	34084532	34097437	
ENSDARG00000023217	1060.633899	1.43425615	0.113731136	3.81826971	0.000134391	0.001530557	crema	cAMP responsive element modulator a [Source:ZFIN;Acc:ZDB-GENE-030131-7031]	2	43159171	43196595	
ENSDARG00000023290	9869.737608	-1.312421009	0.095084433	-3.285721965	0.001017214	0.009704705	fabp3	fatty acid binding protein 3, muscle and heart [Source:ZFIN;Acc:ZDB-GENE-020318-2]	19	42955280	42993306	
ENSDARG00000023299	2067.094127	-1.637770875	0.098588497	-6.469019176	9.86E-11	2.56E-09	snu13b	SNU13 homolog, small nuclear ribonucleoprotein b (U4/U6.U5) [Source:ZFIN;Acc:ZDB-GENE-030131-9670]	12	10443785	10447178	
ENSDARG00000023445	4601.870111	2.11739882	0.097315665	11.48220921	1.62E-30	1.61E-28	atp2b3b	ATPase plasma membrane Ca2+ transporting 3b [Source:ZFIN;Acc:ZDB-GENE-080409-2]	23	19834215	19953089	
ENSDARG00000023532	1401.213341	-1.366057267	0.126474177	-2.894324157	0.003799757	0.032287641	pinx1	PIN2 (TERF1) interacting telomerase inhibitor 1 [Source:ZFIN;Acc:ZDB-GENE-030515-2]	20	19006703	19059240	
ENSDARG00000023537	605.757531	2.832936686	0.153377718	11.95047569	6.46E-33	7.2E-31	ahr1b	aryl hydrocarbon receptor 1b [Source:ZFIN;Acc:ZDB-GENE-050922-1]	22	12979213	13042992	
ENSDARG00000023542	520.9201047	1.641572965	0.143119011	4.482793453	7.37E-06	0.000104892	cdh7a	cadherin 7a [Source:ZFIN;Acc:ZDB-GENE-061019-3]	2	27010439	27094653	
ENSDARG00000023645	1327.224828	-1.627229063	0.127190571	-4.931411648	8.16E-07	1.33E-05	kyat1	kynurenine aminotransferase 1 [Source:ZFIN;Acc:ZDB-GENE-040426-2676]	5	33264842	33280699	
ENSDARG00000023683	684.5540631	2.826864609	0.162376085	11.25082314	2.29E-29	2.2E-27	cacna1fb	calcium channel, voltage-dependent, L type, alpha 1F subunit [Source:ZFIN;Acc:ZDB-GENE-031104-1]	8	22851472	22927224	
ENSDARG00000023768	175.363398	-2.060794604	0.20015182	-5.299949834	1.16E-07	2.08E-06	mfsd4aa	major facilitator superfamily domain containing 4Aa [Source:NCBI gene;Acc:393503]	11	38429319	38458406	
ENSDARG00000023771	1047.801111	1.871520646	0.161707938	5.389473483	7.07E-08	1.31E-06	gabrb3	gamma-aminobutyric acid type A receptor beta3 subunit [Source:ZFIN;Acc:ZDB-GENE-101102-2]	6	38381957	38513578	
ENSDARG00000023820	5487.158481	-1.836299551	0.131902025	-6.34030867	2.29E-10	5.69E-09	faxdc2	fatty acid hydroxylase domain containing 2 [Source:ZFIN;Acc:ZDB-GENE-040426-2907]	21	45839917	45852112	
ENSDARG00000023886	691.2169276	1.682440015	0.131381508	5.194338418	2.05E-07	3.57E-06	cacna2d4b	calcium channel, voltage-dependent, alpha 2/delta subunit 4b [Source:ZFIN;Acc:ZDB-GENE-100422-18]	25	19023737	19090628	
ENSDARG00000023914	715.9882651	1.5197447	0.162247713	3.203402317	0.001358141	0.01264961	mark4a	MAP/microtubule affinity-regulating kinase 4a [Source:ZFIN;Acc:ZDB-GENE-060531-156]	5	36768674	36822220	
ENSDARG00000023916	419.3103034	1.736817184	0.149325668	4.934296914	8.04E-07	1.31E-05	phactr1	phosphatase and actin regulator 1 [Source:ZFIN;Acc:ZDB-GENE-101230-3]	20	52774730	52815590	
ENSDARG00000023933	710.44245	1.519501853	0.122122916	4.253926031	2.1E-05	0.000274043	skila	SKI-like proto-oncogene a [Source:ZFIN;Acc:ZDB-GENE-070912-548]	2	37307326	37353098	
ENSDARG00000023940	626.5405663	2.910470979	0.171956196	11.11021893	1.12E-28	1.03E-26	si:ch73-362m14.4	si:ch73-362m14.4 [Source:ZFIN;Acc:ZDB-GENE-120215-30]	21	43581341	43606502	
ENSDARG00000024017	610.5267954	2.068111675	0.139906615	7.634461582	2.27E-14	7.93E-13	mdga2a	MAM domain containing glycosylphosphatidylinositol anchor 2a [Source:ZFIN;Acc:ZDB-GENE-030616-360]	17	28340138	28564070	
ENSDARG00000024204	2199.27929	-1.323780747	0.106148119	-3.05027306	0.002286334	0.020338036	mcm3	minichromosome maintenance complex component 3 [Source:ZFIN;Acc:ZDB-GENE-020419-4]	20	34770197	34783743	
ENSDARG00000024276	2460.477175	1.458116379	0.113905566	4.021896352	5.77E-05	0.000700734	pcbp4	poly(rC) binding protein 4 [Source:ZFIN;Acc:ZDB-GENE-040426-2122]	22	32289567	32507966	
ENSDARG00000024433	30196.15756	-1.45835083	0.089288511	-5.133368525	2.85E-07	4.87E-06	pvalb4	parvalbumin 4 [Source:ZFIN;Acc:ZDB-GENE-040625-48]	3	61174750	61185746	
ENSDARG00000024478	5071.468413	-1.44143075	0.084590906	-5.218418531	1.8E-07	3.16E-06	mpc2	mitochondrial pyruvate carrier 2 [Source:ZFIN;Acc:ZDB-GENE-030131-330]	9	34221997	34227472	
ENSDARG00000024503	177.1974931	-5.864158054	0.348642479	-13.95170797	3.07E-44	5.55E-42	c6ast3	six-cysteine containing astacin protease 3 [Source:ZFIN;Acc:ZDB-GENE-050320-99]	7	38642114	38644560	
ENSDARG00000024575	413.3292917	1.98753982	0.15436997	6.3972275	1.58E-10	4.01E-09	kif26bb	kinesin family member 26Bb [Source:ZFIN;Acc:ZDB-GENE-041001-188]	20	35013434	35052823	
ENSDARG00000024619	555.7102408	2.611676277	0.166608884	9.673411376	3.91E-22	2.39E-20	foxo6b	forkhead box O6 b [Source:ZFIN;Acc:ZDB-GENE-060503-776]	19	15571290	15620967	
ENSDARG00000024654	612.4964505	-1.456604407	0.13711616	-3.330055382	0.000868287	0.008398796	pgm3	phosphoglucomutase 3 [Source:ZFIN;Acc:ZDB-GENE-041024-13]	4	5784970	5795309	
ENSDARG00000024717	1113.274764	-1.568525996	0.121833736	-4.666408612	3.07E-06	4.61E-05	selenbp1	selenium binding protein 1 [Source:NCBI gene;Acc:393542]	16	38138246	38157299	
ENSDARG00000024740	170.0982451	-2.051545039	0.231186805	-4.548464781	5.4E-06	7.89E-05	gng13a	guanine nucleotide binding protein (G protein), gamma 13a [Source:ZFIN;Acc:ZDB-GENE-081022-188]	1	9227081	9228007	
ENSDARG00000024785	1820.021294	1.300733111	0.10386326	2.89547152	0.003785894	0.032180738	ctnna2	catenin (cadherin-associated protein), alpha 2 [Source:NCBI gene;Acc:567500]	1	42224769	43039672	
ENSDARG00000024865	2076.861393	2.720928082	0.114732725	14.99945265	7.4E-51	1.77E-48	dscama	Down syndrome cell adhesion molecule a [Source:ZFIN;Acc:ZDB-GENE-050310-7]	10	38775408	38974445	

ENSDARG00000025011	3187.92534	1.757127141	0.118496763	6.389433094	1.67E-10	4.2E-09	synj1	synaptojanin 1 [Source:ZFIN;Acc:ZDB-GENE-030131-9180]	10	26515946	26569350	
ENSDARG00000025089	1048.446892	2.101223678	0.160612791	6.856388395	7.06E-12	2.03E-10	loxl4	lysyl oxidase-like 4 [Source:ZFIN;Acc:ZDB-GENE-080722-19]	13	40182487	40238813	
ENSDARG00000025106	2667.14688	1.736328382	0.12737257	5.78090231	7.43E-09	1.55E-07	ppp3cb	protein phosphatase 3, catalytic subunit, beta isozyme [Source:ZFIN;Acc:ZDB-GENE-050417-406]	14	7789093	7888748	
ENSDARG00000025108	724.2434945	1.699481431	0.148298846	4.716701772	2.4E-06	3.68E-05	magixa	MAGI family member, X-linked a [Source:ZFIN;Acc:ZDB-GENE-060503-115]	8	22761854	22838364	
ENSDARG00000025162	241.1121315	1.675697328	0.18869771	3.580845401	0.000342484	0.003607511	asic4a	acid-sensing (proton-gated) ion channel family member 4a [Source:NCBI gene;Acc:407668]	9	11034314	11129565	
ENSDARG00000025187	111.2072854	1.976751873	0.280899897	3.477224035	0.000506634	0.005154542	six6a	SIX homeobox 6a [Source:ZFIN;Acc:ZDB-GENE-040426-1282]	13	31583034	31606247	
ENSDARG00000025189	236.7020873	2.533476326	0.232262721	6.602335148	4.05E-11	1.08E-09	cpne8	copine VIII [Source:ZFIN;Acc:ZDB-GENE-100809-1]	25	35774544	35874406	
ENSDARG00000025206	780.1054178	2.220114153	0.198412141	6.149392612	7.78E-10	1.81E-08	syt2a	synaptotagmin IIa [Source:ZFIN;Acc:ZDB-GENE-060503-315]	23	6232895	6313808	
ENSDARG00000025220	989.3312715	1.337810286	0.110673485	3.05231452	0.00227084	0.020220064	sppl3	signal peptide peptidase 3 [Source:ZFIN;Acc:ZDB-GENE-050913-71]	10	17235370	17433878	
ENSDARG00000025311	59.81635337	-2.331974476	0.337820315	-3.942848949	8.05E-05	0.000954257	cuzd1.2	CUB and zona pellucida-like domains 1, tandem duplicate 2 [Source:ZFIN;Acc:ZDB-GENE-131119-30]	17	21833719	21859164	
ENSDARG00000025350	19149.33746	-1.384859288	0.092343552	-4.167689895	3.08E-05	0.000390674	prdx2	peroxiredoxin 2 [Source:ZFIN;Acc:ZDB-GENE-030326-2]	1	51721851	51729862	
ENSDARG00000025375	7556.170516	-2.254004417	0.114125366	-10.98795527	4.37E-28	3.81E-26	idh1	isocitrate dehydrogenase (NADP(+)) 1 [Source:ZFIN;Acc:ZDB-GENE-031006-1]	9	28103735	28129476	
ENSDARG00000025428	1720.131525	1.817484186	0.098932317	8.263065192	1.42E-16	5.86E-15	socs3a	suppressor of cytokine signaling 3a [Source:ZFIN;Acc:ZDB-GENE-030131-7349]	3	57423137	57425961	
ENSDARG00000025513	1297.260044	1.534376795	0.112298245	4.758549816	1.95E-06	3.03E-05	dip2cb	disco-interacting protein 2 homolog Cb [Source:ZFIN;Acc:ZDB-GENE-070912-589]	2	42496498	42558549	
ENSDARG00000025595	1239.626204	-1.850380061	0.148802083	-5.71483978	1.1E-08	2.25E-07	agmo	alkylglycerol monooxygenase [Source:NCBI gene;Acc:405819]	15	34334722	34418525	
ENSDARG00000025667	261.1313839	2.079536644	0.202518452	5.330559439	9.79E-08	1.77E-06	adgrb2	adhesion G protein-coupled receptor B2 [Source:ZFIN;Acc:ZDB-GENE-060503-573]	19	38766613	38872650	
ENSDARG00000025671	617.7634235	2.186319122	0.138190846	8.584643334	9.11E-18	4.19E-16	grm6b	glutamate receptor, metabotropic 6b [Source:ZFIN;Acc:ZDB-GENE-021120-2]	11	25634041	25679721	
ENSDARG00000025728	6946.544319	2.184439553	0.15258004	7.762742441	8.31E-15	3.03E-13	grin1b	glutamate receptor, ionotropic, N-methyl D-aspartate 1b [Source:ZFIN;Acc:ZDB-GENE-051202-2]	5	29597645	29643930	
ENSDARG00000025854	794.6394454	-1.427637143	0.111466526	-3.836462472	0.000124819	0.001426076	ddx51	DEAD (Asp-Glu-Ala-Asp) box polypeptide 51 [Source:NCBI gene;Acc:445387]	8	49065348	49089382	
ENSDARG00000025974	350.1412057	1.649553019	0.176600275	3.678097444	0.00023498	0.002548936	magi3b	membrane associated guanylate kinase, WW and PDZ domain containing 3b [Source:ZFIN;Acc:ZDB-GENE-060503-301]	6	47529881	47796989	
ENSDARG00000026017	292.3426968	-3.223136788	0.221859569	-10.02046836	1.24E-23	7.46E-22	xpnpep2	X-prolyl aminopeptidase (aminopeptidase P) 2, membrane-bound [Source:ZFIN;Acc:ZDB-GENE-040426-1151]	14	33591626	33613794	
ENSDARG00000026024	1479.639936	-1.402836187	0.10491882	-3.839503585	0.000123283	0.001409169	mospd2	motile sperm domain containing 2 [Source:ZFIN;Acc:ZDB-GENE-041114-1]	9	54984537	55051256	
ENSDARG00000026098	54.46698872	-2.33333615	0.40933545	-3.25731903	0.001124699	0.010641192	si:dkey-13a21.4	si:dkey-13a21.4 [Source:ZFIN;Acc:ZDB-GENE-040724-163]	11	25430851	25435922	
ENSDARG00000026183	1580.648222	-1.456057142	0.111438546	-4.092454174	4.27E-05	0.000527518	polr2eb	polymerase (RNA) II (DNA directed) polypeptide E, b [Source:ZFIN;Acc:ZDB-GENE-040801-83]	22	17606588	17611582	
ENSDARG00000026247	554.2240399	1.803569245	0.159061074	5.051954105	4.37E-07	7.37E-06	insyn1	inhibitory synaptic factor 1 [Source:NCBI gene;Acc:100003007]	18	50275933	50356788	
ENSDARG00000026248	131.8552415	2.450428041	0.287403719	5.046657174	4.5E-07	7.56E-06	slc1a7b	solute carrier family 1 member 7b [Source:ZFIN;Acc:ZDB-GENE-101111-7]	23	41949575	42093537	
ENSDARG00000026359	447.8593641	-1.657650772	0.150353623	-4.374026767	1.22E-05	0.000167215	pbld2	phenazine biosynthesis-like protein domain containing 2 [Source:ZFIN;Acc:ZDB-GENE-050417-456]	13	22849796	22862133	
ENSDARG00000026369	10041.05299	-1.931860591	0.084007933	-11.09253086	1.36E-28	1.24E-26	dbi	diazepam binding inhibitor (GABA receptor modulator, acyl-CoA binding protein) [Source:ZFIN;Acc:ZDB-GENE-040426-18[illegible]	9	907459	914886	
ENSDARG00000026395	488.0198683	1.681645308	0.158919035	4.289261549	1.79E-05	0.000237959	rfx4	regulatory factor X, 4 [Source:NCBI gene;Acc:403016]	18	15334117	15373620	
ENSDARG00000026406	717.3537696	-2.476638593	0.119500135	-12.35679437	4.48E-35	5.45E-33	anxa5a	annexin A5a [Source:ZFIN;Acc:ZDB-GENE-080220-29]	14	46410766	46434196	
ENSDARG00000026611	2136.933864	2.089561595	0.098284862	11.08575187	1.47E-28	1.32E-26	socs3b	suppressor of cytokine signaling 3b [Source:ZFIN;Acc:ZDB-GENE-040426-2528]	12	34258139	34277465	
ENSDARG00000026726	10211.85135	-1.848165835	0.085184657	-9.956791083	2.36E-23	1.55E-21	anxa1a	annexin A1a [Source:ZFIN;Acc:ZDB-GENE-030131-6664]	5	25578181	25583325	
ENSDARG00000026753	318.1403741	1.71925943	0.163804004	4.390975878	1.13E-05	0.000155119	grik4	glutamate receptor, ionotropic, kainate 4 [Source:ZFIN;Acc:ZDB-GENE-070821-5]	15	22722682	23188894	
ENSDARG00000026764	291.2126337	-4.860899826	0.238418412	-16.19379894	5.58E-59	1.75E-56	ahsg1	alpha-2-HS-glycoprotein 1 [Source:ZFIN;Acc:ZDB-GENE-041114-50]	22	37874691	37881714	
ENSDARG00000026784	2938.788942	1.609271768	0.113145434	5.384855115	7.25E-08	1.34E-06	robo1	roundabout, axon guidance receptor, homolog 1 (Drosophila) [Source:ZFIN;Acc:ZDB-GENE-000209-3]	15	39277099	39820491	
ENSDARG00000026820	2059.505879	1.599311958	0.157594867	3.802864702	0.000143032	0.001619422	gucy2d	guanylate cyclase 2D, retinal [Source:ZFIN;Acc:ZDB-GENE-011128-9]	5	38123633	38161033	
ENSDARG00000026840	192.6939857	2.070073611	0.206409082	5.184237048	2.17E-07	3.75E-06	arhgap23b	Rho GTPase activating protein 23b [Source:ZFIN;Acc:ZDB-GENE-081031-78]	12	15477111	15561869	
ENSDARG00000026979	873.021657	-1.794195701	0.146944569	-5.404729858	6.49E-08	1.21E-06	krt1-c5	keratin, type 1, gene c5 [Source:ZFIN;Acc:ZDB-GENE-060316-3]	5	64869176	64883099	
ENSDARG00000027009	2631.58582	-1.568196041	0.094178681	-6.033170518	1.61E-09	3.6E-08	zgc:85777	zgc:85777 [Source:ZFIN;Acc:ZDB-GENE-040426-2210]	19	40861853	40898600	
ENSDARG00000027079	440.6770809	1.937526676	0.184440007	5.083098241	3.71E-07	6.31E-06	znf804a	zinc finger protein 804A [Source:ZFIN;Acc:ZDB-GENE-070912-632]	9	11906770	12034444	
ENSDARG00000027153	100.178045	2.104674419	0.312128061	3.539170477	0.000401387	0.004166751	gabrr3a	gamma-aminobutyric acid (GABA) A receptor, rho 3a [Source:ZFIN;Acc:ZDB-GENE-080722-20]	10	35367722	35410518	
ENSDARG00000027236	2544.345909	2.121254695	0.103581998	10.82480278	2.63E-27	2.18E-25	rs1a	retinoschisin 1a [Source:ZFIN;Acc:ZDB-GENE-040801-171]	11	30244356	30252098	
ENSDARG00000027355	33456.66381	-1.496563128	0.110154197	-4.507891115	6.55E-06	9.38E-05	slc25a4	solute carrier family 25 member 4 [Source:ZFIN;Acc:ZDB-GENE-030131-5275]	1	17676745	17679089	
ENSDARG00000027360	530.2972759	1.698177493	0.171606138	4.068487881	4.73E-05	0.000581096	si:ch211-106h11.3	si:ch211-106h11.3 [Source:ZFIN;Acc:ZDB-GENE-050208-296]	3	5575313	5581867	
ENSDARG00000027394	1375.12563	1.766435115	0.178459341	4.294732402	1.75E-05	0.000233029	si:ch211-150g13.3	si:ch211-150g13.3 [Source:ZFIN;Acc:ZDB-GENE-160728-8]	7	32722227	32749591	
ENSDARG00000027423	1312.628023	1.497207914	0.112624637	4.414734867	1.01E-05	0.00014002	igf1ra	insulin-like growth factor 1a receptor [Source:ZFIN;Acc:ZDB-GENE-020503-1]	18	20881462	21047580	
ENSDARG00000027497	449.5773228	2.514763003	0.163218463	9.280586103	1.69E-20	9.49E-19	mast1a	microtubule associated serine/threonine kinase 1a [Source:ZFIN;Acc:ZDB-GENE-121214-221]	1	51312752	51357850	
ENSDARG00000027564	157.909863	1.848649118	0.30770896	2.757960373	0.005816325	0.047171428	pak6a	p21 protein (Cdc42/Rac)-activated kinase 6a [Source:ZFIN;Acc:ZDB-GENE-130410-1]	17	2162916	2265029	
ENSDARG00000027602	285.4597356	2.000058129	0.207850495	4.811430099	1.5E-06	2.36E-05	lrfn1	leucine rich repeat and fibronectin type III domain containing 1 [Source:NCBI gene;Acc:393587]	15	45994123	46132191	
ENSDARG00000027618	88.84366782	2.005691879	0.290051317	3.467289481	0.000525735	0.005327607	grip2b	glutamate receptor interacting protein 2b [Source:ZFIN;Acc:ZDB-GENE-070705-172]	22	31207226	31466591	
ENSDARG00000027799	182.9136301	-2.933077375	0.229294849	-8.430531169	3.44E-17	1.51E-15	ucmaa	upper zone of growth plate and cartilage matrix associated a [Source:ZFIN;Acc:ZDB-GENE-070410-71]	4	7841627	7850489	
ENSDARG00000027828	6280.849483	1.663250707	0.164317167	4.036405452	5.43E-05	0.000660714	grin1a	glutamate receptor, ionotropic, N-methyl D-aspartate 1a [Source:ZFIN;Acc:ZDB-GENE-051202-1]	21	11468642	11502971	
ENSDARG00000027955	792.0983755	-2.16092256	0.127549613	-9.101733295	8.89E-20	4.67E-18	slc35d1b	solute carrier family 35 member D1b [Source:ZFIN;Acc:ZDB-GENE-040426-1213]	2	5793908	5810326	
ENSDARG00000028000	5016.135658	1.365957201	0.114031903	3.20925278	0.001330804	0.012418037	pfkpa	phosphofructokinase, platelet a [Source:ZFIN;Acc:ZDB-GENE-091112-22]	24	3963684	4004537	
ENSDARG00000028012	161.0383763	-3.278480859	0.265146523	-8.593289611	8.45E-18	3.89E-16	tpmt.2	thiopurine S-methyltransferase, tandem duplicate 2 [Source:ZFIN;Acc:ZDB-GENE-041010-177]	19	47299166	47309383	
ENSDARG00000028027	1597.614905	-1.282278417	0.097778567	-2.886915049	0.003890393	0.033001941	trim63a	tripartite motif containing 63a [Source:ZFIN;Acc:ZDB-GENE-040625-139]	6	15651977	15653494	
ENSDARG00000028087	3335.35147	-1.459878271	0.084351197	-5.451947167	4.98E-08	9.4E-07	aldh2.2	aldehyde dehydrogenase 2 family member, tandem duplicate 2 [Source:ZFIN;Acc:ZDB-GENE-030326-5]	5	69597166	69621227	
ENSDARG00000028099	616.01327	1.65338607	0.154802086	4.220783366	2.43E-05	0.000313543	sh2d3ca	SH2 domain containing 3Ca [Source:ZFIN;Acc:ZDB-GENE-080102-1]	10	10210455	10274162	
ENSDARG00000028106	3193.407122	-1.765628999	0.093198654	-8.21502206	2.12E-16	8.64E-15	glrx	glutaredoxin (thioltransferase) [Source:ZFIN;Acc:ZDB-GENE-041010-11]	21	11776328	11782093	
ENSDARG00000028118	2275.392887	2.467764911	0.09907089	14.81529949	1.17E-49	2.69E-47	dscamb	Down syndrome cell adhesion molecule b [Source:ZFIN;Acc:ZDB-GENE-031118-67]	15	6138423	6404443	
ENSDARG00000028148	958.1617139	2.115188334	0.152622631	7.306834696	2.74E-13	8.78E-12	pax2a	paired box 2a [Source:NCBI gene;Acc:30425]	13	29770837	29805529	
ENSDARG00000028396	6157.549209	1.396439992	0.097957651	4.047054912	5.19E-05	0.000633215	fkbp5	FKBP prolyl isomerase 5 [Source:ZFIN;Acc:ZDB-GENE-030616-630]	6	41096058	41117242	
ENSDARG00000028469	128.1990727	2.266295105	0.298437758	4.24307941	2.2E-05	0.00028689	rps6ka2	ribosomal protein S6 kinase, polypeptide 2 [Source:ZFIN;Acc:ZDB-GENE-090827-1]	15	7086735	7157062	
ENSDARG00000028521	149.6563378	1.887383376	0.273979732	3.23886504	0.001200063	0.011286172	c1ql3b	complement component 1, q subcomponent-like 3b [Source:ZFIN;Acc:ZDB-GENE-040426-1836]	3	31396149	31430489	
ENSDARG00000028583	178.8691853	1.738957629	0.226959884	3.255895347	0.001130354	0.010690662	SLC16A3	si:ch211-234h8.7 [Source:ZFIN;Acc:ZDB-GENE-061009-16]	3	36160979	36167394	SLC16A3
ENSDARG00000028688	533.4372957	-1.401425441	0.126827344	-3.165133229	0.00155012	0.014247364	exosc2	exosome component 2 [Source:ZFIN;Acc:ZDB-GENE-050417-27]	5	72137331	72143292	
ENSDARG00000028740	75.04590043	3.217681827	0.383385949	5.784462979	7.27E-09	1.52E-07	msnb	moesin b [Source:ZFIN;Acc:ZDB-GENE-030717-7]	14	9287683	9336601	
ENSDARG00000028793	1597.358907	1.395923874	0.103490538	3.825701181	0.0001304	0.001486458	nlk2	nemo-like kinase, type 2 [Source:ZFIN;Acc:ZDB-GENE-080320-1]	15	30157602	30301528	
ENSDARG00000028824	426.3300449	-1.746563491	0.272408975	-2.740597999	0.006132749	0.049481882	slc34a1a	solute carrier family 34 member 1a [Source:ZFIN;Acc:ZDB-GENE-101006-3]	14	834619	858987	
ENSDARG00000028889	4910.258567	-1.473986964	0.086908858	-5.453839541	4.93E-08	9.32E-07	ndufb10	NADH:ubiquinone oxidoreductase subunit B10 [Source:ZFIN;Acc:ZDB-GENE-040426-1691]	3	18405136	18410968	
ENSDARG00000029011	2320.212255	-1.322990296	0.093987473	-3.436524944	0.000589228	0.005916086	xpnpep1	X-prolyl aminopeptidase (aminopeptidase P) 1, soluble [Source:ZFIN;Acc:ZDB-GENE-040426-999]	22	27092469	27115241	
ENSDARG00000029057	118.2940122	3.074672007	0.31360673	6.61552132	3.7E-11	9.91E-10	tm6sf2	transmembrane 6 superfamily member 2 [Source:ZFIN;Acc:ZDB-GENE-060503-338]	22	17784414	17823608	
ENSDARG00000029100	1282.274923	-1.52694395	0.110941711	-4.749737001	2.04E-06	3.16E-05	chchd1	coiled-coil-helix-coiled-coil-helix domain containing 1 [Source:ZFIN;Acc:ZDB-GENE-060810-176]	13	21768447	21776678	
ENSDARG00000029114	3040.90406	2.084755613	0.19191118	5.652383629	1.58E-08	3.18E-07	si:dkey-40m6.8	si:dkey-40m6.8 [Source:ZFIN;Acc:ZDB-GENE-100922-162]	16	32059785	32080875	
ENSDARG00000029146	6304.220025	1.682585387	0.143202266	4.766582303	1.87E-06	2.92E-05	lrp1ab	low density lipoprotein receptor-related protein 1Ab [Source:ZFIN;Acc:ZDB-GENE-030131-7126]	23	25452656	25686954	
ENSDARG00000029177	964.5082313	1.803365729	0.127278555	6.31187026	2.76E-10	6.76E-09	lnx2a	ligand of numb-protein X 2a [Source:ZFIN;Acc:ZDB-GENE-060228-2]	24	21646317	21674996	
ENSDARG00000029230	1391.579807	-1.794212594	0.145909341	-5.443192258	5.23E-08	9.85E-07	pnp4b	purine nucleoside phosphorylase 4b [Source:ZFIN;Acc:ZDB-GENE-040426-1887]	16	36807924	36834633	
ENSDARG00000029296	1149.230139	1.698465155	0.177599337	3.932813976	8.4E-05	0.00099079	nexmifb	neurite extension and migration factor b [Source:ZFIN;Acc:ZDB-GENE-140106-18]	14	11103718	11268375	
ENSDARG00000029463	3349.976414	1.381561782	0.096013555	3.974040781	7.07E-05	0.000844228	asap1a	ArfGAP with SH3 domain, ankyrin repeat and PH domain 1a [Source:ZFIN;Acc:ZDB-GENE-070912-129]	2	32263215	32356539	
ENSDARG00000029482	384.4715647	2.597039358	0.176201528	9.063708889	1.26E-19	6.57E-18	ush2a	Usher syndrome 2A (autosomal recessive, mild) [Source:ZFIN;Acc:ZDB-GENE-060503-794]	17	29376854	29771639	
ENSDARG00000029688	121.6144903	2.268468243	0.333879667	3.799177879	0.000145177	0.00164148	hsp70.1	heat shock cognate 70-kd protein, tandem duplicate 1 [Source:ZFIN;Acc:ZDB-GENE-990415-91]	3	26112780	26115559	
ENSDARG00000029747	735.4098911	-4.740338914	0.199517548	-18.74691699	2.05E-78	9.03E-76	mep1a.1	meprin A, alpha (PABA peptide hydrolase), tandem duplicate 1 [Source:ZFIN;Acc:ZDB-GENE-041001-209]	20	35473288	35481584	
ENSDARG00000029761	1410.710081	2.025886134	0.126755408	8.093430886	5.8E-16	2.29E-14	mpp6b	membrane protein, palmitoylated 6b (MAGUK p55 subfamily member 6) [Source:ZFIN;Acc:ZDB-GENE-040426-775]	19	20061892	20106486	
ENSDARG00000029795	802.7475532	-1.992422409	0.151899729	-6.533404733	6.43E-11	1.68E-09	prxl2b	peroxiredoxin like 2B [Source:NCBI gene;Acc:406605]	11	40622310	40647237	
ENSDARG00000029822	2207.724794	-4.825595081	0.144741862	-26.43046759	6.12E-154	7.68E-151	cel.2	carboxyl ester lipase, tandem duplicate 2 [Source:ZFIN;Acc:ZDB-GENE-061110-10]	21	11385031	11393979	
ENSDARG00000029830	217.4221024	-1.764030114	0.214939366	-3.554630905	0.00037851	0.003955446	myf6	myogenic factor 6 [Source:NCBI gene;Acc:404208]	4	21717793	21720943	
ENSDARG00000029832	709.7214609	-1.503544883	0.174804444	-2.880618322	0.003968959	0.033622967	slc26a1	solute carrier family 26 member 1 [Source:ZFIN;Acc:ZDB-GENE-070112-2182]	21	20324562	20329541	
ENSDARG00000029866	103.4750757	2.06415051	0.284982585	3.734089615	0.000188395	0.002081388	slc6a14	solute carrier family 6 member 14 [Source:ZFIN;Acc:ZDB-GENE-130411-2]	11	20994026	21014572	
ENSDARG00000029890	38.15395643	-2.870140873	0.421304154	-4.438932905	9.04E-06	0.000126071	si:dkeyp-86f7.4	si:dkeyp-86f7.4 [Source:ZFIN;Acc:ZDB-GENE-091204-252]	14	9432627	9447115	
ENSDARG00000029905	1984.570532	-1.500707656	0.112788632	-4.439345075	9.02E-06	0.0001259	phyhd1	phytanoyl-CoA dioxygenase domain containing 1 [Source:NCBI gene;Acc:492804]	21	4508959	4525080	
ENSDARG00000029995	13047.63524	-1.354781348	0.094762247	-3.743910239	0.000181178	0.002008727	tnni2b.2	troponin I type 2b (skeletal, fast), tandem duplicate 2 [Source:ZFIN;Acc:ZDB-GENE-040801-9]	7	39385540	39394430	
ENSDARG00000030154	1415.555838	1.387321716	0.11667126	3.31976971	0.000900917	0.008690978	pak7	p21 protein (Cdc42/Rac)-activated kinase 7 [Source:ZFIN;Acc:ZDB-GENE-040426-2085]	20	54685143	54744600	
ENSDARG00000030157	1072.894742	-3.142736583	0.130212495	-16.45569095	7.63E-61	2.49E-58	mybpc2a	myosin binding protein C, fast type a [Source:ZFIN;Acc:ZDB-GENE-060901-1]	3	30257582	30318595	
ENSDARG00000030166	905.5027021	-1.647556723	0.111814136	-5.79136725	6.98E-09	1.46E-07	hyi	hydroxypyruvate isomerase [Source:NCBI gene;Acc:393305]	6	2619893	2627495	
ENSDARG00000030254	26.44269612	-2.82672439	0.536116916	-3.40732466	0.00065603	0.006506132	cbln9	cerebellin 9 [Source:ZFIN;Acc:ZDB-GENE-040724-34]	2	37963266	37967368	
ENSDARG00000030262	131.9614235	-2.044055318	0.235160406	-4.439758096	9.01E-06	0.000125728	si:ch211-220i18.4	si:ch211-220i18.4 [Source:ZFIN;Acc:ZDB-GENE-090312-151]	23	9908940	9925568	
ENSDARG00000030376	1151.930543	2.343467696	0.179217574	7.49629438	6.56E-14	2.23E-12	grin2bb	glutamate receptor, ionotropic, N-methyl D-aspartate 2B, genome duplicate b [Source:ZFIN;Acc:ZDB-GENE-061207-27]	1	45372562	45553602	
ENSDARG00000030463	650.8216823	-1.778494286	0.132086758	-5.893810223	3.77E-09	8.16E-08	tppp3	tubulin polymerization-promoting protein family member 3 [Source:ZFIN;Acc:ZDB-GENE-040426-1909]	7	35229645	35243976	
ENSDARG00000030614	10765.07473	2.063082191	0.134709863	7.891643322	2.98E-15	1.13E-13	syt1a	synaptotagmin Ia [Source:ZFIN;Acc:ZDB-GENE-040718-165]	4	21129752	21315399	
ENSDARG00000030621	242.014025	1.778310772	0.227904305	3.415077092	0.00063764	0.006338769	th	tyrosine hydroxylase [Source:ZFIN;Acc:ZDB-GENE-990621-5]	25	24055341	24074500	
ENSDARG00000030750	132.205364	2.404879625	0.284178511	4.943651868	7.67E-07	1.25E-05	gabrr3b	gamma-aminobutyric acid (GABA) A receptor, rho 3b [Source:ZFIN;Acc:ZDB-GENE-131120-131]	24	25258904	25284835	
ENSDARG00000030765	3431.735755	-1.57431878	0.084801083	-6.772540658	1.27E-11	3.56E-10	hadh	hydroxyacyl-CoA dehydrogenase [Source:ZFIN;Acc:ZDB-GENE-040801-261]	1	49909672	49919042	
ENSDARG00000030781	1611.701389	-1.440782547	0.09628298	-4.577990267	4.69E-06	6.89E-05	acads	acyl-CoA dehydrogenase short chain [Source:ZFIN;Acc:ZDB-GENE-040808-64]	10	43999263	44017665	
ENSDARG00000030915	371.2494039	-3.358521749	0.201248452	-11.71945289	1.01E-31	1.08E-29	cpa1	carboxypeptidase A1 (pancreatic) [Source:ZFIN;Acc:ZDB-GENE-050522-438]	25	18448186	18454016	
ENSDARG00000030980	579.9784301	-1.922339032	0.132007517	-6.987019004	2.81E-12	8.33E-11	csrp1b	cysteine and glycine-rich protein 1b [Source:ZFIN;Acc:ZDB-GENE-070112-252]	6	54838269	54845622	
ENSDARG00000031049	956.7270341	1.641694404	0.118623265	5.409515616	6.32E-08	1.18E-06	igsf21a	immunoglobin superfamily, member 21a [Source:ZFIN;Acc:ZDB-GENE-050809-118]	11	28642913	29082429	
ENSDARG00000031161	194.5884359	2.095791589	0.224101411	4.889713021	1.01E-06	1.63E-05	nr1d4a	nuclear receptor subfamily 1, group D, member 4a [Source:ZFIN;Acc:ZDB-GENE-080403-5]	23	35795759	35821521	
ENSDARG00000031164	6857.302234	-1.628505188	0.079120321	-7.943663233	1.96E-15	7.5E-14	tuba8l2	tubulin, alpha 8 like 2 [Source:ZFIN;Acc:ZDB-GENE-040426-1646]	1	5402476	5419116	
ENSDARG00000031222	1102.768315	1.400154341	0.111855064	3.577436082	0.000346981	0.003651812	lhx2b	LIM homeobox 2b [Source:ZFIN;Acc:ZDB-GENE-051220-1]	8	3085120	3101392	
ENSDARG00000031278	54.97434967	-3.026904226	0.370948749	-5.464108539	4.65E-08	8.8E-07	si:ch73-193i2.2	si:ch73-193i2.2 [Source:ZFIN;Acc:ZDB-GENE-141215-32]	13	3327417	3351708	
ENSDARG00000031293	824.5014657	-1.646650885	0.113455708	-5.69958882	1.2E-08	2.45E-07	dnajc9	DnaJ (Hsp40) homolog, subfamily C, member 9 [Source:ZFIN;Acc:ZDB-GENE-040718-130]	12	9497646	9510403	
ENSDARG00000031299	583.1345967	-1.665370492	0.138250444	-4.812791013	1.49E-06	2.35E-05	dhrs11b	dehydrogenase/reductase (SDR family) member 11b [Source:ZFIN;Acc:ZDB-GENE-040625-155]	15	15403560	15408113	
ENSDARG00000031316	661.5979915	1.56563344	0.162705879	3.476416724	0.000508162	0.005166211	six6b	SIX homeobox 6b [Source:ZFIN;Acc:ZDB-GENE-050522-308]	20	20531002	20533865	
ENSDARG00000031435	9483.877912	-1.37115147	0.092528212	-4.011224939	6.04E-05	0.000729651	TXN	zgc:56493 [Source:ZFIN;Acc:ZDB-GENE-030131-8581]	1	40263671	40270561	TXN

ENSDARG00000031483	4553.882789	1.497104625	0.091409824	5.438196942	5.38E-08	1.01E-06	col9a1b	collagen, type IX, alpha 1b [Source:ZFIN;Acc:ZDB-GENE-040426-2410]	13	39119460	39160018	
ENSDARG00000031494	7422.281902	1.297118543	0.107388467	2.766763972	0.005661573	0.046095083	prrc2a	proline-rich coiled-coil 2A [Source:ZFIN;Acc:ZDB-GENE-010501-4]	19	26739813	26770083	
ENSDARG00000031587	606.8050507	1.594373942	0.131348587	4.525164351	6.03E-06	8.72E-05	flvcr1	feline leukemia virus subgroup C cellular receptor 1 [Source:ZFIN;Acc:ZDB-GENE-041014-359]	20	37844035	37862535	
ENSDARG00000031588	2254.259572	4.113285198	0.630897812	4.93469012	8.03E-07	1.31E-05	si:dkey-239b22.1	si:dkey-239b22.1 [Source:ZFIN;Acc:ZDB-GENE-131119-12]	12	38929663	38974869	
ENSDARG00000031618	400.3360204	1.457304993	0.147199578	3.106700446	0.00189188	0.017082043	unm_sa1261	un-named sa1261 [Source:ZFIN;Acc:ZDB-GENE-120411-10]	3	58506242	58543658	
ENSDARG00000031657	544.4087948	-1.689145178	0.136280843	-5.056801546	4.26E-07	7.19E-06	fahd1	fumarylacetoacetate hydrolase domain containing 1 [Source:ZFIN;Acc:ZDB-GENE-050522-448]	3	18795570	18796559	
ENSDARG00000031683	977.0328542	1.414044605	0.126363624	3.276612296	0.001050605	0.010000471	fosab	v-fos FBJ murine osteosarcoma viral oncogene homolog Ab [Source:ZFIN;Acc:ZDB-GENE-031222-4]	20	46552311	46554440	
ENSDARG00000031712	253.1502931	1.963054487	0.217401166	4.429849678	9.43E-06	0.000131143	grm3	glutamate receptor, metabotropic 3 [Source:ZFIN;Acc:ZDB-GENE-061009-13]	18	8972227	9046805	
ENSDARG00000031720	14218.30072	1.779215908	0.081943413	9.509195205	1.92E-21	1.13E-19	clstn1	calsyntenin 1 [Source:ZFIN;Acc:ZDB-GENE-040426-1064]	23	29580584	29668286	
ENSDARG00000031768	1603.272895	1.726284333	0.100322793	7.239474817	4.5E-13	1.42E-11	roraa	RAR-related orphan receptor A, paralog a [Source:NCBI gene;Acc:564951]	25	33849647	33969717	
ENSDARG00000031915	1650.920117	1.419362757	0.114351051	3.667327533	0.000245099	0.002652964	hif1an	hypoxia inducible factor 1 subunit alpha inhibitor [Source:NCBI gene;Acc:373126]	13	29952633	29980215	
ENSDARG00000031952	2166.913479	1.6686629	0.1096082	6.100482428	1.06E-09	2.43E-08	mb	Danio rerio myoglobin (mb), transcript variant 2, mRNA. [Source:RefSeq mRNA;Acc:NM_001362390]	1	55137943	55145085	
ENSDARG00000032083	4073.076559	1.363949208	0.086480534	4.208452377	2.57E-05	0.000330134	dpysl2b	dihydropyrimidinase like 2b [Source:ZFIN;Acc:ZDB-GENE-031105-1]	10	18952271	18992471	
ENSDARG00000032102	3795.383056	-1.419032419	0.087617564	-4.782516207	1.73E-06	2.71E-05	prdx3	peroxiredoxin 3 [Source:ZFIN;Acc:ZDB-GENE-030826-18]	13	9842276	9851472	
ENSDARG00000032199	183.1244047	1.884158309	0.240729423	3.672830254	0.000239879	0.002597584	gpc3	glypican 3 [Source:NCBI gene;Acc:387365]	14	31176952	31465905	
ENSDARG00000032206	33.02711442	-2.760744677	0.466372507	-3.775404108	0.000159748	0.001792511	cthl	cystathionase (cystathionine gamma-lyase), like [Source:ZFIN;Acc:ZDB-GENE-040905-3]	6	37389683	37403444	
ENSDARG00000032317	3320.290121	1.768544976	0.096289339	7.981620682	1.44E-15	5.6E-14	tox	thymocyte selection-associated high mobility group box [Source:ZFIN;Acc:ZDB-GENE-070912-181]	2	21982911	22108108	
ENSDARG00000032344	1070.613202	1.454671992	0.135106254	3.365291973	0.000764627	0.007485531	pde4a	phosphodiesterase 4A, cAMP-specific [Source:ZFIN;Acc:ZDB-GENE-131125-7]	3	46628885	46754730	
ENSDARG00000032496	844.6702039	-2.035116166	0.118169144	-8.759614636	1.96E-18	9.39E-17	pon1	paraoxonase 1 [Source:ZFIN;Acc:ZDB-GENE-040912-6]	16	24646600	24656265	
ENSDARG00000032553	1923.377764	3.721545043	0.143516617	18.96327481	3.43E-80	1.62E-77	egln3	egl-9 family hypoxia-inducible factor 3 [Source:ZFIN;Acc:ZDB-GENE-040426-2541]	17	9912036	9936915	
ENSDARG00000032565	756.4481615	2.231236368	0.131951952	9.330944704	1.05E-20	5.95E-19	cacng2a	calcium channel, voltage-dependent, gamma subunit 2a [Source:ZFIN;Acc:ZDB-GENE-040426-1401]	3	29714775	29845175	
ENSDARG00000032578	175.351197	1.890853637	0.26945867	3.306086368	0.000946089	0.009095283	pax2b	paired box 2b [Source:ZFIN;Acc:ZDB-GENE-001030-4]	12	45799982	45876387	
ENSDARG00000032584	148.5889672	-2.780105397	0.24355445	-7.308860081	2.69E-13	8.68E-12	thnsl2	threonine synthase-like 2 [Source:NCBI gene;Acc:641568]	5	72094502	72105216	
ENSDARG00000032639	1488.263085	-2.16439814	0.184520354	-6.310404867	2.78E-10	6.82E-09	cd36	CD36 molecule (thrombospondin receptor) [Source:ZFIN;Acc:ZDB-GENE-040718-55]	4	22658472	22671469	
ENSDARG00000032705	1932.680793	3.13863997	0.114379534	18.69775033	5.16E-78	2.23E-75	foxg1b	forkhead box G1b [Source:ZFIN;Acc:ZDB-GENE-040426-2437]	11	43114108	43116249	
ENSDARG00000032725	78999.68163	-1.420448101	0.127847109	-3.288678979	0.001006588	0.009610634	rps27a	ribosomal protein S27a [Source:ZFIN;Acc:ZDB-GENE-030131-10018]	6	6270686	6273519	
ENSDARG00000032737	2505.226811	1.588162549	0.168139821	3.498056242	0.000468662	0.004809441	gria3a	glutamate receptor, ionotropic, AMPA 3a [Source:ZFIN;Acc:ZDB-GENE-020125-5]	5	23353570	23429228	
ENSDARG00000032799	488.7782977	2.414103278	0.183590377	7.702491299	1.33E-14	4.79E-13	kcnd2	potassium voltage-gated channel, Shal-related subfamily, member 2 [Source:ZFIN;Acc:ZDB-GENE-041210-70]	4	10365857	10562174	
ENSDARG00000032836	2123.731055	-1.426045445	0.111184558	-3.831876061	0.00012717	0.00145095	pvalb5	parvalbumin 5 [Source:ZFIN;Acc:ZDB-GENE-050417-336]	3	61208544	61218375	
ENSDARG00000032838	400.5862535	2.170709611	0.188832987	6.199709238	5.66E-10	1.34E-08	si:dkey-206f10.1	si:dkey-206f10.1 [Source:ZFIN;Acc:ZDB-GENE-041014-154]	20	43602810	43644531	
ENSDARG00000032868	4300.980341	1.391567331	0.137005833	2.858034008	0.004262747	0.035749821	pde4ba	phosphodiesterase 4B, cAMP-specific a [Source:ZFIN;Acc:ZDB-GENE-030131-9570]	6	30851545	30954268	
ENSDARG00000032959	380.6025791	1.829031127	0.221433775	3.743923553	0.000181169	0.002008727	kcnc1b	potassium voltage-gated channel, Shaw-related subfamily, member 1b [Source:ZFIN;Acc:ZDB-GENE-080414-3]	25	8063455	8094080	
ENSDARG00000032985	762.0489201	-1.359159312	0.124693579	-2.880335269	0.003972525	0.033630471	mrpl27	mitochondrial ribosomal protein L27 [Source:ZFIN;Acc:ZDB-GENE-050522-129]	12	21684239	21688087	
ENSDARG00000033029	984.629808	3.888045989	0.493944495	5.846903888	5.01E-09	1.07E-07	pkd1b	polycystic kidney disease 1b [Source:ZFIN;Acc:ZDB-GENE-100707-2]	12	21697540	21748008	
ENSDARG00000033175	1436.925262	-1.378442024	0.102232293	-3.701785551	0.000214088	0.002335424	snrpe	small nuclear ribonucleoprotein polypeptide E [Source:NCBI gene;Acc:393979]	11	37720552	37726201	
ENSDARG00000033227	939.6299813	-1.418452375	0.108634125	-3.851942237	0.000117185	0.001342516	lect2l	leukocyte cell-derived chemotaxin 2 like [Source:ZFIN;Acc:ZDB-GENE-070127-4]	14	36885524	36887063	
ENSDARG00000033285	3092.438832	-2.999996317	0.13334697	-14.99843844	7.52E-51	1.78E-48	gsto2	glutathione S-transferase omega 2 [Source:ZFIN;Acc:ZDB-GENE-041114-67]	13	25396896	25402772	
ENSDARG00000033296	519.0103271	1.499870728	0.177350321	2.818549892	0.004824111	0.039963702	gpr37b	G protein-coupled receptor 37b [Source:ZFIN;Acc:ZDB-GENE-041210-264]	4	3980247	3998490	
ENSDARG00000033364	7430.114152	-2.004971987	0.091239725	-11.01463186	3.25E-28	2.85E-26	mgst3b	microsomal glutathione S-transferase 3b [Source:ZFIN;Acc:ZDB-GENE-061215-48]	13	45476181	45482003	
ENSDARG00000033382	107.1947155	2.586026459	0.331654795	4.782160501	1.73E-06	2.72E-05	grifin	galectin-related inter-fiber protein [Source:NCBI gene;Acc:445036]	3	41784542	41795917	
ENSDARG00000033411	131.6515656	2.595735123	0.264785401	6.026522294	1.68E-09	3.75E-08	cabp1b	calcium binding protein 1b [Source:ZFIN;Acc:ZDB-GENE-040718-111]	10	41409255	41450454	
ENSDARG00000033516	865.2984469	1.705976507	0.163530972	4.317081328	1.58E-05	0.000212453	efr3ba	EFR3 homolog Ba (S. cerevisiae) [Source:ZFIN;Acc:ZDB-GENE-030616-64]	17	33232748	33289349	
ENSDARG00000033594	131.501633	-2.14787536	0.242042813	-4.742447609	2.11E-06	3.27E-05	ido1	indoleamine 2,3-dioxygenase 1 [Source:ZFIN;Acc:ZDB-GENE-080228-2]	8	50463227	50482781	
ENSDARG00000033616	1671.086334	1.570373713	0.101198284	5.636199425	1.74E-08	3.49E-07	selenon	selenoprotein N [Source:NCBI gene;Acc:352914]	17	24687338	24695198	
ENSDARG00000033760	491.9315231	1.698235381	0.139302977	5.012350762	5.38E-07	8.96E-06	pmelb	premelanosome protein b [Source:ZFIN;Acc:ZDB-GENE-050912-2]	23	35672542	35690336	
ENSDARG00000033789	4965.312486	-1.582782341	0.098865113	-5.894721815	3.75E-09	8.12E-08	ndufb7	NADH:ubiquinone oxidoreductase subunit B7 [Source:ZFIN;Acc:ZDB-GENE-040426-1886]	1	55780128	55785722	
ENSDARG00000033840	1472.353596	2.031009448	0.105511126	9.77157086	1.49E-22	9.31E-21	fat3a	FAT atypical cadherin 3a [Source:ZFIN;Acc:ZDB-GENE-060929-1254]	15	40665310	40989931	
ENSDARG00000033845	2650.132935	1.892135946	0.200824421	4.442367822	8.9E-06	0.00012449	igsf9ba	immunoglobulin superfamily, member 9Ba [Source:ZFIN;Acc:ZDB-GENE-060503-729]	15	19131702	19250543	
ENSDARG00000034007	2503.013387	1.822129602	0.104100552	7.897456716	2.85E-15	1.08E-13	prom1b	prominin 1 b [Source:ZFIN;Acc:ZDB-GENE-031003-1]	1	22712931	22757145	
ENSDARG00000034076	509.2884541	1.455397495	0.142363709	3.198831344	0.001379859	0.012832839	lrp11	low density lipoprotein receptor-related protein 11 [Source:ZFIN;Acc:ZDB-GENE-030131-7847]	20	1278184	1287799	
ENSDARG00000034165	360.4685623	1.963778672	0.193263607	4.986860616	6.14E-07	1.01E-05	lingo1a	leucine rich repeat and Ig domain containing 1a [Source:ZFIN;Acc:ZDB-GENE-080327-16]	25	7229046	7230905	
ENSDARG00000034268	5148.159475	1.348979744	0.1207696	2.889632356	0.003856926	0.032740176	slit3	slit homolog 3 (Drosophila) [Source:ZFIN;Acc:ZDB-GENE-010306-4]	14	24491818	24761161	
ENSDARG00000034344	3189.237378	1.905219644	0.090386701	10.01496499	1.31E-23	8.79E-22	pcdh19	protocadherin 19 [Source:ZFIN;Acc:ZDB-GENE-030131-4218]	14	40300961	40390757	
ENSDARG00000034420	270.2241839	2.098039276	0.203061034	5.407434678	6.39E-08	1.19E-06	irx6a	iroquois homeobox 6a [Source:ZFIN;Acc:ZDB-GENE-040712-5]	7	35511732	35516251	
ENSDARG00000034423	2193.29264	1.791371686	0.098368749	8.044950165	8.63E-16	3.38E-14	sncga	synuclein, gamma a [Source:ZFIN;Acc:ZDB-GENE-050417-18]	13	22659153	22673166	
ENSDARG00000034434	1243.420337	1.431853731	0.11105716	3.888571722	0.000100836	0.001171255	igf1rb	insulin-like growth factor 1b receptor [Source:ZFIN;Acc:ZDB-GENE-020503-2]	7	15446229	15610129	
ENSDARG00000034493	1283.74081	2.2250405	0.128512304	9.532476371	1.54E-21	9.09E-20	grin2aa	glutamate receptor, ionotropic, N-methyl D-aspartate 2A, a [Source:ZFIN;Acc:ZDB-GENE-070424-129]	3	27071749	27277695	
ENSDARG00000034503	342.9556862	1.435670154	0.159003573	2.740002288	0.006143876	0.049555727	per2	period circadian clock 2 [Source:ZFIN;Acc:ZDB-GENE-011220-2]	2	48309600	48375342	
ENSDARG00000034650	210.3641714	-1.519155513	0.188479072	-2.754446455	0.005879151	0.047619415	fabp7b	fatty acid binding protein 7, brain, b [Source:ZFIN;Acc:ZDB-GENE-040614-4]	20	40369257	40370736	
ENSDARG00000034668	3103.694793	2.096132551	0.10300055	10.64200682	1.9E-26	1.49E-24	celf3a	cugbp, Elav-like family member 3a [Source:NCBI gene;Acc:57922]	19	8816657	8880688	
ENSDARG00000034677	2609.789819	-1.713034496	0.112177765	-6.356290786	2.07E-10	5.15E-09	scel	sciellin [Source:ZFIN;Acc:ZDB-GENE-030616-15]	9	17306162	17334658	
ENSDARG00000034836	1322.646761	-1.446072727	0.113841362	-3.918371298	8.91E-05	0.001045183	si:dkey-222n6.2	si:dkey-222n6.2 [Source:ZFIN;Acc:ZDB-GENE-031118-10]	8	7084974	7093507	
ENSDARG00000034852	1074.940464	1.640299274	0.138502009	4.623032385	3.78E-06	5.62E-05	nt5c2l1	5'-nucleotidase, cytosolic II, like 1 [Source:ZFIN;Acc:ZDB-GENE-031006-8]	10	17460358	17484764	
ENSDARG00000034940	139.2416174	2.507524212	0.258065252	5.841639663	5.17E-09	1.11E-07	slc1a7a	solute carrier family 1 member 7a [Source:ZFIN;Acc:ZDB-GENE-061009-24]	2	5948534	5999636	
ENSDARG00000035136	6388.124124	-1.395446084	0.092120801	-4.292690474	1.77E-05	0.000234934	selenow1	selenoprotein W, 1 [Source:NCBI gene;Acc:352915]	5	61998641	62005081	
ENSDARG00000035167	996.7864303	-1.41014563	0.129968577	-3.155729171	0.001600974	0.014650404	mrpl48	mitochondrial ribosomal protein L48 [Source:ZFIN;Acc:ZDB-GENE-041008-125]	10	36684000	36691681	
ENSDARG00000035253	1600.605293	1.590785549	0.118035199	5.005164154	5.58E-07	9.27E-06	npr3	natriuretic peptide receptor 3 [Source:ZFIN;Acc:ZDB-GENE-060531-91]	5	41260478	41307550	
ENSDARG00000035257	456.984215	-1.570664098	0.170269328	-3.351537845	0.000803641	0.007827777	akr1a1a	aldo-keto reductase family 1, member A1a (aldehyde reductase) [Source:NCBI gene;Acc:445326]	5	41521043	41531629	
ENSDARG00000035326	1309.767072	-1.339366197	0.114916249	-2.953161099	0.003145378	0.027194212	nccrp1	P1, F-box associated domain containing [Source:NCBI gene;Acc:30078]	5	36850650	36854555	
ENSDARG00000035329	5336.245121	-1.380423906	0.082661942	-4.602165101	4.18E-06	6.18E-05	capns1a	calpain, small subunit 1 a [Source:ZFIN;Acc:ZDB-GENE-030113-3]	5	36654817	36665694	
ENSDARG00000035422	175.9712923	1.940565387	0.220612143	4.263434336	2.01E-05	0.000263867	ccn1l1	cellular communication network factor 1, like 1 [Source:ZFIN;Acc:ZDB-GENE-050417-136]	5	32914321	32920149	
ENSDARG00000035519	25546.41061	1.490656452	0.128491388	3.81859407	0.000134214	0.00152924	histh1l	histone H1 like [Source:ZFIN;Acc:ZDB-GENE-050417-145]	5	27432958	27434351	
ENSDARG00000035544	2149.475942	-2.157530284	0.125360253	-9.233630723	2.62E-20	1.43E-18	etnppl	ethanolamine-phosphate phospho-lyase [Source:NCBI gene;Acc:393421]	7	60079302	60092321	
ENSDARG00000035573	1065.583978	-1.844519168	0.11146993	-7.57620616	3.56E-14	1.23E-12	AK6	zgc:86811 [Source:ZFIN;Acc:ZDB-GENE-040625-113]	5	26199537	26203245	AK6
ENSDARG00000035603	751.6922744	-1.791814433	0.12678248	-6.245456249	4.23E-10	1.01E-08	dao.2	D-amino-acid oxidase, tandem duplicate 2 [Source:ZFIN;Acc:ZDB-GENE-040426-2634]	5	20170960	20185665	
ENSDARG00000035606	40.91879551	3.416006434	0.539009338	4.482309048	7.38E-06	0.000105011	aldh3b2	aldehyde dehydrogenase 3 family, member B2 [Source:ZFIN;Acc:ZDB-GENE-060531-79]	5	19477555	19488134	
ENSDARG00000035634	1097.64166	-1.34129865	0.108638371	-3.141603172	0.001680256	0.01532562	rfc5	replication factor C (activator 1) 5 [Source:ZFIN;Acc:ZDB-GENE-040907-1]	5	12528693	12549732	
ENSDARG00000035648	296.6513023	2.342528261	0.185167791	7.250333617	4.16E-13	1.31E-11	irx4a	iroquois homeobox 4a [Source:ZFIN;Acc:ZDB-GENE-040426-1825]	16	378766	383664	
ENSDARG00000035663	267.1730025	-1.811218597	0.218288186	-3.716273491	0.000202183	0.002221002	zgc:103438	zgc:103438 [Source:ZFIN;Acc:ZDB-GENE-041010-131]	19	42567539	42573297	
ENSDARG00000035665	20.1706387	3.869551575	0.864148203	3.32067065	0.000898014	0.008666305	si:dkey-267n13.1	si:dkey-267n13.1 [Source:ZFIN;Acc:ZDB-GENE-141211-35]	19	42553029	42557556	
ENSDARG00000035692	75456.77114	-1.463553271	0.118990006	-3.895732795	9.79E-05	0.001140346	rps3a	ribosomal protein S3A [Source:NCBI gene;Acc:337240]	1	24355515	24359653	
ENSDARG00000035765	232.0495033	1.863413944	0.19652359	4.39343665	1.12E-05	0.000153541	CU633984.1		15	6678521	6727082	
ENSDARG00000035797	20.03493228	7.378936623	1.538572847	4.146008839	3.38E-05	0.000426101	si:dkey-17m8.2	si:dkey-17m8.2 [Source:ZFIN;Acc:ZDB-GENE-110411-234]	16	18142127	18153001	
ENSDARG00000035835	8550.831888	1.366005438	0.09329907	3.922926972	8.75E-05	0.001028009	eef2k	eukaryotic elongation factor 2 kinase [Source:ZFIN;Acc:ZDB-GENE-020916-2]	12	10381957	10409970	
ENSDARG00000035852	365.1765953	2.654306813	0.196925845	8.400658702	4.44E-17	1.92E-15	cart3	cocaine- and amphetamine-regulated transcript 3 [Source:ZFIN;Acc:ZDB-GENE-120223-5]	7	52962632	52963493	
ENSDARG00000035861	263.3935141	1.743172684	0.208206247	3.569406272	0.000357791	0.003754564	si:rp71-39b20.4	si:rp71-39b20.4 [Source:ZFIN;Acc:ZDB-GENE-070912-704]	2	24495804	24505185	
ENSDARG00000035891	5890.250902	3.634863802	0.17302821	15.22794348	2.31E-52	6.03E-50	acana	aggrecan a [Source:ZFIN;Acc:ZDB-GENE-050208-221]	7	48761646	48787379	
ENSDARG00000036028	6309.190471	2.567158685	0.102634217	15.26935881	1.22E-52	3.3E-50	arrdc3b	arrestin domain containing 3b [Source:ZFIN;Acc:ZDB-GENE-040912-182]	10	43761480	43771447	
ENSDARG00000036031	772.2321553	2.013735085	0.201097174	5.041021028	4.63E-07	7.77E-06	sept5b	septin 5b [Source:ZFIN;Acc:ZDB-GENE-040808-43]	10	43936252	43964028	
ENSDARG00000036044	56653.14098	-1.363360182	0.111011429	-3.273178141	0.001063454	0.010115107	rps20	ribosomal protein S20 [Source:ZFIN;Acc:ZDB-GENE-040426-2284]	7	58745987	58750400	
ENSDARG00000036058	3373.178221	1.622312686	0.115484109	5.388730056	7.1E-08	1.31E-06	gnao1b	guanine nucleotide binding protein (G protein), alpha activating activity polypeptide O, b [Source:ZFIN;Acc:ZDB-GENE-040	7	29133321	29144547	
ENSDARG00000036084	34.38535463	-4.20406994	0.539659614	-5.937205333	2.9E-09	6.37E-08	itln2	intelectin 2 [Source:ZFIN;Acc:ZDB-GENE-050411-58]	7	33145529	33148307	
ENSDARG00000036086	927.1610561	1.602194064	0.114960481	5.238270235	1.62E-07	2.86E-06	itga11a	integrin, alpha 11a [Source:ZFIN;Acc:ZDB-GENE-050324-1]	7	33176517	33248773	
ENSDARG00000036107	21687.15629	1.313030063	0.091160335	3.433840646	0.000595094	0.005963055	txnipa	thioredoxin interacting protein a [Source:ZFIN;Acc:ZDB-GENE-030804-10]	19	24488403	24492326	
ENSDARG00000036139	352.9021336	2.317476995	0.232048963	5.677581905	1.37E-08	2.77E-07	lctla	lactase-like a [Source:ZFIN;Acc:ZDB-GENE-040718-233]	7	34227075	34236091	
ENSDARG00000036152	154.2512257	1.762790137	0.220547338	3.458623191	0.000542944	0.005486488	gas2b	growth arrest-specific 2b [Source:ZFIN;Acc:ZDB-GENE-030616-552]	7	32746914	32782430	
ENSDARG00000036156	479.5702864	1.626200898	0.195888757	3.196716892	0.001390013	0.012908143	fnbp1a	formin binding protein 1a [Source:ZFIN;Acc:ZDB-GENE-081105-105]	8	11578318	11640240	
ENSDARG00000036186	2491.230966	-1.544827894	0.119595864	-4.555574709	5.22E-06	7.63E-05	mbpa	myelin basic protein a [Source:ZFIN;Acc:ZDB-GENE-030128-2]	19	21814047	21832630	
ENSDARG00000036237	10725.44112	-1.464233145	0.098219993	-4.726462806	2.28E-06	3.52E-05	slc27a2a	solute carrier family 27 member 2a [Source:ZFIN;Acc:ZDB-GENE-050706-104]	18	62932	73844	
ENSDARG00000036239	48128.30657	-1.553096429	0.092860699	-5.956194971	2.58E-09	5.7E-08	gatm	glycine amidinotransferase (L-arginine:glycine amidinotransferase) [Source:NCBI gene;Acc:266799]	18	35725	46010	
ENSDARG00000036344	3044.044644	1.434609275	0.118155218	3.678291004	0.000234802	0.002548104	calb2b	calbindin 2b [Source:ZFIN;Acc:ZDB-GENE-040426-1668]	7	24681278	24699985	
ENSDARG00000036375	5075.73811	1.750353292	0.150379148	4.989742948	6.05E-07	1E-05	cyfip2	cytoplasmic FMR1 interacting protein 2 [Source:ZFIN;Acc:ZDB-GENE-080724-2]	14	33982007	34044452	
ENSDARG00000036376	2158.298352	1.776181766	0.117670516	6.596229813	4.22E-11	1.12E-09	cldn7a	claudin 7a [Source:NCBI gene;Acc:436612]	7	22616212	22626853	
ENSDARG00000036383	1291.701273	1.49952528	0.158876359	3.144113335	0.001665909	0.015216879	slc24a6a	solute carrier family 24 member 6a [Source:ZFIN;Acc:ZDB-GENE-100922-39]	14	33898976	33936524	
ENSDARG00000036438	14932.7947	-1.457839006	0.082900257	-5.522769407	3.34E-08	6.44E-07	ndufv1	NADH:ubiquinone oxidoreductase core subunit V1 [Source:ZFIN;Acc:ZDB-GENE-040808-73]	19	9212031	9228512	
ENSDARG00000036446	540.0598267	2.174701744	0.206190681	5.697162159	1.22E-08	2.48E-07	PRKAR2B	si:ch211-272n13.7 [Source:ZFIN;Acc:ZDB-GENE-160113-22]	25	3475384	3503458	PRKAR2B
ENSDARG00000036456	6926.220212	-1.277147343	0.088695005	-3.12472322	0.001779724	0.01615655	anxa4	annexin A4 [Source:ZFIN;Acc:ZDB-GENE-030707-1]	10	41159241	41192881	
ENSDARG00000036481	4646.895913	-1.870609577	0.110031863	-7.912340626	2.53E-15	9.59E-14	tcn2	transcobalamin II [Source:ZFIN;Acc:ZDB-GENE-030131-4648]	5	26795465	26806100	
ENSDARG00000036626	304.582343	2.195100282	0.213090154	5.608425629	2.04E-08	4.05E-07	sema6ba	sema domain, transmembrane domain (TM), and cytoplasmic domain, (semaphorin) 6Ba [Source:ZFIN;Acc:ZDB-GENE-131	2	49232559	49342504	
ENSDARG00000036628	43.25527235	-2.515024482	0.438827363	-3.452438494	0.000555544	0.005591304	cd74b	CD74 molecule, major histocompatibility complex, class II invariant chain b [Source:ZFIN;Acc:ZDB-GENE-990910-10]	21	33459524	33463894	
ENSDARG00000036629	63402.77251	-1.467679003	0.108744199	-4.300725982	1.7E-05	0.000227176	rps14	ribosomal protein S14 [Source:ZFIN;Acc:ZDB-GENE-030131-8631]	21	33454147	33458430	
ENSDARG00000036636	131.3936408	-2.653062152	0.234837774	-7.03916634	1.93E-12	5.83E-11	ebpl	EBP like [Source:ZFIN;Acc:ZDB-GENE-030616-542]	1	46660213	46663997	
ENSDARG00000036684	1666.464391	-1.426962178	0.10964433	-3.894065255	9.86E-05	0.001147153	ndufa7	NADH:ubiquinone oxidoreductase subunit A7 [Source:ZFIN;Acc:ZDB-GENE-040801-169]	8	47804284	47811955	
ENSDARG00000036773	1176.668714	-1.668050714	0.125733931	-5.31320946	1.08E-07	1.94E-06	s100s	S100 calcium binding protein S [Source:ZFIN;Acc:ZDB-GENE-040822-31]	19	7595924	7609286	

ENSDARG00000036816	1124.6823	2.249395363	0.117007762	10.67788446	1.29E-26	1.02E-24	pou2f2a	POU class 2 homeobox 2a [Source:ZFIN;Acc:ZDB-GENE-060310-2]	19	6213680	6303099	
ENSDARG00000036830	119177.6197	-2.799913927	0.101213729	-19.73292782	1.12E-86	6.27E-84	krt91	keratin 91 [Source:ZFIN;Acc:ZDB-GENE-040801-181]	19	5378563	5380770	
ENSDARG00000036832	55552.10137	-2.720267113	0.097202778	-17.69771555	4.37E-70	1.74E-67	cyt1l	type I cytokeratin, enveloping layer, like [Source:ZFIN;Acc:ZDB-GENE-061026-4]	19	5356421	5358455	
ENSDARG00000036834	17121.5542	-2.117581814	0.104117784	-10.73382249	7.06E-27	5.7E-25	krt92	keratin 92 [Source:ZFIN;Acc:ZDB-GENE-050417-363]	19	5349656	5352199	
ENSDARG00000036840	22282.10062	-3.582343706	0.086618996	-29.81267193	2.68E-195	5.17E-192	krt15	keratin 15 [Source:ZFIN;Acc:ZDB-GENE-040426-2931]	11	11515778	11518469	
ENSDARG00000036846	947.0688704	-1.764392638	0.1530914	-4.993047522	5.94E-07	9.86E-06	anks4b	ankyrin repeat and sterile alpha motif domain containing 4B [Source:ZFIN;Acc:ZDB-GENE-040704-29]	3	60812851	60827402	
ENSDARG00000036876	550.4767077	-1.490502221	0.132992174	-3.688203642	0.000225843	0.002453	zgc:153284	zgc:153284 [Source:ZFIN;Acc:ZDB-GENE-060825-317]	23	31426493	31436524	
ENSDARG00000036942	1786.493481	-1.671865981	0.100931719	-6.656638655	2.8E-11	7.57E-10	gpd1c	glycerol-3-phosphate dehydrogenase 1c [Source:ZFIN;Acc:ZDB-GENE-040426-2576]	19	317629	325584	
ENSDARG00000036952	244.0388583	1.825364597	0.196512736	4.200056507	2.67E-05	0.000341743	XKR7	zgc:198371 [Source:ZFIN;Acc:ZDB-GENE-091214-2]	23	30859086	30890850	XKR7
ENSDARG00000036966	1738.043487	-1.309199664	0.09893782	-3.125191817	0.001776792	0.016136669	hsd3b7	hydroxy-delta-5-steroid dehydrogenase, 3 beta- and steroid delta-isomerase [Source:ZFIN;Acc:ZDB-GENE-030131-5673]	3	32743946	32778250	
ENSDARG00000036999	310.0482469	1.947370395	0.193942841	4.88479179	1.04E-06	1.66E-05	DISP3	si:dkey-32e23.6 [Source:ZFIN;Acc:ZDB-GENE-081104-407]	8	51050554	51147594	DISP3
ENSDARG00000037000	1243.422993	-2.287761978	0.104399362	-12.33496022	5.87E-35	7.09E-33	fkbp11	FKBP prolyl isomerase 11 [Source:ZFIN;Acc:ZDB-GENE-030616-357]	6	7428613	7439490	
ENSDARG00000037020	8401.399734	1.793198386	0.092856267	8.542217045	1.32E-17	5.97E-16	kif1b	kinesin family member 1B [Source:ZFIN;Acc:ZDB-GENE-030820-1]	23	29396182	29505645	
ENSDARG00000037057	2684.400981	-1.375186876	0.088092268	-4.259021643	2.05E-05	0.000268849	gcdha	glutaryl-CoA dehydrogenase a [Source:ZFIN;Acc:ZDB-GENE-040426-1855]	6	8314451	8330577	
ENSDARG00000037067	200.3180557	-2.436610571	0.203462669	-7.060806675	1.66E-12	5.04E-11	dnajc22	DnaJ (Hsp40) homolog, subfamily C, member 22 [Source:ZFIN;Acc:ZDB-GENE-090313-196]	23	27696694	27702561	
ENSDARG00000037069	232.5292549	1.548287693	0.193162622	2.838477168	0.004532936	0.037763436	zdhhc14	zinc finger DHHC-type containing 14 [Source:ZFIN;Acc:ZDB-GENE-040724-21]	20	4827735	4883673	
ENSDARG00000037099	1727.785337	2.066585018	0.130196074	8.192144249	2.57E-16	1.04E-14	irs2a	insulin receptor substrate 2a [Source:ZFIN;Acc:ZDB-GENE-040426-975]	1	29211540	29228393	
ENSDARG00000037121	1193.312524	-1.867225843	0.11678968	-7.425534906	1.12E-13	3.74E-12	mat2ab	methionine adenosyltransferase II, alpha b [Source:ZFIN;Acc:ZDB-GENE-050327-6]	5	69199198	69212184	
ENSDARG00000037122	2198.497715	2.351795899	0.121839236	11.09491443	1.33E-28	1.21E-26	TENM2	si:dkey-34l15.2 [Source:ZFIN;Acc:ZDB-GENE-081027-5]	14	29200772	29760015	TENM2
ENSDARG00000037188	1194.193956	-1.589576467	0.126658419	-4.654854165	3.24E-06	4.86E-05	rpa2	replication protein A2 [Source:ZFIN;Acc:ZDB-GENE-010131-3]	16	4746588	4770233	
ENSDARG00000037191	1219.306043	-2.487023342	0.111312778	-13.35896351	1.05E-40	1.67E-38	ttr	transthyretin (prealbumin, amyloidosis type I) [Source:ZFIN;Acc:ZDB-GENE-040927-14]	20	6142433	6157942	
ENSDARG00000037259	2818.410481	-1.402020947	0.088676394	-4.533573461	5.8E-06	8.4E-05	ndufb6	NADH:ubiquinone oxidoreductase subunit B [Source:ZFIN;Acc:ZDB-GENE-040426-1781]	1	26099250	26103232	
ENSDARG00000037267	706.7385134	-1.519578686	0.149137751	-3.483884404	0.000494193	0.005042575	zgc:158263	zgc:158263 [Source:ZFIN;Acc:ZDB-GENE-070112-2262]	23	26733232	26751278	
ENSDARG00000037278	315.6140403	-1.515213236	0.156355923	-3.295130914	0.000983758	0.009417742	lrata	lecithin retinol acyltransferase a [Source:ZFIN;Acc:ZDB-GENE-060720-1]	1	25696798	25698039	
ENSDARG00000037285	824.862361	2.831680446	0.219550702	8.342858534	7.25E-17	3.07E-15	mipa	major intrinsic protein of lens fiber a [Source:ZFIN;Acc:ZDB-GENE-040801-41]	23	27068225	27072774	
ENSDARG00000037307	1133.266745	-1.769511376	0.123092363	-6.251495696	4.07E-10	9.77E-09	gnpda1	glucosamine-6-phosphate deaminase 1 [Source:ZFIN;Acc:ZDB-GENE-050417-417]	14	23709134	23715349	
ENSDARG00000037337	385.1196712	2.28074041	0.175980337	7.277747212	3.39E-13	1.08E-11	cnrip1b	cannabinoid receptor interacting protein 1b [Source:ZFIN;Acc:ZDB-GENE-080722-33]	13	1089942	1097017	
ENSDARG00000037373	399.1651836	2.452896438	0.172846341	8.405711276	4.25E-17	1.84E-15	ephb2a	eph receptor B2a [Source:ZFIN;Acc:ZDB-GENE-070713-2]	23	21803410	21946659	
ENSDARG00000037383	86.96250944	2.237212731	0.357290382	3.462765285	0.000534655	0.005409256	pax5	paired box 5 [Source:ZFIN;Acc:ZDB-GENE-001030-2]	1	21725821	21815554	
ENSDARG00000037402	337.4872248	2.132094301	0.228223601	4.960461123	7.03E-07	1.15E-05	lim2.3	lens intrinsic membrane protein 2.3 [Source:ZFIN;Acc:ZDB-GENE-040718-327]	15	19646902	19660757	
ENSDARG00000037415	231.7392815	1.678235499	0.187202456	3.623005348	0.0002912	0.003107741	dlgap1b	discs, large (Drosophila) homolog-associated protein 1b [Source:ZFIN;Acc:ZDB-GENE-091116-98]	24	9162512	9268048	
ENSDARG00000037421	2590.160311	1.317119713	0.113121937	2.803344078	0.005057568	0.041718704	egr1	early growth response 1 [Source:NCBI gene;Acc:30498]	14	21332522	21336385	
ENSDARG00000037487	226.9390501	1.585257477	0.195073512	3.000189375	0.002698118	0.023705096	1-Mar	membrane-associated ring finger (C3HC4) 1 [Source:ZFIN;Acc:ZDB-GENE-081112-1]	1	19708508	19756359	
ENSDARG00000037533	131.664935	-9.946300228	1.213473677	-7.372471603	1.67E-13	5.5E-12	mep1b	meprin A, beta [Source:ZFIN;Acc:ZDB-GENE-041014-209]	20	6493648	6516539	
ENSDARG00000037539	12043.00034	-1.333674605	0.104809957	-3.183615495	0.001454481	0.013461992	tnnc1b	troponin C type 1b (slow) [Source:ZFIN;Acc:ZDB-GENE-040625-62]	23	20110086	20112596	
ENSDARG00000037551	739.1401348	-3.553001679	0.153203389	-16.66413315	2.39E-62	8.33E-60	pm20d1.1	peptidase M20 domain containing 1, tandem duplicate 1 [Source:NCBI gene;Acc:553216]	23	18020928	18030399	
ENSDARG00000037587	781.699189	1.816323714	0.1853944	4.403173529	1.07E-05	0.000147129	SYNPR	si:dkey-30j16.3 [Source:ZFIN;Acc:ZDB-GENE-141212-294]	11	19622925	19694334	SYNPR
ENSDARG00000037588	833.7915544	1.939389503	0.152155331	6.173884926	6.66E-10	1.56E-08	bhlhe23	basic helix-loop-helix family, member e23 [Source:ZFIN;Acc:ZDB-GENE-050913-22]	23	17655790	17657348	
ENSDARG00000037607	460.7734719	1.699452544	0.148198145	4.719711879	2.36E-06	3.63E-05	nol4lb	nucleolar protein 4-like b [Source:ZFIN;Acc:ZDB-GENE-070112-1702]	23	17220986	17346255	
ENSDARG00000037618	2439.251684	1.665884474	0.105884612	6.288774769	3.2E-10	7.78E-09	ddit4	DNA-damage-inducible transcript 4 [Source:NCBI gene;Acc:378866]	12	48340133	48342259	
ENSDARG00000037646	310.1768698	1.808570181	0.167613608	4.824012732	1.41E-06	2.22E-05	rgs11	regulator of G protein signaling 11 [Source:ZFIN;Acc:ZDB-GENE-080220-46]	1	9886991	9914630	
ENSDARG00000037738	267.1139879	3.136940063	0.216500644	9.870363527	5.6E-23	3.56E-21	fbxl3l	F-box and leucine-rich repeat protein 3, like [Source:ZFIN;Acc:ZDB-GENE-130530-598]	14	22367361	22390821	
ENSDARG00000037748	1022.638811	-1.623984443	0.134144681	-4.651577983	3.29E-06	4.93E-05	slc43a1b	solute carrier family 43 member 1b [Source:ZFIN;Acc:ZDB-GENE-070209-173]	1	44591492	44638058	
ENSDARG00000037790	6153.28366	-2.399837926	0.098464238	-14.21671417	7.22E-46	1.39E-43	pvalb8	parvalbumin 8 [Source:ZFIN;Acc:ZDB-GENE-030805-3]	3	61154219	61162750	
ENSDARG00000037794	35.43875709	3.065101326	0.545955909	3.78254231	0.000155235	0.001744983	nptx2a	neuronal pentraxin 2a [Source:ZFIN;Acc:ZDB-GENE-060707-1]	3	61566374	61593274	
ENSDARG00000037838	2190.540547	1.357544999	0.097332489	3.673439375	0.000239307	0.002592515	unkl	unk like zinc finger [Source:ZFIN;Acc:ZDB-GENE-131120-196]	3	45778810	45818361	
ENSDARG00000037859	58.22485434	7.46946944	1.451192589	4.458036438	8.27E-06	0.000116438	il11a	interleukin 11a [Source:ZFIN;Acc:ZDB-GENE-051019-1]	16	12611362	12617155	
ENSDARG00000037860	12169.07234	-1.31958054	0.087626876	-3.647060743	0.000265257	0.002860076	cox6b2	cytochrome c oxidase subunit 6B2 [Source:ZFIN;Acc:ZDB-GENE-040426-1566]	16	12504929	12512593	
ENSDARG00000037873	565.7961739	-3.534073235	0.188391317	-13.45111481	3.03E-41	4.91E-39	cyp3c3	cytochrome P450, family 3, subfamily c, polypeptide 3 [Source:ZFIN;Acc:ZDB-GENE-041114-102]	3	40947961	40955780	
ENSDARG00000037905	3549.776459	2.234157922	0.444780011	2.774760314	0.00552424	0.045093971	cacna1aa	calcium channel, voltage-dependent, P/Q type, alpha 1A subunit, a [Source:ZFIN;Acc:ZDB-GENE-040724-26]	3	33773156	33901483	
ENSDARG00000037921	1842.708531	1.558982066	0.11942514	4.68060634	2.86E-06	4.32E-05	gng13b	guanine nucleotide binding protein (G protein), gamma 13b [Source:ZFIN;Acc:ZDB-GENE-040718-97]	3	35589212	35602233	
ENSDARG00000037925	991.7861603	1.523805802	0.171387019	3.056274658	0.002241059	0.019976141	rgs9a	regulator of G protein signaling 9a [Source:ZFIN;Acc:ZDB-GENE-060929-92]	3	36366053	36403234	
ENSDARG00000037954	766.240248	-2.249926579	0.129378261	-9.66102475	4.41E-22	2.69E-20	tnnt1	troponin T type 1 (skeletal, slow) [Source:ZFIN;Acc:ZDB-GENE-080723-27]	3	32157140	32170878	
ENSDARG00000037995	66.43705035	2.565579065	0.370470092	4.225925651	2.38E-05	0.000307414	gdf3	growth differentiation factor 3 [Source:NCBI gene;Acc:30125]	17	4239437	4252221	
ENSDARG00000038009	151.1702457	-3.7920341	0.303207205	-9.208336912	3.31E-20	1.8E-18	CT573429.1	GTPase IMAP family member 4-like [Source:NCBI gene;Acc:798736]	3	29664357	29671653	
ENSDARG00000038025	1891.54167	1.627925234	0.110974471	5.658285441	1.53E-08	3.08E-07	cbx7a	chromobox homolog 7a [Source:ZFIN;Acc:ZDB-GENE-050417-400]	3	24986145	24994617	
ENSDARG00000038027	627.8673491	-1.444885306	0.144451995	-3.079814196	0.002071298	0.018568444	nol12	nucleolar protein 12 [Source:NCBI gene;Acc:393260]	3	1150348	1156033	
ENSDARG00000038056	3645.58026	2.495091467	0.099026174	15.0979424	1.67E-51	4.11E-49	fgfbp2b	fibroblast growth factor binding protein 2b [Source:ZFIN;Acc:ZDB-GENE-070410-95]	1	22685961	22691182	
ENSDARG00000038147	28.87119498	3.591447843	0.768164443	3.373558703	0.000742032	0.007298492	hbbe3	hemoglobin beta embryonic-3 [Source:ZFIN;Acc:ZDB-GENE-980526-287]	12	20339413	20340543	
ENSDARG00000038153	14059.19498	-3.021567593	0.119262536	-16.95056686	1.91E-64	6.93E-62	lgals2b	lectin, galactoside-binding, soluble, 2b [Source:ZFIN;Acc:ZDB-GENE-040426-1590]	6	603278	609887	
ENSDARG00000038185	311.1513503	-1.82558191	0.222250769	-3.714641416	0.000203492	0.00223343	gh1	growth hormone 1 [Source:ZFIN;Acc:ZDB-GENE-030725-2]	3	22327738	22332913	
ENSDARG00000038248	22.84230938	6.105844136	1.600691927	3.189773153	0.001423845	0.013193037	ggact.2	gamma-glutamylamine cyclotransferase, tandem duplicate 2 [Source:NCBI gene;Acc:447805]	1	2450140	2453174	
ENSDARG00000038363	224.5675186	1.887209628	0.216094127	4.105662837	4.03E-05	0.00050089	drd4a	dopamine receptor D4a [Source:ZFIN;Acc:ZDB-GENE-070112-996]	25	4707173	4733221	
ENSDARG00000038398	190.0603948	2.021251748	0.250550158	4.076037129	4.58E-05	0.000564215	pmm1	phosphomannomutase 1 [Source:ZFIN;Acc:ZDB-GENE-180314-1]	3	3810919	3821845	
ENSDARG00000038424	5894.646778	1.931399345	0.091877499	10.13740417	3.77E-24	2.64E-22	c4b	complement 4B (Chido blood group) [Source:ZFIN;Acc:ZDB-GENE-100805-2]	16	16665203	16701718	
ENSDARG00000038439	7395.186993	-4.243106232	0.137264898	-23.62662477	2.05E-123	1.78E-120	fabp10a	fatty acid binding protein 10a, liver basic [Source:NCBI gene;Acc:171481]	16	52512025	52516915	
ENSDARG00000038489	1020.54464	1.520034273	0.182735488	2.845830762	0.004429573	0.036988261	b3gnt7	UDP-GlcNAc:betaGal beta-1,3-N-acetylglucosaminyltransferase 7 [Source:ZFIN;Acc:ZDB-GENE-021210-4]	22	36656680	36661461	
ENSDARG00000038561	164.8622658	-1.665976181	0.195377649	-3.408661039	0.000652825	0.006479466	klf6b	Kruppel-like factor 6b [Source:ZFIN;Acc:ZDB-GENE-070912-633]	2	48073972	48082182	
ENSDARG00000038574	6190.348411	1.5807205	0.132866339	4.370711974	1.24E-05	0.000169681	scg2b	secretogranin II (chromogranin C), b [Source:ZFIN;Acc:ZDB-GENE-061103-160]	2	47581997	47585197	
ENSDARG00000038608	3572.367872	-1.564087474	0.092400349	-6.104819719	1.03E-09	2.36E-08	sdhc	succinate dehydrogenase complex, subunit C, integral membrane protein [Source:ZFIN;Acc:ZDB-GENE-040801-26]	2	44192652	44199840	
ENSDARG00000038615	828.491385	1.60947304	0.118229664	5.154992552	2.54E-07	4.36E-06	pip4p1a	phosphatidylinositol-4,5-bisphosphate 4-phosphatase 1a [Source:NCBI gene;Acc:556136]	2	44383175	44398611	
ENSDARG00000038643	2190.1322	1.668040106	0.109972695	6.074599773	1.24E-09	2.81E-08	alas2	aminolevulinate, delta-, synthase 2 [Source:NCBI gene;Acc:64607]	8	21353878	21369028	
ENSDARG00000038667	929.031664	-1.405006443	0.121857804	-3.323598717	0.00088864	0.008579132	fggy	FGGY carbohydrate kinase domain containing [Source:NCBI gene;Acc:406567]	2	111919	115367	
ENSDARG00000038694	3040.194793	-1.286545726	0.090197934	-3.176854653	0.001488816	0.01374431	zgc:101744	zgc:101744 [Source:ZFIN;Acc:ZDB-GENE-050320-111]	2	37089362	37103622	
ENSDARG00000038703	1111.07696	-2.076528085	0.157801622	-6.822034335	8.98E-12	2.56E-10	hkdc1	hexokinase domain containing 1 [Source:ZFIN;Acc:ZDB-GENE-030131-9801]	13	23017498	23031803	
ENSDARG00000038716	6829.342262	-1.696140325	0.153647408	-4.530765161	5.88E-06	8.51E-05	casq1a	calsequestrin 1a [Source:ZFIN;Acc:ZDB-GENE-040801-139]	2	38039857	38054482	
ENSDARG00000038742	154.7284524	2.790700688	0.283064448	6.326123613	2.51E-10	6.21E-09	rbp1.2	retinol binding protein 1, cellular, tandem duplicate 2 [Source:ZFIN;Acc:ZDB-GENE-070912-18]	2	39021282	39028052	
ENSDARG00000038847	208.9110463	-1.627934601	0.192410435	-3.263516347	0.001100388	0.010430834	gins3	GINS complex subunit 3 [Source:ZFIN;Acc:ZDB-GENE-040721-1]	25	13399967	13408760	
ENSDARG00000038859	767.1268735	1.89962622	0.155205382	5.796359677	6.78E-09	1.42E-07	rgs20	regulator of G protein signaling 20 [Source:ZFIN;Acc:ZDB-GENE-040718-128]	2	30752501	30784502	
ENSDARG00000038862	505.2664957	1.738220458	0.18773462	3.932255319	8.42E-05	0.000992629	kcnb2	potassium voltage-gated channel, Shab-related subfamily, member 2 [Source:ZFIN;Acc:ZDB-GENE-070912-170]	2	30144979	30177469	
ENSDARG00000038881	4864.89061	-1.463423131	0.099455552	-4.659600431	3.17E-06	4.75E-05	acaa2	acetyl-CoA acyltransferase 2 [Source:ZFIN;Acc:ZDB-GENE-040426-1996]	8	2509625	2529878	
ENSDARG00000038891	244.1901906	-2.099007883	0.182471938	-6.022887122	1.71E-09	3.83E-08	AL954146.1	trypsin I-P1-like [Source:NCBI gene;Acc:560023]	23	37444933	37453984	
ENSDARG00000038900	6597.79989	-1.755555692	0.083065435	-9.095909626	9.38E-20	4.91E-18	acadm	acyl-CoA dehydrogenase medium chain [Source:ZFIN;Acc:ZDB-GENE-040426-1945]	2	26470070	26476150	
ENSDARG00000038918	226.1537409	1.765421413	0.242265173	3.15943643	0.001580746	0.01449171	drd1b	dopamine receptor D1b [Source:ZFIN;Acc:ZDB-GENE-070524-2]	9	54417141	54419308	
ENSDARG00000038974	1551.647223	2.342935962	0.128381786	10.4604867	1.31E-25	9.68E-24	FO904844.1	dihydropyrimidinase like 2a [Source:NCBI gene;Acc:798556]	8	5119360	5155545	
ENSDARG00000039064	929.2245525	-1.825535149	0.151568925	-5.446598975	5.13E-08	9.67E-07	st3gal7	ST3 beta-galactoside alpha-2,3-sialyltransferase 7 [Source:ZFIN;Acc:ZDB-GENE-060322-2]	13	40716319	40726865	
ENSDARG00000039099	2341.626567	-1.784461457	0.167494913	-4.683494229	2.82E-06	4.27E-05	aep1	aerolysin-like protein [Source:NCBI gene;Acc:503710]	7	8373025	8374950	
ENSDARG00000039116	177.311451	-2.560309235	0.331046141	-4.713268157	2.44E-06	3.73E-05	nr5a5	nuclear receptor subfamily 5, group A, member 5 [Source:ZFIN;Acc:ZDB-GENE-030131-4652]	3	53457192	53465249	
ENSDARG00000039123	977.7518211	2.10248424	0.133960695	8.22990834	1.87E-16	7.67E-15	trim9	tripartite motif containing 9 [Source:ZFIN;Acc:ZDB-GENE-030131-8043]	13	36868365	36911118	
ENSDARG00000039145	100.3814152	-2.094076966	0.292723147	-3.737582689	0.000185798	0.002056312	plaub	plasminogen activator, urokinase b [Source:ZFIN;Acc:ZDB-GENE-030619-15]	12	46740584	46766300	
ENSDARG00000039164	57.18637974	5.094012591	0.701383502	5.837052881	5.31E-09	1.13E-07	mhc1uma	major histocompatibility complex class I UMA [Source:ZFIN;Acc:ZDB-GENE-140820-1]	22	3910610	3914222	
ENSDARG00000039196	617.0321501	1.557500245	0.160492415	3.473685943	0.000513362	0.00521063	greb1l	Danio rerio GREB1 like retinoic acid receptor coactivator (greb1l), mRNA. [Source:RefSeq mRNA;Acc:NM_001362307]	2	11685742	11833356	
ENSDARG00000039208	2643.083379	-1.529034151	0.098908563	-5.348719414	8.86E-08	1.62E-06	nasp	nuclear autoantigenic sperm protein (histone-binding) [Source:ZFIN;Acc:ZDB-GENE-030131-5511]	6	33911154	33916788	
ENSDARG00000039264	447.1068962	-1.621856226	0.146141466	-4.255166195	2.09E-05	0.000272812	igfbp5a	insulin-like growth factor binding protein 5a [Source:ZFIN;Acc:ZDB-GENE-070620-8]	6	20910551	20918694	
ENSDARG00000039272	832.6341323	-1.744282011	0.185343629	-4.015687056	5.93E-05	0.000717362	pls1	plastin 1 (I isoform) [Source:ZFIN;Acc:ZDB-GENE-030131-6205]	2	8059957	8111346	
ENSDARG00000039335	3293.982367	1.787311047	0.090586155	8.691295518	3.58E-18	1.69E-16	st6gal2a	ST6 beta-galactoside alpha-2,6-sialyltranferase 2a [Source:NCBI gene;Acc:403116]	9	6009077	6127872	
ENSDARG00000039346	2589.020725	-1.48081742	0.100733097	-4.773182151	1.81E-06	2.83E-05	ndufa5	NADH:ubiquinone oxidoreductase subunit A5 [Source:ZFIN;Acc:ZDB-GENE-050320-17]	4	4930406	4932619	
ENSDARG00000039347	37193.1829	-1.373608165	0.093080893	-4.013800833	5.97E-05	0.000722425	rps24	ribosomal protein S24 [Source:ZFIN;Acc:ZDB-GENE-040109-5]	13	30225262	30235220	
ENSDARG00000039385	1037.409465	1.69717584	0.218894849	3.184980562	0.001447637	0.013403594	dlg4b	discs large MAGUK scaffold protein 4 [Source:HGNC Symbol;Acc:HGNC:2903]	23	44307996	44332790	
ENSDARG00000039393	1347.002632	-3.619002641	0.135168085	-19.3758952	1.23E-83	6.58E-81	si:ch211-240l19.5	si:ch211-240l19.5 [Source:ZFIN;Acc:ZDB-GENE-041210-327]	4	8042805	8043964	
ENSDARG00000039423	951.9984645	-3.264873439	0.174179522	-13.00309825	1.17E-38	1.71E-36	AL831745.1	si:ch211-217g15.3 [Source:NCBI gene;Acc:368914]	13	24585224	24588220	
ENSDARG00000039453	229.1944565	-1.522003552	0.174495071	-2.991508862	0.002776025	0.024312979	dhrs7ca	dehydrogenase/reductase (SDR family) member 7Ca [Source:NCBI gene;Acc:558764]	5	58633692	58650057	
ENSDARG00000039458	619.0877782	2.410423184	0.153815598	9.169571877	4.75E-20	2.55E-18	lhx4	LIM homeobox 4 [Source:ZFIN;Acc:ZDB-GENE-060728-1]	8	14453933	14484599	
ENSDARG00000039500	297.6418494	1.723768859	0.204316267	3.542394685	0.000396512	0.004117849	si:cabz01090165.1	si:cabz01090165.1 [Source:ZFIN;Acc:ZDB-GENE-161017-71]	18	49411417	49447042	
ENSDARG00000039579	2262.447386	-2.021699603	0.115347566	-8.857573981	8.18E-19	4.02E-17	cfd	complement factor D (adipsin) [Source:ZFIN;Acc:ZDB-GENE-050522-411]	13	13681681	13684334	
ENSDARG00000039647	7373.317773	2.081527469	0.128070607	8.444775054	3.05E-17	1.35E-15	slc6a1b	solute carrier family 6 member 1b [Source:ZFIN;Acc:ZDB-GENE-041114-57]	11	997100	1024483	
ENSDARG00000039682	716.0843729	-2.096822822	0.139023309	-7.889488661	3.03E-15	1.14E-13	si:ch211-121a2.2	si:ch211-121a2.2 [Source:ZFIN;Acc:ZDB-GENE-070705-21]	20	7070634	7080470	
ENSDARG00000039730	16328.56312	-4.453724142	0.134744938	-25.63156875	6.79E-145	7.74E-142	zgc:112160	zgc:112160 [Source:ZFIN;Acc:ZDB-GENE-050417-229]	7	35062132	35066457	
ENSDARG00000039747	178.7567674	-2.667278219	0.241845155	-6.893990585	5.42E-12	1.58E-10	BX914200.1		13	45840427	45843939	
ENSDARG00000039806	21.25983656	3.968815826	0.842875049	3.522249035	0.000427902	0.00442371	tbxtb	T-box transcription factor Tb [Source:ZFIN;Acc:ZDB-GENE-081028-48]	13	4439074	4453663	
ENSDARG00000039832	545.126204	-2.288018424	0.158175781	-8.142955986	3.86E-16	1.54E-14	gsta.2	glutathione S-transferase, alpha tandem duplicate 2 [Source:ZFIN;Acc:ZDB-GENE-070822-30]	13	829585	832331	
ENSDARG00000039887	4521.335128	-1.373851039	0.082342791	-4.540179345	5.62E-06	8.18E-05	c1qbp	complement component 1, q subcomponent binding protein [Source:NCBI gene;Acc:334619]	5	3914403	3927763	
ENSDARG00000039900	13.89017147	-7.664706383	1.33081059	-5.00800522	5.5E-07	9.15E-06	si:ch73-168d20.1	si:ch73-168d20.1 [Source:ZFIN;Acc:ZDB-GENE-121214-44]	23	45077358	45119805	
ENSDARG00000039929	12684.15504	-1.715617424	0.090721985	-7.888026495	3.07E-15	1.15E-13	ckmt2b	creatine kinase, mitochondrial 2b (sarcomeric) [Source:ZFIN;Acc:ZDB-GENE-040426-1654]	5	51594209	51609006	

ENSDARG00000039951	190.2273534	1.663626093	0.200728083	3.306094905	0.00094606	0.009095283	si:ch211-87j1.4	si:ch211-87j1.4 [Source:ZFIN;Acc:ZDB-GENE-131127-49]	25	35533790	35542739	
ENSDARG00000039966	1569.245241	1.467910464	0.10996209	4.255197994	2.09E-05	0.000272812	prom1a	prominin 1a [Source:NCBI gene;Acc:322857]	14	46456091	46617228	
ENSDARG00000039979	101.1191632	-2.880472341	0.26886133	-6.994209029	2.67E-12	7.92E-11	tecrl2b	trans-2,3-enoyl-CoA reductase-like 2b [Source:ZFIN;Acc:ZDB-GENE-041010-116]	2	15300088	15324837	
ENSDARG00000040100	914.4149447	-1.963882722	0.179801218	-5.360824196	8.28E-08	1.52E-06	si:dkey-95h12.1	si:dkey-95h12.1 [Source:ZFIN;Acc:ZDB-GENE-060503-85]	18	8869465	8889382	
ENSDARG00000040118	981.9761346	3.881925649	0.169372088	17.01535174	6.32E-65	2.33E-62	zgc:113232	zgc:113232 [Source:ZFIN;Acc:ZDB-GENE-050327-86]	16	38360002	38389883	
ENSDARG00000040123	856.2329335	1.854270986	0.162537575	5.2558369	1.47E-07	2.61E-06	zfpm2a	zinc finger protein, FOG family member 2a [Source:ZFIN;Acc:ZDB-GENE-060130-4]	16	43818896	44127387	
ENSDARG00000040135	167.7676436	2.650506176	0.247799095	6.660662658	2.73E-11	7.37E-10	fosaa	v-fos FBJ murine osteosarcoma viral oncogene homolog Aa [Source:ZFIN;Acc:ZDB-GENE-131121-552]	17	50228998	50234004	
ENSDARG00000040159	807.895396	2.581024438	0.191114997	8.272634095	1.31E-16	5.45E-15	wnt4b	wingless-type MMTV integration site family, member 4b [Source:ZFIN;Acc:ZDB-GENE-000411-1]	16	31802203	31813018	
ENSDARG00000040163	727.1644972	-1.594366223	0.131366125	-4.524501442	6.05E-06	8.74E-05	prim1	DNA primase subunit 1 [Source:ZFIN;Acc:ZDB-GENE-990603-6]	23	17839187	17848907	
ENSDARG00000040237	4049.024857	1.351324098	0.12071659	2.910321588	0.00361057	0.030826166	mef2d	myocyte enhancer factor 2d [Source:ZFIN;Acc:ZDB-GENE-990415-164]	16	29072874	29194517	
ENSDARG00000040274	1093.674853	1.476919038	0.108305704	4.403452648	1.07E-05	0.00014702	scamp5b	secretory carrier membrane protein 5b [Source:ZFIN;Acc:ZDB-GENE-120215-76]	18	50089000	50100149	
ENSDARG00000040282	249.56695	-7.262554754	0.436820112	-14.3366905	1.29E-46	2.57E-44	zgc:92590	zgc:92590 [Source:ZFIN;Acc:ZDB-GENE-041024-15]	16	25126935	25130075	
ENSDARG00000040290	654.53858	-1.552039285	0.120825609	-4.568893056	4.9E-06	7.18E-05	pon3.2	paraoxonase 3, tandem duplicate 2 [Source:ZFIN;Acc:ZDB-GENE-071004-70]	16	24662252	24668620	
ENSDARG00000040298	37739.42808	-1.940473638	0.088597243	-10.61515696	2.53E-26	1.96E-24	apoa4b.1	apolipoprotein A-IV b, tandem duplicate 1 [Source:ZFIN;Acc:ZDB-GENE-030131-1263]	16	23913943	23916263	
ENSDARG00000040358	1110.822639	2.099844214	0.177937235	6.181079612	6.37E-10	1.5E-08	spred3	sprouty related EVH1 domain containing 3 [Source:ZFIN;Acc:ZDB-GENE-061207-31]	18	44596787	44611252	
ENSDARG00000040427	129.6215946	-2.095493704	0.264688974	-4.138796135	3.49E-05	0.000439278	acot19	acyl-CoA thioesterase 19 [Source:ZFIN;Acc:ZDB-GENE-041114-124]	25	31714335	31739309	
ENSDARG00000040430	941.0829883	1.50058822	0.128619592	3.892005967	9.94E-05	0.0011564	nptxra	neuronal pentraxin receptor a [Source:ZFIN;Acc:ZDB-GENE-050208-280]	3	24482999	24501796	
ENSDARG00000040466	5889.193848	-1.972763128	0.33479324	-2.905563826	0.003665921	0.031256208	vil1	villin 1 [Source:ZFIN;Acc:ZDB-GENE-040426-799]	9	44907768	44953664	
ENSDARG00000040479	27.76317485	4.034900233	0.764097292	3.971876703	7.13E-05	0.000851126	cga	glycoprotein hormones, alpha polypeptide [Source:ZFIN;Acc:ZDB-GENE-040715-2]	17	49439219	49443399	
ENSDARG00000040553	444.2622779	-1.45220827	0.158675745	-2.849889062	0.004373448	0.036568261	tmem97	transmembrane protein 97 [Source:ZFIN;Acc:ZDB-GENE-110627-1]	15	23790057	23793641	
ENSDARG00000040557	433.4272694	-1.551358534	0.162540465	-3.39213089	0.000693513	0.006856192	exosc5	exosome component 5 [Source:ZFIN;Acc:ZDB-GENE-060503-675]	18	36060295	36066087	
ENSDARG00000040584	371.8777706	-1.438342802	0.142007931	-3.08674874	0.002023586	0.018179679	eef1akmt1	EEF1A lysine methyltransferase 1 [Source:NCBI gene;Acc:404619]	9	21358941	21363501	
ENSDARG00000040623	1234.363127	2.52491043	0.142625147	10.69173609	1.11E-26	8.84E-25	fosl2	fos-like antigen 2 [Source:ZFIN;Acc:ZDB-GENE-070209-164]	17	41302660	41318547	
ENSDARG00000040627	285.5838937	3.315799042	0.2201403	10.51965059	7.01E-26	5.24E-24	grik1b	glutamate receptor, ionotropic, kainate 1b [Source:ZFIN;Acc:ZDB-GENE-070821-1]	15	42599501	42679838	
ENSDARG00000040683	156.8584223	-2.717582115	0.263129712	-6.527511088	6.69E-11	1.74E-09	MEP1B	si:ch73-40a17.3 [Source:ZFIN;Acc:ZDB-GENE-030131-5797]	2	283764	293793	MEP1B
ENSDARG00000040713	1277.532679	-1.459029685	0.10298056	-4.457440171	8.29E-06	0.000116638	rbbp9	retinoblastoma binding protein 9 [Source:ZFIN;Acc:ZDB-GENE-050208-692]	22	38944615	38951354	
ENSDARG00000040747	3095.688137	-2.253817604	0.192284015	-6.520654376	7E-11	1.82E-09	tm4sf4	transmembrane 4 L six family member 4 [Source:ZFIN;Acc:ZDB-GENE-040801-231]	22	38127467	38137908	
ENSDARG00000040898	236.9678403	-1.723142711	0.228306122	-3.167425846	0.001537949	0.014145865	gpa33b	glycoprotein A33 (transmembrane), paralog b [Source:ZFIN;Acc:ZDB-GENE-070912-194]	9	34307140	34308113	
ENSDARG00000040926	4428.82573	1.587244081	0.087765369	6.691068334	2.22E-11	6.05E-10	nr2f2	nuclear receptor subfamily 2, group F, member 2 [Source:ZFIN;Acc:ZDB-GENE-990415-252]	18	23868650	24023013	
ENSDARG00000040982	267.8452313	2.012268882	0.225538373	4.488233513	7.18E-06	0.000102424	fgf14	fibroblast growth factor 14 [Source:ZFIN;Acc:ZDB-GENE-060506-1]	9	31838044	31915423	
ENSDARG00000041068	3427.759777	-1.345369728	0.120316227	-2.870516614	0.004098017	0.034550137	got2a	glutamic-oxaloacetic transaminase 2a, mitochondrial [Source:ZFIN;Acc:ZDB-GENE-040426-2703]	18	21178721	21188768	
ENSDARG00000041083	678.98589	-2.285908824	0.186473183	-6.895945063	5.35E-12	1.56E-10	anpepa	alanyl (membrane) aminopeptidase a [Source:ZFIN;Acc:ZDB-GENE-030131-4781]	18	21077528	21097663	
ENSDARG00000041115	271.3110495	-1.797440225	0.184837131	-4.314285875	1.6E-05	0.000219429	cnfn	Danio rerio cornifelin (cnfn), mRNA. [Source:RefSeq mRNA;Acc:NM_001004663]	16	11790640	11798994	
ENSDARG00000041119	7557.816768	-1.392538031	0.092831672	-4.228492519	2.35E-05	0.000304712	ceacam1	CEA cell adhesion molecule 1 [Source:ZFIN;Acc:ZDB-GENE-010724-14]	16	11724230	11760596	
ENSDARG00000041140	362.4907189	-1.523029318	0.160284308	-3.263134897	0.001101871	0.010440941	ddb2	damage-specific DNA binding protein 2 [Source:NCBI gene;Acc:100004133]	18	20468157	20482855	
ENSDARG00000041150	2364.948811	1.823481336	0.1499013	5.493490288	3.94E-08	7.56E-07	slc17a6b	solute carrier family 17 member 6b [Source:NCBI gene;Acc:100149756]	7	32811907	32833255	
ENSDARG00000041295	205.6864203	2.059546238	0.298640757	3.547895637	0.000388322	0.004041157	lim2.5	lens intrinsic membrane protein 2.5 [Source:ZFIN;Acc:ZDB-GENE-050320-94]	16	50166421	50181206	
ENSDARG00000041314	4608.474102	-1.659363941	0.111855114	-5.894803725	3.75E-09	8.12E-08	ndufb9	NADH:ubiquinone oxidoreductase subunit B9 [Source:ZFIN;Acc:ZDB-GENE-030131-8364]	16	6386941	6391275	
ENSDARG00000041339	4836.752097	-1.343570919	0.10394894	-3.305189233	0.000949123	0.009120955	zgc:92380	zgc:92380 [Source:ZFIN;Acc:ZDB-GENE-040801-221]	9	48007081	48039964	
ENSDARG00000041372	895.7696808	-2.696430424	0.132819102	-12.77248828	2.34E-37	3.22E-35	C8H9orf64	zgc:136564 [Source:ZFIN;Acc:ZDB-GENE-070410-3]	8	49730163	49743517	C8H9orf64
ENSDARG00000041378	354.0554552	-1.546355247	0.146737123	-3.723360781	0.000196588	0.002163333	sh2d4a	SH2 domain containing 4A [Source:ZFIN;Acc:ZDB-GENE-040426-932]	10	6967293	6976645	
ENSDARG00000041382	361.9759071	1.817049951	0.189188213	4.318714875	1.57E-05	0.000211113	si:dkey-283b15.2	si:dkey-283b15.2 [Source:ZFIN;Acc:ZDB-GENE-100921-93]	16	5650495	5671862	
ENSDARG00000041417	3606.066253	2.030313678	0.119032781	8.655713748	4.9E-18	2.29E-16	atp6ap1a	ATPase H+ transporting accessory protein 1a [Source:ZFIN;Acc:ZDB-GENE-090312-136]	23	4899238	4915118	
ENSDARG00000041492	157.5068722	3.927386519	0.348104486	8.40950529	4.12E-17	1.79E-15	si:ch211-196f5.2	si:ch211-196f5.2 [Source:ZFIN;Acc:ZDB-GENE-041014-306]	24	12740848	12752502	
ENSDARG00000041540	378.212309	-4.095494296	0.230631506	-13.42181886	4.51E-41	7.25E-39	sult1st2	sulfotransferase family 1, cytosolic sulfotransferase 2 [Source:NCBI gene;Acc:791732]	8	46513743	46525092	
ENSDARG00000041554	75.06322599	-2.551032688	0.355503013	-4.362924173	1.28E-05	0.000175168	unc93a	unc-93 homolog A [Source:NCBI gene;Acc:559995]	20	43379029	43389475	
ENSDARG00000041569	21246.62577	-1.438613171	0.07585496	-5.782260898	7.37E-09	1.54E-07	ces2a	carboxylesterase 2a [Source:ZFIN;Acc:ZDB-GENE-061013-99]	18	17408470	17415609	
ENSDARG00000041595	4019.010194	-1.823720359	0.096879048	-8.502564536	1.85E-17	8.33E-16	ces3	carboxylesterase 3 [Source:ZFIN;Acc:ZDB-GENE-030131-9563]	18	17418254	17426306	
ENSDARG00000041609	962.0430753	2.056656805	0.145706374	7.251960064	4.11E-13	1.3E-11	adarb1a	adenosine deaminase RNA specific B1a [Source:ZFIN;Acc:ZDB-GENE-000627-2]	22	12641580	12693837	
ENSDARG00000041685	674.6257235	-2.754237123	0.181829032	-9.64772846	5.03E-22	3.05E-20	si:dkey-105h12.2	si:dkey-105h12.2 [Source:ZFIN;Acc:ZDB-GENE-131118-9]	15	21220744	21239416	
ENSDARG00000041787	1173.846038	-1.405248076	0.144420338	-2.806031895	0.005015573	0.041454031	cx32.3	connexin 32.3 [Source:ZFIN;Acc:ZDB-GENE-030131-1337]	20	40753518	40755614	
ENSDARG00000041811	49252.3785	-1.43219448	0.12220185	-3.536726178	0.000405119	0.004200291	rps25	ribosomal protein S25 [Source:NCBI gene;Acc:393788]	10	39125637	39130935	
ENSDARG00000041848	93.71668172	-3.416569856	0.300552175	-8.040433764	8.95E-16	3.5E-14	rh50	Rh50-like protein [Source:ZFIN;Acc:ZDB-GENE-000427-1]	8	37259398	37263524	
ENSDARG00000041864	1131.914052	2.474789042	0.201189744	7.330339074	2.3E-13	7.47E-12	capn3a	calpain 3a, (p94) [Source:ZFIN;Acc:ZDB-GENE-040912-97]	17	45305645	45346635	
ENSDARG00000041926	231.7279797	2.899739266	0.259056447	7.333302414	2.25E-13	7.33E-12	dlg4a	discs, large homolog 4a (Drosophila) [Source:ZFIN;Acc:ZDB-GENE-130530-613]	5	68529728	68641353	
ENSDARG00000041952	58.72767644	2.655311732	0.418185137	3.958322726	7.55E-05	0.000897476	prox2	prospero homeobox 2 [Source:ZFIN;Acc:ZDB-GENE-081003-3]	17	52493499	52521002	
ENSDARG00000042010	4120.873693	-2.120978625	0.113118103	-9.909807521	3.77E-23	2.44E-21	pklr	pyruvate kinase L/R [Source:ZFIN;Acc:ZDB-GENE-010907-1]	16	54450407	54455573	
ENSDARG00000042033	195.8727566	-2.285183357	0.206428028	-6.22581811	4.79E-10	1.14E-08	mettl11b	methyltransferase like 11B [Source:NCBI gene;Acc:568419]	20	34455645	34466483	
ENSDARG00000042107	420.0671638	1.940777581	0.15959611	5.894740034	3.75E-09	8.12E-08	cngb1b	cyclic nucleotide gated channel subunit beta 1b [Source:ZFIN;Acc:ZDB-GENE-090831-4]	25	17508235	17552299	
ENSDARG00000042115	657.3668719	1.571445097	0.168415246	3.393072255	0.000691134	0.006838059	tmem198a	transmembrane protein 198a [Source:ZFIN;Acc:ZDB-GENE-070912-716]	9	7456076	7499416	
ENSDARG00000042124	786.2448053	-2.052071762	0.152807317	-6.884956685	5.78E-12	1.68E-10	si:dkey-4e7.3	si:dkey-4e7.3 [Source:ZFIN;Acc:ZDB-GENE-070912-542]	9	7072504	7081557	
ENSDARG00000042188	368.3514459	-1.652533527	0.155567595	-4.194533748	2.73E-05	0.000349285	ttc38	tetratricopeptide repeat domain 38 [Source:NCBI gene;Acc:553728]	18	10926197	10959429	
ENSDARG00000042249	1125.105116	1.362903056	0.110087579	3.296494103	0.000978997	0.009375733	FRMD4A	si:ch211-220f12.1 [Source:ZFIN;Acc:ZDB-GENE-050419-154]	18	8503370	8579907	FRMD4A
ENSDARG00000042293	851.4798246	-2.022880051	0.113711791	-8.995373695	2.35E-19	1.21E-17	ca4b	carbonic anhydrase IV b [Source:ZFIN;Acc:ZDB-GENE-080815-5]	15	15390882	15401917	
ENSDARG00000042345	394.7574339	-1.770964767	0.145463318	-5.300063108	1.16E-07	2.08E-06	si:dkeyp-55f12.3	si:dkeyp-55f12.3 [Source:ZFIN;Acc:ZDB-GENE-041001-118]	20	27090290	27092290	
ENSDARG00000042379	420.3195592	-2.861395271	0.187254959	-9.940432451	2.78E-23	1.81E-21	TMEM120A	zgc:103681 [Source:ZFIN;Acc:ZDB-GENE-041010-17]	15	16873690	16884912	TMEM120A
ENSDARG00000042428	3524.389083	-1.719801378	0.095456247	-7.54064192	4.68E-14	1.6E-12	gstt1a	glutathione S-transferase theta 1a [Source:ZFIN;Acc:ZDB-GENE-031001-13]	8	30787111	30791266	
ENSDARG00000042440	137.3472029	1.902118172	0.254617026	3.543039472	0.000395543	0.004109494	p2rx7	purinergic receptor P2X, ligand-gated ion channel, 7 [Source:ZFIN;Acc:ZDB-GENE-030319-6]	8	30758169	30779101	
ENSDARG00000042469	4942.451767	-1.585732965	0.12885432	-4.545699105	5.48E-06	7.99E-05	ndufa12	NADH:ubiquinone oxidoreductase subunit A12 [Source:ZFIN;Acc:ZDB-GENE-050828-1]	4	25829841	25836684	
ENSDARG00000042526	55.59462724	4.792728365	0.65168077	5.819917572	5.89E-09	1.25E-07	sebox	SEBOX homeobox [Source:NCBI gene;Acc:282666]	5	67371650	67382188	
ENSDARG00000042533	2480.427962	-1.718090065	0.101897344	-7.047191202	1.83E-12	5.53E-11	gstm.1	glutathione S-transferase mu, tandem duplicate 1 [Source:ZFIN;Acc:ZDB-GENE-030911-2]	8	25295160	25297928	
ENSDARG00000042559	907.4258114	-1.53394162	0.118440298	-4.508107704	6.54E-06	9.38E-05	tnni1c	troponin I, skeletal, slow c [Source:ZFIN;Acc:ZDB-GENE-060825-192]	18	6625348	6634424	
ENSDARG00000042562	310.7982004	-1.902137776	0.157913515	-5.712859823	1.11E-08	2.27E-07	allc	allantoicase [Source:NCBI gene;Acc:436989]	20	30355436	30370927	
ENSDARG00000042620	5077.511891	-1.574097024	0.118640005	-4.83898348	1.31E-06	2.07E-05	gstr	glutathione S-transferase rho [Source:ZFIN;Acc:ZDB-GENE-090507-1]	19	770300	776076	
ENSDARG00000042641	1861.689654	-2.510400024	0.116216907	-12.9963881	1.28E-38	1.86E-36	cyp51	cytochrome P450, family 51 [Source:NCBI gene;Acc:414331]	19	598657	617246	
ENSDARG00000042644	2854.102834	-1.802075272	0.091858547	-8.731634626	2.51E-18	1.19E-16	sod2	superoxide dismutase 2, mitochondrial [Source:ZFIN;Acc:ZDB-GENE-030131-7742]	20	30937934	30947547	
ENSDARG00000042646	997.9469039	2.572957564	0.143906177	10.93043815	8.24E-28	6.97E-26	zgc:77784	zgc:77784 [Source:ZFIN;Acc:ZDB-GENE-040426-1874]	15	19456112	19602329	
ENSDARG00000042659	207.4034383	-1.601190339	0.191837879	-3.133845846	0.001725315	0.015713761	thyn1	thymocyte nuclear protein 1 [Source:NCBI gene;Acc:393630]	15	19331159	19334448	
ENSDARG00000042703	419.7500427	1.715119487	0.178664452	4.002584046	6.27E-05	0.000754649	parp6a	poly (ADP-ribose) polymerase family, member 6a [Source:ZFIN;Acc:ZDB-GENE-040718-272]	18	887122	898873	
ENSDARG00000042777	2174.561833	-1.765660566	0.124121332	-6.168646062	6.89E-10	1.61E-08	ndufa11	NADH:ubiquinone oxidoreductase subunit A11 [Source:ZFIN;Acc:ZDB-GENE-040426-1631]	11	6411169	6429705	
ENSDARG00000042780	24651.84663	-1.678311642	0.130524491	-5.196815085	2.03E-07	3.53E-06	apoba	apolipoprotein Ba [Source:ZFIN;Acc:ZDB-GENE-070702-4]	17	30704068	30727966	
ENSDARG00000042816	1106.287446	2.695910558	0.149022247	11.38025088	5.24E-30	5.16E-28	mmp9	matrix metallopeptidase 9 [Source:ZFIN;Acc:ZDB-GENE-040426-2132]	8	24281512	24295947	
ENSDARG00000042839	507.5726387	-1.655248654	0.139966193	-4.681478025	2.85E-06	4.3E-05	dph6	diphthamine biosynthesis 6 [Source:NCBI gene;Acc:503603]	17	53156530	53197232	
ENSDARG00000042850	82.99513845	1.946038457	0.308585065	3.065729893	0.002171394	0.019403402	nphs2	NPHS2 stomatin family member, podocin [Source:ZFIN;Acc:ZDB-GENE-051102-2]	22	17251275	17256573	
ENSDARG00000042854	587.2457432	-1.666751591	0.142402292	-4.682168951	2.84E-06	4.29E-05	ephx1	epoxide hydrolase 1, microsomal (xenobiotic) [Source:ZFIN;Acc:ZDB-GENE-040426-913]	20	36628059	36638179	
ENSDARG00000042864	1741.852371	-1.307854664	0.095835063	-3.212338498	0.001316591	0.012303709	rpl7l1	ribosomal protein L7-like 1 [Source:ZFIN;Acc:ZDB-GENE-030131-970]	20	36675083	36679233	
ENSDARG00000042872	324.9720625	2.661940218	0.218196077	7.616728217	2.6E-14	9.07E-13	zdhhc8a	zinc finger DHHC-type containing 8a [Source:ZFIN;Acc:ZDB-GENE-091118-26]	10	428269	454622	
ENSDARG00000042894	998.8016993	-1.603388014	0.120440218	-5.009854898	5.45E-07	9.07E-06	tyms	thymidylate synthetase [Source:ZFIN;Acc:ZDB-GENE-040426-59]	7	71535045	71541329	
ENSDARG00000042920	731.7757177	-3.156943044	0.192146556	-11.22550977	3.06E-29	2.91E-27	sult3st3	sulfotransferase family 3, cytosolic sulfotransferase 3 [Source:ZFIN;Acc:ZDB-GENE-060503-628]	22	15315655	15318280	
ENSDARG00000042927	830.7718707	-1.444591427	0.122911551	-3.61716555	0.000297847	0.003171936	mapre1a	microtubule-associated protein, RP/EB family, member 1a [Source:ZFIN;Acc:ZDB-GENE-030131-6067]	8	23381892	23390368	
ENSDARG00000042934	6762.741123	1.738808689	0.096590357	7.64888664	2.03E-14	7.14E-13	ccn2a	cellular communication network factor 2a [Source:ZFIN;Acc:ZDB-GENE-030131-102]	20	25340814	25343490	
ENSDARG00000042953	2103.943242	-4.649276968	0.16608931	-21.97177512	5.36E-107	3.85E-104	cyp2n13	cytochrome P450, family 2, subfamily N, polypeptide 13 [Source:ZFIN;Acc:ZDB-GENE-041001-158]	20	25515259	25522911	
ENSDARG00000042956	497.4532537	-1.929217744	0.151856226	-6.119062533	9.41E-10	2.17E-08	cyp2ad6	cytochrome P450, family 2, subfamily AD, polypeptide 6 [Source:ZFIN;Acc:ZDB-GENE-041001-157]	20	25525802	25533739	
ENSDARG00000042978	504.2343778	-2.228591767	0.157142458	-7.818331092	5.35E-15	1.98E-13	cyp2p6	cytochrome P450, family 2, subfamily P, polypeptide 6 [Source:ZFIN;Acc:ZDB-GENE-040426-790]	20	25563105	25568441	
ENSDARG00000042980	317.8684197	-2.11500321	0.164930072	-6.76046032	1.38E-11	3.86E-10	cyp2p7	cytochrome P450, family 2, subfamily P, polypeptide 7 [Source:ZFIN;Acc:ZDB-GENE-041001-155]	20	25568694	25615727	
ENSDARG00000042982	1646.981279	-3.653860417	0.158691403	-16.72340383	8.85E-63	3.13E-60	cyp2p8	cytochrome P450, family 2, subfamily P, polypeptide 8 [Source:ZFIN;Acc:ZDB-GENE-061030-1]	20	25575391	25580002	
ENSDARG00000042988	1329.465879	2.519197635	0.152808778	9.941821786	2.74E-23	1.79E-21	slc24a2	solute carrier family 24 member 2 [Source:ZFIN;Acc:ZDB-GENE-060825-277]	1	12009673	12044390	
ENSDARG00000042993	41123.29908	-4.161207934	0.084190454	-37.54829427	0	0	prss1	serine protease 1 [Source:ZFIN;Acc:ZDB-GENE-010131-7]	16	25080764	25085327	
ENSDARG00000043093	872.7904238	1.922465012	0.182981016	5.041315401	4.62E-07	7.77E-06	mpeg1.2	macrophage expressed 1, tandem duplicate 2 [Source:ZFIN;Acc:ZDB-GENE-050522-305]	8	19356072	19359834	
ENSDARG00000043141	321.7292101	1.823489803	0.171057448	4.814112519	1.48E-06	2.33E-05	si:ch211-132f19.7	si:ch211-132f19.7 [Source:ZFIN;Acc:ZDB-GENE-130530-619]	17	33311784	33339626	
ENSDARG00000043171	821.7720089	-1.936233577	0.161783416	-5.786956418	7.17E-09	1.5E-07	cela1.4	chymotrypsin like elastase family member 1, tandem duplicate 4 [Source:ZFIN;Acc:ZDB-GENE-050626-127]	22	7486867	7492548	
ENSDARG00000043173	18.1318397	-3.711343346	0.679682016	-3.989135039	6.63E-05	0.000794921	cela1.3	chymotrypsin like elastase family member 1, tandem duplicate 3 [Source:ZFIN;Acc:ZDB-GENE-040801-12]	22	7480465	7483755	
ENSDARG00000043175	99.11249352	-2.919830842	0.281729779	-6.814440584	9.46E-12	2.69E-10	cela1.1	chymotrypsin like elastase family member 1, tandem duplicate 1 [Source:ZFIN;Acc:ZDB-GENE-050522-187]	22	7462997	7470338	
ENSDARG00000043180	4403.450993	-1.644644616	0.102487914	-6.289957431	3.18E-10	7.73E-09	gpd1b	glycerol-3-phosphate dehydrogenase 1b [Source:ZFIN;Acc:ZDB-GENE-030131-3906]	22	6996046	7006974	
ENSDARG00000043220	45.39769047	2.742609688	0.479370407	3.635204977	0.00027776	0.002987166	kcnh5a	potassium voltage-gated channel, subfamily H (eag-related), member 5a [Source:ZFIN;Acc:ZDB-GENE-061106-2]	20	19964854	20182871	
ENSDARG00000043242	2136.493153	-1.991835219	0.120253278	-8.247885123	1.61E-16	6.63E-15	si:dkey-222f2.1	si:dkey-222f2.1 [Source:ZFIN;Acc:ZDB-GENE-050208-726]	22	5727753	5739360	
ENSDARG00000043279	3162.24537	-1.58967883	0.094210694	-6.259149614	3.87E-10	9.33E-09	aqp12	aquaporin 12 [Source:ZFIN;Acc:ZDB-GENE-040718-310]	22	2874838	2886937	
ENSDARG00000043288	670.8517525	-1.490077674	0.126464476	-3.875220063	0.000106528	0.001228846	impact	impact RWD domain protein [Source:NCBI gene;Acc:449553]	22	2959879	3026838	
ENSDARG00000043313	11857.8927	1.844843676	0.184109728	4.588805201	4.46E-06	6.57E-05	ank2b	ankyrin 2b, neuronal [Source:ZFIN;Acc:ZDB-GENE-041010-165]	7	57886148	58130703	
ENSDARG00000043406	881.7679456	1.470332933	0.117606537	3.999207406	6.36E-05	0.000764029	slc8a1b	solute carrier family 8 member 1b [Source:ZFIN;Acc:ZDB-GENE-060110-3]	17	22311413	22501970	
ENSDARG00000043410	362.437074	2.520166775	0.225815621	6.731893777	1.67E-11	4.66E-10	xkr6b	XK, Kell blood group complex subunit-related family, member 6b [Source:ZFIN;Acc:ZDB-GENE-040724-153]	20	18801525	18915376	
ENSDARG00000043442	500.4445063	-1.824019869	0.150571633	-5.472610315	4.43E-08	8.42E-07	zgc:153665	zgc:153665 [Source:ZFIN;Acc:ZDB-GENE-061027-365]	11	30626768	30636163	
ENSDARG00000043467	724.0473499	-1.426395598	0.131028169	-3.25422846	0.001137008	0.010749543	ndufb11	NADH:ubiquinone oxidoreductase subunit B11 [Source:ZFIN;Acc:ZDB-GENE-050309-25]	8	14180064	14184423	
ENSDARG00000043474	3891.872995	1.722507305	0.13811603	5.231161837	1.68E-07	2.97E-06	atp2b3a	ATPase plasma membrane Ca2+ transporting 3a [Source:ZFIN;Acc:ZDB-GENE-040718-174]	8	14003232	14052349	
ENSDARG00000043482	112.2089274	-1.965401151	0.289385016	-3.336044012	0.000849797	0.008229459	b3gnt3.1	UDP-GlcNAc:betaGal beta-1,3-N-acetylglucosaminyltransferase 3, tandem duplicate 1 [Source:ZFIN;Acc:ZDB-GENE-0406	8	13534385	13541514	

ENSDARG00000043483	1320.8751	2.423539173	0.148403339	9.592366201	8.61E-22	5.17E-20	otx5	orthodenticle homolog 5 [Source:ZFIN;Acc:ZDB-GENE-030508-1]	15	47903864	47909281	
ENSDARG00000043509	51384.16496	-1.641859584	0.139474629	-4.601980929	4.18E-06	6.18E-05	rpl11	ribosomal protein L11 [Source:ZFIN;Acc:ZDB-GENE-040625-147]	19	43780970	43786940	
ENSDARG00000043518	184.3458517	-3.067855282	0.26815749	-7.711346353	1.24E-14	4.48E-13	si:dkey-239i20.2	si:dkey-239i20.2 [Source:ZFIN;Acc:ZDB-GENE-041001-98]	20	15083987	15090862	
ENSDARG00000043584	7.452799086	-4.225425682	1.156302564	-2.789430536	0.005280082	0.043328428	ifit15	interferon-induced protein with tetratricopeptide repeats 15 [Source:ZFIN;Acc:ZDB-GENE-030131-1055]	17	23607195	23609210	
ENSDARG00000043587	842.1284915	-2.958636631	0.153422541	-12.76628984	2.53E-37	3.45E-35	srd5a2a	steroid-5-alpha-reductase, alpha polypeptide 2a [Source:ZFIN;Acc:ZDB-GENE-050417-199]	1	51066671	51073128	
ENSDARG00000043646	2178.688629	1.432142803	0.110741706	3.902258836	9.53E-05	0.001112089	slc6a8	solute carrier family 6 member 8 [Source:ZFIN;Acc:ZDB-GENE-071024-2]	8	9052123	9118958	
ENSDARG00000043661	660.5587675	2.254443659	0.190292833	6.592175007	4.33E-11	1.15E-09	cadpsa	Ca2+-dependent activator protein for secretion a [Source:ZFIN;Acc:ZDB-GENE-030616-525]	11	19780717	20040154	
ENSDARG00000043663	892.1312381	-1.695567358	0.132559905	-5.24719263	1.54E-07	2.73E-06	faub	FAU ubiquitin like and ribosomal protein S30 fusion b [Source:ZFIN;Acc:ZDB-GENE-050417-416]	10	26990095	26992003	
ENSDARG00000043684	1414.343883	-1.855572794	0.130366189	-6.562842719	5.28E-11	1.39E-09	bpnt1	bisphosphate nucleotidase 1 [Source:ZFIN;Acc:ZDB-GENE-040718-46]	20	9128256	9148323	
ENSDARG00000043720	335.0981587	-1.523105456	0.156307578	-3.34664168	0.000817969	0.00795191	cdc45	CDC45 cell division cycle 45 homolog (S. cerevisiae) [Source:ZFIN;Acc:ZDB-GENE-040426-2710]	8	4734005	4760723	
ENSDARG00000043722	3980.853824	-4.664615735	0.102317298	-35.81618967	6.18E-281	2.59E-277	cpa4	carboxypeptidase A4 [Source:ZFIN;Acc:ZDB-GENE-040704-61]	25	16582172	16589461	
ENSDARG00000043729	3574.026526	-4.043796689	0.145270103	-20.95267105	1.77E-97	1.14E-94	plac8.1	placenta associated 8, tandem duplicate 1 [Source:ZFIN;Acc:ZDB-GENE-050809-124]	10	11382365	11385155	
ENSDARG00000043746	2409.776908	2.283457418	0.160652783	7.989014508	1.36E-15	5.29E-14	nrxn3a	neurexin 3a [Source:NCBI gene;Acc:563598]	17	16906523	17447902	
ENSDARG00000043835	842.6598633	1.972010512	0.129067149	7.531045049	5.03E-14	1.72E-12	rab3ab	RAB3A, member RAS oncogene family, b [Source:ZFIN;Acc:ZDB-GENE-041210-268]	11	6819050	6850593	
ENSDARG00000043848	1734.415684	-1.540352782	0.101370666	-5.330464935	9.8E-08	1.77E-06	sod1	superoxide dismutase 1, soluble [Source:NCBI gene;Acc:30553]	10	25694395	25699454	
ENSDARG00000043864	2039.050588	1.797437827	0.103634052	7.69474714	1.42E-14	5.07E-13	nptnb	neuroplastin b [Source:ZFIN;Acc:ZDB-GENE-040426-1821]	18	1158197	1228688	
ENSDARG00000043902	177.0806137	2.644944184	0.238743335	6.890010924	5.58E-12	1.62E-10	gabrr1	gamma-aminobutyric acid (GABA) A receptor, rho 1 [Source:ZFIN;Acc:ZDB-GENE-040724-212]	20	1132092	1151265	
ENSDARG00000043907	631.5625845	1.65569948	0.150359955	4.36086509	1.3E-05	0.000176633	fgf11b	fibroblast growth factor 11b [Source:ZFIN;Acc:ZDB-GENE-100316-2]	10	22161585	22249444	
ENSDARG00000043973	3150.843263	1.578894384	0.08760223	6.608215144	3.89E-11	1.04E-09	znf292b	zinc finger protein 292b [Source:ZFIN;Acc:ZDB-GENE-030131-9559]	20	196541	211920	
ENSDARG00000043986	354.3775026	-1.866424864	0.16868887	-5.136230182	2.8E-07	4.8E-06	setd6	SET domain containing 6, protein lysine methyltransferase [Source:NCBI gene;Acc:322348]	25	17349265	17357165	
ENSDARG00000043997	135.5767721	-5.228074343	0.354017174	-11.94313342	7.05E-33	7.83E-31	cyp2x8	cytochrome P450, family 2, subfamily X, polypeptide 8 [Source:ZFIN;Acc:ZDB-GENE-040704-28]	25	17386763	17395315	
ENSDARG00000044062	3351.274581	1.404815193	0.092630587	4.370210806	1.24E-05	0.000169978	ctbp2a	C-terminal binding protein 2a [Source:ZFIN;Acc:ZDB-GENE-010130-2]	17	8595588	8727748	
ENSDARG00000044093	55658.28699	-1.346875489	0.098054813	-3.53756719	0.000403831	0.004188666	rpl13a	ribosomal protein L13a [Source:ZFIN;Acc:ZDB-GENE-030131-168]	17	24615091	24618279	
ENSDARG00000044204	700.6194789	-2.233378513	0.137070246	-8.998149121	2.3E-19	1.18E-17	endou	endonuclease, polyU-specific [Source:NCBI gene;Acc:791216]	6	39905021	39912440	
ENSDARG00000044280	376.4465195	1.557419889	0.166753731	3.342773113	0.000829457	0.008054235	opn1mw2	opsin 1 (cone pigments), medium-wave-sensitive, 2 [Source:NCBI gene;Acc:360151]	6	41186320	41189460	
ENSDARG00000044319	544.6491278	1.951001375	0.170184572	5.588058672	2.3E-08	4.53E-07	fstl4	follistatin-like 4 [Source:ZFIN;Acc:ZDB-GENE-070127-3]	21	44857293	45117822	
ENSDARG00000044365	1227.292955	-1.353067479	0.112916388	-3.126804588	0.001767173	0.016077499	angptl3	angiopoietin-like 3 [Source:ZFIN;Acc:ZDB-GENE-010817-3]	6	32344197	32349153	
ENSDARG00000044375	1316.961741	1.69222783	0.126053945	5.491520572	3.98E-08	7.62E-07	zgc:158291	zgc:158291 [Source:ZFIN;Acc:ZDB-GENE-061215-70]	21	38312549	38315241	
ENSDARG00000044422	256.5139202	1.992424823	0.207570965	4.781135079	1.74E-06	2.73E-05	acvr2ba	activin A receptor type 2Ba [Source:ZFIN;Acc:ZDB-GENE-980526-549]	24	41167269	41220538	
ENSDARG00000044528	2334.581174	-2.850303487	0.167281005	-11.06104956	1.94E-28	1.73E-26	slc15a1b	solute carrier family 15 member 1b [Source:ZFIN;Acc:ZDB-GENE-030131-4661]	6	12556427	12588044	
ENSDARG00000044532	529.2834132	2.112891974	0.152001661	7.321577726	2.45E-13	7.94E-12	nr4a2b	nuclear receptor subfamily 4, group A, member 2b [Source:ZFIN;Acc:ZDB-GENE-040718-103]	6	12462079	12466550	
ENSDARG00000044550	394.0342463	1.608816918	0.171299671	3.554104424	0.000379269	0.003961723	hif1al2	hypoxia inducible factor 1 subunit alpha, like 2 [Source:ZFIN;Acc:ZDB-GENE-050211-2]	21	27327442	27338639	
ENSDARG00000044632	446.9184561	1.843777305	0.167628453	5.033616248	4.81E-07	8.07E-06	myo7ab	myosin VIIAb [Source:ZFIN;Acc:ZDB-GENE-091110-4]	21	22237964	22309466	
ENSDARG00000044642	353.8709056	-1.8957351	0.163381776	-5.482466422	4.19E-08	8E-07	sc5d	sterol-C5-desaturase [Source:ZFIN;Acc:ZDB-GENE-040912-56]	21	24531679	24535510	
ENSDARG00000044684	3390.684663	1.475360993	0.118820163	4.000676154	6.32E-05	0.00076003	rbp4l	retinol binding protein 4, like [Source:ZFIN;Acc:ZDB-GENE-030131-7591]	21	20338613	20342096	
ENSDARG00000044688	577.2522827	1.906129894	0.135688822	6.677999569	2.42E-11	6.58E-10	dusp4	dual specificity phosphatase 4 [Source:ZFIN;Acc:ZDB-GENE-040426-709]	21	20113470	20120543	
ENSDARG00000044752	268.3671187	-1.804240223	0.1801066	-4.465356757	7.99E-06	0.000113167	p2rx4a	purinergic receptor P2X, ligand-gated ion channel, 4a [Source:ZFIN;Acc:ZDB-GENE-020806-2]	21	15723069	15732401	
ENSDARG00000044755	111.1923351	-1.805201873	0.255044966	-3.157097689	0.00159348	0.014592459	GCNT4	si:dkeyp-87d8.8 [Source:ZFIN;Acc:ZDB-GENE-041111-161]	21	15833222	15841167	GCNT4
ENSDARG00000044815	282.0215625	3.155404975	0.213372699	10.1015968	5.43E-24	3.76E-22	serinc4	serine incorporator 4 [Source:ZFIN;Acc:ZDB-GENE-121031-1]	7	50448478	50477289	
ENSDARG00000044894	826.3141203	-1.61379375	0.117291434	-5.233065429	1.67E-07	2.94E-06	zgc:113307	zgc:113307 [Source:ZFIN;Acc:ZDB-GENE-050522-29]	11	21050326	21061566	
ENSDARG00000044914	4823.34111	-1.782140891	0.127535817	-6.132715585	8.64E-10	2E-08	suclg2	succinate-CoA ligase, GDP-forming, beta subunit [Source:ZFIN;Acc:ZDB-GENE-030114-3]	11	16738179	16975190	
ENSDARG00000044935	4385.836472	-2.577578255	0.108509534	-14.53861426	6.9E-48	1.48E-45	hpdb	4-hydroxyphenylpyruvate dioxygenase b [Source:ZFIN;Acc:ZDB-GENE-040808-62]	8	34731982	34742234	
ENSDARG00000044975	4082.006454	-1.432956756	0.108332723	-3.996546418	6.43E-05	0.000771925	krt94	keratin 94 [Source:ZFIN;Acc:ZDB-GENE-061027-116]	11	11468566	11471857	
ENSDARG00000045038	126.5121623	-1.739146836	0.219140479	-3.372936118	0.000743712	0.007312149	tekt3	tektin 3 [Source:ZFIN;Acc:ZDB-GENE-080130-2]	12	37327867	37343858	
ENSDARG00000045051	1537.48138	1.581532007	0.128535567	4.524288656	6.06E-06	8.74E-05	slc16a3	solute carrier family 16 member 3 [Source:ZFIN;Acc:ZDB-GENE-030131-5487]	12	33350256	33359654	
ENSDARG00000045089	402.3657132	-2.103438456	0.170746912	-6.462421171	1.03E-10	2.66E-09	si:ch211-270n8.1	si:ch211-270n8.1 [Source:ZFIN;Acc:ZDB-GENE-081107-34]	13	20379392	20381485	
ENSDARG00000045142	3680.687773	5.26289816	0.137099229	31.09352393	2.95E-212	8.22E-209	hbae5	hemoglobin, alpha embryonic 5 [Source:ZFIN;Acc:ZDB-GENE-101028-1]	12	20342451	20353541	
ENSDARG00000045143	17630.48128	1.335247464	0.106205207	3.156601022	0.001596196	0.014612003	hbbe2	hemoglobin beta embryonic-2 [Source:ZFIN;Acc:ZDB-GENE-040702-1]	12	20349865	20350629	
ENSDARG00000045150	1477.85729	1.598083261	0.143446518	4.169381506	3.05E-05	0.000388179	csnk1e	casein kinase 1, epsilon [Source:ZFIN;Acc:ZDB-GENE-030131-7873]	12	19305390	19321087	
ENSDARG00000045166	71.09404575	-2.461402066	0.342080198	-4.272103663	1.94E-05	0.000255144	bhlha15	basic helix-loop-helix family, member a15 [Source:ZFIN;Acc:ZDB-GENE-070803-3]	12	17754859	17756905	
ENSDARG00000045262	2582.48835	-1.525642079	0.090344885	-5.818171986	5.95E-09	1.26E-07	gsnb	gelsolin b [Source:ZFIN;Acc:ZDB-GENE-030131-8567]	5	63294865	63302944	
ENSDARG00000045275	293.1870481	2.091110557	0.197967448	5.511565515	3.56E-08	6.84E-07	klhl14	kelch-like family member 14 [Source:ZFIN;Acc:ZDB-GENE-091204-223]	24	35881124	35906239	
ENSDARG00000045299	896.008893	-1.457605242	0.131777039	-3.472571894	0.000515497	0.005230189	vmo1b	vitelline membrane outer layer 1 homolog b [Source:ZFIN;Acc:ZDB-GENE-120215-131]	21	1618181	1625976	
ENSDARG00000045306	1753.753015	-1.549095247	0.098467224	-5.576426574	2.46E-08	4.83E-07	slc51a	solute carrier family 51 alpha subunit [Source:NCBI gene;Acc:447807]	24	24961899	24983047	
ENSDARG00000045378	157.2618602	2.374893973	0.249044161	5.520683423	3.38E-08	6.52E-07	pi15a	peptidase inhibitor 15a [Source:NCBI gene;Acc:561978]	24	14801844	14810634	
ENSDARG00000045408	2955.94713	-1.407093622	0.105162575	-3.871088359	0.000108351	0.001248144	tagln	transgelin [Source:ZFIN;Acc:ZDB-GENE-070912-1]	15	543744	551181	
ENSDARG00000045413	611.502362	-1.462473132	0.146175641	-3.163818046	0.001557141	0.014296196	gcm2	glial cells missing transcription factor 2 [Source:ZFIN;Acc:ZDB-GENE-050127-1]	24	8827603	8831912	
ENSDARG00000045414	1280.879584	-4.381635829	0.174063466	-19.42760252	4.51E-84	2.46E-81	elovl2	ELOVL fatty acid elongase 2 [Source:ZFIN;Acc:ZDB-GENE-060421-5612]	24	8861272	8887825	
ENSDARG00000045442	12097.65552	-4.17567089	0.119019821	-26.68186582	7.64E-157	1.01E-153	cpb1	carboxypeptidase B1 (tissue) [Source:ZFIN;Acc:ZDB-GENE-030131-1132]	24	4755391	4765740	
ENSDARG00000045514	16458.95165	-1.374891949	0.100944997	-3.713823964	0.000204151	0.002239681	atp5f1c	ATP synthase F1 subunit gamma [Source:ZFIN;Acc:ZDB-GENE-030131-8901]	4	25173299	25181552	
ENSDARG00000045548	52.73650537	8.773900687	1.473532394	5.275690387	1.32E-07	2.36E-06	lepb	leptin b [Source:ZFIN;Acc:ZDB-GENE-041210-183]	4	19031768	19033805	
ENSDARG00000045556	288.8949884	1.791117947	0.184918785	4.278191349	1.88E-05	0.000249577	OTUD7A	si:dkey-37f18.2 [Source:ZFIN;Acc:ZDB-GENE-131120-28]	25	34407529	34458452	OTUD7A
ENSDARG00000045592	2662.108418	-1.315726238	0.08697348	-3.630143782	0.000283263	0.003037247	tnni2a.1	troponin I type 2a (skeletal, fast), tandem duplicate 1 [Source:ZFIN;Acc:ZDB-GENE-041114-60]	25	31222069	31236549	
ENSDARG00000045598	1325.954216	-1.99811753	0.107711135	-9.266614163	1.92E-20	1.08E-18	akr1b1.2	aldo-keto reductase family 1, member B1 (aldose reductase), tandem duplicate 2 [Source:ZFIN;Acc:ZDB-GENE-041210-13	4	14906608	14915268	
ENSDARG00000045638	151.8724409	-2.700508066	0.267762101	-6.350816863	2.14E-10	5.32E-09	slc13a1	solute carrier family 13 member 1 [Source:ZFIN;Acc:ZDB-GENE-031222-3]	25	27923846	27932723	
ENSDARG00000045639	13117.55256	2.327026573	0.101024102	13.13574226	2.05E-39	3.05E-37	elavl4	ELAV like neuron-specific RNA binding protein 4 [Source:NCBI gene;Acc:30737]	8	16004154	16117112	
ENSDARG00000045685	1004.258772	1.696886046	0.11547241	6.035087059	1.59E-09	3.56E-08	cntn1b	contactin 1b [Source:ZFIN;Acc:ZDB-GENE-041210-236]	4	13733838	13756716	
ENSDARG00000045750	460.5042953	1.647028454	0.192319249	3.364345772	0.000767254	0.007506807	syt10	synaptotagmin X [Source:ZFIN;Acc:ZDB-GENE-041210-297]	4	10888762	10909086	
ENSDARG00000045789	1069.433562	1.952837186	0.136896983	6.96024969	3.4E-12	1E-10	srgap1b	SLIT-ROBO Rho GTPase activating protein 1b [Source:ZFIN;Acc:ZDB-GENE-041210-103]	4	9279515	9335035	
ENSDARG00000045799	1589.305162	2.315604452	0.112454354	11.69900863	1.29E-31	1.35E-29	acanb	aggrecan b [Source:ZFIN;Acc:ZDB-GENE-100422-16]	25	19213872	19224298	
ENSDARG00000045808	120.7724209	1.87626914	0.253811006	3.452447373	0.000555526	0.005591304	rlbp1b	retinaldehyde binding protein 1b [Source:ZFIN;Acc:ZDB-GENE-040426-1870]	25	19105804	19118127	
ENSDARG00000045811	106.7969446	2.402020597	0.305858566	4.583885345	4.56E-06	6.71E-05	lrtm2b	leucine-rich repeats and transmembrane domains 2b [Source:ZFIN;Acc:ZDB-GENE-131121-544]	25	19041329	19045977	
ENSDARG00000045834	163.9468207	-5.515661759	0.365523733	-12.35394956	4.64E-35	5.52E-33	si:dkey-14d8.7	si:dkey-14d8.7 [Source:ZFIN;Acc:ZDB-GENE-041210-143]	4	5537101	5539842	
ENSDARG00000045835	1685.055067	-5.888534935	0.165529137	-29.53277608	1.09E-191	1.96E-188	si:dkey-14d8.6	si:dkey-14d8.6 [Source:ZFIN;Acc:ZDB-GENE-041210-124]	4	5531583	5533803	
ENSDARG00000045846	478.1074686	-1.395677983	0.132022981	-2.997038702	0.002726161	0.023943096	osgep	O-sialoglycoprotein endopeptidase [Source:NCBI gene;Acc:550447]	4	5327632	5333971	
ENSDARG00000045887	1720.132619	-2.265111791	0.15127357	-8.363072217	6.11E-17	2.6E-15	mmp30	matrix metallopeptidase 30 [Source:ZFIN;Acc:ZDB-GENE-060421-5765]	10	38643304	38663746	
ENSDARG00000045904	442.2863105	1.701553663	0.161877903	4.333844511	1.47E-05	0.000198596	nr2e3	nuclear receptor subfamily 2, group E, member 3 [Source:ZFIN;Acc:ZDB-GENE-041114-63]	25	22274642	22278655	
ENSDARG00000045911	1960.409927	1.396899388	0.099359469	3.994580425	6.48E-05	0.000777986	tulp4a	TUB like protein 4a [Source:ZFIN;Acc:ZDB-GENE-060503-215]	4	331351	353924	
ENSDARG00000045930	1988.69117	1.533615016	0.099164306	5.38111987	7.4E-08	1.36E-06	rbpms2a	RNA binding protein, mRNA processing factor 2a [Source:ZFIN;Acc:ZDB-GENE-040718-106]	25	6122823	6153077	
ENSDARG00000045936	1528.093423	2.114805679	0.127483825	8.744683361	2.24E-18	1.07E-16	pax6b	paired box 6b [Source:ZFIN;Acc:ZDB-GENE-001031-1]	7	15871156	15900606	
ENSDARG00000045944	2705.455186	1.893320229	0.211261471	4.228505213	2.35E-05	0.000304712	slc6a1a	solute carrier family 6 member 1a [Source:ZFIN;Acc:ZDB-GENE-060519-23]	23	4834447	4855122	
ENSDARG00000045947	8788.856467	-2.304097438	0.127808678	-10.20351246	1.91E-24	1.35E-22	hrc	histidine rich calcium binding protein [Source:ZFIN;Acc:ZDB-GENE-041008-189]	12	4377624	4388704	
ENSDARG00000046007	628.4469167	1.494173195	0.141836976	3.484095327	0.000493804	0.005041542	tmcc2	transmembrane and coiled-coil domain family 2 [Source:ZFIN;Acc:ZDB-GENE-091202-2]	22	591809	600016	
ENSDARG00000046087	425.9769314	1.568718483	0.147145982	3.864995	0.000111091	0.001277374	brd2b	bromodomain containing 2b [Source:ZFIN;Acc:ZDB-GENE-070220-1]	16	48714048	48747788	
ENSDARG00000046098	268.0371448	-2.264246037	0.198822316	-6.358672709	2.04E-10	5.08E-09	ebp	EBP cholestenol delta-isomerase [Source:ZFIN;Acc:ZDB-GENE-040718-12]	23	19147128	19153408	
ENSDARG00000046107	2360.88112	2.035436705	0.158376891	6.537801667	6.24E-11	1.63E-09	caskin1	CASK interacting protein 1 [Source:ZFIN;Acc:ZDB-GENE-071115-3]	3	35632773	35801146	
ENSDARG00000046142	340.7291264	-1.752099982	0.189903324	-3.960436105	7.48E-05	0.000891258	asgrl1	asialoglycoprotein receptor-like 1 [Source:ZFIN;Acc:ZDB-GENE-081105-120]	6	39733278	39739640	
ENSDARG00000046150	792.9826316	1.961441661	0.168216069	5.715516161	1.09E-08	2.24E-07	b4galnt4a	beta-1,4-N-acetyl-galactosaminyl transferase 4a [Source:ZFIN;Acc:ZDB-GENE-100422-4]	25	24291156	24610545	
ENSDARG00000051798	1215.014247	1.43947721	0.120164726	3.657289652	0.000254896	0.002754262	brd1b	bromodomain containing 1b [Source:ZFIN;Acc:ZDB-GENE-070209-98]	25	29688671	29714525	
ENSDARG00000051814	1198.410131	1.339690327	0.110786731	3.066164363	0.002168241	0.019382128	ptprz1a	protein tyrosine phosphatase receptor type Z1a [Source:ZFIN;Acc:ZDB-GENE-090406-1]	25	28340171	28443607	
ENSDARG00000051852	1656.001251	1.86837567	0.220623602	3.936005306	8.28E-05	0.000979555	kcnc1a	potassium voltage-gated channel, Shaw-related subfamily, member 1a [Source:ZFIN;Acc:ZDB-GENE-061220-10]	7	33028452	33088144	
ENSDARG00000051874	1583.200228	-1.508923772	0.105030674	-4.845477534	1.26E-06	2.01E-05	stra6	stimulated by retinoic acid 6 [Source:NCBI gene;Acc:724007]	25	22571475	22634675	
ENSDARG00000051876	855.6708871	-1.78766336	0.15975129	-4.930560243	8.2E-07	1.33E-05	ush1c	Usher syndrome 1C [Source:ZFIN;Acc:ZDB-GENE-060312-41]	25	22643954	22704233	
ENSDARG00000051925	1245.845861	-1.599350968	0.133691175	-4.483100474	7.36E-06	0.000104801	cyb5r2	cytochrome b5 reductase 2 [Source:NCBI gene;Acc:751641]	25	16044734	16057539	
ENSDARG00000051940	1720.266504	-2.326771026	0.118697241	-11.17777476	5.24E-29	4.92E-27	ugt2a4	UDP glucuronosyltransferase 2 family, polypeptide A4 [Source:ZFIN;Acc:ZDB-GENE-080721-23]	5	69926766	69948119	
ENSDARG00000051981	474.9862494	2.149583186	0.147304529	7.804126532	5.99E-15	2.2E-13	STX3	zgc:165520 [Source:ZFIN;Acc:ZDB-GENE-070620-13]	1	44826316	44835738	STX3
ENSDARG00000051986	8038.304356	-1.30359862	0.081077727	-3.74453788	0.000180726	0.002006372	ndufs8a	NADH:ubiquinone oxidoreductase core subunit S8a [Source:ZFIN;Acc:ZDB-GENE-040426-2153]	1	44814322	44823071	
ENSDARG00000052016	248.2485218	3.279560805	0.230245352	9.900572526	4.14E-23	2.65E-21	cabp2a	calcium binding protein 2a [Source:ZFIN;Acc:ZDB-GENE-050913-25]	1	44127292	44141442	
ENSDARG00000052057	879.4092222	1.466308306	0.121140249	3.849325978	0.000118463	0.001356316	pcolceb	procollagen C-endopeptidase enhancer b [Source:ZFIN;Acc:ZDB-GENE-080722-44]	7	50849142	50873496	
ENSDARG00000052113	657.4837524	-1.365109852	0.118769123	-3.074114237	0.002111286	0.018906665	hexa	hexosaminidase A (alpha polypeptide) [Source:ZFIN;Acc:ZDB-GENE-050417-283]	25	22216723	22241312	
ENSDARG00000052138	1082.697966	1.992822866	0.172791038	5.745800686	9.15E-09	1.89E-07	slc1a2a	solute carrier family 1 member 2a [Source:ZFIN;Acc:ZDB-GENE-100422-11]	7	49862837	49881742	
ENSDARG00000052139	4653.268795	1.40366637	0.12056307	3.34817593	0.000813454	0.007917215	notch3	notch receptor 3 [Source:ZFIN;Acc:ZDB-GENE-000329-5]	3	53526025	53559581	
ENSDARG00000052150	3456.689471	1.554031659	0.120137487	4.611646825	3.99E-06	5.92E-05	pbx4	pre-B-cell leukemia transcription factor 4 [Source:ZFIN;Acc:ZDB-GENE-000201-18]	3	52999962	53076469	
ENSDARG00000052155	1256.086628	1.40055851	0.115878224	3.456719443	0.000546794	0.00551906	lrrc8ab	leucine rich repeat containing 8 VRAC subunit Ab [Source:ZFIN;Acc:ZDB-GENE-060531-43]	5	64840656	64850497	
ENSDARG00000052242	999.3963327	1.980659479	0.121672522	8.059827004	7.64E-16	3.01E-14	aff2	AF4/FMR2 family, member 2 [Source:ZFIN;Acc:ZDB-GENE-110411-190]	14	20342553	20736925	
ENSDARG00000052271	1592.891361	-1.432197673	0.128836914	-3.35461057	0.000794768	0.007750376	slc22a18	solute carrier family 22 member 18 [Source:ZFIN;Acc:ZDB-GENE-051120-165]	7	39537790	39552314	
ENSDARG00000052336	1414.436127	-2.53878264	0.120310432	-12.79010155	1.86E-37	2.58E-35	ociad2	OCIA domain containing 2 [Source:ZFIN;Acc:ZDB-GENE-041014-253]	20	23238833	23244193	
ENSDARG00000052468	253.7469082	1.482402435	0.174784962	2.759976775	0.005780547	0.046911579	gsk3bb	glycogen synthase kinase 3 beta, genome duplicate b [Source:ZFIN;Acc:ZDB-GENE-090312-2]	1	49370549	49409549	
ENSDARG00000052470	4336.343541	-1.612028207	0.090236267	-6.782508014	1.18E-11	3.33E-10	igfbp2a	insulin-like growth factor binding protein 2a [Source:NCBI gene;Acc:794176]	6	20921542	20952187	
ENSDARG00000052497	727.6618162	-1.385226392	0.135697318	-2.83886519	0.004527428	0.03773007	zgc:110789	zgc:110789 [Source:ZFIN;Acc:ZDB-GENE-050306-38]	18	12034985	12052132	
ENSDARG00000052578	4381.267293	-6.001921371	0.115770628	-43.20544394	0	0	c6ast4	six-cysteine containing astacin protease 4 [Source:ZFIN;Acc:ZDB-GENE-050626-100]	7	38635538	38638809	
ENSDARG00000052604	571.6143878	1.769243403	0.154067165	4.992909443	5.95E-07	9.86E-06	cpeb2	cytoplasmic polyadenylation element binding protein 2 [Source:ZFIN;Acc:ZDB-GENE-130530-639]	23	2136344	2161478	
ENSDARG00000052626	17.3159323	4.094186173	1.061633392	2.914522422	0.00356199	0.030473588	AL954655.1		12	46188692	46204837	
ENSDARG00000052690	6388.175202	1.423115379	0.09397283	4.502528881	6.71E-06	9.61E-05	arrdc3a	arrestin domain containing 3a [Source:ZFIN;Acc:ZDB-GENE-030131-2913]	5	48985861	48995955	

ENSDARG00000052700	718.3409002	2.33942069	0.133469087	10.03543755	1.06E-23	7.22E-22	SLC9B2	si:dkey-162b23.4 [Source:ZFIN;Acc:ZDB-GENE-110411-203]	1	43695033	43727613	SLC9B2
ENSDARG00000052712	14307.42607	-1.369090064	0.080191204	-4.60262531	4.17E-06	6.17E-05	suclg1	succinate-CoA ligase, alpha subunit [Source:ZFIN;Acc:ZDB-GENE-040912-101]	1	43652480	43684584	
ENSDARG00000052721	533.908868	-1.469483316	0.139387654	-3.368184353	0.00075665	0.007419011	prim2	DNA primase subunit 2 [Source:ZFIN;Acc:ZDB-GENE-050522-471]	13	23488282	23612369	
ENSDARG00000052766	425.2366623	1.620377327	0.195854182	3.167547	0.001537309	0.014145865	EVI5L	si:ch211-239f4.1 [Source:ZFIN;Acc:ZDB-GENE-061207-24]	1	45217425	45282774	EVI5L
ENSDARG00000052779	664.7506194	2.514829511	0.496624929	3.05024863	0.00228652	0.020338036	zgc:153932	zgc:153932 [Source:ZFIN;Acc:ZDB-GENE-070209-223]	12	46120836	46145909	
ENSDARG00000052840	2058.988017	-1.666683034	0.091952918	-7.250265151	4.16E-13	1.31E-11	ndufs4	NADH:ubiquinone oxidoreductase subunit S4 [Source:ZFIN;Acc:ZDB-GENE-050522-421]	5	40485503	40501732	
ENSDARG00000052893	203.108051	3.475192057	0.285309132	8.67547434	4.12E-18	1.93E-16	rx3	retinal homeobox gene 3 [Source:NCBI gene;Acc:30474]	21	10756154	10759223	
ENSDARG00000052894	793.7294372	1.596049638	0.154340307	3.861918185	0.0001125	0.001292379	znf532	zinc finger protein 532 [Source:ZFIN;Acc:ZDB-GENE-060531-40]	21	10773513	10816565	
ENSDARG00000052895	102.0110239	2.630489265	0.32799819	4.971031289	6.66E-07	1.1E-05	htra3a	HtrA serine peptidase 3a [Source:ZFIN;Acc:ZDB-GENE-040801-245]	1	40740951	40759629	
ENSDARG00000052905	2265.45314	-1.753636008	0.112410332	-6.704330421	2.02E-11	5.58E-10	zgc:165423	zgc:165423 [Source:ZFIN;Acc:ZDB-GENE-070720-11]	3	39540014	39550089	
ENSDARG00000052948	214.5630646	1.572528995	0.207111113	2.764356715	0.005703515	0.046346368	nppal	natriuretic peptide A-like [Source:ZFIN;Acc:ZDB-GENE-081022-175]	8	48603398	48604351	
ENSDARG00000052958	122.936572	2.270456243	0.27858921	4.560321064	5.11E-06	7.47E-05	nppb	natriuretic peptide B [Source:ZFIN;Acc:ZDB-GENE-130530-642]	8	48609521	48610544	
ENSDARG00000052982	242.6653267	3.056759906	0.239943316	8.571857468	1.02E-17	4.66E-16	gabrr2a	gamma-aminobutyric acid (GABA) A receptor, rho 2a [Source:ZFIN;Acc:ZDB-GENE-060825-164]	17	15845765	15892623	
ENSDARG00000053003	374.2434016	1.483809207	0.153104722	3.159988812	0.001577752	0.014469548	slc35f1	solute carrier family 35 member F1 [Source:ZFIN;Acc:ZDB-GENE-060503-463]	20	41998502	42203629	
ENSDARG00000053026	689.0797154	1.529946034	0.133630322	3.965761856	7.32E-05	0.000872831	kif19	kinesin family member 19 [Source:ZFIN;Acc:ZDB-GENE-080215-2]	12	38770654	38805710	
ENSDARG00000053058	41282.0777	-1.527111255	0.112220816	-4.697089825	2.64E-06	4.02E-05	rps11	ribosomal protein S11 [Source:ZFIN;Acc:ZDB-GENE-040426-2701]	3	37787229	37789244	
ENSDARG00000053068	1470.029707	-1.959108444	0.145912514	-6.573174707	4.93E-11	1.3E-09	cyp8b1	cytochrome P450, family 8, subfamily B, polypeptide 1 [Source:ZFIN;Acc:ZDB-GENE-040808-53]	2	3435888	3437862	
ENSDARG00000053190	637.9984268	1.568364569	0.165838581	3.427215589	0.000609805	0.00609829	galnt16	UDP-N-acetyl-alpha-D-galactosamine:polypeptide N-acetylgalactosaminyltransferase 16 [Source:ZFIN;Acc:ZDB-GENE-13	17	50127648	50186571	
ENSDARG00000053201	85.0954009	3.049818598	0.374072484	5.47973637	4.26E-08	8.12E-07	zgc:172323	zgc:172323 [Source:ZFIN;Acc:ZDB-GENE-080204-113]	16	32736588	32744258	
ENSDARG00000053205	1011.65477	1.582207544	0.112582019	5.17140792	2.32E-07	4.01E-06	ppfia4	PTPRF interacting protein alpha 4 [Source:ZFIN;Acc:ZDB-GENE-070720-14]	11	22374419	22584452	
ENSDARG00000053215	593.9502977	-1.67571291	0.143027976	-4.724340849	2.31E-06	3.56E-05	me1	malic enzyme 1, NADP(+)-dependent, cytosolic [Source:ZFIN;Acc:ZDB-GENE-061013-438]	16	32832213	33001153	
ENSDARG00000053217	4268.777093	-1.366782004	0.099150902	-3.699230143	0.000216254	0.002355991	cox7a2a	cytochrome c oxidase subunit 7A2a [Source:ZFIN;Acc:ZDB-GENE-050522-153]	17	49983157	49995366	
ENSDARG00000053315	1557.471569	-1.41475032	0.151436999	-2.738764794	0.006167048	0.049694723	tmprss3a	transmembrane serine protease 3a [Source:ZFIN;Acc:ZDB-GENE-070912-70]	9	19550367	19585848	
ENSDARG00000053323	518.4737617	1.837462427	0.193294342	4.332576002	1.47E-05	0.000199528	zgc:112285	zgc:112285 [Source:ZFIN;Acc:ZDB-GENE-050913-132]	12	35051636	35070011	
ENSDARG00000053358	9161.76245	1.64913442	0.084186291	7.710690294	1.25E-14	4.5E-13	basp1	brain abundant, membrane attached signal protein 1 [Source:NCBI gene;Acc:569631]	2	42724404	42748657	
ENSDARG00000053364	325.7302284	2.803742346	0.197649177	9.125979563	7.11E-20	3.77E-18	pax10	paired box 10 [Source:ZFIN;Acc:ZDB-GENE-081022-10]	3	32546499	32565883	
ENSDARG00000053365	44708.74796	-1.548227241	0.130635123	-4.196629732	2.71E-05	0.000346422	rpl31	ribosomal protein L31 [Source:ZFIN;Acc:ZDB-GENE-060331-121]	9	56225410	56232296	
ENSDARG00000053375	803.7486389	1.690499306	0.138455731	4.987148611	6.13E-07	1.01E-05	nptxrb	neuronal pentraxin receptor b [Source:ZFIN;Acc:ZDB-GENE-050208-595]	22	25025136	25033105	
ENSDARG00000053380	409.9317318	2.131172312	0.156009052	7.250683832	4.15E-13	1.31E-11	fam189a1	family with sequence similarity 189 member A1 [Source:ZFIN;Acc:ZDB-GENE-040718-129]	7	31145386	31336235	
ENSDARG00000053405	1441.054347	-1.698339828	0.128770957	-5.423115933	5.86E-08	1.1E-06	sord	sorbitol dehydrogenase [Source:ZFIN;Acc:ZDB-GENE-040426-1231]	7	30923283	30926030	
ENSDARG00000053424	156.9346928	-1.557350487	0.203012701	-2.745397132	0.006043771	0.048815054	myl2b	myosin, light chain 2b, regulatory, cardiac, slow [Source:ZFIN;Acc:ZDB-GENE-060331-137]	8	4333914	4359475	
ENSDARG00000053450	20.28454522	7.396440178	1.518799133	4.211511609	2.54E-05	0.000326194	ftr06	finTRIM family, member 6 [Source:ZFIN;Acc:ZDB-GENE-070912-228]	2	42294944	42308424	
ENSDARG00000053452	353.5250773	-1.523094108	0.153061533	-3.417541289	0.000631895	0.006289135	pop5	POP5 homolog, ribonuclease P/MRP subunit [Source:NCBI gene;Acc:541421]	8	41274211	41279326	
ENSDARG00000053454	446.3407903	1.75305371	0.191450916	3.933403529	8.38E-05	0.000989211	cntn3b	contactin 3b [Source:ZFIN;Acc:ZDB-GENE-130530-645]	6	44584775	44711942	
ENSDARG00000053483	861.6010386	-2.014081846	0.146950387	-6.900845025	5.17E-12	1.51E-10	zgc:113054	zgc:113054 [Source:ZFIN;Acc:ZDB-GENE-050320-9]	6	43450221	43474757	
ENSDARG00000053500	605.7882783	-2.13359092	0.145590132	-7.786179646	6.91E-15	2.53E-13	cbsa	cystathionine beta-synthase a [Source:ZFIN;Acc:ZDB-GENE-050417-367]	1	29068649	29093562	
ENSDARG00000053502	341.2897177	3.637704718	0.263267843	10.01909192	1.26E-23	8.46E-22	cryaa	crystallin, alpha A [Source:ZFIN;Acc:ZDB-GENE-020508-1]	1	29096881	29100923	
ENSDARG00000053517	983.2396762	1.893083537	0.124975542	7.146066515	8.93E-13	2.77E-11	EML5	si:dkey-1f12.3 [Source:ZFIN;Acc:ZDB-GENE-030131-1677]	17	43039463	43287290	EML5
ENSDARG00000053525	5.711991857	-6.383348051	1.512131393	-3.560106003	0.000370705	0.003879942	enpp7.2	ectonucleotide pyrophosphatase/phosphodiesterase 7, tandem duplicate 2 [Source:ZFIN;Acc:ZDB-GENE-130531-2]	12	32423375	32426685	
ENSDARG00000053526	741.2521997	-5.348310821	0.205496581	-21.16001545	2.23E-99	1.51E-96	enpp7.1	ectonucleotide pyrophosphatase/phosphodiesterase 7, tandem duplicate 1 [Source:ZFIN;Acc:ZDB-GENE-040724-13]	12	32413524	32421046	
ENSDARG00000053547	2391.068036	1.575318918	0.143646551	4.005100824	6.2E-05	0.000747017	jakmip2	janus kinase and microtubule interacting protein 2 [Source:ZFIN;Acc:ZDB-GENE-091204-293]	21	43029708	43079161	
ENSDARG00000053583	451.8264112	1.770692294	0.172081861	4.478637601	7.51E-06	0.000106773	cers6	ceramide synthase 6 [Source:ZFIN;Acc:ZDB-GENE-070522-1]	9	48870122	48937240	
ENSDARG00000053620	92.00120536	-2.246632146	0.264779284	-4.70819366	2.5E-06	3.82E-05	ebi3	Epstein-Barr virus induced 3 [Source:ZFIN;Acc:ZDB-GENE-070912-615]	2	41936603	41942666	
ENSDARG00000053625	3045.250253	-3.479898351	0.150111472	-16.52037862	2.62E-61	8.88E-59	anxa2b	annexin A2b [Source:ZFIN;Acc:ZDB-GENE-030131-1089]	7	29518396	29534001	
ENSDARG00000053636	351.3816466	-1.45257781	0.161310571	-2.805630207	0.005021829	0.041464777	cracr2b	calcium release activated channel regulator 2B [Source:NCBI gene;Acc:790936]	25	3844582	3867990	
ENSDARG00000053665	559.7913836	1.941697388	0.181429368	5.190435243	2.1E-07	3.64E-06	gabrg2	gamma-aminobutyric acid (GABA) A receptor, gamma 2 [Source:ZFIN;Acc:ZDB-GENE-091118-65]	21	41903760	42007482	
ENSDARG00000053771	273.7557481	1.793491977	0.196581511	4.036452735	5.43E-05	0.000660714	apbb3	amyloid beta (A4) precursor protein-binding, family B, member 3 [Source:ZFIN;Acc:ZDB-GENE-140106-53]	21	40731111	40782393	
ENSDARG00000053774	1008.56093	-3.804686048	0.18345263	-15.28833926	9.15E-53	2.5E-50	alpi.2	alkaline phosphatase, intestinal, tandem duplicate 2 [Source:ZFIN;Acc:ZDB-GENE-050626-142]	22	38778649	38794610	
ENSDARG00000053802	41.35327683	-2.4337424	0.423533424	-3.38519304	0.000711282	0.00701804	cbln5	cerebellin 5 [Source:ZFIN;Acc:ZDB-GENE-070912-577]	2	38034325	38035235	
ENSDARG00000053845	172.4007208	-3.253889838	0.303636979	-7.422975433	1.15E-13	3.81E-12	cbln14	cerebellin 14 [Source:ZFIN;Acc:ZDB-GENE-040724-5]	2	37959040	37960688	
ENSDARG00000053858	3937.930787	-2.107522571	0.102813825	-10.77211718	4.66E-27	3.81E-25	crip1	cysteine-rich protein 1 [Source:ZFIN;Acc:ZDB-GENE-041111-1]	17	37385811	37395496	
ENSDARG00000053862	1234.749907	2.117079578	0.201193706	5.552259068	2.82E-08	5.51E-07	crygmx	crystallin, gamma MX [Source:ZFIN;Acc:ZDB-GENE-030131-7732]	12	30838167	30841679	
ENSDARG00000053875	3728.132111	2.567035089	0.198601468	7.890349996	3.01E-15	1.14E-13	cryba1b	crystallin, beta A1b [Source:ZFIN;Acc:ZDB-GENE-040718-326]	21	39185461	39195314	
ENSDARG00000053906	131.3183807	2.162653118	0.248287977	4.682679902	2.83E-06	4.28E-05	angpt2b	angiopoietin 2b [Source:ZFIN;Acc:ZDB-GENE-010817-2]	19	35799384	35852390	
ENSDARG00000053912	5343.379369	-1.2988194	0.094237574	-3.17091566	0.001519593	0.01400782	fbl	fibrillarin [Source:ZFIN;Acc:ZDB-GENE-040426-1936]	16	42177659	42186093	
ENSDARG00000053973	55714.90767	-2.973213598	0.122359925	-16.12630603	1.67E-58	5.1E-56	fetub	fetuin B [Source:ZFIN;Acc:ZDB-GENE-050208-4]	22	37888249	37900135	
ENSDARG00000053990	10558.70716	-1.462846845	0.103825574	-4.457927151	8.28E-06	0.000116438	hmgb2b	high mobility group box 2b [Source:ZFIN;Acc:ZDB-GENE-040912-122]	23	45025909	45029559	
ENSDARG00000054058	6994.519338	1.38242242	0.130557814	2.929142342	0.003398987	0.029188498	h1-10	H1.10 linker histone [Source:ZFIN;Acc:ZDB-GENE-030131-1228]	6	40769517	40771813	
ENSDARG00000054150	100.303389	2.254034455	0.319114053	3.929737477	8.5E-05	0.001002139	cx23	connexin 23 [Source:NCBI gene;Acc:541419]	20	37294112	37298274	
ENSDARG00000054155	8004.101525	-1.720919715	0.092883788	-7.761523662	8.39E-15	3.05E-13	pcna	proliferating cell nuclear antigen [Source:NCBI gene;Acc:30678]	10	40821804	40826657	
ENSDARG00000054164	494.7582166	-1.574193537	0.134952639	-4.254778136	2.09E-05	0.000273143	mrps17	mitochondrial ribosomal protein S17 [Source:ZFIN;Acc:ZDB-GENE-050522-390]	21	37411547	37418838	
ENSDARG00000054172	4030.033467	-1.76431198	0.101746104	-7.511953265	5.83E-14	1.98E-12	pgrmc1	progesterone receptor membrane component 1 [Source:ZFIN;Acc:ZDB-GENE-041114-91]	21	37436907	37441931	
ENSDARG00000054177	576.2506134	2.958022955	0.158055248	12.38821853	3.03E-35	3.74E-33	adgra1a	adhesion G protein-coupled receptor A1a [Source:ZFIN;Acc:ZDB-GENE-130603-101]	12	29131689	29211598	
ENSDARG00000054184	134.2924887	-2.36531287	0.244882436	-5.575380959	2.47E-08	4.85E-07	btr06	bloodthirsty-related gene family, member 6 [Source:ZFIN;Acc:ZDB-GENE-080516-2]	7	16205609	16214891	
ENSDARG00000054220	408.3939313	-1.591987744	0.166785743	-3.549390569	0.000386124	0.004026795	ap4s1	adaptor related protein complex 4 subunit sigma 1 [Source:ZFIN;Acc:ZDB-GENE-030616-404]	17	28703971	28707970	
ENSDARG00000054253	506.5408974	1.654291358	0.141633009	4.619624793	3.84E-06	5.71E-05	sobpa	sine oculis binding protein homolog (Drosophila) a [Source:NCBI gene;Acc:573683]	13	32740509	32796029	
ENSDARG00000054300	2751.906525	-2.338852537	0.114424596	-11.70074075	1.26E-31	1.34E-29	dhrs1	dehydrogenase/reductase (SDR family) member 1 [Source:ZFIN;Acc:ZDB-GENE-030616-591]	2	38261748	38269675	
ENSDARG00000054321	4855.971725	4.716460636	0.163590367	22.71808973	2.97E-114	2.33E-111	ngs	notochord granular surface [Source:ZFIN;Acc:ZDB-GENE-080722-29]	4	5776347	5784895	
ENSDARG00000054378	1634.287661	1.888919901	0.104876285	8.475890415	2.33E-17	1.04E-15	pcbp3	poly(rC) binding protein 3 [Source:ZFIN;Acc:ZDB-GENE-050522-157]	9	42040557	42127228	
ENSDARG00000054456	1987.873981	2.273144534	0.116508959	10.92743895	8.52E-28	7.18E-26	clip3	CAP-GLY domain containing linker protein 3 [Source:ZFIN;Acc:ZDB-GENE-131127-193]	15	28989573	29006189	
ENSDARG00000054543	731.0798736	1.670801622	0.129859842	5.165581692	2.4E-07	4.13E-06	samsn1a	SAM domain, SH3 domain and nuclear localisation signals 1a [Source:ZFIN;Acc:ZDB-GENE-030131-8639]	15	29564364	29586747	
ENSDARG00000054588	358.5526449	-2.100749502	0.150609725	-7.308621674	2.7E-13	8.69E-12	cox6a2	cytochrome c oxidase subunit 6A2 [Source:ZFIN;Acc:ZDB-GENE-040912-129]	3	31039923	31042102	
ENSDARG00000054683	1071.981027	1.531744181	0.155120079	3.427951968	0.000608153	0.006084197	prdm8b	PR domain containing 8b [Source:ZFIN;Acc:ZDB-GENE-060526-174]	5	39504136	39508958	
ENSDARG00000054786	342.6916155	-3.344857518	0.197944467	-11.84603716	2.26E-32	2.43E-30	faah2b	fatty acid amide hydrolase 2b [Source:NCBI gene;Acc:768298]	7	24508123	24520866	
ENSDARG00000054797	181.5499611	1.63258391	0.207038105	3.055398482	0.002247617	0.020027486	zgc:153441	zgc:153441 [Source:ZFIN;Acc:ZDB-GENE-060825-39]	19	42418227	42424599	
ENSDARG00000054818	38094.45653	-1.281086906	0.092659863	-3.033534646	0.00241707	0.02138557	rpl32	ribosomal protein L32 [Source:ZFIN;Acc:ZDB-GENE-060331-105]	22	31039091	31044066	
ENSDARG00000054837	90.10608822	-2.121445719	0.289086728	-3.879270853	0.00010477	0.001211514	zgc:136870	zgc:136870 [Source:ZFIN;Acc:ZDB-GENE-060312-1]	2	39618951	39621103	
ENSDARG00000054879	1058.069942	1.943849557	0.13410769	7.037997258	1.95E-12	5.87E-11	six3b	SIX homeobox 3b [Source:ZFIN;Acc:ZDB-GENE-990415-128]	12	25600685	25603341	
ENSDARG00000054942	1603.922595	-1.359651508	0.096484149	-3.727570908	0.000193334	0.002129391	lgals2a	lectin, galactoside-binding, soluble, 2a [Source:ZFIN;Acc:ZDB-GENE-050318-2]	3	28953274	28961518	
ENSDARG00000055014	398.8536525	-1.630815516	0.146828716	-4.296268029	1.74E-05	0.000231667	si:dkey-33m11.8	si:dkey-33m11.8 [Source:ZFIN;Acc:ZDB-GENE-141215-49]	11	3575963	3584553	
ENSDARG00000055026	2222.97554	1.715092348	0.145210614	4.924518445	8.46E-07	1.37E-05	ptch2	patched 2 [Source:NCBI gene;Acc:30181]	2	33969212	33993636	
ENSDARG00000055046	705.9112421	-2.101824509	0.13167445	-8.367792747	5.87E-17	2.51E-15	ponzr5	plac8 onzin related protein 5 [Source:ZFIN;Acc:ZDB-GENE-081104-325]	7	22657566	22663971	
ENSDARG00000055052	2008.783108	1.731106937	0.12002928	6.091071572	1.12E-09	2.56E-08	map2	microtubule-associated protein 2 [Source:ZFIN;Acc:ZDB-GENE-041010-118]	9	38883388	38993033	
ENSDARG00000055078	336.7767644	-1.674120934	0.15449709	-4.363324486	1.28E-05	0.000174943	chac2	ChaC, cation transport regulator homolog 2 (E. coli) [Source:NCBI gene;Acc:557614]	12	24056000	24060358	
ENSDARG00000055092	3263.38503	1.258456772	0.090040923	2.870436736	0.004099052	0.034550137	pora	P450 (cytochrome) oxidoreductase a [Source:ZFIN;Acc:ZDB-GENE-050809-121]	10	35275965	35312885	
ENSDARG00000055138	344.4089506	-1.754165743	0.170573578	-4.421351498	9.81E-06	0.000136024	zgc:110410	zgc:110410 [Source:ZFIN;Acc:ZDB-GENE-050522-465]	16	25599379	25608453	
ENSDARG00000055154	888.2739849	1.884824256	0.164460059	5.380177178	7.44E-08	1.37E-06	slc8a4a	solute carrier family 8 member 4a [Source:ZFIN;Acc:ZDB-GENE-060110-2]	21	27047333	27185915	
ENSDARG00000055158	1595.604611	1.34132505	0.111635251	3.057502412	0.002231898	0.019922789	prox1a	prospero homeobox 1a [Source:ZFIN;Acc:ZDB-GENE-980526-397]	17	32822588	32865788	
ENSDARG00000055177	125.2669086	1.832572825	0.281973961	2.952658548	0.003150502	0.027222292	pxdc1a	PX domain containing 1a [Source:ZFIN;Acc:ZDB-GENE-070912-381]	2	32835679	32838894	
ENSDARG00000055185	527.163818	-3.59893713	0.16660255	-15.59962392	7.32E-55	2.02E-52	tm4sf5	transmembrane 4 L six family member 5 [Source:ZFIN;Acc:ZDB-GENE-040718-65]	7	21841037	21848205	
ENSDARG00000055190	2082.232455	-1.514909213	0.108014871	-4.76702151	1.87E-06	2.92E-05	slc17a5	solute carrier family 17 member 5 [Source:ZFIN;Acc:ZDB-GENE-060929-1158]	13	27298673	27313556	
ENSDARG00000055192	53855.03307	-4.293014532	0.142160659	-23.16403537	1.05E-118	8.5E-116	zgc:136930	zgc:136930 [Source:ZFIN;Acc:ZDB-GENE-060312-16]	2	32780138	32790887	
ENSDARG00000055240	1695.82773	-1.993457212	0.161212973	-6.162389982	7.17E-10	1.67E-08	xdh	xanthine dehydrogenase [Source:ZFIN;Acc:ZDB-GENE-070719-9]	17	33340675	33374538	
ENSDARG00000055270	105.1075598	2.957503877	0.31924782	6.131612361	8.7E-10	2.01E-08	si:ch1073-358c10.1	si:ch1073-358c10.1 [Source:ZFIN;Acc:ZDB-GENE-050309-123]	17	27314088	27382868	
ENSDARG00000055302	672.4315468	1.75808688	0.157968492	4.79897522	1.59E-06	2.51E-05	grid2	glutamate receptor, ionotropic, delta 2 [Source:NCBI gene;Acc:448841]	8	28900689	29587703	
ENSDARG00000055305	1165.804454	1.814715236	0.116376611	7.000678505	2.55E-12	7.6E-11	ret	ret proto-oncogene receptor tyrosine kinase [Source:ZFIN;Acc:ZDB-GENE-980526-307]	13	25584556	25632756	
ENSDARG00000055331	110.5387769	-2.16568125	0.284980412	-4.090390781	4.31E-05	0.000531972	aldh9a1a.2	aldehyde dehydrogenase 9 family, member A1a, tandem duplicate 2 [Source:ZFIN;Acc:ZDB-GENE-081104-507]	8	21082361	21091961	
ENSDARG00000055360	2031.183539	-1.745056018	0.112476237	-6.624119327	3.49E-11	9.36E-10	llph	LLP homolog, long-term synaptic facilitation factor [Source:NCBI gene;Acc:100003313]	4	22297839	22300326	
ENSDARG00000055388	4124.949308	-1.457263636	0.08474443	-5.395795726	6.82E-08	1.26E-06	vtna	vitronectin a [Source:ZFIN;Acc:ZDB-GENE-050522-440]	15	28202170	28208926	
ENSDARG00000055395	521.4075536	-1.73146184	0.148550058	-4.924009103	8.48E-07	1.37E-05	foxq1b	forkhead box Q1b [Source:ZFIN;Acc:ZDB-GENE-040426-2090]	20	26683933	26685395	
ENSDARG00000055416	2050.922844	-1.4782657	0.138521634	-3.452642636	0.000555124	0.005591304	serpinb1	serpin peptidase inhibitor, clade B (ovalbumin), member 1 [Source:ZFIN;Acc:ZDB-GENE-040718-400]	20	26880668	26888698	
ENSDARG00000055437	30.59059916	-4.53796975	0.635033924	-5.571308266	2.53E-08	4.96E-07	METTL21C	zgc:172067 [Source:ZFIN;Acc:ZDB-GENE-080204-102]	9	34970418	34974416	METTL21C
ENSDARG00000055439	509.3565589	-1.383114731	0.139285923	-2.750563186	0.005949292	0.048125417	adamtsl7	ADAMTS-like 7 [Source:ZFIN;Acc:ZDB-GENE-070816-2]	1	21576674	21599219	
ENSDARG00000055459	284.6545524	2.312185654	0.187440668	7.000538736	2.55E-12	7.6E-11	dlgap3	discs, large (Drosophila) homolog-associated protein 3 [Source:ZFIN;Acc:ZDB-GENE-060503-413]	19	37458610	37492517	
ENSDARG00000055463	288.7618783	1.759305819	0.174509604	4.351083286	1.35E-05	0.000184201	lrit3a	info leucine-rich repeat, immunoglobulin-like and transmembrane domains 3a [Source:ZFIN;Acc:ZDB-GENE-080723-63]	14	36328425	36345175	
ENSDARG00000055502	3404.719771	1.401401808	0.108359029	3.704368822	0.000211918	0.002316789	cicb	capicua transcriptional repressor b [Source:ZFIN;Acc:ZDB-GENE-030131-1966]	16	11151699	11224444	
ENSDARG00000055514	3319.609313	-2.173446234	0.100892932	-11.63060889	2.88E-31	2.99E-29	icn2	ictacalcin 2 [Source:ZFIN;Acc:ZDB-GENE-070822-9]	16	23487051	23491093	
ENSDARG00000055527	1811.813831	4.192892777	0.137534307	23.2152461	3.19E-119	2.67E-116	cmn	calymmin [Source:ZFIN;Acc:ZDB-GENE-000208-9]	12	13118540	13164249	
ENSDARG00000055539	1408.782871	-2.064929099	0.111407718	-9.558844938	1.19E-21	7.07E-20	epdl2	ependymin-like 2 [Source:ZFIN;Acc:ZDB-GENE-091204-250]	21	28513057	28523548	
ENSDARG00000055559	531.5449318	1.879682232	0.136535267	6.44289386	1.17E-10	3.02E-09	chrna6	cholinergic receptor, nicotinic, alpha 6 [Source:ZFIN;Acc:ZDB-GENE-090312-91]	1	22481723	22512136	
ENSDARG00000055565	272.282748	1.781675758	0.184822136	4.229340562	2.34E-05	0.000303879	cacnb2b	calcium channel, voltage-dependent, beta 2b [Source:ZFIN;Acc:ZDB-GENE-050208-129]	2	31475772	31519662	
ENSDARG00000055589	3105.071291	-2.291540895	0.093319544	-13.83998289	1.46E-43	2.6E-41	s100t	S100 calcium binding protein T [Source:ZFIN;Acc:ZDB-GENE-050320-61]	16	22681818	22683386	
ENSDARG00000055591	682.5760108	-2.19609129	0.152987412	-7.818233354	5.36E-15	1.98E-13	pipox	pipecolic acid oxidase [Source:ZFIN;Acc:ZDB-GENE-110627-2]	15	24150090	24178893	
ENSDARG00000055642	752.3106951	-1.902319827	0.127071583	-7.10087815	1.24E-12	3.81E-11		si:ch211-217a12.1 [Source:ZFIN;Acc:ZDB-GENE-121214-95]	12	14079097	14130716	

ENSDARG00000055656	357.0407305	-2.979290494	0.202777351	-9.760905155	1.66E-22	1.03E-20	pdzd3b	PDZ domain containing 3b [Source:ZFIN;Acc:ZDB-GENE-081022-151]	5	30680804	30697908	
ENSDARG00000055672	336.91741	1.69077756	0.165823568	4.165738124	3.1E-05	0.000393433	map3k10	mitogen-activated protein kinase kinase kinase 10 [Source:ZFIN;Acc:ZDB-GENE-050419-112]	18	36948879	37007294	
ENSDARG00000055698	1416.675124	2.127312447	0.134783045	8.363904002	6.07E-17	2.59E-15	pcnx2	pecanex 2 [Source:ZFIN;Acc:ZDB-GENE-090312-150]	13	23669659	23735251	
ENSDARG00000055708	1111.308224	-1.635017726	0.120002244	-5.291715448	1.21E-07	2.17E-06	timm8b	translocase of inner mitochondrial membrane 8 homolog B (yeast) [Source:ZFIN;Acc:ZDB-GENE-031019-1]	5	30089715	30090506	
ENSDARG00000055715	860.4972645	-1.734018376	0.109646606	-6.694401259	2.17E-11	5.93E-10	capn8	calpain 8 [Source:ZFIN;Acc:ZDB-GENE-050522-279]	22	26400519	26435494	
ENSDARG00000055740	519.1083289	1.976315018	0.15571074	6.270055726	3.61E-10	8.73E-09	9-Mar	membrane-associated ring finger (C3HC4) 9 [Source:ZFIN;Acc:ZDB-GENE-081031-71]	23	36771593	36798620	
ENSDARG00000055751	52.65329082	2.185939521	0.395971632	2.995011326	0.002744347	0.024085959	fosb	FBJ murine osteosarcoma viral oncogene homolog B [Source:ZFIN;Acc:ZDB-GENE-041114-181]	18	36769758	36774965	
ENSDARG00000055752	1185.573136	2.23619453	0.383237994	3.225657553	0.001256836	0.011784778	npas4a	neuronal PAS domain protein 4a [Source:NCBI gene;Acc:724016]	14	30636955	30642819	
ENSDARG00000055825	1843.20652	2.785796675	0.14298444	12.48944764	8.52E-36	1.09E-33	celsr3	cadherin, EGF LAG seven-pass G-type receptor 3 [Source:ZFIN;Acc:ZDB-GENE-070122-3]	8	26125218	26212523	
ENSDARG00000055839	124.6952413	1.899790263	0.257026215	3.500772336	0.000463912	0.004764589	march4l	membrane-associated ring finger (C3HC4) 4, like [Source:NCBI gene;Acc:100000640]	1	29954690	30039332	
ENSDARG00000055855	1092.603341	1.567482749	0.133065431	4.264689516	2E-05	0.000262662	kcnc3a	potassium voltage-gated channel, Shaw-related subfamily, member 3a [Source:ZFIN;Acc:ZDB-GENE-100901-1]	3	31953145	32053929	
ENSDARG00000055868	3205.235288	-1.522052106	0.086395988	-6.042550342	1.52E-09	3.41E-08	rsl1d1	ribosomal L1 domain containing 1 [Source:ZFIN;Acc:ZDB-GENE-130603-67]	12	19384615	19393968	
ENSDARG00000055874	14469.76287	1.439675053	0.157976659	2.783164651	0.005383148	0.044085611	cpe	carboxypeptidase E [Source:ZFIN;Acc:ZDB-GENE-090313-68]	1	20839482	20911297	
ENSDARG00000055876	850.697098	-2.898372084	0.150597922	-12.60556627	1.97E-36	2.56E-34	msmo1	methylsterol monooxygenase 1 [Source:NCBI gene;Acc:406662]	1	20916551	20928772	
ENSDARG00000055900	4437.139141	1.740970346	0.107153195	6.915056023	4.68E-12	1.37E-10	zswim5	zinc finger, SWIM-type containing 5 [Source:ZFIN;Acc:ZDB-GENE-030925-30]	2	27491066	27543942	
ENSDARG00000055926	617.1199214	-1.397840488	0.12560204	-3.167468356	0.001537724	0.014145865	foxi3a	forkhead box I3a [Source:ZFIN;Acc:ZDB-GENE-031126-3]	21	30351256	30352951	
ENSDARG00000055996	58117.86754	-1.317700649	0.105546318	-3.010059051	0.002611969	0.022996505	rps8a	ribosomal protein S8a [Source:NCBI gene;Acc:407690]	2	26498446	26501868	
ENSDARG00000056001	3821.4866	1.618288378	0.094695898	6.529199169	6.61E-11	1.73E-09	fryb	furry homolog b (Drosophila) [Source:ZFIN;Acc:ZDB-GENE-080215-5]	10	33895315	33999781	
ENSDARG00000056008	11035.53052	-1.348585092	0.082284439	-4.236342837	2.27E-05	0.00029517	atp5mc1	ATP synthase membrane subunit c locus 1 [Source:ZFIN;Acc:ZDB-GENE-040426-1966]	3	23599290	23604396	
ENSDARG00000056018	111.0227538	-2.020844829	0.241760673	-4.222542972	2.42E-05	0.000311477	itga6l	integrin, alpha 6, like [Source:ZFIN;Acc:ZDB-GENE-040914-57]	14	41528635	41600040	
ENSDARG00000056019	1155.302218	1.719664345	0.153675204	4.683021902	2.83E-06	4.28E-05	ttbk1a	tau tubulin kinase 1a [Source:ZFIN;Acc:ZDB-GENE-100609-4]	11	30663300	30728241	
ENSDARG00000056036	3030.501256	2.178728737	0.126998351	9.281449143	1.67E-20	9.43E-19	stxbp1b	syntaxin binding protein 1b [Source:ZFIN;Acc:ZDB-GENE-060531-166]	5	28972935	29002322	
ENSDARG00000056037	543.433438	1.494215057	0.145838293	3.388787988	0.000702023	0.006937587	itih6	inter-alpha-trypsin inhibitor heavy chain family member 6 [Source:ZFIN;Acc:ZDB-GENE-050603-1]	23	26039524	26056072	
ENSDARG00000056045	418.7243043	-1.637088967	0.164365271	-3.876055828	0.000106163	0.001225198	si:ch73-139e5.4	si:ch73-139e5.4 [Source:ZFIN;Acc:ZDB-GENE-130531-31]	12	19065679	19091214	
ENSDARG00000056084	512.531784	1.945133947	0.209195552	4.517944762	6.24E-06	8.99E-05	igsf21b	immunoglobin superfamily, member 21b [Source:ZFIN;Acc:ZDB-GENE-070424-59]	23	21638258	21661916	
ENSDARG00000056101	420.3963874	2.4550314	0.158748199	9.165656106	4.92E-20	2.64E-18	kcnd3	potassium voltage-gated channel, Shal-related subfamily, member 3 [Source:ZFIN;Acc:ZDB-GENE-030131-5626]	8	28065803	28232817	
ENSDARG00000056144	78.46713384	-2.956153694	0.334954542	-5.840057237	5.22E-09	1.11E-07	zgc:123244	zgc:123244 [Source:NCBI gene;Acc:556933]	3	24220354	24226028	
ENSDARG00000056167	3825.625603	-1.343330773	0.084717108	-4.052673437	5.06E-05	0.0006197	hspe1	heat shock 10 protein 1 [Source:ZFIN;Acc:ZDB-GENE-000906-2]	9	32301017	32305007	
ENSDARG00000056175	963.5986388	1.70856919	0.172662519	4.103781144	4.06E-05	0.000504567	scrt2	scratch family zinc finger 2 [Source:ZFIN;Acc:ZDB-GENE-030131-4097]	8	28527776	28534245	
ENSDARG00000056178	62.63335974	-2.15460452	0.317581911	-3.635611723	0.000277322	0.002983735	dpt	dermatopontin [Source:ZFIN;Acc:ZDB-GENE-060312-9]	6	29184137	29195642	
ENSDARG00000056181	3204.52224	1.772267058	0.099319027	7.775620476	7.51E-15	2.75E-13	ncam1a	neural cell adhesion molecule 1a [Source:ZFIN;Acc:ZDB-GENE-990415-31]	21	23338059	23746947	
ENSDARG00000056196	1549.382711	-1.545528374	0.12230247	-4.460485316	8.18E-06	0.000115251	slc2a2	solute carrier family 2 member 2 [Source:ZFIN;Acc:ZDB-GENE-030131-1213]	2	25816843	25837986	
ENSDARG00000056203	608.45315	-2.167865334	0.175540351	-6.652973686	2.87E-11	7.74E-10	si:ch211-201h21.5	si:ch211-201h21.5 [Source:ZFIN;Acc:ZDB-GENE-030131-9599]	6	29305190	29312330	
ENSDARG00000056218	1424.443705	1.537243746	0.131207074	4.09462486	4.23E-05	0.000522858	tnika	TRAF2 and NCK interacting kinase a [Source:ZFIN;Acc:ZDB-GENE-030131-3767]	2	25657958	25817227	
ENSDARG00000056247	360.1815147	-1.637342786	0.190824811	-3.339936682	0.000837975	0.008121239	bricd5	BRICHOS domain containing 5 [Source:ZFIN;Acc:ZDB-GENE-050208-85]	12	18551597	18560500	
ENSDARG00000056248	52001.58892	-4.67943875	0.175178619	-21.00392597	6.04E-98	3.99E-95	si:dkey-183i3.5	si:dkey-183i3.5 [Source:ZFIN;Acc:ZDB-GENE-030131-8568]	21	25187210	25197103	
ENSDARG00000056292	2123.258936	2.779860145	0.143815788	12.37597189	3.53E-35	4.34E-33	vsx1	visual system homeobox 1 homolog, chx10-like [Source:NCBI gene;Acc:30598]	17	21063403	21066105	
ENSDARG00000056294	335.437586	1.74517277	0.192156838	3.877940417	0.000105345	0.001216867	SBK1	si:ch211-183d21.3 [Source:ZFIN;Acc:ZDB-GENE-091118-114]	24	37361089	37375262	SBK1
ENSDARG00000056376	831.8282545	3.709043645	0.171715741	15.77632675	4.53E-56	1.32E-53	ankrd37	ankyrin repeat domain 37 [Source:ZFIN;Acc:ZDB-GENE-070308-1]	1	17695426	17698773	
ENSDARG00000056378	1584.416104	-1.380895205	0.097704713	-3.898432278	9.68E-05	0.001128758	ifi30	IFI30 lysosomal thiol reductase [Source:NCBI gene;Acc:336503]	2	24762567	24767470	
ENSDARG00000056427	3478.977823	1.709688983	0.092409504	7.679826731	1.59E-14	5.66E-13	auts2a	activator of transcription and developmental regulator AUTS2 a [Source:ZFIN;Acc:ZDB-GENE-030616-571]	10	27182704	27741793	
ENSDARG00000056464	794.5076813	-1.448411469	0.127534487	-3.516001664	0.000438098	0.004523531	fitm1l	fat storage-inducing transmembrane protein 1 [Source:NCBI gene;Acc:503747]	2	24304854	24308682	
ENSDARG00000056483	3435.973288	1.310895008	0.085273898	3.645840213	0.00026652	0.002871205	ssbp4	single stranded DNA binding protein 4 [Source:ZFIN;Acc:ZDB-GENE-030131-469]	22	20951211	21046843	
ENSDARG00000056490	4322.50774	2.106798229	0.088162453	12.55407701	3.78E-36	4.86E-34	SSBP4	zgc:110158 [Source:ZFIN;Acc:ZDB-GENE-050522-195]	2	41577507	41723487	SSBP4
ENSDARG00000056498	1448.122236	-2.832757115	0.430190825	-4.260335203	2.04E-05	0.000267413	crp2	C-reactive protein 2 [Source:ZFIN;Acc:ZDB-GENE-030131-5826]	24	38109799	38113208	
ENSDARG00000056510	1508.27043	-1.426347576	0.099325645	-4.29242192	1.77E-05	0.000235094	gstk1	glutathione S-transferase kappa 1 [Source:ZFIN;Acc:ZDB-GENE-040718-298]	16	32009203	32013929	
ENSDARG00000056583	2329.142916	-1.308655971	0.112760073	-2.737280688	0.006194941	0.049887461	ndufs6	NADH:ubiquinone oxidoreductase subunit S6 [Source:ZFIN;Acc:ZDB-GENE-040912-86]	19	28281201	28283877	
ENSDARG00000056587	197.5573065	-2.008910989	0.188344232	-5.356739498	8.47E-08	1.55E-06	cyp2r1	cytochrome P450, family 2, subfamily R, polypeptide 1 [Source:ZFIN;Acc:ZDB-GENE-110114-1]	7	27455321	27697234	
ENSDARG00000056605	773.5838682	1.329930172	0.115744047	2.85051527	0.004364845	0.036520662	wbp2	WW domain binding protein 2 [Source:ZFIN;Acc:ZDB-GENE-050522-137]	12	45200744	45218478	
ENSDARG00000056639	1180.80826	1.712290071	0.114355653	6.228726391	4.7E-10	1.12E-08	carmil2	capping protein regulator and myosin 1 linker 2 [Source:ZFIN;Acc:ZDB-GENE-050419-197]	18	22302635	22396371	
ENSDARG00000056650	341.4146279	-1.802332848	0.146607493	-5.472659212	4.43E-08	8.42E-07	zgc:113276	zgc:113276 [Source:ZFIN;Acc:ZDB-GENE-050522-7]	11	29936743	29946927	
ENSDARG00000056652	50.32446614	-2.987142282	0.377417016	-5.265110469	1.4E-07	2.49E-06	zmp:0000001102	zmp:0000001102 [Source:ZFIN;Acc:ZDB-GENE-140106-62]	17	22014345	22021213	
ENSDARG00000056690	1362.298777	1.574533852	0.181313931	3.168724265	0.001531096	0.014103499	mtmr1a	myotubularin related protein 1a [Source:ZFIN;Acc:ZDB-GENE-050522-518]	7	25858380	25878560	
ENSDARG00000056719	471.8768494	-1.548819851	0.19867869	-2.762348859	0.005738712	0.046602207	slc6a19b	solute carrier family 6 member 19b [Source:ZFIN;Acc:ZDB-GENE-030131-4755]	16	18974064	18998549	
ENSDARG00000056742	1503.56963	1.629065853	0.138875382	4.529714642	5.91E-06	8.54E-05	crmp1	collapsin response mediator protein 1 [Source:ZFIN;Acc:ZDB-GENE-050720-1]	1	14348399	14364710	
ENSDARG00000056744	14947.95578	-6.725946162	0.141184449	-40.5564934	0	0	ela2	elastase 2 [Source:ZFIN;Acc:ZDB-GENE-041117-1]	8	21376290	21381734	
ENSDARG00000056750	43.08349324	2.276706093	0.431577826	2.958229123	0.00309412	0.026799696	si:ch211-254p10.2	si:ch211-254p10.2 [Source:ZFIN;Acc:ZDB-GENE-100922-17]	16	19013018	19023116	
ENSDARG00000056753	3216.748844	2.127379114	0.128959767	8.742099462	2.29E-18	1.09E-16	dctn1b	dynactin 1b [Source:ZFIN;Acc:ZDB-GENE-050419-28]	13	11581942	11667661	
ENSDARG00000056765	16779.60738	-5.883841307	0.121396946	-40.23034724	0	0	ela2l	elastase 2 like [Source:ZFIN;Acc:ZDB-GENE-040511-1]	8	21366771	21372485	
ENSDARG00000056791	380.3986699	1.583220006	0.16627613	3.507538979	0.000452272	0.004658384	pde6ga	phosphodiesterase 6G, cGMP-specific, rod, gamma, paralog a [Source:ZFIN;Acc:ZDB-GENE-050522-144]	3	19460991	19466015	
ENSDARG00000056795	934.0830415	1.529319072	0.131363421	4.029425134	5.59E-05	0.000679982	serpine1	serpin peptidase inhibitor, clade E (nexin, plasminogen activator inhibitor type 1), member 1 [Source:ZFIN;Acc:ZDB-GENE-(	7	26265584	26270014	
ENSDARG00000056797	934.5276956	1.611240482	0.201268254	3.036944328	0.002389896	0.021175001	ppp2r2ca	protein phosphatase 2, regulatory subunit B, gamma a [Source:ZFIN;Acc:ZDB-GENE-051120-168]	1	14283692	14306838	
ENSDARG00000056805	189.7147739	2.273349244	0.219823868	5.792588651	6.93E-09	1.45E-07	plch2b	phospholipase C, eta 2b [Source:ZFIN;Acc:ZDB-GENE-101112-1]	11	25752198	25777318	
ENSDARG00000056873	6442.260326	-1.278863516	0.09889501	-2.819793604	0.004805455	0.039861771	and3	actinodin3 [Source:ZFIN;Acc:ZDB-GENE-040724-185]	10	22848556	22854758	
ENSDARG00000056877	2032.659617	2.661573509	0.114736725	14.48161879	1.58E-47	3.31E-45	vamp2	vesicle-associated membrane protein 2 [Source:ZFIN;Acc:ZDB-GENE-030131-8225]	10	22838364	22845485	
ENSDARG00000056909	477.0878937	-1.977803085	0.216772995	-4.510723701	6.46E-06	9.27E-05	LO018309.1	epidermal growth factor receptor-like [Source:NCBI gene;Acc:571431]	24	33552198	33578392	
ENSDARG00000056929	6543.431987	1.331517543	0.092375904	3.588788083	0.000332219	0.003512647	kdm6bb	lysine (K)-specific demethylase 6B, b [Source:ZFIN;Acc:ZDB-GENE-040724-166]	10	22724059	22760659	
ENSDARG00000056998	888.0675664	1.521974294	0.143990962	3.625049014	0.000288907	0.003087206	kirrel1b	kirre like nephrin family adhesion molecule 1b [Source:ZFIN;Acc:ZDB-GENE-070912-173]	2	43961626	44038776	
ENSDARG00000057013	1025.961209	1.925001919	0.146281553	6.323435166	2.56E-10	6.3E-09	cadm3	cell adhesion molecule 3 [Source:ZFIN;Acc:ZDB-GENE-060512-196]	2	44049081	44183613	
ENSDARG00000057035	2321.225936	-2.306520927	0.12430963	-10.51021491	7.75E-26	5.76E-24	stoml3b	stomatin (EPB72)-like 3b [Source:ZFIN;Acc:ZDB-GENE-050417-366]	10	24641229	24648231	
ENSDARG00000057064	1039.605889	-1.416501376	0.14405226	-2.891321353	0.003836256	0.032575734	enpep	glutamyl aminopeptidase [Source:ZFIN;Acc:ZDB-GENE-050309-218]	13	12543634	12581388	
ENSDARG00000057113	2339.350836	2.433917037	0.109135464	13.13887328	1.97E-39	2.95E-37	c6	complement component 6 [Source:ZFIN;Acc:ZDB-GENE-040426-1358]	21	20914888	20932603	
ENSDARG00000057121	827.9346968	-1.445441803	0.118814084	-3.749065656	0.000177495	0.001974864	c7b	complement component 7b [Source:ZFIN;Acc:ZDB-GENE-021120-1]	21	20901505	20915291	
ENSDARG00000057141	411.1871518	2.212915899	0.16929735	7.164411624	7.81E-13	2.43E-11	zgc:110045	zgc:110045 [Source:ZFIN;Acc:ZDB-GENE-050706-149]	13	16230556	16257848	
ENSDARG00000057169	388.3109508	1.64345468	0.197857362	3.252113916	0.001145501	0.010821687	abca4a	ATP-binding cassette, sub-family A (ABC1), member 4a [Source:ZFIN;Acc:ZDB-GENE-050517-3]	24	28561038	28645334	
ENSDARG00000057209	209.6570503	1.932866288	0.199445206	4.677306147	2.91E-06	4.38E-05	plppr5a	phospholipid phosphatase related 5a [Source:ZFIN;Acc:ZDB-GENE-070705-281]	2	20262479	20323901	
ENSDARG00000057408	1358.40946	-2.108079108	0.125880135	-8.802652664	1.34E-18	6.47E-17	CABZ01067232.1	otolin-1-like [Source:NCBI gene;Acc:101886389]	15	45668851	45674213	
ENSDARG00000057427	2625.46994	1.778577927	0.131504818	5.920527792	3.21E-09	7E-08	sv2ba	synaptic vesicle glycoprotein 2Ba [Source:ZFIN;Acc:ZDB-GENE-070209-134]	7	15260093	15306015	
ENSDARG00000057435	322.1049863	-1.683341229	0.153641301	-4.447640213	8.68E-06	0.000121609	amacr	alpha-methylacyl-CoA racemase [Source:ZFIN;Acc:ZDB-GENE-050522-484]	21	19418563	19441617	
ENSDARG00000057468	279.5101568	1.797457785	0.180241048	4.424396069	9.67E-06	0.000134269	kcnc2	potassium voltage-gated channel, Shaw-related subfamily, member 2 [Source:ZFIN;Acc:ZDB-GENE-070410-118]	4	2060524	2196928	
ENSDARG00000057481	1553.552507	1.56190185	0.188982849	2.97329548	0.002946207	0.025686953	RNF157	si:dkey-49c17.3 [Source:ZFIN;Acc:ZDB-GENE-130531-49]	12	31729075	31765822	RNF157
ENSDARG00000057498	918.4630725	-2.381974821	0.113247708	-12.2031152	2.99E-34	3.48E-32	habp2	hyaluronan binding protein 2 [Source:ZFIN;Acc:ZDB-GENE-030131-4721]	12	31713239	31720066	
ENSDARG00000057504	178.3936375	3.993547817	0.313071321	9.561871735	1.16E-21	6.88E-20	si:dkey-202g17.3	si:dkey-202g17.3 [Source:ZFIN;Acc:ZDB-GENE-131127-371]	15	9072821	9089507	
ENSDARG00000057519	579.6870792	1.853680343	0.168277066	5.07306409	3.91E-07	6.62E-06	pcdh1g9	protocadherin 1 gamma 9 [Source:ZFIN;Acc:ZDB-GENE-050609-5]	10	21701551	21820133	
ENSDARG00000057556	43273.31491	-1.37579857	0.100636656	-3.734211602	0.000188304	0.002081295	rpl17	ribosomal protein L17 [Source:ZFIN;Acc:ZDB-GENE-030131-8585]	21	18997503	19067517	
ENSDARG00000057571	8202.469956	-1.804246153	0.130061475	-6.183584757	6.27E-10	1.48E-08	pgam2	phosphoglycerate mutase 2 (muscle) [Source:ZFIN;Acc:ZDB-GENE-040116-6]	21	19001669	19006641	
ENSDARG00000057586	831.8229096	2.148770073	0.131366042	8.744802335	2.23E-18	1.07E-16	sall3b	spalt-like transcription factor 3b [Source:ZFIN;Acc:ZDB-GENE-030131-9140]	19	22062202	22074081	
ENSDARG00000057630	992.2094475	-1.696625676	0.114440908	-6.087208554	1.15E-09	2.62E-08	galm	galactose mutarotase [Source:ZFIN;Acc:ZDB-GENE-040718-66]	13	8306399	8318216	
ENSDARG00000057714	1109.341305	-1.730405264	0.132972388	-5.492909296	3.95E-08	7.58E-07	cmah	cytidine monophospho-N-acetylneuraminic acid hydroxylase [Source:NCBI gene;Acc:431739]	4	2228754	2250264	
ENSDARG00000057729	296.3470838	2.061851719	0.173851196	6.10781948	1.01E-09	2.33E-08	ephb6	eph receptor B6 [Source:ZFIN;Acc:ZDB-GENE-100922-51]	16	12335274	12470034	
ENSDARG00000057738	1223.151568	-1.51237853	0.1183268	-4.330198497	1.49E-05	0.000201369	hells	helicase, lymphoid specific [Source:ZFIN;Acc:ZDB-GENE-030131-9923]	13	8255106	8270718	
ENSDARG00000057741	279.5750001	-1.590437791	0.172563925	-3.421559822	0.00062263	0.006211702	nr1h4	nuclear receptor subfamily 1, group H, member 4 [Source:ZFIN;Acc:ZDB-GENE-040718-313]	18	15644559	15666942	
ENSDARG00000057782	5634.317134	1.658794222	0.094143117	6.997794912	2.6E-12	7.73E-11	baz2ba	bromodomain adjacent to zinc finger domain, 2Ba [Source:ZFIN;Acc:ZDB-GENE-070615-37]	6	11829867	12002935	
ENSDARG00000057790	209.1661474	2.416879642	0.237954821	5.954406112	2.61E-09	5.76E-08	ankrd6a	ankyrin repeat domain 6a [Source:ZFIN;Acc:ZDB-GENE-120525-1]	17	15756828	15777350	
ENSDARG00000057913	3808.1842	1.751682584	0.157985898	4.757909352	1.96E-06	3.04E-05	syt11a	synaptotagmin XIa [Source:ZFIN;Acc:ZDB-GENE-040426-2815]	19	23982466	24034710	
ENSDARG00000058005	2890.378777	-1.614991948	0.111964001	-5.492765041	3.96E-08	7.58E-07	hgd	homogentisate 1,2-dioxygenase [Source:ZFIN;Acc:ZDB-GENE-020802-5]	24	23959107	23975132	
ENSDARG00000058008	907.4354606	1.667190228	0.115977866	5.752737565	8.78E-09	1.82E-07	six3a	SIX homeobox 3a [Source:ZFIN;Acc:ZDB-GENE-990415-127]	13	10258007	10261383	
ENSDARG00000058015	527.8309161	2.246592775	0.152648628	8.166419788	3.18E-16	1.28E-14	ano3	anoctamin 3 [Source:ZFIN;Acc:ZDB-GENE-111031-3]	25	35375848	35468555	
ENSDARG00000058041	4872.520474	-1.435380978	0.090004352	-4.837332492	1.32E-06	2.09E-05	ndufa8	NADH:ubiquinone oxidoreductase subunit A8 [Source:ZFIN;Acc:ZDB-GENE-040426-1688]	10	9410304	9419994	
ENSDARG00000058079	953.6688619	-1.345089468	0.113719051	-3.034579206	0.002408715	0.021319166	txndc17	thioredoxin domain containing 17 [Source:NCBI gene;Acc:445062]	15	16328450	16333015	
ENSDARG00000058103	119.533143	1.813701774	0.253106478	3.214859535	0.001305084	0.012205255	glra4b	glycine receptor, alpha 4b [Source:ZFIN;Acc:ZDB-GENE-020402-2]	5	22515347	22544151	
ENSDARG00000058105	33094.94808	-1.241397346	0.086689954	-2.784605779	0.005359284	0.043918831	rpl36a	ribosomal protein L36A [Source:NCBI gene;Acc:195819]	5	22510639	22515186	
ENSDARG00000058117	4773.227279	1.378201313	0.091935176	4.113782436	3.89E-05	0.000485105	snap25b	synaptosome associated protein 25b [Source:NCBI gene;Acc:30711]	17	12297984	12389259	
ENSDARG00000058147	79.29695117	-2.66950887	0.312637873	-5.340072376	9.29E-08	1.69E-06	dnajc5b	DnaJ (Hsp40) homolog, subfamily C, member 5 beta [Source:ZFIN;Acc:ZDB-GENE-050413-1]	24	24808955	24824899	
ENSDARG00000058154	456.3701201	2.649361152	0.160643013	10.26724486	9.9E-25	7.1E-23	znf385c	zinc finger protein 385C [Source:ZFIN;Acc:ZDB-GENE-091112-1]	3	17744339	17860047	
ENSDARG00000058218	250.411876	-2.173614416	0.24700579	-4.751363983	2.02E-06	3.14E-05	fam151a	family with sequence similarity 151 member A [Source:NCBI gene;Acc:100005645]	2	11069055	11082914	
ENSDARG00000058222	334.6311709	2.463691364	0.193164327	7.577441357	3.52E-14	1.22E-12	mpp4a	membrane protein, palmitoylated 4a (MAGUK p55 subfamily member 4) [Source:ZFIN;Acc:ZDB-GENE-070912-523]	9	13682133	13711991	
ENSDARG00000058232	1911.275063	1.548916165	0.113762854	4.825091348	1.4E-06	2.21E-05	fbrsl1	fibrosin-like 1 [Source:ZFIN;Acc:ZDB-GENE-070705-487]	5	17624463	17753961	
ENSDARG00000058248	881.7072293	1.425207907	0.137866831	3.084192943	0.002041052	0.018316926	si:dkeyp-77h1.4	si:dkeyp-77h1.4 [Source:ZFIN;Acc:ZDB-GENE-050208-18]	16	10346277	10369298	
ENSDARG00000058259	1014.757285	1.3463821	0.110431656	3.136619633	0.001709077	0.015571521	celsr1b	cadherin EGF LAG seven-pass G-type receptor 1b [Source:ZFIN;Acc:ZDB-GENE-050823-4]	25	19753890	19869495	
ENSDARG00000058327	230.0743613	-3.467085198	0.201508436	-12.24308642	1.83E-34	2.16E-32	neu3.3	sialidase 3 (membrane sialidase), tandem duplicate 3 [Source:ZFIN;Acc:ZDB-GENE-061103-511]	21	21803363	21809107	
ENSDARG00000058332	515.1171809	-1.981493915	0.165447613	-5.932354638	2.99E-09	6.55E-08	krt18a.2	keratin 18a, tandem duplicate 2 [Source:ZFIN;Acc:ZDB-GENE-090312-205]	23	10352921	10355552	
ENSDARG00000058369	2871.800518	1.326746856	0.097212339	3.36116649	0.00077614	0.007589368	mex3b	mex-3 RNA binding family member B [Source:ZFIN;Acc:ZDB-GENE-030131-2628]	7	12061787	12065668	
ENSDARG00000058371	155261.5899	-2.492798746	0.112632831	-13.25367332	4.3E-40	6.66E-38	krt5	keratin 5 [Source:ZFIN;Acc:ZDB-GENE-991110-23]	23	10173196	10177458	
ENSDARG00000058445	600.244576	-2.840562355	0.172613187	-10.66293014	1.52E-26	1.2E-24	zgc:136472	zgc:136472 [Source:ZFIN;Acc:ZDB-GENE-060421-2552]	1	9234928	9249943	

ENSDARG00000058460	25.23439033	3.142133999	0.67362368	3.180015881	0.00147267	0.01361528	cacng3a	calcium channel, voltage-dependent, gamma subunit 3a [Source:ZFIN;Acc:ZDB-GENE-090312-124]	1	9082417	9091935	
ENSDARG00000058462	10563.60263	-2.738661702	0.117068484	-14.85166328	6.79E-50	1.59E-47	zgc:158846	zgc:158846 [Source:ZFIN;Acc:ZDB-GENE-070209-50]	6	39336731	39344259	
ENSDARG00000058463	3032.230426	-1.430499706	0.102315918	-4.207553576	2.58E-05	0.00033111	ndufab1a	NADH:ubiquinone oxidoreductase subunit AB1a [Source:ZFIN;Acc:ZDB-GENE-040801-1]	1	9118100	9123465	
ENSDARG00000058476	686.5629042	2.349750802	0.178387613	7.566393109	3.84E-14	1.32E-12	stc1l	stanniocalcin 1, like [Source:ZFIN;Acc:ZDB-GENE-040426-1521]	10	42677891	42685512	
ENSDARG00000058498	320.867383	3.332976888	0.283838142	8.219391763	2.05E-16	8.35E-15	slc9a3.1	solute carrier family 9 member A3, tandem duplicate 1 [Source:ZFIN;Acc:ZDB-GENE-060503-545]	19	10692166	10730488	
ENSDARG00000058557	18.81400222	5.823979238	1.501983087	3.211740052	0.001319337	0.012324777	il11b	interleukin 11b [Source:ZFIN;Acc:ZDB-GENE-051019-2]	19	10429810	10432134	
ENSDARG00000058558	359.820132	2.336572939	0.202520383	6.599695884	4.12E-11	1.1E-09	lzts3b	leucine zipper, putative tumor suppressor family member 3b [Source:ZFIN;Acc:ZDB-GENE-110411-249]	8	1082100	1110352	
ENSDARG00000058570	44.62633538	-2.815533184	0.401804854	-4.518445125	6.23E-06	8.97E-05	ccl27a	chemokine (C-C motif) ligand 27a [Source:ZFIN;Acc:ZDB-GENE-000208-28]	8	1170015	1183948	
ENSDARG00000058606	3046.661437	1.771526733	0.126720001	6.088436902	1.14E-09	2.6E-08	sik1	salt-inducible kinase 1 [Source:ZFIN;Acc:ZDB-GENE-030131-9446]	9	9112159	9122562	
ENSDARG00000058638	891.7106086	-1.615404304	0.155928133	-3.946717572	7.92E-05	0.000939863	si:ch211-71m22.1	si:ch211-71m22.1 [Source:ZFIN;Acc:ZDB-GENE-070705-193]	2	8586415	8611675	
ENSDARG00000058646	2349.00266	2.031595557	0.11455885	9.004939887	2.16E-19	1.11E-17	ptprna	protein tyrosine phosphatase receptor type Na [Source:ZFIN;Acc:ZDB-GENE-030131-3511]	9	7548533	7619622	
ENSDARG00000058649	670.376272	1.809068379	0.195209188	4.144622429	3.4E-05	0.000428472	trim46b	tripartite motif containing 46b [Source:ZFIN;Acc:ZDB-GENE-060503-466]	19	8582685	8604429	
ENSDARG00000058678	73.04562549	-3.646663866	0.363353851	-7.283984623	3.24E-13	1.03E-11	aqp10b	aquaporin 10b [Source:ZFIN;Acc:ZDB-GENE-060503-57]	19	7810028	7816886	
ENSDARG00000058701	267.555687	1.670317426	0.238428619	2.811396669	0.004932693	0.040822793	nos1apb	nitric oxide synthase 1 (neuronal) adaptor protein b [Source:ZFIN;Acc:ZDB-GENE-100824-1]	2	6926100	6952754	
ENSDARG00000058702	54.23152132	2.122900585	0.374226534	3.000590508	0.002694567	0.023682184	vax2	ventral anterior homeobox 2 [Source:NCBI gene;Acc:373869]	7	8844285	8881514	
ENSDARG00000058736	109.4581009	1.995170103	0.263380233	3.778454033	0.000157805	0.001771495	gabra6b	gamma-aminobutyric acid (GABA) A receptor, alpha 6b [Source:ZFIN;Acc:ZDB-GENE-080815-1]	21	42156949	42202792	
ENSDARG00000058771	2146.408466	1.864167002	0.0937982	9.213044667	3.17E-20	1.73E-18	nav1b	neuron navigator 1b [Source:ZFIN;Acc:ZDB-GENE-120215-84]	6	54888493	55022544	
ENSDARG00000058782	78.1608368	2.225200755	0.327393777	3.742284805	0.000182355	0.002020876	si:ch73-242m19.1	si:ch73-242m19.1 [Source:ZFIN;Acc:ZDB-GENE-131121-474]	17	6828550	6932061	
ENSDARG00000058799	1085.072571	-1.698140323	0.134124724	-5.205157586	1.94E-07	3.39E-06	mybpha	myosin binding protein Ha [Source:ZFIN;Acc:ZDB-GENE-040426-1531]	23	6077503	6126003	
ENSDARG00000058803	790.1629876	1.707412247	0.168017487	4.210348933	2.55E-05	0.000327542	grk1a	G protein-coupled receptor kinase 1 a [Source:ZFIN;Acc:ZDB-GENE-050823-1]	1	245076	258138	
ENSDARG00000058815	57.27486024	4.414896508	0.602156402	5.671112187	1.42E-08	2.87E-07	ihhb	Indian hedgehog signaling molecule b [Source:NCBI gene;Acc:30299]	6	7059999	7082108	
ENSDARG00000058824	1712.223045	-1.315695744	0.109767412	-2.876042512	0.004026955	0.034022414	mrpl4	mitochondrial ribosomal protein L4 [Source:NCBI gene;Acc:553771]	11	11319141	11331146	
ENSDARG00000058868	8253.397938	1.377700166	0.082788801	4.562213278	5.06E-06	7.41E-05	apc	APC regulator of WNT signaling pathway [Source:ZFIN;Acc:ZDB-GENE-031112-7]	10	1849874	1893095	
ENSDARG00000058876	377.2279101	1.922718491	0.170860835	5.400409585	6.65E-08	1.23E-06	mmp16b	matrix metallopeptidase 16b (membrane-inserted) [Source:ZFIN;Acc:ZDB-GENE-061009-22]	2	3630307	3678029	
ENSDARG00000058892	331.0997862	1.691595441	0.162897917	4.24557573	2.18E-05	0.000283861	cep170ab	centrosomal protein 170Ab [Source:ZFIN;Acc:ZDB-GENE-050309-106]	6	6445065	6487876	
ENSDARG00000058969	1428.525887	1.55825826	0.135271278	4.126953399	3.68E-05	0.000460666	cntnap2a	contactin associated protein like 2a [Source:ZFIN;Acc:ZDB-GENE-120328-3]	24	17417150	18179535	
ENSDARG00000058990	46.12823788	-2.024005836	0.357345626	-2.865589394	0.004162337	0.034977847	stx1a	syntaxin 1A (brain) [Source:ZFIN;Acc:ZDB-GENE-100802-1]	15	1734444	1746963	
ENSDARG00000059039	86.67233537	3.995762442	0.441604144	6.783818677	1.17E-11	3.31E-10	FO904903.1	major histocompatibility complex class I ULA [Source:NCBI gene;Acc:796419]	22	3914318	3929075	
ENSDARG00000059053	1634.900113	-1.941681777	0.101905775	-9.240710614	2.45E-20	1.36E-18	slc13a4	solute carrier family 13 member 4 [Source:ZFIN;Acc:ZDB-GENE-070424-71]	4	4803698	4832149	
ENSDARG00000059075	587.1825691	-1.502443844	0.135557316	-3.706504817	0.000210139	0.002299348	nip7	NIP7 nucleolar pre-rRNA processing protein [Source:NCBI gene;Acc:553616]	18	5843583	5850683	
ENSDARG00000059128	7388.868702	-1.398308846	0.098036206	-4.062874974	4.85E-05	0.00059438	uqcrh	ubiquinol-cytochrome c reductase hinge protein [Source:ZFIN;Acc:ZDB-GENE-051030-93]	6	3509036	3514753	
ENSDARG00000059227	15138.07072	-3.522841247	0.132998212	-18.96898615	3.08E-80	1.49E-77	fabp1b.1	fatty acid binding protein 1b, tandem duplicate 1 [Source:NCBI gene;Acc:554095]	8	999421	1006174	
ENSDARG00000059255	375.0014558	2.345704161	0.169458106	7.941220371	2E-15	7.64E-14	evx2	even-skipped homeobox 2 [Source:ZFIN;Acc:ZDB-GENE-980526-215]	9	2002701	2007106	
ENSDARG00000059280	725.1131267	1.676628121	0.134785754	5.020026979	5.17E-07	8.63E-06	hoxd3a	homeobox D3a [Source:NCBI gene;Acc:30349]	9	1937049	1959917	
ENSDARG00000059311	4516.139544	2.387034148	0.108922696	12.73411506	3.82E-37	5.19E-35	dpysl5b	dihydropyrimidinase like 5b [Source:ZFIN;Acc:ZDB-GENE-050720-4]	4	798696	815871	
ENSDARG00000059361	76.46930402	-2.43643468	0.322617506	-4.452438729	8.49E-06	0.000119122	slc39a4	solute carrier family 39 member 4 [Source:ZFIN;Acc:ZDB-GENE-060315-8]	19	1119416	1127722	
ENSDARG00000059363	380.3765398	2.007050545	0.163636794	6.154181583	7.55E-10	1.76E-08	tgfbr2a	transforming growth factor beta receptor 2a [Source:ZFIN;Acc:ZDB-GENE-120404-1]	19	917852	932264	
ENSDARG00000059391	2762.323132	-2.493879621	0.117501714	-12.71368369	4.96E-37	6.7E-35	gyg1b	glycogenin 1b [Source:ZFIN;Acc:ZDB-GENE-040625-30]	24	3307857	3326369	
ENSDARG00000059399	103.0703047	3.163066129	0.345204696	6.266039119	3.7E-10	8.94E-09	si:ch211-160o17.6	si:ch211-160o17.6 [Source:ZFIN;Acc:ZDB-GENE-141215-39]	11	2691243	2700397	
ENSDARG00000059466	186.4384567	2.58315626	0.248585104	6.368669056	1.91E-10	4.78E-09	wasf3a	WASP family member 3a [Source:ZFIN;Acc:ZDB-GENE-060531-55]	5	68007172	68022631	
ENSDARG00000059646	6217.039827	1.382951216	0.108686947	3.523433358	0.000425994	0.004405802	nt5dc2	5'-nucleotidase domain containing 2 [Source:ZFIN;Acc:ZDB-GENE-060810-17]	8	23639124	23656730	
ENSDARG00000059705	29.64944691	2.529990262	0.548688774	2.788448269	0.00529612	0.043429572	vwa5b1	von Willebrand factor A domain containing 5B1 [Source:ZFIN;Acc:ZDB-GENE-090313-299]	23	37185247	37198890	
ENSDARG00000059725	3498.876854	-1.29481548	0.089133602	-3.307568338	0.000941097	0.009054231	hypk	huntingtin interacting protein K [Source:ZFIN;Acc:ZDB-GENE-080515-6]	7	50407575	50410524	
ENSDARG00000059729	873.8632887	-1.594085328	0.163617127	-3.63094829	0.000282382	0.003031679	sec14l8	SEC14-like lipid binding 8 [Source:ZFIN;Acc:ZDB-GENE-060526-180]	5	13427826	13469307	
ENSDARG00000059738	2382.232054	1.844064707	0.099601558	8.474412705	2.36E-17	1.05E-15	ptprsa	protein tyrosine phosphatase receptor type Sa [Source:ZFIN;Acc:ZDB-GENE-020107-3]	22	3389347	3595439	
ENSDARG00000059746	2684.632985	1.596277252	0.122274464	4.876547668	1.08E-06	1.73E-05	plod1a	procollagen-lysine, 2-oxoglutarate 5-dioxygenase 1a [Source:ZFIN;Acc:ZDB-GENE-030131-3165]	8	47850073	47895817	
ENSDARG00000059751	287.6587654	2.575801187	0.197683277	7.971342899	1.57E-15	6.04E-14	abtb2a	ankyrin repeat and BTB (POZ) domain containing 2a [Source:ZFIN;Acc:ZDB-GENE-100422-13]	7	49272702	49320952	
ENSDARG00000059773	1465.331724	2.073938918	0.133374272	9.472509957	2.73E-21	1.59E-19	SRCIN1	si:ch211-278a6.1 [Source:ZFIN;Acc:ZDB-GENE-060503-699]	3	37235924	37476475	SRCIN1
ENSDARG00000059774	536.2540517	-1.379926958	0.126041014	-3.01431213	0.002575627	0.022716376	dub	duboraya [Source:ZFIN;Acc:ZDB-GENE-060825-81]	21	22917221	22951646	
ENSDARG00000059775	3862.423021	2.190907137	0.087609444	13.59336479	4.38E-42	7.44E-40	slc32a1	solute carrier family 32 member 1 [Source:ZFIN;Acc:ZDB-GENE-061201-1]	6	60142619	60147517	
ENSDARG00000059781	727.0940289	1.958436614	0.196201881	4.884951204	1.03E-06	1.66E-05	atcayb	ATCAY kinesin light chain interacting caytaxin b [Source:ZFIN;Acc:ZDB-GENE-070912-220]	2	55914699	55970970	
ENSDARG00000059789	781.1467641	1.497966622	0.153855626	3.236583779	0.001209697	0.011368254	parp8	poly (ADP-ribose) polymerase family, member 8 [Source:ZFIN;Acc:ZDB-GENE-060929-432]	5	40844968	40910880	
ENSDARG00000059805	360.881218	2.593634908	0.223202017	7.139876829	9.34E-13	2.89E-11	slc25a38a	solute carrier family 25 member 38a [Source:NCBI gene;Acc:767662]	3	4644031	4663602	
ENSDARG00000059832	1596.277597	1.692307634	0.18143978	3.815633119	0.000135834	0.001546292	adgrb3	adhesion G protein-coupled receptor B3 [Source:ZFIN;Acc:ZDB-GENE-070424-99]	13	38302665	38593701	
ENSDARG00000059885	200.753193	1.58782516	0.211711265	2.776541719	0.005494058	0.044914291	frmd3	FERM domain containing 3 [Source:ZFIN;Acc:ZDB-GENE-070523-1]	8	49617946	49705058	
ENSDARG00000059891	114.6193014	2.528760474	0.312038005	4.899276531	9.62E-07	1.55E-05	AL929217.1	gamma-aminobutyric acid receptor subunit pi-like [Source:NCBI gene;Acc:562831]	13	51140464	51247529	
ENSDARG00000059903	214.6965624	1.943215068	0.206331638	4.571354519	4.85E-06	7.1E-05	hs3st3l	heparan sulfate (glucosamine) 3-O-sulfotransferase 3-like [Source:ZFIN;Acc:ZDB-GENE-070202-6]	12	415711	431249	
ENSDARG00000059908	1812.410297	-1.721899328	0.099319356	-7.268465638	3.64E-13	1.15E-11	si:ch211-217k17.7	si:ch211-217k17.7 [Source:ZFIN;Acc:ZDB-GENE-061009-11]	1	52452010	52461322	
ENSDARG00000059914	456.2230081	-1.471921268	0.157590865	-2.994597876	0.002748069	0.024110197	sdr42e2	short chain dehydrogenase/reductase family 42E, member 2 [Source:ZFIN;Acc:ZDB-GENE-101203-3]	3	58379450	58416968	
ENSDARG00000059939	596.0927407	1.618287543	0.13635401	4.534428737	5.78E-06	8.39E-05	dab1a	DAB adaptor protein 1a [Source:ZFIN;Acc:ZDB-GENE-060528-1]	20	8122693	8443425	
ENSDARG00000059945	10908.0866	2.519494871	0.082262363	18.4713254	3.51E-76	1.47E-73	sv2a	synaptic vesicle glycoprotein 2A [Source:ZFIN;Acc:ZDB-GENE-131001-1]	16	46111849	46202705	
ENSDARG00000059956	401.0652644	-2.314419082	0.205554893	-6.394491825	1.61E-10	4.08E-09	ptk6b	PTK6 protein tyrosine kinase 6b [Source:ZFIN;Acc:ZDB-GENE-060929-1150]	23	17387325	17404011	
ENSDARG00000059960	1655.660564	1.610394067	0.112514358	5.425032674	5.79E-08	1.08E-06	plch2a	phospholipase C, eta 2a [Source:ZFIN;Acc:ZDB-GENE-030131-6367]	8	47342586	47622532	
ENSDARG00000059978	2020.678646	2.081976101	0.144790434	7.472704331	7.86E-14	2.64E-12	cplx4a	complexin 4a [Source:ZFIN;Acc:ZDB-GENE-060526-116]	21	10739846	10749542	
ENSDARG00000060018	853.5214526	1.623796531	0.151274424	4.123608687	3.73E-05	0.000466944	si:ch1073-44g3.1	si:ch1073-44g3.1 [Source:ZFIN;Acc:ZDB-GENE-141216-124]	14	49135264	49137035	
ENSDARG00000060025	177.7527574	2.420295823	0.270070529	5.258981163	1.45E-07	2.57E-06	nsmfa	NMDA receptor synaptonuclear signaling and neuronal migration factor a [Source:ZFIN;Acc:ZDB-GENE-091204-32]	21	13455308	13535601	
ENSDARG00000060041	2097.093051	-1.457702315	0.0957716	-4.779102744	1.76E-06	2.76E-05	lig1	ligase I, DNA, ATP-dependent [Source:ZFIN;Acc:ZDB-GENE-110404-2]	2	44301276	44344321	
ENSDARG00000060072	241.0516399	1.90746299	0.193996327	4.677732845	2.9E-06	4.38E-05	abi3a	ABI family, member 3a [Source:ZFIN;Acc:ZDB-GENE-060929-958]	3	33395942	33417826	
ENSDARG00000060085	459.923076	2.190489266	0.193301168	6.158727745	7.33E-10	1.71E-08	kcnq3	potassium voltage-gated channel, KQT-like subfamily, member 3 [Source:ZFIN;Acc:ZDB-GENE-070912-301]	2	43001512	43083755	
ENSDARG00000060095	148.2222429	1.91296572	0.24961704	3.657465531	0.000254722	0.002753559	kcnb1	potassium voltage-gated channel, Shab-related subfamily, member 1 [Source:ZFIN;Acc:ZDB-GENE-090831-3]	6	46507609	46565466	
ENSDARG00000060106	550.4989178	1.512800323	0.135620598	3.781138941	0.000156113	0.001754065	crb2a	crumbs cell polarity complex component 2a [Source:ZFIN;Acc:ZDB-GENE-060612-1]	21	8478774	8513192	
ENSDARG00000060113	4821.345435	1.603791855	0.11367805	5.311419864	1.09E-07	1.96E-06	znf395a	zinc finger protein 395a [Source:ZFIN;Acc:ZDB-GENE-061215-52]	17	16090160	16108384	
ENSDARG00000060120	952.5873635	-1.460095701	0.123638437	-3.721299892	0.0001982	0.002180112	glyctk	glycerate kinase [Source:NCBI gene;Acc:570678]	6	43019642	43047774	
ENSDARG00000060199	1311.040923	-1.59837019	0.106074553	-5.641034265	1.69E-08	3.39E-07	mrpl18	mitochondrial ribosomal protein L18 [Source:ZFIN;Acc:ZDB-GENE-061013-632]	14	35499678	35505537	
ENSDARG00000060246	1623.735953	1.98076565	0.138445601	7.084122886	1.4E-12	4.27E-11	slc16a6b	solute carrier family 16 member 6b [Source:ZFIN;Acc:ZDB-GENE-110208-3]	3	36313532	36328672	
ENSDARG00000060297	2132.19026	1.423211092	0.113907914	3.715379184	0.000202899	0.002227897	arhgap35b	Rho GTPase activating protein 35b [Source:ZFIN;Acc:ZDB-GENE-060526-44]	5	37864705	37881345	
ENSDARG00000060303	639.4708184	2.138720473	0.177432004	6.417785105	1.38E-10	3.53E-09	slc4a10b	solute carrier family 4 member 10b [Source:ZFIN;Acc:ZDB-GENE-081105-62]	9	51289904	51370293	
ENSDARG00000060309	1291.622548	1.434523879	0.105207229	4.130171302	3.62E-05	0.000454947	srgap3	SLIT-ROBO Rho GTPase activating protein 3 [Source:ZFIN;Acc:ZDB-GENE-060524-4]	6	41554794	41757181	
ENSDARG00000060315	674.1423273	-2.242547427	0.146052202	-8.507556967	1.78E-17	7.99E-16	rabgap1l2	RAB GTPase activating protein 1-like 2 [Source:ZFIN;Acc:ZDB-GENE-070912-414]	2	36898982	36905195	
ENSDARG00000060316	423.0719571	-1.715915065	0.142792449	-5.013675951	5.34E-07	8.91E-06	cish	cytokine inducible SH2-containing protein [Source:ZFIN;Acc:ZDB-GENE-050907-1]	6	41503854	41506749	
ENSDARG00000060330	355.1271031	1.85792633	0.219831169	3.902660096	9.51E-05	0.001110763	si:ch211-284e13.4	si:ch211-284e13.4 [Source:ZFIN;Acc:ZDB-GENE-060526-161]	5	38088594	38094130	
ENSDARG00000060345	1224.073772	-1.522965762	0.112047163	-4.667371747	3.05E-06	4.59E-05	apoda.1	apolipoprotein Da, duplicate 1 [Source:ZFIN;Acc:ZDB-GENE-060929-148]	2	37207799	37210397	
ENSDARG00000060350	4756.784501	-4.554347694	0.139270413	-25.52119737	1.15E-143	1.2E-140	apoda.2	apolipoprotein Da, duplicate 2 [Source:ZFIN;Acc:ZDB-GENE-070912-551]	7	37207461	37215989	
ENSDARG00000060368	3075.980365	2.057767469	0.114492962	9.238711688	2.49E-20	1.38E-18	syn1	synapsin I [Source:ZFIN;Acc:ZDB-GENE-081105-93]	8	31119548	31148319	
ENSDARG00000060372	1982.630824	2.292750275	0.115434147	11.19902827	4.12E-29	3.89E-27	plxna2	plexin A2 [Source:ZFIN;Acc:ZDB-GENE-090311-6]	23	32579495	33038423	
ENSDARG00000060393	272.2317728	1.838463622	0.184609792	4.541815541	5.58E-06	8.12E-05	fibcd1	fibrinogen C domain containing 1 [Source:ZFIN;Acc:ZDB-GENE-070424-245]	5	34066591	34185497	
ENSDARG00000060439	822.0433995	-1.629619793	0.14013336	-4.493004326	7.02E-06	0.000100212	clcn2c	chloride channel 2c [Source:ZFIN;Acc:ZDB-GENE-050506-73]	15	46736794	46779934	
ENSDARG00000060452	1133.54804	1.819919555	0.142466006	5.755194359	8.65E-09	1.8E-07	sdk2b	sidekick cell adhesion molecule 2b [Source:ZFIN;Acc:ZDB-GENE-030131-8203]	12	37463513	37734973	
ENSDARG00000060482	171.2419503	2.160041602	0.222030007	5.224706416	1.74E-07	3.06E-06	mid1	midline 1 [Source:ZFIN;Acc:ZDB-GENE-110411-205]	9	53909566	54001502	
ENSDARG00000060496	776.5955843	1.374244514	0.131871789	2.837942196	0.00454054	0.03779292	cacna1ha	calcium channel, voltage-dependent, T type, alpha 1H subunit a [Source:ZFIN;Acc:ZDB-GENE-130103-1]	3	39759130	39952441	
ENSDARG00000060500	767.1837106	-1.586140501	0.123096706	-4.761626205	1.92E-06	2.99E-05	l2hgdh	L-2-hydroxyglutarate dehydrogenase [Source:ZFIN;Acc:ZDB-GENE-090319-6]	13	36666662	36680531	
ENSDARG00000060532	1828.195057	1.589223379	0.186622201	3.157305917	0.001592342	0.014587363	adam22	ADAM metallopeptidase domain 22 [Source:ZFIN;Acc:ZDB-GENE-070810-1]	16	43152727	43284939	
ENSDARG00000060539	501.5328386	1.756264868	0.215667629	3.506622067	0.000453833	0.004670631	shank1	SH3 and multiple ankyrin repeat domains 1 [Source:ZFIN;Acc:ZDB-GENE-130530-691]	3	30714111	30861177	
ENSDARG00000060597	498.797235	1.842660084	0.195353699	4.31350975	1.61E-05	0.000215569	zgc:158659	zgc:158659 [Source:ZFIN;Acc:ZDB-GENE-070112-492]	23	44190852	44233410	
ENSDARG00000060610	1617.559788	1.435567843	0.102939526	4.231298306	2.32E-05	0.000301714	pcdh7b	protocadherin 7b [Source:ZFIN;Acc:ZDB-GENE-081104-299]	7	63324888	63570206	
ENSDARG00000060631	880.3124949	1.357471145	0.124655593	2.867670312	0.004135062	0.034771922	GARNL3	si:ch211-182d3.1 [Source:NCBI gene;Acc:560594]	5	33356425	33460959	GARNL3
ENSDARG00000060637	915.4752474	2.679153342	0.135395129	12.40187404	2.55E-35	3.19E-33	clstn2	calsyntenin 2 [Source:ZFIN;Acc:ZDB-GENE-070911-2]	2	39108339	39555667	
ENSDARG00000060638	245.5926242	2.195639864	0.212060594	5.638199153	1.72E-08	3.45E-07	CLSTN2	si:ch211-276c2.2 [Source:ZFIN;Acc:ZDB-GENE-121214-77]	15	41555593	41664198	CLSTN2
ENSDARG00000060639	3360.975879	2.350883082	0.113199507	11.3834811	7.9E-33	8.74E-31	apba2b	amyloid beta (A4) precursor protein-binding, family A, member 2b [Source:ZFIN;Acc:ZDB-GENE-030131-5371]	7	30787903	30915965	
ENSDARG00000060671	1160.055565	-4.7542894	0.199285329	-18.8387646	3.63E-79	1.63E-76	lct	lactase [Source:ZFIN;Acc:ZDB-GENE-081104-434]	9	46369932	46399611	
ENSDARG00000060680	1251.075348	2.081635871	0.17453675	6.197181214	5.75E-10	1.36E-08	si:ch73-127m5.1	si:ch73-127m5.1 [Source:ZFIN;Acc:ZDB-GENE-130531-25]	12	35119762	35194800	
ENSDARG00000060711	2716.834249	1.420706592	0.127873181	3.290029925	0.001001767	0.009575537	sv2bb	synaptic vesicle glycoprotein 2Bb [Source:ZFIN;Acc:ZDB-GENE-030131-2789]	25	11034750	11088839	
ENSDARG00000060758	133.685598	2.188091039	0.256446119	4.632907077	3.61E-06	5.37E-05	nphs1	NPHS1 adhesion molecule, nephrin [Source:ZFIN;Acc:ZDB-GENE-051102-1]	15	36553823	36939655	
ENSDARG00000060767	4636.4151	1.266131206	0.096105664	2.769152141	0.005620238	0.045788252	smg7	SMG7 nonsense mediated mRNA decay factor [Source:ZFIN;Acc:ZDB-GENE-030131-9122]	2	36046126	36079907	
ENSDARG00000060808	542.0543111	2.091302489	0.146283426	7.460192295	8.64E-14	2.9E-12	mecom	MDS1 and EVI1 complex locus [Source:ZFIN;Acc:ZDB-GENE-050208-123]	15	35372907	35410860	
ENSDARG00000060854	1011.070209	1.423664421	0.117775837	3.597210032	0.000321649	0.003412386	kctd3	potassium channel tetramerization domain containing 3 [Source:ZFIN;Acc:ZDB-GENE-060503-169]	17	29345606	29370314	
ENSDARG00000060862	2571.305506	1.944294423	0.121036965	7.801702749	6.11E-15	2.24E-13	atxn1b	ataxin 1b [Source:ZFIN;Acc:ZDB-GENE-061218-2]	16	25555309	25568512	
ENSDARG00000060865	262.6318041	1.778312326	0.265227695	2.93450624	0.003340788	0.028718195	scai	suppressor of cancer cell invasion [Source:ZFIN;Acc:ZDB-GENE-060526-57]	5	31995683	32046297	
ENSDARG00000060879	634.2658091	-3.324592698	0.183473263	-12.66992617	8.68E-37	1.15E-34	slc28a1	solute carrier family 28 member 1 [Source:ZFIN;Acc:ZDB-GENE-050419-117]	18	26829086	26850069	
ENSDARG00000060889	257.9901724	-2.971161676	0.194900664	-10.11367345	4.8E-24	3.35E-22	mettl7a	methyltransferase like 7A [Source:ZFIN;Acc:ZDB-GENE-061027-239]	6	39506043	39508375	
ENSDARG00000060893	2482.58378	1.647882749	0.12291351	5.271045871	1.36E-07	2.42E-06	col8a2	collagen, type VIII, alpha 2 [Source:ZFIN;Acc:ZDB-GENE-060503-488]	19	35496282	35596207	
ENSDARG00000060915	832.5163909	1.735276114	0.120389603	6.107471894	1.01E-09	2.33E-08	fbxl16	F-box and leucine-rich repeat protein 16 [Source:ZFIN;Acc:ZDB-GENE-120215-180]	3	28634467	28665291	
ENSDARG00000060925	1330.355064	1.294860509	0.106199331	2.776481787	0.005495071	0.044914291	jarid2a	jumonji, AT rich interactive domain 2a [Source:ZFIN;Acc:ZDB-GENE-061218-1]	16	25407021	25500729	
ENSDARG00000060926	429.9965911	1.717198805	0.14842852	4.831947428	1.35E-06	2.15E-05	rnf19b	ring finger protein 19B [Source:NCBI gene;Acc:562396]	19	35379899	35400819	

ENSDARG00000060927	1945.534428	2.691704021	0.107360098	15.7572883	6.12E-56	1.73E-53	si:ch211-136a13.1	si:ch211-136a13.1 [Source:ZFIN;Acc:ZDB-GENE-050419-214]	18	49225552	49247479	
ENSDARG00000061031	1089.681694	2.091493702	0.166467775	6.556786755	5.5E-11	1.45E-09	dner	delta/notch-like EGF repeat containing [Source:ZFIN;Acc:ZDB-GENE-130502-1]	18	46699731	46763170	
ENSDARG00000061042	320.3232844	1.793776315	0.183591856	4.323592192	1.54E-05	0.000206829	gabbr2	gamma-aminobutyric acid (GABA) B receptor, 2 [Source:ZFIN;Acc:ZDB-GENE-060503-620]	19	34311374	34679738	
ENSDARG00000061047	331.2566266	1.592693702	0.18887294	3.138055141	0.001700729	0.015501087	abcg4a	ATP-binding cassette, sub-family G (WHITE), member 4a [Source:ZFIN;Acc:ZDB-GENE-050517-39]	5	30624183	30672482	
ENSDARG00000061081	1003.133003	1.840074161	0.114885914	7.31224682	2.63E-13	8.49E-12	arpp21	cAMP-regulated phosphoprotein, 21 [Source:ZFIN;Acc:ZDB-GENE-081104-296]	19	42886413	42917226	
ENSDARG00000061099	2629.197136	1.540547195	0.096321819	5.61188732	2E-08	3.98E-07	nfasca	neurofascin homolog (chicken) a [Source:ZFIN;Acc:ZDB-GENE-080229-6]	11	23704410	23884805	
ENSDARG00000061121	2953.567364	2.041703394	0.104031961	10.01330151	1.33E-23	8.92E-22	adgrl3.1	adhesion G protein-coupled receptor L3.1 [Source:ZFIN;Acc:ZDB-GENE-030131-7856]	1	22943551	23157583	
ENSDARG00000061149	251.1620704	1.947758223	0.212381674	4.462523553	8.1E-06	0.000114288	nrn1lb	neuritin 1-like b [Source:ZFIN;Acc:ZDB-GENE-081022-27]	7	29015846	29021757	
ENSDARG00000061191	378.0120023	1.447941007	0.154304212	2.902973298	0.003696382	0.031473159	plekho1a	pleckstrin homology domain containing, family O member 1a [Source:NCBI gene;Acc:563283]	19	42367286	42391390	
ENSDARG00000061201	4115.600682	-1.414850845	0.127998751	-3.241053863	0.001190887	0.011204067	acsf2	acyl-CoA synthetase family member 2 [Source:NCBI gene;Acc:751707]	12	26497167	26538823	
ENSDARG00000061248	623.5953233	1.38509503	0.126174803	3.052075545	0.002272649	0.020228993	xylt1	xylosyltransferase I [Source:ZFIN;Acc:ZDB-GENE-111017-2]	3	26418451	26524934	
ENSDARG00000061272	2250.957285	-1.452559217	0.112610434	-4.018803605	5.85E-05	0.000709112	BX294434.1	solute carrier family 41 member 1 [Source:NCBI gene;Acc:564564]	23	35027177	35082494	
ENSDARG00000061274	225.5379293	-1.931395861	0.197567265	-4.714322798	2.43E-06	3.72E-05	lss	lanosterol synthase (2,3-oxidosqualene-lanosterol cyclase) [Source:ZFIN;Acc:ZDB-GENE-050119-7]	9	38481780	38500391	
ENSDARG00000061304	551.9658914	1.463484225	0.14654411	3.162762568	0.001562797	0.014337637	ppp1r9a	protein phosphatase 1, regulatory subunit 9A [Source:ZFIN;Acc:ZDB-GENE-060503-660]	19	41075488	41160943	
ENSDARG00000061352	1482.383595	1.342217206	0.101041611	3.386893803	0.000706887	0.006980166	bcl11aa	BAF chromatin remodeling complex subunit BCL11A a [Source:ZFIN;Acc:ZDB-GENE-060421-4643]	13	25846528	25925224	
ENSDARG00000061355	2022.465431	-2.864171574	0.178234603	-10.45908899	1.33E-25	9.8E-24	aoc1	amine oxidase copper containing 1 [Source:ZFIN;Acc:ZDB-GENE-061103-112]	24	40001290	40009446	
ENSDARG00000061368	810.9311909	1.768419996	0.124171723	6.188365404	6.08E-10	1.43E-08	klf13	Kruppel-like factor 13 [Source:ZFIN;Acc:ZDB-GENE-060929-1274]	25	34487700	34512102	
ENSDARG00000061421	293.9601737	2.568351203	0.188896094	8.302719064	1.02E-16	4.27E-15	BRSK2	BR serine/threonine kinase 2a [Source:NCBI gene;Acc:100334759]	25	24721849	25058508	BRSK2
ENSDARG00000061454	1875.785883	2.682918911	0.111090475	15.14908375	7.68E-52	1.97E-49	nrxn2a	neurexin 2a [Source:ZFIN;Acc:ZDB-GENE-070206-5]	21	27636288	28340977	
ENSDARG00000061466	224.3824812	1.842201499	0.289941309	2.904730969	0.003675689	0.031318217	cpne5a	copine Va [Source:ZFIN;Acc:ZDB-GENE-081105-9]	8	23916647	24076844	
ENSDARG00000061471	509.6556328	1.545881949	0.138970462	3.928042994	8.56E-05	0.001007805	sema5ba	sema domain, seven thrombospondin repeats (type 1 and type 1-like), transmembrane domain (TM) and short cytoplasmic	9	37481434	37749973	
ENSDARG00000061473	525.8582743	1.602973772	0.150309397	4.011550734	6.03E-05	0.000728995	tbkbp1	TBK1 binding protein 1 [Source:ZFIN;Acc:ZDB-GENE-100203-1]	12	28574863	28678323	
ENSDARG00000061479	1102.904584	2.526044799	0.120075285	12.70906662	5.27E-37	7.07E-35	thsd7aa	thrombospondin, type I, domain containing 7Aa [Source:NCBI gene;Acc:557991]	19	37620342	37842003	
ENSDARG00000061481	465.3815605	-1.556159851	0.167591162	-3.318551186	0.000904857	0.008725634	wu:fj05g07	wu:fj05g07 [Source:ZFIN;Acc:ZDB-GENE-030131-7307]	24	29758272	29777413	
ENSDARG00000061497	151.999247	2.525895358	0.279845004	5.45264463	4.96E-08	9.37E-07	thsd7bb	thrombospondin, type I, domain containing 7Bb [Source:ZFIN;Acc:ZDB-GENE-090708-2]	11	33628104	33772872	
ENSDARG00000061517	1257.766045	1.821965443	0.115812675	7.097370321	1.27E-12	3.89E-11	lrp1bb	low density lipoprotein receptor-related protein 1Bb [Source:ZFIN;Acc:ZDB-GENE-070912-401]	9	36314867	36694998	
ENSDARG00000061576	190.554189	2.013730895	0.236250584	4.290913819	1.78E-05	0.000236571	gldn	gliomedin [Source:ZFIN;Acc:ZDB-GENE-050119-4]	18	39649660	39662370	
ENSDARG00000061585	416.0713588	-1.785117585	0.181232232	-4.332107918	1.48E-05	0.000199845	cyp4v7	cytochrome P450, family 4, subfamily V, polypeptide 7 [Source:ZFIN;Acc:ZDB-GENE-061103-88]	14	29975268	29990757	
ENSDARG00000061634	1005.153014	-1.781173959	0.141432534	-5.523297488	3.33E-08	6.43E-07	aifm4	apoptosis inducing factor mitochondria associated 4 [Source:ZFIN;Acc:ZDB-GENE-070112-2282]	15	25438714	25448626	
ENSDARG00000061647	4086.91394	2.283378876	0.148736308	8.62855137	6.21E-18	2.88E-16	nrxn1a	neurexin 1a [Source:NCBI gene;Acc:565531]	12	24342303	24640339	
ENSDARG00000061665	490.851444	1.913969521	0.143538577	6.367413822	1.92E-10	4.81E-09	hcn2b	hyperpolarization activated cyclic nucleotide-gated potassium channel 2b [Source:ZFIN;Acc:ZDB-GENE-030131-8228]	22	18951096	19007968	
ENSDARG00000061685	82.94122351	2.392124705	0.322119884	4.321759611	1.55E-05	0.000208331	hcn4	hyperpolarization activated cyclic nucleotide-gated potassium channel 4 [Source:ZFIN;Acc:ZDB-GENE-050420-360]	18	1004814	1143998	
ENSDARG00000061728	390.0458403	-1.675078086	0.160829766	-4.197469789	2.7E-05	0.000345316	acot13	acyl-CoA thioesterase 13 [Source:ZFIN;Acc:ZDB-GENE-060503-437]	19	31576849	31579383	
ENSDARG00000061736	6923.904161	1.450408355	0.100812775	4.467770619	7.9E-06	0.000112025	ank3a	ankyrin 3a [Source:ZFIN;Acc:ZDB-GENE-060621-1]	17	20701582	20897407	
ENSDARG00000061746	652.5031167	1.698502905	0.167540863	4.169149492	3.06E-05	0.000388378	arhgef9a	Cdc42 guanine nucleotide exchange factor (GEF) 9a [Source:ZFIN;Acc:ZDB-GENE-070705-271]	5	21939733	22001053	
ENSDARG00000061759	1533.754403	1.388978977	0.10211544	3.809208235	0.000139413	0.001584153	kdm6a	lysine (K)-specific demethylase 6A [Source:ZFIN;Acc:ZDB-GENE-081105-56]	9	33788389	33862124	
ENSDARG00000061778	1122.494143	1.871417334	0.126134213	6.908651631	4.89E-12	1.43E-10	fmn2b	formin 2b [Source:ZFIN;Acc:ZDB-GENE-080225-35]	12	47446158	47541290	
ENSDARG00000061791	243.6016557	3.444137241	0.252341835	9.685818609	3.46E-22	2.13E-20	nyx	nyctalopin [Source:ZFIN;Acc:ZDB-GENE-061026-3]	9	33357011	33361199	
ENSDARG00000061797	119.8077284	-1.720640833	0.257493573	-2.798675032	0.005131275	0.042215711	zgc:154006	zgc:154006 [Source:ZFIN;Acc:ZDB-GENE-061013-134]	2	24386453	24403153	
ENSDARG00000061817	4077.395759	1.818440963	0.128045491	6.39179839	1.64E-10	4.14E-09	kif1aa	kinesin family member 1Aa [Source:ZFIN;Acc:ZDB-GENE-100913-3]	6	27146671	27273027	
ENSDARG00000061835	231.6549418	-1.534906173	0.190857499	-2.802646868	0.005068513	0.041795254	zpld1a	zona pellucida-like domain containing 1a [Source:ZFIN;Acc:ZDB-GENE-110411-81]	10	29112756	29120535	
ENSDARG00000061836	474.8496329	1.59691491	0.138956653	4.295691482	1.74E-05	0.000232147	nfixb	nuclear factor I/Xb [Source:ZFIN;Acc:ZDB-GENE-060421-5000]	3	14768364	15058464	
ENSDARG00000061841	1472.736893	1.329648622	0.101621885	3.243874314	0.001179158	0.011102039	tiparp	TCDD-inducible poly(ADP-ribose) polymerase [Source:ZFIN;Acc:ZDB-GENE-031002-47]	18	34599315	34642086	
ENSDARG00000061858	2321.190594	-2.677230068	0.195988987	-8.557777119	1.15E-17	5.25E-16	zgc:153968	zgc:153968 [Source:ZFIN;Acc:ZDB-GENE-061027-211]	7	21225750	21230668	
ENSDARG00000061885	323.4974819	1.837630739	0.193474373	4.329414424	1.5E-05	0.00020187	satb2	SATB homeobox 2 [Source:ZFIN;Acc:ZDB-GENE-070912-212]	9	32560399	32604414	
ENSDARG00000061909	1878.235267	2.985848193	0.12142324	16.35476196	4.02E-60	1.28E-57	si:ch73-233f7.1	si:ch73-233f7.1 [Source:ZFIN;Acc:ZDB-GENE-141215-5]	14	2382690	2400668	
ENSDARG00000061992	943.8610607	1.399581872	0.118940187	3.359519454	0.000780782	0.007631775	dot1l	DOT1-like histone H3K79 methyltransferase [Source:ZFIN;Acc:ZDB-GENE-060503-341]	22	20782915	20838011	
ENSDARG00000062024	4035.974954	2.096262744	0.096463052	11.36458699	6.28E-30	6.13E-28	kif1ab	kinesin family member 1Ab [Source:ZFIN;Acc:ZDB-GENE-070912-480]	2	22694382	22758590	
ENSDARG00000062045	1024.120524	1.637731893	0.113694712	5.609160534	2.03E-08	4.04E-07	il1rapl1a	interleukin 1 receptor accessory protein-like 1a [Source:NCBI gene;Acc:568868]	9	29716380	29985390	
ENSDARG00000062055	1425.776884	1.784677804	0.107480649	7.300642568	2.86E-13	9.17E-12	rnf38	ring finger protein 38 [Source:ZFIN;Acc:ZDB-GENE-030131-8693]	1	18863060	18933671	
ENSDARG00000062077	309.1988515	-2.206565458	0.189868558	-6.354740736	2.09E-10	5.19E-09	acsbg1	acyl-CoA synthetase bubblegum family member 1 [Source:ZFIN;Acc:ZDB-GENE-110914-219]	25	25993240	26018424	
ENSDARG00000062086	123.7307126	1.708318307	0.241459945	2.933481601	0.003351835	0.028803292	kiaa1549lb	KIAA1549-like b [Source:ZFIN;Acc:ZDB-GENE-050419-18]	18	28102620	28156256	
ENSDARG00000062087	372.4653995	-1.76435496	0.178962802	-4.271026994	1.95E-05	0.000256245	nceh1b.1	neutral cholesterol ester hydrolase 1b, tandem duplicate 1 [Source:ZFIN;Acc:ZDB-GENE-061110-43]	24	26895748	26902697	
ENSDARG00000062123	2835.984737	1.391095727	0.090474401	4.322722468	1.54E-05	0.000207535	agap1	ArfGAP with GTPase domain, ankyrin repeat and PH domain 1 [Source:ZFIN;Acc:ZDB-GENE-130405-1]	6	16043318	16394528	
ENSDARG00000062132	1102.164352	-2.73706481	0.150715135	-11.52548357	9.82E-31	9.94E-29	cyp4v8	cytochrome P450, family 4, subfamily V, polypeptide 8 [Source:ZFIN;Acc:ZDB-GENE-061103-601]	1	17900306	17912645	
ENSDARG00000062165	1067.230881	1.991348755	0.130669729	7.586674935	3.28E-14	1.14E-12	tub	TUB bipartite transcription factor [Source:ZFIN;Acc:ZDB-GENE-121009-1]	7	27898091	28058520	
ENSDARG00000062208	554.2660697	1.893808795	0.146377807	6.106176985	1.02E-09	2.35E-08	prrt1	proline-rich transmembrane protein 1 [Source:ZFIN;Acc:ZDB-GENE-060503-686]	19	26858039	26869103	
ENSDARG00000062267	5220.613055	1.301611906	0.083216747	3.624413543	0.000289618	0.003093489	kdm3b	lysine (K)-specific demethylase 3B [Source:ZFIN;Acc:ZDB-GENE-101007-4]	14	21622154	21661015	
ENSDARG00000062305	166.5841159	2.008621301	0.228332563	4.417334466	9.99E-06	0.0001385	rims3	regulating synaptic membrane exocytosis 3 [Source:ZFIN;Acc:ZDB-GENE-060503-788]	19	25163540	25301711	
ENSDARG00000062325	669.7409282	1.823860669	0.191036762	4.312576586	1.61E-05	0.000216366	shank2b	SH3 and multiple ankyrin repeat domains 2b [Source:ZFIN;Acc:ZDB-GENE-061207-58]	25	14165447	14366077	
ENSDARG00000062346	693.1187486	1.737888614	0.21896434	3.369903118	0.000751946	0.007378655	cacna1ea	calcium channel, voltage-dependent, R type, alpha 1E subunit a [Source:ZFIN;Acc:ZDB-GENE-081104-526]	8	14710542	14828450	
ENSDARG00000062347	1001.55981	2.500193132	0.146519833	10.23884003	1.33E-24	9.47E-23	mtus2a	microtubule associated tumor suppressor candidate 2a [Source:ZFIN;Acc:ZDB-GENE-091204-75]	10	24504292	24622317	
ENSDARG00000062376	1487.731828	2.293589716	0.126742494	10.20644046	1.86E-24	1.32E-22	nlgn3b	neuroligin 3b [Source:ZFIN;Acc:ZDB-GENE-060526-197]	5	23283890	23317477	
ENSDARG00000062415	986.4664592	1.966303184	0.118543792	8.151444894	3.6E-16	1.44E-14	ctnnd2a	catenin (cadherin-associated protein), delta 2a [Source:ZFIN;Acc:ZDB-GENE-030616-60]	24	22118804	22702017	
ENSDARG00000062423	281.9068066	-1.497161341	0.178231394	-2.789415095	0.005280334	0.043328428	zgc:153901	zgc:153901 [Source:ZFIN;Acc:ZDB-GENE-060929-820]	9	24208533	24212884	
ENSDARG00000062429	348.2475941	-1.498077719	0.146405856	-3.402034134	0.000668863	0.006630776	trim109	tripartite motif containing 109 [Source:ZFIN;Acc:ZDB-GENE-060616-101]	16	14064397	14074594	
ENSDARG00000062447	948.2819553	1.844005805	0.122771769	6.874591881	6.22E-12	1.8E-10	hecw2a	HECT, C2 and WW domain containing E3 ubiquitin protein ligase 2a [Source:ZFIN;Acc:ZDB-GENE-090311-22]	9	24159280	24192145	
ENSDARG00000062449	2263.614369	1.694521886	0.153192396	4.533657712	5.8E-06	8.4E-05	xpr1a	xenotropic and polytropic retrovirus receptor 1a [Source:ZFIN;Acc:ZDB-GENE-060503-362]	8	14244476	14375890	
ENSDARG00000062462	592.9933176	2.185019724	0.138146182	8.578012867	9.65E-18	4.43E-16	shisa7a	shisa family member 7a [Source:ZFIN;Acc:ZDB-GENE-100922-37]	16	13636704	13662514	
ENSDARG00000062463	348.8991114	1.570051471	0.160616238	3.549152188	0.000386474	0.004026795	zc3h12b	zinc finger CCCH-type containing 12B [Source:ZFIN;Acc:ZDB-GENE-060526-290]	5	22098591	22106594	
ENSDARG00000062477	2352.50517	2.502863016	0.116815841	12.86523308	7.06E-38	9.96E-36	kiaa1549la	KIAA1549-like a [Source:ZFIN;Acc:ZDB-GENE-061207-55]	25	15354095	15482419	
ENSDARG00000062487	638.0897562	2.063911583	0.139351151	7.634752751	2.26E-14	7.92E-13	si:dkey-6n6.1	si:dkey-6n6.1 [Source:ZFIN;Acc:ZDB-GENE-060503-80]	8	14130245	14151051	
ENSDARG00000062510	2229.246618	1.935595396	0.120588014	7.758610188	8.59E-15	3.12E-13	bcl11ba	BAF chromatin remodeling complex subunit BCL11B a [Source:ZFIN;Acc:ZDB-GENE-061207-62]	17	18117029	18180747	
ENSDARG00000062538	310.7228443	1.631764571	0.168550624	3.748218524	0.000178095	0.001980666		si:dkey-215k6.1 [Source:ZFIN;Acc:ZDB-GENE-060526-251]	5	18431401	18513950	
ENSDARG00000062549	709.7526317	-2.287799001	0.126784648	-10.15737331	3.07E-24	2.16E-22	acmsd	aminocarboxymuconate semialdehyde decarboxylase [Source:ZFIN;Acc:ZDB-GENE-070410-103]	9	23244292	23253870	
ENSDARG00000062562	1128.350391	2.431038332	0.113548167	12.60291883	2.03E-36	2.63E-34	egln2	egl-9 family hypoxia-inducible factor 2 [Source:ZFIN;Acc:ZDB-GENE-060503-757]	15	8335600	8382997	
ENSDARG00000062575	427.1078922	1.515205422	0.156825314	3.28521849	0.001019033	0.009718371	si:ch211-210g13.5	si:ch211-210g13.5 [Source:ZFIN;Acc:ZDB-GENE-030131-5675]	3	17346502	17523082	
ENSDARG00000062621	381.6539209	1.582003041	0.165916492	3.507807063	0.000451817	0.004655603	satb1b	SATB homeobox 1b [Source:ZFIN;Acc:ZDB-GENE-030131-7105]	16	49393200	49505275	
ENSDARG00000062632	164.8101887	-1.97165268	0.232774443	-4.174224049	2.99E-05	0.000380593	duox	dual oxidase [Source:ZFIN;Acc:ZDB-GENE-091117-14]	25	31958911	32018990	
ENSDARG00000062634	433.6467744	1.419260788	0.144560455	2.900245363	0.003728707	0.031705374	kat2b	K(lysine) acetyltransferase 2B [Source:NCBI gene;Acc:563942]	19	20724347	20755146	
ENSDARG00000062646	7885.869076	2.293931776	0.095251521	13.58436863	4.96E-42	8.35E-40	tet3	tet methylcytosine dioxygenase 3 [Source:ZFIN;Acc:ZDB-GENE-060526-109]	5	14306475	14373662	
ENSDARG00000062661	801.6160039	1.41604175	0.133276764	3.121637544	0.001798482	0.01630914	abca4b	ATP-binding cassette, sub-family A (ABC1), member 4b [Source:ZFIN;Acc:ZDB-GENE-050517-4]	2	15203322	15294707	
ENSDARG00000062667	304.2611286	1.687892604	0.194130858	3.543448016	0.000394931	0.004104831	mpp3b	membrane protein, palmitoylated 3b (MAGUK p55 subfamily member 3) [Source:ZFIN;Acc:ZDB-GENE-121214-128]	12	16233077	16276276	
ENSDARG00000062686	377.578693	2.308416753	0.172651516	7.578368188	3.5E-14	1.21E-12	megf11	multiple EGF-like-domains 11 [Source:ZFIN;Acc:ZDB-GENE-060503-252]	18	19143437	19405616	
ENSDARG00000062687	471.3315783	1.889758116	0.199316848	4.464038653	8.04E-06	0.000113674	kcnh7	potassium channel, voltage gated eag related subfamily H, member 7 [Source:ZFIN;Acc:ZDB-GENE-120214-39]	6	10450000	10576657	
ENSDARG00000062693	1729.106312	2.133031374	0.139368147	8.129772826	4.3E-16	1.7E-14	nrxn3b	neurexin 3b [Source:NCBI gene;Acc:570698]	20	5563042	5965307	
ENSDARG00000062720	1268.999191	1.981488411	0.123059551	7.975719083	1.51E-15	5.86E-14	pcdh1a	protocadherin 1a [Source:ZFIN;Acc:ZDB-GENE-091116-32]	10	20920364	21054059	
ENSDARG00000062721	1556.448575	1.544443531	0.115515715	4.71315552	2.44E-06	3.73E-05	dennd4a	DENN/MADD domain containing 4A [Source:ZFIN;Acc:ZDB-GENE-060503-285]	18	19044839	19103929	
ENSDARG00000062744	1700.951445	2.351748574	0.203088063	6.655972543	2.81E-11	7.6E-10	scn1lab	sodium channel, voltage-gated, type I like, alpha b [Source:ZFIN;Acc:ZDB-GENE-060906-1]	6	10248332	10320676	
ENSDARG00000062756	419.0566969	-1.473493122	0.144058877	-3.286802809	0.001013318	0.009671211	pllp	plasmolipin [Source:ZFIN;Acc:ZDB-GENE-050419-195]	18	18861359	18870632	
ENSDARG00000062765	1066.354482	1.823443053	0.113018744	7.285898084	3.2E-13	1.02E-11	nsd3	nuclear receptor binding SET domain protein 3 [Source:ZFIN;Acc:ZDB-GENE-050324-2]	10	20543279	20588889	
ENSDARG00000062790	4166.406946	1.446914209	0.084273407	5.303146315	1.14E-07	2.05E-06	CU639469.1	transforming, acidic coiled-coil containing protein 2 [Source:NCBI gene;Acc:569976]	13	28819768	28902920	
ENSDARG00000062797	3919.160071	2.099264901	0.17458355	6.296497568	3.04E-10	7.43E-09	nell2b	neural EGFL like 2b [Source:ZFIN;Acc:ZDB-GENE-030131-8026]	4	14247693	14328997	
ENSDARG00000062823	373.191163	1.970844735	0.166028188	5.847469316	4.99E-09	1.07E-07	pi4kab	phosphatidylinositol 4-kinase, catalytic, alpha b [Source:ZFIN;Acc:ZDB-GENE-080220-34]	8	1741938	1838315	
ENSDARG00000062830	86.84889125	3.208622463	0.376324467	5.868931348	4.39E-09	9.42E-08	tent5d	terminal nucleotidyltransferase 5D [Source:ZFIN;Acc:ZDB-GENE-091118-44]	14	9343860	9355177	
ENSDARG00000062880	400.1736641	1.62168854	0.179790906	3.457841964	0.000544521	0.005500207	cntn3a.1	contactin 3a, tandem duplicate 1 [Source:ZFIN;Acc:ZDB-GENE-081030-10]	23	11669109	11861393	
ENSDARG00000062892	66.81993973	3.915265739	0.480495172	6.067211299	1.3E-09	2.94E-08	foxe3	forkhead box E3 [Source:ZFIN;Acc:ZDB-GENE-061214-6]	8	19668654	19670305	
ENSDARG00000062933	2605.926977	1.333537464	0.114558553	2.911502062	0.003596955	0.030720373	cmip	c-Maf inducing protein [Source:ZFIN;Acc:ZDB-GENE-050419-50]	18	13324140	13362926	
ENSDARG00000062934	224.4238218	2.575217283	0.27563699	5.714825446	1.1E-08	2.25E-07	gpr12	G protein-coupled receptor 12 [Source:ZFIN;Acc:ZDB-GENE-100209-3]	24	21584074	21588914	
ENSDARG00000062956	1650.043153	2.811772421	0.11000428	16.47001751	6.03E-61	1.99E-58	dagla	diacylglycerol lipase, alpha [Source:ZFIN;Acc:ZDB-GENE-070619-1]	7	13382852	13487378	
ENSDARG00000062973	1062.71777	2.001404168	0.11259154	8.894133285	5.89E-19	2.92E-17	ttll7	tubulin tyrosine ligase-like family, member 7 [Source:ZFIN;Acc:ZDB-GENE-070112-2112]	11	7981243	8126263	
ENSDARG00000062991	354.7641862	2.207587157	0.177596137	6.79962514	1.05E-11	2.98E-10	abi1b	abl-interactor 1b [Source:ZFIN;Acc:ZDB-GENE-060929-1182]	2	9868601	9915870	
ENSDARG00000062998	134.1411499	-1.68649899	0.216050882	-3.177487557	0.001485571	0.013719392	pglyrp2	peptidoglycan recognition protein 2 [Source:ZFIN;Acc:ZDB-GENE-071227-1]	6	8490322	8498908	
ENSDARG00000063007	3890.314552	2.184342423	0.120368485	9.839306571	7.62E-23	4.81E-21	apc2	APC regulator of WNT signaling pathway 2 [Source:ZFIN;Acc:ZDB-GENE-090422-1]	11	7281240	7320211	
ENSDARG00000063008	1068.144994	2.521805351	0.129931878	11.71233244	1.1E-31	1.17E-29	si:dkeyp-27e10.3	si:dkeyp-27e10.3 [Source:ZFIN;Acc:ZDB-GENE-041210-96]	4	9784301	9810999	
ENSDARG00000063018	267.7870555	1.663177078	0.231686655	2.862387902	0.00420462	0.03528591	fam78ba	family with sequence similarity 78 member Ba [Source:ZFIN;Acc:ZDB-GENE-140227-3]	2	10056100	10062575	
ENSDARG00000063019	186.8893749	1.928999694	0.248828003	3.733501382	0.000188836	0.002085339	panx2	pannexin 2 [Source:ZFIN;Acc:ZDB-GENE-050419-55]	18	14906570	14922872	
ENSDARG00000063056	1685.168115	1.90548945	0.151101446	5.99259287	2.07E-09	4.59E-08	tulp4b	TUB like protein 4b [Source:ZFIN;Acc:ZDB-GENE-130530-708]	17	8183393	8211012	
ENSDARG00000063059	155.0966411	-2.763285181	0.241674175	-7.296125789	2.96E-13	9.47E-12	abcg8	ATP-binding cassette, sub-family G (WHITE), member 8 [Source:ZFIN;Acc:ZDB-GENE-050517-41]	13	10926528	10945394	
ENSDARG00000063068	4143.343652	1.933720638	0.098610606	9.468764806	2.83E-21	1.64E-19	syne1b	spectrin repeat containing, nuclear envelope 1b [Source:ZFIN;Acc:ZDB-GENE-071218-4]	17	7844927	8173380	
ENSDARG00000063078	212.2957229	-2.284499274	0.203369665	-6.316080998	2.68E-10	6.59E-09	abcg5	ATP-binding cassette, sub-family G (WHITE), member 5 [Source:ZFIN;Acc:ZDB-GENE-050517-40]	13	10945337	10968607	
ENSDARG00000063101	1479.771168	1.417564852	0.100623739	4.149764795	3.33E-05	0.000419459	man2a2	mannosidase, alpha, class 2A, member 2 [Source:ZFIN;Acc:ZDB-GENE-110331-1]	25	8662469	8742650	
ENSDARG00000063133	5700.799401	2.264739351	0.19052466	6.638192391	3.18E-11	8.53E-10	slc4a10a	solute carrier family 4 member 10a [Source:ZFIN;Acc:ZDB-GENE-100215-3]	11	10669461	10798064	
ENSDARG00000063144	2647.520058	2.257922811	0.176482664	7.127741511	1.02E-12	3.15E-11	shisa7b	shisa family member 7 [Source:ZFIN;Acc:ZDB-GENE-060503-31]	19	10217598	10243148	

ENSDARG00000063150	1482.725979	2.761370994	0.116504567	15.11847165	1.22E-51	3.07E-49	nrxn2b	neurexin 2b [Source:ZFIN;Acc:ZDB-GENE-070206-7]	7	528593	625258	
ENSDARG00000063157	2225.256947	1.87911949	0.13959111	6.297818593	3.02E-10	7.37E-09	mapk8ip2	mitogen-activated protein kinase 8 interacting protein 2 [Source:ZFIN;Acc:ZDB-GENE-070705-326]	18	8255265	8313686	
ENSDARG00000063158	370.8169108	1.812213918	0.17991342	4.514471013	6.35E-06	9.13E-05	SLC24A2	sodium/potassium/calcium exchanger 2-like [Source:NCBI gene;Acc:571043]	7	73332486	73402030	SLC24A2
ENSDARG00000063180	1417.536253	2.693407446	0.116172992	14.57660179	3.96E-48	8.56E-46	dock3	dedicator of cytokinesis 3 [Source:ZFIN;Acc:ZDB-GENE-100624-1]	22	31821815	32220392	
ENSDARG00000063186	121.5437119	2.249165364	0.281329677	4.44021896	8.99E-06	0.000125529	BEND7	si:ch211-220f12.4 [Source:ZFIN;Acc:ZDB-GENE-050419-200]	18	8382131	8398972	BEND7
ENSDARG00000063207	225.1728227	1.766723154	0.238894966	3.209457133	0.001329859	0.012413828	erbb4a	erb-b2 receptor tyrosine kinase 4a [Source:ZFIN;Acc:ZDB-GENE-030918-4]	1	6817292	6925407	
ENSDARG00000063264	1285.670928	2.164123351	0.150095116	7.755904257	8.77E-15	3.18E-13	pcdh9	protocadherin 9 [Source:ZFIN;Acc:ZDB-GENE-091015-1]	6	4872883	4984243	
ENSDARG00000063293	131.1843005	3.579179	0.335610392	7.685039146	1.53E-14	5.45E-13	neto2b	neuropilin (NRP) and tolloid (TLL)-like 2b [Source:ZFIN;Acc:ZDB-GENE-060503-638]	18	7500434	7511763	
ENSDARG00000063299	6633.487056	2.496072297	0.106484517	14.04966978	7.74E-45	1.44E-42	pcloa	piccolo presynaptic cytomatrix protein a [Source:ZFIN;Acc:ZDB-GENE-030131-8115]	4	11375894	11450040	
ENSDARG00000063307	432.5699797	1.724356211	0.190873073	3.79496279	0.000147666	0.001667366	sgsm2	small G protein signaling modulator 2 [Source:ZFIN;Acc:ZDB-GENE-131119-52]	15	20543770	20643827	
ENSDARG00000063332	326.7058487	1.880951219	0.185192195	4.756956513	1.97E-06	3.05E-05	shank3a	SH3 and multiple ankyrin repeat domains 3a [Source:ZFIN;Acc:ZDB-GENE-060503-369]	18	7663802	8030073	
ENSDARG00000063361	269.1529982	2.022713074	0.184708878	5.536891794	3.08E-08	5.98E-07	si:ch211-1e14.1	si:ch211-1e14.1 [Source:ZFIN;Acc:ZDB-GENE-050420-149]	18	12364733	12451772	
ENSDARG00000063372	38.37457596	-2.238205376	0.39416975	-3.14129985	0.001681997	0.01533593	zpld1b	zona pellucida-like domain containing 1b [Source:ZFIN;Acc:ZDB-GENE-121214-104]	15	19783352	19793361	
ENSDARG00000063416	2129.261349	1.395240893	0.124366048	3.178044961	0.001482718	0.013698084	ptprk	protein tyrosine phosphatase receptor type K [Source:ZFIN;Acc:ZDB-GENE-030131-9834]	20	1441548	1560849	
ENSDARG00000063433	2603.124799	1.880143578	0.129556054	6.793534945	1.09E-11	3.1E-10	atp2b2	ATPase plasma membrane Ca2+ transporting 2 [Source:ZFIN;Acc:ZDB-GENE-061016-1]	11	1148701	1291012	
ENSDARG00000063475	572.0620021	2.351281108	0.164648386	8.207071717	2.27E-16	9.22E-15	abcg1	ATP-binding cassette, sub-family G (WHITE), member 1 [Source:ZFIN;Acc:ZDB-GENE-070424-84]	15	5028608	5074983	
ENSDARG00000063481	139.4112692	-1.710846501	0.234081649	-3.036745955	0.002391469	0.021181463	si:ch211-214j24.15	si:ch211-214j24.15 [Source:ZFIN;Acc:ZDB-GENE-110914-85]	4	5304208	5317006	
ENSDARG00000063527	1609.862069	1.627027107	0.18600781	3.370971936	0.000749035	0.007355839	elmo2	engulfment and cell motility 2 [Source:ZFIN;Acc:ZDB-GENE-140106-84]	11	88247	103157	
ENSDARG00000063555	883.8936285	1.412733724	0.117349081	3.517144928	0.000436216	0.0045078		si:ch211-166g5.4 [Source:ZFIN;Acc:ZDB-GENE-121214-319]	12	3766557	3778848	
ENSDARG00000063568	269.1375686	1.964711585	0.192640197	5.00784155	5.5E-07	9.16E-06	syt7a	synaptotagmin VIIa [Source:ZFIN;Acc:ZDB-GENE-090601-5]	25	4231238	4313699	
ENSDARG00000063596	852.2741387	-1.371924713	0.116279292	-3.198546417	0.001381223	0.012840769	bud23	BUD23 rRNA methyltransferase and ribosome maturation factor [Source:ZFIN;Acc:ZDB-GENE-070410-68]	15	1749843	1765137	
ENSDARG00000063614	325.7846449	3.252014784	0.212024935	10.62146197	2.37E-26	1.84E-24	skor2	SKI family transcriptional corepressor 2 [Source:ZFIN;Acc:ZDB-GENE-060825-57]	10	14547941	14556978	
ENSDARG00000063626	11605.99306	-1.302477841	0.088032562	-3.43597681	0.000590422	0.005925698	ddx21	DEAD (Asp-Glu-Ala-Asp) box helicase 21 [Source:ZFIN;Acc:ZDB-GENE-031113-10]	13	319575	330004	
ENSDARG00000063665	1403.883088	-2.156939488	0.157955289	-7.324474507	2.4E-13	7.78E-12	mat2al	methionine adenosyltransferase II, alpha-like [Source:ZFIN;Acc:ZDB-GENE-060421-5255]	10	4969634	4980150	
ENSDARG00000063711	1302.811279	1.538336563	0.155479954	3.462417831	0.000535345	0.005414063	trim3a	tripartite motif containing 3a [Source:ZFIN;Acc:ZDB-GENE-080522-4]	15	32297441	32353828	
ENSDARG00000063713	1295.437634	1.910220703	0.225259014	4.04077371	5.33E-05	0.000649155	syngap1a	synaptic Ras GTPase activating protein 1a [Source:ZFIN;Acc:ZDB-GENE-060503-370]	19	232536	276802	
ENSDARG00000067507	938.1478981	1.541671069	0.185948289	2.913019914	0.003579518	0.03058185	kctd8	potassium channel tetramerization domain containing 8 [Source:ZFIN;Acc:ZDB-GENE-060117-3]	14	5936996	6004780	
ENSDARG00000067509	83.05922385	2.263574094	0.36873871	3.426746528	0.000610859	0.006103301	slc24a4b	solute carrier family 24 member 4b [Source:ZFIN;Acc:ZDB-GENE-081031-75]	20	50115335	50198207	
ENSDARG00000067537	474.0434653	-1.462459098	0.148130922	-3.121961932	0.001796502	0.01630296	pcsk5a	proprotein convertase subtilisin/kexin type 5a [Source:ZFIN;Acc:ZDB-GENE-060531-130]	5	55265274	55349429	
ENSDARG00000067824	1105.626013	1.525945366	0.12007471	4.380151036	1.19E-05	0.000162761	cntnap3	contactin associated protein like 3 [Source:ZFIN;Acc:ZDB-GENE-091113-51]	10	2175583	2393791	
ENSDARG00000067850	398.7448478	1.644015022	0.172268613	3.738435046	0.000185169	0.002050258	jund	JunD proto-oncogene, AP-1 transcription factor subunit [Source:ZFIN;Acc:ZDB-GENE-070725-2]	2	56125174	56131312	
ENSDARG00000067851	460.1406105	-1.940627028	0.137470283	-6.842402654	7.79E-12	2.23E-10	lcn15	lipocalin 15 [Source:ZFIN;Acc:ZDB-GENE-060825-349]	5	65991152	66001200	
ENSDARG00000067859	377.503321	2.991341188	0.184873889	10.77134903	4.7E-27	3.83E-25	scospondin	subcommissural organ spondin [Source:ZFIN;Acc:ZDB-GENE-051114-1]	24	40016889	40171461	
ENSDARG00000067958	938.7724757	-1.519533078	0.112887842	-4.602205762	4.18E-06	6.18E-05	sh3gl1a	SH3-domain GRB2-like 1a [Source:ZFIN;Acc:ZDB-GENE-030131-4524]	2	49457449	49511458	
ENSDARG00000067964	3134.240076	1.844465368	0.199805625	4.226434416	2.37E-05	0.000306878	slc6a5	solute carrier family 6 member 5 [Source:ZFIN;Acc:ZDB-GENE-050105-2]	7	17229282	17252274	
ENSDARG00000068006	455.4200677	-4.746576394	0.215053752	-17.42158112	5.66E-68	2.19E-65	gck	glucokinase (hexokinase 4) [Source:ZFIN;Acc:ZDB-GENE-060825-204]	8	40476811	40490593	
ENSDARG00000068088	1777.433466	-2.540560699	0.134108195	-11.48744638	1.53E-30	1.52E-28	tcnba	transcobalamin beta a [Source:ZFIN;Acc:ZDB-GENE-060531-123]	5	30617915	30620625	
ENSDARG00000068114	355.419433	-1.553616766	0.158042793	-3.502954839	0.000460127	0.004727654	f11r.2	F11 receptor, tandem duplicate 2 [Source:ZFIN;Acc:ZDB-GENE-060531-67]	5	29820266	29825846	
ENSDARG00000068156	521.9753326	-1.363302461	0.126613372	-2.869384604	0.004112714	0.034642022	chchd7	coiled-coil-helix-coiled-coil-helix domain containing 7 [Source:ZFIN;Acc:ZDB-GENE-080204-55]	7	58720601	58729894	
ENSDARG00000068166	212.0708104	2.363578469	0.21746339	6.27038174	3.6E-10	8.72E-09	whrnb	whirlin b [Source:ZFIN;Acc:ZDB-GENE-060526-377]	5	28999474	29122834	
ENSDARG00000068175	138.3197425	-1.682142865	0.220022978	-3.100325567	0.00193308	0.017435232	ing5b	inhibitor of growth family, member 5b [Source:ZFIN;Acc:ZDB-GENE-030616-462]	2	36606019	36608762	
ENSDARG00000068179	488.7002003	1.98721203	0.144398058	6.836740344	8.1E-12	2.32E-10	trib2	tribbles pseudokinase 2 [Source:ZFIN;Acc:ZDB-GENE-091207-3]	20	45404656	45423498	
ENSDARG00000068220	924.6334557	-3.408690613	0.176198623	-13.67031466	1.53E-42	2.64E-40	mbl2	mannose binding lectin 2 [Source:ZFIN;Acc:ZDB-GENE-000427-2]	2	37887277	37889152	
ENSDARG00000068242	423.7766265	2.052096543	0.164399492	6.399633767	1.56E-10	3.95E-09	cngb1a	cyclic nucleotide gated channel subunit beta 1a [Source:ZFIN;Acc:ZDB-GENE-050419-164]	18	45483282	45519748	
ENSDARG00000068290	70.52589756	-2.219865064	0.314863293	-3.874268904	0.000106945	0.001233088	si:dkey-286h2.3	si:dkey-286h2.3 [Source:ZFIN;Acc:ZDB-GENE-161014-2]	7	52135791	52145111	
ENSDARG00000068323	1501.837542	2.223717333	0.134897737	9.071444503	1.17E-19	6.13E-18	astn1	astrotactin 1 [Source:ZFIN;Acc:ZDB-GENE-050320-140]	2	34572690	34984255	
ENSDARG00000068370	361.3682467	2.176564151	0.175886451	6.689339323	2.24E-11	6.11E-10	ralgps1	Ral GEF with PH domain and SH3 binding motif 1 [Source:NCBI gene;Acc:100101642]	8	32914626	33114202	
ENSDARG00000068421	310.2314048	2.016376058	0.287506642	3.535139406	0.00040756	0.004222107	ttc9b	tetratricopeptide repeat domain 9B [Source:ZFIN;Acc:ZDB-GENE-050419-67]	18	36806545	36865386	
ENSDARG00000068428	196.0452001	2.69771405	0.295069742	5.753602644	8.74E-09	1.81E-07	si:ch211-153j24.3	si:ch211-153j24.3 [Source:ZFIN;Acc:ZDB-GENE-041014-38]	20	46544052	46564468	
ENSDARG00000068457	207537.7977	-1.477859224	0.122137417	-3.912472009	9.14E-05	0.001068063	tnnt3b	troponin T type 3b (skeletal, fast) [Source:ZFIN;Acc:ZDB-GENE-030520-2]	7	39444843	39460363	
ENSDARG00000068478	8466.873125	-1.924178506	0.102982132	-8.974163671	2.86E-19	1.46E-17	gpx4a	glutathione peroxidase 4a [Source:ZFIN;Acc:ZDB-GENE-030410-2]	22	17589608	17611742	
ENSDARG00000068483	113.5465633	3.124302946	0.315911048	6.724370552	1.76E-11	4.9E-10	neto1	neuropilin (NRP) and tolloid (TLL)-like 1 [Source:ZFIN;Acc:ZDB-GENE-070912-592]	2	30463825	30480455	
ENSDARG00000068493	184.7368586	-2.391155349	0.258172268	-5.388477076	7.11E-08	1.31E-06	cyp2y3	cytochrome P450, family 2, subfamily Y, polypeptide 3 [Source:ZFIN;Acc:ZDB-GENE-050522-490]	15	18305	20710	
ENSDARG00000068507	5754.14733	3.574196369	0.365236059	7.048034571	1.81E-12	5.5E-11	crybb1	crystallin, beta B1 [Source:ZFIN;Acc:ZDB-GENE-010813-1]	10	44021147	44027391	
ENSDARG00000068516	3968.062619	1.410064473	0.109426589	3.747393343	0.000178682	0.001986311	hapln1b	hyaluronan and proteoglycan link protein 1b [Source:ZFIN;Acc:ZDB-GENE-050920-1]	10	43269918	43334999	
ENSDARG00000068562	3723.699131	1.518210977	0.118243711	4.382566932	1.17E-05	0.000161054	rbm33a	RNA binding motif protein 33a [Source:ZFIN;Acc:ZDB-GENE-140106-90]	7	40900212	40959867	
ENSDARG00000068567	911.27095	2.205518806	0.148208242	8.133952519	4.16E-16	1.65E-14	shha	sonic hedgehog signaling molecule a [Source:NCBI gene;Acc:30269]	7	40884012	40893295	
ENSDARG00000068582	1044.669992	1.492216615	0.115022716	4.279299177	1.87E-05	0.000248469	rnf44	ring finger protein 44 [Source:NCBI gene;Acc:767686]	14	50918769	50975568	
ENSDARG00000068586	1006.395631	-2.074081173	0.167102596	-6.427674953	1.3E-10	3.33E-09	CU302253.1	cadherin related family member 2 [Source:NCBI gene;Acc:100536704]	14	50839282	50892442	
ENSDARG00000068589	2645.189662	3.658503017	0.108202109	24.56978919	2.66E-133	2.47E-130	vimr1	vimentin-related 1 [Source:ZFIN;Acc:ZDB-GENE-130530-722]	21	547098	551014	
ENSDARG00000068624	912.9168131	1.588751733	0.137910273	4.269092648	1.96E-05	0.000258071	sptbn4a	spectrin, beta, non-erythrocytic 4a [Source:ZFIN;Acc:ZDB-GENE-060531-133]	5	22465086	22501663	
ENSDARG00000068680	10060.88471	-3.635138264	0.094680473	-27.83190868	1.78E-170	2.8E-167	ctrl	chymotrypsin-like [Source:ZFIN;Acc:ZDB-GENE-040912-147]	15	37820258	37829160	
ENSDARG00000068701	1103.655302	1.946699771	0.137441743	6.888007605	5.66E-12	1.64E-10	gpr85	G protein-coupled receptor 85 [Source:NCBI gene;Acc:793299]	4	6643421	6647605	
ENSDARG00000068714	329.5707312	2.098198321	0.17167335	6.397022719	1.58E-10	4.01E-09	ksr1a	kinase suppressor of ras 1a [Source:ZFIN;Acc:ZDB-GENE-091113-34]	10	37173029	37256989	
ENSDARG00000068716	757.6877155	1.681253206	0.167317447	4.071620848	4.67E-05	0.000573614	cuedc1a	CUE domain containing 1a [Source:ZFIN;Acc:ZDB-GENE-061027-344]	10	37137464	37167451	
ENSDARG00000068745	4477.469643	2.097408973	0.089849378	12.21387394	2.62E-34	3.06E-32	map4l	microtubule associated protein 4 like [Source:ZFIN;Acc:ZDB-GENE-070912-581]	2	24177006	24252506	
ENSDARG00000068760	1145.320298	-1.699297658	0.215073528	-3.251435291	0.001148239	0.010835319	tnks1bp1	tankyrase 1 binding protein 1 [Source:NCBI gene;Acc:497601]	5	6515741	6567464	
ENSDARG00000068787	1116.485065	2.268888763	0.157676618	8.047412328	8.46E-16	3.32E-14	slc6a17	solute carrier family 6 member 17 [Source:ZFIN;Acc:ZDB-GENE-030131-8187]	8	24854600	24890992	
ENSDARG00000068876	872.9508048	-2.166544288	0.140239638	-8.318220899	8.93E-17	3.76E-15	zgc:153031	zgc:153031 [Source:ZFIN;Acc:ZDB-GENE-060929-1190]	18	15132112	15141119	
ENSDARG00000068883	1010.0027	-1.760154469	0.152828998	-4.973888989	6.56E-07	1.08E-05	tspan13a	tetraspanin 13a [Source:ZFIN;Acc:ZDB-GENE-040718-19]	15	34592215	34599768	
ENSDARG00000068886	893.5764828	-1.298421134	0.107535946	-2.775082625	0.005518769	0.045063958	mrpl14	mitochondrial ribosomal protein L14 [Source:NCBI gene;Acc:394122]	13	42676202	42679041	
ENSDARG00000068894	1211.394764	1.610867542	0.120383402	5.074350212	3.89E-07	6.59E-06	nrip1b	nuclear receptor interacting protein 1b [Source:ZFIN;Acc:ZDB-GENE-030131-4173]	10	38256556	38316134	
ENSDARG00000068934	212.6131723	1.967933744	0.19615578	4.934515541	8.03E-07	1.31E-05	cyp1b1	cytochrome P450, family 1, subfamily B, polypeptide 1 [Source:ZFIN;Acc:ZDB-GENE-030902-1]	13	42011285	42018948	
ENSDARG00000068951	56.02079459	5.074174841	0.77750342	5.240073208	1.61E-07	2.84E-06	si:ch211-219a15.4	si:ch211-219a15.4 [Source:ZFIN;Acc:ZDB-GENE-060503-308]	18	15758375	15768336	
ENSDARG00000068969	48.79676024	2.234294012	0.41588621	2.967864723	0.002998763	0.026108895	zgc:153759	zgc:153759 [Source:ZFIN;Acc:ZDB-GENE-060825-148]	8	22438398	22455390	
ENSDARG00000068989	807.816618	1.714513484	0.177799777	4.018641056	5.85E-05	0.000709112	gabra1	gamma-aminobutyric acid (GABA) A receptor, alpha 1 [Source:ZFIN;Acc:ZDB-GENE-061013-194]	21	42050351	42100471	
ENSDARG00000069018	970.3586083	-2.217191239	0.125226694	-9.719902347	2.48E-22	1.53E-20	cyp7a1	cytochrome P450, family 7, subfamily A, polypeptide 1 [Source:ZFIN;Acc:ZDB-GENE-040426-1296]	2	22150134	22152797	
ENSDARG00000069026	185.6567012	2.061220839	0.283449199	3.743954275	0.000181147	0.002008727	adcy1a	adenylate cyclase 1a [Source:ZFIN;Acc:ZDB-GENE-070705-302]	20	6894886	7000225	
ENSDARG00000069038	210.2982417	1.542838454	0.191269115	2.838087335	0.004538476	0.03779292	si:ch211-242e8.1	si:ch211-242e8.1 [Source:ZFIN;Acc:ZDB-GENE-060503-600]	18	16960966	16971684	
ENSDARG00000069095	502.2714007	-1.847263811	0.162279878	-5.221003483	1.78E-07	3.12E-06	gls2a	glutaminase 2a (liver, mitochondrial) [Source:ZFIN;Acc:ZDB-GENE-081105-57]	8	21166053	21184759	
ENSDARG00000069100	30093.97793	-1.69559026	0.087603164	-7.940241307	2.02E-15	7.69E-14	aldh9a1a.1	aldehyde dehydrogenase 9 family, member A1a, tandem duplicate 1 [Source:NCBI gene;Acc:100005587]	8	21094884	21103522	
ENSDARG00000069113	1318.886049	1.42022217	0.117506669	3.576155908	0.000348684	0.003666657	dbn1	drebrin 1 [Source:ZFIN;Acc:ZDB-GENE-091204-284]	21	36801620	36972127	
ENSDARG00000069135	3083.269057	1.75325175	0.127424448	5.911359717	3.39E-09	7.39E-08	ppp1r15a	protein phosphatase 1, regulatory subunit 15A [Source:ZFIN;Acc:ZDB-GENE-030131-4408]	7	71503356	71508989	
ENSDARG00000069189	54.49650837	8.82083313	1.470795705	5.31741635	1.05E-07	1.9E-06	si:dkey-242h9.3	si:dkey-242h9.3 [Source:ZFIN;Acc:ZDB-GENE-060503-347]	18	19120984	19124312	
ENSDARG00000069245	1018.686401	1.702616317	0.124744212	5.632456263	1.78E-08	3.56E-07	matn3a	matrilin 3a [Source:ZFIN;Acc:ZDB-GENE-040822-21]	20	43760059	43775544	
ENSDARG00000069265	1355.003382	2.107430978	0.123036149	9.000858608	2.24E-19	1.15E-17	matn3b	matrilin 3b [Source:ZFIN;Acc:ZDB-GENE-050221-9]	13	32627444	32635885	
ENSDARG00000069292	275.809915	-1.865320143	0.174137647	-4.969173272	6.72E-07	1.11E-05	acot16	acyl-CoA thioesterase 16 [Source:ZFIN;Acc:ZDB-GENE-041210-201]	4	25651720	25666835	
ENSDARG00000069293	176.7194764	-7.004597527	0.473647898	-12.6773444	7.9E-37	1.05E-34	ahsg2	alpha-2-HS-glycoprotein 2 [Source:ZFIN;Acc:ZDB-GENE-030131-1133]	2	10134345	10141741	
ENSDARG00000069361	907.70212	-1.455597015	0.138907288	-3.279864016	0.001038571	0.009893426	spa17	sperm autoantigenic protein 17 [Source:ZFIN;Acc:ZDB-GENE-061027-337]	15	22267847	22286918	
ENSDARG00000069402	1695.093926	2.438628214	0.110912637	12.97082327	1.79E-38	2.57E-36	lrrc4.1	leucine rich repeat containing 4.1 [Source:ZFIN;Acc:ZDB-GENE-041210-129]	4	19534833	19543042	
ENSDARG00000069409	536.2515467	-1.406695257	0.130496343	-3.116526089	0.001829955	0.016558651	gkup	glucuronokinase with putative uridyl pyrophosphorylase [Source:ZFIN;Acc:ZDB-GENE-080204-36]	15	21262917	21275683	
ENSDARG00000069438	743.3076329	1.796764514	0.143061616	5.569380074	2.56E-08	5.01E-07	neurl1aa	neuralized E3 ubiquitin protein ligase 1Aa [Source:ZFIN;Acc:ZDB-GENE-090311-3]	1	47701924	47771742	
ENSDARG00000069440	1742.94685	1.468471499	0.101510537	4.615003639	3.93E-06	5.83E-05	dachd	dachshund d [Source:ZFIN;Acc:ZDB-GENE-070427-2]	9	30820290	31062704	
ENSDARG00000069446	289.2336971	2.343820433	0.1917937	7.006593195	2.44E-12	7.31E-11	dbh	dopamine beta-hydroxylase (dopamine beta-monooxygenase) [Source:ZFIN;Acc:ZDB-GENE-990621-3]	10	10564261	10607118	
ENSDARG00000069451	58.4858242	2.583216952	0.409969215	3.86179473	0.000112557	0.001292379	gja8a	gap junction protein alpha 8 paralog a [Source:ZFIN;Acc:ZDB-GENE-050616-14]	9	30430188	30432778	
ENSDARG00000069453	74.05422877	1.95144138	0.318749368	2.984920051	0.002836525	0.024790948	zgc:113314	zgc:113314 [Source:ZFIN;Acc:ZDB-GENE-050913-19]	9	30421489	30427117	
ENSDARG00000069467	340.8244729	2.1974235	0.177583563	6.742873479	1.55E-11	4.33E-10	igsf9bb	immunoglobulin superfamily, member 9Bb [Source:ZFIN;Acc:ZDB-GENE-091112-15]	21	24703188	24865454	
ENSDARG00000069560	128.6957469	2.315308564	0.269228075	4.885480701	1.03E-06	1.66E-05	kcnh3	potassium voltage-gated channel, subfamily H (eag-related), member 3 [Source:ZFIN;Acc:ZDB-GENE-070912-23]	9	27909927	28103097	
ENSDARG00000069583	9418.023087	-1.574094537	0.125166729	-4.586638498	4.5E-06	6.64E-05	zgc:114181	zgc:114181 [Source:ZFIN;Acc:ZDB-GENE-050913-105]	16	55003121	55028791	
ENSDARG00000069600	1872.519501	2.528967913	0.135354965	11.29598688	1.37E-29	1.32E-27	zgc:109889	zgc:109889 [Source:ZFIN;Acc:ZDB-GENE-050522-547]	7	23495986	23543642	
ENSDARG00000069630	981.3578203	-1.414531343	0.141755927	-2.9242611	0.00345275	0.029599548	tat	tyrosine aminotransferase [Source:ZFIN;Acc:ZDB-GENE-030131-1144]	18	13162728	13179510	
ENSDARG00000069737	1500.112481	2.09518689	0.133914509	8.178254148	2.88E-16	1.16E-14	pou4f2	POU class 4 homeobox 2 [Source:ZFIN;Acc:ZDB-GENE-040401-1]	1	36436936	36440301	
ENSDARG00000069755	825.3208124	1.493943282	0.11690142	4.225297555	2.39E-05	0.000307955	mtmr11	myotubularin related protein 11 [Source:ZFIN;Acc:ZDB-GENE-130530-726]	16	46000956	46091600	
ENSDARG00000069767	273.821707	2.312778846	0.196041325	6.696439351	2.14E-11	5.86E-10	nectin1a	nectin cell adhesion molecule 1a [Source:ZFIN;Acc:ZDB-GENE-090224-1]	21	22589577	22635262	
ENSDARG00000069828	717.227508	-2.320691073	0.15941793	-8.284457554	1.19E-16	4.96E-15	si:ch211-117n7.7	si:ch211-117n7.7 [Source:ZFIN;Acc:ZDB-GENE-060503-569]	13	9878544	9886579	
ENSDARG00000069846	2622.711246	-1.551577428	0.104784031	-5.263945494	1.41E-07	2.5E-06	zgc:162944	zgc:162944 [Source:ZFIN;Acc:ZDB-GENE-070424-75]	9	21826724	21831588	
ENSDARG00000069878	1018.685215	-1.35504202	0.118588459	-2.993900271	0.00275436	0.024156946	snu13a	SNU13 homolog, small nuclear ribonucleoprotein a (U4/U6.U5) [Source:ZFIN;Acc:ZDB-GENE-040426-879]	3	58158595	58165254	
ENSDARG00000069956	1300.486095	-1.738274722	0.126387693	-5.841349757	5.18E-09	1.11E-07	pdlim2	PDZ and LIM domain 2 (mystique) [Source:ZFIN;Acc:ZDB-GENE-070308-4]	8	38190233	38317914	
ENSDARG00000069981	5538.831619	2.343871747	0.11281706	11.91195506	1.03E-32	1.11E-30	cspg5a	chondroitin sulfate proteoglycan 5a [Source:ZFIN;Acc:ZDB-GENE-080425-4]	16	42319438	42390640	
ENSDARG00000070021	319.6648606	-2.857895105	0.183891765	-10.10319903	5.35E-24	3.71E-22	cyp3c4	cytochrome P450, family 3, subfamily C, polypeptide 4 [Source:ZFIN;Acc:ZDB-GENE-060929-96]	3	40936094	40945724	
ENSDARG00000070029	646.5893741	-1.577342121	0.160964384	-3.586769363	0.0003348	0.003535473	ehhadh	enoyl-CoA, hydratase/3-hydroxyacyl CoA dehydrogenase [Source:NCBI gene;Acc:100000859]	9	12428226	12443726	
ENSDARG00000070038	8655.302983	-3.22087239	0.141915283	-15.64928276	3.36E-55	9.37E-53	rbp2a	retinol binding protein 2a, cellular [Source:ZFIN;Acc:ZDB-GENE-020320-2]	15	5803458	5815006	
ENSDARG00000070039	7.650270163	5.986626801	1.685228003	2.959022039	0.00308617	0.026758536	AL935184.1		1	25709813	25711803	
ENSDARG00000070045	1065.814107	2.347286144	0.176434574	7.636179889	2.24E-14	7.84E-13	celf4	CUGBP, Elav-like family member 4 [Source:NCBI gene;Acc:436835]	21	12079878	12272543	

ENSDARG00000070056	6.488861982	5.75072748	1.646617693	2.885142981	0.003912359	0.033165867	adora1a	adenosine A1 receptor a [Source:ZFIN;Acc:ZDB-GENE-141216-102]	11	14667066	14669026	
ENSDARG00000070057	1903.766643	-2.351997234	0.103457693	-13.06811697	5.01E-39	7.4E-37	si:dkey-69o16.5	si:dkey-69o16.5 [Source:ZFIN;Acc:ZDB-GENE-081104-432]	9	11587629	11595736	
ENSDARG00000070074	1512.303403	1.578359958	0.161961476	3.570972389	0.000355658	0.003733744	lrp8	low density lipoprotein receptor-related protein 8, apolipoprotein e receptor [Source:ZFIN;Acc:ZDB-GENE-050506-134]	6	34511886	34811508	
ENSDARG00000070078	684.400215	-2.442983071	0.194391235	-7.423087117	1.14E-13	3.81E-12	abcb11b	ATP-binding cassette, sub-family B (MDR/TAP), member 11b [Source:ZFIN;Acc:ZDB-GENE-050517-14]	11	13223625	13272085	
ENSDARG00000070080	3700.611758	2.286097668	0.104064672	12.3586385	4.38E-35	5.36E-33	nbeaa	neurobeachin a [Source:ZFIN;Acc:ZDB-GENE-050320-68]	10	34426256	34734995	
ENSDARG00000070107	1101.477108	2.224810097	0.186822086	6.55602409	5.53E-11	1.45E-09	six7	SIX homeobox 7 [Source:ZFIN;Acc:ZDB-GENE-990825-28]	7	7401692	7420301	
ENSDARG00000070145	1716.031799	1.561423641	0.113080064	4.964833051	6.88E-07	1.13E-05	magi1a	membrane associated guanylate kinase, WW and PDZ domain containing 1a [Source:ZFIN;Acc:ZDB-GENE-081105-13]	8	54055390	54072665	
ENSDARG00000070157	857.5168682	-1.972045915	0.144152498	-6.743177751	1.55E-11	4.33E-10	tgm2a	transglutaminase 2, C polypeptide A [Source:ZFIN;Acc:ZDB-GENE-040912-78]	23	43235328	43272125	
ENSDARG00000070162	2938.127545	1.829789326	0.091607073	9.058136029	1.33E-19	6.9E-18	stox2a	storkhead box 2a [Source:ZFIN;Acc:ZDB-GENE-090313-101]	1	39898327	39977551	
ENSDARG00000070170	284.4631252	-1.804589446	0.205440612	-3.916408926	8.99E-05	0.001052249	si:ch211-225p5.8	si:ch211-225p5.8 [Source:ZFIN;Acc:ZDB-GENE-060503-783]	16	41974438	41984133	
ENSDARG00000070178	44.82836413	3.859537801	0.573784215	4.983646684	6.24E-07	1.03E-05	zp3d.2	zona pellucida glycoprotein 3d tandem duplicate 2 [Source:ZFIN;Acc:ZDB-GENE-030131-5363]	16	42048362	42056137	
ENSDARG00000070220	698.6416138	1.830561391	0.154949179	5.360218072	8.31E-08	1.52E-06	pou3f2a	POU class 3 homeobox 2a [Source:ZFIN;Acc:ZDB-GENE-980526-139]	4	5741733	5744457	
ENSDARG00000070387	167.7618909	-1.579243439	0.195566736	-2.961870978	0.003057759	0.026548878	nudt7	nudix (nucleoside diphosphate linked moiety X)-type motif 7 [Source:ZFIN;Acc:ZDB-GENE-131127-212]	25	37282088	37284370	
ENSDARG00000070437	20950.98711	-1.417340203	0.113455758	-3.678440038	0.000234665	0.002547717	rpl22	ribosomal protein L22 [Source:ZFIN;Acc:ZDB-GENE-051113-276]	11	29650011	29658396	
ENSDARG00000070442	2641.31282	2.167796816	0.152573043	7.654018003	1.95E-14	6.89E-13	si:ch211-113g11.6	si:ch211-113g11.6 [Source:ZFIN;Acc:ZDB-GENE-060503-473]	16	29407666	29452039	
ENSDARG00000070467	283.9370783	2.538515407	0.192467708	7.99362878	1.31E-15	5.1E-14	lactbl1b	lactamase, beta-like 1b [Source:ZFIN;Acc:ZDB-GENE-061207-53]	11	28127199	28157129	
ENSDARG00000070484	4149.615803	-1.995880741	0.09146711	-10.88785619	1.32E-27	1.11E-25	zgc:195001	zgc:195001 [Source:ZFIN;Acc:ZDB-GENE-081022-173]	3	30202863	30218504	
ENSDARG00000070522	115.861641	2.629857507	0.300804752	5.418323673	6.02E-08	1.12E-06	cacna1ia	calcium voltage-gated channel subunit alpha1 Ia [Source:ZFIN;Acc:ZDB-GENE-060503-324]	3	29082267	29378430	
ENSDARG00000070537	2960.022413	1.591983208	0.106855692	5.540025033	3.02E-08	5.88E-07	stmn2b	stathmin 2b [Source:ZFIN;Acc:ZDB-GENE-050522-207]	19	31771270	31790551	
ENSDARG00000070543	1135.002534	2.744463304	0.219427863	13.4782671	2.1E-41	3.44E-39	grin2ab	glutamate receptor, ionotropic, N-methyl D-aspartate 2A, b [Source:ZFIN;Acc:ZDB-GENE-070424-223]	1	8763429	8928841	
ENSDARG00000070555	738.6684295	1.416755207	0.123511083	3.374233269	0.000740216	0.007286339	ldlrad4a	low density lipoprotein receptor class A domain containing 4a [Source:ZFIN;Acc:ZDB-GENE-060503-850]	19	12444943	12622855	
ENSDARG00000070558	277.5029275	-3.289086007	0.205328366	-11.14841584	7.29E-29	6.78E-27	si:ch211-93g23.2	si:ch211-93g23.2 [Source:ZFIN;Acc:ZDB-GENE-090312-164]	1	6640437	6646356	
ENSDARG00000070560	5125.367672	-1.278477937	0.081596817	-3.412852942	0.000642866	0.006383143	nap1l4a	nucleosome assembly protein 1-like 4a [Source:ZFIN;Acc:ZDB-GENE-030131-9099]	25	23553532	23583173	
ENSDARG00000070567	4099.522268	1.917657496	0.128403214	7.146686368	8.89E-13	2.76E-11	cadpsb	Ca2+-dependent activator protein for secretion b [Source:ZFIN;Acc:ZDB-GENE-110621-2]	6	41255485	41446053	
ENSDARG00000070584	1548.108082	-1.989171182	0.154252914	-6.412658014	1.43E-10	3.65E-09	clic5b	chloride intracellular channel 5b [Source:ZFIN;Acc:ZDB-GENE-040426-2542]	20	43925266	43968859	
ENSDARG00000070604	1087.136091	-1.310912871	0.103271611	-3.010632524	0.002607042	0.022961175	zgc:162509	zgc:162509 [Source:ZFIN;Acc:ZDB-GENE-050809-100]	16	13965923	13976935	
ENSDARG00000070607	1005.170819	-1.406449269	0.107857765	-3.768382072	0.000164309	0.00183885	mrpl36	mitochondrial ribosomal protein L36 [Source:NCBI gene;Acc:100093703]	19	28284120	28290213	
ENSDARG00000070620	716.7470257	1.493682222	0.160645796	3.073110113	0.002118403	0.018963636	grin2db	glutamate receptor, ionotropic, N-methyl D-aspartate 2D, b [Source:ZFIN;Acc:ZDB-GENE-100920-7]	16	13179146	13388821	
ENSDARG00000070626	1002.320861	1.543946597	0.124517142	4.368447507	1.25E-05	0.00017117	cacng8b	calcium channel, voltage-dependent, gamma subunit 8b [Source:ZFIN;Acc:ZDB-GENE-100921-72]	16	12882381	12953739	
ENSDARG00000070677	379.6161696	2.097254865	0.182243366	6.020821975	1.74E-09	3.88E-08	fezf2	FEZ family zinc finger 2 [Source:NCBI gene;Acc:60634]	11	20056732	20059407	
ENSDARG00000070726	669.6740738	1.568057559	0.176800169	3.212992169	0.001313599	0.012280313	cnga3a	cyclic nucleotide gated channel subunit alpha 3a [Source:ZFIN;Acc:ZDB-GENE-090611-2]	6	38791900	38818582	
ENSDARG00000070775	850.1973624	-3.478105648	0.153616324	-16.1317859	1.53E-58	4.73E-56	cyp2x9	cytochrome P450, family 2, subfamily X, polypeptide 9 [Source:ZFIN;Acc:ZDB-GENE-061027-109]	25	17396263	17403598	
ENSDARG00000070835	128202.8332	-1.339685829	0.123402976	-2.752655074	0.005911414	0.047849858	tnnc2	troponin C type 2 (fast) [Source:ZFIN;Acc:ZDB-GENE-000322-2]	23	20422661	20427299	
ENSDARG00000070858	112.4073907	-2.050062927	0.247808937	-4.237389252	2.26E-05	0.000293951	si:busm1-105l16.2	si:busm1-105l16.2 [Source:ZFIN;Acc:ZDB-GENE-030616-108]	1	46500436	46505310	
ENSDARG00000070864	684.6275574	2.190102402	0.139345798	8.540640758	1.33E-17	6.04E-16	bcl6aa	BCL6A transcription repressor a [Source:ZFIN;Acc:ZDB-GENE-040426-1696]	6	28216487	28222592	
ENSDARG00000070892	98.0935223	-1.962314251	0.268452585	-3.584671211	0.000337503	0.00356102	si:ch211-262h13.5	si:ch211-262h13.5 [Source:ZFIN;Acc:ZDB-GENE-050208-372]	22	38812549	38819603	
ENSDARG00000070929	526.8765708	1.579963366	0.173260651	3.347346115	0.000815893	0.007934802	sox14	SRY-box transcription factor 14 [Source:NCBI gene;Acc:557661]	6	26557979	26559921	
ENSDARG00000070931	147.7411819	2.968528655	0.269303875	7.309693011	2.68E-13	8.64E-12	si:ch211-232m10.6	si:ch211-232m10.6 [Source:ZFIN;Acc:ZDB-GENE-060503-879]	19	10356345	10373630	
ENSDARG00000070941	4.92438598	-6.174245884	1.584631344	-3.265267914	0.001093606	0.010374377	igfbp6a	insulin-like growth factor binding protein 6a [Source:ZFIN;Acc:ZDB-GENE-090521-1]	11	2391469	2397974	
ENSDARG00000070960	410.5252415	-2.392264812	0.156073047	-8.920597388	4.64E-19	2.33E-17	si:ch211-288g17.4	si:ch211-288g17.4 [Source:ZFIN;Acc:ZDB-GENE-060531-62]	19	9344171	9364342	
ENSDARG00000070981	4509.053113	1.570934185	0.126499678	4.513325188	6.38E-06	9.17E-05	ash1l	ash1 (absent, small, or homeotic)-like (Drosophila) [Source:ZFIN;Acc:ZDB-GENE-030131-6101]	19	9032073	9087619	
ENSDARG00000071005	471.1193156	-1.554028378	0.144609657	-3.831199027	0.00012752	0.001454288	ppp1r3ca	protein phosphatase 1, regulatory subunit 3Ca [Source:ZFIN;Acc:ZDB-GENE-040718-70]	17	23311377	23314178	
ENSDARG00000071011	1932.684617	1.533135301	0.137319283	3.882450383	0.000103409	0.001197818	cdk5r2a	cyclin-dependent kinase 5, regulatory subunit 2a (p39) [Source:ZFIN;Acc:ZDB-GENE-050417-22]	6	13742899	13744681	
ENSDARG00000071036	330.0576906	-2.584135061	0.157738663	-10.04278234	9.88E-24	6.74E-22	si:dkey-286j15.3	si:dkey-286j15.3 [Source:ZFIN;Acc:ZDB-GENE-060503-635]	22	29990448	30000826	
ENSDARG00000071082	3916.969823	2.76673171	0.102092407	17.30522141	4.3E-67	1.61E-64	p4ha1b	prolyl 4-hydroxylase, alpha polypeptide I b [Source:ZFIN;Acc:ZDB-GENE-030131-4089]	17	20144626	20167236	
ENSDARG00000071143	62.4465863	2.720802019	0.413000595	4.16658484	3.09E-05	0.000392374	si:dkey-208m12.2	si:dkey-208m12.2 [Source:ZFIN;Acc:ZDB-GENE-060503-716]	22	27189979	27362439	
ENSDARG00000071230	475.3521067	2.232411271	0.161058689	7.651939062	1.98E-14	6.99E-13	lrfn5a	leucine rich repeat and fibronectin type III domain containing 5a [Source:ZFIN;Acc:ZDB-GENE-080327-17]	17	13664442	13780499	
ENSDARG00000071235	1600.133699	1.761224501	0.183439899	4.149721546	3.33E-05	0.000419459	si:ch211-117c9.5	si:ch211-117c9.5 [Source:ZFIN;Acc:ZDB-GENE-110408-2]	23	6795531	6841245	
ENSDARG00000071251	588.8621985	1.553668262	0.177573939	3.117959003	0.001821081	0.016490246	ppp1r18	protein phosphatase 1, regulatory subunit 18 [Source:ZFIN;Acc:ZDB-GENE-060503-350]	19	823945	840530	
ENSDARG00000071272	2949.035434	1.333920451	0.112089705	2.979046562	0.002891468	0.025236004	ncoa6	nuclear receptor coactivator 6 [Source:ZFIN;Acc:ZDB-GENE-080403-12]	23	2703044	2726590	
ENSDARG00000071306	77.74592533	-2.266394031	0.303974809	-4.166115059	3.1E-05	0.000392984	gip	gastric inhibitory polypeptide [Source:ZFIN;Acc:ZDB-GENE-070720-10]	12	20662426	20665865	
ENSDARG00000071325	356.7094449	1.879223625	0.171900426	5.114726267	3.14E-07	5.37E-06	cbarpb	CACN subunit beta associated regulatory protein b [Source:ZFIN;Acc:ZDB-GENE-050208-507]	22	18786797	18824801	
ENSDARG00000071336	1038.991025	-1.490993611	0.130400475	-3.76527472	0.000166366	0.001860113	mif	macrophage migration inhibitory factor [Source:ZFIN;Acc:ZDB-GENE-080708-1]	14	48960078	48970210	
ENSDARG00000071375	566.2113398	2.539365862	0.154987165	9.932215093	3.01E-23	1.95E-21	celf5a	cugbp, Elav-like family member 5a [Source:ZFIN;Acc:ZDB-GENE-050208-746]	22	19665617	20011604	
ENSDARG00000071377	1444.841305	-1.667079609	0.119914712	-5.562950529	2.65E-08	5.19E-07	hsd11b1la	Danio rerio Hydroxysteroid 11-beta-dehydrogenase 1-like protein-like (LOC100006793), mRNA. [Source:RefSeq mRNA;Acc	22	19552987	19562455	
ENSDARG00000071403	3589.458322	2.483563588	0.1944765	7.628497971	2.38E-14	8.29E-13	oaz1a	Danio rerio ornithine decarboxylase antizyme 1a (oaz1a), mRNA. [Source:RefSeq mRNA;Acc:NM_194264]	22	20714624	20720427	
ENSDARG00000071429	1117.143716	-2.601102983	0.143993247	-11.11929216	1.01E-28	9.33E-27	tdo2a	tryptophan 2,3-dioxygenase a [Source:NCBI gene;Acc:100008541]	14	36738063	36750593	
ENSDARG00000071445	10190.25746	-1.468681858	0.100386561	-4.668770918	3.03E-06	4.56E-05	myoz1b	myozenin 1b [Source:ZFIN;Acc:ZDB-GENE-040718-146]	12	26385096	26408876	
ENSDARG00000071468	1294.993051	-1.423504469	0.108989699	-3.885729324	0.000102023	0.001183402	mrpl54	mitochondrial ribosomal protein L54 [Source:NCBI gene;Acc:100034614]	22	15719347	15720347	
ENSDARG00000071475	1955.213873	-1.290350525	0.09354904	-3.103725344	0.001911006	0.017248534	aox5	aldehyde oxidase 5 [Source:ZFIN;Acc:ZDB-GENE-001205-2]	22	14083729	14115316	
ENSDARG00000071495	18.7391058	-3.103967854	0.604848253	-3.4785053	0.000504218	0.005134437	sult3st5	sulfotransferase family 3, cytosolic sulfotransferase 5 [Source:ZFIN;Acc:ZDB-GENE-060503-722]	22	15323930	15327809	
ENSDARG00000071500	288.826017	-1.719337676	0.161183242	-4.462856489	8.09E-06	0.000114175	fam207a	family with sequence similarity 207 member A [Source:ZFIN;Acc:ZDB-GENE-060825-113]	22	12699121	12726556	
ENSDARG00000071567	885.5163826	-1.874471594	0.110605577	-7.906216081	2.65E-15	1.01E-13	TSTD1	si:dkey-34l15.1 [Source:ZFIN;Acc:ZDB-GENE-081107-63]	14	29769332	29774035	TSTD1
ENSDARG00000071579	198.3962228	-2.620151841	0.205805525	-7.872246564	3.48E-15	1.3E-13	urad	ureidoimidazoline (2-oxo-4-hydroxy-4-carboxy-5-) decarboxylase [Source:NCBI gene;Acc:557735]	24	21687276	21689146	
ENSDARG00000071592	1539.120848	-2.301876482	0.1215324	-10.71217621	8.92E-27	7.13E-25	aqp8a.2	aquaporin 8a, tandem duplicate 2 [Source:ZFIN;Acc:ZDB-GENE-070112-1802]	12	20354691	20362061	
ENSDARG00000071601	3323.696228	-2.054281631	0.11528414	-9.145070906	5.96E-20	3.18E-18	pvalb9	parvalbumin 9 [Source:ZFIN;Acc:ZDB-GENE-030805-4]	12	17695986	17698669	
ENSDARG00000071629	11.33495945	6.554789584	1.666472388	3.3332623	0.00085834	0.008308981	BX511034.1		22	7542472	7563593	
ENSDARG00000071652	580.4216989	-1.698895501	0.140692024	-4.967555962	6.78E-07	1.11E-05	rrp8	ribosomal RNA processing 8 [Source:ZFIN;Acc:ZDB-GENE-030131-8898]	15	15221123	15230264	
ENSDARG00000071670	4121.72873	-1.373510933	0.088301302	-4.229959533	2.34E-05	0.000303201	tma7	translation machinery associated 7 homolog [Source:NCBI gene;Acc:768290]	22	5663529	5668138	
ENSDARG00000071684	699.2091898	2.058196299	0.168802595	6.268839067	3.64E-10	8.79E-09	rx1	retinal homeobox gene 1 [Source:NCBI gene;Acc:30472]	22	4997784	5006801	
ENSDARG00000071709	1329.256692	1.483948207	0.172760998	2.801258465	0.005090373	0.041934183	ppp1r9bb	protein phosphatase 1, regulatory subunit 9Bb [Source:ZFIN;Acc:ZDB-GENE-121214-117]	12	9703172	9774747	
ENSDARG00000071823	258.1482394	1.57352037	0.201610381	2.844696625	0.004445374	0.037095523	adarb2	adenosine deaminase RNA specific B2 (inactive) [Source:ZFIN;Acc:ZDB-GENE-060503-160]	24	3537762	3783497	
ENSDARG00000071877	2910.206119	-1.469934739	0.117551641	-3.997687619	6.4E-05	0.000768582	dhrs7cb	dehydrogenase/reductase (SDR family) member 7Cb [Source:NCBI gene;Acc:553684]	12	455552	464007	
ENSDARG00000073695	222.4499023	-1.714266297	0.20541825	-3.477131646	0.000506809	0.005154542	mamdc2b	MAM domain containing 2b [Source:ZFIN;Acc:ZDB-GENE-091117-16]	10	15648857	15672862	
ENSDARG00000073704	1948.121541	1.41672949	0.117839547	3.536414577	0.000405598	0.004203512	si:dkeyp-72g9.4	si:dkeyp-72g9.4 [Source:ZFIN;Acc:ZDB-GENE-031204-23]	21	32476709	32487084	
ENSDARG00000073707	934.2923889	2.608515015	0.139205133	11.55499784	6.96E-31	7.11E-29	cnksr2b	connector enhancer of kinase suppressor of Ras 2b [Source:ZFIN;Acc:ZDB-GENE-030131-8048]	21	25421368	25522906	
ENSDARG00000073728	288.0222672	1.862470804	0.232962043	3.702194536	0.000213743	0.002332676	gal3st3	galactose-3-O-sulfotransferase 3 [Source:ZFIN;Acc:ZDB-GENE-121001-3]	7	7151832	7249789	
ENSDARG00000073741	554.2207658	1.462563027	0.135257131	3.419879029	0.00062649	0.006240287	RAP1GDS1	RAP1, GTP-GDP dissociation stimulator 1 [Source:NCBI gene;Acc:100334870]	7	69528850	69584923	RAP1GDS1
ENSDARG00000073742	23271.24492	-3.250454431	0.087825468	-25.62416658	8.21E-145	8.96E-142	prss59.2	serine protease 59, tandem duplicate 2 [Source:ZFIN;Acc:ZDB-GENE-110408-10]	16	26017360	26018694	
ENSDARG00000073769	298.1061046	2.946453281	0.196609806	9.900082426	4.16E-23	2.66E-21	magi2b	membrane associated guanylate kinase, WW and PDZ domain containing 2b [Source:ZFIN;Acc:ZDB-GENE-141211-6]	25	20743118	20985155	
ENSDARG00000073824	647.3921669	2.019674243	0.162435659	6.277403908	3.44E-10	8.36E-09	RASGRF1	si:ch211-234p18.3 [Source:ZFIN;Acc:ZDB-GENE-090311-28]	18	26337869	26418637	RASGRF1
ENSDARG00000073843	1898.26285	1.706800971	0.118889614	5.945018645	2.76E-09	6.08E-08	myo9ab	myosin IXAb [Source:NCBI gene;Acc:562199]	25	485353	554142	
ENSDARG00000073876	119.7150608	-3.115658446	0.253062952	-8.360206147	6.26E-17	2.66E-15	si:ch1073-165f9.2	si:ch1073-165f9.2 [Source:ZFIN;Acc:ZDB-GENE-141216-228]	11	42474694	42477772	
ENSDARG00000073883	5566.628798	2.002547863	0.108864756	9.209113231	3.29E-20	1.79E-18	clstn3	calsyntenin 3 [Source:ZFIN;Acc:ZDB-GENE-100921-73]	16	12102336	12173647	
ENSDARG00000073914	148.8809724	2.394036196	0.244293309	5.706403512	1.15E-08	2.35E-07	fam163ba	family with sequence similarity 163 member B, genome duplicate a [Source:ZFIN;Acc:ZDB-GENE-091204-96]	10	10636237	10700769	
ENSDARG00000073944	3856.845613	2.108508425	0.099882838	11.09808693	1.28E-28	1.17E-26	si:ch73-386h18.1	si:ch73-386h18.1 [Source:ZFIN;Acc:ZDB-GENE-050309-237]	18	6039141	6083792	
ENSDARG00000073948	79.93054307	2.440557003	0.332299915	4.335110957	1.46E-05	0.000197562	prok1	prokineticin 1 [Source:ZFIN;Acc:ZDB-GENE-070928-34]	8	24714733	24730185	
ENSDARG00000073957	790.1201861	1.446749011	0.118634598	3.765756534	0.000166046	0.001857357	tox3	TOX high mobility group box family member 3 [Source:ZFIN;Acc:ZDB-GENE-090312-209]	7	36898622	36969256	
ENSDARG00000074030	3633.643651	1.878780263	0.121613107	7.226032472	4.97E-13	1.56E-11	myt1a	myelin transcription factor 1a [Source:ZFIN;Acc:ZDB-GENE-030131-3885]	23	30767310	30787932	
ENSDARG00000074064	328.0359835	1.726163857	0.166602451	4.358662519	1.31E-05	0.000178323	rem2	RAS (RAD and GEM)-like GTP binding 2 [Source:ZFIN;Acc:ZDB-GENE-081110-1]	2	38924975	38977298	
ENSDARG00000074070	291.1714227	1.503005586	0.17176058	2.928527529	0.003405717	0.029236284	irx5b	iroquois homeobox 5b [Source:ZFIN;Acc:ZDB-GENE-040628-5]	25	36152215	36157484	
ENSDARG00000074118	163.6072013	-1.888849637	0.221178179	-4.018704022	5.85E-05	0.000709112	ftr12	finTRIM family, member 12 [Source:ZFIN;Acc:ZDB-GENE-070912-196]	2	43144938	43151358	
ENSDARG00000074119	243.557648	2.593353154	0.237996014	6.694873267	2.16E-11	5.92E-10	frmpd2	FERM and PDZ domain containing 2 [Source:ZFIN;Acc:ZDB-GENE-110315-2]	12	2522642	2568460	
ENSDARG00000074153	33.53233936	2.537139068	0.519077558	2.961289781	0.003063535	0.026589836	slitrk5b	SLIT and NTRK-like family, member 5b [Source:ZFIN;Acc:ZDB-GENE-110408-55]	9	53535419	53537989	
ENSDARG00000074191	450.0771747	-2.435318085	0.196837422	-7.291896363	3.06E-13	9.76E-12	zgc:172253	zgc:172253 [Source:ZFIN;Acc:ZDB-GENE-080215-9]	3	1030479	1036544	
ENSDARG00000074217	94.93885396	-1.968917865	0.268358291	-3.610538215	0.000305562	0.003249966	si:dkey-188i13.6	si:dkey-188i13.6 [Source:ZFIN;Acc:ZDB-GENE-030131-7410]	13	37609458	37615029	
ENSDARG00000074221	636.3786041	1.506754538	0.128991048	3.928602393	8.54E-05	0.001005935	ABCA7	zgc:172302 [Source:ZFIN;Acc:ZDB-GENE-081205-5]	11	5588122	5659681	ABCA7
ENSDARG00000074245	3621.875226	1.687514557	0.092760795	7.411693261	1.25E-13	4.13E-12	spen	spen family transcriptional repressor [Source:ZFIN;Acc:ZDB-GENE-050309-70]	23	24436273	24483440	
ENSDARG00000074319	1789.715518	1.900651987	0.106343894	8.469240219	2.47E-17	1.1E-15	sall1a	spalt-like transcription factor 1a [Source:ZFIN;Acc:ZDB-GENE-020228-2]	7	37359004	37392172	
ENSDARG00000074320	194.2575778	1.701399782	0.210866393	3.326275815	0.000880148	0.008510244	KCNAB3	voltage-gated potassium channel subunit beta-2-like [Source:NCBI gene;Acc:100334158]	5	68112194	68190264	KCNAB3
ENSDARG00000074328	1500.720521	1.556203375	0.130839195	4.251045514	2.13E-05	0.000277159	apba1b	amyloid beta (A4) precursor protein-binding, family A, member 1b [Source:ZFIN;Acc:ZDB-GENE-070925-3]	12	15777064	15813279	
ENSDARG00000074329	623.948135	1.786450278	0.136721348	5.752212732	8.81E-09	1.82E-07	arvcfa	ARVCF delta catenin family member a [Source:ZFIN;Acc:ZDB-GENE-091113-43]	10	466926	505370	
ENSDARG00000074345	232.0233439	4.29079986	0.299578265	10.98477507	4.52E-28	3.92E-26	si:ch211-214b16.4	si:ch211-214b16.4 [Source:ZFIN;Acc:ZDB-GENE-081105-53]	3	60338988	60401826	
ENSDARG00000074363	763.6474993	1.74457383	0.153019131	4.865887179	1.14E-06	1.82E-05	TTC9	si:ch211-259k16.3 [Source:ZFIN;Acc:ZDB-GENE-090312-172]	13	36144341	36153572	TTC9
ENSDARG00000074376	206.980868	1.561813673	0.190086742	2.955564746	0.003120972	0.027013625	mdga1	MAM domain containing glycosylphosphatidylinositol anchor 1 [Source:ZFIN;Acc:ZDB-GENE-130530-751]	13	44299676	44630111	
ENSDARG00000074378	1155.348754	2.739727562	0.129189025	13.46652757	2.46E-41	4.01E-39	junba	JunB proto-oncogene, AP-1 transcription factor subunit a [Source:ZFIN;Acc:ZDB-GENE-040426-2172]	1	51732979	51734535	
ENSDARG00000074419	116.089822	2.431151103	0.312367049	4.581632747	4.61E-06	6.78E-05	hcn4l	hyperpolarization activated cyclic nucleotide-gated potassium channel 4l [Source:ZFIN;Acc:ZDB-GENE-110411-3]	25	29211792	29316193	
ENSDARG00000074457	40.68586012	-5.302042635	0.647033592	-6.648870613	2.95E-11	7.95E-10	si:dkey-96f10.1	si:dkey-96f10.1 [Source:ZFIN;Acc:ZDB-GENE-090312-154]	23	12682353	12788262	
ENSDARG00000074466	478.2866748	-1.379894986	0.138531172	-2.742306875	0.006100931	0.049256829	gdpd3a	glycerophosphodiester phosphodiesterase domain containing 3a [Source:ZFIN;Acc:ZDB-GENE-040426-2279]	3	26193592	26204867	
ENSDARG00000074476	113.9781044	2.984005949	0.326662288	6.073569013	1.25E-09	2.83E-08	odam	odontogenic, ameloblast associated [Source:ZFIN;Acc:ZDB-GENE-090313-260]	1	43958198	43963441	
ENSDARG00000074502	3597.9279	1.468173468	0.105419864	4.441036544	8.95E-06	0.000125123	zmiz1a	zinc finger, MIZ-type containing 1a [Source:ZFIN;Acc:ZDB-GENE-080220-27]	13	30421472	30570859	
ENSDARG00000074507	3183.468272	-1.431550187	0.101062273	-4.270141307	1.95E-05	0.000257129	rmdn1	regulator of microtubule dynamics 1 [Source:ZFIN;Acc:ZDB-GENE-070928-17]	24	42141313	42150116	
ENSDARG00000074519	154.5011843	1.790866483	0.275241358	2.873356271	0.004061358	0.034278465	BX248501.1		11	6205969	6224120	
ENSDARG00000074535	433.3525016	2.101287944	0.155666954	7.07464182	1.5E-12	4.56E-11	lingo2a	leucine rich repeat and Ig domain containing 2a [Source:ZFIN;Acc:ZDB-GENE-080327-19]	14	44545092	44591724	
ENSDARG00000074540	428.7448539	1.591665239	0.166503452	3.553471315	0.000380183	0.003969619	gpr63	G protein-coupled receptor 63 [Source:ZFIN;Acc:ZDB-GENE-110411-183]	20	405811	411135	

ENSDARG00000074552	5557.436377	-1.412071871	0.100572196	-4.097274268	4.18E-05	0.000517167	ndufs7	NADH:ubiquinone oxidoreductase core subunit S7 [Source:ZFIN;Acc:ZDB-GENE-041111-261]	11	5842632	5852715	
ENSDARG00000074558	1342.274351	2.374031177	0.120816517	11.37287523	5.70E-30	5.6E-28	cntnap2b	contactin associated protein like 2b [Source:ZFIN;Acc:ZDB-GENE-070912-26]	2	50276755	50372467	
ENSDARG00000074563	249.5818111	1.568335026	0.181803583	3.12609365	0.001771451	0.016104744	gpr37a	G protein-coupled receptor 37a [Source:ZFIN;Acc:ZDB-GENE-131120-175]	25	27493444	27500288	
ENSDARG00000074581	2256.143135	1.854817471	0.138821535	6.157671943	7.38E-10	1.72E-08	add2	adducin 2 (beta) [Source:ZFIN;Acc:ZDB-GENE-080718-4]	5	13781968	13835556	
ENSDARG00000074613	473.8539101	-4.640349755	0.183866798	-19.79884244	3.05E-87	1.82E-84	si:ch211-240l19.6	si:ch211-240l19.6 [Source:ZFIN;Acc:ZDB-GENE-041210-328]	4	8039791	8040436	
ENSDARG00000074635	6927.353338	1.593925505	0.133881913	4.436189256	9.16E-06	0.000127546	abca1a	ATP-binding cassette, sub-family A (ABC1), member 1A [Source:ZFIN;Acc:ZDB-GENE-031006-12]	1	52560549	52628407	
ENSDARG00000074640	62.97398092	2.177086483	0.352315039	3.341005501	0.000834756	0.008096292	FIBCD1	si:dkeyp-51b7.3 [Source:ZFIN;Acc:ZDB-GENE-081105-148]	8	12451739	12468744	FIBCD1
ENSDARG00000074644	125.4657772	2.239389735	0.299869112	4.133102358	3.58E-05	0.000449856	spock1	SPARC (osteonectin), cwcv and kazal like domains proteoglycan 1 [Source:ZFIN;Acc:ZDB-GENE-110428-1]	14	2661646	2933266	
ENSDARG00000074669	149.766742	1.945124366	0.250804629	3.768368914	0.000164318	0.00183885	cacng4b	calcium channel, voltage-dependent, gamma subunit 4b [Source:ZFIN;Acc:ZDB-GENE-120104-6]	3	61078057	61099004	
ENSDARG00000074671	1281.615279	1.470554676	0.111845916	4.207169051	2.59E-05	0.000331505	nptx1l	neuronal pentraxin 1 like [Source:ZFIN;Acc:ZDB-GENE-040426-2418]	3	59453467	59472422	
ENSDARG00000074680	1612.191879	1.909364207	0.121824865	7.464520518	8.36E-14	2.81E-12	rims1a	regulating synaptic membrane exocytosis 1a [Source:ZFIN;Acc:ZDB-GENE-090312-135]	13	27631926	27767443	
ENSDARG00000074684	414.5134911	-1.825477067	0.150771758	-5.475011238	4.37E-08	8.32E-07	mlsl	malate synthase-like [Source:ZFIN;Acc:ZDB-GENE-130530-761]	14	46157379	46173265	
ENSDARG00000074690	270.8403418	2.241052996	0.189582352	6.546247503	5.9E-11	1.55E-09	ppm1j	protein phosphatase, Mg2+/Mn2+ dependent, 1J [Source:ZFIN;Acc:ZDB-GENE-091230-9]	6	48425366	48473694	
ENSDARG00000074698	364.4169668	-2.040114028	0.183042048	-5.682377571	1.33E-08	2.7E-07	sdsl	serine dehydratase-like [Source:ZFIN;Acc:ZDB-GENE-131121-410]	17	14605794	14613711	
ENSDARG00000074723	777.5549342	1.904525706	0.158396732	5.710507388	1.13E-08	2.3E-07	myo10l1	myosin X, like 1 [Source:ZFIN;Acc:ZDB-GENE-080425-1]	6	11101751	11227225	
ENSDARG00000074745	1588.370132	4.057588233	0.154454846	19.79600071	3.22E-87	1.88E-84	zmp:0000000760	zmp:0000000760 [Source:ZFIN;Acc:ZDB-GENE-130530-763]	13	1726953	1786145	
ENSDARG00000074761	259.3469947	1.522951248	0.189150569	2.764735256	0.005696901	0.046307615	bicdl1	BICD family like cargo adaptor 1 [Source:NCBI gene;Acc:100537719]	8	3701042	3773374	
ENSDARG00000074777	2157.163388	2.332312911	0.144220167	9.238048606	2.51E-20	1.38E-18	ank1b	ankyrin 1, erythrocytic b [Source:ZFIN;Acc:ZDB-GENE-091113-6]	21	21027375	21148623	
ENSDARG00000074781	566.3460619	2.864503338	0.1531589	12.17365321	4.29E-34	4.92E-32	ptprt	protein tyrosine phosphatase receptor type T [Source:ZFIN;Acc:ZDB-GENE-101028-4]	6	51020918	51386696	
ENSDARG00000074809	544.5175049	-1.614026271	0.145827848	-4.210624249	2.55E-05	0.00032731	zgc:194392	zgc:194392 [Source:ZFIN;Acc:ZDB-GENE-030131-4774]	17	30700627	30702411	
ENSDARG00000074813	121.6536992	-2.499157233	0.273067446	-5.490062089	4.02E-08	7.68E-07	btr05	bloodthirsty-related gene family, member 5 [Source:ZFIN;Acc:ZDB-GENE-080219-8]	7	16196903	16205471	
ENSDARG00000074839	439.1140973	2.269960621	0.178636757	7.109178654	1.17E-12	3.6E-11	impg1b	interphotoreceptor matrix proteoglycan 1b [Source:ZFIN;Acc:ZDB-GENE-101007-1]	17	36627099	36681740	
ENSDARG00000074860	528.090705	1.800393306	0.145511275	5.500558679	3.79E-08	7.27E-07	slc5a7a	solute carrier family 5 member 7a [Source:ZFIN;Acc:ZDB-GENE-090313-273]	1	34243650	34261993	
ENSDARG00000074866	1475.329535	1.920706172	0.206726191	4.453747084	8.44E-06	0.000118597	ptpn5	protein tyrosine phosphatase non-receptor type 5 [Source:ZFIN;Acc:ZDB-GENE-101028-3]	7	39624728	39661162	
ENSDARG00000074902	465.4033511	2.688251113	0.171514193	9.843215189	7.33E-23	4.64E-21	nol4la	nucleolar protein 4-like a [Source:ZFIN;Acc:ZDB-GENE-081105-118]	8	23252850	23328264	
ENSDARG00000074905	562.2541194	1.742564127	0.133995097	5.541726085	3E-08	5.83E-07	camk1da	calcium/calmodulin-dependent protein kinase 1Da [Source:ZFIN;Acc:ZDB-GENE-080402-7]	4	7888047	7983016	
ENSDARG00000074919	242.1905806	2.580650415	0.230366197	6.861468538	6.82E-12	1.96E-10	BFSP1	beaded filament structural protein 1 [Source:HGNC Symbol;Acc:HGNC:1040]	13	985442	992458	BFSP1
ENSDARG00000074921	1160.034509	2.182731438	0.166615285	7.098577034	1.26E-12	3.87E-11	fam117ba	family with sequence similarity 117 member Ba [Source:ZFIN;Acc:ZDB-GENE-131127-569]	6	12853655	12865827	
ENSDARG00000074949	15.85179578	-2.947113492	0.678830176	-2.868336678	0.004126362	0.034710391	c1qtnf7	C1q and TNF related 7 [Source:ZFIN;Acc:ZDB-GENE-080414-4]	23	2185397	2189062	
ENSDARG00000074983	182.5805739	9.12108496	1.435135862	5.6587569	1.52E-08	3.07E-07	jac9	jacalin 9 [Source:ZFIN;Acc:ZDB-GENE-080218-23]	7	8513993	8515550	
ENSDARG00000074984	92.96724056	2.293493445	0.305910573	4.228338474	2.35E-05	0.000304763		microtubule associated scaffold protein 2 [Source:HGNC Symbol;Acc:HGNC:20595]	15	3219134	3223448	
ENSDARG00000075015	1101.911846	-2.069738838	0.106685113	-10.02706757	1.16E-23	7.84E-22	soul5	heme-binding protein soul5 [Source:ZFIN;Acc:ZDB-GENE-110718-2]	3	53476155	53486169	
ENSDARG00000075017	823.0267559	-1.404822864	0.12394956	-3.266029049	0.001090671	0.010354358	myzap	myocardial zonula adherens protein [Source:ZFIN;Acc:ZDB-GENE-041111-91]	7	52389958	52417777	
ENSDARG00000075041	1662.664494	1.409762764	0.113440734	3.612130762	0.000303691	0.003232806	fmnl2b	formin-like 2b [Source:ZFIN;Acc:ZDB-GENE-041111-182]	6	13390289	13449556	
ENSDARG00000075054	328.5517275	2.758120538	0.196225038	8.959715613	3.26E-19	1.66E-17	rasgrf2a	Ras protein-specific guanine nucleotide-releasing factor 2a [Source:ZFIN;Acc:ZDB-GENE-111128-1]	10	3049636	3106762	
ENSDARG00000075058	286.3639017	1.622066057	0.195017604	3.189794383	0.001423741	0.013193037	gabra6a	gamma-aminobutyric acid (GABA) A receptor, alpha 6a [Source:ZFIN;Acc:ZDB-GENE-040426-1692]	14	22152060	22168786	
ENSDARG00000075116	753.6397624	2.055646487	0.134708164	7.836544219	4.63E-15	1.72E-13	sall2	spalt-like transcription factor 2 [Source:ZFIN;Acc:ZDB-GENE-030829-32]	2	38147761	38157339	
ENSDARG00000075126	311.7972002	2.436050281	0.180699047	7.94719343	1.91E-15	7.3E-14	TMEM8B	si:dkey-84j12.1 [Source:ZFIN;Acc:ZDB-GENE-060526-342]	5	43965078	44265523	TMEM8B
ENSDARG00000075133	2205.485499	2.012197675	0.128325256	7.887751041	3.08E-15	1.15E-13	adgrb1a	adhesion G protein-coupled receptor B1a [Source:ZFIN;Acc:ZDB-GENE-060607-12]	16	37470717	37630250	
ENSDARG00000075139	819.4595196	1.716222	0.126693776	5.653174296	1.58E-08	3.17E-07	hdac5	histone deacetylase 5 [Source:ZFIN;Acc:ZDB-GENE-121219-3]	3	22905341	22976456	
ENSDARG00000075158	2410.012011	1.704587029	0.098057127	7.185479406	6.7E-13	2.09E-11	igdcc3	immunoglobulin superfamily, DCC subclass, member 3 [Source:ZFIN;Acc:ZDB-GENE-080303-24]	7	31494741	31618211	
ENSDARG00000075159	1378.776793	-1.797852666	0.181449464	-4.397106764	1.1E-05	0.000151217	meltf	melanotransferrin [Source:ZFIN;Acc:ZDB-GENE-050309-40]	6	30091811	30109437	
ENSDARG00000075164	2138.99976	-1.305370556	0.107604014	-2.83791044	0.004540992	0.03779292	mylk5	myosin, light chain kinase 5 [Source:ZFIN;Acc:ZDB-GENE-050309-74]	3	31662126	31699298	
ENSDARG00000075189	1151.177698	1.973282024	0.151075181	6.442368737	1.18E-10	3.03E-09	cntnap5b	contactin associated protein-like 5b [Source:ZFIN;Acc:ZDB-GENE-070705-209]	22	13458735	13544244	
ENSDARG00000075222	1121.142258	1.418086021	0.109779737	3.808407938	0.000139864	0.00158785	amer2	APC membrane recruitment protein 2 [Source:NCBI gene;Acc:100101649]	24	25069609	25073032	
ENSDARG00000075261	1361.742127	1.437283185	0.12320721	3.549168794	0.000386449	0.004026795	timp2b	TIMP metallopeptidase inhibitor 2b [Source:ZFIN;Acc:ZDB-GENE-040426-2655]	3	57632965	57666996	
ENSDARG00000075282	2546.68719	2.141396411	0.104386089	10.93437278	7.9E-28	6.7E-26	irs2b	insulin receptor substrate 2b [Source:ZFIN;Acc:ZDB-GENE-081104-462]	9	8429239	8454060	
ENSDARG00000075295	823.3907623	2.168148175	0.158900782	7.351431236	1.96E-13	6.42E-12	tulp1a	TUB like protein 1a [Source:ZFIN;Acc:ZDB-GENE-110411-122]	23	5465806	5498539	
ENSDARG00000075321	100.3672979	2.480017084	0.329844218	4.487018424	7.22E-06	0.000102951	CABZ01071972.1		19	47578811	47587719	
ENSDARG00000075325	2938.96814	-1.336295413	0.103449757	-3.2508091	0.001150771	0.010855131	mrpl16	mitochondrial ribosomal protein L16 [Source:ZFIN;Acc:ZDB-GENE-041122-1]	1	524388	532429	
ENSDARG00000075352	152.8538835	1.628082516	0.224405655	2.798871162	0.005128159	0.042203912	brinp3b	bone morphogenetic protein/retinoic acid inducible neural-specific 3b [Source:ZFIN;Acc:ZDB-GENE-121022-1]	2	6734108	6807174	
ENSDARG00000075362	366.841001	1.959316515	0.166751781	5.752961137	8.77E-09	1.82E-07	whrna	whirlin a [Source:ZFIN;Acc:ZDB-GENE-091118-27]	21	13979033	14175838	
ENSDARG00000075376	720.0485245	2.053853634	0.179040894	5.886105758	3.95E-09	8.54E-08	sgsm1b	small G protein signaling modulator 1b [Source:ZFIN;Acc:ZDB-GENE-091117-39]	10	1638876	1675362	
ENSDARG00000075435	1757.580192	1.309660107	0.101817115	3.04133648	0.002355305	0.020883257	akap11	A kinase (PRKA) anchor protein 11 [Source:ZFIN;Acc:ZDB-GENE-090804-1]	9	17971935	18006632	
ENSDARG00000075444	1377.020092	-2.142341107	0.12810204	-8.917431019	4.77E-19	2.39E-17	cgref1	cell growth regulator with EF-hand domain 1 [Source:ZFIN;Acc:ZDB-GENE-131121-137]	17	32630789	32636533	
ENSDARG00000075491	1555.665993	2.507989343	0.128953419	11.69406248	1.37E-31	1.43E-29	si:ch211-26b3.4	si:ch211-26b3.4 [Source:ZFIN;Acc:ZDB-GENE-081104-60]	5	22871718	22952156	
ENSDARG00000075501	619.0710689	-1.816180659	0.187408284	-4.355093824	1.33E-05	0.000181057	cdnf	cerebral dopamine neurotrophic factor [Source:ZFIN;Acc:ZDB-GENE-070410-37]	4	9587968	9592402	
ENSDARG00000075517	160.8033926	-2.490240713	0.251496077	-5.925502828	3.11E-09	6.81E-08	si:ch211-120k19.1	si:ch211-120k19.1 [Source:ZFIN;Acc:ZDB-GENE-100922-282]	16	16968682	16975264	
ENSDARG00000075541	6.453532305	5.74416815	1.673564155	2.834769217	0.00458588	0.038128577	si:ch73-236c18.3	si:ch73-236c18.3 [Source:ZFIN;Acc:ZDB-GENE-121214-50]	1	58424507	58434252	
ENSDARG00000075542	5740.606852	2.337005678	0.093263874	14.33572953	1.31E-46	2.59E-44	zfhx4	zinc finger homeobox 4 [Source:ZFIN;Acc:ZDB-GENE-070530-3]	24	23241444	23323526	
ENSDARG00000075551	80.23334459	2.125419003	0.317145062	3.548593808	0.000387294	0.004032131	aifm5	apoptosis inducing factor mitochondria associated 5 [Source:ZFIN;Acc:ZDB-GENE-091118-96]	21	39302793	39327223	
ENSDARG00000075560	3670.229576	1.435438533	0.122251377	3.561829289	0.00036828	0.003856567	kmt2cb	lysine (K)-specific methyltransferase 2Cb [Source:ZFIN;Acc:ZDB-GENE-070705-340]	2	12847602	13037544	
ENSDARG00000075648	434.349889	2.124972434	0.162673275	6.915533197	4.66E-12	1.38E-10	sema5bb	sema domain, seven thrombospondin repeats (type 1 and type 1-like), transmembrane domain (TM) and short cytoplasmic	6	4033832	4146073	
ENSDARG00000075670	1567.889011	1.382200889	0.114916261	3.325907795	0.000881311	0.008514928	rereb	arginine-glutamic acid dipeptide (RE) repeats b [Source:ZFIN;Acc:ZDB-GENE-070212-3]	6	40671336	40689042	
ENSDARG00000075698	131.8797422	-2.091483212	0.255750341	-4.267768363	1.97E-05	0.000259471	nkpd1	NTPase, KAP family P-loop domain containing 1 [Source:ZFIN;Acc:ZDB-GENE-141215-19]	11	33284837	33291898	
ENSDARG00000075727	812.2215334	4.147004899	0.191690402	16.41712297	1.44E-60	4.64E-58	map1lc3cl	microtubule-associated protein 1 light chain 3 gamma, like [Source:ZFIN;Acc:ZDB-GENE-140819-1]	14	45406299	45422778	
ENSDARG00000075730	128.6089993	-2.333823704	0.226632175	-5.885411928	3.97E-09	8.57E-08	dnph1	2'-deoxynucleoside 5'-phosphate N-hydrolase 1 [Source:NCBI gene;Acc:561810]	13	4218329	4223955	
ENSDARG00000075754	1192.604607	1.39925264	0.103413317	3.860746864	0.000113041	0.001296227	mri1	methylthioribose-1-phosphate isomerase 1 [Source:NCBI gene;Acc:100002302]	1	55191150	55210619	
ENSDARG00000075766	276.5031589	-1.582077162	0.194636367	-2.990587892	0.00278441	0.024372882	sccpdha.1	saccharopine dehydrogenase a, tandem duplicate 1 [Source:ZFIN;Acc:ZDB-GENE-040718-51]	17	12159529	12175772	
ENSDARG00000075774	956.9579911	1.436679541	0.125410256	3.482008213	0.000497668	0.005073914	foxj3	forkhead box J3 [Source:ZFIN;Acc:ZDB-GENE-101005-1]	11	26228918	26362334	
ENSDARG00000075800	469.2925434	-2.662405629	0.174784853	-9.511153835	1.89E-21	1.11E-19	calml4a	calmodulin-like 4a [Source:ZFIN;Acc:ZDB-GENE-081022-9]	7	34492744	34496196	
ENSDARG00000075806	311.2891747	1.67735839	0.191411051	3.538763236	0.000402006	0.004171459	kirrel3a	kirre like nephrin family adhesion molecule 3a [Source:ZFIN;Acc:ZDB-GENE-091117-44]	10	39476432	39841419	
ENSDARG00000075815	468.3457246	3.168743603	0.193250113	11.22247001	3.16E-29	3E-27	slc12a5a	solute carrier family 12 member 5a [Source:ZFIN;Acc:ZDB-GENE-120927-3]	6	55449300	55585423	
ENSDARG00000075830	685.6166966	2.243135696	0.134826372	9.220271055	2.96E-20	1.62E-18	syt3	synaptotagmin III [Source:ZFIN;Acc:ZDB-GENE-090601-1]	7	30515365	30625219	
ENSDARG00000075833	696.5873902	1.946771401	0.148439696	6.378155062	1.79E-10	4.5E-09	lyve1a	lymphatic vessel endothelial hyaluronic receptor 1a [Source:ZFIN;Acc:ZDB-GENE-031118-138]	7	66822229	66833588	
ENSDARG00000075837	423.1553589	3.127401687	0.190677597	11.15706157	6.61E-29	6.17E-27	si:dkey-1d7.3	si:dkey-1d7.3 [Source:ZFIN;Acc:ZDB-GENE-091118-46]	21	18405585	18445953	
ENSDARG00000075849	3962.412969	1.76548576	0.098604912	7.76316053	8.28E-15	3.02E-13	prr12b	proline rich 12b [Source:ZFIN;Acc:ZDB-GENE-130625-2]	3	32425202	32461604	
ENSDARG00000075914	70.44657185	2.541271451	0.367426125	4.194779159	2.73E-05	0.000349085	kcnj14	potassium inwardly rectifying channel subfamily J member 14 [Source:ZFIN;Acc:ZDB-GENE-100922-48]	16	16824678	16839497	
ENSDARG00000075924	901.6781826	1.504916418	0.139075066	3.630531565	0.000282838	0.00303528	RAPGEF4	zgc:171558 [Source:ZFIN;Acc:ZDB-GENE-080219-4]	6	6850821	6922290	RAPGEF4
ENSDARG00000075928	1018.649825	1.924705616	0.175074031	5.281797724	1.28E-07	2.29E-06	hivep3a	HIVEP zinc finger 3a [Source:ZFIN;Acc:ZDB-GENE-081029-2]	19	15306968	15397946	
ENSDARG00000075931	3733.644434	-2.870282628	0.127927827	-14.6198264	2.1E-48	4.58E-46	acsl5	acyl-CoA synthetase long chain family member 5 [Source:ZFIN;Acc:ZDB-GENE-040912-169]	12	31438579	31461412	
ENSDARG00000075948	167.0078794	2.095815797	0.231143847	4.740839132	2.13E-06	3.29E-05	slco4a1	solute carrier organic anion transporter family, member 4A1 [Source:ZFIN;Acc:ZDB-GENE-060606-4]	23	7067196	7125494	
ENSDARG00000075949	250.2526223	1.696380001	0.198033307	3.516479173	0.000437311	0.00451726	prkd1	protein kinase D1 [Source:ZFIN;Acc:ZDB-GENE-080131-1]	17	28882977	28969194	
ENSDARG00000075956	578.213088	1.690975656	0.166006591	4.162338696	3.15E-05	0.000398937	faxca	failed axon connections homolog, metaxin like GST domain containing a [Source:ZFIN;Acc:ZDB-GENE-100422-7]	4	5750854	5764255	
ENSDARG00000075958	384.6915721	-3.502789292	0.174682209	-14.32767143	1.47E-46	2.88E-44	si:dkey-266f7.9	si:dkey-266f7.9 [Source:ZFIN;Acc:ZDB-GENE-070705-440]	19	8086491	8096984	
ENSDARG00000075960	190.6317978	2.233634141	0.227901519	5.413014112	6.2E-08	1.16E-06	cdh24a	cadherin 24, type 2a [Source:ZFIN;Acc:ZDB-GENE-090312-96]	23	40604951	40744851	
ENSDARG00000076001	545.981192	1.677204749	0.138346299	4.894997222	9.83E-07	1.58E-05	pip5k1ca	phosphatidylinositol-4-phosphate 5-kinase, type I, gamma a [Source:ZFIN;Acc:ZDB-GENE-050208-358]	22	17543607	17586112	
ENSDARG00000076011	1074.400296	-1.283940115	0.102504956	-2.770013534	0.005605396	0.04568216	PPM1K	si:ch211-149b19.3 [Source:ZFIN;Acc:ZDB-GENE-110411-37]	23	43809484	43831195	PPM1K
ENSDARG00000076043	79.15762709	5.574659315	0.736577435	6.21069707	5.28E-10	1.26E-08	si:dkeyp-73d8.9	si:dkeyp-73d8.9 [Source:ZFIN;Acc:ZDB-GENE-070912-685]	2	32642338	32643738	
ENSDARG00000076052	2070.578835	2.572582304	0.146107396	10.7631944	5.14E-27	4.17E-25	sez6l2	seizure related 6 homolog (mouse)-like 2 [Source:ZFIN;Acc:ZDB-GENE-091204-189]	3	15232181	15264732	
ENSDARG00000076055	460.5250824	1.527220182	0.15851475	3.326000766	0.000881017	0.008514928	rpgrip1	RPGR interacting protein 1 [Source:ZFIN;Acc:ZDB-GENE-091204-394]	24	7748751	7777389	
ENSDARG00000076056	285.8092761	-2.095599888	0.169302878	-6.47124195	9.72E-11	2.52E-09	IYD	si:ch211-286f9.2 [Source:ZFIN;Acc:ZDB-GENE-090313-109]	23	39774931	39784368	IYD
ENSDARG00000076076	185.126595	2.411783559	0.235038321	6.006610123	1.89E-09	4.22E-08	crtc1b	CREB regulated transcription coactivator 1b [Source:ZFIN;Acc:ZDB-GENE-041210-266]	11	6902306	6958042	
ENSDARG00000076122	98.51235617	-2.902038907	0.280730781	-6.77531299	1.24E-11	3.5E-10	si:ch211-160b11.4	si:ch211-160b11.4 [Source:ZFIN;Acc:ZDB-GENE-081104-142]	9	34978867	34986827	
ENSDARG00000076170	3105.155366	1.951352788	0.128839064	7.384039887	1.54E-13	5.06E-12	pcsk1nl	proprotein convertase subtilisin/kexin type 1 inhibitor, like [Source:ZFIN;Acc:ZDB-GENE-090427-2]	8	7359294	7379391	
ENSDARG00000076171	1835.58833	1.383388191	0.130079121	2.947346104	0.003205142	0.027627844	znf827	zinc finger protein 827 [Source:ZFIN;Acc:ZDB-GENE-080728-3]	1	36017867	36152131	
ENSDARG00000076192	1794.315775	-1.735760092	0.1095858	-6.71400943	1.89E-11	5.24E-10	ankrd1b	ankyrin repeat domain 1b (cardiac muscle) [Source:ZFIN;Acc:ZDB-GENE-070820-13]	12	16440708	16446373	
ENSDARG00000076213	966.3574051	1.979561927	0.184037687	5.322615956	1.02E-07	1.85E-06	DOCK4	si:dkey-81e3.1 [Source:ZFIN;Acc:ZDB-GENE-120703-34]	25	21517551	21716326	DOCK4
ENSDARG00000076221	430.6848952	-1.549740928	0.154420575	-3.560023831	0.000370821	0.003879942	fthl28	ferritin, heavy polypeptide-like 28 [Source:ZFIN;Acc:ZDB-GENE-030131-7540]	3	32105175	32106672	
ENSDARG00000076223	6274.70829	-1.414682535	0.098011003	-4.230979389	2.33E-05	0.000301986		zgc:92744 [Source:ZFIN;Acc:ZDB-GENE-040718-312]	2	3502430	3509245	
ENSDARG00000076262	1738.905068	1.765701265	0.137117885	5.584255203	2.35E-08	4.62E-07	pou3f2b	POU class 3 homeobox 2b [Source:NCBI gene;Acc:30397]	16	32559821	32563092	
ENSDARG00000076302	812.8378883	1.761072139	0.131350652	5.794201444	6.86E-09	1.44E-07	dtx4a	deltex 4, E3 ubiquitin ligase a [Source:ZFIN;Acc:ZDB-GENE-160728-59]	7	18557939	18601206	
ENSDARG00000076310	358.2037838	2.245821223	0.195108278	6.385281211	1.71E-10	4.3E-09		si:ch73-290k24.5 [Source:ZFIN;Acc:ZDB-GENE-110411-284]	23	45584223	45606426	
ENSDARG00000076360	346.4766285	-1.48997227	0.158827678	-3.084930016	0.002036001	0.018278129	rpl37	ribosomal protein L37 [Source:ZFIN;Acc:ZDB-GENE-040625-39]	2	49860722	49871576	
ENSDARG00000076361	440.0122394	-1.542318451	0.151038758	-3.590591312	0.000329929	0.003492846	CABZ01039859.1	beta-1,4 N-acetylgalactosaminyltransferase 2-like [Source:NCBI gene;Acc:100333855]	25	5304652	5327417	
ENSDARG00000076364	3181.313412	1.712932265	0.199130173	3.580232238	0.000343289	0.00361447	si:dkey-178k16.1	si:dkey-178k16.1 [Source:ZFIN;Acc:ZDB-GENE-090312-61]	23	11864223	12015139	
ENSDARG00000076371	754.2720833	2.668617975	0.144085179	11.58077453	5.16E-31	5.28E-29		shroom family member 2b [Source:NCBI gene;Acc:556551]	11	44178560	44214767	
ENSDARG00000076386	1407.319765	-1.677661858	0.136055728	-4.980766823	6.33E-07	1.04E-05	epdl1	ependymin-like 1 [Source:ZFIN;Acc:ZDB-GENE-090710-3]	7	1045637	1057216	
ENSDARG00000076401	917.2936656	2.527091637	0.134611835	11.34440842	7.91E-30	7.66E-28	cacng3b	calcium channel, voltage-dependent, gamma subunit 3b [Source:ZFIN;Acc:ZDB-GENE-090828-1]	3	35298078	35375978	
ENSDARG00000076443	903.8745699	1.580125906	0.131575775	4.409063183	1.04E-05	0.000143421	si:dkey-183c6.8	si:dkey-183c6.8 [Source:ZFIN;Acc:ZDB-GENE-160728-23]	7	23967332	23996133	
ENSDARG00000076448	966.0253935	-2.185952319	0.144746239	-8.19332045	2.54E-16	1.03E-14	serpinf2a	serpin peptidase inhibitor, clade F (alpha-2 antiplasmin, pigment epithelium derived factor), member 2a [Source:ZFIN;Acc:Z	21	40092301	40104916	
ENSDARG00000076487	1153.647199	-1.645041763	0.136623495	-4.721309181	2.34E-06	3.61E-05	haao	3-hydroxyanthranilate 3,4-dioxygenase [Source:NCBI gene;Acc:492518]	13	8954667	8982782	
ENSDARG00000076508	358.9407334	1.657386067	0.181514237	3.621677722	0.000292699	0.003121082	grm8b	glutamate receptor, metabotropic 8b [Source:ZFIN;Acc:ZDB-GENE-110421-3]	25	26921480	27237323	
ENSDARG00000076530	7.113409408	5.880731039	1.654222129	2.950468956	0.003172919	0.027387705	jac6	jacalin 6 [Source:ZFIN;Acc:ZDB-GENE-090610-2]	7	8489333	8490886	

ENSDARG00000076546	82.44763957	2.897121284	0.351526764	5.396804681	6.78E-08	1.26E-06	trhra	thyrotropin-releasing hormone receptor a [Source:ZFIN;Acc:ZDB-GENE-100922-18]	16	38818864	38826777	
ENSDARG00000076554	850.36021	-1.411035654	0.111000468	-3.70300831	0.000213058	0.002327226	cdkn1a	cyclin-dependent kinase inhibitor 1A [Source:ZFIN;Acc:ZDB-GENE-070705-7]	22	603382	607812	
ENSDARG00000076557	365.5100393	2.461049425	0.203498996	7.179639459	6.99E-13	2.18E-11	bicc2	bicaudal C homolog 2 [Source:ZFIN;Acc:ZDB-GENE-110110-3]	14	2941797	2979878	
ENSDARG00000076571	355.153633	1.573892473	0.185878589	3.087458734	0.002018758	0.018142801	sp5a	Sp5 transcription factor a [Source:ZFIN;Acc:ZDB-GENE-030430-2]	9	3666401	3671911	
ENSDARG00000076620	98.01603962	2.293381669	0.294420787	4.392969948	1.12E-05	0.000153787	abhd8b	abhydrolase domain containing 8b [Source:ZFIN;Acc:ZDB-GENE-130530-805]	11	5933635	5939861	
ENSDARG00000076638	417.7826829	1.514059552	0.173741701	2.958757455	0.003088821	0.026772271	FO082781.1	mex-3 RNA binding family member A [Source:NCBI gene;Acc:100332760]	16	46760625	46766153	
ENSDARG00000076639	137.6479365	2.237926663	0.265554902	4.661659995	3.14E-06	4.71E-05	esamb	endothelial cell adhesion molecule b [Source:ZFIN;Acc:ZDB-GENE-090916-1]	15	19353693	19443997	
ENSDARG00000076679	2215.049624	1.983799619	0.125609369	7.832215272	4.79E-15	1.78E-13	rai1	retinoic acid induced 1 [Source:ZFIN;Acc:ZDB-GENE-101203-2]	3	62000822	62053314	
ENSDARG00000076685	63.90410752	-2.275077732	0.335574269	-3.799688617	0.000144878	0.001639578	ccn4b	cellular communication network factor 4b [Source:ZFIN;Acc:ZDB-GENE-080220-16]	16	31518873	31525290	
ENSDARG00000076689	2854.639649	1.330861508	0.09807728	3.373681518	0.000741701	0.007298096	gramd1a	GRAM domain containing 1A [Source:ZFIN;Acc:ZDB-GENE-060503-688]	16	41902949	41961194	
ENSDARG00000076697	837.734592	2.153833212	0.126259444	9.138589388	6.33E-20	3.36E-18	SIPA1	zmp:0000001168 [Source:ZFIN;Acc:ZDB-GENE-140106-128]	7	72132047	72208248	SIPA1
ENSDARG00000076730	363.7138842	2.216545858	0.198660381	6.123746724	9.14E-10	2.11E-08	syt6a	synaptotagmin VIa [Source:ZFIN;Acc:ZDB-GENE-090601-2]	23	24802899	24856025	
ENSDARG00000076796	2085.729349	1.321036223	0.093487779	3.433991354	0.000594763	0.005962121	dlg3	discs, large homolog 3 (Drosophila) [Source:ZFIN;Acc:ZDB-GENE-050208-93]	14	33070284	33177997	
ENSDARG00000076804	1608.46944	2.212346584	0.143750488	8.433686718	3.35E-17	1.47E-15	ttyh1	tweety family member 1 [Source:ZFIN;Acc:ZDB-GENE-100922-257]	16	13714058	13789908	
ENSDARG00000076819	233.5856751	-2.198308776	0.193497812	-6.192880236	5.91E-10	1.39E-08	nlrc8	NLR family CARD domain containing 8 [Source:ZFIN;Acc:ZDB-GENE-070912-363]	15	41422248	41438821	
ENSDARG00000076836	5970.192749	-1.470412117	0.11307713	-4.160099547	3.18E-05	0.000402057	gpx4b	glutathione peroxidase 4b [Source:ZFIN;Acc:ZDB-GENE-030410-3]	2	56642138	56655769	
ENSDARG00000076843	276.9343465	3.888333212	0.26053793	11.08603729	1.47E-28	1.32E-26	si:dkey-174i8.1	si:dkey-174i8.1 [Source:ZFIN;Acc:ZDB-GENE-160114-17]	13	22104298	22114795	
ENSDARG00000076847	3193.682468	1.600222915	0.096023707	6.250778421	4.08E-10	9.81E-09	tnrc6c1	trinucleotide repeat containing adaptor 6C1 [Source:ZFIN;Acc:ZDB-GENE-030131-7440]	3	34258755	34337969	
ENSDARG00000076853	214.2188979	1.683131761	0.202841626	3.367808547	0.000757682	0.007423331	areg	amphiregulin [Source:ZFIN;Acc:ZDB-GENE-141216-239]	5	6755466	6790840	
ENSDARG00000076904	378.6069626	3.288391452	0.215355104	10.62613054	2.25E-26	1.76E-24	loxl5b	lysyl oxidase-like 5b [Source:ZFIN;Acc:ZDB-GENE-070818-3]	2	56012016	56041890	
ENSDARG00000076950	135.9180511	2.43735634	0.270673515	5.310295478	1.09E-07	1.97E-06	kel	Kell metallo-endopeptidase (Kell blood group) [Source:ZFIN;Acc:ZDB-GENE-110411-118]	16	17263027	17300030	
ENSDARG00000076958	133.740228	10.11519365	1.458487614	6.249757325	4.11E-10	9.87E-09	jac8	jacalin 8 [Source:ZFIN;Acc:ZDB-GENE-080218-18]	7	8543317	8544855	
ENSDARG00000076960	123.2417709	1.992615237	0.263078787	3.773072117	0.00016125	0.001808547	paqr4a	progestin and adipoQ receptor family member IVa [Source:ZFIN;Acc:ZDB-GENE-131127-110]	3	30947964	30962646	
ENSDARG00000076981	221.8692243	-1.910181668	0.185754733	-4.899911046	9.59E-07	1.55E-05	zgc:198329	zgc:198329 [Source:ZFIN;Acc:ZDB-GENE-080226-6]	16	11558868	11569729	
ENSDARG00000076983	8.577182839	-3.997257502	1.046942302	-2.862867893	0.004198256	0.035256077	rnasel4	ribonuclease like 4 [Source:ZFIN;Acc:ZDB-GENE-070713-7]	22	6254194	6258740	
ENSDARG00000077002	707.1499946	1.874532774	0.127051374	6.883300418	5.85E-12	1.69E-10	igsf3	immunoglobulin superfamily, member 3 [Source:ZFIN;Acc:ZDB-GENE-070912-243]	9	20076556	20372977	
ENSDARG00000077007	207.0257029	2.057950806	0.203309558	5.203645193	1.95E-07	3.41E-06	abcc8b	ATP-binding cassette, sub-family C (CFTR/MRP), member 8b [Source:ZFIN;Acc:ZDB-GENE-120215-45]	18	50040849	50083408	
ENSDARG00000077022	126.7092356	2.014780377	0.298406288	3.400666863	0.000672217	0.006661397	fam131a	family with sequence similarity 131 member A [Source:ZFIN;Acc:ZDB-GENE-080204-112]	2	16846772	16867937	
ENSDARG00000077047	820.7191961	2.19960201	0.17141027	6.998425528	2.59E-12	7.71E-11	ptprnb	protein tyrosine phosphatase receptor type Nb [Source:ZFIN;Acc:ZDB-GENE-100505-1]	6	13920479	13987739	
ENSDARG00000077053	2903.994249	2.183898896	0.133863512	8.844074651	9.23E-19	4.51E-17	ppfia3	PTPRF interacting protein alpha 3 [Source:ZFIN;Acc:ZDB-GENE-100311-3]	3	30044011	30123113	
ENSDARG00000077058	426.0830059	-3.704209055	0.198770956	-13.60464881	3.76E-42	6.42E-40	cela1.5	chymotrypsin like elastase family member 1, tandem duplicate 5 [Source:ZFIN;Acc:ZDB-GENE-040801-186]	22	7742211	7754391	
ENSDARG00000077112	556.7584287	1.640393385	0.159771843	4.008174244	6.12E-05	0.000738783	si:ch211-180f4.1	si:ch211-180f4.1 [Source:ZFIN;Acc:ZDB-GENE-080327-27]	8	8845932	8848166	
ENSDARG00000077124	333.2306045	2.04190171	0.180574779	5.769918225	7.93E-09	1.65E-07	si:ch211-255p10.4	si:ch211-255p10.4 [Source:ZFIN;Acc:ZDB-GENE-121214-354]	12	4643996	4672708	
ENSDARG00000077134	747.7607684	1.924143571	0.171889018	5.376396823	7.6E-08	1.4E-06	gpr158a	G protein-coupled receptor 158a [Source:ZFIN;Acc:ZDB-GENE-060130-18]	24	6395385	6501211	
ENSDARG00000077145	705.8451446	1.731874754	0.142688901	5.129163875	2.91E-07	4.98E-06	adcy3a	adenylate cyclase 3a [Source:ZFIN;Acc:ZDB-GENE-111027-6]	16	2515223	2558653	
ENSDARG00000077201	889.4244489	-2.377365683	0.223088267	-6.17408392	6.65E-10	1.56E-08	myo7bb	myosin VIIBb [Source:ZFIN;Acc:ZDB-GENE-070912-482]	2	22880605	22927958	
ENSDARG00000077215	1990.966943	1.400451999	0.114231152	3.505628638	0.00045553	0.004682337	eif4g3a	eukaryotic translation initiation factor 4 gamma, 3a [Source:ZFIN;Acc:ZDB-GENE-120810-2]	11	27828212	27917730	
ENSDARG00000077219	72.1274263	2.318205274	0.365978221	3.601868086	0.000315939	0.003356063	si:ch211-106h4.6	si:ch211-106h4.6 [Source:ZFIN;Acc:ZDB-GENE-070705-16]	2	31614533	31626209	
ENSDARG00000077228	967.499909	2.028529528	0.195009878	5.274243241	1.33E-07	2.38E-06	ntrk3a	neurotrophic tyrosine kinase, receptor, type 3a [Source:ZFIN;Acc:ZDB-GENE-010126-3]	25	11795683	12203961	
ENSDARG00000077231	220.4398382	2.10074234	0.21693697	5.074019159	3.89E-07	6.6E-06	vwf	von Willebrand factor [Source:ZFIN;Acc:ZDB-GENE-070103-1]	18	7150144	7228004	
ENSDARG00000077237	1603.397804	1.275108652	0.098272995	2.799432866	0.005119246	0.042144374	rfx7a	regulatory factor X7a [Source:ZFIN;Acc:ZDB-GENE-100309-3]	7	34506795	34546833	
ENSDARG00000077295	1477.435705	1.360123565	0.119362773	3.017050925	0.002552469	0.022535889	akap6	A kinase (PRKA) anchor protein 6 [Source:ZFIN;Acc:ZDB-GENE-120207-2]	17	8988374	9267175	
ENSDARG00000077329	938.6757248	2.26396372	0.129040238	9.79511306	1.18E-22	7.4E-21	nlgn2a	neuroligin 2a [Source:ZFIN;Acc:ZDB-GENE-090918-2]	7	21940857	22132265	
ENSDARG00000077341	5336.469958	1.745917846	0.127063186	5.870448145	4.35E-09	9.34E-08	ppp1r14c	protein phosphatase 1, regulatory (inhibitor) subunit 14C [Source:ZFIN;Acc:ZDB-GENE-050706-92]	23	39807857	39849155	
ENSDARG00000077349	439.5830548	1.802076826	0.225991941	3.54913907	0.000386493	0.004026795	sorcs3b	sortilin related VPS10 domain containing receptor 3b [Source:ZFIN;Acc:ZDB-GENE-131121-455]	17	20321323	20430177	
ENSDARG00000077352	319.8635547	1.715767599	0.210678053	3.397447385	0.000680177	0.006737614	b4galnt1b	beta-1,4-N-acetyl-galactosaminyl transferase 1b [Source:ZFIN;Acc:ZDB-GENE-100728-3]	6	39222598	39252851	
ENSDARG00000077353	2144.685356	1.300961948	0.0927449	3.245051188	0.001174295	0.011060406	rerea	arginine-glutamic acid dipeptide (RE) repeats a [Source:ZFIN;Acc:ZDB-GENE-060718-1]	23	22785375	22926495	
ENSDARG00000077384	53.62920647	2.633302544	0.43008683	3.797611156	0.000146097	0.001650399	dnah11	dynein, axonemal, heavy chain 11 [Source:ZFIN;Acc:ZDB-GENE-100922-8]	16	19306522	19373310	
ENSDARG00000077403	1071.514239	2.886977639	0.130332388	14.47819433	1.66E-47	3.42E-45	col8a1a	collagen, type VIII, alpha 1a [Source:ZFIN;Acc:ZDB-GENE-070912-274]	9	30090656	30102778	
ENSDARG00000077428	2043.88709	2.104365891	0.113550349	9.725781601	2.34E-22	1.45E-20	camta1a	calmodulin binding transcription activator 1a [Source:ZFIN;Acc:ZDB-GENE-090312-183]	23	30061364	30431333	
ENSDARG00000077465	1228.995381	1.456649608	0.110945838	4.115968801	3.86E-05	0.000481007	sorcs2	sortilin-related VPS10 domain containing receptor 2 [Source:ZFIN;Acc:ZDB-GENE-130530-825]	7	39757608	40057772	
ENSDARG00000077489	440.6153371	2.065764586	0.159041746	6.701162519	2.07E-11	5.7E-10	lrfn2b	leucine rich repeat and fibronectin type III domain containing 2b [Source:ZFIN;Acc:ZDB-GENE-121214-111]	20	25370	76300	
ENSDARG00000077545	1632.458949	1.913372638	0.104544024	8.7367274	2.4E-18	1.14E-16	si:dkey-16p21.7	si:dkey-16p21.7 [Source:ZFIN;Acc:ZDB-GENE-131122-54]	3	32294745	32320543	
ENSDARG00000077549	1396.020262	-1.382790093	0.0995464	-3.845343413	0.000120384	0.00137728	aifm2	apoptosis inducing factor mitochondria associated 2 [Source:ZFIN;Acc:ZDB-GENE-050506-76]	13	7241170	7261679	
ENSDARG00000077560	215.8910618	2.297895371	0.226037015	5.741959438	9.36E-09	1.94E-07	grin2cb	glutamate receptor, ionotropic, N-methyl D-aspartate 2Cb [Source:ZFIN;Acc:ZDB-GENE-100308-2]	12	31909346	32013125	
ENSDARG00000077570	30.20337816	3.641593716	0.656825235	4.021760394	5.78E-05	0.0007008	cdh16	cadherin 16, KSP-cadherin [Source:ZFIN;Acc:ZDB-GENE-140106-140]	7	44857271	44957503	
ENSDARG00000077608	917.457778	2.174289154	0.131462819	8.932481164	4.17E-19	2.1E-17	gpr137bb	G protein-coupled receptor 137Bb [Source:ZFIN;Acc:ZDB-GENE-100721-2]	11	44526892	44543082	
ENSDARG00000077609	135.9740618	2.186575914	0.258090871	4.597512137	4.28E-06	6.31E-05	elfn2a	extracellular leucine-rich repeat and fibronectin type III domain containing 2a [Source:ZFIN;Acc:ZDB-GENE-080327-26]	22	15009168	15010688	
ENSDARG00000077620	1209.289119	-1.427469086	0.101376339	-4.216655411	2.48E-05	0.000319172	cdca7a	cell division cycle associated 7a [Source:ZFIN;Acc:ZDB-GENE-050417-29]	9	2879763	2892250	
ENSDARG00000077654	526.0967722	2.003218135	0.163040042	6.153200922	7.59E-10	1.77E-08	grm8a	glutamate receptor, metabotropic 8a [Source:ZFIN;Acc:ZDB-GENE-110421-2]	4	3478049	3745093	
ENSDARG00000077656	732.3660459	2.112051688	0.155322301	7.159639564	8.09E-13	2.52E-11	arfgef3	ARFGEF family member 3 [Source:ZFIN;Acc:ZDB-GENE-081107-37]	13	2442841	2498321	
ENSDARG00000077686	609.4850121	1.545854979	0.133979326	4.0741732	4.62E-05	0.000567638	aak1b	AP2 associated kinase 1b [Source:ZFIN;Acc:ZDB-GENE-091118-25]	10	41060739	41157135	
ENSDARG00000077688	685.7168142	-4.355041933	0.175685637	-19.09684815	2.68E-81	1.37E-78	cpo	carboxypeptidase O [Source:NCBI gene;Acc:100005630]	1	5576151	5587575	
ENSDARG00000077690	713.8973303	1.575352889	0.185976706	3.09368254	0.001976889	0.017804769	mb21d2	Mab-21 domain containing 2 [Source:ZFIN;Acc:ZDB-GENE-081104-150]	2	5563077	5581772	
ENSDARG00000077710	219.6855062	2.185217463	0.200090868	5.923396068	3.15E-09	6.89E-08	nlgn1	neuroligin 1 [Source:ZFIN;Acc:ZDB-GENE-090918-1]	11	9564154	9985415	
ENSDARG00000077715	302.8377063	1.89448888	0.207284805	4.315265084	1.59E-05	0.000214092	GRIK3	glutamate receptor ionotropic, kainate 3 [Source:NCBI gene;Acc:100334689]	16	3463727	3678976	GRIK3
ENSDARG00000077791	1589.512194	1.674414118	0.106569821	6.328378078	2.48E-10	6.13E-09	brsk2b	BR serine/threonine kinase 2b [Source:ZFIN;Acc:ZDB-GENE-100422-3]	7	49324103	49595067	
ENSDARG00000077792	100.5695704	1.857490955	0.294993102	2.90681697	0.003651268	0.031152437	cdc42ep1b	CDC42 effector protein (Rho GTPase binding) 1b [Source:ZFIN;Acc:ZDB-GENE-120215-219]	3	3519607	3528258	
ENSDARG00000077822	216.3724316	-1.653457476	0.182471475	-3.581148649	0.000342087	0.003604838	si:dkey-6i22.5	si:dkey-6i22.5 [Source:ZFIN;Acc:ZDB-GENE-050506-32]	14	22109241	22118055	
ENSDARG00000077840	3420.064721	1.782174315	0.098181694	7.966600326	1.63E-15	6.26E-14	meis2a	Meis homeobox 2a [Source:ZFIN;Acc:ZDB-GENE-020122-2]	20	10098590	10120442	
ENSDARG00000077855	474.2268648	-2.214356272	0.138463908	-8.770200778	1.78E-18	8.58E-17	znrf2a	zinc and ring finger 2a [Source:ZFIN;Acc:ZDB-GENE-070820-15]	19	1934433	1948236	
ENSDARG00000077862	42.01978253	-2.751915856	0.417190931	-4.199314334	2.68E-05	0.00034269	si:dkey-169i5.4	si:dkey-169i5.4 [Source:ZFIN;Acc:ZDB-GENE-030131-8695]	22	30446987	30457736	
ENSDARG00000077864	272.0434258	1.775362051	0.179572845	4.318783495	1.58E-05	0.000211863	rasgrp3	RAS guanyl releasing protein 3 (calcium and DAG-regulated) [Source:ZFIN;Acc:ZDB-GENE-070424-82]	17	23146976	23201215	
ENSDARG00000077875	543.5107501	-3.213826578	0.1503666	-14.72286114	4.6E-49	1.03E-46	slc2a5	solute carrier family 2 member 5 [Source:ZFIN;Acc:ZDB-GENE-060222-2]	23	22616063	22633136	
ENSDARG00000077880	255.3521885	-2.708998321	0.18429229	-9.273303391	1.8E-20	1.01E-18	si:ch211-255i20.3	si:ch211-255i20.3 [Source:ZFIN;Acc:ZDB-GENE-141216-113]	14	17382803	17393595	
ENSDARG00000077882	125.3942932	2.157376344	0.319119961	3.626775144	0.000286983	0.003070571	ccn5	cellular communication network factor 5 [Source:ZFIN;Acc:ZDB-GENE-060223-1]	23	25993686	26003858	
ENSDARG00000077905	304.5343928	2.114895799	0.216033309	5.160758781	2.46E-07	4.24E-06	csmd3a	CUB and Sushi multiple domains 3a [Source:ZFIN;Acc:ZDB-GENE-050419-118]	16	47531516	48074540	
ENSDARG00000077920	238.7423493	2.51028403	0.316631123	4.769853379	1.84E-06	2.88E-05	csmd3a	CUB and Sushi multiple domains 3a [Source:ZFIN;Acc:ZDB-GENE-050419-118]	19	44123618	44955710	
ENSDARG00000077960	604.021061	-2.173788588	0.18334567	-6.402052399	1.53E-10	3.9E-09	si:ch211-186e20.7	si:ch211-186e20.7 [Source:ZFIN;Acc:ZDB-GENE-110407-6]	5	28797771	28802250	
ENSDARG00000077996	326.3516798	2.013065953	0.166716066	6.076594645	1.23E-09	2.78E-08	cdh24b	cadherin 24, type 2b [Source:ZFIN;Acc:ZDB-GENE-081104-50]	2	38554260	38709083	
ENSDARG00000078052	885.4334318	4.648879558	0.18330786	19.90574517	3.63E-88	2.22E-85	emilin3a	elastin microfibril interfacer 3a [Source:ZFIN;Acc:ZDB-GENE-080612-2]	8	22962690	22965916	
ENSDARG00000078056	529.298396	1.606836451	0.175770248	3.452441233	0.000555538	0.005591304	RNF208	si:dkey-106l3.6 [Source:NCBI gene;Acc:100526811]	10	17102902	17103651	RNF208
ENSDARG00000078057	294.0768438	3.099378447	0.207907887	10.09763734	5.66E-24	3.89E-22	snx8b	sorting nexin 8b [Source:ZFIN;Acc:ZDB-GENE-090313-317]	1	11881559	11900420	
ENSDARG00000078060	302.5871801	1.850400369	0.30003794	2.834309448	0.004592484	0.038170839	syt7b	synaptotagmin VIIb [Source:ZFIN;Acc:ZDB-GENE-090601-6]	7	13039426	13338283	
ENSDARG00000078088	796.7939742	2.547598891	0.130971477	11.81630481	3.22E-32	3.45E-30	si:ch211-186j3.6	si:ch211-186j3.6 [Source:ZFIN;Acc:ZDB-GENE-130530-842]	7	47417529	47850702	
ENSDARG00000078113	1428.166929	-1.59880947	0.106809303	-5.606341891	2.07E-08	4.1E-07	atp5mea	ATP synthase membrane subunit ea [Source:ZFIN;Acc:ZDB-GENE-070928-12]	21	20377752	20381481	
ENSDARG00000078123	469.0720199	2.230160897	0.166506742	7.388054562	1.49E-13	4.92E-12	slitrk3a	SLIT and NTRK-like family, member 3a [Source:ZFIN;Acc:ZDB-GENE-130226-1]	18	1703984	1706599	
ENSDARG00000078125	1366.348803	2.444792018	0.109368861	13.21026848	7.66E-40	1.16E-37	rusc1	RUN and SH3 domain containing 1 [Source:ZFIN;Acc:ZDB-GENE-100922-274]	16	14029283	14055309	
ENSDARG00000078149	303.217574	2.096434949	0.180520533	6.073740926	1.25E-09	2.83E-08	grin2ca	glutamate receptor, ionotropic, N-methyl D-aspartate 2Ca [Source:ZFIN;Acc:ZDB-GENE-070822-3]	3	19005187	19133003	
ENSDARG00000078151	286.7897593	2.196623748	0.199211767	6.006792488	1.89E-09	4.22E-08	si:ch211-264f5.8	si:ch211-264f5.8 [Source:ZFIN;Acc:ZDB-GENE-081104-209]	19	5779084	5812319	
ENSDARG00000078177	26.28379611	-2.409017388	0.489355152	-2.879334939	0.003985148	0.033718929	cxcr3.1	chemokine (C-X-C motif) receptor 3, tandem duplicate 1 [Source:ZFIN;Acc:ZDB-GENE-060130-38]	16	11623956	11642009	
ENSDARG00000078187	2830.423005	2.013642701	0.146140614	6.936078036	4.03E-12	1.19E-10	slc12a5b	solute carrier family 12 member 5b [Source:ZFIN;Acc:ZDB-GENE-080707-1]	8	37280532	37391372	
ENSDARG00000078210	251.5706219	1.973296898	0.192638193	5.052460692	4.36E-07	7.35E-06	tulp1b	TUB like protein 1b [Source:ZFIN;Acc:ZDB-GENE-110411-113]	6	54538948	54562508	
ENSDARG00000078226	254.9298991	1.713841675	0.208713256	3.420202864	0.000625745	0.006235337	cdh12a	cadherin 12, type 2a (N-cadherin 2) [Source:ZFIN;Acc:ZDB-GENE-070912-673]	2	28995776	29116892	
ENSDARG00000078227	1448.129204	1.573745276	0.139904155	4.100988115	4.11E-05	0.000509437	cspg4	chondroitin sulfate proteoglycan 4 [Source:ZFIN;Acc:ZDB-GENE-050519-1]	25	6507141	6629281	
ENSDARG00000078251	834.9626943	1.513817171	0.118720236	4.327966209	1.5E-05	0.000203092	TESK1	si:ch211-113e8.3 [Source:ZFIN;Acc:ZDB-GENE-090312-74]	1	40210840	40227166	TESK1
ENSDARG00000078270	806.2906977	1.398374207	0.119811234	3.325015484	0.000884136	0.00853894	CU467961.1	microtubule associated serine/threonine kinase family member 4 [Source:NCBI gene;Acc:100149322]	5	54945021	55114917	
ENSDARG00000078302	720.5447203	2.369882454	0.179099103	7.648739879	2.03E-14	7.14E-13	brinp1	bone morphogenetic protein/retinoic acid inducible neural-specific 1 [Source:ZFIN;Acc:ZDB-GENE-130530-847]	5	71252856	71460556	
ENSDARG00000078317	438.8679435	2.598139117	0.189942373	8.413810439	3.97E-17	1.73E-15	si:dkey-175m17.7	si:dkey-175m17.7 [Source:ZFIN;Acc:ZDB-GENE-091204-18]	21	27278120	27287062	
ENSDARG00000078324	663.0311934	1.458189237	0.12624386	3.629398177	0.000284083	0.003043432	si:ch211-263k4.2	si:ch211-263k4.2 [Source:ZFIN;Acc:ZDB-GENE-081104-207]	8	48480529	48541957	
ENSDARG00000078327	1067.931638	2.000023042	0.188981475	5.291645864	1.21E-07	2.17E-06	CEP170B	si:ch73-212j7.1 [Source:ZFIN;Acc:ZDB-GENE-081104-258]	20	3108597	3130881	CEP170B
ENSDARG00000078349	93.69407886	-2.409034638	0.26363861	-5.344568609	9.06E-08	1.65E-06	zmp:0000000845	zmp:0000000845 [Source:ZFIN;Acc:ZDB-GENE-130530-848]	22	30656903	30671302	
ENSDARG00000078366	269.0939562	2.553347811	0.197451558	7.86698177	3.63E-15	1.36E-13	robo2	roundabout, axon guidance receptor, homolog 2 (Drosophila) [Source:ZFIN;Acc:ZDB-GENE-001019-1]	15	38859648	38892216	
ENSDARG00000078389	88.70109592	-2.161197376	0.267214048	-4.345570094	1.39E-05	0.000188685	ifi46	interferon alpha inducible protein 46 [Source:ZFIN;Acc:ZDB-GENE-090312-190]	13	37623276	37631092	
ENSDARG00000078416	2100.411503	1.813547982	0.0996948	8.16038531	3.34E-16	1.34E-14	zeb2b	zinc finger E-box binding homeobox 2b [Source:ZFIN;Acc:ZDB-GENE-080717-1]	6	840434	957906	
ENSDARG00000078419	117.4599006	-1.811921773	0.255640198	-3.176033267	0.001493038	0.013773155	filip1a	filamin A interacting protein 1a [Source:ZFIN;Acc:ZDB-GENE-030131-4110]	17	50013594	50022827	
ENSDARG00000078440	644.0913307	2.149661242	0.150351273	7.646501532	2.07E-14	7.25E-13	ccdc88aa	coiled-coil domain containing 88Aa [Source:ZFIN;Acc:ZDB-GENE-100701-2]	6	6301205	6346557	
ENSDARG00000078455	53.26740135	-2.87891862	0.467809065	-4.016422002	5.91E-05	0.000715474	best4	bestrophin 4 [Source:ZFIN;Acc:ZDB-GENE-070912-6]	2	26504969	26509057	
ENSDARG00000078459	3262.531572	1.474671919	0.109139771	4.349211233	1.37E-05	0.00018568	cux1a	cut-like homeobox 1a [Source:ZFIN;Acc:ZDB-GENE-030131-5868]	10	32898533	33156789	
ENSDARG00000078504	538.9454166	1.550017613	0.142112128	3.870307346	0.000108698	0.001251575	si:ch73-60h1.1	si:ch73-60h1.1 [Source:ZFIN;Acc:ZDB-GENE-140106-152]	11	14676236	14751380	
ENSDARG00000078529	1272.723311	2.104271413	0.177799053	6.210783442	5.27E-10	1.26E-08	adgrb1b	adhesion G protein-coupled receptor B1b [Source:ZFIN;Acc:ZDB-GENE-081031-45]	19	92570	103289	
ENSDARG00000078536	439.083465	2.471144043	0.198275853	7.419683325	1.17E-13	3.9E-12	si:ch211-158d24.2	si:ch211-158d24.2 [Source:ZFIN;Acc:ZDB-GENE-081105-115]	8	41533268	41610205	
ENSDARG00000078551	44.42107	8.525231922	1.479155847	5.08751795	3.63E-07	6.17E-06	zgc:171242	zgc:171242 [Source:ZFIN;Acc:ZDB-GENE-071004-79]	5	12515402	12517368	
ENSDARG00000078557	22.45520203	3.189084075	0.726932153	3.011400813	0.002600453	0.022911191	si:ch211-57n23.4	si:ch211-57n23.4 [Source:ZFIN;Acc:ZDB-GENE-140106-153]	19	41551335	41597464	

ENSDARG00000078572	2315.822687	1.492248128	0.092042248	5.348067229	8.89E-08	1.62E-06	stox2b	storkhead box 2b [Source:ZFIN;Acc:ZDB-GENE-130524-1]	14	7168447	7245971	
ENSDARG00000078582	830.2587717	2.114850397	0.132168233	8.435085878	3.31E-17	1.46E-15	myo16	myosin XVI [Source:ZFIN;Acc:ZDB-GENE-070912-470]	9	7909490	8133535	
ENSDARG00000078618	132.6782228	1.990068145	0.265827848	3.724471125	0.000195725	0.002154779	inpp5kb	inositol polyphosphate-5-phosphatase Kb [Source:ZFIN;Acc:ZDB-GENE-100408-3]	10	24302433	24319526	
ENSDARG00000078622	358.792759	2.671478271	0.56601119	2.953083439	0.003146169	0.027194212	scpp5	secretory calcium-binding phosphoprotein 5 [Source:ZFIN;Acc:ZDB-GENE-090313-256]	1	43909933	43915567	
ENSDARG00000078645	391.8999987	1.851410554	0.156506117	5.440110384	5.32E-08	1E-06	si:ch73-62l21.1	si:ch73-62l21.1 [Source:ZFIN;Acc:ZDB-GENE-120215-179]	21	13353263	13376867	
ENSDARG00000078647	188.6743998	1.763625755	0.231055989	3.304938159	0.000949974	0.009125636	samsn1b	SAM domain, SH3 domain and nuclear localisation signals 1b [Source:ZFIN;Acc:ZDB-GENE-041010-135]	10	38417512	38428094	
ENSDARG00000078650	328.6567645	1.585557727	0.163418884	3.583170508	0.000339449	0.003580045	kcna4	potassium voltage-gated channel, shaker-related subfamily, member 4 [Source:ZFIN;Acc:ZDB-GENE-121127-2]	7	32595981	32599669	
ENSDARG00000078671	466.911199	1.548452657	0.156204457	3.511120398	0.000446222	0.00460552	cdk5r2b	cyclin-dependent kinase 5, regulatory subunit 2b (p39) [Source:ZFIN;Acc:ZDB-GENE-040801-251]	9	11532025	11534622	
ENSDARG00000078688	455.9493896	2.103765632	0.186317187	5.924121389	3.14E-09	6.86E-08	frs3	fibroblast growth factor receptor substrate 3 [Source:ZFIN;Acc:ZDB-GENE-131121-119]	6	40587551	40606273	
ENSDARG00000078701	537.8910221	2.574181082	0.178997899	8.794410954	1.44E-18	6.94E-17	prdm13	PR domain containing 13 [Source:ZFIN;Acc:ZDB-GENE-080616-1]	16	32749591	32755900	
ENSDARG00000078755	268.5799189	2.187355932	0.216042281	5.495942406	3.89E-08	7.46E-07	plppr4b	phospholipid phosphatase related 4b [Source:ZFIN;Acc:ZDB-GENE-091118-37]	24	30053651	30096666	
ENSDARG00000078757	41.62770916	-2.759493305	0.430614547	-4.086005265	4.39E-05	0.000541855	si:ch211-212c13.8	si:ch211-212c13.8 [Source:ZFIN;Acc:ZDB-GENE-131118-28]	15	21157585	21165237	
ENSDARG00000078828	326.4708869	2.193950535	0.244470326	4.883826009	1.04E-06	1.67E-05	npb	neuropeptide B [Source:ZFIN;Acc:ZDB-GENE-040107-40]	22	11535131	11537654	
ENSDARG00000078839	122.9401297	2.06984479	0.283798057	3.769739653	0.000163418	0.001830412	LRRTM4	si:dkey-28o19.1 [Source:ZFIN;Acc:ZDB-GENE-091118-30]	10	18291971	18492617	LRRTM4
ENSDARG00000078890	2588.034328	1.558452014	0.098946513	5.643978726	1.66E-08	3.34E-07	wdfy3	WD repeat and FYVE domain containing 3 [Source:ZFIN;Acc:ZDB-GENE-070705-153]	5	40052930	40190949	
ENSDARG00000078898	2062.549965	1.455638445	0.103188731	4.415583394	1.01E-05	0.000139549	pcdh7a	protocadherin 7a [Source:ZFIN;Acc:ZDB-GENE-120206-1]	1	15137901	15203357	
ENSDARG00000078901	465.6510082	2.510297223	0.17496615	8.631939496	6.03E-18	2.8E-16	anks1ab	ankyrin repeat and sterile alpha motif domain containing 1Ab [Source:ZFIN;Acc:ZDB-GENE-110107-1]	6	54358529	54422665	
ENSDARG00000078902	563.4848799	1.504005661	0.129522988	3.891244848	9.97E-05	0.001159497	rims1b	regulating synaptic membrane exocytosis 1b [Source:ZFIN;Acc:ZDB-GENE-101223-4]	1	31374751	31505144	
ENSDARG00000078904	1597.131396	1.464617929	0.104191492	4.459269375	8.22E-06	0.000115841	brd4	bromodomain containing 4 [Source:NCBI gene;Acc:570531]	3	53116172	53224280	
ENSDARG00000078908	487.0454178	1.834727346	0.147303707	5.666709707	1.46E-08	2.94E-07	fbxo41	F-box protein 41 [Source:ZFIN;Acc:ZDB-GENE-090313-350]	1	41666611	41755499	
ENSDARG00000078918	886.5801422	-2.428547606	0.159117735	-8.977928239	2.76E-19	1.41E-17	comtd1	catechol-O-methyltransferase domain containing 1 [Source:ZFIN;Acc:ZDB-GENE-030131-1072]	13	17672527	17685345	
ENSDARG00000079008	731.7701735	1.731017104	0.195669826	3.735972583	0.000186991	0.002068604	si:dkey-7f3.9	si:dkey-7f3.9 [Source:ZFIN;Acc:ZDB-GENE-100922-197]	16	23811554	23835922	
ENSDARG00000079012	839.1072095	1.404029217	0.115637018	3.493943596	0.000475942	0.004874178	cbfa2t3	CBFA2/RUNX1 partner transcriptional co-repressor 3 [Source:ZFIN;Acc:ZDB-GENE-070209-2]	7	55660263	55675617	
ENSDARG00000079097	1962.438176	1.347431885	0.095481515	3.638734529	0.000273981	0.002949056	tanc2a	tetratricopeptide repeat, ankyrin repeat and coiled-coil containing 2a [Source:ZFIN;Acc:ZDB-GENE-060130-180]	3	22578369	22817455	
ENSDARG00000079102	145.9078952	2.035167385	0.251994671	4.107893959	3.99E-05	0.000497143	PRRG3	si:dkey-38k9.5 [Source:ZFIN;Acc:ZDB-GENE-091204-100]	21	37941347	37963141	PRRG3
ENSDARG00000079111	4772.066581	-3.551464959	0.11701514	-21.8045712	2.1E-105	1.46E-102	actc1c	actin alpha cardiac muscle 1c [Source:ZFIN;Acc:ZDB-GENE-040625-46]	25	14087045	14125498	
ENSDARG00000079119	2253.564819	-2.024768836	0.098729538	-10.37955672	3.07E-25	2.24E-23	si:ch211-229d2.5	si:ch211-229d2.5 [Source:ZFIN;Acc:ZDB-GENE-121214-200]	15	19797918	19803478	
ENSDARG00000079124	324.6325922	-1.68291436	0.180351393	-3.786576569	0.000152737	0.001719216	MTERF4	si:dkeyp-94h10.5 [Source:ZFIN;Acc:ZDB-GENE-070912-693]	2	44545912	44557318	MTERF4
ENSDARG00000079148	912.9985629	1.663876175	0.11790191	5.630749953	1.79E-08	3.59E-07	nckap5l	NCK-associated protein 5-like [Source:ZFIN;Acc:ZDB-GENE-090313-304]	23	24600494	24661950	
ENSDARG00000079161	3307.2708	2.285591016	0.10441136	12.31275041	7.73E-35	9.24E-33	bsnb	bassoon (presynaptic cytomatrix protein) b [Source:ZFIN;Acc:ZDB-GENE-120628-1]	11	37431693	37589293	
ENSDARG00000079188	64.73392924	2.536656	0.414436056	3.707824112	0.000209048	0.002289401	nxph2b	neurexophilin 2b [Source:ZFIN;Acc:ZDB-GENE-090708-1]	11	33846440	33857911	
ENSDARG00000079251	466.8117451	2.419960851	0.167099337	8.497704896	1.93E-17	8.67E-16	nlgn2b	neuroligin 2b [Source:ZFIN;Acc:ZDB-GENE-090918-3]	10	22381802	22478129	
ENSDARG00000079264	416.6265711	2.470488397	0.237147935	6.200721926	5.62E-10	1.33E-08	baiap3	BAI1 associated protein 3 [Source:ZFIN;Acc:ZDB-GENE-130530-863]	3	45401472	45539853	
ENSDARG00000079271	521.1946847	-1.689338372	0.168033897	-4.102376857	4.09E-05	0.000507139	dcxr	dicarbonyl/L-xylulose reductase [Source:ZFIN;Acc:ZDB-GENE-030131-7002]	12	3043308	3053882	
ENSDARG00000079274	24830.74537	-6.035469405	0.136615891	-36.85859222	2.13E-297	1.07E-293	prss59.1	serine protease 59, tandem duplicate 1 [Source:ZFIN;Acc:ZDB-GENE-030131-1173]	16	26012569	26013851	
ENSDARG00000079276	873.48101	1.614867252	0.139936235	4.3939102	1.11E-05	0.000153291	ube2ql1	ubiquitin-conjugating enzyme E2Q family-like 1 [Source:ZFIN;Acc:ZDB-GENE-030131-8137]	19	27957119	27966780	
ENSDARG00000079286	1866.137246	1.888871106	0.118751932	7.485108586	7.15E-14	2.42E-12	bcr	BCR activator of RhoGEF and GTPase [Source:ZFIN;Acc:ZDB-GENE-040724-43]	21	17110598	17261078	
ENSDARG00000079295	615.0994043	2.382419876	0.240957762	5.737187566	9.63E-09	1.99E-07	cacna1bb	calcium channel, voltage-dependent, N type, alpha 1B subunit, b [Source:ZFIN;Acc:ZDB-GENE-090514-4]	21	14381227	14630840	
ENSDARG00000079323	2225.446449	1.308354341	0.091898065	3.355395365	0.000792516	0.007731423	rgmb	repulsive guidance molecule BMP co-receptor b [Source:ZFIN;Acc:ZDB-GENE-040527-2]	5	52256885	52277643	
ENSDARG00000079348	463.9571615	2.152726458	0.193721461	5.950432407	2.67E-09	5.89E-08	GRIN2B	glutamate receptor ionotropic, NMDA 2B-like [Source:NCBI gene;Acc:110438919]	3	1303862	1317290	GRIN2B
ENSDARG00000079361	247.5632838	-1.934128831	0.181049575	-5.159519597	2.48E-07	4.26E-06	abcg2b	ATP-binding cassette, sub-family G (WHITE), member 2b [Source:ZFIN;Acc:ZDB-GENE-050517-36]	1	8673061	8691969	
ENSDARG00000079366	1682.920563	1.343461202	0.105175598	3.26559782	0.001092333	0.010366217	ppp1r9ba	protein phosphatase 1, regulatory subunit 9Ba [Source:ZFIN;Acc:ZDB-GENE-091006-1]	3	23247325	23348084	
ENSDARG00000079388	4894.149868	1.617162887	0.100308173	6.152667983	7.62E-10	1.77E-08	agrn	agrin [Source:ZFIN;Acc:ZDB-GENE-030131-1033]	23	23485858	23914687	
ENSDARG00000079391	625.7444071	-1.372967195	0.122415112	-3.046741444	0.002313366	0.0205477	mrpl42	mitochondrial ribosomal protein L42 [Source:ZFIN;Acc:ZDB-GENE-101014-1]	4	1821637	1824836	
ENSDARG00000079396	742.9613689	1.752416442	0.134269313	5.603785586	2.1E-08	4.15E-07	lrfn5b	leucine rich repeat and fibronectin type III domain containing 5b [Source:ZFIN;Acc:ZDB-GENE-080327-20]	20	54383838	54407669	
ENSDARG00000079414	638.9568649	2.515225964	0.150020346	10.10013644	5.52E-24	3.8E-22	sez6b	seizure related 6 homolog b [Source:ZFIN;Acc:ZDB-GENE-110628-3]	15	24212847	24544680	
ENSDARG00000079420	1276.251034	-2.199964393	0.134898694	-8.895300268	5.83E-19	2.9E-17	dpp4	dipeptidyl-peptidase 4 [Source:ZFIN;Acc:ZDB-GENE-081104-277]	22	11628438	11648322	
ENSDARG00000079468	118.9546713	1.871347808	0.295149934	2.952220918	0.003154971	0.027251525	rhobtb1	Rho related BTB domain containing 1 [Source:ZFIN;Acc:ZDB-GENE-130530-871]	12	8003872	8039095	
ENSDARG00000079471	69.585232	-2.387657409	0.323859152	-4.284755888	1.83E-05	0.000242577	si:dkey-88j15.4	si:dkey-88j15.4 [Source:ZFIN;Acc:ZDB-GENE-100922-55]	3	2083860	2091029	
ENSDARG00000079543	946.5889422	-1.692948423	0.19122313	-3.623768851	0.000290341	0.003099895	dpys	dihydropyrimidinase [Source:ZFIN;Acc:ZDB-GENE-070508-1]	16	44298902	44325688	
ENSDARG00000079552	1052.325452	1.642324333	0.109385887	5.872095112	4.3E-09	9.26E-08	maml3	mastermind-like transcriptional coactivator 3 [Source:ZFIN;Acc:ZDB-GENE-090313-292]	1	40336160	40519340	
ENSDARG00000079578	1116.066397	1.680846335	0.150306998	4.529704846	5.91E-06	8.54E-05	rbpms2b	RNA binding protein, mRNA processing factor 2b [Source:ZFIN;Acc:ZDB-GENE-040426-741]	7	48252638	48263633	
ENSDARG00000079581	393.1549826	2.148779048	0.177144038	6.484999773	8.87E-11	2.3E-09	nyap2a	neuronal tyrosine-phosphorylated phosphoinositide-3-kinase adaptor 2a [Source:ZFIN;Acc:ZDB-GENE-131121-129]	15	35698508	35895161	
ENSDARG00000079589	44.86237051	6.093469271	1.092228297	4.66337421	3.11E-06	4.67E-05	si:dkeyp-73d8.6	si:dkeyp-73d8.6 [Source:ZFIN;Acc:ZDB-GENE-070912-683]	2	32621677	32624577	
ENSDARG00000079611	892.6521332	1.433450062	0.11364879	3.813943479	0.000136767	0.001555497	sema4c	sema domain, immunoglobulin domain (Ig), transmembrane domain (TM) and short cytoplasmic domain, (semaphorin) 4C [S	5	14529101	14648433	
ENSDARG00000079613	466.3368107	1.968204174	0.14801814	6.541118354	6.11E-11	1.61E-09	sall3a	spalt-like transcription factor 3a [Source:ZFIN;Acc:ZDB-GENE-020228-4]	16	6632969	6654587	
ENSDARG00000079618	150.305609	1.930147779	0.243864903	3.81419289	0.000136629	0.001554632	sik2a	salt-inducible kinase 2a [Source:ZFIN;Acc:ZDB-GENE-070705-451]	5	57814165	57879235	
ENSDARG00000079647	904.3895772	-1.565571358	0.162728627	-3.475549241	0.000509808	0.005178754	muc13b	mucin 13b, cell surface associated [Source:ZFIN;Acc:ZDB-GENE-080220-61]	12	16106459	16122514	
ENSDARG00000079665	550.7124187	2.703817531	0.15923879	10.69976438	1.02E-26	8.13E-25	gpr158b	G protein-coupled receptor 158b [Source:ZFIN;Acc:ZDB-GENE-130530-875]	2	12104389	12243213	
ENSDARG00000079670	748.7919885	1.954407797	0.215713965	4.424413579	9.67E-06	0.000134269	lrrc7	leucine rich repeat containing 7 [Source:ZFIN;Acc:ZDB-GENE-130530-876]	6	30124197	30210378	
ENSDARG00000079671	580.193534	1.687327219	0.146113401	4.704066947	2.55E-06	3.89E-05	plppr4a	phospholipid phosphatase related 4a [Source:ZFIN;Acc:ZDB-GENE-070705-280]	2	20331445	20371803	
ENSDARG00000079738	1622.583372	1.966596489	0.116914548	8.267546721	1.37E-16	5.66E-15	znf219	zinc finger protein 219 [Source:NCBI gene;Acc:102997060]	7	73593814	73620363	
ENSDARG00000079745	7087.236703	-1.67263261	0.128773971	-5.223358457	1.76E-07	3.08E-06	si:ch211-166a6.5	si:ch211-166a6.5 [Source:ZFIN;Acc:ZDB-GENE-030131-9913]	8	4445232	4508248	
ENSDARG00000079751	3686.946399	1.461000683	0.107902402	4.27238575	1.93E-05	0.000254955	megf8	multiple EGF-like-domains 8 [Source:ZFIN;Acc:ZDB-GENE-090730-1]	16	26380461	26437668	
ENSDARG00000079795	149.608839	-1.820275407	0.220629358	-3.717888747	0.000200895	0.002207819	CABZ01033206.1	V-set and transmembrane domain containing 5 [Source:NCBI gene;Acc:100334955]	15	449097	452347	
ENSDARG00000079840	1498.142603	1.874780707	0.155726843	5.617404747	1.94E-08	3.86E-07	kcnma1a	potassium large conductance calcium-activated channel, subfamily M, alpha member 1a [Source:ZFIN;Acc:ZDB-GENE-07(	13	16521898	16886118	
ENSDARG00000079847	1711.927319	1.885880782	0.11566629	7.658936583	1.87E-14	6.64E-13	zgc:194578	zgc:194578 [Source:ZFIN;Acc:ZDB-GENE-081022-126]	19	10375782	10395683	
ENSDARG00000079849	66.03205485	2.432374523	0.372217795	3.848216133	0.000118981	0.001361853	nrtn	neurturin [Source:ZFIN;Acc:ZDB-GENE-100427-6]	11	6349443	6386572	
ENSDARG00000079850	1913.560361	1.419972246	0.130119574	3.227587012	0.001248391	0.011714338	dchs1b	dachsous cadherin-related 1b [Source:ZFIN;Acc:ZDB-GENE-050208-41]	10	26268790	26430168	
ENSDARG00000079872	200.9351164	2.026076569	0.20815704	4.929338777	8.25E-07	1.34E-05	rapgef4	Rap guanine nucleotide exchange factor (GEF) 4 [Source:ZFIN;Acc:ZDB-GENE-070912-385]	9	2949272	3034125	
ENSDARG00000079878	248.1405068	2.113268178	0.210503261	5.288603	1.23E-07	2.21E-06	tmem151bb	transmembrane protein 151Bb [Source:ZFIN;Acc:ZDB-GENE-131125-107]	17	3183276	3190070	
ENSDARG00000079891	2266.701503	2.175542527	0.12371655	9.501901935	2.06E-21	1.21E-19	dnajc6	DnaJ (Hsp40) homolog, subfamily C, member 6 [Source:ZFIN;Acc:ZDB-GENE-080104-2]	6	31279542	31348999	
ENSDARG00000079901	95.14536192	-2.68199354	0.282515969	-5.953622889	2.62E-09	5.78E-08	si:ch73-15n24.1	si:ch73-15n24.1 [Source:ZFIN;Acc:ZDB-GENE-030131-1323]	19	9295244	9340135	
ENSDARG00000079926	95.06713543	-1.829237147	0.288495052	-2.874354829	0.004048538	0.034193258	syt15	synaptotagmin XV [Source:ZFIN;Acc:ZDB-GENE-090527-2]	12	35067863	35084803	
ENSDARG00000079932	6855.632652	-1.577465023	0.082949004	-6.961687258	3.36E-12	9.95E-11	zgc:152830	zgc:152830 [Source:ZFIN;Acc:ZDB-GENE-090312-55]	8	41229233	41256529	
ENSDARG00000079946	394.4693855	-2.596778389	0.224946336	-7.098485891	1.26E-12	3.87E-11	sqlea	squalene epoxidase a [Source:ZFIN;Acc:ZDB-GENE-071004-98]	16	43401005	43413293	
ENSDARG00000079962	349.5590566	1.782177324	0.164799477	4.746236695	2.07E-06	3.21E-05	doc2d	double C2-like domains, delta [Source:ZFIN;Acc:ZDB-GENE-081104-451]	8	13849999	13955872	
ENSDARG00000079977	1629.336	2.284142985	0.18819762	6.823375277	8.89E-12	2.54E-10	nhsb	Nance-Horan syndrome b (congenital cataracts and dental anomalies) [Source:ZFIN;Acc:ZDB-GENE-090617-2]	11	29999414	30090019	
ENSDARG00000080018	390.1582578	-2.066342453	0.176100733	-6.055298214	1.4E-09	3.15E-08	kif16bb	kinesin family member 16Bb [Source:ZFIN;Acc:ZDB-GENE-070424-241]	13	34627550	34683370	
ENSDARG00000080021	82.7814885	1.923933273	0.313377727	2.948305488	0.003195211	0.027551701	zmat3	zinc finger, matrin-type 3 [Source:ZFIN;Acc:ZDB-GENE-130530-881]	11	34522554	34537304	
ENSDARG00000082187	78.14716925	2.773430774	0.359746879	4.929662712	8.24E-07	1.34E-05	dre-mir-21-1	dre-mir-21-1 [Source:miRBase;Acc:MI0001908]	10	28198894	28199041	
ENSDARG00000082321	41.43545802	2.348316953	0.438439413	3.075264024	0.002103163	0.018840646	AL929108.1		6	52304431	52304621	
ENSDARG00000086027	234.5510713	1.858633507	0.216891334	3.958818869	7.53E-05	0.000896038	si:ch211-168d23.3	si:ch211-168d23.3 [Source:ZFIN;Acc:ZDB-GENE-131127-572]	17	51764310	51789748	
ENSDARG00000086034	1342.853053	1.661884121	0.117725227	5.622279407	1.88E-08	3.76E-07	nectin1b	nectin cell adhesion molecule 1b [Source:ZFIN;Acc:ZDB-GENE-090224-2]	18	42970208	43226503	
ENSDARG00000086104	2803.097024	1.85942723	0.09748524	8.815972862	1.19E-18	5.76E-17	slco5a1a	solute carrier organic anion transporter family member 5A1a [Source:ZFIN;Acc:ZDB-GENE-090316-1]	2	42382472	42415902	
ENSDARG00000086169	319.427085	1.606863113	0.159454755	3.805864004	0.00014131	0.001602088	rskrb	ribosomal protein S6 kinase related b [Source:ZFIN;Acc:ZDB-GENE-120215-252]	21	39645762	39670375	
ENSDARG00000086172	106.1261761	1.977001865	0.292296122	3.342507106	0.000830252	0.008058841	ACVR1C	activin A receptor type 1C [Source:NCBI gene;Acc:100331105]	9	5271524	5318873	ACVR1C
ENSDARG00000086207	623.5458614	2.206019448	0.142605749	8.457018449	2.74E-17	1.21E-15	grin2da	glutamate receptor, ionotropic, N-methyl D-aspartate 2D, a [Source:ZFIN;Acc:ZDB-GENE-041008-124]	19	9909962	10081047	
ENSDARG00000086214	536.3348981	1.556110133	0.179899488	3.091226886	0.001993313	0.017926958	ntrk3b	neurotrophic tyrosine kinase, receptor, type 3b [Source:ZFIN;Acc:ZDB-GENE-010126-4]	7	14632157	14938580	
ENSDARG00000086272	2223.328779	-2.0921208	0.127981815	-8.533406068	1.42E-17	6.42E-16	si:dkey-4p15.5	si:dkey-4p15.5 [Source:ZFIN;Acc:ZDB-GENE-121214-193]	15	19681718	19688389	
ENSDARG00000086302	1778.916172	1.631712201	0.102614827	6.156149375	7.45E-10	1.74E-08	proser1	proline and serine rich 1 [Source:ZFIN;Acc:ZDB-GENE-111003-1]	10	24648903	24659840	
ENSDARG00000086327	1513.485554	1.477111774	0.137328787	3.474229887	0.000512322	0.005202181	srrm4	serine/arginine repetitive matrix 4 [Source:NCBI gene;Acc:796574]	5	15667874	15817204	
ENSDARG00000086345	200.9474209	1.746420295	0.204453104	3.65081419	0.00026141	0.002821006	cux2b	cut-like homeobox 2b [Source:ZFIN;Acc:ZDB-GENE-081104-176]	8	4044578	4327473	
ENSDARG00000086362	218.8435876	2.712748201	0.222438839	7.699861273	1.36E-14	4.88E-13	pou6f2	POU class 6 homeobox 2 [Source:ZFIN;Acc:ZDB-GENE-091118-71]	24	10039165	10272212	
ENSDARG00000086453	72.92268317	3.172837354	0.393652136	5.519688969	3.4E-08	6.55E-07	cx52.9	connexin 52.9 [Source:ZFIN;Acc:ZDB-GENE-040426-2421]	17	53435279	53437459	
ENSDARG00000086551	637.5812111	1.843193997	0.146072465	5.772436276	7.81E-09	1.63E-07	irs4a	insulin receptor substrate 4a [Source:ZFIN;Acc:ZDB-GENE-120215-92]	21	43515673	43527198	
ENSDARG00000086571	291.3597591	-1.64063563	0.198470973	-3.227855535	0.001247219	0.011707724	kcna7	potassium voltage-gated channel, shaker-related subfamily, member 7 [Source:ZFIN;Acc:ZDB-GENE-120215-257]	3	29980603	29983868	
ENSDARG00000086620	20.45699131	-3.132560926	0.610002098	-3.435989496	0.000472307	0.004844865	gckr	glucokinase (hexokinase 4) regulator [Source:ZFIN;Acc:ZDB-GENE-110411-248]	17	5863150	5878032	
ENSDARG00000086647	488.8463086	3.027504754	0.16778049	12.08427005	1.28E-33	1.44E-31	chrng	cholinergic receptor, nicotinic, gamma [Source:ZFIN;Acc:ZDB-GENE-030131-3805]	2	45081489	45097743	
ENSDARG00000086654	39.84306765	2.366789544	0.464151015	2.944708727	0.003232588	0.027845298	cbln11	cerebellin 11 [Source:ZFIN;Acc:ZDB-GENE-111110-2]	7	4291707	4296771	
ENSDARG00000086665	912.3804336	3.403412057	0.208450667	11.52988425	9.33E-31	9.48E-29	si:dkey-175g6.2	si:dkey-175g6.2 [Source:ZFIN;Acc:ZDB-GENE-030131-8061]	10	23022263	23026529	
ENSDARG00000086819	258.1748303	1.637509801	0.202820264	3.143225376	0.001670971	0.015252018	scn1laa	sodium channel, voltage-gated, type I-like, alpha [Source:ZFIN;Acc:ZDB-GENE-040426-751]	9	49906037	50001606	
ENSDARG00000086826	4085.426918	-2.456684104	0.120653036	-12.07333153	1.46E-33	1.64E-31	sult6b1	sulfotransferase family, cytosolic, 6b, member 1 [Source:NCBI gene;Acc:322462]	11	43375326	43388063	
ENSDARG00000086847	18.88055614	4.828914384	1.142337955	3.351822783	0.000802814	0.007822761	irgq1	immunity-related GTPase family, q1 [Source:ZFIN;Acc:ZDB-GENE-051212-3]	16	25806400	25815388	
ENSDARG00000086856	1455.025292	1.785771025	0.12912172	6.085506164	1.16E-09	2.64E-08	stk35	serine/threonine kinase 35 [Source:ZFIN;Acc:ZDB-GENE-061103-553]	23	41739721	41762956	
ENSDARG00000086892	286.1415959	1.971319322	0.199674637	4.864510267	1.15E-06	1.83E-05	erfl1	Ets2 repressor factor like 1 [Source:ZFIN;Acc:ZDB-GENE-090529-3]	16	26234059	26296477	
ENSDARG00000086957	216.7539297	2.910382734	0.222112272	8.600977873	7.9E-18	3.65E-16	si:dkeyp-118a3.2	si:dkeyp-118a3.2 [Source:ZFIN;Acc:ZDB-GENE-060810-149]	1	19303241	19331532	
ENSDARG00000086980	989.0530818	1.69814476	0.116146497	6.010898095	1.84E-09	4.12E-08	mbd6	methyl-CpG binding domain protein 6 [Source:ZFIN;Acc:ZDB-GENE-041027-3]	6	58871600	58910402	
ENSDARG00000086990	754.1637719	1.642461833	0.16950731	3.790171837	0.000150543	0.001695311	carmil3	capping protein regulator and myosin 1 linker 3 [Source:ZFIN;Acc:ZDB-GENE-070912-183]	2	38742338	38890677	
ENSDARG00000086996	806.4699617	-1.485186243	0.131009304	-3.703448752	0.000212688	0.002324199	si:ch73-234b20.5	si:ch73-234b20.5 [Source:ZFIN;Acc:ZDB-GENE-041008-183]	10	4987766	4996049	
ENSDARG00000087017	78.74030755	-3.027777909	0.517089185	-3.921524506	8.8E-05	0.001033045	si:dkey-11k2.7	si:dkey-11k2.7 [Source:ZFIN;Acc:ZDB-GENE-160728-22]	7	24366962	24373662	
ENSDARG00000087040	164.3377092	1.862826501	0.260776505	3.308681894	0.000937363	0.009021757	rhbdl1	rhomboid, veinlet-like 1 (Drosophila) [Source:ZFIN;Acc:ZDB-GENE-120529-2]	24	37598441	37623496	
ENSDARG00000087059	33.77294356	2.530222261	0.498537092	3.069425093	0.002144712	0.019185464	selenou1b	selenoprotein U1b [Source:ZFIN;Acc:ZDB-GENE-060929-88]	12	47778786	47782623	
ENSDARG00000087087	88.73665395	3.130149453	0.373945412	5.696418207	1.22E-08	2.49E-07	col28a1b	collagen, type XXVIII, alpha 1b [Source:ZFIN;Acc:ZDB-GENE-160503-1]	16	47308856	47344147	
ENSDARG00000087093	1989.787288	-2.252772495	0.116129051	-10.78776139	3.93E-27	3.24E-25	si:ch211-157c3.4	si:ch211-157c3.4 [Source:ZFIN;Acc:ZDB-GENE-030131-8454]	3	27642386	27647845	

ENSDARG00000087100	156.0877649	1.785570225	0.241141507	3.257714676	0.001123133	0.010630375	ntng2b	netrin g2b [Source:ZFIN;Acc:ZDB-GENE-110502-1]	21	3979107	4032650	
ENSDARG00000087102	16.07311619	3.527053674	0.880836562	2.868924592	0.0041187	0.034680806	si:ch1073-164k15.3	si:ch1073-164k15.3 [Source:ZFIN;Acc:ZDB-GENE-131119-59]	24	38648885	38657683	
ENSDARG00000087110	567.8550807	1.670150415	0.151859519	4.412962857	1.02E-05	0.000141093	akna	AT-hook transcription factor [Source:ZFIN;Acc:ZDB-GENE-091204-170]	21	13923872	13972745	
ENSDARG00000087120	66.87429284	-2.58816119	0.316455592	-5.018591017	5.21E-07	8.69E-06	slc5a8	solute carrier family 5 member 8 [Source:ZFIN;Acc:ZDB-GENE-141211-63]	4	5850225	5863906	
ENSDARG00000087176	287.0265229	1.996092213	0.291713423	3.414625914	0.000638697	0.006346764	rskra	ribosomal protein S6 kinase related a [Source:ZFIN;Acc:ZDB-GENE-130103-8]	15	16109458	16121496	
ENSDARG00000087178	217.3181499	5.441215585	0.421365044	10.5400665	5.65E-26	4.27E-24	scpp9	secretory calcium-binding phosphoprotein 9 [Source:ZFIN;Acc:ZDB-GENE-090424-3]	1	43969797	43973068	
ENSDARG00000087186	484.9908198	2.078580002	0.170197648	6.337220374	2.34E-10	5.8E-09	si:ch211-232b12.5	si:ch211-232b12.5 [Source:ZFIN;Acc:ZDB-GENE-031002-40]	11	25313810	25324534	
ENSDARG00000087205	772.667043	1.735167474	0.139125951	5.284186525	1.26E-07	2.26E-06	akt3b	v-akt murine thymoma viral oncogene homolog 3b [Source:ZFIN;Acc:ZDB-GENE-110309-3]	1	53735195	53756851	
ENSDARG00000087277	890.884131	-1.382604535	0.111770745	-3.42311877	0.00061907	0.006178641	selenoj	selenoprotein J [Source:ZFIN;Acc:ZDB-GENE-030131-4163]	2	16160906	16172257	
ENSDARG00000087285	29.23551859	-3.078515667	0.530106058	-3.920943058	8.82E-05	0.001035057	tmem182b	transmembrane protein 182b [Source:ZFIN;Acc:ZDB-GENE-131121-565]	6	14301214	14312611	
ENSDARG00000087290	48.32674066	3.441925209	0.53585896	4.557029728	5.19E-06	7.58E-05	si:ch211-202h22.10	si:ch211-202h22.10 [Source:ZFIN;Acc:ZDB-GENE-110914-121]	1	54606354	54608032	
ENSDARG00000087311	20.44211871	3.900231318	0.846370629	3.426668197	0.000611035	0.006103301	lgals9l5	lectin, galactoside-binding, soluble, 9 (galectin 9)-like 5 [Source:ZFIN;Acc:ZDB-GENE-070822-22]	15	30994538	31000306	
ENSDARG00000087318	331.7061185	-2.110444962	0.165952443	-6.691344456	2.21E-11	6.05E-10	jac10	jacalin 10 [Source:ZFIN;Acc:ZDB-GENE-071004-126]	7	8379833	8381432	
ENSDARG00000087330	527.5237367	1.435314863	0.137577818	3.164135539	0.001555443	0.014285834	maza	MYC-associated zinc finger protein a (purine-binding transcription factor) [Source:ZFIN;Acc:ZDB-GENE-110603-4]	3	21096027	21118969	
ENSDARG00000087359	5126.404527	2.404316011	0.126252482	11.12307648	9.69E-29	8.97E-27	c3a.2	complement component c3a, duplicate 2 [Source:ZFIN;Acc:ZDB-GENE-990415-36]	1	56229674	56266731	
ENSDARG00000087403	211.3962455	-1.775076653	0.200361582	-3.868389569	0.000109557	0.00126088	si:ch211-214p13.3	si:ch211-214p13.3 [Source:ZFIN;Acc:ZDB-GENE-060503-779]	9	9251466	9282519	
ENSDARG00000087407	13.87016771	6.847382232	1.542776732	3.790167502	0.000150546	0.001695311	si:ch73-304f21.1	si:ch73-304f21.1 [Source:ZFIN;Acc:ZDB-GENE-131121-162]	22	34661401	34672324	
ENSDARG00000087413	600.4787911	1.544438031	0.175739333	3.097986224	0.001948405	0.01756083	bean1	brain expressed, associated with NEDD4, 1 [Source:ZFIN;Acc:ZDB-GENE-141216-52]	7	44484853	44589233	
ENSDARG00000087440	448.350259	2.963047743	0.172797558	11.36039054	6.58E-30	6.41E-28	ponzr4	plac8 onzin related protein 4 [Source:ZFIN;Acc:ZDB-GENE-050411-6]	7	22680560	22684292	
ENSDARG00000087476	516.1499517	-2.459836784	0.157999043	-9.239529311	2.48E-20	1.37E-18	cbln20	cerebellin 20 [Source:ZFIN;Acc:ZDB-GENE-111109-2]	15	572195	576135	
ENSDARG00000087599	863.3798859	1.910054757	0.196525072	4.630731066	3.64E-06	5.42E-05		si:dkey-237h12.3 [Source:ZFIN;Acc:ZDB-GENE-081027-3]	14	36454087	36725348	
ENSDARG00000087601	278.5867126	2.034048702	0.178338359	5.798240532	6.7E-09	1.41E-07	gpr153	G protein-coupled receptor 153 [Source:ZFIN;Acc:ZDB-GENE-050823-7]	8	46656170	46700278	
ENSDARG00000087616	3352.960793	1.527280315	0.12473945	4.22705339	2.37E-05	0.000306351	maptb	microtubule-associated protein tau b [Source:ZFIN;Acc:ZDB-GENE-081027-2]	3	22158024	22216771	
ENSDARG00000087633	304.4083212	-2.842807251	0.261176245	-7.055799624	1.72E-12	5.22E-11	si:dkey-11o18.5	si:dkey-11o18.5 [Source:ZFIN;Acc:ZDB-GENE-091204-110]	21	14784321	14803554	
ENSDARG00000087646	123.8930829	2.425979874	0.272985572	5.223645577	1.75E-07	3.08E-06	runx1	RUNX family transcription factor 1 [Source:ZFIN;Acc:ZDB-GENE-000605-1]	1	1362932	1402303	
ENSDARG00000087659	58.54603314	2.206488284	0.389129143	3.100482977	0.001932053	0.017432231	si:ch211-219a15.3	si:ch211-219a15.3 [Source:ZFIN;Acc:ZDB-GENE-060503-214]	18	15770461	15771554	
ENSDARG00000087663	164.257992	3.492815934	0.28975525	8.603177811	7.75E-18	3.59E-16	si:ch211-248l17.3	si:ch211-248l17.3 [Source:ZFIN;Acc:ZDB-GENE-120709-105]	24	37999090	38012593	
ENSDARG00000087693	1731.630218	-1.548450172	0.11578854	-4.736653309	2.17E-06	3.36E-05	atox1	antioxidant 1 copper chaperone [Source:ZFIN;Acc:ZDB-GENE-120720-2]	14	25982962	25985762	
ENSDARG00000087704	397.964203	2.049465254	0.15525647	6.759558895	1.38E-11	3.88E-10	gfra3	GDNF family receptor alpha 3 [Source:ZFIN;Acc:ZDB-GENE-110512-1]	14	7452302	7521444	
ENSDARG00000087709	435.910256	1.902863243	0.15015626	6.012824508	1.82E-09	4.07E-08	fat3b	FAT atypical cadherin 3b [Source:ZFIN;Acc:ZDB-GENE-130530-969]	10	25108853	25204034	
ENSDARG00000087782	229.9137852	-1.803786117	0.177958922	-4.516694686	6.28E-06	9.04E-05	rnf152	ring finger protein 152 [Source:NCBI gene;Acc:541544]	2	27867830	27871054	
ENSDARG00000087805	283.4397543	-2.298339281	0.168684331	-7.696857648	1.39E-14	4.99E-13	FP236735.1	claudin 34a [Source:NCBI gene;Acc:568833]	6	30456788	30464567	
ENSDARG00000087867	182.6552345	2.612921563	0.236115859	6.831059836	8.43E-12	2.41E-10	SLC2A13	proton myo-inositol cotransporter-like [Source:NCBI gene;Acc:101882338]	4	75200467	75270523	SLC2A13
ENSDARG00000087873	711.794311	-2.237363142	0.196106257	-6.309656605	2.8E-10	6.85E-09	eevs	2-epi-5-epi-valiolone synthase [Source:ZFIN;Acc:ZDB-GENE-131121-365]	6	43438074	43449013	
ENSDARG00000087921	923.1556376	1.626321552	0.119866726	5.225149407	1.74E-07	3.06E-06	plce1	phospholipase C, epsilon 1 [Source:ZFIN;Acc:ZDB-GENE-061212-4]	12	5251647	5398578	
ENSDARG00000088030	38552.24861	-1.45237829	0.111695405	-4.050106532	5.12E-05	0.000625927	rpl35a	ribosomal protein L35a [Source:ZFIN;Acc:ZDB-GENE-040718-190]	18	45781115	45791224	
ENSDARG00000088079	380.7165356	-3.234840477	0.214158159	-10.43546735	1.71E-25	1.25E-23	tmed6	transmembrane p24 trafficking protein 6 [Source:ZFIN;Acc:ZDB-GENE-131121-182]	25	37285737	37288184	
ENSDARG00000088082	63.01995045	-2.241979888	0.422164774	-2.941931597	0.00326172	0.028076963	si:ch211-214c20.1	si:ch211-214c20.1 [Source:ZFIN;Acc:ZDB-GENE-081103-49]	4	29620959	29638221	
ENSDARG00000088091	43622.28849	-1.692851916	0.103170025	-6.715631968	1.87E-11	5.19E-10	pfn1	profilin 1 [Source:ZFIN;Acc:ZDB-GENE-031002-33]	23	44580254	44605765	
ENSDARG00000088116	1235.963089	-1.827162243	0.127886474	-6.467941576	9.93E-11	2.57E-09	gstm.3	glutathione S-transferase mu tandem duplicate 3 [Source:ZFIN;Acc:ZDB-GENE-050309-24]	8	25302172	25307014	
ENSDARG00000088117	180.7648303	1.690286862	0.217874739	3.168273953	0.001533469	0.014120182	fut8b	fucosyltransferase 8b (alpha (1,6) fucosyltransferase) [Source:ZFIN;Acc:ZDB-GENE-130530-923]	20	50650143	50740404	
ENSDARG00000088136	87.8910641	2.213971861	0.328651915	3.693792137	0.000220934	0.002402805	pcdh2aa15	protocadherin 2 alpha a 15 [Source:ZFIN;Acc:ZDB-GENE-041118-15]	14	2123123	2267515	
ENSDARG00000088140	341.435766	-1.884130872	0.174808692	-5.057705432	4.24E-07	7.16E-06	hsd17b7	hydroxysteroid (17-beta) dehydrogenase 7 [Source:ZFIN;Acc:ZDB-GENE-061013-378]	6	34943351	34958274	
ENSDARG00000088222	169.9109808	1.918906808	0.216097144	4.252285749	2.12E-05	0.000275772	fam78ab	family with sequence similarity 78 member Ab [Source:ZFIN;Acc:ZDB-GENE-080220-25]	5	71983005	71995108	
ENSDARG00000088274	20.89834476	3.599030885	0.797518611	3.258896846	0.001118463	0.010590172	si:ch211-181d7.1	si:ch211-181d7.1 [Source:ZFIN;Acc:ZDB-GENE-121214-14]	15	42693455	42720384	
ENSDARG00000088277	260.0791111	1.964227502	0.196352738	4.910690386	9.08E-07	1.47E-05	susd5	sushi domain containing 5 [Source:ZFIN;Acc:ZDB-GENE-120215-196]	19	42629535	42651615	
ENSDARG00000088318	5934.398187	1.507514054	0.096179132	5.27675851	1.31E-07	2.35E-06	ubap2a	ubiquitin associated protein 2a [Source:ZFIN;Acc:ZDB-GENE-070424-16]	21	11916788	11955313	
ENSDARG00000088357	3863.367372	-1.491566883	0.089111964	-5.516283772	3.46E-08	6.67E-07	acadl	acyl-CoA dehydrogenase long chain [Source:ZFIN;Acc:ZDB-GENE-040426-771]	18	39487486	39505847	
ENSDARG00000088366	2349.655896	-2.838352501	0.142000639	-12.94608608	2.47E-38	3.53E-36	zgc:77938	zgc:77938 [Source:ZFIN;Acc:ZDB-GENE-040426-1910]	14	35901249	35911982	
ENSDARG00000088567	280.1746199	1.679603763	0.245617715	2.76691672	0.005658921	0.046088441	vps72b	vacuolar protein sorting 72 homolog b [Source:ZFIN;Acc:ZDB-GENE-100922-13]	16	29630965	29647738	
ENSDARG00000088589	1036.246502	1.740193575	0.127110743	5.823218082	5.77E-09	1.22E-07	ponzr3	plac8 onzin related protein 3 [Source:ZFIN;Acc:ZDB-GENE-050506-38]	7	22675475	22677211	
ENSDARG00000088625	82.54362029	-4.663889206	0.412263087	-8.887259922	6.26E-19	3.1E-17	si:ch211-142d6.2	si:ch211-142d6.2 [Source:ZFIN;Acc:ZDB-GENE-141216-119]	7	73444325	73445871	
ENSDARG00000088634	477.7426933	2.241646446	0.169627766	7.319830231	2.48E-13	8.03E-12	adcy1b	adenylate cyclase 1b [Source:ZFIN;Acc:ZDB-GENE-100805-1]	2	136346	155270	
ENSDARG00000088638	73.7356151	2.202171611	0.332871569	3.611517845	0.00030441	0.003239084	BX927260.1		22	12933485	12940709	
ENSDARG00000088711	1764.571073	-1.809832012	0.179672079	-4.507278026	6.57E-06	9.4E-05	lgals1l1	lectin, galactoside-binding, soluble, 1 (galectin 1)-like 1 [Source:ZFIN;Acc:ZDB-GENE-030131-4905]	3	28939759	28946756	
ENSDARG00000088717	1624.876432	-1.528473869	0.11364427	-4.650246498	3.32E-06	4.96E-05	ecrg4b	ECRG4 augurin precursor b [Source:NCBI gene;Acc:793804]	6	15268685	15300140	
ENSDARG00000088741	2524.52478	1.50415442	0.090973363	5.541780592	2.99E-08	5.83E-07	BX322530.1		16	14093781	14167673	
ENSDARG00000088789	1387.621315	2.468729871	0.117295097	12.52166465	5.68E-36	7.28E-34	mast1b	microtubule associated serine/threonine kinase 1b [Source:ZFIN;Acc:ZDB-GENE-131025-2]	3	6720858	6768905	
ENSDARG00000088836	55.13667733	-2.491384004	0.385556714	-3.868131325	0.000109673	0.001261637	si:ch211-76m11.5	si:ch211-76m11.5 [Source:ZFIN;Acc:ZDB-GENE-041210-278]	4	43225728	43260960	
ENSDARG00000088885	185.7917786	2.443881757	0.248804961	5.803267559	6.5E-09	1.37E-07	si:ch1073-340i21.3	si:ch1073-340i21.3 [Source:ZFIN;Acc:ZDB-GENE-030131-199]	15	46329149	46336399	
ENSDARG00000088891	114.9714475	-2.705773749	0.267077752	-6.386805845	1.69E-10	4.26E-09	slc23a3	solute carrier family 23 member 3 [Source:ZFIN;Acc:ZDB-GENE-070912-714]	9	7371962	7394412	
ENSDARG00000088900	84.3602647	1.981219741	0.317321289	3.092196382	0.001986814	0.017877742	si:dkey-21o19.2	si:dkey-21o19.2 [Source:ZFIN;Acc:ZDB-GENE-090313-241]	23	24124684	24131962	
ENSDARG00000088911	71.46122145	-2.629979616	0.314074945	-5.189779195	2.11E-07	3.65E-06	cbln17	cerebellin 17 [Source:ZFIN;Acc:ZDB-GENE-111109-4]	10	21867307	21870457	
ENSDARG00000088924	174.5655379	-1.612911228	0.210745747	-2.90829702	0.00363403	0.031015909	si:dkey-185e18.7	si:dkey-185e18.7 [Source:ZFIN;Acc:ZDB-GENE-141215-72]	14	4163773	4177311	
ENSDARG00000089009	2096.784273	1.606367232	0.1061616	5.711737871	1.12E-08	2.29E-07	frmpd1b	FERM and PDZ domain containing 1b [Source:ZFIN;Acc:ZDB-GENE-140328-1]	14	4191136	4273396	
ENSDARG00000089039	17.73952464	3.666525537	0.884371458	3.015164628	0.002568398	0.022660583	zgc:173585	zgc:173585 [Source:ZFIN;Acc:ZDB-GENE-070822-26]	25	35135676	35136267	
ENSDARG00000089042	383.7017501	1.903415332	0.160419018	5.631597463	1.79E-08	3.57E-07	foxb1a	forkhead box B1a [Source:ZFIN;Acc:ZDB-GENE-990616-47]	25	34154171	34156152	
ENSDARG00000089050	46.55752532	-2.165566167	0.425499443	-2.739289527	0.006157212	0.049631403	cuzd1.1	CUB and zona pellucida-like domains 1, tandem duplicate 1 [Source:ZFIN;Acc:ZDB-GENE-131122-16]	17	21870044	21884323	
ENSDARG00000089063	241.994803	2.281678887	0.214775611	5.96752527	2.41E-09	5.34E-08	lactbl1a	lactamase, beta-like 1a [Source:ZFIN;Acc:ZDB-GENE-030131-7625]	23	21964325	21974831	
ENSDARG00000089123	259.4747967	-1.931665715	0.166286283	-5.602781519	2.11E-08	4.17E-07	negaly6	neuromast-expressed gpi-anchored lymphocyte antigen 6 [Source:ZFIN;Acc:ZDB-GENE-131121-604]	16	24698849	24703855	
ENSDARG00000089156	237.2368546	1.623359305	0.210345233	2.963505745	0.003041564	0.026435697	egr3	early growth response 3 [Source:ZFIN;Acc:ZDB-GENE-040718-394]	8	50727220	50733508	
ENSDARG00000089172	150.8838502	2.016643227	0.292798906	3.472155142	0.000516298	0.005235028	si:ch1073-391i24.1	si:ch1073-391i24.1 [Source:ZFIN;Acc:ZDB-GENE-110408-69]	2	59015878	59032808	
ENSDARG00000089177	334.1833269	-2.02705111	0.17942372	-5.724165744	1.04E-08	2.14E-07	cyp46a1.3	cytochrome P450, family 46, subfamily A, polypeptide 1, tandem duplicate 3 [Source:ZFIN;Acc:ZDB-GENE-060519-40]	20	5272655	5291012	
ENSDARG00000089181	63.1309456	2.061348543	0.365332884	2.905154696	0.003670716	0.031286468	adad1	adenosine deaminase domain containing 1 (testis-specific) [Source:ZFIN;Acc:ZDB-GENE-030131-6285]	14	1240235	1267206	
ENSDARG00000089213	579.7787619	1.372393821	0.132098811	2.819055054	0.004816526	0.039914034	adam15	ADAM metallopeptidase domain 15 [Source:ZFIN;Acc:ZDB-GENE-070809-4]	16	28663184	28709874	
ENSDARG00000089262	193.4968269	1.656678567	0.220372237	2.979860689	0.002883795	0.025177787	pbx3a	pre-B-cell leukemia homeobox 3a [Source:ZFIN;Acc:ZDB-GENE-031218-1]	5	5302932	5394468	
ENSDARG00000089271	2610.847663	2.388074742	0.129570475	10.71289387	8.85E-27	7.1E-25	nexmifa	neurite extension and migration factor a [Source:ZFIN;Acc:ZDB-GENE-110411-102]	5	23118470	23132395	
ENSDARG00000089292	303.5611908	2.309933211	0.177166494	7.393797687	1.43E-13	4.72E-12	adgrl1a	adhesion G protein-coupled receptor L1a [Source:ZFIN;Acc:ZDB-GENE-131127-267]	3	47498433	47876427	
ENSDARG00000089310	1607.703357	-1.981439804	0.119349	-8.22327634	1.98E-16	8.1E-15	gc	GC vitamin D binding protein [Source:ZFIN;Acc:ZDB-GENE-040718-307]	5	45677781	45725008	
ENSDARG00000089312	53.79934107	-3.613492564	0.459249996	-5.690784073	1.26E-08	2.57E-07	si:ch73-106l15.4	si:ch73-106l15.4 [Source:ZFIN;Acc:ZDB-GENE-131120-96]	3	5331428	5342333	
ENSDARG00000089314	3982.09197	1.833272881	0.117777624	7.074967634	1.49E-12	4.56E-11	mapta	microtubule-associated protein tau a [Source:ZFIN;Acc:ZDB-GENE-081027-1]	12	4733892	4781821	
ENSDARG00000089326	233.1988676	1.753724884	0.191220007	3.941663305	8.09E-05	0.000958083	arhgap20	Rho GTPase activating protein 20 [Source:ZFIN;Acc:ZDB-GENE-071015-1]	9	29529401	29544720	
ENSDARG00000089368	1279.771678	-1.453619118	0.110688331	-4.098165674	4.16E-05	0.000515433	hopx	HOP homeobox [Source:NCBI gene;Acc:386589]	14	1170968	1178359	
ENSDARG00000089399	2411.548252	-2.560017928	0.1132164	-13.77908089	3.41E-43	5.98E-41	tmem176l.2	transmembrane protein 176l.2 [Source:ZFIN;Acc:ZDB-GENE-080829-12]	16	46639772	46645396	
ENSDARG00000089418	3032.744186	-1.431724966	0.101049055	-4.272429548	1.93E-05	0.000254955	si:dkey-164f24.2	si:dkey-164f24.2 [Source:ZFIN;Acc:ZDB-GENE-030131-7035]	8	32481577	32497815	
ENSDARG00000089429	8233.198172	-2.456705817	0.099534267	-14.63521921	1.67E-48	3.69E-46	si:dkey-205h13.2	si:dkey-205h13.2 [Source:ZFIN;Acc:ZDB-GENE-080225-18]	23	26523998	26536055	
ENSDARG00000089458	564.5440679	1.606427463	0.154360845	3.928635279	8.54E-05	0.001005935	rp1l1a	rp1 like 1a [Source:ZFIN;Acc:ZDB-GENE-120711-1]	17	5768608	5822120	
ENSDARG00000089475	526.4871332	1.645008411	0.147619996	4.369383739	1.25E-05	0.00017053	hbae1.1	hemoglobin, alpha embryonic 1.1 [Source:ZFIN;Acc:ZDB-GENE-980526-80]	3	55120330	55121125	
ENSDARG00000089489	446.9308194	-1.365115928	0.132501777	-2.755554965	0.005859266	0.047473671	fam83e	family with sequence similarity 83 member E [Source:ZFIN;Acc:ZDB-GENE-141215-21]	11	40103720	40128722	
ENSDARG00000089645	901.7192853	-1.591574367	0.109245604	-5.415086267	6.13E-08	1.14E-06	si:ch1073-406l10.2	si:ch1073-406l10.2 [Source:ZFIN;Acc:ZDB-GENE-141212-315]	24	33288455	33291784	
ENSDARG00000089667	100.41915	2.602781009	0.330406639	4.850934635	1.23E-06	1.96E-05	MFAP4	zgc:173915 [Source:ZFIN;Acc:ZDB-GENE-071004-112]	1	59073203	59074515	MFAP4
ENSDARG00000089742	419.6349591	1.990332463	0.188006509	5.267543488	1.38E-07	2.46E-06	fam161a	FAM161 centrosomal protein A [Source:ZFIN;Acc:ZDB-GENE-110310-1]	1	53686995	53700392	
ENSDARG00000089766	494.4859094	1.512083124	0.149720107	3.420269562	0.000625591	0.006235337	nrg3b	neuregulin 3b [Source:ZFIN;Acc:ZDB-GENE-130603-38]	12	29350698	29624638	
ENSDARG00000089856	2171.510214	1.480122404	0.134352148	3.573611683	0.000352091	0.003697839	cita	citron rho-interacting serine/threonine kinase a [Source:ZFIN;Acc:ZDB-GENE-130530-981]	5	2409554	2636078	
ENSDARG00000089858	199.2819726	1.865075286	0.206298913	4.193309955	2.75E-05	0.000350997	cobll1a	cordon-bleu WH2 repeat protein-like 1a [Source:ZFIN;Acc:ZDB-GENE-120215-171]	6	10333920	10365603	
ENSDARG00000089919	368.0501198	-2.958459538	0.186164953	-10.52002273	6.99E-26	5.23E-24	si:ch211-161h7.8	si:ch211-161h7.8 [Source:ZFIN;Acc:ZDB-GENE-091204-302]	24	20960216	20964379	
ENSDARG00000089936	2727.384567	-1.911378784	0.113159185	-8.053953227	8.02E-16	3.15E-14	selenow2b	selenoprotein W, 2b [Source:ZFIN;Acc:ZDB-GENE-030428-2]	3	20103746	20106233	
ENSDARG00000090106	650.0951383	1.951707199	0.134067157	7.098734822	1.26E-12	3.87E-11	slc17a7b	solute carrier family 17 member 7b [Source:ZFIN;Acc:ZDB-GENE-131125-32]	24	38557989	38579066	
ENSDARG00000090164	194.5645002	2.266450038	0.228806843	5.535018191	3.11E-08	6.03E-07	si:ch73-362m14.2	si:ch73-362m14.2 [Source:ZFIN;Acc:ZDB-GENE-120215-235]	21	43536339	43550120	
ENSDARG00000090185	3211.808203	-2.679081465	0.115700041	-14.51236705	1.01E-47	2.15E-45	si:dkeyp-73b11.8	si:dkeyp-73b11.8 [Source:ZFIN;Acc:ZDB-GENE-131120-176]	25	26754578	26758253	
ENSDARG00000090228	6555.820105	-2.463763364	0.122862453	-11.91383799	1E-32	1.09E-30	gsta.1	glutathione S-transferase, alpha tandem duplicate 1 [Source:ZFIN;Acc:ZDB-GENE-040426-2720]	13	844143	852506	
ENSDARG00000090268	165873.5523	-3.138281183	0.111344402	-19.20420916	3.41E-82	1.78E-79	krtt1c19e	keratin type 1 c19e [Source:ZFIN;Acc:ZDB-GENE-050506-95]	19	5339364	5345932	
ENSDARG00000090286	14742.35807	-2.24892104	0.07924553	-15.76014503	5.85E-56	1.67E-53	serpina1	serpin peptidase inhibitor, clade A (alpha-1 antiproteinase, antitrypsin), member 1 [Source:ZFIN;Acc:ZDB-GENE-030131-14	20	10544100	10547411	
ENSDARG00000090297	479.8872159	3.633926137	0.203001195	12.97492925	1.7E-38	2.45E-36	ldlrad2	low density lipoprotein receptor class A domain containing 2 [Source:ZFIN;Acc:ZDB-GENE-110204-1]	11	36477481	36489816	
ENSDARG00000090310	37.20539998	4.196429659	0.717838594	4.452852888	8.47E-06	0.000118959	CABZ01065328.1	CD48 antigen-like [Source:NCBI gene;Acc:100329398]	22	6922006	6923347	
ENSDARG00000090326	301.7312515	1.603867583	0.170148994	3.549051731	0.000386621	0.004026795	MAPK8IP1	mitogen-activated protein kinase 8 interacting protein 1 [Source:HGNC Symbol;Acc:HGNC:6882]	7	72594954	72605673	MAPK8IP1
ENSDARG00000090344	7.460890359	5.9502862	1.63094622	3.035223441	0.002403575	0.021281181	il19l	interleukin 19 like [Source:ZFIN;Acc:ZDB-GENE-071121-1]	11	21096339	21099783	
ENSDARG00000090369	7586.18437	-1.674523221	0.080773427	-8.350806056	6.78E-17	2.87E-15	zgc:86896	zgc:86896 [Source:ZFIN;Acc:ZDB-GENE-040625-80]	3	36275633	36286650	
ENSDARG00000090389	2006.23495	-1.322708689	0.108519692	-2.973733931	0.002942001	0.025659193	ndufv3	NADH:ubiquinone oxidoreductase subunit V3 [Source:ZFIN;Acc:ZDB-GENE-030131-6500]	9	19518674	19528038	
ENSDARG00000090398	63.59128299	2.207388268	0.360817968	3.346253162	0.000819116	0.007959978	PDE1C	phosphodiesterase 1C, calmodulin-dependent b [Source:NCBI gene;Acc:100536162]	2	50477779	50576188	PDE1C
ENSDARG00000090421	264.305643	2.18814482	0.186966122	6.3548669	2.09E-10	5.19E-09	SH2B2	SH2B adaptor protein 2 [Source:NCBI gene;Acc:100535524]	15	2559875	2578139	SH2B2
ENSDARG00000090428	35268.31665	-3.39891001	0.091364195	-26.25656592	6.01E-152	7.19E-149	ctrb1	chymotrypsinogen B1 [Source:ZFIN;Acc:ZDB-GENE-030131-1171]	7	35075847	35079299	
ENSDARG00000090481	1441.830724	1.42825439	0.104401131	4.102009092	4.1E-05	0.000507695	trarg1a	trafficking regulator of GLUT4 (SLC2A4) 1a [Source:ZFIN;Acc:ZDB-GENE-131121-306]	15	24845145	24869826	
ENSDARG00000090496	407.687367	2.147375935	0.182604206	6.283403642	3.31E-10	8.05E-09	thsd7ab	thrombospondin, type I, domain containing 7Ab [Source:ZFIN;Acc:ZDB-GENE-130530-999]	16	46869010	47178134	

ENSDARG00000090560	344.3367175	-1.51680178	0.156630011	-3.299506761	0.000968549	0.009282759	mfap5	microfibril associated protein 5 [Source:ZFIN;Acc:ZDB-GENE-100922-279]	16	12669765	12689080	
ENSDARG00000090618	401.869728	-3.036844001	0.214342955	-9.50273359	2.04E-21	1.2E-19	malrd1	MAM and LDL receptor class A domain containing 1 [Source:ZFIN;Acc:ZDB-GENE-140106-257]	7	41632928	41693004	
ENSDARG00000090624	1309.272734	2.064311215	0.112066742	9.497119275	2.16E-21	1.26E-19	ADGRL3	adhesion G protein-coupled receptor L3 [Source:HGNC Symbol;Acc:HGNC:20974]	7	72882446	73088964	ADGRL3
ENSDARG00000090642	318.9113003	1.568923458	0.168280323	3.380807975	0.00072273	0.007122597	CCDC92B	si:dkey-54n8.4 [Source:ZFIN;Acc:ZDB-GENE-131121-541]	15	25375923	25392589	CCDC92B
ENSDARG00000090646	337.1846169	1.574259068	0.173202048	3.31554433	0.000914648	0.008816662	tnk2a	tyrosine kinase, non-receptor, 2a [Source:ZFIN;Acc:ZDB-GENE-091118-85]	24	26036395	26052319	
ENSDARG00000090656	709.7578728	1.573163023	0.157897515	3.629968603	0.000283456	0.003038011	tomm20a	translocase of outer mitochondrial membrane 20 [Source:ZFIN;Acc:ZDB-GENE-040426-1976]	13	49597258	49707885	
ENSDARG00000090690	1718.864972	1.858143276	0.13684004	6.271141677	3.58E-10	8.68E-09	nell2a	neural EGFL like 2 [Source:HGNC Symbol;Acc:HGNC:7751]	25	581227	691883	
ENSDARG00000090696	58.7706744	2.594546699	0.400877688	3.977638937	6.96E-05	0.000832351	si:dkey-15b23.3	si:dkey-15b23.3 [Source:ZFIN;Acc:ZDB-GENE-141216-304]	7	11614144	11638126	
ENSDARG00000090722	3274.08806	-1.461550259	0.088287087	-5.227834301	1.72E-07	3.02E-06	C20H6orf58	liver-enriched gene 1, tandem duplicate 1 [Source:NCBI gene;Acc:796447]	20	1412193	1421005	C20H6orf58
ENSDARG00000090727	159.2353051	2.52505485	0.31412194	4.854977183	1.2E-06	1.92E-05	celf5b	cugbp, Elav-like family member 5b [Source:ZFIN;Acc:ZDB-GENE-070912-150]	2	53357953	53426522	
ENSDARG00000090830	725.3425636	1.591773513	0.124581439	4.750093743	2.03E-06	3.15E-05	tmem132e	transmembrane protein 132E [Source:NCBI gene;Acc:564044]	5	60518611	60687619	
ENSDARG00000090850	9731.660856	-2.121103779	0.132284462	-8.474946835	2.35E-17	1.05E-15	serpina1l	serine (or cysteine) proteinase inhibitor, clade A (alpha-1 antiproteinase, antitrypsin), member 1, like [Source:ZFIN;Acc:ZDB-	20	10538025	10541197	
ENSDARG00000090883	123.0439297	2.014481075	0.312535184	3.24597398	0.001170495	0.011028755	gabra3	gamma-aminobutyric acid (GABA) A receptor, alpha 3 [Source:ZFIN;Acc:ZDB-GENE-091204-365]	21	44300689	44451712	
ENSDARG00000090899	249.3499153	-5.896550526	0.350018082	-13.98942162	1.81E-44	3.31E-42	sycn.3	syncollin, tandem duplicate 3 [Source:ZFIN;Acc:ZDB-GENE-161220-1]	21	25216397	25219305	
ENSDARG00000090943	566.2318179	-1.885098463	0.138861623	-6.373960249	1.84E-10	4.62E-09	CABZ01033205.1	uncharacterized LOC100002960 [Source:NCBI gene;Acc:100002960]	15	454349	462783	
ENSDARG00000090948	36.70238271	-3.505006561	0.468996819	-5.341201602	9.23E-08	1.68E-06	si:ch73-29c22.1	si:ch73-29c22.1 [Source:ZFIN;Acc:ZDB-GENE-091204-309]	21	4934090	4950610	
ENSDARG00000090969	541.0114007	-2.310821925	0.149520233	-8.766853171	1.84E-18	8.82E-17	cbln18	cerebellin 18 [Source:ZFIN;Acc:ZDB-GENE-111109-3]	15	556956	568645	
ENSDARG00000091043	81.16601704	1.928535572	0.325251088	2.854826953	0.004306028	0.036076642	pcdh2ab6	protocadherin 2 alpha b 6 [Source:ZFIN;Acc:ZDB-GENE-041118-5]	14	2123123	2206476	
ENSDARG00000091059	67.24439653	3.269218272	0.417542895	5.434694977	5.49E-08	1.03E-06	onecut3b	one cut homeobox 3b [Source:ZFIN;Acc:ZDB-GENE-130805-1]	2	57504073	57563888	
ENSDARG00000091072	25.62051136	-2.625716579	0.560236472	-2.901839957	0.00370978	0.031565828	cib3	calcium and integrin binding family member 3 [Source:ZFIN;Acc:ZDB-GENE-121121-1]	2	55249838	55266971	
ENSDARG00000091079	1500.382317	-1.395775033	0.134073698	-2.951921505	0.003158032	0.02726858	si:zfos-1192g2.3	si:zfos-1192g2.3 [Source:ZFIN;Acc:ZDB-GENE-030131-4956]	16	54780544	54782705	
ENSDARG00000091085	14.83775277	4.471186551	1.177114511	2.948894536	0.003189128	0.027515298	lepa	leptin a [Source:ZFIN;Acc:ZDB-GENE-081001-1]	18	10689772	10690365	
ENSDARG00000091130	718.6753751	2.538303526	0.139384969	11.03636594	2.55E-28	2.25E-26	kcnq2b	potassium voltage-gated channel, KQT-like subfamily, member 2b [Source:ZFIN;Acc:ZDB-GENE-120130-1]	6	58406014	58499844	
ENSDARG00000091222	152.9439488	-1.749476082	0.203912216	-3.675483969	0.000237399	0.002574061	si:dkey-19018.2	si:dkey-19018.2 [Source:ZFIN;Acc:ZDB-GENE-030131-9103]	17	30591287	30605734	
ENSDARG00000091293	3919.114651	1.939002947	0.094372648	9.949948088	2.52E-23	1.65E-21	dmxl2	Dmx-like 2 [Source:ZFIN;Acc:ZDB-GENE-031007-6]	18	39669255	39787040	
ENSDARG00000091317	2030.185666	1.440851989	0.125562957	3.511003571	0.000446418	0.004605651	hunk	hormonally up-regulated Neu-associated kinase [Source:ZFIN;Acc:ZDB-GENE-050309-240]	10	33614377	33621739	
ENSDARG00000091359	1098.640227	2.078974965	0.173535776	6.217593791	5.05E-10	1.2E-08	CAMSAP3	calmodulin-regulated spectrin-associated protein 3-like [Source:NCBI gene;Acc:100007608]	1	59172744	59176949	CAMSAP3
ENSDARG00000091408	371.0688889	2.347460569	0.178877905	7.53285079	4.96E-14	1.7E-12	mtcl1	microtubule crosslinking factor 1 [Source:ZFIN;Acc:ZDB-GENE-120508-3]	2	54482603	54509065	
ENSDARG00000091459	193.2003679	1.926267038	0.223998053	4.135156646	3.55E-05	0.000446074	gabra2a	gamma-aminobutyric acid type A receptor alpha2 subunit a [Source:ZFIN;Acc:ZDB-GENE-141216-16]	13	12120664	12151350	
ENSDARG00000091465	728.9841428	-1.363263667	0.117695378	-3.086473503	0.00202546	0.018190007	si:dkey-66a8.7	si:dkey-66a8.7 [Source:ZFIN;Acc:ZDB-GENE-131127-421]	6	37451341	37460178	
ENSDARG00000091509	259.4125541	2.308181689	0.212946049	6.143254101	8.08E-10	1.88E-08	atp6ap1la	ATPase H+ transporting accessory protein 1 like a [Source:ZFIN;Acc:ZDB-GENE-110318-1]	11	42532464	42554290	
ENSDARG00000091560	1514.923756	-2.473047654	0.209998637	-7.014558159	2.31E-12	6.91E-11	slc6a19a.2	solute carrier family 6 member 19a, tandem duplicate 2 [Source:ZFIN;Acc:ZDB-GENE-141210-16]	19	684502	704000	
ENSDARG00000091609	565.1165973	-3.478420309	0.163275916	-15.17933799	4.85E-52	1.25E-49	spink4	serine peptidase inhibitor, Kazal type 4 [Source:ZFIN;Acc:ZDB-GENE-130821-1]	1	12135129	12138612	
ENSDARG00000091655	1008.586018	-1.460946771	0.110104458	-4.186449654	2.83E-05	0.00036122	gcsha	glycine cleavage system protein H (aminomethyl carrier), a [Source:ZFIN;Acc:ZDB-GENE-050320-18]	18	6145307	6155072	
ENSDARG00000091657	289.3378281	-1.504597769	0.172609638	-2.923346422	0.00346291	0.029666384	CT573256.1	serine/threonine-protein kinase VRK1-like [Source:NCBI gene;Acc:794952]	1	16460124	16461398	
ENSDARG00000091679	24.49123696	3.10941024	0.680732628	3.098735324	0.001943486	0.017522784	FO704858.1		3	7245170	7254636	
ENSDARG00000091727	128.4163553	-1.670209753	0.239993492	-2.792616363	0.005228366	0.042944114	CABZ01061592.1	E3 ubiquitin-protein ligase RNF14-like [Source:NCBI gene;Acc:558876]	15	3808996	3816757	
ENSDARG00000091783	272.4538115	3.402344342	0.588113115	4.084833817	4.41E-05	0.000544329	si:dkeyp-71f10.5	si:dkeyp-71f10.5 [Source:ZFIN;Acc:ZDB-GENE-131121-547]	3	30998362	31019832	
ENSDARG00000091831	1763.290499	-1.338503915	0.093339057	-3.626605252	0.000287172	0.003071282	ddx27	DEAD (Asp-Glu-Ala-Asp) box polypeptide 27 [Source:ZFIN;Acc:ZDB-GENE-031001-8]	6	57436314	57476465	
ENSDARG00000091916	713.0290827	-2.922472521	0.145701412	-13.19460459	9.42E-40	1.43E-37	ugt5b4	UDP glucuronosyltransferase 5 family, polypeptide B4 [Source:ZFIN;Acc:ZDB-GENE-070112-2332]	1	9630996	9652696	
ENSDARG00000091931	699.6564488	-1.511689797	0.115651095	-4.424426744	9.67E-06	0.000134269	gpatch4	G patch domain containing 4 [Source:ZFIN;Acc:ZDB-GENE-041008-158]	16	29025292	29030674	
ENSDARG00000091956	162.7600904	2.072128735	0.253968197	4.221507841	2.43E-05	0.000312698	tmem184bb	transmembrane protein 184bb [Source:ZFIN;Acc:ZDB-GENE-070705-124]	3	3313950	3328097	
ENSDARG00000092039	690.5932547	1.738230437	0.200028178	3.690632216	0.000223697	0.002431801	si:dkey-70p6.1	si:dkey-70p6.1 [Source:ZFIN;Acc:ZDB-GENE-040724-140]	8	23040727	23081667	
ENSDARG00000092042	311.2992763	-2.241460135	0.174392496	-7.118770397	1.09E-12	3.36E-11	AL935153.1		8	31438472	31440158	
ENSDARG00000092044	370.7476926	-2.381804994	0.199297988	-6.933361499	4.11E-12	1.21E-10	si:dkey-22f5.9	si:dkey-22f5.9 [Source:ZFIN;Acc:ZDB-GENE-091204-260]	21	30651064	30658520	
ENSDARG00000092067	1690.532923	1.559517213	0.101303814	5.523160417	3.33E-08	6.43E-07	atn1	atrophin 1 [Source:ZFIN;Acc:ZDB-GENE-030131-426]	16	31853919	31868985	
ENSDARG00000092071	355.0990959	-1.601238363	0.171272782	-3.510413959	0.00044741	0.00461398	si:ch211-117n7.6	si:ch211-117n7.6 [Source:ZFIN;Acc:ZDB-GENE-060503-474]	13	9889676	9895628	
ENSDARG00000092123	287.8726932	-1.612494853	0.175939227	-3.481286484	0.000499011	0.005085541	si:dkey-20d21.12	si:dkey-20d21.12 [Source:ZFIN;Acc:ZDB-GENE-081104-365]	8	26382604	26388090	
ENSDARG00000092124	996.7130756	-1.537602132	0.133611922	-4.023609002	5.73E-05	0.000696328	cox14	cytochrome c oxidase assembly factor COX14 [Source:NCBI gene;Acc:568423]	22	5752257	5756384	
ENSDARG00000092142	353.5095885	2.981758883	0.19091222	10.38047165	3.04E-25	2.23E-23	BX663503.1		18	49193824	49214852	
ENSDARG00000092155	25114.92099	-1.550495812	0.076751682	-7.172426645	7.37E-13	2.3E-11	apoc2	apolipoprotein C-II [Source:ZFIN;Acc:ZDB-GENE-030131-2168]	16	23984179	23994642	
ENSDARG00000092170	33387.12292	-1.729092681	0.078900081	-9.240708903	2.45E-20	1.36E-18	apoc1	apolipoprotein C-I [Source:ZFIN;Acc:ZDB-GENE-030131-1074]	16	23972126	23973721	
ENSDARG00000092228	7.250658116	5.910098394	1.636053206	3.001185033	0.002689311	0.023644271	si:dkey-27p18.3	si:dkey-27p18.3 [Source:ZFIN;Acc:ZDB-GENE-060526-287]	5	22406672	22424496	
ENSDARG00000092243	91.33702417	-1.735870939	0.261672332	-2.81218474	0.004920623	0.040736334	BX005065.1		8	45035418	45037752	
ENSDARG00000092272	99.76702546	-1.920500254	0.25616822	-3.593342893	0.000326463	0.00345907	BX511161.1		16	33566997	33592335	
ENSDARG00000092281	1496.385816	-1.273068144	0.099443793	-2.745954615	0.006033511	0.048759549	flnb	filamin B, beta (actin binding protein 278) [Source:ZFIN;Acc:ZDB-GENE-041026-1]	23	19606291	19652915	
ENSDARG00000092283	1356.251822	-2.076490982	0.145743491	-7.386202824	1.51E-13	4.98E-12	cxl34b.11	CX chemokine ligand 34b, duplicate 11 [Source:ZFIN;Acc:ZDB-GENE-091204-33]	24	27470097	27473771	
ENSDARG00000092307	34.80102057	2.56900494	0.53852593	2.913517909	0.003573813	0.03054351	CR456642.2		23	30517199	30698687	
ENSDARG00000092362	339.4455843	2.536142849	0.259730899	5.914363107	3.33E-09	7.27E-08	hsp70.2	heat shock cognate 70-kd protein, tandem duplicate 2 [Source:ZFIN;Acc:ZDB-GENE-110405-1]	3	26119603	26122353	
ENSDARG00000092379	453.0663889	-2.172481082	0.15299826	-7.663362208	1.81E-14	6.42E-13	si:dkeyp-51b9.3	si:dkeyp-51b9.3 [Source:ZFIN;Acc:ZDB-GENE-081104-469]	8	2478862	2481965	
ENSDARG00000092381	117.5583632	-3.333542812	0.261938955	-8.908727662	5.16E-19	2.58E-17	zgc:153759	zgc:153759 [Source:ZFIN;Acc:ZDB-GENE-060825-148]	8	22404981	22476458	
ENSDARG00000092404	68.64682245	-3.587614803	0.390293295	-6.629923801	3.36E-11	9.01E-10	LCT	si:dkey-79p17.3 [Source:ZFIN;Acc:ZDB-GENE-081105-132]	9	46387419	46399611	LCT
ENSDARG00000092419	1846.790844	5.678302734	0.4069156	11.49698545	1.37E-30	1.38E-28	vtg7	vitellogenin 7 [Source:ZFIN;Acc:ZDB-GENE-001201-6]	22	24851227	24858050	
ENSDARG00000092467	9233.798093	1.266594319	0.082824591	3.218782198	0.001287362	0.012057493	si:ch73-46j18.5	si:ch73-46j18.5 [Source:ZFIN;Acc:ZDB-GENE-030131-7892]	10	1708117	1718395	
ENSDARG00000092503	1950.756215	1.509927537	0.122212975	4.172450088	3.01E-05	0.000383374	arid1b	AT rich interactive domain 1B (SWI1-like) [Source:ZFIN;Acc:ZDB-GENE-030131-4459]	20	4897996	5052838	
ENSDARG00000092574	202.6870684	-2.127237004	0.244342577	-4.613346635	3.96E-06	5.87E-05	si:ch211-198d23.1	si:ch211-198d23.1 [Source:ZFIN;Acc:ZDB-GENE-081104-163]	8	38593904	38616712	
ENSDARG00000092578	181.7347486	-1.73648049	0.240966494	-3.056360566	0.002240417	0.019976141	si:ch211-222e20.4	si:ch211-222e20.4 [Source:ZFIN;Acc:ZDB-GENE-091118-60]	10	28428222	28431809	
ENSDARG00000092624	488.6267294	1.76887419	0.151660153	5.069717899	3.98E-07	6.73E-06	sptbn4b	spectrin, beta, non-erythrocytic 4b [Source:ZFIN;Acc:ZDB-GENE-081031-8]	18	46099223	46135927	
ENSDARG00000092638	1047.935216	2.285940611	0.14643318	8.781757028	1.61E-18	7.75E-17	ptprua	protein tyrosine phosphatase receptor type Ua [Source:ZFIN;Acc:ZDB-GENE-060503-232]	19	16222618	16657563	
ENSDARG00000092716	280.9029379	1.624257976	0.172314133	3.622790331	0.000291442	0.003109004	VASH1	vasohibin 1 [Source:NCBI gene;Acc:100001748]	17	53250802	53259501	VASH1
ENSDARG00000092722	483.4464207	1.861893259	0.15554501	5.541118027	3.01E-08	5.85E-07	unc5da	unc-5 netrin receptor Da [Source:ZFIN;Acc:ZDB-GENE-080908-2]	21	16273782	16632808	
ENSDARG00000092730	1747.009633	-2.540455085	0.129090457	-11.93314457	7.95E-33	8.75E-31	enam	enamelin [Source:ZFIN;Acc:ZDB-GENE-071005-4]	1	43901966	43905263	
ENSDARG00000092788	422.0336088	-1.594410972	0.145662973	-4.080727997	4.49E-05	0.00055349	si:dkey-21e2.15	si:dkey-21e2.15 [Source:ZFIN;Acc:ZDB-GENE-050208-780]	22	19289970	19291261	
ENSDARG00000092873	7630.656412	-1.33004306	0.086547792	-3.813419757	0.000137057	0.001558093	zgc:193541	zgc:193541 [Source:ZFIN;Acc:ZDB-GENE-081022-15]	20	20726231	20730631	
ENSDARG00000092890	481.1771837	-1.695751705	0.187772839	-3.705284054	0.000211154	0.002309445	si:ch73-44m9.5	si:ch73-44m9.5 [Source:ZFIN;Acc:ZDB-GENE-081031-86]	22	7738966	7739549	
ENSDARG00000092898	145.6290043	-2.023214831	0.269746117	-3.793251387	0.000148687	0.001676642	si:ch211-157j23.3	si:ch211-157j23.3 [Source:ZFIN;Acc:ZDB-GENE-091117-36]	24	5789582	5798424	
ENSDARG00000092916	80.8626499	-2.15376848	0.324923688	-3.550890633	0.00038393	0.004007079	mlnl	motilin-like [Source:ZFIN;Acc:ZDB-GENE-091204-289]	21	11969603	11981523	
ENSDARG00000092945	273.1425722	1.841975654	0.204079012	4.125733675	3.7E-05	0.000462884	si:ch211-250g4.3	si:ch211-250g4.3 [Source:ZFIN;Acc:ZDB-GENE-060503-506]	19	37509638	37519610	
ENSDARG00000092947	126818.0235	-2.230918661	0.090785769	-13.55849789	7.06E-42	1.17E-39	cyt1	type I cytokeratin, enveloping layer [Source:ZFIN;Acc:ZDB-GENE-991008-6]	19	5362420	5364649	
ENSDARG00000092954	1049.751613	-1.408569236	0.113641485	-3.595247227	0.000324084	0.003436766	mrpl34	mitochondrial ribosomal protein L34 [Source:ZFIN;Acc:ZDB-GENE-070912-582]	2	24317465	24320183	
ENSDARG00000093003	154.1932433	-2.074641849	0.272580183	-3.942479735	8.06E-05	0.000955277	acy3.1	aspartoacylase (aminocyclase) 3, tandem duplicate 1 [Source:NCBI gene;Acc:791456]	1	52499412	52505279	
ENSDARG00000093006	51.03161878	-2.619435537	0.393479223	-4.115682465	3.86E-05	0.000481365	rarres3	retinoic acid receptor responder 3 [Source:ZFIN;Acc:ZDB-GENE-060929-616]	14	45559268	45564908	
ENSDARG00000093019	678.3872804	1.701769298	0.141466776	4.960665096	7.03E-07	1.15E-05	si:dkey-83k24.5	si:dkey-83k24.5 [Source:ZFIN;Acc:ZDB-GENE-081104-440]	8	8936912	8942454	
ENSDARG00000093021	41.83526146	2.368964244	0.452950753	3.02232469	0.002508413	0.022162507	FAM163A	si:dkey-171o17.8 [Source:ZFIN;Acc:ZDB-GENE-070705-312]	22	17286841	17300617	FAM163A
ENSDARG00000093022	1435.503888	-1.663595447	0.115739124	-5.733544744	9.84E-09	2.03E-07	selenoh	selenoprotein H [Source:ZFIN;Acc:ZDB-GENE-030411-2]	8	7315625	7317444	
ENSDARG00000093043	547.5982547	-1.527846077	0.132886978	-3.972142976	7.12E-05	0.000850579	ugt2a7	UDP glucuronosyltransferase 2 family, polypeptide A7 [Source:ZFIN;Acc:ZDB-GENE-141215-33]	5	9377005	9391846	
ENSDARG00000093052	251.9881301	2.348345916	0.218546247	6.169613673	6.85E-10	1.61E-08	c6	complement component 6 [Source:ZFIN;Acc:ZDB-GENE-040426-1358]	21	20795472	20840723	
ENSDARG00000093054	82.75670055	-2.344247616	0.3704557	-3.628632565	0.000284926	0.003049867	BX548073.3		5	42322847	42344441	
ENSDARG00000093062	103.203608	2.187787621	0.306172573	3.879471006	0.000104684	0.001211466	FP016056.1		8	3688979	3691230	
ENSDARG00000093078	218.8835756	-2.786542911	0.23095051	-7.735609284	1.03E-14	3.72E-13	plscr3a	phospholipid scramblase 3a [Source:ZFIN;Acc:ZDB-GENE-060531-17]	5	24234466	24238733	
ENSDARG00000093091	289.6515118	2.432607558	0.209762872	6.829652666	8.51E-12	2.43E-10	iqsec3b	IQ motif and Sec7 domain ArfGEF 3b [Source:ZFIN;Acc:ZDB-GENE-050420-210]	18	6422528	6477662	
ENSDARG00000093105	20.20135949	4.925053865	1.132663665	3.465330429	0.00052958	0.005362242	BX004774.1		8	20842134	20844502	
ENSDARG00000093120	1297.388074	2.031558516	0.116331622	8.867395627	7.49E-19	3.7E-17	si:dkey-117m1.4	si:dkey-117m1.4 [Source:ZFIN;Acc:ZDB-GENE-090313-177]	1	40057412	40086978	
ENSDARG00000093185	220.2018496	2.259826871	0.222287464	5.667557001	1.45E-08	2.93E-07	GUCY1A2	guanylate cyclase 1, soluble, alpha 2 [Source:NCBI gene;Acc:100330875]	15	44366556	44404098	GUCY1A2
ENSDARG00000093193	263.6674174	-2.742567273	0.186649098	-9.336060499	1E-20	5.68E-19	chia.6	chitinase, acidic.6 [Source:ZFIN;Acc:ZDB-GENE-030131-1140]	23	17980875	17993663	
ENSDARG00000093279	109.3431913	-2.956011209	0.305541445	-6.401786855	1.54E-10	3.9E-09	si:dkey-11o1.3	si:dkey-11o1.3 [Source:ZFIN;Acc:ZDB-GENE-100921-37]	16	11603096	11621551	
ENSDARG00000093342	347.4076595	3.523160569	0.22750456	11.09059339	1.39E-28	1.26E-26	si:ch211-13f8.1	si:ch211-13f8.1 [Source:ZFIN;Acc:ZDB-GENE-061207-4]	2	7192966	7209705	
ENSDARG00000093357	500.3902225	3.142691907	0.186481367	11.49011261	1.48E-30	1.48E-28	nyap2b	neuronal tyrosine-phosphorylated phosphoinositide-3-kinase adaptor 2b [Source:ZFIN;Acc:ZDB-GENE-070705-169]	2	5734870	5776030	
ENSDARG00000093359	312.1036583	2.266297978	0.191584688	6.609599084	3.85E-11	1.03E-09	inpp5jb	inositol polyphosphate-5-phosphatase Jb [Source:ZFIN;Acc:ZDB-GENE-081104-46]	20	69232230	69271450	
ENSDARG00000093381	386.3505095	3.729385241	0.206501874	13.21724202	6.98E-40	1.07E-37	tgm2l	transglutaminase 2, like [Source:ZFIN;Acc:ZDB-GENE-050420-97]	18	18803539	18850302	
ENSDARG00000093413	2786.692594	1.298369389	0.096474467	3.092729066	0.001983251	0.017855662	edil3a	EGF-like repeats and discoidin I-like domains 3a [Source:ZFIN;Acc:ZDB-GENE-060503-366]	5	46658045	46980651	
ENSDARG00000093425	11.87844028	6.619152864	1.602569697	3.506339147	0.000454316	0.004673682	BX539332.1		22	24798458	24804287	
ENSDARG00000093453	982.3097079	1.892611872	0.129016066	6.918610224	4.56E-12	1.34E-10	ahdc1	AT hook, DNA binding motif, containing 1 [Source:ZFIN;Acc:ZDB-GENE-060503-584]	19	43439401	43500299	
ENSDARG00000093493	131.6618369	1.790746125	0.26493479	2.984682101	0.002838732	0.024799671	urp1	urotensin-related peptide 1 [Source:ZFIN;Acc:ZDB-GENE-100922-138]	14	33892300	33894915	
ENSDARG00000093584	1873.764786	-2.111815675	0.12569527	-8.845326296	9.13E-19	4.47E-17	zgc:193505	zgc:193505 [Source:ZFIN;Acc:ZDB-GENE-030131-7103]	13	22675802	22678102	
ENSDARG00000093600	177.398173	-1.848886332	0.190798962	-4.449114005	8.62E-06	0.000120845	spp1	osteomodulin [Source:ZFIN;Acc:ZDB-GENE-060503-756]	22	10463890	10470663	
ENSDARG00000093619	275.6772726	-2.716809716	0.215871238	-7.952934052	1.82E-15	6.98E-14	sult3st2	sulfotransferase family 3, cytosolic sulfotransferase 2 [Source:ZFIN;Acc:ZDB-GENE-030131-1135]	22	15336752	15342165	
ENSDARG00000093628	2494.149155	-3.082861085	0.092766804	-22.45265533	1.21E-111	9.17E-109	s100a11	S100 calcium binding protein A11 [Source:ZFIN;Acc:ZDB-GENE-070717-5]	19	7567763	7570475	
ENSDARG00000093671	1544.070011	-1.5487953	0.127917182	-4.290239123	1.78E-05	0.00023704	BX001030.1		24	38098896	38099675	
ENSDARG00000093724	9.823520235	6.349084831	1.594669167	3.354353957	0.000795505	0.007754555	si:dkey-54g2.2	si:dkey-54g2.2 [Source:ZFIN;Acc:ZDB-GENE-041014-140]	20	12936096	12936861	
ENSDARG00000093738	126.9084282	5.780834371	0.586709652	8.148552447	3.68E-16	1.47E-14	pth1a	parathyroid hormone 1a [Source:ZFIN;Acc:ZDB-GENE-040623-1]	25	17860798	17861696	
ENSDARG00000093768	1416.658374	1.828213431	0.129974642	6.372115519	1.86E-10	4.68E-09	prr18	proline rich 18 [Source:ZFIN;Acc:ZDB-GENE-081107-6]	13	4405282	4407074	
ENSDARG00000093774	1921.34913	-2.525648045	0.114240318	-13.3547251	1.11E-40	1.75E-38	rbp2b	retinol binding protein 2b, cellular [Source:ZFIN;Acc:ZDB-GENE-040715-7]	2	39016066	39017838	
ENSDARG00000093780	214.5856839	-1.786983342	0.19728946	-3.988978144	6.64E-05	0.000795067	BX548078.1		15	20960069	20978248	

ENSDARG00000093844	6336.500955	-5.355108219	0.133677879	-32.57912415	8.1E-233	2.54E-229	zgc:136461	zgc:136461 [Source:ZFIN;Acc:ZDB-GENE-060519-7]	7	35068036	35069807	
ENSDARG00000093862	60.82951991	-2.348888108	0.41873874	-3.221311955	0.001276052	0.011960483	si:ch211-274k16.2	si:ch211-274k16.2 [Source:ZFIN;Acc:ZDB-GENE-110411-199]	22	6211114	6229275	
ENSDARG00000093975	18.65276893	3.148723581	0.755595419	2.843748821	0.004458618	0.037193677	si:dkey-222h21.9	si:dkey-222h21.9 [Source:ZFIN;Acc:ZDB-GENE-081031-10]	1	58228542	58238120	
ENSDARG00000094005	24.78580235	5.207122623	1.121761058	3.750462359	0.000176509	0.001965638	si:dkey-98j1.5	si:dkey-98j1.5 [Source:ZFIN;Acc:ZDB-GENE-090313-358]	23	23254976	23256726	
ENSDARG00000094021	16.32258254	7.081308511	1.538173409	3.953590977	7.7E-05	0.000914553	BX649641.5		18	18696207	18701764	
ENSDARG00000094041	13140.05684	-2.45690878	0.09645708	-15.10421811	1.52E-51	3.78E-49	krt17	keratin 17 [Source:ZFIN;Acc:ZDB-GENE-060503-86]	19	5367330	5372572	
ENSDARG00000094077	353.9879448	-1.549388129	0.192134822	-2.85938865	0.004244584	0.035609393	si:dkey-21e2.16	si:dkey-21e2.16 [Source:ZFIN;Acc:ZDB-GENE-050208-698]	22	19311411	19313738	
ENSDARG00000094159	40.64121519	2.236075031	0.439976058	2.809414303	0.004963173	0.041061505	BX072561.2		8	22844700	22845798	
ENSDARG00000094265	261.4867823	-1.844797735	0.180146547	-4.689502801	2.74E-06	4.16E-05	si:dkey-183n20.15	si:dkey-183n20.15 [Source:ZFIN;Acc:ZDB-GENE-041001-160]	20	25473056	25486384	
ENSDARG00000094282	202.9670085	1.550366757	0.196668399	2.798450379	0.005134846	0.042231247	dnah11	dynein, axonemal, heavy chain 11 [Source:ZFIN;Acc:ZDB-GENE-100922-8]	16	19181163	19349318	
ENSDARG00000094312	1288.19204	-1.537468675	0.116391161	-4.617779143	3.88E-06	5.76E-05	ndufa9b	NADH:ubiquinone oxidoreductase subunit A9b [Source:ZFIN;Acc:ZDB-GENE-091116-31]	10	8128374	8129456	
ENSDARG00000094316	123.0171284	4.254092972	0.397404591	8.188362804	2.65E-16	1.07E-14	si:rp71-80o10.4	si:rp71-80o10.4 [Source:ZFIN;Acc:ZDB-GENE-090313-413]	23	43300892	43307237	
ENSDARG00000094324	2107.797051	1.46878616	0.108228391	4.33145272	1.48E-05	0.000200333	efemp2a	EGF containing fibulin extracellular matrix protein 2a [Source:ZFIN;Acc:ZDB-GENE-041212-8]	14	30669662	30704075	
ENSDARG00000094433	116.7380606	-2.313629864	0.301543902	-4.356346976	1.32E-05	0.000180121	caspbl	caspase b, like [Source:ZFIN;Acc:ZDB-GENE-090311-53]	1	58105762	58115298	
ENSDARG00000094512	196.4947986	1.626569348	0.2150432	2.913690593	0.003571837	0.03054351	gabrz	gamma-aminobutyric acid (GABA) A receptor, zeta [Source:ZFIN;Acc:ZDB-GENE-080303-26]	12	29054907	29105525	
ENSDARG00000094522	888.2909678	1.869500057	0.151867902	5.725370835	1.03E-08	2.13E-07	si:ch211-14c7.2	si:ch211-14c7.2 [Source:ZFIN;Acc:ZDB-GENE-060503-856]	18	46194037	46208581	
ENSDARG00000094526	503.9888238	2.488733641	0.166881367	8.920909922	4.62E-19	2.33E-17	ksr2	kinase suppressor of ras 2 [Source:ZFIN;Acc:ZDB-GENE-030131-8047]	5	12272632	12525441	
ENSDARG00000094550	8.536128418	-6.968203855	1.44577248	-4.128038082	3.66E-05	0.000458728	BX649490.2		22	30497494	30499478	
ENSDARG00000094557	5694.605867	-1.557330032	0.108315075	-5.145452096	2.67E-07	4.58E-06	nupr1b	nuclear protein 1b [Source:ZFIN;Acc:ZDB-GENE-030131-4653]	3	15505275	15506158	
ENSDARG00000094577	474.6779535	1.710627424	0.236559726	3.004008487	0.002664479	0.023442371	cdc42se2	CDC42 small effector 2 [Source:NCBI gene;Acc:794234]	10	5159475	5221537	
ENSDARG00000094605	52.60028752	2.170175838	0.383895316	3.048163888	0.002302443	0.020457921	si:ch211-154e10.1	si:ch211-154e10.1 [Source:ZFIN;Acc:ZDB-GENE-081104-136]	5	69981453	70065490	
ENSDARG00000094666	17.86340904	7.21345258	1.529179511	4.06325911	4.84E-05	0.000593692	CU571324.1		5	36505473	36506619	
ENSDARG00000094677	564.798299	1.444815355	0.147140031	3.023075036	0.002502202	0.022115412	si:dkey-92j12.5	si:dkey-92j12.5 [Source:ZFIN;Acc:ZDB-GENE-090313-354]	1	23161369	23240046	
ENSDARG00000094803	41.80969054	-2.076055677	0.375136922	-2.868434468	0.004125086	0.034710391	CR392026.2		2	25814552	25816832	
ENSDARG00000094833	513.5178293	-1.586964033	0.140255833	-4.184952741	2.85E-05	0.000363424	coa5	cytochrome C oxidase assembly factor 5 [Source:NCBI gene;Acc:751633]	9	7081577	7089303	
ENSDARG00000094844	12.82089786	6.732525385	1.550623402	3.696916593	0.000218234	0.002375495	BX323457.3		9	25066661	25067730	
ENSDARG00000094860	226.6563472	2.25999974	0.229701162	5.485386881	4.13E-08	7.88E-07	gpr186	G protein-coupled receptor 186 [Source:ZFIN;Acc:ZDB-GENE-060503-489]	19	43547465	43552252	
ENSDARG00000094923	43.37331302	-3.458654017	0.492574345	-4.991437418	5.99E-07	9.93E-06	CU469568.2		25	29393632	29395206	
ENSDARG00000094929	468.9655721	-4.227859696	0.180718658	-17.8612421	2.36E-71	9.57E-69	apoa4b.2	apolipoprotein A-IV b, tandem duplicate 2 [Source:ZFIN;Acc:ZDB-GENE-030131-3143]	16	23921610	23922868	
ENSDARG00000094946	110.249123	-1.756646674	0.25351699	-2.984599475	0.002839499	0.024799671	si:ch211-132b12.2	si:ch211-132b12.2 [Source:ZFIN;Acc:ZDB-GENE-050420-178]	18	40572294	40583104	
ENSDARG00000094959	5.298313847	-6.273969872	1.543102756	-3.417769719	0.000631365	0.006286352	si:ch73-379f5.5	si:ch73-379f5.5 [Source:ZFIN;Acc:ZDB-GENE-091204-256]	3	8224622	8227209	
ENSDARG00000094965	2263.330641	1.727799915	0.095980928	7.582755527	3.38E-14	1.17E-12	nfil3-5	nuclear factor, interleukin 3 regulated, member 5 [Source:ZFIN;Acc:ZDB-GENE-030131-7677]	6	8172227	8180042	
ENSDARG00000094975	150.7468656	-2.003271357	0.221986013	-4.519525089	6.2E-06	8.93E-05	si:ch211-214p16.2	si:ch211-214p16.2 [Source:NCBI gene;Acc:368902]	17	25587625	25588430	
ENSDARG00000095002	1050.685784	-3.274203774	0.150775925	-15.08333489	2.08E-51	5.08E-49	TNNC2	si:rp71-17i16.4 [Source:ZFIN;Acc:ZDB-GENE-090312-215]	23	20408227	20420243	TNNC2
ENSDARG00000095022	686.6793713	2.504564564	0.162826698	9.240281712	2.46E-20	1.36E-18	thsd7ba	thrombospondin, type I, domain containing 7Ba [Source:ZFIN;Acc:ZDB-GENE-070621-2]	9	10152820	10532591	
ENSDARG00000095025	40.8288752	3.150886421	0.513312857	4.190205628	2.79E-05	0.000355472	CR388166.2		21	25747063	25748913	
ENSDARG00000095082	234.4815364	1.989339751	0.202873296	4.876638611	1.08E-06	1.73E-05	ITLN1	si:ch211-194p6.12 [Source:ZFIN;Acc:ZDB-GENE-160728-9]	7	33172066	33176106	ITLN1
ENSDARG00000095157	590.2639712	-1.393895419	0.120788576	-3.261032072	0.001110075	0.010514713	zgc:163080	zgc:163080 [Source:ZFIN;Acc:ZDB-GENE-070410-137]	13	852289	856701	
ENSDARG00000095193	22.34672508	7.535558903	1.528144053	4.276795039	1.9E-05	0.000250882	si:dkey-23c22.7	si:dkey-23c22.7 [Source:ZFIN;Acc:ZDB-GENE-081105-139]	8	22404981	22436971	
ENSDARG00000095230	158.8710686	-1.741662119	0.219923659	-3.372361672	0.000745265	0.007321683	CR769772.3		22	25432849	25435548	
ENSDARG00000095325	13.90996131	5.394237431	1.558670823	2.819220945	0.004814037	0.039906585	si:dkey-49n23.3	si:dkey-49n23.3 [Source:ZFIN;Acc:ZDB-GENE-050208-799]	22	30839702	30841615	
ENSDARG00000095362	91.55985559	-5.304170616	0.44090381	-9.762153365	1.64E-22	1.02E-20	si:ch211-77g15.32	si:ch211-77g15.32 [Source:ZFIN;Acc:ZDB-GENE-070912-320]	2	47986662	47988542	
ENSDARG00000095400	507.480451	-1.435502993	0.150128454	-2.900869087	0.003721293	0.031653059	si:ch73-52f15.5	si:ch73-52f15.5 [Source:ZFIN;Acc:ZDB-GENE-091204-341]	10	26667475	26677986	
ENSDARG00000095402	99.45743658	3.089731881	0.337135197	6.1984981	5.7E-10	1.35E-08	BX897741.1		5	67161442	67164419	
ENSDARG00000095462	207.0714134	-4.237682537	0.234741014	-13.79257286	2.83E-43	4.99E-41	cela1.2	chymotrypsin like elastase family member 1, tandem duplicate 2 [Source:ZFIN;Acc:ZDB-GENE-050208-732]	22	7472420	7476352	
ENSDARG00000095464	1439.878997	-1.391131836	0.125384387	-3.119462035	0.001811816	0.016424123	gstt2	glutathione S-transferase theta 2 [Source:NCBI gene;Acc:393493]	5	10046643	10056341	
ENSDARG00000095633	929.3119694	-3.533905438	0.152833261	-16.57954177	9.8E-62	3.37E-59	si:ch211-133l5.7	si:ch211-133l5.7 [Source:ZFIN;Acc:ZDB-GENE-081104-117]	8	20832584	20838342	
ENSDARG00000095705	717.9146424	-1.450940922	0.142483431	-3.164865686	0.001551546	0.014255251	CDPF1	si:ch211-239e6.4 [Source:NCBI gene;Acc:563220]	4	18597162	18606573	CDPF1
ENSDARG00000095802	583.6028697	2.218460715	0.136846455	8.903852943	5.39E-19	2.69E-17	pcare2	photoreceptor cilium actin regulator 2 [Source:ZFIN;Acc:ZDB-GENE-110411-72]	20	106481	113317	
ENSDARG00000095807	1356.56609	-2.060888636	0.10678873	-9.934462522	2.95E-23	1.91E-21	hp	haptoglobin [Source:ZFIN;Acc:ZDB-GENE-030131-1259]	8	50143194	50147948	
ENSDARG00000095831	228.4594126	1.580670721	0.201561052	2.880867687	0.003965821	0.033607721	si:ch211-175f12.2	si:ch211-175f12.2 [Source:ZFIN;Acc:ZDB-GENE-110411-220]	16	4010967	4031386	
ENSDARG00000095832	728.4714564	-2.092326803	0.131149881	-8.328843288	8.16E-17	3.45E-15	CU682777.2		23	44788132	44794174	
ENSDARG00000095833	471.4210606	2.168243648	0.146523822	7.973062879	1.55E-15	5.98E-14	alk	ALK receptor tyrosine kinase [Source:ZFIN;Acc:ZDB-GENE-031027-1]	17	47187870	47305034	
ENSDARG00000095897	4939.310297	-1.330448313	0.088234566	-3.745111768	0.000180313	0.002002676	atp5f1e	ATP synthase F1 subunit epsilon [Source:ZFIN;Acc:ZDB-GENE-110411-22]	6	49893488	49902140	
ENSDARG00000095906	1371.634819	1.919202691	0.213526362	4.304867465	1.67E-05	0.000223324	CR848047.1		16	29212421	29227002	
ENSDARG00000095911	34.39793168	8.15873309	1.496138519	4.784806352	1.71E-06	2.69E-05	si:ch211-149b19.4	si:ch211-149b19.4 [Source:ZFIN;Acc:ZDB-GENE-110411-90]	23	43834376	43843790	
ENSDARG00000095930	1418.22118	-1.798096486	0.125323274	-6.36830225	1.91E-10	4.78E-09	myha	myosin, heavy chain a [Source:ZFIN;Acc:ZDB-GENE-060531-50]	5	32246872	32257316	
ENSDARG00000095939	12.70185904	-3.316926876	0.791221687	-2.928290405	0.003408315	0.029238594	si:ch73-226l13.2	si:ch73-226l13.2 [Source:ZFIN;Acc:ZDB-GENE-110411-18]	11	29975830	29981972	
ENSDARG00000095949	148.6677512	5.207825859	0.48574961	8.662540886	4.61E-18	2.16E-16	si:dkey-22i16.9	si:dkey-22i16.9 [Source:ZFIN;Acc:ZDB-GENE-090313-261]	1	43973880	43983405	
ENSDARG00000095965	118.2164594	1.924022751	0.26650844	3.467142546	0.000526023	0.00532837	BX005108.3		9	45551068	45552324	
ENSDARG00000096003	4196.290387	-1.431744645	0.095166739	-4.536717851	5.71E-06	8.3E-05	atp5md	ATP synthase membrane subunit DAPIT [Source:ZFIN;Acc:ZDB-GENE-110914-234]	1	49432137	49434892	
ENSDARG00000096110	154.6799277	-3.755998407	0.286934861	-9.604961907	7.62E-22	4.6E-20	zgc:123278	zgc:123278 [Source:ZFIN;Acc:ZDB-GENE-051127-21]	25	25381548	25384045	
ENSDARG00000096242	465.3720017	-2.487271979	0.144669151	-10.28050531	8.63E-25	6.21E-23	si:ch211-76l23.4	si:ch211-76l23.4 [Source:ZFIN;Acc:ZDB-GENE-030131-8310]	3	28822065	28828569	
ENSDARG00000096269	62.93795397	-2.788956869	0.404454396	-4.423136167	9.73E-06	0.00013498	CR388364.1		6	39491846	39506586	
ENSDARG00000096307	133.2262147	1.93167076	0.289628899	3.216774172	0.001296406	0.01212862	cacna1ib	calcium voltage-gated channel subunit alpha1 Ib [Source:ZFIN;Acc:ZDB-GENE-120215-18]	6	515181	543693	
ENSDARG00000096338	22.80259871	-2.946210382	0.542291437	-3.588864309	0.000332122	0.003512647	CR792438.1		3	28695762	28698856	
ENSDARG00000096375	108.5738566	2.031915036	0.277977189	3.712229189	0.000205442	0.002251876	7-Mar	membrane-associated ring finger (C3HC4) 7 [Source:ZFIN;Acc:ZDB-GENE-080204-121]	6	11765341	11812068	
ENSDARG00000096398	128.1110873	2.323470181	0.33034413	4.006337814	6.17E-05	0.000743832	si:ch211-276a17.5	si:ch211-276a17.5 [Source:ZFIN;Acc:ZDB-GENE-120215-248]	6	8339298	8346939	
ENSDARG00000096508	1855.140394	-1.745383634	0.125185117	-5.954251222	2.61E-09	5.76E-08	BX322618.1		20	12203762	12208117	
ENSDARG00000096519	65.97012416	2.647249862	0.380178285	4.332835217	1.47E-05	0.000199401	irbpl	interphotoreceptor retinoid-binding protein like [Source:ZFIN;Acc:ZDB-GENE-070301-4]	12	2660733	2663471	
ENSDARG00000096527	39.91562649	8.371207791	1.478893722	4.984271474	6.22E-07	1.03E-05	BX548015.2		1	30853969	30857925	
ENSDARG00000096556	7.648995123	5.986286461	1.654504813	3.013763647	0.002580287	0.022749488	si:dkey-19f4.2	si:dkey-19f4.2 [Source:ZFIN;Acc:ZDB-GENE-121214-262]	20	7799615	7801342	
ENSDARG00000096616	4458.390756	-1.50547771	0.147362787	-3.430158456	0.000603229	0.006042158	si:ch211-255p10.3	si:ch211-255p10.3 [Source:ZFIN;Acc:ZDB-GENE-121214-339]	12	4677193	4683325	
ENSDARG00000096637	130.1525866	-1.734332735	0.21671277	-3.388506985	0.000702742	0.006941966	si:dkey-11c5.11	si:dkey-11c5.11 [Source:ZFIN;Acc:ZDB-GENE-121214-253]	12	27236871	27242533	
ENSDARG00000096655	641.2336339	3.202609857	0.163054468	13.50842998	1.39E-41	2.3E-39	BX901920.1		20	14657141	14661432	
ENSDARG00000096687	228.8168097	1.673565576	0.2214069	3.042206795	0.002348505	0.020830326	rgs7b	regulator of G protein signaling 7b [Source:ZFIN;Acc:ZDB-GENE-130531-78]	12	47573586	47601845	
ENSDARG00000096712	545.1560333	-2.397608585	0.145857224	-9.582032011	9.52E-22	5.69E-20	si:dkey-193p11.2	si:dkey-193p11.2 [Source:ZFIN;Acc:ZDB-GENE-030131-8407]	12	25885910	25887864	
ENSDARG00000096713	82.99205112	1.92272688	0.312283754	2.954770681	0.003129015	0.027064581	CR388040.1		2	6958513	6959134	
ENSDARG00000096721	1923.422769	-2.724124129	0.123652944	-13.94325177	3.46E-44	6.2E-42	si:rp71-1c10.11	si:rp71-1c10.11 [Source:ZFIN;Acc:ZDB-GENE-130603-61]	12	28964034	28971002	
ENSDARG00000096722	114.7607916	-2.194296694	0.265731672	-4.494370894	6.98E-06	9.96E-05	si:dkey-16i5.8	si:dkey-16i5.8 [Source:ZFIN;Acc:ZDB-GENE-030131-1207]	12	19401854	19402950	
ENSDARG00000096739	222.196546	-4.571108658	0.246327131	-14.49742318	1.26E-47	2.65E-45	si:dkey-219e21.2	si:dkey-219e21.2 [Source:ZFIN;Acc:ZDB-GENE-141215-26]	12	22607761	22618080	
ENSDARG00000096756	83.36066589	2.31638046	0.352086028	3.738803467	0.000184898	0.00204816	si:ch211-196c10.13	si:ch211-196c10.13 [Source:ZFIN;Acc:ZDB-GENE-130603-71]	8	23142946	23143712	
ENSDARG00000096770	616.6366509	1.425974848	0.132769844	3.208370483	0.001334894	0.012451572	CU571074.2		20	46957091	46959768	
ENSDARG00000096784	60.4213656	2.489573037	0.415466469	3.585302665	0.000336688	0.003553908	CU929359.2		12	2578020	2606520	
ENSDARG00000096794	13.10242826	5.304786589	1.552939397	2.772024844	0.005570878	0.045430351	BX119915.2		20	39458596	39473280	
ENSDARG00000096809	200.3941697	-1.719631968	0.187389934	-3.840291496	0.000122888	0.001405294	si:ch211-168b3.2	si:ch211-168b3.2 [Source:ZFIN;Acc:ZDB-GENE-130603-47]	2	5927255	5933351	
ENSDARG00000096843	52.9108869	2.134258442	0.385315576	2.943712931	0.003243007	0.027925457	zgc:193593	zgc:193593 [Source:ZFIN;Acc:ZDB-GENE-080723-21]	17	24917350	24919297	
ENSDARG00000096849	7850.256364	-1.408968217	0.107785742	-3.794270094	0.000148078	0.001671275	si:dkey-16p21.8	si:dkey-16p21.8 [Source:ZFIN;Acc:ZDB-GENE-131121-428]	3	32325143	32337695	
ENSDARG00000096920	475.463623	2.393563792	0.267059936	5.218168672	1.81E-07	3.16E-06	si:zfos-375h5.1	si:zfos-375h5.1 [Source:ZFIN;Acc:ZDB-GENE-131121-284]	5	3611498	3627110	
ENSDARG00000096927	16.09578478	-3.093363758	0.677426707	-3.090170103	0.002000419	0.017984425	si:ch211-210b2.3	si:ch211-210b2.3 [Source:ZFIN;Acc:ZDB-GENE-131122-13]	15	656069	660289	
ENSDARG00000096978	48.8229054	4.607344216	0.690551365	5.223860814	1.75E-07	3.08E-06	CU972512.1		3	25951407	25974077	
ENSDARG00000096990	515.1035969	-2.608570721	0.1592162	-10.10305939	5.35E-24	3.71E-22	si:ch1073-340i21.2	si:ch1073-340i21.2 [Source:ZFIN;Acc:ZDB-GENE-131121-430]	15	46344655	46355392	
ENSDARG00000096991	385.4405767	1.75085043	0.224284199	3.347763388	0.000814665	0.007925936	CR847968.1	cadherin-12 [Source:NCBI gene;Acc:570372]	12	40205995	40518280	
ENSDARG00000097026	158.819068	-1.696492174	0.199975392	-3.482889405	0.000496033	0.005059297	si:dkey-16m19.1	si:dkey-16m19.1 [Source:ZFIN;Acc:ZDB-GENE-030131-7281]	22	27134809	27136451	
ENSDARG00000097054	169.1067376	-2.773268604	0.362817997	-4.887487991	1.02E-06	1.64E-05	CU927934.1		2	14009630	14010658	
ENSDARG00000097080	268.456426	1.99767562	0.314231559	3.174969514	0.001498522	0.013818667	si:ch73-181m17.1	si:ch73-181m17.1 [Source:ZFIN;Acc:ZDB-GENE-131122-49]	12	38983989	39031459	
ENSDARG00000097118	20559.23107	-2.977005911	0.106070256	-18.63864568	1.56E-77	6.64E-75	CU693494.2		2	342478	344595	
ENSDARG00000097137	573.9450825	2.17671476	0.139640018	8.42677322	3.55E-17	1.56E-15	CR753876.1		19	35368602	35369837	
ENSDARG00000097166	34.26236221	-2.905191143	0.480763595	-3.962844032	7.41E-05	0.000882732	BX936386.1		3	39259430	39264373	
ENSDARG00000097193	473.573501	-1.59251956	0.15768576	-3.757597144	0.000171553	0.001914694	si:dkey-28n18.9	si:dkey-28n18.9 [Source:ZFIN;Acc:ZDB-GENE-131127-205]	6	40422224	40429414	
ENSDARG00000097196	1028.730296	2.034659308	0.200048124	5.172052048	2.32E-07	4E-06	CR847531.1		21	42043974	42047227	
ENSDARG00000097231	109.3886562	-1.849009811	0.299318874	-2.836472688	0.004561488	0.037950915	ugt1b2	UDP glucuronosyltransferase 1 family, polypeptide B2 [Source:ZFIN;Acc:ZDB-GENE-080227-11]	11	30314885	30329055	
ENSDARG00000097275	13.70696547	5.36567803	1.535960869	2.842310712	0.004478782	0.037337065	si:ch211-147g22.4	si:ch211-147g22.4 [Source:ZFIN;Acc:ZDB-GENE-131121-226]	25	10843985	10857650	
ENSDARG00000097279	644.9140199	2.27155257	0.143674032	8.850260203	8.73E-19	4.28E-17	CU856222.1		5	63739863	63741137	
ENSDARG00000097285	692.7779066	-1.725542782	0.142859701	-5.078708544	3.8E-07	6.45E-06	ANPEP	si:ch211-147g22.5 [Source:ZFIN;Acc:ZDB-GENE-131127-23]	25	10830269	10841591	ANPEP
ENSDARG00000097369	182.6856655	2.600680904	0.229966628	6.960492125	3.39E-12	1E-10	FP017274.1		17	27400703	27401354	
ENSDARG00000097397	20.01736914	-2.814581874	0.612049011	-2.964765635	0.003029136	0.026355058	ugt5a4	UDP glucuronosyltransferase 5 family, polypeptide A4 [Source:ZFIN;Acc:ZDB-GENE-100406-1]	25	29381862	29383370	
ENSDARG00000097416	201.5588947	-2.370595308	0.190328338	-7.201215137	5.97E-13	1.87E-11	si:ch73-306e8.2	si:ch73-306e8.2 [Source:ZFIN;Acc:ZDB-GENE-131121-409]	17	20616016	20630315	
ENSDARG00000097503	459.7941491	-1.671662054	0.187166732	-3.588576062	0.000332489	0.003514024	si:ch73-288o11.5	si:ch73-288o11.5 [Source:ZFIN;Acc:ZDB-GENE-131121-416]	17	20605013	20611312	
ENSDARG00000097513	490.6833699	-2.294501761	0.141315607	-9.160359461	5.17E-20	2.77E-18	CT573383.1	annexin A2-A-like [Source:NCBI gene;Acc:100006250]	25	34035541	34050402	
ENSDARG00000097539	292.1462887	4.628027957	0.378856378	9.576262047	1.01E-21	6E-20	si:ch211-39f2.3	si:ch211-39f2.3 [Source:ZFIN;Acc:ZDB-GENE-131126-52]	12	38878830	38913459	
ENSDARG00000097543	238.6093535	1.764081702	0.189912327	4.023339165	5.74E-05	0.00069679	cbx6b	chromobox homolog 6b [Source:ZFIN;Acc:ZDB-GENE-110613-1]	8	24458204	24494794	
ENSDARG00000097553	96.71410424	2.398354686	0.322145288	4.340757841	1.42E-05	0.000192761	BX908750.1	uncharacterized LOC110439470 [Source:NCBI gene;Acc:110439470]	2	12798366	12802906	
ENSDARG00000097572	56.36863223	3.664419937	0.494332211	5.389937938	7.05E-08	1.3E-06	ptprt	protein tyrosine phosphatase receptor type T [Source:ZFIN;Acc:ZDB-GENE-101028-4]	6	51009981	51101834	

ENSDARG00000097615	147.5119442	2.055515451	0.268577938	3.93001547	8.49E-05	0.001001451	si:ch211-108d22.2	si:ch211-108d22.2 [Source:ZFIN;Acc:ZDB-GENE-131127-97]	3	14157090	14189022	
ENSDARG00000097635	1997.592182	-2.798319255	0.118880303	-15.12714226	1.07E-51	2.72E-49	wu:fb18f06	wu:fb18f06 [Source:ZFIN;Acc:ZDB-GENE-030131-261]	15	46356879	46358766	
ENSDARG00000097653	34.6228541	2.888024793	0.541905491	3.484048091	0.000493891	0.005041542	si:dkey-23k10.3	si:dkey-23k10.3 [Source:ZFIN;Acc:ZDB-GENE-131126-11]	15	36358144	36369743	
ENSDARG00000097693	967.073245	-1.508771791	0.149801857	-3.396298296	0.000683039	0.00676063	si:ch211-248a14.8	si:ch211-248a14.8 [Source:ZFIN;Acc:ZDB-GENE-131126-33]	17	20173882	20189395	
ENSDARG00000097725	233.8367403	-2.476057203	0.204105464	-7.231835827	4.77E-13	1.5E-11	si:dkey-96g2.1	si:dkey-96g2.1 [Source:ZFIN;Acc:ZDB-GENE-131121-563]	15	42043102	42048022	
ENSDARG00000097762	236.8837254	-3.3345186	0.212925208	-10.96403108	5.69E-28	4.88E-26	CU693494.3		2	335949	338458	
ENSDARG00000097804	890.9450918	-1.755797522	0.117679172	-6.422525821	1.34E-10	3.44E-09	lypc	ly6 domain containing, pigment cell [Source:ZFIN;Acc:ZDB-GENE-131127-361]	15	39957809	39963009	
ENSDARG00000097824	816.2926852	-1.360351559	0.111377706	-3.235401159	0.001214719	0.011411179	smco4	single-pass membrane protein with coiled-coil domains 4 [Source:ZFIN;Acc:ZDB-GENE-131121-542]	15	41238247	41245962	
ENSDARG00000097827	8958.928909	1.395669801	0.106665122	3.7094581	0.000207703	0.002275671	lrp1aa	low density lipoprotein receptor-related protein 1Aa [Source:ZFIN;Acc:ZDB-GENE-030131-7889]	11	1628538	1864288	
ENSDARG00000097875	130.8331297	1.992569185	0.283621665	3.499624001	0.000465915	0.004783202	CR786577.1		14	44013419	44208429	
ENSDARG00000097892	114.0006372	2.232361812	0.272434747	4.523511878	6.08E-06	8.77E-05	BX470264.1		2	9862833	9863388	
ENSDARG00000097897	2592.014277	2.22736171	0.099687964	12.31203507	7.8E-35	9.28E-33	sgip1a	SH3GL interacting endocytic adaptor 1a [Source:ZFIN;Acc:ZDB-GENE-131121-25]	6	30709926	30839763	
ENSDARG00000097959	1047.36227	-2.17379416	0.128339899	-9.145980086	5.91E-20	3.16E-18	si:dkey-248g15.3	si:dkey-248g15.3 [Source:ZFIN;Acc:ZDB-GENE-131127-95]	17	2549503	2555215	
ENSDARG00000097973	232.2338927	10.91221063	1.45292159	6.822261226	8.96E-12	2.56E-10	si:ch1073-190k2.1	si:ch1073-190k2.1 [Source:ZFIN;Acc:ZDB-GENE-131127-449]	15	46313082	46315233	
ENSDARG00000098011	1303.507361	-2.10892132	0.117441301	-9.442345306	3.65E-21	2.1E-19	BX000438.2		15	30922926	30924032	
ENSDARG00000098044	282.0358403	1.757534117	0.189027538	4.007533117	6.14E-05	0.000740434	BX663503.2		18	49124810	49156951	
ENSDARG00000098057	1554.500687	2.732182813	0.118164174	14.65912007	1.18E-48	2.62E-46	dscaml1	Down syndrome cell adhesion molecule like 1 [Source:ZFIN;Acc:ZDB-GENE-110601-1]	15	12071037	12229874	
ENSDARG00000098063	51.59603919	-2.583486183	0.399888634	-3.95981793	7.5E-05	0.000893145	alp3	alkaline phosphatase 3 [Source:ZFIN;Acc:ZDB-GENE-120919-1]	15	44618623	44637492	
ENSDARG00000098070	132.167427	1.77337877	0.241713253	3.199571225	0.001376322	0.01280469	CABZ01085140.1	transmembrane protein 88B [Source:NCBI gene;Acc:101887175]	22	44365	50878	
ENSDARG00000098081	347.4474589	1.667515989	0.171710191	3.887457039	0.0001013	0.001175557	GABRR1	gamma-aminobutyric acid receptor subunit rho-1-like [Source:NCBI gene;Acc:100330554]	17	53273684	53282539	GABRR1
ENSDARG00000098082	414.2741103	1.581516962	0.207411678	2.803684758	0.005052228	0.041688352	CU459093.1		11	45389377	45390076	
ENSDARG00000098096	170.9022563	2.715890767	0.25074074	6.843286692	7.74E-12	2.22E-10	slc12a1	solute carrier family 12 member 1 [Source:ZFIN;Acc:ZDB-GENE-080130-3]	18	5273953	5300028	
ENSDARG00000098113	266.8059246	1.806428701	0.180419703	4.469737433	7.83E-06	0.000111062	WSCD1	WSC domain containing 1a [Source:NCBI gene;Acc:100329926]	17	3281440	3303805	WSCD1
ENSDARG00000098130	277.7493017	-1.515396904	0.163175809	-3.158537447	0.001585629	0.014531173	bola3	bolA family member 3 [Source:ZFIN;Acc:ZDB-GENE-040801-32]	7	65296901	65306513	
ENSDARG00000098161	402.1636798	1.609214116	0.156833891	3.884454517	0.00010256	0.001189079	cntn4	contactin 4 [Source:ZFIN;Acc:ZDB-GENE-060929-776]	6	45494227	45838103	
ENSDARG00000098162	1687.192982	-2.378435657	0.146429243	-9.413663759	4.79E-21	2.75E-19	zgc:77748	zgc:77748 [Source:ZFIN;Acc:ZDB-GENE-040426-1864]	3	13469704	13473197	
ENSDARG00000098164	471.6793404	1.471930165	0.168221914	2.805402419	0.00502538	0.041480451	aipl2	aryl hydrocarbon receptor interacting protein like 2 [Source:ZFIN;Acc:ZDB-GENE-141216-23]	14	40874608	40903014	
ENSDARG00000098175	172.6080442	1.77929671	0.226911883	3.434358313	0.000593958	0.005956433	dgkab	diacylglycerol kinase, alpha b [Source:ZFIN;Acc:ZDB-GENE-121105-5]	11	2480293	2504750	
ENSDARG00000098239	1390.409159	-1.592941051	0.158063883	-3.751274745	0.000175938	0.001961019	zgc:85932	zgc:85932 [Source:ZFIN;Acc:ZDB-GENE-040426-2460]	15	43333121	43389922	
ENSDARG00000098240	6781.087263	1.678822087	0.096261235	7.051873876	1.77E-12	5.36E-11	meis2a	Meis homeobox 2a [Source:ZFIN;Acc:ZDB-GENE-020122-2]	17	52822422	52921236	
ENSDARG00000098263	115.8709901	3.032697241	0.319372101	6.364667531	1.96E-10	4.89E-09	CU929294.1	transmembrane protein 132A [Source:NCBI gene;Acc:100002045]	1	44983825	44997770	
ENSDARG00000098290	939.8207422	2.082754542	0.388617902	2.786167429	0.005333532	0.043722069	unc80	unc-80 homolog (C. elegans) [Source:ZFIN;Acc:ZDB-GENE-140106-167]	6	16509818	16667886	
ENSDARG00000098315	1481.524849	-1.620344649	0.118542293	-5.233108249	1.67E-07	2.94E-06	cyp1a	cytochrome P450, family 1, subfamily A [Source:ZFIN;Acc:ZDB-GENE-011219-1]	18	5588068	5598958	
ENSDARG00000098349	572.3216841	1.358609348	0.12929493	2.773576266	0.005544384	0.04524369	hdac4	histone deacetylase 4 [Source:ZFIN;Acc:ZDB-GENE-061013-95]	9	45884031	46276626	
ENSDARG00000098355	10746.44973	-1.348794881	0.088165465	-3.956139534	7.62E-05	0.000905284	atp5pd	ATP synthase peripheral stalk subunit d [Source:ZFIN;Acc:ZDB-GENE-040426-1658]	3	48549294	48559906	
ENSDARG00000098376	333.9573577	1.771358999	0.163022444	4.731612296	2.23E-06	3.44E-05	si:ch211-207l14.1	si:ch211-207l14.1 [Source:ZFIN;Acc:ZDB-GENE-120215-234]	6	9175886	9187959	
ENSDARG00000098392	1729.545898	2.457042783	0.173283859	8.408416052	4.16E-17	1.8E-15	ly6pge	lymphocyte antigen 6 family member pge [Source:ZFIN;Acc:ZDB-GENE-141216-463]	13	28903959	28914437	
ENSDARG00000098401	731.5912952	-1.397383779	0.12117966	-3.279294387	0.00104067	0.009909659	fam136a	family with sequence similarity 136 member A [Source:NCBI gene;Acc:406709]	10	7671260	7672949	
ENSDARG00000098415	141.1581409	-1.637835199	0.225902826	-2.82349367	0.004750336	0.039443663	CT956093.1		14	21025026	21025388	
ENSDARG00000098475	2517.32275	1.520166178	0.105009771	4.953502637	7.29E-07	1.19E-05	arr3b	arrestin 3b, retinal (X-arrestin) [Source:ZFIN;Acc:ZDB-GENE-030616-74]	14	32837914	32850768	
ENSDARG00000098478	61.30716188	-5.081067923	0.481397158	-8.477548857	2.3E-17	1.03E-15	FO704758.1	uncharacterized LOC100329294 [Source:NCBI gene;Acc:100329294]	8	547317	554540	
ENSDARG00000098481	533.0605875	-1.936676975	0.146051601	-6.413329039	1.42E-10	3.64E-09	anxa1d	annexin A1d [Source:ZFIN;Acc:ZDB-GENE-050417-72]	5	64994085	65004791	
ENSDARG00000098507	769.6837358	-1.451935578	0.121930336	-3.706506447	0.000210138	0.002299348	lyar	Ly1 antibody reactive homolog (mouse) [Source:ZFIN;Acc:ZDB-GENE-040426-1414]	14	32964	34751	
ENSDARG00000098511	456.6552822	2.391522015	0.222579204	6.251806045	4.06E-10	9.76E-09	ntrk2b	neurotrophic tyrosine kinase, receptor, type 2b [Source:ZFIN;Acc:ZDB-GENE-010126-2]	5	55626693	55689656	
ENSDARG00000098526	528.1309172	1.576450683	0.160955359	3.581432057	0.000341716	0.003602442	gpr75	G protein-coupled receptor 75 [Source:ZFIN;Acc:ZDB-GENE-100316-3]	13	35745572	35748676	
ENSDARG00000098528	194.3454878	2.066862173	0.230196488	4.634571886	3.58E-06	5.33E-05	rtn4rl1b	reticulon 4 receptor-like 1b [Source:ZFIN;Acc:ZDB-GENE-040310-3]	15	26399538	26402301	
ENSDARG00000098551	218.5780102	1.676509192	0.219447023	3.08279047	0.002050695	0.018396888	CABZ01085700.1		4	3287819	3290445	
ENSDARG00000098566	149.8621373	1.805845397	0.253541312	3.178359338	0.001481111	0.013688276	si:ch73-233f7.4	si:ch73-233f7.4 [Source:ZFIN;Acc:ZDB-GENE-141215-59]	14	2359230	2361692	
ENSDARG00000098586	169.1194038	-2.772457688	0.251746111	-7.04065568	1.91E-12	5.78E-11	zgc:194887	zgc:194887 [Source:ZFIN;Acc:ZDB-GENE-081022-162]	17	450956	456458	
ENSDARG00000098589	3839.39084	-1.861032117	0.153289907	-5.617017692	1.94E-08	3.87E-07	cyb5a	cytochrome b5 type A (microsomal) [Source:ZFIN;Acc:ZDB-GENE-040426-2148]	24	2836307	2843107	
ENSDARG00000098597	98.82458405	2.494644546	0.297140282	5.030097344	4.9E-07	8.21E-06	CU570791.3	NACHT and WD repeat domain-containing protein 2-like [Source:NCBI gene;Acc:108179119]	18	3022188	3037446	
ENSDARG00000098612	287.0820268	1.867033507	0.236776285	3.661825791	0.000250424	0.002709438	chrm2a	cholinergic receptor, muscarinic 2a [Source:ZFIN;Acc:ZDB-GENE-030314-1]	4	1146346	1284672	
ENSDARG00000098631	2236.943915	-2.140184482	0.168339408	-6.77312873	1.26E-11	3.55E-10	CABZ01044277.1		13	1535511	1543927	
ENSDARG00000098644	2790.763043	1.829192793	0.097814987	8.477154841	2.31E-17	1.03E-15	si:ch211-126j24.1	si:ch211-126j24.1 [Source:ZFIN;Acc:ZDB-GENE-050208-133]	22	38520209	38607504	
ENSDARG00000098645	1123.064009	-2.220670578	0.132299983	-9.226536165	2.8E-20	1.53E-18	rgn	regucalcin [Source:NCBI gene;Acc:403070]	6	9130989	9139986	
ENSDARG00000098683	1988.03702	1.572406569	0.097444546	5.874177618	4.25E-09	9.15E-08	zmiz2	zinc finger, MIZ-type containing 2 [Source:ZFIN;Acc:ZDB-GENE-090313-285]	10	45248326	45341821	
ENSDARG00000098702	59.72883306	2.313286541	0.439705633	2.986740316	0.002819691	0.024661	zgc:165603	zgc:165603 [Source:ZFIN;Acc:ZDB-GENE-070620-21]	2	53204750	53238469	
ENSDARG00000098736	65.75781051	2.298513792	0.382260755	3.396932001	0.000681459	0.006747654	si:dkey-201i2.4	si:dkey-201i2.4 [Source:ZFIN;Acc:ZDB-GENE-141215-24]	12	44009628	44018667	
ENSDARG00000098742	116.3232984	2.070553844	0.259299371	4.128640348	3.65E-05	0.000457757	lrtm1	leucine rich repeats and transmembrane domains 1 [Source:ZFIN;Acc:ZDB-GENE-161221-2]	8	53639625	53649294	
ENSDARG00000098746	2683.626481	-2.7968448	0.144765905	-12.41207175	2.25E-35	2.85E-33	dhrs13l1	dehydrogenase/reductase (SDR family) member 13 like 1 [Source:ZFIN;Acc:ZDB-GENE-040426-1907]	14	4151374	4161770	
ENSDARG00000098751	509.0564347	1.824675813	0.181482146	4.544115399	5.52E-06	8.05E-05	lzts3a	leucine zipper, putative tumor suppressor family member 3a [Source:ZFIN;Acc:ZDB-GENE-060313-2]	5	2742232	2752573	
ENSDARG00000098777	2079.025543	-1.610373208	0.122529808	-4.981426312	6.31E-07	1.04E-05	CU693379.1	crystallin beta-gamma domain containing 2 [Source:NCBI gene;Acc:569677]	19	48251273	48286941	
ENSDARG00000098788	9114.138276	1.685635774	0.090603427	7.56743753	3.81E-14	1.31E-12	pcdh2g28	protocadherin 2 gamma 28 [Source:ZFIN;Acc:ZDB-GENE-050610-19]	14	1887677	2032753	
ENSDARG00000098800	40.15592254	4.324021292	0.716268828	4.640745431	3.47E-06	5.18E-05	znf1156	zinc finger protein 1156 [Source:ZFIN;Acc:ZDB-GENE-050208-787]	22	1911269	1919599	
ENSDARG00000098803	942.2487277	-2.487742669	0.13767614	-10.80610387	3.22E-27	2.66E-25	cyp2aa4	cytochrome P450, family 2, subfamily AA, polypeptide 4 [Source:ZFIN;Acc:ZDB-GENE-040625-72]	23	42362337	42372651	
ENSDARG00000098805	1429.227666	-2.634188966	0.207177037	-7.88788657	3.07E-15	1.15E-13	vwa11	von Willebrand factor A domain containing 11 [Source:ZFIN;Acc:ZDB-GENE-141211-58]	1	44074445	44084071	
ENSDARG00000098809	2941.365623	2.451648956	0.13814742	10.50797009	7.94E-26	5.88E-24	snap91a	synaptosome associated protein 91a [Source:ZFIN;Acc:ZDB-GENE-100422-6]	16	33005688	33059269	
ENSDARG00000098810	563.4429018	1.929346804	0.222805121	4.171119592	3.03E-05	0.000385425	VSTM2B	zgc:194678 [Source:ZFIN;Acc:ZDB-GENE-030131-8082]	7	45040526	45076131	VSTM2B
ENSDARG00000098816	246.2108022	1.673534449	0.227380671	2.9621447	0.003055042	0.026534465	kcnc3b	potassium voltage-gated channel, Shaw-related subfamily, member 3b [Source:ZFIN;Acc:ZDB-GENE-100901-2]	24	37080771	37170289	
ENSDARG00000098824	103.3092844	2.346807845	0.299453219	4.497556743	6.87E-06	9.83E-05	cdh22	cadherin 22 [Source:ZFIN;Acc:ZDB-GENE-140106-113]	6	56638688	56877366	
ENSDARG00000098832	822.1050245	1.462926296	0.114430268	4.045488157	5.22E-05	0.000636846	fam168a	family with sequence similarity 168 member A [Source:ZFIN;Acc:ZDB-GENE-060825-128]	18	2837563	2935695	
ENSDARG00000098834	5666.458977	1.424932696	0.133407152	3.185231757	0.001446381	0.013396906	sox4b	SRY-box transcription factor 4b [Source:ZFIN;Acc:ZDB-GENE-040426-1274]	16	68069	71108	
ENSDARG00000098838	204.9934763	-2.007842273	0.187641664	-5.371100698	7.83E-08	1.44E-06	sdr42e1	short chain dehydrogenase/reductase family 42E, member 1 [Source:NCBI gene;Acc:565964]	18	501116	506082	
ENSDARG00000098849	113.3268343	-1.720509004	0.253248443	-2.845067851	0.004440196	0.037064639	SLC25A29	solute carrier family 25 member 29 [Source:NCBI gene;Acc:100329459]	17	706630	722218	SLC25A29
ENSDARG00000098852	3024.726994	-1.570005181	0.09349974	-6.096329064	1.09E-09	2.48E-08	smdt1b	single-pass membrane protein with aspartate-rich tail 1b [Source:ZFIN;Acc:ZDB-GENE-141212-9]	1	54062510	54063520	
ENSDARG00000098856	1727.089812	1.872260912	0.130389867	6.689637277	2.24E-11	6.1E-10		ryanodine receptor 2a (cardiac) [Source:NCBI gene;Acc:100126126]	12	47162456	47456979	
ENSDARG00000098871	345.7398694	1.635341218	0.164055434	3.872722782	0.000107626	0.00124037	gga3b	golgi associated, gamma adaptin ear containing, ARF binding protein 3b [Source:ZFIN;Acc:ZDB-GENE-131126-18]	3	6490520	6519760	
ENSDARG00000098880	5305.144414	2.525023016	0.108990199	13.99229493	1.74E-44	3.21E-42	pclob	piccolo presynaptic cytomatrix protein b [Source:ZFIN;Acc:ZDB-GENE-030131-8100]	18	9615147	9722291	
ENSDARG00000098890	1183.359482	-1.371792365	0.122062452	-3.045919189	0.002319702	0.020596687	cyp2aa9	cytochrome P450, family 2, subfamily AA, polypeptide 9 [Source:ZFIN;Acc:ZDB-GENE-080724-3]	23	42414652	42428299	
ENSDARG00000098903	209.7073679	1.679153355	0.200806283	3.382131996	0.000719256	0.007093925	tfe3a	transcription factor binding to IGHM enhancer 3a [Source:ZFIN;Acc:ZDB-GENE-010919-2]	8	7778770	7809451	
ENSDARG00000098909	196.7253235	2.201335018	0.231253567	5.194882109	2.05E-07	3.57E-06	SHC3	SHC (Src homology 2 domain containing) transforming protein 3 [Source:NCBI gene;Acc:100332111]	21	3093419	3117074	SHC3
ENSDARG00000098936	6991.131311	1.72333762	0.105640536	6.847159674	7.53E-12	2.17E-10	kif5aa	kinesin family member 5A, a [Source:ZFIN;Acc:ZDB-GENE-070912-141]	9	265988	296169	
ENSDARG00000098966	35.44573595	-2.228059572	0.437283175	-2.80838514	0.004979064	0.041165839	CABZ01064972.1	uncharacterized LOC100538212 [Source:NCBI gene;Acc:100538212]	22	5483588	5518117	
ENSDARG00000098995	241.3549283	3.745915273	0.284173289	9.662819761	4.34E-22	2.65E-20	cyp2k6	cytochrome P450, family 2, subfamily K, polypeptide 6 [Source:NCBI gene;Acc:393481]	3	12816362	12826387	
ENSDARG00000099003	843.409602	-1.709527704	0.121972749	-5.81710021	5.99E-09	1.26E-07	cyp2aa1	cytochrome P450, family 2, subfamily AA, polypeptide 1 [Source:ZFIN;Acc:ZDB-GENE-070424-33]	23	42434348	42451859	
ENSDARG00000099008	292.978044	1.846887919	0.170601053	4.964142401	6.9E-07	1.13E-05	EGFLAM	EGF like, fibronectin type III and laminin G domains [Source:NCBI gene;Acc:799405]	5	7919878	7969760	EGFLAM
ENSDARG00000099034	172.7130287	2.0003306	0.266698006	3.75079895	0.000176272	0.001963872	unc5a	unc-5 netrin receptor A [Source:ZFIN;Acc:ZDB-GENE-080219-19]	14	51164056	51619388	
ENSDARG00000099035	584.3770387	1.931099221	0.217731957	4.276355348	1.9E-05	0.000251245	pcdh1g33	protocadherin 1 gamma 33 [Source:ZFIN;Acc:ZDB-GENE-071004-76]	10	21783213	21820002	
ENSDARG00000099096	230.6165365	2.702720999	0.311143698	5.47245858	4.44E-08	8.42E-07	gabrb4	gamma-aminobutyric acid type A receptor beta4 subunit [Source:ZFIN;Acc:ZDB-GENE-070424-211]	21	37787805	37889727	
ENSDARG00000099111	963.843799	-3.042516178	0.165630824	-12.33173948	6.11E-35	7.34E-33	zgc:175280	zgc:175280 [Source:ZFIN;Acc:ZDB-GENE-080219-39]	5	67812062	67834257	
ENSDARG00000099113	570.2376166	1.690194666	0.152022042	4.540096004	5.62E-06	8.18E-05	UNC13B	unc-13 homolog Bb (C. elegans) [Source:NCBI gene;Acc:100329499]	10	16911951	17019944	UNC13B
ENSDARG00000099117	2324.026516	-1.385112713	0.091582223	-4.205103344	2.61E-05	0.000334376	si:dkey-33i11.4	si:dkey-33i11.4 [Source:NCBI gene;Acc:569335]	16	45228101	45235947	
ENSDARG00000099185	10101.14872	-4.845477733	0.141003777	-27.27216121	9.08E-164	1.27E-160	chia.2	chitinase, acidic.2 [Source:ZFIN;Acc:ZDB-GENE-040426-2014]	11	24001993	24008739	
ENSDARG00000099200	27205.9666	-1.921869321	0.075583108	-12.1967639	3.23E-34	3.74E-32	zgc:123103	zgc:123103 [Source:ZFIN;Acc:ZDB-GENE-051030-105]	19	42547699	42551338	
ENSDARG00000099203	10451.71933	1.412362572	0.147844027	2.789173022	0.005284283	0.043346659	atp1b2a	ATPase Na+/K+ transporting subunit beta 2a [Source:ZFIN;Acc:ZDB-GENE-001127-2]	23	44732863	44763861	
ENSDARG00000099247	32.72716301	-2.546001407	0.473187486	-3.267206873	0.001086143	0.010315274	si:dkey-68o6.5	si:dkey-68o6.5 [Source:ZFIN;Acc:ZDB-GENE-030131-5416]	3	24595922	24603705	
ENSDARG00000099276	671.4378585	-4.246194684	0.229186767	-14.16397086	1.53E-45	2.93E-43	ugt5b2	UDP glucuronosyltransferase 5 family, polypeptide B2 [Source:ZFIN;Acc:ZDB-GENE-100406-4]	1	9635978	9652725	
ENSDARG00000099283	2304.910852	2.033810428	0.116754555	8.854561816	8.4E-19	4.13E-17	epb41a	erythrocyte membrane protein band 4.1a [Source:ZFIN;Acc:ZDB-GENE-070705-81]	19	13639486	13774502	
ENSDARG00000099295	168.8380771	-4.24275434	0.266217698	-12.18083683	3.93E-34	4.53E-32	BX511034.4		22	7439255	7442462	
ENSDARG00000099304	126.886331	1.904233307	0.25298721	3.57422538	0.000351266	0.003690722	si:ch211-51f19.1	si:ch211-51f19.1 [Source:ZFIN;Acc:ZDB-GENE-141216-485]	14	7305686	7306983	
ENSDARG00000099306	3585.331389	-1.593695254	0.110077078	-5.393450349	6.91E-08	1.28E-06	zgc:175088	zgc:175088 [Source:ZFIN;Acc:ZDB-GENE-080204-124]	11	41078191	41084363	
ENSDARG00000099315	294.8832424	-1.933176977	0.159720599	-5.842558694	5.14E-09	1.1E-07	nsdhl	NAD(P) dependent steroid dehydrogenase-like [Source:ZFIN;Acc:ZDB-GENE-050417-163]	14	14646717	14659127	
ENSDARG00000099321	417.0768212	2.175330897	0.194565518	6.040797507	1.53E-09	3.44E-08	btbd17a	BTB (POZ) domain containing 17a [Source:ZFIN;Acc:ZDB-GENE-130531-68]	12	36092218	36107156	
ENSDARG00000099336	487.2931504	-1.678020554	0.153057695	-4.429836447	9.43E-06	0.000131143	mvda	mevalonate (diphospho) decarboxylase a [Source:NCBI gene;Acc:492781]	18	31056645	31068755	
ENSDARG00000099351	2915.617879	1.965889639	0.116624649	8.282036854	1.21E-16	5.04E-15	igfbp1a	insulin-like growth factor binding protein 1a [Source:ZFIN;Acc:ZDB-GENE-021231-1]	20	6808774	6812688	
ENSDARG00000099364	10.72361422	6.475988723	1.597380865	3.42810462	0.000607811	0.006083201	BX511034.5		22	7522728	7539414	
ENSDARG00000099370	46.75771799	-2.49090316	0.40276614	-3.701659633	0.000214194	0.002335569	AL954861.2		6	52756334	52757276	
ENSDARG00000099401	17.89103176	-2.899715412	0.62304192	-3.049097261	0.002295302	0.020401687	ccl33.3	chemokine (C-C motif) ligand 33, duplicate 3 [Source:ZFIN;Acc:ZDB-GENE-081104-131]	25	12841940	12844002	
ENSDARG00000099406	820.3093174	1.762762099	0.146742171	5.197974763	2.01E-07	3.51E-06	lrit1b	leucine-rich repeat, immunoglobulin-like and transmembrane domains 1b [Source:ZFIN;Acc:ZDB-GENE-060616-45]	12	48972915	48976282	
ENSDARG00000099420	48417.27384	-1.926053238	0.111504961	-8.305040669	9.98E-17	4.2E-15	nme2b.2	NME/NM23 nucleoside diphosphate kinase 2b, tandem duplicate 2 [Source:ZFIN;Acc:ZDB-GENE-000210-33]	19	48111285	48114444	
ENSDARG00000099446	1025.140801	2.443908578	0.187763876	7.690023278	1.47E-14	5.25E-13	slit1b	slit homolog 1b (Drosophila) [Source:ZFIN;Acc:ZDB-GENE-030722-4]	22	34843282	34937464	
ENSDARG00000099461	294.7200127	-2.390640922	0.186992953	-7.436862728	1.03E-13	3.45E-12	asb10	ankyrin repeat and SOCS box containing 10 [Source:ZFIN;Acc:ZDB-GENE-060929-1106]	24	34085940	34107618	
ENSDARG00000099470	311.4904895	3.161429671	0.215013537	10.05252831	8.95E-24	6.12E-22	muc5.3	mucin 5.3 [Source:ZFIN;Acc:ZDB-GENE-040426-1562]	7	72291181	72337942	
ENSDARG00000099525	821.1445485	-2.087118351	0.178613921	-6.086414469	1.15E-09	2.63E-08	si:ch1073-13h15.3	si:ch1073-13h15.3 [Source:ZFIN;Acc:ZDB-GENE-121214-245]	12	3220285	3237561	
ENSDARG00000099554	192.6231911	-1.573301749	0.205101633	-2.795208108	0.005186632	0.042643183	sec16b	SEC16 homolog B, endoplasmic reticulum export factor [Source:ZFIN;Acc:ZDB-GENE-050208-274]	6	23891230	23931442	

ENSDARG00000099633	760.5622855	-2.606200263	0.179668021	-8.939822762	3.9E-19	1.98E-17	sec14l7	SEC14-like lipid binding 7 [Source:ZFIN;Acc:ZDB-GENE-141216-145]	10	44924684	44939658	
ENSDARG00000099648	433.9014162	1.90418742	0.151739978	5.958794997	2.54E-09	5.62E-08	LO018363.1		21	45590391	45613564	
ENSDARG00000099663	5013.90586	-1.651381497	0.097245531	-6.698318077	2.11E-11	5.8E-10	cox5ab	cytochrome c oxidase subunit 5Ab [Source:ZFIN;Acc:ZDB-GENE-030131-5162]	18	808911	812753	
ENSDARG00000099695	505.0989668	1.833268835	0.156365276	5.328989006	9.88E-08	1.79E-06	si:dkey-61n16.5	si:dkey-61n16.5 [Source:ZFIN;Acc:ZDB-GENE-100922-235]	3	13911950	13921173	
ENSDARG00000099729	3599.219649	1.338004425	0.094773193	3.566456044	0.000361841	0.00379377	pcdh10a	protocadherin 10a [Source:ZFIN;Acc:ZDB-GENE-020731-1]	1	12763461	12797410	
ENSDARG00000099755	2240.98729	1.521077557	0.10518814	4.953767189	7.28E-07	1.19E-05	pfklb	phosphofructokinase, liver b [Source:ZFIN;Acc:ZDB-GENE-050417-62]	11	44910615	44931962	
ENSDARG00000099764	14.86816766	6.943264287	1.561843884	3.805287038	0.00014164	0.001605102	CR855389.1		15	32707770	32709645	
ENSDARG00000099802	568.1278434	1.880009542	0.151469247	5.80982316	6.25E-09	1.32E-07	pnp5b	purine nucleoside phosphorylase 5b [Source:ZFIN;Acc:ZDB-GENE-040912-54]	2	58186846	58201173	
ENSDARG00000099811	3.635612275	-5.736709166	1.706455411	-2.775759117	0.005507299	0.044984941	si:dkey-203a12.7	si:dkey-203a12.7 [Source:ZFIN;Acc:ZDB-GENE-141216-159]	14	15543331	15544198	
ENSDARG00000099812	521.0811658	1.731490375	0.139077776	5.259577748	1.44E-07	2.56E-06	si:dkey-31f5.11	si:dkey-31f5.11 [Source:ZFIN;Acc:ZDB-GENE-030131-7826]	4	18985529	19028861	
ENSDARG00000099821	151.6565833	-1.961359441	0.225860478	-4.256430551	2.08E-05	0.000271557	tymp	thymidine phosphorylase [Source:ZFIN;Acc:ZDB-GENE-100415-1]	4	9543459	9549693	
ENSDARG00000099841	209.9267871	1.615550457	0.194439917	3.165761785	0.001546774	0.014221823	prkcab	protein kinase C, alpha, b [Source:ZFIN;Acc:ZDB-GENE-131121-412]	3	56163276	56342119	
ENSDARG00000099849	505.3883173	1.788361156	0.140935743	5.593763071	2.22E-08	4.39E-07	ebf3a	EBF transcription factor 1a [Source:NCBI gene;Acc:565310]	14	35013656	35246931	
ENSDARG00000099850	1043.309191	1.612211205	0.121522801	5.037829944	4.71E-07	7.9E-06	iqsec3a	IQ motif and Sec7 domain ArfGEF 3a [Source:ZFIN;Acc:ZDB-GENE-041210-343]	4	26496489	26766355	
ENSDARG00000099874	790.2967344	1.518235339	0.128072617	4.046417967	5.2E-05	0.000634631	agap2	ArfGAP with GTPase domain, ankyrin repeat and PH domain 2 [Source:ZFIN;Acc:ZDB-GENE-061103-343]	23	36675207	36724575	
ENSDARG00000099985	439.7486469	2.161292125	0.153839935	7.548703953	4.4E-14	1.51E-12	ccn1l2	cellular communication network factor 1, like 2 [Source:ZFIN;Acc:ZDB-GENE-060404-5]	8	52619365	52631064	
ENSDARG00000100007	537.5230612	2.400932385	0.152646535	9.177623198	4.41E-20	2.37E-18	arhgap39	Rho GTPase activating protein 39 [Source:ZFIN;Acc:ZDB-GENE-041014-247]	20	52389858	52447533	
ENSDARG00000100012	234.7440883	1.85960147	0.214922101	3.999595515	6.35E-05	0.000763143	CR855860.1		19	25155542	25157191	
ENSDARG00000100055	804.835881	1.956463945	0.122275512	7.822203538	5.19E-15	1.92E-13	tox2	TOX high mobility group box family member 2 [Source:ZFIN;Acc:ZDB-GENE-040426-2095]	6	57900237	58149165	
ENSDARG00000100086	73.13781599	-3.08118325	0.391139663	-5.320818745	1.03E-07	1.87E-06	adipoqb	adiponectin, C1Q and collagen domain containing, b [Source:ZFIN;Acc:ZDB-GENE-060825-220]	2	4490604	4496358	
ENSDARG00000100093	1233.972816	1.596520878	0.207843291	2.870051162	0.004104054	0.034580684	ppp2r2ca	protein phosphatase 2, regulatory subunit B, gamma a [Source:ZFIN;Acc:ZDB-GENE-051120-168]	14	52522717	52552875	
ENSDARG00000100095	3931.994535	-1.847194092	0.143323004	-5.91108245	3.4E-09	7.4E-08	anxa1b	annexin A1b [Source:ZFIN;Acc:ZDB-GENE-030131-6554]	5	65022287	65037525	
ENSDARG00000100097	372.1874712	1.678022938	0.201032145	3.372709064	0.000744326	0.007315315	mafgb	v-maf avian musculoaponeurotic fibrosarcoma oncogene homolog Gb [Source:ZFIN;Acc:ZDB-GENE-040426-2403]	11	45299447	45328509	
ENSDARG00000100104	177.9156734	-1.831978427	0.214202263	-3.884078601	0.000102719	0.00119037	zgc:101785	zgc:101785 [Source:ZFIN;Acc:ZDB-GENE-041010-152]	19	48290786	48308400	
ENSDARG00000100116	974.7023434	-1.463027365	0.121602547	-3.807711087	0.000140259	0.00159161	mrps24	mitochondrial ribosomal protein S24 [Source:NCBI gene;Acc:553711]	10	45053577	45058886	
ENSDARG00000100128	487.0950692	1.965401713	0.15055283	6.41237837	1.43E-10	3.65E-09	CU861477.1		2	59527149	59565832	
ENSDARG00000100145	1878.908361	2.20615977	0.132458131	9.105970029	8.55E-20	4.51E-18	CABZ01092282.1		2	18866	22535	
ENSDARG00000100166	2548.400232	2.062495112	0.124193948	8.555127925	1.18E-17	5.36E-16	si:dkeyp-9d4.3	si:dkeyp-9d4.3 [Source:ZFIN;Acc:ZDB-GENE-110914-175]	1	8022320	8101495	
ENSDARG00000100184	157.2266476	-2.345221382	0.318353129	-4.225563568	2.38E-05	0.00030775	ly97.2	lymphocyte antigen 97, tandem duplicate 2 [Source:ZFIN;Acc:ZDB-GENE-170621-2]	2	51774062	51777238	
ENSDARG00000100244	5511.470379	1.510610219	0.128077158	3.986739157	6.7E-05	0.000802221	ebf3a	EBF transcription factor 3a [Source:NCBI gene;Acc:567751]	12	42436328	42579702	
ENSDARG00000100246	587.0861625	-2.054332529	0.153405587	-6.872843101	6.29E-12	1.82E-10	FP016234.1		3	5514677	5523012	
ENSDARG00000100255	262.2669585	2.203364643	0.211828949	5.68083186	1.34E-08	2.72E-07	si:ch73-29l19.1	si:ch73-29l19.1 [Source:ZFIN;Acc:ZDB-GENE-141222-33]	6	36795225	36819371	
ENSDARG00000100267	503.6873456	1.923669152	0.147673018	6.254826804	3.98E-10	9.59E-09	GRM7	glutamate metabotropic receptor 7 [Source:HGNC Symbol;Acc:HGNC:4599]	22	33535575	33679277	GRM7
ENSDARG00000100274	1297.522963	1.525118845	0.128503937	3.79136404	0.000149822	0.001688678	WDR7	WD repeat domain 7 [Source:NCBI gene;Acc:572502]	21	1885698	1972236	WDR7
ENSDARG00000100313	1323.499224	2.096736741	0.109250778	10.03870876	1.03E-23	7.01E-22	pip5k1cb	phosphatidylinositol-4-phosphate 5-kinase, type I, gamma b [Source:ZFIN;Acc:ZDB-GENE-110408-21]	2	56508451	56635782	
ENSDARG00000100315	881.6654812	-4.658226967	0.179867427	-20.33846279	5.87E-92	3.69E-89	slc15a1a	solute carrier family 15 member 1a [Source:ZFIN;Acc:ZDB-GENE-091204-9]	9	1137430	1162804	
ENSDARG00000100339	171.073408	-2.521957745	0.24802522	-6.136302372	8.45E-10	1.96E-08	arf4b	ADP-ribosylation factor 4b [Source:ZFIN;Acc:ZDB-GENE-050417-205]	11	42466834	42472941	
ENSDARG00000100402	1272.506032	1.422887315	0.107090269	3.948886464	7.85E-05	0.00093227	fgd1	FYVE, RhoGEF and PH domain containing 1 [Source:ZFIN;Acc:ZDB-GENE-081104-33]	8	7737062	7772319	
ENSDARG00000100406	10973.89237	-1.411435074	0.103088505	-3.99108586	6.58E-05	0.000789163	zgc:112265	zgc:112265 [Source:ZFIN;Acc:ZDB-GENE-050626-133]	11	37201483	37229533	
ENSDARG00000100422	1601.490417	1.705286154	0.104272279	6.763889321	1.34E-11	3.78E-10	ptpro	protein tyrosine phosphatase receptor type O [Source:ZFIN;Acc:ZDB-GENE-070424-54]	4	190547	211714	
ENSDARG00000100424	173.488975	2.100679429	0.316244172	3.480473398	0.000500529	0.005098931	si:ch73-233f7.8	si:ch73-233f7.8 [Source:ZFIN;Acc:ZDB-GENE-141215-62]	14	2346509	2348917	
ENSDARG00000100466	50.82993919	2.230032232	0.423191979	2.90655847	0.003654286	0.031167595	nrl	neural retina leucine zipper [Source:ZFIN;Acc:ZDB-GENE-050729-1]	20	661037	673579	
ENSDARG00000100476	501.0527988	-2.110258881	0.148530169	-7.474972174	7.72E-14	2.6E-12	si:dkeyp-110c7.4	si:dkeyp-110c7.4 [Source:ZFIN;Acc:ZDB-GENE-070705-532]	5	37068223	37074101	
ENSDARG00000100486	108.4481149	2.266983212	0.314527448	4.02821191	5.62E-05	0.000683169	foxo6a	forkhead box O6 a [Source:ZFIN;Acc:ZDB-GENE-110914-126]	16	7315144	7362806	
ENSDARG00000100494	2474.152218	1.379658915	0.091215726	4.162209004	3.15E-05	0.000398962	pbx1a	pre-B-cell leukemia homeobox 1a [Source:ZFIN;Acc:ZDB-GENE-000405-1]	2	18698388	18830794	
ENSDARG00000100506	404.0551675	1.609135729	0.157737861	3.861696401	0.000112602	0.001292379	crb1	crumbs cell polarity complex component 1 [Source:ZFIN;Acc:ZDB-GENE-050208-382]	22	23493170	23545828	
ENSDARG00000100588	28695.26739	-1.35062908	0.122127189	-2.871015736	0.004091552	0.034510093	rpl36	ribosomal protein L36 [Source:NCBI gene;Acc:405888]	22	3153876	3159456	
ENSDARG00000100592	495.6958947	2.113044588	0.148474508	7.496536633	6.55E-14	2.23E-12	slc8a2b	solute carrier family 8 member 2b [Source:ZFIN;Acc:ZDB-GENE-050419-209]	15	42767803	42981467	
ENSDARG00000100594	710.1512886	2.001913312	0.134415435	7.453856094	9.07E-14	3.04E-12	sez6l	seizure related 6 homolog (mouse)-like [Source:ZFIN;Acc:ZDB-GENE-091204-162]	10	44581378	44672402	
ENSDARG00000100621	55.31542279	2.595576652	0.422918104	3.77277926	0.000161439	0.001809863	CABZ01080550.1		25	8269947	8292650	
ENSDARG00000100635	1435.4462	-3.53978871	0.114358794	-22.20894977	2.81E-109	2.08E-106	chia.1	chitinase, acidic.1 [Source:ZFIN;Acc:ZDB-GENE-040426-1994]	11	23993298	24000171	
ENSDARG00000100697	10807.13127	1.987247034	0.123773978	7.976208327	1.51E-15	5.85E-14	FQ323156.1		16	51192655	51214029	
ENSDARG00000100712	1040.565427	-1.512203858	0.108714215	-4.711470888	2.46E-06	3.76E-05	si:dkey-19b23.12	si:dkey-19b23.12 [Source:ZFIN;Acc:ZDB-GENE-141216-440]	7	20503344	20508771	
ENSDARG00000100722	1441.575673	1.898790051	0.144877578	6.203789873	5.51E-10	1.31E-08	si:ch211-160f23.7	si:ch211-160f23.7 [Source:ZFIN;Acc:ZDB-GENE-141216-470]	17	27176243	27183448	
ENSDARG00000100774	98.88559844	-1.851649924	0.26023125	-3.272665843	0.001065384	0.010129618	cmc4	C-x(9)-C motif containing 4 homolog (S. cerevisiae) [Source:ZFIN;Acc:ZDB-GENE-120216-1]	21	44557006	44557513	
ENSDARG00000100792	842.9235481	-3.989293478	0.481120874	-6.213185994	5.19E-10	1.24E-08	zgc:154142	zgc:154142 [Source:ZFIN;Acc:ZDB-GENE-070615-2]	1	50794669	50838160	
ENSDARG00000100794	39.41553381	2.277760443	0.465423745	2.745370122	0.006044269	0.048815054	mmp13b	matrix metallopeptidase 13b [Source:ZFIN;Acc:ZDB-GENE-030131-6152]	15	33825750	33834577	
ENSDARG00000100818	29.61532264	-3.980023153	0.622085411	-4.790376207	1.66E-06	2.61E-05	KYAT3	si:ch73-97h19.2 [Source:ZFIN;Acc:ZDB-GENE-120214-33]	6	25213934	25228496	KYAT3
ENSDARG00000100828	15.349711	5.535193847	1.515451519	2.992635389	0.0027658	0.024232263	CU929150.2	phospholipid scramblase 2-like [Source:NCBI gene;Acc:100536708]	2	45100963	45113236	
ENSDARG00000100851	1235.545332	1.832626215	0.141143074	5.899164512	3.65E-09	7.94E-08	gphnb	gephyrin b [Source:ZFIN;Acc:ZDB-GENE-070705-4]	20	50852356	50999404	
ENSDARG00000100854	2436.017899	1.475402982	0.09154818	5.19292661	2.07E-07	3.6E-06	ago4	argonaute RISC component 4 [Source:ZFIN;Acc:ZDB-GENE-030131-5357]	19	47800550	47832853	
ENSDARG00000100865	143.2682599	-2.553909922	0.249046503	-6.239436824	4.39E-10	1.05E-08	si:dkey-269o24.1	si:dkey-269o24.1 [Source:ZFIN;Acc:ZDB-GENE-041210-347]	4	56932843	56954002	
ENSDARG00000100867	986.0116677	1.377606495	0.121095687	3.118248924	0.001819291	0.016479977	rictora	RPTOR independent companion of MTOR, complex 2 a [Source:ZFIN;Acc:ZDB-GENE-060526-49]	5	8533929	8614217	
ENSDARG00000100876	550.0581983	1.476846013	0.160614576	2.968883795	0.002988836	0.026031496	sez6a	seizure related 6 homolog a [Source:ZFIN;Acc:ZDB-GENE-030131-8208]	10	36828069	36927594	
ENSDARG00000100897	1294.371872	1.476991725	0.118828558	4.014116914	5.97E-05	0.000721805	pdgfrb	platelet-derived growth factor receptor, beta polypeptide [Source:ZFIN;Acc:ZDB-GENE-030805-2]	14	3186485	3268155	
ENSDARG00000100908	596.875908	1.856690299	0.156742067	5.465605478	4.61E-08	8.73E-07	lcor	ligand dependent nuclear receptor corepressor [Source:ZFIN;Acc:ZDB-GENE-061201-6]	22	34784075	34818128	
ENSDARG00000100912	71.49216076	-3.328197698	0.34692525	-6.710949105	1.93E-11	5.35E-10	zgc:162297	zgc:162297 [Source:ZFIN;Acc:ZDB-GENE-070410-62]	7	46009862	46019756	
ENSDARG00000100917	392.2107572	-2.953672082	0.183531057	-10.64491273	1.84E-26	1.45E-24	slc30a8	solute carrier family 30 member 8 [Source:ZFIN;Acc:ZDB-GENE-060315-10]	19	46343385	46362232	
ENSDARG00000100955	351.2086163	-2.618594373	0.234223018	-6.910483796	4.83E-12	1.41E-10	stk24a	serine/threonine kinase 24a (STE20 homolog, yeast) [Source:ZFIN;Acc:ZDB-GENE-070424-52]	9	1162216	1176354	
ENSDARG00000100956	135.2673703	3.614532653	0.321167284	8.140719141	3.93E-16	1.57E-14	si:ch211-276i12.4	si:ch211-276i12.4 [Source:ZFIN;Acc:ZDB-GENE-141216-218]	14	45645024	45671346	
ENSDARG00000100958	842.2683079	2.509503711	0.137799274	10.95436623	6.33E-28	5.39E-26	HECW1	si:ch211-188f17.1 [Source:ZFIN;Acc:ZDB-GENE-131120-111]	24	673161	736994	HECW1
ENSDARG00000100964	23.2329961	7.587244302	1.543825409	4.266832418	1.98E-05	0.000260425	si:ch211-57b15.2	si:ch211-57b15.2 [Source:ZFIN;Acc:ZDB-GENE-141219-31]	21	29189471	29197700	
ENSDARG00000101021	71.87323278	-1.817326817	0.295468739	-2.766204035	0.005671304	0.046144375	slc22a5	solute carrier family 22 member 5 [Source:ZFIN;Acc:ZDB-GENE-091012-1]	21	45659525	45670911	
ENSDARG00000101043	14849.44406	-1.772469115	0.086415413	-8.939020127	3.93E-19	1.99E-17	ppl	periplakin [Source:ZFIN;Acc:ZDB-GENE-041008-196]	3	36459790	36481986	
ENSDARG00000101054	1056.232954	2.120668569	0.130313045	8.599818764	7.98E-18	3.68E-16	syn2b	synapsin IIb [Source:ZFIN;Acc:ZDB-GENE-051127-49]	11	675296	762721	
ENSDARG00000101063	915.0387915	-1.373787902	0.132519222	-2.820631583	0.004792921	0.039770948	CABZ01058650.1	thiosulfate sulfurtransferase/rhodanese-like domain-containing protein 1 [Source:NCBI gene;Acc:561325]	5	4533244	4547917	
ENSDARG00000101081	1312.103941	1.38654421	0.108319659	3.568550827	0.000358961	0.003765268	ptprub	protein tyrosine phosphatase receptor type Ub [Source:NCBI gene;Acc:335096]	16	34916162	35330001	
ENSDARG00000101088	18.03140876	3.710289529	0.865902164	3.130018196	0.001747955	0.015914187	si:ch73-359m17.5	si:ch73-359m17.5 [Source:ZFIN;Acc:ZDB-GENE-141212-366]	19	48339349	48340774	
ENSDARG00000101090	498.4933681	2.287386312	0.148767244	8.653694705	4.99E-18	2.33E-16	pappaa	pregnancy-associated plasma protein A, pappalysin 1a [Source:ZFIN;Acc:ZDB-GENE-121113-1]	5	70345518	70519104	
ENSDARG00000101109	56.03356337	-2.016503923	0.368755632	-2.756578704	0.005840955	0.047340595	cldn15b	claudin 15b [Source:ZFIN;Acc:ZDB-GENE-060421-7652]	5	53908609	53955218	
ENSDARG00000101135	4823.376982	3.905279049	0.120236113	24.16311522	5.44E-129	4.87E-126	si:dkey-85k7.7	si:dkey-85k7.7 [Source:ZFIN;Acc:ZDB-GENE-040128-7]	7	21859337	21874346	
ENSDARG00000101160	866.9801113	-1.548997155	0.127859442	-4.293755294	1.76E-05	0.000233933	apoa4a	apolipoprotein A-IV a [Source:ZFIN;Acc:ZDB-GENE-050417-469]	19	10845953	10850834	
ENSDARG00000101164	264.5324909	-1.505979118	0.172090048	-2.94019975	0.003280007	0.028224704	nansb	N-acetylneuraminic acid synthase b [Source:ZFIN;Acc:ZDB-GENE-041114-125]	25	5288665	5299452	
ENSDARG00000101170	166.9190454	-1.974535709	0.205810191	-4.73511883	2.19E-06	3.38E-05	rab42b	RAB42, member RAS oncogene family [Source:ZFIN;Acc:ZDB-GENE-050417-172]	16	35401543	35405887	
ENSDARG00000101181	731.6812277	-2.366947104	0.142467818	-9.594778134	8.41E-22	5.06E-20	s100w	S100 calcium binding protein W [Source:ZFIN;Acc:ZDB-GENE-080407-2]	16	23403602	23406155	
ENSDARG00000101199	28149.28322	-1.593217579	0.088816429	-6.679142459	2.4E-11	6.54E-10	rbp4	retinol binding protein 4, plasma [Source:ZFIN;Acc:ZDB-GENE-000210-19]	12	5115929	5120339	
ENSDARG00000101215	573.4272448	2.381135183	0.14918829	9.257664828	2.09E-20	1.17E-18	sgip1b	SH3GL interacting endocytic adaptor 1b [Source:ZFIN;Acc:ZDB-GENE-131121-110]	2	14276639	14390627	
ENSDARG00000101225	200.9971386	1.959112129	0.206482883	4.644995815	3.4E-06	5.08E-05	cngb3.1	cyclic nucleotide gated channel subunit beta 3, tandem duplicate 1 [Source:ZFIN;Acc:ZDB-GENE-090611-1]	24	31402894	31425799	
ENSDARG00000101234	1304.66679	1.857417774	0.145232873	5.903744487	3.55E-09	7.73E-08	nsmfb	NMDA receptor synaptonuclear signaling and neuronal migration factor b [Source:NCBI gene;Acc:569891]	5	28398449	28491518	
ENSDARG00000101259	419.1588419	-1.520948582	0.14085799	-3.698395687	0.000216966	0.002362723	hgh1	HGH1 homolog (S. cerevisiae) [Source:NCBI gene;Acc:436795]	19	2876106	2889941	
ENSDARG00000101282	791.9170538	1.457121096	0.137918764	3.314422804	0.000918325	0.008848708	zswim6	zinc finger, SWIM-type containing 6 [Source:ZFIN;Acc:ZDB-GENE-060602-4]	10	44296863	44341288	
ENSDARG00000101297	497.5248816	1.676358036	0.196460053	3.442725518	0.000575883	0.005789047	tmem266	transmembrane protein 266 [Source:ZFIN;Acc:ZDB-GENE-070112-2382]	25	32787363	32869794	
ENSDARG00000101317	615.5253754	1.738485732	0.127623779	5.78642741	7.19E-09	1.5E-07	grinaa	glutamate receptor, ionotropic, N-methyl D-aspartate-associated protein 1a (glutamate binding) [Source:ZFIN;Acc:ZDB-GEI	19	46259619	46303622	
ENSDARG00000101318	225.0343754	2.377161947	0.270986476	5.08203202	3.73E-07	6.34E-06	pcdh2ab9	protocadherin 2 alpha b 9 [Source:ZFIN;Acc:ZDB-GENE-041118-8]	14	2118775	2285955	
ENSDARG00000101324	296192.8239	-1.692413069	0.092477234	-7.487389456	7.03E-14	2.38E-12	apoa1b	apolipoprotein A-Ib [Source:ZFIN;Acc:ZDB-GENE-050302-172]	15	102925	108414	
ENSDARG00000101343	36.12086197	-2.534479261	0.476440602	-3.220714719	0.001278714	0.01198096	CR293511.2		11	45136052	45143840	
ENSDARG00000101348	115.0518493	2.612876509	0.287298527	5.613939351	1.98E-08	3.94E-07	plppr5b	phospholipid phosphatase related 5b [Source:ZFIN;Acc:ZDB-GENE-070620-15]	24	30120815	30199070	
ENSDARG00000101367	228.830729	-1.512117122	0.179271274	-2.856660244	0.004281238	0.035880923	si:ch73-193i22.1	si:ch73-193i22.1 [Source:ZFIN;Acc:ZDB-GENE-121214-266]	9	46692166	46701326	
ENSDARG00000101368	720.3644251	1.506559274	0.152911951	3.312751357	0.000923831	0.00889834	cngb3.2	cyclic nucleotide gated channel subunit beta 3, tandem duplicate 2 [Source:ZFIN;Acc:ZDB-GENE-091118-102]	24	31430890	31452923	
ENSDARG00000101385	719.1911725	2.548293379	0.206723177	7.48969421	6.9E-14	2.34E-12	f8	coagulation factor VIII, procoagulant component [Source:ZFIN;Acc:ZDB-GENE-090629-2]	14	1007169	1040736	
ENSDARG00000101423	4354.254479	-2.218628222	0.122080749	-9.98214899	1.82E-23	1.21E-21	cyp2y3	cytochrome P450, family 2, subfamily Y, polypeptide 3 [Source:ZFIN;Acc:ZDB-GENE-050522-490]	15	16175	32151	
ENSDARG00000101449	439.0279094	2.084099794	0.151444971	7.158374326	8.16E-13	2.54E-11	grik5	glutamate receptor, ionotropic, kainate 5 [Source:ZFIN;Acc:ZDB-GENE-070821-6]	16	11483811	11555934	
ENSDARG00000101455	50.03580873	-2.3574852	0.348531441	-3.894871567	9.83E-05	0.001143874	si:ch211-284k5.2	si:ch211-284k5.2 [Source:ZFIN;Acc:ZDB-GENE-131127-514]	25	1035656	1040972	
ENSDARG00000101458	3453.157374	1.474886724	0.151760844	3.129178198	0.00175296	0.015953967	r3hdm1	R3H domain containing 1 [Source:ZFIN;Acc:ZDB-GENE-030131-7119]	22	12361317	12393949	
ENSDARG00000101478	343.9160724	1.685058687	0.193829285	3.53434048	0.000408794	0.004231398	plppr2a	phospholipid phosphatase related 2a [Source:ZFIN;Acc:ZDB-GENE-040426-1171]	3	14317802	14373165	
ENSDARG00000101576	216.6998301	4.531373397	0.344735699	10.24371251	1.26E-24	9.03E-23	tbxta	T-box transcription factor Ta [Source:NCBI gene;Acc:30399]	19	14187540	14191592	
ENSDARG00000101589	112.0220874	1.953865736	0.288181976	3.309942384	0.000933152	0.008984673	cacna1db	calcium channel, voltage-dependent, L type, alpha 1D subunit, b [Source:ZFIN;Acc:ZDB-GENE-040525-3]	8	53464216	53465017	
ENSDARG00000101628	414.8480285	-1.518368256	0.162303408	-3.193822373	0.001404025	0.013023809	ascl1b	achaete-scute family bHLH transcription factor 1b [Source:NCBI gene;Acc:30478]	7	49715750	49717195	
ENSDARG00000101642	54.27302647	3.590200924	0.490181808	5.284163711	1.26E-07	2.26E-06	si:ch211-155m12.1	si:ch211-155m12.1 [Source:NCBI gene;Acc:567653]	5	9037650	9044893	
ENSDARG00000101667	122.8934191	2.077812719	0.255677984	4.215508513	2.49E-05	0.000320634	si:ch73-233f7.6	si:ch73-233f7.6 [Source:ZFIN;Acc:ZDB-GENE-141215-55]	14	2353398	2355833	
ENSDARG00000101695	134.9354036	-2.277218938	0.271901171	-4.697364602	2.64E-06	4.01E-05	si:dkey-26m3.3	si:dkey-26m3.3 [Source:ZFIN;Acc:ZDB-GENE-070705-445]	4	46842926	47096170	
ENSDARG00000101707	1088.058721	-1.727069099	0.124769713	-5.827288402	5.63E-09	1.2E-07	si:ch211-156b7.4	si:ch211-156b7.4 [Source:ZFIN;Acc:ZDB-GENE-141212-376]	3	26342768	26343648	
ENSDARG00000101716	505.9700799	1.64105535	0.200184729	3.202318942	0.00136326	0.012687869	bace1	beta-secretase 1 [Source:ZFIN;Acc:ZDB-GENE-040426-1835]	15	93131	97205	
ENSDARG00000101726	5734.143925	-2.558308594	0.142034592	-10.9713315	5.25E-28	4.51E-26	ces2b	carboxylesterase 2b [Source:ZFIN;Acc:ZDB-GENE-041014-96]	25	30385691	30429750	

ENSDARG00000101733	2059.008227	1.302455291	0.094430865	3.20292831	0.001360378	0.012665749	fam168b	family with sequence similarity 168 member B [Source:NCBI gene;Acc:561051]	24	31334209	31347202	
ENSDARG00000101749	151.2648848	-1.774277841	0.213044282	-3.63435167	0.000278681	0.002994503	zgc:92161	zgc:92161 [Source:ZFIN;Acc:ZDB-GENE-040912-22]	3	49043917	49058326	
ENSDARG00000101757	69.63769268	2.182611608	0.430557839	2.746696265	0.006019886	0.048665104	si:dkey-27n6.4	si:dkey-27n6.4 [Source:ZFIN;Acc:ZDB-GENE-041014-42]	4	71578099	71610565	
ENSDARG00000101762	923.9000044	-1.482046494	0.106134303	-4.541853876	5.58E-06	8.12E-05	fah	fumarylacetoacetate hydrolase (fumarylacetoacetase) [Source:ZFIN;Acc:ZDB-GENE-030131-1091]	7	10592152	10632375	
ENSDARG00000101776	760.2310752	1.797288124	0.145784263	5.468958783	4.53E-08	8.58E-07	syt12	synaptotagmin XII [Source:ZFIN;Acc:ZDB-GENE-081022-147]	25	7461624	7489371	
ENSDARG00000101777	381.3154815	1.731547047	0.15797398	4.630807228	3.64E-06	5.42E-05	pdlim5a	PDZ and LIM domain 5a [Source:NCBI gene;Acc:436927]	5	7722017	7844265	
ENSDARG00000101789	958.3396754	2.274296389	0.118484606	10.75495318	5.62E-27	4.55E-25	cyp1c2	cytochrome P450, family 1, subfamily C, polypeptide 2 [Source:ZFIN;Acc:ZDB-GENE-050705-1]	17	996509	998695	
ENSDARG00000101861	438.8148563	-2.3752003	0.235474998	-5.840111731	5.22E-09	1.11E-07	cyp2k19	cytochrome P450, family 2, subfamily k, polypeptide 19 [Source:ZFIN;Acc:ZDB-GENE-091211-1]	3	12732382	12740310	
ENSDARG00000101866	443.9473579	1.660246382	0.143980725	4.58565812	4.53E-06	6.66E-05	asic1b	acid-sensing (proton-gated) ion channel 1b [Source:NCBI gene;Acc:407672]	22	7044625	7168571	
ENSDARG00000101919	503.8365011	1.99226103	0.160939028	6.165446884	7.03E-10	1.64E-08	foxj1a	forkhead box J1a [Source:NCBI gene;Acc:767737]	3	60716904	60733722	
ENSDARG00000101956	28.92154689	7.906817401	1.492873861	4.62652444	3.72E-06	5.53E-05	CU571315.2		3	19507415	19517462	
ENSDARG00000101959	326.6597763	1.554106726	0.163870134	3.381377138	0.000721235	0.00711065	etv1	ETS variant transcription factor 1 [Source:ZFIN;Acc:ZDB-GENE-070104-1]	15	34172371	34214440	
ENSDARG00000101964	7.967766476	6.041853341	1.679027939	3.002840646	0.002674724	0.023524267		zgc:171592 [Source:ZFIN;Acc:ZDB-GENE-080220-23]	15	37331585	37340879	
ENSDARG00000102055	6786.043567	-1.276355036	0.081618349	-3.385942483	0.000709342	0.007001654	hadhb	hydroxyacyl-CoA dehydrogenase trifunctional multienzyme complex subunit beta [Source:ZFIN;Acc:ZDB-GENE-030131-85	20	47927720	47953524	
ENSDARG00000102059	40.16062612	-2.776917861	0.437269238	-4.063669944	4.83E-05	0.000592937	si:ch211-117l17.6	si:ch211-117l17.6 [Source:ZFIN;Acc:ZDB-GENE-110914-213]	22	24287774	24297510	
ENSDARG00000102064	366.664216	1.637552555	0.186564953	3.417322201	0.000632404	0.006291704	kcna2b	potassium voltage-gated channel, shaker-related subfamily, member 2b [Source:ZFIN;Acc:ZDB-GENE-080204-89]	23	5009964	5032587	
ENSDARG00000102067	190.7406763	2.507843649	0.281744236	5.351817207	8.71E-08	1.59E-06	grm5b	glutamate receptor, metabotropic 5b [Source:ZFIN;Acc:ZDB-GENE-090821-6]	15	43656241	43768776	
ENSDARG00000102115	3420.328563	-1.599581599	0.090195625	-6.647568544	2.98E-11	8.01E-10	ndufc2	NADH:ubiquinone oxidoreductase subunit C2 [Source:ZFIN;Acc:ZDB-GENE-050320-87]	15	11427620	11443214	
ENSDARG00000102125	764.0140428	1.741230845	0.138500939	5.351810968	8.71E-08	1.59E-06	iqsec2a	IQ motif and Sec7 domain ArfGEF 2a [Source:ZFIN;Acc:ZDB-GENE-170302-3]	8	22567363	22739757	
ENSDARG00000102160	249.163989	1.770281276	0.225182627	3.420695839	0.000624611	0.006228992	si:dkeyp-61b2.1	si:dkeyp-61b2.1 [Source:ZFIN;Acc:ZDB-GENE-141216-430]	11	40467618	40504170	
ENSDARG00000102161	324.6254598	2.519172853	0.182573519	8.320882787	8.73E-17	3.68E-15	sdk2a	sidekick cell adhesion molecule 2a [Source:ZFIN;Acc:ZDB-GENE-091008-1]	3	51030667	51142942	
ENSDARG00000102176	337.6324962	-1.550693198	0.150996498	-3.647059414	0.000265259	0.002860076	pla1a	phospholipase A1 member A [Source:NCBI gene;Acc:334017]	9	21214940	21226132	
ENSDARG00000102185	504.6063322	1.558123948	0.155454287	3.590276991	0.000330327	0.003495587	PCDH8	si:ch211-199f5.1 [Source:ZFIN;Acc:ZDB-GENE-120215-215]	9	53707240	53719543	PCDH8
ENSDARG00000102204	920.1216495	2.004982997	0.127871532	7.859317725	3.86E-15	1.44E-13	jph3	junctophilin 3 [Source:ZFIN;Acc:ZDB-GENE-060503-415]	25	12640211	12696137	
ENSDARG00000102206	522.3286872	1.850938658	0.174505655	4.876281286	1.08E-06	1.73E-05	stc2b	stanniocalcin 2b [Source:ZFIN;Acc:ZDB-GENE-120203-5]	21	41511469	41531434	
ENSDARG00000102229	1467.593421	1.537994788	0.150852836	3.566355155	0.000361981	0.00379377	mapk8ip1a	mitogen-activated protein kinase 8 interacting protein 1a [Source:ZFIN;Acc:ZDB-GENE-101025-1]	25	13801716	13842618	
ENSDARG00000102251	88.11762159	2.039618076	0.311244267	3.34019992	0.000837181	0.008116678	pdzph1	PDZ and pleckstrin homology domains 1 [Source:ZFIN;Acc:ZDB-GENE-090313-385]	10	5644356	5655109	
ENSDARG00000102277	1230.877784	2.258840338	0.138143071	9.112584017	8.04E-20	4.25E-18	lama1	laminin, alpha 1 [Source:ZFIN;Acc:ZDB-GENE-060118-1]	24	41690545	41784173	
ENSDARG00000102288	496.7854961	2.627223788	0.164823519	9.872521819	5.48E-23	3.49E-21	CABZ01056321.1	photoreceptor cilium actin regulator 1 [Source:NCBI gene;Acc:100537793]	17	47083446	47090440	
ENSDARG00000102291	22163.50518	-1.315256922	0.100100419	-3.14940661	0.001636024	0.01494934	eef1da	eukaryotic translation elongation factor 1 delta a (guanine nucleotide exchange protein) [Source:ZFIN;Acc:ZDB-GENE-0404	2	50999477	51032888	
ENSDARG00000102310	225.3298687	3.401809346	0.263971807	9.098734355	9.14E-20	4.79E-18	si:ch211-156l18.8	si:ch211-156l18.8 [Source:ZFIN;Acc:ZDB-GENE-141215-43]	11	12052791	12092178	
ENSDARG00000102312	128.5611578	2.011607265	0.328537089	3.07912652	0.002076085	0.018604716	scn5lab	sodium channel, voltage gated, type V-like, alpha b [Source:ZFIN;Acc:ZDB-GENE-060908-3]	24	40905100	41143256	
ENSDARG00000102334	130.7949062	1.689124896	0.241216334	2.856874924	0.004278344	0.035868641	SH3RF3	si:ch211-124n19.2 [Source:ZFIN;Acc:ZDB-GENE-081104-105]	9	55893789	55946377	SH3RF3
ENSDARG00000102364	1821.791508	1.994507408	0.179309642	5.546313049	2.92E-08	5.69E-07	si:dkey-202l22.6	si:dkey-202l22.6 [Source:ZFIN;Acc:ZDB-GENE-131121-181]	15	12004777	12011390	
ENSDARG00000102389	68.44364082	-2.53324632	0.324962565	-4.718224448	2.38E-06	3.65E-05	ved	ventrally expressed dharma/bozozok antagonist [Source:ZFIN;Acc:ZDB-GENE-030813-1]	10	44997062	45002196	
ENSDARG00000102411	8.212260274	6.088539096	1.608492911	3.16354462	0.001558605	0.0143044	CR847850.1	zinc finger protein 271-like [Source:NCBI gene;Acc:100534733]	3	7435802	7436932	
ENSDARG00000102412	3012.056743	-1.377739696	0.091969984	-4.107206255	4E-05	0.000498378	eci2	enoyl-CoA delta isomerase 2 [Source:ZFIN;Acc:ZDB-GENE-040718-392]	24	2933687	2972039	
ENSDARG00000102415	14903.30925	-2.591939922	0.112652919	-14.13136859	2.43E-45	4.63E-43	scinla	scinderin like a [Source:ZFIN;Acc:ZDB-GENE-030131-2005]	6	24090950	24103666	
ENSDARG00000102424	242.4127275	1.641197173	0.200026081	3.205567839	0.001347963	0.012564133	ARHGAP44	si:ch211-114m9.1 [Source:ZFIN;Acc:ZDB-GENE-120215-49]	3	48638594	48716422	ARHGAP44
ENSDARG00000102430	216.9215546	2.62067374	0.222512157	7.283528962	3.25E-13	1.03E-11	opn6a	opsin 6, group member a [Source:ZFIN;Acc:ZDB-GENE-061201-30]	11	44858979	44876005	
ENSDARG00000102442	405.6027734	-2.671493799	0.198108681	-8.437256735	3.25E-17	1.43E-15	folr	folate receptor [Source:ZFIN;Acc:ZDB-GENE-070615-23]	15	47341856	47350232	
ENSDARG00000102443	426.6559318	2.963340447	0.171314049	11.46047544	2.08E-30	2.06E-28	shank2a	SH3 and multiple ankyrin repeat domains 2a [Source:ZFIN;Acc:ZDB-GENE-120215-55]	18	50880096	50911958	
ENSDARG00000102445	1165.110168	1.492755569	0.11431246	4.310602457	1.63E-05	0.00021819	si:dkey-22o22.2	si:dkey-22o22.2 [Source:ZFIN;Acc:ZDB-GENE-100922-84]	16	40594128	40841363	
ENSDARG00000102453	15982.59479	2.271381579	0.148473264	8.563033788	1.1E-17	5.03E-16	slc1a2b	solute carrier family 1 member 2b [Source:ZFIN;Acc:ZDB-GENE-030131-7779]	25	37366698	37407757	
ENSDARG00000102472	220.4731008	1.548785637	0.189088819	2.902263809	0.003704764	0.031533836	btbd17b	BTB (POZ) domain containing 17b [Source:ZFIN;Acc:ZDB-GENE-091204-214]	3	12884117	12890779	
ENSDARG00000102499	8.941250788	-4.031925801	1.038979593	-2.918176469	0.00352085	0.030142177	BX511227.4		14	31633146	31635433	
ENSDARG00000102517	125.3572582	2.507138363	0.313614562	4.80570275	1.54E-06	2.43E-05	crtac1b	cartilage acidic protein 1b [Source:ZFIN;Acc:ZDB-GENE-090312-28]	13	40064481	40141630	
ENSDARG00000102555	859.3827531	-1.769594409	0.153710384	-5.00678217	5.53E-07	9.2E-06	si:dkey-33i11.9	si:dkey-33i11.9 [Source:ZFIN;Acc:ZDB-GENE-060503-262]	16	45172793	45178430	
ENSDARG00000102558	305.8738062	1.902605774	0.194069331	4.650944941	3.3E-06	4.95E-05	pde6ha	phosphodiesterase 6H, cGMP-specific, cone, gamma, paralog a [Source:ZFIN;Acc:ZDB-GENE-040426-1754]	6	344423	346084	
ENSDARG00000102584	203.4808226	2.019572032	0.247340768	4.122134978	3.75E-05	0.000469551	cpne7	copine VII [Source:ZFIN;Acc:ZDB-GENE-131125-70]	7	67486807	67594818	
ENSDARG00000102585	271.4119799	1.644603211	0.184818255	3.487768092	0.00048707	0.004975968	fam160a1b	family with sequence similarity 160 member A1b [Source:ZFIN;Acc:ZDB-GENE-110411-10]	23	45668597	45682136	
ENSDARG00000102593	61.39096718	8.993235078	1.473196173	5.425777792	5.77E-08	1.08E-06	si:ch211-214b16.2	si:ch211-214b16.2 [Source:ZFIN;Acc:ZDB-GENE-141212-1]	3	60314274	60316118	
ENSDARG00000102687	475.3537391	-1.609088494	0.148334551	-4.106180857	4.02E-05	0.000500099	CABZ01085139.1		22	56644	57177	
ENSDARG00000102690	1055.143613	1.654210203	0.113448341	5.766591196	8.09E-09	1.68E-07	rims2b	regulating synaptic membrane exocytosis 2b [Source:ZFIN;Acc:ZDB-GENE-060503-893]	19	13099541	13186822	
ENSDARG00000102713	80.12384069	2.159618225	0.368084638	3.150411899	0.001630404	0.014908844	si:ch211-205a14.1	si:ch211-205a14.1 [Source:ZFIN;Acc:ZDB-GENE-050420-271]	4	71274311	71294485	
ENSDARG00000102715	27.90255392	3.127389309	0.621068945	3.425367386	0.000613969	0.006130161	UST	uronyl 2-sulfotransferase [Source:HGNC Symbol;Acc:HGNC:17223]	17	6969846	6984338	UST
ENSDARG00000102720	26.49991031	-3.6808427	0.55643923	-4.817853518	1.45E-06	2.29E-05	zgc:123284	zgc:123284 [Source:ZFIN;Acc:ZDB-GENE-051113-92]	3	49097775	49100456	
ENSDARG00000102730	1014.52215	1.865771043	0.134509234	6.436517516	1.22E-10	3.14E-09	mapk10	mitogen-activated protein kinase 10 [Source:ZFIN;Acc:ZDB-GENE-051120-117]	21	9576176	9713292	
ENSDARG00000102744	2478.55847	-2.065422029	0.098558425	-10.81005539	3.08E-27	2.56E-25	mgst3a	microsomal glutathione S-transferase 3a [Source:ZFIN;Acc:ZDB-GENE-040426-2767]	20	48588679	48604653	
ENSDARG00000102755	983.972015	2.086123559	0.139000747	7.813796579	5.55E-15	2.05E-13	EML6	EMAP like 6 [Source:NCBI gene;Acc:100000931]	11	42929547	43002262	EML6
ENSDARG00000102756	11.00374004	-6.342977235	1.362171129	-3.922397944	8.77E-05	0.001029787	nr0b2b	nuclear receptor subfamily 0, group B, member 2b [Source:ZFIN;Acc:ZDB-GENE-080403-9]	19	14153486	14155781	
ENSDARG00000102769	395.6937797	1.412289197	0.1492428	2.762539955	0.005735354	0.046590008	CDH18	cadherin-18 [Source:NCBI gene;Acc:101886358]	12	40810418	41114078	CDH18
ENSDARG00000102798	3582.605207	-1.327593902	0.099213617	-3.301904618	0.000960307	0.009210824	mcm2	minichromosome maintenance complex component 2 [Source:ZFIN;Acc:ZDB-GENE-020419-24]	22	5632029	5655680	
ENSDARG00000102805	438.844842	-3.517459497	0.216986792	-11.60190203	4.03E-31	4.15E-29	cyp2aa12	cytochrome P450, family 2, subfamily AA, polypeptide 12 [Source:ZFIN;Acc:ZDB-GENE-071004-47]	23	42292748	42306332	
ENSDARG00000102824	2906.454114	1.527744184	0.101989297	5.174505569	2.29E-07	3.95E-06	pcdh10b	protocadherin 10b [Source:ZFIN;Acc:ZDB-GENE-030721-4]	14	42978695	43000836	
ENSDARG00000102879	1807.914386	2.365921224	0.136655792	9.995340911	1.6E-23	1.07E-21	myt1b	myelin transcription factor 1b [Source:ZFIN;Acc:ZDB-GENE-090313-67]	23	7762033	7826849	
ENSDARG00000102896	1047.002049	1.494037421	0.107737219	4.585577995	4.53E-06	6.66E-05	jmjd6	jumonji domain containing 6, arginine demethylase and lysine hydroxylase [Source:NCBI gene;Acc:266962]	3	60575113	60589292	
ENSDARG00000102946	34.03948787	-2.78265919	0.453157558	-3.933861763	8.36E-05	0.000987872	si:ch73-167f10.1	si:ch73-167f10.1 [Source:ZFIN;Acc:ZDB-GENE-131121-334]	3	49379774	49382896	
ENSDARG00000102967	152.6954901	2.353875778	0.269612	5.021570916	5.13E-07	8.56E-06	CT573282.1		11	25475283	25476185	
ENSDARG00000102981	1241.877669	-2.115580821	0.105990864	-10.52525454	6.61E-26	4.97E-24	cyp2k16	cytochrome P450, family 2, subfamily K, polypeptide16 [Source:ZFIN;Acc:ZDB-GENE-041010-37]	3	12764894	12780758	
ENSDARG00000102987	560.0340861	1.794514195	0.142242654	5.585625488	2.33E-08	4.59E-07	capn7	calpain 7 [Source:ZFIN;Acc:ZDB-GENE-111111-1]	16	36167907	36196700	
ENSDARG00000103013	673.5146052	1.542983471	0.1744543	3.112468264	0.0018553	0.016769851	pcdh1g22	protocadherin 1 gamma 22 [Source:ZFIN;Acc:ZDB-GENE-030131-2695]	10	21730585	21820133	
ENSDARG00000103034	524.2134671	-3.201183971	0.161646646	-13.6172573	3.16E-42	5.44E-40	CABZ01068499.1	cysteine-rich venom protein natrin-1-like [Source:NCBI gene;Acc:100536500]	16	49005321	49017878	
ENSDARG00000103037	16.5115485	7.094121541	1.602220608	3.803547096	0.000142639	0.001615694	CR848788.1		16	5607138	5612480	
ENSDARG00000103057	7081.988077	1.563792054	0.104012788	5.420410954	5.95E-08	1.11E-06	zfhx3	zinc finger homeobox 3 [Source:ZFIN;Acc:ZDB-GENE-030131-7577]	7	68214935	68373495	
ENSDARG00000103067	872.4277176	2.906136968	0.180921842	10.5356929	5.91E-26	4.46E-24	adgrd2	adhesion G protein-coupled receptor D2 [Source:ZFIN;Acc:ZDB-GENE-081104-190]	8	52788997	52826181	
ENSDARG00000103069	248.0721227	2.531521221	0.251218013	6.096382992	1.08E-09	2.48E-08	CABZ01099795.1		15	47180744	47193564	
ENSDARG00000103071	988.1172536	2.29448446	0.121772282	10.63037034	2.15E-26	1.68E-24	si:ch211-244c8.4	si:ch211-244c8.4 [Source:ZFIN;Acc:ZDB-GENE-141216-488]	10	35537974	35542071	
ENSDARG00000103109	3179.35316	1.368874974	0.109644107	3.364293664	0.000767398	0.007506807	chl1b	cell adhesion molecule L1-like b [Source:ZFIN;Acc:ZDB-GENE-091105-1]	6	44812444	44977428	
ENSDARG00000103123	392.0310313	2.406834841	0.174299452	8.071366992	6.95E-16	2.74E-14	CR854980.1	neuronal tyrosine-phosphorylated phosphoinositide-3-kinase adapter 1 [Source:NCBI gene;Acc:101885998]	5	38517486	38533861	
ENSDARG00000103156	79.32948768	1.934177449	0.302112648	3.092149415	0.001987128	0.017877742	gpc6a	glypican 6a [Source:ZFIN;Acc:ZDB-GENE-071004-15]	1	2712956	3036019	
ENSDARG00000103211	611.8922156	1.541781761	0.127833037	4.238198298	2.25E-05	0.000293045	si:dkey-19b23.8	si:dkey-19b23.8 [Source:ZFIN;Acc:ZDB-GENE-030131-4634]	7	20600674	20611039	
ENSDARG00000103260	82.67317569	9.420731743	1.471252123	5.72351374	1.04E-08	2.15E-07	si:ch211-57b15.1	si:ch211-57b15.1 [Source:ZFIN;Acc:ZDB-GENE-141210-17]	21	29179887	29186128	
ENSDARG00000103266	869.4904935	-2.210718398	0.179700397	-6.737427503	1.61E-11	4.49E-10	ogal	O-GlcNAcase like [Source:ZFIN;Acc:ZDB-GENE-110914-153]	11	45110865	45126501	
ENSDARG00000103295	4644.397139	-2.932361417	0.413655637	-4.671425324	2.99E-06	4.51E-05	cyp3a65	cytochrome P450, family 3, subfamily A, polypeptide 65 [Source:ZFIN;Acc:ZDB-GENE-050604-1]	1	59338175	59348118	
ENSDARG00000103298	494.8249612	-2.241882557	0.155777188	-7.972172143	1.56E-15	6.01E-14	ephx5	epoxide hydrolase 5 [Source:ZFIN;Acc:ZDB-GENE-030131-1050]	13	33689740	33695296	
ENSDARG00000103312	87.92139985	-1.76906109	0.27748174	-2.771573693	0.005578604	0.04547858	C16H1orf232	testis development-related protein-like [Source:NCBI gene;Acc:101883133]	16	51326237	51331520	C16H1orf232
ENSDARG00000103316	927.9088038	1.387392876	0.11932917	3.246422276	0.001168654	0.011015536	si:ch211-212k18.5	si:ch211-212k18.5 [Source:ZFIN;Acc:ZDB-GENE-030131-379]	7	19552381	19558842	
ENSDARG00000103347	489.6897176	-1.526785279	0.141621328	-3.719674744	0.000199479	0.002193226	cyp2aa3	cytochrome P450, family 2, subfamily AA, polypeptide 3 [Source:ZFIN;Acc:ZDB-GENE-031030-8]	23	42482137	42493876	
ENSDARG00000103358	208.9607873	1.693741073	0.200694433	3.456703125	0.000546827	0.00551906	CU467856.1		6	39524924	39525505	
ENSDARG00000103361	1643.723708	1.320206445	0.100217651	3.195110265	0.001397774	0.012970622	tshz3b	teashirt zinc finger homeobox 3b [Source:ZFIN;Acc:ZDB-GENE-100319-2]	7	46727891	46782264	
ENSDARG00000103379	2091.980552	1.45982058	0.109722893	4.190744208	2.78E-05	0.00035481	pax6a	paired box 6a [Source:NCBI gene;Acc:30567]	25	15029041	15049781	
ENSDARG00000103390	275.9414097	1.838164836	0.230878282	3.630332089	0.000283057	0.003036328	cacna2d2a	calcium channel, voltage-dependent, alpha 2/delta subunit 2a [Source:ZFIN;Acc:ZDB-GENE-050320-71]	6	53503273	53885514	
ENSDARG00000103398	62.48749669	-2.261208105	0.323730654	-3.895856291	9.79E-05	0.001140294	fabp1b.2	fatty acid binding protein 1b, liver, tandem duplicate 2 [Source:ZFIN;Acc:ZDB-GENE-100318-6]	8	1009831	1013283	
ENSDARG00000103413	603.4202151	2.25389095	0.135351485	9.263961559	1.97E-20	1.1E-18	zgc:109949	zgc:109949 [Source:ZFIN;Acc:ZDB-GENE-050522-166]	3	7617353	7639107	
ENSDARG00000103476	1378.662087	1.483106375	0.10827828	4.461710824	8.13E-06	0.000114658	trpm1b	transient receptor potential cation channel, subfamily M, member 1b [Source:ZFIN;Acc:ZDB-GENE-070424-31]	25	34576067	34615463	
ENSDARG00000103479	2039.685173	1.307476622	0.11164898	2.753958167	0.005887929	0.047675134	ptprfa	protein tyrosine phosphatase receptor type Fa [Source:ZFIN;Acc:ZDB-GENE-020107-2]	6	2951645	3276052	
ENSDARG00000103498	4990.480282	-2.523775258	0.087494931	-17.41558319	6.28E-68	2.39E-65	epd	ependymin [Source:NCBI gene;Acc:30199]	5	37837245	37839310	
ENSDARG00000103503	786.4053877	-1.627445098	0.14151738	-4.433696388	9.26E-06	0.000128959	dgat1a	diacylglycerol O-acyltransferase 1a [Source:ZFIN;Acc:ZDB-GENE-030131-4600]	19	1370504	1393308	
ENSDARG00000103504	35.45983705	2.723448844	0.514493772	3.349795348	0.000808713	0.007874128	CR855311.4		16	31646390	31651183	
ENSDARG00000103515	116.774169	1.73922357	0.267995516	2.758343052	0.00580952	0.047131464	vcana	versican a [Source:ZFIN;Acc:ZDB-GENE-011023-1]	5	46424437	46486028	
ENSDARG00000103519	926.3720929	1.589919505	0.125739301	4.691607957	2.71E-06	4.12E-05	adamts1	ADAM metallopeptidase with thrombospondin type 1 motif, 1 [Source:ZFIN;Acc:ZDB-GENE-070814-1]	1	673163	681116	
ENSDARG00000103535	265.4503904	2.068461849	0.293979416	3.634478437	0.000278544	0.002994313	SORCS3	zmp:0000001327 [Source:NCBI gene;Acc:100534837]	1	48256950	48490034	SORCS3
ENSDARG00000103554	1830.614898	1.584077697	0.102628642	5.691176296	1.26E-08	2.56E-07	notch1a	notch receptor 1a [Source:NCBI gene;Acc:30718]	21	4766244	4849056	
ENSDARG00000103576	302.2706474	1.884283811	0.171589138	5.15349526	2.56E-07	4.39E-06		EPH receptor B3 [Source:HGNC Symbol;Acc:HGNC:3394]	22	35930526	35941371	
ENSDARG00000103588	636.3856254	2.349883482	0.142996098	9.440002231	3.73E-21	2.15E-19	prrt2	proline-rich transmembrane protein 2 [Source:ZFIN;Acc:ZDB-GENE-121210-3]	12	5081759	5095774	
ENSDARG00000103590	686.7773318	-2.090217656	0.155075133	-7.030254535	2.06E-12	6.19E-11	cyp2aa6	cytochrome P450, family 2, subfamily AA, polypeptide 6 [Source:ZFIN;Acc:ZDB-GENE-030131-2932]	23	42316065	42330488	
ENSDARG00000103594	1419.474193	-1.509211373	0.103189917	-4.934700849	8.03E-07	1.31E-05	npm3	nucleophosmin/nucleoplasmin, 3 [Source:ZFIN;Acc:ZDB-GENE-050320-50]	13	11975954	11986754	
ENSDARG00000103639	1691.944593	-2.481409627	0.156118392	-9.489014129	2.33E-21	1.36E-19	si:dkey-36i7.3	si:dkey-36i7.3 [Source:ZFIN;Acc:ZDB-GENE-131127-316]	15	12251044	12270857	
ENSDARG00000103648	1992.320312	1.58582676	0.094958485	6.169293454	6.86E-10	1.61E-08	znf536	zinc finger protein 536 [Source:ZFIN;Acc:ZDB-GENE-030616-624]	7	46252993	46514996	
ENSDARG00000103650	108.5182619	2.678532187	0.301666497	5.564198226	2.63E-08	5.16E-07	si:ch73-329n5.1	si:ch73-329n5.1 [Source:ZFIN;Acc:ZDB-GENE-131121-475]	12	44119432	44307963	
ENSDARG00000103651	737.8379054	2.254762529	0.138626141	9.051413511	1.41E-19	7.32E-18	map3k12	mitogen-activated protein kinase kinase kinase 12 [Source:ZFIN;Acc:ZDB-GENE-040426-2436]	11	219124	235602	
ENSDARG00000103658	1462.973271	1.600028165	0.119345964	5.027636824	4.97E-07	8.31E-06	hivep1	HIVEP zinc finger 1 [Source:ZFIN;Acc:ZDB-GENE-070912-512]	19	3383025	3488860	
ENSDARG00000103659	78.50317861	-1.967339665	0.293417838	-3.296799099	0.000977934	0.009369133	bco1l	beta-carotene oxygenase 1, like [Source:ZFIN;Acc:ZDB-GENE-040426-1191]	7	65240227	65252938	

**ENSDARG00000103666**	197.516565	-1.656610128	0.187876549	-3.494902013	0.000474236	0.004859542	dapk2b	death-associated protein kinase 2b [Source:ZFIN;Acc:ZDB-GENE-060810-178]	25	26441054	26604529	
**ENSDARG00000103687**	3071.315557	-5.278753224	0.148518538	-28.80955655	1.63E-182	2.72E-179	sycn.2	syncollin, tandem duplicate 2 [Source:ZFIN;Acc:ZDB-GENE-141212-321]	21	25226558	25227191	
**ENSDARG00000103691**	2045.982324	1.50872709	0.098382578	5.170906295	2.33E-07	4.02E-06	rfx7b	regulatory factor X7b [Source:ZFIN;Acc:ZDB-GENE-170623-1]	25	388258	408173	
**ENSDARG00000103697**	3710.657705	1.760627131	0.090345587	8.419084523	3.79E-17	1.66E-15	arnt2	aryl-hydrocarbon receptor nuclear translocator 2 [Source:NCBI gene;Acc:64277]	7	10701732	10877536	
**ENSDARG00000103739**	1262.693047	1.645052223	0.10617964	6.075102775	1.24E-09	2.81E-08	bahcc1a	BAH domain and coiled-coil containing 1a [Source:ZFIN;Acc:ZDB-GENE-170301-1]	3	57038033	57174041	
**ENSDARG00000103760**	842.2478056	1.413800459	0.132377891	3.125903097	0.0017726	0.01610824	cfhl2	complement factor H like 2 [Source:ZFIN;Acc:ZDB-GENE-041114-123]	22	23734001	23748284	
**ENSDARG00000103785**	1467.958358	-2.076894632	0.123588237	-8.713568997	2.94E-18	1.39E-16	sult2st2	sulfotransferase family 2, cytosolic sulfotransferase 2 [Source:ZFIN;Acc:ZDB-GENE-061117-5]	12	648334	656540	
**ENSDARG00000103786**	55.84400028	2.274608548	0.379499241	3.358659018	0.000783216	0.007652595	klhl13	kelch-like family member 13 [Source:ZFIN;Acc:ZDB-GENE-030131-2671]	5	37379825	37440750	
**ENSDARG00000103792**	145.3891575	1.937370107	0.25316109	3.702662623	0.000213349	0.002329387	CABZ01076737.1		5	3184755	3209345	
**ENSDARG00000103810**	56.73674667	2.646550293	0.403939647	4.076228478	4.58E-05	0.000564028	cacna2d4a	calcium channel, voltage-dependent, alpha 2/delta subunit 4a [Source:ZFIN;Acc:ZDB-GENE-041210-213]	4	20237461	20243021	
**ENSDARG00000103824**	1691.74645	-1.619494485	0.152573311	-4.06030701	4.9E-05	0.000600076	hint1	histidine triad nucleotide binding protein 1 [Source:ZFIN;Acc:ZDB-GENE-040927-8]	10	7636811	7640797	
**ENSDARG00000103834**	1406.100063	1.404679773	0.127189543	3.18170631	0.001464102	0.013541056	rbm14b	RNA binding motif protein 14b [Source:ZFIN;Acc:ZDB-GENE-040426-2455]	7	6941583	7043133	
**ENSDARG00000103837**	1458.164329	-1.51503325	0.136554538	-3.771630413	0.000162184	0.001817406	CU633479.2	myosin-7 [Source:NCBI gene;Acc:100329813]	24	40763020	40774028	
**ENSDARG00000103847**	55.43088951	2.430770719	0.398365328	3.591604532	0.000328648	0.00348076	CR339059.1		3	9737490	9738985	
**ENSDARG00000103849**	3245.908366	-1.923448029	0.172717881	-5.346568776	8.96E-08	1.63E-06	mdh1ab	malate dehydrogenase 1Ab, NAD (soluble) [Source:ZFIN;Acc:ZDB-GENE-030131-7655]	17	24439532	24444402	
**ENSDARG00000103851**	3996.724885	2.305502724	0.111758385	11.68147454	1.59E-31	1.65E-29	tnrc6c2	trinucleotide repeat containing adaptor 6C2 [Source:ZFIN;Acc:ZDB-GENE-061207-48]	6	18714213	18751696	
**ENSDARG00000103878**	3134.665906	-3.633148455	0.191118181	-13.77759273	3.48E-43	6.06E-41	anpepb	alanyl (membrane) aminopeptidase b [Source:ZFIN;Acc:ZDB-GENE-030131-1253]	25	10811551	10827771	
**ENSDARG00000103906**	120.2602498	2.718084452	0.292150207	5.880825721	4.08E-09	8.8E-08	pcdh2ab10	protocadherin 2 alpha b 10 [Source:ZFIN;Acc:ZDB-GENE-041118-9]	14	2123123	2193248	
**ENSDARG00000103917**	2195.570254	-1.861369745	0.195317508	-4.410100002	1.03E-05	0.000142893	znf185	zinc finger protein 185 with LIM domain [Source:ZFIN;Acc:ZDB-GENE-061103-355]	14	14620830	14643190	
**ENSDARG00000103937**	12731.69314	2.105955066	0.118299089	9.348804582	8.86E-21	5.05E-19	ndrg4	NDRG family member 4 [Source:ZFIN;Acc:ZDB-GENE-060512-226]	25	13332280	13381854	
**ENSDARG00000103949**	44.51486373	4.455613454	0.664707635	5.198696795	2.01E-07	3.5E-06	zbtb12.1	zinc finger and BTB domain containing 12, tandem duplicate 1 [Source:ZFIN;Acc:ZDB-GENE-060503-875]	19	26937543	26943936	
**ENSDARG00000103957**	393.0036776	-1.47339136	0.155254587	-3.049129619	0.002295054	0.020401687	aamdc	adipogenesis associated, Mth938 domain containing [Source:NCBI gene;Acc:553802]	18	3096612	3103827	
**ENSDARG00000103971**	74.79752061	2.492227425	0.437802874	3.408445933	0.00065334	0.006482014	si:ch73-173p19.2	si:ch73-173p19.2 [Source:ZFIN;Acc:ZDB-GENE-141212-282]	24	33439243	33461313	
**ENSDARG00000104005**	251.0871174	2.253170546	0.206852016	6.05829506	1.38E-09	3.1E-08	slc4a3	solute carrier family 4 member 3 [Source:ZFIN;Acc:ZDB-GENE-141006-1]	9	46644633	46684539	
**ENSDARG00000104011**	18161.46206	-1.328197161	0.10355973	-3.16915814	0.001528812	0.014087631	rps17	ribosomal protein S17 [Source:ZFIN;Acc:ZDB-GENE-040426-1852]	7	54092291	54098631	
**ENSDARG00000104027**	12.09677456	6.645178621	1.608594524	3.5093857	0.000449143	0.004629955	si:ch211-207b24.4	si:ch211-207b24.4 [Source:ZFIN;Acc:ZDB-GENE-100921-9]	3	48206469	48215187	
**ENSDARG00000104028**	1883.22486	-1.503333242	0.10826712	-4.648994479	3.34E-06	4.99E-05	si:dkey-74k8.3	si:dkey-74k8.3 [Source:ZFIN;Acc:ZDB-GENE-141222-20]	14	21052504	21064199	
**ENSDARG00000104068**	11988.15881	-1.879284986	0.081187472	-10.8303038	2.47E-27	2.07E-25	gstp1	glutathione S-transferase pi 1 [Source:ZFIN;Acc:ZDB-GENE-020806-4]	14	3507326	3514055	
**ENSDARG00000104080**	93.53649528	3.118035806	0.387263501	5.469236843	4.52E-08	8.57E-07	pcdh2ab12	protocadherin 2 alpha b 12 [Source:ZFIN;Acc:ZDB-GENE-041118-11]	14	2123123	2187202	
**ENSDARG00000104085**	2512.119602	-1.348107045	0.10855967	-3.206596386	0.001343153	0.012523954	abcd3a	ATP-binding cassette, sub-family D (ALD), member 3a [Source:ZFIN;Acc:ZDB-GENE-040426-2868]	24	31310640	31332087	
**ENSDARG00000104101**	953.9783628	2.155870465	0.149426488	7.735378669	1.03E-14	3.72E-13	RIMBP2	si:ch73-287m6.1 [Source:ZFIN;Acc:ZDB-GENE-100920-2]	21	18313152	18386881	RIMBP2
**ENSDARG00000104113**	83.79678119	-2.180893743	0.351872897	-3.356023586	0.000790718	0.007716881	zgc:136767	zgc:136767 [Source:ZFIN;Acc:ZDB-GENE-060421-7499]	3	6325895	6351893	
**ENSDARG00000104181**	9486.99276	-1.237553352	0.08266726	-2.873608647	0.004058115	0.034262609	tmsb1	thymosin beta 1 [Source:ZFIN;Acc:ZDB-GENE-030131-8575]	14	12066695	12071679	
**ENSDARG00000104193**	158.8427884	-2.369082983	0.283503072	-4.829164542	1.37E-06	2.17E-05	CABZ01019904.1		3	8158039	8199772	
**ENSDARG00000104204**	368.0111496	2.662876017	0.186028516	8.938823228	3.93E-19	1.99E-17	slc1a8a	solute carrier family 1 member 8a [Source:ZFIN;Acc:ZDB-GENE-101111-8]	11	15068307	15090564	
**ENSDARG00000104207**	466.3948164	-2.463159019	0.184430318	-7.933397472	2.13E-15	8.11E-14	pcyt1bb	phosphate cytidylyltransferase 1, choline, beta b [Source:ZFIN;Acc:ZDB-GENE-051120-48]	21	40618615	40630650	
**ENSDARG00000104227**	1220.189861	-1.474025164	0.103782515	-4.567485805	4.94E-06	7.23E-05	nop10	NOP10 ribonucleoprotein homolog (yeast) [Source:NCBI gene;Acc:445391]	7	1505507	1508912	
**ENSDARG00000104231**	210.7278574	1.914323169	0.204260922	4.476251074	7.6E-06	0.000107851	opn9	opsin 9 [Source:ZFIN;Acc:ZDB-GENE-141216-390]	16	17163193	17188294	
**ENSDARG00000104256**	1708.293724	-2.063147121	0.130696742	-8.134457733	4.14E-16	1.65E-14	bco1	beta-carotene oxygenase 1 [Source:ZFIN;Acc:ZDB-GENE-010508-1]	7	65261354	65290474	
**ENSDARG00000104264**	1245.812163	1.894831686	0.157835035	5.66941103	1.43E-08	2.9E-07	XKR4	zgc:123035 [Source:ZFIN;Acc:ZDB-GENE-051030-45]	2	27670284	27775236	XKR4
**ENSDARG00000104282**	187.6700008	2.436358444	0.239264902	6.003214138	1.93E-09	4.3E-08	dcc	DCC netrin 1 receptor [Source:NCBI gene;Acc:569360]	5	31748	201600	
**ENSDARG00000104301**	190.4451983	-1.776539736	0.225268702	-3.447171	0.00056649	0.005699184	zgc:193726	zgc:193726 [Source:ZFIN;Acc:ZDB-GENE-081022-35]	4	20042807	20043484	
**ENSDARG00000104359**	2945.077692	-1.418301846	0.086678083	-4.825924072	1.39E-06	2.21E-05	anxa1c	annexin A1c [Source:ZFIN;Acc:ZDB-GENE-030131-5274]	5	65009764	65021736	
**ENSDARG00000104370**	169.4880472	2.097923126	0.225321761	4.872690152	1.1E-06	1.76E-05	esm1	endothelial cell-specific molecule 1 [Source:ZFIN;Acc:ZDB-GENE-060929-748]	21	9987313	9991502	
**ENSDARG00000104387**	579.6289791	1.84748516	0.136463062	6.210363083	5.29E-10	1.26E-08	SLC4A5	si:ch211-48m9.1 [Source:ZFIN;Acc:ZDB-GENE-060531-64]	10	44811700	44901444	SLC4A5
**ENSDARG00000104399**	71.29860332	-2.508654208	0.300361386	-5.02279679	5.09E-07	8.52E-06	tppp	tubulin polymerization promoting protein [Source:ZFIN;Acc:ZDB-GENE-101115-2]	22	5478353	5479326	
**ENSDARG00000104403**	81.1962252	2.137415262	0.401161635	2.835304187	0.004578207	0.038077394	oscp1a	organic solute carrier partner 1a [Source:ZFIN;Acc:ZDB-GENE-080204-2]	19	3826782	3843327	
**ENSDARG00000104418**	950.0057106	1.379218454	0.136338534	2.78144734	0.005411711	0.044305074	BX248318.1		11	28078148	28086924	
**ENSDARG00000104480**	395.0056568	2.065319161	0.174887895	6.091440238	1.12E-09	2.56E-08	hcn1	hyperpolarization activated cyclic nucleotide-gated potassium channel 1 [Source:ZFIN;Acc:ZDB-GENE-110520-1]	21	10253976	10488379	
**ENSDARG00000104487**	453.1100217	-1.501998383	0.145082528	-3.460088476	0.000539998	0.005458916	mthfs	5,10-methenyltetrahydrofolate synthetase (5-formyltetrahydrofolate cyclo-ligase) [Source:ZFIN;Acc:ZDB-GENE-130103-5]	7	10502631	10560964	
**ENSDARG00000104497**	624.1259717	1.988620384	0.194278229	5.088683317	3.61E-07	6.14E-06	pcdh1g29	protocadherin 1 gamma 29 [Source:ZFIN;Acc:ZDB-GENE-050609-22]	10	21796477	21820133	
**ENSDARG00000104517**	4817.450115	-1.433702411	0.08275983	-5.240494217	1.6E-07	2.83E-06	uqcr10	ubiquinol-cytochrome c reductase, complex III subunit X [Source:ZFIN;Acc:ZDB-GENE-170406-3]	21	5882300	5885723	
**ENSDARG00000104519**	29.37956266	3.039748276	0.633824625	3.218158772	0.001290164	0.012079225	KDELR3	KDEL endoplasmic reticulum protein retention receptor 3 [Source:HGNC Symbol;Acc:HGNC:6306]	3	301479	302222	KDELR3
**ENSDARG00000104537**	6462.56674	-1.474975803	0.091464641	-5.192999158	2.07E-07	3.6E-06	cox7c	cytochrome c oxidase subunit 7C [Source:ZFIN;Acc:ZDB-GENE-030131-8062]	5	47726197	47727819	
**ENSDARG00000104540**	3026.668307	-2.720865346	0.106903562	-16.09736215	2.66E-58	7.96E-56	cyp2aa8	cytochrome P450, family 2, subfamily AA, polypeptide 8 [Source:ZFIN;Acc:ZDB-GENE-041010-183]	23	42336142	42409339	
**ENSDARG00000104549**	421.6646753	1.636077906	0.160042978	3.974419325	7.06E-05	0.000843288	arhgap44	Rho GTPase activating protein 44 [Source:ZFIN;Acc:ZDB-GENE-120206-2]	12	48480632	48538302	
**ENSDARG00000104593**	329.2175288	-2.267746505	0.207987556	-6.095299788	1.09E-09	2.5E-08	cyp2k18	cytochrome P450, family 2, subfamily K, polypeptide 18 [Source:ZFIN;Acc:ZDB-GENE-040426-1584]	3	12710350	12715432	
**ENSDARG00000104664**	2302.359819	1.368325038	0.109308988	3.369576874	0.000752837	0.007384509	dclk1b	doublecortin-like kinase 1b [Source:ZFIN;Acc:ZDB-GENE-080516-7]	15	32821392	32891268	
**ENSDARG00000104682**	32111.29017	-1.392140382	0.098266001	-3.990600806	6.59E-05	0.000790401	tpm2	tropomyosin 2 (beta) [Source:ZFIN;Acc:ZDB-GENE-040625-114]	10	6318227	6375394	
**ENSDARG00000104687**	1463.991749	2.089688595	0.142175976	7.664365169	1.8E-14	6.38E-13	slc16a9b	solute carrier family 16 member 9b [Source:ZFIN;Acc:ZDB-GENE-040801-69]	12	7589193	7607114	
**ENSDARG00000104693**	5379.861991	1.326460381	0.083692255	3.900723905	9.59E-05	0.001118645	il6st	interleukin 6 signal transducer [Source:ZFIN;Acc:ZDB-GENE-080104-6]	10	5788620	5821584	
**ENSDARG00000104696**	810.6361277	-1.427380767	0.115466319	-3.701345715	0.000214459	0.002337445	apooa	apolipoprotein O, a [Source:NCBI gene;Acc:641319]	5	4382378	4418555	
**ENSDARG00000104721**	87.74780229	-3.313516042	0.34454009	-6.714794912	1.88E-11	5.22E-10	si:dkey-203a12.9	si:dkey-203a12.9 [Source:ZFIN;Acc:ZDB-GENE-141216-334]	14	15620640	15625472	
**ENSDARG00000104722**	1102.90378	-1.816307799	0.111178053	-7.342346588	2.1E-13	6.86E-12	myl2a	myosin, light chain 2a, regulatory, cardiac, slow [Source:ZFIN;Acc:ZDB-GENE-120917-2]	5	1589458	1611199	
**ENSDARG00000104742**	417.2349929	-1.653770291	0.139937271	-4.67188109	2.98E-06	4.5E-05	hnf4b	hepatic nuclear factor 4, beta [Source:ZFIN;Acc:ZDB-GENE-030131-2730]	7	69019851	69047484	
**ENSDARG00000104767**	300.7018104	2.052693235	0.22472606	4.684339843	2.81E-06	4.26E-05	CR381531.2	inactive dipeptidyl peptidase 10-like [Source:NCBI gene;Acc:101886435]	23	33777519	33901337	
**ENSDARG00000104773**	2536.837404	1.619987261	0.113814438	5.4473516	5.11E-08	9.63E-07	junbb	JunB proto-oncogene, AP-1 transcription factor subunit b [Source:ZFIN;Acc:ZDB-GENE-040426-2666]	3	7654655	7656059	
**ENSDARG00000104775**	1104.228021	-1.756856483	0.119346366	-6.341680193	2.27E-10	5.64E-09	ly6m2	lymphocyte antigen 6 family member M2 [Source:ZFIN;Acc:ZDB-GENE-131121-523]	21	5472330	5481824	
**ENSDARG00000104786**	700.9382286	1.683062957	0.150558043	4.536874586	5.71E-06	8.3E-05	nlgn3a	neuroligin 3a [Source:ZFIN;Acc:ZDB-GENE-071219-1]	14	14224730	14534457	
**ENSDARG00000104810**	318.7766688	1.483948565	0.162888739	2.971037579	0.002967955	0.025858601	akt3a	v-akt murine thymoma viral oncogene homolog 3a [Source:ZFIN;Acc:ZDB-GENE-050419-180]	13	11175208	11378355	
**ENSDARG00000104818**	2175.919743	-2.797169834	0.150746522	-11.92179967	9.11E-33	9.99E-31	ces2b	carboxylesterase 2b [Source:ZFIN;Acc:ZDB-GENE-041014-96]	25	30380705	30383617	
**ENSDARG00000104829**	586.6624757	-3.812282064	0.1680818	-16.73162744	7.71E-63	2.76E-60	zgc:112146	zgc:112146 [Source:ZFIN;Acc:ZDB-GENE-050417-237]	3	49074008	49079318	
**ENSDARG00000104890**	117.6744727	2.471457712	0.311792731	4.719345788	2.37E-06	3.63E-05	si:ch211-76m11.3	si:ch211-76m11.3 [Source:ZFIN;Acc:ZDB-GENE-041210-276]	4	71423591	71439440	
**ENSDARG00000104899**	289.8716814	2.471862695	0.516458437	2.849915095	0.00437309	0.036568261	CR931813.2		8	7828414	7848287	
**ENSDARG00000104919**	523.1234543	2.189670697	0.204204223	5.825886848	5.68E-09	1.21E-07	si:ch211-153b23.3	si:ch211-153b23.3 [Source:ZFIN;Acc:ZDB-GENE-141216-408]	14	11430796	11438060	
**ENSDARG00000104929**	330.3604891	1.853323206	0.201892869	4.226613892	2.37E-05	0.000306791	si:ch211-153f2.7	si:ch211-153f2.7 [Source:ZFIN;Acc:ZDB-GENE-141216-200]	19	45970692	45980732	
**ENSDARG00000104930**	330.7664709	1.853028705	0.219398296	3.888037063	0.000101058	0.001173294	TMEM275	si:dkey-225f23.5 [Source:ZFIN;Acc:ZDB-GENE-131121-130]	2	19100422	19109304	TMEM275
**ENSDARG00000104956**	67.88336864	2.635986933	0.393313422	4.15949938	3.19E-05	0.000402912	si:dkey-165o8.2	si:dkey-165o8.2 [Source:ZFIN;Acc:ZDB-GENE-131122-2]	4	75690971	75708006	
**ENSDARG00000104980**	232.2946609	2.136043377	0.20684045	5.492365633	3.97E-08	7.59E-07	PPEF2	protein phosphatase with EF-hand domain 2a [Source:NCBI gene;Acc:100537120]	5	52881744	52921532	PPEF2
**ENSDARG00000104995**	300.5346412	-3.469424942	0.222374174	-11.10481897	1.19E-28	1.09E-26	ugt5b1	UDP glucuronosyltransferase 5 family, polypeptide B1 [Source:ZFIN;Acc:ZDB-GENE-100406-3]	1	9645275	9652725	
**ENSDARG00000105029**	265.2543048	2.501995606	0.192235017	7.813329904	5.57E-15	2.05E-13	TPPP	tubulin polymerization-promoting protein [Source:NCBI gene;Acc:100333482]	16	52875093	52879741	TPPP
**ENSDARG00000105083**	642.7203823	1.651818008	0.166474082	3.915432366	9.02E-05	0.001056025	chd5	chromodomain helicase DNA binding protein 5 [Source:ZFIN;Acc:ZDB-GENE-120314-2]	11	29574743	29650930	
**ENSDARG00000105088**	1757.98632	2.007648577	0.106378378	9.472306302	2.74E-21	1.59E-19	tenm4	teneurin transmembrane protein 4 [Source:NCBI gene;Acc:30156]	15	10000553	10455569	
**ENSDARG00000105109**	396.3630511	2.116769495	0.166726948	6.698194304	2.11E-11	5.8E-10	FP102783.1		14	3571256	3576820	
**ENSDARG00000105120**	245.5749203	1.535300345	0.190132987	2.815399643	0.004871661	0.040344296	gab2	GRB2-associated binding protein 2 [Source:ZFIN;Acc:ZDB-GENE-130530-756]	15	9444982	9593532	
**ENSDARG00000105136**	168.3344168	1.705630529	0.217291763	3.247387372	0.001164698	0.010982371	ccdc50	coiled-coil domain containing 50 [Source:ZFIN;Acc:ZDB-GENE-150115-1]	22	24318131	24362712	
**ENSDARG00000105151**	103.2240254	-2.285856616	0.315581522	-4.074562433	4.61E-05	0.000566967	cyp2k21	cytochrome P450, family 2, subfamily k, polypeptide 21 [Source:ZFIN;Acc:ZDB-GENE-091113-17]	3	12744083	12749198	
**ENSDARG00000105178**	850.715411	2.484769279	0.140573775	10.56220679	4.46E-26	3.4E-24	erc2	ELKS/RAB6-interacting/CAST family member 2 [Source:ZFIN;Acc:ZDB-GENE-130530-700]	11	37310881	37425407	
**ENSDARG00000105184**	151.8934251	2.376679291	0.278882362	4.936415776	7.96E-07	1.3E-05	nbeab	neurobeachin b [Source:ZFIN;Acc:ZDB-GENE-120329-2]	15	32895659	33325046	
**ENSDARG00000105188**	1049.477203	3.882025991	0.152933187	18.8450005	3.23E-79	1.47E-76	vimr2	vimentin-related 2 [Source:ZFIN;Acc:ZDB-GENE-160113-76]	14	33788835	33824329	
**ENSDARG00000105201**	2676.282652	2.220176001	0.190335275	6.410666648	1.45E-10	3.69E-09	adcyap1r1a	adenylate cyclase activating polypeptide 1a (pituitary) receptor type I [Source:ZFIN;Acc:ZDB-GENE-050315-1]	2	50862527	50899928	
**ENSDARG00000105210**	143.4694558	-2.524686657	0.248762542	-6.129084555	8.84E-10	2.04E-08	etf1a	eukaryotic translation termination factor 1a [Source:ZFIN;Acc:ZDB-GENE-080218-14]	14	2589465	2602445	
**ENSDARG00000105265**	434.0904849	-2.178883104	0.14007855	-8.415871689	3.9E-17	1.7E-15	zgc:171534	zgc:171534 [Source:ZFIN;Acc:ZDB-GENE-080218-6]	13	330611	337318	
**ENSDARG00000105285**	2928.050838	1.408139685	0.143360369	2.846949184	0.004414041	0.036870828	FAT4	FAT atypical cadherin 4 [Source:NCBI gene;Acc:101882648]	14	353099	451555	FAT4
**ENSDARG00000105296**	1465.179205	1.477443748	0.127811016	3.735544585	0.000187309	0.002071213	AP3B2	adaptor related protein complex 3 beta 2 subunit [Source:NCBI gene;Acc:100000165]	25	5702094	5740373	AP3B2
**ENSDARG00000105344**	507.2057224	3.816002699	0.596588254	4.720177916	2.36E-06	3.62E-05	pkd1l2a	polycystic kidney disease 1 like 2a [Source:ZFIN;Acc:ZDB-GENE-050208-89]	7	65177132	65236649	
**ENSDARG00000105351**	439.3737209	-4.261325671	0.225241822	-14.47921902	1.64E-47	3.4E-45	si:ch211-113d11.6	si:ch211-113d11.6 [Source:ZFIN;Acc:ZDB-GENE-160113-62]	14	46233704	46238186	
**ENSDARG00000105411**	4027.993698	-3.229125532	0.08742494	-25.49759305	2.1E-143	2.1E-140	si:ch211-113d11.5	si:ch211-113d11.5 [Source:ZFIN;Acc:ZDB-GENE-030131-6391]	14	46221004	46228094	
**ENSDARG00000105416**	412.6030989	-2.531031755	0.154053143	-9.938335102	2.84E-23	1.84E-21	adgrg4a	adhesion G protein-coupled receptor G4a [Source:ZFIN;Acc:ZDB-GENE-121129-2]	14	31721171	31751154	
**ENSDARG00000105420**	66.25962968	2.608903546	0.373277278	4.310210241	1.63E-05	0.000218461	CR361564.1		16	17704917	17706631	
**ENSDARG00000105422**	661.7846968	8.169783799	0.558103129	12.84670059	8.98E-38	1.26E-35	si:dkey-1k23.3	si:dkey-1k23.3 [Source:ZFIN;Acc:ZDB-GENE-160113-121]	5	15841523	15851953	
**ENSDARG00000105435**	52.51644052	2.886454082	0.437731766	4.309612027	1.64E-05	0.000218936	camta1	calmodulin binding transcription activator 1 [Source:ZFIN;Acc:ZDB-GENE-160114-49]	11	41110669	41157954	
**ENSDARG00000105441**	417.8479801	2.248422129	0.171478701	7.280333504	3.33E-13	1.06E-11	pcdh11	protocadherin 11 [Source:ZFIN;Acc:ZDB-GENE-140106-21]	14	26995114	27121854	
**ENSDARG00000105443**	220.2784241	3.911393867	0.280914214	10.36399628	3.62E-25	2.63E-23	si:ch211-63p21.8	si:ch211-63p21.8 [Source:ZFIN;Acc:ZDB-GENE-130530-652]	7	4299907	4315859	
**ENSDARG00000105447**	499.3978442	3.927767743	0.19736643	14.83417286	8.81E-50	2.05E-47	si:ch211-129p13.1	si:ch211-129p13.1 [Source:ZFIN;Acc:ZDB-GENE-160113-47]	18	43365890	43440181	
**ENSDARG00000105452**	1190.925425	2.006699753	0.114112638	8.82198302	1.12E-18	5.47E-17	plxna1a	plexin A1a [Source:ZFIN;Acc:ZDB-GENE-140106-137]	23	34321237	34753811	
**ENSDARG00000105463**	201.1308791	2.738536221	0.293215487	5.92921007	3.04E-09	6.67E-08	CR352285.1		11	37297501	37298097	
**ENSDARG00000105489**	48.84375808	7.214055155	1.457462723	4.263611725	2.01E-05	0.000263795	loc564660	hypothetical protein LOC564660 [Source:ZFIN;Acc:ZDB-GENE-060130-8]	7	8502801	8504355	
**ENSDARG00000105522**	426.9931871	-1.783632253	0.165896325	-4.723626367	2.32E-06	3.57E-05	si:ch211-196f2.3	si:ch211-196f2.3 [Source:ZFIN;Acc:ZDB-GENE-160728-62]	7	26100024	26110819	
**ENSDARG00000105527**	136.3203528	2.137914434	0.276380941	4.117195738	3.84E-05	0.000478692	SMIM36	si:ch73-256g18.2 [Source:ZFIN;Acc:ZDB-GENE-160728-114]	12	32179211	32183255	SMIM36
**ENSDARG00000105528**	193.6799535	-1.683811214	0.182531458	-3.74626501	0.000179487	0.001994379	si:dkey-86l18.10	si:dkey-86l18.10 [Source:ZFIN;Acc:ZDB-GENE-160728-106]	7	41295974	41302753	
**ENSDARG00000105529**	1559.936049	2.20653228	0.13253852	9.103257502	8.77E-20	4.61E-18	vgf	VGF nerve growth factor inducible [Source:ZFIN;Acc:ZDB-GENE-121009-2]	7	26224211	26230166	
**ENSDARG00000105545**	141.1361326	-1.742357406	0.260700299	-2.847551035	0.004405703	0.036813432	si:dkey-165a24.10	si:dkey-165a24.10 [Source:ZFIN;Acc:ZDB-GENE-160728-16]	7	22776174	22777850	
**ENSDARG00000105549**	208.8988005	2.800948765	0.238368686	7.555307681	4.18E-14	1.43E-12	RIC3	si:ch211-235p24.2 [Source:ZFIN;Acc:ZDB-GENE-160728-28]	7	28047279	28058442	RIC3
**ENSDARG00000105631**	180.4930059	1.806127588	0.241881231	3.332741382	0.000859948	0.008321343	CABP4	si:cabz01076231.1 [Source:ZFIN;Acc:ZDB-GENE-160728-151]	7	808916	823495	CABP4

ENSDARG00000105636	86.35915018	-2.187590738	0.278172652	-4.269257707	1.96E-05	0.000258015	BX323797.3		7	38193343	38194702	
ENSDARG00000105648	949.333402	1.597587947	0.112471763	5.313226448	1.08E-07	1.94E-06	si:cabz01102082.1	si:cabz01102082.1 [Source:ZFIN;Acc:ZDB-GENE-160728-40]	7	1521834	1527551	
ENSDARG00000105674	14.47054102	3.810475182	0.994971748	2.824678377	0.00473281	0.039311137	ms4a17a.2	membrane-spanning 4-domains, subfamily A, member 17a.2 [Source:ZFIN;Acc:ZDB-GENE-070705-367]	4	76671012	76688961	
ENSDARG00000105731	101.1003511	9.712091221	1.465378078	5.945285626	2.76E-09	6.07E-08	CR788316.1	verrucotoxin subunit beta-like [Source:NCBI gene;Acc:103908715]	4	72463842	72476526	
ENSDARG00000105744	511.5917703	1.526387319	0.152383048	3.45436928	0.000551582	0.00556481	ptprr	protein tyrosine phosphatase receptor type R [Source:ZFIN;Acc:ZDB-GENE-101029-2]	4	74226447	74271898	
ENSDARG00000105749	15.54653506	7.011712496	1.534464665	3.917791418	8.94E-05	0.001047211	CR788316.2		4	72486529	72488135	
ENSDARG00000105829	42.88277366	7.026798564	1.478971299	4.074993592	4.6E-05	0.000566349	CR788316.4	neoverrucotoxin subunit beta-like [Source:NCBI gene;Acc:100004951]	4	72500840	72513569	
ENSDARG00000105972	62.11014268	2.988267627	0.411363446	4.833360001	1.34E-06	2.13E-05	CABZ01050944.1	protein shisa-9-like [Source:NCBI gene;Acc:100535636]	3	24793921	24909245	
ENSDARG00000106138	122.8814721	-1.684509731	0.24626989	-2.77951044	0.00544409	0.044541106	BX855618.1		4	20037521	20039635	
ENSDARG00000106473	144.2887139	2.546428355	0.272711078	5.670574027	1.42E-08	2.88E-07	KCNH2	potassium voltage-gated channel subfamily H member 2 [Source:HGNC Symbol;Acc:HGNC:6251]	24	40473032	40500312	KCNH2
ENSDARG00000106522	86.28806362	2.261733941	0.364317171	3.463284302	0.000533624	0.005401009	LRRTM4	leucine rich repeat transmembrane neuronal 4 [Source:HGNC Symbol;Acc:HGNC:19411]	8	5844174	5850472	LRRTM4
ENSDARG00000106529	1253.504202	-1.412025085	0.13904948	-2.963154437	0.003045038	0.026456728	dut	deoxyuridine triphosphatase [Source:ZFIN;Acc:ZDB-GENE-041010-93]	18	5308392	5318966	
ENSDARG00000106631	212.3570616	2.042022946	0.209529235	4.973162559	6.59E-07	1.08E-05	tmem63bb	transmembrane protein 63Bb [Source:NCBI gene;Acc:569678]	11	43470176	43473824	
ENSDARG00000106873	390.2595781	1.788550614	0.153244921	5.145688406	2.67E-07	4.57E-06	LO018188.1	synaptopodin [Source:NCBI gene;Acc:100536545]	14	49604362	49665640	
ENSDARG00000107086	105.1111969	1.949201919	0.33971937	2.794076533	0.005204816	0.042764676	PIEZO2	piezo type mechanosensitive ion channel component 2 [Source:HGNC Symbol;Acc:HGNC:26270]	2	54086436	54270366	PIEZO2
ENSDARG00000107296	78.91076504	2.006583542	0.34021751	2.95864708	0.003089927	0.026772617	INSYN2A	family with sequence similarity 196 member A [Source:NCBI gene;Acc:100333552]	12	44610912	44656412	INSYN2A
ENSDARG00000107353	307.7357153	3.291476498	0.23358765	9.809921449	1.02E-22	6.42E-21	BX957322.3	protocadherin-10 [Source:NCBI gene;Acc:100006777]	10	21511495	21521507	
ENSDARG00000107441	1341.0162	1.55342809	0.104824586	5.279563822	1.29E-07	2.31E-06	TANC2	tetratricopeptide repeat, ankyrin repeat and coiled-coil containing 2b [Source:NCBI gene;Acc:335127]	12	3313504	3453589	TANC2
ENSDARG00000107526	93.4874273	-1.760146355	0.26002505	-2.923358174	0.003462779	0.029666384	smkr1	small lysine rich protein 1 [Source:NCBI gene;Acc:103910385]	4	4232562	4233275	
ENSDARG00000107858	160.5653819	2.116620181	0.225204848	4.958242208	7.11E-07	1.16E-05	CABZ01073424.1		21	2721752	2738317	
ENSDARG00000107989	59.03808815	2.213056169	0.370990037	3.269780989	0.001076308	0.010229611	FAM163B	protein FAM163B-like [Source:NCBI gene;Acc:794939]	5	65871688	65875446	FAM163B
ENSDARG00000108006	401.3581409	-1.483266654	0.136248857	-3.546940963	0.000389732	0.004054149	CU693481.1	fibronectin type III domain-containing protein 7-like [Source:NCBI gene;Acc:101886366]	23	1644754	1660708	
ENSDARG00000108475	1094.428049	-1.561437397	0.104859433	-5.354190636	8.59E-08	1.57E-06	otos	otospiralin [Source:ZFIN;Acc:ZDB-GENE-120601-1]	24	26302781	26304386	
ENSDARG00000108781	90.86223847	2.500759638	0.313419939	4.788334919	1.68E-06	2.64E-05	C17H14orf132	chromosome 17 C14orf132 homolog [Source:NCBI gene;Acc:103909003]	17	2902113	2945592	C17H14orf132
ENSDARG00000108787	264.8667838	2.472871105	0.197922004	7.441674357	9.94E-14	3.33E-12	TRIM67	tripartite motif containing 67 [Source:NCBI gene;Acc:100332164]	20	4060839	4132099	TRIM67
ENSDARG00000109310	1528.604441	-1.598880416	0.110260492	-5.431505004	5.59E-08	1.05E-06	im:7150988	im:7150988 [Source:ZFIN;Acc:ZDB-GENE-050309-169]	14	3363368	3381303	
ENSDARG00000109358	423.5060609	1.771676314	0.24036242	3.210469903	0.001325181	0.012374769	trhde.2	thyrotropin releasing hormone degrading enzyme, tandem duplicate 2 [Source:ZFIN;Acc:ZDB-GENE-070705-89]	4	23223881	23408029	
ENSDARG00000109361	49.09225253	2.232787006	0.411919324	2.992787503	0.002764422	0.024232263	BX005294.2		14	2035749	2036604	
ENSDARG00000109502	230.069383	1.885275069	0.19833957	4.463431414	8.07E-06	0.000113933	PHF21B	PHD finger protein 21B [Source:NCBI gene;Acc:101883360]	4	74828055	74892355	PHF21B
ENSDARG00000109611	142.56938	-1.810783631	0.232150453	-3.492492138	0.000478536	0.004898746	FP236810.1		5	9408901	9409816	
ENSDARG00000109726	346.4486748	2.104190182	0.234404503	4.710618477	2.47E-06	3.78E-05	PLCB1	si:ch1073-140o9.2 [Source:ZFIN;Acc:ZDB-GENE-120709-104]	17	3841012	3986236	PLCB1
ENSDARG00000109820	1976.226542	2.452352871	0.113723771	12.77088205	2.38E-37	3.27E-35	NPAS3	neuronal PAS domain protein 3 [Source:NCBI gene;Acc:570491]	17	9308425	9894157	NPAS3
ENSDARG00000109963	442.5250684	1.837424579	0.239612321	3.49491451	0.000474214	0.004859542	TCERG1L	transcription elongation regulator 1 like [Source:NCBI gene;Acc:110437752]	12	41991635	42199254	TCERG1L
ENSDARG00000109996	438.8001852	-1.432231937	0.140255759	-3.081741109	0.002057937	0.018455265	CABZ01101996.1		19	43123331	43134843	
ENSDARG00000110019	346.9975734	-3.635435926	0.186600028	-14.12344868	2.72E-45	5.14E-43	CABZ01079267.1		6	60004871	60024255	
ENSDARG00000110137	7.558777351	5.966147995	1.662968814	2.986314568	0.00282362	0.024686761	si:dkey-240e12.6	si:dkey-240e12.6 [Source:ZFIN;Acc:ZDB-GENE-050208-367]	22	25352530	25354059	
ENSDARG00000110142	53.65387885	2.163911348	0.392725179	2.963678957	0.003039852	0.026429975	CABZ01113818.1		25	222921	225964	
ENSDARG00000110206	97.147894	1.969965686	0.282786025	3.430034024	0.000603506	0.006042518	CABZ01085887.1		2	243778	250511	
ENSDARG00000110266	517.4712758	1.747020787	0.151927225	4.916964609	8.79E-07	1.42E-05	si:ch211-200p22.4	si:ch211-200p22.4 [Source:ZFIN;Acc:ZDB-GENE-081104-61]	21	30563115	30583086	
ENSDARG00000110341	360.1492672	-1.437388264	0.143974602	-3.037954322	0.002381901	0.021111612	hsd11b1lb	hydroxysteroid (11-beta) dehydrogenase 1-like b [Source:ZFIN;Acc:ZDB-GENE-101130-4]	2	53459873	53481912	
ENSDARG00000110439	537.2951071	-1.952364158	0.171911618	-5.539847548	3.03E-08	5.88E-07	ap1s3a	adaptor related protein complex 1 subunit sigma 3a [Source:ZFIN;Acc:ZDB-GENE-050417-222]	15	43154744	43164591	
ENSDARG00000110470	964.7603567	1.590565384	0.119203221	4.95427372	7.26E-07	1.19E-05	ptpro	protein tyrosine phosphatase receptor type O [Source:ZFIN;Acc:ZDB-GENE-070424-54]	4	184863	193762	
ENSDARG00000110485	137.537426	1.630075977	0.228952076	2.7519994	0.005923263	0.04793031	FP236318.3		25	207422	210730	
ENSDARG00000110528	9856.98735	1.811328324	0.173917646	4.665014407	3.09E-06	4.64E-05	sypa	synaptophysin a [Source:ZFIN;Acc:ZDB-GENE-031104-2]	8	22930627	22953964	
ENSDARG00000110545	442.1418128	1.825983026	0.193207748	4.27510301	1.91E-05	0.000252396	CABZ01113373.1		18	50852359	50862939	
ENSDARG00000110642	62.82810357	3.325997644	0.434962583	5.347580995	8.91E-08	1.62E-06	cacna1db	calcium channel, voltage-dependent, L type, alpha 1D subunit, b [Source:ZFIN;Acc:ZDB-GENE-040525-3]	8	53423408	53436880	
ENSDARG00000110836	1079.915286	-1.368347171	0.10971856	-3.357200203	0.000787361	0.007690095	tmem14ca	transmembrane protein 14Ca [Source:NCBI gene;Acc:751728]	24	8736497	8741830	
ENSDARG00000110874	178.255944	2.151981394	0.234512971	4.912228901	9E-07	1.46E-05	ELFN2	protein phosphatase 1 regulatory subunit 29-like [Source:NCBI gene;Acc:796777]	3	33570583	33574576	ELFN2
ENSDARG00000110940	1846.047702	2.200464352	0.179945135	6.671279834	2.54E-11	6.88E-10	ptprsa	protein tyrosine phosphatase receptor type Sa [Source:ZFIN;Acc:ZDB-GENE-020107-3]	2	58777364	58795694	
ENSDARG00000110961	254.757037	2.413632896	0.192978598	7.325335099	2.38E-13	7.74E-12	zgc:110045	zgc:110045 [Source:ZFIN;Acc:ZDB-GENE-050706-149]	13	16219082	16226312	
ENSDARG00000110964	51.74224078	3.011490497	0.456369531	4.40759157	1.05E-05	0.000144319	BX957352.1		25	35881415	35881531	
ENSDARG00000111106	534.4666733	1.518443896	0.157514717	3.291399731	0.000996901	0.00953629	arl3l1	ADP ribosylation factor like GTPase 3, like 1 [Source:ZFIN;Acc:ZDB-GENE-040426-1649]	21	33172526	33187913	
ENSDARG00000111110	2277.993508	2.486097112	0.115915424	12.82052954	1.26E-37	1.75E-35	BSN	bassoon presynaptic cytomatrix protein [Source:HGNC Symbol;Acc:HGNC:1117]	8	53794022	53803429	BSN
ENSDARG00000111235	2595.704035	1.657167544	0.125738905	5.226445567	1.73E-07	3.04E-06	nrsn1	neurensin 1 [Source:ZFIN;Acc:ZDB-GENE-030131-7491]	16	221739	229482	
ENSDARG00000111357	38.80584223	-3.27878263	0.444893224	-5.122088871	3.02E-07	5.17E-06	tnfsf14	TNF superfamily member 14 [Source:ZFIN;Acc:ZDB-GENE-101109-3]	3	47245764	47258444	
ENSDARG00000111704	177.8427181	2.321561851	0.264402369	4.998298081	5.78E-07	9.6E-06	CABZ01096573.1		1	58762742	58766424	
ENSDARG00000111795	51.395975	2.866619073	0.46839914	3.985103543	6.75E-05	0.000807382	CR855311.6		16	31639110	31644545	
ENSDARG00000111837	227.0408044	2.769131075	0.216604211	8.167574721	3.15E-16	1.27E-14	trhde.2	thyrotropin releasing hormone degrading enzyme, tandem duplicate 2 [Source:ZFIN;Acc:ZDB-GENE-070705-89]	4	23440404	23643272	
ENSDARG00000111856	4.439098458	-6.026263909	1.679547077	-2.99263056	0.002765843	0.024232263	BX649490.4	CD276 antigen homolog [Source:NCBI gene;Acc:101886973]	22	30496360	30497431	
ENSDARG00000111902	58.65019448	2.106904943	0.378296195	2.926027168	0.00343321	0.029442088	FO704772.1		7	5296	5449	
ENSDARG00000111980	74.4976568	2.075042741	0.337850224	3.182009847	0.001462568	0.013531858	LO018585.2		19	5849008	5865766	
ENSDARG00000112034	97.65398044	1.951142747	0.302554131	3.143710987	0.001668201	0.015232272	LO017711.1		1	48581374	48615105	
ENSDARG00000112048	5.981778946	5.630874433	1.669984987	2.773003631	0.00555415	0.045308652	BX855613.1		4	50995331	50995399	
ENSDARG00000112056	242.5590104	1.649517772	0.204497241	3.176168881	0.00149234	0.013771779	CABZ01066921.1		9	993477	1001460	
ENSDARG00000112132	6.872947649	5.833047914	1.638976724	2.948820349	0.003189893	0.027515298	BX470182.1		16	40217043	40220920	
ENSDARG00000112310	56.76216295	2.657213982	0.401084242	4.131835183	3.6E-05	0.000452118	CABZ01084617.1		1	58735326	58744037	
ENSDARG00000112349	132.7848874	2.076626889	0.251944576	4.273268773	1.93E-05	0.000254348	CABZ01015105.1		18	7264961	7269671	
ENSDARG00000112431	298.0357204	1.999948794	0.192255322	5.201150132	1.98E-07	3.46E-06	LO018598.1		18	50978940	51015718	
ENSDARG00000112435	1150.6346	1.725036918	0.111671582	6.49258211	8.44E-11	2.19E-09	CR769778.1	protein unc-13 homolog C-like [Source:NCBI gene;Acc:110437876]	18	2377067	2433011	
ENSDARG00000112452	3905.682643	1.352402405	0.110348207	3.193548996	0.001405355	0.013031331	crebbpb	CREB binding protein b [Source:ZFIN;Acc:ZDB-GENE-050302-102]	3	9634777	9722603	
ENSDARG00000112454	275.6476983	-1.768630188	0.16360943	-4.697957727	2.63E-06	4.01E-05	FO744833.2	poly [ADP-ribose] polymerase 14 [Source:NCBI gene;Acc:562648]	10	21913105	21955231	
ENSDARG00000112667	716.7725733	-1.432112885	0.119156268	-3.626438563	0.000287357	0.003071956	tp53rk	TP53 regulating kinase [Source:ZFIN;Acc:ZDB-GENE-050522-123]	11	2458264	2466879	
ENSDARG00000112767	323.4315854	1.655212326	0.220938943	2.965580973	0.003021118	0.026294412	CABZ01077951.1		6	45346808	45376758	
ENSDARG00000112895	85.35478609	2.26234315	0.323045178	3.907636562	9.32E-05	0.001089152	FO834857.1	homeobox protein TGIF2-like [Source:NCBI gene;Acc:100535338]	6	1808039	1820606	
ENSDARG00000112909	1695.341471	1.392174894	0.10020049	3.913901947	9.08E-05	0.001062249	ZNF462	zinc finger protein 462 [Source:HGNC Symbol;Acc:HGNC:21684]	21	349091	370877	ZNF462
ENSDARG00000113027	9.777042858	-4.643712196	1.107771524	-3.28922717	0.001004629	0.009599236	BX005073.3		7	47143303	47148440	
ENSDARG00000113064	123.0132279	1.727468125	0.255886807	2.842929391	0.004470097	0.037277047	CR392045.1	potassium channel subfamily T member 2-like [Source:NCBI gene;Acc:565691]	3	46919522	47105267	
ENSDARG00000113076	269.2125454	1.734106296	0.202033027	3.63359549	0.000279499	0.003002012	si:ch211-182p11.1	si:ch211-182p11.1 [Source:ZFIN;Acc:ZDB-GENE-131127-323]	12	43848634	44024244	
ENSDARG00000113112	924.996851	2.965224028	0.133073988	14.76790512	2.36E-49	5.38E-47	NRXN2	neurexin 2 [Source:HGNC Symbol;Acc:HGNC:8009]	7	427503	506340	NRXN2
ENSDARG00000113218	249.6321757	-3.267643927	0.260140731	-8.716989146	2.86E-18	1.35E-16	FP236810.2	UDP-glucuronosyltransferase 2B20-like [Source:NCBI gene;Acc:571561]	5	9360394	9370190	
ENSDARG00000113250	1608.70084	1.801884313	0.120248765	6.668545086	2.58E-11	7E-10	C21H5orf24	zgc:77058 [Source:NCBI gene;Acc:405836]	21	45733871	45739886	C21H5orf24
ENSDARG00000113257	126.8426316	-1.923999546	0.264844995	-3.488831448	0.000485137	0.004962275	FO704821.1	zmp:0000000930 [Source:NCBI gene;Acc:100151586]	16	43353106	43356018	
ENSDARG00000113274	1296.989656	-2.36759365	0.186514279	-7.332380447	2.26E-13	7.37E-12	AL929536.3		13	835390	840787	
ENSDARG00000113552	39.2405994	3.117588608	0.556019373	3.808479901	0.000139824	0.00158785	fam78bb	family with sequence similarity 78 member Bb [Source:ZFIN;Acc:ZDB-GENE-030131-7811]	8	19642199	19649617	
ENSDARG00000113564	40.93060607	-2.215453008	0.397607211	-3.056918922	0.002236248	0.019954515	znf1107	zinc finger protein 1107 [Source:ZFIN;Acc:ZDB-GENE-131122-103]	4	44804898	44892744	
ENSDARG00000113657	473.9792532	1.375523988	0.136003254	2.761139738	0.005760003	0.046759969	LO018363.2		21	45581985	45588720	
ENSDARG00000113702	623.3955565	1.9814839	0.140644503	6.978473246	2.98E-12	8.84E-11	RIMS2	regulating synaptic membrane exocytosis 2 [Source:HGNC Symbol;Acc:HGNC:17283]	16	44435453	44649053	RIMS2
ENSDARG00000113737	250.8249784	2.150110271	0.243666344	4.72002104	2.36E-06	3.62E-05	LO018287.1		14	4478416	4556911	
ENSDARG00000113771	564.0274463	2.444397781	0.175178907	8.245272254	1.65E-16	6.77E-15	GRIK2	glutamate receptor, ionotropic, kainate 2 [Source:NCBI gene;Acc:556013]	16	1802307	2105556	GRIK2
ENSDARG00000113971	66.41039405	2.421003087	0.451134713	3.149842043	0.001633588	0.014932514	LO018181.1	gastrula zinc finger protein XlCGF49.1-like [Source:NCBI gene;Acc:100149352]	4	73973242	73983880	
ENSDARG00000113977	368.8115016	-3.548218803	0.177111064	-14.38768841	6.18E-47	1.25E-44	fthl29	ferritin, heavy polypeptide-like 29 [Source:ZFIN;Acc:ZDB-GENE-080722-16]	3	32125452	32127583	
ENSDARG00000114142	109.0987331	2.385614942	0.320003552	4.329998626	1.49E-05	0.000201443	si:dkeyp-4f2.1	si:dkeyp-4f2.1 [Source:ZFIN;Acc:ZDB-GENE-081103-33]	4	71691806	71904377	
ENSDARG00000114258	341.3188575	-1.690780457	0.150897947	-4.577798924	4.7E-06	6.89E-05	si:dkey-30c15.13	si:dkey-30c15.13 [Source:ZFIN;Acc:ZDB-GENE-060503-73]	18	7392147	7400075	
ENSDARG00000114495	76.05542567	2.623235531	0.375369929	4.324362198	1.53E-05	0.00020633	ATP8A1	ATPase phospholipid transporting 8A1 [Source:HGNC Symbol;Acc:HGNC:13531]	14	45028062	45179545	ATP8A1
ENSDARG00000114502	13.25072044	6.78220954	1.583809828	3.650823121	0.000261401	0.002821006	CR388079.3		22	27599775	27622854	
ENSDARG00000114572	137.07476	-1.782885957	0.214243267	-3.654191653	0.000257994	0.00278653	asb18	ankyrin repeat and SOCS box containing 18 [Source:ZFIN;Acc:ZDB-GENE-090313-391]	9	23885441	23894392	
ENSDARG00000115424	148.2437468	2.05984874	0.295354559	3.588394723	0.00033272	0.003514988	CABZ01083778.1		17	189276	200316	
ENSDARG00000115501	217.0690132	2.760141748	0.224499679	7.840286246	4.5E-15	1.67E-13	shisa6	shisa family member 6 [Source:NCBI gene;Acc:796884]	12	9059	11570	
ENSDARG00000115505	92.56017921	2.008879952	0.329014748	3.066366955	0.002166772	0.019375899	MGAT5B	mannosyl (alpha-1,6-)-glycoprotein beta-1,6-N-acetyl-glucosaminyltransferase, isozyme B [Source:NCBI gene;Acc:101884	3	59117136	59167824	MGAT5B
ENSDARG00000115528	151.154413	1.766584394	0.232972017	3.290456958	0.001000248	0.009564657	FO082780.1		8	44828432	44835944	
ENSDARG00000115545	374.6660576	2.733443589	0.226631442	7.648733873	2.03E-14	7.14E-13	clec19a	C-type lectin domain containing 19A [Source:ZFIN;Acc:ZDB-GENE-080917-46]	3	31177972	31188131	
ENSDARG00000115552	33.84375379	2.747526068	0.518422071	3.370855847	0.000749351	0.007356061	CABZ01117752.1		2	56937548	56976287	
ENSDARG00000115557	7031.793832	-1.267745158	0.086436527	-3.097592729	0.001950993	0.017577849	cox7b	cytochrome c oxidase subunit 7B [Source:ZFIN;Acc:ZDB-GENE-030131-6602]	21	37442458	37445458	
ENSDARG00000115603	64.64188393	2.147515805	0.352899334	3.251680279	0.00114725	0.010831611	cacna1db	calcium channel, voltage-dependent, L type, alpha 1D subunit, b [Source:ZFIN;Acc:ZDB-GENE-040525-3]	8	53452681	53456702	
ENSDARG00000115613	134.2628169	3.966617952	0.357604227	8.295813445	1.08E-16	4.52E-15	CU468826.3	U2 small nuclear ribonucleoprotein auxiliary factor 35 kDa subunit-related protein 1-like [Source:NCBI gene;Acc:100331497	4	4376498	4387012	
ENSDARG00000115741	59.84285134	2.732797718	0.389650486	4.447056481	8.71E-06	0.000121872	LO017974.1		6	475264	504283	
ENSDARG00000115868	121.3530734	2.042566318	0.274439355	3.798895081	0.000145343	0.001642614	sp5l	Sp5 transcription factor-like [Source:ZFIN;Acc:ZDB-GENE-030131-2981]	23	28023091	28025943	
ENSDARG00000115869	119.3045782	1.935529663	0.262854923	3.559110294	0.000372113	0.003891839	ATP9A	ATPase phospholipid transporting 9A (putative) [Source:NCBI gene;Acc:101886226]	23	38952529	39063597	ATP9A
ENSDARG00000115870	94.21202492	2.009996269	0.299891811	3.367868784	0.000757516	0.007423331	CABZ01117706.1		3	4997545	4998034	
ENSDARG00000115956	6066.561456	-4.039608968	0.189426596	-16.04636852	6.06E-58	1.79E-55	CABZ01044281.1		13	1542493	1552927	
ENSDARG00000116223	47.28332216	2.367060263	0.423718912	3.226337611	0.001253854	0.011761203	BX901973.1		3	17855981	17879354	
ENSDARG00000116341	183.6551718	2.197900331	0.218727713	5.47667379	4.33E-08	8.25E-07	FP103009.1	desumoylating isopeptidase 2-like [Source:NCBI gene;Acc:794838]	13	36537426	36545258	
ENSDARG00000116378	104.4243991	-1.838701069	0.244174049	-3.434849333	0.000592883	0.005948028	ms4a17a.17	membrane-spanning 4-domains, subfamily A, member 17A.17 [Source:ZFIN;Acc:ZDB-GENE-070424-27]	4	76492691	76507576	
ENSDARG00000116393	118.5040586	2.240346289	0.319875919	3.877585696	0.000105498	0.001218082	CABZ01067581.1		17	51499789	51501572	

**ENSDARG00000116445**	135.0816194	-1.855790602	0.23932677	-3.575824811	0.000349125	0.003669764	CABZ01046954.1		25	33266586	33275666	
**ENSDARG00000116497**	136.3434585	2.634619103	0.295295094	5.535544396	3.1E-08	6.01E-07	phyhip	phytanoyl-CoA 2-hydroxylase interacting protein [Source:NCBI gene;Acc:100002455]	8	52453146	52473054	
**ENSDARG00000116586**	240.9405308	-2.078332991	0.200133744	-5.388061857	7.12E-08	1.31E-06	fthl27	ferritin, heavy polypeptide-like 27 [Source:ZFIN;Acc:ZDB-GENE-040718-72]	3	32139666	32140446	
**ENSDARG00000116666**	213.6089047	1.796460129	0.221631662	3.593620702	0.000326114	0.003456841	CABZ01078320.1		18	642889	644970	
**ENSDARG00000116713**	945.3486921	4.066107799	0.419701564	7.305447636	2.76E-13	8.86E-12	si:ch73-265d7.2	si:ch73-265d7.2 [Source:ZFIN;Acc:ZDB-GENE-100922-146]	23	44633858	44642913	
**ENSDARG00000116725**	260.0277664	2.03109792	0.195721366	5.268192952	1.38E-07	2.45E-06	CABZ01114053.1		25	172331	173165	
**ENSDARG00000116727**	156.1298401	2.74470082	0.281320583	6.201824275	5.58E-10	1.32E-08	CABZ01081294.2	transmembrane protein 200C-like [Source:NCBI gene;Acc:101883651]	2	58631830	58640644	
**ENSDARG00000116769**	691.264189	1.489332292	0.128990643	3.793548752	0.000148509	0.001675387	ZNF521	zinc finger protein 521 [Source:HGNC Symbol;Acc:HGNC:24605]	2	1264971	1279387	ZNF521
**ENSDARG00000116835**	1901.74487	-1.591417023	0.119244399	-4.959704835	7.06E-07	1.16E-05	park7	parkinson protein 7 [Source:NCBI gene;Acc:449674]	11	41459408	41475995	
**ENSDARG00000117001**	181.2370328	-1.527841679	0.187226192	-2.819272629	0.004813262	0.039906585	CABZ01086812.1		14	69716	83154	
**ENSDARG00000117035**	14.71687453	6.930862033	1.547614641	3.832260225	0.000126971	0.001449344	BX005336.4		7	21836676	21836866	
**ENSDARG00000117109**	112.0188762	1.85761984	0.289370309	2.963745122	0.003039199	0.026429975	CR626884.6	si:dkey-83h2.2 [Source:NCBI gene;Acc:101885518]	15	4018362	4027367	
**ENSDARG00000117297**	123.4346727	1.827340477	0.258678954	3.198329301	0.001382263	0.012845684	CABZ01026153.1		23	5871506	5922464	
**ENSDARG00000117317**	8.772749446	6.183781277	1.605509551	3.228745212	0.001243346	0.011675732	CABZ01044022.1		15	46288890	46289904	
**ENSDARG00000117337**	56.30929751	-2.275060545	0.386400022	-3.299845945	0.000967379	0.009275089	CABZ01071874.1		23	44013682	44017082	
**ENSDARG00000117373**	97.76028549	2.608918096	0.306415524	5.250772137	1.51E-07	2.68E-06	CABZ01100278.1		2	58995304	59013775	
**ENSDARG00000117378**	68.37373347	-2.906735939	0.343916847	-5.54417719	2.95E-08	5.76E-07	CABZ01078989.1		19	1135052	1142130	
**ENSDARG00000117388**	269.9787013	2.917017794	0.221627094	8.649744741	5.16E-18	2.4E-16	CABZ01053592.1		15	3169974	3201297	
**ENSDARG00000117411**	121.318394	2.705763225	0.302666078	5.635792537	1.74E-08	3.49E-07	CT997819.5	uncharacterized LOC101887009 [Source:NCBI gene;Acc:101887009]	23	42626534	42643126	
**ENSDARG00000117442**	82.5286677	1.947710293	0.304397676	3.113395297	0.001849481	0.016723281	CR848683.2		15	35414466	35661368	
**ENSDARG00000117446**	26.67035878	3.960928396	0.761056534	3.890549863	0.000100017	0.001162285	CABZ01089980.1		3	314376	322398	
**ENSDARG00000117450**	136.473368	-2.016721467	0.256422166	-3.965029563	7.34E-05	0.000875099	FO834828.2		1	40683491	40692718	
**ENSDARG00000117485**	150.9534159	-2.193957055	0.289842857	-4.119325447	3.8E-05	0.000474761	FO681323.1		1	40693323	40705186	
**ENSDARG00000117519**	200.1219121	1.973985304	0.227256185	4.28584729	1.82E-05	0.000241516	CABZ01117357.1	protein unc-79 homolog [Source:NCBI gene;Acc:101884319]	17	53388239	53393653	
**ENSDARG00000117543**	35.75870847	-2.179044748	0.413457839	-2.85166863	0.004349041	0.036400561	CU682640.1		23	3913304	3915754	
**ENSDARG00000117614**	236.7535642	1.850393029	0.21254054	4.001086238	6.31E-05	0.000759078	CR550304.1		13	22778060	22784433	
**ENSDARG00000117625**	30.4630087	7.979559003	1.495619906	4.666666293	3.06E-06	4.6E-05	CABZ01038493.1		3	58210305	58212028	
**ENSDARG00000117626**	181.454782	2.183257998	0.239437042	4.941833513	7.74E-07	1.26E-05	CABZ01102517.1	reelin-like [Source:NCBI gene;Acc:110438115]	18	50782247	50786520	
**ENSDARG00000117654**	15.14523577	3.899913736	0.992407245	2.922100531	0.003476793	0.029775156	BX545917.6		8	32435738	32446891	
**ENSDARG00000117668**	81.55627645	2.337167922	0.351933831	3.799486731	0.000144996	0.001640174	FO704648.2		5	3660658	3670238	
**ENSDARG00000117719**	165.7229505	2.105014863	0.265269924	4.165624385	3.11E-05	0.000393433	CABZ01075114.1		12	49033723	49038796	
**ENSDARG00000117734**	29.0651708	7.914324034	1.493585505	4.629345966	3.67E-06	5.46E-05	CABZ01044023.1		15	46291409	46295532	
**ENSDARG00000117740**	377.2749697	2.133495195	0.202018224	5.610856148	2.01E-08	4E-07	CABZ01113369.1		18	50866261	50872907	
**ENSDARG00000117755**	72.06734749	1.961585566	0.333965695	2.879294428	0.00398566	0.033718929	CU467655.2	cadherin-related family member 5-like [Source:NCBI gene;Acc:100537321]	18	7000194	7012242	
**ENSDARG00000117758**	253.5299969	1.815389759	0.19871231	4.103368137	4.07E-05	0.000505219	CABZ01050280.1		2	54430632	54447713	
**ENSDARG00000117772**	68.71865914	2.633808382	0.412688667	3.958936872	7.53E-05	0.00089602	CABZ01102518.1		18	50786633	50790250	
