# Supplementary material for: Succinate supplementation ameliorates musculoskeletal defects caused by PLOD3 mutations in a BCARD syndrome model
Source: Genome Med. 2026 Mar 13;18:29. doi: 10.1186/s13073-026-01608-y (PMC12994257; doi:10.1186/s13073-026-01608-y)
Supplement: Supplementary file 5 — Additional file 5: Table S10. RPKM values. [file 13073_2026_1608_MOESM5_ESM.pdf]

Table S10

Table S10: RPKM values

ensembl	external_gene_name	description	chromosome_name	start_position	end_position	hgnc_symbol	X7050.DC.1_wt1_read_counts_new.txt	X7050.DC.2_wt2_read_counts_new.txt	X7050.DC.4_wt3_read_counts_new.txt	X6931.DC.6_mgt1_read_counts_new.txt	X6931.DC.9_mgt2_read_counts_new.txt	X6931.DC.10_mgt3_read_counts_new.txt
ENSDARG00000000001	slc35a5	solute carrier family 35 member A5 [Source:NCBI gene;Acc:368418]	9	34112067	34121839		2.3	1.71	1.9	2.93	2.8	2.67
ENSDARG00000000002	ccdc80	coiled-coil domain containing 80 [Source:NCBI gene;Acc:368419]	9	34089156	34113209		6.54	7.4	6.03	13.13	13.58	13.17
ENSDARG00000000018	nrf1	nuclear respiratory factor 1 [Source:NCBI gene;Acc:64604]	4	15081385	15103696		10.19	5.62	6.75	11.14	10.81	11.07
ENSDARG00000000019	ube2h	ubiquitin-conjugating enzyme E2H (UBC8 homolog, yeast) [Source:ZFIN;Acc:ZDB-GENE-030616-67]	4	15011341	15059876		41.26	26.04	23.98	50.56	51.54	53.3
ENSDARG00000000068	slc9a3r1a	SLC9A3 regulator 1a [Source:ZFIN;Acc:ZDB-GENE-031006-7]	12	33484458	33537126		24.07	25.85	15.02	12.8	12.73	11.51
ENSDARG00000000069	dap	death-associated protein [Source:ZFIN;Acc:ZDB-GENE-000511-3]	24	22070290	22103585		31.34	39.47	35.72	21.12	21.24	21.23
ENSDARG00000000086	itsn1	intersectin 1 (SH3 domain protein) [Source:ZFIN;Acc:ZDB-GENE-030616-226]	1	46999441	47089818		9.48	5.34	4.79	16.49	16.22	17.27
ENSDARG00000000103	myh10	myosin, heavy chain 10, non-muscle [Source:ZFIN;Acc:ZDB-GENE-030616-162]	6	7553085	7658285		35.38	28.61	24.82	39.74	40.54	40.93
ENSDARG00000000142	tmem267	transmembrane protein 267 [Source:NCBI gene;Acc:368645]	10	13279079	13287169		4.94	2.4	3.06	5.55	6.58	6.46
ENSDARG00000000151	thraa	thyroid hormone receptor alpha a [Source:NCBI gene;Acc:30670]	3	34688022	34753605		3.18	2.59	2.12	3.25	3.37	3.27
ENSDARG00000000161	itsn2a	intersectin 2a [Source:ZFIN;Acc:ZDB-GENE-041014-84]	20	25359064	25436389		3.88	1.81	1.86	3.86	3.32	3.74
ENSDARG00000000175	hoxb2a	homeobox B2a [Source:NCBI gene;Acc:30338]	3	23752150	23754831		3.02	2.11	2.39	4.6	5.28	5.36
ENSDARG00000000183	ptpn4b	protein tyrosine phosphatase non-receptor type 4b [Source:ZFIN;Acc:ZDB-GENE-030131-6259]	22	11778053	11833334		5.99	1.79	1.92	6.43	6.32	6.38
ENSDARG00000000189	sema6e	sema domain, transmembrane domain (TM), and cytoplasmic domain, (semaphorin) 6E [Source:ZFIN;Acc:ZDB-GENE-040724-119]	16	30002605	30271444		7.23	3.68	3.15	10.06	9.29	9.52
ENSDARG00000000212	krt97	keratin 97 [Source:ZFIN;Acc:ZDB-GENE-040718-78]	22	11756040	11759106		500.31	545.83	533.72	153.96	165.51	179.31
ENSDARG00000000229	usp49	ubiquitin specific peptidase 49 [Source:ZFIN;Acc:ZDB-GENE-050208-484]	22	567733	584610		3.21	1.59	1.54	3.97	3.64	3.32
ENSDARG00000000241	slc40a1	solute carrier family 40 member 1 [Source:NCBI gene;Acc:58153]	9	41103127	41113878		20.76	22.02	16.73	16.12	16.49	16.26
ENSDARG00000000324	dcaf8	DDB1 and CUL4 associated factor 8 [Source:ZFIN;Acc:ZDB-GENE-070912-578]	2	38002717	38020603		16.66	9.12	9.03	12.71	12.55	14.49
ENSDARG00000000349	cfap300	cilia and flagella associated protein 300 [Source:ZFIN;Acc:ZDB-GENE-040724-203]	21	22828500	22832715		0.75	0.68	0.91	0.69	0.7	0.78
ENSDARG00000000369	prkag3b	protein kinase, AMP-activated, gamma 3b non-catalytic subunit [Source:ZFIN;Acc:ZDB-GENE-060421-6938]	9	14090329	14108896		5.1	6.98	7.03	2.8	3.22	3.07
ENSDARG00000000370	triob	trio Rho guanine nucleotide exchange factor b [Source:ZFIN;Acc:ZDB-GENE-030616-399]	16	8751815	8927425		10.34	4.36	3.98	11.77	11.73	12.45
ENSDARG00000000380	pde6a	phosphodiesterase 6A, cGMP-specific, rod, alpha [Source:ZFIN;Acc:ZDB-GENE-030616-42]	14	25005403	25035010		8.79	2.05	1.71	3.82	3.97	3.77
ENSDARG00000000394	matr3l1.1	matrin 3-like 1.1 [Source:ZFIN;Acc:ZDB-GENE-030616-40]	14	25079782	25099988		29.39	19.14	20.01	31.38	30.96	31.45
ENSDARG00000000423	si:ch73-314g15.3	si:ch73-314g15.3 [Source:ZFIN;Acc:ZDB-GENE-030616-19]	7	32629674	32645200		4.18	3.42	3.39	2.93	3.34	3.15
ENSDARG00000000442	slc39a13	solute carrier family 39 member 13 [Source:NCBI gene;Acc:368686]	7	32693890	32716019		3.81	4.06	3.81	8.87	8.7	7.82
ENSDARG00000000460	nitr2b	novel immune-type receptor 2b [Source:ZFIN;Acc:ZDB-GENE-001106-6]	7	17443567	17446703		0.2	0.37	0.17	0.22	0.14	0.16
ENSDARG00000000472	cntn2	contactin 2 [Source:ZFIN;Acc:ZDB-GENE-990630-12]	11	23933016	23987682		7.06	1.73	2.59	12.44	13.4	14.07
ENSDARG00000000474	tnni1a	troponin I type 1a (skeletal, slow) [Source:ZFIN;Acc:ZDB-GENE-030616-266]	23	5723090	5729484		1.27	1.6	1.89	0.65	0.53	0.63
ENSDARG00000000476	pms1	PMS1 homolog 1, mismatch repair system component [Source:ZFIN;Acc:ZDB-GENE-030616-528]	9	40948569	41025062		2.5	2.38	2.52	1.91	1.7	1.64
ENSDARG00000000489	upf3b	UPF3B regulator of nonsense mediated mRNA decay [Source:ZFIN;Acc:ZDB-GENE-040426-1630]	14	33339322	33351103		16.4	15.96	14.6	15.4	15.87	15.76
ENSDARG00000000503	stx1b	syntaxin 1B [Source:ZFIN;Acc:ZDB-GENE-000330-4]	19	5158306	5254699		58.46	12.75	13.53	85.29	85.16	88.5
ENSDARG00000000516	ankar	ankyrin and armadillo repeat containing [Source:ZFIN;Acc:ZDB-GENE-030616-533]	9	41045874	41077934		0.09	0.06	0.09	0.14	0.13	0.14
ENSDARG00000000529	ofd1	OFD1 centriole and centriolar satellite protein [Source:NCBI gene;Acc:327216]	9	54716436	54743987		4.69	3.38	3.55	3.16	3.23	3.63
ENSDARG00000000540	asdurf	asnsd1 upstream reading frame [Source:ZFIN;Acc:ZDB-GENE-170731-1]	9	41079669	41090343		3	3.05	2.85	2.36	2.45	2.29
ENSDARG00000000542	gtf2e1	general transcription factor IIE, polypeptide 1, alpha [Source:ZFIN;Acc:ZDB-GENE-041210-221]	9	27688274	27720612		2.56	2.43	2.55	2.5	2.47	2.63
ENSDARG00000000551	slc1a4	solute carrier family 1 member 4 [Source:ZFIN;Acc:ZDB-GENE-030616-566]	13	24429918	24448278		6.77	5.94	5.81	15.3	15.81	16.14
ENSDARG00000000563	ttn.1	titin, tandem duplicate 1 [Source:ZFIN;Acc:ZDB-GENE-030616-413]	9	42732992	42873700		2.93	4.05	3.01	13.13	7.08	7.36
ENSDARG00000000567	znf281a	zinc finger protein 281a [Source:ZFIN;Acc:ZDB-GENE-131126-34]	3	38762891	38783951		2.19	1.03	1.02	3.71	3.31	3.42
ENSDARG00000000568	ell	elongation factor RNA polymerase II [Source:ZFIN;Acc:ZDB-GENE-030131-3946]	22	20847580	20924747		6.4	6.58	4.74	9.51	9.2	9.62
ENSDARG00000000588	grasp	GRP1 (general receptor for phosphoinositides 1)-associated scaffold protein [Source:ZFIN;Acc:ZDB-GENE-060312-30]	23	32199588	32236122		2.92	3.02	2.39	3.81	3.67	3.53
ENSDARG00000000606	dnah6	dynein, axonemal, heavy chain 6 [Source:ZFIN;Acc:ZDB-GENE-030616-623]	1	22002649	22235421		0.49	0.31	0.32	0.89	0.87	0.95
ENSDARG00000000631	ptpn4a	protein tyrosine phosphatase non-receptor type 4a [Source:ZFIN;Acc:ZDB-GENE-030616-449]	9	28939912	29029980		6.43	3.68	3.01	7.78	7.44	7.47
ENSDARG00000000638	opn1mw4	opsin 1 (cone pigments), medium-wave-sensitive, 4 [Source:NCBI gene;Acc:30504]	6	41200500	41202554		0	0	0	0	0	0
ENSDARG00000000651	osgepl1	O-sialoglycoprotein endopeptidase-like 1 [Source:NCBI gene;Acc:368635]	9	41040294	41045873		3.01	2.12	2.57	1.61	1.73	1.74
ENSDARG00000000656	psmb9a	proteasome 20S subunit beta 9a [Source:ZFIN;Acc:ZDB-GENE-990415-140]	19	7120380	7122373		0.36	0.28	0.55	0.22	0.2	0.19
ENSDARG00000000666	creg2	cellular repressor of E1A-stimulated genes 2 [Source:ZFIN;Acc:ZDB-GENE-041114-173]	9	7228083	7238839		13.4	12.88	12.79	20.91	25.38	22.84
ENSDARG00000000690	sypl2b	synaptophysin-like 2b [Source:ZFIN;Acc:ZDB-GENE-050417-309]	8	25079470	25090458		4.01	4.87	4.64	5.15	5.24	5.82
ENSDARG00000000729	daxx	death-domain associated protein [Source:ZFIN;Acc:ZDB-GENE-010110-3]	19	7014455	7033670		8.15	4.98	4.16	6.53	6.26	6.78
ENSDARG00000000730	slc6a22.1	solute carrier family 6 member 22, tandem duplicate 1 [Source:ZFIN;Acc:ZDB-GENE-030616-629]	6	41118910	41138855		6.31	7.9	6.97	6.77	7.09	6.11
ENSDARG00000000747	lcmt2	leucine carboxyl methyltransferase 2 [Source:ZFIN;Acc:ZDB-GENE-110714-2]	9	27720267	27734078		1.56	1.02	1.41	1.32	1.18	1.08
ENSDARG00000000760	znf511	zinc finger protein 511 [Source:NCBI gene;Acc:368916]	13	24665334	24669258		3.66	4.69	3.7	4.1	3.15	3.86
ENSDARG00000000767	spi1b	Spi-1 proto-oncogene b [Source:ZFIN;Acc:ZDB-GENE-980526-164]	7	32644673	32659353		1.69	2.44	2.58	2.36	2.76	2.7
ENSDARG00000000779	scfd1	sec1 family domain containing 1 [Source:ZFIN;Acc:ZDB-GENE-030116-3]	17	28771028	28797395		17.64	17.78	18.02	17.29	17.36	16.55
ENSDARG00000000796	nr4a1	nuclear receptor subfamily 4, group A, member 1 [Source:ZFIN;Acc:ZDB-GENE-040704-11]	23	32149269	32194397		3.73	4.72	4.01	4.29	3.26	3.37
ENSDARG00000000804	rassf6	Ras association domain family member 6 [Source:ZFIN;Acc:ZDB-GENE-030616-513]	10	13209580	13236012		5.87	7.14	5.23	4.21	3.97	3.99
ENSDARG00000000830	telo2	TEL2, telomere maintenance 2, homolog (S. cerevisiae) [Source:NCBI gene;Acc:777634]	24	37799818	37823488		4.65	3.78	3.48	4.04	4.37	4.41
ENSDARG00000000837	snx9a	sorting nexin 9a [Source:ZFIN;Acc:ZDB-GENE-030616-66]	17	33158294	33201840		7.01	7.32	6.36	8.33	8.43	7.93
ENSDARG00000000853	dstyk	dual serine/threonine and tyrosine protein kinase [Source:NCBI gene;Acc:402922]	22	472327	506522		12.67	9.92	10.41	18.38	18.67	20.56
ENSDARG00000000857	mapk14a	mitogen-activated protein kinase 14a [Source:NCBI gene;Acc:65237]	8	23826985	23860719		9.97	8.91	6.76	12.96	13.89	12.72
ENSDARG00000000861	scrib	scribble planar cell polarity protein [Source:NCBI gene;Acc:368473]	7	41160225	41275897		13.33	9.32	7.37	17.22	16.35	15.79
ENSDARG00000000887	zgc:56699	zgc:56699 [Source:ZFIN;Acc:ZDB-GENE-040426-2099]	23	32101202	32107608		1.85	2.07	2.73	3.6	3.15	3.66
ENSDARG00000000906	skap2	src kinase associated phosphoprotein 2 [Source:NCBI gene;Acc:368564]	16	20934353	20977706		3.86	5.28	4.45	2.77	2.93	2.83
ENSDARG00000000935	unk	unk zinc finger [Source:ZFIN;Acc:ZDB-GENE-040426-707]	12	36416173	36471889		1.45	0.84	0.82	1.9	1.81	1.96
ENSDARG00000000943	dzip1	DAZ interacting zinc finger protein 1 [Source:NCBI gene;Acc:402875]	6	7249531	7280299		6.14	4.27	4.16	6.39	6.64	6.43
ENSDARG00000000966	ncor2	nuclear receptor corepressor 2 [Source:ZFIN;Acc:ZDB-GENE-030616-81]	8	43379436	43574935		11.08	6.18	5.1	18.64	17.88	18.26
ENSDARG00000001014	myh9b	myosin, heavy chain 9b, non-muscle [Source:ZFIN;Acc:ZDB-GENE-030131-998]	3	5102656	5228841		30.49	30.99	24.7	46.28	46.82	48.05
ENSDARG00000001015	znf384l	zinc finger protein 384 like [Source:ZFIN;Acc:ZDB-GENE-030616-498]	19	6960388	6988866		6.07	3.38	3.27	9.29	9.33	8.85
ENSDARG00000001057	bysl	bystin-like [Source:ZFIN;Acc:ZDB-GENE-040426-1287]	22	550828	557965		63.66	64.55	67.86	34.85	36.81	35.8
ENSDARG00000001127	slc17a6a	solute carrier family 17 member 6a [Source:NCBI gene;Acc:494492]	25	35250976	35271284		16.3	3.7	4.99	20.95	21.98	21.4
ENSDARG00000001129	dicer1	dicer 1, ribonuclease type III [Source:NCBI gene;Acc:324724]	17	19419989	19466319		6.68	4.22	3.31	11.09	10.03	10.89
ENSDARG00000001154	rimbp2	RIMS binding protein 2 [Source:ZFIN;Acc:ZDB-GENE-040724-96]	8	43941313	44015210		6.17	0.98	1.54	5.72	5.76	6.15
ENSDARG00000001162	matr3l1.1	matrin 3-like 1.1 [Source:ZFIN;Acc:ZDB-GENE-030616-40]	14	25063318	25078580		19.89	10.78	12.67	17.82	18.54	18.15
ENSDARG00000001169	hsd17b8	hydroxysteroid (17-beta) dehydrogenase 8 [Source:ZFIN;Acc:ZDB-GENE-010110-1]	19	7173613	7179694		3.41	4.22	4.72	2.49	2.39	2.26
ENSDARG00000001210	arl14ep	ADP-ribosylation factor-like 14 effector protein [Source:ZFIN;Acc:ZDB-GENE-060825-216]	7	32624684	32629458		19.84	23.37	23.54	18.72	16.19	19.09
ENSDARG00000001220	mycbp2	MYC binding protein 2 [Source:NCBI gene;Acc:368439]	9	17045875	17286330		10.54	6.82	5.3	17.76	17	16.92
ENSDARG00000001234	map4k2	mitogen-activated protein kinase kinase kinase kinase 2 [Source:ZFIN;Acc:ZDB-GENE-030616-163]	7	17648931	17690983		4.34	4.16	3.8	3.68	3.73	3.46
ENSDARG00000001241	puf60b	poly-U binding splicing factor b [Source:NCBI gene;Acc:415211]	7	41146560	41158143		44.29	30.4	32.09	39.74	37.92	39.49
ENSDARG00000001244	srrm1	serine/arginine repetitive matrix 1 [Source:ZFIN;Acc:ZDB-GENE-040426-2789]	17	25426091	25440344		25.95	11.09	13.47	41.43	40.3	40.41
ENSDARG00000001249	pcgf1	polycomb group ring finger 1 [Source:NCBI gene;Acc:368900]	14	5394630	5407555		3.48	3.32	3.17	3.25	2.46	3.33
ENSDARG00000001259	gas2a	growth arrest-specific 2a [Source:ZFIN;Acc:ZDB-GENE-091013-1]	25	35304903	35343303		1.42	1.63	1.7	1.94	2.34	2.78
ENSDARG00000001303	psmb8a	proteasome 20S subunit beta 8A [Source:ZFIN;Acc:ZDB-GENE-990415-141]	19	7105838	7144548		0.14	0.13	0.1	0.01	0.08	0.06
ENSDARG00000001313	g2e3	G2/M-phase specific E3 ubiquitin protein ligase [Source:ZFIN;Acc:ZDB-GENE-030616-400]	17	28798725	28811747		4.55	4.07	3.88	3.71	3.89	3.68
ENSDARG00000001354	slc6a22.2	solute carrier family 6 member 22, tandem duplicate 2 [Source:ZFIN;Acc:ZDB-GENE-030616-628]	6	41144155	41161017		4.8	6.23	5.39	2.73	2.59	2.71
ENSDARG00000001414	mctp2a	multiple C2 domains, transmembrane 2a [Source:ZFIN;Acc:ZDB-GENE-040724-63]	18	23373683	23456101		3.4	3	2.43	2.29	2.29	1.81
ENSDARG00000001431	actn3b	actinin alpha 3b [Source:ZFIN;Acc:ZDB-GENE-030131-2040]	7	6969909	7133475		67.06	91.9	76.28	87.14	71.36	73.56
ENSDARG00000001437	slc2a1a	solute carrier family 2 member 1a [Source:ZFIN;Acc:ZDB-GENE-030131-3158]	23	22200467	22212635		15.73	3.9	3.55	6.07	6.78	6.47
ENSDARG00000001442	inppl1b	inositol polyphosphate phosphatase-like 1b [Source:NCBI gene;Acc:368433]	14	32793272	32824408		2.71	2.4	1.97	1.97	2.43	2.09
ENSDARG00000001447	mier1b	mesoderm induction early response 1b, transcriptional regulator [Source:ZFIN;Acc:ZDB-GENE-070705-170]	2	5811482	5836116		5.08	3.29	3.42	4.22	4.27	4.72
ENSDARG00000001452	adam8a	ADAM metallopeptidase domain 8a [Source:ZFIN;Acc:ZDB-GENE-030616-622]	13	24671481	24679636		1.46	1.52	1.54	3.67	3.57	4.07
ENSDARG00000001463	tdh2	L-threonine dehydrogenase 2 [Source:ZFIN;Acc:ZDB-GENE-070620-14]	17	25414033	25425321		10.61	1.21	1.62	10.45	9.67	10.56
ENSDARG00000001470	mhc1zea	major histocompatibility complex class I ZEA [Source:ZFIN;Acc:ZDB-GENE-070705-176]	1	47116121	47122141		2.73	2.5	2.09	4.04	3.81	3.44
ENSDARG00000001498	skap1	src kinase associated phosphoprotein 1 [Source:ZFIN;Acc:ZDB-GENE-090721-1]	12	27156943	27206904		0.39	0.33	0.27	0.57	0.59	0.54
ENSDARG00000001549	sp3a	sp3a transcription factor [Source:ZFIN;Acc:ZDB-GENE-070410-111]	9	2762270	2777286		2.16	1.34	1.23	3.03	3.26	3.18
ENSDARG00000001557	tomm34	translocase of outer mitochondrial membrane 34 [Source:ZFIN;Acc:ZDB-GENE-030131-2081]	6	52226682	52235118		5.87	6.05	5.38	5.39	5.38	4.37
ENSDARG00000001558	kifc1	kinesin family member C1 [Source:ZFIN;Acc:ZDB-GENE-000208-21]	19	6990970	7000603		11.69	9.44	9.6	9.18	8.69	9.01
ENSDARG00000001559	csmd2	CUB and Sushi multiple domains 2 [Source:ZFIN;Acc:ZDB-GENE-030616-39]	19	36114846	36675023		1.85	0.36	0.6	3.4	3.05	3.21
ENSDARG00000001572	prf1.3	perforin 1.3 [Source:ZFIN;Acc:ZDB-GENE-060503-461]	19	5259503	5265155		0.11	0.07	0.08	0.01	0.07	0.03
ENSDARG00000001578	echs1	enoyl CoA hydratase, short chain, 1, mitochondrial [Source:ZFIN;Acc:ZDB-GENE-030616-617]	13	24579108	24582236		55.13	78.04	70.62	48.73	48.74	47.42
ENSDARG00000001621	nfyal	nuclear transcription factor Y, alpha, like [Source:ZFIN;Acc:ZDB-GENE-040718-491]	8	25019542	25034411		3.93	2.84	3.59	4.49	5.45	5.45
ENSDARG00000001634	kirrel1a	kirre like nephrin family adhesion molecule 1a [Source:ZFIN;Acc:ZDB-GENE-040426-2126]	7	19070464	19146926		4.42	2.84	2.57	8.37	8.19	7.95
ENSDARG00000001656	psmb13a	proteasome 20S subunit beta 13a [Source:ZFIN;Acc:ZDB-GENE-001208-2]	19	7111779	7144585		0.11	0.11	0.04	0.02	0.05	0.03
ENSDARG00000001676	gpm6bb	glycoprotein M6Bb [Source:ZFIN;Acc:ZDB-GENE-030710-10]	9	54751201	54840209		19.32	8.17	12.21	26.07	26.37	25.74
ENSDARG00000001686	uckl1a	uridine-cytidine kinase 1-like 1a [Source:ZFIN;Acc:ZDB-GENE-040724-238]	8	23009662	23028536		2.41	0.46	0.21	1.84	1.91	1.59
ENSDARG00000001710	flot1a	flotillin 1a [Source:ZFIN;Acc:ZDB-GENE-020430-1]	19	6938289	6957404		31.57	16.16	22.05	39.35	40.77	42.51
ENSDARG00000001712	si:ch211-247n2.1	si:ch211-247n2.1 [Source:ZFIN;Acc:ZDB-GENE-141216-101]	11	8303941	8350945		0.73	0.65	0.45	0.8	0.76	0.88
ENSDARG00000001721	oc90	otoconin 90 [Source:ZFIN;Acc:ZDB-GENE-070912-300]	2	42942552	42960353		0.17	0.08	0.17	0.51	0.55	0.71
ENSDARG00000001729	strn3	striatin, calmodulin binding protein 3 [Source:ZFIN;Acc:ZDB-GENE-030616-405]	17	28706946	28742980		22.65	17.15	15.47	26.23	24.31	24.85
ENSDARG00000001733	gulp1a	GULP PTB domain containing engulfment adaptor 1a [Source:NCBI gene;Acc:368404]	9	42546504	42650623		5.79	5.69	5.55	7.36	7.88	6.98
ENSDARG00000001734	fkbp8	FKBP prolyl isomerase 8 [Source:ZFIN;Acc:ZDB-GENE-040426-1849]	22	20925849	20950448		15.11	13.51	13.98	11.34	12.57	11.95
ENSDARG00000001754	noc2l	NOC2-like nucleolar associated transcriptional repressor [Source:ZFIN;Acc:ZDB-GENE-000607-75]	23	23126130	23179417		16.29	19.43	18.19	9.35	8.85	8.34
ENSDARG00000001760	tnxba	tenascin XBa [Source:ZFIN;Acc:ZDB-GENE-070103-5]	16	16621242	16649764		3.67	3.84	2.95	9.09	8.76	8.41
ENSDARG00000001767	slc29a2	solute carrier family 29 member 2 [Source:ZFIN;Acc:ZDB-GENE-050220-15]	21	27407481	27413294		2.76	2.03	2	2.07	2.07	2.15
ENSDARG00000001769	ptpra	protein tyrosine phosphatase receptor type A [Source:ZFIN;Acc:ZDB-GENE-020107-1]	21	15756549	15816317		9.51	6.36	5.57	11.27	11.51	11.45
ENSDARG00000001771	otol1a	otolin 1a [Source:NCBI gene;Acc:553759]	2	6303639	6316167		0.99	0.76	0.43	0.53	0.39	0.37
ENSDARG00000001776	stim2b	stromal interaction molecule 2b [Source:ZFIN;Acc:ZDB-GENE-120404-3]	7	62320134	62439872		0.74	0.82	0.72	1.37	1.11	0.88
ENSDARG00000001777	nup155	nucleoporin 155 [Source:ZFIN;Acc:ZDB-GENE-040426-711]	10	1597270	1636452		8.68	7.58	6.05	9.65	9.15	9.02
ENSDARG00000001781	tspan11	tetraspanin 11 [Source:ZFIN;Acc:ZDB-GENE-041210-93]	4	11172964	11198055		2.36	1.59	1.86	3.8	3.73	3.88
ENSDARG00000001782	prkacba	protein kinase, cAMP-dependent, catalytic, beta a [Source:ZFIN;Acc:ZDB-GENE-040426-1351]	11	8129536	8150894		3.2	5.46	5.05	2.9	2.8	2.63
ENSDARG00000001785	irx2a	iroquois homeobox 2a [Source:ZFIN;Acc:ZDB-GENE-040426-1446]	16	555746	563732		3.23	1.72	1.6	3.93	3.54	3.98
ENSDARG00000001788	atp5po	ATP synthase peripheral stalk subunit OSCP [Source:ZFIN;Acc:ZDB-GENE-050522-147]	9	33145522	33148873		262.44	314.79	338.05	177.49	183.95	178.04
ENSDARG00000001803	kcnh6a	potassium voltage-gated channel, subfamily H (eag-related), member 6a [Source:ZFIN;Acc:ZDB-GENE-040702-5]	3	31728871	31784082		1.25	0.39	0.32	2.31	2.08	2.06
ENSDARG00000001804	zgc:112148	zgc:112148 [Source:ZFIN;Acc:ZDB-GENE-050417-235]	3	56861059	56871984		3.35	4.8	4.12	2	2.04	2.13
ENSDARG00000001807	tnfrsf21	tumor necrosis factor receptor superfamily, member 21 [Source:ZFIN;Acc:ZDB-GENE-030131-5971]	20	35797998	35841570		6.07	3.14	2.69	9.03	8.74	9.46
ENSDARG00000001817	zmp:0000000521	zmp:0000000521 [Source:ZFIN;Acc:ZDB-GENE-130530-524]	2	55199721	55215510		4.03	1.74	2.52	4.57	5.11	3.97
ENSDARG00000001818	c3b.2	complement component c3b, tandem duplicate 2 [Source:ZFIN;Acc:ZDB-GENE-030131-3063]	22	26129250	26175237		12.73	16.42	13.58	9.66	10.29	10.67
ENSDARG00000001825	cfap43	cilia and flagella associated protein 43 [Source:ZFIN;Acc:ZDB-GENE-080815-3]	13	24717880	24743151		0.5	0.46	0.47	0.85	0.91	0.69
ENSDARG00000001829	zgc:112982	zgc:112982 [Source:ZFIN;Acc:ZDB-GENE-050306-55]	24	24101811	24118451		28.02	12.17	15.14	27.44	27.18	28.3
ENSDARG00000001835	nalcn	sodium leak channel, non-selective [Source:ZFIN;Acc:ZDB-GENE-050410-12]	9	31589425	31747106		4.41	1.07	1.31	5.9	5.34	6.22
ENSDARG00000001857	aff4	AF4/FMR2 family, member 4 [Source:ZFIN;Acc:ZDB-GENE-100910-5]	21	43178817	43246873		8.57	5.55	4.39	12.83	12.58	12.66
ENSDARG00000001859	dbx1b	developing brain homeobox 1b [Source:NCBI gene;Acc:30416]	25	14507567	14510482		3.59	1.51	2.87	2.4	2.57	2.78
ENSDARG00000001870	atp1a1a.4	ATPase Na+/K+ transporting subunit alpha 1a, tandem duplicate 4 [Source:ZFIN;Acc:ZDB-GENE-001212-4]	1	1689775	1706159		68.1	95.54	73.38	41.41	43.04	41.59
ENSDARG00000001873	phgdh	phosphoglycerate dehydrogenase [Source:ZFIN;Acc:ZDB-GENE-030131-647]	9	48185936	48214216		21.06	19.24	21.96	41.33	44.67	45.68
ENSDARG00000001879	nav2b	neuron navigator 2b [Source:ZFIN;Acc:ZDB-GENE-061207-13]	25	14510773	14637660		4.05	2.97	2.15	4.8	4.57	4.5
ENSDARG00000001880	stx3a	syntaxin 3A [Source:ZFIN;Acc:ZDB-GENE-040426-1489]	14	21699129	21751346		8.97	4.85	4.74	8.88	8.26	9.55
ENSDARG00000001881	cacnb3a	calcium channel, voltage-dependent, beta 3a [Source:ZFIN;Acc:ZDB-GENE-060221-2]	23	14097508	14180307		2.53	0.64	1.26	3.5	3.28	3.14
ENSDARG00000001882	kbtbd12	kelch repeat and BTB (POZ) domain containing 12 [Source:NCBI gene;Acc:559380]	6	40715637	40722480		5.12	7.28	6.7	6.29	4.99	5.33
ENSDARG00000001888	ppm1ba	protein phosphatase, Mg2+/Mn2+ dependent, 1Ba [Source:ZFIN;Acc:ZDB-GENE-991102-16]	13	10682427	10731325		4.47	2.84	2.99	5.6	5.74	5.57
ENSDARG00000001889	tuba1a	tubulin, alpha 1a [Source:ZFIN;Acc:ZDB-GENE-090507-4]	23	35708730	35711398		206.93	82.61	98.63	155.37	133.58	142.83
ENSDARG00000001890	stt3b	STT3 oligosaccharyltransferase complex catalytic subunit B [Source:ZFIN;Acc:ZDB-GENE-030131-2638]	16	39385483	39477746		8.55	6.85	5.08	14.3	13.36	12.58
ENSDARG00000001891	syt14b	synaptotagmin XIVb [Source:ZFIN;Acc:ZDB-GENE-041014-36]	20	9241794	9278252		3.21	1.22	2.43	4.74	5.04	5.25
ENSDARG00000001897	man2b1	mannosidase, alpha, class 2B, member 1 [Source:ZFIN;Acc:ZDB-GENE-050327-52]	11	31258116	31276080		12.2	8.38	7.39	10.39	11.2	10.87
ENSDARG00000001898	manea	mannosidase, endo-alpha [Source:ZFIN;Acc:ZDB-GENE-040912-87]	17	15658867	15667285		3.2	3.35	2.83	2.5	2.15	2.48
ENSDARG00000001906	si:dkey-247m21.3	si:dkey-247m21.3 [Source:ZFIN;Acc:ZDB-GENE-091204-249]	21	20229926	20308457		0.23	0.16	0.18	0.29	0.19	0.19
ENSDARG00000001909	vill	villin-like [Source:ZFIN;Acc:ZDB-GENE-081222-1]	24	20418959	20444844		4.76	2.68	2.55	4.96	5.24	5.06
ENSDARG00000001910	rorab	RAR-related orphan receptor A, paralog b [Source:NCBI gene;Acc:554722]	7	29538628	29625509		20.67	8.74	8.12	23.45	22.78	23.14
ENSDARG00000001912	drd4-rs	dopamine receptor D4 related sequence [Source:ZFIN;Acc:ZDB-GENE-051113-172]	4	19946294	20027571		1.88	1	1.06	1.76	1.92	1.78
ENSDARG00000001913	palmda	palmdelphin a [Source:ZFIN;Acc:ZDB-GENE-070705-48]	2	20406399	20428140		1.63	1.71	1.03	2.1	1.72	1.74
ENSDARG00000001929	pink1	PTEN induced kinase 1 [Source:ZFIN;Acc:ZDB-GENE-041212-53]	23	37466967	37480785		6.56	5.49	4.92	5.61	5.71	5.7
ENSDARG00000001930	klhl15	kelch-like family member 15 [Source:NCBI gene;Acc:445186]	24	16962969	16980337		3.74	1.77	2.04	4.19	3.67	4.13
ENSDARG00000001933	prkag2b	protein kinase, AMP-activated, gamma 2 non-catalytic subunit b [Source:ZFIN;Acc:ZDB-GENE-070912-442]	2	13056802	13148453		2.27	3.39	2.53	3.09	3.22	2.72
ENSDARG00000001937	LO018422.1	solute carrier family 2, facilitated glucose transporter member 1-like [Source:NCBI gene;Acc:100334818]	17	48433817	48458789		0.01	0.07	0.04	0.99	1.07	1.02
ENSDARG00000001939	b3gat3	beta-1,3-glucuronyltransferase 3 (glucuronosyltransferase I) [Source:ZFIN;Acc:ZDB-GENE-020419-3]	7	25015151	25029261		4.82	3.62	3.69	5.17	4.7	4.98
ENSDARG00000001940	dnajc1	DnaJ (Hsp40) homolog, subfamily C, member 1 [Source:ZFIN;Acc:ZDB-GENE-061103-529]	2	21174998	21182561		19.24	21.51	20.62	18.19	17.4	17.99
ENSDARG00000001950	ak1	adenylate kinase 1 [Source:ZFIN;Acc:ZDB-GENE-040822-37]	5	71699893	71722257		645.55	843.62	1229.89	838.28	922.36	872.13
ENSDARG00000001953	pfkfb3	6-phosphofructo-2-kinase/fructose-2,6-biphosphatase 3 [Source:ZFIN;Acc:ZDB-GENE-040426-2724]	4	25472505	25485404		3.26	2.91	2.55	23.37	21.09	20.23
ENSDARG00000001968	dock5	dedicator of cytokinesis 5 [Source:ZFIN;Acc:ZDB-GENE-080108-4]	8	6651253	6825588		2.5	2.37	1.7	2.21	2.29	1.73
ENSDARG00000001969	txlng	taxilin gamma [Source:ZFIN;Acc:ZDB-GENE-060616-122]	11	12782296	12802123		4.23	3.42	3.25	4.78	5.02	4.78
ENSDARG00000001971	cthrc1b	collagen triple helix repeat containing 1b [Source:ZFIN;Acc:ZDB-GENE-050420-46]	16	47454586	47483142		4.54	4.38	4.22	4.06	4.92	4.34
ENSDARG00000001975	hsd11b2	hydroxysteroid (11-beta) dehydrogenase 2 [Source:ZFIN;Acc:ZDB-GENE-030131-6030]	7	35112052	35126374		7.73	7.08	6.97	22.26	23.33	21.9
ENSDARG00000001976	fkbp16	FKBP prolyl isomerase 16 [Source:ZFIN;Acc:ZDB-GENE-050208-116]	18	25653599	25745636		12.81	6.69	5.6	8.56	8.4	8.61
ENSDARG00000001993	myhb	myosin, heavy chain b [Source:ZFIN;Acc:ZDB-GENE-081124-1]	6	40745437	40756138		26.19	21.04	17.64	67.35	71.92	68.87
ENSDARG00000001994	stxbp1a	syntaxin binding protein 1a [Source:ZFIN;Acc:ZDB-GENE-050626-106]	21	13861589	13921022		79.46	19.65	20.55	114.11	111.23	116.48
ENSDARG00000001999	adamts18	ADAM metallopeptidase with thrombospondin type 1 motif, 18 [Source:ZFIN;Acc:ZDB-GENE-070815-1]	7	42460302	42547992		3.29	1.73	1.05	3.5	2.89	3.04
ENSDARG00000002002	lmcd1	LIM and cysteine-rich domains 1 [Source:ZFIN;Acc:ZDB-GENE-040426-1067]	22	39096911	39108026		4.22	4.64	5.85	4.98	5.24	5.13
ENSDARG00000002006	rxrbb	retinoid x receptor, beta b [Source:NCBI gene;Acc:30486]	16	18534834	18572875		2.9	1.96	2.1	3.59	3.58	3.36
ENSDARG00000002013	grb10a	growth factor receptor-bound protein 10a [Source:ZFIN;Acc:ZDB-GENE-040822-32]	19	23136170	23249822		3.98	3.45	2.74	6.06	6.26	5.59
ENSDARG00000002016	ppil2	peptidylprolyl isomerase (cyclophilin)-like 2 [Source:ZFIN;Acc:ZDB-GENE-040426-1096]	5	12908781	12979986		12.88	12.58	11.77	10.16	9.56	9.28
ENSDARG00000002020	rictorb	RPTOR independent companion of MTOR, complex 2b [Source:ZFIN;Acc:ZDB-GENE-070810-5]	21	21195487	21242387		2.67	1.3	1.24	3.34	3.2	3.45
ENSDARG00000002021	pygb	phosphorylase, glycogen; brain [Source:ZFIN;Acc:ZDB-GENE-040928-2]	17	21126495	21152173		20.38	25.71	23.15	18.72	19.32	17.53
ENSDARG00000002026	qkib	QKI, KH domain containing, RNA binding b [Source:ZFIN;Acc:ZDB-GENE-081028-54]	13	3667230	3843692		7.24	3.64	4.82	14.79	15.42	16.82
ENSDARG00000002031	tox4a	TOX high mobility group box family member 4 a [Source:ZFIN;Acc:ZDB-GENE-070912-576]	2	38068069	38080075		9.26	5.36	4.12	10.09	9.98	10.3
ENSDARG00000002037	pfkfb2b	6-phosphofructo-2-kinase/fructose-2,6-biphosphatase 2b [Source:ZFIN;Acc:ZDB-GENE-120222-1]	11	18784847	18800299		5.23	1.7	2.53	3.68	3.71	3.86
ENSDARG00000002060	brf2	BRF2 RNA polymerase III transcription initiation factor subunit [Source:NCBI gene;Acc:445142]	10	41279007	41302909		1.16	0.81	0.91	0.8	0.61	0.69
ENSDARG00000002071	adss2	adenylosuccinate synthase 2 [Source:NCBI gene;Acc:550502]	13	11489953	11536951		17.82	21.82	20.09	16.2	14.74	14.37
ENSDARG00000002077	mnat1	MNAT1 component of CDK activating kinase [Source:ZFIN;Acc:ZDB-GENE-041010-203]	13	31648271	31681951		7.71	6.59	6.19	6.03	4.8	5.72
ENSDARG00000002082	csnk2a1	casein kinase 2, alpha 1 polypeptide [Source:ZFIN;Acc:ZDB-GENE-990415-27]	16	31434524	31452507		25.44	20.11	18.69	31	31.08	31.01

ENSDARG00000002084	lamb2	laminin, beta 2 (laminin S) [Source:ZFIN;Acc:ZDB-GENE-081030-4]	23	20180159	20258490		10.15	13.37	10.44	25.15	20.76	20.9
ENSDARG00000002097	si:dkeyp-52c3.7	si:dkeyp-52c3.7 [Source:ZFIN;Acc:ZDB-GENE-110411-213]	3	1749793	1761980		0.14	0.08	0.03	0.21	0.35	0.21
ENSDARG00000002127	stam	signal transducing adaptor molecule (SH3 domain and ITAM motif) 1 [Source:ZFIN;Acc:ZDB-GENE-031118-123]	2	31437547	31452606		17.55	11.53	10.75	17.83	16.26	17.04
ENSDARG00000002128	cwf19l1	CWF19 like cell cycle control factor 1 [Source:NCBI gene;Acc:387304]	4	17642731	17653566		7.43	5.66	6.19	6.53	6.33	6.48
ENSDARG00000002131	celf2	cugbp, Elav-like family member 2 [Source:NCBI gene;Acc:373117]	4	23886726	24031924		26.16	11.88	13.27	33.35	33.43	35.35
ENSDARG00000002165	psme1	proteasome activator subunit 1 [Source:ZFIN;Acc:ZDB-GENE-991110-17]	24	12945803	12954482		6.48	10.59	7.34	4.01	3.62	3.8
ENSDARG00000002167	cacnb1	calcium channel, voltage-dependent, beta 1 subunit [Source:ZFIN;Acc:ZDB-GENE-040718-399]	3	16157120	16227683		7.11	4.62	4.83	10.48	8.48	8.72
ENSDARG00000002168	tra2b	transformer 2 beta homolog [Source:ZFIN;Acc:ZDB-GENE-040426-1094]	9	12579077	12595003		21.83	13.52	15.24	22.05	19.69	21.39
ENSDARG00000002172	aplnra	apelin receptor a [Source:NCBI gene;Acc:561935]	8	40325690	40327397		10.05	8.26	7.31	13.07	14.21	13.23
ENSDARG00000002174	itfg2	integrin alpha FG-GAP repeat containing 2 [Source:ZFIN;Acc:ZDB-GENE-041008-161]	4	17539134	17553996		6.62	5.74	6.52	3.66	3.64	3.92
ENSDARG00000002184	igbp1	immunoglobulin (CD79A) binding protein 1 [Source:ZFIN;Acc:ZDB-GENE-040426-2928]	5	36530836	36537975		13.42	9.9	11.6	12.55	12.79	12.22
ENSDARG00000002190	chmp2bb	charged multivesicular body protein 2Bb [Source:NCBI gene;Acc:405840]	1	33558555	33569245		30.21	31.94	29.49	27.09	26.66	26.36
ENSDARG00000002192	aspn	asporin (LRR class 1) [Source:ZFIN;Acc:ZDB-GENE-041105-7]	22	10479965	10487490		14.52	15.29	15.85	10.03	10.22	9.47
ENSDARG00000002193	rho	rhodopsin [Source:NCBI gene;Acc:30295]	8	54076195	54077740		1318.28	297.93	222.43	634.23	649.09	654.7
ENSDARG00000002194	rhd	Rh blood group, D antigen [Source:ZFIN;Acc:ZDB-GENE-051213-1]	13	45496766	45523026		0.48	0.77	0.74	1.05	1.72	1.29
ENSDARG00000002196	bach1b	BTB and CNC homology 1, basic leucine zipper transcription factor 1 b [Source:ZFIN;Acc:ZDB-GENE-050522-220]	10	25369254	25385009		4.55	4.55	3.2	6.14	6.03	5.61
ENSDARG00000002197	pygl	phosphorylase, glycogen, liver [Source:ZFIN;Acc:ZDB-GENE-041205-1]	13	36846789	36863039		57.05	76.31	75.21	54.59	63.42	59.26
ENSDARG00000002204	hspb11	heat shock protein, alpha-crystallin-related, b11 [Source:NCBI gene;Acc:796767]	21	20383837	20384878		10.84	17.05	16.13	11.21	11.94	13.49
ENSDARG00000002210	stk25a	serine/threonine kinase 25a [Source:ZFIN;Acc:ZDB-GENE-041010-92]	6	27041594	27057702		6.83	4.04	5.22	5.2	5.15	5.41
ENSDARG00000002213	invs	inversin [Source:NCBI gene;Acc:245946]	16	27442549	27502702		1.5	1.44	1.27	2.59	2.27	2.37
ENSDARG00000002215	rtca	RNA 3'-terminal phosphate cyclase [Source:ZFIN;Acc:ZDB-GENE-030131-9687]	22	16282144	16291041		9.65	6.15	6.46	9.67	8.76	9.63
ENSDARG00000002216	tbx3a	T-box transcription factor 3a [Source:ZFIN;Acc:ZDB-GENE-070209-80]	5	72316803	72324371		8.56	4.96	4.32	11.94	12.04	12.19
ENSDARG00000002217	scocb	short coiled-coil protein b [Source:NCBI gene;Acc:791709]	14	48932235	48939560		20.01	10.35	11.46	25.58	27.13	27.22
ENSDARG00000002219	si:dkey-103j14.5	si:dkey-103j14.5 [Source:ZFIN;Acc:ZDB-GENE-090313-170]	13	23233956	23270576		1.59	1.88	1.07	1.22	1.61	1.44
ENSDARG00000002220	C5H9orf78	zgc:103692 [Source:ZFIN;Acc:ZDB-GENE-041010-15]	5	33295678	33298352	C5H9orf78	18.4	21.76	19.62	15.85	15.18	15.46
ENSDARG00000002230	sypb	synaptophysin b [Source:ZFIN;Acc:ZDB-GENE-040718-205]	8	49403668	49432061		48.99	12.41	14.98	44.56	45	45.4
ENSDARG00000002231	plpp2a	phospholipid phosphatase 2a [Source:ZFIN;Acc:ZDB-GENE-140703-2]	2	52232630	52306803		6.32	6.56	5.98	9.62	8.32	9.7
ENSDARG00000002235	mmp14a	matrix metallopeptidase 14a (membrane-inserted) [Source:ZFIN;Acc:ZDB-GENE-030901-1]	7	24124289	24149670		27.04	30.7	25.76	38.1	37.99	39
ENSDARG00000002240	psmb6	proteasome 20S subunit beta 6 [Source:ZFIN;Acc:ZDB-GENE-990415-216]	23	44536926	44546152		50.33	64.21	74.98	41.93	43.89	42.73
ENSDARG00000002241	kcna2a	potassium voltage-gated channel, shaker-related subfamily, member 2a [Source:ZFIN;Acc:ZDB-GENE-131113-1]	6	49412754	49414235		1.51	0.48	0.56	1.09	1.6	1.48
ENSDARG00000002249	tbxas1	thromboxane A synthase 1 (platelet) [Source:ZFIN;Acc:ZDB-GENE-030131-8805]	18	12655766	12834323		6.68	6.19	5.83	4.95	4.37	5.34
ENSDARG00000002255	epb41l3a	erythrocyte membrane protein band 4.1-like 3a [Source:ZFIN;Acc:ZDB-GENE-040822-8]	24	41931585	41986895		13.41	3.36	3.95	16.7	16.5	17.84
ENSDARG00000002259	ca15c	carbonic anhydrase XV c [Source:ZFIN;Acc:ZDB-GENE-061013-737]	12	4338298	4346085		0.07	0.08	0.1	0.72	0.76	0.79
ENSDARG00000002264	mrpl41	mitochondrial ribosomal protein L41 [Source:NCBI gene;Acc:553805]	5	25072894	25075179		24.48	22.65	21.41	18.91	19.6	21
ENSDARG00000002267	dcaf13	ddb1 and cul4 associated factor 13 [Source:NCBI gene;Acc:393097]	16	44653266	44673851		13.71	15.41	15.12	9.44	10.08	10.03
ENSDARG00000002271	zfand5b	zinc finger, AN1-type domain 5b [Source:ZFIN;Acc:ZDB-GENE-040426-875]	10	15603082	15613302		25.63	23.04	20.13	36.06	36.04	34.39
ENSDARG00000002285	mcoln1a	mucolipin 1a [Source:ZFIN;Acc:ZDB-GENE-040426-2704]	1	45671687	45700574		4.45	3.16	3.01	5.09	5.15	5.45
ENSDARG00000002293	si:ch211-197g15.6	si:ch211-197g15.6 [Source:ZFIN;Acc:ZDB-GENE-050208-457]	22	17460871	17463643		1.28	1.68	1.25	0.41	0.44	0.51
ENSDARG00000002295	si:dkey-21p1.3	si:dkey-21p1.3 [Source:ZFIN;Acc:ZDB-GENE-081105-162]	8	14855471	14883692		0	0.04	0.05	1.3	1.22	1.36
ENSDARG00000002298	ankrd22	ankyrin repeat domain 22 [Source:ZFIN;Acc:ZDB-GENE-040426-1120]	12	17154655	17158635		2.23	2.52	2.32	2.19	2	2.71
ENSDARG00000002299	arsh	arylsulfatase H [Source:ZFIN;Acc:ZDB-GENE-081104-120]	9	55576475	55594965		3.53	2.98	2.06	3.46	3.24	3.12
ENSDARG00000002300	p2rx2	purinergic receptor P2X, ligand-gated ion channel, 2 [Source:ZFIN;Acc:ZDB-GENE-030319-2]	5	12807902	12824607		0.73	0.54	0.57	0.8	0.65	0.6
ENSDARG00000002304	gins2	GINS complex subunit 2 [Source:ZFIN;Acc:ZDB-GENE-050419-19]	18	30481030	30499489		7.08	8.23	7.44	2.7	3.24	2.91
ENSDARG00000002305	me3	malic enzyme 3, NADP(+)-dependent, mitochondrial [Source:ZFIN;Acc:ZDB-GENE-041111-294]	9	34246384	34260214		35.8	35.08	33.62	31.53	29.57	31.36
ENSDARG00000002311	fabp11b	fatty acid binding protein 11b [Source:ZFIN;Acc:ZDB-GENE-050522-391]	16	16148761	16152199		0.58	0.51	0.34	1.44	1.56	2.52
ENSDARG00000002330	lhx8a	LIM homeobox 8a [Source:ZFIN;Acc:ZDB-GENE-031008-2]	2	11205795	11210168		1.74	0.93	1.63	1.51	1.7	1.89
ENSDARG00000002332	frmd6	FERM domain containing 6 [Source:ZFIN;Acc:ZDB-GENE-050522-354]	13	36958086	36997761		4.66	4.36	3.56	7.59	7.39	7.02
ENSDARG00000002333	fam102bb	family with sequence similarity 102 member Bb [Source:ZFIN;Acc:ZDB-GENE-041014-330]	20	33752967	33790003		13.69	5.01	8.79	13.94	15.54	16.06
ENSDARG00000002336	dlc	deltaC [Source:NCBI gene;Acc:30120]	15	20463448	20468302		10.77	5.74	6.39	9.77	10.13	10.57
ENSDARG00000002339	sgf29	SAGA complex associated factor 29 [Source:ZFIN;Acc:ZDB-GENE-041010-62]	3	15489725	15496610		8.35	7.07	8.72	7.41	6.88	7.65
ENSDARG00000002344	tubb4b	tubulin, beta 4B class IVb [Source:ZFIN;Acc:ZDB-GENE-030131-8625]	14	5861435	5865146		411.26	359.27	389.13	414.59	434.16	428.28
ENSDARG00000002347	cyp11a1	cytochrome P450, family 11, subfamily A, polypeptide 1 [Source:ZFIN;Acc:ZDB-GENE-010306-1]	25	22319940	22335283		0.06	0.06	0.07	0.12	0.16	0.07
ENSDARG00000002353	tagapa	T cell activation RhoGTPase activating protein a [Source:ZFIN;Acc:ZDB-GENE-040426-1877]	17	45404758	45411170		0.41	0.24	0.36	0.27	0.35	0.34
ENSDARG00000002355	shpk	sedoheptulokinase [Source:ZFIN;Acc:ZDB-GENE-060526-357]	5	42124706	42132825		1.99	2.11	1.6	1.64	1.65	1.52
ENSDARG00000002362	ubald1a	UBA-like domain containing 1a [Source:NCBI gene;Acc:794012]	12	20059859	20120711		12.95	8.3	8.42	13.38	13.97	13.39
ENSDARG00000002365	cers5	ceramide synthase 5 [Source:ZFIN;Acc:ZDB-GENE-030131-1740]	22	5758090	5822147		10	8.56	8.15	9.64	9.88	9.02
ENSDARG00000002369	UBB	si:ch211-202a12.4 [Source:ZFIN;Acc:ZDB-GENE-030131-8486]	5	42064144	42068490	UBB	112.96	120.71	114.62	79.65	81.16	79.5
ENSDARG00000002377	mief2	mitochondrial elongation factor 2 [Source:ZFIN;Acc:ZDB-GENE-060929-864]	1	8521323	8532009		3.64	3.04	3.06	6.39	6.34	6.01
ENSDARG00000002385	atm	ATM serine/threonine kinase [Source:ZFIN;Acc:ZDB-GENE-040809-1]	15	6514964	6650993		2.45	2.18	1.45	2.31	2.32	2.27
ENSDARG00000002391	tlcd1	TLC domain containing 1 [Source:ZFIN;Acc:ZDB-GENE-041010-173]	21	26080215	26089964		13.11	15.89	16.95	7.1	8.39	7.39
ENSDARG00000002394	ugt5d1	UDP glucuronosyltransferase 5 family, polypeptide D1 [Source:ZFIN;Acc:ZDB-GENE-091118-36]	10	25726694	25728744		5.81	9.35	6.47	2.12	2.81	2.43
ENSDARG00000002396	cry-dash	cryptochrome DASH [Source:NCBI gene;Acc:402986]	24	20194022	20208474		8.69	9.14	8.91	4.16	3.51	3.96
ENSDARG00000002401	gale	UDP-galactose-4-epimerase [Source:ZFIN;Acc:ZDB-GENE-060421-6479]	17	24877392	24890932		3.67	4.15	3.91	3.57	3.76	3.44
ENSDARG00000002402	ercc3	excision repair cross-complementation group 3 [Source:NCBI gene;Acc:324323]	6	9595414	9630081		8.83	7.86	7.47	8.45	8.86	8.19
ENSDARG00000002403	nusap1	nucleolar and spindle associated protein 1 [Source:NCBI gene;Acc:567446]	13	33251721	33256667		23.74	16.51	17.2	13.94	12.72	13.96
ENSDARG00000002405	si:ch211-225b11.1	si:ch211-225b11.1 [Source:ZFIN;Acc:ZDB-GENE-060531-39]	5	20786772	20814588		2.01	2.02	1.74	2.91	3.02	2.74
ENSDARG00000002406	zmynd10	zinc finger, MYND-type containing 10 [Source:NCBI gene;Acc:393368]	1	22691256	22703383		0.93	0.69	0.78	1.52	1.46	2.25
ENSDARG00000002411	pde4cb	phosphodiesterase 4C, cAMP-specific b [Source:ZFIN;Acc:ZDB-GENE-030131-4075]	11	6684968	6794144		2.24	2.12	1.63	2.34	2.31	2.1
ENSDARG00000002445	prdm1a	PR domain containing 1a, with ZNF domain [Source:ZFIN;Acc:ZDB-GENE-030131-2193]	16	7430998	7443388		4.3	3.8	3.2	6.31	6.06	7.23
ENSDARG00000002463	arhgef37	Rho guanine nucleotide exchange factor (GEF) 37 [Source:ZFIN;Acc:ZDB-GENE-100514-3]	14	24840669	24873654		3.18	2.53	2.15	2.37	2.45	2.49
ENSDARG00000002467	rdh14b	retinol dehydrogenase 14b [Source:ZFIN;Acc:ZDB-GENE-030131-6605]	20	9421448	9428021		8.86	7.15	7.13	7.13	6.84	7.78
ENSDARG00000002479	ercc6l	excision repair cross-complementation group 6-like [Source:NCBI gene;Acc:100005291]	5	23913585	23926519		5.32	4.39	4.12	3.91	3.63	3.44
ENSDARG00000002483	wnt7ab	wingless-type MMTV integration site family, member 7Ab [Source:ZFIN;Acc:ZDB-GENE-100922-44]	23	4414164	4433220		2.57	2.01	1.57	1.5	1.15	1.25
ENSDARG00000002487	noc3l	NOC3-like DNA replication regulator [Source:NCBI gene;Acc:321289]	12	5399680	5418340		11.27	10.85	11.66	5.43	6.32	6.16
ENSDARG00000002494	itgb6	integrin, beta 6 [Source:ZFIN;Acc:ZDB-GENE-100812-2]	11	10975012	11020857		3.05	2.81	2.31	3.57	3.11	2.94
ENSDARG00000002506	C7H16orf87	zgc:92818 [Source:NCBI gene;Acc:436785]	7	41873080	41881238	C7H16orf87	23.93	22.24	24.26	21.03	20.54	22.36
ENSDARG00000002507	itga10	integrin, alpha 10 [Source:ZFIN;Acc:ZDB-GENE-100922-54]	16	14276364	14353567		1.93	1.47	1.35	2.8	2.65	2.49
ENSDARG00000002508	ankrd33ab	ankyrin repeat domain 33Ab [Source:ZFIN;Acc:ZDB-GENE-100729-1]	6	39279104	39286029		0.14	0.02	0.01	0.2	0.2	0.12
ENSDARG00000002509	zgc:153911	zgc:153911 [Source:ZFIN;Acc:ZDB-GENE-061013-174]	13	35974362	35976986		0.85	0.86	0.72	0.94	0.79	1.14
ENSDARG00000002510	dmbx1b	diencephalon/mesencephalon homeobox 1b [Source:NCBI gene;Acc:550288]	6	3563890	3573716		2.27	0.93	1.17	2.45	2.63	2.56
ENSDARG00000002523	hsdl2	hydroxysteroid dehydrogenase like 2 [Source:NCBI gene;Acc:322347]	10	11260170	11283239		20.24	25.1	23.43	15.11	14.97	14.67
ENSDARG00000002533	adora2b	adenosine A2b receptor [Source:ZFIN;Acc:ZDB-GENE-060511-1]	5	62781626	62801879		2.11	1.39	1.68	2.79	2.07	2.4
ENSDARG00000002536	tm9sf4	transmembrane 9 superfamily protein member 4 [Source:ZFIN;Acc:ZDB-GENE-040426-1575]	23	7548797	7578892		22.06	19.62	19.95	25.84	25.47	24.65
ENSDARG00000002538	smu1a	SMU1 DNA replication regulator and spliceosomal factor a [Source:NCBI gene;Acc:393168]	14	6228996	6245748		15.52	12.35	12.71	15.68	15.39	16.04
ENSDARG00000002546	rundc3ab	RUN domain containing 3Ab [Source:NCBI gene;Acc:393118]	12	9817440	9840819		15.3	5.93	8.25	17.29	16.67	18.12
ENSDARG00000002549	eif3eb	eukaryotic translation initiation factor 3, subunit E, b [Source:NCBI gene;Acc:561049]	19	22622355	22664398		2.95	2.5	2.99	3.61	3.57	3.43
ENSDARG00000002552	mapkapk2a	MAPK activated protein kinase 2a [Source:ZFIN;Acc:ZDB-GENE-030131-5532]	11	21154678	21218172		13.25	14.49	13.24	16.97	15.62	15.47
ENSDARG00000002564	syngr1a	synaptogyrin 1a [Source:ZFIN;Acc:ZDB-GENE-041010-169]	3	21251627	21280373		34.53	11.02	15.45	35.88	36.81	39.04
ENSDARG00000002571	tmod2	tropomodulin 2 [Source:ZFIN;Acc:ZDB-GENE-040912-185]	18	39327010	39382869		12.05	10.18	9.73	14.06	14.03	13.67
ENSDARG00000002576	cabp5a	calcium binding protein 5a [Source:ZFIN;Acc:ZDB-GENE-040426-1655]	12	3848109	3861759		3.94	0.5	0.27	1.84	1.96	2.31
ENSDARG00000002582	tbx15	T-box transcription factor 15 [Source:ZFIN;Acc:ZDB-GENE-020529-1]	9	21030930	21067971		2.01	2.33	1.87	2.56	2.58	2.58
ENSDARG00000002587	dpysl3	dihydropyrimidinase like 3 [Source:NCBI gene;Acc:553166]	21	42970983	43022048		34.55	13.71	15.37	46.12	46.4	46.76
ENSDARG00000002589	mylpfb	myosin light chain, phosphorylatable, fast skeletal muscle b [Source:ZFIN;Acc:ZDB-GENE-040912-115]	12	15002757	15010996		1323.94	1838.36	2316.59	1127.5	1096.85	1137.35
ENSDARG00000002591	ruvbl1	RuvB-like AAA ATPase 1 [Source:NCBI gene;Acc:317679]	6	40832635	40841032		27.62	24.03	22.59	20.82	22.05	21.53
ENSDARG00000002593	slc45a2	solute carrier family 45 member 2 [Source:ZFIN;Acc:ZDB-GENE-050208-97]	21	19445942	19484289		9.06	11.95	12.81	14.51	16.95	15.01
ENSDARG00000002597	pabpc1l	poly(A) binding protein, cytoplasmic 1-like [Source:ZFIN;Acc:ZDB-GENE-030131-5836]	8	45334255	45352219		0.04	0.03	0.06	0.01	0	0.05
ENSDARG00000002600	pcsk1	proprotein convertase subtilisin/kexin type 1 [Source:ZFIN;Acc:ZDB-GENE-071009-1]	21	11684830	11722343		12.13	2.09	2.78	17.09	19.42	19.65
ENSDARG00000002601	irx7	iroquois homeobox 7 [Source:ZFIN;Acc:ZDB-GENE-020103-1]	23	37815645	37816969		5.86	1.26	1.76	4.79	5.47	5.23
ENSDARG00000002607	unm_sa1614	un-named sa1614 [Source:ZFIN;Acc:ZDB-GENE-120411-1]	11	26476153	26562346		3.43	1.09	1.2	3.94	4.72	4.44
ENSDARG00000002609	rnf145a	ring finger protein 145a [Source:ZFIN;Acc:ZDB-GENE-111012-3]	14	34966598	35012162		4.01	0.99	1.22	4.77	4.48	5.03
ENSDARG00000002613	rpa3	replication protein A3 [Source:ZFIN;Acc:ZDB-GENE-040426-977]	19	25465025	25467185		16.41	19.68	17.88	7.64	7.87	9.11
ENSDARG00000002632	anxa11b	annexin A11b [Source:ZFIN;Acc:ZDB-GENE-030707-5]	12	48815988	48837308		41.6	44.45	35.92	50.76	52.1	51.42
ENSDARG00000002634	b4galt1	UDP-Gal:betaGlcNAc beta 1,4- galactosyltransferase, polypeptide 1 [Source:ZFIN;Acc:ZDB-GENE-050417-236]	1	12141876	12161148		11.43	9.34	8.77	8.68	7.27	7.74
ENSDARG00000002635	FAM184A	zgc:85722 [Source:ZFIN;Acc:ZDB-GENE-040516-7]	17	15309432	15382704	FAM184A	11.43	2.73	4.7	10.98	11.29	12.1
ENSDARG00000002642	sh3gl1b	SH3-domain GRB2-like 1b [Source:ZFIN;Acc:ZDB-GENE-031001-6]	8	18842278	18880399		13.74	16.31	15.65	14.73	14.97	14.47
ENSDARG00000002644	rgs5a	regulator of G protein signaling 5a [Source:ZFIN;Acc:ZDB-GENE-030131-7570]	6	35387367	35439406		16.91	20.87	19	20.38	22.26	21.43
ENSDARG00000002656	stxbp5a	syntaxin binding protein 5a (tomosyn) [Source:ZFIN;Acc:ZDB-GENE-041001-161]	20	31745949	31905968		4.49	0.86	1.03	5.92	5.91	6.2
ENSDARG00000002659	mapre1b	microtubule-associated protein, RP/EB family, member 1b [Source:ZFIN;Acc:ZDB-GENE-040426-2256]	23	9794730	9807578		21.74	17.47	18.31	27.68	26.05	26.29
ENSDARG00000002663	optn	optineurin [Source:NCBI gene;Acc:336159]	4	8016457	8027318		11.78	13.89	11.82	9.1	8.37	8.92
ENSDARG00000002666	kcnj1b	potassium inwardly rectifying channel subfamily J member 1b [Source:ZFIN;Acc:ZDB-GENE-070620-9]	10	39304422	39307819		0.95	0.5	0.83	1.38	1.18	0.92
ENSDARG00000002670	ATG14	zgc:113944 [Source:ZFIN;Acc:ZDB-GENE-050913-155]	17	10748366	10766679	ATG14	8.82	6.07	6.81	7.83	6.98	7.18
ENSDARG00000002682	tbcela	tubulin folding cofactor E-like a [Source:ZFIN;Acc:ZDB-GENE-040426-1719]	15	20940904	20956454		5.37	3.25	3.21	5.21	5.18	5.39
ENSDARG00000002685	fam113	family with sequence similarity 113 [Source:ZFIN;Acc:ZDB-GENE-041212-23]	7	24830576	24838857		0	0.05	0.01	0.02	0	0.05
ENSDARG00000002690	rabif	RAB interacting factor [Source:ZFIN;Acc:ZDB-GENE-040718-216]	11	24815667	24817637		8.11	7.08	6.09	7.77	8.46	7.87
ENSDARG00000002696	gnb3b	guanine nucleotide binding protein (G protein), beta polypeptide 3b [Source:ZFIN;Acc:ZDB-GENE-040426-2280]	19	5106226	5135345		333.81	84.16	62.42	123.66	139.81	130.13
ENSDARG00000002701	rasl12	RAS-like, family 12 [Source:NCBI gene;Acc:393366]	7	13756215	13782279		0.55	0.5	0.52	0.72	0.73	0.52
ENSDARG00000002707	her11	hairy-related 11 [Source:ZFIN;Acc:ZDB-GENE-040824-4]	14	30538482	30540967		0.1	0.02	0.07	0.1	0.22	0.11
ENSDARG00000002710	ncl	nucleolin [Source:ZFIN;Acc:ZDB-GENE-030131-6986]	22	36673312	36690751		200.13	172.18	147.33	135.69	139.54	138.01
ENSDARG00000002720	utp15	UTP15 small subunit processome component [Source:NCBI gene;Acc:325106]	18	7037349	7056760		10.87	11.24	9.82	7.45	7.58	7.63
ENSDARG00000002722	ankrd2	ankyrin repeat domain 2 (stretch responsive muscle) [Source:ZFIN;Acc:ZDB-GENE-090313-383]	13	40729618	40741005		0.1	0.16	0.16	0.66	0.29	0.28
ENSDARG00000002728	kiss1ra	KISS1 receptor a [Source:ZFIN;Acc:ZDB-GENE-070912-521]	2	33796207	33821163		0.92	0.17	0.36	0.55	0.69	0.58
ENSDARG00000002731	sdc2	syndecan 2 [Source:ZFIN;Acc:ZDB-GENE-021206-3]	19	47191005	47276297		63.82	64.13	50.37	94.53	95.3	95.96
ENSDARG00000002732	spon2b	spondin 2b, extracellular matrix protein [Source:ZFIN;Acc:ZDB-GENE-990415-161]	14	17376940	17382534		10.45	10.58	11.75	5.52	5.93	5.49
ENSDARG00000002745	tdh	L-threonine dehydrogenase [Source:ZFIN;Acc:ZDB-GENE-040426-2379]	20	18992679	18998022		93.15	98.41	93.52	76.12	77.46	78.95
ENSDARG00000002748	sema6d	sema domain, transmembrane domain (TM), and cytoplasmic domain, (semaphorin) 6D [Source:ZFIN;Acc:ZDB-GENE-040426-1933	19	390298	417381		6.23	2.46	1.88	6.63	6.66	6.95
ENSDARG00000002750	phf20a	PHD finger protein 20, a [Source:ZFIN;Acc:ZDB-GENE-070912-270]	11	25044082	25063586		4.29	2.34	2.94	3.76	3.73	4.22
ENSDARG00000002754	pdcd2l	programmed cell death 2-like [Source:ZFIN;Acc:ZDB-GENE-040426-967]	25	30442389	30454772		6.02	5.91	5.93	2.58	2.51	2.37
ENSDARG00000002758	dedd1	death effector domain-containing 1 [Source:ZFIN;Acc:ZDB-GENE-000616-2]	16	10841163	10863041		24.66	7.97	12.8	26.57	27.87	28.17
ENSDARG00000002760	gdf5	growth differentiation factor 5 [Source:ZFIN;Acc:ZDB-GENE-990415-39]	6	52637308	52661824		1.79	1.4	1.29	3.41	3.95	3.63
ENSDARG00000002764	miox	myo-inositol oxygenase [Source:NCBI gene;Acc:571850]	18	6732195	6742287		28.51	38.92	32.95	30.47	32.7	33.1
ENSDARG00000002768	pvalb2	parvalbumin 2 [Source:NCBI gene;Acc:58028]	12	17710425	17712498		6290.13	8493.78	11706.35	5647.26	5815.89	5711.68
ENSDARG00000002771	SLC4A5	si:ch211-48m9.1 [Source:ZFIN;Acc:ZDB-GENE-060531-64]	5	28045764	28109416	SLC4A5	2.38	0.18	0.15	2.52	2.45	2.37
ENSDARG00000002779	pdx1	pancreatic and duodenal homeobox 1 [Source:ZFIN;Acc:ZDB-GENE-990415-122]	24	21684189	21686706		2.25	3.07	3.08	2.59	2.45	2.42
ENSDARG00000002787	tle3a	TLE family member 3, transcriptional corepressor a [Source:NCBI gene;Acc:30206]	18	20225961	20257511		7.09	4.43	4.07	13.06	12.02	12.2
ENSDARG00000002790	ap2m1a	adaptor related protein complex 2 subunit mu 1a [Source:NCBI gene;Acc:321051]	2	9789811	9818711		123.06	94.08	104.78	110.57	116.39	116.99
ENSDARG00000002791	atp1a1a.1	ATPase Na+/K+ transporting subunit alpha 1a, tandem duplicate 1 [Source:ZFIN;Acc:ZDB-GENE-001212-1]	1	1712140	1793371		158.37	127.9	100.86	168.02	173.45	177.98
ENSDARG00000002792	arcn1a	archain 1a [Source:ZFIN;Acc:ZDB-GENE-021031-2]	5	30502249	30516646		20.13	19.04	18.01	23.57	24.2	22.08
ENSDARG00000002795	meis3	myeloid ecotropic viral integration site 3 [Source:ZFIN;Acc:ZDB-GENE-010406-2]	15	6838039	6863317		6.19	4.77	4.55	9.67	10.08	9.87
ENSDARG00000002797	BX908804.1		3	3693417	3695256		0.03	0	0	0.01	0.01	0
ENSDARG00000002798	cog8	component of oligomeric golgi complex 8 [Source:ZFIN;Acc:ZDB-GENE-050320-74]	18	5850837	5859484		4.76	3.72	3.21	4	4.24	4.38
ENSDARG00000002816	rasgrf2b	Ras protein-specific guanine nucleotide-releasing factor 2b [Source:NCBI gene;Acc:553520]	5	51443009	51581775		1.91	0.32	0.28	1.99	1.8	2.08
ENSDARG00000002826	cep57l1	centrosomal protein 57, like 1 [Source:ZFIN;Acc:ZDB-GENE-040808-27]	20	32141562	32149020		4.06	3.29	3	2.88	2.53	3.22
ENSDARG00000002830	trmt2a	tRNA methyltransferase 2 homolog A [Source:ZFIN;Acc:ZDB-GENE-030131-6876]	5	24532401	24543526		5.63	6.53	5.78	3.68	3.57	3.72
ENSDARG00000002831	col4a4	collagen, type IV, alpha 4 [Source:ZFIN;Acc:ZDB-GENE-101001-1]	15	35951888	36055401		0.64	0.57	0.35	1.35	1.29	1.39
ENSDARG00000002840	si:dkey-28b4.8	si:dkey-28b4.8 [Source:ZFIN;Acc:ZDB-GENE-090501-2]	1	44651727	44704261		10.37	3.13	3.25	14.87	14.87	15.05
ENSDARG00000002847	fndc1	fibronectin type III domain containing 1 [Source:ZFIN;Acc:ZDB-GENE-041210-332]	20	31002188	31067306		1.96	2.17	1.84	1.54	1.41	1.58
ENSDARG00000002872	zgc:113210	zgc:113210 [Source:ZFIN;Acc:ZDB-GENE-050320-83]	3	52647144	52661242		5.75	4.39	4.48	5.65	4.98	5.09
ENSDARG00000002877	reps1	RALBP1 associated Eps domain containing 1 [Source:ZFIN;Acc:ZDB-GENE-030131-9536]	20	39223235	39259880		2.99	2.75	2.34	6.31	6.45	6.23
ENSDARG00000002880	vps50	VPS50 EARP/GARPII complex subunit [Source:ZFIN;Acc:ZDB-GENE-060503-325]	19	40379874	40686675		4.76	3.08	3.08	4.75	4.71	4.92
ENSDARG00000002894	aven	apoptosis, caspase activation inhibitor [Source:ZFIN;Acc:ZDB-GENE-041001-171]	20	29231556	29269362		5.56	6.1	6.85	5.35	4.88	5.36
ENSDARG00000002897	si:ch211-160o17.2	si:ch211-160o17.2 [Source:ZFIN;Acc:ZDB-GENE-141219-3]	11	2616816	2632747		3.84	2.75	2.72	4.95	4.99	5.04
ENSDARG00000002909	tjp3	tight junction protein 3 [Source:ZFIN;Acc:ZDB-GENE-030828-10]	22	17657612	17688873		9.04	7.37	5.47	6.41	6.67	6.55
ENSDARG00000002912	adipor1a	adiponectin receptor 1a [Source:ZFIN;Acc:ZDB-GENE-040718-169]	11	24800156	24815193		13.63	8.4	8.35	17.43	18.65	19.01
ENSDARG00000002916	sec31b	SEC31 homolog B, COPII coat complex component [Source:ZFIN;Acc:ZDB-GENE-060418-2]	12	48419226	48467781		5.61	3.85	3.08	6.37	6.74	6.32
ENSDARG00000002917	gls2b	glutaminase 2b (liver, mitochondrial) [Source:ZFIN;Acc:ZDB-GENE-050208-408]	22	10098446	10110988		2.37	3.62	2.88	1.93	1.94	1.79
ENSDARG00000002933	gbx2	gastrulation brain homeobox 2 [Source:ZFIN;Acc:ZDB-GENE-020509-2]	6	16031189	16034286		6.45	2.43	1.87	8.93	8.76	9.72
ENSDARG00000002937	meis1a	Meis homeobox 1 a [Source:ZFIN;Acc:ZDB-GENE-020122-3]	1	51475094	51525086		11.5	13	11.49	12.42	13.52	13.52
ENSDARG00000002945	bgnb	biglycan b [Source:ZFIN;Acc:ZDB-GENE-040426-21]	23	20014030	20051369		5.48	4.48	4.25	15.05	15.68	15.15
ENSDARG00000002947	abcd3b	ATP-binding cassette, sub-family D (ALD), member 3b [Source:ZFIN;Acc:ZDB-GENE-050517-29]	2	15109528	15125512		6.34	5.55	4.91	4.55	4.13	4.41
ENSDARG00000002949	ppp6c	protein phosphatase 6, catalytic subunit [Source:ZFIN;Acc:ZDB-GENE-040426-949]	5	31983098	31995401		31.49	30.23	29.21	38.3	38.99	38.77
ENSDARG00000002952	smo	smoothened, frizzled class receptor [Source:ZFIN;Acc:ZDB-GENE-980526-89]	4	14971239	14979937		6.4	4.41	3.9	9.42	9.46	9.4
ENSDARG00000002956	si:dkey-32n7.4	si:dkey-32n7.4 [Source:ZFIN;Acc:ZDB-GENE-110411-175]	12	4114401	4145033		0.4	0.35	0.41	1.31	1.25	1.22
ENSDARG00000002959	gpr34b	G protein-coupled receptor 34b [Source:ZFIN;Acc:ZDB-GENE-040823-1]	9	33417969	33419321		0	0	0	0.02	0	0
ENSDARG00000002967	pdap1b	pdgfa associated protein 1b [Source:ZFIN;Acc:ZDB-GENE-040426-942]	3	40277704	40284744		21.86	25.74	22.58	22.04	23.2	21.77
ENSDARG00000002968	a1cf	apobec1 complementation factor [Source:ZFIN;Acc:ZDB-GENE-060824-3]	12	6160446	6177894		16.6	22.8	12.77	13.15	14.49	12.91
ENSDARG00000002970	ss18	SS18 subunit of BAF chromatin remodeling complex [Source:ZFIN;Acc:ZDB-GENE-030131-4867]	2	1348281	1364678		29.26	16.1	16.11	35.82	36.76	37.44
ENSDARG00000002971	phc1	polyhomeotic homolog 1 [Source:ZFIN;Acc:ZDB-GENE-081022-39]	16	31942527	31960652		3.03	2.32	1.88	3.44	3.31	3.24
ENSDARG00000002986	gda	guanine deaminase [Source:ZFIN;Acc:ZDB-GENE-050522-434]	5	25708369	25723079		9.18	15.31	10.5	1.4	1.52	1.65
ENSDARG00000002988	tnnt2d	troponin T2d, cardiac [Source:ZFIN;Acc:ZDB-GENE-050626-97]	25	20215964	20226584		79.67	141.2	126.76	88.4	82.33	89.37
ENSDARG00000002991	cep135	centrosomal protein 135 [Source:NCBI gene;Acc:553303]	20	25711718	25735315		4.12	2.27	2.71	3.24	3.17	3.18
ENSDARG00000002994	itpkca	inositol-trisphosphate 3-kinase Ca [Source:ZFIN;Acc:ZDB-GENE-030131-6009]	21	21279159	21298995		13.18	11.19	8.53	11.31	10.91	10.22
ENSDARG00000003008	prkcea	protein kinase C, epsilon a [Source:ZFIN;Acc:ZDB-GENE-030131-8191]	12	25640480	25770232		6.55	2.63	3.16	8.74	8.9	9.04
ENSDARG00000003017	ube2al	ubiquitin conjugating enzyme E2 A, like [Source:ZFIN;Acc:ZDB-GENE-030131-4195]	9	8968702	8972763		19.74	18.87	18.52	25.74	26.38	26.23
ENSDARG00000003020	lrrc4.2	leucine rich repeat containing 4.2 [Source:ZFIN;Acc:ZDB-GENE-030131-7997]	18	10296228	10298162		5.23	1.36	1.88	6.5	6.17	6.4
ENSDARG00000003021	hdac8	histone deacetylase 8 [Source:NCBI gene;Acc:406740]	7	51656099	51710015		6.05	4.66	4.84	5.25	5.11	4.58
ENSDARG00000003022	limk1b	LIM domain kinase 1b [Source:ZFIN;Acc:ZDB-GENE-110411-106]	21	40805336	40835069		2.85	3.35	3.36	2.72	3.36	3.28

ENSDARG00000003027	scnm1	sodium channel modifier 1 [Source:NCBI gene;Acc:321696]	16	29685557	29690212		2.74	2.86	2.87	1.94	1.74	1.93
ENSDARG00000003032	eif4a1b	eukaryotic translation initiation factor 4A1B [Source:ZFIN;Acc:ZDB-GENE-040120-6]	5	24208413	24219344		256.68	286.31	313.52	221.97	229.84	220.93
ENSDARG00000003035	hspa9	heat shock protein 9 [Source:ZFIN;Acc:ZDB-GENE-030828-12]	14	12142813	12168897		49.58	52.14	46.11	54.48	54.77	52.45
ENSDARG00000003041	CABZ01111496.1	E3 ubiquitin-protein ligase pellino homolog 2-like [Source:NCBI gene;Acc:100334245]	20	54174978	54192130		5.91	3.14	4.01	13.54	13.37	14.6
ENSDARG00000003046	sorbs2a	sorbin and SH3 domain containing 2a [Source:ZFIN;Acc:ZDB-GENE-070308-2]	1	17747656	17803614		3.04	0.66	1.16	4.88	4.36	4.73
ENSDARG00000003054	tacr3l	tachykinin receptor 3-like [Source:ZFIN;Acc:ZDB-GENE-061207-19]	1	52518176	52538517		0.56	0.2	0.29	0.48	0.55	0.67
ENSDARG00000003058	fgfr1op	FGFR1 oncogene partner [Source:ZFIN;Acc:ZDB-GENE-090417-1]	20	30578163	30610170		7.28	6.03	6.58	6.33	6.56	6.17
ENSDARG00000003061	cd276	CD276 molecule [Source:ZFIN;Acc:ZDB-GENE-070112-452]	18	50433722	50524042		5.78	6.1	6.22	8.04	7.73	8.1
ENSDARG00000003068	gorasp1a	golgi reassembly stacking protein 1a [Source:ZFIN;Acc:ZDB-GENE-030826-23]	2	23808640	23820280		7.05	5.45	3.6	8.26	8.56	7.8
ENSDARG00000003069	btbd7	BTB (POZ) domain containing 7 [Source:ZFIN;Acc:ZDB-GENE-030131-4477]	20	27114874	27190393		1.82	0.97	0.99	2.81	2.82	2.44
ENSDARG00000003077	gpx9	glutathione peroxidase 9 [Source:ZFIN;Acc:ZDB-GENE-130603-13]	12	45676667	45688953		0.03	0.1	0.18	0.87	0.88	1.19
ENSDARG00000003081	mybphb	myosin binding protein Hb [Source:ZFIN;Acc:ZDB-GENE-030131-9794]	6	55032439	55082533		110.5	185.98	207.07	168.08	160.35	155.97
ENSDARG00000003084	spire2	spire-type actin nucleation factor 2 [Source:NCBI gene;Acc:492470]	25	34887999	34911606		1.03	0.31	0.4	1.56	1.22	1.48
ENSDARG00000003085	stambpl1	STAM binding protein-like 1 [Source:ZFIN;Acc:ZDB-GENE-100215-4]	12	17117405	17152139		1.51	0.73	1.1	1.77	1.88	1.9
ENSDARG00000003087	ubxn6	UBX domain protein 6 [Source:ZFIN;Acc:ZDB-GENE-030131-5680]	22	22260210	22274393		25.12	21.54	20.62	19.08	21.37	21.22
ENSDARG00000003090	or103-4	odorant receptor, family C, subfamily 103, member 4 [Source:ZFIN;Acc:ZDB-GENE-000329-12]	15	31438568	31445759		0	0	0	0	0	0
ENSDARG00000003091	oclnb	occludin b [Source:ZFIN;Acc:ZDB-GENE-041212-43]	5	26212621	26220871		19.38	15.27	12.09	13.6	12.13	11.76
ENSDARG00000003098	kdm5bb	lysine (K)-specific demethylase 5Bb [Source:NCBI gene;Acc:415256]	8	28305213	28342487		28.79	16.22	15.05	45.51	45.7	48.31
ENSDARG00000003109	rcan1a	regulator of calcineurin 1a [Source:ZFIN;Acc:ZDB-GENE-040801-135]	9	35843732	35865528		15.14	12.82	11.25	16.08	15.96	15.34
ENSDARG00000003113	ada	adenosine deaminase [Source:NCBI gene;Acc:436919]	23	25946448	25976656		110.05	180.3	137.07	27.69	27.49	28.78
ENSDARG00000003127	zgc:123105	Danio rerio zgc:123105 (zgc:123105), transcript variant 2, mRNA. [Source:RefSeq mRNA;Acc:NM_001365567]	21	32454896	32467799		14.22	7.1	7.02	17.47	18.36	18.51
ENSDARG00000003132	apip	APAF1 interacting protein [Source:NCBI gene;Acc:447941]	7	49901483	49908518		35.47	40.53	45.57	23.95	23.36	24.92
ENSDARG00000003142	dachc	dachshund c [Source:ZFIN;Acc:ZDB-GENE-020402-5]	1	30585140	30689004		3.52	2.28	2.09	5.83	6.38	5.91
ENSDARG00000003144	pxmp2	peroxisomal membrane protein 2 [Source:ZFIN;Acc:ZDB-GENE-040912-184]	5	20115774	20123002		5.68	7.82	6.25	5.36	6.23	4.99
ENSDARG00000003146	si:dkey-27m7.4	si:dkey-27m7.4 [Source:ZFIN;Acc:ZDB-GENE-090313-297]	13	17702522	17708031		2.98	2.75	2.03	2.21	2.26	2.1
ENSDARG00000003151	skp1	S-phase kinase-associated protein 1 [Source:ZFIN;Acc:ZDB-GENE-040426-1707]	21	45341786	45349548		192.33	172.65	193.87	157.22	159.04	163.79
ENSDARG00000003153	nudt21	nudix hydrolase 21 [Source:NCBI gene;Acc:394092]	18	22721551	22735002		33.61	31.32	31.14	26.61	27.23	27.42
ENSDARG00000003157	nfkbil1	nuclear factor of kappa light polypeptide gene enhancer in B-cells inhibitor-like 1 [Source:ZFIN;Acc:ZDB-GENE-040116-7]	10	8034688	8041948		2.42	1.76	1.48	2.31	2.19	2.25
ENSDARG00000003165	nr2f6b	nuclear receptor subfamily 2, group F, member 6b [Source:ZFIN;Acc:ZDB-GENE-040426-2351]	11	6115621	6134583		12.41	9.58	8.89	12.08	12.78	13.32
ENSDARG00000003167	mlst8	MTOR associated protein, LST8 homolog (S. cerevisiae) [Source:NCBI gene;Acc:792689]	12	18542954	18571563		5.84	5.28	5.24	5.49	5.56	5.55
ENSDARG00000003169	magi1b	membrane associated guanylate kinase, WW and PDZ domain containing 1b [Source:ZFIN;Acc:ZDB-GENE-030131-5110]	11	18873113	19091767		10.66	4.72	4.37	14.81	14.21	15.45
ENSDARG00000003175	stx5al	syntaxin 5A, like [Source:ZFIN;Acc:ZDB-GENE-030131-1825]	21	26536950	26554325		8.13	8.48	8.84	8.21	9.29	9.09
ENSDARG00000003181	sult1st4	sulfotransferase family 1, cytosolic sulfotransferase 4 [Source:ZFIN;Acc:ZDB-GENE-040426-2054]	8	53171002	53179683		5.7	9.22	8.62	5.45	5.4	5.53
ENSDARG00000003189	psmd1	proteasome 26S subunit, non-ATPase 1 [Source:ZFIN;Acc:ZDB-GENE-040426-810]	22	37631034	37716627		38.46	35.35	29.01	40.93	37.27	37.51
ENSDARG00000003193	rassf7b	Ras association domain family member 7b [Source:ZFIN;Acc:ZDB-GENE-030429-1]	7	49654588	49692001		3.35	3.17	2.67	2.25	2.52	2.3
ENSDARG00000003197	wdr21	WD repeat domain 21 [Source:ZFIN;Acc:ZDB-GENE-040109-2]	16	3185541	3189799		0.96	0.43	0.54	0.93	1.11	0.65
ENSDARG00000003200	foxm1	forkhead box M1 [Source:ZFIN;Acc:ZDB-GENE-040426-1275]	4	5807964	5831522		2.8	1.7	1.6	2.61	2.47	2.27
ENSDARG00000003203	rhcga	Rh family, C glycoprotein a [Source:ZFIN;Acc:ZDB-GENE-070424-81]	7	14291323	14302716		26.96	22.7	19.05	42.84	39.58	35.55
ENSDARG00000003205	ngly1	N-glycanase 1 [Source:NCBI gene;Acc:553627]	19	20761108	20777351		9.17	7.55	8.41	8.38	8.01	8.06
ENSDARG00000003206	chchd6a	coiled-coil-helix-coiled-coil-helix domain containing 6a [Source:NCBI gene;Acc:449542]	23	34047413	34185019		14.92	15.53	18.19	12.19	11.13	13.41
ENSDARG00000003207	mterf3	mitochondrial transcription termination factor 3 [Source:ZFIN;Acc:ZDB-GENE-040718-359]	19	44058557	44065581		5.15	4.54	5.3	4.44	4.84	4.37
ENSDARG00000003208	pomk	protein O-mannose kinase [Source:NCBI gene;Acc:492773]	13	9548849	9558423		2.67	1.86	1.73	2.43	2.4	2.63
ENSDARG00000003210	ano2a	anoctamin 2a [Source:ZFIN;Acc:ZDB-GENE-091204-400]	18	7283283	7304547		8.05	1.77	2.03	8.39	9.05	9.07
ENSDARG00000003213	naa15a	N(alpha)-acetyltransferase 15, NatA auxiliary subunit a [Source:ZFIN;Acc:ZDB-GENE-030131-2392]	14	46118834	46155077		28.63	26.62	23.13	28.46	28.79	27.98
ENSDARG00000003216	anxa2a	annexin A2a [Source:ZFIN;Acc:ZDB-GENE-030131-4282]	25	34013093	34027440		81.67	88.62	87.98	84.18	83.64	85.39
ENSDARG00000003217	urb2	URB2 ribosome biogenesis homolog [Source:ZFIN;Acc:ZDB-GENE-030131-2621]	13	24231327	24263682		1.81	1.77	1.64	1.76	1.78	1.7
ENSDARG00000003219	bin2a	bridging integrator 2a [Source:ZFIN;Acc:ZDB-GENE-041114-126]	23	33739215	33753361		23.6	38.95	31.21	13.27	14.56	12.95
ENSDARG00000003229	gdf9	growth differentiation factor 9 [Source:ZFIN;Acc:ZDB-GENE-050221-7]	14	22457230	22464183		0	0	0.01	0.02	0.06	0.04
ENSDARG00000003235	nup88	nucleoporin 88 [Source:ZFIN;Acc:ZDB-GENE-050522-297]	5	59089699	59111966		12.04	9.88	10.99	11.08	10.7	10.86
ENSDARG00000003244	gbp3	guanylate binding protein 3 [Source:ZFIN;Acc:ZDB-GENE-070912-176]	2	43894368	43918874		4.7	4.28	4.68	4.75	4.84	4.6
ENSDARG00000003250	pik3cd	phosphatidylinositol-4,5-bisphosphate 3-kinase, catalytic subunit delta [Source:ZFIN;Acc:ZDB-GENE-040426-1128]	23	29756603	29812667		1.71	0.72	0.63	1.36	1.36	1.52
ENSDARG00000003251	zbtb22b	zinc finger and BTB domain containing 22b [Source:ZFIN;Acc:ZDB-GENE-010110-4]	19	7001170	7007154		4.24	2.33	2.49	4.64	4.8	4.59
ENSDARG00000003257	zgc:101559	zgc:101559 [Source:ZFIN;Acc:ZDB-GENE-041114-122]	9	17429170	17434157		3.02	2.66	2.49	3.45	3.28	3.26
ENSDARG00000003259	loxa	lysyl oxidase a [Source:ZFIN;Acc:ZDB-GENE-040426-2398]	10	7738540	7756876		3.41	5.4	5.01	9.99	12.46	11.44
ENSDARG00000003269	clcn3	chloride channel 3 [Source:ZFIN;Acc:ZDB-GENE-030131-1699]	7	7511914	7643941		7.86	4.57	4.08	14.83	13.96	14.04
ENSDARG00000003270	dhps	deoxyhypusine synthase [Source:ZFIN;Acc:ZDB-GENE-040426-2001]	3	13423935	13437817		9.58	8.85	9.04	7.44	8.35	7.46
ENSDARG00000003281	pik3ip1	phosphoinositide-3-kinase interacting protein 1 [Source:NCBI gene;Acc:386643]	5	20866572	20879527		19.21	11.22	11.2	28.15	31.15	32.98
ENSDARG00000003287	wdr12	WD repeat domain 12 [Source:NCBI gene;Acc:321652]	6	9630523	9646275		12.32	14.44	13.63	9.47	8.57	8.59
ENSDARG00000003290	dab1b	DAB adaptor protein 1b [Source:ZFIN;Acc:ZDB-GENE-060421-7958]	2	1522721	1569250		3.07	0.53	0.75	4.88	4.79	5.02
ENSDARG00000003293	sox9a	SRY-box transcription factor 9a [Source:ZFIN;Acc:ZDB-GENE-001103-1]	12	1947593	1951284		12.95	11.4	10.44	41.47	42.86	45.95
ENSDARG00000003303	stc1	stanniocalcin 1 [Source:ZFIN;Acc:ZDB-GENE-060825-79]	8	50322769	50346246		0.86	0.56	0.44	1.09	1.29	1.07
ENSDARG00000003307	stx16	syntaxin 16 [Source:ZFIN;Acc:ZDB-GENE-060810-113]	6	49722970	49740750		5.72	3.57	3.79	6.34	6.55	6.24
ENSDARG00000003308	emc2	ER membrane protein complex subunit 2 [Source:NCBI gene;Acc:407073]	16	38659475	38729606		59.04	43.07	41.52	54.03	54.15	55.68
ENSDARG00000003311	pank2	pantothenate kinase 2 [Source:ZFIN;Acc:ZDB-GENE-070112-1952]	13	15182200	15226538		27.95	9.01	14.45	20.9	19.75	21.42
ENSDARG00000003313	eps8l1b	eps8 like 1b [Source:ZFIN;Acc:ZDB-GENE-050506-143]	24	38658309	38694952		2.65	2.26	1.96	2.47	2.39	2.33
ENSDARG00000003320	fam91a1	family with sequence similarity 91 member A1 [Source:NCBI gene;Acc:393413]	19	33093395	33137720		3.87	1.42	1.6	9.24	9.17	9.34
ENSDARG00000003326	cacng5a	calcium channel, voltage-dependent, gamma subunit 5a [Source:ZFIN;Acc:ZDB-GENE-120104-4]	6	21395051	21455822		2.18	0.57	0.54	1.91	1.75	1.93
ENSDARG00000003328	slf2	SMC5-SMC6 complex localization factor 2 [Source:ZFIN;Acc:ZDB-GENE-081107-61]	13	9612395	9657951		2.47	1.75	1.63	1.95	1.93	2.26
ENSDARG00000003344	trpc2b	transient receptor potential cation channel subfamily C member 2b [Source:ZFIN;Acc:ZDB-GENE-050712-3]	21	30746348	30765533		0.73	0.25	0.36	0.5	0.45	0.51
ENSDARG00000003373	commd2	COMM domain containing 2 [Source:ZFIN;Acc:ZDB-GENE-040718-118]	22	38037530	38045244		23.04	26.71	28.49	16.79	17.4	17.56
ENSDARG00000003375	gng12a	guanine nucleotide binding protein (G protein), gamma 12a [Source:ZFIN;Acc:ZDB-GENE-040426-2555]	2	10287133	10338813		6.52	8.8	7.14	8.81	9.12	9.16
ENSDARG00000003380	si:dkey-275b16.2	si:dkey-275b16.2 [Source:ZFIN;Acc:ZDB-GENE-090312-204]	13	30655172	30662403		0	0	0	0	0	0
ENSDARG00000003381	si:ch211-266g18.6	si:ch211-266g18.6 [Source:ZFIN;Acc:ZDB-GENE-131121-599]	17	15388479	15410985		4.56	5.13	5.07	3.65	3.47	3.55
ENSDARG00000003382	cwc25	CWC25 spliceosome associated protein homolog [Source:ZFIN;Acc:ZDB-GENE-040426-2817]	2	5709424	5723786		16.63	10.93	11.85	13.73	13.76	14.41
ENSDARG00000003383	asb8	ankyrin repeat and SOCS box containing 8 [Source:ZFIN;Acc:ZDB-GENE-030131-5411]	23	27495091	27506161		8.22	5.63	5.16	8.4	8.41	8.17
ENSDARG00000003395	col4a3	collagen, type IV, alpha 3 [Source:ZFIN;Acc:ZDB-GENE-070802-4]	15	36054864	36112794		1.02	0.88	0.56	1.42	1.37	1.53
ENSDARG00000003398	rbpja	recombination signal binding protein for immunoglobulin kappa J region a [Source:ZFIN;Acc:ZDB-GENE-031117-1]	1	14454663	14464020		4.99	4.29	3.96	5.74	5.7	5.33
ENSDARG00000003399	fgf8a	fibroblast growth factor 8a [Source:NCBI gene;Acc:30538]	13	28495419	28501985		2.99	3.04	2.93	3.01	3.4	3.16
ENSDARG00000003403	tenm1	teneurin transmembrane protein 1 [Source:ZFIN;Acc:ZDB-GENE-060531-26]	5	21295747	21524251		4.44	1.51	1.6	7.53	6.87	7.93
ENSDARG00000003411	foxa2	forkhead box A2 [Source:NCBI gene;Acc:30126]	17	42565998	42568498		4.03	2.58	2.36	5.06	4.9	4.89
ENSDARG00000003414	capslb	calcyphosine-like b [Source:ZFIN;Acc:ZDB-GENE-050417-298]	21	22423286	22428328		1.57	1.72	1.34	0.99	1.1	1.97
ENSDARG00000003420	chrna5	cholinergic receptor, nicotinic, alpha 5 [Source:ZFIN;Acc:ZDB-GENE-050417-440]	18	40486457	40509006		4.56	2.28	3.51	5.62	6.49	6.98
ENSDARG00000003421	igf2bp2a	insulin-like growth factor 2 mRNA binding protein 2a [Source:ZFIN;Acc:ZDB-GENE-070912-44]	9	12468500	12575776		5.41	3.69	3.56	7.71	8.51	8.44
ENSDARG00000003429	hnrnpdl	heterogeneous nuclear ribonucleoprotein D-like [Source:ZFIN;Acc:ZDB-GENE-040426-2717]	10	5017216	5024520		67.76	40.94	46.8	60.17	59.16	57.37
ENSDARG00000003444	dhrs7	dehydrogenase/reductase (SDR family) member 7 [Source:ZFIN;Acc:ZDB-GENE-060825-21]	13	31536709	31544365		16.36	20.79	20.98	12.01	13.55	13.34
ENSDARG00000003446	ippk	inositol 1,3,4,5,6-pentakisphosphate 2-kinase [Source:NCBI gene;Acc:541511]	22	10516332	10539180		5.22	4.21	3.66	4.73	4.93	4.89
ENSDARG00000003449	pde10a	phosphodiesterase 10A [Source:ZFIN;Acc:ZDB-GENE-040426-1115]	13	4505079	4620715		2.83	1.39	1.81	7.39	8.29	8.27
ENSDARG00000003462	fech	ferrochelatase [Source:ZFIN;Acc:ZDB-GENE-000928-1]	21	1647990	1670264		1.53	1.8	1.6	1.84	2.86	2.01
ENSDARG00000003469	neurod4	neuronal differentiation 4 [Source:ZFIN;Acc:ZDB-GENE-030730-1]	23	28343988	28347039		33.83	5.37	5.45	29.99	30.13	33.24
ENSDARG00000003475	sult1st5	sulfotransferase family 1, cytosolic sulfotransferase 5 [Source:ZFIN;Acc:ZDB-GENE-050809-2]	23	24489077	24493811		1.63	2.25	1.49	0.85	0.86	0.73
ENSDARG00000003486	ppp1caa	protein phosphatase 1, catalytic subunit, alpha isozyme a [Source:ZFIN;Acc:ZDB-GENE-040516-3]	3	30888126	30909487		31.98	19.86	17.08	41.24	40.3	40.08
ENSDARG00000003487	nup93	nucleoporin 93 [Source:NCBI gene;Acc:30172]	18	17537311	17580522		10.34	7.24	8.05	9.69	9.05	8.8
ENSDARG00000003495	madd	MAP-kinase activating death domain [Source:ZFIN;Acc:ZDB-GENE-030131-8076]	7	34317401	34413398		11.94	3.48	4.07	17.36	17.01	17.48
ENSDARG00000003512	anks1b	ankyrin repeat and sterile alpha motif domain containing 1B [Source:NCBI gene;Acc:565408]	4	17886838	18202291		7.71	2.99	3.25	9.44	9.23	9.59
ENSDARG00000003517	impa1	inositol monophosphatase 1 [Source:ZFIN;Acc:ZDB-GENE-040718-245]	2	26682913	26688908		15.1	19.68	17.86	12.86	11.79	12.98
ENSDARG00000003519	aprt	adenine phosphoribosyltransferase [Source:ZFIN;Acc:ZDB-GENE-040426-1492]	7	55530532	55539805		5.56	7.23	6.34	5.96	5.52	5.25
ENSDARG00000003520	tbc1d14	TBC1 domain family, member 14 [Source:ZFIN;Acc:ZDB-GENE-030131-6136]	7	39679944	39705763		3.02	2.43	2.54	2.71	2.73	2.64
ENSDARG00000003523	itln3	intelectin 3 [Source:ZFIN;Acc:ZDB-GENE-090527-4]	14	16345003	16352209		6.83	10.03	7.92	1.4	1.1	0.74
ENSDARG00000003526	psma5	proteasome 20S subunit alpha 5 [Source:ZFIN;Acc:ZDB-GENE-040625-96]	11	36342997	36350993		45.37	55.16	52.91	32.72	31.97	32.09
ENSDARG00000003527	mak16	MAK16 homolog (S. cerevisiae) [Source:NCBI gene;Acc:192312]	8	45394914	45416187		16.56	19.18	15.43	12.83	12.4	12.14
ENSDARG00000003531	tcea1	transcription elongation factor A (SII), 1 [Source:ZFIN;Acc:ZDB-GENE-030131-8049]	2	30739680	30748070		19.04	12.28	12.72	17.39	17.12	16.83
ENSDARG00000003533	col8a1b	collagen, type VIII, alpha 1b [Source:ZFIN;Acc:ZDB-GENE-060503-140]	22	28647130	28668442		0.97	1.16	0.89	1.51	2.02	1.86
ENSDARG00000003544	adarb1b	adenosine deaminase RNA specific B1b [Source:ZFIN;Acc:ZDB-GENE-030219-15]	9	45227028	45541367		2.64	1.34	1.36	5.18	4.67	4.83
ENSDARG00000003553	gnrhr2	gonadotropin releasing hormone receptor 2 [Source:ZFIN;Acc:ZDB-GENE-090128-3]	7	52364748	52369218		0.2	0.04	0.06	0.32	0.16	0.27
ENSDARG00000003564	dohh	deoxyhypusine hydroxylase/monooxygenase [Source:NCBI gene;Acc:321732]	8	20852995	20858676		19.32	21.18	22.97	16.86	16.67	16.89
ENSDARG00000003570	hsp90b1	heat shock protein 90, beta (grp94), member 1 [Source:ZFIN;Acc:ZDB-GENE-031002-1]	4	9836465	9843836		137.2	140.89	123.25	145.08	146.85	141.22
ENSDARG00000003573	dctn5	dynactin 5 [Source:ZFIN;Acc:ZDB-GENE-040718-201]	3	35550870	35554809		15.11	14.5	15.94	17.12	15.02	16.02
ENSDARG00000003576	klhl32	kelch-like family member 32 [Source:ZFIN;Acc:ZDB-GENE-070209-47]	16	32238020	32276030		6.07	2.81	3.47	4.3	4.09	4.35
ENSDARG00000003584	pla2g7	phospholipase A2, group VII (platelet-activating factor acetylhydrolase, plasma) [Source:ZFIN;Acc:ZDB-GENE-040426-2248]	20	35461736	35471079		6.92	10.68	8.73	8.44	7.29	8.43
ENSDARG00000003592	surf1	SURF1 cytochrome c oxidase assembly factor [Source:ZFIN;Acc:ZDB-GENE-041108-2]	5	33281105	33283718		10.07	9.48	10.61	7.25	6.53	6.68
ENSDARG00000003599	rpl3	ribosomal protein L3 [Source:ZFIN;Acc:ZDB-GENE-030131-1291]	3	29867213	29871364		1885.94	2072.13	2342.55	1329.82	1429.04	1436.1
ENSDARG00000003607	nr2f6a	nuclear receptor subfamily 2, group F, member 6a [Source:ZFIN;Acc:ZDB-GENE-030131-4362]	2	24374305	24386382		7.21	5.05	4.61	11.02	10.85	10.67
ENSDARG00000003615	slc26a3.2	solute carrier family 26 member 3, tandem duplicate 2 [Source:ZFIN;Acc:ZDB-GENE-041210-179]	4	18804317	18823032		8.43	14.02	10.22	0.97	1.22	1.35
ENSDARG00000003616	arih1	ariadne ubiquitin-conjugating enzyme E2 binding protein homolog 1 (Drosophila) [Source:NCBI gene;Acc:327005]	18	18879733	18901017		14.68	8.3	9.02	15.78	16.6	16.84
ENSDARG00000003631	clockb	clock circadian regulator b [Source:ZFIN;Acc:ZDB-GENE-030408-2]	1	19433004	19466453		15.49	9.46	9.17	15.06	14.85	14.97
ENSDARG00000003632	zgc:172270	zgc:172270 [Source:ZFIN;Acc:ZDB-GENE-080204-67]	11	36243774	36249902		2.62	3.78	3.4	3.11	2.85	1.85
ENSDARG00000003635	mogat3b	monoacylglycerol O-acyltransferase 3b [Source:ZFIN;Acc:ZDB-GENE-110302-2]	5	38596630	38604470		13.69	17.57	14.69	7.57	7.47	7.36
ENSDARG00000003641	tfg	trafficking from ER to golgi regulator [Source:ZFIN;Acc:ZDB-GENE-030131-8410]	9	30112423	30128760		31.87	29.78	24.87	46.45	44.22	45.28
ENSDARG00000003642	zfyve19	zinc finger, FYVE domain containing 19 [Source:ZFIN;Acc:ZDB-GENE-040718-109]	17	2108984	2126517		16.72	13.1	15.4	11.61	11.86	12.91
ENSDARG00000003655	tmem59l	transmembrane protein 59-like [Source:ZFIN;Acc:ZDB-GENE-040426-2651]	22	21128113	21150845		50.7	16.89	19.14	59.18	59.14	60.2
ENSDARG00000003675	cryl1	crystallin, lambda 1 [Source:ZFIN;Acc:ZDB-GENE-060810-7]	9	21383620	21401302		6.33	8.65	7.6	4.48	4.31	4.08
ENSDARG00000003680	runx1t1	RUNX1 partner transcriptional co-repressor 1 [Source:ZFIN;Acc:ZDB-GENE-060929-164]	19	33553586	33785914		10.1	3.34	3.39	17.51	17.56	18.66
ENSDARG00000003681	eif5	eukaryotic translation initiation factor 5 [Source:ZFIN;Acc:ZDB-GENE-030131-975]	20	18702624	18715649		41.24	39.59	40.34	32.4	31.91	31.21
ENSDARG00000003684	obsl1a	obscurin like cytoskeletal adaptor 1a [Source:ZFIN;Acc:ZDB-GENE-060503-649]	6	21157195	21189295		3.09	4.05	3.18	3.26	2.49	2.5
ENSDARG00000003693	hars	histidyl-tRNA synthetase [Source:ZFIN;Acc:ZDB-GENE-040912-152]	14	7377552	7395997		53.69	56.34	54.55	50.21	50.11	50.66
ENSDARG00000003695	vdac3	voltage-dependent anion channel 3 [Source:ZFIN;Acc:ZDB-GENE-040426-2380]	8	2412583	2434287		202.72	212.48	248.22	221.04	231.68	230.41
ENSDARG00000003697	slc5a8l	solute carrier family 5 member 8, like [Source:NCBI gene;Acc:556156]	6	49063289	49078702		1.51	1.6	1.24	3.1	3.66	3.78
ENSDARG00000003699	atp7a	ATPase copper transporting alpha [Source:ZFIN;Acc:ZDB-GENE-060825-45]	14	22397251	22443502		2.6	2.39	2.02	5.46	5.59	5.56
ENSDARG00000003701	cldng	claudin g [Source:NCBI gene;Acc:81586]	1	33645044	33647169		0.19	0.17	0.38	0.36	1.03	0.33
ENSDARG00000003705	prnprs3	prion protein, related sequence 3 [Source:ZFIN;Acc:ZDB-GENE-041221-3]	25	3677650	3685280		30.58	17.2	18.37	42.37	44.4	43.98
ENSDARG00000003706	ryr2b	ryanodine receptor 2b (cardiac) [Source:ZFIN;Acc:ZDB-GENE-061226-3]	17	20009132	20118244		0.13	0.11	0.07	0.39	0.36	0.34
ENSDARG00000003732	mitfa	melanocyte inducing transcription factor a [Source:ZFIN;Acc:ZDB-GENE-990910-11]	6	43390387	43433277		1.4	1.46	1.58	1.36	1.62	1.68
ENSDARG00000003749	polb	polymerase (DNA directed), beta [Source:NCBI gene;Acc:554204]	8	2456854	2469761		14.12	11.19	11.98	12.26	11.48	11.8
ENSDARG00000003750	pfas	phosphoribosylformylglycinamidine synthase [Source:ZFIN;Acc:ZDB-GENE-030131-4420]	20	25445826	25473275		10.48	11.61	10.21	9.63	9.7	9.06
ENSDARG00000003751	lats1	large tumor suppressor kinase 1 [Source:ZFIN;Acc:ZDB-GENE-050523-2]	20	1240461	1268869		3.39	1.84	1.47	4.27	4.04	4.07
ENSDARG00000003754	tspan2a	tetraspanin 2a [Source:ZFIN;Acc:ZDB-GENE-050522-511]	6	49107048	49159207		14.58	8.42	9.02	14.98	15.34	16.86
ENSDARG00000003757	cnpy1	canopy FGF signaling regulator 1 [Source:NCBI gene;Acc:402819]	2	29985664	29996036		8.47	10.93	9.31	7.69	7.81	7.35
ENSDARG00000003769	tada2b	transcriptional adaptor 2B [Source:NCBI gene;Acc:554156]	7	39738505	39743452		2.87	1.73	1.86	3.33	3.34	3.02
ENSDARG00000003776	pip4k2aa	phosphatidylinositol-5-phosphate 4-kinase, type II, alpha a [Source:ZFIN;Acc:ZDB-GENE-080721-18]	24	17068724	17127833		4.57	2.65	2.78	7.06	7.16	7
ENSDARG00000003779	ctnnd2b	catenin (cadherin-associated protein), delta 2b [Source:ZFIN;Acc:ZDB-GENE-070912-532]	2	30554256	30693742		5.19	1.52	1.5	8.96	8.66	8.91
ENSDARG00000003789	dennd5b	DENN/MADD domain containing 5B [Source:NCBI gene;Acc:323528]	4	16649542	16706978		15.7	11.7	9.87	19.64	19.5	19.21
ENSDARG00000003795	idh2	isocitrate dehydrogenase (NADP(+)) 2 [Source:ZFIN;Acc:ZDB-GENE-031118-95]	18	26096814	26101800		643.61	682.47	797.23	548.31	566.14	560.33
ENSDARG00000003797	asb2a.1	ankyrin repeat and SOCS box containing 2a, tandem duplicate 1 [Source:ZFIN;Acc:ZDB-GENE-030131-5355]	20	27295701	27311675		1.82	2.86	2.59	2.67	2.37	2.11
ENSDARG00000003803	LHX3	LIM homeobox 3 [Source:HGNC Symbol;Acc:HGNC:6595]	5	71802014	71819807	LHX3	3.91	0.52	1.06	6.78	6.7	7.06
ENSDARG00000003808	aqp3a	aquaporin 3a [Source:ZFIN;Acc:ZDB-GENE-040426-2826]	5	43006422	43011814		129.07	159.88	139.75	107.04	102.71	92.16
ENSDARG00000003811	plxnb2a	plexin b2a [Source:ZFIN;Acc:ZDB-GENE-030131-8917]	4	14449233	14531687		9.15	7.3	4.63	10.57	10.15	10.27
ENSDARG00000003818	prkag1	protein kinase, AMP-activated, gamma 1 non-catalytic subunit [Source:ZFIN;Acc:ZDB-GENE-040426-2188]	6	2206495	2222707		12.81	11.89	10.32	13.52	13.63	13.78
ENSDARG00000003820	nr1d2a	nuclear receptor subfamily 1, group D, member 2a [Source:ZFIN;Acc:ZDB-GENE-040504-1]	16	50089417	50111057		13.87	5.05	7.84	35.81	41.8	35.32
ENSDARG00000003822	ankrd16	ankyrin repeat domain 16 [Source:ZFIN;Acc:ZDB-GENE-041210-253]	4	11458078	11462702		4.48	4.53	4.52	3.11	2.71	2.96
ENSDARG00000003827	snapc1b	small nuclear RNA activating complex, polypeptide 1b [Source:ZFIN;Acc:ZDB-GENE-040426-716]	13	37586491	37600600		4.39	4.97	4.22	3.52	3.73	3.99
ENSDARG00000003829	galnt2	UDP-N-acetyl-alpha-D-galactosamine:polypeptide N-acetylgalactosaminyltransferase 2 [Source:ZFIN;Acc:ZDB-GENE-041111-110]	13	24068577	24218795		5.81	4.29	3.99	8.34	8.39	8.26
ENSDARG00000003835	stom	stomatin [Source:ZFIN;Acc:ZDB-GENE-980526-486]	21	17541634	17570251		29.25	29.22	27.92	29.79	30.08	28.89
ENSDARG00000003841	usb1	U6 snRNA biogenesis 1 [Source:NCBI gene;Acc:445066]	25	13677299	13686751		3.04	2.73	2.45	2.98	3.36	3.38
ENSDARG00000003845	chm	CHM Rab escort protein [Source:ZFIN;Acc:ZDB-GENE-030318-2]	21	34686764	34793909		12.52	11.01	11.82	10.23	9.86	10.09
ENSDARG00000003846	mrpl21	mitochondrial ribosomal protein L21 [Source:ZFIN;Acc:ZDB-GENE-061027-183]	7	72577898	72585111		21.38	22.39	20.8	13.79	12.92	14.1
ENSDARG00000003854	acsl1b	acyl-CoA synthetase long chain family member 1b [Source:ZFIN;Acc:ZDB-GENE-040801-88]	14	937194	970853		6.36	8.21	5.31	5.83	5.87	5.02
ENSDARG00000003860	cbx3a	chromobox homolog 3a (HP1 gamma homolog, Drosophila) [Source:ZFIN;Acc:ZDB-GENE-030131-1158]	19	18131512	18136410		25.26	22.2	22.94	20.38	18.96	20.48
ENSDARG00000003865	pard6b	par-6 partitioning defective 6 homolog beta (C. elegans) [Source:ZFIN;Acc:ZDB-GENE-090312-133]	23	19066696	19103855		2.29	2.13	1.39	3.65	3.46	3.41
ENSDARG00000003866	tm9sf2	transmembrane 9 superfamily member 2 [Source:ZFIN;Acc:ZDB-GENE-030131-6302]	1	29798140	29824375		39.84	32.31	29.74	46.43	45.74	44.19
ENSDARG00000003867	cdk20	cyclin-dependent kinase 20 [Source:NCBI gene;Acc:494112]	8	7494941	7504201		2.83	1.01	1.34	2.97	3.68	3.16
ENSDARG00000003869	decr1	2,4-dienoyl CoA reductase 1, mitochondrial [Source:ZFIN;Acc:ZDB-GENE-040718-142]	16	36748538	36760156		12.48	14.28	13.48	11.73	10.05	9.86
ENSDARG00000003872	ccdc102a	coiled-coil domain containing 102A [Source:NCBI gene;Acc:325319]	7	28724910	28837318		11.03	7.53	7.79	12.6	13.5	13.54
ENSDARG00000003877	pbrm1	polybromo 1 [Source:ZFIN;Acc:ZDB-GENE-080926-4]	11	3909756	3959889		6.99	2.02	2.55	5.95	5.84	6.36
ENSDARG00000003899	zdhhc23b	zinc finger DHHC-type containing 23b [Source:ZFIN;Acc:ZDB-GENE-040808-13]	24	21122836	21131607		1.37	1.83	1.86	1.11	1.35	1.65
ENSDARG00000003900	gpr61l	G protein-coupled receptor 61-like [Source:ZFIN;Acc:ZDB-GENE-131127-443]	6	42031836	42036685		0.24	0.17	0.21	0.38	0.26	0.32
ENSDARG00000003902	ctsl.1	cathepsin L.1 [Source:ZFIN;Acc:ZDB-GENE-040718-61]	17	27456804	27461321		32.84	24.57	22.84	23.65	22.06	21.24
ENSDARG00000003903	hapln2	hyaluronan and proteoglycan link protein 2 [Source:ZFIN;Acc:ZDB-GENE-030131-8089]	16	29303971	29308947		0.42	0.84	0.62	0.81	0.69	0.54
ENSDARG00000003904	sycp1	synaptonemal complex protein 1 [Source:ZFIN;Acc:ZDB-GENE-040910-6]	6	49052741	49061086		0.17	0.11	0.09	0.1	0.07	0.09
ENSDARG00000003909	ftr01	finTRIM family, member 1 [Source:ZFIN;Acc:ZDB-GENE-070705-491]	2	11670270	11677705		1.45	2.34	1.7	0.38	0.25	0.26
ENSDARG00000003910	med13b	mediator complex subunit 13b [Source:NCBI gene;Acc:564707]	15	20060643	20125331		5.18	3.01	2.67	8.03	7.31	7.94
ENSDARG00000003920	setb	SET nuclear proto-oncogene b [Source:ZFIN;Acc:ZDB-GENE-030131-433]	21	13224239	13231101		55.66	50.49	46.56	45.65	44.54	42.61
ENSDARG00000003925	cx28.6	connexin 28.6 [Source:ZFIN;Acc:ZDB-GENE-040406-4]	17	14711765	14712599		1.57	1.65	1.31	1.21	0.59	0.77
ENSDARG00000003927	cldn12	claudin 12 [Source:ZFIN;Acc:ZDB-GENE-010328-14]	16	43227724	43233509		8.87	6.51	6.12	11.5	12.31	11.9
ENSDARG00000003931	cndp2	carnosine dipeptidase 2 [Source:ZFIN;Acc:ZDB-GENE-030131-5499]	19	20801060	20823099		48.9	79.98	63.05	22.03	22.84	23.04
ENSDARG00000003934	nxnl2	nucleoredoxin like 2 [Source:ZFIN;Acc:ZDB-GENE-041212-55]	10	2582254	2585625		8.53	4.11	4.67	7.3	8.13	7.29
ENSDARG00000003938	rpa1	replication protein A1 [Source:NCBI gene;Acc:327491]	15	26498339	26538989		38.56	38.63	38.52	23.34	23.01	21.85
ENSDARG00000003940	mtfr1l	mitochondrial fission regulator 1-like [Source:NCBI gene;Acc:334296]	17	24718272	24727255		11.92	8.35	8.49	13.77	13.13	12.22
ENSDARG00000003941	rrs1	ribosome biogenesis regulator 1 homolog [Source:ZFIN;Acc:ZDB-GENE-030131-9345]	24	23837519	23839647		14.08	17.46	17.33	8.32	8.49	8.64
ENSDARG00000003944	nmur1b	neuromedin U receptor 1b [Source:ZFIN;Acc:ZDB-GENE-131121-154]	2	13376099	13382726		0.21	0.28	0.13	0.19	0.24	0.08
ENSDARG00000003952	pfn2	profilin 2 [Source:ZFIN;Acc:ZDB-GENE-040115-4]	22	35179575	35194243		119.83	128.62	105.01	156.1	153.75	149.58
ENSDARG00000003961	parp3	poly (ADP-ribose) polymerase family, member 3 [Source:ZFIN;Acc:ZDB-GENE-030131-7435]	6	42038358	42049350		7.3	7.96	6.4	5.52	6.03	5.62
ENSDARG00000003963	traf4a	tnf receptor-associated factor 4a [Source:ZFIN;Acc:ZDB-GENE-040308-1]	15	15516612	15593263		12.05	6.82	5.8	12.38	12.23	12.32
ENSDARG00000003965	tlx1	T cell leukemia homeobox 1 [Source:ZFIN;Acc:ZDB-GENE-021021-2]	13	28267997	28272299		0.46	0.19	0.24	0.72	0.61	0.79

ENSDARG00000003968	larp6b	La ribonucleoprotein 6, translational regulator b [Source:ZFIN;Acc:ZDB-GENE-041008-244]		11	6446235	6452492		0.05	0	0.01	0.03	0.06	0.03
ENSDARG00000003970	trim35-13	tripartite motif containing 35-13 [Source:ZFIN;Acc:ZDB-GENE-110920-5]		3	52607039	52614747		2.61	3.71	3.3	2.62	2.51	2.7
ENSDARG00000003971	isl2a	ISL LIM homeobox 2a [Source:NCBI gene;Acc:30157]		25	32746014	32751982		9.7	4.8	5.36	15.76	15.14	17.85
ENSDARG00000003973	chordc1a	cysteine and histidine-rich domain (CHORD) containing 1a [Source:ZFIN;Acc:ZDB-GENE-050626-79]		15	43961814	43978141		10.91	11.68	9.58	8.06	9.52	7.81
ENSDARG00000003974	spoplb	speckle type BTB/POZ protein like b [Source:NCBI gene;Acc:558170]		11	33818179	33842715		12.21	7.92	8.46	10.91	12.2	11.13
ENSDARG00000003977	zgc:153896	zgc:153896 [Source:ZFIN;Acc:ZDB-GENE-060825-119]		20	25879826	25887231		3.15	6.49	4.73	0.72	0.86	0.78
ENSDARG00000003984	LTN1	si:dkey-175g6.5 [Source:ZFIN;Acc:ZDB-GENE-091204-127]		10	23099365	23143104	LTN1	3.05	2.75	2.57	2.78	2.79	2.87
ENSDARG00000003989	crhr1	corticotropin releasing hormone receptor 1 [Source:ZFIN;Acc:ZDB-GENE-081231-1]		3	21669545	21889616		0.61	0.16	0.19	1.04	1.26	1.1
ENSDARG00000003991	fhl2b	four and a half LIM domains 2b [Source:ZFIN;Acc:ZDB-GENE-041010-121]		6	15071399	15101477		4.84	6.28	6.18	4.36	4.08	4.3
ENSDARG00000003994	syt9a	synaptotagmin IXa [Source:ZFIN;Acc:ZDB-GENE-031002-18]		18	28988373	29060842		4.33	0.78	1.3	4.14	4.34	4.39
ENSDARG00000003998	phyhipla	phytanoyl-CoA 2-hydroxylase interacting protein-like a [Source:ZFIN;Acc:ZDB-GENE-061215-64]		17	20963477	20979077		5.02	1.16	2.13	5.23	5.7	5.4
ENSDARG00000004015	adcyap1a	adenylate cyclase activating polypeptide 1a [Source:ZFIN;Acc:ZDB-GENE-020809-4]		7	71547747	71556734		2.48	2.4	2.47	2.67	2.47	2.47
ENSDARG00000004017	spag1a	sperm associated antigen 1a [Source:NCBI gene;Acc:564953]		19	11984725	12018446		7.79	5.53	4.89	5.84	6.87	6.4
ENSDARG00000004018	CHST13	si:ch211-236h17.3 [Source:ZFIN;Acc:ZDB-GENE-030131-738]		23	34990693	35025117	CHST13	1.94	3.06	2.77	5.09	4.49	5.08
ENSDARG00000004023	isl1	ISL LIM homeobox 1 [Source:NCBI gene;Acc:30147]		5	40728613	40734045		26.89	6.96	8.08	33.19	31.6	35.27
ENSDARG00000004034	arhgdig	Rho GDP dissociation inhibitor (GDI) gamma [Source:ZFIN;Acc:ZDB-GENE-040426-1493]		3	27713610	27769232		29.1	27.55	24.48	34.02	33.59	31.83
ENSDARG00000004037	cog2	component of oligomeric golgi complex 2 [Source:ZFIN;Acc:ZDB-GENE-040426-2671]		13	23996070	24018916		5.7	4.98	5.14	5.85	6.7	6.53
ENSDARG00000004046	phf6	PHD finger protein 6 [Source:ZFIN;Acc:ZDB-GENE-030131-5278]		14	31488579	31507744		8.78	7.08	8.49	7.6	7.46	8.44
ENSDARG00000004049	marcksa	myristoylated alanine-rich protein kinase C substrate a [Source:ZFIN;Acc:ZDB-GENE-050522-145]		20	3312859	3319736		13.22	5.36	6.6	14.29	13.46	14.32
ENSDARG00000004051	CR354540.1	cytochrome P450, family 7, subfamily A, polypeptide 1b [Source:NCBI gene;Acc:559097]		7	58270474	58289847		0.5	0.74	0.52	0.54	0.47	0.41
ENSDARG00000004055	uhrf1bp1l	UHRF1 binding protein 1-like [Source:ZFIN;Acc:ZDB-GENE-041210-177]		4	18207655	18281160		5.25	2.44	2.37	6.22	5.51	5.13
ENSDARG00000004059	galk2	galactokinase 2 [Source:ZFIN;Acc:ZDB-GENE-041114-143]		25	32390794	32400209		2.1	1.97	1.88	1.88	2.1	1.95
ENSDARG00000004060	bhlhe40	basic helix-loop-helix family, member e40 [Source:ZFIN;Acc:ZDB-GENE-030131-3133]		11	35759652	35763323		42.29	17.93	13.66	42.31	43.56	43.73
ENSDARG00000004074	bach2b	BTB and CNC homology 1, basic leucine zipper transcription factor 2b [Source:ZFIN;Acc:ZDB-GENE-041001-139]		20	23972948	24122881		3.44	1.16	1.37	6.8	5.9	6.26
ENSDARG00000004078	acsl4a	acyl-CoA synthetase long chain family member 4a [Source:ZFIN;Acc:ZDB-GENE-040426-1565]		14	28438947	28459446		17.59	16.85	15.91	21.81	23.23	21.9
ENSDARG00000004082	cx39.9	connexin 39.9 [Source:ZFIN;Acc:ZDB-GENE-040406-2]		5	23630384	23635908		2.99	5.05	4.04	5.14	5	5.14
ENSDARG00000004094	acsbg2	acyl-CoA synthetase bubblegum family member 2 [Source:ZFIN;Acc:ZDB-GENE-030131-7099]		8	20140321	20192151		18.81	10.59	8.05	21.52	19.24	20.84
ENSDARG00000004105	tie1	tyrosine kinase with immunoglobulin-like and EGF-like domains 1 [Source:NCBI gene;Acc:30746]		6	33979523	34008901		3.15	3.33	2.86	5.32	5.61	6
ENSDARG00000004109	zgc:162154	zgc:162154 [Source:ZFIN;Acc:ZDB-GENE-070424-57]	KN149697.1		4287	11359		0	0	0	0	0	0
ENSDARG00000004111	esr1	estrogen receptor 1 [Source:NCBI gene;Acc:259252]		20	26359756	26383375		0.14	0.05	0.04	0.1	0.27	0.11
ENSDARG00000004114	tti1	TELO2 interacting protein 1 [Source:ZFIN;Acc:ZDB-GENE-080819-3]		23	43424518	43448409		2.02	1.44	1.68	2.08	2.05	2.13
ENSDARG00000004115	mgat4b	alpha-1,3-mannosyl-glycoprotein 4-beta-N-acetylglucosaminyltransferase B [Source:NCBI gene;Acc:431727]		14	16483926	16754262		6.35	4.14	3.97	6.34	6.55	6.12
ENSDARG00000004130	mvk	mevalonate kinase [Source:ZFIN;Acc:ZDB-GENE-041114-37]		5	19494198	19509695		3.74	3.53	3.14	2	1.79	1.69
ENSDARG00000004131	rttn	rotatin [Source:ZFIN;Acc:ZDB-GENE-090609-5]		24	15130979	15263142		0.49	0.38	0.37	0.61	0.55	0.52
ENSDARG00000004132	usp19	ubiquitin specific peptidase 19 [Source:ZFIN;Acc:ZDB-GENE-030131-3055]		23	20145300	20177527		9.89	6.91	5.58	10.84	10.86	10.41
ENSDARG00000004141		zgc:92630 [Source:ZFIN;Acc:ZDB-GENE-040718-449]		15	15415623	15437530		13.83	24.68	21.57	4.9	4.46	4.38
ENSDARG00000004150	grm2a	glutamate receptor, metabotropic 2a [Source:ZFIN;Acc:ZDB-GENE-030131-8097]		6	42051480	42112191		5.46	1.37	2.05	4.72	4.57	5.15
ENSDARG00000004151	rab23	RAB23, member RAS oncogene family [Source:ZFIN;Acc:ZDB-GENE-050522-328]		13	1381953	1395002		9.06	6.22	7.1	10.24	10.24	9.98
ENSDARG00000004154	orai1b	ORAI calcium release-activated calcium modulator 1b [Source:NCBI gene;Acc:402892]		10	41914084	41921417		3.8	2.8	2.65	4.34	4.1	3.15
ENSDARG00000004158	tmem63c	transmembrane protein 63C [Source:ZFIN;Acc:ZDB-GENE-120928-2]		17	44780166	44923661		2.98	0.89	1.28	3.27	3.47	3.28
ENSDARG00000004160	reep3b	receptor accessory protein 3b [Source:NCBI gene;Acc:393130]		12	8822717	8882186		23.8	28.95	25.6	23.77	22.63	21.68
ENSDARG00000004161	ik	IK cytokine [Source:ZFIN;Acc:ZDB-GENE-030131-960]		21	43987153	44037106		56.27	43.82	45.79	41.01	43.42	42.73
ENSDARG00000004167	zgc:110366	zgc:110366 [Source:ZFIN;Acc:ZDB-GENE-050417-302]		2	7132281	7148449		3.68	3.86	3.87	2.55	2.68	2.29
ENSDARG00000004169	stmn1a	stathmin 1a [Source:ZFIN;Acc:ZDB-GENE-031006-14]		19	17205143	17210928		12.71	13.3	10.6	5.13	5.03	5.13
ENSDARG00000004171	rabac1	Rab acceptor 1 (prenylated) [Source:ZFIN;Acc:ZDB-GENE-040625-146]		16	10828787	10837245		10.21	10.86	9.9	15.16	14.7	14.65
ENSDARG00000004173	copa	coatomer protein complex, subunit alpha [Source:ZFIN;Acc:ZDB-GENE-020905-2]		2	37176814	37206080		43.33	35.82	30.87	50.09	51.25	50.23
ENSDARG00000004174	cnot1	CCR4-NOT transcription complex, subunit 1 [Source:NCBI gene;Acc:448949]		25	17313568	17346929		18.68	11.07	8.84	27.51	25.75	27.01
ENSDARG00000004176	ulk4	unc-51 like kinase 4 [Source:ZFIN;Acc:ZDB-GENE-100922-243]		16	5898878	6182429		0.62	0.45	0.36	0.67	0.65	0.71
ENSDARG00000004177	fam169ab	family with sequence similarity 169 member Ab [Source:ZFIN;Acc:ZDB-GENE-091118-95]		21	15870752	15898689		12.9	11.67	11.06	17.08	20.26	19.99
ENSDARG00000004184	nf1b	neurofibromin 1b [Source:ZFIN;Acc:ZDB-GENE-091111-4]		10	37268854	37394957		3.14	1.75	1.61	4.79	4.85	4.79
ENSDARG00000004187	zgc:122979	zgc:122979 [Source:ZFIN;Acc:ZDB-GENE-051127-45]		21	5169154	5174054		8.81	2.2	3.21	10.99	11.23	11.89
ENSDARG00000004189	cbx1a	chromobox homolog 1a (HP1 beta homolog Drosophila) [Source:ZFIN;Acc:ZDB-GENE-030131-4945]		3	24050043	24071582		41.48	36.72	37.08	35.03	33	33.85
ENSDARG00000004196	tnfsf10l	TNF superfamily member 10, like [Source:ZFIN;Acc:ZDB-GENE-010801-1]		7	22911610	22956889		8.26	7.51	4.81	8.6	7.85	7.01
ENSDARG00000004199	spam1	sperm adhesion molecule 1 [Source:ZFIN;Acc:ZDB-GENE-050506-63]		25	27531874	27541621		1.43	2.75	2.76	1.03	0.87	0.75
ENSDARG00000004204	efhc2	EF-hand domain (C-terminal) containing 2 [Source:NCBI gene;Acc:567323]		9	33758720	33781845		1	0.94	1.42	1.08	1.35	1.49
ENSDARG00000004211	ccdc106a	coiled-coil domain containing 106a [Source:ZFIN;Acc:ZDB-GENE-050220-4]		16	17639896	17660594		12.9	9.23	9.51	13.96	13.47	13.67
ENSDARG00000004218	rnd1b	Rho family GTPase 1b [Source:ZFIN;Acc:ZDB-GENE-060315-7]		23	26979174	27006062		5.82	1.96	2.1	7.75	7.61	8.16
ENSDARG00000004219	slc6a3	solute carrier family 6 member 3 [Source:ZFIN;Acc:ZDB-GENE-010316-1]		16	248239	263658		3.76	0.84	1.27	3.23	3.6	3.92
ENSDARG00000004227	pde3a	phosphodiesterase 3A, cGMP-inhibited [Source:ZFIN;Acc:ZDB-GENE-041210-23]		4	2752852	2868112		3.85	1.98	1.9	8.94	9.51	9.11
ENSDARG00000004232	dlb	deltaB [Source:NCBI gene;Acc:30141]		5	36693859	36701760		10.36	5	6.32	8.06	7.45	8.05
ENSDARG00000004243	asic4b	acid-sensing (proton-gated) ion channel family member 4b [Source:NCBI gene;Acc:407667]		6	13606410	13688991		1.6	0.37	0.43	1.07	1	1
ENSDARG00000004246	slit2	slit homolog 2 (Drosophila) [Source:ZFIN;Acc:ZDB-GENE-010306-3]		1	23783349	23912788		6.44	3.15	2.52	9.4	8.87	8.83
ENSDARG00000004251	dhfr	dihydrofolate reductase [Source:ZFIN;Acc:ZDB-GENE-010406-5]		5	26138313	26146488		7.15	9.92	8.68	4.54	4.15	3.62
ENSDARG00000004256	wnt11f2	wnt 11, family member 2 [Source:NCBI gene;Acc:791595]		5	36513605	36520117		0.69	0.67	0.77	1.05	1.16	1.4
ENSDARG00000004260	cul5b	cullin 5b [Source:ZFIN;Acc:ZDB-GENE-091113-45]		21	22124736	22142972		1.53	0.7	1.03	1.39	1.37	1.39
ENSDARG00000004261	tmed9	transmembrane p24 trafficking protein 9 [Source:ZFIN;Acc:ZDB-GENE-050417-434]		14	26404640	26411918		36.52	46.25	39.57	48.47	45.03	44.93
ENSDARG00000004262	cyp46a1.2	cytochrome P450, family 46, subfamily A, polypeptide 1, tandem duplicate 2 [Source:ZFIN;Acc:ZDB-GENE-040426-1184]		20	5237286	5254610		0.81	1.43	1.11	1.3	1.34	0.98
ENSDARG00000004270	atl3	atlastin 3 [Source:ZFIN;Acc:ZDB-GENE-041010-109]		14	30773764	30794824		10.08	11.01	9.95	10.61	10.69	10.88
ENSDARG00000004274	zgc:112496	zgc:112496 [Source:ZFIN;Acc:ZDB-GENE-050320-25]		24	37631632	37640705		4.43	4.92	5.24	2.11	2.3	2.01
ENSDARG00000004280	seh1l	SEH1-like (S. cerevisiae) [Source:NCBI gene;Acc:334749]		19	12406583	12431988		11.08	10.09	9.49	10.05	10.49	10.7
ENSDARG00000004282	zgc:77375	zgc:77375 [Source:ZFIN;Acc:ZDB-GENE-040426-1827]		11	457423	472547		10.45	7.09	6.2	15.43	16.35	16.95
ENSDARG00000004291	hecw1b	HECT, C2 and WW domain containing E3 ubiquitin protein ligase 1b [Source:ZFIN;Acc:ZDB-GENE-060503-2]		2	49884696	49978227		5	1.18	1.54	7.25	7.23	7.64
ENSDARG00000004296	ambp	alpha-1-microglobulin/bikunin precursor [Source:ZFIN;Acc:ZDB-GENE-040426-1608]		10	10801564	10824286		58.3	91.34	103.53	64.29	68.46	70.5
ENSDARG00000004297	irf2bpl	interferon regulatory factor 2 binding protein-like [Source:ZFIN;Acc:ZDB-GENE-040426-1968]		17	11327164	11329959		23.91	11.95	11.94	49.79	47.73	49.68
ENSDARG00000004301	rhogb	ras homolog family member Gb [Source:ZFIN;Acc:ZDB-GENE-030131-8877]		21	30937690	30951909		5.66	7.64	6.86	5.81	5.86	6.01
ENSDARG00000004302	slc45a1	solute carrier family 45 member 1 [Source:ZFIN;Acc:ZDB-GENE-070112-2152]		11	40649412	40674070		4.48	1.45	1.6	6.53	6.84	6.12
ENSDARG00000004305	vangl1	VANGL planar cell polarity protein 1 [Source:ZFIN;Acc:ZDB-GENE-040621-2]		9	33009284	33055440		11.81	3.74	3.57	12.52	12.76	13.2
ENSDARG00000004306	klhl18	kelch-like family member 18 [Source:ZFIN;Acc:ZDB-GENE-030131-5785]		2	32743807	32756706		5.54	3.08	3.13	6.23	6.6	6.26
ENSDARG00000004307	lypd6	LY6/PLAUR domain containing 6 [Source:NCBI gene;Acc:447932]		9	23071149	23118350		6.67	4.87	5.44	9.27	9.15	8.83
ENSDARG00000004308	fam8a1b	family with sequence similarity 8 member A1b [Source:ZFIN;Acc:ZDB-GENE-050410-14]		16	25593339	25597909		7.69	6.64	7.47	7.07	6.7	7.49
ENSDARG00000004311	ldlrap1a	low density lipoprotein receptor adaptor protein 1a [Source:ZFIN;Acc:ZDB-GENE-030328-13]		19	29337789	29363779		6.59	8.86	7.6	8.44	9.19	8.94
ENSDARG00000004318	cbwd	COBW domain containing [Source:ZFIN;Acc:ZDB-GENE-040426-2388]		8	30425700	30450072		8.61	10.48	9.42	7.41	6.61	6.09
ENSDARG00000004322	ush1ga	Usher syndrome 1Ga (autosomal recessive) [Source:ZFIN;Acc:ZDB-GENE-131120-173]		3	56882832	56896702		1.06	0.27	0.34	0.77	0.72	0.77
ENSDARG00000004325	casp9	caspase 9, apoptosis-related cysteine peptidase [Source:ZFIN;Acc:ZDB-GENE-030825-5]		23	24973773	24985272		4.55	3.85	4.46	5.7	6.3	7.21
ENSDARG00000004328	LIN28A	si:ch1073-284b18.2 [Source:ZFIN;Acc:ZDB-GENE-110914-157]		16	4654333	4686245	LIN28A	0.23	0.38	0.38	0.66	0.92	0.54
ENSDARG00000004336	exoc4	exocyst complex component 4 [Source:ZFIN;Acc:ZDB-GENE-041210-112]		4	15605844	15816540		11.44	7.6	7.69	11.33	12.07	11.92
ENSDARG00000004343	nelfe	negative elongation factor complex member E [Source:ZFIN;Acc:ZDB-GENE-040718-69]		19	26922780	26932829		11.69	10.49	12.26	7.82	7.97	7.89
ENSDARG00000004358	gnb3a	guanine nucleotide binding protein (G protein), beta polypeptide 3a [Source:ZFIN;Acc:ZDB-GENE-040718-134]		16	12022543	12049762		31.47	4.69	4.79	43.46	41.09	47.88
ENSDARG00000004363	lhfpl6	LHFPL tetraspan subfamily member 6 [Source:NCBI gene;Acc:494110]		10	31938796	32041846		3.17	3.48	3.15	4.86	5.33	5.14
ENSDARG00000004372	fmnl3	formin-like 3 [Source:NCBI gene;Acc:407694]		23	25354856	25448872		2.76	2.87	2.26	4.55	4.39	4.58
ENSDARG00000004377	ppp1r13ba	protein phosphatase 1, regulatory subunit 13Ba [Source:ZFIN;Acc:ZDB-GENE-030131-9805]		17	14614145	14691576		6.29	4.29	3.9	7.47	7.15	7.47
ENSDARG00000004378	yrk	Yes-related kinase [Source:ZFIN;Acc:ZDB-GENE-030131-9517]		19	43359075	43432415		3.93	4.19	3.75	5.16	5.13	4.58
ENSDARG00000004386	ELAPOR2	si:dkey-153k10.9 [Source:ZFIN;Acc:ZDB-GENE-030131-3893]		4	9669717	9690419	ELAPOR2	15.66	3.8	5.17	18.63	18.43	20.31
ENSDARG00000004392	hdr	hematopoietic death receptor [Source:ZFIN;Acc:ZDB-GENE-030826-5]		5	27404946	27415081		5.37	7.89	6.18	6.12	5.73	5.3
ENSDARG00000004396	b3gnt5b	UDP-GlcNAc:betaGal beta-1,3-N-acetylglucosaminyltransferase 5b [Source:NCBI gene;Acc:791470]		2	7749394	7757273		3.11	3.6	3.19	1.67	1.5	1.63
ENSDARG00000004402	elovl6	ELOVL fatty acid elongase 6 [Source:NCBI gene;Acc:317738]		14	36231126	36268710		18.21	9.18	11.04	16.68	16.67	16.08
ENSDARG00000004405	snx10a	sorting nexin 10a [Source:NCBI gene;Acc:403027]		19	18118321	18130567		0.65	0.6	0.54	1.46	1.51	1.96
ENSDARG00000004406	spra	sepiapterin reductase a [Source:ZFIN;Acc:ZDB-GENE-050522-412]		5	72129708	72136548		4.4	4.34	4.14	5.68	5.55	5.06
ENSDARG00000004415	tcf7l2	transcription factor 7 like 2 [Source:ZFIN;Acc:ZDB-GENE-991110-8]		12	30977736	31103906		6	2.59	2.48	11.8	11.13	11.65
ENSDARG00000004427	dpy30	dpy-30 histone methyltransferase complex regulatory subunit [Source:ZFIN;Acc:ZDB-GENE-040718-136]		1	51039558	51042328		37.56	39.11	40.57	27.22	25.62	28.32
ENSDARG00000004433	btk	Bruton agammaglobulinemia tyrosine kinase [Source:ZFIN;Acc:ZDB-GENE-070531-1]		14	38891722	38929885		1.08	0.93	0.93	1.59	1.41	1.08
ENSDARG00000004436	fam92a1	family with sequence similarity 92 member A1 [Source:NCBI gene;Acc:445210]		19	33258572	33275035		3.24	4.05	3.93	3.41	3.3	3.59
ENSDARG00000004445	grm5a	glutamate receptor, metabotropic 5a [Source:ZFIN;Acc:ZDB-GENE-090821-9]		10	25219728	25272236		1.81	0.4	0.51	1.83	1.9	1.8
ENSDARG00000004447	aplnr2	apelin receptor 2 [Source:ZFIN;Acc:ZDB-GENE-030131-4347]		7	38685545	38689562		0.69	0.92	0.72	3.27	4.27	4.53
ENSDARG00000004451	tnfrsfa	tumor necrosis factor receptor superfamily, member a [Source:ZFIN;Acc:ZDB-GENE-010802-1]		21	16172931	16219400		7.15	7.9	7.39	11.05	10.46	11.56
ENSDARG00000004452	tardbpl	TAR DNA binding protein, like [Source:ZFIN;Acc:ZDB-GENE-031030-1]		23	29357790	29362845		52.48	32.57	39.3	62.73	61.37	62.73
ENSDARG00000004455	p2rx5	purinergic receptor P2X, ligand-gated ion channel, 5 [Source:ZFIN;Acc:ZDB-GENE-030319-5]		5	42097608	42120927		2.85	4.59	4.82	2.86	2.79	2.81
ENSDARG00000004459	unc119.2	unc-119 lipid binding chaperone B homolog 2 [Source:ZFIN;Acc:ZDB-GENE-030131-7635]		3	32594357	32619634		2.34	1.07	0.98	0.75	0.74	1.04
ENSDARG00000004460	desi2	desumoylating isopeptidase 2 [Source:NCBI gene;Acc:445138]		13	11550454	11570568		8.86	6.27	6.26	8.44	7.63	7.41
ENSDARG00000004470	prkcsh	protein kinase C substrate 80K-H [Source:ZFIN;Acc:ZDB-GENE-040426-770]		3	46762703	46795783		13.17	13.24	12.7	12.17	11.46	11.45
ENSDARG00000004472	atat1	alpha tubulin acetyltransferase 1 [Source:NCBI gene;Acc:406389]		19	27479563	27515651		5.78	2.56	2.63	7.41	6.17	7.26
ENSDARG00000004473	tbx21	T-box transcription factor 21 [Source:ZFIN;Acc:ZDB-GENE-080104-3]		12	28749189	28770817		1.23	0.94	1.14	1.55	1.53	1.71
ENSDARG00000004492	pcyt1ab	phosphate cytidylyltransferase 1, choline, alpha b [Source:ZFIN;Acc:ZDB-GENE-050522-114]		18	46010081	46020138		15.28	9.54	11.53	12.68	13.06	13.17
ENSDARG00000004497	tspan33a	tetraspanin 33a [Source:NCBI gene;Acc:436890]		4	14957360	14968641		12.65	13.97	12.23	9.93	10.33	10.63
ENSDARG00000004498	xkr8.1	XK, Kell blood group complex subunit-related family, member 8, tandem duplicate 1 [Source:ZFIN;Acc:ZDB-GENE-110106-1]		19	24982672	24988640		0.89	0.77	0.56	0.34	0.56	0.37
ENSDARG00000004502	rcor3	REST corepressor 3 [Source:ZFIN;Acc:ZDB-GENE-050208-371]		22	38276024	38294242		3.88	1.55	1.78	8.37	8.39	8.36
ENSDARG00000004517	ppat	phosphoribosyl pyrophosphate amidotransferase [Source:ZFIN;Acc:ZDB-GENE-041210-323]		20	25625872	25639508		15.79	18.16	18.74	18.23	20.04	18.64
ENSDARG00000004525	C2H1orf35	zgc:63882 [Source:ZFIN;Acc:ZDB-GENE-040426-1286]		2	3044992	3062793	C2H1orf35	4.22	3.01	3.21	3.57	3.72	3.44
ENSDARG00000004527	pin4	protein (peptidylprolyl cis/trans isomerase) NIMA-interacting, 4 (parvulin) [Source:NCBI gene;Acc:553574]		14	28432737	28437426		22.75	28.43	29.98	12.99	15.08	16.12
ENSDARG00000004529	zgc:173742	zgc:173742 [Source:ZFIN;Acc:ZDB-GENE-030131-6489]		18	14277003	14332060		0.48	0.42	0.4	0.48	0.57	0.51
ENSDARG00000004537	kmt2a	lysine (K)-specific methyltransferase 2A [Source:ZFIN;Acc:ZDB-GENE-080521-3]		15	23382199	23420170		5.79	2.88	2.34	9.7	9.6	9.69
ENSDARG00000004539	ptgs2a	prostaglandin-endoperoxide synthase 2a [Source:ZFIN;Acc:ZDB-GENE-020530-2]		2	20916497	20923864		5.16	5.07	4.32	11.77	11.84	11.33
ENSDARG00000004561	prkcg	protein kinase C, gamma [Source:ZFIN;Acc:ZDB-GENE-090206-1]		16	13020776	13109749		1.69	0.7	0.71	1.63	1.21	1.49
ENSDARG00000004574	gtf3c2	general transcription factor IIIC, polypeptide 2, beta [Source:ZFIN;Acc:ZDB-GENE-041014-349]		20	38543314	38584161		1.97	1.47	1.53	1.58	1.64	1.32
ENSDARG00000004576	plk4	polo-like kinase 4 (Drosophila) [Source:NCBI gene;Acc:368390]		17	43410753	43428397		3.63	2.62	2.49	2.89	2.82	2.92
ENSDARG00000004577	TMEM178B	zgc:194665 [Source:ZFIN;Acc:ZDB-GENE-080722-38]		25	19999623	20003528	TMEM178B	11.61	3.53	7.41	11.98	10.81	10.96
ENSDARG00000004580	nmnat2	nicotinamide nucleotide adenylyltransferase 2 [Source:NCBI gene;Acc:336257]		2	36026183	36040820		24.36	11.1	17.82	25.86	28.82	28.38
ENSDARG00000004581	sel1l	SEL1L adaptor subunit of ERAD E3 ubiquitin ligase [Source:ZFIN;Acc:ZDB-GENE-041001-122]		20	26987416	27003298		11.49	10.22	8.54	12.36	11.69	11.36
ENSDARG00000004586	ntrk1	neurotrophic tyrosine kinase, receptor, type 1 [Source:ZFIN;Acc:ZDB-GENE-980526-118]		16	45796602	45854882		0.67	0.38	0.41	0.96	1.15	0.93
ENSDARG00000004587	kat5a	K(lysine) acetyltransferase 5a [Source:ZFIN;Acc:ZDB-GENE-040718-57]		5	23597140	23605948		10.53	6.1	5.46	10.68	11.4	11.61
ENSDARG00000004588	sox4a	SRY-box transcription factor 4a [Source:ZFIN;Acc:ZDB-GENE-030131-8290]		19	28786149	28789409		54.72	20.55	26.51	88.09	87.53	96.05
ENSDARG00000004592	gpr22a	G protein-coupled receptor 22a [Source:NCBI gene;Acc:504167]		4	2619132	2623487		3.67	1.86	2.38	3.89	4.3	4.09
ENSDARG00000004594	znf800a	zinc finger protein 800a [Source:ZFIN;Acc:ZDB-GENE-041008-151]		25	26895962	26913274		2.58	1.31	1.1	2.89	2.68	2.84
ENSDARG00000004597	lrrc4ba	leucine rich repeat containing 4Ba [Source:ZFIN;Acc:ZDB-GENE-080327-25]		3	30369765	30434016		2.35	0.63	0.71	3.17	3.33	3.1
ENSDARG00000004598	or102-2	odorant receptor, family C, subfamily 102, member 2 [Source:ZFIN;Acc:ZDB-GENE-010308-4]		15	31466978	31470101		0	0	0.01	0	0	0
ENSDARG00000004616	mtmr2	myotubularin related protein 2 [Source:NCBI gene;Acc:30644]		5	23608026	23619911		9.49	9.31	10.78	8.85	9.52	9.29
ENSDARG00000004618	stx2a	syntaxin 2a [Source:ZFIN;Acc:ZDB-GENE-040801-80]		21	17951286	17956739		3.01	2.95	2.17	2.88	3.31	2.9
ENSDARG00000004621	gpm6ab	glycoprotein M6Ab [Source:ZFIN;Acc:ZDB-GENE-030710-8]		1	38649141	38756904		75.65	25.04	38.51	103.13	101.45	104.03
ENSDARG00000004627	panx3	pannexin 3 [Source:ZFIN;Acc:ZDB-GENE-091204-114]		10	30997238	31016806		1.08	1.2	1.14	0.81	0.85	0.85
ENSDARG00000004632	egln1b	egl-9 family hypoxia-inducible factor 1b [Source:ZFIN;Acc:ZDB-GENE-040718-338]		11	31558006	31603692		6.06	5.71	6.33	13.84	14.74	14.6
ENSDARG00000004633	epn3b	epsin 3b [Source:ZFIN;Acc:ZDB-GENE-121214-173]		1	57331813	57340192		1.46	0.26	0.76	2.48	2.51	3.44
ENSDARG00000004634	osbp	oxysterol binding protein [Source:ZFIN;Acc:ZDB-GENE-090312-18]		1	52662203	52699063		12.26	10.68	7.87	12.35	13.42	12.87
ENSDARG00000004635	epha7	eph receptor A7 [Source:ZFIN;Acc:ZDB-GENE-030131-3745]		20	24817118	24963092		1.47	0.73	0.72	2.21	2.25	2.19
ENSDARG00000004636	ccdc9	coiled-coil domain containing 9 [Source:ZFIN;Acc:ZDB-GENE-060929-520]		15	26903267	26931541		10.24	7.55	6.94	8.73	8.68	8.16
ENSDARG00000004643	cdhr1a	cadherin-related family member 1a [Source:NCBI gene;Acc:449008]		13	29480010	29505946		5.26	1.05	0.85	4.21	4.78	4.64
ENSDARG00000004647	spice1	spindle and centriole associated protein 1 [Source:ZFIN;Acc:ZDB-GENE-041212-64]		24	21330753	21343982		1.01	0.74	0.68	0.76	0.93	1.22
ENSDARG00000004648	kctd16a	potassium channel tetramerization domain containing 16a [Source:ZFIN;Acc:ZDB-GENE-060117-1]		14	23811808	23812725		3.36	0.45	1.02	3.61	3.15	3.46
ENSDARG00000004658	zgc:101810	zgc:101810 [Source:ZFIN;Acc:ZDB-GENE-041121-7]		7	7378920	7398350		41.65	49.62	47.7	33.02	32.26	29.99
ENSDARG00000004672	ptk2ab	protein tyrosine kinase 2ab [Source:ZFIN;Acc:ZDB-GENE-020114-1]		16	30735426	30870170		5.79	3.56	3.27	6.61	6.4	6.53
ENSDARG00000004680	dnajb6a	DnaJ heat shock protein family (Hsp40) member B6a [Source:ZFIN;Acc:ZDB-GENE-040718-45]		2	30103131	30116467		41.61	28.03	26.08	38.91	39.57	39.86
ENSDARG00000004687	acaa1	acetyl-CoA acyltransferase 1 [Source:ZFIN;Acc:ZDB-GENE-040704-48]		24	20325312	20332460		21.68	30.03	25.58	11.53	11.55	11.17
ENSDARG00000004692	nabp1a	nucleic acid binding protein 1a [Source:NCBI gene;Acc:494100]		9	24214660	24218367		8.19	5.93	6.45	9.19	8.54	9.57
ENSDARG00000004695	six4a	SIX homeobox 4a [Source:ZFIN;Acc:ZDB-GENE-010201-2]		13	31636732	31647323		4.85	4.91	5.26	4.81	4.78	4.97
ENSDARG00000004696	eefsec	eukaryotic elongation factor, selenocysteine-tRNA-specific [Source:ZFIN;Acc:ZDB-GENE-051120-72]		6	40822587	40832245		6.1	6.5	6.72	5.35	5.78	4.79
ENSDARG00000004697	rxrgb	retinoid X receptor, gamma b [Source:NCBI gene;Acc:436617]		20	33875256	33920055		4.96	3	2.47	5.17	4.79	5.23
ENSDARG00000004702	irf2bp2a	interferon regulatory factor 2 binding protein 2a [Source:NCBI gene;Acc:335866]		13	49443226	49444636		46.06	27.65	30.9	60.39	60.93	59.52
ENSDARG00000004706	syf2	SYF2 pre-mRNA-splicing factor [Source:NCBI gene;Acc:445043]		13	45431660	45443008		43.84	39.7	41.54	27.72	28.02	30.42
ENSDARG00000004712	tbr1b	T-box brain transcription factor 1b [Source:ZFIN;Acc:ZDB-GENE-000323-1]		9	51427826	51436377		3.09	0.92	1.21	3.1	3	3.13
ENSDARG00000004713	mad2l1	MAD2 mitotic arrest deficient-like 1 (yeast) [Source:ZFIN;Acc:ZDB-GENE-030515-3]		7	57629370	57637779		15.54	18.73	20.03	13.39	16.12	14.06
ENSDARG00000004714	tcf12	transcription factor 12 [Source:ZFIN;Acc:ZDB-GENE-040516-11]		7	52516706	52709935		12.74	10.39	9.11	19.98	20.46	20.85
ENSDARG00000004721	mpp5b	membrane protein, palmitoylated 5b (MAGUK p55 subfamily member 5) [Source:ZFIN;Acc:ZDB-GENE-060531-2]		20	51006362	51081372		4.16	0.95	0.57	1.82	2.9	2.29
ENSDARG00000004724	tcea3	transcription elongation factor A (SII), 3 [Source:ZFIN;Acc:ZDB-GENE-040426-1860]		16	34111919	34135244		8.77	10.15	8.25	5.71	5.2	5.84
ENSDARG00000004726	trappc11	trafficking protein particle complex 11 [Source:NCBI gene;Acc:323003]		14	7320948	7375049		12.69	10.03	9.67	12.83	14.05	13.11
ENSDARG00000004735	hnrnpub	heterogeneous nuclear ribonucleoprotein Ub [Source:ZFIN;Acc:ZDB-GENE-030131-6422]		20	35058634	35070476		45.25	27.33	27.16	49.99	47.17	45.52
ENSDARG00000004745	lmbr1l	limb development membrane protein 1-like [Source:NCBI gene;Acc:321314]		23	27703749	27727512		7.35	5.72	4.39	5.29	5.43	5.09
ENSDARG00000004748	zgc:100868	zgc:100868 [Source:ZFIN;Acc:ZDB-GENE-040801-33]		3	39472342	39488639		14.2	11.79	7.8	32.55	27.43	21.34
ENSDARG00000004753	mylkb	myosin light chain kinase b [Source:ZFIN;Acc:ZDB-GENE-041111-150]		6	13458257	13498745		3.44	3.72	2.84	3.62	3.52	3.47
ENSDARG00000004754	hspa4a	heat shock protein 4a [Source:ZFIN;Acc:ZDB-GENE-040426-2832]		21	43137500	43171249		37.07	32.12	25.14	29.42	28.83	26.52
ENSDARG00000004757	ybx1	Y box binding protein 1 [Source:ZFIN;Acc:ZDB-GENE-000629-3]		8	47143829	47152086		633.41	742.48	735.1	703.05	711.52	686.95
ENSDARG00000004763	hhla2a.2	HERV-H LTR-associating 2a, tandem duplicate 2 [Source:ZFIN;Acc:ZDB-GENE-091204-324]		10	35439952	35452191		0.5	0.6	0.56	1.06	0.99	1.05
ENSDARG00000004771	ankrd10b	ankyrin repeat domain 10b [Source:ZFIN;Acc:ZDB-GENE-061013-49]		9	8929764	8967936		7.68	4.78	6.62	5.68	5.79	5.53
ENSDARG00000004774	wdr36	WD repeat domain 36 [Source:ZFIN;Acc:ZDB-GENE-030131-464]		5	55866769	55914268		11.18	12.13	11.77	9.21	7.76	7.86
ENSDARG00000004782	fgfr3	fibroblast growth factor receptor 3 [Source:NCBI gene;Acc:58129]		13	13213567	13294872		4.41	3.3	2.66	15.11	14.61	14.09
ENSDARG00000004785	cops2	COP9 signalosome subunit 2 [Source:NCBI gene;Acc:415145]		25	32366071	32381663		27.62	29.04	28.68	26.93	26.25	27.22
ENSDARG00000004789	lrp1ba	low density lipoprotein receptor-related protein 1Ba [Source:ZFIN;Acc:ZDB-GENE-050208-608]		22	14445713	14739491		1.13	0.37	0.38	1.43	1.42	1.5
ENSDARG00000004794	ccdc146	coiled-coil domain containing 146 [Source:ZFIN;Acc:ZDB-GENE-041210-217]		4	20177526	20199224		0.96	0.49	0.6	0.92	0.98	0.86
ENSDARG00000004796	mgrn1b	mahogunin, ring finger 1b [Source:ZFIN;Acc:ZDB-GENE-110401-3]		3	54849083	54875874		8.74	5.27	5.37	8.44	8.81	8.76
ENSDARG00000004806	grwd1	glutamate-rich WD repeat containing 1 [Source:ZFIN;Acc:ZDB-GENE-030131-9844]		16	13860299	13870899		16.8	16.95	16.58	15.29	14.12	14.74
ENSDARG00000004823	asz1	ankyrin repeat, SAM and basic leucine zipper domain containing 1 [Source:ZFIN;Acc:ZDB-GENE-040426-988]		18	20642378	20674121		0.19	0.15	0.25	0.25	0.24	0.17
ENSDARG00000004830	flot2a	flotillin 2a [Source:NCBI gene;Acc:245698]		5	42835082	42872712		91.77	83.31	75.89	114.13	119.59	120.36
ENSDARG00000004836	dnajc5ab	DnaJ (Hsp40) homolog, subfamily C, member 5ab [Source:ZFIN;Acc:ZDB-GENE-081021-2]		23	17472702	17509656		10.42	4.25	4.31	16.48	16.27	17.7
ENSDARG00000004840	rassf1	Ras association domain family member 1 [Source:ZFIN;Acc:ZDB-GENE-040912-14]		22	10645088	10658520		11.06	6.25	6	9.87	9.88	10.02
ENSDARG00000004843	foxp1a	forkhead box P1a [Source:ZFIN;Acc:ZDB-GENE-060929-624]		23	10634837	10723009		6.9	5.51	3.98	6.6	7.34	7.37

ENSDARG00000004851	hinfp	histone H4 transcription factor [Source:ZFIN;Acc:ZDB-GENE-040426-2764]	5	30596477	30608624		1.5	1.11	1.05	1.57	1.13	1.37
ENSDARG00000004861	esrrga	estrogen-related receptor gamma a [Source:ZFIN;Acc:ZDB-GENE-030821-2]	17	29797876	29902187		11.45	3.66	3.9	19.16	17.04	18.12
ENSDARG00000004864	foxr1	forkhead box R1 [Source:ZFIN;Acc:ZDB-GENE-070928-26]	5	58679071	58684455		0	0	0	0	0	0
ENSDARG00000004866	mindy1	MINDY lysine 48 deubiquitinase 1 [Source:NCBI gene;Acc:563470]	19	7453996	7478597		11.27	10.23	10.69	8.84	9.13	9.63
ENSDARG00000004869	penka	proenkephalin a [Source:ZFIN;Acc:ZDB-GENE-030729-31]	2	11508691	11512819		5.97	5.13	5.76	5.87	6.28	5.74
ENSDARG00000004870	psen1	presenilin 1 [Source:NCBI gene;Acc:30221]	17	51206225	51224800		10.3	7.94	6.88	12.26	11.89	12.87
ENSDARG00000004871	mst1rb	macrophage stimulating 1 receptor b [Source:ZFIN;Acc:ZDB-GENE-071218-3]	11	35196587	35238756		0.18	0.18	0.12	0.56	0.42	0.49
ENSDARG00000004874	spata6l	spermatogenesis associated 6-like [Source:ZFIN;Acc:ZDB-GENE-040426-1369]	7	69478914	69494504		0.27	0.39	0.27	0.33	0.51	0.62
ENSDARG00000004875	tmc6b	transmembrane channel-like 6b [Source:ZFIN;Acc:ZDB-GENE-040718-460]	11	45219558	45244641		9.92	9.48	8.63	9.84	9.53	10.68
ENSDARG00000004877	rock2b	rho-associated, coiled-coil containing protein kinase 2b [Source:ZFIN;Acc:ZDB-GENE-060125-2]	20	33601046	33705044		6.53	5.71	4.95	6.48	6.39	6.44
ENSDARG00000004880	acsm3	acyl-CoA synthetase medium chain family member 3 [Source:ZFIN;Acc:ZDB-GENE-080220-22]	2	44725550	44746723		2.06	2.7	2	3.25	3.15	3.27
ENSDARG00000004889	aga	aspartylglucosaminidase [Source:ZFIN;Acc:ZDB-GENE-040426-2311]	14	36428441	36437249		9.32	7.19	9.13	5.01	4.48	5
ENSDARG00000004891	pex19	peroxisomal biogenesis factor 19 [Source:ZFIN;Acc:ZDB-GENE-050417-424]	2	37134281	37146572		28.88	26.18	25.75	23.72	25.49	25.28
ENSDARG00000004898	zp2l2	zona pellucida glycoprotein 2, like 2 [Source:ZFIN;Acc:ZDB-GENE-060818-4]	6	9945303	9952103		0	0	0.02	0.06	0.08	0.06
ENSDARG00000004904	gys2	glycogen synthase 2 [Source:ZFIN;Acc:ZDB-GENE-030826-31]	4	16783972	16824556		21.71	31.16	28.5	7.35	7.67	7.6
ENSDARG00000004906	stip1	stress-induced phosphoprotein 1 [Source:ZFIN;Acc:ZDB-GENE-041121-17]	5	66807179	66823794		33.07	29.33	26	31.72	30.11	29.48
ENSDARG00000004925	immp2l	inner mitochondrial membrane peptidase subunit 2 [Source:NCBI gene;Acc:445299]	25	21296588	21507676		2.32	2.59	2.74	2.21	2.35	2.08
ENSDARG00000004930	lmo7a	LIM domain 7a [Source:ZFIN;Acc:ZDB-GENE-030219-74]	9	21893757	21970067		12.52	10.02	7.4	13.39	12.13	12.62
ENSDARG00000004931	slc8a3	solute carrier family 8 member 3 [Source:ZFIN;Acc:ZDB-GENE-050810-1]	13	36196778	36264861		1.84	1.27	1.02	3.02	2.57	2.42
ENSDARG00000004932	anos1b	anosmin 1b [Source:ZFIN;Acc:ZDB-GENE-000201-13]	22	38824012	38913871		3.48	3.94	2.74	7.79	7.51	8.16
ENSDARG00000004937	skp2	S-phase kinase-associated protein 2, E3 ubiquitin protein ligase [Source:ZFIN;Acc:ZDB-GENE-030219-158]	21	22347120	22359336		2.84	2.62	2.38	1.93	2.27	2.09
ENSDARG00000004939	mtdhb	metadherin b [Source:ZFIN;Acc:ZDB-GENE-090929-2]	16	41717313	41732459		16.92	13.17	13.98	9.82	9.4	7.44
ENSDARG00000004948	bccip	BRCA2 and CDKN1A interacting protein [Source:NCBI gene;Acc:541348]	12	45186533	45197038		22.23	28.78	31.67	12.92	13.34	14.14
ENSDARG00000004952	rsad2	radical S-adenosyl methionine domain containing 2 [Source:NCBI gene;Acc:570456]	17	35357451	35361322		0.28	0.28	0.17	0.14	0.05	0.12
ENSDARG00000004953	mxb	myxovirus (influenza) resistance B [Source:NCBI gene;Acc:360143]	1	7574351	7603734		0.07	0.11	0.1	0.05	0.17	0.09
ENSDARG00000004954	grna	granulin a [Source:ZFIN;Acc:ZDB-GENE-030131-8434]	3	29914427	29941357		1.28	1.24	1.28	1.67	1.76	1.8
ENSDARG00000004956	zmat2	zinc finger, matrin-type 2 [Source:ZFIN;Acc:ZDB-GENE-030131-3197]	21	30293564	30300049		49.23	51.15	45.5	38.74	37.33	40.68
ENSDARG00000004964	cyp4t8	cytochrome P450, family 4, subfamily T, polypeptide 8 [Source:ZFIN;Acc:ZDB-GENE-031219-3]	16	4615661	4640578		4.67	5.52	4.07	1.32	1.77	1.83
ENSDARG00000004979	elovl5	ELOVL fatty acid elongase 5 [Source:ZFIN;Acc:ZDB-GENE-040407-2]	13	2394264	2417409		6.79	9.15	7.93	8.94	8.9	8.56
ENSDARG00000004988	ppp3ca	protein phosphatase 3, catalytic subunit, alpha isozyme [Source:ZFIN;Acc:ZDB-GENE-091113-24]	21	28958471	29075815		16.64	4.01	6.26	22.81	23.6	23.44
ENSDARG00000005002	brf1b	BRF1 RNA polymerase III transcription initiation factor subunit b [Source:ZFIN;Acc:ZDB-GENE-030131-6248]	20	20901791	21018334		2.78	1.41	1.38	1.76	1.89	1.92
ENSDARG00000005004	slc5a12	solute carrier family 5 member 12 [Source:NCBI gene;Acc:393339]	25	35474987	35524166		3.25	6.07	3.41	4.51	5.71	6.13
ENSDARG00000005010	si:dkeyp-2e4.8	si:dkeyp-2e4.8 [Source:ZFIN;Acc:ZDB-GENE-061207-81]	13	44818447	44819254		2.08	1.79	1.77	2.36	2.97	2.86
ENSDARG00000005015	tspy	testis specific protein Y-linked [Source:ZFIN;Acc:ZDB-GENE-030131-9158]	8	25708067	25716074		14.38	9.71	9.17	13.58	12.76	13.69
ENSDARG00000005023	fkbp9	FKBP prolyl isomerase 9 [Source:ZFIN;Acc:ZDB-GENE-040801-23]	19	42591150	42607451		20.86	25.17	24.14	36.45	36.81	36.41
ENSDARG00000005026	tshz1	teashirt zinc finger homeobox 1 [Source:NCBI gene;Acc:369196]	19	21266008	21416407		4.6	1.82	1.96	6.66	6.6	6.6
ENSDARG00000005034	crppa	CDP-L-ribitol pyrophosphorylase A [Source:NCBI gene;Acc:798716]	15	34495056	34568705		9.77	10.16	11.29	6.89	6.85	6.58
ENSDARG00000005049	rab20	RAB20, member RAS oncogene family [Source:ZFIN;Acc:ZDB-GENE-030131-9143]	9	8887528	8895243		7.36	9.71	7.8	5.89	6.59	7.37
ENSDARG00000005050	wnt2ba	wingless-type MMTV integration site family, member 2Ba [Source:ZFIN;Acc:ZDB-GENE-030717-2]	6	48253449	48271687		1.03	1.22	0.7	1.11	0.99	1.14
ENSDARG00000005057	dimt1l	DIM1 dimethyladenosine transferase 1-like (S. cerevisiae) [Source:ZFIN;Acc:ZDB-GENE-040801-75]	8	17184602	17193041		8.26	10.09	8.26	5.04	5.47	5.35
ENSDARG00000005058	ncapd2	non-SMC condensin I complex, subunit D2 [Source:ZFIN;Acc:ZDB-GENE-050506-59]	2	44749201	44777679		8.47	6.07	6.31	6.21	6.35	6.2
ENSDARG00000005078	eif4e3	eukaryotic translation initiation factor 4E family member 3 [Source:NCBI gene;Acc:447850]	23	10724042	10745319		14.26	13.8	13.69	15.34	15.56	16.27
ENSDARG00000005085	ggctb	gamma-glutamylcyclotransferase b [Source:ZFIN;Acc:ZDB-GENE-040426-1939]	16	53709011	53709101		23.13	29.01	28.98	20.86	25.21	24.29
ENSDARG00000005092	cep89	centrosomal protein 89 [Source:NCBI gene;Acc:393852]	7	38395197	38500305		1.84	1.33	1.3	1.7	1.53	1.7
ENSDARG00000005095	glcea	glucuronic acid epimerase a [Source:ZFIN;Acc:ZDB-GENE-040630-9]	25	7918695	7925269		2.99	1.18	1.13	2.4	3.02	3.14
ENSDARG00000005098	zgc:86764	zgc:86764 [Source:ZFIN;Acc:ZDB-GENE-040718-435]	21	20309085	20322816		3.79	2.66	3.12	2.5	2.49	2.34
ENSDARG00000005104	limk2	LIM domain kinase 2 [Source:ZFIN;Acc:ZDB-GENE-040718-398]	5	20823409	20863374		12.37	10.99	10.25	17.89	17.81	17.86
ENSDARG00000005108	oclna	occludin a [Source:ZFIN;Acc:ZDB-GENE-040426-2685]	10	2924775	2943474		23.02	17.19	14.24	19.33	17.7	15.55
ENSDARG00000005112	cdh17	cadherin 17, LI cadherin (liver-intestine) [Source:ZFIN;Acc:ZDB-GENE-030910-3]	16	26774182	26802798		34.47	55.47	37.88	25.54	27.93	25.92
ENSDARG00000005115	calub	calumenin b [Source:NCBI gene;Acc:394057]	18	7015283	7032227		11.47	14.07	12.94	19.28	18.33	18.12
ENSDARG00000005122	atp2a2b	ATPase sarcoplasmic/endoplasmic reticulum Ca2+ transporting 2b [Source:ZFIN;Acc:ZDB-GENE-030131-867]	21	17051478	17108649		14.19	13.18	7.5	24.43	23.03	21.76
ENSDARG00000005126	large1	LARGE xylosyl- and glucuronyltransferase 1 [Source:NCBI gene;Acc:446213]	4	75013307	75057322		3.71	1.7	1.81	5.68	5.67	5.85
ENSDARG00000005128	rnaseh2b	ribonuclease H2, subunit B [Source:ZFIN;Acc:ZDB-GENE-040426-1090]	9	29671553	29679172		11.89	14.6	12.53	7.04	6.41	6.49
ENSDARG00000005134	psmd11b	proteasome 26S subunit, non-ATPase 11b [Source:NCBI gene;Acc:322265]	12	28346722	28363166		7.13	6.04	5.66	7.62	7.06	6.85
ENSDARG00000005139	hhatlb	hedgehog acyltransferase like, b [Source:ZFIN;Acc:ZDB-GENE-050506-130]	24	20559009	20572170		16.53	21.03	17.77	15.3	13.38	14.6
ENSDARG00000005141	camkvb	CaM kinase-like vesicle-associated b [Source:NCBI gene;Acc:393422]	11	35364445	35441348		4.03	0.87	1.18	4.21	4.06	3.77
ENSDARG00000005150	tbx20	T-box transcription factor 20 [Source:NCBI gene;Acc:57936]	16	42509011	42523744		1.92	1.18	1.28	3.13	2.83	2.63
ENSDARG00000005154	aspa	aspartoacylase [Source:NCBI gene;Acc:557232]	5	62340799	62356975		5.54	4.01	3.24	5.59	5.38	6.03
ENSDARG00000005159	cyth1a	cytohesin 1a [Source:ZFIN;Acc:ZDB-GENE-030131-657]	3	56408840	56541751		9.36	4.55	5	11.6	11.26	11.25
ENSDARG00000005162	tpm3	tropomyosin 3 [Source:ZFIN;Acc:ZDB-GENE-030826-16]	19	10985369	11031394		94.19	122.72	126.3	77.96	82.71	80.33
ENSDARG00000005163	efr3a	EFR3 homolog A (S. cerevisiae) [Source:ZFIN;Acc:ZDB-GENE-040426-1681]	2	42871831	42936509		6.3	3.43	3.79	5.79	6.14	5.84
ENSDARG00000005172	dynll2b	dynein, light chain, LC8-type 2b [Source:ZFIN;Acc:ZDB-GENE-050320-132]	21	39557433	39566854		29.84	18.93	21.17	26.87	27.34	26.17
ENSDARG00000005176	zgc:101040	zgc:101040 [Source:ZFIN;Acc:ZDB-GENE-040801-107]	18	40479684	40481028		6.32	9.8	6.97	7.07	7.6	8.14
ENSDARG00000005179	dglucy	D-glutamate cyclase [Source:ZFIN;Acc:ZDB-GENE-070725-6]	17	38738840	38778901		4.25	4.13	3.32	2.56	2.73	2.86
ENSDARG00000005185	cpn1	carboxypeptidase N, polypeptide 1 [Source:ZFIN;Acc:ZDB-GENE-030131-9116]	12	31616412	31631297		0.08	0.18	0.11	0.57	0.66	0.68
ENSDARG00000005186	sp4	sp4 transcription factor [Source:ZFIN;Acc:ZDB-GENE-031202-3]	19	2364552	2563324		4.36	2.78	2.3	8.34	7.92	8.47
ENSDARG00000005191	ahcy	adenosylhomocysteinase [Source:ZFIN;Acc:ZDB-GENE-031219-6]	6	49926115	49966288		630.63	851.21	952.02	583	634.36	617.19
ENSDARG00000005210	coro2a	coronin, actin binding protein, 2A [Source:ZFIN;Acc:ZDB-GENE-030131-2258]	1	26605065	26654311		5.41	5.33	4.46	3.94	3.96	3.53
ENSDARG00000005216	zgc:158328	zgc:158328 [Source:ZFIN;Acc:ZDB-GENE-070112-1202]	15	35253247	35339302		1.25	1.76	1.62	3.59	4.02	3.6
ENSDARG00000005218	wrnip1	WRN helicase interacting protein 1 [Source:ZFIN;Acc:ZDB-GENE-040426-2415]	2	374365	389867		2.38	1.89	1.89	1.68	1.79	1.5
ENSDARG00000005220	pdcd6	programmed cell death 6 [Source:ZFIN;Acc:ZDB-GENE-040426-1307]	24	12629649	12689571		15.79	22.65	22.16	21.8	20.89	21.68
ENSDARG00000005221	desmb	desmin b [Source:ZFIN;Acc:ZDB-GENE-061027-102]	6	13871641	13891727		12.91	18.05	15.81	5.56	5.11	5.65
ENSDARG00000005230	ssr2	signal sequence receptor, beta [Source:ZFIN;Acc:ZDB-GENE-010413-1]	19	11231083	11238721		133.73	185.11	174.15	135.51	136.05	136.25
ENSDARG00000005232	ccdc85ca	coiled-coil domain containing 85C, a [Source:NCBI gene;Acc:751667]	17	17914369	18025691		7.19	4.19	4.24	8.52	8.48	8.65
ENSDARG00000005236	srcap	Snf2-related CREBBP activator protein [Source:ZFIN;Acc:ZDB-GENE-041008-127]	12	27061720	27092286		6.99	4.08	3.24	7.86	7.32	7.62
ENSDARG00000005246	asb3	ankyrin repeat and SOCS box containing 3 [Source:ZFIN;Acc:ZDB-GENE-030131-6142]	12	24060894	24077141		4.18	2.84	2.74	3.78	3.68	3.98
ENSDARG00000005251	rpe	ribulose-5-phosphate-3-epimerase [Source:ZFIN;Acc:ZDB-GENE-030131-6837]	9	24025444	24031461		23.36	36.59	37.3	19.64	19.48	18.13
ENSDARG00000005254	kdelr2a	KDEL endoplasmic reticulum protein retention receptor 2a [Source:NCBI gene;Acc:373129]	3	18376269	18384501		31.22	36.51	32.52	40.71	40.48	39.6
ENSDARG00000005271	zc3h12a	zinc finger CCCH-type containing 12A [Source:ZFIN;Acc:ZDB-GENE-110411-6]	16	3982590	4001743		1.86	1.55	1.12	1.6	1.49	1.34
ENSDARG00000005274	rad52	RAD52 homolog, DNA repair protein [Source:ZFIN;Acc:ZDB-GENE-050731-10]	25	21098675	21115213		1.26	0.86	0.98	0.91	1.02	0.97
ENSDARG00000005284	fbxl15	F-box and leucine-rich repeat protein 15 [Source:NCBI gene;Acc:405878]	12	45661810	45669050		3.81	2.68	2.41	2.72	2.75	3.01
ENSDARG00000005314	CALHM1	si:dkeyp-80c12.5 [Source:ZFIN;Acc:ZDB-GENE-090312-195]	1	49501171	49505449	CALHM1	0.41	0.32	0.45	0.63	0.57	0.39
ENSDARG00000005315	celf1	cugbp, Elav-like family member 1 [Source:NCBI gene;Acc:58137]	7	38535934	38570940		11.23	7.86	6.88	17.46	16.3	15.46
ENSDARG00000005316	stam2	signal transducing adaptor molecule (SH3 domain and ITAM motif) 2 [Source:ZFIN;Acc:ZDB-GENE-041114-64]	9	38043337	38065147		14.02	13.1	9.53	16.73	16.64	16.21
ENSDARG00000005320	nipsnap1	nipsnap homolog 1 (C. elegans) [Source:NCBI gene;Acc:30231]	5	12032802	12063381		7.22	10.66	8.47	4.29	4.16	3.48
ENSDARG00000005327	ftr66	finTRIM family, member 66 [Source:ZFIN;Acc:ZDB-GENE-070912-661]	2	37762924	37770331		0.1	0.11	0.11	0.03	0.03	0.09
ENSDARG00000005332	lipib	lipase, member Ib [Source:ZFIN;Acc:ZDB-GENE-091118-47]	24	24086249	24101132		1.52	0.2	0.21	1.25	1.73	1.76
ENSDARG00000005335	slc22a4	solute carrier family 22 member 4 [Source:ZFIN;Acc:ZDB-GENE-040426-1517]	21	45897814	45903536		0.86	1.5	1.46	1.11	1.1	1.28
ENSDARG00000005343	ccdc85al	coiled-coil domain containing 85A, like [Source:ZFIN;Acc:ZDB-GENE-040912-92]	6	6736925	6775821		4.26	1.68	1.87	4.4	3.9	4.5
ENSDARG00000005350	dip2ba	disco-interacting protein 2 homolog Ba [Source:NCBI gene;Acc:405899]	23	27358727	27442544		7.95	5.96	4.7	11.35	11.38	11.73
ENSDARG00000005355	larp6a	La ribonucleoprotein 6, translational regulator a [Source:ZFIN;Acc:ZDB-GENE-030131-6403]	18	173603	194844		19.81	15.56	14.76	17.21	18.46	17.47
ENSDARG00000005356	tyro3	TYRO3 protein tyrosine kinase [Source:NCBI gene;Acc:30709]	17	10501647	10553538		3.15	2.31	1.47	3.6	3.05	3.21
ENSDARG00000005359	sucla2	succinate-CoA ligase, ADP-forming, beta subunit [Source:ZFIN;Acc:ZDB-GENE-040426-1963]	6	10037252	10056640		95.77	111.58	121.36	79.77	75.59	74.95
ENSDARG00000005364	grin3ba	glutamate receptor, ionotropic, N-methyl-D-aspartate 3Ba [Source:ZFIN;Acc:ZDB-GENE-070912-354]	2	26060528	26156784		0.68	0.41	0.33	0.9	0.81	0.86
ENSDARG00000005368	mcamb	melanoma cell adhesion molecule b [Source:ZFIN;Acc:ZDB-GENE-030219-213]	15	23271862	23342752		9.77	11.15	9.22	15.21	16.44	16.12
ENSDARG00000005371	gprc6a	G protein-coupled receptor, class C, group 6, member A [Source:NCBI gene;Acc:494131]	16	32152612	32159033		1.21	1.32	0.94	0.38	0.32	0.37
ENSDARG00000005372	camk4	calcium/calmodulin-dependent protein kinase IV [Source:ZFIN;Acc:ZDB-GENE-050417-76]	5	55763276	55848511		6.63	3.59	4.74	5.53	6.47	5.64
ENSDARG00000005374	tubgcp4	tubulin, gamma complex associated protein 4 [Source:ZFIN;Acc:ZDB-GENE-081022-124]	7	52929964	52950123		4.32	2.83	3.28	3.98	3.77	3.47
ENSDARG00000005382	prdm9	PR domain containing 9 [Source:NCBI gene;Acc:393876]	12	9781524	9796237		4.28	3.68	3.75	4.34	4.54	4.36
ENSDARG00000005392	slc5a5	solute carrier family 5 member 5 [Source:ZFIN;Acc:ZDB-GENE-060503-585]	8	13364950	13382351		0.11	0.25	0.25	0.17	0.21	0.12
ENSDARG00000005394	dnmt3aa	DNA (cytosine-5-)-methyltransferase 3 alpha a [Source:ZFIN;Acc:ZDB-GENE-050314-5]	20	46967269	47051996		3.09	1.72	1.7	6.52	6.2	5.44
ENSDARG00000005397	trim3b	tripartite motif containing 3b [Source:ZFIN;Acc:ZDB-GENE-070112-1132]	10	26129830	26179805		9.1	3.86	4.04	10.23	10.91	10.84
ENSDARG00000005414	grapa	GRB2 related adaptor protein a [Source:ZFIN;Acc:ZDB-GENE-030131-4375]	3	39630930	39663982		4.11	1.95	2.2	3.18	3.69	3.17
ENSDARG00000005416	map3k5	mitogen-activated protein kinase kinase kinase 5 [Source:ZFIN;Acc:ZDB-GENE-030827-3]	20	3005472	3087162		9.57	5.37	5.33	10.32	9.93	11
ENSDARG00000005423	pgam1a	phosphoglycerate mutase 1a [Source:ZFIN;Acc:ZDB-GENE-030131-1827]	13	31393192	31397987		64.1	93.07	78.22	104.68	112.85	113.94
ENSDARG00000005425	wdfy1	WD repeat and FYVE domain containing 1 [Source:ZFIN;Acc:ZDB-GENE-041024-4]	15	43208593	43238220		7.92	5.22	5.12	8.33	9.16	9.29
ENSDARG00000005448	mtfmt	mitochondrial methionyl-tRNA formyltransferase [Source:ZFIN;Acc:ZDB-GENE-060929-120]	7	52753067	52761572		4.34	5.23	4.93	4.2	3.4	3.81
ENSDARG00000005451	gdi2	GDP dissociation inhibitor 2 [Source:ZFIN;Acc:ZDB-GENE-030131-2485]	4	11464255	11477630		130.1	118.08	105.62	127	130.79	128.42
ENSDARG00000005453	foxp2	forkhead box P2 [Source:ZFIN;Acc:ZDB-GENE-041203-2]	4	6423041	6623645		7	2.88	2.73	13.12	13.21	14.1
ENSDARG00000005454	tacc3	transforming, acidic coiled-coil containing protein 3 [Source:ZFIN;Acc:ZDB-GENE-050522-327]	13	35880573	35892305		10.47	7.71	8.8	8.36	8.58	8.08
ENSDARG00000005456	katnb1	katanin p80 (WD repeat containing) subunit B 1 [Source:NCBI gene;Acc:406291]	7	52158818	52285349		8.89	6.87	7.14	9.8	9.61	9.98
ENSDARG00000005458	csnk1g2a	casein kinase 1, gamma 2a [Source:ZFIN;Acc:ZDB-GENE-030131-6445]	2	23039041	23078748		14.68	10.62	9.98	17.24	17.23	16.7
ENSDARG00000005462	kif4	kinesin family member 4 [Source:ZFIN;Acc:ZDB-GENE-040426-1545]	21	44735181	44772738		4.54	2.92	2.78	3.29	3.37	3.23
ENSDARG00000005463	slc30a1a	solute carrier family 30 member 1a [Source:ZFIN;Acc:ZDB-GENE-070112-1840]	20	13894123	13913790		8.06	6.43	8.56	9.8	8.8	9.55
ENSDARG00000005464	dnase1l1	deoxyribonuclease I-like 1 [Source:ZFIN;Acc:ZDB-GENE-040426-2170]	23	19701587	19711988		6.29	7.15	6.16	5.98	5.71	5.65
ENSDARG00000005468	irf2bp1	interferon regulatory factor 2 binding protein 1 [Source:NCBI gene;Acc:556852]	18	46361544	46369516		11.17	10.21	7.75	15.64	15.96	15.43
ENSDARG00000005476	nav3	neuron navigator 3 [Source:NCBI gene;Acc:567779]	4	20812900	20987564		4.55	2.58	2.03	7.32	6.94	7.12
ENSDARG00000005479	tenm3	Teneurin-3 [Source:UniProtKB/Swiss-Prot;Acc:Q9W7R4]	1	39387876	39779913		7.04	3.4	3.42	11.64	11.77	11.83
ENSDARG00000005481	nfkbiaa	nuclear factor of kappa light polypeptide gene enhancer in B-cells inhibitor, alpha a [Source:ZFIN;Acc:ZDB-GENE-040426-2227]	20	16881883	16884737		17.23	19.88	18.89	21.33	21.29	20.67
ENSDARG00000005482	rapgef2	Rap guanine nucleotide exchange factor (GEF) 2 [Source:ZFIN;Acc:ZDB-GENE-030131-6959]	14	47779846	48033073		7.99	3.38	3	11.83	11.88	12.2
ENSDARG00000005485	ucmab	upper zone of growth plate and cartilage matrix associated b [Source:NCBI gene;Acc:405870]	25	7982979	7988936		45.08	58.2	59.35	58.27	60.46	61.25
ENSDARG00000005500	dgcr6	DiGeorge syndrome critical region gene 6 [Source:ZFIN;Acc:ZDB-GENE-040912-125]	8	1632622	1649242		1.58	1.7	1.68	1.35	1.32	1.41
ENSDARG00000005510	gdf6b	growth differentiation factor 6b [Source:NCBI gene;Acc:30180]	19	22999181	23002883		0.53	0.44	0.58	0.67	0.7	0.84
ENSDARG00000005513	naca	nascent polypeptide associated complex subunit alpha [Source:NCBI gene;Acc:195823]	23	17865554	17876777		139.99	193.23	184.04	146.19	139.28	148.59
ENSDARG00000005518	slc5a9	solute carrier family 5 member 9 [Source:NCBI gene;Acc:564815]	8	15254564	15268932		2.25	4.29	1.82	2.48	2.57	2.59
ENSDARG00000005522	galr1a	galanin receptor 1a [Source:ZFIN;Acc:ZDB-GENE-060503-290]	19	21919856	21924241		0.39	0.22	0.24	0.37	0.21	0.26
ENSDARG00000005525	acy3.2	aspartoacylase (aminocyclase) 3, tandem duplicate 2 [Source:NCBI gene;Acc:436619]	1	52489962	52498146		6.43	8.56	7.4	3.48	3.15	3.1
ENSDARG00000005526	igfn1.1	immunoglobulin-like and fibronectin type III domain containing 1, tandem duplicate 1 [Source:ZFIN;Acc:ZDB-GENE-050327-30]	6	46878889	46941245		17.77	32.58	21.93	15.72	11.24	11.32
ENSDARG00000005536	ubr7	ubiquitin protein ligase E3 component n-recognin 7 [Source:ZFIN;Acc:ZDB-GENE-030131-1535]	20	27093042	27107709		20.64	19.99	20.15	14.39	13.9	13.96
ENSDARG00000005540	xpo7	exportin 7 [Source:ZFIN;Acc:ZDB-GENE-060628-2]	10	20070178	20128254		9.73	6.65	5.94	12.94	13.43	13.48
ENSDARG00000005541	wif1	wnt inhibitory factor 1 [Source:NCBI gene;Acc:30476]	4	12931763	12956718		20.13	21.58	23.59	38.24	44.46	43.04
ENSDARG00000005544	btbd3a	BTB (POZ) domain containing 3a [Source:ZFIN;Acc:ZDB-GENE-090312-7]	1	51191049	51197601		3.26	1.75	1.55	3.34	3.88	3.5
ENSDARG00000005547	rint1	RAD50 interactor 1 [Source:ZFIN;Acc:ZDB-GENE-041210-174]	4	18500012	18512550		4.54	4.39	3.81	4.93	4.73	4.91
ENSDARG00000005549	znf710b	zinc finger protein 710b [Source:ZFIN;Acc:ZDB-GENE-120503-2]	7	30209911	30227878		12.55	6.49	6.28	14.59	14.75	14.54
ENSDARG00000005551	hnrnph1l	heterogeneous nuclear ribonucleoprotein H1, like [Source:ZFIN;Acc:ZDB-GENE-030131-275]	21	30169610	30181764		53.83	38.61	41.32	60.6	58.03	56.78
ENSDARG00000005559	pou4f1	POU class 4 homeobox 1 [Source:ZFIN;Acc:ZDB-GENE-000523-2]	6	4539953	4543005		6.71	2.45	2.43	8.6	8.8	9.82
ENSDARG00000005560	ywhah	tyrosine 3-monooxygenase/tryptophan 5-monooxygenase activation protein, eta polypeptide [Source:ZFIN;Acc:ZDB-GENE-040426	10	17166035	17170086		112.82	49.93	49.93	111.88	108.04	112.84
ENSDARG00000005561	gfm2	G elongation factor, mitochondrial 2 [Source:NCBI gene;Acc:558184]	5	34578479	34600710		6.93	5.37	5.32	4.79	4.94	5.03
ENSDARG00000005565	entpd8	ectonucleoside triphosphate diphosphohydrolase 8 [Source:ZFIN;Acc:ZDB-GENE-040724-142]	6	41038757	41060423		1.42	2.4	1.91	0.77	0.89	0.89
ENSDARG00000005574	vsx2	visual system homeobox 2 [Source:NCBI gene;Acc:796163]	17	31652640	31659670		11.07	3.38	3.62	11.76	11.58	13.14
ENSDARG00000005576	mphosph8	M-phase phosphoprotein 8 [Source:ZFIN;Acc:ZDB-GENE-050309-191]	9	21462467	21488976		29.17	25.21	25	21.2	21.97	21.05
ENSDARG00000005578	ugp2a	UDP-glucose pyrophosphorylase 2a [Source:ZFIN;Acc:ZDB-GENE-090311-46]	1	50997156	51015627		33.29	46.57	33.24	27.99	24.97	23.8
ENSDARG00000005586	zbtb20	zinc finger and BTB domain containing 20 [Source:ZFIN;Acc:ZDB-GENE-070112-1992]	5	67533180	67661102		0.53	0.19	0.29	1.36	1.42	1.54
ENSDARG00000005590	mtf2	metal response element binding transcription factor 2 [Source:ZFIN;Acc:ZDB-GENE-060512-94]	2	10603550	10641998		2.72	1.47	1.33	3.16	2.94	3
ENSDARG00000005593	rxrga	retinoid x receptor, gamma a [Source:NCBI gene;Acc:30464]	2	20725684	20776841		4.15	4.51	2.86	3.61	3.69	3.34
ENSDARG00000005595	adgb	androglobin [Source:ZFIN;Acc:ZDB-GENE-041001-205]	20	31929377	32007209		1.13	0.59	0.97	0.91	0.89	0.91
ENSDARG00000005606	paxip1	PAX interacting (with transcription-activation domain) protein 1 [Source:NCBI gene;Acc:449658]	2	29902294	29923630		6.97	4.37	3.92	5.55	5.59	5.5
ENSDARG00000005612	aph1b	APH1B gamma secretase subunit [Source:NCBI gene;Acc:386808]	7	29176924	29189839		14.8	12.71	14.05	14.34	14.12	14.42
ENSDARG00000005616	bfb	complement component bfb [Source:ZFIN;Acc:ZDB-GENE-990415-34]	21	27437867	27443995		3.17	5.56	4.2	2.27	2.73	2.27
ENSDARG00000005619	nek2	NIMA-related kinase 2 [Source:ZFIN;Acc:ZDB-GENE-040426-752]	20	13883131	13894916		1.09	0.92	0.94	0.68	0.67	0.66
ENSDARG00000005625	maco1a	macoilin 1a [Source:NCBI gene;Acc:393289]	19	29303847	29333867		8.64	5.75	6.45	8.4	8.39	8.78
ENSDARG00000005626	RHOU	si:ch211-133l11.10 [Source:ZFIN;Acc:ZDB-GENE-141219-14]	1	53980120	53988017	RHOU	2.06	1.14	1.18	2.65	2.6	2.45
ENSDARG00000005627	zmp:0000000527	zmp:0000000527 [Source:ZFIN;Acc:ZDB-GENE-130530-530]	17	53356451	53360342		0.72	0.31	0.35	0.92	0.72	0.72
ENSDARG00000005629	smyd2b	SET and MYND domain containing 2b [Source:NCBI gene;Acc:568616]	20	572037	592746		9.8	15.84	15.95	8.77	8.23	9.36
ENSDARG00000005640	adat3	adenosine deaminase tRNA specific 3 [Source:NCBI gene;Acc:368634]	9	41035925	41040492		1.71	2.57	1.66	1.41	0.95	1.51
ENSDARG00000005643	gcat	glycine C-acetyltransferase [Source:ZFIN;Acc:ZDB-GENE-060518-3]	3	25990052	25998243		25.53	34.39	31.49	21.78	18.6	19.97
ENSDARG00000005645	robo3	roundabout, axon guidance receptor, homolog 3 (Drosophila) [Source:ZFIN;Acc:ZDB-GENE-000209-4]	10	31298972	31563049		6.12	3.91	3.67	11.36	11.4	11.76
ENSDARG00000005648	olfml2a	olfactomedin-like 2A [Source:ZFIN;Acc:ZDB-GENE-081105-107]	8	41281801	41342918		3.75	4.94	3.99	5.09	4.94	5.18
ENSDARG00000005651	hrasb	HRas proto-oncogene, GTPase b [Source:ZFIN;Acc:ZDB-GENE-050522-466]	7	49638413	49654492		15.72	6.94	9.7	17.6	17.75	18.3
ENSDARG00000005652	man1a1	mannosidase, alpha, class 1A, member 1 [Source:ZFIN;Acc:ZDB-GENE-041014-321]	20	41021054	41242638		4.83	3.11	2.31	6.21	6	5.8
ENSDARG00000005670	ttbk2a	tau tubulin kinase 2a [Source:ZFIN;Acc:ZDB-GENE-120928-3]	17	45216084	45254585		2.66	0.82	0.96	4.91	4.76	4.69
ENSDARG00000005673	f3b	coagulation factor IIIb [Source:ZFIN;Acc:ZDB-GENE-050417-234]	2	15100742	15106968		4.82	4.56	3.94	6.88	7.79	8.44
ENSDARG00000005675	sec61a1l	SEC61 translocon alpha 1 subunit, like [Source:NCBI gene;Acc:266796]	6	42377911	42388608		8.84	8.58	7.39	10.21	10.42	9.41
ENSDARG00000005679	trip10a	thyroid hormone receptor interactor 10a [Source:NCBI gene;Acc:492762]	3	54431093	54450351		3.54	3.27	2.82	3.84	3.76	4.04
ENSDARG00000005690	slc25a23a	solute carrier family 25 member 23a [Source:ZFIN;Acc:ZDB-GENE-121214-154]	1	54974191	54997746		4.53	3.24	2.75	5.86	5.69	5.6
ENSDARG00000005699	ddx19	DEAD/H (Asp-Glu-Ala-Asp/His) box polypeptide 19 (DBP5 homolog, yeast) [Source:ZFIN;Acc:ZDB-GENE-020419-30]	11	3523358	3535581		16.92	17.54	17.64	16.11	15.92	14.92
ENSDARG00000005704	eps8l3b	EPS8-like 3b [Source:ZFIN;Acc:ZDB-GENE-040426-1335]	8	25308003	25329967		6.27	6.77	3.98	3.75	4.21	4.48
ENSDARG00000005713	ethe1	ETHE1 persulfide dioxygenase [Source:ZFIN;Acc:ZDB-GENE-040426-2503]	16	54303491	54320489		9.67	11.78	9.59	14.46	15.23	15.7
ENSDARG00000005716	olfml2ba	olfactomedin-like 2Ba [Source:ZFIN;Acc:ZDB-GENE-041014-329]	20	33792156	33806508		3.47	3.84	3.89	5.41	5.57	4.97
ENSDARG00000005738	rpf1	ribosome production factor 1 homolog [Source:NCBI gene;Acc:415250]	11	16094661	16115804		16.26	19.88	17.04	12.92	13.79	12.57
ENSDARG00000005739	gpm6ba	glycoprotein M6Ba [Source:ZFIN;Acc:ZDB-GENE-030710-9]	1	28961653	29045426		22.54	5.35	9.12	29.74	30.16	29.99
ENSDARG00000005746	cand2	cullin-associated and neddylation-dissociated 2 (putative) [Source:ZFIN;Acc:ZDB-GENE-060503-645]	22	31045277	31060579		5.63	8.08	8.04	5.39	5.59	5.75
ENSDARG00000005754	ptprfb	protein tyrosine phosphatase receptor type Fb [Source:NCBI gene;Acc:799867]	2	17578320	17947389		18.19	11.73	7.44	26.48	25.23	26.47
ENSDARG00000005762	col14a1a	collagen, type XIV, alpha 1a [Source:ZFIN;Acc:ZDB-GENE-030131-9889]	16	14684916	15050842		1.76	1.03	1	3.13	2.97	2.58
ENSDARG00000005772	tsr2	TSR2 ribosome maturation factor [Source:NCBI gene;Acc:317639]	8	8341082	8349653		18.93	20.53	21.47	12.95	12.6	14.02
ENSDARG00000005774	ddx3xb	DEAD-box helicase 3 X-linked b [Source:ZFIN;Acc:ZDB-GENE-980526-150]	6	59832786	59850404		34.76	27.87	24.58	51.5	48.89	48.9
ENSDARG00000005775	scn8aa	sodium channel, voltage gated, type VIII, alpha subunit a [Source:ZFIN;Acc:ZDB-GENE-000828-1]	23	27251626	27345425		17.78	3.5	6.54	23.54	25.48	25.66

ENSDARG00000005776	guk1b	guanylate kinase 1b [Source:ZFIN;Acc:ZDB-GENE-020916-1]	8	18230948	18239498		19.74	6.55	5.21	11.94	13.42	13.87
ENSDARG00000005780	npy8br	neuropeptide Y receptor Y8b [Source:ZFIN;Acc:ZDB-GENE-980526-208]	8	51634803	51635930		1.9	0.84	1.08	2.21	2.2	2.53
ENSDARG00000005783	ncanb	neurocan b [Source:ZFIN;Acc:ZDB-GENE-050208-586]	22	17864872	18116635		7.75	1.68	2.11	9.59	8.77	9.63
ENSDARG00000005786	ppm1f	protein phosphatase, Mg2+/Mn2+ dependent, 1F [Source:ZFIN;Acc:ZDB-GENE-051128-2]	5	13181029	13206878		0.67	0.36	0.32	0.79	0.79	0.72
ENSDARG00000005789	enpp1	ectonucleotide pyrophosphatase/phosphodiesterase 1 [Source:ZFIN;Acc:ZDB-GENE-040724-172]	20	13403281	13521010		6.65	8.25	7.13	9.05	9.43	8.92
ENSDARG00000005791	rpl28	ribosomal protein L28 [Source:ZFIN;Acc:ZDB-GENE-040930-10]	16	51883725	51889734		348.35	375.52	440.45	266.74	268.26	275.92
ENSDARG00000005799	smim14	small integral membrane protein 14 [Source:ZFIN;Acc:ZDB-GENE-040426-22]	1	23297623	23308308		6.56	7.52	6.89	6.29	5.64	5.59
ENSDARG00000005800	ampd3a	adenosine monophosphate deaminase 3a [Source:ZFIN;Acc:ZDB-GENE-110407-2]	7	66836476	66868543		3.1	2.63	1.97	4.76	4.29	4.29
ENSDARG00000005814	klhl10a	kelch-like family member 10a [Source:ZFIN;Acc:ZDB-GENE-131015-1]	17	4386649	4396692		0	0	0.01	0	0.01	0.01
ENSDARG00000005821	ncf2	neutrophil cytosolic factor 2 [Source:ZFIN;Acc:ZDB-GENE-070209-29]	7	41329748	41338923		0.53	0.47	0.52	0.59	0.64	0.79
ENSDARG00000005823	slc39a10	solute carrier family 39 member 10 [Source:NCBI gene;Acc:393644]	9	24920677	24951662		6.1	5.18	3.89	6.35	6.43	6.93
ENSDARG00000005827	eif2s1a	eukaryotic translation initiation factor 2, subunit 1 alpha a [Source:ZFIN;Acc:ZDB-GENE-030131-526]	17	34244345	34254233		25.07	29.44	25.21	20.92	19.96	19.59
ENSDARG00000005828	faima	Fas apoptotic inhibitory molecule a [Source:ZFIN;Acc:ZDB-GENE-040718-323]	9	23748133	23755337		3.47	2.2	2.98	3.37	3.33	3.51
ENSDARG00000005834	gatad2b	GATA zinc finger domain containing 2B [Source:ZFIN;Acc:ZDB-GENE-060503-655]	19	41701660	41724246		7.4	2.87	2.78	8.98	9.94	9.35
ENSDARG00000005840	got2b	glutamic-oxaloacetic transaminase 2b, mitochondrial [Source:ZFIN;Acc:ZDB-GENE-030131-7917]	7	51774502	51793881		113.74	137.12	155.76	89.63	87.46	87.14
ENSDARG00000005841	tnni2a.2	troponin I type 2a (skeletal, fast), tandem duplicate 2 [Source:ZFIN;Acc:ZDB-GENE-040426-970]	25	31238606	31248602		11.13	11.95	11.94	6.63	7.2	6.96
ENSDARG00000005842	cd9a	CD9 molecule a [Source:ZFIN;Acc:ZDB-GENE-030131-1175]	18	7122577	7143920		16.62	11.9	14.13	18.01	20.43	19.43
ENSDARG00000005846		Sp5 transcription factor b [Source:NCBI gene;Acc:799504]	6	3730843	3813374		0.18	0.19	0.21	0.04	0.12	0.12
ENSDARG00000005853	slc25a6	solute carrier family 25 member 6 [Source:ZFIN;Acc:ZDB-GENE-070912-446]	9	34866576	34871900		20.47	9.46	11.76	15.3	17.5	17.04
ENSDARG00000005861	ralgapa2	Ral GTPase activating protein, alpha subunit 2 (catalytic) [Source:ZFIN;Acc:ZDB-GENE-131122-56]	17	41475606	41798856		6.2	3.8	3.06	9.06	9.21	9.21
ENSDARG00000005865	or131-1	odorant receptor, family H, subfamily 131, member 1 [Source:ZFIN;Acc:ZDB-GENE-070806-45]	15	18221859	18223769		0.11	0.05	0.06	0.06	0.03	0.08
ENSDARG00000005867	gon4l	gon-4-like (C. elegans) [Source:ZFIN;Acc:ZDB-GENE-070117-2447]	16	28994709	29024769		5.87	3.3	2.83	5.51	5.48	5.29
ENSDARG00000005870	mmadhc	metabolism of cobalamin associated D [Source:ZFIN;Acc:ZDB-GENE-040704-50]	9	23049500	23056240		22.95	25.3	25.18	18.12	17.15	18
ENSDARG00000005883	rfx1a	regulatory factor X, 1a (influences HLA class II expression) [Source:ZFIN;Acc:ZDB-GENE-070424-94]	3	19152222	19200651		5.39	1.09	2.03	6.37	6.44	7.05
ENSDARG00000005891	cyb5r3	cytochrome b5 reductase 3 [Source:ZFIN;Acc:ZDB-GENE-030131-3281]	4	11804675	11810799		19.93	25.17	21.8	15.13	14	15.19
ENSDARG00000005894	slc7a9	solute carrier family 7 member 9 [Source:ZFIN;Acc:ZDB-GENE-080116-2]	7	38507006	38527833		2.54	1.41	1.06	0.98	0.78	0.91
ENSDARG00000005897	dera	deoxyribose-phosphate aldolase (putative) [Source:ZFIN;Acc:ZDB-GENE-041010-200]	4	12755136	12766418		11.04	12.86	11.01	8.62	9.01	8.83
ENSDARG00000005908	clk4b	CDC-like kinase 4b [Source:ZFIN;Acc:ZDB-GENE-050227-19]	21	32280175	32301109		8.94	5.24	7.02	8.54	8.46	8.33
ENSDARG00000005913	tgm1l3	transglutaminase 1 like 3 [Source:ZFIN;Acc:ZDB-GENE-120718-1]	23	40234602	40250886		0.7	0.45	0.44	0.67	0.51	0.92
ENSDARG00000005915	tcf3a	transcription factor 3a [Source:ZFIN;Acc:ZDB-GENE-990415-51]	2	57310523	57379216		6.05	3.95	3.91	8.58	8.61	8.51
ENSDARG00000005916	srpk3	SRSF protein kinase 3 [Source:ZFIN;Acc:ZDB-GENE-101025-2]	8	8291492	8339372		8.39	6.33	6.39	10.67	11.37	10.58
ENSDARG00000005924	serpina10a	serpin peptidase inhibitor, clade A (alpha-1 antiproteinase, antitrypsin), member 10a [Source:ZFIN;Acc:ZDB-GENE-041014-246]	20	27371394	27381691		54.36	43.46	50.4	53.79	58.56	57.3
ENSDARG00000005926	ak2	adenylate kinase 2 [Source:NCBI gene;Acc:321793]	19	35418683	35428914		82.53	97.1	115.38	65.14	68.88	68.8
ENSDARG00000005927	tbc1d17	TBC1 domain family, member 17 [Source:ZFIN;Acc:ZDB-GENE-050522-79]	3	30166946	30186298		12.23	10.47	9.68	12.31	11.49	12.01
ENSDARG00000005941	clul1	clusterin-like 1 (retinal) [Source:ZFIN;Acc:ZDB-GENE-031118-57]	2	31271082	31364659		8.56	7.39	5.44	13.07	13.95	13.56
ENSDARG00000005942	vmn2r1	vomeronasal 2, receptor 1 [Source:ZFIN;Acc:ZDB-GENE-050419-65]	18	34225520	34242558		0.89	0.45	0.35	0.48	0.48	0.54
ENSDARG00000005943	htra4	HtrA serine peptidase 4 [Source:ZFIN;Acc:ZDB-GENE-131120-192]	5	26930062	26950374		7.83	8.25	6.92	1.49	1.11	1.09
ENSDARG00000005945	sart1	spliceosome associated factor 1, recruiter of U4/U6.U5 tri-snRNP [Source:ZFIN;Acc:ZDB-GENE-040718-422]	21	27302752	27324771		36.35	28.23	28.78	29.92	30.89	29.86
ENSDARG00000005948	ankmy2a	ankyrin repeat and MYND domain containing 2a [Source:ZFIN;Acc:ZDB-GENE-030131-6321]	15	34573311	34590820		7.32	7.24	7.16	5.94	5.5	5.91
ENSDARG00000005972	ric8b	RIC8 guanine nucleotide exchange factor B [Source:NCBI gene;Acc:334503]	18	15287065	15329471		12.1	10.03	10.72	10.13	10.47	9.67
ENSDARG00000005980	sfswap	splicing factor SWAP [Source:ZFIN;Acc:ZDB-GENE-040426-931]	21	15125497	15282452		25.55	10.15	14.47	22.85	23.84	23.89
ENSDARG00000005989	rgl1	ral guanine nucleotide dissociation stimulator-like 1 [Source:NCBI gene;Acc:402933]	8	19283143	19342111		6.75	7.84	5.96	16.72	17.71	18.03
ENSDARG00000005993	prc1b	protein regulator of cytokinesis 1b [Source:ZFIN;Acc:ZDB-GENE-040426-777]	7	50353297	50367642		4.81	4.64	4.69	3.78	3.85	4.08
ENSDARG00000006003	mycla	MYCL proto-oncogene, bHLH transcription factor a [Source:NCBI gene;Acc:405873]	13	35904335	35908275		8.33	5.29	5.47	6.69	6.02	6.25
ENSDARG00000006008	dct	dopachrome tautomerase [Source:ZFIN;Acc:ZDB-GENE-000508-1]	9	53327332	53352682		10.15	12.42	13.38	20.65	19.15	20.93
ENSDARG00000006010	bmi1a	bmi1 polycomb ring finger oncogene 1a [Source:NCBI gene;Acc:321505]	24	17260001	17267235		8.05	7.81	8.32	9.57	10.27	9.55
ENSDARG00000006011	klhl42	kelch-like family, member 42 [Source:ZFIN;Acc:ZDB-GENE-041210-190]	4	17634311	17642168		0.95	0.69	0.58	0.84	0.74	0.66
ENSDARG00000006019	tktb	transketolase b [Source:ZFIN;Acc:ZDB-GENE-030909-13]	23	20113378	20126257		103.98	121.59	127.46	145.39	161.74	153.23
ENSDARG00000006025	tp53i11b	tumor protein p53 inducible protein 11b [Source:ZFIN;Acc:ZDB-GENE-030131-6328]	18	27439680	27570030		6.67	3.73	3.99	7.87	8.31	8.06
ENSDARG00000006029	lta4h	leukotriene A4 hydrolase [Source:ZFIN;Acc:ZDB-GENE-040426-2477]	4	7653607	7673165		43.68	77.41	61.93	19.3	21.8	21.58
ENSDARG00000006031	abat	4-aminobutyrate aminotransferase [Source:ZFIN;Acc:ZDB-GENE-031006-4]	3	27832500	27880229		57.05	51.46	45.13	62.92	72.98	68.22
ENSDARG00000006038	aebp2	AE binding protein 2 [Source:NCBI gene;Acc:406815]	4	2923199	2945306		6.31	4.82	4.52	8.22	7.84	8.56
ENSDARG00000006040	cyp20a1	cytochrome P450, family 20, subfamily A, polypeptide 1 [Source:ZFIN;Acc:ZDB-GENE-040426-2646]	6	9952678	9969458		13.63	15.86	17.18	13.04	13.42	12.86
ENSDARG00000006044	usta	uronyl 2-sulfotransferase a [Source:ZFIN;Acc:ZDB-GENE-041001-163]	20	31309364	31427390		1.72	1.9	1.73	3.27	3.59	3
ENSDARG00000006052	malt2	MALT paracaspase 2 [Source:ZFIN;Acc:ZDB-GENE-020801-2]	22	15706062	15717897		1.25	1.22	1.24	1.85	2.34	1.96
ENSDARG00000006060	crybg1b	crystallin beta-gamma domain containing 1b [Source:ZFIN;Acc:ZDB-GENE-030131-7179]	4	861444	890220		6.18	4.14	2.5	5.23	5.06	5.01
ENSDARG00000006062	akap1b	A kinase (PRKA) anchor protein 1b [Source:ZFIN;Acc:ZDB-GENE-030131-6844]	15	30836981	30857350		8.97	8.34	6.17	10.26	9.87	9.69
ENSDARG00000006065	znf385b	zinc finger protein 385B [Source:NCBI gene;Acc:368405]	9	43243212	43538328		4.39	2.34	2.36	5.43	5.85	6.16
ENSDARG00000006074	uck2a	uridine-cytidine kinase 2a [Source:NCBI gene;Acc:100005763]	8	21114338	21133119		8.1	4.72	5.02	10.42	10.16	9.68
ENSDARG00000006079	gpr37l1a	G protein-coupled receptor 37 like 1a [Source:ZFIN;Acc:ZDB-GENE-110411-20]	22	696931	701105		3.89	1.32	1.96	4.84	5.16	5.1
ENSDARG00000006092	rad21a	RAD21 cohesin complex component a [Source:NCBI gene;Acc:322275]	16	48409066	48430272		82.09	60.08	58.11	75.88	76.09	78.39
ENSDARG00000006093	cdk15	cyclin-dependent kinase 15 [Source:NCBI gene;Acc:791619]	6	9747108	9792004		4.17	2.25	2.29	2.58	3.22	3.53
ENSDARG00000006094	igf2r	insulin-like growth factor 2 receptor [Source:ZFIN;Acc:ZDB-GENE-041014-300]	20	42563618	42660656		3.38	2.7	1.86	5.37	5.16	5.15
ENSDARG00000006109	pimr185	Pim proto-oncogene, serine/threonine kinase, related 185 [Source:ZFIN;Acc:ZDB-GENE-081104-450]	8	47817454	47819141		0.27	0.32	0.14	0.02	0.1	0.15
ENSDARG00000006110	chrd	chordin [Source:NCBI gene;Acc:30161]	15	46790676	46828958		0.91	0.8	0.67	1.7	1.79	1.53
ENSDARG00000006112	myof	myoferlin [Source:ZFIN;Acc:ZDB-GENE-040426-1138]	12	10517473	10567188		5.73	5.4	4.53	5.63	5.69	5.22
ENSDARG00000006120	tbx2b	T-box transcription factor 2b [Source:NCBI gene;Acc:30253]	15	27364394	27371340		26.43	14.18	11.57	27.04	28.09	31.23
ENSDARG00000006124	kdm5c	lysine (K)-specific demethylase 5C [Source:ZFIN;Acc:ZDB-GENE-060810-94]	8	36983559	37026607		29.69	19.02	17.12	39.96	39.55	38.92
ENSDARG00000006125	csnk1db	casein kinase 1, delta b [Source:NCBI gene;Acc:406533]	12	33320504	33336787		41.09	29.63	26.69	53.16	55.34	53.69
ENSDARG00000006128	cep170aa	centrosomal protein 170Aa [Source:NCBI gene;Acc:557463]	13	10980755	11072964		3.78	1.9	1.76	6.86	6.06	6.22
ENSDARG00000006137	star	steroidogenic acute regulatory protein [Source:NCBI gene;Acc:63999]	8	44703854	44707937		6.14	8.3	7.55	8.93	8.8	9.02
ENSDARG00000006151	pde11al	phosphodiesterase 11a, like [Source:ZFIN;Acc:ZDB-GENE-031118-106]	16	21144669	21185778		1.4	0.69	0.86	1.39	1.37	1.23
ENSDARG00000006169	lrrk2	leucine-rich repeat kinase 2 [Source:ZFIN;Acc:ZDB-GENE-071218-6]	25	35603107	35664817		4.69	2.54	2.8	5.24	5.32	5.55
ENSDARG00000006174	lrrc23	leucine rich repeat containing 23 [Source:ZFIN;Acc:ZDB-GENE-041010-153]	23	20127883	20133994		1.08	1	0.97	0.57	1.12	1.05
ENSDARG00000006181	hif1aa	hypoxia inducible factor 1 subunit alpha a [Source:ZFIN;Acc:ZDB-GENE-080917-55]	13	31757331	31783663		6.75	2.14	2.56	6.16	6.01	5.71
ENSDARG00000006192	gatad2ab	GATA zinc finger domain containing 2Ab [Source:ZFIN;Acc:ZDB-GENE-040426-2372]	22	18315490	18387919		2.46	1.8	1.39	3.79	3.67	3.43
ENSDARG00000006196	sav1	salvador family WW domain containing protein 1 [Source:ZFIN;Acc:ZDB-GENE-040912-28]	13	36787077	36798204		14.4	16.55	14.29	15.33	15.45	15.31
ENSDARG00000006200	eif4g1a	eukaryotic translation initiation factor 4 gamma, 1a [Source:ZFIN;Acc:ZDB-GENE-070112-702]	2	16798652	16842699		16.88	16.95	11.4	24.67	23.45	23.42
ENSDARG00000006202	erbb3a	erb-b2 receptor tyrosine kinase 3a [Source:ZFIN;Acc:ZDB-GENE-030916-3]	6	7466223	7531427		9.8	5.72	3.64	7.54	6.52	6.5
ENSDARG00000006206	pou4f3	POU class 4 homeobox 3 [Source:NCBI gene;Acc:30534]	9	54290896	54292162		4.77	1.85	1.88	4.06	4.37	5.09
ENSDARG00000006207	gpx1b	glutathione peroxidase 1b [Source:ZFIN;Acc:ZDB-GENE-040912-60]	6	42338309	42341219		17.53	20.83	24.35	11.72	11.44	12.9
ENSDARG00000006215	akr1b1.1	aldo-keto reductase family 1, member B1 (aldose reductase), tandem duplicate 1 [Source:ZFIN;Acc:ZDB-GENE-040625-7]	4	14899720	14910386		7.96	12.45	9.54	6.05	6.16	6.25
ENSDARG00000006219	gnl3	guanine nucleotide binding protein-like 3 (nucleolar) [Source:NCBI gene;Acc:321897]	11	3959495	3971035		33.79	41.22	37.78	20.32	20.1	19.79
ENSDARG00000006220	ugt1a1	UDP glucuronosyltransferase 1 family, polypeptide A1 [Source:ZFIN;Acc:ZDB-GENE-071004-4]	9	9969987	10003782		15.57	28.87	21.03	5.79	6.14	5.2
ENSDARG00000006225	ddx39aa	DEAD (Asp-Glu-Ala-Asp) box polypeptide 39Aa [Source:ZFIN;Acc:ZDB-GENE-030131-4275]	1	45205920	45215343		62.96	60.85	69.18	51.85	54.2	52.48
ENSDARG00000006235	brms1la	BRMS1 like transcriptional repressor a [Source:NCBI gene;Acc:792017]	17	12753579	12764360		11.75	11.37	9.25	13.58	13.53	15.06
ENSDARG00000006240	slc27a1a	solute carrier family 27 member 1a [Source:ZFIN;Acc:ZDB-GENE-050320-112]	3	52545014	52603683		10.23	11.76	9.99	13.23	13.06	12.45
ENSDARG00000006242	ptp4a1	protein tyrosine phosphatase 4A1 [Source:ZFIN;Acc:ZDB-GENE-041121-11]	11	11267493	11274919		52.84	34.15	39.29	78.5	79.08	81.52
ENSDARG00000006251	ell2	elongation factor, RNA polymerase II, 2 [Source:ZFIN;Acc:ZDB-GENE-030131-1717]	21	11749097	11774964		10.17	11.48	7.79	11.59	11	10.85
ENSDARG00000006253	mier3a	mesoderm induction early response 1, family member 3 a [Source:ZFIN;Acc:ZDB-GENE-050913-97]	10	2695464	2713240		10.98	7.43	7.58	12.66	12.63	12.49
ENSDARG00000006257	vldlr	very low density lipoprotein receptor [Source:ZFIN;Acc:ZDB-GENE-040426-803]	10	15255012	15305767		14.53	4.76	5.25	21.26	21.62	22.54
ENSDARG00000006260	tuba8l4	tubulin, alpha 8 like 4 [Source:ZFIN;Acc:ZDB-GENE-040426-860]	6	6972953	6976107		226.31	210.93	202.05	218.88	218.95	214.35
ENSDARG00000006266	stat1a	signal transducer and activator of transcription 1a [Source:ZFIN;Acc:ZDB-GENE-980526-499]	22	12798569	12826962		12.42	12.46	10.11	12.03	11.67	12.46
ENSDARG00000006272	mpp5a	membrane protein, palmitoylated 5a (MAGUK p55 subfamily member 5) [Source:NCBI gene;Acc:252845]	17	34186632	34239514		3.03	1.87	1.73	4.1	4.27	4.3
ENSDARG00000006279	rragd	ras-related GTP binding D [Source:ZFIN;Acc:ZDB-GENE-070620-6]	17	15788100	15820304		0.92	0.84	0.68	0.92	0.84	0.92
ENSDARG00000006283	waslb	WASP like actin nucleation promoting factor b [Source:ZFIN;Acc:ZDB-GENE-040426-718]	4	4079418	4111767		11.6	8.84	6.16	10.81	10.63	10.75
ENSDARG00000006288	prdm5	PR domain containing 5 [Source:ZFIN;Acc:ZDB-GENE-040708-1]	23	1926562	2037659		1.5	1.57	1.53	3.27	3.66	3.35
ENSDARG00000006290	ndufs5	NADH:ubiquinone oxidoreductase subunit S5 [Source:ZFIN;Acc:ZDB-GENE-050522-437]	19	35357196	35361556		145.52	155.66	184.49	103.82	99.93	102.53
ENSDARG00000006292	maats1	MYCBP-associated, testis expressed 1 [Source:ZFIN;Acc:ZDB-GENE-070912-489]	9	9441453	9501931		0.44	0.38	0.37	0.66	0.57	0.54
ENSDARG00000006299	arhgef7a	Rho guanine nucleotide exchange factor (GEF) 7a [Source:ZFIN;Acc:ZDB-GENE-060322-11]	9	21535885	21570566		5.49	1.56	2.3	6.59	6.84	6.96
ENSDARG00000006300	psmd9	proteasome 26S subunit, non-ATPase 9 [Source:ZFIN;Acc:ZDB-GENE-030131-431]	7	72003301	72011167		15.5	19.06	20.23	13.21	13.7	14.12
ENSDARG00000006301	RAPH1	wu:fj59g11 [Source:ZFIN;Acc:ZDB-GENE-030131-8202]	9	13814963	13963078	RAPH1	6.7	3.61	2.87	11.72	10.38	11.07
ENSDARG00000006307	shisa4	shisa family member 4 [Source:ZFIN;Acc:ZDB-GENE-050417-419]	11	24877820	24888595		4.18	1.62	1.89	5.15	5.69	5.1
ENSDARG00000006308	ptpn18	protein tyrosine phosphatase non-receptor type 18 [Source:ZFIN;Acc:ZDB-GENE-050320-12]	8	9641252	9684872		0.7	0.77	0.42	0.67	0.62	0.48
ENSDARG00000006314	itgav	integrin, alpha V [Source:ZFIN;Acc:ZDB-GENE-060616-382]	9	11597500	11655031		41.96	40.64	30.72	45.36	38.41	37.22
ENSDARG00000006316	rpl23a	ribosomal protein L23a [Source:ZFIN;Acc:ZDB-GENE-030131-7479]	21	26071874	26074640		811.54	906.56	989.29	610.85	651.55	649.54
ENSDARG00000006332	snx14	sorting nexin 14 [Source:NCBI gene;Acc:555970]	20	851583	894374		13.11	9.02	7.98	13.26	14.06	14.22
ENSDARG00000006341	tas1r3	taste receptor, type 1, member 3 [Source:ZFIN;Acc:ZDB-GENE-070917-3]	11	40731602	40742507		0.59	0.37	0.3	0.12	0.09	0.13
ENSDARG00000006345	med17	mediator complex subunit 17 [Source:NCBI gene;Acc:567560]	15	436776	446010		13.68	10.48	10.84	13.53	14.93	15.06
ENSDARG00000006350	nkx1.2la	NK1 transcription factor related 2-like,a [Source:ZFIN;Acc:ZDB-GENE-040615-1]	13	40770628	40775530		0.1	0.09	0.06	0.23	0.31	0.19
ENSDARG00000006353	itga5	integrin, alpha 5 (fibronectin receptor, alpha polypeptide) [Source:ZFIN;Acc:ZDB-GENE-031116-52]	23	33447625	33558161		2.26	2.53	1.89	3.94	3.68	3.65
ENSDARG00000006356	slc18a3a	solute carrier family 18 member 3a [Source:NCBI gene;Acc:559347]	13	29422049	29424454		13.27	6.24	7.8	14.09	15.51	16.12
ENSDARG00000006368	crlf1b	cytokine receptor-like factor 1b [Source:ZFIN;Acc:ZDB-GENE-080108-1]	22	21152847	21165192		2.19	2.63	2.63	2.62	2.42	2.26
ENSDARG00000006370	atp6v1h	ATPase H+ transporting V1 subunit H [Source:ZFIN;Acc:ZDB-GENE-021219-2]	2	30786773	30846927		64.42	48.93	57.28	47.63	50.74	50.55
ENSDARG00000006372	ugt5c2	UDP glucuronosyltransferase 5 family, polypeptide C2 [Source:ZFIN;Acc:ZDB-GENE-060825-206]	18	40462445	40469527		0.34	0.34	0.29	0.25	0.45	0.2
ENSDARG00000006383	stxbp5l	syntaxin binding protein 5-like [Source:NCBI gene;Acc:567605]	9	27415754	27649406		5.68	1.17	1.93	7.63	7.91	7.51
ENSDARG00000006385	triobpb	TRIO and F-actin binding protein b [Source:ZFIN;Acc:ZDB-GENE-061215-80]	3	1167026	1180520		10.91	11	10.96	10.56	10.34	10.02
ENSDARG00000006389	smad2	SMAD family member 2 [Source:NCBI gene;Acc:30639]	10	14871136	14943281		6.52	4.55	3.5	10.5	10.78	10.97
ENSDARG00000006392	exosc9	exosome component 9 [Source:NCBI gene;Acc:450057]	1	19083501	19092155		8.77	10.29	10.17	7.49	6.4	6.78
ENSDARG00000006395	rbm25b	RNA binding motif protein 25b [Source:ZFIN;Acc:ZDB-GENE-030131-5547]	20	51131933	51186565		30.89	17.04	16.33	28.47	26.8	27.77
ENSDARG00000006396	nrcama	neuronal cell adhesion molecule a [Source:ZFIN;Acc:ZDB-GENE-041210-235]	4	13696537	13728375		7.54	1.21	2.15	8.52	7.88	8.95
ENSDARG00000006397	laptm4a	lysosomal protein transmembrane 4 alpha [Source:ZFIN;Acc:ZDB-GENE-030131-1024]	20	43775793	43786515		46.67	38.1	38.02	53.98	52.54	50.67
ENSDARG00000006399	ywhae1	tyrosine 3-monooxygenase/tryptophan 5-monooxygenase activation protein, epsilon polypeptide 1 [Source:ZFIN;Acc:ZDB-GENE-0	15	24746444	24754895		123.54	104.57	101.52	132.79	132.54	135.05
ENSDARG00000006408	hmgb3b	high mobility group box 3b [Source:ZFIN;Acc:ZDB-GENE-050417-290]	21	34119759	34129776		37.35	44.78	39.48	27.62	30.01	28.92
ENSDARG00000006409	mapk12b	mitogen-activated protein kinase 12b [Source:ZFIN;Acc:ZDB-GENE-041210-123]	4	5523690	5530441		0.39	0.45	0.49	0.71	0.57	0.54
ENSDARG00000006413	rpl38	ribosomal protein L38 [Source:ZFIN;Acc:ZDB-GENE-030131-8752]	12	38556462	38561842		306.68	446.92	419.06	243	260.97	270.1
ENSDARG00000006420	atrnl1b	attractin-like 1b [Source:ZFIN;Acc:ZDB-GENE-030131-8092]	12	29842836	30032188		7.31	3.57	3.04	12.59	12.26	13.41
ENSDARG00000006422	esyt3	extended synaptotagmin-like protein 3 [Source:ZFIN;Acc:ZDB-GENE-050809-35]	9	23783856	23807032		6.05	4.21	3.34	4.62	4.46	4.06
ENSDARG00000006427	fabp2	fatty acid binding protein 2, intestinal [Source:ZFIN;Acc:ZDB-GENE-991019-5]	1	25848231	25851575		524.07	1009.16	698.38	46.22	42.36	48.85
ENSDARG00000006434	polr1e	RNA polymerase I subunit E [Source:ZFIN;Acc:ZDB-GENE-050522-251]	1	19637312	19648227		9.42	11.54	11.31	5.52	5.34	5.16
ENSDARG00000006447	slc19a3a	solute carrier family 19 member 3a [Source:ZFIN;Acc:ZDB-GENE-110411-41]	15	35139457	35156791		0.89	0.97	0.93	0.3	0.2	0.16
ENSDARG00000006456	pdgfrl	platelet-derived growth factor receptor-like [Source:ZFIN;Acc:ZDB-GENE-040426-901]	14	30359124	30366196		25.54	32.36	35.84	20.95	21.99	21.4
ENSDARG00000006467	pcdh8	protocadherin 8 [Source:ZFIN;Acc:ZDB-GENE-980526-242]	9	53654866	53666031		3.18	0.46	0.36	3.8	3.86	4.44
ENSDARG00000006468	grap2a	GRB2 related adaptor protein 2a [Source:ZFIN;Acc:ZDB-GENE-050417-275]	3	29445473	29468859		1.78	1.74	1.67	1.84	1.94	2.15
ENSDARG00000006474	saxo2	stabilizer of axonemal microtubules 2 [Source:ZFIN;Acc:ZDB-GENE-051127-27]	7	12371061	12380200		1.71	1.67	1.45	1.48	2.17	2.21
ENSDARG00000006477	ddi2	DNA-damage inducible protein 2 [Source:NCBI gene;Acc:386644]	6	40554551	40563509		10.74	9	7.35	14.31	13.31	13.69
ENSDARG00000006487	anp32a	acidic (leucine-rich) nuclear phosphoprotein 32 family, member A [Source:NCBI gene;Acc:335907]	7	33345624	33351532		130.14	108.55	105.91	121.61	127.07	124.3
ENSDARG00000006491	agpat9l	1-acylglycerol-3-phosphate O-acyltransferase 9, like [Source:NCBI gene;Acc:567414]	5	8919698	8957068		8.34	12.05	10.25	5.63	5.69	5.15
ENSDARG00000006494	asb7	ankyrin repeat and SOCS box containing 7 [Source:ZFIN;Acc:ZDB-GENE-040426-2429]	7	9840994	9873652		1.92	1.14	0.8	2.37	2.18	2.13
ENSDARG00000006497	rtn1a	reticulon 1a [Source:ZFIN;Acc:ZDB-GENE-030131-2426]	13	31424586	31470439		123.86	75.94	89.32	122.83	127.84	127.85
ENSDARG00000006501	cyp2x10.2	cytochrome P450, family 2, subfamily X, polypeptide 10.2 [Source:ZFIN;Acc:ZDB-GENE-060818-12]	7	51950614	51953850		0.41	0.57	0.64	0.19	0.22	0.18
ENSDARG00000006508	pip5k1bb	phosphatidylinositol-4-phosphate 5-kinase, type I, beta b [Source:ZFIN;Acc:ZDB-GENE-040912-141]	8	11234168	11324143		5.03	4.61	4.55	4.53	4.7	4.32
ENSDARG00000006514	her6	hairy-related 6 [Source:ZFIN;Acc:ZDB-GENE-980526-144]	6	36551083	36552844		25.3	22.48	25.09	21.13	22.64	22.02
ENSDARG00000006522	otop2	otopetrin 2 [Source:ZFIN;Acc:ZDB-GENE-050104-2]	3	56905103	56918442		1.11	1.14	0.65	0.35	0.51	0.31
ENSDARG00000006524	supt6h	SPT6 homolog, histone chaperone and transcription elongation factor [Source:NCBI gene;Acc:337866]	21	26028947	26058547		14.44	9.35	8.18	17.67	17.67	18.19
ENSDARG00000006526	fn1b	fibronectin 1b [Source:ZFIN;Acc:ZDB-GENE-030131-6545]	1	5504128	5543065		42.24	46.7	20.88	232.55	185.06	180.95
ENSDARG00000006527	brd3a	bromodomain containing 3a [Source:ZFIN;Acc:ZDB-GENE-030131-6141]	21	18266319	18275337		13.23	8.47	8.72	15.24	14.75	15.07
ENSDARG00000006544	c1qtnf6b	C1q and TNF related 6b [Source:ZFIN;Acc:ZDB-GENE-080411-1]	6	214269	218882		1.79	2.33	2.65	2.83	2.52	2.71
ENSDARG00000006545	rgp1	GP1 homolog, RAB6A GEF complex partner 1 [Source:ZFIN;Acc:ZDB-GENE-040718-352]	7	24627076	24645540		1.66	1.6	1.63	1.81	1.88	1.79
ENSDARG00000006546	ak4	adenylate kinase 4 [Source:ZFIN;Acc:ZDB-GENE-040426-2505]	6	31350101	31364551		6.94	5.99	5.51	8.41	8.44	8.65
ENSDARG00000006553	rras	RAS related [Source:ZFIN;Acc:ZDB-GENE-041010-217]	3	32410746	32416121		12.31	15.35	12.03	17.94	16.91	16.26
ENSDARG00000006560	irf4a	interferon regulatory factor 4a [Source:ZFIN;Acc:ZDB-GENE-070912-330]	2	854444	879800		0.63	0.39	0.37	0.45	0.45	0.36
ENSDARG00000006566	nlk1	nemo-like kinase, type 1 [Source:ZFIN;Acc:ZDB-GENE-040701-1]	3	21066315	21094525		10.8	4.15	4.97	16.25	15.77	16.91
ENSDARG00000006567	dus4l	dihydrouridine synthase 4-like (S. cerevisiae) [Source:ZFIN;Acc:ZDB-GENE-040801-233]	4	2637947	2642535		3.72	5.1	5.2	2.49	2.54	3.31
ENSDARG00000006568	kcnmb2	potassium large conductance calcium-activated channel, subfamily M, beta member 2 [Source:ZFIN;Acc:ZDB-GENE-050320-97]	2	7818368	7829400		2.61	3.11	3.25	2.43	2.52	2.35
ENSDARG00000006577	nadka	NAD kinase a [Source:ZFIN;Acc:ZDB-GENE-050417-39]	8	53051701	53062017		8.46	8.25	8.22	6.8	4.96	4.68
ENSDARG00000006580	cldnd	claudin d [Source:NCBI gene;Acc:81583]	21	25777425	25778201		0.09	0.15	0.08	0.06	0.03	0.29
ENSDARG00000006584	phf8	PHD finger protein 8 [Source:NCBI gene;Acc:566534]	23	27591366	27608257		11.72	6.02	5.76	15.42	14.99	15.09
ENSDARG00000006585	DMWD	zmp:0000000529 [Source:ZFIN;Acc:ZDB-GENE-130530-532]	15	47883585	47895200	DMWD	1.83	0.8	0.79	2.18	2.12	2.15
ENSDARG00000006588	zgc:111983	zgc:111983 [Source:ZFIN;Acc:ZDB-GENE-050417-335]	1	45173421	45177401		80.63	71.88	57.29	160.39	142.92	109.87
ENSDARG00000006598	sgk2b	serum/glucocorticoid regulated kinase 2b [Source:ZFIN;Acc:ZDB-GENE-060929-44]	16	26530426	26537114		3.58	6.14	5.15	21.6	20.26	21.07
ENSDARG00000006600	lsm14aa	LSM14A mRNA processing body assembly factor a [Source:ZFIN;Acc:ZDB-GENE-040426-1567]	25	35889102	35909626		13.88	10.83	9.06	18.12	18.1	18.42
ENSDARG00000006601	kaznb	kazrin, periplakin interacting protein b [Source:ZFIN;Acc:ZDB-GENE-061215-31]	11	25857941	26180714		4.49	2.24	2.4	4.52	4.41	4.89
ENSDARG00000006602	chrna2a	cholinergic receptor, nicotinic, alpha 2a (neuronal) [Source:ZFIN;Acc:ZDB-GENE-040108-2]	17	6079881	6101820		0.48	0.22	0.41	0.74	0.89	1.01
ENSDARG00000006603	csrp1a	cysteine and glycine-rich protein 1a [Source:ZFIN;Acc:ZDB-GENE-030909-4]	23	5765514	5818992		12.81	18.01	17.38	15.13	16.66	15.65
ENSDARG00000006604	nectin3b	nectin cell adhesion molecule 3b [Source:NCBI gene;Acc:541491]	21	21448996	21515466		2.66	1.73	1.45	3.97	3.95	4.34
ENSDARG00000006607	gpr27	G protein-coupled receptor 27 [Source:ZFIN;Acc:ZDB-GENE-071022-3]	6	43903209	43907321		3.6	0.87	1.19	4.79	5.27	4.83
ENSDARG00000006609	map2k2a	mitogen-activated protein kinase kinase 2a [Source:ZFIN;Acc:ZDB-GENE-041027-1]	22	20388621	20403194		13.03	12.63	11.19	15.11	15.36	14.36
ENSDARG00000006615	hnf1ba	HNF1 homeobox Ba [Source:NCBI gene;Acc:792391]	15	15997149	16012963		4.87	6.06	3.37	6.01	6.45	6.43
ENSDARG00000006617	napga	N-ethylmaleimide-sensitive factor attachment protein, gamma a [Source:ZFIN;Acc:ZDB-GENE-050320-46]	24	947473	982443		7.43	4.61	5.6	7.29	8.29	8.47
ENSDARG00000006621	pspc1	paraspeckle component 1 [Source:NCBI gene;Acc:692285]	1	27808916	27861148		18.05	12.03	13.7	17.86	17.17	17.7
ENSDARG00000006624	ppp2r2ab	protein phosphatase 2, regulatory subunit B, alpha b [Source:ZFIN;Acc:ZDB-GENE-030131-5474]	10	18877362	18906672		12.46	9.65	10.91	14.08	13.58	12.79
ENSDARG00000006636	slitrk2	SLIT and NTRK-like family, member 2 [Source:ZFIN;Acc:ZDB-GENE-080327-7]	14	19258702	19261733		3.93	1.27	1.59	4.51	4.82	4.59
ENSDARG00000006639	lnpk	lunapark, ER junction formation factor [Source:NCBI gene;Acc:337509]	6	10836147	10863669		3.84	2.46	2.18	4.4	3.63	3.97
ENSDARG00000006640	eomesa	eomesodermin homolog a [Source:ZFIN;Acc:ZDB-GENE-001228-1]	19	860830	874888		6.22	3.34	3.96	6.21	5.13	6.19
ENSDARG00000006642	drg2	developmentally regulated GTP binding protein 2 [Source:ZFIN;Acc:ZDB-GENE-040808-29]	3	40153819	40162867		16.74	22.36	21.73	13.49	13.52	13.72
ENSDARG00000006666	bud13	BUD13 homolog [Source:ZFIN;Acc:ZDB-GENE-030131-6333]	21	22697059	22705347		9.18	6.42	7.19	7.59	7.58	7.54
ENSDARG00000006672	fam49a	family with sequence similarity 49 member A [Source:ZFIN;Acc:ZDB-GENE-031002-50]	20	35255403	35298023		7.33	6.43	5.38	9.34	9.25	10.46
ENSDARG00000006677	ripk1l	receptor (TNFRSF)-interacting serine-threonine kinase 1, like [Source:ZFIN;Acc:ZDB-GENE-070705-132]	2	904362	920580		3.23	3.42	2.12	3.38	3.33	3.35
ENSDARG00000006678	pth2ra	parathyroid hormone 2 receptor a [Source:ZFIN;Acc:ZDB-GENE-991123-10]	9	29122039	29321665		0.55	0.17	0.35	0.36	0.38	0.53
ENSDARG00000006683	arhgef6	Rac/Cdc42 guanine nucleotide exchange factor (GEF) 6 [Source:ZFIN;Acc:ZDB-GENE-101013-2]	14	31799787	31856819		3.99	3.33	2.71	5.01	5.06	4.66
ENSDARG00000006691	rpl12	ribosomal protein L12 [Source:ZFIN;Acc:ZDB-GENE-030131-5297]	5	32882688	32887020		724.75	918.05	1094.06	631.27	648.37	657.31
ENSDARG00000006712	wipf2b	WAS/WASL interacting protein family, member 2b [Source:ZFIN;Acc:ZDB-GENE-040625-182]	19	5604241	5621298		6.04	3.85	3.67	10.53	10.57	10.8

ENSDARG00000006719	specc1la	sperm antigen with calponin homology and coiled-coil domains 1-like a [Source:NCBI gene;Acc:561775]	8	30600386	30664631		5.06	4.19	3.73	5.97	6.14	6.05
ENSDARG00000006747	tmem178b	transmembrane protein 178B [Source:NCBI gene;Acc:436669]	4	12111154	12239160		4.46	1.32	1.64	7.34	7.38	7.41
ENSDARG00000006754	mllt3	MLLT3 super elongation complex subunit [Source:ZFIN;Acc:ZDB-GENE-050417-356]	7	18779555	18881358		3.44	2.44	2.87	3.66	4	4.06
ENSDARG00000006757	klhl41b	kelch-like family member 41b [Source:NCBI gene;Acc:321064]	6	3680651	3692280		28.64	42.12	43.65	35.82	33.73	33.81
ENSDARG00000006758	fam234b	family with sequence similarity 234 member B [Source:ZFIN;Acc:ZDB-GENE-030131-3308]	3	1191370	1204341		6.7	2.54	3.16	9.14	9.78	9.1
ENSDARG00000006760	slc24a3	solute carrier family 24 member 3 [Source:ZFIN;Acc:ZDB-GENE-090312-67]	13	27073901	27210001		16.8	7.52	5.87	14.9	14.88	14.59
ENSDARG00000006766	snd1	staphylococcal nuclease and tudor domain containing 1 [Source:NCBI gene;Acc:324404]	4	19396945	19694108		31.89	33.43	27.1	32.66	33.5	34.56
ENSDARG00000006773	elovl4a	ELOVL fatty acid elongase 4a [Source:ZFIN;Acc:ZDB-GENE-040426-1767]	16	5156420	5167280		14.04	4.28	6.41	18.82	19.05	19.1
ENSDARG00000006775	cass4	Cas scaffold protein family member 4 [Source:ZFIN;Acc:ZDB-GENE-110131-9]	11	27745618	27778831		0.9	1.02	0.87	1.56	1.39	1.69
ENSDARG00000006782	rb1	retinoblastoma 1 [Source:ZFIN;Acc:ZDB-GENE-040428-1]	21	22960735	23017478		6.18	5.37	4.2	5.86	5.41	4.85
ENSDARG00000006786	copz2	coatomer protein complex, subunit zeta 2 [Source:ZFIN;Acc:ZDB-GENE-000406-5]	3	24094581	24111412		11.28	9.03	7.29	15.05	13.92	13.41
ENSDARG00000006791	arntl1a	aryl hydrocarbon receptor nuclear translocator-like 1a [Source:ZFIN;Acc:ZDB-GENE-000509-1]	25	17893784	17918810		9.43	5.32	5.92	9.76	10.82	10.16
ENSDARG00000006797	nt5dc1	5'-nucleotidase domain containing 1 [Source:ZFIN;Acc:ZDB-GENE-030131-8274]	20	474288	530663		8.5	4.04	4.27	8.54	8.99	8.42
ENSDARG00000006811	sult1st6	sulfotransferase family 1, cytosolic sulfotransferase 6 [Source:ZFIN;Acc:ZDB-GENE-040718-343]	12	15197857	15205087		29.34	56.81	32.75	4.2	4.71	4.45
ENSDARG00000006812	wdr26b	WD repeat domain 26b [Source:NCBI gene;Acc:564794]	20	46699021	46734051		9.09	5.8	5.91	11.79	11.61	11.96
ENSDARG00000006816	gtf2f2b	general transcription factor IIF, polypeptide 2b [Source:ZFIN;Acc:ZDB-GENE-050417-374]	9	18829360	18905317		0.04	0	0.02	0.02	0.02	0.05
ENSDARG00000006818	urod	uroporphyrinogen decarboxylase [Source:NCBI gene;Acc:30617]	2	27556168	27575803		8.95	12.73	10.59	11.6	14.59	12.42
ENSDARG00000006832	galnt9	polypeptide N-acetylgalactosaminyltransferase 9 [Source:ZFIN;Acc:ZDB-GENE-060526-21]	5	16797209	16996482		4.41	1.58	2.83	4.96	5.42	4.67
ENSDARG00000006837	mycn	MYCN proto-oncogene, bHLH transcription factor [Source:NCBI gene;Acc:252851]	20	33294428	33299384		24.24	15.54	17.22	21.99	24.56	24.63
ENSDARG00000006838	eef1a2	eukaryotic translation elongation factor 1 alpha 2 [Source:ZFIN;Acc:ZDB-GENE-040718-64]	23	15850938	15878879		1.01	0.63	0.83	0.27	0.19	0.42
ENSDARG00000006840	gnmt	glycine N-methyltransferase [Source:ZFIN;Acc:ZDB-GENE-040227-1]	17	48920799	48936123		31.42	51.87	50.14	32.63	36.73	35.51
ENSDARG00000006848	parp9	poly(ADP-ribose) polymerase family member 9 [Source:ZFIN;Acc:ZDB-GENE-081104-179]	9	23754275	23765480		0.37	0.58	0.36	0.34	0.35	0.48
ENSDARG00000006849	asic2	acid-sensing (proton-gated) ion channel 2 [Source:NCBI gene;Acc:407669]	3	37824268	38625940		5.5	1.39	2.16	5.01	5.01	5.15
ENSDARG00000006859	nkain1	sodium/potassium transporting ATPase interacting 1 [Source:NCBI gene;Acc:393517]	19	43017628	43035187		5.97	2.95	3.24	7.73	6.86	7.45
ENSDARG00000006862	kifap3b	kinesin-associated protein 3b [Source:ZFIN;Acc:ZDB-GENE-030131-2219]	2	23222939	23293501		11.9	5.56	6.76	13.32	12.1	12.87
ENSDARG00000006863	si:ch1073-349o24.2	si:ch1073-349o24.2 [Source:ZFIN;Acc:ZDB-GENE-081104-83]	9	7732714	7767156		0.58	0.44	0.42	0.74	0.69	0.68
ENSDARG00000006865	glra4a	glycine receptor, alpha 4a [Source:ZFIN;Acc:ZDB-GENE-010410-3]	14	39002116	39074539		2.95	2.95	3.43	3.07	2.65	3.06
ENSDARG00000006868	trh	thyrotropin-releasing hormone [Source:NCBI gene;Acc:266721]	8	54220551	54223316		4.37	1.47	1.7	6.39	7.18	8.16
ENSDARG00000006869	stx17	syntaxin 17 [Source:ZFIN;Acc:ZDB-GENE-041114-164]	16	27383717	27405460		4.95	4.9	4.82	3.32	3.48	3.68
ENSDARG00000006877	enpp4	ectonucleotide pyrophosphatase/phosphodiesterase 4 [Source:NCBI gene;Acc:550586]	17	5599014	5610514		1.74	0.91	1.05	1.88	2.53	1.82
ENSDARG00000006878	phf21aa	PHD finger protein 21Aa [Source:ZFIN;Acc:ZDB-GENE-050208-99]	7	38821438	38861741		6.89	2.09	3.12	7.55	7.82	8.44
ENSDARG00000006889	mpped2a	metallophosphoesterase domain containing 2a [Source:ZFIN;Acc:ZDB-GENE-050417-241]	7	16348835	16461232		5.91	3.52	3.15	10.79	10.64	10.11
ENSDARG00000006891	kcns3a	potassium voltage-gated channel, delayed-rectifier, subfamily S, member 3a [Source:ZFIN;Acc:ZDB-GENE-080723-45]	13	32575777	32577386		2.3	1.67	1.93	4	3.92	4.05
ENSDARG00000006892	myo3b	myosin IIIB [Source:ZFIN;Acc:ZDB-GENE-070706-2]	9	3699397	3866707		3.4	1.64	1.38	2.4	2.71	2.68
ENSDARG00000006894	oprk1	opioid receptor, kappa 1 [Source:ZFIN;Acc:ZDB-GENE-030729-1]	2	30878864	30886567		2.03	0.31	0.54	1.36	2.04	2.38
ENSDARG00000006896	lhx6	LIM homeobox 6 [Source:ZFIN;Acc:ZDB-GENE-041025-1]	10	9372702	9395220		2.66	1.24	1.19	3.15	3.16	3.31
ENSDARG00000006899	far1	fatty acyl CoA reductase 1 [Source:ZFIN;Acc:ZDB-GENE-040426-2908]	25	16079913	16129678		4.27	3.1	2.85	5.69	5.68	5.46
ENSDARG00000006900	impdh2	IMP (inosine 5'-monophosphate) dehydrogenase 2 [Source:NCBI gene;Acc:317745]	8	26059677	26079140		28.72	39.52	39.34	31.53	31.61	27.88
ENSDARG00000006901	si:ch1073-459j12.1	si:ch1073-459j12.1 [Source:ZFIN;Acc:ZDB-GENE-030131-8546]	8	44759576	44800841		11.27	15.48	13.93	21.33	22.77	22.13
ENSDARG00000006911	wnt8b	wingless-type MMTV integration site family, member 8b [Source:NCBI gene;Acc:30144]	13	29991090	30006260		0.67	0.13	0.26	0.72	0.71	0.66
ENSDARG00000006915	arl8ba	ADP-ribosylation factor-like 8Ba [Source:NCBI gene;Acc:337424]	6	42951960	42971184		22.87	24.63	24.24	17.58	17.23	18.18
ENSDARG00000006921	lrp5	low density lipoprotein receptor-related protein 5 [Source:ZFIN;Acc:ZDB-GENE-050518-2]	25	10640623	10769039		5.68	5.62	3.85	12.82	12.71	13.23
ENSDARG00000006923	cacna1ab	calcium channel, voltage-dependent, P/Q type, alpha 1A subunit, b [Source:ZFIN;Acc:ZDB-GENE-090514-3]	11	30817943	31026335		2.68	0.61	1.1	6.48	6.54	6.41
ENSDARG00000006924	fbxo38	F-box protein 38 [Source:ZFIN;Acc:ZDB-GENE-030131-2797]	14	38656776	38690871		2.92	1.68	1.65	3.68	3.74	3.26
ENSDARG00000006939	tbx16l	T-box transcription factor 16, like [Source:NCBI gene;Acc:30254]	5	42280372	42297333		0.01	0.04	0.04	0.27	0.34	0.26
ENSDARG00000006944	gdpd3b	glycerophosphodiester phosphodiesterase domain containing 3b [Source:ZFIN;Acc:ZDB-GENE-121210-2]	12	3893032	3904011		1.65	3.09	1.96	1.45	0.81	1.2
ENSDARG00000006963	cse1l	CSE1 chromosome segregation 1-like (yeast) [Source:NCBI gene;Acc:30707]	11	5468629	5493191		11.65	9.24	9.15	10.32	10.86	9.9
ENSDARG00000006978	zak	sterile alpha motif and leucine zipper containing kinase AZK [Source:ZFIN;Acc:ZDB-GENE-070912-386]	9	2896009	2945008		1.83	1.5	1.25	1.54	1.81	1.75
ENSDARG00000006981	ccdc113	coiled-coil domain containing 113 [Source:NCBI gene;Acc:450041]	25	13662606	13668229		1.05	1.43	1.01	1.44	1.28	1.57
ENSDARG00000006983	celf3b	cugbp, Elav-like family member 3b [Source:ZFIN;Acc:ZDB-GENE-110408-50]	16	1334431	1359903		24.03	5.17	9.59	26.18	28.89	30.27
ENSDARG00000006990	creld1b	cysteine-rich with EGF-like domains 1b [Source:ZFIN;Acc:ZDB-GENE-161219-1]	8	54202554	54210276		15.93	6.82	7.56	16.36	15.48	14.77
ENSDARG00000007009	hoxa11b	homeobox A11b [Source:NCBI gene;Acc:30382]	16	20904754	20907378		2.31	3.2	3.1	6.17	5.15	5.79
ENSDARG00000007018	ms4a17a.6	membrane-spanning 4-domains, subfamily A, member 17A.6 [Source:ZFIN;Acc:ZDB-GENE-070209-265]	4	76735113	76742479		0.71	0.73	0.56	1.32	1.11	0.83
ENSDARG00000007024	uox	urate oxidase [Source:NCBI gene;Acc:436604]	11	8160894	8168011		46.81	77.51	64.26	28.61	30.55	36.04
ENSDARG00000007025	ttc9c	tetratricopeptide repeat domain 9C [Source:ZFIN;Acc:ZDB-GENE-040426-1053]	14	45350008	45363735		7.34	5.24	6.83	4.89	5.45	5.08
ENSDARG00000007034	hnrpkl	heterogeneous nuclear ribonucleoprotein K, like [Source:ZFIN;Acc:ZDB-GENE-040426-1923]	5	55602720	55625247		12.04	8.06	8.84	14.68	14.51	14.97
ENSDARG00000007040	tmigd1	transmembrane and immunoglobulin domain containing 1 [Source:ZFIN;Acc:ZDB-GENE-080303-6]	21	39925379	39931400		1.22	0.5	0.48	0.95	1.07	0.83
ENSDARG00000007045	cnot4a	CCR4-NOT transcription complex, subunit 4a [Source:ZFIN;Acc:ZDB-GENE-090313-262]	25	20004835	20024860		6.34	5.79	5.21	8.89	9.46	9.94
ENSDARG00000007065	mrps18b	mitochondrial ribosomal protein S18B [Source:ZFIN;Acc:ZDB-GENE-050417-278]	19	30423117	30428901		18.74	22.27	20.39	15.64	14.69	15.28
ENSDARG00000007067	si:dkey-19a16.16	si:dkey-19a16.16 [Source:ZFIN;Acc:ZDB-GENE-141212-322]	22	6084771	6085666		0	0	0	0.06	0	0
ENSDARG00000007077	ankrd50l	ankyrin repeat domain 50-like [Source:ZFIN;Acc:ZDB-GENE-041008-117]	9	32859967	32875568		4.29	4.37	3.3	3.3	3.35	3.14
ENSDARG00000007080	rhcgl1	Rh family, C glycoprotein, like 1 [Source:ZFIN;Acc:ZDB-GENE-060209-2]	6	36381709	36391687		5.25	3.33	2.82	1.68	1.93	1.51
ENSDARG00000007081	pias4a	protein inhibitor of activated STAT, 4a [Source:NCBI gene;Acc:406712]	22	20406410	20419660		7.74	4.86	4.38	9.72	10.47	10.51
ENSDARG00000007086	aqp10a	aquaporin 10a [Source:ZFIN;Acc:ZDB-GENE-040718-40]	16	22288777	22294265		1.99	3.6	2.77	2.45	2.32	2.59
ENSDARG00000007092	xab2	XPA binding protein 2 [Source:ZFIN;Acc:ZDB-GENE-040426-685]	3	53317040	53350547		11.96	9.89	10.26	9.24	10.32	10.22
ENSDARG00000007097	her13	hairy-related 13 [Source:ZFIN;Acc:ZDB-GENE-050228-1]	15	7187228	7190183		4.33	1.98	3	3.83	3.09	4.08
ENSDARG00000007098	itm2ba	integral membrane protein 2Ba [Source:ZFIN;Acc:ZDB-GENE-030131-2352]	6	10015525	10026858		184.67	174.8	173.85	185.85	189.07	188.05
ENSDARG00000007099	cx43.4	connexin 43.4 [Source:NCBI gene;Acc:30276]	9	46405587	46415847		17.26	13.23	12.76	21.37	20.56	20.51
ENSDARG00000007108	lipia	lipase, member Ia [Source:NCBI gene;Acc:445105]	21	34945033	34972872		7.73	6.04	5.25	5.4	4.29	4.78
ENSDARG00000007125	asb6	ankyrin repeat and SOCS box containing 6 [Source:ZFIN;Acc:ZDB-GENE-050522-338]	8	6903327	6918721		4.18	2.63	2.56	4.73	4.53	3.93
ENSDARG00000007127	acat2	acetyl-CoA acetyltransferase 2 [Source:ZFIN;Acc:ZDB-GENE-990714-22]	20	30922595	30931146		14.55	15.25	14.04	11.48	11.24	11.16
ENSDARG00000007129	slc6a16a	solute carrier family 6 member 16a [Source:ZFIN;Acc:ZDB-GENE-061009-43]	3	16566361	16591648		4.37	3.99	3.18	3.9	4.22	4.17
ENSDARG00000007130	mrto4	MRT4 homolog, ribosome maturation factor [Source:ZFIN;Acc:ZDB-GENE-050417-142]	11	41846059	41853874		31.71	41.53	32.88	17.98	16.62	17.28
ENSDARG00000007135	cnot3a	CCR4-NOT transcription complex, subunit 3a [Source:ZFIN;Acc:ZDB-GENE-040927-6]	16	32110195	32128345		15.82	10.31	9.3	16.97	16.09	17.62
ENSDARG00000007136	gtf2h4	general transcription factor IIH, polypeptide 4 [Source:ZFIN;Acc:ZDB-GENE-030131-6779]	19	27283072	27336092		1.96	0.93	0.95	2.19	2.32	2.35
ENSDARG00000007141	psmc3	proteasome 26S subunit, ATPase 3 [Source:ZFIN;Acc:ZDB-GENE-030131-666]	7	38680034	38685337		63.81	70.46	72.32	51.34	48.51	50.4
ENSDARG00000007149	l1cama	L1 cell adhesion molecule, paralog a [Source:NCBI gene;Acc:30634]	23	28582865	28681775		14.52	3.85	4.55	21.9	20.08	22.49
ENSDARG00000007151	kcnk2b	potassium channel, subfamily K, member 2b [Source:ZFIN;Acc:ZDB-GENE-051120-69]	20	591505	605512		1.66	0.38	0.54	1.73	1.69	1.98
ENSDARG00000007169	itgb1bp1	integrin beta 1 binding protein 1 [Source:ZFIN;Acc:ZDB-GENE-051113-264]	17	35225614	35238359		13.67	13.75	16.67	11.63	12.46	11.97
ENSDARG00000007171	aig1	androgen-induced 1 (H. sapiens) [Source:ZFIN;Acc:ZDB-GENE-041014-358]	20	37661229	37736703		4.81	2.98	3.53	6.72	6.67	6.39
ENSDARG00000007172	plxna3	plexin A3 [Source:NCBI gene;Acc:567422]	8	9222684	9570511		10.09	6.09	6.6	13.13	12.44	12.61
ENSDARG00000007175	recql	RecQ helicase-like [Source:ZFIN;Acc:ZDB-GENE-050809-134]	4	17424855	17538270		4.82	3.28	3.61	4.62	4.84	4.95
ENSDARG00000007179	sash1a	SAM and SH3 domain containing 1a [Source:ZFIN;Acc:ZDB-GENE-030131-9960]	20	31428819	31743817		4.74	3.15	2.52	7	6.8	7.43
ENSDARG00000007180	slc30a4	solute carrier family 30 member 4 [Source:ZFIN;Acc:ZDB-GENE-040426-1408]	18	5510251	5541784		10.9	11.41	10	11.77	11.97	12.2
ENSDARG00000007181	nadk2	NAD kinase 2, mitochondrial [Source:ZFIN;Acc:ZDB-GENE-040801-202]	21	22330005	22346723		6.42	6.89	6.3	9.59	9.47	9.32
ENSDARG00000007184	zbtb16a	zinc finger and BTB domain containing 16a [Source:NCBI gene;Acc:323269]	21	23159385	23331619		3.5	1.04	1.1	6.06	6.13	6.44
ENSDARG00000007186	ppp1r8a	protein phosphatase 1, regulatory subunit 8a [Source:ZFIN;Acc:ZDB-GENE-060503-681]	19	15521997	15532515		3.77	2.12	1.92	3.1	3.19	3.05
ENSDARG00000007195	grm2b	glutamate receptor, metabotropic 2b [Source:ZFIN;Acc:ZDB-GENE-060201-5]	11	36158134	36216651		7.25	1.6	2.77	6.85	6.69	7.08
ENSDARG00000007196	rae1	ribonucleic acid export 1 [Source:NCBI gene;Acc:393973]	6	22326624	22334059		20.92	22	21.37	17.73	18.87	16.99
ENSDARG00000007198	mta1	metastasis associated 1 [Source:ZFIN;Acc:ZDB-GENE-131121-353]	17	37510808	37618891		4.44	3.13	2.64	5.64	5.05	5.32
ENSDARG00000007199	CR846090.1		7	25702443	25709533		0	0	0	0	0	0
ENSDARG00000007207	cnpy2	canopy FGF signaling regulator 2 [Source:ZFIN;Acc:ZDB-GENE-060315-4]	11	2540139	2549140		2.97	3.31	2.85	3.62	3.82	3.53
ENSDARG00000007216	abce1	ATP-binding cassette, sub-family E (OABP), member 1 [Source:ZFIN;Acc:ZDB-GENE-040426-1995]	23	43695036	43718067		37.18	43.78	43.55	29.11	30.83	29.09
ENSDARG00000007217	wdr55	WD repeat domain 55 [Source:NCBI gene;Acc:445394]	21	34814444	34843004		2.04	2.07	1.95	1.63	1.61	1.54
ENSDARG00000007219	actn1	actinin, alpha 1 [Source:ZFIN;Acc:ZDB-GENE-081113-4]	13	36321088	36391502		22.74	21.68	17.57	29.82	28.02	27.79
ENSDARG00000007220	ncam1b	neural cell adhesion molecule 1b [Source:ZFIN;Acc:ZDB-GENE-010822-2]	15	18368809	18574716		8	2.49	2.37	11.2	11.77	11.78
ENSDARG00000007221	pbk	PDZ binding kinase [Source:NCBI gene;Acc:360218]	20	39359729	39368079		7.47	5.52	5.38	4.56	4.31	4.11
ENSDARG00000007231	ryk	receptor like tyrosine kinase [Source:ZFIN;Acc:ZDB-GENE-070209-277]	6	27514465	27615701		7.44	7	5.99	8.75	9.15	8.64
ENSDARG00000007241	mycb	MYC proto-oncogene, bHLH transcription factor b [Source:NCBI gene;Acc:393141]	2	32016256	32018371		23.33	17.6	16.85	20.22	19.52	20.88
ENSDARG00000007244	acp2	acid phosphatase 2, lysosomal [Source:ZFIN;Acc:ZDB-GENE-050306-40]	7	39360797	39371386		19.92	19.92	21.82	18.77	18.44	19.93
ENSDARG00000007245	rundc3aa	RUN domain containing 3Aa [Source:ZFIN;Acc:ZDB-GENE-130530-533]	3	21018053	21037840		2.13	1.14	1.23	2.32	1.91	1.76
ENSDARG00000007247	ric8a	RIC8 guanine nucleotide exchange factor A [Source:NCBI gene;Acc:449546]	25	10458990	10483226		16.57	4.13	4.36	10.96	12.14	12.16
ENSDARG00000007250	use1	unconventional SNARE in the ER 1 homolog (S. cerevisiae) [Source:NCBI gene;Acc:393294]	22	11070842	11079068		7.34	5.44	5.85	7.55	6.95	8.26
ENSDARG00000007257	rab5ab	RAB5A, member RAS oncogene family, b [Source:ZFIN;Acc:ZDB-GENE-040122-3]	16	49647402	49661406		30.28	26.69	22.22	30.79	27.87	29.75
ENSDARG00000007264	nipsnap2		15	404891	413541		70.8	71.42	89.24	59.85	59.17	56.91
ENSDARG00000007271	mynn	myoneurin [Source:NCBI gene;Acc:327167]	24	26328787	26343203		14.62	9.49	10.72	10.54	10.04	10.28
ENSDARG00000007275	si:ch211-251b21.1	si:ch211-251b21.1 [Source:ZFIN;Acc:ZDB-GENE-060809-5]	8	47633438	47665026		189.12	204.14	210.49	196.8	214.94	205.73
ENSDARG00000007276	ela3l	elastase 3 like [Source:ZFIN;Acc:ZDB-GENE-060710-2]	5	26111596	26118855		252.69	336.8	245.09	13.33	14.61	14.49
ENSDARG00000007277	myf5	myogenic factor 5 [Source:ZFIN;Acc:ZDB-GENE-000616-6]	4	21741228	21745107		2.21	3.28	2.98	1.21	1.55	1.39
ENSDARG00000007278	ect2	epithelial cell transforming 2 [Source:ZFIN;Acc:ZDB-GENE-040831-2]	11	10388303	10456553		1.23	0.87	0.64	1.19	1.06	1.11
ENSDARG00000007279	yipf5	Yip1 domain family, member 5 [Source:NCBI gene;Acc:393265]	21	40930177	40938382		17.13	16.94	16.09	22.18	22.07	19.74
ENSDARG00000007285	mrpl57	mitochondrial ribosomal protein L57 [Source:NCBI gene;Acc:393748]	17	26608510	26610862		13.67	17.03	17.38	11.29	10.48	11.92
ENSDARG00000007289	metrnlb	meteorin like, glial cell differentiation regulator b [Source:ZFIN;Acc:ZDB-GENE-121214-223]	1	57371447	57382010		1.31	2.03	1.55	2.9	2.48	2.5
ENSDARG00000007294	aco2	aconitase 2, mitochondrial [Source:ZFIN;Acc:ZDB-GENE-030131-1390]	12	19106571	19119176		126.02	134.93	112.41	136.69	128.69	127.28
ENSDARG00000007302	sh3gl3b	SH3-domain GRB2-like 3b [Source:ZFIN;Acc:ZDB-GENE-040121-4]	25	19522954	19537323		5.47	7.36	5.7	3.14	3.51	3.35
ENSDARG00000007315	tmpoa	thymopoietin a [Source:ZFIN;Acc:ZDB-GENE-030131-6003]	4	16857722	16876336		10.97	9.2	9.08	8.37	9.14	8.1
ENSDARG00000007320	rpl7	ribosomal protein L7 [Source:ZFIN;Acc:ZDB-GENE-030131-8654]	2	30177476	30182353		1611.04	2110.17	2332.87	1172.35	1215.66	1215.79
ENSDARG00000007323	chmp4bb	charged multivesicular body protein 4Bb [Source:NCBI gene;Acc:406766]	8	45361735	45375108		38.49	50.56	38.54	41.24	37.53	39.35
ENSDARG00000007329	tbx16	T-box transcription factor 16 [Source:ZFIN;Acc:ZDB-GENE-990615-5]	8	51733907	51753604		0.08	0.04	0.09	0.22	0.15	0.18
ENSDARG00000007344	tcap	titin-cap (telethonin) [Source:ZFIN;Acc:ZDB-GENE-070501-5]	3	21240594	21242460		5.85	7.5	9.19	31.21	31.4	33.03
ENSDARG00000007347	degs1	delta(4)-desaturase, sphingolipid 1 [Source:ZFIN;Acc:ZDB-GENE-030131-5283]	13	502230	514843		20.88	20.59	21.41	24.14	25.78	23.06
ENSDARG00000007349	dmrt1	doublesex and mab-3 related transcription factor 1 [Source:NCBI gene;Acc:402923]	5	44944778	44991344		0.24	0.22	0.16	0.58	0.48	0.79
ENSDARG00000007356	fgf20a	fibroblast growth factor 20a [Source:ZFIN;Acc:ZDB-GENE-060110-1]	1	16393077	16394814		3.5	2.2	2.8	2.8	3.25	2.73
ENSDARG00000007358	cxcr3.1	chemokine (C-X-C motif) receptor 3, tandem duplicate 1 [Source:ZFIN;Acc:ZDB-GENE-060130-38]	16	11853212	11859309		0.29	0.19	0.29	0.2	0.13	0.22
ENSDARG00000007359	UBL4A	zgc:56596 [Source:NCBI gene;Acc:393270]	8	50953776	50962889	UBL4A	16.03	15.17	14.78	16.57	16.63	16.81
ENSDARG00000007362	sft2d1	SFT2 domain containing 1 [Source:ZFIN;Acc:ZDB-GENE-040426-1350]	13	4368504	4400816		10.31	12.85	11.07	13.85	14.38	14.22
ENSDARG00000007366	AMDHD1	si:ch73-71d17.1 [Source:NCBI gene;Acc:402826]	7	19389304	19401501	AMDHD1	6.57	10.1	7.91	2.91	3.28	3.08
ENSDARG00000007369	tcf7l1b	transcription factor 7 like 1b [Source:NCBI gene;Acc:30556]	8	52106818	52230115		4.17	3.1	2.51	6.43	6.52	6.97
ENSDARG00000007370	dysf	dysferlin, limb girdle muscular dystrophy 2B (autosomal recessive) [Source:ZFIN;Acc:ZDB-GENE-070501-1]	7	25558275	25697285		2.6	3.93	3.01	6.79	6.72	5.82
ENSDARG00000007371	slc26a3.1	solute carrier family 26 member 3 [Source:ZFIN;Acc:ZDB-GENE-041210-180]	4	18824959	18835544		0.13	0.26	0.22	0.03	0.02	0
ENSDARG00000007377	odc1	ornithine decarboxylase 1 [Source:ZFIN;Acc:ZDB-GENE-010816-1]	17	51743908	51760243		7.44	6.12	5.47	14.36	15.6	15.98
ENSDARG00000007382	ubtd1a	ubiquitin domain containing 1a [Source:ZFIN;Acc:ZDB-GENE-070424-93]	13	31371536	31378683		6.72	1.47	1.29	4.73	5.47	5.35
ENSDARG00000007383	kcnk6	potassium channel, subfamily K, member 6 [Source:ZFIN;Acc:ZDB-GENE-050309-213]	18	46171104	46183462		2.92	3.63	3.18	6.89	7.64	7.78
ENSDARG00000007385	cct7	chaperonin containing TCP1, subunit 7 (eta) [Source:ZFIN;Acc:ZDB-GENE-020419-7]	7	60148452	60156409		160.67	172.36	186.6	122.77	122.79	126.09
ENSDARG00000007387	irf2a	interferon regulatory factor 2a [Source:ZFIN;Acc:ZDB-GENE-030131-4135]	1	39859782	39880189		7.82	7.74	6.44	7.15	7.24	7.53
ENSDARG00000007396	rnd3b	Rho family GTPase 3b [Source:ZFIN;Acc:ZDB-GENE-040718-331]	9	23026324	23033818		7.04	9.47	8.8	11.04	11.53	11.48
ENSDARG00000007398	lrrk1	leucine-rich repeat kinase 1 [Source:ZFIN;Acc:ZDB-GENE-101019-2]	7	9526268	9717020		1.53	0.91	0.65	1.96	1.83	1.6
ENSDARG00000007405	strap	serine/threonine kinase receptor associated protein [Source:ZFIN;Acc:ZDB-GENE-040426-1110]	4	16876831	16883051		32.89	28.98	26.71	30.88	31.93	30.4
ENSDARG00000007406	phox2a	paired like homeobox 2A [Source:ZFIN;Acc:ZDB-GENE-000223-14]	15	47440477	47449248		2.32	1.35	1.35	6.64	6.41	7.3
ENSDARG00000007407	barx1	BARX homeobox 1 [Source:NCBI gene;Acc:553644]	11	27543093	27545719		4.93	4.52	4.84	6.69	7.99	7.87
ENSDARG00000007409	ppifa	peptidylprolyl isomerase Fa [Source:ZFIN;Acc:ZDB-GENE-040912-52]	13	30572172	30575799		7.93	9.32	7.98	5.65	5.64	5.41
ENSDARG00000007412	slc2a1b	solute carrier family 2 member 1b [Source:ZFIN;Acc:ZDB-GENE-090915-1]	6	48068695	48094342		9.12	9.65	7.06	22.46	23.41	21.77
ENSDARG00000007413	arid2	AT rich interactive domain 2 (ARID, RFX-like) [Source:ZFIN;Acc:ZDB-GENE-030131-6311]	4	1647098	1684347		5.77	4.27	3.49	11.47	10.67	11.21
ENSDARG00000007418	chmp4c	charged multivesicular body protein 4C [Source:ZFIN;Acc:ZDB-GENE-050417-389]	24	11070110	11076400		8.33	9.26	8.06	3.94	2.88	3.7
ENSDARG00000007421	ftcd	formimidoyltransferase cyclodeaminase [Source:ZFIN;Acc:ZDB-GENE-030131-9643]	22	12770877	12786532		55.01	80.96	80.29	29.77	31.67	32.47
ENSDARG00000007425	apol1	apolipoprotein L, 1 [Source:ZFIN;Acc:ZDB-GENE-030131-3217]	2	37971353	37974725		7.65	8.33	8.27	5.79	6.7	5.51
ENSDARG00000007429	ndor1	NADPH dependent diflavin oxidoreductase 1 [Source:NCBI gene;Acc:393621]	5	32490238	32498736		4.61	4.18	4.29	4.04	3.63	4.09
ENSDARG00000007430	prdm12b	PR domain containing 12b [Source:ZFIN;Acc:ZDB-GENE-041114-172]	5	63509761	63515210		4.03	1.18	1.49	3.94	3.76	3.86
ENSDARG00000007436	avpr2aa	arginine vasopressin receptor 2a, duplicate a [Source:ZFIN;Acc:ZDB-GENE-090313-344]	23	25038995	25050521		1.69	1.29	1.62	2.03	2.37	2.08
ENSDARG00000007438	ube2ib	ubiquitin-conjugating enzyme E2Ib [Source:NCBI gene;Acc:30622]	24	37257596	37272116		74.81	79.13	87.4	70.15	70.13	69.16
ENSDARG00000007443	fbxl18	F-box and leucine-rich repeat protein 18 [Source:ZFIN;Acc:ZDB-GENE-050913-65]	1	8638162	8646941		2.57	1.95	1.99	2.22	2.53	2.9
ENSDARG00000007444	ift81	intraflagellar transport 81 homolog [Source:ZFIN;Acc:ZDB-GENE-040714-3]	8	11642070	11681559		3.39	2.16	2.46	3.68	3.69	3.56
ENSDARG00000007449	slc25a39	solute carrier family 25 member 39 [Source:NCBI gene;Acc:393458]	3	29872819	29891500		13.41	9.94	11	11.77	13.17	13.26
ENSDARG00000007461	srgap1a	SLIT-ROBO Rho GTPase activating protein 1a [Source:ZFIN;Acc:ZDB-GENE-050420-284]	18	12867400	13056801		4.19	3.92	3.07	6.6	6.89	6.77
ENSDARG00000007465	tdrd1	tudor domain containing 1 [Source:NCBI gene;Acc:553522]	12	30344076	30359540		0.27	0.27	0.28	0.43	0.41	0.4
ENSDARG00000007467	ifit10	interferon-induced protein with tetratricopeptide repeats 10 [Source:ZFIN;Acc:ZDB-GENE-121214-248]	12	17025726	17028255		0.09	0.15	0.06	0.02	0	0.04
ENSDARG00000007477	fbxo8	F-box protein 8 [Source:ZFIN;Acc:ZDB-GENE-060421-7091]	1	38346043	38361496		6.93	5.58	4.26	5.23	6.51	5.64
ENSDARG00000007480	rpe65a	retinoid isomerohydrolase RPE65 a [Source:NCBI gene;Acc:393724]	2	1428696	1443692		11.36	5.05	4.01	27.73	27.5	28.75
ENSDARG00000007485	epc2	enhancer of polycomb homolog 2 (Drosophila) [Source:ZFIN;Acc:ZDB-GENE-040426-802]	9	25347814	25366541		9.47	6.2	4.74	9.73	9.33	10.31
ENSDARG00000007490	adrb1	adrenoceptor beta 1 [Source:ZFIN;Acc:ZDB-GENE-081022-145]	12	30442230	30443562		3.11	1.52	1.76	2.17	1.8	2.95
ENSDARG00000007494	st3gal8	ST3 beta-galactoside alpha-2,3-sialyltransferase 8 [Source:ZFIN;Acc:ZDB-GENE-050417-286]	11	26568492	26590429		3.94	4.77	5.11	8.2	8.72	8.37
ENSDARG00000007496	ufl1	UFM1-specific ligase 1 [Source:NCBI gene;Acc:393578]	20	418513	438646		14.1	13.43	13.19	11.58	13.1	12.82
ENSDARG00000007523	kmt2e	lysine (K)-specific methyltransferase 2E [Source:ZFIN;Acc:ZDB-GENE-030131-4120]	4	22424451	22472653		10.89	5.92	5.29	21.33	19.39	21.37
ENSDARG00000007526	ndufs2	NADH:ubiquinone oxidoreductase core subunit S2 [Source:ZFIN;Acc:ZDB-GENE-050522-273]	7	19043750	19068539		132.45	144.38	151.37	89.56	90.45	87.66
ENSDARG00000007531	zcchc9	zinc finger, CCHC domain containing 9 [Source:ZFIN;Acc:ZDB-GENE-031030-9]	10	42898103	42903716		8.37	8.75	9.64	5.32	5.18	5.25
ENSDARG00000007534	itln1	intelectin 1 [Source:ZFIN;Acc:ZDB-GENE-060929-564]	1	15204633	15224338		0.13	0.13	0.02	0.06	0.07	0.04
ENSDARG00000007553	opn4.1	opsin 4.1 [Source:NCBI gene;Acc:352918]	2	32843133	32846324		6.75	1	1.05	6.78	7.11	7.75
ENSDARG00000007560	sema3bl	sema domain, immunoglobulin domain (Ig), short basic domain, secreted, (semaphorin) 3bl [Source:ZFIN;Acc:ZDB-GENE-030131-2	8	26522013	26573963		6.72	5.63	4.79	15.37	14.95	17.25
ENSDARG00000007561	cdh23	cadherin-related 23 [Source:ZFIN;Acc:ZDB-GENE-040513-7]	13	43050562	43213012		0.45	0.21	0.2	0.87	0.82	0.93
ENSDARG00000007566	appbp2	amyloid beta precursor protein (cytoplasmic tail) binding protein 2 [Source:ZFIN;Acc:ZDB-GENE-030131-1715]	15	23922264	23942861		14.98	9.76	10.81	19.88	19.63	19.91
ENSDARG00000007576	crybb1l1	crystallin, beta B1, like 1 [Source:ZFIN;Acc:ZDB-GENE-100112-3]	14	46301973	46310549		1.12	5	7.02	63.13	54.08	75.61
ENSDARG00000007582	olfcb1	olfactory receptor C family, b1 [Source:ZFIN;Acc:ZDB-GENE-030131-2664]	20	98179	106012		0.17	0.09	0.17	0.2	0.12	0.15
ENSDARG00000007597	bloc1s4	biogenesis of lysosomal organelles complex-1, subunit 4, cappuccino [Source:ZFIN;Acc:ZDB-GENE-040801-215]	19	25679538	25693728		7.95	7.37	6.5	7.54	7.14	7.08
ENSDARG00000007601	zmynd8	zinc finger, MYND-type containing 8 [Source:ZFIN;Acc:ZDB-GENE-041119-1]	11	18560667	18604542		3.21	1.2	0.94	3.11	3	3.02
ENSDARG00000007603	stxbp2	syntaxin binding protein 2 [Source:ZFIN;Acc:ZDB-GENE-040426-2482]	3	53240562	53274285		15.06	14.15	12.1	14.68	14.06	13.81
ENSDARG00000007614	wwox	WW domain containing oxidoreductase [Source:NCBI gene;Acc:393887]	25	36960724	37071036		5.94	4.6	4.52	4.05	3.74	4.8
ENSDARG00000007624	gins1	GINS complex subunit 1 (Psf1 homolog) [Source:ZFIN;Acc:ZDB-GENE-041010-184]	13	7164345	7170351		6.46	9.1	6.91	2.48	2.78	3.01
ENSDARG00000007630	mkrn2	makorin, ring finger protein, 2 [Source:NCBI gene;Acc:170783]	11	543365	575786		5.09	4.3	4.25	5.48	5.03	4.97
ENSDARG00000007639	cnot4b	CCR4-NOT transcription complex, subunit 4b [Source:ZFIN;Acc:ZDB-GENE-040426-1164]	4	12243698	12286067		7.31	7.17	5.74	9.03	9.2	8.72
ENSDARG00000007654	nsfa	N-ethylmaleimide-sensitive factor a [Source:ZFIN;Acc:ZDB-GENE-030616-37]	3	34766989	34801041		61.4	16.42	24.93	48.36	53.5	52.3
ENSDARG00000007655	crybb1l3	crystallin, beta B1, like 3 [Source:ZFIN;Acc:ZDB-GENE-070820-11]	15	28096152	28104403		0.46	0.3	0.46	1.09	0.97	1.73
ENSDARG00000007657	ccnh	cyclin H [Source:ZFIN;Acc:ZDB-GENE-050320-13]	5	47963940	47975440		6.32	6.47	5.36	4.58	4.68	5.06
ENSDARG00000007663	amph	amphiphysin [Source:ZFIN;Acc:ZDB-GENE-040426-1711]	2	31891335	31937005		36.85	7.68	10.94	35.25	36.6	36.53

ENSDARG00000007671	ghrb	growth hormone receptor b [Source:ZFIN;Acc:ZDB-GENE-071119-4]	21	20742876	20765338		5.48	6.48	4.95	7.78	7.74	7.88
ENSDARG00000007678	ttyh3b	tweety family member 3b [Source:NCBI gene;Acc:798421]	1	11781544	11850353		21.13	5.98	6.96	33.11	32.1	33.54
ENSDARG00000007682	ppdpfa	pancreatic progenitor cell differentiation and proliferation factor a [Source:NCBI gene;Acc:338304]	8	23213320	23216035		91.59	94.07	92.74	53.7	55.16	55.3
ENSDARG00000007691	cul1b	cullin 1b [Source:ZFIN;Acc:ZDB-GENE-040426-2887]	24	17364745	17400472		11.09	8.26	7.98	11.69	13.13	12.3
ENSDARG00000007693	nfkbiab	nuclear factor of kappa light polypeptide gene enhancer in B-cells inhibitor, alpha b [Source:ZFIN;Acc:ZDB-GENE-030131-1819]	17	12942021	12949754		11.78	11.65	12.06	13.22	13.82	13.15
ENSDARG00000007697	fabp7a	fatty acid binding protein 7, brain, a [Source:ZFIN;Acc:ZDB-GENE-000627-1]	17	15433518	15435887		355.82	268.9	290.99	344.7	332.42	360.97
ENSDARG00000007708	nsa2	NSA2 ribosome biogenesis homolog (S. cerevisiae) [Source:ZFIN;Acc:ZDB-GENE-030131-488]	21	3789839	3796461		476.53	542.96	578.45	322.26	338.34	355.26
ENSDARG00000007709	adamts8a	ADAM metallopeptidase with thrombospondin type 1 motif, 8a [Source:ZFIN;Acc:ZDB-GENE-060526-202]	5	30155834	30176970		2.51	2.8	2.29	11.8	12.91	13.42
ENSDARG00000007711	rab3il1	RAB3A interacting protein (rabin3)-like 1 [Source:ZFIN;Acc:ZDB-GENE-110921-5]	25	3099073	3118114		5.7	5.66	5.12	5.79	5.89	5.84
ENSDARG00000007715	lgsn	lengsin, lens protein with glutamine synthetase domain [Source:ZFIN;Acc:ZDB-GENE-060312-26]	11	11263451	11266882		0.15	0.71	1.65	3.82	3.46	3.9
ENSDARG00000007720	sub1b	SUB1 regulator of transcription b [Source:ZFIN;Acc:ZDB-GENE-040718-209]	5	41143563	41153469		9.13	10.4	9.6	5.8	6.26	6.62
ENSDARG00000007722	nudt8	nudix (nucleoside diphosphate linked moiety X)-type motif 8 [Source:ZFIN;Acc:ZDB-GENE-061013-219]	15	499848	506010		5.63	5.06	4.59	4.22	3.83	4.48
ENSDARG00000007723	efnb1	ephrin-B1 [Source:ZFIN;Acc:ZDB-GENE-010618-2]	5	36271016	36328688		13.23	7.83	5.64	14.68	12.96	14.39
ENSDARG00000007727	rgl2	ral guanine nucleotide dissociation stimulator-like 2 [Source:ZFIN;Acc:ZDB-GENE-010131-1]	16	45364153	45398408		3.8	3.56	2.72	3.81	3.56	3.7
ENSDARG00000007734	stn1	STN1 subunit of CST complex [Source:NCBI gene;Acc:393360]	1	47499888	47513484		4.81	3.74	3.96	4.56	3.8	3.47
ENSDARG00000007737	zyg11	zyg-11 homolog (C. elegans) [Source:NCBI gene;Acc:555349]	20	16170848	16212016		11.09	7.14	7.32	14.06	13.1	13.46
ENSDARG00000007739	atp1a1a.2	ATPase Na+/K+ transporting subunit alpha 1a, tandem duplicate 2 [Source:ZFIN;Acc:ZDB-GENE-001212-2]	1	1789357	1796832		44.49	46.43	45.51	27.03	32.2	28.1
ENSDARG00000007743	il15l	interleukin 15, like [Source:ZFIN;Acc:ZDB-GENE-041111-173]	15	20352123	20363786		0.73	1.09	0.95	0.89	0.86	0.98
ENSDARG00000007744	tsr1	TSR1 ribosome maturation factor [Source:ZFIN;Acc:ZDB-GENE-030131-3762]	15	25635326	25656331		32.48	37.86	35.98	20.71	19.69	19.77
ENSDARG00000007745	uqcrfs1	ubiquinol-cytochrome c reductase, Rieske iron-sulfur polypeptide 1 [Source:ZFIN;Acc:ZDB-GENE-040426-2060]	7	45400716	45406089		60.46	67.66	69.55	49.43	48.92	48.25
ENSDARG00000007751	lmnl3	lamin L3 [Source:ZFIN;Acc:ZDB-GENE-020424-4]	23	45845159	45860097		0.66	0.37	0.54	0.53	0.41	0.39
ENSDARG00000007753	cpne2	copine II [Source:ZFIN;Acc:ZDB-GENE-030131-7896]	18	17660158	17704546		6.69	2.93	3.82	6.56	7.01	6.79
ENSDARG00000007755	wdr61	WD repeat domain 61 [Source:NCBI gene;Acc:393827]	25	6280991	6292560		13.96	14.69	17.37	11.99	11.05	11.25
ENSDARG00000007760	spg21	SPG21 abhydrolase domain containing, maspardin [Source:NCBI gene;Acc:406704]	7	13625929	13648663		11.59	12.17	12.19	10.17	9.62	8.66
ENSDARG00000007764	klhdc10	kelch domain containing 10 [Source:ZFIN;Acc:ZDB-GENE-040426-776]	4	14991789	15003893		7.63	4.64	5.27	7.05	6.96	7.45
ENSDARG00000007769	sult5a1	sulfotransferase family 5A, member 1 [Source:ZFIN;Acc:ZDB-GENE-060929-978]	7	56602521	56606752		1.04	1.43	0.98	3.28	2.99	1.65
ENSDARG00000007774	vash2	vasohibin 2 [Source:ZFIN;Acc:ZDB-GENE-041014-355]	20	37866171	37916522		3.38	1.88	1.55	5.53	5.19	5.33
ENSDARG00000007783	blk	BLK proto-oncogene, Src family tyrosine kinase [Source:ZFIN;Acc:ZDB-GENE-040724-106]	20	19175031	19198334		0.12	0.12	0.15	0.19	0.11	0.14
ENSDARG00000007786	tmx2b	thioredoxin-related transmembrane protein 2b [Source:NCBI gene;Acc:415203]	1	44569282	44581937		19.42	15.5	15.55	19	18.01	18.56
ENSDARG00000007787	zgc:112965	zgc:112965 [Source:ZFIN;Acc:ZDB-GENE-050522-10]	3	10728970	10751491		0.21	0.32	0.12	0.29	0.47	0.27
ENSDARG00000007788	atp2b1b	ATPase plasma membrane Ca2+ transporting 1b [Source:ZFIN;Acc:ZDB-GENE-080409-1]	25	19609713	19666107		16.21	3.9	3.12	14.66	16.18	16.41
ENSDARG00000007791	ppp2r1ba	protein phosphatase 2, regulatory subunit A, beta a [Source:ZFIN;Acc:ZDB-GENE-040426-2700]	5	57773222	57798096		61.95	49.64	55.27	64.64	65.33	63.99
ENSDARG00000007795	cst3	cystatin C (amyloid angiopathy and cerebral hemorrhage) [Source:ZFIN;Acc:ZDB-GENE-030131-373]	13	33675556	33683913		71.92	77.47	66.12	69.31	75.84	73.04
ENSDARG00000007804	gas6	growth arrest-specific 6 [Source:ZFIN;Acc:ZDB-GENE-030131-7773]	1	312784	338445		11.65	11.65	8.91	11.48	12.27	12.65
ENSDARG00000007807	cyth3a	cytohesin 3a [Source:ZFIN;Acc:ZDB-GENE-120612-1]	12	17504559	17562703		5.35	2.68	2.78	6.79	6.3	6.38
ENSDARG00000007808	zdhhc16a	zinc finger DHHC-type containing 16a [Source:NCBI gene;Acc:394017]	13	31379784	31389661		5.29	3.15	3.09	5.89	5.34	5.71
ENSDARG00000007812	sp3b	Sp3b transcription factor [Source:ZFIN;Acc:ZDB-GENE-110815-3]	6	10721765	10740365		8.95	6.26	5.29	11.4	11.74	11.88
ENSDARG00000007813	rnpepl1	arginyl aminopeptidase like 1 [Source:ZFIN;Acc:ZDB-GENE-030131-5522]	18	45862414	45887894		2.41	1.99	1.7	3.34	3.6	3.28
ENSDARG00000007818	palm1b	paralemmin 1b [Source:ZFIN;Acc:ZDB-GENE-040426-1147]	2	26237322	26284245		31.97	15.71	16.83	35.84	37.56	38.49
ENSDARG00000007823	atf3	activating transcription factor 3 [Source:ZFIN;Acc:ZDB-GENE-040426-728]	20	37794633	37800737		2.05	1.43	1.67	11.23	12.01	10.82
ENSDARG00000007824	camta1b	calmodulin binding transcription activator 1b [Source:ZFIN;Acc:ZDB-GENE-080229-1]	11	39672874	39926872		7.42	1.79	2.7	15.72	15.78	16.44
ENSDARG00000007825	map2k1	mitogen-activated protein kinase kinase 1 [Source:ZFIN;Acc:ZDB-GENE-040426-2759]	18	19419120	19451165		26.43	19.46	20.87	21.23	23.36	21.47
ENSDARG00000007836	ctsla	cathepsin La [Source:ZFIN;Acc:ZDB-GENE-030131-106]	5	44804791	44810920		631.32	996.11	1031.24	922.13	974.54	956.35
ENSDARG00000007842	MCHR2	si:dkey-165n16.5 [Source:ZFIN;Acc:ZDB-GENE-100922-121]	16	32820327	32837806	MCHR2	0.24	0.03	0.09	0.26	0.22	0.21
ENSDARG00000007856	rtn4rl1a	reticulon 4 receptor-like 1a [Source:ZFIN;Acc:ZDB-GENE-091118-38]	10	35952532	35959571		2.28	0.53	0.38	1.94	2.37	2.36
ENSDARG00000007863	tra2a	transformer 2 alpha homolog [Source:ZFIN;Acc:ZDB-GENE-040426-1391]	19	20177208	20191378		21.5	12.31	15.85	22.28	22.18	22.5
ENSDARG00000007864	pimr195	Pim proto-oncogene, serine/threonine kinase, related 195 [Source:ZFIN;Acc:ZDB-GENE-141216-224]	14	21005372	21007653		0	0	0	0	0	0
ENSDARG00000007867	dazap2	DAZ associated protein 2 [Source:ZFIN;Acc:ZDB-GENE-030131-3036]	23	33718602	33729552		23.43	17.64	14.21	35.21	35.77	36.24
ENSDARG00000007869	ehd3	EH-domain containing 3 [Source:ZFIN;Acc:ZDB-GENE-041014-352]	20	38201644	38241859		9.08	6.1	6.78	12.54	12.75	11.36
ENSDARG00000007885	fancl	FA complementation group L [Source:ZFIN;Acc:ZDB-GENE-040426-1045]	13	26703897	26780797		0.28	0.35	0.33	0.36	0.42	0.39
ENSDARG00000007886	slc35b2	solute carrier family 35 member B2 [Source:ZFIN;Acc:ZDB-GENE-050213-1]	20	51237646	51267192		5.7	3.71	4.07	6.46	6.54	5.57
ENSDARG00000007891	meox1	mesenchyme homeobox 1 [Source:NCBI gene;Acc:436723]	12	27462225	27469267		3.96	5.08	5.69	5.43	6	5.67
ENSDARG00000007892	pdcd2	programmed cell death 2 [Source:ZFIN;Acc:ZDB-GENE-040426-1236]	13	24380132	24383805		3.01	3.74	3.85	2.58	2.29	2.37
ENSDARG00000007901	crnkl1	crooked neck pre-mRNA splicing factor 1 [Source:ZFIN;Acc:ZDB-GENE-040426-694]	20	49081967	49103317		18.5	14.44	15.34	16.68	16.65	17.35
ENSDARG00000007906	lrp2b	low density lipoprotein receptor-related protein 2b [Source:ZFIN;Acc:ZDB-GENE-111115-1]	12	16132612	16229402		0.81	0.07	0.03	0.54	0.5	0.45
ENSDARG00000007912	si:ch211-196i2.1	si:ch211-196i2.1 [Source:ZFIN;Acc:ZDB-GENE-091204-150]	21	14253404	14310159		6.32	5.47	3.88	9.1	7.8	8.38
ENSDARG00000007916	xpnpep3	X-prolyl aminopeptidase 3, mitochondrial [Source:ZFIN;Acc:ZDB-GENE-040426-2076]	3	25059502	25086986		6.84	6.83	7	5.8	5.51	6.04
ENSDARG00000007918	ttc27	tetratricopeptide repeat domain 27 [Source:ZFIN;Acc:ZDB-GENE-030102-1]	17	22760126	22947117		6.67	6.09	5.6	6.05	5.74	5.99
ENSDARG00000007923	ptpn1	protein tyrosine phosphatase non-receptor type 1 [Source:ZFIN;Acc:ZDB-GENE-980605-23]	8	28398780	28408410		8.98	8.15	6.58	11.47	10.62	10.63
ENSDARG00000007941	hmx4	H6 family homeobox 4 [Source:ZFIN;Acc:ZDB-GENE-060825-142]	1	40909750	40911332		17.86	12.21	12.64	14.64	15.81	14.52
ENSDARG00000007943	gabpb2a	GA binding protein transcription factor subunit beta 2a [Source:ZFIN;Acc:ZDB-GENE-030131-5733]	19	7432710	7441948		3.74	2.88	3.02	3.85	3.78	3.85
ENSDARG00000007944	lhx1b	LIM homeobox 1b [Source:ZFIN;Acc:ZDB-GENE-980526-116]	5	56268436	56274174		2.96	0.67	0.96	3.66	3.18	3.35
ENSDARG00000007950	itga11b	integrin, alpha 11b [Source:ZFIN;Acc:ZDB-GENE-071009-4]	25	1343161	1426876		0.84	0.75	0.54	1.9	1.86	2.15
ENSDARG00000007955	iars1	isoleucyl-tRNA synthetase 1 [Source:ZFIN;Acc:ZDB-GENE-030131-6325]	11	1337620	1392468		19	22.08	19.08	32.32	34.83	34.14
ENSDARG00000007959	hibadhb	3-hydroxyisobutyrate dehydrogenase b [Source:ZFIN;Acc:ZDB-GENE-040426-1582]	16	20871015	20889516		32.22	37.39	39.91	29.04	28.98	27.83
ENSDARG00000007960	hnrnpaba	heterogeneous nuclear ribonucleoprotein A/Ba [Source:ZFIN;Acc:ZDB-GENE-030131-185]	14	6962271	6968589		87.95	70.1	66.21	116.32	111.23	116.65
ENSDARG00000007971	cks1b	CDC28 protein kinase regulatory subunit 1B [Source:ZFIN;Acc:ZDB-GENE-040801-112]	19	7835025	7838232		9.39	8.85	9.43	5.53	6.33	6.38
ENSDARG00000007975	fth1b	ferritin, heavy polypeptide 1b [Source:ZFIN;Acc:ZDB-GENE-040912-30]	25	3058924	3066067		15.98	11.3	11.3	13.68	13.49	13.68
ENSDARG00000007976	si:ch211-220f16.2	si:ch211-220f16.2 [Source:ZFIN;Acc:ZDB-GENE-070912-199]	18	29968205	30021479		4.29	2.36	2.24	4.13	4.34	4.71
ENSDARG00000007982	onecut1	one cut homeobox 1 [Source:ZFIN;Acc:ZDB-GENE-040426-1469]	18	38755023	38760271		3.27	1.58	1.51	7.87	6.91	7.78
ENSDARG00000007988	masp2	mannan-binding lectin serine peptidase 2 [Source:ZFIN;Acc:ZDB-GENE-060130-154]	23	28770225	28796157		5.45	8.08	8.6	5.52	5.88	5.32
ENSDARG00000007989	nedd8	NEDD8 ubiquitin like modifier [Source:ZFIN;Acc:ZDB-GENE-030616-588]	7	24205034	24207788		43.72	53.86	50.21	32.05	32.5	34.17
ENSDARG00000007990	wt1b	WT1 transcription factor b [Source:ZFIN;Acc:ZDB-GENE-050420-319]	18	45594085	45617146		1.44	1.92	1.63	2.1	2.31	1.93
ENSDARG00000007996	pou2f1b	POU class 2 homeobox 1b [Source:ZFIN;Acc:ZDB-GENE-030131-2422]	9	34319333	34378674		4.24	1.93	1.97	7.71	6.75	7.74
ENSDARG00000008010	zgc:194189	zgc:194189 [Source:ZFIN;Acc:ZDB-GENE-081022-71]	23	29823286	29824146		0.56	0.48	0.69	0.79	0.88	0.8
ENSDARG00000008014	nudt17	nudix (nucleoside diphosphate linked moiety X)-type motif 17 [Source:NCBI gene;Acc:556651]	16	43000828	43011501		7.74	7.25	8.86	6.83	6.45	6.6
ENSDARG00000008019	mxg	myxovirus (influenza virus) resistance G [Source:ZFIN;Acc:ZDB-GENE-030721-10]	25	19695652	19715136		0.01	0.06	0.02	0.05	0.06	0.02
ENSDARG00000008020	med31	mediator complex subunit 31 [Source:NCBI gene;Acc:436690]	21	40045224	40049642		14.95	15	16.21	12.73	13.26	14.21
ENSDARG00000008022	kif18a	kinesin family member 18A [Source:ZFIN;Acc:ZDB-GENE-040426-862]	7	31976764	32021853		2.27	1.6	1.66	1.68	1.75	1.72
ENSDARG00000008026	niban2b	niban apoptosis regulator 2b [Source:ZFIN;Acc:ZDB-GENE-030131-6218]	5	28915389	28970059		4.62	5.34	3.85	4.45	4.17	4.15
ENSDARG00000008029	mep1a.2	meprin A, alpha (PABA peptide hydrolase), tandem duplicate 2 [Source:ZFIN;Acc:ZDB-GENE-041001-208]	20	35484070	35496459		9.43	14.75	9.91	0.31	0.36	0.47
ENSDARG00000008030	myl9b	myosin, light chain 9b, regulatory [Source:ZFIN;Acc:ZDB-GENE-040426-2296]	6	1787160	1799561		18.2	25.04	22.4	35.54	41.04	38.7
ENSDARG00000008032	sart3	spliceosome associated factor 3, U4/U6 recycling protein [Source:NCBI gene;Acc:558581]	5	20453874	20481389		13.43	7.86	7.92	15.05	15.89	15.46
ENSDARG00000008034	skib	v-ski avian sarcoma viral oncogene homolog b [Source:ZFIN;Acc:ZDB-GENE-990715-10]	11	25782721	25829712		12.19	7.19	6.61	15.07	15.47	15.34
ENSDARG00000008049	si:dkey-42i9.4	si:dkey-42i9.4 [Source:ZFIN;Acc:ZDB-GENE-030131-8398]	22	10540446	10541712		40.73	39.52	42.7	59.62	62.8	65.1
ENSDARG00000008060	scara3	scavenger receptor class A, member 3 [Source:ZFIN;Acc:ZDB-GENE-041014-317]	20	39283849	39300730		2.52	3.45	3	3.94	3.82	3.88
ENSDARG00000008068	tma16	translation machinery associated 16 homolog [Source:NCBI gene;Acc:553647]	1	19759370	19764038		4.87	6.55	5.93	3.62	3.96	3.51
ENSDARG00000008075	illr4	immune-related, lectin-like receptor 4 [Source:ZFIN;Acc:ZDB-GENE-050311-5]	19	40167363	40175800		0	0.07	0.02	0.26	0.19	0.22
ENSDARG00000008095	olfcd1	olfactory receptor C family, d1 [Source:ZFIN;Acc:ZDB-GENE-100721-9]	18	33212589	33216757		0	0.01	0.01	0	0	0
ENSDARG00000008097	srsf9	serine and arginine rich splicing factor 9 [Source:ZFIN;Acc:ZDB-GENE-040426-2397]	10	1686975	1697074		25.78	20.16	24.21	26.5	27.9	28.81
ENSDARG00000008098	ifit9	interferon-induced protein with tetratricopeptide repeats 9 [Source:ZFIN;Acc:ZDB-GENE-121214-233]	12	17033494	17035339		0.04	0.01	0	0.05	0	0
ENSDARG00000008100	slc7a10a	solute carrier family 7 member 10a [Source:ZFIN;Acc:ZDB-GENE-080116-1]	7	38315647	38340674		14.93	8.92	10.83	20.94	19.47	19.02
ENSDARG00000008105	mettl2a	methyltransferase like 2A [Source:NCBI gene;Acc:100006618]	3	19665319	19682819		7.98	7.95	8.01	7.3	6.59	6.72
ENSDARG00000008107	src	v-src avian sarcoma (Schmidt-Ruppin A-2) viral oncogene homolog [Source:NCBI gene;Acc:325084]	23	39346774	39450700		5.72	3.4	3.24	7.35	6.85	7.11
ENSDARG00000008119	si:ch211-195d17.2	si:ch211-195d17.2 [Source:ZFIN;Acc:ZDB-GENE-030131-513]	20	29676874	29683783		11.87	8.33	9.23	9.4	7.77	9.46
ENSDARG00000008127	pcdh15b	protocadherin-related 15b [Source:ZFIN;Acc:ZDB-GENE-050214-1]	12	6566233	6905375		2.2	0.57	0.55	3.12	3.14	3.23
ENSDARG00000008131	sox1b	SRY-box transcription factor 1b [Source:NCBI gene;Acc:562710]	1	46194333	46196107		6.22	2.07	2.87	10.46	8.82	10.04
ENSDARG00000008133	foxl1	forkhead box L1 [Source:ZFIN;Acc:ZDB-GENE-040426-1181]	18	17483557	17485419		3.38	2.82	2.64	5.13	5.5	6.39
ENSDARG00000008140	kcnab1a	potassium voltage-gated channel, shaker-related subfamily, beta member 1 a [Source:ZFIN;Acc:ZDB-GENE-050327-79]	18	34362608	34536067		0.94	0.45	0.45	1.77	1.44	1.63
ENSDARG00000008141	rbl1	retinoblastoma-like 1 (p107) [Source:ZFIN;Acc:ZDB-GENE-030131-8179]	6	51549203	51573975		4.91	4.48	3.86	5	5.36	5.03
ENSDARG00000008142	stxbp3	syntaxin binding protein 3 [Source:ZFIN;Acc:ZDB-GENE-040426-2833]	23	1661743	1698431		12.99	13.93	12.7	11.64	11.17	11.16
ENSDARG00000008153	serinc5	serine incorporator 5 [Source:NCBI gene;Acc:406835]	5	51947150	51998708		9.65	5.15	4.9	13.09	11.61	12.07
ENSDARG00000008155	sms	spermine synthase [Source:ZFIN;Acc:ZDB-GENE-040625-150]	21	25399133	25416391		25.1	16.72	18.05	18.42	17.17	18.52
ENSDARG00000008164	btr33	bloodthirsty-related gene family, member 33 [Source:ZFIN;Acc:ZDB-GENE-090512-4]	24	7708652	7714562		0.77	0.54	0.61	0.41	0.35	0.33
ENSDARG00000008165	caspa	caspase a [Source:ZFIN;Acc:ZDB-GENE-000616-3]	16	41993551	42004544		6.77	8.83	7.57	5.61	6.29	6.18
ENSDARG00000008170	dbnla	drebrin-like a [Source:ZFIN;Acc:ZDB-GENE-040704-42]	10	42358426	42372811		7.01	6.22	4.95	5.56	4.47	3.56
ENSDARG00000008174	hoxb1a	homeobox B1a [Source:NCBI gene;Acc:30337]	3	23768898	23771142		0.24	0.21	0.13	0.28	0.38	0.23
ENSDARG00000008182	si:ch73-111e15.1	si:ch73-111e15.1 [Source:ZFIN;Acc:ZDB-GENE-110411-28]	10	22110842	22117121		0.22	0.34	0.05	0.18	0.03	0.19
ENSDARG00000008184	mia3	MIA SH3 domain ER export factor 3 [Source:ZFIN;Acc:ZDB-GENE-041014-270]	20	51758426	51831816		7.05	6.57	4.76	8.91	8.65	9.13
ENSDARG00000008186	syk	spleen tyrosine kinase [Source:ZFIN;Acc:ZDB-GENE-040702-3]	10	5533843	5581487		2.77	1.46	1.73	3.11	2.76	2.8
ENSDARG00000008188	sf1	splicing factor 1 [Source:ZFIN;Acc:ZDB-GENE-030131-2492]	7	22809905	22820816		31.9	23.43	22.17	47.51	46.35	46.36
ENSDARG00000008191	tmeff1a	transmembrane protein with EGF-like and two follistatin-like domains 1a [Source:ZFIN;Acc:ZDB-GENE-070912-294]	2	42176922	42187118		2.55	0.72	1.63	3.21	3.56	3.1
ENSDARG00000008192	pank1a	pantothenate kinase 1a [Source:ZFIN;Acc:ZDB-GENE-040426-2846]	17	23554932	23575465		6.87	5.33	4.79	5.31	5.45	5.05
ENSDARG00000008200	ugp2b	UDP-glucose pyrophosphorylase 2b [Source:ZFIN;Acc:ZDB-GENE-030131-6352]	17	24445818	24474703		7.06	7.85	7.1	8.02	9.25	8.34
ENSDARG00000008203	pax1a	paired box 1a [Source:ZFIN;Acc:ZDB-GENE-070112-182]	17	42274240	42278340		1.69	1.62	1.73	2.3	2.04	2.87
ENSDARG00000008209	myt1la	myelin transcription factor 1-like, a [Source:ZFIN;Acc:ZDB-GENE-041001-172]	20	30445971	30574029		13.21	2.9	3.57	20.2	20.92	21.54
ENSDARG00000008212	kcnk13a	potassium channel, subfamily K, member 13a [Source:ZFIN;Acc:ZDB-GENE-080219-46]	17	16336278	16342388		0.7	0.35	0.3	0.84	0.79	0.8
ENSDARG00000008218	znf410	zinc finger protein 410 [Source:ZFIN;Acc:ZDB-GENE-040426-1817]	20	54349333	54360183		14.39	10.75	14.05	10.36	10.89	10.7
ENSDARG00000008219	crygm4	crystallin, gamma M4 [Source:ZFIN;Acc:ZDB-GENE-120312-2]	6	72040	72996		0.18	0.04	0.08	0.27	0.25	0.23
ENSDARG00000008224	vps35	VPS35 retromer complex component [Source:ZFIN;Acc:ZDB-GENE-030131-2042]	7	41757462	41812355		26.69	18.18	18.88	25.41	26.31	24.89
ENSDARG00000008235	cog5	component of oligomeric golgi complex 5 [Source:ZFIN;Acc:ZDB-GENE-041210-47]	4	2548783	2637710		4.93	3.78	3.96	4.56	4.62	4.69
ENSDARG00000008237	sars1	seryl-tRNA synthetase 1 [Source:NCBI gene;Acc:445405]	23	34030550	34043651		51.57	53.07	51.27	53.93	58.41	56.96
ENSDARG00000008238	manba	mannosidase, beta A, lysosomal [Source:ZFIN;Acc:ZDB-GENE-040426-721]	13	11714869	11743973		1.11	1.15	0.86	0.87	0.86	0.77
ENSDARG00000008239	mocs3	molybdenum cofactor synthesis 3 [Source:NCBI gene;Acc:393095]	17	42812265	42842863		2.86	2.53	3.25	2.97	2.8	2.57
ENSDARG00000008243	cct8	chaperonin containing TCP1, subunit 8 (theta) [Source:ZFIN;Acc:ZDB-GENE-040426-876]	10	25325808	25343521		46.13	48.65	52.43	38.45	40.64	39.79
ENSDARG00000008247	vezf1a	vascular endothelial zinc finger 1a [Source:ZFIN;Acc:ZDB-GENE-040108-4]	10	37083654	37126562		8.06	3.33	3.39	9.43	8.41	8.12
ENSDARG00000008249	ptchd4	patched domain containing 4 [Source:NCBI gene;Acc:564097]	20	36066408	36107422		1.51	0.45	0.71	2.31	2.31	2.11
ENSDARG00000008255	cnot6a	CCR4-NOT transcription complex, subunit 6a [Source:ZFIN;Acc:ZDB-GENE-030131-2768]	21	32757601	32781612		14.75	8.51	6.53	20.86	19.33	19.69
ENSDARG00000008263	mfsd4ab	major facilitator superfamily domain containing 4Ab [Source:NCBI gene;Acc:558146]	23	18103080	18114890		2.19	3.66	2.26	7.15	8.62	8.79
ENSDARG00000008275	klhl24b	kelch-like family member 24b [Source:ZFIN;Acc:ZDB-GENE-040426-1185]	24	25760986	25788841		15.57	13.42	10.94	13.62	12.58	11.62
ENSDARG00000008278	rcor2	REST corepressor 2 [Source:NCBI gene;Acc:402934]	7	24979833	24995640		6.92	2.66	3.11	10.44	9.92	10.64
ENSDARG00000008279	map2k7	mitogen-activated protein kinase kinase 7 [Source:ZFIN;Acc:ZDB-GENE-090312-186]	1	45839927	45856178		3.33	2.16	1.76	5.56	5.64	5.99
ENSDARG00000008287	fam114a1	family with sequence similarity 114 member A1 [Source:ZFIN;Acc:ZDB-GENE-070410-52]	1	18575747	18592068		6.68	8.83	7.41	8.28	7.55	7.15
ENSDARG00000008292	eif2s3	eukaryotic translation initiation factor 2, subunit 3 gamma [Source:NCBI gene;Acc:327341]	24	16983269	17002950		179.34	195.87	216.86	179.49	192.93	192.22
ENSDARG00000008305	hand2	heart and neural crest derivatives expressed 2 [Source:NCBI gene;Acc:58150]	1	38193147	38195012		7.92	9.6	9.28	15.12	15.83	14.98
ENSDARG00000008306	rdh5	retinol dehydrogenase 5 (11-cis/9-cis) [Source:ZFIN;Acc:ZDB-GENE-050208-411]	22	10110358	10121880		1.23	1.16	0.96	1.49	1.25	1.45
ENSDARG00000008310	ip6k2a	inositol hexakisphosphate kinase 2a [Source:ZFIN;Acc:ZDB-GENE-030131-760]	6	42409082	42418225		59.05	24.31	24.98	41.16	39.98	41.11
ENSDARG00000008322	ambra1a	autophagy/beclin-1 regulator 1a [Source:ZFIN;Acc:ZDB-GENE-111104-2]	7	39255781	39360325		2.6	1.7	1.39	2.86	2.89	2.97
ENSDARG00000008329	asic1a	acid-sensing (proton-gated) ion channel 1a [Source:NCBI gene;Acc:791696]	8	6967108	7023196		4.2	0.92	1.42	5.32	6	6.31
ENSDARG00000008333	znfl2a	zinc finger-like gene 2a [Source:ZFIN;Acc:ZDB-GENE-030828-7]	2	118225	122164		4.66	6.78	7.14	4.8	7.09	5.69
ENSDARG00000008363	mcl1b	MCL1 apoptosis regulator, BCL2 family member b [Source:ZFIN;Acc:ZDB-GENE-030825-1]	16	29586004	29587969		27.05	29.49	26.64	100.73	107.06	103.65
ENSDARG00000008370	csnk1da	casein kinase 1, delta a [Source:NCBI gene;Acc:322106]	3	36173203	36210344		43.74	28.77	26.37	46.29	44.75	45.07
ENSDARG00000008372	spred2b	sprouty related EVH1 domain containing 2b [Source:NCBI gene;Acc:793290]	13	5870644	5937034		9.77	8.44	7.61	12.96	14.7	14.63
ENSDARG00000008377	epn2	epsin 2 [Source:ZFIN;Acc:ZDB-GENE-071212-1]	3	39656188	39690218		6.44	5.46	3.73	11.95	11.54	11
ENSDARG00000008380	brd7	bromodomain containing 7 [Source:ZFIN;Acc:ZDB-GENE-040426-2687]	7	37742299	37755346		26.55	19.72	21.94	19.61	19.25	19.78
ENSDARG00000008383	arpc1a	actin related protein 2/3 complex, subunit 1A [Source:ZFIN;Acc:ZDB-GENE-040116-1]	3	40796110	40804455		74.84	83.31	79.79	73.14	74.65	72.75
ENSDARG00000008384	hdac6	histone deacetylase 6 [Source:ZFIN;Acc:ZDB-GENE-030131-3232]	8	7393435	7444390		5.86	4.61	3.62	5.65	5.11	5.15
ENSDARG00000008388	mmp14b	matrix metallopeptidase 14b (membrane-inserted) [Source:ZFIN;Acc:ZDB-GENE-030901-2]	2	38161318	38183641		19.06	21.78	17.23	24.95	25.47	26.47
ENSDARG00000008395	snupn	snurportin 1 [Source:ZFIN;Acc:ZDB-GENE-030131-3464]	25	6432578	6448050		2.55	2.22	2.45	1.66	1.92	1.96
ENSDARG00000008396	ftr24	finTRIM family, member 24 [Source:ZFIN;Acc:ZDB-GENE-050731-2]	2	47916204	47923803		2.31	2.49	2.36	2.06	2.26	2.09
ENSDARG00000008398	cacna1c	calcium channel, voltage-dependent, L type, alpha 1C subunit [Source:ZFIN;Acc:ZDB-GENE-020129-1]	4	20318127	20474972		3.5	1.01	1.41	6.09	6.21	6.2
ENSDARG00000008403	phospho1	phosphoethanolamine/phosphocholine phosphatase 1 [Source:NCBI gene;Acc:100002812]	3	15805917	15811037		10.62	9.17	7.13	7.91	6.1	7.16
ENSDARG00000008407	tspan7b	tetraspanin 7b [Source:ZFIN;Acc:ZDB-GENE-040927-5]	22	11440705	11493236		44.29	17.79	24.66	57.05	58.94	59.87
ENSDARG00000008413	atp11a	ATPase phospholipid transporting 11A [Source:ZFIN;Acc:ZDB-GENE-090311-52]	1	46263473	46401385		9.47	3.71	3.21	10.6	10.63	10.49
ENSDARG00000008414	exoc3l2a	exocyst complex component 3-like 2a [Source:ZFIN;Acc:ZDB-GENE-060526-343]	5	36752943	36763394		2.59	3.34	2.41	4.57	4.82	4.81
ENSDARG00000008433	unc45b	unc-45 myosin chaperone B [Source:NCBI gene;Acc:266640]	8	11204059	11229523		6.76	10.33	8.37	9.89	8.26	7.84
ENSDARG00000008434	bcl2l1	BCL2 like 1 [Source:ZFIN;Acc:ZDB-GENE-010730-1]	23	18953870	18982499		20.1	17.25	15.98	27.76	28.02	29.45
ENSDARG00000008439	atxn10	ataxin 10 [Source:ZFIN;Acc:ZDB-GENE-060503-180]	4	18720785	18742106		4.04	4.01	3.35	2	2.16	2.01
ENSDARG00000008447	fkbp4	FKBP prolyl isomerase 4 [Source:ZFIN;Acc:ZDB-GENE-030131-514]	18	6536293	6550378		47.99	41.42	36.19	34.7	33.78	32.42
ENSDARG00000008450	pcolce2a	procollagen C-endopeptidase enhancer 2a [Source:ZFIN;Acc:ZDB-GENE-070705-25]	2	8031234	8050498		0.31	0.41	0.36	0.62	0.65	0.57
ENSDARG00000008454	cpeb1b	cytoplasmic polyadenylation element binding protein 1b [Source:NCBI gene;Acc:30702]	7	48240707	48251235		0.08	0.04	0.02	0.29	0.34	0.32
ENSDARG00000008457	faah2a	fatty acid amide hydrolase 2a [Source:NCBI gene;Acc:436973]	1	40237276	40258430		7.62	11.33	7.43	4.23	4.68	4.51
ENSDARG00000008461	anapc5	anaphase promoting complex subunit 5 [Source:ZFIN;Acc:ZDB-GENE-030131-1574]	8	40185600	40205712		2.64	2.76	3.02	1.89	2.1	2.03
ENSDARG00000008472	apex2	APEX nuclease (apurinic/apyrimidinic endonuclease) 2 [Source:ZFIN;Acc:ZDB-GENE-040426-835]	8	22509508	22515042		2.22	1.47	1.83	1.48	1.34	1.6
ENSDARG00000008473	asb14a	ankyrin repeat and SOCS box containing 14a [Source:ZFIN;Acc:ZDB-GENE-140106-2]	11	42585138	42599018		0	0.01	0	0.01	0.03	0.02
ENSDARG00000008480	trmt61a	tRNA methyltransferase 61A [Source:ZFIN;Acc:ZDB-GENE-040718-5]	13	15701596	15750103		3.79	4.81	3.99	2.16	2.61	2.49
ENSDARG00000008485	lrp3	low density lipoprotein receptor-related protein 3 [Source:ZFIN;Acc:ZDB-GENE-080116-3]	7	38278860	38310177		2.62	0.79	0.89	3.46	3.67	3.78
ENSDARG00000008487	dmd	dystrophin [Source:ZFIN;Acc:ZDB-GENE-010426-1]	1	10824351	11075405		5.71	4.5	3.93	9.36	8.17	8.08
ENSDARG00000008490	ltv1	LTV1 ribosome biogenesis factor [Source:NCBI gene;Acc:436587]	1	53865677	53880639		23.41	25.38	23.14	16.22	16.27	17.02
ENSDARG00000008491	si:ch211-107o10.3	si:ch211-107o10.3 [Source:ZFIN;Acc:ZDB-GENE-030131-4716]	7	30178253	30191357		11.55	20.48	15.55	5.23	5.71	5.57
ENSDARG00000008494	myl6	myosin, light chain 6, alkali, smooth muscle and non-muscle [Source:ZFIN;Acc:ZDB-GENE-041010-28]	6	39874177	39893526		17.8	22.7	22.04	13.69	15.03	13.71
ENSDARG00000008502	pno1	partner of NOB1 homolog [Source:NCBI gene;Acc:393520]	13	1125185	1130096		96.81	111.29	119.69	67.02	73.56	75.47
ENSDARG00000008503	glcci1a	glucocorticoid induced 1a [Source:ZFIN;Acc:ZDB-GENE-031010-41]	19	25352289	25427255		4.6	2.17	2.05	4.48	4.33	4.48
ENSDARG00000008540	sox21b	SRY-box transcription factor 21b [Source:NCBI gene;Acc:406246]	9	53276356	53277722		8.38	2.89	4.13	10.62	9.75	11.43
ENSDARG00000008541	chia.4	chitinase, acidic.4 [Source:ZFIN;Acc:ZDB-GENE-030131-9279]	23	17926279	17933143		0.05	0.1	0.1	0.04	0.02	0.03
ENSDARG00000008542	yod1	YOD1 deubiquitinase [Source:NCBI gene;Acc:550411]	11	37708384	37720266		6.66	4.38	4.26	8.2	7.92	8.51
ENSDARG00000008548	arhgap12a	Rho GTPase activating protein 12a [Source:ZFIN;Acc:ZDB-GENE-050809-38]	2	43749221	43797218		8.08	3.99	3.05	6.38	6.27	6.59

ENSDARG00000008551	pole3	polymerase (DNA directed), epsilon 3 (p17 subunit) [Source:ZFIN;Acc:ZDB-GENE-040426-1773]	5	32888119	32890807		18.68	18.63	17.79	16.25	15.77	15.69
ENSDARG00000008553	zgc:136872	zgc:136872 [Source:ZFIN;Acc:ZDB-GENE-060421-5204]	17	3124129	3166898		5.37	8.45	7.05	2.93	3.02	3.18
ENSDARG00000008560	brap	BRCA1 associated protein [Source:ZFIN;Acc:ZDB-GENE-040718-168]	10	3336776	3359189		7.26	5.07	5.68	6.42	6.31	7.06
ENSDARG00000008568	slc25a24	solute carrier family 25 member 24 [Source:NCBI gene;Acc:447867]	20	34653916	34670236		15.02	18.47	15.37	10.68	10.84	10.2
ENSDARG00000008573	fam20b	FAM20B glycosaminoglycan xylosylkinase [Source:NCBI gene;Acc:557060]	20	15857041	15922986		10.61	6.81	6.02	10.64	10.36	11.39
ENSDARG00000008575	lyst	lysosomal trafficking regulator [Source:ZFIN;Acc:ZDB-GENE-070702-1]	13	49867268	50002852		2.49	1.66	1.43	4.78	4.64	4.59
ENSDARG00000008582	mtmr8	myotubularin related protein 8 [Source:NCBI gene;Acc:393365]	5	22027588	22052852		4.45	4.33	3.88	4.43	4.5	4.8
ENSDARG00000008593	nbas	NBAS subunit of NRZ tethering complex [Source:NCBI gene;Acc:556592]	20	32838467	33174899		2.54	1.49	1.09	3.13	2.77	2.76
ENSDARG00000008623	cops3	COP9 signalosome subunit 3 [Source:HGNC Symbol;Acc:HGNC:2239]	12	1455147	1467915		28.12	25.84	26.97	24.07	23.93	24.61
ENSDARG00000008637	mttp	microsomal triglyceride transfer protein [Source:NCBI gene;Acc:406207]	1	12105901	12126625		18.38	29.89	20.56	10.76	11.74	10.61
ENSDARG00000008639	kifap3a	kinesin-associated protein 3a [Source:ZFIN;Acc:ZDB-GENE-040912-74]	20	34420623	34453623		13.92	5.68	7.07	13.84	13.87	14.05
ENSDARG00000008660	coro1b	coronin, actin binding protein, 1B [Source:ZFIN;Acc:ZDB-GENE-040123-3]	7	18617982	18656069		17.67	10.3	11.91	21.33	21.98	21.65
ENSDARG00000008662	fign	fidgetin [Source:NCBI gene;Acc:553599]	9	50599362	50755669		0.82	0.33	0.42	1.37	1.43	1.65
ENSDARG00000008678	snx3	sorting nexin 3 [Source:ZFIN;Acc:ZDB-GENE-030131-921]	20	32406011	32432671		25.29	28.13	26.96	26.46	26.16	25.83
ENSDARG00000008697	epas1a	endothelial PAS domain protein 1a [Source:ZFIN;Acc:ZDB-GENE-030131-4490]	12	25775734	25830268		19.8	23.47	19.21	17.68	18.54	17.27
ENSDARG00000008703	apeh	acylaminoacyl-peptide hydrolase [Source:ZFIN;Acc:ZDB-GENE-030131-9870]	8	53747925	53773539		13.11	12.23	12.4	10.65	11.5	11.71
ENSDARG00000008706	paip2b	poly(A) binding protein interacting protein 2B [Source:ZFIN;Acc:ZDB-GENE-040426-1736]	1	41465771	41472087		91.42	86.73	89.39	78.38	78.88	82.62
ENSDARG00000008720	lmo3	LIM domain only 3 [Source:NCBI gene;Acc:553774]	4	12612145	12679293		17.91	5.67	9.21	18.58	18.13	18.59
ENSDARG00000008722	prkcba	protein kinase C, beta a [Source:ZFIN;Acc:ZDB-GENE-040426-1354]	1	9004719	9064912		17.43	10	10.32	17.86	17.01	17.82
ENSDARG00000008727	ranbp3a	RAN binding protein 3a [Source:ZFIN;Acc:ZDB-GENE-040426-1132]	11	6295370	6316718		5.64	2.94	3.04	7.15	6.96	6.94
ENSDARG00000008732	zgc:66479	zgc:66479 [Source:ZFIN;Acc:ZDB-GENE-031030-10]	6	46736251	46742455		22.01	36.19	29.79	31.34	29.79	30.93
ENSDARG00000008735	atp6ap2	ATPase H+ transporting accessory protein 2 [Source:ZFIN;Acc:ZDB-GENE-040426-1960]	22	11123487	11137268		54.06	41.5	48.9	53.03	54.42	54.5
ENSDARG00000008740	esf1	ESF1, nucleolar pre-rRNA processing protein, homolog (S. cerevisiae) [Source:ZFIN;Acc:ZDB-GENE-030131-4917]	1	51202984	51219965		17.92	17.57	16.47	10.84	11.51	11.04
ENSDARG00000008748	ube2na	ubiquitin-conjugating enzyme E2Na [Source:ZFIN;Acc:ZDB-GENE-040426-2873]	18	15778110	15793163		58.51	54.05	49.13	69.7	66.16	65.99
ENSDARG00000008757	entpd6	ectonucleoside triphosphate diphosphohydrolase 6 [Source:ZFIN;Acc:ZDB-GENE-050417-412]	17	21102301	21123575		17.93	16.55	15.08	18.8	18.69	17.77
ENSDARG00000008765	tmed5	transmembrane p24 trafficking protein 5 [Source:ZFIN;Acc:ZDB-GENE-040426-1302]	20	14789148	14806671		7.99	10.37	9.72	9.91	10.14	9.82
ENSDARG00000008767	tle2b	TLE family member 2, transcriptional corepressor b [Source:ZFIN;Acc:ZDB-GENE-050208-677]	22	21672656	21755966		3.13	1.52	1.58	4.97	4.41	4.82
ENSDARG00000008772	cacng1a	calcium channel, voltage-dependent, gamma subunit 1a [Source:ZFIN;Acc:ZDB-GENE-030131-732]	6	21470261	21492752		7.13	11.6	10.45	15.48	14.79	13.39
ENSDARG00000008784	prpf4ba	pre-mRNA processing factor 4Ba [Source:ZFIN;Acc:ZDB-GENE-040426-947]	2	32812402	32826108		14.54	6.47	7.6	13.31	12.45	12.33
ENSDARG00000008785	dldh	dihydrolipoamide dehydrogenase [Source:ZFIN;Acc:ZDB-GENE-040120-4]	25	26877705	26893006		148.93	183.48	180.08	123.38	128.89	126.42
ENSDARG00000008788	camk1gb	calcium/calmodulin-dependent protein kinase IGb [Source:ZFIN;Acc:ZDB-GENE-040426-1694]	20	39558508	39597901		8.84	2.11	1.56	4.92	5.55	5.38
ENSDARG00000008790	actr3b	actin-related protein 3B [Source:ZFIN;Acc:ZDB-GENE-070424-10]	24	7294929	7321934		2.02	1.29	1.08	2.14	1.98	2.22
ENSDARG00000008796	her5	hairy-related 5 [Source:ZFIN;Acc:ZDB-GENE-990415-90]	14	30543008	30544666		0.12	0.03	0.03	0.02	0.08	0.07
ENSDARG00000008803	marcksb	myristoylated alanine-rich protein kinase C substrate b [Source:ZFIN;Acc:ZDB-GENE-030131-1921]	17	15534815	15539701		78.84	40.7	55.35	69.19	64.65	71
ENSDARG00000008805	orc3	origin recognition complex, subunit 3 [Source:ZFIN;Acc:ZDB-GENE-030131-6247]	17	49313504	49339681		3.68	4.44	4.25	2.81	2.75	2.43
ENSDARG00000008807	bokb	BCL2 family apoptosis regulator BOK b [Source:NCBI gene;Acc:394160]	2	22640215	22650435		1.02	0.81	0.94	0.61	0.55	0.38
ENSDARG00000008808	eml2	EMAP like 2 [Source:ZFIN;Acc:ZDB-GENE-050706-71]	15	28874441	28908027		11.64	8.67	9.37	10.17	10.26	10.18
ENSDARG00000008811	cmtm3	CKLF-like MARVEL transmembrane domain containing 3 [Source:ZFIN;Acc:ZDB-GENE-040801-206]	7	44608224	44621281		2.96	3.93	3.67	2.92	3.53	3.57
ENSDARG00000008814	glud1a	glutamate dehydrogenase 1a [Source:ZFIN;Acc:ZDB-GENE-030114-2]	13	22679084	22699024		33.96	44.64	40.72	38.81	39.69	37.66
ENSDARG00000008818	hsf1	heat shock transcription factor 1 [Source:ZFIN;Acc:ZDB-GENE-000616-16]	19	3056450	3082268		5.13	3.51	3.12	6.77	6.14	6.05
ENSDARG00000008829	chga	chromogranin A [Source:ZFIN;Acc:ZDB-GENE-041010-161]	20	27020201	27045991		21.56	15.58	17.8	13.21	13.98	14.47
ENSDARG00000008832	neu1	neuraminidase 1 [Source:ZFIN;Acc:ZDB-GENE-060503-592]	19	26810208	26823709		15.52	14.09	12.6	15.05	17.65	16.26
ENSDARG00000008835	si:dkey-46g23.5	si:dkey-46g23.5 [Source:ZFIN;Acc:ZDB-GENE-030131-9306]	3	3432960	3448095		0	0.01	0.02	0.02	0.04	0.03
ENSDARG00000008840	hmbsa	hydroxymethylbilane synthase a [Source:ZFIN;Acc:ZDB-GENE-040426-1375]	5	30518036	30534426		6.19	8.59	7.77	6.89	6.68	6.07
ENSDARG00000008849	ptprq	protein tyrosine phosphatase receptor type Q [Source:ZFIN;Acc:ZDB-GENE-050419-183]	18	6319447	6406609		0.4	0.26	0.21	0.77	0.74	0.7
ENSDARG00000008852	elp4	elongator acetyltransferase complex subunit 4 [Source:NCBI gene;Acc:550331]	7	15905691	16034324		1.15	1.05	1	0.9	0.82	0.88
ENSDARG00000008858	cyp7b1	cytochrome P450, family 7, subfamily B, polypeptide 1 [Source:ZFIN;Acc:ZDB-GENE-070912-293]	2	42135719	42152135		6.05	6.55	5.85	5.66	6.1	4.82
ENSDARG00000008859	mylipa	myosin regulatory light chain interacting protein a [Source:NCBI gene;Acc:335886]	19	26340736	26378655		12.42	7.17	6.06	12.96	14.44	13.03
ENSDARG00000008861	tfap2e	transcription factor AP-2 epsilon [Source:NCBI gene;Acc:393794]	19	47429722	47456787		4.31	1.66	1.75	5.9	4.94	5.64
ENSDARG00000008866	cabp4	calcium binding protein 4 [Source:ZFIN;Acc:ZDB-GENE-081104-291]	8	13794785	13823098		4.46	1.84	1.5	2	2.03	1.57
ENSDARG00000008867	rap1b	RAP1B, member of RAS oncogene family [Source:NCBI gene;Acc:321107]	4	18852821	18939338		26.98	32.41	28.48	33.82	32.55	31.92
ENSDARG00000008880	usp28	ubiquitin specific peptidase 28 [Source:ZFIN;Acc:ZDB-GENE-041008-142]	21	23050310	23110930		6.92	9.38	8.92	8.84	8.56	8.48
ENSDARG00000008884	hprt1	hypoxanthine phosphoribosyltransferase 1 [Source:ZFIN;Acc:ZDB-GENE-040426-1918]	14	31509922	31521543		15.58	16.64	14.4	16.07	16.55	16.22
ENSDARG00000008886	msx1b	muscle segment homeobox 1b [Source:NCBI gene;Acc:30511]	1	49352900	49355313		9.55	12.51	13.09	12.13	11.83	11.84
ENSDARG00000008890	ctns	cystinosin, lysosomal cystine transporter [Source:ZFIN;Acc:ZDB-GENE-050522-352]	11	6281647	6293709		4.35	4.83	4.88	4.69	4.11	4.28
ENSDARG00000008904	smarca2	SWI/SNF related, matrix associated, actin dependent regulator of chromatin, subfamily a, member 2 [Source:ZFIN;Acc:ZDB-GENE-(	5	45138934	45284136		7.74	7.16	5.84	9.85	9.73	10.27
ENSDARG00000008906	si:ch211-241e1.5	si:ch211-241e1.5 [Source:ZFIN;Acc:ZDB-GENE-030131-6298]	2	9379357	942319		4.34	3.68	3.66	3.62	3.48	3.81
ENSDARG00000008912	slc8a2a	solute carrier family 8 member 2a [Source:ZFIN;Acc:ZDB-GENE-060531-155]	5	36870737	36892723		1.3	0.53	0.61	1.22	1.31	1.74
ENSDARG00000008920	mrpl53	mitochondrial ribosomal protein L53 [Source:ZFIN;Acc:ZDB-GENE-040912-93]	19	32158010	32164532		35.96	44.33	40.54	23.14	26.28	24.55
ENSDARG00000008931	renbp	renin binding protein [Source:ZFIN;Acc:ZDB-GENE-041212-34]	8	37030974	37043900		2.39	2.23	3.05	3.35	3.88	3.36
ENSDARG00000008936	sec11a	SEC11 homolog A, signal peptidase complex subunit [Source:ZFIN;Acc:ZDB-GENE-040718-227]	18	26788083	26797723		32.12	39.64	31.18	30.67	28.65	30.47
ENSDARG00000008946	ccdc127b	coiled-coil domain containing 127b [Source:ZFIN;Acc:ZDB-GENE-060503-656]	19	7292445	7295926		5.43	7.53	6.63	3.48	3.79	2.76
ENSDARG00000008947	rtf1	RTF1 homolog, Paf1/RNA polymerase II complex component [Source:ZFIN;Acc:ZDB-GENE-030131-6778]	13	33229446	33246154		36.28	29.54	27.9	28.94	30.96	30.45
ENSDARG00000008948	pla2g3	phospholipase A2 group III [Source:ZFIN;Acc:ZDB-GENE-051113-96]	10	3520256	3532997		0.07	0.02	0.02	0.04	0.02	0.11
ENSDARG00000008953	pofut1	protein O-fucosyltransferase 1 [Source:ZFIN;Acc:ZDB-GENE-040303-2]	23	7692042	7709134		6.55	7.22	6.29	6.31	6.23	5.77
ENSDARG00000008966	tbl1xr1b	transducin (beta)-like 1 X-linked receptor 1b [Source:ZFIN;Acc:ZDB-GENE-040426-1192]	2	7764871	7800702		4.62	3.01	2.65	6.52	6.18	5.99
ENSDARG00000008969	fgb	fibrinogen beta chain [Source:ZFIN;Acc:ZDB-GENE-030131-9261]	1	10051763	10059496		92.07	145.5	148.82	177.53	200.88	196.4
ENSDARG00000008976	mdn1	midasin AAA ATPase 1 [Source:ZFIN;Acc:ZDB-GENE-041001-138]	20	23877299	23946296		5.33	4.28	3.23	5.34	5.15	5
ENSDARG00000008979	golga1	golgin A1 [Source:ZFIN;Acc:ZDB-GENE-040426-1237]	8	41467922	41519234		9.23	6.58	5.89	7.93	8.02	8.71
ENSDARG00000008982	casq2	calsequestrin 2 [Source:ZFIN;Acc:ZDB-GENE-010724-12]	9	33097289	33107237		30.35	36.37	35.3	33.87	35.44	35.68
ENSDARG00000008983	sh3glb2a	SH3-domain GRB2-like endophilin B2a [Source:ZFIN;Acc:ZDB-GENE-061201-8]	5	2757821	2789783		2.14	0.68	0.88	2.54	3.01	2.73
ENSDARG00000008986	e2f7	E2F transcription factor 7 [Source:NCBI gene;Acc:567941]	4	2530841	2545345		2.22	1.47	1.39	1.56	1.5	1.67
ENSDARG00000009001	pdia6	protein disulfide isomerase family A, member 6 [Source:ZFIN;Acc:ZDB-GENE-030131-879]	20	31217495	31233122		53.3	61.55	57.85	62.06	60.33	59.1
ENSDARG00000009014	col11a1b	collagen, type XI, alpha 1b [Source:ZFIN;Acc:ZDB-GENE-070912-607]	2	15389193	15563194		22.22	26.04	16.48	52.53	51.4	52.53
ENSDARG00000009018	rhbg	Rh family, B glycoprotein (gene/pseudogene) [Source:NCBI gene;Acc:337596]	16	29265796	29277164		137.18	128.64	94.5	63.32	56.82	51.38
ENSDARG00000009020	cnr1	cannabinoid receptor 1 [Source:ZFIN;Acc:ZDB-GENE-040312-3]	20	902412	915234		1.89	0.56	1	2.82	3.19	2.59
ENSDARG00000009021	chrna1	cholinergic receptor, nicotinic, alpha 1 (muscle) [Source:NCBI gene;Acc:30725]	6	10812337	10828923		14.96	19.1	17.72	25.79	22.99	23.1
ENSDARG00000009023	ankrd28b	ankyrin repeat domain 28b [Source:ZFIN;Acc:ZDB-GENE-070622-4]	16	20055878	20154859		6.32	5.11	4.04	6.95	6.87	6.79
ENSDARG00000009026	ank2a	ankyrin 2a, neuronal [Source:ZFIN;Acc:ZDB-GENE-111215-3]	1	14073891	14158563		4.22	1.43	1.95	6.56	6.79	6.66
ENSDARG00000009031	tnikb	TRAF2 and NCK interacting kinase b [Source:ZFIN;Acc:ZDB-GENE-051113-320]	24	26491904	26632182		9.32	6.21	4.63	12.72	12.41	12.11
ENSDARG00000009040	si:ch73-141c7.1	si:ch73-141c7.1 [Source:NCBI gene;Acc:393762]	3	18023975	18030938		5.46	5.99	5.72	7.73	7.63	9.05
ENSDARG00000009046	nphp1	nephronophthisis 1 [Source:ZFIN;Acc:ZDB-GENE-060929-1234]	17	22072337	22091141		3.18	1.66	1.92	2.42	2.41	2.35
ENSDARG00000009074	cbr4	carbonyl reductase 4 [Source:NCBI gene;Acc:402954]	20	23650832	23657304		5.13	5.33	4.9	4.3	4.07	4.27
ENSDARG00000009079	BX296551.1	sodium-dependent lysophosphatidylcholine symporter 1-like [Source:NCBI gene;Acc:570014]	23	16696410	16709231		0.19	0.29	0.3	0.18	0.22	0.33
ENSDARG00000009087	cd74a	CD74 molecule, major histocompatibility complex, class II invariant chain a [Source:ZFIN;Acc:ZDB-GENE-000901-1]	14	3038473	3052361		0.52	0.83	0.59	0.2	0.27	0.29
ENSDARG00000009094	gata2b	GATA binding protein 2b [Source:ZFIN;Acc:ZDB-GENE-040718-440]	6	40794015	40803800		0.74	0.86	0.86	0.65	0.56	0.57
ENSDARG00000009106	serinc1	serine incorporator 1 [Source:ZFIN;Acc:ZDB-GENE-030131-4415]	20	40457599	40472519		87.94	53.29	62.84	83.79	86.83	88.46
ENSDARG00000009113	pigc	phosphatidylinositol glycan anchor biosynthesis, class C [Source:ZFIN;Acc:ZDB-GENE-030131-2714]	20	14810886	14815492		3.11	2.25	2.26	4.28	4.01	4.34
ENSDARG00000009123	sele	selectin E [Source:ZFIN;Acc:ZDB-GENE-041014-221]	20	33969430	33976937		0.87	1.07	1.18	2.19	2.36	2.04
ENSDARG00000009131	slc10a4	solute carrier family 10 member 4 [Source:ZFIN;Acc:ZDB-GENE-041014-249]	20	23431485	23440955		1.1	0.63	0.69	2.19	2.9	2.65
ENSDARG00000009133	myo1eb	myosin IEb [Source:ZFIN;Acc:ZDB-GENE-040426-1347]	16	38201840	38238909		11.64	11.23	8.86	9.77	9.41	9.05
ENSDARG00000009134	hlx1	H2.0-like homeo box 1 (Drosophila) [Source:ZFIN;Acc:ZDB-GENE-030131-5304]	20	52195525	52199311		7.15	9.01	8.82	7.12	7.85	8.43
ENSDARG00000009136	tp53bp2a	tumor protein p53 binding protein, 2a [Source:ZFIN;Acc:ZDB-GENE-040516-8]	13	646700	664472		19.81	16.01	14.04	24.81	26.31	28.1
ENSDARG00000009142	ppp1r13bb	protein phosphatase 1, regulatory subunit 13Bb [Source:ZFIN;Acc:ZDB-GENE-030131-4327]	17	20731052	20814177		19.63	8.11	10.1	23.46	23.79	26.8
ENSDARG00000009153	pla2g1b	phospholipase A2, group IB (pancreas) [Source:ZFIN;Acc:ZDB-GENE-070117-1]	8	39724138	39733667		10.7	13.87	12.37	0.34	0.29	0.64
ENSDARG00000009160	kmo	kynurenine 3-monooxygenase [Source:NCBI gene;Acc:368242]	13	42272515	42306348		11.6	13.06	9.97	6.47	6.56	6.98
ENSDARG00000009161	ftr55	finTRIM family, member 55 [Source:ZFIN;Acc:ZDB-GENE-070424-161]	19	15423057	15434813		0.17	0.25	0.21	0.19	0.05	0.14
ENSDARG00000009169	mob1ba	MOB kinase activator 1Ba [Source:ZFIN;Acc:ZDB-GENE-040426-919]	5	42951852	42962707		13.58	16.11	14.77	16.12	16.14	15.73
ENSDARG00000009170	ctr9	CTR9 homolog, Paf1/RNA polymerase II complex component [Source:ZFIN;Acc:ZDB-GENE-030131-3782]	7	66655729	66693712		19.06	13.96	11.78	16.94	16.87	16.66
ENSDARG00000009178	zfand1	zinc finger, AN1-type domain 1 [Source:ZFIN;Acc:ZDB-GENE-060810-269]	24	11083146	11087183		7.44	8.7	8.15	6.16	7.05	6.45
ENSDARG00000009194	col16a1	collagen, type XVI, alpha 1 [Source:ZFIN;Acc:ZDB-GENE-060503-351]	19	38473758	38611814		17.36	19.08	12.25	24.58	22.31	23.97
ENSDARG00000009196	anxa3a	annexin A3a [Source:ZFIN;Acc:ZDB-GENE-040912-58]	5	39087364	39095623		12.11	12.23	11.24	8.78	7.36	7.95
ENSDARG00000009208	prkcda	protein kinase C, delta a [Source:ZFIN;Acc:ZDB-GENE-030131-6503]	6	40523370	40544207		11.09	8.33	8.28	7.89	7.54	7.34
ENSDARG00000009209	slc9a6a	solute carrier family 9 member A6 [Source:ZFIN;Acc:ZDB-GENE-070424-104]	14	31618982	31647900		3.84	2.2	2.02	4.93	5.04	5.19
ENSDARG00000009212	ppiaa	peptidylprolyl isomerase Aa (cyclophilin A) [Source:ZFIN;Acc:ZDB-GENE-030131-8556]	8	45754871	45760087		723.24	889.12	1054.62	606.34	629.89	656.2
ENSDARG00000009214	mfsd13a	major facilitator superfamily domain containing 13A [Source:ZFIN;Acc:ZDB-GENE-041212-83]	12	33894396	33915123		6.46	5.13	3.5	8.18	8.11	7.76
ENSDARG00000009215	zgc:112437	zgc:112437 [Source:ZFIN;Acc:ZDB-GENE-050417-293]	21	25773530	25774605		3.23	3.49	4.58	10.33	10.83	10.22
ENSDARG00000009217	tom1l2	target of myb1 like 2 membrane trafficking protein [Source:ZFIN;Acc:ZDB-GENE-061013-3]	12	18381982	18408588		13.54	7.06	6.35	15.45	16.52	16.77
ENSDARG00000009218	mars2	methionyl-tRNA synthetase 2, mitochondrial [Source:ZFIN;Acc:ZDB-GENE-111201-1]	14	23679049	23684814		2.37	2.59	2.56	1.66	1.88	1.74
ENSDARG00000009242	fev	FEV transcription factor, ETS family member [Source:NCBI gene;Acc:791175]	9	11548233	11551608		1.5	1.24	1.73	2.37	1.89	2.62
ENSDARG00000009250	etfb	electron transfer flavoprotein subunit beta [Source:ZFIN;Acc:ZDB-GENE-040426-1931]	16	50214819	50229274		128.56	174.74	172.77	85.8	88.04	87.67
ENSDARG00000009252	napepld	N-acyl phosphatidylethanolamine phospholipase D [Source:ZFIN;Acc:ZDB-GENE-030131-3856]	4	20480399	20486005		3.17	3.13	3.33	1.91	1.74	1.89
ENSDARG00000009253	rtraf	RNA transcription, translation and transport factor [Source:NCBI gene;Acc:393240]	17	14784258	14794741		13.15	15.35	15.44	12.29	12.69	12
ENSDARG00000009262	FAR2	si:dkey-97m3.1 [Source:ZFIN;Acc:ZDB-GENE-060503-367]	4	1988460	2036620	FAR2	5.26	3.33	3.14	5.55	5.12	4.71
ENSDARG00000009266	uevld	UEV and lactate/malate dehyrogenase domains [Source:NCBI gene;Acc:445225]	25	24181067	24202576		4.24	3.7	3.73	3.23	2.96	2.48
ENSDARG00000009267	zgc:136439	zgc:136439 [Source:ZFIN;Acc:ZDB-GENE-060421-2858]	9	41080029	41083181		9.4	14.04	10.83	5.44	5.15	4.66
ENSDARG00000009273	ppm1da	protein phosphatase, Mg2+/Mn2+ dependent, 1Da [Source:ZFIN;Acc:ZDB-GENE-040426-815]	15	26933196	26939404		14.49	9.77	10.06	13.47	12.78	13.25
ENSDARG00000009280	smyd1a	SET and MYND domain containing 1a [Source:ZFIN;Acc:ZDB-GENE-030131-9825]	5	72106837	72126424		27.97	38.91	50.24	31.08	30.2	29.05
ENSDARG00000009281	dnm1b	dynamin 1b [Source:ZFIN;Acc:ZDB-GENE-100920-3]	5	63668735	63737734		15.37	3.5	4.86	21.05	22.62	24.64
ENSDARG00000009285	rpl15	ribosomal protein L15 [Source:ZFIN;Acc:ZDB-GENE-040801-183]	19	17385561	17395587		357.94	450.8	552.46	296.74	320.04	309.24
ENSDARG00000009299	dusp8a	dual specificity phosphatase 8a [Source:ZFIN;Acc:ZDB-GENE-040426-1842]	25	22979743	23052707		13.26	4.42	4.47	20.51	22.15	21.81
ENSDARG00000009311	pvalb6	parvalbumin 6 [Source:ZFIN;Acc:ZDB-GENE-040912-95]	3	24297482	24434905		23.8	9	10.27	23.01	21.53	24.1
ENSDARG00000009313	napsa	napsin A aspartic peptidase [Source:ZFIN;Acc:ZDB-GENE-030131-8690]	3	25121156	25133244		5.17	6.16	5.45	5.66	5.63	5.74
ENSDARG00000009315	clgn	calmegin [Source:ZFIN;Acc:ZDB-GENE-060929-708]	1	40572757	40608392		22.89	13.32	13.38	20.42	21.37	20.84
ENSDARG00000009336	aif1l	allograft inflammatory factor 1-like [Source:ZFIN;Acc:ZDB-GENE-030131-9646]	5	34549365	34575502		17.38	10.65	11.79	17.85	18.38	19.08
ENSDARG00000009341	nrap	nebulin-related anchoring protein [Source:ZFIN;Acc:ZDB-GENE-060616-224]	12	30586599	30618440		11.55	14.41	13.04	5.15	4.45	4.46
ENSDARG00000009342	txndc5	thioredoxin domain containing 5 [Source:ZFIN;Acc:ZDB-GENE-040426-1951]	24	7941373	7957581		24.41	29.07	24.51	34.46	34.13	33.12
ENSDARG00000009346	caprin1a	cell cycle associated protein 1a [Source:ZFIN;Acc:ZDB-GENE-040801-226]	7	26998169	27021303		15.18	9.8	11.04	15.99	14.92	15.68
ENSDARG00000009350	atg3	autophagy related 3 [Source:NCBI gene;Acc:336632]	6	7109954	7123210		29.07	35.71	35.43	32.74	30.1	29.3
ENSDARG00000009351	fgf6a	fibroblast growth factor 6a [Source:ZFIN;Acc:ZDB-GENE-980526-559]	25	16945348	16951890		4.15	4.31	3.82	4.55	4.29	4.61
ENSDARG00000009372	diras1b	DIRAS family, GTP-binding RAS-like 1b [Source:ZFIN;Acc:ZDB-GENE-070912-620]	2	37059304	37059966		4.1	1.21	1.44	4.57	3.54	3.64
ENSDARG00000009386	mpp4l	membrane protein, palmitoylated 4-like [Source:ZFIN;Acc:ZDB-GENE-091118-7]	21	18976623	18993110		0.25	0.09	0.06	0.51	0.94	0.88
ENSDARG00000009387	robo4	roundabout, axon guidance receptor, homolog 4 (Drosophila) [Source:ZFIN;Acc:ZDB-GENE-020809-1]	10	31244619	31284790		1.23	0.51	0.58	2.83	2.92	2.9
ENSDARG00000009390	npl	N-acetylneuraminate pyruvate lyase (dihydrodipicolinate synthase) [Source:NCBI gene;Acc:100148292]	8	19489854	19500354		5.77	6.34	6.77	6.77	6.37	7.39
ENSDARG00000009401	vcanb	versican b [Source:ZFIN;Acc:ZDB-GENE-030131-2185]	10	43186678	43263885		18.41	24.45	18.9	27.47	28.02	27.81
ENSDARG00000009402	si:ch211-210b2.2	si:ch211-210b2.2 [Source:ZFIN;Acc:ZDB-GENE-131118-33]	15	665156	669476		1.29	1.27	1.05	0.75	0.64	0.61
ENSDARG00000009418	mef2cb	myocyte enhancer factor 2cb [Source:ZFIN;Acc:ZDB-GENE-040901-7]	5	48179340	48307804		4.21	2.73	2.11	6.81	5.9	6
ENSDARG00000009430	tmem30c	transmembrane protein 30C [Source:ZFIN;Acc:ZDB-GENE-040426-1127]	9	29994439	30007477		2.06	2.82	2.12	1.83	1.9	1.99
ENSDARG00000009436	ubxn7	UBX domain protein 7 [Source:ZFIN;Acc:ZDB-GENE-040704-8]	2	16710883	16737363		8.81	5.22	4.54	8.93	8.93	9.08
ENSDARG00000009438	myog	myogenin [Source:ZFIN;Acc:ZDB-GENE-980526-265]	11	22592935	22605981		2.23	2.74	2.42	2.17	2.46	2.28
ENSDARG00000009443	zgc:92137	zgc:92137 [Source:ZFIN;Acc:ZDB-GENE-040801-179]	17	42983983	42988356		70.59	104.71	82.26	3.86	3.43	3.12
ENSDARG00000009447	atp5mc3b	ATP synthase membrane subunit c locus 3b [Source:ZFIN;Acc:ZDB-GENE-020814-1]	6	10830844	10835922		644.3	838.09	1039.4	469.47	495.23	492.32
ENSDARG00000009459	slc35e4	solute carrier family 35 member E4 [Source:ZFIN;Acc:ZDB-GENE-050417-407]	10	7901012	7913591		1.43	0.6	0.75	1.45	1.53	1.55
ENSDARG00000009466	rgs9bp	regulator of G protein signaling 9 binding protein [Source:ZFIN;Acc:ZDB-GENE-030728-1]	7	41303572	41312197		2.63	0.9	0.83	1.43	1.64	1.67
ENSDARG00000009470	hnf1a	HNF1 homeobox a [Source:ZFIN;Acc:ZDB-GENE-021206-12]	8	39802506	39817167		1.74	3.02	2.14	1.48	1.6	1.56
ENSDARG00000009471	nid2b	nidogen 2b (osteonidogen) [Source:ZFIN;Acc:ZDB-GENE-060724-13]	20	9643499	9674877		0.02	0.03	0.03	0.05	0.02	0.02
ENSDARG00000009472	med19b	mediator complex subunit 19b [Source:NCBI gene;Acc:445021]	1	44582369	44591489		8.81	5.2	6.05	6.63	6.98	5.77
ENSDARG00000009473	ppardb	peroxisome proliferator-activated receptor delta b [Source:ZFIN;Acc:ZDB-GENE-000112-47]	8	23703464	23726040		6.35	4.75	4.14	6.68	6.4	6.86
ENSDARG00000009477	prkar2ab	protein kinase, cAMP-dependent, regulatory, type II, alpha, B [Source:ZFIN;Acc:ZDB-GENE-040426-2427]	22	110158	130695		4.61	0.91	0.73	4.39	3.86	4.51
ENSDARG00000009480	pdcl	phosducin-like [Source:ZFIN;Acc:ZDB-GENE-030131-2365]	16	51950510	51966846		31.37	18.32	17.47	31.84	30.66	32.81
ENSDARG00000009482	slc38a8a	solute carrier family 38 member 8a [Source:ZFIN;Acc:ZDB-GENE-121005-5]	18	21622414	21640389		0.06	0.11	0.15	0.14	0.12	0.25
ENSDARG00000009484	arf1	ADP-ribosylation factor 1 [Source:ZFIN;Acc:ZDB-GENE-010724-5]	2	3062737	3077027		22.11	23.97	20.83	24.33	24.38	23.83
ENSDARG00000009488	gipc2	GIPC PDZ domain containing family, member 2 [Source:ZFIN;Acc:ZDB-GENE-040426-921]	2	9560740	9590850		12.05	12.34	9.56	9.62	11.18	10.61
ENSDARG00000009493	MAP3K13	si:ch211-45c16.2 [Source:ZFIN;Acc:ZDB-GENE-081105-15]	9	52694971	52814314	MAP3K13	2.82	0.65	0.67	4.49	4.18	4.4
ENSDARG00000009494	xrcc1	X-ray repair complementing defective repair in Chinese hamster cells 1 [Source:ZFIN;Acc:ZDB-GENE-040822-30]	16	54209504	54248267		5.38	3.64	3.99	3.94	4.05	3.77
ENSDARG00000009499	syne1a	spectrin repeat containing, nuclear envelope 1a [Source:ZFIN;Acc:ZDB-GENE-030131-4172]	20	26095530	26359434		6.7	2.72	2.75	8.3	8.19	8.91
ENSDARG00000009505	prelid3b	PRELI domain containing 3 [Source:ZFIN;Acc:ZDB-GENE-031002-13]	6	60112200	60122081		49.54	48.24	51.1	43.14	44.29	44.15
ENSDARG00000009511	tnfa	tumor necrosis factor a (TNF superfamily, member 2) [Source:ZFIN;Acc:ZDB-GENE-050317-1]	19	27019432	27024019		0	0	0.08	0.02	0.02	0
ENSDARG00000009524	rnf150b	ring finger protein 150b [Source:NCBI gene;Acc:792211]	23	43770149	43787147		3.92	2.83	3	4.72	4.77	4.43
ENSDARG00000009525	tial1	TIA1 cytotoxic granule-associated RNA binding protein-like 1 [Source:ZFIN;Acc:ZDB-GENE-040426-1547]	13	25472005	25484752		31.97	17.11	21.01	36.68	35.91	36.95
ENSDARG00000009534	wls	Wnt ligand secretion mediator [Source:ZFIN;Acc:ZDB-GENE-040426-2161]	2	10342177	10386738		31.88	31.4	31.93	38.56	38.66	37.27
ENSDARG00000009544	cldnb	claudin b [Source:ZFIN;Acc:ZDB-GENE-010328-2]	21	25765734	25766840		63.42	55.14	54.01	50.6	49.73	45.91
ENSDARG00000009549	ubr4	ubiquitin protein ligase E3 component n-recognin 4 [Source:ZFIN;Acc:ZDB-GENE-090313-341]	23	21278948	21376847		9.67	6.31	4.73	15.49	13.63	14.48
ENSDARG00000009550	foxi3b	forkhead box I3b [Source:ZFIN;Acc:ZDB-GENE-031126-4]	14	34514336	34516587		12.47	11.63	10.33	5.39	6.29	5.77
ENSDARG00000009553	gng3	guanine nucleotide binding protein (G protein), gamma 3 [Source:ZFIN;Acc:ZDB-GENE-010705-1]	21	26697536	26707052		122.17	66.62	78.08	111.37	105.87	115.64
ENSDARG00000009557	wrap73	WD repeat containing, antisense to TP73 [Source:ZFIN;Acc:ZDB-GENE-030131-6313]	8	48816082	48847772		3.13	3.18	2.87	3	2.95	2.9
ENSDARG00000009567	spegb	striated muscle enriched protein kinase b [Source:ZFIN;Acc:ZDB-GENE-081104-517]	9	41612642	41681053		3.76	3.25	2.69	7.1	5.85	5.93
ENSDARG00000009568	ca10b	carbonic anhydrase Xb [Source:ZFIN;Acc:ZDB-GENE-080815-2]	3	34919810	34981673		3.23	1.53	1.34	2.51	2.8	3.03
ENSDARG00000009585	papola	poly(A) polymerase alpha [Source:ZFIN;Acc:ZDB-GENE-030131-3507]	20	4712776	4738133		37.24	26.72	24.87	42.71	41.22	42.34
ENSDARG00000009594	nr1d2b	nuclear receptor subfamily 1, group D, member 2b [Source:ZFIN;Acc:ZDB-GENE-990415-244]	19	17400263	17407115		46.94	24.09	24.88	35.61	37.55	37.48
ENSDARG00000009603	ccnc	cyclin C [Source:ZFIN;Acc:ZDB-GENE-030131-7368]	4	5764486	5775507		9.74	7.96	7.02	10.2	9.75	10.07
ENSDARG00000009610	rgs12a	regulator of G protein signaling 12a [Source:ZFIN;Acc:ZDB-GENE-030131-2614]	7	18474563	18547420		2.89	2.12	1.53	3.21	3.31	3.61
ENSDARG00000009612	chia.3	chitinase, acidic.3 [Source:ZFIN;Acc:ZDB-GENE-040426-2891]	11	24013769	24016813		3.36	5.46	3.2	0.2	0.22	0.41
ENSDARG00000009624	CABZ01027646.1	urotensin 2 receptor [Source:NCBI gene;Acc:559133]	12	28117365	28181720		0.56	0.32	0.26	0.6	0.63	0.57
ENSDARG00000009626	nrbp2b	nuclear receptor binding protein 2b [Source:ZFIN;Acc:ZDB-GENE-080724-1]	24	33226331	33276139		8.5	3.44	4.05	7.22	7.13	7.02
ENSDARG00000009629	unc119.1	unc-119 homolog 1 [Source:NCBI gene;Acc:338230]	8	39848504	39859688		3.49	2.09	2	4.78	4.5	5
ENSDARG00000009637	rcvrn3	recoverin 3 [Source:ZFIN;Acc:ZDB-GENE-040426-1661]	19	10339538	10352579		50.52	13.81	10.16	50.36	56.83	52.62
ENSDARG00000009640	psmb1	proteasome 20S subunit beta 1 [Source:ZFIN;Acc:ZDB-GENE-040618-2]	13	1142724	1151410		127.42	130.2	151.84	76.97	80.95	80.39
ENSDARG00000009653	lhfpl2a	LHFPL tetraspan subfamily member 2a [Source:NCBI gene;Acc:402962]	21	300647	308852		5.45	5.78	6.86	7.59	7.11	6.35
ENSDARG00000009657	fgfr1op2	FGFR1 oncogene partner 2 [Source:NCBI gene;Acc:335511]	4	1722899	1730920		13.19	16.54	15.19	9.26	10.26	10.78
ENSDARG00000009673	si:ch211-197g15.6	si:ch211-197g15.6 [Source:ZFIN;Acc:ZDB-GENE-050208-457]	22	17493170	17499513		0.03	0	0.01	0.03	0.03	0.04
ENSDARG00000009677	dlg1	discs, large homolog 1 (Drosophila) [Source:NCBI gene;Acc:114446]	6	29860776	30082922		7.91	3.55	3.39	8.62	8.42	8.34
ENSDARG00000009681	med27	mediator complex subunit 27 [Source:NCBI gene;Acc:393633]	8	11884671	11988065		8.67	7.35	8.85	7.34	8.22	7.83

ENSDARG00000009685	ppa2	inorganic pyrophosphatase 2 [Source:ZFIN;Acc:ZDB-GENE-040426-1857]	1	26389153	26398361		4.36	4.32	3.9	3.24	3.28	3.47
ENSDARG00000009689	daam1b	dishevelled associated activator of morphogenesis 1b [Source:ZFIN;Acc:ZDB-GENE-030131-4212]	20	21815613	21994901		13.59	10.81	9.05	14.58	14.39	13.88
ENSDARG00000009693	llgl1	LLGL scribble cell polarity complex component 1 [Source:ZFIN;Acc:ZDB-GENE-060825-69]	3	39970355	40054749		5.23	3.96	3.48	7.02	6.77	6.69
ENSDARG00000009702	ascl1b	si:ch211-210p4.6 [Source:NCBI gene;Acc:560440]	7	49695904	49715154		0.69	1.05	0.77	0.61	0.59	0.59
ENSDARG00000009724	rpn2	ribophorin II [Source:ZFIN;Acc:ZDB-GENE-030131-7928]	11	15225534	15297210		53.43	58.3	53.95	60.19	60.52	62.56
ENSDARG00000009727	twf2b	twinfilin actin-binding protein 2b [Source:ZFIN;Acc:ZDB-GENE-050522-238]	11	17965098	17981427		1.53	1.53	1.91	1.24	1.17	1.31
ENSDARG00000009733	kif21b	kinesin family member 21B [Source:ZFIN;Acc:ZDB-GENE-130530-537]	11	2855430	2974067		2.2	0.99	1.07	2.56	2.67	2.64
ENSDARG00000009735	gigyf2	GRB10 interacting GYF protein 2 [Source:NCBI gene;Acc:561830]	2	48203278	48255843		15.02	12.07	10.01	15.94	15.44	16.11
ENSDARG00000009738	cacnb4a	calcium channel, voltage-dependent, beta 4a subunit [Source:ZFIN;Acc:ZDB-GENE-080320-2]	9	38074082	38130085		2.45	0.52	0.88	2.78	2.73	2.74
ENSDARG00000009740	ppp1r7	protein phosphatase 1, regulatory (inhibitor) subunit 7 [Source:NCBI gene;Acc:560323]	2	16696052	16707806		17.2	12.82	12.42	19.06	18.08	19.86
ENSDARG00000009743	efhc1	EF-hand domain (C-terminal) containing 1 [Source:ZFIN;Acc:ZDB-GENE-040426-1300]	20	48097252	48157755		1.1	0.98	1.31	1.83	2.1	2.09
ENSDARG00000009745	fbxw2	F-box and WD repeat domain containing 2 [Source:ZFIN;Acc:ZDB-GENE-050522-303]	5	32380026	32396929		7.32	4.78	4.55	8.25	8.13	7.95
ENSDARG00000009748	dffb	DNA fragmentation factor, beta polypeptide (caspase-activated DNase) [Source:ZFIN;Acc:ZDB-GENE-030826-4]	8	22277198	22283112		4.14	3.8	3.36	4.94	3.76	4.44
ENSDARG00000009753	sf3b6	splicing factor 3b, subunit 6 [Source:ZFIN;Acc:ZDB-GENE-040625-36]	20	35433192	35437940		28.56	34.61	29.72	22.7	23.19	23.87
ENSDARG00000009754	zc3h11a	zinc finger CCCH-type containing 11A [Source:ZFIN;Acc:ZDB-GENE-030131-3272]	11	24034345	24044586		25.71	12.44	12.96	26.95	28.19	28.69
ENSDARG00000009764	srek1	splicing regulatory glutamine/lysine-rich protein 1 [Source:ZFIN;Acc:ZDB-GENE-040801-132]	5	55160348	55201172		9.77	3.94	5.12	9.59	9.48	9
ENSDARG00000009771	ppme1	protein phosphatase methylesterase 1 [Source:ZFIN;Acc:ZDB-GENE-030131-7064]	21	25380675	25395223		35.74	24.89	28.01	36.18	37.42	38.66
ENSDARG00000009779	mcl1a	MCL1 apoptosis regulator, BCL2 family member a [Source:ZFIN;Acc:ZDB-GENE-000511-7]	19	348729	353231		63.85	63.01	71.15	65.47	69.85	66.62
ENSDARG00000009782	myh11a	myosin, heavy chain 11a, smooth muscle [Source:ZFIN;Acc:ZDB-GENE-050531-1]	6	8499865	8580858		17.84	18.73	13.96	13.64	14.74	14.01
ENSDARG00000009783	slc49a4	solute carrier family 49 member 4 [Source:ZFIN;Acc:ZDB-GENE-040912-167]	9	37366973	37453128		9.58	7.08	6.94	8.23	7.71	8.3
ENSDARG00000009796	klc1a	kinesin light chain 1a [Source:ZFIN;Acc:ZDB-GENE-040426-1599]	13	15816573	15904342		31.52	10.95	15.65	31.35	34.74	33.25
ENSDARG00000009806	smfn	small fragment nuclease [Source:ZFIN;Acc:ZDB-GENE-040426-1709]	10	39893439	39905253		9.14	12.61	11.49	6.1	5.96	6.55
ENSDARG00000009822	her4.4	hairy-related 4, tandem duplicate 4 [Source:ZFIN;Acc:ZDB-GENE-081031-104]	23	21462021	21463788		1.93	0.67	1.31	2.5	1.82	2.35
ENSDARG00000009823	pou3f1	POU class 3 homeobox 1 [Source:NCBI gene;Acc:30398]	16	33593116	33595985		21.15	6.5	8.88	21.23	21.63	22.69
ENSDARG00000009825	mmp23bb	matrix metallopeptidase 23bb [Source:ZFIN;Acc:ZDB-GENE-050417-448]	18	45990394	46005179		5.25	3.57	3.68	3.22	2.77	3.35
ENSDARG00000009830	hdlbpa	high density lipoprotein binding protein a [Source:ZFIN;Acc:ZDB-GENE-030131-2032]	6	26874839	26895750		244.58	275.58	210.9	247.8	250.1	240.59
ENSDARG00000009837	agk	acylglycerol kinase [Source:NCBI gene;Acc:334270]	18	20532618	20540709		12.24	12.3	8.85	10.19	10.01	10.93
ENSDARG00000009839	fam117bb	family with sequence similarity 117 member Bb [Source:ZFIN;Acc:ZDB-GENE-070912-554]	9	13120419	13212790		3.83	3.34	3.03	5.82	5.96	5.49
ENSDARG00000009844	dusp23a	dual specificity phosphatase 23a [Source:ZFIN;Acc:ZDB-GENE-030131-2554]	20	43113465	43118411		4.73	4.86	3.78	1.99	1.65	1.95
ENSDARG00000009851	wdr41	WD repeat domain 41 [Source:ZFIN;Acc:ZDB-GENE-050706-152]	21	7605803	7630091		14.77	8.69	11.05	11.64	11.77	12.86
ENSDARG00000009862	dcps	decapping enzyme, scavenger [Source:ZFIN;Acc:ZDB-GENE-040311-1]	10	39283985	39294725		2.64	1.96	2.32	2.97	2.47	2.39
ENSDARG00000009864	ddx55	DEAD (Asp-Glu-Ala-Asp) box polypeptide 55 [Source:NCBI gene;Acc:286745]	10	3397871	3416258		6.82	7.96	7.8	4.61	4.84	4.52
ENSDARG00000009868	dcaf11	ddb1 and cul4 associated factor 11 [Source:ZFIN;Acc:ZDB-GENE-050809-116]	24	12963119	12983829		21.13	17.76	18.68	25.66	24.98	27.12
ENSDARG00000009870	mapk8b	mitogen-activated protein kinase 8b [Source:NCBI gene;Acc:65236]	12	2505519	2522487		5.53	3.38	2.88	9.84	10.07	8.95
ENSDARG00000009871	snrpc	small nuclear ribonucleoprotein polypeptide C [Source:NCBI gene;Acc:192330]	6	54430413	54433995		29.43	25.14	27.2	30.34	26.65	28.02
ENSDARG00000009874	cyp2k6	cytochrome P450, family 2, subfamily k, polypeptide 31 [Source:NCBI gene;Acc:100001426]	3	8261132	8272632		0	0	0	0	0	0
ENSDARG00000009881	ier5	immediate early response 5 [Source:ZFIN;Acc:ZDB-GENE-030616-127]	22	16497670	16499153		54.75	49.38	50.68	55.84	57.66	60.24
ENSDARG00000009885	sult3st1	sulfotransferase family 3, cytosolic sulfotransferase 1 [Source:ZFIN;Acc:ZDB-GENE-061207-52]	3	29655677	29671653		0.28	0.17	0.2	0.21	0.09	0.17
ENSDARG00000009886	rnf114	ring finger protein 114 [Source:ZFIN;Acc:ZDB-GENE-040813-1]	23	3503596	3511630		7.92	9.17	8.61	7.5	6.57	7.61
ENSDARG00000009899	znf385a	zinc finger protein 385A [Source:ZFIN;Acc:ZDB-GENE-090313-255]	23	28185883	28294763		5.16	4.29	3.02	6.19	5.89	6.38
ENSDARG00000009901	slc38a5a	solute carrier family 38 member 5a [Source:ZFIN;Acc:ZDB-GENE-081105-92]	8	25615781	25625800		8.06	2.92	2.04	14.54	15.19	14.52
ENSDARG00000009922	dmbx1a	diencephalon/mesencephalon homeobox 1a [Source:NCBI gene;Acc:142987]	2	10183640	10192487		7.1	1.23	2	9.97	9.93	10.57
ENSDARG00000009930	cadm2a	cell adhesion molecule 2a [Source:ZFIN;Acc:ZDB-GENE-040426-1614]	10	23216678	23379146		8.43	2.13	3.9	8.53	10.12	9.68
ENSDARG00000009939	micu2	mitochondrial calcium uptake 2 [Source:ZFIN;Acc:ZDB-GENE-030131-3374]	5	36599107	36609700		16.68	21.98	17.36	13.11	12.59	12.73
ENSDARG00000009941	erc1a	ELKS/RAB6-interacting/CAST family member 1a [Source:ZFIN;Acc:ZDB-GENE-091214-5]	25	21176937	21253162		11.88	2.57	3.96	11.61	13.62	13.48
ENSDARG00000009942	cdc6	cell division cycle 6 homolog (S. cerevisiae) [Source:ZFIN;Acc:ZDB-GENE-050506-131]	11	11781973	11792766		3.36	3.14	2.73	2.21	2.11	2.09
ENSDARG00000009949	fndc4b	fibronectin type III domain containing 4b [Source:ZFIN;Acc:ZDB-GENE-120711-3]	17	5846202	5928942		4.29	2.41	3.57	5.6	5.55	5.55
ENSDARG00000009950	edf1	endothelial differentiation-related factor 1 [Source:NCBI gene;Acc:793036]	5	64397454	64406058		136.11	162.11	171.81	106.53	109.77	115.04
ENSDARG00000009953	med14	mediator complex subunit 14 [Source:ZFIN;Acc:ZDB-GENE-030131-8867]	9	34397516	34428152		3.52	2.21	2.02	4.99	4.83	4.93
ENSDARG00000009958	cbl	Cbl proto-oncogene, E3 ubiquitin protein ligase [Source:ZFIN;Acc:ZDB-GENE-041114-207]	15	23208042	23265405		4.63	3.09	2.48	8.37	7.88	7.55
ENSDARG00000009961	rundc3b	RUN domain containing 3b [Source:NCBI gene;Acc:492775]	16	43077909	43118095		0.11	0.23	0.25	0.28	0.46	0.31
ENSDARG00000009978	icn	ictacalcin [Source:ZFIN;Acc:ZDB-GENE-030131-8599]	16	23431189	23432552		825.75	1015.63	992.3	338.14	322.84	345.28
ENSDARG00000009982	fam199x	family with sequence similarity 199, X-linked [Source:NCBI gene;Acc:445309]	14	10338417	10371638		7.28	3.88	4.04	9.03	8.59	8.59
ENSDARG00000010002	gna11a	guanine nucleotide binding protein (G protein), alpha 11a (Gq class) [Source:ZFIN;Acc:ZDB-GENE-050208-597]	22	21852070	21897279		9.46	5.16	4.75	10.93	12.27	9.67
ENSDARG00000010008	vim	vimentin [Source:ZFIN;Acc:ZDB-GENE-011212-3]	24	32176155	32192681		88.69	86.41	105.6	78.4	88.34	89.27
ENSDARG00000010010	trim13	tripartite motif containing 13 [Source:NCBI gene;Acc:553630]	9	29642857	29654998		15.53	3.82	6.35	10.26	9.96	11.47
ENSDARG00000010018	qki2	QKI, KH domain containing, RNA binding 2 [Source:NCBI gene;Acc:393815]	12	34953038	35019519		8.89	7.88	7.07	20.05	20.55	21.86
ENSDARG00000010021	grpel1	GrpE-like 1, mitochondrial [Source:ZFIN;Acc:ZDB-GENE-051120-111]	7	39744244	39751540		15.09	18.78	16.72	14.31	15.06	13.67
ENSDARG00000010023	CU856539.1	hippocampus abundant transcript 1 protein-like [Source:NCBI gene;Acc:557306]	10	2499383	2527342		19.34	4.26	5.66	18.95	20.21	20.6
ENSDARG00000010029	spon1a	spondin 1a [Source:ZFIN;Acc:ZDB-GENE-000427-9]	18	28564640	28880191		2.09	0.54	0.88	2.61	2.7	2.83
ENSDARG00000010031	ssh1b	slingshot protein phosphatase 1b [Source:ZFIN;Acc:ZDB-GENE-030131-5909]	5	31914906	31928913		5.58	1.1	1.85	5.32	4.6	4.78
ENSDARG00000010032	cmtm7	CKLF-like MARVEL transmembrane domain containing 7 [Source:ZFIN;Acc:ZDB-GENE-041111-174]	16	41495670	41503925		7.08	8.3	8.56	9.87	10.29	9.57
ENSDARG00000010035	engase	endo-beta-N-acetylglucosaminidase [Source:NCBI gene;Acc:561239]	3	36136530	36153591		3.28	2.41	2.67	2.67	3.14	2.83
ENSDARG00000010042	dnm1a	dynamin 1a [Source:NCBI gene;Acc:100307098]	5	1278092	1421927		7	1.3	1.86	13.52	13.4	13.55
ENSDARG00000010047	neu3.2	sialidase 3 (membrane sialidase), tandem duplicate 2 [Source:ZFIN;Acc:ZDB-GENE-040801-262]	21	21796663	21799292		1.52	1.41	0.78	8.07	8.18	8.63
ENSDARG00000010051	ufd1l	ubiquitin recognition factor in ER associated degradation 1 [Source:ZFIN;Acc:ZDB-GENE-040718-150]	8	4745340	4776395		22.01	24.52	26.24	19	18.25	19.48
ENSDARG00000010052	ndrg3b	ndrg family member 3b [Source:ZFIN;Acc:ZDB-GENE-030131-5606]	23	14829468	14990921		10.43	6.24	5.9	9.7	10.25	9.89
ENSDARG00000010059	itpkb	inositol-trisphosphate 3-kinase B [Source:ZFIN;Acc:ZDB-GENE-041014-166]	20	38386282	38449903		4.38	3.87	3.21	3.66	3.52	3.07
ENSDARG00000010070	adam9	ADAM metallopeptidase domain 9 [Source:ZFIN;Acc:ZDB-GENE-040912-127]	8	45850139	45939691		8.67	7.8	6.73	11.06	10.91	11.11
ENSDARG00000010075	PTCHD3	patched domain containing 3 [Source:HGNC Symbol;Acc:HGNC:24776]	24	32560395	32563875	PTCHD3	0.55	0.06	0.18	0.39	0.59	0.36
ENSDARG00000010078	nup133	nucleoporin 133 [Source:NCBI gene;Acc:406852]	1	53919110	53941906		8.31	7.55	6.58	8.84	7.95	8.22
ENSDARG00000010083	rbfox3a	RNA binding fox-1 homolog 3a [Source:ZFIN;Acc:ZDB-GENE-111213-1]	12	32729470	33251465		3.83	1.35	1.63	10.27	9.7	10.39
ENSDARG00000010085	p4ha2	procollagen-proline, 2-oxoglutarate 4-dioxygenase (proline 4-hydroxylase), alpha polypeptide 2 [Source:ZFIN;Acc:ZDB-GENE-0308	21	43097798	43131752		10.88	9.77	8.49	43.47	45.53	45.96
ENSDARG00000010096	slc1a6	solute carrier family 1 member 6 [Source:ZFIN;Acc:ZDB-GENE-071004-45]	11	6152643	6175057		7.99	2.02	3.15	6.82	6.4	6.52
ENSDARG00000010097	f9a	coagulation factor IXa [Source:ZFIN;Acc:ZDB-GENE-030714-2]	14	32430982	32438200		0.36	0.46	0.53	0.18	0.15	0.14
ENSDARG00000010098	pank1b	pantothenate kinase 1b [Source:ZFIN;Acc:ZDB-GENE-070209-259]	12	16953415	16964840		17.99	16.07	14.26	20.77	21.07	20.65
ENSDARG00000010108	bri3bp	bri3 binding protein [Source:ZFIN;Acc:ZDB-GENE-030131-5254]	5	18904968	18911114		13.32	5.41	6.6	15.74	16.8	16.4
ENSDARG00000010113	ndufb8	NADH:ubiquinone oxidoreductase subunit B8 [Source:ZFIN;Acc:ZDB-GENE-040426-1858]	12	48469755	48477031		146.9	180.72	203.47	107.36	101.64	102.75
ENSDARG00000010130	smpdl3b	sphingomyelin phosphodiesterase acid like 3B [Source:ZFIN;Acc:ZDB-GENE-041008-132]	16	4710977	4721629		11.7	20.61	16.36	5.78	5.34	4.85
ENSDARG00000010132	dacha	dachshund a [Source:ZFIN;Acc:ZDB-GENE-020402-3]	21	34364456	34658266		7.29	4.62	4.4	10.37	10.18	10.49
ENSDARG00000010137	ldb1a	LIM domain binding 1a [Source:NCBI gene;Acc:30579]	13	28705143	28748749		16.33	8.26	8.72	22.05	22.21	22.75
ENSDARG00000010144	plppr3a	phospholipid phosphatase related 3a [Source:ZFIN;Acc:ZDB-GENE-070912-555]	2	26179096	26209425		9.37	2.07	2.8	12.31	11.83	12.22
ENSDARG00000010145	tor4aa	torsin family 4, member Aa [Source:NCBI gene;Acc:550133]	10	3322663	3332362		3.64	3.92	3.75	4.68	4.74	4.5
ENSDARG00000010146	cpa2	carboxypeptidase A2 (pancreatic) [Source:ZFIN;Acc:ZDB-GENE-040801-182]	25	16590636	16600811		1.27	1.54	1.29	3.26	3.31	3.63
ENSDARG00000010149	atp5fa1	ATP synthase F1 subunit alpha [Source:ZFIN;Acc:ZDB-GENE-060201-1]	21	5080789	5092539		714.45	787.51	737.65	635.99	627.71	650.78
ENSDARG00000010154	vstm4a	V-set and transmembrane domain containing 4a [Source:ZFIN;Acc:ZDB-GENE-060810-69]	13	30951155	30967784		3.59	4.2	4.16	2.64	2.49	2.34
ENSDARG00000010155	abi1a	abl-interactor 1a [Source:ZFIN;Acc:ZDB-GENE-040426-1701]	24	5935377	6012786		7.71	6.01	4.72	11.59	11.3	10.81
ENSDARG00000010160	rps15a	ribosomal protein S15a [Source:NCBI gene;Acc:336764]	3	26345732	26351656		419.43	549.89	623.88	343.03	338.08	357.94
ENSDARG00000010169	myd88	MYD88 innate immune signal transduction adaptor [Source:NCBI gene;Acc:403145]	24	20188914	20192996		4.47	5.14	4.53	6.96	6.9	7.24
ENSDARG00000010181	asap2a	ArfGAP with SH3 domain, ankyrin repeat and PH domain 2a [Source:ZFIN;Acc:ZDB-GENE-041210-256]	17	35097024	35223482		5.2	2.37	2.23	5.89	6.07	6.29
ENSDARG00000010183	si:ch73-265h17.1	si:ch73-265h17.1 [Source:ZFIN;Acc:ZDB-GENE-131121-358]	25	4979446	5002358		0.14	0.13	0.18	0.2	0.39	0.24
ENSDARG00000010186	myo3a	myosin IIIA [Source:ZFIN;Acc:ZDB-GENE-041026-4]	24	6161037	6260333		2.22	0.62	0.55	2.24	2.19	2.37
ENSDARG00000010192	pax3a	paired box 3a [Source:ZFIN;Acc:ZDB-GENE-980526-52]	2	47370323	47431205		1.48	0.7	0.93	1.09	1.24	1.15
ENSDARG00000010194	eif4bb	eukaryotic translation initiation factor 4Bb [Source:ZFIN;Acc:ZDB-GENE-030131-8563]	6	39064824	39080701		65.31	59.44	46.36	51.71	50.04	49.32
ENSDARG00000010207	smad3b	SMAD family member 3b [Source:ZFIN;Acc:ZDB-GENE-030128-4]	18	19772874	19806209		4.87	3	2.85	4.95	5.13	4.95
ENSDARG00000010231	ppm1na	protein phosphatase, Mg2+/Mn2+ dependent, 1Na (putative) [Source:ZFIN;Acc:ZDB-GENE-040426-2731]	15	9053059	9069172		3.03	0.39	0.4	1.48	1.56	1.92
ENSDARG00000010238	rbm22	RNA binding motif protein 22 [Source:NCBI gene;Acc:406495]	21	33211306	33229950		17.29	12.02	11.2	20.99	20.65	22.65
ENSDARG00000010244	rpl22l1	ribosomal protein L22-like 1 [Source:ZFIN;Acc:ZDB-GENE-060804-3]	24	26475182	26479841		253.56	348.87	329.05	207.3	216.97	232.57
ENSDARG00000010246	prmt1	protein arginine methyltransferase 1 [Source:ZFIN;Acc:ZDB-GENE-030131-693]	3	32356833	32363021		40.92	39.96	41.79	40.63	38.54	37.13
ENSDARG00000010248	wdr54	WD repeat domain 54 [Source:ZFIN;Acc:ZDB-GENE-040801-151]	5	43072951	43083464		6.01	3.09	3.66	5.96	6.46	6.11
ENSDARG00000010250	nqo1	NAD(P)H dehydrogenase, quinone 1 [Source:ZFIN;Acc:ZDB-GENE-030131-1226]	7	56703254	56722320		56.18	72.22	68.03	32.47	32.4	29.09
ENSDARG00000010252	jak3	Janus kinase 3 (a protein tyrosine kinase, leukocyte) [Source:ZFIN;Acc:ZDB-GENE-070725-4]	8	13389115	13412597		0.37	0.44	0.49	0.35	0.64	0.55
ENSDARG00000010255	bin2b	bridging integrator 2b [Source:ZFIN;Acc:ZDB-GENE-030131-5598]	6	38879961	38894366		1.95	2.15	1.43	1.94	1.46	1.47
ENSDARG00000010266	igf2bp3	insulin-like growth factor 2 mRNA binding protein 3 [Source:NCBI gene;Acc:30967]	19	20201254	20247336		12.12	11.02	8.21	19.92	20.04	19.37
ENSDARG00000010267	dpydb	dihydropyrimidine dehydrogenase b [Source:NCBI gene;Acc:405829]	2	19824190	20120904		14.12	19	10.69	12.94	14.47	14.34
ENSDARG00000010270	fezf1	FEZ family zinc finger 1 [Source:NCBI gene;Acc:558801]	25	28272908	28276091		3.13	0.42	0.57	2.96	2.57	2.64
ENSDARG00000010276	ptgs2b	prostaglandin-endoperoxide synthase 2b [Source:ZFIN;Acc:ZDB-GENE-041014-323]	20	34121818	34127415		2.15	1.85	1.34	3.33	3.01	3.05
ENSDARG00000010279	scamp2	secretory carrier membrane protein 2 [Source:ZFIN;Acc:ZDB-GENE-040426-2702]	25	3015676	3034668		11.19	11.2	8.08	12.66	13.03	12.26
ENSDARG00000010280	clasp1a	cytoplasmic linker associated protein 1a [Source:ZFIN;Acc:ZDB-GENE-081104-520]	9	38163876	38291809		7.95	3.32	3.8	9.78	9.1	8.91
ENSDARG00000010281	xpo4	exportin 4 [Source:NCBI gene;Acc:324342]	9	21306902	21357841		5.8	4.76	4.2	6.22	5.5	5.84
ENSDARG00000010294	fmodb	fibromodulin b [Source:ZFIN;Acc:ZDB-GENE-080327-28]	8	37489495	37495786		23.49	22.58	16	16.31	13.79	13.02
ENSDARG00000010296	kcnh6b	potassium voltage-gated channel, subfamily H (eag-related), member 6b [Source:ZFIN;Acc:ZDB-GENE-121214-55]	12	9320446	9347327		0.35	0.07	0.02	0.36	0.36	0.45
ENSDARG00000010300	traf3ip1	TNF receptor-associated factor 3 interacting protein 1 [Source:NCBI gene;Acc:393572]	9	45605410	45667487		4.11	2.05	2.78	4.81	5.83	5.36
ENSDARG00000010301	b4galt6	UDP-Gal:betaGlcNAc beta 1,4- galactosyltransferase, polypeptide 6 [Source:ZFIN;Acc:ZDB-GENE-040426-789]	20	6163932	6196989		5.31	2.07	2.1	8.83	8.33	8.13
ENSDARG00000010312	cp	ceruloplasmin [Source:ZFIN;Acc:ZDB-GENE-010522-1]	22	38173960	38199988		53.22	83.52	61	81.72	94.81	82.45
ENSDARG00000010316	qars1	glutaminyl-tRNA synthetase 1 [Source:ZFIN;Acc:ZDB-GENE-040426-1011]	8	46353203	46386060		18.13	21.65	19.72	17.77	16.51	14.55
ENSDARG00000010317	gpr183a	G protein-coupled receptor 183a [Source:NCBI gene;Acc:556770]	9	31105389	31108285		0.49	0.95	1.11	0.94	1.37	0.97
ENSDARG00000010318	srpx	sushi-repeat containing protein X-linked [Source:ZFIN;Acc:ZDB-GENE-050327-43]	9	30294073	30316790		11.21	12.43	13.4	13.55	13.23	13.6
ENSDARG00000010331	radx	RPA1 related single stranded DNA binding protein [Source:ZFIN;Acc:ZDB-GENE-081104-212]	5	22836364	22842238		1.86	1.38	1.02	1.47	1.48	1.35
ENSDARG00000010332	zgc:56231	zgc:56231 [Source:ZFIN;Acc:ZDB-GENE-040426-1914]	7	58751504	58759890		0.03	0	0.01	0.04	0.02	0.01
ENSDARG00000010347	acer1	alkaline ceramidase 1 [Source:NCBI gene;Acc:550266]	8	20232421	20245892		1.02	0.82	0.76	1.3	0.7	1.16
ENSDARG00000010350	c1ql3a	complement component 1, q subcomponent-like 3a [Source:ZFIN;Acc:ZDB-GENE-080417-1]	24	31940504	31942686		2.78	1.23	1.19	4.53	4.25	3.77
ENSDARG00000010376	slc5a3a	solute carrier family 5 member 3a [Source:ZFIN;Acc:ZDB-GENE-090312-3]	1	2025085	2027100		0.01	0.01	0.01	0.03	0	0.04
ENSDARG00000010381	rnf2	ring finger protein 2 [Source:NCBI gene;Acc:327035]	2	23351454	23361933		36.98	30.76	32.12	35	35.35	36.36
ENSDARG00000010384	lpar5a	lysophosphatidic acid receptor 5a [Source:ZFIN;Acc:ZDB-GENE-030131-1193]	2	48282590	48284160		0.45	0.54	0.54	0.52	0.71	0.56
ENSDARG00000010385	sept4a	septin 4a [Source:ZFIN;Acc:ZDB-GENE-051120-87]	21	39512437	39546737		8.86	1.55	1.37	7.17	6.62	7.16
ENSDARG00000010400	lrrfip2	leucine rich repeat (in FLII) interacting protein 2 [Source:ZFIN;Acc:ZDB-GENE-030131-7781]	13	42621205	42674001		12.29	11	9.92	11.19	10.94	10.69
ENSDARG00000010407	ppp4r4	protein phosphatase 4, regulatory subunit 4 [Source:ZFIN;Acc:ZDB-GENE-041001-164]	20	27298783	27372450		1.82	0.76	1.17	2.31	2.52	2.74
ENSDARG00000010408	igsf9b	immunoglobulin superfamily, member 9b [Source:ZFIN;Acc:ZDB-GENE-060810-28]	8	4856440	4892913		4.31	1.69	1.57	5.34	4.85	5.61
ENSDARG00000010411	epn1	epsin 1 [Source:ZFIN;Acc:ZDB-GENE-040426-2680]	16	12730311	12756528		18.78	11.87	11.27	22.56	22.73	22.19
ENSDARG00000010415	sirt4	sirtuin 4 [Source:ZFIN;Acc:ZDB-GENE-041010-65]	6	40877394	40885496		1.89	2.5	2.31	1.69	1.79	2.22
ENSDARG00000010420	ndrg1b	N-myc downstream regulated 1b [Source:ZFIN;Acc:ZDB-GENE-040426-1648]	16	31511739	31517227		19.22	5.36	4.46	32.11	38.32	41.33
ENSDARG00000010423	npsn	nephrosin [Source:ZFIN;Acc:ZDB-GENE-040420-2]	7	38653481	38659477		6.99	10.27	9.81	10.4	11.89	11.1
ENSDARG00000010425	scara5	scavenger receptor class A, member 5 (putative) [Source:NCBI gene;Acc:564500]	20	1354695	1383916		5.38	7.7	7.05	6.47	7.83	8.07
ENSDARG00000010432	eaf2	ELL associated factor 2 [Source:ZFIN;Acc:ZDB-GENE-040625-179]	9	23003208	23007941		7.96	9.09	7.13	8.97	9.29	8.96
ENSDARG00000010434	clu	clusterin [Source:ZFIN;Acc:ZDB-GENE-040426-1774]	20	39260475	39273987		34.15	33.97	38.16	73.28	77.73	78.64
ENSDARG00000010437	tent5c	terminal nucleotidyltransferase 5C [Source:NCBI gene;Acc:327154]	9	20780813	20784047		1.63	2.28	1.87	3.33	3.54	3.31
ENSDARG00000010442	rnf11a	ring finger protein 11a [Source:ZFIN;Acc:ZDB-GENE-040426-1277]	1	52130164	52137313		12.62	8.85	8.41	10.93	11.61	11.63
ENSDARG00000010445	trabd	TraB domain containing [Source:ZFIN;Acc:ZDB-GENE-030131-1301]	18	14885545	14901480		7.16	8.3	7.06	8.9	8.77	7.41
ENSDARG00000010454	guca1a	guanylate cyclase activator 1A [Source:ZFIN;Acc:ZDB-GENE-011128-5]	4	22343093	22350976		0.81	0.19	0.29	1.06	1.16	1.32
ENSDARG00000010472	atp1a2a	ATPase Na+/K+ transporting subunit alpha 2 [Source:ZFIN;Acc:ZDB-GENE-001212-6]	2	44203075	44255537		13.02	22.19	17.89	39.96	27.26	27.06
ENSDARG00000010477	p2rx3a	purinergic receptor P2X, ligand-gated ion channel, 3a [Source:ZFIN;Acc:ZDB-GENE-000427-3]	14	21942383	21959712		1.48	1.05	1.07	2.25	2.5	2.16
ENSDARG00000010478	hsp90aa1.1	heat shock protein 90, alpha (cytosolic), class A member 1, tandem duplicate 1 [Source:NCBI gene;Acc:30591]	20	53982317	54012043		36.42	54.59	52.13	44.56	38.81	36.9
ENSDARG00000010481	bzw1a	basic leucine zipper and W2 domains 1a [Source:NCBI gene;Acc:325226]	22	14117078	14126065		45.92	42.7	42.66	60.57	61.11	62.08
ENSDARG00000010482	prg4a	proteoglycan 4a [Source:ZFIN;Acc:ZDB-GENE-030131-8295]	2	20793982	20812059		0.39	0.4	0.58	1.05	0.83	0.85
ENSDARG00000010487	sae1	SUMO1 activating enzyme subunit 1 [Source:NCBI gene;Acc:415148]	15	23722620	23739104		20.71	18.96	21.7	15.05	14.38	14.56
ENSDARG00000010514	cxcr5	chemokine (C-X-C motif) receptor 5 [Source:ZFIN;Acc:ZDB-GENE-070302-8]	18	43891051	43894087		0	0	0	0	0	0
ENSDARG00000010515	dvl1a	dishevelled segment polarity protein 1a [Source:ZFIN;Acc:ZDB-GENE-090312-40]	23	29966466	29992458		3.23	2.91	1.83	4.81	4.46	4.54
ENSDARG00000010516	rpl21	ribosomal protein L21 [Source:ZFIN;Acc:ZDB-GENE-030131-8512]	24	21621654	21627293		442.15	557.94	612.3	363.76	378.45	378.62
ENSDARG00000010519	per3	period circadian clock 3 [Source:ZFIN;Acc:ZDB-GENE-000804-1]	11	39959039	40021080		11.04	5.75	4.99	10.32	11.02	11.3
ENSDARG00000010524	si:ch211-282j22.3	si:ch211-282j22.3 [Source:ZFIN;Acc:ZDB-GENE-091113-49]	21	13762409	13784859		5.8	4.51	2.98	7.55	7.05	7.13
ENSDARG00000010531	scamp4	secretory carrier membrane protein 4 [Source:ZFIN;Acc:ZDB-GENE-050913-40]	22	22302614	22315323		8.67	9.66	8.61	13.54	12.71	12.78
ENSDARG00000010553	mmgt1	membrane magnesium transporter 1 [Source:NCBI gene;Acc:402891]	14	31615577	31619408		17.4	14.16	13.43	23.94	23.42	23.42
ENSDARG00000010555	pdha1b	pyruvate dehydrogenase E1 alpha 1 subunit b [Source:ZFIN;Acc:ZDB-GENE-040718-96]	24	24273371	24282427		5.29	1.34	1.41	2.34	2.04	1.81
ENSDARG00000010556	mmp25b	matrix metallopeptidase 25b [Source:ZFIN;Acc:ZDB-GENE-100308-4]	12	4260779	4278329		1.35	1.27	1.56	1.35	2.27	1.77
ENSDARG00000010563	spopla	speckle type BTB/POZ protein like a [Source:NCBI gene;Acc:541318]	9	10050890	10068105		7.35	5.07	4.99	7.24	7.05	6.91
ENSDARG00000010565	aqp4	aquaporin 4 [Source:ZFIN;Acc:ZDB-GENE-040724-152]	20	18163355	18186784		2.68	2.86	1.77	2.13	1.92	1.55
ENSDARG00000010571	ezh2	enhancer of zeste 2 polycomb repressive complex 2 subunit [Source:NCBI gene;Acc:768133]	24	17345521	17364456		7	4.39	4.55	5.8	4.98	5.59
ENSDARG00000010572	slc25a25a	solute carrier family 25 member 25a [Source:NCBI gene;Acc:406541]	8	2606068	2616326		5.58	6.9	6.75	17.01	20.21	17.37
ENSDARG00000010583	pard3ab	par-3 family cell polarity regulator alpha, b [Source:ZFIN;Acc:ZDB-GENE-030925-47]	2	43204919	43389248		5.87	4.53	3.87	7.34	7.37	7.6
ENSDARG00000010586	tex9	testis expressed 9 [Source:ZFIN;Acc:ZDB-GENE-110623-3]	7	34496113	34506046		2.59	2.42	2.03	3.33	2.76	3.1
ENSDARG00000010591	foxn4	forkhead box N4 [Source:NCBI gene;Acc:30315]	5	19385503	19400166		4.94	1.9	1.56	4.65	4.02	4.12
ENSDARG00000010596	slc7a6os	solute carrier family 7 member 6 opposite strand [Source:NCBI gene;Acc:492472]	7	28612671	28623292		25.38	16.48	18.31	21.24	21.03	19.16
ENSDARG00000010601	mtmr10	myotubularin related protein 10 [Source:NCBI gene;Acc:555239]	7	30725473	30771147		5.31	3.57	3.16	5.28	5.47	4.84
ENSDARG00000010602	zgc:112294	zgc:112294 [Source:NCBI gene;Acc:553704]	5	11407504	11414648		2.75	0.68	0.91	1.84	2.07	2.59
ENSDARG00000010603	kctd9b	potassium channel tetramerization domain containing 9b [Source:ZFIN;Acc:ZDB-GENE-040718-238]	10	42542517	42555822		10.05	6.09	6.72	9.55	10.79	9.57
ENSDARG00000010610	sarm1	sterile alpha and TIR motif containing 1 [Source:ZFIN;Acc:ZDB-GENE-040219-1]	15	28188362	28200049		6.32	1.56	2.03	6.23	6.01	5.85
ENSDARG00000010619	themis	thymocyte selection associated [Source:NCBI gene;Acc:555433]	20	1423192	1439256		0.29	0.26	0.28	0.35	0.37	0.38
ENSDARG00000010625	clic2	chloride intracellular channel 2 [Source:ZFIN;Acc:ZDB-GENE-040718-299]	14	33882973	33892311		6.76	8.2	6.81	10.54	11.08	11.28
ENSDARG00000010630	hoxb6a	homeobox B6a [Source:NCBI gene;Acc:30341]	3	23703704	23705746		6.17	7.16	5.97	8.15	8.12	9.01
ENSDARG00000010641	slc20a1b	solute carrier family 20 member 1b [Source:NCBI gene;Acc:321541]	10	17713022	17728165		22.6	16.4	11.51	47.3	47.29	46.35
ENSDARG00000010654	arhgap42b	Rho GTPase activating protein 42b [Source:ZFIN;Acc:ZDB-GENE-120217-5]	18	41857403	42071876		0.81	0.59	0.66	1.31	1.15	1.35
ENSDARG00000010655	ppm1k	protein phosphatase, Mg2+/Mn2+ dependent, 1K [Source:ZFIN;Acc:ZDB-GENE-050306-8]	1	50594175	50611031		12.12	5.55	5.96	13.9	14.68	14.21
ENSDARG00000010658	insig1	insulin induced gene 1 [Source:NCBI gene;Acc:334189]	7	41007773	41014773		6.72	6.31	5.82	19.28	22.21	21.46
ENSDARG00000010662	fbxo36a	F-box protein 36a [Source:ZFIN;Acc:ZDB-GENE-060929-986]	18	41226444	41232297		0.05	0.1	0.15	0.56	0.08	0.34
ENSDARG00000010673	trim16	tripartite motif containing 16 [Source:ZFIN;Acc:ZDB-GENE-090512-6]	12	46634736	46679477		6.54	6.75	6.11	3.77	3.88	3.15
ENSDARG00000010680	gngt2a	guanine nucleotide binding protein (G protein), gamma transducing activity polypeptide 2a [Source:ZFIN;Acc:ZDB-GENE-030131-75	3	23092762	23096141		42.65	23.6	18.53	25.35	22.47	27.42
ENSDARG00000010681	tjap1	tight junction associated protein 1 (peripheral) [Source:ZFIN;Acc:ZDB-GENE-081107-47]	13	3979341	4134141		3.06	2.24	1.94	4.99	4.67	4.74
ENSDARG00000010700	il17d	interleukin 17d [Source:ZFIN;Acc:ZDB-GENE-061031-4]	9	21363623	21365350		0.07	0.07	0.09	0.48	0.57	0.31
ENSDARG00000010701	crygs4	crystallin, gamma S4 [Source:ZFIN;Acc:ZDB-GENE-050223-2]	9	22359919	22360742		0.03	0.03	0	0	0.11	0
ENSDARG00000010710	msi1	musashi RNA-binding protein 1 [Source:ZFIN;Acc:ZDB-GENE-050320-86]	8	39607466	39647216		18.62	7	7.92	18.49	18.78	19.82
ENSDARG00000010712	npffr1l3	neuropeptide FF receptor 1 like 3 [Source:ZFIN;Acc:ZDB-GENE-070424-44]	10	518546	532628		0.62	0.44	0.42	0.84	0.8	0.44
ENSDARG00000010717	chchd10	coiled-coil-helix-coiled-coil-helix domain containing 10 [Source:ZFIN;Acc:ZDB-GENE-040426-1753]	21	15704556	15708626		61.25	64.75	66.8	33.56	32.74	33.31

ENSDARG00000010721	6-Sep	septin 6 [Source:ZFIN;Acc:ZDB-GENE-030131-1414]	14	33297781	33328031		27.21	18.33	18.4	31.08	31.39	30.49
ENSDARG00000010727	tfyh2l	tweety homolog 2, like [Source:NCBI gene;Acc:406540]	3	51563695	51657722		6.02	3.96	3.44	7.96	8.47	8.46
ENSDARG00000010728	scin	scinderin [Source:ZFIN;Acc:ZDB-GENE-060503-6]	19	31347675	31372896		6.9	8.8	7.94	6.99	8.26	8.21
ENSDARG00000010729	CABZ01073795.1	interferon-induced protein 44 [Source:NCBI gene;Acc:795887]	10	4367430	4375190		0.02	0.04	0	0.01	0.03	0.03
ENSDARG00000010732	xrcc4	X-ray repair complementing defective repair in Chinese hamster cells 4 [Source:ZFIN;Acc:ZDB-GENE-040426-1755]	10	43088270	43184354		2.74	2.58	3.07	2.46	2.64	2.72
ENSDARG00000010738	zgc:101716	zgc:101716 [Source:ZFIN;Acc:ZDB-GENE-041114-135]	16	44678302	44687969		3.51	3	3.28	2.35	3.02	2.89
ENSDARG00000010745	dnaja2b	DnaJ heat shock protein family (Hsp40) member A2b [Source:ZFIN;Acc:ZDB-GENE-030131-2884]	18	6857071	6869356		42.41	23.8	25.97	51.24	50.82	49.8
ENSDARG00000010752	acsl4b	acyl-CoA synthetase long chain family member 4b [Source:ZFIN;Acc:ZDB-GENE-030131-6493]	5	23518399	23581355		1.87	1.66	1.48	3.06	2.98	3.14
ENSDARG00000010756	nod2	nucleotide-binding oligomerization domain containing 2 [Source:ZFIN;Acc:ZDB-GENE-061108-4]	7	37555568	37563965		1.4	1.17	1.19	1.49	1.39	1.41
ENSDARG00000010758	capn12	calpain 12 [Source:ZFIN;Acc:ZDB-GENE-050419-245]	18	36664654	36729637		9.51	10.25	9.25	13.09	11	10.83
ENSDARG00000010764	flj13639	flj13639 [Source:ZFIN;Acc:ZDB-GENE-030131-8104]	15	43166511	43198413		2.14	1.98	2.17	1.05	0.84	1.1
ENSDARG00000010767	epha4l	eph receptor A4, like [Source:ZFIN;Acc:ZDB-GENE-990415-61]	15	41945310	42039192		0.9	0.77	0.6	1.28	1.05	1.12
ENSDARG00000010770	sox19a	SRY-box transcription factor 19a [Source:NCBI gene;Acc:30038]	5	24199180	24201437		18.08	6.89	9.49	12.97	12.62	14.37
ENSDARG00000010773	pnpla6	patatin-like phospholipase domain containing 6 [Source:ZFIN;Acc:ZDB-GENE-050107-4]	1	45866692	45920632		12.58	5.73	6.73	13.13	12.12	12.18
ENSDARG00000010775	tlk2	tousled-like kinase 2 [Source:NCBI gene;Acc:407732]	3	19685873	19717934		8.06	4.57	4.2	8.77	8.57	9.08
ENSDARG00000010782	zgc:55621	zgc:55621 [Source:ZFIN;Acc:ZDB-GENE-040426-762]	15	34946928	34960914		0	0	0	0	0	0
ENSDARG00000010784	ppp1r12a	protein phosphatase 1, regulatory subunit 12A [Source:NCBI gene;Acc:445393]	4	21488990	21584158		6.26	3.22	2.61	8.31	7.44	7.62
ENSDARG00000010785	thbs1b	thrombospondin 1b [Source:ZFIN;Acc:ZDB-GENE-020708-1]	20	29036328	29052391		28.21	26.08	19.16	59.81	63.23	63.72
ENSDARG00000010789	homer3b	homer scaffold protein 3b [Source:ZFIN;Acc:ZDB-GENE-040426-739]	11	13423776	13505942		7.98	6.85	5.93	8.73	9	8.59
ENSDARG00000010791	dla	deltaA [Source:NCBI gene;Acc:30131]	1	54013457	54026352		8.69	3.54	4.78	7.2	7.21	6.99
ENSDARG00000010792	cdc25b	cell division cycle 25B [Source:ZFIN;Acc:ZDB-GENE-000330-5]	13	14915344	14929236		4.82	3.34	3.04	5.26	5.12	5.21
ENSDARG00000010794	tfeb	transcription factor EB [Source:ZFIN;Acc:ZDB-GENE-090807-3]	11	22287954	22361306		1.92	1.53	1.06	1.99	1.82	1.79
ENSDARG00000010816	slc7a14a	solute carrier family 7 member 14a [Source:NCBI gene;Acc:566270]	2	25860344	25990784		4.99	1.4	1.32	10.45	9.81	9.88
ENSDARG00000010823	memo1	mediator of cell motility 1 [Source:NCBI gene;Acc:327360]	13	35510893	35527697		9.03	7.74	7.84	14.05	13.22	13.55
ENSDARG00000010831	churc1	churchill domain containing 1 [Source:NCBI gene;Acc:492508]	20	25929440	25934439		14.45	17.39	16.14	13.14	11.95	12.24
ENSDARG00000010841	fshb	follicle stimulating hormone subunit beta [Source:ZFIN;Acc:ZDB-GENE-040806-1]	7	32605705	32612560		0.02	0.01	0.03	0.01	0	0
ENSDARG00000010844	kras	v-Ki-ras2 Kirsten rat sarcoma viral oncogene homolog [Source:ZFIN;Acc:ZDB-GENE-040808-67]	25	31099908	31118923		22.79	17.75	17.32	21.31	22.59	21.81
ENSDARG00000010862	amt	aminomethyltransferase [Source:ZFIN;Acc:ZDB-GENE-041010-114]	8	26396552	26408670		20.26	29.07	22.69	9.67	9.5	10.67
ENSDARG00000010873	DDX17	si:dkey-156n14.5 [Source:ZFIN;Acc:ZDB-GENE-030131-18]	6	22104565	22119697	DDX17	63.53	34.77	43.07	71.05	75.03	75.39
ENSDARG00000010878	cdkn1ca	cyclin-dependent kinase inhibitor 1Ca [Source:ZFIN;Acc:ZDB-GENE-040123-1]	7	48663031	48667056		8.86	8.08	8.79	7.03	7.66	8.63
ENSDARG00000010905	mettl13	methyltransferase like 13 [Source:NCBI gene;Acc:557908]	20	14926881	14941169		2.83	2.25	2.27	3	2.76	3.03
ENSDARG00000010918	nprl3	NPR3-like, GATOR1 complex subunit [Source:ZFIN;Acc:ZDB-GENE-030131-1348]	3	55045346	55079742		3.6	2.29	2.51	4.96	4.97	4.45
ENSDARG00000010925	HTRA3	HtrA serine peptidase 3b [Source:NCBI gene;Acc:573378]	1	61817629	61845282	HTRA3	6.48	9.24	8.99	8.89	10.2	10.42
ENSDARG00000010933	cacna1fa	calcium channel, voltage-dependent, L type, alpha 1F subunit a [Source:ZFIN;Acc:ZDB-GENE-091204-7]	8	44850710	44868020		4.61	0.94	0.91	4.82	5.14	4.8
ENSDARG00000010934	syt14a	synaptotagmin XIVa [Source:ZFIN;Acc:ZDB-GENE-090313-200]	13	31844011	31888487		3.39	0.35	0.83	2.64	2.6	2.59
ENSDARG00000010936	abcb4	ATP-binding cassette, sub-family B (MDR/TAP), member 4 [Source:ZFIN;Acc:ZDB-GENE-080204-52]	16	28629242	28658341		18.21	21.92	13.75	5.36	5.61	5.9
ENSDARG00000010940	BX957278.1		7	19817306	19825746		5	4.29	3.09	5.97	5.95	5.55
ENSDARG00000010941	pomgnt2	protein O-linked mannose N-acetylglucosaminyltransferase 2 (beta 1,4-) [Source:NCBI gene;Acc:497644]	16	8139515	8162380		6.08	2.55	2.69	6.77	7.12	7.25
ENSDARG00000010945	rapgef6	Rap guanine nucleotide exchange factor (GEF) 6 [Source:ZFIN;Acc:ZDB-GENE-030131-6972]	21	45041684	45086170		7.24	3.56	4.54	11.34	12.43	11.79
ENSDARG00000010946	cbsb	cystathionine beta-synthase b [Source:ZFIN;Acc:ZDB-GENE-021030-3]	9	9180620	9228941		5.56	5.24	4.47	10.95	11.46	11.21
ENSDARG00000010948	kif11	kinesin family member 11 [Source:ZFIN;Acc:ZDB-GENE-020426-1]	17	19481049	19497006		15.15	11.26	12.23	11.78	11.13	12.26
ENSDARG00000010953	ndel1a	nudE neurodevelopment protein 1-like 1a [Source:NCBI gene;Acc:394244]	12	26467847	26487363		18.96	13.41	15.79	18.29	19.32	19.3
ENSDARG00000010956	sh3glb1b	SH3-domain GRB2-like endophilin B1b [Source:ZFIN;Acc:ZDB-GENE-030131-6851]	2	16173099	16210623		11.56	11.39	13.04	10.31	11.14	11.41
ENSDARG00000010957	mpp2b	membrane protein, palmitoylated 2b (MAGUK p55 subfamily member 2) [Source:ZFIN;Acc:ZDB-GENE-040704-70]	12	10199219	10300101		13.73	5.05	7.89	11.79	13.93	13.76
ENSDARG00000010958	glmnb	glomulin, FKBP associated protein b [Source:ZFIN;Acc:ZDB-GENE-080723-14]	6	28970513	28980829		11.92	2.4	1.88	6.89	7.04	8.04
ENSDARG00000010962	fkbp7	FKBP prolyl isomerase 7 [Source:ZFIN;Acc:ZDB-GENE-030616-182]	9	42728269	42730672		15.17	23.76	24.44	23.32	23.12	22.44
ENSDARG00000010965	psma8	proteasome 20S subunit alpha 8 [Source:ZFIN;Acc:ZDB-GENE-040426-2194]	20	18306122	18313864		115.73	149.38	155.77	76.37	80.23	79.76
ENSDARG00000010977	rab34b	RAB34, member RAS oncogene family b [Source:ZFIN;Acc:ZDB-GENE-091118-61]	21	26062452	26071773		2.48	2.82	2.42	2.78	2.47	2.96
ENSDARG00000010978	trmt1	tRNA methyltransferase 1 [Source:ZFIN;Acc:ZDB-GENE-060810-40]	11	31236001	31258356		4.38	4.53	3.86	3.5	3.78	3.32
ENSDARG00000010981	zdhhc3a	zinc finger DHHC-type containing 3a [Source:ZFIN;Acc:ZDB-GENE-040718-484]	16	39196727	39233451		3.83	3.19	2.78	3.56	3.76	2.89
ENSDARG00000011000	gtf2a1	general transcription factor IIA, 1 [Source:ZFIN;Acc:ZDB-GENE-030131-2694]	20	16437741	16453603		20.28	13.77	15.04	19.79	19.75	21.24
ENSDARG00000011020	hnrnpa1a	heterogeneous nuclear ribonucleoprotein A1a [Source:ZFIN;Acc:ZDB-GENE-040426-1546]	11	2240075	2250767		231.29	142.93	132.78	250.93	222.52	240.86
ENSDARG00000011023	pdlim3a	PDZ and LIM domain 3a [Source:ZFIN;Acc:ZDB-GENE-050505-1]	1	17721853	17735861		1.29	1.42	1.4	1.77	1.72	1.98
ENSDARG00000011026	zgc:92907	zgc:92907 [Source:ZFIN;Acc:ZDB-GENE-040718-161]	14	30967442	30971264		5.93	6.17	6.82	4.75	4.41	4.26
ENSDARG00000011027	fgfr1a	fibroblast growth factor receptor 1a [Source:NCBI gene;Acc:30705]	8	51430597	51524453		5.35	4.51	3.5	7.64	7.93	7.71
ENSDARG00000011042	pgbd5	piggyBac transposable element derived 5 [Source:ZFIN;Acc:ZDB-GENE-040724-229]	13	24022963	24056275		5.53	1.42	1.82	5.62	5.1	5.49
ENSDARG00000011049	slc17a9b	solute carrier family 17 member 9b [Source:ZFIN;Acc:ZDB-GENE-040718-380]	23	17522867	17549145		3.17	2.75	2.09	3.59	3.65	3.49
ENSDARG00000011055	fbxo9	F-box protein 9 [Source:NCBI gene;Acc:325469]	13	2426993	2440631		27.69	27.75	28.01	18.19	18.64	18.72
ENSDARG00000011065	camk2b1	calcium/calmodulin-dependent protein kinase (CaM kinase) II beta 1 [Source:ZFIN;Acc:ZDB-GENE-030131-9588]	5	20931252	21030996		28.19	13.54	12.29	31.27	30.76	29.68
ENSDARG00000011066	glra3	glycine receptor, alpha 3 [Source:ZFIN;Acc:ZDB-GENE-020402-1]	1	38416106	38538284		4.4	0.72	0.88	3.74	4.05	3.87
ENSDARG00000011072	ddx11	DEAD/H (Asp-Glu-Ala-Asp/His) box helicase 11 [Source:NCBI gene;Acc:492353]	18	6502441	6534516		1.44	1.35	1.27	0.93	0.8	1
ENSDARG00000011076	zgc:91968	zgc:91968 [Source:NCBI gene;Acc:431714]	19	4990320	4999232		9.29	12.46	15.83	10.04	9.34	9.2
ENSDARG00000011079	wdr3	WD repeat domain 3 [Source:ZFIN;Acc:ZDB-GENE-030131-9830]	9	20833894	20872565		16.96	17.79	17.69	13.36	14.75	14.1
ENSDARG00000011083	si:dkey-42i23.2	si:dkey-42i23.2 [Source:ZFIN;Acc:ZDB-GENE-131121-582]	15	37795286	37799496		0.43	0.28	0.3	0.29	0.33	0.23
ENSDARG00000011088	si:ch211-288d18.1	si:ch211-288d18.1 [Source:ZFIN;Acc:ZDB-GENE-060503-323]	7	48999723	49056247		4.91	1.49	2.05	6.12	6.22	6.28
ENSDARG00000011091	drd2b	dopamine receptor D2b [Source:ZFIN;Acc:ZDB-GENE-030910-2]	5	58075301	58173627		2.61	0.78	1.52	2.96	3.21	2.55
ENSDARG00000011094	ccna2	cyclin A2 [Source:ZFIN;Acc:ZDB-GENE-020418-1]	14	46354648	46374870		4.92	3.88	4.22	3.82	4.04	3.36
ENSDARG00000011113	chrna10a	cholinergic receptor, nicotinic, alpha 10a [Source:ZFIN;Acc:ZDB-GENE-060503-725]	15	38179904	38193383		2.03	0.18	0.15	1.6	1.61	1.87
ENSDARG00000011122	pptc7a	PTC7 protein phosphatase homolog a [Source:NCBI gene;Acc:492506]	8	4368534	4402181		7.37	4.14	3.85	11.29	11.62	11.64
ENSDARG00000011125	snrpb	small nuclear ribonucleoprotein polypeptides B and B1 [Source:ZFIN;Acc:ZDB-GENE-040426-1819]	6	57478337	57485411		122.37	137.47	138.04	116.57	117.34	116.9
ENSDARG00000011141	dpysl5a	dihydropyrimidinase like 5a [Source:ZFIN;Acc:ZDB-GENE-030131-3136]	17	36898874	36936856		23.84	6.33	7.58	24.13	22.73	23.38
ENSDARG00000011146	uqcrb	ubiquinol-cytochrome c reductase binding protein [Source:ZFIN;Acc:ZDB-GENE-050522-542]	19	44049501	44055008		0.63	0.66	0.4	1.29	1.09	0.75
ENSDARG00000011152	rpp14	ribonuclease P/MRP 14 subunit [Source:ZFIN;Acc:ZDB-GENE-050522-182]	22	10158502	10164533		7.96	9.15	8.76	6.95	6.69	6.8
ENSDARG00000011157	arhgef18a	rho/rac guanine nucleotide exchange factor (GEF) 18a [Source:ZFIN;Acc:ZDB-GENE-040516-14]	2	37504244	37537904		9.66	8.74	7.08	6.82	7.05	6.66
ENSDARG00000011163	sema3fa	sema domain, immunoglobulin domain (Ig), short basic domain, secreted, (semaphorin) 3Fa [Source:ZFIN;Acc:ZDB-GENE-050513-1	6	42818963	42916343		6.24	4.32	3.49	9.04	8.4	8.87
ENSDARG00000011166	cahz	carbonic anhydrase [Source:NCBI gene;Acc:30331]	2	29477383	29485408		27.34	26.29	25.94	36.99	42.43	35.82
ENSDARG00000011170	ndrg2	NDRG family member 2 [Source:NCBI gene;Acc:494050]	7	3930742	4036875		28.21	24.89	21.3	34.12	31.68	32.36
ENSDARG00000011171	TENM2	si:ch211-12m10.1 [Source:ZFIN;Acc:ZDB-GENE-060503-100]	21	31434251	32008843	TENM2	3.69	0.75	1.02	5.85	5.46	6.03
ENSDARG00000011175	atp6v1d	ATPase H+ transporting V1 subunit D [Source:ZFIN;Acc:ZDB-GENE-040426-727]	20	51088606	51100669		63.42	55.41	60.05	54.52	57.54	59.54
ENSDARG00000011188	acvr2aa	activin A receptor type 2Aa [Source:ZFIN;Acc:ZDB-GENE-980526-227]	9	25380758	25444091		12.82	6.3	5.82	15.38	15.13	14.96
ENSDARG00000011190	fgfr1b	fibroblast growth factor receptor 1b [Source:ZFIN;Acc:ZDB-GENE-060503-14]	10	42589391	42661445		2.96	3	2.59	6.49	6.26	6.75
ENSDARG00000011196	dnajc11a	DnaJ (Hsp40) homolog, subfamily C, member 11a [Source:ZFIN;Acc:ZDB-GENE-030131-1673]	23	30707837	30724909		23.31	25.5	22.09	19.07	18.65	18.04
ENSDARG00000011201	rplp2l	ribosomal protein, large P2, like [Source:ZFIN;Acc:ZDB-GENE-070327-2]	7	12959846	12968701		1587.06	1849.98	2188.67	1256.96	1283.23	1311.86
ENSDARG00000011219	akt2	v-akt murine thymoma viral oncogene homolog 2 [Source:ZFIN;Acc:ZDB-GENE-031007-5]	18	40711188	40753583		24.29	20.93	17.28	32.64	33.28	36.42
ENSDARG00000011222	ccdc28b	coiled-coil domain containing 28B [Source:ZFIN;Acc:ZDB-GENE-060208-3]	13	33282095	33293654		12.8	2.86	4.3	11.23	11.96	12.79
ENSDARG00000011233	pcyt1aa	phosphate cytidylyltransferase 1, choline, alpha a [Source:ZFIN;Acc:ZDB-GENE-050417-108]	2	9755422	9779959		6.52	5.96	5.66	9.18	8.57	7.86
ENSDARG00000011235	otx2b	orthodenticle homeobox 2b [Source:NCBI gene;Acc:30501]	17	44244659	44249720		19.25	6.24	7.1	21.24	21.27	22.68
ENSDARG00000011239	ppp1r14aa	protein phosphatase 1, regulatory (inhibitor) subunit 14Aa [Source:ZFIN;Acc:ZDB-GENE-050417-413]	5	36730726	36739509		9.24	9.98	9.8	6.89	7.06	5.64
ENSDARG00000011240	ift46	intraflagellar transport 46 homolog (Chlamydomonas) [Source:NCBI gene;Acc:565922]	10	29735900	29744921		8.77	7.21	8.51	7.58	8.73	9.06
ENSDARG00000011245	esrp1	epithelial splicing regulatory protein 1 [Source:NCBI gene;Acc:560190]	16	26645663	26676685		19.63	13.76	9.38	8.67	7.69	7.6
ENSDARG00000011246	krr1	KRR1 small subunit processome component homolog [Source:ZFIN;Acc:ZDB-GENE-060623-10]	4	2258935	2265271		12.86	13.47	13.28	8.72	9.04	9.73
ENSDARG00000011247	mpc3	RNA-binding region (RNP1, RRM) containing 3 [Source:NCBI gene;Acc:565672]	24	28934875	28968355		3.26	1.69	2.3	3.09	3.01	3.13
ENSDARG00000011257	enpp2	ectonucleotide pyrophosphatase/phosphodiesterase 2 [Source:ZFIN;Acc:ZDB-GENE-040426-1156]	16	9609721	9662205		4.53	5.23	4.68	5.26	5.69	5.78
ENSDARG00000011259	trpm1a	transient receptor potential cation channel, subfamily M, member 1a [Source:ZFIN;Acc:ZDB-GENE-070112-1372]	7	29312376	29341364		2.77	0.16	0.19	2.6	2.45	2.79
ENSDARG00000011262	traip	TRAF-interacting protein [Source:ZFIN;Acc:ZDB-GENE-040801-30]	11	35162684	35171768		1.38	1.21	1.16	0.81	0.95	0.87
ENSDARG00000011272	uqcc2	ubiquinol-cytochrome c reductase complex assembly factor 2 [Source:NCBI gene;Acc:393731]	6	40573365	40577109		31.44	41.65	36.23	26.2	28.13	29.18
ENSDARG00000011273	tlx2	T cell leukemia homeobox 2 [Source:ZFIN;Acc:ZDB-GENE-020208-1]	14	5656510	5690363		2.52	1.14	1.27	3.42	3.2	3.32
ENSDARG00000011283	si:dkey-110k5.10	si:dkey-110k5.10 [Source:ZFIN;Acc:ZDB-GENE-060531-70]	5	23721515	23729590		0.67	0.51	0.7	0.56	0.78	0.57
ENSDARG00000011298	atf7a	activating transcription factor 7a [Source:ZFIN;Acc:ZDB-GENE-050721-2]	23	27446929	27479558		3.29	3.41	2.43	5.63	6.16	6.63
ENSDARG00000011299	bzf1	basic leucine zipper nuclear factor 1 [Source:ZFIN;Acc:ZDB-GENE-010724-1]	1	494297	505620		9.8	7.38	7.21	9.96	10.06	9.83
ENSDARG00000011301	nae1	nedd8 activating enzyme E1 subunit 1 [Source:NCBI gene;Acc:573336]	7	44773912	44794322		22.58	22.73	25.56	14.21	14.69	14.99
ENSDARG00000011312	stk3	serine/threonine kinase 3 (STE20 homolog, yeast) [Source:NCBI gene;Acc:324125]	19	32807236	32820107		13.4	11.32	11.33	14.8	14.18	13.19
ENSDARG00000011317	adra1ba	adrenoceptor alpha 1Ba [Source:ZFIN;Acc:ZDB-GENE-120510-1]	21	29227224	29247942		0.36	0.63	0.31	0.63	0.91	0.69
ENSDARG00000011326	ankrd45	ankyrin repeat domain 45 [Source:ZFIN;Acc:ZDB-GENE-050522-311]	2	35615547	35731776		1.01	0.95	0.91	0.96	1.25	1.36
ENSDARG00000011333	arhgap33	Rho GTPase activating protein 33 [Source:ZFIN;Acc:ZDB-GENE-121105-12]	16	44788331	44878255		1.99	0.99	0.93	2.69	2.5	2.43
ENSDARG00000011334	ncaldb	neurocalcin delta b [Source:NCBI gene;Acc:445319]	19	12297100	12324514		29.79	6.78	4.14	15.56	18.49	19.41
ENSDARG00000011360	nfatc2ip	nuclear factor of activated T cells 2 interacting protein [Source:ZFIN;Acc:ZDB-GENE-030131-9520]	3	15476536	15487111		8.19	6.05	6.76	7.76	7.22	7.58
ENSDARG00000011370	fyna	FYN proto-oncogene, Src family tyrosine kinase a [Source:NCBI gene;Acc:373872]	17	15502964	15528597		20.99	9.01	12.52	21.15	20.75	22.29
ENSDARG00000011371	zgc:64106	zgc:64106 [Source:ZFIN;Acc:ZDB-GENE-040426-1370]	21	11984805	11996769		13.55	14.84	12.67	9.56	10.29	9.88
ENSDARG00000011373	mknk2a	MAPK interacting serine/threonine kinase 2a [Source:ZFIN;Acc:ZDB-GENE-030131-6099]	2	23006792	23024952		3.97	3.71	3.77	6.07	7.05	6.47
ENSDARG00000011376	dnase1l4.2	deoxyribonuclease 1 like 4, tandem duplicate 2 [Source:ZFIN;Acc:ZDB-GENE-040426-1884]	22	5682635	5700482		3.57	5.28	4.3	2.57	2.58	2.21
ENSDARG00000011400	tnnc1a	troponin C type 1a (slow) [Source:ZFIN;Acc:ZDB-GENE-030523-1]	23	4890693	4895876		3.24	4.33	4.27	5.8	5.53	5.04
ENSDARG00000011404	fen1	flap structure-specific endonuclease 1 [Source:NCBI gene;Acc:386707]	24	11908480	11921184		9.67	12.24	12.46	5.56	5.7	5.08
ENSDARG00000011405	rps9	ribosomal protein S9 [Source:ZFIN;Acc:ZDB-GENE-010724-15]	16	51921812	31927522		336.28	430.15	512.06	204.3	219.31	221.02
ENSDARG00000011407	col2a1b	collagen, type II, alpha 1b [Source:ZFIN;Acc:ZDB-GENE-050302-9]	11	32007	55224		103.62	104.41	66.67	205.86	201.25	206.91
ENSDARG00000011410	aadat	aminoadipate aminotransferase [Source:ZFIN;Acc:ZDB-GENE-111229-1]	7	7675685	7695955		5.2	5.44	4.28	5.97	6.13	5.25
ENSDARG00000011418	sigmar1	sigma non-opioid intracellular receptor 1 [Source:NCBI gene;Acc:393952]	14	14488625	14494239		20.15	28.23	25.72	17.22	18.17	17.72
ENSDARG00000011422	ryr1a	ryanodine receptor 1a (skeletal) [Source:ZFIN;Acc:ZDB-GENE-020108-2]	10	33406819	33540336		4.49	5.19	3.9	7.03	6.07	6.34
ENSDARG00000011434	ptger2a	prostaglandin E receptor 2a (subtype EP2) [Source:ZFIN;Acc:ZDB-GENE-040426-1321]	17	14886828	14894144		2.05	1.91	1.98	2.87	2.81	2.92
ENSDARG00000011445	elmsan1a	ELM2 and Myb/SANT-like domain containing 1a [Source:ZFIN;Acc:ZDB-GENE-041001-151]	20	25886491	25902141		6.05	5.29	3.53	4.92	4.69	4.78
ENSDARG00000011449	fes	FES proto-oncogene, tyrosine kinase [Source:ZFIN;Acc:ZDB-GENE-100812-10]	7	14211322	14266466		1.97	1.81	1.73	1.33	1.24	1.32
ENSDARG00000011459	gsna	gelsolin a [Source:ZFIN;Acc:ZDB-GENE-030131-9653]	21	17572421	17603218		17.85	19.33	18.49	22.88	22.73	21.88
ENSDARG00000011462	otub1b	OTU deubiquitinase, ubiquitin aldehyde binding 1b [Source:ZFIN;Acc:ZDB-GENE-040718-204]	7	24815809	24828887		34.08	29.92	32.78	27.12	28.13	28.82
ENSDARG00000011463	CABZ01044048.1	coagulation factor IX-like [Source:NCBI gene;Acc:100004411]	15	46605482	46624720		0.89	1.16	1.05	0.76	0.71	0.8
ENSDARG00000011466	afg1la	AFG1 like ATPase a [Source:ZFIN;Acc:ZDB-GENE-091006-2]	17	6765621	6789670		6.04	6.94	6.82	5.25	4.95	4.7
ENSDARG00000011473	calcrla	calcitonin receptor-like a [Source:NCBI gene;Acc:445508]	9	42360679	42418470		5.32	4.49	4.55	6.78	6.53	5.98
ENSDARG00000011487	gnaq	guanine nucleotide binding protein (G protein), q polypeptide [Source:ZFIN;Acc:ZDB-GENE-081104-25]	5	55588389	55600689		7.39	3.88	4.89	6.25	6.55	6.57
ENSDARG00000011488	sirt2	sirtuin 2 (silent mating type information regulation 2, homolog) 2 (S. cerevisiae) [Source:NCBI gene;Acc:322309]	15	6786757	6807963		26.73	28.34	25.43	21.58	20.07	20.98
ENSDARG00000011490	scube3	signal peptide, CUB domain, EGF-like 3 [Source:ZFIN;Acc:ZDB-GENE-060717-1]	23	41313084	41585776		4.06	2.29	2.08	5.27	5.56	5.91
ENSDARG00000011493	dcun1d3	defective in cullin neddylation 1 domain containing 3 [Source:ZFIN;Acc:ZDB-GENE-070928-2]	24	39518774	39535255		4.81	2.95	3.18	5.74	5.74	6.18
ENSDARG00000011496	ppm1bb	protein phosphatase, Mg2+/Mn2+ dependent, 1Bb [Source:ZFIN;Acc:ZDB-GENE-041114-185]	12	25432626	25487244		14.15	9.88	9.17	15.63	15.77	15.1
ENSDARG00000011498	si:ch211-212o1.2	si:ch211-212o1.2 [Source:ZFIN;Acc:ZDB-GENE-041111-310]	18	18000695	18060544		5.53	4.79	4.02	5.19	4.83	5.39
ENSDARG00000011506	lpcat1	lysophosphatidylcholine acyltransferase 1 [Source:NCBI gene;Acc:555969]	19	28291062	28334164		3.4	3.07	3.34	2.77	2.93	3
ENSDARG00000011510	ncc2	regulator of chromosome condensation 2 [Source:NCBI gene;Acc:406455]	23	21520769	21535040		30.43	29.69	21.53	25.58	23.59	23.37
ENSDARG00000011515	orai1a	ORAI calcium release-activated calcium modulator 1a [Source:ZFIN;Acc:ZDB-GENE-110721-1]	8	40275830	40288223		0.98	1.41	1.13	1.56	1.66	1.56
ENSDARG00000011519	myl4	myosin, light chain 4, alkali; atrial, embryonic [Source:ZFIN;Acc:ZDB-GENE-050626-112]	3	22222244	22242868		15.65	19.99	19.54	21.92	23.51	21.63
ENSDARG00000011521	upb1	ureidopropionase, beta [Source:ZFIN;Acc:ZDB-GENE-030131-1380]	8	30699429	30730675		15.88	21.66	17.49	10.99	12.55	11.95
ENSDARG00000011533	sema6d	sema domain, transmembrane domain (TM), and cytoplasmic domain, (semaphorin) 6D, like [Source:ZFIN;Acc:ZDB-GENE-050417-	18	40354998	40387862		10.83	5.74	4.82	14.63	13.88	13.92
ENSDARG00000011537	ugt2a5	UDP glucuronosyltransferase 2 family, polypeptide A5 [Source:ZFIN;Acc:ZDB-GENE-060929-796]	1	69868891	69876834		3.35	4.36	3.88	3.57	3.77	3.57
ENSDARG00000011545	arrb2b	arrestin, beta 2b [Source:ZFIN;Acc:ZDB-GENE-040426-1332]	7	25920792	26005691		13.43	10.29	10.2	18.8	19.56	19.26
ENSDARG00000011549	c1ql4a	complement component 1, q subcomponent-like 4 [Source:ZFIN;Acc:ZDB-GENE-090313-185]	23	28399313	28423222		2.07	0.84	1.27	2.59	2.97	2.63
ENSDARG00000011553	atp5pb	ATP synthase peripheral stalk-membrane subunit b [Source:ZFIN;Acc:ZDB-GENE-041010-33]	8	25329032	25338709		181.55	212.32	225.21	117.92	118.11	118.9
ENSDARG00000011555	spag7	sperm associated antigen 7 [Source:NCBI gene;Acc:394100]	5	68059924	68074592		12.13	11.58	11.27	9.62	9.23	9.67
ENSDARG00000011564	sfpq	splicing factor proline/glutamine-rich [Source:ZFIN;Acc:ZDB-GENE-040426-2452]	19	40025928	40055391		62.36	42.22	38.41	64.17	59.68	62.53
ENSDARG00000011570	pou6f1	POU class 6 homeobox 1 [Source:NCBI gene;Acc:30334]	23	33681677	33710029		4.96	2.22	2.25	6.04	5.91	6.01
ENSDARG00000011571	calcrlb	calcitonin receptor-like b [Source:ZFIN;Acc:ZDB-GENE-080924-1]	6	11680726	11704680		1.12	1.85	1.45	1.57	1.73	2.22
ENSDARG00000011573	abcb11a	ATP-binding cassette, sub-family B (MDR/TAP), member 11a [Source:ZFIN;Acc:ZDB-GENE-050517-13]	9	48755638	48817119		1.89	2.71	1.82	1.78	1.94	1.91
ENSDARG00000011579	hoxb10a	homeobox B10a [Source:NCBI gene;Acc:58056]	3	23669267	23671863		2.85	3.51	3.66	5.67	6.39	6.57
ENSDARG00000011581	si:dkey-23k10.5	si:dkey-23k10.5 [Source:ZFIN;Acc:ZDB-GENE-030131-4787]	15	36349009	36357889		2.01	3.06	1.96	0.82	0.68	0.83
ENSDARG00000011582	sox5	SRY-box transcription factor 5 [Source:ZFIN;Acc:ZDB-GENE-000607-13]	4	16982249	17055950		9.6	4.93	4.83	18.72	17.1	17.17
ENSDARG00000011583	cry1b	cryptochrome circadian regulator 1b [Source:ZFIN;Acc:ZDB-GENE-010426-3]	18	15106518	15129696		4.82	3.69	3.45	5.91	7.08	6.4
ENSDARG00000011594	smarcb1b	SWI/SNF related, matrix associated, actin dependent regulator of chromatin, subfamily b, member 1b [Source:ZFIN;Acc:ZDB-GENE	21	15592426	15621361		6.85	3.8	5.13	6.23	6.43	6.24
ENSDARG00000011597	atxn2l	ataxin 2-like [Source:ZFIN;Acc:ZDB-GENE-030131-3246]	5	15394428	15425689		6.54	5.08	4.56	10.9	11.06	10.06
ENSDARG00000011600	epha4b	eph receptor A4b [Source:ZFIN;Acc:ZDB-GENE-030826-6]	2	40294313	40514151		10.29	2.72	3.55	15.58	15.8	15.93
ENSDARG00000011602	CRACD	si:dkeyp-117h8.2 [Source:NCBI gene;Acc:570321]	20	25659839	25709349	CRACD	10.04	3.46	3.79	11.51	11.3	12.06
ENSDARG00000011605	dennd6b	DENN/MADD domain containing 6B [Source:ZFIN;Acc:ZDB-GENE-030131-9382]	4	20071128	20081621		4.14	2.1	3.19	6	6.29	6.64
ENSDARG00000011609	FOXK2	si:ch211-262e15.1 [Source:ZFIN;Acc:ZDB-GENE-030131-5310]	3	11779689	11828206	FOXK2	15.63	9.47	9.38	23.43	24.68	23.95
ENSDARG00000011611	actr1	actin related protein 1, centractin [Source:ZFIN;Acc:ZDB-GENE-040426-2695]	10	42521943	42540839		45.2	28.97	26.8	47.82	48.21	48.8
ENSDARG00000011613	rbm39a	RNA binding motif protein 39a [Source:ZFIN;Acc:ZDB-GENE-040426-2852]	11	24339362	24348715		18.04	8.79	10.62	20.78	21.3	21.34
ENSDARG00000011615	mybpc3	myosin binding protein C, cardiac [Source:ZFIN;Acc:ZDB-GENE-070112-2362]	7	31838320	31899925		28.71	37.56	36.42	22.1	22.04	21.95
ENSDARG00000011618	slc26a2	solute carrier family 26 member 2 [Source:ZFIN;Acc:ZDB-GENE-030717-3]	14	25438015	25452503		2.75	2.16	1.75	4.46	4.56	4.21
ENSDARG00000011635	scxb	scleraxis bHLH transcription factor b [Source:ZFIN;Acc:ZDB-GENE-090806-1]	16	31396647	31402154		1.63	1.86	1.84	0.93	0.99	0.61
ENSDARG00000011640	syt5b	synaptotagmin Vb [Source:ZFIN;Acc:ZDB-GENE-050522-134]	24	7686464	7699356		75.08	16.1	18.58	74.42	76.65	80.01
ENSDARG00000011648	snrpd1	small nuclear ribonucleoprotein D1 polypeptide [Source:ZFIN;Acc:ZDB-GENE-020419-14]	19	1872794	1877746		37.1	38.03	38.13	28.34	29.57	30.39
ENSDARG00000011652	fth52p	fthTRIM family, member 52, pseudogene [Source:ZFIN;Acc:ZDB-GENEP-110922-3]	9	3153466	3166159		2.04	1.93	1.67	0.85	1.21	1.31
ENSDARG00000011661	twf1b	twinfilin actin-binding protein 1b [Source:ZFIN;Acc:ZDB-GENE-040711-3]	4	14193080	14207471		13.78	12.43	10.04	9.69	9.96	9.63
ENSDARG00000011662	chuk	component of inhibitor of nuclear factor kappa B kinase complex [Source:NCBI gene;Acc:393287]	13	25286538	25379820		13.17	11.23	10.89	13.56	14.24	13.39
ENSDARG00000011665	aldoaa	aldolase a, fructose-bisphosphate, a [Source:ZFIN;Acc:ZDB-GENE-030131-8369]	3	39566999	39577052		283.18	278.68	329.25	405.91	433.3	416.05
ENSDARG00000011671	pde6b	phosphodiesterase 6B, cGMP-specific, rod, beta [Source:ZFIN;Acc:ZDB-GENE-090421-2]	21	20356458	20376607		8.59	1.28	1.07	4.3	4.68	4.77
ENSDARG00000011672	sema3b	sema domain, immunoglobulin domain (Ig), short basic domain, secreted, (semaphorin) 3B [Source:ZFIN;Acc:ZDB-GENE-081105-7	8	26586484	26609259		13.31	10.45	5.56	11.46	9.02	9.15
ENSDARG00000011673	si:ch211-246m6.5	si:ch211-246m6.5 [Source:ZFIN;Acc:ZDB-GENE-050208-577]	22	14145510	14161309		5.04	5.95	6.01	9	8.15	8.62
ENSDARG00000011683	prrfdc1	phosphoribosyl transferase domain containing 1 [Source:ZFIN;Acc:ZDB-GENE-040426-1298]	2	12255568	12280508		4.66	4.63	4.42	5.97	6.88	6.6
ENSDARG00000011693	ttc36	tetratricopeptide repeat domain 36 [Source:NCBI gene;Acc:492516]	18	42781622	42830563		22.4	36.48	32.41	11.4	10.02	11
ENSDARG00000011696	esrrgb	estrogen-related receptor gamma b [Source:ZFIN;Acc:ZDB-GENE-040616-4]	20	46790056	46817223		3.01	0.98	1.07	1.05	1.5	1.51
ENSDARG00000011701	ctsll	cathepsin L, like [Source:ZFIN;Acc:ZDB-GENE-041010-76]	4	24305877	24309667		0.4	0.26	0.42	0.46	0.31	0.38
ENSDARG00000011703	clocka	clock circadian regulator a [Source:ZFIN;Acc:ZDB-GENE-990630-14]	20	22067337	22176003		1.63	0.64	0.74	2.2	2.32	2.24
ENSDARG00000011723	C2H5orf22	zgc:56556 [Source:NCBI gene;Acc:393302]	2	27957642	27975530	C2H5orf22	2.62	2.3	2.18	1.58	1.73	2.03

ENSDARG00000011724	marveld1	MARVEL domain containing 1 [Source:NCBI gene;Acc:497317]	13	39532050	39533365		5.01	7.13	6.06	11.34	12.98	12.4
ENSDARG00000011727	b9d1	B9 protein domain 1 [Source:NCBI gene;Acc:554150]	3	39691525	39696066		5.98	4.86	5.56	6.42	6.28	7.27
ENSDARG00000011740	u2af2b	U2 small nuclear RNA auxiliary factor 2b [Source:ZFIN;Acc:ZDB-GENE-040426-1881]	19	10299002	10324573		27.04	21.75	20.36	28.84	28.67	29.01
ENSDARG00000011743	pigo	phosphatidylinositol glycan anchor biosynthesis, class O [Source:ZFIN;Acc:ZDB-GENE-091204-80]	10	17747880	17759878		3.64	2.42	1.75	4.98	4.32	4.66
ENSDARG00000011764	ints13	integrator complex subunit 13 [Source:ZFIN;Acc:ZDB-GENE-030131-6224]	4	20486041	20499668		8.08	5.93	5.98	6.46	6.73	6.62
ENSDARG00000011769	cpm	carboxypeptidase M [Source:ZFIN;Acc:ZDB-GENE-041210-191]	4	23740655	23759213		8.26	8.68	8.95	9.14	10.02	9.27
ENSDARG00000011770	dhrs12	dehydrogenase/reductase (SDR family) member 12 [Source:ZFIN;Acc:ZDB-GENE-060929-1134]	9	23808546	23812636		5.61	5.9	5.64	5.41	4.92	4.77
ENSDARG00000011777	cttn	cortactin [Source:ZFIN;Acc:ZDB-GENE-040715-3]	18	50914231	50921148		34.56	28.35	26.87	37.18	37.66	34.99
ENSDARG00000011783	stag2a	stromal antigen 2a [Source:ZFIN;Acc:ZDB-GENE-030131-3432]	5	23488055	23517747		9.94	7.3	6.13	10.56	9.94	10.75
ENSDARG00000011785	tbx6	T-box transcription factor 6 [Source:NCBI gene;Acc:192294]	12	3912544	3923070		0.4	0.14	0.21	1.33	0.96	1.57
ENSDARG00000011797	tent5bb	terminal nucleotidyltransferase 5Bb [Source:ZFIN;Acc:ZDB-GENE-060503-431]	19	14573998	14588414		4.14	4.64	4.03	4.27	4.42	4.06
ENSDARG00000011801	wars2	tryptophanyl tRNA synthetase 2, mitochondrial [Source:ZFIN;Acc:ZDB-GENE-050306-26]	9	21072359	21098413		3.46	2.33	2.87	2.56	1.97	2.07
ENSDARG00000011809	mical1	microtubule associated monooxygenase, calponin and LIM domain containing 1 [Source:NCBI gene;Acc:568573]	23	39461989	39528988		3.17	2.18	1.86	3.41	3.04	3.36
ENSDARG00000011818	batf	basic leucine zipper transcription factor, ATF-like [Source:NCBI gene;Acc:564589]	20	46572550	46646556		0.23	0.14	0.14	0.3	0.21	0.22
ENSDARG00000011819	pcnx1	pecanex 1 [Source:ZFIN;Acc:ZDB-GENE-081113-6]	13	36053985	36134401		5.22	3.28	3.18	8.65	8.61	8.52
ENSDARG00000011821	plod2	procollagen-lysine, 2-oxoglutarate 5-dioxygenase 2 [Source:ZFIN;Acc:ZDB-GENE-070326-1]	24	5237709	5314707		4.15	4.64	3.85	13.24	14.93	14.38
ENSDARG00000011824	pbxip1b	pre-B-cell leukemia homeobox interacting protein 1b [Source:ZFIN;Acc:ZDB-GENE-070112-2032]	16	22750174	22775528		9.77	10.25	8.97	7.61	7.06	7.26
ENSDARG00000011825	cetn3	centrin 3 [Source:ZFIN;Acc:ZDB-GENE-050522-152]	10	43705795	43759532		3.55	2.61	2.72	2.63	2.94	3.06
ENSDARG00000011837	C13H9orf72	zgc:100846 [Source:ZFIN;Acc:ZDB-GENE-040801-47]	13	12761707	12778947	C13H9orf72	5.11	3.19	3.45	6.7	6.33	6.08
ENSDARG00000011841	atp5l	ATP synthase, H+ transporting, mitochondrial F0 complex, subunit g [Source:ZFIN;Acc:ZDB-GENE-030131-5177]	5	30699260	30701891		526.26	637.45	680.15	391.84	412.43	400.76
ENSDARG00000011845	chn2	chimerin 2 [Source:ZFIN;Acc:ZDB-GENE-091020-5]	16	20318400	20492799		2.12	1.84	1.66	1.7	1.86	1.19
ENSDARG00000011851	map2k5	mitogen-activated protein kinase kinase 5 [Source:ZFIN;Acc:ZDB-GENE-030131-1517]	7	33970698	34062301		2.43	2.03	1.75	2.74	2.62	2.86
ENSDARG00000011855	aak1a	AP2 associated kinase 1a [Source:ZFIN;Acc:ZDB-GENE-081105-101]	8	48965767	49044259		3.74	1.32	1.07	3.1	3.36	2.88
ENSDARG00000011862	inaa	internexin neuronal intermediate filament protein, alpha a [Source:ZFIN;Acc:ZDB-GENE-060531-65]	13	28675686	28681036		34	8.38	6.84	10.04	12.78	11.16
ENSDARG00000011863	ptk7a	protein tyrosine kinase 7a [Source:ZFIN;Acc:ZDB-GENE-050522-216]	22	38394502	38459454		10.23	8.94	7.5	15.86	15.44	15.54
ENSDARG00000011869	galnt8a.2	polypeptide N-acetylgalactosaminyltransferase 8a, tandem duplicate 2 [Source:ZFIN;Acc:ZDB-GENE-131122-40]	25	16706101	16724913		0.07	0.1	0.08	0.08	0.06	0.05
ENSDARG00000011870	sp8a	sp8 transcription factor a [Source:ZFIN;Acc:ZDB-GENE-030131-9849]	19	2315208	2318391		6.49	5.3	5.12	8.4	9.07	7.88
ENSDARG00000011876	ednraa	endothelin receptor type Aa [Source:ZFIN;Acc:ZDB-GENE-010906-2]	1	36612660	36702355		4.91	6.35	6.2	10.19	11.08	10.76
ENSDARG00000011879	foxn1	forkhead box N1 [Source:ZFIN;Acc:ZDB-GENE-021008-1]	15	28129683	28161224		1.06	0.59	0.48	0.48	0.2	0.19
ENSDARG00000011884	MFN1	mitofusin 1a [Source:NCBI gene;Acc:100329462]	2	5338759	5356686	MFN1	7.8	0.61	0.62	4.55	5.86	6.05
ENSDARG00000011885	mrpl19	mitochondrial ribosomal protein L19 [Source:ZFIN;Acc:ZDB-GENE-040801-55]	20	25440514	25445246		30.05	35.97	36.8	26.93	27.35	28.58
ENSDARG00000011886	pdca	phosducin a [Source:ZFIN;Acc:ZDB-GENE-031023-1]	2	20882954	20890429		5.65	1.29	1.04	2.74	2.77	3.07
ENSDARG00000011890	cry4	cryptochrome circadian regulator 4 [Source:ZFIN;Acc:ZDB-GENE-010426-7]	22	819656	834106		10.39	8.62	10.49	21.01	23.61	22.98
ENSDARG00000011897	tsg101b	tumor susceptibility 101b [Source:ZFIN;Acc:ZDB-GENE-130530-542]	7	49619375	49637278		6.04	5.59	4.76	6.3	6.22	6.16
ENSDARG00000011909	itpr2	inositol 1,4,5-trisphosphate receptor, type 2 [Source:ZFIN;Acc:ZDB-GENE-050419-192]	18	15937610	16042504		5.14	4.38	2.64	4.01	4.2	3.99
ENSDARG00000011921	txnl1	thioredoxin-like 1 [Source:ZFIN;Acc:ZDB-GENE-040426-701]	21	917593	929448		23.49	23.92	24.22	21.02	21.31	20.76
ENSDARG00000011925	spns1	spinster homolog 1 (Drosophila) [Source:NCBI gene;Acc:799873]	3	15455319	15475080		22.64	29.77	26.66	24.14	25.56	23.9
ENSDARG00000011926	tpgs2	tubulin polyglutamylase complex subunit 2 [Source:ZFIN;Acc:ZDB-GENE-040704-49]	21	12023796	12036134		3.03	1.87	2.49	2.41	3.32	3.47
ENSDARG00000011929	plp1b	proteolipid protein 1b [Source:ZFIN;Acc:ZDB-GENE-030710-6]	5	23268121	23280098		6.74	5.26	6.07	5.94	6.85	6.25
ENSDARG00000011932	prkcbp1l	protein kinase C binding protein 1, like [Source:ZFIN;Acc:ZDB-GENE-030131-2199]	23	9809540	9864166		10.73	5.36	5.27	14.97	14.36	16.29
ENSDARG00000011934	gyg1a	glycogenin 1a [Source:ZFIN;Acc:ZDB-GENE-040426-2910]	2	24936766	24944819		13.97	15.83	14.61	17.36	18.44	17.43
ENSDARG00000011941	bmpr2a	bone morphogenetic protein receptor, type II a (serine/threonine kinase) [Source:ZFIN;Acc:ZDB-GENE-070618-1]	6	12797648	12868401		8.14	2.83	2.84	11.11	10.65	11.66
ENSDARG00000011945	slc35e1	solute carrier family 35 member E1 [Source:ZFIN;Acc:ZDB-GENE-040426-2033]	11	5499661	5515922		5.58	3.57	3.22	5.63	5.58	5.89
ENSDARG00000011948	insra	insulin receptor a [Source:ZFIN;Acc:ZDB-GENE-020503-3]	2	37544951	37632896		9.53	6.15	4.54	11.23	11.41	11.27
ENSDARG00000011956	dlx4a	distal-less homeobox 4a [Source:NCBI gene;Acc:30561]	3	34670076	34681421		8.16	7.6	8.91	8.49	7.53	7.43
ENSDARG00000011958	ctnnbl1	catenin, beta like 1 [Source:ZFIN;Acc:ZDB-GENE-040426-1850]	2	53720028	53815532		20.84	18.51	17.61	17.71	16.13	16.88
ENSDARG00000011961	csrp2	cysteine and glycine-rich protein 2 [Source:ZFIN;Acc:ZDB-GENE-040426-1863]	4	2511629	2525934		12.04	16.04	15.7	10.91	12.22	12.34
ENSDARG00000011973	slc35d1a	solute carrier family 35 member D1a [Source:ZFIN;Acc:ZDB-GENE-040826-5]	6	34847276	34860574		3.12	2.97	2.5	5.34	5.65	5.34
ENSDARG00000011975	fmn1	formin 1 [Source:ZFIN;Acc:ZDB-GENE-041001-198]	20	29436601	29469228		0.18	0.2	0.15	0.28	0.33	0.28
ENSDARG00000011983	zgc:136908	zgc:136908 [Source:ZFIN;Acc:ZDB-GENE-060312-22]	1	55786986	55810730		12.87	18.24	20.53	9.74	8.54	8.9
ENSDARG00000011989	crx	cone-rod homeobox [Source:ZFIN;Acc:ZDB-GENE-010403-1]	5	36932718	36936443		37.38	8.06	5.82	58.59	52.73	62.87
ENSDARG00000011998	bfsp2	beaded filament structural protein 2, phakinin [Source:ZFIN;Acc:ZDB-GENE-041212-58]	2	32761130	32769759		0.38	2.31	4.57	10.48	8.45	11.46
ENSDARG00000011999	ybey	ybeY metalloendoribonuclease [Source:ZFIN;Acc:ZDB-GENE-030131-437]	9	711638	715241		9.55	11.17	11.87	7.28	7.08	6.58
ENSDARG00000012002	slc38a7	solute carrier family 38 member 7 [Source:NCBI gene;Acc:445254]	7	51795667	51816589		3.11	2.97	2.82	3.67	3.08	3.09
ENSDARG00000012005	mob2b	MOB kinase activator 2b [Source:ZFIN;Acc:ZDB-GENE-060616-319]	18	49969568	50040161		4.51	3.2	2.74	6.47	6.43	6.22
ENSDARG00000012013	cpa6	carboxypeptidase A6 [Source:ZFIN;Acc:ZDB-GENE-091204-331]	24	18880254	18919649		0.62	0.68	0.62	0.75	0.6	0.65
ENSDARG00000012016	hpgd	15-hydroxyprostaglandin dehydrogenase [Source:ZFIN;Acc:ZDB-GENE-080728-2]	1	38398696	38415158		10.7	12.87	12.58	10.91	11.92	11.41
ENSDARG00000012019	glra1	glycine receptor, alpha 1 [Source:NCBI gene;Acc:30676]	14	25816874	25911567		4.28	1.49	2.13	7.89	8.02	7.93
ENSDARG00000012021	kdsr	3-ketodihydrosphingosine reductase [Source:ZFIN;Acc:ZDB-GENE-040426-853]	2	13223755	13254821		6.41	5.88	5.14	7.67	7.57	7.48
ENSDARG00000012025	hs6st2	heparan sulfate 6-O-sulfotransferase 2 [Source:NCBI gene;Acc:378450]	14	31081862	31087830		9.44	8.43	7.36	11.94	11.75	11.96
ENSDARG00000012030	dnaaf1	dynein, axonemal, assembly factor 1 [Source:NCBI gene;Acc:386722]	7	29012033	29017892		0.59	0.45	0.59	0.83	1.09	0.92
ENSDARG00000012035	zgc:100832	zgc:100832 [Source:ZFIN;Acc:ZDB-GENE-040801-53]	23	16814968	16823262		3.99	4.89	4.63	3.24	3.62	3.22
ENSDARG00000012039	ttc26	tetratricopeptide repeat domain 26 [Source:NCBI gene;Acc:494074]	6	7688491	7711349		2.32	1.25	1.88	2.85	2.81	2.7
ENSDARG00000012040	rnf19a	ring finger protein 19A, RBR E3 ubiquitin protein ligase [Source:ZFIN;Acc:ZDB-GENE-030131-9414]	19	12030832	12078583		5.69	4.75	3.92	10.49	10.64	10.31
ENSDARG00000012044	polr3gla	polymerase (RNA) III (DNA directed) polypeptide G like a [Source:ZFIN;Acc:ZDB-GENE-040912-67]	19	24545731	24555949		4.92	7.48	5.64	3.23	2.77	2.59
ENSDARG00000012051	mettl21a	methyltransferase like 21A [Source:NCBI gene;Acc:541441]	22	11657139	11661724		2.81	3.17	3.21	2.31	2.19	2.26
ENSDARG00000012057	mtnr1al	melatonin receptor type 1A like [Source:NCBI gene;Acc:30660]	14	26075799	26094172		0.57	0.21	0.2	0.43	0.24	0.42
ENSDARG00000012060	thbs3b	thrombospondin 3b [Source:NCBI gene;Acc:571317]	19	24419693	24443867		7.95	11.34	9.22	6.15	6.2	6.2
ENSDARG00000012066	dcn	decorin [Source:ZFIN;Acc:ZDB-GENE-010102-1]	4	16368762	16412084		71.6	87.36	78.9	99.41	105.22	98.44
ENSDARG00000012071	angptl1a	angiopoietin-like 1a [Source:ZFIN;Acc:ZDB-GENE-040724-269]	20	15982482	16007636		6.2	6.63	5.81	7.13	7.65	7.33
ENSDARG00000012072	dmac2l	distal membrane arm assembly complex 2 like [Source:ZFIN;Acc:ZDB-GENE-040426-959]	13	36680564	36691709		10.69	9.77	12.1	8.72	8.68	10.11
ENSDARG00000012073	kif15	kinesin family member 15 [Source:ZFIN;Acc:ZDB-GENE-050622-16]	25	35068166	35095129		5.38	4.94	5.25	3.25	3.18	3.07
ENSDARG00000012076	apoa1a	apolipoprotein A-Ia [Source:NCBI gene;Acc:30355]	5	37978501	37981521		2402.59	3433.46	3825.67	2279.32	2484.19	2476.08
ENSDARG00000012078	meis1b	Meis homeobox 1 b [Source:ZFIN;Acc:ZDB-GENE-020122-1]	13	5490551	5569562		52.32	39.86	38.69	60.88	63.46	63.57
ENSDARG00000012113	c19h1orf109	c19h1orf109 homolog (H. sapiens) [Source:NCBI gene;Acc:286778]	19	47783137	47787275		6.56	6.82	9.04	4.24	2.84	3.37
ENSDARG00000012125	cnga1b	cyclic nucleotide gated channel subunit alpha 1b [Source:ZFIN;Acc:ZDB-GENE-110408-39]	23	43849190	43866354		5.16	1.28	0.97	2.91	3.49	3.58
ENSDARG00000012126	zgc:109965	zgc:109965 [Source:ZFIN;Acc:ZDB-GENE-050913-21]	21	13066705	13085242		8.48	3.41	3.39	10.3	9.75	11.8
ENSDARG00000012135	fbxl2	F-box and leucine-rich repeat protein 2 [Source:ZFIN;Acc:ZDB-GENE-030925-12]	19	42660158	42682947		7.22	3.7	3.92	6.49	7.34	7.09
ENSDARG00000012137	cyp46a1.1	cytochrome P450, family 46, subfamily A, polypeptide 1, tandem duplicate 1 [Source:ZFIN;Acc:ZDB-GENE-050522-464]	20	5106568	5120937		2.6	4.42	3.21	1.1	1.02	0.83
ENSDARG00000012138	sgce	sarcoglycan, epsilon [Source:ZFIN;Acc:ZDB-GENE-030724-1]	19	41038668	41069701		22.98	11.96	14.45	26.85	28.45	28.09
ENSDARG00000012140	ccnl1b	cyclin L1b [Source:NCBI gene;Acc:326088]	2	36664173	36674005		12.29	8.82	10.49	12.49	11.18	11.67
ENSDARG00000012141	papolg	poly(A) polymerase gamma [Source:ZFIN;Acc:ZDB-GENE-040426-1256]	13	25824345	25842074		19.06	10.12	11.79	25.28	24.38	25.52
ENSDARG00000012144	emc7	ER membrane protein complex subunit 7 [Source:NCBI gene;Acc:561549]	20	29217835	29225133		31.95	29.71	28.1	31.33	29.45	30.33
ENSDARG00000012177	rab4b	RAB4B, member RAS oncogene family [Source:NCBI gene;Acc:445498]	15	8813755	8856785		1.65	1.24	0.95	2.7	2.75	2.65
ENSDARG00000012184	zgc:162872	zgc:162872 [Source:ZFIN;Acc:ZDB-GENE-081022-1]	15	46685434	46718795		4.54	3.07	3.2	5.77	6.45	6.63
ENSDARG00000012192	cant1a	calcium activated nucleotidase 1a [Source:ZFIN;Acc:ZDB-GENE-040426-2441]	3	57747716	57765169		11.73	10.11	8.95	9.22	8.66	9.3
ENSDARG00000012194	scp2a	sterol carrier protein 2a [Source:ZFIN;Acc:ZDB-GENE-040426-1846]	2	6005199	6039757		65.91	106.21	87.8	28.5	30.59	32.37
ENSDARG00000012196	fer	fer (fps/fes related) tyrosine kinase [Source:ZFIN;Acc:ZDB-GENE-060526-234]	5	57243568	57311037		4.21	2.46	2.38	4.25	4.1	3.94
ENSDARG00000012199	gpt2	glutamic pyruvate transaminase (alanine aminotransferase) 2 [Source:ZFIN;Acc:ZDB-GENE-030729-8]	7	41887429	41901688		4.3	3.77	2.75	4.81	4.87	5.01
ENSDARG00000012204	CDK18	si:dkey-166c18.1 [Source:ZFIN;Acc:ZDB-GENE-141215-51]	11	38280454	38420819	CDK18	5.24	2.53	2.62	5.98	6.27	6.39
ENSDARG00000012215	umps	uridine monophosphate synthetase [Source:ZFIN;Acc:ZDB-GENE-040426-785]	9	11576502	11587070		11.34	11.92	10.63	6.55	7.24	6.18
ENSDARG00000012219	dpf2	D4, zinc and double PHD fingers family 2 [Source:ZFIN;Acc:ZDB-GENE-041024-2]	5	37744625	37757818		7	4.95	4.47	8.17	7.54	7.4
ENSDARG00000012222	nup35	nucleoporin 35 [Source:NCBI gene;Acc:337461]	9	12259469	12401871		3.9	3.63	3.2	3.17	3.48	3.37
ENSDARG00000012234	psme3	proteasome activator subunit 3 [Source:ZFIN;Acc:ZDB-GENE-991110-19]	12	27206736	27212880		13.94	16.42	13.38	12.76	11.86	11.24
ENSDARG00000012244	ube2e3	ubiquitin-conjugating enzyme E2E 3 (UBC4/5 homolog, yeast) [Source:ZFIN;Acc:ZDB-GENE-030131-408]	9	43797902	43883267		21.92	14.16	12.46	22.75	22.25	22.79
ENSDARG00000012247	def6a	DEF6 guanine nucleotide exchange factor a [Source:NCBI gene;Acc:394015]	8	14185295	14209852		1.37	1.21	1.35	0.86	0.93	1.04
ENSDARG00000012248	rgma	repulsive guidance molecule BMP co-receptor a [Source:ZFIN;Acc:ZDB-GENE-040527-1]	18	24919614	24932329		21.59	10.86	9.83	25.88	24.38	25.25
ENSDARG00000012261	eftud2	elongation factor Tu GTP binding domain containing 2 [Source:ZFIN;Acc:ZDB-GENE-040426-1569]	3	16389020	16413635		18.18	14.07	13.04	15.41	13.95	14.84
ENSDARG00000012269	clcn1b	chloride channel, voltage-sensitive 1b [Source:ZFIN;Acc:ZDB-GENE-100921-11]	16	17477152	17541890		0.49	1.06	0.69	0.89	0.76	0.66
ENSDARG00000012271	pacc1	proton activated chloride channel 1 [Source:NCBI gene;Acc:393429]	17	45378908	45387134		2.95	3.48	3.43	3.01	2.88	2.94
ENSDARG00000012274	eif4e1c	eukaryotic translation initiation factor 4E family member 1c [Source:ZFIN;Acc:ZDB-GENE-050417-398]	13	18311410	18324469		17.98	20.17	20.65	21.39	20.18	19.02
ENSDARG00000012275	katnal2	katanin p60 subunit A-like 2 [Source:ZFIN;Acc:ZDB-GENE-051113-156]	10	14499201	14521190		0.68	0.71	0.7	0.63	0.7	0.71
ENSDARG00000012281	zgc:65851	zgc:65851 [Source:ZFIN;Acc:ZDB-GENE-030131-9669]	5	13671614	13685047		22.09	12.98	11.34	20.87	22.33	21.71
ENSDARG00000012295	bcs1l	BC1 (ubiquinol-cytochrome c reductase) synthesis-like [Source:NCBI gene;Acc:394157]	9	38399912	38407176		7.32	7.35	6.58	6.09	5.71	5.87
ENSDARG00000012297	cnga3b	cyclic nucleotide gated channel subunit alpha 3b [Source:ZFIN;Acc:ZDB-GENE-090312-121]	1	10771457	10776547		2.71	0.8	0.69	4.01	4.48	4.3
ENSDARG00000012306	syt13	synaptotagmin XIII [Source:ZFIN;Acc:ZDB-GENE-050417-135]	7	38685654	38737718		5.94	1.34	1.82	6.39	7.44	7.38
ENSDARG00000012311	myoz2a	myozenin 2a [Source:ZFIN;Acc:ZDB-GENE-040426-1880]	7	69829442	69857692		8.58	12.97	12.84	5.77	5.63	5.6
ENSDARG00000012314	usp25	ubiquitin specific peptidase 25 [Source:ZFIN;Acc:ZDB-GENE-040426-2847]	10	38168158	38243851		7.25	5.45	4.92	8.9	8.4	9.07
ENSDARG00000012325	zgc:110269	zgc:110269 [Source:NCBI gene;Acc:550274]	2	16449627	16458472		7.86	8.42	7.8	5.22	4.91	5.98
ENSDARG00000012340	ptpn11b	protein tyrosine phosphatase non-receptor type 11b [Source:ZFIN;Acc:ZDB-GENE-040426-1158]	23	23918421	23995856		4.39	2.09	2.04	5.6	5.31	5.03
ENSDARG00000012341	capn9	calpain 9 [Source:ZFIN;Acc:ZDB-GENE-010724-2]	1	53881844	53907092		21.13	15.26	13.07	7.12	6.03	5.85
ENSDARG00000012347	nacad	NAC alpha domain containing [Source:ZFIN;Acc:ZDB-GENE-091020-7]	16	27624395	27640995		6.05	2.22	2.47	4.94	4.99	5.03
ENSDARG00000012353	vipr2	vasoactive intestinal peptide receptor 2 [Source:ZFIN;Acc:ZDB-GENE-010406-4]	24	27268001	27346951		3.41	2.03	2.09	2.83	2.9	2.91
ENSDARG00000012355	galnt8a.1	polypeptide N-acetylgalactosaminyltransferase 8a, tandem duplicate 1 [Source:ZFIN;Acc:ZDB-GENE-091015-4]	25	16708584	16742636		0.05	0.08	0.1	0.07	0.13	0.1
ENSDARG00000012366	fbp2	fructose-1,6-bisphosphatase 2 [Source:ZFIN;Acc:ZDB-GENE-040822-23]	5	44814064	44829719		34.37	66.15	47.86	30.66	24.45	36.54
ENSDARG00000012367	trim46a	tripartite motif containing 46a [Source:ZFIN;Acc:ZDB-GENE-110825-1]	16	23353541	23379464		5.35	1.19	1.9	6.89	8.01	6.75
ENSDARG00000012368	ttc21b	tetratricopeptide repeat domain 21B [Source:ZFIN;Acc:ZDB-GENE-031010-34]	9	49808483	49860756		5.27	2.73	2.9	4.94	5.69	5.87
ENSDARG00000012369	rdh10b	retinol dehydrogenase 10b [Source:NCBI gene;Acc:378722]	2	30182431	30186307		5.73	7.51	7.58	5.34	6.82	6.18
ENSDARG00000012378	naa35	N(alpha)-acetyltransferase 35, NatC auxiliary subunit [Source:NCBI gene;Acc:321587]	8	49936585	49969184		17.35	12.64	12.77	14.53	14.98	14.85
ENSDARG00000012381	hsc70	heat shock cognate 70 [Source:ZFIN;Acc:ZDB-GENE-040426-1221]	15	37834594	37850969		49.53	66.45	70.39	26.1	24.75	25.07
ENSDARG00000012387	pdha1a	pyruvate dehydrogenase E1 alpha 1 subunit a [Source:ZFIN;Acc:ZDB-GENE-040426-2719]	5	24031314	24044877		92.07	96.55	88.17	83.4	82.37	83.8
ENSDARG00000012388	cox4i1l	cytochrome c oxidase subunit 4I1, like [Source:ZFIN;Acc:ZDB-GENE-130814-2]	24	28231602	28245872		5.42	10.09	9.11	28.42	31.68	30.94
ENSDARG00000012389	adar	adenosine deaminase RNA specific [Source:ZFIN;Acc:ZDB-GENE-000616-5]	16	23531583	23557714		7.7	4.4	3.52	8.25	8.01	8.18
ENSDARG00000012390	kcnk5b	potassium channel, subfamily K, member 5b [Source:ZFIN;Acc:ZDB-GENE-040426-1297]	20	9223514	9239037		1.63	1.58	1.4	2.78	2.23	2.4
ENSDARG00000012395	mmp13a	matrix metallopeptidase 13a [Source:ZFIN;Acc:ZDB-GENE-031202-2]	10	38593645	38602561		1.21	1.55	1.49	1.94	2.06	2.06
ENSDARG00000012397	eya4	EYA transcriptional coactivator and phosphatase 4 [Source:ZFIN;Acc:ZDB-GENE-050327-93]	23	31439713	31512497		10.18	11.63	9.76	9.35	8.65	9.18
ENSDARG00000012399	adssl	adenylosuccinate synthase, like [Source:ZFIN;Acc:ZDB-GENE-020419-28]	21	26522571	26531535		25	23.19	19.39	20.97	21.29	19.43
ENSDARG00000012403	ercc6l2	excision repair cross-complementation group 6-like 2 [Source:ZFIN;Acc:ZDB-GENE-110411-274]	8	29898715	29930821		2.41	1.5	1.47	2.35	2.54	2.49
ENSDARG00000012405	col1a1a	collagen, type I, alpha 1a [Source:ZFIN;Acc:ZDB-GENE-030131-9102]	3	23221047	23239458		435.85	567.88	479.29	657.67	628.52	599.55
ENSDARG00000012407	mgat1a	alpha-1,3-mannosyl-glycoprotein 2-beta-N-acetylglucosaminyltransferase a [Source:ZFIN;Acc:ZDB-GENE-040426-1515]	15	34867518	34878388		6.9	6.34	5.98	7.36	7.04	6.53
ENSDARG00000012409	rnf207b	ring finger protein 207b [Source:NCBI gene;Acc:568322]	23	21499585	21515182		2.57	3.5	2.4	3.64	2.96	3.28
ENSDARG00000012422	col11a2	collagen, type XI, alpha 2 [Source:ZFIN;Acc:ZDB-GENE-000208-23]	19	7179740	7225206		35.98	29.84	16.94	107.84	109.24	112.36
ENSDARG00000012426	neflb	neurofilament, light polypeptide b [Source:ZFIN;Acc:ZDB-GENE-060312-44]	8	6873853	6877390		11.02	9.88	10.38	9.09	8.22	9.2
ENSDARG00000012432	fam76b	family with sequence similarity 76 member B [Source:NCBI gene;Acc:335015]	5	23585215	23596339		19.03	13.1	14.98	20.03	20.81	20.04
ENSDARG00000012435	spic	Spi-C transcription factor (Spi-1/PU.1 related) [Source:ZFIN;Acc:ZDB-GENE-040912-47]	4	17807837	17812131		0.19	0.27	0.25	0.11	0.26	0.2
ENSDARG00000012446	zgc:113057	zgc:113057 [Source:ZFIN;Acc:ZDB-GENE-050508-3]	7	9008372	9046302		0.04	0.03	0.02	0.02	0.02	0.02
ENSDARG00000012449	si:ch211-169p10.1	si:ch211-169p10.1 [Source:ZFIN;Acc:ZDB-GENE-081104-59]	8	7975745	8034978		2.63	1.29	1.6	3.51	3.4	3.77
ENSDARG00000012450	vmp1	vacuole membrane protein 1 [Source:NCBI gene;Acc:336789]	15	17343319	17373352		21.91	25.15	22.06	21.37	20.6	20.46
ENSDARG00000012453	rnls	renalase, FAD-dependent amine oxidase [Source:ZFIN;Acc:ZDB-GENE-040718-351]	12	17201522	17323341		7.21	8.85	8.74	5.31	4.64	5.86
ENSDARG00000012458		zgc:77849 [Source:NCBI gene;Acc:322387]	1	8694196	8702684		63.66	49.81	51.03	80.34	82.55	82.11
ENSDARG00000012460	rassf4a	Ras association domain family member 4a [Source:ZFIN;Acc:ZDB-GENE-050102-3]	13	30664819	30700460		0.85	0.34	0.64	0.62	0.5	0.74
ENSDARG00000012467	spint1b	serine peptidase inhibitor, Kunitz type 1 b [Source:ZFIN;Acc:ZDB-GENE-071218-1]	20	3218893	3238110		16.14	16.55	13.06	15.5	14.99	13
ENSDARG00000012468	aacs	acetoacetyl-CoA synthetase [Source:NCBI gene;Acc:393984]	5	18827314	18897482		9.01	7.19	5.58	8.48	9.16	8.99
ENSDARG00000012485	aurka	aurora kinase A [Source:ZFIN;Acc:ZDB-GENE-040801-161]	6	60125033	60139655		7.71	5.61	6.25	5.97	6.07	5.33
ENSDARG00000012495	mphosph10	M-phase phosphoprotein 10 (U3 small nucleolar ribonucleoprotein) [Source:ZFIN;Acc:ZDB-GENE-030131-2146]	7	30772991	30779575		14.24	17.07	14.25	11.39	9.83	9.35
ENSDARG00000012496		si:ch211-213o11.11 [Source:ZFIN;Acc:ZDB-GENE-141216-4]	11	36231248	36237676		0.36	0.16	0.11	0.72	0.77	0.88
ENSDARG00000012499	per1b	period circadian clock 1b [Source:ZFIN;Acc:ZDB-GENE-040419-1]	7	50822465	50847296		9.48	5.33	4.1	12.06	12.92	13.3
ENSDARG00000012504	rlbp1a	retinaldehyde binding protein 1a [Source:ZFIN;Acc:ZDB-GENE-040426-1662]	7	13867722	13884610		27.95	11.03	9.4	19.37	22.92	20.36
ENSDARG00000012505	u2af2a	U2 small nuclear RNA auxiliary factor 2a [Source:ZFIN;Acc:ZDB-GENE-050706-131]	16	12812214	12821768		20.45	13.14	12.05	22.19	21.87	23.52
ENSDARG00000012506	rnpep	arginyl aminopeptidase (aminopeptidase B) [Source:ZFIN;Acc:ZDB-GENE-040718-241]	11	24758967	24783118		7.01	7.06	6.78	4.74	5.05	5.12
ENSDARG00000012513	sdcbp2	syndecan binding protein (syntenin) 2 [Source:ZFIN;Acc:ZDB-GENE-030131-3727]	23	16648621	16682186		36.09	32.25	30.83	49.15	51.85	54.47
ENSDARG00000012519	hcfc1b	host cell factor C1b [Source:ZFIN;Acc:ZDB-GENE-030131-2411]	8	7506827	7567815		8.06	5.92	4.54	13.19	11.64	11.87
ENSDARG00000012534	slc6a6a	solute carrier family 6 member 6a [Source:ZFIN;Acc:ZDB-GENE-081104-1]	8	7144066	7195929		2.07	0.85	1.04	1.93	1.63	1.8
ENSDARG00000012535	epx	eosinophil peroxidase [Source:ZFIN;Acc:ZDB-GENE-130425-2]	25	3119810	3139805		0.4	0.54	0.45	0.14	0.15	0.2
ENSDARG00000012539	dnase1	deoxyribonuclease I [Source:ZFIN;Acc:ZDB-GENE-040718-423]	3	27778975	27784457		0.39	0.33	0.49	0.05	0.18	0.13
ENSDARG00000012553	rap1aa	RAP1A, member of RAS oncogene family a [Source:ZFIN;Acc:ZDB-GENE-040625-167]	8	28267546	28274552		29.28	29.51	26.96	31.58	30.24	30.03
ENSDARG00000012555	ak5	adenylate kinase 5 [Source:ZFIN;Acc:ZDB-GENE-030131-8256]	2	8845840	9059955		5.87	1.59	2.76	5.26	5.1	4.96
ENSDARG00000012563	pdss2	prenyl (decaprenyl) diphosphate synthase, subunit 2 [Source:ZFIN;Acc:ZDB-GENE-040718-43]	13	32660544	32726178		6.85	8.2	7.54	5.95	6.23	5.28
ENSDARG00000012572	polr3f	polymerase (RNA) III (DNA directed) polypeptide F [Source:ZFIN;Acc:ZDB-GENE-040426-972]	22	25034740	25043103		7.5	6.97	7.42	6.23	5.19	5.78
ENSDARG00000012574	slkb	STE20-like kinase b [Source:ZFIN;Acc:ZDB-GENE-030131-1684]	1	49606518	49649122		7.2	3.15	3.17	6.87	7.24	7.2
ENSDARG00000012577	waca	WW domain containing adaptor with coiled-coil a [Source:NCBI gene;Acc:323904]	12	23371542	23416061		20.72	11.85	11.08	24.7	23.91	24.72
ENSDARG00000012581	AL590149.1		17	33206780	33231714		2.45	0.69	0.67	2.93	3.35	2.92
ENSDARG00000012586	fmnl2a	formin-like 2a [Source:ZFIN;Acc:ZDB-GENE-081105-70]	9	4730398	4856767		2.54	2.49	2.24	5.47	5.95	5.72
ENSDARG00000012588	ptdss1a	phosphatidylserine synthase 1a [Source:NCBI gene;Acc:393931]	16	53278406	53302453		11.99	9.51	8.72	12.39	13.31	12.23
ENSDARG00000012591	pdcd10b	programmed cell death 10b [Source:NCBI gene;Acc:405869]	2	36721357	36731963		23.48	26.5	24.07	20.9	19.91	20.74
ENSDARG00000012593	col5a1	procollagen, type V, alpha 1 [Source:ZFIN;Acc:ZDB-GENE-041105-6]	21	6556635	6725780		48.91	60.5	39.44	110.37	107.88	111.31
ENSDARG00000012600	si:dkey-97a13.6	si:dkey-97a13.6 [Source:ZFIN;Acc:ZDB-GENE-070912-617]	2	41949186	41964402		1.11	0.72	0.65	0.83	0.77	0.64
ENSDARG00000012603	eif3ja	eukaryotic translation initiation factor 3, subunit Ja [Source:NCBI gene;Acc:394189]	25	31900825	31907672		12.88	13.85	12.47	14.67	13.13	14.26
ENSDARG00000012609	hpxa	hemopexin a [Source:NCBI gene;Acc:327588]	9	32840114	32847642		0.1	0.12	0.31	0.91	1.49	1.2
ENSDARG00000012610	saga	S-antigen; retina and pineal gland (arrestin) a [Source:ZFIN;Acc:ZDB-GENE-040426-1538]	2	5480125	5505094		33.03	6.79	6.23	20.9	23.63	24.92
ENSDARG00000012625	prkag2a	protein kinase, AMP-activated, gamma 2 non-catalytic subunit a [Source:ZFIN;Acc:ZDB-GENE-060929-160]	24	28252520	28381437		4.9	4.37	4.02	5.03	5.24	4.93
ENSDARG00000012627	cdc34b	cell division cycle 34 homolog (S. cerevisiae) b [Source:ZFIN;Acc:ZDB-GENE-040718-439]	2	49007592	49031303		13.92	9.64	9.53	18.39	18.54	18.59
ENSDARG00000012632	zgc:92749	zgc:92749 [Source:ZFIN;Acc:ZDB-GENE-040718-309]	12	48613795	48671612		2.07	1.2	1.89	0.8	0.75	0.96
ENSDARG00000012640	cideb	cell death inducing DFFA like effector b [Source:ZFIN;Acc:ZDB-GENE-080514-2]	7	24015507	24022340		17.43	27.57	26.37	7.76	9.65	7.94
ENSDARG00000012642	si:ch211-266d19.4	si:ch211-266d19.4 [Source:ZFIN;Acc:ZDB-GENE-091204-74]	24	2318351	2341088		5.21	2.87	2.47	5.93	5.77	5.71
ENSDARG00000012649	smad4b	SMAD family member 4b [Source:ZFIN;Acc:ZDB-GENE-060130-140]	5	6875272	6922168		6.84	3.84	3.33	9.16	9.33	9.79
ENSDARG00000012656	atf6	activating transcription factor 6 [Source:ZFIN;Acc:ZDB-GENE-041014-328]	20	33809434	33909872		10.66	6.91	5.17	11.17	11.67	10.89
ENSDARG00000012667	tfap2b	transcription factor AP-2 beta [Source:ZFIN;Acc:ZDB-GENE-050417-394]	20	47686629	47704973		12.58	3.27	4.21	13.88	13.37	13.31
ENSDARG00000012671	inhbaa	inhibin subunit beta Aa [Source:ZFIN;Acc:ZDB-GENE-000210-21]	24	3050020	3062610		1.68	1.21	0.99	3.67	3.4	4.15
ENSDARG00000012672	gtf2e2	general transcription factor IIE, polypeptide 2, beta [Source:ZFIN;Acc:ZDB-GENE-030131-6794]	1	22851261	22876174		4.48	4.65	4.52	3.58	3.45	3.99
ENSDARG00000012674	rpusd4	RNA pseudouridine synthase D4 [Source:NCBI gene;Acc:445101]	11	17964689	18081864		2.19	2.85	2.49	1.54	1.66	1.8
ENSDARG00000012682	pfn2l	profilin 2 like [Source:ZFIN;Acc:ZDB-GENE-030131-34]	2	10147029	10151839		72.79	63.28	60.65	81.71	78.82	74.17
ENSDARG00000012684	atp2b1a	ATPase plasma membrane Ca2+ transporting 1a [Source:ZFIN;Acc:ZDB-GENE-030925-29]	4	16076719	16124419		10.55	6.15	5.11	12.2	11.49	12.1

ENSDARG00000012688	eif1	eukaryotic translation initiation factor 1 [Source:ZFIN;Acc:ZDB-GENE-030131-956]	19	5418006	5421610	169.41	155.08	152.3	246.09	254.6	265.85
ENSDARG00000012694	c3a.1	complement component c3a, duplicate 1 [Source:ZFIN;Acc:ZDB-GENE-990415-35]	1	56130222	56176976	91.51	139.97	123.88	174.18	181.49	189.24
ENSDARG00000012699	bbx	BBX high mobility group box domain containing [Source:ZFIN;Acc:ZDB-GENE-091204-306]	10	28470307	28525611	2.85	1.84	1.43	3.73	3.84	4.12
ENSDARG00000012718	wee2	WEE1 homolog 2 (S. pombe) [Source:NCBI gene;Acc:327471]	18	20560442	20565982	0.02	0.06	0.05	0.03	0.07	0.02
ENSDARG00000012723	rbm14a	RNA binding motif protein 14a [Source:ZFIN;Acc:ZDB-GENE-050522-496]	21	27370671	27378961	14.57	10.28	8.14	21.48	20.94	20.54
ENSDARG00000012726	calhm5.2	calcium homeostasis modulator family member 5, tandem duplicate 2 [Source:ZFIN;Acc:ZDB-GENE-140107-2]	16	32205660	32208728	1.61	1.54	1.35	1.41	0.9	0.62
ENSDARG00000012729	hcls1	hematopoietic cell-specific Lyn substrate 1 [Source:ZFIN;Acc:ZDB-GENE-030131-7191]	4	22300330	22311610	17.8	21.42	15.53	12.9	11.41	11.58
ENSDARG00000012741	maco1b	macoilin 1b [Source:NCBI gene;Acc:393435]	13	45524475	45572062	15.07	8.19	8.13	17.07	17.21	16.99
ENSDARG00000012745	poc1bl	POC1 centriolar protein homolog B (Chlamydomonas), like [Source:ZFIN;Acc:ZDB-GENE-050306-41]	19	34169055	34218156	6.23	4.79	3.6	4.62	4.78	4.75
ENSDARG00000012747	rer1	retention in endoplasmic reticulum sorting receptor 1 [Source:ZFIN;Acc:ZDB-GENE-040426-1494]	8	48942470	48961405	7.69	7.43	7.29	8.51	8.43	7.8
ENSDARG00000012763	arl13b	ADP-ribosylation factor-like 13b [Source:NCBI gene;Acc:286784]	1	33668236	33694795	6.28	3.71	3.53	5.48	5.98	6.1
ENSDARG00000012776	smarca1	SWI/SNF related, matrix associated, actin dependent regulator of chromatin, subfamily a, member 1 [Source:ZFIN;Acc:ZDB-GENE-	5	21852714	21888368	5.24	3.74	3.84	3.87	4.89	4.68
ENSDARG00000012777	nucks1b	nuclear casein kinase and cyclin-dependent kinase substrate 1b [Source:ZFIN;Acc:ZDB-GENE-030131-4544]	23	18118535	18130264	74.1	60.87	66.77	48.89	45.95	46.41
ENSDARG00000012788	foxa3	forkhead box A3 [Source:ZFIN;Acc:ZDB-GENE-980526-423]	18	46350574	46354269	3.74	3.69	2.45	6.62	7.1	6.53
ENSDARG00000012789	plek2	pleckstrin 2 [Source:ZFIN;Acc:ZDB-GENE-080219-2]	13	33123484	33134611	1.22	1.52	1.2	1.45	1.19	1.45
ENSDARG00000012790	tmem161a	transmembrane protein 161A [Source:NCBI gene;Acc:492692]	22	18230864	18241390	7.13	7.38	5.56	7.82	7.57	7.04
ENSDARG00000012796	hnmt	histamine N-methyltransferase [Source:NCBI gene;Acc:445242]	9	10138196	10145880	14.26	23.29	17.35	4.76	5.63	5.17
ENSDARG00000012801	pfkpb	phosphofructokinase, platelet b [Source:ZFIN;Acc:ZDB-GENE-070912-634]	2	48119494	48171441	7.98	2.23	2.28	10.64	11.23	10.95
ENSDARG00000012816	dipk2aa	divergent protein kinase domain 2Aa [Source:ZFIN;Acc:ZDB-GENE-041114-148]	2	8261109	8312199	6.92	2.99	3.09	10	11.03	9.84
ENSDARG00000012818	csnk2a2a	casein kinase 2, alpha prime polypeptide a [Source:ZFIN;Acc:ZDB-GENE-990415-28]	7	56751746	56766973	15.63	11.23	12.8	17.01	18.14	17.8
ENSDARG00000012820	nop56	NOP56 ribonucleoprotein homolog [Source:ZFIN;Acc:ZDB-GENE-040109-1]	21	11958758	11970199	53.4	58.21	56.01	37.84	38.71	37.1
ENSDARG00000012823	dlgap4b	discs, large (Drosophila) homolog-associated protein 4b [Source:ZFIN;Acc:ZDB-GENE-101215-2]	6	1831111	1874664	2.92	1.27	1.26	4.69	5.12	5.08
ENSDARG00000012824	itga3b	integrin, alpha 3b [Source:ZFIN;Acc:ZDB-GENE-050411-55]	12	5587347	5663189	15.47	14.19	10.42	13.85	12.89	12.87
ENSDARG00000012827	mettl3	methyltransferase like 3 [Source:NCBI gene;Acc:100004398]	7	21876548	21887104	5.83	4.23	4.61	3.79	3.9	3.66
ENSDARG00000012829	asah2	N-acylsphingosine amidohydrolase 2 [Source:NCBI gene;Acc:493602]	12	6117682	6142673	12.67	18.91	12.94	1.3	1	1.04
ENSDARG00000012833	foxn3	forkhead box N3 [Source:ZFIN;Acc:ZDB-GENE-060512-39]	17	16564921	16734329	3.23	1.53	1.39	4.02	4.06	3.71
ENSDARG00000012840	alg9	ALG9 alpha-1,2-mannosyltransferase [Source:ZFIN;Acc:ZDB-GENE-040426-1270]	5	57743815	57763179	11.22	5.17	6.85	12.01	11.23	11.64
ENSDARG00000012848	arih2	ariadne homolog 2 (Drosophila) [Source:ZFIN;Acc:ZDB-GENE-040426-2158]	8	26034623	26057401	23.58	16.64	18	31.53	29.72	31.26
ENSDARG00000012860	tmprss4b	transmembrane serine protease 4b [Source:ZFIN;Acc:ZDB-GENE-030131-5862]	5	37890521	37913074	0.1	0.12	0.15	0.11	0.17	0.15
ENSDARG00000012866	picalma	phosphatidylinositol binding clathrin assembly protein a [Source:ZFIN;Acc:ZDB-GENE-040426-761]	10	29137482	29225165	11.11	9.72	8.63	13.51	13.81	13.37
ENSDARG00000012871	npepl1	aminopeptidase like 1 [Source:ZFIN;Acc:ZDB-GENE-050417-177]	6	49742164	49760876	30.64	29.92	27.88	31.51	30.28	30.31
ENSDARG00000012874	snap23.1	synaptosome associated protein 23.1 [Source:ZFIN;Acc:ZDB-GENE-040426-883]	17	33415319	33433097	12.51	13.71	11.14	11.66	11.53	11.66
ENSDARG00000012881	slc4a1a	solute carrier family 4 member 1a (Diego blood group) [Source:ZFIN;Acc:ZDB-GENE-010525-1]	3	20065861	20092124	13.82	16.93	17.99	46.44	54.92	47.24
ENSDARG00000012887	znf143a	zinc finger protein 143a [Source:ZFIN;Acc:ZDB-GENE-041114-154]	7	67220374	67252108	15.38	14.92	17.67	11.06	11.95	11.32
ENSDARG00000012892	ammecr1	AMMECR nuclear protein 1 [Source:ZFIN;Acc:ZDB-GENE-040426-1533]	21	44709280	44731865	17.86	11.93	11.87	27.04	26.81	26.13
ENSDARG00000012896	anos1a	anosmin 1a [Source:ZFIN;Acc:ZDB-GENE-000201-9]	1	31942961	31979510	24.34	7.32	7.1	27.28	25.98	28.41
ENSDARG00000012899	ddx49	DEAD (Asp-Glu-Ala-Asp) box polypeptide 49 [Source:ZFIN;Acc:ZDB-GENE-031030-3]	11	6869312	6880725	6.53	7.18	7.03	5.03	4.38	4.38
ENSDARG00000012903	slc34a2a	solute carrier family 34 member 2a [Source:ZFIN;Acc:ZDB-GENE-000524-1]	1	14432434	14442902	8.74	14.55	11.19	0.81	0.95	1.12
ENSDARG00000012914	arhgef4	Rho guanine nucleotide exchange factor (GEF) 4 [Source:ZFIN;Acc:ZDB-GENE-070912-575]	2	16198735	16359042	9.76	5.16	4.69	9.58	9.02	9.18
ENSDARG00000012915	ech1	enoyl CoA hydratase 1, peroxisomal [Source:ZFIN;Acc:ZDB-GENE-041010-170]	15	6926987	6946286	14.95	22.97	19.99	8.96	9.01	8.33
ENSDARG00000012927	tcea2	transcription elongation factor A (SII), 2 [Source:ZFIN;Acc:ZDB-GENE-040426-985]	23	8718950	8733092	6.57	4.03	5.15	5.93	5.76	6.47
ENSDARG00000012929	eif2d	eukaryotic translation initiation factor 2D [Source:ZFIN;Acc:ZDB-GENE-040426-1226]	8	37447606	37461835	8.05	8.6	7.77	7.43	7.95	7.81
ENSDARG00000012932	mat2b	methionine adenosyltransferase II, beta [Source:NCBI gene;Acc:541347]	21	32061725	32082149	4.64	3.76	3.75	3.3	3.13	3.18
ENSDARG00000012942	itgb5	integrin, beta 5 [Source:ZFIN;Acc:ZDB-GENE-030131-6022]	9	22677503	22748015	9.55	7.6	7.17	11.76	11.45	11.43
ENSDARG00000012944	myhz2	myosin, heavy polypeptide 2, fast muscle specific [Source:ZFIN;Acc:ZDB-GENE-020604-1]	5	32206378	32217579	746.64	893.51	1047.64	766.48	730.95	732.95
ENSDARG00000012945	tnfsf13b	TNF superfamily member 13b [Source:ZFIN;Acc:ZDB-GENE-080204-44]	9	5325015	5337923	0.53	0.25	0.28	0.46	0.43	0.33
ENSDARG00000012946	syap1	synapse associated protein 1 [Source:ZFIN;Acc:ZDB-GENE-040426-917]	23	45860676	45867848	27.3	19.83	19.14	21.08	21.1	20.68
ENSDARG00000012949	scml2	Scm polycomb group protein like 2 [Source:ZFIN;Acc:ZDB-GENE-130530-546]	11	30106182	30158609	7.35	3.87	3.07	9.11	9.05	9.46
ENSDARG00000012953	mob1bb	MOB kinase activator 1Bb [Source:ZFIN;Acc:ZDB-GENE-050522-58]	21	5960443	5972819	9.35	7.28	6.87	7.56	7.43	7.18
ENSDARG00000012968	rhoub	ras homolog family member Ub [Source:ZFIN;Acc:ZDB-GENE-050417-308]	15	8990577	9002155	12.16	13.52	10.47	17.5	17.5	16.14
ENSDARG00000012972	cfl1l	cofilin 1 (non-muscle), like [Source:ZFIN;Acc:ZDB-GENE-040426-2770]	13	33606739	33609427	397.01	459.43	475.59	230.8	235.01	240.26
ENSDARG00000012981	osbpl7	oxysterol binding protein-like 7 [Source:ZFIN;Acc:ZDB-GENE-041010-212]	12	28773589	28794957	18.92	6.55	4.97	11.86	11.71	11.52
ENSDARG00000012982	nf1a	neurofibromin 1a [Source:ZFIN;Acc:ZDB-GENE-030131-4907]	15	30861647	30984804	4.95	2.68	2.02	6.31	6.12	6.2
ENSDARG00000012986	adal	adenosine deaminase-like [Source:NCBI gene;Acc:619263]	25	20567821	20574859	1.15	1.44	1.31	1.1	1.1	1.38
ENSDARG00000012987	gpia	glucose-6-phosphate isomerase a [Source:ZFIN;Acc:ZDB-GENE-020513-2]	25	35913614	35938996	94.49	72.39	65.35	133.59	148.28	143.9
ENSDARG00000013000	ppfia2	PTPRF interacting protein alpha 2 [Source:ZFIN;Acc:ZDB-GENE-030131-7905]	4	21962316	22207873	9.34	1.8	2.76	11.95	12.7	12.93
ENSDARG00000013004	tdg.1	thymine DNA glycosylase, tandem duplicate 1 [Source:ZFIN;Acc:ZDB-GENE-050522-44]	25	18964782	18978083	9.89	6.05	7.08	11.81	11.1	11.51
ENSDARG00000013005	opcml	opioid binding protein/cell adhesion molecule-like [Source:ZFIN;Acc:ZDB-GENE-040927-3]	10	30617826	30785501	3.19	1.05	1.52	4.47	5.32	4.46
ENSDARG00000013006	pibf1	progesterone immunomodulatory binding factor 1 [Source:ZFIN;Acc:ZDB-GENE-040801-94]	1	34616054	34685329	0	0	0.01	0	0	0
ENSDARG00000013009	ubiad1	UbiA prenyltransferase domain containing 1 [Source:NCBI gene;Acc:558410]	8	53918234	53926228	13.21	8.73	7.35	12.24	13.13	12.78
ENSDARG00000013014	or101-1	odorant receptor, family B, subfamily 101, member 1 [Source:ZFIN;Acc:ZDB-GENE-990415-190]	21	40313877	40317054	0.28	0.15	0.23	0.16	0.23	0.23
ENSDARG00000013016	pole4	polymerase (DNA-directed), epsilon 4, accessory subunit [Source:ZFIN;Acc:ZDB-GENE-050522-309]	11	6197276	6206625	8.23	8.23	8.52	5.4	6.36	5.99
ENSDARG00000013020	dtnbb	dystrobrevin, beta b [Source:ZFIN;Acc:ZDB-GENE-070117-3]	17	37070096	37156520	8.93	1.83	2.75	10.28	9.58	10.1
ENSDARG00000013022	si:ch211-59h6.1	si:ch211-59h6.1 [Source:ZFIN;Acc:ZDB-GENE-091204-418]	22	7675388	7702958	0	0	0	0.09	0.18	0.06
ENSDARG00000013023	nsun3	NOP2/Sun RNA methyltransferase 3 [Source:NCBI gene;Acc:553534]	1	33697170	33722780	1.65	1.65	1.45	1.4	1.2	1.5
ENSDARG00000013024	erap1a	endoplasmic reticulum aminopeptidase 1a [Source:ZFIN;Acc:ZDB-GENE-091113-7]	10	2899108	2921297	1.75	1.61	1.4	2.3	2.2	1.94
ENSDARG00000013025	ints6	integrator complex subunit 6 [Source:ZFIN;Acc:ZDB-GENE-070906-1]	9	23825440	23841704	2.85	2.06	1.75	3.08	2.94	2.92
ENSDARG00000013031	mta2	metastasis associated 1 family, member 2 [Source:ZFIN;Acc:ZDB-GENE-030131-4803]	7	17907883	17944488	13.06	8.38	6.72	18.92	17.13	17.57
ENSDARG00000013044	ndufv2	NADH:ubiquinone oxidoreductase core subunit V2 [Source:ZFIN;Acc:ZDB-GENE-040426-1713]	2	54641644	54690184	124.27	144.18	164.96	82.79	84.79	84.42
ENSDARG00000013050	abcc12	ATP-binding cassette, sub-family C (CFTR/MRP), member 12 [Source:ZFIN;Acc:ZDB-GENE-050517-25]	7	38158604	38183331	2.99	3.77	2.67	2.13	2.13	2.22
ENSDARG00000013051	zgc:158412	zgc:158412 [Source:NCBI gene;Acc:566090]	5	30384554	30391820	0.73	0.45	0.53	0.76	0.57	0.73
ENSDARG00000013056	itpk1a	inositol-tetrakisphosphate 1-kinase a [Source:NCBI gene;Acc:406290]	20	27049523	27086143	17.07	24.11	17.47	7.55	7.69	7.37
ENSDARG00000013057	hoxb5a	homeobox B5a [Source:NCBI gene;Acc:30317]	3	23707691	23710095	14.43	9.05	9.62	17.46	18.44	18.34
ENSDARG00000013058	ascc2	activating signal cointegrator 1 complex subunit 2 [Source:ZFIN;Acc:ZDB-GENE-040426-1223]	5	18012154	18035253	6.47	6.09	5.42	6.44	6.55	6.43
ENSDARG00000013063	oxct1a	3-oxoacid CoA transferase 1a [Source:ZFIN;Acc:ZDB-GENE-040723-3]	8	31716872	31818916	30.64	24.52	25.5	18.31	19.39	19.85
ENSDARG00000013072	mmp15b	matrix metallopeptidase 15b [Source:ZFIN;Acc:ZDB-GENE-070817-6]	2	28453338	28521429	3.88	3.31	2.73	6.55	6.69	6.42
ENSDARG00000013075	mrpl11	mitochondrial ribosomal protein L11 [Source:ZFIN;Acc:ZDB-GENE-040718-294]	14	30557361	30620537	24.43	28.67	31.49	18.71	19.68	18.99
ENSDARG00000013076	bmi1b	bmi1 polycomb ring finger oncogene 1b [Source:NCBI gene;Acc:394249]	2	21164930	21170517	8.35	5.29	6.11	9.13	9.21	9.72
ENSDARG00000013078	ywhaba	tyrosine 3-monooxygenase/tryptophan 5-monooxygenase activation protein, beta polypeptide a [Source:NCBI gene;Acc:323055]	6	52212574	52225289	135.95	96.99	97.51	131.99	131.18	131.39
ENSDARG00000013079	tubgcp2	tubulin, gamma complex associated protein 2 [Source:ZFIN;Acc:ZDB-GENE-031118-150]	12	9183626	9218258	8.52	6.7	6.93	6.48	5.74	5.9
ENSDARG00000013082	uap1l1	UDP-N-acetylglucosamine pyrophosphorylase 1, like 1 [Source:NCBI gene;Acc:393264]	5	31811662	31817395	10.29	11.38	11.44	9.98	8.98	9.26
ENSDARG00000013087	ndrg3a	ndrg family member 3a [Source:ZFIN;Acc:ZDB-GENE-980526-124]	11	25064519	25140425	40.23	22.51	24.95	39.86	41.62	42.14
ENSDARG00000013095	gclc	glutamate-cysteine ligase, catalytic subunit [Source:ZFIN;Acc:ZDB-GENE-030131-5056]	13	2357637	2392664	14.34	14.47	11.29	12.73	12.79	12.75
ENSDARG00000013110	dmtn	dematin actin binding protein [Source:ZFIN;Acc:ZDB-GENE-030131-9438]	10	20108557	20146544	10.16	3.13	4.73	11.2	11.86	11.84
ENSDARG00000013117	hiat1a	hippocampus abundant transcript 1a [Source:ZFIN;Acc:ZDB-GENE-030131-834]	2	37656078	37684641	7.07	4.41	3.61	8.32	7.68	7.92
ENSDARG00000013122	maf1	MAF1 homolog, negative regulator of RNA polymerase III [Source:NCBI gene;Acc:406750]	11	24046179	24056609	11.26	7.8	7.58	11.56	11.9	12.29
ENSDARG00000013125	dlx1a	distal-less homeobox 1a [Source:NCBI gene;Acc:30568]	9	3388099	3390109	11.03	2.82	3.67	7.74	7.75	8.72
ENSDARG00000013128	pdk1	pyruvate dehydrogenase kinase, isozyme 1 [Source:ZFIN;Acc:ZDB-GENE-091204-402]	9	3135062	3149896	12.22	11.9	13.39	14.15	12.76	12.38
ENSDARG00000013134	prph2la	peripherin 2-like a [Source:ZFIN;Acc:ZDB-GENE-130530-548]	17	39873584	39886628	2.61	0.71	0.55	3.45	3.26	4.12
ENSDARG00000013144	atp1b1a	ATPase Na+/K+ transporting subunit beta 1a [Source:ZFIN;Acc:ZDB-GENE-001127-3]	6	29217392	29227487	107.05	138.58	105.95	142.28	146.03	144.68
ENSDARG00000013150	dhx16	DEAH (Asp-Glu-Ala-His) box polypeptide 16 [Source:ZFIN;Acc:ZDB-GENE-030131-8589]	15	34899645	34934784	7.82	6.79	6.22	6.23	6.77	6.31
ENSDARG00000013153	dennd3b	DENN/MADD domain containing 3b [Source:ZFIN;Acc:ZDB-GENE-091230-7]	16	30871614	30903930	0.05	0.05	0.05	0.05	0.11	0.09
ENSDARG00000013161	zgc:55558	zgc:55558 [Source:ZFIN;Acc:ZDB-GENE-040426-740]	3	16662209	16683623	6.03	4.43	4.58	7.04	6.85	7.27
ENSDARG00000013168	jag1b	jagged canonical Notch ligand 1b [Source:NCBI gene;Acc:140423]	13	35339182	35390095	3.96	3.9	2.65	4.3	4.26	4
ENSDARG00000013174	nkd3	NKD inhibitor of WNT signaling pathway 3 [Source:ZFIN;Acc:ZDB-GENE-060503-63]	19	44009473	44036792	6.75	6.32	7	4.54	5.3	5.29
ENSDARG00000013203	si:ch211-1f22.1	si:ch211-1f22.1 [Source:ZFIN;Acc:ZDB-GENE-121214-79]	1	56943492	56948694	0.14	0.14	0.16	0.21	0.15	0.29
ENSDARG00000013207	zeb1b	zinc finger E-box binding homeobox 1b [Source:ZFIN;Acc:ZDB-GENE-010621-1]	12	26750274	26851726	7.31	5.68	5.33	9.56	9.09	8.78
ENSDARG00000013210	htr2cl2	5-hydroxytryptamine (serotonin) receptor 2C, G protein-coupled-like 2 [Source:ZFIN;Acc:ZDB-GENE-120215-109]	21	43609452	43616586	0.41	0.26	0.2	0.46	0.56	0.46
ENSDARG00000013221	pde4ca	phosphodiesterase 4C, cAMP-specific a [Source:ZFIN;Acc:ZDB-GENE-070912-373]	2	24781026	24866649	0.79	0.48	0.44	1.05	1.09	1.16
ENSDARG00000013222	slc35a3a	solute carrier family 35 member A3a [Source:ZFIN;Acc:ZDB-GENE-081105-80]	8	19621511	19638362	5.04	4.93	4	3.98	3.79	4.05
ENSDARG00000013224	phc2b	polyhomeotic homolog 2b (Drosophila) [Source:ZFIN;Acc:ZDB-GENE-040426-1113]	6	48154954	48199276	7.76	3.52	3.72	10.79	10.09	10.06
ENSDARG00000013227	glulc	glutamate-ammonia ligase (glutamine synthase) c [Source:ZFIN;Acc:ZDB-GENE-060929-540]	21	14768411	14775724	1.26	0.75	1.02	0.69	1.06	0.76
ENSDARG00000013236	dhcr24	24-dehydrocholesterol reductase [Source:ZFIN;Acc:ZDB-GENE-041212-73]	20	7149100	7176810	5.63	5.27	5.22	3.26	2.81	3.14
ENSDARG00000013240	zgc:172271	zgc:172271 [Source:ZFIN;Acc:ZDB-GENE-080204-110]	6	40884615	40899618	3.08	4.22	4.52	2.69	2.63	2.51
ENSDARG00000013245	wbp1la	WW domain binding protein 1-like a [Source:ZFIN;Acc:ZDB-GENE-030131-1961]	13	28580357	28596765	5.99	6.14	4.3	7.55	6.89	6.87
ENSDARG00000013250	tars1	threonyl-tRNA synthetase 1 [Source:ZFIN;Acc:ZDB-GENE-041010-218]	5	44346691	44371177	37.62	42.22	38.55	36.66	38.22	37.22
ENSDARG00000013251	hsf4	heat shock transcription factor 4 [Source:ZFIN;Acc:ZDB-GENE-050306-18]	18	22739732	22753637	0	0.08	0.11	0.46	0.59	0.63
ENSDARG00000013252	tmc5	transmembrane channel like 5 [Source:ZFIN;Acc:ZDB-GENE-120411-6]	3	26734162	26751819	2.29	2.64	2.48	1.72	1.74	1.68
ENSDARG00000013266	colec11	collectin sub-family member 11 [Source:NCBI gene;Acc:492459]	17	36516695	36529449	0.97	1.58	1.41	1.17	1.18	1.09
ENSDARG00000013274	eif4eb	eukaryotic translation initiation factor 4eb [Source:ZFIN;Acc:ZDB-GENE-041121-14]	13	12671513	12681979	8.85	11.28	9.77	8.6	7.62	6.79
ENSDARG00000013279	znf76	zinc finger protein 76 [Source:ZFIN;Acc:ZDB-GENE-061027-379]	22	737211	753392	4.69	3.3	3.55	5.35	5.38	5.26
ENSDARG00000013292	tmem179aa	transmembrane protein 179a, genome duplicate A [Source:NCBI gene;Acc:445310]	20	27393668	27396568	10.32	6.08	8.03	10.18	9.78	9.96
ENSDARG00000013293	crispld1b	cysteine-rich secretory protein LCCL domain containing 1b [Source:ZFIN;Acc:ZDB-GENE-040426-1204]	2	30379650	30395993	8.91	11.04	9.25	9.21	9.88	9.71
ENSDARG00000013295	slc2a3a	solute carrier family 2 member 3a [Source:ZFIN;Acc:ZDB-GENE-040718-390]	19	9685165	9712530	22.68	11.7	11.56	35.22	41.55	39.83
ENSDARG00000013302	gpx8	glutathione peroxidase 8 (putative) [Source:NCBI gene;Acc:393191]	8	40177361	40183197	2.7	3.46	3.57	5.19	4.74	4.96
ENSDARG00000013307	rpl19	ribosomal protein L19 [Source:NCBI gene;Acc:406489]	3	16229911	16232442	994.04	1203.7	1358.78	913.07	942.7	941.8
ENSDARG00000013310	map3k15	mitogen-activated protein kinase kinase kinase 15 [Source:ZFIN;Acc:ZDB-GENE-091204-246]	24	25913162	25989500	2.59	2.61	2.17	3.73	3.75	3.48
ENSDARG00000013312	cadps2	Ca++-dependent secretion activator 2 [Source:ZFIN;Acc:ZDB-GENE-030903-1]	25	27937585	28269953	2.45	1.34	1.37	2.34	2.51	2.61
ENSDARG00000013317	pygmb	phosphorylase, glycogen, muscle b [Source:ZFIN;Acc:ZDB-GENE-040426-1206]	7	22823889	22852189	26.92	8.61	9.85	45.6	47.56	45.42
ENSDARG00000013329	ttc5	tetratricopeptide repeat domain 5 [Source:ZFIN;Acc:ZDB-GENE-050417-137]	7	19528265	19538406	12.82	8.8	9.93	8.39	8.98	8.7
ENSDARG00000013333	ndufa10	NADH:ubiquinone oxidoreductase subunit A10 [Source:ZFIN;Acc:ZDB-GENE-030131-670]	9	23740506	23747409	118.46	152.84	150.78	83.27	85.14	86.14
ENSDARG00000013335	anxa6	annexin A6 [Source:ZFIN;Acc:ZDB-GENE-030707-3]	14	49251331	49360686	25.6	34.75	39.02	41.3	40.77	40.63
ENSDARG00000013343	kif26ba	kinesin family member 26Ba [Source:ZFIN;Acc:ZDB-GENE-070521-4]	17	11507131	11718491	2.77	2.15	1.7	7.48	7.37	7.24
ENSDARG00000013346	tasorb	transcription activation suppressor b [Source:ZFIN;Acc:ZDB-GENE-090311-29]	23	19473935	19486571	9.61	6.09	5.11	13.33	13.51	13.63
ENSDARG00000013351	cirbpb	cold inducible RNA binding protein b [Source:ZFIN;Acc:ZDB-GENE-030131-5841]	22	18542010	18546649	412.83	304.99	351.06	465.47	468.8	468.15
ENSDARG00000013360	sh3gl3a	SH3-domain GRB2-like 3a [Source:ZFIN;Acc:ZDB-GENE-070112-242]	7	12840556	12909352	2.69	1.71	1.81	4.51	4.86	4.72
ENSDARG00000013371	isoc2	isochorismatase domain containing 2 [Source:NCBI gene;Acc:492617]	16	12793911	12809886	22.65	31.91	29.82	14.59	14.87	16.15
ENSDARG00000013379	ppp6r3	protein phosphatase 6, regulatory subunit 3 [Source:ZFIN;Acc:ZDB-GENE-030131-3251]	25	10602193	10630592	21.95	15.83	15.18	21.99	21.73	21.36
ENSDARG00000013382	cwc15	CWC15 spliceosome associated protein homolog [Source:NCBI gene;Acc:415140]	5	23576328	23583446	1.34	1.63	0.79	2.07	1.71	2.28
ENSDARG00000013389	gabra4	gamma-aminobutyric acid (GABA) A receptor, subunit alpha 4 [Source:ZFIN;Acc:ZDB-GENE-050417-360]	14	4610439	4682114	1.39	0.32	0.19	1.47	1.38	1.36
ENSDARG00000013390	CABZ01088367.1		6	59029485	59048088	3.86	0.85	0.76	3.29	4.19	4.25
ENSDARG00000013393	guca1b	guanylate cyclase activator 1B [Source:ZFIN;Acc:ZDB-GENE-011128-6]	23	19227296	19230627	2.99	1.1	0.84	1.59	1.64	2.02
ENSDARG00000013397	bnip2	BCL2 interacting protein 2 [Source:ZFIN;Acc:ZDB-GENE-040109-6]	25	34246625	34268439	8.29	8.95	7.48	9.26	8.87	9.52
ENSDARG00000013409	bmp2a	bone morphogenetic protein 2a [Source:ZFIN;Acc:ZDB-GENE-980526-388]	17	4227254	4231532	5.27	4.78	4.83	4.06	3.76	4.48
ENSDARG00000013414	lin7a	lin-7 homolog A (C. elegans) [Source:ZFIN;Acc:ZDB-GENE-040426-1665]	4	21751762	21851473	23.64	8.46	11.02	23.68	22.96	23.32
ENSDARG00000013415	lmna	lamin A [Source:ZFIN;Acc:ZDB-GENE-020424-3]	16	30520357	30570161	3.72	3.61	3.48	3.11	3.47	3.3
ENSDARG00000013422	slc8a1a	solute carrier family 8 member 1a [Source:ZFIN;Acc:ZDB-GENE-050809-1]	11	30535943	30612166	0.88	0.76	0.57	2.17	2.27	1.98
ENSDARG00000013430	bhmt	betaine-homocysteine methyltransferase [Source:NCBI gene;Acc:322228]	21	40408	45920	1171.6	1779.17	1988.66	1901.29	2091.01	1995.45
ENSDARG00000013434	hira	histone cell cycle regulator a [Source:ZFIN;Acc:ZDB-GENE-030131-6296]	5	18047111	18072844	6.35	4.48	4.39	6.73	6.93	6.92
ENSDARG00000013438	sycp3	synaptonemal complex protein 3 [Source:ZFIN;Acc:ZDB-GENE-040910-2]	25	19578048	19585010	0.4	0.32	0.36	0.39	0.29	0.17
ENSDARG00000013441	hey2	hes-related family bHLH transcription factor with YRPW motif 2 [Source:NCBI gene;Acc:58146]	20	39588897	39596338	2.85	2.7	3.07	3.21	3.39	3.24
ENSDARG00000013443	atp6v1ba	ATPase, H+ transporting, lysosomal, V1 subunit B, member a [Source:ZFIN;Acc:ZDB-GENE-030711-3]	17	21372291	21418340	81.77	57.42	60.48	72.39	73.74	67.47
ENSDARG00000013453	tdrd9	tudor domain containing 9 [Source:NCBI gene;Acc:553222]	13	31402067	31423346	0.41	0.5	0.54	0.64	0.74	0.47
ENSDARG00000013457	mtrex	Mtr4 exosome RNA helicase [Source:ZFIN;Acc:ZDB-GENE-040426-2854]	10	8197827	8266094	13.77	11.5	10.89	10.63	10.75	10.65
ENSDARG00000013460	wfikkn2a	info WAP, follistatin/kazal, immunoglobulin, kunitz and netrin domain containing 2a [Source:ZFIN;Acc:ZDB-GENE-120914-1]	3	18097700	18104658	3.76	3.07	3.07	6.79	6.92	7.87
ENSDARG00000013461	supt7l	SPT7 like, STAGA complex gamma subunit [Source:ZFIN;Acc:ZDB-GENE-050227-17]	17	40860016	40879108	3.52	2.42	2.16	3.04	3.14	2.9
ENSDARG00000013472	nedd1	NEDD1 gamma-tubulin ring complex targeting factor [Source:ZFIN;Acc:ZDB-GENE-040426-2906]	25	19541019	19556986	3.83	3.83	3.51	4.25	4.25	3.72
ENSDARG00000013475	cct4	chaperonin containing TCP1, subunit 4 (delta) [Source:ZFIN;Acc:ZDB-GENE-040426-1421]	1	53701801	53714885	186.75	182.43	201.09	154.96	167.31	158.39
ENSDARG00000013476	arhgef39	Rho guanine nucleotide exchange factor (GEF) 39 [Source:ZFIN;Acc:ZDB-GENE-041114-28]	4	12401146	12477278	1.37	1.52	1.12	1.32	1.08	0.96
ENSDARG00000013477	gata1a	GATA binding protein 1a [Source:ZFIN;Acc:ZDB-GENE-980526-268]	11	25411914	25418856	1.36	1.61	1.54	2.07	2.66	2.08
ENSDARG00000013478	rrp15	ribosomal RNA processing 15 homolog [Source:NCBI gene;Acc:327053]	16	4196932	4203022	21.58	23.37	23.35	12.76	12.64	11.44
ENSDARG00000013491	map3k9	mitogen-activated protein kinase kinase kinase 9 [Source:ZFIN;Acc:ZDB-GENE-081113-5]	13	36164639	36184701	3.27	0.69	0.79	4.44	3.9	3.69
ENSDARG00000013492	zbtb34	zinc finger and BTB domain containing 34 [Source:ZFIN;Acc:ZDB-GENE-050624-2]	8	33132502	33154677	1.49	1.37	1.37	2.85	2.53	2.33
ENSDARG00000013500	abca2	ATP-binding cassette, sub-family A (ABC1), member 2 [Source:ZFIN;Acc:ZDB-GENE-050517-1]	5	25088622	25236340	4.71	1.54	1.4	5.91	5.98	6.14
ENSDARG00000013505	ube2kb	ubiquitin-conjugating enzyme E2Kb (UBC1 homolog, yeast) [Source:ZFIN;Acc:ZDB-GENE-980605-9]	1	23311617	23322969	18.65	15.17	15.04	18.18	16.59	18.27
ENSDARG00000013522	pck1	phosphoenolpyruvate carboxykinase 1 (soluble) [Source:ZFIN;Acc:ZDB-GENE-030909-11]	6	60055168	60062097	85.83	135.28	134.48	212.36	263.59	248.96
ENSDARG00000013524	cyp2ae1	cytochrome P450, family 2, subfamily AE, polypeptide 1 [Source:ZFIN;Acc:ZDB-GENE-090312-107]	23	42643960	42666469	0.78	1	0.92	1.53	1.37	1.29
ENSDARG00000013526	svep1	sushi, von Willebrand factor type A, EGF and pentraxin domain containing 1 [Source:ZFIN;Acc:ZDB-GENE-110726-1]	7	24251367	24357169	2.38	2.49	1.67	6.42	6.29	6.28
ENSDARG00000013528	mcm9	minichromosome maintenance 9 homologous recombination repair factor [Source:NCBI gene;Acc:555610]	20	41801913	41839744	1.16	0.71	0.67	0.55	0.57	0.63
ENSDARG00000013533	hoxb4a	homeobox B4a [Source:NCBI gene;Acc:30340]	3	23710839	23723982	6.14	4.12	3.56	10.36	9.7	10.37
ENSDARG00000013539	ikzf1	IKAROS family zinc finger 1 (Ikaros) [Source:ZFIN;Acc:ZDB-GENE-980526-304]	13	15957508	15994472	1.6	1.17	1.17	1.74	1.8	1.57
ENSDARG00000013542	lpgat1	lysophosphatidylglycerol acyltransferase 1 [Source:ZFIN;Acc:ZDB-GENE-030131-2906]	20	13781779	13874340	4.81	3.44	2.91	9.41	7.91	8.92
ENSDARG00000013561	pgm1	phosphoglucomutase 1 [Source:ZFIN;Acc:ZDB-GENE-040426-1245]	6	32010183	32025225	83	96.3	95.66	85.67	92.03	88.03
ENSDARG00000013575	rfx2	regulatory factor X, 2 (influences HLA class II expression) [Source:NCBI gene;Acc:503590]	8	20072987	20138251	4.38	3.18	2.44	6.68	7.1	7.09
ENSDARG00000013576	gadd45bb	growth arrest and DNA-damage-inducible, beta b [Source:ZFIN;Acc:ZDB-GENE-050223-1]	2	36939749	36941988	4.38	4.66	4.38	6.6	5.94	6.76
ENSDARG00000013582	csnk2a2b	casein kinase 2, alpha prime polypeptide b [Source:ZFIN;Acc:ZDB-GENE-090406-4]	25	13639232	13659249	15.43	9.35	8.11	20.16	20	19.62
ENSDARG00000013596	brd1a	bromodomain containing 1a [Source:ZFIN;Acc:ZDB-GENE-041008-168]	4	27130412	27150414	5.02	1.9	3.16	5.87	5.99	5.84
ENSDARG00000013598	tnfb	tumor necrosis factor b (TNF superfamily, member 2) [Source:ZFIN;Acc:ZDB-GENE-050601-2]	15	34699585	34702661	0.37	0.56	0.58	0.68	0.58	0.75
ENSDARG00000013607	sema3gb	sema domain, immunoglobulin domain (Ig), short basic domain, secreted, (semaphorin) 3Gb [Source:ZFIN;Acc:ZDB-GENE-050513-	8	23485079	23553859	9.14	6.99	5.5	12.27	12.16	12.18
ENSDARG00000013613	anxa13l	annexin A13, like [Source:ZFIN;Acc:ZDB-GENE-050522-310]	2	9821757	9842240	10.51	10.84	10.36	10.25	11.4	11.42
ENSDARG00000013615	pbx3b	pre-B-cell leukemia homeobox 3b [Source:ZFIN;Acc:ZDB-GENE-000405-3]	8	33895773	34065573	23.9	8.27	10.71	36.76	38.42	38.76
ENSDARG00000013623	vdac2	voltage-dependent anion channel 2 [Source:ZFIN;Acc:ZDB-GENE-030131-845]	13	17714296	17729474	663.96	760.45	777.18	627.95	639.84	623.86
ENSDARG00000013628	cd164	CD164 molecule, sialomucin [Source:ZFIN;Acc:ZDB-GENE-030131-1598]	20	32118559	32139062	25.04	32.24	28.34	34.71	33.99	31.51
ENSDARG00000013647	cntn3a.2	contactin 3a, tandem duplicate 2 [Source:ZFIN;Acc:ZDB-GENE-090312-57]	23	11014693	11130683	1.13	0.3	0.44	1.41	1.39	1.27
ENSDARG00000013649	casr	calcium-sensing receptor [Source:ZFIN;Acc:ZDB-GENE-050119-8]	5	67768162	67783593	0.01	0.01	0.02	0.04	0.06	0.04
ENSDARG00000013653	adgrl4	adhesion G protein-coupled receptor L4 [Source:NCBI gene;Acc:406676]	11	7383177	7411436	0.91	1.21	1.13	1.7	1.59	2.06
ENSDARG00000013655	tpd52l2b	tpd52 like 2b [Source:ZFIN;Acc:ZDB-GENE-030131-822]	23	17431345	17451264	29.31	30.6	28.4	29.9	30.73	28.91
ENSDARG00000013659	brox	BRO1 domain and CAAX motif containing [Source:NCBI gene;Acc:492786]	20	51700633	51727860	15.78	8.64	9.25	16.24	17.07	15.97
ENSDARG00000013667	tbck	TBC1 domain containing kinase [Source:ZFIN;Acc:ZDB-GENE-070112-1062]	1	50198607	50293948	6.48	4.25	3.96	6.05	6.15	6.49
ENSDARG00000013669	napba	N-ethylmaleimide-sensitive factor attachment protein, beta a [Source:ZFIN;Acc:ZDB-GENE-050522-230]	17	4297076	4318428	24.9	5.54	7.24	36.43	37.67	36.93
ENSDARG00000013670	hyou1	hypoxia up-regulated 1 [Source:NCBI gene;Acc:327133]	10	29770120	29791466	20.83	21.48	15.92	21.45	20.01	18.83
ENSDARG00000013681	si:dkey-42i9.6	si:dkey-42i9.6 [Source:ZFIN;Acc:ZDB-GENE-030131-6596]	22	10560686	10570749	25.03	25.39	20.89	34.82	33.58	33.92
ENSDARG00000013685	vsig8b	V-set and immunoglobulin domain containing 8b [Source:ZFIN;Acc:ZDB-GENE-081104-51]	8	6576940	6609288	7.05	5.75	3.73	4.49	4.33	4.39

**ENSDARG00000013687**	cilp2	cartilage intermediate layer protein 2 [Source:ZFIN;Acc:ZDB-GENE-050208-436]	22	18469004	18485470		3.85	5.12	3.87	8.23	8.23	8.3
**ENSDARG00000013694**	stimate	STIM activating enhance [Source:ZFIN;Acc:ZDB-GENE-040426-1198]	11	18270313	18283886		5.55	4.1	4.66	6.36	6.08	5.79
**ENSDARG00000013704**	cers2a	ceramide synthase 2a [Source:ZFIN;Acc:ZDB-GENE-020808-2]	19	8758336	8812963		0.7	0.67	0.61	1.23	1.26	1.06
**ENSDARG00000013705**	ccm2	CCM2 scaffold protein [Source:NCBI gene;Acc:436586]	20	18774129	18794789		3.89	3.62	3.08	5.94	5.74	5.31
**ENSDARG00000013708**	usp9	ubiquitin specific peptidase 9 [Source:ZFIN;Acc:ZDB-GENE-061019-1]	9	33261330	33321040		19.31	14.01	10.91	28.85	27.28	27.7
**ENSDARG00000013711**	zgc:77486	zgc:77486 [Source:ZFIN;Acc:ZDB-GENE-040426-2056]	8	19268474	19280856		2.69	3.84	3.48	3.64	4.03	3.58
**ENSDARG00000013719**	gtse1	G-2 and S-phase expressed 1 [Source:ZFIN;Acc:ZDB-GENE-050522-493]	25	19594160	19608460		2.16	1.46	1.45	1.8	1.61	1.67
**ENSDARG00000013721**	g6pca.2	glucose-6-phosphatase a, catalytic subunit, tandem duplicate 2 [Source:ZFIN;Acc:ZDB-GENE-050309-17]	12	6041575	6048286		35.62	46.78	43.56	60.27	68.28	61.79
**ENSDARG00000013726**	ap4b1	adaptor related protein complex 4 subunit beta 1 [Source:ZFIN;Acc:ZDB-GENE-040426-1284]	23	354418	367693		4.93	3.86	3.33	5.13	5.37	5.32
**ENSDARG00000013729**	srsf6a	serine and arginine rich splicing factor 6a [Source:ZFIN;Acc:ZDB-GENE-040426-2824]	23	4260458	4271984		74.31	52.16	46.99	68.95	69.01	68.68
**ENSDARG00000013730**	slc4a4a	solute carrier family 4 member 4a [Source:ZFIN;Acc:ZDB-GENE-060526-274]	5	45746457	45877387		10.46	5.75	4.83	14.4	13.88	15.03
**ENSDARG00000013732**	vta1	vesicle (multivesicular body) trafficking 1 [Source:ZFIN;Acc:ZDB-GENE-040426-2154]	20	37300296	37379578		8.13	7.17	6.5	7.18	6.41	6.96
**ENSDARG00000013734**	polr3glb	polymerase (RNA) III (DNA directed) polypeptide G like b [Source:ZFIN;Acc:ZDB-GENE-041010-32]	16	42829735	42843397		14.59	10.87	10.3	10.12	10.9	12.61
**ENSDARG00000013741**	lancl1	LanC antibiotic synthetase component C-like 1 (bacterial) [Source:NCBI gene;Acc:494154]	1	7517449	7539716		4.13	2.36	2.71	3.77	4.23	4.48
**ENSDARG00000013749**	slc25a55b	solute carrier family 25 member 55b [Source:ZFIN;Acc:ZDB-GENE-040801-184]	23	409578	419441		22.03	28.77	33.44	9.69	7.75	8.98
**ENSDARG00000013750**	eno1b	enolase 1b, (alpha) [Source:ZFIN;Acc:ZDB-GENE-040426-1651]	6	40661703	40669406		55.4	20.12	21.75	33.81	35.78	33.37
**ENSDARG00000013752**	tnni2a.3	troponin I type 2a (skeletal, fast), tandem duplicate 3 [Source:ZFIN;Acc:ZDB-GENE-040718-80]	25	31264155	31275944		1.22	1.17	1.5	2.09	2.18	1.78
**ENSDARG00000013755**	actn3a	actinin alpha 3a [Source:ZFIN;Acc:ZDB-GENE-000329-9]	21	27382893	27404978		324.88	498.98	582.57	403.55	366.4	348.72
**ENSDARG00000013763**	rrbp1a	ribosome binding protein 1a [Source:ZFIN;Acc:ZDB-GENE-030131-9795]	13	33611703	33639050		9.3	11.92	7.22	14.65	14.21	13.5
**ENSDARG00000013771**	ctss2.2	cathepsin S, ortholog 2, tandem duplicate 2 [Source:ZFIN;Acc:ZDB-GENE-050626-55]	16	29514473	29519977		10.27	11.81	10.32	8.45	7.53	6.59
**ENSDARG00000013775**	si:dkey-246g23.4	si:dkey-246g23.4 [Source:ZFIN;Acc:ZDB-GENE-050419-234]	18	14564085	14579787		2.29	2.6	2.03	1.47	1.9	2
**ENSDARG00000013776**	thop1	thimet oligopeptidase 1 [Source:ZFIN;Acc:ZDB-GENE-040718-47]	11	7183025	7212097		27.63	24.18	23.23	20.73	19.53	20.37
**ENSDARG00000013777**	ppp1r13l	protein phosphatase 1, regulatory subunit 13 like [Source:ZFIN;Acc:ZDB-GENE-050327-33]	18	35842933	35899617		11.62	10.76	7.94	10.05	9.97	9.61
**ENSDARG00000013784**	zgc:77158	zgc:77158 [Source:ZFIN;Acc:ZDB-GENE-040426-1799]	25	16880990	16941619		1.74	2.1	1.71	4.03	4.08	4.04
**ENSDARG00000013787**	gucy1a1	guanylate cyclase 1 soluble subunit alpha 1 [Source:ZFIN;Acc:ZDB-GENE-050417-230]	1	25783801	25799051		6.18	4.59	4.03	5.17	5.36	5.61
**ENSDARG00000013789**	sim1b	SIM bHLH transcription factor 1b [Source:ZFIN;Acc:ZDB-GENE-041210-5]	4	929557	948842		1.07	0.79	0.37	1.71	1.8	1.59
**ENSDARG00000013794**	klf11b	Kruppel-like factor 11b [Source:ZFIN;Acc:ZDB-GENE-061103-82]	17	32365941	32370047		5.46	6.03	4.88	9.11	9.61	9.37
**ENSDARG00000013795**	hps4	HPS4 biogenesis of lysosomal organelles complex 3 subunit 2 [Source:ZFIN;Acc:ZDB-GENE-040426-1068]	8	39767915	39793396		1.3	1.44	1.38	1.71	1.87	2
**ENSDARG00000013800**	snrpd3	small nuclear ribonucleoprotein D3 polypeptide [Source:ZFIN;Acc:ZDB-GENE-040625-55]	8	30742898	30745598		0.04	0.03	0.06	0	0.08	0.15
**ENSDARG00000013802**	pcid2	PCI domain containing 2 [Source:NCBI gene;Acc:492509]	1	152007	157563		10.34	8.85	9.03	6.79	7.17	6.56
**ENSDARG00000013804**	capns1b	calpain, small subunit 1 b [Source:ZFIN;Acc:ZDB-GENE-030131-5206]	15	29113616	29122830		40.79	50.25	48.21	35.26	32.96	31.62
**ENSDARG00000013813**	fam219ab	family with sequence similarity 219 member Ab [Source:ZFIN;Acc:ZDB-GENE-050522-213]	5	26082434	26093976		21.23	6	9.29	26.03	29.6	27.73
**ENSDARG00000013822**	anapc16	anaphase promoting complex subunit 16 [Source:ZFIN;Acc:ZDB-GENE-050417-430]	12	48319221	48327599		40.29	52.45	61.06	37.06	35.14	39.92
**ENSDARG00000013828**	snx13	sorting nexin 13 [Source:ZFIN;Acc:ZDB-GENE-050306-12]	19	2064173	2115040		5.53	2.96	2.57	5.88	5.98	5.66
**ENSDARG00000013830**	osbpl2b	oxysterol binding protein-like 2b [Source:ZFIN;Acc:ZDB-GENE-030131-6138]	23	9508538	9551967		13.73	13.48	12.05	10.9	11	11.05
**ENSDARG00000013834**	arhgef3	Rho guanine nucleotide exchange factor (GEF) 3 [Source:ZFIN;Acc:ZDB-GENE-050208-773]	22	38968246	38993218		0.85	0.62	0.76	1.75	1.88	1.96
**ENSDARG00000013837**	spo11	SPO11 initiator of meiotic double stranded breaks [Source:ZFIN;Acc:ZDB-GENE-040426-1900]	23	6544453	6574123		0.04	0.05	0.07	0.03	0.12	0.1
**ENSDARG00000013838**	sulf2b	sulfatase 2b [Source:ZFIN;Acc:ZDB-GENE-030131-775]	23	15203822	15330230		26.04	7.99	8.25	34.19	34.42	35.76
**ENSDARG00000013839**	aldh3b1	aldehyde dehydrogenase 3 family, member B1 [Source:ZFIN;Acc:ZDB-GENE-021120-3]	3	34120191	34128952		9.82	9.83	8.16	5.65	6.33	5.27
**ENSDARG00000013841**	abl2	c-abl oncogene 2, non-receptor tyrosine kinase [Source:ZFIN;Acc:ZDB-GENE-020809-2]	8	14915332	14985276		2.35	1.58	1.2	4.04	4.1	3.85
**ENSDARG00000013842**	tbc1d19	TBC1 domain family, member 19 [Source:ZFIN;Acc:ZDB-GENE-040426-1265]	7	62248514	62312788		3.42	2.18	2.21	2.56	2.41	2.44
**ENSDARG00000013843**	sept5a	septin 5a [Source:ZFIN;Acc:ZDB-GENE-030131-7868]	8	4588398	4618653		12.61	7.83	10.14	9	9.67	10.36
**ENSDARG00000013845**	ttc14	tetratricopeptide repeat domain 14 [Source:ZFIN;Acc:ZDB-GENE-050327-26]	22	37588528	37611681		9.61	4.25	5.57	10.26	9.55	9.45
**ENSDARG00000013847**	egfra	epidermal growth factor receptor a (erythroblastic leukemia viral (v-erb-b) oncogene homolog, avian) [Source:ZFIN;Acc:ZDB-GENE-(	2	3696038	3820159		3.1	2.47	1.68	5.48	4.83	5.37
**ENSDARG00000013853**	lmo4a	LIM domain only 4a [Source:ZFIN;Acc:ZDB-GENE-010702-1]	5	32924669	32962023		13.8	10.32	12.19	12.38	13.38	12.9
**ENSDARG00000013855**	slc12a3	solute carrier family 12 member 3 [Source:ZFIN;Acc:ZDB-GENE-030131-9505]	18	17583479	17598755		0.28	0.27	0.12	3.64	3.77	3.85
**ENSDARG00000013856**	amy2a	amylase alpha 2A [Source:ZFIN;Acc:ZDB-GENE-040426-2393]	2	15612755	15626972		103	149.72	103.24	2.86	2.87	2.77
**ENSDARG00000013858**	cdc14aa	cell division cycle 14Aa [Source:ZFIN;Acc:ZDB-GENE-040426-1214]	2	7075220	7105532		3.7	0.95	0.67	1.16	1.57	1.66
**ENSDARG00000013860**	ptpmt1	protein tyrosine phosphatase mitochondrial 1 [Source:ZFIN;Acc:ZDB-GENE-070112-272]	7	38577869	38582618		6.58	7.34	7.61	4.96	5.19	5.08
**ENSDARG00000013861**	armc1	armadillo repeat containing 1 [Source:ZFIN;Acc:ZDB-GENE-041212-33]	24	24701780	24724284		8.57	7.86	6.87	10.57	10.63	10.65
**ENSDARG00000013863**	fam133b	family with sequence similarity 133 member B [Source:NCBI gene;Acc:790942]	19	40335271	40347943		34.35	30.05	29.34	16.84	18	19.21
**ENSDARG00000013871**	slc5a1	solute carrier family 5 member 1 [Source:ZFIN;Acc:ZDB-GENE-040426-1524]	10	17135631	17159761		4.99	9.84	6.55	3.46	3.86	3.52
**ENSDARG00000013880**	spata20	spermatogenesis associated 20 [Source:ZFIN;Acc:ZDB-GENE-030131-7269]	12	27589607	27665591		3.93	3.78	3.2	4.77	6.03	5.68
**ENSDARG00000013881**	pi4k2b	phosphatidylinositol 4-kinase type 2 beta [Source:NCBI gene;Acc:564750]	1	15258641	15285455		10.1	10.24	9.15	7.37	7.94	8.29
**ENSDARG00000013892**	sorl1	sortilin-related receptor, L(DLR class) A repeats containing [Source:ZFIN;Acc:ZDB-GENE-050208-22]	15	21541010	21669618		8.24	4.24	3.68	12.36	11.89	12.31
**ENSDARG00000013907**	si:ch211-253p14.2	si:ch211-253p14.2 [Source:ZFIN;Acc:ZDB-GENE-131127-524]	25	29610121	29611476		0.07	0.07	0.06	0.07	0.04	0.05
**ENSDARG00000013921**	frya	furry homolog a (Drosophila) [Source:ZFIN;Acc:ZDB-GENE-060510-4]	15	31806303	31910235		5.65	3.1	2.51	6.19	6.31	6.14
**ENSDARG00000013926**	slc16a9a	solute carrier family 16 member 9a [Source:ZFIN;Acc:ZDB-GENE-040426-1364]	17	20653773	20666616		1.34	1.36	1.18	8.88	8.87	9.06
**ENSDARG00000013928**	plekhb2	pleckstrin homology domain containing, family B (evectins) member 2 [Source:ZFIN;Acc:ZDB-GENE-040426-754]	22	16432404	16443202		7.28	4.56	4.96	8.65	8.59	8.06
**ENSDARG00000013931**	eif3m	eukaryotic translation initiation factor 3, subunit M [Source:NCBI gene;Acc:334626]	18	45645357	45658076		211.34	265.15	308.86	152.51	159.94	161.43
**ENSDARG00000013933**	XRCC2	X-ray repair complementing defective repair in Chinese hamster cells 2 [Source:NCBI gene;Acc:553395]	24	7322116	7330156	XRCC2	0.25	0.34	0.23	0.2	0.11	0.12
**ENSDARG00000013938**	psmb3	proteasome 20S subunit beta 3 [Source:ZFIN;Acc:ZDB-GENE-040426-2682]	6	16478099	16493516		74.1	100.66	102.91	70.02	69.05	66.58
**ENSDARG00000013946**	ivns1abpb	influenza virus NS1A binding protein b [Source:NCBI gene;Acc:334334]	2	23379401	23391266		15.7	11.05	12.08	20.38	22.36	22.66
**ENSDARG00000013950**	pdf	peptide deformylase, mitochondrial [Source:ZFIN;Acc:ZDB-GENE-050913-42]	18	14688643	14691727		5.37	6.62	6.3	4.62	4.17	3.91
**ENSDARG00000013961**	slc31a1	solute carrier family 31 member 1 [Source:ZFIN;Acc:ZDB-GENE-040415-3]	5	57328535	57354915		11.74	15.66	11.11	15.06	16.46	15.11
**ENSDARG00000013963**	mipb	major intrinsic protein of lens fiber b [Source:ZFIN;Acc:ZDB-GENE-050706-86]	23	6637611	6641223		0.43	0.75	0.8	20.01	16.92	25.57
**ENSDARG00000013965**	cul2	cullin 2 [Source:ZFIN;Acc:ZDB-GENE-030131-8032]	24	2283959	2343366		15.01	11.86	12.36	13.61	13.7	13.45
**ENSDARG00000013966**	psma6b	proteasome 20S subunit alpha 6b [Source:ZFIN;Acc:ZDB-GENE-040718-329]	20	16884386	16906767		40.45	51.39	56.68	29.49	30.56	29.35
**ENSDARG00000013968**	psap	prosaposin [Source:ZFIN;Acc:ZDB-GENE-020108-1]	13	5004924	5028964		180.41	175.41	177.27	161.02	168.84	167.33
**ENSDARG00000013973**	pex3	peroxisomal biogenesis factor 3 [Source:ZFIN;Acc:ZDB-GENE-040426-979]	20	37760493	37769303		5.54	6.19	5	5.18	4.64	4.66
**ENSDARG00000013976**	anxa13	annexin A13 [Source:ZFIN;Acc:ZDB-GENE-010406-1]	24	6360423	6375774		28.1	30.25	32.54	34.46	36.36	37.35
**ENSDARG00000013979**	ndfip1	Nedd4 family interacting protein 1 [Source:ZFIN;Acc:ZDB-GENE-030131-2767]	14	23717165	23726768		59.58	47.73	45.87	58.09	58.49	58.15
**ENSDARG00000013990**	ube2q2	ubiquitin-conjugating enzyme E2Q family member 2 [Source:ZFIN;Acc:ZDB-GENE-040426-1919]	25	29427852	29463951		7.83	4.59	4.15	11.01	10.58	10.89
**ENSDARG00000013997**	ern1	endoplasmic reticulum to nucleus signaling 1 [Source:ZFIN;Acc:ZDB-GENE-050522-431]	3	55420176	55481157		4.77	2.48	2.45	3.31	2.77	3.01
**ENSDARG00000014003**	stard8	StAR-related lipid transfer (START) domain containing 8 [Source:ZFIN;Acc:ZDB-GENE-030131-9447]	5	35786141	35867256		2.96	2.67	2.6	4.54	4.67	4.7
**ENSDARG00000014004**	eif2b4	eukaryotic translation initiation factor 2B, subunit 4 delta [Source:ZFIN;Acc:ZDB-GENE-030131-955]	20	19512622	19532600		9.44	7.92	9.51	8.23	8.81	8.53
**ENSDARG00000014005**	lrrc45	leucine rich repeat containing 45 [Source:ZFIN;Acc:ZDB-GENE-121214-317]	12	2993523	3019847		5.75	4.09	4.4	4.36	4.04	4.44
**ENSDARG00000014008**	cwc22	CWC22 spliceosome associated protein homolog [Source:NCBI gene;Acc:566033]	9	43552515	43644261		12.32	9.01	8.18	9.84	9.99	9.81
**ENSDARG00000014013**	lbr	lamin B receptor [Source:ZFIN;Acc:ZDB-GENE-030804-11]	20	36393912	36416922		11.1	7.8	6.83	12.03	11.74	11.31
**ENSDARG00000014015**	pdia7	protein disulfide isomerase family A, member 7 [Source:ZFIN;Acc:ZDB-GENE-040426-2238]	16	30422481	30434279		26.21	28.07	27.74	26.76	26.88	25.44
**ENSDARG00000014017**	rrm1	ribonucleotide reductase M1 polypeptide [Source:NCBI gene;Acc:30740]	21	30772043	30787414		31.49	28.92	30.15	20.69	20.33	19.26
**ENSDARG00000014018**	lhx1a	LIM homeobox 1a [Source:NCBI gene;Acc:30463]	15	27703409	27710575		17.29	3.78	7.85	28.86	28.34	28.9
**ENSDARG00000014024**	ms4a17a.4	membrane-spanning 4-domains, subfamily A, member 17A.4 [Source:ZFIN;Acc:ZDB-GENE-070822-16]	4	76752707	76762432		0.2	0.27	0.31	0.73	0.71	0.87
**ENSDARG00000014031**	abcc2	ATP-binding cassette, sub-family C (CFTR/MRP), member 2 [Source:ZFIN;Acc:ZDB-GENE-040426-1523]	13	681628	711576		25.15	37.06	25.97	10.56	11.78	11.28
**ENSDARG00000014039**	si:dkeyp-93d12.1	si:dkeyp-93d12.1 [Source:ZFIN;Acc:ZDB-GENE-041014-303]	20	42537768	42561740		0.07	0.07	0.02	0.12	0.08	0.05
**ENSDARG00000014041**	smarcad1a	SWI/SNF-related, matrix-associated actin-dependent regulator of chromatin, subfamily a, containing DEAD/H box 1 a [Source:NCBI	8	29635968	29652415		6.13	4.53	4.79	4.51	4.24	3.64
**ENSDARG00000014047**	cldn7b	claudin 7b [Source:NCBI gene;Acc:60635]	10	22144768	22150419		30.23	28.48	24.1	31.99	30.82	28.92
**ENSDARG00000014050**	ngfb	nerve growth factor b (beta polypeptide) [Source:NCBI gene;Acc:58133]	6	49169420	49215827		2.3	1.86	2.61	2.75	2.57	2.3
**ENSDARG00000014053**	olfm1b	olfactomedin 1b [Source:ZFIN;Acc:ZDB-GENE-040801-228]	21	6751405	6813561		34.99	10.24	15.35	41.97	42.25	43.25
**ENSDARG00000014057**	gabrr2b	gamma-aminobutyric acid (GABA) A receptor, rho 2b [Source:ZFIN;Acc:ZDB-GENE-041014-174]	20	1158393	1176631		0.67	0.29	0.62	0.7	0.7	0.87
**ENSDARG00000014058**	rab36	RAB36, member RAS oncogene family [Source:ZFIN;Acc:ZDB-GENE-040910-5]	10	17223681	17232372		1.48	1.03	1.06	1.59	1.83	1.81
**ENSDARG00000014059**	cldn5b	claudin 5b [Source:ZFIN;Acc:ZDB-GENE-041010-140]	10	43994471	43996117		11.56	10.99	10.81	17.4	18.92	19.22
**ENSDARG00000014068**	pgam1b	phosphoglycerate mutase 1b [Source:ZFIN;Acc:ZDB-GENE-030131-5376]	1	54707667	54718863		60.99	32.52	35.48	41.32	41.9	40.76
**ENSDARG00000014081**	fam184a	family with sequence similarity 184 member A [Source:ZFIN;Acc:ZDB-GENE-041014-214]	20	41549200	41799828		4.3	1	1.32	4.03	4.18	4.33
**ENSDARG00000014083**	wdr82	WD repeat domain 82 [Source:NCBI gene;Acc:321614]	6	41452979	41461390		13.7	13.98	12.97	15.38	15.52	15.17
**ENSDARG00000014085**	atp6v1e1a	ATPase H+ transporting V1 subunit E1a [Source:ZFIN;Acc:ZDB-GENE-041212-51]	25	16601839	16613912		10.55	11.84	9.67	9.97	9.42	9.45
**ENSDARG00000014091**	osr1	odd-skipped related transcription factor 1 [Source:NCBI gene;Acc:450059]	13	32144370	32150443		9.84	11.68	10.48	9.18	9.45	10.34
**ENSDARG00000014098**	pkd2	polycystic kidney disease 2 [Source:NCBI gene;Acc:432387]	1	50538839	50557030		5.21	5.21	4.81	8.35	9.84	9.15
**ENSDARG00000014101**	pyroxd2	pyridine nucleotide-disulphide oxidoreductase domain 2 [Source:ZFIN;Acc:ZDB-GENE-050506-147]	13	40284872	40316367		4.18	5.63	3.93	5.03	4.52	5.1
**ENSDARG00000014103**	dkk1a	dickkopf WNT signaling pathway inhibitor 1a [Source:ZFIN;Acc:ZDB-GENE-090313-406]	13	41022502	41026398		2.65	3.47	3.16	4.62	4.62	4.38
**ENSDARG00000014106**	cfl2	cofilin 2 (muscle) [Source:ZFIN;Acc:ZDB-GENE-040426-1815]	17	10007071	10025234		38.44	61.13	53.31	48.69	44.2	45.05
**ENSDARG00000014113**	wasla	WASP like actin nucleation promoting factor a [Source:ZFIN;Acc:ZDB-GENE-070209-220]	25	27744293	27812810		9.98	8.99	6.92	15.04	14.98	16.17
**ENSDARG00000014137**	picalmb	phosphatidylinositol binding clathrin assembly protein b [Source:ZFIN;Acc:ZDB-GENE-030131-6795]	15	19726229	19772372		13.47	5.68	6.34	16.77	16.91	16.97
**ENSDARG00000014138**	acot8	acyl-CoA thioesterase 8 [Source:ZFIN;Acc:ZDB-GENE-041010-174]	11	1512571	1520946		4.48	4.9	5.13	2.57	3.31	3.27
**ENSDARG00000014139**	srp72	signal recognition particle 72 [Source:ZFIN;Acc:ZDB-GENE-030131-5546]	14	1203608	1228690		36.01	33.74	27.58	33.8	33.54	35.38
**ENSDARG00000014148**	med22	mediator complex subunit 22 [Source:NCBI gene;Acc:415234]	5	33287611	33294747		5.06	6.34	5.2	5.38	5.42	5.46
**ENSDARG00000014159**	mtdha	metadherin a [Source:ZFIN;Acc:ZDB-GENE-030616-615]	19	32895379	32914305		28.55	24.85	18.62	32.6	33.9	33.23
**ENSDARG00000014161**	ercc4	excision repair cross-complementation group 4 [Source:ZFIN;Acc:ZDB-GENE-030131-5533]	12	19958845	19975373		2.8	1.49	1.55	1.97	1.94	1.8
**ENSDARG00000014165**	ssr3	signal sequence receptor, gamma [Source:ZFIN;Acc:ZDB-GENE-030131-9134]	18	34542525	34549758		94.89	133.59	143.26	90.96	97.35	98.37
**ENSDARG00000014169**	SYT2	synaptotagmin 2 [Source:HGNC Symbol;Acc:HGNC:11510]	6	55174744	55192813	SYT2	15.63	4.46	6.81	15.91	16.04	17.71
**ENSDARG00000014171**	bloc1s1	biogenesis of lysosomal organelles complex-1, subunit 1 [Source:ZFIN;Acc:ZDB-GENE-050208-569]	22	10123729	10127944		16	18.76	17.74	13.15	13.66	13.03
**ENSDARG00000014179**	pfkma	phosphofructokinase, muscle a [Source:ZFIN;Acc:ZDB-GENE-040912-135]	23	27553786	27571667		36.74	61.65	60.81	35.41	30.02	30.66
**ENSDARG00000014180**	ildr2	immunoglobulin-like domain containing receptor 2 [Source:ZFIN;Acc:ZDB-GENE-050706-83]	9	34274501	34300707		5.19	3.55	3.3	6.21	7.05	6.96
**ENSDARG00000014181**	foxp1b	forkhead box P1b [Source:NCBI gene;Acc:569047]	6	43602143	43890462		2.86	1.89	1.59	4.33	4.48	4.38
**ENSDARG00000014190**	sst2	somatostatin 2 [Source:ZFIN;Acc:ZDB-GENE-010219-2]	2	5726353	5728843		13	12.04	10.33	10.72	11.66	10.59
**ENSDARG00000014196**	myl1	myosin, light chain 1, alkali; skeletal, fast [Source:ZFIN;Acc:ZDB-GENE-030131-8109]	9	38994232	39005317		850.11	1257.41	1381.14	890.41	863.3	847.94
**ENSDARG00000014201**	otpa	orthopedia homeobox a [Source:ZFIN;Acc:ZDB-GENE-070216-1]	21	7582036	7586992		10.63	1.77	3.27	28.78	28.61	31.55
**ENSDARG00000014208**	zgc:55413	zgc:55413 [Source:ZFIN;Acc:ZDB-GENE-040426-2909]	15	35036368	35126632		0.06	0.02	0.03	0.04	0.05	0.02
**ENSDARG00000014209**	odf3l2a	outer dense fiber of sperm tails 3-like 2a [Source:ZFIN;Acc:ZDB-GENE-050417-380]	2	41926707	41934019		0.4	0.32	0.6	0.75	0.96	0.7
**ENSDARG00000014215**	cdh13	cadherin 13, H-cadherin (heart) [Source:ZFIN;Acc:ZDB-GENE-060503-286]	18	13792490	14134035		10.35	3.04	3.79	11.04	11.01	11.84
**ENSDARG00000014218**	mecp2	methyl CpG binding protein 2 [Source:ZFIN;Acc:ZDB-GENE-030131-7190]	8	7616573	7637640		6	3.11	2.77	5.92	7.28	7.18
**ENSDARG00000014222**	zgc:86598	zgc:86598 [Source:ZFIN;Acc:ZDB-GENE-040625-181]	19	3135271	3234924		13.9	11.29	9.37	14.74	15.56	15.5
**ENSDARG00000014230**	dlst	dihydrolipoamide S-succinyltransferase [Source:ZFIN;Acc:ZDB-GENE-030326-1]	17	52524355	52553447		112.84	131.17	116.34	104.96	105.64	103.54
**ENSDARG00000014232**	prkg3	protein kinase cGMP-dependent 3 [Source:ZFIN;Acc:ZDB-GENE-030131-4745]	20	616114	641511		4.76	6.14	4.42	1.49	1.52	1.63
**ENSDARG00000014233**	sept8b	septin 8b [Source:NCBI gene;Acc:571702]	14	49040190	49063157		13.92	7.46	8.16	10.49	11.64	12.38
**ENSDARG00000014239**	esyt1b	extended synaptotagmin-like protein 1b [Source:ZFIN;Acc:ZDB-GENE-040724-209]	6	52263236	52336619		4.15	3.64	3.28	5.43	5.79	5.53
**ENSDARG00000014244**	rbmx	RNA binding motif protein X-linked [Source:NCBI gene;Acc:321860]	14	31855592	31862720		42.82	26.62	29.33	38.01	38.29	37.87
**ENSDARG00000014246**	jag2a	jagged canonical Notch ligand 2a [Source:ZFIN;Acc:ZDB-GENE-130530-549]	13	33488032	33551699		1.9	1.52	1.21	2.18	1.83	1.98
**ENSDARG00000014248**	pdlim3b	PDZ and LIM domain 3b [Source:ZFIN;Acc:ZDB-GENE-060130-104]	14	29782508	29800107		5.38	7.6	6.96	10.39	9.11	9.14
**ENSDARG00000014259**	eya1	EYA transcriptional coactivator and phosphatase 1 [Source:ZFIN;Acc:ZDB-GENE-990712-18]	24	13869092	14015459		7.85	6.89	6.27	10.64	10.48	9.88
**ENSDARG00000014273**	camk2d2	calcium/calmodulin-dependent protein kinase (CaM kinase) II delta 2 [Source:NCBI gene;Acc:436815]	1	14163506	14234076		43.74	11.11	16.45	41.19	44.56	43.22
**ENSDARG00000014274**	rfc2	replication factor C (activator 1) 2 [Source:ZFIN;Acc:ZDB-GENE-050306-29]	21	25206289	25213616		10.9	12.79	10.56	6.33	6.27	5.35
**ENSDARG00000014280**	dlgap1a	discs, large (Drosophila) homolog-associated protein 1a [Source:NCBI gene;Acc:569898]	2	29491314	29692825		1.3	0.22	0.35	1.5	1.51	2.02
**ENSDARG00000014287**	eno2	enolase 2 [Source:ZFIN;Acc:ZDB-GENE-040704-27]	19	5072918	5090732		53.19	16.39	21.42	52.92	58.29	57.06
**ENSDARG00000014292**	med15	mediator complex subunit 15 [Source:NCBI gene;Acc:406346]	21	18929319	18946648		6.41	4.18	4.14	7.76	7.66	7.73
**ENSDARG00000014302**	brinp2	bone morphogenetic protein/retinoic acid inducible neural-specific 2 [Source:ZFIN;Acc:ZDB-GENE-070912-621]	2	34380982	34555945		5.37	1.81	2.48	6.19	5.88	5.75
**ENSDARG00000014303**	vac14	vac14 homolog (S. cerevisiae) [Source:NCBI gene;Acc:447912]	25	17589906	17613025		3.56	2.7	2.78	3.48	3.52	3.52
**ENSDARG00000014309**	spaw	southpaw [Source:ZFIN;Acc:ZDB-GENE-030219-1]	5	68878124	68885081		0.11	0.04	0.05	0.17	0.29	0.14
**ENSDARG00000014311**	pgghg	protein-glucosylgalactosylhydroxylysine glucosidase [Source:NCBI gene;Acc:777617]	25	4635355	4660125		4.37	4.19	4.17	4.28	4.72	4.77
**ENSDARG00000014313**	atp5pf	ATP synthase peripheral stalk subunit F6 [Source:ZFIN;Acc:ZDB-GENE-040426-2534]	1	610082	614638		92.66	120.35	116.44	66.61	63.54	65.95
**ENSDARG00000014320**	gucy2c	guanylate cyclase 2C [Source:ZFIN;Acc:ZDB-GENE-091118-67]	3	3765831	3798755		3.75	3.87	2.61	4.79	4.69	4.95
**ENSDARG00000014321**	en1a	engrailed homeobox 1a [Source:NCBI gene;Acc:30244]	9	778745	785444		0.66	0.77	0.66	0.69	0.63	0.72
**ENSDARG00000014324**	sh2d5	SH2 domain containing 5 [Source:ZFIN;Acc:ZDB-GENE-050327-67]	11	37638873	37651879		0.41	0.56	0.46	0.72	0.7	0.5
**ENSDARG00000014329**	npm1a	nucleophosmin 1a [Source:ZFIN;Acc:ZDB-GENE-021028-1]	10	22034477	22045119		79.92	118.88	104.1	67.8	62.41	61.43
**ENSDARG00000014333**	ormdl1	ORMDL sphingolipid biosynthesis regulator 1 [Source:NCBI gene;Acc:368632]	9	41024973	41034174		11.55	11.03	10.42	12.54	12.2	12.62
**ENSDARG00000014340**	rab11al	RAB11a, member RAS oncogene family, like [Source:ZFIN;Acc:ZDB-GENE-031118-188]	16	46725087	46741779		7.75	8.74	6.36	7.39	7.08	5.54
**ENSDARG00000014348**	stk17b	serine/threonine kinase 17b (apoptosis-inducing) [Source:ZFIN;Acc:ZDB-GENE-040426-1499]	9	24192370	24201159		6.05	6.57	5.32	6.85	6.54	7.55
**ENSDARG00000014356**	krt95	kertain 95 [Source:ZFIN;Acc:ZDB-GENE-040718-88]	11	12188120	12198914		0.03	0	0.02	0.01	0	0
**ENSDARG00000014357**	amh	anti-Mullerian hormone [Source:ZFIN;Acc:ZDB-GENE-050706-192]	22	20747182	20761759		0.02	0.03	0.09	0.11	0.05	0.06
**ENSDARG00000014358**	optc	opticin [Source:ZFIN;Acc:ZDB-GENE-030131-1798]	11	23205889	23219367		0.44	0.68	0.26	1.65	1.17	1.43
**ENSDARG00000014361**	tmbim4	transmembrane BAX inhibitor motif containing 4 [Source:ZFIN;Acc:ZDB-GENE-040426-2152]	4	12971255	12978925		25.67	23.5	23.24	28.28	25.82	26.36
**ENSDARG00000014366**	luc7l3	LUC7-like 3 pre-mRNA splicing factor [Source:ZFIN;Acc:ZDB-GENE-060616-375]	3	58805790	58823762		57.2	29.48	37.22	51.09	48.42	49.39
**ENSDARG00000014373**	ddx4	DEAD (Asp-Glu-Ala-Asp) box polypeptide 4 [Source:ZFIN;Acc:ZDB-GENE-990415-272]	10	6884123	6905574		0.97	1.31	1.14	1.94	1.49	1.75
**ENSDARG00000014378**	slc12a4	solute carrier family 12 member 4 [Source:ZFIN;Acc:ZDB-GENE-090218-27]	7	35282885	35314347		5.56	5.89	4.57	7.97	7.86	7.68
**ENSDARG00000014386**	galnt6	UDP-N-acetyl-alpha-D-galactosamine:polypeptide N-acetylgalactosaminyltransferase 6 (GalNAc-T6) [Source:ZFIN;Acc:ZDB-GENE-	23	26763338	26784771		7.67	5.87	4.88	5.58	4.68	4.53
**ENSDARG00000014395**	selenoo2	selenoprotein O2 [Source:ZFIN;Acc:ZDB-GENE-041210-110]	4	5378338	5387478		0.49	0.28	0.21	0.53	0.66	0.58
**ENSDARG00000014402**	smc1b	structural maintenance of chromosomes 1B [Source:ZFIN;Acc:ZDB-GENE-091217-1]	25	16554817	16581233		0.13	0.21	0.09	0.13	0.14	0.18
**ENSDARG00000014420**	elavl3	ELAV like neuron-specific RNA binding protein 3 [Source:ZFIN;Acc:ZDB-GENE-980526-76]	3	46800190	46818001		56.7	13.08	16.93	90.91	88.69	92.81
**ENSDARG00000014427**	mxe	myxovirus (influenza virus) resistance E [Source:NCBI gene;Acc:337663]	9	32824639	32837860		0.03	0.07	0.03	0.14	0.11	0.12
**ENSDARG00000014428**	ppp2r5d	protein phosphatase 2, regulatory subunit B', delta [Source:ZFIN;Acc:ZDB-GENE-040426-2568]	13	4232568	4303641		14.24	10.13	8.52	14.63	14.92	14.23
**ENSDARG00000014430**	rabgef1	RAB guanine nucleotide exchange factor (GEF) 1 [Source:ZFIN;Acc:ZDB-GENE-040426-1486]	10	32854901	32880298		7.01	5.02	3.85	7.85	7.75	7.78
**ENSDARG00000014439**	dgkza	diacylglycerol kinase, zeta a [Source:ZFIN;Acc:ZDB-GENE-111104-3]	7	38962207	39157394		2.36	1.49	1.42	2.84	2.75	2.75
**ENSDARG00000014462**	rab3c	RAB3C, member RAS oncogene family [Source:ZFIN;Acc:ZDB-GENE-040718-263]	8	17055292	17067364		12.82	3.88	4.3	11.62	10.76	11.46
**ENSDARG00000014463**	usf1l	upstream transcription factor 1, like [Source:ZFIN;Acc:ZDB-GENE-040426-2269]	5	29803380	29817848		15.57	16.48	16.78	12.5	14.56	13.04
**ENSDARG00000014465**	arhgef25b	Rho guanine nucleotide exchange factor (GEF) 25b [Source:ZFIN;Acc:ZDB-GENE-080225-17]	6	39256170	39275915		5.74	4.1	3.53	5.89	5.28	5.42
**ENSDARG00000014474**	yipf1	Yip1 domain family, member 1 [Source:ZFIN;Acc:ZDB-GENE-040822-35]	2	26598801	26604318		5.1	5.54	5.01	4.97	5.14	5.2
**ENSDARG00000014477**	sstr3	somatostatin receptor 3 [Source:ZFIN;Acc:ZDB-GENE-061009-35]	3	29600917	29602479		0.8	0.46	0.5	0.42	0.83	0.68
**ENSDARG00000014479**	ptf1a	pancreas associated transcription factor 1a [Source:NCBI gene;Acc:368662]	2	29710857	29711761		4.27	4.35	4.39	2.3	2.12	3.08
**ENSDARG00000014480**	nr6a1b	nuclear receptor subfamily 6, group A, member 1b [Source:NCBI gene;Acc:497167]	21	8198652	8259645		0.98	0.74	1.06	1.36	1.16	1.34
**ENSDARG00000014488**	ca2	carbonic anhydrase II [Source:ZFIN;Acc:ZDB-GENE-031219-5]	24	32650227	32665283		33.03	31.27	26.97	31.08	33.6	34.77
**ENSDARG00000014490**	tac1	tachykinin precursor 1 [Source:ZFIN;Acc:ZDB-GENE-060503-904]	19	25649626	25653185		12.64	6.09	7.37	18.67	20.46	21.61
**ENSDARG00000014496**	trpv6	transient receptor potential cation channel, subfamily V, member 6 [Source:ZFIN;Acc:ZDB-GENE-040624-12]	16	12294284	12319822		16.91	21.69	20.27	6.98	6.31	5.99
**ENSDARG00000014498**	ythdf2	YTH N(6)-methyladenosine RNA binding protein 2 [Source:NCBI gene;Acc:393220]	17	27407918	27419499		9.85	7.64	7.26	11.4	11.04	11.55
**ENSDARG00000014499**	nutf2l	nuclear transport factor 2, like [Source:ZFIN;Acc:ZDB-GENE-020416-1]	19	20778011	20783758		30.65	39.85	38.28	19.38	18	18.61
**ENSDARG00000014506**	tmem126a	transmembrane protein 126A [Source:ZFIN;Acc:ZDB-GENE-040426-1770]	10	29269998	29272757		23.32	27.51	30.74	15.18	14.34	13.96
**ENSDARG00000014517**	usp5	ubiquitin specific peptidase 5 (isopeptidase T) [Source:ZFIN;Acc:ZDB-GENE-040426-2584]	16	11965371	11986321		32.27	26.27	25.98	31.88	27.99	29.65
**ENSDARG00000014522**	cdh6	cadherin 6 [Source:ZFIN;Acc:ZDB-GENE-050320-92]	2	28049046	28102264		20.23	12.89	11.21	21.91	20.08	20.93

ENSDARG00000014527	pdk3a	pyruvate dehydrogenase kinase, isozyme 3a [Source:ZFIN;Acc:ZDB-GENE-061220-3]	24	24944352	24959686	7.86	5.17	5.68	13.32	14.41	14.41
ENSDARG00000014531	atg4a	autophagy related 4A, cysteine peptidase [Source:ZFIN;Acc:ZDB-GENE-050522-430]	21	43485512	43510481	2.25	1.85	1.61	2.54	2.79	3.16
ENSDARG00000014532	aida	axin interactor, dorsalization associated [Source:NCBI gene;Acc:393763]	20	51730658	51753885	6.72	6.35	5.98	7.39	6.97	7.21
ENSDARG00000014536	pfdn1	prefoldin subunit 1 [Source:NCBI gene;Acc:503766]	21	30116424	30160411	26.53	27.8	28.23	30.83	31.22	30.84
ENSDARG00000014545	golim4b	golgi integral membrane protein 4b [Source:ZFIN;Acc:ZDB-GENE-050327-74]	2	36701322	36721289	1.66	2.06	2.05	2.16	2.37	2.1
ENSDARG00000014550	rfx3	regulatory factor X, 3 (influences HLA class II expression) [Source:NCBI gene;Acc:570989]	10	600164	641609	5.69	1.95	2.17	10.12	9.94	10.05
ENSDARG00000014554	ppp1r3cb	protein phosphatase 1, regulatory subunit 3Cb [Source:NCBI gene;Acc:393807]	12	16281312	16288285	11.72	12.36	11.47	9.21	10.03	8.99
ENSDARG00000014556	serpinb1l3	serpin peptidase inhibitor, clade B (ovalbumin), member 1, like 3 [Source:ZFIN;Acc:ZDB-GENE-030131-7059]	24	42148140	42157913	32.44	47.44	30.21	24.04	24.62	23.98
ENSDARG00000014565	eif4e1b	eukaryotic translation initiation factor 4E family member 1B [Source:NCBI gene;Acc:30738]	5	67785991	67799821	0.06	0.05	0.03	0.06	0.02	0.03
ENSDARG00000014569	hnrnpr	heterogeneous nuclear ribonucleoprotein R [Source:ZFIN;Acc:ZDB-GENE-040426-2766]	19	72661	82516	29.15	17.69	18.86	28.44	26.68	27.75
ENSDARG00000014571	ctnnb1	catenin (cadherin-associated protein), beta 1 [Source:NCBI gene;Acc:30265]	16	6182501	6205790	115.39	80.79	74.88	169.32	164.99	168.79
ENSDARG00000014572	chpt1	choline phosphotransferase 1 [Source:NCBI gene;Acc:322605]	4	17707008	17725008	47.15	72.25	53.57	29.45	29.17	29.17
ENSDARG00000014577	rhpn2	rhophilin, Rho GTPase binding protein 2 [Source:NCBI gene;Acc:321330]	7	38349665	38393421	8.78	7.7	6.88	8.17	8.36	7.44
ENSDARG00000014582	exoc3	exocyst complex component 3 [Source:ZFIN;Acc:ZDB-GENE-030131-5947]	2	50104811	50126337	12.64	7.92	7.74	11.17	10.67	11.31
ENSDARG00000014587	slc38a5b	solute carrier family 38 member 5b [Source:ZFIN;Acc:ZDB-GENE-040718-395]	8	23579974	23612462	60.57	64.96	48.88	54.99	58.53	54.21
ENSDARG00000014588	adcy2b	adenylate cyclase 2b (brain) [Source:ZFIN;Acc:ZDB-GENE-060503-69]	19	27608927	27777228	3.37	1.83	1.84	4.54	4.67	4.97
ENSDARG00000014591	ilf2	interleukin enhancer binding factor 2 [Source:NCBI gene;Acc:406517]	19	37501652	37508571	81.2	65.91	71.4	87.2	82.35	86.47
ENSDARG00000014592	dennd1a	DENN/MADD domain containing 1A [Source:ZFIN;Acc:ZDB-GENE-060404-6]	21	8427059	8462109	5.94	3.05	3.41	6.39	7.42	7.9
ENSDARG00000014599	slc5a6b	solute carrier family 5 member 6 [Source:ZFIN;Acc:ZDB-GENE-050913-86]	17	6535694	6558596	0.87	0.87	0.8	0.62	0.64	0.84
ENSDARG00000014623	si:ch211-212d10.1	si:ch211-212d10.1 [Source:ZFIN;Acc:ZDB-GENE-050208-355]	22	18530395	18537415	0.29	0.39	0.34	0.22	0.21	0.24
ENSDARG00000014624	si:dkeyp-94g1.1	si:dkeyp-94g1.1 [Source:ZFIN;Acc:ZDB-GENE-030131-7910]	2	48545804	48561471	0	0.06	0.06	0.1	0.1	0.06
ENSDARG00000014626	dlx3b	distal-less homeobox 3b [Source:NCBI gene;Acc:30585]	12	5708400	5711920	19.97	19.25	17.55	18.21	17.19	16.63
ENSDARG00000014634	mbtps1	membrane-bound transcription factor peptidase, site 1 [Source:ZFIN;Acc:ZDB-GENE-030131-4909]	18	21645309	21685055	13.95	10.55	8.79	14.15	14.33	14.53
ENSDARG00000014646	aoc2	amine oxidase copper containing 2 [Source:ZFIN;Acc:ZDB-GENE-050320-133]	3	32924185	32934359	53.93	65.33	47.6	38.41	39.17	37.35
ENSDARG00000014651	svilb	supervillin b [Source:ZFIN;Acc:ZDB-GENE-081104-411]	2	48817958	48826707	5.61	6.29	4.47	7.6	6.83	8.19
ENSDARG00000014652	ankrd13b	ankyrin repeat domain 13B [Source:ZFIN;Acc:ZDB-GENE-050913-151]	15	28482862	28539650	2.63	0.81	0.91	2.58	2.94	2.96
ENSDARG00000014655	itpr1a	inositol 1,4,5-trisphosphate receptor, type 1a [Source:ZFIN;Acc:ZDB-GENE-070604-3]	6	39923052	39991554	2.13	1.95	1.8	1.97	2.09	1.95
ENSDARG00000014657	casp23	caspase 23, apoptosis-related cysteine peptidase [Source:ZFIN;Acc:ZDB-GENE-071004-27]	7	19600262	19604900	0.23	0.18	0.15	0.08	0.09	0.24
ENSDARG00000014670	colgalt2	collagen beta(1-O)galactosyltransferase 2 [Source:ZFIN;Acc:ZDB-GENE-070222-1]	8	19364568	19395110	6.04	7.21	6.89	10.32	10.63	11.44
ENSDARG00000014673	fzd9b	frizzled class receptor 9b [Source:ZFIN;Acc:ZDB-GENE-000906-4]	18	41648812	41650648	11.13	10.36	9.95	11.09	11.27	10.84
ENSDARG00000014674	acsl3b	acyl-CoA synthetase long chain family member 3b [Source:ZFIN;Acc:ZDB-GENE-030131-492]	2	47471647	47516648	10.48	5.61	5.56	11.07	10.69	11.02
ENSDARG00000014675	efna3a	ephrin-A3a [Source:ZFIN;Acc:ZDB-GENE-060503-186]	19	8144556	8339860	2.12	0.67	1.01	2.49	2.2	2.42
ENSDARG00000014676	bckdhb	branched chain keto acid dehydrogenase E1 subunit beta [Source:ZFIN;Acc:ZDB-GENE-030124-2]	16	5013868	5105322	32.01	34.61	34.92	25.51	24.43	24.53
ENSDARG00000014680	znf710a	zinc finger protein 710a [Source:ZFIN;Acc:ZDB-GENE-030131-5559]	18	26048128	26094678	7.9	3.73	4.19	8.85	9.89	9.67
ENSDARG00000014682	tmem26b	transmembrane protein 26b [Source:ZFIN;Acc:ZDB-GENE-080204-66]	12	8064029	8070969	0.67	0.77	0.65	0.75	0.7	0.43
ENSDARG00000014685	esco2	establishment of sister chromatid cohesion N-acetyltransferase 2 [Source:NCBI gene;Acc:445395]	20	39344889	39359154	9.93	9.65	9.67	5.61	5.86	6.18
ENSDARG00000014690	rps4x	ribosomal protein S4 X-linked [Source:NCBI gene;Acc:449547]	7	51633815	51639699	832.7	944.51	1121.77	627.38	646.82	653.49
ENSDARG00000014692	znf622	zinc finger protein 622 [Source:ZFIN;Acc:ZDB-GENE-050927-1]	2	41563073	41571454	16.04	16.59	15.63	15.94	17.25	15.23
ENSDARG00000014704	pgm2l1	phosphoglucomutase 2-like 1 [Source:ZFIN;Acc:ZDB-GENE-041008-205]	15	5116179	5148793	2.05	1.65	1.7	2.19	2.26	2.55
ENSDARG00000014717	dync1h1	dynein, cytoplasmic 1, heavy chain 1 [Source:ZFIN;Acc:ZDB-GENE-030131-7050]	17	918005	945883	18.58	15.89	14.06	39.97	38.32	40.15
ENSDARG00000014722	her1	hairy-related 1 [Source:ZFIN;Acc:ZDB-GENE-980526-125]	5	68788658	68795063	0	0.03	0	0	0	0.01
ENSDARG00000014727	acox1	acyl-CoA oxidase 1, palmitoyl [Source:ZFIN;Acc:ZDB-GENE-041010-219]	6	23122789	23138502	24.45	28.2	18.43	14.08	14.3	13.69
ENSDARG00000014731	cacybp	calcyclin binding protein [Source:ZFIN;Acc:ZDB-GENE-040426-2579]	2	35595454	35601370	27.66	32.15	26.5	23.43	21.16	23.03
ENSDARG00000014746	rbfox1	RNA binding fox-1 homolog 1 [Source:NCBI gene;Acc:449554]	3	27982269	28428198	20.66	7.9	7.84	27.62	26.03	27.09
ENSDARG00000014761	mfsd6l	major facilitator superfamily domain containing 6-like [Source:NCBI gene;Acc:445192]	3	25849560	25852579	0.95	1.35	0.73	1.07	0.87	0.85
ENSDARG00000014763	arf2a	ADP-ribosylation factor 2a [Source:ZFIN;Acc:ZDB-GENE-040122-4]	3	37558491	37588855	89.66	80.38	80.99	106.17	103.7	98.67
ENSDARG00000014770	usp4	ubiquitin specific peptidase 4 (proto-oncogene) [Source:ZFIN;Acc:ZDB-GENE-041008-187]	6	42345005	42369186	11.78	9.73	7.35	14.41	13.42	12.43
ENSDARG00000014775	zgc:113220	zgc:113220 [Source:ZFIN;Acc:ZDB-GENE-050320-64]	22	2409175	2421040	6.65	4.87	5.23	7.31	6.61	6.86
ENSDARG00000014790	g3bp2	GTPase activating protein (SH3 domain) binding protein 2 [Source:ZFIN;Acc:ZDB-GENE-070112-1972]	1	26444986	26466002	12.1	6.55	6.89	14.47	14.6	14.19
ENSDARG00000014792	lrrc4bb	leucine rich repeat containing 4Bb [Source:ZFIN;Acc:ZDB-GENE-091116-44]	24	38369351	38384432	10.36	2.37	3.22	13.3	12.9	13.11
ENSDARG00000014793	ehd1b	EH-domain containing 1b [Source:ZFIN;Acc:ZDB-GENE-030131-2069]	10	27068251	27093034	29.26	36.77	27.99	25.34	25.95	25.38
ENSDARG00000014794	uqcrc2a	ubiquinol-cytochrome c reductase core protein 2a [Source:ZFIN;Acc:ZDB-GENE-040718-405]	3	58167288	58185030	100.55	111.13	113.77	84.66	86.55	85.11
ENSDARG00000014796	wnt11	wingless-type MMTV integration site family, member 11 [Source:ZFIN;Acc:ZDB-GENE-980526-249]	10	32104305	32123204	22.58	26.65	25.08	27.21	29.33	30.19
ENSDARG00000014803	cryba1l2	crystallin, beta A1, like 2 [Source:ZFIN;Acc:ZDB-GENE-041010-40]	1	44421920	44434707	0.16	0.4	0.63	5.23	4.6	6.54
ENSDARG00000014804	cacna2d1a	calcium channel, voltage-dependent, alpha 2/delta subunit 1a [Source:ZFIN;Acc:ZDB-GENE-041210-215]	4	19768679	19884440	6.11	6.14	4.61	8.64	7.53	7.88
ENSDARG00000014805	fhl5	four and a half LIM domains 5 [Source:ZFIN;Acc:ZDB-GENE-040822-3]	17	15605572	15611744	7.62	10.56	10.03	1.8	1.5	1.57
ENSDARG00000014806	hacd2	3-hydroxyacyl-CoA dehydratase 2 [Source:ZFIN;Acc:ZDB-GENE-030131-6053]	9	38029475	38042939	31.46	26.45	20.64	28	27.27	26.94
ENSDARG00000014817	ranbp1	RAN binding protein 1 [Source:ZFIN;Acc:ZDB-GENE-040426-2548]	5	19006701	19014589	41.77	51.83	48.58	28.87	26.99	27.09
ENSDARG00000014825	si:dkeyp-69b9.6	si:dkeyp-69b9.6 [Source:ZFIN;Acc:ZDB-GENE-100922-227]	16	13616239	13623928	11.49	5.54	5.05	13.43	13	13.64
ENSDARG00000014828	l3mbtl1a	L3MBTL histone methyl-lysine binding protein 1a [Source:ZFIN;Acc:ZDB-GENE-090312-120]	23	4299887	4318418	6.07	1.47	2.18	7.46	7.77	7.98
ENSDARG00000014840	prph2b	peripherin 2b (retinal degeneration, slow) [Source:ZFIN;Acc:ZDB-GENE-000616-9]	13	3252950	3258772	83.06	14.48	8.03	44.9	51.8	48.59
ENSDARG00000014849	pih1d2	PIH1 domain containing 2 [Source:NCBI gene;Acc:494086]	15	18157665	18162647	0.33	0.14	0.23	1.09	1.95	1.39
ENSDARG00000014854	reep1	receptor accessory protein 1 [Source:ZFIN;Acc:ZDB-GENE-110411-267]	17	43486436	43504103	3.58	1.7	1.95	5.21	4.54	5
ENSDARG00000014858	drd2l	dopamine receptor D2 like [Source:ZFIN;Acc:ZDB-GENE-030910-1]	16	54248706	54291748	0.45	0.15	0.26	0.52	0.42	0.42
ENSDARG00000014866	hprt1l	hypoxanthine phosphoribosyltransferase 1, like [Source:ZFIN;Acc:ZDB-GENE-040625-17]	25	37073189	37084032	10.23	4.9	5.53	11.41	12	12.24
ENSDARG00000014867	rpl8	ribosomal protein L8 [Source:NCBI gene;Acc:393686]	9	33323104	33329787	1165.79	1402.45	1775.09	889.7	941.03	921.78
ENSDARG00000014871	syngr3a	synaptogyrin 3a [Source:ZFIN;Acc:ZDB-GENE-050522-541]	3	40170216	40180201	32.45	9.13	15.67	30.83	30.66	30.46
ENSDARG00000014875	abi2b	abl-interactor 2b [Source:ZFIN;Acc:ZDB-GENE-060519-5]	6	9970733	10010348	21.33	13.15	11.21	27.65	29.74	30.63
ENSDARG00000014891	robo2	roundabout, axon guidance receptor, homolog 2 (Drosophila) [Source:ZFIN;Acc:ZDB-GENE-001019-1]	15	39096736	39228226	11.13	3.62	3.49	23.48	23.33	25.26
ENSDARG00000014898	ncbp2	nuclear cap binding protein subunit 2 [Source:NCBI gene;Acc:192325]	22	38930725	38934989	18.34	22.96	21.27	16.18	15.02	16.31
ENSDARG00000014907	htra1b	HtrA serine peptidase 1b [Source:NCBI gene;Acc:565082]	12	11388724	11457625	5.8	5.75	5.16	6.76	6.91	7.75
ENSDARG00000014910	panx1b	pannexin 1b [Source:ZFIN;Acc:ZDB-GENE-070705-192]	5	37840914	37852508	3.76	1.61	2.19	4.83	4.74	5.02
ENSDARG00000014914	lamp2	lysosomal-associated membrane protein 2 [Source:ZFIN;Acc:ZDB-GENE-030729-9]	14	33473804	33481618	24.45	19.86	18.04	26.51	26.87	25.29
ENSDARG00000014915	ndufab1b	NADH:ubiquinone oxidoreductase subunit AB1b [Source:ZFIN;Acc:ZDB-GENE-030131-4437]	3	35535121	35541381	43.13	46.91	49.24	28.78	29.01	29.83
ENSDARG00000014916	slc10a2	solute carrier family 10 member 2 [Source:ZFIN;Acc:ZDB-GENE-040426-1328]	9	27339505	27344683	5.42	2.49	2.19	2.2	2.78	2.84
ENSDARG00000014927	uts1	urotensin 1 [Source:ZFIN;Acc:ZDB-GENE-041014-348]	20	38724575	38727396	1.79	0.8	1	4.96	5.72	6.36
ENSDARG00000014929	p2ry11	purinergic receptor P2Y11 [Source:ZFIN;Acc:ZDB-GENE-111221-4]	3	54572725	54577985	0.18	0.27	0.22	0.78	0.27	0.27
ENSDARG00000014939	kcnn2	potassium calcium-activated channel subfamily N member 2 [Source:ZFIN;Acc:ZDB-GENE-091116-41]	10	17592273	17674345	2.23	0.88	1.42	2.03	2.54	2.57
ENSDARG00000014941	tprkb	Tp53rk binding protein [Source:NCBI gene;Acc:492501]	4	28371732	28375127	6.64	7.98	7.09	4.09	3.6	3.8
ENSDARG00000014943	kif23	kinesin family member 23 [Source:ZFIN;Acc:ZDB-GENE-991019-4]	18	20435821	20458840	5.45	3.34	3.14	4.58	4.59	4.27
ENSDARG00000014945	znf598	zinc finger protein 598 [Source:NCBI gene;Acc:407728]	3	40185253	40202607	15.1	13.76	11.93	14.54	14.57	13.43
ENSDARG00000014946	angpt2a	angiopoietin 2a [Source:ZFIN;Acc:ZDB-GENE-121101-1]	13	6295173	6306886	1.02	1.44	1.35	1.27	1.49	1.43
ENSDARG00000014951	svopb	SV2 related protein b [Source:ZFIN;Acc:ZDB-GENE-070705-359]	5	31931834	31942621	0.28	0.35	0.36	0.43	0.42	0.37
ENSDARG00000014953	cnih3	cornichon family AMPA receptor auxiliary protein 3 [Source:ZFIN;Acc:ZDB-GENE-041001-180]	20	36233873	36298140	5.83	2.14	3.07	6.35	5.96	6.86
ENSDARG00000014954	snx33	sorting nexin 33 [Source:ZFIN;Acc:ZDB-GENE-041111-209]	25	6451038	6481191	1.02	0.88	0.57	1.75	1.53	1.5
ENSDARG00000014956	diablob	diablo, IAP-binding mitochondrial protein b [Source:ZFIN;Acc:ZDB-GENE-070112-202]	21	21523082	21610857	12.09	14.44	15.8	9.87	10.4	10.64
ENSDARG00000014962	ppp3cca	protein phosphatase 3, catalytic subunit, gamma isozyme, a [Source:ZFIN;Acc:ZDB-GENE-030829-36]	5	27924347	27994679	14.99	7.9	7.46	14.41	14.46	14.41
ENSDARG00000014963	triap1	TP53 regulated inhibitor of apoptosis 1 [Source:ZFIN;Acc:ZDB-GENE-050417-161]	10	1681518	1684882	10.36	11.81	11.03	8.14	6.51	6.46
ENSDARG00000014966	ccdc82	coiled-coil domain containing 82 [Source:ZFIN;Acc:ZDB-GENE-030131-5749]	21	40685895	40709239	11.31	7.86	7.69	9.44	9.6	9.91
ENSDARG00000014967	g6pcb	glucose-6-phosphatase b, catalytic subunit [Source:ZFIN;Acc:ZDB-GENE-081223-2]	3	32906082	32912787	0.2	0.18	0.26	0.86	0.8	0.84
ENSDARG00000014969	ankhb	ANKH inorganic pyrophosphate transport regulator b [Source:NCBI gene;Acc:323738]	2	31948352	32009716	5.23	2.54	2.53	4.75	4.94	5.21
ENSDARG00000014973	ntng1a	netrin g1a [Source:ZFIN;Acc:ZDB-GENE-091118-13]	24	29381946	29529252	5.98	1.4	1.38	8.17	7.76	8.58
ENSDARG00000014975	irge1	immunity-related GTPase family, e1 [Source:ZFIN;Acc:ZDB-GENE-051209-1]	16	25975426	25978063	1.16	0.99	0.52	0.37	0.05	0.1
ENSDARG00000014976	lims2	LIM and senescent cell antigen-like domains 2 [Source:ZFIN;Acc:ZDB-GENE-050522-56]	9	94884	100579	11.92	16.68	18.41	12.23	11.72	11.1
ENSDARG00000014986	acvr1l	activin A receptor, type 1 like [Source:ZFIN;Acc:ZDB-GENE-990415-9]	2	41524238	41543762	11.45	8.17	7.41	12.36	12.35	12.29
ENSDARG00000014994	tbp	TATA box binding protein [Source:NCBI gene;Acc:368882]	13	24381344	24396199	3.87	2.93	2.92	4.01	4.17	4.14
ENSDARG00000014995	jagn1b	jagunal homolog 1b [Source:NCBI gene;Acc:445190]	22	28639026	28645676	5.52	7.27	7.12	3.94	4.02	4.12
ENSDARG00000015002	cdh4	cadherin 4, type 1, R-cadherin (retinal) [Source:ZFIN;Acc:ZDB-GENE-991207-1]	11	20371179	20826753	6.07	1.37	1.75	7.13	6.74	7.36
ENSDARG00000015003	arhgap4b	Rho GTPase activating protein 4b [Source:ZFIN;Acc:ZDB-GENE-090311-38]	23	18615992	18668836	7.93	2.62	3.43	10.69	11.43	11.13
ENSDARG00000015006	dnm1l	dynamin 1-like [Source:NCBI gene;Acc:393896]	25	20229573	20258634	27.27	19.44	20.39	26.12	26.83	26.74
ENSDARG00000015010	ccdc114	coiled-coil domain containing 114 [Source:ZFIN;Acc:ZDB-GENE-041114-110]	11	40713723	40728380	1.43	1.74	1.86	2.04	2.14	1.89
ENSDARG00000015015	tspan18b	tetraspanin 18b [Source:ZFIN;Acc:ZDB-GENE-040718-137]	18	27732285	27813164	21.9	13.89	15.65	28.58	30.07	31.21
ENSDARG00000015016	kif26ab	kinesin family member 26Ab [Source:ZFIN;Acc:ZDB-GENE-100716-6]	17	45815353	45989466	5	3.56	3.07	6.8	6.49	7.06
ENSDARG00000015025	l1camb	L1 cell adhesion molecule, paralog b [Source:NCBI gene;Acc:30656]	23	597082	637347	6.15	1.66	2.35	17.35	17.51	17.96
ENSDARG00000015033	slc23a1	solute carrier family 23 member 1 [Source:ZFIN;Acc:ZDB-GENE-080804-2]	21	30091417	30111134	4.46	7.61	5.15	2.64	2.81	2.99
ENSDARG00000015050	calm3a	calmodulin 3a (phosphorylase kinase, delta) [Source:ZFIN;Acc:ZDB-GENE-030131-5590]	12	24952902	24958792	290.24	306.12	299.11	218.01	214.87	221.29
ENSDARG00000015053	grip1	glutamate receptor interacting protein 1 [Source:ZFIN;Acc:ZDB-GENE-041210-125]	4	13035870	13255813	7	2.57	2.91	10.83	10.13	10.64
ENSDARG00000015057	ube2d4	ubiquitin-conjugating enzyme E2D 4 (putative) [Source:ZFIN;Acc:ZDB-GENE-070424-86]	8	52284075	52297799	7.91	3.86	3.34	10.1	9.92	9.45
ENSDARG00000015059	daam1a	dishevelled associated activator of morphogenesis 1a [Source:ZFIN;Acc:ZDB-GENE-030911-6]	17	14467015	14591764	13.07	10.32	9.33	13.12	12.95	14.11
ENSDARG00000015064	esrrd	estrogen-related receptor delta [Source:ZFIN;Acc:ZDB-GENE-040616-3]	18	48983506	48992363	0.75	0.19	0.14	0.57	0.73	0.86
ENSDARG00000015065	c4	complement component 4 [Source:ZFIN;Acc:ZDB-GENE-030131-5697]	15	32351886	32383529	0.37	0.4	0.38	0.72	0.88	0.86
ENSDARG00000015068	txk	TXK tyrosine kinase [Source:ZFIN;Acc:ZDB-GENE-130530-553]	23	44049509	44060455	0.15	0.12	0.18	0.06	0.07	0.13
ENSDARG00000015070	pola2	polymerase (DNA directed), alpha 2 [Source:ZFIN;Acc:ZDB-GENE-030131-778]	8	17174796	17184482	9.05	9.79	9.89	5.41	4.97	5.75
ENSDARG00000015072	dmrt2a	doublesex and mab-3 related transcription factor 2a [Source:ZFIN;Acc:ZDB-GENE-990621-7]	5	45007962	45013409	3.88	3.37	3.39	2.43	2.67	2.83
ENSDARG00000015076	gja8b	gap junction protein alpha 8 paralog b [Source:ZFIN;Acc:ZDB-GENE-010607-3]	1	47446032	47448850	0.07	0.11	0.19	3.98	3.5	3.9
ENSDARG00000015088	dnajb11	DnaJ heat shock protein family (Hsp40) member B11 [Source:ZFIN;Acc:ZDB-GENE-031113-9]	9	32641026	32648725	21.2	25.58	20.54	20.93	18.48	19.57
ENSDARG00000015110	slc18a2	solute carrier family 18 member 2 [Source:ZFIN;Acc:ZDB-GENE-080514-1]	17	23782324	23811332	1.49	0.44	0.7	2.31	2.29	2.32
ENSDARG00000015111	ddx39ab	DEAD (Asp-Glu-Ala-Asp) box polypeptide 39Ab [Source:ZFIN;Acc:ZDB-GENE-040426-2902]	3	5619058	5644028	113.11	95.87	108.09	85.47	84.58	85.14
ENSDARG00000015123	dnase1l4.1	deoxyribonuclease 1 like 4, tandem duplicate 1 [Source:ZFIN;Acc:ZDB-GENE-040808-35]	22	5667371	5682494	41.38	52.17	53.4	24.28	23.39	21.83
ENSDARG00000015126	gorasp2	golgi reassembly stacking protein 2 [Source:ZFIN;Acc:ZDB-GENE-040426-1659]	6	3864040	3875115	24.47	21.88	18.85	26.98	27.09	27.73
ENSDARG00000015128	rpl27	ribosomal protein L27 [Source:NCBI gene;Acc:325618]	3	29893966	29899230	825	1105.17	1258.98	693.44	711.88	724.24
ENSDARG00000015134	camkk1b	calcium/calmodulin-dependent protein kinase kinase 1, alpha b [Source:ZFIN;Acc:ZDB-GENE-080722-13]	15	15314189	15331501	6.12	1.84	1.03	6.33	7.39	8.72
ENSDARG00000015144	sh2d4bb	SH2 domain containing 4Bb [Source:ZFIN;Acc:ZDB-GENE-130531-42]	12	29289387	29305533	0.42	0.51	0.48	0.22	0.24	0.29
ENSDARG00000015149	wasa	WASP actin nucleation promoting factor a [Source:ZFIN;Acc:ZDB-GENE-081104-419]	8	25629722	25643932	0.6	0.63	0.75	0.8	0.78	0.79
ENSDARG00000015153	wdr91	WD repeat domain 91 [Source:NCBI gene;Acc:402898]	21	1586379	1611000	7.69	4.52	4.86	6.38	6.71	7.17
ENSDARG00000015156	fbln2	fibulin 2 [Source:ZFIN;Acc:ZDB-GENE-060711-1]	11	27274355	27383732	11.54	12.46	11.32	13.09	13.47	13.19
ENSDARG00000015158	slc50a1	solute carrier family 50 member 1 [Source:NCBI gene;Acc:503533]	16	23303859	23314878	1.26	1.95	1.36	1.8	2.21	1.47
ENSDARG00000015161	zgc:92664	zgc:92664 [Source:ZFIN;Acc:ZDB-GENE-040718-119]	7	26073114	26076970	9.4	10.41	9.5	9.12	8.12	9.48
ENSDARG00000015164	mknk2b	MAPK interacting serine/threonine kinase 2b [Source:ZFIN;Acc:ZDB-GENE-030829-2]	8	20438884	20450375	32.44	23.33	19.54	118.78	142.54	141.47
ENSDARG00000015174	atp6v0a1b	ATPase H+ transporting V0 subunit a1b [Source:ZFIN;Acc:ZDB-GENE-050522-215]	24	7596009	7632187	44.63	10.85	16.04	49.35	50.02	52.98
ENSDARG00000015176	ror1	receptor tyrosine kinase-like orphan receptor 1 [Source:ZFIN;Acc:ZDB-GENE-030131-6104]	6	31772182	31987940	2.18	1.94	1.39	2.84	2.51	2.62
ENSDARG00000015184	mpp3a	membrane protein, palmitoylated 3a (MAGUK p55 subfamily member 3) [Source:ZFIN;Acc:ZDB-GENE-130530-555]	3	38647987	38692920	7.71	2.31	3.31	7.45	7.13	7.04
ENSDARG00000015199	cblb	Cbl proto-oncogene B, E3 ubiquitin protein ligase [Source:ZFIN;Acc:ZDB-GENE-030616-417]	10	28587446	28733002	2.37	2.21	1.66	4.32	4.12	3.75
ENSDARG00000015201	pcmt	protein-L-isoaspartate (D-aspartate) O-methyltransferase [Source:NCBI gene;Acc:30751]	20	1293988	1314537	16.56	14.97	17.54	13.11	13.34	14
ENSDARG00000015206	hnrnpua	heterogeneous nuclear ribonucleoprotein Ua [Source:ZFIN;Acc:ZDB-GENE-030131-3731]	17	11470572	11495466	20.39	13.01	11.46	18.91	18.77	18.82
ENSDARG00000015208	rbb4l	retinoblastoma binding protein 4, like [Source:NCBI gene;Acc:322129]	11	12744575	12770546	15.74	15.81	16.04	11.97	11.93	11.82
ENSDARG00000015221	nsrp1	nuclear speckle splicing regulatory protein 1 [Source:NCBI gene;Acc:323926]	21	40402508	40413191	11.94	11.16	9.79	10.9	9.41	9.81
ENSDARG00000015222	cbll1	Cbl proto-oncogene-like 1, E3 ubiquitin protein ligase [Source:ZFIN;Acc:ZDB-GENE-040426-691]	4	18836791	18841071	3.3	2.49	2.23	4.63	4.02	4.32
ENSDARG00000015224	cd2ap	CD2-associated protein [Source:ZFIN;Acc:ZDB-GENE-030131-8078]	20	35857399	35980452	14.26	12.85	9.05	13.52	13.45	12.49
ENSDARG00000015228	ero1a	endoplasmic reticulum oxidoreductase alpha [Source:NCBI gene;Acc:393321]	17	15018219	15029639	2.37	2.6	1.82	9.23	10.5	10.34
ENSDARG00000015236	pycr3	pyrroline-5-carboxylate reductase 3 [Source:NCBI gene;Acc:560682]	20	52529044	52544761	4.79	5.35	4.57	3.66	3.12	3.6
ENSDARG00000015239	prpf19	pre-mRNA processing factor 19 [Source:ZFIN;Acc:ZDB-GENE-030131-263]	14	16287968	16301402	24	20.7	18.83	20.39	18.71	19.36
ENSDARG00000015240	cdkl5	cyclin-dependent kinase-like 5 [Source:ZFIN;Acc:ZDB-GENE-081022-110]	11	30161168	30232133	4.95	1.18	1.48	7.1	7.12	7.04
ENSDARG00000015252	st3gal3b	ST3 beta-galactoside alpha-2,3-sialyltransferase 3b [Source:ZFIN;Acc:ZDB-GENE-050411-13]	2	17266759	17393971	3.67	3.32	2.87	5.16	5.41	5.29
ENSDARG00000015254	fzr1a	fizzy/cell division cycle 20 related 1a [Source:ZFIN;Acc:ZDB-GENE-040426-712]	8	20863025	20876562	17.29	13.12	13.61	18.89	21.32	20.48
ENSDARG00000015263	adma	adrenomedullin a [Source:ZFIN;Acc:ZDB-GENE-120221-5]	7	66874770	66877058	5.14	7.01	6.8	22.26	27.41	26.1
ENSDARG00000015266	mab21l2	mab-21-like 2 [Source:NCBI gene;Acc:117234]	1	24076243	24078099	50.76	25.99	34.92	66.8	67.73	72.19
ENSDARG00000015273	alpi.1	alkaline phosphatase, intestinal, tandem duplicate 1 [Source:ZFIN;Acc:ZDB-GENE-050327-78]	22	38762693	38779462	3.62	3.32	3.37	4.77	5.46	5.1
ENSDARG00000015278	plxnc1	plexin C1 [Source:ZFIN;Acc:ZDB-GENE-030131-1620]	4	18281954	18315912	1.95	2.02	2.14	2.54	2.86	2.79
ENSDARG00000015290	sacm1la	SAC1 like phosphatidylinositide phosphatase a [Source:NCBI gene;Acc:558940]	19	26967357	27006764	9.52	6.67	6.49	8.6	8.22	7.58
ENSDARG00000015292	ube2g1a	ubiquitin-conjugating enzyme E2G 1a (UBC7 homolog, yeast) [Source:ZFIN;Acc:ZDB-GENE-030131-6065]	5	30986512	30995108	11.87	9.13	8.19	13.09	12.47	12.44
ENSDARG00000015293	fam110a	family with sequence similarity 110 member A [Source:ZFIN;Acc:ZDB-GENE-030521-6]	8	49206057	49207319	4.98	4.97	2.35	2.88	3.43	2.62
ENSDARG00000015310	pitpnbl	phosphatidylinositol transfer protein, beta, like [Source:ZFIN;Acc:ZDB-GENE-040426-2791]	3	15332268	15352438	37.93	33.29	35.7	35.45	34.28	33.97
ENSDARG00000015312	klf12a	Kruppel-like factor 12a [Source:ZFIN;Acc:ZDB-GENE-030131-9188]	1	34496855	34558804	2.34	2.18	1.94	3.93	4.33	4.57
ENSDARG00000015314	zc4h2	zinc finger, C4H2 domain containing [Source:NCBI gene;Acc:323487]	5	22073710	22082918	63.47	33.02	42.5	67.15	66.72	73.55
ENSDARG00000015315	psmc5	proteasome 26S subunit, ATPase 5 [Source:ZFIN;Acc:ZDB-GENE-030131-6547]	12	10163585	10175487	77.06	92.1	100.99	59.44	59.83	58.6
ENSDARG00000015325	u2af1	U2 small nuclear RNA auxiliary factor 1 [Source:ZFIN;Acc:ZDB-GENE-020419-1]	9	9230446	9242777	88.73	92.37	92.96	64.24	60.57	61.41
ENSDARG00000015337	comta	catechol-O-methyltransferase a [Source:NCBI gene;Acc:561372]	8	8459192	8480579	21.53	25.64	21.75	12.12	14	12.97
ENSDARG00000015343	pgd	phosphogluconate dehydrogenase [Source:ZFIN;Acc:ZDB-GENE-040426-2807]	23	29381441	29394505	42.51	51.69	43.46	30	31.3	30.39
ENSDARG00000015349	mfge8a	milk fat globule-EGF factor 8 protein a [Source:ZFIN;Acc:ZDB-GENE-050327-9]	7	48788147	48805181	18.57	9.52	14.01	15.39	15.89	15.77
ENSDARG00000015352	lsg1	large 60S subunit nuclear export GTPase 1 [Source:NCBI gene;Acc:323464]	11	34219991	34232906	16.51	18.59	17.41	11.96	12.94	12.12
ENSDARG00000015355	fosl1a	FOS-like antigen 1a [Source:ZFIN;Acc:ZDB-GENE-061207-7]	14	30739476	30747686	0.56	0.38	0.38	3.95	3.95	4.33
ENSDARG00000015356	fcho1	FCH and mu domain containing endocytic adaptor 1 [Source:NCBI gene;Acc:100913078]	8	20037368	20072776	2.67	1.51	1.27	2.72	2.52	2.63
ENSDARG00000015374	st3gal3a	ST3 beta-galactoside alpha-2,3-sialyltransferase 3a [Source:ZFIN;Acc:ZDB-GENE-040426-1322]	6	3334392	3405495	8.67	8.01	8.02	8.96	9.5	9.35
ENSDARG00000015385	ndufs3	NADH:ubiquinone oxidoreductase core subunit S3 [Source:ZFIN;Acc:ZDB-GENE-050417-274]	7	38588866	38594602	105.05	128.45	135.57	71.1	75.3	77.86
ENSDARG00000015392	dhx15	DEAH (Asp-Glu-Ala-His) box helicase 15 [Source:ZFIN;Acc:ZDB-GENE-030131-650]	7	71278625	71331690	42.71	29.06	28.57	36.86	37.12	36.73
ENSDARG00000015394	thoc7	THO complex 7 [Source:NCBI gene;Acc:445035]	11	19603251	19611356	21.38	22.86	23.01	13.67	15.64	15.74
ENSDARG00000015399	foxf1	forkhead box F1 [Source:NCBI gene;Acc:566407]	18	30847237	30850365	4.6	5.06	5.01	9.22	8.03	9.09
ENSDARG00000015404	arl3l2	ADP ribosylation factor like GTPase 3, like 2 [Source:ZFIN;Acc:ZDB-GENE-040426-1678]	14	909769	917065	20.11	4.64	4.34	13	13.12	13.43
ENSDARG00000015422	ppil1	peptidylprolyl isomerase (cyclophilin)-like 1 [Source:ZFIN;Acc:ZDB-GENE-051009-1]	6	40647788	40657672	18.88	21.66	23.75	11.98	10.86	12.12
ENSDARG00000015425	slc24a4a	solute carrier family 24 member 4a [Source:ZFIN;Acc:ZDB-GENE-050417-291]	17	38784362	38887424	3.22	1.16	1.54	3.95	4.74	3.78
ENSDARG00000015427	hdac1	histone deacetylase 1 [Source:ZFIN;Acc:ZDB-GENE-020419-32]	19	29808471	29821601	48.47	37.85	40.56	42.11	41	41.56
ENSDARG00000015445	lim2.4	lens intrinsic membrane protein 2.4 [Source:ZFIN;Acc:ZDB-GENE-040718-104]	19	10537443	10554352	0.9	0.98	1.47	6.77	5.74	8.18
ENSDARG00000015449	fut8a	fucosyltransferase 8a (alpha (1,6) fucosyltransferase) [Source:ZFIN;Acc:ZDB-GENE-031118-20]	17	33719415	33906488	16.73	8.88	7.23	20.54	20.54	21.01
ENSDARG00000015460	racgap1	Rac GTPase activating protein 1 [Source:ZFIN;Acc:ZDB-GENE-030131-1917]	23	33757173	33775309	3.06	2.45	2.36	2.8	2.62	2.29
ENSDARG00000015471	chd1l	chromodomain helicase DNA binding protein 1-like [Source:NCBI gene;Acc:393283]	6	36844127	36877587	2.84	2.34	2.1	2.77	2.6	2.83
ENSDARG00000015472	gpc4	glypican 4 [Source:ZFIN;Acc:ZDB-GENE-011119-1]	14	31090151	31151148	24.4	22.36	18.5	33.9	34.68	33.24
ENSDARG00000015474	ppp2r5ea	protein phosphatase 2, regulatory subunit B', epsilon isoform a [Source:ZFIN;Acc:ZDB-GENE-030904-7]	20	20229586	20270191	9.09	6.54	5.7	8.97	8.78	9.11
ENSDARG00000015476	iqch	IQ motif containing H [Source:NCBI gene;Acc:567336]	18	19820008	19880934	0.13	0.09	0.18	0.37	0.45	0.31
ENSDARG00000015482	zgc:113518	zgc:113518 [Source:ZFIN;Acc:ZDB-GENE-050220-9]	2	176711	184051	4.61	3.14	3.76	3.58	3.67	3.9
ENSDARG00000015495	klf3	Kruppel-like factor 3 (basic) [Source:ZFIN;Acc:ZDB-GENE-011116-1]	1	18594146	18615063	7.99	7.01	4.71	9.65	9.67	9.1
ENSDARG00000015502	adam10b	ADAM metallopeptidase domain 10b [Source:ZFIN;Acc:ZDB-GENE-071115-1]	25	150570	160547	18.43	11.35	11.57	32.23	29.83	33.32
ENSDARG00000015506	pibf1	progesterone immunomodulatory binding factor 1 [Source:ZFIN;Acc:ZDB-GENE-040801-94]	1	34593490	34685007	3.38	2.41	1.72	2.91	2.65	2.73

ENSDARG00000015515	mc4r	melanocortin 4 receptor [Source:NCBI gene;Acc:286833]	2	27932781	27933864		0.89	0.38	0.31	0.72	0.89	0.89
ENSDARG00000015524	prps1a	phosphoribosyl pyrophosphate synthetase 1A [Source:ZFIN;Acc:ZDB-GENE-011212-5]	5	23136544	23147665		31.18	40.22	40.09	39.82	39.09	37.9
ENSDARG00000015527	CU929145.1	myeloid-associated differentiation marker-like protein 2 [Source:NCBI gene;Acc:101882206]	24	39120725	39121900		0.04	0.12	0.02	0.08	0	0.02
ENSDARG00000015531	slc4a8	solute carrier family 4 member 8 [Source:ZFIN;Acc:ZDB-GENE-131119-88]	6	40354424	40405934		7.77	1.81	3.05	7.11	7.81	7.76
ENSDARG00000015536	sox6	SRY-box transcription factor 6 [Source:ZFIN;Acc:ZDB-GENE-081120-6]	7	27250012	27454645		4.75	3.75	2.61	7.63	7.01	6.96
ENSDARG00000015537	gad2	glutamate decarboxylase 2 [Source:ZFIN;Acc:ZDB-GENE-030909-9]	24	6133147	6158933		29.01	4.69	8.41	34.7	36.36	35.81
ENSDARG00000015538	znf207a	zinc finger protein 207, a [Source:ZFIN;Acc:ZDB-GENE-040426-2273]	3	34234029	34245025		28.26	18.79	16.24	28.4	24.89	26.05
ENSDARG00000015540	psen2	presenilin 2 [Source:NCBI gene;Acc:58026]	1	51048975	51062405		13.58	12.13	10.17	16.19	16.98	17.16
ENSDARG00000015543	s100a1	S100 calcium binding protein A1 [Source:ZFIN;Acc:ZDB-GENE-040916-1]	16	29385565	29387215		1.17	2.27	1.17	0.86	1.16	0.78
ENSDARG00000015546	alpl	alkaline phosphatase, biomineralization associated [Source:ZFIN;Acc:ZDB-GENE-040420-1]	11	27968834	28010171		16.24	16.69	12.8	15.4	17.09	16.77
ENSDARG00000015551	fth1a	ferritin, heavy polypeptide 1a [Source:ZFIN;Acc:ZDB-GENE-000831-2]	7	12927944	12936708		971.22	1285.09	1289.82	667.29	723.14	722.8
ENSDARG00000015552	phactr4a	phosphatase and actin regulator 4a [Source:NCBI gene;Acc:569383]	19	15142527	15229421		5.91	5.5	4.09	7.62	7.55	7.62
ENSDARG00000015554	zic2a	zic family member 2 (odd-paired homolog, Drosophila), a [Source:ZFIN;Acc:ZDB-GENE-000710-4]	9	31282161	31287010		18.4	11.31	10.79	18.52	19.18	19.25
ENSDARG00000015557	zgc:100829	zgc:100829 [Source:ZFIN;Acc:ZDB-GENE-040801-54]	21	28747069	28794078		12.24	11.11	9.96	11.89	12.75	12.79
ENSDARG00000015559	fstl1a	follistatin-like 1a [Source:ZFIN;Acc:ZDB-GENE-030131-4148]	1	47459723	47485360		13.95	20.69	20.02	21.57	23.82	22.38
ENSDARG00000015563	zgc:123305	zgc:123305 [Source:ZFIN;Acc:ZDB-GENE-051120-171]	6	1567446	1591002		2.33	1.74	1.48	2.74	2.81	2.98
ENSDARG00000015566	dnmt3ab	DNA (cytosine-5-)-methyltransferase 3 alpha b [Source:ZFIN;Acc:ZDB-GENE-050314-3]	17	36936918	37054959		5.82	3.25	2.9	8.97	8.64	8.91
ENSDARG00000015567	zic1	zic family member 1 (odd-paired homolog, Drosophila) [Source:ZFIN;Acc:ZDB-GENE-000208-4]	24	4970580	4973765		20.32	11.93	12.91	25.09	25.75	26.37
ENSDARG00000015575	cyp3c1	cytochrome P450, family 3, subfamily c, polypeptide 1 [Source:ZFIN;Acc:ZDB-GENE-030131-3060]	3	40969242	40976582		25.33	31.39	28.42	22.26	22.03	22.78
ENSDARG00000015576	itprip	inositol 1,4,5-trisphosphate receptor interacting protein [Source:NCBI gene;Acc:550570]	13	25403413	25408387		2.56	1.94	2.05	1.78	1.85	1.78
ENSDARG00000015589	ankrd33bb	ankyrin repeat domain 33Bb [Source:ZFIN;Acc:ZDB-GENE-070912-531]	2	30531726	30548220		1.28	0.14	0.18	0.76	1.29	0.98
ENSDARG00000015592	c1ql2	complement component 1, q subcomponent-like 2 [Source:ZFIN;Acc:ZDB-GENE-081105-160]	9	844221	849069		1.7	0.37	0.4	1.73	2.02	2.31
ENSDARG00000015607	si:ch211-220f21.2	si:ch211-220f21.2 [Source:ZFIN;Acc:ZDB-GENE-160113-17]	7	6085485	6279138		0	0.01	0	0	0	0
ENSDARG00000015610	tubg1	tubulin, gamma 1 [Source:ZFIN;Acc:ZDB-GENE-040426-836]	3	37059803	37082618		11.71	7.21	8.53	10.25	10.17	9.83
ENSDARG00000015611	rasl11b	RAS-like, family 11, member B [Source:NCBI gene;Acc:393109]	20	23022985	23026223		5.61	4.55	4.99	10.27	11.57	11.28
ENSDARG00000015623	ada2b	adenosine deaminase 2b [Source:ZFIN;Acc:ZDB-GENE-041210-77]	4	5225552	5240128		0.25	0.23	0.25	0.56	0.53	0.41
ENSDARG00000015627	rgs6	regulator of G protein signaling 6 [Source:ZFIN;Acc:ZDB-GENE-030131-31]	20	28511941	28642061		5.33	2.25	3.09	4.52	5.52	4.71
ENSDARG00000015628	psmd10	proteasome 26S subunit, non-ATPase 10 [Source:ZFIN;Acc:ZDB-GENE-050112-1]	14	28464736	28469244		7.11	7.62	8.31	6.83	4.44	6.18
ENSDARG00000015638	gemin2	gem (nuclear organelle) associated protein 2 [Source:ZFIN;Acc:ZDB-GENE-030131-3756]	17	13088594	13098319		5.69	6.48	6.72	5.1	4.66	5.07
ENSDARG00000015649	rap2c	RAP2C, member of RAS oncogene family [Source:ZFIN;Acc:ZDB-GENE-041024-1]	14	30975163	30983011		16.84	15.79	13.32	25.84	25.11	26.05
ENSDARG00000015653	tnip1	TNFAIP3 interacting protein 1 [Source:ZFIN;Acc:ZDB-GENE-070112-1502]	14	49376011	49432800		22.96	12.62	11.78	25.11	24.56	25.81
ENSDARG00000015654	ca15a	carbonic anhydrase XVa [Source:ZFIN;Acc:ZDB-GENE-070424-7]	12	4320041	4331117		21.67	26.35	25.52	14.31	12.59	12.54
ENSDARG00000015657	zgc:77112	zgc:77112 [Source:ZFIN;Acc:ZDB-GENE-040426-2219]	8	52442622	52447101		2.65	0.66	0.59	2.15	2.29	2.1
ENSDARG00000015662	pla2g12b	phospholipase A2, group XIIB [Source:ZFIN;Acc:ZDB-GENE-040426-2771]	13	4671698	4683595		97.63	162.93	143.66	75.92	71.12	70.37
ENSDARG00000015674	msx3	muscle segment homeobox 3 [Source:NCBI gene;Acc:30526]	13	24662238	24664668		10.62	12.99	13.17	10.01	10.06	10.49
ENSDARG00000015676	nmd3	NMD3 ribosome export adaptor [Source:NCBI gene;Acc:541444]	15	1653779	1672461		18.47	22.31	23.14	13.94	13.38	13.59
ENSDARG00000015678	si:dkeyp-75b4.9	si:dkeyp-75b4.9 [Source:ZFIN;Acc:ZDB-GENE-090313-396]	1	9986675	9988840		0	0	0	1.62	1.71	1.38
ENSDARG00000015681	gsk3ab	glycogen synthase kinase 3 alpha b [Source:ZFIN;Acc:ZDB-GENE-990714-3]	16	11242443	11292148		18.37	12.05	10.52	30.07	29.4	29.06
ENSDARG00000015686	bmp6	bone morphogenetic protein 6 [Source:ZFIN;Acc:ZDB-GENE-050306-42]	24	7861373	7935809		2.25	2.23	2.2	3.79	3.47	3.71
ENSDARG00000015707	dvl3a	dishevelled segment polarity protein 3a [Source:ZFIN;Acc:ZDB-GENE-010319-1]	2	9702053	9744081		7.47	5.82	5	9.87	9.26	9.77
ENSDARG00000015709	hsd17b12a	hydroxysteroid (17-beta) dehydrogenase 12a [Source:NCBI gene;Acc:327417]	25	30196039	30230559		10.58	8.07	6.33	10.68	11.21	10.46
ENSDARG00000015722	kctd5a	potassium channel tetramerization domain containing 5a [Source:ZFIN;Acc:ZDB-GENE-030131-727]	3	45261387	45281350		12.8	9.89	8.91	15.36	16.18	15.57
ENSDARG00000015731	ano5a	anoctamin 5a [Source:ZFIN;Acc:ZDB-GENE-091013-2]	25	35189555	35244242		4.8	4.44	2.94	4.57	4.48	4.69
ENSDARG00000015732	cax2	cation/H+ exchanger protein 2 [Source:ZFIN;Acc:ZDB-GENE-100825-2]	25	18889332	18906871		3.99	3.42	3.85	3.85	3.55	3.99
ENSDARG00000015734	aicda	activation-induced cytidine deaminase [Source:ZFIN;Acc:ZDB-GENE-041122-2]	16	12660477	12665652		0	0	0.02	0.03	0	0
ENSDARG00000015747	aarsd1	alanyl-tRNA synthetase domain containing 1 [Source:NCBI gene;Acc:445178]	12	6049355	6063328		10.8	11.81	10.68	8.07	8.21	7.16
ENSDARG00000015749	hps3	HPS3 biogenesis of lysosomal organelles complex 2 subunit 1 [Source:ZFIN;Acc:ZDB-GENE-061110-115]	22	38202614	38224321		4.91	4.71	4.32	5.15	5.22	5.36
ENSDARG00000015752	zap70	zeta chain of T cell receptor associated protein kinase 70 [Source:ZFIN;Acc:ZDB-GENE-050522-257]	8	20393250	20414364		0.26	0.27	0.31	0.16	0.2	0.21
ENSDARG00000015757	tmem50a	transmembrane protein 50A [Source:ZFIN;Acc:ZDB-GENE-040426-2937]	13	45485185	45495562		32.45	32.54	32.77	31.64	30.03	30.47
ENSDARG00000015759	tspan7	tetraspanin 7 [Source:ZFIN;Acc:ZDB-GENE-030131-5435]	14	41469478	41478392		28.87	36.85	33.47	31.89	32.33	30.47
ENSDARG00000015765	iah1	isoamyl acetate hydrolyzing esterase 1 (putative) [Source:NCBI gene;Acc:449926]	17	35243753	35247374		3.16	3.88	3.96	3.94	3.77	3.41
ENSDARG00000015780	ankrd46b	ankyrin repeat domain 46b [Source:NCBI gene;Acc:402888]	7	50910959	50917255		15.47	8.75	10.18	12.44	14.17	14.25
ENSDARG00000015790	atp1b3a	ATPase Na+/K+ transporting subunit beta 3a [Source:ZFIN;Acc:ZDB-GENE-990415-167]	2	16547706	16565690		45.47	40.04	39.8	47.75	48	50.29
ENSDARG00000015793	creb3l1	cAMP responsive element binding protein 3-like 1 [Source:ZFIN;Acc:ZDB-GENE-030219-181]	7	38897836	38954874		2.13	2.01	1.79	3.02	2.92	3.1
ENSDARG00000015803	smcr8b	Smith-Magenis syndrome chromosome region, candidate 8b [Source:NCBI gene;Acc:407723]	1	8534698	8540050		0.64	0.83	0.72	0.76	0.58	0.83
ENSDARG00000015805	cgnl1	cingulin-like 1 [Source:ZFIN;Acc:ZDB-GENE-071009-2]	7	52431747	52498175		14.76	13.46	12.98	14.83	14.56	14.89
ENSDARG00000015807	rab22a	RAB22A, member RAS oncogene family [Source:ZFIN;Acc:ZDB-GENE-041114-161]	6	49551614	49580086		3.38	3.2	2.88	4.7	5.03	4.52
ENSDARG00000015822	sesn3	sestrin 3 [Source:ZFIN;Acc:ZDB-GENE-040426-2924]	15	35043007	35092735		10.61	11.45	9.23	13.87	13.48	13.62
ENSDARG00000015823	vps26b	VPS26, retromer complex component B [Source:NCBI gene;Acc:406684]	15	19324697	19331078		13.3	14.25	12.54	12.89	11.91	12.49
ENSDARG00000015824	lemd3	LEM domain containing 3 [Source:ZFIN;Acc:ZDB-GENE-030131-490]	4	12917164	12930086		11.07	7.36	7.13	14.13	13.32	14.66
ENSDARG00000015829	mov10a	Mov10 RISC complex RNA helicase a [Source:ZFIN;Acc:ZDB-GENE-091230-8]	6	48348415	48376245		8.89	9.33	8.62	6.87	6.85	7.19
ENSDARG00000015835	eif4e2rs1	eukaryotic translation initiation factor 4E family member 2 related sequence 1 [Source:ZFIN;Acc:ZDB-GENE-040426-1728]	23	44427188	44440798		11.64	10.1	11.29	13.53	13.03	13
ENSDARG00000015851	pnn	pinin, desmosome associated protein [Source:ZFIN;Acc:ZDB-GENE-060519-3]	17	13099476	13110121		49.58	30.94	33.62	41.6	39.45	39.16
ENSDARG00000015854	chata	choline O-acetyltransferase a [Source:NCBI gene;Acc:100170938]	13	29406814	29421331		5.38	2.34	2.87	6.64	7.5	7.15
ENSDARG00000015856	slc25a40	solute carrier family 25 member 40 [Source:NCBI gene;Acc:436633]	16	43126252	43139257		3.33	3.11	2.23	2.9	2.5	3
ENSDARG00000015860	mtmr9	myotubularin related protein 9 [Source:ZFIN;Acc:ZDB-GENE-040724-223]	20	18943406	18983769		10.04	5.98	6.47	8.74	9.61	9.5
ENSDARG00000015862	rpl5b	ribosomal protein L5b [Source:ZFIN;Acc:ZDB-GENE-040625-93]	6	29086594	29097393		1157.67	1375.51	1577.8	898.73	922.24	938.81
ENSDARG00000015863	socs6b	suppressor of cytokine signaling 6b [Source:ZFIN;Acc:ZDB-GENE-030131-1670]	2	30423428	30430345		6.78	4.29	4.28	6.53	6.27	6.68
ENSDARG00000015866	apoa2	apolipoprotein A-II [Source:ZFIN;Acc:ZDB-GENE-030131-1046]	16	23978978	23980354		3505.87	5530.05	6189.29	2638.13	2800.03	2632.78
ENSDARG00000015869	slc22a16	solute carrier family 22 member 16 [Source:ZFIN;Acc:ZDB-GENE-041001-166]	20	31287356	31305503		6.47	9.84	8.39	2.67	2.31	2.45
ENSDARG00000015876	npas1	neuronal PAS domain protein 1 [Source:ZFIN;Acc:ZDB-GENE-060503-934]	15	8424711	8517555		2.11	0.58	0.69	3.21	3.35	3
ENSDARG00000015879	pax8	paired box 8 [Source:ZFIN;Acc:ZDB-GENE-001030-3]	5	72407526	72415578		4	1.45	3.12	9.68	10.63	9.65
ENSDARG00000015887	b2ml	beta-2-microglobulin, like [Source:ZFIN;Acc:ZDB-GENE-040426-2136]	8	50942240	50950549		30.75	31.41	24.03	29.68	30.03	29.99
ENSDARG00000015889	zc3h15	zinc finger CCCH-type containing 15 [Source:NCBI gene;Acc:334307]	9	11664797	11676491		35.73	41.25	39.87	26.88	25.26	24.67
ENSDARG00000015890	mafa	v-maf avian musculoaponeurotic fibrosarcoma oncogene homolog a (paralog a) [Source:ZFIN;Acc:ZDB-GENE-010605-2]	18	29402623	29413756		4.14	4.04	2.89	5.22	4.97	4.69
ENSDARG00000015891	ptprea	protein tyrosine phosphatase receptor type Ea [Source:ZFIN;Acc:ZDB-GENE-100714-1]	12	43376436	43576437		2.55	1.77	1.39	4.17	4.08	4.33
ENSDARG00000015901	ptbp2b	polypyrimidine tract binding protein 2b [Source:ZFIN;Acc:ZDB-GENE-060810-49]	2	19777146	19795593		12.93	5.04	6.2	15.61	15.3	16.51
ENSDARG00000015902	stat6	signal transducer and activator of transcription 6, interleukin-4 induced [Source:ZFIN;Acc:ZDB-GENE-030131-9359]	23	27078866	27123180		10.82	10.11	7.98	11.25	11.45	10.62
ENSDARG00000015905	lztr1	leucine-zipper-like transcription regulator 1 [Source:ZFIN;Acc:ZDB-GENE-030131-9564]	5	12724046	12743212		9.24	6.94	6.41	9.67	9.95	9.91
ENSDARG00000015906	mxtx2	mix-type homeobox gene 2 [Source:ZFIN;Acc:ZDB-GENE-000710-6]	12	29236274	29237815		0.03	0	0.04	0.05	0.04	0.02
ENSDARG00000015907	dcbld1	discoidin, CUB and LCCL domain containing 1 [Source:ZFIN;Acc:ZDB-GENE-050913-77]	20	42296256	42378994		2.36	1.83	1.34	2.17	2.06	2.15
ENSDARG00000015915	slc25a36b	solute carrier family 25 member 36b [Source:ZFIN;Acc:ZDB-GENE-080219-28]	15	41767675	41807371		11.15	11.38	9.42	7.52	8.46	8.24
ENSDARG00000015917	rnft1	ring finger protein, transmembrane 1 [Source:NCBI gene;Acc:406509]	10	28160265	28169169		10.09	11.29	11.09	12.03	11.73	11.93
ENSDARG00000015918	dlat	dihydrolipoamide S-acetyltransferase (E2 component of pyruvate dehydrogenase complex) [Source:ZFIN;Acc:ZDB-GENE-030131-2	21	23019372	23035120		33.05	38.56	31.75	50.32	46.53	45.35
ENSDARG00000015921	pwp1	PWP1 homolog, endonuclein [Source:ZFIN;Acc:ZDB-GENE-040426-1049]	4	1825839	1839352		10.95	12.91	13.51	6.23	6.22	5.94
ENSDARG00000015922	stk17al	serine/threonine kinase 17a like [Source:ZFIN;Acc:ZDB-GENE-061103-427]	3	16841942	16872909		4.29	4.19	3.28	3.6	4.28	3.45
ENSDARG00000015930	rwdd1	RWD domain containing 1 [Source:ZFIN;Acc:ZDB-GENE-040718-270]	16	32197554	32204470		42.08	55.26	47.22	37.45	35.17	35.64
ENSDARG00000015931	snap91b	synaptosome associated protein 91b [Source:ZFIN;Acc:ZDB-GENE-041210-81]	4	5260941	5302866		41.19	9.78	11.06	49.91	50.82	52.39
ENSDARG00000015937	kptn	kaptin (actin binding protein) [Source:ZFIN;Acc:ZDB-GENE-040801-258]	5	36913423	36924509		7.26	7.01	6.94	6.06	5.78	5.74
ENSDARG00000015946	slc44a1a	solute carrier family 44 member 1a [Source:ZFIN;Acc:ZDB-GENE-050417-204]	1	52632856	52652163		6.29	5.26	5.55	6.42	6.07	5.97
ENSDARG00000015947	matn4	matrilin 4 [Source:ZFIN;Acc:ZDB-GENE-050208-64]	6	52790049	52846194		141.34	160.25	148.27	153.94	165	160.46
ENSDARG00000015955	cldnc	claudin c [Source:ZFIN;Acc:ZDB-GENE-010328-3]	21	25754268	25756119		9.97	12.48	12.04	14.32	15.21	14.33
ENSDARG00000015964	zgc:153311	zgc:153311 [Source:ZFIN;Acc:ZDB-GENE-060825-194]	6	30484005	30485009		0.69	0.87	1.01	0.84	1.01	1.1
ENSDARG00000015966	yaf2	YY1 associated factor 2 [Source:ZFIN;Acc:ZDB-GENE-041210-115]	4	13905522	13921206		11.15	9.52	8.93	11.47	10.48	11.09
ENSDARG00000015968	rabgef1l	RAB guanine nucleotide exchange factor (GEF) 1, like [Source:ZFIN;Acc:ZDB-GENE-040426-813]	15	1718668	1733259		1.71	0.81	0.79	2.45	2.36	2.58
ENSDARG00000015970	phip	pleckstrin homology domain interacting protein [Source:ZFIN;Acc:ZDB-GENE-050208-261]	23	31276162	31346387		6.01	2.64	2.67	6.9	7.02	6.93
ENSDARG00000015971	rapgef1b	Rap guanine nucleotide exchange factor (GEF) 1b [Source:ZFIN;Acc:ZDB-GENE-040426-1001]	21	4116437	4219783		4.81	3.27	2.63	6.51	6.51	6.39
ENSDARG00000015978	COX5B	zgc:86599 [Source:ZFIN;Acc:ZDB-GENE-040625-180]	10	42374770	42378524	COX5B	682.17	809.75	855.43	506.73	525.81	532.27
ENSDARG00000015985	abcc9	ATP-binding cassette, sub-family C (CFTR/MRP), member 9 [Source:ZFIN;Acc:ZDB-GENE-050517-23]	4	14660769	14713773		4.86	2.88	2.64	8.56	7.03	7.47
ENSDARG00000015989	zdhhc16b	zinc finger DHHC-type containing 16b [Source:NCBI gene;Acc:393316]	12	2849584	2869584		1.96	1.31	1.53	1.6	1.96	1.76
ENSDARG00000015990	hcfc1a	host cell factor C1a [Source:ZFIN;Acc:ZDB-GENE-030912-10]	11	25436440	25461336		12.21	8.01	6.95	16.68	15.7	15.47
ENSDARG00000015997	mfsd5	major facilitator superfamily domain containing 5 [Source:NCBI gene;Acc:436986]	23	35786942	35790260		3.09	2.79	2.49	4.22	4.13	3.78
ENSDARG00000015998	wdhd1	WD repeat and HMG-box DNA binding protein 1 [Source:ZFIN;Acc:ZDB-GENE-030131-1665]	17	15148797	15189502		4.37	3.58	3.25	2.57	2.4	2.38
ENSDARG00000016010	nup107	nucleoporin 107 [Source:ZFIN;Acc:ZDB-GENE-041210-245]	4	22730795	22749553		14.84	13.66	12.6	12.38	13.49	12.4
ENSDARG00000016011	gpcpd1	glycerophosphocholine phosphodiesterase 1 [Source:ZFIN;Acc:ZDB-GENE-060503-472]	20	45669176	45722895		18.07	16.16	16.35	20.07	21.16	20.95
ENSDARG00000016016	dbnlb	drebrin-like b [Source:ZFIN;Acc:ZDB-GENE-050522-293]	8	52314542	52350684		9.57	7.29	7.1	13.13	14.18	13.14
ENSDARG00000016022	zic5	zic family member 5 (odd-paired homolog, Drosophila) [Source:ZFIN;Acc:ZDB-GENE-040622-1]	9	31274108	31314324		7.98	4.93	5.29	6.38	7.38	7.13
ENSDARG00000016038	hacd3	3-hydroxyacyl-CoA dehydratase 3 [Source:NCBI gene;Acc:393441]	18	18982077	18995283		15.66	17.56	15.35	11.15	11.45	11.96
ENSDARG00000016044	gins4	GINS complex subunit 4 (Sld5 homolog) [Source:ZFIN;Acc:ZDB-GENE-040801-59]	8	52415603	52431522		4.27	5.21	4.85	2.24	2.61	2.36
ENSDARG00000016048	gpat3	glycerol-3-phosphate acyltransferase 3 [Source:NCBI gene;Acc:436958]	21	19301562	19316985		15.23	19.52	18.18	19.11	19.26	19.31
ENSDARG00000016056	mhc1laa	major histocompatibility complex class I LAA [Source:ZFIN;Acc:ZDB-GENE-050417-464]	8	44442220	44448423		3.92	5.02	3.9	4.25	4.56	4.38
ENSDARG00000016058	fibpa	fibroblast growth factor (acidic) intracellular binding protein a [Source:ZFIN;Acc:ZDB-GENE-040426-1363]	14	30713736	30724165		12.03	10.89	11.3	11.73	13.29	12.75
ENSDARG00000016059	rab5b	RAB5B, member RAS oncogene family [Source:ZFIN;Acc:ZDB-GENE-040426-2593]	9	27750490	27790405		3.29	2.87	2.71	2.76	3.12	3.17
ENSDARG00000016065	tlr3	toll-like receptor 3 [Source:ZFIN;Acc:ZDB-GENE-040219-7]	1	17892944	17898227		0.58	0.6	0.81	0.79	0.74	0.63
ENSDARG00000016074	cryz	crystallin, zeta (quinone reductase) [Source:ZFIN;Acc:ZDB-GENE-050306-24]	2	11100613	11120220		4.36	5.98	5.39	2.66	2.64	2.77
ENSDARG00000016080	nob1	NIN1 (RPN12) binding protein 1 homolog [Source:ZFIN;Acc:ZDB-GENE-030131-3112]	18	5861755	5875433		13.76	17.08	15.82	12.66	13.08	12.16
ENSDARG00000016081	cldn15la	claudin 15-like a [Source:ZFIN;Acc:ZDB-GENE-010328-12]	2	5508542	5517438		86.71	151.26	120.13	28.48	30.24	28.86
ENSDARG00000016084	spsb4a	splA/ryanodine receptor domain and SOCS box containing 4a [Source:ZFIN;Acc:ZDB-GENE-070911-3]	2	39584066	39675829		4.48	3.11	2.72	8.72	8.34	8.61
ENSDARG00000016085	mbnl3	muscleblind-like splicing regulator 3 [Source:ZFIN;Acc:ZDB-GENE-081002-3]	14	30986754	31080183		4.4	2.4	2.09	8.15	7.43	8.01
ENSDARG00000016086	smurf1	SMAD specific E3 ubiquitin protein ligase 1 [Source:ZFIN;Acc:ZDB-GENE-040426-2744]	3	40690212	40768548		4.37	2.49	2.24	7.17	7.6	6.95
ENSDARG00000016088	rtn2a	reticulon 2a [Source:ZFIN;Acc:ZDB-GENE-060420-1]	15	9010891	9031996		18.06	26.88	26.64	17	14.81	14.68
ENSDARG00000016089	bicral	BRD4 interacting chromatin remodeling complex associated protein like [Source:ZFIN;Acc:ZDB-GENE-100716-3]	12	34876125	34887943		4.23	1.94	2.25	5.93	6.42	6.37
ENSDARG00000016093	qtrt2	queuine tRNA-ribosyltransferase accessory subunit 2 [Source:NCBI gene;Acc:402798]	24	21058779	21090584		1.64	1.69	1.51	1.39	1.26	1.12
ENSDARG00000016095	htr6	5-hydroxytryptamine (serotonin) receptor 6 [Source:ZFIN;Acc:ZDB-GENE-030131-7839]	23	39730965	39738055		0.21	0.08	0.11	0.26	0.13	0.12
ENSDARG00000016112	bbs12	Bardet-Biedl syndrome 12 [Source:ZFIN;Acc:ZDB-GENE-070626-3]	14	1351214	1366544		0.85	0.48	0.57	0.72	0.82	0.87
ENSDARG00000016117	exd2	exonuclease 3'-5' domain containing 2 [Source:ZFIN;Acc:ZDB-GENE-030131-6387]	17	51796668	51818675		4.66	2.8	2.41	4.89	4.3	4.83
ENSDARG00000016122	ptrhd1	peptidyl-tRNA hydrolase domain containing 1 [Source:ZFIN;Acc:ZDB-GENE-081110-2]	20	36797612	36800002		5.96	7.32	6.99	4.23	4.22	3.52
ENSDARG00000016128	ap3m2	adaptor related protein complex 3 subunit mu 2 [Source:ZFIN;Acc:ZDB-GENE-021022-2]	5	14375108	14390445		13.47	7.08	8.46	13.78	14.6	14.56
ENSDARG00000016132	keap1a	kelch-like ECH-associated protein 1a [Source:ZFIN;Acc:ZDB-GENE-030131-556]	2	41835025	41861040		5.56	6.69	5.72	3.78	4.28	3.7
ENSDARG00000016138	nme6	NME/NM23 nucleoside diphosphate kinase 6 [Source:NCBI gene;Acc:58120]	20	19562383	19575565		5.87	7.28	6.92	3.35	4.33	3.66
ENSDARG00000016139	mkks	McKusick-Kaufman syndrome [Source:ZFIN;Acc:ZDB-GENE-040426-757]	13	35472803	35481843		1.21	0.75	0.72	0.72	0.63	0.73
ENSDARG00000016141	slc6a2	solute carrier family 6 member 2 [Source:ZFIN;Acc:ZDB-GENE-110408-4]	7	35348471	35380186		2.47	2.11	2.09	2.14	2.48	2.16
ENSDARG00000016143	xiap	X-linked inhibitor of apoptosis [Source:ZFIN;Acc:ZDB-GENE-030825-7]	14	28473173	28483419		16.26	15.87	16.19	18.57	20.34	19.86
ENSDARG00000016153	dag1	dystroglycan 1 [Source:ZFIN;Acc:ZDB-GENE-021223-1]	22	33131891	33194802		16.84	14.76	10.2	29.89	26.19	26.47
ENSDARG00000016154	zfp36l1a	zinc finger protein 36, C3H type-like 1a [Source:ZFIN;Acc:ZDB-GENE-030131-9860]	17	37792617	37795865		40.38	34.66	27.43	54.93	57.58	54.95
ENSDARG00000016156	fam53b	family with sequence similarity 53 member B [Source:NCBI gene;Acc:406586]	12	46883785	46937956		33.09	33.86	34.94	28.25	31.16	29.4
ENSDARG00000016160	gmpr	guanosine monophosphate reductase [Source:ZFIN;Acc:ZDB-GENE-050522-48]	19	32472996	32487469		14.03	18.48	17.03	26.57	25.57	24.64
ENSDARG00000016161	ccdc170	coiled-coil domain containing 170 [Source:ZFIN;Acc:ZDB-GENE-070112-162]	17	7821890	7842209		0.26	0.27	0.29	0.64	0.77	0.53
ENSDARG00000016163	usp33	ubiquitin specific peptidase 33 [Source:NCBI gene;Acc:406508]	2	8717155	8777496		14.48	8.63	8.85	16.01	16.73	16.18
ENSDARG00000016173	cct3	chaperonin containing TCP1, subunit 3 (gamma) [Source:ZFIN;Acc:ZDB-GENE-020419-5]	16	30408697	30421275		153.32	160.99	181.38	119.9	123.15	119.18
ENSDARG00000016177	eif4enif1	eukaryotic translation initiation factor 4E nuclear import factor 1 [Source:ZFIN;Acc:ZDB-GENE-050320-113]	6	40922572	40947671		5.16	2.81	2.42	7.39	6.82	6.67
ENSDARG00000016181	trim33	tripartite motif containing 33 [Source:NCBI gene;Acc:324053]	8	10956468	11004739		9.83	5.88	5.26	12.67	12.69	13.48
ENSDARG00000016188	SBNO2	si:ch73-63e15.2 [Source:ZFIN;Acc:ZDB-GENE-091204-463]	22	17691381	17781213	SBNO2	2.75	2.16	1.66	9.89	9.6	9.57
ENSDARG00000016200	trib3	tribbles pseudokinase 3 [Source:ZFIN;Acc:ZDB-GENE-040426-2609]	11	24058530	24063196		8.3	5.86	5.05	17.34	19.21	19.47
ENSDARG00000016207	zgc:112408	zgc:112408 [Source:ZFIN;Acc:ZDB-GENE-050417-63]	24	24064562	24071024		0.37	0.39	0.26	0.25	0.57	0.44
ENSDARG00000016212	nup214	nucleoporin 214 [Source:ZFIN;Acc:ZDB-GENE-110203-3]	5	71924175	71948555		2.67	2.22	2.11	4.81	4.74	5.04
ENSDARG00000016213	zmp:0000001048	zmp:0000001048 [Source:ZFIN;Acc:ZDB-GENE-140106-8]	3	53511936	53522208		1.85	1.58	0.98	2.8	3.02	2.51
ENSDARG00000016227	mhc1lba	major histocompatibility complex class I LBA [Source:ZFIN;Acc:ZDB-GENE-140820-3]	8	44462599	44463985		0	0	0	0.05	0.03	0.08
ENSDARG00000016231	bcap29	B cell receptor associated protein 29 [Source:ZFIN;Acc:ZDB-GENE-050522-468]	4	2655358	2666310		8.39	8.61	7.81	5.83	5.98	5.63
ENSDARG00000016233	sdr16c5a	short chain dehydrogenase/reductase family 16C, member 5a [Source:ZFIN;Acc:ZDB-GENE-040426-2049]	2	11483072	11504778		13.41	18.56	16.66	11.26	11.5	12.36
ENSDARG00000016238	arl6ip5b	ADP-ribosylation factor-like 6 interacting protein 5b [Source:ZFIN;Acc:ZDB-GENE-040718-223]	23	4253957	4259712		34.36	31.5	33.87	23.16	24.2	25.17
ENSDARG00000016239	psmd13	proteasome 26S subunit, non-ATPase 13 [Source:ZFIN;Acc:ZDB-GENE-040426-1004]	25	10485103	10496779		53.05	59.71	67.15	49.33	48.01	50.03
ENSDARG00000016253	ralab	v-ral simian leukemia viral oncogene homolog Ab (ras related) [Source:ZFIN;Acc:ZDB-GENE-041114-195]	2	21608352	21618629		9.74	9.58	8.82	9.7	10.04	9.97
ENSDARG00000016255	chmp4ba	charged multivesicular body protein 4Ba [Source:NCBI gene;Acc:393164]	6	49901465	49920320		29.34	26.34	26.56	30.24	31.28	31.92
ENSDARG00000016256	nudt3a	nudix (nucleoside diphosphate linked moiety X)-type motif 3a [Source:ZFIN;Acc:ZDB-GENE-040426-792]	23	3710946	3721444		11.29	8.6	8.38	12.07	12.11	11.07
ENSDARG00000016260	fxr2	fragile X mental retardation, autosomal homolog 2 [Source:ZFIN;Acc:ZDB-GENE-040426-943]	7	22718251	22761090		18.77	22.65	19.59	28.28	25.25	24.41
ENSDARG00000016263	zdhhc5a	zinc finger DHHC-type containing 5a [Source:ZFIN;Acc:ZDB-GENE-090312-92]	1	44523516	44566433		6.23	4.02	2.9	8.99	8.47	8.71
ENSDARG00000016280	eif1ad	eukaryotic translation initiation factor 1A domain containing [Source:NCBI gene;Acc:393440]	14	8450841	8456679		14.68	15.64	13.8	11.04	11.44	11.37
ENSDARG00000016290	clca1	chloride channel accessory 1 [Source:ZFIN;Acc:ZDB-GENE-030131-6221]	15	14109652	14120431		1.57	1.85	1.51	0.09	0.02	0.08
ENSDARG00000016301	zgc:65894	zgc:65894 [Source:ZFIN;Acc:ZDB-GENE-030131-7741]	1	56217388	56223913		89.52	28.02	34.39	90.26	85.72	89.17
ENSDARG00000016302	upf1	UPF1 RNA helicase and ATPase [Source:ZFIN;Acc:ZDB-GENE-040426-2836]	2	56168619	56237133		9.67	6.5	5.91	12.92	13.25	12.73
ENSDARG00000016304	tfdp1b	transcription factor Dp-1, b [Source:ZFIN;Acc:ZDB-GENE-040426-1746]	1	227241	243798		3.59	2.57	2.34	2.96	2.86	2.78
ENSDARG00000016307	mrpl46	mitochondrial ribosomal protein L46 [Source:ZFIN;Acc:ZDB-GENE-040718-224]	7	14536083	14543964		10.6	12.93	12.21	6.82	7.1	7.11
ENSDARG00000016311	dock9b	dedicator of cytokinesis 9b [Source:ZFIN;Acc:ZDB-GENE-090916-2]	6	12593986	12788539		3.65	2.66	2.66	4.53	4.5	4.5
ENSDARG00000016318	med7	mediator complex subunit 7 [Source:NCBI gene;Acc:326098]	14	34064249	34065937		12.31	11.47	12.51	8.87	8.38	7.96
ENSDARG00000016319	c9	complement component 9 [Source:ZFIN;Acc:ZDB-GENE-050522-442]	5	32345187	32355289		132.48	211.81	181.8	135.81	138.93	151
ENSDARG00000016336	tmco6	transmembrane and coiled-coil domains 6 [Source:ZFIN;Acc:ZDB-GENE-070410-47]	14	8456870	8472393		2.69	2.44	2.57	2.52	2.32	2.13
ENSDARG00000016337	lctlb	lactase-like b [Source:ZFIN;Acc:ZDB-GENE-060503-93]	18	19463425	19484919		0.06	0.09	0.19	0.81	0.62	1.11
ENSDARG00000016343	zgc:92518	zgc:92518 [Source:ZFIN;Acc:ZDB-GENE-040718-53]	1	466780	471925		4.25	5.39	4.95	5.73	4.84	5.11
ENSDARG00000016345	ncoa5	nuclear receptor coactivator 5 [Source:ZFIN;Acc:ZDB-GENE-040912-155]	6	55428924	55437087		36.47	17.97	19.77	43.52	41.97	44.09
ENSDARG00000016348	dip2bb	disco-interacting protein 2 homolog Bb [Source:ZFIN;Acc:ZDB-GENE-100729-4]	6	39531135	39605734		5.76	1.51	2	8.88	8.67	9.2
ENSDARG00000016350	cap1	CAP, adenylate cyclase-associated protein 1 (yeast) [Source:ZFIN;Acc:ZDB-GENE-030131-6459]	19	43715911	43737071		12.86	13	11.08	9.34	9.62	9.22
ENSDARG00000016357	fmo5	flavin containing dimethylaniline monoxygenase 5 [Source:ZFIN;Acc:ZDB-GENE-030131-6606]	20	15093609	15104709		13.36	15.75	14.55	8.27	7.74	7.49
ENSDARG00000016360	kpna1	karyopherin alpha 1 (importin alpha 5) [Source:ZFIN;Acc:ZDB-GENE-091116-39]	24	20941360	20956781		20.49	12.92	13.66	24.69	24.18	23.83
ENSDARG00000016363	her8a	hairy-related 8a [Source:ZFIN;Acc:ZDB-GENE-030131-2376]	7	26429941	26436480		1.83	1.19	1	2.88	2.98	2.91
ENSDARG00000016364	gna15.1	guanine nucleotide binding protein (G protein), alpha 15 (Gq class), tandem duplicate 1 [Source:ZFIN;Acc:ZDB-GENE-040801-146]	22	22021936	22036307		8.12	6.61	5.18	6.57	6.75	5.68
ENSDARG00000016368	zbtb8os	zinc finger and BTB domain containing 8 opposite strand [Source:NCBI gene;Acc:550382]	13	44857087	44888950		13.34	20.94	16.98	8.37	8.64	8.07
ENSDARG00000016375	asns	asparagine synthetase [Source:ZFIN;Acc:ZDB-GENE-040426-1091]	19	25656812	25678002		7.46	9.16	7.82	9.85	10.08	9.68
ENSDARG00000016391	calcoco1b	calcium binding and coiled-coil domain 1b [Source:ZFIN;Acc:ZDB-GENE-031118-126]	11	2111582	2131280		2.06	3.77	2.54	2.76	2.31	2.9
ENSDARG00000016393	arf2b	ADP-ribosylation factor 2b [Source:ZFIN;Acc:ZDB-GENE-030131-5234]	24	36392040	36399271		0.07	0.04	0.01	0.05	0.05	0.05
ENSDARG00000016396	fam219aa	family with sequence similarity 219 member Aa [Source:NCBI gene;Acc:445197]	21	13093136	13123185		4.91	2.7	2.78	8.26	8.45	8.13
ENSDARG00000016399	riok1	RIO kinase 1 (yeast) [Source:ZFIN;Acc:ZDB-GENE-040426-1927]	2	21063660	21076570		7.51	7.24	6.73	7.67	6.51	6.87
ENSDARG00000016404	ptch1	patched 1 [Source:ZFIN;Acc:ZDB-GENE-980526-196]	8	29962635	30068277		6.06	4.77	3.94	15.64	15.97	15.76

ENSDARG00000016412	agt	angiotensinogen [Source:ZFIN;Acc:ZDB-GENE-030131-1205]	13	23988442	23995876		64.71	107.11	106.1	75.22	92.67	90.11
ENSDARG00000016415	dhtkd1	dehydrogenase E1 and transketolase domain containing 1 [Source:NCBI gene;Acc:494076]	25	8031999	8055229		29.08	31.45	25.09	22.46	21.23	20.6
ENSDARG00000016421	crygm1	crystallin, gamma M1 [Source:ZFIN;Acc:ZDB-GENE-050516-5]	9	22011912	22012819		0	0	0.1	0	0	0.04
ENSDARG00000016427	fignl1	fidgetin-like 1 [Source:ZFIN;Acc:ZDB-GENE-030131-1862]	13	15933168	15943904		2.81	2.4	2.7	1.89	1.9	1.62
ENSDARG00000016429	farp2	FERM, RhoGEF and pleckstrin domain protein 2 [Source:ZFIN;Acc:ZDB-GENE-030131-2993]	6	26948093	27038629		3.23	2.4	1.81	4.1	4.08	4.07
ENSDARG00000016439	slc7a1a	solute carrier family 7 member 1a [Source:ZFIN;Acc:ZDB-GENE-091116-69]	10	24445698	24493323		4.01	4.66	3.07	5.68	5.93	4.72
ENSDARG00000016443	eif3c	eukaryotic translation initiation factor 3, subunit C [Source:NCBI gene;Acc:334234]	12	3944229	3962372		192.65	170.13	151.29	157.27	162.34	162.9
ENSDARG00000016447	ythdf1	YTH N(6)-methyladenosine RNA binding protein 1 [Source:ZFIN;Acc:ZDB-GENE-040426-2786]	8	23034718	23120252		11.74	9.07	7.44	18.22	17.88	18.01
ENSDARG00000016448	vtg3	vitellogenin 3, phosvitinless [Source:ZFIN;Acc:ZDB-GENE-991019-2]	11	7359496	7380688		0.04	0	0.01	0.04	0.07	0.1
ENSDARG00000016454	esr2a	estrogen receptor 2a [Source:ZFIN;Acc:ZDB-GENE-030116-2]	20	21583639	21635659		0.33	0.25	0.18	0.26	0.19	0.17
ENSDARG00000016457	irf9	interferon regulatory factor 9 [Source:ZFIN;Acc:ZDB-GENE-040912-165]	12	13282797	13306883		3.86	4.63	4.71	7.61	7.09	7.47
ENSDARG00000016460	fut9a	fucosyltransferase 9a [Source:ZFIN;Acc:ZDB-GENE-080723-75]	17	15631619	15657029		2.75	0.36	0.86	3.25	3.71	3.57
ENSDARG00000016463	trappc6b	trafficking protein particle complex 6b [Source:NCBI gene;Acc:450008]	23	32021803	32027198		11.52	12.2	12.01	10.71	10.08	10.29
ENSDARG00000016464	cdc42bpab	CDC42 binding protein kinase alpha (DMPK-like) b [Source:ZFIN;Acc:ZDB-GENE-080219-17]	20	35085224	35206280		3.69	2.32	2	5.05	5.06	4.78
ENSDARG00000016470	anxa5b	annexin A5b [Source:ZFIN;Acc:ZDB-GENE-030131-9076]	1	22654875	22665098		38.8	57.96	34.21	36.26	35.32	38.24
ENSDARG00000016474	galcb	galactosylceramidase b [Source:ZFIN;Acc:ZDB-GENE-040426-2115]	17	18889637	18898115		7.6	8.33	7.41	8.97	9.34	9
ENSDARG00000016477	eif4a2	eukaryotic translation initiation factor 4A, isoform 2 [Source:ZFIN;Acc:ZDB-GENE-040426-2802]	2	7715810	7727248		45.35	41.54	43.71	44.01	49.15	46.34
ENSDARG00000016479	ugt5a1	UDP glucuronosyltransferase 5 family, polypeptide A1 [Source:ZFIN;Acc:ZDB-GENE-051120-60]	25	29407671	29415382		1.13	1.58	1.16	1.09	1.13	0.89
ENSDARG00000016480	slc17a7a	solute carrier family 17 member 7a [Source:ZFIN;Acc:ZDB-GENE-050105-5]	3	16612574	16654088		17.14	3.8	3.79	15.56	14.26	15.55
ENSDARG00000016481	ptpn2a	protein tyrosine phosphatase non-receptor type 2a [Source:ZFIN;Acc:ZDB-GENE-040426-1196]	16	10055492	10071887		4.22	4.17	3.9	4.13	3.6	4.09
ENSDARG00000016483	baiap2l2a	BAR/IMD domain containing adaptor protein 2 like 2a [Source:ZFIN;Acc:ZDB-GENE-040108-10]	3	24437363	24458281		2.46	3.09	1.91	1.1	1.09	1.06
ENSDARG00000016484	dkc1	dyskeratosis congenita 1, dyskerin [Source:ZFIN;Acc:ZDB-GENE-031118-120]	21	43702010	43716471		42.9	51.15	49.4	27.82	25.7	25.87
ENSDARG00000016490	rab11fip3	RAB11 family interacting protein 3 (class II) [Source:NCBI gene;Acc:573631]	24	37722048	37794593		5.3	2.59	2.45	6.19	6.04	5.96
ENSDARG00000016494	ddc	dopa decarboxylase [Source:ZFIN;Acc:ZDB-GENE-040426-2656]	16	8298542	8342749		6.47	7.48	4.62	4.16	4.32	4.18
ENSDARG00000016496	cdk8	cyclin-dependent kinase 8 [Source:NCBI gene;Acc:373868]	24	21514283	21539344		8.78	7.04	6.52	8.4	8.88	8.79
ENSDARG00000016505	apbb1ip	amyloid beta (A4) precursor protein-binding, family B, member 1 interacting protein [Source:NCBI gene;Acc:393607]	24	6040029	6078222		2.82	2.93	1.72	3.19	2.64	2.94
ENSDARG00000016513	setd3	SET domain containing 3, actin histidine methyltransferase [Source:NCBI gene;Acc:337193]	17	18031304	18070787		13.26	7.92	8.95	22.78	21.51	25.04
ENSDARG00000016514	gtf2h2	general transcription factor IIH, polypeptide 2 [Source:ZFIN;Acc:ZDB-GENE-030131-1959]	5	26221011	26233068		5.12	4.39	4.72	4.16	4.33	3.98
ENSDARG00000016516	rbm8a	RNA binding motif protein 8A [Source:NCBI gene;Acc:503767]	16	45922175	45930077		88.58	91.83	88.5	81.09	79.04	78.33
ENSDARG00000016519	ctdnep1a	CTD nuclear envelope phosphatase 1a [Source:NCBI gene;Acc:492343]	5	24047292	24056796		5.1	3.72	4.42	4.67	5.18	4.85
ENSDARG00000016526	gata3	GATA binding protein 3 [Source:NCBI gene;Acc:30458]	4	25054510	25064510		17.64	8.95	7.32	26.93	24.55	26.77
ENSDARG00000016527	helz2	helicase with zinc finger 2, transcriptional coactivator [Source:ZFIN;Acc:ZDB-GENE-120920-1]	6	58569808	58615281		0.11	0.05	0.09	0.07	0.13	0.1
ENSDARG00000016528	wdcp	WD repeat and coiled coil containing [Source:NCBI gene;Acc:541407]	20	44498056	44508391		5.66	2.28	1.51	4.05	4.68	4.05
ENSDARG00000016531	klhl4	kelch-like family member 4 [Source:ZFIN;Acc:ZDB-GENE-070410-63]	14	26796684	26843597		3.16	1.53	2.05	4.5	5.16	4.93
ENSDARG00000016532	suco	SUN domain containing ossification factor [Source:ZFIN;Acc:ZDB-GENE-030131-2941]	20	14683699	14781904		9.7	6.2	4.47	16.6	17.11	18.89
ENSDARG00000016536	npas2	neuronal PAS domain protein 2 [Source:ZFIN;Acc:ZDB-GENE-030408-3]	5	22791686	22830724		2.09	1.66	1.22	2.13	2.26	2.2
ENSDARG00000016538	zgc:55888	zgc:55888 [Source:ZFIN;Acc:ZDB-GENE-040426-834]	4	9467049	9476418		0.95	0.27	0.47	1.35	1.43	1.36
ENSDARG00000016541	sash3	SAM and SH3 domain containing 3 [Source:ZFIN;Acc:ZDB-GENE-130411-1]	14	33568838	33585809		1.06	1.31	0.89	1.25	1.22	1.33
ENSDARG00000016543	mrm3a	mitochondrial rRNA methyltransferase 3a [Source:NCBI gene;Acc:550390]	15	16185118	16192208		2.91	2.82	2.15	2.16	1.73	2.26
ENSDARG00000016545	CRAT	zgc:154046 [Source:ZFIN;Acc:ZDB-GENE-061103-289]	8	44926629	44976455	CRAT	5.74	8.03	7.42	6.16	5.04	5.42
ENSDARG00000016548	eif5b	eukaryotic translation initiation factor 5B [Source:ZFIN;Acc:ZDB-GENE-050208-20]	1	45925150	45959721		16.36	15.21	13.18	14.12	13.72	13.94
ENSDARG00000016551	iqsec1b	IQ motif and Sec7 domain ArfGEF 1b [Source:ZFIN;Acc:ZDB-GENE-120813-1]	11	26704181	27057572		5.6	3.05	2.57	10.08	10.18	10.04
ENSDARG00000016570	prlra	prolactin receptor a [Source:ZFIN;Acc:ZDB-GENE-080103-2]	21	11105007	11135942		7.3	3.15	2.48	5.69	5.52	5.97
ENSDARG00000016573	mroh1	maestro heat-like repeat family member 1 [Source:ZFIN;Acc:ZDB-GENE-090806-2]	16	31258283	31351419		9.6	5.21	5.66	8.37	8.63	8.79
ENSDARG00000016584	rgs7a	regulator of G protein signaling 7a [Source:ZFIN;Acc:ZDB-GENE-040718-276]	17	19626479	19731406		3.59	1.23	2.35	2.85	3.07	3.39
ENSDARG00000016594	haus4	HAUS augmin-like complex, subunit 4 [Source:NCBI gene;Acc:436698]	5	1493767	1515857		2.67	2.6	2.5	1.65	1.66	1.73
ENSDARG00000016598	ckmt1	creatine kinase, mitochondrial 1 [Source:ZFIN;Acc:ZDB-GENE-030131-611]	25	21829777	21847553		111.46	129.79	108.74	102.21	112.62	108.14
ENSDARG00000016605	pimr110	Pim proto-oncogene, serine/threonine kinase, related 110 [Source:ZFIN;Acc:ZDB-GENE-041014-282]	20	42881104	42885820		0	0	0	0	0.01	0.02
ENSDARG00000016607	echdc2	enoyl CoA hydratase domain containing 2 [Source:ZFIN;Acc:ZDB-GENE-030219-147]	20	16205080	16223245		11.67	13.48	13.68	8.63	8.33	7.69
ENSDARG00000016611	scrn3	secernin 3 [Source:NCBI gene;Acc:326058]	9	2560219	2573121		6.12	4.61	4.68	4.66	4.42	4.6
ENSDARG00000016613	mcat	malonyl CoA:ACP acyltransferase (mitochondrial) [Source:ZFIN;Acc:ZDB-GENE-041111-231]	4	18846624	18851365		1.07	1.25	0.98	0.54	0.91	0.82
ENSDARG00000016623	si:ch211-195b13.1	si:ch211-195b13.1 [Source:ZFIN;Acc:ZDB-GENE-030131-7626]	19	24872159	24889227		13.82	6.91	7.41	12.09	13.09	13.4
ENSDARG00000016630	tprb	translocated promoter region b, nuclear basket protein [Source:ZFIN;Acc:ZDB-GENE-030131-6410]	20	34039281	34069956		12.69	8.91	7.51	12.18	11.2	11.93
ENSDARG00000016649	akr7a3	aldo-keto reductase family 7, member A3 (aflatoxin aldehyde reductase) [Source:ZFIN;Acc:ZDB-GENE-040718-62]	11	41838036	41850086		5.58	7.03	7	3.24	3.1	3.19
ENSDARG00000016651	znf106a	zinc finger protein 106a [Source:ZFIN;Acc:ZDB-GENE-040718-21]	17	45347726	45378473		8.47	9.1	6.63	12	10.18	10.59
ENSDARG00000016656	mgme1	mitochondrial genome maintenance exonuclease 1 [Source:ZFIN;Acc:ZDB-GENE-041212-68]	13	33655404	33658885		2.39	2.59	3.46	2.43	2.4	2.42
ENSDARG00000016664	rnf20	ring finger protein 20, E3 ubiquitin protein ligase [Source:ZFIN;Acc:ZDB-GENE-130404-2]	14	21065434	21098343		9.94	5.88	5.78	8.72	8.88	8.6
ENSDARG00000016667	gabbr1b	gamma-aminobutyric acid (GABA) B receptor, 1b [Source:ZFIN;Acc:ZDB-GENE-060503-5]	19	27040533	27261102		10.29	2.2	3.5	11.39	12.01	12.41
ENSDARG00000016674	FP102888.1	tripartite motif containing 35-4 [Source:NCBI gene;Acc:100006932]	3	823084	829433		0.47	0.38	0.38	0.54	0.25	0.43
ENSDARG00000016676	gnao1a	guanine nucleotide binding protein (G protein), alpha activating activity polypeptide O, a [Source:ZFIN;Acc:ZDB-GENE-040426-1757	18	22793465	22975655		46.19	12.25	13.8	61.89	61.98	65.23
ENSDARG00000016687	acadvl	acyl-CoA dehydrogenase very long chain [Source:ZFIN;Acc:ZDB-GENE-030131-899]	7	20298333	20333524		65.97	79.59	75.15	53.63	54.95	54.88
ENSDARG00000016691	cd9b	CD9 molecule b [Source:ZFIN;Acc:ZDB-GENE-040426-2768]	4	4234994	4261673		37.17	40.05	37.12	31.8	28.39	29.37
ENSDARG00000016695	p2rx1	purinergic receptor P2X, ligand-gated ion channel, 1 [Source:ZFIN;Acc:ZDB-GENE-030319-1]	5	31439390	31470426		2.02	1.83	1.71	1.56	1.44	1.29
ENSDARG00000016699	trpa1a	transient receptor potential cation channel, subfamily A, member 1a [Source:ZFIN;Acc:ZDB-GENE-050105-6]	2	30116486	30135446		0.31	0.19	0.16	0.37	0.37	0.36
ENSDARG00000016704	pbld	phenazine biosynthesis like protein domain containing [Source:ZFIN;Acc:ZDB-GENE-050522-217]	13	22839821	22843805		11.54	19.75	14.25	3.62	3.23	3.29
ENSDARG00000016706	atic	5-aminoimidazole-4-carboxamide ribonucleotide formyltransferase/IMP cyclohydrolase [Source:ZFIN;Acc:ZDB-GENE-011212-4]	1	5437390	5504130		30.76	44.84	48.6	50.8	55.31	47.83
ENSDARG00000016710	rchy1	ring finger and CHY zinc finger domain containing 1 [Source:NCBI gene;Acc:321875]	5	9147497	9161478		13.54	13.44	15.78	12.38	12.34	12.4
ENSDARG00000016718	mmp11b	matrix metallopeptidase 11b [Source:ZFIN;Acc:ZDB-GENE-070817-2]	21	15636540	15674802		7.03	9.78	8.93	15.9	13.48	12.24
ENSDARG00000016721	sdha	succinate dehydrogenase complex, subunit A, flavoprotein (Fp) [Source:NCBI gene;Acc:393884]	19	7276060	7291733		88.03	93.34	88.44	74.94	74	71.88
ENSDARG00000016724	zgc:162339	zgc:162339 [Source:ZFIN;Acc:ZDB-GENE-070410-76]	15	21707783	21719970		1.23	1.07	1	1.17	0.91	1.13
ENSDARG00000016725	gadd45gb.1	growth arrest and DNA-damage-inducible, gamma b, tandem duplicate 1 [Source:ZFIN;Acc:ZDB-GENE-040426-2321]	21	20715020	20716561		19.61	9.87	16.15	11.26	11.7	12.58
ENSDARG00000016730	rfxank	regulatory factor X-associated ankyrin-containing protein [Source:ZFIN;Acc:ZDB-GENE-050208-596]	22	18160330	18164835		1.55	1.9	1.93	1.58	1.35	1.31
ENSDARG00000016733	psat1	phosphoserine aminotransferase 1 [Source:ZFIN;Acc:ZDB-GENE-030131-5723]	5	69486184	69499595		17.15	20.12	23.17	46.88	48.05	51.85
ENSDARG00000016739	lrrc4cb	leucine rich repeat containing 4C, genome duplicate b [Source:ZFIN;Acc:ZDB-GENE-080327-5]	7	50053339	50113581		2.7	0.56	0.9	3.55	3.81	3.79
ENSDARG00000016742	rgs7bpb	regulator of G protein signaling 7 binding protein b [Source:NCBI gene;Acc:337556]	21	20933354	20939488		7.01	3.6	5.21	5.99	5.44	5.82
ENSDARG00000016743	angel2	angel homolog 2 (Drosophila) [Source:NCBI gene;Acc:562704]	20	37920096	37934315		5.98	4.56	4.2	5.54	5.83	5.81
ENSDARG00000016745	slc35f6	solute carrier family 35 member F6 [Source:ZFIN;Acc:ZDB-GENE-040718-297]	20	25904199	25910643		18.52	18.99	17.96	16.68	16.46	17.24
ENSDARG00000016749	slco1c1	solute carrier organic anion transporter family, member 1C1 [Source:ZFIN;Acc:ZDB-GENE-041210-50]	4	2674898	2727573		3.38	1.76	1.86	3.51	3.72	3.95
ENSDARG00000016750	abcc6a	ATP-binding cassette, sub-family C (CFTR/MRP), member 6a [Source:ZFIN;Acc:ZDB-GENE-050517-18]	6	11026354	11067995		0.7	0.48	0.48	1.2	1.14	1.2
ENSDARG00000016753	ipo9	importin 9 [Source:ZFIN;Acc:ZDB-GENE-040426-2953]	11	24851671	24876621		7.25	6.12	5.25	7.17	6.79	6.25
ENSDARG00000016754	tbca	tubulin cofactor a [Source:ZFIN;Acc:ZDB-GENE-040426-962]	21	7545062	7570765		69.4	99.45	93.45	49.92	52.16	56.54
ENSDARG00000016763	znf292a	zinc finger protein 292a [Source:ZFIN;Acc:ZDB-GENE-071004-18]	17	49415354	49438873		7.08	3.5	4.39	7.74	8.21	8.19
ENSDARG00000016765	scamp3	secretory carrier membrane protein 3 [Source:ZFIN;Acc:ZDB-GENE-041114-33]	19	8985197	9001483		13.61	8.4	7.17	15.86	15.13	16.58
ENSDARG00000016769	sgtb	small glutamine-rich tetratricopeptide repeat (TPR)-containing, beta [Source:ZFIN;Acc:ZDB-GENE-040704-72]	10	15381972	15405564		5.62	3.51	4.19	5.91	6.1	7.27
ENSDARG00000016771	tfa	transferrin-a [Source:ZFIN;Acc:ZDB-GENE-980526-35]	2	16780643	16794710		369.41	580.18	557.13	166.88	174.47	185.82
ENSDARG00000016773	cishb	cytokine inducible SH2-containing protein b [Source:ZFIN;Acc:ZDB-GENE-080204-120]	11	17959507	17964525		3.23	3.29	3.17	1.42	1.83	1.67
ENSDARG00000016774	plekho2	pleckstrin homology domain containing, family O member 2 [Source:ZFIN;Acc:ZDB-GENE-120406-4]	7	13527396	13572981		1.6	1.44	1.24	2.13	2.26	2.19
ENSDARG00000016775	aqr	aquarius intron-binding spliceosomal factor [Source:ZFIN;Acc:ZDB-GENE-040426-1189]	17	51020114	51121525		11.4	8.79	8.05	8.55	8.5	7.96
ENSDARG00000016782	huwe1	HECT, UBA and WWE domain containing E3 ubiquitin protein ligase 1 [Source:ZFIN;Acc:ZDB-GENE-081104-387]	23	28494189	28568978		13.83	11.01	8.79	23.49	21.85	22.3
ENSDARG00000016783	srsf6b	serine and arginine rich splicing factor 6b [Source:ZFIN;Acc:ZDB-GENE-041219-1]	11	1522127	1530865		43.68	20.21	23.53	53.36	52.15	52.85
ENSDARG00000016788	zeb1a	zinc finger E-box binding homeobox 1a [Source:ZFIN;Acc:ZDB-GENE-070912-124]	2	43807230	43852207		5.62	3.13	2.78	7.78	8.31	9.09
ENSDARG00000016789	zgc:152891	zgc:152891 [Source:ZFIN;Acc:ZDB-GENE-061013-229]	21	31194980	31226045		0.12	0.07	0.14	0.26	0.29	0.27
ENSDARG00000016793	crybb1l2	crystallin, beta B1, like 2 [Source:ZFIN;Acc:ZDB-GENE-070822-18]	1	44439661	44448387		4.23	15.76	29.48	93.54	82.77	110.2
ENSDARG00000016794	mtmr6	myotubularin related protein 6 [Source:ZFIN;Acc:ZDB-GENE-030131-5557]	24	25032340	25059539		8.42	5.86	5.64	8.81	8.41	9.47
ENSDARG00000016809	prkacab	protein kinase, cAMP-dependent, catalytic, alpha, genome duplicate b [Source:ZFIN;Acc:ZDB-GENE-040801-208]	1	55843552	55873178		14.17	7.58	7.69	19.19	19.71	20.3
ENSDARG00000016811	ints3	integrator complex subunit 3 [Source:NCBI gene;Acc:560575]	19	26640096	26673855		8.09	5.74	5.1	8.2	6.89	7.6
ENSDARG00000016815	casc1	cancer susceptibility candidate 1 [Source:NCBI gene;Acc:566485]	4	17245001	17251758		0.47	0.69	0.57	0.88	0.84	0.89
ENSDARG00000016818	abcg2d	ATP-binding cassette, sub-family G (WHITE), member 2d [Source:ZFIN;Acc:ZDB-GENE-050517-38]	1	50566256	50590626		0.56	0.65	0.65	0.89	0.79	0.88
ENSDARG00000016825	vtg6	vitellogenin 6 [Source:ZFIN;Acc:ZDB-GENE-001201-5]	22	24811396	24818066		0.01	0.01	0	0.12	0.25	0.08
ENSDARG00000016830	rimkla	ribosomal modification protein rimK-like family member A [Source:NCBI gene;Acc:447815]	8	26868105	26887308		35.66	16.71	16.1	70.66	72.7	77
ENSDARG00000016835	tcirg1a	T cell immune regulator 1, ATPase H+ transporting V0 subunit a3a [Source:ZFIN;Acc:ZDB-GENE-090311-31]	1	44870880	44901163		5.41	3.49	3.8	4.92	5.4	5.8
ENSDARG00000016837	glipr2l	GLI pathogenesis-related 2, like [Source:ZFIN;Acc:ZDB-GENE-041010-53]	16	27614989	27622183		3	4.69	3.68	6.41	4.96	5.2
ENSDARG00000016854	neurod2	neuronal differentiation 2 [Source:NCBI gene;Acc:114435]	12	14920088	14922955		6.36	2.78	3.92	4.23	4.96	4.62
ENSDARG00000016855	sf3b5	splicing factor 3b, subunit 5 [Source:ZFIN;Acc:ZDB-GENE-040718-181]	13	23897975	23901732		49.48	56.33	62.78	30.37	28.9	28.8
ENSDARG00000016856	pon2	paraoxonase 2 [Source:ZFIN;Acc:ZDB-GENE-030131-7116]	16	24669883	24672919		18.32	24.8	20.23	14.79	14.68	13.48
ENSDARG00000016858	smad7	SMAD family member 7 [Source:ZFIN;Acc:ZDB-GENE-030128-3]	21	3419459	3452683		3.27	2.05	2.13	5.03	4.94	4.7
ENSDARG00000016864	farsb	phenylalanyl-tRNA synthetase subunit beta [Source:ZFIN;Acc:ZDB-GENE-021206-2]	15	42264891	42283830		24.51	24.4	26.18	30.51	32.82	34.06
ENSDARG00000016866	fam102ab	family with sequence similarity 102 member Ab [Source:ZFIN;Acc:ZDB-GENE-060929-412]	5	31723404	31773209		8.41	7.47	5.5	6.82	6.81	6.67
ENSDARG00000016867	rnf128a	ring finger protein 128a [Source:ZFIN;Acc:ZDB-GENE-030131-1044]	5	23677420	23696926		29.39	34.1	30.54	20.68	20.84	19.31
ENSDARG00000016868	rhobtb4	Rho related BTB domain containing 4 [Source:ZFIN;Acc:ZDB-GENE-060315-11]	5	43427841	43502675		3.43	2.11	2.1	4.13	4.23	4.11
ENSDARG00000016871	smarce1	SWI/SNF related, matrix associated, actin dependent regulator of chromatin, subfamily e, member 1 [Source:ZFIN;Acc:ZDB-GENE-(	3	34985895	34995062		67.62	39.77	38.88	86.04	83.37	91.57
ENSDARG00000016875	gys1	glycogen synthase 1 (muscle) [Source:ZFIN;Acc:ZDB-GENE-040426-1243]	3	16722014	16750036		14.76	21.06	18.06	31.37	29.95	29.7
ENSDARG00000016886	dnajb9b	DnaJ heat shock protein family (Hsp40) member B9b [Source:ZFIN;Acc:ZDB-GENE-050522-76]	4	17821390	17823424		3.79	5.1	3.43	6.3	6.08	6.17
ENSDARG00000016889	eif3g	eukaryotic translation initiation factor 3, subunit G [Source:NCBI gene;Acc:393974]	3	54581987	54589823		163.27	168.06	167.7	135.47	146.65	145.95
ENSDARG00000016897	fbxo15	F-box protein 15 [Source:ZFIN;Acc:ZDB-GENE-080416-4]	24	15655069	15669210		0.35	0.28	0.39	0.52	0.38	0.39
ENSDARG00000016903	wdr5	WD repeat domain 5 [Source:ZFIN;Acc:ZDB-GENE-040426-2082]	21	18274825	18290978		17.39	15.07	14.78	19.56	19.23	18.41
ENSDARG00000016904	bckdk	branched chain ketoacid dehydrogenase kinase [Source:ZFIN;Acc:ZDB-GENE-021204-1]	5	61508418	61554010		15.82	13.9	13.45	17.99	17.24	17.8
ENSDARG00000016908	zp3e	zona pellucida glycoprotein 3e [Source:ZFIN;Acc:ZDB-GENE-071004-38]	3	31875772	31879201		0.01	0	0.06	0.04	0.09	0.04
ENSDARG00000016918	ace2	angiotensin I converting enzyme 2 [Source:ZFIN;Acc:ZDB-GENE-041114-6]	11	29981678	29996357		57.92	103.78	70.65	12.06	12.45	12.77
ENSDARG00000016923	si:dkey-94f20.4	si:dkey-94f20.4 [Source:ZFIN;Acc:ZDB-GENE-121214-276]	12	4573696	4576816		4.92	0.46	0.34	2.53	4.25	3.33
ENSDARG00000016934	ttyh2	tweety family member 2 [Source:ZFIN;Acc:ZDB-GENE-040718-193]	12	38563073	38687993		1.57	0.78	0.65	2.78	2.79	2.89
ENSDARG00000016936	hmcn1	hemicentin 1 [Source:ZFIN;Acc:ZDB-GENE-041014-322]	20	34157308	34292607		1.15	1.19	0.88	2.78	2.66	2.74
ENSDARG00000016939	itgb2	integrin, beta 2 [Source:ZFIN;Acc:ZDB-GENE-110411-4]	9	44961138	44983666		2.13	2.7	2.22	2.87	3.04	3.44
ENSDARG00000016951	neurog3	neurogenin 3 [Source:ZFIN;Acc:ZDB-GENE-010608-4]	13	25761279	25762391		0.1	0.04	0.09	0.15	0.16	0.18
ENSDARG00000016963	slc16a1a	solute carrier family 16 member 1a [Source:ZFIN;Acc:ZDB-GENE-110408-16]	6	48978202	49049019		1.52	1.35	1.49	2.04	2.23	1.5
ENSDARG00000016964	MCUR1	zgc:165532 [Source:ZFIN;Acc:ZDB-GENE-050417-213]	7	41313568	41318214	MCUR1	5.82	7.1	7.13	5.06	5.5	5.82
ENSDARG00000016968	tmem214	transmembrane protein 214 [Source:ZFIN;Acc:ZDB-GENE-110307-2]	4	764100	779796		19.8	19.62	17.55	22.19	21.39	21.12
ENSDARG00000016977	snx27b	sorting nexin 27b [Source:ZFIN;Acc:ZDB-GENE-040426-1529]	16	23495600	23524374		7.92	4.18	4.21	9.22	8.91	8.4
ENSDARG00000016979	pmpcb	peptidase (mitochondrial processing) beta [Source:ZFIN;Acc:ZDB-GENE-050220-10]	4	1761029	1771308		16.72	17.06	17.22	13.93	14.04	15.67
ENSDARG00000016981	bcar3	BCAR3 adaptor protein, NSP family member [Source:ZFIN;Acc:ZDB-GENE-080513-4]	8	15084386	15229979		6.43	4.32	3.68	6.1	5.93	5.43
ENSDARG00000016994	ssrp1b	structure specific recognition protein 1b [Source:ZFIN;Acc:ZDB-GENE-041008-209]	1	44710955	44737931		9.54	6.09	6.12	11.47	11.61	12.26
ENSDARG00000016999	lin28a	lin-28 homolog A (C. elegans) [Source:NCBI gene;Acc:394066]	19	15440841	15473157		0.27	0.22	0.26	1.15	1.15	1.11
ENSDARG00000017004	myo10	myosin X [Source:ZFIN;Acc:ZDB-GENE-070912-277]	2	42605252	42705284		6.83	6.05	5.23	8.41	8.41	8.02
ENSDARG00000017006	dennd5a	DENN/MADD domain containing 5A [Source:ZFIN;Acc:ZDB-GENE-071211-1]	7	64978738	65093209		7.16	3.89	3.3	11.11	11.92	13.03
ENSDARG00000017007	nr4a2a	nuclear receptor subfamily 4, group A, member 2a [Source:ZFIN;Acc:ZDB-GENE-990415-184]	9	5040985	5046315		5.43	0.68	0.98	8.02	7.03	8.47
ENSDARG00000017010	scamp2l	secretory carrier membrane protein 2, like [Source:ZFIN;Acc:ZDB-GENE-030131-6432]	7	57088920	57106722		3.22	2.86	2.69	2.66	2.02	1.99
ENSDARG00000017014	ywhae2	tyrosine 3-monooxygenase/tryptophan 5-monooxygenase activation protein, epsilon polypeptide 2 [Source:ZFIN;Acc:ZDB-GENE-0	7	72012397	72029683		21.92	16.76	14.42	26.32	25.31	24.53
ENSDARG00000017023	ak7b	adenylate kinase 7b [Source:ZFIN;Acc:ZDB-GENE-050309-170]	17	18877918	18888959		1.38	0.77	1.08	1.21	1.19	1.35
ENSDARG00000017034	sqor	sulfide quinone oxidoreductase [Source:ZFIN;Acc:ZDB-GENE-050417-436]	25	32473277	32495363		13.34	15.67	13.96	38.56	49.68	47.78
ENSDARG00000017036	svilc	supervillin c [Source:ZFIN;Acc:ZDB-GENE-041008-237]	3	39112898	39171968		8.38	11.33	10.73	9.03	8.14	7.74
ENSDARG00000017037	ikbkg	inhibitor of nuclear factor kappa B kinase regulatory subunit gamma [Source:ZFIN;Acc:ZDB-GENE-050327-68]	23	18574221	18597709		17.62	12.77	13.69	14.23	14.61	15.1
ENSDARG00000017038	zgc:152670	zgc:152670 [Source:ZFIN;Acc:ZDB-GENE-060929-954]	2	44348473	44362943		0.48	0.29	0.22	0.48	0.51	0.52
ENSDARG00000017047	slc27a4	solute carrier family 27 member 4 [Source:ZFIN;Acc:ZDB-GENE-050417-248]	10	10738880	10782810		7.44	6.01	4.19	5.29	5.49	4.87
ENSDARG00000017049	adsl	adenylosuccinate lyase [Source:ZFIN;Acc:ZDB-GENE-030131-6363]	3	24207243	24218622		31.81	47.35	57.49	33.84	36.73	35.31
ENSDARG00000017055	zmynd12	zinc finger, MYND-type containing 12 [Source:ZFIN;Acc:ZDB-GENE-041114-133]	8	26902358	26918516		0.29	0.24	0.23	0.32	0.33	0.22
ENSDARG00000017058	large2	LARGE xylosyl- and glucuronyltransferase 2 [Source:NCBI gene;Acc:446214]	18	45114392	45264117		5.71	3.92	2.43	5.87	5.53	5.79
ENSDARG00000017067	chordc1b	cysteine and histidine-rich domain (CHORD) containing 1b [Source:NCBI gene;Acc:393310]	10	25204626	25213025		14.23	17.29	14.85	12.59	11.8	12.95
ENSDARG00000017078	mespaa	mesoderm posterior aa [Source:ZFIN;Acc:ZDB-GENE-000406-8]	7	15308042	15333427		0	0	0	0	0.04	0
ENSDARG00000017084	bud31	BUD31 homolog [Source:NCBI gene;Acc:445383]	3	40284598	40287841		29.15	31.26	31.69	25.38	25.04	28.81
ENSDARG00000017086	asb12a	ankyrin repeat and SOCS box-containing 12a [Source:ZFIN;Acc:ZDB-GENE-030131-1647]	5	22021142	22027357		10.51	10.07	10.49	9.48	9.27	9.7
ENSDARG00000017093	emc8	ER membrane protein complex subunit 8 [Source:ZFIN;Acc:ZDB-GENE-040426-2692]	18	30500690	30504652		13.53	15.48	14.05	14.66	14.88	14.73
ENSDARG00000017105	vaspb	vasodilator stimulated phosphoprotein b [Source:ZFIN;Acc:ZDB-GENE-030131-7118]	18	40761913	40856354		35.96	35.5	26.23	37.22	34.96	33.02
ENSDARG00000017107	nr2e1	nuclear receptor subfamily 2, group E, member 1 [Source:ZFIN;Acc:ZDB-GENE-040801-127]	20	32437396	32446406		4.71	1.94	3.02	3.25	3.82	3.61
ENSDARG00000017108	kcna1b	potassium voltage-gated channel, shaker-related subfamily, member 1b [Source:ZFIN;Acc:ZDB-GENE-140515-1]	4	74551606	74556277		1.29	0.24	0.61	2.21	2.17	1.96
ENSDARG00000017111	fam8a1a	family with sequence similarity 8 member A1a [Source:ZFIN;Acc:ZDB-GENE-141216-66]	19	32201991	32206819		2.35	1.21	1.85	1.87	2.01	1.27
ENSDARG00000017115	kctd10	potassium channel tetramerization domain containing 10 [Source:ZFIN;Acc:ZDB-GENE-040426-2843]	5	19436483	19444949		6.17	5.19	4.27	8.62	8.64	8.63
ENSDARG00000017119	vps37a	VPS37A subunit of ESCRT-I [Source:ZFIN;Acc:ZDB-GENE-030131-8312]	1	16463079	16480213		11.45	8.13	7.72	12.27	11.55	12.95
ENSDARG00000017121	mafba	v-maf avian musculoaponeurotic fibrosarcoma oncogene homolog Ba [Source:NCBI gene;Acc:30210]	23	3405665	3409140		7.89	5.96	5.12	16.74	16.4	16.77
ENSDARG00000017126	ilvbl	ilvB (bacterial acetolactate synthase)-like [Source:NCBI gene;Acc:393639]	18	44768829	44794015		5.41	8.16	4.61	5.38	5.59	5.58
ENSDARG00000017127	pdzk1ip1	PDZK1 interacting protein 1 [Source:ZFIN;Acc:ZDB-GENE-030616-128]	22	16555939	16566815		2.84	5.01	2.41	4.55	4.38	4.55
ENSDARG00000017128	myofl	myoferlin like [Source:ZFIN;Acc:ZDB-GENE-111116-2]	13	29238850	29288902		3.89	3.57	2.48	4.53	4.34	4.31
ENSDARG00000017140	zgc:63587	zgc:63587 [Source:ZFIN;Acc:ZDB-GENE-040426-1175]	8	4621243	4694458		29.24	35.91	32.2	31.67	31.84	30.58
ENSDARG00000017141	pla2g4ab	phospholipase A2, group IVAb (cytosolic, calcium-dependent) [Source:ZFIN;Acc:ZDB-GENE-041014-325]	20	34129465	34148190		0.18	0.32	0.22	0.18	0.3	0.39
ENSDARG00000017143	brd9	bromodomain containing 9 [Source:NCBI gene;Acc:393245]	16	52926139	52966865		15.84	9.55	9.7	13.11	13.04	13.34
ENSDARG00000017154	cables2b	Cdk5 and Abl enzyme substrate 2b [Source:ZFIN;Acc:ZDB-GENE-090313-249]	23	9088191	9137648		4.38	1.67	1.35	3.4	3.62	3.15
ENSDARG00000017155	wnt10a	wingless-type MMTV integration site family, member 10a [Source:NCBI gene;Acc:30171]	9	11379782	11409691		0.88	0.92	0.92	1.16	1.26	0.92
ENSDARG00000017162	kif3ca	kinesin family member 3Ca [Source:ZFIN;Acc:ZDB-GENE-111214-2]	20	47383841	47422469		15.41	4.38	4.94	16.35	16.16	17.01
ENSDARG00000017163	sinup	siaz-interacting nuclear protein [Source:ZFIN;Acc:ZDB-GENE-050913-2]	4	20176143	20177868		0.04	0	0.03	0.15	0.21	0.36
ENSDARG00000017165	slc3a1	solute carrier family 3 member 1 [Source:ZFIN;Acc:ZDB-GENE-090313-225]	13	10649069	10672538		2.34	2.12	1.64	1.62	1.71	1.42
ENSDARG00000017168	nr2f1b	nuclear receptor subfamily 2, group F, member 1b [Source:NCBI gene;Acc:393564]	10	43797130	43808197		5.62	2.35	2.11	6.74	6.51	6.65
ENSDARG00000017173	si:dkey-29p10.4	si:dkey-29p10.4 [Source:ZFIN;Acc:ZDB-GENE-050420-117]	18	27337994	27358871		5.34	3.47	3.78	5.7	5.87	5.93
ENSDARG00000017174	dlx2b	distal-less homeobox 2b [Source:NCBI gene;Acc:30557]	1	30977936	30979707		3.79	2.47	3.31	3.85	3.72	3.38
ENSDARG00000017180	npc1	Niemann-Pick disease, type C1 [Source:ZFIN;Acc:ZDB-GENE-030131-3161]	2	3823813	3866971		23.63	27.69	21.42	36.57	37.25	36.25
ENSDARG00000017188	zpax2	zona pellucida protein AX 2 [Source:ZFIN;Acc:ZDB-GENE-040724-123]	20	14096858	14114151		0.1	0.03	0.06	0.09	0.09	0.11
ENSDARG00000017193	cnn1a	calponin 1, basic, smooth muscle, a [Source:ZFIN;Acc:ZDB-GENE-090312-141]	1	52214895	52222989		1.87	3.26	1.08	0.26	0.1	0.29
ENSDARG00000017195	foxf2a	forkhead box F2a [Source:ZFIN;Acc:ZDB-GENE-110407-5]	2	713080	716426		2.94	2.69	2.52	6.24	5.8	7.14
ENSDARG00000017199	elp2	elongator acetyltransferase complex subunit 2 [Source:NCBI gene;Acc:560338]	19	34691645	34742440		5.19	5.13	5.48	4.4	4.31	4.22

ENSDARG00000017211	plxnb1b	plexin b1b [Source:ZFIN;Acc:ZDB-GENE-090812-4]	6	21874932	21988375		3.76	2.52	2.1	5.38	4.9	5.34
ENSDARG00000017219	pabpc1a	poly(A) binding protein, cytoplasmic 1a [Source:ZFIN;Acc:ZDB-GENE-030131-3238]	16	54829284	54851247		442.06	402.28	318.89	410.11	422.19	426.09
ENSDARG00000017220	otud7b	OTU deubiquitinase 7B [Source:ZFIN;Acc:ZDB-GENE-060616-1]	16	45930962	45982657		4.4	2.68	2.48	5.06	5.3	5.01
ENSDARG00000017229	megf10	multiple EGF-like-domains 10 [Source:NCBI gene;Acc:564571]	10	16069987	16161040		1.93	2.01	1.63	2.84	2.96	2.79
ENSDARG00000017230	fbxw11b	F-box and WD repeat domain containing 11b [Source:ZFIN;Acc:ZDB-GENE-040426-2903]	10	21444654	21470593		14.69	11.63	11.81	23.74	23.13	23.51
ENSDARG00000017234	npy8ar	neuropeptide Y receptor Y8a [Source:ZFIN;Acc:ZDB-GENE-990415-175]	10	42423318	42463859		0.4	0.06	0.17	0.85	0.58	0.56
ENSDARG00000017235	eif5a	eukaryotic translation initiation factor 5A [Source:ZFIN;Acc:ZDB-GENE-040426-2229]	24	26481300	26485345		297.61	359.91	338	352.95	352.41	353.62
ENSDARG00000017240	traf5	Tnf receptor-associated factor 5 [Source:ZFIN;Acc:ZDB-GENE-071120-3]	22	38301365	38322451		1.15	0.53	0.51	0.6	0.77	0.74
ENSDARG00000017242	garem	GRB2 associated, regulator of MAPK1 [Source:NCBI gene;Acc:393138]	7	41340520	41350398		4.58	4.14	3.7	3.72	4.34	4.3
ENSDARG00000017244	chd6	chromodomain helicase DNA binding protein 6 [Source:ZFIN;Acc:ZDB-GENE-130530-559]	6	51575924	51666739		3.7	1.95	1.52	4.79	4.1	4.12
ENSDARG00000017246	prx	periaxin [Source:ZFIN;Acc:ZDB-GENE-030131-5790]	18	46211210	46241856		7.94	11.69	7.54	18.41	15.24	14.71
ENSDARG00000017251	smtlb	somatolactin beta [Source:ZFIN;Acc:ZDB-GENE-060123-1]	10	39314578	39321367		1.99	0.4	1.3	1.12	1.13	1.41
ENSDARG00000017254	kcnk1b	potassium channel, subfamily K, member 1b [Source:ZFIN;Acc:ZDB-GENE-090312-78]	13	35856463	35872596		1.91	0.59	0.7	2.25	2.57	2.65
ENSDARG00000017255	tmed1b	transmembrane p24 trafficking protein 1b [Source:ZFIN;Acc:ZDB-GENE-070112-1112]	1	58840889	58850686		12.15	12.88	13.83	13.74	13.4	12.55
ENSDARG00000017261	gdpd1	glycerophosphodiester phosphodiesterase domain containing 1 [Source:ZFIN;Acc:ZDB-GENE-040822-22]	15	16886196	16894875		14.57	13.78	10.9	13.28	13.59	12.37
ENSDARG00000017266	scml4	Scm polycomb group protein like 4 [Source:ZFIN;Acc:ZDB-GENE-041210-338]	20	32523576	32576703		2.03	0.7	0.94	2.25	2.28	2.24
ENSDARG00000017272	eapp	e2f-associated phosphoprotein [Source:ZFIN;Acc:ZDB-GENE-030131-5529]	17	9951618	9962578		4.48	4.43	4.62	4.07	3.59	4
ENSDARG00000017274	opn1sw2	opsin 1 (cone pigments), short-wave-sensitive 2 [Source:NCBI gene;Acc:30435]	11	25459697	25475403		475.37	134.22	90.61	177.05	189.12	177.89
ENSDARG00000017294	gnai2b	guanine nucleotide binding protein (G protein), alpha inhibiting activity polypeptide 2b [Source:ZFIN;Acc:ZDB-GENE-030131-5861]	6	53050166	53144336		16.1	6.94	7.8	15.69	16.2	16.86
ENSDARG00000017299	fabp11a	fatty acid binding protein 11a [Source:ZFIN;Acc:ZDB-GENE-040912-132]	19	32166702	32169161		67.01	92.74	98.89	77.35	79.47	87.67
ENSDARG00000017311	gpsm2	G protein signaling modulator 2 [Source:ZFIN;Acc:ZDB-GENE-040426-1169]	2	45491032	45510704		5.87	4.13	3.66	7.1	7.1	6.81
ENSDARG00000017312	lmbrd2b	LMBR1 domain containing 2b [Source:NCBI gene;Acc:335257]	21	22400951	22422939		11.97	5.09	6.98	13.98	14.24	15.37
ENSDARG00000017314	cela1.6	chymotrypsin like elastase family member 1, tandem duplicate 6 [Source:ZFIN;Acc:ZDB-GENE-040808-55]	22	7439186	7447391		266.77	367.05	264.9	35.26	37.89	38.54
ENSDARG00000017315	larp7	La ribonucleoprotein 7, transcriptional regulator [Source:NCBI gene;Acc:334956]	7	59572379	59589566		3.57	3.18	2.69	3.43	3.14	3.11
ENSDARG00000017316	bag5	BCL2 associated athanogene 5 [Source:ZFIN;Acc:ZDB-GENE-050913-58]	13	15762278	15799391		5.41	3.19	3.39	5.08	5.92	6.19
ENSDARG00000017320	f11r.1	F11 receptor, tandem duplicate 1 [Source:ZFIN;Acc:ZDB-GENE-030131-2416]	5	29831195	29848772		37.45	37.93	30.53	28.84	29.77	25.67
ENSDARG00000017321	kdr	kinase insert domain receptor (a type III receptor tyrosine kinase) [Source:NCBI gene;Acc:554230]	20	22220988	22277544		1.44	1.25	1.02	3.77	4.12	3.7
ENSDARG00000017329	cdkl1	cyclin-dependent kinase-like 1 (CDC2-related kinase) [Source:NCBI gene;Acc:445316]	13	36693152	36703164		3.01	2.21	2.91	5.1	5.89	6.29
ENSDARG00000017337	C11H6orf120	zgc:77929 [Source:NCBI gene;Acc:402944]	11	11332652	11336986	C11H6orf120	10.13	11.52	10.98	8.22	9.8	8.85
ENSDARG00000017338	kidins220b	kinase D-interacting substrate 220b [Source:NCBI gene;Acc:335881]	20	29743904	29783644		51.55	13.43	14.72	65.12	66.28	70.25
ENSDARG00000017340	uts2b	urotensin 2, beta [Source:NCBI gene;Acc:402853]	11	40024182	40030139		0.08	0.27	0.24	1.07	1.59	1.25
ENSDARG00000017354	epha2a	eph receptor A2 a [Source:ZFIN;Acc:ZDB-GENE-990415-63]	6	45874595	45905746		3.67	2.98	2.52	4.73	4.49	4.51
ENSDARG00000017355	rsph9	radial spoke head component 9 [Source:NCBI gene;Acc:558005]	4	5645251	5652030		1.66	1.72	1.8	1.82	1.67	1.92
ENSDARG00000017359	mettl8	methyltransferase like 8 [Source:ZFIN;Acc:ZDB-GENE-041114-19]	9	3519191	3535024		1.96	1.44	1.36	1.42	1.17	1.85
ENSDARG00000017365	slc23a2	solute carrier family 23 member 2 [Source:ZFIN;Acc:ZDB-GENE-090521-3]	8	42917515	42986689		1.07	0.5	0.71	1.76	1.6	1.63
ENSDARG00000017366	prdm4	PR domain containing 4 [Source:ZFIN;Acc:ZDB-GENE-041210-131]	4	14917502	14926637		5.24	3.31	2.74	6.78	5.8	6.52
ENSDARG00000017367	rhbdf1b	rhomboid 5 homolog 1b (Drosophila) [Source:ZFIN;Acc:ZDB-GENE-130531-6]	12	20227395	20303438		2.93	1.83	1.93	2.25	2.17	2.57
ENSDARG00000017369	sema3d	sema domain, immunoglobulin domain (Ig), short basic domain, secreted, (semaphorin) 3D [Source:NCBI gene;Acc:30250]	18	9171778	9238675		13.22	14.27	12.74	16.92	18.19	16.97
ENSDARG00000017385	b9d2	B9 domain containing 2 [Source:NCBI gene;Acc:436667]	21	21611867	21615651		3.12	2.58	3.26	2.95	2.66	3.57
ENSDARG00000017386	sobpb	sine oculis binding protein homolog (Drosophila) b [Source:NCBI gene;Acc:100006088]	4	818928	858434		2.65	2.3	2.31	5.68	6.42	6.22
ENSDARG00000017388	gstt1b	glutathione S-transferase theta 1b [Source:ZFIN;Acc:ZDB-GENE-040426-1491]	21	15713097	15720534		20.86	34.38	29.99	12.83	14.01	13.59
ENSDARG00000017391	unc13ba	unc-13 homolog Ba (C. elegans) [Source:ZFIN;Acc:ZDB-GENE-100921-10]	5	19094153	19237700		3.52	0.52	0.69	3.47	3.39	3.64
ENSDARG00000017397	smarcc1a	SWI/SNF related, matrix associated, actin dependent regulator of chromatin, subfamily c, member 1a [Source:ZFIN;Acc:ZDB-GENE	16	42423530	42461263		24.63	17.46	15.2	35.63	32.11	33.54
ENSDARG00000017398	slc44a5a	solute carrier family 44 member 5a [Source:ZFIN;Acc:ZDB-GENE-070705-333]	2	11216814	11468654		0.67	0.2	0.27	1.16	1.11	1.15
ENSDARG00000017400	klf1	Kruppel-like factor 1 (erythroid) [Source:ZFIN;Acc:ZDB-GENE-980526-55]	6	8129543	8140958		0.7	0.97	0.64	1.53	1.81	1.65
ENSDARG00000017422	apmap	adipocyte plasma membrane associated protein [Source:NCBI gene;Acc:266637]	13	6219551	6239216		20.54	19.11	15.1	21.48	21.59	22.38
ENSDARG00000017423	patl1	PAT1 homolog 1, processing body mRNA decay factor [Source:NCBI gene;Acc:100009659]	1	52763979	52790782		17.24	12.51	10.87	17.98	19.59	19.57
ENSDARG00000017425	crocc2	ciliary rootlet coiled-coil, rootletin family member 2 [Source:ZFIN;Acc:ZDB-GENE-131121-625]	6	27361978	27450504		0.68	0.39	0.57	0.79	0.94	1
ENSDARG00000017427	phf20l1	PHD finger protein 20 like 1 [Source:ZFIN;Acc:ZDB-GENE-030131-5837]	16	31601181	31622805		8.09	5.39	4.2	7.71	7.63	7.98
ENSDARG00000017429	sphkap	SPHK1 interactor, AKAP domain containing [Source:NCBI gene;Acc:100034387]	18	46395909	46464501		5.64	1.14	1.2	6.84	6.85	6.63
ENSDARG00000017431	mindy2	MINDY lysine 48 deubiquitinase 2 [Source:ZFIN;Acc:ZDB-GENE-030616-328]	7	30493684	30537778		7.12	3.91	3.43	8.32	8.45	7.71
ENSDARG00000017437	adcy3b	adenylate cyclase 3b [Source:ZFIN;Acc:ZDB-GENE-041014-276]	20	46651916	46693942		0.42	0.15	0.2	0.41	0.37	0.5
ENSDARG00000017439	eif4ba	eukaryotic translation initiation factor 4Ba [Source:ZFIN;Acc:ZDB-GENE-060629-1]	23	10396842	10453077		7.41	5.93	5.4	6.72	6.46	6.5
ENSDARG00000017441	mylz3	myosin, light polypeptide 3, skeletal muscle [Source:ZFIN;Acc:ZDB-GENE-000322-6]	1	7538909	7554463		2607.76	3777.84	4799.99	2678.44	2629.41	2557.84
ENSDARG00000017444	hal	histidine ammonia-lyase [Source:ZFIN;Acc:ZDB-GENE-070410-5]	25	7954140	7974494		18.67	23.91	21.15	32.66	38.05	33.77
ENSDARG00000017445	eif3i	eukaryotic translation initiation factor 3, subunit I [Source:NCBI gene;Acc:406263]	19	29822500	29832876		202.79	227.89	265.21	182.89	185.07	189.95
ENSDARG00000017446	camk1db	calcium/calmodulin-dependent protein kinase 1Db [Source:ZFIN;Acc:ZDB-GENE-070112-1872]	25	7987920	8030425		5.5	1.91	2.7	5.42	5.61	5.7
ENSDARG00000017454	nup50	nucleoporin 50 [Source:ZFIN;Acc:ZDB-GENE-030131-1689]	4	74928675	74972270		8.9	6.72	5.87	10.27	9.84	9.63
ENSDARG00000017466	ncam2	neural cell adhesion molecule 2 [Source:ZFIN;Acc:ZDB-GENE-010822-1]	9	35170335	35557397		12.75	4.07	5.13	14.69	15.91	15.73
ENSDARG00000017474	zgc:110699	zgc:110699 [Source:ZFIN;Acc:ZDB-GENE-050522-487]	7	38811800	38822387		0.88	1	0.89	0.75	0.8	0.8
ENSDARG00000017489	ifi45	interferon alpha inducible protein 45 [Source:ZFIN;Acc:ZDB-GENE-051030-98]	13	37605896	37608441		4.34	5.06	5.27	1.65	1.49	1.51
ENSDARG00000017490	cel.1	carboxyl ester lipase, tandem duplicate 1 [Source:ZFIN;Acc:ZDB-GENE-030131-1201]	21	11401247	11411365		73.48	111.35	95.8	5.04	4.76	5.01
ENSDARG00000017494	tgfbr1a	transforming growth factor, beta receptor 1 a [Source:ZFIN;Acc:ZDB-GENE-051120-75]	2	23856045	23931536		3	2.05	1.97	2.64	3.02	3.05
ENSDARG00000017500	rock2a	rho-associated, coiled-coil containing protein kinase 2a [Source:ZFIN;Acc:ZDB-GENE-030115-3]	13	32815241	32899322		10.49	9.09	7.84	11.27	11.28	11.53
ENSDARG00000017504	si:ch211-1o7.3	si:ch211-1o7.3 [Source:ZFIN;Acc:ZDB-GENE-130530-561]	7	8722566	8763156		0.96	1.17	1.33	1.19	1.34	1.31
ENSDARG00000017522	ctdp1	CTD (carboxy-terminal domain, RNA polymerase II, polypeptide A) phosphatase, subunit 1 [Source:ZFIN;Acc:ZDB-GENE-040720-1]	19	11632834	11846958		3.96	1.96	1.72	4.14	3.12	3.31
ENSDARG00000017525	ccnt1	cyclin T1 [Source:ZFIN;Acc:ZDB-GENE-030131-6397]	23	35650771	35664505		3.76	1.87	1.82	4.25	4.35	4.22
ENSDARG00000017532	kif25	kinesin family member 25 [Source:ZFIN;Acc:ZDB-GENE-041001-167]	20	30902138	30920356		3.79	1.23	0.85	3.88	3.17	3.72
ENSDARG00000017537	prkag3a	protein kinase, AMP-activated, gamma 3a non-catalytic subunit [Source:ZFIN;Acc:ZDB-GENE-091204-419]	1	6171585	6185532		1.69	1.28	1.57	1.37	1.43	1.57
ENSDARG00000017542	fgf1a	fibroblast growth factor 1a [Source:NCBI gene;Acc:393733]	14	23731949	23737674		1.47	1.58	1.33	1.37	1.52	1.65
ENSDARG00000017562	banp	BTG3 associated nuclear protein [Source:NCBI gene;Acc:553659]	25	30460378	30550350		6.87	4.53	4.41	6.82	7.35	7.54
ENSDARG00000017565	itk	IL2 inducible T cell kinase [Source:ZFIN;Acc:ZDB-GENE-990415-261]	14	34045284	34074510		0.17	0.05	0.13	0.1	0.06	0.09
ENSDARG00000017569	reep3a	receptor accessory protein 3a [Source:ZFIN;Acc:ZDB-GENE-051127-47]	17	19607387	19626357		6.79	6.93	6.1	8.83	8.64	8.56
ENSDARG00000017571	mccc2	methylcrotonoyl-CoA carboxylase 2 (beta) [Source:ZFIN;Acc:ZDB-GENE-040426-2493]	5	43808838	43819663		57.82	68.02	65.35	38.78	38.05	37.79
ENSDARG00000017579	si:ch211-195b15.7	si:ch211-195b15.7 [Source:ZFIN;Acc:ZDB-GENE-131121-183]	3	32458720	32472155		1.25	0.36	0.31	0.53	0.67	0.59
ENSDARG00000017590	nit2	nitrilase family, member 2 [Source:NCBI gene;Acc:402904]	6	37431169	37441520		15.79	25.26	16.64	9.69	9.9	10.34
ENSDARG00000017591	fat1a	FAT atypical cadherin 1a [Source:ZFIN;Acc:ZDB-GENE-050425-1]	1	17376922	17524922		10.77	7.03	4.61	20.8	18.65	19.07
ENSDARG00000017595	ccdc6b	coiled-coil domain containing 6b [Source:ZFIN;Acc:ZDB-GENE-040426-869]	12	7609928	7639149		3.74	1.99	1.86	5.1	4.97	5.2
ENSDARG00000017602	ccng2	cyclin G2 [Source:ZFIN;Acc:ZDB-GENE-021016-1]	21	5800306	5809022		13.42	11	11.96	18.88	18.9	19.06
ENSDARG00000017605	rpp40	ribonuclease P/MRP 40 subunit [Source:ZFIN;Acc:ZDB-GENE-040625-161]	2	23613040	23620223		4.85	5.53	5.95	3.22	3.04	3.36
ENSDARG00000017606	sys1	SYS1 golgi trafficking protein [Source:ZFIN;Acc:ZDB-GENE-030131-8283]	6	54438355	54444929		12.32	11.9	13.23	11.04	12.31	10.47
ENSDARG00000017612	pnpo	pyridoxamine 5'-phosphate oxidase [Source:ZFIN;Acc:ZDB-GENE-060602-2]	12	28799988	28807184		10.61	9.95	9.74	10.63	11.7	11.98
ENSDARG00000017615	tlx3b	T cell leukemia homeobox 3b [Source:ZFIN;Acc:ZDB-GENE-021021-1]	10	22051054	22057001		1.69	0.71	1.19	1.53	1.81	1.84
ENSDARG00000017617	alg6	ALG6 alpha-1,3-glucosyltransferase [Source:ZFIN;Acc:ZDB-GENE-040808-45]	6	32065094	32087111		3.32	4.12	3.69	3.33	3.12	2.96
ENSDARG00000017624	krt4	keratin 4 [Source:ZFIN;Acc:ZDB-GENE-000607-83]	6	39308498	39313027		6199.67	5881.76	5302.84	2147.6	1873.05	1810.09
ENSDARG00000017634	pdcb	phosducin b [Source:ZFIN;Acc:ZDB-GENE-031023-2]	20	34084331	34090740		55.95	14.49	11.24	30.19	32.47	32.16
ENSDARG00000017636	rnf167	ring finger protein 167 [Source:ZFIN;Acc:ZDB-GENE-110126-2]	5	68037629	68058168		9.01	7.23	6.22	9.7	9.63	10.54
ENSDARG00000017649	cdh7b	cadherin 7b [Source:ZFIN;Acc:ZDB-GENE-171002-1]	24	31493	39567		0.07	0.02	0.03	0.1	0.15	0.17
ENSDARG00000017653	rgs13	regulator of G protein signaling 13 [Source:ZFIN;Acc:ZDB-GENE-070410-138]	2	6475567	6482240		1.21	1.69	1.59	1.45	1.43	1.56
ENSDARG00000017657	zgc:112163	zgc:112163 [Source:NCBI gene;Acc:550418]	6	40874206	40875253		2.31	2.2	2.64	2.2	2.29	1.93
ENSDARG00000017658	gmppb	GDP-mannose pyrophosphorylase B [Source:NCBI gene;Acc:445097]	22	39084107	39097217		8.55	7.99	7.35	9.59	9.05	9.34
ENSDARG00000017659	sephs2	selenophosphate synthetase 2 [Source:ZFIN;Acc:ZDB-GENE-030327-5]	23	18558209	18568522		12.92	12.09	9.44	11.14	10.15	10.59
ENSDARG00000017661	braf	B-Raf proto-oncogene, serine/threonine kinase [Source:ZFIN;Acc:ZDB-GENE-040805-1]	4	12074776	12102036		6.77	2.84	3.38	10.62	10.99	11.36
ENSDARG00000017665	snrkb	SNF related kinase b [Source:ZFIN;Acc:ZDB-GENE-040426-1724]	25	13498188	13526319		6.63	3.91	4.26	7.78	7.42	8.32
ENSDARG00000017673	nova2	NOVA alternative splicing regulator 2 [Source:ZFIN;Acc:ZDB-GENE-080211-1]	15	28685625	28766824		26.6	6.87	7.77	53.89	50.92	55.59
ENSDARG00000017675	utp4	UTP4 small subunit processome component [Source:ZFIN;Acc:ZDB-GENE-040426-2466]	18	14619544	14633308		15.27	18.3	18.4	10.49	11.23	10.35
ENSDARG00000017676	mmp2	matrix metallopeptidase 2 [Source:ZFIN;Acc:ZDB-GENE-030131-9123]	7	35410050	35432901		66.54	82.97	73.28	87.53	90.47	83.32
ENSDARG00000017687	dnajc5gb	DnaJ (Hsp40) homolog, subfamily C, member 5 gamma b [Source:ZFIN;Acc:ZDB-GENE-040426-1238]	17	6490089	6514962		4.12	4.87	3.78	4.11	3.82	3.54
ENSDARG00000017696	utp25	UTP25 small subunit processor component [Source:NCBI gene;Acc:286787]	13	31925254	31938640		10.18	9.94	9.83	6.94	6.65	5.72
ENSDARG00000017703	paqr3a	progestin and adipoQ receptor family member IIIa [Source:ZFIN;Acc:ZDB-GENE-040426-1667]	5	39154617	39171302		3.45	2.73	2.12	4.39	4.15	3.88
ENSDARG00000017708	lrrc40	leucine rich repeat containing 40 [Source:NCBI gene;Acc:334130]	6	30113750	30122554		5.46	5.81	5.29	5.04	5.06	5.03
ENSDARG00000017710	ppp1r14ab	protein phosphatase 1, regulatory (inhibitor) subunit 14Ab [Source:ZFIN;Acc:ZDB-GENE-040426-1783]	15	20204628	20233105		5.34	3.92	3.04	3.95	5.11	3.78
ENSDARG00000017720	si:ch211-106h11.1	si:ch211-106h11.1 [Source:ZFIN;Acc:ZDB-GENE-061009-2]	3	5647772	5664123		0.57	0.19	0.61	4.34	3.9	4.3
ENSDARG00000017722	chrm4b	cholinergic receptor, muscarinic 4b [Source:ZFIN;Acc:ZDB-GENE-090410-7]	25	7882847	7887274		0.17	0.21	0.36	0.15	0.09	0.13
ENSDARG00000017728	phb2b	prohibitin 2b [Source:ZFIN;Acc:ZDB-GENE-040718-430]	6	29707129	29714290		37.13	44.49	36.73	28.83	27.48	27.22
ENSDARG00000017739	ak5l	adenylate kinase 5, like [Source:ZFIN;Acc:ZDB-GENE-050410-2]	5	64177484	64203101		6.32	5.31	4.61	3.14	3.65	3.4
ENSDARG00000017740	sec63	SEC63 homolog, protein translocation regulator [Source:ZFIN;Acc:ZDB-GENE-040718-328]	20	32501748	32521973		14.03	12.12	11.04	14.48	15.03	14.28
ENSDARG00000017741	g3bp1	GTPase activating protein (SH3 domain) binding protein 1 [Source:ZFIN;Acc:ZDB-GENE-030131-7452]	14	25923373	25935167		24.43	21.67	19.76	25.17	23.18	23.25
ENSDARG00000017742	grm6a	glutamate receptor, metabotropic 6a [Source:ZFIN;Acc:ZDB-GENE-060208-1]	8	8532407	8624008		1.54	0.21	0.22	1.78	1.58	1.95
ENSDARG00000017744	smc2	structural maintenance of chromosomes 2 [Source:ZFIN;Acc:ZDB-GENE-030131-105]	1	19535120	19561801		15.72	13.38	13.11	9.96	9.32	9.29
ENSDARG00000017748	arhgap29b	Rho GTPase activating protein 29b [Source:NCBI gene;Acc:378998]	2	15128027	15198117		4.16	3.78	3.24	5.41	5.24	5.59
ENSDARG00000017757	pik3cg	phosphatidylinositol-4,5-bisphosphate 3-kinase, catalytic subunit gamma [Source:ZFIN;Acc:ZDB-GENE-040426-2532]	4	3343171	3353595		2.35	2.4	2.19	2.18	2.69	2.38
ENSDARG00000017762	srrt	serrate RNA effector molecule homolog (Arabidopsis) [Source:NCBI gene;Acc:192311]	5	4253929	4297459		22.84	15.28	13.44	20.87	19.67	19.28
ENSDARG00000017772	mdh1aa	malate dehydrogenase 1Aa, NAD (soluble) [Source:ZFIN;Acc:ZDB-GENE-040204-1]	1	50968908	50990889		251.3	305.39	321.49	163.96	174.71	173.28
ENSDARG00000017773	slc16a12a	solute carrier family 16 member 12a [Source:ZFIN;Acc:ZDB-GENE-080721-24]	17	23578316	23596969		6.68	9.77	8.8	5.6	6.69	6.26
ENSDARG00000017775	atp5mc3a	ATP synthase membrane subunit c locus 3a [Source:ZFIN;Acc:ZDB-GENE-040426-857]	9	2330798	2341127		90.12	106.45	110.53	85.68	84.34	83.32
ENSDARG00000017780	rorcb	RAR-related orphan receptor C b [Source:ZFIN;Acc:ZDB-GENE-040724-215]	2	49417900	49454451		2.65	1.46	1.47	4.79	4.53	4.91
ENSDARG00000017781	hsd17b10	hydroxysteroid (17-beta) dehydrogenase 10 [Source:ZFIN;Acc:ZDB-GENE-041010-201]	23	18372048	18381361		48.68	61.33	64.21	32.51	35.58	33.62
ENSDARG00000017785	zgc:158689	zgc:158689 [Source:ZFIN;Acc:ZDB-GENE-070112-1902]	16	5678071	5710821		2.36	2.2	2.3	4.39	4.36	4.7
ENSDARG00000017790	chrnb2	cholinergic receptor, nicotinic, beta 2 [Source:ZFIN;Acc:ZDB-GENE-070821-3]	16	22618620	22678294		4.47	1.18	1.31	5.83	6.07	5.75
ENSDARG00000017794	sdr16c5b	short chain dehydrogenase/reductase family 16C, member 5b [Source:ZFIN;Acc:ZDB-GENE-040426-2861]	7	58699718	58718472		53.31	82.61	73.32	26.49	27.54	28.01
ENSDARG00000017798	bcor	BCL6 corepressor [Source:ZFIN;Acc:ZDB-GENE-040408-1]	22	11144153	11200846		9.12	5.66	4.62	21.95	21.11	21.4
ENSDARG00000017799	tgm1	transglutaminase 1, K polypeptide [Source:ZFIN;Acc:ZDB-GENE-030131-9032]	23	1730663	1748141		3.94	3.25	2.73	2.65	2.63	2.33
ENSDARG00000017803	gsk3ba	glycogen synthase kinase 3 beta, genome duplicate a [Source:ZFIN;Acc:ZDB-GENE-990714-4]	9	9577743	9671244		15.27	9.96	7.08	25.39	24.15	24.04
ENSDARG00000017809	stard3	StAR-related lipid transfer (START) domain containing 3 [Source:NCBI gene;Acc:63998]	19	4968947	4985996		5.91	5.32	5.06	7.31	8.03	7.19
ENSDARG00000017811	si:ch211-210c8.6	si:ch211-210c8.6 [Source:ZFIN;Acc:ZDB-GENE-030131-3630]	23	33752275	33757742		14.23	19.09	17.02	9.64	8.79	9.85
ENSDARG00000017813	pigs	phosphatidylinositol glycan anchor biosynthesis, class S [Source:ZFIN;Acc:ZDB-GENE-060929-436]	21	39583458	39604628		7.91	5.59	6.12	6.76	6.68	6.07
ENSDARG00000017821	gata5	GATA binding protein 5 [Source:ZFIN;Acc:ZDB-GENE-980526-340]	23	7379728	7391028		7.23	9.98	7.76	9.94	9.71	9.23
ENSDARG00000017834	micall2b	mical-like 2b [Source:ZFIN;Acc:ZDB-GENE-030131-5803]	1	9459760	9486214		1.74	1.5	1.2	1.27	1.2	1.23
ENSDARG00000017835	brf1a	BRF1 RNA polymerase III transcription initiation factor subunit a [Source:ZFIN;Acc:ZDB-GENE-030131-6334]	13	33368140	33449639		1.39	1.21	0.99	1.61	1.85	1.66
ENSDARG00000017841	cand1	cullin-associated and neddylation-dissociated 1 [Source:ZFIN;Acc:ZDB-GENE-040426-2872]	4	13412030	13425447		25.92	20.05	19.24	25.29	25.43	24.16
ENSDARG00000017842	syvn1	synovial apoptosis inhibitor 1, synoviolin [Source:NCBI gene;Acc:335226]	14	26452740	26465729		14.62	11.54	10.6	14.12	13.19	12.77
ENSDARG00000017843	srsf1b	serine and arginine rich splicing factor 1b [Source:NCBI gene;Acc:393565]	21	39567505	39577517		29.51	13.47	17.82	28.69	28.16	27.36
ENSDARG00000017844	copz1	coatomer protein complex, subunit zeta 1 [Source:ZFIN;Acc:ZDB-GENE-000406-6]	23	36340520	36354106		50.12	63.86	60.57	41	42.76	43.05
ENSDARG00000017853	prepl	prolyl endopeptidase like [Source:ZFIN;Acc:ZDB-GENE-090313-224]	13	10621257	10647310		3.53	2.45	2.39	2.55	2.41	2.81
ENSDARG00000017860	rgs5b	regulator of G protein signaling 5b [Source:ZFIN;Acc:ZDB-GENE-050417-359]	2	6885852	6889119		5.35	5.25	5.39	4.88	4.23	5.4
ENSDARG00000017874	dnajc3b	DnaJ (Hsp40) homolog, subfamily C, member 3b [Source:ZFIN;Acc:ZDB-GENE-000831-4]	7	20779026	20796935		4.73	4.66	4.21	6	6.47	6.22
ENSDARG00000017880	kcnip3b	Kv channel interacting protein 3b, calsenilin [Source:ZFIN;Acc:ZDB-GENE-040426-1752]	10	20653716	20706270		1.83	0.86	0.74	1.09	1.28	1.16
ENSDARG00000017882	rdh1	retinol dehydrogenase 1 [Source:ZFIN;Acc:ZDB-GENE-030912-15]	9	48673093	48701035		8.44	13.73	12.42	4.34	4.14	4.15
ENSDARG00000017884	bgna	biglycan a [Source:ZFIN;Acc:ZDB-GENE-040704-74]	8	14104496	14126646		2.49	2.96	2.36	1.43	1.15	1.64
ENSDARG00000017886	zbtb11	zinc finger and BTB domain containing 11 [Source:ZFIN;Acc:ZDB-GENE-030131-5055]	6	29144776	29159888		7.96	6.04	6.03	6.86	7.29	6.69
ENSDARG00000017891	tcp1	t-complex 1 [Source:ZFIN;Acc:ZDB-GENE-990714-24]	23	2421313	2443881		146.14	136.95	129.92	118.67	119.67	116.2
ENSDARG00000017901	tln2a	talin 2a [Source:ZFIN;Acc:ZDB-GENE-040724-263]	7	29863548	29946044		6.04	5.39	4.5	8.12	6.83	6.8
ENSDARG00000017905	casp3a	caspase 3, apoptosis-related cysteine peptidase a [Source:ZFIN;Acc:ZDB-GENE-011210-1]	1	17527342	17536307		30.97	24.11	25.63	30.59	28.88	30.79
ENSDARG00000017910	pdss1	prenyl (decaprenyl) diphosphate synthase, subunit 1 [Source:ZFIN;Acc:ZDB-GENE-030131-4430]	24	6015299	6024466		11.02	14.38	14.17	10.34	10.22	10.62
ENSDARG00000017917	her7	hairy and enhancer of split related-7 [Source:ZFIN;Acc:ZDB-GENE-000427-6]	5	68807170	68808485		0.23	0.09	0.16	0.17	0.15	0.34
ENSDARG00000017927	cx36.7	connexin 36.7 [Source:ZFIN;Acc:ZDB-GENE-050616-5]	7	12835048	12836225		0.08	0.04	0.1	0.08	0.18	0.19
ENSDARG00000017928	xrcc3	X-ray repair complementing defective repair in Chinese hamster cells 3 [Source:ZFIN;Acc:ZDB-GENE-050320-116]	20	20896622	20901558		1.69	1.77	2.22	1.54	1.37	1.38
ENSDARG00000017929	ncoa2	nuclear receptor coactivator 2 [Source:NCBI gene;Acc:81884]	24	14240196	14445137		9.36	5.87	4.39	14.73	13.8	13.62
ENSDARG00000017931	ints6l	integrator complex subunit 6 like [Source:NCBI gene;Acc:393406]	14	31566517	31608159		6.17	2.65	2.56	4.88	4.8	5.13
ENSDARG00000017947	ptk6a	PTK6 protein tyrosine kinase 6a [Source:ZFIN;Acc:ZDB-GENE-081105-117]	8	23227100	23240156		1.1	0.68	0.55	0.84	0.81	0.93
ENSDARG00000017953	tp73	tumor protein p73 [Source:ZFIN;Acc:ZDB-GENE-030814-2]	8	48848200	48935476		1.48	1.15	1.01	0.89	0.95	0.76
ENSDARG00000017960	sfxn2	sideroflexin 2 [Source:ZFIN;Acc:ZDB-GENE-040426-2831]	12	33975065	33988885		27.8	38.4	38.54	23.72	24.31	23.33
ENSDARG00000017982	cyp39a1	cytochrome P450, family 39, subfamily A, polypeptide 1 [Source:ZFIN;Acc:ZDB-GENE-041014-339]	20	39190854	39219537		0.39	0.3	0.52	0.38	0.36	0.34
ENSDARG00000017983	rsph14	radial spoke head 14 homolog [Source:ZFIN;Acc:ZDB-GENE-081104-375]	8	44566708	44586985		0.51	0.44	0.54	0.67	0.44	0.36
ENSDARG00000017984	zgc:172106	zgc:172106 [Source:ZFIN;Acc:ZDB-GENE-080204-42]	1	55293424	55299173		0.02	0	0.02	0.08	0.07	0.1
ENSDARG00000017985	C1H4orf33	zgc:77739 [Source:NCBI gene;Acc:393870]	1	12394205	12400783	C1H4orf33	5.2	7.93	6.88	1.93	2.09	2
ENSDARG00000017986	vps13d	vacuolar protein sorting 13 homolog D [Source:ZFIN;Acc:ZDB-GENE-050912-1]	23	21696560	21763598		3.23	2.37	1.59	3.57	3.28	3.13
ENSDARG00000017988	dse	dermatan sulfate epimerase [Source:ZFIN;Acc:ZDB-GENE-041001-202]	20	535458	557724		3.79	4.78	3.78	7.07	7.68	6.82
ENSDARG00000017994	tssk6	testis-specific serine kinase 6 [Source:ZFIN;Acc:ZDB-GENE-060216-3]	22	18386253	18387059		0	0	0	0	0.03	0
ENSDARG00000018000	ubp1	upstream binding protein 1 [Source:ZFIN;Acc:ZDB-GENE-060503-870]	19	42806812	42841889		5.2	2.45	2.96	10.63	11.69	11.65
ENSDARG00000018002	eci1	enoyl-CoA delta isomerase 1 [Source:ZFIN;Acc:ZDB-GENE-030131-6033]	3	27770110	27777671		25.67	41.97	37.92	18.32	16.28	16.3
ENSDARG00000018004	nkx2.5	NK2 homeobox 5 [Source:ZFIN;Acc:ZDB-GENE-980526-321]	14	24241241	24244034		0.25	0.14	0.2	0.47	0.52	0.49
ENSDARG00000018006	vrk1	VRK serine/threonine kinase 1 [Source:NCBI gene;Acc:406694]	17	18773961	18797245		2.93	3	2.81	2.78	2.33	2.11
ENSDARG00000018008	mdh1b	malate dehydrogenase 1B, NAD (soluble) [Source:NCBI gene;Acc:767794]	1	6218632	6225285		0.61	0.44	0.52	0.73	0.65	0.57
ENSDARG00000018010	crtap	cartilage associated protein [Source:ZFIN;Acc:ZDB-GENE-040426-2619]	19	42609132	42617723		22.17	27.39	27.6	39.71	39.07	39.12
ENSDARG00000018022	msh2	mutS homolog 2 (E. coli) [Source:ZFIN;Acc:ZDB-GENE-040426-2932]	12	24906106	24928497		12.46	11.72	13.33	7.77	6.89	6.46
ENSDARG00000018025	tbx2a	T-box transcription factor 2a [Source:ZFIN;Acc:ZDB-GENE-990917-4]	5	56504717	56513825		10.09	4.97	4.6	12.21	11.16	13.03
ENSDARG00000018032	scn8ab	sodium channel, voltage gated, type VIII, alpha subunit b [Source:ZFIN;Acc:ZDB-GENE-060906-2]	6	39392442	39489190		6.12	1.97	2.29	8.47	8.46	8.79
ENSDARG00000018040	msantd4	Myb/SANT-like DNA-binding domain containing 4 with coiled-coils [Source:ZFIN;Acc:ZDB-GENE-121023-2]	15	44472356	44493753		2.97	1.15	1.62	3.61	3.86	3.58
ENSDARG00000018047	brms1lb	BRMS1 like transcriptional repressor b [Source:ZFIN;Acc:ZDB-GENE-081031-54]	20	16830415	16849362		2.42	1.23	1.43	2.28	2.09	2.23
ENSDARG00000018048	zgc:171704	zgc:171704 [Source:ZFIN;Acc:ZDB-GENE-070820-16]	16	28792158	28796865		0.2	0.11	0.16	0.82	0.85	0.87
ENSDARG00000018049	sf3b2	splicing factor 3b, subunit 2 [Source:ZFIN;Acc:ZDB-GENE-070928-1]	7	6756206	6800689		39.75	30.78	27.11	39.98	38.71	40.65
ENSDARG00000018060	pik3r2	phosphoinositide-3-kinase, regulatory subunit 2 (beta) [Source:ZFIN;Acc:ZDB-GENE-040309-1]	2	24700922	24755347		6.66	5.9	4.09	7.79	7.88	7.26
ENSDARG00000018061	neil1	nei-like DNA glycosylase 1 [Source:ZFIN;Acc:ZDB-GENE-040426-994]	25	26827140	26833100		5.55	6.43	5.4	3.77	3.48	4.03
ENSDARG00000018065	ntm	neurotrimin [Source:ZFIN;Acc:ZDB-GENE-060421-6020]	10	29963518	30589172		2.25	0.47	1.19	3.24	3.7	3.39
ENSDARG00000018066	ptchd1	patched domain containing 1 [Source:ZFIN;Acc:ZDB-GENE-061220-1]	24	25347503	25364775		0.95	0.2	0.3	1.25	1.11	1.35
ENSDARG00000018069	rdh12	retinol dehydrogenase 12 [Source:ZFIN;Acc:ZDB-GENE-040718-9]	13	33055548	33065830		11.99	8.8	11.12	7.24	7.24	8.77
ENSDARG00000018073	mrps22	mitochondrial ribosomal protein S22 [Source:ZFIN;Acc:ZDB-GENE-070615-31]	15	6948644	6966245		15.49	17.33	15.04	12.15	11.55	12.32
ENSDARG00000018077	rbp1.1	retinol binding protein 1, cellular, tandem duplicate 1 [Source:ZFIN;Acc:ZDB-GENE-050421-2]	2	39028337	39036604		1.14	0.9	0.86	1.58	1.29	1.4
ENSDARG00000018096	scap	SREBF chaperone [Source:ZFIN;Acc:ZDB-GENE-040107-41]	16	41060161	41103782		3.59	2.69	1.82	5.27	4.93	5.45
ENSDARG00000018105	casq1b	calsequestrin 1b [Source:ZFIN;Acc:ZDB-GENE-060929-1070]	7	73717581	73752955		40.45	59.5	68.59	43.09	39.5	40.74
ENSDARG00000018106	eif2b3	eukaryotic translation initiation factor 2B, subunit 3 gamma [Source:ZFIN;Acc:ZDB-GENE-040426-1039]	2	34008798	34072218		11.15	10.5	11.44	7.78	7.95	8.8

ENSDARG00000018107	rab3da	RAB3D, member RAS oncogene family, a [Source:ZFIN;Acc:ZDB-GENE-080722-14]	1	52178916	52201279		6.66	5.44	6.15	5.76	5.41	5.54
ENSDARG00000018109	mafga	v-maf avian musculoaponeurotic fibrosarcoma oncogene homolog Ga [Source:ZFIN;Acc:ZDB-GENE-040624-7]	3	55401951	55404985		3	0.39	1.14	3.33	3.17	2.95
ENSDARG00000018110	pak4	p21 protein (Cdc42/Rac)-activated kinase 4 [Source:ZFIN;Acc:ZDB-GENE-040704-69]	15	25452092	25486010		4.74	3.85	3.21	6.5	6.76	6.1
ENSDARG00000018111	kdm7aa	lysine (K)-specific demethylase 7Aa [Source:NCBI gene;Acc:321248]	4	22484830	22519525		5.13	2.53	2.15	10.56	10.89	10.39
ENSDARG00000018117	CR392347.1		1	41949303	41950304		0.32	0.09	0.22	0.19	0.26	0.12
ENSDARG00000018121	cenpj	centromere protein J [Source:ZFIN;Acc:ZDB-GENE-030131-6323]	9	29569375	29579908		0.73	0.59	0.56	0.4	0.41	0.43
ENSDARG00000018124	psmd3	proteasome 26S subunit, non-ATPase 3 [Source:ZFIN;Acc:ZDB-GENE-040426-1444]	19	48049202	48059334		47.52	44.21	44.55	51.7	50.49	51.94
ENSDARG00000018145	mid1ip1l	MID1 interacting protein 1, like [Source:NCBI gene;Acc:322977]	14	41466368	41469036		13.59	13.54	17.04	22.18	22.62	23.48
ENSDARG00000018146	gpx1a	glutathione peroxidase 1a [Source:ZFIN;Acc:ZDB-GENE-030410-1]	11	36048579	36156971		19.19	26.14	28.87	13.47	15.93	12.69
ENSDARG00000018149	slc38a4	solute carrier family 38 member 4 [Source:ZFIN;Acc:ZDB-GENE-041010-14]	4	1516716	1566572		19.47	20.09	18.52	32.55	32.04	31.46
ENSDARG00000018153	nt5c1ba	5'-nucleotidase, cytosolic IB a [Source:ZFIN;Acc:ZDB-GENE-070424-103]	13	32446169	32453932		2.77	1.38	1.84	2.51	2.94	2.9
ENSDARG00000018159	ano10b	anoctamin 10b [Source:ZFIN;Acc:ZDB-GENE-061013-169]	25	13528257	13558212		5.12	4.35	3.94	4.68	4.21	3.87
ENSDARG00000018162	ctnnal1	catenin (cadherin-associated protein), alpha-like 1 [Source:ZFIN;Acc:ZDB-GENE-060503-507]	19	34751687	34873566		3.45	3.44	3.06	3.82	4.69	3.93
ENSDARG00000018165	depdc1a	DEP domain containing 1a [Source:NCBI gene;Acc:100003959]	8	16710634	16725959		1.34	0.7	0.8	1.49	1.28	1.39
ENSDARG00000018174	gnai2a	guanine nucleotide binding protein (G protein), alpha inhibiting activity polypeptide 2a [Source:ZFIN;Acc:ZDB-GENE-030131-8365]	11	34921492	35042194		34.21	33.18	32.84	44.61	48.18	45.06
ENSDARG00000018178	pgm2	phosphoglucomutase 2 [Source:ZFIN;Acc:ZDB-GENE-040426-2218]	1	18779168	18803919		11.7	13.43	10.93	13.03	12.59	13.67
ENSDARG00000018179	acvrl1	activin A receptor like type 1 [Source:ZFIN;Acc:ZDB-GENE-021003-1]	23	27835647	27857104		7.83	8.11	6.77	11.4	12.85	12.41
ENSDARG00000018181	dnajc28	DnaJ (Hsp40) homolog, subfamily C, member 28 [Source:ZFIN;Acc:ZDB-GENE-050417-130]	9	35872698	35875051		5.76	6.49	6.44	3.33	3.22	3.11
ENSDARG00000018190	get3	guided entry of tail-anchored proteins factor 3, ATPase [Source:NCBI gene;Acc:325704]	1	52137528	52152993		16.89	12.34	11.48	18.06	17.87	16.74
ENSDARG00000018192	ubr5	ubiquitin protein ligase E3 component n-recognin 5 [Source:ZFIN;Acc:ZDB-GENE-030131-6559]	16	52542550	52646789		17.67	12.11	10.73	26.11	25.27	25.16
ENSDARG00000018206	nck2a	NCK adaptor protein 2a [Source:ZFIN;Acc:ZDB-GENE-040426-686]	9	6393368	6502491		3.12	1.97	1.93	4.04	3.64	3.98
ENSDARG00000018228	htr2cl1	5-hydroxytryptamine (serotonin) receptor 2C, G protein-coupled-like 1 [Source:ZFIN;Acc:ZDB-GENE-081104-48]	7	23292133	23494310		2.48	1.42	1.31	2.82	2.82	2.83
ENSDARG00000018241	mfap1	microfibril associated protein 1 [Source:ZFIN;Acc:ZDB-GENE-040426-1075]	7	50399627	50407729		27.95	24.91	24.1	22.22	22.35	24.38
ENSDARG00000018257	ncoa1	nuclear receptor coactivator 1 [Source:ZFIN;Acc:ZDB-GENE-041001-175]	20	36682051	36792710		2.08	1.39	1.11	3.71	3.79	3.91
ENSDARG00000018259	atp1a3a	ATPase Na+/K+ transporting subunit alpha 3a [Source:ZFIN;Acc:ZDB-GENE-001212-7]	19	6328234	6385594		66.91	12.11	11.84	90.52	83.87	91.04
ENSDARG00000018260	bmp7a	bone morphogenetic protein 7a [Source:ZFIN;Acc:ZDB-GENE-000208-25]	11	7107798	7147540		0.25	0.17	0.25	0.33	0.41	0.32
ENSDARG00000018263	pdia2	protein disulfide isomerase family A, member 2 [Source:ZFIN;Acc:ZDB-GENE-040426-705]	1	9954489	9966195		51.73	87.65	63.79	8.43	9.22	8.77
ENSDARG00000018264	trim101	tripartite motif containing 101 [Source:ZFIN;Acc:ZDB-GENE-040801-100]	23	17454691	17470146		13.95	19.21	21.41	7.6	7.44	7.1
ENSDARG00000018266	mthfd1a	methylenetetrahydrofolate dehydrogenase (NADP+ dependent) 1a, methenyltetrahydrofolate cyclohydrolase, formyltetrahydrofolate	20	27646772	27687457		9.1	13.62	13.75	6.7	7.45	7.2
ENSDARG00000018270	olfm1a	olfactomedin 1a [Source:ZFIN;Acc:ZDB-GENE-040718-194]	5	64732036	64775757		24.81	7.46	11.58	19.04	18.52	19.76
ENSDARG00000018272	wdr33	WD repeat domain 33 [Source:ZFIN;Acc:ZDB-GENE-040924-3]	2	5410432	5443745		12.56	8.82	7.99	14.99	14.99	14.94
ENSDARG00000018281	cfap161	cilia and flagella associated protein 161 [Source:NCBI gene;Acc:550471]	18	7090920	7097403		0.42	0.5	0.79	0.84	0.85	0.94
ENSDARG00000018283	cyba	cytochrome b-245, alpha polypeptide [Source:ZFIN;Acc:ZDB-GENE-040426-1526]	18	31073265	31075619		8.86	9.71	9.61	10.23	9.44	8.67
ENSDARG00000018285	pdpk1b	3-phosphoinositide dependent protein kinase 1b [Source:ZFIN;Acc:ZDB-GENE-040426-1820]	24	37301852	37326236		18.03	13.35	11.5	22.35	22.94	24.04
ENSDARG00000018296	rev1	REV1 DNA directed polymerase [Source:ZFIN;Acc:ZDB-GENE-081105-180]	9	21793565	21816606		4.14	2.21	2.27	4.05	3.85	3.94
ENSDARG00000018301	si:ch73-186j5.2	si:ch73-186j5.2 [Source:ZFIN;Acc:ZDB-GENE-070705-214]	18	22123072	22127985		0.04	0.04	0.19	0.02	0.02	0.12
ENSDARG00000018303	etv4	ETS variant transcription factor 4 [Source:NCBI gene;Acc:30700]	12	27536095	27568694		13.53	12.44	11.39	14.5	14.7	15.31
ENSDARG00000018312	rell1	RELT like 1 [Source:ZFIN;Acc:ZDB-GENE-161213-2]	7	61230013	61279523		4.92	5.62	4.25	4.2	4	4
ENSDARG00000018319	arglu1b	arginine and glutamate rich 1b [Source:NCBI gene;Acc:393131]	1	7662091	7673376		44.5	20.86	27.22	42.96	41.75	41.72
ENSDARG00000018321	lbx1a	ladybird homeobox 1a [Source:ZFIN;Acc:ZDB-GENE-040724-40]	13	28305798	28308138		2.56	1.67	1.91	4.83	5.47	4.81
ENSDARG00000018325	taf5	TAF5 RNA polymerase II, TATA box binding protein (TBP)-associated factor [Source:ZFIN;Acc:ZDB-GENE-051120-180]	1	49415281	49432147		9.21	6.43	5.83	10.04	10.27	10.04
ENSDARG00000018327	illr3	immune-related, lectin-like receptor 3 [Source:ZFIN;Acc:ZDB-GENE-050311-4]	19	3266914	3272998		0	0.02	0.01	0.02	0.06	0.11
ENSDARG00000018328	rhocb	ras homolog family member Cb [Source:NCBI gene;Acc:436718]	8	27555314	27624329		47.83	53.41	58.22	40.86	42.42	44.39
ENSDARG00000018329	gc2	guanylyl cyclase 2 [Source:ZFIN;Acc:ZDB-GENE-011128-8]	7	51267478	51300277		1.19	0.33	0.27	0.89	1.06	1.07
ENSDARG00000018334	rpl35	ribosomal protein L35 [Source:NCBI gene;Acc:192299]	21	5873227	5881344		863.97	1289.2	1385.56	881.06	942.71	947.28
ENSDARG00000018342	ggt1b	gamma-glutamyltransferase 1b [Source:ZFIN;Acc:ZDB-GENE-110408-13]	10	41624711	41664427		3.75	4.97	3.91	1.63	2.1	1.49
ENSDARG00000018351	hpda	4-hydroxyphenylpyruvate dioxygenase a [Source:NCBI gene;Acc:394142]	5	2815021	2852285		24.3	35.3	29.28	17.87	23.27	22.01
ENSDARG00000018361	sult1st3	sulfotransferase family 1, cytosolic sulfotransferase 3 [Source:NCBI gene;Acc:368270]	8	46559332	46581448		5.79	9.7	6.76	1.79	2.01	1.92
ENSDARG00000018382	prkcha	protein kinase C, eta, a [Source:ZFIN;Acc:ZDB-GENE-080402-5]	13	31716820	31748970		0.66	0.57	0.44	0.56	0.46	0.66
ENSDARG00000018383	frzb	frizzled related protein [Source:ZFIN;Acc:ZDB-GENE-990715-1]	9	44832032	44839148		3.74	2.91	2.95	7.47	8.08	8.51
ENSDARG00000018393	kank2	KN motif and ankyrin repeat domains 2 [Source:NCBI gene;Acc:571549]	3	55161627	55214130		4.77	4.65	4.05	9.31	9.94	10.02
ENSDARG00000018397	hpca	hippocalcin [Source:ZFIN;Acc:ZDB-GENE-040426-1683]	19	32622177	32710922		23.14	10.99	14.92	26.53	27.12	27.09
ENSDARG00000018399	rps6kb1a	ribosomal protein S6 kinase b, polypeptide 1a [Source:ZFIN;Acc:ZDB-GENE-091113-52]	10	28172344	28193642		8.35	8.41	8.59	12.14	13.25	12.35
ENSDARG00000018404	krt18a.1	keratin 18a, tandem duplicate 1 [Source:NCBI gene;Acc:352912]	23	10347851	10351754		108.58	116.07	116.05	210.87	238.02	242.84
ENSDARG00000018411	mknk1	MAPK interacting serine/threonine kinase 1 [Source:ZFIN;Acc:ZDB-GENE-080220-11]	11	13176568	13189751		3.32	2.43	2.09	4.66	4.86	4.59
ENSDARG00000018418	pth3r	parathyroid hormone 3 receptor [Source:ZFIN;Acc:ZDB-GENE-991123-12]	12	9257457	9294819		1.41	2.02	1.95	1.93	2.09	1.87
ENSDARG00000018423	sulf2a	sulfatase 2a [Source:ZFIN;Acc:ZDB-GENE-040426-759]	11	24907469	24993225		8.66	2.81	4.59	9.48	8.98	8.94
ENSDARG00000018425	ddx61	DEAD (Asp-Glu-Ala-Asp) box helicase 61 [Source:ZFIN;Acc:ZDB-GENE-030131-6611]	16	25682577	25699657		24.16	21.12	20.08	25.29	25.02	25.16
ENSDARG00000018426	aldh7a1	aldehyde dehydrogenase 7 family, member A1 [Source:ZFIN;Acc:ZDB-GENE-030131-6129]	10	16006905	16028082		123.44	147.35	141.93	72.19	73.81	71.72
ENSDARG00000018432	m6pr	mannose-6-phosphate receptor (cation dependent) [Source:ZFIN;Acc:ZDB-GENE-040426-2285]	16	17578607	17586883		8.13	9.06	7.36	8	7.14	7.89
ENSDARG00000018441	heg1	heart development protein with EGF-like domains 1 [Source:NCBI gene;Acc:406202]	9	38684871	38707510		1.49	1.47	1.38	2.67	2.33	2.56
ENSDARG00000018459	msrb2	methionine sulfoxide reductase B2 [Source:NCBI gene;Acc:405857]	24	17040211	17049270		20.1	34.27	32.79	19.8	22.07	19.22
ENSDARG00000018460	mbnl2	muscleblind-like splicing regulator 2 [Source:ZFIN;Acc:ZDB-GENE-030131-9582]	1	2101541	2217861		11.84	6.65	5.97	15.04	14.46	14.21
ENSDARG00000018461	zgc:56095	zgc:56095 [Source:ZFIN;Acc:ZDB-GENE-040426-1948]	24	7741521	7746478		43.78	63.09	57.86	32.12	32.62	33.15
ENSDARG00000018476	pak2b	p21 protein (Cdc42/Rac)-activated kinase 2b [Source:ZFIN;Acc:ZDB-GENE-030131-3759]	22	2895511	2929156		13.83	9.67	9.22	16.18	16.04	16.82
ENSDARG00000018478	agxtb	alanine--glyoxylate and serine--pyruvate aminotransferase b [Source:ZFIN;Acc:ZDB-GENE-010302-3]	2	22676743	22688651		76.51	124.85	117.56	122.55	142.93	149.17
ENSDARG00000018485	cyp2v1	cytochrome P450, family 2, subfamily V, polypeptide 1 [Source:ZFIN;Acc:ZDB-GENE-040912-139]	20	25533064	25558418		11.66	13.69	12.56	9.76	9.29	9.08
ENSDARG00000018491	pdia4	protein disulfide isomerase family A, member 4 [Source:ZFIN;Acc:ZDB-GENE-030131-5493]	24	17334678	17341458		43.04	50.11	45.58	46.37	45.35	42.11
ENSDARG00000018492	znf503	zinc finger protein 503 [Source:NCBI gene;Acc:324913]	13	17468161	17471044		18.81	17.8	15.29	39.79	36.91	40.25
ENSDARG00000018494	smn1	survival of motor neuron 1, telomeric [Source:NCBI gene;Acc:30432]	5	43798345	43805610		11.51	9.51	10.37	9.29	9.26	8.57
ENSDARG00000018506	ftr06	finTRIM family, member 6 [Source:ZFIN;Acc:ZDB-GENE-070912-228]	2	42247560	42250254		0.02	0.06	0.06	0	0.02	0.02
ENSDARG00000018508	zdhhc8b	zinc finger DHHC-type containing 8b [Source:ZFIN;Acc:ZDB-GENE-030407-3]	5	17881074	18007077		4.85	4.34	3.27	7.38	6.8	7.02
ENSDARG00000018516	stk38b	serine/threonine kinase 38b [Source:ZFIN;Acc:ZDB-GENE-081031-2]	22	883678	898645		4.73	4.54	4.86	4.56	4.73	5.45
ENSDARG00000018521	or115-1	odorant receptor, family F, subfamily 115, member 1 [Source:ZFIN;Acc:ZDB-GENE-010309-2]	21	40202617	40204435		0.23	0.13	0.15	0.18	0.1	0.16
ENSDARG00000018524	midn	midnolin [Source:NCBI gene;Acc:323713]	22	18725101	18749313		15.07	8.25	6.83	26.11	24.5	24.57
ENSDARG00000018529	lipf	lipase, gastric [Source:ZFIN;Acc:ZDB-GENE-040426-2737]	12	17158642	17201028		30.1	47.98	46.88	28.35	28.93	28.05
ENSDARG00000018530	mapkapk2b	MAPK activated protein kinase 2b [Source:ZFIN;Acc:ZDB-GENE-071018-1]	8	37465380	37477845		5.13	0.98	1.07	2.79	2.67	2.79
ENSDARG00000018534	slc6a9	solute carrier family 6 member 9 [Source:ZFIN;Acc:ZDB-GENE-050105-3]	2	33335911	33422798		15.05	9.35	7.97	15.96	14.42	14.49
ENSDARG00000018542	hapln4	hyaluronan and proteoglycan link protein 4 [Source:ZFIN;Acc:ZDB-GENE-060503-243]	22	17828267	17831253		0.18	0.05	0.15	0.84	0.55	0.92
ENSDARG00000018553	ifih1	interferon induced with helicase C domain 1 [Source:ZFIN;Acc:ZDB-GENE-081104-438]	9	51147380	51217396		0.73	0.56	0.58	0.75	0.61	0.63
ENSDARG00000018559	kdm7ab	lysine (K)-specific demethylase 7Ab [Source:NCBI gene;Acc:503902]	25	17244532	17310301		5.93	2.64	2.46	7.08	6.46	6.86
ENSDARG00000018562	cope	coatomer protein complex, subunit epsilon [Source:ZFIN;Acc:ZDB-GENE-041114-59]	2	9513160	9527210		68.78	79.68	81.5	67.24	65.43	66.71
ENSDARG00000018566	flnca	filamin C, gamma a (actin binding protein 280) [Source:ZFIN;Acc:ZDB-GENE-070503-1]	25	35019693	35041057		3.5	4.1	2.72	10.18	9.06	8.62
ENSDARG00000018569	tnfrsf1a	tumor necrosis factor receptor superfamily, member 1a [Source:ZFIN;Acc:ZDB-GENE-040426-2252]	16	17060758	17072561		4.43	5.03	3.93	8.44	8.67	7.82
ENSDARG00000018574	sf3b4	splicing factor 3b, subunit 4 [Source:ZFIN;Acc:ZDB-GENE-020419-22]	19	7424347	7430858		14.38	12.92	11.14	16.72	16.35	15.61
ENSDARG00000018587	zgc:152658	zgc:152658 [Source:ZFIN;Acc:ZDB-GENE-060929-68]	1	40140498	40146782		0.12	0.06	0.11	0.03	0.02	0.03
ENSDARG00000018593	parp16	poly (ADP-ribose) polymerase family, member 16 [Source:ZFIN;Acc:ZDB-GENE-040426-2289]	25	31868268	31882618		11.13	10.54	9.99	10.99	11.32	11.84
ENSDARG00000018600	retsat	retinol saturase (all-trans-retinol 13,14-reductase) [Source:NCBI gene;Acc:325922]	3	39181387	39190317		13.14	17.09	15.42	9.63	9.13	9.2
ENSDARG00000018602	rab5aa	RAB5A, member RAS oncogene family, a [Source:ZFIN;Acc:ZDB-GENE-030131-139]	19	20708460	20722368		32.74	23.06	19.07	38.5	38.73	38.55
ENSDARG00000018611	lbx1b	ladybird homeobox 1b [Source:ZFIN;Acc:ZDB-GENE-050309-27]	1	31531990	31534089		0.84	0.74	0.98	1.19	1.61	1.26
ENSDARG00000018616	agtr1a	angiotensin II receptor, type 1a [Source:ZFIN;Acc:ZDB-GENE-070912-546]	2	24931677	24932753		0.79	0.81	0.42	1.33	1.43	1.14
ENSDARG00000018618	cpsf6	cleavage and polyadenylation specific factor 6 [Source:NCBI gene;Acc:327069]	4	2041067	2059302		12.38	8.05	7.38	16.73	16.82	17.65
ENSDARG00000018619	cers4b	ceramide synthase 4b [Source:ZFIN;Acc:ZDB-GENE-110719-2]	2	56356652	56387041		6.83	2.2	3.36	8.37	8.39	9.21
ENSDARG00000018621	slc6a19a.1	solute carrier family 6 member 19a, tandem duplicate 1 [Source:ZFIN;Acc:ZDB-GENE-070620-2]	19	663796	677713		1.94	3.58	1.68	2.85	2.47	2.56
ENSDARG00000018623	rad54l	RAD54 like [Source:ZFIN;Acc:ZDB-GENE-040426-968]	6	3473657	3491357		3.76	2.11	2.04	1.93	1.84	1.73
ENSDARG00000018627	arl5a	ADP-ribosylation factor-like 5A [Source:ZFIN;Acc:ZDB-GENE-040718-28]	9	22902865	22918413		9.94	8.4	8.31	7.8	7.12	7.15
ENSDARG00000018637	sec61g	SEC61 translocon gamma subunit [Source:ZFIN;Acc:ZDB-GENE-040718-203]	24	18444189	18446604		133.04	177.43	213.56	107.84	117.44	119.46
ENSDARG00000018643	igf2a	insulin-like growth factor 2a [Source:ZFIN;Acc:ZDB-GENE-991111-3]	7	48870496	48876480		7.05	8.01	7.56	6.77	7.69	8
ENSDARG00000018650	gtpbp3	GTP binding protein 3, mitochondrial [Source:NCBI gene;Acc:553807]	11	6009984	6037759		1.69	1.3	1.65	0.75	0.64	0.96
ENSDARG00000018653	acot7	acyl-CoA thioesterase 7 [Source:ZFIN;Acc:ZDB-GENE-040912-42]	8	46758264	46894362		7.96	6.14	6.55	7.58	6.81	8.71
ENSDARG00000018658	aar2	AAR2 splicing factor [Source:ZFIN;Acc:ZDB-GENE-040704-68]	23	4216196	4225830		7.13	6.28	7.64	6.72	6.78	6.8
ENSDARG00000018671	si:ch211-262i1.4	si:ch211-262i1.4 [Source:ZFIN;Acc:ZDB-GENE-140106-10]	11	14295011	14300253		0.04	0.02	0.05	0.06	0.04	0
ENSDARG00000018681	saal1	serum amyloid A-like 1 [Source:NCBI gene;Acc:393104]	25	8180539	8201983		5.19	4.23	4.72	3.65	3.85	3.54
ENSDARG00000018687	itga2b	integrin, alpha 2b [Source:ZFIN;Acc:ZDB-GENE-051031-1]	3	21350509	21382491		0.27	0.19	0.22	0.24	0.47	0.45
ENSDARG00000018688	elk3	ETS transcription factor ELK3 [Source:ZFIN;Acc:ZDB-GENE-030716-2]	4	7677318	7713665		6.38	6.67	5.6	9.42	8.47	8.94
ENSDARG00000018691	phf2	PHD finger protein 2 [Source:NCBI gene;Acc:503778]	11	27644524	27702835		12.3	5.18	5.01	15.55	15.25	15.64
ENSDARG00000018693	cdh2	cadherin 2, type 1, N-cadherin (neuronal) [Source:NCBI gene;Acc:30291]	20	17581881	17819238		29.37	16.09	13.21	50.53	46.52	50.29
ENSDARG00000018698	carm1	coactivator-associated arginine methyltransferase 1 [Source:NCBI gene;Acc:445251]	3	34149337	34172214		22.81	15.56	14.63	21.75	20.23	19.36
ENSDARG00000018716	dgkh	diacylglycerol kinase, eta [Source:ZFIN;Acc:ZDB-GENE-070912-328]	9	17787864	17960341		5.71	2.37	2.68	6.41	7.98	7.52
ENSDARG00000018721	npnta	nephronectin a [Source:ZFIN;Acc:ZDB-GENE-090312-201]	1	50085440	50188006		13.26	9.11	7.38	15.11	13.48	13.69
ENSDARG00000018726	slco3a1	solute carrier organic anion transporter family, member 3A1 [Source:ZFIN;Acc:ZDB-GENE-030131-8662]	18	25073984	25177230		5.03	2.86	1.9	4.86	4.43	3.74
ENSDARG00000018738	zw10	zw10 kinetochore protein [Source:ZFIN;Acc:ZDB-GENE-040426-800]	21	23036314	23046606		10.23	9.74	8.79	8.05	9.5	9.32
ENSDARG00000018742	psme4b	proteasome activator subunit 4b [Source:NCBI gene;Acc:553346]	12	23991276	24053079		6.83	4.8	4.37	8.79	8.25	7.98
ENSDARG00000018743	scamp5a	secretory carrier membrane protein 5a [Source:NCBI gene;Acc:324467]	25	31837228	31863374		30.66	8.87	10.57	39.9	38.49	37.74
ENSDARG00000018744	prl2	prolactin 2 [Source:ZFIN;Acc:ZDB-GENE-041210-160]	4	14343706	14348811		0.36	0.07	0.16	0.31	0.3	0.56
ENSDARG00000018750	npr1b	natriuretic peptide receptor 1b [Source:ZFIN;Acc:ZDB-GENE-100805-4]	16	28908478	28991376		2.61	2.78	2.03	3.05	3.01	3.01
ENSDARG00000018757	klf5l	Kruppel-like factor 5 like [Source:ZFIN;Acc:ZDB-GENE-060312-34]	21	38128877	38153824		5.67	4.87	3.23	7.98	7.71	8.37
ENSDARG00000018765	pknox1.1	pbx/knotted 1 homeobox 1.1 [Source:ZFIN;Acc:ZDB-GENE-020122-5]	9	19529951	19550058		10.3	5.75	6.15	10.57	11.71	11.22
ENSDARG00000018773	hivep2b	HIVEP zinc finger 2b [Source:ZFIN;Acc:ZDB-GENE-130530-562]	17	6393719	6399920		2.7	1.12	1.2	2.79	2.83	2.81
ENSDARG00000018782	kdm4aa	lysine (K)-specific demethylase 4A, genome duplicate a [Source:ZFIN;Acc:ZDB-GENE-110609-4]	6	3280939	3319142		7.21	4.21	3.63	7.33	7.38	7.55
ENSDARG00000018787	efna1b	ephrin-A1b [Source:ZFIN;Acc:ZDB-GENE-041007-5]	16	23282655	23298149		12.38	7.68	7.98	16.12	16.21	16.41
ENSDARG00000018788	st8sia2	ST8 alpha-N-acetyl-neuraminide alpha-2,8-sialyltransferase 2 [Source:ZFIN;Acc:ZDB-GENE-020919-4]	18	25027495	25051846		1.41	0.35	0.72	1.85	2.17	1.95
ENSDARG00000018797	crygmxl1	crystallin, gamma MX, like 1 [Source:ZFIN;Acc:ZDB-GENE-050522-392]	9	22056172	22057658		0.23	0.39	0.7	1.09	0.91	0.8
ENSDARG00000018809	abhd3	abhydrolase domain containing 3 [Source:ZFIN;Acc:ZDB-GENE-040912-90]	2	11848510	11912347		2.14	0.92	1.1	2.57	2.72	2.5
ENSDARG00000018814	esrp2	epithelial splicing regulatory protein 2 [Source:NCBI gene;Acc:572992]	7	34794829	34821836		12.77	10.04	6.46	8.84	8.23	7.75
ENSDARG00000018817	bdnf	brain-derived neurotrophic factor [Source:ZFIN;Acc:ZDB-GENE-000412-1]	7	31924983	31941670		5.2	2.67	3.37	6.98	6.26	6.55
ENSDARG00000018820	flncb	filamin C, gamma b (actin binding protein 280) [Source:ZFIN;Acc:ZDB-GENE-041008-175]	4	10066840	10130349		5.4	6.92	5.65	11.25	10.81	11.02
ENSDARG00000018825	tmem248	transmembrane protein 248 [Source:NCBI gene;Acc:541403]	5	60886103	60906496		3.28	2.85	2.75	4.54	5.26	4.25
ENSDARG00000018840	trpc4apa	transient receptor potential cation channel, subfamily C, member 4 associated protein a [Source:ZFIN;Acc:ZDB-GENE-000330-3]	11	25157374	25180330		14.03	10.6	9.17	13.22	14.48	13.86
ENSDARG00000018846	dgat2	diacylglycerol O-acyltransferase 2 [Source:NCBI gene;Acc:565316]	10	32501961	32524771		28.99	44.74	38.18	19.56	22.65	21.41
ENSDARG00000018853	si:dkey-199f5.8	si:dkey-199f5.8 [Source:ZFIN;Acc:ZDB-GENE-030131-4733]	16	41740365	41787421		7	6.29	4.73	9.88	10.11	9.56
ENSDARG00000018854	scaf4b	SR-related CTD-associated factor 4b [Source:ZFIN;Acc:ZDB-GENE-050506-42]	15	42285643	42347209		6.44	3.31	3.58	8.13	7.84	7.8
ENSDARG00000018856	dclk1a	doublecortin-like kinase 1a [Source:ZFIN;Acc:ZDB-GENE-061013-124]	10	34737322	34871737		7.74	2.03	2.01	7.71	8.09	8.21
ENSDARG00000018871	henmt1	HEN methyltransferase 1 [Source:NCBI gene;Acc:550540]	20	33744065	33749174		0.2	0.31	0.23	0.24	0.32	0.35
ENSDARG00000018881	apobec2a	apolipoprotein B mRNA editing enzyme, catalytic polypeptide-like 2a [Source:ZFIN;Acc:ZDB-GENE-050306-15]	22	641342	651719		19.13	28.37	31.81	14.33	14.25	12.63
ENSDARG00000018882	jak2b	Janus kinase 2b [Source:ZFIN;Acc:ZDB-GENE-980526-123]	5	66043820	66121852		5.16	3.99	3.92	5.81	6.1	5.79
ENSDARG00000018890	snrpa	small nuclear ribonucleoprotein polypeptide A [Source:ZFIN;Acc:ZDB-GENE-030131-2841]	18	35402759	35407790		24.9	22.52	23.73	21.88	21.91	21
ENSDARG00000018891	rnaseh2a	ribonuclease H2, subunit A [Source:ZFIN;Acc:ZDB-GENE-040426-976]	1	51712869	51720633		13.58	13.91	14.52	8.72	7.2	7.89
ENSDARG00000018896	mmp24	matrix metallopeptidase 24 [Source:ZFIN;Acc:ZDB-GENE-040724-262]	6	52362395	52428826		7.25	2.1	2.62	13.99	13.42	14.48
ENSDARG00000018898	zfand5a	zinc finger, AN1-type domain 5a [Source:ZFIN;Acc:ZDB-GENE-040426-1979]	5	25680842	25694362		36.2	31.35	28.32	41.64	38.92	38.99
ENSDARG00000018902	pes	pescadillo [Source:NCBI gene;Acc:30228]	5	12836913	12865386		32.25	38.59	34.06	22.7	22.28	22.34
ENSDARG00000018903	aimp2	aminoacyl tRNA synthetase complex interacting multifunctional protein 2 [Source:ZFIN;Acc:ZDB-GENE-040426-2652]	3	61108356	61116295		53.54	57.46	64.64	56.28	58.85	58.75
ENSDARG00000018904	cstf3	cleavage stimulation factor, 3' pre-RNA, subunit 3 [Source:ZFIN;Acc:ZDB-GENE-040426-1997]	18	45734297	45761868		34.63	25.68	26.16	34.64	35.84	35.27
ENSDARG00000018907	kat6a	K(lysine) acetyltransferase 6A [Source:ZFIN;Acc:ZDB-GENE-021022-3]	5	14394323	14443124		9.39	5.31	4.45	16.34	14.92	16.18
ENSDARG00000018914	hnrnpk	heterogeneous nuclear ribonucleoprotein K [Source:ZFIN;Acc:ZDB-GENE-040426-1926]	8	49744801	49766241		18.1	11.54	12.56	26.23	25.35	26.48
ENSDARG00000018918	rad17	RAD17 checkpoint clamp loader component [Source:ZFIN;Acc:ZDB-GENE-040718-409]	5	26188264	26199505		2.56	2.28	2.23	2.19	2.49	2.5
ENSDARG00000018921	lratd1	LRAT domain containing 1 [Source:ZFIN;Acc:ZDB-GENE-040426-1247]	20	32756763	32758949		14.07	9.37	11.64	16.71	17.73	15.62
ENSDARG00000018923	fat2	FAT atypical cadherin 2 [Source:ZFIN;Acc:ZDB-GENE-111031-1]	14	26103330	26177156		4.52	3.15	1.63	3.77	3.46	2.97
ENSDARG00000018936	zcchc17	zinc finger, CCHC domain containing 17 [Source:ZFIN;Acc:ZDB-GENE-040325-2]	19	42996968	43004356		9.77	9.77	9.14	10.6	9.94	9.74
ENSDARG00000018944	hoga1	4-hydroxy-2-oxoglutarate aldolase 1 [Source:NCBI gene;Acc:405806]	22	34710036	34732882		14.4	21.68	21.51	8.73	7.27	7.76
ENSDARG00000018953	gclm	glutamate-cysteine ligase, modifier subunit [Source:ZFIN;Acc:ZDB-GENE-030131-5906]	8	15269423	15277252		5.97	7.25	6.17	6.06	5.14	5.88
ENSDARG00000018956	tmem39b	transmembrane protein 39B [Source:NCBI gene;Acc:334110]	13	33268657	33281310		8.82	6.43	6.28	10.18	9.84	10.93
ENSDARG00000018958	nrarpb	NOTCH regulated ankyrin repeat protein b [Source:NCBI gene;Acc:353225]	5	66025336	66028729		3.89	1.94	2.04	4.21	4.36	3.92
ENSDARG00000018961	gtpbp4	GTP binding protein 4 [Source:ZFIN;Acc:ZDB-GENE-030131-5982]	2	42361373	42375275		143.54	152.38	148.18	111.97	116.62	114.9
ENSDARG00000018966	cyb5r1	cytochrome b5 reductase 1 [Source:ZFIN;Acc:ZDB-GENE-030131-8497]	11	24786378	24799675		17.2	21.53	20.29	9.9	10.88	10.26
ENSDARG00000018967	gabbr1a	gamma-aminobutyric acid (GABA) B receptor, 1a [Source:ZFIN;Acc:ZDB-GENE-030904-5]	15	34717514	34845414		0.55	0.1	0.21	0.97	1.06	0.92
ENSDARG00000018968	acvr1ba	activin A receptor type 1Ba [Source:ZFIN;Acc:ZDB-GENE-980526-527]	23	27800519	27822920		14.07	8.39	7.87	18.68	18.19	17.43
ENSDARG00000018971	b3gnt5a	UDP-GlcNAc:betaGal beta-1,3-N-acetylglucosaminyltransferase 5a [Source:NCBI gene;Acc:336526]	11	10541258	10550377		7.76	9.36	7.46	11.85	11.65	11.8
ENSDARG00000018973	clptm1	CLPTM1 regulator of GABA type A receptor forward trafficking [Source:NCBI gene;Acc:795480]	15	17074185	17095655		29.66	22.81	19.1	28.11	27.33	28.03
ENSDARG00000018976	sptlc2a	serine palmitoyltransferase, long chain base subunit 2a [Source:ZFIN;Acc:ZDB-GENE-050522-23]	17	17804727	17856118		9.42	11.37	9.58	7.86	8.14	8.5
ENSDARG00000018980	zgc:91890	zgc:91890 [Source:NCBI gene;Acc:431729]	16	5597600	5605341		3.77	3.22	3.16	4.38	4.17	3.98
ENSDARG00000018984	eya2	EYA transcriptional coactivator and phosphatase 2 [Source:ZFIN;Acc:ZDB-GENE-040912-24]	6	55909795	55997350		9.27	8.06	6.98	15.59	14.9	13.85
ENSDARG00000018985	castor2	cytosolic arginine sensor for mTORC1 subunit 2 [Source:ZFIN;Acc:ZDB-GENE-030131-5505]	5	61361815	61403813		10.78	7.42	8.88	19.32	19.41	19.99
ENSDARG00000018989	hspa4b	heat shock protein 4b [Source:ZFIN;Acc:ZDB-GENE-030131-6018]	14	23687678	23708159		63.48	56.46	59.3	50.05	52.75	50.85
ENSDARG00000018997	cplx2l	complexin 2, like [Source:ZFIN;Acc:ZDB-GENE-040718-160]	1	12262980	12278581		46.95	22.27	26.1	51.96	49.21	51.57
ENSDARG00000019000	smc3	structural maintenance of chromosomes 3 [Source:ZFIN;Acc:ZDB-GENE-030131-3196]	22	29858535	29906764		43.26	26.04	28.45	39.13	41.33	41.48
ENSDARG00000019001	ak7a	adenylate kinase 7a [Source:ZFIN;Acc:ZDB-GENE-040724-122]	20	4742400	4766649		1.83	1.43	1.66	3.22	3.14	3.28
ENSDARG00000019004	smarcd1	SWI/SNF related, matrix associated, actin dependent regulator of chromatin, subfamily d, member 1 [Source:ZFIN;Acc:ZDB-GENE-(	22	7009062	7025393		28.65	18.59	18.89	32.72	32.76	31.39
ENSDARG00000019008	erp44	endoplasmic reticulum protein 44 [Source:ZFIN;Acc:ZDB-GENE-040426-1902]	16	27417200	27442344		32.04	31.16	28.82	35.56	37.68	33.98
ENSDARG00000019013	barhl1b	BarH-like homeobox 1b [Source:ZFIN;Acc:ZDB-GENE-060118-2]	21	17478493	17482465		8.93	2.59	4.44	11.49	12.7	11.27
ENSDARG00000019025	podn	podocan [Source:ZFIN;Acc:ZDB-GENE-100922-117]	23	41889093	41931897		0.25	0.22	0.18	0.1	0.09	0.04
ENSDARG00000019033	tmem59	transmembrane protein 59 [Source:ZFIN;Acc:ZDB-GENE-070912-397]	2	26612574	26620902		39.72	33.2	28.78	32.75	31.98	33.71
ENSDARG00000019038	sgsm3	small G protein signaling modulator 3 [Source:NCBI gene;Acc:406635]	3	33461075	33494163		8.51	4.18	4.88	10.97	12.4	12.23
ENSDARG00000019060	snx12	sorting nexin 12 [Source:ZFIN;Acc:ZDB-GENE-040426-787]	21	30470809	30488267		15.79	13.83	12.74	21.41	20.52	20.29
ENSDARG00000019062	arpc5b	actin related protein 2/3 complex, subunit 5B [Source:ZFIN;Acc:ZDB-GENE-040120-7]	20	34151670	34155608		35.79	41.4	37.89	43.18	42.54	41.38
ENSDARG00000019063	fat1b	FAT atypical cadherin 1b [Source:ZFIN;Acc:ZDB-GENE-130530-563]	14	30156449	30226882		2.09	0.65	0.62	2.58	2.69	2.8
ENSDARG00000019081	DHDH	zgc:101723 [Source:NCBI gene;Acc:449558]	18	951092	968706	DHDH	7.83	9.61	12.4	4.97	5.16	4.66
ENSDARG00000019093	si:ch73-160i9.2	si:ch73-160i9.2 [Source:ZFIN;Acc:ZDB-GENE-130530-564]	19	10518448	10519589		0.21	0.36	0.17	0.15	0.1	0.21
ENSDARG00000019096	myl7	myosin, light chain 7, regulatory [Source:ZFIN;Acc:ZDB-GENE-991019-3]	8	40457107	40464935		23.65	36.22	29.87	35.11	36.86	34.46
ENSDARG00000019098	cd82a	CD82 molecule a [Source:ZFIN;Acc:ZDB-GENE-030131-2818]	7	26709251	26739674		16.1	20.34	17.96	20.21	20.37	20.09
ENSDARG00000019103	kdm4ab	lysine (K)-specific demethylase 4A, genome duplicate b [Source:ZFIN;Acc:ZDB-GENE-030131-6279]	2	17450228	17492486		7.33	4.03	3.87	9.36	8.89	9.36
ENSDARG00000019113	mob3a	MOB kinase activator 3A [Source:ZFIN;Acc:ZDB-GENE-030131-4091]	8	20415824	20429843		4.21	4.64	3.9	4.78	5.31	5
ENSDARG00000019116	ing1	inhibitor of growth family, member 1 [Source:ZFIN;Acc:ZDB-GENE-060421-4388]	9	8973080	8979468		6.71	5.77	5.94	7.77	7.97	8.09

ENSDARG00000019117	parvb	parvin, beta [Source:ZFIN;Acc:ZDB-GENE-030131-4411]	25	5035343	5063523		19.47	23.7	25.8	16.38	15.08	16.45
ENSDARG00000019122	he1.2	hatching enzyme 1, tandem dupliate 2 [Source:ZFIN;Acc:ZDB-GENE-030131-2100]	22	14098028	14099399		0.21	1.27	0.3	1.49	0	0.15
ENSDARG00000019125	klhl40b	kelch-like family member 40b [Source:NCBI gene;Acc:795319]	24	20575259	20581672		2.46	3.72	3.28	5.27	3.85	4.65
ENSDARG00000019128	tpm4b	tropomyosin 4b [Source:ZFIN;Acc:ZDB-GENE-030131-2964]	2	55300425	55337585		13.99	15.73	13.09	18.8	21.8	20.29
ENSDARG00000019130	plk2b	polo-like kinase 2b (Drosophila) [Source:ZFIN;Acc:ZDB-GENE-070720-17]	8	17078692	17086195		10.51	7.54	7.33	13.08	13.06	13.15
ENSDARG00000019135	ankef1a	ankyrin repeat and EF-hand domain containing 1a [Source:ZFIN;Acc:ZDB-GENE-041001-196]	20	34868933	34902205		0.36	0.41	0.4	0.35	0.44	0.52
ENSDARG00000019137	tram1	translocation associated membrane protein 1 [Source:ZFIN;Acc:ZDB-GENE-021011-1]	24	14214831	14234824		18.29	27.47	20.43	20.66	20.19	20.26
ENSDARG00000019142	fam185a	family with sequence similarity 185 member A [Source:ZFIN;Acc:ZDB-GENE-131121-241]	25	18711804	18721297		1.71	1.94	2.25	1.41	1.35	1.43
ENSDARG00000019144	rab4a	RAB4a, member RAS oncogene family [Source:NCBI gene;Acc:403031]	13	24295896	24311628		11.68	6.81	7.23	11.73	13.11	12.58
ENSDARG00000019156	haus5	HAUS augmin-like complex, subunit 5 [Source:ZFIN;Acc:ZDB-GENE-041114-150]	8	20897849	20914829		3.05	2.51	2.37	2.22	2.09	1.9
ENSDARG00000019160	sh3bp5la	SH3-binding domain protein 5-like, a [Source:ZFIN;Acc:ZDB-GENE-040426-2187]	15	34854025	34866219		8.29	5.38	4.99	6.64	6.74	6.73
ENSDARG00000019179	lrit1a	leucine-rich repeat, immunoglobulin-like and transmembrane domains 1a [Source:ZFIN;Acc:ZDB-GENE-040924-5]	13	29462249	29467558		4.96	0.41	0.4	5.03	5.03	5.48
ENSDARG00000019181	rpsa	ribosomal protein SA [Source:NCBI gene;Acc:394027]	6	7709662	7720332		447.09	474.38	531.21	342.27	365.43	358.1
ENSDARG00000019185	slain1a	SLAIN motif family, member 1a [Source:ZFIN;Acc:ZDB-GENE-030131-7751]	9	17348745	17386456		4.59	1.62	1.75	5.16	4.43	5.35
ENSDARG00000019191	12-Sep	septin 12 [Source:ZFIN;Acc:ZDB-GENE-120215-68]	3	28502419	28627447		11.12	10.9	9.05	11.05	10.99	11.52
ENSDARG00000019195	vps9d1	VPS9 domain containing 1 [Source:ZFIN;Acc:ZDB-GENE-050306-57]	18	14633974	14668398		3.44	2.59	3	4.73	4.99	5.31
ENSDARG00000019205	fam120c	family with sequence similarity 120C [Source:ZFIN;Acc:ZDB-GENE-090313-228]	23	18386989	18415872		10.64	8.38	6.73	16.13	16.2	15.72
ENSDARG00000019207	abo	ABO blood group (transferase A, alpha 1-3-N-acetylgalactosaminyltransferase; transferase B, alpha 1-3-galactosyltransferase) [Sou	5	25025053	25037458		1.64	1.56	1.48	0.82	0.99	1.09
ENSDARG00000019208	camsap1a	calmodulin regulated spectrin-associated protein 1a [Source:ZFIN;Acc:ZDB-GENE-060518-4]	21	4712134	4764124		5.34	1.5	1.24	7.61	7.28	8.01
ENSDARG00000019213	CTBP1	zgc:136929 [Source:ZFIN;Acc:ZDB-GENE-060421-4235]	14	21140612	21199502	CTBP1	14.92	9.12	8.92	17.32	16.25	16.56
ENSDARG00000019222	mrpl24	mitochondrial ribosomal protein L24 [Source:NCBI gene;Acc:436674]	19	7495791	7506134		30.9	40.38	37.82	20.48	20.68	20.73
ENSDARG00000019223	arglu1a	arginine and glutamate rich 1a [Source:NCBI gene;Acc:406834]	9	26086135	26096626		37.32	13.32	20.45	38.63	34.35	36.08
ENSDARG00000019228	mogat2	monoacylglycerol O-acyltransferase 2 [Source:ZFIN;Acc:ZDB-GENE-041010-186]	10	32534814	32610776		10.84	17.61	12.84	3.21	3.79	3.39
ENSDARG00000019230	rpl7a	ribosomal protein L7a [Source:ZFIN;Acc:ZDB-GENE-031001-9]	5	33284144	33287691		1759.01	2128.08	2595.05	1236.3	1356.68	1357.99
ENSDARG00000019231	sptan1	spectrin alpha, non-erythrocytic 1 [Source:ZFIN;Acc:ZDB-GENE-051113-60]	21	13150937	13217905		75.45	43.88	36.52	88.74	88.32	93.61
ENSDARG00000019233	nln	neurolysin (metallopeptidase M3 family) [Source:ZFIN;Acc:ZDB-GENE-041010-216]	10	15408501	15451217		1.54	1.69	1.5	2.43	2.67	2.41
ENSDARG00000019235	sema3aa	sema domain, immunoglobulin domain (Ig), short basic domain, secreted, (semaphorin) 3Aa [Source:NCBI gene;Acc:30266]	4	11311315	11368490		21.77	14.75	11.3	26.1	25.25	26.65
ENSDARG00000019236	gsr	glutathione reductase [Source:ZFIN;Acc:ZDB-GENE-050522-116]	14	38852969	38873145		12.5	17.9	14.58	7.72	7.39	7.16
ENSDARG00000019239	cul1a	cullin 1a [Source:ZFIN;Acc:ZDB-GENE-030131-2603]	2	50229183	50272077		16.58	9.97	9.33	17.96	16.47	17.27
ENSDARG00000019250	fancf	FA complementation group F [Source:ZFIN;Acc:ZDB-GENE-060306-3]	25	35294670	35296165		1.65	1.28	1.68	0.97	1.31	1.1
ENSDARG00000019253	rhag	Rh associated glycoprotein [Source:ZFIN;Acc:ZDB-GENE-030131-8229]	20	14014813	14054083		53.72	75.23	62.61	78.87	79.15	78.37
ENSDARG00000019260	dhrs9	dehydrogenase/reductase (SDR family) member 9 [Source:ZFIN;Acc:ZDB-GENE-030131-1249]	9	48706038	48736388		24.03	42.3	31.33	7.46	7.24	8.71
ENSDARG00000019265	zbtb39	zinc finger and BTB domain containing 39 [Source:ZFIN;Acc:ZDB-GENE-130822-1]	23	36660750	36670369		2.12	0.94	0.93	2.94	2.69	2.74
ENSDARG00000019274	rasd1	RAS, dexamethasone-induced 1 [Source:ZFIN;Acc:ZDB-GENE-040426-1473]	12	1398404	1400009		18.9	19.17	23.65	24.62	26.04	27.82
ENSDARG00000019278	GNB4	guanine nucleotide binding protein (G protein), beta polypeptide 4a [Source:NCBI gene;Acc:100332205]	2	5300405	5311253	GNB4	17.82	8.8	11.88	16.01	20.41	19.23
ENSDARG00000019280	lars1b	leucyl-tRNA synthetase 1b [Source:ZFIN;Acc:ZDB-GENE-030114-7]	21	41026612	41070182		9.17	9.04	8.08	8.45	8.71	8.17
ENSDARG00000019293	tfdp1a	transcription factor Dp-1, a [Source:ZFIN;Acc:ZDB-GENE-040426-2883]	9	34950942	34968429		13.09	11.33	10.53	11.14	11.16	11.18
ENSDARG00000019294	cbln8	cerebellin 8 [Source:ZFIN;Acc:ZDB-GENE-080204-13]	7	73453486	73455918		0.03	0.08	0.17	0.03	0	0.03
ENSDARG00000019299	znf687b	zinc finger protein 687b [Source:NCBI gene;Acc:100006039]	19	7819480	7832439		7.04	3.02	2.78	8.7	8.57	8.85
ENSDARG00000019300	ints7	integrator complex subunit 7 [Source:NCBI gene;Acc:286776]	20	13020489	13142357		4.41	2.7	2.62	4.04	4.36	4.18
ENSDARG00000019302	mid1ip1b	MID1 interacting protein 1b [Source:NCBI gene;Acc:393805]	22	11436447	11438627		27.93	26.43	26.32	19.9	20.46	20.76
ENSDARG00000019304	phactr3b	phosphatase and actin regulator 3b [Source:ZFIN;Acc:ZDB-GENE-050522-156]	11	1422737	1509773		7.09	2.55	2.96	9.28	8.42	9.31
ENSDARG00000019307	dusp5	dual specificity phosphatase 5 [Source:ZFIN;Acc:ZDB-GENE-010625-1]	22	29911326	29922872		4.38	2.12	2.82	5.23	4.81	5.38
ENSDARG00000019311	fbxo30b	F-box protein 30b [Source:ZFIN;Acc:ZDB-GENE-040426-1125]	23	1561931	1571682		2.09	1.84	1.32	2.73	2.21	2.14
ENSDARG00000019312	rab39ba	RAB39B, member RAS oncogene family a [Source:ZFIN;Acc:ZDB-GENE-070620-7]	21	30540465	30545121		7.86	3.13	5.25	8.87	9.75	9.94
ENSDARG00000019316	fer1l6	fer-1 like family member 6 [Source:ZFIN;Acc:ZDB-GENE-070912-312]	9	14044310	14084743		1.01	0.75	0.56	0.89	0.9	0.79
ENSDARG00000019326	prpsap2	phosphoribosyl pyrophosphate synthetase-associated protein 2 [Source:ZFIN;Acc:ZDB-GENE-040426-2077]	3	39600562	39613226		13.86	12.17	11.62	13.55	13.87	14.35
ENSDARG00000019328	plxna4	plexin A4 [Source:NCBI gene;Acc:325938]	4	15217929	15420452		10.82	3.54	3.4	16.32	15.34	15.78
ENSDARG00000019332	ndufb4	NADH:ubiquinone oxidoreductase subunit B4 [Source:ZFIN;Acc:ZDB-GENE-040426-1973]	24	23971056	23977285		106.14	135.18	158.53	88.32	82.62	85.12
ENSDARG00000019335	hes6	hes family bHLH transcription factor 6 [Source:ZFIN;Acc:ZDB-GENE-030828-5]	2	48288461	48304696		19.15	8.76	8.77	17.19	15.77	17.44
ENSDARG00000019338	adprhl2	ADP-ribosylhydrolase like 2 [Source:NCBI gene;Acc:796446]	17	31611854	31619266		11.16	6.18	9.03	8.99	9.66	9.51
ENSDARG00000019339	uxt	ubiquitously-expressed, prefoldin-like chaperone [Source:ZFIN;Acc:ZDB-GENE-040912-118]	8	8699085	8704430		8.69	9.51	8.95	6.78	7.47	7.65
ENSDARG00000019341	gpc1a	glypican 1a [Source:NCBI gene;Acc:553367]	22	15507218	15548201		13.64	9.41	11.63	14.03	15.03	14.23
ENSDARG00000019342	chrnd	cholinergic receptor, nicotinic, delta (muscle) [Source:ZFIN;Acc:ZDB-GENE-030131-6665]	24	25683071	25691020		9.32	12.52	10.56	11.29	10.75	10.02
ENSDARG00000019345	mark3a	MAP/microtubule affinity-regulating kinase 3a [Source:ZFIN;Acc:ZDB-GENE-030131-6232]	13	15580758	15668714		11.15	6.99	5.59	13.33	13.59	13.94
ENSDARG00000019353	sparc	secreted protein, acidic, cysteine-rich (osteonectin) [Source:ZFIN;Acc:ZDB-GENE-030131-9]	14	25937948	25956860		361.78	476.21	473.54	292.74	295.81	286.15
ENSDARG00000019355	coa7	cytochrome c oxidase assembly factor 7 [Source:NCBI gene;Acc:555268]	20	16163142	16171297		9.54	11.45	10.63	8.84	8.58	9.26
ENSDARG00000019357	fabp1a	fatty acid binding protein 1a, liver [Source:NCBI gene;Acc:791610]	5	72108241	72111389		0.24	0.2	0.41	0.08	0	0.08
ENSDARG00000019360	sec23b	SEC23 homolog B, coat complex II component [Source:ZFIN;Acc:ZDB-GENE-030131-5479]	6	6779114	6796687		13.02	13.07	9.38	16.21	15.58	14.99
ENSDARG00000019362	ptbp1a	polypyrimidine tract binding protein 1a [Source:ZFIN;Acc:ZDB-GENE-050522-492]	2	26288301	26300619		10.97	11.44	9.3	15.27	15	14.48
ENSDARG00000019364	mbip	MAP3K12 binding inhibitory protein 1 [Source:ZFIN;Acc:ZDB-GENE-060810-43]	17	38295847	38308379		5.14	3.52	5.08	3.54	3.99	3.34
ENSDARG00000019365	krt99	keratin 99 [Source:ZFIN;Acc:ZDB-GENE-050417-50]	11	11565996	11575070		6.75	6.11	5.91	0.88	0.73	0.71
ENSDARG00000019367	tgfb3	transforming growth factor, beta 3 [Source:ZFIN;Acc:ZDB-GENE-030723-4]	17	52059020	52091999		18.38	17.33	16.93	31.32	32.78	32.6
ENSDARG00000019371	flt1	fms-related tyrosine kinase 1 (vascular endothelial growth factor/vascular permeability factor receptor) [Source:ZFIN;Acc:ZDB-GENE	24	21750810	21808819		1.22	0.9	0.78	3.08	3.12	2.74
ENSDARG00000019383	cdc42bpb	CDC42 binding protein kinase beta (DMPK-like) [Source:ZFIN;Acc:ZDB-GENE-030131-3647]	20	18391860	18536474		12.48	9.73	7.54	19	17.83	18.02
ENSDARG00000019392	stat5a	signal transducer and activator of transcription 5a [Source:ZFIN;Acc:ZDB-GENE-030820-2]	3	17251542	17331919		7.34	6.31	4.18	7.4	6.69	7.54
ENSDARG00000019396	rergla	RERG/RAS-like a [Source:NCBI gene;Acc:436767]	4	12381735	12388535		4.17	3.41	3.27	3.65	4.67	3.57
ENSDARG00000019398	psma6a	proteasome 20S subunit alpha 6a [Source:ZFIN;Acc:ZDB-GENE-020326-1]	17	12955390	12966907		42.31	48.68	52.67	33.99	33.8	33.13
ENSDARG00000019404	atp5f1d	ATP synthase F1 subunit delta [Source:ZFIN;Acc:ZDB-GENE-030131-7649]	22	18766061	18779232		216.46	247.56	267.65	179.96	186.1	180.93
ENSDARG00000019406	C7H11orf68	zgc:63470 [Source:NCBI gene;Acc:393420]	7	756942	763406	C7H11orf68	5.97	5.19	6.29	4.67	4.78	4.91
ENSDARG00000019417	gadd45ga	growth arrest and DNA-damage-inducible, gamma a [Source:ZFIN;Acc:ZDB-GENE-040426-1882]	8	31367667	31369161		16	9.83	10.08	26.02	24.67	27.35
ENSDARG00000019418	kcnj2a	potassium inwardly rectifying channel subfamily J member 2a [Source:ZFIN;Acc:ZDB-GENE-120319-1]	12	36259018	36260532		9.08	7.82	6.11	6.62	5.35	5.12
ENSDARG00000019420	etnk1	ethanolamine kinase 1 [Source:ZFIN;Acc:ZDB-GENE-030328-21]	4	16885854	16906923		21.44	10.98	10.64	25.81	25.42	24.43
ENSDARG00000019426	trioa	trio Rho guanine nucleotide exchange factor a [Source:NCBI gene;Acc:557667]	19	41841147	42045372		5.73	2.55	2.2	9.94	10.2	10.57
ENSDARG00000019438	rnf13	ring finger protein 13 [Source:ZFIN;Acc:ZDB-GENE-040426-772]	22	35131890	35177782		6.18	4.56	4.6	9.69	8.86	9.68
ENSDARG00000019442	CABZ01086041.1	angiomotin like 1 [Source:NCBI gene;Acc:101884950]	15	2820768	2841677		1.4	1.57	0.9	2.27	1.95	2.08
ENSDARG00000019444	ssr4	signal sequence receptor, delta [Source:ZFIN;Acc:ZDB-GENE-030131-4900]	23	18569291	18572685		99.6	145.56	144.09	90.51	96.54	96.62
ENSDARG00000019451	pcsk2	proprotein convertase subtilisin/kexin type 2 [Source:ZFIN;Acc:ZDB-GENE-090608-1]	13	912123	977652		5.81	1.46	1.72	12.19	14	14.28
ENSDARG00000019457	tfe3b	transcription factor binding to IGHM enhancer 3b [Source:ZFIN;Acc:ZDB-GENE-010919-3]	11	25504215	25519837		4.53	3.95	3.65	4.1	4.43	4.21
ENSDARG00000019459	elf2a	E74-like factor 2a (ets domain transcription factor) [Source:ZFIN;Acc:ZDB-GENE-040822-41]	14	46047761	46070802		9.45	5.93	5.58	9.81	9.47	8.97
ENSDARG00000019484	zdhhc7	zinc finger DHHC-type containing 7 [Source:ZFIN;Acc:ZDB-GENE-040718-346]	18	5813996	5830414		5.53	3.95	2.85	6.98	6.1	5.99
ENSDARG00000019487	tmx1	thioredoxin-related transmembrane protein 1 [Source:ZFIN;Acc:ZDB-GENE-030131-2327]	13	36923052	36934915		4.66	4.64	3.8	4.56	4.38	4.4
ENSDARG00000019492	shbg	sex hormone-binding globulin [Source:ZFIN;Acc:ZDB-GENE-030131-1324]	12	22511015	22524388		20.61	31.34	25.38	12.11	14.19	13.88
ENSDARG00000019496	esd	esterase D/formylglutathione hydrolase [Source:ZFIN;Acc:ZDB-GENE-050417-328]	9	25171662	25181697		32.99	48.07	48.64	18.16	18.77	18.11
ENSDARG00000019498	cry5	cryptochrome circadian regulator 5 [Source:ZFIN;Acc:ZDB-GENE-010426-8]	10	39264802	39283883		0.14	0.12	0.16	0.13	0.15	0.18
ENSDARG00000019501	pcnx3	pecanex 3 [Source:ZFIN;Acc:ZDB-GENE-091112-9]	21	27575758	27619701		1.19	0.57	0.48	1.88	1.54	1.7
ENSDARG00000019503	zgc:103759	zgc:103759 [Source:ZFIN;Acc:ZDB-GENE-041010-100]	23	18702516	18707568		1	1.26	1.26	0.44	0.64	0.4
ENSDARG00000019507	mcm5	minichromosome maintenance complex component 5 [Source:ZFIN;Acc:ZDB-GENE-021209-1]	3	25999477	26011440		38.2	36.93	38.64	19.79	19.96	18.69
ENSDARG00000019516	sp7	Sp7 transcription factor [Source:ZFIN;Acc:ZDB-GENE-040629-2]	6	58622831	58674160		0.61	0.68	0.4	0.63	0.63	0.76
ENSDARG00000019521	mpx	myeloid-specific peroxidase [Source:ZFIN;Acc:ZDB-GENE-030131-9460]	10	7778870	7785945		2.82	3.73	2.68	4.1	4.57	4.62
ENSDARG00000019525	tbcelb	tubulin folding cofactor E-like b [Source:ZFIN;Acc:ZDB-GENE-100921-13]	5	63248266	63286077		9.18	6.36	6.14	7.38	7.44	7.85
ENSDARG00000019526	agfg1b	ArfGAP with FG repeats 1b [Source:ZFIN;Acc:ZDB-GENE-040426-1520]	2	16460321	16479291		9.14	5.31	5.11	8.5	8.1	7.99
ENSDARG00000019529	parp1	poly (ADP-ribose) polymerase 1 [Source:ZFIN;Acc:ZDB-GENE-030131-3955]	20	43648369	43689779		25.1	19.79	20.8	16.35	15.99	14.85
ENSDARG00000019530	hdgfl2	HDGF like 2 [Source:NCBI gene;Acc:80965]	22	22234415	22259175		26.35	16.78	14.27	21.52	20.22	19.69
ENSDARG00000019532	fads2	fatty acid desaturase 2 [Source:NCBI gene;Acc:140615]	25	4133488	4148719		14.29	17.07	14.9	3.02	3.14	3.44
ENSDARG00000019541	gpt2l	glutamic pyruvate transaminase (alanine aminotransferase) 2, like [Source:NCBI gene;Acc:403086]	2	3533117	3573000		21.8	27.66	19.97	23.58	28.77	25.1
ENSDARG00000019549	cds1	CDP-diacylglycerol synthase (phosphatidate cytidylyltransferase) 1 [Source:ZFIN;Acc:ZDB-GENE-070705-78]	5	40009958	40040488		16.12	12.54	10.74	9.91	9.84	9.28
ENSDARG00000019556	clcn7	chloride channel 7 [Source:ZFIN;Acc:ZDB-GENE-061103-196]	3	27664864	27709312		3.71	3.12	2.51	3.56	3.49	3.58
ENSDARG00000019564	asap2b	ArfGAP with SH3 domain, ankyrin repeat and PH domain 2b [Source:ZFIN;Acc:ZDB-GENE-030131-5060]	20	29634653	29675458		9.92	9.66	6.86	10.93	10.35	9.59
ENSDARG00000019566	neurod1	neuronal differentiation 1 [Source:NCBI gene;Acc:30169]	9	44291721	44295071		56.25	18.36	26.08	62.08	62.21	68.13
ENSDARG00000019572	taf7	TAF7 RNA polymerase II, TATA box binding protein (TBP)-associated factor [Source:ZFIN;Acc:ZDB-GENE-020419-18]	14	40852497	40874102		20.41	20.42	21.84	16.73	16.84	18.48
ENSDARG00000019579	ldb2a	LIM domain binding 2a [Source:ZFIN;Acc:ZDB-GENE-990415-136]	14	46678711	46832825		5.43	2.77	2.98	6.75	7	6.8
ENSDARG00000019581	uso1	USO1 vesicle transport factor [Source:ZFIN;Acc:ZDB-GENE-040426-688]	1	26467071	26509316		15.26	13.24	12.25	14.87	15.37	15.32
ENSDARG00000019585	gstk2	glutathione S-transferase kappa 2 [Source:ZFIN;Acc:ZDB-GENE-100922-14]	16	30933044	31006689		0	0	0	0	0	0.03
ENSDARG00000019588	chico	chico [Source:ZFIN;Acc:ZDB-GENE-000210-28]	2	52406565	52455525		5.7	3.94	3.54	9.77	11.33	11.8
ENSDARG00000019591	igl4v9	immunoglobulin light 4 variable 9 [Source:ZFIN;Acc:ZDB-GENE-051013-8]	25	37318550	37319117		0	0	0	0	0	0
ENSDARG00000019595	senp8	SUMO peptidase family member, NEDD8 specific [Source:ZFIN;Acc:ZDB-GENE-060929-1186]	7	56830881	56835543		3	1.8	1.66	2.67	2.49	2.04
ENSDARG00000019596	nrd1a	nardilysin a (N-arginine dibasic convertase) [Source:ZFIN;Acc:ZDB-GENE-070912-417]	2	36828243	36854500		15.61	13.18	11.85	16.97	16.97	16.98
ENSDARG00000019601	col12a1b	collagen, type XII, alpha 1b [Source:ZFIN;Acc:ZDB-GENE-120215-116]	20	49451764	49681850		20.16	19.43	11.01	38.45	34.05	34.61
ENSDARG00000019613	ip6k2b	inositol hexakisphosphate kinase 2b [Source:ZFIN;Acc:ZDB-GENE-040718-85]	6	40089743	40162742		38.08	20.46	21.45	26.93	28.42	27.02
ENSDARG00000019614	jade3	jade family PHD finger 3 [Source:NCBI gene;Acc:322209]	6	37478765	37513133		6.93	4.81	4.88	5.41	5.18	5.71
ENSDARG00000019636	yif1a	Yip1 interacting factor homolog A (S. cerevisiae) [Source:NCBI gene;Acc:334982]	14	30753666	30759503		8.13	11.09	10.32	15.3	13.81	14.55
ENSDARG00000019644	ldhba	lactate dehydrogenase Ba [Source:NCBI gene;Acc:30497]	4	16828752	16836006		395.63	427.92	485.58	315.35	331.47	331.49
ENSDARG00000019646	twist3	twist3 [Source:ZFIN;Acc:ZDB-GENE-000210-7]	23	2669	9339		6.51	9.41	9.52	5.43	5.22	5.17
ENSDARG00000019651	zgc:162239	zgc:162239 [Source:ZFIN;Acc:ZDB-GENE-070424-28]	21	40786804	40799404		0.21	0.21	0.31	0.68	0.33	0.22
ENSDARG00000019658	pou2f2a	POU class 2 homeobox 2a [Source:ZFIN;Acc:ZDB-GENE-060310-2]	16	10918252	10991240		2.31	0.77	0.76	5.93	5.56	5.86
ENSDARG00000019660	rxfp2b	relaxin family peptide receptor 2b [Source:ZFIN;Acc:ZDB-GENE-110408-27]	15	31735931	31772657		0.56	0.1	0.29	0.56	0.92	0.76
ENSDARG00000019686	fgl2b	fibrinogen-like 2b [Source:ZFIN;Acc:ZDB-GENE-120709-53]	25	21267579	21273875		1.86	2.05	1.96	0.27	0.47	0.41
ENSDARG00000019692	colq	collagen-like tail subunit (single strand of homotrimer) of asymmetric acetylcholinesterase [Source:ZFIN;Acc:ZDB-GENE-110411-1	16	20198064	20238985		1.74	1.62	1.34	1.87	1.92	1.8
ENSDARG00000019693	clcn5a	chloride channel, voltage-sensitive 5a [Source:ZFIN;Acc:ZDB-GENE-091113-15]	21	30662263	30696595		5.84	4.9	3.36	7.87	7.25	7.9
ENSDARG00000019702	aldocb	aldolase C, fructose-bisphosphate, b [Source:NCBI gene;Acc:369193]	21	39608637	39639954		205.83	75.98	96.47	240.12	269.61	255.98
ENSDARG00000019706	zar1	zygote arrest 1 [Source:NCBI gene;Acc:369189]	20	23423814	23426339		0.23	0.14	0.12	0.34	0.3	0.17
ENSDARG00000019707	spout1	SPOUT domain containing methyltransferase 1 [Source:ZFIN;Acc:ZDB-GENE-040912-53]	21	3796196	3814491		5.65	6.75	6.92	4.43	3.62	3.71
ENSDARG00000019709	rhoua	ras homolog family member Ua [Source:ZFIN;Acc:ZDB-GENE-040618-3]	13	24371815	24379271		5.72	5.88	5.29	5.95	6.73	6.66
ENSDARG00000019713	oatx	organic anion transporter X [Source:ZFIN;Acc:ZDB-GENE-070115-1]	21	44091342	44104600		4.62	8.51	7.18	9.07	9.85	8.81
ENSDARG00000019715	metap1d	methionyl aminopeptidase type 1D (mitochondrial) [Source:NCBI gene;Acc:554090]	1	30946231	30974165		4.39	4.4	6.26	2.92	3.54	3.23
ENSDARG00000019717	pbx2	pre-B-cell leukemia homeobox 2 [Source:ZFIN;Acc:ZDB-GENE-000405-5]	16	48616220	48643191		12.57	11.22	7.78	15.34	15.55	14.88
ENSDARG00000019726	celsr2	cadherin, EGF LAG seven-pass G-type receptor 2 [Source:ZFIN;Acc:ZDB-GENE-070122-2]	22	413349	465663		3.4	1.19	1.46	5.92	5.83	6.36
ENSDARG00000019728	bmpr1aa	bone morphogenetic protein receptor, type IAa [Source:ZFIN;Acc:ZDB-GENE-000502-1]	13	22555342	22619790		9.83	8.99	8.28	10.06	10.29	9.82
ENSDARG00000019732	mtch2	mitochondrial carrier homolog 2 [Source:ZFIN;Acc:ZDB-GENE-991008-9]	7	38667066	38678090		45.27	50.8	46.77	40.59	38.3	36.86
ENSDARG00000019741	ccndx	cyclin Dx [Source:ZFIN;Acc:ZDB-GENE-080917-47]	3	30153966	30158395		0.49	0.72	0.51	0.38	0.3	0.41
ENSDARG00000019742	tlr4ba	toll-like receptor 4b, duplicate a [Source:ZFIN;Acc:ZDB-GENE-040219-8]	13	18514788	18517453		0.13	0.12	0.08	0.08	0.08	0.1
ENSDARG00000019743	dctn1a	dynactin 1a [Source:ZFIN;Acc:ZDB-GENE-070117-2205]	7	1062972	1108935		4.91	3.18	3.2	6.26	6.12	6.11
ENSDARG00000019746	rfng	RFNG O-fucosylpeptide 3-beta-N-acetylglucosaminyltransferase [Source:NCBI gene;Acc:414848]	12	3054442	3077395		3.46	2.38	2.09	3.82	3.52	3.88
ENSDARG00000019747	hsd3b2	hydroxy-delta-5-steroid dehydrogenase, 3 beta- and steroid delta-isomerase 2 [Source:ZFIN;Acc:ZDB-GENE-030828-2]	20	1272526	1276175		0.24	0.15	0.17	0.14	0.13	0.49
ENSDARG00000019752	rom1a	retinal outer segment membrane protein 1a [Source:ZFIN;Acc:ZDB-GENE-040426-1765]	5	67126774	67145505		12.3	2.65	1.77	6.83	7.47	6.72
ENSDARG00000019753	kcnn3	potassium intermediate/small conductance calcium-activated channel, subfamily N, member 3 [Source:ZFIN;Acc:ZDB-GENE-10092	16	23598908	23719649		2.9	1.42	1.48	4.44	4.17	4.39
ENSDARG00000019763	acp5a	acid phosphatase 5a, tartrate resistant [Source:ZFIN;Acc:ZDB-GENE-040426-2864]	6	8355122	8362516		0.63	0.99	1.4	2.33	2.07	1.66
ENSDARG00000019765	zgc:158803	zgc:158803 [Source:ZFIN;Acc:ZDB-GENE-070424-15]	1	55169130	55183088		12.94	12.17	10.49	15.41	15.41	15.31
ENSDARG00000019772	si:ch1073-280e3.1	si:ch1073-280e3.1 [Source:ZFIN;Acc:ZDB-GENE-110411-261]	15	478524	496549		1.38	1.31	1.58	2.05	2.4	2.38
ENSDARG00000019774	zhx3	zinc fingers and homeoboxes 3 [Source:ZFIN;Acc:ZDB-GENE-031107-1]	23	2872855	2901167		5.47	3.73	3.94	7.2	7.35	8.07
ENSDARG00000019778	rps6	ribosomal protein S6 [Source:ZFIN;Acc:ZDB-GENE-040801-8]	1	45043767	45049603		1029.54	1205.09	1439.15	784.25	834.36	833.08
ENSDARG00000019782	impg2a	interphotoreceptor matrix proteoglycan 2a [Source:ZFIN;Acc:ZDB-GENE-140106-217]	9	30188764	30190513		16.73	2.77	1.68	11.55	11.8	12.6
ENSDARG00000019791	prmt3	protein arginine methyltransferase 3 [Source:ZFIN;Acc:ZDB-GENE-041105-1]	7	17106160	17223955		5.83	5.83	6.21	3.38	4.02	3.6
ENSDARG00000019797	qrich1	glutamine-rich 1 [Source:ZFIN;Acc:ZDB-GENE-030131-8204]	11	17997403	18107447		3.04	1.78	1.76	2.78	2.73	2.97
ENSDARG00000019808	evpla	envoplakin a [Source:ZFIN;Acc:ZDB-GENE-030829-19]	6	23057311	23082678		14.75	12.08	9.03	5.2	4.35	4.41
ENSDARG00000019810	nfe2l3	nuclear factor, erythroid 2-like 3 [Source:ZFIN;Acc:ZDB-GENE-040426-2334]	19	18143593	18152942		7.44	5.62	4.22	8.67	7.45	7.28
ENSDARG00000019815	fn1a	fibronectin 1a [Source:ZFIN;Acc:ZDB-GENE-000426-1]	9	15362300	15424639		10.23	11.63	7.49	31.58	28.44	29.84
ENSDARG00000019834	edrf1	erythroid differentiation regulatory factor 1 [Source:ZFIN;Acc:ZDB-GENE-131121-620]	17	8754020	8796333		2.62	2.12	2.08	2.39	2.37	2.5
ENSDARG00000019835	nkx2.1	NK2 homeobox 1 [Source:ZFIN;Acc:ZDB-GENE-010404-1]	17	38262408	38264625		3.42	0.66	1.05	2.9	1.99	2.35
ENSDARG00000019838	ugdh	UDP-glucose 6-dehydrogenase [Source:ZFIN;Acc:ZDB-GENE-011022-1]	1	23283532	23294753		11.55	17.79	12.9	10.73	10.1	9.14
ENSDARG00000019842	cnot3b	CCR4-NOT transcription complex, subunit 3b [Source:ZFIN;Acc:ZDB-GENE-030131-5838]	19	10252052	10295173		5.17	4.27	3.19	7.64	7.54	7.65
ENSDARG00000019845	pdlim1	PDZ and LIM domain 1 (elfin) [Source:ZFIN;Acc:ZDB-GENE-030131-5227]	13	25762908	25774183		20.77	25.5	21.82	25.46	27.83	26.32
ENSDARG00000019856	atp1a1b	ATPase Na+/K+ transporting subunit alpha 1b [Source:ZFIN;Acc:ZDB-GENE-001212-5]	9	35901554	35922201		79.76	33.28	54.62	103.02	109.05	112.24
ENSDARG00000019861	fgl2a	fibrinogen-like 2a [Source:ZFIN;Acc:ZDB-GENE-030131-9506]	4	20178694	20181964		2.59	3.39	3.01	2.61	2.73	2.5
ENSDARG00000019874	hsph1	heat shock 105/110 protein 1 [Source:ZFIN;Acc:ZDB-GENE-120410-4]	15	31564137	31588162		1.59	1	1.24	1.92	2.14	2.11
ENSDARG00000019877	rbks	ribokinase [Source:ZFIN;Acc:ZDB-GENE-040625-112]	17	40999835	41081806		5.64	9.7	8.78	3.5	2.67	3.62
ENSDARG00000019881	bsg	basigin [Source:ZFIN;Acc:ZDB-GENE-030131-9638]	22	18929412	18946453		205.23	216.13	195.78	259.73	269.61	266.97
ENSDARG00000019897	gpat4	glycerol-3-phosphate acyltransferase 4 [Source:ZFIN;Acc:ZDB-GENE-060421-5102]	10	41362390	41400049		13.33	6.19	6.84	12.71	12.31	12.33
ENSDARG00000019902	rcvrn2	recoverin 2 [Source:ZFIN;Acc:ZDB-GENE-030131-7590]	16	13911692	13921589		82.33	25.69	18.92	71.88	76.64	78.25
ENSDARG00000019907	dscc1	DNA replication and sister chromatid cohesion 1 [Source:NCBI gene;Acc:447872]	16	14572008	14587332		5.48	5.8	5.74	2.57	3.04	2.44
ENSDARG00000019917	epb41l3b	erythrocyte membrane protein band 4.1-like 3b [Source:ZFIN;Acc:ZDB-GENE-021106-1]	2	58513535	58581663		57.95	68.31	70.16	47.34	47.39	46.38
ENSDARG00000019920	lft1	lefty1 [Source:ZFIN;Acc:ZDB-GENE-990630-10]	20	35073981	35076387		0.07	0.02	0.06	0.05	0	0.23
ENSDARG00000019924	cmpk	cytidylate kinase [Source:NCBI gene;Acc:406383]	2	10063747	10076929		46.63	56.35	49.33	42.81	37.4	38.96
ENSDARG00000019930	tal1	T-cell acute lymphocytic leukemia 1 [Source:NCBI gene;Acc:30766]	22	16535575	16545330		9.23	3.45	5.08	11.09	11.83	11.82
ENSDARG00000019932	slc5a11	solute carrier family 5 member 11 [Source:ZFIN;Acc:ZDB-GENE-041114-9]	3	18655673	18675688		3.07	4.36	3.18	2.5	2.2	1.9
ENSDARG00000019941	sgta	small glutamine-rich tetratricopeptide repeat (TPR)-containing, alpha [Source:ZFIN;Acc:ZDB-GENE-030131-8811]	11	14771908	14813063		34.68	32.07	27.88	35.41	36.38	34.71
ENSDARG00000019945	ptprdb	protein tyrosine phosphatase receptor type Db [Source:ZFIN;Acc:ZDB-GENE-070615-38]	1	19122920	19237612		6.77	1.19	1.72	9.55	8.76	9.52
ENSDARG00000019949	serpinh1b	serpin peptidase inhibitor, clade H (heat shock protein 47), member 1b [Source:ZFIN;Acc:ZDB-GENE-990415-93]	15	29382454	29388012		78.5	96.72	88.05	143.08	139.76	132.99
ENSDARG00000019950	carf	calcium responsive transcription factor [Source:ZFIN;Acc:ZDB-GENE-070912-172]	9	13229585	13251327		5.33	3.04	3.65	5.56	5.78	5.96
ENSDARG00000019951	sec62	SEC62 homolog, preprotein translocation factor [Source:ZFIN;Acc:ZDB-GENE-050913-83]	2	37245382	37265903		26.13	23.04	21.87	25.97	27.06	26.05
ENSDARG00000019961	znf513a	zinc finger protein 513a [Source:ZFIN;Acc:ZDB-GENE-030131-9895]	20	19423823	19432766		4.29	2.24	2.64	5.25	4.72	5.12
ENSDARG00000019962	dop1a	DOP1 leucine zipper like protein A [Source:ZFIN;Acc:ZDB-GENE-050309-65]	16	33062570	33097398		4.56	2.3	1.93	6.97	6.47	6.82
ENSDARG00000019963	sfxn1	sideroflexin 1 [Source:ZFIN;Acc:ZDB-GENE-040801-45]	14	49078606	49110280		4.16	4.56	3.61	10.48	10.73	10.2
ENSDARG00000019969	smarcd3b	SWI/SNF related, matrix associated, actin dependent regulator of chromatin, subfamily d, member 3b [Source:ZFIN;Acc:ZDB-GENE	7	41436488	41489997		10.91	5.51	6.13	14.35	13.28	12.97
ENSDARG00000019973	stk38a	serine/threonine kinase 38a [Source:ZFIN;Acc:ZDB-GENE-040426-2811]	8	25590841	25605537		8.79	6.79	6.14	10.81	10.78	9.5

ENSDARG00000019976	idi1	isopentenyl-diphosphate delta isomerase 1 [Source:ZFIN;Acc:ZDB-GENE-050913-44]	24	3469373	3477103		13.7	16.76	13.89	13.69	13.2	12.97
ENSDARG00000019986	grhprb	glyoxylate reductase/hydroxypyruvate reductase b [Source:ZFIN;Acc:ZDB-GENE-040426-1847]	1	19835803	19845539		88.78	133.19	137.36	67.75	69.04	75.11
ENSDARG00000019990	cabp1a	calcium binding protein 1a [Source:ZFIN;Acc:ZDB-GENE-041010-36]	8	39890186	39952727		3.36	1.07	1.28	2.82	2.89	3.05
ENSDARG00000019995	bmp4	bone morphogenetic protein 4 [Source:ZFIN;Acc:ZDB-GENE-980528-2059]	17	50564070	50586135		5.37	5.78	5.15	8.34	8.81	7.92
ENSDARG00000019997	slc30a7	solute carrier family 30 member 7 [Source:NCBI gene;Acc:327439]	22	16123090	16154771		14.1	11.59	10.07	12.53	14.61	13.45
ENSDARG00000020000	sh3bp4a	SH3-domain binding protein 4a [Source:NCBI gene;Acc:403082]	22	13886821	13944737		3.38	2.29	2.19	4.74	5.02	4.87
ENSDARG00000020001	rad23aa	RAD23 homolog A, nucleotide excision repair protein a [Source:ZFIN;Acc:ZDB-GENE-040808-59]	1	52111273	52128425		23.68	19.01	18.13	24.61	26.02	26.6
ENSDARG00000020007	col1a2	collagen, type I, alpha 2 [Source:ZFIN;Acc:ZDB-GENE-030131-8415]	19	40983221	41002980		206.03	235.65	155.12	314.93	283.34	286.3
ENSDARG00000020008	vcp	valosin containing protein [Source:NCBI gene;Acc:327197]	5	45103387	45120980		134.7	118.75	113.91	116.43	112.39	109.52
ENSDARG00000020009	bmp2k	BMP2 inducible kinase [Source:ZFIN;Acc:ZDB-GENE-041212-72]	5	39099172	39152767		4.74	3.76	2.69	5.78	5.97	5.87
ENSDARG00000020015	mrps33	mitochondrial ribosomal protein S33 [Source:ZFIN;Acc:ZDB-GENE-040715-6]	4	12102267	12106039		10.63	12.93	11	7.47	7.95	7.36
ENSDARG00000020024	fam151b	family with sequence similarity 151 member B [Source:ZFIN;Acc:ZDB-GENE-040801-38]	5	26162401	26170253		2.36	2.05	2.18	1.55	1.4	1.57
ENSDARG00000020028	cps1	carbamoyl-phosphate synthase 1, mitochondrial [Source:ZFIN;Acc:ZDB-GENE-081105-17]	9	39026311	39139722		0.09	0.06	0.02	1.02	0.65	1.22
ENSDARG00000020031	cldn11a	claudin 11a [Source:ZFIN;Acc:ZDB-GENE-040718-369]	2	25997426	26001173		20.52	25.89	26.9	33.31	38.82	40.53
ENSDARG00000020041	vps35l	VPS35 endosomal protein sorting factor like [Source:NCBI gene;Acc:563454]	12	11167870	11198776		8.7	8.16	8.01	8.38	8.82	8.99
ENSDARG00000020043	cnot8	CCR4-NOT transcription complex, subunit 8 [Source:ZFIN;Acc:ZDB-GENE-040426-2845]	21	36442085	36453594		9.68	8.21	8.61	10.17	10.06	9.92
ENSDARG00000020046	ahr1a	aryl hydrocarbon receptor 1a [Source:ZFIN;Acc:ZDB-GENE-020531-2]	16	20012190	20038263		0.74	0.94	0.76	0.21	0.11	0.23
ENSDARG00000020053	nkd1	NKD inhibitor of WNT signaling pathway 1 [Source:NCBI gene;Acc:751108]	7	37582281	37622370		6.72	5.72	5.2	10.09	9.83	9.57
ENSDARG00000020054	aox6	aldehyde oxidase 6 [Source:ZFIN;Acc:ZDB-GENE-050208-742]	22	14051245	14083583		3.14	2.88	2.6	2.31	2.39	2.06
ENSDARG00000020057	bmpr2b	bone morphogenetic protein receptor, type II b (serine/threonine kinase) [Source:ZFIN;Acc:ZDB-GENE-070618-2]	9	12959760	13111163		5.39	2.94	2.7	8.89	9.69	9.13
ENSDARG00000020072	thbs4b	thrombospondin 4b [Source:NCBI gene;Acc:252850]	21	5129513	5162849		27.2	36.67	34.53	43.94	48.43	47.9
ENSDARG00000020077	nol10	nucleolar protein 10 [Source:NCBI gene;Acc:393206]	17	51682429	51720803		12.48	11.29	10.7	6.41	6.94	7.31
ENSDARG00000020079	neil3	nei-like DNA glycosylase 3 [Source:ZFIN;Acc:ZDB-GENE-041114-18]	14	36414856	36428437		2.33	1.5	1.82	1.42	1.64	1.44
ENSDARG00000020080	ek1	eph-like kinase 1 [Source:NCBI gene;Acc:30311]	24	27548474	27715238		2.74	1.02	1.02	2.68	2.72	2.71
ENSDARG00000020084	tg	thyroglobulin [Source:ZFIN;Acc:ZDB-GENE-030519-1]	16	31530762	31598771		0.69	0.17	0.17	1.49	1.53	1.38
ENSDARG00000020085	slc33a1	solute carrier family 33 member 1 [Source:ZFIN;Acc:ZDB-GENE-040426-1241]	18	34158178	34171280		6.42	6.11	5.65	5.72	5.64	5.15
ENSDARG00000020086	nuak1a	NUAK family, SNF1-like kinase, 1a [Source:ZFIN;Acc:ZDB-GENE-041210-122]	4	14360372	14372843		7.31	6.62	6.02	12.73	12.68	12.97
ENSDARG00000020101	psmc2	proteasome 26S subunit, ATPase 2 [Source:ZFIN;Acc:ZDB-GENE-040426-1327]	4	14926948	14932359		75.43	80.3	89.21	63.14	62.17	60.24
ENSDARG00000020102	kctd16b	potassium channel tetramerization domain containing 16b [Source:ZFIN;Acc:ZDB-GENE-060117-2]	21	40944530	40959709		3.34	0.83	1.06	2.19	2.62	2.2
ENSDARG00000020107	usp2a	ubiquitin specific peptidase 2a [Source:ZFIN;Acc:ZDB-GENE-041212-59]	15	20900627	20939579		18.07	18.57	17.18	20.3	20.41	19.93
ENSDARG00000020114	slc20a1a	solute carrier family 20 member 1a [Source:NCBI gene;Acc:406458]	8	31057115	31075015		4.8	8.43	5.16	11.3	11.61	12.01
ENSDARG00000020122	endou2	endonuclease, polyU-specific 2 [Source:NCBI gene;Acc:553584]	21	41863540	41873584		0.44	0.65	0.8	3.53	2.84	3.7
ENSDARG00000020123	coq8aa	coenzyme Q8A, genome duplicate a [Source:NCBI gene;Acc:437001]	20	38458084	38519126		27.01	30.39	29.04	22.87	23.92	24.57
ENSDARG00000020131	fnbp1l	formin binding protein 1-like [Source:ZFIN;Acc:ZDB-GENE-040801-155]	8	14986833	15080573		14.22	12.01	11.9	14.39	13.53	14.64
ENSDARG00000020133	jdp2b	Jun dimerization protein 2b [Source:NCBI gene;Acc:436766]	20	46586678	46595151		1.93	1.32	1.69	2.53	2.92	2.98
ENSDARG00000020134	sipa1l1	signal-induced proliferation-associated 1 like 1 [Source:ZFIN;Acc:ZDB-GENE-030131-9909]	20	28650025	28768114		3.14	1.68	1.46	4.79	4.59	4.59
ENSDARG00000020136	ptges	prostaglandin E synthase [Source:ZFIN;Acc:ZDB-GENE-050407-2]	5	33339762	33347993		5.71	6.86	7.13	10.57	11.31	10.5
ENSDARG00000020143	pah	phenylalanine hydroxylase [Source:ZFIN;Acc:ZDB-GENE-031006-2]	4	17391680	17409533		50.44	71.89	76.64	23.69	24.09	23.99
ENSDARG00000020149	acoxl	acyl-CoA oxidase-like [Source:ZFIN;Acc:ZDB-GENE-081107-18]	13	47427160	47556447		3.06	4.34	3.34	3.22	3.29	3.29
ENSDARG00000020158	spag6	sperm associated antigen 6 [Source:ZFIN;Acc:ZDB-GENE-040704-53]	24	17269849	17279904		0.63	0.54	0.71	0.67	0.77	0.92
ENSDARG00000020164	efnb2a	ephrin-B2a [Source:NCBI gene;Acc:30219]	9	26103814	26127305		16.8	9.86	9.32	18.81	17.79	18.93
ENSDARG00000020176	zbtb22a	zinc finger and BTB domain containing 22a [Source:ZFIN;Acc:ZDB-GENE-101105-1]	19	192741	205104		3.94	2.18	2.06	6.68	6.84	7.79
ENSDARG00000020178	nova1	NOVA alternative splicing regulator 1 [Source:ZFIN;Acc:ZDB-GENE-060526-113]	5	36611128	36626646		32.28	8.28	13.04	45.06	46.55	47.01
ENSDARG00000020187	GCA	zgc:92027 [Source:ZFIN;Acc:ZDB-GENE-040930-3]	6	13993500	14010554	GCA	31.77	42.73	39.17	27.51	26.17	24.43
ENSDARG00000020191	ppef1	protein phosphatase, EF-hand calcium binding domain 1 [Source:ZFIN;Acc:ZDB-GENE-080303-27]	11	30253968	30280958		1.46	0.29	0.65	1.86	1.72	1.66
ENSDARG00000020197	rpl5a	ribosomal protein L5a [Source:ZFIN;Acc:ZDB-GENE-030131-5161]	2	10687276	10703621		979.34	1180.73	1361.91	767.98	798.04	797.4
ENSDARG00000020201	cdc73	cell division cycle 73, Paf1/RNA polymerase II complex component, homolog (S. cerevisiae) [Source:ZFIN;Acc:ZDB-GENE-040426-	2	11946554	12003175		12.49	10.47	9.63	12.42	12.64	12.98
ENSDARG00000020204	nf2a	neurofibromin 2a (merlin) [Source:NCBI gene;Acc:561184]	5	11724428	11809710		0.97	0.94	0.95	1.02	0.82	0.88
ENSDARG00000020212	slc1a1	solute carrier family 1 member 1 [Source:ZFIN;Acc:ZDB-GENE-040718-414]	7	69496293	69521481		10.93	8.82	7.98	7.87	7.84	8.48
ENSDARG00000020217	cpsf4	cleavage and polyadenylation specific factor 4 [Source:ZFIN;Acc:ZDB-GENE-990415-180]	3	40289418	40295255		12.1	12.51	11.88	10.53	10.81	11.26
ENSDARG00000020218	amfra	autocrine motility factor receptor a [Source:ZFIN;Acc:ZDB-GENE-040426-2190]	25	36197733	36217563		11.94	8.7	6.89	14.44	14.14	14.48
ENSDARG00000020219	dld	deltaD [Source:NCBI gene;Acc:30138]	13	6075572	6081803		4.74	3.3	3.19	4.82	4.73	4.81
ENSDARG00000020224		si:ch211-225b10.3 [Source:ZFIN;Acc:ZDB-GENE-130531-53]	12	30360184	30367008		1.64	1.41	0.97	1.09	1.27	0.68
ENSDARG00000020228	usf2	upstream transcription factor 2, c-fos interacting [Source:ZFIN;Acc:ZDB-GENE-030131-8475]	19	10558535	10577420		16.87	12.11	11.85	19.07	18.42	18.81
ENSDARG00000020231	mapre3a	microtubule-associated protein, RP/EB family, member 3a [Source:ZFIN;Acc:ZDB-GENE-050913-88]	17	36867281	36896560		14.71	4.24	6.34	13.63	14.63	14.67
ENSDARG00000020232	eif6	eukaryotic translation initiation factor 6 [Source:NCBI gene;Acc:386850]	6	52339548	52348651		34.56	46.79	49.95	26.14	25.25	25.22
ENSDARG00000020235	sept9a	septin 9a [Source:ZFIN;Acc:ZDB-GENE-030131-9187]	3	34452336	34586493		8.43	9.58	8.42	10.35	10.43	10.02
ENSDARG00000020237	mc1r	melanocortin 1 receptor [Source:ZFIN;Acc:ZDB-GENE-030502-1]	18	31226288	31227615		2.29	2.61	3.1	1.84	1.71	1.37
ENSDARG00000020239	lpin1	lipin 1 [Source:ZFIN;Acc:ZDB-GENE-080722-2]	17	30442518	30470271		18.62	17.83	14.34	16.85	15.7	16.39
ENSDARG00000020241	icmt	isoprenylcysteine carboxyl methyltransferase [Source:ZFIN;Acc:ZDB-GENE-050417-90]	23	21492151	21498974		9.62	5.77	5.81	8.31	7.56	8.81
ENSDARG00000020242	fermt2	fermitin family member 2 [Source:NCBI gene;Acc:553051]	17	50701748	50787754		7.53	7.39	7	13	13.59	12.44
ENSDARG00000020250	rhoj	ras homolog family member J [Source:ZFIN;Acc:ZDB-GENE-040724-272]	20	20186706	20222219		2.38	2.44	1.82	3.39	3.71	3.34
ENSDARG00000020252	btbd10b	BTB (POZ) domain containing 10b [Source:ZFIN;Acc:ZDB-GENE-080104-5]	7	66112194	66133786		20.38	15.53	12.82	21.77	21.64	20.37
ENSDARG00000020258	ewsr1a	EWS RNA-binding protein 1a [Source:ZFIN;Acc:ZDB-GENE-030131-2317]	10	7975585	7988417		75.03	39.84	47.73	93.71	86.03	86.8
ENSDARG00000020261	rab2a	RAB2A, member RAS oncogene family [Source:ZFIN;Acc:ZDB-GENE-011212-2]	2	21821683	21847935		88.2	69.32	67.12	100.38	100.15	100.38
ENSDARG00000020265	angptl6	angiopoietin-like 6 [Source:ZFIN;Acc:ZDB-GENE-030131-9735]	3	54524227	54544612		1.16	1.93	1.65	2.01	2.05	2.18
ENSDARG00000020278	si:dkeyp-120h9.1	si:dkeyp-120h9.1 [Source:ZFIN;Acc:ZDB-GENE-070705-534]	19	31743104	31765615		4.25	3.85	3	4.4	4.62	4.36
ENSDARG00000020279	efcab7	EF-hand calcium binding domain 7 [Source:ZFIN;Acc:ZDB-GENE-040625-115]	6	32029231	32045983		4.77	3.04	3.75	4.2	4.37	4.6
ENSDARG00000020284	si:ch211-278j3.3	si:ch211-278j3.3 [Source:ZFIN;Acc:ZDB-GENE-041014-147]	20	19373428	19422496		3.99	1.7	2.28	6.34	6.87	6.56
ENSDARG00000020289	pif1	PIF1 5'-to-3' DNA helicase homolog (S. cerevisiae) [Source:NCBI gene;Acc:326729]	2	17096161	17115256		0.66	0.43	0.49	0.76	0.75	0.71
ENSDARG00000020292	si:ch211-254n4.3	si:ch211-254n4.3 [Source:ZFIN;Acc:ZDB-GENE-081105-89]	8	27361687	27551540		4.64	2.48	4.03	5.38	5.67	5.4
ENSDARG00000020298	btg2	B-cell translocation gene 2 [Source:ZFIN;Acc:ZDB-GENE-000210-15]	22	336256	339623		15.73	10.42	8.63	25.24	22.31	24.26
ENSDARG00000020301	os9	OS9 endoplasmic reticulum lectin [Source:ZFIN;Acc:ZDB-GENE-060929-32]	6	39202685	39218609		29.87	22.23	18.95	24.13	23.99	26.85
ENSDARG00000020311	cnih1	cornichon family AMPA receptor auxiliary protein 1 [Source:ZFIN;Acc:ZDB-GENE-040426-1944]	13	36592194	36606941		16.65	14.5	13.45	21.28	20.88	19.78
ENSDARG00000020326	tyk2	tyrosine kinase 2 [Source:ZFIN;Acc:ZDB-GENE-071116-4]	3	46479705	46555542		1.66	1.01	0.91	3.36	3.31	3.24
ENSDARG00000020328	plekha6	pleckstrin homology domain containing, family A member 6 [Source:ZFIN;Acc:ZDB-GENE-140106-14]	11	23342996	23501467		13.33	8.42	6.89	13.78	13.59	13.53
ENSDARG00000020332	nkx2.9	NK2 transcription factor related, locus 9 (Drosophila) [Source:ZFIN;Acc:ZDB-GENE-030131-4340]	17	38255105	38256390		0.18	0.06	0.12	0.16	0.42	0.4
ENSDARG00000020334	ptpn11a	protein tyrosine phosphatase non-receptor type 11a [Source:ZFIN;Acc:ZDB-GENE-030131-5911]	10	3428194	3455639		7.18	6.07	5.1	8.24	8.23	8.14
ENSDARG00000020345	clasp2	cytoplasmic linker associated protein 2 [Source:NCBI gene;Acc:404604]	19	42693855	42802298		20.21	9.42	9.74	31.62	31.18	32.11
ENSDARG00000020354	lmx1a	LIM homeobox transcription factor 1, alpha [Source:ZFIN;Acc:ZDB-GENE-041014-332]	20	33922339	33940260		0.12	0.18	0.09	0.27	0.26	0.16
ENSDARG00000020363	snrnp25	small nuclear ribonucleoprotein 25 [Source:ZFIN;Acc:ZDB-GENE-050417-179]	3	46377305	46379900		8.46	10.59	8.63	7.12	5.93	7.16
ENSDARG00000020364	fbp1b	fructose-1,6-bisphosphatase 1b [Source:ZFIN;Acc:ZDB-GENE-021206-11]	5	35458190	35462126		84.8	148.42	138.75	103.26	117.45	111.45
ENSDARG00000020371	g6pc3	glucose 6 phosphatase, catalytic, 3 [Source:NCBI gene;Acc:790919]	12	3301149	3310677		3.24	3.21	2.31	3.31	2.56	2.76
ENSDARG00000020377	eif4g2a	eukaryotic translation initiation factor 4, gamma 2a [Source:ZFIN;Acc:ZDB-GENE-030131-2173]	7	66634167	66654025		31.58	27.61	24.1	36.44	35.16	34.46
ENSDARG00000020387	nipsnap3a	nipsnap homolog 3A (C. elegans) [Source:ZFIN;Acc:ZDB-GENE-040426-1037]	4	6520310	6527010		11.05	16.07	16.21	4.47	5.04	5.13
ENSDARG00000020395	mical2b	microtubule associated monooxygenase, calponin and LIM domain containing 2b [Source:NCBI gene;Acc:569564]	25	16194450	16309747		3.81	3.92	3.43	4.21	4.17	4.24
ENSDARG00000020397	snx15	sorting nexin 15 [Source:ZFIN;Acc:ZDB-GENE-040426-1377]	7	19953756	19966406		4.77	3.81	3.56	4.31	4.03	4.11
ENSDARG00000020405	napab	N-ethylmaleimide-sensitive factor attachment protein, alpha b [Source:ZFIN;Acc:ZDB-GENE-030131-5290]	18	44673990	44696815		21.29	28.18	27.79	20.27	19.16	19.13
ENSDARG00000020417	emx3	empty spiracles homeobox 3 [Source:ZFIN;Acc:ZDB-GENE-990415-53]	14	26363537	26377044		2.89	2.41	1.88	3.15	2.89	3.4
ENSDARG00000020427	nceh1a	neutral cholesterol ester hydrolase 1a [Source:ZFIN;Acc:ZDB-GENE-040711-2]	2	25131349	25143373		0.01	0.06	0.07	0.06	0.04	0.08
ENSDARG00000020440	snx5	sorting nexin 5 [Source:ZFIN;Acc:ZDB-GENE-040426-2857]	13	33645413	33654931		16.91	19.49	17.74	17.21	16.68	16.32
ENSDARG00000020443	xkr6a	XK, Kell blood group complex subunit-related family, member 6a [Source:ZFIN;Acc:ZDB-GENE-131121-106]	17	5662379	5732299		0.63	0.11	0.36	1.18	1.28	1.27
ENSDARG00000020447	tmtc4	transmembrane O-mannosyltransferase targeting cadherins 4 [Source:ZFIN;Acc:ZDB-GENE-070912-148]	9	31494941	31525126		3.62	2.17	2.85	3.61	3.55	3.3
ENSDARG00000020448	adi1	acireductone dioxygenase 1 [Source:NCBI gene;Acc:324079]	17	51224421	51229737		19.54	22.92	21.47	19.11	19.11	17.63
ENSDARG00000020449	yipf3	Yip1 domain family, member 3 [Source:NCBI gene;Acc:334511]	13	3938526	3950673		24.59	20.72	19.84	24.32	24	24.84
ENSDARG00000020450	cacng8a	calcium channel, voltage-dependent, gamma subunit 8a [Source:ZFIN;Acc:ZDB-GENE-050417-239]	19	9813287	9829965		5.32	1.2	1.71	5.83	5.55	6.51
ENSDARG00000020454	psmd8	proteasome 26S subunit, non-ATPase 8 [Source:ZFIN;Acc:ZDB-GENE-040625-136]	18	44615591	44623675		44.82	54.4	52.65	38.03	36.29	35.55
ENSDARG00000020455	si:ch211-171h4.3	si:ch211-171h4.3 [Source:ZFIN;Acc:ZDB-GENE-081031-85]	19	10415484	10425140		0.96	1.38	1.07	1.06	1.06	1
ENSDARG00000020465	ewsr1b	EWS RNA-binding protein 1b [Source:ZFIN;Acc:ZDB-GENE-030131-1600]	5	24873196	24882360		38.44	27.82	26.95	41.75	39.77	42.31
ENSDARG00000020467	safb	scaffold attachment factor B [Source:ZFIN;Acc:ZDB-GENE-030131-698]	22	15695746	15704710		82.98	43.5	52.42	75.69	80.07	77.35
ENSDARG00000020469	map3k7	mitogen-activated protein kinase kinase kinase 7 [Source:ZFIN;Acc:ZDB-GENE-041001-135]	20	24149794	24183382		8.57	6.03	6.09	9.22	8.97	8.8
ENSDARG00000020473	hddc3	HD domain containing 3 [Source:NCBI gene;Acc:550533]	7	50395002	50397968		8.94	9.62	8.53	7.49	6.88	7.15
ENSDARG00000020475	znrf1	zinc and ring finger 1 [Source:NCBI gene;Acc:393844]	25	13406069	13471290		8.1	4.05	3.44	10.32	9.48	10.12
ENSDARG00000020482	nono	non-POU domain containing, octamer-binding [Source:ZFIN;Acc:ZDB-GENE-030131-713]	5	22590326	22602979		18.44	12.79	11.88	19.73	18.78	19.59
ENSDARG00000020483	lta	lymphotoxin alpha (TNF superfamily, member 1) [Source:ZFIN;Acc:ZDB-GENE-050601-3]	15	34675660	34681272		0.02	0	0	0	0.02	0.05
ENSDARG00000020488	arhgap17a	Rho GTPase activating protein 17a [Source:ZFIN;Acc:ZDB-GENE-050417-56]	12	20412293	20482061		3.52	3.19	2.29	3.96	4.1	3.93
ENSDARG00000020491	lgi1a	leucine-rich, glioma inactivated 1a [Source:ZFIN;Acc:ZDB-GENE-030131-1731]	13	421231	430143		14.65	4.51	6.55	17.65	17.26	18.67
ENSDARG00000020494	znf330	zinc finger protein 330 [Source:ZFIN;Acc:ZDB-GENE-040426-2697]	1	53266504	53277660		12.6	14.11	12.45	12.6	12.07	12.06
ENSDARG00000020497	rab7a	RAB7a, member RAS oncogene family [Source:ZFIN;Acc:ZDB-GENE-040426-1352]	11	16176603	16215470		18.5	15.55	12.37	20.93	21.73	22.78
ENSDARG00000020504	h3f3b.1	H3 histone, family 3B.1 [Source:ZFIN;Acc:ZDB-GENE-050417-65]	3	45776370	45778123		268.48	270.03	267.16	230.97	236.73	242.74
ENSDARG00000020509	srd5a1	steroid-5-alpha-reductase, alpha polypeptide 1 (3-oxo-5 alpha-steroid delta 4-dehydrogenase alpha 1) [Source:ZFIN;Acc:ZDB-GEN	19	27836913	27858033		1.18	0.83	0.56	0.9	0.89	0.66
ENSDARG00000020521	exoc6	exocyst complex component 6 [Source:ZFIN;Acc:ZDB-GENE-040426-2511]	12	8968275	9087566		5.09	2.49	2.8	5.47	6.03	5.73
ENSDARG00000020527	nup62l	nucleoporin 62 like [Source:ZFIN;Acc:ZDB-GENE-030131-8491]	14	10624911	10656211		21.13	17.79	17.21	17.91	19.94	18.09
ENSDARG00000020529	hacl1	2-hydroxyacyl-CoA lyase 1 [Source:ZFIN;Acc:ZDB-GENE-040426-2058]	16	20167811	20194385		8.49	11.66	9.51	5.79	5.26	6.11
ENSDARG00000020541	ism1	isthmin 1 [Source:NCBI gene;Acc:497617]	13	34765141	34793487		7.71	9.03	8.53	11.47	11.57	11.03
ENSDARG00000020544	rem1	RAS (RAD and GEM)-like GTP-binding 1 [Source:ZFIN;Acc:ZDB-GENE-040317-1]	11	24313931	24332052		2.38	1.38	1.15	2.21	2.29	2.21
ENSDARG00000020548	si:dkey-77f5.10	si:dkey-77f5.10 [Source:ZFIN;Acc:ZDB-GENE-131121-133]	15	934660	941773		0.02	0	0.1	0	0	0.08
ENSDARG00000020573	ddx3xa	DEAD-box helicase 3 X-linked a [Source:ZFIN;Acc:ZDB-GENE-030131-1565]	9	33334501	33354738		23.81	19.3	16.85	32.64	33.04	31.94
ENSDARG00000020574	atp2a1	ATPase sarcoplasmic/endoplasmic reticulum Ca2+ transporting 1 [Source:ZFIN;Acc:ZDB-GENE-020905-1]	3	26081343	26157066		850.59	1274.67	1115.77	1922.73	1624.95	1593
ENSDARG00000020581	otofb	otoferlin b [Source:ZFIN;Acc:ZDB-GENE-110406-5]	17	50313650	50374005		6.99	6.96	6.33	3.51	3.86	3.83
ENSDARG00000020594	txlnba	taxilin beta a [Source:ZFIN;Acc:ZDB-GENE-030131-322]	20	36845787	36887516		8.63	12.58	9.9	15.78	14.15	15.13
ENSDARG00000020595	gnl3l	guanine nucleotide binding protein-like 3 (nucleolar)-like [Source:ZFIN;Acc:ZDB-GENE-040723-1]	11	25485774	25502491		17.35	19.35	19.16	13.56	13.61	13.8
ENSDARG00000020596	miga1	mitoguardin 1 [Source:NCBI gene;Acc:450070]	2	8663108	8688759		7.14	7.46	6.4	6.14	6.4	6.55
ENSDARG00000020600	rnf144aa	ring finger protein 144aa [Source:NCBI gene;Acc:570968]	17	35285876	35352690		1.27	0.83	0.65	2.59	2.3	2.21
ENSDARG00000020602	grk7a	G protein-coupled receptor kinase 7a [Source:NCBI gene;Acc:566120]	8	16536453	16544343		51.64	9.57	6.85	23.97	26.39	25.78
ENSDARG00000020606	nfe2	nuclear factor, erythroid 2 [Source:ZFIN;Acc:ZDB-GENE-030124-1]	23	36311581	36316352		1.31	1.67	1.75	1.91	1.84	2.06
ENSDARG00000020607	emc3	ER membrane protein complex subunit 3 [Source:NCBI gene;Acc:406826]	6	42369999	42377307		19.08	15.42	14.5	25.89	26.13	25.43
ENSDARG00000020609	snap25a	synaptosome associated protein 25a [Source:NCBI gene;Acc:30712]	20	34913069	34956230		122.32	56.9	80.78	115.19	122.96	123.23
ENSDARG00000020610	tnnt2a	troponin T type 2a (cardiac) [Source:ZFIN;Acc:ZDB-GENE-000626-1]	23	5653224	5685172		13.01	22.79	16.49	22.72	18.72	20.99
ENSDARG00000020611	C18H19orf47	si:ch211-160d20.3 [Source:ZFIN;Acc:ZDB-GENE-030131-8906]	18	36782930	36800041	C18H19orf47	4.43	2.63	2.49	6.33	6.63	5.62
ENSDARG00000020618	gatd3a	glutamine amidotransferase like class 1 domain containing 3A [Source:NCBI gene;Acc:550355]	9	32797816	32804953		16.78	21.14	21.28	12.3	10.48	11.25
ENSDARG00000020620	ap1b1	adaptor related protein complex 1 subunit beta 1 [Source:ZFIN;Acc:ZDB-GENE-061025-1]	5	24756496	24812899		14.41	10.63	8.91	19.06	18.72	18.58
ENSDARG00000020623	baxa	BCL2 associated X, apoptosis regulator a [Source:ZFIN;Acc:ZDB-GENE-000511-6]	3	37776489	37785873		11.59	12	12.24	12.69	13.05	13.68
ENSDARG00000020625	jak1	Janus kinase 1 [Source:NCBI gene;Acc:30280]	6	31368071	31429161		13.7	12.7	10.91	23.29	23.47	23.07
ENSDARG00000020642	gldc1	glycosyltransferase-like domain containing 1 [Source:NCBI gene;Acc:550132]	9	25840720	25913984		0.88	0.75	0.65	1.23	1.11	0.98
ENSDARG00000020645	slc7a3a	solute carrier family 7 member 3a [Source:ZFIN;Acc:ZDB-GENE-040426-2081]	21	30502002	30537411		11.23	6.38	5.37	23.81	24.18	25.53
ENSDARG00000020647	trpc4apb	transient receptor potential cation channel, subfamily C, member 4 associated protein b [Source:ZFIN;Acc:ZDB-GENE-000330-2]	23	18759542	18777957		9.45	7.8	8.12	8.41	9.16	8.41
ENSDARG00000020655	INSM2	INSM transcriptional repressor 2 [Source:NCBI gene;Acc:792891]	17	12924930	12926456	INSM2	4.56	0.87	1.41	5.26	4.71	4.72
ENSDARG00000020656	prkg1a	protein kinase cGMP-dependent 1a [Source:ZFIN;Acc:ZDB-GENE-040426-1308]	12	40815012	40955599		3.02	2.85	2.38	5.55	5.68	5.09
ENSDARG00000020676	dpp3	dipeptidyl-peptidase 3 [Source:ZFIN;Acc:ZDB-GENE-030131-1247]	21	27336550	27354672		15.28	13.33	12.46	14.09	14	13.7
ENSDARG00000020679	clpp	caseinolytic mitochondrial matrix peptidase proteolytic subunit [Source:ZFIN;Acc:ZDB-GENE-030131-7860]	3	34129505	34136778		19.54	22.88	21.36	17.58	17.6	18.01
ENSDARG00000020693	sesn1	sestrin 1 [Source:ZFIN;Acc:ZDB-GENE-040718-408]	20	32152355	32231157		39.37	34.69	31.94	48.98	51.99	51.89
ENSDARG00000020699	slc9a8	solute carrier family 9 member 8 [Source:ZFIN;Acc:ZDB-GENE-041212-7]	23	3949548	4019928		2.82	2.33	2.37	2.51	2.5	2.5
ENSDARG00000020702	odf2b	outer dense fiber of sperm tails 2b [Source:ZFIN;Acc:ZDB-GENE-041008-119]	8	31016180	31031534		2.1	1.02	1.37	1.39	1.48	1.61
ENSDARG00000020708	mdkb	midkine b [Source:ZFIN;Acc:ZDB-GENE-010131-6]	25	7872135	7880678		235.93	118.44	173.54	215.15	223.65	232.38
ENSDARG00000020711	rrm2	ribonucleotide reductase regulatory subunit M2 [Source:NCBI gene;Acc:100330864]	20	29492469	29499426		4.3	3.77	3.96	1.95	1.97	1.63
ENSDARG00000020718	slc25a22a	solute carrier family 25 member 22a [Source:ZFIN;Acc:ZDB-GENE-110408-61]	25	4460039	4482506		14.85	7.98	8.65	15.3	15.64	14.92
ENSDARG00000020730	smpd4	sphingomyelin phosphodiesterase 4 [Source:NCBI gene;Acc:793294]	21	18907102	18926900		2.56	1.79	2.05	2.88	2.58	2.73
ENSDARG00000020735	efcab6	EF-hand calcium binding domain 6 [Source:ZFIN;Acc:ZDB-GENE-070828-2]	25	18978648	19008227		0.37	0.22	0.24	0.49	0.55	0.49
ENSDARG00000020736	zgc:153921	zgc:153921 [Source:ZFIN;Acc:ZDB-GENE-060929-816]	3	1039518	1045311		0.12	0.08	0.05	0.04	0.03	0.2
ENSDARG00000020741	fga	fibrinogen alpha chain [Source:ZFIN;Acc:ZDB-GENE-031010-21]	1	10063501	10071539		91.44	142.67	126.25	189.72	218.91	216.85
ENSDARG00000020744	bdkrb1	bradykinin receptor B1 [Source:ZFIN;Acc:ZDB-GENE-030814-1]	17	2833685	2834764		1.94	2.71	2.45	2.46	2.7	2.27
ENSDARG00000020746	gfi1aa	growth factor independent 1A transcription repressor a [Source:ZFIN;Acc:ZDB-GENE-050522-534]	2	10766744	10785866		1.81	2.15	2	0.91	1.16	0.86
ENSDARG00000020749	caprin2	caprin family member 2 [Source:NCBI gene;Acc:407729]	4	16615026	16628802		0.64	0.15	0.23	0.4	0.39	0.33
ENSDARG00000020758	tmem178	transmembrane protein 178 [Source:NCBI gene;Acc:767702]	11	30513656	30531106		8.86	3.63	4.68	6.91	7.8	7.15
ENSDARG00000020759	elf1	E74-like ETS transcription factor 1 [Source:ZFIN;Acc:ZDB-GENE-980526-335]	14	40745833	40850481		8.24	6.67	5.22	9.8	9.48	8.85
ENSDARG00000020761	arrdc2	arrestin domain containing 2 [Source:ZFIN;Acc:ZDB-GENE-030131-7146]	11	13524504	13533782		19.81	15.08	13.1	39.29	42.55	40.3
ENSDARG00000020764	slmapb	sarcolemma associated protein b [Source:ZFIN;Acc:ZDB-GENE-040426-809]	23	19590006	19604413		14.56	6.71	8.23	7.56	8.33	7.88
ENSDARG00000020771	tnr	tenascin R (restrictin, janusin) [Source:ZFIN;Acc:ZDB-GENE-030804-1]	2	35240485	35474115		9.06	1.43	1.66	10.49	10.2	10.77
ENSDARG00000020777	cep85l	centrosomal protein 85, like [Source:ZFIN;Acc:ZDB-GENE-041014-309]	20	41859277	41963921		4.44	2.74	2.36	5.59	5.84	5.39
ENSDARG00000020785	lama4	laminin, alpha 4 [Source:ZFIN;Acc:ZDB-GENE-040724-213]	20	218886	259965		17.86	20.2	15.79	28.1	29.97	30.13
ENSDARG00000020786	sla2	Src-like-adaptor 2 [Source:ZFIN;Acc:ZDB-GENE-080204-98]	23	14808221	14830627		0.68	0.28	0.37	0.37	0.3	0.17
ENSDARG00000020794	neurod6b	neuronal differentiation 6b [Source:NCBI gene;Acc:114415]	2	50608099	50629155		19.7	9.84	16.88	17.08	17.88	18.09
ENSDARG00000020795	rac3b	Rac family small GTPase 3b [Source:ZFIN;Acc:ZDB-GENE-080220-21]	12	3022882	3042029		28.43	11.66	15.43	27.05	26.05	27.28
ENSDARG00000020798	necap1	NECAP endocytosis associated 1 [Source:ZFIN;Acc:ZDB-GENE-040426-1682]	19	10396042	10408029		36.93	12.69	14.39	34.13	34.98	36
ENSDARG00000020811	efemp2b	EGF containing fibulin extracellular matrix protein 2b [Source:ZFIN;Acc:ZDB-GENE-030219-125]	21	25588436	25604609		3.71	1.67	2	3.87	4.32	4.25
ENSDARG00000020812	si:ch73-177h5.2	si:ch73-177h5.2 [Source:ZFIN;Acc:ZDB-GENE-081104-247]	4	1921011	1930024		0.09	0.03	0.06	0.1	0.09	0.01
ENSDARG00000020814	faimb	Fas apoptotic inhibitory molecule b [Source:ZFIN;Acc:ZDB-GENE-040718-91]	6	16400049	16406210		14.4	5.81	3.81	11.44	10.12	11.16
ENSDARG00000020820	srp68	signal recognition particle 68 [Source:ZFIN;Acc:ZDB-GENE-040831-3]	6	23026170	23057363		11.45	12.07	10.73	13.1	12.9	12.64
ENSDARG00000020822	ift22	intraflagellar transport 22 homolog (Chlamydomonas) [Source:NCBI gene;Acc:550583]	5	30702084	30704390		3.37	2.34	3.35	3.34	3.67	3.94
ENSDARG00000020834	si:dkey-230p4.1	si:dkey-230p4.1 [Source:ZFIN;Acc:ZDB-GENE-081104-380]	9	12905591	12953824		3.8	2.01	1.81	2.79	2.73	2.74
ENSDARG00000020840	mcrs1	microspherule protein 1 [Source:ZFIN;Acc:ZDB-GENE-040426-2434]	11	3364193	3381343		6.35	4.61	5.45	5.66	5.51	5.62
ENSDARG00000020841	rbm7	RNA binding motif protein 7 [Source:ZFIN;Acc:ZDB-GENE-030131-6724]	18	47301086	47306847		12.85	11.38	8.38	13.8	15.96	13.9
ENSDARG00000020845	tns1b	tensin 1b [Source:ZFIN;Acc:ZDB-GENE-030131-6933]	9	47436010	47654204		4.02	3.96	3.44	7.51	8	7.71
ENSDARG00000020847	atp6v0a1a	ATPase H+ transporting V0 subunit a1a [Source:ZFIN;Acc:ZDB-GENE-030131-3027]	3	16922226	16973125		29.13	21.06	17.61	21.87	20.27	21.21
ENSDARG00000020850	eef1a1l1	eukaryotic translation elongation factor 1 alpha 1, like 2 [Source:ZFIN;Acc:ZDB-GENE-050708-188]	19	43119014	43137740		1850.31	1924.34	1918.54	1531.65	1555.16	1596.55
ENSDARG00000020853	galr2a	galanin receptor 2a [Source:ZFIN;Acc:ZDB-GENE-130603-37]	12	36390422	36394468		0.2	0.1	0.23	0.14	0.09	0.22
ENSDARG00000020857	ccdc149b	coiled-coil domain containing 149b [Source:NCBI gene;Acc:436983]	1	40034061	40053012		5.65	2.61	3.01	4.62	5.11	5.26
ENSDARG00000020860	trim25l	tripartite motif containing 25, like [Source:ZFIN;Acc:ZDB-GENE-040914-87]	6	4160579	4176841		1.72	0.89	0.64	0.85	1.08	0.81
ENSDARG00000020866	apoa4b.2	apolipoprotein A-IV b, tandem duplicate 2 [Source:ZFIN;Acc:ZDB-GENE-030131-3143]	16	23947196	23948538		159.37	273.97	242.25	67.27	71.31	71.69
ENSDARG00000020871	ptprz1b	protein tyrosine phosphatase receptor type Z1b [Source:ZFIN;Acc:ZDB-GENE-050506-100]	4	4834085	4926061		16.14	5.19	7.33	16.95	17.84	18.63
ENSDARG00000020872	nfat5a	nuclear factor of activated T cells 5a [Source:ZFIN;Acc:ZDB-GENE-030131-6322]	25	37094476	37121335		0.2	0.09	0.12	0.5	0.41	0.41
ENSDARG00000020876	pdk2a	pyruvate dehydrogenase kinase, isozyme 2a [Source:ZFIN;Acc:ZDB-GENE-120910-1]	12	28559408	28570989		17.4	19.47	15.78	42.92	45.51	46.5

**ENSDARG00000020884**	gli2b	GLI family zinc finger 2b [Source:ZFIN;Acc:ZDB-GENE-050523-1]	11	43661735	43713750		2.83	2.24	2.15	3.96	3.75	3.94
**ENSDARG00000020887**	armc1l	armadillo repeat containing 1, like [Source:ZFIN;Acc:ZDB-GENE-030131-9819]	23	31912882	31919155		7.85	8.43	7.44	6.1	4.82	4.4
**ENSDARG00000020890**	tmod4	tropomodulin 4 (muscle) [Source:ZFIN;Acc:ZDB-GENE-991019-9]	16	29650698	29684858		69.78	102.65	114.34	39.61	38.99	37.2
**ENSDARG00000020891**	LO018197.1	presenilin associated rhomboid like [Source:NCBI gene;Acc:792889]	15	45544589	45558281		7.36	7.09	6.09	9.87	9.81	9.53
**ENSDARG00000020893**	slc25a55a	solute carrier family 25 member 55a [Source:ZFIN;Acc:ZDB-GENE-040426-2745]	6	49095636	49107046		66.5	39.32	42.43	54.41	50.14	49.2
**ENSDARG00000020895**	bag1	BCL2 associated athanogene 1 [Source:ZFIN;Acc:ZDB-GENE-050309-89]	2	30904069	30908300		17.33	16.34	15.66	16.06	14.26	14.51
**ENSDARG00000020898**	cyb5r4	cytochrome b5 reductase 4 [Source:NCBI gene;Acc:553685]	16	3067134	3096745		4.46	3.51	2.74	3.64	3.77	3.9
**ENSDARG00000020901**	gabrp	gamma-aminobutyric acid (GABA) A receptor, pi [Source:ZFIN;Acc:ZDB-GENE-081028-62]	14	34547554	34557058		8.89	4.42	6.57	9.24	9.22	11.18
**ENSDARG00000020913**	ddx56	DEAD (Asp-Glu-Ala-Asp) box helicase 56 [Source:ZFIN;Acc:ZDB-GENE-040825-3]	10	2856377	2875735		9.11	10.19	10.25	6.21	6.66	5.94
**ENSDARG00000020924**	myo1ca	myosin Ic, paralog a [Source:NCBI gene;Acc:567536]	5	37785152	37817140		8	8.73	7.96	6.16	5.93	6.24
**ENSDARG00000020926**	creb3l3l	cAMP responsive element binding protein 3-like 3 like [Source:ZFIN;Acc:ZDB-GENE-040426-2942]	8	31375119	31384620		36.48	31.35	31.69	38.5	36.6	36.24
**ENSDARG00000020929**	fam49ba	family with sequence similarity 49 member Ba [Source:ZFIN;Acc:ZDB-GENE-030131-9537]	2	32215272	32262287		9.01	10.36	10.42	7	7.38	6.76
**ENSDARG00000020944**	ezra	ezrin a [Source:ZFIN;Acc:ZDB-GENE-050522-18]	17	45413324	45452116		9.28	12.12	7.22	7.72	7.8	7.78
**ENSDARG00000020946**	auts2b	activator of transcription and developmental regulator AUTS2 b [Source:ZFIN;Acc:ZDB-GENE-131127-20]	15	20004766	20024205		6.74	2.78	2.87	8.75	8.8	8.61
**ENSDARG00000020948**	gmcl1	germ cell-less, spermatogenesis associated [Source:ZFIN;Acc:ZDB-GENE-030131-865]	10	7673446	7702029		9.47	5.21	6.58	9.63	10.31	10.15
**ENSDARG00000020952**	si:ch211-214j8.1	si:ch211-214j8.1 [Source:ZFIN;Acc:ZDB-GENE-060526-100]	5	26248380	26261125		9.12	7.4	4.92	2.83	3.49	2.7
**ENSDARG00000020953**	dnajb6b	DnaJ heat shock protein family (Hsp40) member B6b [Source:ZFIN;Acc:ZDB-GENE-040426-1122]	7	40536928	40578733		5.14	4.99	5.12	5.12	5.07	5.31
**ENSDARG00000020956**	pck2	phosphoenolpyruvate carboxykinase 2 (mitochondrial) [Source:ZFIN;Acc:ZDB-GENE-040426-2266]	24	12920019	12938922		54.08	90.41	58.03	78.15	103.22	97.15
**ENSDARG00000020957**	pth1r	parathyroid hormone 1 receptor [Source:ZFIN;Acc:ZDB-GENE-991123-8]	2	2290148	2451181		1.7	0.65	0.73	2.17	1.75	1.77
**ENSDARG00000020960**	hgs	hepatocyte growth factor-regulated tyrosine kinase substrate [Source:ZFIN;Acc:ZDB-GENE-030131-6107]	3	50076793	50118140		17.74	13.05	9.64	20.86	20.19	20.43
**ENSDARG00000020964**	thap12b	THAP domain containing 12b [Source:ZFIN;Acc:ZDB-GENE-040718-429]	15	19900197	19908909		4.3	3.23	3.86	3.63	3.55	3.46
**ENSDARG00000020976**	ubald1b	UBA-like domain containing 1b [Source:ZFIN;Acc:ZDB-GENE-050522-446]	3	54833658	54846444		5.06	4.97	4.27	5.5	5.01	5.23
**ENSDARG00000020979**	ripor3	RIPOR family member 3 [Source:NCBI gene;Acc:503535]	6	51676227	51763503		2.03	2.76	2.74	2.7	3.04	2.66
**ENSDARG00000020981**	slc35a3b	solute carrier family 35 member A3b [Source:ZFIN;Acc:ZDB-GENE-050417-460]	2	9990491	10040264		1.51	1.22	0.94	1.36	1.4	1.29
**ENSDARG00000020982**	prickle2a	prickle homolog 2a [Source:ZFIN;Acc:ZDB-GENE-061110-112]	8	54081819	54105734		3.34	0.92	0.91	5	4.7	5.11
**ENSDARG00000020984**	slc16a10	solute carrier family 16 member 10 [Source:NCBI gene;Acc:566499]	20	122123	147578		26.6	39.14	34.22	35.2	36.29	35.48
**ENSDARG00000020986**	ctu1	cytosolic thiouridylase subunit 1 homolog (S. pombe) [Source:NCBI gene;Acc:393098]	8	3431671	3439484		6.27	5.61	5.58	5.2	5.29	5.63
**ENSDARG00000021004**	c5	complement component 5 [Source:ZFIN;Acc:ZDB-GENE-120510-2]	5	63458263	63509581		13.83	21.91	18.32	20.07	21.45	21.17
**ENSDARG00000021021**	trim35-28	tripartite motif containing 35-28 [Source:ZFIN;Acc:ZDB-GENE-071004-19]	16	26439518	26446321		6.49	6.6	4.98	7.21	7.13	6.15
**ENSDARG00000021022**	ift57	intraflagellar transport 57 homolog (Chlamydomonas) [Source:NCBI gene;Acc:414929]	2	42200390	42210526		5.92	4.71	5.15	6.85	7.34	7.71
**ENSDARG00000021032**	foxd3	forkhead box D3 [Source:ZFIN;Acc:ZDB-GENE-980526-143]	6	32092032	32093830		8.4	8.57	8.76	14.91	15.08	13.89
**ENSDARG00000021033**	herpud2	HERPUD family member 2 [Source:NCBI gene;Acc:393157]	16	42481447	42495383		7.42	6.32	5.64	10.36	9.68	10.06
**ENSDARG00000021046**	rhbdd1	rhomboid domain containing 1 [Source:ZFIN;Acc:ZDB-GENE-050417-85]	15	35933094	35944902		2.19	1.58	2.07	3.99	3.4	3.46
**ENSDARG00000021048**	clptm1l	CLPTM1 like [Source:NCBI gene;Acc:436653]	19	1856250	1871415		13.23	12.66	13.89	12.61	11.37	11.46
**ENSDARG00000021055**	isl1l	islet1, like [Source:ZFIN;Acc:ZDB-GENE-040624-1]	10	8680730	8687894		0.18	0.09	0.11	0.06	0.09	0.05
**ENSDARG00000021059**	alas1	aminolevulinate, delta-, synthase 1 [Source:ZFIN;Acc:ZDB-GENE-001229-2]	11	260763	270210		57.34	67.85	51.51	50.56	50.86	50.33
**ENSDARG00000021082**	sec31a	SEC31 homolog A, COPII coat complex component [Source:NCBI gene;Acc:266638]	10	5092511	5135788		4.67	4.94	3.15	9.57	9.23	8.82
**ENSDARG00000021086**	znf367	zinc finger protein 367 [Source:NCBI gene;Acc:550421]	8	1210468	1219815		7.06	5.24	4.86	5	5.22	4.61
**ENSDARG00000021097**	nelfcd	negative elongation factor complex member C/D [Source:ZFIN;Acc:ZDB-GENE-040426-720]	6	49781884	49801173		8.35	7.83	7.77	7	6.6	7.66
**ENSDARG00000021107**	sf3a2	splicing factor 3a, subunit 2 [Source:ZFIN;Acc:ZDB-GENE-040426-897]	2	57825432	57837838		40.48	35.66	39.99	26.18	26.81	29.95
**ENSDARG00000021110**	poc1b	POC1 centriolar protein B [Source:NCBI gene;Acc:386932]	25	18366978	18435481		3.27	3.43	2.86	2.25	1.96	2.37
**ENSDARG00000021112**	c1d	C1D nuclear receptor corepressor [Source:NCBI gene;Acc:474329]	13	4656504	4664403		5.42	7.95	5.76	3.86	3.89	4.03
**ENSDARG00000021113**	ptmaa	prothymosin alpha a [Source:NCBI gene;Acc:335707]	6	29754141	29795029		387.24	370.94	346.96	364.14	360.53	379.61
**ENSDARG00000021115**	spata6	spermatogenesis associated 6 [Source:ZFIN;Acc:ZDB-GENE-050417-351]	8	15287424	15305528		1.42	1.24	0.98	1.47	1.8	1.72
**ENSDARG00000021120**	ndc1	NDC1 transmembrane nucleoporin [Source:NCBI gene;Acc:335236]	2	26576853	26590628		6.45	5.95	6.1	6.82	6.5	6.01
**ENSDARG00000021124**	cfl1	cofilin 1 [Source:ZFIN;Acc:ZDB-GENE-030131-215]	14	30795559	30801555		327.17	368.12	310.08	304.2	307.93	302.54
**ENSDARG00000021135**	dhrs4	dehydrogenase/reductase (SDR family) member 4 [Source:ZFIN;Acc:ZDB-GENE-040426-1498]	7	24039464	24047316		10.34	17.85	14.89	7.95	8.43	8.22
**ENSDARG00000021137**	adgrv1	adhesion G protein-coupled receptor V1 [Source:NCBI gene;Acc:415105]	5	48683447	48940127		2	0.79	0.78	3.4	3.34	3.43
**ENSDARG00000021140**	pabpc1b	poly A binding protein, cytoplasmic 1 b [Source:ZFIN;Acc:ZDB-GENE-050308-1]	19	12121944	12145765		43.41	40.46	33.98	61.22	63.22	62.56
**ENSDARG00000021141**	plekhf2	pleckstrin homology domain containing, family F (with FYVE domain) member 2 [Source:NCBI gene;Acc:393214]	16	40425873	40459104		15.06	12.05	11.98	11.09	11.83	11.82
**ENSDARG00000021143**	rtn1b	reticulon 1b [Source:ZFIN;Acc:ZDB-GENE-030710-3]	20	20637866	20696957		19.04	6.99	10.8	20.18	20.59	21.92
**ENSDARG00000021147**	nipal3	NIPA like domain containing 3 [Source:ZFIN;Acc:ZDB-GENE-040718-379]	17	26928418	26935339		9.3	9.11	8.12	7.89	7.25	7.15
**ENSDARG00000021149**	cbr1l	carbonyl reductase 1-like [Source:ZFIN;Acc:ZDB-GENE-030131-9642]	21	21633213	21641194		52.05	86.56	67.97	19.03	18.86	18.78
**ENSDARG00000021150**	valopa	vertebrate ancient long opsin a [Source:ZFIN;Acc:ZDB-GENE-000427-4]	13	51502950	51527140		1.41	0.71	1.16	1.23	1.17	1.33
**ENSDARG00000021151**	ptpreb	protein tyrosine phosphatase receptor type Eb [Source:ZFIN;Acc:ZDB-GENE-060503-263]	17	29319587	29343657		8.22	2.67	3.89	9.26	9.39	9.77
**ENSDARG00000021154**	zgc:92040	zgc:92040 [Source:ZFIN;Acc:ZDB-GENE-040718-87]	12	3926168	3940768		12.14	17.11	13.74	10.13	9.82	9.7
**ENSDARG00000021163**	thrb	thyroid hormone receptor beta [Source:NCBI gene;Acc:30607]	19	17514631	17658253		6.37	1.2	2.07	8.97	9.09	8.91
**ENSDARG00000021172**	cyp2ad2	cytochrome P450, family 2, subfamily AD, polypeptide 2 [Source:ZFIN;Acc:ZDB-GENE-020812-2]	20	25534669	25542183		20.11	27.83	22.34	3.79	3.51	4.73
**ENSDARG00000021177**	cept1b	choline/ethanolamine phosphotransferase 1b [Source:ZFIN;Acc:ZDB-GENE-071004-33]	22	866681	881725		2.93	1.93	1.97	4.62	4.32	4.27
**ENSDARG00000021184**	rbfox1l	RNA binding fox-1 homolog 1, like [Source:NCBI gene;Acc:407613]	16	31900127	31919568		19.82	26.14	26.49	22.2	18.02	18.19
**ENSDARG00000021186**	pth2rb	parathyroid hormone 2 receptor b [Source:ZFIN;Acc:ZDB-GENE-050208-334]	22	11898661	11955907		0.51	0.15	0.1	0.52	0.47	0.51
**ENSDARG00000021193**	coro1cb	coronin, actin binding protein, 1Cb [Source:ZFIN;Acc:ZDB-GENE-030131-418]	5	31880586	31904562		16.39	8.61	11.09	18.37	19.43	19.28
**ENSDARG00000021195**	st8sia6	ST8 alpha-N-acetyl-neuraminide alpha-2,8-sialyltransferase 6 [Source:ZFIN;Acc:ZDB-GENE-060322-9]	3	16265924	16285259		9.89	10.65	8.99	11.54	10.9	10.91
**ENSDARG00000021200**	nebl	nebulette [Source:ZFIN;Acc:ZDB-GENE-050320-151]	2	21076585	21082695		0.69	0.2	0.5	0.55	0.78	0.51
**ENSDARG00000021201**	noto	notochord homeobox [Source:ZFIN;Acc:ZDB-GENE-990415-75]	13	14976108	14978104		0.19	0.14	0.24	0.07	0.24	0.17
**ENSDARG00000021208**	serpind1	serpin peptidase inhibitor, clade D (heparin cofactor), member 1 [Source:ZFIN;Acc:ZDB-GENE-030711-2]	8	1766206	1781733		6.25	9.74	7.08	3.58	3.92	3.16
**ENSDARG00000021209**	kctd9a	potassium channel tetramerization domain containing 9a [Source:ZFIN;Acc:ZDB-GENE-050320-147]	8	51586483	51599169		5.01	2.6	2.01	5.14	4.99	5.23
**ENSDARG00000021220**	hmgcl	3-hydroxy-3-methylglutaryl-CoA lyase [Source:ZFIN;Acc:ZDB-GENE-040426-958]	17	24855384	24866964		15.74	15.24	15.32	12.41	11.09	11.57
**ENSDARG00000021225**	prkci	protein kinase C, iota [Source:NCBI gene;Acc:117507]	2	37365551	37401600		18.77	15.1	15.81	20.79	21.94	19.46
**ENSDARG00000021232**	nkx2.7	NK2 transcription factor related 7 [Source:ZFIN;Acc:ZDB-GENE-990415-179]	8	50284859	50287949		0.84	0.65	0.58	0.9	0.82	1.07
**ENSDARG00000021233**	pgls	6-phosphogluconolactonase [Source:ZFIN;Acc:ZDB-GENE-040822-9]	3	52684556	52700610		6.1	6.12	4.65	3.65	3.75	3.55
**ENSDARG00000021239**	apaf1	apoptotic peptidase activating factor 1 [Source:NCBI gene;Acc:58131]	4	17843717	17874048		5.97	4.74	4.47	6.42	6.57	6.33
**ENSDARG00000021241**	zgc:165604	zgc:165604 [Source:ZFIN;Acc:ZDB-GENE-070615-34]	21	27558381	27570216		1.46	0.31	0.26	1.8	1.56	1.72
**ENSDARG00000021242**	mvp	major vault protein [Source:NCBI gene;Acc:373081]	3	15708419	15734530		37.12	40.54	26.28	49.9	55.91	48.97
**ENSDARG00000021250**	slc25a48	solute carrier family 25 member 48 [Source:NCBI gene;Acc:436640]	14	25590603	25599002		8.45	11.11	10.59	7.69	8.44	8.46
**ENSDARG00000021252**	cct6a	chaperonin containing TCP1, subunit 6A (zeta 1) [Source:ZFIN;Acc:ZDB-GENE-011018-2]	21	41819288	41839684		111.37	120.56	127.97	90.84	95.92	93.69
**ENSDARG00000021255**	arhgap22	Rho GTPase activating protein 22 [Source:ZFIN;Acc:ZDB-GENE-090313-87]	13	31108334	31139766		2.24	0.87	1.45	2.85	2.84	2.97
**ENSDARG00000021257**	eif3d	eukaryotic translation initiation factor 3, subunit D [Source:NCBI gene;Acc:336498]	3	29640837	29654925		112.72	114.71	123.94	92.97	99.52	96.87
**ENSDARG00000021265**	mybpc2b	myosin binding protein C, fast type b [Source:ZFIN;Acc:ZDB-GENE-050320-80]	24	38301080	38348805		16.29	20.42	18.74	18.99	18.63	20.27
**ENSDARG00000021287**	rab7b	RAB7b, member RAS oncogene family [Source:ZFIN;Acc:ZDB-GENE-180906-1]	8	50968990	50981175		14.37	12.56	11.38	17.55	19.23	18.15
**ENSDARG00000021288**	mier3b	mesoderm induction early response 1, family member 3 b [Source:ZFIN;Acc:ZDB-GENE-070424-65]	8	17143501	17157074		8.56	4.51	4.91	8.94	10.46	10.29
**ENSDARG00000021305**	slc30a2	solute carrier family 30 member 2 [Source:ZFIN;Acc:ZDB-GENE-041014-347]	20	38595935	38617766		1.32	0.21	0.48	3.6	4.25	3.24
**ENSDARG00000021309**	rhoca	ras homolog family member Ca [Source:ZFIN;Acc:ZDB-GENE-040426-1337]	6	48382697	48418021		23.1	28.58	27.33	30.65	31.98	30.48
**ENSDARG00000021324**	nsun4	NOP2/Sun RNA methyltransferase 4 [Source:ZFIN;Acc:ZDB-GENE-041212-77]	2	10160484	10169433		2.64	3.07	3	2.6	2.21	2.61
**ENSDARG00000021336**	pax4	paired box 4 [Source:ZFIN;Acc:ZDB-GENE-041210-244]	4	19700308	19704628		0.11	0.37	0.13	0.21	0.44	0.68
**ENSDARG00000021339**	cpa5	carboxypeptidase A5 [Source:ZFIN;Acc:ZDB-GENE-020514-1]	25	18459924	18470695		604.78	829.41	712.88	70.54	73.65	75.47
**ENSDARG00000021343**	wsb1	WD repeat and SOCS box containing 1 [Source:NCBI gene;Acc:323226]	15	31161783	31177324		25.3	18.12	19.75	61.89	66.31	62.44
**ENSDARG00000021345**	prph2lb	peripherin 2-like b [Source:ZFIN;Acc:ZDB-GENE-041001-147]	20	27384138	27390258		3.41	0.85	0.57	2.46	2.61	2.64
**ENSDARG00000021346**	pdhb	pyruvate dehydrogenase E1 beta subunit [Source:ZFIN;Acc:ZDB-GENE-040426-2173]	22	10201826	10212502		181.85	176.38	178.31	145.7	140.72	143.76
**ENSDARG00000021351**	nefma	neurofilament, medium polypeptide a [Source:ZFIN;Acc:ZDB-GENE-050522-205]	21	19013850	19018455		30.15	16.86	14.73	22.27	23.48	24.03
**ENSDARG00000021352**	gria1a	glutamate receptor, ionotropic, AMPA 1a [Source:ZFIN;Acc:ZDB-GENE-020125-1]	14	34125855	34276574		4.4	0.97	1.73	6.2	6.3	6.7
**ENSDARG00000021366**	fbp1a	fructose-1,6-bisphosphatase 1a [Source:ZFIN;Acc:ZDB-GENE-030131-7171]	5	44833313	44843738		28.77	32.69	32.79	17.9	16.23	17.16
**ENSDARG00000021369**	mc3r	melanocortin 3 receptor [Source:ZFIN;Acc:ZDB-GENE-030502-3]	8	28654289	28655338		1.03	0.04	0.19	0.27	0.6	0.38
**ENSDARG00000021370**	actr6	actin related protein 6 [Source:ZFIN;Acc:ZDB-GENE-030131-3543]	25	19585318	19594092		8.76	8.63	8.59	7.39	7.5	6.62
**ENSDARG00000021372**	tob1b	transducer of ERBB2, 1b [Source:ZFIN;Acc:ZDB-GENE-040426-2151]	12	847187	850457		58.26	48.88	41.98	80.47	80.18	73.99
**ENSDARG00000021374**	cab39l1	calcium binding protein 39, like 1 [Source:ZFIN;Acc:ZDB-GENE-040426-2897]	14	31476973	31489410		13.77	14.9	11.54	13.26	13.07	13.32
**ENSDARG00000021378**	phf21ab	PHD finger protein 21Ab [Source:ZFIN;Acc:ZDB-GENE-030131-6456]	25	7718967	7764083		3.9	1.54	1.98	6.04	4.93	5.65
**ENSDARG00000021380**	ppm1db	protein phosphatase, Mg2+/Mn2+ dependent, 1Db [Source:ZFIN;Acc:ZDB-GENE-041114-27]	5	56914891	56924747		7.62	3.05	3.61	7.31	7.32	6.47
**ENSDARG00000021383**	naalad2	N-acetylated alpha-linked acidic dipeptidase 2 [Source:ZFIN;Acc:ZDB-GENE-040426-1025]	15	43906043	43942064		9.1	12.36	9.08	7.19	7.38	8.06
**ENSDARG00000021389**	jag2b	jagged canonical Notch ligand 2b [Source:ZFIN;Acc:ZDB-GENE-011128-3]	20	21391181	21475620		3.68	2.57	2.14	5.79	5.92	6.46
**ENSDARG00000021392**	chrnb5b	cholinergic receptor, nicotinic, beta 5b [Source:ZFIN;Acc:ZDB-GENE-120809-5]	14	41490262	41515188		0.35	0.04	0.1	0.38	0.43	0.39
**ENSDARG00000021398**	mul1a	mitochondrial E3 ubiquitin protein ligase 1a [Source:NCBI gene;Acc:553180]	2	44960876	44971551		1.79	1.58	1.75	2.44	2.51	2.5
**ENSDARG00000021399**	yipf2	Yip1 domain family, member 2 [Source:ZFIN;Acc:ZDB-GENE-040724-124]	3	34174004	34180464		17.51	17.11	16.78	14.04	13.99	15.08
**ENSDARG00000021402**	mcm3ap	minichromosome maintenance complex component 3 associated protein [Source:ZFIN;Acc:ZDB-GENE-040715-1]	9	38457806	38483789		3.89	2.43	1.86	4.7	4.4	4.46
**ENSDARG00000021404**	zgc:110319	zgc:110319 [Source:ZFIN;Acc:ZDB-GENE-050417-345]	13	18331509	18365319		6.13	5.56	5.91	5.32	5.48	5.4
**ENSDARG00000021405**	chd3	chromodomain helicase DNA binding protein 3 [Source:ZFIN;Acc:ZDB-GENE-121031-4]	7	20673287	20758828		6.68	3.62	3.63	10.88	10.58	11.16
**ENSDARG00000021408**	poglut3	protein O-glucosyltransferase 3 [Source:ZFIN;Acc:ZDB-GENE-060810-142]	21	35041399	35056093		3.92	4.34	3.52	3.37	3.25	3.06
**ENSDARG00000021432**	odf3l2b	outer dense fiber of sperm tails 3-like 2b [Source:ZFIN;Acc:ZDB-GENE-080723-64]	22	21305682	21311540		0.16	0.41	0.42	0.44	0.34	0.35
**ENSDARG00000021433**	asmtl	acetylserotonin O-methyltransferase-like [Source:ZFIN;Acc:ZDB-GENE-030131-2854]	9	34872733	34882516		18.07	25.12	25.67	12.77	12.66	12.05
**ENSDARG00000021439**	ncoa4	nuclear receptor coactivator 4 [Source:ZFIN;Acc:ZDB-GENE-040426-689]	13	3915260	3936555		28.89	26.46	23.76	38.4	39.7	38.12
**ENSDARG00000021442**	cdh11	cadherin 11, type 2, OB-cadherin (osteoblast) [Source:ZFIN;Acc:ZDB-GENE-980526-170]	7	43760149	43857938		21.76	16.87	13.8	32.03	32.35	33.28
**ENSDARG00000021443**	zfp36l1b	zinc finger protein 36, C3H type-like 1b [Source:ZFIN;Acc:ZDB-GENE-030131-2391]	20	9575374	9580446		18.07	19.75	13.51	37.23	37.71	36.17
**ENSDARG00000021444**	prf1.2	perforin 1.2 [Source:ZFIN;Acc:ZDB-GENE-111212-3]	19	24161712	24163845		0	0	0	0.04	0.01	0.01
**ENSDARG00000021455**	strbp	spermatid perinuclear RNA binding protein [Source:ZFIN;Acc:ZDB-GENE-030729-18]	10	9834896	10018794		8.2	3.9	4.25	8.84	8.43	8.43
**ENSDARG00000021462**	daw1	dynein assembly factor with WDR repeat domains 1 [Source:NCBI gene;Acc:556760]	18	46382484	46394080		1.57	1.5	1.72	3.01	2.42	2.97
**ENSDARG00000021466**	ireb2	iron-responsive element binding protein 2 [Source:ZFIN;Acc:ZDB-GENE-051205-1]	25	6228496	6261693		10.32	6.94	6.04	15.91	14.78	15.65
**ENSDARG00000021480**	olfml2bb	olfactomedin-like 2Bb [Source:ZFIN;Acc:ZDB-GENE-070912-266]	2	20604775	20615639		2.79	3.55	2.9	1.92	1.96	2
**ENSDARG00000021483**	cdc14b	cell division cycle 14B [Source:ZFIN;Acc:ZDB-GENE-040426-820]	8	1222132	1267247		22.27	17.68	18.22	17.99	17.99	18.47
**ENSDARG00000021488**	cyth1b	cytohesin 1b [Source:ZFIN;Acc:ZDB-GENE-030131-4311]	12	34051434	34192955		3.69	4.03	3.99	5.99	6.5	6.03
**ENSDARG00000021494**	hnf4a	hepatocyte nuclear factor 4, alpha [Source:ZFIN;Acc:ZDB-GENE-030131-1077]	23	25832689	25865856		5.4	7.32	5.27	5.13	5.54	5.46
**ENSDARG00000021497**	gpr132a	G protein-coupled receptor 132a [Source:ZFIN;Acc:ZDB-GENE-131127-108]	17	39760145	39761086		0	0.1	0.02	0.05	0	0.03
**ENSDARG00000021509**	tab2	TGF-beta activated kinase 1 (MAP3K7) binding protein 2 [Source:NCBI gene;Acc:494163]	20	1329509	1350296		5.06	5.18	4.26	8.67	8.29	7.78
**ENSDARG00000021521**	rgs14a	regulator of G protein signaling 14a [Source:ZFIN;Acc:ZDB-GENE-030131-5579]	14	872016	899192		0.22	0.23	0.16	0.29	0.34	0.27
**ENSDARG00000021539**	ephb2b	eph receptor B2b [Source:ZFIN;Acc:ZDB-GENE-050522-415]	11	28166165	28424186		5.37	2.01	1.81	7.25	7.03	7.19
**ENSDARG00000021547**	vrk2	VRK serine/threonine kinase 2 [Source:ZFIN;Acc:ZDB-GENE-040426-1046]	13	26780004	26799244		2.79	1.97	2.57	1.27	1.21	1.36
**ENSDARG00000021550**	rad23b	RAD23 homolog B, nucleotide excision repair protein [Source:ZFIN;Acc:ZDB-GENE-040426-1487]	21	382400	410449		77.88	74.02	73.29	68.33	69.44	69.45
**ENSDARG00000021555**	prkn	parkin RBR E3 ubiquitin protein ligase [Source:ZFIN;Acc:ZDB-GENE-050417-109]	13	3354895	3516473		3.75	3.64	3.18	2.8	2.57	3.02
**ENSDARG00000021556**	ubl3b	ubiquitin-like 3b [Source:ZFIN;Acc:ZDB-GENE-040630-4]	14	30854553	30876708		13.88	6.02	5.29	22.52	21	23.61
**ENSDARG00000021557**	wdr45b	WD repeat domain 45B [Source:NCBI gene;Acc:393210]	12	42592	46985		26.09	20.06	26.41	22.84	25.78	25.05
**ENSDARG00000021566**	OTOP1	otopetrin 1 [Source:HGNC Symbol;Acc:HGNC:19656]	14	49602	58161	OTOP1	1.93	1.79	1.91	1.48	1.66	1.58
**ENSDARG00000021569**	gdap2	ganglioside induced differentiation associated protein 2 [Source:NCBI gene;Acc:447824]	9	20806334	20853467		5.84	5.43	4.79	6.89	6.45	6.75
**ENSDARG00000021572**	cgas	cyclic GMP-AMP synthase [Source:ZFIN;Acc:ZDB-GENE-090313-23]	13	27328098	27332360		0.08	0.19	0.18	0.12	0.24	0.08
**ENSDARG00000021573**	slc16a7	solute carrier family 16 member 7 [Source:ZFIN;Acc:ZDB-GENE-051113-248]	23	9867483	9889330		2.12	2.86	2.03	0.84	0.61	0.54
**ENSDARG00000021579**	orc5	origin recognition complex, subunit 5 [Source:ZFIN;Acc:ZDB-GENE-040426-2275]	4	22353465	22363709		4.87	5.22	5.3	2.84	2.79	2.74
**ENSDARG00000021582**	dynlrb1	dynein, light chain, roadblock-type 1 [Source:ZFIN;Acc:ZDB-GENE-040426-989]	11	26604224	26608636		3.7	3.61	3.78	5.43	5.75	5.68
**ENSDARG00000021584**	cntn5	contactin 5 [Source:NCBI gene;Acc:327429]	18	42150947	42395161		3.99	1.03	1.12	5.46	5.23	5.75
**ENSDARG00000021589**	tmem60	transmembrane protein 60 [Source:ZFIN;Acc:ZDB-GENE-040801-222]	25	21277478	21280584		6.39	5.12	5.58	5.21	5.62	6.22
**ENSDARG00000021590**	magi2a	membrane associated guanylate kinase, WW and PDZ domain containing 2a [Source:ZFIN;Acc:ZDB-GENE-050810-4]	4	22755465	23083187		3.6	1.56	1.65	4.77	4.77	4.9
**ENSDARG00000021595**	lhfpl3	LHFPL tetraspan subfamily member 3 [Source:NCBI gene;Acc:445050]	4	22365061	22419743		23.44	8.95	12.68	22.65	22.92	23.61
**ENSDARG00000021607**	negr1	neuronal growth regulator 1 [Source:ZFIN;Acc:ZDB-GENE-040822-27]	6	36942966	37164417		17.95	5.16	7.13	18.33	19.74	19.51
**ENSDARG00000021611**	si:dkey-151g10.3	si:dkey-151g10.3 [Source:ZFIN;Acc:ZDB-GENE-090312-81]	23	25172682	25222673		4.82	1.88	2.36	7.37	7.49	7.13
**ENSDARG00000021633**	sh3bgr	SH3 domain binding glutamate-rich protein [Source:ZFIN;Acc:ZDB-GENE-040801-27]	15	5923851	5958803		59.35	81.22	83.17	49.36	44.66	49.52
**ENSDARG00000021647**	gnai1	guanine nucleotide binding protein (G protein), alpha inhibiting activity polypeptide 1 [Source:ZFIN;Acc:ZDB-GENE-040426-1310]	4	22680442	22714063		37.36	27.74	32.34	45.58	48.41	47.1
**ENSDARG00000021664**	fzd3a	frizzled class receptor 3a [Source:ZFIN;Acc:ZDB-GENE-990415-225]	20	35208855	35246150		6.17	2.02	1.7	8.46	7.94	8.23
**ENSDARG00000021669**	sec61a1	SEC61 translocon alpha 1 subunit [Source:NCBI gene;Acc:192298]	6	40065201	40071899		86.16	103.09	93.21	109.82	114.82	110.76
**ENSDARG00000021677**	phf11	PHD finger protein 11 [Source:ZFIN;Acc:ZDB-GENE-081104-417]	9	29616854	29634354		0.57	0.45	0.49	0.46	0.44	0.42
**ENSDARG00000021681**	asrgl1	asparaginase and isoaspartyl peptidase 1 [Source:NCBI gene;Acc:541402]	13	27768898	27782764		7.43	10.35	8.1	6.99	7.28	7.86
**ENSDARG00000021688**	mxa	myxovirus (influenza) resistance A [Source:NCBI gene;Acc:360142]	1	7562316	7570181		0.15	0.11	0.16	0.2	0.1	0.13
**ENSDARG00000021697**	morn3	MORN repeat containing 3 [Source:ZFIN;Acc:ZDB-GENE-050417-152]	10	41926888	41940302		1.52	1.75	1.5	2.77	2.3	3.02
**ENSDARG00000021702**	pdcd4a	programmed cell death 4a [Source:ZFIN;Acc:ZDB-GENE-040426-1920]	1	26046148	26071535		63.58	60.99	57.62	45.17	48.42	49.71
**ENSDARG00000021720**	col7a1	collagen, type VII, alpha 1 [Source:ZFIN;Acc:ZDB-GENE-030131-2427]	6	40184235	40312091		9.44	7.36	3.21	4.38	2.84	2.92
**ENSDARG00000021735**	cacna1ba	calcium channel, voltage-dependent, N type, alpha 1B subunit, a [Source:ZFIN;Acc:ZDB-GENE-060531-44]	5	29296396	29488245		2.76	0.59	1.28	4.69	4.96	4.89
**ENSDARG00000021739**	klhl24a	kelch-like family member 24a [Source:ZFIN;Acc:ZDB-GENE-061013-572]	2	44571200	44604455		6.17	4.68	4.37	4.76	5.41	5.04
**ENSDARG00000021753**	ccdc25	coiled-coil domain containing 25 [Source:NCBI gene;Acc:393359]	20	39338700	39344130		25.45	26.48	23.17	15.79	15.85	16.02
**ENSDARG00000021787**	abcb5	ATP-binding cassette, sub-family B (MDR/TAP), member 5 [Source:ZFIN;Acc:ZDB-GENE-030131-6414]	16	19537038	19568795		21.79	12.29	7.5	4.4	3.57	3.09
**ENSDARG00000021789**	myoc	myocilin [Source:ZFIN;Acc:ZDB-GENE-050425-2]	20	15015557	15021628		18.51	28.72	26.48	25.42	23.74	21.91
**ENSDARG00000021794**	hmmr	hyaluronan-mediated motility receptor (RHAMM) [Source:ZFIN;Acc:ZDB-GENE-030131-731]	14	28545198	28562971		10.09	5.61	6.11	5.82	6.17	6.43
**ENSDARG00000021805**	tle5	TLE family member 5, transcriptional modulator [Source:ZFIN;Acc:ZDB-GENE-040426-1409]	22	21794037	21845685		8.51	4.05	4.26	15.22	13.9	14.72
**ENSDARG00000021806**	zfp36l2	zinc finger protein 36, C3H type-like 2 [Source:ZFIN;Acc:ZDB-GENE-030131-5873]	12	25376639	25380028		20.08	23.05	17.05	33.25	36.11	34.86
**ENSDARG00000021808**	nmt2	N-myristoyltransferase 2 [Source:NCBI gene;Acc:556483]	16	28578352	28591541		13.54	8.16	9.66	14.9	15.54	15.86
**ENSDARG00000021811**	calm1a	calmodulin 1a [Source:ZFIN;Acc:ZDB-GENE-030131-8308]	17	26713111	26721007		242.63	177.32	176.04	226.34	230.05	236.25
**ENSDARG00000021812**	cherp	calcium homeostasis endoplasmic reticulum protein [Source:ZFIN;Acc:ZDB-GENE-040407-1]	22	4962370	4990045		11.28	9.36	8.04	15.97	15	15.85
**ENSDARG00000021827**	katna1	katanin p60 (ATPase containing) subunit A 1 [Source:NCBI gene;Acc:553631]	20	23860858	23876291		2.58	2.63	2.34	2.05	2.14	2.5
**ENSDARG00000021833**	ahr2	aryl hydrocarbon receptor 2 [Source:ZFIN;Acc:ZDB-GENE-990714-16]	22	13046270	13165186		12.6	12.73	9.62	16.55	15.87	15.83
**ENSDARG00000021838**	rps23	ribosomal protein S23 [Source:ZFIN;Acc:ZDB-GENE-080220-50]	11	42556395	42561215		1690.53	2111.54	2482.13	1357.26	1426.57	1419.09
**ENSDARG00000021846**	hid1a	HID1 domain containing a [Source:ZFIN;Acc:ZDB-GENE-030131-8263]	3	56921990	56933578		6.36	2.24	2.73	6.18	6.83	7.47
**ENSDARG00000021849**	si:dkey-256h2.1	si:dkey-256h2.1 [Source:ZFIN;Acc:ZDB-GENE-030131-9466]	16	47359754	47381652		0.24	0.16	0.21	0.21	0.25	0.27
**ENSDARG00000021853**	ntpcr	nucleoside-triphosphatase, cancer-related [Source:ZFIN;Acc:ZDB-GENE-040801-200]	13	48747449	48756082		4.97	5.31	4.22	5.04	4.83	4.4
**ENSDARG00000021855**	arnt	aryl hydrocarbon receptor nuclear translocator [Source:ZFIN;Acc:ZDB-GENE-060126-7]	16	4570417	4610278		4.94	2.64	2.87	6.64	6.94	7
**ENSDARG00000021859**	erap1b	endoplasmic reticulum aminopeptidase 1b [Source:ZFIN;Acc:ZDB-GENE-040426-934]	5	26234425	26247973		1.88	1.85	1.71	1.13	1.14	1.02
**ENSDARG00000021864**	rplp1	ribosomal protein, large, P1 [Source:ZFIN;Acc:ZDB-GENE-030131-8663]	19	1852078	1855368		1429.67	1819.84	2010.97	932.91	1000.02	1033.26
**ENSDARG00000021866**	upk1a	uroplakin 1a [Source:ZFIN;Acc:ZDB-GENE-060421-2603]	16	44768361	44780744		0.1	0.1	0.17	0.17	0.22	0.06
**ENSDARG00000021869**	rcan2	regulator of calcineurin 2 [Source:ZFIN;Acc:ZDB-GENE-040516-17]	20	39055432	39188476		13.52	13.66	11.69	12.15	14.54	13.13
**ENSDARG00000021882**	fbxl3b	F-box and leucine-rich repeat protein 3b [Source:ZFIN;Acc:ZDB-GENE-041010-178]	6	59935950	59942335		13.19	9.62	9.81	16.69	16.49	17
**ENSDARG00000021885**	cacul1	CDK2 associated cullin domain 1 [Source:ZFIN;Acc:ZDB-GENE-170113-2]	13	50328815	50341004		0.07	0.1	0.09	0.28	0.27	0.4
**ENSDARG00000021889**	gja3	gap junction protein, alpha 3 [Source:ZFIN;Acc:ZDB-GENE-031217-3]	9	21407987	21409466		0.28	0.17	0.32	1.21	0.95	1.26
**ENSDARG00000021891**	zbtb7b	zinc finger and BTB domain containing 7B [Source:ZFIN;Acc:ZDB-GENE-040426-1134]	16	54804815	54810464		3.52	2.45	2	3.48	3.26	2.78
**ENSDARG00000021895**	disc1	DISC1 scaffold protein [Source:ZFIN;Acc:ZDB-GENE-090122-1]	13	49057081	49144881		1.62	1.63	1.01	3.1	3.44	3.45
**ENSDARG00000021896**	asap3	ArfGAP with SH3 domain, ankyrin repeat and PH domain 3 [Source:ZFIN;Acc:ZDB-GENE-050913-39]	17	27186948	27235797		2.74	2.62	2.18	4.23	3.97	4.25
**ENSDARG00000021899**	b4galt7	xylosylprotein beta 1,4-galactosyltransferase, polypeptide 7 (galactosyltransferase I) [Source:ZFIN;Acc:ZDB-GENE-040727-3]	14	26379484	26392475		2.03	1.89	1.6	2.75	2.67	2.62
**ENSDARG00000021905**	derl1	derlin 1 [Source:ZFIN;Acc:ZDB-GENE-040426-2793]	16	25643445	25663846		19.9	22.3	19.95	21.57	21.71	22.15
**ENSDARG00000021909**	VSTM2A	zgc:110852 [Source:ZFIN;Acc:ZDB-GENE-050306-31]	24	402493	495025	VSTM2A	3.31	1.35	1.81	3.91	3.89	3.54
**ENSDARG00000021913**	ak9	adenylate kinase 9 [Source:ZFIN;Acc:ZDB-GENE-041014-337]	20	33708318	33737831		0.53	0.52	0.48	0.89	0.87	0.85

ENSDARG00000021916	vax1	ventral anterior homeobox 1 [Source:NCBI gene;Acc:373870]	17	21432693	21441464		3.15	0.52	0.8	4.18	4	3.8
ENSDARG00000021924	hsp70.3	heat shock cognate 70-kd protein, tandem duplicate 3 [Source:ZFIN;Acc:ZDB-GENE-110713-1]	3	26123736	26127091		6.06	3.12	2.54	9.98	18.76	13.51
ENSDARG00000021938	smad9	SMAD family member 9 [Source:ZFIN;Acc:ZDB-GENE-031014-1]	10	34960751	35002459		0.8	0.92	0.9	1.75	1.85	1.85
ENSDARG00000021945	ddx23	DEAD (Asp-Glu-Ala-Asp) box polypeptide 23 [Source:ZFIN;Acc:ZDB-GENE-030131-6215]	23	14193547	14216506		23.39	17.19	18.83	20.35	21.08	21.83
ENSDARG00000021948	tnc	tenascin C [Source:ZFIN;Acc:ZDB-GENE-980526-104]	5	28560847	28679135		22.04	19.28	13.98	40.67	38.22	39.33
ENSDARG00000021959	zgc:101100	zgc:101100 [Source:ZFIN;Acc:ZDB-GENE-040801-82]	8	20488322	20491779		1.62	0.78	0.92	1.77	1.88	2.13
ENSDARG00000021973	st13	ST13 Hsp70 interacting protein [Source:ZFIN;Acc:ZDB-GENE-030131-5395]	3	25087605	25100726		61.33	51.28	46.53	47.07	52.97	50.51
ENSDARG00000021974	sfr1	SWI5-dependent homologous recombination repair protein 1 [Source:NCBI gene;Acc:568747]	13	24742724	24745288		0.68	1	0.9	0.52	0.88	0.52
ENSDARG00000021975	ginm1	glycoprotein integral membrane 1 [Source:NCBI gene;Acc:555775]	20	23855025	23860808		26.14	26.74	27.94	34.83	35.13	35.9
ENSDARG00000021979	mical3a		18	10884996	10921940		1.23	0.55	0.65	2.27	2.19	2.06
ENSDARG00000021982	sh3bp2	SH3-domain binding protein 2 [Source:ZFIN;Acc:ZDB-GENE-091204-268]	10	15224660	15241223		0.62	0.91	0.71	0.84	1.05	0.86
ENSDARG00000021984	ndufa2	NADH:ubiquinone oxidoreductase subunit A2 [Source:ZFIN;Acc:ZDB-GENE-050522-377]	21	44005521	44009169		122.83	155.19	178.03	93.55	91.79	101.55
ENSDARG00000021985	ercc2	excision repair cross-complementation group 2 [Source:ZFIN;Acc:ZDB-GENE-040426-997]	15	23680018	23692359		5.9	4.46	4.79	5.08	5.54	5.77
ENSDARG00000021987	plecb	plectin b [Source:ZFIN;Acc:ZDB-GENE-100917-2]	16	5251768	5497474		20.5	20.01	13.85	24.84	23.05	22.62
ENSDARG00000021991	erlin1	ER lipid raft associated 1 [Source:NCBI gene;Acc:541490]	13	24701406	24717618		24.91	27.24	21.97	23.62	22.58	22.63
ENSDARG00000021996	ppp2r2aa	protein phosphatase 2, regulatory subunit B, alpha a [Source:ZFIN;Acc:ZDB-GENE-130530-565]	8	5233450	5267442		9.23	7.51	5.81	10.7	10.87	11.23
ENSDARG00000021997	cfap206	cilia and flagella associated protein 206 [Source:NCBI gene;Acc:402910]	20	2648405	2667902		0.55	0.34	0.44	0.61	1	0.79
ENSDARG00000022000	traf3	TNF receptor-associated factor 3 [Source:ZFIN;Acc:ZDB-GENE-040801-257]	17	29227502	29249258		3.76	1.98	1.99	3.61	4.34	3.97
ENSDARG00000022007	zgc:66426	zgc:66426 [Source:ZFIN;Acc:ZDB-GENE-040426-1639]	13	24679674	24688122		0.31	0.37	0.25	0.3	0.44	0.42
ENSDARG00000022020	focad	focadhesin [Source:ZFIN;Acc:ZDB-GENE-080213-1]	7	18878973	18983431		1.9	1.83	1.43	2.23	1.97	1.75
ENSDARG00000022045	map1ab	microtubule-associated protein 1Ab [Source:ZFIN;Acc:ZDB-GENE-100426-3]	25	19356587	19433244		6.52	2.77	2.97	6.14	6.95	6.6
ENSDARG00000022048	tlr4bb	toll-like receptor 4b, duplicate b [Source:ZFIN;Acc:ZDB-GENE-040219-9]	13	18523661	18528862		0.01	0.02	0.01	0	0	0
ENSDARG00000022085	si:dkey-34e4.1	si:dkey-34e4.1 [Source:ZFIN;Acc:ZDB-GENE-070705-456]	5	33537789	33607062		4.55	2.57	2.12	4.19	3.3	3.75
ENSDARG00000022098	opn4a	opsin 4a (melanopsin) [Source:NCBI gene;Acc:571624]	13	22421151	22464121		0.62	0	0.12	0.6	0.52	0.59
ENSDARG00000022101	obscnb	obscurin, cytoskeletal calmodulin and titin-interacting RhoGEF b [Source:ZFIN;Acc:ZDB-GENE-070119-5]	24	35933869	36002202		3.84	4.58	3.32	8.36	4.85	4.83
ENSDARG00000022129	rbm4.3	RNA binding motif protein 4.3 [Source:ZFIN;Acc:ZDB-GENE-040426-1938]	7	22792132	22795637		47.69	37.5	31.99	69.25	64.38	66.25
ENSDARG00000022139	ocstamp	osteoclast stimulatory transmembrane protein [Source:ZFIN;Acc:ZDB-GENE-050327-65]	8	28695914	28703318		0.06	0.06	0.1	0.16	0.21	0.15
ENSDARG00000022160	ttc23	tetratricopeptide repeat domain 23 [Source:ZFIN;Acc:ZDB-GENE-050419-126]	18	20825375	20853748		1.74	1.67	1.7	1.26	1.47	1.4
ENSDARG00000022165	mgst1.2	microsomal glutathione S-transferase 1.2 [Source:ZFIN;Acc:ZDB-GENE-040704-59]	4	12718965	12725513		30.34	39.76	37.09	15.95	16.79	15.06
ENSDARG00000022176	IGLON5	zgc:110372 [Source:ZFIN;Acc:ZDB-GENE-050417-297]	16	50434668	50824469	IGLON5	5.55	1.47	2.54	11	10.89	10.9
ENSDARG00000022177	ddx59	DEAD (Asp-Glu-Ala-Asp) box polypeptide 59 [Source:ZFIN;Acc:ZDB-GENE-050208-665]	22	22417751	22430191		2.57	1.54	1.84	2.25	2.5	2.28
ENSDARG00000022183	gsto1	glutathione S-transferase omega 1 [Source:ZFIN;Acc:ZDB-GENE-040718-365]	13	25380432	25389051		7.1	11.24	8.69	1.61	1.53	1.41
ENSDARG00000022187	usp6nl	USP6 N-terminal like [Source:ZFIN;Acc:ZDB-GENE-041210-192]	4	23771034	23873924		3.68	2.22	1.75	4.83	4.97	4.77
ENSDARG00000022203	gtf2ird1	GTF2I repeat domain containing 1 [Source:ZFIN;Acc:ZDB-GENE-010328-18]	5	59494079	59550943		6.18	2.59	2.96	7.23	6.98	7.32
ENSDARG00000022213	uck2b	uridine-cytidine kinase 2b [Source:NCBI gene;Acc:327057]	2	6051916	6065416		16.74	15.2	16.1	16.42	15.82	17.76
ENSDARG00000022218	uck1	uridine-cytidine kinase 1 [Source:NCBI gene;Acc:447928]	21	4227260	4244514		3.5	1.82	2.64	3.51	3.01	3.49
ENSDARG00000022232	ppan	peter pan homolog [Source:ZFIN;Acc:ZDB-GENE-030114-4]	3	54553931	54567961		14.93	19.27	17.26	11.43	10.85	12.21
ENSDARG00000022254	prkcbb	protein kinase C, beta b [Source:NCBI gene;Acc:393953]	3	35005062	35266060		5.56	1.86	1.91	5.52	5.25	5.44
ENSDARG00000022260	si:ch211-57i17.1	si:ch211-57i17.1 [Source:ZFIN;Acc:ZDB-GENE-041014-259]	20	46741074	46759519		12.95	11.45	11.67	12.89	13.85	13.7
ENSDARG00000022261	pdzk1	PDZ domain containing 1 [Source:ZFIN;Acc:ZDB-GENE-031222-1]	9	5862040	5892075		38.66	64.4	40.5	12.55	13.85	13.46
ENSDARG00000022271	trim35-34	tripartite motif containing 35-34 [Source:ZFIN;Acc:ZDB-GENE-081030-23]	22	36692891	36699718		0.05	0	0.01	0	0.04	0.05
ENSDARG00000022280	brd2a	bromodomain containing 2a [Source:ZFIN;Acc:ZDB-GENE-990415-248]	19	7152966	7170674		15.28	9.97	9.45	16.59	15.74	16.88
ENSDARG00000022295	hnf1bb	HNF1 homeobox Bb [Source:NCBI gene;Acc:324283]	21	38817785	38840667		1.37	1.88	0.75	2.03	2.22	2.16
ENSDARG00000022303	higd1a	HIG1 hypoxia inducible domain family, member 1A [Source:ZFIN;Acc:ZDB-GENE-030826-15]	2	2168547	2175629		3.39	3.84	3.57	7.99	8.24	7.89
ENSDARG00000022309	dspa	desmoplakin a [Source:ZFIN;Acc:ZDB-GENE-030131-2743]	2	2110271	2162791		24.67	21.83	16.39	20.8	21.25	20.69
ENSDARG00000022315	atp6v1g1	ATPase H+ transporting V1 subunit G1 [Source:ZFIN;Acc:ZDB-GENE-030131-6965]	5	57320113	57325557		205.08	183.82	183.69	154.27	156.54	158.08
ENSDARG00000022319	or115-5	odorant receptor, family F, subfamily 115, member 5 [Source:ZFIN;Acc:ZDB-GENE-070806-7]	21	40287100	40289652		0.02	0	0	0	0	0
ENSDARG00000022334	si:ch211-156l18.7	si:ch211-156l18.7 [Source:ZFIN;Acc:ZDB-GENE-130530-566]	11	12175162	12184543		0	0.01	0	0.02	0.01	0.01
ENSDARG00000022340	ufc1	ubiquitin-fold modifier conjugating enzyme 1 [Source:NCBI gene;Acc:445256]	5	57714902	57718850		8.2	9.39	7.01	7.88	7.32	7.27
ENSDARG00000022350	nek1	NIMA-related kinase 1 [Source:ZFIN;Acc:ZDB-GENE-040730-1]	20	23670494	23714017		5.38	2.52	2.44	5.4	5.48	5.32
ENSDARG00000022372	kng1	kininogen 1 [Source:ZFIN;Acc:ZDB-GENE-041010-56]	22	36839080	36876133		34.2	47.08	41.83	27.96	30.4	30.65
ENSDARG00000022378	mtmr1b	myotubularin related protein 1b [Source:ZFIN;Acc:ZDB-GENE-050327-48]	21	34088110	34118530		1.57	1.55	1.56	1.14	1.17	0.99
ENSDARG00000022399	ntmt1	N-terminal Xaa-Pro-Lys N-methyltransferase 1 [Source:NCBI gene;Acc:405834]	5	32499070	32505280		11.39	12.75	12.89	11.31	10.99	10.39
ENSDARG00000022410	rrp12	ribosomal RNA processing 12 homolog [Source:ZFIN;Acc:ZDB-GENE-050706-182]	13	31343326	31370184		25.79	28.34	25.84	16.93	16.95	15.94
ENSDARG00000022413	ing5a	inhibitor of growth family, member 5a [Source:ZFIN;Acc:ZDB-GENE-031016-1]	22	2949111	3092136		13.82	10.59	10.51	10.08	11.81	12.38
ENSDARG00000022418	faf1	Fas (TNFRSF6) associated factor 1 [Source:ZFIN;Acc:ZDB-GENE-040426-2863]	8	16274778	16406440		13.07	10.65	9.57	12.88	12.89	13.23
ENSDARG00000022424	slc26a5	solute carrier family 26 member 5 [Source:ZFIN;Acc:ZDB-GENE-030131-1566]	4	14932537	14954327		8.68	9.76	9.59	19.79	22.31	21.71
ENSDARG00000022430	ppp1r8b	protein phosphatase 1, regulatory subunit 8b [Source:ZFIN;Acc:ZDB-GENE-040426-1271]	16	4513597	4531657		5.22	3.86	3.06	5.63	5.62	5.47
ENSDARG00000022437	cd81b	CD81 molecule b [Source:ZFIN;Acc:ZDB-GENE-040808-52]	18	27318589	27336157		12.98	12.44	14.75	10.48	10.51	9.86
ENSDARG00000022438	cox6a1	cytochrome c oxidase subunit 6A1 [Source:ZFIN;Acc:ZDB-GENE-030131-7715]	8	39760258	39765996		245.62	303.16	334.11	171.49	172.21	165.1
ENSDARG00000022456	eno1a	enolase 1a, (alpha) [Source:ZFIN;Acc:ZDB-GENE-030131-6048]	23	22656477	22678882		169.31	134.14	161.06	237.29	271.53	254.61
ENSDARG00000022466	clcn5b	chloride channel, voltage-sensitive 5b [Source:ZFIN;Acc:ZDB-GENE-110407-11]	7	22853788	22900344		0.91	0.86	0.58	1.45	1.45	1.39
ENSDARG00000022493	rad21l1	RAD21 cohesin complex component like 1 [Source:ZFIN;Acc:ZDB-GENE-061103-505]	8	49117506	49140645		0.07	0.04	0.07	0.1	0.07	0.15
ENSDARG00000022503	pkd2l1	polycystic kidney disease 2-like 1 [Source:ZFIN;Acc:ZDB-GENE-030616-558]	13	25455319	25471376		1.25	0.61	0.91	3.72	3.94	3.98
ENSDARG00000022509	cox4i2	cytochrome c oxidase subunit 4I2 [Source:ZFIN;Acc:ZDB-GENE-040426-1775]	23	18944388	18951148		36.18	26.56	28.24	30.92	33.78	37.01
ENSDARG00000022512	zgc:109913	zgc:109913 [Source:NCBI gene;Acc:553549]	6	55282629	55286872		0.47	0.36	0.26	0.35	0.43	0.63
ENSDARG00000022518	pex5la	peroxisomal biogenesis factor 5-like a [Source:ZFIN;Acc:ZDB-GENE-070705-298]	6	29476933	29612269		1.02	0.48	0.54	1.21	1.4	1.44
ENSDARG00000022525	mchr1b	melanin-concentrating hormone receptor 1b [Source:ZFIN;Acc:ZDB-GENE-030502-5]	3	25503442	25512817		0.2	0.08	0.05	0.19	0.38	0.23
ENSDARG00000022531	ntn1b	netrin 1b [Source:ZFIN;Acc:ZDB-GENE-990415-168]	3	25708549	25814097		4.13	1.39	1.99	11.27	10.16	10.82
ENSDARG00000022532	chrnb1l	cholinergic receptor, nicotinic, beta 1 (muscle) like [Source:ZFIN;Acc:ZDB-GENE-060811-1]	7	22585447	22608687		0.08	0.06	0.08	0.14	0.07	0.12
ENSDARG00000022550	gxylt1b	glucoside xylosyltransferase 1b [Source:NCBI gene;Acc:556315]	4	13892773	13902188		1.78	1.32	1.54	1.83	1.7	1.75
ENSDARG00000022560	clcnk	chloride channel K [Source:ZFIN;Acc:ZDB-GENE-040426-1379]	23	24272421	24291927		0.44	0.41	0.36	1.67	1.67	1.66
ENSDARG00000022564	C18H11orf58	zgc:56106 [Source:NCBI gene;Acc:393163]	18	27309404	27316599	C18H11orf58	11.81	13.05	11.56	8.92	8.68	8.78
ENSDARG00000022569	nkx6.1	NK6 homeobox 1 [Source:ZFIN;Acc:ZDB-GENE-040718-178]	21	19070921	19073831		3.94	1.31	2.17	6.81	6.15	7.02
ENSDARG00000022570	nmur3	neuromedin U receptor 3 [Source:ZFIN;Acc:ZDB-GENE-130530-567]	11	28107632	28126139		0.41	0.16	0.15	0.5	0.52	0.56
ENSDARG00000022599	dnpep	aspartyl aminopeptidase [Source:ZFIN;Acc:ZDB-GENE-040426-852]	6	13896264	13920394		21.72	29.79	27.21	13.31	13.8	13.18
ENSDARG00000022614	ddx43	DEAD (Asp-Glu-Ala-Asp) box polypeptide 43 [Source:ZFIN;Acc:ZDB-GENE-080204-77]	13	27332656	27354033		3.29	3.84	3.1	1.21	0.7	0.95
ENSDARG00000022615	si:ch211-241e1.3	si:ch211-241e1.3 [Source:ZFIN;Acc:ZDB-GENE-030131-230]	2	944341	985417		2.61	2.37	2.39	7.19	7.06	6.36
ENSDARG00000022623	fbxo44	F-box protein 44 [Source:ZFIN;Acc:ZDB-GENE-041212-85]	23	16748806	16757755		10.66	13.3	11.21	10.74	11.69	11.34
ENSDARG00000022631	cyp2p9	cytochrome P450, family 2, subfamily P, polypeptide 9 [Source:ZFIN;Acc:ZDB-GENE-040120-2]	20	25581627	25584869		14.76	25.97	17.18	6.93	6.04	6.41
ENSDARG00000022648	otud3	OTU deubiquitinase 3 [Source:ZFIN;Acc:ZDB-GENE-040426-2509]	23	39611688	39626681		7.37	5.84	6.39	5.38	5.91	5.63
ENSDARG00000022650	cyp2ad3	cytochrome P450, family 2, subfamily AD, polypeptide 3 [Source:ZFIN;Acc:ZDB-GENE-041001-156]	20	25548425	25551676		42.47	49.26	46.9	23.78	23.37	25.08
ENSDARG00000022652	psmf1	proteasome inhibitor subunit 1 [Source:ZFIN;Acc:ZDB-GENE-040718-282]	6	51796296	51811095		8.01	5.3	5.6	5.52	6.37	6.33
ENSDARG00000022659	snx4	sorting nexin 4 [Source:ZFIN;Acc:ZDB-GENE-030131-1923]	9	38588081	38621202		16.01	14.9	14.77	17.23	16.33	15.93
ENSDARG00000022660	armc2	armadillo repeat containing 2 [Source:ZFIN;Acc:ZDB-GENE-041014-7]	20	32228210	32270866		0.83	0.56	0.48	0.93	0.65	0.92
ENSDARG00000022668	grapb	GRB2 related adaptor protein b [Source:ZFIN;Acc:ZDB-GENE-050913-4]	1	8165340	8192418		0.98	0.32	0.58	0.73	0.84	0.77
ENSDARG00000022670	he1.3	hatching enzyme 1, tandem duplicate 3 [Source:ZFIN;Acc:ZDB-GENE-040518-1]	22	14102397	14103633		0	0	0	0	0.05	0
ENSDARG00000022684	fkbp1aa	FKBP prolyl isomerase 1Aa [Source:ZFIN;Acc:ZDB-GENE-030131-7275]	8	49169768	49172500		326.54	366.88	410.44	283.04	293	291.54
ENSDARG00000022689	itgb1b.2	integrin, beta 1b.2 [Source:ZFIN;Acc:ZDB-GENE-040426-2598]	2	43638983	43653328		13.81	17.32	14.76	7.28	6.06	5.61
ENSDARG00000022690	chic2	cysteine-rich hydrophobic domain 2 [Source:ZFIN;Acc:ZDB-GENE-040426-778]	1	14406098	14416435		9.21	6.95	6.88	9.75	9.7	9.59
ENSDARG00000022698	lad1	ladinin [Source:ZFIN;Acc:ZDB-GENE-030616-268]	23	5687972	5719453		5.88	7.46	5.99	2.55	2.66	3.12
ENSDARG00000022712	stat3	signal transducer and activator of transcription 3 (acute-phase response factor) [Source:ZFIN;Acc:ZDB-GENE-980526-68]	3	17030665	17107445		9.31	7.66	6.62	23.43	24.46	24.36
ENSDARG00000022718	casp8ap2	caspase 8 associated protein 2 [Source:ZFIN;Acc:ZDB-GENE-030826-8]	20	23947004	23956014		4.49	3.14	2.83	3.99	4.58	3.72
ENSDARG00000022727	epha2b	eph receptor A2 b [Source:ZFIN;Acc:ZDB-GENE-030219-187]	23	24351304	24394719		10.64	8.48	6.31	11.32	11.81	10.68
ENSDARG00000022730	aasdh	aminoadipate-semialdehyde dehydrogenase [Source:NCBI gene;Acc:570338]	20	25645459	25659372		1.99	2.58	2.62	3.11	3.52	3.17
ENSDARG00000022763	hpcal1	hippocalcin-like 1 [Source:ZFIN;Acc:ZDB-GENE-040426-1242]	20	31232923	31308805		4.38	4.16	3.95	6.36	6.19	6.19
ENSDARG00000022767	apobb.1	apolipoprotein Bb, tandem duplicate 1 [Source:ZFIN;Acc:ZDB-GENE-030131-9732]	20	31269778	31285980		395.08	650.06	502.59	504.61	545.94	497.38
ENSDARG00000022768	gid8a	GID complex subunit 8 homolog a (S. cerevisiae) [Source:NCBI gene;Acc:393685]	8	23147218	23154955		4.14	2.63	2.84	4.1	3.63	3.54
ENSDARG00000022772	OSBP2	si:ch211-106a19.1 [Source:ZFIN;Acc:ZDB-GENE-060526-3]	5	24571750	24712405	OSBP2	6.4	3.68	3.38	6.11	6.34	6.66
ENSDARG00000022788	cops7a	COP9 constitutive photomorphogenic homolog subunit 7A [Source:ZFIN;Acc:ZDB-GENE-030131-8680]	16	21751922	21777545		29.9	27.74	29.1	24.69	23.85	25.93
ENSDARG00000022795	golt1ba	golgi transport 1Ba [Source:ZFIN;Acc:ZDB-GENE-041210-157]	4	16787040	16792196		4.08	4.68	4.58	4.78	4.49	4.05
ENSDARG00000022807	pitpnc1b	phosphatidylinositol transfer protein cytoplasmic 1b [Source:ZFIN;Acc:ZDB-GENE-041114-158]	16	13424534	13453818		7.83	8.81	8.1	7.94	7.62	6.46
ENSDARG00000022810	fzr1b	fizzy/cell division cycle 20 related 1b [Source:ZFIN;Acc:ZDB-GENE-131120-69]	2	6126086	6149293		12.63	6.41	8.81	10.72	10.62	11.79
ENSDARG00000022813	dnd1	DND microRNA-mediated repression inhibitor 1 [Source:NCBI gene;Acc:373074]	14	7397804	7409364		0.04	0.05	0.05	0.19	0.15	0.13
ENSDARG00000022817	pvalb3	parvalbumin 3 [Source:ZFIN;Acc:ZDB-GENE-040426-945]	12	17705218	17707517		23.29	29.5	37.06	18.12	18.03	18.18
ENSDARG00000022832	bnip4	BCL2 interacting protein 4 [Source:ZFIN;Acc:ZDB-GENE-051113-212]	17	20231801	20236309		2.27	1.79	2.07	6.05	4.58	8.59
ENSDARG00000022841	ptk2ba	protein tyrosine kinase 2 beta, a [Source:ZFIN;Acc:ZDB-GENE-080207-1]	17	6113680	6202816		0.44	0.33	0.39	0.57	0.64	0.53
ENSDARG00000022845	lias	lipoic acid synthetase [Source:NCBI gene;Acc:393528]	1	23274710	23283468		9.75	10.77	10.71	9.46	10.37	9.61
ENSDARG00000022853	eogt	EGF domain-specific O-linked N-acetylglucosamine (GlcNAc) transferase [Source:ZFIN;Acc:ZDB-GENE-120813-8]	11	17726649	17755444		1.75	1.27	1.31	2.24	2.02	2.26
ENSDARG00000022858	wnt7ba	wingless-type MMTV integration site family, member 7Ba [Source:ZFIN;Acc:ZDB-GENE-041210-178]	4	18702129	18713979		0.21	0.33	0.24	0.17	0.24	0.45
ENSDARG00000022891	zgc:175214	zgc:175214 [Source:ZFIN;Acc:ZDB-GENE-080303-32]	1	49481203	49498116		17.62	9.78	11.11	16.41	15.99	17.01
ENSDARG00000022895	mcf2a	MCF.2 cell line derived transforming sequence a [Source:ZFIN;Acc:ZDB-GENE-030616-52]	14	32440639	32503363		1.29	0.44	0.56	0.99	0.98	0.89
ENSDARG00000022904	skilb	SKI-like proto-oncogene b [Source:ZFIN;Acc:ZDB-GENE-080723-40]	24	26373341	26386627		8.59	3.58	3.64	9.96	11.17	10.89
ENSDARG00000022905	znf1163	zinc finger protein 1163 [Source:ZFIN;Acc:ZDB-GENE-060503-151]	22	2715815	2718913		3.43	1.59	1.88	2.35	3.29	2.57
ENSDARG00000022918	tbcc	tubulin folding cofactor C [Source:ZFIN;Acc:ZDB-GENE-041008-103]	12	34891529	34892823		8.63	8.26	9.52	5.15	5.82	5.25
ENSDARG00000022938	taar11	trace amine-associated receptor 11 [Source:ZFIN;Acc:ZDB-GENE-041014-52]	20	46131812	46132911		0.06	0.04	0.09	0	0.07	0.11
ENSDARG00000022939	ino80e	INO80 complex subunit E [Source:ZFIN;Acc:ZDB-GENE-040426-1818]	16	10413551	10422490		11.66	11.18	9.98	7.33	7.72	8.31
ENSDARG00000022944	ecd	ecdysoneless homolog (Drosophila) [Source:ZFIN;Acc:ZDB-GENE-030131-6304]	17	20188704	20202725		5.76	4.45	4.21	4.27	4.31	5.14
ENSDARG00000022951	pth2	parathyroid hormone 2 [Source:NCBI gene;Acc:402812]	17	33495194	33499023		1.83	1.1	1.12	1.16	0.87	1.49
ENSDARG00000022952	zgc:66448	zgc:66448 [Source:ZFIN;Acc:ZDB-GENE-030131-5612]	16	25107344	25115472		12.57	7.9	9.31	10.62	10.9	10.57
ENSDARG00000022968	fxr1	fragile X mental retardation, autosomal homolog 1 [Source:ZFIN;Acc:ZDB-GENE-030131-5431]	22	37542741	37565410		28.89	33.34	34.64	35.73	36.8	37.07
ENSDARG00000022970	tm9sf1	transmembrane 9 superfamily member 1 [Source:ZFIN;Acc:ZDB-GENE-040801-64]	24	11887885	11908115		6.01	5.32	4.22	6.34	6.28	6.03
ENSDARG00000022971	epha6	eph receptor A6 [Source:ZFIN;Acc:ZDB-GENE-090311-43]	1	33969015	34201195		3.44	1.03	1.31	3.11	3.53	3.29
ENSDARG00000022974	wdr13	WD repeat domain 13 [Source:ZFIN;Acc:ZDB-GENE-050522-274]	8	6920834	6943190		15.75	11.99	12.16	12.64	11.2	11.53
ENSDARG00000022978	tmpob	thymopoietin b [Source:ZFIN;Acc:ZDB-GENE-050522-8]	25	3294150	3302666		30.35	13.92	14.16	25.93	25.98	25.78
ENSDARG00000022983	ccdc28a	coiled-coil domain containing 28A [Source:ZFIN;Acc:ZDB-GENE-030131-5007]	17	33409038	33416020		0.25	0.21	0.09	0.26	0.24	0.26
ENSDARG00000022995	clic4	chloride intracellular channel 4 [Source:ZFIN;Acc:ZDB-GENE-030326-3]	17	25444323	25480375		16.04	15.56	13.92	28.24	29.11	28.42
ENSDARG00000022996	setx	senataxin [Source:ZFIN;Acc:ZDB-GENE-070402-1]	21	3928947	3973165		3.55	3.48	2.7	3.5	3.56	3.52
ENSDARG00000023002	dtl	denticleless E3 ubiquitin protein ligase homolog (Drosophila) [Source:NCBI gene;Acc:192314]	20	13141408	13174165		2	1.73	1.76	1.38	1.6	1.21
ENSDARG00000023003	ccdc12	coiled-coil domain containing 12 [Source:ZFIN;Acc:ZDB-GENE-040801-108]	16	16529748	16554130		12.94	16.37	14.39	10.24	9.4	10.19
ENSDARG00000023026	pkp2	plakophilin 2 [Source:ZFIN;Acc:ZDB-GENE-041210-167]	4	16710001	16725144		1.76	1.43	1.07	1.72	1.79	1.7
ENSDARG00000023028	cdv3	carnitine deficiency-associated gene expressed in ventricle 3 [Source:NCBI gene;Acc:334102]	24	9881219	9893794		45.02	46.76	40.28	41.28	40.82	40.71
ENSDARG00000023031	hoxa2b	homeobox A2b [Source:NCBI gene;Acc:30325]	16	20926673	20929183		1.97	1.67	1.71	4.04	5.05	4.42
ENSDARG00000023036	si:dkey-23a23.2	si:dkey-23a23.2 [Source:ZFIN;Acc:ZDB-GENE-060503-158]	4	29032643	29050583		0.81	0.55	0.57	0.73	0.81	0.73
ENSDARG00000023040	zbtb1	zinc finger and BTB domain containing 1 [Source:ZFIN;Acc:ZDB-GENE-041001-126]	20	27712714	27720098		2.54	2.21	2.04	2.54	2.62	2.34
ENSDARG00000023053	niban1b	niban apoptosis regulator 1b [Source:ZFIN;Acc:ZDB-GENE-070912-688]	2	23295415	23349116		1.03	1.07	0.89	1.51	1.49	1.43
ENSDARG00000023058	foxo3a	forkhead box O3A [Source:ZFIN;Acc:ZDB-GENE-050116-1]	17	6793001	6810785		29.16	8.69	9.14	29.39	28.87	29.6
ENSDARG00000023062	ccn1	cellular communication network factor 1 [Source:ZFIN;Acc:ZDB-GENE-040426-3]	23	45456490	45465320		4.53	5.03	4.4	15.36	15.53	15.83
ENSDARG00000023082	krt1-19d	keratin, type 1, gene 19d [Source:ZFIN;Acc:ZDB-GENE-060316-1]	19	5324653	5332784		169.53	147.17	170.98	41.06	46.91	44.32
ENSDARG00000023104	scaper	S-phase cyclin A-associated protein in the ER [Source:ZFIN;Acc:ZDB-GENE-060303-5]	25	32525131	32739724		6.94	3.97	3.37	8.49	7.76	8.52
ENSDARG00000023110	mapk7	mitogen-activated protein kinase 7 [Source:ZFIN;Acc:ZDB-GENE-050320-10]	12	4220353	4238827		5.25	3.06	3.25	4.9	4.9	4.99
ENSDARG00000023111	plg	plasminogen [Source:ZFIN;Acc:ZDB-GENE-030131-1411]	20	42688488	42702884		69.11	99.19	87.51	62.81	67.63	69.45
ENSDARG00000023117	gopc	golgi-associated PDZ and coiled-coil motif containing [Source:ZFIN;Acc:ZDB-GENE-030131-5086]	20	42246948	42281434		9.15	5.64	5.84	8.5	8.66	9.05
ENSDARG00000023151	ucp1	uncoupling protein 1 [Source:ZFIN;Acc:ZDB-GENE-010503-1]	1	53374454	53388914		64.23	93.84	79.47	65.24	69.14	71.55
ENSDARG00000023152	wdr73	WD repeat domain 73 [Source:NCBI gene;Acc:550339]	21	21819878	21824285		4.25	3.71	3.39	3.74	3.17	3.93
ENSDARG00000023157	cbln10	cerebellin 10 [Source:ZFIN;Acc:ZDB-GENE-030131-4615]	2	37953571	37956768		4.79	7.99	5.24	6.24	6.76	5.52
ENSDARG00000023160	snrnp40	small nuclear ribonucleoprotein 40 (U5) [Source:ZFIN;Acc:ZDB-GENE-040426-978]	19	43004408	43016395		15.31	14.15	15.57	14.37	14.69	13.12
ENSDARG00000023168	olfcv1	olfactory receptor C family, v1 [Source:ZFIN;Acc:ZDB-GENE-050419-263]	18	33265514	33268439		0.07	0.02	0.03	0	0.01	0.01
ENSDARG00000023174	fez1	fasciculation and elongation protein zeta 1 (zygin I) [Source:ZFIN;Acc:ZDB-GENE-040426-2723]	10	31738501	31782616		56.29	17.83	25.87	57.09	59.58	63.46
ENSDARG00000023176	tdo2b	tryptophan 2,3-dioxygenase b [Source:NCBI gene;Acc:334082]	11	34084532	34097437		7.96	8.05	5.93	1.49	1.3	1.63
ENSDARG00000023181	pcp4l1	Purkinje cell protein 4 like 1 [Source:ZFIN;Acc:ZDB-GENE-040724-35]	2	37995176	38000276		25.78	16.48	12.79	17.48	19.12	20.33
ENSDARG00000023185	si:rp71-1g18.13	si:rp71-1g18.13 [Source:ZFIN;Acc:ZDB-GENE-040724-111]	2	37975026	37990505		3.24	3.88	3.27	1.83	1.63	2.04
ENSDARG00000023188	lcp1	lymphocyte cytosolic protein 1 (L-plastin) [Source:NCBI gene;Acc:30583]	9	56251000	56272465		11.87	13.21	13.41	14.15	14.51	14.25
ENSDARG00000023190	kpna4	karyopherin alpha 4 (importin alpha 3) [Source:ZFIN;Acc:ZDB-GENE-030131-5584]	15	1596508	1622485		37.68	24.15	23.56	39.57	40.47	40.85
ENSDARG00000023203	mhc1lda	major histocompatibility complex class I LDA [Source:ZFIN;Acc:ZDB-GENE-140820-5]	25	10953351	10961324		0	0	0	0.03	0.01	0.07
ENSDARG00000023214	mob2a	MOB kinase activator 2a [Source:ZFIN;Acc:ZDB-GENE-040718-56]	25	22863028	22889519		9.91	10.21	9.3	12.97	12.15	12.37
ENSDARG00000023217	crema	cAMP responsive element modulator a [Source:ZFIN;Acc:ZDB-GENE-030131-7031]	2	43159171	43196595		11.95	5.96	5.72	20.28	21.6	22.39
ENSDARG00000023220	selenot2	selenoprotein T, 2 [Source:NCBI gene;Acc:352917]	14	17580571	17588345		31.36	25.34	23.75	36.62	37.03	35.97
ENSDARG00000023228	vsnl1a	visinin-like 1a [Source:ZFIN;Acc:ZDB-GENE-041001-211]	20	35382482	35429737		36.47	13.79	18.25	33.75	34.57	33.1
ENSDARG00000023236	kcnh1b	potassium voltage-gated channel, subfamily H (eag-related), member 1b [Source:ZFIN;Acc:ZDB-GENE-121128-4]	22	37902976	37952513		0.71	0.28	0.38	1.38	1.5	1.55
ENSDARG00000023237	syne3	spectrin repeat containing, nuclear envelope family member 3 [Source:ZFIN;Acc:ZDB-GENE-070209-271]	17	15899039	15966746		6.21	6.08	4.84	6.07	5.66	5.22
ENSDARG00000023267	atad1a	ATPase family AAA domain containing 1a [Source:NCBI gene;Acc:368672]	5	16677264	16791223		2.98	1.69	1.79	3.05	3.1	2.93
ENSDARG00000023272	tfap2d	transcription factor AP-2 delta (activating enhancer binding protein 2 delta) [Source:NCBI gene;Acc:566838]	20	47717389	47732703		4.74	1.43	1.79	4.15	4.24	4.87
ENSDARG00000023279	psmd4b	proteasome 26S subunit, non-ATPase 4b [Source:ZFIN;Acc:ZDB-GENE-050417-96]	16	9762261	9777928		33.51	33.37	34.3	27.78	29.09	28.62
ENSDARG00000023287	hsd17b3	hydroxysteroid (17-beta) dehydrogenase 3 [Source:ZFIN;Acc:ZDB-GENE-040426-1339]	8	29852353	29876413		0.46	0.21	0.24	0.71	0.73	0.57
ENSDARG00000023290	fabp3	fatty acid binding protein 3, muscle and heart [Source:ZFIN;Acc:ZDB-GENE-020318-2]	19	42955280	42993306		303.42	345.65	375.33	191.34	196.6	203.85
ENSDARG00000023298	rps27.1	ribosomal protein S27, isoform 1 [Source:ZFIN;Acc:ZDB-GENE-050522-549]	19	8743260	8748571		1093.5	1295.94	1580.82	854.97	883.27	913.76
ENSDARG00000023299	snu13b	SNU13 homolog, small nuclear ribonucleoprotein b (U4/U6.U5) [Source:ZFIN;Acc:ZDB-GENE-030131-9670]	12	10443785	10447178		64.86	88.4	91.01	40.11	37.32	40.04
ENSDARG00000023302	gpbar1	G protein-coupled bile acid receptor 1 [Source:ZFIN;Acc:ZDB-GENE-120315-1]	9	47961482	47970760		0.22	0.13	0.07	0.17	0.14	0.15
ENSDARG00000023303	zgc:66427	zgc:66427 [Source:ZFIN;Acc:ZDB-GENE-040426-1913]	2	37866880	37874647		8.64	6.58	5.97	9.56	8.78	8.86
ENSDARG00000023309	spryd4	SPRY domain containing 4 [Source:ZFIN;Acc:ZDB-GENE-040718-180]	8	21159122	21162762		15.02	20.62	22.37	14.26	14.6	14.45
ENSDARG00000023316	sim1a	SIM bHLH transcription factor 1a [Source:NCBI gene;Acc:260351]	16	1465624	1503023		0.71	0.48	0.31	1.31	1.28	1.33
ENSDARG00000023318	fmn2a	formin 2a [Source:ZFIN;Acc:ZDB-GENE-101107-1]	17	19753559	19828505		1.77	0.97	0.91	1.59	1.57	1.74
ENSDARG00000023323	ywhaqb	tyrosine 3-monooxygenase/tryptophan 5-monooxygenase activation protein, theta polypeptide b [Source:ZFIN;Acc:ZDB-GENE-030	17	32374876	32527320		117.61	86.89	94.88	108.87	110.1	110.06
ENSDARG00000023330	anp32b	acidic (leucine-rich) nuclear phosphoprotein 32 family, member B [Source:ZFIN;Acc:ZDB-GENE-030131-719]	1	26660369	26664840		72.64	68.32	72.15	60.23	60.87	61.45
ENSDARG00000023362	nr5a1b	nuclear receptor subfamily 5, group A, member 1b [Source:ZFIN;Acc:ZDB-GENE-040702-6]	21	8276989	8317588		0.24	0.05	0.1	0.56	0.44	0.62
ENSDARG00000023369	mxd	myxovirus (influenza virus) resistance D [Source:ZFIN;Acc:ZDB-GENE-030721-8]	25	19718504	19731255		0.21	0.2	0.18	0.35	0.2	0.29

ENSDARG00000023394	slc37a2	solute carrier family 37 member 2 [Source:NCBI gene;Acc:406287]	5	58455488	58483036		14.01	12.68	12.05	17.5	17.47	17.33
ENSDARG00000023396	atg5	ATG5 autophagy related 5 homolog (S. cerevisiae) [Source:ZFIN;Acc:ZDB-GENE-040801-149]	16	7380463	7416532		9.94	9.16	7.3	9.37	9.07	9.4
ENSDARG00000023443	tjp2b	tight junction protein 2b (zona occludens 2) [Source:ZFIN;Acc:ZDB-GENE-040718-58]	8	11425048	11542289		16.46	12.52	12.47	17.79	18.37	18.82
ENSDARG00000023445	atp2b3b	ATPase plasma membrane Ca2+ transporting 3b [Source:ZFIN;Acc:ZDB-GENE-080409-2]	23	19834215	19953089		12.3	3.07	3.34	18.88	18.48	19.38
ENSDARG00000023448	galnt14	UDP-N-acetyl-alpha-D-galactosamine:polypeptide N-acetylgalactosaminyltransferase 14 (GalNAc-T14) [Source:ZFIN;Acc:ZDB-GEN	20	38031439	38119890		4.14	2.69	3.23	3.9	4.49	4.27
ENSDARG00000023472	ctnnb2	catenin, beta 2 [Source:ZFIN;Acc:ZDB-GENE-040426-2575]	19	47975916	47997424		61.74	56.19	53.56	88.46	86.02	82.13
ENSDARG00000023479	lrp13	low-density lipoprotein receptor related-protein 13 [Source:ZFIN;Acc:ZDB-GENE-130729-1]	5	9179578	9216758		0.01	0.03	0.02	0.01	0.02	0.02
ENSDARG00000023484	rflna	refilin A [Source:ZFIN;Acc:ZDB-GENE-030616-80]	8	43340995	43363590		4.54	4.9	3.35	6.5	6.78	6.23
ENSDARG00000023495	ift74	intraflagellar transport 74 [Source:NCBI gene;Acc:436658]	5	457729	478627		17.08	10.01	13.06	21.6	22.61	20.72
ENSDARG00000023498	gmppab	GDP-mannose pyrophosphorylase Ab [Source:NCBI gene;Acc:393469]	6	13506841	13514230		5.63	6.42	5.4	7.98	6.37	6.81
ENSDARG00000023508	ankrd49	ankyrin repeat domain 49 [Source:ZFIN;Acc:ZDB-GENE-050417-46]	15	2850965	2857961		1.79	1.57	1.51	2.06	1.95	1.73
ENSDARG00000023519	BX323555.1		21	8058252	8059239		0	0	0	0.02	0.03	0.07
ENSDARG00000023526	ggt1a	gamma-glutamyltransferase 1a [Source:ZFIN;Acc:ZDB-GENE-040426-1388]	8	40042632	40075983		0.09	0.17	0.13	0.59	0.57	0.65
ENSDARG00000023532	pinx1	PIN2 (TERF1) interacting telomerase inhibitor 1 [Source:ZFIN;Acc:ZDB-GENE-030515-2]	20	19006703	19059240		19.1	26.62	21.29	13.44	11.8	12.52
ENSDARG00000023536	nnt	nicotinamide nucleotide transhydrogenase [Source:ZFIN;Acc:ZDB-GENE-040426-2592]	21	19763546	19828423		124.19	119.76	107.52	125.5	117.39	118.56
ENSDARG00000023537	ahr1b	aryl hydrocarbon receptor 1b [Source:ZFIN;Acc:ZDB-GENE-050922-1]	22	12979213	13042992		4.74	0.7	0.56	6.23	5.79	6.24
ENSDARG00000023542	cdh7a	cadherin 7a [Source:ZFIN;Acc:ZDB-GENE-061019-3]	2	27010439	27094653		5.27	1.59	1.64	7.19	7.07	6.34
ENSDARG00000023546	kcnn1b	potassium intermediate/small conductance calcium-activated channel, subfamily N, member 1b [Source:ZFIN;Acc:ZDB-GENE-0605	8	13244371	13315567		1.4	0.74	0.7	2.41	2.23	2.3
ENSDARG00000023578	lpp	LIM domain containing preferred translocation partner in lipoma [Source:ZFIN;Acc:ZDB-GENE-040426-918]	6	28294113	28690670		3.6	4.44	2.54	6.39	6.02	6
ENSDARG00000023583	coq9	coenzyme Q9 homolog (S. cerevisiae) [Source:ZFIN;Acc:ZDB-GENE-061207-37]	25	14381405	14398281		27.25	36.08	38.94	21.33	21.47	20.83
ENSDARG00000023584	cdc7	cell division cycle 7 homolog (S. cerevisiae) [Source:ZFIN;Acc:ZDB-GENE-041114-113]	2	10863942	10877765		2.04	1.76	1.78	1.79	1.49	1.63
ENSDARG00000023587	kcnk5a	potassium channel, subfamily K, member 5a [Source:ZFIN;Acc:ZDB-GENE-051113-112]	17	48683769	48705993		2.16	2.83	2.3	1.46	1.28	1.28
ENSDARG00000023591	nrbf2b	nuclear receptor binding factor 2b [Source:ZFIN;Acc:ZDB-GENE-040718-357]	12	8569685	8579727		9.84	7.01	6.55	8.98	9.29	9.3
ENSDARG00000023600	sh3gl2a	SH3 domain containing GRB2 like 2a, endophilin A1 [Source:ZFIN;Acc:ZDB-GENE-040121-3]	1	26740718	26782573		50.37	19.69	22.94	44.25	46.46	45.91
ENSDARG00000023609	AL845324.1		11	38614476	38876352		3.87	0.87	1.33	3.5	4.06	3.74
ENSDARG00000023627	ggps1	geranylgeranyl diphosphate synthase 1 [Source:ZFIN;Acc:ZDB-GENE-030131-8742]	13	49727333	49769372		7.55	5.65	4.84	10.2	9.53	9.21
ENSDARG00000023645	kyat1	kynurenine aminotransferase 1 [Source:ZFIN;Acc:ZDB-GENE-040426-2676]	5	33264842	33280699		4.8	7.11	5.5	2.79	2.74	2.76
ENSDARG00000023648	idh3g	isocitrate dehydrogenase (NAD(+)) 3 non-catalytic subunit gamma [Source:ZFIN;Acc:ZDB-GENE-050417-435]	23	25121998	25135105		24.31	26.83	24.97	22.7	21.4	22.16
ENSDARG00000023656	he1.1	hatching enzyme 1, tandem duplicate 1 [Source:ZFIN;Acc:ZDB-GENE-021211-3]	22	14093306	14094746		2.17	5.36	1.97	11.1	1.06	1.44
ENSDARG00000023659	gabpb1	GA binding protein transcription factor subunit beta 1 [Source:ZFIN;Acc:ZDB-GENE-061027-46]	18	81867	92046		4.88	2.28	3.39	5.58	6.1	6.31
ENSDARG00000023672	timm8a	translocase of inner mitochondrial membrane 8 homolog A (yeast) [Source:NCBI gene;Acc:445243]	14	38878482	38881818		13.76	19.05	18.52	10.18	10.76	9.43
ENSDARG00000023678	ercc5	excision repair cross-complementation group 5 [Source:ZFIN;Acc:ZDB-GENE-050327-28]	1	474085	494280		4.29	3.11	3.5	4.14	4.27	4.11
ENSDARG00000023683	cacna1fb	calcium channel, voltage-dependent, L type, alpha 1F subunit [Source:ZFIN;Acc:ZDB-GENE-031104-1]	8	22851472	22927224		3.97	0.45	0.3	3.74	3.47	3.58
ENSDARG00000023694	spon1b	spondin 1b [Source:ZFIN;Acc:ZDB-GENE-000427-10]	25	15647750	15800834		17.96	12.78	12.21	24.8	24.99	26.38
ENSDARG00000023703	kctd6b	potassium channel tetramerization domain containing 6b [Source:NCBI gene;Acc:436601]	23	19670085	19673769		11.41	9.54	9.52	10.11	9.73	10.61
ENSDARG00000023712	mao	monoamine oxidase [Source:NCBI gene;Acc:404730]	9	33660964	33725972		17.36	24.05	15.06	13.03	13.26	12.99
ENSDARG00000023713	aqp1a.1	aquaporin 1a (Colton blood group), tandem duplicate 1 [Source:ZFIN;Acc:ZDB-GENE-030131-7764]	2	2737422	2749333		41.03	51.64	52.96	63.94	67.82	68.38
ENSDARG00000023722	hcrtr2	hypocretin (orexin) receptor 2 [Source:ZFIN;Acc:ZDB-GENE-070228-3]	13	2020215	2060713		0.58	0.14	0.17	0.45	0.69	0.56
ENSDARG00000023724	cdc42se1	CDC42 small effector 1 [Source:NCBI gene;Acc:791998]	16	38311887	38333976		7.98	10.32	7.14	11.19	11	11.26
ENSDARG00000023759	zgc:73226	zgc:73226 [Source:NCBI gene;Acc:402792]	5	6350328	6377865		1.7	0.82	0.73	1.84	1.86	1.86
ENSDARG00000023766	gnsa	glucosamine (N-acetyl)-6-sulfatase a [Source:ZFIN;Acc:ZDB-GENE-050107-5]	4	4509996	4524284		12.73	9.51	10.78	17.31	17.81	18.38
ENSDARG00000023768	mfsd4aa	major facilitator superfamily domain containing 4Aa [Source:NCBI gene;Acc:393503]	11	38429319	38458406		2.56	2.72	2.91	0.89	0.86	1
ENSDARG00000023771	gabrb3	gamma-aminobutyric acid type A receptor beta3 subunit [Source:ZFIN;Acc:ZDB-GENE-101102-2]	6	38381957	38513578		8.59	1.65	2.35	10.32	9.8	9.8
ENSDARG00000023797	ryr1b	ryanodine receptor 1b (skeletal) [Source:ZFIN;Acc:ZDB-GENE-070705-417]	18	35491473	35736591		7.87	10.73	8.35	20.02	14.01	13.72
ENSDARG00000023814	dyrk1ab	dual-specificity tyrosine-(Y)-phosphorylation regulated kinase 1A, b [Source:ZFIN;Acc:ZDB-GENE-030131-5677]	15	19884242	19896816		5.7	2.54	2.34	6.91	6.63	6.82
ENSDARG00000023815	uvrag	UV radiation resistance associated gene [Source:ZFIN;Acc:ZDB-GENE-040426-924]	10	32264857	32494499		2.65	1.73	1.65	2.4	2.58	2.55
ENSDARG00000023820	faxdc2	fatty acid hydroxylase domain containing 2 [Source:ZFIN;Acc:ZDB-GENE-040426-2907]	21	45839917	45852112		59.63	96.24	77.87	28.75	35.86	34.51
ENSDARG00000023840	pfkfb2a	6-phosphofructo-2-kinase/fructose-2,6-biphosphatase 2a [Source:ZFIN;Acc:ZDB-GENE-040426-966]	23	42765700	42810664		1.89	1.59	1.19	1.41	1.69	1.5
ENSDARG00000023852	lsm4	LSM4 homolog, U6 small nuclear RNA and mRNA degradation associated [Source:ZFIN;Acc:ZDB-GENE-040426-1652]	11	6859876	6868474		38.95	44.17	43.06	44.01	40.13	41.58
ENSDARG00000023858	ccdc174	coiled-coil domain containing 174 [Source:ZFIN;Acc:ZDB-GENE-050320-54]	6	40429773	40435025		9.13	8	7.98	8.2	8.33	8.57
ENSDARG00000023861	dnase1l1l	deoxyribonuclease I-like 1-like [Source:ZFIN;Acc:ZDB-GENE-040718-100]	8	25351863	25358860		0.05	0.29	0.45	1.76	1.22	2.17
ENSDARG00000023868	si:dkey-172h23.2	si:dkey-172h23.2 [Source:ZFIN;Acc:ZDB-GENE-141212-258]	7	15087193	15257223		2.5	1.4	1.02	2.45	2.57	2.55
ENSDARG00000023878	stk24b	serine/threonine kinase 24b (STE20 homolog, yeast) [Source:ZFIN;Acc:ZDB-GENE-040426-2841]	6	12482599	12551359		16.8	12.39	11.06	19.59	20.37	21.62
ENSDARG00000023886	cacna2d4b	calcium channel, voltage-dependent, alpha 2/delta subunit 4b [Source:ZFIN;Acc:ZDB-GENE-100422-18]	25	19023737	19090628		5.28	1.01	0.89	3.88	4.17	4.31
ENSDARG00000023900	casd1	CAS1 domain containing 1 [Source:NCBI gene;Acc:692268]	19	41006975	41034224		9.31	5.12	5.65	10.6	11.4	11
ENSDARG00000023903	atf2	activating transcription factor 2 [Source:ZFIN;Acc:ZDB-GENE-030911-8]	9	2343096	2382588		1.5	0.61	0.68	2.35	2.34	2.25
ENSDARG00000023914	mark4a	MAP/microtubule affinity-regulating kinase 4a [Source:ZFIN;Acc:ZDB-GENE-060531-156]	5	36768674	36822220		7.8	2.22	3.03	9.69	10.72	10.43
ENSDARG00000023916	phactr1	phosphatase and actin regulator 1 [Source:ZFIN;Acc:ZDB-GENE-101230-3]	20	52774730	52815590		7	2.02	2.17	9.25	9.73	9.55
ENSDARG00000023920	llgl2	LLGL scribble cell polarity complex component 2 [Source:NCBI gene;Acc:795670]	12	33734849	33789218		5.14	4.25	3.3	5.84	5.64	5.6
ENSDARG00000023927	dnajc24	DnaJ (Hsp40) homolog, subfamily C, member 24 [Source:ZFIN;Acc:ZDB-GENE-040426-1153]	25	14870043	14899109		1.74	1.8	2.07	1.91	1.6	1.55
ENSDARG00000023933	skila	SKI-like proto-oncogene a [Source:ZFIN;Acc:ZDB-GENE-070912-548]	2	37307326	37353098		6.26	2.97	2.99	11.65	11.91	11.24
ENSDARG00000023940	si:ch73-362m14.4	si:ch73-362m14.4 [Source:ZFIN;Acc:ZDB-GENE-120215-30]	21	43581341	43606502		5.35	1.15	1.46	12.22	13.22	14.73
ENSDARG00000023950	znhit3	zinc finger, HIT-type containing 3 [Source:NCBI gene;Acc:393243]	5	3927989	3937715		5.67	6	7.11	2.76	2.67	3.45
ENSDARG00000023952	fam167ab	family with sequence similarity 167 member Ab [Source:NCBI gene;Acc:567341]	20	18740518	18760027		0.6	0.43	0.4	0.52	0.66	0.63
ENSDARG00000023958	rnf181	ring finger protein 181 [Source:NCBI gene;Acc:393276]	5	26756021	26765188		3.82	4	2.9	4.11	3.19	4.69
ENSDARG00000023963	tpm4a	tropomyosin 4a [Source:ZFIN;Acc:ZDB-GENE-040426-2185]	22	15577827	15602760		34.48	48.97	45.82	61.31	66.4	66.18
ENSDARG00000023967	atp6v1c1a	ATPase H+ transporting V1 subunit C1a [Source:NCBI gene;Acc:368907]	16	52748263	52767591		50.29	36.95	42	45.92	47.55	51.32
ENSDARG00000023988	dctn4	dynactin 4 [Source:ZFIN;Acc:ZDB-GENE-050417-306]	14	49462855	49490735		10.31	7.3	6.79	13.75	13.58	13.88
ENSDARG00000023989	pef1	penta-EF-hand domain containing 1 [Source:NCBI gene;Acc:445249]	19	37120491	37134211		10.73	13.57	11.92	9.54	10.52	11.02
ENSDARG00000023999	heatr5a	HEAT repeat containing 5a [Source:NCBI gene;Acc:368728]	17	28624321	28670042		4.95	4.06	3.34	5.97	5.45	5.84
ENSDARG00000024006	rprmb	reprimo, TP53 dependent G2 arrest mediator candidate b [Source:NCBI gene;Acc:393779]	6	13333133	13334222		4.55	3.17	3.01	4.35	4.19	4.65
ENSDARG00000024017	mdga2a	MAM domain containing glycosylphosphatidylinositol anchor 2a [Source:ZFIN;Acc:ZDB-GENE-030616-360]	17	28340138	28564070		4.34	0.91	1.03	5.37	5.53	5.72
ENSDARG00000024026	sdf2	stromal cell-derived factor 2 [Source:ZFIN;Acc:ZDB-GENE-030131-8823]	21	26021496	26028205		6.56	7.04	6.58	7.22	7.38	6.94
ENSDARG00000024030	angptl2a	angiopoietin-like 2a [Source:NCBI gene;Acc:569092]	5	4806851	4830356		3.43	4.53	3.79	7.06	8.32	7.9
ENSDARG00000024032	coch	coagulation factor C homolog, cochlin (Limulus polyphemus) [Source:ZFIN;Acc:ZDB-GENE-030616-403]	17	28743661	28749640		13.93	17.42	17.71	10.88	12.25	11.74
ENSDARG00000024047	tmem38a	transmembrane protein 38A [Source:NCBI gene;Acc:393874]	11	7158723	7179516		149.42	209.8	232.69	211.5	197.26	201
ENSDARG00000024090	dnajc4	DnaJ (Hsp40) homolog, subfamily C, member 4 [Source:ZFIN;Acc:ZDB-GENE-030131-3093]	14	8475007	8496669		2.82	2.46	2.49	2.52	2.27	2.44
ENSDARG00000024092	lmbr1	limb development membrane protein 1 [Source:ZFIN;Acc:ZDB-GENE-090128-4]	7	40658003	40708140		6.15	4.53	4.5	7.33	7.35	6.8
ENSDARG00000024101	adra1d	adrenoceptor alpha 1D [Source:ZFIN;Acc:ZDB-GENE-090312-203]	1	41957210	41982582		1.13	0.89	0.53	1.64	1.65	1.31
ENSDARG00000024109	naa40	N(alpha)-acetyltransferase 40, NatD catalytic subunit, homolog (S. cerevisiae) [Source:NCBI gene;Acc:550325]	7	25000060	25011597		15.15	10.07	11.59	14.61	15.28	14.17
ENSDARG00000024112	gmppaa	GDP-mannose pyrophosphorylase Aa [Source:NCBI gene;Acc:431743]	9	10770880	10780660		7.36	7.21	7.44	6.43	6.74	6.29
ENSDARG00000024116	vamp8	vesicle-associated membrane protein 8 (endobrevin) [Source:ZFIN;Acc:ZDB-GENE-030131-5215]	5	28271412	28279364		18.13	18.54	12.38	13.56	12.92	13.16
ENSDARG00000024124	sde2	SDE2 telomere maintenance homolog (S. pombe) [Source:NCBI gene;Acc:550448]	22	26277618	26289549		14.82	11.37	13.04	12.95	14.87	15.06
ENSDARG00000024139	p2rx8	purinergic receptor P2X, ligand-gated ion channel, 8 [Source:ZFIN;Acc:ZDB-GENE-030319-7]	15	16343073	16371103		0.8	0.32	0.38	0.71	0.69	0.75
ENSDARG00000024141	cav3	caveolin 3 [Source:ZFIN;Acc:ZDB-GENE-050522-426]	6	41996066	42003780		43.73	61.58	66.44	42	35.33	39.33
ENSDARG00000024160	hao1	hydroxyacid oxidase (glycolate oxidase) 1 [Source:ZFIN;Acc:ZDB-GENE-060519-2]	17	4030493	4063581		37.28	61.37	62.18	45.82	51.04	46.03
ENSDARG00000024167	kcnh1a	potassium voltage-gated channel, subfamily H (eag-related), member 1a [Source:ZFIN;Acc:ZDB-GENE-061226-1]	17	25677458	25765938		0.78	0.13	0.36	0.89	1.14	1.14
ENSDARG00000024184	narf	nuclear prelamin A recognition factor [Source:NCBI gene;Acc:436614]	12	33460794	33474029		10.19	9.44	10.44	13.17	13.95	13.7
ENSDARG00000024189	pnocb	prepronociceptin b [Source:ZFIN;Acc:ZDB-GENE-040114-1]	20	34717403	34734022		1.68	0.99	1.63	3.49	4.41	5.2
ENSDARG00000024195	znf395b	zinc finger protein 395b [Source:ZFIN;Acc:ZDB-GENE-041001-193]	20	34737738	34754617		26.46	14.33	15.26	22.65	24.33	23.35
ENSDARG00000024204	mcm3	minichromosome maintenance complex component 3 [Source:ZFIN;Acc:ZDB-GENE-020419-4]	20	34770197	34783743		24.38	22.46	21.53	12.78	12.12	10.89
ENSDARG00000024208	opn7a	opsin 7, group member a [Source:ZFIN;Acc:ZDB-GENE-140106-15]	20	6241855	6278817		0.05	0	0.03	0.07	0.08	0.14
ENSDARG00000024209	wasf2	WASP family member 2 [Source:ZFIN;Acc:ZDB-GENE-040426-865]	19	43523690	43543128		15.57	16.95	14.39	19.14	19.39	18.99
ENSDARG00000024211	ifng1	interferon gamma 1 [Source:ZFIN;Acc:ZDB-GENE-040629-1]	4	13507584	13509946		0	0	0	0	0	0
ENSDARG00000024219	cdk5rap2	CDK5 regulatory subunit associated protein 2 [Source:ZFIN;Acc:ZDB-GENE-030131-6392]	21	4647784	4695583		10.77	5.22	4.36	8.46	8.89	9.52
ENSDARG00000024229	ubl7a	ubiquitin-like 7a (bone marrow stromal cell-derived) [Source:ZFIN;Acc:ZDB-GENE-050417-285]	25	31743651	31763897		6.59	3.47	3.76	5.65	6.18	6.34
ENSDARG00000024276	pcbp4	poly(rC) binding protein 4 [Source:ZFIN;Acc:ZDB-GENE-040426-2122]	22	32289567	32507966		9.55	5.92	5.25	19.16	20.69	22.53
ENSDARG00000024277	pigm	phosphatidylinositol glycan anchor biosynthesis, class M [Source:NCBI gene;Acc:393361]	24	42102204	42108655		2.23	2.02	1.46	2.64	2.5	2.68
ENSDARG00000024278	adh8b	alcohol dehydrogenase 8b [Source:ZFIN;Acc:ZDB-GENE-030529-2]	13	12633223	12646752		56.69	94.51	78.07	50.73	55.58	52.83
ENSDARG00000024295	slc11a2	solute carrier family 11 member 2 [Source:ZFIN;Acc:ZDB-GENE-021115-1]	11	281833	287670		23.25	27.3	22.27	34.97	34.83	32.44
ENSDARG00000024299	c1qtnf4	C1q and TNF related 4 [Source:ZFIN;Acc:ZDB-GENE-050417-198]	7	38600150	38634845		17.78	6.58	9.28	15.12	16.23	16.62
ENSDARG00000024306	zbed4	zinc finger, BED-type containing 4 [Source:ZFIN;Acc:ZDB-GENE-041210-305]	4	27112399	27129697		5.71	2.94	2.83	6.83	6.16	6.57
ENSDARG00000024311	nsmce4a	NSE4 homolog A, SMC5-SMC6 complex component [Source:ZFIN;Acc:ZDB-GENE-050522-111]	13	28785814	28796637		5.73	5.8	6.15	5.16	5.09	4.97
ENSDARG00000024314	herpud1	homocysteine-inducible, endoplasmic reticulum stress-inducible, ubiquitin-like domain member 1 [Source:ZFIN;Acc:ZDB-GENE-05(	18	17600570	17611849		4.85	4.43	3.46	5.11	5.2	4.9
ENSDARG00000024317	trap1	TNF receptor-associated protein 1 [Source:ZFIN;Acc:ZDB-GENE-030131-4257]	3	9588705	9626674		18.74	17.72	17.98	14.45	13.64	14.06
ENSDARG00000024324	arhgap1	Rho GTPase activating protein 1 [Source:ZFIN;Acc:ZDB-GENE-050417-305]	7	38762043	38785745		24.31	13.48	14.95	26.06	26.82	26.59
ENSDARG00000024325	cert1a	ceramide transporter 1a [Source:NCBI gene;Acc:796455]	5	50912729	50970974		2	1.8	1.65	1.98	2.23	2.18
ENSDARG00000024365	crlf1a	cytokine receptor-like factor 1a [Source:ZFIN;Acc:ZDB-GENE-040718-397]	2	41876595	41920632		3.66	4.11	3.36	4.82	4.95	5.28
ENSDARG00000024381	msto1	misato mitochondrial distribution and morphology regulator 1 [Source:NCBI gene;Acc:334306]	19	776208	791016		5.28	4.54	4.22	4.14	4.45	4.77
ENSDARG00000024398	get4	guided entry of tail-anchored proteins factor 4 [Source:ZFIN;Acc:ZDB-GENE-030131-5399]	12	11262954	11306766		22.49	18.17	21.1	21.47	21.47	21.66
ENSDARG00000024416	twistnb	TWIST neighbor [Source:NCBI gene;Acc:402856]	19	2395114	2404599		2.85	2.89	2.39	2.54	2.26	2.33
ENSDARG00000024431	ets1	v-ets avian erythroblastosis virus E26 oncogene homolog 1 [Source:ZFIN;Acc:ZDB-GENE-021115-5]	18	48279202	48296793		5.2	5.02	4.69	9.69	11.31	9.1
ENSDARG00000024433	pvalb4	parvalbumin 4 [Source:ZFIN;Acc:ZDB-GENE-040625-48]	3	61174750	61185746		351.28	466.68	500.59	232.93	241.8	242.74
ENSDARG00000024443	gnpda2	glucosamine-6-phosphate deaminase 2 [Source:ZFIN;Acc:ZDB-GENE-091117-41]	14	4276387	4293280		10.97	13.13	12.61	11.26	10.34	10.46
ENSDARG00000024465	mrps18a	mitochondrial ribosomal protein S18A [Source:ZFIN;Acc:ZDB-GENE-041210-109]	4	5642696	5645166		19.77	23.47	21.83	17.61	17.56	17.17
ENSDARG00000024471	nsl1	NSL1 component of MIS12 kinetochore complex [Source:ZFIN;Acc:ZDB-GENE-041014-362]	20	37814345	37820939		2.34	2.57	2.02	1.57	1.19	1.27
ENSDARG00000024478	mpc2	mitochondrial pyruvate carrier 2 [Source:ZFIN;Acc:ZDB-GENE-030131-330]	9	34221997	34227472		94.29	118.43	116.64	60.82	57.21	58.25
ENSDARG00000024479	pigk	phosphatidylinositol glycan anchor biosynthesis, class K [Source:ZFIN;Acc:ZDB-GENE-040625-162]	2	9061885	9138497		2.83	2.06	2.01	3.09	3.31	2.29
ENSDARG00000024488	top2a	DNA topoisomerase II alpha [Source:ZFIN;Acc:ZDB-GENE-030131-2453]	12	10706772	10740236		24.56	17.72	15.76	16.19	15.38	16.2
ENSDARG00000024492	col9a2	procollagen, type IX, alpha 2 [Source:ZFIN;Acc:ZDB-GENE-030131-9786]	19	38422059	38467554		84.58	92.93	70.28	165.02	170.16	174.57
ENSDARG00000024503	c6ast3	six-cysteine containing astacin protease 3 [Source:ZFIN;Acc:ZDB-GENE-050320-99]	7	38642114	38644560		6.79	9.18	9.21	0.26	0.14	0.24
ENSDARG00000024522	prf1.6	perforin 1.6 [Source:ZFIN;Acc:ZDB-GENE-070410-126]	8	29670527	29677302		0	0.01	0.02	0.03	0.01	0.01
ENSDARG00000024537	emilin1a	elastin microfibril interfacer 1a [Source:ZFIN;Acc:ZDB-GENE-041001-191]	20	34845672	34865031		7.4	9.8	8.86	9.22	9.69	9.9
ENSDARG00000024540	tspan36	tetraspanin 36 [Source:NCBI gene;Acc:437021]	8	29726620	29734951		27.37	42.81	45.26	41.57	44.08	46.48
ENSDARG00000024546	pla2g4aa	phospholipase A2, group IVAa (cytosolic, calcium-dependent) [Source:NCBI gene;Acc:30554]	2	20925899	20957362		3.08	2.71	2.3	5.28	6.05	5.87
ENSDARG00000024548	cryba4	crystallin, beta A4 [Source:ZFIN;Acc:ZDB-GENE-050522-143]	10	44042033	44051808		10.11	17.26	29.48	100.99	74.97	109.37
ENSDARG00000024560	slc4a1b	solute carrier family 4 member 1b (Diego blood group) [Source:ZFIN;Acc:ZDB-GENE-110215-2]	12	10053852	10100504		6.93	6.55	5.69	4.49	4.18	3.91
ENSDARG00000024561	nolc1	nucleolar and coiled-body phosphoprotein 1 [Source:ZFIN;Acc:ZDB-GENE-030131-6349]	13	4973211	4992395		14.53	15.04	10.65	14.43	13.25	13.85
ENSDARG00000024564	sccpdhb	saccharopine dehydrogenase b [Source:ZFIN;Acc:ZDB-GENE-041010-211]	20	34967410	34985555		16.46	17.25	16.76	16.26	17.6	17.95
ENSDARG00000024575	kif26bb	kinesin family member 26Bb [Source:ZFIN;Acc:ZDB-GENE-041001-188]	20	35013434	35052823		1.67	0.45	0.46	2.43	2.38	2.59
ENSDARG00000024588	gpc5b	glypican 5b [Source:ZFIN;Acc:ZDB-GENE-060503-64]	22	15438513	15501417		3.85	2.26	2.5	3.03	2.81	3.27
ENSDARG00000024598	homezb	homeobox and leucine zipper encoding b [Source:ZFIN;Acc:ZDB-GENE-030131-9225]	7	24006875	24013888		9.32	5.51	5.43	10.04	9.42	9.31
ENSDARG00000024602	ACBD3	zgc:162964 [Source:ZFIN;Acc:ZDB-GENE-030131-9798]	17	33375469	33386590	ACBD3	5.53	6.3	5.38	4.79	3.97	4.34
ENSDARG00000024619	foxo6b	forkhead box O6 b [Source:ZFIN;Acc:ZDB-GENE-060503-776]	19	15571290	15620967		4.24	0.59	0.8	6.03	5.68	5.65
ENSDARG00000024631	nlrc5	NLR family, CARD domain containing 5 [Source:ZFIN;Acc:ZDB-GENE-080429-2]	18	17624295	17657802		0.08	0.08	0.08	0.12	0.16	0.14
ENSDARG00000024642	pip5k1ab	phosphatidylinositol-4-phosphate 5-kinase, type I, alpha, b [Source:ZFIN;Acc:ZDB-GENE-041111-123]	16	9726038	9761973		3.22	2.03	2	5.66	5.35	5.31
ENSDARG00000024651	snrpa1	small nuclear ribonucleoprotein polypeptide A' [Source:ZFIN;Acc:ZDB-GENE-041114-82]	7	9290929	9305581		16.03	14.76	15	17.46	16.99	16.69
ENSDARG00000024654	pgm3	phosphoglucomutase 3 [Source:ZFIN;Acc:ZDB-GENE-041024-13]	4	5784970	5795309		5.66	7.77	6.46	3.77	3.29	3.47
ENSDARG00000024669	oser1	oxidative stress responsive serine-rich 1 [Source:ZFIN;Acc:ZDB-GENE-040426-1325]	6	52715708	52723901		15.02	9.33	9.15	12.52	11.57	13.29
ENSDARG00000024681	pane1	proliferation associated nuclear element [Source:NCBI gene;Acc:402852]	1	55161083	55166611		8.38	7.25	9.62	3.96	5.12	4.78
ENSDARG00000024687	polr3g	polymerase (RNA) III (DNA directed) polypeptide G [Source:ZFIN;Acc:ZDB-GENE-081104-312]	5	48666485	48675195		0.05	0.04	0.04	0.02	0.01	0.06
ENSDARG00000024693	lysmd3	LysM, putative peptidoglycan-binding, domain containing 3 [Source:NCBI gene;Acc:415194]	5	48672553	48680580		10.57	11.76	11.75	11.37	11.66	11.83
ENSDARG00000024694	myo1b	myosin IB [Source:ZFIN;Acc:ZDB-GENE-030131-695]	9	16127209	16239130		16.13	17.49	12.47	17.09	17.42	17.45
ENSDARG00000024708	slc25a23b	solute carrier family 25 member 23b [Source:ZFIN;Acc:ZDB-GENE-040724-220]	3	34220194	34230565		5.08	1.73	2.56	3.66	4.33	4.43
ENSDARG00000024717	selenbp1	selenium binding protein 1 [Source:NCBI gene;Acc:393542]	16	38138246	38157299		15.08	21.65	18.09	9.14	8.52	9.57
ENSDARG00000024728	hdhd2	haloacid dehalogenase-like hydrolase domain containing 2 [Source:NCBI gene;Acc:541346]	10	14525184	14540443		1.01	1.24	1.06	0.85	0.77	0.94
ENSDARG00000024740	gng13a	guanine nucleotide binding protein (G protein), gamma 13a [Source:ZFIN;Acc:ZDB-GENE-081022-188]	1	9227081	9228007		7.36	11.16	10.21	3.36	2.73	4.38
ENSDARG00000024744	dpp6b	dipeptidyl-peptidase 6b [Source:ZFIN;Acc:ZDB-GENE-070705-88]	2	12406713	12632761		10.77	3.27	5.17	11.8	13.08	13.65
ENSDARG00000024746	hsp90aa1.2	heat shock protein 90, alpha (cytosolic), class A member 1, tandem duplicate 2 [Source:ZFIN;Acc:ZDB-GENE-031001-3]	20	53967350	53981626		87.17	49.67	51.9	67.58	81.98	74.26
ENSDARG00000024759	inhbab	inhibin subunit beta Ab [Source:ZFIN;Acc:ZDB-GENE-050525-2]	2	21485492	21504467		0.57	0.2	0.24	0.5	0.47	0.43
ENSDARG00000024771	slc24a5	solute carrier family 24 member 5 [Source:ZFIN;Acc:ZDB-GENE-031210-1]	18	5213338	5227420		1.77	2.19	1.82	2.85	3.55	3.16
ENSDARG00000024775	si:ch211-129c21.1	si:ch211-129c21.1 [Source:ZFIN;Acc:ZDB-GENE-030131-5796]	22	22316855	22340688		6.82	6.5	5.13	5.41	5.59	5.19
ENSDARG00000024785	ctnna2	catenin (cadherin-associated protein), alpha 2 [Source:NCBI gene;Acc:567500]	1	42224769	43039672		3.07	1.13	1.14	3.83	3.59	3.97
ENSDARG00000024789	mxc	myxovirus (influenza virus) resistance C [Source:NCBI gene;Acc:360145]	9	32805688	32813177		0.47	0.52	0.64	0.64	0.59	0.53
ENSDARG00000024815	ogfrl2	opioid growth factor receptor-like 2 [Source:ZFIN;Acc:ZDB-GENE-030616-523]	11	20091255	20096018		0.72	1.15	0.54	0.71	0.6	0.6
ENSDARG00000024818	rilpl2	Rab interacting lysosomal protein-like 2 [Source:NCBI gene;Acc:100004718]	5	69795204	69804433		4.03	4.96	4.25	4.72	4.39	4.12
ENSDARG00000024827	rnf150a	ring finger protein 150a [Source:ZFIN;Acc:ZDB-GENE-060213-1]	1	53279861	53299024		1.94	2.1	1.75	2.19	2.36	1.96
ENSDARG00000024828	tmem134	transmembrane protein 134 [Source:ZFIN;Acc:ZDB-GENE-040426-1345]	1	39878634	39895918		1.99	1.96	2.11	2.15	2	2.23
ENSDARG00000024829	tnn	tenascin N [Source:ZFIN;Acc:ZDB-GENE-990415-262]	2	35509418	35567110		69.26	100.46	89.38	120.16	123.3	127.74
ENSDARG00000024831	crhbp	corticotropin releasing hormone binding protein [Source:ZFIN;Acc:ZDB-GENE-040801-196]	21	7823146	7832351		14.66	7.8	10.88	18.26	20.44	19.59
ENSDARG00000024844	max	myc associated factor X [Source:NCBI gene;Acc:30467]	20	28832762	28842604		36.9	21.54	22.61	39.04	39.53	39.3
ENSDARG00000024846	cops8	COP9 signalosome subunit 8 [Source:NCBI gene;Acc:393198]	9	23895711	23906205		24.04	25.45	27.57	17.82	18.83	17.63
ENSDARG00000024847	col5a2b	collagen, type V, alpha 2b [Source:ZFIN;Acc:ZDB-GENE-110406-2]	6	11438972	11495601		3.77	2.69	1.78	2.78	3.15	2.91
ENSDARG00000024865	dscama	Down syndrome cell adhesion molecule a [Source:ZFIN;Acc:ZDB-GENE-050310-7]	10	38775408	38974445		4.65	1.19	1.33	11.19	10.83	11.83
ENSDARG00000024874	dock4b	dedicator of cytokinesis 4b [Source:ZFIN;Acc:ZDB-GENE-060130-74]	4	6833583	6994283		3.12	1.7	1.57	4.74	4.71	4.83
ENSDARG00000024877	ptgr1	prostaglandin reductase 1 [Source:ZFIN;Acc:ZDB-GENE-041212-80]	7	24375070	24390879		6.92	8.41	7.91	4.92	5.25	5.01
ENSDARG00000024894	tbx5a	T-box transcription factor 5a [Source:NCBI gene;Acc:30071]	5	72264192	72289648		1.19	1.02	1.04	3.47	3.01	2.88
ENSDARG00000024895	fam50a	family with sequence similarity 50 member A [Source:NCBI gene;Acc:550329]	23	33987382	34005304		18.92	20.75	19.35	15.04	14.06	16.29
ENSDARG00000024904	stil	STIL centriolar assembly protein [Source:NCBI gene;Acc:192317]	22	15959844	15973184		0.86	0.54	0.54	0.69	0.76	0.66
ENSDARG00000024920	tlcd5a	TLC domain containing 5a [Source:ZFIN;Acc:ZDB-GENE-000607-58]	15	22435460	22444746		4.51	2.1	2.85	5.19	4.7	5.16
ENSDARG00000024933	spast	spastin [Source:NCBI gene;Acc:405851]	1	51021279	51038885		8.96	6.23	5.45	9.29	8.28	8.01
ENSDARG00000024940	rnf144b	ring finger protein 144B [Source:ZFIN;Acc:ZDB-GENE-040426-1292]	16	43368572	43392455		9.86	7.74	7.53	8.04	8.31	7.5
ENSDARG00000024954	tmed4	transmembrane p24 trafficking protein 4 [Source:ZFIN;Acc:ZDB-GENE-040625-140]	23	33977724	33986762		15.18	12.15	11.1	15.44	14.9	15.06
ENSDARG00000024964	patj	PATJ crumbs cell polarity complex component [Source:ZFIN;Acc:ZDB-GENE-030616-129]	22	16569123	16759151		4.59	3.49	2.8	4.93	5.17	5.2
ENSDARG00000024966	MARK4	si:dkey-31m14.7 [Source:ZFIN;Acc:ZDB-GENE-121214-92]	15	23550890	23641516	MARK4	10.65	5.49	5.73	16.71	16.55	16.98
ENSDARG00000024967	fancg	FA complementation group G [Source:ZFIN;Acc:ZDB-GENE-050417-103]	5	41496490	41509339		1.17	0.94	1.01	0.84	0.95	1.01
ENSDARG00000025011	synj1	synaptojanin 1 [Source:ZFIN;Acc:ZDB-GENE-030131-9180]	10	26515946	26569350		8.31	3.46	2.7	14.7	13.54	14.03
ENSDARG00000025012	tpi1a	triosephosphate isomerase 1a [Source:NCBI gene;Acc:192309]	19	5094218	5103313		18.92	8.69	10.82	14.59	15.64	15.13
ENSDARG00000025013	gna14a	guanine nucleotide binding protein (G protein), alpha 14a [Source:ZFIN;Acc:ZDB-GENE-081105-76]	8	51958968	51986997		2.35	1.86	1.68	1.11	1.73	1.08
ENSDARG00000025017	prdm8	PR domain containing 8 [Source:ZFIN;Acc:ZDB-GENE-040426-2747]	13	31292700	31296358		1.76	0.91	1.22	3.06	3.24	3.06
ENSDARG00000025024	pnoca	prepronociceptin a [Source:ZFIN;Acc:ZDB-GENE-101229-1]	17	16110285	16133249		3.67	2.74	3.31	6.77	7.59	8.15
ENSDARG00000025032	nr3c1	nuclear receptor subfamily 3, group C, member 1 (glucocorticoid receptor) [Source:ZFIN;Acc:ZDB-GENE-050522-503]	14	23746710	23801389		14.14	10.3	7.96	16.24	16.7	16.47
ENSDARG00000025033	stx5a	syntaxin 5A [Source:ZFIN;Acc:ZDB-GENE-040718-18]	14	8594367	8616663		8.68	7.26	6.42	10.01	9.44	9.49

ENSDARG00000025034	syngr3b	synaptogyrin 3b [Source:ZFIN;Acc:ZDB-GENE-070822-6]	1	8415983	8428761		8.19	2.33	3.4	11.19	10.62	9.99
ENSDARG00000025043	trip13	thyroid hormone receptor interactor 13 [Source:NCBI gene;Acc:393554]	16	52966812	52991487		1.97	1.65	1.9	1.47	1.47	1.39
ENSDARG00000025046	AL935186.1		4	77709678	77712195		0.02	0.01	0.02	0.05	0.03	0.06
ENSDARG00000025071	brd8	bromodomain containing 8 [Source:ZFIN;Acc:ZDB-GENE-030722-10]	14	7697694	7740058		19.66	13.04	12.91	18.55	18.49	18.93
ENSDARG00000025073	rpl18a	ribosomal protein L18a [Source:NCBI gene;Acc:394035]	4	391297	398451		630.64	782.32	1012.87	622.22	638.94	625.43
ENSDARG00000025074	slc25a10	solute carrier family 25 member 10 [Source:ZFIN;Acc:ZDB-GENE-040426-1095]	1	56421754	56435182		18.9	19.03	16.53	18.54	18.2	18.76
ENSDARG00000025076	marveld2a	MARVEL domain containing 2a [Source:ZFIN;Acc:ZDB-GENE-050411-59]	10	2943676	2971407		2.31	2.26	1.86	2.88	2.58	2.66
ENSDARG00000025081	grnb	granulin b [Source:ZFIN;Acc:ZDB-GENE-030131-7393]	24	36339242	36364591		14.05	14.79	11.2	12.89	13.3	13.37
ENSDARG00000025089	loxl4	lysyl oxidase-like 4 [Source:ZFIN;Acc:ZDB-GENE-080722-19]	13	40182487	40238813		1.95	1.3	0.87	5.92	6.44	6.55
ENSDARG00000025091	ezrb	ezrin b [Source:ZFIN;Acc:ZDB-GENE-050803-1]	20	13564931	13625588		22.22	17.9	14.51	18.68	17.12	17.13
ENSDARG00000025094	edem1	ER degradation enhancer, mannosidase alpha-like 1 [Source:ZFIN;Acc:ZDB-GENE-040426-808]	6	42932757	42949184		9.55	8.15	6.01	10.09	9.83	9.42
ENSDARG00000025106	ppp3cb	protein phosphatase 3, catalytic subunit, beta isozyme [Source:ZFIN;Acc:ZDB-GENE-050417-406]	14	7789093	7888748		21.06	5.3	6.66	26.57	27.4	27.42
ENSDARG00000025108	magixa	MAGI family member, X-linked a [Source:ZFIN;Acc:ZDB-GENE-060503-115]	8	22761854	22838364		3.13	1.03	1.33	5.16	5.16	5.32
ENSDARG00000025131	lbx2	ladybird homeobox 2 [Source:ZFIN;Acc:ZDB-GENE-001206-2]	14	5383060	5391183		2.22	2.17	2.21	3.23	3.02	3.48
ENSDARG00000025147	cd63	CD63 molecule [Source:ZFIN;Acc:ZDB-GENE-030131-180]	23	32066168	32085939		128.53	152.83	162.34	152.44	160.7	151.7
ENSDARG00000025150	si:dkey-181c13.1	si:dkey-181c13.1 [Source:ZFIN;Acc:ZDB-GENE-110411-174]	10	23502026	23505767		0	0	0	0.04	0.05	0
ENSDARG00000025162	asic4a	acid-sensing (proton-gated) ion channel family member 4a [Source:NCBI gene;Acc:407668]	9	11034314	11229565		1.66	0.49	0.5	1.96	2.28	2.21
ENSDARG00000025168	mpp7b	membrane protein, palmitoylated 7b (MAGUK p55 subfamily member 7) [Source:ZFIN;Acc:ZDB-GENE-141212-293]	2	3881000	3894103		0.23	0	0	0.07	0.07	0.1
ENSDARG00000025174	zgc:103482	zgc:103482 [Source:ZFIN;Acc:ZDB-GENE-041010-115]	21	20396858	20402686		0.57	0.31	0.44	0.52	0.61	0.35
ENSDARG00000025187	six6a	SIX homeobox 6a [Source:ZFIN;Acc:ZDB-GENE-040426-1282]	13	31583034	31606247		2.36	0.55	0.62	2.65	3.17	3.56
ENSDARG00000025189	cpne8	copine VIII [Source:ZFIN;Acc:ZDB-GENE-100809-1]	25	35774544	35874406		3.45	0.29	0.49	2.99	3.19	3.18
ENSDARG00000025206	syt2a	synaptotagmin IIa [Source:ZFIN;Acc:ZDB-GENE-060503-315]	23	6232895	6313808		7.51	1.8	2.96	14.33	14.7	16.4
ENSDARG00000025212	ints11	integrator complex subunit 11 [Source:NCBI gene;Acc:553648]	23	37057603	37085968		2.24	1.7	1.81	2.43	2.42	2.62
ENSDARG00000025218	myo5ab	myosin VAb [Source:ZFIN;Acc:ZDB-GENE-050411-72]	25	7612606	7650335		4.56	5.11	3.14	2.58	2.32	1.87
ENSDARG00000025220	sppl3	signal peptide peptidase 3 [Source:ZFIN;Acc:ZDB-GENE-050913-71]	10	17235370	17433878		5.61	2.96	2.76	10.16	9.73	9.55
ENSDARG00000025226	znf668	zinc finger protein 668 [Source:ZFIN;Acc:ZDB-GENE-041001-46]	12	28955766	28960633		2.53	1.83	1.51	2.64	2.73	2.45
ENSDARG00000025233	lipt1	lipoyltransferase 1 [Source:ZFIN;Acc:ZDB-GENE-060929-112]	1	45922699	45924004		3.55	2.75	3.2	2.27	2.62	2.09
ENSDARG00000025254	s100a10b	S100 calcium binding protein A10b [Source:ZFIN;Acc:ZDB-GENE-040426-1937]	16	23397785	23399484		119.15	152.79	147.56	101.07	106.43	104.91
ENSDARG00000025269	pdcd6ip	programmed cell death 6 interacting protein [Source:ZFIN;Acc:ZDB-GENE-040426-2678]	19	42847306	42875185		27.76	24.77	20.03	31.23	30.72	31.81
ENSDARG00000025275	gbgt1l1	globoside alpha-1,3-N-acetylgalactosaminyltransferase 1, like 1 [Source:ZFIN;Acc:ZDB-GENE-040426-1117]	5	23702295	23715861		0.87	0.73	0.73	0.91	0.87	1.17
ENSDARG00000025285	panx1a	pannexin 1a [Source:ZFIN;Acc:ZDB-GENE-040426-766]	15	2475894	2489934		7.34	6.89	6.43	8.53	7.92	7.6
ENSDARG00000025299	tspan9a	tetraspanin 9a [Source:ZFIN;Acc:ZDB-GENE-060503-632]	18	11016203	11317187		3.26	1.61	1.55	3.51	3.63	3.62
ENSDARG00000025301	gfap	glial fibrillary acidic protein [Source:NCBI gene;Acc:30646]	3	31792441	31804481		26.59	34.6	40.1	85.94	90.31	92.46
ENSDARG00000025302	dixdc1a	DIX domain containing 1a [Source:NCBI gene;Acc:360137]	15	18130109	18155991		17.37	7.64	9.81	15.96	15.44	16.47
ENSDARG00000025309	dpf3	D4, zinc and double PHD fingers, family 3 [Source:NCBI gene;Acc:562738]	20	28434196	28501429		8.95	3.68	4.74	8.93	8.44	8.72
ENSDARG00000025311	cuzd1.2	CUB and zona pellucida-like domains 1, tandem duplicate 2 [Source:ZFIN;Acc:ZDB-GENE-131119-30]	17	21833719	21859164		0.97	0.99	0.75	0.27	0.24	0.19
ENSDARG00000025319	fynb	FYN proto-oncogene, Src family tyrosine kinase b [Source:NCBI gene;Acc:574422]	20	328412	396013		6.14	3.51	3.98	6.76	6.84	6.29
ENSDARG00000025320	col21a1	collagen, type XXI, alpha 1 [Source:ZFIN;Acc:ZDB-GENE-110607-3]	13	1799003	1866711		2.21	2.39	1.79	4.16	4.99	4.16
ENSDARG00000025325	ccsapa	centriole, cilia and spindle-associated protein a [Source:ZFIN;Acc:ZDB-GENE-111116-1]	1	53954230	53963933		7.66	2.75	3.48	7.48	8.11	8.46
ENSDARG00000025326	myl12.2	myosin, light chain 12, genome duplicate 2 [Source:ZFIN;Acc:ZDB-GENE-030131-9028]	2	30910134	30912922		48.31	43.42	46.08	37.74	36.57	34.62
ENSDARG00000025332	rbm28	RNA binding motif protein 28 [Source:ZFIN;Acc:ZDB-GENE-040426-960]	18	10692945	10713414		14.53	16.81	14.1	12.61	11.33	11.79
ENSDARG00000025335	nek10	NIMA-related kinase 10 [Source:ZFIN;Acc:ZDB-GENE-130530-570]	16	50114739	50157187		0.32	0.21	0.27	0.33	0.37	0.39
ENSDARG00000025338	hagh	hydroxyacylglutathione hydrolase [Source:NCBI gene;Acc:336977]	3	18774511	18792534		20.96	24.96	20.52	23.3	27.33	26.24
ENSDARG00000025340	tusc2b	tumor suppressor 2, mitochondrial calcium regulator b [Source:ZFIN;Acc:ZDB-GENE-040718-99]	22	10660140	10663935		21.26	24.6	24.94	20.84	20.71	19.73
ENSDARG00000025346	znf277	zinc finger protein 277 [Source:ZFIN;Acc:ZDB-GENE-040426-1633]	25	21716263	21743922		5.63	5.71	5.75	6.08	6.56	7.25
ENSDARG00000025348	igfbp5b	insulin-like growth factor binding protein 5b [Source:ZFIN;Acc:ZDB-GENE-040319-2]	9	46820636	46842179		12.69	12.18	8.22	18.6	17.24	16.62
ENSDARG00000025350	prdx2	peroxiredoxin 2 [Source:ZFIN;Acc:ZDB-GENE-030326-2]	1	51721851	51729862		429.57	568.19	601.05	293.96	320.13	298.43
ENSDARG00000025372	admp	anti-dorsalizing morphogenic protein [Source:ZFIN;Acc:ZDB-GENE-011214-1]	2	53483322	53500424		0.01	0	0	0	0.03	0
ENSDARG00000025375	idh1	isocitrate dehydrogenase (NADP(+)) 1 [Source:ZFIN;Acc:ZDB-GENE-031006-1]	9	28103735	28129476		48.76	73.1	57.42	19.3	18.31	18
ENSDARG00000025383	arl2bp	ADP-ribosylation factor-like 2 binding protein [Source:NCBI gene;Acc:393976]	18	18870625	18875308		6.13	4.44	4.59	5.51	5.09	5.22
ENSDARG00000025386	lsm14b	LSM family member 14B [Source:ZFIN;Acc:ZDB-GENE-040426-779]	23	553396	567183		0.24	0.09	0.07	0.12	0.11	0.2
ENSDARG00000025391	pfdn2	prefoldin subunit 2 [Source:ZFIN;Acc:ZDB-GENE-060519-27]	7	7477398	7493758		31.98	39.51	35.58	27.44	26.92	24.61
ENSDARG00000025397	srsf3a	serine and arginine rich splicing factor 3a [Source:ZFIN;Acc:ZDB-GENE-030616-631]	6	41080003	41085692		0.88	0.52	0.51	2.07	1.75	1.6
ENSDARG00000025403	ftr83	finTRIM family, member 83 [Source:ZFIN;Acc:ZDB-GENE-031002-11]	5	30741730	30753167		6.05	4.3	3.56	3.47	3.23	2.88
ENSDARG00000025404	ube2g2	ubiquitin-conjugating enzyme E2G 2 (UBC7 homolog, yeast) [Source:ZFIN;Acc:ZDB-GENE-050417-288]	6	8598428	8609395		17.29	18.34	16.98	17.15	15.54	17.47
ENSDARG00000025420	fzd5	frizzled class receptor 5 [Source:ZFIN;Acc:ZDB-GENE-990415-218]	9	28232522	28234444		6.57	3.18	2.23	8.58	7.15	7.7
ENSDARG00000025421	tdrd3	tudor domain containing 3 [Source:NCBI gene;Acc:402828]	11	42726712	42819730		4.74	3.14	2.96	4.38	4.5	4.05
ENSDARG00000025428	socs3a	suppressor of cytokine signaling 3a [Source:ZFIN;Acc:ZDB-GENE-030131-7349]	3	57423137	57425961		7.74	7.23	6.63	32.87	33.05	33.48
ENSDARG00000025436	msrb1a	methionine sulfoxide reductase B1a [Source:NCBI gene;Acc:352916]	3	18798431	18805294		5.39	6.93	5.71	6.11	5.35	5.23
ENSDARG00000025465	spon2a	spondin 2a, extracellular matrix protein [Source:ZFIN;Acc:ZDB-GENE-990415-160]	14	21861099	21901815		1.27	1.56	1.41	4.83	4.64	4.71
ENSDARG00000025467	aatf	apoptosis antagonizing transcription factor [Source:ZFIN;Acc:ZDB-GENE-030131-9654]	5	56277866	56306081		9.7	11.47	11.22	7.63	6.85	6.71
ENSDARG00000025468	bnip3la	BCL2 interacting protein 3 like a [Source:ZFIN;Acc:ZDB-GENE-030131-2283]	8	5202589	5220125		24.02	15.95	14.82	21.93	19.49	21.56
ENSDARG00000025478	gipr	gastric inhibitory polypeptide receptor [Source:ZFIN;Acc:ZDB-GENE-050516-4]	15	28940352	28974120		1.33	0.52	0.61	1.17	1.2	0.96
ENSDARG00000025495	nhlh2	nescient helix loop helix 2 [Source:ZFIN;Acc:ZDB-GENE-040426-1809]	9	32978302	32981660		15.86	7.8	9.54	18.2	18.24	18.93
ENSDARG00000025500	mtx1a	metaxin 1a [Source:ZFIN;Acc:ZDB-GENE-060606-2]	16	42938859	42965192		2.29	2	1.35	2.89	3.19	3.17
ENSDARG00000025504	gucy2f	guanylate cyclase 2F, retinal [Source:ZFIN;Acc:ZDB-GENE-011128-7]	15	29362714	29381134		1.95	0.45	0.43	0.9	0.96	0.94
ENSDARG00000025505	si:ch73-335d12.2	si:ch73-335d12.2 [Source:ZFIN;Acc:ZDB-GENE-060503-423]	7	3783875	3850090		0.02	0.04	0	0.02	0	0
ENSDARG00000025507	wdr24	WD repeat domain 24 [Source:NCBI gene;Acc:406336]	24	37640626	37660664		5.08	3.58	3.56	4.01	4.25	4.32
ENSDARG00000025513	dip2cb	disco-interacting protein 2 homolog Cb [Source:ZFIN;Acc:ZDB-GENE-070912-589]	2	42496498	42558549		6.51	2.37	2.62	9.6	9.96	9.95
ENSDARG00000025518	zgc:101679	zgc:101679 [Source:ZFIN;Acc:ZDB-GENE-041010-181]	1	45332641	45341851		9.63	5.61	7.65	9.13	9.32	8.44
ENSDARG00000025522	sgk1	serum/glucocorticoid regulated kinase 1 [Source:NCBI gene;Acc:324140]	23	31640239	31693309		10.74	9.17	8.32	25.5	27.54	25.82
ENSDARG00000025535	clint1a	clathrin interactor 1a [Source:ZFIN;Acc:ZDB-GENE-030131-2590]	14	35279343	35390283		11.98	12.64	10.26	12.84	13.06	13.1
ENSDARG00000025547	si:ch73-343l4.6	si:ch73-343l4.6 [Source:ZFIN;Acc:ZDB-GENE-090313-158]	1	55903811	55905967		0	0	0.06	0	0	0
ENSDARG00000025549	ndufaf1	NADH:ubiquinone oxidoreductase complex assembly factor 1 [Source:ZFIN;Acc:ZDB-GENE-050306-9]	13	33246150	33251757		18.61	22.4	21.03	13.3	11.85	12.62
ENSDARG00000025554	wdr83os	WD repeat domain 83 opposite strand [Source:ZFIN;Acc:ZDB-GENE-040426-1674]	11	30744266	30747836		34.03	38.45	40.26	29.88	29.15	30.17
ENSDARG00000025555	ormdl3	ORMDL sphingolipid biosynthesis regulator 3 [Source:NCBI gene;Acc:450067]	12	22230412	22238004		1.56	1.24	0.99	1.67	2.11	1.76
ENSDARG00000025559	pex11g	peroxisomal biogenesis factor 11 gamma [Source:ZFIN;Acc:ZDB-GENE-050913-79]	11	5554163	5563498		3.85	6.04	4.75	2.48	2.24	3.18
ENSDARG00000025566	slc25a3b	solute carrier family 25 member 3b [Source:ZFIN;Acc:ZDB-GENE-040426-1916]	25	3306620	3321365		296.72	337.21	353.1	313.55	316.96	311.34
ENSDARG00000025567	nf2b	neurofibromin 2b (merlin) [Source:ZFIN;Acc:ZDB-GENE-040622-3]	21	25801345	25819707		5.67	5.76	5.1	7.43	7.81	7.94
ENSDARG00000025576	mkln1	muskelin 1, intracellular mediator containing kelch motifs [Source:ZFIN;Acc:ZDB-GENE-030131-3942]	4	11690923	11730768		4.53	2.77	2.91	4.45	4.46	4.37
ENSDARG00000025577	zgc:66447	zgc:66447 [Source:NCBI gene;Acc:326023]	14	12020936	12051654		4.99	3.87	4.15	4.6	4.55	4.04
ENSDARG00000025581	rpl10	ribosomal protein L10 [Source:NCBI gene;Acc:336712]	23	19712557	19715859		556.66	670.1	856.53	432.52	470.73	462.34
ENSDARG00000025593	plekhj1	pleckstrin homology domain containing, family J member 1 [Source:NCBI gene;Acc:406577]	2	57848844	57901998		3.35	2.49	2.13	4.22	3.86	4.09
ENSDARG00000025595	agmo	alkylglycerol monooxygenase [Source:NCBI gene;Acc:405819]	15	34334722	34418525		13.91	22.97	19.27	6.61	8.51	8.71
ENSDARG00000025600	or102-5	odorant receptor, family C, subfamily 102, member 5 [Source:ZFIN;Acc:ZDB-GENE-010308-1]	15	31481939	31482892		0.05	0	0	0.05	0	0
ENSDARG00000025601	trim35-14	tripartite motif containing 35-14 [Source:ZFIN;Acc:ZDB-GENE-110920-6]	4	1461079	1466395		0.04	0	0	0.04	0.01	0.05
ENSDARG00000025605	nvl	nuclear VCP like [Source:ZFIN;Acc:ZDB-GENE-040426-2871]	13	516258	554033		11.12	8.79	9.62	10.24	10.66	10.96
ENSDARG00000025607	ifnphi1	interferon phi 1 [Source:ZFIN;Acc:ZDB-GENE-030721-3]	3	22362687	22366562		0.03	0.17	0.19	0.17	0.6	0.36
ENSDARG00000025608	casp6b.1	caspase 6b.1, apoptosis-related cysteine peptidase [Source:ZFIN;Acc:ZDB-GENE-041010-48]	3	32953075	32958574		0.76	0.77	0.65	0.58	0.49	0.34
ENSDARG00000025615	prr15la	proline rich 15 like a [Source:NCBI gene;Acc:445084]	3	24189804	24197270		5.15	4.2	3.78	2.81	2.74	2.74
ENSDARG00000025641	gli2a	GLI family zinc finger 2a [Source:ZFIN;Acc:ZDB-GENE-990706-8]	9	37141836	37297485		3.85	3.64	3.04	6.01	6.04	5.39
ENSDARG00000025658	or111-2	odorant receptor, family D, subfamily 111, member 2 [Source:ZFIN;Acc:ZDB-GENE-010308-19]	15	31331356	31364729		0.17	0.11	0.14	0.2	0.14	0.15
ENSDARG00000025667	adgrb2	adhesion G protein-coupled receptor B2 [Source:ZFIN;Acc:ZDB-GENE-060503-573]	19	38766613	38872650		3.33	0.68	0.9	4.88	4.15	4.61
ENSDARG00000025670	fstl3	follistatin-like 3 (secreted glycoprotein) [Source:ZFIN;Acc:ZDB-GENE-050913-111]	22	18884971	18897231		5.5	6.69	7.05	6.11	7.5	7.17
ENSDARG00000025671	grm6b	glutamate receptor, metabotropic 6b [Source:ZFIN;Acc:ZDB-GENE-021120-2]	11	25634041	25679721		4.9	0.95	0.8	5.33	5.33	5.54
ENSDARG00000025672	antxr1a	ANTXR cell adhesion molecule 1a [Source:ZFIN;Acc:ZDB-GENE-110411-15]	8	50066802	50133064		2.04	2.61	2.85	2.82	3.19	3.01
ENSDARG00000025679	comtb	catechol-O-methyltransferase b [Source:NCBI gene;Acc:565370]	11	25619892	25631442		3.42	3.12	3.24	4.19	4.31	4.42
ENSDARG00000025693	tmem9b	TMEM9 domain family, member B [Source:ZFIN;Acc:ZDB-GENE-030131-4656]	7	28556166	28568238		6.16	5.02	5.76	7.43	7.2	7.14
ENSDARG00000025699	mbd1b	methyl-CpG binding domain protein 1b [Source:ZFIN;Acc:ZDB-GENE-030131-6318]	11	25560072	25576199		11	7.64	7.25	12.26	11.21	11.63
ENSDARG00000025703	dmgdh	dimethylglycine dehydrogenase [Source:ZFIN;Acc:ZDB-GENE-080227-8]	21	51521	73786		29.67	45.13	40.5	50.76	52.6	48.13
ENSDARG00000025718	cxxc1b	CXXC finger protein 1b [Source:ZFIN;Acc:ZDB-GENE-030728-4]	11	25539698	25550845		9.73	7.16	7.95	9.18	9.55	10.09
ENSDARG00000025728	grin1b	glutamate receptor, ionotropic, N-methyl D-aspartate 1b [Source:ZFIN;Acc:ZDB-GENE-051202-2]	5	29597645	29643930		54.82	6.21	8.77	47.05	44.29	47.83
ENSDARG00000025754	ube2f	ubiquitin-conjugating enzyme E2F (putative) [Source:NCBI gene;Acc:406606]	1	6646529	6664123		11.23	10.01	10.11	13.32	12.66	12.97
ENSDARG00000025757	tspan35	tetraspanin 35 [Source:ZFIN;Acc:ZDB-GENE-040426-1362]	3	14611299	14638399		9.11	11.44	10.91	8.18	8.55	8.85
ENSDARG00000025766	DTX3	zgc:66440 [Source:ZFIN;Acc:ZDB-GENE-040426-1640]	23	27041111	27061072	DTX3	3.61	1.5	1.84	4.5	4.21	4.36
ENSDARG00000025783	si:ch211-125e6.11	si:ch211-125e6.11 [Source:ZFIN;Acc:ZDB-GENE-070912-35]	9	33207574	33209659		0	0	0	0.1	0.08	0
ENSDARG00000025788	chp2	calcineurin-like EF-hand protein 2 [Source:ZFIN;Acc:ZDB-GENE-030131-5810]	15	20406143	20412286		9.42	10.4	10.07	9	9.15	10.7
ENSDARG00000025789	chd4b	chromodomain helicase DNA binding protein 4b [Source:ZFIN;Acc:ZDB-GENE-030131-4532]	16	31761825	31791165		34.69	21.69	18.55	39.07	36.33	38.24
ENSDARG00000025797	abhd2a	abhydrolase domain containing 2a [Source:NCBI gene;Acc:393888]	7	13824150	13858955		0.9	0.56	0.63	0.83	1.09	0.81
ENSDARG00000025808	taf5l	TAF5-like RNA polymerase II, p300/CBP-associated factor (PCAF)-associated factor [Source:ZFIN;Acc:ZDB-GENE-030131-5987]	1	53911834	53918839		4.2	2.91	2.35	3.76	3.42	3.36
ENSDARG00000025820	chek2	checkpoint kinase 2 [Source:NCBI gene;Acc:336998]	5	10055980	10082244		3.74	3.85	3.22	2.72	2.1	2.09
ENSDARG00000025824	dmtf1	cyclin D binding myb-like transcription factor 1 [Source:NCBI gene;Acc:436938]	4	9652216	9667410		4.28	2.45	3.27	4.59	5.01	5.03
ENSDARG00000025826	gna12a	guanine nucleotide binding protein (G protein) alpha 12a [Source:ZFIN;Acc:ZDB-GENE-050221-1]	3	41647637	41706238		6.07	6.64	5.7	9.66	9.63	9.19
ENSDARG00000025847	sox12	SRY-box transcription factor 12 [Source:ZFIN;Acc:ZDB-GENE-040724-33]	11	24187610	24191928		14.57	4.49	6.57	14.85	13.73	16.73
ENSDARG00000025850	rps21	ribosomal protein S21 [Source:NCBI gene;Acc:394166]	23	9220436	9228578		756.27	1017.29	1146.67	625.21	651.39	650.54
ENSDARG00000025854	ddx51	DEAD (Asp-Glu-Ala-Asp) box polypeptide 51 [Source:NCBI gene;Acc:445387]	8	49065348	49089382		10.59	11.66	10.88	5.74	5.56	5.75
ENSDARG00000025855	camk2n1a	calcium/calmodulin-dependent protein kinase II inhibitor 1a [Source:ZFIN;Acc:ZDB-GENE-050522-319]	2	44943396	44946094		31.33	14.7	20.36	41.59	43.45	44.86
ENSDARG00000025858	zgc:56525	zgc:56525 [Source:ZFIN;Acc:ZDB-GENE-040426-1060]	8	49092039	49115695		11.46	10.62	10.36	12.39	12.28	12.95
ENSDARG00000025859	lmf2b	lipase maturation factor 2b [Source:ZFIN;Acc:ZDB-GENE-070206-11]	4	9478500	9502846		13.01	11	7.43	11.08	9.93	10.62
ENSDARG00000025866	ankdd1b	ankyrin repeat and death domain containing 1B [Source:ZFIN;Acc:ZDB-GENE-060526-136]	5	45918680	45938325		1.3	1.03	1.15	1.07	1.35	1.78
ENSDARG00000025875	kctd2	potassium channel tetramerization domain containing 2 [Source:ZFIN;Acc:ZDB-GENE-060825-85]	12	26594013	26609347		3.46	1.57	1.47	5.1	5.19	5.49
ENSDARG00000025889	usp14	ubiquitin specific peptidase 14 (tRNA-guanine transglycosylase) [Source:ZFIN;Acc:ZDB-GENE-030131-7676]	2	2790573	2813259		21.25	22.82	25.79	19.03	18.34	17.84
ENSDARG00000025891	shox	short stature homeobox [Source:ZFIN;Acc:ZDB-GENE-051030-21]	9	34641237	34647695		21.69	8.2	11.24	24.66	25.86	28.38
ENSDARG00000025899	pnx	posterior neuron-specific homeobox [Source:NCBI gene;Acc:352939]	10	2799285	2801857		0.11	0.3	0.07	0.08	0.03	0.06
ENSDARG00000025902	plekhg5a	pleckstrin homology domain containing, family G (with RhoGef domain) member 5a [Source:ZFIN;Acc:ZDB-GENE-060503-766]	11	24464356	24538860		3.6	1.98	1.83	4.38	4.67	4.5
ENSDARG00000025903	lgals9l1	lectin, galactoside-binding, soluble, 9 (galectin 9)-like 1 [Source:ZFIN;Acc:ZDB-GENE-030131-9543]	15	31032255	31043183		2.66	2.98	2.35	1.9	2.13	2.12
ENSDARG00000025904	tecrb	trans-2,3-enoyl-CoA reductase b [Source:ZFIN;Acc:ZDB-GENE-030131-5154]	1	55752593	55777154		60.68	63.55	60.59	57.86	57.63	56.2
ENSDARG00000025912	bscl2l	BSCL2 lipid droplet biogenesis associated, seipin, like [Source:ZFIN;Acc:ZDB-GENE-050320-130]	14	6159162	6175947		11.54	17.11	17.08	10.56	10.5	11.07
ENSDARG00000025914	si:dkey-190g11.3	si:dkey-190g11.3 [Source:ZFIN;Acc:ZDB-GENE-081104-341]	8	17069577	17075398		0.49	0.21	0.32	0.99	1.28	1.26
ENSDARG00000025920	tiam1b	TIAM Rac1 associated GEF 1b [Source:ZFIN;Acc:ZDB-GENE-120221-1]	15	42397125	42551156		2.27	1.35	1.36	3.61	4.11	4.15
ENSDARG00000025943	crygs3	crystallin, gamma S3 [Source:ZFIN;Acc:ZDB-GENE-050223-3]	9	22351443	22366295		0	0.02	0.04	0.05	0	0
ENSDARG00000025948	mlh1	mutL homolog 1, colon cancer, nonpolyposis type 2 (E. coli) [Source:ZFIN;Acc:ZDB-GENE-040426-1600]	13	42602406	42617639		4.67	4.7	4.82	2.71	2.72	2.67
ENSDARG00000025949	irak1	interleukin-1 receptor-associated kinase 1 [Source:ZFIN;Acc:ZDB-GENE-071116-5]	8	7571095	7603700		1.61	1.25	1.43	1.71	1.9	1.84
ENSDARG00000025953	rhoq	ras homolog family member Q [Source:ZFIN;Acc:ZDB-GENE-030131-5721]	12	25110016	25150279		9.02	8.26	9.05	9.92	9.77	10.08
ENSDARG00000025972	mcts1	MCTS1 re-initiation and release factor [Source:NCBI gene;Acc:394046]	14	33507608	33513079		47.4	61.02	52.53	29.64	32.03	32.35
ENSDARG00000025974	magi3b	membrane associated guanylate kinase, WW and PDZ domain containing 3b [Source:ZFIN;Acc:ZDB-GENE-060503-301]	6	47529881	47796989		1.83	0.62	0.47	2.12	2.29	2.49
ENSDARG00000025977	blmh	bleomycin hydrolase [Source:ZFIN;Acc:ZDB-GENE-030131-8485]	15	28634580	28785512		25.92	25.06	23.61	18.12	18.63	17.84
ENSDARG00000025983	l3mbtl3	L3MBTL histone methyl-lysine binding protein 3 [Source:ZFIN;Acc:ZDB-GENE-040724-127]	20	2134816	2206310		1.06	0.82	0.77	1.65	1.71	1.82
ENSDARG00000026017	xpnpep2	X-prolyl aminopeptidase (aminopeptidase P) 2, membrane-bound [Source:ZFIN;Acc:ZDB-GENE-040426-1151]	14	33591626	33613794		3.49	5.17	3.55	0.53	0.74	0.63
ENSDARG00000026024	mospd2	motile sperm domain containing 2 [Source:ZFIN;Acc:ZDB-GENE-041114-1]	9	54984537	55051256		14.74	18.85	16.68	8.76	9.48	9.1
ENSDARG00000026028	ankrd44	ankyrin repeat domain 44 [Source:ZFIN;Acc:ZDB-GENE-070912-605]	9	32109911	32158288		4.02	1.91	2.15	5.43	5.18	5.28
ENSDARG00000026035	ube2b	ubiquitin-conjugating enzyme E2B (RAD6 homolog) [Source:ZFIN;Acc:ZDB-GENE-040718-247]	21	45366229	45375285		11.49	8.6	8.23	15.2	15.72	16.3
ENSDARG00000026048	tsc1a	TSC complex subunit 1a [Source:ZFIN;Acc:ZDB-GENE-030131-9111]	5	29652198	29672144		6.35	4.93	3.91	6.03	6	5.89
ENSDARG00000026049	mxf	myxovirus (influenza virus) resistance F [Source:ZFIN;Acc:ZDB-GENE-030721-9]	15	5086338	5106171		0.24	0.35	0.41	0.31	0.37	0.37
ENSDARG00000026052	ccdc3a	coiled-coil domain containing 3a [Source:ZFIN;Acc:ZDB-GENE-041210-326]	4	8001874	8014463		1.63	1.77	1.27	2.42	2.69	2.28
ENSDARG00000026053	hltf	helicase-like transcription factor [Source:ZFIN;Acc:ZDB-GENE-030131-3306]	2	24944040	24962820		2.77	1.66	1.99	2	2.03	2.33
ENSDARG00000026068	trappc5	trafficking protein particle complex 5 [Source:ZFIN;Acc:ZDB-GENE-040718-185]	1	45663727	45669444		21.94	18.87	19.08	17.43	16.06	17.47
ENSDARG00000026070	cd82b	CD82 molecule b [Source:ZFIN;Acc:ZDB-GENE-030131-2857]	18	27571448	27626391		19.1	20.14	17.47	16.7	17.39	17.56
ENSDARG00000026072	pdcd5	programmed cell death 5 [Source:ZFIN;Acc:ZDB-GENE-040426-980]	18	31089957	31105391		36.91	45.85	41.71	33.88	35.54	36.21
ENSDARG00000026081	lhcgr	luteinizing hormone/choriogonadotropin receptor [Source:ZFIN;Acc:ZDB-GENE-040806-3]	13	47573405	47618847		0.05	0.07	0.1	0.17	0.17	0.12
ENSDARG00000026084	si:dkey-43k4.5	si:dkey-43k4.5 [Source:ZFIN;Acc:ZDB-GENE-120215-82]	6	21633286	21678263		0.62	0.37	0.48	0.56	0.71	0.66
ENSDARG00000026086		zgc:162324 [Source:ZFIN;Acc:ZDB-GENE-070410-73]	6	46258820	46309060		0.16	0.07	0.17	0.16	0.38	0.28
ENSDARG00000026090	adprm	ADP-ribose/CDP-alcohol diphosphatase, manganese-dependent [Source:NCBI gene;Acc:393393]	12	288108	290815		1.96	2.04	2.07	2.18	2.29	2.3
ENSDARG00000026098	si:dkey-13a21.4	si:dkey-13a21.4 [Source:ZFIN;Acc:ZDB-GENE-040724-163]	11	25430851	25435922		1.37	2.1	1.55	0.29	0.44	0.74
ENSDARG00000026109	slc48a1b	solute carrier family 48 member 1b [Source:NCBI gene;Acc:792763]	6	38896158	38902498		11.41	15.35	15.89	10.92	10.8	10.44
ENSDARG00000026137	sfxn5b	sideroflexin 5b [Source:ZFIN;Acc:ZDB-GENE-050706-107]	14	45306073	45335712		10.12	9.23	10.66	11.15	11.43	11.01
ENSDARG00000026149	slc46a1	solute carrier family 46 member 1 [Source:NCBI gene;Acc:393255]	15	28175638	28186529		2.14	2.31	1.89	4.11	4.73	4.15
ENSDARG00000026165	col11a1a	collagen, type XI, alpha 1a [Source:ZFIN;Acc:ZDB-GENE-070209-167]	24	28764021	28893303		119.07	116.85	63.37	189.71	179.06	181.3
ENSDARG00000026166	emilin1b	elastin microfibril interfacer 1b [Source:ZFIN;Acc:ZDB-GENE-060818-36]	17	12439966	12498219		14.03	17.71	15.36	11.9	12.03	12.89
ENSDARG00000026170	hps1	HPS1 biogenesis of lysosomal organelles complex 3 subunit 1 [Source:ZFIN;Acc:ZDB-GENE-051113-152]	13	40686133	40716702		2.07	2.04	1.9	3.19	2.87	2.93
ENSDARG00000026178	ube3d	ubiquitin protein ligase E3D [Source:ZFIN;Acc:ZDB-GENE-091218-3]	16	4770266	4796565		2.53	2.5	2.84	1.91	2.09	1.71
ENSDARG00000026180	prpf8	pre-mRNA processing factor 8 [Source:ZFIN;Acc:ZDB-GENE-030131-577]	15	28248052	28262709		28.24	19.73	15.87	28.93	28.2	29.27
ENSDARG00000026183	polr2eb	polymerase (RNA) II (DNA directed) polypeptide E, b [Source:ZFIN;Acc:ZDB-GENE-040801-83]	22	17606588	17611582		30.44	38.76	43.5	20.15	20.24	20.76
ENSDARG00000026185	yju2	YJU2 splicing factor homolog [Source:NCBI gene;Acc:393117]	2	52049239	52063440		12.96	12.95	12	10.3	9.85	9.71
ENSDARG00000026198	enoph1	enolase-phosphatase 1 [Source:NCBI gene;Acc:431773]	10	5025696	5037449		13.92	13.85	15.04	14.17	14.2	14.12
ENSDARG00000026224	fanci	FA complementation group I [Source:ZFIN;Acc:ZDB-GENE-030131-2905]	25	8447565	8479171		2.08	1.78	1.75	1.17	1.04	1.26
ENSDARG00000026225	nsd2	nuclear receptor binding SET domain protein 2 [Source:ZFIN;Acc:ZDB-GENE-030131-2581]	13	12993851	13030851		8.33	4.88	4.08	7.61	7.13	7.27
ENSDARG00000026229	prnpa	prion protein a [Source:ZFIN;Acc:ZDB-GENE-041217-6]	8	43053519	43056975		66.25	10.5	5.16	19.33	21.93	21.93
ENSDARG00000026236	zgc:56585	zgc:56585 [Source:ZFIN;Acc:ZDB-GENE-040426-1084]	21	34835811	34844316		5.5	6.67	7.29	3.59	4.54	3.93
ENSDARG00000026246	CR847944.1	adenosine receptor A1-like [Source:NCBI gene;Acc:571410]	6	54481941	54486410		0.05	0.07	0.06	0.02	0.05	0.05
ENSDARG00000026247	insyn1	inhibitory synaptic factor 1 [Source:NCBI gene;Acc:100003007]	18	50275933	50356788		5.37	1.56	2.06	8.44	8.8	8.64
ENSDARG00000026248	slc1a7b	solute carrier family 1 member 7b [Source:ZFIN;Acc:ZDB-GENE-101111-7]	23	41949575	42093537		3.33	0.34	0.45	2.47	2.85	3.43
ENSDARG00000026281	lyrm1	LYR motif containing 1 [Source:NCBI gene;Acc:436779]	24	39509721	39518630		2.82	1.86	2.07	1.68	1.63	1.86
ENSDARG00000026294	erbb2	erb-b2 receptor tyrosine kinase 2 [Source:ZFIN;Acc:ZDB-GENE-031118-121]	12	9360316	9438227		6.06	5.13	4.02	7.41	7.34	6.68
ENSDARG00000026296	zgc:112994	zgc:112994 [Source:ZFIN;Acc:ZDB-GENE-050522-519]	23	38159715	38182757		1.31	1.44	1	0.91	0.91	0.84
ENSDARG00000026313	adgre5a	adhesion G protein-coupled receptor E5a [Source:ZFIN;Acc:ZDB-GENE-130530-571]	3	52467879	52471761		0.07	0.04	0	0	0	0.04
ENSDARG00000026322	dhrs13a.1	dehydrogenase/reductase (SDR family) member 13a, tandem duplicate 1 [Source:ZFIN;Acc:ZDB-GENE-041114-58]	10	36795861	36808348		9.51	13.09	13.08	9.37	9.65	9.41
ENSDARG00000026325	mmp11a	matrix metallopeptidase 11a [Source:ZFIN;Acc:ZDB-GENE-070817-3]	8	30797363	30825415		7.21	9.56	10.45	5.24	5.08	6.02
ENSDARG00000026329	arhgap29a	Rho GTPase activating protein 29a [Source:ZFIN;Acc:ZDB-GENE-030131-9510]	24	28448387	28557301		2.78	2.45	2.07	4.19	4.14	4.01
ENSDARG00000026333	xkr7	XK, Kell blood group complex subunit-related family, member 7 [Source:ZFIN;Acc:ZDB-GENE-051113-336]	23	8989168	9015026		2.17	0.79	0.9	2.38	2.56	2.64
ENSDARG00000026335	susd4	sushi domain containing 4 [Source:NCBI gene;Acc:560304]	17	45529453	45552631		3.25	1.07	1.79	3.05	3.01	3.07
ENSDARG00000026338	styxl1	serine/threonine/tyrosine interacting-like 1 [Source:ZFIN;Acc:ZDB-GENE-040912-45]	10	35257651	35262368		0.07	0.15	0.08	0.02	0.02	0.18
ENSDARG00000026348	csad	cysteine sulfinic acid decarboxylase [Source:ZFIN;Acc:ZDB-GENE-041114-36]	23	36424768	36441693		12.83	16.87	15.2	19.15	23.24	23.06

ENSDARG00000026350	wasb	WASP actin nucleation promoting factor b [Source:ZFIN;Acc:ZDB-GENE-030131-7098]	8	23560183	23578660		1.43	1.47	1.48	2.07	2.62	2.41
ENSDARG00000026359	pbld2	phenazine biosynthesis-like protein domain containing 2 [Source:ZFIN;Acc:ZDB-GENE-050417-456]	13	22849796	22862133		8.68	12.69	12.55	5.96	4.72	5.6
ENSDARG00000026369	dbi	diazepam binding inhibitor (GABA receptor modulator, acyl-CoA binding protein) [Source:ZFIN;Acc:ZDB-GENE-040426-1861]	9	907459	914886		126.36	192.35	177.02	63.48	67.42	66.15
ENSDARG00000026376	aco1	aconitase 1, soluble [Source:ZFIN;Acc:ZDB-GENE-031118-76]	7	71463106	71486162		18.87	21.36	13.48	17.56	17.47	17.09
ENSDARG00000026383	si:dkey-27j5.5	si:dkey-27j5.5 [Source:ZFIN;Acc:ZDB-GENE-121214-264]	1	57277945	57280585		1.22	0.36	0.67	1.38	1.23	1.55
ENSDARG00000026387	pip4p1b	phosphatidylinositol-4,5-bisphosphate 4-phosphatase 1b [Source:NCBI gene;Acc:553253]	1	10318077	10325025		4.02	2.17	2.11	2.84	3.71	3.91
ENSDARG00000026395	rfx4	regulatory factor X, 4 [Source:NCBI gene;Acc:403016]	18	15334117	15373620		2.3	1.04	1.32	4.93	5.27	5.33
ENSDARG00000026400	rad51d	RAD51 paralog D [Source:ZFIN;Acc:ZDB-GENE-040426-2490]	5	60873055	60885935		1.24	1.13	1.2	1.12	1.06	0.95
ENSDARG00000026403	hephl1b	hephaestin-like 1b [Source:ZFIN;Acc:ZDB-GENE-091204-388]	10	24394023	24416471		0.28	0.14	0.3	0.19	0.29	0.26
ENSDARG00000026406	anxa5a	annexin A5a [Source:ZFIN;Acc:ZDB-GENE-080220-29]	14	46410766	46434196		18.25	23.7	22.36	5.53	5.68	5.63
ENSDARG00000026417	ccr12b.2	chemokine (C-C motif) receptor 12b, tandem duplicate 2 [Source:ZFIN;Acc:ZDB-GENE-070301-2]	24	264839	265858		0.14	0.18	0.25	0.39	0.46	0.55
ENSDARG00000026444	apoob	apolipoprotein O, b [Source:ZFIN;Acc:ZDB-GENE-041001-40]	24	21978230	21989406		9.19	5.86	6.44	10.78	11.31	10.69
ENSDARG00000026448	med29	mediator complex subunit 29 [Source:NCBI gene;Acc:797134]	15	15232855	15237211		12.71	12.45	13.31	10.37	10.02	12.41
ENSDARG00000026453	zgc:66474	zgc:66474 [Source:ZFIN;Acc:ZDB-GENE-030131-5711]	3	15646192	15668433		4.07	2.54	2.37	3.85	4.05	3.94
ENSDARG00000026454	mis12	MIS12 kinetochore complex component [Source:ZFIN;Acc:ZDB-GENE-040426-1372]	5	58990583	58996324		2.43	1.93	2.04	1.1	1.54	1.22
ENSDARG00000026473	six1b	SIX homeobox 1b [Source:NCBI gene;Acc:404627]	20	20499869	20505918		7.27	8.33	8.87	6.19	6.63	6.51
ENSDARG00000026476	tia1l	cytotoxic granule-associated RNA binding protein 1, like [Source:ZFIN;Acc:ZDB-GENE-030131-2167]	5	24510619	24517768		33.8	33.8	31.46	39.38	38.48	37.81
ENSDARG00000026482	arhgap12b	Rho GTPase activating protein 12b [Source:ZFIN;Acc:ZDB-GENE-040426-1727]	12	26621906	26726048		7.46	3.15	2.63	7.56	7.42	7.64
ENSDARG00000026484	rab15	RAB15, member RAS oncogene family [Source:ZFIN;Acc:ZDB-GENE-040718-2]	20	28782903	28800999		8.56	4.28	6.25	7.99	8.63	9.1
ENSDARG00000026489	khsrp	KH-type splicing regulatory protein [Source:ZFIN;Acc:ZDB-GENE-030131-4357]	1	54960189	54972236		12.8	9.34	9.45	20.83	20.74	19.3
ENSDARG00000026499	ppm1e	protein phosphatase, Mg2+/Mn2+ dependent, 1E [Source:ZFIN;Acc:ZDB-GENE-070326-2]	10	32683089	32788832		8.96	3.1	2.7	9.63	9.7	9.34
ENSDARG00000026500	xkr9	XK, Kell blood group complex subunit-related family, member 9 [Source:ZFIN;Acc:ZDB-GENE-060421-6224]	24	14202327	14212521		1.2	1.97	2.23	2.02	2.9	1.74
ENSDARG00000026505	si:ch211-285c6.3	si:ch211-285c6.3 [Source:ZFIN;Acc:ZDB-GENE-040724-148]	7	3483334	3510563		0.01	0	0	0.01	0	0
ENSDARG00000026513	hoxb6b	homeobox B6b [Source:NCBI gene;Acc:58053]	12	27127139	27128768		4.96	4.18	4.4	6.22	5.77	6.7
ENSDARG00000026519	ralgps2	Ral GEF with PH domain and SH3 binding motif 2 [Source:ZFIN;Acc:ZDB-GENE-040426-1212]	20	15933028	16156419		1.71	0.86	0.84	2.51	2.3	2.24
ENSDARG00000026531	alcama	activated leukocyte cell adhesion molecule a [Source:NCBI gene;Acc:30194]	10	28731749	28835812		30.73	23.56	23.2	42.06	42.63	41.46
ENSDARG00000026540	ppp4r2a	protein phosphatase 4, regulatory subunit 2a [Source:NCBI gene;Acc:326723]	23	10805188	10827419		8.99	7.49	7.22	8.66	7.46	8.44
ENSDARG00000026548	cyp2u1	cytochrome P450, family 2, subfamily U, polypeptide 1 [Source:ZFIN;Acc:ZDB-GENE-070730-1]	1	49923402	49932040		3.39	2.34	2.69	2.7	2.43	2.51
ENSDARG00000026569	pimr196	Pim proto-oncogene, serine/threonine kinase, related 196 [Source:ZFIN;Acc:ZDB-GENE-070705-328]	2	17569644	17575580		0	0	0	0.02	0	0.01
ENSDARG00000026577	cdk2	cyclin-dependent kinase 2 [Source:ZFIN;Acc:ZDB-GENE-040426-2741]	11	3301277	3308569		9.05	9.06	8.44	5.91	5.72	5.22
ENSDARG00000026582	iscub	iron-sulfur cluster assembly enzyme b [Source:ZFIN;Acc:ZDB-GENE-050417-332]	5	31860043	31868847		5.28	4.56	4.39	3.28	3.28	4.23
ENSDARG00000026595	pafah1b1b	platelet-activating factor acetylhydrolase 1b, regulatory subunit 1b [Source:NCBI gene;Acc:394247]	21	39423974	39454192		40.26	22.34	25.89	42.48	43.11	41.89
ENSDARG00000026599	en2a	engrailed homeobox 2a [Source:NCBI gene;Acc:30243]	7	40988901	40993456		5.31	2.21	2.34	4.47	4.57	4.69
ENSDARG00000026611	socs3b	suppressor of cytokine signaling 3b [Source:ZFIN;Acc:ZDB-GENE-040426-2528]	12	34258139	34327465		6.53	6.12	6.39	36.58	35.96	35.97
ENSDARG00000026616	zgc:113425	zgc:113425 [Source:ZFIN;Acc:ZDB-GENE-050320-128]	14	46190807	46198373		0.32	0.18	0.41	0.42	0.25	0.32
ENSDARG00000026629	gmds	GDP-mannose 4,6-dehydratase [Source:ZFIN;Acc:ZDB-GENE-050419-45]	20	26709009	26822550		9.84	9.09	8.23	8.97	7.89	7.92
ENSDARG00000026630	mark3b	MAP/microtubule affinity-regulating kinase 3b [Source:ZFIN;Acc:ZDB-GENE-060929-80]	20	43792866	43917689		10.38	5.36	4.72	13.06	13.25	13.68
ENSDARG00000026634	ehmt1b	euchromatic histone-lysine N-methyltransferase 1b [Source:ZFIN;Acc:ZDB-GENE-080515-3]	21	14644247	14692119		11.57	4.59	4.68	12.57	12.14	12.06
ENSDARG00000026635	asb1	ankyrin repeat and SOCS box containing 1 [Source:ZFIN;Acc:ZDB-GENE-040718-234]	9	45680802	45697644		0.7	0.72	1.03	1.16	0.87	0.94
ENSDARG00000026654	gosr1	golgi SNAP receptor complex member 1 [Source:ZFIN;Acc:ZDB-GENE-050417-133]	15	24795473	24837655		15.24	14.32	12.38	12.08	12.67	11.22
ENSDARG00000026655	tspo	translocator protein [Source:ZFIN;Acc:ZDB-GENE-041010-125]	6	40578219	40583253		22.6	27	27.94	19.57	20.05	21.37
ENSDARG00000026663	tlr19	toll-like receptor 19 [Source:ZFIN;Acc:ZDB-GENE-040220-3]	16	22722352	22729491		0.03	0.07	0.07	0.02	0.05	0.05
ENSDARG00000026664	uri1	URI1 prefoldin like chaperone [Source:ZFIN;Acc:ZDB-GENE-050913-30]	18	14693682	14705999		21.86	17.05	15.85	17.25	18.25	18.52
ENSDARG00000026680	slc25a14	solute carrier family 25 member 14 [Source:ZFIN;Acc:ZDB-GENE-040426-749]	21	38585686	38619305		2.43	1.58	1.3	3.54	3.04	2.81
ENSDARG00000026682	zgc:101583	zgc:101583 [Source:ZFIN;Acc:ZDB-GENE-041212-75]	14	38882652	38889840		1.72	1.65	1.27	2.22	2.32	1.83
ENSDARG00000026701	gtf2h1	general transcription factor IIH, polypeptide 1 [Source:ZFIN;Acc:ZDB-GENE-040912-164]	25	3196855	3217115		7.15	4.38	3.99	6.07	6.05	5.77
ENSDARG00000026704	ftr72	finTRIM family, member 72 [Source:ZFIN;Acc:ZDB-GENE-041114-86]	15	41752335	41756507		0.18	0.25	0.21	0.23	0.53	0.38
ENSDARG00000026712	rab5c	RAB5C, member RAS oncogene family [Source:ZFIN;Acc:ZDB-GENE-031118-30]	3	17615599	17651899		40.47	33.99	29.24	48.92	47.43	49.33
ENSDARG00000026723	syncripl	synaptotagmin binding, cytoplasmic RNA interacting protein, like [Source:ZFIN;Acc:ZDB-GENE-030131-3104]	17	50261603	50285417		25.61	16.67	15.48	22.48	23.08	21.4
ENSDARG00000026726	anxa1a	annexin A1a [Source:ZFIN;Acc:ZDB-GENE-030131-6664]	5	25578181	25583325		172.99	210.75	218.95	80.64	83.03	79.53
ENSDARG00000026736	il11ra	interleukin 11 receptor, alpha [Source:ZFIN;Acc:ZDB-GENE-030131-5942]	10	13305610	13396720		1.17	1.34	0.98	2.3	2.4	1.82
ENSDARG00000026753	grik4	glutamate receptor, ionotropic, kainate 4 [Source:ZFIN;Acc:ZDB-GENE-070821-5]	15	22722682	23188894		1.21	0.5	0.53	2.27	2.32	2.36
ENSDARG00000026759	ldlrb	low density lipoprotein receptor b [Source:ZFIN;Acc:ZDB-GENE-040426-1254]	6	102506	109626		8.68	4.71	4.83	8.4	8.13	7.44
ENSDARG00000026762	fam126a	family with sequence similarity 126 member A [Source:NCBI gene;Acc:402926]	19	2602903	2669023		9.36	4.99	4.73	9.13	9.22	8.88
ENSDARG00000026764	ahsg1	alpha-2-HS-glycoprotein 1 [Source:ZFIN;Acc:ZDB-GENE-041114-50]	22	37874691	37881714		7.54	9.81	8.04	0.37	0.34	0.53
ENSDARG00000026766	bcl2l10	BCL2 like 10 [Source:ZFIN;Acc:ZDB-GENE-030825-2]	18	39106722	39119005		25.19	18.35	21.76	32.83	35.51	36.62
ENSDARG00000026767	nol11	nucleolar protein 11 [Source:NCBI gene;Acc:321700]	6	22957709	23002373		7.79	8.02	7.61	7.31	7.4	6.7
ENSDARG00000026771	tmem41ab	transmembrane protein 41ab [Source:NCBI gene;Acc:406356]	1	30422143	30438036		4.22	7.31	5.75	5.38	4.79	4.89
ENSDARG00000026784	robo1	roundabout, axon guidance receptor, homolog 1 (Drosophila) [Source:ZFIN;Acc:ZDB-GENE-000209-3]	15	39277099	39820491		5.65	3.71	3.02	13.97	13.22	14.59
ENSDARG00000026787	aqp7	aquaporin 7 [Source:ZFIN;Acc:ZDB-GENE-030131-6461]	21	12010505	12021593		2.77	3.46	3.12	2.6	2.9	2.82
ENSDARG00000026796	grm1a	glutamate receptor, metabotropic 1a [Source:ZFIN;Acc:ZDB-GENE-030131-7892]	20	32047385	32112818		5.59	1.69	1.74	5.57	5.8	6.06
ENSDARG00000026799	suv39h1a	suppressor of variegation 3-9 homolog 1a [Source:NCBI gene;Acc:445198]	8	23395469	23405105		0.13	0.19	0.23	0.08	0.13	0.15
ENSDARG00000026801	stau2	staufen double-stranded RNA binding protein 2 [Source:NCBI gene;Acc:393899]	24	13017586	13160880		84.96	72.37	57.92	92.6	93.97	92.37
ENSDARG00000026806	taf8	TAF8 RNA polymerase II, TATA box binding protein (TBP)-associated factor [Source:NCBI gene;Acc:393256]	23	32392461	32423348		1.84	1.49	1.67	1.9	2.03	1.92
ENSDARG00000026811	extl3	exostosin-like glycosyltransferase 3 [Source:ZFIN;Acc:ZDB-GENE-041124-2]	20	49904818	50014936		1.96	0.88	1.17	3.94	3.63	3.69
ENSDARG00000026820	gucy2d	guanylate cyclase 2D, retinal [Source:ZFIN;Acc:ZDB-GENE-011128-9]	5	38123633	38151033		22.71	4.89	3.19	16	16.83	16.8
ENSDARG00000026829	cotl1	coactosin-like F-actin binding protein 1 [Source:ZFIN;Acc:ZDB-GENE-030131-8325]	18	13203831	13216672		202.01	225.77	198.3	218.73	207.2	198.21
ENSDARG00000026834	cdr2l	cerebellar degeneration-related protein 2-like [Source:NCBI gene;Acc:336227]	3	59951010	59981476		13.63	6.8	7.36	17.08	18.92	18.82
ENSDARG00000026835	slc25a32b	solute carrier family 25 member 32b [Source:ZFIN;Acc:ZDB-GENE-050306-39]	16	47428721	47450004		7.42	6.95	7	5.47	6.21	5.24
ENSDARG00000026839	med21	mediator complex subunit 21 [Source:NCBI gene;Acc:406732]	4	1757462	1759555		14.48	12.75	14.72	12.85	12.55	12.44
ENSDARG00000026840	arhgap23b	Rho GTPase activating protein 23b [Source:ZFIN;Acc:ZDB-GENE-081031-78]	12	15477111	15561869		2.07	0.27	0.26	1.51	1.49	1.54
ENSDARG00000026842	acin1b	apoptotic chromatin condensation inducer 1b [Source:ZFIN;Acc:ZDB-GENE-030826-12]	7	24069421	24097070		25.52	13.87	13.61	25.54	24.88	25.25
ENSDARG00000026845	rhoaa	ras homolog gene family, member Aa [Source:NCBI gene;Acc:406411]	8	14170567	14183265		50.18	47.14	42.13	52.1	53.72	52.65
ENSDARG00000026855	cacna2d4a	calcium channel, voltage-dependent, alpha 2/delta subunit 4a [Source:ZFIN;Acc:ZDB-GENE-041210-213]	4	20271463	20292853		1.44	0.23	0.4	1.47	1.37	1.4
ENSDARG00000026862	aktip	akt interacting protein [Source:NCBI gene;Acc:378716]	7	36467315	36484078		17.13	16.1	15.11	19.02	18.12	17.64
ENSDARG00000026864	si:ch211-226m16.3	si:ch211-226m16.3 [Source:ZFIN;Acc:ZDB-GENE-040724-8]	23	33294633	33309622		0	0.02	0	0	0	0
ENSDARG00000026865	fam107b	family with sequence similarity 107 member B [Source:ZFIN;Acc:ZDB-GENE-031030-12]	18	12276567	12327478		34.37	24.28	21.43	31.78	32.38	34.29
ENSDARG00000026871	uchl1	ubiquitin carboxyl-terminal esterase L1 (ubiquitin thiolesterase) [Source:ZFIN;Acc:ZDB-GENE-030131-3844]	1	22647756	22652424		14.64	12.05	13.76	14.1	14.65	14.78
ENSDARG00000026875	tlcd3ba	TLC domain containing 3Ba [Source:ZFIN;Acc:ZDB-GENE-030131-2046]	3	21044154	21062778		10.46	2.92	2.66	8.59	8.08	8.35
ENSDARG00000026882	palm1a	paralemmin 1a [Source:ZFIN;Acc:ZDB-GENE-050417-409]	11	14199802	14267855		14.22	11.15	14.25	15.22	13.74	15.03
ENSDARG00000026892	gpatch11	G patch domain containing 11 [Source:NCBI gene;Acc:436666]	13	50235706	50247689		10.15	10	8.61	8.83	8.76	9.34
ENSDARG00000026895	tor1l3	torsin family 1 like 3 [Source:ZFIN;Acc:ZDB-GENE-040426-1859]	23	37566097	37575030		0.32	0.55	0.67	0.11	0.16	0.23
ENSDARG00000026902	cryzl1	crystallin, zeta (quinone reductase)-like 1 [Source:ZFIN;Acc:ZDB-GENE-040718-378]	1	47089501	47105214		7.07	6.08	7.3	5.43	5.47	6
ENSDARG00000026904	cbln13	cerebellin 13 [Source:ZFIN;Acc:ZDB-GENE-070912-288]	2	37874771	37878304		1.07	1.49	1.68	0.72	0.8	0.77
ENSDARG00000026907	slc48a1a	solute carrier family 48 member 1a [Source:NCBI gene;Acc:436697]	23	33357163	33361425		7.22	2.95	2.84	9.23	9.62	9.63
ENSDARG00000026908	tmed2	transmembrane p24 trafficking protein 2 [Source:ZFIN;Acc:ZDB-GENE-030131-269]	10	3419832	3427630		67.09	80.65	78.89	75.01	73.96	71.31
ENSDARG00000026925	nos2a	nitric oxide synthase 2a, inducible [Source:ZFIN;Acc:ZDB-GENE-040305-1]	5	43782267	43796971		0.04	0.01	0.02	0.03	0.01	0.01
ENSDARG00000026926	rom1b	retinal outer segment membrane protein 1b [Source:ZFIN;Acc:ZDB-GENE-040426-1073]	14	45675306	45701004		21.87	4.69	3.3	10.66	10.49	10.1
ENSDARG00000026941	fbxl4	F-box and leucine-rich repeat protein 4 [Source:ZFIN;Acc:ZDB-GENE-041114-187]	16	32566218	32582798		3.35	2.32	1.99	3.56	3.18	4.19
ENSDARG00000026967	ap2s1	adaptor related protein complex 2 subunit sigma 1 [Source:ZFIN;Acc:ZDB-GENE-040426-2174]	15	8767650	8799977		47.02	37.04	37.97	49.34	48.68	48.85
ENSDARG00000026972	si:ch211-119o8.6	si:ch211-119o8.6 [Source:ZFIN;Acc:ZDB-GENE-070912-17]	2	38985489	38992313		0.03	0	0.01	0.01	0.02	0.01
ENSDARG00000026979	krt1-c5	keratin, type 1, gene c5 [Source:ZFIN;Acc:ZDB-GENE-060316-3]	5	64869176	64883099		15.88	18.15	20.99	6.79	8.58	7.66
ENSDARG00000026985	lims1	LIM and senescent cell antigen-like domains 1 [Source:ZFIN;Acc:ZDB-GENE-050522-236]	11	33598213	33618612		12.09	12.53	9.58	19.11	19.24	19.62
ENSDARG00000026986	rexo1	REX1, RNA exonuclease 1 homolog [Source:ZFIN;Acc:ZDB-GENE-030131-1650]	8	20455134	20471546		3.98	2.25	2.19	3.82	3.63	3.9
ENSDARG00000026988	tbc1d22b	TBC1 domain family, member 22B [Source:ZFIN;Acc:ZDB-GENE-091118-28]	8	10339869	10449671		1.29	0.68	0.79	1.58	1.34	1.49
ENSDARG00000026990	klhl6	kelch-like family member 6 [Source:ZFIN;Acc:ZDB-GENE-030616-325]	2	36646451	36659156		0.41	0.44	0.48	0.59	0.61	0.63
ENSDARG00000027009	zgc:85777	zgc:85777 [Source:ZFIN;Acc:ZDB-GENE-040426-2210]	19	40861853	40898600		43.24	55.33	49.5	24.76	23.7	23.47
ENSDARG00000027016	gbf1	golgi brefeldin A resistant guanine nucleotide exchange factor 1 [Source:ZFIN;Acc:ZDB-GENE-081107-54]	13	7575563	7749222		5.54	3.93	2.94	8.97	8.53	8.51
ENSDARG00000027017	ppp2r5a	protein phosphatase 2, regulatory subunit B', alpha isoform [Source:ZFIN;Acc:ZDB-GENE-040724-174]	20	13175379	13311918		1.32	0.88	0.56	1.02	0.85	0.9
ENSDARG00000027041	pcdh17	protocadherin 17 [Source:ZFIN;Acc:ZDB-GENE-090608-3]	11	32576300	32723851		20.02	5.32	7.42	22	22.62	23.33
ENSDARG00000027046	scyl3	SCY1-like, kinase-like 3 [Source:ZFIN;Acc:ZDB-GENE-030131-2559]	20	34012571	34028969		9.78	8.07	7.89	8.17	8.01	8.58
ENSDARG00000027063	arpc1b	actin related protein 2/3 complex, subunit 1B [Source:ZFIN;Acc:ZDB-GENE-030131-7414]	3	40809011	40820612		19.7	24.7	25.29	25.59	28.31	27.08
ENSDARG00000027065	slc38a3a	solute carrier family 38 member 3a [Source:ZFIN;Acc:ZDB-GENE-070615-25]	11	34824099	34914857		7.08	5.06	4.86	11.97	12.66	11.78
ENSDARG00000027070	acbd4	acyl-CoA binding domain containing 4 [Source:ZFIN;Acc:ZDB-GENE-040426-2074]	12	15568761	15620090		2.82	2.69	2.36	2.13	2.45	2.2
ENSDARG00000027078	cnppd1	cyclin Pas1/PHO80 domain containing 1 [Source:ZFIN;Acc:ZDB-GENE-030131-6808]	1	6161930	6172328		12.12	10.62	9.07	12.99	12.94	12.38
ENSDARG00000027079	znf804a	zinc finger protein 804A [Source:ZFIN;Acc:ZDB-GENE-070912-632]	9	11906770	12034444		4.41	0.7	1.03	4.77	4.51	4.37
ENSDARG00000027082	hmbox1a	homeobox containing 1a [Source:ZFIN;Acc:ZDB-GENE-050913-89]	17	16298122	16324565		3.17	1.25	1.45	3.63	3.63	3.23
ENSDARG00000027087	tgfb2	transforming growth factor, beta 2 [Source:ZFIN;Acc:ZDB-GENE-030723-3]	16	2745006	2807527		6.49	6.22	5.81	10.82	11.11	11.75
ENSDARG00000027088	ptgdsb.1	prostaglandin D2 synthase b, tandem duplicate 1 [Source:ZFIN;Acc:ZDB-GENE-030131-8436]	24	17027310	17029374		42.19	60.23	60.68	53.52	49.22	47.48
ENSDARG00000027099	psmc4	proteasome 26S subunit, ATPase 4 [Source:ZFIN;Acc:ZDB-GENE-030131-5083]	16	41794715	41805240		65.96	74.77	78.72	51.33	52.6	51.77
ENSDARG00000027109	zfr	zinc finger RNA binding protein [Source:NCBI gene;Acc:321659]	5	41124098	41142768		32.24	16.71	15.24	42.27	42.19	43.68
ENSDARG00000027112	ephb4b	eph receptor B4b [Source:NCBI gene;Acc:30691]	23	44349252	44401223		5.33	5.93	5.03	7.36	7.42	7.4
ENSDARG00000027129	tmem218	transmembrane protein 218 [Source:NCBI gene;Acc:393369]	10	31222433	31230014		1.71	0.61	1.12	2.3	2.16	2.57
ENSDARG00000027131	tmem169a	transmembrane protein 169a [Source:ZFIN;Acc:ZDB-GENE-090107-1]	11	30348273	30352333		10.73	3.99	5.69	8.97	10.06	9.74
ENSDARG00000027140	nup160	nucleoporin 160 [Source:ZFIN;Acc:ZDB-GENE-040426-1603]	25	34681018	34723976		13.87	11.13	10.13	14.27	15.63	15.56
ENSDARG00000027141	ube2l3b	ubiquitin-conjugating enzyme E2L 3b [Source:ZFIN;Acc:ZDB-GENE-030131-662]	5	13337507	13353905		29.02	25.98	20	31.66	29.58	30.29
ENSDARG00000027143	aptx	aprataxin [Source:NCBI gene;Acc:405797]	1	52653626	52659711		6.03	7.14	6.59	5.44	4.62	4.83
ENSDARG00000027153	gabrr3a	gamma-aminobutyric acid (GABA) A receptor, rho 3a [Source:ZFIN;Acc:ZDB-GENE-080722-20]	10	35367722	35410518		1.71	0.54	0.26	2.27	2.17	2.46
ENSDARG00000027154	tpd52l2a	tpd52 like 2a [Source:ZFIN;Acc:ZDB-GENE-030131-7047]	8	23194212	23212965		13.5	10.25	10.81	9.64	9.51	10.54
ENSDARG00000027159	fbxo28	F-box protein 28 [Source:ZFIN;Acc:ZDB-GENE-030131-4665]	13	482911	490569		18.02	11.21	13.43	20.38	22.4	20.66
ENSDARG00000027161	iglv3v2	immunoglobulin light 3 variable 2 [Source:ZFIN;Acc:ZDB-GENE-060503-410]	24	39700552	39701027		0	0	0	0	0	0
ENSDARG00000027169	kpna7	karyopherin alpha 7 (importin alpha 8) [Source:ZFIN;Acc:ZDB-GENE-040426-2023]	10	25214270	25217347		0.05	0.03	0.03	0.03	0.09	0.03
ENSDARG00000027172	phactr2	phosphatase and actin regulator 2 [Source:ZFIN;Acc:ZDB-GENE-040724-11]	13	23919480	23956361		4.47	3.74	3.7	5.33	5.53	5.85
ENSDARG00000027182	rnf25	ring finger protein 25 [Source:ZFIN;Acc:ZDB-GENE-040426-1581]	3	32671146	32680126		19.56	18.42	18.15	16.91	17.61	18.72
ENSDARG00000027183	namptb	nicotinamide phosphoribosyltransferase b [Source:ZFIN;Acc:ZDB-GENE-030131-2931]	11	19729771	19775182		7.37	4.53	5.33	9.11	9.04	9.5
ENSDARG00000027187	kat8	K(lysine) acetyltransferase 8 [Source:ZFIN;Acc:ZDB-GENE-030131-7510]	3	32681274	32689328		9.9	8.42	8.29	7.87	8.47	8.49
ENSDARG00000027192	hcn3	hyperpolarization activated cyclic nucleotide-gated potassium channel 3 [Source:ZFIN;Acc:ZDB-GENE-060503-193]	19	9186175	9208113		1.42	0.56	0.66	0.97	1.2	1.09
ENSDARG00000027196	si:dkey-16l2.17	si:dkey-16l2.17 [Source:ZFIN;Acc:ZDB-GENE-141212-262]	3	32689707	32696592		1.11	0.9	1	2.37	2.63	2.44
ENSDARG00000027199	smad1	SMAD family member 1 [Source:NCBI gene;Acc:30628]	1	35896488	35929143		7.65	7.07	5.57	10.37	10.41	10.32
ENSDARG00000027200	gabarapl2	GABA(A) receptor-associated protein like 2 [Source:ZFIN;Acc:ZDB-GENE-040718-336]	25	34835553	34845302		37.88	34.65	40.22	33.45	36.98	39.3
ENSDARG00000027222	adgrg1	adhesion G protein-coupled receptor G1 [Source:ZFIN;Acc:ZDB-GENE-030131-3671]	7	28668979	28696556		3.38	3.08	2.85	4.03	4.04	4.2
ENSDARG00000027234	ift88	intraflagellar transport 88 homolog [Source:ZFIN;Acc:ZDB-GENE-030131-574]	9	21365043	21383489		6.53	3.42	3.98	5.71	5.99	5.62
ENSDARG00000027236	rs1a	retinoschisin 1a [Source:ZFIN;Acc:ZDB-GENE-040801-171]	11	30244356	30252098		52.19	12.1	12.01	71.72	66.93	75
ENSDARG00000027249	btg1	B-cell translocation gene 1, anti-proliferative [Source:ZFIN;Acc:ZDB-GENE-010726-1]	4	16536173	16545085		48.56	35.93	30.97	101.88	109.44	105.6
ENSDARG00000027279	numb	NUMB endocytic adaptor protein [Source:ZFIN;Acc:ZDB-GENE-060422-1]	17	51821906	51893123		2.1	1.96	1.37	2.62	2.78	2.5
ENSDARG00000027285	kcnf1b	potassium voltage-gated channel, subfamily F, member 1b [Source:ZFIN;Acc:ZDB-GENE-090312-39]	13	32993911	32995365		2.57	1.25	1.59	1.77	2	2.57
ENSDARG00000027290	nrp1b	neuropilin 1b [Source:ZFIN;Acc:ZDB-GENE-040611-1]	2	43469241	43507278		7.36	4.22	4.25	10.88	10.63	11.1
ENSDARG00000027310	lsp1a	lymphocyte specific protein 1 a [Source:ZFIN;Acc:ZDB-GENE-131127-171]	25	31323970	31394515		22.02	27.2	24.24	19.47	20.95	20.62
ENSDARG00000027316	tcp11l2	t-complex 11, testis-specific-like 2 [Source:ZFIN;Acc:ZDB-GENE-040426-1956]	4	16725960	16742893		28.33	23.15	19.19	28.88	27.24	30.57
ENSDARG00000027322	lin7c	lin-7 homolog C (C. elegans) [Source:ZFIN;Acc:ZDB-GENE-041011-2]	7	31902333	31922432		18.56	13.34	10.84	18.41	18.97	18.33
ENSDARG00000027345	mpzl2b	myelin protein zero-like 2b [Source:ZFIN;Acc:ZDB-GENE-030131-8774]	5	37948283	37963763		11.02	12.89	10.22	8.77	8.82	8.11
ENSDARG00000027353	zmym2	zinc finger, MYM-type 2 [Source:ZFIN;Acc:ZDB-GENE-030131-2408]	9	21430311	21460164		9.82	6.35	5.7	15.1	14.5	15.42
ENSDARG00000027355	slc25a4	solute carrier family 25 member 4 [Source:ZFIN;Acc:ZDB-GENE-030131-5275]	1	17676745	17679089		439.88	681.48	807.73	368.19	355.52	353.04
ENSDARG00000027357	cd2bp2	CD2 (cytoplasmic tail) binding protein 2 [Source:ZFIN;Acc:ZDB-GENE-040426-925]	3	32832042	32842383		14.61	18.53	15.34	17.07	15.73	14.99
ENSDARG00000027360	si:ch211-106h11.3	si:ch211-106h11.3 [Source:ZFIN;Acc:ZDB-GENE-050208-296]	3	5575313	5611867		3.84	4.3	3.21	14.43	18.07	16.97
ENSDARG00000027381	ccdc171	coiled-coil domain containing 171 [Source:ZFIN;Acc:ZDB-GENE-110411-270]	23	45155156	45339439		0.54	0.4	0.37	0.54	0.58	0.59
ENSDARG00000027391	ccnd3	cyclin D3 [Source:ZFIN;Acc:ZDB-GENE-041010-110]	22	537717	547760		0.13	0.22	0.19	0.21	0.23	0.16
ENSDARG00000027394	si:ch211-150g13.3	si:ch211-150g13.3 [Source:ZFIN;Acc:ZDB-GENE-160728-8]	7	32722227	32749591		5.07	1.04	1.61	6.32	5.87	6.2
ENSDARG00000027397	vangl2	VANGL planar cell polarity protein 2 [Source:NCBI gene;Acc:245949]	7	5324061	5375214		8.36	5.68	4.74	15.19	14.22	14.53
ENSDARG00000027403	zgpat	zinc finger, CCCH-type with G patch domain [Source:NCBI gene;Acc:393457]	11	12860260	12875188		4.02	3.57	3.77	3.88	3.42	3.56
ENSDARG00000027418	trappc4	trafficking protein particle complex 4 [Source:ZFIN;Acc:ZDB-GENE-040426-1734]	15	9315177	9321970		13.27	14.13	12.61	11.67	11.72	13.27
ENSDARG00000027419	gad1b	glutamate decarboxylase 1b [Source:ZFIN;Acc:ZDB-GENE-030909-3]	6	3827751	3863452		43.29	9.59	14.67	35.99	39.45	39.26
ENSDARG00000027423	igf1ra	insulin-like growth factor 1a receptor [Source:ZFIN;Acc:ZDB-GENE-020503-1]	18	20881462	21047580		4.02	2.07	1.74	7.11	7.42	7.32
ENSDARG00000027424	slc25a3a	solute carrier family 25 member 3a [Source:ZFIN;Acc:ZDB-GENE-040426-1673]	4	16844973	16853474		49.73	12.47	12	21.06	24	21.6
ENSDARG00000027428	rpp30	ribonuclease P/MRP 30 subunit [Source:ZFIN;Acc:ZDB-GENE-050320-45]	12	16446228	16452200		3.34	4.08	3.59	3.08	2.85	2.6
ENSDARG00000027461	mettl26	methyltransferase like 26 [Source:NCBI gene;Acc:386899]	3	18046776	18061783		3.03	2.88	2.43	3.77	4.76	4.69
ENSDARG00000027465	rmnd1	required for meiotic nuclear division 1 homolog [Source:ZFIN;Acc:ZDB-GENE-050522-21]	17	7795559	7818991		5.67	5.73	5.76	4.02	4.7	4.28
ENSDARG00000027469	hsd17b1	hydroxysteroid (17-beta) dehydrogenase 1 [Source:ZFIN;Acc:ZDB-GENE-040901-5]	3	18038972	18047150		0.02	0.02	0.01	0.06	0.01	0.02
ENSDARG00000027472	gphb5	glycoprotein hormone subunit beta 5 [Source:ZFIN;Acc:ZDB-GENE-090319-4]	13	37273010	37289109		0.22	0.48	0.54	0.26	0.59	0.43
ENSDARG00000027491	uros	uroporphyrinogen III synthase [Source:ZFIN;Acc:ZDB-GENE-040323-2]	12	48674381	48682751		7.98	6.42	6.99	7.06	6.24	6.86
ENSDARG00000027495	elovl4b	ELOVL fatty acid elongase 4b [Source:ZFIN;Acc:ZDB-GENE-030131-7672]	23	40410644	40438581		21.91	5.55	4.79	15.94	14.86	14.62
ENSDARG00000027497	mast1a	microtubule associated serine/threonine kinase 1a [Source:ZFIN;Acc:ZDB-GENE-121214-221]	1	51312752	51377850		1.44	0.44	0.38	3.07	3.02	3.39
ENSDARG00000027500	oxsr1b	oxidative stress responsive kinase 1b [Source:ZFIN;Acc:ZDB-GENE-040426-723]	24	20064088	20152965		9	10.13	8.14	10.22	10.44	9.45
ENSDARG00000027501	usp20	ubiquitin specific peptidase 20 [Source:NCBI gene;Acc:393962]	5	33301005	33330923		6.85	4.77	5.26	6.54	7.17	7.54
ENSDARG00000027523	trpc7a	transient receptor potential cation channel, subfamily C, member 7a [Source:ZFIN;Acc:ZDB-GENE-091113-40]	10	21395298	21428998		0.48	0.21	0.21	0.37	0.37	0.4
ENSDARG00000027529	hmox1a	heme oxygenase 1a [Source:ZFIN;Acc:ZDB-GENE-030131-3102]	3	26011482	26017831		14.35	22.45	24.95	17.12	18	19.18
ENSDARG00000027538	noxred1	NADP-dependent oxidoreductase domain containing 1 [Source:ZFIN;Acc:ZDB-GENE-130530-573]	17	45154497	45164291		0.08	0.08	0.03	0.13	0.09	0.08
ENSDARG00000027547	TPGS1	zgc:92789 [Source:ZFIN;Acc:ZDB-GENE-040718-280]	2	57879347	57891504	TPGS1	11.4	8.45	10.27	10.88	12.38	12.33
ENSDARG00000027552	mapk1	mitogen-activated protein kinase 1 [Source:ZFIN;Acc:ZDB-GENE-030722-2]	5	13104851	13167120		11.18	6.54	7.8	13.21	14.83	14.01
ENSDARG00000027564	pak6a	p21 protein (Cdc42/Rac)-activated kinase 6a [Source:ZFIN;Acc:ZDB-GENE-130410-1]	17	2162916	2265029		1.23	0.28	0.65	2.25	2.17	2.5
ENSDARG00000027572	as3mt	arsenite methyltransferase [Source:ZFIN;Acc:ZDB-GENE-060312-15]	1	31706668	31722875		12.36	13.03	13.12	8.79	8.85	10.07
ENSDARG00000027582	angptl7	angiopoietin-like 7 [Source:ZFIN;Acc:ZDB-GENE-041010-175]	8	46217861	46221296		28.02	38.56	36.86	53.61	52.88	56.53
ENSDARG00000027584	ttpa	tocopherol (alpha) transfer protein [Source:ZFIN;Acc:ZDB-GENE-030131-4631]	2	26647472	26653148		4.84	6.01	5.44	2.75	3.54	2.89
ENSDARG00000027586	top3b	DNA topoisomerase III beta [Source:ZFIN;Acc:ZDB-GENE-070705-37]	5	13211452	13251907		1.24	0.84	0.8	1.49	1.57	1.36
ENSDARG00000027587	htr7b	5-hydroxytryptamine (serotonin) receptor 7b [Source:ZFIN;Acc:ZDB-GENE-091118-105]	10	20158838	20175118		0.19	0.08	0.23	0.11	0.21	0.22
ENSDARG00000027589	fzd7b	frizzled class receptor 7b [Source:ZFIN;Acc:ZDB-GENE-990415-229]	6	9703721	9707599		17.42	18.56	18.06	23.08	25.6	26.89
ENSDARG00000027590	2-Sep	septin 2 [Source:ZFIN;Acc:ZDB-GENE-030131-911]	2	22592139	22615366		39.48	39.37	39.3	48.41	47.85	51.11

ENSDARG00000027595	selenot1b	selenoprotein T, 1b [Source:NCBI gene;Acc:352921]	18	41527877	41539256		16.65	21.47	22.07	16.01	16.51	16.24
ENSDARG00000027599	fam221a	family with sequence similarity 221 member A [Source:ZFIN;Acc:ZDB-GENE-050320-27]	19	20158657	20163233		0.39	0.47	0.58	0.21	0.47	0.41
ENSDARG00000027600	pdlim5b	PDZ and LIM domain 5b [Source:ZFIN;Acc:ZDB-GENE-040426-908]	10	1187653	1276046		10.56	13.11	12.72	14.07	15.53	14.95
ENSDARG00000027602	lrfn1	leucine rich repeat and fibronectin type III domain containing 1 [Source:NCBI gene;Acc:393587]	15	45994123	46132191		3.98	0.83	1.1	4.57	5.43	5.79
ENSDARG00000027609	hpn	hepsin [Source:ZFIN;Acc:ZDB-GENE-141212-373]	16	45249055	45288599		1.64	1.94	1.29	1.24	1.28	1.19
ENSDARG00000027611	cavin2a	caveolae associated protein 2a [Source:ZFIN;Acc:ZDB-GENE-090313-215]	9	24220315	24244383		14.55	12.17	10.26	9.71	9.09	9.3
ENSDARG00000027612	gatad1	GATA zinc finger domain containing 1 [Source:NCBI gene;Acc:678617]	19	43969363	43976522		8.61	6.34	5.95	8.62	9.76	9.1
ENSDARG00000027618	grip2b	glutamate receptor interacting protein 2b [Source:ZFIN;Acc:ZDB-GENE-070705-172]	22	31207226	31466591		0.59	0.12	0.12	0.65	0.7	0.67
ENSDARG00000027620	bco2l	beta-carotene 15, 15-dioxygenase 2, like [Source:ZFIN;Acc:ZDB-GENE-010509-1]	5	26121393	26130527		0.7	0.68	0.61	0.35	0.61	0.32
ENSDARG00000027638	adra1ab	adrenoceptor alpha 1Ab [Source:ZFIN;Acc:ZDB-GENE-060503-384]	10	18999841	19011934		0.78	0.18	0.63	0.86	0.54	0.85
ENSDARG00000027639	zgc:165539	zgc:165539 [Source:ZFIN;Acc:ZDB-GENE-030131-5478]	10	21541925	21545117		0	0	0	0.01	0.01	0.01
ENSDARG00000027649	cpsf3	cleavage and polyadenylation specific factor 3 [Source:ZFIN;Acc:ZDB-GENE-030131-3275]	20	29619820	29633507		15.99	13.77	14.06	14.21	13.44	13.02
ENSDARG00000027657	crhb	corticotropin releasing hormone b [Source:ZFIN;Acc:ZDB-GENE-041114-75]	24	24847683	24881996		5.23	2	2.5	4.52	4.39	5.14
ENSDARG00000027658	irf10	interferon regulatory factor 10 [Source:ZFIN;Acc:ZDB-GENE-040426-2084]	23	642001	653333		0.31	0.37	0.46	0.18	0.4	0.25
ENSDARG00000027676	si:ch211-133l5.8	si:ch211-133l5.8 [Source:ZFIN;Acc:ZDB-GENE-081104-118]	8	20861358	20862443		2	2.3	2.37	1.75	2.12	2.11
ENSDARG00000027687	tmem230a	transmembrane protein 230a [Source:ZFIN;Acc:ZDB-GENE-040822-20]	10	19017146	19021576		2.15	2.5	2.33	2.18	2.38	2.18
ENSDARG00000027689	pold1	polymerase (DNA directed), delta 1, catalytic subunit [Source:ZFIN;Acc:ZDB-GENE-060429-1]	3	16687830	16719244		11.56	10	9.4	7.39	6.83	5.77
ENSDARG00000027699	zgc:103510	zgc:103510 [Source:ZFIN;Acc:ZDB-GENE-041212-27]	8	3345133	3413202		0.29	0.59	0.4	0.44	0.33	0.25
ENSDARG00000027710	btbd16	BTB (POZ) domain containing 16 [Source:ZFIN;Acc:ZDB-GENE-100513-3]	12	11601559	11649690		0.18	0.11	0.17	0.25	0.32	0.12
ENSDARG00000027724	dgkb	diacylglycerol kinase, beta [Source:ZFIN;Acc:ZDB-GENE-070126-1]	15	34215796	34326461		0.66	0.09	0.14	0.45	0.59	0.43
ENSDARG00000027734	srsf5b	serine and arginine rich splicing factor 5b [Source:ZFIN;Acc:ZDB-GENE-040718-354]	20	28869969	28884829		47.01	17.31	23.5	57.25	57.65	56.48
ENSDARG00000027738	si:ch211-13c6.2	si:ch211-13c6.2 [Source:ZFIN;Acc:ZDB-GENE-060503-666]	19	42047427	42068989		12.91	7.28	7.73	15.05	15.12	15.37
ENSDARG00000027740	adcyap1b	adenylate cyclase activating polypeptide 1b [Source:ZFIN;Acc:ZDB-GENE-041010-89]	2	31296644	31302619		22.2	10.89	16.18	25	25.76	27.9
ENSDARG00000027744	gadd45ba	growth arrest and DNA-damage-inducible, beta a [Source:ZFIN;Acc:ZDB-GENE-040426-1971]	11	6456146	6457748		27.71	21.8	19.07	23.13	23.3	22.57
ENSDARG00000027749	hirip3	HIRA interacting protein 3 [Source:ZFIN;Acc:ZDB-GENE-030131-5753]	3	15193521	15210491		16.31	17.18	14.24	11.52	12.41	11.54
ENSDARG00000027750	dpp7	dipeptidyl-peptidase 7 [Source:ZFIN;Acc:ZDB-GENE-050306-16]	5	29159777	29177272		17.22	19.49	19.47	15.15	16.88	16.34
ENSDARG00000027777	tnfaip3	tumor necrosis factor, alpha-induced protein 3 [Source:ZFIN;Acc:ZDB-GENE-100212-2]	13	1182257	1208930		4.62	1.77	2.34	5.67	6.7	6.51
ENSDARG00000027797	adcy6b	adenylate cyclase 6b [Source:ZFIN;Acc:ZDB-GENE-090127-2]	23	26797035	26888217		4.46	3.02	2.81	3.88	3.74	4.21
ENSDARG00000027799	ucmaa	upper zone of growth plate and cartilage matrix associated a [Source:ZFIN;Acc:ZDB-GENE-070410-71]	4	7841627	7850489		9.85	11.38	14.37	2.51	2.34	2.04
ENSDARG00000027803	sbds	SBDS ribosome maturation factor [Source:ZFIN;Acc:ZDB-GENE-040426-1116]	15	1891689	1898233		16.25	20.38	17.3	15	14.29	14.7
ENSDARG00000027807	frk	fyn-related Src family tyrosine kinase [Source:ZFIN;Acc:ZDB-GENE-110411-7]	20	441942	463786		5.67	6.06	3.7	4.38	4.07	4.18
ENSDARG00000027813	nus1	NUS1 dehydrodolichyl diphosphate synthase subunit [Source:NCBI gene;Acc:436629]	20	42221158	42241456		6.26	5.62	5.34	8.64	8.74	7.93
ENSDARG00000027822	tmtops2b	teleost multiple tissue opsin 2b [Source:ZFIN;Acc:ZDB-GENE-110411-43]	9	6118362	6208067		0.64	0.46	0.68	1.62	2.13	1.72
ENSDARG00000027824	top1mt	DNA topoisomerase I mitochondrial [Source:ZFIN;Acc:ZDB-GENE-040624-2]	24	42074143	42075487		62.21	65.34	76.16	68.87	72.67	69.79
ENSDARG00000027825	naa50	N(alpha)-acetyltransferase 50, NatE catalytic subunit [Source:NCBI gene;Acc:445229]	24	21174523	21183891		15.66	16.05	14.34	16.24	16.53	16.54
ENSDARG00000027826	pudp	pseudouridine 5'-phosphatase [Source:ZFIN;Acc:ZDB-GENE-050522-36]	1	32341484	32385665		2.86	3.2	3.08	3.08	2.95	3.03
ENSDARG00000027828	grin1a	glutamate receptor, ionotropic, N-methyl D-aspartate 1a [Source:ZFIN;Acc:ZDB-GENE-051202-1]	21	11468642	11502971		47.22	7.99	11.97	42.93	42.93	43.39
ENSDARG00000027851	ccdc191	coiled-coil domain containing 191 [Source:ZFIN;Acc:ZDB-GENE-050102-4]	24	21092156	21120182		0.86	0.69	0.62	0.76	0.59	0.81
ENSDARG00000027867	paplna	papilin a, proteoglycan-like sulfated glycoprotein [Source:ZFIN;Acc:ZDB-GENE-070815-4]	17	51138092	51199558		9.89	8.22	5.3	19.41	16.53	15.2
ENSDARG00000027887	zfyve21	zinc finger, FYVE domain containing 21 [Source:ZFIN;Acc:ZDB-GENE-030131-1296]	13	43639867	43659541		14.51	12.47	13.92	16.47	17.22	18.27
ENSDARG00000027899	sem1	SEM1 26S proteasome complex subunit [Source:ZFIN;Acc:ZDB-GENE-030131-988]	19	41401133	41405315		57.36	63.56	61.95	43.47	45.02	47.6
ENSDARG00000027903	adamtsl3	ADAMTS-like 3 [Source:ZFIN;Acc:ZDB-GENE-121030-8]	7	12389866	12789251		1.33	0.95	0.85	2	1.94	1.96
ENSDARG00000027907	si:ch211-216p19.6	si:ch211-216p19.6 [Source:ZFIN;Acc:ZDB-GENE-160728-7]	7	24187581	24193080		0.32	0.36	0.38	0.58	0.48	0.65
ENSDARG00000027915	zc2hc1a	zinc finger, C2HC-type containing 1A [Source:NCBI gene;Acc:324412]	24	22740543	22756566		14.88	9.32	8.9	16.06	15.33	14.96
ENSDARG00000027916	fntb	farnesyltransferase, CAAX box, beta [Source:ZFIN;Acc:ZDB-GENE-030131-2220]	20	28803642	28827760		10.95	10.89	12.25	9.86	10.24	10.74
ENSDARG00000027930	naprt	nicotinate phosphoribosyltransferase [Source:NCBI gene;Acc:393864]	20	52273446	52314299		10.13	12.93	12.8	8.64	8.73	8.62
ENSDARG00000027933	glis1b	GLIS family zinc finger 1b [Source:ZFIN;Acc:ZDB-GENE-081105-172]	8	18044257	18165677		0.76	0.72	0.68	1.25	1.17	0.99
ENSDARG00000027938	rad18	RAD18 E3 ubiquitin protein ligase [Source:ZFIN;Acc:ZDB-GENE-040426-745]	6	41808673	41887398		3.01	3.37	3.68	2.26	2.19	1.97
ENSDARG00000027940	kcnf1a	potassium voltage-gated channel, subfamily F, member 1a [Source:ZFIN;Acc:ZDB-GENE-041001-217]	20	33532296	33537243		0.9	1.14	0.86	2.24	2.28	2.25
ENSDARG00000027957	fgf12a	fibroblast growth factor 12a [Source:ZFIN;Acc:ZDB-GENE-050221-6]	2	5587495	5681658		6.35	2.76	3.98	7.56	7.39	7.45
ENSDARG00000027963	camkva	CaM kinase-like vesicle-associated a [Source:ZFIN;Acc:ZDB-GENE-100819-2]	6	42545797	42598328		5.5	1.37	2.1	4.24	4.36	4.83
ENSDARG00000027966	ap1s3b	adaptor related protein complex 1 subunit sigma 3b [Source:ZFIN;Acc:ZDB-GENE-040912-62]	2	47587238	47620806		13.86	17.06	15.15	9.21	9.33	9.17
ENSDARG00000027978	yy1b	YY1 transcription factor b [Source:ZFIN;Acc:ZDB-GENE-040426-2127]	20	54419592	54435287		9.87	8.45	9.08	9.18	9.03	10.08
ENSDARG00000027984	gstz1	glutathione S-transferase zeta 1 [Source:ZFIN;Acc:ZDB-GENE-040718-184]	17	45106424	45114519		12.87	15.45	16.33	9.52	10.65	10.8
ENSDARG00000027986	slc35d1b	solute carrier family 35 member D1b [Source:ZFIN;Acc:ZDB-GENE-040426-1213]	2	5793908	5810326		12.22	20.14	16.82	5.81	5.6	5.39
ENSDARG00000027992	hao2	hydroxyacid oxidase 2 (long chain) [Source:ZFIN;Acc:ZDB-GENE-040426-1239]	9	21146862	21164617		13.34	20.39	16.99	12.99	13.18	12.67
ENSDARG00000028000	pfkpa	phosphofructokinase, platelet a [Source:ZFIN;Acc:ZDB-GENE-091112-22]	24	3963684	4004537		27.17	10.26	12.24	38.95	39.22	40.19
ENSDARG00000028012	tpmt.2	thiopurine S-methyltransferase, tandem duplicate 2 [Source:ZFIN;Acc:ZDB-GENE-041010-177]	19	47299166	47309383		3.11	7.9	5.89	0.78	0.84	1.26
ENSDARG00000028017	tp53inp1	tumor protein p53 inducible nuclear protein 1 [Source:ZFIN;Acc:ZDB-GENE-031018-3]	16	40560622	40569769		18.51	12	12.11	27.86	27.49	29.28
ENSDARG00000028027	trim63a	tripartite motif containing 63a [Source:ZFIN;Acc:ZDB-GENE-040625-139]	6	15651977	15653494		22.65	33.36	30.44	18.34	17.52	17.51
ENSDARG00000028048	rdh8a	retinol dehydrogenase 8a [Source:ZFIN;Acc:ZDB-GENE-021115-3]	3	53687645	53722182		10.48	9.48	9.24	9.47	10.1	10.04
ENSDARG00000028053	bmp1b	bone morphogenetic protein 1b [Source:ZFIN;Acc:ZDB-GENE-060818-2]	10	40848354	40939706		2.61	1.44	1.41	1.67	1.51	1.53
ENSDARG00000028058	traf6	TNF receptor-associated factor 6 [Source:NCBI gene;Acc:554561]	7	48719924	48738807		3.29	3.4	2.87	3.98	4.26	3.78
ENSDARG00000028066	diras1a	DIRAS family, GTP-binding RAS-like 1a [Source:ZFIN;Acc:ZDB-GENE-030131-5788]	11	7324704	7341807		30.16	10.91	16.41	29.99	33.04	31.94
ENSDARG00000028067	bnip3lb	BCL2 interacting protein 3 like b [Source:ZFIN;Acc:ZDB-GENE-040325-1]	10	18911152	18935945		13.67	12.65	12.43	36.79	43.06	41.45
ENSDARG00000028071	bmp1a	bone morphogenetic protein 1a [Source:ZFIN;Acc:ZDB-GENE-060818-1]	8	51165392	51293301		17.15	17.38	13.22	35.78	34.79	37.57
ENSDARG00000028082	mapkapk5	MAPK activated protein kinase 5 [Source:ZFIN;Acc:ZDB-GENE-040718-25]	5	69553686	69571572		7.13	6.79	6.24	6.6	6.83	6.83
ENSDARG00000028086	zbtb2b	zinc finger and BTB domain containing 2b [Source:ZFIN;Acc:ZDB-GENE-040426-2753]	20	26394324	26407233		7.41	5.81	5.81	7.43	8.22	7.42
ENSDARG00000028087	aldh2.2	aldehyde dehydrogenase 2 family member, tandem duplicate 2 [Source:ZFIN;Acc:ZDB-GENE-030326-5]	5	69597166	69621227		34.21	45.21	42.56	21.72	21.68	21.56
ENSDARG00000028088	galk1	galactokinase 1 [Source:ZFIN;Acc:ZDB-GENE-041010-79]	12	33790121	33805366		3.98	4.45	4.33	4.12	4.03	3.51
ENSDARG00000028096	cldn23a	claudin 23a [Source:ZFIN;Acc:ZDB-GENE-091204-28]	10	15046837	15048781		23.16	16.58	11.26	15.4	14.28	11.98
ENSDARG00000028098	fut9d	fucosyltransferase 9d [Source:ZFIN;Acc:ZDB-GENE-050309-15]	8	13503377	13505486		15.87	13.61	11.75	10.8	8.63	8.2
ENSDARG00000028099	sh2d3ca	SH2 domain containing 3Ca [Source:ZFIN;Acc:ZDB-GENE-080102-1]	10	10210455	10274162		6.4	2.15	2.58	9.14	10.83	10.47
ENSDARG00000028106	glrx	glutaredoxin (thioltransferase) [Source:ZFIN;Acc:ZDB-GENE-041010-11]	21	11776328	11782093		44.57	68.82	65.99	27.07	28.23	25.44
ENSDARG00000028118	dscamb	Down syndrome cell adhesion molecule b [Source:ZFIN;Acc:ZDB-GENE-031118-67]	15	6138423	6404443		5.45	1.6	1.55	11.71	11.58	12.23
ENSDARG00000028119	sumo3a	small ubiquitin like modifier 3a [Source:NCBI gene;Acc:406398]	6	11070261	11073880		66.29	50.57	49.79	69.39	65.47	66.35
ENSDARG00000028131	hccsa.1	holocytochrome c synthase a [Source:ZFIN;Acc:ZDB-GENE-040718-202]	2	14998612	15033163		5.3	5.62	5.83	3.59	4.03	3.87
ENSDARG00000028148	pax2a	paired box 2a [Source:NCBI gene;Acc:30425]	13	29770837	29805529		2.86	1.03	1.39	7.18	6.85	7.46
ENSDARG00000028159	tead1a	TEA domain family member 1a [Source:ZFIN;Acc:ZDB-GENE-040426-2101]	25	16356083	16431599		2.84	2.05	1.88	4.14	3.75	4.49
ENSDARG00000028163	prg4b	proteoglycan 4b [Source:ZFIN;Acc:ZDB-GENE-041014-324]	20	34029820	34039638		6.16	9.1	7.55	6.89	7.37	6.82
ENSDARG00000028164	cst14a.1	cystatin 14a, tandem duplicate 1 [Source:ZFIN;Acc:ZDB-GENE-141031-1]	24	20916751	20918412		3.54	4.44	4.04	2.4	2.71	2.51
ENSDARG00000028173	slc4a2a	solute carrier family 4 member 2a [Source:ZFIN;Acc:ZDB-GENE-051101-2]	2	32391897	32457923		2.62	2.97	2.07	5.31	4.93	5.36
ENSDARG00000028192	cradd	CASP2 and RIPK1 domain containing adaptor with death domain [Source:ZFIN;Acc:ZDB-GENE-041010-168]	4	18383253	18416566		1.83	2.14	2.31	2.07	2.55	2
ENSDARG00000028198	ube2v2	ubiquitin-conjugating enzyme E2 variant 2 [Source:NCBI gene;Acc:406836]	24	35522301	35534273		60.6	52.7	50.92	70.41	69.56	64.74
ENSDARG00000028201	commd8	COMM domain containing 8 [Source:ZFIN;Acc:ZDB-GENE-061103-124]	13	12378074	12389748		10.29	11.12	12.46	8.35	9.58	9.67
ENSDARG00000028213	ttn.2	titin, tandem duplicate 2 [Source:ZFIN;Acc:ZDB-GENE-030113-2]	9	42894845	43073960		2.95	4.06	2.81	11.83	6.56	6.8
ENSDARG00000028222	itgb8	integrin, beta 8 [Source:ZFIN;Acc:ZDB-GENE-071213-3]	19	2275019	2307722		0.98	0.37	0.6	1.57	1.84	1.73
ENSDARG00000028228	zbtb18	zinc finger and BTB domain containing 18 [Source:NCBI gene;Acc:100003157]	13	11436130	11445034		10.17	3.87	4.62	11.83	11.81	12.61
ENSDARG00000028257	cyb561a3b	cytochrome b561 family, member A3b [Source:ZFIN;Acc:ZDB-GENE-091204-222]	21	16102938	16114061		0.25	0.53	0.36	0.64	0.79	0.48
ENSDARG00000028259	aldh3a2a	aldehyde dehydrogenase 3 family, member A2a [Source:ZFIN;Acc:ZDB-GENE-040718-74]	15	20830146	20839910		4.49	5.84	5.29	4.16	4.02	4.27
ENSDARG00000028275	sult1st1	sulfotransferase family 1, cytosolic sulfotransferase 1 [Source:NCBI gene;Acc:323424]	8	46499098	46509194		29.45	43.51	34.38	43.61	41.06	45.7
ENSDARG00000028276	si:ch211-196h16.12	si:ch211-196h16.12 [Source:ZFIN;Acc:ZDB-GENE-121214-351]	1	55002583	55007223		4.23	4.36	5.81	4.54	4.59	3.95
ENSDARG00000028295	mkrn4	makorin, ring finger protein, 4 [Source:ZFIN;Acc:ZDB-GENE-030131-5954]	8	23725957	23731213		4.79	4.61	4.4	4.23	4.32	4.69
ENSDARG00000028306	prph	peripherin [Source:ZFIN;Acc:ZDB-GENE-990415-207]	11	3320598	3334250		10.14	5.7	6.79	9.7	10.06	9.87
ENSDARG00000028321	synj2bp	synaptojanin 2 binding protein [Source:ZFIN;Acc:ZDB-GENE-030131-6531]	13	36557205	36566260		10.57	12.98	12.3	8.67	9.34	8.6
ENSDARG00000028322	pex14	peroxisomal biogenesis factor 14 [Source:ZFIN;Acc:ZDB-GENE-060130-169]	23	28809002	28925757		5.29	4.68	4.12	4.82	4.71	4.05
ENSDARG00000028327	gmfb	glia maturation factor, beta [Source:ZFIN;Acc:ZDB-GENE-040426-2114]	13	36585399	36593618		31.85	32.96	28.67	30.91	29.55	30.64
ENSDARG00000028333	nitr9	novel immune-type receptor 9 [Source:ZFIN;Acc:ZDB-GENE-041001-6]	7	17255705	17309337		0.01	0.02	0.05	0.01	0.03	0.04
ENSDARG00000028335	hmga1a	high mobility group AT-hook 1a [Source:ZFIN;Acc:ZDB-GENE-010502-1]	23	3751451	3759692		275.56	210.28	223.15	255.75	242.85	246.21
ENSDARG00000028336	dhdhl	dihydrodiol dehydrogenase (dimeric), like [Source:ZFIN;Acc:ZDB-GENE-040426-2180]	18	970246	977075		24.41	33.3	26.21	15.47	17.23	17.61
ENSDARG00000028346	tesca	tescalcin a [Source:NCBI gene;Acc:406633]	5	11840905	11864424		2.88	4.44	3.11	2.61	2.55	2.68
ENSDARG00000028348	pax3b	paired box 3b [Source:ZFIN;Acc:ZDB-GENE-080917-53]	15	42163356	42206890		1.03	1.42	1.25	1.4	1.52	1.12
ENSDARG00000028354	stxbp6l	syntaxin binding protein 6 (amisyn), like [Source:ZFIN;Acc:ZDB-GENE-040724-16]	20	9828769	9848953		3.03	1.34	2.14	2.76	2.79	3.13
ENSDARG00000028367	sult2st3	sulfotransferase family 2, cytosolic sulfotransferase 3 [Source:ZFIN;Acc:ZDB-GENE-061117-4]	16	44983613	45001842		2.03	2.21	2.54	2.5	2.23	2.5
ENSDARG00000028379	trub2	TruB pseudouridine (psi) synthase family member 2 [Source:ZFIN;Acc:ZDB-GENE-040704-41]	6	41023580	41028698		8.43	7.13	8.57	6.32	6.37	6.46
ENSDARG00000028386	htatip2	HIV-1 Tat interactive protein 2 [Source:ZFIN;Acc:ZDB-GENE-001219-1]	7	17063761	17105323		5.5	5.31	4.53	3.87	3.88	3.93
ENSDARG00000028389	rab44	RAB44, member RAS oncogene family [Source:ZFIN;Acc:ZDB-GENE-040724-117]	6	41057943	41079209		0.23	0.28	0.21	0.41	0.38	0.4
ENSDARG00000028393	daam2	dishevelled associated activator of morphogenesis 2 [Source:ZFIN;Acc:ZDB-GENE-070709-1]	17	48831579	48892781		2.85	2.34	1.82	3.11	2.93	3.06
ENSDARG00000028396	fkbp5	FKBP prolyl isomerase 5 [Source:ZFIN;Acc:ZDB-GENE-030616-630]	6	41096058	41117242		13.26	13.72	14.03	50.05	52.62	46.22
ENSDARG00000028401	rhogc	ras homolog gene family, member Gc [Source:ZFIN;Acc:ZDB-GENE-030131-3402]	5	36595149	36597636		15.11	17.98	17.02	15.39	15.04	14.67
ENSDARG00000028431	adnp2b	ADNP homeobox 2b [Source:ZFIN;Acc:ZDB-GENE-030131-5930]	19	20785724	20792473		10.94	6.3	6.6	10.97	10.06	10.32
ENSDARG00000028442	cplane2	ciliogenesis and planar polarity effector 2 [Source:NCBI gene;Acc:447911]	6	41446541	41451844		2.99	2.55	2.7	2.1	1.52	2.07
ENSDARG00000028448	edem2	ER degradation enhancer, mannosidase alpha-like 2 [Source:ZFIN;Acc:ZDB-GENE-060519-1]	23	6948941	6982986		2.99	2.47	2.63	2.86	2.68	3.08
ENSDARG00000028469	rps6ka2	ribosomal protein S6 kinase, polypeptide 2 [Source:ZFIN;Acc:ZDB-GENE-090827-1]	13	7086735	7157062		2.17	0.27	0.42	1.87	2.72	2.21
ENSDARG00000028475	lin9	lin-9 DREAM MuvB core complex component [Source:NCBI gene;Acc:560867]	20	43691208	43715242		3.62	3	3.21	3.1	2.87	2.87
ENSDARG00000028478	si:ch211-173n18.3	si:ch211-173n18.3 [Source:ZFIN;Acc:ZDB-GENE-120215-62]	6	39499623	39503381		5.33	7.92	5.49	3.96	4.06	4.49
ENSDARG00000028485	cabp5b	calcium binding protein 5b [Source:ZFIN;Acc:ZDB-GENE-050522-146]	3	15296824	15317392		6.34	3.7	2.89	3.64	3.27	4.08
ENSDARG00000028507	itgb4	integrin, beta 4 [Source:ZFIN;Acc:ZDB-GENE-030131-7209]	8	13106760	13159587		7.49	7.51	3.97	9.81	7.29	7.54
ENSDARG00000028517	hbp1	HMG-box transcription factor 1 [Source:ZFIN;Acc:ZDB-GENE-050522-414]	25	3449959	3470910		9.16	9.48	8.81	15.66	15.28	17.79
ENSDARG00000028521	c1ql3b	complement component 1, q subcomponent-like 3b [Source:ZFIN;Acc:ZDB-GENE-040426-1836]	3	31396149	31430489		3.98	0.85	1.62	6.02	6.45	6.34
ENSDARG00000028523	inip	ints3 and nabp interacting protein [Source:NCBI gene;Acc:393188]	10	11319971	11323134		13.12	15.52	14	10.61	9.79	8.96
ENSDARG00000028533	macf1a	microtubule actin crosslinking factor 1a [Source:ZFIN;Acc:ZDB-GENE-030131-3606]	19	35039902	35340027		12.51	7.44	6.07	17.77	16.67	17.49
ENSDARG00000028539	morc2	MORC family CW-type zinc finger 2 [Source:ZFIN;Acc:ZDB-GENE-040822-40]	25	3708087	3745393		14.47	11.51	10.29	16.85	16.38	16.91
ENSDARG00000028541	si:ch211-121a2.4	si:ch211-121a2.4 [Source:ZFIN;Acc:ZDB-GENE-070705-23]	20	7122527	7133546		2.5	1.72	2.09	2.61	1.79	2.55
ENSDARG00000028542	si:ch211-114c12.2	si:ch211-114c12.2 [Source:ZFIN;Acc:ZDB-GENE-030131-8522]	5	24128908	24135569		22.09	11.02	12.35	30.9	30.71	31.67
ENSDARG00000028546	ndufs1	NADH:ubiquinone oxidoreductase core subunit S1 [Source:ZFIN;Acc:ZDB-GENE-030131-478]	6	13206516	13231951		0	0	0.01	0.03	0	0.03
ENSDARG00000028552	plppr3b	phospholipid phosphatase related 3b [Source:ZFIN;Acc:ZDB-GENE-120514-1]	11	14147913	14161975		7.53	1.7	2.83	6.52	7.2	7.14
ENSDARG00000028559	march5l	membrane-associated ring finger (C3HC4) 5, like [Source:NCBI gene;Acc:326067]	10	42500895	42519773		16.4	19	20.55	15.16	15.32	15.59
ENSDARG00000028581	jkamp	jnk1/mapk8 associated membrane protein [Source:ZFIN;Acc:ZDB-GENE-040718-382]	17	14443406	14451718		6.31	5.05	4.56	7.04	7.42	7.05
ENSDARG00000028583	SLC16A3	si:ch211-234h8.7 [Source:ZFIN;Acc:ZDB-GENE-061009-16]	3	36160979	36167394	SLC16A3	2.8	0.87	1.06	4.21	5	3.95
ENSDARG00000028586	gpr135	G protein-coupled receptor 135 [Source:ZFIN;Acc:ZDB-GENE-060420-3]	17	14452329	14458667		2.13	0.87	0.86	2.97	3.27	3.07
ENSDARG00000028614	mospd1	motile sperm domain containing 1 [Source:ZFIN;Acc:ZDB-GENE-040426-2691]	14	31550759	31562795		22.18	17.93	22.23	21.98	21.75	25.29
ENSDARG00000028618	krt18b	keratin 18b [Source:ZFIN;Acc:ZDB-GENE-040426-1508]	6	39360377	39365467		148.27	194.87	207.35	117.65	132.64	128.89
ENSDARG00000028625	jph2	junctophilin 2 [Source:ZFIN;Acc:ZDB-GENE-030131-9848]	23	35721607	35756115		73.5	102.53	93.07	82.05	81.37	79.29
ENSDARG00000028628	gdap1l1	ganglioside induced differentiation associated protein 1-like 1 [Source:ZFIN;Acc:ZDB-GENE-080812-2]	23	35759843	35782365		4.05	1.67	2.27	6.04	6.87	5.89
ENSDARG00000028659	ms4a17c.2	membrane-spanning 4-domains, subfamily A, member 17c.2 [Source:ZFIN;Acc:ZDB-GENE-080829-10]	5	33498253	33500331		0	0.03	0	0	0	0
ENSDARG00000028661	cntfr	ciliary neurotrophic factor receptor [Source:ZFIN;Acc:ZDB-GENE-030616-82]	10	13460130	13772861		13.83	11.05	9.85	18.57	19.09	19.44
ENSDARG00000028663	tek	TEK tyrosine kinase, endothelial [Source:NCBI gene;Acc:30747]	5	480119	496781		1.46	1.29	1.34	3.24	3.34	3.48
ENSDARG00000028664	ahsa1a	AHA1, activator of heat shock protein ATPase homolog 1a [Source:ZFIN;Acc:ZDB-GENE-030131-1869]	20	26966725	26974248		0.05	0.05	0.07	0.23	0.05	0.11
ENSDARG00000028676	nuak1b	NUAK family, SNF1-like kinase, 1b [Source:ZFIN;Acc:ZDB-GENE-131120-18]	25	5935938	5963535		7.92	7.08	5.94	18.65	19.94	19.39
ENSDARG00000028688	exosc2	exosome component 2 [Source:ZFIN;Acc:ZDB-GENE-050417-27]	5	72137331	72143292		10.15	11.85	12.11	6.02	6.38	6.09
ENSDARG00000028699	crkl	v-crk avian sarcoma virus CT10 oncogene homolog-like [Source:ZFIN;Acc:ZDB-GENE-040426-2951]	21	18950575	18958079		13.77	9.06	7.69	19.74	20.06	19.55
ENSDARG00000028715	mindy3	MINDY lysine 48 deubiquitinase 3 [Source:NCBI gene;Acc:777708]	16	28270037	28371330		9.31	4.56	5.51	9.19	8.95	9.24
ENSDARG00000028721	mapk14b	mitogen-activated protein kinase 14b [Source:NCBI gene;Acc:266756]	11	2710530	2760373		5.41	4.28	3.64	6.88	6.95	6.95
ENSDARG00000028725	adra1aa	adrenoceptor alpha 1Aa [Source:ZFIN;Acc:ZDB-GENE-030131-2831]	8	5024468	5080889		0.65	0.47	0.49	1.09	0.83	1.16
ENSDARG00000028731	stat4	signal transducer and activator of transcription 4 [Source:ZFIN;Acc:ZDB-GENE-030616-264]	9	41156287	41208343		1.28	1.11	0.71	1.42	1.12	1.12
ENSDARG00000028740	msnb	moesin b [Source:ZFIN;Acc:ZDB-GENE-030717-7]	14	9287683	9336601		0.7	0.12	0.11	1.45	1.5	1.47
ENSDARG00000028748	tm9sf3	transmembrane 9 superfamily member 3 [Source:ZFIN;Acc:ZDB-GENE-040426-2714]	13	9855380	9875538		15.48	13.35	11.63	20.11	20.01	18.9
ENSDARG00000028780	gzmk	granzyme K [Source:ZFIN;Acc:ZDB-GENE-091204-352]	21	19517492	19520320		0.02	0.03	0.01	0.12	0.09	0.06
ENSDARG00000028784	si:dkey-24p1.6	si:dkey-24p1.6 [Source:ZFIN;Acc:ZDB-GENE-060503-67]	15	38299563	38331933		0.23	0.27	0.17	0.18	0.17	0.24
ENSDARG00000028786	chst12a	carbohydrate (chondroitin 4) sulfotransferase 12a [Source:ZFIN;Acc:ZDB-GENE-081022-13]	3	41726360	41732947		4.08	1.84	1.74	5.08	4.04	4.49
ENSDARG00000028793	nlk2	nemo-like kinase, type 2 [Source:ZFIN;Acc:ZDB-GENE-080320-1]	15	30157602	30301528		9.76	4.67	4.35	15.44	16.01	16.8
ENSDARG00000028800	CYTH2	si:dkey-283b15.4 [Source:ZFIN;Acc:ZDB-GENE-100921-55]	16	5612547	5636376	CYTH2	8.1	3.23	4.28	10.42	10.67	10.95
ENSDARG00000028804	ankrd9	ankyrin repeat domain 9 [Source:ZFIN;Acc:ZDB-GENE-041114-3]	17	29276544	29277731		9.08	12.99	11.55	17.3	13.1	13.34
ENSDARG00000028815	zgc:162945	zgc:162945 [Source:ZFIN;Acc:ZDB-GENE-030131-3975]	7	30270410	30272871		5.37	4.64	5.12	6.65	6.59	7.44
ENSDARG00000028816	tmed3	transmembrane p24 trafficking protein 3 [Source:ZFIN;Acc:ZDB-GENE-030131-8335]	7	30175656	30177691		16.14	20.67	18.81	15.37	14.75	15.36
ENSDARG00000028824	slc34a1a	solute carrier family 34 member 1a [Source:ZFIN;Acc:ZDB-GENE-101006-3]	14	834619	858987		2.18	3.99	1.77	1.24	1.05	1.17
ENSDARG00000028845	calcr	calcitonin receptor [Source:ZFIN;Acc:ZDB-GENE-060503-420]	19	40716975	40826024		3.19	0.97	1.47	2.34	2.7	2.9
ENSDARG00000028848	lsm12a	LSM12 homolog a [Source:NCBI gene;Acc:407986]	3	22984098	22994433		8.04	7.23	7.45	9.72	9.78	9.09
ENSDARG00000028850	btr16	bloodthirsty-related gene family, member 16 [Source:ZFIN;Acc:ZDB-GENE-060929-1066]	15	18198878	18209723		0.28	0.37	0.31	0.17	0.17	0.3
ENSDARG00000028857	sgsm1a	small G protein signaling modulator 1a [Source:ZFIN;Acc:ZDB-GENE-060526-379]	5	20030414	20093196		3.68	0.96	1.43	4.23	3.87	4.69
ENSDARG00000028862	rdh20	retinol dehydrogenase 20 [Source:ZFIN;Acc:ZDB-GENE-131127-122]	6	46458416	46474483		0.19	0.08	0.04	0.23	0.19	0.09
ENSDARG00000028878	vipr1a	vasoactive intestinal peptide receptor 1a [Source:ZFIN;Acc:ZDB-GENE-070912-506]	2	21229909	21300115		1.85	1.69	1.67	2.49	2.51	2.56
ENSDARG00000028889	ndufb10	NADH:ubiquinone oxidoreductase subunit B10 [Source:ZFIN;Acc:ZDB-GENE-040426-1691]	3	18405136	18410968		157.24	210.55	215.27	102.06	103.39	106.87
ENSDARG00000028894	mrgbp	MRG/MORF4L binding protein [Source:ZFIN;Acc:ZDB-GENE-040426-1698]	23	15899693	15916391		11.88	9.21	10.52	11.21	10.54	11.71
ENSDARG00000028896	plpp2b	phospholipid phosphatase 2b [Source:ZFIN;Acc:ZDB-GENE-030131-7425]	22	21549419	21584670		7.35	7.18	6.33	6.52	6.45	6.21
ENSDARG00000028899	tekt4	tektin 4 [Source:ZFIN;Acc:ZDB-GENE-070410-98]	1	54613757	54622227		1.42	1.1	1.27	1.34	1.38	1.06
ENSDARG00000028900	odf2a	outer dense fiber of sperm tails 2a [Source:ZFIN;Acc:ZDB-GENE-091117-46]	21	13127742	13148318		1.22	0.65	0.77	1.01	1.15	1.23
ENSDARG00000028912	si:dkey-10h3.2	si:dkey-10h3.2 [Source:ZFIN;Acc:ZDB-GENE-060503-142]	7	5699624	5711197		0.04	0	0.01	0.03	0.03	0.06
ENSDARG00000028937	taf1b	TATA box binding protein (Tbp)-associated factor, RNA polymerase I, B [Source:NCBI gene;Acc:449647]	20	29513470	29533010		2.69	2.13	2.37	2.91	2.64	2.59
ENSDARG00000028943	mtss1la	MTSS I-BAR domain containing 2a [Source:ZFIN;Acc:ZDB-GENE-030219-89]	18	14772474	14836862		13.36	8.98	8.36	20.22	21.37	22.77
ENSDARG00000028949	ZBTB26	si:ch211-236k19.4 [Source:ZFIN;Acc:ZDB-GENE-060526-122]	5	64861368	64865521	ZBTB26	2.13	1.12	1.53	1.93	2.13	2.05
ENSDARG00000028957	maff	v-maf avian musculoaponeurotic fibrosarcoma oncogene homolog F [Source:ZFIN;Acc:ZDB-GENE-040426-1280]	12	19340786	19346678		30.92	25.06	23.53	32.82	36.31	38.88
ENSDARG00000028971	cpsf2	cleavage and polyadenylation specific factor 2 [Source:ZFIN;Acc:ZDB-GENE-040718-79]	20	50052116	50089898		7.38	5.08	4.73	6.36	6.66	6.07
ENSDARG00000028973	tekt2	tektin 2 (testicular) [Source:ZFIN;Acc:ZDB-GENE-040113-1]	19	10636369	10648523		1.48	1.68	1.7	1.07	0.89	1.17
ENSDARG00000028976	pus3	pseudouridylate synthase 3 [Source:ZFIN;Acc:ZDB-GENE-030131-9433]	24	36317544	36329800		1.78	1.96	1.64	1.92	1.56	1.66
ENSDARG00000028982	pcca	propionyl-CoA carboxylase subunit alpha [Source:ZFIN;Acc:ZDB-GENE-040718-246]	1	29862074	29953746		19.89	23.6	20.8	20.74	21.25	21.12
ENSDARG00000029003	eif1axa	eukaryotic translation initiation factor 1A X-linked a [Source:ZFIN;Acc:ZDB-GENE-041010-185]	5	24002598	24008997		31.43	39.48	34	25.7	23.66	23.37
ENSDARG00000029011	xpnpep1	X-prolyl aminopeptidase (aminopeptidase P) 1, soluble [Source:ZFIN;Acc:ZDB-GENE-040426-999]	22	27092469	27115241		28.59	30.23	29.68	16.19	17.01	15.57
ENSDARG00000029018	cdkn1ba	cyclin-dependent kinase inhibitor 1Ba [Source:ZFIN;Acc:ZDB-GENE-040812-3]	4	18592743	18595525		11.64	9.02	9.41	21.92	23.13	21.37
ENSDARG00000029019	epb41b	erythrocyte membrane protein band 4.1b [Source:ZFIN;Acc:ZDB-GENE-030130-1]	16	26449615	26488169		2.71	3.33	3.12	8.39	9.61	8.82
ENSDARG00000029036	rab32a	RAB32a, member RAS oncogene family [Source:ZFIN;Acc:ZDB-GENE-031006-10]	20	32009271	32045182		2.69	4.01	3.25	3.46	2.79	3.18
ENSDARG00000029039	zdhhc23a	zinc finger DHHC-type containing 23a [Source:ZFIN;Acc:ZDB-GENE-060526-38]	5	24282570	24294160		1.76	2.81	2.02	4.45	3.93	3.86

ENSDARG00000029043	nup42	nucleoporin 42 [Source:NCBI gene;Acc:406491]	19	619200	627153		3.12	2.52	2.87	2.61	2.37	2.69
ENSDARG00000029045	peli2	pellino E3 ubiquitin protein ligase family member 2 [Source:ZFIN;Acc:ZDB-GENE-040718-360]	17	44030692	44080733		8.77	7.63	5.8	7.55	7.96	7.74
ENSDARG00000029051	gbe1a	glucan (1,4-alpha-), branching enzyme 1a [Source:ZFIN;Acc:ZDB-GENE-110411-171]	10	24048663	24298042		0.69	0.85	0.8	0.63	0.65	0.75
ENSDARG00000029057	tm6sf2	transmembrane 6 superfamily member 2 [Source:ZFIN;Acc:ZDB-GENE-060503-338]	22	17784414	17823608		0.16	0.05	0.1	0.84	0.92	0.85
ENSDARG00000029058	rbbp4	retinoblastoma binding protein 4 [Source:NCBI gene;Acc:321726]	19	30999833	31007417		45.74	43.42	42.94	34.65	33.24	30.42
ENSDARG00000029063	clpxa	caseinolytic mitochondrial matrix peptidase chaperone subunit a [Source:ZFIN;Acc:ZDB-GENE-040912-143]	7	13639473	13679937		19.77	18.42	15.17	22.26	23.89	23.28
ENSDARG00000029064	uqcrq	ubiquinol-cytochrome c reductase, complex III subunit VII [Source:ZFIN;Acc:ZDB-GENE-040718-199]	14	38875078	38878419		77.13	91.37	101.59	58.22	59.53	63.3
ENSDARG00000029069	tnni2a.4	troponin I type 2a (skeletal, fast), tandem duplicate 4 [Source:ZFIN;Acc:ZDB-GENE-040625-119]	25	31276842	31288658		985.26	1348.1	1730.32	1019.2	1016.4	1017.26
ENSDARG00000029071	creld2	cysteine-rich with EGF-like domains 2 [Source:NCBI gene;Acc:393495]	4	27081972	27099285		4.7	7.19	5.03	5.35	5.38	5.85
ENSDARG00000029072	klf6a	Kruppel-like factor 6a [Source:ZFIN;Acc:ZDB-GENE-021115-9]	24	4141484	4148915		28.22	25.89	22.06	39.31	37.39	38.13
ENSDARG00000029075	pfkfb4b	6-phosphofructo-2-kinase/fructose-2,6-biphosphatase 4b [Source:ZFIN;Acc:ZDB-GENE-031031-4]	6	39993605	40029423		16.02	14.6	17.57	39.36	39.83	41.54
ENSDARG00000029088	guf1	GUF1 homolog, GTPase [Source:ZFIN;Acc:ZDB-GENE-030131-4289]	14	4295933	4321874		5.24	3.92	3.64	3.87	3.86	3.65
ENSDARG00000029100	chchd1	coiled-coil-helix-coiled-coil-helix domain containing 1 [Source:ZFIN;Acc:ZDB-GENE-060810-176]	13	21768447	21776678		19.07	25.38	23.38	11.99	10.57	11.86
ENSDARG00000029105	ftr51	finTRIM family, member 51 [Source:ZFIN;Acc:ZDB-GENE-081104-54]	4	76468839	76494299		2.95	2.75	2.55	1.73	2.05	1.87
ENSDARG00000029107	ube2d1a	ubiquitin-conjugating enzyme E2D 1a [Source:ZFIN;Acc:ZDB-GENE-040426-1609]	17	21051022	21057674		17.34	18.37	16.92	15.3	15.17	14.34
ENSDARG00000029112	gem	GTP binding protein overexpressed in skeletal muscle [Source:ZFIN;Acc:ZDB-GENE-060825-251]	16	26766423	26769327		6.19	1.8	2.06	5.3	6.07	6.4
ENSDARG00000029114	si:dkey-40m6.8	si:dkey-40m6.8 [Source:ZFIN;Acc:ZDB-GENE-100922-162]	16	32059785	32080875		8.22	1.5	2.51	11.6	11.02	12.15
ENSDARG00000029124	adamts15a	ADAM metallopeptidase with thrombospondin type 1 motif, 15a [Source:ZFIN;Acc:ZDB-GENE-060526-203]	5	30179010	30209068		3.89	4.32	3.73	6.48	7.27	6.81
ENSDARG00000029133	tubgcp3	tubulin, gamma complex associated protein 3 [Source:ZFIN;Acc:ZDB-GENE-030616-538]	1	46404968	46452686		4.68	2.88	2.23	6.08	5.74	5.45
ENSDARG00000029146	lrp1ab	low density lipoprotein receptor-related protein 1Ab [Source:ZFIN;Acc:ZDB-GENE-030131-7126]	23	25452656	25686954		6.28	4.5	3.09	16.98	15.81	16.7
ENSDARG00000029150	hsp90ab1	heat shock protein 90, alpha (cytosolic), class A member 1, tandem duplicate 2 [Source:ZFIN;Acc:ZDB-GENE-031001-3]	20	51197758	51213829		858.13	773.32	671.48	621	648.01	660.28
ENSDARG00000029157	med23	mediator complex subunit 23 [Source:NCBI gene;Acc:445483]	20	2499622	2584115		6.37	4.28	4	8.91	8.04	8.96
ENSDARG00000029168	ppfibp2b	PPFIA binding protein 2b [Source:ZFIN;Acc:ZDB-GENE-040718-54]	25	15933411	16042941		2.97	2.81	2.47	2.3	2.22	2.18
ENSDARG00000029170	si:ch211-200p22.4	si:ch211-200p22.4 [Source:ZFIN;Acc:ZDB-GENE-081104-61]	7	23748971	23848012		6.15	4.08	3.24	8.96	9.24	8.72
ENSDARG00000029177	lnx2a	ligand of numb-protein X 2a [Source:ZFIN;Acc:ZDB-GENE-060228-2]	24	21646317	21674996		8.98	2.52	2.75	12.05	12.07	13.4
ENSDARG00000029179	foxd1	forkhead box D1 [Source:ZFIN;Acc:ZDB-GENE-040426-2094]	5	34997763	34999553		5.66	5.7	4.56	9.2	9.58	10.64
ENSDARG00000029204	tyrp1a	tyrosinase-related protein 1a [Source:ZFIN;Acc:ZDB-GENE-070718-2]	7	74084203	74090168		11.5	11.75	14.05	16.38	14.81	15.65
ENSDARG00000029215	ube2z	ubiquitin-conjugating enzyme E2Z [Source:NCBI gene;Acc:436602]	3	23581614	23596840		6.37	4.9	4.88	7.28	7.24	6.97
ENSDARG00000029219	avpr2ab	arginine vasopressin receptor 2a, duplicate b [Source:ZFIN;Acc:ZDB-GENE-110411-48]	23	18527028	18543359		2.52	1.58	1.24	1.36	1.53	1.17
ENSDARG00000029230	pnp4b	purine nucleoside phosphorylase 4b [Source:ZFIN;Acc:ZDB-GENE-040426-1887]	16	36807924	36834633		19.82	32.35	25.28	9.76	11.87	12.14
ENSDARG00000029232	FO704779.1		25	7591293	7601561		0.84	0.57	0.59	0.4	0.3	0.33
ENSDARG00000029234	stxbp5b	syntaxin binding protein 5b (tomosyn) [Source:ZFIN;Acc:ZDB-GENE-090821-5]	17	7229384	7351488		3.2	1.82	1.58	3.6	3.64	3.13
ENSDARG00000029239	syt9b	synaptotagmin IXb [Source:ZFIN;Acc:ZDB-GENE-040822-6]	25	15841670	15905903		9.5	2.48	3.29	8.29	8.75	9.4
ENSDARG00000029242	fbxo3	F-box protein 3 [Source:ZFIN;Acc:ZDB-GENE-050522-269]	7	38703338	38714562		7.16	5.28	6.01	6.31	5.93	5.64
ENSDARG00000029248	fubp1	far upstream element (FUSE) binding protein 1 [Source:ZFIN;Acc:ZDB-GENE-040426-2159]	2	8649293	8663104		55	33.44	33.36	63.84	61.96	60.87
ENSDARG00000029250	poli	polymerase (DNA directed) iota [Source:ZFIN;Acc:ZDB-GENE-050417-376]	5	18912950	18924834		4.49	3.89	3.53	3.22	3.3	3.16
ENSDARG00000029252	ssb	small RNA binding exonuclease protection factor La [Source:ZFIN;Acc:ZDB-GENE-030131-223]	6	3716666	3728694		34.19	38.17	37.28	23.8	24.07	23.96
ENSDARG00000029259	zgc:136493	zgc:136493 [Source:ZFIN;Acc:ZDB-GENE-060512-216]	16	25394763	25400257		4	5.29	5.05	3.53	3.78	3.48
ENSDARG00000029263	hoxb3a	homeobox B3a [Source:NCBI gene;Acc:30339]	3	23697997	23771945		5.86	3.03	3.42	10.6	11	10.41
ENSDARG00000029290	stx11b.1	syntaxin 11b, tandem duplicate 1 [Source:ZFIN;Acc:ZDB-GENE-040426-1893]	20	26538137	26541177		13.3	16.66	16.95	16.78	13.76	15.63
ENSDARG00000029291	ipmkb	inositol polyphosphate multikinase b [Source:ZFIN;Acc:ZDB-GENE-061215-5]	12	7193779	7235103		4.18	2.67	2.07	4.93	5.14	4.46
ENSDARG00000029292	zgc:55943	zgc:55943 [Source:ZFIN;Acc:ZDB-GENE-040426-2013]	2	9958081	9971728		15.72	12.03	14.48	14.35	13.45	14.76
ENSDARG00000029296	nexmifb	neurite extension and migration factor b [Source:ZFIN;Acc:ZDB-GENE-140106-18]	14	11103718	11268375		4.27	0.93	1.44	5.16	5.28	5.31
ENSDARG00000029305	baiap2l1a	BAR/IMD domain containing adaptor protein 2 like 1a [Source:ZFIN;Acc:ZDB-GENE-031201-1]	12	17815725	17863467		5.78	4.99	3.55	2.7	2.72	2.32
ENSDARG00000029307	rmc1	regulator of MON1-CCZ1 [Source:ZFIN;Acc:ZDB-GENE-041212-46]	24	33462800	33480652		6.41	5.29	5.16	4.7	4.64	4.87
ENSDARG00000029308	prr15lb	proline rich 15 like b [Source:NCBI gene;Acc:550400]	12	28808645	28818734		1.52	2.73	1.92	2.41	3.02	2.36
ENSDARG00000029331	atxn7l3	ataxin 7-like 3 [Source:NCBI gene;Acc:368510]	3	20025709	20040636		7.14	3.39	3.68	7.07	7.47	7.7
ENSDARG00000029353	serpine2	serpin peptidase inhibitor, clade E (nexin, plasminogen activator inhibitor type 1), member 2 [Source:ZFIN;Acc:ZDB-GENE-040426-8	15	43241915	43284021		30.6	30.47	32.7	28.27	30.55	30.15
ENSDARG00000029356	mboat1	membrane bound O-acyltransferase domain containing 1 [Source:ZFIN;Acc:ZDB-GENE-041114-98]	16	6395311	6424818		5.39	3.18	3.12	3.7	2.85	2.83
ENSDARG00000029370	ankrd6b	ankyrin repeat domain 6b [Source:ZFIN;Acc:ZDB-GENE-030916-4]	20	1203238	1239603		7.52	3.75	3.67	6.1	6.8	7.26
ENSDARG00000029372	rasa4	RAS p21 protein activator 4 [Source:ZFIN;Acc:ZDB-GENE-060526-349]	5	59278193	59391081		1.68	1.19	1.26	2.17	2.26	2.45
ENSDARG00000029381	aldh3a2b	aldehyde dehydrogenase 3 family, member A2b [Source:ZFIN;Acc:ZDB-GENE-040912-103]	21	39029713	39058490		7.17	9.99	5.6	5.12	5.67	5.79
ENSDARG00000029388	si:dkey-90m5.4	si:dkey-90m5.4 [Source:ZFIN;Acc:ZDB-GENE-041014-250]	20	23440632	23444653		5.84	8.52	7.2	4.07	5.41	5.17
ENSDARG00000029402	sort1a	sortilin 1a [Source:NCBI gene;Acc:406511]	11	36280523	36341189		5.52	5.04	4.72	6.5	6.88	6.9
ENSDARG00000029406	h2ax	H2A.X variant histone [Source:NCBI gene;Acc:394048]	5	30534702	30535327		19.25	16.5	19.48	14.6	13.41	14.04
ENSDARG00000029415	mtx2	metaxin 2 [Source:ZFIN;Acc:ZDB-GENE-021210-2]	6	10871659	10879963		23.46	24.8	28.37	21.33	20.64	20.79
ENSDARG00000029431	si:ch211-147k10.5	si:ch211-147k10.5 [Source:ZFIN;Acc:ZDB-GENE-120411-9]	25	30074947	30114384		1.84	0.92	0.55	2.06	2.41	2.42
ENSDARG00000029432	tph1a	tryptophan hydroxylase 1 (tryptophan 5-monooxygenase) a [Source:ZFIN;Acc:ZDB-GENE-030317-1]	25	8139220	8160030		3.6	2.15	2.84	3.93	5.29	4.38
ENSDARG00000029439	atp2a2a	ATPase sarcoplasmic/endoplasmic reticulum Ca2+ transporting 2a [Source:ZFIN;Acc:ZDB-GENE-040426-702]	8	11687254	11744994		13.28	12.98	9.71	12.46	11.98	12.32
ENSDARG00000029443	zgc:92242	zgc:92242 [Source:ZFIN;Acc:ZDB-GENE-040718-371]	14	8043441	8080416		19.39	20.33	17.67	15.88	14.67	15.54
ENSDARG00000029445	eif1b	eukaryotic translation initiation factor 1B [Source:ZFIN;Acc:ZDB-GENE-040426-1109]	24	32517915	32522655		87.49	88.61	88.11	88.03	87.63	83.81
ENSDARG00000029457	cacna1sa	calcium channel, voltage-dependent, L type, alpha 1S subunit, a [Source:ZFIN;Acc:ZDB-GENE-090514-1]	22	912450	968484		2.95	3.33	2.79	4.66	3.83	3.55
ENSDARG00000029461	cd209	CD209 molecule [Source:ZFIN;Acc:ZDB-GENE-061207-22]	10	24971473	25028527		0.06	0.05	0.01	0.08	0.04	0.16
ENSDARG00000029463	asap1a	ArfGAP with SH3 domain, ankyrin repeat and PH domain 1a [Source:ZFIN;Acc:ZDB-GENE-070912-129]	2	32263215	32356539		7.77	3.94	3.38	12.9	13	12.91
ENSDARG00000029472	afdna	afadin, adherens junction formation factor a [Source:ZFIN;Acc:ZDB-GENE-050419-52]	20	43120132	43353097		5.61	4.21	3.47	9.74	9.37	10
ENSDARG00000029473	gstm.2	glutathione S-transferase mu tandem duplicate 2 [Source:ZFIN;Acc:ZDB-GENE-080218-30]	8	25299069	25301802		0.69	0.46	0.55	1.08	1.11	1.5
ENSDARG00000029474	camk1b	calcium/calmodulin-dependent protein kinase Ib [Source:ZFIN;Acc:ZDB-GENE-141014-1]	11	4026229	4132662		4.44	2.5	2.37	5.86	6.42	5.42
ENSDARG00000029476	ldlra	low density lipoprotein receptor a [Source:ZFIN;Acc:ZDB-GENE-031217-1]	3	19299309	19323689		10.19	11.18	9.09	8.35	7.46	8.14
ENSDARG00000029479	zgc:86609	zgc:86609 [Source:ZFIN;Acc:ZDB-GENE-050102-7]	8	8686130	8698607		9.2	14.58	12.74	8.18	8.31	7.22
ENSDARG00000029480	specc1	sperm antigen with calponin homology and coiled-coil domains 1 [Source:ZFIN;Acc:ZDB-GENE-060526-207]	5	62804252	62988463		2.79	1.38	1.59	2.94	3.54	3.54
ENSDARG00000029482	ush2a	Usher syndrome 2A (autosomal recessive, mild) [Source:ZFIN;Acc:ZDB-GENE-060503-794]	17	29376854	29771639		0.43	0.12	0.12	0.94	0.9	1.05
ENSDARG00000029493	f9b	coagulation factor IXb [Source:ZFIN;Acc:ZDB-GENE-060421-7346]	10	26747755	26757486		5.62	8.22	7.62	9	11.48	9.43
ENSDARG00000029497	tfcp2l1	transcription factor CP2-like 1 [Source:ZFIN;Acc:ZDB-GENE-040704-58]	9	38292919	38311750		2.87	2.65	2.65	1.68	2.05	2.06
ENSDARG00000029500	rpl34	ribosomal protein L34 [Source:NCBI gene;Acc:394097]	7	59649399	59664545		366.16	492.21	550.46	332.21	345.43	361.95
ENSDARG00000029501	gsk3aa	glycogen synthase kinase 3 alpha a [Source:ZFIN;Acc:ZDB-GENE-060503-796]	19	6059673	6083784		5.24	3.01	2.94	7	7.39	7.2
ENSDARG00000029510	timm17a	translocase of inner mitochondrial membrane 17 homolog A (yeast) [Source:ZFIN;Acc:ZDB-GENE-031030-6]	11	24900123	24906264		44.53	45.1	42.79	42.58	42.76	42.15
ENSDARG00000029511	kcns3b	potassium voltage-gated channel, delayed-rectifier, subfamily S, member 3b [Source:ZFIN;Acc:ZDB-GENE-051120-78]	20	14114258	14118296		0.03	0.07	0.01	0.07	0.02	0.02
ENSDARG00000029524	impdh1b	IMP (inosine 5'-monophosphate) dehydrogenase 1b [Source:NCBI gene;Acc:338306]	4	18960914	18985481		19.46	18.61	18.09	22.4	21.92	22.43
ENSDARG00000029533	rpl18	ribosomal protein L18 [Source:ZFIN;Acc:ZDB-GENE-040801-165]	16	16969086	16991808		1402	1761.31	2108.94	1112.21	1155.16	1186.7
ENSDARG00000029544	id2b	inhibitor of DNA binding 2b [Source:ZFIN;Acc:ZDB-GENE-030131-15]	20	29784927	29787210		8.53	6.93	7.7	7.45	7.61	8.47
ENSDARG00000029546	grem1a	gremlin 1a, DAN family BMP antagonist [Source:ZFIN;Acc:ZDB-GENE-040625-1]	20	29469753	29471577		0.09	0.18	0.14	0.09	0.24	0.11
ENSDARG00000029556	kansl3	KAT8 regulatory NSL complex subunit 3 [Source:NCBI gene;Acc:555951]	8	51304099	51340931		5.64	3.95	3.67	7.49	7.5	7.66
ENSDARG00000029569	snapc1a	small nuclear RNA activating complex, polypeptide 1a [Source:ZFIN;Acc:ZDB-GENE-040426-1031]	20	20314574	20324792		0.03	0.08	0	0.08	0.15	0.12
ENSDARG00000029581	tbc1d7	TBC1 domain family, member 7 [Source:NCBI gene;Acc:393750]	20	52825215	52846212		10.2	7.63	9.14	11.92	11.39	12.39
ENSDARG00000029587	msra	methionine sulfoxide reductase A [Source:ZFIN;Acc:ZDB-GENE-041014-344]	20	38910214	39046181		5.86	5.34	5.52	6.96	7.19	7.26
ENSDARG00000029596	trim55a	tripartite motif containing 55a [Source:ZFIN;Acc:ZDB-GENE-040718-50]	2	42114310	42128714		5.8	8.88	10.21	7.03	6.53	6.69
ENSDARG00000029600	wdr32	WD repeat domain 32 [Source:NCBI gene;Acc:405882]	13	9559461	9572205		4.25	3.24	3.4	5.35	5.2	5.47
ENSDARG00000029609	macrod1	mono-ADP ribosylhydrolase 1 [Source:ZFIN;Acc:ZDB-GENE-040912-100]	14	45789454	45967981		1.21	1.34	1.2	1.01	0.89	0.95
ENSDARG00000029612	gpkow	G patch domain and KOW motifs [Source:NCBI gene;Acc:368735]	8	36591791	36618073		14.95	11.19	11.81	13.14	11.44	11.18
ENSDARG00000029615	zgc:77056	zgc:77056 [Source:ZFIN;Acc:ZDB-GENE-030131-5248]	16	39242339	39246079		16.38	13.96	14.14	12.94	12.41	11.87
ENSDARG00000029621	sgms1	sphingomyelin synthase 1 [Source:ZFIN;Acc:ZDB-GENE-061103-295]	12	6065661	6114296		4.82	5.92	5.41	6.26	6.73	6.68
ENSDARG00000029635	olfcq13	olfactory receptor C family, q13 [Source:ZFIN;Acc:ZDB-GENE-060503-830]	18	33570170	33577224		0.05	0.01	0.02	0.05	0.05	0.01
ENSDARG00000029639	tomm70a	translocase of outer mitochondrial membrane 70 homolog A (S. cerevisiae) [Source:ZFIN;Acc:ZDB-GENE-030131-8173]	6	28946041	28961660		36.77	32.86	30.39	34.45	36	35.82
ENSDARG00000029660	commd5	COMM domain containing 5 [Source:ZFIN;Acc:ZDB-GENE-040801-71]	14	10374530	10387377		5.31	4.61	5.27	5.79	5.34	6.02
ENSDARG00000029663	rab1ab	RAB1A, member RAS oncogene family b [Source:ZFIN;Acc:ZDB-GENE-030616-564]	13	24402406	24411961		53.15	50.26	42.56	60.87	58.02	57.64
ENSDARG00000029668	crim1	cysteine rich transmembrane BMP regulator 1 (chordin-like) [Source:NCBI gene;Acc:404210]	17	38927889	39185336		4.7	2.81	2.33	7.12	6.99	7.31
ENSDARG00000029671	xpr1b	xenotropic and polytropic retrovirus receptor 1b [Source:NCBI gene;Acc:558034]	2	6999369	7057330		11.67	9.03	7.8	13.55	13.75	14.16
ENSDARG00000029688	hsp70.1	heat shock cognate 70-kd protein, tandem duplicate 1 [Source:ZFIN;Acc:ZDB-GENE-990415-91]	3	26112780	26115559		0.7	0.28	0.35	1.67	2.84	1.68
ENSDARG00000029689	tkta	transketolase a [Source:ZFIN;Acc:ZDB-GENE-130530-578]	11	36989696	37035835		31.65	13.61	9.04	27.75	27.5	29.41
ENSDARG00000029692	rufy3	RUN and FYVE domain containing 3 [Source:ZFIN;Acc:ZDB-GENE-050327-58]	5	42912966	42933814		30.15	17.55	21.89	27.66	27.12	28.52
ENSDARG00000029695	pgp	phosphoglycolate phosphatase [Source:ZFIN;Acc:ZDB-GENE-030131-6240]	12	18565553	18568834		8.36	8.36	8.46	12.32	12.23	12.09
ENSDARG00000029710	lrrc30a	leucine rich repeat containing 30a [Source:ZFIN;Acc:ZDB-GENE-050506-25]	24	41678257	41679356		1.26	2.09	1.57	3.05	2.58	2.68
ENSDARG00000029718	p2rx3b	purinergic receptor P2X, ligand-gated ion channel, 3b [Source:ZFIN;Acc:ZDB-GENE-030319-3]	1	44746154	44758388		2.62	1.4	1.78	3.51	3.72	3.46
ENSDARG00000029720	si:dkeyp-9d4.2	si:dkeyp-9d4.2 [Source:ZFIN;Acc:ZDB-GENE-090312-156]	1	8017069	8020589		0.16	0.03	0.03	0.18	0.09	0.09
ENSDARG00000029722	hmgb2a	high mobility group box 2a [Source:ZFIN;Acc:ZDB-GENE-051120-126]	1	38169512	38171787		224.66	200.9	191.26	156.28	148.22	157.78
ENSDARG00000029724	pqbp1	polyglutamine binding protein 1 [Source:ZFIN;Acc:ZDB-GENE-030616-158]	8	36582728	36589390		21.05	18.89	20.05	15.45	15.22	16.58
ENSDARG00000029729	clybl	citrate lyase beta like [Source:ZFIN;Acc:ZDB-GENE-030516-6]	9	31075896	31259145		10.56	11.16	12.03	9.26	9.95	9.97
ENSDARG00000029747	mep1a.1	meprin A, alpha (PABA peptide hydrolase), tandem duplicate 1 [Source:ZFIN;Acc:ZDB-GENE-041001-209]	20	35473288	35481584		10.86	17.29	11.94	0.81	0.61	0.79
ENSDARG00000029751	wbp1lb	WW domain binding protein 1-like b [Source:ZFIN;Acc:ZDB-GENE-050522-112]	1	31674297	31699955		8.34	8.88	7.15	8.67	8.93	8.36
ENSDARG00000029761	mpp6b	membrane protein, palmitoylated 6b (MAGUK p55 subfamily member 6) [Source:ZFIN;Acc:ZDB-GENE-040426-775]	19	20061892	20106486		18.47	3.78	4.05	21.33	19.98	23.68
ENSDARG00000029764	mef2ca	myocyte enhancer factor 2ca [Source:ZFIN;Acc:ZDB-GENE-980526-253]	10	43544814	43655449		12.22	12.14	9.59	19.05	16.59	16.99
ENSDARG00000029766	nr1i2	nuclear receptor subfamily 1, group I, member 2 [Source:ZFIN;Acc:ZDB-GENE-030903-3]	9	9502610	9562644		6.26	5.69	4.35	6.45	6.34	6.49
ENSDARG00000029768	waplb	WAPL cohesin release factor b [Source:ZFIN;Acc:ZDB-GENE-080220-45]	12	11650146	11763658		12.5	7.88	7.87	13.21	13.01	13.28
ENSDARG00000029783	dedd	death effector domain containing [Source:ZFIN;Acc:ZDB-GENE-040718-386]	8	14058185	14080534		4.55	2.29	2.44	5.67	5.45	5.8
ENSDARG00000029795	prxl2b	peroxiredoxin like 2B [Source:NCBI gene;Acc:406605]	11	40622310	40647237		13.79	19.93	14.54	6.15	5.65	5.78
ENSDARG00000029803	oxgr1b	oxoglutarate (alpha-ketoglutarate) receptor 1b [Source:ZFIN;Acc:ZDB-GENE-070912-324]	9	29323163	29328009		0.02	0	0.02	0.02	0	0
ENSDARG00000029818	srsf5a	serine and arginine rich splicing factor 5a [Source:ZFIN;Acc:ZDB-GENE-030131-7336]	13	36526806	36535128		49.16	28.98	36.46	52.38	52.58	53.75
ENSDARG00000029822	cel.2	carboxyl ester lipase, tandem duplicate 2 [Source:ZFIN;Acc:ZDB-GENE-061110-10]	21	11385031	11393979		37.65	64.48	48.3	2.59	2.74	2.75
ENSDARG00000029830	myf6	myogenic factor 6 [Source:NCBI gene;Acc:404208]	4	21717793	21720943		8.45	10.42	10.11	4.86	3.08	4.34
ENSDARG00000029832	slc26a1	solute carrier family 26 member 1 [Source:ZFIN;Acc:ZDB-GENE-070112-2182]	21	20324562	20329541		5.43	9.3	6.06	3.71	3.58	3.69
ENSDARG00000029838	si:dkey-23a13.11	si:dkey-23a13.11 [Source:ZFIN;Acc:ZDB-GENE-160113-14]	7	5920665	5942776		0.01	0	0	0.02	0	0.01
ENSDARG00000029841	tmprss9	transmembrane serine protease 9 [Source:ZFIN;Acc:ZDB-GENE-050208-573]	22	10757848	10770418		2.03	1.72	1.48	1.38	1.46	1.31
ENSDARG00000029848	zgc:161973	zgc:161973 [Source:ZFIN;Acc:ZDB-GENE-070410-13]	2	22659787	22677019		0.84	0.41	0.34	0.47	0.26	0.59
ENSDARG00000029856	cnn3b	calponin 3, acidic b [Source:ZFIN;Acc:ZDB-GENE-050522-263]	2	15048410	15075295		13.59	12.7	13.63	10.3	11.01	11.14
ENSDARG00000029859	armh1	armadillo like helical domain containing 1 [Source:ZFIN;Acc:ZDB-GENE-081104-194]	8	18214081	18229209		0.54	0.35	0.45	0.74	0.86	1.19
ENSDARG00000029865	rassf2a	Ras association domain family member 2a [Source:ZFIN;Acc:ZDB-GENE-040912-124]	8	42998944	43049069		8.23	5.78	6.65	9.51	9.55	10.39
ENSDARG00000029866	slc6a14	solute carrier family 6 member 14 [Source:ZFIN;Acc:ZDB-GENE-130411-2]	11	20994026	21014572		0.58	0.39	0.31	1.87	2.24	1.82
ENSDARG00000029874	khk	ketohexokinase [Source:ZFIN;Acc:ZDB-GENE-040718-381]	17	12408188	12423175		4.7	4.71	5.33	4.74	4.53	4
ENSDARG00000029881	kcnh2a	potassium voltage-gated channel, subfamily H (eag-related), member 2a [Source:ZFIN;Acc:ZDB-GENE-070912-699]	2	24080694	24168670		0.59	0.27	0.27	0.67	0.46	0.59
ENSDARG00000029885	rab41	RAB41, member RAS oncogene family [Source:ZFIN;Acc:ZDB-GENE-040426-2686]	14	33280718	33297318		37.23	27.26	27.34	38.74	41.38	43.58
ENSDARG00000029890	si:dkeyp-86f7.4	si:dkeyp-86f7.4 [Source:ZFIN;Acc:ZDB-GENE-091204-252]	14	9432627	9447115		1.82	1.68	1.55	0.34	0.33	0.23
ENSDARG00000029894	slc2a15a	solute carrier family 2 member 15a [Source:ZFIN;Acc:ZDB-GENE-060531-109]	13	28417297	28442086		8.36	12.96	11.25	9.82	9.27	8.97
ENSDARG00000029898	cnga1a	cyclic nucleotide gated channel subunit alpha 1a [Source:ZFIN;Acc:ZDB-GENE-090312-66]	1	43894238	43899422		5.07	1.07	0.9	2.58	2.18	2.26
ENSDARG00000029905	phyhd1	phytanoyl-CoA dioxygenase domain containing 1 [Source:NCBI gene;Acc:492804]	21	4508959	4525080		30.88	41.62	41.65	18.27	19.84	21.84
ENSDARG00000029907	trim54	tripartite motif containing 54 [Source:ZFIN;Acc:ZDB-GENE-041014-342]	20	38730291	38758797		0.38	0.25	0.35	0.33	0.44	0.45
ENSDARG00000029911	casc3	casc3 exon junction complex subunit [Source:NCBI gene;Acc:403025]	3	23428953	23461954		12.52	8.65	6.92	17.33	16.46	16.25
ENSDARG00000029928	ap3m1	adaptor related protein complex 3 subunit mu 1 [Source:ZFIN;Acc:ZDB-GENE-021015-2]	13	25199849	25209083		11.84	14.65	12.1	10.23	10.41	9.28
ENSDARG00000029930	SPDEF	SAM pointed domain containing ETS transcription factor [Source:NCBI gene;Acc:100003543]	6	54280365	54290227	SPDEF	1.51	0.71	0.89	1.15	1.28	1.28
ENSDARG00000029931	atp13a1	ATPase 13A1 [Source:ZFIN;Acc:ZDB-GENE-040426-2804]	3	52899801	52952026		8.92	6.83	5.29	8.64	8.55	8.44
ENSDARG00000029936	grtp1a	growth hormone regulated TBC protein 1a [Source:NCBI gene;Acc:406711]	1	184915	192033		20.62	11.26	12.68	11.39	11.64	13
ENSDARG00000029944	parpbp	PARP1 binding protein [Source:NCBI gene;Acc:678635]	4	17337763	17353972		1.53	1.23	1.05	1.29	1.59	1.34
ENSDARG00000029952	ampd2b	adenosine monophosphate deaminase 2b [Source:ZFIN;Acc:ZDB-GENE-070713-5]	8	25247245	25293690		6.68	2.72	3.26	6.91	6.84	7.27
ENSDARG00000029955	glb1l	galactosidase, beta 1-like [Source:ZFIN;Acc:ZDB-GENE-050309-196]	9	12847040	12869049		1.96	2.65	2.13	2.46	2.4	2.67
ENSDARG00000029993	si:dkey-42l23.9	si:dkey-42l23.9 [Source:ZFIN;Acc:ZDB-GENE-131127-442]	15	37773126	37774544		0	0	0	0.03	0	0
ENSDARG00000029995	tnni2b.2	troponin I type 2b (skeletal, fast), tandem duplicate 2 [Source:ZFIN;Acc:ZDB-GENE-040801-9]	7	39385540	39394430		85.02	142.77	156.54	81.78	79.33	77.5
ENSDARG00000029997	BX511121.1		17	7643531	7672760		0.17	0.17	0.22	0.18	0.2	0.23
ENSDARG00000030006	slc6a7	solute carrier family 6 member 7 [Source:ZFIN;Acc:ZDB-GENE-070112-1492]	21	30794351	30830901		2.54	1.93	1.41	3	2.34	2.79
ENSDARG00000030012	lrrfip1a	leucine rich repeat (in FLII) interacting protein 1a [Source:ZFIN;Acc:ZDB-GENE-030131-99]	9	24065855	24115589		7.99	5.94	5.2	6.3	6.02	5.95
ENSDARG00000030020	agfg1a	ArfGAP with FG repeats 1a [Source:ZFIN;Acc:ZDB-GENE-030131-5808]	15	35156414	35212462		7.04	5.26	5.02	9.43	10.17	9.96
ENSDARG00000030022	nup188	nucleoporin 188 [Source:ZFIN;Acc:ZDB-GENE-030131-7184]	21	4540127	4606386		11.91	9.06	9.37	11.09	11.23	10.62
ENSDARG00000030025	pimr135	Pim proto-oncogene, serine/threonine kinase, related 135 [Source:ZFIN;Acc:ZDB-GENE-131119-42]	15	31013578	31018413		0	0	0	0	0	0
ENSDARG00000030038	C20H12orf57	gene rich cluster, C10 gene [Source:NCBI gene;Acc:323559]	20	14458930	14463012	C20H12orf57	13.94	10.84	12.27	11.17	11.26	12.49
ENSDARG00000030053	eef1db	eukaryotic translation elongation factor 1 delta b (guanine nucleotide exchange protein) [Source:ZFIN;Acc:ZDB-GENE-030131-6544	20	52546186	52582645		110.74	113.73	122.52	99.14	105.88	108.15
ENSDARG00000030058	tbx4	T-box transcription factor 4 [Source:ZFIN;Acc:ZDB-GENE-991124-5]	15	27384798	27411695		1.23	0.69	0.46	1.35	1.87	1.67
ENSDARG00000030064	plekha1a	pleckstrin homology domain containing, family A (phosphoinositide binding specific) member 1a [Source:ZFIN;Acc:ZDB-GENE-040	17	21887823	21913328		12.69	10.49	9.79	13.36	14.37	15.16
ENSDARG00000030078	glipr1b	GLI pathogenesis-related 1b [Source:ZFIN;Acc:ZDB-GENE-040426-1459]	18	7345417	7355225		4.37	1.38	2.28	4.97	5.91	5.37
ENSDARG00000030087	nfkbib	nuclear factor of kappa light polypeptide gene enhancer in B-cells inhibitor, beta [Source:ZFIN;Acc:ZDB-GENE-070705-529]	5	37032038	37038083		24.62	21.01	19.33	25.07	27.23	24.92
ENSDARG00000030092	tram2	translocation associated membrane protein 2 [Source:ZFIN;Acc:ZDB-GENE-040426-2024]	20	48116476	48139941		7.49	11.1	11.21	9.97	9.91	8.81
ENSDARG00000030094	malb	mal, T cell differentiation protein b [Source:ZFIN;Acc:ZDB-GENE-050417-175]	17	22102791	22106541		6.32	8.21	7.21	5.65	4.89	5.47
ENSDARG00000030095	ccdc78	coiled-coil domain containing 78 [Source:ZFIN;Acc:ZDB-GENE-060301-1]	24	37902687	37921940		0.18	0.18	0.12	0.29	0.32	0.29
ENSDARG00000030097	sdcbp	syndecan binding protein (syntenin) [Source:ZFIN;Acc:ZDB-GENE-081104-57]	7	58259455	58269812		14.13	4.77	7.16	9.87	11.36	10.9
ENSDARG00000030104	sh3bp4	SH3-domain binding protein 4 [Source:ZFIN;Acc:ZDB-GENE-081104-509]	9	23369294	23383131		2.61	2.63	2.4	2.22	2.2	2.02
ENSDARG00000030106	stmn4	stathmin-like 4 [Source:ZFIN;Acc:ZDB-GENE-040426-2732]	20	34773646	34868814		47	28.96	35.9	55.83	61.17	63.64
ENSDARG00000030107	si:ch211-207i1.2	si:ch211-207i1.2 [Source:ZFIN;Acc:ZDB-GENE-041014-149]	20	21638397	21806170		2.93	1.21	0.92	2.2	2.19	2.47
ENSDARG00000030108	sec22ba	SEC22 homolog B, vesicle trafficking protein a [Source:NCBI gene;Acc:114464]	2	9970942	9983661		5.03	9.58	7.44	6.58	6.93	6.73
ENSDARG00000030110	myod1	myogenic differentiation 1 [Source:NCBI gene;Acc:30513]	25	31421253	31423493		1.22	1.55	1.25	4.89	4.16	3.78
ENSDARG00000030114	vps4a	vacuolar protein sorting 4 homolog A [Source:ZFIN;Acc:ZDB-GENE-060929-388]	25	34938317	34948058		7.99	4.91	4.72	7.74	7.28	7.39
ENSDARG00000030116	susd6	sushi domain containing 6 [Source:ZFIN;Acc:ZDB-GENE-030131-8832]	20	28885990	28932038		9.09	5.99	4.83	14.36	13.67	13.59
ENSDARG00000030125	sox7	SRY-box transcription factor 7 [Source:ZFIN;Acc:ZDB-GENE-040109-4]	20	19066596	19080593		2	1.34	1.13	2.45	2.38	2.33
ENSDARG00000030129	si:ch211-87m7.2	si:ch211-87m7.2 [Source:ZFIN;Acc:ZDB-GENE-120215-153]	8	595122	622722		3.28	2.46	2.3	3.1	3.34	3.09
ENSDARG00000030133	fkbp6	FKBP prolyl isomerase 6 [Source:ZFIN;Acc:ZDB-GENE-050302-4]	5	55984270	55989773		0.02	0.09	0.1	0.09	0.15	0.16
ENSDARG00000030139	sdhdb	succinate dehydrogenase complex, subunit D, integral membrane protein b [Source:NCBI gene;Acc:445500]	15	21684435	21692630		47.5	59.4	59.71	38.01	37.88	37.34
ENSDARG00000030153	gnptab	N-acetylglucosamine-1-phosphate transferase subunits alpha and beta [Source:NCBI gene;Acc:553365]	4	17671164	17705108		7.2	6.44	5	6.88	6.92	6.58
ENSDARG00000030154	pak7	p21 protein (Cdc42/Rac)-activated kinase 7 [Source:ZFIN;Acc:ZDB-GENE-040426-2085]	20	54685143	54744600		4.7	2.36	2.67	9.29	8.58	9.02
ENSDARG00000030155	ssbp3b	single stranded DNA binding protein 3b [Source:ZFIN;Acc:ZDB-GENE-040426-2801]	8	18010568	18036639		14.43	8.38	8.77	22.31	21.52	22.39
ENSDARG00000030156	naglu	N-acetylglucosaminidase, alpha [Source:ZFIN;Acc:ZDB-GENE-100726-2]	24	36303637	36316104		1.65	1.98	1.48	1.93	2.29	2.29
ENSDARG00000030157	mybpc2a	myosin binding protein C, fast type a [Source:ZFIN;Acc:ZDB-GENE-060901-1]	3	30257582	30318595		8.91	10.14	8.34	1.51	1.36	1.38
ENSDARG00000030161	ppp1r14bb	protein phosphatase 1, regulatory (inhibitor) subunit 14Bb [Source:ZFIN;Acc:ZDB-GENE-030616-595]	7	60359347	60396578		11.05	9.49	7.88	13.28	12.04	11.98
ENSDARG00000030163	cdk21	cyclin-dependent kinase 21 [Source:ZFIN;Acc:ZDB-GENE-131121-149]	3	39175860	39180048		0.25	0.27	0.25	0.18	0.19	0.1
ENSDARG00000030166	hyi	hydroxypyruvate isomerase [Source:NCBI gene;Acc:393305]	6	2619893	2627495		18.97	24.72	23.7	10.39	10.04	11.02
ENSDARG00000030173	traf2a	Tnf receptor-associated factor 2a [Source:ZFIN;Acc:ZDB-GENE-071120-2]	5	28755475	28767636		5.87	2.76	3.71	6.24	5.37	6.28
ENSDARG00000030176	zgc:92429	zgc:92429 [Source:ZFIN;Acc:ZDB-GENE-040801-194]	7	23727768	23745984		5.79	7.67	8.06	5.61	5.71	5.72
ENSDARG00000030177	uchl3	ubiquitin carboxyl-terminal esterase L3 (ubiquitin thiolesterase) [Source:ZFIN;Acc:ZDB-GENE-050522-158]	9	21971260	21976670		21.24	25	23.66	14.63	15.26	15.41

ENSDARG00000030213	sap30l	sap30-like [Source:NCBI gene;Acc:327079]	21	45875792	45878872		50.3	47.31	54.09	42.44	44	44.49
ENSDARG00000030215	matn1	matrilin 1 [Source:ZFIN;Acc:ZDB-GENE-050307-3]	19	43314700	43338280		147.58	155.27	170.04	280.06	321.72	301.06
ENSDARG00000030224	ppp2ca	protein phosphatase 2, catalytic subunit, alpha isozyme [Source:ZFIN;Acc:ZDB-GENE-050417-441]	14	49118860	49133426		25.44	18.42	18.82	28.95	29.66	30.39
ENSDARG00000030236	tmem30aa	transmembrane protein 30Aa [Source:ZFIN;Acc:ZDB-GENE-040704-26]	17	49998159	50010121		17.2	11.25	10.72	24.62	25.62	24
ENSDARG00000030237	pgrmc2	progesterone receptor membrane component 2 [Source:ZFIN;Acc:ZDB-GENE-040426-2102]	17	26803470	26813630		17.23	15.87	14.2	18.82	18.76	18.11
ENSDARG00000030239	dennd6aa	DENN/MADD domain containing 6Aa [Source:ZFIN;Acc:ZDB-GENE-041010-63]	11	42422371	42459902		1.04	0.66	0.45	1.58	1.76	1.52
ENSDARG00000030241	vbp1	von Hippel-Lindau binding protein 1 [Source:ZFIN;Acc:ZDB-GENE-050522-494]	7	23886756	23894879		27.59	32.77	34.15	22.24	23.31	25.49
ENSDARG00000030250	dennd2db	DENN/MADD domain containing 2Db [Source:ZFIN;Acc:ZDB-GENE-041114-34]	22	835728	856886		0.91	0.97	1.14	0.9	1.09	1.12
ENSDARG00000030251	klhl36	kelch-like family member 36 [Source:NCBI gene;Acc:553604]	18	13195745	13203401		3.21	2.46	2.47	3.42	4.22	3.66
ENSDARG00000030254	cbln9	cerebellin 9 [Source:ZFIN;Acc:ZDB-GENE-040724-34]	2	37963266	37967368		0.9	1.64	1	0.35	0.16	0.24
ENSDARG00000030262	si:ch211-220i18.4	si:ch211-220i18.4 [Source:ZFIN;Acc:ZDB-GENE-090312-151]	23	9908940	9925568		2.7	3.77	2.93	0.98	1.2	1.13
ENSDARG00000030263	mfsd2b	major facilitator superfamily domain containing 2B [Source:ZFIN;Acc:ZDB-GENE-041210-308]	20	44507667	44557037		3.69	1.37	1.39	2.91	2.91	3.05
ENSDARG00000030265	scdb	stearoyl-CoA desaturase b [Source:ZFIN;Acc:ZDB-GENE-050522-12]	13	30007554	30028103		15.51	6.1	8.65	14.94	15.62	15.84
ENSDARG00000030267	gtf3aa	general transcription factor IIIAa [Source:ZFIN;Acc:ZDB-GENE-041006-1]	5	67980187	67993086		8.54	8.9	7.65	7.21	7.68	7.58
ENSDARG00000030269	grtp1b	growth hormone regulated TBC protein 1b [Source:ZFIN;Acc:ZDB-GENE-050417-346]	9	34389976	34396264		1.11	1.36	1.05	0.53	0.62	0.75
ENSDARG00000030270	tnnt3a	troponin T type 3a (skeletal, fast) [Source:ZFIN;Acc:ZDB-GENE-000322-3]	25	31405266	31418457		144.65	245.25	264.46	213.71	198.26	207.95
ENSDARG00000030278	idh3a	isocitrate dehydrogenase (NAD(+)) 3 catalytic subunit alpha [Source:ZFIN;Acc:ZDB-GENE-040426-1007]	25	25766033	25829109		66	66.24	66.82	57.64	57.58	54.67
ENSDARG00000030289	jag1a	jagged canonical Notch ligand 1a [Source:NCBI gene;Acc:140421]	1	51104984	51157660		0.94	0.53	0.45	1.77	1.84	1.67
ENSDARG00000030297	sox13	SRY-box transcription factor 13 [Source:ZFIN;Acc:ZDB-GENE-100519-1]	11	37768298	37831776		6.04	3.52	2.75	6.52	6.03	6.09
ENSDARG00000030307	hspa12b	heat shock protein 12B [Source:ZFIN;Acc:ZDB-GENE-070226-1]	7	60111458	60145018		3.56	4.06	4.22	6.72	6.82	7.21
ENSDARG00000030311	tmc2b	transmembrane channel-like 2b [Source:NCBI gene;Acc:567304]	5	25624819	25645593		0.85	0.67	0.8	0.45	0.43	0.51
ENSDARG00000030326	efna1a	ephrin-A1a [Source:ZFIN;Acc:ZDB-GENE-040426-1135]	19	8481802	8502321		12.05	8.42	8	16.11	15.01	16.54
ENSDARG00000030335	zc3h10	zinc finger CCCH-type containing 10 [Source:ZFIN;Acc:ZDB-GENE-040426-2359]	6	52242139	52256744		3	1.92	2.09	3.34	3.51	3.24
ENSDARG00000030340	guk1a	guanylate kinase 1a [Source:ZFIN;Acc:ZDB-GENE-040625-130]	2	3026802	3045863		15.26	15.95	12.78	20.17	20.69	21.19
ENSDARG00000030347	mespba	mesoderm posterior ba [Source:ZFIN;Acc:ZDB-GENE-000406-9]	7	15308219	15316731		0	0	0	0	0	0
ENSDARG00000030349	cryba2a	crystallin, beta A2a [Source:ZFIN;Acc:ZDB-GENE-040625-8]	6	13780205	13783634		4.97	18.3	34.77	64.74	56.87	71.12
ENSDARG00000030350	mnx2b	motor neuron and pancreas homeobox 2b [Source:ZFIN;Acc:ZDB-GENE-040415-2]	1	5449777	5455502		0.66	0.94	0.95	3.83	3.69	3.53
ENSDARG00000030357	zgc:66313	zgc:66313 [Source:ZFIN;Acc:ZDB-GENE-040426-1606]	17	43863708	43888569		0.49	0.62	0.6	0.98	1.02	0.85
ENSDARG00000030364	angpt1	angiopoietin 1 [Source:ZFIN;Acc:ZDB-GENE-010817-1]	19	46812272	46957968		2.3	2.07	2.08	4.99	5.04	5.25
ENSDARG00000030367	metrn	meteorin, glial cell differentiation regulator [Source:ZFIN;Acc:ZDB-GENE-050102-1]	24	37941618	37955993		8.11	6.54	6.06	10.97	11.2	11.52
ENSDARG00000030368	naa15b	N(alpha)-acetyltransferase 15, NatA auxiliary subunit b [Source:ZFIN;Acc:ZDB-GENE-030131-6031]	1	40613292	40641467		21.98	17.8	17.43	18.74	18.74	18.7
ENSDARG00000030376	grin2bb	glutamate receptor, ionotropic, N-methyl D-aspartate 2B, genome duplicate b [Source:ZFIN;Acc:ZDB-GENE-061207-27]	1	45372562	45553602		2.91	0.54	0.85	4.94	4.75	4.8
ENSDARG00000030394	prf1.1	perforin 1.1 [Source:ZFIN;Acc:ZDB-GENE-111212-2]	3	30980852	30988333		0.01	0.03	0.07	0.02	0.01	0.02
ENSDARG00000030402	twist1a	twist family bHLH transcription factor 1a [Source:ZFIN;Acc:ZDB-GENE-000210-6]	19	2229162	2231188		12.28	14.03	15.21	15.65	15.84	15.24
ENSDARG00000030408	rps26l	ribosomal protein S26, like [Source:ZFIN;Acc:ZDB-GENE-040426-1706]	6	49520124	49526510		351.11	427.83	522.38	319.68	332.42	319.25
ENSDARG00000030411	crygn2	crystallin, gamma N2 [Source:NCBI gene;Acc:445034]	24	41305391	41312459		58.3	70.52	111.46	201.54	173.45	225.97
ENSDARG00000030417	pkd1a	polycystic kidney disease 1a [Source:ZFIN;Acc:ZDB-GENE-100707-1]	1	54282042	54425791		0.88	0.88	0.79	2.67	2.73	2.84
ENSDARG00000030440	rsrp1	arginine/serine-rich protein 1 [Source:NCBI gene;Acc:724003]	13	45468403	45475289		76.28	27.8	38.42	84.8	80.79	74.89
ENSDARG00000030441	ppa1b	inorganic pyrophosphatase 1b [Source:ZFIN;Acc:ZDB-GENE-050417-375]	13	30152217	30161684		58.31	75.02	65.42	46.87	43.32	42.79
ENSDARG00000030448	sppl2	signal peptide peptidase-like 2 [Source:ZFIN;Acc:ZDB-GENE-030131-4265]	2	37480669	37503009		12.66	8.83	8.81	10.63	12.32	13.02
ENSDARG00000030449	crabp2b	cellular retinoic acid binding protein 2, b [Source:ZFIN;Acc:ZDB-GENE-050208-52]	19	8973042	8980038		7.85	7.37	6.76	13.27	12.74	11.96
ENSDARG00000030463	tppp3	tubulin polymerization-promoting protein family member 3 [Source:ZFIN;Acc:ZDB-GENE-040426-1909]	7	35229645	35243976		5.12	7.19	6.75	2.91	2.46	2.9
ENSDARG00000030465	tmem263	transmembrane protein 263 [Source:NCBI gene;Acc:406415]	18	15259388	15269272		7.91	8.92	6.86	9.52	10.08	9.4
ENSDARG00000030472	slc16a5a	solute carrier family 16 member 5a [Source:ZFIN;Acc:ZDB-GENE-121108-4]	12	26566422	26587231		2.38	2.04	1.66	3.15	3.02	3.37
ENSDARG00000030478	zgc:66484	zgc:66484 [Source:ZFIN;Acc:ZDB-GENE-030131-5768]	9	44897806	44905897		7.11	9.11	5.1	3.71	3.46	3.72
ENSDARG00000030479	hmgb1b	high mobility group box 1b [Source:ZFIN;Acc:ZDB-GENE-030131-8480]	15	31513065	31516583		110.68	78.68	87.46	101.35	95.54	103.93
ENSDARG00000030490	sptb	spectrin, beta, erythrocytic [Source:ZFIN;Acc:ZDB-GENE-000906-1]	17	38566717	38599022		8.92	10.74	8.27	20.03	18.6	17.08
ENSDARG00000030494	hjv	hemojuvelin BMP co-receptor [Source:ZFIN;Acc:ZDB-GENE-030131-651]	16	42883799	42894628		4.23	6.23	5.77	5.94	5.86	6.04
ENSDARG00000030508	CCDC134	si:dkey-167k11.5 [Source:ZFIN;Acc:ZDB-GENE-091204-218]	3	431250	440125	CCDC134	0	0	0	0	0	0
ENSDARG00000030512	tsnaxip1	translin-associated factor X interacting protein 1 [Source:ZFIN;Acc:ZDB-GENE-070424-39]	18	21931741	21950751		1.73	0.91	1.31	1.68	1.92	1.7
ENSDARG00000030514	acsl1a	acyl-CoA synthetase long chain family member 1a [Source:ZFIN;Acc:ZDB-GENE-050809-115]	1	17537451	17587552		8.86	7.02	6.86	8.49	8.19	8.31
ENSDARG00000030530	slc22a2	solute carrier family 22 member 2 [Source:ZFIN;Acc:ZDB-GENE-040426-2167]	20	42668875	42688487		4.99	6.06	5.56	3.47	3.86	3.59
ENSDARG00000030532	arhgef12a	Rho guanine nucleotide exchange factor (GEF) 12a [Source:ZFIN;Acc:ZDB-GENE-121017-3]	15	22448456	22538843		5.68	4.25	3.44	6.7	6.87	6.69
ENSDARG00000030537	psmc1a	proteasome 26S subunit, ATPase 1a [Source:ZFIN;Acc:ZDB-GENE-030131-8730]	17	8899016	8941421		45.58	44.12	45.97	28.41	28.28	28.87
ENSDARG00000030547	rnd1a	Rho family GTPase 1a [Source:ZFIN;Acc:ZDB-GENE-040630-6]	23	17800717	17815253		8.78	2.87	3.61	8.91	8.42	9.15
ENSDARG00000030560	helz	helicase with zinc finger [Source:NCBI gene;Acc:406550]	3	60957512	61034549		9.93	8.58	5.97	18.16	17.7	18.16
ENSDARG00000030563	zgc:77880	zgc:77880 [Source:ZFIN;Acc:ZDB-GENE-040426-1901]	1	10305611	10315206		5.17	2.48	4.05	5.27	5.24	5.32
ENSDARG00000030573	nudt1	nudix (nucleoside diphosphate linked moiety X)-type motif 1 [Source:ZFIN;Acc:ZDB-GENE-040426-2757]	3	42121166	42125655		3.01	2.86	2.91	1.58	1.69	2.2
ENSDARG00000030583	foxk2	forkhead box K2 [Source:ZFIN;Acc:ZDB-GENE-030131-2861]	12	32370	42214		17.38	11.17	11.61	27.44	27.99	28.57
ENSDARG00000030588	slc10a1	solute carrier family 10 member 1 [Source:ZFIN;Acc:ZDB-GENE-041001-190]	20	28861435	28867748		1.44	2.47	2.13	1.35	1.02	1.27
ENSDARG00000030591	lrrc42	leucine rich repeat containing 42 [Source:NCBI gene;Acc:326793]	2	26604773	26611033		3.75	2.47	2.74	2.58	3.46	2.34
ENSDARG00000030598	nampta	nicotinamide phosphoribosyltransferase a [Source:ZFIN;Acc:ZDB-GENE-110420-1]	4	3358383	3385452		21.72	15.66	16.45	19.76	17.72	18.18
ENSDARG00000030602	rps19	ribosomal protein S19 [Source:ZFIN;Acc:ZDB-GENE-040426-1716]	16	24188114	24195361		193.97	233.6	290.02	141.24	150.73	147.33
ENSDARG00000030604	phkg1a	phosphorylase kinase, gamma 1a (muscle) [Source:ZFIN;Acc:ZDB-GENE-061103-385]	15	5722525	5742531		6.13	4.72	5.26	7.26	7.74	7.25
ENSDARG00000030608	rhogd	ras homolog gene family, member Gd [Source:ZFIN;Acc:ZDB-GENE-040426-1504]	14	12169979	12176369		1.76	1.92	1.78	1.81	2.02	1.95
ENSDARG00000030614	syt1a	synaptotagmin Ia [Source:ZFIN;Acc:ZDB-GENE-040718-165]	4	21129752	21315399		56.22	11.78	15.5	76.89	76.32	79.65
ENSDARG00000030616	nfe2l1a	nuclear factor, erythroid 2-like 1a [Source:ZFIN;Acc:ZDB-GENE-040630-5]	3	24072720	24093144		4.34	5.54	4.65	5.17	4.47	4.65
ENSDARG00000030621	th	tyrosine hydroxylase [Source:ZFIN;Acc:ZDB-GENE-990621-5]	25	24055341	24074500		4.73	1.05	1.56	5.26	6.32	6.76
ENSDARG00000030626	lrit2	leucine-rich repeat, immunoglobulin-like and transmembrane domains 2 [Source:NCBI gene;Acc:553557]	13	29470442	29474304		3.99	0.85	0.61	2.42	2.5	2.62
ENSDARG00000030630	mfsd2aa	major facilitator superfamily domain containing 2aa [Source:NCBI gene;Acc:492810]	13	35925490	35953596		2.49	1.49	1.42	2.61	2.31	2.24
ENSDARG00000030632	ccdc3b	coiled-coil domain containing 3b [Source:ZFIN;Acc:ZDB-GENE-050522-184]	11	4148808	4175773		5.46	6.45	4.9	4.52	4.38	4.17
ENSDARG00000030633	plekhm3	pleckstrin homology domain containing, family M, member 3 [Source:ZFIN;Acc:ZDB-GENE-070912-382]	9	28140089	28208549		1.89	0.8	0.8	2.08	1.79	2.08
ENSDARG00000030634	pimr189	Pim proto-oncogene, serine/threonine kinase, related 189 [Source:ZFIN;Acc:ZDB-GENE-081104-447]	8	47209659	47212686		0.06	0.02	0.02	0.02	0.06	0.04
ENSDARG00000030636	tex30	testis expressed 30 [Source:ZFIN;Acc:ZDB-GENE-041210-219]	9	27393468	27398369		2.95	3.1	2.89	1.72	1.64	2
ENSDARG00000030641	letmd1	LETM1 domain containing 1 [Source:ZFIN;Acc:ZDB-GENE-080723-8]	23	32079046	32100106		5.79	5.32	4.76	4.61	4.52	4.28
ENSDARG00000030644	gnai3	guanine nucleotide binding protein (G protein), alpha inhibiting activity polypeptide 3 [Source:ZFIN;Acc:ZDB-GENE-070713-6]	8	25145464	25220957		7.2	6.41	5.83	6.54	7.15	7.54
ENSDARG00000030650	r3hdml	R3H domain containing-like [Source:ZFIN;Acc:ZDB-GENE-090313-275]	23	25821055	25827370		0.43	0.82	1.01	1.56	1.25	1.9
ENSDARG00000030656	3-Sep	septin 3 [Source:NCBI gene;Acc:554123]	1	45616067	45636362		24.91	9.59	13.85	22.38	22.03	24.89
ENSDARG00000030665	TCERG1	transcription elongation regulator 1b (CA150) [Source:NCBI gene;Acc:797473]	21	41782307	41818359	TCERG1	7.34	5	4.94	9.81	10.99	10.65
ENSDARG00000030687	phka2	phosphorylase kinase, alpha 2 (liver) [Source:ZFIN;Acc:ZDB-GENE-130422-1]	11	33870357	33919309		9.59	6.07	5.99	14.46	14.21	15.12
ENSDARG00000030694	atp6v1e1b	ATPase H+ transporting V1 subunit E1b [Source:ZFIN;Acc:ZDB-GENE-020419-11]	4	5240126	5247335		132.87	113.27	119.87	101.56	99.78	108.31
ENSDARG00000030700	ctps1a	CTP synthase 1a [Source:NCBI gene;Acc:322089]	19	16015881	16031503		7.27	9.36	9.26	6.46	6.01	5.82
ENSDARG00000030703	otx2a	orthodenticle homeobox 2a [Source:NCBI gene;Acc:30462]	1	50921266	50929957		2.24	1.32	1.32	1.85	1.34	1.78
ENSDARG00000030716	ing4	inhibitor of growth family, member 4 [Source:ZFIN;Acc:ZDB-GENE-050522-47]	19	9512982	9522548		37.71	27.5	29	47.01	47.1	47.79
ENSDARG00000030722	xirp1	xin actin binding repeat containing 1 [Source:NCBI gene;Acc:497637]	2	23749614	23768832		2.36	2.32	1.7	2.79	2.69	2.85
ENSDARG00000030723	cldn11b	claudin 11b [Source:ZFIN;Acc:ZDB-GENE-010328-13]	24	26432541	26441318		6.56	7.43	7.66	6.54	6.06	6.13
ENSDARG00000030733	b3gat2	beta-1,3-glucuronyltransferase 2 (glucuronosyltransferase S) [Source:ZFIN;Acc:ZDB-GENE-040801-191]	13	27951688	28009574		10.03	3.34	5.12	11.12	9.75	11.13
ENSDARG00000030743	sptlc3	serine palmitoyltransferase, long chain base subunit 3 [Source:ZFIN;Acc:ZDB-GENE-041210-281]	13	34804243	34862484		7.84	9.07	7.26	7.61	7.36	6.8
ENSDARG00000030750	gabrr3b	gamma-aminobutyric acid (GABA) A receptor, rho 3b [Source:ZFIN;Acc:ZDB-GENE-131120-131]	24	25258904	25284835		3.45	0.4	0.46	3.43	2.5	3.41
ENSDARG00000030753	prrg4	proline rich Gla (G-carboxyglutamic acid) 4 (transmembrane) [Source:ZFIN;Acc:ZDB-GENE-050417-112]	18	45666489	45676686		4.64	5.42	4.61	3.91	3.7	3.72
ENSDARG00000030756	dnmt1	DNA (cytosine-5-)-methyltransferase 1 [Source:ZFIN;Acc:ZDB-GENE-990714-15]	3	54591218	54607166		21.12	16.54	14.35	13.58	12.71	13.1
ENSDARG00000030758	guca1c	guanylate cyclase activator 1C [Source:ZFIN;Acc:ZDB-GENE-030829-1]	15	2884711	2903475		2.92	0.68	0.49	1.96	1.87	1.98
ENSDARG00000030759	melk	maternal embryonic leucine zipper kinase [Source:NCBI gene;Acc:30724]	1	21861387	21881818		4.79	2.97	2.91	2.5	3.09	2.64
ENSDARG00000030761	syt16	synaptotagmin XVI [Source:ZFIN;Acc:ZDB-GENE-090313-210]	13	37543498	37545487		6.58	1.26	2.25	4.95	5.33	5.58
ENSDARG00000030765	hadh	hydroxyacyl-CoA dehydrogenase [Source:ZFIN;Acc:ZDB-GENE-040801-261]	1	49909672	49919042		52.05	75.56	73.64	34.72	33.84	33.47
ENSDARG00000030775	sybl1	synaptobrevin-like 1 [Source:ZFIN;Acc:ZDB-GENE-040426-1055]	14	9033315	9056067		7.94	5.24	5.71	8.31	8.41	8.2
ENSDARG00000030778	BX323854.1		24	39990695	39992254		0.04	0.01	0	0.03	0.02	0.19
ENSDARG00000030781	acads	acyl-CoA dehydrogenase short chain [Source:ZFIN;Acc:ZDB-GENE-040808-64]	10	43999263	44017665		26.56	32.66	30.47	15.46	15.7	16.18
ENSDARG00000030782	exoc3l2b	exocyst complex component 3-like 2b [Source:ZFIN;Acc:ZDB-GENE-100728-5]	15	20139031	20190052		4.26	5.21	2.58	5.41	5.4	4.78
ENSDARG00000030786	mpi	mannose phosphate isomerase [Source:ZFIN;Acc:ZDB-GENE-050904-6]	25	3035384	3047644		7.97	4.4	5.02	10.85	10.85	12.45
ENSDARG00000030789	ddx18	DEAD (Asp-Glu-Ala-Asp) box polypeptide 18 [Source:ZFIN;Acc:ZDB-GENE-030131-9685]	9	38314466	38352220		24.26	27.86	28.43	15.5	17.06	16.09
ENSDARG00000030803	C10H21orf91	zgc:110006 [Source:NCBI gene;Acc:553573]	10	35059856	35108683	C10H21orf91	8.22	4.17	4.12	6.27	6.4	6.33
ENSDARG00000030804	fam120b	family with sequence similarity 120B [Source:ZFIN;Acc:ZDB-GENE-040801-119]	13	6086730	6125334		1.41	1.1	1.4	1.78	1.46	1.8
ENSDARG00000030805	znhit2	zinc finger, HIT-type containing 2 [Source:ZFIN;Acc:ZDB-GENE-061103-46]	10	26982254	26985231		4.65	3.25	3.93	3.81	3.39	3.01
ENSDARG00000030824	esyt2a	extended synaptotagmin-like protein 2a [Source:ZFIN;Acc:ZDB-GENE-070912-588]	2	42419841	42492445		5.6	6.23	4.28	7.36	7.15	7.24
ENSDARG00000030830	cmtr1	cap methyltransferase 1 [Source:NCBI gene;Acc:393102]	23	35361724	35396978		7.26	5.78	5.23	7.25	7.54	7.27
ENSDARG00000030832	otofa	otoferlin a [Source:NCBI gene;Acc:557476]	20	31076488	31212556		5.79	2.17	2.71	4.22	4.52	4.4
ENSDARG00000030839	tescb	tescalcin b [Source:ZFIN;Acc:ZDB-GENE-040426-1903]	5	32141790	32148529		3.08	2.32	2.43	2.86	3.1	2.95
ENSDARG00000030844	klf11a	Kruppel-like factor 11a [Source:ZFIN;Acc:ZDB-GENE-030131-3568]	20	29502065	29505863		9.58	7.66	7.68	8.6	8.73	9.02
ENSDARG00000030867	opn7c	opsin 7, group member c [Source:ZFIN;Acc:ZDB-GENE-060526-333]	8	25858224	25874426		0.72	0.78	0.91	0.98	0.9	0.88
ENSDARG00000030871	siah1	siah E3 ubiquitin protein ligase 1 [Source:NCBI gene;Acc:80955]	18	18439332	18472257		10.67	7.04	5.86	12.58	12.31	12.7
ENSDARG00000030872	cetp	cholesteryl ester transfer protein, plasma [Source:ZFIN;Acc:ZDB-GENE-041114-54]	18	17611627	17621826		5.41	8.74	8.44	8.25	7.93	8.6
ENSDARG00000030881	baxb	BCL2 associated X, apoptosis regulator b [Source:ZFIN;Acc:ZDB-GENE-050227-21]	3	32074806	32079916		3.9	5.16	4.32	3.55	3.18	3.58
ENSDARG00000030887	phf23a	PHD finger protein 23a [Source:NCBI gene;Acc:541372]	5	24240318	24245218		9.09	7.59	7.1	9.03	9.31	9.06
ENSDARG00000030896	foxq1a	forkhead box Q1a [Source:ZFIN;Acc:ZDB-GENE-070424-74]	2	720779	722170		10.1	9.04	7.22	8.08	6.97	7.5
ENSDARG00000030905	cited2	Cbp/p300-interacting transactivator, with Glu/Asp-rich carboxy-terminal domain, 2 [Source:ZFIN;Acc:ZDB-GENE-041010-141]	20	36915143	36917670		16.65	13.98	13.98	25	27.18	26.3
ENSDARG00000030913	cluha	clustered mitochondria (cluA/CLU1) homolog a [Source:NCBI gene;Acc:562666]	15	25321572	25367309		15.12	14.98	9.78	16.29	14.34	13.97
ENSDARG00000030914	tmem120a	transmembrane protein 120A [Source:NCBI gene;Acc:100005623]	10	35265083	35275350		5.01	4.95	4.05	7.15	6.49	6.7
ENSDARG00000030915	cpa1	carboxypeptidase A1 (pancreatic) [Source:ZFIN;Acc:ZDB-GENE-050522-438]	25	18448186	18454016		7.82	11.51	9.95	1.03	1.57	1.66
ENSDARG00000030932	fgf10a	fibroblast growth factor 10a [Source:ZFIN;Acc:ZDB-GENE-030715-1]	21	19635486	19683168		2.78	2.67	3.33	2.78	2.59	2.49
ENSDARG00000030933	ksr1b	kinase suppressor of ras 1b [Source:ZFIN;Acc:ZDB-GENE-040724-45]	15	31070603	31147322		2.66	2.97	1.97	3.14	2.54	2.46
ENSDARG00000030938	fermt3b	fermitin family member 3b [Source:ZFIN;Acc:ZDB-GENE-040426-1366]	14	45970033	45997630		1.17	1.42	1.67	1.67	1.65	1.55
ENSDARG00000030945	si:ch211-259g3.4	si:ch211-259g3.4 [Source:ZFIN;Acc:ZDB-GENE-030131-8118]	15	16122984	16177603		3.88	2.16	1.82	4.46	4.72	5.08
ENSDARG00000030949	srprb	SRP receptor subunit beta [Source:ZFIN;Acc:ZDB-GENE-040718-311]	15	2510761	2519640		37.21	44.09	41.8	25.63	24.8	23.97
ENSDARG00000030954	ftr56	finTRIM family, member 56 [Source:ZFIN;Acc:ZDB-GENE-030131-2801]	19	12398544	12404590		1.03	0.86	1	0.78	0.53	0.62
ENSDARG00000030957	yipf4	Yip1 domain family, member 4 [Source:NCBI gene;Acc:405827]	17	22577472	22585596		26.3	22.73	21.43	26.37	24.56	25.04
ENSDARG00000030961	ak8	adenylate kinase 8 [Source:NCBI gene;Acc:553671]	5	29671681	29711948		0.56	0.56	0.31	0.99	1.21	1.19
ENSDARG00000030964	pde12	phosphodiesterase 12 [Source:ZFIN;Acc:ZDB-GENE-060519-25]	11	42506052	42525450		4.96	3.38	4	3.99	3.95	4.32
ENSDARG00000030967	si:ch211-51a6.2	si:ch211-51a6.2 [Source:ZFIN;Acc:ZDB-GENE-090313-118]	13	21779975	21822894		1.46	1.54	0.99	1.19	1.21	1.14
ENSDARG00000030972	dnaja1	DnaJ heat shock protein family (Hsp40) member A1 [Source:ZFIN;Acc:ZDB-GENE-030131-2642]	1	45002971	45014434		19.01	15.55	15.2	15.1	14.67	14.32
ENSDARG00000030975	ccdc80l1	coiled-coil domain containing 80 like 1 [Source:ZFIN;Acc:ZDB-GENE-120328-4]	6	16406723	16423323		2.96	4.66	3.67	3.32	3.6	3.22
ENSDARG00000030980	csrp1b	cysteine and glycine-rich protein 1b [Source:ZFIN;Acc:ZDB-GENE-070112-252]	6	54838269	54845622		12.38	22.64	23.72	7.77	8.44	8.73
ENSDARG00000030981	tmem127	transmembrane protein 127 [Source:NCBI gene;Acc:553545]	8	4803906	4813669		9.88	7.08	5.29	14.53	14.02	14.57
ENSDARG00000030999	mybl1	v-myb avian myeloblastosis viral oncogene homolog-like 1 [Source:ZFIN;Acc:ZDB-GENE-041111-281]	24	23791758	23817821		1.87	1	0.98	2.79	2.83	2.66
ENSDARG00000031015	dtna	dystrobrevin, alpha [Source:ZFIN;Acc:ZDB-GENE-070117-2]	24	35713659	35767501		6.43	4.22	4.6	10.39	10.49	10.27
ENSDARG00000031020	pip4k2ca	phosphatidylinositol-5-phosphate 4-kinase, type II, gamma a [Source:NCBI gene;Acc:373124]	23	36616717	36645135		24.72	18.42	16.81	25.31	26.04	27.2
ENSDARG00000031044	lipg	lipase, endothelial [Source:ZFIN;Acc:ZDB-GENE-040426-699]	8	32376936	32386042		3.76	3.08	3.58	5.6	5.46	5.4
ENSDARG00000031046	nr1h5	nuclear receptor subfamily 1, group H, member 5 [Source:ZFIN;Acc:ZDB-GENE-080403-3]	8	10903325	10932206		2.53	3.03	3.16	4.22	4.74	4.29
ENSDARG00000031048	gspt1	G1 to S phase transition 1 [Source:ZFIN;Acc:ZDB-GENE-040822-36]	12	19362335	19383945		7.14	7.57	7.42	7.4	7.24	6.77
ENSDARG00000031049	igsf21a	immunoglobin superfamily, member 21a [Source:ZFIN;Acc:ZDB-GENE-050809-118]	11	28642913	29082429		8.3	2.56	2.59	10.96	10.33	11.42
ENSDARG00000031075	cadm1a	cell adhesion molecule 1a [Source:ZFIN;Acc:ZDB-GENE-080505-2]	21	23953181	24412427		22.32	6.68	9.61	23.81	24.97	25.29
ENSDARG00000031095	veph1	ventricular zone expressed PH domain-containing 1 [Source:NCBI gene;Acc:405756]	18	41313999	41465606		1.26	0.68	0.84	1.53	1.73	1.53
ENSDARG00000031098	timm50	translocase of inner mitochondrial membrane 50 homolog (S. cerevisiae) [Source:NCBI gene;Acc:393638]	15	20475694	20528494		11.76	12.25	12.09	9.36	9.31	8.34
ENSDARG00000031100	ivns1abpa	influenza virus NS1A binding protein a [Source:NCBI gene;Acc:387591]	20	34320635	34341183		45.9	36.3	32.44	48.24	48.52	49.12
ENSDARG00000031108	lrba	LPS-responsive vesicle trafficking, beach and anchor containing [Source:ZFIN;Acc:ZDB-GENE-090311-19]	1	23919418	24349789		6.85	5.51	3.5	7.64	7.33	7.24
ENSDARG00000031116	dnal4a	dynein, axonemal, light chain 4a [Source:ZFIN;Acc:ZDB-GENE-040801-122]	3	24511959	24518496		2.33	1.41	1.51	2.78	2.73	2.6
ENSDARG00000031119	baiap2l1b	BAR/IMD domain containing adaptor protein 2 like 1b [Source:ZFIN;Acc:ZDB-GENE-050417-371]	3	61401311	61494840		2.75	2.48	2.58	2.58	3.13	2.58
ENSDARG00000031126	notum1a	notum, palmitoleoyl-protein carboxylesterase a [Source:NCBI gene;Acc:570510]	3	57948377	57979190		5.13	6.16	6.44	8.37	9.27	9.76
ENSDARG00000031136	moxd1	monooxygenase, DBH-like 1 [Source:NCBI gene;Acc:570584]	20	25225112	25271878		1.45	1.45	1.26	3.43	3.71	3.47
ENSDARG00000031138	irx3b	iroquois homeobox 3b [Source:ZFIN;Acc:ZDB-GENE-030131-525]	25	36488164	36492779		3.66	1.85	2.32	6.69	6.8	7.33
ENSDARG00000031150	gba3	glucosidase, beta, acid 3 (gene/pseudogene) [Source:ZFIN;Acc:ZDB-GENE-050522-351]	7	70338270	70344448		0.93	1.27	0.74	0.71	0.59	0.59
ENSDARG00000031161	nr1d4a	nuclear receptor subfamily 1, group D, member 4a [Source:ZFIN;Acc:ZDB-GENE-080403-5]	23	35795759	35821521		3.76	0.72	0.58	3.34	3.7	4.19
ENSDARG00000031164	tuba8l2	tubulin, alpha 8 like 2 [Source:ZFIN;Acc:ZDB-GENE-040426-1646]	1	5402476	5419116		109.85	141.9	137.93	62.3	61.22	60.78
ENSDARG00000031198	hmg20a	high mobility group 20A [Source:ZFIN;Acc:ZDB-GENE-030131-5107]	25	7241084	7336346		8.69	4.21	4.4	8.59	7.02	7.48
ENSDARG00000031200	ppp2r5cb	protein phosphatase 2, regulatory subunit B', gamma b [Source:ZFIN;Acc:ZDB-GENE-041014-4]	20	53898193	53963515		6.67	4.36	4	8.31	8.42	8.05
ENSDARG00000031202	alg11	ALG11 alpha-1,2-mannosyltransferase [Source:NCBI gene;Acc:407614]	1	29750937	29761086		3.82	3.32	3.17	3.24	3.98	3.73
ENSDARG00000031203	commd1	copper metabolism (Murr1) domain containing 1 [Source:ZFIN;Acc:ZDB-GENE-060512-329]	1	53490669	53504603		8.53	6.75	7.57	10.38	9.41	9.58
ENSDARG00000031214	arcn1b	archain 1b [Source:ZFIN;Acc:ZDB-GENE-040121-7]	15	18102280	18115624		26.69	28.5	25.84	28.55	29.72	28.76
ENSDARG00000031216	ssu72	SSU72 homolog, RNA polymerase II CTD phosphatase [Source:NCBI gene;Acc:393701]	22	359273	367569		44.47	38.49	41.86	35.76	36.83	36.83
ENSDARG00000031222	lhx2b	LIM homeobox 2b [Source:ZFIN;Acc:ZDB-GENE-051220-1]	8	3085120	3101392		8.37	2.85	3.07	10.65	10.42	10.77
ENSDARG00000031228	podxl	podocalyxin-like [Source:ZFIN;Acc:ZDB-GENE-030131-2805]	4	11732156	11751037		20.08	26.28	21.98	22.34	23.58	21.97
ENSDARG00000031240	kidins220a	kinase D-interacting substrate 220a [Source:ZFIN;Acc:ZDB-GENE-041014-170]	17	34809643	34963575		3.29	2.78	2.18	4.27	3.7	3.71
ENSDARG00000031243	snai3	snail family zinc finger 3 [Source:ZFIN;Acc:ZDB-GENE-061013-757]	7	55112922	55120300		0.43	0.63	0.79	0.29	0.37	0.48
ENSDARG00000031246	hbegfb	heparin-binding EGF-like growth factor b [Source:ZFIN;Acc:ZDB-GENE-070820-6]	21	30161295	30168949		2.29	2.21	2.08	3.26	3.95	3.55
ENSDARG00000031248	enkur	enkurin, TRPC channel interacting protein [Source:ZFIN;Acc:ZDB-GENE-070718-1]	24	6673200	6678640		3.26	3.83	3.33	3.34	3.26	3.41
ENSDARG00000031261	sap30bp	SAP30 binding protein [Source:ZFIN;Acc:ZDB-GENE-030131-457]	8	13064750	13098192		16.52	14.18	15.08	11.61	12.06	12.03
ENSDARG00000031269	rnf123	ring finger protein 123 [Source:ZFIN;Acc:ZDB-GENE-050208-529]	22	34350349	34591295		0.81	0.46	0.44	1.8	1.55	1.73
ENSDARG00000031278	si:ch73-193i2.2	si:ch73-193i2.2 [Source:ZFIN;Acc:ZDB-GENE-141215-32]	13	3327417	3351708		0.73	0.86	1.02	0.13	0.15	0.19
ENSDARG00000031283	vamp1	vesicle-associated membrane protein 1 [Source:ZFIN;Acc:ZDB-GENE-040426-2725]	19	9460432	9472935		35.1	11.64	11.39	34.99	34	36.52
ENSDARG00000031293	dnajc9	DnaJ (Hsp40) homolog, subfamily C, member 9 [Source:ZFIN;Acc:ZDB-GENE-040718-130]	12	9497646	9510403		19.74	19.85	20.05	9.02	8.48	8.46
ENSDARG00000031299	dhrs11b	dehydrogenase/reductase (SDR family) member 11b [Source:ZFIN;Acc:ZDB-GENE-040625-155]	15	15403560	15408113		11.42	14.84	12.04	5.84	5.52	5.86
ENSDARG00000031302	smap1	small ArfGAP 1 [Source:ZFIN;Acc:ZDB-GENE-060920-2]	13	28009727	28263868		28.36	19.94	17.19	32.02	32.05	33.48
ENSDARG00000031307	zic4	zic family member 4 [Source:ZFIN;Acc:ZDB-GENE-040622-4]	24	4977862	4983423		3.63	2.38	2.8	4.11	4.15	4.57
ENSDARG00000031316	six6b	SIX homeobox 6b [Source:ZFIN;Acc:ZDB-GENE-050522-308]	20	20531002	20533865		14.13	3.99	3.4	13.37	13.81	17.27
ENSDARG00000031317	ppdpfb	pancreatic progenitor cell differentiation and proliferation factor b [Source:NCBI gene;Acc:336303]	23	17427838	17429775		266.97	271.71	305.87	447.79	507.11	505.53
ENSDARG00000031325	tmed1a	transmembrane p24 trafficking protein 1a [Source:ZFIN;Acc:ZDB-GENE-040801-229]	3	47222096	47235997		2	2.71	2.66	1.78	1.6	1.82
ENSDARG00000031336	hsd20b2	hydroxysteroid (20-beta) dehydrogenase 2 [Source:ZFIN;Acc:ZDB-GENE-030804-21]	5	38200807	38214807		5.33	5.59	5.24	11.36	10.93	11.07

ENSDARG00000031337	hoxa10b	homeobox A10b [Source:NCBI gene;Acc:30391]	16	20910186	20913003		3.51	4.62	4.62	7.31	7.7	7.82
ENSDARG00000031343	rab6bb	RAB6B, member RAS oncogene family b [Source:ZFIN;Acc:ZDB-GENE-050320-28]	2	22795494	22803665		26.33	13.63	16.5	27.15	27.55	28.27
ENSDARG00000031345	RTKN	si:dkey-40c11.1 [Source:ZFIN;Acc:ZDB-GENE-060531-142]	5	43617262	43682930	RTKN	6.42	3.59	3.05	7.9	9.04	8.47
ENSDARG00000031346	rbm17	RNA binding motif protein 17 [Source:ZFIN;Acc:ZDB-GENE-040426-817]	4	25497104	25515591		19.03	14.91	17.14	16.21	15.36	17.74
ENSDARG00000031348	mc5ra	melanocortin 5a receptor [Source:ZFIN;Acc:ZDB-GENE-021223-3]	19	12762887	12803408		2.31	1.17	1.35	2.23	2.15	2.12
ENSDARG00000031359	cmpk2	cytidine monophosphate (UMP-CMP) kinase 2, mitochondrial [Source:ZFIN;Acc:ZDB-GENE-040724-24]	17	35362851	35375448		0.04	0.07	0.1	0.02	0.06	0.06
ENSDARG00000031366	rtn4ip1	reticulon 4 interacting protein 1 [Source:NCBI gene;Acc:393323]	17	25550235	25563847		9.5	9.67	10.63	7.29	6.7	7.65
ENSDARG00000031372	efna2a	ephrin-A2a [Source:NCBI gene;Acc:30218]	2	26303627	26444503		4.54	1.74	1.99	6.05	6.13	5.95
ENSDARG00000031382	reep2	receptor accessory protein 2 [Source:NCBI gene;Acc:568927]	14	21596716	21618005		8.68	3.26	4.78	8.9	9.77	9.69
ENSDARG00000031383	klhl38a	kelch-like family member 38a [Source:NCBI gene;Acc:559083]	19	33084066	33087246		0.57	0.67	0.67	0.98	1.13	1.21
ENSDARG00000031387	cers4a	ceramide synthase 4a [Source:ZFIN;Acc:ZDB-GENE-020808-1]	22	4707663	4733106		7.92	3.58	3.85	11.62	11.08	10.92
ENSDARG00000031388	dctn2	dynactin 2 (p50) [Source:NCBI gene;Acc:394141]	6	58832155	58867662		53	43.72	45.73	46.27	47.42	49.84
ENSDARG00000031420	wt1a	WT1 transcription factor a [Source:ZFIN;Acc:ZDB-GENE-980526-558]	25	15178225	15214161		1.62	2.25	1.48	2.61	3.28	2.59
ENSDARG00000031422	igfbp2b	insulin-like growth factor binding protein 2b [Source:NCBI gene;Acc:798920]	9	46745348	46799620		9.77	11.5	11.05	9.55	9.61	8.31
ENSDARG00000031426	csrnp1a	cysteine-serine-rich nuclear protein 1a [Source:ZFIN;Acc:ZDB-GENE-070912-475]	2	23790748	23805139		2.2	2.02	2.02	6.07	6.12	6.17
ENSDARG00000031427	calm2a	calmodulin 2a (phosphorylase kinase, delta) [Source:ZFIN;Acc:ZDB-GENE-030804-3]	13	8777633	8785610		70.94	59.98	65.51	70.22	67.25	68.54
ENSDARG00000031434	rcor1	REST corepressor 1 [Source:ZFIN;Acc:ZDB-GENE-050506-79]	17	29251662	29271359		13.22	11.58	9.82	13.56	13.79	13.07
ENSDARG00000031435	TXN	zgc:56493 [Source:ZFIN;Acc:ZDB-GENE-030131-8581]	1	40263671	40270561	TXN	180.95	259.78	280.06	139.2	140.41	145.79
ENSDARG00000031438	kcnj11l	potassium inwardly rectifying channel subfamily J member 11, like [Source:ZFIN;Acc:ZDB-GENE-050222-2]	25	7670683	7676816		11.66	2.25	3.57	10.15	10.44	11.38
ENSDARG00000031463	syt6b	synaptotagmin VIb [Source:ZFIN;Acc:ZDB-GENE-090601-3]	11	24620742	24664031		2.46	0.61	0.86	2.18	2.19	2.23
ENSDARG00000031483	col9a1b	collagen, type IX, alpha 1b [Source:ZFIN;Acc:ZDB-GENE-040426-2410]	13	39119460	39160018		10.8	14.16	14.11	53.28	52.44	56.84
ENSDARG00000031489	ognb	osteoglycin, paralog b [Source:ZFIN;Acc:ZDB-GENE-080723-61]	23	19679557	19686791		0.28	0.26	0.35	0.06	0.12	0.16
ENSDARG00000031494	prrc2a	proline-rich coiled-coil 2A [Source:ZFIN;Acc:ZDB-GENE-010501-4]	19	26739813	26770083		14.16	9.25	7.4	28.23	27.05	27.88
ENSDARG00000031495	seta	SET nuclear proto-oncogene a [Source:ZFIN;Acc:ZDB-GENE-030131-2221]	8	2585256	2591654		124.63	113.65	115.2	93.52	91.32	92.89
ENSDARG00000031496	ap1ar	adaptor related protein complex 1 associated regulatory protein [Source:ZFIN;Acc:ZDB-GENE-130530-580]	14	4796168	4814464		1.23	1.05	0.84	1.91	1.87	1.59
ENSDARG00000031506	flvcr2b	feline leukemia virus subgroup C cellular receptor family, member 2b [Source:ZFIN;Acc:ZDB-GENE-041210-312]	20	46311242	46349064		4.14	4.16	3.82	4.03	3.85	4.23
ENSDARG00000031511	psmb2	proteasome 20S subunit beta 2 [Source:ZFIN;Acc:ZDB-GENE-040718-353]	19	47394270	47423628		42.37	47.41	54.73	35.71	34.36	34.71
ENSDARG00000031533	ap5m1	adaptor related protein complex 5 subunit mu 1 [Source:ZFIN;Acc:ZDB-GENE-050522-231]	17	44441042	44454022		3.38	2.5	2.17	3.46	3.36	3.29
ENSDARG00000031534	CU571315.1		3	19468777	19477473		1.22	1.75	1.2	1.03	1.02	1.11
ENSDARG00000031540	tmem200a	transmembrane protein 200A [Source:NCBI gene;Acc:556369]	20	2227860	2241954		3.24	0.99	1.12	3.81	4.03	4.09
ENSDARG00000031548	ephb3a	eph receptor B3a [Source:NCBI gene;Acc:30313]	2	7923964	8017579		9.91	5.75	5.42	15.48	15.36	16.16
ENSDARG00000031560	srpk1b	SRSF protein kinase 1b [Source:ZFIN;Acc:ZDB-GENE-070112-2292]	11	2763168	2826562		16.13	11.22	9.89	14.27	13.47	13.23
ENSDARG00000031562	tada2a	transcriptional adaptor 2A [Source:ZFIN;Acc:ZDB-GENE-060929-508]	15	15786160	15819598		8.57	4.01	5.47	8.15	9.1	8.9
ENSDARG00000031587	flvcr1	feline leukemia virus subgroup C cellular receptor 1 [Source:ZFIN;Acc:ZDB-GENE-041014-359]	20	37844035	37862535		5.12	2.19	1.88	8.35	8.18	8.46
ENSDARG00000031588	si:dkey-239b22.1	si:dkey-239b22.1 [Source:ZFIN;Acc:ZDB-GENE-131119-12]	12	38929663	38974869		0.52	0.37	0.13	8.31	5.39	3.82
ENSDARG00000031598	calb1	calbindin 1 [Source:ZFIN;Acc:ZDB-GENE-030131-8107]	16	36765934	36798783		9.16	3.77	4.23	8.9	8.7	8.78
ENSDARG00000031600	rd3	retinal degeneration 3, GUCY2D regulator [Source:ZFIN;Acc:ZDB-GENE-040724-103]	20	13969414	13997835		8.54	2.13	2.5	8.01	8.7	7.01
ENSDARG00000031616	g6pca.1	glucose-6-phosphatase a, catalytic subunit, tandem duplicate 1 [Source:ZFIN;Acc:ZDB-GENE-031001-4]	12	6023490	6033854		7.52	10.8	11.12	29.66	37.65	37.19
ENSDARG00000031618	unm_sa1261	un-named sa1261 [Source:ZFIN;Acc:ZDB-GENE-120411-10]	3	58506242	58543658		9.03	2.27	2.36	8.37	8.81	8.72
ENSDARG00000031632	chst10	carbohydrate sulfotransferase 10 [Source:NCBI gene;Acc:445322]	1	28031697	28047242		4.7	3.08	3.34	4.95	4.81	4.89
ENSDARG00000031637	pan3	poly(A) specific ribonuclease subunit PAN3 [Source:ZFIN;Acc:ZDB-GENE-070928-13]	24	21720304	21748700		5.2	3.76	3.01	6.51	5.94	6.22
ENSDARG00000031647	stat2	signal transducer and activator of transcription 2 [Source:ZFIN;Acc:ZDB-GENE-080321-1]	6	39123544	39160422		3.77	3.03	2.56	3.65	3.58	3.97
ENSDARG00000031649	cort	cortistatin [Source:ZFIN;Acc:ZDB-GENE-030813-3]	23	28731379	28732313		19.36	9.03	11.48	20.3	19.16	20.06
ENSDARG00000031657	fahd1	fumarylacetoacetate hydrolase domain containing 1 [Source:ZFIN;Acc:ZDB-GENE-050522-448]	3	18795570	18796559		13.5	19.87	16.94	8.05	7.93	7.23
ENSDARG00000031658	si:ch211-207d6.2	si:ch211-207d6.2 [Source:ZFIN;Acc:ZDB-GENE-070912-185]	2	29795833	29896572		3.21	1.81	1.23	3.23	3.26	3.15
ENSDARG00000031664	sox21a	SRY-box transcription factor 21a [Source:NCBI gene;Acc:30543]	6	7414215	7415343		6	5.07	6.2	3.58	3.35	3.97
ENSDARG00000031678	col5a2a	collagen, type V, alpha 2a [Source:ZFIN;Acc:ZDB-GENE-030616-13]	9	42653188	42696408		84.84	109	85.26	111.79	114.86	118.68
ENSDARG00000031680	ercc1	excision repair cross-complementation group 1 [Source:ZFIN;Acc:ZDB-GENE-040426-2606]	9	32674676	32684008		6.68	7.53	7.26	5.4	5.29	5.21
ENSDARG00000031681	atp6v0b	ATPase H+ transporting V0 subunit b [Source:ZFIN;Acc:ZDB-GENE-030131-443]	2	33681199	33687214		48.41	29.73	29.57	54.95	52.4	55.15
ENSDARG00000031683	fosab	v-fos FBJ murine osteosarcoma viral oncogene homolog Ab [Source:ZFIN;Acc:ZDB-GENE-031222-4]	20	46552311	46554440		13.47	5.52	5.74	22.13	18.82	20.22
ENSDARG00000031693	kif9	kinesin family member 9 [Source:ZFIN;Acc:ZDB-GENE-160728-129]	7	41275201	41295511		1.09	1.12	1.36	1.09	1.41	1.34
ENSDARG00000031698	mylk2	myosin light chain kinase 2 [Source:ZFIN;Acc:ZDB-GENE-080616-2]	23	32039386	32060885		0.54	0.63	0.5	0.88	0.95	0.98
ENSDARG00000031702	prkg1b	protein kinase cGMP-dependent 1b [Source:ZFIN;Acc:ZDB-GENE-080225-40]	12	6195191	6451609		4.55	6.48	5.24	4.34	3.9	3.93
ENSDARG00000031709	mllt1b	MLLT1 super elongation complex subunit b [Source:ZFIN;Acc:ZDB-GENE-060421-7142]	2	22970140	23004355		2.2	1.58	1.63	1.51	1.7	1.49
ENSDARG00000031712	grm3	glutamate receptor, metabotropic 3 [Source:ZFIN;Acc:ZDB-GENE-061009-13]	18	8972227	9046805		1.95	0.47	0.75	3.28	3.34	3.19
ENSDARG00000031720	clstn1	calsyntenin 1 [Source:ZFIN;Acc:ZDB-GENE-040426-1064]	23	29580584	29668286		59.17	16.06	16.61	76.39	75.99	76.19
ENSDARG00000031731	si:ch73-27e22.6	si:ch73-27e22.6 [Source:ZFIN;Acc:ZDB-GENE-070705-234]	22	8596974	8601775		0.42	0.59	0.22	0.23	0.29	0.45
ENSDARG00000031737	pthlha	parathyroid hormone-like hormone a [Source:ZFIN;Acc:ZDB-GENE-050119-9]	4	612363	626550		1.13	1.27	1.01	1.22	1.43	1.63
ENSDARG00000031745	mhc2a	major histocompatibility complex class II integral membrane alpha chain gene [Source:ZFIN;Acc:ZDB-GENE-990415-150]	8	36472444	36475328		0.1	0.25	0.13	0.01	0.03	0.02
ENSDARG00000031749	C3AR1	C3a anaphylatoxin chemotactic receptor [Source:NCBI gene;Acc:794255]	10	1899595	1900662	C3AR1	0.22	0.43	0.24	0.59	0.49	0.37
ENSDARG00000031751	npr1a	natriuretic peptide receptor 1a [Source:ZFIN;Acc:ZDB-GENE-060503-539]	19	26423590	26629117		2.11	1.49	1.67	4.08	4.51	4.13
ENSDARG00000031756	mef2aa	myocyte enhancer factor 2aa [Source:ZFIN;Acc:ZDB-GENE-990415-163]	18	23187419	23356077		5.62	4.07	3.18	9.49	9.18	8.95
ENSDARG00000031757	tmc4	transmembrane channel-like 4 [Source:ZFIN;Acc:ZDB-GENE-030131-7489]	16	32029090	32050153		2.31	2.47	1.96	1.01	1.28	1.02
ENSDARG00000031761	dab2	DAB adaptor protein 2 [Source:ZFIN;Acc:ZDB-GENE-040303-1]	5	32323775	32338872		10.75	9.72	6.39	8.17	8.49	8.65
ENSDARG00000031763	smad6b	SMAD family member 6b [Source:ZFIN;Acc:ZDB-GENE-050419-198]	18	19648275	19699061		12.5	12.62	12.35	14.51	14.9	14.52
ENSDARG00000031768	roraa	RAR-related orphan receptor A, paralog a [Source:NCBI gene;Acc:564951]	25	33849647	33969717		4.98	1.56	1.59	7.06	7.21	6.98
ENSDARG00000031770	kat7b	K(lysine) acetyltransferase 7b [Source:ZFIN;Acc:ZDB-GENE-030131-1901]	12	5981333	5998938		13.71	9.66	11.15	14.89	15.12	15.74
ENSDARG00000031774	pus7	pseudouridine synthase 7 [Source:ZFIN;Acc:ZDB-GENE-060620-1]	25	235992	244424		10.12	9.11	8.31	8.5	8.61	8.55
ENSDARG00000031775	ube2s	ubiquitin-conjugating enzyme E2S [Source:NCBI gene;Acc:565498]	16	13672768	13680692		12.41	10.44	9.82	13.76	12.89	12.45
ENSDARG00000031776	fthl27	ferritin, heavy polypeptide-like 27 [Source:ZFIN;Acc:ZDB-GENE-040718-72]	3	32099507	32100999		114.49	134.45	142.39	79.22	86.16	92.2
ENSDARG00000031777	pparaa	peroxisome proliferator-activated receptor alpha a [Source:ZFIN;Acc:ZDB-GENE-041210-169]	4	18609539	18635005		4.98	3.71	2.26	3.68	4.18	3.33
ENSDARG00000031782	crybg1a	crystallin beta-gamma domain containing 1a [Source:ZFIN;Acc:ZDB-GENE-041008-100]	17	25481466	25550249		10.1	10.07	7.94	9.71	9.33	9.05
ENSDARG00000031783	adcy8	adenylate cyclase 8 (brain) [Source:ZFIN;Acc:ZDB-GENE-070912-197]	2	42776224	42871286		4.11	1.28	1.82	6.04	5.38	5.32
ENSDARG00000031795	abcf1	ATP-binding cassette, sub-family F (GCN20), member 1 [Source:ZFIN;Acc:ZDB-GENE-050517-31]	19	30428913	30447743		38.92	42.51	36.92	31.61	30.77	27.99
ENSDARG00000031796	ache	acetylcholinesterase [Source:NCBI gene;Acc:114549]	7	26077153	26087807		39.51	33.76	32.84	38.32	34.71	35.54
ENSDARG00000031809	rbm24b	RNA binding motif protein 24b [Source:ZFIN;Acc:ZDB-GENE-060312-43]	16	53455638	53475519		7	9.09	5.6	10.09	8.88	8.71
ENSDARG00000031814	dhrs13b.1	dehydrogenase/reductase (SDR family) member 13b.1 [Source:ZFIN;Acc:ZDB-GENE-040801-253]	15	24563504	24569062		3.06	3.83	3.96	3.06	3.27	3.51
ENSDARG00000031817	trim2a	tripartite motif containing 2a [Source:ZFIN;Acc:ZDB-GENE-050327-99]	1	25322445	25370281		2.96	1.77	1.11	2.36	2.81	2.44
ENSDARG00000031819	eif4ebp2	eukaryotic translation initiation factor 4E binding protein 2 [Source:ZFIN;Acc:ZDB-GENE-031118-83]	13	30163515	30168897		100.98	104.43	84.07	101.41	102.14	103.6
ENSDARG00000031836	vps37c	VPS37C subunit of ESCRT-I [Source:ZFIN;Acc:ZDB-GENE-061110-126]	1	44949909	44963259		6.85	4.49	3.32	9.09	9.3	9.42
ENSDARG00000031845	mboat2a	membrane bound O-acyltransferase domain containing 2a [Source:ZFIN;Acc:ZDB-GENE-041210-257]	17	34969123	35076730		0.62	0.32	0.24	0.98	0.93	0.8
ENSDARG00000031848	pparg	peroxisome proliferator-activated receptor gamma [Source:ZFIN;Acc:ZDB-GENE-990415-213]	11	609516	669558		7.54	9.37	5.03	5.57	5.48	5.14
ENSDARG00000031875	trpa1b	transient receptor potential cation channel, subfamily A, member 1b [Source:ZFIN;Acc:ZDB-GENE-050106-1]	24	13635108	13715761		1.18	0.51	0.7	0.79	0.96	0.84
ENSDARG00000031876	pard6ga	par-6 family cell polarity regulator gamma a [Source:ZFIN;Acc:ZDB-GENE-050923-1]	16	25388503	25393699		0.24	0.04	0.15	0.17	0.03	0.21
ENSDARG00000031885	psmb12	proteasome 20S subunit beta 12 [Source:ZFIN;Acc:ZDB-GENE-001208-1]	19	7115223	7119456		0.05	0.26	0.27	0.22	0.21	0.11
ENSDARG00000031886	ift140	intraflagellar transport 140 homolog (Chlamydomonas) [Source:ZFIN;Acc:ZDB-GENE-040724-165]	24	37825634	37901493		3.27	2.01	2.12	3.26	3.51	3.6
ENSDARG00000031888	mapk8a	mitogen-activated protein kinase 8a [Source:ZFIN;Acc:ZDB-GENE-080218-17]	13	31147518	31167461		3.93	1.47	1.94	5.48	5.44	5.47
ENSDARG00000031890	tcp11l1	t-complex 11, testis-specific-like 1 [Source:ZFIN;Acc:ZDB-GENE-050327-11]	25	15273370	15285590		4.91	3.14	3.22	4.16	4.19	4.84
ENSDARG00000031891	tbx1	T-box transcription factor 1 [Source:NCBI gene;Acc:368206]	5	15202495	15215483		7.38	6.59	5.88	10.24	10.83	10.1
ENSDARG00000031894	lef1	lymphoid enhancer-binding factor 1 [Source:ZFIN;Acc:ZDB-GENE-990714-26]	1	49814461	49905357		4.99	3.03	2.84	6.96	6.79	7.06
ENSDARG00000031907	ptbp1b	polypyrimidine tract binding protein 1b [Source:ZFIN;Acc:ZDB-GENE-030131-9796]	11	14321113	14342507		12.36	12.3	11.5	15.67	16.56	16.21
ENSDARG00000031911	tns3.2	tensin 3, tandem duplicate 2 [Source:ZFIN;Acc:ZDB-GENE-081031-95]	20	6590220	6666641		7.12	8.35	6.77	10.31	10.63	10.93
ENSDARG00000031915	hif1an	hypoxia inducible factor 1 subunit alpha inhibitor [Source:NCBI gene;Acc:373126]	13	29952633	29980215		8.21	6.89	7.43	24.44	26.04	27.65
ENSDARG00000031929	stard14	START domain containing 14 [Source:ZFIN;Acc:ZDB-GENE-040426-963]	14	33270387	33278084		7.3	7.2	6.35	6.26	6.25	5.16
ENSDARG00000031930	erc1b	ELKS/RAB6-interacting/CAST family member 1b [Source:ZFIN;Acc:ZDB-GENE-041210-51]	4	8376362	8515824		0	0.03	0	0	0	0
ENSDARG00000031952	mb	Danio rerio myoglobin (mb), transcript variant 2, mRNA. [Source:RefSeq mRNA;Acc:NM_001362390]	1	55137943	55145085		13.58	12.4	12.28	50	57.77	52.08
ENSDARG00000031954	rbck1	RanBP-type and C3HC4-type zinc finger containing 1 [Source:ZFIN;Acc:ZDB-GENE-040704-3]	22	10145098	10165446		3.02	2.7	2.38	2.54	2.22	2.43
ENSDARG00000031956	tmem63a	transmembrane protein 63A [Source:ZFIN;Acc:ZDB-GENE-041210-107]	4	5665182	5691257		4.34	4.15	3.5	5.15	4.94	4.94
ENSDARG00000031968	cpped1	calcineurin-like phosphoesterase domain containing 1 [Source:NCBI gene;Acc:492772]	12	19866865	19932623		4.32	5.11	4.64	4.99	5.08	5.15
ENSDARG00000031971	poglut2	protein O-glucosyltransferase 2 [Source:NCBI gene;Acc:393905]	9	27398323	27410659		4.69	4.77	5.17	6.76	6.08	6.43
ENSDARG00000031976	nos2b	nitric oxide synthase 2b, inducible [Source:ZFIN;Acc:ZDB-GENE-080916-1]	15	30323491	30348677		0.45	0.22	0.24	0.6	0.51	0.54
ENSDARG00000031981	pcbd1	pterin-4 alpha-carbinolamine dehydratase/dimerization cofactor of hepatocyte nuclear factor 1 alpha [Source:ZFIN;Acc:ZDB-GENE-	13	25720969	25726534		42.01	59.51	64.99	38.16	41.79	44.06
ENSDARG00000031983	six4b	SIX homeobox 4b [Source:ZFIN;Acc:ZDB-GENE-010201-1]	20	20484827	20492161		3.49	3.45	3.65	5.02	4.31	4.72
ENSDARG00000031985	ptpa	protein phosphatase 2 phosphatase activator [Source:ZFIN;Acc:ZDB-GENE-040801-11]	5	32793886	32814417		5.02	4.02	3.99	5.53	5.19	5.44
ENSDARG00000032005	ccdc65	coiled-coil domain containing 65 [Source:NCBI gene;Acc:368824]	6	7421898	7427512		3.31	1.58	2.85	5.52	6.69	6.14
ENSDARG00000032010	slc15a2	solute carrier family 15 member 2 [Source:NCBI gene;Acc:556684]	9	37320222	37339656		6.28	5.59	4.57	3.94	3.08	3.65
ENSDARG00000032013	pafah1b1a	platelet-activating factor acetylhydrolase 1b, regulatory subunit 1a [Source:NCBI gene;Acc:394246]	15	25273185	25319663		36.61	23.54	19.72	49.79	47.75	47.46
ENSDARG00000032037	bet1l	Bet1 golgi vesicular membrane trafficking protein-like [Source:NCBI gene;Acc:445493]	25	7667634	7670655		4.03	4.95	4.56	4.39	4.6	3.7
ENSDARG00000032039	mxd1	MAX dimerization protein 1 [Source:ZFIN;Acc:ZDB-GENE-060526-246]	5	13706959	13766651		6.47	3.68	3.83	11.97	13.47	12.8
ENSDARG00000032043	ftr16	finTRIM family, member 16 [Source:ZFIN;Acc:ZDB-GENE-070912-126]	2	43742574	43745175		0.36	0.32	0.25	0.42	0.57	0.35
ENSDARG00000032049	enah	ENAH actin regulator [Source:ZFIN;Acc:ZDB-GENE-010323-11]	20	36419947	36617313		7.62	8.11	6.65	10.82	10.64	10.86
ENSDARG00000032056	arl6	ADP-ribosylation factor-like 6 [Source:NCBI gene;Acc:494134]	1	34203817	34233938		6.36	2.81	3.23	6.67	6.59	6.27
ENSDARG00000032072	cdk16	cyclin-dependent kinase 16 [Source:ZFIN;Acc:ZDB-GENE-030131-2939]	8	8362441	8446668		10.81	7.28	6.14	11.71	11.67	10.9
ENSDARG00000032077	BX323082.1	major facilitator superfamily domain containing 12b [Source:NCBI gene;Acc:564425]	8	20564621	20580582		0.15	0.17	0.17	0.15	0.12	0.15
ENSDARG00000032079	acsl3a	acyl-CoA synthetase long chain family member 3a [Source:ZFIN;Acc:ZDB-GENE-050420-181]	15	42995051	43078542		3.73	2.08	1.46	2.6	2.7	2.49
ENSDARG00000032083	dpysl2b	dihydropyrimidinase like 2b [Source:ZFIN;Acc:ZDB-GENE-031105-1]	10	18952271	18992471		46.66	20.31	19.03	70.47	66.99	68.65
ENSDARG00000032087	sgsh	N-sulfoglucosamine sulfohydrolase (sulfamidase) [Source:ZFIN;Acc:ZDB-GENE-030131-4958]	22	11520249	11532760		6.22	6.1	6.16	5.75	5.62	6.69
ENSDARG00000032098	c8g	complement component 8, gamma polypeptide [Source:ZFIN;Acc:ZDB-GENE-040426-1898]	24	17031129	17039638		8.56	13.44	13.58	8.37	6.56	7.18
ENSDARG00000032102	prdx3	peroxiredoxin 3 [Source:ZFIN;Acc:ZDB-GENE-030826-18]	13	9842276	9851472		59.84	78.14	78.17	39.85	40.92	38.32
ENSDARG00000032103	mapk6	mitogen-activated protein kinase 6 [Source:ZFIN;Acc:ZDB-GENE-050420-7]	18	39170127	39288894		6.75	4.13	3.94	9.6	9.18	9.53
ENSDARG00000032114	slain2	SLAIN motif family, member 2 [Source:ZFIN;Acc:ZDB-GENE-041014-251]	20	23446243	23472319		6.36	4.35	3.35	7.78	7.18	8.37
ENSDARG00000032116	cnot7	CCR4-NOT transcription complex, subunit 7 [Source:NCBI gene;Acc:768119]	14	30413312	30425984		1.2	1.11	0.98	1.28	1.07	1.08
ENSDARG00000032117	ddx1	DEAD (Asp-Glu-Ala-Asp) box helicase 1 [Source:ZFIN;Acc:ZDB-GENE-041001-215]	20	33175190	33189652		20.14	16.91	17.36	16.26	16.77	16.54
ENSDARG00000032126	scg5	secretogranin V [Source:ZFIN;Acc:ZDB-GENE-040426-1687]	20	29471151	29475172		5.41	2.85	4	3.7	4.16	3.82
ENSDARG00000032129	gtf2f1	general transcription factor IIF, polypeptide 1 [Source:ZFIN;Acc:ZDB-GENE-030131-4557]	3	33921971	33941875		21.42	16.11	17.64	15.41	14.86	14.8
ENSDARG00000032131	drd3	dopamine receptor D3 [Source:ZFIN;Acc:ZDB-GENE-021119-1]	24	20968308	21006319		1.61	0.54	0.68	1.56	1.56	1.51
ENSDARG00000032155	ppm1aa	protein phosphatase, Mg2+/Mn2+ dependent, 1Aa [Source:ZFIN;Acc:ZDB-GENE-991102-15]	13	31545530	31580190		5.03	3.67	3.07	7	6.69	6.76
ENSDARG00000032156	zgc:171776	zgc:171776 [Source:ZFIN;Acc:ZDB-GENE-071004-36]	9	8396755	8399119		0	0.03	0.05	0	0.03	0
ENSDARG00000032157	grk6	G protein-coupled receptor kinase 6 [Source:ZFIN;Acc:ZDB-GENE-101006-5]	21	36623162	36737293		0.97	0.61	0.39	2.24	2.16	2.09
ENSDARG00000032161	srgap2	SLIT-ROBO Rho GTPase activating protein 2 [Source:NCBI gene;Acc:556198]	11	21405697	21586157		5.12	3.49	3.03	7.63	7.08	7.02
ENSDARG00000032175	puf60a	poly-U binding splicing factor a [Source:ZFIN;Acc:ZDB-GENE-030131-2891]	2	32650729	32671854		11.66	8.24	10.15	12.41	12.75	12.75
ENSDARG00000032188	lrrc8aa	leucine rich repeat containing 8 VRAC subunit Aa [Source:ZFIN;Acc:ZDB-GENE-030131-5853]	21	4256291	4304945		5.03	5.17	4.79	8.41	8.29	6.91
ENSDARG00000032197	klf12b	Kruppel-like factor 12b [Source:ZFIN;Acc:ZDB-GENE-071004-22]	9	30720048	30783892		3.14	1.94	1.83	4.52	5.27	4.29
ENSDARG00000032199	gpc3	glypican 3 [Source:NCBI gene;Acc:387365]	14	31176952	31465905		1.97	0.47	0.6	2.22	2.97	2.88
ENSDARG00000032206	cthl	cystathionase (cystathionine gamma-lyase), like [Source:ZFIN;Acc:ZDB-GENE-040905-3]	6	37389683	37403444		0.52	0.82	0.61	0.1	0.2	0.13
ENSDARG00000032218	acss3	acyl-CoA synthetase short chain family member 3 [Source:ZFIN;Acc:ZDB-GENE-060503-816]	4	21866851	21958295		2.28	2.72	2.2	1.98	1.98	2
ENSDARG00000032221	phactr4b	phosphatase and actin regulator 4b [Source:NCBI gene;Acc:393643]	16	34199297	34285106		5.18	3.64	3.78	6.64	7.36	6.74
ENSDARG00000032234	wnt2bb	wingless-type MMTV integration site family, member 2Bb [Source:ZFIN;Acc:ZDB-GENE-060824-6]	8	27743550	27768785		1.55	1.74	1.56	1.88	2.03	1.69
ENSDARG00000032237	anapc4	anaphase promoting complex subunit 4 [Source:ZFIN;Acc:ZDB-GENE-041212-10]	20	23475381	23494712		4.84	4.52	4.3	3.75	3.72	3.79
ENSDARG00000032238	dnm3a	dynamin 3a [Source:ZFIN;Acc:ZDB-GENE-040724-76]	20	14817630	14925281		1.17	0.98	1.12	1.63	1.39	1.17
ENSDARG00000032242	tnnt2c	troponin T2c, cardiac [Source:ZFIN;Acc:ZDB-GENE-030520-1]	4	12031958	12033866		6.7	8.75	8.64	12.73	12.24	14.65
ENSDARG00000032246	tmtopsb	teleost multiple tissue opsin b [Source:ZFIN;Acc:ZDB-GENE-091118-50]	24	26134029	26197398		1.45	0.7	0.54	1.45	1.16	1.27
ENSDARG00000032256	txndc16	thioredoxin domain containing 16 [Source:ZFIN;Acc:ZDB-GENE-100712-1]	17	14918101	14966384		1.57	0.97	0.93	1.58	1.37	1.71
ENSDARG00000032261	rngtt	RNA guanylyltransferase and 5'-phosphatase [Source:NCBI gene;Acc:405803]	20	943879	1120056		7.35	4.64	3.91	7.54	6.97	6.8
ENSDARG00000032263	zgc:110224	zgc:110224 [Source:ZFIN;Acc:ZDB-GENE-050417-120]	21	30721733	30742852		1.63	1.2	1.35	0.99	0.88	0.83
ENSDARG00000032264	mybl2b	v-myb avian myeloblastosis viral oncogene homolog-like 2b [Source:ZFIN;Acc:ZDB-GENE-041007-1]	11	1551603	1574656		3.99	3.22	2.76	3.01	3.1	2.93
ENSDARG00000032272	sat1b	spermidine/spermine N1-acetyltransferase 1b [Source:ZFIN;Acc:ZDB-GENE-041001-39]	24	21973929	21978844		4.59	4.77	4.97	4.26	4.28	4.37
ENSDARG00000032277	rars2	arginyl-tRNA synthetase 2, mitochondrial [Source:ZFIN;Acc:ZDB-GENE-040426-1244]	20	2681992	2713190		2.64	2.32	2.48	2.47	2.71	2.89
ENSDARG00000032285	zgc:113294	zgc:113294 [Source:ZFIN;Acc:ZDB-GENE-050320-124]	15	808647	822471		2.25	1.75	1.36	1.22	1.29	1.35
ENSDARG00000032296	pomp	proteasome maturation protein [Source:ZFIN;Acc:ZDB-GENE-040801-10]	21	40712757	40717678		59.86	64.81	64.67	49.06	47.17	49.29
ENSDARG00000032298	tbc1d20	TBC1 domain family, member 20 [Source:ZFIN;Acc:ZDB-GENE-050626-61]	22	10129788	10141469		5.4	2.52	2.9	5.77	6.44	6.62
ENSDARG00000032317	tox	thymocyte selection-associated high mobility group box [Source:ZFIN;Acc:ZDB-GENE-070912-181]	2	21982911	22108108		10.93	4.19	4.03	18.89	18.15	20.03
ENSDARG00000032318	mfsd6a	major facilitator superfamily domain containing 6a [Source:NCBI gene;Acc:100034426]	22	12911236	12925741		2.4	2.34	1.56	1.9	2.18	2
ENSDARG00000032319	mest	mesoderm specific transcript [Source:ZFIN;Acc:ZDB-GENE-991111-5]	4	15996234	16001215		6.12	5.5	5.66	5.47	5.93	5.36
ENSDARG00000032324	epb41l5	erythrocyte membrane protein band 4.1 like 5 [Source:ZFIN;Acc:ZDB-GENE-030616-450]	9	28870357	28939796		7.1	4.49	4.52	7.95	8.4	8.79
ENSDARG00000032326	mecr	mitochondrial trans-2-enoyl-CoA reductase [Source:NCBI gene;Acc:550550]	16	4695075	4707668		6.81	8.05	7.65	4.29	4.75	4.88
ENSDARG00000032327	usp36	ubiquitin specific peptidase 36 [Source:ZFIN;Acc:ZDB-GENE-030131-5450]	3	57583656	57630791		12.11	10.51	7.64	16.26	16.7	16.75
ENSDARG00000032340	serhl	serine hydrolase like [Source:NCBI gene;Acc:322648]	1	45650285	45662774		7.51	10.21	9.08	6.72	5.48	5.76
ENSDARG00000032344	pde4a	phosphodiesterase 4A, cAMP-specific [Source:ZFIN;Acc:ZDB-GENE-131125-7]	3	46628885	46754730		3.88	1.21	1.51	5.06	5.01	5.18
ENSDARG00000032353	tiprl	TIP41, TOR signaling pathway regulator-like (S. cerevisiae) [Source:ZFIN;Acc:ZDB-GENE-040426-1611]	10	15970	22780		10.95	9.25	10.61	8.01	8.04	8.12
ENSDARG00000032369	btbd6b	BTB (POZ) domain containing 6b [Source:NCBI gene;Acc:553533]	20	20927827	20932782		18.98	10.4	13.51	17	17.51	18.41
ENSDARG00000032372	ccdc180	coiled-coil domain containing 180 [Source:ZFIN;Acc:ZDB-GENE-130530-582]	5	51127953	51148298		0.38	0.41	0.43	0.33	0.35	0.42
ENSDARG00000032373	rnf145b	ring finger protein 145b [Source:NCBI gene;Acc:327056]	21	34001712	34032650		9.72	7.34	6.78	11.79	12.26	12.46
ENSDARG00000032380	ompb	olfactory marker protein b [Source:ZFIN;Acc:ZDB-GENE-030103-1]	21	22188010	22189719		3.53	1.84	2.36	1.74	1.71	1.9
ENSDARG00000032389	tspan12	tetraspanin 12 [Source:ZFIN;Acc:ZDB-GENE-040426-1285]	4	10574822	10599062		5.79	7.92	8.76	6.55	5.73	5.94
ENSDARG00000032405	ckap4	cytoskeleton associated protein 4 [Source:ZFIN;Acc:ZDB-GENE-040426-2417]	4	77975221	77979432		64.55	60.24	61.18	58.8	59.77	57.38
ENSDARG00000032426	coq8ab	coenzyme Q8A, genome duplicate b [Source:ZFIN;Acc:ZDB-GENE-030131-5543]	17	6467398	6487813		4.74	4.61	4.8	2.65	2.4	2.16
ENSDARG00000032430	ppp2r1bb	protein phosphatase 2, regulatory subunit A, beta b [Source:ZFIN;Acc:ZDB-GENE-040927-20]	15	21744680	21772839		58.02	46.3	52.51	56.42	59.9	56.49
ENSDARG00000032431	mpv17	mitochondrial inner membrane protein MPV17 [Source:NCBI gene;Acc:394140]	20	38671894	38716529		11.32	16.56	14	7.81	9	7.59
ENSDARG00000032435	itga9	integrin, alpha 9 [Source:ZFIN;Acc:ZDB-GENE-040426-1129]	13	47863695	48058813		2.12	2.41	1.81	4.44	4.35	5.2
ENSDARG00000032458	mark2b	MAP/microtubule affinity-regulating kinase 2b [Source:ZFIN;Acc:ZDB-GENE-080215-4]	7	24888910	24977171		10.85	6.28	5.41	12.74	13.08	11.95
ENSDARG00000032465	slc1a8b	solute carrier family 1 member 8b [Source:ZFIN;Acc:ZDB-GENE-070912-552]	2	37110504	37122351		6.69	0.83	0.52	2.16	2.49	2.31
ENSDARG00000032469	ampd3b	adenosine monophosphate deaminase 3b [Source:ZFIN;Acc:ZDB-GENE-030131-5929]	18	16762806	16795262		7.96	8.82	8.77	5.17	6.35	5.49
ENSDARG00000032482	si:dkey-40c11.2	si:dkey-40c11.2 [Source:ZFIN;Acc:ZDB-GENE-060526-300]	5	43530388	43608280		15.69	17.79	15.7	22.95	24.21	24.8
ENSDARG00000032496	pon1	paraoxonase 1 [Source:ZFIN;Acc:ZDB-GENE-040912-6]	16	24646600	24656265		5.82	8.56	7.43	2.65	2.62	2.67
ENSDARG00000032516	rrp36	ribosomal RNA processing 36 [Source:ZFIN;Acc:ZDB-GENE-030131-9751]	22	15343953	15352546		9.44	12.82	10.33	8.23	7.74	7.97
ENSDARG00000032532	arpp19a	cAMP-regulated phosphoprotein 19a [Source:ZFIN;Acc:ZDB-GENE-040426-1281]	18	38885309	38897664		22.43	16.75	14.72	31.08	30.17	29.38
ENSDARG00000032535	praf2	PRA1 domain family, member 2 [Source:ZFIN;Acc:ZDB-GENE-050417-190]	8	44893628	44904723		8.36	5.15	5.93	11.2	11.29	10.07
ENSDARG00000032552	5-Mar	membrane-associated ring finger (C3HC4) 5 [Source:ZFIN;Acc:ZDB-GENE-070424-47]	13	42361599	42400647		10.37	6.45	6.02	11.74	11.6	9.99
ENSDARG00000032553	egln3	egl-9 family hypoxia-inducible factor 3 [Source:ZFIN;Acc:ZDB-GENE-040426-2541]	17	9912036	9936915		1.73	2.28	1.66	33.71	38	33.44
ENSDARG00000032565	cacng2a	calcium channel, voltage-dependent, gamma subunit 2a [Source:ZFIN;Acc:ZDB-GENE-040426-1401]	3	29714775	29845175		5.28	1.69	1.4	9.75	9.66	10
ENSDARG00000032571	il20ra	interleukin 20 receptor, alpha [Source:ZFIN;Acc:ZDB-GENE-040724-249]	20	2589414	2609270		4.35	4.44	3.2	1.81	2.12	1.78
ENSDARG00000032575	ywhaz	tyrosine 3-monooxygenase/tryptophan 5-monooxygenase activation protein, zeta polypeptide [Source:ZFIN;Acc:ZDB-GENE-03013	19	12164428	12193622		166.9	170.05	170.74	151.15	144.09	149.4

**ENSDARG00000032577**	clcn6	chloride channel 6 [Source:ZFIN;Acc:ZDB-GENE-030131-2056]	8	48629495	48669127		2.62	1.27	1.37	2.95	3.07	3.32
**ENSDARG00000032578**	pax2b	paired box 2b [Source:ZFIN;Acc:ZDB-GENE-001030-4]	12	45799982	45876387		1.7	0.34	0.64	2.59	2.66	2.22
**ENSDARG00000032584**	thnsl2	threonine synthase-like 2 [Source:NCBI gene;Acc:641568]	5	72094502	72105216		2.26	3.05	2.77	0.56	0.72	0.44
**ENSDARG00000032601**	macrod2	mono-ADP ribosylhydrolase 2 [Source:ZFIN;Acc:ZDB-GENE-040426-1490]	13	33703784	34558921		23.03	18.03	18.87	16.61	16.83	17.13
**ENSDARG00000032603**	st7l	suppression of tumorigenicity 7 like [Source:NCBI gene;Acc:558013]	6	48290125	48317562		5.43	4.02	4.95	3.81	4.09	4.13
**ENSDARG00000032606**	sept8a	septin 8a [Source:NCBI gene;Acc:321590]	21	43328685	43374893		36.04	32.08	34.42	34.66	37.38	36.34
**ENSDARG00000032614**	msi2b	musashi RNA-binding protein 2b [Source:ZFIN;Acc:ZDB-GENE-040426-1268]	15	30424645	30832897		9.56	3.73	4.13	12.22	12.88	13.01
**ENSDARG00000032617**	fgfrl1a	fibroblast growth factor receptor like 1a [Source:ZFIN;Acc:ZDB-GENE-040128-2]	14	17442913	17563773		14.16	13.9	12.54	21.06	20.67	20.58
**ENSDARG00000032618**	mgst1.1	microsomal glutathione S-transferase 1.1 [Source:ZFIN;Acc:ZDB-GENE-041010-30]	4	12715695	12718367		8.62	11	8.53	7.2	8.28	6.98
**ENSDARG00000032619**	tob1a	transducer of ERBB2, 1a [Source:ZFIN;Acc:ZDB-GENE-031030-4]	3	18185616	18189283		34.61	29.43	25.08	57.82	58.7	53.78
**ENSDARG00000032623**	rcan3	regulator of calcineurin 3 [Source:ZFIN;Acc:ZDB-GENE-040718-445]	17	26879725	26926577		9.19	3.12	4.06	11.27	11.83	12.04
**ENSDARG00000032627**	SCYL2	si:ch211-244b2.1 [Source:ZFIN;Acc:ZDB-GENE-041210-205]	4	26053044	26082119	SCYL2	3.54	2	1.62	4.32	4.2	3.86
**ENSDARG00000032630**	neb	nebulin [Source:ZFIN;Acc:ZDB-GENE-041111-216]	9	22805138	22892838		31.09	44.71	32.36	64.23	42.46	43.73
**ENSDARG00000032631**	ltb4r	leukotriene B4 receptor [Source:ZFIN;Acc:ZDB-GENE-070705-164]	7	5016717	5039562		0.93	0.91	0.96	0.89	1.06	1
**ENSDARG00000032637**	si:dkey-261m9.12	si:dkey-261m9.12 [Source:ZFIN;Acc:ZDB-GENE-131126-58]	25	35141528	35142127		0	0.12	0.17	0.2	0.04	0.04
**ENSDARG00000032639**	cd36	CD36 molecule (thrombospondin receptor) [Source:ZFIN;Acc:ZDB-GENE-040718-55]	4	22658472	22671469		28.35	36.42	21.92	8.32	9.28	8.78
**ENSDARG00000032650**	fuk	fucose kinase [Source:ZFIN;Acc:ZDB-GENE-110819-1]	25	7571920	7591229		2.13	1.62	1.74	2.9	2.77	2.79
**ENSDARG00000032651**	ppp1r10	protein phosphatase 1, regulatory subunit 10 [Source:ZFIN;Acc:ZDB-GENE-020419-15]	19	30408317	30422239		12.79	6.52	5.97	13.66	12.96	13.5
**ENSDARG00000032653**	sinhcaf	SIN3-HDAC complex associated factor [Source:ZFIN;Acc:ZDB-GENE-041210-166]	4	16631483	16644708		4.57	2.36	2.56	6.04	6.3	6.42
**ENSDARG00000032704**	qrsl1	glutaminyl-tRNA amidotransferase subunit QRSL1 [Source:NCBI gene;Acc:327329]	17	25563979	25588544		1.74	1.39	1.45	1.35	1.4	1.23
**ENSDARG00000032705**	foxg1b	forkhead box G1b [Source:ZFIN;Acc:ZDB-GENE-040426-2437]	11	43114108	43116249		21.51	3.38	2.85	36.04	37.24	38.23
**ENSDARG00000032708**	arl4cb	ADP-ribosylation factor-like 4Cb [Source:ZFIN;Acc:ZDB-GENE-040426-2382]	9	23356774	23358597		16.32	14.86	17.85	15.83	16.24	16.66
**ENSDARG00000032714**	gria1b	glutamate receptor, ionotropic, AMPA 1b [Source:ZFIN;Acc:ZDB-GENE-020125-2]	21	26152332	26250744		3.29	0.93	1.47	3.56	3.67	3.64
**ENSDARG00000032725**	rps27a	ribosomal protein S27a [Source:ZFIN;Acc:ZDB-GENE-030131-10018]	6	6270686	6273519		1597.21	2034.25	2601.27	1144.03	1188.75	1202.62
**ENSDARG00000032737**	gria3a	glutamate receptor, ionotropic, AMPA 3a [Source:ZFIN;Acc:ZDB-GENE-020125-5]	5	23353570	23429228		33.68	5.25	7.88	27.34	25.85	27.55
**ENSDARG00000032743**	si:dkey-11p23.7	si:dkey-11p23.7 [Source:ZFIN;Acc:ZDB-GENE-100922-120]	16	41517188	41521289		0.07	0.28	0.09	0.11	0.17	0.16
**ENSDARG00000032746**	tnfsf10l3	tumor necrosis factor (ligand) superfamily, member 10 like 3 [Source:ZFIN;Acc:ZDB-GENE-070606-1]	21	30701902	30714665		2.23	2.37	2.78	1.73	2.02	1.21
**ENSDARG00000032761**	pde4d	phosphodiesterase 4D, cAMP-specific [Source:ZFIN;Acc:ZDB-GENE-081105-16]	8	16990120	17044714		3.44	0.96	1.28	3.94	4.12	3.93
**ENSDARG00000032765**	net1	neuroepithelial cell transforming 1 [Source:ZFIN;Acc:ZDB-GENE-041121-18]	4	11573568	11603149		23.61	23.57	20.16	45.61	48.6	46.59
**ENSDARG00000032769**	slc38a9	solute carrier family 38 member 9 [Source:NCBI gene;Acc:562137]	10	6907715	6944407		3.59	3.77	3.67	7.41	6.72	7.6
**ENSDARG00000032780**	pigf	phosphatidylinositol glycan anchor biosynthesis, class F [Source:ZFIN;Acc:ZDB-GENE-040426-1801]	12	25097754	25110974		10.53	9.93	9.31	11.15	9.99	11.33
**ENSDARG00000032795**	pgap6	post-glycosylphosphatidylinositol attachment to proteins 6 [Source:ZFIN;Acc:ZDB-GENE-090312-158]	1	9919909	9940494		4.52	2.83	2.49	5.71	5.49	4.99
**ENSDARG00000032799**	kcnd2	potassium voltage-gated channel, Shal-related subfamily, member 2 [Source:ZFIN;Acc:ZDB-GENE-041210-70]	4	10365857	10562174		1.53	0.35	0.51	2.95	3.21	3.27
**ENSDARG00000032801**	grk5	G protein-coupled receptor kinase 5 [Source:ZFIN;Acc:ZDB-GENE-091204-274]	10	2715548	2755378		4.31	3.1	2.56	1.83	2.1	1.17
**ENSDARG00000032802**	ktn1	kinectin 1 [Source:ZFIN;Acc:ZDB-GENE-030408-4]	17	43926523	44002032		43.6	46.05	34.76	42.3	43.56	43.8
**ENSDARG00000032808**	tdrd7a	tudor domain containing 7 a [Source:NCBI gene;Acc:406379]	1	12195700	12228116		0.14	0.11	0.15	0.37	0.21	0.21
**ENSDARG00000032816**	tm7sf2	transmembrane 7 superfamily member 2 [Source:ZFIN;Acc:ZDB-GENE-041212-19]	10	26972755	26978376		8.01	8.57	7.11	4.24	3.93	4.46
**ENSDARG00000032820**	rxfp2a	relaxin family peptide receptor 2a [Source:ZFIN;Acc:ZDB-GENE-040426-1225]	10	33744098	33867426		0.72	0.32	0.68	1.23	1.24	1.27
**ENSDARG00000032831**	htra1a	HtrA serine peptidase 1a [Source:NCBI gene;Acc:431766]	17	21919131	21959556		9.77	12.23	12.05	7.45	7.39	7.45
**ENSDARG00000032836**	pvalb5	parvalbumin 5 [Source:ZFIN;Acc:ZDB-GENE-050417-336]	3	61208544	61218375		40.45	34.22	37.65	17.11	19.22	18.22
**ENSDARG00000032838**	si:dkey-206f10.1	si:dkey-206f10.1 [Source:ZFIN;Acc:ZDB-GENE-041014-154]	20	43602810	43644531		3.36	0.55	0.35	2.68	2.65	2.84
**ENSDARG00000032849**	ndrg1a	N-myc downstream regulated 1a [Source:ZFIN;Acc:ZDB-GENE-030826-34]	19	34959967	34979837		54.22	45.48	35.47	79.9	90.97	84.74
**ENSDARG00000032856**	ccdc120	coiled-coil domain containing 120 [Source:ZFIN;Acc:ZDB-GENE-061027-93]	11	25583950	25618583		2.32	1.39	1.12	4.21	4.12	3.85
**ENSDARG00000032859**	lratd2	LRAT domain containing 2 [Source:ZFIN;Acc:ZDB-GENE-060929-1130]	19	33139164	33140403		2.62	2.37	2.65	3.35	3.2	3.45
**ENSDARG00000032862**	ugt5g1	UDP glucuronosyltransferase 5 family, polypeptide G1 [Source:ZFIN;Acc:ZDB-GENE-080305-10]	7	22688781	22696987		1.75	2.87	2.41	2.2	2.1	2.17
**ENSDARG00000032865**	pacsin1a	protein kinase C and casein kinase substrate in neurons 1a [Source:ZFIN;Acc:ZDB-GENE-050522-155]	23	3653386	3703569		36.12	8.91	12.43	33.67	35.16	35.23
**ENSDARG00000032866**	erh	ERH mRNA splicing and mitosis factor [Source:NCBI gene;Acc:30475]	20	33493924	33497128		80.24	79.24	80.9	76.4	72.97	77.87
**ENSDARG00000032868**	pde4ba	phosphodiesterase 4B, cAMP-specific a [Source:ZFIN;Acc:ZDB-GENE-030131-9570]	6	30851545	30954268		17.42	6.58	8.69	27.37	27.67	26.78
**ENSDARG00000032876**	trit1	tRNA isopentenyltransferase 1 [Source:ZFIN;Acc:ZDB-GENE-060503-297]	19	30722767	30800082		4.64	3.25	3.11	3.53	3.59	3.6
**ENSDARG00000032885**	si:ch211-226m7.4	si:ch211-226m7.4 [Source:ZFIN;Acc:ZDB-GENE-080919-1]	11	28620683	28631342		0.71	0.57	0.66	1.31	1.4	1.11
**ENSDARG00000032896**	xgb	x globin [Source:ZFIN;Acc:ZDB-GENE-050125-1]	17	1664632	1703259		0.64	0.34	0.59	0.99	0.82	1.22
**ENSDARG00000032919**	chchd3a	coiled-coil-helix-coiled-coil-helix domain containing 3a [Source:ZFIN;Acc:ZDB-GENE-030131-5005]	4	15490215	15603588		32.17	35.9	32.02	26.98	28.03	29.46
**ENSDARG00000032929**	cryba1l1	crystallin, beta A1, like 1 [Source:ZFIN;Acc:ZDB-GENE-050417-249]	14	46313135	46321016		4.71	13.88	24.36	85.56	71.64	98.5
**ENSDARG00000032932**	cnksr1	connector enhancer of kinase suppressor of Ras 1 [Source:ZFIN;Acc:ZDB-GENE-040426-1802]	17	26990531	27048640		6.15	6.38	4.68	5.8	5.56	5.74
**ENSDARG00000032933**	dtx2	deltex 2, E3 ubiquitin ligase [Source:ZFIN;Acc:ZDB-GENE-081022-150]	5	59553636	59595597		3.94	2.5	2.07	4.7	4.62	4.67
**ENSDARG00000032951**	nrbp2a	nuclear receptor binding protein 2a [Source:ZFIN;Acc:ZDB-GENE-070713-4]	2	32676002	32738535		8.73	7.36	6.35	10.4	10.5	10.9
**ENSDARG00000032959**	kcnc1b	potassium voltage-gated channel, Shaw-related subfamily, member 1b [Source:ZFIN;Acc:ZDB-GENE-080414-3]	25	8063455	8094080		6.36	1.2	2.05	7.61	7.43	8.68
**ENSDARG00000032963**	her12	hairy-related 12 [Source:ZFIN;Acc:ZDB-GENE-040824-5]	23	21444922	21446985		4.89	2.39	2.78	3.47	2.81	3.14
**ENSDARG00000032970**	cox4i1	cytochrome c oxidase subunit 4I1 [Source:ZFIN;Acc:ZDB-GENE-030131-5175]	18	30507839	30510241		535.21	786.26	754.48	462.38	469.33	478.31
**ENSDARG00000032976**	cmlc1	cardiac myosin light chain-1 [Source:ZFIN;Acc:ZDB-GENE-030522-1]	5	64167620	64168415		24.06	35.18	30.74	35.39	42	36.24
**ENSDARG00000032985**	mrpl27	mitochondrial ribosomal protein L27 [Source:ZFIN;Acc:ZDB-GENE-050522-129]	12	21684239	21688087		29.91	34.77	36.41	17.35	19.69	19.55
**ENSDARG00000032990**	plxnb3	plexin B3 [Source:ZFIN;Acc:ZDB-GENE-081104-53]	8	8166285	8282555		2.94	1.42	1.08	4.34	4.07	3.83
**ENSDARG00000032993**	ccl34b.3	chemokine (C-C motif) ligand 34b, duplicate 3 [Source:ZFIN;Acc:ZDB-GENE-130603-64]	24	27407955	27409599		0	0	0	0.07	0.08	0
**ENSDARG00000032997**	rnf185	ring finger protein 185 [Source:NCBI gene;Acc:406310]	5	1055042	1070794		24.79	21.95	25.65	21.61	20.98	20.99
**ENSDARG00000033009**	h3f3c	H3 histone, family 3C [Source:ZFIN;Acc:ZDB-GENE-030131-8175]	5	36944533	36949476		294.07	186.69	205.49	273.94	272.59	280.38
**ENSDARG00000033012**	pcnt	pericentrin [Source:ZFIN;Acc:ZDB-GENE-030829-26]	6	11272286	11362871		5.04	2.68	2.23	4.93	4.63	4.96
**ENSDARG00000033020**	ppcdc	phosphopantothenoylcysteine decarboxylase [Source:ZFIN;Acc:ZDB-GENE-040426-1749]	18	18975539	18979520		2.74	3.32	3.41	1.96	1.78	1.66
**ENSDARG00000033029**	pkd1b	polycystic kidney disease 1b [Source:ZFIN;Acc:ZDB-GENE-100707-2]	12	21697540	21748008		0.38	0.1	0.25	3.43	3.46	3.84
**ENSDARG00000033042**	ptprja	protein tyrosine phosphatase receptor type Ja [Source:ZFIN;Acc:ZDB-GENE-030131-8301]	7	39553046	39611109		0.48	0.41	0.44	0.76	0.67	0.74
**ENSDARG00000033046**	ccni2	cyclin I family, member 2 [Source:ZFIN;Acc:ZDB-GENE-081106-2]	21	43379878	43398457		0.36	0.2	0.22	1.2	1	1.29
**ENSDARG00000033056**	zgc:171927	zgc:171927 [Source:ZFIN;Acc:ZDB-GENE-070822-21]	15	31282220	31287970		0.92	0.5	0.64	0.46	0.44	0.43
**ENSDARG00000033071**	trim35-10	tripartite motif containing 35-10 [Source:ZFIN;Acc:ZDB-GENE-091113-36]	3	8290595	8297250		0.12	0.05	0.01	0.15	0.15	0.28
**ENSDARG00000033089**	CR735102.1		21	38458072	38463146		15.21	12.44	10.59	14.18	13.35	14.99
**ENSDARG00000033099**	kif7	kinesin family member 7 [Source:NCBI gene;Acc:544651]	7	14393059	14446512		2.69	2.07	2.28	2.71	2.44	2.63
**ENSDARG00000033104**	tmc2a	transmembrane channel-like 2a [Source:NCBI gene;Acc:571240]	5	55221593	55251994		1.57	0.95	1.19	0.9	0.88	0.95
**ENSDARG00000033126**	prkrip1	PRKR interacting protein 1 [Source:NCBI gene;Acc:550595]	5	59234421	59248274		16.37	13.79	13.48	11.11	10.53	11.19
**ENSDARG00000033134**	ndufaf7	NADH:ubiquinone oxidoreductase complex assembly factor 7 [Source:NCBI gene;Acc:767796]	20	3277620	3288089		5.6	6.15	6.79	6.79	7.25	6.31
**ENSDARG00000033138**	lyrm2	LYR motif containing 2 [Source:NCBI gene;Acc:569076]	20	1196386	1199009		20.52	19.67	18.81	18.96	18.57	20.59
**ENSDARG00000033140**	desi1a	desumoylating isopeptidase 1a [Source:ZFIN;Acc:ZDB-GENE-040426-1657]	3	24973025	24980067		17.28	11.56	13.36	29.46	31.13	29.65
**ENSDARG00000033144**	psme2	proteasome activator subunit 2 [Source:ZFIN;Acc:ZDB-GENE-991110-16]	12	13319132	13331980		2.92	3.12	2.23	1.85	1.96	1.47
**ENSDARG00000033160**	nr1d1	nuclear receptor subfamily 1, group d, member 1 [Source:ZFIN;Acc:ZDB-GENE-050105-1]	3	23488652	23496223		31.74	12.52	8.82	19.3	20.37	19.74
**ENSDARG00000033161**	sst1.2	somatostatin 1, tandem duplicate 2 [Source:ZFIN;Acc:ZDB-GENE-100910-2]	15	36115955	36120277		7	9.06	8.47	5.78	5.93	5.44
**ENSDARG00000033170**	sult2st1	sulfotransferase family 2, cytosolic sulfotransferase 1 [Source:ZFIN;Acc:ZDB-GENE-030219-114]	12	630295	645972		17.75	22.36	24.24	18.67	22.78	20.29
**ENSDARG00000033171**	trappc6bl	trafficking protein particle complex 6b-like [Source:ZFIN;Acc:ZDB-GENE-040426-1602]	15	14076004	14083140		9.13	7.51	7.55	8.15	6.94	8
**ENSDARG00000033172**	nr2f5	nuclear receptor subfamily 2, group F, member 5 [Source:NCBI gene;Acc:30427]	16	46294337	46327301		10.01	9.26	7.44	13.36	12.91	14.02
**ENSDARG00000033175**	snrpe	small nuclear ribonucleoprotein polypeptide E [Source:NCBI gene;Acc:393979]	11	37720552	37726201		55.14	79.29	70.08	39.52	38.29	39.06
**ENSDARG00000033179**	tmem243b	transmembrane protein 243, mitochondrial b [Source:ZFIN;Acc:ZDB-GENE-040426-2205]	4	9612574	9619557		4.43	3.87	4.44	4.54	4.89	4.29
**ENSDARG00000033182**	arl4ab	ADP-ribosylation factor-like 4ab [Source:ZFIN;Acc:ZDB-GENE-030219-167]	19	31339305	31341850		7.57	7.89	8.15	8.79	9.82	9.87
**ENSDARG00000033184**	prkar2aa	protein kinase, cAMP-dependent, regulatory, type II, alpha A [Source:ZFIN;Acc:ZDB-GENE-030131-1007]	8	26329482	26369115		45.06	40	34.72	55.11	53.19	52.6
**ENSDARG00000033201**	crip2l	cysteine-rich protein 2-like [Source:ZFIN;Acc:ZDB-GENE-041010-43]	20	9428965	9462449		19.42	13.03	15.35	17.27	18.17	18.2
**ENSDARG00000033227**	lect2l	leukocyte cell-derived chemotaxin 2 like [Source:ZFIN;Acc:ZDB-GENE-070127-4]	14	36885524	36887063		24.05	40.43	40.41	20.34	20	21.27
**ENSDARG00000033231**	mcm6l	MCM6 minichromosome maintenance deficient 6, like [Source:ZFIN;Acc:ZDB-GENE-050913-141]	2	50199738	50225495		0.13	0.15	0.13	0.32	0.33	0.28
**ENSDARG00000033234**	stmn2a	stathmin 2a [Source:ZFIN;Acc:ZDB-GENE-041010-85]	16	16165029	16182319		78.62	34.67	51.56	75.66	72.22	75.44
**ENSDARG00000033251**	osbpl3a	oxysterol binding protein-like 3a [Source:ZFIN;Acc:ZDB-GENE-040912-77]	19	19988869	20033047		3.53	3.79	3.1	3.43	2.86	3.18
**ENSDARG00000033259**	dis3l2	DIS3 like 3'-5' exoribonuclease 2 [Source:ZFIN;Acc:ZDB-GENE-070705-171]	2	5841108	5884404		3.69	2.61	2.48	3.89	3.93	3.74
**ENSDARG00000033273**	primpol	primase and polymerase (DNA-directed) [Source:NCBI gene;Acc:556571]	1	21563311	21573013		3.87	3.71	3.38	2.78	2.7	2.7
**ENSDARG00000033285**	gsto2	glutathione S-transferase omega 2 [Source:ZFIN;Acc:ZDB-GENE-041114-67]	13	25396896	25402772		81.39	135.19	99.82	20.6	19.17	19.88
**ENSDARG00000033296**	gpr37b	G protein-coupled receptor 37b [Source:ZFIN;Acc:ZDB-GENE-041210-264]	4	3980247	3998490		8.33	3.13	2.05	9.87	10.48	9.29
**ENSDARG00000033307**	igf2b	insulin-like growth factor 2b [Source:ZFIN;Acc:ZDB-GENE-030131-2935]	25	24039364	24046870		13.03	14.32	13.13	19.64	21.83	20.95
**ENSDARG00000033320**	sar1ab	secretion associated, Ras related GTPase 1Ab [Source:ZFIN;Acc:ZDB-GENE-050417-437]	13	30144937	30150592		12.86	17.87	15.34	13.69	13.68	12.39
**ENSDARG00000033327**	unc5b	unc-5 netrin receptor B [Source:ZFIN;Acc:ZDB-GENE-041213-1]	13	29127960	29227803		2.03	1.03	1.16	3.38	3.2	3.04
**ENSDARG00000033345**	cntln	centlein, centrosomal protein [Source:ZFIN;Acc:ZDB-GENE-090313-409]	1	26821963	27014935		0.59	0.5	0.48	0.61	0.58	0.63
**ENSDARG00000033355**	zgc:101699	zgc:101699 [Source:NCBI gene;Acc:492789]	5	1877464	1901838		0.05	0.07	0.1	0.23	0.17	0.34
**ENSDARG00000033361**	asl	argininosuccinate lyase [Source:ZFIN;Acc:ZDB-GENE-040426-1152]	21	31253048	31277336		1.42	1.58	1.63	1.36	1.37	1.21
**ENSDARG00000033364**	mgst3b	microsomal glutathione S-transferase 3b [Source:ZFIN;Acc:ZDB-GENE-061215-48]	13	45476181	45482003		149.3	241.12	216.65	76.23	81.34	74.54
**ENSDARG00000033367**	rrm2b	ribonucleotide reductase M2 b [Source:ZFIN;Acc:ZDB-GENE-030616-614]	19	32916372	32928486		18.77	15.64	18.08	15.85	15.56	16.32
**ENSDARG00000033370**	ppil3	peptidylprolyl isomerase (cyclophilin)-like 3 [Source:ZFIN;Acc:ZDB-GENE-040625-159]	5	64851986	64856033		4.61	6.01	7.14	3.33	3.24	4.15
**ENSDARG00000033376**	chchd4a	coiled-coil-helix-coiled-coil-helix domain containing 4a [Source:NCBI gene;Acc:445144]	11	34779610	34783938		10.44	12.63	10.9	8	7.58	7.25
**ENSDARG00000033380**	gyg2	glycogenin 2 [Source:ZFIN;Acc:ZDB-GENE-080723-74]	1	33336923	33353778		0.53	0.66	0.44	0.4	0.27	0.31
**ENSDARG00000033382**	grifin	galectin-related inter-fiber protein [Source:NCBI gene;Acc:445036]	3	41784542	41795917		0.55	0.27	0.22	1.64	1.78	2.54
**ENSDARG00000033411**	cabp1b	calcium binding protein 1b [Source:ZFIN;Acc:ZDB-GENE-040718-111]	10	41409255	41450454		3.96	0.45	0.47	3.65	3.94	3.75
**ENSDARG00000033413**	acot18	acyl-CoA thioesterase 18 [Source:ZFIN;Acc:ZDB-GENE-041210-254]	4	25691642	25704064		5.72	8.22	6.5	5.68	5.45	5
**ENSDARG00000033418**	gpatch1	G patch domain containing 1 [Source:ZFIN;Acc:ZDB-GENE-040914-65]	7	38265363	38277255		10.5	7.33	6.56	8.88	8.67	8.86
**ENSDARG00000033436**	mboat7	membrane bound O-acyltransferase domain containing 7 [Source:NCBI gene;Acc:393509]	16	32014552	32026333		9.97	8.91	8.64	9.21	9.08	8.32
**ENSDARG00000033437**	rps6ka1	ribosomal protein S6 kinase a, polypeptide 1 [Source:ZFIN;Acc:ZDB-GENE-060929-516]	17	27064158	27172352		4.04	3.4	2.73	4.68	4.97	4.65
**ENSDARG00000033440**	metap1	methionyl aminopeptidase 1 [Source:NCBI gene;Acc:503783]	13	12606821	12633160		41.05	45.03	46.94	43.26	44.22	41.71
**ENSDARG00000033443**	mdm2	MDM2 proto-oncogene [Source:NCBI gene;Acc:30637]	4	23125689	23136195		12.93	14.11	15.21	14.17	13.48	13.96
**ENSDARG00000033444**	map4k6	mitogen-activated protein kinase kinase kinase kinase 6 [Source:ZFIN;Acc:ZDB-GENE-060512-339]	21	34033580	34075107		4.27	1.27	1.93	4.58	4.18	5.1
**ENSDARG00000033446**	tap2t	transporter associated with antigen processing, subunit type t, teleost specific [Source:ZFIN;Acc:ZDB-GENE-130531-48]	12	26546820	26559650		3.37	3.39	3.2	3.01	3.01	2.26
**ENSDARG00000033447**	nxph1	neurexophilin 1 [Source:ZFIN;Acc:ZDB-GENE-040718-231]	19	37848830	37928041		10.95	2.88	4.24	12.52	12.66	12.39
**ENSDARG00000033450**	nkap	NFKB activating protein [Source:ZFIN;Acc:ZDB-GENE-030131-8064]	14	33418500	33425233		23.56	25.56	23.5	21.13	20.32	22.86
**ENSDARG00000033451**	atpsckmt	fATP synthase c subunit lysine N-methyltransferase [Source:ZFIN;Acc:ZDB-GENE-041114-90]	2	30480907	30483209		5.63	6.66	6.74	3.1	3.84	3.78
**ENSDARG00000033462**	pimr111	Pim proto-oncogene, serine/threonine kinase, related 111 [Source:ZFIN;Acc:ZDB-GENE-070705-107]	14	33391851	33396934		0	0	0	0	0	0
**ENSDARG00000033466**	tagln2	transgelin 2 [Source:ZFIN;Acc:ZDB-GENE-020802-2]	5	37087583	37095460		88.76	119.88	117.11	73.27	75.18	73.33
**ENSDARG00000033489**	ube2j1	ubiquitin-conjugating enzyme E2, J1 [Source:ZFIN;Acc:ZDB-GENE-040426-2853]	20	1177751	1191910		7.93	5.83	4.17	9.18	8.4	8.03
**ENSDARG00000033498**	rorb	RAR-related orphan receptor B [Source:ZFIN;Acc:ZDB-GENE-061204-2]	5	25366677	25406807		9.38	3.07	2.76	9.39	8.51	8.77
**ENSDARG00000033501**	zgc:64189	zgc:64189 [Source:ZFIN;Acc:ZDB-GENE-040426-1397]	14	28013626	28052551		0.12	0.05	0.06	0.3	0.37	0.33
**ENSDARG00000033516**	efr3ba	EFR3 homolog Ba (S. cerevisiae) [Source:ZFIN;Acc:ZDB-GENE-030616-64]	17	33232748	33289349		5.33	1.35	1.92	7.12	7.5	7.18
**ENSDARG00000033533**	ccdc115	coiled-coil domain containing 115 [Source:NCBI gene;Acc:503608]	23	19198244	19201328		11.53	9.71	9.68	7.27	8.43	8.25
**ENSDARG00000033537**	p4ha1a	prolyl 4-hydroxylase, alpha polypeptide I a [Source:ZFIN;Acc:ZDB-GENE-040724-91]	12	9542124	9585670		7.69	8.28	6.6	21.83	21.53	21.15
**ENSDARG00000033539**	paics	phosphoribosylaminoimidazole carboxylase, phosphoribosylaminoimidazole succinocarboxamide synthetase [Source:ZFIN;Acc:ZDE	20	25618114	25631256		150.43	215.56	243.6	206.51	213.87	235.55
**ENSDARG00000033544**	adamts15b	ADAM metallopeptidase with thrombospondin type 1 motif, 15b [Source:ZFIN;Acc:ZDB-GENE-070814-5]	15	17603488	17619306		7.62	9.06	8.62	5.99	6.14	6.13
**ENSDARG00000033551**	fbxw4	F-box and WD repeat domain containing 4 [Source:ZFIN;Acc:ZDB-GENE-000516-1]	13	28512530	28572642		6.38	4.84	5.58	4.83	4.62	5.1
**ENSDARG00000033566**	cyp17a1	cytochrome P450, family 17, subfamily A, polypeptide 1 [Source:ZFIN;Acc:ZDB-GENE-040213-2]	13	28601195	28610965		3.15	2.14	1.51	2.86	2.32	2.14
**ENSDARG00000033567**	fkbp1ab	FKBP prolyl isomerase 1Ab [Source:ZFIN;Acc:ZDB-GENE-040927-9]	23	16688420	16692312		16.51	12.57	13.89	19.1	15.58	16.42
**ENSDARG00000033579**	mtrf1	mitochondrial translational release factor 1 [Source:ZFIN;Acc:ZDB-GENE-050220-8]	6	59984772	59990769		7.26	5.1	5.98	3.85	4.24	3.2
**ENSDARG00000033587**	CABZ01088134.1	enoyl-[acyl-carrier-protein] reductase, mitochondrial-like [Source:NCBI gene;Acc:101884755]	20	2039518	2054547		0.23	0.38	0.43	0.8	0.68	0.47
**ENSDARG00000033594**	ido1	indoleamine 2,3-dioxygenase 1 [Source:ZFIN;Acc:ZDB-GENE-080228-2]	8	50463227	50482781		2.61	4.07	2.93	1.06	1.04	1.11
**ENSDARG00000033596**	polr2c	polymerase (RNA) II (DNA directed) polypeptide C [Source:ZFIN;Acc:ZDB-GENE-030131-6609]	25	14369493	14378835		14.28	13.99	15.78	13.1	14.3	12.92
**ENSDARG00000033597**	api5	apoptosis inhibitor 5 [Source:ZFIN;Acc:ZDB-GENE-030131-395]	25	30131055	30152039		29.41	22.33	22.59	30.87	31.09	29.71
**ENSDARG00000033599**	pdia8	protein disulfide isomerase family A, member 8 [Source:ZFIN;Acc:ZDB-GENE-040801-20]	19	26714949	26730250		6.5	2.83	4.01	6.33	5.98	5.68
**ENSDARG00000033604**	lrpap1	low density lipoprotein receptor-related protein associated protein 1 [Source:ZFIN;Acc:ZDB-GENE-030131-5871]	1	41131481	41141446		22.76	25.1	19.64	24.2	22.23	22.56
**ENSDARG00000033609**	map1lc3a	microtubule-associated protein 1 light chain 3 alpha [Source:ZFIN;Acc:ZDB-GENE-040426-2232]	11	26609110	26615608		13.88	12.32	10.31	17.06	16.12	18.34
**ENSDARG00000033610**	morn5	MORN repeat containing 5 [Source:NCBI gene;Acc:554117]	10	9397305	9410068		0.2	0.2	0.08	0.18	0.3	0.11
**ENSDARG00000033614**	rasgef1ba	RasGEF domain family, member 1Ba [Source:NCBI gene;Acc:327572]	5	39706345	39805874		10.11	5.04	5.01	11.19	11.85	11.9
**ENSDARG00000033616**	selenon	selenoprotein N [Source:NCBI gene;Acc:352914]	17	24687338	24695198		5.72	4.58	4.64	17.95	18.97	18.87
**ENSDARG00000033635**	NPFFR2	neuropeptide FF receptor 2a [Source:NCBI gene;Acc:560955]	5	45613024	45651548	NPFFR2	1.44	0.26	0.37	1.24	1.11	0.99
**ENSDARG00000033647**	smarcb1a	SWI/SNF related, matrix associated, actin dependent regulator of chromatin, subfamily b, member 1a [Source:NCBI gene;Acc:7997	8	30929704	30944491		24.25	20.04	21.32	25.27	23.44	25.51
**ENSDARG00000033655**	stmn1b	stathmin 1b [Source:ZFIN;Acc:ZDB-GENE-050417-397]	16	34523515	34527206		281.13	159.49	176.28	239.59	247.21	264.31
**ENSDARG00000033662**	scd	stearoyl-CoA desaturase (delta-9-desaturase) [Source:ZFIN;Acc:ZDB-GENE-031106-3]	12	48390715	48404672		0.57	0.22	0.36	0.84	0.53	1.02
**ENSDARG00000033666**	pi4k2a	phosphatidylinositol 4-kinase type 2 alpha [Source:NCBI gene;Acc:406667]	1	54737353	54755556		5.25	4.79	4.15	6.52	6.22	6.77
**ENSDARG00000033683**	tpma	alpha-tropomyosin [Source:NCBI gene;Acc:30324]	7	29951997	29962080		1990.9	2776.32	3562.73	1717.09	1714.31	1706.38
**ENSDARG00000033684**	oxgr1a.1	oxoglutarate (alpha-ketoglutarate) receptor 1a, tandem duplicate 1 [Source:ZFIN;Acc:ZDB-GENE-060421-6734]	1	2048047	2051599		0.09	0.2	0.22	0.46	0.24	0.31
**ENSDARG00000033706**	adora2aa	adenosine A2a receptor a [Source:ZFIN;Acc:ZDB-GENE-080723-28]	8	30664077	30684625		0.32	0.16	0.15	0.61	0.61	0.57
**ENSDARG00000033707**	jade1	jade family PHD finger 1 [Source:NCBI gene;Acc:327437]	17	26828027	26850542		4.96	3.08	3.1	4.47	4.23	4.59
**ENSDARG00000033726**	tshba	thyroid stimulating hormone subunit beta a [Source:ZFIN;Acc:ZDB-GENE-030513-3]	6	49081141	49085296		0.64	0.19	0.17	0.59	0.56	0.53
**ENSDARG00000033727**	il12ba	interleukin 12Ba [Source:ZFIN;Acc:ZDB-GENE-050525-3]	14	34951202	34958756		0.17	0.08	0.12	0.06	0.07	0.12
**ENSDARG00000033735**	ncf1	neutrophil cytosolic factor 1 [Source:ZFIN;Acc:ZDB-GENE-031006-6]	5	61432541	61445697		1.74	1.93	1.88	2.86	3.23	2.56
**ENSDARG00000033738**	zgc:153867	zgc:153867 [Source:ZFIN;Acc:ZDB-GENE-030131-9170]	23	28374458	28388513		136.45	166.82	161	124.94	131.83	126.94
**ENSDARG00000033742**	nt5c1bb	5'-nucleotidase, cytosolic IB b [Source:ZFIN;Acc:ZDB-GENE-030131-7205]	20	9277299	9301208		6.17	6.12	4.58	5.01	4.9	4.54
**ENSDARG00000033754**	rbm48	RNA binding motif protein 48 [Source:NCBI gene;Acc:402897]	19	40177246	40182642		7.64	8.68	9.39	5.27	6	6.79
**ENSDARG00000033757**	ncaph2	non-SMC condensin II complex, subunit H2 [Source:NCBI gene;Acc:562678]	4	18441988	18455497		2.43	2.41	2.35	1.69	1.71	1.75
**ENSDARG00000033760**	pmelb	premelanosome protein b [Source:ZFIN;Acc:ZDB-GENE-050912-2]	23	35672542	35690336		2.26	1.81	1.92	8.2	8.35	8.17
**ENSDARG00000033768**	trappc13	trafficking protein particle complex 13 [Source:NCBI gene;Acc:321203]	10	15340768	15382125		12.22	10.48	12.01	10.97	11.71	11.46
**ENSDARG00000033771**	hspbap1	hspb associated protein 1 [Source:NCBI gene;Acc:445320]	9	37341240	37367477		1.03	0.84	1.11	0.51	0.48	0.76
**ENSDARG00000033789**	ndufb7	NADH:ubiquinone oxidoreductase subunit B7 [Source:ZFIN;Acc:ZDB-GENE-040426-1886]	1	55780128	55785722		165.86	223.43	241	101.16	104.8	110.1
**ENSDARG00000033802**	cyp27a7	cytochrome P450, family 27, subfamily A, polypeptide 7 [Source:ZFIN;Acc:ZDB-GENE-081104-519]	9	38408991	38419140		7.52	7.03	6.41	4.4	3.48	3.72
**ENSDARG00000033804**	snx27a	sorting nexin 27a [Source:ZFIN;Acc:ZDB-GENE-060503-371]	19	8612839	8647894		18.18	13.01	11.49	16.3	15.68	15.85
**ENSDARG00000033832**	ampd1	adenosine monophosphate deaminase 1 (isoform M) [Source:ZFIN;Acc:ZDB-GENE-040426-1906]	8	11080571	11112058		142.74	206.12	219.19	247.3	242.18	227.94
**ENSDARG00000033840**	fat3a	FAT atypical cadherin 3a [Source:ZFIN;Acc:ZDB-GENE-060929-1254]	15	40665310	40989931		1.72	0.61	0.63	3.44	3.4	3.53
**ENSDARG00000033845**	igsf9ba	immunoglobulin superfamily, member 9Ba [Source:ZFIN;Acc:ZDB-GENE-060503-729]	15	19131702	19250543		5.93	1.49	2.55	10.52	9.29	10.84
**ENSDARG00000033852**	mad1l1	mitotic arrest deficient 1 like 1 [Source:ZFIN;Acc:ZDB-GENE-040426-1081]	1	9739370	9858508		4.67	4.4	4.34	3.08	2.91	3.01
**ENSDARG00000033854**	abrab	actin binding Rho activating protein b [Source:ZFIN;Acc:ZDB-GENE-040822-7]	19	46768266	46777963		1.17	2.17	1.68	1.41	1.41	1.17
**ENSDARG00000033855**	cnot9	CCR4-NOT transcription complex subunit 9 [Source:NCBI gene;Acc:406632]	22	12752286	12763350		19.73	16.22	18.49	19.59	19.21	17.97
**ENSDARG00000033861**	sepsecs	Sep (O-phosphoserine) tRNA:Sec (selenocysteine) tRNA synthase [Source:NCBI gene;Acc:393123]	7	71437142	71456117		3.73	4.25	3.97	2.2	2.58	2.16
**ENSDARG00000033871**	derl3	derlin 3 [Source:ZFIN;Acc:ZDB-GENE-040426-1324]	8	30921403	30929638		9.64	11.87	10.89	6.68	7.55	7.04
**ENSDARG00000033889**	spty2d1	SPT2 chromatin protein domain containing 1 [Source:NCBI gene;Acc:337191]	25	24233010	24248000		5.85	3.46	3.98	5.73	5.78	5.69
**ENSDARG00000033899**	ap2m1b	adaptor related protein complex 2 subunit mu 1b [Source:NCBI gene;Acc:394001]	18	45955042	45983382		95.3	57.32	60.51	96.17	100.89	100.74
**ENSDARG00000033901**	msi2a	musashi RNA-binding protein 2a [Source:ZFIN;Acc:ZDB-GENE-030826-25]	10	37500234	37784024		8.05	3.85	4.76	12.14	12.26	11.6
**ENSDARG00000033916**	si:ch211-243j20.2	si:ch211-243j20.2 [Source:ZFIN;Acc:ZDB-GENE-041014-345]	20	38620666	38670696		6.85	3.87	4.12	7.4	7.29	7.69
**ENSDARG00000033928**	ndfip2	Nedd4 family interacting protein 2 [Source:ZFIN;Acc:ZDB-GENE-040426-2662]	1	5354489	5394791		13.23	7.56	6.19	20.47	19.37	19.94
**ENSDARG00000033949**	fbxo16	F-box protein 16 [Source:ZFIN;Acc:ZDB-GENE-041015-747]	20	35208020	35254641		0.42	0.49	0.54	2.05	2.29	2.18
**ENSDARG00000033950**	lamb2l	laminin, beta 2-like [Source:ZFIN;Acc:ZDB-GENE-030131-4205]	23	20261570	20325731		6.94	5.15	3.76	7.08	7.19	7.21

ENSDARG00000033953	entpd2a.2	ectonucleoside triphosphate diphosphohydrolase 2a, tandem duplicate 2 [Source:ZFIN;Acc:ZDB-GENE-040724-67]	5	29563819	29570141		2.47	2.13	1.81	1.36	1.28	1.26
ENSDARG00000033955	CABZ01088025.1	calcitonin receptor-like 2 [Source:NCBI gene;Acc:100537117]	23	45734011	45746228		0.4	0.44	0.34	0.46	0.59	0.56
ENSDARG00000033956	oxtr	oxytocin receptor [Source:ZFIN;Acc:ZDB-GENE-110805-2]	6	41956355	41974205		0.88	0.54	0.69	0.77	0.91	0.82
ENSDARG00000033957	trmt44	tRNA methyltransferase 44 homolog [Source:ZFIN;Acc:ZDB-GENE-041010-189]	7	59677273	59719911		3.68	2.82	2.89	3.34	3.48	3.07
ENSDARG00000033963	nitr1i	novel immune-type receptor 1i [Source:ZFIN;Acc:ZDB-GENE-001106-3]	7	17520427	17522093		0	0	0	0	0	0
ENSDARG00000033965	nup58	nucleoporin 58 [Source:ZFIN;Acc:ZDB-GENE-040426-2326]	24	25008211	25030243		9.3	6.56	5.43	9.91	10.06	9.81
ENSDARG00000033967	trmt2b	tRNA methyltransferase 2 homolog B [Source:NCBI gene;Acc:606657]	13	30033036	30034754		2.37	2.62	2.09	1.54	1.59	1.45
ENSDARG00000033971	prrx1a	paired related homeobox 1a [Source:ZFIN;Acc:ZDB-GENE-020905-5]	2	23165250	23172708		19.71	26	25	23.08	24.27	24.37
ENSDARG00000033973	mrps23	mitochondrial ribosomal protein S23 [Source:ZFIN;Acc:ZDB-GENE-040801-134]	15	30350061	30354786		11.13	14.22	11.68	11.39	10.59	10.45
ENSDARG00000033978	nxn	nucleoredoxin [Source:NCBI gene;Acc:553621]	15	16193809	16323750		11.71	7.35	8.43	14.16	13.48	13.78
ENSDARG00000033987	rsrc1	arginine/serine-rich coiled-coil 1 [Source:ZFIN;Acc:ZDB-GENE-040426-2374]	15	2190229	2415435		4.34	4.8	4.52	4.45	5.19	4.8
ENSDARG00000033993	slc19a1	solute carrier family 19 member 1 [Source:ZFIN;Acc:ZDB-GENE-110401-1]	6	20647155	20670145		3.26	2.45	2.64	2.93	2.97	2.99
ENSDARG00000033999	cyp26a1	cytochrome P450, family 26, subfamily A, polypeptide 1 [Source:NCBI gene;Acc:30381]	12	8954761	8958353		6.89	6.22	6.06	6.71	7.19	7.14
ENSDARG00000034001	tango6	transport and golgi organization 6 homolog (Drosophila) [Source:ZFIN;Acc:ZDB-GENE-050419-237]	18	17032312	17075098		0.77	0.44	0.55	1.14	1.05	0.95
ENSDARG00000034007	prom1b	prominin 1 b [Source:ZFIN;Acc:ZDB-GENE-031003-1]	1	22712931	22757145		17.43	4.69	5.17	23.06	24.08	24
ENSDARG00000034013	rgmd	RGM domain family, member D [Source:ZFIN;Acc:ZDB-GENE-030131-561]	22	19640309	19646421		26.19	25.99	24.63	34.22	36.18	33.98
ENSDARG00000034042	dnal1	dynein, axonemal, light chain 1 [Source:NCBI gene;Acc:445048]	17	33708682	33716688		1.51	1.52	1.22	1.73	1.68	2.24
ENSDARG00000034043	irx5a	iroquois homeobox 5a [Source:ZFIN;Acc:ZDB-GENE-010716-2]	7	35706765	35710263		20.34	10.27	8.44	23.47	21.46	24.27
ENSDARG00000034048	zgc:92275	zgc:92275 [Source:ZFIN;Acc:ZDB-GENE-040718-356]	21	11415224	11434315		3.36	1.56	1.69	2.57	3.08	2.8
ENSDARG00000034056	csnk1g2b	casein kinase 1, gamma 2b [Source:ZFIN;Acc:ZDB-GENE-060825-285]	8	20495889	20559955		12	8.78	7.7	15.07	14.63	13.74
ENSDARG00000034060	elac2	elaC ribonuclease Z 2 [Source:ZFIN;Acc:ZDB-GENE-041111-227]	3	10535957	10582384		5.93	5	4.82	5.02	5.16	4.69
ENSDARG00000034063	unm_sa911	un-named sa911 [Source:ZFIN;Acc:ZDB-GENE-120727-3]	17	53349626	53353653		1.37	1.6	1.4	1.49	1.72	1.27
ENSDARG00000034076	lrp11	low density lipoprotein receptor-related protein 11 [Source:ZFIN;Acc:ZDB-GENE-030131-7847]	20	1278184	1287799		6.11	2.87	2.32	9.41	9.72	9.79
ENSDARG00000034080	plcd1b	phospholipase C, delta 1b [Source:ZFIN;Acc:ZDB-GENE-030131-9435]	2	21387902	21438492		12.78	13.4	12.65	9.68	9.28	9.22
ENSDARG00000034086	zgc:65895	zgc:65895 [Source:ZFIN;Acc:ZDB-GENE-040426-1466]	6	59967994	59972559		19.29	6.73	9.3	28.64	27.79	30.51
ENSDARG00000034093	dclk2a	doublecortin-like kinase 2a [Source:ZFIN;Acc:ZDB-GENE-050420-170]	1	37195465	37310490		1.54	0.63	0.59	2.16	1.9	1.94
ENSDARG00000034095	si:dkey-59l11.10	si:dkey-59l11.10 [Source:ZFIN;Acc:ZDB-GENE-120703-17]	4	38818194	38829098		0.17	0.21	0.11	0.09	0.06	0.03
ENSDARG00000034105	mtus1b	microtubule associated tumor suppressor 1b [Source:ZFIN;Acc:ZDB-GENE-070619-5]	1	16548733	16619858		11.45	7.31	7.48	10.47	10.82	10.96
ENSDARG00000034107	actl6b	actin-like 6B [Source:ZFIN;Acc:ZDB-GENE-050522-191]	23	44472414	44494488		1.75	0.72	0.7	1.4	1.52	1.33
ENSDARG00000034117	rarga	retinoic acid receptor gamma a [Source:NCBI gene;Acc:30606]	23	35847200	35963735		27.65	22.48	21.12	29.77	31.95	30.96
ENSDARG00000034138	shisa2a	shisa family member 2a [Source:ZFIN;Acc:ZDB-GENE-030925-31]	5	22067570	22070229		0.52	0.62	0.72	1.1	1.04	0.82
ENSDARG00000034146	ccnk	cyclin K [Source:NCBI gene;Acc:569432]	20	5074118	5094423		4.09	3.08	3.19	6.51	5.67	5.76
ENSDARG00000034160	slc4a1ap	solute carrier family 4 member 1 adaptor protein [Source:ZFIN;Acc:ZDB-GENE-080521-2]	17	40879331	40903365		3.98	2.9	2.58	4.33	4.11	4.15
ENSDARG00000034165	lingo1a	leucine rich repeat and Ig domain containing 1a [Source:ZFIN;Acc:ZDB-GENE-080327-16]	25	7229046	7230905		5.83	1.13	1.63	6.75	7.84	7.44
ENSDARG00000034168	serpini1	serpin peptidase inhibitor, clade I (neuroserpin), member 1 [Source:ZFIN;Acc:ZDB-GENE-060503-390]	18	5137241	5170417		12.79	3.97	5.74	12.83	13.84	13.44
ENSDARG00000034173	prkcq	protein kinase C, theta [Source:ZFIN;Acc:ZDB-GENE-041210-195]	4	25410293	25441127		1.24	1.23	0.97	1.28	1.01	1.19
ENSDARG00000034174	mrrf	mitochondrial ribosome recycling factor [Source:ZFIN;Acc:ZDB-GENE-040704-12]	10	9305050	9346360		12.75	12.74	12.85	8.06	8.26	8.29
ENSDARG00000034178	cpsf1	cleavage and polyadenylation specific factor 1 [Source:ZFIN;Acc:ZDB-GENE-040709-2]	19	11897631	11975164		6.62	4.57	4.76	5.93	6.34	6.39
ENSDARG00000034181	esr2b	estrogen receptor 2b [Source:ZFIN;Acc:ZDB-GENE-030116-1]	13	37022601	37072558		0.54	0.58	0.41	1.17	1.01	1.22
ENSDARG00000034187	calm1b	calmodulin 1b [Source:ZFIN;Acc:ZDB-GENE-030804-2]	20	16743056	16775258		125.66	76.86	85.28	103.35	108.4	110.22
ENSDARG00000034189	oxsr1a	oxidative stress responsive kinase 1a [Source:ZFIN;Acc:ZDB-GENE-070620-25]	2	23677179	23726605		4.99	3.23	2.79	6.84	6.9	6.63
ENSDARG00000034195	top2b	DNA topoisomerase II beta [Source:ZFIN;Acc:ZDB-GENE-041008-136]	19	17688350	17774884		35.41	21.31	23.44	34.89	34.42	35.13
ENSDARG00000034207	cx44.2	connexin 44.2 [Source:ZFIN;Acc:ZDB-GENE-010619-1]	6	21001264	21028431		1.41	2.13	2.14	2.6	2.71	2.4
ENSDARG00000034210	si:dkey-240h12.4	si:dkey-240h12.4 [Source:ZFIN;Acc:ZDB-GENE-070705-406]	19	11167289	11208824		6.82	6.47	6.01	8.43	7.87	7.59
ENSDARG00000034211	capn2l	calpain 2, (m/II) large subunit, like [Source:ZFIN;Acc:ZDB-GENE-050417-341]	22	26443235	26476078		6.09	4.47	4.22	11.29	10.51	9.97
ENSDARG00000034215	rab42a	RAB42, member RAS oncogene family a [Source:ZFIN;Acc:ZDB-GENE-040426-1983]	19	16032383	16036147		12.56	4.43	6.55	14.03	14.24	13.48
ENSDARG00000034222	hs1bp3	HCLS1 binding protein 3 [Source:ZFIN;Acc:ZDB-GENE-131121-391]	17	48947762	48961698		2.04	3.08	2.71	1.68	1.91	1.72
ENSDARG00000034227	si:dkey-243i1.1	si:dkey-243i1.1 [Source:ZFIN;Acc:ZDB-GENE-030131-6399]	15	26848909	26887028		4.37	1.22	1.66	3.27	2.89	3.62
ENSDARG00000034229	kcnip3a	Kv channel interacting protein 3a, calsenilin [Source:ZFIN;Acc:ZDB-GENE-040426-1791]	8	42764234	42907824		16.15	5.14	6.68	10.73	11.25	12.05
ENSDARG00000034240	capza1a	capping actin protein of muscle Z-line subunit alpha 1a [Source:ZFIN;Acc:ZDB-GENE-030131-5237]	6	48322250	48347724		43.09	42.66	43.24	41.47	38.87	41.66
ENSDARG00000034256	cdk10	cyclin-dependent kinase 10 [Source:ZFIN;Acc:ZDB-GENE-050417-94]	25	34956368	34973211		5.4	4.76	4.78	4.41	4.9	4.68
ENSDARG00000034262	pld2	phospholipase D2 [Source:ZFIN;Acc:ZDB-GENE-060216-4]	5	38388443	38438613		6.27	5.36	4.21	5.71	6.28	5.96
ENSDARG00000034268	slit3	slit homolog 3 (Drosophila) [Source:ZFIN;Acc:ZDB-GENE-010306-4]	14	24491818	24761161		9.59	9.24	7.06	28.2	26.99	29.15
ENSDARG00000034270	ogdha	oxoglutarate (alpha-ketoglutarate) dehydrogenase a (lipoamide) [Source:ZFIN;Acc:ZDB-GENE-030131-2143]	8	45762484	45838481		32.17	33.38	26.87	46.35	41.22	42.05
ENSDARG00000034291	rpl37	ribosomal protein L37 [Source:ZFIN;Acc:ZDB-GENE-040625-39]	21	5194602	5205653		521.1	704.41	802.99	427.6	452.1	440.76
ENSDARG00000034293	hif1ab	hypoxia inducible factor 1 subunit alpha b [Source:ZFIN;Acc:ZDB-GENE-040426-706]	20	20326611	20355577		31.6	22.61	15.75	45.36	43.76	44.87
ENSDARG00000034300	sema3c	sema domain, immunoglobulin domain (Ig), short basic domain, secreted, (semaphorin) 3C [Source:ZFIN;Acc:ZDB-GENE-050513-5]	4	22613625	22652944		3.87	4.15	3.81	5.55	5.83	5.02
ENSDARG00000034307	chrne	cholinergic receptor, nicotinic, epsilon [Source:ZFIN;Acc:ZDB-GENE-040808-32]	5	38440099	38451082		9.5	12.16	10.35	14.3	12.25	12.18
ENSDARG00000034309	cox10	cytochrome c oxidase assembly factor heme A:farnesyltransferase COX10 [Source:ZFIN;Acc:ZDB-GENE-041111-239]	12	36891483	36960525		12.3	12.86	11.94	11.39	11.83	10.97
ENSDARG00000034313	ppp5c	protein phosphatase 5, catalytic subunit [Source:ZFIN;Acc:ZDB-GENE-050327-75]	15	2727670	2754056		15.83	15.14	13.13	14.81	14.34	14.08
ENSDARG00000034321	palmdb	palmdelphin b [Source:ZFIN;Acc:ZDB-GENE-090713-3]	24	29964976	29997190		9.74	4.43	4.13	3.81	4.23	4.58
ENSDARG00000034326	ing3	inhibitor of growth family, member 3 [Source:ZFIN;Acc:ZDB-GENE-040109-3]	4	10604110	10615355		24.1	19.14	21.63	21.86	22.62	23.12
ENSDARG00000034330	si:dkey-88n24.7	si:dkey-88n24.7 [Source:ZFIN;Acc:ZDB-GENE-041014-91]	7	3848298	3850107		0.1	0.04	0.03	0	0.02	0
ENSDARG00000034344	pcdh19	protocadherin 19 [Source:ZFIN;Acc:ZDB-GENE-030131-4218]	14	40300961	40390757		9.02	2.76	2.75	14.24	13.66	14.15
ENSDARG00000034368	hexb	hexosaminidase B (beta polypeptide) [Source:ZFIN;Acc:ZDB-GENE-030131-2333]	5	34600714	34616599		33.83	25.79	21.88	29.76	29.66	29.11
ENSDARG00000034375	chst11	carbohydrate (chondroitin 4) sulfotransferase 11 [Source:NCBI gene;Acc:404232]	4	9103182	9173552		3.52	1.63	1.92	6.2	6.22	6.01
ENSDARG00000034396	mars1	methionyl-tRNA synthetase 1 [Source:ZFIN;Acc:ZDB-GENE-030219-83]	6	58921240	58975019		13.35	14.67	11.67	13.95	13.87	13.36
ENSDARG00000034403	zgc:173443	zgc:173443 [Source:ZFIN;Acc:ZDB-GENE-071004-101]	10	8077969	8079737		0.95	1.1	1.36	1.1	0.85	1.44
ENSDARG00000034409	pik3r3b	phosphoinositide-3-kinase, regulatory subunit 3b (gamma) [Source:ZFIN;Acc:ZDB-GENE-040426-743]	6	33537267	33558977		7.61	3.3	3.71	11.13	11.18	11.56
ENSDARG00000034420	irx6a	iroquois homeobox 6a [Source:ZFIN;Acc:ZDB-GENE-040712-5]	7	35511732	35516251		3.58	0.63	0.88	4.49	4.13	4.67
ENSDARG00000034423	sncga	synuclein, gamma a [Source:ZFIN;Acc:ZDB-GENE-050417-18]	13	22659153	22673166		16.46	13.03	13.67	63.1	61.48	63.82
ENSDARG00000034424	atp1b2b	ATPase Na+/K+ transporting subunit beta 2b [Source:ZFIN;Acc:ZDB-GENE-010718-1]	5	38475531	38506981		18.99	7.08	5.97	17.34	17.67	17.56
ENSDARG00000034427	jpt2	Jupiter microtubule associated homolog 2 [Source:ZFIN;Acc:ZDB-GENE-030131-599]	3	15893039	15902229		26.73	33.07	25.31	29.69	30.15	27.91
ENSDARG00000034430	zgc:172121	zgc:172121 [Source:ZFIN;Acc:ZDB-GENE-030131-9545]	6	29288006	29292887		1.65	1.25	1.38	1.09	1.05	0.73
ENSDARG00000034434	igf1rb	insulin-like growth factor 1b receptor [Source:ZFIN;Acc:ZDB-GENE-020503-2]	7	15446229	15610129		1.83	1.04	0.91	3.42	3.66	3.63
ENSDARG00000034443	mpped2	metallophosphoesterase domain containing 2b [Source:ZFIN;Acc:ZDB-GENE-030131-8903]	25	14694876	14739282		3.82	2.26	1.97	4.73	5.21	5.14
ENSDARG00000034453	unc119a	unc-119 homolog a (C. elegans) [Source:ZFIN;Acc:ZDB-GENE-090930-2]	15	28106498	28125224		16.47	7.59	6.58	9.77	10.52	10.36
ENSDARG00000034457	si:ch211-163l21.7	si:ch211-163l21.7 [Source:NCBI gene;Acc:567726]	8	23781028	23783633		0.37	0.58	0.38	0.33	0.56	0.45
ENSDARG00000034470	aldoab	aldolase a, fructose-bisphosphate, b [Source:ZFIN;Acc:ZDB-GENE-040426-2299]	12	4052180	4070058		407.1	529.91	603.89	467.62	447.23	448.03
ENSDARG00000034473	ttyh3a	tweety family member 3a [Source:ZFIN;Acc:ZDB-GENE-120508-1]	3	41955358	42086648		5.47	2.59	2.79	7.03	6.36	6.32
ENSDARG00000034493	grin2aa	glutamate receptor, ionotropic, N-methyl D-aspartate 2A, a [Source:ZFIN;Acc:ZDB-GENE-070424-129]	3	27071749	27277695		4.24	0.77	0.87	4.84	5.29	5.5
ENSDARG00000034497	fbxl14a	F-box and leucine-rich repeat protein 14a [Source:ZFIN;Acc:ZDB-GENE-030131-5920]	25	21064019	21066136		8.56	7.89	7	9.32	9.1	8.42
ENSDARG00000034503	per2	period circadian clock 2 [Source:ZFIN;Acc:ZDB-GENE-011220-2]	2	48309600	48375342		1.42	0.67	0.56	2.26	2.32	2.17
ENSDARG00000034504	lmo1	LIM domain only 1 [Source:NCBI gene;Acc:280646]	7	28109364	28148310		21.84	6.37	11.35	25.19	25.55	26.94
ENSDARG00000034518	trdmt1	tRNA aspartic acid methyltransferase 1 [Source:ZFIN;Acc:ZDB-GENE-041008-138]	24	32151629	32196412		3.64	2.78	3.41	2.42	2.12	2.1
ENSDARG00000034522	rab6ba	RAB6B, member RAS oncogene family a [Source:ZFIN;Acc:ZDB-GENE-050809-136]	6	27667359	27900013		14.14	6.45	9.15	14.27	13.76	15.11
ENSDARG00000034528	chodl	chondrolectin [Source:NCBI gene;Acc:550240]	24	24014880	24028235		5.77	2.05	4.1	4.57	5.81	5.79
ENSDARG00000034534	atp6v1aa	ATPase H+ transporting V1 subunit Aa [Source:ZFIN;Acc:ZDB-GENE-040426-1143]	5	24245682	24259992		109.13	65.85	66.47	86.2	88.38	89.67
ENSDARG00000034541	tgfbr2b	transforming growth factor beta receptor 2b [Source:ZFIN;Acc:ZDB-GENE-980526-375]	16	39505163	39605661		18.96	21.44	20.36	20.74	20.75	19.9
ENSDARG00000034555	pard3bb	par-3 family cell polarity regulator beta b [Source:ZFIN;Acc:ZDB-GENE-070912-290]	9	14569211	15208129		2.54	2.8	2.47	3.64	3.92	4.13
ENSDARG00000034559	srpx2	sushi-repeat containing protein X-linked 2 [Source:ZFIN;Acc:ZDB-GENE-110411-231]	14	35442511	35462148		0.47	0.83	0.65	0.75	0.97	0.66
ENSDARG00000034568	fitm2	fat storage inducing transmembrane protein 2 [Source:NCBI gene;Acc:552928]	23	25796348	25798099		7.5	8.75	6.99	8.69	8.33	8.31
ENSDARG00000034572	gpr143	G protein-coupled receptor 143 [Source:ZFIN;Acc:ZDB-GENE-040426-1476]	9	55056310	55090829		4.53	6.5	7.4	5.87	7.25	7.26
ENSDARG00000034577	si:dkey-23o4.6	si:dkey-23o4.6 [Source:ZFIN;Acc:ZDB-GENE-100922-3]	16	38109941	38118003		1.33	0.21	0.32	1	1.34	1.44
ENSDARG00000034588	scn4ab	sodium channel, voltage-gated, type IV, alpha, b [Source:NCBI gene;Acc:564977]	3	22242269	22316844		4.9	3.98	2.93	7.75	7.21	7.09
ENSDARG00000034598	dicp1.16	diverse immunoglobulin domain-containing protein 1.16 [Source:ZFIN;Acc:ZDB-GENE-120306-9]	KN149948.1	3740	47183		0	0	0	0	0	0
ENSDARG00000034600	tmem165	transmembrane protein 165 [Source:ZFIN;Acc:ZDB-GENE-030131-3222]	20	22186297	22193190		20.2	19.01	16.41	21.12	21.44	20.95
ENSDARG00000034605	zgc:153169	zgc:153169 [Source:ZFIN;Acc:ZDB-GENE-060929-764]	13	24396666	24402100		3.89	5.01	4.22	2.98	2.01	1.92
ENSDARG00000034610	insb	preproinsulin b [Source:ZFIN;Acc:ZDB-GENE-040113-2]	14	28563081	28568152		0.11	0.3	0.21	0.16	0.07	0.12
ENSDARG00000034616	mlf2	myeloid leukemia factor 2 [Source:ZFIN;Acc:ZDB-GENE-050913-67]	16	31827502	31837482		15.51	9.08	11.12	20.05	19.38	19.62
ENSDARG00000034624	nuf2	UF2 component of NDC80 kinetochore complex [Source:NCBI gene;Acc:393280]	11	429776	438518		9.44	9.97	9.8	5.82	5.73	5.21
ENSDARG00000034633	emc9	ER membrane protein complex subunit 9 [Source:ZFIN;Acc:ZDB-GENE-040426-693]	12	13311939	13318969		14.2	13.88	13.16	13.14	12.46	12.55
ENSDARG00000034643	fhl3a	four and a half LIM domains 3a [Source:ZFIN;Acc:ZDB-GENE-030131-4741]	16	33655890	33736831		8.67	12.85	11.44	11.07	11.6	11.55
ENSDARG00000034650	fabp7b	fatty acid binding protein 7, brain, b [Source:ZFIN;Acc:ZDB-GENE-040614-4]	20	40369257	40370736		8.35	10.8	9.31	4.22	5.27	4.79
ENSDARG00000034667	CABZ01090041.1	lipoprotein lipase-like [Source:NCBI gene;Acc:100331214]	22	15633013	15643059		0.96	0.75	0.75	0.38	0.65	0.56
ENSDARG00000034668	celf3a	cugbp, Elav-like family member 3a [Source:NCBI gene;Acc:57922]	19	8816657	8880688		30.33	6.81	7.27	41.23	38.91	42.68
ENSDARG00000034670	ube2e2	ubiquitin-conjugating enzyme E2E 2 [Source:ZFIN;Acc:ZDB-GENE-040801-237]	16	50006145	50078469		11.84	6.73	7.67	13.56	13.04	13.42
ENSDARG00000034677	scel	sciellin [Source:ZFIN;Acc:ZDB-GENE-030616-15]	9	17306162	17334658		32.57	34.92	29.34	14.12	12.4	13.32
ENSDARG00000034685	tmem198b	transmembrane protein 198b [Source:NCBI gene;Acc:564608]	6	13779857	13870158		9.06	4.91	4.77	8.91	8.8	8.95
ENSDARG00000034693	mysm1	Myb-like, SWIRM and MPN domains 1 [Source:NCBI gene;Acc:561225]	20	9093366	9123296		3.74	2.44	2.78	2.92	3.01	3.32
ENSDARG00000034700	vegfab	vascular endothelial growth factor Ab [Source:ZFIN;Acc:ZDB-GENE-030131-4605]	4	5568688	5597802		4.13	3.65	3.99	9.39	10.22	9.17
ENSDARG00000034705	pvalb7	parvalbumin 7 [Source:NCBI gene;Acc:402807]	22	29165330	29242347		14.19	16.35	15.91	9.3	9.74	9.22
ENSDARG00000034707	ftr85	finTRIM family, member 85 [Source:ZFIN;Acc:ZDB-GENE-070705-22]	20	7084154	7104637		1.31	1.78	1.37	1.12	0.89	0.9
ENSDARG00000034714	esyt1a	extended synaptotagmin-like protein 1a [Source:ZFIN;Acc:ZDB-GENE-090311-55]	23	25308018	25340070		2.4	1.78	1.73	3.27	3.26	3.25
ENSDARG00000034717	def6c	DEF6 guanine nucleotide exchange factor c [Source:ZFIN;Acc:ZDB-GENE-060503-87]	4	18759289	18775548		5.02	5.75	4.72	3.5	3.3	2.91
ENSDARG00000034718	tfpia	tissue factor pathway inhibitor a [Source:ZFIN;Acc:ZDB-GENE-030711-1]	9	42420636	42484448		18.66	24.91	19.28	26.06	26.68	24.88
ENSDARG00000034734	med6	mediator complex subunit 6 [Source:NCBI gene;Acc:415174]	13	36135122	36141486		17.48	15.31	17.12	13.67	11.93	13.55
ENSDARG00000034753	tspan3a	tetraspanin 3a [Source:ZFIN;Acc:ZDB-GENE-030131-7787]	18	26899316	26920132		41.43	29.55	30.27	46.96	45.65	47.23
ENSDARG00000034757	zdhhc2	zinc finger DHHC-type palmitoyltransferase 2 [Source:ZFIN;Acc:ZDB-GENE-050320-58]	14	30426956	30452218		2.6	1.03	1.57	1.78	1.81	1.87
ENSDARG00000034768	phf3	PHD finger protein 3 [Source:ZFIN;Acc:ZDB-GENE-090313-207]	13	37653851	37673576		8.29	6.09	4.36	8.82	8.88	8.87
ENSDARG00000034771	rab13	RAB13, member RAS oncogene family [Source:NCBI gene;Acc:373105]	16	23796612	23809611		11.44	14.95	13.96	14.05	12.54	11.91
ENSDARG00000034773	ncapd3	non-SMC condensin II complex, subunit D3 [Source:ZFIN;Acc:ZDB-GENE-040426-737]	1	21540453	21563040		4.97	3.9	4.02	3.44	3.41	3.56
ENSDARG00000034777	txn2	thioredoxin 2 [Source:ZFIN;Acc:ZDB-GENE-040426-1795]	3	5265101	5277041		28.49	30.62	28.26	31.55	29.73	29.55
ENSDARG00000034785	dachb	dachshund b [Source:ZFIN;Acc:ZDB-GENE-020402-4]	1	10378706	10496809		3.74	2.57	2.71	3.97	3.95	4.19
ENSDARG00000034801	mylka	myosin, light chain kinase a [Source:ZFIN;Acc:ZDB-GENE-030131-8207]	9	4024250	4075620		7.11	7.86	7.01	12.12	13.63	13.44
ENSDARG00000034808	kcnip1b	Kv channel interacting protein 1 b [Source:ZFIN;Acc:ZDB-GENE-041212-57]	10	22003750	22031921		17.27	7.05	10.01	13.15	13.48	14.28
ENSDARG00000034817	asah1b	N-acylsphingosine amidohydrolase (acid ceramidase) 1b [Source:ZFIN;Acc:ZDB-GENE-040426-1512]	1	16654254	16665044		12.73	16.59	15.02	10.09	10.18	9.43
ENSDARG00000034823	copg2	coatomer protein complex, subunit gamma 2 [Source:NCBI gene;Acc:30187]	4	15983541	15996191		44.16	37.43	33.58	41	42.02	41.42
ENSDARG00000034825	uspl1	ubiquitin specific peptidase like 1 [Source:NCBI gene;Acc:445403]	10	36439293	36455985		10.3	4.69	6.4	11.25	11.73	11.83
ENSDARG00000034836	si:dkey-222n6.2	si:dkey-222n6.2 [Source:ZFIN;Acc:ZDB-GENE-031118-10]	8	7084974	7093507		30.52	29.5	24.9	13.34	13.11	14.15
ENSDARG00000034852	nt5c2l1	5'-nucleotidase, cytosolic II, like 1 [Source:ZFIN;Acc:ZDB-GENE-031006-8]	10	17460358	17484764		2.76	3.09	3.8	13.67	15.17	15.07
ENSDARG00000034855	ccnb3	cyclin B3 [Source:ZFIN;Acc:ZDB-GENE-060929-684]	7	22981441	23006732		2.05	2.02	2.23	2.08	1.83	1.68
ENSDARG00000034862	f7	coagulation factor VII [Source:ZFIN;Acc:ZDB-GENE-010814-1]	1	119960	125246		7.76	10.66	9.46	6.56	7.6	8.91
ENSDARG00000034871	mid2	midline 2 [Source:ZFIN;Acc:ZDB-GENE-090220-1]	14	28281744	28385565		6.14	3.07	3.18	9.51	10.1	10.83
ENSDARG00000034883	acbd5a	acyl-CoA binding domain containing 5a [Source:NCBI gene;Acc:553674]	24	5915895	5933082		7.67	6.03	5.65	7.52	8.01	8.32
ENSDARG00000034885	bet1	Bet1 golgi vesicular membrane trafficking protein [Source:ZFIN;Acc:ZDB-GENE-040625-3]	19	40899910	40906161		25.33	26.52	21.63	19.97	21.38	19.34
ENSDARG00000034893	rarab	retinoic acid receptor, alpha b [Source:NCBI gene;Acc:555364]	3	33065613	33259621		9.14	5.9	4.83	11.35	11.58	11.9
ENSDARG00000034895	tgfb1b	transforming growth factor, beta 1b [Source:ZFIN;Acc:ZDB-GENE-091028-1]	21	21621042	21629828		1.63	1.53	1.6	2.68	2.29	2.16
ENSDARG00000034896	ldb2b	LIM domain binding 2b [Source:ZFIN;Acc:ZDB-GENE-990415-137]	1	22805398	22834824		9.9	3.9	4.75	10.9	10.46	11.4
ENSDARG00000034897	rps10	ribosomal protein S10 [Source:ZFIN;Acc:ZDB-GENE-040426-1481]	6	54174696	54180699		955.81	1112.34	1316.46	676.62	710.05	700.64
ENSDARG00000034907	paqr7b	progestin and adipoQ receptor family member VII, b [Source:NCBI gene;Acc:368256]	16	34522977	34532888		10.1	2.78	4.89	8.83	8.64	9.2
ENSDARG00000034910	si:ch211-152f2.2	si:ch211-152f2.2 [Source:ZFIN;Acc:ZDB-GENE-121214-75]	1	56873359	56878634		0	0	0	0	0	0
ENSDARG00000034916	hat1	histone acetyltransferase 1 [Source:ZFIN;Acc:ZDB-GENE-040912-99]	9	3282369	3314374		29.57	31.09	33.74	20.37	19.31	19.39
ENSDARG00000034930	cx52.6	connexin 52.6 [Source:ZFIN;Acc:ZDB-GENE-040310-6]	20	23960525	23964383		0.22	0.05	0.03	0.21	0.37	0.3
ENSDARG00000034933	chchd3b	coiled-coil-helix-coiled-coil-helix domain containing 3b [Source:ZFIN;Acc:ZDB-GENE-030131-4476]	25	3358701	3369328		7.79	10.89	9.97	7.78	6.45	6.58
ENSDARG00000034940	slc1a7a	solute carrier family 1 member 7a [Source:ZFIN;Acc:ZDB-GENE-061009-24]	2	5948534	5999636		1.84	0.37	0.35	2.99	2.59	2.69
ENSDARG00000034941	si:dkey-224e22.2	si:dkey-224e22.2 [Source:ZFIN;Acc:ZDB-GENE-061207-59]	18	44746594	44765515		3.4	4.5	3.75	3.54	3.63	4.16
ENSDARG00000034943	tm9sf5	transmembrane 9 superfamily protein member 5 [Source:ZFIN;Acc:ZDB-GENE-101013-1]	14	31865099	31884097		10.38	8.34	7.32	8.2	7.32	7.27
ENSDARG00000034956	myclb	MYCL proto-oncogene, bHLH transcription factor b [Source:NCBI gene;Acc:567762]	19	30805513	30811161		8.29	6.36	5.78	9.92	9.56	9.74
ENSDARG00000034957	hddc2	HD domain containing 2 [Source:NCBI gene;Acc:554129]	20	39685572	39689791		11.63	13.82	14.99	10.23	10.86	13.28
ENSDARG00000034975	gpr157	G protein-coupled receptor 157 [Source:ZFIN;Acc:ZDB-GENE-050809-126]	23	22597624	22605915		1.83	2.26	2.11	2.44	2.41	2.21
ENSDARG00000034982	zcchc8	zinc finger, CCHC domain containing 8 [Source:ZFIN;Acc:ZDB-GENE-060526-6]	5	67250959	67267707		3.96	2.51	2.5	3.71	3.88	3.58
ENSDARG00000034988	asb4	ankyrin repeat and SOCS box containing 4 [Source:ZFIN;Acc:ZDB-GENE-050417-100]	19	41169996	41182251		1.53	2	2.55	2.14	2.66	2.08
ENSDARG00000034989	retsatl	retinol saturase (all-trans-retinol 13,14-reductase) like [Source:NCBI gene;Acc:677660]	9	44994214	44998722		0	0.05	0.03	0.11	0.09	0.06
ENSDARG00000035005	bag2	BCL2 associated athanogene 2 [Source:ZFIN;Acc:ZDB-GENE-040426-1399]	13	1404924	1408775		9.86	13.14	10.98	11.78	10.6	11.75
ENSDARG00000035009	trim35-27	tripartite motif containing 35-27 [Source:ZFIN;Acc:ZDB-GENE-030131-6185]	14	757435	763744		1.42	1.11	1.22	0.92	1	0.79
ENSDARG00000035018	thy1	Thy-1 cell surface antigen [Source:ZFIN;Acc:ZDB-GENE-030131-8561]	5	29794058	29803293		15	22.11	22.6	16.6	15.06	14.17
ENSDARG00000035023	khdc4	KH domain containing 4, pre-mRNA splicing factor [Source:NCBI gene;Acc:321686]	16	54785264	54800202		14.62	6.18	8.5	16.92	15.67	15.83
ENSDARG00000035028	lpcat4	lysophosphatidylcholine acyltransferase 4 [Source:ZFIN;Acc:ZDB-GENE-030131-5777]	7	1473929	1495966		5.72	3.69	2.84	2.99	2.68	2.38
ENSDARG00000035043	pfdn5	prefoldin 5 [Source:ZFIN;Acc:ZDB-GENE-030131-6858]	6	39661588	39666722		41.78	56.83	54.92	39.72	40.15	42.08
ENSDARG00000035048	or115-10	odorant receptor, family F, subfamily 115, member 10 [Source:ZFIN;Acc:ZDB-GENE-010309-3]	21	40235905	40237971		0	0	0	0	0	0
ENSDARG00000035054	pdcd10a	programmed cell death 10a [Source:NCBI gene;Acc:393527]	18	5086318	5111449		16.78	17.6	14.7	13.35	12.27	13.51
ENSDARG00000035056	fgf13a	fibroblast growth factor 13a [Source:ZFIN;Acc:ZDB-GENE-041114-101]	14	32183978	32405387		6.71	2.41	3.09	8.35	8.53	8.81
ENSDARG00000035066	ubtf	upstream binding transcription factor [Source:ZFIN;Acc:ZDB-GENE-030616-252]	3	20041907	20063782		26.54	14.48	15.76	30.82	31.19	32.3
ENSDARG00000035074	rtel1	regulator of telomere elongation helicase 1 [Source:NCBI gene;Acc:503732]	11	12811906	12859263		3.01	2.47	2.31	2.74	2.68	2.44
ENSDARG00000035080	hsf2bp	heat shock transcription factor 2 binding protein [Source:ZFIN;Acc:ZDB-GENE-030131-1458]	1	29102011	29139183		0.02	0.09	0.03	0.03	0.12	0.03
ENSDARG00000035088	si:ch211-254c8.3	si:ch211-254c8.3 [Source:ZFIN;Acc:ZDB-GENE-100922-130]	3	1930066	1970820		0.45	0.03	0.25	0.46	1.13	1.35
ENSDARG00000035095	tdgf1	teratocarcinoma-derived growth factor 1 [Source:ZFIN;Acc:ZDB-GENE-990415-198]	10	1955475	1961930		0.37	0.5	0.38	0.74	0.87	0.92
ENSDARG00000035101	dicp2.1	diverse immunoglobulin domain-containing protein 2.1 [Source:ZFIN;Acc:ZDB-GENE-120306-16]	14	17029813	17038117		0.05	0	0	0.07	0	0
ENSDARG00000035120	gldc	glycine dehydrogenase (decarboxylating) [Source:ZFIN;Acc:ZDB-GENE-030131-340]	5	66170479	66202748		16.25	22.37	16.14	46.21	58.57	60.63
ENSDARG00000035122	camsap1b	calmodulin regulated spectrin-associated protein 1b [Source:NCBI gene;Acc:561094]	5	65613292	65662996		4.05	1.77	1.53	4.63	4.44	4.7
ENSDARG00000035127	rxrab	retinoid x receptor, alpha b [Source:NCBI gene;Acc:793011]	5	64476118	64511428		3.31	2.75	2.15	3.8	3.39	2.95
ENSDARG00000035129	zgc:101858	zgc:101858 [Source:ZFIN;Acc:ZDB-GENE-040927-13]	5	64856666	64861300		20.57	23.63	24.53	12.92	13.78	13.81
ENSDARG00000035131	surf4l	surfeit 4, like [Source:ZFIN;Acc:ZDB-GENE-040718-172]	5	63629000	63644938		9.36	9.35	6.65	12.04	12.05	10.61
ENSDARG00000035132	rgs3b	regulator of G protein signaling 3b [Source:ZFIN;Acc:ZDB-GENE-060531-24]	5	63767376	63864536		2.31	1.26	1.26	3.67	3.33	3.38
ENSDARG00000035133	pho	phoenix [Source:ZFIN;Acc:ZDB-GENE-030131-5935]	5	62319217	62335694		2.74	2	1.83	2.44	2.45	2.66
ENSDARG00000035136	selenow1	selenoprotein W, 1 [Source:NCBI gene;Acc:352915]	5	61998641	62005081		197.08	227.84	236.79	115.64	126.01	118.29

ENSDARG00000035137	ehd2a	EH-domain containing 2a [Source:ZFIN;Acc:ZDB-GENE-060531-86]	5	61976511	61992423		0.04	0.03	0	0.06	0.21	0.04
ENSDARG00000035146	polr2j	polymerase (RNA) II (DNA directed) polypeptide J [Source:ZFIN;Acc:ZDB-GENE-050522-120]	5	61459422	61465028		24.69	24.57	23.14	21.63	21.24	21.06
ENSDARG00000035147	lrwd1	leucine-rich repeats and WD repeat domain containing 1 [Source:NCBI gene;Acc:327124]	5	61475346	61504874		2.6	3.11	2.24	1.89	1.98	1.73
ENSDARG00000035148	orai2	ORAI calcium release-activated calcium modulator 2 [Source:ZFIN;Acc:ZDB-GENE-060531-127]	5	61556172	61571447		2.36	1.76	2.9	3.32	3.77	3.56
ENSDARG00000035149	pex12	peroxisomal biogenesis factor 12 [Source:ZFIN;Acc:ZDB-GENE-040426-929]	5	61583655	61588998		2.15	2.05	1.91	2.46	2.45	2.8
ENSDARG00000035150	drll.2	draculin-like, tandem duplicate 2 [Source:ZFIN;Acc:ZDB-GENE-060531-125]	5	61615663	61624693		0.12	0.2	0.2	0.46	0.32	0.41
ENSDARG00000035151	drll.1	draculin-like, tandem duplicate 1 [Source:ZFIN;Acc:ZDB-GENE-060531-124]	5	61630696	61638125		0.15	0.25	0.07	0.34	0.54	0.47
ENSDARG00000035152	ap2b1	adaptor related protein complex 2 subunit beta 1 [Source:ZFIN;Acc:ZDB-GENE-030131-6564]	5	61657010	61724082		40.77	20.66	20.23	42.73	44.28	45.23
ENSDARG00000035161	pkdcca	protein kinase domain containing, cytoplasmic a [Source:ZFIN;Acc:ZDB-GENE-081107-30]	13	3109064	3155244		4.99	3.96	3.49	8.4	8.57	9.09
ENSDARG00000035163	tlcd5b	TLC domain containing 5b [Source:NCBI gene;Acc:436974]	5	58836354	58840971		7.44	1.9	1.68	3.54	4.28	3.99
ENSDARG00000035167	mrpl48	mitochondrial ribosomal protein L48 [Source:ZFIN;Acc:ZDB-GENE-041008-125]	10	36684000	36691681		28.47	39.25	36.87	17.79	18.54	21.97
ENSDARG00000035169	layna	layilin a [Source:ZFIN;Acc:ZDB-GENE-070705-452]	5	57886049	57898008		1.53	0.98	1.42	1.74	2.14	1.52
ENSDARG00000035171	btg4	B-cell translocation gene 4 [Source:ZFIN;Acc:ZDB-GENE-031008-1]	5	57924611	57926693		0	0	0.03	0.04	0.02	0
ENSDARG00000035175	schip1	schwannomin interacting protein 1 [Source:ZFIN;Acc:ZDB-GENE-050417-466]	15	1372343	1436707		14.47	7.97	9.01	20.39	21.48	21.66
ENSDARG00000035178	gna14	guanine nucleotide binding protein (G protein), alpha 14 [Source:ZFIN;Acc:ZDB-GENE-040808-7]	5	55543580	55560937		7.46	7.7	6.25	7.63	6.29	5.81
ENSDARG00000035181	slc25a46	solute carrier family 25 member 46 [Source:NCBI gene;Acc:436831]	5	55920892	55933420		6.98	4.82	4.8	6.61	6.29	7.11
ENSDARG00000035184	tmem150ab	transmembrane protein 150Ab [Source:ZFIN;Acc:ZDB-GENE-040426-1576]	5	55934129	55952128		3.63	1.64	1.48	4.22	4.46	3.95
ENSDARG00000035185	fzd9a	frizzled class receptor 9a [Source:ZFIN;Acc:ZDB-GENE-060531-77]	5	56023186	56027776		1.12	1.89	1.71	1.49	1.4	1.57
ENSDARG00000035187	abl1	c-abl oncogene 1, non-receptor tyrosine kinase [Source:ZFIN;Acc:ZDB-GENE-100812-9]	5	72145468	72168783		8.45	8.08	6.92	12.67	12.3	13.02
ENSDARG00000035188	rab14l	RAB14, member RAS oncogene family, like [Source:ZFIN;Acc:ZDB-GENE-040426-1207]	5	72172299	72178739		25.89	17.16	18.5	26.79	28.13	25.98
ENSDARG00000035193	fam81b	family with sequence similarity 81 member B [Source:ZFIN;Acc:ZDB-GENE-130530-586]	5	50788329	50796702		0.35	0.18	0.3	0.36	0.35	0.33
ENSDARG00000035198	gcnt4a	glucosaminyl (N-acetyl) transferase 4a [Source:NCBI gene;Acc:324510]	5	51026563	51030105		6.88	6.78	6.78	6.25	5.98	5.8
ENSDARG00000035214	olfcq17	olfactory receptor C family, q17 [Source:ZFIN;Acc:ZDB-GENE-060503-359]	18	33675664	33679254		0	0	0	0	0	0
ENSDARG00000035221	zgc:110249	zgc:110249 [Source:ZFIN;Acc:ZDB-GENE-050522-331]	3	12977	46058		1.94	1.8	1.52	0.92	1.35	1.21
ENSDARG00000035253	npr3	natriuretic peptide receptor 3 [Source:ZFIN;Acc:ZDB-GENE-060531-91]	5	41260478	41307550		4.71	4.01	4.59	16.87	17.56	18.42
ENSDARG00000035256	eef2l2	eukaryotic translation elongation factor 2, like 2 [Source:ZFIN;Acc:ZDB-GENE-030131-8112]	5	41485277	41494831		144.18	181.74	186.69	154.34	161.44	152.67
ENSDARG00000035257	akr1a1a	aldo-keto reductase family 1, member A1a (aldehyde reductase) [Source:NCBI gene;Acc:445326]	5	41521043	41531629		8.63	11.73	15.27	5.91	6.1	6.57
ENSDARG00000035264	riok2	RIO kinase 2 (yeast) [Source:ZFIN;Acc:ZDB-GENE-040426-2913]	5	41639693	41645058		15.75	17.67	17.3	11.35	10.85	11.45
ENSDARG00000035273	tmtc2b	transmembrane O-mannosyltransferase targeting cadherins 2b [Source:ZFIN;Acc:ZDB-GENE-081031-100]	18	11858397	11909472		5.37	2.28	2.29	6.15	6.31	6.3
ENSDARG00000035282	emc6	ER membrane protein complex subunit 6 [Source:NCBI gene;Acc:337220]	5	42122810	42123794		31.22	28.22	31.62	35.71	40.53	37.71
ENSDARG00000035285	ncor1	nuclear receptor corepressor 1 [Source:ZFIN;Acc:ZDB-GENE-040426-993]	5	41878252	41996414		9	6.96	5.21	13.52	13.33	13.44
ENSDARG00000035290	dmrt3a	doublesex and mab-3 related transcription factor 3a [Source:NCBI gene;Acc:450035]	5	45000597	45003863		0.96	0.5	0.84	1.68	1.83	2.09
ENSDARG00000035308	grb10b	growth factor receptor-bound protein 10b [Source:ZFIN;Acc:ZDB-GENE-091020-12]	16	8364085	8520474		0.51	0.46	0.31	1.28	1.47	1.19
ENSDARG00000035309	entpd3	ectonucleoside triphosphate diphosphohydrolase 3 [Source:ZFIN;Acc:ZDB-GENE-030131-6186]	16	8258203	8280885		11.36	12.73	11.22	15.28	12.47	12.36
ENSDARG00000035322	myh7bb	myosin, heavy chain 7B, cardiac muscle, beta b [Source:ZFIN;Acc:ZDB-GENE-090311-5]	23	18722715	18757078		13.36	16.03	13.15	15.83	14.78	14.96
ENSDARG00000035324	hnrnpl	heterogeneous nuclear ribonucleoprotein L [Source:ZFIN;Acc:ZDB-GENE-040426-710]	5	36899691	36911903		70.72	54.22	63.04	65.63	65.4	62.92
ENSDARG00000035325	srsf7a	serine and arginine rich splicing factor 7a [Source:ZFIN;Acc:ZDB-GENE-040426-1798]	5	36895545	36899144		39	27.59	29.26	41.24	41.82	40.9
ENSDARG00000035326	nccrp1	P1, F-box associated domain containing [Source:NCBI gene;Acc:30078]	5	36850650	36854555		18.74	23.8	23.07	11.36	13.13	13.24
ENSDARG00000035327	ckma	creatine kinase, muscle a [Source:ZFIN;Acc:ZDB-GENE-980526-109]	5	36832079	36837846		2088.15	3043.84	3351.13	3708.27	3743.37	3578.81
ENSDARG00000035329	capns1a	calpain, small subunit 1 a [Source:ZFIN;Acc:ZDB-GENE-030113-3]	5	36654817	36665694		76.68	93.27	90.03	48.86	48.22	46.3
ENSDARG00000035330	taf1	TAF1 RNA polymerase II, TATA box binding protein (TBP)-associated factor [Source:ZFIN;Acc:ZDB-GENE-030131-5576]	5	36551470	36592394		12.48	7.15	6.38	11.04	10.5	11.06
ENSDARG00000035332	cfap298	cilia and flagella associated protein 298 [Source:NCBI gene;Acc:792532]	10	33573838	33579162		7.41	6.99	6.73	7.53	8.22	7.95
ENSDARG00000035333	mis18a	MIS18 kinetochore protein A [Source:ZFIN;Acc:ZDB-GENE-091204-314]	10	33588715	33594110		3.98	4.3	3.78	2.39	2.75	3
ENSDARG00000035338	tppp2	tubulin polymerization-promoting protein family member 2 [Source:ZFIN;Acc:ZDB-GENE-140106-24]	5	37056818	37065741		22.37	17.39	20.35	27.88	28.71	29.48
ENSDARG00000035339	ftr99	finTRIM family, member 99 [Source:ZFIN;Acc:ZDB-GENE-141212-215]	5	37040853	37047166		0.82	0.35	0.2	0.64	0.67	0.77
ENSDARG00000035350	ins	preproinsulin [Source:NCBI gene;Acc:30262]	5	37854685	37857696		42.76	50	49.17	38.37	37.37	37.55
ENSDARG00000035352	pafah1b2	platelet-activating factor acetylhydrolase 1b, catalytic subunit 2 [Source:ZFIN;Acc:ZDB-GENE-060526-43]	5	37966505	37971008		17.77	6.78	8.91	16.76	14.74	14.91
ENSDARG00000035357	gnb2	guanine nucleotide binding protein (G protein), beta polypeptide 2 [Source:ZFIN;Acc:ZDB-GENE-050320-65]	5	38263240	38297391		26.27	10.35	13.53	30.67	32.49	32.89
ENSDARG00000035360	mink1	misshapen-like kinase 1 [Source:ZFIN;Acc:ZDB-GENE-060526-45]	5	38301906	38384755		8.29	5.35	4.33	10.95	11.08	10.75
ENSDARG00000035377	fgf5	fibroblast growth factor 5 [Source:ZFIN;Acc:ZDB-GENE-050201-6]	5	39537195	39556884		0.61	0.48	0.67	0.97	1.08	0.73
ENSDARG00000035388	tmem174	transmembrane protein 174 [Source:ZFIN;Acc:ZDB-GENE-080819-2]	5	35105085	35108893		1.83	3.59	2.27	2.56	2.58	2.85
ENSDARG00000035389	fcho2	FCH and mu domain containing endocytic adaptor 2 [Source:NCBI gene;Acc:553949]	5	35123662	35201482		8.09	6.43	4.97	9.2	9.2	9.22
ENSDARG00000035390	tnpo1	transportin 1 [Source:ZFIN;Acc:ZDB-GENE-080819-1]	5	35202063	35252761		8.25	3.71	4.16	12.01	11.39	11.64
ENSDARG00000035391	ptcd2	pentatricopeptide repeat domain 2 [Source:NCBI gene;Acc:550275]	5	35253457	35264517		2.34	1.54	1.9	2.64	2.38	2.32
ENSDARG00000035398	enc1	ectodermal-neural cortex 1 [Source:ZFIN;Acc:ZDB-GENE-060531-140]	5	34622320	34638624		2.97	1.65	1.46	4.38	4.24	4.13
ENSDARG00000035399	ankra2	ankyrin repeat, family A (RFXANK-like), 2 [Source:ZFIN;Acc:ZDB-GENE-040426-2141]	5	34981584	34987273		17.14	13.53	15.64	13.62	13.76	15.49
ENSDARG00000035400	btf3	basic transcription factor 3 [Source:ZFIN;Acc:ZDB-GENE-030131-8731]	5	34987448	34997630		675.46	820.06	979.63	569.65	584.73	590.01
ENSDARG00000035406	ttc33	tetratricopeptide repeat domain 33 [Source:NCBI gene;Acc:436995]	5	35421766	35456269		8.86	9.69	9.02	5.62	6.75	7.45
ENSDARG00000035415	ptger4b	prostaglandin E receptor 4 (subtype EP4) b [Source:ZFIN;Acc:ZDB-GENE-060531-102]	5	35398745	35401568		2.44	2.05	1.73	3.27	3.25	3.67
ENSDARG00000035420	ophn1	oligophrenin 1 [Source:ZFIN;Acc:ZDB-GENE-040718-464]	5	35682681	35743741		2.88	2.02	1.8	3.11	3.32	3.02
ENSDARG00000035422	ccn1l1	cellular communication network factor 1, like 1 [Source:ZFIN;Acc:ZDB-GENE-050417-136]	5	32914321	32920149		0.83	0.78	0.6	3.84	3.39	3.52
ENSDARG00000035427	surf6	surfeit 6 [Source:ZFIN;Acc:ZDB-GENE-040426-1333]	5	33275150	33281156		0.74	0.8	0.62	0.69	0.85	0.92
ENSDARG00000035433	arpc5lb	actin related protein 2/3 complex, subunit 5-like, b [Source:ZFIN;Acc:ZDB-GENE-060531-161]	5	32060881	32074637		15.97	12.96	13.42	14.9	14.83	17.01
ENSDARG00000035434	zmat5	zinc finger, matrin-type 5 [Source:NCBI gene;Acc:445314]	5	32076109	32080192		12.2	12.88	13.46	11.08	12.18	12.97
ENSDARG00000035435	m17	IL-6 subfamily cytokine M17 [Source:ZFIN;Acc:ZDB-GENE-060526-368]	5	32155141	32158242		0.42	0.11	0.16	0.46	0.3	0.11
ENSDARG00000035438	myhc4	myosin heavy chain 4 [Source:ZFIN;Acc:ZDB-GENE-030131-6206]	5	32221755	32233862		9.51	14.03	10.21	17.15	14.8	15.09
ENSDARG00000035452	camkk1a	calcium/calmodulin-dependent protein kinase kinase 1, alpha a [Source:ZFIN;Acc:ZDB-GENE-050327-62]	5	31283576	31432727		2.35	1.01	1.36	3.35	3.66	3.52
ENSDARG00000035458	atp2a1l	ATPase sarcoplasmic/endoplasmic reticulum Ca2+ transporting 1, like [Source:ZFIN;Acc:ZDB-GENE-041229-2]	12	13244149	13263658		142.59	218.77	183.21	195.43	167.07	158.05
ENSDARG00000035459	spns3	spinster homolog 3 (Drosophila) [Source:NCBI gene;Acc:568387]	5	30956981	30984271		0.92	1	0.74	0.88	0.81	0.95
ENSDARG00000035468	slc25a25b	solute carrier family 25 member 25b [Source:NCBI gene;Acc:569227]	5	31779911	31806613		4.95	1.91	1.86	4.06	3.85	3.76
ENSDARG00000035470	sh3glb2b	SH3-domain GRB2-like endophilin B2b [Source:ZFIN;Acc:ZDB-GENE-040426-833]	5	31640881	31689911		5.22	2.65	3.61	5.71	5.84	6.09
ENSDARG00000035471	si:dkey-220k22.1	si:dkey-220k22.1 [Source:ZFIN;Acc:ZDB-GENE-060526-255]	5	31480342	31591000		7.31	5.24	4.49	8.71	8.21	8.02
ENSDARG00000035505	nelfb	negative elongation factor complex member B [Source:ZFIN;Acc:ZDB-GENE-040426-765]	5	29548441	29559927		8.31	5.52	6.96	7.04	6.93	7.49
ENSDARG00000035506	entpd2a.1	ectonucleoside triphosphate diphosphohydrolase 2a, tandem duplicate 1 [Source:ZFIN;Acc:ZDB-GENE-040724-187]	5	29571803	29587438		0.8	0.77	0.56	0.66	0.69	0.54
ENSDARG00000035507	ddx31	DEAD (Asp-Glu-Ala-Asp) box polypeptide 31 [Source:ZFIN;Acc:ZDB-GENE-030131-3973]	5	29714786	29745566		3.17	2.74	2.41	2.5	1.9	2.36
ENSDARG00000035508	barhl1a	BarH-like homeobox 1a [Source:ZFIN;Acc:ZDB-GENE-050417-212]	5	29746355	29750377		6.68	2.35	3.72	5.86	5.76	6.34
ENSDARG00000035514	adam28	ADAM metallopeptidase domain 28 [Source:ZFIN;Acc:ZDB-GENE-060929-532]	5	27897504	27920706		2.7	2.67	1.96	2.46	2.19	2.05
ENSDARG00000035515	tm2d2	TM2 domain containing 2 [Source:NCBI gene;Acc:436913]	5	26921033	26926527		14.15	13.51	13.94	12.86	14.46	13.1
ENSDARG00000035519	histh1l	histone H1 like [Source:ZFIN;Acc:ZDB-GENE-050417-145]	5	27432958	27434351		354.28	176.79	167.43	570.33	643.58	755.13
ENSDARG00000035521	sfrp1a	secreted frizzled-related protein 1a [Source:ZFIN;Acc:ZDB-GENE-040310-5]	5	27488975	27538745		16.88	17.2	13.51	16	17.06	15.99
ENSDARG00000035532	zgc:110329	zgc:110329 [Source:ZFIN;Acc:ZDB-GENE-050417-334]	5	28134688	28149767		8.54	4.01	5.67	9.39	9.38	10.4
ENSDARG00000035533	tacr1a	tachykinin receptor 1a [Source:ZFIN;Acc:ZDB-GENE-060526-185]	5	28160503	28213627		1.07	0.66	0.69	1.53	1.87	1.3
ENSDARG00000035535	rasa1a	RAS p21 protein activator (GTPase activating protein) 1a [Source:ZFIN;Acc:ZDB-GENE-030131-4694]	5	47862636	47967526		10.15	8.38	6.92	10.43	10.49	10.11
ENSDARG00000035538	atp6v0a2a	ATPase H+ transporting V0 subunit a2a [Source:ZFIN;Acc:ZDB-GENE-091113-4]	10	7988746	8033468		5.25	1.57	2.52	5.89	4.82	5.23
ENSDARG00000035540	brwd3	bromodomain and WD repeat domain containing 3 [Source:ZFIN;Acc:ZDB-GENE-100921-43]	5	22974019	23020606		7.78	4.23	4.02	8.64	8.53	8.44
ENSDARG00000035544	etnppl	ethanolamine-phosphate phospho-lyase [Source:NCBI gene;Acc:393421]	7	60079302	60092321		14.27	24.77	19.24	6.58	6.44	7.02
ENSDARG00000035546	jmjd7	jumonji domain containing 7 [Source:ZFIN;Acc:ZDB-GENE-050417-86]	17	10711641	10738045		4.65	5.68	6.72	3.57	3.82	3.75
ENSDARG00000035549	uprt	uracil phosphoribosyltransferase (FUR1) homolog (S. cerevisiae) [Source:NCBI gene;Acc:404623]	5	23107804	23118290		25.55	22.4	22.85	22.14	23.11	23.44
ENSDARG00000035550	cfap73	cilia and flagella associated protein 73 [Source:ZFIN;Acc:ZDB-GENE-060526-194]	5	23190642	23194639		0.23	0.04	0.07	0.36	0.31	0.37
ENSDARG00000035551	iqcd	IQ motif containing D [Source:ZFIN;Acc:ZDB-GENE-060526-200]	5	23196067	23200880		1.43	0.98	0.87	0.9	1.02	1.06
ENSDARG00000035552	agtr2	angiotensin II receptor, type 2 [Source:ZFIN;Acc:ZDB-GENE-050306-56]	5	23242370	23249494		4.56	5.71	6.67	6.83	7.94	7.22
ENSDARG00000035553	cx27.5	connexin 27.5 [Source:ZFIN;Acc:ZDB-GENE-010619-2]	5	23622177	23628166		1.7	1.94	2.24	1.16	1.29	1.38
ENSDARG00000035555	gbgt1l3	globoside alpha-1,3-N-acetylgalactosaminyltransferase 1, like 3 [Source:ZFIN;Acc:ZDB-GENE-040912-46]	5	23849647	23855447		7.98	7.38	5.99	4.12	3.53	3.74
ENSDARG00000035556	rps6ka3a	ribosomal protein S6 kinase a, polypeptide 3a [Source:ZFIN;Acc:ZDB-GENE-030219-119]	5	24009668	24029228		13.39	13.78	11.96	13.77	14.33	13.34
ENSDARG00000035557	gabarapa	GABA(A) receptor-associated protein a [Source:ZFIN;Acc:ZDB-GENE-030131-5174]	5	24059598	24061806		37.5	38.2	37.22	42.4	41.24	44.06
ENSDARG00000035558	gps2	G protein pathway suppressor 2 [Source:ZFIN;Acc:ZDB-GENE-040426-1176]	5	24063046	24074538		12.63	9.83	10.19	11.59	13.93	12.41
ENSDARG00000035559	tp53	tumor protein p53 [Source:NCBI gene;Acc:30590]	5	24086227	24097805		15.44	15.95	14.83	18.72	17.66	16.94
ENSDARG00000035560	capga	capping protein (actin filament), gelsolin-like a [Source:ZFIN;Acc:ZDB-GENE-060531-6]	5	24116428	24127432		0.49	0.51	0.73	0.51	0.64	0.58
ENSDARG00000035562	mpdu1a	mannose-P-dolichol utilization defect 1a [Source:ZFIN;Acc:ZDB-GENE-040912-9]	5	24179307	24185865		3.81	4.54	3.73	3.84	3.3	3.77
ENSDARG00000035563	znf703	zinc finger protein 703 [Source:NCBI gene;Acc:114429]	5	24329983	24333684		11.11	9.72	8.46	20.37	20.18	21.3
ENSDARG00000035564	dgcr8	DGCR8 microprocessor complex subunit [Source:ZFIN;Acc:ZDB-GENE-030131-3421]	5	24520437	24532386		11.65	5.68	5.91	11.59	11.04	11.26
ENSDARG00000035565	atp6v0a2b	ATPase H+ transporting V0 subunit a2b [Source:ZFIN;Acc:ZDB-GENE-060526-4]	5	24543862	24567869		3.98	4.69	3.81	3.57	3.73	3.98
ENSDARG00000035569	cyp1d1	cytochrome P450, family 1, subfamily D, polypeptide 1 [Source:ZFIN;Acc:ZDB-GENE-041114-179]	5	25603761	25621386		3.22	4.02	3.45	4.6	4.35	4.28
ENSDARG00000035570	C5H9orf85	zgc:101016 [Source:ZFIN;Acc:ZDB-GENE-040822-14]	5	25726479	25733745	C5H9orf85	5.55	5.32	4.68	3.77	4.09	3.97
ENSDARG00000035571	abhd17b	abhydrolase domain containing 17B [Source:ZFIN;Acc:ZDB-GENE-040718-244]	5	25733774	25757578		3.13	2.37	2.38	3.51	3.51	3.45
ENSDARG00000035573	AK6	zgc:86811 [Source:ZFIN;Acc:ZDB-GENE-040625-113]	5	26199537	26203245	AK6	25.73	35.92	31.86	12.36	13.34	12.7
ENSDARG00000035577	cds2	CDP-diacylglycerol synthase (phosphatidate cytidylyltransferase) 2 [Source:ZFIN;Acc:ZDB-GENE-030717-4]	5	21144269	21172199		24.19	13.25	15.49	27.97	29.28	30.45
ENSDARG00000035578	hs3st1l2	heparan sulfate (glucosamine) 3-O-sulfotransferase 1-like 2 [Source:ZFIN;Acc:ZDB-GENE-070202-3]	5	21100356	21141090		3.98	2.13	2.8	3.69	3.53	3.33
ENSDARG00000035579	si:dkey-174n20.1	si:dkey-174n20.1 [Source:ZFIN;Acc:ZDB-GENE-030131-7890]	5	20922100	20928198		6.02	2.66	3.34	3.92	4.05	4.44
ENSDARG00000035595	ficd	FIC domain containing [Source:NCBI gene;Acc:334648]	5	20485624	20491272		4.06	2.98	2.79	4	4.89	4.38
ENSDARG00000035596	ISCU	si:ch211-191d15.2 [Source:ZFIN;Acc:ZDB-GENE-040724-113]	5	20443888	20446082	ISCU	39.65	43.09	40.18	24.33	25.58	26.07
ENSDARG00000035598	coro1ca	coronin, actin binding protein, 1Ca [Source:NCBI gene;Acc:317741]	5	20319519	20419863		17.33	18.37	14.35	20.84	20.05	20.47
ENSDARG00000035601	dao.3	D-amino-acid oxidase, tandem duplicate 3 [Source:ZFIN;Acc:ZDB-GENE-040426-1894]	5	20197138	20205075		0.4	0.43	0.63	0.42	0.57	0.43
ENSDARG00000035602	dao.1	D-amino-acid oxidase, tandem duplicate 1 [Source:ZFIN;Acc:ZDB-GENE-050913-127]	5	20188438	20195350		5.68	15.11	15.53	21	20.91	19.32
ENSDARG00000035603	dao.2	D-amino-acid oxidase, tandem duplicate 2 [Source:ZFIN;Acc:ZDB-GENE-040426-2634]	5	20170960	20185665		13.56	21.59	18.14	8.13	7.53	7.66
ENSDARG00000035605	tchp	trichoplein, keratin filament binding [Source:NCBI gene;Acc:678595]	5	19867794	19879252		8.67	6.41	7	6.61	6.84	7.17
ENSDARG00000035606	aldh3b2	aldehyde dehydrogenase 3 family, member B2 [Source:ZFIN;Acc:ZDB-GENE-060531-79]	5	19477555	19488134		0.38	0.09	0.07	1.11	1.23	1.06
ENSDARG00000035607	ankle2	ankyrin repeat and LEM domain containing 2 [Source:ZFIN;Acc:ZDB-GENE-030131-6090]	5	18943043	18962794		4.29	2.35	2.2	3.93	4.42	4.06
ENSDARG00000035608	pgam5	PGAM family member 5, serine/threonine protein phosphatase, mitochondrial [Source:NCBI gene;Acc:492357]	5	18925367	18936235		13.56	11.89	10.15	15.51	15.98	15.06
ENSDARG00000035609	mtnr1c	melatonin receptor 1C [Source:NCBI gene;Acc:30661]	5	22633188	22665855		0.26	0.1	0.1	0.51	0.57	0.59
ENSDARG00000035610	zgc:113208	zgc:113208 [Source:ZFIN;Acc:ZDB-GENE-050417-450]	5	22605530	22621417		9.95	9	10.64	8.12	8.46	9.74
ENSDARG00000035618	coq5	coenzyme Q5, methyltransferase [Source:NCBI gene;Acc:447802]	5	16128214	16139600		7.53	9.85	8.49	5.88	6.02	5.43
ENSDARG00000035622	xbp1	X-box binding protein 1 [Source:ZFIN;Acc:ZDB-GENE-011210-2]	5	15944411	15948920		86.38	101.03	85.62	85.58	89.34	90.06
ENSDARG00000035625	snrnp27	small nuclear ribonucleoprotein 27 (U4/U6.U5) [Source:NCBI gene;Acc:436976]	5	13767743	13778726		35.91	36.82	40.02	28.85	29.16	30.35
ENSDARG00000035629	parvab	parvin, alpha b [Source:ZFIN;Acc:ZDB-GENE-070424-60]	7	65673885	65706839		3.84	4.98	5.14	4	4.06	3.44
ENSDARG00000035630	ypel1	yippee-like 1 [Source:ZFIN;Acc:ZDB-GENE-040808-41]	5	13037106	13086616		13.98	6.63	7.16	16.63	16.87	17.23
ENSDARG00000035631	sdf2l1	stromal cell-derived factor 2-like 1 [Source:ZFIN;Acc:ZDB-GENE-040808-46]	5	13308531	13315726		14.82	14.14	13.69	19.8	17.72	16.54
ENSDARG00000035632	ccl19a.2	chemokine (C-C motif) ligand 19a, tandem duplicate 2 [Source:ZFIN;Acc:ZDB-GENE-081022-55]	5	13373593	13376424		0.04	0	0	0	0.21	0.2
ENSDARG00000035633	tctn2	tectonic family member 2 [Source:ZFIN;Acc:ZDB-GENE-030131-751]	5	13394543	13427349		1.76	0.9	1.08	1.82	1.8	1.7
ENSDARG00000035634	rfc5	replication factor C (activator 1) 5 [Source:ZFIN;Acc:ZDB-GENE-040907-1]	5	12528693	12549732		16.31	18.67	18.87	9.96	9.63	10.59
ENSDARG00000035646	pimr123	Pim proto-oncogene, serine/threonine kinase, related 123 [Source:ZFIN;Acc:ZDB-GENE-060531-12]	5	9134187	9135912		0	0	0	0	0	0
ENSDARG00000035648	irx4a	iroquois homeobox 4a [Source:ZFIN;Acc:ZDB-GENE-040426-1825]	16	378766	383664		4.29	0.74	0.73	4.66	5.17	5.31
ENSDARG00000035649	ext1c	exostosin-1c [Source:NCBI gene;Acc:108179064]	19	47311020	47336408		8.46	5.86	5.05	13.37	14.17	14.21
ENSDARG00000035650	evla	Enah/Vasp-like a [Source:ZFIN;Acc:ZDB-GENE-000607-65]	17	30897292	30975978		6.04	4.03	3.9	6.16	5.89	5.99
ENSDARG00000035652	sat1a.1	spermidine/spermine N1-acetyltransferase 1a, duplicate 1 [Source:NCBI gene;Acc:565700]	5	4332220	4342866		46.18	56.67	44.22	30.39	28.54	30.56
ENSDARG00000035655	rad21b	RAD21 cohesin complex component b [Source:ZFIN;Acc:ZDB-GENE-060503-223]	19	44068250	44091405		18.65	10.19	11.67	16.48	16.31	16.5
ENSDARG00000035657	BX649384.1		19	43517049	43542568		0.25	0.42	0.14	0	0	0.09
ENSDARG00000035660	si:ch211-193k19.1	si:ch211-193k19.1 [Source:ZFIN;Acc:ZDB-GENE-060503-110]	19	43669122	43707261		4.7	3.08	2.55	6.5	6.74	6.59
ENSDARG00000035663	zgc:103438	zgc:103438 [Source:ZFIN;Acc:ZDB-GENE-041010-131]	19	42567539	42573297		4.17	6.21	4.14	2.09	1.67	2.21
ENSDARG00000035665	si:dkey-267n13.1	si:dkey-267n13.1 [Source:ZFIN;Acc:ZDB-GENE-141211-35]	19	42553029	42557556		0.03	0.04	0.01	0.4	0.64	0.57
ENSDARG00000035676	ptp4a2b	protein tyrosine phosphatase 4A2b [Source:ZFIN;Acc:ZDB-GENE-041114-111]	19	35455172	35492784		11.99	9.95	9.5	15.2	15.2	14.83
ENSDARG00000035677	bmp8a	bone morphogenetic protein 8a [Source:ZFIN;Acc:ZDB-GENE-030912-13]	19	35019304	35035857		1.1	1.36	0.83	0.91	0.87	0.82
ENSDARG00000035678	zgc:91910	zgc:91910 [Source:ZFIN;Acc:ZDB-GENE-040808-48]	19	35013129	35017047		44.6	51.6	51.79	43.95	40.96	42.97
ENSDARG00000035679		si:rp71-45k5.4 [Source:ZFIN;Acc:ZDB-GENE-060503-941]	19	35001253	35010342		10.81	12.23	11.92	8.89	8.55	8.66
ENSDARG00000035680	zgc:113424	zgc:113424 [Source:ZFIN;Acc:ZDB-GENE-050227-9]	19	34998029	34999379		0	0	0	0.02	0	0
ENSDARG00000035682	FP102018.1	latent transforming growth factor beta binding protein 3 [Source:NCBI gene;Acc:562912]	5	37517800	37585469		5	4.44	3.95	7.63	7.35	7.52
ENSDARG00000035686	mon1ba	MON1 secretory trafficking family member Ba [Source:ZFIN;Acc:ZDB-GENE-081104-318]	8	9684479	9700640		0.43	0.57	0.57	0.24	0.24	0.33
ENSDARG00000035692	rps3a	ribosomal protein S3A [Source:NCBI gene;Acc:337240]	1	24355515	24359653		858.88	1145.75	1403.13	603.71	644.86	637.46
ENSDARG00000035694	stm	starmaker [Source:NCBI gene;Acc:386700]	19	3137518	3167878		77.08	54.42	69.85	60.69	65.02	67.59
ENSDARG00000035695	scxa	scleraxis bHLH transcription factor a [Source:ZFIN;Acc:ZDB-GENE-060503-414]	19	3001473	3009570		3.6	3.23	3.5	4.59	5.03	4.67
ENSDARG00000035697	spinb	spindlin b [Source:ZFIN;Acc:ZDB-GENE-050417-414]	8	622640	632241		2.84	2.99	3.42	2.75	2.78	2.19
ENSDARG00000035699	srp19	signal recognition particle 19 [Source:ZFIN;Acc:ZDB-GENE-040426-2657]	8	537809	544787		11.75	14.1	12.39	10.12	9.09	10.3
ENSDARG00000035700	zgc:101664	zgc:101664 [Source:ZFIN;Acc:ZDB-GENE-041010-187]	8	532228	537716		6.3	5.66	4.71	7.04	5.77	6.64
ENSDARG00000035706	dock6	dedicator of cytokinesis 6 [Source:ZFIN;Acc:ZDB-GENE-120411-11]	3	55218597	55328548		2.98	2.26	1.99	4.62	4.68	4.47
ENSDARG00000035715	marcksl1b	MARCKS-like 1b [Source:NCBI gene;Acc:406504]	19	29798064	29800812		569.09	279.35	277.52	586.4	583.46	617.81
ENSDARG00000035718	cbln6	cerebellin 6 [Source:ZFIN;Acc:ZDB-GENE-111110-3]	7	73447724	73449051		0.06	0.1	0.05	0.02	0.02	0
ENSDARG00000035719	arl5c	ADP-ribosylation factor-like 5C [Source:ZFIN;Acc:ZDB-GENE-040426-1866]	3	16134376	16142057		5.04	6.06	6.55	7.32	8.15	7.01
ENSDARG00000035732	arntl1b	aryl hydrocarbon receptor nuclear translocator-like 1b [Source:ZFIN;Acc:ZDB-GENE-030408-1]	7	66048102	66105828		3.18	2.46	2	5.94	6.08	6.08
ENSDARG00000035735	gsx1	GS homeobox 1 [Source:ZFIN;Acc:ZDB-GENE-041008-135]	5	67909644	67911111		5.23	1.47	2.32	4.07	3.8	4.5
ENSDARG00000035741	slc25a11	solute carrier family 25 member 11 [Source:ZFIN;Acc:ZDB-GENE-040625-79]	5	68076913	68093169		65.67	74.31	72.63	60.76	57.87	57.42
ENSDARG00000035751	ipo7	importin 7 [Source:ZFIN;Acc:ZDB-GENE-030131-458]	7	65121743	65174007		55.65	47.61	37.45	47.86	48.97	49.59
ENSDARG00000035763	stx18	syntaxin 18 [Source:NCBI gene;Acc:554130]	14	20351	25513		11.18	12.2	11.96	10.42	10.54	12.18
ENSDARG00000035765	CU633984.1		15	6678521	6727082		5.73	1.13	1.23	5.54	6.41	5.57
ENSDARG00000035771		si:ch1073-157b13.1 [Source:ZFIN;Acc:ZDB-GENE-110411-13]	23	44236281	44251006		3.62	2.86	2.83	3.66	3.45	3.68
ENSDARG00000035781	ctdnep1b	CTD nuclear envelope phosphatase 1b [Source:NCBI gene;Acc:492799]	7	69449814	69467790		4.63	4.07	3.68	4.33	4.69	4.48
ENSDARG00000035796	cdh31	cadherin 31 [Source:ZFIN;Acc:ZDB-GENE-110407-4]	7	55149001	55198275		0.06	0.02	0.02	0.07	0.07	0.03
ENSDARG00000035797	si:dkey-17m8.2	si:dkey-17m8.2 [Source:ZFIN;Acc:ZDB-GENE-110411-234]	16	18142127	18153001		0	0	0	0.67	0.41	0.31
ENSDARG00000035798	gngt1	guanine nucleotide binding protein (G protein), gamma transducing activity polypeptide 1 [Source:ZFIN;Acc:ZDB-GENE-030131-75[illegible]	19	40856534	40859468		109.31	50.34	40.58	39.74	42.5	49.33
ENSDARG00000035808	clcn4	chloride channel, voltage-sensitive 4 [Source:ZFIN;Acc:ZDB-GENE-061013-353]	11	44144612	44163164		10.27	3.96	4.84	11.99	12.54	12.1
ENSDARG00000035809	col1a1b	collagen, type I, alpha 1b [Source:ZFIN;Acc:ZDB-GENE-030131-4400]	12	3095699	3133483		286.65	330.6	209.01	439.29	413.54	396.43
ENSDARG00000035810	rgcc	regulator of cell cycle [Source:ZFIN;Acc:ZDB-GENE-040704-31]	9	17438765	17449872		34.35	42.29	42.58	41.06	40.71	42.41
ENSDARG00000035819	sirt3	sirtuin 3 [Source:ZFIN;Acc:ZDB-GENE-070112-1762]	7	57465814	57509768		1.99	1.6	1.55	1.8	1.81	2.09
ENSDARG00000035820	drd4b	dopamine receptor D4b [Source:ZFIN;Acc:ZDB-GENE-070508-3]	7	57432318	57470497		1.37	0.64	0.78	1.06	1.22	1.11
ENSDARG00000035821	si:ch211-173p18.3	si:ch211-173p18.3 [Source:ZFIN;Acc:ZDB-GENE-060503-672]	19	40115977	40165799		12.72	7.8	8.18	12.33	12.86	13.34
ENSDARG00000035823	zmym4	zinc finger, MYM-type 4 [Source:ZFIN;Acc:ZDB-GENE-030131-5459]	19	40069524	40113935		6.93	4.11	4.07	5.75	5.65	6.33
ENSDARG00000035832	pyyb	peptide YYb [Source:ZFIN;Acc:ZDB-GENE-060215-2]	12	10304435	10315039		3.8	3.83	2.97	9	10.11	9.81
ENSDARG00000035835	eef2k	eukaryotic elongation factor 2 kinase [Source:ZFIN;Acc:ZDB-GENE-020916-2]	12	10381957	10409970		32.23	28.51	27.3	90.96	100.81	101.11
ENSDARG00000035837	mosmob	modulator of smoothened b [Source:NCBI gene;Acc:767723]	12	10412009	10421955		3.14	2.15	2.64	3.61	3.67	4.08
ENSDARG00000035852	cart3	cocaine- and amphetamine-regulated transcript 3 [Source:ZFIN;Acc:ZDB-GENE-120223-5]	7	52962632	52963493		3.97	2.94	2.54	20.16	23.62	26.22
ENSDARG00000035853	cdkn2aip	CDKN2A interacting protein [Source:ZFIN;Acc:ZDB-GENE-041010-188]	7	52949847	52960714		2.33	2.32	2.38	2.04	2.23	2.49
ENSDARG00000035858	cnn2	calponin 2 [Source:ZFIN;Acc:ZDB-GENE-030131-542]	2	24548249	24554432		30.48	40.18	34.3	57.17	57.2	60.87
ENSDARG00000035859	angptl4	angiopoietin-like 4 [Source:ZFIN;Acc:ZDB-GENE-041111-222]	2	24536762	24542518		23.6	26.78	27.5	60.6	60.8	59.24
ENSDARG00000035860	rps28	ribosomal protein S28 [Source:NCBI gene;Acc:406307]	2	24507597	24509698		352.66	466.06	478.37	310.72	333.19	343.02

ENSDARG00000035861	si:rp71-39b20.4	si:rp71-39b20.4 [Source:ZFIN;Acc:ZDB-GENE-070912-704]	2	24495804	24505185		2.98	0.73	0.47	2.57	2.63	2.96
ENSDARG00000035862	insl3	insulin-like 3 (Leydig cell) [Source:ZFIN;Acc:ZDB-GENE-050310-5]	2	24487320	24488652		0.2	0	0.11	0.1	0	0.26
ENSDARG00000035868	spire1a	spire-type actin nucleation factor 1a [Source:NCBI gene;Acc:557962]	19	32979132	33072157		6.97	2.89	3.08	7.36	7.38	7.64
ENSDARG00000035869	azin1b	antizyme inhibitor 1b [Source:ZFIN;Acc:ZDB-GENE-030121-1]	19	32930476	32944050		140.38	125.05	125.57	167.12	179.56	176.8
ENSDARG00000035870	laptm4b	lysosomal protein transmembrane 4 beta [Source:ZFIN;Acc:ZDB-GENE-030616-616]	19	32864577	32888758		30.12	9.73	8.45	27.32	29.69	28.52
ENSDARG00000035871	rpl30	ribosomal protein L30 [Source:ZFIN;Acc:ZDB-GENE-030131-8657]	19	32855139	32860780		1148.5	1439.6	1858.41	879.39	921.95	902.17
ENSDARG00000035872	hsd17b12b	hydroxysteroid (17-beta) dehydrogenase 12b [Source:NCBI gene;Acc:322626]	7	50228015	50272912		15.78	20.43	16.28	11.16	11.58	10.65
ENSDARG00000035873	fjx1	four-jointed box kinase 1 [Source:ZFIN;Acc:ZDB-GENE-100422-10]	7	49797528	49801183		3.83	2.65	3.59	3.8	4.26	3.79
ENSDARG00000035875	tmem54b	transmembrane protein 54b [Source:ZFIN;Acc:ZDB-GENE-060503-898]	19	32603627	32619257		0.65	0.51	0.46	0.47	0.59	0.53
ENSDARG00000035879	fuca1.2	alpha-L-fucosidase 1, tandem duplicate 2 [Source:ZFIN;Acc:ZDB-GENE-040801-78]	19	32488572	32493866		5.94	5.91	6.39	8.37	7.29	7.44
ENSDARG00000035880	atp6v1c1b	ATPase H+ transporting V1 subunit C1b [Source:NCBI gene;Acc:449655]	19	32947910	32966762		26.38	27.23	26.42	20.61	20.4	21.88
ENSDARG00000035882	rida	reactive intermediate imine deaminase A homolog [Source:ZFIN;Acc:ZDB-GENE-040718-315]	19	32822269	32884513		25.54	40.86	37.38	25.51	25.09	25.18
ENSDARG00000035883	nt5c1aa	5'-nucleotidase, cytosolic IAa [Source:ZFIN;Acc:ZDB-GENE-070705-423]	19	32766423	32804535		1.94	1.39	1.21	1.64	1.55	1.75
ENSDARG00000035887	zgc:91944	zgc:91944 [Source:ZFIN;Acc:ZDB-GENE-040718-417]	19	32571727	32600868		5.13	4.12	3.28	3.85	3.84	3.96
ENSDARG00000035889	zbtb8b	zinc finger and BTB domain containing 8B [Source:ZFIN;Acc:ZDB-GENE-050417-42]	19	32505747	32518556		10.48	7.4	7.31	10.29	9.84	10.66
ENSDARG00000035890	fuca1.1	alpha-L-fucosidase 1, tandem duplicate 1 [Source:ZFIN;Acc:ZDB-GENE-030131-7434]	19	32495419	32500373		36.4	36.91	34.41	32.5	30.96	31.49
ENSDARG00000035891	acana	aggrecan a [Source:ZFIN;Acc:ZDB-GENE-050208-221]	7	48761646	48787379		3.96	2.97	1.77	38.2	41.58	39.55
ENSDARG00000035895	fndc5b	fibronectin type III domain containing 5b [Source:NCBI gene;Acc:555635]	19	29383486	29437109		5.23	1.89	3.35	6.54	6.92	6.79
ENSDARG00000035899	lingo1b	leucine rich repeat and Ig domain containing 1b [Source:NCBI gene;Acc:447837]	7	48460239	48484917		6.69	2.12	2.87	5.93	7.22	6.65
ENSDARG00000035904	crabp1b	cellular retinoic acid binding protein 1b [Source:ZFIN;Acc:ZDB-GENE-040624-3]	7	48319916	48342750		12.73	15.19	17.15	13.52	13.93	14.88
ENSDARG00000035905	slc25a44b	solute carrier family 25 member 44b [Source:ZFIN;Acc:ZDB-GENE-050320-89]	7	48297842	48304787		4.07	3.39	3.47	4.68	4.12	4.13
ENSDARG00000035907	fam49al	family with sequence similarity 49 member A, like [Source:ZFIN;Acc:ZDB-GENE-040801-125]	19	30633453	30684874		24.99	7.25	10.77	26.27	27.88	28.34
ENSDARG00000035909	mfsd2ab	major facilitator superfamily domain containing 2ab [Source:NCBI gene;Acc:445176]	19	30867845	30884054		10.14	7.11	7.55	11.89	10.48	11.43
ENSDARG00000035910	znf281b	zinc finger protein 281b [Source:ZFIN;Acc:ZDB-GENE-050220-1]	12	16087077	16102422		6.5	2.87	3.41	7.8	7.92	8.13
ENSDARG00000035913	yars1	tyrosyl-tRNA synthetase 1 [Source:NCBI gene;Acc:368235]	19	30884706	30895273		18.49	24.56	22.88	21.59	21.69	21.13
ENSDARG00000035914	tmem167a	transmembrane protein 167A [Source:NCBI gene;Acc:541340]	10	43110788	43117644		23.37	26.7	30.26	20.15	18.91	19.95
ENSDARG00000035918	bzw2	basic leucine zipper and W2 domains 2 [Source:NCBI gene;Acc:393892]	19	31007901	31042709		16.3	16.47	15.98	16.24	15.95	16.02
ENSDARG00000035924	dync1li2	dynein, cytoplasmic 1, light intermediate chain 2 [Source:ZFIN;Acc:ZDB-GENE-050417-156]	7	44674956	44704910		15.3	12.47	12.58	16.99	16.14	16.38
ENSDARG00000035942	hrh3	histamine receptor H3 [Source:ZFIN;Acc:ZDB-GENE-040724-204]	7	41748693	41753237		1.42	0.59	0.75	2.02	1.97	1.59
ENSDARG00000035944	sync	syncoilin, intermediate filament protein [Source:ZFIN;Acc:ZDB-GENE-060503-747]	19	30990129	30997794		0.69	0.81	0.79	1.52	1.57	1.61
ENSDARG00000035949	tmem106bb	transmembrane protein 106Bb [Source:NCBI gene;Acc:566699]	19	31392350	31402429		25.72	17.12	18.22	25.96	27.26	27.53
ENSDARG00000035952	cdr2a	cerebellar degeneration-related protein 2a [Source:ZFIN;Acc:ZDB-GENE-101203-5]	3	58430678	58455289		5.04	1.59	1.77	4.69	4.21	4.02
ENSDARG00000035954	tdp2b	tyrosyl-DNA phosphodiesterase 2b [Source:NCBI gene;Acc:553516]	19	31568992	31576321		2.82	2.66	2.72	2.12	1.93	2.08
ENSDARG00000035957	gmnn	geminin DNA replication inhibitor [Source:ZFIN;Acc:ZDB-GENE-030429-30]	19	31541607	31592425		3.81	3.81	3.13	2.53	2.03	2.11
ENSDARG00000035958	tnni2b.1	troponin I type 2b (skeletal, fast), tandem duplicate 1 [Source:ZFIN;Acc:ZDB-GENE-050417-49]	7	39399747	39406763		67.79	92.5	81.04	60.22	61.09	62.34
ENSDARG00000035961	kcng2	potassium voltage-gated channel, subfamily G, member 2 [Source:ZFIN;Acc:ZDB-GENE-100701-1]	19	11508960	11546516		1.85	1.25	1.71	1.46	1.95	1.93
ENSDARG00000035963	ccdc96	coiled-coil domain containing 96 [Source:ZFIN;Acc:ZDB-GENE-081022-156]	7	39730429	39738460		0.92	0.77	0.65	0.79	0.91	0.91
ENSDARG00000035966	pgr	progesterone receptor [Source:ZFIN;Acc:ZDB-GENE-990415-214]	18	41820039	41838419		0.26	0.14	0.16	0.71	0.77	0.8
ENSDARG00000035970	ptprn2	protein tyrosine phosphatase receptor type N2 [Source:ZFIN;Acc:ZDB-GENE-030131-3758]	7	40228422	40499386		20.51	4.99	7.22	18.73	20.43	21.15
ENSDARG00000035978	ube3c	ubiquitin protein ligase E3C [Source:ZFIN;Acc:ZDB-GENE-040426-1211]	7	40583939	40630698		7.03	5.45	5.29	7.04	6.87	6.95
ENSDARG00000035984	mnx1	motor neuron and pancreas homeobox 1 [Source:ZFIN;Acc:ZDB-GENE-040409-1]	7	40638210	40641006		1.11	0.91	0.97	2.21	1.99	2.63
ENSDARG00000035985	fam210aa	family with sequence similarity 210 member Aa [Source:NCBI gene;Acc:562734]	19	12630414	12648408		8.2	7.86	7.09	9.01	8.41	7.92
ENSDARG00000035986	ptpn2b	protein tyrosine phosphatase non-receptor type 2b [Source:ZFIN;Acc:ZDB-GENE-030909-8]	19	12362787	12392819		15.38	13.81	11.46	12.73	13.09	13.73
ENSDARG00000035987	psmg2	proteasome (prosome, macropain) assembly chaperone 2 [Source:NCBI gene;Acc:573107]	19	12350069	12360121		12.07	15.22	15.17	10.1	8.05	9.55
ENSDARG00000035990	cited4a	Cbp/p300-interacting transactivator, with Glu/Asp-rich carboxy-terminal domain, 4a [Source:ZFIN;Acc:ZDB-GENE-030131-7661]	19	15851226	15855427		28.22	23.17	19.52	32.69	33.02	32.88
ENSDARG00000035993	sumo3b	small ubiquitin like modifier 3b [Source:NCBI gene;Acc:436950]	9	12648705	12652984		40.36	36.61	35.69	33.92	31.1	28.32
ENSDARG00000036005	pitpnb	phosphatidylinositol transfer protein, beta [Source:ZFIN;Acc:ZDB-GENE-040426-895]	10	4046448	4085216		12.84	13.11	11.15	14.01	15.01	13.23
ENSDARG00000036008	caly	calcyon neuron-specific vesicular protein [Source:ZFIN;Acc:ZDB-GENE-090313-405]	1	49266886	49295059		14.99	5.32	6.05	18.56	18.78	18.38
ENSDARG00000036028	arrdc3b	arrestin domain containing 3b [Source:ZFIN;Acc:ZDB-GENE-040912-182]	10	43761480	43771447		20.18	11.37	10.86	82.04	94.74	91.46
ENSDARG00000036031	sept5b	septin 5b [Source:ZFIN;Acc:ZDB-GENE-040808-43]	10	43936252	43964028		22.41	3.31	5.54	24.88	22.78	25.61
ENSDARG00000036036	mdka	midkine a [Source:ZFIN;Acc:ZDB-GENE-990621-1]	7	39166460	39178198		136.3	130.97	135.4	169.9	172.71	164.32
ENSDARG00000036038	harbi1	harbinger transposase derived 1 [Source:NCBI gene;Acc:445279]	7	38808027	38810706		5.59	5.82	5.93	4.64	5.02	5.97
ENSDARG00000036040	atg13	ATG13 autophagy related 13 homolog (S. cerevisiae) [Source:NCBI gene;Acc:393405]	7	38793832	38807827		23.77	16.88	18.65	26.09	26.08	28.5
ENSDARG00000036041	f2	coagulation factor II (thrombin) [Source:ZFIN;Acc:ZDB-GENE-030131-4606]	7	38750871	38760158		65.08	105.27	89.25	60.16	66.15	65.03
ENSDARG00000036043	znf408	zinc finger protein 408 [Source:ZFIN;Acc:ZDB-GENE-130530-589]	7	38744924	38750197		1.29	0.89	1.03	1.22	0.78	0.87
ENSDARG00000036044	rps20	ribosomal protein S20 [Source:ZFIN;Acc:ZDB-GENE-040426-2284]	7	58745987	58750400		2276.27	3071.36	3618.66	1691.05	1806.74	1806.57
ENSDARG00000036045	penkb	proenkephalin b [Source:ZFIN;Acc:ZDB-GENE-030729-3]	7	58686860	58696086		5.14	4.9	3.8	7.05	8.36	7.94
ENSDARG00000036051	irx4b	iroquois homeobox 4b [Source:ZFIN;Acc:ZDB-GENE-040712-4]	19	28114503	28262897		0.95	0.3	0.24	1.12	1.17	1.11
ENSDARG00000036055	thap11	THAP domain containing 11 [Source:NCBI gene;Acc:406390]	7	29041465	29043075		7.51	5.23	6.28	6.58	5.92	6.37
ENSDARG00000036056	vrk3	VRK serine/threonine kinase 3 [Source:ZFIN;Acc:ZDB-GENE-030131-246]	7	29065915	29080556		5.21	5.66	5.04	4.43	3.85	4.09
ENSDARG00000036057	tradd	tnfrsf1a-associated via death domain [Source:NCBI gene;Acc:58130]	7	29115890	29124578		7.01	8.57	8.48	8.03	7.27	7.54
ENSDARG00000036058	gnao1b	guanine nucleotide binding protein (G protein), alpha activating activity polypeptide O, b [Source:ZFIN;Acc:ZDB-GENE-040426-169	7	29133321	29144547		51.29	11.21	13.28	52.56	49.53	51.79
ENSDARG00000036059	tmppe	transmembrane protein with metallophosphoesterase domain [Source:NCBI gene;Acc:569483]	7	29153018	29161923		3.07	1.75	1.63	3.99	3.93	4.06
ENSDARG00000036060	exosc6	exosome component 6 [Source:NCBI gene;Acc:326786]	7	29163592	29164818		10.97	11.04	12.45	8.71	8.95	8.4
ENSDARG00000036061	ogfod1	2-oxoglutarate and iron-dependent oxygenase domain containing 1 [Source:ZFIN;Acc:ZDB-GENE-030131-3294]	7	29096194	29110094		8.98	10.19	9.78	6.11	5.7	6
ENSDARG00000036063	ppp1r11	protein phosphatase 1, regulatory (inhibitor) subunit 11 [Source:NCBI gene;Acc:550290]	19	27448625	27453644		20.25	16.14	15.93	16.13	17.05	16.52
ENSDARG00000036064	cnep1r1	CTD nuclear envelope phosphatase 1 regulatory subunit 1 [Source:NCBI gene;Acc:550572]	7	38258749	38264380		15.1	15.26	14.42	13.07	12.83	12.26
ENSDARG00000036065	mgat1b	alpha-1,3-mannosyl-glycoprotein 2-beta-N-acetylglucosaminyltransferase b [Source:ZFIN;Acc:ZDB-GENE-061103-589]	19	27366932	27395531		3.79	3.56	3.11	4.59	4.77	4.62
ENSDARG00000036066	si:dkey-78a14.4	si:dkey-78a14.4 [Source:ZFIN;Acc:ZDB-GENE-110408-8]	7	38249858	38257848		0.51	0.71	0.71	0.86	0.83	0.77
ENSDARG00000036069	ddx39b	DEAD (Asp-Glu-Ala-Asp) box polypeptide 39B [Source:ZFIN;Acc:ZDB-GENE-040426-1259]	19	27339848	27358545		8.79	4.65	5.29	8.96	8.81	8.61
ENSDARG00000036070	znrd1	zinc ribbon domain containing 1 [Source:ZFIN;Acc:ZDB-GENE-050320-131]	19	27334527	27339872		1.08	1.15	0.93	0.67	1.17	0.38
ENSDARG00000036071	gtf2h4	general transcription factor IIH, polypeptide 4 [Source:ZFIN;Acc:ZDB-GENE-030131-6779]	19	27316467	27334394		0.04	0.02	0.01	0.04	0.04	0.02
ENSDARG00000036073	cebpg	CCAAT enhancer binding protein gamma [Source:ZFIN;Acc:ZDB-GENE-020111-5]	7	38089650	38095324		22.67	20.34	20.73	30.16	30.32	31.64
ENSDARG00000036074	cebpa	CCAAT enhancer binding protein alpha [Source:ZFIN;Acc:ZDB-GENE-020111-2]	7	38084986	38087865		16.21	17.84	13.99	19.95	20.63	21.22
ENSDARG00000036076	heatr3	HEAT repeat containing 3 [Source:ZFIN;Acc:ZDB-GENE-040426-1876]	7	37898618	37919475		9.3	10.14	10.57	6.89	7.3	7.34
ENSDARG00000036080	cd81a	CD81 molecule a [Source:ZFIN;Acc:ZDB-GENE-000831-5]	7	32983440	33023404		141.31	152.16	149.98	205.16	208.26	204.54
ENSDARG00000036082	tph1b	tryptophan hydroxylase 1b [Source:ZFIN;Acc:ZDB-GENE-030805-6]	7	33106332	33114884		1.19	1.29	1.24	2.25	2.15	2.44
ENSDARG00000036084	itln2	intelectin 2 [Source:ZFIN;Acc:ZDB-GENE-050411-58]	7	33145529	33148307		1.15	1.18	1.17	0.11	0.04	0.11
ENSDARG00000036086	itga11a	integrin, alpha 11a [Source:ZFIN;Acc:ZDB-GENE-050324-1]	7	33176517	33248773		1.62	1.77	1.67	7.05	6.85	7.33
ENSDARG00000036090	pard6gb	par-6 family cell polarity regulator gamma b [Source:ZFIN;Acc:ZDB-GENE-010319-35]	19	25699329	25772980		4.02	3.37	2.72	6.7	6.05	5.57
ENSDARG00000036092	sdhaf3	succinate dehydrogenase complex assembly factor 3 [Source:NCBI gene;Acc:449654]	19	25526354	25548630		9.95	11.91	10.56	7	5.89	6.48
ENSDARG00000036094	pias1a	protein inhibitor of activated STAT, 1a [Source:ZFIN;Acc:ZDB-GENE-120727-5]	7	33882597	33949246		0.86	0.46	0.43	1.3	1.54	1.46
ENSDARG00000036096	smad3a	SMAD family member 3a [Source:ZFIN;Acc:ZDB-GENE-000509-3]	7	34102661	34149263		6.27	3.58	2.97	6.87	6.32	6.43
ENSDARG00000036100	eya3	EYA transcriptional coactivator and phosphatase 3 [Source:ZFIN;Acc:ZDB-GENE-060503-864]	19	24891747	24917208		4.47	2.58	2.41	5.44	5.28	4.86
ENSDARG00000036102	ctdsp2	CTD (carboxy-terminal domain, RNA polymerase II, polypeptide A) small phosphatase 2 [Source:ZFIN;Acc:ZDB-GENE-030131-184]	23	24669035	24682244		59.21	47.34	50.17	62.24	63.9	65.66
ENSDARG00000036104	myo1g	myosin IG [Source:ZFIN;Acc:ZDB-GENE-060503-562]	19	24640467	24745894		0.19	0.17	0.19	0.36	0.3	0.4
ENSDARG00000036105	si:dkeyp-92c9.2	si:dkeyp-92c9.2 [Source:ZFIN;Acc:ZDB-GENE-060503-468]	19	24596324	24597277		2.13	0.99	0.9	3.28	2.84	2.66
ENSDARG00000036106	rgs18	regulator of G protein signaling 18 [Source:ZFIN;Acc:ZDB-GENE-061013-722]	2	6535865	6548838		0.91	1.2	1.21	0.93	1.05	0.9
ENSDARG00000036107	txnipa	thioredoxin interacting protein a [Source:ZFIN;Acc:ZDB-GENE-030804-10]	19	24488403	24492326		86.96	81.46	81.71	258.55	290.03	277.25
ENSDARG00000036109	mtx1b	metaxin 1b [Source:ZFIN;Acc:ZDB-GENE-021210-1]	19	24448584	24484758		4.74	5.37	5.57	5.53	7.34	6.44
ENSDARG00000036116	eif3hb	eukaryotic translation initiation factor 3, subunit H, b [Source:NCBI gene;Acc:567151]	19	45856171	45960227		5.59	4.55	5.26	6.85	6.44	7.13
ENSDARG00000036117	mchr2	melanin-concentrating hormone receptor 2 [Source:ZFIN;Acc:ZDB-GENE-030502-6]	24	34806822	34823043		0.53	0.51	0.52	0.63	1.08	0.92
ENSDARG00000036125	nitr13	novel immune-type receptor 13 [Source:ZFIN;Acc:ZDB-GENE-070108-2]	14	41576849	41581315		0.01	0	0.06	0.03	0	0
ENSDARG00000036135	bbox1	butyrobetaine (gamma), 2-oxoglutarate dioxygenase (gamma-butyrobetaine hydroxylase) 1 [Source:ZFIN;Acc:ZDB-GENE-050417-2	7	34296789	34315291		3.84	5.36	4.08	6.42	7.12	6.51
ENSDARG00000036136	tipin	timeless interacting protein [Source:NCBI gene;Acc:445058]	7	34236238	34245115		1.18	1.56	1.33	0.87	0.78	0.84
ENSDARG00000036139	lctla	lactase-like a [Source:ZFIN;Acc:ZDB-GENE-040718-233]	7	34227075	34236091		0.15	0.56	0.95	5.36	4.42	5.68
ENSDARG00000036140	crybgx	crystallin beta gamma X [Source:ZFIN;Acc:ZDB-GENE-070410-2]	7	34222433	34225011		4.58	15.3	26.07	36.91	33.57	42.79
ENSDARG00000036144	tssc4	tumor suppressing subtransferable candidate 4 [Source:NCBI gene;Acc:559769]	7	32897723	32899843		16.24	14.79	13.96	12.32	11.07	12.15
ENSDARG00000036147	ano5b	anoctamin 5b [Source:ZFIN;Acc:ZDB-GENE-061215-108]	7	32834142	32895668		7.6	7.17	7.11	7.16	7.38	7.39
ENSDARG00000036152	gas2b	growth arrest-specific 2b [Source:ZFIN;Acc:ZDB-GENE-030616-552]	7	32746914	32782430		0.41	0.26	0.23	1.18	1.11	1.1
ENSDARG00000036155	gla	galactosidase, alpha [Source:ZFIN;Acc:ZDB-GENE-041010-207]	14	38932441	38946808		0.68	0.53	0.7	2.06	2.02	2.12
ENSDARG00000036156	fnbp1a	formin binding protein 1a [Source:ZFIN;Acc:ZDB-GENE-081105-105]	8	11578318	11640240		6.56	1.63	2.37	7.71	8.05	9.58
ENSDARG00000036158	nudcd1	NudC domain containing 1 [Source:NCBI gene;Acc:553654]	19	22759116	22843480		7.27	7.99	7.89	6.46	5.97	6.14
ENSDARG00000036159	trhrb	thyrotropin-releasing hormone receptor b [Source:ZFIN;Acc:ZDB-GENE-060503-382]	19	22727940	22731812		0.47	0.12	0.2	0.53	0.63	0.52
ENSDARG00000036161	hnrnpa0l	heterogeneous nuclear ribonucleoprotein A0, like [Source:ZFIN;Acc:ZDB-GENE-030131-618]	14	38849432	38850933		257.73	227.99	217.34	272.69	264.48	274.18
ENSDARG00000036162	hnrnpa0b	heterogeneous nuclear ribonucleoprotein A0b [Source:ZFIN;Acc:ZDB-GENE-030131-6154]	14	38845674	38848166		177.33	127.92	116.96	194.71	179.58	183.45
ENSDARG00000036164	cars1	cysteinyl-tRNA synthetase 1 [Source:ZFIN;Acc:ZDB-GENE-030328-29]	7	31796237	31831044		15.67	13.08	12.81	15.1	15.4	15.33
ENSDARG00000036168	nfatc1	nuclear factor of activated T cells 1 [Source:ZFIN;Acc:ZDB-GENE-060503-677]	19	22216778	22304534		1.63	1.73	1.19	3.32	3.65	3.32
ENSDARG00000036171	rnasel3	ribonuclease like 3 [Source:NCBI gene;Acc:798787]	14	38654300	38656463		8.99	15.25	13.83	23.26	22.77	23.04
ENSDARG00000036175	pcdh1b	protocadherin 1b [Source:ZFIN;Acc:ZDB-GENE-091015-2]	14	37545341	37831941		10.66	5.09	4	12.46	12.31	12.81
ENSDARG00000036179	myo1ea	myosin IEa [Source:ZFIN;Acc:ZDB-GENE-030131-5127]	7	30632229	30722952		5.14	4.64	3.76	5.81	5.76	6
ENSDARG00000036180	ccnb2	cyclin B2 [Source:ZFIN;Acc:ZDB-GENE-030429-12]	7	30626378	30631119		2.1	2.11	2.18	1.49	1.21	1.41
ENSDARG00000036186	mbpa	myelin basic protein a [Source:ZFIN;Acc:ZDB-GENE-030128-2]	19	21814047	21832630		29.51	33.17	27.1	14.47	12.83	14.69
ENSDARG00000036189	spata4	spermatogenesis associated 4 [Source:ZFIN;Acc:ZDB-GENE-050417-153]	14	36393854	36397876		1.35	2.23	1.69	1.97	2.03	2.39
ENSDARG00000036190	txnl4a	thioredoxin-like 4A [Source:ZFIN;Acc:ZDB-GENE-041010-25]	19	20793237	20796910		22.96	25.6	23.19	16.73	16.34	16.88
ENSDARG00000036194	pitx2	paired-like homeodomain 2 [Source:NCBI gene;Acc:30164]	14	36218072	36231211		6.45	6.73	6.52	6.85	6.61	7.34
ENSDARG00000036212	tbc1d5	TBC1 domain family, member 5 [Source:ZFIN;Acc:ZDB-GENE-040426-1197]	19	20404153	20446756		8.61	5.89	5.85	8.83	8.33	8.93
ENSDARG00000036214	dazl	deleted in azoospermia-like [Source:NCBI gene;Acc:58039]	19	20389104	20403871		0.07	0.09	0.12	0.12	0.26	0.13
ENSDARG00000036215	lcp2b	lymphocyte cytosolic protein 2b [Source:ZFIN;Acc:ZDB-GENE-081107-21]	14	34501245	34510631		0.22	0.17	0.12	0.16	0.12	0.05
ENSDARG00000036222	npy	neuropeptide Y [Source:NCBI gene;Acc:30281]	19	20110699	20114149		1.64	1.25	1.62	1.35	1.28	1.47
ENSDARG00000036227	npvf	neuropeptide VF precursor [Source:ZFIN;Acc:ZDB-GENE-070424-226]	19	19976990	19980132		2.19	1.4	1.66	0.9	0.55	1.08
ENSDARG00000036232	trpm7	transient receptor potential cation channel, subfamily M, member 7 [Source:ZFIN;Acc:ZDB-GENE-021115-2]	18	113928	158541		5.75	4.08	3.86	7.34	7.05	6.49
ENSDARG00000036235	zbtb3	zinc finger and BTB domain containing 3 [Source:ZFIN;Acc:ZDB-GENE-050506-105]	14	35405518	35408771		4.07	2.14	2.35	3.4	3.75	3.65
ENSDARG00000036237	slc27a2a	solute carrier family 27 member 2a [Source:ZFIN;Acc:ZDB-GENE-050706-104]	18	62932	73844		81.86	114.72	110.69	54.33	51.93	60.08
ENSDARG00000036239	gatm	glycine amidinotransferase (L-arginine:glycine amidinotransferase) [Source:NCBI gene;Acc:266799]	18	35725	46010		440	720.18	761.86	326.31	359.89	343.09
ENSDARG00000036245	wipf3	WAS/WASL interacting protein family member 3 [Source:ZFIN;Acc:ZDB-GENE-030804-15]	19	19437648	19446529		1.24	1.4	1.5	1.94	1.96	1.74
ENSDARG00000036247	tmem144b	transmembrane protein 144b [Source:ZFIN;Acc:ZDB-GENE-041010-58]	14	35884853	35892767		0.06	0	0.03	0.03	0.02	0.05
ENSDARG00000036252	rras2	RAS related 2 [Source:ZFIN;Acc:ZDB-GENE-050417-352]	7	27834130	27887883		3.37	3.21	2.91	5.24	4.87	5.22
ENSDARG00000036254	hoxa13b	homeobox A13b [Source:NCBI gene;Acc:30438]	16	20895904	20897836		3.44	3.18	3.4	2.64	3.31	3.41
ENSDARG00000036257	rasal2	RAS protein activator like 2 [Source:ZFIN;Acc:ZDB-GENE-070912-180]	2	35732652	35851661		5.79	3.6	3.08	7.4	7.57	7.63
ENSDARG00000036272	gcgrb	glucagon receptor b [Source:ZFIN;Acc:ZDB-GENE-050516-2]	1	56344651	56363108		2.17	2.21	2.23	2.96	3.17	3.25
ENSDARG00000036279	lamc1	laminin, gamma 1 [Source:NCBI gene;Acc:286832]	2	35880600	35979178		34.49	32.82	21.05	52.3	47.16	45.6
ENSDARG00000036281	CC2D1A	coiled-coil and C2 domain containing 1A [Source:NCBI gene;Acc:100001297]	1	55215787	55238610	CC2D1A	8.65	4.99	5.14	9.08	9.95	10.19
ENSDARG00000036282	rnaset2	ribonuclease T2 [Source:NCBI gene;Acc:791890]	13	7049210	7065999		3.47	3.7	2.97	5.32	5.21	4.87
ENSDARG00000036291	nucb2b	nucleobindin 2b [Source:ZFIN;Acc:ZDB-GENE-030131-4775]	18	38191346	38210092		7	11.76	8.32	6.55	5.87	6.15
ENSDARG00000036292	cdx4	caudal type homeobox 4 [Source:ZFIN;Acc:ZDB-GENE-980526-330]	14	32961718	32965169		0.59	0.31	0.34	0.66	0.91	0.71
ENSDARG00000036293	gnb1l	guanine nucleotide binding protein (G protein), beta polypeptide 1-like [Source:ZFIN;Acc:ZDB-GENE-050320-77]	5	15222011	15283509		1.06	0.69	0.89	0.91	1.09	0.99
ENSDARG00000036295	golga7ba	golgin A7 family, member Ba [Source:ZFIN;Acc:ZDB-GENE-050417-14]	1	54908895	54931913		14.47	6.21	6.68	18.26	18.1	19.51
ENSDARG00000036298	rps13	ribosomal protein S13 [Source:ZFIN;Acc:ZDB-GENE-040625-52]	7	27041315	27044013		802.2	905.8	989.79	687.2	700.92	696
ENSDARG00000036299	nucb2a	nucleobindin 2a [Source:ZFIN;Acc:ZDB-GENE-030826-14]	7	27021368	27038532		21.71	25.9	23.73	17.53	18.73	18.58
ENSDARG00000036304	dnaaf3l	dynein, axonemal, assembly factor 3 like [Source:ZFIN;Acc:ZDB-GENE-150903-1]	24	36903172	36910224		1.19	0.59	0.54	1.12	1.31	1.2
ENSDARG00000036305	phf23b	PHD finger protein 23b [Source:NCBI gene;Acc:100004597]	7	26616241	26627253		6.43	4.48	4.36	7	7.05	7.11
ENSDARG00000036308	nod1	nucleotide-binding oligomerization domain containing 1 [Source:ZFIN;Acc:ZDB-GENE-071116-9]	16	53710416	53734405		1.04	1	0.83	0.85	1.07	1.06
ENSDARG00000036316	rpl39	ribosomal protein L39 [Source:ZFIN;Acc:ZDB-GENE-040625-51]	14	33331773	33348221		439.41	559.99	633.59	395.22	400.53	397.12
ENSDARG00000036329	ndufa1	NADH:ubiquinone oxidoreductase subunit A1 [Source:ZFIN;Acc:ZDB-GENE-040625-168]	14	33413980	33415439		57.38	71.31	62.2	51.25	50.44	50.82
ENSDARG00000036335	c1galt1c1	C1GALT1-specific chaperone 1 [Source:ZFIN;Acc:ZDB-GENE-030131-2772]	14	33515515	33521071		9.66	8.6	7.94	8.66	8.27	7.6
ENSDARG00000036337	cers3b	ceramide synthase 3b [Source:ZFIN;Acc:ZDB-GENE-080220-32]	18	7309150	7325818		3.04	3.48	2.79	2.46	2.78	2.44
ENSDARG00000036338	vps11	VPS11 core subunit of CORVET and HOPS complexes [Source:ZFIN;Acc:ZDB-GENE-050731-5]	10	29751029	29768556		10.14	6.3	5.82	7.69	7.69	8.03
ENSDARG00000036344	calb2b	calbindin 2b [Source:ZFIN;Acc:ZDB-GENE-040426-1668]	7	24681278	24699985		103.61	19.99	23.9	81.94	77.69	82.5
ENSDARG00000036345	cmtr2	cap methyltransferase 2 [Source:NCBI gene;Acc:406742]	7	24714840	24719655		3.29	2.22	2.21	3.63	3.82	3.75
ENSDARG00000036359	riox2	ribosomal oxygenase 2 [Source:ZFIN;Acc:ZDB-GENE-040426-1283]	10	23088	28198		8.17	7.83	8.18	7.14	7.09	6.74
ENSDARG00000036371	acta1a	actin alpha 1, skeletal muscle a [Source:ZFIN;Acc:ZDB-GENE-050417-267]	1	53945934	53953532		116	141.64	165.82	120.16	112.29	117.22
ENSDARG00000036375	cyfip2	cytoplasmic FMR1 interacting protein 2 [Source:ZFIN;Acc:ZDB-GENE-080724-2]	14	33982007	34044452		20.15	5.01	7.03	26.91	27.79	28.14
ENSDARG00000036376	cldn7a	claudin 7a [Source:NCBI gene;Acc:436612]	7	22616212	22626853		6.19	8.24	8.83	36.09	41.92	41.21
ENSDARG00000036382	ponzr6	plac8 onzin related protein 6 [Source:ZFIN;Acc:ZDB-GENE-081104-326]	7	22767678	22773132		5.81	4.68	6.04	3.64	3.24	4.29
ENSDARG00000036383	slc24a6a	solute carrier family 24 member 6a [Source:ZFIN;Acc:ZDB-GENE-100922-39]	14	33898976	33936524		7.56	2.01	2.8	9.11	9.84	8.87
ENSDARG00000036386	rbm4.1	RNA binding motif protein 4.1 [Source:NCBI gene;Acc:573001]	7	22801465	22806803		29.3	15.4	17.77	23.25	22.7	22.69
ENSDARG00000036394	pop7	POP7 homolog, ribonuclease P/MRP subunit [Source:ZFIN;Acc:ZDB-GENE-041010-88]	7	21887307	21888521		17.42	11.23	13.41	15.15	15.57	16.82
ENSDARG00000036412	si:ch211-81a5.5	si:ch211-81a5.5 [Source:ZFIN;Acc:ZDB-GENE-060503-892]	19	9091673	9107063		0.68	0.35	0.54	0.65	0.86	0.77
ENSDARG00000036414	si:ch73-335l21.1	si:ch73-335l21.1 [Source:ZFIN;Acc:ZDB-GENE-130530-591]	7	20147202	20225261		7.64	6.05	4.73	8.78	8.88	8.98
ENSDARG00000036415	glb1	galactosidase, beta 1 [Source:ZFIN;Acc:ZDB-GENE-050410-9]	1	52462068	52478746		5.5	4.69	3.81	5.9	6.26	5.55
ENSDARG00000036420	si:dkey-33c9.6	si:dkey-33c9.6 [Source:ZFIN;Acc:ZDB-GENE-130530-592]	7	20383841	20436781		0.17	0.23	0.24	0.26	0.33	0.28
ENSDARG00000036422	ntn5	netrin 5 [Source:ZFIN;Acc:ZDB-GENE-130530-593]	7	20431633	20453661		3.4	5.1	3.8	6.73	6.89	6.33
ENSDARG00000036424	pcdh20	protocadherin 20 [Source:NCBI gene;Acc:100007362]	11	42910904	42918971		2.17	1	1.07	3.05	2.96	3.09
ENSDARG00000036426	zgc:103586	zgc:103586 [Source:ZFIN;Acc:ZDB-GENE-041114-88]	7	20518218	20521608		5.84	7.58	6.65	6.38	6.49	5.61
ENSDARG00000036427	slc3a2a	solute carrier family 3 member 2a [Source:ZFIN;Acc:ZDB-GENE-000831-3]	7	20524064	20530447		60.31	79.46	78.15	69.32	76.4	71.08
ENSDARG00000036428	EMP3	si:dkey-264d12.4 [Source:ZFIN;Acc:ZDB-GENE-141216-123]	11	40134856	40172295	EMP3	2.54	3.17	2.97	6.88	7.51	7.25
ENSDARG00000036430	sat2a	spermidine/spermine N1-acetyltransferase family member 2a [Source:ZFIN;Acc:ZDB-GENE-041212-21]	7	20645121	20651736		0.01	0.04	0.01	0.15	0.05	0.06
ENSDARG00000036438	ndufv1	NADH:ubiquinone oxidoreductase core subunit V1 [Source:ZFIN;Acc:ZDB-GENE-040808-73]	19	9212031	9228512		150.52	181.09	186.5	93.3	89.6	89.76
ENSDARG00000036442	atp11c	ATPase phospholipid transporting 11C [Source:ZFIN;Acc:ZDB-GENE-030616-51]	14	32507811	32631519		5.5	3.89	2.87	6.22	5.97	5.76
ENSDARG00000036445	serpinh2	serine (or cysteine) peptidase inhibitor, clade H, member 2 [Source:ZFIN;Acc:ZDB-GENE-050417-12]	7	21180747	21313684		2.92	3.6	3.03	4.52	4.33	4
ENSDARG00000036446	PRKAR2B	si:ch211-272n13.7 [Source:ZFIN;Acc:ZDB-GENE-160113-22]	25	3475384	3503458	PRKAR2B	7.56	1.63	2.61	13.35	11.56	14.33
ENSDARG00000036456	anxa4	annexin A4 [Source:ZFIN;Acc:ZDB-GENE-030707-1]	10	41159241	41192881		108.12	146.9	135.39	75.85	78.65	82.57
ENSDARG00000036457	cacng6a	calcium channel, voltage-dependent, gamma subunit 6a [Source:ZFIN;Acc:ZDB-GENE-060503-440]	19	9786722	9798248		5.3	7.2	6.43	6.3	5.43	5.38
ENSDARG00000036458	prlhr2b	prolactin releasing hormone receptor 2b [Source:ZFIN;Acc:ZDB-GENE-091117-9]	10	41252525	41260567		0.46	0.04	0.11	0.22	0.39	0.19
ENSDARG00000036460	tmtops3a	teleost multiple tissue opsin 3a [Source:ZFIN;Acc:ZDB-GENE-130828-1]	14	31766144	31780794		0.21	0.11	0.12	0.38	0.15	0.21
ENSDARG00000036462	rab11fip1b	RAB11 family interacting protein 1 (class I) b [Source:ZFIN;Acc:ZDB-GENE-091204-322]	10	41318583	41352608		14.55	15.5	13.88	10.57	11.05	10.03
ENSDARG00000036471	dand5	DAN domain family, member 5 [Source:ZFIN;Acc:ZDB-GENE-040421-2]	1	51827046	51829291		0.04	0	0	0.07	0	0
ENSDARG00000036473	snapc2	small nuclear RNA activating complex, polypeptide 2 [Source:ZFIN;Acc:ZDB-GENE-120328-2]	7	26116103	26125092		2.79	2.32	1.8	2.74	2.73	2.71
ENSDARG00000036479	ITPRIP	si:dkeyp-80c12.4 [Source:ZFIN;Acc:ZDB-GENE-090313-403]	1	49510209	49512544	ITPRIP	0.31	0.41	0.33	0.19	0.4	0.35
ENSDARG00000036481	tcn2	transcobalamin II [Source:ZFIN;Acc:ZDB-GENE-030131-4648]	5	26795465	26806100		53.7	81.95	70.87	25.78	29.93	29.3
ENSDARG00000036482	hexim1	HEXIM P-TEFb complex subunit 1 [Source:NCBI gene;Acc:562319]	12	15565344	15567104		43.88	34.99	34.28	35.39	34.6	36.04
ENSDARG00000036483	c1ql3b	complement component 1, q subcomponent-like 3b [Source:ZFIN;Acc:ZDB-GENE-040426-1836]	2	3595333	3597975		1.26	0.74	1.17	0.9	0.55	1.05

ENSDARG00000036489	MAF 1.00	zgc:112356 [Source:ZFIN;Acc:ZDB-GENE-050913-28]	7	40081630	40092016	MAF 1.00	18.56	12.87	12.71	27.06	24.67	27.29
ENSDARG00000036493	si:ch73-174h16.4	si:ch73-174h16.4 [Source:ZFIN;Acc:ZDB-GENE-160728-69]	7	40093662	40102043		2.48	1.97	2.03	2.39	1.99	2.06
ENSDARG00000036494	gstcd	glutathione S-transferase, C-terminal domain containing [Source:ZFIN;Acc:ZDB-GENE-050320-41]	1	49951707	50065603		2.42	2.78	2.03	2	2.11	2.14
ENSDARG00000036499	zgc:162171	zgc:162171 [Source:ZFIN;Acc:ZDB-GENE-070424-24]	7	23865336	23873173		0.74	0.81	0.57	1.02	0.75	1.03
ENSDARG00000036500	fam122b	family with sequence similarity 122B [Source:ZFIN;Acc:ZDB-GENE-040801-204]	14	31529958	31546577		9.12	5.19	5.81	8.6	9.02	9.1
ENSDARG00000036501	rab39bb	RAB39B, member RAS oncogene family b [Source:ZFIN;Acc:ZDB-GENE-061215-104]	7	23875269	23884894		7.04	2.23	2.79	6.16	6.49	6.68
ENSDARG00000036505	syt4	synaptotagmin IV [Source:ZFIN;Acc:ZDB-GENE-030131-7297]	7	23907692	23916118		18.23	5.55	9.2	20.85	21.85	21.98
ENSDARG00000036510	ccnt2b	cyclin T2b [Source:ZFIN;Acc:ZDB-GENE-030131-183]	22	12161738	12177012		11.55	7.53	6.47	12.59	13.26	13.29
ENSDARG00000036541	rhbdf1a	rhomboid 5 homolog 1a (Drosophila) [Source:NCBI gene;Acc:798402]	3	54896331	54993027		10.29	9.78	7.52	11.97	11.63	11.48
ENSDARG00000036542	pknox1.2	pbx/knotted 1 homeobox 1.2 [Source:ZFIN;Acc:ZDB-GENE-020123-1]	1	46963993	46984142		6.98	3.72	4.4	11.11	11.85	12
ENSDARG00000036545	tmprss3b	transmembrane serine protease 3b [Source:ZFIN;Acc:ZDB-GENE-030616-522]	1	46948010	46957351		0.73	0.82	0.71	0.6	0.51	0.55
ENSDARG00000036546	pdxkb	pyridoxal (pyridoxine, vitamin B6) kinase b [Source:ZFIN;Acc:ZDB-GENE-030616-521]	1	46888033	46924801		4.48	2.53	2.54	6.14	5.82	5.92
ENSDARG00000036548	s1pr2	sphingosine-1-phosphate receptor 2 [Source:NCBI gene;Acc:170457]	3	54616015	54669185		8.41	8.39	7.71	12.75	13.49	12.2
ENSDARG00000036549	agpat3	1-acylglycerol-3-phosphate O-acyltransferase 3 [Source:ZFIN;Acc:ZDB-GENE-040426-2765]	1	46837849	46875493		68.31	54.79	36.04	52.47	54.22	52.73
ENSDARG00000036558	col18a1a	collagen type XVIII alpha 1 chain a [Source:ZFIN;Acc:ZDB-GENE-030516-3]	9	41821613	41979505		37.45	35.78	20.3	53.39	44.68	43.28
ENSDARG00000036560	gpr185b	G protein-coupled receptor 185 b [Source:ZFIN;Acc:ZDB-GENE-081104-390]	7	23561035	23563092		2.21	0.5	0.99	2.84	2.65	2.04
ENSDARG00000036567	aanat1	arylalkylamine N-acetyltransferase 1 [Source:ZFIN;Acc:ZDB-GENE-040329-1]	6	23616893	23619428		4.71	1.16	1.15	2.61	3.34	3.17
ENSDARG00000036569	bach2a	BTB and CNC homology 1, basic leucine zipper transcription factor 2a [Source:ZFIN;Acc:ZDB-GENE-120525-2]	17	15559046	15742138		0.2	0.17	0.09	0.45	0.5	0.44
ENSDARG00000036571	sema3e	sema domain, immunoglobulin domain (Ig), short basic domain, secreted, (semaphorin) 3E [Source:ZFIN;Acc:ZDB-GENE-030131-7	18	9493720	9576778		14.84	15.56	16.68	13.63	14.48	14.26
ENSDARG00000036575	si:ch73-71d17.2	si:ch73-71d17.2 [Source:ZFIN;Acc:ZDB-GENE-140106-25]	7	19397129	19420334		1.68	0.81	0.8	1.73	1.98	1.84
ENSDARG00000036577	atp6v0cb	ATPase H+ transporting V0 subunit cb [Source:ZFIN;Acc:ZDB-GENE-030131-4127]	24	39158283	39175750		210.37	85.43	117.46	214.84	228.79	225.76
ENSDARG00000036584	st8sia5	ST8 alpha-N-acetyl-neuraminide alpha-2,8-sialyltransferase 5 [Source:ZFIN;Acc:ZDB-GENE-060322-8]	21	5209476	5240595		19.7	4.93	9.09	27	26.76	28.43
ENSDARG00000036587	cbr1	carbonyl reductase 1 [Source:ZFIN;Acc:ZDB-GENE-030902-2]	1	47165842	47173403		2.9	2.55	2.06	2.18	2.25	2.06
ENSDARG00000036588	mhc1zba	major histocompatibility complex class I ZBA [Source:ZFIN;Acc:ZDB-GENE-040426-2149]	1	47155424	47161996		22.6	26.39	20.06	15.93	13.45	13.83
ENSDARG00000036592	setd4	SET domain containing 4 [Source:ZFIN;Acc:ZDB-GENE-050808-2]	1	47106268	47114319		3.9	4.61	4.14	2.85	2.51	2.83
ENSDARG00000036593	kdm2ba	lysine (K)-specific demethylase 2Ba [Source:ZFIN;Acc:ZDB-GENE-040426-2195]	8	40238647	40276193		13.48	5.55	6.46	15.28	15.78	16.04
ENSDARG00000036613	tab1	TGF-beta activated kinase 1/MAP3K7 binding protein 1 [Source:ZFIN;Acc:ZDB-GENE-060628-1]	6	181142	185297		27.24	23.27	29.22	28.77	33.77	32.96
ENSDARG00000036616	gpr182	G protein-coupled receptor 182 [Source:ZFIN;Acc:ZDB-GENE-030131-2361]	23	36653376	36655640		4.37	7.44	7.73	9.47	9.72	9.83
ENSDARG00000036619	mgat3b	beta-1,4-mannosyl-glycoprotein 4-beta-N-acetylglucosaminyltransferase b [Source:ZFIN;Acc:ZDB-GENE-130820-2]	6	202367	205291		9.87	4.94	5.36	15.92	15.81	16.87
ENSDARG00000036625	polr2f	polymerase (RNA) II (DNA directed) polypeptide F [Source:ZFIN;Acc:ZDB-GENE-040718-279]	3	1511199	1520657		39.35	49.41	51.78	28.7	27.24	30.28
ENSDARG00000036626	sema6ba	sema domain, transmembrane domain (TM), and cytoplasmic domain, (semaphorin) 6Ba [Source:ZFIN;Acc:ZDB-GENE-131024-1]	2	49232559	49342504		2.13	0.52	0.81	4.59	4.09	3.9
ENSDARG00000036628	cd74b	CD74 molecule, major histocompatibility complex, class II invariant chain b [Source:ZFIN;Acc:ZDB-GENE-990910-10]	21	33459524	33463894		1.48	1.37	1.75	0.26	0.58	0.28
ENSDARG00000036629	rps14	ribosomal protein S14 [Source:ZFIN;Acc:ZDB-GENE-030131-8631]	21	33454147	33458430		2711.06	3663.73	4264.15	1863.42	1969.82	2013.38
ENSDARG00000036630	plp2	proteolipid protein 2 [Source:ZFIN;Acc:ZDB-GENE-030710-11]	8	49310785	49345388		36.36	43.68	37.87	40.75	39.65	39.9
ENSDARG00000036636	ebpl	EBP like [Source:ZFIN;Acc:ZDB-GENE-030616-542]	1	46660213	46663997		4.07	5.73	5.02	1.09	1.29	1.09
ENSDARG00000036637	arl11	ADP-ribosylation factor-like 11 [Source:ZFIN;Acc:ZDB-GENE-030131-3410]	1	46652615	46654528		2.9	2.55	2.73	1.52	1.34	1.41
ENSDARG00000036645	rcbtb1	regulator of chromosome condensation (RCC1) and BTB (POZ) domain containing protein 1 [Source:ZFIN;Acc:ZDB-GENE-030131-7	1	46635236	46650041		4.6	2.74	3.05	4.69	4.55	4.55
ENSDARG00000036650	cdadc1	cytidine and dCMP deaminase domain containing 1 [Source:NCBI gene;Acc:492807]	1	46617006	46632966		4.37	3.03	2.57	3.16	3.62	3.29
ENSDARG00000036658	cab39l	calcium binding protein 39-like [Source:ZFIN;Acc:ZDB-GENE-041114-204]	1	46598502	46617283		23.93	21.41	20.86	25.4	24.84	25.44
ENSDARG00000036670	aplnrb	apelin receptor b [Source:NCBI gene;Acc:565492]	10	8629275	8631361		6.21	4.12	3.58	8.72	7.83	8.81
ENSDARG00000036671	tnni4b.2	troponin I4b, tandem duplicate 2 [Source:ZFIN;Acc:ZDB-GENE-040625-81]	25	20089986	20099773		15.19	18.37	17.94	19.23	20.48	20.26
ENSDARG00000036675	hnrnpa1b	heterogeneous nuclear ribonucleoprotein A1b [Source:ZFIN;Acc:ZDB-GENE-030912-14]	23	36306539	36310418		148.39	115.42	100.25	146.34	134.74	133.27
ENSDARG00000036678	trmt10a	tRNA methyltransferase 10A [Source:ZFIN;Acc:ZDB-GENE-130530-594]	7	18702534	18712554		1.44	1.59	1.51	1.12	1.14	1.18
ENSDARG00000036684	ndufa7	NADH:ubiquinone oxidoreductase subunit A7 [Source:ZFIN;Acc:ZDB-GENE-040801-169]	8	47804284	47811955		79.5	104.2	112.91	51.91	57.24	55.5
ENSDARG00000036685	ccnt2a	cyclin T2a [Source:ZFIN;Acc:ZDB-GENE-030131-5779]	9	23234233	23242684		9.21	5.44	6.29	11.07	10.96	10.51
ENSDARG00000036687	bcl9	BCL9 transcription coactivator [Source:ZFIN;Acc:ZDB-GENE-090313-135]	1	47331644	47411186		8.08	5.62	4.55	15.79	15.39	15.06
ENSDARG00000036695	calcoco1a	calcium binding and coiled-coil domain 1a [Source:NCBI gene;Acc:334971]	23	35981839	36003441		22.35	18.22	16.41	19.04	17.81	19.55
ENSDARG00000036698	znf865	zinc finger protein 865 [Source:ZFIN;Acc:ZDB-GENE-060503-896]	19	10201170	10214265		8.27	4.81	3.75	12.05	11.71	11.88
ENSDARG00000036700	si:ch211-114n24.6	si:ch211-114n24.6 [Source:ZFIN;Acc:ZDB-GENE-120214-26]	6	6989454	6993046		32.61	34.63	26.23	37.97	37.18	36.04
ENSDARG00000036704	tmem18	transmembrane protein 18 [Source:NCBI gene;Acc:449559]	23	35642061	35649000		14.93	18.17	19.34	15.04	12.69	14.55
ENSDARG00000036721	tomm40l	translocase of outer mitochondrial membrane 40 homolog, like [Source:ZFIN;Acc:ZDB-GENE-040426-2319]	19	10831362	10845087		3.87	3.17	2.37	4.49	4.49	4.85
ENSDARG00000036722	slc9a3.2	solute carrier family 9 member A3, tandem duplicate 2 [Source:ZFIN;Acc:ZDB-GENE-060503-640]	19	10738150	10771558		1.59	2.08	1.97	1.99	1.51	1.87
ENSDARG00000036728	si:dkey-211g8.1	si:dkey-211g8.1 [Source:ZFIN;Acc:ZDB-GENE-060503-436]	19	10536093	10537982		0	0	0.02	0	0.08	0.05
ENSDARG00000036743	nitr7a	novel immune-type receptor 7a [Source:ZFIN;Acc:ZDB-GENE-041001-9]	7	17374560	17376777		0	0	0	0	0.09	0
ENSDARG00000036748	nitr8	novel immune-type receptor 8 [Source:ZFIN;Acc:ZDB-GENE-041001-7]	7	17332123	17337345		0	0.03	0.02	0	0.02	0.04
ENSDARG00000036754	hmgn3	high mobility group nucleosomal binding domain 3 [Source:ZFIN;Acc:ZDB-GENE-041114-180]	23	31358088	31372807		51.09	47.53	50.88	66.97	62.45	69.87
ENSDARG00000036755	prmt9	protein arginine methyltransferase 9 [Source:ZFIN;Acc:ZDB-GENE-080728-4]	1	36746078	36772147		2.9	2.26	2.58	2.42	2.33	2.51
ENSDARG00000036764	hax1	HCLS1 associated protein X-1 [Source:ZFIN;Acc:ZDB-GENE-040718-26]	19	7799036	7805132		21.43	24.99	23.63	18.4	20.18	19.56
ENSDARG00000036767	urgcp	upregulator of cell proliferation [Source:ZFIN;Acc:ZDB-GENE-120215-160]	19	7682109	7690975		1.01	0.87	0.58	3.57	3.43	2
ENSDARG00000036772	pygo2	pygopus homolog 2 (Drosophila) [Source:ZFIN;Acc:ZDB-GENE-050809-108]	19	7627070	7633062		9.79	6.14	5.12	12.01	10.74	11.7
ENSDARG00000036773	s100s	S100 calcium binding protein S [Source:ZFIN;Acc:ZDB-GENE-040822-31]	19	7595924	7609286		23.97	23.14	22.74	8.59	10.3	10.52
ENSDARG00000036774	rgs12b	regulator of G protein signaling 12b [Source:ZFIN;Acc:ZDB-GENE-031006-13]	1	41201586	41287914		6.34	4.25	4.1	5.61	5.7	5.78
ENSDARG00000036775	dtd2	D-aminoacyl-tRNA deacylase 2 [Source:NCBI gene;Acc:445510]	17	31717192	31719071		5.36	7.57	6.12	4.37	4.07	3.4
ENSDARG00000036776	aldh8a1	aldehyde dehydrogenase 8 family, member A1 [Source:NCBI gene;Acc:447801]	23	31752604	31763753		15.08	20.2	18.29	14.38	16.53	14.14
ENSDARG00000036778	hbs1l	HBS1-like translational GTPase [Source:ZFIN;Acc:ZDB-GENE-030131-2987]	23	31764061	31810391		7.78	8.15	7.27	7.13	7.11	7.46
ENSDARG00000036781	si:dkey-170l10.1	si:dkey-170l10.1 [Source:ZFIN;Acc:ZDB-GENE-030131-5201]	17	31606635	31611692		1.23	1.34	1.4	1.29	1.02	1.38
ENSDARG00000036784	commd7	COMM domain containing 7 [Source:ZFIN;Acc:ZDB-GENE-040801-96]	23	17102960	17114092		19.52	19.67	18.64	11.56	13.25	13.49
ENSDARG00000036785	si:ch211-226m16.2	si:ch211-226m16.2 [Source:ZFIN;Acc:ZDB-GENE-030131-2302]	23	33337734	33350990		8.91	9.57	7.3	10.64	9.84	9.69
ENSDARG00000036787	tap2a	transporter associated with antigen processing, subunit type a [Source:ZFIN;Acc:ZDB-GENE-030616-245]	19	7124337	7132315		0.01	0.01	0.03	0.06	0.06	0.05
ENSDARG00000036791	dnmt3bb.1	DNA (cytosine-5-)-methyltransferase 3 beta, duplicate b.1 [Source:ZFIN;Acc:ZDB-GENE-050314-1]	23	17011454	17101360		7.09	6.11	4.75	6.94	6.46	5.86
ENSDARG00000036793	ttll5	tubulin tyrosine ligase-like family, member 5 [Source:ZFIN;Acc:ZDB-GENE-100922-236]	17	51940768	52053880		0.77	0.52	0.4	0.93	1	0.93
ENSDARG00000036809	ANPEP	si:ch211-106j24.1 [Source:ZFIN;Acc:ZDB-GENE-130530-595]	7	15373412	15413188	ANPEP	3.9	4.89	4.01	3.76	3.55	3.31
ENSDARG00000036811	ifrd2	interferon-related developmental regulator 2 [Source:ZFIN;Acc:ZDB-GENE-040801-140]	8	26410197	26427311		20.48	23.21	19.25	19.08	17.49	18.1
ENSDARG00000036816	pou2f2a	POU class 2 homeobox 2a [Source:ZFIN;Acc:ZDB-GENE-060310-2]	19	6213680	6303099		2.73	1.04	1.09	6.92	6.62	7.11
ENSDARG00000036818	hyal3	hyaluronidase 3 [Source:ZFIN;Acc:ZDB-GENE-081104-366]	8	26373149	26381305		0.69	0.4	0.5	0.35	0.42	0.84
ENSDARG00000036820	mgll	monoglyceride lipase [Source:ZFIN;Acc:ZDB-GENE-031006-9]	8	26292560	26326501		19.84	13.94	11.23	16.06	15.13	14.55
ENSDARG00000036826	ankrd52a	ankyrin repeat domain 52a [Source:NCBI gene;Acc:553206]	23	32362085	32390742		4.81	2.77	2.56	8.28	7.85	7.51
ENSDARG00000036830	krt91	keratin 91 [Source:ZFIN;Acc:ZDB-GENE-040801-181]	19	5378563	5380770		2065.35	2616.57	2852.88	542.15	538.37	520.6
ENSDARG00000036831	rnf41	ring finger protein 41 [Source:NCBI gene;Acc:406837]	23	32308814	32334208		4.93	2.93	2.87	7.81	7.93	7.44
ENSDARG00000036832	cyt1l	type I cytokeratin, enveloping layer, like [Source:ZFIN;Acc:ZDB-GENE-061026-4]	19	5356421	5358455		1145.36	1349.35	1486.54	293.02	281.09	303.21
ENSDARG00000036833	upp2	uridine phosphorylase 2 [Source:ZFIN;Acc:ZDB-GENE-040426-830]	9	16097812	16109001		0.88	1.43	1.13	0.74	0.44	0.86
ENSDARG00000036834	krt92	keratin 92 [Source:ZFIN;Acc:ZDB-GENE-050417-363]	19	5349656	5352199		277.91	294.97	328.86	91.94	102.01	99.16
ENSDARG00000036840	krt15	keratin 15 [Source:ZFIN;Acc:ZDB-GENE-040426-2931]	11	11515778	11518469		520.88	615.78	638.74	68.6	72.82	72
ENSDARG00000036844	tsen54	TSEN54 tRNA splicing endonuclease subunit [Source:ZFIN;Acc:ZDB-GENE-050522-454]	3	60761811	60795831		9.86	7.28	7.12	7.03	6.8	6.39
ENSDARG00000036846	anks4b	ankyrin repeat and sterile alpha motif domain containing 4B [Source:ZFIN;Acc:ZDB-GENE-040704-29]	3	60812851	60827402		4.13	5.71	4.03	1.98	1.92	1.92
ENSDARG00000036848	slc43a2a	solute carrier family 43 member 2a [Source:ZFIN;Acc:ZDB-GENE-040426-964]	15	28368644	28405742		34.37	39.91	38.17	56.02	61.19	61.33
ENSDARG00000036852	DXO	decapping exoribonuclease [Source:HGNC Symbol;Acc:HGNC:2992]	3	60851049	60856157	DXO	4.92	3.03	2.77	4.49	5.13	4.44
ENSDARG00000036855	plagx	pleiomorphic adenoma gene X [Source:ZFIN;Acc:ZDB-GENE-030131-839]	23	32011768	32020198		9.26	9.99	7.46	13.49	12.48	12.45
ENSDARG00000036863	MYO1D	myosin 1D [Source:NCBI gene;Acc:559562]	12	28377745	28537615	MYO1D	6.87	7.43	5.63	5.35	6.23	5.7
ENSDARG00000036864	slc34a2b	solute carrier family 34 member 2b [Source:ZFIN;Acc:ZDB-GENE-030709-1]	23	1078072	1104995		3.93	3.99	2.44	8.2	8.24	8.28
ENSDARG00000036865	slc2a12	solute carrier family 2 member 12 [Source:NCBI gene;Acc:393510]	23	31608259	31633310		4.31	5.2	4.83	5.46	6.04	6.14
ENSDARG00000036868	tbpl1	TBP-like 1 [Source:NCBI gene;Acc:403035]	23	31596194	31605592		4.08	2.93	3.5	3.55	3.17	2.92
ENSDARG00000036869	tcf21	transcription factor 21 [Source:NCBI gene;Acc:558148]	23	31554269	31555696		2.17	3.18	2.96	2.87	2.45	3.31
ENSDARG00000036870	arih1l	ariadne homolog, ubiquitin-conjugating enzyme E2 binding protein, 1 like [Source:NCBI gene;Acc:405859]	7	13491452	13514737		4.01	3.18	2.79	4.62	4.96	4.63
ENSDARG00000036875	rps12	ribosomal protein S12 [Source:ZFIN;Acc:ZDB-GENE-030131-8951]	23	31431956	31435922		2343.87	2862.86	3116.86	1671.47	1788.86	1811.45
ENSDARG00000036876	zgc:153284	zgc:153284 [Source:ZFIN;Acc:ZDB-GENE-060825-317]	23	31426493	31436524		13.16	14.21	14.87	6.96	6.61	7.52
ENSDARG00000036878	sh3bgrl2	SH3 domain binding glutamate-rich protein like 2 [Source:NCBI gene;Acc:431744]	23	31405497	31423729		9.36	3.41	3.95	9.5	10.31	10.94
ENSDARG00000036888	zgc:153935	zgc:153935 [Source:ZFIN;Acc:ZDB-GENE-061103-148]	25	27714932	27735763		5.58	3.76	3.43	5.76	5.73	5.86
ENSDARG00000036893	f13a1b	coagulation factor XIII, A1 polypeptide b [Source:ZFIN;Acc:ZDB-GENE-030131-9634]	7	8267985	8344243		1.61	1.36	1.36	4.17	4.5	4.75
ENSDARG00000036894	aimp1b	aminoacyl tRNA synthetase complex interacting multifunctional protein 1b [Source:ZFIN;Acc:ZDB-GENE-041212-13]	23	45402128	45407724		26.09	32.31	40.99	40.66	39.87	38.57
ENSDARG00000036897	sgms2b	sphingomyelin synthase 2b [Source:ZFIN;Acc:ZDB-GENE-040801-162]	23	45304830	45398655		3.74	2.6	1.79	2.62	2.36	2.13
ENSDARG00000036903	irak1bp1	interleukin-1 receptor-associated kinase 1 binding protein 1 [Source:NCBI gene;Acc:553809]	23	31245395	31248952		3.33	2.42	3.49	2.69	2.47	2.73
ENSDARG00000036911	scamp1	secretory carrier membrane protein 1 [Source:ZFIN;Acc:ZDB-GENE-040426-1803]	21	3715820	3770636		8.93	3.94	3.79	11.21	10.43	11.78
ENSDARG00000036912	edn1	endothelin 1 [Source:ZFIN;Acc:ZDB-GENE-000920-1]	19	3376430	3381422		2.8	3.62	3.49	4.78	4.66	4.78
ENSDARG00000036913	st6galnac6	ST6 (alpha-N-acetyl-neuraminyl-2,3-beta-galactosyl-1,3)-N-acetylgalactosaminide alpha-2,6-sialyltransferase 6 [Source:ZFIN;Acc:Z	21	3820779	3826706		3.64	1.01	1.51	3.37	3.92	3.77
ENSDARG00000036915	dolpp1	dolichyldiphosphatase 1 [Source:ZFIN;Acc:ZDB-GENE-041024-11]	21	3897680	3918450		8.9	5.39	5.53	9.99	10.83	10.96
ENSDARG00000036924	tatdn1	TatD DNase domain containing 1 [Source:NCBI gene;Acc:100151626]	19	167612	169846		10.03	9.4	11.09	5.26	5.28	5.34
ENSDARG00000036929	rnf139	ring finger protein 139 [Source:ZFIN;Acc:ZDB-GENE-080401-4]	19	170705	175977		8.38	7.66	6.72	9.77	9.28	9.47
ENSDARG00000036930	tbx18	T-box transcription factor 18 [Source:ZFIN;Acc:ZDB-GENE-020529-2]	23	31107685	31118371		2.8	2.99	2.51	4.26	4.43	4.01
ENSDARG00000036940	ctss1	cathepsin S, ortholog 1 [Source:ZFIN;Acc:ZDB-GENE-040426-1583]	19	310494	317532		0.64	0.42	0.46	1.02	0.83	0.98
ENSDARG00000036942	gpd1c	glycerol-3-phosphate dehydrogenase 1c [Source:ZFIN;Acc:ZDB-GENE-040426-2576]	19	317629	325584		25.5	34.33	33.52	13.7	15.31	14.38
ENSDARG00000036943	golph3l	golgi phosphoprotein 3-like [Source:ZFIN;Acc:ZDB-GENE-051113-168]	19	329808	340641		4.8	3.78	2.71	3.19	3.29	3.16
ENSDARG00000036944	ensaa	endosulfine alpha a [Source:NCBI gene;Acc:569530]	19	342094	344188		21.34	6.1	6.2	18.05	17.95	18.5
ENSDARG00000036952	XKR7	zgc:198371 [Source:ZFIN;Acc:ZDB-GENE-091214-2]	23	30859086	30890850	XKR7	2.58	0.86	1.09	4.69	4.45	4.99
ENSDARG00000036956	asxl1	ASXL transcriptional regulator 1 [Source:ZFIN;Acc:ZDB-GENE-091116-9]	23	30730121	30752553		14.18	6.96	6.48	18.39	17.54	17.64
ENSDARG00000036965	rnf24	ring finger protein 24 [Source:ZFIN;Acc:ZDB-GENE-041114-40]	1	41770842	41817784		6.24	4.31	4.26	9.96	10.13	10.31
ENSDARG00000036966	hsd3b7	hydroxy-delta-5-steroid dehydrogenase, 3 beta- and steroid delta-isomerase [Source:ZFIN;Acc:ZDB-GENE-030131-5673]	3	32743946	32778250		16.07	23.71	20.92	12.32	12.08	12.23
ENSDARG00000036967	smox	spermine oxidase [Source:ZFIN;Acc:ZDB-GENE-031201-3]	1	41849152	41865986		22.98	19.35	21.66	36.75	40.66	39.91
ENSDARG00000036968	si:ch1073-416d2.3	si:ch1073-416d2.3 [Source:ZFIN;Acc:ZDB-GENE-131127-199]	17	53292215	53295507		2.21	2.24	1.45	1.37	0.91	1.29
ENSDARG00000036974	ripply3	ripply transcriptional repressor 3 [Source:NCBI gene;Acc:654448]	10	116115	120580		0.13	0.11	0.15	0.32	0.2	0.12
ENSDARG00000036985	plxnb2b	plexin b2b [Source:ZFIN;Acc:ZDB-GENE-080902-1]	25	5604512	5681121		5.37	3.18	2.75	6.11	6.22	6.56
ENSDARG00000036987	has2	hyaluronan synthase 2 [Source:ZFIN;Acc:ZDB-GENE-020828-1]	16	15369634	15387459		0.57	0.7	0.55	1.16	1.36	1.18
ENSDARG00000036990	dctd	dCMP deaminase [Source:ZFIN;Acc:ZDB-GENE-050417-116]	1	39813229	39859626		3.77	5.09	3.79	2.27	2.42	2.07
ENSDARG00000036993	erbb3b	erb-b2 receptor tyrosine kinase 3b [Source:ZFIN;Acc:ZDB-GENE-050107-1]	23	25232411	25287666		5.43	4.82	3.19	5.02	5	4.76
ENSDARG00000036994	tbc1d16	TBC1 domain family, member 16 [Source:ZFIN;Acc:ZDB-GENE-041010-179]	3	18437397	18502030		1.78	1.4	1.36	2.08	1.89	1.87
ENSDARG00000036995	lsm6	LSM6 homolog, U6 small nuclear RNA and mRNA degradation associated [Source:ZFIN;Acc:ZDB-GENE-040625-50]	1	36194761	36204000		34.86	38.28	40.9	25.67	23.49	26.26
ENSDARG00000036998	arf3b	ADP-ribosylation factor 3b [Source:ZFIN;Acc:ZDB-GENE-030616-356]	6	7444899	7452559		28.44	8.77	14.62	36.32	35.82	35.83
ENSDARG00000036999	DISP3	si:dkey-32e23.6 [Source:ZFIN;Acc:ZDB-GENE-081104-407]	8	51050554	51147594	DISP3	1.9	0.41	0.59	2.64	2.73	2.57
ENSDARG00000037000	fkbp11	FKBP prolyl isomerase 11 [Source:ZFIN;Acc:ZDB-GENE-030616-357]	6	7428613	7439490		14.16	20.27	19.69	5.58	5.35	5.73
ENSDARG00000037002	zgc:152968	zgc:152968 [Source:ZFIN;Acc:ZDB-GENE-061013-448]	1	35985813	36002774		0.04	0.02	0.06	0.08	0.06	0.06
ENSDARG00000037008	bscl2	BSCL2 lipid droplet biogenesis associated, seipin [Source:ZFIN;Acc:ZDB-GENE-051113-140]	21	26678484	26691959		9.72	6.66	7.47	9.46	10.35	9.53
ENSDARG00000037009	banf1	barrier to autointegration factor 1 [Source:NCBI gene;Acc:334717]	21	26709801	26715270		84.91	105.11	98.11	67.47	62.28	61.46
ENSDARG00000037012	slc3a2b	solute carrier family 3 member 2b [Source:ZFIN;Acc:ZDB-GENE-040122-2]	21	26720803	26725478		54.19	41.21	44.48	78.3	83.91	86.02
ENSDARG00000037017	ube4b	ubiquitination factor E4B, UFD2 homolog (S. cerevisiae) [Source:ZFIN;Acc:ZDB-GENE-020205-1]	23	29507315	29553691		11.79	8.9	6.94	17.03	16.2	16
ENSDARG00000037018	gab1	GRB2-associated binding protein 1 [Source:ZFIN;Acc:ZDB-GENE-040426-2674]	1	35494837	35609659		5.16	5.18	3.71	7.46	7.12	7.25
ENSDARG00000037020	kif1b	kinesin family member 1B [Source:ZFIN;Acc:ZDB-GENE-030820-1]	23	29396182	29505645		15.17	6.93	6.06	29.8	30.07	31.75
ENSDARG00000037030	casz1	castor zinc finger 1 [Source:ZFIN;Acc:ZDB-GENE-060130-108]	23	28954137	29004753		4.07	2.51	1.97	8.22	7.27	7.43
ENSDARG00000037033	ptger2b	prostaglandin E receptor 2b (subtype EP2) [Source:ZFIN;Acc:ZDB-GENE-040724-267]	20	9683994	9694666		0.74	0.5	0.38	0.49	0.88	0.83
ENSDARG00000037038	psmc6	proteasome 26S subunit, ATPase 6 [Source:ZFIN;Acc:ZDB-GENE-030131-304]	20	9763364	9776334		57.52	57.1	63.93	47.77	43.94	46.2
ENSDARG00000037040	cgrrf1	cell growth regulator with ring finger domain 1 [Source:ZFIN;Acc:ZDB-GENE-040724-100]	20	9776390	9780917		2.78	2.76	2.89	2.66	2.05	2.28
ENSDARG00000037042	ddhd1b	DDHD domain containing 1b [Source:ZFIN;Acc:ZDB-GENE-040724-18]	20	9781932	9823810		8.24	4.8	5.58	8.36	8.94	8.45
ENSDARG00000037046	rad51b	RAD51 paralog B [Source:ZFIN;Acc:ZDB-GENE-040426-2750]	20	9474841	9568005		0.68	0.54	0.48	0.61	0.7	0.82
ENSDARG00000037056	zte38	zebrafish testis-expressed 38 [Source:ZFIN;Acc:ZDB-GENE-060312-39]	2	6092058	6104035		0.03	0.08	0.07	0.03	0.04	0.05
ENSDARG00000037057	gcdha	glutaryl-CoA dehydrogenase a [Source:ZFIN;Acc:ZDB-GENE-040426-1855]	6	8314451	8330577		17.74	20.23	19.72	10.6	10.62	10.14
ENSDARG00000037059	slc44a2	solute carrier family 44 member 2 [Source:ZFIN;Acc:ZDB-GENE-030131-3065]	6	8263548	8311044		8.79	4.29	4.2	11.78	12.13	11.81
ENSDARG00000037060	kmt2d	lysine (K)-specific methyltransferase 2D [Source:ZFIN;Acc:ZDB-GENE-060223-2]	23	27756984	27793877		6.13	3.07	2.45	9.07	8.05	8.96
ENSDARG00000037061	aldh9a1b	aldehyde dehydrogenase 9 family, member A1b [Source:NCBI gene;Acc:399481]	2	6067371	6092062		11.24	15.98	15.61	10.55	11.89	12.36
ENSDARG00000037062	dhh	desert hedgehog signaling molecule [Source:NCBI gene;Acc:558147]	23	27740592	27749062		0.47	0.54	0.39	0.96	1.08	1.01
ENSDARG00000037064	galca	galactosylceramidase a [Source:NCBI gene;Acc:449649]	20	4769762	4793450		10.95	9.13	7.33	7.23	6.69	7.09
ENSDARG00000037066	lgals8a	galectin 8a [Source:ZFIN;Acc:ZDB-GENE-040724-22]	20	4793516	4814098		5.04	4.16	3.87	4.85	4.3	4.5
ENSDARG00000037067	dnajc22	DnaJ (Hsp40) homolog, subfamily C, member 22 [Source:ZFIN;Acc:ZDB-GENE-090313-196]	23	27696694	27702561		3.49	5.79	4.49	1.27	1.25	1.33
ENSDARG00000037068	ikzf4	IKAROS family zinc finger 4 [Source:ZFIN;Acc:ZDB-GENE-180319-1]	23	27683014	27695173		3.08	0.74	1.58	2.5	2.84	2.59
ENSDARG00000037069	zdhhc14	zinc finger DHHC-type containing 14 [Source:ZFIN;Acc:ZDB-GENE-040724-21]	20	4827735	4883673		1.89	1.39	1.03	5.03	4.61	4.72
ENSDARG00000037070	tmem242	transmembrane protein 242 [Source:NCBI gene;Acc:569632]	20	4885298	4896355		7.71	8.74	7.75	6.2	6.88	6.04
ENSDARG00000037071	rps26	ribosomal protein S26 [Source:ZFIN;Acc:ZDB-GENE-030131-8606]	23	27675581	27677516		595.35	674.17	772.73	541.51	562.88	557.09
ENSDARG00000037073	si:dkey-60a16.1	si:dkey-60a16.1 [Source:ZFIN;Acc:ZDB-GENE-040724-244]	20	5065268	5070324		2.73	1.66	2.46	2.71	2.76	2.59
ENSDARG00000037079	cfap36	cilia and flagella associated protein 36 [Source:NCBI gene;Acc:678564]	13	233482	238375		26.72	20.65	24.97	23.63	25.71	27.46
ENSDARG00000037091	asah1a	N-acylsphingosine amidohydrolase (acid ceramidase) 1a [Source:ZFIN;Acc:ZDB-GENE-041010-191]	14	30272891	30278408		6.11	6.35	5.38	5.84	5.87	5.81
ENSDARG00000037097	slc7a2	solute carrier family 7 member 2 [Source:NCBI gene;Acc:494041]	14	30367602	30390145		6.09	6.16	5.23	6.92	7.99	7.65
ENSDARG00000037099	irs2a	insulin receptor substrate 2a [Source:ZFIN;Acc:ZDB-GENE-040426-975]	1	29211540	29228393		5.66	2.96	2.59	14.46	17.71	15.13
ENSDARG00000037100	ankrd10a	ankyrin repeat domain 10a [Source:ZFIN;Acc:ZDB-GENE-040426-700]	1	29140884	29164911		3.93	2.07	2.42	3.88	3.65	3.81
ENSDARG00000037101	mtmr7a	myotubularin related protein 7a [Source:ZFIN;Acc:ZDB-GENE-060421-7754]	14	30398546	30412141		4.09	2.55	3.04	4.33	4.62	4.49
ENSDARG00000037108	pfdn6	prefoldin subunit 6 [Source:ZFIN;Acc:ZDB-GENE-040426-1589]	14	30550397	30557637		12.46	15.29	13.32	11.39	11.49	12.19
ENSDARG00000037109	pwp2h	PWP2 small subunit processome component [Source:ZFIN;Acc:ZDB-GENE-021031-3]	1	28898945	28950366		12.44	14.84	13.04	8.64	8.44	7.85
ENSDARG00000037116	cxcl12a	chemokine (C-X-C motif) ligand 12a (stromal cell-derived factor 1) [Source:ZFIN;Acc:ZDB-GENE-030318-1]	13	30804367	30819715		23.68	30.26	27.28	45.72	47.46	46.28
ENSDARG00000037121	mat2ab	methionine adenosyltransferase II, alpha b [Source:ZFIN;Acc:ZDB-GENE-050327-6]	5	69199198	69212184		20.05	22.23	18.53	7.83	7.43	7.45
ENSDARG00000037122	TENM2	si:dkey-34l15.2 [Source:ZFIN;Acc:ZDB-GENE-081027-5]	14	29200772	29760015	TENM2	3.38	0.98	1.17	7.59	7.11	7.64
ENSDARG00000037140	pfkfb1	6-phosphofructo-2-kinase/fructose-2,6-biphosphatase 1 [Source:ZFIN;Acc:ZDB-GENE-030131-5664]	23	26017227	26037969		4.27	5.81	5.3	3.24	3.04	2.99
ENSDARG00000037142	C14H4orf47	zgc:153146 [Source:NCBI gene;Acc:751702]	14	29775402	29795189	C14H4orf47	0.58	0.69	0.65	0.26	0.53	0.67
ENSDARG00000037144	cpb2	carboxypeptidase B2 (plasma) [Source:ZFIN;Acc:ZDB-GENE-050522-259]	1	29759678	29764060		6.31	10.89	9.51	5.62	5.34	5.24
ENSDARG00000037145	slc8a4b	solute carrier family 8 member 4b [Source:ZFIN;Acc:ZDB-GENE-050809-103]	7	60161002	60245616		2.86	0.85	0.87	2.88	2.99	2.8
ENSDARG00000037153	atp6ap1b	ATPase H+ transporting accessory protein 1b [Source:ZFIN;Acc:ZDB-GENE-020423-2]	23	26079467	26094178		18.86	21.03	18.33	15.51	16.41	15.95
ENSDARG00000037154	hivep3b	HIVEP zinc finger 3b [Source:ZFIN;Acc:ZDB-GENE-100922-42]	16	34321394	34401412		2.23	1.37	1.18	3.68	3.65	3.68
ENSDARG00000037155	si:dkey-78k11.9	si:dkey-78k11.9 [Source:ZFIN;Acc:ZDB-GENE-060503-929]	23	26009266	26010376		0.25	0.54	0.66	0.32	0.21	0.24
ENSDARG00000037158	rcc1	regulator of chromosome condensation 1 [Source:ZFIN;Acc:ZDB-GENE-040426-2216]	16	34185613	34195002		7.84	8.6	6.46	7.48	7.64	6.76
ENSDARG00000037159	oprd1b	opioid receptor, delta 1b [Source:ZFIN;Acc:ZDB-GENE-030131-8473]	16	34160835	34174260		1.03	0.83	1.04	1.08	0.98	0.93
ENSDARG00000037172	ddrgk1	DDRGK domain containing 1 [Source:NCBI gene;Acc:393263]	5	1515938	1525112		3.69	3.04	3	2.95	2.71	2.81

ENSDARG00000037174	nek12	NIMA-related kinase 12 [Source:ZFIN;Acc:ZDB-GENE-050410-11]	14	28426602	28432699		0.75	0.68	0.58	0.95	1.48	1.44
ENSDARG00000037176	ggh	gamma-glutamyl hydrolase (conjugase, folylpolygammaglutamyl hydrolase) [Source:ZFIN;Acc:ZDB-GENE-040426-2615]	7	58179513	58186877		5.64	6.91	5.71	4.56	4.22	4.74
ENSDARG00000037177	zc3h13	zinc finger CCCH-type containing 13 [Source:ZFIN;Acc:ZDB-GENE-040426-1619]	1	29766725	29797072		19.45	13.57	11.54	18.71	18.01	18.94
ENSDARG00000037178	zic2b	zic family member 2 (odd-paired homolog, Drosophila) b [Source:ZFIN;Acc:ZDB-GENE-030219-92]	1	29831759	29860895		5.25	3.55	3.47	4.84	5.04	5.28
ENSDARG00000037186	tpbgb	trophoblast glycoprotein b [Source:ZFIN;Acc:ZDB-GENE-091218-2]	16	4834527	4836218		5.75	6.45	5.28	8.1	8.05	7.88
ENSDARG00000037188	rpa2	replication protein A2 [Source:ZFIN;Acc:ZDB-GENE-010131-3]	16	4746588	4770233		19.16	22.39	25.14	11.03	9.8	11.38
ENSDARG00000037191	ttr	transthyretin (prealbumin, amyloidosis type I) [Source:ZFIN;Acc:ZDB-GENE-040927-14]	20	6142433	6157942		40.24	57.72	54.08	13.74	12.59	14.25
ENSDARG00000037192	zgc:113364	zgc:113364 [Source:ZFIN;Acc:ZDB-GENE-050410-5]	14	28530153	28534563		1.99	1.61	1.39	1.77	1.92	1.01
ENSDARG00000037195	tshr	thyroid stimulating hormone receptor [Source:ZFIN;Acc:ZDB-GENE-110524-4]	20	6035427	6079923		0.16	0.13	0.19	0.17	0.1	0.15
ENSDARG00000037196	arid5b	AT-rich interaction domain 5B [Source:NCBI gene;Acc:100536827]	12	8168272	8385702		5.05	2.7	2.39	7.46	6.69	6.36
ENSDARG00000037199	C9H2orf76	zgc:101851 [Source:NCBI gene;Acc:447937]	9	900392	904227	C9H2orf76	8.76	9.05	10.96	6.77	7.83	7.45
ENSDARG00000037229	denr	density-regulated protein [Source:NCBI gene;Acc:436970]	10	44437080	44444714		19.55	20.53	20.52	16.88	17.11	16.92
ENSDARG00000037238	smad5	SMAD family member 5 [Source:NCBI gene;Acc:30641]	14	26467120	26498325		9.91	8.64	7.31	12	12.06	11.4
ENSDARG00000037244	gpr137	G protein-coupled receptor 137 [Source:ZFIN;Acc:ZDB-GENE-040718-443]	14	26437891	26450701		2.66	2.83	2.31	3.91	3.91	3.91
ENSDARG00000037245	trmo	tRNA methyltransferase O [Source:ZFIN;Acc:ZDB-GENE-051023-15]	1	26670828	26676391		1.75	2.15	1.66	1.43	1.43	1.49
ENSDARG00000037246	pimr212	Pim proto-oncogene, serine/threonine kinase, related 212 [Source:ZFIN;Acc:ZDB-GENE-070424-268]	14	26395314	26400501		0	0	0	0	0	0
ENSDARG00000037256	si:ch211-145b13.5	si:ch211-145b13.5 [Source:ZFIN;Acc:ZDB-GENE-090313-35]	1	26011479	26023678		1.72	1.57	1.31	1.41	1.2	1
ENSDARG00000037259	ndufb6	NADH:ubiquinone oxidoreductase subunit B [Source:ZFIN;Acc:ZDB-GENE-040426-1781]	1	26099250	26103232		101.35	123.35	125.25	63.35	63.68	64.62
ENSDARG00000037260	toporsa	topoisomerase I binding, arginine/serine-rich a [Source:ZFIN;Acc:ZDB-GENE-030131-6401]	1	26105141	26109961		2.14	1.79	1.91	1.84	1.96	2.11
ENSDARG00000037261	mtap	methylthioadenosine phosphorylase [Source:NCBI gene;Acc:393526]	1	26110985	26127944		16.72	20.96	19.14	15.82	15.33	14.98
ENSDARG00000037262	cdkn2a/b	cyclin-dependent kinase inhibitor 2A/B (p15, inhibits CDK4) [Source:ZFIN;Acc:ZDB-GENE-081104-306]	1	26127818	26131088		2.97	1.98	2.16	2.08	1.19	1.8
ENSDARG00000037266	myoz2b	myozenin 2b [Source:ZFIN;Acc:ZDB-GENE-040426-1853]	1	25917591	25936679		9.5	13.41	10.23	7.78	7.75	7.54
ENSDARG00000037267	zgc:158263	zgc:158263 [Source:ZFIN;Acc:ZDB-GENE-070112-2262]	23	26733232	26751278		10.77	9.3	7.57	4.52	3.81	3.62
ENSDARG00000037276	map9	microtubule-associated protein 9 [Source:ZFIN;Acc:ZDB-GENE-080225-36]	1	25737767	25749888		1.53	1.32	1.06	1.62	1.64	1.65
ENSDARG00000037278	lrata	lecithin retinol acyltransferase a [Source:ZFIN;Acc:ZDB-GENE-060720-1]	1	25696798	25698039		8.82	9.3	8.2	4.4	3.87	4.18
ENSDARG00000037281	fgg	fibrinogen gamma chain [Source:ZFIN;Acc:ZDB-GENE-040426-1998]	1	25669799	25679383		101.96	176.39	177.27	182.15	196.84	200.06
ENSDARG00000037283	plrg1	pleiotropic regulator 1 [Source:ZFIN;Acc:ZDB-GENE-040426-2787]	1	25650917	25666604		14.76	13.69	13.03	11.77	13.16	12.6
ENSDARG00000037284	ptges3a	prostaglandin E synthase 3a (cytosolic) [Source:ZFIN;Acc:ZDB-GENE-040426-2200]	23	27051919	27059998		47.99	47.53	44.61	50.01	51.14	52.65
ENSDARG00000037285	mipa	major intrinsic protein of lens fiber a [Source:ZFIN;Acc:ZDB-GENE-040801-41]	23	27068225	27072774		0.69	0.96	1.45	11.59	9.43	14.24
ENSDARG00000037286	si:dkey-30k22.7	si:dkey-30k22.7 [Source:ZFIN;Acc:ZDB-GENE-090313-302]	1	25642558	25648568		0.89	0.73	0.36	1.45	1.63	1.42
ENSDARG00000037291	dpf2l	D4, zinc and double PHD fingers family 2, like [Source:ZFIN;Acc:ZDB-GENE-030131-5132]	14	24267892	24277805		11.78	5.79	6.23	14.07	13.76	14.43
ENSDARG00000037292	chrm1a	cholinergic receptor, muscarinic 1a [Source:ZFIN;Acc:ZDB-GENE-090410-9]	14	24258562	24262815		0.16	0.05	0.07	0.11	0.12	0.17
ENSDARG00000037307	gnpda1	glucosamine-6-phosphate deaminase 1 [Source:ZFIN;Acc:ZDB-GENE-050417-417]	14	23709134	23715349		8.8	12.09	10.92	4.49	4.18	5.05
ENSDARG00000037309	gatb	glutamyl-tRNA(Gln) amidotransferase, subunit B [Source:NCBI gene;Acc:541317]	1	24520503	24558838		3.72	4.05	3.92	2.64	2.59	2.94
ENSDARG00000037318	ddost	dolichyl-diphosphooligosaccharide--protein glycosyltransferase subunit (non-catalytic) [Source:NCBI gene;Acc:406408]	23	24227296	24234371		69.46	73.04	61.43	70.51	69.06	65.77
ENSDARG00000037320	fam131c	family with sequence similarity 131 member C [Source:ZFIN;Acc:ZDB-GENE-050706-119]	23	24300651	24343363		1.78	1.43	1.31	2.62	2.81	3.03
ENSDARG00000037324	klhl17	kelch-like family member 17 [Source:ZFIN;Acc:ZDB-GENE-030131-8131]	23	23182037	23214599		4.32	1.57	2	5.77	6.02	6.49
ENSDARG00000037334	wdr92	WD repeat domain 92 [Source:NCBI gene;Acc:550284]	13	1131748	1141171		4.77	4.11	4.29	4.31	4.3	4.48
ENSDARG00000037337	cnrip1b	cannabinoid receptor interacting protein 1b [Source:ZFIN;Acc:ZDB-GENE-080722-33]	13	1089942	1097017		11.76	1.95	2.51	14.14	14.64	15.59
ENSDARG00000037350	rpl9	ribosomal protein L9 [Source:ZFIN;Acc:ZDB-GENE-030131-8646]	1	23269503	23274504		6.66	6.53	4.53	9.83	8.53	8.4
ENSDARG00000037353	frs2b	fibroblast growth factor receptor substrate 2b [Source:ZFIN;Acc:ZDB-GENE-040718-406]	25	34727928	34740627		5.76	4.45	4.31	8.16	7.83	7.24
ENSDARG00000037358	polr3e	polymerase (RNA) III (DNA directed) polypeptide E [Source:ZFIN;Acc:ZDB-GENE-030131-8462]	12	1014418	1034460		15.06	11.87	12.44	11.32	10.5	11.17
ENSDARG00000037359	si:ch1073-272o11.3	si:ch1073-272o11.3 [Source:ZFIN;Acc:ZDB-GENE-121214-325]	12	1000323	1010416		0.67	0.79	0.71	0.8	1.01	0.97
ENSDARG00000037361	kdelr2b	KDEL endoplasmic reticulum protein retention receptor 2b [Source:ZFIN;Acc:ZDB-GENE-030131-4189]	12	982796	990149		30.32	36.69	30.61	32.02	31.67	31.51
ENSDARG00000037362	scube2	signal peptide, CUB domain, EGF-like 2 [Source:NCBI gene;Acc:503728]	7	28569266	28611145		3.01	1.84	1.6	4.49	4.92	5.18
ENSDARG00000037363	dennd2b	DENN domain containing 2B [Source:ZFIN;Acc:ZDB-GENE-030131-2547]	7	28464794	28549535		6.82	6.18	4.81	8.15	8.89	9.41
ENSDARG00000037373	ephb2a	eph receptor B2a [Source:ZFIN;Acc:ZDB-GENE-070713-2]	23	21803410	21946659		1.28	0.24	0.26	1.9	1.68	1.96
ENSDARG00000037378	qdprb1	quinoid dihydropteridine reductase b1 [Source:ZFIN;Acc:ZDB-GENE-050522-320]	1	22845225	22851481		10.1	10.24	10.5	8.13	7.91	7.21
ENSDARG00000037383	pax5	paired box 5 [Source:ZFIN;Acc:ZDB-GENE-001030-2]	1	21725821	21815554		1.05	0.19	0.42	1.68	1.94	2.28
ENSDARG00000037390	gsg1l	gsg1-like [Source:NCBI gene;Acc:664746]	3	28718817	28750495		1.83	0.78	1.19	1.95	2.22	2.28
ENSDARG00000037392	med19a	mediator complex subunit 19a [Source:NCBI gene;Acc:767651]	14	22102203	22108718		18.58	15.65	16.07	12.26	12.29	13.47
ENSDARG00000037393	slc43a1a	solute carrier family 43 member 1a [Source:ZFIN;Acc:ZDB-GENE-030131-6327]	14	22076596	22098011		6.3	7.27	5.89	8.35	8.11	6.99
ENSDARG00000037397	ssrp1a	structure specific recognition protein 1a [Source:ZFIN;Acc:ZDB-GENE-031118-9]	14	21980446	22100118		23.56	22.29	22.46	14.64	14.71	14.02
ENSDARG00000037398	zgc:113229	zgc:113229 [Source:ZFIN;Acc:ZDB-GENE-050417-475]	14	21934331	21944409		1.19	0.73	0.62	0.58	0.32	0.54
ENSDARG00000037402	lim2.3	lens intrinsic membrane protein 2.3 [Source:ZFIN;Acc:ZDB-GENE-040718-327]	15	19646902	19660757		0.67	0.63	0.93	4.29	4.08	5.6
ENSDARG00000037403	hspa8b	heat shock protein family A (Hsp70) member 8b [Source:ZFIN;Acc:ZDB-GENE-121214-31]	15	19661498	19677511		2.83	2.67	2.78	2.5	2.67	2.46
ENSDARG00000037405	zgc:112083	zgc:112083 [Source:ZFIN;Acc:ZDB-GENE-050417-284]	15	19990068	20003872		3.21	2.11	1.97	2.83	2.7	2.91
ENSDARG00000037406	wdr19	WD repeat domain 19 [Source:ZFIN;Acc:ZDB-GENE-090313-277]	1	21495850	21538673		3.56	1.91	2.27	3.34	3.5	3.31
ENSDARG00000037408	tbc1d10c	TBC1 domain family, member 10C [Source:ZFIN;Acc:ZDB-GENE-130125-1]	14	21905492	21923281		0.36	0.15	0.24	0.4	0.31	0.37
ENSDARG00000037409	tmem144a	transmembrane protein 144a [Source:ZFIN;Acc:ZDB-GENE-040822-12]	1	21313165	21321482		2.97	2.9	2.43	3	3.43	2.91
ENSDARG00000037410	med28	mediator complex subunit 28 [Source:NCBI gene;Acc:493610]	1	21309386	21312596		11.89	10.33	9.41	12.3	11.36	10.34
ENSDARG00000037411	npy1r	neuropeptide Y receptor Y1 [Source:ZFIN;Acc:ZDB-GENE-080221-1]	1	21242836	21297748		0.54	0.09	0.29	0.49	0.71	0.53
ENSDARG00000037415	dlgap1b	discs, large (Drosophila) homolog-associated protein 1b [Source:ZFIN;Acc:ZDB-GENE-091116-98]	24	9162512	9268048		2.09	0.65	0.61	2.6	2.92	2.69
ENSDARG00000037421	egr1	early growth response 1 [Source:NCBI gene;Acc:30498]	14	21332522	21336385		20.54	9.23	7.75	28.88	26.74	30.4
ENSDARG00000037423	smim19	small integral membrane protein 19 [Source:NCBI gene;Acc:565058]	14	21238682	21245773		5.26	5.23	5.51	4.83	5.01	4.64
ENSDARG00000037425	s100a10a	S100 calcium binding protein A10a [Source:ZFIN;Acc:ZDB-GENE-041010-35]	19	8506178	8608414		44.06	57.66	41.08	26.01	29.74	30.03
ENSDARG00000037427	si:ch211-39a7.1	si:ch211-39a7.1 [Source:ZFIN;Acc:ZDB-GENE-060503-606]	19	8680580	8733037		1.24	0.19	0.66	1.5	1.87	1.62
ENSDARG00000037429	tll1	tolloid-like 1 [Source:NCBI gene;Acc:474335]	1	20733102	20826023		1.86	1.54	1.15	2.22	2.67	2.68
ENSDARG00000037432	haus7	HAUS augmin-like complex, subunit 7 [Source:ZFIN;Acc:ZDB-GENE-050506-15]	23	19822434	19831739		1.44	1.42	1.45	1.29	1.23	1.2
ENSDARG00000037433	fmr1	FMRP translational regulator 1 [Source:ZFIN;Acc:ZDB-GENE-020731-6]	14	20156477	20191281		14.03	8.99	7.38	16.85	16.49	17.02
ENSDARG00000037455	ugt8	UDP glycosyltransferase 8 [Source:ZFIN;Acc:ZDB-GENE-060616-129]	1	20556067	20593778		6.2	7.15	5.47	6.5	6.37	6.3
ENSDARG00000037473	cd79a	CD79a molecule, immunoglobulin-associated alpha [Source:ZFIN;Acc:ZDB-GENE-100922-201]	16	26132951	26140768		0.13	1.14	0.58	0.14	0.19	0.14
ENSDARG00000037475	foxb2	forkhead box B2 [Source:ZFIN;Acc:ZDB-GENE-081104-320]	8	38505383	38506339		0.82	0.1	0.75	1.18	0.9	0.87
ENSDARG00000037476	sorbs3	sorbin and SH3 domain containing 3 [Source:ZFIN;Acc:ZDB-GENE-050327-1]	8	38318357	38406868		4.82	4.93	3.64	4.61	4.18	4.15
ENSDARG00000037484	tmem192	transmembrane protein 192 [Source:NCBI gene;Acc:797335]	1	19649545	19665774		7.07	6.8	7.53	6.78	6.64	6.58
ENSDARG00000037487	1-Mar	membrane-associated ring finger (C3HC4) 1 [Source:ZFIN;Acc:ZDB-GENE-081112-1]	1	19708508	19756359		6.8	1.27	1.21	4.49	5.44	5.18
ENSDARG00000037488	canx	calnexin [Source:ZFIN;Acc:ZDB-GENE-040426-2797]	14	16432738	16476863		51.94	40.93	26.63	73.01	69.07	68.86
ENSDARG00000037491	bmp15	bone morphogenetic protein 15 [Source:ZFIN;Acc:ZDB-GENE-030131-6115]	7	51768343	51775688		0.07	0.05	0.03	0.03	0.01	0.05
ENSDARG00000037495	rtn4rl2b	reticulon 4 receptor-like 2b [Source:ZFIN;Acc:ZDB-GENE-040310-2]	14	17197132	17200874		3.25	1.88	2.37	2.1	2.17	1.76
ENSDARG00000037496	gria4a	glutamate receptor, ionotropic, AMPA 4a [Source:ZFIN;Acc:ZDB-GENE-020125-7]	15	44499187	44810137		6.66	1.27	2.78	8.93	8.8	9.89
ENSDARG00000037498	gria3b	glutamate receptor, ionotropic, AMPA 3b [Source:ZFIN;Acc:ZDB-GENE-030616-53]	14	12533560	12837432		9.3	1.62	3.74	9.61	9.09	9.54
ENSDARG00000037501	aasdhppt	aminoadipate-semialdehyde dehydrogenase-phosphopantetheinyl transferase [Source:ZFIN;Acc:ZDB-GENE-050913-36]	15	44440136	44465679		5.34	4.65	5.36	4.04	3.53	3.74
ENSDARG00000037503	thoc2	THO complex 2 [Source:ZFIN;Acc:ZDB-GENE-030616-54]	14	12391871	12486627		15.3	7.8	8.56	18.64	18.72	19.05
ENSDARG00000037506	prps1b	phosphoribosyl pyrophosphate synthetase 1B [Source:ZFIN;Acc:ZDB-GENE-030131-4011]	14	12076821	12106603		11.45	10.63	8.83	9.68	9.6	11.08
ENSDARG00000037507	kctd12b	potassium channel tetramerisation domain containing 12b [Source:ZFIN;Acc:ZDB-GENE-050417-104]	14	12109535	12129130		0.89	0.39	0.39	0.97	0.83	0.95
ENSDARG00000037514	hdac3	histone deacetylase 3 [Source:NCBI gene;Acc:393965]	14	12178915	12205865		13.64	11.55	11.79	16.23	16.06	15.3
ENSDARG00000037517	slc35c2	solute carrier family 35 member C2 [Source:ZFIN;Acc:ZDB-GENE-030131-2202]	23	20431140	20511954		6.97	7.12	5.24	9.07	8.84	9.54
ENSDARG00000037526	CR749762.1	olfactory receptor 2G3-like [Source:NCBI gene;Acc:101885849]	23	20373314	20374396		0.13	0.04	0.04	0.02	0.07	0.11
ENSDARG00000037527	si:rp71-17i16.5	si:rp71-17i16.5 [Source:ZFIN;Acc:ZDB-GENE-090312-117]	23	20357247	20363261		0.1	0.11	0.08	0.13	0.05	0.07
ENSDARG00000037528	si:ch211-191a24.4	si:ch211-191a24.4 [Source:ZFIN;Acc:ZDB-GENE-030131-4489]	20	6533260	6540933		4.79	5.43	4.59	3.35	3.75	4.13
ENSDARG00000037532	mcm3l	MCM3 minichromosome maintenance deficient 3 (S. cerevisiae), like [Source:ZFIN;Acc:ZDB-GENE-040121-2]	20	6516523	6532462		0.14	0.03	0.12	0.26	0.33	0.36
ENSDARG00000037533	mep1b	meprin A, beta [Source:ZFIN;Acc:ZDB-GENE-041014-209]	20	6493648	6516539		1.76	3.58	2	0	0	0.01
ENSDARG00000037537	ccnq	cyclin Q [Source:NCBI gene;Acc:553650]	23	19968956	19977214		3.88	3.71	3.49	4.2	4.3	4.24
ENSDARG00000037539	tnnc1b	troponin C type 1b (slow) [Source:ZFIN;Acc:ZDB-GENE-040625-62]	23	20110086	20112596		253.18	392.2	452.48	225.57	226.38	231.61
ENSDARG00000037545	mtnr1aa	melatonin receptor 1A a [Source:NCBI gene;Acc:30667]	1	18251335	18336878		1.67	0.38	0.68	1.47	1.48	1.72
ENSDARG00000037550	cyp21a2	cytochrome P450, family 21, subfamily A, polypeptide 2 [Source:ZFIN;Acc:ZDB-GENE-070103-6]	16	16605517	16619897		0.46	0.78	0.45	0.82	0.97	0.94
ENSDARG00000037551	pm20d1.1	peptidase M20 domain containing 1, tandem duplicate 1 [Source:NCBI gene;Acc:553216]	23	18020928	18030399		12.58	20.29	16.25	2.34	1.96	2.03
ENSDARG00000037553	il1rapl2	interleukin 1 receptor accessory protein-like 2 [Source:ZFIN;Acc:ZDB-GENE-040219-5]	14	9717678	10321851		1.62	0.36	0.51	1.13	1.35	1.31
ENSDARG00000037554	arhgef3l	Rho guanine nucleotide exchange factor (GEF) 3, like [Source:ZFIN;Acc:ZDB-GENE-050522-85]	23	20669149	20684086		1.33	0.68	1.17	2.11	2.27	1.98
ENSDARG00000037555	atoh8	atonal bHLH transcription factor 8 [Source:NCBI gene;Acc:561606]	14	9507291	9522364		8.37	13.17	11.85	12.98	12.29	12.92
ENSDARG00000037556	st3gal5	ST3 beta-galactoside alpha-2,3-sialyltransferase 5 [Source:ZFIN;Acc:ZDB-GENE-060322-1]	14	9481443	9499474		4.51	3.97	4.81	5.92	5.62	5.46
ENSDARG00000037558	asb12b	ankyrin repeat and SOCS box-containing 12b [Source:ZFIN;Acc:ZDB-GENE-040822-25]	14	9265741	9281232		0.82	1.46	1.51	0.65	0.82	0.72
ENSDARG00000037559	uba1	ubiquitin-like modifier activating enzyme 1 [Source:ZFIN;Acc:ZDB-GENE-040426-2009]	23	20640484	20664621		55.15	41.15	39.55	59.75	58.25	57.47
ENSDARG00000037560	mtm1	myotubularin 1 [Source:ZFIN;Acc:ZDB-GENE-051113-128]	7	25825667	25854387		8.21	6.09	5.45	8.03	7.86	7.18
ENSDARG00000037570	polr1d	RNA polymerase I and III subunit D [Source:NCBI gene;Acc:445412]	14	9077314	9085349		17.14	19.59	18.46	11.2	13.06	12.51
ENSDARG00000037574	rps6kal	ribosomal protein S6 kinase a, like [Source:NCBI gene;Acc:337670]	14	8940326	9002405		12.6	4.92	6.91	15.04	15.62	15.26
ENSDARG00000037576	DRD5	D(1) dopamine receptor-like [Source:NCBI gene;Acc:567498]	1	14372680	14373891	DRD5	0.13	0.08	0.21	0.16	0.26	0.04
ENSDARG00000037587	SYNPR	si:dkey-30j16.3 [Source:ZFIN;Acc:ZDB-GENE-141212-294]	11	19622925	19694334	SYNPR	4.45	1.31	0.77	5	4.87	4.92
ENSDARG00000037588	bhlhe23	basic helix-loop-helix family, member e23 [Source:ZFIN;Acc:ZDB-GENE-050913-22]	23	17655790	17657348		19.48	3.31	4.41	20.3	19.57	20.73
ENSDARG00000037589	gid8b	GID complex subunit 8 homolog b (S. cerevisiae) [Source:NCBI gene;Acc:566067]	23	17509794	17523384		8.56	7.45	7.4	6.63	6.86	6.73
ENSDARG00000037593	prickle2b	prickle homolog 2b [Source:ZFIN;Acc:ZDB-GENE-030724-6]	11	19217557	19471758		3.59	2.19	1.7	4.36	4.39	4.23
ENSDARG00000037598	prf1.5	perforin 1.5 [Source:ZFIN;Acc:ZDB-GENE-081105-3]	8	29701428	29706882		1.95	1.82	1.68	1.6	1.44	1.24
ENSDARG00000037603	zgc:162144	zgc:162144 [Source:ZFIN;Acc:ZDB-GENE-030131-7630]	14	8619126	8625845		2.73	1.1	0.63	1.7	1.99	2.24
ENSDARG00000037607	nol4lb	nucleolar protein 4-like b [Source:ZFIN;Acc:ZDB-GENE-070112-1702]	23	17220986	17346255		7.23	2.25	1.91	9.45	8.65	9.34
ENSDARG00000037613	lgals8b	galectin 8b [Source:ZFIN;Acc:ZDB-GENE-120727-7]	17	48906344	48915427		5.63	1.86	4.29	4.34	4.02	4.64
ENSDARG00000037618	ddit4	DNA-damage-inducible transcript 4 [Source:NCBI gene;Acc:378866]	12	48340133	48342259		16.24	12.95	13.76	57.11	61.08	54.63
ENSDARG00000037619	GNA12	guanine nucleotide-binding protein subunit alpha-12-like [Source:NCBI gene;Acc:797518]	1	11659861	11668782	GNA12	0.33	0.24	0.48	0.34	0.27	0.18
ENSDARG00000037628	trpt1	tRNA phosphotransferase 1 [Source:NCBI gene;Acc:503604]	14	7129816	7141550		2.29	2.62	2.11	2.21	2.22	2.36
ENSDARG00000037633	leng1	leukocyte receptor cluster (LRC) member 1 [Source:ZFIN;Acc:ZDB-GENE-040912-159]	16	32051572	32058276		9.47	8.13	8.51	8.15	7.78	8.62
ENSDARG00000037639	nkx3-2	NK3 homeobox 2 [Source:ZFIN;Acc:ZDB-GENE-030127-1]	14	212867	215051		3.93	4.59	4.54	7.73	7.7	8.26
ENSDARG00000037640	aurkb	aurora kinase B [Source:NCBI gene;Acc:791894]	14	240487	246342		12.99	10.98	11.65	8.36	9.59	8.77
ENSDARG00000037645	zgc:173570	zgc:173570 [Source:ZFIN;Acc:ZDB-GENE-080218-15]	23	16823385	16854489		2.92	3.21	3.07	2.5	2.78	2.63
ENSDARG00000037646	rgs11	regulator of G protein signaling 11 [Source:ZFIN;Acc:ZDB-GENE-080220-46]	1	9886991	9914630		7.34	1.41	1.23	6.24	6.09	6.49
ENSDARG00000037647	rnf175	ring finger protein 175 [Source:ZFIN;Acc:ZDB-GENE-040426-1104]	1	10037444	10048514		15.8	13.85	17.58	17.76	17.58	15.99
ENSDARG00000037652	C1H7orf26	zgc:56409 [Source:NCBI gene;Acc:393295]	1	9873295	9885326	C1H7orf26	4.56	3.88	3.03	4.73	5.43	5.03
ENSDARG00000037654	pmm2	phosphomannomutase 2 [Source:ZFIN;Acc:ZDB-GENE-030722-6]	1	9860381	9866856		7.26	10.58	8.15	6.91	5.44	5.03
ENSDARG00000037655	pls3	plastin 3 (T isoform) [Source:ZFIN;Acc:ZDB-GENE-040718-10]	14	13453130	13525458		40.23	43.82	34.58	38.55	36.99	34.98
ENSDARG00000037675	npm1b	nucleophosmin 1b [Source:ZFIN;Acc:ZDB-GENE-080723-7]	14	5588029	5642461		5.82	4.24	4.25	5.9	5.46	5.96
ENSDARG00000037677	fgf24	fibroblast growth factor 24 [Source:ZFIN;Acc:ZDB-GENE-030708-1]	14	5506983	5546497		3.46	3.06	3.29	3.42	3.27	2.87
ENSDARG00000037679	MPC2	zgc:103678 [Source:ZFIN;Acc:ZDB-GENE-040912-107]	1	10683843	10696603	MPC2	34.17	34.27	36.84	35.1	35.44	33.59
ENSDARG00000037691	pcdh2ab2	protocadherin 2 alpha b 2 [Source:ZFIN;Acc:ZDB-GENE-041118-2]	14	2123564	2217377		0	0	0	0.1	0.11	0.03
ENSDARG00000037695	pcdh2aa1	protocadherin 2 alpha a 1 [Source:ZFIN;Acc:ZDB-GENE-041118-13]	14	2123323	2327853		0	0	0	0.02	0.04	0.03
ENSDARG00000037706	gss	glutathione synthetase [Source:ZFIN;Acc:ZDB-GENE-041010-208]	23	14743087	14769523		5.8	7.92	6.33	4.72	4.37	5.29
ENSDARG00000037708	exosc10	exosome component 10 [Source:ZFIN;Acc:ZDB-GENE-040426-781]	23	14771979	14784890		12.57	10.4	9.44	10.92	10.94	10.69
ENSDARG00000037713	pcnp	PEST proteolytic signal containing nuclear protein [Source:ZFIN;Acc:ZDB-GENE-041010-130]	1	7556965	7561222		29.7	19.19	22.62	28.13	26.52	27.42
ENSDARG00000037738	fbxl3l	F-box and leucine-rich repeat protein 3, like [Source:ZFIN;Acc:ZDB-GENE-130530-598]	14	22367361	22390821		0.22	0.19	0.13	1.85	1.86	1.95
ENSDARG00000037739	zgc:112980	zgc:112980 [Source:ZFIN;Acc:ZDB-GENE-050306-1]	1	8539671	8553298		3.29	2.94	2.97	3.96	3.66	3.44
ENSDARG00000037746	actb1	actin, beta 1 [Source:NCBI gene;Acc:57934]	1	8649099	8653385		1019.16	1005.16	942.78	1006.2	978.82	972.49
ENSDARG00000037747	fscn1b	fascin actin-bundling protein 1b [Source:ZFIN;Acc:ZDB-GENE-120507-1]	1	8662530	8670486		8.29	2.15	3.88	6.41	5.75	6.3
ENSDARG00000037748	slc43a1b	solute carrier family 43 member 1b [Source:ZFIN;Acc:ZDB-GENE-070209-173]	14	44591492	44638058		7.23	9.81	7.92	4.28	3.71	3.7
ENSDARG00000037758	tlr2	toll-like receptor 2 [Source:ZFIN;Acc:ZDB-GENE-040219-6]	1	8984068	8988504		0.07	0.04	0.04	0.02	0.02	0.09
ENSDARG00000037760	uncx4.1	Unc4.1 homeobox (C. elegans) [Source:NCBI gene;Acc:553947]	1	9290103	9294115		8.3	4.43	5.1	6.78	7.09	6.51
ENSDARG00000037773	rybpa	RING1 and YY1 binding protein a [Source:NCBI gene;Acc:368248]	23	10762684	10786403		14.48	6.47	9.07	15.25	15.15	15.46
ENSDARG00000037777	stk16	serine/threonine kinase 16 [Source:ZFIN;Acc:ZDB-GENE-050417-102]	1	5442439	5434261		6.9	5.3	5.17	6.65	7.08	6.38
ENSDARG00000037781	acss2	acyl-CoA synthetase short chain family member 2 [Source:ZFIN;Acc:ZDB-GENE-040718-388]	23	9230431	9291892		8.25	7.24	6.72	8.25	8.96	8.12
ENSDARG00000037782	sox8b	SRY-box transcription factor 8b [Source:ZFIN;Acc:ZDB-GENE-031114-1]	3	62397718	62403550		0.78	0.41	0.4	0.72	0.88	0.76
ENSDARG00000037783	proza	protein Z, vitamin K-dependent plasma glycoprotein a [Source:ZFIN;Acc:ZDB-GENE-061013-403]	3	62382661	62393457		33.15	18.8	19.23	42.54	46.03	44.64
ENSDARG00000037789	pvalb1	parvalbumin 1 [Source:ZFIN;Acc:ZDB-GENE-040625-57]	3	61193890	61205711		9169.39	11789.79	14790.97	7862.2	8233.04	8277.44
ENSDARG00000037790	pvalb8	parvalbumin 8 [Source:ZFIN;Acc:ZDB-GENE-030805-3]	3	61154219	61162750		214.49	270.11	288.04	68.08	75.38	72.14
ENSDARG00000037794	nptx2a	neuronal pentraxin 2a [Source:ZFIN;Acc:ZDB-GENE-060707-1]	3	61566374	61593274		0.44	0.05	0.06	0.51	0.69	0.66
ENSDARG00000037804	phlda3	pleckstrin homology-like domain, family A, member 3 [Source:NCBI gene;Acc:368779]	23	5756442	5759242		5.72	6.03	5.7	7.92	8.75	8.49
ENSDARG00000037805	lgals3bpa	lectin, galactoside-binding, soluble, 3 binding protein a [Source:NCBI gene;Acc:677742]	3	57699246	57711711		0.49	0.41	0.41	0.14	0.37	0.27
ENSDARG00000037810	adtrp1	androgen dependent TFPI regulating protein 1 [Source:ZFIN;Acc:ZDB-GENE-050417-220]	23	4362463	4367415		13	13.59	10.71	10.51	12.01	9.15
ENSDARG00000037813	zgc:113278	zgc:113278 [Source:ZFIN;Acc:ZDB-GENE-050327-66]	23	4226341	4243553		6.69	4.72	2.92	5.93	5.41	5.2
ENSDARG00000037815	b4galt5	UDP-Gal:betaGlcNAc beta 1,4- galactosyltransferase, polypeptide 5 [Source:NCBI gene;Acc:724080]	23	4022620	4071748		0.4	0.12	0.11	0.55	0.42	0.53
ENSDARG00000037833	mitfb	melanocyte inducing transcription factor b [Source:ZFIN;Acc:ZDB-GENE-010919-1]	23	711075	764135		0.62	0.61	0.46	0.65	1.08	0.82
ENSDARG00000037835	polr3k	polymerase (RNA) III (DNA directed) polypeptide K [Source:ZFIN;Acc:ZDB-GENE-040718-291]	3	46380086	46384147		15.26	17.51	17.3	10.39	11.04	11.66
ENSDARG00000037836	igfals	insulin-like growth factor binding protein, acid labile subunit [Source:ZFIN;Acc:ZDB-GENE-061009-25]	3	45845007	45848257		0.64	0.72	0.51	0.4	0.22	0.23
ENSDARG00000037837	ogfr	opioid growth factor receptor [Source:ZFIN;Acc:ZDB-GENE-030131-377]	23	578218	588248		6.21	3.11	3.8	7.43	7.37	7.84
ENSDARG00000037838	unkl	unk like zinc finger [Source:ZFIN;Acc:ZDB-GENE-131120-196]	3	45778810	45818361		6.51	3.05	2.93	9.91	10.74	10.56
ENSDARG00000037845	col9a3	collagen, type IX, alpha 3 [Source:ZFIN;Acc:ZDB-GENE-030131-9799]	23	495514	524780		92.78	106.05	84.79	185.99	205.02	206.02
ENSDARG00000037846	hm13	histocompatibility (minor) 13 [Source:ZFIN;Acc:ZDB-GENE-020802-3]	23	480423	493384		24.19	23.29	19.19	27.26	25.81	24.04
ENSDARG00000037852	zgc:101663	zgc:101663 [Source:NCBI gene;Acc:492796]	23	383782	397300		3.95	3.99	2.96	3.56	3.18	3.04
ENSDARG00000037855	taf11	TAF11 RNA polymerase II, TATA box binding protein (TBP)-associated factor [Source:ZFIN;Acc:ZDB-GENE-040718-283]	23	309063	315437		7.27	5.41	6.1	6.74	6.74	7.05
ENSDARG00000037859	il11a	interleukin 11a [Source:ZFIN;Acc:ZDB-GENE-051019-1]	16	12611362	12617155		0.03	0	0.03	3.49	3.87	4.33
ENSDARG00000037860	cox6b2	cytochrome c oxidase subunit 6B2 [Source:ZFIN;Acc:ZDB-GENE-040426-1566]	16	12504929	12512593		174.43	227.7	242.17	126.74	127.24	129.71
ENSDARG00000037861	slc2a3b	solute carrier family 2 member 3b [Source:ZFIN;Acc:ZDB-GENE-121031-5]	16	12474861	12496632		0.85	0.97	0.66	2.23	2.82	2.4
ENSDARG00000037865	cln3	CLN3 lysosomal/endosomal transmembrane protein, battenin [Source:ZFIN;Acc:ZDB-GENE-041114-174]	3	39579393	39599339		10.01	12.74	14.69	7.93	8.42	9.12
ENSDARG00000037867	atp5mf	ATP synthase membrane subunit f [Source:ZFIN;Acc:ZDB-GENE-050309-87]	3	40296785	40301467		121.29	154.01	160.78	100.98	95.37	98.2
ENSDARG00000037870	actb2	actin, beta 2 [Source:NCBI gene;Acc:57935]	3	40526107	40529804		2049.81	2027.19	1627.8	2305.18	2311.42	2332.2
ENSDARG00000037871	wipi2	WD repeat domain, phosphoinositide interacting 2 [Source:ZFIN;Acc:ZDB-GENE-040718-488]	3	40821657	40837218		31.9	23.3	24.14	35.54	36.72	38.64
ENSDARG00000037872	foxk1	forkhead box K1 [Source:ZFIN;Acc:ZDB-GENE-030131-6402]	3	40901743	40933415		11.36	9.89	7.99	15.31	14.67	16.36
ENSDARG00000037873	cyp3c3	cytochrome P450, family 3, subfamily c, polypeptide 3 [Source:ZFIN;Acc:ZDB-GENE-041114-102]	3	40947961	40955780		8.15	12.85	9.25	1.11	1.5	1.27
ENSDARG00000037874	cyp3c3	cytochrome P450, family 3, subfamily c, polypeptide 3 [Source:ZFIN;Acc:ZDB-GENE-041114-102]	3	40958870	40965596		0.91	0.99	0.7	0.86	0.51	1.1
ENSDARG00000037879	lfng	LFNG O-fucosylpeptide 3-beta-N-acetylglucosaminyltransferase [Source:NCBI gene;Acc:30158]	3	41917499	41926869		21.99	16.61	18.32	26.23	28.79	27.41
ENSDARG00000037883	prcp	prolylcarboxypeptidase (angiotensinase C) [Source:ZFIN;Acc:ZDB-GENE-040718-447]	15	11218246	11303691		3.93	4.33	4.09	4.3	4.32	4.36
ENSDARG00000037884	rab30	RAB30, member RAS oncogene family [Source:ZFIN;Acc:ZDB-GENE-040718-250]	15	11308462	11341635		5.4	2.52	2.91	6.81	6.48	6.61
ENSDARG00000037889	wnt9b	wingless-type MMTV integration site family, member 9B [Source:ZFIN;Acc:ZDB-GENE-080201-1]	3	37574885	37581620		1.07	1.18	1.08	2.32	1.86	1.63
ENSDARG00000037891	mlx	MAX dimerization protein MLX [Source:ZFIN;Acc:ZDB-GENE-050522-316]	3	37135516	37148594		8.16	10.51	9.39	8	8.16	7.43
ENSDARG00000037892	psmc3ip	PSMC3 interacting protein [Source:ZFIN;Acc:ZDB-GENE-040625-125]	3	37112693	37135569		4.41	4.81	4.24	2.44	1.98	2.07
ENSDARG00000037894	ezh1	enhancer of zeste 1 polycomb repressive complex 2 subunit [Source:ZFIN;Acc:ZDB-GENE-050114-1]	3	36859701	36892651		4.77	2.02	2.52	5.74	6.31	6.49
ENSDARG00000037895	ramp2	receptor (G protein-coupled) activity modifying protein 2 [Source:ZFIN;Acc:ZDB-GENE-080204-79]	3	36836711	36846496		6.44	8.45	7.79	8.31	8.96	9.88
ENSDARG00000037905	cacna1aa	calcium channel, voltage-dependent, P/Q type, alpha 1A subunit, a [Source:ZFIN;Acc:ZDB-GENE-040724-26]	3	33773156	33901483		10.28	1.81	4.22	19.02	19.75	19.44

ENSDARG00000037906	alkbh7	alkB homolog 7 [Source:ZFIN;Acc:ZDB-GENE-050417-78]	3	33918510	33922150		7.33	8.37	7.86	4.37	4.22	5.19
ENSDARG00000037909	timm29	translocase of inner mitochondrial membrane 29 [Source:ZFIN;Acc:ZDB-GENE-050522-380]	3	34180835	34182587		15.27	16.82	17.14	10.32	10.43	10.81
ENSDARG00000037913	nmrk1	nicotinamide riboside kinase 1 [Source:ZFIN;Acc:ZDB-GENE-050320-8]	3	34597741	34599662		1.92	0.89	0.79	2.22	2.72	1.97
ENSDARG00000037914	znfl1	zinc finger-like gene 1 [Source:ZFIN;Acc:ZDB-GENE-030828-6]	3	34652144	34656745		0.03	0.01	0.03	0.01	0.03	0.02
ENSDARG00000037916	cdk5r1a	cyclin-dependent kinase 5, regulatory subunit 1a (p35) [Source:ZFIN;Acc:ZDB-GENE-080723-17]	3	34821327	34822320		11.54	2.43	4.46	10.47	11.6	11.93
ENSDARG00000037917	itga3a	integrin, alpha 3a [Source:ZFIN;Acc:ZDB-GENE-090805-3]	3	34846201	34917041		4.96	5.46	4.05	4.44	3.54	3.35
ENSDARG00000037919	rbbp6	retinoblastoma binding protein 6 [Source:ZFIN;Acc:ZDB-GENE-040930-5]	3	35406395	35421194		13.01	6.8	6.24	16.11	15.09	15.48
ENSDARG00000037921	gng13b	guanine nucleotide binding protein (G protein), gamma 13b [Source:ZFIN;Acc:ZDB-GENE-040718-97]	3	35589212	35602233		38.7	9.63	10.37	40.67	36.55	42.92
ENSDARG00000037924	gna13b	guanine nucleotide binding protein (G protein), alpha 13b [Source:ZFIN;Acc:ZDB-GENE-030131-8277]	3	36332579	36364903		8.26	6.55	5.91	9.51	10.16	9.71
ENSDARG00000037925	rgs9a	regulator of G protein signaling 9a [Source:ZFIN;Acc:ZDB-GENE-060929-92]	3	36366053	36403234		14.26	2.61	1.64	8.19	8.6	8
ENSDARG00000037928	ddx42	DEAD (Asp-Glu-Ala-Asp) box helicase 42 [Source:ZFIN;Acc:ZDB-GENE-050706-53]	3	31879442	31893008		12.88	8.44	8.36	13.51	13.3	14.15
ENSDARG00000037929	strada	STE20 related adaptor alpha [Source:ZFIN;Acc:ZDB-GENE-030131-6482]	3	31897029	31917390		7.48	5.2	5.62	7.26	6.58	6.77
ENSDARG00000037930	rnf113a	ring finger protein 113A [Source:ZFIN;Acc:ZDB-GENE-040825-1]	3	31919042	31924643		7.29	7.77	8.25	6.22	6.56	5.57
ENSDARG00000037932	lin7b	lin-7 homolog B (C. elegans) [Source:ZFIN;Acc:ZDB-GENE-041011-1]	3	31933893	31944709		25.75	12.61	16.84	27.41	29.59	27.38
ENSDARG00000037935	aldh16a1	aldehyde dehydrogenase 16 family, member A1 [Source:ZFIN;Acc:ZDB-GENE-070112-2062]	3	32080403	32098515		6.95	8.3	5.8	6.81	6.88	6.49
ENSDARG00000037941	syt5a	synaptotagmin Va [Source:ZFIN;Acc:ZDB-GENE-040718-110]	3	32142382	32151167		39.79	11.19	8.07	20.79	20.66	22.03
ENSDARG00000037943	cpt1cb	carnitine palmitoyltransferase 1Cb [Source:ZFIN;Acc:ZDB-GENE-050417-155]	3	32228901	32290096		3.8	3.54	2.41	3.33	3.01	2.27
ENSDARG00000037946	prl	prolactin [Source:ZFIN;Acc:ZDB-GENE-030513-1]	3	32341917	32345900		2.73	1.09	1.95	5.96	6.75	5.42
ENSDARG00000037954	tnnt1	troponin T type 1 (skeletal, slow) [Source:ZFIN;Acc:ZDB-GENE-080723-27]	3	32157140	32170878		18.78	23.74	20.81	6.21	5.94	6.89
ENSDARG00000037955	pih1d1	PIH1 domain containing 1 [Source:NCBI gene;Acc:553260]	3	32172534	32180833		2.66	2.17	2.28	2.47	2.23	2.71
ENSDARG00000037958	nosip	nitric oxide synthase interacting protein [Source:NCBI gene;Acc:492793]	3	32532000	32536378		12.6	10.08	10.73	8.1	7.85	8.56
ENSDARG00000037960	lrrc17	leucine rich repeat containing 17 [Source:ZFIN;Acc:ZDB-GENE-030131-8774]	4	20141191	20156085		17.05	21.65	18.47	19.88	20.21	18.68
ENSDARG00000037961	rcn3	reticulocalbin 3, EF-hand calcium binding domain [Source:ZFIN;Acc:ZDB-GENE-040625-175]	3	32535986	32541072		21.18	33.4	28.33	49.22	47.86	49.15
ENSDARG00000037962	psmb7	proteasome 20S subunit beta 7 [Source:ZFIN;Acc:ZDB-GENE-001208-4]	21	8341774	8352095		109.43	120.58	148.35	83.22	86.86	83.72
ENSDARG00000037963	nek6	NIMA-related kinase 6 [Source:ZFIN;Acc:ZDB-GENE-040724-133]	21	8357602	8370500		8.74	8.45	8.51	8.07	7.99	7.51
ENSDARG00000037964	lhx2a	LIM homeobox 2a [Source:ZFIN;Acc:ZDB-GENE-091118-109]	21	8407545	8422363		0.49	0.15	0.17	0.33	0.31	0.41
ENSDARG00000037965	srrm2	serine/arginine repetitive matrix 2 [Source:ZFIN;Acc:ZDB-GENE-050522-399]	3	32631372	32650278		16.18	11.1	9.56	17.84	18.67	19.29
ENSDARG00000037966	thoc6	THO complex 6 [Source:NCBI gene;Acc:100004014]	3	32651697	32658436		4.91	4.9	4.96	3.79	3.57	3.7
ENSDARG00000037967	si:dkey-16l2.16	si:dkey-16l2.16 [Source:ZFIN;Acc:ZDB-GENE-141219-36]	3	32661765	32669219		7.82	8.65	6.92	9.22	9.43	9.01
ENSDARG00000037968	fus	FUS RNA binding protein [Source:ZFIN;Acc:ZDB-GENE-040426-1010]	3	32698424	32711280		32.8	14.17	18.14	51.24	50.59	50.09
ENSDARG00000037980	elob	elongin B [Source:ZFIN;Acc:ZDB-GENE-020415-1]	3	31069276	31079246		51.04	51.13	51.59	57.77	56.69	56.23
ENSDARG00000037995	gdf3	growth differentiation factor 3 [Source:NCBI gene;Acc:30125]	17	4239437	4252221		0.33	0.12	0.18	1.19	1.17	1.32
ENSDARG00000037997	tubb5	tubulin, beta 5 [Source:ZFIN;Acc:ZDB-GENE-031110-4]	16	10318893	10327620		176.91	71.38	66.96	131.46	113.09	125.42
ENSDARG00000037998	flot1b	flotillin 1b [Source:ZFIN;Acc:ZDB-GENE-020430-2]	16	10287588	10316359		69.48	76.11	74.17	70.6	70.89	68.68
ENSDARG00000038000	crls1	cardiolipin synthase 1 [Source:ZFIN;Acc:ZDB-GENE-040426-2519]	17	4368859	4386084		13.88	16.58	17.33	8.57	10.4	10.5
ENSDARG00000038005	med25	mediator complex subunit 25 [Source:NCBI gene;Acc:406793]	3	30454353	30488100		9.89	5.56	4.68	9.9	9.56	9.97
ENSDARG00000038006	osr2	odd-skipped related transciption factor 2 [Source:NCBI gene;Acc:550389]	16	54402310	54405976		12	14.64	11.6	6.37	6.58	7.22
ENSDARG00000038009	CT573429.1	GTPase IMAP family member 4-like [Source:NCBI gene;Acc:798736]	3	29664357	29671653		2.77	4.51	3.5	0.45	0.2	0.51
ENSDARG00000038010	rac2	Rac family small GTPase 2 [Source:ZFIN;Acc:ZDB-GENE-040625-27]	3	29510818	29544039		6.69	9.09	9.04	5.43	5.3	4.93
ENSDARG00000038011	cyth4a	cytohesin 4a [Source:ZFIN;Acc:ZDB-GENE-090624-6]	3	29487300	29508959		0.56	0.24	0.32	0.18	0.33	0.34
ENSDARG00000038012	fam83fa	family with sequence similarity 83 member Fa [Source:ZFIN;Acc:ZDB-GENE-090624-5]	3	29469283	29486498		0.35	0.37	0.38	0.33	0.4	0.42
ENSDARG00000038018	prph2a	peripherin 2a (retinal degeneration, slow) [Source:ZFIN;Acc:ZDB-GENE-000616-8]	12	34896956	34901874		85.14	18.89	11.8	42.56	48.12	44.35
ENSDARG00000038024	flr	fleer [Source:NCBI gene;Acc:797829]	3	28831450	28853935		1.76	1.13	1.42	2.43	2.61	3.02
ENSDARG00000038025	cbx7a	chromobox homolog 7a [Source:ZFIN;Acc:ZDB-GENE-050417-400]	3	24986145	24994617		8.72	7.6	8.3	31.5	34.34	34.4
ENSDARG00000038027	nol12	nucleolar protein 12 [Source:NCBI gene;Acc:393260]	3	1150348	1156033		9.53	11.21	13.53	6.45	6.1	6
ENSDARG00000038028	ndufa6	NADH:ubiquinone oxidoreductase subunit A6 [Source:ZFIN;Acc:ZDB-GENE-040426-1124]	3	1182315	1186100		86.4	95.7	128.44	72.67	72.94	72.94
ENSDARG00000038030	rbx1	ring-box 1, E3 ubiquitin protein ligase [Source:ZFIN;Acc:ZDB-GENE-041008-106]	3	25101960	25107802		82.46	101.57	119.95	76.81	79.87	76.29
ENSDARG00000038040	tpk1	thiamin pyrophosphokinase 1 [Source:ZFIN;Acc:ZDB-GENE-041010-176]	24	18286427	18445254		2.69	3.44	3.64	3.58	2.96	3.33
ENSDARG00000038052	si:ch211-149k23.9	si:ch211-149k23.9 [Source:ZFIN;Acc:ZDB-GENE-100922-15]	16	13898399	13905633		2.16	1.65	1.23	1.36	1.74	2.23
ENSDARG00000038056	fgfbp2b	fibroblast growth factor binding protein 2b [Source:ZFIN;Acc:ZDB-GENE-070410-95]	1	22685961	22691182		15.41	13.32	14.07	100.53	108.14	106.13
ENSDARG00000038059	grb2b	growth factor receptor-bound protein 2b [Source:ZFIN;Acc:ZDB-GENE-040426-1975]	3	25432419	25492495		6.61	4.6	4.28	8.58	8.96	8.66
ENSDARG00000038064	chmp3	charged multivesicular body protein 3 [Source:NCBI gene;Acc:406621]	17	43468732	43476280		13.42	14.46	13.19	10.66	10.31	10.43
ENSDARG00000038066	kpna2	karyopherin alpha 2 (RAG cohort 1, importin alpha 1) [Source:ZFIN;Acc:ZDB-GENE-040718-22]	3	25369965	25377163		34.58	31.26	32.36	28.89	27.22	27.22
ENSDARG00000038067	smurf2	SMAD specific E3 ubiquitin protein ligase 2 [Source:NCBI gene;Acc:563633]	3	25281947	25369557		7.76	4.33	4.37	10.48	10.61	11.22
ENSDARG00000038068	ddx5	DEAD (Asp-Glu-Ala-Asp) box helicase 5 [Source:ZFIN;Acc:ZDB-GENE-030131-925]	3	25269861	25275403		135.43	73.18	96.08	154.77	147.46	145.93
ENSDARG00000038069	mgat3a	beta-1,4-mannosyl-glycoprotein 4-beta-N-acetylglucosaminyltransferase a [Source:ZFIN;Acc:ZDB-GENE-050522-249]	3	25237399	25268751		2.43	3.33	2.79	1.58	1.45	1.37
ENSDARG00000038072	ptx4	pentraxin 4, long [Source:ZFIN;Acc:ZDB-GENE-120215-70]	3	27642078	27663447		0.08	0.04	0.12	0.06	0.05	0.03
ENSDARG00000038074	ergic3	ERGIC and golgi 3 [Source:NCBI gene;Acc:393990]	6	50393047	50414197		10.86	7.88	9.14	11.58	11.34	11.13
ENSDARG00000038075	cyc1	cytochrome c-1 [Source:ZFIN;Acc:ZDB-GENE-031105-2]	6	50381347	50390089		112.4	120.67	112.11	105.89	108.66	108.09
ENSDARG00000038076	romo1	reactive oxygen species modulator 1 [Source:NCBI gene;Acc:393770]	6	50401620	50404732		188.9	239.45	226.45	154.12	155.87	156.85
ENSDARG00000038089	TMEM114	transmembrane protein 114 [Source:NCBI gene;Acc:100150208]	3	27929505	27952302	TMEM114	0.58	0.41	0.3	0.43	0.42	0.52
ENSDARG00000038094	clec16a	C-type lectin domain containing 16A [Source:ZFIN;Acc:ZDB-GENE-040426-951]	3	26823788	26906656		4.55	3.04	2.91	5.72	5.8	5.83
ENSDARG00000038095	socs1a	suppressor of cytokine signaling 1a [Source:ZFIN;Acc:ZDB-GENE-040801-205]	3	26813058	26814953		3.39	3.51	3.52	2.63	2.67	3.24
ENSDARG00000038097	pigq	phosphatidylinositol glycan anchor biosynthesis, class Q [Source:ZFIN;Acc:ZDB-GENE-030131-9793]	3	26792546	26806032		7.01	4.22	4.61	6.1	6.45	6.39
ENSDARG00000038099	gskip	gsk3b interacting protein [Source:NCBI gene;Acc:406744]	17	43013171	43019060		18.85	18.87	16.61	21.05	21	22.58
ENSDARG00000038106	slc37a4a	solute carrier family 37 member 4a [Source:ZFIN;Acc:ZDB-GENE-040426-2230]	15	9294620	9315566		18.01	31.27	28.26	18.52	23.99	20.52
ENSDARG00000038107	sgcg	sarcoglycan, gamma [Source:ZFIN;Acc:ZDB-GENE-030724-2]	15	9327252	9353696		2.45	4.65	3.45	4.14	3.12	3.53
ENSDARG00000038112	si:dkeyp-59c12.1	si:dkeyp-59c12.1 [Source:ZFIN;Acc:ZDB-GENE-050419-167]	18	25225524	25226704		1.06	0.47	0.94	0.89	0.74	0.58
ENSDARG00000038113	rprd1b	regulation of nuclear pre-mRNA domain containing 1B [Source:ZFIN;Acc:ZDB-GENE-030131-355]	23	43405205	43454523		6.78	6.15	6.98	4.28	4.68	4.55
ENSDARG00000038114	xpa	xeroderma pigmentosum, complementation group A [Source:ZFIN;Acc:ZDB-GENE-040426-1205]	1	12068800	12097818		3.01	2.3	2.12	2.38	2.06	2.4
ENSDARG00000038116	gnrhr4	gonadotropin releasing hormone receptor 4 [Source:ZFIN;Acc:ZDB-GENE-050419-76]	18	25391717	25402715		0.42	0.12	0.09	0.33	0.7	0.49
ENSDARG00000038121	si:ch211-13k12.2	si:ch211-13k12.2 [Source:ZFIN;Acc:ZDB-GENE-061207-5]	18	25563292	25646286		4.57	3.38	2.94	5.52	5.83	5.73
ENSDARG00000038123	myl9a	myosin, light chain 9a, regulatory [Source:ZFIN;Acc:ZDB-GENE-041010-120]	23	42810055	42823356		24.79	34.82	34.46	23.97	22.67	22.27
ENSDARG00000038133	zgc:113411	zgc:113411 [Source:ZFIN;Acc:ZDB-GENE-050417-474]	3	24640946	24649894		1.53	1.37	1.39	2.02	2.57	2.24
ENSDARG00000038139	pdgfbb	platelet-derived growth factor beta polypeptide b [Source:ZFIN;Acc:ZDB-GENE-131121-332]	3	24275597	24297419		1.15	0.93	0.66	1.56	1.5	1.59
ENSDARG00000038141	atf4b	activating transcription factor 4b [Source:ZFIN;Acc:ZDB-GENE-070928-23]	3	24197934	24200819		76.63	45.71	45.74	91.05	87.05	89.54
ENSDARG00000038147	hbbe3	hemoglobin beta embryonic-3 [Source:ZFIN;Acc:ZDB-GENE-980526-287]	12	20339413	20340543		0	0.09	0.19	3.62	2.24	1.08
ENSDARG00000038151	zgc:92360	zgc:92360 [Source:ZFIN;Acc:ZDB-GENE-040718-470]	6	584632	598165		16.78	19	15.68	15.97	15.63	14.36
ENSDARG00000038153	lgals2b	lectin, galactoside-binding, soluble, 2b [Source:ZFIN;Acc:ZDB-GENE-040426-1590]	6	603278	609887		529.67	965.61	759.51	133.17	151.06	147.56
ENSDARG00000038154	isca2	iron-sulfur cluster assembly 2 [Source:ZFIN;Acc:ZDB-GENE-131121-486]	17	43032529	43036291		24.45	22.87	24.17	15.95	15.21	16.81
ENSDARG00000038156	dusp11	dual specificity phosphatase 11 (RNA/RNP complex 1-interacting) [Source:ZFIN;Acc:ZDB-GENE-050417-226]	10	42134256	42147318		3.74	3.64	3.09	3.59	3.71	3.62
ENSDARG00000038159	tcf7l1a	transcription factor 7 like 1a [Source:NCBI gene;Acc:30523]	10	42195732	42297889		5.35	5.45	4.51	9.36	9.37	8.97
ENSDARG00000038166	hyal6	hyaluronoglucosaminidase 6 [Source:ZFIN;Acc:ZDB-GENE-070112-2372]	25	27596076	27633115		0.89	0.1	0.1	0.11	0.16	0.32
ENSDARG00000038176	cyb561	cytochrome b561 [Source:ZFIN;Acc:ZDB-GENE-070713-3]	3	22817155	22829710		2.01	1.15	0.86	3.94	3.56	3.79
ENSDARG00000038185	gh1	growth hormone 1 [Source:ZFIN;Acc:ZDB-GENE-030725-2]	3	22327738	22332913		11.11	7.25	10.57	2.85	3.62	3.82
ENSDARG00000038186	zgc:103564	zgc:103564 [Source:NCBI gene;Acc:492461]	3	22335030	22341106		1.32	1.07	0.99	1.06	0.76	0.58
ENSDARG00000038191	smtla	somatolactin alpha [Source:ZFIN;Acc:ZDB-GENE-060123-2]	18	48168755	48178125		0.13	0	0.06	0	0.04	0.04
ENSDARG00000038199	cdab	cytidine deaminase b [Source:ZFIN;Acc:ZDB-GENE-040426-1911]	11	3585934	3593118		5.9	9.89	6.54	4.47	5.2	6.42
ENSDARG00000038205	her2	hairy-related 2 [Source:ZFIN;Acc:ZDB-GENE-980526-274]	11	41966117	41966854		9.12	5.36	7.57	3.53	4.1	4.27
ENSDARG00000038207	aldh4a1	aldehyde dehydrogenase 4 family, member A1 [Source:NCBI gene;Acc:394133]	11	41936435	41960276		50.83	55.21	50.39	33.19	34.02	33.49
ENSDARG00000038213	slc35b1	solute carrier family 35 member B1 [Source:NCBI gene;Acc:447844]	3	20963162	20982706		13.27	12.82	11.74	13.35	13.29	12.3
ENSDARG00000038222	tyw3	tRNA-yW synthesizing protein 3 homolog (S. cerevisiae) [Source:NCBI gene;Acc:436884]	2	11119315	11126221		4.43	5.2	4.1	3.21	2.93	2.96
ENSDARG00000038225	nras	NRAS proto-oncogene, GTPase [Source:NCBI gene;Acc:30380]	8	11114754	11131695		6.83	8.71	6.82	10.22	9.1	9.07
ENSDARG00000038226	CABZ01074994.1	steroidogenic acute regulatory protein, mitochondrial-like [Source:NCBI gene;Acc:100331467]	12	47945664	47959911		0	0.05	0	0.03	0	0.08
ENSDARG00000038228	selenow2a	selenoprotein W, 2a [Source:ZFIN;Acc:ZDB-GENE-030428-1]	3	20108221	20118342		53.12	45.91	46.88	50.28	48.29	48.52
ENSDARG00000038235	pkdccb	protein kinase domain containing, cytoplasmic b [Source:ZFIN;Acc:ZDB-GENE-130530-599]	17	31151215	31164219		0.6	0.52	0.44	1.26	1.94	1.5
ENSDARG00000038239	tnpo2	transportin 2 (importin 3, karyopherin beta 2b) [Source:ZFIN;Acc:ZDB-GENE-040822-16]	3	18807006	18839529		12.89	10.45	8.47	15.62	15.69	15.03
ENSDARG00000038242	zgc:113333	zgc:113333 [Source:ZFIN;Acc:ZDB-GENE-050320-57]	3	18740950	18756076		6.66	6.14	4.56	5.92	5.91	5.78
ENSDARG00000038243	e4f1	E4F transcription factor 1 [Source:NCBI gene;Acc:561158]	3	18714143	18737126		6.21	4.39	5.21	5.11	6.95	6.81
ENSDARG00000038248	ggact.2	gamma-glutamylamine cyclotransferase, tandem duplicate 2 [Source:NCBI gene;Acc:447805]	1	2450140	2453174		0	0.01	0	0.85	0.38	0.11
ENSDARG00000038258	proca	protein C (inactivator of coagulation factors Va and VIIIa), a [Source:ZFIN;Acc:ZDB-GENE-060824-5]	2	5456541	5475910		3.54	5.67	4.54	3.38	4.04	3.64
ENSDARG00000038270	PIGG	si:ch73-49o8.1 [Source:ZFIN;Acc:ZDB-GENE-141215-6]	14	3717745	3944017	PIGG	1.71	1.06	0.85	1.92	1.7	1.71
ENSDARG00000038271	zgc:113293	zgc:113293 [Source:ZFIN;Acc:ZDB-GENE-050417-453]	2	205763	211064		0.12	0.03	0.01	0.02	0.05	0.06
ENSDARG00000038278	ptgir	prostaglandin I2 receptor [Source:ZFIN;Acc:ZDB-GENE-120511-1]	15	9117330	9184449		1.09	0.9	0.84	1.87	1.89	1.53
ENSDARG00000038281	natd1	zgc:110779 [Source:NCBI gene;Acc:100002945]	3	15817644	15824100		14.17	9.04	10.28	18.49	18.39	19.39
ENSDARG00000038283	wnt6a	wingless-type MMTV integration site family, member 6a [Source:ZFIN;Acc:ZDB-GENE-111114-1]	6	13726844	13734490		1.56	1.52	1.18	1.33	1.38	1.61
ENSDARG00000038288	cd151	CD151 molecule [Source:ZFIN;Acc:ZDB-GENE-041010-137]	25	3809733	3830272		5.97	7.75	8.29	7.32	7.76	7.63
ENSDARG00000038290	thoc5	THO complex 5 [Source:NCBI gene;Acc:325064]	25	3759553	3787221		8.05	6.17	6.02	7.24	8	7.36
ENSDARG00000038293	zgc:103559	zgc:103559 [Source:ZFIN;Acc:ZDB-GENE-041212-22]	6	27126620	27139396		3.91	6.63	6.93	3.75	3.91	3.24
ENSDARG00000038296	tmem86b	transmembrane protein 86B [Source:ZFIN;Acc:ZDB-GENE-060825-132]	3	16762483	16770318		16.53	29.99	22.53	13.87	15.03	14.92
ENSDARG00000038297	aspdh	aspartate dehydrogenase domain containing [Source:NCBI gene;Acc:447919]	3	16747317	16760923		10.31	13.99	12.55	7.51	7.49	7.62
ENSDARG00000038300	rnf34a	ring finger protein 34a [Source:ZFIN;Acc:ZDB-GENE-030131-6135]	8	40210398	40238509		2.58	1.33	1.25	1.73	2.08	1.63
ENSDARG00000038302	ccar2	cell cycle and apoptosis regulator 2 [Source:ZFIN;Acc:ZDB-GENE-091204-207]	10	2876020	2898402		7.07	4.72	4.77	6.59	6.15	6.17
ENSDARG00000038305	vgll3	vestigial-like family member 3 [Source:ZFIN;Acc:ZDB-GENE-130530-600]	9	19007959	19016295		0.46	0.42	0.52	1.05	1.37	1.25
ENSDARG00000038306	tsc22d1	TSC22 domain family, member 1 [Source:ZFIN;Acc:ZDB-GENE-030131-7785]	9	18732505	18814737		29.86	16.67	17.77	34.75	34.26	35.39
ENSDARG00000038308	ykt6	YKT6 v-SNARE homolog (S. cerevisiae) [Source:NCBI gene;Acc:394067]	10	2841205	2850936		8.63	7.02	8.07	10.03	11.46	11.23
ENSDARG00000038309	dnajc15	DnaJ (Hsp40) homolog, subfamily C, member 15 [Source:ZFIN;Acc:ZDB-GENE-040801-216]	9	18219940	18278580		10.04	9.55	9.3	8.92	9.15	8.43
ENSDARG00000038312	rcl1	RNA terminal phosphate cyclase-like 1 [Source:NCBI gene;Acc:445388]	5	1833741	1869982		13.51	16.06	15.07	9.72	9.74	10.31
ENSDARG00000038321	zgc:172053	zgc:172053 [Source:ZFIN;Acc:ZDB-GENE-080219-25]	9	33080438	33081978		0.16	0.07	0.09	1.04	0.78	0.95
ENSDARG00000038348	pigh	phosphatidylinositol glycan anchor biosynthesis, class H [Source:ZFIN;Acc:ZDB-GENE-040718-303]	20	28399593	28404443		6.4	4.87	4.14	6.66	7.06	6.77
ENSDARG00000038352	slc27a1b	solute carrier family 27 member 1b [Source:ZFIN;Acc:ZDB-GENE-061013-672]	7	58370526	58371503		0	0	0	0	0	0
ENSDARG00000038359	enosf1	enolase superfamily member 1 [Source:ZFIN;Acc:ZDB-GENE-060929-780]	2	57108823	57134010		4.73	5.72	5.34	4.52	3.91	3.92
ENSDARG00000038360	thg1l	tRNA-histidine guanylyltransferase 1-like [Source:ZFIN;Acc:ZDB-GENE-041114-170]	14	6535426	6540646		4.01	4.43	4.07	2.74	3.05	3.11
ENSDARG00000038363	drd4a	dopamine receptor D4a [Source:ZFIN;Acc:ZDB-GENE-070112-996]	25	4707173	4733221		3.73	0.74	0.45	3.02	2.89	2.98
ENSDARG00000038378	saga	S-antigen; retina and pineal gland (arrestin) b [Source:ZFIN;Acc:ZDB-GENE-050913-98]	15	45640906	45663081		157.15	71	74.59	53.86	58.71	58.24
ENSDARG00000038384	th2	tyrosine hydroxylase 2 [Source:ZFIN;Acc:ZDB-GENE-050201-1]	4	17380578	17391091		0.42	0.13	0.3	0.33	0.29	0.38
ENSDARG00000038386	ascl1a	achaete-scute family bHLH transcription factor 1a [Source:NCBI gene;Acc:30466]	4	17417111	17419193		7.76	3.86	5.43	7.04	6.83	6.7
ENSDARG00000038392	si:dkeyp-52c3.7	si:dkeyp-52c3.7 [Source:ZFIN;Acc:ZDB-GENE-110411-213]	3	4047789	4214905		0.33	0.38	0.19	0.13	0.16	0.05
ENSDARG00000038397	rpap2	RNA polymerase II associated protein 2 [Source:NCBI gene;Acc:554138]	2	10790875	10821053		3.86	4.21	4.29	2.28	2.6	2.21
ENSDARG00000038398	pmm1	phosphomannomutase 1 [Source:ZFIN;Acc:ZDB-GENE-180314-1]	3	3810919	3821845		4.2	1.62	2.65	10.34	12.76	12.57
ENSDARG00000038405	zgc:153921	zgc:153921 [Source:ZFIN;Acc:ZDB-GENE-060929-816]	3	1037946	1061723		0.18	0.1	0.1	0.05	0.02	0.08
ENSDARG00000038414	b3galt6	UDP-Gal:betaGal beta 1,3-galactosyltransferase polypeptide 6 [Source:ZFIN;Acc:ZDB-GENE-101104-13]	11	2685112	2690933		2.19	1.1	1.2	2.45	2.49	2.76
ENSDARG00000038422	entpd4	ectonucleoside triphosphate diphosphohydrolase 4 [Source:ZFIN;Acc:ZDB-GENE-040718-116]	8	50150834	50180226		6.64	3.51	3.37	8.97	8.78	7.82
ENSDARG00000038424	c4b	complement 4B (Chido blood group) [Source:ZFIN;Acc:ZDB-GENE-100805-2]	16	16665203	16701718		5.18	7.67	6.72	36.76	38.32	36.85
ENSDARG00000038428	sulf1	sulfatase 1 [Source:ZFIN;Acc:ZDB-GENE-030131-9242]	24	19414561	19588659		4.36	3.74	2.76	8.11	8.11	7.77
ENSDARG00000038429	csrnp1b	cysteine-serine-rich nuclear protein 1b [Source:ZFIN;Acc:ZDB-GENE-030131-1515]	24	19707609	19719240		13.76	9.23	9.15	24.61	23.74	23.55
ENSDARG00000038432	actr10	actin related protein 10 [Source:ZFIN;Acc:ZDB-GENE-040426-768]	20	54274431	54286860		30.3	20.49	20.61	29.18	29.18	28.23
ENSDARG00000038439	fabp10a	fatty acid binding protein 10a, liver basic [Source:NCBI gene;Acc:171481]	16	52512025	52516915		513.27	798.07	752.27	51.44	49.02	66.16
ENSDARG00000038442	plcg1	phospholipase C, gamma 1 [Source:ZFIN;Acc:ZDB-GENE-030421-3]	23	2789422	2827819		10.16	7.82	6.7	17.29	17.67	17.54
ENSDARG00000038445	spire1b	spire-type actin nucleation factor 1b [Source:ZFIN;Acc:ZDB-GENE-120608-4]	16	52786644	52821023		3.55	1.14	1.73	4.44	4.64	4.65
ENSDARG00000038446	nrp2b	neuropilin 2b [Source:ZFIN;Acc:ZDB-GENE-040611-3]	9	14367570	14504834		17.6	8.29	7.41	24.26	21.19	22.96
ENSDARG00000038456	mrps35	mitochondrial ribosomal protein S35 [Source:NCBI gene;Acc:503880]	25	2333258	2355107		20.3	24.01	25.25	16.62	17.98	16.98
ENSDARG00000038465	stmn3	stathmin-like 3 [Source:ZFIN;Acc:ZDB-GENE-081124-2]	6	58543336	58563582		6.18	3.25	4.2	5.74	6.37	5.23
ENSDARG00000038467	igsf8	immunoglobulin superfamily, member 8 [Source:ZFIN;Acc:ZDB-GENE-040426-2444]	6	58698475	58772272		9.08	3.55	3.79	10.48	11.19	10.9
ENSDARG00000038473	ube2d3	ubiquitin-conjugating enzyme E2D 3 [Source:ZFIN;Acc:ZDB-GENE-030131-551]	13	11746412	11763191		59.83	74.02	85.65	59.33	56.55	56.2
ENSDARG00000038475	acy1	aminoacylase 1 [Source:ZFIN;Acc:ZDB-GENE-040426-755]	22	36755263	36774243		13	14.27	13.43	15.27	16.1	17.45
ENSDARG00000038476	si:dkeyp-68b7.7	si:dkeyp-68b7.7 [Source:ZFIN;Acc:ZDB-GENE-070912-672]	2	57892666	57916317		2.99	2.46	2.68	1.93	2.4	2.47
ENSDARG00000038489	b3gnt7	UDP-GlcNAc:betaGal beta-1,3-N-acetylglucosaminyltransferase 7 [Source:ZFIN;Acc:ZDB-GENE-021210-4]	22	36656680	36661461		7.5	4.95	3.36	19.09	14.63	14.65
ENSDARG00000038500	cep41	centrosomal protein 41 [Source:NCBI gene;Acc:431741]	25	18436301	18445279		2.44	1.76	2.43	2.45	2.44	2.66
ENSDARG00000038506	polr2h	info polymerase (RNA) II (DNA directed) polypeptide H [Source:ZFIN;Acc:ZDB-GENE-050417-66]	22	36521139	36530902		2.89	3.14	3.07	2.39	2.24	2.12
ENSDARG00000038508	chrnb3b	cholinergic receptor, nicotinic, beta 3b (neuronal) [Source:ZFIN;Acc:ZDB-GENE-030102-4]	14	41518257	41530140		1.98	0.44	0.35	1.52	1.33	1.7
ENSDARG00000038516	snap29	synaptosome associated protein 29 [Source:ZFIN;Acc:ZDB-GENE-041111-226]	8	1839695	1856656		3.99	3.13	3.3	6.3	5.8	5.95
ENSDARG00000038524	pik3r1	phosphoinositide-3-kinase, regulatory subunit 1 (alpha) [Source:ZFIN;Acc:ZDB-GENE-030131-1280]	5	54765702	54792239		27.69	19.22	17.48	29.32	29.24	28.42
ENSDARG00000038537	gga1	golgi-associated, gamma adaptin ear containing, ARF binding protein 1 [Source:ZFIN;Acc:ZDB-GENE-040822-13]	6	22119189	22146258		9.84	7.93	6.84	11.23	10.79	10.92
ENSDARG00000038541	ccr12a	chemokine (C-C motif) receptor 12a [Source:ZFIN;Acc:ZDB-GENE-060503-97]	2	49984159	49985351		1.28	0.76	1.32	2.41	1.78	2.08
ENSDARG00000038545	wdr48b	WD repeat domain 48b [Source:NCBI gene;Acc:334682]	2	49805892	49834052		7.75	5.18	5.61	5.77	6.82	6.85
ENSDARG00000038553	BX890608.1		2	48263750	48264602		0.3	0.13	0.28	0.28	0.12	0.14
ENSDARG00000038556	zgc:112185	zgc:112185 [Source:NCBI gene;Acc:541326]	24	37681082	37688398		4	4.8	4.11	3.1	2.57	3.63
ENSDARG00000038557	anks3	ankyrin repeat and sterile alpha motif domain containing 3 [Source:ZFIN;Acc:ZDB-GENE-080204-51]	24	37661140	37680917		1.76	1.62	1.53	2.74	2.94	2.84
ENSDARG00000038559	h1-0	H1.0 linker histone [Source:ZFIN;Acc:ZDB-GENE-030131-337]	24	37567029	37568359		280.25	171.44	204.74	496.54	547.04	614.38
ENSDARG00000038561	klf6b	Kruppel-like factor 6b [Source:ZFIN;Acc:ZDB-GENE-070912-633]	2	48073972	48082182		3.83	3.48	3.57	1.51	1.56	1.46
ENSDARG00000038562	pitrm1	pitrilysin metallopeptidase 1 [Source:NCBI gene;Acc:406808]	2	48089057	48119409		14.04	15.58	14.07	10.45	9.73	10.23
ENSDARG00000038566	gnptg	N-acetylglucosamine-1-phosphate transferase subunit gamma [Source:ZFIN;Acc:ZDB-GENE-040625-18]	24	37338169	37351737		3.14	3.81	3.56	2.92	2.95	3.1
ENSDARG00000038567	tsr3	TSR3 ribosome maturation factor [Source:ZFIN;Acc:ZDB-GENE-040718-63]	24	37329119	37338739		8.71	9.49	9.97	7.02	5.57	6.01
ENSDARG00000038569	fzd8b	frizzled class receptor 8b [Source:ZFIN;Acc:ZDB-GENE-000328-4]	2	47955160	47957673		1.48	0.54	0.7	1.24	1.52	1.21
ENSDARG00000038570	BX548047.1		2	47942191	47943492		0.14	0.07	0.06	0.14	0	0
ENSDARG00000038574	scg2b	secretogranin II (chromogranin C), b [Source:ZFIN;Acc:ZDB-GENE-061103-160]	2	47581997	47585197		66.53	18.03	22.77	77	85.23	86.96
ENSDARG00000038576	ube2d1b	ubiquitin-conjugating enzyme E2D 1b [Source:ZFIN;Acc:ZDB-GENE-030131-2735]	12	7237666	7253270		19.82	23.63	19.35	22.63	22.84	22.85
ENSDARG00000038577	cox6c	cytochrome c oxidase subunit 6C [Source:ZFIN;Acc:ZDB-GENE-040724-95]	2	52235441	52241529		361.41	463.85	481.02	296.26	291.07	292.85
ENSDARG00000038583	abraa	actin binding Rho activating protein a [Source:ZFIN;Acc:ZDB-GENE-120730-1]	6	52064304	52072829		6.32	11.54	8.03	9.06	8.12	7.82
ENSDARG00000038585	eif4e2	eukaryotic translation initiation factor 4E family member 2 [Source:ZFIN;Acc:ZDB-GENE-050327-59]	2	45116819	45135618		16.09	14.45	14.16	9.05	9.6	9.56
ENSDARG00000038587	CU929150.1	uncharacterized LOC100004199 [Source:NCBI gene;Acc:100004199]	2	45159855	45162467		0.06	0.11	0.05	0.21	0.29	0.34
ENSDARG00000038593	rspo4	R-spondin 4 [Source:ZFIN;Acc:ZDB-GENE-110103-2]	6	51824596	51843277		0.65	1.61	1.07	0.69	0.72	0.94
ENSDARG00000038608	sdhc	succinate dehydrogenase complex, subunit C, integral membrane protein [Source:ZFIN;Acc:ZDB-GENE-040801-26]	2	44192652	44199840		82.52	110.84	113.95	49.56	53.37	51.99
ENSDARG00000038609	mpz	myelin protein zero [Source:ZFIN;Acc:ZDB-GENE-010724-4]	2	44259482	44283554		5.49	3.48	3.28	3.48	3.12	3.8
ENSDARG00000038612	pih1d3	PIH1 domain containing 3 [Source:ZFIN;Acc:ZDB-GENE-040722-2]	2	44400770	44406125		1.62	1.6	1.21	1.85	1.72	1.42
ENSDARG00000038615	pip4p1a	phosphatidylinositol-4,5-bisphosphate 4-phosphatase 1a [Source:NCBI gene;Acc:556136]	2	44383175	44398611		5.28	1.96	1.75	7.84	7.62	7.55
ENSDARG00000038618	cpt2	carnitine palmitoyltransferase 2 [Source:NCBI gene;Acc:100005717]	8	21137446	21142602		12.8	16.2	12.29	8.25	7.55	7.7
ENSDARG00000038635	magoh	mago homolog, exon junction complex subunit [Source:NCBI gene;Acc:100005893]	8	21133307	21135732		54.56	67.52	59.46	45.23	40.93	43.99
ENSDARG00000038639	elovl6l	ELOVL family member 6, elongation of long chain fatty acids like [Source:ZFIN;Acc:ZDB-GENE-031110-3]	13	21666521	21672131		3.47	2.91	2.07	2.32	1.9	1.98
ENSDARG00000038643	alas2	aminolevulinate, delta-, synthase 2 [Source:NCBI gene;Acc:64607]	8	21353878	21369028		5.52	6.09	6.47	25.83	29.13	26.4
ENSDARG00000038646	ftr11	finTRIM family, member 11 [Source:ZFIN;Acc:ZDB-GENE-070912-195]	2	42708674	42713902		0.01	0	0	0.01	0	0.02
ENSDARG00000038655	ajap1	adherens junctions associated protein 1 [Source:ZFIN;Acc:ZDB-GENE-041210-353]	8	21588067	21676306		5.91	1.21	2.31	5.98	6.49	6.03

ENSDARG00000038658	cavin4a	caveolae associated protein 4a [Source:NCBI gene;Acc:552940]	2	42191592	42198600		33.38	48.45	50.61	35.67	35.03	35.27
ENSDARG00000038666	igfbp1b	insulin-like growth factor binding protein 1b [Source:ZFIN;Acc:ZDB-GENE-081210-3]	2	124433	127945		6.72	7.31	7.67	9.75	12.63	13.43
ENSDARG00000038667	fggy	FGGY carbohydrate kinase domain containing [Source:NCBI gene;Acc:406567]	2	111919	115367		13.14	16.54	16.51	7.68	9.11	8.64
ENSDARG00000038668	gbp1	guanylate binding protein 1 [Source:ZFIN;Acc:ZDB-GENE-040718-32]	2	42020915	42035307		1.79	2.44	2.11	2.26	1.86	1.91
ENSDARG00000038669	gbp2	guanylate binding protein 2 [Source:ZFIN;Acc:ZDB-GENE-070912-618]	2	42005217	42017282		1.03	1.63	1.42	1.96	1.87	2
ENSDARG00000038672	tcf7	transcription factor 7 [Source:ZFIN;Acc:ZDB-GENE-050222-4]	21	45267589	45337118		2.14	2.08	2.01	2.34	2.5	2.68
ENSDARG00000038681	MFAP4	wu:fj11g02 [Source:ZFIN;Acc:ZDB-GENE-030131-7370]	1	59094416	59096190	MFAP4	0.02	0.37	0.24	0.86	0.56	0.67
ENSDARG00000038682	zgc:171687	zgc:171687 [Source:ZFIN;Acc:ZDB-GENE-071004-9]	1	58912051	58913813		0.51	0.58	0.56	0.3	0.34	0.23
ENSDARG00000038683	MFAP4	si:zfos-2330d3.1 [Source:ZFIN;Acc:ZDB-GENE-110408-35]	1	59075795	59104145	MFAP4	0.07	0.3	0.26	0.27	0.42	0.36
ENSDARG00000038686	SHLD2	si:ch211-134m17.9 [Source:ZFIN;Acc:ZDB-GENE-090313-30]	13	22698215	22715102	SHLD2	1.57	1	1.14	1.19	1.52	1.47
ENSDARG00000038687	nfkb2	nuclear factor of kappa light polypeptide gene enhancer in B-cells 2 (p49/p100) [Source:ZFIN;Acc:ZDB-GENE-030131-6701]	13	22717366	22735547		7.93	7.19	6.82	8.72	8.6	8.4
ENSDARG00000038693	gng7	guanine nucleotide binding protein (G protein), gamma 7 [Source:ZFIN;Acc:ZDB-GENE-040718-93]	2	36990024	37043905		23.32	15.21	16.88	25.84	26.42	27.01
ENSDARG00000038694	zgc:101744	zgc:101744 [Source:ZFIN;Acc:ZDB-GENE-050320-111]	2	37089362	37103622		51.59	77.97	81.01	44.19	44.69	43.94
ENSDARG00000038695	elavl1a	ELAV like RNA binding protein 1a [Source:ZFIN;Acc:ZDB-GENE-990415-245]	2	37123338	37134198		32.21	23.52	23.25	33.85	31.72	31.91
ENSDARG00000038700	gpr160	G protein-coupled receptor 160 [Source:ZFIN;Acc:ZDB-GENE-081022-189]	2	37295088	37296159		3.74	3.8	3.77	1.42	2.21	1.29
ENSDARG00000038703	hkdc1	hexokinase domain containing 1 [Source:ZFIN;Acc:ZDB-GENE-030131-9801]	13	23017498	23031803		10.28	14.58	9.97	3.96	3.91	3.94
ENSDARG00000038709	MYADM	si:dkeyp-66d1.7 [Source:ZFIN;Acc:ZDB-GENE-070912-662]	2	37750016	37755128	MYADM	7.27	8.43	6.53	10.45	10.75	10.95
ENSDARG00000038713	tep1	telomerase-associated protein 1 [Source:ZFIN;Acc:ZDB-GENE-030131-6153]	2	37897079	37906118		4.38	4.71	2.62	2.72	2.99	2.73
ENSDARG00000038716	casq1a	calsequestrin 1a [Source:ZFIN;Acc:ZDB-GENE-040801-139]	2	38039857	38054482		39.39	71.16	82.08	38.57	27.57	30.32
ENSDARG00000038720	zp3f.1	zona pellucida glycoprotein 3f, tandem duplicate 1 [Source:ZFIN;Acc:ZDB-GENE-030616-590]	2	38282647	38284749		0.02	0.02	0.02	0	0	0
ENSDARG00000038728	ch25hl2	cholesterol 25-hydroxylase like 2 [Source:NCBI gene;Acc:100136851]	21	7900107	7901424		0.87	0.77	0.62	0.75	0.99	0.78
ENSDARG00000038729	s100z	S100 calcium binding protein Z [Source:ZFIN;Acc:ZDB-GENE-050522-69]	21	7863243	7882905		5.73	4.71	6.47	3.86	4.22	3.47
ENSDARG00000038731	slc25a36a	solute carrier family 25 member 36a [Source:NCBI gene;Acc:436940]	2	39731284	39759059		10.42	5.58	6.55	17.12	17.48	17.57
ENSDARG00000038737	phf20b	PHD finger protein 20, b [Source:ZFIN;Acc:ZDB-GENE-040822-33]	23	14992800	15036789		8.71	5.72	4.91	8.42	7.81	7.7
ENSDARG00000038742	rbp1.2	retinol binding protein 1, cellular, tandem duplicate 2 [Source:ZFIN;Acc:ZDB-GENE-070912-18]	2	39021282	39028052		11.88	1.85	1.15	16.15	13.8	11.92
ENSDARG00000038743	copb2	coatomer protein complex, subunit beta 2 [Source:ZFIN;Acc:ZDB-GENE-010724-7]	2	38997357	39015130		34.54	36.43	33.59	38.26	41.09	38.23
ENSDARG00000038754	plk3	polo-like kinase 3 (Drosophila) [Source:ZFIN;Acc:ZDB-GENE-030131-6134]	2	35603637	35611789		10.59	7.76	6.51	12.4	11.56	12.17
ENSDARG00000038768	mrpl12	mitochondrial ribosomal protein L12 [Source:ZFIN;Acc:ZDB-GENE-050417-187]	3	50054187	50066528		44.51	51.16	51.1	31.93	30.52	30.9
ENSDARG00000038770	zgc:103625	zgc:103625 [Source:NCBI gene;Acc:447931]	3	50069204	50075190		4.61	0.82	0.98	3.25	3.72	3.72
ENSDARG00000038780	ubtfl	upstream binding transcription factor, like [Source:ZFIN;Acc:ZDB-GENE-040426-1159]	2	32357226	32387441		14.96	9.21	10.11	12.74	11.56	12.54
ENSDARG00000038784	faim2a	Fas apoptotic inhibitory molecule 2a [Source:ZFIN;Acc:ZDB-GENE-050320-88]	2	32494212	32505150		13.62	6.9	8.05	17.48	15.67	15.97
ENSDARG00000038785	abcf2a	ATP-binding cassette, sub-family F (GCN20), member 2a [Source:ZFIN;Acc:ZDB-GENE-030131-8714]	2	32505554	32513538		17.71	17.64	18.32	20.9	20.62	20.94
ENSDARG00000038786	smarcd3a	SWI/SNF related, matrix associated, actin dependent regulator of chromatin, subfamily d, member 3a [Source:ZFIN;Acc:ZDB-GENE	2	32515846	32558795		7.74	5.6	5.32	13.05	11.96	11.28
ENSDARG00000038787	tmub1	transmembrane and ubiquitin-like domain containing 1 [Source:NCBI gene;Acc:406589]	2	32561183	32574944		12.35	13.05	13.35	12.87	11.36	11.77
ENSDARG00000038788	dnai1.2	dynein, axonemal, intermediate chain 1, paralog 2 [Source:ZFIN;Acc:ZDB-GENE-070112-1302]	21	17956024	17982845		1.47	1.47	1.53	1.69	2	1.57
ENSDARG00000038789	tmem53	transmembrane protein 53 [Source:NCBI gene;Acc:436910]	2	32846602	32850621		5.25	3.96	3.98	4.41	5.04	5.31
ENSDARG00000038792	klf17	Kruppel-like factor 17 [Source:ZFIN;Acc:ZDB-GENE-010129-1]	2	33326522	33328688		4.85	5.13	4.25	4.66	4.75	5.16
ENSDARG00000038793	ccdc24	coiled-coil domain containing 24 [Source:ZFIN;Acc:ZDB-GENE-050327-18]	2	33422411	33455274		0.39	0.34	0.41	0.83	0.55	0.86
ENSDARG00000038794	zgc:113531	zgc:113531 [Source:ZFIN;Acc:ZDB-GENE-050320-52]	2	33457310	33466371		17.35	19.71	20.1	17.42	19.24	18.75
ENSDARG00000038801	klhl20	kelch-like family member 20 [Source:ZFIN;Acc:ZDB-GENE-040426-1935]	2	34112100	34126879		7.3	5.02	5.06	7.32	7.34	7.91
ENSDARG00000038802	cenpl	centromere protein L [Source:NCBI gene;Acc:445401]	2	34128738	34138400		1.11	0.93	0.99	0.8	1.04	0.94
ENSDARG00000038805	pigp	phosphatidylinositol glycan anchor biosynthesis, class P [Source:ZFIN;Acc:ZDB-GENE-030131-4041]	10	110868	116036		17.63	15.96	17.63	10.86	10.42	9.12
ENSDARG00000038812	e2f5	E2F transcription factor 5 [Source:ZFIN;Acc:ZDB-GENE-030828-3]	2	31676790	31686403		0.28	0.15	0.22	0.32	0.4	0.5
ENSDARG00000038814	myrip	Danio rerio myosin VIIA and Rab interacting protein (myrip), mRNA. [Source:RefSeq mRNA;Acc:NM_001353925]	24	11112113	11325849		8.48	1.63	2.73	8.27	7.68	7.69
ENSDARG00000038822	mrc1b	mannose receptor, C type 1b [Source:ZFIN;Acc:ZDB-GENE-070705-13]	2	31515853	31540922		2.16	2.93	2.57	6.34	6.72	6.93
ENSDARG00000038826	jph1b	junctophilin 1b [Source:ZFIN;Acc:ZDB-GENE-060616-389]	2	30290719	30324610		20.15	29.24	25.28	38.26	33.75	31.26
ENSDARG00000038834	etfdh	electron transfer flavoprotein dehydrogenase [Source:ZFIN;Acc:ZDB-GENE-040912-168]	14	48070872	48103207		25.2	32.22	28	18.95	18.95	18.33
ENSDARG00000038835	ppid	peptidylprolyl isomerase D [Source:ZFIN;Acc:ZDB-GENE-040625-34]	14	48045193	48065365		37.23	37.8	34.38	30.94	30.33	30.59
ENSDARG00000038845	ldhd	lactate dehydrogenase D [Source:ZFIN;Acc:ZDB-GENE-030131-6140]	25	13473938	13490744		7.77	5.97	4.9	5.08	5.31	4.68
ENSDARG00000038847	gins3	GINS complex subunit 3 [Source:ZFIN;Acc:ZDB-GENE-040721-1]	25	13399967	13408760		4.54	5.29	5.55	2.35	2.73	2.08
ENSDARG00000038855	chmp5a	charged multivesicular body protein 5a [Source:ZFIN;Acc:ZDB-GENE-060503-60]	2	30894595	30901120		10.87	5.2	6.22	9.78	9.92	10.09
ENSDARG00000038858	setd7	SET domain containing 7, histone lysine methyltransferase [Source:NCBI gene;Acc:436729]	14	46199941	46213571		15.14	12.06	13.75	11.55	13.33	13.88
ENSDARG00000038859	rgs20	regulator of G protein signaling 20 [Source:ZFIN;Acc:ZDB-GENE-040718-128]	2	30752501	30784502		4.35	0.86	1.17	5.15	5.16	5.24
ENSDARG00000038862	kcnb2	potassium voltage-gated channel, Shab-related subfamily, member 2 [Source:ZFIN;Acc:ZDB-GENE-070912-170]	2	30144979	30177469		5.07	1.09	0.66	3.96	3.79	4.03
ENSDARG00000038863	babam2	BRISC and BRCA1 A complex member 2 [Source:NCBI gene;Acc:550474]	17	41081512	41266561		3.93	3.8	3.9	2.99	3.4	3.26
ENSDARG00000038865	acox3	acyl-CoA oxidase 3, pristanoyl [Source:ZFIN;Acc:ZDB-GENE-040426-2163]	7	61764040	61806065		6.24	8.74	5.73	4.53	4.7	5.13
ENSDARG00000038866	trappc8	trafficking protein particle complex 8 [Source:ZFIN;Acc:ZDB-GENE-130530-601]	20	6308564	6476705		6.73	4.4	3.85	7.47	7.68	7.78
ENSDARG00000038867	shhb	sonic hedgehog signaling molecule b [Source:NCBI gene;Acc:30444]	2	30050541	30055432		3.29	1.85	2.33	4.53	4.64	4.82
ENSDARG00000038868	en2b	engrailed homeobox 2b [Source:NCBI gene;Acc:30238]	2	29976419	29979956		5.21	3.92	3.52	4.44	4.36	4.8
ENSDARG00000038869	htr5aa	5-hydroxytryptamine (serotonin) receptor 5A, genome duplicate a [Source:ZFIN;Acc:ZDB-GENE-060531-129]	2	29925561	29940519		0.08	0.06	0.1	0.09	0.06	0.06
ENSDARG00000038870	slu7	SLU7 homolog, splicing factor [Source:NCBI gene;Acc:492495]	14	46003973	46018728		26.65	24.68	24.39	20.99	21.91	21.66
ENSDARG00000038876	zgc:101569	zgc:101569 [Source:ZFIN;Acc:ZDB-GENE-041010-72]	24	7828097	7854229		14.82	18.36	18.58	10.31	10.41	10.73
ENSDARG00000038878	il12a	interleukin 12a [Source:ZFIN;Acc:ZDB-GENE-060724-1]	15	1440364	1448705		0.05	0.05	0.06	0.02	0.14	0.05
ENSDARG00000038879	ift80	intraflagellar transport 80 homolog (Chlamydomonas) [Source:ZFIN;Acc:ZDB-GENE-041212-50]	15	1494493	1534258		5.74	3	3.94	6.06	6.42	6.69
ENSDARG00000038881	acaa2	acetyl-CoA acyltransferase 2 [Source:ZFIN;Acc:ZDB-GENE-040426-1996]	8	2509625	2529878		70.4	88.6	74.05	40.5	39.75	39.69
ENSDARG00000038882	smc4	structural maintenance of chromosomes 4 [Source:ZFIN;Acc:ZDB-GENE-020419-21]	15	1534644	1573129		13.09	10.66	9.35	10.32	10.06	10.22
ENSDARG00000038891	AL954146.1	trypsin I-P1-like [Source:NCBI gene;Acc:560023]	23	37444933	37453984		12.93	13.18	12.82	3.66	4.75	3.98
ENSDARG00000038894	tmx3a	thioredoxin related transmembrane protein 3a [Source:ZFIN;Acc:ZDB-GENE-050522-396]	2	27318174	27330181		5.15	1.87	1.79	5.07	4.19	4.9
ENSDARG00000038898	zgc:113691	zgc:113691 [Source:ZFIN;Acc:ZDB-GENE-050220-7]	2	26591968	26596794		0.51	0.71	0.56	1.11	1.02	1.04
ENSDARG00000038900	acadm	acyl-CoA dehydrogenase medium chain [Source:ZFIN;Acc:ZDB-GENE-040426-1945]	2	26470070	26476150		80.79	111.82	102.87	42.26	43.66	43.5
ENSDARG00000038901	tmem256	transmembrane protein 256 [Source:NCBI gene;Acc:550283]	7	22293894	22309032		15.21	21.32	22.22	11.87	12.22	11.8
ENSDARG00000038905	hectd3	HECT domain containing 3 [Source:ZFIN;Acc:ZDB-GENE-031118-179]	2	26479676	26495044		14.22	12.93	10.91	15.05	14.77	15.04
ENSDARG00000038909	msl2a	MSL complex subunit 2a [Source:ZFIN;Acc:ZDB-GENE-070912-449]	2	25124498	25129409		5.22	2.74	2.28	5.02	5.24	5.53
ENSDARG00000038910	pccb	propionyl-CoA carboxylase subunit beta [Source:ZFIN;Acc:ZDB-GENE-040426-2467]	2	25108569	25120197		44.94	50.4	52.15	38.79	38.48	37.09
ENSDARG00000038917	rad50	RAD50 homolog, double strand break repair protein [Source:ZFIN;Acc:ZDB-GENE-050506-110]	9	54312035	54361357		6.48	6.88	6.52	3.72	4.32	4.18
ENSDARG00000038918	drd1b	dopamine receptor D1b [Source:ZFIN;Acc:ZDB-GENE-070524-2]	9	54417141	54419308		4.89	0.63	1.13	3.97	4.29	4.06
ENSDARG00000038938	itprid2	ITPR interacting domain containing 2 [Source:ZFIN;Acc:ZDB-GENE-090824-1]	9	44304980	44420380		3.57	3.12	1.89	4.13	3.92	3.8
ENSDARG00000038941	ppp1r1c	protein phosphatase 1, regulatory (inhibitor) subunit 1C [Source:ZFIN;Acc:ZDB-GENE-040718-273]	9	44430705	44489978		2.13	1.74	1.99	2.46	2.82	2.73
ENSDARG00000038954	ctnnbip1	catenin, beta interacting protein 1 [Source:ZFIN;Acc:ZDB-GENE-000906-3]	23	29711569	29751730		22.31	12	14.92	25.4	25.61	24.74
ENSDARG00000038955	pimr68	Pim proto-oncogene, serine/threonine kinase, related 68 [Source:ZFIN;Acc:ZDB-GENE-070912-431]	2	24352497	24355324		0.41	0.27	0.37	0.19	0.25	0.24
ENSDARG00000038956	fdx1b	ferredoxin 1b [Source:ZFIN;Acc:ZDB-GENE-060526-382]	5	22177033	22226645		0.81	1.06	0.94	0.8	0.62	1.04
ENSDARG00000038957	flvcr2a	feline leukemia virus subgroup C cellular receptor family, member 2a [Source:ZFIN;Acc:ZDB-GENE-111020-14]	17	51906053	51929012		1.2	1.08	0.52	1.07	0.78	0.87
ENSDARG00000038964	traf4b	tnf receptor-associated factor 4b [Source:ZFIN;Acc:ZDB-GENE-040305-2]	21	39005905	39024754		4.92	4.29	4.07	3.22	3.25	3.3
ENSDARG00000038967	cul3a	cullin 3a [Source:ZFIN;Acc:ZDB-GENE-030131-3376]	2	47644041	47681454		28.55	22.38	23.21	25.32	27.75	27.1
ENSDARG00000038968	ccr6b	chemokine (C-C motif) receptor 6b [Source:ZFIN;Acc:ZDB-GENE-030131-8888]	17	51642687	51651631		0.17	0.25	0.35	0.14	0.15	0.2
ENSDARG00000038969	zgc:113142	zgc:113142 [Source:ZFIN;Acc:ZDB-GENE-050220-2]	17	51627209	51638206		0.32	0.56	0.55	0.43	0.34	0.4
ENSDARG00000038974	FO904844.1	dihydropyrimidinase like 2a [Source:NCBI gene;Acc:798555]	8	5119360	5155545		21.87	4.65	5.54	35.22	33.26	37.06
ENSDARG00000038976	pdcd7	programmed cell death 7 [Source:ZFIN;Acc:ZDB-GENE-040728-3]	7	13684012	13737618		2.86	1.85	1.64	2.55	2.57	2.78
ENSDARG00000038977	slc25a24l	solute carrier family 25 member 24, like [Source:ZFIN;Acc:ZDB-GENE-040923-1]	2	9529488	9544161		0.12	0.08	0.01	0.23	0.18	0.15
ENSDARG00000038978	dnajb4	DnaJ heat shock protein family (Hsp40) member B4 [Source:ZFIN;Acc:ZDB-GENE-040801-192]	2	9552456	9560307		9.69	4.75	5.94	6.92	7.7	8.88
ENSDARG00000038979	si:dkey-24c2.9	si:dkey-24c2.9 [Source:ZFIN;Acc:ZDB-GENE-070912-476]	2	23823622	23837423		0.49	1.05	0.89	0.77	0.71	0.71
ENSDARG00000038980	txndc12	thioredoxin domain containing 12 (endoplasmic reticulum) [Source:ZFIN;Acc:ZDB-GENE-050522-497]	2	9592319	9607879		14.05	17.33	14.89	21.46	21.15	20.44
ENSDARG00000038981	zgc:153615	zgc:153615 [Source:ZFIN;Acc:ZDB-GENE-061110-79]	2	9629558	9646857		21.98	6.31	9.05	19.45	20.62	21.52
ENSDARG00000038985	cdyl	chromodomain protein, Y-like [Source:ZFIN;Acc:ZDB-GENE-070912-561]	2	23622788	23677422		2.73	2.3	2.32	3.63	3.57	3.57
ENSDARG00000038987	si:dkey-31m5.5	si:dkey-31m5.5 [Source:ZFIN;Acc:ZDB-GENE-070912-517]	2	33726862	33728796		0	0	0	0	0	0
ENSDARG00000038990	wnt3	wingless-type MMTV integration site family, member 3 [Source:ZFIN;Acc:ZDB-GENE-081003-1]	12	22258962	22304841		3.15	3.06	2.59	2.42	2.49	2.09
ENSDARG00000038991	nsfb	N-ethylmaleimide-sensitive factor b [Source:ZFIN;Acc:ZDB-GENE-050808-1]	12	22310365	22355452		7.59	7.72	6.75	7.13	7.66	7.27
ENSDARG00000038996	egln1a	egl-9 family hypoxia-inducible factor 1a [Source:ZFIN;Acc:ZDB-GENE-110408-34]	13	49175624	49206713		1.41	1.57	1.25	3.92	4.85	4.81
ENSDARG00000039005	trappc1	trafficking protein particle complex 1 [Source:ZFIN;Acc:ZDB-GENE-040718-242]	23	44546319	44550533		24.96	32.98	37.57	20.39	20.81	20.65
ENSDARG00000039007	eno3	enolase 3, (beta, muscle) [Source:ZFIN;Acc:ZDB-GENE-031006-5]	23	44611864	44625579		216.61	274.79	299.38	229.95	246.12	240
ENSDARG00000039008		zgc:85858 [Source:ZFIN;Acc:ZDB-GENE-040426-2333]	23	44644911	44653668		17.73	16.06	15.98	17	17.32	16.3
ENSDARG00000039020	fbxo5	F-box protein 5 [Source:NCBI gene;Acc:445392]	13	46941930	46958802		1.63	0.97	0.93	1.09	1.06	1.02
ENSDARG00000039022	stk25b	serine/threonine kinase 25b [Source:ZFIN;Acc:ZDB-GENE-040426-2537]	2	22617675	22636721		34.3	24.94	24.86	30.96	30.25	30.88
ENSDARG00000039034	marcksl1a	MARCKS-like 1a [Source:NCBI gene;Acc:406407]	13	46177478	46180510		154.03	130.78	119.08	218.32	223.71	226.12
ENSDARG00000039041	sfrp5	secreted frizzled-related protein 5 [Source:ZFIN;Acc:ZDB-GENE-011108-2]	13	39911551	39947335		6.54	7.52	6.38	9.08	9.01	9.1
ENSDARG00000039051	hhatla	hedgehog acyltransferase like, a [Source:ZFIN;Acc:ZDB-GENE-040426-1865]	2	21333529	21352101		16.67	24.47	27.93	18.71	16.57	16.22
ENSDARG00000039052	klhl40a	kelch-like family member 40a [Source:NCBI gene;Acc:553257]	2	21326957	21335131		1.25	2.01	1.76	2.34	2.11	1.77
ENSDARG00000039062	morn4	MORN repeat containing 4 [Source:ZFIN;Acc:ZDB-GENE-050417-7]	13	40746281	40754499		4.13	1.9	1.78	5.84	5.52	5.56
ENSDARG00000039063	CR847936.1	piggyBac transposable element-derived protein 4-like [Source:NCBI gene;Acc:100330143]	1	14658801	14660280		1.23	1.12	0.97	2.39	2.42	2.37
ENSDARG00000039064	st3gal7	ST3 beta-galactoside alpha-2,3-sialyltransferase 7 [Source:ZFIN;Acc:ZDB-GENE-060322-2]	13	40716319	40726865		8.51	13.56	10.06	4.37	4.96	4.21
ENSDARG00000039065	trpc2a	transient receptor potential cation channel subfamily C member 2a [Source:ZFIN;Acc:ZDB-GENE-130530-602]	15	38339386	38361787		0.27	0.16	0.18	0.13	0.2	0.21
ENSDARG00000039066	klhl31	kelch-like family member 31 [Source:NCBI gene;Acc:407079]	13	2338761	2350762		31.11	46.25	36.54	46.09	42.22	43.25
ENSDARG00000039067	srd5a2b	steroid-5-alpha-reductase, alpha polypeptide 2b [Source:ZFIN;Acc:ZDB-GENE-050417-182]	13	35484229	35497586		1.57	1.12	1.29	1.88	1.74	1.54
ENSDARG00000039069	slx4ip	SLX4 interacting protein [Source:NCBI gene;Acc:393383]	13	35391609	35472531		0.88	0.45	0.47	0.53	0.55	0.55
ENSDARG00000039070	im:7138239	im:7138239 [Source:ZFIN;Acc:ZDB-GENE-081007-2]	2	53501750	53525896		0.05	0	0	0.06	0.14	0.15
ENSDARG00000039077	tyr	tyrosinase [Source:ZFIN;Acc:ZDB-GENE-991026-3]	15	43799258	43818697		5.44	7.02	7.16	9.97	10.11	9.56
ENSDARG00000039082	zgc:123010	zgc:123010 [Source:ZFIN;Acc:ZDB-GENE-051120-15]	13	40247379	40282770		4.29	5.74	4.45	5.46	6.04	5.95
ENSDARG00000039093	got1	glutamic-oxaloacetic transaminase 1, soluble [Source:ZFIN;Acc:ZDB-GENE-040426-2003]	13	40437550	40456995		78.73	84.13	69.99	77.43	78.58	76.36
ENSDARG00000039095	nkx2.3	NK2 homeobox 3 [Source:ZFIN;Acc:ZDB-GENE-990415-178]	13	40409215	40411917		5.23	4.91	3.84	4.26	4.42	4.92
ENSDARG00000039096	si:dkey-24p1.1	si:dkey-24p1.1 [Source:ZFIN;Acc:ZDB-GENE-060503-631]	15	38101135	38129845		0.03	0.01	0	0.04	0.02	0.06
ENSDARG00000039098	ca8	carbonic anhydrase VIII [Source:ZFIN;Acc:ZDB-GENE-050417-26]	2	21855036	21878527		6.2	5.05	5.86	4.8	4.49	4.82
ENSDARG00000039099	aep1	aerolysin-like protein [Source:NCBI gene;Acc:503710]	7	8373025	8374950		49.02	53.96	43	22.51	15.18	19.5
ENSDARG00000039100	jac7	jacalin 7 [Source:ZFIN;Acc:ZDB-GENE-080219-24]	7	8358547	8362727		0.08	0.05	0.09	0.01	0.05	0.03
ENSDARG00000039108	trim35-9	tripartite motif containing 35-9 [Source:ZFIN;Acc:ZDB-GENE-091112-11]	3	8256231	8285123		0.88	0.79	0.58	0.29	0.4	0.38
ENSDARG00000039116	nr5a5	nuclear receptor subfamily 5, group A, member 5 [Source:ZFIN;Acc:ZDB-GENE-030131-4652]	3	53457192	53465249		3.17	4.56	1.89	0.7	0.82	0.69
ENSDARG00000039117	tefa	TEF transcription factor, PAR bZIP family member a [Source:ZFIN;Acc:ZDB-GENE-990415-264]	12	19144833	19153177		24.94	20.54	20.02	23.17	25.22	25.15
ENSDARG00000039123	trim9	tripartite motif containing 9 [Source:ZFIN;Acc:ZDB-GENE-030131-8043]	13	36868365	36911118		6	1.66	1.99	10.6	10.24	11.17
ENSDARG00000039125	map4k5	mitogen-activated protein kinase kinase kinase kinase 5 [Source:ZFIN;Acc:ZDB-GENE-030131-6497]	13	36705595	36761454		9.21	8.39	6.37	12.16	11.99	12.01
ENSDARG00000039130	cdkn3	cyclin-dependent kinase inhibitor 3 [Source:ZFIN;Acc:ZDB-GENE-060427-2]	13	36612930	36621926		5.67	4.35	4.89	3.22	3.18	4.1
ENSDARG00000039131	atp1a1a.3	ATPase Na+/K+ transporting subunit alpha 1a, tandem duplicate 3 [Source:ZFIN;Acc:ZDB-GENE-001212-3]	1	1805294	1815660		15.54	18.58	18.45	15.2	16.8	15.8
ENSDARG00000039133	lamb4	laminin, beta 4 [Source:NCBI gene;Acc:286831]	25	31633322	31702970		12.38	10.42	5.52	7.56	4.59	4.34
ENSDARG00000039136	cox16	cytochrome c oxidase assembly factor COX16 [Source:ZFIN;Acc:ZDB-GENE-050417-60]	13	36311894	36319308		6.25	5.85	5.28	6.91	6.22	6.8
ENSDARG00000039142	arpc5a	actin related protein 2/3 complex, subunit 5A [Source:ZFIN;Acc:ZDB-GENE-030131-8448]	2	20967673	20975677		11.49	11.94	10.12	17.51	16.2	15.64
ENSDARG00000039145	plaub	plasminogen activator, urokinase b [Source:ZFIN;Acc:ZDB-GENE-030619-15]	12	46740584	46766300		0.81	0.65	0.73	0.28	0.23	0.15
ENSDARG00000039150	lgmn	legumain [Source:ZFIN;Acc:ZDB-GENE-021030-1]	13	36037970	36050327		44.51	50.05	44.78	39.44	43.03	43.66
ENSDARG00000039152	fbxo36b	F-box protein 36b [Source:ZFIN;Acc:ZDB-GENE-060825-271]	2	16482338	16485408		0.26	0.07	0.35	0.56	0.54	0.64
ENSDARG00000039162	rhebl1	Ras homolog, mTORC1 binding like 1 [Source:ZFIN;Acc:ZDB-GENE-000510-1]	6	21684296	21696868		10.74	12.82	10.77	10.71	9.78	10.01
ENSDARG00000039164	mhc1uma	major histocompatibility complex class I UMA [Source:ZFIN;Acc:ZDB-GENE-140820-1]	22	3910610	3914222		0.11	0.06	0.01	1.71	1.7	1.76
ENSDARG00000039173	ctslb	cathepsin Lb [Source:ZFIN;Acc:ZDB-GENE-980526-285]	12	16617138	16619757		0.03	0.26	0.1	0.14	0.04	0.11
ENSDARG00000039174	olfm3b	olfactomedin 3b [Source:ZFIN;Acc:ZDB-GENE-070912-606]	2	15341689	15362923		0.38	0.19	0.21	0.27	0.2	0.42
ENSDARG00000039181	trpm3	transient receptor potential cation channel, subfamily M, member 3 [Source:ZFIN;Acc:ZDB-GENE-060531-95]	5	25945246	26063332		3.98	1.18	1.8	4.9	4.53	4.74
ENSDARG00000039185	nfyba	nuclear transcription factor Y, beta a [Source:ZFIN;Acc:ZDB-GENE-050417-13]	4	9177997	9183602		39.64	32.62	36.27	39.43	37.02	37.84
ENSDARG00000039196	greb1l	Danio rerio GREB1 like retinoic acid receptor coactivator (greb1l), mRNA. [Source:RefSeq mRNA;Acc:NM_001362307]	2	11685742	11833356		0.79	0.66	0.52	2.18	2.2	2.65
ENSDARG00000039203	MFF	zgc:110130 [Source:NCBI gene;Acc:553588]	2	11651176	11662923	MFF	6.9	3.39	4.34	5.82	4.91	5.75
ENSDARG00000039208	nasp	nuclear autoantigenic sperm protein (histone-binding) [Source:ZFIN;Acc:ZDB-GENE-030131-5511]	6	33911154	33916788		31.57	32.74	35.43	16.38	15.93	15.85
ENSDARG00000039211	zgc:77439	zgc:77439 [Source:ZFIN;Acc:ZDB-GENE-031010-24]	7	26297169	26308242		14.5	17.05	15.68	11.56	11.04	11
ENSDARG00000039213	prpf38a	pre-mRNA processing factor 38A [Source:NCBI gene;Acc:445089]	6	33926829	33931696		13.83	15.27	12.74	12.63	10.99	12.68
ENSDARG00000039215	arrb2a	arrestin, beta 2a [Source:ZFIN;Acc:ZDB-GENE-030131-29]	10	22890791	22907418		8.52	4.92	5.43	11.09	9.66	9.62
ENSDARG00000039217	orc1	origin recognition complex, subunit 1 [Source:ZFIN;Acc:ZDB-GENE-030131-6960]	6	33931095	33950917		1.88	1.8	1.59	1.31	1.15	1.21
ENSDARG00000039218	gpr101	G protein-coupled receptor 101 [Source:ZFIN;Acc:ZDB-GENE-070914-1]	14	31890001	31893996		2.16	0.29	0.61	2.21	2.72	1.91
ENSDARG00000039220	st6galnac5a	ST6 (alpha-N-acetyl-neuraminyl-2,3-beta-galactosyl-1,3)-N-acetylgalactosaminide alpha-2,6-sialyltransferase 5a [Source:ZFIN;Acc:	2	9160947	9260002		2.28	0.63	1.15	2.62	2.76	2.58
ENSDARG00000039222	mpl	MPL proto-oncogene, thrombopoietin receptor [Source:ZFIN;Acc:ZDB-GENE-060421-1]	6	33949280	33980881		0.27	0.38	0.4	0.49	0.54	0.5
ENSDARG00000039225	vps13a	vacuolar protein sorting 13 homolog A [Source:ZFIN;Acc:ZDB-GENE-030926-1]	8	51992344	52091696		9.56	7.06	5.67	13.6	12.9	13.09
ENSDARG00000039229	cep78	centrosomal protein 78 [Source:ZFIN;Acc:ZDB-GENE-070905-1]	8	51933983	51954598		0.92	0.42	0.55	0.88	0.78	1.07
ENSDARG00000039234	zgc:171579	zgc:171579 [Source:ZFIN;Acc:ZDB-GENE-080204-32]	13	38213694	38222714		0.04	0.03	0.15	0.08	0.16	0.23
ENSDARG00000039240	cacng5b	calcium channel, voltage-dependent, gamma subunit 5b [Source:ZFIN;Acc:ZDB-GENE-120104-3]	3	56366395	56394018		0.66	0.29	0.34	0.86	0.87	0.86
ENSDARG00000039243	zgc:152791	zgc:152791 [Source:ZFIN;Acc:ZDB-GENE-060901-4]	13	37617518	37620290		0.35	0.58	0.86	0.19	0.26	0.25
ENSDARG00000039255	klhl21	kelch-like family member 21 [Source:NCBI gene;Acc:404613]	23	24926407	24939148		14.42	12.67	11.68	15.07	15.54	15.59
ENSDARG00000039256	spega	striated muscle enriched protein kinase a [Source:NCBI gene;Acc:570504]	6	21095918	21155195		3.58	1.19	1.74	4.46	4.26	4.51
ENSDARG00000039263	zbtb48	zinc finger and BTB domain containing 48 [Source:ZFIN;Acc:ZDB-GENE-030131-4450]	23	24941299	24955155		3.06	2.12	2.29	2.32	1.78	1.94
ENSDARG00000039264	igfbp5a	insulin-like growth factor binding protein 5a [Source:ZFIN;Acc:ZDB-GENE-070620-8]	6	20910551	20918694		7.06	7.75	6.48	3.25	3.23	2.91
ENSDARG00000039265	arhgap4a	Rho GTPase activating protein 4a [Source:ZFIN;Acc:ZDB-GENE-040426-1229]	23	24989387	25015393		0.68	0.63	0.55	0.77	0.55	0.9
ENSDARG00000039266	rbm25a	RNA binding motif protein 25a [Source:ZFIN;Acc:ZDB-GENE-090312-142]	13	32997020	33022372		11	3.04	5.27	8.42	8.68	8.79
ENSDARG00000039268	emc4	ER membrane protein complex subunit 4 [Source:NCBI gene;Acc:402956]	7	1457379	1468521		25.65	21.99	15.59	25.34	23.58	23.86
ENSDARG00000039269	arg2	arginase 2 [Source:ZFIN;Acc:ZDB-GENE-030131-1334]	13	33024547	33034374		16.22	16.34	15.45	15.35	16.96	16.99
ENSDARG00000039270	vti1b	vesicle transport through interaction with t-SNAREs 1B [Source:ZFIN;Acc:ZDB-GENE-040426-1148]	13	33040436	33054856		6.56	6.04	5.9	6.35	6.3	6.64
ENSDARG00000039272	pls1	plastin 1 (I isoform) [Source:ZFIN;Acc:ZDB-GENE-030131-6205]	2	8059957	8111346		6.6	8.49	5.34	2.84	2.55	2.98
ENSDARG00000039273	fbln5	fibulin 5 [Source:ZFIN;Acc:ZDB-GENE-041010-54]	13	33155428	33170733		4.72	4.86	4.64	8.35	7.27	8.12
ENSDARG00000039279	golga5	golgin A5 [Source:NCBI gene;Acc:406720]	13	33211014	33227411		16.99	14.06	13.84	14.92	16.52	16.91
ENSDARG00000039299	rdh14a	retinol dehydrogenase 14a [Source:ZFIN;Acc:ZDB-GENE-041010-124]	13	32454262	32456842		6.29	5.04	5.36	4.32	4.03	4.16
ENSDARG00000039302	terfa	telomeric repeat binding factor a [Source:ZFIN;Acc:ZDB-GENE-020419-38]	4	77973876	77996958		13.64	11.86	13.37	10.5	10.63	11.04
ENSDARG00000039304	six1a	SIX homeobox 1a [Source:NCBI gene;Acc:494168]	13	31619961	31622195		7.35	7.78	7.25	9.02	9.32	9.67
ENSDARG00000039309	hook2	hook microtubule-tethering protein 2 [Source:ZFIN;Acc:ZDB-GENE-040426-1636]	3	7771420	7801208		2.81	2.23	1.97	2.12	1.9	1.95
ENSDARG00000039310	parla	presenilin associated, rhomboid-like a [Source:NCBI gene;Acc:541485]	2	5543901	5561019		1.98	2.35	2.2	1.33	1.58	1.42
ENSDARG00000039319	vps37b	VPS37B subunit of ESCRT-I [Source:ZFIN;Acc:ZDB-GENE-060526-254]	5	27999886	28016805		11.64	12.04	9.34	15.64	15.18	16.05
ENSDARG00000039325	pmepa1	prostate transmembrane protein, androgen induced 1 [Source:ZFIN;Acc:ZDB-GENE-040718-320]	6	60076456	60104628		1.51	1.84	2.35	5.42	6.21	5.95
ENSDARG00000039328	ccndbp1	cyclin D-type binding-protein 1 [Source:NCBI gene;Acc:553373]	6	60043053	60049272		14.23	12.68	13.11	19.89	19.91	19.85

ENSDARG00000039335	st6gal2a	ST6 beta-galactosamide alpha-2,6-sialyltranferase 2a [Source:NCBI gene;Acc:403116]	9	6009077	6127872		10.07	4.25	3.85	18.93	18.71	19.25
ENSDARG00000039338	gadd45gip1	growth arrest and DNA-damage-inducible, gamma interacting protein 1 [Source:ZFIN;Acc:ZDB-GENE-091204-282]	3	14691312	14695033		24.44	23.4	23.26	17.6	18.17	17.3
ENSDARG00000039340	tmem209	transmembrane protein 209 [Source:ZFIN;Acc:ZDB-GENE-030131-2879]	4	5094007	5108941		7.6	7	6	6.96	7.16	6.69
ENSDARG00000039343	ahcyl2b	adenosylhomocysteinase like 2b [Source:ZFIN;Acc:ZDB-GENE-040115-5]	4	5025625	5092335		33.6	19.72	18.43	44.21	45.93	45.56
ENSDARG00000039345	drg1	developmentally regulated GTP binding protein 1 [Source:ZFIN;Acc:ZDB-GENE-030131-8767]	10	17681074	17691171		31.3	29.12	26.23	23.2	22.62	22.85
ENSDARG00000039346	ndufa5	NADH:ubiquinone oxidoreductase subunit A5 [Source:ZFIN;Acc:ZDB-GENE-050320-17]	4	4930406	4932619		94.73	124.89	125.66	57.59	60.2	65.08
ENSDARG00000039347	rps24	ribosomal protein S24 [Source:ZFIN;Acc:ZDB-GENE-040109-5]	13	30225262	30235220		235.95	323.43	322.83	159.96	166.81	181.21
ENSDARG00000039349	qrfpra	pyroglutamylated RFamide peptide receptor a [Source:ZFIN;Acc:ZDB-GENE-090313-105]	13	30215765	30225311		0.1	0.03	0.01	0.13	0.22	0.14
ENSDARG00000039350	ssbp1	single-stranded DNA binding protein 1 [Source:ZFIN;Acc:ZDB-GENE-050417-340]	4	4752622	4756349		14.71	15.89	14.81	10.54	9.96	9.83
ENSDARG00000039351	ccl19b	chemokine (C-C motif) ligand 19b [Source:ZFIN;Acc:ZDB-GENE-091204-19]	10	17776981	17780102		7.47	8.02	7.52	6.1	5.19	7.95
ENSDARG00000039352	pald1b	phosphatase domain containing paladin 1b [Source:ZFIN;Acc:ZDB-GENE-070424-88]	13	30169233	30214368		1.54	1.08	1.52	1.98	2.33	2.3
ENSDARG00000039354	ska1	spindle and kinetochore associated complex subunit 1 [Source:NCBI gene;Acc:436865]	10	17543276	17550931		3.15	3.48	3.12	2.77	2.84	2.71
ENSDARG00000039356	zgc:194209	zgc:194209 [Source:ZFIN;Acc:ZDB-GENE-081022-75]	4	4526248	4535189		5.51	6.41	5.5	5.73	5	5.83
ENSDARG00000039363	dnajb12a	DnaJ heat shock protein family (Hsp40) member B12a [Source:ZFIN;Acc:ZDB-GENE-030131-2725]	13	30035253	30046753		5.86	5.9	5.84	5.85	5.93	5.73
ENSDARG00000039365	cuedc2	CUE domain containing 2 [Source:NCBI gene;Acc:334351]	13	29925397	29936259		5.35	2.67	3.16	3.2	3.35	3.51
ENSDARG00000039373	epha3	eph receptor A3 [Source:ZFIN;Acc:ZDB-GENE-010724-10]	9	16449398	16618607		3.02	2.32	2.08	4.36	4.47	4.33
ENSDARG00000039374	apool	apolipoprotein O-like [Source:ZFIN;Acc:ZDB-GENE-050327-76]	5	22851857	22868650		11.66	13	9.62	13.2	13.15	13.05
ENSDARG00000039378	rcn2	reticulocalbin 2 [Source:ZFIN;Acc:ZDB-GENE-050706-62]	18	26967414	26977940		2.76	3	2.36	3.92	3.7	3.84
ENSDARG00000039381	ch25hl1.1	cholesterol 25-hydroxylase like 1, tandem duplicate 1 [Source:NCBI gene;Acc:336673]	18	26853891	26854959		16.71	10.78	6.64	9.65	7.73	7.74
ENSDARG00000039385	dlg4b	discs large MAGUK scaffold protein 4 [Source:HGNC Symbol;Acc:HGNC:2903]	23	44307996	44332790		20.37	3.62	6.71	22.04	23.04	23.56
ENSDARG00000039390	sdhaf4	succinate dehydrogenase complex assembly factor 4 [Source:ZFIN;Acc:ZDB-GENE-040724-116]	13	28264834	28265919		14.2	16.15	11.54	9.36	9.06	8.69
ENSDARG00000039392	wnk1b	WNK lysine deficient protein kinase 1b [Source:ZFIN;Acc:ZDB-GENE-030131-2656]	4	8045564	8155571		6.04	5.04	4.4	10.7	10.2	10.29
ENSDARG00000039393	si:ch211-240l19.5	si:ch211-240l19.5 [Source:ZFIN;Acc:ZDB-GENE-041210-327]	4	8042805	8043964		38.44	59.63	48.61	6.35	5.37	6.18
ENSDARG00000039399	nt5c2a	5'-nucleotidase, cytosolic IIa [Source:ZFIN;Acc:ZDB-GENE-030131-3566]	13	28634862	28674422		11.33	10.93	9.88	14.93	14.65	14.65
ENSDARG00000039400	polr1c	RNA polymerase I and III subunit C [Source:ZFIN;Acc:ZDB-GENE-040426-1495]	13	28690355	28694924		8.95	8.73	7.4	7.05	7.59	8.15
ENSDARG00000039406	prom2	prominin 2 [Source:ZFIN;Acc:ZDB-GENE-080723-16]	13	28747934	28774347		4.59	4.21	3.76	4.49	4.22	4.17
ENSDARG00000039411	cep68	centrosomal protein 68 [Source:ZFIN;Acc:ZDB-GENE-030616-565]	13	24421515	24429095		2.64	1.18	1.94	2.37	2.74	3.09
ENSDARG00000039412	dmrta2	DMRT-like family A2 [Source:NCBI gene;Acc:474350]	8	16257220	16259063		5.16	2.71	2.47	4.9	5.61	5.72
ENSDARG00000039422	fuom	fucose mutarotase [Source:NCBI gene;Acc:447896]	13	24584401	24586483		2.46	3	2.83	2.33	3.55	2.75
ENSDARG00000039423	AL831745.1	si:ch211-217g15.3 [Source:NCBI gene;Acc:368914]	13	24585224	24588220		11.19	19.4	12.97	2.2	2.44	2.2
ENSDARG00000039424	snrpb2	small nuclear ribonucleoprotein polypeptide B2 [Source:ZFIN;Acc:ZDB-GENE-060616-2]	1	51702087	51710340		15.14	16.71	16.23	12.01	10.12	11.17
ENSDARG00000039429	adka	adenosine kinase a [Source:ZFIN;Acc:ZDB-GENE-030425-3]	13	24918096	25199260		31.87	41.66	29.37	20.48	20.23	18.58
ENSDARG00000039430	msl2b	MSL complex subunit 2b [Source:ZFIN;Acc:ZDB-GENE-030131-1621]	24	26941197	26945390		12.25	5.85	6.63	12.59	13.15	13.29
ENSDARG00000039434	oprm1	opioid receptor, mu 1 [Source:ZFIN;Acc:ZDB-GENE-010125-1]	13	23843712	23882977		2.68	1.04	1.13	2.08	1.78	2.18
ENSDARG00000039435	rgs17	regulator of G protein signaling 17 [Source:ZFIN;Acc:ZDB-GENE-040718-229]	13	23728176	23756700		17.09	5.82	9.55	13.09	13.14	14.54
ENSDARG00000039436	il13ra2	interleukin 13 receptor, alpha 2 [Source:ZFIN;Acc:ZDB-GENE-030521-10]	5	37101393	37117778		7.12	7.09	5.98	5.04	4.95	4.92
ENSDARG00000039439	il22ra2	interleukin 22 receptor, alpha 2 [Source:ZFIN;Acc:ZDB-GENE-060512-349]	13	23623346	23630027		0	0	0.02	0	0.03	0.02
ENSDARG00000039443	zgc:110353	zgc:110353 [Source:ZFIN;Acc:ZDB-GENE-050417-310]	8	24246659	24252933		1.27	1	1.02	1.27	1.21	1.11
ENSDARG00000039452	hk1	hexokinase 1 [Source:ZFIN;Acc:ZDB-GENE-040426-2848]	13	22972888	23007813		35.74	15.55	16.09	49.77	51.37	48.14
ENSDARG00000039453	dhrs7ca	dehydrogenase/reductase (SDR family) member 7Ca [Source:NCBI gene;Acc:558764]	3	58633692	58650057		2.89	4.4	3.76	1.86	2	1.92
ENSDARG00000039455	tspan15	tetraspanin 15 [Source:ZFIN;Acc:ZDB-GENE-050417-295]	13	22924024	22961610		3.29	4.28	3.74	4.32	4.61	3.83
ENSDARG00000039456	acbd6	acyl-CoA binding domain containing 6 [Source:NCBI gene;Acc:324090]	8	14381272	14441812		7.04	6.32	6.05	6.88	6.72	6.68
ENSDARG00000039458	lhx4	LIM homeobox 4 [Source:ZFIN;Acc:ZDB-GENE-060728-1]	8	14453933	14484599		4.51	1.35	1.23	9.55	8.31	9.98
ENSDARG00000039459	qsox1	quiescin Q6 sulfhydryl oxidase 1 [Source:ZFIN;Acc:ZDB-GENE-030131-7019]	8	14503610	14554785		21.97	31.59	30.57	22.24	22.48	23.51
ENSDARG00000039466	hnrnph3	heterogeneous nuclear ribonucleoprotein H3 (2H9) [Source:ZFIN;Acc:ZDB-GENE-060421-3878]	13	22863516	22873184		9.06	7.57	7.41	8.67	8.07	8.63
ENSDARG00000039477	terb2	telomere repeat binding bouquet formation protein 2 [Source:NCBI gene;Acc:541341]	7	30929459	30932094		0.12	0.25	0.13	0.17	0.06	0.23
ENSDARG00000039482	calhm2.1	calcium homeostasis modulator family member 2, tandem duplicate 1 [Source:ZFIN;Acc:ZDB-GENE-030616-561]	13	25421531	25426955		1.41	2.05	1.71	1.26	1.06	1.01
ENSDARG00000039483	si:dkey-51a16.9	si:dkey-51a16.9 [Source:ZFIN;Acc:ZDB-GENE-030616-560]	13	25428677	25438298		4.75	2.99	3.59	4.33	4.35	4.81
ENSDARG00000039486	bag3	BCL2 associated athanogene 3 [Source:ZFIN;Acc:ZDB-GENE-040801-40]	13	25486608	25498661		6.29	7.02	5.77	7.99	8.54	7.91
ENSDARG00000039489	git1	G protein-coupled receptor kinase interacting ArfGAP 1 [Source:ZFIN;Acc:ZDB-GENE-100408-5]	15	28431621	28480576		4.74	2.92	2.51	5.88	6.09	5.96
ENSDARG00000039490	pitpnaa	phosphatidylinositol transfer protein, alpha a [Source:ZFIN;Acc:ZDB-GENE-040426-744]	15	28410664	28425011		15.66	19.9	16.84	16	14.58	13.31
ENSDARG00000039493	naa20	N(alpha)-acetyltransferase 20, NatB catalytic subunit [Source:NCBI gene;Acc:541516]	20	49105429	49117438		40.82	37.33	35.21	33.33	31.84	35.51
ENSDARG00000039497	ccdc85a	coiled-coil domain containing 85A [Source:ZFIN;Acc:ZDB-GENE-080220-55]	13	27000382	27038407		5.25	1.15	1.64	4.61	5.47	4.7
ENSDARG00000039499	soul2	heme-binding protein soul2 [Source:ZFIN;Acc:ZDB-GENE-030131-2524]	13	27040887	27043334		38.88	39.47	31.38	22.87	21.24	19.16
ENSDARG00000039500	si:cabz01090165.1	si:cabz01090165.1 [Source:ZFIN;Acc:ZDB-GENE-161017-71]	18	49411417	49447042		3.14	0.64	0.92	3.81	3.17	3.64
ENSDARG00000039501	ugt2a6	UDP glucuronosyltransferase 2 family, polypeptide A6 [Source:ZFIN;Acc:ZDB-GENE-081104-3]	5	69856153	69865574		2.63	2.58	2.59	1.91	2.02	1.62
ENSDARG00000039502	eef1a1a	eukaryotic translation elongation factor 1 alpha 1a [Source:ZFIN;Acc:ZDB-GENE-030131-8278]	13	27314795	27325885		37.27	13.9	16.03	16.47	18.81	19.19
ENSDARG00000039512	ap1m3	adaptor related protein complex 1 subunit mu 3 [Source:ZFIN;Acc:ZDB-GENE-040801-159]	6	34038963	34073164		3.33	3.7	3.12	2.97	3.3	2.64
ENSDARG00000039513	C18H19orf54	zgc:153119 [Source:NCBI gene;Acc:768129]	18	35407921	35413608	C18H19orf54	1.11	1.14	0.85	1.3	1.42	1.09
ENSDARG00000039516	c8a	complement component 8, alpha polypeptide [Source:ZFIN;Acc:ZDB-GENE-040801-239]	2	1486822	1499584		15.64	25.47	23.65	19.19	19.84	19.7
ENSDARG00000039517	c8b	complement component 8, beta polypeptide [Source:ZFIN;Acc:ZDB-GENE-070912-163]	2	1478225	1518360		9.33	14.91	12.65	14.93	15.99	15.81
ENSDARG00000039521	ssx2ipa	synovial sarcoma, X breakpoint 2 interacting protein a [Source:ZFIN;Acc:ZDB-GENE-040426-1756]	2	1988036	2008463		6.75	4.37	4.36	4.15	4.26	4.54
ENSDARG00000039522	tubb2	tubulin, beta 2A class IIa [Source:ZFIN;Acc:ZDB-GENE-050522-384]	2	2010277	2020044		33.78	20.64	28.08	21.89	19.26	21.48
ENSDARG00000039534	rrh	retinal pigment epithelium-derived rhodopsin homolog [Source:ZFIN;Acc:ZDB-GENE-040912-94]	13	12528043	12540539		1.32	0.42	0.35	1.09	1.32	1.14
ENSDARG00000039535	dapp1	dual adaptor of phosphotyrosine and 3-phosphoinositides [Source:ZFIN;Acc:ZDB-GENE-050417-180]	13	12456412	12477757		0.67	0.52	0.34	1.52	1.17	1.04
ENSDARG00000039543	mrps6	mitochondrial ribosomal protein S6 [Source:ZFIN;Acc:ZDB-GENE-060825-59]	13	4995433	5000739		30.75	35.06	37.2	23.71	20.63	22.95
ENSDARG00000039563	loxl3b	lysyl oxidase-like 3b [Source:NCBI gene;Acc:336266]	13	15234521	15404571		6.4	6.81	6.59	14.68	16.18	15.48
ENSDARG00000039569	emx1	empty spiracles homeobox 1 [Source:NCBI gene;Acc:378964]	13	15004398	15010683		2.84	1.96	1.85	3.61	2.81	3.13
ENSDARG00000039576	fstl1b	follistatin-like 1b [Source:ZFIN;Acc:ZDB-GENE-030131-3029]	9	9735493	9842149		37.01	44.35	35.96	54.07	54.55	52.36
ENSDARG00000039577	ptk2bb	protein tyrosine kinase 2 beta, b [Source:ZFIN;Acc:ZDB-GENE-020507-1]	20	19827379	19864131		11.61	12.39	8.88	7.91	7.51	7.38
ENSDARG00000039578	pa2g4a	proliferation-associated 2G4, a [Source:ZFIN;Acc:ZDB-GENE-030616-161]	6	7533601	7551140		87.42	100.49	90.6	62.08	58.09	58.08
ENSDARG00000039579	cfd	complement factor D (adipsin) [Source:ZFIN;Acc:ZDB-GENE-050522-411]	13	13681681	13684334		69.79	90.75	89	26.86	32.92	30.41
ENSDARG00000039601	ier3ip1	immediate early response 3 interacting protein 1 [Source:NCBI gene;Acc:554110]	10	14542726	14545658		17.99	21.8	17.93	21.85	23.08	20.35
ENSDARG00000039602	atoh1c	atonal bHLH transcription factor 1c [Source:ZFIN;Acc:ZDB-GENE-090805-1]	12	11768114	11768889		1.07	1.9	1.23	0.67	0.6	0.44
ENSDARG00000039605	mat1a	methionine adenosyltransferase I, alpha [Source:ZFIN;Acc:ZDB-GENE-030131-6127]	13	18628920	18637244		89.05	141.68	122.78	104.15	113.67	102.09
ENSDARG00000039613	poll	polymerase (DNA directed), lambda [Source:ZFIN;Acc:ZDB-GENE-040426-2712]	1	31657842	31672343		6.68	4.24	5.66	5.21	4.43	5.53
ENSDARG00000039615	fgf8b	fibroblast growth factor 8 b [Source:NCBI gene;Acc:65089]	1	31638274	31669901		0.95	1.04	0.88	1.12	1.11	1.03
ENSDARG00000039616	cenpk	centromere protein K [Source:ZFIN;Acc:ZDB-GENE-090313-204]	1	31508739	31516112		2.78	1.89	2.48	1.93	1.27	1.4
ENSDARG00000039626	nrgna	neurogranin (protein kinase C substrate, RC3) a [Source:ZFIN;Acc:ZDB-GENE-090710-4]	5	58372164	58382655		30.82	16.88	18.02	15.23	15.5	15.06
ENSDARG00000039647	slc6a1b	solute carrier family 6 member 1b [Source:ZFIN;Acc:ZDB-GENE-041114-57]	11	997100	1024483		56.2	10.05	12.78	65.58	63.99	67.81
ENSDARG00000039649	mtif3	mitochondrial translational initiation factor 3 [Source:ZFIN;Acc:ZDB-GENE-060228-1]	5	67971627	67978743		9.94	11	10.3	5.86	6.07	6.54
ENSDARG00000039650	itm2cb	integral membrane protein 2Cb [Source:ZFIN;Acc:ZDB-GENE-030131-7806]	2	48284464	48298985		15.23	5.4	7.01	17.27	18.26	17.96
ENSDARG00000039652	si:dkey-235d18.5	si:dkey-235d18.5 [Source:ZFIN;Acc:ZDB-GENE-041014-243]	20	52584532	52725696		2.15	1.91	1.64	4.64	4.29	4.12
ENSDARG00000039657	fank1	fibronectin type III and ankyrin repeat domains 1 [Source:ZFIN;Acc:ZDB-GENE-130613-3]	12	45132906	45149516		0.05	0	0.06	0.05	0	0.04
ENSDARG00000039661	slc36a4	solute carrier family 36 member 4 [Source:ZFIN;Acc:ZDB-GENE-050309-239]	15	41169211	41235027		4.74	2.61	3.11	4.33	4.24	4.08
ENSDARG00000039666	tsta3	tissue specific transplantation antigen P35B [Source:ZFIN;Acc:ZDB-GENE-040722-1]	20	52458765	52488233		6.31	5.93	6.33	8.2	8.97	8.03
ENSDARG00000039667	mrpl38	mitochondrial ribosomal protein L38 [Source:ZFIN;Acc:ZDB-GENE-040426-2373]	12	45238292	45257211		30.83	32.13	30.44	23.81	23.99	23.68
ENSDARG00000039669	GFUS	zgc:100864 [Source:ZFIN;Acc:ZDB-GENE-040801-35]	20	52492192	52525680	GFUS	13.72	10.24	8.94	9.63	8.39	8.31
ENSDARG00000039677	dsc2l	desmocollin 2 like [Source:ZFIN;Acc:ZDB-GENE-031116-55]	20	7262434	7293837		12.32	11.91	8.51	10.05	9.68	9.75
ENSDARG00000039681	samm50l	sorting and assembly machinery component 50 homolog, like [Source:NCBI gene;Acc:492471]	4	9011448	9025993		9.95	8.81	8.34	9.47	10.07	9.92
ENSDARG00000039682	si:ch211-121a2.2	si:ch211-121a2.2 [Source:ZFIN;Acc:ZDB-GENE-070705-21]	20	7070634	7080470		11.12	14.09	13.78	5.04	4.27	3.95
ENSDARG00000039684	sirt5	sirtuin 5 [Source:NCBI gene;Acc:436878]	20	7052870	7069612		9.57	10.5	11.74	10.22	10.47	9.91
ENSDARG00000039687	zgc:194221	zgc:194221 [Source:ZFIN;Acc:ZDB-GENE-081022-77]	22	2680895	2682759		0.06	0	0.01	0.34	0.27	0.29
ENSDARG00000039697	shtn1	shootin 1 [Source:NCBI gene;Acc:407656]	17	21311401	21368775		3.65	4.31	3.37	2.27	1.84	2.18
ENSDARG00000039699	fam204a	family with sequence similarity 204 member A [Source:ZFIN;Acc:ZDB-GENE-040718-271]	13	19766896	19799824		11.56	10.78	11	9.04	8.58	8.72
ENSDARG00000039701	emx2	empty spiracles homeobox 2 [Source:ZFIN;Acc:ZDB-GENE-990415-54]	13	19322686	19327657		12.31	6.53	6.86	13.11	12.96	13.64
ENSDARG00000039719	mrps26	mitochondrial ribosomal protein S26 [Source:ZFIN;Acc:ZDB-GENE-040718-115]	13	9303554	9311253		21.53	23.31	24.7	14.75	14.88	14.9
ENSDARG00000039724	OSBPL8	oxysterol binding protein-like 8 [Source:NCBI gene;Acc:101883298]	4	2396024	2440175	OSBPL8	0.55	0.25	0.2	0.92	1.2	0.72
ENSDARG00000039726	zgc:123321	zgc:123321 [Source:ZFIN;Acc:ZDB-GENE-051023-1]	13	9432501	9436539		6.11	10.66	11.15	8.05	6.95	6.34
ENSDARG00000039729	asap1b	ArfGAP with SH3 domain, ankyrin repeat and PH domain 1b [Source:ZFIN;Acc:ZDB-GENE-091112-16]	24	10897768	11057305		5.41	1.79	1.81	5.77	5.62	6.24
ENSDARG00000039730	zgc:112160	zgc:112160 [Source:ZFIN;Acc:ZDB-GENE-050417-229]	7	35062132	35066457		372.41	548.12	396.9	29.15	27.64	30.8
ENSDARG00000039747	BX914200.1		13	45840427	45843939		3.83	6.49	4.7	1.37	1.27	0.93
ENSDARG00000039750	ldlrap1b	low density lipoprotein receptor adaptor protein 1b [Source:ZFIN;Acc:ZDB-GENE-070112-1012]	13	45582391	45645167		3.4	2.25	2	3.54	3.71	3.61
ENSDARG00000039752	si:dkey-167k11.5	si:dkey-167k11.5 [Source:ZFIN;Acc:ZDB-GENE-091204-218]	3	431208	454166		2.18	1.46	1.27	2.44	2.24	2.21
ENSDARG00000039754	xpc	xeroderma pigmentosum, complementation group C [Source:ZFIN;Acc:ZDB-GENE-030131-8461]	8	26007308	26032245		3.17	2.4	2.41	2.6	2.65	2.74
ENSDARG00000039756	prop1	PROP paired-like homeobox 1 [Source:ZFIN;Acc:ZDB-GENE-081107-40]	13	8677166	8683552		0.12	0	0	0.12	0.08	0.23
ENSDARG00000039757	mcfd2	multiple coagulation factor deficiency 2 [Source:ZFIN;Acc:ZDB-GENE-041010-8]	13	8682176	8692860		7.78	10.32	8.94	6.52	6.81	6.41
ENSDARG00000039770	dync2li1	dynein, cytoplasmic 2, light intermediate chain 1 [Source:NCBI gene;Acc:393452]	13	10970614	10981704		2.38	1.96	2.57	2.31	2.99	3.01
ENSDARG00000039806	tbxtb	T-box transcription factor Tb [Source:ZFIN;Acc:ZDB-GENE-081028-48]	13	4439074	4453663		0.03	0.01	0.04	0.57	0.55	0.56
ENSDARG00000039820	mea1	male-enhanced antigen 1 [Source:ZFIN;Acc:ZDB-GENE-070424-117]	13	4225173	4232690		16.34	15.23	14.9	14.09	14.38	14.16
ENSDARG00000039824	zgc:172182	zgc:172182 [Source:NCBI gene;Acc:100136847]	9	48386928	48407408		2.38	1.28	1.45	2.34	2.86	2.72
ENSDARG00000039827	bbs5	Bardet-Biedl syndrome 5 [Source:NCBI gene;Acc:393269]	9	48284606	48296469		5.82	4.07	3.75	5.29	5.09	6.42
ENSDARG00000039828	zp3b	zona pellucida glycoprotein 3b [Source:ZFIN;Acc:ZDB-GENE-031124-2]	2	6253246	6258796		0.29	0.09	0.18	0.32	0.27	0.37
ENSDARG00000039830	gng5	guanine nucleotide binding protein (G protein), gamma 5 [Source:ZFIN;Acc:ZDB-GENE-030131-9966]	2	6243144	6249163		48.56	58.42	55.97	50.48	48.19	51.61
ENSDARG00000039832	gsta.2	glutathione S-transferase, alpha tandem duplicate 2 [Source:ZFIN;Acc:ZDB-GENE-070822-30]	13	829585	832331		12.91	21.78	17.08	5.04	6.02	5.12
ENSDARG00000039843	znf800b	zinc finger protein 800b [Source:ZFIN;Acc:ZDB-GENE-040426-1166]	4	3455146	3475397		3.01	2.74	3.02	2.9	3.15	3.1
ENSDARG00000039850	dusp22b	dual specificity phosphatase 22b [Source:NCBI gene;Acc:100002272]	2	881954	898899		1.65	1.56	1.84	2.11	2.09	1.84
ENSDARG00000039851	dhdds	dehydrodolichyl diphosphate synthase [Source:ZFIN;Acc:ZDB-GENE-040426-2236]	16	51154918	51178268		9.63	10.76	10.85	6.59	7.37	7.69
ENSDARG00000039852	ftr93	finTRIM family, member 93 [Source:ZFIN;Acc:ZDB-GENE-131126-26]	2	696244	707152		0.32	0.31	0.3	0.57	0.55	0.29
ENSDARG00000039863	lifrb	LIF receptor subunit alpha b [Source:ZFIN;Acc:ZDB-GENE-080107-2]	21	21244046	21261636		1.38	1.48	1.24	3.4	3.28	3.72
ENSDARG00000039871	rpz4	rapunzel 4 [Source:ZFIN;Acc:ZDB-GENE-050417-472]	16	46497149	46503818		9.34	9.97	7.8	9.03	8.75	8.73
ENSDARG00000039872	lamtor2	late endosomal/lysosomal adaptor, MAPK and MTOR activator 2 [Source:NCBI gene;Acc:445155]	16	46684855	46691206		15.7	16.35	18.88	16.67	15.81	14.92
ENSDARG00000039878	ambra1b	autophagy/beclin-1 regulator 1b [Source:ZFIN;Acc:ZDB-GENE-050208-235]	25	7888019	7918526		3.12	1.77	1.83	5.5	4.96	5.04
ENSDARG00000039880	arpp19b	cAMP-regulated phosphoprotein 19b [Source:ZFIN;Acc:ZDB-GENE-040426-930]	25	7653902	7663608		38.94	32.4	33.12	26.34	26.4	24.89
ENSDARG00000039881	cemip	cell migration inducing hyaluronidase 1 [Source:ZFIN;Acc:ZDB-GENE-130103-7]	7	11197022	11342611		7.83	6.06	4.07	11.07	10.08	10.12
ENSDARG00000039882	ap3s2	adaptor related protein complex 3 subunit sigma 2 [Source:ZFIN;Acc:ZDB-GENE-040718-274]	25	10762016	10803532		14.78	12.28	13.72	14.3	14.44	14.02
ENSDARG00000039887	c1qbp	complement component 1, q subcomponent binding protein [Source:NCBI gene;Acc:334619]	5	3914403	3927763		105.7	122.2	117.14	61.52	63.13	63.4
ENSDARG00000039888	CR855393.1		7	3158104	3160910		0	0	0.02	0	0.02	0
ENSDARG00000039891	BX323555.2	proteinase-activated receptor 1-like [Source:NCBI gene;Acc:108180716]	21	7982302	7983300		0	0.02	0.06	0.05	0.03	0.07
ENSDARG00000039892	gnpnat1	glucosamine-phosphate N-acetyltransferase 1 [Source:ZFIN;Acc:ZDB-GENE-050522-450]	17	15213496	15219940		8.2	9.8	7.31	5.6	6.45	5.63
ENSDARG00000039899	zbtb7a	zinc finger and BTB domain containing 7a [Source:ZFIN;Acc:ZDB-GENE-050208-583]	22	20372533	20386982		2.6	1.14	1.17	2.32	2.18	2.45
ENSDARG00000039900	si:ch73-168d20.1	si:ch73-168d20.1 [Source:ZFIN;Acc:ZDB-GENE-121214-44]	23	45077358	45119805		0.24	0.18	0.12	0	0	0
ENSDARG00000039901	sh3gl2b	SH3 domain containing GRB2 like 2b, endophilin A1 [Source:ZFIN;Acc:ZDB-GENE-170303-2]	23	45043301	45049044		3.99	0.71	1.62	5.38	4.07	5.53
ENSDARG00000039910	rbm42	RNA binding motif protein 42 [Source:NCBI gene;Acc:445360]	16	44881829	44900306		12.47	9.11	7.64	10.89	9.76	11.54
ENSDARG00000039913	tmem147	transmembrane protein 147 [Source:NCBI gene;Acc:436757]	16	45019449	45042236		39.19	37.19	36.89	34.18	34.14	33.94
ENSDARG00000039914	gapdhs	glyceraldehyde-3-phosphate dehydrogenase, spermatogenic [Source:NCBI gene;Acc:406367]	16	45044642	45069882		600.91	323.89	378.2	762.23	818.5	810.04
ENSDARG00000039915	bola1	bolA family member 1 [Source:ZFIN;Acc:ZDB-GENE-040801-76]	16	46205693	46209855		9.2	10.37	9.72	5.89	5.69	6.9
ENSDARG00000039929	ckmt2b	creatine kinase, mitochondrial 2b (sarcomeric) [Source:ZFIN;Acc:ZDB-GENE-040426-1654]	5	51594209	51609006		184.65	227.43	238.76	99.9	97.55	91.82
ENSDARG00000039931	slc25a33	solute carrier family 25 member 33 [Source:NCBI gene;Acc:406436]	23	29861243	29878643		19.5	23.03	22.63	28.38	28.76	27.94
ENSDARG00000039934	hlcs	holocarboxylase synthetase (biotin-(proprionyl-CoA-carboxylase (ATP-hydrolysing)) ligase) [Source:ZFIN;Acc:ZDB-GENE-030131-53	10	53587	57270		7.7	7.93	8.31	10.26	9.96	9.83
ENSDARG00000039935	CABZ01064671.1		3	8506061	8510201		0.39	0.41	0.24	0.78	0.85	0.59
ENSDARG00000039937	ccdc172	coiled-coil domain containing 172 [Source:ZFIN;Acc:ZDB-GENE-090313-1]	13	20524921	20534948		0.26	0.15	0.15	0.11	0.07	0.09
ENSDARG00000039943	tent5ba	terminal nucleotidyltransferase 5ba [Source:ZFIN;Acc:ZDB-GENE-061110-129]	16	34046733	34056443		10.02	11.74	10.03	13.58	14.68	15.06
ENSDARG00000039949	fibina	fin bud initiation factor a [Source:ZFIN;Acc:ZDB-GENE-111031-2]	25	35502552	35503409		18.77	21.88	22.14	14.51	13.07	13.48
ENSDARG00000039951	si:ch211-87j1.4	si:ch211-87j1.4 [Source:ZFIN;Acc:ZDB-GENE-131127-49]	25	35533790	35542739		2.66	1.11	1.25	5.13	5.08	5.05
ENSDARG00000039958	utp11	UTP11 small subunit processome component [Source:ZFIN;Acc:ZDB-GENE-030131-8093]	16	33645721	33650578		12.6	12.48	10.74	9.76	8.91	8.97
ENSDARG00000039959	gdnfa	glial cell derived neurotrophic factor a [Source:NCBI gene;Acc:79379]	10	1396940	1414161		1.97	0.67	0.97	2.37	2.22	2.18
ENSDARG00000039960	st7	suppressor of tumorigenicity 7 protein homolog [Source:NCBI gene;Acc:110437773]	18	50948003	50956280		1.39	1.12	0.98	2.25	1.96	2.27
ENSDARG00000039963	fgfbp1b	fibroblast growth factor binding protein 1b [Source:ZFIN;Acc:ZDB-GENE-081106-1]	14	46441617	46442459		0.46	0.63	0.47	0.67	0.53	0.88
ENSDARG00000039964	fgfbp2a	fibroblast growth factor binding protein 2a [Source:ZFIN;Acc:ZDB-GENE-030131-8319]	14	46449070	46451249		1.97	2.2	1.81	2.45	2.37	2.58
ENSDARG00000039966	prom1a	prominin 1a [Source:NCBI gene;Acc:322857]	14	46456091	46617228		6.22	1.92	2.16	7.66	7.7	7.68
ENSDARG00000039969	akirin1	akirin 1 [Source:ZFIN;Acc:ZDB-GENE-040426-2178]	16	33121106	33141934		12.69	11.67	11.4	13.09	12.4	12.45
ENSDARG00000039970	cnr2	cannabinoid receptor 2 [Source:ZFIN;Acc:ZDB-GENE-040702-7]	16	33109496	33119626		0.05	0.01	0.02	0.03	0.02	0.01
ENSDARG00000039974	rai14	retinoic acid induced 14 [Source:ZFIN;Acc:ZDB-GENE-040718-235]	21	19547806	19608698		3.24	2.63	2.07	3.68	3.39	3.31
ENSDARG00000039979	tecrl2b	trans-2,3-enoyl-CoA reductase-like 2b [Source:ZFIN;Acc:ZDB-GENE-041010-116]	2	15300088	15324837		1.6	2.59	2.5	0.45	0.55	0.41
ENSDARG00000039980	ppt1	palmitoyl-protein thioesterase 1 (ceroid-lipofuscinosis, neuronal 1, infantile) [Source:ZFIN;Acc:ZDB-GENE-040426-2653]	19	43736269	43757568		6.51	8.28	8.25	8.04	7.79	8.43
ENSDARG00000039987	hivep2a	HIVEP zinc finger 2a [Source:ZFIN;Acc:ZDB-GENE-041014-354]	20	37511067	37629084		5.3	2.33	2.03	6.6	6.75	6.76
ENSDARG00000039989	snrnp48	small nuclear ribonucleoprotein 48 (U11/U12) [Source:ZFIN;Acc:ZDB-GENE-030131-4482]	16	36974523	36979597		5.02	3.54	3.33	4.01	4.52	3.88
ENSDARG00000039996	xkr8.3	XK, Kell blood group complex subunit-related family, member 8, tandem duplicate 3 [Source:ZFIN;Acc:ZDB-GENE-041010-107]	19	25072053	25081711		2.86	3.34	3.51	3.18	2.81	3.23
ENSDARG00000039997	ptp4a3a	protein tyrosine phosphatase 4A3a [Source:ZFIN;Acc:ZDB-GENE-040426-2220]	19	25088150	25149598		11.45	14.15	14.94	21.52	21.73	22.13
ENSDARG00000039999	slc13a5b	solute carrier family 13 member 5b [Source:ZFIN;Acc:ZDB-GENE-130530-605]	15	26573676	26593684		0.18	0.29	0.17	0.58	0.72	0.54
ENSDARG00000040001	fryl	furry homolog, like [Source:ZFIN;Acc:ZDB-GENE-040914-58]	20	23315242	23422345		7.29	5.18	3.92	10.92	10.2	10.89
ENSDARG00000040002	pnpla7b	patatin-like phospholipase domain containing 7b [Source:ZFIN;Acc:ZDB-GENE-050309-66]	5	25044311	25072607		6.16	6.11	5.51	6.95	7.13	7.46
ENSDARG00000040004	PKHD1L1	si:ch211-244a23.1 [Source:ZFIN;Acc:ZDB-GENE-060503-789]	19	22930177	22997652	PKHD1L1	0.44	0.26	0.18	0.49	0.51	0.45
ENSDARG00000040005	scfd2	sec1 family domain containing 2 [Source:ZFIN;Acc:ZDB-GENE-050320-143]	20	22799641	23021279		3.76	3.98	3.6	2.83	3.39	3.12
ENSDARG00000040008	neurod6a	neuronal differentiation 6a [Source:NCBI gene;Acc:114414]	24	32411753	32474949		7.01	2.61	3.98	6.02	5.98	5.84
ENSDARG00000040009	palld	palladin, cytoskeletal associated protein [Source:ZFIN;Acc:ZDB-GENE-041014-252]	20	23498255	23649028		10.25	12.81	10.99	23.8	24.2	23.52
ENSDARG00000040021	ebag9	estrogen receptor binding site associated antigen 9 [Source:ZFIN;Acc:ZDB-GENE-040426-1093]	16	39185103	39195318		18.95	19.34	20.4	19.85	21.46	21.46
ENSDARG00000040024	gpd1l	glycerol-3-phosphate dehydrogenase 1 like [Source:NCBI gene;Acc:449663]	16	39248644	39267227		11.2	9.66	9.48	10.54	10.84	9.97
ENSDARG00000040027	osbpl10b	oxysterol binding protein-like 10b [Source:ZFIN;Acc:ZDB-GENE-081002-4]	16	39271123	39386073		3.06	2.13	2.31	3.06	3.43	3.29
ENSDARG00000040031	tardbp	TAR DNA binding protein [Source:ZFIN;Acc:ZDB-GENE-030131-3777]	6	45937777	45949121		69.34	44.53	46.9	84.63	80.64	81.52
ENSDARG00000040033	trmt11	tRNA methyltransferase 11 homolog [Source:ZFIN;Acc:ZDB-GENE-040426-953]	16	40043673	40074145		3.01	3.48	2.87	2.15	2.18	2.38
ENSDARG00000040036	tcf19l	transcription factor 19 (SC1), like [Source:ZFIN;Acc:ZDB-GENE-060324-2]	19	6834380	6840506		1.28	0.86	0.94	0.73	0.81	1.09
ENSDARG00000040039	pttg1ipb	PTTG1 interacting protein b [Source:ZFIN;Acc:ZDB-GENE-041010-10]	9	12655278	12659140		14.76	18.79	16.27	18.5	20.67	19.34
ENSDARG00000040041	mcm4	minichromosome maintenance complex component 4 [Source:ZFIN;Acc:ZDB-GENE-030131-9544]	24	35536664	35561672		27.78	24.37	21.94	18.8	17.56	17.14
ENSDARG00000040045	cldn1	claudin 1 [Source:ZFIN;Acc:ZDB-GENE-010328-11]	2	68789	71801		61.12	65.39	76.77	55.04	56.98	55.6
ENSDARG00000040046	snai2	snail family zinc finger 2 [Source:ZFIN;Acc:ZDB-GENE-030326-6]	24	35387517	35393533		11.81	12.3	10.66	16.91	17.65	16.14
ENSDARG00000040048	GALNTL6	si:ch211-15e22.3 [Source:ZFIN;Acc:ZDB-GENE-090312-115]	1	37752171	38132612	GALNTL6	4.55	1.4	1.87	4.6	4.45	4.65
ENSDARG00000040059	gbp	glycogen synthase kinase binding protein [Source:ZFIN;Acc:ZDB-GENE-990714-7]	16	40954481	40955839		16.53	11.41	9.57	17.6	19.11	18.24
ENSDARG00000040064	acp6	acid phosphatase 6, lysophosphatidic [Source:ZFIN;Acc:ZDB-GENE-050208-290]	9	30478768	30490414		2.59	3.54	3.27	2.77	3.09	2.93
ENSDARG00000040065	gja5a	gap junction protein, alpha 5a [Source:ZFIN;Acc:ZDB-GENE-040407-3]	9	30464641	30477311		0.37	0.39	0.15	0.98	0.99	1.09
ENSDARG00000040070	coil	coilin p80 [Source:ZFIN;Acc:ZDB-GENE-000330-8]	3	36119782	36127234		3.41	3.35	2.91	2.96	2.73	2.92
ENSDARG00000040072	scpep1	serine carboxypeptidase 1 [Source:ZFIN;Acc:ZDB-GENE-040426-890]	3	36127287	36138174		6.92	6.9	6.52	5.03	5.32	5.28

**ENSDARG00000040076**	pycard	PYD and CARD domain containing [Source:NCBI gene;Acc:57923]	16	41984032	41990421		48.02	53.12	49.14	31.24	28.7	28.02
**ENSDARG00000040080**	fli1b	Fli-1 proto-oncogene, ETS transcription factor b [Source:ZFIN;Acc:ZDB-GENE-031114-3]	16	42018041	42046881		1.95	2.15	1.71	3.8	3.72	3.63
**ENSDARG00000040081**	zp3d.1	zona pellucida glycoprotein 3d tandem duplicate 1 [Source:ZFIN;Acc:ZDB-GENE-030131-5827]	16	42057918	42066617		0.2	0.14	0.19	0.27	0.15	0.16
**ENSDARG00000040085**	ccsapb	centriole, cilia and spindle-associated protein b [Source:NCBI gene;Acc:100003801]	13	24287093	24294103		7.68	4.15	4.12	6.65	6.69	6.73
**ENSDARG00000040087**	epb41l4a	erythrocyte membrane protein band 4.1 like 4A [Source:NCBI gene;Acc:30470]	10	1745475	1827225		10.88	5.05	4.59	11.09	12.57	11.33
**ENSDARG00000040100**	si:dkey-95h12.1	si:dkey-95h12.1 [Source:ZFIN;Acc:ZDB-GENE-060503-85]	18	8869465	8889382		5.36	5.64	4.34	2.1	1.69	1.41
**ENSDARG00000040110**	kif20bb	kinesin family member 20Bb [Source:ZFIN;Acc:ZDB-GENE-030131-9684]	12	16452575	16479291		3.79	2.71	2.87	2.64	2.61	2.52
**ENSDARG00000040111**	pi4kb	phosphatidylinositol 4-kinase, catalytic, beta [Source:NCBI gene;Acc:563201]	16	38159758	38200523		5.32	4.28	3.19	8.27	7.87	7.98
**ENSDARG00000040116**	znf366	zinc finger protein 366 [Source:ZFIN;Acc:ZDB-GENE-110408-46]	8	955162	979735		0.35	0.33	0.31	0.33	0.43	0.41
**ENSDARG00000040118**	zgc:113232	zgc:113232 [Source:ZFIN;Acc:ZDB-GENE-050327-86]	16	38360002	38389883		0.29	0.66	1.01	16.71	16.56	17.37
**ENSDARG00000040121**	psma2	proteasome 20S subunit alpha 2 [Source:ZFIN;Acc:ZDB-GENE-050522-479]	16	43335696	43344859		27.31	33.99	32.59	22.56	22.4	23.67
**ENSDARG00000040123**	zfpm2a	zinc finger protein, FOG family member 2a [Source:ZFIN;Acc:ZDB-GENE-060130-4]	16	43818896	44127387		5.15	1.04	1.44	5.78	6.1	6.5
**ENSDARG00000040130**	si:ch211-215k15.4	si:ch211-215k15.4 [Source:ZFIN;Acc:ZDB-GENE-131127-215]	16	42464009	42470156		0.17	0.51	0.27	0.19	0.08	0.11
**ENSDARG00000040131**	zfyve26	zinc finger, FYVE domain containing 26 [Source:ZFIN;Acc:ZDB-GENE-030131-3286]	13	33066605	33114934		3.54	3.05	2.22	4.04	4.11	4.2
**ENSDARG00000040133**	ackr4b	atypical chemokine receptor 4b [Source:ZFIN;Acc:ZDB-GENE-051107-10]	24	11381400	11390669		1.5	2.22	1.81	3.02	3.54	3.32
**ENSDARG00000040135**	fosaa	v-fos FBJ murine osteosarcoma viral oncogene homolog Aa [Source:ZFIN;Acc:ZDB-GENE-131121-552]	17	50228998	50234004		2.51	0.56	0.64	5.25	4.6	5.48
**ENSDARG00000040137**	jdp2a	Jun dimerization protein 2a [Source:ZFIN;Acc:ZDB-GENE-131127-459]	17	50195267	50220228		2.39	2.32	1.77	2.67	3.21	3.37
**ENSDARG00000040141**	si:ch73-138e16.5	si:ch73-138e16.5 [Source:ZFIN;Acc:ZDB-GENE-070705-204]	22	1300587	1307143		0.39	0.15	0.21	0.74	0.49	0.41
**ENSDARG00000040152**	nek4	NIMA-related kinase 4 [Source:ZFIN;Acc:ZDB-GENE-040426-1392]	11	3991911	4024087		2.27	1.55	1.33	2.13	2.05	2.19
**ENSDARG00000040156**	grm4	glutamate receptor, metabotropic 4 [Source:ZFIN;Acc:ZDB-GENE-030131-5781]	6	46981486	47246948		8.31	2.77	2.96	7.93	8.54	8.69
**ENSDARG00000040157**	glt8d1	glycosyltransferase 8 domain containing 1 [Source:NCBI gene;Acc:492466]	11	3971716	3987885		5.75	7.22	6.39	7.91	6.11	6.75
**ENSDARG00000040158**	cdc42l	cell division cycle 42, like [Source:ZFIN;Acc:ZDB-GENE-030131-6069]	16	31815244	31824525		45.55	53.48	43.97	49.07	46.7	47.96
**ENSDARG00000040159**	wnt4b	wingless-type MMTV integration site family, member 4b [Source:ZFIN;Acc:ZDB-GENE-000411-1]	16	31802203	31813018		1.63	1.39	2.17	13.08	14.77	15.86
**ENSDARG00000040161**	zgc:92287	zgc:92287 [Source:ZFIN;Acc:ZDB-GENE-040718-350]	23	18032569	18057958		10.85	10.24	12.1	11.31	9.7	10.03
**ENSDARG00000040162**	fgfbp3	fibroblast growth factor binding protein 3 [Source:ZFIN;Acc:ZDB-GENE-050208-135]	17	23298928	23302321		30	17.72	23.88	39.54	43.23	49.94
**ENSDARG00000040163**	prim1	DNA primase subunit 1 [Source:ZFIN;Acc:ZDB-GENE-990603-6]	23	17839187	17848907		12.06	12.45	12.67	6.25	5.14	5.55
**ENSDARG00000040177**	rgs16	regulator of G protein signaling 16 [Source:ZFIN;Acc:ZDB-GENE-050417-393]	6	35443430	35446110		19.67	6.17	6.24	12.45	12.28	14.47
**ENSDARG00000040178**	havcr1	hepatitis A virus cellular receptor 1 [Source:ZFIN;Acc:ZDB-GENE-040718-131]	21	29100972	29110607		1.35	1.24	1.54	1.43	1.69	1.64
**ENSDARG00000040179**	zgc:101562	zgc:101562 [Source:ZFIN;Acc:ZDB-GENE-041010-74]	20	2642855	2647566		1.51	1.93	2.25	1.92	1.69	1.52
**ENSDARG00000040180**	slc35a1	solute carrier family 35 member A1 [Source:ZFIN;Acc:ZDB-GENE-080716-17]	20	2669017	2679683		20.47	17.34	16.04	18.44	19.53	17.26
**ENSDARG00000040184**	syncrip	synaptotagmin binding, cytoplasmic RNA interacting protein [Source:ZFIN;Acc:ZDB-GENE-030131-4862]	20	2731436	2745385		44.3	37.69	32.63	63.69	61.34	64.04
**ENSDARG00000040186**	fam210ab	family with sequence similarity 210 member Ab [Source:NCBI gene;Acc:503762]	16	30604387	30612730		9.34	12.35	10.8	7.33	7.82	6.91
**ENSDARG00000040190**	qdpra	quinoid dihydropteridine reductase a [Source:ZFIN;Acc:ZDB-GENE-070705-197]	14	46873229	46897067		10.87	15.4	12.55	8.18	8.11	7.7
**ENSDARG00000040192**	nenf	neudesin neurotrophic factor [Source:ZFIN;Acc:ZDB-GENE-050320-129]	17	45395846	45402961		9.72	12.49	10.82	8.06	6.95	8.4
**ENSDARG00000040198**	fstl5	follistatin-like 5 [Source:ZFIN;Acc:ZDB-GENE-070127-1]	14	47000351	47391084		20.09	5.13	6.74	18.36	20.13	19.89
**ENSDARG00000040200**	BX465228.1	GTPase IMAP family member 7-like [Source:NCBI gene;Acc:100005907]	3	1880882	1888204		6.41	4.54	4.5	3.57	5.47	5.73
**ENSDARG00000040214**	scrt1b	scratch family zinc finger 1b [Source:ZFIN;Acc:ZDB-GENE-050327-72]	16	31186083	31188715		7.87	2.43	3.29	6.32	7.14	6.47
**ENSDARG00000040224**	krcp	kelch repeat-containing protein [Source:ZFIN;Acc:ZDB-GENE-030131-2126]	7	24881680	24888634		9.79	9.2	8.78	5.67	5.95	5.65
**ENSDARG00000040230**	si:dkey-37m8.11	si:dkey-37m8.11 [Source:ZFIN;Acc:ZDB-GENE-120214-18]	22	36923240	36926342		0.62	0.73	0.54	0.13	0.55	0.27
**ENSDARG00000040237**	mef2d	myocyte enhancer factor 2d [Source:ZFIN;Acc:ZDB-GENE-990415-164]	16	29072874	29194517		12.14	6.47	4.92	19.87	19.11	20.07
**ENSDARG00000040245**	kpnb3	karyopherin (importin) beta 3 [Source:ZFIN;Acc:ZDB-GENE-030424-2]	1	2260407	2299553		43.8	39.3	34.99	39.81	39.79	36.11
**ENSDARG00000040246**	rap2ab	RAP2A, member of RAS oncogene family b [Source:ZFIN;Acc:ZDB-GENE-090312-116]	1	2222363	2240119		6.81	2.11	2.9	8.85	7.85	7.76
**ENSDARG00000040247**	oxgr1a.3	oxoglutarate (alpha-ketoglutarate) receptor 1a, tandem duplicate 3 [Source:ZFIN;Acc:ZDB-GENE-090312-23]	1	2060430	2061419		0	0	0	0.02	0	0.02
**ENSDARG00000040248**	crata	carnitine O-acetyltransferase a [Source:ZFIN;Acc:ZDB-GENE-040927-17]	5	32815745	32833358		8.87	7.82	6.71	6.85	7.41	7.76
**ENSDARG00000040249**	tlr18	toll-like receptor 18 [Source:ZFIN;Acc:ZDB-GENE-040220-2]	16	29387675	29405009		0.61	0.75	0.64	0.84	0.81	0.82
**ENSDARG00000040250**	si:ch211-132g1.1	si:ch211-132g1.1 [Source:ZFIN;Acc:ZDB-GENE-090313-24]	1	1915967	1923253		0.1	0.3	0.23	0.17	0.15	0.14
**ENSDARG00000040251**	ctsk	cathepsin K [Source:ZFIN;Acc:ZDB-GENE-001205-4]	16	29492749	29502787		15.04	21.33	19.98	14	15.48	15.01
**ENSDARG00000040252**	atp1a1a.5	ATPase Na+/K+ transporting subunit alpha 1a, tandem duplicate 5 [Source:ZFIN;Acc:ZDB-GENE-020501-1]	1	1838164	1850104		3.24	4.18	4.08	3.43	3.47	3.56
**ENSDARG00000040253**	onecutl	one cut domain, family member, like [Source:ZFIN;Acc:ZDB-GENE-020531-1]	16	29521736	29528198		6.87	3.65	3.39	10.32	10.03	10.56
**ENSDARG00000040254**	hormad1	HORMA domain containing 1 [Source:NCBI gene;Acc:436630]	16	29551023	29557338		0.06	0.04	0.18	0.08	0.09	0.12
**ENSDARG00000040255**	ephx2	epoxide hydrolase 2, cytoplasmic [Source:ZFIN;Acc:ZDB-GENE-041212-70]	17	6016243	6076266		18.16	25.96	22.15	13.04	15.31	15.11
**ENSDARG00000040257**	slc38a11	solute carrier family 38 member 11 [Source:NCBI gene;Acc:550337]	9	50000504	50032969		0.62	0.86	1.16	0.65	0.69	1.02
**ENSDARG00000040258**	si:ch73-340m8.2	si:ch73-340m8.2 [Source:ZFIN;Acc:ZDB-GENE-110411-209]	17	5829851	5860222		3.85	3.04	2.22	3.22	2.73	2.7
**ENSDARG00000040261**	runx2a	RUNX family transcription factor 2a [Source:ZFIN;Acc:ZDB-GENE-040629-3]	17	5351922	5516693		0.28	0.13	0.09	0.4	0.47	0.59
**ENSDARG00000040265**	mpdu1b	mannose-P-dolichol utilization defect 1b [Source:ZFIN;Acc:ZDB-GENE-040625-135]	7	26466826	26473483		6.59	7.16	5.4	8.98	7.66	7.39
**ENSDARG00000040266**	sox19b	SRY-box transcription factor 19b [Source:NCBI gene;Acc:64812]	7	26494583	26497947		2.83	1.1	1.4	4.3	4.52	4.54
**ENSDARG00000040274**	scamp5b	secretory carrier membrane protein 5b [Source:ZFIN;Acc:ZDB-GENE-120215-76]	18	50089000	50100149		17.98	4.02	3.71	14.64	14.23	14.89
**ENSDARG00000040277**	fbxo32	F-box protein 32 [Source:ZFIN;Acc:ZDB-GENE-040426-1040]	16	25259313	25272633		3.48	4.6	4.13	4.47	4.36	4.21
**ENSDARG00000040278**	klhl38b	kelch-like family member 38b [Source:ZFIN;Acc:ZDB-GENE-120727-8]	16	25245857	25250414		1.43	1.9	1.49	1.66	1.62	1.86
**ENSDARG00000040280**	LRATD2	zgc:110182 [Source:ZFIN;Acc:ZDB-GENE-050417-381]	16	25231963	25233515	LRATD2	4.72	4.39	5.3	5.81	6.92	5.55
**ENSDARG00000040282**	zgc:92590	zgc:92590 [Source:ZFIN;Acc:ZDB-GENE-041024-15]	16	25126935	25130075		11.37	15.26	12.89	0.08	0.09	0.2
**ENSDARG00000040284**	si:dkey-79d12.5	si:dkey-79d12.5 [Source:ZFIN;Acc:ZDB-GENE-131127-429]	16	24819118	24832038		1.04	1.22	0.73	3.07	3.28	2.95
**ENSDARG00000040286**	ubl7b	ubiquitin-like 7b (bone marrow stromal cell-derived) [Source:ZFIN;Acc:ZDB-GENE-040426-1334]	18	50523870	50533716		6.65	6.08	6.92	5.67	6.31	6.38
**ENSDARG00000040287**	ywhabl	tyrosine 3-monooxygenase/tryptophan 5-monooxygenase activation protein, beta polypeptide like [Source:ZFIN;Acc:ZDB-GENE-03	16	24681177	24695891		272.7	205	210.07	252.1	253.29	247.92
**ENSDARG00000040290**	pon3.2	paraoxonase 3, tandem duplicate 2 [Source:ZFIN;Acc:ZDB-GENE-071004-70]	16	24662252	24668620		15.39	19.86	17.64	8.63	8.64	8.74
**ENSDARG00000040291**	cadm4	cell adhesion molecule 4 [Source:ZFIN;Acc:ZDB-GENE-041114-138]	16	24213078	24518040		29.34	11.44	14.37	31.38	31.77	33.82
**ENSDARG00000040295**	apoeb	apolipoprotein Eb [Source:NCBI gene;Acc:30314]	16	23960744	23963461		340.87	416.46	403.93	494.77	560.04	524.23
**ENSDARG00000040298**	apoa4b.1	apolipoprotein A-IV b, tandem duplicate 1 [Source:ZFIN;Acc:ZDB-GENE-030131-1263]	16	23913943	23916263		446.92	782.38	698.46	250.19	271.8	263.07
**ENSDARG00000040300**	gtpbp10	GTP-binding protein 10 (putative) [Source:NCBI gene;Acc:436727]	16	23891443	23902439		7.49	6.34	6.27	5.26	4.99	5.05
**ENSDARG00000040302**	osgin2	oxidative stress induced growth inhibitor family member 2 [Source:ZFIN;Acc:ZDB-GENE-081028-57]	16	23880004	23885482		1.26	0.23	0.57	1.11	0.86	0.96
**ENSDARG00000040303**	nbn	nibrin [Source:NCBI gene;Acc:544655]	16	23837847	23977525		3	2.3	2.07	2.48	2.47	2.34
**ENSDARG00000040306**	otomp	otolith matrix protein [Source:NCBI gene;Acc:565818]	2	41504752	41518340		65.06	56.89	75.62	37.22	38.9	39.35
**ENSDARG00000040309**	pxdc1b	PX domain containing 1b [Source:ZFIN;Acc:ZDB-GENE-091204-294]	24	11451077	11467549		0.96	1.55	1.1	1.89	1.63	1.83
**ENSDARG00000040310**	prpf4bb	pre-mRNA processing factor 4Bb [Source:ZFIN;Acc:ZDB-GENE-040426-2799]	24	11469811	11506064		8.96	6.22	5.99	7.38	7.7	7.44
**ENSDARG00000040314**	psph	phosphoserine phosphatase [Source:ZFIN;Acc:ZDB-GENE-050809-127]	15	413543	419597		16.55	18.29	18.3	19.58	19.07	18.37
**ENSDARG00000040319**	CABZ01041494.1	C5a anaphylatoxin chemotactic receptor [Source:NCBI gene;Acc:100000959]	18	46798159	46799199		0.6	0.75	0.73	1.22	1.4	1.42
**ENSDARG00000040321**	rx2	retinal homeobox gene 2 [Source:NCBI gene;Acc:30473]	2	55833425	55861351		8.98	2.12	1.34	6.06	6.04	6.84
**ENSDARG00000040326**	dhx29	DEAH (Asp-Glu-Ala-His) box polypeptide 29 [Source:ZFIN;Acc:ZDB-GENE-130530-606]	10	8154874	8197049		7.73	5.05	4.04	6.44	5.78	6.06
**ENSDARG00000040332**	crfb2	cytokine receptor family member b2 [Source:ZFIN;Acc:ZDB-GENE-070905-2]	9	19073556	19092322		1.49	0.74	0.75	1.44	1.09	1.41
**ENSDARG00000040334**	mat2aa	methionine adenosyltransferase II, alpha a [Source:ZFIN;Acc:ZDB-GENE-030131-2049]	10	7810455	7821686		39.33	29.86	27.16	33.69	31.87	31.64
**ENSDARG00000040338**	hvcn1	hydrogen voltage-gated channel 1 [Source:NCBI gene;Acc:436618]	10	8401929	8412964		10.41	11.69	10.87	9.26	10.9	10.83
**ENSDARG00000040350**	rpp38	ribonuclease P/MRP 38 subunit [Source:ZFIN;Acc:ZDB-GENE-040718-258]	16	28592495	28594181		4.29	3.85	3.18	2.89	3.5	3.46
**ENSDARG00000040352**	crot	carnitine O-octanoyltransferase [Source:ZFIN;Acc:ZDB-GENE-050522-35]	16	28601974	28627568		14.05	14.71	11.84	11.17	11.45	10.9
**ENSDARG00000040358**	spred3	sprouty related EVH1 domain containing 3 [Source:ZFIN;Acc:ZDB-GENE-061207-31]	18	44596787	44611252		5.9	1.45	2.24	11.01	10.5	10.87
**ENSDARG00000040362**	ehd2b	EH-domain containing 2b [Source:ZFIN;Acc:ZDB-GENE-030131-9347]	18	44649804	44663244		11.59	13.14	11.33	14.69	15.07	14.03
**ENSDARG00000040374**	tmem182a	transmembrane protein 182a [Source:NCBI gene;Acc:503742]	9	6915611	6927587		23.41	39.44	35.11	33.76	26.08	26.74
**ENSDARG00000040380**	arhgef1a	Rho guanine nucleotide exchange factor (GEF) 1a [Source:ZFIN;Acc:ZDB-GENE-030722-5]	19	11039517	11106315		4.18	3.32	2.56	6.06	6.05	5.82
**ENSDARG00000040387**	mmd	monocyte to macrophage differentiation-associated [Source:ZFIN;Acc:ZDB-GENE-051120-39]	3	11548516	11576337		14.74	8.53	9.41	18.06	18.57	18.03
**ENSDARG00000040396**	bcl7bb	BAF chromatin remodeling complex subunit BCL7B b [Source:NCBI gene;Acc:81595]	18	41549764	41559418		8.51	9.62	8.27	7.4	8.39	6.52
**ENSDARG00000040401**	slc25a20	solute carrier family 25 member 20 [Source:ZFIN;Acc:ZDB-GENE-040426-1869]	22	105376	109686		40.02	55.52	52.27	39.32	37.75	36.11
**ENSDARG00000040407**	usp22	ubiquitin specific peptidase 22 [Source:NCBI gene;Acc:692275]	12	1469090	1526051		6.37	2.56	3.29	6.74	7.37	6.69
**ENSDARG00000040427**	acot19	acyl-CoA thioesterase 19 [Source:ZFIN;Acc:ZDB-GENE-041114-124]	25	31714335	31739309		2.22	2.82	2.14	0.63	0.73	1
**ENSDARG00000040430**	nptxra	neuronal pentraxin receptor a [Source:ZFIN;Acc:ZDB-GENE-050208-280]	3	24482999	24501796		18.29	5.02	5.84	20	21.7	21.06
**ENSDARG00000040432**	klf2b	Kruppel-like factor 2b [Source:ZFIN;Acc:ZDB-GENE-011109-2]	2	55665095	55673225		6.27	4.9	3.79	6.25	6.02	5.79
**ENSDARG00000040433**	cyp2k22	cytochrome P450, family 2, subfamily K, polypeptide 22 [Source:ZFIN;Acc:ZDB-GENE-040426-822]	3	8229466	8246315		0.01	0.07	0.01	0.03	0.01	0.02
**ENSDARG00000040439**	rsl24d1	ribosomal L24 domain containing 1 [Source:NCBI gene;Acc:406266]	25	469855	475217		318.93	400.85	430.78	251.78	259.88	271.07
**ENSDARG00000040440**	snrpd2	small nuclear ribonucleoprotein D2 polypeptide [Source:ZFIN;Acc:ZDB-GENE-040914-10]	18	40875873	40884087		58.98	70.44	69.66	40.18	40.6	44.23
**ENSDARG00000040442**	prtgb	protogenin homolog b (Gallus gallus) [Source:NCBI gene;Acc:572241]	25	418932	463592		0.13	0.02	0.04	0.16	0.19	0.18
**ENSDARG00000040443**	polr2i	polymerase (RNA) II (DNA directed) polypeptide I [Source:ZFIN;Acc:ZDB-GENE-041010-106]	18	40909821	40913301		10.42	8.7	9.22	7.68	7.08	6.86
**ENSDARG00000040445**	si:ch211-219a4.3	si:ch211-219a4.3 [Source:ZFIN;Acc:ZDB-GENE-070424-157]	9	23765587	23779525		1.27	1.54	1.43	1.17	1.09	0.98
**ENSDARG00000040452**	fam214a	family with sequence similarity 214 member A [Source:NCBI gene;Acc:555595]	18	38774584	38857612		5.74	4.17	3.41	6.27	6.04	6.65
**ENSDARG00000040453**	wdfy2	WD repeat and FYVE domain containing 2 [Source:ZFIN;Acc:ZDB-GENE-041111-211]	9	23813593	23824290		10.41	7.13	7.91	9.53	10.02	9.26
**ENSDARG00000040455**	unc50	unc-50 homolog (C. elegans) [Source:NCBI gene;Acc:393217]	6	3984841	3998199		12.11	11.75	12.12	12.27	13.93	12.95
**ENSDARG00000040463**	slc25a51b	solute carrier family 25 member 51b [Source:ZFIN;Acc:ZDB-GENE-140328-2]	14	4178236	4184702		8.19	8.81	9.13	7.14	7.21	7.13
**ENSDARG00000040465**	irf2	interferon regulatory factor 2 [Source:ZFIN;Acc:ZDB-GENE-041212-38]	14	4084120	4121052		4.82	4.79	3.52	4.37	3.53	3.47
**ENSDARG00000040466**	vil1	villin 1 [Source:ZFIN;Acc:ZDB-GENE-040426-799]	9	44907768	44953664		28.37	40.97	21.02	9.97	11.3	10.72
**ENSDARG00000040469**	enpp6	ectonucleotide pyrophosphatase/phosphodiesterase 6 [Source:NCBI gene;Acc:791915]	14	4047215	4076480		2.37	2.92	2.52	4.44	4.51	4.18
**ENSDARG00000040474**	snx10b	sorting nexin 10b [Source:NCBI gene;Acc:569469]	16	21033633	21038015		0.75	0.7	0.68	0.42	0.55	0.54
**ENSDARG00000040478**	lrp2bp	LRP2 binding protein [Source:NCBI gene;Acc:100073340]	14	3944826	3961332		0.29	0.14	0.17	0.44	0.58	0.44
**ENSDARG00000040479**	cga	glycoprotein hormones, alpha polypeptide [Source:ZFIN;Acc:ZDB-GENE-040715-2]	17	49439219	49443399		0.21	0.01	0.04	0.58	0.8	0.6
**ENSDARG00000040482**	osbpl3b	oxysterol binding protein-like 3b [Source:ZFIN;Acc:ZDB-GENE-090109-1]	16	21242491	21418775		1.85	1.22	1.15	2.02	2.22	2.04
**ENSDARG00000040485**	gsdmeb	gasdermin Eb [Source:ZFIN;Acc:ZDB-GENE-030131-7662]	16	21425114	21441226		3.67	3.29	3.04	2.34	2	2.56
**ENSDARG00000040487**	zgc:113176	zgc:113176 [Source:ZFIN;Acc:ZDB-GENE-050327-60]	17	49276061	49306686		2.57	1.37	1.69	3.07	2.9	2.8
**ENSDARG00000040490**	dusp19a	dual specificity phosphatase 19a [Source:ZFIN;Acc:ZDB-GENE-081104-382]	9	44713033	44718628		0.98	1.31	1.02	0.71	0.69	0.65
**ENSDARG00000040492**	mthfd1b	methylenetetrahydrofolate dehydrogenase (NADP+ dependent) 1b [Source:ZFIN;Acc:ZDB-GENE-020905-4]	17	49354343	49412313		23.48	32.45	29.65	17.63	19.39	18.04
**ENSDARG00000040497**	BX004816.1	GTPase IMAP family member 4-like [Source:NCBI gene;Acc:571124]	3	2971852	2979129		0.01	0	0	0	0	0
**ENSDARG00000040503**	sb:cb81	sb:cb81 [Source:ZFIN;Acc:ZDB-GENE-030131-9939]	18	49309953	49318823		5.89	3.92	3.83	11.8	12.58	12.75
**ENSDARG00000040505**	yif1b	Yip1 interacting factor homolog B (S. cerevisiae) [Source:NCBI gene;Acc:492462]	18	49248389	49267856		3.47	3.2	3.14	4.78	4.51	4.42
**ENSDARG00000040510**	ca15b	carbonic anhydrase XVb [Source:ZFIN;Acc:ZDB-GENE-040426-2222]	12	4294923	4301376		0.2	0.1	0.1	0.12	0.07	0.07
**ENSDARG00000040513**	zgc:92313	zgc:92313 [Source:ZFIN;Acc:ZDB-GENE-040718-339]	12	4241458	4249000		13.71	13.21	12.47	13.47	13.12	11.92
**ENSDARG00000040523**	smpd2a	sphingomyelin phosphodiesterase 2a [Source:ZFIN;Acc:ZDB-GENE-051014-1]	23	5565261	5587934		4.65	5.32	4.9	4.66	4.57	4.63
**ENSDARG00000040526**	s1pr5a	sphingosine-1-phosphate receptor 5a [Source:ZFIN;Acc:ZDB-GENE-041114-188]	3	19364948	19368435		3.13	4.03	3.21	4.68	4.3	3.91
**ENSDARG00000040527**	siae	sialic acid acetylesterase [Source:ZFIN;Acc:ZDB-GENE-040426-1032]	18	36582815	36598693		4.58	5.34	4.8	3.52	3.5	3.35
**ENSDARG00000040528**	lgals3bpb	lectin, galactoside-binding, soluble, 3 binding protein b [Source:NCBI gene;Acc:405809]	3	57741933	57744323		6.83	8.2	9.03	12.22	14.44	12.11
**ENSDARG00000040534**	epcam	epithelial cell adhesion molecule [Source:ZFIN;Acc:ZDB-GENE-040426-2209]	13	8840772	8843363		141.52	144.4	139.75	146.94	151.89	134.68
**ENSDARG00000040535**	csgalnact1a	chondroitin sulfate N-acetylgalactosaminyltransferase 1a [Source:ZFIN;Acc:ZDB-GENE-121129-1]	10	8298984	8358396		4.11	4.53	3.97	7.01	7.05	6.59
**ENSDARG00000040536**	ftr90	finTRIM family, member 90 [Source:ZFIN;Acc:ZDB-GENE-131122-80]	15	41711727	41714329		0	0	0	0.06	0.04	0.19
**ENSDARG00000040538**	spsb4b	splA/ryanodine receptor domain and SOCS box containing 4b [Source:ZFIN;Acc:ZDB-GENE-040801-213]	15	41670742	41689981		6.14	4.52	4.23	6.84	6.03	5.97
**ENSDARG00000040547**	b3gat1a	beta-1,3-glucuronyltransferase 1 (glucuronosyltransferase P) a [Source:ZFIN;Acc:ZDB-GENE-050419-21]	18	36105930	36135799		7.95	2.05	2.97	9.35	10.34	9.76
**ENSDARG00000040553**	tmem97	transmembrane protein 97 [Source:ZFIN;Acc:ZDB-GENE-110627-1]	15	23790057	23793641		8.92	10.73	8.47	5	4.27	4.98
**ENSDARG00000040555**	bckdha	branched chain keto acid dehydrogenase E1 subunit alpha [Source:ZFIN;Acc:ZDB-GENE-050522-376]	18	36066389	36081908		39.86	43.69	41.6	30.37	28.19	28.15
**ENSDARG00000040556**	ift20	intraflagellar transport 20 homolog (Chlamydomonas) [Source:NCBI gene;Acc:414930]	15	23784842	23790172		9.38	8.07	7.51	9.47	8.11	8.53
**ENSDARG00000040557**	exosc5	exosome component 5 [Source:ZFIN;Acc:ZDB-GENE-060503-675]	18	36060295	36066087		12.95	16.02	17.54	7.7	6.77	8.86
**ENSDARG00000040559**	rad1	RAD1 homolog (S. pombe) [Source:ZFIN;Acc:ZDB-GENE-040426-1873]	15	23776417	23784600		1.17	1.16	1.07	1.01	1.08	0.89
**ENSDARG00000040564**	hnrnpul1	heterogeneous nuclear ribonucleoprotein U-like 1 [Source:ZFIN;Acc:ZDB-GENE-040426-2432]	18	35807588	35837673		45.63	36.85	38.6	34.45	35.53	35.36
**ENSDARG00000040565**	ckmb	creatine kinase, muscle b [Source:ZFIN;Acc:ZDB-GENE-040426-2128]	15	23643718	23647078		1809.82	2513.12	3009.47	3084.92	3030.5	2862.83
**ENSDARG00000040566**	phykpl	5-phosphohydroxy-L-lysine phospho-lyase [Source:ZFIN;Acc:ZDB-GENE-051127-33]	15	23443665	23451449		6.96	7.7	8.18	4.91	5.08	5.28
**ENSDARG00000040568**	pdzd3a	PDZ domain containing 3a [Source:ZFIN;Acc:ZDB-GENE-080430-1]	15	23452862	23467750		5.98	8.36	5.49	3.44	3.65	3.91
**ENSDARG00000040571**	ube4a	ubiquitination factor E4A (UFD2 homolog, yeast) [Source:ZFIN;Acc:ZDB-GENE-070410-21]	15	23423672	23443937		9.43	6.26	5.15	9.98	10.29	10.14
**ENSDARG00000040582**	C18H3orf33	c18h3orf33 homolog (H. sapiens) [Source:ZFIN;Acc:ZDB-GENE-061121-2]	18	34146007	34156765	C18H3orf33	1.31	1.34	1.26	1.1	1.22	1.32
**ENSDARG00000040584**	eef1akmt1	EEF1A lysine methyltransferase 1 [Source:NCBI gene;Acc:404619]	9	21358941	21363501		6.02	9.06	8.34	4.25	4.53	4.29
**ENSDARG00000040594**	olfcq3	olfactory receptor C family, q3 [Source:ZFIN;Acc:ZDB-GENE-050419-41]	18	33373811	33378068		0.11	0.09	0.05	0.06	0.1	0.1
**ENSDARG00000040601**	CU570684.1		3	58828341	58831683		0	0	0	0	0	0
**ENSDARG00000040606**	tfap2c	transcription factor AP-2 gamma (activating enhancer binding protein 2 gamma) [Source:ZFIN;Acc:ZDB-GENE-041212-61]	6	56141852	56179707		12.66	9.03	5.43	12.24	11.12	11.28
**ENSDARG00000040607**	rtf2	replication termination factor 2 [Source:ZFIN;Acc:ZDB-GENE-030131-4825]	6	56076595	56140968		9.2	8.07	7.65	8.51	8.4	8.67
**ENSDARG00000040608**	gcnt7	glucosaminyl (N-acetyl) transferase family member 7 [Source:ZFIN;Acc:ZDB-GENE-120919-3]	6	56097971	56111829		3.85	3.01	2.67	2.36	2.7	2.45
**ENSDARG00000040610**	si:ch73-352p4.8	si:ch73-352p4.8 [Source:ZFIN;Acc:ZDB-GENE-140106-36]	4	5796761	5807645		6.15	5.51	5.9	6.94	7.1	7.27
**ENSDARG00000040614**	sestd1	SEC14 and spectrin domains 1 [Source:NCBI gene;Acc:360133]	9	43149854	43213229		16.63	12.47	14.07	15.98	15.3	16.21
**ENSDARG00000040615**	ilkap	integrin-linked kinase-associated serine/threonine phosphatase [Source:ZFIN;Acc:ZDB-GENE-070410-122]	15	46722510	46736432		4.09	2.8	3.49	2.98	3.16	3.4
**ENSDARG00000040620**	psmg1	proteasome (prosome, macropain) assembly chaperone 1 [Source:NCBI gene;Acc:436987]	10	206996	210703		13.43	14.17	16.12	10.56	11.88	11.57
**ENSDARG00000040621**	lrrc32	leucine rich repeat containing 32 [Source:ZFIN;Acc:ZDB-GENE-090630-1]	10	252425	257043		2.65	3.42	3.04	6.09	6.2	5.27
**ENSDARG00000040623**	fosl2	fos-like antigen 2 [Source:ZFIN;Acc:ZDB-GENE-070209-164]	17	41302660	41318547		4.42	2.01	1.46	14.3	13.09	13.1
**ENSDARG00000040627**	grik1b	glutamate receptor, ionotropic, kainate 1b [Source:ZFIN;Acc:ZDB-GENE-070821-1]	15	42599501	42679838		4.01	0.37	0.29	4.58	4.14	4.75
**ENSDARG00000040628**	zgc:110333	zgc:110333 [Source:ZFIN;Acc:ZDB-GENE-050417-331]	15	42560354	42575208		21.48	19.14	17.89	17.25	15.04	13.3
**ENSDARG00000040632**	olfcu1	olfactory receptor C family, u1 [Source:ZFIN;Acc:ZDB-GENE-050419-175]	18	33333411	33337703		0.12	0.04	0.05	0.04	0.05	0.04
**ENSDARG00000040633**	PRMT8	protein arginine N-methyltransferase 8-B-like [Source:NCBI gene;Acc:556054]	18	11506561	11549906	PRMT8	3.2	1.05	1.5	3.76	2.9	3.46
**ENSDARG00000040635**	rnf11b	ring finger protein 11b [Source:ZFIN;Acc:ZDB-GENE-050913-69]	8	16424722	16464453		9.57	5.16	5.05	10.27	9.74	9.58
**ENSDARG00000040640**	si:ch73-44m9.3	si:ch73-44m9.3 [Source:ZFIN;Acc:ZDB-GENE-081104-290]	22	7758462	7809904		0.6	0.31	0.29	0.4	0.45	0.46
**ENSDARG00000040643**	ccr10	chemokine (C-C motif) receptor 10 [Source:ZFIN;Acc:ZDB-GENE-040718-424]	24	36673686	36680261		0.05	0.03	0.02	0	0.05	0
**ENSDARG00000040644**	pxylp1	2-phosphoxylose phosphatase 1 [Source:NCBI gene;Acc:436703]	15	41815703	41845709		5.76	3.47	3.4	7.59	7.83	8.33
**ENSDARG00000040649**	prickle1a	prickle homolog 1a [Source:ZFIN;Acc:ZDB-GENE-030724-5]	25	314608	348784		1.73	2.07	1.56	3.13	3.02	2.82
**ENSDARG00000040650**	gps1	G protein pathway suppressor 1 [Source:ZFIN;Acc:ZDB-GENE-041111-240]	3	36234060	36246975		21.69	22.67	23.56	18.65	18.71	17.98
**ENSDARG00000040657**	wipi1	WD repeat domain, phosphoinositide interacting 1 [Source:ZFIN;Acc:ZDB-GENE-040426-1404]	3	36284986	36306403		11.24	8.06	7.55	13.82	14.52	15.06
**ENSDARG00000040666**	nifk	nucleolar protein interacting with the FHA domain of MKI67 [Source:NCBI gene;Acc:317644]	9	38158570	38162095		20.23	26.99	22.91	14.48	13.5	13.36
**ENSDARG00000040668**	lrrc51	leucine rich repeat containing 51 [Source:ZFIN;Acc:ZDB-GENE-060929-962]	15	44053244	44062824		0.44	0.53	0.36	0.41	0.6	0.54
**ENSDARG00000040674**	ctdsp1	CTD (carboxy-terminal domain, RNA polymerase II, polypeptide A) small phosphatase 1 [Source:ZFIN;Acc:ZDB-GENE-050522-523]	9	41690153	41741420		12.5	14.76	13.44	17.13	16.96	16.64
**ENSDARG00000040678**	ccdc93	coiled-coil domain containing 93 [Source:ZFIN;Acc:ZDB-GENE-060421-8315]	9	38353452	38368138		9.49	9.6	10.01	10.39	10.66	11.21
**ENSDARG00000040683**	MEP1B	si:ch73-40a17.3 [Source:ZFIN;Acc:ZDB-GENE-030131-5797]	2	283764	293793	MEP1B	2.68	2.7	1.69	0.46	0.4	0.49
**ENSDARG00000040684**	plcd4b	phospholipase C, delta 4b [Source:ZFIN;Acc:ZDB-GENE-080512-3]	9	38369872	38390990		4.15	3.33	3.46	3.99	4.46	4.3
**ENSDARG00000040695**	fkbp14	FKBP prolyl isomerase 14 [Source:ZFIN;Acc:ZDB-GENE-040801-210]	16	20294976	20305104		6.41	8.27	6.77	11.39	11.41	10.6
**ENSDARG00000040698**	plekha8	pleckstrin homology domain containing, family A (phosphoinositide binding specific) member 8 [Source:NCBI gene;Acc:492502]	16	20265499	20294236		4.72	3.28	2.86	5.61	5.5	4.99
**ENSDARG00000040700**	nab1b	NGFI-A binding protein 1b (EGR1 binding protein 1) [Source:ZFIN;Acc:ZDB-GENE-081105-85]	9	41367848	41401564		2.22	1.16	1.61	3.58	3.23	4.04
**ENSDARG00000040701**	si:ch211-113j13.2	si:ch211-113j13.2 [Source:ZFIN;Acc:ZDB-GENE-131127-162]	15	37419552	37436082		0.04	0	0.01	0.04	0.02	0.01
**ENSDARG00000040703**	BX936415.1		6	40174216	40182520		1.96	0.64	0.84	2.13	2.63	1.72
**ENSDARG00000040705**	glsb	glutaminase b [Source:ZFIN;Acc:ZDB-GENE-030616-550]	9	41273231	41351426		5.88	6.76	5.75	7.16	6.98	7.08
**ENSDARG00000040707**	chchd4b	coiled-coil-helix-coiled-coil-helix domain containing 4b [Source:ZFIN;Acc:ZDB-GENE-041010-149]	6	40060409	40063464		2.78	2.8	3.05	2.01	1.67	1.95
**ENSDARG00000040712**	adprh	ADP-ribosylarginine hydrolase [Source:ZFIN;Acc:ZDB-GENE-040801-190]	12	15148618	15196223		5.23	7.47	6.71	5.72	5.72	5.23
**ENSDARG00000040713**	rbbp9	retinoblastoma binding protein 9 [Source:ZFIN;Acc:ZDB-GENE-050208-692]	22	38944615	38951354		9.44	11.72	11.92	6.03	5.64	5.85
**ENSDARG00000040717**	macc1	MET transcriptional regulator MACC1 [Source:ZFIN;Acc:ZDB-GENE-091006-3]	16	19637384	19677375		1.99	1.96	1.67	2.41	2.27	2.05
**ENSDARG00000040725**	ZFP36L1	zgc:114130 [Source:ZFIN;Acc:ZDB-GENE-050913-48]	15	14034254	14038227	ZFP36L1	4.15	3.26	2.97	4.51	5.16	4.62
**ENSDARG00000040727**	tfb1m	transcription factor B1, mitochondrial [Source:ZFIN;Acc:ZDB-GENE-060929-1010]	17	49220126	49280428		3.34	3.42	3.25	1.69	1.61	1.83
**ENSDARG00000040730**	wdr75	WD repeat domain 75 [Source:NCBI gene;Acc:368478]	9	41120158	41156972		11.03	11.1	9.25	8.14	8.55	7.58
**ENSDARG00000040732**	elavl2	ELAV like neuron-specific RNA binding protein 2 [Source:ZFIN;Acc:ZDB-GENE-040704-9]	22	38252686	38274995		0.01	0.05	0.05	0.16	0.09	0.12

ENSDARG00000040735	crygm6	crystallin, gamma M6 [Source:ZFIN;Acc:ZDB-GENE-050516-7]	9	22000812	22001778		0	0	0	0	0	0
ENSDARG00000040736	crygm7	crystallin, gamma M7 [Source:ZFIN;Acc:ZDB-GENE-050516-8]	9	21993092	21994057		0	0	0.02	0	0.18	0.11
ENSDARG00000040738	zgc:153846	zgc:153846 [Source:ZFIN;Acc:ZDB-GENE-060929-898]	9	22017368	22018304		0.04	0.07	0.03	0	0	0.04
ENSDARG00000040741	kcnab1b	potassium voltage-gated channel, shaker-related subfamily, beta member 1 b [Source:ZFIN;Acc:ZDB-GENE-070912-690]	2	44426609	44510752		5.73	2	1.75	4.53	4.95	5.47
ENSDARG00000040747	tm4sf4	transmembrane 4 L six family member 4 [Source:ZFIN;Acc:ZDB-GENE-040801-231]	22	38127467	38137908		49.74	72.36	41.02	15.06	17.04	16.07
ENSDARG00000040764	id1	inhibitor of DNA binding 1, HLH protein [Source:ZFIN;Acc:ZDB-GENE-990415-96]	11	18704191	18705303		255.73	274.55	309.4	210.12	224.43	228.97
ENSDARG00000040765	drd6b	dopamine receptor D6b [Source:ZFIN;Acc:ZDB-GENE-121214-145]	12	5458462	5460203		0.27	0.02	0.03	0.22	0.16	0.34
ENSDARG00000040777	inhbb	inhibin subunit beta B [Source:ZFIN;Acc:ZDB-GENE-990415-1]	9	36967294	36974879		6.21	5.57	5.19	6.17	6.36	6.38
ENSDARG00000040778	ralba	v-ral simian leukemia viral oncogene homolog Ba (ras related) [Source:ZFIN;Acc:ZDB-GENE-040801-267]	9	36896257	36924388		12.4	10.05	9.16	13.46	13.3	12.82
ENSDARG00000040779	med16	mediator complex subunit 16 [Source:ZFIN;Acc:ZDB-GENE-090306-4]	11	14104417	14143702		4.35	3.02	2.62	4.6	4.69	4.55
ENSDARG00000040781	sult3st4	sulfotransferase family 3, cytosolic sulfotransferase 4 [Source:ZFIN;Acc:ZDB-GENE-050913-17]	22	15331214	15336381		0.18	0.09	0.15	0.09	0.07	0.18
ENSDARG00000040799	sst1.1	somatostatin 1, tandem duplicate 1 [Source:ZFIN;Acc:ZDB-GENE-030131-4743]	15	36156986	36158851		17.82	5.78	8.5	15.12	19.87	18.96
ENSDARG00000040803	lactb	lactamase, beta [Source:ZFIN;Acc:ZDB-GENE-020111-1]	25	33256012	33266553		6.02	4.51	4.58	7.24	7.67	7.31
ENSDARG00000040812	ncf4	neutrophil cytosolic factor 4 [Source:ZFIN;Acc:ZDB-GENE-041124-1]	22	29204885	29252049		0.72	0.66	0.81	0.86	0.86	1.07
ENSDARG00000040815	tsku	tsukushi small leucine rich proteoglycan homolog (Xenopus laevis) [Source:NCBI gene;Acc:325958]	15	29343280	29354020		6.84	7.87	6.59	6.31	6.6	6.07
ENSDARG00000040816	uts2r3	urotensin-2 receptor 3 [Source:ZFIN;Acc:ZDB-GENE-100922-123]	16	16757219	16761164		0.8	0.8	0.77	1.32	1.12	1.43
ENSDARG00000040822	fundc1	FUN14 domain containing 1 [Source:NCBI gene;Acc:436984]	9	33783549	33785200		40.29	34.55	35.84	43.36	42.92	45.61
ENSDARG00000040838	dcun1d2a	DCN1, defective in cullin neddylation 1, domain containing 2a [Source:ZFIN;Acc:ZDB-GENE-020416-2]	9	34938316	34945582		16.98	19.27	20.95	17.34	15.94	15.83
ENSDARG00000040841	adra2a	adrenoceptor alpha 2A [Source:NCBI gene;Acc:266750]	22	29584800	29586608		1.53	1	1.15	2.08	2.4	1.8
ENSDARG00000040851	plekhh2	pleckstrin homology domain containing, family H (with MyTH4 domain) member 2 [Source:ZFIN;Acc:ZDB-GENE-081028-42]	13	8847421	8892514		1	1.09	0.8	1.66	1.6	1.66
ENSDARG00000040853	shoc2	SHOC2 leucine rich repeat scaffold protein [Source:NCBI gene;Acc:555476]	22	29596646	29640181		8.61	6.03	4.85	10.78	10.54	10.25
ENSDARG00000040854	tsg101a	tumor susceptibility 101a [Source:ZFIN;Acc:ZDB-GENE-030217-1]	25	25132703	25142387		24.23	15.94	15.82	21.74	20.14	20.75
ENSDARG00000040861	tcf25	transcription factor 25 (basic helix-loop-helix) [Source:ZFIN;Acc:ZDB-GENE-040905-2]	18	31117136	31206658		10.24	11.87	10.91	8.7	8.35	8.64
ENSDARG00000040864	si:dkey-286j15.1	si:dkey-286j15.1 [Source:ZFIN;Acc:ZDB-GENE-060503-447]	22	30009926	30033583		23.65	22.88	21.94	19.9	19.37	20.57
ENSDARG00000040869	upp1	uridine phosphorylase 1 [Source:ZFIN;Acc:ZDB-GENE-050306-2]	16	16210208	16212615		6.65	7.76	6.34	6.17	6.7	6.35
ENSDARG00000040874	add3a	adducin 3 (gamma) a [Source:ZFIN;Acc:ZDB-GENE-030131-2721]	22	30046365	30270469		16.49	15.37	12.43	15.14	15.21	15.34
ENSDARG00000040881	hnrnph1	heterogeneous nuclear ribonucleoprotein H1 [Source:ZFIN;Acc:ZDB-GENE-040426-1856]	14	6991142	7014588		10.12	7.94	8.51	14.35	14.86	14.6
ENSDARG00000040884	mxi1	max interactor 1, dimerization protein [Source:NCBI gene;Acc:30485]	22	30330574	30361078		18.07	7.77	7.66	22.67	22.58	23.74
ENSDARG00000040890	fdps	farnesyl diphosphate synthase (farnesyl pyrophosphate synthetase, dimethylallyltranstransferase, geranyltranstransferase) [Source:Z	16	13993351	14025096		7.78	10.34	7.7	7	6.58	6.7
ENSDARG00000040898	gpa33b	glycoprotein A33 (transmembrane), paralog b [Source:ZFIN;Acc:ZDB-GENE-070912-194]	9	34307140	34308113		10.75	20.21	12.31	6.58	7.09	6.29
ENSDARG00000040901	si:dkey-11d18.4	si:dkey-11d18.4 [Source:ZFIN;Acc:ZDB-GENE-091204-474]	18	22695407	22696357		0.05	0	0.04	0	0.03	0
ENSDARG00000040902	si:ch73-113g13.1	si:ch73-113g13.1 [Source:ZFIN;Acc:ZDB-GENE-091204-462]	18	22716708	22717673		0	0	0	0	0.03	0.03
ENSDARG00000040907	gcgb	glucagon b [Source:ZFIN;Acc:ZDB-GENE-040712-2]	9	51265283	51271597		11.68	10.3	12.13	28.02	28.81	28.94
ENSDARG00000040910	ildr1b	immunoglobulin-like domain containing receptor 1b [Source:ZFIN;Acc:ZDB-GENE-040912-72]	9	34260604	34270066		4.96	4.18	3.53	3.53	3.39	3.1
ENSDARG00000040911	meox2a	mesenchyme homeobox 2a [Source:ZFIN;Acc:ZDB-GENE-080613-1]	15	34428308	34468599		0.67	1.02	0.66	0.73	0.67	0.75
ENSDARG00000040912	kdelr3	KDEL endoplasmic reticulum protein retention receptor 3 [Source:NCBI gene;Acc:393613]	5	162315	180860		5.37	6.46	5.58	8.49	8.55	8.38
ENSDARG00000040917	cbfb	core-binding factor subunit beta [Source:ZFIN;Acc:ZDB-GENE-980526-440]	18	22994113	23072665		8	5.92	5.73	11.04	10.79	10.94
ENSDARG00000040920	si:dkey-49n23.1	si:dkey-49n23.1 [Source:ZFIN;Acc:ZDB-GENE-050208-762]	22	30811775	30935510		5.52	5.64	5.27	10.3	11.43	11.06
ENSDARG00000040921	zmp:0000000606	zmp:0000000606 [Source:ZFIN;Acc:ZDB-GENE-130530-609]	22	30631072	30635832		0.14	0.15	0.12	0.13	0.16	0.21
ENSDARG00000040925	wnt10b	wingless-type MMTV integration site family, member 10b [Source:NCBI gene;Acc:30308]	23	27641471	27647769		1.2	0.88	1.34	0.9	1.07	1.32
ENSDARG00000040926	nr2f2	nuclear receptor subfamily 2, group F, member 2 [Source:ZFIN;Acc:ZDB-GENE-990415-252]	18	23868650	24023013		20.45	9.45	9	38.87	36.31	37.68
ENSDARG00000040930	deptor	DEP domain containing MTOR-interacting protein [Source:ZFIN;Acc:ZDB-GENE-061013-453]	16	14588141	14675907		16.22	13.73	11.35	13.13	13.05	13.3
ENSDARG00000040938	ssuh2.1	ssu-2 homolog, tandem duplicate 1 [Source:ZFIN;Acc:ZDB-GENE-050208-558]	22	30945349	30948457		0.6	0.52	1.04	0.46	0.93	0.88
ENSDARG00000040942	pnp6	purine nucleoside phosphorylase 6 [Source:ZFIN;Acc:ZDB-GENE-040426-1800]	18	21257261	21271373		10.74	9.82	7.51	6.43	8.43	8.05
ENSDARG00000040944	rtd5	rtf-dependent gene 5 [Source:ZFIN;Acc:ZDB-GENE-031030-7]	16	13568697	13595027		29.98	37.75	40.45	23.51	26.87	25.01
ENSDARG00000040946	olig2	oligodendrocyte lineage transcription factor 2 [Source:ZFIN;Acc:ZDB-GENE-030131-4013]	9	32751428	32753535		7.7	4.07	5.41	7.91	8.07	8.01
ENSDARG00000040948	olig1	oligodendrocyte transcription factor 1 [Source:ZFIN;Acc:ZDB-GENE-050107-2]	9	32728653	32730487		2.31	2.27	2.4	1.86	2.22	2.45
ENSDARG00000040955	dharma	dharma [Source:ZFIN;Acc:ZDB-GENE-990415-22]	15	29011290	29012493		0	0	0	0	0	0
ENSDARG00000040959	rabl3	RAB, member of RAS oncogene family-like 3 [Source:NCBI gene;Acc:403048]	9	32635546	32670526		4.58	5.46	4.55	3.78	3.75	4.12
ENSDARG00000040965	zgc:101731	zgc:101731 [Source:ZFIN;Acc:ZDB-GENE-040912-57]	15	29123031	29138266		1.82	1.5	1.83	1.34	1.7	1.8
ENSDARG00000040966	zgc:113149	zgc:113149 [Source:ZFIN;Acc:ZDB-GENE-050320-56]	15	29140126	29152619		5.76	2.86	2.71	5.91	5.98	5.81
ENSDARG00000040971	zgc:92606	zgc:92606 [Source:ZFIN;Acc:ZDB-GENE-040718-462]	16	12265158	12276225		54.06	54.98	55.99	110.17	124.71	137.92
ENSDARG00000040982	fgf14	fibroblast growth factor 14 [Source:ZFIN;Acc:ZDB-GENE-060506-1]	9	31838044	31915423		10.95	1.82	3.12	13.78	13.86	13.31
ENSDARG00000040984	hspa13	heat shock protein 70 family, member 13 [Source:ZFIN;Acc:ZDB-GENE-070410-58]	15	29549115	29556757		1.92	1.93	1.73	4.44	3.94	3.97
ENSDARG00000040985	itgbl1	integrin, beta-like 1 [Source:ZFIN;Acc:ZDB-GENE-050522-410]	9	31752628	31833565		20.59	28.7	30.8	22.02	24.71	22.33
ENSDARG00000040988	tpi1b	triosephosphate isomerase 1b [Source:NCBI gene;Acc:560753]	16	12236339	12254994		198.51	234.22	256.98	229.07	241.25	240.45
ENSDARG00000040990	usp37	ubiquitin specific peptidase 37 [Source:NCBI gene;Acc:768201]	9	711638	746317		3.12	2.39	1.99	3.06	3.09	2.94
ENSDARG00000040991	cttnbp2	cortactin binding protein 2 [Source:ZFIN;Acc:ZDB-GENE-030131-8134]	18	20726149	20822045		10.55	4.64	4.23	12.67	12.42	13.19
ENSDARG00000041003	or102-4	odorant receptor, family C, subfamily 102, member 4 [Source:ZFIN;Acc:ZDB-GENE-070804-1]	15	31476727	31477915		0.02	0	0	0	0.07	0
ENSDARG00000041005	or102-1	odorant receptor, family C, subfamily 102, member 1 [Source:ZFIN;Acc:ZDB-GENE-010308-5]	15	31462664	31464048		0.03	0	0.01	0.02	0	0
ENSDARG00000041006	st3gal4	ST3 beta-galactoside alpha-2,3-sialyltransferase 4 [Source:ZFIN;Acc:ZDB-GENE-050309-174]	18	48696094	48775049		6.01	7.04	6.58	7.55	7.35	6.85
ENSDARG00000041007	AL845428.1	uncharacterized LOC101883115 [Source:NCBI gene;Acc:101883115]	15	31454930	31457948		0.03	0.03	0.05	0.02	0.03	0
ENSDARG00000041011	gemin7	gem (nuclear organelle) associated protein 7 [Source:ZFIN;Acc:ZDB-GENE-120409-2]	18	48758916	48760465		8.98	11.39	13.59	9.12	9.68	10.39
ENSDARG00000041019	or111-11	odorant receptor, family D, subfamily 111, member 11 [Source:ZFIN;Acc:ZDB-GENE-010308-10]	15	31417886	31419805		0.5	0.25	0.26	0.51	0.43	0.61
ENSDARG00000041022	pdcd4b	programmed cell death 4b [Source:ZFIN;Acc:ZDB-GENE-030131-9847]	22	29655981	29699981		16.55	8.87	7.8	13.77	14.53	14.57
ENSDARG00000041024	or111-6	odorant receptor, family D, subfamily 111, member 6 [Source:ZFIN;Acc:ZDB-GENE-010308-14]	15	31389403	31390985		0.06	0.31	0.18	0.05	0.25	0.2
ENSDARG00000041030	or111-1	odorant receptor, family D, subfamily 111, member 1 [Source:ZFIN;Acc:ZDB-GENE-000329-16]	15	31350122	31352129		0.26	0.14	0.22	0.17	0.26	0.36
ENSDARG00000041032	or107-1	odorant receptor, family D, subfamily 107, member 1 [Source:ZFIN;Acc:ZDB-GENE-010308-21]	15	31344472	31345885		0.26	0.2	0.2	0.12	0.17	0.1
ENSDARG00000041033	or119-2	odorant receptor, family F, subfamily 119, member 2 [Source:ZFIN;Acc:ZDB-GENE-010308-22]	15	31332552	31333781		0.48	0.48	0.64	0.8	0.39	0.94
ENSDARG00000041034	zgc:152857	zgc:152857 [Source:ZFIN;Acc:ZDB-GENE-060929-72]	15	31327455	31328963		0.15	0.19	0.2	0.3	0.2	0.16
ENSDARG00000041040	piga	phosphatidylinositol glycan anchor biosynthesis, class A [Source:ZFIN;Acc:ZDB-GENE-040426-1086]	9	30374181	30385278		2.56	1.72	2.01	2.7	2.75	2.6
ENSDARG00000041041	cxcr3.2	chemokine (C-X-C motif) receptor 3, tandem duplicate 2 [Source:NCBI gene;Acc:791973]	16	11818126	11825303		1.13	1.52	1.7	3.45	3.53	2.94
ENSDARG00000041051	mid1ip1a	MID1 interacting protein 1a [Source:NCBI gene;Acc:30600]	9	30263188	30264415		51.32	49.14	38	27.93	29.01	26.82
ENSDARG00000041060	lgals9l3	lectin, galactoside-binding, soluble, 9 (galectin 9)-like 3 [Source:ZFIN;Acc:ZDB-GENE-040718-159]	15	31054200	31067589		7.97	9.8	7.53	5.64	5.55	5.79
ENSDARG00000041062	calb2a	calbindin 2a [Source:ZFIN;Acc:ZDB-GENE-040426-1677]	18	21198762	21218851		97.37	34.59	36.91	73	75.65	73.91
ENSDARG00000041065	hspb1	heat shock protein, alpha-crystallin-related, 1 [Source:NCBI gene;Acc:368243]	5	3568016	3584815		12.43	15.63	17.53	16.32	18.82	19.43
ENSDARG00000041068	got2a	glutamic-oxaloacetic transaminase 2a, mitochondrial [Source:ZFIN;Acc:ZDB-GENE-040426-2703]	18	21178721	21188768		39.09	46.58	35.65	22.02	22.09	21.63
ENSDARG00000041071	jagn1a	jagunal homolog 1a [Source:NCBI gene;Acc:449830]	9	30108641	30111105		6.55	3.93	4.88	8.49	7.88	8.22
ENSDARG00000041072	mier1a	mesoderm induction early response 1a, transcriptional regulator [Source:ZFIN;Acc:ZDB-GENE-030131-409]	6	34823721	34838413		3.51	2.51	2.76	2.71	2.55	2.61
ENSDARG00000041073	si:dkey-12e7.4	si:dkey-12e7.4 [Source:ZFIN;Acc:ZDB-GENE-050419-83]	18	21171465	21177674		2.49	1.84	1.29	1.71	2.34	1.93
ENSDARG00000041078	chka	choline kinase alpha [Source:ZFIN;Acc:ZDB-GENE-050208-149]	18	21122818	21145374		19.9	9.53	10.94	19.27	20.6	20.82
ENSDARG00000041080	thap3	THAP domain containing, apoptosis associated protein 3 [Source:ZFIN;Acc:ZDB-GENE-050327-4]	23	30725716	30727596		1.44	1.12	0.82	1.57	1.91	1.83
ENSDARG00000041081	kmt5b	lysine methyltransferase 5B [Source:NCBI gene;Acc:572849]	18	21113216	21120218		18.78	10.12	10.71	17.85	17.42	18.33
ENSDARG00000041083	anpepa	alanyl (membrane) aminopeptidase a [Source:ZFIN;Acc:ZDB-GENE-030131-4781]	18	21077528	21097663		6.72	5.77	9	2.19	1.89	2.07
ENSDARG00000041085	arpin	actin related protein 2/3 complex inhibitor [Source:NCBI gene;Acc:550477]	18	21071023	21076006		6.95	11.63	8.23	6.36	6.9	7.4
ENSDARG00000041086	LO018380.1	sodium/potassium/calcium exchanger 3-like [Source:NCBI gene;Acc:101886560]	7	72040939	72067475		3.27	1.02	1.3	3.46	3.94	3.97
ENSDARG00000041092	pgpep1l	pyroglutamyl-peptidase I like [Source:ZFIN;Acc:ZDB-GENE-050419-171]	18	20869923	20875997		0.01	0.02	0.02	0.07	0.04	0.03
ENSDARG00000041098	barx2	BARX homeobox 2 [Source:ZFIN;Acc:ZDB-GENE-081120-4]	18	47313715	47346327		9.68	8.16	7.77	7.49	5.95	6.5
ENSDARG00000041107	cftr	CF transmembrane conductance regulator [Source:NCBI gene;Acc:559080]	18	20677090	20722810		0.67	0.63	0.48	2.03	2.03	1.99
ENSDARG00000041108	ctsh	cathepsin H [Source:ZFIN;Acc:ZDB-GENE-030131-3539]	18	26422124	26428525		11.17	12.59	10.98	10.27	9.9	9.83
ENSDARG00000041110	dnajc3a	DnaJ (Hsp40) homolog, subfamily C, member 3a [Source:ZFIN;Acc:ZDB-GENE-030131-1264]	9	29484043	29497916		16.51	21.7	18.34	11.65	11.26	11.21
ENSDARG00000041113	wdr18	WD repeat domain 18 [Source:NCBI gene;Acc:445819]	11	13642157	13748151		7.9	8.35	9.13	6.18	6.02	6.2
ENSDARG00000041115	cnfn	Danio rerio cornifelin (cnfn), mRNA. [Source:RefSeq mRNA;Acc:NM_001004663]	16	11790640	11798994		9.37	14	11.03	4.4	5.48	4.77
ENSDARG00000041117	wnt2	wingless-type MMTV integration site family member 2 [Source:NCBI gene;Acc:30127]	18	20613241	20636549		2.43	2.74	2.93	2.07	2.13	2.19
ENSDARG00000041119	ceacam1	CEA cell adhesion molecule 1 [Source:ZFIN;Acc:ZDB-GENE-010724-14]	16	11724230	11760596		50.98	62.76	61.42	31.14	34.44	30.76
ENSDARG00000041133	rapsn	receptor-associated protein of the synapse, 43kD [Source:ZFIN;Acc:ZDB-GENE-030313-1]	18	20494413	20511015		12.68	18.75	15.3	21.09	18.79	20.21
ENSDARG00000041137	dhrs13a.3	dehydrogenase/reductase (SDR family) member 13a, duplicate 3 [Source:ZFIN;Acc:ZDB-GENE-041114-134]	15	28006768	28085563		2.39	2.39	2.21	2.41	2.21	2.23
ENSDARG00000041140	ddb2	damage-specific DNA binding protein 2 [Source:NCBI gene;Acc:100004133]	18	20468157	20482855		2.67	3.07	3.04	1.45	1.26	1.62
ENSDARG00000041141	cryba1a	crystallin, beta A1a [Source:ZFIN;Acc:ZDB-GENE-040718-107]	15	28086193	28107505		0.2	0.13	0.09	0.52	0.54	0.59
ENSDARG00000041145	paqr5a	progestin and adipoQ receptor family member Va [Source:NCBI gene;Acc:393156]	18	20459888	20466061		1.3	0.77	0.98	0.98	1.15	1.07
ENSDARG00000041150	slc17a6b	solute carrier family 17 member 6b [Source:NCBI gene;Acc:100149756]	7	32811907	32833255		10.35	2.7	3.74	15.62	16	14.98
ENSDARG00000041155	morf4l1	mortality factor 4 like 1 [Source:ZFIN;Acc:ZDB-GENE-040718-348]	18	20034023	20045307		32.1	33.15	33.12	30.46	27.99	29.88
ENSDARG00000041159	si:ch211-63p21.4	si:ch211-63p21.4 [Source:ZFIN;Acc:ZDB-GENE-160113-20]	7	4219234	4238510		0	0.03	0	0.01	0.02	0.01
ENSDARG00000041165	rab32b	RAB32b, member RAS oncogene family [Source:ZFIN;Acc:ZDB-GENE-090821-4]	17	7364911	7371564		0.62	0.39	0.18	0.3	0.36	0.22
ENSDARG00000041170	aagab	alpha and gamma adaptin binding protein [Source:ZFIN;Acc:ZDB-GENE-040718-120]	18	19812608	19819812		11.4	11.47	11.08	9.9	9.8	9.96
ENSDARG00000041171	crygs2	crystallin, gamma S2 [Source:ZFIN;Acc:ZDB-GENE-050126-3]	9	27733334	27738110		0	0	0	0.04	0.07	0.09
ENSDARG00000041173	adra1bb	adrenoceptor alpha 1Bb [Source:ZFIN;Acc:ZDB-GENE-041114-51]	14	43554490	43572769		0.88	0.82	0.7	0.3	0.44	0.58
ENSDARG00000041179	crygm5	crystallin, gamma M5 [Source:ZFIN;Acc:ZDB-GENE-041024-6]	9	22345047	22352280		0.26	0.9	1.28	1.63	1.43	1.28
ENSDARG00000041182	rpl4	ribosomal protein L4 [Source:ZFIN;Acc:ZDB-GENE-030131-9034]	18	19451070	19456269		1119.68	1352.11	1620.81	886.32	923.61	903.53
ENSDARG00000041198	alg12	ALG12 alpha-1,6-mannosyltransferase [Source:ZFIN;Acc:ZDB-GENE-041210-295]	4	27100528	27109748		4.37	3.92	4.29	3.58	3.28	3.61
ENSDARG00000041203	drap1	DR1-associated protein 1 (negative cofactor 2 alpha) [Source:ZFIN;Acc:ZDB-GENE-030131-400]	7	742517	753231		36.46	29.59	34.11	37.51	37.14	37.33
ENSDARG00000041205	slc6a1l	solute carrier family 6 member 1, like [Source:ZFIN;Acc:ZDB-GENE-041210-296]	4	26972862	27079859		0.94	0.12	0.21	0.56	0.54	0.62
ENSDARG00000041215	cetn4	centrin 4 [Source:ZFIN;Acc:ZDB-GENE-030826-21]	14	1348065	1355544		29.52	31.7	33.07	20.99	19.61	23.13
ENSDARG00000041220	xpo6	exportin 6 [Source:NCBI gene;Acc:333980]	3	15085294	15131438		7.83	5.52	4.71	12.23	12.01	11.87
ENSDARG00000041221	ftr53	finTRIM family, member 53 [Source:ZFIN;Acc:ZDB-GENE-030616-515]	9	17415241	17417666		0.42	0.3	0.35	0.39	0.28	0.31
ENSDARG00000041221	zmp:0000000608	zmp:0000000608 [Source:ZFIN;Acc:ZDB-GENE-130530-611]	16	21867161	21867872		0.15	0.22	0.03	0.22	0.5	0.24
ENSDARG00000041223	antkmt	adenine nucleotide translocase lysine methyltransferase [Source:ZFIN;Acc:ZDB-GENE-040625-59]	3	15132629	15144182		2.48	2.97	2.82	2.31	2.33	2.37
ENSDARG00000041226	cdc40	cell division cycle 40 homolog (S. cerevisiae) [Source:ZFIN;Acc:ZDB-GENE-050116-3]	20	53368611	53401999		17.42	13.19	15.32	15.37	15.77	15.6
ENSDARG00000041232	rps29	ribosomal protein S29 [Source:ZFIN;Acc:ZDB-GENE-040622-5]	20	54079341	54085949		1194.48	1575.24	1618.75	1103.24	1171.69	1186.76
ENSDARG00000041237	med18	mediator of RNA polymerase II transcription, subunit 18 homolog (yeast) [Source:NCBI gene;Acc:393306]	16	7376471	7379328		5.73	4.13	4.15	4.58	4.99	4.53
ENSDARG00000041239	wdr20b	WD repeat domain 20b [Source:ZFIN;Acc:ZDB-GENE-041014-234]	20	54036981	54051196		3.63	1.74	1.95	4.05	4.34	4.21
ENSDARG00000041243	setdb1a	SET domain bifurcated histone lysine methyltransferase 1a [Source:NCBI gene;Acc:553292]	19	9111550	9147533		6.41	4.66	4.39	6.08	6.33	6.39
ENSDARG00000041248	si:dkey-241l7.6	si:dkey-241l7.6 [Source:ZFIN;Acc:ZDB-GENE-041014-235]	20	54013212	54034543		0	0	0.02	0	0	0
ENSDARG00000041252	gemin4	gem (nuclear organelle) associated protein 4 [Source:ZFIN;Acc:ZDB-GENE-050522-188]	15	24583682	24588649		3.02	3.21	3.34	2.41	2.3	2.43
ENSDARG00000041257	smtnl1	smoothelin-like 1 [Source:ZFIN;Acc:ZDB-GENE-050306-23]	14	17108003	17121713		2.58	4.49	3.79	7.26	8.71	9.05
ENSDARG00000041292	si:ch211-231m23.4	si:ch211-231m23.4 [Source:ZFIN;Acc:ZDB-GENE-131127-603]	16	50163352	50164281		0.07	0	0.09	0.13	0.17	0.08
ENSDARG00000041294	noxo1a	NADPH oxidase organizer 1a [Source:ZFIN;Acc:ZDB-GENE-030131-9700]	3	62161184	62169060		0.78	0.66	0.64	3.15	2.37	2.17
ENSDARG00000041295	lim2.5	lens intrinsic membrane protein 2.5 [Source:ZFIN;Acc:ZDB-GENE-050320-94]	16	50166421	50181206		0.59	0.72	1.48	6.23	5.08	7.52
ENSDARG00000041299	zgc:110796	zgc:110796 [Source:NCBI gene;Acc:503755]	5	6617401	6620005		5.88	2.71	2.89	5.99	6.4	7.11
ENSDARG00000041300	drc3	dynein regulatory complex subunit 3 [Source:ZFIN;Acc:ZDB-GENE-061103-349]	3	62126981	62133955		1.89	1.03	1.54	1.65	1.87	2.31
ENSDARG00000041301	crybb3	crystallin, beta B3 [Source:NCBI gene;Acc:553184]	5	6489260	6508250		0.47	0.25	0.25	0.21	0.25	0.14
ENSDARG00000041304	trak1a	trafficking protein, kinesin binding 1a [Source:ZFIN;Acc:ZDB-GENE-100922-182]	16	5786770	5873866		4	3.7	2.79	5.16	4.68	4.98
ENSDARG00000041306	mrps25	mitochondrial ribosomal protein S25 [Source:ZFIN;Acc:ZDB-GENE-050417-52]	8	53143211	53152093		4.88	5.37	5.56	3.71	4.13	3.9
ENSDARG00000041314	ndufb9	NADH:ubiquinone oxidoreductase subunit B9 [Source:ZFIN;Acc:ZDB-GENE-030131-8364]	16	6386941	6391275		181.01	221.08	258.52	103.27	100.63	105.83
ENSDARG00000041317	rangap1a	RAN GTPase activating protein 1a [Source:ZFIN;Acc:ZDB-GENE-060929-492]	3	4502066	4530195		27.84	27.95	32.15	21.65	24.02	23.98
ENSDARG00000041323	csdc2a	cold shock domain containing C2, RNA binding a [Source:ZFIN;Acc:ZDB-GENE-040718-442]	12	19099264	19103490		23.11	12.2	15.57	21.75	22.92	22.44
ENSDARG00000041332	tmem33	transmembrane protein 33 [Source:ZFIN;Acc:ZDB-GENE-030131-5425]	14	46342882	46354499		9.62	7.28	6.51	9.95	9.54	9.16
ENSDARG00000041333	si:dkey-125e8.4	si:dkey-125e8.4 [Source:ZFIN;Acc:ZDB-GENE-160113-103]	7	2455344	2456229		0	0	0	0	0	0
ENSDARG00000041339	zgc:92380	zgc:92380 [Source:ZFIN;Acc:ZDB-GENE-040801-221]	9	48007081	48039964		72.75	93.38	80.7	44	49.82	45.73
ENSDARG00000041340	mrpl51	mitochondrial ribosomal protein L51 [Source:NCBI gene;Acc:797002]	16	4540981	4547193		15.73	17.86	15.51	12.39	12.95	13.61
ENSDARG00000041348	cyp19a1a	cytochrome P450, family 19, subfamily A, polypeptide 1a [Source:NCBI gene;Acc:30390]	18	39620535	39636348		0.15	0.06	0.02	0.06	0.07	0.09
ENSDARG00000041350	ankrd39	ankyrin repeat domain 39 [Source:ZFIN;Acc:ZDB-GENE-081104-153]	8	51573124	51579286		4.19	4.57	5.24	3.75	3.72	4.32
ENSDARG00000041358	lgi3	leucine-rich repeat LGI family, member 3 [Source:ZFIN;Acc:ZDB-GENE-060217-4]	8	51375308	51404806		1.65	0.92	0.94	2.52	2.51	2.94
ENSDARG00000041359	zgc:161969	zgc:161969 [Source:ZFIN;Acc:ZDB-GENE-030131-2597]	6	16468776	16475879		12.56	9.96	10.8	10.54	11.49	11.08
ENSDARG00000041361	ttk	ttk protein kinase [Source:NCBI gene;Acc:317763]	16	5110455	5143176		6.03	4.15	4.34	4.56	4.57	4.46
ENSDARG00000041362	chmp7	charged multivesicular body protein 7 [Source:NCBI gene;Acc:393754]	8	51350453	51367298		9.69	10.69	10.83	8.12	8.68	8.39
ENSDARG00000041363	dctn3	dynactin 3 (p22) [Source:ZFIN;Acc:ZDB-GENE-040704-65]	16	5145132	5154032		24.04	25.69	24.25	24.89	24.04	25.98
ENSDARG00000041372	C8H9orf64	zgc:136564 [Source:ZFIN;Acc:ZDB-GENE-070410-3]	8	49730163	49743517	C8H9orf64	20.04	29.75	30.59	6.99	6.41	5.57
ENSDARG00000041375	si:ch211-57i17.5	si:ch211-57i17.5 [Source:ZFIN;Acc:ZDB-GENE-041014-39]	20	46765733	46773258		0.71	0.53	0.63	0.2	0.45	0.19
ENSDARG00000041376	rmi1	RMI1, RecQ mediated genome instability 1, homolog (S. cerevisiae) [Source:NCBI gene;Acc:393149]	8	49766338	49769987		1.95	1.49	1.32	1.43	1.66	1.93
ENSDARG00000041378	sh2d4a	SH2 domain containing 4A [Source:ZFIN;Acc:ZDB-GENE-040426-932]	10	6967293	6976645		17.38	19.87	18.72	9.57	8.65	8.66
ENSDARG00000041379	si:ch211-245j22.3	si:ch211-245j22.3 [Source:ZFIN;Acc:ZDB-GENE-050419-38]	18	15554381	15559817		1.55	1.41	1.63	0.45	0.76	0.54
ENSDARG00000041381	arntl2	aryl hydrocarbon receptor nuclear translocator-like 2 [Source:ZFIN;Acc:ZDB-GENE-000509-2]	18	15569627	15610856		1.93	1.09	1.35	4.28	4.18	3.96
ENSDARG00000041382	si:dkey-283b15.2	si:dkey-283b15.2 [Source:ZFIN;Acc:ZDB-GENE-100921-93]	16	5650495	5671862		5.82	1.68	1.1	6.16	6.81	6.88
ENSDARG00000041389	si:dkey-81n2.1	si:dkey-81n2.1 [Source:ZFIN;Acc:ZDB-GENE-070705-492]	7	4987530	4990000		0	0	0	0	0	0
ENSDARG00000041391	tmed10	transmembrane p24 trafficking protein 10 [Source:ZFIN;Acc:ZDB-GENE-030131-4915]	20	46536598	46541843		78.5	71.38	61.28	88.29	87.65	85.03
ENSDARG00000041394	dnajb1b	DnaJ heat shock protein family (Hsp40) member B1b [Source:ZFIN;Acc:ZDB-GENE-030131-5455]	1	55745878	55750208		13.66	15.32	14.41	13.39	14.86	13.44
ENSDARG00000041397	eif2b2	eukaryotic translation initiation factor 2B, subunit 2 beta [Source:ZFIN;Acc:ZDB-GENE-040426-2605]	20	46523078	46534179		28.86	28.3	36.99	22.21	24.17	23.27
ENSDARG00000041399	adgre7	adhesion G protein-coupled receptor E7 [Source:ZFIN;Acc:ZDB-GENE-121214-45]	1	55643198	55646413		0	0	0.01	0.01	0	0
ENSDARG00000041400	ndufa3	NADH:ubiquinone oxidoreductase subunit A3 [Source:ZFIN;Acc:ZDB-GENE-040801-18]	16	5717760	5721386		53.73	70.47	74.1	42.37	41	42.47
ENSDARG00000041402	zc3h14	zinc finger CCCH-type containing 14 [Source:NCBI gene;Acc:562846]	20	46442580	46523077		12.87	10.76	11.12	11.81	12.83	12.19
ENSDARG00000041407	rmdn3	regulator of microtubule dynamics 3 [Source:ZFIN;Acc:ZDB-GENE-041014-256]	20	46442282	46467280		11.07	7.59	9.26	11.25	12.05	11.79
ENSDARG00000041411	rad51	RAD51 recombinase [Source:ZFIN;Acc:ZDB-GENE-040426-2286]	20	46427993	46442137		4.52	4.85	4.59	3.95	4.26	3.77
ENSDARG00000041413	adgrg11	adhesion G protein-coupled receptor G11 [Source:ZFIN;Acc:ZDB-GENE-041210-320]	20	46371458	46423475		0.51	0.43	0.37	0.2	0.27	0.24
ENSDARG00000041414	bmf2	BCL2 modifying factor 2 [Source:ZFIN;Acc:ZDB-GENE-041210-319]	20	46351606	46362606		2.7	0.97	1.82	3.81	4.17	3.92
ENSDARG00000041417	atp6ap1a	ATPase H+ transporting accessory protein 1a [Source:ZFIN;Acc:ZDB-GENE-090312-136]	23	4899238	4915118		28.49	6.22	7.59	38.34	38.11	38.76
ENSDARG00000041421	taz	tafazzin [Source:ZFIN;Acc:ZDB-GENE-030131-684]	23	4915595	4925724		6.53	6.77	5.96	5.71	6.11	6.23
ENSDARG00000041426	yrdc	yrdC N(6)-threonylcarbamoyltransferase domain containing [Source:ZFIN;Acc:ZDB-GENE-070410-65]	16	4178949	4190726		2.41	2.42	2.01	2.84	2.78	2.17
ENSDARG00000041429	ints14	integrator complex subunit 14 [Source:ZFIN;Acc:ZDB-GENE-030131-5804]	18	18995247	19006634		7.86	6.05	5.57	6.49	6.13	6.06
ENSDARG00000041430	bmp2b	bone morphogenetic protein 2b [Source:ZFIN;Acc:ZDB-GENE-980526-474]	20	45893173	45899149		6.99	6.96	7.34	6.87	8.3	7.54
ENSDARG00000041431	slc24a1	solute carrier family 24 member 1 [Source:ZFIN;Acc:ZDB-GENE-060503-191]	18	19006063	19039960		1.73	0.32	0.33	1.36	1.4	1.2
ENSDARG00000041433	si:dkey-7c18.24	si:dkey-7c18.24 [Source:ZFIN;Acc:ZDB-GENE-041014-63]	20	46039591	46043405		6.66	6.42	5.02	5.92	4.82	5.68
ENSDARG00000041435	uba52	ubiquitin A-52 residue ribosomal protein fusion product 1 [Source:ZFIN;Acc:ZDB-GENE-051027-7]	2	56339058	56348727		1725.91	2221.19	2681.5	1491.36	1540.8	1495.64
ENSDARG00000041449	spred1	sprouty related EVH1 domain containing 1 [Source:ZFIN;Acc:ZDB-GENE-040426-2338]	17	52639492	52659119		17.08	9.68	9.36	23.47	23.2	23.54
ENSDARG00000041450	rab11a	RAB11a, member RAS oncogene family [Source:ZFIN;Acc:ZDB-GENE-041114-53]	18	19131773	19139152		51.75	41.09	33.81	57.5	56.81	56.48
ENSDARG00000041454	taar12b	trace amine associated receptor 12b [Source:ZFIN;Acc:ZDB-GENE-041014-69]	20	46100634	46101648		0	0	0	0.07	0.18	0.05
ENSDARG00000041462	pimr183	Pim proto-oncogene, serine/threonine kinase, related 183 [Source:ZFIN;Acc:ZDB-GENE-141222-29]	8	48673596	48675775		0	0	0	0	0	0.02

ENSDARG00000041481	smg5	SMG5 nonsense mediated mRNA decay factor [Source:ZFIN;Acc:ZDB-GENE-050913-144]	16	45693413	45734213		13.65	9.06	8.04	14.03	14.2	14.96
ENSDARG00000041483	paqr6	progestin and adipoQ receptor family member VI [Source:ZFIN;Acc:ZDB-GENE-090714-1]	16	45739193	45782607		11.89	11.4	9.01	9.12	9.23	9.39
ENSDARG00000041490	afp4	antifreeze protein type IV [Source:ZFIN;Acc:ZDB-GENE-030131-5217]	16	45904153	45910050		3.89	7.72	9.16	9.47	11.37	10.93
ENSDARG00000041492	si:ch211-196f5.2	si:ch211-196f5.2 [Source:ZFIN;Acc:ZDB-GENE-041014-306]	24	12740848	12752502		0	0.74	0.2	10.04	8.77	9.27
ENSDARG00000041493	ipo4	importin 4 [Source:ZFIN;Acc:ZDB-GENE-041014-307]	24	12754013	12832499		6.54	7.71	6.42	7.07	6.34	5.96
ENSDARG00000041502	tgfb1a	transforming growth factor, beta 1a [Source:ZFIN;Acc:ZDB-GENE-030618-1]	15	2776506	2803313		5.51	5.36	4.75	8.75	9.24	9.22
ENSDARG00000041503	med4	mediator complex subunit 4 [Source:NCBI gene;Acc:550582]	9	25320680	25328527		5.52	4.24	4.04	5.41	5.11	5.49
ENSDARG00000041505	itm2bb	integral membrane protein 2Bb [Source:ZFIN;Acc:ZDB-GENE-040426-2139]	9	25330905	25346583		95.17	137.45	127.36	77.72	82.29	80.88
ENSDARG00000041511	pex10	peroxisomal biogenesis factor 10 [Source:ZFIN;Acc:ZDB-GENE-041010-71]	8	47319804	47329755		1.86	1.67	1.56	1.83	1.59	2.37
ENSDARG00000041515	rasal1b	RAS protein activator like 1b (GAP1 like) [Source:ZFIN;Acc:ZDB-GENE-141212-314]	8	1651821	1682945		1.96	0.48	1.03	2.08	2.82	2.69
ENSDARG00000041530	ppih	peptidylprolyl isomerase H (cyclophilin H) [Source:ZFIN;Acc:ZDB-GENE-040426-127]	8	47152704	47206399		4.83	6.26	5.87	3.94	3.56	3.92
ENSDARG00000041533	ccdc130	coiled-coil domain containing 130 [Source:NCBI gene;Acc:402887]	22	26239424	26265355		6.37	5.6	5.23	5.36	5.85	5.47
ENSDARG00000041538	mrps28	mitochondrial ribosomal protein S28 [Source:ZFIN;Acc:ZDB-GENE-061110-52]	22	26290209	26290863		17.34	21.11	23.02	14.78	16.2	16.19
ENSDARG00000041540	sult1st2	sulfotransferase family 1, cytosolic sulfotransferase 2 [Source:NCBI gene;Acc:791732]	8	46513743	46525092		7.27	11.79	9.25	0.61	0.76	1.14
ENSDARG00000041546	gypc	glycophorin C (Gerbich blood group) [Source:ZFIN;Acc:ZDB-GENE-030131-7930]	9	23665777	23718614		5.76	7.95	5.75	6.72	6.13	6.35
ENSDARG00000041550	pimr138	Pim proto-oncogene, serine/threonine kinase, related 138 [Source:ZFIN;Acc:ZDB-GENE-041014-153]	20	43396318	43403699		0.01	0	0.02	0.03	0.01	0.05
ENSDARG00000041554	unc93a	unc-93 homolog A [Source:NCBI gene;Acc:559995]	20	43379029	43389475		2.01	2.48	1.38	0.42	0.55	0.37
ENSDARG00000041560	PHAF1	si:dkey-102f14.5 [Source:ZFIN;Acc:ZDB-GENE-060503-705]	18	17493859	17507010	PHAF1	6.45	3.47	4.31	8.75	9.94	10.66
ENSDARG00000041565	tnfaip1	tumor necrosis factor, alpha-induced protein 1 (endothelial) [Source:ZFIN;Acc:ZDB-GENE-030131-5610]	15	20529197	20541185		5.31	5.69	4.86	5.48	5.8	5.96
ENSDARG00000041569	ces2a	carboxylesterase 2a [Source:ZFIN;Acc:ZDB-GENE-061013-99]	18	17408470	17415609		234.36	372.82	360.8	180.87	185.7	184.57
ENSDARG00000041570	mrpl39	mitochondrial ribosomal protein L39 [Source:ZFIN;Acc:ZDB-GENE-030131-6520]	1	532766	591331		13.19	13.53	13.06	10.06	10.05	9.58
ENSDARG00000041572	zfpm1	zinc finger protein, FOG family member 1 [Source:NCBI gene;Acc:407655]	18	17270427	17399376		2.38	2.35	1.61	3.75	4.02	3.37
ENSDARG00000041575	trmt10c	tRNA methyltransferase 10C, mitochondrial RNase P subunit [Source:ZFIN;Acc:ZDB-GENE-041114-12]	1	508885	513786		6.96	8.39	7.61	5.32	5.12	5.15
ENSDARG00000041576	nudt18	nudix (nucleoside diphosphate linked moiety X)-type motif 18 [Source:ZFIN;Acc:550541]	8	45941942	45952598		3.75	2.63	2.9	2.45	2.59	2.48
ENSDARG00000041581	znf143b	zinc finger protein 143b [Source:NCBI gene;Acc:393954]	18	16924865	16937109		10.86	5.67	7.43	9.57	8.65	8.84
ENSDARG00000041586	dhx40	DEAH (Asp-Glu-Ala-His) box polypeptide 40 [Source:ZFIN;Acc:ZDB-GENE-080507-1]	15	17258246	17285188		1.29	1.21	1.15	1.67	1.9	1.85
ENSDARG00000041589	adprhl1	ADP-ribosylhydrolase like 1 [Source:NCBI gene;Acc:450012]	1	200294	206208		2.62	2.8	2.74	1.6	1.85	2
ENSDARG00000041592	dcun1d2b	DCN1, defective in cullin neddylation 1, domain containing 2b [Source:ZFIN;Acc:ZDB-GENE-040625-171]	1	208181	214911		23.15	20.72	21.85	19.3	19.74	18.91
ENSDARG00000041595	ces3	carboxylesterase 3 [Source:ZFIN;Acc:ZDB-GENE-030131-9563]	18	17418254	17426306		42.01	75.64	82.35	30.63	30.58	29.78
ENSDARG00000041599	zgc:91860	zgc:91860 [Source:ZFIN;Acc:ZDB-GENE-040704-34]	18	17428258	17461796		18.97	6.5	8.63	22.77	24.74	26.17
ENSDARG00000041600	wdr83	WD repeat domain containing 83 [Source:NCBI gene;Acc:436702]	22	26264543	26274177		6.71	6.64	6.79	5.88	5.72	5.6
ENSDARG00000041602	zgc:92335	zgc:92335 [Source:ZFIN;Acc:ZDB-GENE-040718-152]	22	12595144	12604606		11.73	14.02	15.42	13.99	16.6	15.87
ENSDARG00000041607	eif4ebp3l	eukaryotic translation initiation factor 4E binding protein 3, like [Source:NCBI gene;Acc:324490]	14	6972868	6987649		50.88	70.39	62.22	81.78	89.98	97
ENSDARG00000041609	adarb1a	adenosine deaminase RNA specific B1a [Source:ZFIN;Acc:ZDB-GENE-000627-2]	22	12641580	12693837		4.32	0.94	1.22	5.9	6.12	6.34
ENSDARG00000041616	b3gnt9	UDP-GlcNAc:betaGal beta-1,3-N-acetylglucosaminyltransferase 9 [Source:ZFIN;Acc:ZDB-GENE-060503-611]	18	17511995	17513426		1.53	1.57	1.13	1.89	2.39	1.41
ENSDARG00000041617	nme5	NME/NM23 family member 5 [Source:ZFIN;Acc:ZDB-GENE-040718-221]	14	6954579	6961746		1.83	1.76	2.03	2.04	1.78	2.24
ENSDARG00000041619	rack1	receptor for activated C kinase 1 [Source:NCBI gene;Acc:30722]	14	6946482	6953765		1382.79	1590.42	1903.31	1136.83	1208.41	1189.21
ENSDARG00000041621	bbs2	Bardet-Biedl syndrome 2 [Source:NCBI gene;Acc:259187]	18	17516569	17531483		10.23	5.1	5.92	6.84	7.57	7.22
ENSDARG00000041623	mt2	metallothionein 2 [Source:NCBI gene;Acc:100174951]	18	17534627	17535979		42.01	22.81	24.24	67.87	73.49	70.19
ENSDARG00000041631	psme3ip1	proteasome activator subunit 3 interacting protein 1 [Source:ZFIN;Acc:ZDB-GENE-030131-6692]	18	17707688	17724295		25.33	22.48	22.36	17.52	18.88	20.29
ENSDARG00000041644	si:dkey-194e6.1	si:dkey-194e6.1 [Source:ZFIN;Acc:ZDB-GENE-100922-43]	23	40872688	40895168		5.73	11.87	7.1	7.24	7.79	6.83
ENSDARG00000041645	sb:cb37	sb:cb37 [Source:ZFIN;Acc:ZDB-GENE-030131-9800]	15	21137046	21155657		8.03	10.87	10.15	14.23	13.31	14.82
ENSDARG00000041660	oprd1a	opioid receptor, delta 1a [Source:ZFIN;Acc:ZDB-GENE-990415-199]	19	14921000	14951756		1.87	0.27	0.57	2.25	2.01	1.93
ENSDARG00000041665	mkrn1	makorin, ring finger protein, 1 [Source:NCBI gene;Acc:170782]	4	12292274	12307834		15.35	11.57	13.52	16.55	17.94	18.57
ENSDARG00000041685	si:dkey-105h12.2	si:dkey-105h12.2 [Source:ZFIN;Acc:ZDB-GENE-131118-9]	15	21220744	21239416		6.29	7.23	4.99	1.34	1.06	1.27
ENSDARG00000041689	usf1	upstream transcription factor 1 [Source:ZFIN;Acc:ZDB-GENE-040426-1072]	15	21252532	21262567		10.5	6.6	7.66	11.41	10.8	11.69
ENSDARG00000041691	bhlhe41	basic helix-loop-helix family, member e41 [Source:ZFIN;Acc:ZDB-GENE-050419-146]	18	16125852	16130165		16.67	8.28	7.16	12.76	13.65	14.95
ENSDARG00000041699	piwil1	piwi-like RNA-mediated gene silencing 1 [Source:NCBI gene;Acc:368200]	8	44224693	44299247		0.62	0.74	0.41	0.68	0.73	0.6
ENSDARG00000041702	rfx6	regulatory factor X, 6 [Source:NCBI gene;Acc:556925]	20	42506612	42534504		0.07	0.07	0.04	0.15	0.12	0.11
ENSDARG00000041703	rrbp1b	ribosome binding protein 1b [Source:ZFIN;Acc:ZDB-GENE-030429-36]	22	25086567	25137256		9.15	11.83	9.81	20.48	22.13	21.24
ENSDARG00000041705	si:busm1-228j01.6	si:busm1-228j01.6 [Source:ZFIN;Acc:ZDB-GENE-030616-396]	8	44373764	44378410		0.02	0.12	0.02	0	0.04	0
ENSDARG00000041706	vgll2a	vestigial-like family member 2a [Source:ZFIN;Acc:ZDB-GENE-050913-38]	20	42431542	42439896		1.76	1.52	0.96	1.6	1.35	1.42
ENSDARG00000041708	kat14	lysine acetyltransferase 14 [Source:ZFIN;Acc:ZDB-GENE-040718-452]	22	25066110	25078718		7.67	4.49	4.65	8.92	8.48	8.35
ENSDARG00000041715	bco2b	beta-carotene oxygenase 2b [Source:ZFIN;Acc:ZDB-GENE-060421-7448]	15	21673454	21678634		0.33	0.12	0.16	0.45	0.28	0.41
ENSDARG00000041720	arpc5la	actin related protein 2/3 complex, subunit 5-like, a [Source:ZFIN;Acc:ZDB-GENE-040426-1453]	8	41453691	41462732		16.93	14.51	14.06	19.82	19.07	19.46
ENSDARG00000041723	TUBB4B	zgc:55461 [Source:ZFIN;Acc:ZDB-GENE-040426-2879]	5	33018764	33022014	TUBB4B	41.52	37.93	46.22	26.13	28.02	25.95
ENSDARG00000041724	glipr2	GLI pathogenesis-related 2 [Source:ZFIN;Acc:ZDB-GENE-030131-4424]	5	33028083	33036670		2.14	2.29	1.76	1.66	1.78	1.53
ENSDARG00000041728	man1a2	mannosidase, alpha, class 1A, member 2 [Source:ZFIN;Acc:ZDB-GENE-070912-390]	9	20554178	20767773		6.45	3.76	3.5	7.56	7.6	7.63
ENSDARG00000041729	sntb1	syntrophin, basic 1 [Source:ZFIN;Acc:ZDB-GENE-040704-13]	16	15164983	15263099		10.27	7.07	6.23	10.39	10.69	11.31
ENSDARG00000041734	tbc1d32	TBC1 domain family, member 32 [Source:NCBI gene;Acc:566474]	20	40766645	40840993		2.45	0.99	1.23	2.29	2.24	2.22
ENSDARG00000041735	tmem237a	transmembrane protein 237a [Source:NCBI gene;Acc:557237]	9	13714379	13728937		26.38	5.45	4.45	9.41	10.05	10.17
ENSDARG00000041736	hsdl1	hydroxysteroid dehydrogenase like 1 [Source:NCBI gene;Acc:494064]	20	40761717	40766618		2.09	1.85	1.78	1.59	1.74	1.73
ENSDARG00000041747	sspn	sarcospan (Kras oncogene-associated gene) [Source:ZFIN;Acc:ZDB-GENE-051127-43]	18	16111185	16123222		19.15	29.81	28	24.28	23.57	23.21
ENSDARG00000041750	ccdc92	coiled-coil domain containing 92 [Source:ZFIN;Acc:ZDB-GENE-051113-120]	8	43094799	43158486		5.1	1.69	2.14	5.55	6.01	6.54
ENSDARG00000041776	ndst3	N-deacetylase/N-sulfotransferase (heparan glucosaminyl) 3 [Source:ZFIN;Acc:ZDB-GENE-090312-199]	1	20147222	20273227		5.96	2.51	3	6.85	6.19	6.44
ENSDARG00000041779	trdn	triadin [Source:ZFIN;Acc:ZDB-GENE-041014-193]	20	40150612	40231379		9.34	13.27	12.21	14.59	11.21	10.54
ENSDARG00000041787	cx32.3	connexin 32.3 [Source:ZFIN;Acc:ZDB-GENE-030131-1337]	20	40753518	40755614		18.63	31.37	23.42	12.96	15.13	13.93
ENSDARG00000041791	mgat4c	mgat4 family member C [Source:ZFIN;Acc:ZDB-GENE-091204-429]	18	16387745	16590056		3.2	0.59	1.07	3.08	3.08	3.29
ENSDARG00000041792	cx28.1	connexin 28.1 [Source:ZFIN;Acc:ZDB-GENE-070320-1]	20	40739964	40774857		0.09	0.06	0.05	0.29	0.26	0.46
ENSDARG00000041797	cx28.9	connexin 28.9 [Source:ZFIN;Acc:ZDB-GENE-041114-200]	20	40744754	40750953		8.25	13.3	9.42	5.88	6.76	6.87
ENSDARG00000041799	cx43	connexin 43 [Source:NCBI gene;Acc:30236]	20	40715163	40720530		27.97	16.68	19.83	50.67	49.88	52.9
ENSDARG00000041802	rnf141	ring finger protein 141 [Source:NCBI gene;Acc:414846]	18	16749091	16756144		11.56	7.54	9.01	12.9	12.74	12.93
ENSDARG00000041811	rps25	ribosomal protein S25 [Source:NCBI gene;Acc:393788]	10	39125637	39130935		1952.94	2426.77	2972.6	1282.38	1379.53	1420.98
ENSDARG00000041828	si:dkeyp-28d2.4	si:dkeyp-28d2.4 [Source:ZFIN;Acc:ZDB-GENE-091204-68]	14	12894282	12918330		0.13	0.08	0.09	0.1	0.11	0.14
ENSDARG00000041830	tsn	translin [Source:ZFIN;Acc:ZDB-GENE-030131-6039]	9	22929675	22939610		7.24	5.46	5.77	6.77	6.54	5.98
ENSDARG00000041835	ccl38a.5	chemokine (C-C motif) ligand 38, duplicate 5 [Source:ZFIN;Acc:ZDB-GENE-041014-167]	20	38279523	38280392		0.04	0.11	0.04	0.18	0.2	0.19
ENSDARG00000041839	tsc22d2	TSC22 domain family 2 [Source:ZFIN;Acc:ZDB-GENE-031001-12]	22	35205017	35263902		6.45	5.52	4.28	16.46	17.28	16.1
ENSDARG00000041848	rh50	Rh50-like protein [Source:ZFIN;Acc:ZDB-GENE-000427-1]	8	37259398	37263524		1.28	2.53	2.08	0.24	0.33	0.31
ENSDARG00000041853	rbm39b	RNA binding motif protein 39b [Source:ZFIN;Acc:ZDB-GENE-050327-97]	8	37228611	37249991		10.82	7.11	8.2	10.67	11.33	11.26
ENSDARG00000041858	ren	renin [Source:ZFIN;Acc:ZDB-GENE-040630-3]	8	37111643	37132922		0.31	0.21	0.1	0.85	0.68	1.19
ENSDARG00000041864	capn3a	calpain 3a, (p94) [Source:ZFIN;Acc:ZDB-GENE-040912-97]	17	45305645	45346635		0.12	1.57	2.1	13.57	11.14	17.05
ENSDARG00000041869	tnfrsf19	tumor necrosis factor receptor superfamily, member 19 [Source:ZFIN;Acc:ZDB-GENE-030131-6760]	15	8235531	8309217		7.81	7.5	6.25	8.96	9.5	9.53
ENSDARG00000041870	ift172	intraflagellar transport 172 [Source:NCBI gene;Acc:432389]	20	38837238	38900420		6.58	4.22	4.06	6.54	7.01	7.19
ENSDARG00000041873	si:dkey-221h15.4	si:dkey-221h15.4 [Source:ZFIN;Acc:ZDB-GENE-041014-340]	20	38831588	38836161		0.85	0.58	0.68	0.28	0.22	0.34
ENSDARG00000041875	ube2v1	ubiquitin-conjugating enzyme E2 variant 1 [Source:ZFIN;Acc:ZDB-GENE-051030-102]	11	2416064	2429303		62.05	51.69	55.71	57.11	57.76	56.39
ENSDARG00000041878	rab11ba	RAB11B, member RAS oncogene family, a [Source:ZFIN;Acc:ZDB-GENE-040426-2860]	22	17499905	17506843		31.24	24.74	22.54	37.04	39.87	37.38
ENSDARG00000041887	sf3a1	splicing factor 3a, subunit 1 [Source:ZFIN;Acc:ZDB-GENE-030616-156]	8	36554816	36570665		18.83	14.54	12.54	21.72	22.18	23.65
ENSDARG00000041895	cad	carbamoyl-phosphate synthetase 2, aspartate transcarbamylase, and dihydroorotase [Source:ZFIN;Acc:ZDB-GENE-021030-4]	20	38788319	38827623		23.36	23.51	19.38	17.64	17.9	17.52
ENSDARG00000041896	dnajc5ga	DnaJ (Hsp40) homolog, subfamily C, member 5 gamma a [Source:ZFIN;Acc:ZDB-GENE-030131-1583]	20	38763953	38787341		7.48	5	4.63	10.32	9.94	10.13
ENSDARG00000041904	ankzf1	ankyrin repeat and zinc finger peptidyl tRNA hydrolase 1 [Source:ZFIN;Acc:ZDB-GENE-050309-80]	9	12834414	12886108		5.55	5.71	4.6	4.43	4.87	4.45
ENSDARG00000041908	usp39	ubiquitin specific peptidase 39 [Source:ZFIN;Acc:ZDB-GENE-031030-966]	5	68826514	68843111		11.6	9.1	9.58	10.49	9.67	9.6
ENSDARG00000041910	znf512	zinc finger protein 512 [Source:ZFIN;Acc:ZDB-GENE-041014-346]	20	38524827	38538994		1.12	0.42	0.68	1.28	1.52	1.47
ENSDARG00000041911	si:ch211-245h14.1	si:ch211-245h14.1 [Source:ZFIN;Acc:ZDB-GENE-041014-350]	20	38517441	38525467		0.43	0.52	0.48	0.28	0.38	0.37
ENSDARG00000041915	stum	stum, mechanosensory transduction mediator homolog [Source:NCBI gene;Acc:562918]	20	38322444	38359333		3.05	2.03	2.4	3.43	3.17	3.35
ENSDARG00000041917	ccl38a.4	chemokine (C-C motif) ligand 38, duplicate 4 [Source:ZFIN;Acc:ZDB-GENE-041014-163]	20	38285671	38286553		0	0.07	0.06	0.11	0	0.11
ENSDARG00000041919	ccl38.1	chemokine (C-C motif) ligand 38, duplicate 1 [Source:ZFIN;Acc:ZDB-GENE-041014-320]	20	39332579	39333799		0.48	0.58	0.47	0.93	0.54	0.69
ENSDARG00000041921	hsbp1b	heat shock factor binding protein 1b [Source:ZFIN;Acc:ZDB-GENE-040426-1721]	18	14334123	14337458		50.39	52.68	51.16	38.93	37.64	41.81
ENSDARG00000041923	ccl38.6	chemokine (C-C motif) ligand 38, duplicate 6 [Source:ZFIN;Acc:ZDB-GENE-041014-165]	20	38257766	38291952		0.26	0.25	0.19	0.14	0.27	0.37
ENSDARG00000041925	cryba2b	crystallin, beta A2b [Source:ZFIN;Acc:ZDB-GENE-040718-324]	9	11554089	11560432		19.58	29.84	45.29	70.24	64.5	84.51
ENSDARG00000041926	dlg4a	discs, large homolog 4a (Drosophila) [Source:ZFIN;Acc:ZDB-GENE-130530-613]	5	68529728	68641353		1.55	0.29	0.57	4.24	4.19	4.9
ENSDARG00000041944	adat2	adenosine deaminase tRNA specific 2 [Source:NCBI gene;Acc:100034610]	20	37733181	37760481		1.89	2.01	2.24	1.18	1.25	1.75
ENSDARG00000041947	styk1b	serine/tyrosine kinase 1b [Source:ZFIN;Acc:ZDB-GENE-030131-3435]	16	31965340	31976269		4.77	4.46	3.82	3.31	2.8	2.8
ENSDARG00000041951	selenoo1	selenoprotein O1 [Source:NCBI gene;Acc:558648]	18	14863407	14879135		6.19	5.8	5.4	4.87	3.84	4.41
ENSDARG00000041952	prox2	prospero homeobox 2 [Source:ZFIN;Acc:ZDB-GENE-081003-3]	17	52493499	52521002		0.47	0.08	0.15	1.11	0.79	1
ENSDARG00000041959	cxcr4b	chemokine (C-X-C motif), receptor 4b [Source:ZFIN;Acc:ZDB-GENE-010614-1]	9	10692905	10694930		4.5	4.88	3.9	5.96	5.67	5.12
ENSDARG00000041960	si:ch211-173a9.6	si:ch211-173a9.6 [Source:ZFIN;Acc:ZDB-GENE-030131-4623]	17	52581012	52587598		6.15	4.88	3.46	3.05	4.59	4.13
ENSDARG00000041974	brk1	BRICK1 subunit of SCAR/WAVE actin nucleating complex [Source:NCBI gene;Acc:415187]	11	36041617	36156935		34.27	26.06	28.88	36.66	33.65	35.4
ENSDARG00000041976	il17a/f3	interleukin 17a/f3 [Source:ZFIN;Acc:ZDB-GENE-041001-192]	20	34792166	34794465		0.02	0.08	0.08	0.17	0.17	0.12
ENSDARG00000041980	calhm6	calcium homeostasis modulator family member 6 [Source:ZFIN;Acc:ZDB-GENE-061201-60]	16	32226664	32233463		0.34	0.26	0.49	0.55	0.4	0.46
ENSDARG00000041982	adamts6	ADAM metallopeptidase with thrombospondin type 1 motif, 6 [Source:ZFIN;Acc:ZDB-GENE-091113-9]	10	11593998	11761927		0.84	0.57	0.46	1.47	1.4	1.74
ENSDARG00000041991	rrp9	ribosomal RNA processing 9, U3 small nucleolar RNA binding protein [Source:ZFIN;Acc:ZDB-GENE-060427-1]	11	36215698	36230163		12.82	14.11	15.01	8.6	8.06	8.48
ENSDARG00000041998	zgc:113337	zgc:113337 [Source:ZFIN;Acc:ZDB-GENE-050306-20]	9	9322424	9348058		4.45	5.86	4.98	4.15	4.11	4.58
ENSDARG00000042000	si:ch211-63o20.7	si:ch211-63o20.7 [Source:ZFIN;Acc:ZDB-GENE-041001-194]	20	34702410	34711528		1.77	1.18	1.45	0.99	1.3	1.06
ENSDARG00000042004	nfya	nuclear transcription factor Y, alpha [Source:ZFIN;Acc:ZDB-GENE-010501-1]	11	36254284	36279664		20.93	15.05	17.06	24.26	23.79	23.88
ENSDARG00000042005	elp3	elongator acetyltransferase complex subunit 3 [Source:NCBI gene;Acc:541509]	20	34671386	34700654		7.72	7.37	7.55	6.83	7.46	7.06
ENSDARG00000042010	pklr	pyruvate kinase L/R [Source:ZFIN;Acc:ZDB-GENE-010907-1]	16	54450407	54455573		67.46	86.47	81.51	23.42	26.47	28.73
ENSDARG00000042014	cyp11c1	cytochrome P450, family 11, subfamily C, polypeptide 1 [Source:ZFIN;Acc:ZDB-GENE-070828-1]	15	54459135	54471235		4.82	0.86	1.24	3.6	4	4.05
ENSDARG00000042018	fhl2a	four and a half LIM domains 2a [Source:ZFIN;Acc:ZDB-GENE-040808-49]	9	6578580	6601988		18.62	23.81	20.71	26.77	28.6	27.54
ENSDARG00000042021	mapk12a	mitogen-activated protein kinase 12a [Source:NCBI gene;Acc:30681]	18	14844669	14860435		16.01	18.23	15.92	13.67	13.53	13.07
ENSDARG00000042025	C9H2orf49	si:dkeyp-118h3.6 [Source:NCBI gene;Acc:553393]	9	6607514	6618067	C9H2orf49	6.35	4.83	4.85	5.77	6.6	6.13
ENSDARG00000042027	prrx1b	paired related homeobox 1b [Source:ZFIN;Acc:ZDB-GENE-030131-9033]	20	34511678	34521041		8.85	10.41	10.49	8.52	8.35	9.33
ENSDARG00000042030	kpna5	karyopherin alpha 5 (importin alpha 6) [Source:NCBI gene;Acc:553205]	16	32167430	32184460		16.64	12.08	11.6	22.01	21.64	21.12
ENSDARG00000042031	gorab	golgin, rab6-interacting [Source:NCBI gene;Acc:393356]	20	34501152	34507495		2.48	2.38	2.04	2.81	2.53	2.82
ENSDARG00000042032	pou3f3a	POU class 3 homeobox 3a [Source:NCBI gene;Acc:30386]	9	6658594	6661666		9.94	4.35	4.12	10.92	11.15	11.35
ENSDARG00000042033	mettl11b	methyltransferase like 11B [Source:NCBI gene;Acc:568419]	20	34455645	34466483		4.82	7.92	6.25	2.04	2.14	1.73
ENSDARG00000042041	tal2	T-cell acute lymphocytic leukemia 2 [Source:NCBI gene;Acc:394239]	5	9348284	9349045		21.49	4.04	7.05	20.48	18.47	21.38
ENSDARG00000042045	hmg20b	high mobility group 20B [Source:ZFIN;Acc:ZDB-GENE-030131-4258]	22	20168352	20181597		5.45	4.9	5.17	5.37	5.29	5.36
ENSDARG00000042050	zgc:171971	zgc:171971 [Source:ZFIN;Acc:ZDB-GENE-070928-37]	12	7268392	7280551		5.46	2.91	3.28	5.63	5.3	5.21
ENSDARG00000042055	niban1a	niban apoptosis regulator 1a [Source:ZFIN;Acc:ZDB-GENE-041014-181]	20	34400715	34419523		6.51	4.29	3.27	2.88	2.47	2.3
ENSDARG00000042056	mau2	MAU2 sister chromatid cohesion factor [Source:ZFIN;Acc:ZDB-GENE-050522-383]	2	58887599	58902477		10.58	8.39	7.76	15.23	15.43	15.56
ENSDARG00000042057	trmt1l	tRNA methyltransferase 1-like [Source:ZFIN;Acc:ZDB-GENE-041014-189]	20	34388425	34399442		2.77	2.36	2.41	3.07	3.58	3.19
ENSDARG00000042063	si:ch211-145c1.1	si:ch211-145c1.1 [Source:ZFIN;Acc:ZDB-GENE-070912-81]	9	8288268	8314028		0.05	0.01	0	0.03	0.03	0.04
ENSDARG00000042065	eef2a.2	eukaryotic translation elongation factor 2a, tandem duplicate 2 [Source:ZFIN;Acc:ZDB-GENE-050208-348]	22	20145036	20155026		1.33	1	0.92	1.05	0.76	0.92
ENSDARG00000042069	pebp1	phosphatidylethanolamine binding protein 1 [Source:ZFIN;Acc:ZDB-GENE-040426-2621]	5	15350954	15357819		11.48	12.15	13.44	12.41	10.9	11.63
ENSDARG00000042072	fbxl17	F-box and leucine-rich repeat protein 17 [Source:ZFIN;Acc:ZDB-GENE-111013-3]	8	1284784	1304505		1.1	0.67	0.58	1.35	1.1	1.28
ENSDARG00000042087	dmap1	DNA methyltransferase 1 associated protein 1 [Source:ZFIN;Acc:ZDB-GENE-040426-748]	18	18354485	18367736		6.83	3.89	3.92	5.63	5.58	5.54
ENSDARG00000042090	si:ch73-55i23.1	si:ch73-55i23.1 [Source:NCBI gene;Acc:100148818]	5	1911814	1923875		0.4	0.77	0.49	0.32	0.4	0.56
ENSDARG00000042091	btbd2a	BTB (POZ) domain containing 2a [Source:ZFIN;Acc:ZDB-GENE-030829-3]	22	20159067	20166664		16.78	7.13	9.83	16.54	16.28	15.65
ENSDARG00000042094	eef2a.1	eukaryotic translation elongation factor 2a, tandem duplicate 1 [Source:ZFIN;Acc:ZDB-GENE-050522-500]	22	20135443	20143425		4.03	3.01	3.11	5.26	5.17	4.97
ENSDARG00000042106	mnx2a	motor neuron and pancreas homeobox 2a [Source:ZFIN;Acc:ZDB-GENE-040415-1]	9	7724152	7728223		1.04	1.06	0.88	1.51	1.59	1.72
ENSDARG00000042107	cngb1b	cyclic nucleotide gated channel subunit beta 1b [Source:ZFIN;Acc:ZDB-GENE-090831-4]	25	17508235	17552299		4.87	0.68	0.55	3.01	3.24	3.32
ENSDARG00000042112	dio1	iodothyronine deiodinase 1 [Source:ZFIN;Acc:ZDB-GENE-030328-41]	8	18039570	18042089		3.89	5.45	4.14	3.4	4.05	3.87
ENSDARG00000042115	bin1a	bridging integrator 1a [Source:ZFIN;Acc:ZDB-GENE-070912-715]	9	7410798	7444753		8.91	2.26	3.12	9.85	9.41	9.9
ENSDARG00000042115	tmem198a	transmembrane protein 198a [Source:ZFIN;Acc:ZDB-GENE-070912-716]	9	7456076	7499416		10.02	2.48	3.54	12.12	12.24	12.23
ENSDARG00000042116	trim35-40	tripartite motif containing 35-40 [Source:ZFIN;Acc:ZDB-GENE-030131-6081]	25	17495615	17503393		6.4	6.54	6.08	5.51	5.22	5.39
ENSDARG00000042120	C20H1orf112	si:dkey-97o5.1 [Source:NCBI gene;Acc:553598]	20	33994580	34010235	C20H1orf112	2.8	1.99	2.05	1.94	1.9	1.85
ENSDARG00000042122	acot11b	acyl-CoA thioesterase 11b [Source:ZFIN;Acc:ZDB-GENE-051127-37]	8	17993788	18010735		2.44	3.01	2.98	3.08	2.76	2.7
ENSDARG00000042123	nudt3b	nudix (nucleoside diphosphate linked moiety X)-type motif 3b [Source:ZFIN;Acc:ZDB-GENE-041010-128]	6	54154807	54165141		7.43	3.56	4.1	8.93	8.54	8.12
ENSDARG00000042124	si:dkey-4e7.3	si:dkey-4e7.3 [Source:ZFIN;Acc:ZDB-GENE-070912-542]	9	7072504	7081557		11.23	20.07	15.74	5.11	6.4	6.05
ENSDARG00000042126	elmo3	engulfment and cell motility 3 [Source:ZFIN;Acc:ZDB-GENE-050309-39]	25	17423515	17471260		6.73	6.28	4.85	6.53	6.86	6.65
ENSDARG00000042128	pacsin1b	protein kinase C and casein kinase substrate in neurons 1b [Source:NCBI gene;Acc:619246]	6	54187643	54271549		15.77	10.45	11.29	11.78	11.73	11.91
ENSDARG00000042129	zp3a.1	zona pellucida glycoprotein 3a, tandem duplicate 1 [Source:ZFIN;Acc:ZDB-GENE-040727-1]	20	33991801	33994699		0	0	0.01	0	0	0.02
ENSDARG00000042130	zp3a.2	zona pellucida glycoprotein 3a, tandem duplicate 2 [Source:ZFIN;Acc:ZDB-GENE-040727-2]	20	33987465	33989427		0.05	0.13	0.1	0.28	0.27	0.26
ENSDARG00000042133	si:dkey-51e6.1	si:dkey-51e6.1 [Source:ZFIN;Acc:ZDB-GENE-041014-198]	20	33981946	33985743		11.08	17.29	16.33	9.88	10.27	9.89
ENSDARG00000042138	selp	selectin P [Source:ZFIN;Acc:ZDB-GENE-110107-2]	20	33948642	33961697		0.01	0.02	0.03	0.07	0.1	0.08
ENSDARG00000042139	fpgt	fucose-1-phosphate guanylyltransferase [Source:ZFIN;Acc:ZDB-GENE-050522-489]	8	17983369	17987549		1.21	1.34	1.53	1.23	1.06	0.93
ENSDARG00000042141	myo6b	myosin VIb [Source:ZFIN;Acc:ZDB-GENE-030318-3]	17	36687094	36842267		6.04	4.77	3.78	4.16	4.51	4.42
ENSDARG00000042145	lhx8b	LIM homeobox 8b [Source:ZFIN;Acc:ZDB-GENE-081105-153]	8	17921429	17926814		0.09	0.09	0.19	0.06	0.18	0.27
ENSDARG00000042147	rbbp5	retinoblastoma binding protein 5 [Source:ZFIN;Acc:ZDB-GENE-040426-886]	22	770001	784110		15.24	11.73	11.56	14.72	13.9	14.56
ENSDARG00000042151	skia	v-ski avian sarcoma viral oncogene homolog a [Source:ZFIN;Acc:ZDB-GENE-990715-9]	8	17395929	17516448		10.13	8.38	6.28	15.56	14.59	13.85
ENSDARG00000042157	ppil6	peptidylprolyl isomerase (cyclophilin)-like 6 [Source:ZFIN;Acc:ZDB-GENE-050522-70]	20	33739059	33743295		0.58	0.39	0.35	0.87	0.68	0.6
ENSDARG00000042159	RFESD	Rieske domain-containing protein-like [Source:NCBI gene;Acc:559859]	8	32501405	32506569	RFESD	3.58	2.94	2.76	1.93	1.76	1.9
ENSDARG00000042172	c7a	complement component 7a [Source:ZFIN;Acc:ZDB-GENE-081104-392]	8	31872992	31899329		5.58	6.76	6.26	16.73	16.69	17.16
ENSDARG00000042180	foxl2a	forkhead box L2a [Source:ZFIN;Acc:ZDB-GENE-060512-241]	15	7054754	7058214		3.63	3.87	3.66	2.36	2.07	2.88
ENSDARG00000042182	drc1	dynein regulatory complex subunit 1 homolog (Chlamydomonas) [Source:NCBI gene;Acc:100034551]	20	33519435	33530425		1.11	0.61	0.79	0.96	1.3	1.17
ENSDARG00000042186	papln b	papilin b, proteoglycan-like sulfated glycoprotein [Source:ZFIN;Acc:ZDB-GENE-030131-6023]	20	33499378	33512443		0.74	0.17	0.18	0.59	0.54	0.61
ENSDARG00000042187	smek1	SMEK homolog 1, suppressor of mek1 (Dictyostelium) [Source:NCBI gene;Acc:558578]	20	33467161	33487729		16.58	10.49	10.95	19.3	19.31	18.85
ENSDARG00000042188	ttc38	tetratricopeptide repeat domain 38 [Source:NCBI gene;Acc:553728]	18	10926197	10959429		6.94	8.33	6.72	3.31	3.18	3.24
ENSDARG00000042189	tspan33b	tetraspanin 33b [Source:ZFIN;Acc:ZDB-GENE-060503-607]	18	10967461	10995410		0.19	0.16	0.3	1.1	0.93	0.95
ENSDARG00000042210	sema3ab	sema domain, immunoglobulin domain (Ig), short basic domain, secreted, (semaphorin) 3Ab [Source:NCBI gene;Acc:30267]	18	9323211	9439922		7.75	5.91	5.2	8.7	8.73	9.02
ENSDARG00000042213	ELAPOR2	si:ch211-233h19.2 [Source:ZFIN;Acc:ZDB-GENE-061009-14]	18	8917766	8955573	ELAPOR2	0.96	0.37	0.39	1.03	1.03	1.09
ENSDARG00000042214	tmem243a	transmembrane protein 243, mitochondrial a [Source:ZFIN;Acc:ZDB-GENE-050417-264]	18	8912113	8916000		1.88	2.16	2.04	1.82	2.34	2.22
ENSDARG00000042215	pias4b	protein inhibitor of activated STAT, 4b [Source:ZFIN;Acc:ZDB-GENE-040426-1298]	2	57447105	57473991		0.22	0.14	0.16	0.25	0.13	0.17
ENSDARG00000042221	mthfd1l	methylenetetrahydrofolate dehydrogenase (NADP+ dependent) 1 like [Source:ZFIN;Acc:ZDB-GENE-041001-133]	20	26478197	26532167		13.71	20.65	18.78	19.37	19.28	17.56
ENSDARG00000042231	stx11b.2	syntaxin 11b, tandem duplicate 2 [Source:ZFIN;Acc:ZDB-GENE-050417-148]	20	26535342	26537171		1.3	1.26	1.24	1.24	1.2	1.04
ENSDARG00000042232	specc1lb	sperm antigen with calponin homology and coiled-coil domains 1-like b [Source:ZFIN;Acc:ZDB-GENE-090423-1]	21	13233377	13284660		2.9	2.15	1.72	3.28	3.38	3.4
ENSDARG00000042233	fgf16	fibroblast growth factor 16 [Source:NCBI gene;Acc:692065]	14	10751249	10762629		1.21	1.22	1.45	1.63	1.95	1.89
ENSDARG00000042236	atrx	ATRX chromatin remodeler [Source:ZFIN;Acc:ZDB-GENE-030912-11]	14	10656975	10737898		33.04	18.53	18.81	27.37	26.17	25.98
ENSDARG00000042245	myl13	myosin, light chain 13 [Source:ZFIN;Acc:ZDB-GENE-040426-1593]	2	2470687	2481947		450.43	590.84	645.54	344.74	379.26	378.35
ENSDARG00000042249	FRMD4A	si:ch211-220f12.1 [Source:ZFIN;Acc:ZDB-GENE-050419-154]	18	8503370	8579907	FRMD4A	7.09	3.99	3.55	13.61	13.06	12.76
ENSDARG00000042252	eif4h	eukaryotic translation initiation factor 4h [Source:ZFIN;Acc:ZDB-GENE-010328-19]	15	4585266	4616928		42.19	41.64	37.89	38.59	37.18	37.82
ENSDARG00000042259	tgfbr1b	transforming growth factor, beta receptor 1 b [Source:ZFIN;Acc:ZDB-GENE-091027-1]	24	119680	150447		10.06	5.51	5.41	16.89	16.02	16.6
ENSDARG00000042261	cdan1	codanin 1 [Source:ZFIN;Acc:ZDB-GENE-110411-280]	17	38447473	38473470		1.01	0.76	0.67	1.17	1.11	0.95

ENSDARG00000042270	arsa	arylsulfatase A [Source:ZFIN;Acc:ZDB-GENE-050320-118]	18	8231138	8243538		4.64	2.69	3.17	3.64	3.98	3.85
ENSDARG00000042272	tmem231	transmembrane protein 231 [Source:NCBI gene;Acc:393358]	25	34845115	34859819		3.8	3.22	3.96	4.09	3.95	4.13
ENSDARG00000042277	efnb3b	ephrin-B3b [Source:ZFIN;Acc:ZDB-GENE-010618-3]	7	20965880	21089475		12.43	5.84	5.76	15.38	14.42	15.13
ENSDARG00000042282	itga6a	integrin, alpha 6a [Source:ZFIN;Acc:ZDB-GENE-050309-23]	9	3429662	3486488		2.25	1.42	1.28	3.3	3.54	3.44
ENSDARG00000042285	atp8b3	ATPase phospholipid transporting 8B3 [Source:ZFIN;Acc:ZDB-GENE-050208-424]	22	20473229	20515347		0.59	0.27	0.29	0.63	0.62	0.6
ENSDARG00000042291	dlx6a	distal-less homeobox 6a [Source:NCBI gene;Acc:30586]	19	41419519	41467041		4.55	2.4	3.51	4.72	3.81	4.52
ENSDARG00000042293	ca4b	carbonic anhydrase IV b [Source:ZFIN;Acc:ZDB-GENE-080815-5]	15	15390882	15401917		11.61	19.47	18.01	6.3	6.46	6.01
ENSDARG00000042296	dlx5a	distal-less homeobox 5a [Source:NCBI gene;Acc:30569]	19	41470216	41472228		14.18	10.88	12.1	14.92	15.48	15.5
ENSDARG00000042308	arhgef18b	rho/rac guanine nucleotide exchange factor (GEF) 18b [Source:ZFIN;Acc:ZDB-GENE-070705-462]	22	10814204	10891213		1.88	1.42	1.02	2.03	2.03	1.86
ENSDARG00000042319	cdca4	cell division cycle associated 4 [Source:ZFIN;Acc:ZDB-GENE-030131-1695]	20	26939742	26946248		20.97	8.61	12.22	23.52	22.35	22.86
ENSDARG00000042322	si:dkey-177p2.6	si:dkey-177p2.6 [Source:ZFIN;Acc:ZDB-GENE-030131-5926]	20	26947800	26962841		8.37	5.62	4.85	10.7	10.39	10.74
ENSDARG00000042329	BCR	si:dkey-91m11.5 [Source:ZFIN;Acc:ZDB-GENE-060503-932]	8	30501170	30593651	BCR	3.86	2.57	2.06	6.42	6.5	6.11
ENSDARG00000042332	plin2	perilipin 2 [Source:ZFIN;Acc:ZDB-GENE-050913-12]	15	17030473	17048822		1.17	1.47	1.65	2.29	2.53	2.26
ENSDARG00000042336	impdh1a	IMP (inosine 5'-monophosphate) dehydrogenase 1a [Source:NCBI gene;Acc:431724]	18	8805575	8842818		19.22	5.81	3.96	11.28	10.41	11.64
ENSDARG00000042337	chrac1	chromatin accessibility complex subunit 1 [Source:ZFIN;Acc:ZDB-GENE-050227-18]	19	41515759	41518951		17.45	17.57	16.87	11.04	11.01	11.44
ENSDARG00000042338	lpar2a	lysophosphatidic acid receptor 2a [Source:ZFIN;Acc:ZDB-GENE-040801-97]	3	53081027	53092509		2.13	2.33	2.56	1.94	1.9	2.18
ENSDARG00000042341	tmem251	transmembrane protein 251 [Source:NCBI gene;Acc:619265]	20	27087539	27090853		7.98	8.29	7.64	8.84	9	8.74
ENSDARG00000042344	trmt6	tRNA methyltransferase 6 [Source:ZFIN;Acc:ZDB-GENE-051120-141]	20	45754323	45772306		5.82	5.89	5.01	4.32	5.06	4.55
ENSDARG00000042345	si:dkeyp-55f12.3	si:dkeyp-55f12.3 [Source:ZFIN;Acc:ZDB-GENE-041001-118]	20	27090908	27092290		14.9	22.18	21.78	8.77	8.07	9.39
ENSDARG00000042350	syt1b	synaptotagmin Ib [Source:ZFIN;Acc:ZDB-GENE-060503-166]	18	7387090	7393668		1.38	0.94	1.45	1.23	0.74	1.05
ENSDARG00000042357	cldnk	claudin k [Source:ZFIN;Acc:ZDB-GENE-040801-201]	3	50123507	50127668		6.41	7.23	8.5	6.25	7.25	7.32
ENSDARG00000042368	kif26aa	kinesin family member 26Aa [Source:ZFIN;Acc:ZDB-GENE-041001-148]	20	27426507	27552885		1.26	0.64	0.65	2.11	2.29	2.16
ENSDARG00000042370	ptafr	platelet-activating factor receptor [Source:ZFIN;Acc:ZDB-GENE-131127-236]	17	27971144	27976031		0.23	0.56	0.46	1.03	1.26	1.36
ENSDARG00000042379	TMEM120A	zgc:103681 [Source:ZFIN;Acc:ZDB-GENE-041010-17]	15	16873690	16884912	TMEM120A	6.82	12.34	8.52	1.93	2	1.89
ENSDARG00000042383	zbtb25	zinc finger and BTB domain containing 25 [Source:ZFIN;Acc:ZDB-GENE-041001-125]	20	27707766	27711970		3.78	0.94	1.65	3.1	3.1	3.21
ENSDARG00000042387	zgc:77752	zgc:77752 [Source:ZFIN;Acc:ZDB-GENE-040426-1867]	18	7594012	7609173		1.87	0.62	0.29	1.03	1.07	1.19
ENSDARG00000042390	syndig1l	synapse differentiation inducing 1-like [Source:NCBI gene;Acc:555204]	20	27822044	27864964		7.11	1.48	2.18	5.76	5.7	5.58
ENSDARG00000042396	odf3b	tripartite motif containing 35-30 [Source:ZFIN;Acc:ZDB-GENE-060503-557]	18	7611298	7622892		0.19	0.26	0.27	0.14	0.22	0.18
ENSDARG00000042401	derl2	derlin 2 [Source:ZFIN;Acc:ZDB-GENE-050522-90]	5	58996765	59012599		2.64	2.78	2.74	2.94	2.8	2.82
ENSDARG00000042409	RALGDS	si:ch211-251j10.3 [Source:ZFIN;Acc:ZDB-GENE-081105-110]	8	30949968	30979494	RALGDS	4.21	3.23	2.72	4.77	4.88	4.51
ENSDARG00000042410	rab40b	RAB40B, member RAS oncogene family [Source:ZFIN;Acc:ZDB-GENE-040718-257]	12	32039177	32066470		8.68	5.24	6.31	8.57	9.16	8.42
ENSDARG00000042418	galnt17	polypeptide N-acetylgalactosaminyltransferase 17 [Source:ZFIN;Acc:ZDB-GENE-110419-5]	15	16521785	16546472		2.11	0.47	0.82	2.24	2.05	2.14
ENSDARG00000042428	gstt1a	glutathione S-transferase theta 1a [Source:ZFIN;Acc:ZDB-GENE-031001-13]	8	30787111	30791266		48.33	71.93	69.74	27.71	31.13	28.77
ENSDARG00000042440	p2rx7	purinergic receptor P2X, ligand-gated ion channel, 7 [Source:ZFIN;Acc:ZDB-GENE-030319-6]	8	30758169	30779101		0.76	0.43	0.65	2.9	2.75	2.61
ENSDARG00000042452	p2rx4b	purinergic receptor P2X, ligand-gated ion channel, 4b [Source:ZFIN;Acc:ZDB-GENE-030319-4]	8	30743263	30755977		0.05	0.05	0.03	0.03	0.06	0.06
ENSDARG00000042456	mad2l2	mitotic arrest deficient 2 like 2 [Source:NCBI gene;Acc:550258]	8	22345282	22348391		5.47	4.36	3.99	5.12	5.11	6.08
ENSDARG00000042458	rfc4	replication factor C (activator 1) 4 [Source:ZFIN;Acc:ZDB-GENE-040824-3]	11	45426609	45435014		9.1	9.61	11.54	6.89	6.5	5.41
ENSDARG00000042467	tlk1a	tousled-like kinase 1a [Source:ZFIN;Acc:ZDB-GENE-071008-2]	9	3548529	3609216		12.09	8.98	8.23	12.47	13.98	13.27
ENSDARG00000042469	ndufa12	NADH:ubiquinone oxidoreductase subunit A12 [Source:ZFIN;Acc:ZDB-GENE-050828-1]	4	25829841	25836684		256.08	289.64	362.22	156.19	143.53	143.58
ENSDARG00000042470	s1pr3a	sphingosine-1-phosphate receptor 3a [Source:ZFIN;Acc:ZDB-GENE-050208-347]	22	21475648	21476836		1.09	1.08	1.15	3.44	3.2	3.58
ENSDARG00000042477	nr2c2	nuclear receptor subfamily 2, group C, member 2 [Source:ZFIN;Acc:ZDB-GENE-080403-8]	8	53120278	53142068		7.41	3.35	3.54	8.99	9.08	9.52
ENSDARG00000042484	tle2a	TLE family member 2, transcriptional corepressor a [Source:ZFIN;Acc:ZDB-GENE-030131-2826]	22	21618121	21667832		12.11	6.12	6.46	16.09	16.16	17.06
ENSDARG00000042485	foxd5	forkhead box D5 [Source:ZFIN;Acc:ZDB-GENE-980605-4]	8	30452945	30454096		0.26	0.22	0.28	0.67	0.7	0.75
ENSDARG00000042489	thap12a	THAP domain containing 12a [Source:ZFIN;Acc:ZDB-GENE-040426-2045]	10	32058692	32064405		8.87	8.17	8.22	6.27	6.25	5.71
ENSDARG00000042492	si:dkey-250d21.1	si:dkey-250d21.1 [Source:ZFIN;Acc:ZDB-GENE-030131-8276]	10	32066355	32072789		11.94	7.95	8.16	10.4	10.36	10.21
ENSDARG00000042496	parp12a	poly (ADP-ribose) polymerase family, member 12a [Source:ZFIN;Acc:ZDB-GENE-050419-6]	18	12839126	12858016		2.49	2.28	1.97	3.07	3.28	2.56
ENSDARG00000042508	ubr3	ubiquitin protein ligase E3 component n-recognin 3 [Source:NCBI gene;Acc:553247]	9	3898132	4018452		4.91	3.77	3.32	6.26	6.18	6.02
ENSDARG00000042509	glod4	glyoxalase domain containing 4 [Source:ZFIN;Acc:ZDB-GENE-040912-38]	15	16178775	16183583		9.8	12.59	11.63	9.19	7.27	8.6
ENSDARG00000042510	acot20	acyl-CoA thioesterase 20 [Source:ZFIN;Acc:ZDB-GENE-041001-182]	20	28935413	28938730		8.86	10.87	9.78	6.1	6.26	6.2
ENSDARG00000042513	acot21	acyl-CoA thioesterase 21 [Source:ZFIN;Acc:ZDB-GENE-041001-181]	20	28946709	28956303		0.14	0.1	0.13	0.21	0.06	0.04
ENSDARG00000042515	gpr176	G protein-coupled receptor 176 [Source:ZFIN;Acc:ZDB-GENE-041001-183]	20	28960340	28974309		2.33	0.8	1.41	2.31	2.53	2.47
ENSDARG00000042518	hipk2	homeodomain interacting protein kinase 2 [Source:ZFIN;Acc:ZDB-GENE-031125-4]	18	12464461	12654011		8.63	5.32	4.64	13.82	13.45	13.88
ENSDARG00000042520	UTP14C	si:dkey-251i10.3 [Source:ZFIN;Acc:ZDB-GENE-060526-271]	5	67474119	67499279	UTP14C	16.36	17.55	15.35	9.5	9.12	9.65
ENSDARG00000042521	si:dkey-251i10.1	si:dkey-251i10.1 [Source:ZFIN;Acc:ZDB-GENE-060531-122]	5	67448664	67459538		8.47	7.44	6.63	3.81	3.62	3.5
ENSDARG00000042522	katnbl1	katanin p80 subunit B-like 1 [Source:ZFIN;Acc:ZDB-GENE-030131-4155]	20	29209615	29216236		6.34	5.56	4.41	5.29	5.83	6.6
ENSDARG00000042525	ebf2	EBF transcription factor 2 [Source:NCBI gene;Acc:30692]	5	67390115	67441054		2.31	1.03	1.15	3.01	2.52	2.85
ENSDARG00000042526	sebox	SEBOX homeobox [Source:NCBI gene;Acc:282666]	5	67371650	67382188		0.25	0.01	0.02	0.56	0.51	0.53
ENSDARG00000042527	unga	uracil DNA glycosylase a [Source:ZFIN;Acc:ZDB-GENE-040426-900]	5	67351219	67365750		4.38	4.28	4.05	2.77	2.43	2.48
ENSDARG00000042529	gnat2	guanine nucleotide binding protein (G protein), alpha transducing activity polypeptide 2 [Source:ZFIN;Acc:ZDB-GENE-011128-10]	8	25225590	25247284		483.1	82.2	52.91	153.13	159.66	154.72
ENSDARG00000042530	nup205	nucleoporin 205 [Source:ZFIN;Acc:ZDB-GENE-040715-8]	18	12236044	12270848		10.57	7.81	6.94	9.98	10.17	10.17
ENSDARG00000042533	gstm.1	glutathione S-transferase mu, tandem duplicate 1 [Source:ZFIN;Acc:ZDB-GENE-030911-2]	8	25295160	25297928		58.64	80.42	69.53	32.31	30.39	30
ENSDARG00000042534	rsrc2	arginine/serine-rich coiled-coil 2 [Source:NCBI gene;Acc:406566]	5	67275888	67292690		28.32	13.35	19.86	24.24	24.21	24.2
ENSDARG00000042535	actc1a	actin alpha cardiac muscle 1a [Source:ZFIN;Acc:ZDB-GENE-040520-4]	20	29476852	29483514		145.3	220.55	257.41	171.67	183.91	174.63
ENSDARG00000042536	si:dkey-48j7.3	si:dkey-48j7.3 [Source:ZFIN;Acc:ZDB-GENE-081104-418]	8	25569903	25572071		0	0	0	0	0	0.03
ENSDARG00000042539	ywhaqa	tyrosine 3-monooxygenase/tryptophan 5-monooxygenase activation protein, theta polypeptide a [Source:ZFIN;Acc:ZDB-GENE-040	20	29565906	29585336		122.05	85.51	83.86	102.48	103.71	101.61
ENSDARG00000042544	degs2	delta(4)-desaturase, sphingolipid 2 [Source:ZFIN;Acc:ZDB-GENE-080228-1]	17	30894431	30900380		11.23	12.96	9.82	7.55	7.11	5.97
ENSDARG00000042545	sema3ga	sema domain, immunoglobulin domain (Ig), short basic domain, secreted, (semaphorin) 3Ga [Source:ZFIN;Acc:ZDB-GENE-050513-	8	25650229	25707421		2.99	1.84	1.86	3.05	3.72	3.55
ENSDARG00000042548	tpd52l1	tpd52 like 1 [Source:ZFIN;Acc:ZDB-GENE-050522-121]	20	39691793	39735987		8.6	10.03	8.84	5.31	5.61	5.98
ENSDARG00000042551	mboat2b	membrane bound O-acyltransferase domain containing 2b [Source:ZFIN;Acc:ZDB-GENE-041001-185]	20	29690901	29743160		6.09	2.15	3	7.89	7.95	8.06
ENSDARG00000042552	cacna1sb	calcium channel, voltage-dependent, L type, alpha 1S subunit, b [Source:ZFIN;Acc:ZDB-GENE-051227-1]	8	25767610	25808403		7.04	10.62	8.6	18.06	14.55	14.01
ENSDARG00000042553	pex7	peroxisomal biogenesis factor 7 [Source:ZFIN;Acc:ZDB-GENE-050320-105]	20	39520628	39582316		7.22	7.35	7.64	4.57	4.35	3.96
ENSDARG00000042555	dym	dymeclin [Source:ZFIN;Acc:ZDB-GENE-091204-348]	21	3476207	3613702		3.97	2.49	2.24	4.4	4.23	4.05
ENSDARG00000042557	rnf144ab	ring finger protein 144ab [Source:NCBI gene;Acc:437000]	20	29836276	29864530		7.27	3.04	3.78	9.02	10.04	9.59
ENSDARG00000042559	tnni1c	troponin I, skeletal, slow c [Source:ZFIN;Acc:ZDB-GENE-060825-192]	18	6625348	6634424		31.66	39.18	34.62	17.19	18.39	16.27
ENSDARG00000042561	lpar2b	lysophosphatidic acid receptor 2b [Source:ZFIN;Acc:ZDB-GENE-051120-99]	1	59192197	59216197		8.35	7.87	7.05	9.22	9.08	7.51
ENSDARG00000042562	allc	allantoicase [Source:NCBI gene;Acc:436989]	20	30355436	30370927		2.94	4.8	5.06	1.7	1.87	1.81
ENSDARG00000042563	mis18bp1	MIS18 binding protein 1 [Source:ZFIN;Acc:ZDB-GENE-030616-151]	17	28611746	28623360		2.44	1.76	1.81	1.66	1.47	1.57
ENSDARG00000042566	rps7	ribosomal protein S7 [Source:NCBI gene;Acc:393725]	20	30372352	30377221		1681.8	2306.06	2477.89	1334.95	1410.96	1437.99
ENSDARG00000042571	rnaseh1	ribonuclease H1 [Source:ZFIN;Acc:ZDB-GENE-040718-407]	20	30378803	30385570		12.38	15.17	13.82	8.71	9.84	9.47
ENSDARG00000042577	batf3	basic leucine zipper transcription factor, ATF-like 3 [Source:ZFIN;Acc:ZDB-GENE-041014-356]	20	37812699	37813863		0.48	0.55	0.58	0.66	0.44	0.65
ENSDARG00000042590	rbm19	RNA binding motif protein 19 [Source:ZFIN;Acc:ZDB-GENE-031125-3]	6	45843193	45869164		7	7.03	5.93	5.02	4.95	5.45
ENSDARG00000042608	ica1	islet cell autoantigen 1 [Source:ZFIN;Acc:ZDB-GENE-040718-490]	16	47207691	47232146		3.61	1	1.48	3.79	4.32	4.17
ENSDARG00000042613	crp3	C-reactive protein 3 [Source:ZFIN;Acc:ZDB-GENE-060503-220]	24	38092608	38097305		24.04	33.56	32.33	26.16	22.69	24.45
ENSDARG00000042616	sema3h	sema domain, immunoglobulin domain (Ig), short basic domain, secreted, (semaphorin) 3H [Source:ZFIN;Acc:ZDB-GENE-990715-8]	8	25913787	25951806		1.89	0.61	0.79	2.25	2.43	2.15
ENSDARG00000042620	gstr	glutathione S-transferase rho [Source:ZFIN;Acc:ZDB-GENE-090507-1]	19	770300	776076		59.22	84.73	64.83	34.04	34.34	33.67
ENSDARG00000042621	cryaba	crystallin, alpha B, a [Source:ZFIN;Acc:ZDB-GENE-991119-2]	15	18119618	18124678		0.02	0.05	0.1	0.02	0	0
ENSDARG00000042623	acbd3	acyl-Coenzyme A binding domain containing 3 [Source:ZFIN;Acc:ZDB-GENE-040426-1930]	20	30702531	30721862		9.2	6.24	5.93	10.84	10.08	9.79
ENSDARG00000042627	nhsl1b	NHS-like 1b [Source:ZFIN;Acc:ZDB-GENE-040910-1]	20	30725778	30889820		4.95	3.13	2.55	5.18	4.95	4.49
ENSDARG00000042630	hebp2	heme binding protein 2 [Source:ZFIN;Acc:ZDB-GENE-040426-914]	20	30894093	30901034		11.37	10.64	9.41	8.66	7.55	8.03
ENSDARG00000042637	tert	telomerase reverse transcriptase [Source:ZFIN;Acc:ZDB-GENE-080405-1]	19	627899	642878		2.81	2.39	2.57	2.55	2.72	2.62
ENSDARG00000042641	cyp51	cytochrome P450, family 51 [Source:NCBI gene;Acc:414331]	19	598657	617246		23.42	30.66	25.01	6.86	6.57	6.43
ENSDARG00000042642	wtap	WT1 associated protein [Source:NCBI gene;Acc:334058]	20	30931414	30938246		28.46	17.19	19.57	25.37	25.5	25.22
ENSDARG00000042643	FUT9	si:ch1073-440p11.2 [Source:ZFIN;Acc:ZDB-GENE-140106-39]	19	591490	592667	FUT9	0.02	0.02	0	0	0	0
ENSDARG00000042644	sod2	superoxide dismutase 2, mitochondrial [Source:ZFIN;Acc:ZDB-GENE-030131-7742]	20	30937934	30947547		60.83	81.96	78.78	29.97	32.58	31.33
ENSDARG00000042646	zgc:77784	zgc:77784 [Source:ZFIN;Acc:ZDB-GENE-040426-1874]	15	19456112	19602329		3.28	0.59	0.71	5.31	4.88	5.63
ENSDARG00000042652	rreb1b	ras responsive element binding protein 1b [Source:ZFIN;Acc:ZDB-GENE-070912-273]	2	21000334	21062305		5.43	5.91	3.89	6.7	6.61	6.73
ENSDARG00000042656	nrn1b	neuritin 1b [Source:ZFIN;Acc:ZDB-GENE-041010-98]	2	20991985	20994126		1.17	0.6	0.29	0.83	0.9	0.87
ENSDARG00000042658	acad8	acyl-CoA dehydrogenase family, member 8 [Source:ZFIN;Acc:ZDB-GENE-040426-828]	15	19335646	19348383		16.43	17.66	15.73	11.73	11.26	12.35
ENSDARG00000042659	thyn1	thymocyte nuclear protein 1 [Source:NCBI gene;Acc:393630]	15	19331159	19334448		5.22	6.18	6.49	3.15	2.43	2.91
ENSDARG00000042660	fam32a	family with sequence similarity 32 member A [Source:NCBI gene;Acc:431750]	22	15720381	15721823		52.86	58.49	53.06	39.89	42.63	44.24
ENSDARG00000042661	tsta3l2	tissue specific transplantation antigen P35B, like 2 [Source:ZFIN;Acc:ZDB-GENE-050417-317]	20	48739154	48775067		0	0	0	0	0	0
ENSDARG00000042663	si:dkeyp-70f9.7	si:dkeyp-70f9.7 [Source:ZFIN;Acc:ZDB-GENE-070705-550]	22	15747337	15748440		0.07	0	0.03	0	0	0
ENSDARG00000042667	klf2a	Kruppel-like factor 2a [Source:ZFIN;Acc:ZDB-GENE-011109-1]	22	15898221	15900919		23.69	23.46	19.72	16.14	15.86	15.58
ENSDARG00000042670	eps15l1a	epidermal growth factor receptor pathway substrate 15-like 1a [Source:ZFIN;Acc:ZDB-GENE-060503-274]	22	15912253	15957079		9.66	4.61	4.1	13.73	13.91	13.8
ENSDARG00000042671	odr4	odr-4 GPCR localization factor homolog [Source:ZFIN;Acc:ZDB-GENE-080502-1]	2	20866898	20879820		5	4.31	4.8	4.18	4.83	4.47
ENSDARG00000042677	cadm1b	cell adhesion molecule 1b [Source:ZFIN;Acc:ZDB-GENE-080327-34]	15	18863872	18988585		3.28	1.02	1.36	4.16	4.76	4.9
ENSDARG00000042683	si:dkey-15j16.3	si:dkey-15j16.3 [Source:ZFIN;Acc:ZDB-GENE-030131-4680]	20	23838814	23842631		0	0	0	0	0	0.01
ENSDARG00000042684	serpinc1	serpin peptidase inhibitor, clade C (antithrombin), member 1 [Source:ZFIN;Acc:ZDB-GENE-030131-264]	22	16022211	16028191		45.01	75.88	66.95	49.58	56.46	52.6
ENSDARG00000042688	bora	bora aurora kinase A activator [Source:NCBI gene;Acc:555606]	1	30723380	30735478		1.01	0.72	0.55	0.76	0.93	0.63
ENSDARG00000042689	zbtb37	zinc finger and BTB domain containing 37 [Source:ZFIN;Acc:ZDB-GENE-050208-621]	22	16032160	16034137		8.08	5.39	5.04	7.71	6.99	6.43
ENSDARG00000042690	s1pr1	sphingosine-1-phosphate receptor 1 [Source:NCBI gene;Acc:64617]	22	16038495	16042243		30.61	12.44	17.38	29.47	28.36	28.62
ENSDARG00000042698	si:dkey-15j16.6	si:dkey-15j16.6 [Source:ZFIN;Acc:ZDB-GENE-130531-45]	20	23847380	23852174		0.02	0.05	0.04	0.09	0.14	0.09
ENSDARG00000042703	parp6a	poly (ADP-ribose) polymerase family, member 6a [Source:ZFIN;Acc:ZDB-GENE-040718-272]	18	887122	898873		8.28	1.7	2.31	8.29	9.23	9.41
ENSDARG00000042707	cx30.3	connexin 30.3 [Source:ZFIN;Acc:ZDB-GENE-040406-1]	9	21401189	21405066		12.36	11.3	9.7	7.61	6.26	7.06
ENSDARG00000042708	tuba8l	tubulin, alpha 8 like [Source:ZFIN;Acc:ZDB-GENE-030131-9167]	8	28345352	28349859		171.32	210.16	223.11	146.47	152.11	141.72
ENSDARG00000042717	adipor1b	adiponectin receptor 1b [Source:ZFIN;Acc:ZDB-GENE-040426-2896]	8	28358161	28365547		13.79	10.62	10.13	15.44	15.21	12.95
ENSDARG00000042722	blnk	B cell linker [Source:ZFIN;Acc:ZDB-GENE-040702-4]	13	50518815	50567658		0.29	0.35	0.21	0.64	0.46	0.57
ENSDARG00000042723	gfra2a	GDNF family receptor alpha 2a [Source:ZFIN;Acc:ZDB-GENE-040630-1]	8	42059495	42238543		3.62	1.82	1.83	4.41	4.57	4.73
ENSDARG00000042724	supt5h	SPT5 homolog, DSIF elongation factor subunit [Source:NCBI gene;Acc:64275]	15	20363859	20395249		20.1	13.72	13.02	22.31	21.1	23.65
ENSDARG00000042725	cebpb	CCAAT enhancer binding protein beta [Source:ZFIN;Acc:ZDB-GENE-020111-3]	8	28448267	28449782		21.51	22.9	21.81	44.62	48.29	46.14
ENSDARG00000042726	grb7	growth factor receptor bound protein 7 [Source:ZFIN;Acc:ZDB-GENE-091118-23]	24	36414829	36442590		2.05	1.59	1.51	1.44	1.47	1.51
ENSDARG00000042727	exo5	exonuclease 5 [Source:NCBI gene;Acc:641427]	15	25084821	25094026		2.38	2.03	2.54	1.63	1.41	1.88
ENSDARG00000042728	plaa	phospholipase A2-activating protein [Source:ZFIN;Acc:ZDB-GENE-030826-13]	7	61977050	62003838		10.45	8.01	6.81	10.64	9.62	9.47
ENSDARG00000042732	tmem189	transmembrane protein 189 [Source:ZFIN;Acc:ZDB-GENE-041010-150]	8	28452738	28466870		8.34	8.22	6.15	10.97	10.72	9.97
ENSDARG00000042737	slc52a3	solute carrier family 52 member 3 [Source:NCBI gene;Acc:678609]	8	28467893	28477739		0.85	0.96	0.69	0.4	0.45	0.54
ENSDARG00000042742	stx6	syntaxin 6 [Source:ZFIN;Acc:ZDB-GENE-030131-858]	22	16482631	16494406		10.87	7.18	8.16	9.48	9.44	9.51
ENSDARG00000042747	coasy	CoA synthase [Source:ZFIN;Acc:ZDB-GENE-040912-137]	24	36275660	36301072		10.39	8.83	8.61	10.01	8.78	9.46
ENSDARG00000042751	riok3	RIO kinase 3 (yeast) [Source:ZFIN;Acc:ZDB-GENE-040801-133]	24	36239617	36253217		25.93	21.6	21.45	30.45	33.28	34.11
ENSDARG00000042752	si:ch211-282j17.11	si:ch211-282j17.11 [Source:ZFIN;Acc:ZDB-GENE-041210-284]	7	3755739	3762912		0	0	0	0	0	0
ENSDARG00000042753	cts12	cathepsin 12 [Source:ZFIN;Acc:ZDB-GENE-050208-336]	22	16406920	16419126		3.63	1.75	1.72	1.98	1.66	2.03
ENSDARG00000042759	cnstb	consortin, connexin sorting protein b [Source:ZFIN;Acc:ZDB-GENE-041001-189]	20	34991620	35012093		4.73	3.43	3.46	5.63	6.21	5.91
ENSDARG00000042777	ndufa11	NADH:ubiquinone oxidoreductase subunit A11 [Source:ZFIN;Acc:ZDB-GENE-040426-1631]	11	6411169	6429705		105.24	135.77	161.01	55.92	60.13	62.05
ENSDARG00000042780	apoba	apolipoprotein Ba [Source:ZFIN;Acc:ZDB-GENE-070702-4]	17	30704068	30727966		42.12	68.93	53.22	26.91	27.71	22.95
ENSDARG00000042784	gdf7	growth differentiation factor 7 [Source:ZFIN;Acc:ZDB-GENE-990714-1]	17	30835076	30839338		1.45	1.99	1.82	2.04	2.04	1.55
ENSDARG00000042791	git2b	G protein-coupled receptor kinase interacting ArfGAP 2b [Source:ZFIN;Acc:ZDB-GENE-070112-2192]	10	40284003	40317479		4.62	2.58	2.66	6.74	6.44	6.36
ENSDARG00000042793	tpp1	tripeptidyl peptidase I [Source:NCBI gene;Acc:798347]	17	30843881	30855792		13.34	16.22	14.02	13.12	12.97	12.54
ENSDARG00000042796	yy1a	YY1 transcription factor a [Source:ZFIN;Acc:ZDB-GENE-030131-1117]	17	30857008	30863252		32.67	22.4	23.65	28.95	29.21	31.39
ENSDARG00000042799	atpaf1	ATP synthase mitochondrial F1 complex assembly factor 1 [Source:NCBI gene;Acc:550606]	22	17192767	17202113		16.77	20.07	18.47	12.79	12.42	12.87
ENSDARG00000042807	slc16a4	solute carrier family 16 member 4 [Source:ZFIN;Acc:ZDB-GENE-070112-602]	8	24745041	24779395		6.59	8.19	6.53	9.56	9.19	9.36
ENSDARG00000042810	or128-10	odorant receptor, family E, subfamily 128, member 10 [Source:ZFIN;Acc:ZDB-GENE-070806-114]	15	5155137	5157572		0.17	0.06	0.12	0.21	0.09	0.15
ENSDARG00000042811	fgf1b	fibroblast growth factor 1b [Source:ZFIN;Acc:ZDB-GENE-071004-7]	21	44112914	44116241		0.22	0.37	0.23	0.92	0.81	0.91
ENSDARG00000042815	alkal2b	ALK and LTK ligand 2b [Source:ZFIN;Acc:ZDB-GENE-111115-2]	17	30661348	30666037		0.71	0.66	0.59	0.8	0.99	0.75
ENSDARG00000042816	mmp9	matrix metallopeptidase 9 [Source:ZFIN;Acc:ZDB-GENE-040426-2132]	8	24281512	24295947		1.8	1.75	1.49	15.89	14.19	12.49
ENSDARG00000042821	agps	alkylglycerone phosphate synthase [Source:ZFIN;Acc:ZDB-GENE-031118-14]	9	1589672	1639965		6.87	5.58	5.73	8.56	8.88	8.57
ENSDARG00000042823	spcs2	signal peptidase complex subunit 2 [Source:NCBI gene;Acc:541342]	15	4988189	4999810		47.72	56.54	50.77	40.62	42.47	39.53
ENSDARG00000042824	nfe2l2a	nuclear factor, erythroid 2-like 2a [Source:ZFIN;Acc:ZDB-GENE-030723-2]	9	1654284	1683368		31.08	31.63	27.49	34.64	36.23	33.65
ENSDARG00000042825	rnf169	ring finger protein 169 [Source:NCBI gene;Acc:795748]	15	4969128	4985077		3.54	2.32	2.25	4.65	4.49	4.5
ENSDARG00000042826	egr2b	early growth response 2b [Source:NCBI gene;Acc:30190]	12	8483838	8504278		2.45	1.53	1.55	3.08	3.21	3.8
ENSDARG00000042827	adoa	2-aminoethanethiol (cysteamine) dioxygenase a [Source:ZFIN;Acc:ZDB-GENE-041212-62]	12	8474868	8476513		9.66	8.08	9.04	12.08	12.33	12.69
ENSDARG00000042829	si:dkey-30j22.1	si:dkey-30j22.1 [Source:ZFIN;Acc:ZDB-GENE-041001-184]	20	35576739	35706379		0.7	0.44	0.26	0.65	0.57	0.46
ENSDARG00000042833	rffl	ring finger and FYVE-like domain containing E3 ubiquitin protein ligase [Source:ZFIN;Acc:ZDB-GENE-040426-2274]	15	4549240	4580763		7.26	7.81	7.12	6.74	7.48	6.74
ENSDARG00000042835	tfdp2	transcription factor Dp-2 [Source:ZFIN;Acc:ZDB-GENE-030219-104]	15	4421797	4528387		8.4	5.86	5.77	6.51	6.42	6.01
ENSDARG00000042837	atp1b3b	ATPase Na+/K+ transporting subunit beta 3b [Source:ZFIN;Acc:ZDB-GENE-001127-1]	15	4353161	4415917		138.61	94.67	99.55	126.14	125.6	126.49
ENSDARG00000042839	dph6	diphthamine biosynthesis 6 [Source:NCBI gene;Acc:503603]	17	53156530	53197232		6.04	6.18	6.62	3	2.62	2.66
ENSDARG00000042840	eml1	EMAP like 1 [Source:ZFIN;Acc:ZDB-GENE-060929-156]	17	30976484	31082381		6.49	2.19	2.04	6.73	6.65	7.3
ENSDARG00000042845	oxt	oxytocin [Source:ZFIN;Acc:ZDB-GENE-030407-1]	5	72087619	72093253		4.2	0.43	1.03	3.81	3.74	4.06
ENSDARG00000042846	disp2	dispatched homolog 2 (Drosophila) [Source:NCBI gene;Acc:405793]	17	31185276	31202280		6.74	1.89	3.7	6.04	5.91	6.45
ENSDARG00000042848	pcmtl	l-isoaspartyl protein carboxyl methyltransferase, like [Source:ZFIN;Acc:ZDB-GENE-040426-1738]	17	31214167	31221774		3.33	3.23	3.39	2.91	2.93	3.36
ENSDARG00000042850	nphs2	NPHS2 stomatin family member, podocin [Source:ZFIN;Acc:ZDB-GENE-051102-2]	22	17251275	17256573		0.74	0.37	0.3	1.96	1.69	1.55
ENSDARG00000042851	srp9	signal recognition particle 9 [Source:ZFIN;Acc:ZDB-GENE-040426-1106]	20	36623807	36626569		19.9	23.8	20.71	17.34	19.1	17.38
ENSDARG00000042853	ivd	isovaleryl-CoA dehydrogenase [Source:ZFIN;Acc:ZDB-GENE-030616-262]	17	31346560	31360956		30.27	38.13	36.2	26.31	25.18	26.27
ENSDARG00000042854	ephx1	epoxide hydrolase 1, microsomal (xenobiotic) [Source:ZFIN;Acc:ZDB-GENE-040426-913]	20	36628059	36638179		7.05	9.38	7.45	3.6	3.75	3.43
ENSDARG00000042856	itpka	inositol-trisphosphate 3-kinase A [Source:ZFIN;Acc:ZDB-GENE-030616-116]	17	31363000	31394615		7.36	6.12	5.17	6.46	6.83	7.35
ENSDARG00000042857	si:ch211-246m6.4	si:ch211-246m6.4 [Source:ZFIN;Acc:ZDB-GENE-050208-498]	22	14127472	14128716		4.07	5.54	5.36	2.93	2.95	2.47
ENSDARG00000042859	slc5a6a	solute carrier family 5 member 6a [Source:ZFIN;Acc:ZDB-GENE-041001-177]	20	36646761	36671660		3.52	3.34	2.89	3.43	3.56	3.71
ENSDARG00000042861	ltk	leukocyte receptor tyrosine kinase [Source:ZFIN;Acc:ZDB-GENE-030616-115]	17	31400584	31483469		3.16	3.43	2.01	5.05	5.49	5.66
ENSDARG00000042864	rpl7l1	ribosomal protein L7-like 1 [Source:ZFIN;Acc:ZDB-GENE-030131-970]	20	36675083	36679233		22.93	28.92	28.1	15.81	15.02	16.06
ENSDARG00000042866	cx35.4	connexin 35.4 [Source:ZFIN;Acc:ZDB-GENE-050417-174]	17	24848976	24853918		7.19	7.28	6.78	6.84	6.66	6.5
ENSDARG00000042872	zdhhc8a	zinc finger DHHC-type containing 8a [Source:ZFIN;Acc:ZDB-GENE-091118-26]	10	428269	454622		4.23	0.57	0.83	6.68	5.24	6.31
ENSDARG00000042873	slc25a27	solute carrier family 25 member 27 [Source:ZFIN;Acc:ZDB-GENE-040426-1290]	20	36801804	36809059		2.21	3.06	1.82	1.28	1.13	1.25
ENSDARG00000042874	phlda2	pleckstrin homology-like domain, family A, member 2 [Source:NCBI gene;Acc:553622]	25	23524796	23526086		13.81	18.93	18.37	13.96	13.23	14.11
ENSDARG00000042876	abracl	ABRA C-terminal like [Source:NCBI gene;Acc:336650]	20	36806398	36818195		17.18	23.5	20.58	17.69	17.57	16.87
ENSDARG00000042877	heca	hdc homolog, cell cycle regulator [Source:ZFIN;Acc:ZDB-GENE-041001-173]	20	36820965	36839074		5.53	2.53	2.71	5.34	6.12	6.2
ENSDARG00000042880	si:ch211-222f23.6	si:ch211-222f23.6 [Source:ZFIN;Acc:ZDB-GENE-070912-208]	9	1485234	1499721		0.13	0.01	0	0.03	0.14	0.11
ENSDARG00000042881	nthl1	nth-like DNA glycosylase 1 [Source:ZFIN;Acc:ZDB-GENE-120511-4]	1	54178703	54191626		4.18	4.1	3.96	2.65	3.34	2.69
ENSDARG00000042887	TNPO2	transportin 2 [Source:HGNC Symbol;Acc:HGNC:19998]	1	54137089	54146461	TNPO2	18.84	12.05	11.13	26.74	27.04	25.67
ENSDARG00000042892	paip1	poly(A) binding protein interacting protein 1 [Source:NCBI gene;Acc:445110]	5	8684652	8711157		13.56	10.82	13.26	14.63	14.03	13.73
ENSDARG00000042894	tyms	thymidylate synthetase [Source:ZFIN;Acc:ZDB-GENE-040426-59]	7	71535045	71541329		23.69	25.69	26.96	12.83	11.29	11.19
ENSDARG00000042899	ELAPOR1	si:ch211-163l21.8 [Source:ZFIN;Acc:ZDB-GENE-041008-93]	8	23783066	23809686	ELAPOR1	1.72	2.12	1.56	1.22	1.37	1.34
ENSDARG00000042900	gtpbp1l	GTP binding protein 1, like [Source:ZFIN;Acc:ZDB-GENE-041007-2]	22	14836040	14854420		2.81	3.64	2.86	2.21	2.02	1.77
ENSDARG00000042902	afg1lb	AFG1 like ATPase b [Source:NCBI gene;Acc:555926]	20	32337522	32405451		2.03	2.27	2.24	1.63	2.08	1.74
ENSDARG00000042904	foxo3b	forkhead box O3b [Source:ZFIN;Acc:ZDB-GENE-990708-6]	20	32286765	32332736		31.49	20.27	20.12	46.52	47.35	48.42
ENSDARG00000042905	rpl10a	ribosomal protein L10a [Source:NCBI gene;Acc:323305]	8	23738122	23742985		1069.63	1291.45	1624.51	750	818.1	815.18

ENSDARG00000042908	hsh2d	hematopoietic SH2 domain containing [Source:NCBI gene;Acc:548337]	22	15570965	15578402		0.41	0.71	0.53	0.99	1.11	1.03
ENSDARG00000042920	sult3st3	sulfotransferase family 3, cytosolic sulfotransferase 3 [Source:ZFIN;Acc:ZDB-GENE-060503-628]	22	15315655	15318280		19.58	34.56	23.23	4.68	4.85	3.63
ENSDARG00000042922	gpr173	G protein-coupled receptor 173 [Source:NCBI gene;Acc:57926]	8	23415023	23416362		5.93	1.1	2.76	10.38	10.39	11.11
ENSDARG00000042925	mfng	MFNG O-fucosylpeptide 3-beta-N-acetylglucosaminyltransferase [Source:NCBI gene;Acc:493633]	22	15251527	15280638		1.81	1.5	1.79	2.29	2.46	2.25
ENSDARG00000042927	mapre1a	microtubule-associated protein, RP/EB family, member 1a [Source:ZFIN;Acc:ZDB-GENE-030131-6067]	8	23381892	23390368		5.96	7.96	6.92	3.68	3.49	3.95
ENSDARG00000042929	tmem120b	transmembrane protein 120B [Source:NCBI gene;Acc:798580]	10	41939963	41959934		4.04	2.98	3.07	4.29	3.95	4.44
ENSDARG00000042933	bloc1s6	biogenesis of lysosomal organelles complex-1, subunit 6, pallidin [Source:ZFIN;Acc:ZDB-GENE-070112-1862]	18	5506025	5509616		32.82	41.31	46.92	32.51	28.15	31.91
ENSDARG00000042934	ccn2a	cellular communication network factor 2a [Source:ZFIN;Acc:ZDB-GENE-030131-102]	20	25340814	25343490		27.67	27.37	24.71	110.38	120.6	122.69
ENSDARG00000042936	fam228a	family with sequence similarity 228 member A [Source:ZFIN;Acc:ZDB-GENE-041014-83]	20	25350435	25355985		0.99	0.84	0.8	0.85	0.97	1.29
ENSDARG00000042937	si:dkey-256e7.5	si:dkey-256e7.5 [Source:ZFIN;Acc:ZDB-GENE-090313-282]	1	17618015	17650223		0.65	0.64	0.45	0.56	0.89	0.55
ENSDARG00000042940	nab1a	NGFI-A binding protein 1a (EGR1 binding protein 1) [Source:ZFIN;Acc:ZDB-GENE-050208-574]	22	12870275	12890921		3.18	1.11	1.65	2.85	3.01	2.81
ENSDARG00000042946	hook1	hook microtubule-tethering protein 1 [Source:NCBI gene;Acc:570393]	20	25486021	25513483		7.21	4.27	4.02	5.87	6.3	6.11
ENSDARG00000042947	mstna	myostatin a [Source:ZFIN;Acc:ZDB-GENE-050208-735]	22	12790837	12795340		0	0	0	0	0	0
ENSDARG00000042948	dnajc5aa	DnaJ (Hsp40) homolog, subfamily C, member 5aa [Source:ZFIN;Acc:ZDB-GENE-031113-20]	8	23165749	23179849		27.75	7.16	11.13	31.8	34.68	34.56
ENSDARG00000042953	cyp2n13	cytochrome P450, family 2, subfamily N, polypeptide 13 [Source:ZFIN;Acc:ZDB-GENE-041001-158]	20	25515259	25522911		31.97	62	44.66	2.79	2.51	3.33
ENSDARG00000042954	slc17a9a	solute carrier family 17 member 9a [Source:ZFIN;Acc:ZDB-GENE-081021-1]	8	23152244	23165413		0.38	0.28	0.3	0.21	0.19	0.22
ENSDARG00000042956	cyp2ad6	cytochrome P450, family 2, subfamily AD, polypeptide 6 [Source:ZFIN;Acc:ZDB-GENE-041001-157]	20	25525802	25533739		11.22	10.65	9.81	3.2	3.59	4.15
ENSDARG00000042961	zgc:100920	zgc:100920 [Source:ZFIN;Acc:ZDB-GENE-040801-156]	8	23093155	23103547		10.02	7.54	7.22	15.47	16.49	19.47
ENSDARG00000042962	dgcr2	DiGeorge syndrome critical region gene 2 [Source:ZFIN;Acc:ZDB-GENE-040718-404]	10	42044220	42087230		0.42	0.29	0.29	0.77	0.76	0.69
ENSDARG00000042969	znf1035	zinc finger protein 1035 [Source:ZFIN;Acc:ZDB-GENE-050309-241]	16	25011994	25038855		12.81	6.78	7.12	14.6	13.86	13.84
ENSDARG00000042970	gng8	guanine nucleotide binding protein (G protein), gamma 8 [Source:ZFIN;Acc:ZDB-GENE-050522-312]	21	25231160	25235926		4.43	4.32	3.98	2.65	3.82	3.33
ENSDARG00000042972	zgc:136605	zgc:136605 [Source:ZFIN;Acc:ZDB-GENE-060312-11]	8	22955262	22959401		0.07	0.02	0	0.04	0	0.06
ENSDARG00000042975	auh	AU RNA binding protein/enoyl-CoA hydratase [Source:ZFIN;Acc:ZDB-GENE-040801-95]	10	5422575	5475690		27.91	34.63	32.46	23.83	23.5	22.31
ENSDARG00000042977	nfil3	nuclear factor, interleukin 3 regulated [Source:NCBI gene;Acc:445509]	10	5414224	5419256		11.2	9.28	11.42	17.19	16.31	16.61
ENSDARG00000042978	cyp2p6	cytochrome P450, family 2, subfamily P, polypeptide 6 [Source:ZFIN;Acc:ZDB-GENE-040426-790]	20	25563105	25568441		5.99	8.37	6.63	2.02	2.1	2.38
ENSDARG00000042980	cyp2p7	cytochrome P450, family 2, subfamily P, polypeptide 7 [Source:ZFIN;Acc:ZDB-GENE-041001-155]	20	25568694	25615727		4.69	5.68	4.87	1.69	1.77	1.49
ENSDARG00000042982	cyp2p8	cytochrome P450, family 2, subfamily P, polypeptide 8 [Source:ZFIN;Acc:ZDB-GENE-061030-1]	20	25575391	25580002		25.52	45.04	32.37	3.83	3.99	4.67
ENSDARG00000042983	has1	hyaluronan synthase 1 [Source:ZFIN;Acc:ZDB-GENE-040218-2]	16	24940377	24945337		0.05	0.04	0.08	0.12	0.06	0.24
ENSDARG00000042984	fpr1	formyl peptide receptor 1 [Source:ZFIN;Acc:ZDB-GENE-121227-2]	16	24971717	24974700		2.17	2.56	2.56	2.26	2.14	2.04
ENSDARG00000042988	slc24a2	solute carrier family 24 member 2 [Source:ZFIN;Acc:ZDB-GENE-060825-277]	1	12009673	12044390		7.39	1.43	0.96	8.87	9.29	9.56
ENSDARG00000042989	si:dkeyp-84f3.5	si:dkeyp-84f3.5 [Source:ZFIN;Acc:ZDB-GENE-050208-162]	16	24978203	24992830		5.57	3.16	3.29	6.68	6.4	6.24
ENSDARG00000042990	cyp2p10	cytochrome P450, family 2, subfamily P, polypeptide 10 [Source:ZFIN;Acc:ZDB-GENE-040120-1]	20	25586099	25593607		2.65	1.9	2.1	2.48	2.27	2.49
ENSDARG00000042992	crygs1	crystallin, gamma S1 [Source:ZFIN;Acc:ZDB-GENE-050223-4]	22	11710467	11714158		0.08	0.11	0.16	0.41	0.19	0.07
ENSDARG00000042993	prss1	serine protease 1 [Source:ZFIN;Acc:ZDB-GENE-010131-7]	16	25080764	25085327		1415.46	2158.37	2107.75	154.11	167.87	163.63
ENSDARG00000042995	sptlc1	serine palmitoyltransferase, long chain base subunit 1 [Source:ZFIN;Acc:ZDB-GENE-050522-61]	10	5234327	5265160		12.03	11.33	10.67	10.59	9.91	10.05
ENSDARG00000043002	vmo1a	vitelline membrane outer layer 1 homolog a [Source:ZFIN;Acc:ZDB-GENE-131121-600]	3	5403848	5413018		0.34	0.33	0.28	0.37	0.37	0.17
ENSDARG00000043003	pcyt2	phosphate cytidylyltransferase 2, ethanolamine [Source:ZFIN;Acc:ZDB-GENE-041010-132]	22	11596432	11614973		15.26	12.88	11.54	17.45	18.48	17.49
ENSDARG00000043004	si:dkeyp-117h8.4	si:dkeyp-117h8.4 [Source:ZFIN;Acc:ZDB-GENE-030804-4]	20	25639470	25645150		2.66	2.1	2.09	1.83	1.68	1.84
ENSDARG00000043006	gnav1	guanine nucleotide binding protein (G protein) alpha v1 [Source:ZFIN;Acc:ZDB-GENE-081031-92]	22	11538392	11594414		2.92	3.67	3.32	3.98	4.6	3.89
ENSDARG00000043009	fam43a	family with sequence similarity 43 member A [Source:ZFIN;Acc:ZDB-GENE-030131-6098]	11	34235356	34237802		29.39	18.28	14.62	28.9	29.85	29.62
ENSDARG00000043010	camk2d1	calcium/calmodulin-dependent protein kinase (CaM kinase) II delta 1 [Source:NCBI gene;Acc:445208]	7	57725708	57880131		14.2	11.52	10.37	6.92	6.61	6.2
ENSDARG00000043011	chst14	carbohydrate (N-acetylgalactosamine 4-0) sulfotransferase 14 [Source:NCBI gene;Acc:404732]	20	25739979	25748407		2.61	2.41	1.83	2.96	3.12	3.49
ENSDARG00000043012	napgb	N-ethylmaleimide-sensitive factor attachment protein, gamma b [Source:ZFIN;Acc:ZDB-GENE-040718-171]	2	54364273	54387550		47.89	15.02	19.64	37.35	38.32	41.49
ENSDARG00000043019	exoc1	exocyst complex component 1 [Source:ZFIN;Acc:ZDB-GENE-030131-1057]	20	25853012	25878607		21.64	17.31	18.72	20.79	21.88	22.35
ENSDARG00000043021	slc26a11	solute carrier family 26 member 11 [Source:ZFIN;Acc:ZDB-GENE-030131-5349]	22	11497366	11520405		1.48	1.1	1.12	1.27	1.21	1.39
ENSDARG00000043026	ttbk2b	tau tubulin kinase 2b [Source:ZFIN;Acc:ZDB-GENE-030131-8246]	20	25911342	25928355		5.33	1.8	2.08	6.96	6.37	6.28
ENSDARG00000043031	ints10	integrator complex subunit 10 [Source:NCBI gene;Acc:323368]	14	712115	730053		6.05	3.37	3.78	5.08	5.46	5.09
ENSDARG00000043035	capn3b	calpain 3b [Source:ZFIN;Acc:ZDB-GENE-041001-149]	20	25955118	26001290		8.48	14.48	15.19	13.43	12.34	11.71
ENSDARG00000043046	fbxl5	F-box and leucine-rich repeat protein 5 [Source:NCBI gene;Acc:322181]	23	45869010	45885777		9.51	6.36	6.16	18.07	18.27	19.3
ENSDARG00000043055	hiat1b	hippocampus abundant transcript 1b [Source:ZFIN;Acc:ZDB-GENE-040426-2935]	22	10713713	10735248		5.55	3.53	2.77	8.68	8.34	8.33
ENSDARG00000043066	nog2	noggin 2 [Source:NCBI gene;Acc:30185]	24	38814734	38816725		7.08	5.47	4.69	10.45	10.88	10.95
ENSDARG00000043074	grp	gastrin-releasing peptide [Source:ZFIN;Acc:ZDB-GENE-060526-117]	21	10765886	10773344		4.87	6.2	6.84	5.15	4.78	3.5
ENSDARG00000043077	nisch	nischarin [Source:ZFIN;Acc:ZDB-GENE-050208-570]	22	10361170	10397680		6.14	4.09	3.79	6.08	5.99	6.15
ENSDARG00000043079	MMP23B	matrix metallopeptidase 23ba [Source:NCBI gene;Acc:101882601]	8	53928472	53946034	MMP23B	3.68	3.65	3.21	3.2	3.13	3.42
ENSDARG00000043081	ctsz	cathepsin Z [Source:ZFIN;Acc:ZDB-GENE-041010-139]	6	49771372	49781362		33.84	41	39.78	23.39	22.82	24.96
ENSDARG00000043084	abhd17ab	abhydrolase domain containing 17Ab [Source:ZFIN;Acc:ZDB-GENE-081105-136]	8	19232238	19246342		3.41	2.62	1.78	6.19	5.08	4.99
ENSDARG00000043085	alox5b.1	arachidonate 5-lipoxygenase b, tandem duplicate 1 [Source:ZFIN;Acc:ZDB-GENE-131219-3]	15	942531	952891		0.13	0.11	0.08	0.13	0.31	0.36
ENSDARG00000043089	alox5b.2	arachidonate 5-lipoxygenase b, tandem duplicate 2 [Source:ZFIN;Acc:ZDB-GENE-060623-19]	15	963292	976353		0	0.04	0.05	0.35	0.29	0.49
ENSDARG00000043093	mpeg1.2	macrophage expressed 1, tandem duplicate 2 [Source:ZFIN;Acc:ZDB-GENE-050522-305]	8	19356072	19359834		3.5	2.87	2	14.6	12.15	10.66
ENSDARG00000043094	gnas	GNAS complex locus [Source:ZFIN;Acc:ZDB-GENE-090417-2]	6	49811831	49873020		10.36	5.89	5.69	13.89	13.19	13.15
ENSDARG00000043095	kctd6a	potassium channel tetramerization domain containing 6a [Source:ZFIN;Acc:ZDB-GENE-050208-474]	22	10214994	10220609		10.37	5.58	6.99	12.79	11.9	13.08
ENSDARG00000043102	lxn	latexin [Source:ZFIN;Acc:ZDB-GENE-041212-18]	15	1179876	1198886		23.41	28.4	34.27	22.06	27.21	26
ENSDARG00000043105	qtrt1	queuine tRNA-ribosyltransferase 1 [Source:NCBI gene;Acc:393985]	3	47114328	47128096		5.67	4.95	5.14	4.27	4.8	4.31
ENSDARG00000043126	blf	bloody fingers [Source:ZFIN;Acc:ZDB-GENE-050721-1]	22	9917007	9923692		1.82	2.46	2.22	3.38	3.21	2.98
ENSDARG00000043128	cldne	claudin e [Source:ZFIN;Acc:ZDB-GENE-010328-5]	15	2638884	2640966		62.31	48.86	38.59	47.24	43.32	37.34
ENSDARG00000043130	notch2	notch receptor 2 [Source:ZFIN;Acc:ZDB-GENE-000329-4]	8	19548985	19619792		8.99	8.92	6.11	14.32	13.47	13.31
ENSDARG00000043131	BX664625.1	natural killer cell receptor 2B4-like [Source:NCBI gene;Acc:100007935]	22	9751117	9755111		0.24	0.15	0.13	0.2	0.32	0.32
ENSDARG00000043133	cldnf	claudin f [Source:ZFIN;Acc:ZDB-GENE-010328-6]	15	2650787	2652640		5.13	4.29	4.13	6.46	5.85	5.27
ENSDARG00000043134	cux1b	cut-like homeobox 1b [Source:ZFIN;Acc:ZDB-GENE-040801-260]	15	2520319	2543834		11.48	9.3	11.74	9.49	10.01	9.32
ENSDARG00000043135	pomca	proopiomelanocortin a [Source:ZFIN;Acc:ZDB-GENE-030513-2]	17	33226955	33230039		5.4	1.26	2.57	6.74	7.05	6.49
ENSDARG00000043137	cdca8	cell division cycle associated 8 [Source:NCBI gene;Acc:492815]	19	47771659	47782916		4.26	4.39	4.69	3.15	2.95	2.8
ENSDARG00000043141	si:ch211-132f19.7	si:ch211-132f19.7 [Source:ZFIN;Acc:ZDB-GENE-130530-619]	17	33311784	33339626		3.1	0.5	0.4	2.11	2.27	2.09
ENSDARG00000043142	or108-3	odorant receptor, family D, subfamily 108, member 3 [Source:ZFIN;Acc:ZDB-GENE-070806-66]	10	36178713	36180091		0.06	0.02	0.07	0.02	0	0.04
ENSDARG00000043148	slc1a3b	solute carrier family 1 member 3b [Source:ZFIN;Acc:ZDB-GENE-090708-3]	10	36558804	36592486		18.61	7.04	10.78	27.42	27.28	28.37
ENSDARG00000043154	ucp2	uncoupling protein 2 [Source:NCBI gene;Acc:555812]	10	36662640	36673388		46.28	62.98	76.52	81.43	89.05	83.96
ENSDARG00000043157	dnajb13	DnaJ heat shock protein family (Hsp40) member B13 [Source:ZFIN;Acc:ZDB-GENE-040910-4]	10	36673387	36682522		0.86	0.87	0.77	0.54	0.76	0.73
ENSDARG00000043168	cela1.5	chymotrypsin like elastase family member 1, tandem duplicate 5 [Source:ZFIN;Acc:ZDB-GENE-040801-186]	22	7497319	7508894		0	0	0.02	0.13	0.06	0.05
ENSDARG00000043171	cela1.4	chymotrypsin like elastase family member 1, tandem duplicate 4 [Source:ZFIN;Acc:ZDB-GENE-050626-127]	22	7486867	7492548		17.5	24.41	17.8	6.66	8.24	7.51
ENSDARG00000043173	cela1.3	chymotrypsin like elastase family member 1, tandem duplicate 3 [Source:ZFIN;Acc:ZDB-GENE-040801-12]	22	7480465	7483755		0.42	0.77	0.95	0.05	0.11	0.11
ENSDARG00000043175	cela1.1	chymotrypsin like elastase family member 1, tandem duplicate 1 [Source:ZFIN;Acc:ZDB-GENE-050522-187]	22	7462997	7470338		1.9	3.14	3.35	0.49	0.73	0.53
ENSDARG00000043177	lsm1	LSM1, U6 small nuclear RNA associated [Source:ZFIN;Acc:ZDB-GENE-040801-68]	8	44613135	44618213		5.84	8.21	6.93	6.39	5.93	7.61
ENSDARG00000043179	bag4	BCL2 associated athanogene 4 [Source:ZFIN;Acc:ZDB-GENE-030131-5484]	8	44599405	44611357		7.23	5.15	3.61	7.63	6.53	7.46
ENSDARG00000043180	gpd1b	glycerol-3-phosphate dehydrogenase 1b [Source:ZFIN;Acc:ZDB-GENE-030131-3906]	22	6996046	7006974		63.21	95.49	103.52	41.53	42.46	45.76
ENSDARG00000043182	hhla2a.1	HERV-H LTR-associating 2a, tandem duplicate 1 [Source:ZFIN;Acc:ZDB-GENE-050327-45]	10	35417099	35437624		14.3	16.41	14.5	18.07	18.8	19.09
ENSDARG00000043185	mllt1a	MLLT1 super elongation complex subunit a [Source:ZFIN;Acc:ZDB-GENE-081105-45]	8	20194155	20230802		3.72	2.45	2.15	5.5	5.23	5.79
ENSDARG00000043196	rnasel2	ribonuclease like 2 [Source:ZFIN;Acc:ZDB-GENE-050208-420]	22	6293563	6294749		0.07	0.26	0.02	0.02	0.08	0.03
ENSDARG00000043198	si:rp71-1i20.2	si:rp71-1i20.2 [Source:ZFIN;Acc:ZDB-GENE-040724-207]	22	6393227	6404910		0.13	0.07	0.03	0.25	0.42	0.34
ENSDARG00000043209	nsun5	NOP2/Sun RNA methyltransferase 5 [Source:ZFIN;Acc:ZDB-GENE-040426-2476]	10	35152928	35163612		6.04	6.58	5.24	4.7	5.62	5.29
ENSDARG00000043210	nfic	nuclear factor I/C [Source:ZFIN;Acc:ZDB-GENE-080305-2]	8	20624510	20795631		1.01	0.72	0.69	1.9	1.48	1.46
ENSDARG00000043211	ripk4	receptor-interacting serine-threonine kinase 4 [Source:ZFIN;Acc:ZDB-GENE-040426-2042]	10	35114153	35153388		2.68	1.87	1.27	2.46	2.43	2.04
ENSDARG00000043213	adam17a	ADAM metallopeptidase domain 17a [Source:ZFIN;Acc:ZDB-GENE-030131-2862]	17	35247497	35279422		11.13	8.23	7.16	11.42	11.05	10.68
ENSDARG00000043220	kcnh5a	potassium voltage-gated channel, subfamily H (eag-related), member 5a [Source:ZFIN;Acc:ZDB-GENE-061106-2]	20	19964854	20182871		0.24	0.04	0.07	0.49	0.44	0.66
ENSDARG00000043226	nfixa	nuclear factor I/Xa [Source:ZFIN;Acc:ZDB-GENE-080722-15]	1	51896147	52086496		1.86	0.48	0.62	2.04	2.2	2.03
ENSDARG00000043232	CT573139.1		10	34937548	34951865		1.35	0.63	0.88	2.36	2.28	1.77
ENSDARG00000043236	ccna1	cyclin A1 [Source:ZFIN;Acc:ZDB-GENE-040311-2]	10	34905980	34916208		0.02	0.02	0	0.05	0.05	0.07
ENSDARG00000043238	atg16l2	ATG16 autophagy related 16-like 2 (S. cerevisiae) [Source:ZFIN;Acc:ZDB-GENE-060503-197]	15	5542068	5563551		1.08	0.43	0.4	0.96	1.27	1.59
ENSDARG00000043241	arrb1	arrestin, beta 1 [Source:ZFIN;Acc:ZDB-GENE-060824-1]	15	5390734	5467477		8.15	2.67	3.48	8.61	8.75	8.43
ENSDARG00000043242	si:dkey-222f2.1	si:dkey-222f2.1 [Source:ZFIN;Acc:ZDB-GENE-050208-726]	22	5727753	5739360		37.88	42.5	49.37	14.86	15.83	16.44
ENSDARG00000043243	prkchb	protein kinase C, eta, b [Source:ZFIN;Acc:ZDB-GENE-041001-104]	20	20360337	20410127		0.85	0.94	0.65	1.22	1.1	0.85
ENSDARG00000043247	gtf3c3	general transcription factor IIIC, polypeptide 3 [Source:ZFIN;Acc:ZDB-GENE-040914-80]	1	6243906	6272146		6.34	3.91	5.05	5.22	5.06	5.16
ENSDARG00000043249	irf1b	interferon regulatory factor 1b [Source:ZFIN;Acc:ZDB-GENE-041114-13]	21	45626136	45633317		1.2	1.13	1.12	1.82	1.94	1.94
ENSDARG00000043250	ppm1ab	protein phosphatase, Mg2+/Mn2+ dependent, 1Ab [Source:ZFIN;Acc:ZDB-GENE-991102-14]	20	20600971	20611063		2.93	1.35	1.47	4.69	4.91	4.41
ENSDARG00000043257	ckbb	creatine kinase, brain b [Source:ZFIN;Acc:ZDB-GENE-020103-2]	20	20816747	20821789		496.94	201.72	184.66	357.08	362.93	386.47
ENSDARG00000043260	xylb	xylulokinase homolog (H. influenzae) [Source:ZFIN;Acc:ZDB-GENE-040426-1373]	24	41233755	41267184		3.83	5.01	3.94	3.5	3.17	3.17
ENSDARG00000043271	morc3b	MORC family CW-type zinc finger 3b [Source:ZFIN;Acc:ZDB-GENE-040801-98]	1	47178529	47202163		0.03	0.04	0.11	0.09	0.05	0.08
ENSDARG00000043275	mgmt	O-6-methylguanine-DNA methyltransferase [Source:ZFIN;Acc:ZDB-GENE-120614-1]	17	20214213	20218391		6.36	6.07	6.6	3.19	2.93	3.29
ENSDARG00000043279	aqp12	aquaporin 12 [Source:ZFIN;Acc:ZDB-GENE-040718-310]	22	2874838	2886937		78.89	106.52	101.46	44.34	47.25	49.1
ENSDARG00000043281	stap2b	signal transducing adaptor family member 2b [Source:ZFIN;Acc:ZDB-GENE-040426-2540]	8	18650414	18667693		6.25	6.98	6.37	7.2	6.45	5.94
ENSDARG00000043283	prrg1	proline rich Gla (G-carboxyglutamic acid) 1 [Source:ZFIN;Acc:ZDB-GENE-041010-20]	8	18588551	18601241		3.53	1.84	2.33	4.78	5.13	5.27
ENSDARG00000043285	zbtb21	zinc finger and BTB domain containing 21 [Source:ZFIN;Acc:ZDB-GENE-050411-7]	10	32646402	32648803		2.96	1.69	1.99	4.98	4.94	5.06
ENSDARG00000043287	atg101	autophagy related 101 [Source:NCBI gene;Acc:567680]	10	32657496	32663759		15.51	13.97	17.54	11.88	12.54	13.22
ENSDARG00000043288	impact	impact RWD domain protein [Source:NCBI gene;Acc:449553]	22	2959879	3026838		11.41	12.98	14.29	6.68	6.43	6.68
ENSDARG00000043299	nmu	neuromedin U [Source:ZFIN;Acc:ZDB-GENE-041001-111]	20	22045089	22062715		0.8	0.7	0.72	0.47	0.56	0.68
ENSDARG00000043301	gtf3c6	general transcription factor IIIC, polypeptide 6, alpha [Source:ZFIN;Acc:ZDB-GENE-040426-1009]	16	17252487	17253389		8.09	7.26	8.35	5.07	5.02	4.8
ENSDARG00000043304	nop2	NOP2 nucleolar protein homolog (yeast) [Source:ZFIN;Acc:ZDB-GENE-050309-7]	16	17234540	17251898		17.33	20.71	15.72	11.51	12.45	11.25
ENSDARG00000043307	srd5a3	steroid 5 alpha-reductase 3 [Source:NCBI gene;Acc:560717]	20	22194318	22198982		3.56	5.17	4.68	3.89	3.93	3.49
ENSDARG00000043309	obscna	obscurin, cytoskeletal calmodulin and titin-interacting RhoGEF a [Source:ZFIN;Acc:ZDB-GENE-040812-4]	8	18369177	18398787		1.55	1.25	1.11	1.83	2.01	1.86
ENSDARG00000043313	ank2b	ankyrin 2b, neuronal [Source:ZFIN;Acc:ZDB-GENE-041010-165]	7	57886148	58130703		22.47	4.89	7.98	31.22	30.41	32.96
ENSDARG00000043317	kita	KIT proto-oncogene, receptor tyrosine kinase a [Source:NCBI gene;Acc:30256]	20	22335511	22378401		4.07	3.34	3.15	3.84	3.47	3.38
ENSDARG00000043322	gsx2	GS homeobox 2 [Source:ZFIN;Acc:ZDB-GENE-041001-114]	20	22483048	22484621		2.59	0.63	1.13	2.05	1.82	2.51
ENSDARG00000043323	lnx1	ligand of numb-protein X 1 [Source:ZFIN;Acc:ZDB-GENE-030131-9439]	20	22666548	22740675		12.8	6.89	5.49	15.28	15.83	16.63
ENSDARG00000043328	fip1l1a	FIP1 like 1a (S. cerevisiae) [Source:ZFIN;Acc:ZDB-GENE-041014-8]	20	22739792	22798794		0.68	0.63	0.42	0.73	0.83	1.03
ENSDARG00000043332	prkcz	protein kinase C, zeta [Source:ZFIN;Acc:ZDB-GENE-070511-1]	8	17554313	17771755		8.44	2.94	3.6	9.23	10.36	9.79
ENSDARG00000043334	ccdc6a	coiled-coil domain containing 6a [Source:ZFIN;Acc:ZDB-GENE-030131-7848]	17	20673963	20695998		20.63	16.68	14.98	18.17	18.59	18.87
ENSDARG00000043339	abraxas1	abraxas 1, BRCA1 A complex subunit [Source:NCBI gene;Acc:449820]	21	19319804	19329076		3.05	3.37	3.34	2.33	2.93	2.77
ENSDARG00000043341	npy4r	neuropeptide Y receptor Y4 [Source:ZFIN;Acc:ZDB-GENE-980526-393]	17	20639180	20640505		0.21	0.12	0.14	0.14	0.28	0.24
ENSDARG00000043342	gpx3	glutathione peroxidase 3 [Source:ZFIN;Acc:ZDB-GENE-070222-3]	14	49439887	49460409		3.28	3.39	3	5.74	6.5	6.18
ENSDARG00000043357	tbl2	transducin (beta)-like 2 [Source:ZFIN;Acc:ZDB-GENE-030131-9012]	10	33253952	33265566		8.12	7.65	6.57	7.31	7.65	7.59
ENSDARG00000043359	rmnd5b	required for meiotic nuclear division 5 homolog B [Source:ZFIN;Acc:ZDB-GENE-040426-1744]	14	49220026	49245819		18.14	13.18	14.68	18.92	19.85	19.49
ENSDARG00000043360	dph3	diphthamide biosynthesis 3 [Source:ZFIN;Acc:ZDB-GENE-050522-545]	6	15127507	15131196		2.24	2.98	3.26	1.9	2.44	2.22
ENSDARG00000043361	nck2b	NCK adaptor protein 2b [Source:ZFIN;Acc:ZDB-GENE-040801-235]	6	15145123	15246534		2.83	2.74	2.55	2.56	2.52	2.66
ENSDARG00000043371	mdh2	malate dehydrogenase 2, NAD (mitochondrial) [Source:ZFIN;Acc:ZDB-GENE-040426-2143]	10	33382858	33394568		140.37	172.4	182.2	105.7	105.16	104.82
ENSDARG00000043388	atraid	all-trans retinoic acid-induced differentiation factor [Source:NCBI gene;Acc:553468]	20	19512727	19516640		6.28	4.66	4.78	5.15	4.66	5.45
ENSDARG00000043394	si:dkey-71h2.2	si:dkey-71h2.2 [Source:ZFIN;Acc:ZDB-GENE-041014-145]	20	19276932	19366022		7.67	6.91	5.7	3.95	4.26	4.31
ENSDARG00000043395	crip3	cysteine-rich protein 3 [Source:ZFIN;Acc:ZDB-GENE-130530-620]	17	21964472	21982835		6.4	7.36	5.9	7.48	7.03	7.05
ENSDARG00000043396	fndc4a	fibronectin type III domain containing 4a [Source:ZFIN;Acc:ZDB-GENE-041014-146]	20	19212962	19271872		3.73	1.01	1.42	2.95	3.2	2.87
ENSDARG00000043398	nitr2a	novel immune-type receptor 2a [Source:ZFIN;Acc:ZDB-GENE-020225-28]	7	17433836	17438419		0	0.04	0.05	0.11	0.05	0
ENSDARG00000043402	zgc:171480	zgc:171480 [Source:ZFIN;Acc:ZDB-GENE-080204-83]	8	15251448	15253800		17.94	21.97	18.25	17.21	16.11	15.88
ENSDARG00000043403	paox1	polyamine oxidase (exo-N4-amino) 1 [Source:ZFIN;Acc:ZDB-GENE-081104-436]	8	15239549	15250265		2.74	3.11	3.34	4.63	4.73	4.9
ENSDARG00000043404	rpp21	ribonuclease P 21 subunit [Source:ZFIN;Acc:ZDB-GENE-040801-37]	22	15429099	15437242		3.54	3.93	3.8	2.46	2.88	2.88
ENSDARG00000043406	slc8a1b	solute carrier family 8 member 1b [Source:ZFIN;Acc:ZDB-GENE-060110-3]	17	22311413	22501970		4.01	2.02	1.83	7.39	7.42	6.88
ENSDARG00000043410	xkr6b	XK, Kell blood group complex subunit-related family, member 6b [Source:ZFIN;Acc:ZDB-GENE-040724-153]	20	18801525	18915376		2.58	0.5	0.92	5.48	5.88	5.49
ENSDARG00000043416	tnfaip2a	tumor necrosis factor, alpha-induced protein 2a [Source:ZFIN;Acc:ZDB-GENE-041210-301]	20	18661624	18685352		5.97	5.1	5.77	7.4	6.56	7.5
ENSDARG00000043417	eipr1	EARP complex and GARP complex interacting protein 1 [Source:NCBI gene;Acc:450045]	17	51262556	51317306		2.31	1.89	1.85	1.9	1.97	2.02
ENSDARG00000043431	b3gntl1	UDP-GlcNAc:betaGal beta-1,3-N-acetylglucosaminyltransferase-like 1 [Source:ZFIN;Acc:ZDB-GENE-050913-133]	1	57347888	57365052		4.08	4.41	4.46	2.57	2.6	2.68
ENSDARG00000043436	si:dkey-5n18.1	si:dkey-5n18.1 [Source:ZFIN;Acc:ZDB-GENE-041014-144]	20	17038903	17041025		8.28	11.08	14.61	12.65	10.56	14.46
ENSDARG00000043442	zgc:153665	zgc:153665 [Source:ZFIN;Acc:ZDB-GENE-061027-365]	11	30626768	30636163		3.17	3.4	3.42	1.42	1.12	1.38
ENSDARG00000043443	kcnj13	potassium inwardly rectifying channel subfamily J member 13 [Source:ZFIN;Acc:ZDB-GENE-070129-1]	15	40217256	40267485		2.58	1.96	1.76	2.04	1.93	1.88
ENSDARG00000043445	si:ch211-152f2.3	si:ch211-152f2.3 [Source:ZFIN;Acc:ZDB-GENE-121214-85]	1	56900580	56904810		0	0.02	0	0	0	0
ENSDARG00000043446	efhd1	EF-hand domain family, member D1 [Source:ZFIN;Acc:ZDB-GENE-030131-7461]	15	40188076	40212204		20.43	14.4	15.93	17.73	19.68	20.23
ENSDARG00000043447	gpr55a	G protein-coupled receptor 55a [Source:ZFIN;Acc:ZDB-GENE-051113-260]	15	40051218	40065296		0.28	0.27	0.17	0.15	0.18	0.07
ENSDARG00000043448	itm2ca	integral membrane protein 2Ca [Source:ZFIN;Acc:ZDB-GENE-040718-284]	15	40008370	40028883		37.03	11.66	18.88	49.41	54.64	52.94
ENSDARG00000043451	cab39	calcium binding protein 39 [Source:ZFIN;Acc:ZDB-GENE-040625-158]	15	39977461	40002930		5.78	3.64	3.56	6.02	6.14	6.35
ENSDARG00000043453	rps5	ribosomal protein S5 [Source:ZFIN;Acc:ZDB-GENE-020419-12]	15	39964943	39971756		1285.04	1563	1953.03	1006.31	1055.9	1065.67
ENSDARG00000043457	gapdh	glyceraldehyde-3-phosphate dehydrogenase [Source:NCBI gene;Acc:317743]	16	17189839	17200120		390.63	572.52	652.84	367.6	399.01	382.41
ENSDARG00000043460	wu:fj39g12	wu:fj39g12 [Source:ZFIN;Acc:ZDB-GENE-030131-7901]	16	17204251	17207754		10.22	7.08	8.46	5.94	6.14	5.99
ENSDARG00000043465	atp6v1b2	ATPase H+ transporting V1 subunit B2 [Source:ZFIN;Acc:ZDB-GENE-030711-4]	10	19554604	19566474		45.36	22.48	33.22	45.98	46.87	48.39
ENSDARG00000043466	kcnk3a	potassium channel, subfamily K, member 3a [Source:ZFIN;Acc:ZDB-GENE-040724-171]	20	13360656	13394780		1.45	1.18	0.92	1.54	2.02	1.24
ENSDARG00000043467	ndufb11	NADH:ubiquinone oxidoreductase subunit B11 [Source:ZFIN;Acc:ZDB-GENE-050309-25]	8	14180064	14184423		24.48	30.37	25.5	15.17	13.65	13.42
ENSDARG00000043468	sytl3	synaptotagmin-like 3 [Source:ZFIN;Acc:ZDB-GENE-040724-150]	20	13533544	13565720		0.1	0.11	0.11	0.19	0.12	0.13
ENSDARG00000043470	rsph3	radial spoke head 3 [Source:ZFIN;Acc:ZDB-GENE-040724-173]	20	13632526	13640598		0.97	0.86	0.93	1.49	1.54	1.5
ENSDARG00000043474	atp2b3a	ATPase plasma membrane Ca2+ transporting 3a [Source:ZFIN;Acc:ZDB-GENE-040718-174]	8	14003232	14052349		24.96	5.44	7.2	27.63	28.46	29.12
ENSDARG00000043475	TAGAP	si:ch211-122h15.4 [Source:ZFIN;Acc:ZDB-GENE-121214-340]	20	13647987	13673566	TAGAP	0.89	0.54	0.45	1.19	1.07	1.03
ENSDARG00000043479	opn8c	opsin 8, group member c [Source:ZFIN;Acc:ZDB-GENE-040724-51]	20	13750537	13774826		0.01	0	0.01	0.03	0.04	0.06
ENSDARG00000043480	rbbp8	retinoblastoma binding protein 8 [Source:NCBI gene;Acc:503536]	24	36204028	36223943		1.74	1.28	1.26	1.42	1.27	1.17
ENSDARG00000043482	b3gnt3.1	UDP-GlcNAc:betaGal beta-1,3-N-acetylglucosaminyltransferase 3, tandem duplicate 1 [Source:ZFIN;Acc:ZDB-GENE-040625-129]	8	13534385	13541514		2.95	3.07	2.41	1.21	0.65	0.98
ENSDARG00000043483	otx5	orthodenticle homolog 5 [Source:ZFIN;Acc:ZDB-GENE-030508-1]	15	47903864	47909281		22.83	4.65	3.56	29.01	27.12	33.26
ENSDARG00000043484	ube2d2	ubiquitin-conjugating enzyme E2D 2 (UBC4/5 homolog, yeast) [Source:ZFIN;Acc:ZDB-GENE-120312-1]	14	7892021	7901635		17.2	12.65	10.39	28.42	27.89	24.85
ENSDARG00000043485	si:ch211-22i13.2	si:ch211-22i13.2 [Source:ZFIN;Acc:ZDB-GENE-041014-143]	20	14003060	14012859		20.52	17.46	16.98	14	14.89	15.97
ENSDARG00000043492	irf1a	interferon regulatory factor 1a [Source:ZFIN;Acc:ZDB-GENE-060421-4031]	13	311380	318827		0.46	0.42	0.41	0.47	0.69	0.38
ENSDARG00000043493	cltca	clathrin, heavy chain a (Hc) [Source:ZFIN;Acc:ZDB-GENE-030131-2299]	10	28311031	28368913		31.66	22.59	17.02	56.24	52.47	54.15
ENSDARG00000043497	scrn2	secernin 2 [Source:NCBI gene;Acc:558306]	20	14632492	14680917		7.82	3.89	4.51	6.47	6.82	6.49
ENSDARG00000043502	ccdc124	coiled-coil domain containing 124 [Source:NCBI gene;Acc:393537]	8	13351698	13364879		51.15	56.42	58.57	37.02	39.29	37.9
ENSDARG00000043509	rpl11	ribosomal protein L11 [Source:ZFIN;Acc:ZDB-GENE-040625-147]	19	43780970	43786940		1839.29	2370.88	3175.68	1161.86	1249.13	1218.93
ENSDARG00000043510	vamp4	vesicle-associated membrane protein 4 [Source:ZFIN;Acc:ZDB-GENE-040625-92]	20	14968031	15006337		7.23	4.18	5	7.79	8.41	8
ENSDARG00000043511	prdx6	peroxiredoxin 6 [Source:ZFIN;Acc:ZDB-GENE-040426-1778]	20	15164482	15184869		46.02	56.03	52.49	34.1	30.82	30.92
ENSDARG00000043514	si:dkey-239i20.4	si:dkey-239i20.4 [Source:ZFIN;Acc:ZDB-GENE-030131-2644]	20	15114957	15134513		1.79	1.05	0.8	2.62	2.87	2.8

ENSDARG00000043518	si:dkey-239i20.2	si:dkey-239i20.2 [Source:ZFIN;Acc:ZDB-GENE-041001-98]	20	15083987	15090862		1.98	3.41	2.15	0.46	0.35	0.53
ENSDARG00000043527	plpp6	phospholipid phosphatase 6 [Source:NCBI gene;Acc:553687]	20	15142972	15161669		2.16	1.3	0.96	2.44	2.71	2.25
ENSDARG00000043531	jun	Jun proto-oncogene, AP-1 transcription factor subunit [Source:ZFIN;Acc:ZDB-GENE-030131-7859]	20	15552657	15554606		42.03	38.85	37.95	78.63	84.92	82.81
ENSDARG00000043536	si:dkey-86e18.1	si:dkey-86e18.1 [Source:ZFIN;Acc:ZDB-GENE-041014-18]	20	15565926	15578475		5.57	4.61	4.68	3.39	3.96	3.09
ENSDARG00000043542	zpr1	ZPR1 zinc finger [Source:ZFIN;Acc:ZDB-GENE-040426-2110]	10	29820172	29831944		13.76	15.03	13.56	9.79	9.54	9.33
ENSDARG00000043548	atg4da	autophagy related 4D, cysteine peptidase a [Source:ZFIN;Acc:ZDB-GENE-041111-102]	1	51386649	51406531		4.68	3.12	3.23	4.9	4.51	4.47
ENSDARG00000043550	ENKD1	si:ch211-220d9.3 [Source:ZFIN;Acc:ZDB-GENE-081104-180]	8	2438638	2454525	ENKD1	2.49	0.9	0.88	2.05	2.53	2.47
ENSDARG00000043553	ches1	checkpoint suppressor 1 [Source:ZFIN;Acc:ZDB-GENE-040217-1]	20	16477570	16548912		2.75	1.52	1.84	4.63	4.42	4.6
ENSDARG00000043554	mrpl40	mitochondrial ribosomal protein L40 [Source:ZFIN;Acc:ZDB-GENE-040912-37]	8	2530065	2535826		24.14	28.19	29.26	16.87	17.19	17.45
ENSDARG00000043555	tmem30ab	transmembrane protein 30Ab [Source:ZFIN;Acc:ZDB-GENE-030131-5884]	20	16639848	16660851		21.8	16.63	16.09	20.19	23.35	23.7
ENSDARG00000043557	kcnk13b	potassium channel, subfamily K, member 13b [Source:ZFIN;Acc:ZDB-GENE-040724-105]	20	16703050	16709138		0.56	0.11	0.21	0.23	0.27	0.26
ENSDARG00000043559	gle1	GLE1 RNA export mediator [Source:NCBI gene;Acc:445408]	8	2543525	2574781		5.73	4.51	4.2	5.52	5.48	5.24
ENSDARG00000043561	psmc1b	proteasome 26S subunit, ATPase 1b [Source:ZFIN;Acc:ZDB-GENE-040625-69]	20	16721933	16740957		47.16	48.11	50.37	30.02	32.35	31.79
ENSDARG00000043562	zgc:65997	zgc:65997 [Source:ZFIN;Acc:ZDB-GENE-040426-1502]	17	23549117	23552068		5.44	5.34	5.57	2.95	2.78	3.26
ENSDARG00000043566	nepro	nucleolus and neural progenitor protein [Source:NCBI gene;Acc:550343]	9	27381318	27391908		2.51	2.19	2.5	1.93	1.7	1.63
ENSDARG00000043569	dram2a	DNA-damage regulated autophagy modulator 2a [Source:ZFIN;Acc:ZDB-GENE-081104-360]	8	12975963	12989137		0.32	0.73	0.82	0.5	0.46	0.46
ENSDARG00000043571	KIF2A	zgc:103670 [Source:ZFIN;Acc:ZDB-GENE-040912-111]	8	12925385	12948930	KIF2A	14.41	8.97	9.67	13.19	14.08	12.97
ENSDARG00000043581	gadd45aa	growth arrest and DNA-damage-inducible, alpha, a [Source:ZFIN;Acc:ZDB-GENE-040426-1501]	2	10280645	10284572		5.22	5.8	4.67	3.43	4.33	4.05
ENSDARG00000043584	ifit15	interferon-induced protein with tetratricopeptide repeats 15 [Source:ZFIN;Acc:ZDB-GENE-030131-1055]	17	23607195	23609210		0.05	0.15	0.21	0.01	0	0.03
ENSDARG00000043586	fas	Fas cell surface death receptor [Source:ZFIN;Acc:ZDB-GENE-061019-2]	17	23619924	23631411		0.73	0.55	0.5	0.93	0.75	1.44
ENSDARG00000043587	srd5a2a	steroid-5-alpha-reductase, alpha polypeptide 2a [Source:ZFIN;Acc:ZDB-GENE-050417-199]	1	51066671	51073128		13.15	21.92	17.11	3.69	3.54	2.97
ENSDARG00000043589	ca4a	carbonic anhydrase IV a [Source:NCBI gene;Acc:555196]	5	2241723	2282256		6.23	4.33	3.85	11.26	11.54	12.35
ENSDARG00000043593	rapgef1a	Rap guanine nucleotide exchange factor (GEF) 1a [Source:ZFIN;Acc:ZDB-GENE-081105-26]	8	11779547	11835607		1.57	0.84	0.73	2.62	2.73	3.01
ENSDARG00000043608	eif4ebp1	eukaryotic translation initiation factor 4E binding protein 1 [Source:ZFIN;Acc:ZDB-GENE-030131-2332]	21	21013147	21020708		59.65	66.13	62.56	50.54	47.4	49
ENSDARG00000043624	slc66a1	solute carrier family 66 member 1 [Source:ZFIN;Acc:ZDB-GENE-050306-4]	8	10937523	10949847		10.57	14.71	15.69	16.66	18.4	18.66
ENSDARG00000043627	tbc1d9	TBC1 domain family, member 9 (with GRAM domain) [Source:ZFIN;Acc:ZDB-GENE-060810-33]	14	1365097	1392173		3.54	2.38	2.1	3.94	4.05	4.22
ENSDARG00000043631	bcas2	BCAS2 pre-mRNA processing factor [Source:NCBI gene;Acc:493614]	8	11008763	11016766		43.84	52.83	51.56	34.52	33.17	36.54
ENSDARG00000043635	cmc2	C-x(9)-C motif containing 2 [Source:NCBI gene;Acc:368857]	18	29885190	29898369		3.15	3.28	3.14	2.26	2.55	2.29
ENSDARG00000043640	cenpn	centromere protein N [Source:ZFIN;Acc:ZDB-GENE-030616-408]	18	29898955	29906010		2.28	2.48	2.23	1.44	1.59	1.21
ENSDARG00000043643	ehbp1	EH domain binding protein 1 [Source:ZFIN;Acc:ZDB-GENE-091006-4]	17	24109012	24315315		6.25	4.19	3.62	8.38	7.82	8.03
ENSDARG00000043644	vkorc1l1	vitamin K epoxide reductase complex, subunit 1-like 1 [Source:NCBI gene;Acc:553717]	5	2002804	2047801		6.62	6.04	5.1	9.44	10.41	8.31
ENSDARG00000043646	slc6a8	solute carrier family 6 member 8 [Source:ZFIN;Acc:ZDB-GENE-071024-2]	8	9052123	9118958		12.05	10.94	8.98	35.93	37.91	35.41
ENSDARG00000043648	pnck	pregnancy up-regulated nonubiquitous CaM kinase [Source:ZFIN;Acc:ZDB-GENE-081105-127]	8	9149436	9167115		1	0.53	0.63	0.64	0.6	0.78
ENSDARG00000043658	cxadr	CXADR Ig-like cell adhesion molecule [Source:NCBI gene;Acc:791793]	10	27096043	27158240		19.73	16.48	11.49	19.44	19.01	18.13
ENSDARG00000043661	cadpsa	Ca2+-dependent activator protein for secretion a [Source:ZFIN;Acc:ZDB-GENE-030616-525]	11	19780717	20040154		4.08	0.66	1.03	5.32	5.25	6
ENSDARG00000043662	cnih2	cornichon family AMPA receptor auxiliary protein 2 [Source:NCBI gene;Acc:541397]	10	27028019	27049170		12.53	2.71	4.41	10.43	10.76	11.22
ENSDARG00000043663	faub	FAU ubiquitin like and ribosomal protein S30 fusion b [Source:ZFIN;Acc:ZDB-GENE-050417-416]	10	26990095	26992003		24.92	37.26	29.66	13.77	14.79	13.43
ENSDARG00000043664	BDKRB2	si:dkey-63b1.1 [Source:ZFIN;Acc:ZDB-GENE-041210-292]	20	10287061	10288156	BDKRB2	0.19	0.36	0.29	0.56	0.59	0.47
ENSDARG00000043665	glrx5	glutaredoxin 5 homolog (S. cerevisiae) [Source:NCBI gene;Acc:406294]	20	10484015	10487951		33.81	39.28	35.46	24.36	27.13	26.21
ENSDARG00000043666	abt1	activator of basal transcription 1 [Source:ZFIN;Acc:ZDB-GENE-050522-127]	8	8483664	8489886		7.67	8.65	8.26	5.16	5.11	5.67
ENSDARG00000043667	zgc:100997	zgc:100997 [Source:ZFIN;Acc:ZDB-GENE-040801-124]	20	10498704	10520821		1.19	1.02	0.88	1.25	1	1.1
ENSDARG00000043673	si:dkey-174m14.3	si:dkey-174m14.3 [Source:ZFIN;Acc:ZDB-GENE-030131-8158]	20	9355912	9421263		7.71	1.94	2.53	8.2	7.85	7.41
ENSDARG00000043680	ylpm1	YLP motif containing 1 [Source:ZFIN;Acc:ZDB-GENE-030131-2196]	20	9172301	9199721		9.78	6.48	5.26	13.84	12.56	12.8
ENSDARG00000043684	bpnt1	bisphosphate nucleotidase 1 [Source:ZFIN;Acc:ZDB-GENE-040718-46]	20	9128256	9148323		16.18	25.57	19.96	8.04	8.54	8.99
ENSDARG00000043685	nabp1b	nucleic acid binding protein 1b [Source:ZFIN;Acc:ZDB-GENE-050913-74]	6	8698677	8704702		0.02	0.06	0	0.02	0	0.04
ENSDARG00000043687	si:ch211-59d15.9	si:ch211-59d15.9 [Source:ZFIN;Acc:ZDB-GENE-041014-27]	20	9124369	9125663		5.53	4.48	3.59	2.75	2.26	2.82
ENSDARG00000043697	nefmb	neurofilament, medium polypeptide b [Source:ZFIN;Acc:ZDB-GENE-070103-4]	8	6863462	6866890		22.58	19.73	19.14	18.94	18.5	19.21
ENSDARG00000043701	gpd1a	glycerol-3-phosphate dehydrogenase 1a [Source:ZFIN;Acc:ZDB-GENE-050417-209]	8	7033049	7052493		14.3	20.26	22.86	17.3	17.6	18.43
ENSDARG00000043705	mob3c	MOB kinase activator 3C [Source:ZFIN;Acc:ZDB-GENE-040704-32]	11	13159988	13174477		2.2	3.29	2.39	2.57	2.53	2.54
ENSDARG00000043710	parvaa	parvin, alpha a [Source:ZFIN;Acc:ZDB-GENE-040721-2]	25	16312258	16345430		4.71	6.06	5.08	6.04	6.71	6.13
ENSDARG00000043713	asf1bb	anti-silencing function 1Bb histone chaperone [Source:NCBI gene;Acc:792029]	1	55878113	55888970		12.91	9.49	9.23	10.92	10.63	11.53
ENSDARG00000043716	cldn5a	claudin 5a [Source:ZFIN;Acc:ZDB-GENE-040426-2442]	8	4697764	4699320		7.44	5.44	4.98	13.88	14.19	15.08
ENSDARG00000043719	c3a.6	complement component c3a, duplicate 6 [Source:ZFIN;Acc:ZDB-GENE-041212-2]	1	56036644	56080165		10.96	15.26	14.12	14.21	15.2	15.26
ENSDARG00000043720	cdc45	CDC45 cell division cycle 45 homolog (S. cerevisiae) [Source:ZFIN;Acc:ZDB-GENE-040426-2710]	8	4734005	4760723		4.12	4.62	5.12	2.34	2.41	2.17
ENSDARG00000043722	cpa4	carboxypeptidase A4 [Source:ZFIN;Acc:ZDB-GENE-040704-61]	25	16582172	16589461		93.57	137.7	125.94	6.64	7.34	7.19
ENSDARG00000043727	cwc27	CWC27 spliceosome associated cyclophilin [Source:NCBI gene;Acc:394078]	10	11398111	11501424		11.03	10.85	10.98	7.6	6.79	8.17
ENSDARG00000043728	srek1ip1	SREK1-interacting protein 1 [Source:NCBI gene;Acc:553315]	10	11394528	11397590		11.33	11.92	11.83	7.03	7.33	7.72
ENSDARG00000043729	plac8.1	placenta associated 8, tandem duplicate 1 [Source:ZFIN;Acc:ZDB-GENE-050809-124]	10	11382365	11385155		112.3	237.42	168.85	17.74	16.27	16
ENSDARG00000043732	cops4	COP9 constitutive photomorphogenic homolog subunit 4 (Arabidopsis) [Source:NCBI gene;Acc:325592]	10	11355841	11372541		10.08	8.79	9.97	8.88	9.23	8.57
ENSDARG00000043734	trmu	tRNA 5-methylaminomethyl-2-thiouridylate methyltransferase [Source:NCBI gene;Acc:553625]	4	28338642	28353538		4.15	5.11	4.62	6.14	5.85	5.85
ENSDARG00000043736	si:ch211-282j17.8	si:ch211-282j17.8 [Source:ZFIN;Acc:ZDB-GENE-041210-290]	7	3622558	3723144		0.02	0.03	0	0	0.07	0.02
ENSDARG00000043740	efcab11	EF-hand calcium binding domain 11 [Source:NCBI gene;Acc:550510]	17	16423721	16533069		0.75	0.94	0.66	1.41	1.77	1.37
ENSDARG00000043746	nrxn3a	neurexin 3a [Source:NCBI gene;Acc:563598]	17	16906523	17447902		5.5	1.11	1.61	9.3	8.58	9.21
ENSDARG00000043757	ptbp3	polypyrimidine tract binding protein 3 [Source:ZFIN;Acc:ZDB-GENE-091116-1]	10	11146321	11261565		7.45	4.8	4.28	11.63	12.05	11.79
ENSDARG00000043767	si:ch211-282j17.3	si:ch211-282j17.3 [Source:ZFIN;Acc:ZDB-GENE-041210-287]	7	3654800	3657284		0	0	0	0	0	0
ENSDARG00000043768	si:ch211-282j17.2	si:ch211-282j17.2 [Source:ZFIN;Acc:ZDB-GENE-041210-286]	7	3643979	3649414		0.02	0.07	0.08	0.06	0.02	0.04
ENSDARG00000043769	si:ch211-282j17.1	si:ch211-282j17.1 [Source:ZFIN;Acc:ZDB-GENE-041210-285]	7	3632351	3639171		0	0	0	0.12	0	0
ENSDARG00000043770	si:dkey-192d15.2	si:dkey-192d15.2 [Source:ZFIN;Acc:ZDB-GENE-040724-74]	7	3597413	3611842		1.54	1.48	1.36	1.12	0.99	0.96
ENSDARG00000043781	psmb10	proteasome 20S subunit beta 10 [Source:ZFIN;Acc:ZDB-GENE-040718-278]	4	77207693	77218637		1.06	0.98	1.18	0.86	0.88	1.03
ENSDARG00000043792	si:rp71-7i19.2	si:rp71-7i19.2 [Source:ZFIN;Acc:ZDB-GENE-081103-31]	4	30045493	30057608		0.05	0.06	0.03	0.04	0.07	0.03
ENSDARG00000043795	arhgdia	Rho GDP dissociation inhibitor (GDI) alpha [Source:ZFIN;Acc:ZDB-GENE-040426-2816]	3	59899452	59947208		57.66	61.52	50.74	81.49	75.99	73.57
ENSDARG00000043796	ms4a17a.7	membrane-spanning 4-domains, subfamily A, member 17A.7 [Source:ZFIN;Acc:ZDB-GENE-080829-2]	4	76705830	76718322		7.87	9.5	9.73	18.81	20.96	20.76
ENSDARG00000043797	cdc5l	CDC5 cell division cycle 5-like (S. pombe) [Source:ZFIN;Acc:ZDB-GENE-040426-821]	17	5061135	5105922		33.27	24.58	27.99	26.63	27.9	27.63
ENSDARG00000043798	ms4a17a.1	membrane-spanning 4-domains, subfamily A, member 17A.1 [Source:ZFIN;Acc:ZDB-GENE-050417-307]	4	76692834	76701249		0.68	0.47	0.39	1.91	1.54	1.68
ENSDARG00000043799	KLHL29	zmp:0000000619 [Source:ZFIN;Acc:ZDB-GENE-130530-622]	17	4578552	4854851	KLHL29	0.68	0.3	0.4	1.4	1.29	1.32
ENSDARG00000043802	ms4a17a.8	membrane-spanning 4-domains, subfamily A, member 17A.8 [Source:ZFIN;Acc:ZDB-GENE-070705-365]	4	76637548	76655373		4.9	3.76	4.73	10.11	8.83	8.78
ENSDARG00000043806	postna	periostin, osteoblast specific factor a [Source:ZFIN;Acc:ZDB-GENE-091113-23]	10	25762196	25817854		36.13	37.38	29.24	20.41	18.92	17.28
ENSDARG00000043814	st6galnac1.1	ST6 (alpha-N-acetyl-neuraminyl-2,3-beta-galactosyl-1,3)-N-acetylgalactosaminide alpha-2,6-sialyltransferase 1, tandem duplicate 1	12	20678187	20685624		5.72	5.88	3.91	4.7	5.75	5.55
ENSDARG00000043816	st6galnac1.2	ST6 (alpha-N-acetyl-neuraminyl-2,3-beta-galactosyl-1,3)-N-acetylgalactosaminide alpha-2,6-sialyltransferase 1, tandem duplicate 2	12	20691310	20700872		0.15	0.06	0.05	0.29	0.36	0.34
ENSDARG00000043818	CASKIN2	si:ch211-119c20.2 [Source:ZFIN;Acc:ZDB-GENE-041001-137]	12	20699534	20785149	CASKIN2	6.36	3.38	3.24	5.29	4.92	4.69
ENSDARG00000043820	nme2a	NME/NM23 nucleoside diphosphate kinase 2a [Source:ZFIN;Acc:ZDB-GENE-030131-7656]	12	20788243	20796658		27.01	10.99	9.83	12.28	10.84	11.87
ENSDARG00000043821	klf7b	Kruppel-like factor 7b [Source:ZFIN;Acc:ZDB-GENE-041014-171]	9	28341109	28399071		10.09	4.65	5.03	12.93	12.92	12.51
ENSDARG00000043835	rab3ab	RAB3A, member RAS oncogene family, b [Source:ZFIN;Acc:ZDB-GENE-041210-268]	11	6819050	6850593		29.16	6.84	7.84	38.22	39.28	40.18
ENSDARG00000043842	C20H1orf198	si:ch73-111k22.2 [Source:NCBI gene;Acc:568408]	20	3916406	3927624	C20H1orf198	11.48	7.33	6.39	16.13	16.05	15.21
ENSDARG00000043843	akap7	A kinase (PRKA) anchor protein 7 [Source:ZFIN;Acc:ZDB-GENE-040724-128]	20	2460864	2479450		54.04	75.31	71.91	58.03	61.16	60.46
ENSDARG00000043847	tmem244	transmembrane protein 244 [Source:ZFIN;Acc:ZDB-GENE-040724-54]	20	2118356	2134620		4.39	1.57	1.24	4.58	4.66	4.68
ENSDARG00000043848	sod1	superoxide dismutase 1, soluble [Source:NCBI gene;Acc:30553]	10	25694395	25699454		33.77	47.93	41.82	20.69	21.3	20.77
ENSDARG00000043850	ftr54	finTRIM family, member 54 [Source:ZFIN;Acc:ZDB-GENE-070620-19]	10	25819996	25823258		0.11	0.09	0.08	0.13	0.04	0.11
ENSDARG00000043854	ppil4	peptidylprolyl isomerase (cyclophilin)-like 4 [Source:ZFIN;Acc:ZDB-GENE-030131-6251]	20	2982381	3004126		10.02	12.26	12.32	7.82	8.1	8.48
ENSDARG00000043856	amd1	adenosylmethionine decarboxylase 1 [Source:ZFIN;Acc:ZDB-GENE-040426-760]	20	2950005	2978968		35.91	24.9	20.72	28.39	26.85	26.8
ENSDARG00000043857	ufm1	ubiquitin-fold modifier 1 [Source:NCBI gene;Acc:322756]	10	25947946	25962058		21.26	24.87	23.36	17.35	17.19	16.79
ENSDARG00000043858	cdk19	cyclin-dependent kinase 19 [Source:ZFIN;Acc:ZDB-GENE-040724-19]	20	2879700	2949028		3.16	1.56	1.53	3.09	2.75	2.67
ENSDARG00000043864	nptnb	neuroplastin b [Source:ZFIN;Acc:ZDB-GENE-040426-1821]	18	1158197	1228688		15.96	3.47	3.41	15.49	16.06	17.16
ENSDARG00000043865	timm9	translocase of inner mitochondrial membrane 9 homolog [Source:NCBI gene;Acc:282678]	17	11372531	11377195		10.93	12.13	12.69	7.62	8.56	7.86
ENSDARG00000043873	arid4a	AT rich interactive domain 4A (RBP1-like) [Source:ZFIN;Acc:ZDB-GENE-050809-105]	17	11377970	11418553		16.22	9.69	7.94	16.86	16.64	16.54
ENSDARG00000043877	mmachc	metabolism of cobalamin associated C [Source:NCBI gene;Acc:555267]	20	1385674	1393020		2.82	2.83	3.1	1.23	1.47	1.51
ENSDARG00000043884	nup43	nucleoporin 43 [Source:ZFIN;Acc:ZDB-GENE-040426-2533]	20	1316495	1327859		10.15	10.69	10.87	6.46	6.14	5.69
ENSDARG00000043894	si:ch211-214k5.3	si:ch211-214k5.3 [Source:ZFIN;Acc:ZDB-GENE-041210-206]	10	24935765	24937070		0.06	0	0.16	0	0.21	0.07
ENSDARG00000043898	timm10b	translocase of inner mitochondrial membrane 10 homolog B (yeast) [Source:NCBI gene;Acc:550527]	10	24762216	24765988		8.86	10.41	10.87	7.34	6.58	7.27
ENSDARG00000043899	psma6l	proteasome 20S subunit alpha 6, like [Source:ZFIN;Acc:ZDB-GENE-010502-2]	15	47920629	47929455		3.13	2.25	2.58	1.52	1.55	1.6
ENSDARG00000043901	ugt5g2	UDP glucuronosyltransferase 5 family, polypeptide G2 [Source:ZFIN;Acc:ZDB-GENE-081028-66]	10	22082596	22086626		0.01	0	0	0	0.02	0.01
ENSDARG00000043902	gabrr1	gamma-aminobutyric acid (GABA) A receptor, rho 1 [Source:ZFIN;Acc:ZDB-GENE-040724-212]	20	1132092	1151265		7.12	0.61	0.48	4.46	4.38	4.9
ENSDARG00000043904	pnrc1	proline-rich nuclear receptor coactivator 1 [Source:ZFIN;Acc:ZDB-GENE-040724-189]	20	1121458	1125395		6.96	3.25	3.25	5.67	4.68	5.54
ENSDARG00000043905	rnf14	ring finger protein 14 [Source:ZFIN;Acc:ZDB-GENE-040625-65]	21	41837610	41862079		6.75	7.97	8.27	6.44	6.72	6.07
ENSDARG00000043907	fgf11b	fibroblast growth factor 11b [Source:ZFIN;Acc:ZDB-GENE-100316-2]	10	22161585	22249444		3.29	0.71	0.85	3.16	3.28	3.62
ENSDARG00000043912	mei4	meiosis-specific, MEI4 homolog (S. cerevisiae) [Source:NCBI gene;Acc:568897]	20	812012	823276		0.16	0.13	0.04	0.08	0.15	0.08
ENSDARG00000043918	FO904943.1	neuromedin-K receptor-like [Source:NCBI gene;Acc:100333270]	23	43995820	44007014		0.16	0.11	0.14	0.36	0.48	0.67
ENSDARG00000043923	sox9b	SRY-box transcription factor 9b [Source:ZFIN;Acc:ZDB-GENE-001103-2]	3	62521041	62527696		12.11	9.45	7.96	20.44	20.05	19.59
ENSDARG00000043928	gpn1	GPN-loop GTPase 1 [Source:ZFIN;Acc:ZDB-GENE-040801-154]	17	12658411	12675267		10.41	9.74	10.82	7.78	9.39	8.05
ENSDARG00000043932	stmn4l	stathmin-like 4, like [Source:ZFIN;Acc:ZDB-GENE-040718-301]	17	12698532	12711195		9.47	4.46	6.35	16.68	18.82	20.61
ENSDARG00000043933	il17a/f1	interleukin 17a/f1 [Source:ZFIN;Acc:ZDB-GENE-061031-3]	17	12710430	12712776		0	0	0.05	0	0	0
ENSDARG00000043934	il17a/f2	interleukin 17a/f2 [Source:ZFIN;Acc:ZDB-GENE-061031-2]	17	12730025	12733858		0	0	0	0	0.03	0
ENSDARG00000043938	lrrc57	leucine rich repeat containing 57 [Source:NCBI gene;Acc:436900]	17	12737389	12747835		10.24	10.47	8.24	11.81	11.77	11.13
ENSDARG00000043960	rpf2	ribosome production factor 2 homolog [Source:ZFIN;Acc:ZDB-GENE-040426-2501]	20	150890	154989		24.97	26.63	28.2	15.55	15.69	15.5
ENSDARG00000043962	fgf18b	fibroblast growth factor 18b [Source:ZFIN;Acc:ZDB-GENE-050221-4]	10	21471316	21484065		0.43	0.26	0.27	0.68	0.77	0.56
ENSDARG00000043963	si:ch211-241j12.3	si:ch211-241j12.3 [Source:ZFIN;Acc:ZDB-GENE-040724-72]	20	159277	182841		2.43	1.84	1.91	2.24	2.1	2.14
ENSDARG00000043970	lrpprc	leucine-rich pentatricopeptide repeat containing [Source:ZFIN;Acc:ZDB-GENE-030131-7887]	13	10799380	10922808		24.85	24.78	22.03	21.65	20.99	19.88
ENSDARG00000043972	ppp2r3c	protein phosphatase 2, regulatory subunit B'', gamma [Source:NCBI gene;Acc:393100]	17	13007288	13019643		9.34	8.38	8.63	8.14	7.32	9.12
ENSDARG00000043973	znf292b	zinc finger protein 292b [Source:ZFIN;Acc:ZDB-GENE-030131-9559]	20	196541	211920		6.89	3.45	3.25	13.81	13.52	13.39
ENSDARG00000043976	etf1b	eukaryotic translation termination factor 1b [Source:ZFIN;Acc:ZDB-GENE-021029-1]	10	21434649	21444731		34.52	34.26	29.86	38.04	38.47	36.14
ENSDARG00000043982	tube1	tubulin, epsilon 1 [Source:ZFIN;Acc:ZDB-GENE-040426-1174]	20	262995	318105		0	0	0	0	0	0
ENSDARG00000043986	setd6	SET domain containing 6, protein lysine methyltransferase [Source:NCBI gene;Acc:322348]	25	17349265	17357165		5.59	7.84	6.13	2.65	2.37	2.76
ENSDARG00000043997	cyp2x8	cytochrome P450, family 2, subfamily X, polypeptide 8 [Source:ZFIN;Acc:ZDB-GENE-040704-28]	25	17386763	17395315		2.44	4.56	3.56	0.1	0.22	0.13
ENSDARG00000044001	lgals3b	lectin, galactoside binding soluble 3b [Source:ZFIN;Acc:ZDB-GENE-030131-4324]	17	10824659	10838434		72.6	85.75	84.52	53.77	57.21	52.13
ENSDARG00000044002	cyp2x7	cytochrome P450, family 2, subfamily X, polypeptide 7 [Source:ZFIN;Acc:ZDB-GENE-110114-2]	25	17372290	17378881		3	4.3	3.79	1.84	2.31	1.68
ENSDARG00000044010	loxl2a	lysyl oxidase-like 2a [Source:NCBI gene;Acc:565508]	10	20407888	20445549		8.59	10.47	9.53	30.47	35.21	34.22
ENSDARG00000044011	xkrx	XK related X-linked [Source:ZFIN;Acc:ZDB-GENE-041114-108]	14	41318412	41341284		8.58	7.45	5.58	7.53	6.33	5.16
ENSDARG00000044015	gfra2b	GDNF family receptor alpha 2b [Source:ZFIN;Acc:ZDB-GENE-091204-26]	10	19723025	19801821		5.44	2.11	3.26	4.72	4.83	4.51
ENSDARG00000044016	myo6a	myosin VIa [Source:ZFIN;Acc:ZDB-GENE-040819-2]	20	715739	777219		26.76	20.49	15.84	19.5	19.66	20.21
ENSDARG00000044023	nkl.4	NK-lysin tandem duplicate 4 [Source:ZFIN;Acc:ZDB-GENE-030131-7503]	17	8854108	8862424		0.19	0.3	0.23	0	0	0.04
ENSDARG00000044034	tmem86a	transmembrane protein 86A [Source:ZFIN;Acc:ZDB-GENE-050320-81]	25	24250247	24262450		9.04	5.03	4.63	6.77	7.37	7.02
ENSDARG00000044038	zbtb2a	zinc finger and BTB domain containing 2a [Source:ZFIN;Acc:ZDB-GENE-040426-726]	17	7786246	7792376		0.6	0.87	0.79	0.9	0.72	0.76
ENSDARG00000044039	stx11a	syntaxin 11a [Source:ZFIN;Acc:ZDB-GENE-040426-2564]	17	7727464	7733037		1.42	2.11	1.89	1.72	1.61	1.5
ENSDARG00000044044	rassf2b	Ras association domain family member 2b [Source:ZFIN;Acc:ZDB-GENE-041221-6]	10	20641001	20650302		0.27	0.31	0.39	0.33	0.6	0.5
ENSDARG00000044047	slc18b1	solute carrier family 18 member B1 [Source:ZFIN;Acc:ZDB-GENE-050417-386]	17	8211992	8228712		2.76	4.62	2.55	1.56	1.84	1.48
ENSDARG00000044048	prnpb	prion protein b [Source:ZFIN;Acc:ZDB-GENE-041221-2]	10	20628698	20632779		1.39	1.41	1.71	1.04	1.45	1.29
ENSDARG00000044053	vsnl1b	visinin-like 1b [Source:ZFIN;Acc:ZDB-GENE-080225-39]	17	6715575	6759149		78.8	24.21	41.78	63.97	68.45	68.13
ENSDARG00000044056	ahi1	Abelson helper integration site 1 [Source:ZFIN;Acc:ZDB-GENE-060803-1]	17	8229868	8268406		4.95	2.23	2.46	5.51	5.57	6.23
ENSDARG00000044059	lft2	lefty2 [Source:ZFIN;Acc:ZDB-GENE-990630-11]	17	8308182	8312923		0	0.02	0.12	0.14	0.14	0.21
ENSDARG00000044061	adora4a	adenosine A4 receptor a [Source:ZFIN;Acc:ZDB-GENE-131127-239]	6	54496808	54504476		0.02	0.08	0.08	0.12	0.11	0.13
ENSDARG00000044062	ctbp2a	C-terminal binding protein 2a [Source:ZFIN;Acc:ZDB-GENE-010130-2]	17	8595588	8727748		9.95	4.15	4.09	14.78	14.24	15.45
ENSDARG00000044072	TATDN3	TatD DNase domain containing 3-like [Source:NCBI gene;Acc:571912]	17	6452511	6462588	TATDN3	5.6	6.09	6.58	4.14	4.71	4.99
ENSDARG00000044073	fuca2	alpha-L-fucosidase 2 [Source:ZFIN;Acc:ZDB-GENE-040822-39]	17	6438051	6451801		32.61	31.28	29.23	28.14	28.12	28.51
ENSDARG00000044074	loxl2b	lysyl oxidase-like 2b [Source:NCBI gene;Acc:791144]	5	27434601	27490499		7.6	8.46	7.77	13.84	16.55	15.19
ENSDARG00000044083	peli1b	pellino E3 ubiquitin protein ligase 1b [Source:ZFIN;Acc:ZDB-GENE-061027-372]	17	24479880	24521382		8.47	5.36	5.65	11.05	9.87	10.43
ENSDARG00000044090	zmpste24	zinc metallopeptidase, STE24 homolog [Source:ZFIN;Acc:ZDB-GENE-030131-6312]	17	24604373	24613061		12.66	11.44	10.97	12.01	11.48	11.57
ENSDARG00000044091	pitpnab	phosphatidylinositol transfer protein, alpha b [Source:ZFIN;Acc:ZDB-GENE-110408-51]	10	24323751	24371312		44.35	12.25	19.7	37.43	35.66	38.74
ENSDARG00000044092	atp5if1b	ATP synthase inhibitory factor subunit 1b [Source:NCBI gene;Acc:558368]	17	24613255	24614724		135.33	181.25	141.16	101.35	103.8	113.56
ENSDARG00000044093	rpl13a	ribosomal protein L13a [Source:ZFIN;Acc:ZDB-GENE-030131-168]	17	24615091	24618279		490.24	653.96	735.8	365.21	379.23	369.71
ENSDARG00000044094	gfpt2	glutamine-fructose-6-phosphate transaminase 2 [Source:ZFIN;Acc:ZDB-GENE-071113-1]	21	32390707	32436679		13.59	15.63	10.53	8.53	7	5.86
ENSDARG00000044098	egr2a	early growth response 2a [Source:ZFIN;Acc:ZDB-GENE-030723-6]	17	43663504	43677471		0.78	0.32	0.51	0.64	0.67	0.7
ENSDARG00000044102	znf593	zinc finger protein 593 [Source:ZFIN;Acc:ZDB-GENE-040426-1789]	17	24684778	24686816		18.39	24.92	22.84	11.51	13.03	13.82
ENSDARG00000044114	cyb561d2	cytochrome b561 family, member D2 [Source:ZFIN;Acc:ZDB-GENE-041010-205]	22	10639440	10641873		2.39	2.88	2.11	2.71	2.93	2.9
ENSDARG00000044125	txn	thioredoxin [Source:ZFIN;Acc:ZDB-GENE-040718-162]	7	24357231	24364826		22.88	27.38	26.18	28.12	28.21	29.87
ENSDARG00000044129	si:ch211-214p16.1	si:ch211-214p16.1 [Source:NCBI gene;Acc:368901]	17	25585487	25586858		5.87	7.11	7.23	4.89	6.28	4.78
ENSDARG00000044132	ogna	osteoglycin, paralog a [Source:ZFIN;Acc:ZDB-GENE-050208-650]	22	10454603	10459880		27.67	33.28	32.38	37.12	40.04	37.47
ENSDARG00000044134	iars2	isoleucyl-tRNA synthetase 2, mitochondrial [Source:ZFIN;Acc:ZDB-GENE-030616-608]	17	25590127	25611557		4.56	4.17	3.48	4.11	4.1	3.99
ENSDARG00000044135	cenpp	centromere protein P [Source:NCBI gene;Acc:445161]	22	10440991	10514343		1.89	2.06	1.99	1.17	1.24	1.17
ENSDARG00000044136	rab3gap2	RAB3 GTPase activating protein subunit 2 (non-catalytic) [Source:ZFIN;Acc:ZDB-GENE-030616-610]	17	25609826	25630848		9.63	7.32	6.48	10.24	10.45	10.19
ENSDARG00000044142	acss1	acyl-CoA synthetase short chain family member 1 [Source:ZFIN;Acc:ZDB-GENE-050320-139]	17	25833947	25853922		5.38	6.66	6.43	5.41	6.59	5.67
ENSDARG00000044143	nol8	nucleolar protein 8 [Source:ZFIN;Acc:ZDB-GENE-050208-491]	22	10424995	10440688		5.3	5.19	4.5	3.26	3.39	3.36
ENSDARG00000044144	wapla	WAPL cohesin release factor a [Source:ZFIN;Acc:ZDB-GENE-131010-1]	17	25856671	25885472		4.62	3.06	2.56	5.23	5.62	5.43
ENSDARG00000044153	ppp1cb	protein phosphatase 1, catalytic subunit, beta isozyme [Source:ZFIN;Acc:ZDB-GENE-030616-609]	17	25635953	25649079		92.73	65.76	72.31	97.42	106.44	112.73
ENSDARG00000044155	mafaa	v-maf avian musculoaponeurotic fibrosarcoma oncogene homolog Aa [Source:NCBI gene;Acc:100000492]	6	32699359	32703317		4.05	4.45	3.21	9.15	7.72	7.21
ENSDARG00000044161	grid1b	glutamate receptor, ionotropic, delta 1b [Source:ZFIN;Acc:ZDB-GENE-121214-84]	12	12112384	12833736		2.47	0.68	0.88	2.12	2.05	2.02
ENSDARG00000044167	padi2	peptidyl arginine deiminase, type II [Source:ZFIN;Acc:ZDB-GENE-031116-67]	6	47843760	47865858		27.64	33.53	27.66	21.73	19.09	17.48
ENSDARG00000044169	prelid1b	PRELI domain containing 1b [Source:ZFIN;Acc:ZDB-GENE-050522-169]	23	45512825	45521759		6.75	3.44	2.72	8.04	9.1	8.53
ENSDARG00000044175	oxtrl	oxytocin receptor like [Source:ZFIN;Acc:ZDB-GENE-110805-1]	6	46668717	46687079		0.12	0.14	0.1	0.11	0.17	0.18
ENSDARG00000044179	rbms2a	RNA binding motif, single stranded interacting protein 2a [Source:ZFIN;Acc:ZDB-GENE-030912-2]	11	1867613	1920517		2.66	2.28	1.79	6.59	6.59	6.12
ENSDARG00000044182	stau1	staufen double-stranded RNA binding protein 1 [Source:ZFIN;Acc:ZDB-GENE-030131-6372]	6	46431848	46454823		38.82	35.79	31.75	57.9	53.84	55.61
ENSDARG00000044183	prkab1a	protein kinase, AMP-activated, beta 1 non-catalytic subunit, a [Source:ZFIN;Acc:ZDB-GENE-040718-377]	10	17936441	17946163		14.08	7.17	9.35	13.17	13.16	14.08
ENSDARG00000044191	gxylt2	glucoside xylosyltransferase 2 [Source:ZFIN;Acc:ZDB-GENE-070424-64]	6	44163727	44193740		5.28	4.43	4.27	7.8	8.66	7.76
ENSDARG00000044194	tcta	T cell leukemia translocation altered [Source:NCBI gene;Acc:436858]	6	43015916	43019222		21.96	18.48	19.76	18.53	18.1	18.71
ENSDARG00000044199	gnat1	guanine nucleotide binding protein (G protein), alpha transducing activity polypeptide 1 [Source:ZFIN;Acc:ZDB-GENE-011128-11]	6	42918933	42930302		93.17	24.16	19.39	33.92	36.67	36.35
ENSDARG00000044203	sumf1	sulfatase modifying factor 1 [Source:ZFIN;Acc:ZDB-GENE-060421-3113]	6	39913853	39920105		4.87	5.18	5.71	4.28	4.47	4.53
ENSDARG00000044204	endou	endonuclease, polyU-specific [Source:NCBI gene;Acc:791216]	6	39905021	39912440		11.79	19.82	17.52	4.92	6.02	5.23
ENSDARG00000044216	fto	FTO alpha-ketoglutarate dependent dioxygenase [Source:ZFIN;Acc:ZDB-GENE-061108-1]	7	36110972	36358735		2.59	1.23	1.49	2.49	2.94	2.74
ENSDARG00000044225		si:dkey-27b3.2 [Source:ZFIN;Acc:ZDB-GENE-091117-10]	10	8660654	8672820		30.2	22.51	23	25.42	27.04	26.3
ENSDARG00000044235	BX005421.1		3	3971436	3992554		0.07	0.03	0.07	0.04	0.04	0.04
ENSDARG00000044241	dram2b	DNA-damage regulated autophagy modulator 2b [Source:ZFIN;Acc:ZDB-GENE-040625-141]	22	856869	866196		13.81	10.35	9.51	14.38	13.53	13.94
ENSDARG00000044246	snx18b	sorting nexin 18b [Source:ZFIN;Acc:ZDB-GENE-080213-6]	10	9092815	9097996		1.42	1.44	1.41	2.23	1.92	2.31
ENSDARG00000044251	rasgef1bb	RasGEF domain family, member 1Bb [Source:NCBI gene;Acc:445085]	10	9159279	9184667		9.61	7.37	7.13	16.05	16.25	15.61
ENSDARG00000044253	paqr3b	progestin and adipoQ receptor family member IIIb [Source:ZFIN;Acc:ZDB-GENE-050913-128]	10	9222476	9235446		3.59	3.86	3.64	4.16	3.67	3.86

ENSDARG00000044254	anxa3b	annexin A3b [Source:ZFIN;Acc:ZDB-GENE-041114-128]	10	9247593	9269733		17.26	19.88	20.8	17.12	18.61	19.16
ENSDARG00000044255	rbm18	RNA binding motif protein 18 [Source:NCBI gene;Acc:393766]	10	9346383	9370944		5.67	3.83	4.13	5.89	5.01	5.7
ENSDARG00000044261	si:ch211-243g18.2	si:ch211-243g18.2 [Source:ZFIN;Acc:ZDB-GENE-030131-2830]	10	9550419	9571012		33.48	33.47	37.99	22.51	25.03	23.61
ENSDARG00000044264	pimr71	Pim proto-oncogene, serine/threonine kinase, related 71 [Source:ZFIN;Acc:ZDB-GENE-120215-21]	6	9457966	9459440		0	0.03	0.27	0.11	0.15	0.06
ENSDARG00000044265	cdc37l1	cell division cycle 37-like 1 [Source:NCBI gene;Acc:569357]	10	10972795	10998499		3.73	3.24	3.01	2.85	2.81	2.41
ENSDARG00000044267	sumo1	small ubiquitin like modifier 1 [Source:NCBI gene;Acc:406438]	6	9677420	9688511		38.09	43.16	43.44	37.68	40.61	39.08
ENSDARG00000044276	spp1	secreted phosphoprotein 1 [Source:ZFIN;Acc:ZDB-GENE-050706-97]	10	15120711	15128771		6.85	5.51	6.11	2.81	3.66	4.04
ENSDARG00000044278	synpr	synaptoporin [Source:ZFIN;Acc:ZDB-GENE-030616-625]	6	41223204	41229787		1.65	1.42	1.83	1.75	1.54	1.43
ENSDARG00000044279	opn1mw3	opsin 1 (cone pigments), medium-wave-sensitive, 3 [Source:NCBI gene;Acc:360152]	6	41191482	41193419		0.03	0.02	0.01	0	0	0.02
ENSDARG00000044280	opn1mw2	opsin 1 (cone pigments), medium-wave-sensitive, 2 [Source:NCBI gene;Acc:360151]	6	41186320	41189460		6.98	1.86	2.23	7.98	8.84	7.76
ENSDARG00000044281	erbin	erbb2 interacting protein [Source:ZFIN;Acc:ZDB-GENE-061103-403]	10	15454745	15599149		5.48	3.78	2.98	8.24	8.41	8.36
ENSDARG00000044282	smc5	structural maintenance of chromosomes 5 [Source:ZFIN;Acc:ZDB-GENE-061013-288]	10	15617741	15644974		7.94	6.43	6.48	4.62	4.6	5.14
ENSDARG00000044290	rph3al	rabphilin 3A-like (without C2 domains) [Source:ZFIN;Acc:ZDB-GENE-090605-1]	5	60934345	61035016		0	0	0	0	0	0
ENSDARG00000044295	pip5k1ba	phosphatidylinositol-4-phosphate 5-kinase, type I, beta a [Source:ZFIN;Acc:ZDB-GENE-041010-81]	10	15881309	15919839		1.63	1.68	1.54	1.57	1.28	1.55
ENSDARG00000044298	phax	phosphorylated adaptor for RNA export [Source:ZFIN;Acc:ZDB-GENE-040822-42]	10	16029260	16035413		15.31	12.59	12.93	11.85	11.11	11.62
ENSDARG00000044299	lmnb1	lamin B1 [Source:ZFIN;Acc:ZDB-GENE-020424-2]	10	16036246	16056908		28.51	23.16	21.73	28.02	25.89	25.8
ENSDARG00000044301	atf1	activating transcription factor 1 [Source:ZFIN;Acc:ZDB-GENE-030131-4334]	6	39505565	39521832		6.46	6.01	6.12	7.5	7.49	8.04
ENSDARG00000044304	prrc1	proline-rich coiled-coil 1 [Source:NCBI gene;Acc:474330]	10	16165533	16187800		3.77	3.85	3.22	6.07	5.79	5.97
ENSDARG00000044318	itgb7	integrin, beta 7 [Source:ZFIN;Acc:ZDB-GENE-100728-2]	6	39703232	39727244		0.85	0.83	0.86	1.16	1.58	1.18
ENSDARG00000044319	fstl4	follistatin-like 4 [Source:ZFIN;Acc:ZDB-GENE-070127-3]	21	44857293	45117822		4.1	0.96	1.34	6	5.94	6.28
ENSDARG00000044325	zgc:193690	zgc:193690 [Source:ZFIN;Acc:ZDB-GENE-080721-12]	23	37095792	37113430		0.34	0.69	0.66	0.81	0.76	0.63
ENSDARG00000044326	BX950188.1		6	36465347	36542102		0.1	0.07	0.09	0.31	0.38	0.39
ENSDARG00000044327	tmem185	transmembrane protein 185 [Source:NCBI gene;Acc:393409]	21	43460631	43474012		6.24	4.61	5.11	7.05	7.08	7.28
ENSDARG00000044328	ankrd46a	ankyrin repeat domain 46a [Source:ZFIN;Acc:ZDB-GENE-030131-2643]	21	43475746	43485351		12.18	6.27	6.78	16.47	16.75	17.81
ENSDARG00000044332	zranb2	zinc finger, RAN-binding domain containing 2 [Source:ZFIN;Acc:ZDB-GENE-040426-2743]	6	37301341	37307487		33.05	20.32	23.62	27.92	26.54	26.49
ENSDARG00000044336	si:ch211-218m3.16	si:ch211-218m3.16 [Source:ZFIN;Acc:ZDB-GENE-091116-19]	21	40292801	40296052		0.12	0.06	0.02	0.05	0.06	0.05
ENSDARG00000044337	or115-6	odorant receptor, family F, subfamily 115, member 6 [Source:ZFIN;Acc:ZDB-GENE-070806-8]	21	40280027	40282140		0.1	0.02	0.01	0.02	0.08	0.11
ENSDARG00000044338	or115-7	odorant receptor, family F, subfamily 115, member 7 [Source:ZFIN;Acc:ZDB-GENE-070806-9]	21	40268274	40270488		0.1	0.08	0.09	0.08	0.07	0.1
ENSDARG00000044339	rp2	RP2 activator of ARL3 GTPase [Source:NCBI gene;Acc:406755]	6	37513911	37525879		6.77	3.61	4	5.75	6.58	6.82
ENSDARG00000044341	chst7	carbohydrate (N-acetylglucosamine 6-O) sulfotransferase 7 [Source:ZFIN;Acc:ZDB-GENE-131121-150]	6	37603968	37622576		6.38	2.93	2.87	9.09	9.11	9.31
ENSDARG00000044343	or115-13	odorant receptor, family F, subfamily 115, member 13 [Source:ZFIN;Acc:ZDB-GENE-070806-14]	21	40219818	40221641		0	0	0	0	0	0.02
ENSDARG00000044345	cyfip1	cytoplasmic FMR1 interacting protein 1 [Source:NCBI gene;Acc:336613]	6	37655078	37730907		22.39	22.72	18.52	24.63	24.96	24.36
ENSDARG00000044355	CT030712.1	uncharacterized LOC100534830 [Source:NCBI gene;Acc:100534830]	9	24708653	24710074		0.05	0.06	0.03	0.08	0.06	0.02
ENSDARG00000044356	tp63	tumor protein p63 [Source:ZFIN;Acc:ZDB-GENE-030819-1]	6	28795780	28914225		11.68	11.8	7.19	12.78	10.77	10.32
ENSDARG00000044357	tbc1d23	TBC1 domain family, member 23 [Source:NCBI gene;Acc:798995]	6	28914233	28943056		16.97	11.25	10.57	18.35	18.76	19.48
ENSDARG00000044362	unc119b	unc-119 homolog b (C. elegans) [Source:ZFIN;Acc:ZDB-GENE-050201-2]	21	39121453	39177564		24.15	8.26	8.92	18.26	20.07	20.56
ENSDARG00000044365	angptl3	angiopoietin-like 3 [Source:ZFIN;Acc:ZDB-GENE-010817-3]	6	32344197	32349153		11.26	15.92	13.94	7.72	7.62	8.43
ENSDARG00000044373	atg4c	autophagy related 4C, cysteine peptidase [Source:ZFIN;Acc:ZDB-GENE-040625-84]	6	32300018	32315494		3.47	3.66	3.32	3.84	3.74	4.14
ENSDARG00000044375	zgc:158291	zgc:158291 [Source:ZFIN;Acc:ZDB-GENE-061215-70]	21	38312549	38315241		20.28	5.27	4.18	20.19	19.98	21.88
ENSDARG00000044380	rbmx2	RNA binding motif protein X-linked 2 [Source:ZFIN;Acc:ZDB-GENE-050626-88]	21	38638979	38647743		8.46	5.77	5.89	5.86	5.67	6.46
ENSDARG00000044381	siah2l	seven in absentia homolog 2 (Drosophila)-like [Source:NCBI gene;Acc:378728]	21	38659302	38717854		10.59	4.97	6.68	14.04	14.29	14.85
ENSDARG00000044387	cldn2	claudin 2 [Source:ZFIN;Acc:ZDB-GENE-040912-26]	21	38002879	38009507		3.6	2.46	1.75	3.93	3.9	4.2
ENSDARG00000044400	wdr78	WD repeat domain 78 [Source:ZFIN;Acc:ZDB-GENE-050522-322]	6	30689239	30701207		0.5	0.4	0.51	0.65	0.65	0.58
ENSDARG00000044402	nop16	NOP16 nucleolar protein homolog (yeast) [Source:NCBI gene;Acc:436807]	21	36612440	36619599		28.63	32.24	25.56	15.96	16.53	15.3
ENSDARG00000044404	pars2	prolyl-tRNA synthetase 2, mitochondrial [Source:ZFIN;Acc:ZDB-GENE-030131-4219]	6	30684696	30689126		3.16	3.08	2.76	2.29	2.38	1.99
ENSDARG00000044405	ttc4	tetratricopeptide repeat domain 4 [Source:ZFIN;Acc:ZDB-GENE-040426-1021]	6	30676995	30683637		8.12	9.57	9.19	6.76	6.57	6.96
ENSDARG00000044414	csmd1a	CUB and Sushi multiple domains 1a [Source:ZFIN;Acc:ZDB-GENE-050417-402]	13	6342410	6582725		0.64	0.13	0.3	1.01	1.31	1.21
ENSDARG00000044415	tlr5a	toll-like receptor 5a [Source:ZFIN;Acc:ZDB-GENE-040219-14]	20	51478939	51482591		0.71	0.8	0.61	0.93	1.06	0.88
ENSDARG00000044417	disp1	dispatched homolog 1 (Drosophila) [Source:NCBI gene;Acc:619201]	20	51489195	51559419		3.8	3.42	3.08	5.2	4.98	4.88
ENSDARG00000044420	dnajc19	DnaJ (Hsp40) homolog, subfamily C, member 19 [Source:NCBI gene;Acc:393734]	22	37534094	37541020		11.18	17.24	16.14	11.53	10.99	11.89
ENSDARG00000044422	acvr2ba	activin A receptor type 2Ba [Source:ZFIN;Acc:ZDB-GENE-980526-549]	24	41167269	41220538		1.21	0.99	0.76	4.12	5.29	4.69
ENSDARG00000044431	ppig	peptidylprolyl isomerase G (cyclophilin G) [Source:ZFIN;Acc:ZDB-GENE-040426-2822]	6	3693441	3708386		21.74	15.41	15.41	14.36	13.68	14.8
ENSDARG00000044433	sned1	sushi, nidogen and EGF-like domains 1 [Source:ZFIN;Acc:ZDB-GENE-100913-1]	6	27451879	27510625		1.44	1.19	1.16	2.38	2.24	2.1
ENSDARG00000044436	rps6ka4	ribosomal protein S6 kinase, polypeptide 4 [Source:ZFIN;Acc:ZDB-GENE-060526-284]	5	67079540	67115872		4.9	3.39	3.17	5.71	5.51	5.59
ENSDARG00000044437	cdca5	cell division cycle associated 5 [Source:ZFIN;Acc:ZDB-GENE-030131-235]	5	67175283	67180800		5	3.95	4.93	3.78	3.18	3.44
ENSDARG00000044441	MYO7B	si:ch73-194h10.2 [Source:ZFIN;Acc:ZDB-GENE-100921-21]	6	28051978	28111704	MYO7B	4.65	5.54	3.38	3.54	4.05	3.84
ENSDARG00000044457	gfi1ab	growth factor independent 1A transcription repressor b [Source:ZFIN;Acc:ZDB-GENE-040116-8]	6	29004062	29007493		3.51	3.3	3.58	2.66	2.21	2.18
ENSDARG00000044484	cdc23	CDC23 (cell division cycle 23, yeast, homolog) [Source:ZFIN;Acc:ZDB-GENE-040426-866]	21	34849406	34880924		3.53	2.46	3.28	3.3	3.14	3.27
ENSDARG00000044485	sall4	spalt-like transcription factor 4 [Source:ZFIN;Acc:ZDB-GENE-060328-2]	23	38940700	38952596		0.95	1.11	1.12	2.49	2.55	2.7
ENSDARG00000044488	pak1ip1	PAK1 interacting protein 1 [Source:NCBI gene;Acc:336470]	24	36158745	36175365		15.85	19.74	18.21	15.43	13.98	13.4
ENSDARG00000044490	tlr9	toll-like receptor 9 [Source:ZFIN;Acc:ZDB-GENE-040219-10]	8	53730368	53735399		0.01	0	0	0.01	0.01	0.01
ENSDARG00000044491	kif20a	kinesin family member 20A [Source:ZFIN;Acc:ZDB-GENE-050522-252]	21	34879268	34926738		5.29	4.27	3.88	4.29	3.9	4.16
ENSDARG00000044492	ublcp1	ubiquitin-like domain containing CTD phosphatase 1 [Source:NCBI gene;Acc:792145]	21	35304974	35325541		6.43	3.55	4.15	6.02	5.26	5.65
ENSDARG00000044501	viml	vimentin like [Source:ZFIN;Acc:ZDB-GENE-040426-1743]	2	50391331	50409763		0.96	1.37	0.94	1.29	1.37	1.11
ENSDARG00000044511	etv5b	ETS variant transcription factor 5b [Source:ZFIN;Acc:ZDB-GENE-991228-4]	6	14030903	14040161		12.48	8.13	7.57	9.48	9.87	10.28
ENSDARG00000044512	ca4c	carbonic anhydrase IV c [Source:ZFIN;Acc:ZDB-GENE-080815-6]	21	31275327	31290582		0.54	0.65	0.7	0.77	0.88	0.76
ENSDARG00000044513	tpst1l	tyrosylprotein sulfotransferase 1, like [Source:ZFIN;Acc:ZDB-GENE-040316-1]	21	31232873	31241254		7.18	4.18	4.9	12.58	12.97	14.33
ENSDARG00000044514	st6gal1	ST6 beta-galactosamide alpha-2,6-sialyltranferase 1 [Source:ZFIN;Acc:ZDB-GENE-060322-3]	21	31150438	31184035		4.23	0.94	1.65	4.11	4.24	4.38
ENSDARG00000044521	eef1b2	eukaryotic translation elongation factor 1 beta 2 [Source:ZFIN;Acc:ZDB-GENE-030131-7310]	6	13189563	13206255		147.32	176.71	200	106.11	113.72	112.94
ENSDARG00000044524	def6b	DEF6 guanine nucleotide exchange factor b [Source:ZFIN;Acc:ZDB-GENE-110408-59]	22	978867	996521		0.35	0.17	0.2	0.82	0.76	0.93
ENSDARG00000044525	pparda	peroxisome proliferator-activated receptor delta a [Source:ZFIN;Acc:ZDB-GENE-990415-212]	22	997838	1018752		2.97	3.65	2.94	4.16	4.7	4.89
ENSDARG00000044526	camk1ga	calcium/calmodulin-dependent protein kinase IGa [Source:ZFIN;Acc:ZDB-GENE-030131-7594]	22	1049367	1069407		11.07	1.99	1.15	4.92	5.41	5.57
ENSDARG00000044528	slc15a1b	solute carrier family 15 member 1b [Source:ZFIN;Acc:ZDB-GENE-030131-4661]	6	12556427	12588044		26.15	43.76	28.44	6.22	7.01	7.09
ENSDARG00000044532	nr4a2b	nuclear receptor subfamily 4, group A, member 2b [Source:ZFIN;Acc:ZDB-GENE-040718-103]	6	12462079	12466550		4.7	1.25	1.17	7.8	6.68	6.83
ENSDARG00000044534	CR631122.1	protein RD3-like [Source:NCBI gene;Acc:100149591]	21	26555989	26558268		0.63	0.04	0.16	0.19	0.55	0.36
ENSDARG00000044540	fkbp2	FKBP prolyl isomerase 2 [Source:ZFIN;Acc:ZDB-GENE-040912-126]	21	26991198	26996035		47.2	59.6	49.38	35.88	38.38	41.2
ENSDARG00000044541	ppp1r14ba	protein phosphatase 1, regulatory (inhibitor) subunit 14Ba [Source:ZFIN;Acc:ZDB-GENE-060825-331]	21	26996276	27010837		43.2	15.14	22.29	35.44	33.27	36.8
ENSDARG00000044544	zgc:110782	zgc:110782 [Source:ZFIN;Acc:ZDB-GENE-050320-51]	21	27192763	27195323		1.28	1.6	1.34	1.37	1.13	1.4
ENSDARG00000044545	zgc:77262	zgc:77262 [Source:ZFIN;Acc:ZDB-GENE-040426-1811]	21	27197711	27201495		16.8	10.09	9.6	29.64	25.29	25.99
ENSDARG00000044549	zgc:112416	zgc:112416 [Source:ZFIN;Acc:ZDB-GENE-050417-347]	6	11369135	11396429		0.34	0.37	0.35	0.2	0.09	0.19
ENSDARG00000044550	hif1al2	hypoxia inducible factor 1 subunit alpha, like 2 [Source:ZFIN;Acc:ZDB-GENE-050211-2]	21	27327442	27338639		1.33	1.18	1.55	5.45	5.78	5.83
ENSDARG00000044551	atg9a	ATG9 autophagy related 9 homolog A (S. cerevisiae) [Source:ZFIN;Acc:ZDB-GENE-070209-65]	6	11249706	11267002		6.39	3.35	3.33	6.6	6.44	6.69
ENSDARG00000044556	pacs1a	phosphofurin acidic cluster sorting protein 1a [Source:ZFIN;Acc:ZDB-GENE-050208-244]	21	27513859	27555345		6.01	3.85	3.03	5.65	5.49	6.23
ENSDARG00000044561	ccr7	chemokine (C-C motif) receptor 7 [Source:ZFIN;Acc:ZDB-GENE-060522-2]	6	10923105	10927766		0.04	0.06	0.05	0.22	0.33	0.21
ENSDARG00000044562	cycsb	cytochrome c, somatic b [Source:NCBI gene;Acc:415158]	6	10910589	10912424		286.1	355.9	337.71	234.71	227.69	231.39
ENSDARG00000044565	ola1	Obg-like ATPase 1 [Source:NCBI gene;Acc:326864]	6	10741266	10753507		104.45	127.84	132.59	84.73	84.49	85.83
ENSDARG00000044566	fabp6	fatty acid binding protein 6, ileal (gastrotropin) [Source:ZFIN;Acc:ZDB-GENE-040625-49]	21	30043054	30047353		223.44	136.59	95.81	65.54	69.81	73.3
ENSDARG00000044567	ttc1	tetratricopeptide repeat domain 1 [Source:ZFIN;Acc:ZDB-GENE-041114-80]	21	30007566	30019626		10.45	11.48	10.31	9.5	9.77	9.63
ENSDARG00000044569	cldn19	claudin 19 [Source:ZFIN;Acc:ZDB-GENE-050417-242]	11	39013763	39044595		9.87	7.8	9.05	14.95	15.82	16.96
ENSDARG00000044573	cdc42	cell division cycle 42 [Source:ZFIN;Acc:ZDB-GENE-030131-8783]	11	39120306	39148199		45.59	37.93	37.71	60.3	58.21	59.72
ENSDARG00000044574	rbms3	RNA binding motif, single stranded interacting protein [Source:ZFIN;Acc:ZDB-GENE-060929-244]	16	39643268	39900665		6.28	3.83	3.46	10.14	10.19	10.63
ENSDARG00000044575	vars1	valyl-tRNA synthetase 1 [Source:ZFIN;Acc:ZDB-GENE-010601-1]	16	10429770	10475814		14.55	14.36	10.49	17.54	18.35	16.78
ENSDARG00000044584	tex261	testis expressed 261 [Source:ZFIN;Acc:ZDB-GENE-050522-400]	7	8694557	8712148		6.9	7.94	6.91	7.17	6.05	5.37
ENSDARG00000044588	emp2	epithelial membrane protein 2 [Source:NCBI gene;Acc:445506]	3	27027781	27056180		18.13	23.34	19.2	29.27	30.7	29.38
ENSDARG00000044596	pgap2	post-GPI attachment to proteins 2 [Source:NCBI gene;Acc:541417]	21	30973697	30994577		3.71	2.77	2.8	4.24	3.48	3.71
ENSDARG00000044597	ncbp3	nuclear cap binding subunit 3 [Source:NCBI gene;Acc:393112]	21	30996076	31013817		12.94	9.13	10.28	10.25	10.08	10.66
ENSDARG00000044600	plppr2b	phospholipid phosphatase related 2b [Source:ZFIN;Acc:ZDB-GENE-121030-5]	6	7795602	7842078		4.14	1.03	1.15	3.74	3.57	3.94
ENSDARG00000044601	rtn4a	reticulon 4a [Source:ZFIN;Acc:ZDB-GENE-030710-1]	6	6231012	6258451		96.56	112.5	96.78	93.41	88.55	86.11
ENSDARG00000044605	stx12l	syntaxin 12, like [Source:ZFIN;Acc:ZDB-GENE-110715-1]	16	7626535	7641367		9.33	2.95	4.68	8.87	9.44	9.62
ENSDARG00000044612	c1qb	complement component 1, q subcomponent, B chain [Source:ZFIN;Acc:ZDB-GENE-040801-223]	21	22712307	22715297		0.22	0.49	0.68	0.55	0.92	0.71
ENSDARG00000044613	c1qa	complement component 1, q subcomponent, A chain [Source:ZFIN;Acc:ZDB-GENE-050522-86]	21	22721957	22724980		0.22	0.39	0.6	0.5	0.67	0.43
ENSDARG00000044615	zak	sterile alpha motif and leucine zipper containing kinase AZK [Source:ZFIN;Acc:ZDB-GENE-070912-386]	6	6924637	6967588		3.59	5.13	4.14	3.87	3.68	3.76
ENSDARG00000044619	birc2	baculoviral IAP repeat containing 2 [Source:ZFIN;Acc:ZDB-GENE-030825-6]	21	22833905	22851230		25.69	19.28	17.88	37.01	39.44	40.1
ENSDARG00000044622	polq	polymerase (DNA directed), theta [Source:ZFIN;Acc:ZDB-GENE-040724-232]	21	22851198	22896108		0.6	0.37	0.35	0.4	0.44	0.36
ENSDARG00000044625	pcf11	PCF11 cleavage and polyadenylation factor subunit [Source:ZFIN;Acc:ZDB-GENE-041114-175]	21	22878834	22888375		17.51	8.57	9.53	22.41	22.36	23.48
ENSDARG00000044626	ccdc90b	coiled-coil domain containing 90B [Source:ZFIN;Acc:ZDB-GENE-040724-162]	21	22888889	22892124		7.37	10.2	7.5	4.66	4.59	4.77
ENSDARG00000044627	alg8	ALG8 alpha-1,3-glucosyltransferase [Source:ZFIN;Acc:ZDB-GENE-030131-5812]	21	22892836	22901407		3.58	3.58	3.07	3.19	3.71	3.65
ENSDARG00000044628	dtd1	D-aminoacyl-tRNA deacylase 1 [Source:NCBI gene;Acc:445046]	6	6802142	6809598		2.84	2.48	2.62	2.27	2.24	2.28
ENSDARG00000044629	guca1d	guanylate cyclase activator 1d [Source:ZFIN;Acc:ZDB-GENE-040724-231]	21	22906479	22910530		0.25	0.15	0.05	0.92	1.11	0.56
ENSDARG00000044632	myo7ab	myosin VIIAb [Source:ZFIN;Acc:ZDB-GENE-091110-4]	21	22237964	22309466		2.22	0.57	0.47	2.31	2.45	2.83
ENSDARG00000044642	sc5d	sterol-C5-desaturase [Source:ZFIN;Acc:ZDB-GENE-040912-56]	21	24531679	24535510		8.03	10.73	8.44	3.46	3.64	3.37
ENSDARG00000044655	st14b	suppression of tumorigenicity 14b [Source:ZFIN;Acc:ZDB-GENE-061103-613]	21	25275693	25295087		8.44	6.96	6.36	6.04	5.63	5.35
ENSDARG00000044669	hp1bp3	heterochromatin protein 1, binding protein 3 [Source:ZFIN;Acc:ZDB-GENE-060810-79]	11	37612214	37631714		64.99	38.05	37.68	63.38	64.1	69.03
ENSDARG00000044672	parapinopsinb	parapinopsin b [Source:ZFIN;Acc:ZDB-GENE-030616-134]	11	36777613	36788797		0.09	0.09	0.04	0.16	0.09	0.2
ENSDARG00000044684	rbp4l	retinol binding protein 4, like [Source:ZFIN;Acc:ZDB-GENE-030131-7591]	21	20338613	20342096		51.82	18.07	14.29	57.17	63.68	61.96
ENSDARG00000044685	nr0b2a	nuclear receptor subfamily 0, group B, member 2a [Source:ZFIN;Acc:ZDB-GENE-080403-1]	16	54875530	54880550		13.59	20.07	18.56	16.08	18.11	16.59
ENSDARG00000044688	dusp4	dual specificity phosphatase 4 [Source:ZFIN;Acc:ZDB-GENE-040426-709]	21	20113470	20120543		5.02	2.5	2.21	11.68	12.36	11.83
ENSDARG00000044691	ppp1r3b	protein phosphatase 1, regulatory subunit 3B [Source:NCBI gene;Acc:327285]	21	19916456	19919934		3.54	5.2	4.11	3.59	4.16	4.17
ENSDARG00000044692	eri1	exoribonuclease 1 [Source:ZFIN;Acc:ZDB-GENE-050522-39]	21	19907759	19916021		5.72	6.61	5.76	5.15	5.21	4.84
ENSDARG00000044694	fybb	FYN binding protein b [Source:ZFIN;Acc:ZDB-GENE-091204-85]	21	19844195	19866351		0.94	1.1	0.95	1.57	1.39	1.35
ENSDARG00000044695	ccdc80l2	coiled-coil domain containing 80 like 2 [Source:ZFIN;Acc:ZDB-GENE-091118-63]	21	19834072	19841870		14.67	21.31	20.04	12.2	12.37	13
ENSDARG00000044704	or137-2	odorant receptor, family H, subfamily 137, member 2 [Source:ZFIN;Acc:ZDB-GENE-070806-31]	6	52924103	52925772		0.04	0.01	0.05	0.01	0	0.04
ENSDARG00000044718	vav2	vav 2 guanine nucleotide exchange factor [Source:ZFIN;Acc:ZDB-GENE-091204-420]	21	18021087	18262287		3.81	1.97	1.8	4.17	4.23	4.15
ENSDARG00000044748	or115-12	odorant receptor, family F, subfamily 115, member 12 [Source:ZFIN;Acc:ZDB-GENE-070806-13]	21	40225915	40227326		0.39	0.11	0.34	0.45	0.22	0.12
ENSDARG00000044751	ddt	D-dopachrome tautomerase [Source:ZFIN;Acc:ZDB-GENE-040625-160]	21	15709061	15711765		47.7	68.49	56.93	39.18	40.29	39.01
ENSDARG00000044752	p2rx4a	purinergic receptor P2X, ligand-gated ion channel, 4a [Source:ZFIN;Acc:ZDB-GENE-020806-2]	21	15723069	15732401		4.3	6.62	5.19	2.36	2.41	2.1
ENSDARG00000044753	idh3b	isocitrate dehydrogenase (NAD(+)) 3 non-catalytic subunit beta [Source:ZFIN;Acc:ZDB-GENE-040625-174]	21	15733880	15749719		78.22	81.36	80.84	55.92	54.41	55.12
ENSDARG00000044754	gnrh2	gonadotropin-releasing hormone 2 [Source:ZFIN;Acc:ZDB-GENE-030516-1]	21	15824182	15828029		2.1	0.98	1.57	3.64	3.88	4.67
ENSDARG00000044755	GCNT4	si:dkeyp-87d8.8 [Source:ZFIN;Acc:ZDB-GENE-041111-161]	21	15833222	15841167	GCNT4	2.52	2.49	1.77	0.89	0.78	0.8
ENSDARG00000044760	gnaia	guanine nucleotide binding protein (G protein), alpha inhibiting activity polypeptide a [Source:ZFIN;Acc:ZDB-GENE-030131-2229]	25	20997671	21031007		11.84	11.79	9.83	12.27	12.29	12.37
ENSDARG00000044766	mitd1	MIT, microtubule interacting and transport, domain containing 1 [Source:ZFIN;Acc:ZDB-GENE-040426-923]	9	7275185	7287375		2.16	1.8	1.53	1.88	1.77	1.73
ENSDARG00000044767	tspan10	tetraspanin 10 [Source:ZFIN;Acc:ZDB-GENE-041111-201]	3	15176737	15186292		0.96	1.33	1.91	1.35	1.69	1.35
ENSDARG00000044769	kctd13	potassium channel tetramerization domain containing 13 [Source:NCBI gene;Acc:100000647]	3	15213980	15227666		11.13	6.38	6.93	11.28	11.35	11.83
ENSDARG00000044774	pou5f3	POU domain, class 5, transcription factor 3 [Source:NCBI gene;Acc:30333]	21	13685853	13690712		0.13	0.12	0.1	0.35	0.2	0.33
ENSDARG00000044775	fut7	fucosyltransferase 7 (alpha (1,3) fucosyltransferase) [Source:ZFIN;Acc:ZDB-GENE-060929-997]	21	13676890	13681447		0.2	0.31	0.2	0.59	0.37	0.44
ENSDARG00000044776	clic3	chloride intracellular channel 3 [Source:ZFIN;Acc:ZDB-GENE-010507-2]	21	13663656	13672195		1.97	1.44	1.22	1.4	1.1	1
ENSDARG00000044781	bace2	beta-secretase 2 [Source:ZFIN;Acc:ZDB-GENE-041010-68]	15	6459847	6493615		6.18	8.39	7.72	7.1	8.07	7.35
ENSDARG00000044795	entpd2b	ectonucleoside triphosphate diphosphohydrolase 2b [Source:ZFIN;Acc:ZDB-GENE-061013-2]	21	11509461	11518864		0.37	0.19	0.25	0.2	0.47	0.11
ENSDARG00000044797	cd8a	CD8a molecule [Source:ZFIN;Acc:ZDB-GENE-060210-2]	21	11560153	11563406		0	0	0	0	0	0
ENSDARG00000044801	wdr38	WD repeat domain 38 [Source:ZFIN;Acc:ZDB-GENE-050417-160]	21	5862476	5867786		0.69	0.33	0.45	0.5	0.34	0.49
ENSDARG00000044802	aadacl4	arylacetamide deacetylase-like 4 [Source:ZFIN;Acc:ZDB-GENE-080919-2]	11	28617564	28619811		0.29	0.36	0.25	0.3	0.36	0.22
ENSDARG00000044803	dhrs3b	dehydrogenase/reductase (SDR family) member 3b [Source:ZFIN;Acc:ZDB-GENE-041010-172]	11	28590733	28614608		11.76	11.74	10.29	9.65	9	9.27
ENSDARG00000044807	dck	deoxycytidine kinase [Source:ZFIN;Acc:ZDB-GENE-041024-3]	21	5974590	5986172		6.41	4.78	5.12	3.78	4.1	2.75
ENSDARG00000044808	slc4a4b	solute carrier family 4 member 4b [Source:ZFIN;Acc:ZDB-GENE-091030-1]	21	5993188	6104568		9.41	5.7	4.09	7.83	8.12	8.12
ENSDARG00000044809	fpgs	folylpolyglutamate synthase [Source:ZFIN;Acc:ZDB-GENE-040426-2781]	21	6114305	6134570		7.62	8.2	6.73	7.06	6.89	7.05
ENSDARG00000044811	cdk9	cyclin-dependent kinase 9 (CDC2-related kinase) [Source:ZFIN;Acc:ZDB-GENE-030131-321]	21	6136981	6150568		25.16	20.1	18.37	20.97	19.5	20.73
ENSDARG00000044812	ttc17	tetratricopeptide repeat domain 17 [Source:NCBI gene;Acc:569538]	7	50295715	50349430		4.59	3.11	2.4	5.54	5.42	5.37
ENSDARG00000044813	vps33b	VPS33B late endosome and lysosome associated [Source:NCBI gene;Acc:541534]	7	50376097	50395395		6.71	6.01	6.18	6.25	6.82	6.41
ENSDARG00000044815	serinc4	serine incorporator 4 [Source:ZFIN;Acc:ZDB-GENE-121031-1]	7	50448478	50477289		1.74	0.23	0.23	2.62	3.02	2.72
ENSDARG00000044820	cstf1	cleavage stimulation factor, 3' pre-RNA, subunit 1 [Source:ZFIN;Acc:ZDB-GENE-030131-2958]	11	27817301	27827533		7.9	6.39	5.75	7.26	7.4	7.18
ENSDARG00000044827	wnt7aa	wingless-type MMTV integration site family, member 7Aa [Source:ZFIN;Acc:ZDB-GENE-051129-1]	11	27486584	27501027		8.9	5.75	6.15	11.61	10.24	10.94
ENSDARG00000044847	zgc:171599	zgc:171599 [Source:ZFIN;Acc:ZDB-GENE-080219-33]	17	49631844	49637023		5.25	3.71	2.97	5.12	5.41	5.22
ENSDARG00000044852	wbp2nl	WBP2 N-terminal like [Source:ZFIN;Acc:ZDB-GENE-030131-4012]	3	1223824	1228130		47.44	42.02	40.76	51.74	55.06	53.32
ENSDARG00000044861	opn1lw2	opsin 1 (cone pigments), long-wave-sensitive, 2 [Source:NCBI gene;Acc:436716]	11	25481046	25483206		823.98	217.53	152.13	354.8	375.87	357.98
ENSDARG00000044862	opn1lw1	opsin 1 (cone pigments), long-wave-sensitive, 1 [Source:NCBI gene;Acc:30413]	11	25477643	25480037		0.18	0.07	0.06	0.29	0.28	0.28
ENSDARG00000044874	zgc:92107	zgc:92107 [Source:ZFIN;Acc:ZDB-GENE-041114-4]	11	24994705	25043214		6.27	5.17	3.72	6.66	6.84	6.86
ENSDARG00000044875	crygm2e	crystallin, gamma M2e [Source:ZFIN;Acc:ZDB-GENE-081105-116]	9	22080122	22080989		0	0.21	0.3	0.14	0.35	0.37
ENSDARG00000044894	zgc:113307	zgc:113307 [Source:ZFIN;Acc:ZDB-GENE-050522-29]	11	21050326	21061566		12.17	17.34	18.22	8.04	8.13	7.52
ENSDARG00000044895	fmoda	fibromodulin a [Source:ZFIN;Acc:ZDB-GENE-041008-23]	11	21025895	21031773		65.57	93.64	88.89	73.57	74.41	75.21
ENSDARG00000044899	tmem183a	transmembrane protein 183A [Source:ZFIN;Acc:ZDB-GENE-050327-5]	11	22363572	22372072		8.75	6.91	6.28	9.57	9.41	8.08
ENSDARG00000044900	prlhr2a	prolactin releasing hormone receptor 2a [Source:ZFIN;Acc:ZDB-GENE-060209-1]	8	37905666	37950194		0.4	0.29	0.44	0.38	0.33	0.4
ENSDARG00000044902	atp2b4	ATPase plasma membrane Ca2+ transporting 4 [Source:ZFIN;Acc:ZDB-GENE-061027-60]	11	22920587	23080970		17.12	9.43	7.58	28.95	27.68	27.92
ENSDARG00000044914	suclg2	succinate-CoA ligase, GDP-forming, beta subunit [Source:ZFIN;Acc:ZDB-GENE-030114-3]	11	16738179	16975190		53.53	79.16	58.81	27.02	28.19	26.27
ENSDARG00000044923	ube2w	ubiquitin-conjugating enzyme E2W (putative) [Source:NCBI gene;Acc:553013]	2	30193747	30200206		6.05	5.39	5.27	6.21	5.93	5.99
ENSDARG00000044924	gdf11	growth differentiation factor 11 [Source:ZFIN;Acc:ZDB-GENE-040427-2]	11	15613218	15756385		0.84	0.32	0.44	1.52	1.06	1.34
ENSDARG00000044925	cdk5rap1	CDK5 regulatory subunit associated protein 1 [Source:ZFIN;Acc:ZDB-GENE-070424-90]	11	15480695	15587134		2	2.85	2.31	1.91	1.94	1.76
ENSDARG00000044935	hpdb	4-hydroxyphenylpyruvate dioxygenase b [Source:ZFIN;Acc:ZDB-GENE-040808-62]	8	34731982	34742234		68.8	100.76	110.77	23.96	25.74	22.56
ENSDARG00000044938	cbx2	chromobox homolog 2 (Drosophila Pc class) [Source:ZFIN;Acc:ZDB-GENE-030131-5502]	3	10152092	10164759		8.97	6.78	7.39	9.77	10.65	10.15
ENSDARG00000044942	mrpl45	mitochondrial ribosomal protein L45 [Source:ZFIN;Acc:ZDB-GENE-040426-1041]	11	12096199	12104917		28.73	33	36.59	19.64	20.14	21.59
ENSDARG00000044943	npepps	aminopeptidase puromycin sensitive [Source:ZFIN;Acc:ZDB-GENE-060524-3]	11	12108355	12158412		33.73	28.1	24.07	37.31	36.87	35.27
ENSDARG00000044949	rnf8	ring finger protein 8, E3 ubiquitin protein ligase [Source:NCBI gene;Acc:404050]	20	51363686	51386564		13.71	10.74	12.12	11.49	12.18	13.07
ENSDARG00000044954	slit1a	slit homolog 1a (Drosophila) [Source:ZFIN;Acc:ZDB-GENE-030722-3]	13	555321	638485		8.64	6.15	5.68	18.58	19.13	18.97
ENSDARG00000044956	zgc:114123	zgc:114123 [Source:ZFIN;Acc:ZDB-GENE-050913-53]	23	2728095	2745986		0.04	0.04	0.02	0.08	0.01	0.07
ENSDARG00000044968	vcla	vinculin a [Source:ZFIN;Acc:ZDB-GENE-050506-61]	13	25211939	25284716		5.98	7.63	5.77	7.27	6.47	6.43
ENSDARG00000044972	bcap31	B cell receptor associated protein 31 [Source:ZFIN;Acc:ZDB-GENE-030616-63]	8	8973425	9029175		43.52	51.59	52.08	37.85	39.16	36.37
ENSDARG00000044973	krt98	keratin 98 [Source:ZFIN;Acc:ZDB-GENE-070822-8]	11	11504014	11513985		0.64	0.75	0.5	0.4	0.34	0.38
ENSDARG00000044975	krt94	keratin 94 [Source:ZFIN;Acc:ZDB-GENE-061027-116]	11	11468566	11471857		52.96	65.04	70.72	31.56	36.1	34.85
ENSDARG00000044976	krt93	keratin 93 [Source:ZFIN;Acc:ZDB-GENE-060421-4592]	11	11454545	11458208		1.16	0.99	0.36	0.17	0.1	0.3
ENSDARG00000044980	kpna6	karyopherin alpha 6 (importin alpha 7) [Source:ZFIN;Acc:ZDB-GENE-130530-626]	19	29861859	29887629		4.96	3.69	3.55	6.49	7.14	7.05
ENSDARG00000044982	dhrs3a	dehydrogenase/reductase (SDR family) member 3a [Source:ZFIN;Acc:ZDB-GENE-040801-217]	23	21663631	21674679		8.61	11.32	10.27	9.08	8.61	8.27
ENSDARG00000045003	plvapb	plasmalemma vesicle associated protein b [Source:ZFIN;Acc:ZDB-GENE-060616-96]	11	6043742	6048490		19.75	30.25	29.14	34.24	37.99	40.1
ENSDARG00000045006	ptprga	protein tyrosine phosphatase receptor type Ga [Source:ZFIN;Acc:ZDB-GENE-101101-4]	11	4953964	5463254		0.8	0.39	0.48	1.11	1.06	1.06
ENSDARG00000045011	tapbp.2	TAP binding protein (tapasin), tandem duplicate 2 [Source:ZFIN;Acc:ZDB-GENE-060503-136]	19	7065988	7070691		0.37	0.53	0.34	0.58	0.7	0.6

ENSDARG00000045014	tuba2	tubulin, alpha 2 [Source:ZFIN;Acc:ZDB-GENE-040426-1970]	11	3336524	3343463		71.3	27.43	40.95	64.24	62.27	64.77
ENSDARG00000045015	cyp27b1	cytochrome P450, family 27, subfamily B, polypeptide 1 [Source:ZFIN;Acc:ZDB-GENE-111005-2]	11	329687	339847		1.05	1.3	1.18	1.57	1.77	1.64
ENSDARG00000045016	npffl	neuropeptide FF-amide peptide precursor like [Source:ZFIN;Acc:ZDB-GENE-030806-1]	11	276057	279328		3.86	2.62	4.18	4.54	5.25	4.9
ENSDARG00000045017	pdrg1	p53 and DNA-damage regulated 1 [Source:ZFIN;Acc:ZDB-GENE-050417-276]	11	355305	360317		5.19	4.07	4.79	3.31	3.53	4.07
ENSDARG00000045019	aamp	angio-associated, migratory cell protein [Source:ZFIN;Acc:ZDB-GENE-040426-2370]	6	59913147	59922078		51.39	57.79	61.69	45.55	51.82	51.76
ENSDARG00000045023	lhfpl5a	LHFPL tetraspan subfamily member 5a [Source:ZFIN;Acc:ZDB-GENE-110131-8]	11	2829812	2838699		0.6	0.57	0.68	0.18	0.48	0.58
ENSDARG00000045025	ift52	intraflagellar transport 52 homolog (Chlamydomonas) [Source:ZFIN;Acc:ZDB-GENE-040614-3]	11	1533097	1546015		8.46	6.1	7.26	7.26	8.64	8.71
ENSDARG00000045027	rab9a	RAB9A, member RAS oncogene family [Source:ZFIN;Acc:ZDB-GENE-060825-293]	9	54686686	54704498		5.91	4.48	4.51	6.7	6.13	6.88
ENSDARG00000045036	cdc42ep4b	CDC42 effector protein (Rho GTPase binding) 4b [Source:ZFIN;Acc:ZDB-GENE-091008-2]	12	37407105	37449396		10.43	10.33	8.13	12.02	11.28	10.86
ENSDARG00000045038	tekt3	tektin 3 [Source:ZFIN;Acc:ZDB-GENE-080130-2]	12	37327867	37343858		2.16	2.45	2.34	0.92	1.02	0.98
ENSDARG00000045039	kcnj16	potassium inwardly rectifying channel subfamily J member 16 [Source:ZFIN;Acc:ZDB-GENE-130603-81]	12	36267761	36268723		0.89	0.86	0.99	1.68	2.33	2.61
ENSDARG00000045051	slc16a3	solute carrier family 16 member 3 [Source:ZFIN;Acc:ZDB-GENE-030131-5487]	12	33350256	33359654		6.66	4.98	3.82	16.94	18.48	18.05
ENSDARG00000045064	ablim1b	actin binding LIM protein 1b [Source:ZFIN;Acc:ZDB-GENE-050327-91]	12	30046320	30220346		13.09	10.58	8.73	13.23	12.48	12.58
ENSDARG00000045065	vti1a	vesicle transport through interaction with t-SNAREs 1A [Source:ZFIN;Acc:ZDB-GENE-050809-135]	12	31161564	31422536		12.59	8.98	10.34	9.39	9.71	9.65
ENSDARG00000045066	entpd1	ectonucleoside triphosphate diphosphohydrolase 1 [Source:ZFIN;Acc:ZDB-GENE-040801-58]	12	30740797	30777743		20.92	28.8	27.46	20.01	19.17	20.32
ENSDARG00000045067	kcnk1a	potassium channel, subfamily K, member 1a [Source:ZFIN;Acc:ZDB-GENE-070620-26]	12	30705769	30712111		2.38	1.8	1.51	2.18	2.31	2.26
ENSDARG00000045070	itgb3b	integrin beta 3b [Source:ZFIN;Acc:ZDB-GENE-071207-2]	12	26434478	26461744		2.38	3.27	3	4.3	4.53	4.68
ENSDARG00000045071	chad	chondroadherin [Source:ZFIN;Acc:ZDB-GENE-040426-1130]	12	26512569	26525699		1.38	1.62	1.52	5.05	5.63	5.61
ENSDARG00000045074	arl4d	ADP-ribosylation factor-like 4D [Source:ZFIN;Acc:ZDB-GENE-030131-2293]	12	27243059	27245757		22.83	15.53	17.62	23.48	22.33	25.4
ENSDARG00000045075	tmem106a	transmembrane protein 106A [Source:ZFIN;Acc:ZDB-GENE-030131-5129]	12	27231212	27237032		9.88	13.1	11.32	9.16	10.11	9.99
ENSDARG00000045087	cdk5r1b	cyclin-dependent kinase 5, regulatory subunit 1b (p35) [Source:ZFIN;Acc:ZDB-GENE-040718-220]	12	28367557	28370484		38.68	11.94	18.17	42.51	43.91	45.62
ENSDARG00000045089	si:ch211-270n8.1	si:ch211-270n8.1 [Source:ZFIN;Acc:ZDB-GENE-081107-34]	13	20379392	20381485		11.16	19.27	15.64	4.88	5.32	6.31
ENSDARG00000045091	mrpl10	mitochondrial ribosomal protein L10 [Source:NCBI gene;Acc:541330]	12	28796622	28799642		9.42	10.17	10.28	6.65	6.89	6.04
ENSDARG00000045092	cdk5rap3	CDK5 regulatory subunit associated protein 3 [Source:ZFIN;Acc:ZDB-GENE-040625-90]	12	28825735	28837752		5.14	7.96	7.48	5.06	5.04	4.9
ENSDARG00000045094	si:dkey-222b8.1	si:dkey-222b8.1 [Source:ZFIN;Acc:ZDB-GENE-060503-248]	19	23932259	23970686		3.66	1.98	2.42	6.59	6.15	6.07
ENSDARG00000045095	elac1	elaC ribonuclease Z 1 [Source:NCBI gene;Acc:445109]	5	6854345	6864127		6	5.86	5.73	3.68	3.94	4.39
ENSDARG00000045105	foxn2b	forkhead box N2b [Source:ZFIN;Acc:ZDB-GENE-050417-1]	12	24796517	24832297		3.67	2.95	2.09	4.99	4.28	4.68
ENSDARG00000045123	oplah	5-oxoprolinase, ATP-hydrolysing [Source:ZFIN;Acc:ZDB-GENE-121214-293]	12	13652361	13721912		13.17	13.63	10.8	7.59	7.71	7.93
ENSDARG00000045127	FAM53C	zgc:175066 [Source:ZFIN;Acc:ZDB-GENE-091027-2]	21	30256952	30284404	FAM53C	3.31	1.49	1.56	4.88	4.67	4.82
ENSDARG00000045129	fkbp10b	FKBP prolyl isomerase 10b [Source:ZFIN;Acc:ZDB-GENE-030131-3101]	12	13905286	13957037		8.24	10.44	10.04	14.46	14.24	14.25
ENSDARG00000045130	klhl11	kelch-like family member 11 [Source:ZFIN;Acc:ZDB-GENE-030131-5524]	12	13961268	13966184		7.42	3.81	5.07	7.45	7.62	6.88
ENSDARG00000045131	id4	inhibitor of DNA binding 4 [Source:ZFIN;Acc:ZDB-GENE-051113-208]	16	9977858	9980402		21.59	16.98	19.06	23.91	21.9	24.98
ENSDARG00000045132	vdac1	voltage-dependent anion channel 1 [Source:ZFIN;Acc:ZDB-GENE-030131-6514]	21	45190582	45210092		153.29	121.89	141.12	125.72	138.18	131.76
ENSDARG00000045137	CABZ01084564.1	histamine H3 receptor-like [Source:NCBI gene;Acc:100331077]	2	1174778	1188707		0.17	0.1	0.17	0.28	0.42	0.31
ENSDARG00000045139	ca7	carbonic anhydrase VII [Source:ZFIN;Acc:ZDB-GENE-040426-1786]	7	44802353	44813763		1.97	1.34	1.16	0.95	1.17	1.16
ENSDARG00000045140	lcmt1	leucine carboxyl methyltransferase 1 [Source:ZFIN;Acc:ZDB-GENE-040912-75]	12	20378376	20409794		5.55	5.57	5.28	4.92	5.12	4.32
ENSDARG00000045141	aqp8a.1	aquaporin 8a, tandem duplicate 1 [Source:ZFIN;Acc:ZDB-GENE-040912-106]	12	20366109	20373058		47.16	74.99	69.16	44.16	49.55	46.86
ENSDARG00000045142	hbae5	hemoglobin, alpha embryonic 5 [Source:ZFIN;Acc:ZDB-GENE-101028-1]	12	20342451	20353541		1.57	2.81	2.1	134.83	125.24	121.11
ENSDARG00000045143	hbbe2	hemoglobin beta embryonic-2 [Source:ZFIN;Acc:ZDB-GENE-040702-1]	12	20349865	20350629		196.84	286.22	324.53	1009.68	1110.03	1023.21
ENSDARG00000045144	zgc:163057	zgc:163057 [Source:ZFIN;Acc:ZDB-GENE-070410-131]	12	20336070	20337274		2.03	2.06	2.3	6.37	7	6.31
ENSDARG00000045145	shisa9a	shisa family member 9a [Source:NCBI gene;Acc:541382]	12	19792281	19865585		6.57	1.59	2.46	5.25	5.36	6.06
ENSDARG00000045146	tomm22	translocase of outer mitochondrial membrane 22 homolog (yeast) [Source:ZFIN;Acc:ZDB-GENE-030131-9810]	12	19348538	19353771		41.76	43.18	40.2	40.52	41.75	40.83
ENSDARG00000045147	tmem184ba	transmembrane protein 184ba [Source:ZFIN;Acc:ZDB-GENE-050417-219]	12	19320657	19340220		9.57	5.06	4.18	11.66	11.68	11.99
ENSDARG00000045150	csnk1e	casein kinase 1, epsilon [Source:ZFIN;Acc:ZDB-GENE-030131-7873]	12	19305390	19321087		6.56	2.02	2.67	9.42	9.97	9.6
ENSDARG00000045153	slc16a8	solute carrier family 16 member 8 [Source:ZFIN;Acc:ZDB-GENE-121128-1]	12	19191787	19198771		2.53	0.64	1.45	3.27	2.89	3.23
ENSDARG00000045155	phf5a	PHD finger protein 5A [Source:ZFIN;Acc:ZDB-GENE-021219-3]	12	19138452	19143692		7.88	10.16	9.58	5.56	5.3	5.85
ENSDARG00000045156	rgs9b	regulator of G protein signaling 9b [Source:ZFIN;Acc:ZDB-GENE-040426-1708]	12	18681423	18717659		3.64	1.02	1.67	3.13	3.27	3.28
ENSDARG00000045157	si:ch211-147h1.4	si:ch211-147h1.4 [Source:ZFIN;Acc:ZDB-GENE-040724-228]	12	18663154	18666282		0.01	0.03	0.02	0	0.01	0
ENSDARG00000045159	zdhhc4	zinc finger DHHC-type containing 4 [Source:ZFIN;Acc:ZDB-GENE-030131-9031]	12	18571455	18578432		6.85	8.75	9.23	7.67	6.71	7.51
ENSDARG00000045160	msrb1b	methionine sulfoxide reductase B1b [Source:ZFIN;Acc:ZDB-GENE-040912-88]	12	18529311	18531943		0	0	0	0	0	0
ENSDARG00000045164	nptx2b	neuronal pentraxin IIb [Source:ZFIN;Acc:ZDB-GENE-110328-1]	12	17889465	17897134		3.65	0.56	0.62	1.72	2.36	2.29
ENSDARG00000045166	bhlha15	basic helix-loop-helix family, member a15 [Source:ZFIN;Acc:ZDB-GENE-070803-3]	12	17754859	17756905		2.61	3.26	1.93	0.71	0.62	0.58
ENSDARG00000045167	dlgap5	discs, large (Drosophila) homolog-associated protein 5 [Source:ZFIN;Acc:ZDB-GENE-040912-160]	12	17633354	17655683		8.77	5.61	5.08	7.27	6.97	7.53
ENSDARG00000045172	minpp1b	multiple inositol-polyphosphate phosphatase 1b [Source:ZFIN;Acc:ZDB-GENE-040426-888]	12	17482607	17492885		2.37	2.93	2.26	1.69	1.27	1.74
ENSDARG00000045175	pofut2	protein O-fucosyltransferase 2 [Source:ZFIN;Acc:ZDB-GENE-030131-3595]	9	40851088	40873934		4.25	3.74	3.94	5.54	6.51	6
ENSDARG00000045180	acta2	actin alpha 2, smooth muscle [Source:ZFIN;Acc:ZDB-GENE-030131-1229]	12	17100021	17116481		74.61	89.72	58.9	43.05	44.69	41.74
ENSDARG00000045190	ch25h	cholesterol 25-hydroxylase [Source:NCBI gene;Acc:494109]	12	17042754	17043874		0.23	0.18	0.18	0.71	0.54	0.61
ENSDARG00000045199	CAPN1	zgc:55262 [Source:ZFIN;Acc:ZDB-GENE-040426-2814]	7	6684123	6754012	CAPN1	13.78	14.24	11.06	10.73	11.51	11.14
ENSDARG00000045200	dusp19b	dual specificity phosphatase 19b [Source:ZFIN;Acc:ZDB-GENE-040502-1]	7	5885800	6680628		1.97	1.46	1.55	1.93	2.17	2.06
ENSDARG00000045219	dkk1b	dickkopf WNT signaling pathway inhibitor 1b [Source:ZFIN;Acc:ZDB-GENE-990708-5]	12	6465557	6467675		4.71	5.7	6.99	5.95	6.37	6.63
ENSDARG00000045224	glipr1a	GLI pathogenesis-related 1a [Source:ZFIN;Acc:ZDB-GENE-110309-2]	4	2252123	2258796		0.76	0.66	0.72	1.25	1.45	1.6
ENSDARG00000045228	stub1	STIP1 homology and U-Box containing protein 1 [Source:ZFIN;Acc:ZDB-GENE-030131-2963]	24	39656834	39667465		17.57	14.84	14.94	17.31	17.73	18.97
ENSDARG00000045230	cox6b1	cytochrome c oxidase subunit 6B1 [Source:ZFIN;Acc:ZDB-GENE-040718-448]	24	39606053	39610585		115.07	148.11	131.57	161.16	177.19	178.58
ENSDARG00000045248	h3f3d	H3 histone, family 3D [Source:ZFIN;Acc:ZDB-GENE-040426-1023]	24	37688729	37693028		468.95	332.15	331.2	519.99	517.35	549.91
ENSDARG00000045249	nif3l1	NIF3 NGG1 interacting factor 3-like 1 (S. cerevisiae) [Source:ZFIN;Acc:ZDB-GENE-050706-80]	12	3871452	3909738		5.88	5.98	5.54	5.43	5.42	5.36
ENSDARG00000045254	zmp:0000000624	zmp:0000000624 [Source:ZFIN;Acc:ZDB-GENE-130530-627]	24	37700259	37708345		10.85	14.21	10.16	7.35	5.91	6.14
ENSDARG00000045257	decr2	2,4-dienoyl CoA reductase 2, peroxisomal [Source:NCBI gene;Acc:406623]	24	37709191	37721739		4.96	6.46	5.71	4.13	4.13	4.54
ENSDARG00000045262	gsnb	gelsolin b [Source:ZFIN;Acc:ZDB-GENE-030131-8567]	5	63294865	63302944		31.41	31.5	30.59	15.05	14.11	14.76
ENSDARG00000045265	gsg1l2b	gsg1-like 2b [Source:ZFIN;Acc:ZDB-GENE-121214-171]	12	387850	408076		2.73	0.5	1.08	3.11	3.55	3.8
ENSDARG00000045268	tectb	tectorin beta [Source:ZFIN;Acc:ZDB-GENE-041212-16]	12	31476169	31484677		3.17	2.13	2.52	4.48	4.8	5.45
ENSDARG00000045269	pts	6-pyruvoyltetrahydropterin synthase [Source:ZFIN;Acc:ZDB-GENE-070615-3]	12	312448	314899		40.54	57.3	61	49.4	43.52	49.11
ENSDARG00000045273	tmem204	transmembrane protein 204 [Source:ZFIN;Acc:ZDB-GENE-040724-12]	24	37854002	37877978		8.41	10.17	9.7	15.55	16.78	17.15
ENSDARG00000045275	klhl14	kelch-like family member 14 [Source:ZFIN;Acc:ZDB-GENE-091204-223]	24	35881124	35906239		3.62	0.79	1.11	5.29	5.4	5.93
ENSDARG00000045277	rdh12l	retinol dehydrogenase 12, like [Source:ZFIN;Acc:ZDB-GENE-040801-48]	24	32573480	32582880		1.94	2.9	1.89	1.52	1.59	1.74
ENSDARG00000045289	gpr137c	G protein-coupled receptor 137c [Source:ZFIN;Acc:ZDB-GENE-041114-145]	17	14965570	15012127		5.23	4.52	4.09	7.2	8.08	7.7
ENSDARG00000045294	sh3glb1a	SH3-domain GRB2-like endophilin B1a [Source:ZFIN;Acc:ZDB-GENE-050417-89]	24	28383278	28397902		9.25	10.44	10.73	13.29	12.33	12.15
ENSDARG00000045296	cops9	COP9 signalosome subunit 9 [Source:NCBI gene;Acc:393820]	24	26284514	26302375		45.03	44.49	46.63	49.69	53.42	52.99
ENSDARG00000045297	phb2a	prohibitin 2a [Source:ZFIN;Acc:ZDB-GENE-030131-3141]	2	5178172	5199431		54.2	52.09	49.58	44.98	44.54	43.64
ENSDARG00000045298	wipf2a	WAS/WASL interacting protein family, member 2a [Source:ZFIN;Acc:ZDB-GENE-121219-6]	11	11844123	11890001		8.91	8.54	5.68	9.36	8.29	8.77
ENSDARG00000045299	vmo1b	vitelline membrane outer layer 1 homolog b [Source:ZFIN;Acc:ZDB-GENE-120215-131]	21	1618181	1625976		17.1	25.74	21.09	10.61	12.14	11.92
ENSDARG00000045301	st8sia3	ST8 alpha-N-acetyl-neuraminide alpha-2,8-sialyltransferase 3 [Source:ZFIN;Acc:ZDB-GENE-060228-4]	21	1780790	1799265		2.19	0.23	0.82	2.44	2.71	2.5
ENSDARG00000045302	smpx	small muscle protein X-linked [Source:ZFIN;Acc:ZDB-GENE-040704-24]	24	25467465	25500712		4.98	5.56	5.21	3.95	4.14	3.83
ENSDARG00000045304	mtfr1	mitochondrial fission regulator 1 [Source:NCBI gene;Acc:494035]	24	24726956	24733255		3.53	3.91	2.79	3.51	3.88	3.8
ENSDARG00000045305	pde7a	phosphodiesterase 7A [Source:ZFIN;Acc:ZDB-GENE-031222-10]	24	24733307	24797455		8.57	4.69	4.85	13.05	12.08	13.33
ENSDARG00000045306	slc51a	solute carrier family 51 alpha subunit [Source:NCBI gene;Acc:447807]	24	24961899	24983047		24	31.91	29.89	13.81	15.04	14.15
ENSDARG00000045308	pola1	polymerase (DNA directed), alpha 1 [Source:ZFIN;Acc:ZDB-GENE-030114-9]	24	23840821	23932525		8.79	7.1	6.61	5.23	4.95	4.56
ENSDARG00000045316	map7d2b	MAP7 domain containing 2b [Source:ZFIN;Acc:ZDB-GENE-091118-82]	24	24118193	24163201		17.08	5.27	6.68	17.43	18.36	19.16
ENSDARG00000045331	pkp4	plakophilin 4 [Source:ZFIN;Acc:ZDB-GENE-120411-14]	6	12197186	12296170		4.21	2.01	1.74	5.63	5.46	5.39
ENSDARG00000045342	si:ch211-140b10.6	si:ch211-140b10.6 [Source:ZFIN;Acc:ZDB-GENE-091204-382]	24	21676921	21681424		4.55	3.44	3.7	4.32	4.35	4.31
ENSDARG00000045343	usp46	ubiquitin specific peptidase 46 [Source:NCBI gene;Acc:100148916]	20	23083800	23111727		1.46	0.5	0.79	1.67	1.88	1.96
ENSDARG00000045351	ccdc58	coiled-coil domain containing 58 [Source:NCBI gene;Acc:393780]	24	20922544	20926812		11.64	13.33	12.97	10.09	8.68	8.45
ENSDARG00000045352	cst14a.2	cystatin 14a, tandem duplicate 2 [Source:ZFIN;Acc:ZDB-GENE-040426-1065]	24	20920563	20921714		44.55	59.51	51.84	36.79	36.82	38.3
ENSDARG00000045355	eloca	elongin C paralog a [Source:ZFIN;Acc:ZDB-GENE-040912-120]	24	12983434	12988849		11.41	13.66	13.74	11.69	10.33	11.2
ENSDARG00000045359	elocb	elongin C paralog b [Source:ZFIN;Acc:ZDB-GENE-040718-138]	2	30244376	30250311		13.94	16.76	14.96	10.96	11.44	11.94
ENSDARG00000045360	flj11011l	hypothetical protein FLJ11011-like (H. sapiens) [Source:NCBI gene;Acc:692325]	24	12989727	13016024		4.75	3.81	3.57	6.86	6.44	6.7
ENSDARG00000045364	trappc3	trafficking protein particle complex 3 [Source:ZFIN;Acc:ZDB-GENE-040801-120]	19	35722902	35733401		23.36	17.7	15.16	19.52	20.04	21.35
ENSDARG00000045367	tuba1b	tubulin, alpha 1b [Source:ZFIN;Acc:ZDB-GENE-030822-1]	23	35713557	35716519		52.43	26.23	27.53	42.96	46.12	53.15
ENSDARG00000045371	prdm14	PR domain containing 14 [Source:ZFIN;Acc:ZDB-GENE-030131-2709]	24	14451404	14456103		0.28	0.27	0.22	0.44	0.89	0.62
ENSDARG00000045372	ngdn	neuroguidin, EIF4E binding protein [Source:NCBI gene;Acc:386642]	24	14541013	14557158		4.12	5.18	4.96	3.04	2.32	2.71
ENSDARG00000045373	pabpn1	poly(A) binding protein, nuclear 1 [Source:ZFIN;Acc:ZDB-GENE-040426-2401]	24	14559328	14577559		48.66	36.59	37.84	45.22	41.99	41.94
ENSDARG00000045378	pi15a	peptidase inhibitor 15a [Source:NCBI gene;Acc:561978]	24	14801844	14810634		0.57	0.79	0.72	4.6	5.67	5.65
ENSDARG00000045383	gpr22b	G protein-coupled receptor 22b [Source:ZFIN;Acc:ZDB-GENE-141212-234]	25	3378135	3393705		1.08	0.43	0.55	1.79	1.69	1.62
ENSDARG00000045391	cd226	CD226 molecule [Source:ZFIN;Acc:ZDB-GENE-070112-1322]	24	15114092	15133886		0.37	0.58	0.55	0.34	0.43	0.53
ENSDARG00000045398	mtrr	5-methyltetrahydrofolate-homocysteine methyltransferase reductase [Source:ZFIN;Acc:ZDB-GENE-101115-3]	24	16851213	16905018		1.87	1.94	1.51	1.62	1.51	1.47
ENSDARG00000045399	cct5	chaperonin containing TCP1, subunit 5 (epsilon) [Source:ZFIN;Acc:ZDB-GENE-030131-977]	24	16905188	16912897		154.64	143.8	143.6	138.37	137.13	138.18
ENSDARG00000045401	mllt10	MLLT10 histone lysine methyltransferase DOT1L cofactor [Source:ZFIN;Acc:ZDB-GENE-041114-176]	24	17140938	17216312		5.7	3.07	3.11	9.47	9.25	9.81
ENSDARG00000045402	commd3	COMM domain containing 3 [Source:ZFIN;Acc:ZDB-GENE-041219-3]	24	17256793	17259643		8.42	9.58	8.73	8.19	9.71	9.1
ENSDARG00000045408	tagln	transgelin [Source:ZFIN;Acc:ZDB-GENE-070912-1]	15	543744	551181		79.16	114.33	110.61	52.39	61.17	59.19
ENSDARG00000045411	acad11	acyl-CoA dehydrogenase family, member 11 [Source:ZFIN;Acc:ZDB-GENE-040426-814]	24	11350330	11446166		7.26	9.7	6.6	4.31	4.01	4.24
ENSDARG00000045413	gcm2	glial cells missing transcription factor 2 [Source:ZFIN;Acc:ZDB-GENE-050127-1]	24	8827603	8831912		8.08	9.2	8.87	3.82	4.58	4.96
ENSDARG00000045414	elovl2	ELOVL fatty acid elongase 2 [Source:ZFIN;Acc:ZDB-GENE-060421-5612]	24	8861272	8887825		9.72	18.1	12.47	0.89	1.07	1.02
ENSDARG00000045415	gnal	guanine nucleotide binding protein (G protein), alpha activating activity polypeptide, olfactory type [Source:ZFIN;Acc:ZDB-GENE-04	24	8904135	8973300		5.84	2.28	2.64	6.37	5.95	6.1
ENSDARG00000045416	mppe1	metallophosphoesterase 1 [Source:NCBI gene;Acc:550396]	24	8979452	9002100		4.97	3.4	3.82	4.29	4.54	4.97
ENSDARG00000045417	fam49bb	family with sequence similarity 49 member Bb [Source:ZFIN;Acc:ZDB-GENE-040426-2590]	24	10792773	10897557		7.9	6.25	6.16	5.98	5.7	5.96
ENSDARG00000045420	epdr1	ependymin related 1 [Source:ZFIN;Acc:ZDB-GENE-040718-113]	2	31833997	31836902		8.11	4.13	5.18	9.21	8.67	9.35
ENSDARG00000045421	stard3nl	STARD3 N-terminal like [Source:ZFIN;Acc:ZDB-GENE-040718-4]	2	31838442	31853345		6.02	4.15	3.69	7.88	7.58	7.46
ENSDARG00000045422	otulina	OTU deubiquitinase with linear linkage specificity a [Source:ZFIN;Acc:ZDB-GENE-040426-2157]	24	10303071	10309934		2.29	1.74	2.51	2.39	2.88	3.51
ENSDARG00000045423	si:ch211-146l10.8	si:ch211-146l10.8 [Source:ZFIN;Acc:ZDB-GENE-160113-6]	24	10027902	10032784		0	0	0	0	0	0.01
ENSDARG00000045424	zgc:173856	zgc:173856 [Source:ZFIN;Acc:ZDB-GENE-030131-5358]	24	10018653	10021341		0	0	0.01	0.04	0.01	0.06
ENSDARG00000045427		si:dkey-96n2.3 [Source:ZFIN;Acc:ZDB-GENE-091204-118]	24	9604442	9605320		0	0.05	0	0.05	0	0.11
ENSDARG00000045428	mcee	methylmalonyl CoA epimerase [Source:ZFIN;Acc:ZDB-GENE-050522-368]	7	30779761	30783043		11.4	11.64	10.37	7.02	7.46	7.17
ENSDARG00000045437	slc35b3	solute carrier family 35 member B3 [Source:ZFIN;Acc:ZDB-GENE-060312-46]	24	8393290	8412526		5.7	5.75	5.93	5.8	4.81	4.56
ENSDARG00000045442	cpb1	carboxypeptidase B1 (tissue) [Source:ZFIN;Acc:ZDB-GENE-030131-1132]	24	4755391	4765740		174.02	269.54	223.95	17.03	18.04	20.45
ENSDARG00000045443	agtr1b	angiotensin II receptor, type 1b [Source:ZFIN;Acc:ZDB-GENE-110411-117]	24	4767806	4782052		1.51	1.44	1.84	2.72	2.45	2.83
ENSDARG00000045444	fzd8a	frizzled class receptor 8a [Source:ZFIN;Acc:ZDB-GENE-000328-3]	24	4447635	4450238		9.82	6.84	5.54	13.19	13.16	13.86
ENSDARG00000045447	slc35g2b	solute carrier family 35 member G2b [Source:ZFIN;Acc:ZDB-GENE-050320-142]	24	3413854	3419998		15.56	4.8	7.77	14.94	15.62	16.3
ENSDARG00000045453	f13a1a.1	coagulation factor XIII, A1 polypeptide a, tandem duplicate 1 [Source:ZFIN;Acc:ZDB-GENE-060929-1250]	24	2495197	2515623		0	0	0	0.01	0	0.01
ENSDARG00000045463	ralaa	v-ral simian leukemia viral oncogene homolog Aa (ras related) [Source:ZFIN;Acc:ZDB-GENE-040426-1344]	24	1011158	1023184		5.63	3.56	4.38	4.8	4.89	5.15
ENSDARG00000045465	lancl2	LanC lantibiotic synthetase component C-like 2 (bacterial) [Source:ZFIN;Acc:ZDB-GENE-070209-191]	24	166892	188943		9.61	5.8	6.77	14.6	14.6	14.54
ENSDARG00000045481	gsap	gamma-secretase activating protein [Source:ZFIN;Acc:ZDB-GENE-041210-216]	4	20198294	20222182		2.9	2.9	3.24	4.31	4.68	4.21
ENSDARG00000045482	stk38l	serine/threonine kinase 38 like [Source:ZFIN;Acc:ZDB-GENE-040426-798]	4	20224099	20235904		16.47	8.03	9.97	11.2	11.39	12.6
ENSDARG00000045483	lrtm2a	leucine-rich repeats and transmembrane domains 2a [Source:ZFIN;Acc:ZDB-GENE-041210-218]	4	20255160	20265726		14.83	6.15	5.65	15.76	15.69	16.17
ENSDARG00000045484	dcp1b	decapping mRNA 1B [Source:ZFIN;Acc:ZDB-GENE-040819-1]	4	20302832	20314796		3.84	3.55	3.33	4.26	4.41	4.47
ENSDARG00000045485	rassf8b	Ras association domain family member 8b [Source:ZFIN;Acc:ZDB-GENE-040426-1338]	4	20507105	20521441		5.88	6.02	6.91	8.3	8.61	8.83
ENSDARG00000045486	pawr	PRKC, apoptosis, WT1, regulator [Source:ZFIN;Acc:ZDB-GENE-041010-108]	4	21323006	21466825		12.2	14.67	14.15	10.54	12.68	12.68
ENSDARG00000045487	rps16	ribosomal protein S16 [Source:ZFIN;Acc:ZDB-GENE-050506-107]	4	21648010	21652812		259.13	330.55	388.07	233.98	237.63	236.98
ENSDARG00000045490	ndufb2	NADH:ubiquinone oxidoreductase subunit B2 [Source:ZFIN;Acc:ZDB-GENE-040426-1762]	4	22480169	22483131		26.16	34.65	44.65	40.68	42.13	42.57
ENSDARG00000045493	slc35e3	solute carrier family 35 member E3 [Source:NCBI gene;Acc:563675]	4	23117557	23122395		5.48	8.02	6.98	7.31	6.51	7.63
ENSDARG00000045506	gramd4b	GRAM domain containing 4b [Source:ZFIN;Acc:ZDB-GENE-030131-4780]	25	19870603	19936598		6.33	3.67	3.17	6.69	6.76	6.91
ENSDARG00000045510	tbc1d22a	TBC1 domain family, member 22a [Source:ZFIN;Acc:ZDB-GENE-041114-144]	4	27626869	27897315		2.63	2.05	1.96	2.67	2.76	2.92
ENSDARG00000045513	taf3	TAF3 RNA polymerase II, TATA box binding protein (TBP)-associated facto [Source:ZFIN;Acc:ZDB-GENE-030131-6406]	4	25084228	25168458		10.93	8.69	7.39	14.03	13.19	13.81
ENSDARG00000045514	atp5f1c	ATP synthase F1 subunit gamma [Source:ZFIN;Acc:ZDB-GENE-030131-8901]	4	25173299	25181552		372.92	458.85	522.66	255.26	259	257.58
ENSDARG00000045515	kin	Kin17 DNA and RNA binding protein [Source:ZFIN;Acc:ZDB-GENE-030131-3689]	4	25181572	25188160		8.34	8.4	7.92	6.99	6.31	7
ENSDARG00000045516	itih2	inter-alpha-trypsin inhibitor heavy chain 2 [Source:ZFIN;Acc:ZDB-GENE-040426-1942]	4	25187912	25215968		48.86	81.59	77.73	44.58	48.88	49.84
ENSDARG00000045517	itih5	inter-alpha-trypsin inhibitor heavy chain 5 [Source:ZFIN;Acc:ZDB-GENE-041210-193]	4	25220674	25245817		1.23	1.81	1.59	1	0.87	1
ENSDARG00000045518	tmem110l	transmembrane protein 110, like [Source:ZFIN;Acc:ZDB-GENE-040426-912]	4	25251855	25271455		23.85	36.57	34.15	24.95	23.66	25.57
ENSDARG00000045519	sfmbt2	Scm like with four mbt domains 2 [Source:ZFIN;Acc:ZDB-GENE-041210-196]	4	25300910	25359034		3.79	2.98	2.82	3.58	3.68	3.71
ENSDARG00000045522	acot15	acyl-CoA thioesterase 15 [Source:ZFIN;Acc:ZDB-GENE-041210-203]	4	25627147	25647986		11.33	15.14	14.35	15.67	17.86	18.57
ENSDARG00000045524	lamb1b	laminin, beta 1b [Source:ZFIN;Acc:ZDB-GENE-041210-197]	4	25704579	25751361		8.2	9.91	8.19	12.76	14.05	13.35
ENSDARG00000045525	tmcc3	transmembrane and coiled-coil domain family 3 [Source:ZFIN;Acc:ZDB-GENE-030131-1811]	4	25754725	25812329		8.83	8.69	6.45	9.62	9.6	9.24
ENSDARG00000045527	nr2c1	nuclear receptor subfamily 2, group C, member 1 [Source:ZFIN;Acc:ZDB-GENE-041210-198]	4	25838345	25859305		5.98	4.36	4.79	5.66	5.3	5.62
ENSDARG00000045528	fgd6	FYVE, RhoGEF and PH domain containing 6 [Source:ZFIN;Acc:ZDB-GENE-041210-200]	4	25859445	25911609		1.99	1.71	1.4	2.59	2.7	2.58
ENSDARG00000045530	si:ch211-244b2.4	si:ch211-244b2.4 [Source:ZFIN;Acc:ZDB-GENE-030131-8699]	4	26095462	26108053		1.1	1.62	1.02	0.75	1.04	0.85
ENSDARG00000045540	ppp6r2a	protein phosphatase 6, regulatory subunit 2a [Source:ZFIN;Acc:ZDB-GENE-030131-5428]	4	20082475	20101972		6.09	5.2	4.22	8.09	7.98	7.57
ENSDARG00000045541	gcc1	GRIP and coiled-coil domain containing 1 [Source:ZFIN;Acc:ZDB-GENE-040801-118]	4	20063279	20069493		9.84	7.18	6.36	7.85	7.64	8.06
ENSDARG00000045542	lamtor4	late endosomal/lysosomal adaptor, MAPK and MTOR activator 4 [Source:NCBI gene;Acc:561019]	4	20051478	20054583		27.96	27.52	30.64	20.35	20.22	20.87
ENSDARG00000045543	atp6v1f	ATPase H+ transporting V1 subunit F [Source:ZFIN;Acc:ZDB-GENE-040718-259]	4	20049260	20063234		57.51	55.41	55.76	49.7	44.75	46.8
ENSDARG00000045544	hgfa	hepatocyte growth factor a [Source:ZFIN;Acc:ZDB-GENE-041014-2]	4	19710657	19742303		0.66	1.01	0.71	1.55	1.48	1.54
ENSDARG00000045548	lepb	leptin b [Source:ZFIN;Acc:ZDB-GENE-041210-183]	4	19031768	19033805		0.04	0	0	4.55	3.81	3.55
ENSDARG00000045549	bik	BCL2 interacting killer [Source:ZFIN;Acc:ZDB-GENE-041210-181]	4	18843015	18846611		3.36	3.06	3.55	2.66	2.54	2.09
ENSDARG00000045552	si:dkey-202b22.6	si:dkey-202b22.6 [Source:ZFIN;Acc:ZDB-GENE-041210-176]	4	18494857	18499761		0.51	0.44	0.41	0.68	0.65	0.64
ENSDARG00000045553	hsd17b2	hydroxysteroid (17-beta) dehydrogenase 2 [Source:ZFIN;Acc:ZDB-GENE-040928-1]	25	35942867	35950535		1.29	1.21	1.23	1.77	1.76	1.64
ENSDARG00000045554	snx20	sorting nexin 20 [Source:ZFIN;Acc:ZDB-GENE-100806-1]	25	35957603	35963241		1.1	1.95	1.95	0.92	1.15	1.21
ENSDARG00000045555	sco2	SCO cytochrome c oxidase assembly protein 2 [Source:NCBI gene;Acc:606683]	4	18453702	18455480		7.3	9.23	9.21	4.94	4.79	4.87
ENSDARG00000045556	OTUD7A	si:dkey-37f18.2 [Source:ZFIN;Acc:ZDB-GENE-131120-28]	25	34407529	34458452	OTUD7A	2.64	0.65	0.83	3.53	3.3	3.65
ENSDARG00000045557	socs2	suppressor of cytokine signaling 2 [Source:ZFIN;Acc:ZDB-GENE-041210-171]	4	18432182	18436899		6.5	4.25	3.43	5.76	5.1	5.4
ENSDARG00000045560	mybpc1	myosin binding protein C, slow type [Source:ZFIN;Acc:ZDB-GENE-041114-196]	4	17726841	17793152		8.65	10.26	7.95	5.88	5.63	6.19
ENSDARG00000045561	dram1	DNA-damage regulated autophagy modulator 1 [Source:ZFIN;Acc:ZDB-GENE-041010-147]	4	17662413	17669881		1.36	1.81	2.15	1.18	1.13	1.38
ENSDARG00000045562	pus1	pseudouridine synthase 1 [Source:ZFIN;Acc:ZDB-GENE-041212-26]	21	14826583	14832369		8.93	8.54	6.83	5.06	4.76	4.48
ENSDARG00000045563	washc3	WASH complex subunit 3 [Source:NCBI gene;Acc:393142]	4	17655872	17662078		5.19	3.9	3.96	5.48	6.73	5.99
ENSDARG00000045565	noc4l	nucleolar complex associated 4 homolog [Source:NCBI gene;Acc:554096]	21	14817273	14826066		11.74	13.62	14.32	8.49	8.43	8.71
ENSDARG00000045567	phpt1	phosphohistidine phosphatase 1 [Source:ZFIN;Acc:ZDB-GENE-040801-85]	21	14806342	14815974		9.19	11.37	9.59	9.05	8.29	9.05
ENSDARG00000045568	bcat1	branched chain amino-acid transaminase 1, cytosolic [Source:ZFIN;Acc:ZDB-GENE-030131-9358]	4	17279966	17297420		4.5	2.22	2.58	7.26	7.8	8.36
ENSDARG00000045574	lrmp	lymphoid-restricted membrane protein [Source:NCBI gene;Acc:567234]	4	17252596	17278928		7.86	1.59	1.26	4.21	4.83	4.74
ENSDARG00000045580	lum	lumican [Source:ZFIN;Acc:ZDB-GENE-040625-24]	4	16349042	16354292		16.01	21.9	21.89	23.28	22.63	22.99
ENSDARG00000045586	zgc:172145	zgc:172145 [Source:ZFIN;Acc:ZDB-GENE-080516-9]	25	31424850	31433512		1.61	2.2	1.78	1.62	2.1	1.77

ENSDARG00000045589	kcnj8	potassium inwardly rectifying channel subfamily J member 8 [Source:ZFIN;Acc:ZDB-GENE-060308-1]	4	14717502	14719854		1.75	0.99	1.13	2.29	2.79	2.52
ENSDARG00000045592	tnni2a.1	troponin I type 2a (skeletal, fast), tandem duplicate 1 [Source:ZFIN;Acc:ZDB-GENE-041114-60]	25	31222069	31236549		65.97	100.31	95.51	53.95	53.91	52.3
ENSDARG00000045594	shisal1a	shisa like 1a [Source:NCBI gene;Acc:560435]	4	14826299	14863510		6.35	3.47	4.61	9.33	9.85	10.56
ENSDARG00000045595	si:dkey-14k9.3	si:dkey-14k9.3 [Source:ZFIN;Acc:ZDB-GENE-030131-2893]	4	14895506	14899113		4.64	2.91	3.24	3.37	4.1	3.99
ENSDARG00000045596	rassf8a	Ras association domain family member 8a [Source:ZFIN;Acc:ZDB-GENE-091014-4]	25	31122806	31162816		5.92	6.83	5.73	6.37	7.41	7.05
ENSDARG00000045598	akr1b1.2	aldo-keto reductase family 1, member B1 (aldose reductase), tandem duplicate 2 [Source:ZFIN;Acc:ZDB-GENE-041210-132]	4	14906608	14915268		26.31	30.41	28.91	9.95	9.53	10.74
ENSDARG00000045601	cax1	cation/H+ exchanger protein 1 [Source:ZFIN;Acc:ZDB-GENE-030131-3044]	4	14981854	14990926		2.35	3.54	3.22	3.05	3.38	3.01
ENSDARG00000045605	ticrr	TopBP1-interacting, checkpoint, and replication regulator [Source:NCBI gene;Acc:445411]	25	30316839	30337989		2.22	1.44	1.12	1.89	1.6	1.63
ENSDARG00000045606	alkbh3	alkB homolog 3, alpha-ketoglutarate-dependent dioxygenase [Source:ZFIN;Acc:ZDB-GENE-040801-254]	25	30233638	30264796		2.41	2.05	2.07	2.06	2.34	2.1
ENSDARG00000045608	tafa5b	TAFA chemokine like family member 5b [Source:ZFIN;Acc:ZDB-GENE-131127-365]	25	29911977	29988352		2.12	1.11	1.5	1.89	1.81	1.92
ENSDARG00000045611	nfybb	nuclear transcription factor Y, beta b [Source:ZFIN;Acc:ZDB-GENE-050306-25]	25	28823952	28846945		18.31	20.03	21.8	17.18	21.06	19.32
ENSDARG00000045612	lrrc10	leucine rich repeat containing 10 [Source:ZFIN;Acc:ZDB-GENE-071119-6]	25	28672539	28674739		0.4	0.92	0.57	0.85	1.18	0.83
ENSDARG00000045618	clta	clathrin, light chain A [Source:ZFIN;Acc:ZDB-GENE-040426-1986]	1	12348213	12361930		107.2	81.63	95.25	86.13	89.33	88.77
ENSDARG00000045620	nansa	N-acetylneuraminic acid synthase a [Source:ZFIN;Acc:ZDB-GENE-030131-1500]	1	12335816	12344792		24.31	32.27	31.43	20.3	17.82	18.4
ENSDARG00000045626	nek8	NIMA-related kinase 8 [Source:NCBI gene;Acc:171094]	15	15479442	15514013		0.96	0.71	0.84	1.7	2.05	1.64
ENSDARG00000045628	rab34a	RAB34, member RAS oncogene family a [Source:ZFIN;Acc:ZDB-GENE-041010-197]	15	15459974	15469096		5.06	5.52	4.73	5.11	5.26	5.14
ENSDARG00000045633	asb15a	ankyrin repeat and SOCS box containing 15a [Source:ZFIN;Acc:ZDB-GENE-110421-5]	25	27822188	27843066		0.84	1.23	1.15	1.23	0.88	0.94
ENSDARG00000045634	lmod2a	leiomodin 2 (cardiac) a [Source:ZFIN;Acc:ZDB-GENE-040912-41]	25	27816115	27819838		0.17	0.11	0.15	0.2	0.25	0.14
ENSDARG00000045636	rbl2	retinoblastoma-like 2 (p130) [Source:ZFIN;Acc:ZDB-GENE-061109-2]	25	36010815	36044583		3.8	1.67	1.37	4.24	4.12	4.22
ENSDARG00000045638	slc13a1	solute carrier family 13 member 1 [Source:ZFIN;Acc:ZDB-GENE-031222-3]	25	27923846	27932723		1.88	2.92	1.8	0.48	0.55	0.44
ENSDARG00000045639	elavl4	ELAV like neuron-specific RNA binding protein 4 [Source:NCBI gene;Acc:30737]	8	16004154	16117112		35.46	9.12	10.35	66.5	65.16	67.56
ENSDARG00000045641	usp3	ubiquitin specific peptidase 3 [Source:ZFIN;Acc:ZDB-GENE-030131-5142]	25	26738450	26753196		3.68	2.53	3.34	4.67	4.58	4.59
ENSDARG00000045644	ca12	carbonic anhydrase XII [Source:ZFIN;Acc:ZDB-GENE-080815-4]	25	26798673	26810914		0.58	0.68	0.29	0.34	0.43	0.36
ENSDARG00000045658	msrb3	methionine sulfoxide reductase B3 [Source:ZFIN;Acc:ZDB-GENE-040625-74]	4	12890668	12914307		3.62	4.95	4.01	3.1	3.23	2.88
ENSDARG00000045660	hic1l	hypermethylated in cancer 1 like [Source:ZFIN;Acc:ZDB-GENE-030620-1]	25	25564558	25575717		8.61	6.64	5.27	10.88	10.14	10.11
ENSDARG00000045661	irf7	interferon regulatory factor 7 [Source:ZFIN;Acc:ZDB-GENE-040426-1518]	25	25546789	25550955		0.75	0.72	0.8	1.59	1.6	1.88
ENSDARG00000045666	ANKRD50	ankyrin repeat domain 50 [Source:HGNC Symbol;Acc:HGNC:29223]	14	467104	470505	ANKRD50	5.74	3.97	3.78	7.33	6.47	7.61
ENSDARG00000045669	si:dkey-39a18.1	si:dkey-39a18.1 [Source:ZFIN;Acc:ZDB-GENE-041210-127]	4	13428993	13434027		0.13	0.2	0.09	0.3	0.35	0.3
ENSDARG00000045671	ifng1r	interferon gamma related [Source:ZFIN;Acc:ZDB-GENE-060210-1]	4	13517446	13518381		0	0	0	0	0	0
ENSDARG00000045672	il26	interleukin 26 [Source:ZFIN;Acc:ZDB-GENE-060209-4]	4	13535054	13538714		0	0.25	0.06	0.4	0.2	0.08
ENSDARG00000045673	il22	interleukin 22 [Source:ZFIN;Acc:ZDB-GENE-060209-3]	4	13544595	13548806		0.14	0	0.12	0.04	0.2	0.12
ENSDARG00000045675	mdm1	Mdm1 nuclear protein [Source:NCBI gene;Acc:557148]	4	13551400	13567516		2.63	0.93	1.23	1.77	1.9	2.29
ENSDARG00000045676	calua	calumenin a [Source:NCBI gene;Acc:415241]	4	13568469	13576215		24.79	28.35	26.64	52.97	57.38	55.02
ENSDARG00000045677	opn1sw1	opsin 1 (cone pigments), short-wave-sensitive 1 [Source:NCBI gene;Acc:30582]	4	13577665	13580348		1922.92	421.63	303.47	647.14	661.14	650.23
ENSDARG00000045680	tnpo3	transportin 3 [Source:ZFIN;Acc:ZDB-GENE-040426-708]	4	13586455	13605158		14.65	12.54	11.13	16.57	16.38	15.6
ENSDARG00000045681	irf5	interferon regulatory factor 5 [Source:ZFIN;Acc:ZDB-GENE-040426-2587]	4	13605600	13614936		2.31	2.42	1.87	2.15	2	1.98
ENSDARG00000045683	ccdc87	coiled-coil domain containing 87 [Source:ZFIN;Acc:ZDB-GENE-041210-118]	4	13615731	13624294		0.14	0.1	0.11	0.19	0.24	0.33
ENSDARG00000045685	cntn1b	contactin 1b [Source:ZFIN;Acc:ZDB-GENE-041210-236]	4	13733838	13756716		7.01	1.75	1.75	8.02	7.35	7.77
ENSDARG00000045687	pdzrn4	PDZ domain containing ring finger 4 [Source:ZFIN;Acc:ZDB-GENE-041210-237]	4	13810811	13861445		6.66	2.08	3.63	7.61	7.06	8.41
ENSDARG00000045691	zcrb1	zinc finger CCHC-type and RNA binding motif 1 [Source:NCBI gene;Acc:550251]	4	13923927	13931508		4.1	3.46	3.61	3.45	2.79	2.83
ENSDARG00000045692	pphln1	periphilin 1 [Source:ZFIN;Acc:ZDB-GENE-030131-1873]	4	13901458	13963440		13.34	11.47	11.32	11.98	10.98	11.73
ENSDARG00000045694	prickle1b	prickle homolog 1b [Source:ZFIN;Acc:ZDB-GENE-030131-2152]	4	13963499	13995766		10.8	9.45	8.55	12.39	11.81	11.19
ENSDARG00000045695	myca	MYC proto-oncogene, bHLH transcription factor a [Source:NCBI gene;Acc:30686]	24	10413484	10416284		20.82	16.18	14.44	19	17.77	17.86
ENSDARG00000045696	mrpl23	mitochondrial ribosomal protein L23 [Source:NCBI gene;Acc:415142]	25	23777469	23815949		34.84	41.9	42.45	30.49	32.86	32
ENSDARG00000045704	dclre1c	DNA cross-link repair 1C, PSO2 homolog (S. cerevisiae) [Source:NCBI gene;Acc:566285]	4	9603306	9637398		4.06	2.53	3.17	3.48	2.9	2.95
ENSDARG00000045705	meig1	meiosis/spermiogenesis associated 1 [Source:NCBI gene;Acc:541343]	4	9609905	9611875		4.15	4.17	4.62	3.14	3.48	3.4
ENSDARG00000045706	tspan9b	tetraspanin 9b [Source:NCBI gene;Acc:431733]	4	9696965	9722568		8.77	8.08	7.28	13.68	14.04	13.68
ENSDARG00000045708	adm2a	adrenomedullin 2a [Source:ZFIN;Acc:ZDB-GENE-041210-100]	4	9880647	9891874		1.86	2.06	1.81	4.2	4.52	4.1
ENSDARG00000045733	clapin1	cytokine induced apoptosis inhibitor 1 [Source:NCBI gene;Acc:445283]	25	14398650	14406731		28.33	32.61	34.52	22.18	20.62	20.77
ENSDARG00000045737	guca1g	guanylate cyclase activator 1g [Source:ZFIN;Acc:ZDB-GENE-050120-1]	25	14092871	14096311		5.01	1.58	1.37	3.65	3.61	3.66
ENSDARG00000045747	cped1	cadherin-like and PC-esterase domain containing 1 [Source:ZFIN;Acc:ZDB-GENE-041210-247]	4	10616626	10704585		6.4	6.28	5.26	5.64	5.51	5.6
ENSDARG00000045748	stab2	stabilin 2 [Source:ZFIN;Acc:ZDB-GENE-041210-336]	4	10721795	10763547		2.32	2.69	2.64	4.5	4.47	4.83
ENSDARG00000045749	ppfibp1a	PPFIA binding protein 1a [Source:ZFIN;Acc:ZDB-GENE-041210-298]	4	10811490	10835620		9.23	10.58	10.23	9.05	8.69	9.79
ENSDARG00000045750	syt10	synaptotagmin X [Source:ZFIN;Acc:ZDB-GENE-041210-297]	4	10888762	10909086		2.52	0.63	0.95	3.11	3.43	3.61
ENSDARG00000045752	si:ch211-161n3.4	si:ch211-161n3.4 [Source:ZFIN;Acc:ZDB-GENE-110913-46]	4	10949389	10958074		0.01	0	0	0.02	0.03	0.02
ENSDARG00000045753	tmtc2a	transmembrane O-mannosyltransferase targeting cadherins 2a [Source:ZFIN;Acc:ZDB-GENE-041210-299]	4	10972585	11024844		2.34	1.2	1.61	3.13	3.33	3.34
ENSDARG00000045754	mettl25	methyltransferase like 25 [Source:ZFIN;Acc:ZDB-GENE-041210-90]	4	11035103	11053543		1.82	1.52	1.41	1.61	1.5	1.57
ENSDARG00000045755	ccdc59	coiled-coil domain containing 59 [Source:NCBI gene;Acc:564486]	4	11053301	11055737		7.12	7.62	5.53	4.92	4.58	5.91
ENSDARG00000045758	cracr2ab	calcium release activated channel regulator 2Ab [Source:ZFIN;Acc:ZDB-GENE-041210-94]	4	11135048	11148941		0.27	0.1	0.11	0.51	0.5	0.44
ENSDARG00000045760	prmt8b	protein arginine methyltransferase 8b [Source:NCBI gene;Acc:564110]	4	11144600	11165961		3.63	1.17	1.68	2.78	2.93	2.96
ENSDARG00000045765	asb13a.2	ankyrin repeat and SOCS box containing 13a, tandem duplicate 2 [Source:ZFIN;Acc:ZDB-GENE-041210-88]	4	11483335	11524888		1.36	0.66	1.05	0.63	0.86	0.66
ENSDARG00000045768	cry1a	cryptochrome circadian regulator 1a [Source:ZFIN;Acc:ZDB-GENE-010426-2]	4	12013043	12026507		8.68	5.07	3.99	8.33	9.41	9.04
ENSDARG00000045773	PYURF	zgc:162634 [Source:ZFIN;Acc:ZDB-GENE-050506-109]	25	17847054	17849574	PYURF	19.6	25	26.03	14.91	15.84	15.47
ENSDARG00000045776	cnbpa	CCHC-type zinc finger, nucleic acid binding protein a [Source:ZFIN;Acc:ZDB-GENE-030131-5045]	23	4699144	4705253		91.68	100.97	97.82	80.97	79.18	77.5
ENSDARG00000045785	C25H12orf29	zgc:103499 [Source:NCBI gene;Acc:494063]	25	17925424	17934675	C25H12orf29	6.29	4.99	4.96	5.31	5.29	5.06
ENSDARG00000045788	avpr1ab	arginine vasopressin receptor 1Ab [Source:ZFIN;Acc:ZDB-GENE-041210-105]	4	9207722	9214288		0.99	0.66	0.68	1.22	1.43	1.4
ENSDARG00000045789	srgap1b	SLIT-ROBO Rho GTPase activating protein 1b [Source:ZFIN;Acc:ZDB-GENE-041210-103]	4	9279515	9335035		5.64	1.63	1.32	7.77	8.19	7.38
ENSDARG00000045790	KICS2	si:ch211-125a15.1 [Source:NCBI gene;Acc:555715]	4	9339067	9350585	KICS2	6.42	5.6	5.57	4.64	4.76	4.45
ENSDARG00000045792	si:dkey-106n21.1	si:dkey-106n21.1 [Source:ZFIN;Acc:ZDB-GENE-131120-167]	25	18159795	18194521		0.82	1.44	1.2	0.73	0.61	0.54
ENSDARG00000045797	C25H12orf73	si:ch211-68a17.7 [Source:ZFIN;Acc:ZDB-GENE-100427-3]	25	18951554	18953322	C25H12orf73	11.97	15.57	14.94	7.24	9.18	9.79
ENSDARG00000045799	acanb	aggrecan b [Source:ZFIN;Acc:ZDB-GENE-100422-16]	25	19213872	19224298		3.22	2.29	2.03	13.78	14.95	14.99
ENSDARG00000045801	fbxl14b	F-box and leucine-rich repeat protein 14b [Source:ZFIN;Acc:ZDB-GENE-030131-3556]	4	8609517	8611841		13.25	8.22	7.57	17.42	18.35	17.19
ENSDARG00000045802	hapln3	hyaluronan and proteoglycan link protein 3 [Source:ZFIN;Acc:ZDB-GENE-040426-2089]	25	19201231	19205875		9.7	11.15	12.49	9.22	10.06	10.13
ENSDARG00000045803	mfge8b	milk fat globule-EGF factor 8 protein b [Source:ZFIN;Acc:ZDB-GENE-070209-137]	25	19149241	19192303		1.61	0.78	0.63	2.19	2.04	2.13
ENSDARG00000045804	abhd2b	abhydrolase domain containing 2b [Source:NCBI gene;Acc:559290]	25	19120529	19139259		3.85	1.44	2.33	2.22	1.98	2.59
ENSDARG00000045808	rbp1b	retinaldehyde binding protein 1b [Source:ZFIN;Acc:ZDB-GENE-040426-1870]	25	19105804	19118127		0.81	0.42	0.39	1.89	2.23	1.99
ENSDARG00000045811	lrtm2b	leucine-rich repeats and transmembrane domains 2b [Source:ZFIN;Acc:ZDB-GENE-131121-544]	25	19041329	19045977		2.81	0.39	0.43	3.44	2.43	3.02
ENSDARG00000045814	samm50	SAMM50 sorting and assembly machinery component [Source:NCBI gene;Acc:393111]	25	19008497	19021823		21.06	24.24	21.33	21.88	23.25	21.9
ENSDARG00000045815	mcm10	minichromosome maintenance 10 replication initiation factor [Source:NCBI gene;Acc:553396]	4	7851129	7869731		2.31	2.02	1.81	1.16	1.25	1.19
ENSDARG00000045822	tnnt2e	troponin T2e, cardiac [Source:ZFIN;Acc:ZDB-GENE-041210-27]	4	7508316	7544687		83.1	104.38	101.51	105.35	111.14	108.92
ENSDARG00000045824	zgc:101783	zgc:101783 [Source:ZFIN;Acc:ZDB-GENE-040912-48]	25	19733704	19752145		1.69	1.67	1.39	1.79	1.67	1.86
ENSDARG00000045827	lymS5b	LYR motif containing 5b [Source:NCBI gene;Acc:561760]	25	19557628	19561261		0.71	1.18	1.31	0.76	0.61	0.8
ENSDARG00000045832	cart2	cocaine- and amphetamine-regulated transcript 2 [Source:ZFIN;Acc:ZDB-GENE-050417-23]	25	19441920	19443421		5.46	2.32	3.29	7.8	9.26	9.54
ENSDARG00000045834	si:dkey-14d8.7	si:dkey-14d8.7 [Source:ZFIN;Acc:ZDB-GENE-041210-143]	4	5537101	5539842		9.46	11.73	8.9	0.16	0.21	0.55
ENSDARG00000045835	si:dkey-14d8.6	si:dkey-14d8.6 [Source:ZFIN;Acc:ZDB-GENE-041210-124]	4	5531583	5533803		93.01	132.54	103.92	2.41	2.6	3.1
ENSDARG00000045836	mapk11	mitogen-activated protein kinase 11 [Source:ZFIN;Acc:ZDB-GENE-040625-75]	4	5506946	5522437		7.42	3.66	4.86	6.34	5.66	6.85
ENSDARG00000045837	si:dkey-14d8.20	si:dkey-14d8.20 [Source:ZFIN;Acc:ZDB-GENE-041210-146]	4	5392094	5494298		0.67	0.31	0.38	0.57	0.74	0.61
ENSDARG00000045842	zgc:113263	zgc:113263 [Source:ZFIN;Acc:ZDB-GENE-050306-34]	4	5341592	5368158		2.94	1.8	1.89	5.36	5.85	6.05
ENSDARG00000045843	apex1	APEX nuclease (multifunctional DNA repair enzyme) 1 [Source:NCBI gene;Acc:406730]	4	5333988	5340530		26.34	18.3	18.2	16.45	15.15	15.4
ENSDARG00000045846	osgep	O-sialoglycoprotein endopeptidase [Source:NCBI gene;Acc:550447]	4	5327632	5333971		7.19	8.9	9.34	4.63	4.76	4.74
ENSDARG00000045847	si:ch211-214j24.10	si:ch211-214j24.10 [Source:ZFIN;Acc:ZDB-GENE-030131-6301]	4	5317483	5325336		26.89	29.27	26.1	30.81	33.17	31.78
ENSDARG00000045850	ccdc167	coiled-coil domain containing 167 [Source:NCBI gene;Acc:556080]	4	5255041	5273838		14.84	16.28	15.27	10.55	10.75	11.15
ENSDARG00000045851	galnt8b.1	polypeptide N-acetylgalactosaminyltransferase 8b, tandem duplicate 1 [Source:ZFIN;Acc:ZDB-GENE-041210-83]	4	5205265	5213425		0.66	0.3	0.53	0.85	0.69	0.55
ENSDARG00000045853	rad51ap1	RAD51 associated protein 1 [Source:ZFIN;Acc:ZDB-GENE-050419-96]	4	5196469	5203343		1.62	1.53	1.64	1.32	1.18	1.29
ENSDARG00000045854	fgf23	fibroblast growth factor 23 [Source:ZFIN;Acc:ZDB-GENE-050201-4]	4	5193198	5195027		0.88	1	1.21	0.59	0.48	0.64
ENSDARG00000045856	fgf6b	fibroblast growth factor 6b [Source:ZFIN;Acc:ZDB-GENE-050201-5]	4	5180650	5183337		1.95	1.6	1.63	2.51	1.99	2.32
ENSDARG00000045857	cebpz	CCAAT enhancer binding protein zeta [Source:ZFIN;Acc:ZDB-GENE-050417-261]	20	3290231	3310017		14.22	15.56	15.16	11.64	11.2	11.34
ENSDARG00000045858	tigarb	TP53 induced glycolysis regulatory phosphatase b [Source:NCBI gene;Acc:393160]	4	5156117	5161877		6.83	8.17	7.77	4.59	4.94	5.29
ENSDARG00000045863	asb15b	ankyrin repeat and SOCS box containing 15b [Source:ZFIN;Acc:ZDB-GENE-041210-265]	4	4120570	4129187		0.53	0.51	0.57	0.94	0.65	0.67
ENSDARG00000045864	lmod2b	leiomodin 2 (cardiac) b [Source:ZFIN;Acc:ZDB-GENE-041210-356]	4	4115778	4119425		2.42	2.5	2.25	2.38	2.38	2.15
ENSDARG00000045870	plekha5	pleckstrin homology domain containing, family A member 5 [Source:ZFIN;Acc:ZDB-GENE-041210-25]	4	2948063	3145359		10.84	4.97	4.72	12.29	12.13	12.79
ENSDARG00000045873	frs2a	fibroblast growth factor receptor substrate 2a [Source:ZFIN;Acc:ZDB-GENE-060503-462]	4	2092683	2099581		2.78	1.73	2.13	3.66	3.35	2.76
ENSDARG00000045874	yeats4	YEATS domain containing 4 [Source:ZFIN;Acc:ZDB-GENE-040718-252]	4	2059692	2062435		16.56	15.39	15.19	16.57	14.07	17.22
ENSDARG00000045877	ube2nb	ubiquitin-conjugating enzyme E2Nb [Source:ZFIN;Acc:ZDB-GENE-040426-1291]	4	1809562	1818315		47.75	45.25	49	42.52	42.86	42.46
ENSDARG00000045878	nudt4b	nudix (nucleoside diphosphate linked moiety X)-type motif 4b [Source:ZFIN;Acc:ZDB-GENE-040912-79]	4	1788748	1801619		7.61	4.9	4.82	8.79	9.14	8.39
ENSDARG00000045880	gas2l3	growth arrest-specific 2 like 3 [Source:ZFIN;Acc:ZDB-GENE-030131-2560]	4	1689312	1720648		5.41	2.29	1.92	5.91	5.74	6.22
ENSDARG00000045885	scaf11	SR-related CTD-associated factor 11 [Source:ZFIN;Acc:ZDB-GENE-041210-22]	4	1619584	1643101		10.87	9.65	8.43	11.76	12.26	12.54
ENSDARG00000045886	slc38a2	solute carrier family 38 member 2 [Source:NCBI gene;Acc:556537]	4	1600034	1617897		16.13	8.66	8.39	18.1	17.08	17.76
ENSDARG00000045887	mmp30	matrix metallopeptidase 30 [Source:ZFIN;Acc:ZDB-GENE-060421-5765]	10	38643304	38663746		23.23	28.36	20.46	7.61	6.34	6.67
ENSDARG00000045888	acat1	acetyl-CoA acetyltransferase 1 [Source:NCBI gene;Acc:445290]	10	38663917	38698966		32.73	39.85	38.39	23.26	25.37	24.83
ENSDARG00000045893	kctd15a	potassium channel tetramerization domain containing 15a [Source:NCBI gene;Acc:449991]	25	20357731	20381271		6.16	2.78	2.97	7.64	6.97	7.13
ENSDARG00000045898	si:ch211-152c2.3	si:ch211-152c2.3 [Source:ZFIN;Acc:ZDB-GENE-030131-9914]	4	842010	845033		34.41	38.98	52.45	26.37	26.91	28.72
ENSDARG00000045900	agbl5	ATP/GTP binding protein-like 5 [Source:NCBI gene;Acc:445479]	4	737539	760560		7.45	3.92	3.6	9.79	9.97	11.12
ENSDARG00000045904	nr2e3	nuclear receptor subfamily 2, group E, member 3 [Source:ZFIN;Acc:ZDB-GENE-041114-63]	25	22274642	22278655		8.11	1.93	1.63	7.22	7.65	8.67
ENSDARG00000045909	dynlt1	dynein, light chain, Tctex-type 1 [Source:ZFIN;Acc:ZDB-GENE-041210-9]	4	386435	390442		6.51	3.65	3.47	7.21	7.71	8.92
ENSDARG00000045910	tmem181	transmembrane protein 181 [Source:ZFIN;Acc:ZDB-GENE-060503-309]	4	357810	386168		9.31	7.76	7.88	10.28	10.23	10.4
ENSDARG00000045911	tulp4a	TUB like protein 4a [Source:ZFIN;Acc:ZDB-GENE-060503-215]	4	331351	353924		5.96	3.3	3.14	10.96	11.75	11.85
ENSDARG00000045913	mrps10	mitochondrial ribosomal protein S10 [Source:NCBI gene;Acc:565937]	4	325028	330748		17.12	20.47	25.51	12.91	12.29	12.42
ENSDARG00000045914	si:ch211-51e12.7	si:ch211-51e12.7 [Source:ZFIN;Acc:ZDB-GENE-030131-8279]	4	308688	317971		75.5	44.39	44.95	77.9	71.93	77.59
ENSDARG00000045926	crabp1a	cellular retinoic acid binding protein 1a [Source:ZFIN;Acc:ZDB-GENE-020320-3]	25	6306885	6337380		27.56	14.09	24.36	17.84	17.26	20.04
ENSDARG00000045927	slc25a44a	solute carrier family 25 member 44a [Source:ZFIN;Acc:ZDB-GENE-041114-36]	25	6266009	6275207		3.81	3.6	2.66	4.13	3.31	4
ENSDARG00000045928	psma4	proteasome 20S subunit alpha 4 [Source:ZFIN;Acc:ZDB-GENE-040426-1932]	25	6211677	6223567		60.85	65.75	71.23	41.15	40.2	40.45
ENSDARG00000045929	oaz2a	ornithine decarboxylase antizyme 2a [Source:ZFIN;Acc:ZDB-GENE-121005-1]	25	6186823	6206443		23.3	26.27	19.05	36.28	36.11	36.26
ENSDARG00000045930	rbpms2a	RNA binding protein, mRNA processing factor 2a [Source:ZFIN;Acc:ZDB-GENE-040718-106]	25	6122823	6153077		10.53	4.01	3.54	14.61	14.78	15.07
ENSDARG00000045932	cpeb1a	cytoplasmic polyadenylation element binding protein 1a [Source:ZFIN;Acc:ZDB-GENE-080723-42]	25	6065011	6082509		0.42	0.29	0.28	0.92	0.75	0.75
ENSDARG00000045935	immp1l	inner mitochondrial membrane peptidase subunit 1 [Source:ZFIN;Acc:ZDB-GENE-070522-4]	7	16033273	16064685		1.09	1.04	1.04	0.98	1	0.84
ENSDARG00000045936	pax6b	paired box 6b [Source:ZFIN;Acc:ZDB-GENE-001031-1]	7	15871156	15900606		7.77	1.6	1.24	8.25	7.94	8.72
ENSDARG00000045944	slc6a1a	solute carrier family 6 member 1a [Source:ZFIN;Acc:ZDB-GENE-060519-23]	23	4834447	4855122		38.31	5.83	10.67	40.39	43.79	41.39
ENSDARG00000045945	syn2a	synapsin IIa [Source:ZFIN;Acc:ZDB-GENE-040718-341]	23	4751959	4775446		45.45	13.7	18.32	42.57	45.74	47.83
ENSDARG00000045946	sec24d	SEC24 homolog D, COPII coat complex component [Source:ZFIN;Acc:ZDB-GENE-070117-2501]	7	69905307	69951096		8.65	11.43	7.13	15.21	15.61	14.17
ENSDARG00000045947	hrc	histidine rich calcium binding protein [Source:ZFIN;Acc:ZDB-GENE-041008-189]	12	4377624	4388704		134.9	183.32	193.04	59.6	45.82	49.88
ENSDARG00000045951	kat5b	K(lysine) acetyltransferase 5b [Source:ZFIN;Acc:ZDB-GENE-030131-985]	5	66733289	66749535		7.71	3.75	5.13	7.15	7.06	7.88
ENSDARG00000045955	prlrb	prolactin receptor b [Source:ZFIN;Acc:ZDB-GENE-070705-155]	5	66282256	66301142		1.41	1	1.23	1.83	1.86	1.92
ENSDARG00000045956	kcnk10b	potassium channel, subfamily K, member 10b [Source:ZFIN;Acc:ZDB-GENE-050420-31]	17	2727807	2765952		1.23	0.26	0.67	1.89	1.99	2.17
ENSDARG00000045957	gpr65	G protein-coupled receptor 65 [Source:ZFIN;Acc:ZDB-GENE-120709-81]	17	2810776	2817035		0.1	0.19	0.15	0.19	0.14	0.18
ENSDARG00000045958	egfl6	EGF-like-domain, multiple 6 [Source:ZFIN;Acc:ZDB-GENE-040718-157]	9	54629993	54681473		13.68	16.15	14.47	17.02	15.16	14.28
ENSDARG00000045959	csf3r	colony stimulating factor 3 receptor (granulocyte) [Source:ZFIN;Acc:ZDB-GENE-080104-4]	16	3103182	3127749		0.82	0.6	0.64	1.17	1.5	0.9
ENSDARG00000045968	spg11	SPG11 vesicle trafficking associated, spatacsin [Source:ZFIN;Acc:ZDB-GENE-101017-1]	25	1088693	1126311		4.03	2.57	2.39	4.43	4.54	4.71
ENSDARG00000045976	sidt2	SID1 transmembrane family, member 2 [Source:ZFIN;Acc:ZDB-GENE-030131-7356]	15	47525073	47554887		27.73	26.14	27.57	32.14	33.72	35.69
ENSDARG00000045978	chchd6b	coiled-coil-helix-coiled-coil-helix domain containing 6b [Source:ZFIN;Acc:ZDB-GENE-041114-95]	6	1494817	1514787		31.31	31.18	36	24.93	26.56	24.02
ENSDARG00000045979	zgc:153704	zgc:153704 [Source:NCBI gene;Acc:767759]	5	4482437	4493524		33.26	47.57	57.19	28.98	31.33	31.71
ENSDARG00000045982	si:ch73-55i23.1	phospholipase A2, group IVC (cytosolic, calcium-independent)-like [Source:NCBI gene;Acc:100148581]	5	1933131	1947624		0.01	0.01	0.02	0	0	0.01
ENSDARG00000045983	zbtb43	zinc finger and BTB domain containing 43 [Source:NCBI gene;Acc:557590]	5	4921092	4931266		3.1	1.44	1.48	3.71	3.16	3.49
ENSDARG00000045989	arf4a	ADP-ribosylation factor 4a [Source:ZFIN;Acc:ZDB-GENE-040801-109]	11	42494531	42502940		8.52	10.93	8.22	10.55	10.53	9.65
ENSDARG00000045999	saa	serum amyloid A [Source:ZFIN;Acc:ZDB-GENE-040927-15]	7	12950507	12956434		0.19	0.36	0.28	0.55	0.98	0.58
ENSDARG00000046002	necap2	NECAP endocytosis associated 2 [Source:ZFIN;Acc:ZDB-GENE-050522-67]	22	341990	347424		18.44	17.63	17.88	19.24	18.55	18.92
ENSDARG00000046006	med20	mediator complex subunit 20 [Source:NCBI gene;Acc:445191]	22	558287	567923		3.43	3.37	4.2	2.22	2.2	2.33
ENSDARG00000046007	tmcc2	transmembrane and coiled-coil domain family 2 [Source:ZFIN;Acc:ZDB-GENE-091202-2]	22	591809	600016		8.27	2.7	3.26	11.8	11.16	11.36
ENSDARG00000046010	kdm2bb	lysine (K)-specific demethylase 2Bb [Source:ZFIN;Acc:ZDB-GENE-080225-13]	10	41826331	41907213		4.81	2.51	2.58	4.87	4.95	5.55
ENSDARG00000046012	slc47a1	solute carrier family 47 member 1 [Source:ZFIN;Acc:ZDB-GENE-050327-20]	21	39941184	39976240		1.54	1.66	1.39	1.44	1.19	1.32
ENSDARG00000046013	rasl11a	RAS-like, family 11, member A [Source:NCBI gene;Acc:550538]	7	51314734	51321126		4.39	4.76	4.05	4.47	3.73	4.45
ENSDARG00000046014	kcna6a	potassium voltage-gated channel, shaker-related, subfamily, member 6 a [Source:ZFIN;Acc:ZDB-GENE-080722-21]	25	19947096	19951331		1.02	1.59	1.2	1.62	1.2	1.2
ENSDARG00000046019	snai1b	snail family zinc finger 1b [Source:ZFIN;Acc:ZDB-GENE-980526-514]	23	2502181	2513300		1.13	1.37	1.12	3.52	3.76	3.87
ENSDARG00000046021	CU914487.1	peptidase inhibitor 16-like [Source:NCBI gene;Acc:101883528]	23	41679586	41692463		3.74	4.92	5.53	9.94	13.43	11.25
ENSDARG00000046022	tmed7	transmembrane p24 trafficking protein 7 [Source:ZFIN;Acc:ZDB-GENE-040426-2570]	8	1239	9428		17.48	12.09	10.84	16.92	17.08	17.6
ENSDARG00000046024	pym1	PYM homolog 1, exon junction complex associated factor [Source:NCBI gene;Acc:393566]	8	387822	389507		10.24	8.43	9.97	9.53	10.56	11.44
ENSDARG00000046030	zgc:110339	zgc:110339 [Source:ZFIN;Acc:ZDB-GENE-050417-323]	25	10547228	10553685		63.1	81.83	89.15	61.49	63.16	56.96
ENSDARG00000046053	slc27a6	solute carrier family 27 member 6 [Source:ZFIN;Acc:ZDB-GENE-060825-27]	10	16501699	16540603		2.47	2.26	1.71	2.64	2.78	2.3
ENSDARG00000046054	CT027815.1	long-chain fatty acid transport protein 6-like [Source:NCBI gene;Acc:568883]	10	16473923	16495597		1.51	1.14	1.06	2.07	2.42	2.61
ENSDARG00000046071	C21H18orf25	zgc:77838 [Source:ZFIN;Acc:ZDB-GENE-040426-1889]	21	5023049	5066462	C21H18orf25	7.38	4.36	4.43	7.36	7.39	7.79
ENSDARG00000046072	snx16	sorting nexin 16 [Source:ZFIN;Acc:ZDB-GENE-040426-1832]	24	42117231	42121054		19.97	15.96	16.18	20.5	21.69	21.44
ENSDARG00000046074	stk11	serine/threonine kinase 11 [Source:ZFIN;Acc:ZDB-GENE-050417-383]	2	59041081	59063573		18.65	14.69	16.28	18.84	19.05	19.77
ENSDARG00000046079	cacng6b	calcium channel, voltage-dependent, gamma subunit 6b [Source:ZFIN;Acc:ZDB-GENE-100922-272]	16	12836143	12866551		6.69	11.01	11.64	7.65	5.16	5.91
ENSDARG00000046081	bloc1s2	biogenesis of lysosomal organelles complex-1, subunit 2 [Source:ZFIN;Acc:ZDB-GENE-050809-129]	20	7584211	7593106		14.22	13.46	11.97	11.44	11.77	11.96
ENSDARG00000046087	brd2b	bromodomain containing 2b [Source:ZFIN;Acc:ZDB-GENE-070220-1]	16	48714048	48747788		2.2	0.87	0.79	3.52	3.24	3.27
ENSDARG00000046090	dhrs11a	dehydrogenase/reductase (SDR family) member 11a [Source:NCBI gene;Acc:791578]	5	4054704	4142955		5.69	4.25	3.19	3.62	2.97	3.11
ENSDARG00000046091	si:ch211-283g2.1	si:ch211-283g2.1 [Source:ZFIN;Acc:ZDB-GENE-060531-57]	5	4150814	4204601		1.84	2.97	1.83	1.23	0.97	0.95
ENSDARG00000046092	si:ch211-283g2.3	si:ch211-283g2.3 [Source:ZFIN;Acc:ZDB-GENE-060526-152]	5	4232196	4245186		0	0	0	0	0	0
ENSDARG00000046098	ebp	EBP cholestenol delta-isomerase [Source:ZFIN;Acc:ZDB-GENE-040718-12]	23	19147128	19153408		2.03	3.72	2.67	0.91	0.97	0.82
ENSDARG00000046106	rab10	RAB10, member RAS oncogene family [Source:ZFIN;Acc:ZDB-GENE-040718-304]	20	47434709	47471595		75.88	61.14	57.2	81.31	83.31	83.93
ENSDARG00000046107	caskin1	CASK interacting protein 1 [Source:ZFIN;Acc:ZDB-GENE-071115-3]	3	35632773	35801146		3.69	0.83	1.19	5.54	5.55	5.86
ENSDARG00000046124	rab11fip1a	RAB11 family interacting protein 1 (class I) a [Source:ZFIN;Acc:ZDB-GENE-051113-116]	8	37998387	38022298		9.71	10.28	8.63	6.86	7.15	7.29
ENSDARG00000046127	tk2	thymidine kinase 2 [Source:ZFIN;Acc:ZDB-GENE-040718-243]	7	44599313	44605056		3.22	2.47	2.51	3.27	3.36	3.48
ENSDARG00000046132	dynlt3	dynein, light chain, Tctex-type 3 [Source:ZFIN;Acc:ZDB-GENE-040927-24]	11	29790626	29794308		29.26	21.42	22.11	28.59	28.78	31.81
ENSDARG00000046133	b3galnt2	beta-1,3-N-acetylgalactosaminyltransferase 2 [Source:NCBI gene;Acc:553716]	13	49774315	49819027		2.16	1.82	1.74	2.78	2.45	2.38
ENSDARG00000046140	zgc:112038	zgc:112038 [Source:ZFIN;Acc:ZDB-GENE-050522-271]	3	21200763	21218133		2.41	1.86	1.99	1.49	0.98	0.94
ENSDARG00000046141	zgc:162025	zgc:162025 [Source:NCBI gene;Acc:571775]	8	39816523	39822917		5.09	6.31	5.89	4.73	3.89	4.9
ENSDARG00000046142	asgr1	asialoglycoprotein receptor-like 1 [Source:ZFIN;Acc:ZDB-GENE-081105-120]	8	39733278	39739640		5.71	9.11	6.41	3.12	2.81	3.41
ENSDARG00000046143	prkab1b	protein kinase, AMP-activated, beta 1 non-catalytic subunit, b [Source:ZFIN;Acc:ZDB-GENE-030131-7172]	8	39674707	39703689		44.76	57.58	58.27	36.7	37.75	37.94
ENSDARG00000046148	tnfaip8l2b	tumor necrosis factor, alpha-induced protein 8-like 2b [Source:NCBI gene;Acc:393345]	16	29698565	29714540		6.9	7.53	6.94	4.31	3.94	3.99
ENSDARG00000046150	b4galnt4a	beta-1,4-N-acetyl-galactosaminyl transferase 4a [Source:ZFIN;Acc:ZDB-GENE-100422-4]	25	24291156	24610545		3.45	0.71	1.01	4.78	4.31	4.57
ENSDARG00000046157	RPS17	zgc:114188 [Source:ZFIN;Acc:ZDB-GENE-050320-15]	25	192116	196950	RPS17	1732.93	1885.2	2466.6	1385.6	1386	1390.21
ENSDARG00000051710	CR339041.1	major histocompatibility complex class I-related gene protein-like [Source:NCBI gene;Acc:101881672]	25	10882597	10907786		0	0	0	0.06	0.07	0.05

ENSDARG00000051711	CR339041.2		25	10929790	10935577		0.06	0.02	0.02	0.11	0.01	0.12
ENSDARG00000051712	mhc1lfa	major histocompatibility complex class I LFA [Source:ZFIN;Acc:ZDB-GENE-140820-7]	25	10947743	10951126		0	0	0	0.04	0.07	0.04
ENSDARG00000051713	mhc1lga	major histocompatibility complex class I LGA [Source:ZFIN;Acc:ZDB-GENE-140820-8]	25	11002640	11006571		0.06	0.08	0.02	0.01	0.03	0.06
ENSDARG00000051718	si:ch211-113a14.24	si:ch211-113a14.24 [Source:ZFIN;Acc:ZDB-GENE-121214-122]	25	36368458	36369057		0.04	0.27	0.1	0.04	0.09	0.04
ENSDARG00000051729	nfatc3b	nuclear factor of activated T cells 3b [Source:ZFIN;Acc:ZDB-GENE-100922-25]	25	36219436	36248053		2.49	2.56	1.96	2.89	2.98	2.91
ENSDARG00000051730	slc7a10b	solute carrier family 7 member 10b [Source:ZFIN;Acc:ZDB-GENE-121105-2]	25	36266782	36286186		3.9	0.9	1.95	1.99	1.83	1.98
ENSDARG00000051731	faap24	FA core complex associated protein 24 [Source:ZFIN;Acc:ZDB-GENE-070410-53]	25	36287745	36292346		1.32	0.91	0.82	0.56	0.67	0.6
ENSDARG00000051735	si:ch211-113a14.18	si:ch211-113a14.18 [Source:ZFIN;Acc:ZDB-GENE-121214-116]	25	36339867	36340516		0.04	0	0	0.07	0	0
ENSDARG00000051736	hist1h2a4	histone cluster 1 H2A family member 4 [Source:ZFIN;Acc:ZDB-GENE-050731-6]	25	36328524	36329005		0.1	0.19	0	0	0	0.2
ENSDARG00000051737		zgc:173552 [Source:ZFIN;Acc:ZDB-GENE-080220-24]	25	36329623	36330033		0.06	0.06	0.19	0.23	0.06	0.06
ENSDARG00000051746	ada2a	adenosine deaminase 2a [Source:NCBI gene;Acc:373884]	25	16689633	16700924		7.17	5.34	6.8	6.58	7.55	8.57
ENSDARG00000051748	ccnd2a	cyclin D2, a [Source:ZFIN;Acc:ZDB-GENE-070424-30]	25	16973945	17239079		16.51	17.87	16.15	15.89	18.01	16.06
ENSDARG00000051749	tigara	TP53 induced glycolysis regulatory phosphatase a [Source:NCBI gene;Acc:664696]	25	16962972	16969922		1.67	2.1	1.96	1.51	1.45	1.58
ENSDARG00000051754	rpgrip1l	RPGRIP1 like [Source:ZFIN;Acc:ZDB-GENE-081104-81]	25	36045072	36086409		1.55	0.72	0.81	2.21	2.21	2.12
ENSDARG00000051756	pdhx	pyruvate dehydrogenase complex, component X [Source:ZFIN;Acc:ZDB-GENE-040426-1539]	25	37428187	37435076		27.93	30.23	33.13	29.2	32.22	30.19
ENSDARG00000051760	igl4v10	immunoglobulin light 4 variable 10 [Source:ZFIN;Acc:ZDB-GENE-051013-5]	25	37320928	37322081		0	0	0.03	0	0	0
ENSDARG00000051761	trim44	tripartite motif containing 44 [Source:ZFIN;Acc:ZDB-GENE-050522-37]	25	37332636	37338048		6.89	5.17	4.98	6.26	6.31	7.32
ENSDARG00000051762	CABZ01080568.1		25	34662358	34670413		16.38	20.15	22.38	18.81	18.88	16
ENSDARG00000051763	sntb2	syntrophin, beta 2 [Source:ZFIN;Acc:ZDB-GENE-131121-511]	25	34915576	34938157		5.38	5.02	4.02	6.88	6.7	6.34
ENSDARG00000051768	fhod1	formin homology 2 domain containing 1 [Source:ZFIN;Acc:ZDB-GENE-110623-4]	7	34549198	34618314		1.66	1.41	0.91	2.21	2.31	2.18
ENSDARG00000051783	rplp0	ribosomal protein, large, P0 [Source:NCBI gene;Acc:58101]	5	1950660	1963498		1947.57	2144.33	2281.94	1771.95	1852.39	1860.32
ENSDARG00000051785	dhx33	DEAH (Asp-Glu-Ala-His) box polypeptide 33 [Source:NCBI gene;Acc:796505]	5	1965296	1991634		1.23	1.46	0.92	1.93	1.87	1.76
ENSDARG00000051793	zgc:113426	zgc:113426 [Source:ZFIN;Acc:ZDB-GENE-050913-149]	25	19670273	19680133		0.55	0.75	0.63	0.6	0.39	0.37
ENSDARG00000051795	si:dkeyp-110c12.3	si:dkeyp-110c12.3 [Source:ZFIN;Acc:ZDB-GENE-131126-68]	25	19680962	19693901		0	0	0	0	0	0
ENSDARG00000051798	brd1b	bromodomain containing 1b [Source:ZFIN;Acc:ZDB-GENE-070209-98]	25	29688671	29714525		3.9	1.7	1.45	6.14	5.43	5.79
ENSDARG00000051800	fnbp4	formin binding protein 4 [Source:ZFIN;Acc:ZDB-GENE-130530-633]	25	28640459	28668776		34.6	12.97	16.32	36.69	35.36	38.2
ENSDARG00000051804	BX664721.1		7	73701410	73702063		0.28	0.2	0.31	0	0	0
ENSDARG00000051805	CU469568.1		25	29373572	29375149		5.86	7.61	6.5	6.43	5.34	5.44
ENSDARG00000051809	btr12	bloodthirsty-related gene family, member 12 [Source:ZFIN;Acc:ZDB-GENE-060929-548]	7	73670137	73685576		6.94	4.63	3.07	3.5	3.3	3.38
ENSDARG00000051814	ptprz1a	protein tyrosine phosphatase receptor type Z1a [Source:ZFIN;Acc:ZDB-GENE-090406-1]	25	28340171	28443607		6.25	2.01	1.97	6.94	6.46	7.15
ENSDARG00000051816	aass	aminoadipate-semialdehyde synthase [Source:ZFIN;Acc:ZDB-GENE-061220-8]	25	28279577	28336106		6.92	6.49	4.54	6.87	6.1	5.69
ENSDARG00000051819	swap70a	switching B cell complex subunit SWAP70a [Source:ZFIN;Acc:ZDB-GENE-040426-1182]	7	67183718	67214972		2.5	2.22	1.27	2.5	2.05	2.03
ENSDARG00000051823	hyal4	hyaluronidase 4 [Source:ZFIN;Acc:ZDB-GENE-061220-9]	25	27541796	27564590		4.36	3.62	3.49	5.36	5.55	5.51
ENSDARG00000051824	tmeff2a	transmembrane protein with EGF-like and two follistatin-like domains 2a [Source:ZFIN;Acc:ZDB-GENE-070912-622]	9	24249184	24413008		16.55	8.36	9.36	16.74	16.8	17.92
ENSDARG00000051836	si:dkeyp-19e1.3	si:dkeyp-19e1.3 [Source:ZFIN;Acc:ZDB-GENE-081104-56]	25	24121611	24179999		0.65	0.58	0.38	0.91	0.77	0.86
ENSDARG00000051851	ctu2	cytosolic thiouridylase subunit 2 homolog (S. pombe) [Source:NCBI gene;Acc:445166]	7	55292959	55325712		4.38	4.67	4.81	4.4	3.32	3.9
ENSDARG00000051852	kcnc1a	potassium voltage-gated channel, Shaw-related subfamily, member 1a [Source:ZFIN;Acc:ZDB-GENE-061220-10]	7	33028452	33088144		7.78	1.31	2.48	9.86	9.11	9.36
ENSDARG00000051853	galns	galactosamine (N-acetyl)-6-sulfatase [Source:ZFIN;Acc:ZDB-GENE-070112-1152]	7	55543351	55633475		4.91	5.22	5.68	5.55	6.14	4.82
ENSDARG00000051854	cdt1	chromatin licensing and DNA replication factor 1 [Source:ZFIN;Acc:ZDB-GENE-070111-1]	7	55518519	55530612		3.21	2.55	2.45	2.43	1.81	2.21
ENSDARG00000051857	tes	testis derived transcript (3 LIM domains) [Source:ZFIN;Acc:ZDB-GENE-040718-59]	25	18475032	18496410		10.72	11.13	11.36	13.94	14.21	13.83
ENSDARG00000051858	ccdc33	coiled-coil domain containing 33 [Source:ZFIN;Acc:ZDB-GENE-101011-1]	25	22335914	22363378		0.21	0.1	0.09	0.1	0.12	0.17
ENSDARG00000051861	pkp3a	plakophilin 3a [Source:ZFIN;Acc:ZDB-GENE-060512-114]	25	22151271	22191983		26.49	22.04	15.33	14.95	12.95	12.91
ENSDARG00000051873	ahcyl2a	adenosylhomocysteinase like 2a [Source:ZFIN;Acc:ZDB-GENE-070112-1522]	25	21800397	21822426		10.45	12.17	11.82	9.27	9.35	8.58
ENSDARG00000051874	stra6	stimulated by retinoic acid 6 [Source:NCBI gene;Acc:724007]	25	22571475	22634675		9.73	14.19	12.23	6.44	6.19	6.25
ENSDARG00000051875	islr2	immunoglobulin superfamily containing leucine-rich repeat 2 [Source:ZFIN;Acc:ZDB-GENE-050320-95]	25	22635166	22639133		84.13	25.92	40.5	85.38	90.33	92.76
ENSDARG00000051876	ush1c	Usher syndrome 1C [Source:ZFIN;Acc:ZDB-GENE-060312-41]	25	22643954	22704233		10.14	13.21	9.36	4.06	4.59	4.62
ENSDARG00000051879	abcc8	ATP-binding cassette, sub-family C (CFTR/MRP), member 8 [Source:ZFIN;Acc:ZDB-GENE-050517-22]	25	22719852	22849186		4.91	1.57	2.03	5.26	5.06	5.15
ENSDARG00000051880	kcnj11	potassium inwardly rectifying channel subfamily J member 11 [Source:ZFIN;Acc:ZDB-GENE-060308-2]	25	22853718	22854863		6.76	7.93	7.85	7.46	6.49	6.87
ENSDARG00000051886	ankrd11	ankyrin repeat domain 11 [Source:ZFIN;Acc:ZDB-GENE-030131-3932]	7	56253914	56405541		10.85	5.5	3.9	14.17	14.18	14.17
ENSDARG00000051888	ist1	IST1 factor associated with ESCRT-III [Source:ZFIN;Acc:ZDB-GENE-030131-179]	7	56472568	56484798		21.31	17.92	17.4	22.33	22.16	23.22
ENSDARG00000051889	dhodh	dihydroorotate dehydrogenase [Source:ZFIN;Acc:ZDB-GENE-030131-3157]	7	56517402	56529522		7.29	7.85	7.77	4.72	4.39	4.55
ENSDARG00000051890	hp	haptoglobin [Source:ZFIN;Acc:ZDB-GENE-030131-1259]	7	56577522	56585738		0	0.01	0.01	0	0.01	0.03
ENSDARG00000051891	rsph10b	radial spoke head 10 homolog B [Source:NCBI gene;Acc:767792]	12	17620067	17633727		0.94	0.98	0.88	1.12	1.05	1.15
ENSDARG00000051892	kcng4b	potassium voltage-gated channel, subfamily G, member 4b [Source:ZFIN;Acc:ZDB-GENE-130530-634]	7	56651759	56655773		1.55	0.41	0.51	1.17	0.75	1.07
ENSDARG00000051896	fbn2a	fibrillin 2a [Source:ZFIN;Acc:ZDB-GENE-090112-4]	10	16313851	16474216		1.48	1.73	1.27	1.91	1.58	1.58
ENSDARG00000051899	exoc3l1	exocyst complex component 3-like 1 [Source:NCBI gene;Acc:567832]	25	14425834	14433503		3.08	2.69	2.29	3.78	4.13	3.97
ENSDARG00000051902	prmt7	protein arginine methyltransferase 7 [Source:NCBI gene;Acc:393475]	25	14408936	14424406		6.04	5.42	5.22	5.59	5.48	5.16
ENSDARG00000051912	hpxb	hemopexin b [Source:ZFIN;Acc:ZDB-GENE-060929-800]	16	2394193	2414063		3.56	4.69	3.8	6.95	6.72	6.47
ENSDARG00000051914	slc14a2	solute carrier family 14 member 2 [Source:ZFIN;Acc:ZDB-GENE-030131-4833]	5	52167986	52177850		1.09	0.69	0.57	1.79	1.58	1.24
ENSDARG00000051915	pde5ab	phosphodiesterase 5A, cGMP-specific, b [Source:ZFIN;Acc:ZDB-GENE-060824-4]	7	57539146	57619470		3.91	4.11	3.44	3.28	3.83	3.86
ENSDARG00000051916	cdk7	cyclin-dependent kinase 7 [Source:ZFIN;Acc:ZDB-GENE-010320-2]	5	52000163	52010213		13.31	13.3	12	10.2	10.84	9.52
ENSDARG00000051920	nsmaf	neutral sphingomyelinase (N-SMase) activation associated factor [Source:ZFIN;Acc:ZDB-GENE-050417-342]	7	58139372	58178980		1.81	1.26	1.29	1.9	1.69	1.93
ENSDARG00000051921	slc30a5	solute carrier family 30 member 5 [Source:NCBI gene;Acc:436594]	5	54716670	54729578		11.14	10.66	10.57	10.98	10.87	10.69
ENSDARG00000051923	ccnb1	cyclin B1 [Source:ZFIN;Acc:ZDB-GENE-000406-10]	5	54711027	54714789		12.73	10.1	11.41	10.25	9.46	9.39
ENSDARG00000051925	cyb5r2	cytochrome b5 reductase 2 [Source:NCBI gene;Acc:751641]	25	16044734	16057539		7.26	13.58	10.87	4.94	5.68	5.78
ENSDARG00000051926	plag1	pleiomorphic adenoma gene 1 [Source:ZFIN;Acc:ZDB-GENE-060302-2]	7	58730201	58743802		1.97	1.96	1.65	2.43	2.25	1.89
ENSDARG00000051931	edc3	enhancer of mRNA decapping 3 homolog (S. cerevisiae) [Source:NCBI gene;Acc:504054]	25	20668272	20691609		4.26	3.29	2.98	4.74	4.66	4.54
ENSDARG00000051934	kxd1	KxDL motif containing 1 [Source:NCBI gene;Acc:445075]	25	20694177	20701924		4.65	4.87	4.5	2.83	3.1	3.72
ENSDARG00000051936	ergic2	ERGIC and golgi 2 [Source:NCBI gene;Acc:393378]	25	20715950	20726987		9.24	10.32	9.52	11.19	10.7	10.15
ENSDARG00000051939	pcxb	pyruvate carboxylase b [Source:ZFIN;Acc:ZDB-GENE-000831-1]	7	60457138	60831105		16.73	28.02	16.96	27.12	32.39	27.78
ENSDARG00000051940	ugt2a4	UDP glucuronosyltransferase 2 family, polypeptide A4 [Source:ZFIN;Acc:ZDB-GENE-080721-23]	5	69926766	69948119		8.23	13.72	12.02	3.23	3.84	3.37
ENSDARG00000051953	ythdc1	YTH domain containing 1 [Source:ZFIN;Acc:ZDB-GENE-041114-114]	5	69878693	69903372		27.8	12.41	13.88	28.95	31.22	32.89
ENSDARG00000051955	brms1	BRMS1 transcriptional repressor and anoikis regulator [Source:ZFIN;Acc:ZDB-GENE-030616-596]	7	60396957	60417382		4.47	3.46	3.45	5.02	4.67	4.58
ENSDARG00000051956	isca1	iron-sulfur cluster assembly 1 [Source:NCBI gene;Acc:573999]	5	69426654	69437422		18.01	22	24.37	27.01	31.64	31.63
ENSDARG00000051957	selenom	selenoprotein M [Source:NCBI gene;Acc:352913]	5	69278089	69287567		15.24	15.16	18.04	20.8	18.67	18.19
ENSDARG00000051959	mmp15a	matrix metallopeptidase 15a [Source:ZFIN;Acc:ZDB-GENE-070817-4]	25	17581393	17587860		0	0	0	0	0	0
ENSDARG00000051962	mmp15a	matrix metallopeptidase 15a [Source:ZFIN;Acc:ZDB-GENE-070817-4]	25	17559399	17590971		3.47	3.52	3.09	4.17	4.07	4.01
ENSDARG00000051965	btr01	bloodthirsty-related gene family, member 1 [Source:ZFIN;Acc:ZDB-GENE-090511-2]	1	45056371	45076434		3.47	2.32	2.14	2.25	1.5	1.77
ENSDARG00000051970	smu1b	SMU1 DNA replication regulator and spliceosomal factor b [Source:ZFIN;Acc:ZDB-GENE-130530-635]	1	45012391	45042210		1.59	0.48	0.83	1.01	1.22	1.08
ENSDARG00000051975	cd99	CD99 molecule [Source:ZFIN;Acc:ZDB-GENE-061103-391]	1	33353174	33380340		8.87	7.93	6.43	11.8	11.29	10.54
ENSDARG00000051981	STX3	zgc:165520 [Source:ZFIN;Acc:ZDB-GENE-070620-13]	1	44826316	44835738	STX3	6.77	1.67	1.47	9.26	9.53	9.54
ENSDARG00000051986	ndufs8a	NADH:ubiquinone oxidoreductase core subunit S8a [Source:ZFIN;Acc:ZDB-GENE-040426-2153]	1	44814322	44823071		119.91	154.55	156.25	86.4	86.13	83.98
ENSDARG00000051989	tmem187	transmembrane protein 187 [Source:ZFIN;Acc:ZDB-GENE-090313-366]	1	44800298	44801179		3.79	3.99	2.8	3.7	3.43	3.21
ENSDARG00000051993	si:dkey-9i23.4	si:dkey-9i23.4 [Source:ZFIN;Acc:ZDB-GENE-090313-363]	1	44767657	44770055		0.03	0.06	0.14	0.11	0.19	0.03
ENSDARG00000052000	cav2	caveolin 2 [Source:ZFIN;Acc:ZDB-GENE-040625-164]	25	18556588	18561795		10.62	12.51	9.76	10.42	9.68	9.74
ENSDARG00000052010	ciao2b	cytosolic iron-sulfur assembly component 2B [Source:ZFIN;Acc:ZDB-GENE-040718-148]	7	44965773	44970994		8.26	9.61	8.04	7.19	7.3	7.19
ENSDARG00000052011	rrad	Ras-related associated with diabetes [Source:ZFIN;Acc:ZDB-GENE-030131-5607]	7	44960188	44963154		8.34	4.17	4.91	14	13.43	14.29
ENSDARG00000052012	rtn4rl2a	reticulon 4 receptor-like 2 a [Source:ZFIN;Acc:ZDB-GENE-040310-4]	1	44491077	44495902		2.03	0.51	0.78	2.49	1.99	2.45
ENSDARG00000052016	cabp2a	calcium binding protein 2a [Source:ZFIN;Acc:ZDB-GENE-050913-25]	1	44127292	44141442		5.95	0.49	0.39	6.16	5.38	5.68
ENSDARG00000052019	si:ch73-109d9.1	si:ch73-109d9.1 [Source:ZFIN;Acc:ZDB-GENE-110411-256]	1	44011472	44025066		6.79	3.7	4.47	5.05	5.98	5.51
ENSDARG00000052020	cmtm4	CKLF-like MARVEL transmembrane domain containing 4 [Source:ZFIN;Acc:ZDB-GENE-040718-195]	7	44628681	44672095		5.25	4.4	3.83	7.35	7.01	7.93
ENSDARG00000052025	pomgnt1	protein O-linked mannose N-acetylglucosaminyltransferase 1 (beta 1,2-) [Source:ZFIN;Acc:ZDB-GENE-070112-991]	6	33076839	33095766		6.59	5.07	4.9	6.78	6.69	6.62
ENSDARG00000052027	tspan1	tetraspanin 1 [Source:ZFIN;Acc:ZDB-GENE-070502-5]	6	33104544	33129312		1.37	1.03	1.49	0.64	0.66	0.9
ENSDARG00000052030	akr1a1b	aldo-keto reductase family 1, member A1b (aldehyde reductase) [Source:NCBI gene;Acc:799805]	6	33918813	33925432		12.1	14.11	13.6	8.11	8.64	8.97
ENSDARG00000052035	nxnl1	nucleoredoxin like 1 [Source:NCBI gene;Acc:567911]	1	58005222	58009216		10.05	2.63	1.98	4.33	3.73	4.09
ENSDARG00000052037	trim35-1	tripartite motif containing 35-1 [Source:ZFIN;Acc:ZDB-GENE-050522-17]	1	58039551	58049515		0.85	1	0.9	0.95	0.8	0.84
ENSDARG00000052039	caspb	caspase b [Source:ZFIN;Acc:ZDB-GENE-020812-1]	1	58050905	58064738		50.15	57.93	50.92	39.12	32.26	35.44
ENSDARG00000052045	ggt5a	gamma-glutamyltransferase 5a [Source:ZFIN;Acc:ZDB-GENE-080226-1]	8	39998467	40031011		1.99	2.04	1.61	3.03	2.82	3.1
ENSDARG00000052056	parietopsin	parietopsin [Source:ZFIN;Acc:ZDB-GENE-101129-2]	25	18909607	18920440		0.12	0.12	0.02	0.06	0.13	0
ENSDARG00000052057	pcolceb	procollagen C-endopeptidase enhancer b [Source:ZFIN;Acc:ZDB-GENE-080722-44]	7	50849142	50873496		3.93	3.43	3.01	11.66	12.74	11.83
ENSDARG00000052059	PAQR9	si:dkey-37f13.4 [Source:NCBI gene;Acc:101886606]	7	50876581	50883433	PAQR9	2.09	1.53	1.1	1.91	2.03	1.95
ENSDARG00000052061	col4a6	collagen, type IV, alpha 6 [Source:ZFIN;Acc:ZDB-GENE-101001-2]	7	50920437	51102479		36.51	29.91	16.01	34.84	29.78	30.76
ENSDARG00000052063	col4a5	collagen, type IV, alpha 5 (Alport syndrome) [Source:ZFIN;Acc:ZDB-GENE-030131-2281]	7	51103232	51194430		74.25	63.52	38.92	65.26	58.58	57.42
ENSDARG00000052064	si:dkeyp-93a5.2	si:dkeyp-93a5.2 [Source:ZFIN;Acc:ZDB-GENE-131127-18]	25	18723618	18739924		0.02	0	0.06	0	0	0
ENSDARG00000052072	dnajb9a	DnaJ heat shock protein family (Hsp40) member B9a [Source:ZFIN;Acc:ZDB-GENE-050626-115]	25	20264117	20268027		5.12	2.82	3.3	4.31	4.63	4.3
ENSDARG00000052073	blcap	bladder cancer associated protein [Source:NCBI gene;Acc:57918]	6	51769760	51771634		57.4	61.05	59.63	50.29	48.06	51.39
ENSDARG00000052074	si:ch211-122f10.4	si:ch211-122f10.4 [Source:ZFIN;Acc:ZDB-GENE-140106-45]	7	51816321	51825070		4.59	4.03	4.09	4.43	4.01	4.25
ENSDARG00000052082	gabarapb	GABA(A) receptor-associated protein b [Source:ZFIN;Acc:ZDB-GENE-101102-9]	7	69470142	69478567		89.89	82.19	82.09	95.12	100.58	105.78
ENSDARG00000052088	cxcr1	chemokine (C-X-C motif) receptor 1 [Source:ZFIN;Acc:ZDB-GENE-070302-4]	9	48085039	48091741		0.03	0.05	0.02	0.07	0.04	0.04
ENSDARG00000052089	CCKAR	cholecystokinin A receptor [Source:NCBI gene;Acc:569038]	7	62229075	62244744	CCKAR	1.66	1.64	1.31	0.7	0.65	0.9
ENSDARG00000052091	rbpjb	recombination signal binding protein for immunoglobulin kappa J region b [Source:ZFIN;Acc:ZDB-GENE-070319-1]	7	62080456	62206933		3.36	2.4	2.37	3.8	3.7	3.79
ENSDARG00000052094	notch1b	notch receptor 1b [Source:ZFIN;Acc:ZDB-GENE-990415-183]	5	65679595	65782785		3.59	2.13	1.65	6.14	5.7	6.42
ENSDARG00000052097	trim35-31	tripartite motif containing 35-31 [Source:ZFIN;Acc:ZDB-GENE-061009-3]	3	5538485	5555500		0.06	0.13	0.06	0.1	0.14	0.09
ENSDARG00000052099	agxta	alanine--glyoxylate and serine--pyruvate aminotransferase a [Source:ZFIN;Acc:ZDB-GENE-040718-16]	6	27112884	27123327		18.82	26.08	19.63	21.95	24.91	24.73
ENSDARG00000052103	dtymk	deoxythymidylate kinase (thymidylate kinase) [Source:ZFIN;Acc:ZDB-GENE-990603-11]	6	27104962	27108844		8.91	11.22	11.5	8.2	8.1	7.91
ENSDARG00000052104	atg4b	autophagy related 4B, cysteine peptidase [Source:NCBI gene;Acc:448859]	6	27090800	27105013		9.77	10.79	12.2	12.13	12.9	12.46
ENSDARG00000052109	zgc:114041	zgc:114041 [Source:ZFIN;Acc:ZDB-GENE-050706-122]	5	66132394	66146759		0.95	0.54	0.64	0.84	0.8	0.51
ENSDARG00000052110	mboat4	membrane bound O-acyltransferase domain containing 4 [Source:NCBI gene;Acc:795709]	5	66157117	66160415		0.28	0.37	0.27	0.09	0.08	0.1
ENSDARG00000052113	hexa	hexosaminidase A (alpha polypeptide) [Source:ZFIN;Acc:ZDB-GENE-050417-283]	25	22216723	22241312		8.74	9.6	9.86	5.08	5.24	5.08
ENSDARG00000052114	crtc3	CREB regulated transcription coactivator 3 [Source:ZFIN;Acc:ZDB-GENE-070712-2]	7	50548074	50633285		2.94	2.21	1.45	3.96	3.93	3.52
ENSDARG00000052115	ehf	ets homologous factor [Source:ZFIN;Acc:ZDB-GENE-060312-7]	25	10410620	10436906		0.35	0.52	0.21	0.38	0.5	0.3
ENSDARG00000052118	adamtsl5	ADAMTS like 5 [Source:ZFIN;Acc:ZDB-GENE-161017-76]	7	50475703	50517023		2.53	2.27	1.88	5.29	5.6	5.43
ENSDARG00000052121	rag2	recombination activating gene 2 [Source:NCBI gene;Acc:30658]	25	9010425	9013963		0.43	0.17	0.32	0.17	0.17	0.19
ENSDARG00000052122	rag1	recombination activating 1 [Source:NCBI gene;Acc:30663]	25	9003230	9009902		1.88	0.61	0.85	0.65	0.63	0.63
ENSDARG00000052124	accs	1-aminocyclopropane-1-carboxylate synthase homolog (Arabidopsis)(non-functional) [Source:ZFIN;Acc:ZDB-GENE-050327-39]	25	8921425	8949639		2.69	1.56	1.52	3.23	2.67	2.47
ENSDARG00000052129	boka	BCL2 family apoptosis regulator BOK a [Source:NCBI gene;Acc:445218]	6	27062512	27089014		5.17	4.88	4.17	3.88	3.7	3.76
ENSDARG00000052131	gli3	GLI family zinc finger 3 [Source:ZFIN;Acc:ZDB-GENE-041111-162]	24	11528270	11821505		3.73	3.04	2.31	3.8	3.37	3.38
ENSDARG00000052138	slc1a2a	solute carrier family 1 member 2a [Source:ZFIN;Acc:ZDB-GENE-100422-11]	7	49862837	49881742		13.18	2.49	1.57	10.16	11.32	11.28
ENSDARG00000052139	notch3	notch receptor 3 [Source:ZFIN;Acc:ZDB-GENE-000329-5]	3	53526025	53559581		8.21	4.44	3.37	14.41	13.72	13.81
ENSDARG00000052142	acvr1bb	activin A receptor type 1Bb [Source:ZFIN;Acc:ZDB-GENE-131120-9]	6	2174082	2198683		11.85	6.24	5	12.71	12.35	12.98
ENSDARG00000052148	ptgs1	prostaglandin-endoperoxide synthase 1 [Source:ZFIN;Acc:ZDB-GENE-020530-1]	5	64900223	64946436		3.81	3.95	3.23	7.09	7.32	6.76
ENSDARG00000052150	pbx4	pre-B-cell leukemia transcription factor 4 [Source:ZFIN;Acc:ZDB-GENE-000201-18]	3	52999962	53076469		12.44	8.13	6.21	29.41	28.18	27.94
ENSDARG00000052154	lix1	limb and CNS expressed 1 [Source:ZFIN;Acc:ZDB-GENE-060526-123]	5	64814482	64831207		9.1	6.13	7.83	13.74	15.96	16.05
ENSDARG00000052155	lrrc8ab	leucine rich repeat containing 8 VRAC subunit Ab [Source:ZFIN;Acc:ZDB-GENE-060531-43]	5	64840656	64850497		3.11	1.49	1.67	5.52	5.83	5.69
ENSDARG00000052157	il12rb2	interleukin 12 receptor, beta 2a [Source:ZFIN;Acc:ZDB-GENE-080107-3]	6	34908597	34925690		0.54	0.33	0.43	0.9	0.99	0.86
ENSDARG00000052158	il23r	interleukin 23 receptor [Source:ZFIN;Acc:ZDB-GENE-080107-5]	6	34868156	34895175		0.45	0.39	0.26	0.1	0.22	0.18
ENSDARG00000052164	znf507	zinc finger protein 507 [Source:ZFIN;Acc:ZDB-GENE-040426-1233]	7	47243564	47266151		2.66	1.53	1.36	2.68	2.74	2.8
ENSDARG00000052165	LHX6	si:ch211-236k19.2 [Source:ZFIN;Acc:ZDB-GENE-060531-41]	5	64893630	64900552	LHX6	0.15	0	0.05	0.03	0.07	0.13
ENSDARG00000052170	uap1	UDP-N-acetylglucosamine pyrophosphorylase 1 [Source:ZFIN;Acc:ZDB-GENE-030131-1233]	6	35033457	35052388		5.35	4.79	4.47	4	4	3.29
ENSDARG00000052176	shfl	shiftless antiviral inhibitor of ribosomal frameshifting [Source:NCBI gene;Acc:797287]	3	53491105	53508580		0.71	0.97	0.96	0.65	0.39	0.41
ENSDARG00000052178	eif2s1b	eukaryotic translation initiation factor 2, subunit 1 alpha b [Source:ZFIN;Acc:ZDB-GENE-030131-283]	20	51104367	51123265		112.52	125.85	139.27	106.02	112.12	116.52
ENSDARG00000052182	rbfox2	RNA binding fox-1 homolog 2 [Source:ZFIN;Acc:ZDB-GENE-040426-2464]	6	296130	342216		28.68	15.73	16.4	47.4	46.26	45
ENSDARG00000052188	znf541	zinc finger protein 541 [Source:ZFIN;Acc:ZDB-GENE-060526-239]	5	61953086	61970674		0.05	0.04	0.04	0.09	0.1	0.15
ENSDARG00000052190	fdx2	ferredoxin 2 [Source:NCBI gene;Acc:767726]	6	149405	152955		7.86	7.85	8.5	6.3	7.66	6.73
ENSDARG00000052192	s1pr5b	sphingosine-1-phosphate receptor 5b [Source:ZFIN;Acc:ZDB-GENE-061201-27]	6	140121	141697		0.56	0.95	0.9	0.94	0.68	0.7
ENSDARG00000052207	c3a.3	complement component c3a, duplicate 3 [Source:ZFIN;Acc:ZDB-GENE-990415-37]	1	56274613	56320569		18.06	27.48	23.56	17.92	17.76	17.42
ENSDARG00000052215	btr02	bloodthirsty-related gene family, member 2 [Source:ZFIN;Acc:ZDB-GENE-060825-228]	3	50127726	50147160		2.05	2.48	1.98	2.03	1.56	1.49
ENSDARG00000052222	rasd2	RASD family member 2 [Source:ZFIN;Acc:ZDB-GENE-050913-57]	3	50175166	50177658		0.73	0.11	0.12	0.82	0.4	0.4
ENSDARG00000052223	rcvrna	recoverin a [Source:ZFIN;Acc:ZDB-GENE-050913-106]	3	50441444	50443607		108.18	27.93	19.35	49.44	48.7	49.96
ENSDARG00000052242	aff2	AF4/FMR2 family, member 2 [Source:ZFIN;Acc:ZDB-GENE-110411-190]	14	20342553	20736925		1.55	0.55	0.56	3.02	2.8	3.12
ENSDARG00000052244	zgc:158564	zgc:158564 [Source:ZFIN;Acc:ZDB-GENE-070112-1662]	7	39664055	39678530		11.38	7.18	6.13	12.82	12.6	12.37
ENSDARG00000052247	alkbh4	alkB homolog 4, lysine demthylase [Source:ZFIN;Acc:ZDB-GENE-060526-275]	5	61467618	61472766		7.51	7.79	8.85	5.06	5.37	4.99
ENSDARG00000052255	trim35-3	tripartite motif containing 35-3 [Source:ZFIN;Acc:ZDB-GENE-070912-56]	3	4444233	4456956		0.06	0.03	0.07	0.09	0.11	0.12
ENSDARG00000052263	rab1aa	RAB1A, member RAS oncogene family a [Source:ZFIN;Acc:ZDB-GENE-090312-110]	1	55124296	55131439		19.97	19.99	17.94	21.45	23.08	21
ENSDARG00000052271	slc22a18	solute carrier family 22 member 18 [Source:ZFIN;Acc:ZDB-GENE-051120-165]	7	39537790	39552314		18.01	27.88	21.19	12.56	12.06	12.32
ENSDARG00000052277	cabp2b	calcium binding protein 2b [Source:ZFIN;Acc:ZDB-GENE-081028-55]	14	46223458	46301887		1.13	0.76	0.82	0.59	0.75	0.68
ENSDARG00000052279	osgn1	oxidative stress induced growth inhibitor 1 [Source:ZFIN;Acc:ZDB-GENE-040426-864]	6	50695381	50704689		7.82	6.55	6.07	7.8	7.29	7.45
ENSDARG00000052288	zmp:0000000634	zmp:0000000634 [Source:ZFIN;Acc:ZDB-GENE-130530-637]	6	22679610	22686016		0.06	0.07	0.05	0.01	0.01	0.02
ENSDARG00000052290	rab33ba	RAB33B, member RAS oncogene family a [Source:ZFIN;Acc:ZDB-GENE-050809-122]	14	46177524	46189621		1.32	0.84	0.75	1.94	1.85	1.77
ENSDARG00000052296	pimr198	Pim proto-oncogene, serine/threonine kinase, related 198 [Source:ZFIN;Acc:ZDB-GENE-131119-89]	2	17540720	17542659		0	0	0	0	0	0
ENSDARG00000052300	zgc:171489	zgc:171489 [Source:ZFIN;Acc:ZDB-GENE-080303-25]	3	58455428	58468835		1.22	1.05	0.57	1.53	1.37	1.16
ENSDARG00000052304	uqcrc1	ubiquinol-cytochrome c reductase core protein 1 [Source:ZFIN;Acc:ZDB-GENE-040426-1792]	6	22337081	22351939		179.86	225.19	223.35	148.99	149.37	147.47
ENSDARG00000052319	pimr196	Pim proto-oncogene, serine/threonine kinase, related 196 [Source:ZFIN;Acc:ZDB-GENE-070705-328]	2	17524278	17575605		0	0	0	0	0	0.03
ENSDARG00000052322	tlr5b	toll-like receptor 5b [Source:ZFIN;Acc:ZDB-GENE-040219-15]	20	51466046	51476849		2.01	2.92	2.47	3.66	3.7	3.64
ENSDARG00000052330	slc4a2b	solute carrier family 4 member 2b [Source:ZFIN;Acc:ZDB-GENE-030429-14]	24	33301129	33366188		6.29	5.67	4.17	6.03	5.9	5.92
ENSDARG00000052331	abcf2b	ATP-binding cassette, sub-family F (GCN20), member 2b [Source:ZFIN;Acc:ZDB-GENE-050506-58]	7	41403690	41420226		2.12	2.32	2.79	2.28	2.11	2.16
ENSDARG00000052332	ftr98	finTRIM family, member 98 [Source:ZFIN;Acc:ZDB-GENE-081105-171]	8	39827198	39838660		0.1	0.2	0.18	0.15	0.13	0.17
ENSDARG00000052334	ociad1	OCIA domain containing 1 [Source:NCBI gene;Acc:393272]	20	23245620	23254876		28.26	35.51	30.58	25.62	23.71	24.61
ENSDARG00000052336	ociad2	OCIA domain containing 2 [Source:ZFIN;Acc:ZDB-GENE-041014-253]	20	23238833	23244193		22.9	41.08	33.29	8.65	8.63	8.73
ENSDARG00000052341	sgcb	sarcoglycan, beta (dystrophin-associated glycoprotein) [Source:ZFIN;Acc:ZDB-GENE-030131-6695]	20	23173710	23181762		32.54	26.97	27.64	36.98	34.07	36.69
ENSDARG00000052343	spata18	spermatogenesis associated 18 [Source:NCBI gene;Acc:497395]	20	23160512	23171430		1.62	0.98	1.06	1.18	1.49	1.76
ENSDARG00000052344	tbl3	transducin (beta)-like 3 [Source:ZFIN;Acc:ZDB-GENE-041114-104]	3	62173396	62194512		19.8	18.7	19.2	14.52	14.35	14.75
ENSDARG00000052348	smarca5	SWI/SNF related, matrix associated, actin dependent regulator of chromatin, subfamily a, member 5 [Source:ZFIN;Acc:ZDB-GENE-	1	52792439	52820954		36.88	23.42	25.8	30.41	28.9	28.33
ENSDARG00000052351	si:dkey-156n14.3	si:dkey-156n14.3 [Source:ZFIN;Acc:ZDB-GENE-030131-4816]	6	22025806	22042654		9.52	5.17	5.32	9.39	10.24	9.98
ENSDARG00000052360	cacng1b	calcium channel, voltage-dependent, gamma subunit 1b [Source:ZFIN;Acc:ZDB-GENE-131127-98]	3	61043774	61065055		0.94	0.78	0.94	0.62	0.58	0.65
ENSDARG00000052361	il15	interleukin 15 [Source:ZFIN;Acc:ZDB-GENE-060213-2]	1	53234693	53260082		4.72	4.54	4.48	3.34	4.12	3.69
ENSDARG00000052368	cltb	clathrin, light chain B [Source:ZFIN;Acc:ZDB-GENE-101005-2]	14	51023954	51039702		10.77	9.32	7.88	10.37	10.25	10.57
ENSDARG00000052371	higd2a	HIG1 hypoxia inducible domain family, member 2A [Source:ZFIN;Acc:ZDB-GENE-050417-119]	14	51015205	51018162		28.64	34.87	38.9	27.2	25.16	25.64
ENSDARG00000052374	faf2	Fas associated factor family member 2 [Source:ZFIN;Acc:ZDB-GENE-050208-312]	14	50994138	51014292		17.91	14.46	11.92	19.17	17.89	18.91
ENSDARG00000052375	zgc:66455	zgc:66455 [Source:ZFIN;Acc:ZDB-GENE-040426-1642]	1	53454741	53468160		0.06	0.18	0.12	0.15	0.14	0.18
ENSDARG00000052376	b3gnt2a	UDP-GlcNAc:betaGal beta-1,3-N-acetylglucosaminyltransferase 2a [Source:ZFIN;Acc:ZDB-GENE-090312-192]	1	53475074	53476758		1.37	1.34	1.24	0.82	1.11	1.2
ENSDARG00000052377	psmd12	proteasome 26S subunit, non-ATPase 12 [Source:ZFIN;Acc:ZDB-GENE-030131-617]	6	21525543	21534301		33.24	37.84	35.69	27.93	25.79	25.76
ENSDARG00000052379	ccdc22	coiled-coil domain containing 22 [Source:NCBI gene;Acc:556259]	8	23615132	23638031		8.65	6.48	6.71	8.58	9.31	9.62

ENSDARG00000052386	cldnd1b	claudin domain containing 1b [Source:ZFIN;Acc:ZDB-GENE-040808-4]	1	52481332	52486995		10.28	7.98	8.35	14.36	15.18	13.78
ENSDARG00000052387	pou2f3	POU class 2 homeobox 3 [Source:ZFIN;Acc:ZDB-GENE-060512-299]	5	58550291	58602307		1.92	1.63	1.37	1.84	1.97	2.05
ENSDARG00000052388	pcgf5b	polycomb group ring finger 5b [Source:NCBI gene;Acc:692252]	12	16405598	16435150		0.8	0.8	0.88	0.98	0.69	0.79
ENSDARG00000052390	tmed8	transmembrane p24 trafficking protein 8 [Source:ZFIN;Acc:ZDB-GENE-050522-283]	17	45136353	45155181		4.83	2.75	3.34	5.33	5.09	5.64
ENSDARG00000052396	pkz	protein kinase containing Z-DNA binding domains [Source:ZFIN;Acc:ZDB-GENE-050301-2]	13	50190664	50200348		0.25	0.47	0.23	0.34	0.16	0.27
ENSDARG00000052400	gpr6	G protein-coupled receptor 6 [Source:ZFIN;Acc:ZDB-GENE-170413-1]	20	53278746	53279747		0.44	0.3	0.28	0.56	0.7	0.84
ENSDARG00000052402	dnmt3ba	DNA (cytosine-5-)-methyltransferase 3 beta, duplicate a [Source:ZFIN;Acc:ZDB-GENE-050314-4]	8	23355484	23381548		8.4	5.61	5.52	6.99	6.55	6.14
ENSDARG00000052405	pak6b	p21 protein (Cdc42/Rac)-activated kinase 6b [Source:ZFIN;Acc:ZDB-GENE-041014-121]	20	53521648	53568849		2.04	2.32	1.65	2.11	1.71	1.89
ENSDARG00000052408	mgat2	alpha-1,6-mannosyl-glycoprotein 2-beta-N-acetylglucosaminyltransferase [Source:ZFIN;Acc:ZDB-GENE-070410-54]	20	54067041	54075136		9.93	7.21	7	9.38	9.66	9.66
ENSDARG00000052411	si:dkey-71l1.1	si:dkey-71l1.1 [Source:ZFIN;Acc:ZDB-GENE-070705-489]	5	57693472	57715272		2.31	0.8	0.98	2.15	1.75	2.16
ENSDARG00000052413	crfb12	cytokine receptor family member B12 [Source:ZFIN;Acc:ZDB-GENE-060421-4184]	5	57673141	57686487		2.24	1.53	1.22	1.13	1.04	1.11
ENSDARG00000052419	ankrd12	ankyrin repeat domain 12 [Source:ZFIN;Acc:ZDB-GENE-040426-846]	2	54696042	54786748		18.82	8.24	8.68	19.49	21.69	21.67
ENSDARG00000052423	ppp1r12c	protein phosphatase 1, regulatory subunit 12C [Source:ZFIN;Acc:ZDB-GENE-010724-11]	6	21588205	21616659		9.01	4.72	5.39	9.64	9.97	10.03
ENSDARG00000052426	si:ch211-256e16.3	si:ch211-256e16.3 [Source:ZFIN;Acc:ZDB-GENE-131127-256]	3	58133227	58154489		4.21	2.06	2.58	5.47	5.97	5.51
ENSDARG00000052427	bicdl2	BICD family like cargo adaptor 2 [Source:NCBI gene;Acc:777640]	5	57658898	57671298		6.16	5.71	5.09	4.38	4.54	4.33
ENSDARG00000052428	si:dkey-43k4.3	si:dkey-43k4.3 [Source:ZFIN;Acc:ZDB-GENE-120215-146]	6	21576164	21582444		0.68	0.93	0.42	0.67	0.79	0.68
ENSDARG00000052429	syngr2a	synaptogyrin 2a [Source:ZFIN;Acc:ZDB-GENE-070112-1292]	5	57766627	57779801		3.57	4.05	3.15	4.67	3.94	4.62
ENSDARG00000052433	inha	inhibin subunit alpha [Source:ZFIN;Acc:ZDB-GENE-060503-554]	6	21068783	21091948		0.04	0	0	0	0	0
ENSDARG00000052435	spred2a	sprouty related EVH1 domain containing 2a [Source:ZFIN;Acc:ZDB-GENE-110830-2]	1	51444549	51474974		5.03	2.98	2.52	7.5	7.09	7.2
ENSDARG00000052437	mia	MIA SH3 domain containing [Source:ZFIN;Acc:ZDB-GENE-081022-66]	5	57653459	57655092		28.98	40.04	38.76	37.59	41.89	40.95
ENSDARG00000052438	actr2a	actin related protein 2a [Source:NCBI gene;Acc:406820]	1	51407520	51428106		14.19	13.54	10.33	16.05	15.85	15.76
ENSDARG00000052450	hspb2	heat shock protein, alpha-crystallin-related, b2 [Source:ZFIN;Acc:ZDB-GENE-050417-260]	5	57629207	57641257		6.01	6.27	7.2	4.18	4.11	4.53
ENSDARG00000052454	rhpn1	rhophilin, Rho GTPase binding protein 1 [Source:ZFIN;Acc:ZDB-GENE-081031-79]	20	52233981	52271262		0.97	0.34	0.45	1.13	1.33	1.75
ENSDARG00000052455	porcnl	porcupine O-acyltransferase like [Source:ZFIN;Acc:ZDB-GENE-030131-5931]	8	22544290	22558773		3.21	2.3	2.27	3.55	3.6	3.29
ENSDARG00000052462	pisd	phosphatidylserine decarboxylase [Source:ZFIN;Acc:ZDB-GENE-061215-46]	5	57489253	57528943		11.89	6.78	6.73	10.45	9.83	11.21
ENSDARG00000052465	dusp10	dual specificity phosphatase 10 [Source:ZFIN;Acc:ZDB-GENE-041014-271]	20	51917506	51946052		1.63	2.02	1.84	1.43	1.15	1.56
ENSDARG00000052468	gsk3bb	glycogen synthase kinase 3 beta, genome duplicate b [Source:ZFIN;Acc:ZDB-GENE-090312-2]	1	49370549	49409549		4.66	1.75	1.66	6.24	6.42	6.73
ENSDARG00000052470	igfbp2a	insulin-like growth factor binding protein 2a [Source:NCBI gene;Acc:794176]	6	20921542	20952187		56.35	79.41	83.46	36.33	36.79	35.47
ENSDARG00000052471	taf1a	TATA box binding protein (TBP)-associated factor, RNA polymerase I, A [Source:ZFIN;Acc:ZDB-GENE-041014-272]	20	51813432	51874288		3.07	2.66	2.63	3.05	2.27	2.38
ENSDARG00000052480	pdcd11	programmed cell death 11 [Source:ZFIN;Acc:ZDB-GENE-030131-4076]	1	49435017	49481173		16.96	15.59	13.13	13.25	13.61	13.19
ENSDARG00000052482	arhgef11	Rho guanine nucleotide exchange factor (GEF) 11 [Source:ZFIN;Acc:ZDB-GENE-070604-1]	7	5380320	5487593		11.05	5.16	4.25	12.26	12.43	13.39
ENSDARG00000052494	pcdh18b	protocadherin 18b [Source:ZFIN;Acc:ZDB-GENE-080804-1]	14	42172257	42179789		14.68	9.9	8.37	21.93	21.48	22.02
ENSDARG00000052496	arhgap45a	Rho GTPase activating protein 45a [Source:ZFIN;Acc:ZDB-GENE-140106-47]	2	56679214	56757069		0.57	0.56	0.39	0.96	1.02	0.91
ENSDARG00000052497	zgc:110789	zgc:110789 [Source:ZFIN;Acc:ZDB-GENE-050306-38]	18	12034985	12052132		7.21	10.67	8.96	4.61	5.42	5.25
ENSDARG00000052511	lin28b	lin-28 homolog B (C. elegans) [Source:ZFIN;Acc:ZDB-GENE-140811-1]	20	47491247	47531869		6.26	4.11	5.4	9.72	11.12	9.64
ENSDARG00000052512	tsga10	testis specific, 10 [Source:ZFIN;Acc:ZDB-GENE-061013-482]	1	49651012	49674006		1.18	0.36	0.5	0.93	1.02	1.27
ENSDARG00000052515	calcoco2	calcium binding and coiled-coil domain 2 [Source:ZFIN;Acc:ZDB-GENE-050522-346]	12	20641102	20652923		1.69	1.96	1.66	1.25	1.17	1.21
ENSDARG00000052518	stx4	syntaxin 4 [Source:ZFIN;Acc:ZDB-GENE-030131-2455]	12	20627164	20642019		5.04	6.26	5.2	5.33	5.66	5.15
ENSDARG00000052520	sgms2a	sphingomyelin synthase 2a [Source:ZFIN;Acc:ZDB-GENE-070730-2]	1	49933652	49950643		3.12	3.51	3.6	2.52	2.96	2.95
ENSDARG00000052522	snx11	sorting nexin 11 [Source:ZFIN;Acc:ZDB-GENE-041111-253]	12	20605191	20616160		4.48	3.66	3.25	4.89	4.74	4.67
ENSDARG00000052527	mul1b	mitochondrial E3 ubiquitin protein ligase 1b [Source:ZFIN;Acc:ZDB-GENE-050208-236]	23	37287542	37291840		2.82	2.23	2.04	2.98	3.07	2.87
ENSDARG00000052528	CR762407.1		23	37277073	37280713		2.42	1.21	1.76	2.08	1.97	4.2
ENSDARG00000052535	ubxn10	UBX domain protein 10 [Source:NCBI gene;Acc:751736]	23	37090889	37094205		0.39	0.39	0.46	0.55	0.4	0.44
ENSDARG00000052536	tia1	TIA1 cytotoxic granule-associated RNA binding protein [Source:ZFIN;Acc:ZDB-GENE-030131-1506]	5	56950861	56964547		20.95	12.94	15.25	22.02	20.21	21.43
ENSDARG00000052537	cptp	ceramide-1-phosphate transfer protein [Source:NCBI gene;Acc:791641]	23	37086159	37093123		9.56	9.7	8.37	5.65	4.95	5.01
ENSDARG00000052553	lig3	ligase III, DNA, ATP-dependent [Source:ZFIN;Acc:ZDB-GENE-030131-8453]	5	60847823	60873092		12.79	9.25	9.37	11.86	12.28	11.65
ENSDARG00000052556	fgfr1lb	fibroblast growth factor receptor like 1b [Source:ZFIN;Acc:ZDB-GENE-050201-3]	14	41600340	41678749		6.6	7.82	7.27	11.88	12.17	11.95
ENSDARG00000052558	porcn	porcupine O-acyltransferase [Source:ZFIN;Acc:ZDB-GENE-081105-87]	8	21146262	21155964		5.91	1.82	3.04	7.53	6.18	6.09
ENSDARG00000052565	cenpi	centromere protein I [Source:ZFIN;Acc:ZDB-GENE-060810-127]	14	41244877	41272122		3.55	2.68	2.94	3.4	3.29	2.99
ENSDARG00000052567	tmem35	transmembrane protein 35 [Source:NCBI gene;Acc:791920]	14	41276307	41285392		25.19	12.07	14.35	26.24	26.7	27.21
ENSDARG00000052575	arl13a	ADP-ribosylation factor-like 13A [Source:ZFIN;Acc:ZDB-GENE-040426-1790]	14	41293110	41308561		8.92	2.42	1.99	6.26	7.64	7.36
ENSDARG00000052578	c6ast4	six-cysteine containing astacin protease 4 [Source:ZFIN;Acc:ZDB-GENE-050626-100]	7	38635538	38638809		106.79	159.26	139.86	3.27	3.1	3.12
ENSDARG00000052594	nkiras2	NFKB inhibitor interacting Ras-like 2 [Source:NCBI gene;Acc:445039]	3	17867126	17871860		8	6.62	7.12	7.76	8.28	8.94
ENSDARG00000052604	cpeb2	cytoplasmic polyadenylation element binding protein 2 [Source:ZFIN;Acc:ZDB-GENE-130530-639]	23	2136344	2161478		8.6	2.69	3.75	16.41	14.78	15.81
ENSDARG00000052606	dpp9	dipeptidyl-peptidase 9 [Source:ZFIN;Acc:ZDB-GENE-061013-777]	8	47677208	47723544		11.94	10.34	9.74	14.71	14.72	14.45
ENSDARG00000052609	CU468164.1	uncharacterized LOC100005318 [Source:NCBI gene;Acc:100005318]	14	9056071	9066756		0.05	0.03	0.09	0.05	0	0.04
ENSDARG00000052610	olig4	oligodendrocyte transcription factor 4 [Source:ZFIN;Acc:ZDB-GENE-030131-3580]	13	45967179	45968256		2.16	1.37	2.64	1.57	1.43	1.55
ENSDARG00000052615	tnmd	tenomodulin [Source:ZFIN;Acc:ZDB-GENE-080220-31]	14	40641350	40722928		14.04	17.86	15.11	10.59	11.01	10.12
ENSDARG00000052617	raver1	ribonucleoprotein, PTB-binding 1 [Source:ZFIN;Acc:ZDB-GENE-050522-99]	3	46559639	46587006		1.26	0.74	0.63	1.62	1.58	1.56
ENSDARG00000052618	ahrrb	aryl-hydrocarbon receptor repressor b [Source:ZFIN;Acc:ZDB-GENE-051018-2]	2	50133594	50198328		0.54	0.26	0.2	0.26	0.38	0.18
ENSDARG00000052623	bsdc1	BSD domain containing 1 [Source:NCBI gene;Acc:794882]	13	45980163	46000113		27.84	24.43	23.67	28.46	29.61	29.28
ENSDARG00000052624	spryd7a	SPRY domain containing 7a [Source:ZFIN;Acc:ZDB-GENE-040426-2713]	1	46710647	46718353		11.71	10.46	9.81	12.65	12.32	11.72
ENSDARG00000052625	fkbp1b	FKBP prolyl isomerase 1B [Source:ZFIN;Acc:ZDB-GENE-040426-1785]	20	44464345	44499245		34.15	39.7	40.09	22.91	23.41	24.05
ENSDARG00000052626	AL954655.1		12	46188692	46204837		0.07	0	0.01	0.25	0.13	0.06
ENSDARG00000052633	si:ch211-106n13.3	si:ch211-106n13.3 [Source:ZFIN;Acc:ZDB-GENE-030131-9088]	2	49987088	50053006		4.85	3.42	2.92	3.32	3.42	2.96
ENSDARG00000052638	fam210b	family with sequence similarity 210 member B [Source:ZFIN;Acc:ZDB-GENE-050913-122]	6	59350457	59357256		14	14.27	14.26	10.75	10.85	11.08
ENSDARG00000052641	kpna3	karyopherin alpha 3 (importin alpha 4) [Source:ZFIN;Acc:ZDB-GENE-030616-541]	1	46666696	46706639		19.5	16.89	14.72	21.9	21	19.91
ENSDARG00000052642	shisa9b	shisa family member 9b [Source:NCBI gene;Acc:566015]	3	46043406	46115232		1.61	0.27	0.78	1.8	2.2	2.31
ENSDARG00000052644	ca10a	carbonic anhydrase Xa [Source:ZFIN;Acc:ZDB-GENE-051030-123]	12	21298317	21553637		3.83	2.3	2.82	3.58	3.8	3.63
ENSDARG00000052646	ftr69	finTRIM family, member 69 [Source:ZFIN;Acc:ZDB-GENE-090508-10]	13	46196354	46200240		0.09	0.06	0.04	0.08	0.05	0.03
ENSDARG00000052648	hs3st4	heparan sulfate (glucosamine) 3-O-sulfotransferase 4 [Source:ZFIN;Acc:ZDB-GENE-070202-8]	3	44541322	44753325		2.51	0.62	0.88	2.76	2.32	2.77
ENSDARG00000052649	ube2ia	ubiquitin-conjugating enzyme E2Ia [Source:NCBI gene;Acc:114445]	3	45364849	45373511		21.4	14.21	14.37	24.36	24.33	23.47
ENSDARG00000052652	fermt1	fermitin family member 1 [Source:ZFIN;Acc:ZDB-GENE-030131-3264]	20	45775935	45812152		9.55	11.62	9.55	8.55	8.31	8.63
ENSDARG00000052654	thrab	thyroid hormone receptor alpha b [Source:ZFIN;Acc:ZDB-GENE-060813-1]	12	21799130	22039350		9.06	5.56	4.68	9.75	10.66	10.03
ENSDARG00000052656	si:ch211-193e13.5	si:ch211-193e13.5 [Source:ZFIN;Acc:ZDB-GENE-081104-158]	10	8527196	8530065		0.26	0.26	0.43	0.63	0.7	0.64
ENSDARG00000052658	pptc7b	PTC7 protein phosphatase homolog b [Source:ZFIN;Acc:ZDB-GENE-081105-111]	10	8437930	8468949		15.65	14	11.88	12.77	12.19	12.5
ENSDARG00000052673	sept7b	septin 7b [Source:ZFIN;Acc:ZDB-GENE-030131-37]	16	47389539	47427016		25.2	20.57	19.81	27.57	26.44	27
ENSDARG00000052674	csnk1a1	casein kinase 1, alpha 1 [Source:ZFIN;Acc:ZDB-GENE-020419-19]	14	38762977	38783619		53.3	44.97	44.44	74.09	78.09	75.46
ENSDARG00000052677	si:ch211-138g9.2	si:ch211-138g9.2 [Source:ZFIN;Acc:ZDB-GENE-090311-41]	1	46026457	46030577		0	0	0	0.01	0	0
ENSDARG00000052680	si:dkey-182g1.2	si:dkey-182g1.2 [Source:ZFIN;Acc:ZDB-GENE-060503-619]	22	8753092	8770357		2.52	2.68	2.22	4.29	3.94	4.46
ENSDARG00000052688	paqr5b	progestin and adipoQ receptor family member Vb [Source:NCBI gene;Acc:100007661]	7	33457148	33475194		3.91	2.64	2.16	1.8	1.9	1.3
ENSDARG00000052690	arrdc3a	arrestin domain containing 3a [Source:ZFIN;Acc:ZDB-GENE-030131-2913]	5	48985861	48995955		24.4	17.05	15.69	55.95	62.13	60.64
ENSDARG00000052693	si:dkey-172m14.1	si:dkey-172m14.1 [Source:ZFIN;Acc:ZDB-GENE-060526-221]	5	49019182	49028962		9.6	9.39	8.38	8.74	9.71	8.96
ENSDARG00000052695	nr2f1a	nuclear receptor subfamily 2, group F, member 1a [Source:NCBI gene;Acc:30418]	5	49744713	49751549		19.01	8.14	9.77	31.51	28.45	30.7
ENSDARG00000052696	bdh2	3-hydroxybutyrate dehydrogenase, type 2 [Source:NCBI gene;Acc:550507]	1	43714149	43727576		0.04	0.01	0.01	0.04	0	0.01
ENSDARG00000052697	fam172a	family with sequence similarity 172 member A [Source:NCBI gene;Acc:393390]	5	49768223	50084310		7.66	7.85	7.14	7.18	7.79	6.69
ENSDARG00000052700	SLC9B2	si:dkey-162b23.4 [Source:ZFIN;Acc:ZDB-GENE-110411-203]	1	43695033	43727613	SLC9B2	2.45	0.65	0.69	4.7	4.44	4.72
ENSDARG00000052701	smpd2b	sphingomyelin phosphodiesterase 2b [Source:ZFIN;Acc:ZDB-GENE-130530-640]	6	54680730	54705050		2.39	2.12	2.01	2.23	2.72	2.49
ENSDARG00000052702	capn1a	calpain 1, (mu/l) large subunit a [Source:ZFIN;Acc:ZDB-GENE-040426-1263]	13	42679767	42724645		9.8	9.97	8.64	10.53	10.3	10.67
ENSDARG00000052703	cisd2	CDGSH iron sulfur domain 2 [Source:NCBI gene;Acc:393354]	1	43686251	43692141		5.39	5.93	5.04	5.44	5.22	4.99
ENSDARG00000052705	pkp1b	plakophilin 1b [Source:ZFIN;Acc:ZDB-GENE-030131-417]	6	54711306	54754042		9.15	7.15	4.75	3.78	3.86	4.14
ENSDARG00000052708	tnni1b	troponin I type 1b (skeletal, slow) [Source:ZFIN;Acc:ZDB-GENE-041212-37]	6	54805136	54826061		4.71	7.57	6.1	9.75	9.61	10.23
ENSDARG00000052712	suclg1	succinate-CoA ligase, alpha subunit [Source:ZFIN;Acc:ZDB-GENE-040912-101]	1	43652480	43684584		220.73	278.78	277.56	142.09	148.36	148.26
ENSDARG00000052713	lrrtm1	leucine rich repeat transmembrane neuronal 1 [Source:ZFIN;Acc:ZDB-GENE-050506-80]	1	42776553	42779075		15.54	3.81	6.54	17.76	18.23	17.83
ENSDARG00000052721	prim2	DNA primase subunit 2 [Source:ZFIN;Acc:ZDB-GENE-050522-471]	13	23488282	23612369		7.42	7.22	7.96	3.45	3.9	3.83
ENSDARG00000052728	sltm	SAFB-like, transcription modulator [Source:ZFIN;Acc:ZDB-GENE-040426-2647]	7	30532636	30560450		15.36	7.49	9.04	15.69	16.98	17.08
ENSDARG00000052731	ankrd31	ankyrin repeat domain 31 [Source:ZFIN;Acc:ZDB-GENE-050417-473]	5	51001985	51011386		0.04	0.03	0.06	0.05	0.01	0.03
ENSDARG00000052734	hmgcra	3-hydroxy-3-methylglutaryl-CoA reductase a [Source:ZFIN;Acc:ZDB-GENE-040401-2]	5	50972485	50992775		15.2	12.77	8.26	7.11	7	6.95
ENSDARG00000052739	egf	epidermal growth factor [Source:ZFIN;Acc:ZDB-GENE-070922-1]	14	36279826	36325558		0.5	0.37	0.32	0.99	1.17	1.05
ENSDARG00000052741	card11	caspase recruitment domain family, member 11 [Source:ZFIN;Acc:ZDB-GENE-100219-1]	3	41558682	41632059		1.07	1.04	0.88	1.22	1.39	1.09
ENSDARG00000052746	grinab	glutamate receptor, ionotropic, N-methyl D-aspartate-associated protein 1b (glutamate binding) [Source:ZFIN;Acc:ZDB-GENE-0404	16	53489676	53516433		31.47	28.69	28.75	49.73	50.83	52.09
ENSDARG00000052747	gpatch3	G patch domain containing 3 [Source:ZFIN;Acc:ZDB-GENE-070112-902]	16	53590586	53601602		21.51	9.11	12.15	21.74	19.14	21.01
ENSDARG00000052748	capn1b	calpain 1, (mu/l) large subunit b [Source:ZFIN;Acc:ZDB-GENE-060503-491]	22	26299016	26321360		9.81	8.56	9.72	10.54	10.06	11.46
ENSDARG00000052758	pimr130	Pim proto-oncogene, serine/threonine kinase, related 130 [Source:ZFIN;Acc:ZDB-GENE-041014-24]	20	39373480	39378581		0.01	0.01	0	0.01	0	0
ENSDARG00000052764	chrnb3a	cholinergic receptor, nicotinic, beta polypeptide 3a [Source:ZFIN;Acc:ZDB-GENE-040108-1]	1	22388799	22412042		7.16	2.49	3.32	7.28	6.62	7.75
ENSDARG00000052765	gria2b	glutamate receptor, ionotropic, AMPA 2b [Source:ZFIN;Acc:ZDB-GENE-020125-4]	14	35748206	35813662		21.46	4.71	10.6	19.93	20.24	20
ENSDARG00000052766	EVI5L	si:ch211-239f4.1 [Source:ZFIN;Acc:ZDB-GENE-061207-24]	1	45217425	45282774	EVI5L	4.86	0.95	1.49	5.25	5.01	5.1
ENSDARG00000052769	glrbb	glycine receptor, beta b [Source:ZFIN;Acc:ZDB-GENE-040801-106]	14	35691889	35732221		24.82	8.41	12.28	23.66	24.81	23.92
ENSDARG00000052775	opn3	opsin 3 [Source:ZFIN;Acc:ZDB-GENE-080227-16]	13	42245221	42265585		2.8	0.56	0.69	2.85	2.48	2.63
ENSDARG00000052779	zgc:153932	zgc:153932 [Source:ZFIN;Acc:ZDB-GENE-070209-223]	12	46120836	46145909		1.71	1.33	0.66	10.32	7.5	5.19
ENSDARG00000052782	glrba	glycine receptor, beta a [Source:ZFIN;Acc:ZDB-GENE-010410-2]	1	21423010	21483832		5.07	2.04	2.37	3.2	3.2	3.48
ENSDARG00000052783	cdc42ep3	CDC42 effector protein (Rho GTPase binding) 3 [Source:ZFIN;Acc:ZDB-GENE-040912-89]	13	42124566	42143637		14.4	15.92	13.63	12.13	11.4	12.7
ENSDARG00000052787	zdhhc12b	zinc finger DHHC-type containing 12b [Source:ZFIN;Acc:ZDB-GENE-070705-356]	5	31819436	31826573		6.28	1.13	1.98	5.43	5.47	5.95
ENSDARG00000052799	azin1a	antizyme inhibitor 1a [Source:ZFIN;Acc:ZDB-GENE-050417-20]	16	52706059	52736549		7.87	5.18	5.57	11.15	12.03	12.22
ENSDARG00000052816	shmt1	serine hydroxymethyltransferase 1 (soluble) [Source:ZFIN;Acc:ZDB-GENE-040426-1558]	3	40260929	40276071		24.38	28.92	26.78	19.1	22.84	21.24
ENSDARG00000052818	smcr8a	Smith-Magenis syndrome chromosome region, candidate 8a [Source:NCBI gene;Acc:558696]	3	40255408	40258950		5.51	2.97	3.39	4.73	5.31	5.48
ENSDARG00000052826	runx3	RUNX family transcription factor 3 [Source:ZFIN;Acc:ZDB-GENE-000605-2]	13	45092766	45225980		1.74	1.37	0.95	2.43	2.48	2.24
ENSDARG00000052827	top3a	DNA topoisomerase III alpha [Source:ZFIN;Acc:ZDB-GENE-071212-2]	3	40235288	40254634		2.7	1.94	2	1.71	1.83	1.97
ENSDARG00000052833	zfand3	zinc finger, AN1-type domain 3 [Source:ZFIN;Acc:ZDB-GENE-050706-134]	13	44702196	44759100		33.56	22.91	20.95	41.63	42.18	44.21
ENSDARG00000052840	ndufs4	NADH:ubiquinone oxidoreductase subunit S4 [Source:ZFIN;Acc:ZDB-GENE-050522-421]	5	40485503	40501732		79.31	108.52	109.13	46.88	46.06	46.72
ENSDARG00000052842	ppifb	peptidylprolyl isomerase Fb [Source:ZFIN;Acc:ZDB-GENE-051030-126]	12	48803098	48811125		56.07	73.6	67.75	49.27	55.83	52.06
ENSDARG00000052846	fsta	follistatin a [Source:NCBI gene;Acc:100004116]	5	40504910	40510399		6.44	5.53	5.49	13.66	14.03	14.63
ENSDARG00000052851	golph3	golgi phosphoprotein 3 [Source:ZFIN;Acc:ZDB-GENE-041010-209]	5	41082534	41103644		13.63	10.61	10.14	17.35	17.04	16.41
ENSDARG00000052855	ctso	cathepsin O [Source:ZFIN;Acc:ZDB-GENE-080724-8]	14	36752033	36763302		8.12	5.47	6.04	7.86	8.11	7.74
ENSDARG00000052856	khdrbs1a	KH domain containing, RNA binding, signal transduction associated 1a [Source:ZFIN;Acc:ZDB-GENE-000210-25]	13	45009957	45022527		292.38	225.15	225.33	290.95	280.09	289.23
ENSDARG00000052859	chp1	calcineurin-like EF-hand protein 1 [Source:ZFIN;Acc:ZDB-GENE-030131-4086]	13	45035756	45063686		15.59	17.24	13.84	16.92	16.69	17.16
ENSDARG00000052866	htt	huntingtin [Source:ZFIN;Acc:ZDB-GENE-990415-131]	1	41293964	41374117		4.81	2.53	2.13	7	6.97	7.28
ENSDARG00000052873	CR933791.1		3	2329708	2335791		0.01	0	0.01	0	0	0
ENSDARG00000052876	CR933791.2	GTPase IMAP family member 7-like [Source:NCBI gene;Acc:799889]	3	2200895	2209415		0.22	0.52	0.37	0.18	0.24	0.13
ENSDARG00000052881	dnajb5	DnaJ heat shock protein family (Hsp40) member B5 [Source:ZFIN;Acc:ZDB-GENE-070705-308]	5	41542436	41560874		3.11	1.79	2.25	2.48	2.82	2.79
ENSDARG00000052893	rx3	retinal homeobox gene 3 [Source:NCBI gene;Acc:30474]	21	10756154	10759223		4.7	0.29	0.49	6.16	5.08	6.82
ENSDARG00000052894	znf532	zinc finger protein 532 [Source:ZFIN;Acc:ZDB-GENE-060531-40]	21	10773513	10816565		3.07	1.27	1.66	5.82	5.85	6.44
ENSDARG00000052895	htra3a	HtrA serine peptidase 3a [Source:ZFIN;Acc:ZDB-GENE-040801-245]	1	40740951	40759629		0.19	0.2	0.09	1.19	1.13	1.23
ENSDARG00000052897	atxn2	ataxin 2 [Source:ZFIN;Acc:ZDB-GENE-060526-217]	5	41645479	41709234		6.44	3.34	2.65	6.92	6.51	6.44
ENSDARG00000052898	kcnk3b	potassium channel, subfamily K, member 3b [Source:ZFIN;Acc:ZDB-GENE-120709-43]	17	45454943	45459207		0.59	0.17	0.36	1.11	1.16	1.1
ENSDARG00000052899	CU550714.1		1	40724140	40752950		0.64	0.86	0.78	0.19	0.17	0.12
ENSDARG00000052900	zgc:153642	zgc:153642 [Source:ZFIN;Acc:ZDB-GENE-061013-527]	1	40733921	40735381		0.03	0.07	0.09	0.02	0.02	0.05
ENSDARG00000052903	rtkn2b	rhotekin 2b [Source:ZFIN;Acc:ZDB-GENE-040912-64]	21	11256425	11327830		2.38	2.53	2	2.22	2.26	2.52
ENSDARG00000052905	zgc:165423	zgc:165423 [Source:ZFIN;Acc:ZDB-GENE-070720-11]	3	39540014	39550089		42.6	34.35	37.89	14.09	15.65	13.96
ENSDARG00000052907	bcl7a	BAF chromatin remodeling complex subunit BCL7A [Source:NCBI gene;Acc:57952]	5	41793001	41799882		19.97	13.63	14.78	20.82	19.65	20.25
ENSDARG00000052910	wnt8a	wingless-type MMTV integration site family, member 8a [Source:NCBI gene;Acc:30122]	14	34490445	34494899		0	0	0	0.01	0	0
ENSDARG00000052912	si:dkey-57h24.6	si:dkey-57h24.6 [Source:ZFIN;Acc:ZDB-GENE-130530-641]	11	26669724	26683712		0.32	0.16	0.25	0.26	0.26	0.25
ENSDARG00000052917	si:ch211-202f3.3	si:ch211-202f3.3 [Source:ZFIN;Acc:ZDB-GENE-080917-19]	17	45577326	45599190		6.37	6.54	4.12	4.98	4.23	3.21
ENSDARG00000052919	adrb3b	adrenoceptor beta 3b [Source:ZFIN;Acc:ZDB-GENE-081022-154]	10	41199660	41214668		0	0.04	0	0.01	0.03	0.06
ENSDARG00000052923	zgc:113162	zgc:113162 [Source:ZFIN;Acc:ZDB-GENE-050522-530]	21	13383413	13432544		3.29	2.41	2.42	4.19	4.35	4.59
ENSDARG00000052928	arf6b	ADP-ribosylation factor 6b [Source:ZFIN;Acc:ZDB-GENE-030131-9056]	17	45731865	45734459		29.56	30.29	30.41	27.96	26.56	27.01
ENSDARG00000052942	aspg	asparaginase homolog (S. cerevisiae) [Source:ZFIN;Acc:ZDB-GENE-070820-14]	17	45737992	45775010		8.33	8.28	7.52	10.81	10.57	10.59
ENSDARG00000052948	nppal	natriuretic peptide A-like [Source:ZFIN;Acc:ZDB-GENE-081022-175]	8	48603398	48604351		2.99	1.9	2.42	7.98	8.92	9.42
ENSDARG00000052950	arhgap23a	Rho GTPase activating protein 23a [Source:ZFIN;Acc:ZDB-GENE-121019-1]	3	39313094	39363065		7.16	4.62	3.58	10.67	10.99	10.95
ENSDARG00000052954	rab25b	RAB25, member RAS oncogene family b [Source:ZFIN;Acc:ZDB-GENE-050706-113]	16	46695777	46714052		4.54	4.25	3.09	4.32	4.67	3.82
ENSDARG00000052957	plcd3a	phospholipase C, delta 3a [Source:NCBI gene;Acc:569040]	3	39260082	39305291		3.52	2.84	2.77	5.32	5.04	5.19
ENSDARG00000052958	nppb	natriuretic peptide B [Source:ZFIN;Acc:ZDB-GENE-040801-65]	8	48609521	48610544		0.75	0.97	0.59	5.46	4.63	5.07
ENSDARG00000052960	nppa	natriuretic peptide A [Source:ZFIN;Acc:ZDB-GENE-030131-95]	8	48613040	48617664		4.51	7	5.36	15.61	16.6	14.57
ENSDARG00000052966	nmt1a	N-myristoyltransferase 1a [Source:ZFIN;Acc:ZDB-GENE-050522-477]	3	39238096	39259919		31.72	28.92	28.49	28.23	27.21	26.73
ENSDARG00000052975	ubqln4	ubiquilin 4 [Source:ZFIN;Acc:ZDB-GENE-030131-6269]	16	46666074	46681170		26.32	23.52	19.02	38.96	36.55	38.2
ENSDARG00000052978	mbnl1	muscleblind-like splicing regulator 1 [Source:ZFIN;Acc:ZDB-GENE-060929-704]	2	47708853	47829287		3	2.08	2.03	4.86	5.5	5.29
ENSDARG00000052982	gabrr2a	gamma-aminobutyric acid (GABA) A receptor, rho 2a [Source:ZFIN;Acc:ZDB-GENE-060825-164]	17	15845765	15892623		6.51	0.41	0.62	5.72	5.41	6.53
ENSDARG00000052986	xcr1b.1	chemokine (C motif) receptor 1b, duplicate 1 [Source:ZFIN;Acc:ZDB-GENE-070302-7]	2	50718112	50719170		0.02	0	0	0	0	0.02
ENSDARG00000052994	slc25a28	solute carrier family 25 member 28 [Source:NCBI gene;Acc:100535877]	13	40334727	40354313		14.56	12.24	12.61	14.36	13.62	13.27
ENSDARG00000052997	sema4e	semaphorin 4e [Source:NCBI gene;Acc:796377]	2	49569017	49603681		6.73	5.47	4.49	5.65	4.79	4.65
ENSDARG00000053003	slc35f1	solute carrier family 35 member F1 [Source:ZFIN;Acc:ZDB-GENE-060503-463]	20	41998502	42203629		3.89	1.53	1.65	6.2	5.83	6.13
ENSDARG00000053005	cd22	cd22 molecule [Source:ZFIN;Acc:ZDB-GENE-050522-521]	16	44906324	44917748		0.13	0.08	0.22	0.35	0.43	0.46
ENSDARG00000053012	lpcat2	lysophosphatidylcholine acyltransferase 2 [Source:NCBI gene;Acc:553683]	7	35385472	35409027		1.98	1.97	1.3	3.1	2.6	3.06
ENSDARG00000053019	rnf2	ring finger protein, transmembrane 2 [Source:NCBI gene;Acc:553554]	5	11916928	11944398		4.53	1.82	2.92	4.15	4.28	4.61
ENSDARG00000053021	grsf1	G-rich RNA sequence binding factor 1 [Source:ZFIN;Acc:ZDB-GENE-080825-196]	5	42943972	42950963		14.79	19.09	18.51	12.97	11.58	11.79
ENSDARG00000053026	kif19	kinesin family member 19 [Source:ZFIN;Acc:ZDB-GENE-080215-2]	12	38770654	38805710		2.27	1.1	1.11	4.05	4.3	4.68
ENSDARG00000053027	si:dkey-33i11.1	si:dkey-33i11.1 [Source:ZFIN;Acc:ZDB-GENE-030131-4630]	16	45303096	45327616		0.08	0.02	0.03	0.05	0.05	0.04
ENSDARG00000053036	olfcr1	olfactory receptor C family, r1 [Source:ZFIN;Acc:ZDB-GENE-060503-302]	18	33970385	33979693		0.04	0.04	0.03	0.02	0.04	0.02
ENSDARG00000053047	eif2s2	eukaryotic translation initiation factor 2, subunit 2 beta [Source:ZFIN;Acc:ZDB-GENE-030131-3085]	6	49995781	50011926		230.78	269.63	258.77	155.21	158.71	157.04
ENSDARG00000053058	rps11	ribosomal protein S11 [Source:ZFIN;Acc:ZDB-GENE-040426-2701]	3	37787229	37789244		1040.24	1308.86	1554.43	649.52	687.95	689.33
ENSDARG00000053062	GJE1	gap junction epsilon-1 protein-like [Source:NCBI gene;Acc:796202]	17	6382275	6385314	GJE1	0.1	0.1	0.12	0.09	0.07	0.09
ENSDARG00000053065	tubb1	tubulin, beta 1 class VI [Source:ZFIN;Acc:ZDB-GENE-110408-5]	6	49881864	49888418		1.74	2.53	1.65	4.27	6.02	4.44
ENSDARG00000053068	cyp8b1	cytochrome P450, family 8, subfamily B, polypeptide 1 [Source:ZFIN;Acc:ZDB-GENE-040808-53]	2	3435888	3437862		18	22.7	30.02	8.94	10.27	8.84
ENSDARG00000053070	gosr2	golgi SNAP receptor complex member 2 [Source:ZFIN;Acc:ZDB-GENE-030131-3290]	3	37649436	37660860		15.24	21.21	19.05	16.4	15.32	17.19
ENSDARG00000053074	gipc3	GIPC PDZ domain containing family, member 3 [Source:ZFIN;Acc:ZDB-GENE-060616-326]	22	3303671	3328241		2.42	2.78	2.91	1.94	1.4	1.94
ENSDARG00000053082	thada	THADA armadillo repeat containing [Source:ZFIN;Acc:ZDB-GENE-050320-49]	13	8892784	8953947		1.4	1.16	0.96	1.34	1.44	1.26
ENSDARG00000053087	mthfr	methylenetetrahydrofolate reductase (NAD(P)H) [Source:ZFIN;Acc:ZDB-GENE-041111-215]	8	47219107	47252552		8.74	9.28	7.11	9.72	9.56	9.36

ENSDARG00000053091	DAB2	si:ch211-204c21.1 [Source:ZFIN;Acc:ZDB-GENE-030429-35]	5	43860564	43959972	DAB2	6.52	6.25	5.55	16.3	18.09	17.91
ENSDARG00000053097	hsf2	heat shock transcription factor 2 [Source:ZFIN;Acc:ZDB-GENE-011128-1]	20	40475576	40487284		14.11	8.41	9.07	10.8	11.08	10.95
ENSDARG00000053101	prpf38b	pre-mRNA processing factor 38B [Source:NCBI gene;Acc:406810]	2	45655294	45663945		27.6	17.14	17.55	20.86	20.26	20.06
ENSDARG00000053104	fam102ba	family with sequence similarity 102 member Ba [Source:ZFIN;Acc:ZDB-GENE-060616-312]	2	45665493	45691128		4.79	5.72	4.95	2.75	2.86	2.65
ENSDARG00000053106	cep44	centrosomal protein 44 [Source:ZFIN;Acc:ZDB-GENE-060929-392]	1	38362348	38374799		1.29	1.44	1.03	1.56	1.3	1.26
ENSDARG00000053110	pkib	protein kinase (cAMP-dependent, catalytic) inhibitor beta [Source:ZFIN;Acc:ZDB-GENE-041014-312]	20	40371897	40451115		9.44	5.44	6.45	10	11.9	11.46
ENSDARG00000053113	ly75	lymphocyte antigen 75 [Source:ZFIN;Acc:ZDB-GENE-050309-166]	11	11120532	11193205		2.78	2.24	1.77	2.87	3.14	3.02
ENSDARG00000053114	si:ch211-272h9.3	si:ch211-272h9.3 [Source:ZFIN;Acc:ZDB-GENE-070705-165]	7	5007310	5012639		0	0	0	0	0.02	0
ENSDARG00000053119	smpdl3a	sphingomyelin phosphodiesterase acid like 3A [Source:ZFIN;Acc:ZDB-GENE-050327-61]	20	40337423	40367493		2.17	1.92	1.46	1.62	1.86	1.65
ENSDARG00000053122	clvs2	clavesin 2 [Source:NCBI gene;Acc:566769]	20	40269891	40319890		11.52	2.72	4.68	12.82	12.95	12.01
ENSDARG00000053124	si:dkey-148a17.6	si:dkey-148a17.6 [Source:ZFIN;Acc:ZDB-GENE-160728-65]	7	34789353	34791992		0.58	0.59	0.73	0.99	0.94	0.82
ENSDARG00000053127	helb	helicase (DNA) B [Source:ZFIN;Acc:ZDB-GENE-030729-16]	4	12769951	12781182		1.67	1.32	1.33	1.48	1.92	1.79
ENSDARG00000053129	carhsp1	calcium regulated heat stable protein 1 [Source:ZFIN;Acc:ZDB-GENE-040426-1479]	3	27798094	27831815		17.19	17.17	15.62	18.63	19.21	21.05
ENSDARG00000053130	pcp4a	Purkinje cell protein 4a [Source:ZFIN;Acc:ZDB-GENE-031118-105]	10	38969429	39011514		57.69	38.36	44.42	49.77	53.6	54.96
ENSDARG00000053131	irak3	interleukin-1 receptor-associated kinase 3 [Source:ZFIN;Acc:ZDB-GENE-060503-710]	4	12782134	12790990		0.12	0.07	0.18	0.29	0.37	0.53
ENSDARG00000053133	b3gnt7l	UDP-GlcNAc:betaGal beta-1,3-N-acetylglucosaminyltransferase 7, like [Source:ZFIN;Acc:ZDB-GENE-040718-143]	8	22289320	22290886		6.29	5.99	5.78	5.44	5.51	6.08
ENSDARG00000053135	tas1r2.2	taste receptor, type 1, member 2, tandem duplicate 2 [Source:ZFIN;Acc:ZDB-GENE-070917-2]	8	46587593	46597751		0.03	0.01	0.03	0	0	0.01
ENSDARG00000053136	b2m	beta-2-microglobulin [Source:NCBI gene;Acc:30400]	4	12785964	12795489		0.81	1.12	0.9	0.84	0.52	0.66
ENSDARG00000053138	lrrc47	leucine rich repeat containing 47 [Source:ZFIN;Acc:ZDB-GENE-060503-289]	8	22327251	22337944		12.63	11.33	11.64	10.51	11.3	11.36
ENSDARG00000053152	pcmtd1	protein-L-isoaspartate (D-aspartate) O-methyltransferase domain containing 1 [Source:ZFIN;Acc:ZDB-GENE-030131-5387]	24	35183595	35219631		5.24	3.15	2.68	5.82	5.62	6.31
ENSDARG00000053155	alg3	ALG3 alpha-1,3- mannosyltransferase [Source:ZFIN;Acc:ZDB-GENE-050522-334]	2	44972720	44981344		9.12	8.88	8.26	6.79	7.18	6.46
ENSDARG00000053158	prss56	serine protease 56 [Source:ZFIN;Acc:ZDB-GENE-070912-579]	2	45049290	45058240		0.28	0.23	0.47	0.55	0.61	0.93
ENSDARG00000053159	zgc:110626	zgc:110626 [Source:ZFIN;Acc:ZDB-GENE-050417-447]	5	46277593	46285575		9.07	9.96	9.46	10.28	9.98	10.2
ENSDARG00000053165	zgc:113377	zgc:113377 [Source:ZFIN;Acc:ZDB-GENE-050913-148]	17	50478455	50490475		1.21	1.16	1.01	0.94	1	0.75
ENSDARG00000053166	chic1	cysteine-rich hydrophobic domain 1 [Source:ZFIN;Acc:ZDB-GENE-030131-7763]	14	32951842	32959851		4.35	3.26	3.38	5.72	6.23	5.33
ENSDARG00000053179	cttnbp2nla	CTTNBP2 N-terminal like a [Source:ZFIN;Acc:ZDB-GENE-140106-51]	6	48206535	48242713		3.73	3.3	2.98	4.14	3.9	4.08
ENSDARG00000053186	vstm4b	V-set and transmembrane domain containing 4b [Source:ZFIN;Acc:ZDB-GENE-080303-17]	12	2399130	2421670		2.96	3.94	3.65	3.28	3.98	4.17
ENSDARG00000053190	galnt16	UDP-N-acetyl-alpha-D-galactosamine:polypeptide N-acetylgalactosaminyltransferase 16 [Source:ZFIN;Acc:ZDB-GENE-131120-18	17	50127648	50186571		6.48	1.8	2.47	8.71	9.07	8.11
ENSDARG00000053194	pdzd11	PDZ domain containing 11 [Source:NCBI gene;Acc:405893]	14	33264303	33268494		20.54	21.01	19.09	16.49	16.05	17.05
ENSDARG00000053196	mtor	mechanistic target of rapamycin kinase [Source:ZFIN;Acc:ZDB-GENE-030131-2974]	8	46077609	46321918		7.97	5.4	4.36	10.29	9.83	10.06
ENSDARG00000053200	dis3l	DIS3 like exosome 3'-5' exoribonuclease [Source:NCBI gene;Acc:560362]	7	34241319	34256374		4.85	4.08	3.75	4.39	4.44	4.22
ENSDARG00000053201	zgc:172323	zgc:172323 [Source:ZFIN;Acc:ZDB-GENE-080204-113]	16	32736588	32744258		0.88	0.1	0.08	0.86	1.02	1.25
ENSDARG00000053204	snx22	sorting nexin 22 [Source:ZFIN;Acc:ZDB-GENE-060825-154]	25	5992789	6011047		2.9	2.87	3.3	5.14	4.97	5.73
ENSDARG00000053205	ppfia4	PTPRF interacting protein alpha 4 [Source:ZFIN;Acc:ZDB-GENE-070720-14]	11	22374419	22584452		3.88	1.37	1.2	5.3	5.2	5.18
ENSDARG00000053209	smad6a	SMAD family member 6a [Source:ZFIN;Acc:ZDB-GENE-011015-1]	7	34182963	34192834		10.32	9.07	9.16	12.33	12.91	12.71
ENSDARG00000053215	me1	malic enzyme 1, NADP(+)-dependent, cytosolic [Source:ZFIN;Acc:ZDB-GENE-061013-438]	16	32832213	33001153		5.78	8.6	7.42	3.82	3.15	3.22
ENSDARG00000053217	cox7a2a	cytochrome c oxidase subunit 7A2a [Source:ZFIN;Acc:ZDB-GENE-050522-153]	17	49983157	49995366		141.46	189.82	209	106.26	102.42	106.62
ENSDARG00000053222	asb5b	ankyrin repeat and SOCS box containing 5b [Source:ZFIN;Acc:ZDB-GENE-050417-271]	1	38804961	38816685		6.06	5.31	5.32	7.95	8.25	8.51
ENSDARG00000053232	itgb1b.1	integrin, beta 1b.1 [Source:ZFIN;Acc:ZDB-GENE-060803-3]	2	43619555	43635777		41.76	44.1	34.34	33.14	31.39	28.39
ENSDARG00000053240	kif6	kinesin family member 6 [Source:ZFIN;Acc:ZDB-GENE-061027-130]	17	48745740	48805159		0.41	0.31	0.33	0.48	0.54	0.43
ENSDARG00000053241	sts	steroid sulfatase (microsomal), isozyme S [Source:ZFIN;Acc:ZDB-GENE-030717-5]	1	32051581	32077473		1.58	1.39	1.19	1.35	1.11	1.11
ENSDARG00000053246	depdc7b	DEP domain containing 7, paralog b [Source:ZFIN;Acc:ZDB-GENE-030131-9492]	25	5972690	5982502		0	0.01	0	0.03	0.03	0
ENSDARG00000053248	inab	internexin neuronal intermediate filament protein, alpha b [Source:ZFIN;Acc:ZDB-GENE-990415-83]	1	31864404	31867717		16.56	7.27	7.44	19.66	21.48	21.33
ENSDARG00000053254	mylpfa	myosin light chain, phosphorylatable, fast skeletal muscle a [Source:ZFIN;Acc:ZDB-GENE-990712-15]	3	32813048	32818607		2626.09	3593.92	4203.44	3209.98	3049.8	2957.88
ENSDARG00000053260	zgc:113090	zgc:113090 [Source:ZFIN;Acc:ZDB-GENE-050522-532]	3	32870185	32873641		1.74	1.29	0.94	1.89	1.71	1.66
ENSDARG00000053262	atp1b4	ATPase Na+/K+ transporting subunit beta 4 [Source:ZFIN;Acc:ZDB-GENE-070412-1]	14	33458294	33469953		54.8	28.7	40.91	72.16	77.66	76.85
ENSDARG00000053263	zgc:113372	zgc:113372 [Source:ZFIN;Acc:ZDB-GENE-050913-150]	17	49637257	49642366		6.14	3.8	3.91	4.23	4.85	3.92
ENSDARG00000053269	slc2a15b	solute carrier family 2 member 15b [Source:ZFIN;Acc:ZDB-GENE-050508-1]	1	31573225	31598417		7.13	11.3	11.41	7.78	8.82	7.72
ENSDARG00000053272	RAP1GAP	si:dkey-166d12.2 [Source:ZFIN;Acc:ZDB-GENE-131119-39]	6	47869397	47953829	RAP1GAP	3.65	1.73	2.16	3.38	3.46	3.31
ENSDARG00000053279	apln	apelin [Source:NCBI gene;Acc:798375]	14	33722950	33757715		20.97	8.75	9.62	22.72	24.68	23.97
ENSDARG00000053291	pnrc2	proline-rich nuclear receptor coactivator 2 [Source:NCBI gene;Acc:565522]	16	33101851	33105677		54.21	39.19	43.34	78.52	82.09	77.89
ENSDARG00000053293	ftr14	finTRIM family, member 14 [Source:ZFIN;Acc:ZDB-GENE-060512-201]	2	43131147	43135700		2.2	2.76	2.72	1.43	1.96	1.53
ENSDARG00000053298	nkx2.2a	NK2 homeobox 2a [Source:NCBI gene;Acc:30697]	17	42208623	42218652		1.98	0.76	1.11	2.29	1.92	2.22
ENSDARG00000053301	insm1b	insulinoma-associated 1b [Source:NCBI gene;Acc:323882]	17	41463942	41466145		9.86	3.94	5.24	15.75	15.49	16.72
ENSDARG00000053311	tespa1	thymocyte expressed, positive selection associated 1 [Source:ZFIN;Acc:ZDB-GENE-120727-10]	6	46697710	46722893		0.53	0.31	0.4	0.58	0.52	0.43
ENSDARG00000053315	tmprss3a	transmembrane serine protease 3a [Source:ZFIN;Acc:ZDB-GENE-070912-70]	9	19550367	19585848		13.43	21.78	26.45	13.88	12.42	10.62
ENSDARG00000053318	lrrc6	leucine rich repeat containing 6 [Source:NCBI gene;Acc:432388]	2	43098237	43124747		0.86	0.61	0.86	0.99	1.17	1.09
ENSDARG00000053323	zgc:112285	zgc:112285 [Source:ZFIN;Acc:ZDB-GENE-050913-132]	12	35051636	35070011		0.79	1.6	2.04	8.38	7.64	10.52
ENSDARG00000053326	gna11b	guanine nucleotide binding protein (G protein), alpha 11b (Gq class) [Source:ZFIN;Acc:ZDB-GENE-041121-9]	2	52469854	52550135		7.5	4.41	4.03	11.17	11.34	11.53
ENSDARG00000053340	si:ch211-138g9.3	si:ch211-138g9.3 [Source:ZFIN;Acc:ZDB-GENE-090311-27]	1	46238774	46244523		0.01	0	0.01	0	0	0.01
ENSDARG00000053344	adgrd1	adhesion G protein-coupled receptor D1 [Source:ZFIN;Acc:ZDB-GENE-041212-24]	8	43814821	43923788		0.8	0.97	0.73	1.46	1.64	1.61
ENSDARG00000053345	si:dkey-56i24.1	si:dkey-56i24.1 [Source:ZFIN;Acc:ZDB-GENE-081105-8]	8	44420108	44421418		1.49	1.42	0.71	0.7	0.88	1.45
ENSDARG00000053358	basp1	brain abundant, membrane attached signal protein 1 [Source:NCBI gene;Acc:569631]	2	42724404	42748657		102.4	32.25	31.94	136.56	132.29	141.19
ENSDARG00000053362	cilp	cartilage intermediate layer protein, nucleotide pyrophosphohydrolase [Source:ZFIN;Acc:ZDB-GENE-050208-76]	7	31432140	31441420		14.42	20.04	16.59	37.38	38.67	37.95
ENSDARG00000053364	pax10	paired box 10 [Source:ZFIN;Acc:ZDB-GENE-081022-10]	3	32546499	32565883		7.28	0.81	0.72	6.6	7.01	7.99
ENSDARG00000053365	rpl31	ribosomal protein L31 [Source:ZFIN;Acc:ZDB-GENE-060331-121]	9	56225410	56232296		786.13	1041.25	1310.11	517.02	536.67	587.29
ENSDARG00000053366	ftr12	finTRIM family, member 12 [Source:ZFIN;Acc:ZDB-GENE-070912-196]	2	42715995	42722040		0.24	0.36	0.36	0.4	0.55	0.45
ENSDARG00000053370	eif3jb	eukaryotic translation initiation factor 3, subunit Jb [Source:NCBI gene;Acc:406823]	7	31425976	31432103		16.49	20.78	18.2	13.25	13.66	13.93
ENSDARG00000053375	nptxrb	neuronal pentraxin receptor b [Source:ZFIN;Acc:ZDB-GENE-050208-595]	22	25025136	25033105		10.23	3.38	4.15	16.65	17.05	16.02
ENSDARG00000053380	fam189a1	family with sequence similarity 189 member A1 [Source:ZFIN;Acc:ZDB-GENE-040718-129]	7	31145386	31336235		2.3	0.75	0.68	4.38	4.29	4.05
ENSDARG00000053381	plpp1a	phospholipid phosphatase 1a [Source:ZFIN;Acc:ZDB-GENE-080225-26]	10	8265972	8295342		22.21	28.23	28.48	21.71	20.75	20.59
ENSDARG00000053383	rprma	reprimo, TP53 dependent G2 arrest mediator candidate a [Source:NCBI gene;Acc:553689]	9	4609746	4610777		3.66	2.47	2.37	2.8	2.53	2.58
ENSDARG00000053386	wdr27	WD repeat domain 27 [Source:ZFIN;Acc:ZDB-GENE-051120-93]	13	35528607	35610118		0.16	0.11	0.15	0.14	0.24	0.15
ENSDARG00000053387	CU207281.1		22	35431169	35435494		0.09	0.04	0.04	0	0.05	0.05
ENSDARG00000053389	jade2	jade family PHD finger 2 [Source:ZFIN;Acc:ZDB-GENE-120215-66]	21	45386033	45410625		1.97	0.99	1.1	1.9	1.9	2.11
ENSDARG00000053395	cdkn2aipnl	CDKN2A interacting protein N-terminal like [Source:ZFIN;Acc:ZDB-GENE-041010-154]	21	45377211	45382195		19.87	20.51	21.73	13.86	14.33	13.69
ENSDARG00000053404	PPP2CA	zgc:56064 [Source:ZFIN;Acc:ZDB-GENE-040426-877]	21	45351660	45363956	PPP2CA	41.77	40.85	41.5	59.25	64.57	61.83
ENSDARG00000053405	sord	sorbitol dehydrogenase [Source:ZFIN;Acc:ZDB-GENE-040426-1231]	7	30923283	30926030		22.98	32.13	35.18	12.9	15.79	13.63
ENSDARG00000053424	myl2b	myosin, light chain 2b, regulatory, cardiac, slow [Source:ZFIN;Acc:ZDB-GENE-060331-137]	8	4333914	4359475		2.35	2.8	2.76	1.42	1.22	1.19
ENSDARG00000053425	serinc2l	serine incorporator 2, like [Source:ZFIN;Acc:ZDB-GENE-100921-58]	16	34459066	34477805		5.67	4.47	3.41	9.33	9.47	9.21
ENSDARG00000053431	si:ch211-257p13.3	si:ch211-257p13.3 [Source:ZFIN;Acc:ZDB-GENE-110914-108]	16	16554687	16590780		5.97	1.82	3.08	7.76	7.51	8.02
ENSDARG00000053446	sub1a	SUB1 regulator of transcription a [Source:ZFIN;Acc:ZDB-GENE-050522-151]	10	8091144	8098099		100.07	130.99	116.61	68.9	73.62	78.02
ENSDARG00000053447	ppp4r2b	protein phosphatase 4, regulatory subunit 2b [Source:NCBI gene;Acc:100005419]	6	44197099	44209390		9.5	7.22	7.48	8.85	9.32	9.03
ENSDARG00000053448	si:ch211-251f6.6	si:ch211-251f6.6 [Source:ZFIN;Acc:ZDB-GENE-030131-3219]	10	8058850	8079830		20.15	32.1	34.65	19.48	20.77	19.8
ENSDARG00000053449	pyya	peptide YYa [Source:NCBI gene;Acc:30211]	3	33340939	33344780		10.92	9.06	10.98	16.75	16.89	16.99
ENSDARG00000053450	ftr06	finTRIM family, member 6 [Source:ZFIN;Acc:ZDB-GENE-070912-228]	2	42294944	42308424		0.01	0	0	0.26	0.18	0.18
ENSDARG00000053452	pop5	POP5 homolog, ribonuclease P/MRP subunit [Source:NCBI gene;Acc:541421]	8	41274211	41279326		10.49	12.68	12.16	5.9	5.3	6.39
ENSDARG00000053453	mpp2a	membrane protein, palmitoylated 2a (MAGUK p55 subfamily member 2) [Source:ZFIN;Acc:ZDB-GENE-071004-82]	3	33345347	33379440		7.24	2.11	2.91	8.68	9.3	10.01
ENSDARG00000053454	cntn3b	contactin 3b [Source:ZFIN;Acc:ZDB-GENE-130530-645]	6	44584775	44711942		1.63	0.45	0.67	2.71	2.66	2.37
ENSDARG00000053455	ccdc103	coiled-coil domain containing 103 [Source:NCBI gene;Acc:437026]	3	33420214	33422738		0.66	0.62	0.62	0.85	0.89	1.01
ENSDARG00000053456	GK3P	zgc:172295 [Source:ZFIN;Acc:ZDB-GENE-080204-72]	1	28723817	28831848	GK3P	13.31	16.09	13.43	11.07	10.89	11.01
ENSDARG00000053457	rpl23	ribosomal protein L23 [Source:NCBI gene;Acc:336812]	3	33423986	33427803		595.68	754.63	918.48	487.28	504.09	496.28
ENSDARG00000053459	rybpb	RING1 and YY1 binding protein b [Source:NCBI gene;Acc:497613]	6	44006709	44044385		6.48	3.21	3.41	9.31	9.09	9.34
ENSDARG00000053460	si:dkey-283b1.7	si:dkey-283b1.7 [Source:ZFIN;Acc:ZDB-GENE-120215-126]	3	33432191	33437796		0.64	0.11	0.12	0.49	0.29	0.43
ENSDARG00000053462	c3a.5	complement component c3a, duplicate 5 [Source:ZFIN;Acc:ZDB-GENE-090311-30]	1	56107522	56122485		0.05	0.02	0.03	0.03	0.1	0.07
ENSDARG00000053463	poglut1	protein O-glucosyltransferase 1 [Source:ZFIN;Acc:ZDB-GENE-051120-24]	1	28874389	28890028		4.95	6.43	5.28	4.46	4.17	3.96
ENSDARG00000053466	timmdc1	translocase of inner mitochondrial membrane domain containing 1 [Source:NCBI gene;Acc:550526]	1	28832671	28861226		6.03	6.27	6.64	5.14	4.58	4.84
ENSDARG00000053467	gtpbp1	GTP binding protein 1 [Source:ZFIN;Acc:ZDB-GENE-030909-12]	3	33440615	33459764		15.73	10.13	9.44	20.74	20.24	19.91
ENSDARG00000053468	adam10a	ADAM metallopeptidase domain 10a [Source:ZFIN;Acc:ZDB-GENE-040917-2]	7	30406150	30492261		17.29	16.95	15.54	20.1	20.06	19.87
ENSDARG00000053474	tdrkh	tudor and KH domain containing [Source:ZFIN;Acc:ZDB-GENE-050327-81]	12	33561418	33582382		5.47	3.82	3.95	4.37	4.69	4.02
ENSDARG00000053475	ngb	neuroglobin [Source:NCBI gene;Acc:100000711]	17	44961908	44968407		11.1	4.8	5.63	9.02	9.12	9.05
ENSDARG00000053476	lipca	lipase, hepatic a [Source:ZFIN;Acc:ZDB-GENE-040426-1361]	7	30392613	30406629		0.58	0.87	0.38	1.25	1.66	1.78
ENSDARG00000053479	gdf6a	growth differentiation factor 6a [Source:NCBI gene;Acc:566470]	16	39125589	39131666		3.76	4.35	4.69	3.8	4.58	4.14
ENSDARG00000053480	aqp9b	aquaporin 9b [Source:ZFIN;Acc:ZDB-GENE-070911-1]	7	30371893	30390376		48.91	16.39	11.58	31.4	36.69	33.39
ENSDARG00000053481	entpd5a	ectonucleoside triphosphate diphosphohydrolase 5a [Source:ZFIN;Acc:ZDB-GENE-100419-1]	17	45030407	45040813		4.79	3.45	2.59	3.18	3.09	3.41
ENSDARG00000053483	zgc:113054	zgc:113054 [Source:ZFIN;Acc:ZDB-GENE-050320-9]	6	43450221	43474757		11.79	14.21	12.19	5.11	3.88	4.29
ENSDARG00000053484	syne2b	spectrin repeat containing, nuclear envelope 2b [Source:ZFIN;Acc:ZDB-GENE-071218-5]	13	37075322	37254777		7.98	4.81	4.27	9.9	9.38	9.4
ENSDARG00000053485	aldh6a1	aldehyde dehydrogenase 6 family, member A1 [Source:ZFIN;Acc:ZDB-GENE-030131-9192]	17	45071531	45104750		100.48	117.39	108.01	71.07	75.26	72.08
ENSDARG00000053487	osbp2	oxysterol binding protein 2 [Source:ZFIN;Acc:ZDB-GENE-091113-18]	10	7916720	7974155		8.69	2.35	2.89	7.16	8.16	8.39
ENSDARG00000053488	si:ch73-22a13.3	si:ch73-22a13.3 [Source:ZFIN;Acc:ZDB-GENE-141216-64]	14	6182346	6193966		0.33	0.25	0.28	0.43	0.29	0.27
ENSDARG00000053493	aldh1a2	aldehyde dehydrogenase 1 family, member A2 [Source:ZFIN;Acc:ZDB-GENE-011010-3]	7	30326184	30367650		24.31	30.43	25.58	15.64	17.46	17.76
ENSDARG00000053496	gemin8	gem (nuclear organelle) associated protein 8 [Source:ZFIN;Acc:ZDB-GENE-041114-189]	1	29058268	29061327		10.47	12.51	14.23	10.11	10.5	9.63
ENSDARG00000053498	ednrbb	endothelin receptor type Bb [Source:ZFIN;Acc:ZDB-GENE-081105-182]	9	53799614	53817274		0.06	0.06	0.05	0.08	0.17	0.18
ENSDARG00000053499	isl2b	ISL LIM homeobox 2b [Source:NCBI gene;Acc:30151]	7	29992889	29997444		7.42	2.45	2.86	8.22	8.43	8.56
ENSDARG00000053500	cbsa	cystathionine beta-synthase a [Source:ZFIN;Acc:ZDB-GENE-050417-367]	1	29068649	29093562		5.49	7.31	6.15	2.09	2.29	1.86
ENSDARG00000053502	cryaa	crystallin, alpha A [Source:ZFIN;Acc:ZDB-GENE-020508-1]	1	29096881	29100923		0.2	0.49	0.7	9.68	8.21	12.51
ENSDARG00000053505	fbxo22	F-box protein 22 [Source:ZFIN;Acc:ZDB-GENE-040724-68]	7	29962559	29970437		5.83	5.05	5.03	9.04	9.24	8.67
ENSDARG00000053509	kazald3	Kazal-type serine peptidase inhibitor domain 3 [Source:ZFIN;Acc:ZDB-GENE-050208-155]	8	42609777	42624454		7.69	9.19	9.95	6.43	6.29	6.81
ENSDARG00000053512	crybb2	crystallin, beta B2 [Source:ZFIN;Acc:ZDB-GENE-060130-1]	8	42629748	42636278		0	0.05	0.03	0.08	0.09	0.02
ENSDARG00000053517	EML5	si:dkey-1f12.3 [Source:ZFIN;Acc:ZDB-GENE-030131-1677]	17	43039463	43287290	EML5	3.84	0.9	1	5.06	4.76	4.58
ENSDARG00000053518	adhfe1	alcohol dehydrogenase iron containing 1 [Source:ZFIN;Acc:ZDB-GENE-040426-2070]	2	42095487	42109575		8.93	9.95	9.48	5.66	5.96	6.01
ENSDARG00000053520	LO017829.1	testis expressed 47 [Source:NCBI gene;Acc:100006770]	12	48542998	48552716		1.53	1.88	2.09	7.81	5.91	7.3
ENSDARG00000053525	enpp7.2	ectonucleotide pyrophosphatase/phosphodiesterase 7, tandem duplicate 2 [Source:ZFIN;Acc:ZDB-GENE-130531-2]	12	32423375	32426685		0.11	0.19	0.14	0	0	0
ENSDARG00000053526	enpp7.1	ectonucleotide pyrophosphatase/phosphodiesterase 7, tandem duplicate 1 [Source:ZFIN;Acc:ZDB-GENE-040724-13]	12	32413524	32421046		13.84	22.75	16.2	0.68	0.54	0.72
ENSDARG00000053527	snx24	sorting nexin 24 [Source:ZFIN;Acc:ZDB-GENE-050913-45]	10	7727281	7734939		3.92	3.44	2.7	3.74	3.95	3.83
ENSDARG00000053528	nog3	noggin 3 [Source:NCBI gene;Acc:30173]	12	32388860	32390444		5.25	6.37	6.53	7.46	7.11	6.77
ENSDARG00000053530	cyp4f3	cytochrome P450, family 4, subfamily F, polypeptide 3 [Source:ZFIN;Acc:ZDB-GENE-070410-108]	12	48559480	48566778		0.27	0.21	0.22	0.53	0.36	0.29
ENSDARG00000053535	lmo7b	LIM domain 7b [Source:ZFIN;Acc:ZDB-GENE-060825-242]	1	34365845	34450784		1.56	0.89	0.96	1.79	1.5	1.56
ENSDARG00000053542	kctd12.2	potassium channel tetramerisation domain containing 12.2 [Source:ZFIN;Acc:ZDB-GENE-060117-4]	1	34295925	34297403		13.83	13.77	13.93	13.04	14.99	15.41
ENSDARG00000053544	hspa4l	heat shock protein 4 like [Source:ZFIN;Acc:ZDB-GENE-030131-2412]	17	43430365	43466317		16.08	6.24	7.26	9.05	9.33	8.67
ENSDARG00000053547	jakmip2	janus kinase and microtubule interacting protein 2 [Source:ZFIN;Acc:ZDB-GENE-091204-293]	21	43029708	43079161		10.15	2.45	3.3	11.58	11.64	11.81
ENSDARG00000053554	wdr76	WD repeat domain 76 [Source:ZFIN;Acc:ZDB-GENE-030131-4513]	7	30201611	30209790		1.26	1.28	1.4	0.6	0.68	0.73
ENSDARG00000053558	rtkn2a	rhotekin 2a [Source:NCBI gene;Acc:449666]	17	43610894	43623370		1.83	0.96	0.99	1.23	1.09	1.02
ENSDARG00000053559	tspan3b	tetraspanin 3b [Source:ZFIN;Acc:ZDB-GENE-070424-42]	7	30075245	30082931		7.33	2.51	3.19	8.05	7.61	7.93
ENSDARG00000053563	ms4a17a.12	membrane-spanning 4-domains, subfamily A, member 17A.12 [Source:ZFIN;Acc:ZDB-GENE-080829-4]	4	76755294	76774721		0.33	0.2	0.17	0.63	0.65	0.35
ENSDARG00000053568	pstpip1b	proline-serine-threonine phosphatase interacting protein 1b [Source:ZFIN;Acc:ZDB-GENE-051127-25]	7	30051488	30067228		0.26	0.16	0.17	0.29	0.33	0.3
ENSDARG00000053569	sox3	SRY-box transcription factor 3 [Source:NCBI gene;Acc:30529]	14	32742701	32744464		21.07	8.43	10.7	17.33	16.96	18.36
ENSDARG00000053570	AL590151.1		14	32773672	32789588		0.75	0.9	0.67	0.78	0.61	0.63
ENSDARG00000053571	adob	2-aminoethanethiol (cysteamine) dioxygenase b [Source:ZFIN;Acc:ZDB-GENE-040426-2267]	17	43659940	43661745		8.42	7.64	5.56	15.52	16.09	15.29
ENSDARG00000053574	fancd2	FA complementation group D2 [Source:ZFIN;Acc:ZDB-GENE-040116-5]	6	42291308	42336995		4.37	3.66	3.48	3.88	4.52	4.05
ENSDARG00000053575	gpat2	glycerol-3-phosphate acyltransferase 2, mitochondrial [Source:ZFIN;Acc:ZDB-GENE-050309-118]	8	41035230	41173369		0.25	0.18	0.15	0.21	0.34	0.38
ENSDARG00000053580	htr1fb	5-hydroxytryptamine (serotonin) receptor 1Fb [Source:ZFIN;Acc:ZDB-GENE-090312-140]	1	33634186	33635304		0.1	0.04	0	0.06	0.14	0.15
ENSDARG00000053583	cers6	ceramide synthase 6 [Source:ZFIN;Acc:ZDB-GENE-070522-1]	9	48870122	48937240		1.42	0.35	0.49	1.95	1.96	2
ENSDARG00000053586	creb1a	cAMP responsive element binding protein 1a [Source:ZFIN;Acc:ZDB-GENE-040426-750]	1	33547532	33557915		18.32	9.97	10.88	21.67	22.2	22.3
ENSDARG00000053609	gatd1	glutamine amidotransferase like class 1 domain containing 1 [Source:NCBI gene;Acc:100148360]	25	3801227	3808642		4.89	4.31	4.47	4.11	4.35	4.38
ENSDARG00000053617	camk2a	calcium/calmodulin-dependent protein kinase II alpha [Source:ZFIN;Acc:ZDB-GENE-051113-72]	21	43897128	43952958		10.66	3.92	5.23	8.17	8.91	8.53
ENSDARG00000053619	sra1	steroid receptor RNA activator 1 [Source:ZFIN;Acc:ZDB-GENE-040625-6]	21	43882274	43891419		10.76	13.73	13.95	11.08	10.78	9.7
ENSDARG00000053620	ebi3	Epstein-Barr virus induced 3 [Source:ZFIN;Acc:ZDB-GENE-070912-615]	2	41936603	41942666		1.8	3.46	3.15	0.88	0.88	1.07
ENSDARG00000053624	csf1rb	colony stimulating factor 1 receptor, b [Source:ZFIN;Acc:ZDB-GENE-091118-1]	21	43854594	43877428		0.76	0.68	0.51	1.45	1.64	1.4
ENSDARG00000053625	anxa2b	annexin A2b [Source:ZFIN;Acc:ZDB-GENE-030131-1089]	7	29518396	29534001		54.56	99.6	69.32	10.01	10.96	9.78
ENSDARG00000053630	si:dkey-182o15.5	si:dkey-182o15.5 [Source:ZFIN;Acc:ZDB-GENE-160728-6]	7	29509255	29515727		0	0	0	0.05	0	0
ENSDARG00000053636	cracr2b	calcium release activated channel regulator 2B [Source:NCBI gene;Acc:790936]	25	3844582	3867990		5.27	6.09	5.1	2.81	2.49	3
ENSDARG00000053644	got1l1	glutamic-oxaloacetic transaminase 1 like 1 [Source:ZFIN;Acc:ZDB-GENE-060929-556]	8	37755099	37772150		0.55	0.5	0.57	0.68	0.79	0.65
ENSDARG00000053646	adrb3a	adrenoceptor beta 3a [Source:ZFIN;Acc:ZDB-GENE-080917-21]	8	37700090	37738751		2.74	2.82	2.34	2.97	3.22	2.83
ENSDARG00000053648	or135-1	odorant receptor, family H, subfamily 135, member 1 [Source:ZFIN;Acc:ZDB-GENE-070806-32]	8	37527575	37531221		0.06	0.01	0.02	0.01	0.03	0.03
ENSDARG00000053650	foxb1b	forkhead box B1b [Source:ZFIN;Acc:ZDB-GENE-990415-77]	7	29461060	29463253		1.45	0.61	0.56	1.67	1.58	1.81
ENSDARG00000053652	ndufaf6	NADH:ubiquinone oxidoreductase complex assembly factor 6 [Source:ZFIN;Acc:ZDB-GENE-100112-1]	16	40487732	40508624		8.24	9.12	9.8	5.15	6	6.29
ENSDARG00000053656	lypla2	lysophospholipase 2 [Source:ZFIN;Acc:ZDB-GENE-040426-1715]	19	43884120	43901467		18.29	14.17	12.55	21.24	20.6	19.76
ENSDARG00000053665	gabrg2	gamma-aminobutyric acid (GABA) A receptor, gamma 2 [Source:ZFIN;Acc:ZDB-GENE-091118-65]	21	41903760	42007482		10.5	1.69	2.53	10.83	11.5	10.91
ENSDARG00000053666	myb	v-myb avian myeloblastosis viral oncogene homolog [Source:ZFIN;Acc:ZDB-GENE-991110-14]	23	31815423	31830043		6.61	5.61	5.54	6.07	5.62	5.39
ENSDARG00000053668	stag2b	stromal antigen 2b [Source:ZFIN;Acc:ZDB-GENE-030131-2785]	14	28486213	28527422		18.22	9.91	8.46	24.2	25.15	24.35
ENSDARG00000053684	aldob	aldolase b, fructose-bisphosphate [Source:NCBI gene;Acc:321664]	14	21106444	21121199		344.29	450.46	421.77	260.39	277.43	311.13
ENSDARG00000053685	gtf2a2	general transcription factor IIA, 2 [Source:ZFIN;Acc:ZDB-GENE-050522-513]	7	29351499	29356084		15.55	15.68	15.08	14.81	15.03	16.32
ENSDARG00000053691	maea	macrophage erythroblast attacher [Source:NCBI gene;Acc:321575]	14	21123867	21141068		19.03	13.91	17.17	20.78	21.74	21.31
ENSDARG00000053695	zgc:162948	zgc:162948 [Source:ZFIN;Acc:ZDB-GENE-070424-46]	4	77253851	77260727		1.71	1.76	1.81	2.19	1.99	2.36
ENSDARG00000053702	il2rgb	interleukin 2 receptor, gamma b [Source:ZFIN;Acc:ZDB-GENE-080402-2]	14	30961549	30967284		0.48	0.16	0.12	0.47	0.53	0.53
ENSDARG00000053708	ghrhrb	growth hormone releasing hormone receptor b [Source:ZFIN;Acc:ZDB-GENE-110127-2]	6	32882821	32961129		1.23	0.65	0.77	0.84	0.85	0.67
ENSDARG00000053709	si:ch73-89b15.3	si:ch73-89b15.3 [Source:ZFIN;Acc:ZDB-GENE-141212-224]	4	2267641	2290268		2.79	3.71	2.53	2.69	2.31	2.67
ENSDARG00000053711	GPR68	ovarian cancer G-protein coupled receptor 1-like [Source:NCBI gene;Acc:799203]	17	38724473	38725534	GPR68	0.52	0.22	0.19	0.29	0.29	0.37
ENSDARG00000053713	ccdc88c	coiled-coil domain containing 88C [Source:ZFIN;Acc:ZDB-GENE-100419-3]	17	38602602	38716245		4.76	3.4	2.96	4.9	4.83	4.88
ENSDARG00000053716	zgc:153990	zgc:153990 [Source:ZFIN;Acc:ZDB-GENE-061013-144]	5	36666715	36675023		6.84	6.67	6.53	6.05	6.01	5.12
ENSDARG00000053724	adcyap1r1a	adenylate cyclase activating polypeptide 1a (pituitary) receptor type I [Source:ZFIN;Acc:ZDB-GENE-050315-1]	6	32966750	33023745		6.36	1.99	3.86	7.42	7	6.81
ENSDARG00000053728	ttll3	tubulin tyrosine ligase-like family, member 3 [Source:NCBI gene;Acc:724000]	6	40329308	40352215		1.13	0.59	0.63	1.05	0.84	0.87
ENSDARG00000053731	AL954695.1		1	26573435	26575260		0	0	0	0.02	0	0
ENSDARG00000053744	dcdc2b	doublecortin domain containing 2B [Source:ZFIN;Acc:ZDB-GENE-051113-124]	13	33304187	33314758		0.99	0.58	1.09	1.26	2.04	1.67
ENSDARG00000053746	CABZ01084566.1	oxysterol-binding protein-related protein 1-like [Source:NCBI gene;Acc:100331149]	2	1202347	1217192		9.77	1.95	2.12	4.46	4.72	5.05
ENSDARG00000053753	mff	mitochondrial fission factor [Source:NCBI gene;Acc:393508]	15	35226673	35252548		10.63	6.97	5.38	14.33	13.4	15.01
ENSDARG00000053761	si:dkey-187j14.4	si:dkey-187j14.4 [Source:ZFIN;Acc:ZDB-GENE-131119-26]	7	2228276	2234151		3.11	2.7	2.21	3.32	2.64	2.08
ENSDARG00000053771	apbb3	amyloid beta (A4) precursor protein-binding, family B, member 3 [Source:ZFIN;Acc:ZDB-GENE-140106-53]	21	40731111	40782393		4.69	1.4	1.59	6.37	6.76	8.03
ENSDARG00000053773	vgll2b	vestigial-like family member 2b [Source:ZFIN;Acc:ZDB-GENE-050809-113]	17	27434626	27447527		6.05	8.4	6.08	6.26	4.15	5.35
ENSDARG00000053774	alpi.2	alkaline phosphatase, intestinal, tandem duplicate 2 [Source:ZFIN;Acc:ZDB-GENE-050626-142]	22	38778649	38794610		7.38	11.74	8.76	0.99	0.79	1.2
ENSDARG00000053779	si:ch211-218m3.13	si:ch211-218m3.13 [Source:ZFIN;Acc:ZDB-GENE-091113-55]	21	40276569	40279077		0.15	0.03	0.02	0.04	0.09	0.05
ENSDARG00000053792	si:rp71-1g18.1	si:rp71-1g18.1 [Source:ZFIN;Acc:ZDB-GENE-030131-5470]	2	38055529	38066173		6.93	5.58	6.2	5.72	6.06	5.53

ENSDARG00000053800	si:ch211-276c2.4	si:ch211-276c2.4 [Source:ZFIN;Acc:ZDB-GENE-030131-423]	15	41693635	41708657		7.85	4.12	4.43	8.26	7.77	7.61
ENSDARG00000053802	cbln5	cerebellin 5 [Source:ZFIN;Acc:ZDB-GENE-070912-577]	2	38034325	38035235		2.51	2.9	2.04	0.4	0.81	0.65
ENSDARG00000053803	klhl43	kelch-like family member 43 [Source:ZFIN;Acc:ZDB-GENE-141212-285]	19	43790932	43819610		19.81	30.13	31.02	19.59	16.79	16.39
ENSDARG00000053804	osbpl2a	oxysterol binding protein-like 2a [Source:ZFIN;Acc:ZDB-GENE-091116-46]	23	30937757	30960506		13.53	8.57	9.62	16.83	17.37	17.1
ENSDARG00000053805	trub1	TruB pseudouridine (psi) synthase family member 1 [Source:ZFIN;Acc:ZDB-GENE-080723-73]	12	30036425	30043906		5.6	6.36	5.84	4.78	3.96	3.25
ENSDARG00000053810	hnrnpc	heterogeneous nuclear ribonucleoprotein C [Source:ZFIN;Acc:ZDB-GENE-040426-2043]	2	38025260	38034190		40.18	36.06	37.63	37.24	37.38	37.09
ENSDARG00000053813	or115-11	odorant receptor, family F, subfamily 115, member 11 [Source:ZFIN;Acc:ZDB-GENE-070806-12]	21	40232910	40234525		0.46	0.2	0.27	0.31	0.16	0.14
ENSDARG00000053817	or115-2	odorant receptor, family F, subfamily 115, member 2 [Source:ZFIN;Acc:ZDB-GENE-070806-6]	21	40296480	40297710		0	0	0	0	0	0
ENSDARG00000053820	pcmtd2a	protein-L-isoaspartate (D-aspartate) O-methyltransferase domain containing 2a [Source:ZFIN;Acc:ZDB-GENE-060312-14]	23	30755804	30764319		7.14	5.87	5.87	5.65	6.51	6.96
ENSDARG00000053828	ubxn11	UBX domain protein 11 [Source:ZFIN;Acc:ZDB-GENE-100922-125]	16	41668474	41681746		0.99	0.96	0.81	0.77	0.96	0.87
ENSDARG00000053829	pax9	paired box 9 [Source:ZFIN;Acc:ZDB-GENE-980526-399]	17	38236469	38291065		5.95	5.93	5.56	4.74	5.39	5.31
ENSDARG00000053831	vtnb	vitronectin b [Source:ZFIN;Acc:ZDB-GENE-041116-1]	21	39067512	39081107		38.46	59.32	63.33	48.08	53.1	50.4
ENSDARG00000053833	mtnr1ba	melatonin receptor type 1Ba [Source:NCBI gene;Acc:30669]	15	41027466	41064007		1.01	0.16	0.32	1.01	1.37	0.88
ENSDARG00000053835	plekhh1	pleckstrin homology domain containing, family H (with MyTH4 domain) member 1 [Source:NCBI gene;Acc:565914]	17	37932433	38023273		1.11	1.03	0.86	2.18	2.19	2.1
ENSDARG00000053836	si:ch211-284o19.8	si:ch211-284o19.8 [Source:ZFIN;Acc:ZDB-GENE-070912-289]	2	37851124	37864665		0.51	0.83	1.05	0.52	0.68	0.79
ENSDARG00000053840	ei24	EI24 autophagy associated transmembrane protein [Source:ZFIN;Acc:ZDB-GENE-050417-457]	10	39199547	39207900		8.28	5.97	5.16	10.09	10.78	10.55
ENSDARG00000053845	cbln14	cerebellin 14 [Source:ZFIN;Acc:ZDB-GENE-040724-5]	2	37959040	37960688		5.92	9.67	5.89	1.35	0.69	1.25
ENSDARG00000053853	slc13a2	solute carrier family 13 member 2 [Source:ZFIN;Acc:ZDB-GENE-040426-2389]	21	39100289	39118502		41.69	62.95	50.36	56.6	64.48	69.28
ENSDARG00000053854	uts2a	urotensin 2, alpha [Source:ZFIN;Acc:ZDB-GENE-040630-11]	23	30048849	30055719		0.03	0.16	0.22	0.48	0.82	0.85
ENSDARG00000053857	ccdc187	coiled-coil domain containing 187 [Source:ZFIN;Acc:ZDB-GENE-070410-81]	23	30013240	30045733		8.18	7.85	7.67	5.79	6.12	6.32
ENSDARG00000053858	crip1	cysteine-rich protein 1 [Source:ZFIN;Acc:ZDB-GENE-041111-1]	17	37385811	37395496		88.08	129.48	115.03	35.59	39.84	40.02
ENSDARG00000053859	ism2b	isthmin 2b [Source:NCBI gene;Acc:553163]	20	5302186	5328637		1.4	0.79	0.88	1.91	1.49	1.4
ENSDARG00000053862	crygmx	crystallin, gamma MX [Source:ZFIN;Acc:ZDB-GENE-030131-7732]	12	30838167	30841679		7.5	8.38	10.56	50.07	46.34	71.37
ENSDARG00000053864	elmsan1b	ELM2 and Myb/SANT-like domain containing 1b [Source:ZFIN;Acc:ZDB-GENE-131121-13]	17	37301860	37365668		4.6	3.55	2.41	9.09	9.13	9.35
ENSDARG00000053868	etv2	ETS variant transcription factor 2 [Source:ZFIN;Acc:ZDB-GENE-050622-14]	16	42007090	42014272		1.36	1.56	1.47	1.82	1.83	2.4
ENSDARG00000053875	cryba1b	crystallin, beta A1b [Source:ZFIN;Acc:ZDB-GENE-040718-326]	21	39185461	39195314		1.5	6.39	9.82	64.98	54.33	77.21
ENSDARG00000053876	klhl26	kelch-like family member 26 [Source:NCBI gene;Acc:558035]	11	6881001	6895981		0.58	0.31	0.38	0.53	0.49	0.55
ENSDARG00000053877	cpdb	carboxypeptidase D, b [Source:ZFIN;Acc:ZDB-GENE-060929-1270]	21	39197418	39226333		0	0.01	0.02	0.02	0.03	0.05
ENSDARG00000053878	inpp5l	inositol polyphosphate-5-phosphatase L [Source:ZFIN;Acc:ZDB-GENE-051127-29]	8	38421333	38477817		7.09	6.11	5.45	6.66	6.86	6.73
ENSDARG00000053884	med13a	mediator complex subunit 13a [Source:ZFIN;Acc:ZDB-GENE-070627-13]	10	28029578	28118035		7.41	4.66	3.68	12.96	12.02	12.37
ENSDARG00000053890	tmem62	transmembrane protein 62 [Source:ZFIN;Acc:ZDB-GENE-040426-1837]	17	37253706	37281313		4.89	2.62	1.97	5.78	5.75	5.45
ENSDARG00000053896	slc30a1b	solute carrier family 30 member 1b [Source:ZFIN;Acc:ZDB-GENE-070424-43]	17	37215820	37221044		1.14	0.95	1.25	0.71	0.86	0.86
ENSDARG00000053906	angpt2b	angiopoietin 2b [Source:ZFIN;Acc:ZDB-GENE-010817-2]	19	35799384	35852390		0.48	0.48	0.46	2.77	2.96	2.76
ENSDARG00000053911	si:dkey-42l23.3	si:dkey-42l23.3 [Source:ZFIN;Acc:ZDB-GENE-131127-385]	15	37778574	37779978		0	0	0	0	0	0
ENSDARG00000053912	fbl	fibrillarin [Source:ZFIN;Acc:ZDB-GENE-040426-1936]	16	42177659	42186093		103.31	116.51	109.95	66.48	62.38	58.55
ENSDARG00000053917	alg10	ALG10 alpha-1,2-mannosyltransferase [Source:ZFIN;Acc:ZDB-GENE-110310-8]	4	28374628	28380308		3.34	1.95	2.17	4.32	3.81	4.68
ENSDARG00000053918	srfa	serum response factor a [Source:ZFIN;Acc:ZDB-GENE-990714-29]	22	35089031	35124655		2.25	1.67	1.27	3.84	3.4	3.5
ENSDARG00000053928	si:dkey-117a8.3	si:dkey-117a8.3 [Source:ZFIN;Acc:ZDB-GENE-131121-476]	15	37682403	37683768		0	0	0	0	0.13	0.02
ENSDARG00000053929	opn4b	opsin 4b [Source:ZFIN;Acc:ZDB-GENE-080415-3]	12	26084882	26153101		2.32	0.43	0.75	2.12	1.8	1.68
ENSDARG00000053931	si:dkey-42l23.5	si:dkey-42l23.5 [Source:ZFIN;Acc:ZDB-GENE-131121-596]	15	37751113	37752236		0	0	0	0.12	0	0.05
ENSDARG00000053934	hdhd3	haloacid dehalogenase-like hydrolase domain containing 3 [Source:NCBI gene;Acc:553464]	6	41014791	41021880		1.08	1.15	1.18	0.77	0.84	1.06
ENSDARG00000053938	si:ch211-137j23.8	si:ch211-137j23.8 [Source:ZFIN;Acc:ZDB-GENE-131121-611]	15	37619004	37620167		0	0.03	0	0.03	0.1	0.09
ENSDARG00000053939	tgfa	transforming growth factor, alpha [Source:ZFIN;Acc:ZDB-GENE-040724-208]	6	40992409	41009381		2.88	1.58	0.86	2.09	1.93	2.19
ENSDARG00000053940	btr32	bloodthirsty-related gene family, member 32 [Source:ZFIN;Acc:ZDB-GENE-060421-3827]	23	46157638	46180005		0	0	0	0	0.01	0
ENSDARG00000053950	lin37	lin-37 DREAM MuvB core complex component [Source:ZFIN;Acc:ZDB-GENE-050417-446]	15	37589698	37599675		6.25	5.67	5.12	4.66	4.29	4.15
ENSDARG00000053953	myadml2	myeloid-associated differentiation marker-like 2 [Source:ZFIN;Acc:ZDB-GENE-120807-3]	3	57991074	57991985		0.4	0.73	0.79	1.11	0.6	0.7
ENSDARG00000053956	sptbn2	spectrin, beta, non-erythrocytic 2 [Source:ZFIN;Acc:ZDB-GENE-030131-787]	5	37649206	37709139		19.79	11.59	9.95	23.18	23.37	23.92
ENSDARG00000053959	cdc42ep2	CDC42 effector protein (Rho GTPase binding) 2 [Source:ZFIN;Acc:ZDB-GENE-050320-104]	5	37720777	37730189		3.1	2.28	2.31	2.67	2.99	2.33
ENSDARG00000053961	slc2a11a	solute carrier family 2 member 11a [Source:ZFIN;Acc:ZDB-GENE-080723-20]	6	40975655	40992683		0.27	0.56	0.31	0.54	0.68	0.58
ENSDARG00000053963	npm2b	nucleophosmin/nucleoplasmin, 2b [Source:ZFIN;Acc:ZDB-GENE-070410-125]	10	44552548	44560302		0	0.01	0.03	0.07	0.05	0.03
ENSDARG00000053966	cyp17a2	cytochrome P450, family 17, subfamily A, polypeptide 2 [Source:ZFIN;Acc:ZDB-GENE-100822-1]	23	45560534	45597854		0.93	1.16	0.59	1.79	1.95	1.97
ENSDARG00000053973	fetub	fetuin B [Source:ZFIN;Acc:ZDB-GENE-050208-4]	22	37888249	37900135		526.66	784.83	647.66	112.98	119.73	138.16
ENSDARG00000053979	chmp2a	charged multivesicular body protein 2A [Source:NCBI gene;Acc:798901]	12	27027311	27031060		37.83	44.11	36.69	35.93	34.44	34.76
ENSDARG00000053987	htr1b	5-hydroxytryptamine (serotonin) receptor 1B [Source:ZFIN;Acc:ZDB-GENE-081022-141]	17	36588281	36589605		2.7	0.5	1.1	3.32	2.85	2.38
ENSDARG00000053990	hmgb2b	high mobility group box 2b [Source:ZFIN;Acc:ZDB-GENE-040912-122]	23	45025909	45029559		365.41	350.52	395.86	186.1	176.17	189.89
ENSDARG00000053992	sfi1	SFI1 centrin binding protein [Source:ZFIN;Acc:ZDB-GENE-080206-4]	6	40898877	40922971		1.86	1.05	1.21	1.23	1.25	1.12
ENSDARG00000053995	gpank1	G patch domain and ankyrin repeats 1 [Source:ZFIN;Acc:ZDB-GENE-070928-32]	1	26036123	26045584		4.26	3.89	4.24	3.45	4	3.86
ENSDARG00000054003	rargb	retinoic acid receptor, gamma b [Source:NCBI gene;Acc:100034753]	11	2089461	2096469		3.25	2.72	3.06	5.77	5.23	5.15
ENSDARG00000054007	ppp1r2	protein phosphatase 1, regulatory (inhibitor) subunit 2 [Source:ZFIN;Acc:ZDB-GENE-040426-1814]	22	37804781	37834312		11.39	9.58	9.39	11.2	10.13	11.18
ENSDARG00000054010	scaf1	SR-related CTD-associated factor 1 [Source:ZFIN;Acc:ZDB-GENE-120215-214]	3	32477782	32490621		19.25	12.3	10.09	23.83	22.34	22.75
ENSDARG00000054023	ttll6	tubulin tyrosine ligase-like family, member 6 [Source:NCBI gene;Acc:564075]	12	27096835	27115412		0.86	0.64	0.78	1.28	1.41	1.53
ENSDARG00000054025	hoxb8b	homeobox B8b [Source:NCBI gene;Acc:30420]	12	27117609	27120171		3.66	3.57	4.46	4.81	5.41	5.38
ENSDARG00000054026	mustn1a	musculoskeletal, embryonic nuclear protein 1a [Source:ZFIN;Acc:ZDB-GENE-111129-3]	6	40841156	40842768		1.93	1.54	2	1.26	1.57	0.91
ENSDARG00000054030	hoxb5b	homeobox B5b [Source:NCBI gene;Acc:58052]	12	27129659	27131735		11.85	9.06	9.71	14.54	13.33	13.67
ENSDARG00000054031	mxd4	MAX dimerization protein 4 [Source:ZFIN;Acc:ZDB-GENE-050208-44]	1	26071869	26096572		3.32	2.17	1.76	5.54	5.98	5.77
ENSDARG00000054032	rab24	RAB24, member RAS oncogene family [Source:ZFIN;Acc:ZDB-GENE-050706-110]	21	38732945	38739728		6.62	5.2	5.73	5.54	5.59	5.71
ENSDARG00000054033	hoxb1b	homeobox B1b [Source:NCBI gene;Acc:30374]	12	27141140	27142834		4.58	4.4	3.72	6.1	6.77	6.36
ENSDARG00000054036	rnf34b	ring finger protein 34b [Source:ZFIN;Acc:ZDB-GENE-071004-31]	10	41765616	41817109		3.76	0.86	1.04	3.18	4.24	3.55
ENSDARG00000054048	plin1	perilipin 1 [Source:ZFIN;Acc:ZDB-GENE-050419-213]	18	25229634	25279323		0	0	0.01	0.12	0.11	0.08
ENSDARG00000054050	vsig8a	V-set and immunoglobulin domain containing 8a [Source:ZFIN;Acc:ZDB-GENE-050417-338]	10	19525839	19551118		3.63	2.05	2.05	2.22	2.49	2.22
ENSDARG00000054054	zgc:153219	zgc:153219 [Source:ZFIN;Acc:ZDB-GENE-060929-144]	7	33130639	33134907		0.42	0.3	0.36	0.39	0.35	0.49
ENSDARG00000054055	ostn	osteocrin [Source:ZFIN;Acc:ZDB-GENE-121211-2]	15	36228835	36249087		1.08	1.21	1.07	0.95	0.93	1.14
ENSDARG00000054058	h1-10	H1.10 linker histone [Source:ZFIN;Acc:ZDB-GENE-030131-1228]	6	40769517	40771813		47.09	31.54	29.22	91.17	111.42	119.79
ENSDARG00000054060	pof1b	POF1B actin binding protein [Source:ZFIN;Acc:ZDB-GENE-030131-4287]	21	38509465	38582061		13.51	12.3	10.1	6.72	7.06	6.24
ENSDARG00000054063	arpc4	actin related protein 2/3 complex, subunit 4 [Source:ZFIN;Acc:ZDB-GENE-010724-9]	6	40743367	40778294		27.12	29.46	27.03	25.55	24.06	23.05
ENSDARG00000054076	tatdn3	TatD DNase domain containing 3 [Source:NCBI gene;Acc:553592]	20	37820990	37827383		1.69	2.03	2.06	2.09	1.83	1.67
ENSDARG00000054087	irs1	insulin receptor substrate 1 [Source:ZFIN;Acc:ZDB-GENE-030131-872]	15	35888549	35930070		1.54	1.06	0.72	2.94	2.81	2.91
ENSDARG00000054096	rbm41	RNA binding motif protein 41 [Source:ZFIN;Acc:ZDB-GENE-041114-107]	21	37996822	38031138		1.77	1.14	1.44	1.75	2.09	1.98
ENSDARG00000054103	ripply1	ripply transcriptional repressor 1 [Source:NCBI gene;Acc:654446]	21	37966933	37973819		0.54	0.32	0.36	0.52	0.7	0.69
ENSDARG00000054122	tmem30b	transmembrane protein 30B [Source:ZFIN;Acc:ZDB-GENE-040704-17]	17	28102487	28111225		5.77	4.93	4.02	4.4	3.98	3.6
ENSDARG00000054124	htr1d	5-hydroxytryptamine (serotonin) receptor 1D, G protein-coupled [Source:ZFIN;Acc:ZDB-GENE-090409-3]	17	28166859	28198099		2.31	0.73	1.1	2.1	1.96	1.84
ENSDARG00000054127	slc26a6l	solute carrier family 26 member 6, like [Source:ZFIN;Acc:ZDB-GENE-080220-10]	6	40723554	40732692		1.73	3.07	1.92	2.78	2.77	3.12
ENSDARG00000054128	im:7136021	im:7136021 [Source:ZFIN;Acc:ZDB-GENE-041008-74]	17	28607417	28612851		2.05	1.97	1.65	1.51	1.61	1.53
ENSDARG00000054136	ccdc105	coiled-coil domain containing 105 [Source:ZFIN;Acc:ZDB-GENE-131121-219]	15	34988148	35000993		1.49	1.08	1.45	0.64	0.66	0.58
ENSDARG00000054137	adgrg6	adhesion G protein-coupled receptor G6 [Source:NCBI gene;Acc:561970]	20	37383034	37469640		6.85	7.02	6.96	8.82	10.12	9.48
ENSDARG00000054150	cx23	connexin 23 [Source:NCBI gene;Acc:541419]	20	37294112	37298274		0.37	0.2	0.4	1.93	1.84	2.1
ENSDARG00000054152	nmbr	neuromedin B receptor [Source:ZFIN;Acc:ZDB-GENE-041014-369]	20	37263663	37287107		0.47	0.28	0.32	0.59	0.72	0.72
ENSDARG00000054153	ftr50	finTRIM family, member 50 [Source:ZFIN;Acc:ZDB-GENE-080215-17]	3	4576089	4591643		0	0	0.01	0.04	0.01	0.03
ENSDARG00000054154	bms1	BMS1 ribosome biogenesis factor [Source:ZFIN;Acc:ZDB-GENE-060720-2]	12	29240124	29270362		18.35	19.57	15.76	11.92	11.42	11.12
ENSDARG00000054155	pcna	proliferating cell nuclear antigen [Source:NCBI gene;Acc:30678]	10	40821804	40826657		155.06	179.63	192.87	77.79	78.5	74.06
ENSDARG00000054160	zgc:113625	zgc:113625 [Source:ZFIN;Acc:ZDB-GENE-050320-47]	10	40786196	40819136		0.44	0.26	0.26	0.98	0.85	0.72
ENSDARG00000054162	nfatc4	nuclear factor of activated T cells 4 [Source:ZFIN;Acc:ZDB-GENE-070912-659]	2	37775655	37803614		1.12	1.14	1.02	1.94	1.87	2.07
ENSDARG00000054164	mrps17	mitochondrial ribosomal protein S17 [Source:ZFIN;Acc:ZDB-GENE-050522-390]	21	37411547	37418838		18.09	26.65	22.92	11.37	10.98	11.49
ENSDARG00000054170	ltb4r2b	leukotriene B4 receptor 2b [Source:ZFIN;Acc:ZDB-GENE-070912-658]	2	37804235	37812185		0.91	0.76	0.82	0.7	0.39	0.53
ENSDARG00000054172	pgrmc1	progesterone receptor membrane component 1 [Source:ZFIN;Acc:ZDB-GENE-041114-91]	21	37436907	37441931		37.7	63.03	52.51	22.71	23.34	23.11
ENSDARG00000054177	adgra1a	adhesion G protein-coupled receptor A1a [Source:ZFIN;Acc:ZDB-GENE-130603-101]	12	29131689	29211598		2.1	0.35	0.37	4.07	3.62	3.68
ENSDARG00000054181	valopb	vertebrate ancient long opsin b [Source:ZFIN;Acc:ZDB-GENE-040724-41]	12	28995942	29012892		1.05	0.23	0.14	1.1	1.35	0.82
ENSDARG00000054184	btr06	bloodthirsty-related gene family, member 6 [Source:ZFIN;Acc:ZDB-GENE-080516-2]	7	16205609	16214891		1.31	1.83	1.52	0.39	0.39	0.54
ENSDARG00000054186	ncs1b	neuronal calcium sensor 1b [Source:ZFIN;Acc:ZDB-GENE-050930-1]	8	32516160	32599858		2.46	1.43	1.82	1.85	1.93	1.75
ENSDARG00000054191	pgk1	phosphoglycerate kinase 1 [Source:ZFIN;Acc:ZDB-GENE-040426-2711]	21	37445871	37463555		139.87	149.07	144.8	187.91	203.89	190.6
ENSDARG00000054196	slc38a8b	solute carrier family 38 member 8b [Source:ZFIN;Acc:ZDB-GENE-121005-6]	7	29163762	29176207		0.18	0.22	0.21	0.69	0.69	0.71
ENSDARG00000054199	mthfd2l	methylenetetrahydrofolate dehydrogenase (NADP+ dependent) 2 like [Source:ZFIN;Acc:ZDB-GENE-030131-5707]	8	32389838	32403826		1.22	0.28	0.85	1.26	0.86	1.01
ENSDARG00000054201	rnf40	ring finger protein 40 [Source:ZFIN;Acc:ZDB-GENE-030131-6607]	12	28910762	28933282		25.8	18.53	18.25	23.57	23.99	24.74
ENSDARG00000054202	hbl4	hexose-binding lectin 4 [Source:ZFIN;Acc:ZDB-GENE-070912-287]	2	37879812	37883256		0.85	1.8	1.07	4.71	5.44	4.07
ENSDARG00000054203	npffr1l1	neuropeptide FF receptor 1 like 1 [Source:ZFIN;Acc:ZDB-GENE-091118-18]	10	40951697	40968095		0.48	0.22	0.4	0.6	0.42	0.59
ENSDARG00000054207	CU682777.1	ST8 alpha-N-acetyl-neuraminide alpha-2,8-sialyltransferase 7.1, pseudogene [Source:NCBI gene;Acc:567285]	23	44824083	44838825		0.48	0.22	0.32	0.22	0.22	0.19
ENSDARG00000054208	phkg2	phosphorylase kinase, gamma 2 (testis) [Source:ZFIN;Acc:ZDB-GENE-040426-825]	12	28888975	28901184		8.55	9.87	9.46	11.04	11.1	10.51
ENSDARG00000054211	st8sia7.1	ST8 alpha-N-acetyl-neuraminide alpha-2,8-sialyltransferase 7.1 [Source:ZFIN;Acc:ZDB-GENE-090504-4]	23	44797665	44819100		2.6	3.09	2.27	1.66	1.56	1.75
ENSDARG00000054213	hectd1	HECT domain containing 1 [Source:ZFIN;Acc:ZDB-GENE-030616-153]	17	28670132	28701283		20.54	15.53	13.09	28.39	26.89	26.93
ENSDARG00000054218	si:ch73-269m23.5	si:ch73-269m23.5 [Source:ZFIN;Acc:ZDB-GENE-110411-68]	23	44772661	44786844		2.03	3.12	2.2	1.83	2.22	1.58
ENSDARG00000054220	ap4s1	adaptor related protein complex 4 subunit sigma 1 [Source:ZFIN;Acc:ZDB-GENE-030616-404]	17	28703971	28707970		11.34	13.43	14.98	5.85	5.94	7.42
ENSDARG00000054222	fbxl8	F-box and leucine-rich repeat protein 8 [Source:ZFIN;Acc:ZDB-GENE-051120-81]	7	29108956	29115772		2.46	3.11	2.27	1.48	1.91	1.78
ENSDARG00000054224	ipo11	importin 11 [Source:ZFIN;Acc:ZDB-GENE-040711-1]	8	32028367	32354721		3.7	3.43	3.02	3.61	3.41	3.46
ENSDARG00000054237	taar16a	trace amine associated receptor 16a [Source:ZFIN;Acc:ZDB-GENE-100514-1]	10	40593443	40594447		0	0	0	0	0	0
ENSDARG00000054239	ghrl	ghrelin/obestatin prepropeptide [Source:ZFIN;Acc:ZDB-GENE-070622-2]	6	40437987	40439615		0.5	0.77	0.53	0.2	0.39	0.7
ENSDARG00000054250	crbn	cereblon [Source:NCBI gene;Acc:445491]	6	40456798	40466861		10	5.6	6.46	7.79	7.77	7.78
ENSDARG00000054251	slc66a3	solute carrier family 66 member 3 [Source:ZFIN;Acc:ZDB-GENE-040912-40]	13	32807419	32812903		5.28	5.77	4.63	8.96	9.15	8.6
ENSDARG00000054252	taar18d	trace amine associated receptor 18d [Source:ZFIN;Acc:ZDB-GENE-060413-12]	10	40531123	40532157		0.04	0.04	0.04	0	0	0
ENSDARG00000054253	sobpa	sine oculis binding protein homolog (Drosophila) a [Source:NCBI gene;Acc:573683]	13	32740509	32796029		3.54	1.66	1.42	6.84	6.36	6.48
ENSDARG00000054254	taar18a	trace amine associated receptor 18a [Source:ZFIN;Acc:ZDB-GENE-100514-5]	10	40523707	40524741		0	0	0	0	0	0
ENSDARG00000054255	tctex1d2	Tctex1 domain containing 2 [Source:ZFIN;Acc:ZDB-GENE-081104-219]	22	35472653	35479229		6.01	5.77	5.55	5.54	6.15	6.34
ENSDARG00000054259	nat10	N-acetyltransferase 10 [Source:ZFIN;Acc:ZDB-GENE-040426-1543]	18	38213778	38245062		5.55	5.85	5.2	3.7	4.41	4.08
ENSDARG00000054261	taar20c	trace amine associated receptor 20c [Source:ZFIN;Acc:ZDB-GENE-060414-7]	10	40378761	40380136		0.03	0	0	0	0.02	0.02
ENSDARG00000054264	cog4	component of oligomeric golgi complex 4 [Source:NCBI gene;Acc:569049]	7	29024282	29040707		5.5	4.81	4.79	5.15	5.06	4.8
ENSDARG00000054271	cdkn1bb	cyclin-dependent kinase inhibitor 1Bb [Source:ZFIN;Acc:ZDB-GENE-030521-13]	25	2282265	2287189		18.89	12.38	13.8	28.79	29.06	28.81
ENSDARG00000054272	caprin1b	cell cycle associated protein 1b [Source:ZFIN;Acc:ZDB-GENE-040426-2020]	18	38246940	38271298		81.89	74.28	69.87	68.67	70.93	71.76
ENSDARG00000054288	zgc:63972	zgc:63972 [Source:NCBI gene;Acc:572862]	5	33940215	33959868		2.85	3.23	1.96	2.35	2.49	2.35
ENSDARG00000054290	acin1a	apoptotic chromatin condensation inducer 1a [Source:ZFIN;Acc:ZDB-GENE-030616-583]	2	38197820	38225472		48.1	28.52	23.94	42.08	42.1	43.77
ENSDARG00000054292	C2H14orf119	si:ch211-14a17.11 [Source:ZFIN;Acc:ZDB-GENE-030616-582]	2	38225427	38230008	C2H14orf119	11.6	12.41	13.47	9.16	8.44	9.64
ENSDARG00000054300	dhrs1	dehydrogenase/reductase (SDR family) member 1 [Source:ZFIN;Acc:ZDB-GENE-030616-591]	2	38261748	38269675		51.54	85.29	70.19	20.69	22.14	19.67
ENSDARG00000054301	kti12	KTI12 chromatin associated homolog [Source:NCBI gene;Acc:751662]	18	26770967	26781692		2.83	2.38	2.35	2.39	2.03	2.48
ENSDARG00000054302	ehd4	EH-domain containing 4 [Source:ZFIN;Acc:ZDB-GENE-060810-22]	17	29195962	29213710		4.77	5.7	5.03	7.26	7.63	6.39
ENSDARG00000054304	homeza	homeobox and leucine zipper encoding a [Source:ZFIN;Acc:ZDB-GENE-030616-592]	2	38271392	38275658		4.87	4.1	3.56	9	10.61	9.63
ENSDARG00000054307	zgc:77151	zgc:77151 [Source:ZFIN;Acc:ZDB-GENE-030131-9097]	21	36985209	37027252		6.29	4.85	4.72	9.23	9.11	8.68
ENSDARG00000054312	slc38a6	solute carrier family 38 member 6 [Source:NCBI gene;Acc:336741]	13	31687973	31707870		3.72	3.63	3.68	4.41	3.67	4.3
ENSDARG00000054313	zp3f.2	zona pellucida glycoprotein 3f, tandem duplicate 2 [Source:ZFIN;Acc:ZDB-GENE-030616-589]	2	38285414	38288024		0.06	0.03	0.01	0.03	0	0.03
ENSDARG00000054318	stk33	serine/threonine kinase 33 [Source:ZFIN;Acc:ZDB-GENE-040724-242]	7	28374947	28403930		0.43	0.4	0.28	0.37	0.3	0.51
ENSDARG00000054319	oxct1b	3-oxoacid CoA transferase 1b [Source:ZFIN;Acc:ZDB-GENE-060929-212]	21	20771082	20785888		26.27	41.43	33.45	24.11	25.4	26.78
ENSDARG00000054320	ap2a1	adaptor related protein complex 2 subunit alpha 1 [Source:ZFIN;Acc:ZDB-GENE-090302-2]	3	32365811	32401481		45.56	24.1	22.2	53.7	52.43	50.62
ENSDARG00000054321	ngs	notochord granular surface [Source:ZFIN;Acc:ZDB-GENE-080722-29]	4	5776347	5784895		0.27	1.93	2.97	90	84.67	89.05
ENSDARG00000054323	pparab	peroxisome proliferator-activated receptor alpha b [Source:ZFIN;Acc:ZDB-GENE-990415-211]	25	2198781	2254112		4.51	3.83	2.86	4.83	5.92	5.63
ENSDARG00000054324	rerglb	RERG/RAS-like b [Source:ZFIN;Acc:ZDB-GENE-040724-73]	7	28406134	28413224		2.39	3.09	2.71	3.95	4.04	4.03
ENSDARG00000054331	ccn4a	cellular communication network factor 4a [Source:ZFIN;Acc:ZDB-GENE-070705-178]	19	34950200	34958185		0.26	0.24	0.32	0.53	0.74	0.59
ENSDARG00000054332	trim66	tripartite motif containing 66 [Source:ZFIN;Acc:ZDB-GENE-040724-145]	7	28423996	28463106		1.64	0.55	0.64	1.11	1.13	1.29
ENSDARG00000054334	dctpp1	dCTP pyrophosphatase 1 [Source:ZFIN;Acc:ZDB-GENE-040426-1741]	1	24557414	24558749		5.31	7.04	6.27	3.76	4.35	3.83
ENSDARG00000054337	ap1g2	adaptor related protein complex 1 subunit gamma 2 [Source:ZFIN;Acc:ZDB-GENE-030616-587]	2	38287358	38312757		5.72	4.2	3.96	5.2	5.2	5.17
ENSDARG00000054340	sla1a	Src like adaptor 1a [Source:ZFIN;Acc:ZDB-GENE-050904-3]	19	34913855	34927201		0.87	1.48	0.88	1.17	1.43	0.97
ENSDARG00000054343	slc7a8b	solute carrier family 7 member 8b [Source:ZFIN;Acc:ZDB-GENE-030616-586]	2	38318000	38337122		1.07	0.35	0.62	1.39	1.55	1.49
ENSDARG00000054344	gabpb2b	GA binding protein transcription factor subunit beta 2b [Source:ZFIN;Acc:ZDB-GENE-050208-136]	16	38337783	38347556		4.75	4.79	4.44	4.5	5.38	4.9
ENSDARG00000054352	lrrc9	leucine rich repeat containing 9 [Source:ZFIN;Acc:ZDB-GENE-080917-56]	13	31473190	31514150		0.32	0.14	0.2	0.45	0.34	0.39
ENSDARG00000054355	dcaf7	ddb1 and cul4 associated factor 7 [Source:ZFIN;Acc:ZDB-GENE-030131-9511]	3	31717261	31726949		53.17	29.92	29.6	55.68	55.42	55.11
ENSDARG00000054362	ccdc47	coiled-coil domain containing 47 [Source:NCBI gene;Acc:447812]	3	31705454	31716157		17.86	15.8	14.11	20.01	20.54	20.22
ENSDARG00000054363	si:zfos-464b6.2	si:zfos-464b6.2 [Source:ZFIN;Acc:ZDB-GENE-030131-5337]	18	49273989	49286521		2.49	3.26	3.11	3.79	4.3	4.22
ENSDARG00000054373	b3gnt3.2	UDP-GlcNAc:betaGal beta-1,3-N-acetylglucosaminyltransferase 3, tendem duplicate 2 [Source:ZFIN;Acc:ZDB-GENE-081104-528]	8	13572531	13574764		0.02	0.02	0.03	0.02	0.04	0
ENSDARG00000054374	alg13	ALG13 UDP-N-acetylglucosaminyltransferase subunit [Source:ZFIN;Acc:ZDB-GENE-060307-1]	21	34233492	34261784		0.03	0.05	0.02	0.06	0.04	0.09
ENSDARG00000054378	pcbp3	poly(rC) binding protein 3 [Source:ZFIN;Acc:ZDB-GENE-050522-157]	9	42040557	42127228		10.9	2.88	2.77	14.61	13.47	14.46
ENSDARG00000054389	rhof	ras homolog family member F [Source:ZFIN;Acc:ZDB-GENE-050522-280]	10	41969428	41980797		2.49	2.52	2.82	2.69	3.34	2.93
ENSDARG00000054400	si:ch211-198n5.11	si:ch211-198n5.11 [Source:ZFIN;Acc:ZDB-GENE-050411-52]	8	16676894	16688511		5.82	6.21	6.08	3.59	3.68	3.45
ENSDARG00000054414	spata17	spermatogenesis associated 17 [Source:ZFIN;Acc:ZDB-GENE-111214-1]	17	30205258	30274315		0.55	0.68	0.82	0.64	0.66	0.69
ENSDARG00000054418	ssuh2.2	ssu-2 homolog, tandem duplicate 2 [Source:ZFIN;Acc:ZDB-GENE-060421-4694]	22	30959733	30973791		0.26	0.34	0.46	0.61	0.92	0.7
ENSDARG00000054420	rpe65c	retinoid isomerohydrolase RPE65 c [Source:NCBI gene;Acc:100004076]	8	16702106	16712255		1.74	1.1	1.62	0.9	0.92	0.96
ENSDARG00000054423	slc7a6	solute carrier family 7 member 6 [Source:NCBI gene;Acc:57957]	7	28623294	28647959		4.58	4.16	3.15	9.73	10.34	9.77
ENSDARG00000054433	yipf6	Yip1 domain family, member 6 [Source:NCBI gene;Acc:415186]	5	35744623	35754882		12.89	13.34	14.07	13.58	12.44	12.88
ENSDARG00000054438	fzd2	frizzled class receptor 2 [Source:ZFIN;Acc:ZDB-GENE-990415-224]	3	31621774	31624213		31.24	36.33	34.87	32.84	33.77	32.49
ENSDARG00000054442	cbx1b	chromobox homolog 1b (HP1 beta homolog Drosophila) [Source:ZFIN;Acc:ZDB-GENE-040625-68]	12	28870462	28881681		28.4	13.9	18.78	23.86	23.75	24.67
ENSDARG00000054446	ccdc43	coiled-coil domain containing 43 [Source:NCBI gene;Acc:393631]	3	31600593	31605331		5.41	6.42	4.84	6.71	6.35	6
ENSDARG00000054447	slc29a1b	solute carrier family 29 member 1b [Source:ZFIN;Acc:ZDB-GENE-130415-4]	11	43419809	43458612		3.63	5.22	3.37	4.88	4.92	4.6
ENSDARG00000054448	kif1c	kinesin family member 1C [Source:ZFIN;Acc:ZDB-GENE-090312-119]	23	44667616	44677315		3.47	3.63	3	6.29	5.6	5.84
ENSDARG00000054451	loxl1	lysyl oxidase-like 1 [Source:ZFIN;Acc:ZDB-GENE-060503-693]	18	50124297	50152689		9.13	10.61	9.33	24.99	28.06	25.2
ENSDARG00000054454	epha4a	eph receptor A4a [Source:NCBI gene;Acc:64271]	2	40067808	40136024		9.66	5.09	4.58	14.06	13.22	13.35
ENSDARG00000054456	clip3	CAP-GLY domain containing linker protein 3 [Source:ZFIN;Acc:ZDB-GENE-131127-193]	15	28989573	29006189		17.84	3.79	4.39	25.85	27.44	27.4
ENSDARG00000054458	slmapa	sarcolemma associated protein a [Source:ZFIN;Acc:ZDB-GENE-061013-184]	11	42304585	42418374		14.35	13.1	10.92	12.9	12.67	12.78
ENSDARG00000054473	tinf2	TERF1 (TRF1)-interacting nuclear factor 2 [Source:ZFIN;Acc:ZDB-GENE-030131-6168]	7	24023653	24040061		3.7	2.47	2.35	2.49	1.88	1.94
ENSDARG00000054501	stambpa	STAM binding protein a [Source:NCBI gene;Acc:797422]	10	42111288	42131408		8.65	6.62	7.3	9.99	9.63	10.5
ENSDARG00000054510	adrb2b	adrenoceptor beta 2, surface b [Source:ZFIN;Acc:ZDB-GENE-070410-32]	21	35078766	35082715		1	1.41	0.92	1.1	1.11	0.91
ENSDARG00000054530	rars1	arginyl-tRNA synthetase 1 [Source:ZFIN;Acc:ZDB-GENE-030131-9014]	21	35327328	35378321		21.63	25.16	23.72	15.67	15.28	14.62
ENSDARG00000054533	araf	A-Raf proto-oncogene, serine/threonine kinase [Source:ZFIN;Acc:ZDB-GENE-050102-2]	5	26856253	26869392		6.21	4.35	3.89	6.28	6.12	6.28
ENSDARG00000054534	ACOT12	acyl-CoA thioesterase 12 [Source:NCBI gene;Acc:100536873]	10	42903091	42923385	ACOT12	0.38	0.38	0.32	0.2	0.25	0.15
ENSDARG00000054537	nhsl1a	NHS-like 1a [Source:ZFIN;Acc:ZDB-GENE-050411-25]	17	30522764	30591223		5.1	2.75	2.17	6.18	6.46	6.25
ENSDARG00000054539	lrp10	low density lipoprotein receptor-related protein 10 [Source:ZFIN;Acc:ZDB-GENE-140418-2]	7	24153070	24162868		10.39	9.33	6.94	13.82	12.79	13.13
ENSDARG00000054540	imp4	IMP U3 small nucleolar ribonucleoprotein 4 [Source:ZFIN;Acc:ZDB-GENE-040718-474]	5	26847190	26852040		13.39	17.6	17.15	10.58	8.82	8.69
ENSDARG00000054542	il12bb	interleukin 12B, b [Source:ZFIN;Acc:ZDB-GENE-030131-7937]	21	35289469	35302253		0.03	0	0.06	0.03	0	0.06
ENSDARG00000054543	samsn1a	SAM domain, SH3 domain and nuclear localisation signals 1a [Source:ZFIN;Acc:ZDB-GENE-030131-8639]	15	29564364	29586747		9.39	2.41	2.09	9.17	9.84	10.11

ENSDARG00000054548	si:ch73-285p12.4	si:ch73-285p12.4 [Source:ZFIN;Acc:ZDB-GENE-110411-145]	23	43750646	43757389		0.06	0.06	0.02	0.06	0	0.12
ENSDARG00000054560	her15.2	hairy and enhancer of split-related 15, tandem duplicate 2 [Source:NCBI gene;Acc:359836]	11	41994287	41996957		2.69	1.57	2.07	1.54	1.38	1.43
ENSDARG00000054562	her15.1	hairy and enhancer of split-related 15, tandem duplicate 1 [Source:ZFIN;Acc:ZDB-GENE-030707-2]	11	41981959	41984487		5.23	3.2	4.61	1.83	2.13	2.14
ENSDARG00000054570	smg1	SMG1 nonsense mediated mRNA decay associated PI3K related kinase [Source:ZFIN;Acc:ZDB-GENE-061013-767]	3	31118026	31158437		11.28	7.17	5.51	15.5	15.7	15.81
ENSDARG00000054572	wdr95	WD40 repeat domain 95 [Source:ZFIN;Acc:ZDB-GENE-131127-243]	15	31526225	31562960		0.53	0.42	0.57	0.35	0.51	0.38
ENSDARG00000054574	znhit1	zinc finger, HIT-type containing 1 [Source:ZFIN;Acc:ZDB-GENE-050417-272]	5	38604877	38611407		13.2	13.91	12.64	10.55	10.36	10.39
ENSDARG00000054575	waif2	Wnt-activated inhibitory factor 2 [Source:ZFIN;Acc:ZDB-GENE-120209-3]	10	36150911	36156658		1.5	0.56	0.86	2.08	1.99	2.15
ENSDARG00000054578	arl6ip1	ADP-ribosylation factor-like 6 interacting protein 1 [Source:ZFIN;Acc:ZDB-GENE-040426-1087]	3	31104891	31115996		92.88	97.79	105.96	104.03	105.07	103.92
ENSDARG00000054583	mfsd6b	major facilitator superfamily domain containing 6b [Source:NCBI gene;Acc:447825]	9	41482831	41507712		2.69	2.18	1.94	2.53	2.94	2.66
ENSDARG00000054584	FO681288.1	zinc/cadmium resistance protein-like [Source:NCBI gene;Acc:100002134]	18	48943875	48953002		0.74	0.92	0.83	1.38	1.51	1.34
ENSDARG00000054588	cox6a2	cytochrome c oxidase subunit 6A2 [Source:ZFIN;Acc:ZDB-GENE-040912-129]	3	31039923	31042102		15.17	19.17	17.3	6.09	5.48	5.72
ENSDARG00000054597	cnot6l	CCR4-NOT transcription complex, subunit 6-like [Source:NCBI gene;Acc:553665]	5	38786481	38824466		15.44	16.24	14.55	13.7	13.15	12.56
ENSDARG00000054606	mrpl1	mitochondrial ribosomal protein L1 [Source:ZFIN;Acc:ZDB-GENE-060526-311]	5	38825225	38834564		11.74	15.04	12.29	8.91	8.91	8.55
ENSDARG00000054609	slco2b1	solute carrier organic anion transporter family, member 2B1 [Source:ZFIN;Acc:ZDB-GENE-051023-6]	21	40139036	40174677		7.16	8.95	8.04	13.66	14.72	13.81
ENSDARG00000054610	coro1a	coronin, actin binding protein, 1A [Source:ZFIN;Acc:ZDB-GENE-030131-9512]	3	30926364	30941362		8.64	10.9	12.7	10.86	12.02	11.08
ENSDARG00000054616	cldni	claudin i [Source:ZFIN;Acc:ZDB-GENE-010328-9]	3	30921246	30927002		120.63	149.05	131.81	107.49	105.88	100.93
ENSDARG00000054619	fras1	Fraser extracellular matrix complex subunit 1 [Source:ZFIN;Acc:ZDB-GENE-060526-68]	5	38837429	39086768		6.95	6.24	4.52	9.29	7.81	7.92
ENSDARG00000054632	fli1a	Fli-1 proto-oncogene, ETS transcription factor a [Source:ZFIN;Acc:ZDB-GENE-980526-426]	18	48423973	48487875		5.89	6.32	4.78	10.12	10.37	9.75
ENSDARG00000054641	tent5ab	terminal nucleotidyltransferase 5Ab [Source:ZFIN;Acc:ZDB-GENE-071004-35]	23	40275400	40286059		7.96	1.74	1.1	3.01	3.61	3.32
ENSDARG00000054645	si:ch211-51c14.1	si:ch211-51c14.1 [Source:ZFIN;Acc:ZDB-GENE-120215-48]	3	30665195	30685401		11.91	15.51	13.78	13.82	13.25	12.82
ENSDARG00000054649	rac1l	Rac family small GTPase 1, like [Source:ZFIN;Acc:ZDB-GENE-060315-6]	1	57170145	57172294		0.49	0.56	0.56	0.25	0.41	0.31
ENSDARG00000054655	si:ch211-1f22.5	si:ch211-1f22.5 [Source:ZFIN;Acc:ZDB-GENE-121214-105]	1	56823437	56827882		0	0	0	0	0	0
ENSDARG00000054658	slc34a1b	solute carrier family 34 member 1b [Source:ZFIN;Acc:ZDB-GENE-101006-4]	21	33006650	33032716		0.22	0.32	0.16	0.51	0.44	0.42
ENSDARG00000054666	pgpep1	pyroglutamyl-peptidase I [Source:ZFIN;Acc:ZDB-GENE-050522-88]	2	56139941	56162607		3.34	3.53	2.52	4.84	5.2	4.67
ENSDARG00000054667	adra2db	adrenergic, alpha-2D-, receptor b [Source:NCBI gene;Acc:266755]	21	32820175	32821422		1.53	0.28	0.65	1.05	1.21	1.22
ENSDARG00000054680	chrna9	cholinergic receptor, nicotinic, alpha 9 [Source:ZFIN;Acc:ZDB-GENE-090312-63]	1	23406403	23417937		0.96	0.93	1.19	0.38	0.55	0.46
ENSDARG00000054683	prdm8b	PR domain containing 8b [Source:ZFIN;Acc:ZDB-GENE-060526-174]	5	39504136	39508958		16.15	3.35	4.48	14.6	15.13	16.57
ENSDARG00000054690	si:dkey-166k12.1	si:dkey-166k12.1 [Source:ZFIN;Acc:ZDB-GENE-120215-78]	19	42469058	42485064		2.69	1.73	1.75	3.57	4.06	4.2
ENSDARG00000054696	psmb4	proteasome 20S subunit beta 4 [Source:ZFIN;Acc:ZDB-GENE-050417-333]	19	42456922	42462491		111.85	118.39	129.3	77.17	78.13	79.05
ENSDARG00000054700	prlh2r	prolactin releasing hormone 2 receptor [Source:ZFIN;Acc:ZDB-GENE-120411-41]	17	31237866	31238986		0.25	0.27	0.3	0.13	0.16	0.24
ENSDARG00000054707	dhx8	DEAH (Asp-Glu-Ala-His) box polypeptide 8 [Source:ZFIN;Acc:ZDB-GENE-050809-39]	12	27569125	27588341		10.15	7.69	7.67	8.97	8.68	8.87
ENSDARG00000054718	or118-2	odorant receptor, family F, subfamily 118, member 2 [Source:ZFIN;Acc:ZDB-GENE-070806-81]	15	31320017	31323932		0.01	0.01	0	0	0	0
ENSDARG00000054719	or118-3	odorant receptor, family F, subfamily 118, member 3 [Source:ZFIN;Acc:ZDB-GENE-070806-82]	15	31317358	31319276		0	0	0	0	0	0
ENSDARG00000054720	or118-1	odorant receptor, family F, subfamily 118, member 1 [Source:ZFIN;Acc:ZDB-GENE-070806-80]	15	31311716	31313932		0	0	0	0	0	0
ENSDARG00000054723	si:ch211-242b18.1	si:ch211-242b18.1 [Source:ZFIN;Acc:ZDB-GENE-030131-1518]	20	34537736	34644082		12.05	8.1	6.82	14.76	13.29	13.01
ENSDARG00000054734	or116-1	odorant receptor, family F, subfamily 116, member 1 [Source:ZFIN;Acc:ZDB-GENE-070806-77]	15	31296517	31298174		0.13	0.03	0.02	0.1	0.06	0.09
ENSDARG00000054741	prkg2	protein kinase cGMP-dependent 2 [Source:ZFIN;Acc:ZDB-GENE-060531-80]	5	39679767	39702345		1.87	0.38	0.63	0.99	1.16	0.96
ENSDARG00000054744	si:ch211-244e12.7	si:ch211-244e12.7 [Source:ZFIN;Acc:ZDB-GENE-131127-278]	15	31276869	31277691		0.03	0.14	0.31	0.63	0.38	0.75
ENSDARG00000054746	uggt1	UDP-glucose glycoprotein glucosyltransferase 1 [Source:ZFIN;Acc:ZDB-GENE-061103-619]	2	40830768	40890264		4.58	3.89	2.86	4.11	4.15	3.61
ENSDARG00000054748	cuedc1b	CUE domain containing 1b [Source:ZFIN;Acc:ZDB-GENE-070424-29]	15	31188016	31237473		5.42	3.41	3.24	7.26	6.25	7.12
ENSDARG00000054749	lmo4b	LIM domain only 4b [Source:ZFIN;Acc:ZDB-GENE-030131-3570]	6	25933736	25952870		30.32	18.14	20.72	30.07	30.97	30.93
ENSDARG00000054753	col10a1a	collagen, type X, alpha 1a [Source:ZFIN;Acc:ZDB-GENE-030131-8373]	17	15542077	15546862		161.11	155.23	131.59	95.64	102.15	103.49
ENSDARG00000054754	hs6st1a	heparan sulfate 6-O-sulfotransferase 1a [Source:NCBI gene;Acc:553162]	2	40901997	41124013		5.96	2.48	2.63	7.17	6.24	6.92
ENSDARG00000054755	alox5ap	arachidonate 5-lipoxygenase-activating protein [Source:ZFIN;Acc:ZDB-GENE-030131-9322]	10	36458563	36463715		0.21	0.42	0.23	0.74	0.72	0.45
ENSDARG00000054771	ghra	growth hormone receptor a [Source:ZFIN;Acc:ZDB-GENE-070509-1]	8	31465222	31606514		3.68	3.37	2.43	4.97	4.34	4.1
ENSDARG00000054777	C8H5orf51	si:dkey-46a10.3 [Source:NCBI gene;Acc:325885]	8	31704624	31716302	C8H5orf51	17.45	14.13	14.07	15.85	16.67	16.65
ENSDARG00000054778	pogza	pogo transposable element derived with ZNF domain a [Source:ZFIN;Acc:ZDB-GENE-040914-76]	19	42432625	42456001		2.89	1.73	1.62	1.97	1.79	1.96
ENSDARG00000054786	faah2b	fatty acid amide hydrolase 2b [Source:NCBI gene;Acc:768298]	7	24508123	24520866		6.88	9.63	7.11	0.98	1.13	1.24
ENSDARG00000054789	lysmd1	LysM, putative peptidoglycan-binding, domain containing 1 [Source:ZFIN;Acc:ZDB-GENE-060929-1094]	16	29690708	29696789		2.72	2.96	2.58	3.54	2.83	3.4
ENSDARG00000054793	emc10	ER membrane protein complex subunit 10 [Source:NCBI gene;Acc:565829]	3	30241237	30248301		30.98	25.37	24.75	32.14	33.4	31.35
ENSDARG00000054794	plcxd3	phosphatidylinositol-specific phospholipase C, X domain containing 3 [Source:NCBI gene;Acc:541487]	8	31821396	31862854		8	2.19	3.75	7.21	7.81	7.79
ENSDARG00000054797	zgc:153441	zgc:153441 [Source:ZFIN;Acc:ZDB-GENE-060825-39]	19	42418227	42424599		4.56	1.2	1.05	4.46	5.06	4.66
ENSDARG00000054799	rfc1	replication factor C (activator 1) 1 [Source:ZFIN;Acc:ZDB-GENE-070410-99]	1	23243093	23274038		6.05	4.92	4.6	4.1	4.15	4.09
ENSDARG00000054804	anp32e	acidic (leucine-rich) nuclear phosphoprotein 32 family, member E [Source:NCBI gene;Acc:406562]	19	42328423	42341815		155.53	76.34	76.6	150.21	152.07	152.17
ENSDARG00000054805	btr09	bloodthirsty-related gene family, member 9 [Source:ZFIN;Acc:ZDB-GENE-080722-46]	7	24520518	24527246		0.56	0.52	0.71	0.29	0.3	0.28
ENSDARG00000054807	sec13	SEC13 homolog, nuclear pore and COPII coat complex component [Source:ZFIN;Acc:ZDB-GENE-040426-2649]	22	31059919	31083489		60.93	79.75	85.59	47.64	48.1	42.56
ENSDARG00000054814	ptp4a3b	protein tyrosine phosphatase 4A3b [Source:ZFIN;Acc:ZDB-GENE-030131-2635]	16	31453980	31505802		14.14	10.61	10.08	23.24	23.1	22.63
ENSDARG00000054818	rpl32	ribosomal protein L32 [Source:ZFIN;Acc:ZDB-GENE-060331-105]	22	31039091	31044066		1643.97	2187.54	2306.11	1189.45	1272.22	1306.47
ENSDARG00000054821	nubpl	nucleotide binding protein-like [Source:ZFIN;Acc:ZDB-GENE-040718-140]	17	31719034	31726868		4.96	4.61	5.61	4.69	4.05	4.61
ENSDARG00000054823	id3	inhibitor of DNA binding 3 [Source:ZFIN;Acc:ZDB-GENE-020515-1]	17	27271399	27273296		38.97	43.85	47.1	40.6	40.64	40.76
ENSDARG00000054827	ssuh2.4	ssu-2 homolog, tandem duplicate 4 [Source:ZFIN;Acc:ZDB-GENE-070424-72]	22	31004735	31020690		2.05	1.73	1.79	1.49	2.19	1.56
ENSDARG00000054829	pik3c3	phosphatidylinositol 3-kinase, catalytic subunit type 3 [Source:ZFIN;Acc:ZDB-GENE-050410-13]	7	24593209	24621689		9.83	8.2	8.37	9.77	9.32	9.32
ENSDARG00000054833	nucb1	nucleobindin 1 [Source:ZFIN;Acc:ZDB-GENE-060825-222]	3	30140580	30153242		33.99	37.11	32.33	31.27	29.91	29.22
ENSDARG00000054835	ugt5f1	UDP glucuronosyltransferase 5 family, polypeptide F1 [Source:ZFIN;Acc:ZDB-GENE-100406-6]	18	1249617	1270220		1.78	1.59	1.77	2.54	2.91	2.56
ENSDARG00000054837	zgc:136870	zgc:136870 [Source:ZFIN;Acc:ZDB-GENE-060312-1]	2	39618951	39621103		2.15	2.6	1.94	0.57	0.72	0.83
ENSDARG00000054842	hsd17b14	hydroxysteroid (17-beta) dehydrogenase 14 [Source:ZFIN;Acc:ZDB-GENE-040801-24]	3	29969143	29977495		6.6	6.89	5.4	5.95	5.18	5.51
ENSDARG00000054846	CU657980.1		23	39951553	39952406		1.27	1.86	1.75	2.17	1.86	1.96
ENSDARG00000054847	xcr1a.1	chemokine (C motif) receptor 1a, duplicate 1 [Source:ZFIN;Acc:ZDB-GENE-070302-6]	23	39958166	39959784		0	0.11	0	0	0.09	0.06
ENSDARG00000054848	pdk4	pyruvate dehydrogenase kinase, isozyme 4 [Source:ZFIN;Acc:ZDB-GENE-060503-754]	19	41190258	41213718		9.63	11.68	8.87	24.68	23.11	24.97
ENSDARG00000054849	bcat2	branched chain amino-acid transaminase 2, mitochondrial [Source:ZFIN;Acc:ZDB-GENE-040718-425]	3	29949984	29968055		19.59	17.38	16.43	15.75	13.58	14.67
ENSDARG00000054856	CSKMT	zgc:113305 [Source:NCBI gene;Acc:553815]	7	24828870	24831653	CSKMT	2.98	3.28	3.48	2.44	1.99	2.17
ENSDARG00000054858	tp53bp2b	tumor protein p53 binding protein, 2b [Source:ZFIN;Acc:ZDB-GENE-050208-453]	22	28803739	28849279		7.34	2.74	2.52	8.47	8.53	8.3
ENSDARG00000054864	aplp2	amyloid beta (A4) precursor-like protein 2 [Source:ZFIN;Acc:ZDB-GENE-061009-28]	18	44364530	44527124		20.81	12.2	8.62	33.01	29.66	30.11
ENSDARG00000054867	hibch	3-hydroxyisobutyryl-CoA hydrolase [Source:NCBI gene;Acc:798364]	9	40874194	40924943		11.31	14.46	12.72	11.68	9.98	9.95
ENSDARG00000054874	gpsm1b	G protein signaling modulator 1b [Source:ZFIN;Acc:ZDB-GENE-110203-5]	5	71715703	71765780		10.46	4.78	4.78	11.34	11.24	11.96
ENSDARG00000054878	six2b	SIX homeobox 2b [Source:ZFIN;Acc:ZDB-GENE-080723-23]	12	25610019	25612170		3.73	3.05	2.98	3.61	3.97	4.26
ENSDARG00000054879	six3b	SIX homeobox 3b [Source:ZFIN;Acc:ZDB-GENE-990415-128]	12	25600685	25603341		20.12	4.21	4.48	23.05	20.46	24.65
ENSDARG00000054882	FP015808.1		2	35615832	35621867		0.42	0.35	0.66	0.49	0.78	0.74
ENSDARG00000054890	rgra	retinal G protein coupled receptor a [Source:ZFIN;Acc:ZDB-GENE-040924-6]	13	29446226	29455859		8.87	3.86	5.04	11.72	11.68	12.56
ENSDARG00000054898	ms4a17a.16	membrane-spanning 4-domains, subfamily A, member 17A.16 [Source:ZFIN;Acc:ZDB-GENE-060825-240]	4	76509294	76517613		2.38	2.42	2.11	1.13	1.33	1.33
ENSDARG00000054903	mta3	metastasis associated 1 family, member 3 [Source:ZFIN;Acc:ZDB-GENE-030131-6485]	12	25223799	25284510		13.98	9.65	8.5	13.94	14.38	14.8
ENSDARG00000054906	ier5l	immediate early response 5-like [Source:NCBI gene;Acc:326935]	5	32791245	32793481		38.77	23.92	22.84	47.06	47.63	48.97
ENSDARG00000054907	cox7a2l	cytochrome c oxidase subunit 7A2 like [Source:ZFIN;Acc:ZDB-GENE-030131-7691]	12	25197383	25201576		115.28	133.18	130.28	98.31	101.12	105.18
ENSDARG00000054911	tmsb	thymosin, beta [Source:NCBI gene;Acc:402820]	21	26389391	26393520		19.14	19.64	16.11	17.8	17.79	19.33
ENSDARG00000054914	dgat1b	diacylglycerol O-acyltransferase 1b [Source:ZFIN;Acc:ZDB-GENE-040718-158]	16	30948451	31000727		6.31	6.81	6.47	4.67	4.47	3.89
ENSDARG00000054916	eif4ebp3	eukaryotic translation initiation factor 4E binding protein 3 [Source:ZFIN;Acc:ZDB-GENE-041114-44]	21	26401332	26406304		20.38	24.72	21.03	15.02	16.6	17.54
ENSDARG00000054929	zgc:110540	zgc:110540 [Source:ZFIN;Acc:ZDB-GENE-050327-77]	21	26485849	26490186		11.4	10.88	9.95	7.59	6.98	6.49
ENSDARG00000054930	tpra1	transmembrane protein, adipocyte asscociated 1 [Source:NCBI gene;Acc:570573]	6	1535303	1553314		7.15	5.99	6.02	10.12	9.84	9.85
ENSDARG00000054931	ppp2r5b	protein phosphatase 2, regulatory subunit B', beta [Source:ZFIN;Acc:ZDB-GENE-101208-1]	7	25049577	25100304		12.47	2.77	4.74	14.56	15.63	14.74
ENSDARG00000054934	zgc:101765	zgc:101765 [Source:ZFIN;Acc:ZDB-GENE-041010-156]	7	25126629	25129581		1.75	2.57	1.86	1.58	1.76	2.19
ENSDARG00000054937	badb	BCL2 associated agonist of cell death b [Source:ZFIN;Acc:ZDB-GENE-000616-1]	7	25129520	25133853		7.24	8.13	7.46	4.96	4.74	5.51
ENSDARG00000054941	ldlrad4b	low density lipoprotein receptor class A domain containing 4b [Source:ZFIN;Acc:ZDB-GENE-091230-3]	16	30621684	30655980		2.57	1.76	1.8	4.1	4.14	3.39
ENSDARG00000054942	lgals2a	lectin, galactoside-binding, soluble, 2a [Source:ZFIN;Acc:ZDB-GENE-050318-2]	3	28953274	28961518		15.29	23.77	22.38	12.28	11.82	12.51
ENSDARG00000054946	mc5rb	melanocortin 5b receptor [Source:ZFIN;Acc:ZDB-GENE-021223-4]	16	30582989	30584279		0.39	0.2	0.26	0.36	0.48	0.44
ENSDARG00000054949	mc2r	melanocortin 2 receptor [Source:ZFIN;Acc:ZDB-GENE-030502-2]	16	30575901	30577136		0.04	0.15	0.06	0.08	0.06	0.06
ENSDARG00000054950	eef2kmt	eukaryotic elongation factor 2 lysine methyltransferase [Source:ZFIN;Acc:ZDB-GENE-041010-160]	3	28869610	28872756		2.2	2	1.53	1.38	1.87	1.59
ENSDARG00000054957	znf1015	zinc finger protein 1015 [Source:ZFIN;Acc:ZDB-GENE-050913-139]	4	73573395	73587205		1.52	1.09	1.15	1.24	1.2	1.19
ENSDARG00000054963	alg1	ALG1 chitobiosyldiphosphodolichol beta-mannosyltransferase [Source:ZFIN;Acc:ZDB-GENE-030131-6093]	3	28860283	28869640		5.12	5.07	5.08	4.73	5.12	4.64
ENSDARG00000054968	cd40	CD40 molecule, TNF receptor superfamily member 5 [Source:ZFIN;Acc:ZDB-GENE-090313-95]	23	37823560	37835794		2.61	2.15	1.59	2.38	2.42	2.87
ENSDARG00000054973	itsn2b	intersectin 2b [Source:ZFIN;Acc:ZDB-GENE-050309-102]	17	30473507	30521043		13.33	10.69	9.01	10.71	10.94	11.04
ENSDARG00000054975	cxcr2	chemokine (C-X-C motif) receptor 2 [Source:ZFIN;Acc:ZDB-GENE-100414-1]	9	48108174	48109197		0.07	0.09	0.19	0.21	0	0.12
ENSDARG00000054978	kifc3	kinesin family member C3 [Source:ZFIN;Acc:ZDB-GENE-030131-3724]	18	45526483	45594749		11.05	4.77	4.92	10.62	11.07	11.44
ENSDARG00000054980	ebna1bp2	EBNA1 binding protein 2 [Source:ZFIN;Acc:ZDB-GENE-030131-6989]	2	13694450	13721149		6.65	8.27	6.74	4.33	4.34	4.42
ENSDARG00000054984	zbtb41	zinc finger and BTB domain containing 41 [Source:ZFIN;Acc:ZDB-GENE-130530-649]	2	13721888	13800596		5.34	2.77	3.05	4.52	4.41	4.73
ENSDARG00000055009	col4a1	collagen, type IV, alpha 1 [Source:ZFIN;Acc:ZDB-GENE-081105-114]	9	8634229	8670158		11.42	13.11	8.75	20.38	20.41	21.03
ENSDARG00000055011	si:dkey-33m11.7	si:dkey-33m11.7 [Source:ZFIN;Acc:ZDB-GENE-141216-115]	11	3563091	3574311		0.17	0.12	0.26	0.33	0.36	0.11
ENSDARG00000055014	si:dkey-33m11.8	si:dkey-33m11.8 [Source:ZFIN;Acc:ZDB-GENE-141215-49]	11	3575963	3584553		9.02	8.64	7.42	3.31	3.63	3.62
ENSDARG00000055022	pds5a	PDS5 cohesin associated factor A [Source:NCBI gene;Acc:393967]	1	23323002	23370618		20.06	15.06	15.21	17.53	17.53	17.11
ENSDARG00000055026	ptch2	patched 2 [Source:NCBI gene;Acc:30181]	2	33969212	33993636		6.29	4.49	3.11	17.4	16.06	17.15
ENSDARG00000055027	pomt2	protein-O-mannosyltransferase 2 [Source:NCBI gene;Acc:563878]	17	31696657	31712041		5.03	4.26	3.9	4.69	4.94	5.44
ENSDARG00000055036	itih3a	inter-alpha-trypsin inhibitor heavy chain 3a [Source:ZFIN;Acc:ZDB-GENE-050522-262]	11	3614326	3629216		5.92	8.68	9.2	5.11	6.57	5.44
ENSDARG00000055043	depdc7a	DEP domain containing 7, paralog a [Source:NCBI gene;Acc:692297]	18	45708744	45732585		4.25	3.98	3.75	2.41	2.54	2.53
ENSDARG00000055045	casp3b	caspase 3, apoptosis-related cysteine peptidase b [Source:ZFIN;Acc:ZDB-GENE-070607-1]	14	4125363	4145594		1.51	1.46	1.07	1.97	1.87	1.67
ENSDARG00000055046	ponzr5	plac8 onzin related protein 5 [Source:ZFIN;Acc:ZDB-GENE-081104-325]	7	22657566	22663971		13.51	15.91	17.38	4.94	5.29	5.59
ENSDARG00000055052	map2	microtubule-associated protein 2 [Source:ZFIN;Acc:ZDB-GENE-041010-118]	9	38883388	38993033		8.53	2.74	3.18	13.51	12.62	14
ENSDARG00000055053	itih1	inter-alpha-trypsin inhibitor heavy chain 1 [Source:ZFIN;Acc:ZDB-GENE-130530-650]	11	3630627	3647014		1.88	1.78	1.87	5.35	5.54	5.29
ENSDARG00000055054	bcl9l	bcl9 like [Source:NCBI gene;Acc:449532]	18	43896153	43993634		6.57	3.79	2.95	8.9	8.66	9.09
ENSDARG00000055064	prdx5	peroxiredoxin 5 [Source:ZFIN;Acc:ZDB-GENE-050522-159]	21	26657404	26664825		72.74	92.03	101.93	51.38	57	56.35
ENSDARG00000055066	zdhhc20a	zinc finger DHHC-type containing 20a [Source:ZFIN;Acc:ZDB-GENE-070424-38]	10	35554219	35580086		3.01	2.05	2.23	4.2	4.27	4.34
ENSDARG00000055070	borcs7	BLOC-1 related complex subunit 7 [Source:NCBI gene;Acc:664759]	13	28612313	28615893		5.93	4.73	3.98	4.58	4.81	4.93
ENSDARG00000055075	svila	supervillin a [Source:ZFIN;Acc:ZDB-GENE-990706-5]	12	23812530	23935333		6.38	5.55	4.82	6.32	6.63	6.66
ENSDARG00000055076	nxf1	nuclear RNA export factor 1 [Source:ZFIN;Acc:ZDB-GENE-030131-2585]	21	26665014	26677834		17.69	9.01	9.77	22.23	22.24	23.37
ENSDARG00000055078	chac2	ChaC, cation transport regulator homolog 2 (E. coli) [Source:NCBI gene;Acc:557614]	12	24056000	24060358		5.05	6.18	5.27	2.39	2.35	2.55
ENSDARG00000055080	rbm4.2	RNA binding motif protein 4.2 [Source:ZFIN;Acc:ZDB-GENE-030131-3019]	7	22796083	22801248		18.77	13.84	11.65	28.79	26.97	25.88
ENSDARG00000055086	ggnbp2	gametogenetin binding protein 2 [Source:NCBI gene;Acc:552967]	5	4016271	4052298		12.58	9.33	9.09	12.57	13.42	12.91
ENSDARG00000055092	pora	P450 (cytochrome) oxidoreductase a [Source:ZFIN;Acc:ZDB-GENE-050809-121]	10	35275965	35312885		12	9.58	9.78	30.73	32.15	31.49
ENSDARG00000055093	cdh27	cadherin 27 [Source:ZFIN;Acc:ZDB-GENE-141215-13]	11	7001948	7030047		3.34	2.29	1.39	3.64	3.61	2.79
ENSDARG00000055099	fam184b	family with sequence similarity 184 member B [Source:ZFIN;Acc:ZDB-GENE-090313-380]	1	23506525	23557877		16.33	6.7	8.48	13.22	12.05	13.06
ENSDARG00000055100	cxcl12b	chemokine (C-X-C motif) ligand 12b (stromal cell-derived factor 1) [Source:ZFIN;Acc:ZDB-GENE-030721-1]	22	26929554	26945493		9.96	10.71	9.47	14.98	15.99	15.4
ENSDARG00000055106	znf148	zinc finger protein 148 [Source:NCBI gene;Acc:560300]	9	38624478	38641307		3.44	2.34	1.95	4.52	4.58	4.53
ENSDARG00000055108	gde1	glycerophosphodiester phosphodiesterase 1 [Source:ZFIN;Acc:ZDB-GENE-040426-881]	22	26839241	26852516		9.71	8.43	10.02	10.12	10.61	8.92
ENSDARG00000055113	pom121	POM121 transmembrane nucleoporin [Source:ZFIN;Acc:ZDB-GENE-030131-6557]	10	35169644	35186310		4.21	2.88	2.51	5.59	5.04	5.95
ENSDARG00000055115	dtnbp1a	dystrobrevin binding protein 1a [Source:NCBI gene;Acc:394109]	16	25504109	25535430		11.17	9.36	9.92	10.9	11.67	11.39
ENSDARG00000055118	mylipb	myosin regulatory light chain interacting protein b [Source:NCBI gene;Acc:565911]	16	25535993	25548738		7.56	9.14	8.6	8.09	8.1	7.08
ENSDARG00000055120	ctsba	cathepsin Ba [Source:ZFIN;Acc:ZDB-GENE-040426-2650]	17	32622931	32631514		43.93	48.56	45.01	69.41	69.56	67.32
ENSDARG00000055123	kcnk2a	potassium channel, subfamily K, member 2a [Source:ZFIN;Acc:ZDB-GENE-061226-2]	17	32657202	32698517		0.42	0.38	0.38	0.36	0.38	0.3
ENSDARG00000055124	ccnl1a	cyclin L1a [Source:ZFIN;Acc:ZDB-GENE-050419-206]	18	41512102	41526488		13.04	6.13	8.01	15.4	16.38	15.97
ENSDARG00000055128	armc4	armadillo repeat containing 4 [Source:ZFIN;Acc:ZDB-GENE-100316-7]	12	23026760	23129458		2.85	1.58	2.06	2.18	1.65	2.1
ENSDARG00000055129	pim3	Pim-3 proto-oncogene, serine/threonine kinase [Source:ZFIN;Acc:ZDB-GENE-050809-111]	25	29662411	29666902		7.62	4.24	4.98	14.57	14.94	14.2
ENSDARG00000055132	lrfn4a	leucine rich repeat and fibronectin type III domain containing 4a [Source:ZFIN;Acc:ZDB-GENE-091113-56]	21	26908627	26918903		6.09	1.4	2.51	5.98	6.34	5.61
ENSDARG00000055133	cenpf	centromere protein F [Source:ZFIN;Acc:ZDB-GENE-041111-205]	17	32720413	32743249		5.28	3.83	3.2	4.24	4.25	4.22
ENSDARG00000055136	si:dkey-101k6.5	si:dkey-101k6.5 [Source:ZFIN;Acc:ZDB-GENE-081105-143]	9	38564900	38587275		2.25	2.37	1.92	3.12	3.69	2.99
ENSDARG00000055138	zgc:110410	zgc:110410 [Source:ZFIN;Acc:ZDB-GENE-050522-465]	16	25599379	25608453		2.74	5.54	4.21	1.84	2.02	2.01
ENSDARG00000055151	smyd2a	SET and MYND domain containing 2a [Source:NCBI gene;Acc:541423]	17	32745763	32779556		11.53	13.65	13.05	12.54	12.41	11.75
ENSDARG00000055154	slc8a4a	solute carrier family 8 member 4a [Source:ZFIN;Acc:ZDB-GENE-060110-2]	21	27047333	27185915		7.18	1.24	1.74	7.67	7.82	7.01
ENSDARG00000055158	prox1a	prospero homeobox 1a [Source:ZFIN;Acc:ZDB-GENE-980526-397]	17	32822588	32865788		16.12	5.88	4.96	18.54	17.89	19.4
ENSDARG00000055159	cyp27a1.4	cytochrome P450, family 27, subfamily A, polypeptide 1, gene 4 [Source:ZFIN;Acc:ZDB-GENE-030131-1060]	9	38446504	38457413		0.96	1.2	1.09	0.61	0.5	0.54
ENSDARG00000055160	chadla	chondroadherin-like a [Source:ZFIN;Acc:ZDB-GENE-121205-4]	3	25108968	25119839		0.26	0.12	0.21	0.57	0.41	0.51
ENSDARG00000055162	zhx2a	zinc fingers and homeoboxes 2a [Source:ZFIN;Acc:ZDB-GENE-031107-2]	16	25608778	25643226		6.44	4.58	4.43	6.98	7.28	6.78
ENSDARG00000055163	epoa	erythropoietin a [Source:NCBI gene;Acc:100004455]	7	21890473	21918432		0.14	0.15	0.17	0.39	0.64	0.64
ENSDARG00000055172	si:ch211-256m1.8	si:ch211-256m1.8 [Source:ZFIN;Acc:ZDB-GENE-131127-417]	3	25154078	25165189		10.04	15.08	12.08	7.51	7.62	8.59
ENSDARG00000055175	c1qtnf6a	C1q and TNF related 6a [Source:ZFIN;Acc:ZDB-GENE-080108-3]	3	25216790	25222662		3.36	3.82	3.4	4.36	3.92	4.08
ENSDARG00000055177	pxdc1a	PX domain containing 1a [Source:ZFIN;Acc:ZDB-GENE-070912-381]	2	32835679	32838894		5.12	1.15	1.45	6.05	5.2	7.66
ENSDARG00000055185	tm4sf5	transmembrane 4 L six family member 5 [Source:ZFIN;Acc:ZDB-GENE-040718-65]	7	21841037	21848205		9.1	15.33	12.49	1.52	1.39	1.76
ENSDARG00000055186	ccr9a	chemokine (C-C motif) receptor 9a [Source:ZFIN;Acc:ZDB-GENE-060130-125]	2	32796873	32799083		0.62	0.57	0.7	0.9	1.26	0.93
ENSDARG00000055190	slc17a5	solute carrier family 17 member 5 [Source:ZFIN;Acc:ZDB-GENE-060929-1158]	13	27298673	27313556		24.62	32.46	28.34	14.88	13.39	14.99
ENSDARG00000055192	zgc:136930	zgc:136930 [Source:ZFIN;Acc:ZDB-GENE-060312-16]	2	32780138	32790887		1206.49	1474.57	1472.68	121.94	86.55	97.76
ENSDARG00000055206	dtnbp1b	dystrobrevin binding protein 1b [Source:ZFIN;Acc:ZDB-GENE-060503-889]	19	26161709	26235219		9.24	5.29	6.98	6.86	6.19	6.71
ENSDARG00000055216	tuba1c	tubulin, alpha 1c [Source:ZFIN;Acc:ZDB-GENE-061114-1]	23	35690657	35694461		305.85	81.7	96.81	216.08	182.36	202.12
ENSDARG00000055223	ablim2	actin binding LIM protein family, member 2 [Source:ZFIN;Acc:ZDB-GENE-100316-5]	12	22680115	22815847		5.09	3.51	3.89	9.99	9.79	9.19
ENSDARG00000055225	dock8	dedicator of cytokinesis 8 [Source:ZFIN;Acc:ZDB-GENE-060503-743]	8	30325902	30424182		8.33	6.06	4.84	8.68	8.06	8.04
ENSDARG00000055226	slc7a7	solute carrier family 7 member 7 [Source:ZFIN;Acc:ZDB-GENE-051127-5]	7	19562045	19587542		12.57	20.35	17.27	10.99	12.35	10.79
ENSDARG00000055229	ncs1a	neuronal calcium sensor 1a [Source:ZFIN;Acc:ZDB-GENE-021220-1]	5	32402026	32445835		2.76	1.27	1.81	3.46	3.8	3.76
ENSDARG00000055232	fancc	FA complementation group C [Source:ZFIN;Acc:ZDB-GENE-060510-2]	8	30112624	30156117		0.81	0.82	0.68	0.83	0.77	0.74
ENSDARG00000055238	kif17	kinesin family member 17 [Source:ZFIN;Acc:ZDB-GENE-080724-12]	11	37652870	37677206		0.43	0.24	0.27	0.28	0.36	0.34
ENSDARG00000055240	xdh	xanthine dehydrogenase [Source:ZFIN;Acc:ZDB-GENE-070719-9]	17	33340675	33374538		9.25	12.34	8.16	3.38	3.65	3.42
ENSDARG00000055245	zmp:0000000650	zmp:0000000650 [Source:ZFIN;Acc:ZDB-GENE-130530-653]	4	20536120	20545465		1.05	1.6	1.16	0.71	0.57	0.64
ENSDARG00000055250	cntd2	cyclin N-terminal domain containing 2 [Source:ZFIN;Acc:ZDB-GENE-030131-5453]	18	40698550	40708537		0.12	0.03	0.12	0.17	0.27	0.21
ENSDARG00000055252	snap23.2	synaptosome associated protein 23.2 [Source:ZFIN;Acc:ZDB-GENE-051030-75]	17	33433566	33441020		1.13	2.36	1.81	6.34	6.25	6.13
ENSDARG00000055253	slc12a10.3	solute carrier family 12 member 10, tandem duplicate 3 [Source:ZFIN;Acc:ZDB-GENE-060503-518]	7	4384863	4420201		0.91	0.83	0.67	1.66	1.38	1.62
ENSDARG00000055255	pitpnm3	PITPNM family member 3 [Source:ZFIN;Acc:ZDB-GENE-030131-9216]	15	26678834	26844591		2.79	1.41	1.35	4.23	4.25	4.52
ENSDARG00000055270	si:ch1073-358c10.1	si:ch1073-358c10.1 [Source:ZFIN;Acc:ZDB-GENE-050309-123]	17	27314088	27382868		0.17	0.12	0.09	1.16	1.19	0.99
ENSDARG00000055271	nkd2a	Danio rerio NKD inhibitor of WNT signaling pathway 2a (nkd2a), mRNA. [Source:RefSeq mRNA;Acc:NM_001098197]	16	52999778	53054838		5.66	6.04	5.19	6.4	7.52	7.41
ENSDARG00000055276	rel	v-rel avian reticuloendotheliosis viral oncogene homolog [Source:ZFIN;Acc:ZDB-GENE-040718-255]	13	25795511	25819825		6.88	6.09	5.32	7.08	6.41	6.05
ENSDARG00000055278	cfb	complement factor B [Source:ZFIN;Acc:ZDB-GENE-980526-487]	21	27416284	27431397		25.36	41.31	34.71	90.6	101.29	105.7
ENSDARG00000055279	b3gat1b	beta-1,3-glucuronyltransferase 1 (glucuronosyltransferase P) b [Source:ZFIN;Acc:ZDB-GENE-131101-1]	10	39178450	39198942		0.18	0.08	0.12	0.19	0.27	0.16
ENSDARG00000055283	id2a	inhibitor of DNA binding 2a [Source:ZFIN;Acc:ZDB-GENE-020910-1]	17	34805897	34808165		68.03	41.19	50.04	63.02	62.51	63.56

ENSDARG00000055284	afap1	actin filament associated protein 1 [Source:ZFIN;Acc:ZDB-GENE-100316-6]	12	22823219	22916314		6.12	4.48	4.15	8.3	7.79	7.94
ENSDARG00000055288	prelid3a	PRELI domain containing 3A [Source:ZFIN;Acc:ZDB-GENE-130530-654]	16	25275767	25285582		4.53	2.34	3.09	5.18	4.4	4.38
ENSDARG00000055290	mpeg1.1	macrophage expressed 1, tandem duplicate 1 [Source:ZFIN;Acc:ZDB-GENE-030131-7347]	8	29710110	29713621		4.07	5.92	5.49	6.72	6.81	7.34
ENSDARG00000055291	rab18a	RAB18A, member RAS oncogene family [Source:ZFIN;Acc:ZDB-GENE-030131-3980]	12	22921477	22933524		15.61	10.9	11.55	14.52	13.63	14.81
ENSDARG00000055292	atg9b	autophagy related 9B [Source:ZFIN;Acc:ZDB-GENE-091111-3]	24	33802528	33839355		4.59	3.2	2.74	5.88	5.75	5.73
ENSDARG00000055294	atoh1a	atonal bHLH transcription factor 1a [Source:ZFIN;Acc:ZDB-GENE-990415-17]	8	29593986	29595615		2.37	1.88	1.82	1.7	1.65	1.34
ENSDARG00000055295	cyb561d1	cytochrome b561 family, member D1 [Source:ZFIN;Acc:ZDB-GENE-040912-157]	11	36408636	36415700		1.42	0.67	0.86	1.1	1.62	1.2
ENSDARG00000055300	atxn7l2a	ataxin 7-like 2a [Source:ZFIN;Acc:ZDB-GENE-060519-13]	11	36379293	36407866		2.28	1.17	1.25	3.27	2.76	3.35
ENSDARG00000055302	grid2	glutamate receptor, ionotropic, delta 2 [Source:NCBI gene;Acc:448841]	8	28900689	29587703		4.45	1.22	1.62	6.5	6.25	6.92
ENSDARG00000055305	ret	ret proto-oncogene receptor tyrosine kinase [Source:ZFIN;Acc:ZDB-GENE-980526-307]	13	25584556	25632756		5.51	1.69	1.87	8.45	8.39	8.75
ENSDARG00000055307	sypl2a	synaptophysin-like 2a [Source:ZFIN;Acc:ZDB-GENE-040426-1434]	11	36355348	36376073		23.49	30.12	21.66	25.56	25.5	24.43
ENSDARG00000055313	slc12a10.1	solute carrier family 12 member 10, tandem duplicate 1 [Source:ZFIN;Acc:ZDB-GENE-060503-330]	7	4425348	4461104		0.76	0.24	0.22	0.87	0.79	0.73
ENSDARG00000055314	mcmbp	minichromosome maintenance complex binding protein [Source:NCBI gene;Acc:321119]	13	25531004	25548882		3.94	3.73	3.79	2.46	2.38	2.17
ENSDARG00000055317	c1ql4b	complement component 1, q subcomponent-like 4b [Source:ZFIN;Acc:ZDB-GENE-131127-59]	6	39812475	39831089		2.49	0.79	1.2	3.3	3.66	3.03
ENSDARG00000055331	aldh9a1a.2	aldehyde dehydrogenase 9 family, member A1a, tandem duplicate 2 [Source:ZFIN;Acc:ZDB-GENE-081104-507]	8	21082361	21091961		1.2	1.72	1.82	0.72	0.5	0.39
ENSDARG00000055332	si:dkey-28d5.11	si:dkey-28d5.11 [Source:ZFIN;Acc:ZDB-GENE-060503-448]	7	4911404	4913261		0.02	0	0	0	0	0
ENSDARG00000055334	zmp:0000000652	zmp:0000000652 [Source:ZFIN;Acc:ZDB-GENE-130530-655]	22	8701238	8702901		0	0	0	0	0.08	0
ENSDARG00000055338	CAPN1	si:dkeyp-50d11.2 [Source:ZFIN;Acc:ZDB-GENE-091113-22]	21	27482149	27507786	CAPN1	1.78	2.09	1.99	1.13	1.09	1.03
ENSDARG00000055339	si:ch73-1a9.4	si:ch73-1a9.4 [Source:ZFIN;Acc:ZDB-GENE-050506-64]	10	39091353	39123213		20.99	22.51	21.89	17.43	18.6	17.56
ENSDARG00000055342	slc16a13	solute carrier family 16 member 13 [Source:ZFIN;Acc:ZDB-GENE-120907-2]	7	20030888	20055983		1.4	1.39	1.04	1.35	1.31	1.18
ENSDARG00000055344	mipep	mitochondrial intermediate peptidase [Source:ZFIN;Acc:ZDB-GENE-060503-662]	15	8192715	8235529		7.67	8.19	6.6	6.4	5.85	5.62
ENSDARG00000055346	si:dkey-83f18.2	si:dkey-83f18.2 [Source:ZFIN;Acc:ZDB-GENE-070705-501]	7	4657987	4660749		0	0	0	0	0	0
ENSDARG00000055349	pknox2	pbx/knotted 1 homeobox 2 [Source:ZFIN;Acc:ZDB-GENE-031118-112]	10	31019607	31220558		5.04	2.46	2.49	5.89	5.71	6.3
ENSDARG00000055350	sun1	Sad1 and UNC84 domain containing 1 [Source:ZFIN;Acc:ZDB-GENE-050522-551]	3	13190012	13258898		23.5	14.63	11.67	20.52	18.4	19.76
ENSDARG00000055357	leo1	LEO1 homolog, Paf1/RNA polymerase II complex component [Source:NCBI gene;Acc:404599]	18	39292911	39321484		26.31	19.63	20.4	22.67	24.08	23.59
ENSDARG00000055359	sp100.1	SP110 nuclear body protein, tandem duplicate 1 [Source:ZFIN;Acc:ZDB-GENE-141212-239]	3	24537023	24555259		0.44	0.23	0.21	0.4	0.37	0.38
ENSDARG00000055360	llph	LLP homolog, long-term synaptic facilitation factor [Source:NCBI gene;Acc:100003313]	4	22297839	22300326		57.76	83.11	70.13	29.2	31.01	32.75
ENSDARG00000055365	si:dkey-25e12.3	si:dkey-25e12.3 [Source:NCBI gene;Acc:678527]	20	26543683	26551233		0.89	1.05	0.89	2.12	1.95	1.6
ENSDARG00000055370	zgc:163121	zgc:163121 [Source:ZFIN;Acc:ZDB-GENE-070424-80]	2	27877908	27900535		1.68	0.65	0.63	1.27	1.22	1.03
ENSDARG00000055373	sema3fb	sema domain, immunoglobulin domain (Ig), short basic domain, secreted, (semaphorin) 3Fb [Source:ZFIN;Acc:ZDB-GENE-050513-2	11	35447417	35575830		5.94	4.34	3.2	6.42	5.7	5.84
ENSDARG00000055374	irf4b	interferon regulatory factor 4b [Source:ZFIN;Acc:ZDB-GENE-041001-131]	20	26556174	26563832		0.03	0.03	0.04	0.01	0.02	0.07
ENSDARG00000055377	gnb5b	guanine nucleotide binding protein (G protein), beta 5b [Source:NCBI gene;Acc:393719]	18	39067575	39105519		45.96	11.04	10.04	23.77	25.21	24.61
ENSDARG00000055381	bambia	BMP and activin membrane-bound inhibitor (Xenopus laevis) homolog a [Source:ZFIN;Acc:ZDB-GENE-010416-1]	12	23424108	23430356		19.11	21.26	22.39	19.83	21.03	20.16
ENSDARG00000055383		zgc:66160 [Source:ZFIN;Acc:ZDB-GENE-030131-5510]	3	27595037	27601812		8.79	7.31	7.33	9.29	9.64	9.55
ENSDARG00000055388	vtna	vitronectin a [Source:ZFIN;Acc:ZDB-GENE-050522-440]	15	28202170	28208926		41.86	72.01	68.85	33.8	35.43	35.23
ENSDARG00000055389	si:dkey-67c22.2	si:dkey-67c22.2 [Source:ZFIN;Acc:ZDB-GENE-030131-5845]	9	35832294	35842375		13.26	9.2	7.45	14.36	14.22	14.57
ENSDARG00000055395	foxq1b	forkhead box Q1b [Source:ZFIN;Acc:ZDB-GENE-040426-2090]	20	26683933	26685395		12.6	12.41	11.65	5.4	4.21	5.13
ENSDARG00000055398	foxc1b	forkhead box C1b [Source:NCBI gene;Acc:79375]	20	26702377	26704331		10.73	10.25	10.23	22.38	21.78	24.22
ENSDARG00000055400	si:dkey-56f14.7	si:dkey-56f14.7 [Source:ZFIN;Acc:ZDB-GENE-131121-78]	16	24626448	24646067		14.75	7.32	8.32	13.67	13.78	14.91
ENSDARG00000055412	mylk4b	myosin light chain kinase family, member 4b [Source:ZFIN;Acc:ZDB-GENE-041001-128]	20	26827265	26866111		2.7	5.18	3.47	6.27	5.52	5.47
ENSDARG00000055415	zp2l1	zona pellucida glycoprotein 2, like 1 [Source:ZFIN;Acc:ZDB-GENE-060818-3]	9	35631440	35633827		0.03	0.1	0.08	0.14	0.1	0.13
ENSDARG00000055416	serpinb1	serpin peptidase inhibitor, clade B (ovalbumin), member 1 [Source:ZFIN;Acc:ZDB-GENE-040718-400]	20	26880668	26888698		20.55	23.02	16.57	9.48	9.9	9.47
ENSDARG00000055426	rwdd2b	RWD domain containing 2B [Source:ZFIN;Acc:ZDB-GENE-041001-119]	20	26905158	26915916		3.77	3.9	3.37	4.07	2.86	3.55
ENSDARG00000055429	kat6b	K(lysine) acetyltransferase 6B [Source:ZFIN;Acc:ZDB-GENE-000607-52]	13	24871050	24906307		7.17	3.92	3.56	10.64	10.91	11.6
ENSDARG00000055431	BX248515.1	serpin peptidase inhibitor, clade B (ovalbumin), member 1-like [Source:NCBI gene;Acc:569077]	20	26899745	26902268		0.38	0.28	0.33	0.61	0.52	0.49
ENSDARG00000055433	vps26bl	vacuolar protein sorting 26 homolog B, like [Source:NCBI gene;Acc:436688]	10	31800212	31805923		7.29	4.97	4.21	9.15	8.96	8.5
ENSDARG00000055436	ftr97	finTRIM family, member 97 [Source:ZFIN;Acc:ZDB-GENE-061026-1]	20	26931496	26937453		1.03	1.26	1.01	1.28	1.26	1.18
ENSDARG00000055437	METTL21C	zgc:172067 [Source:ZFIN;Acc:ZDB-GENE-080204-102]	9	34970418	34974416	METTL21C	0.87	1.2	0.93	0.04	0.02	0.13
ENSDARG00000055439	adamtsl7	ADAMTS-like 7 [Source:ZFIN;Acc:ZDB-GENE-070816-2]	1	21576674	21599219		5.64	6.64	7.57	3.69	3.74	3.68
ENSDARG00000055443	zcchc7	zinc finger, CCHC domain containing 7 [Source:ZFIN;Acc:ZDB-GENE-030102-2]	1	21610471	21714025		3.86	3.15	2.79	3.58	3.48	3.54
ENSDARG00000055445	slc22a15	solute carrier family 22 member 15 [Source:ZFIN;Acc:ZDB-GENE-070928-5]	17	19499157	19511206		4.01	2.45	3.23	4.44	4.09	4.34
ENSDARG00000055455	gpm6aa	glycoprotein M6Aa [Source:ZFIN;Acc:ZDB-GENE-030710-7]	14	36348549	36378494		171.45	39.92	69.66	189.56	196.7	198
ENSDARG00000055459	dlgap3	discs, large (Drosophila) homolog-associated protein 3 [Source:ZFIN;Acc:ZDB-GENE-060503-413]	19	37458610	37492517		1.59	0.38	0.47	2.9	2.83	2.94
ENSDARG00000055463	lrit3a	info leucine-rich repeat, immunoglobulin-like and transmembrane domains 3a [Source:ZFIN;Acc:ZDB-GENE-080723-63]	14	36328425	36345175		6.09	0.91	0.93	4.01	4.15	4.51
ENSDARG00000055467	si:dkeyp-3f10.14	si:dkeyp-3f10.14 [Source:ZFIN;Acc:ZDB-GENE-081104-36]	6	52911338	52913806		0.21	0.12	0.11	0.19	0.11	0.13
ENSDARG00000055470	cdc16	cell division cycle 16 homolog (S. cerevisiae) [Source:ZFIN;Acc:ZDB-GENE-051113-132]	9	34920001	34937256		5.03	3.83	4.24	4.94	4.59	4.59
ENSDARG00000055472	nubp1	nucleotide binding protein 1 (MinD homolog, E. coli) [Source:NCBI gene;Acc:503919]	3	26931710	26978793		4.79	4.84	5.08	4.62	4.52	4.28
ENSDARG00000055474	dexi	Dexi homolog (mouse) [Source:NCBI gene;Acc:445307]	3	26905024	26906351		6.74	7.13	7.39	5.42	5.29	4.86
ENSDARG00000055475	rps27.2	ribosomal protein S27, isoform 2 [Source:ZFIN;Acc:ZDB-GENE-040426-1735]	16	23788616	23800484		605.71	755.38	824.04	464.92	476.55	478.73
ENSDARG00000055476	CR352329.1	tripartite motif-containing protein 16-like [Source:NCBI gene;Acc:100006839]	24	6353394	6359270		0.15	0.1	0.08	0.12	0.06	0.08
ENSDARG00000055477	pelo	pelota mRNA surveillance and ribosome rescue factor [Source:NCBI gene;Acc:394111]	12	13188616	13205854		12.4	13.83	14.32	10.43	10.34	9.77
ENSDARG00000055479	mmp20b	matrix metallopeptidase 20b (enamelysin) [Source:ZFIN;Acc:ZDB-GENE-100603-3]	15	25556922	25571865		0.14	0.14	0.1	0.1	0.24	0.21
ENSDARG00000055481	atf7b	activating transcription factor 7b [Source:ZFIN;Acc:ZDB-GENE-030131-3028]	6	39613357	39631181		16.83	7.41	6.96	16.18	16.74	17.24
ENSDARG00000055490	galnt12	UDP-N-acetyl-alpha-D-galactosamine:polypeptide N-acetylgalactosaminyltransferase 12 [Source:ZFIN;Acc:ZDB-GENE-060503-70	19	34230108	34259666		6.71	5.99	5	7.59	8.01	8.38
ENSDARG00000055493	hic1	hypermethylated in cancer 1 [Source:ZFIN;Acc:ZDB-GENE-040713-2]	15	25681044	25690267		4.06	4.96	4.33	4.93	5.23	5.21
ENSDARG00000055498	si:ch1073-184j22.1	si:ch1073-184j22.1 [Source:ZFIN;Acc:ZDB-GENE-081104-74]	2	5371492	5389696		4.03	4.41	4.61	7.02	6.23	7.12
ENSDARG00000055502	cicb	capicua transcriptional repressor b [Source:ZFIN;Acc:ZDB-GENE-030131-1966]	16	11151699	11224444		6.69	3.12	2.63	10.09	9.84	10.97
ENSDARG00000055504	si:ch211-212k18.7	si:ch211-212k18.7 [Source:ZFIN;Acc:ZDB-GENE-030131-1312]	7	19482084	19491627		26.63	39.02	24.05	33.09	30.58	30.48
ENSDARG00000055506	si:ch211-37e10.1	si:ch211-37e10.1 [Source:ZFIN;Acc:ZDB-GENE-131127-547]	24	6094164	6096557		0.4	0.25	0.24	0.23	0.17	0.19
ENSDARG00000055510	ypel3	yippee-like 3 [Source:NCBI gene;Acc:368214]	3	26186153	26191960		15.48	12.24	11.05	17.05	16.87	17.22
ENSDARG00000055514	icn2	ictacalcin 2 [Source:ZFIN;Acc:ZDB-GENE-070822-9]	16	23487051	23491093		245.03	275.5	286.36	90.52	80.52	82.74
ENSDARG00000055518	pygma	phosphorylase, glycogen, muscle A [Source:ZFIN;Acc:ZDB-GENE-050522-482]	21	28445052	28472239		31.49	48.03	46.15	71.21	57.44	57.35
ENSDARG00000055520	ftr61	finTRIM family, member 61 [Source:ZFIN;Acc:ZDB-GENE-070424-55]	24	6034990	6038073		0.03	0.04	0.02	0.04	0.03	0.04
ENSDARG00000055523	slc22a6l	solute carrier family 22 member 6, like [Source:NCBI gene;Acc:404609]	21	28478663	28494322		3.05	4.83	3.61	9.78	9.58	9.93
ENSDARG00000055524	rnf7	ring finger protein 7 [Source:ZFIN;Acc:ZDB-GENE-050220-12]	18	41757263	41766098		82.83	93.14	121.35	66.14	66.36	68.27
ENSDARG00000055525	lgalslb	lectin, galactoside-binding-like b [Source:ZFIN;Acc:ZDB-GENE-030616-570]	13	24552254	24554028		10.42	3.2	5.18	11.64	10.84	11.41
ENSDARG00000055527	cmn	calymmin [Source:ZFIN;Acc:ZDB-GENE-000208-9]	12	13118540	13164249		0.3	0.88	0.64	18.87	18.45	19.21
ENSDARG00000055530	sertad2b	SERTA domain containing 2b [Source:ZFIN;Acc:ZDB-GENE-030616-569]	13	24500963	24524545		4.29	3.59	2.53	6.55	6.06	6.49
ENSDARG00000055534	grk7b	G protein-coupled receptor kinase 7b [Source:NCBI gene;Acc:373871]	18	41740742	41754020		7.17	1.11	1.26	1.96	1.99	2.16
ENSDARG00000055536	otub1a	OTU deubiquitinase, ubiquitin aldehyde binding 1a [Source:ZFIN;Acc:ZDB-GENE-040718-108]	21	28502340	28513055		18.66	15.8	16.73	16.7	16.1	17.69
ENSDARG00000055538	avl9	AVL9 homolog (S. cerevisiase) [Source:ZFIN;Acc:ZDB-GENE-060825-261]	12	14166036	14211293		6.98	3.73	4.33	8.08	7.23	8.18
ENSDARG00000055539	epdl2	ependymin-like 2 [Source:ZFIN;Acc:ZDB-GENE-091204-250]	21	28513057	28523548		57.3	62.67	58.28	18.18	21.14	19.54
ENSDARG00000055540	pfkfb4a	6-phosphofructo-2-kinase/fructose-2,6-biphosphatase 4a [Source:ZFIN;Acc:ZDB-GENE-051120-51]	11	34555036	34577106		10.31	6.22	6.48	11.71	12.91	12.44
ENSDARG00000055543	appb	amyloid beta (A4) precursor protein b [Source:ZFIN;Acc:ZDB-GENE-020220-1]	9	35026415	35155089		81.36	35.94	35.95	93.79	96.3	98.58
ENSDARG00000055548	npat	nuclear protein, ataxia-telangiectasia locus [Source:ZFIN;Acc:ZDB-GENE-050506-54]	15	25528290	25542592		6.91	4.53	4.26	5.93	5.49	5.62
ENSDARG00000055550	casp21	caspase 21, apoptosis-related cysteine peptidase [Source:ZFIN;Acc:ZDB-GENE-190425-1]	21	8533533	8538300		1.01	1.35	1.09	0.67	0.49	0.49
ENSDARG00000055552	dvl1b	dishevelled segment polarity protein 1b [Source:ZFIN;Acc:ZDB-GENE-071004-1]	11	24360744	24428319		6.73	1.72	2.79	8.16	8.25	8.39
ENSDARG00000055554	wnt1	wingless-type MMTV integration site family, member 1 [Source:NCBI gene;Acc:30128]	23	27652906	27658054		1.44	0.71	1.02	1.5	1.23	1.19
ENSDARG00000055559	chrna6	cholinergic receptor, nicotinic, alpha 6 [Source:ZFIN;Acc:ZDB-GENE-090312-91]	1	22481723	22512136		3.9	0.82	0.82	4.04	4.17	4.12
ENSDARG00000055560	p2ry8	P2Y receptor family member 8 [Source:ZFIN;Acc:ZDB-GENE-070912-447]	9	34884933	34885902		0.29	0.12	0.18	0.17	0.13	0.15
ENSDARG00000055561	c1galt1b	core 1 synthase, glycoprotein-N-acetylgalactosamine 3-beta-galactosyltransferase, 1b [Source:NCBI gene;Acc:337131]	6	39190402	39199070		9.18	8.56	6.6	8.69	8.09	7.66
ENSDARG00000055563	drosha	drosha ribonuclease III [Source:ZFIN;Acc:ZDB-GENE-070209-23]	12	13344896	13390841		4.22	2.53	2.19	4.8	4.61	4.78
ENSDARG00000055565	cacnb2b	calcium channel, voltage-dependent, beta 2b [Source:ZFIN;Acc:ZDB-GENE-050208-129]	2	31475772	31519662		6.59	1.17	0.92	5.04	5.04	4.5
ENSDARG00000055566	mastl	microtubule associated serine/threonine kinase-like [Source:NCBI gene;Acc:445215]	24	5893134	5908241		2.4	1.7	1.82	2.03	2.29	2.22
ENSDARG00000055569	ghdc	GH3 domain containing [Source:ZFIN;Acc:ZDB-GENE-050208-165]	12	13527422	13549538		1.4	1.89	1.36	1.26	1.48	1.05
ENSDARG00000055570	gpr61	G protein-coupled receptor 61 [Source:ZFIN;Acc:ZDB-GENE-081104-239]	8	25173317	25177767		2.42	0.7	1.25	2.6	3.01	2.75
ENSDARG00000055574	FOXN2	forkhead box N2a [Source:NCBI gene;Acc:100330010]	13	48482256	48551810	FOXN2	1.54	0.62	0.6	2.73	2.26	2.26
ENSDARG00000055575	pcolce2b	procollagen C-endopeptidase enhancer 2b [Source:ZFIN;Acc:ZDB-GENE-040426-1177]	24	5871589	5910811		5.74	5.88	5.81	10.83	11.01	11.7
ENSDARG00000055585	c1galt1a	core 1 synthase, glycoprotein-N-acetylgalactosamine 3-beta-galactosyltransferase, 1a [Source:NCBI gene;Acc:557675]	23	28092083	28136739		2.85	1.07	1.51	2.86	2.51	2.75
ENSDARG00000055588	stat5b	signal transducer and activator of transcription 5b [Source:ZFIN;Acc:ZDB-GENE-040818-1]	12	13560082	13650472		4.28	3.42	3.1	5.28	4.84	5.02
ENSDARG00000055589	s100t	S100 calcium binding protein T [Source:ZFIN;Acc:ZDB-GENE-050320-61]	16	22681818	22683386		82.02	99.95	95.68	25.98	28.55	26.85
ENSDARG00000055591	pipox	pipecolic acid oxidase [Source:ZFIN;Acc:ZDB-GENE-110627-2]	15	24150090	24178893		8.5	14.77	10.95	3.82	3.95	3.64
ENSDARG00000055592	capn2b	calpain 2, (m/II) large subunit b [Source:ZFIN;Acc:ZDB-GENE-050522-84]	22	26328063	26353916		17.63	15.26	12.83	10.01	8.93	9.06
ENSDARG00000055595	clul1	clusterin-like 1 (retinal) [Source:ZFIN;Acc:ZDB-GENE-031118-57]	2	31363286	31376607		16	2.92	1.67	4.97	5.35	5.75
ENSDARG00000055607	cgna	cingulin a [Source:ZFIN;Acc:ZDB-GENE-100921-78]	16	22520712	22585309		2.21	2.01	1.73	1.94	1.71	2.13
ENSDARG00000055610	exoc2	exocyst complex component 2 [Source:ZFIN;Acc:ZDB-GENE-040426-1160]	20	26572456	26624042		15.15	11.77	11.93	14.25	15.22	15.09
ENSDARG00000055613	C16H1orf43	zgc:123238 [Source:ZFIN;Acc:ZDB-GENE-051023-3]	16	22345513	22361219	C16H1orf43	11.5	7.29	8.75	9.98	11.55	12
ENSDARG00000055618	acta1b	actin alpha 1, skeletal muscle b [Source:ZFIN;Acc:ZDB-GENE-030131-55]	13	24279021	24284442		619.24	961.61	1058.3	588.02	609.59	598.89
ENSDARG00000055620	acad9	acyl-CoA dehydrogenase family, member 9 [Source:ZFIN;Acc:ZDB-GENE-060616-196]	11	26685982	26701611		11.84	13.59	11.94	8.91	9.05	8.55
ENSDARG00000055622	zgc:153345	zgc:153345 [Source:ZFIN;Acc:ZDB-GENE-061103-487]	10	33393829	33403522		0.1	0.03	0.05	0.16	0.1	0.14
ENSDARG00000055629	dipk2ab	divergent protein kinase domain 2Ab [Source:ZFIN;Acc:ZDB-GENE-070830-2]	24	5653162	5713799		7.01	4.42	4.72	9.69	8.9	9.11
ENSDARG00000055630	foxh1	forkhead box H1 [Source:NCBI gene;Acc:57930]	12	13722385	13730501		0.01	0.01	0.01	0	0.02	0.01
ENSDARG00000055632	smtnl	smoothelin, like [Source:ZFIN;Acc:ZDB-GENE-040212-1]	15	24644016	24671419		5.74	6.98	6.15	15.2	16.41	16.77
ENSDARG00000055635	crk	v-crk avian sarcoma virus CT10 oncogene homolog [Source:ZFIN;Acc:ZDB-GENE-040801-148]	15	24737599	24744920		17.42	13.61	12.43	21.16	21.66	20.04
ENSDARG00000055638	ankrd33aa	ankyrin repeat domain 33Aa [Source:ZFIN;Acc:ZDB-GENE-080520-2]	23	27862807	27875140		13.01	2.41	1.41	6.19	6.43	7.25
ENSDARG00000055639	ruvbl2	RuvB-like AAA ATPase 2 [Source:NCBI gene;Acc:317678]	5	30759232	30770807		26.81	25.57	26.62	19.3	19.8	20.07
ENSDARG00000055642		si:ch211-217a12.1 [Source:ZFIN;Acc:ZDB-GENE-121214-95]	12	14079097	14130716		6.35	11.03	9.87	4.1	3.49	3.78
ENSDARG00000055644	prss60.2	serine protease 60.2 [Source:ZFIN;Acc:ZDB-GENE-050320-109]	6	39098397	39105480		4.96	4.23	4.51	5.64	5.62	5.54
ENSDARG00000055647	ftr82	finTRIM family, member 82 [Source:ZFIN;Acc:ZDB-GENE-030219-207]	5	30707133	30715225		20.07	23.52	20.77	15.8	17.21	17.22
ENSDARG00000055648	cpda	carboxypeptidase D, a [Source:ZFIN;Acc:ZDB-GENE-081112-2]	15	24756860	24794610		4.44	3.24	2.31	6.75	6.32	6.32
ENSDARG00000055652	aclyb	ATP citrate lyase b [Source:ZFIN;Acc:ZDB-GENE-090909-2]	12	13973058	14078070		1.26	1.47	1.27	1.38	1.52	1.4
ENSDARG00000055653	nubp2	nucleotide binding protein 2 (MinD homolog, E. coli) [Source:NCBI gene;Acc:641290]	24	39178348	39186185		12.19	12.84	14.34	9.31	9.79	11.22
ENSDARG00000055655	birc5b	baculoviral IAP repeat containing 5b [Source:ZFIN;Acc:ZDB-GENE-030826-2]	23	28322986	28325846		0.15	0.03	0.15	0.23	0.09	0.19
ENSDARG00000055656	pdzd3b	PDZ domain containing 3b [Source:ZFIN;Acc:ZDB-GENE-081022-151]	5	30680804	30697908		4.99	8.84	5.95	1.28	1.35	1.17
ENSDARG00000055659	gpr161	G protein-coupled receptor 161 [Source:NCBI gene;Acc:368421]	9	34148714	34162374		2.03	2.06	1.68	2.2	2.24	2.08
ENSDARG00000055672	map3k10	mitogen-activated protein kinase kinase kinase 10 [Source:ZFIN;Acc:ZDB-GENE-050419-112]	18	36948879	37007294		2.55	0.84	0.92	4.11	3.9	3.63
ENSDARG00000055676	armt1	acidic residue methyltransferase 1 [Source:NCBI gene;Acc:541518]	20	26387179	26391958		6.82	5.77	6.22	7.69	6.99	6.77
ENSDARG00000055678	akap12b	A kinase (PRKA) anchor protein 12b [Source:ZFIN;Acc:ZDB-GENE-030131-9753]	20	26407758	26467454		30.65	34.41	31.81	37.36	38.66	38.51
ENSDARG00000055679	mto1	mitochondrial tRNA translation optimization 1 [Source:ZFIN;Acc:ZDB-GENE-070209-253]	12	14214755	14309962		7.88	7.92	6.26	8.2	8.29	8.41
ENSDARG00000055683	pimr191	Pim proto-oncogene, serine/threonine kinase, related 191 [Source:ZFIN;Acc:ZDB-GENE-131119-3]	15	25080041	25083200		0.14	0.07	0.24	0.2	0.05	0.14
ENSDARG00000055690	CU655961.1	THAP domain-containing protein 6-like [Source:NCBI gene;Acc:100535121]	15	44106641	44117290		1.72	1.18	0.99	1.35	1.74	1.61
ENSDARG00000055698	pcnx2	pecanex 2 [Source:ZFIN;Acc:ZDB-GENE-090312-150]	13	23669659	23735251		3.45	0.74	0.94	4.91	5.19	4.93
ENSDARG00000055705	f5	coagulation factor V [Source:ZFIN;Acc:ZDB-GENE-030131-5531]	9	34127005	34147996		6.94	9.81	6.7	16.31	18.42	18.1
ENSDARG00000055708	timm8b	translocase of inner mitochondrial membrane 8 homolog B (yeast) [Source:ZFIN;Acc:ZDB-GENE-031019-1]	5	30089715	30090506		37.06	51.34	55.39	24.73	23.84	21.49
ENSDARG00000055712	sdhda	succinate dehydrogenase complex, subunit D, integral membrane protein a [Source:NCBI gene;Acc:436762]	5	30081241	30088077		1.06	1.31	1.02	0.96	1.34	0.92
ENSDARG00000055713	fmnl1a	formin-like 1a [Source:ZFIN;Acc:ZDB-GENE-120215-83]	3	37196258	37235300		0.37	0.52	0.39	0.75	0.84	0.88
ENSDARG00000055715	capn8	calpain 8 [Source:ZFIN;Acc:ZDB-GENE-050522-279]	22	26400519	26435494		11.09	12.82	12.01	5.13	5	5.06
ENSDARG00000055719	ino80c	INO80 complex subunit C [Source:ZFIN;Acc:ZDB-GENE-040625-97]	16	26850481	26855945		12.62	11.31	11.65	10.99	11.25	12.99
ENSDARG00000055722	bco2a	beta-carotene oxygenase 2a [Source:ZFIN;Acc:ZDB-GENE-030131-8039]	5	30052714	30080332		2.57	2.09	1.67	1.53	1.5	1.49
ENSDARG00000055723	hsp70l	heat shock cognate 70-kd protein, like [Source:ZFIN;Acc:ZDB-GENE-050321-1]	8	4798158	4800911		15.84	6.3	4.91	18.73	34.96	24.55
ENSDARG00000055730	trim35-29	tripartite motif containing 35-29 [Source:ZFIN;Acc:ZDB-GENE-041114-35]	16	26846495	26849318		0.01	0	0	0	0	0.04
ENSDARG00000055731	si:dkeyp-69e1.8	si:dkeyp-69e1.8 [Source:ZFIN;Acc:ZDB-GENE-070912-676]	3	26064091	26070801		7.13	7.33	6.29	6.02	4.68	4.92
ENSDARG00000055737	ube3a	ubiquitin protein ligase E3A [Source:ZFIN;Acc:ZDB-GENE-041114-190]	6	38773376	38790064		16.14	14.14	13.18	17.52	16.39	17.36
ENSDARG00000055739	ribc1	RIB43A domain with coiled-coils 1 [Source:ZFIN;Acc:ZDB-GENE-081031-7]	23	28685266	28692581		1.11	0.6	0.8	0.52	0.72	0.73
ENSDARG00000055740	9-Mar	membrane-associated ring finger (C3HC4) 9 [Source:ZFIN;Acc:ZDB-GENE-081031-71]	23	36771593	36798620		9.94	2.47	2.89	13.34	14.35	15.34
ENSDARG00000055743	ankrd54	ankyrin repeat domain 54 [Source:NCBI gene;Acc:436851]	3	26035550	26044759		14.59	11.7	12.03	15.41	14.65	14.9
ENSDARG00000055746	si:ch211-238g23.1	si:ch211-238g23.1 [Source:ZFIN;Acc:ZDB-GENE-050420-173]	18	36914221	36920453		0	0.01	0	0.04	0.01	0
ENSDARG00000055750	foxp3a	forkhead box P3a [Source:ZFIN;Acc:ZDB-GENE-061116-2]	8	25716308	25729543		0.18	0.06	0.04	0.11	0.2	0.07
ENSDARG00000055751	fosb	FBJ murine osteosarcoma viral oncogene homolog B [Source:ZFIN;Acc:ZDB-GENE-041114-181]	18	36769758	36774965		0.35	0.1	0.13	0.82	0.74	0.61
ENSDARG00000055752	npas4a	neuronal PAS domain protein 4a [Source:NCBI gene;Acc:724016]	14	30636955	30642819		6.23	1.06	1.96	11.56	8.4	9.2
ENSDARG00000055753	suv39h1b	suppressor of variegation 3-9 homolog 1b [Source:ZFIN;Acc:ZDB-GENE-030131-5105]	8	25750963	25771474		3.48	3.06	3.11	2.55	2.33	2.33
ENSDARG00000055754	smc1a	structural maintenance of chromosomes 1A [Source:ZFIN;Acc:ZDB-GENE-090506-9]	23	28693278	28713982		6.52	1.7	2.14	4.12	4.71	5.49
ENSDARG00000055757	tmem9	transmembrane protein 9 [Source:ZFIN;Acc:ZDB-GENE-030131-6083]	8	25761654	25766675		7.3	5.65	6.05	5.81	5.19	5.86
ENSDARG00000055759	efhd2	EF-hand domain family, member D2 [Source:ZFIN;Acc:ZDB-GENE-060531-152]	8	25818622	25846188		14.84	14.96	12.17	16.8	16.41	15.87
ENSDARG00000055760	srm	spermidine synthase [Source:ZFIN;Acc:ZDB-GENE-040426-1183]	23	28718863	28727106		10.65	11.02	9.18	12	10.9	10.4
ENSDARG00000055763	tmem115	transmembrane protein 115 [Source:ZFIN;Acc:ZDB-GENE-050913-157]	8	25892319	25900351		5.6	4.68	4.27	6.59	6.84	7.19
ENSDARG00000055770	dffa	DNA fragmentation factor, alpha polypeptide [Source:ZFIN;Acc:ZDB-GENE-040718-376]	23	28803007	28808480		8.77	8.99	8.81	6.33	7.48	7.34
ENSDARG00000055781	ptger3	prostaglandin E receptor 3 (subtype EP3) [Source:ZFIN;Acc:ZDB-GENE-120508-5]	6	37308716	37311049		1.01	0.72	0.99	1.21	1.82	1.61
ENSDARG00000055784	ptpn3	protein tyrosine phosphatase non-receptor type 3 [Source:ZFIN;Acc:ZDB-GENE-030131-2934]	16	26547152	26600624		8.52	8.42	5.96	9.62	9.26	9.62
ENSDARG00000055786	prss23	serine protease 23 [Source:ZFIN;Acc:ZDB-GENE-030131-9149]	14	30802465	30808174		8.47	7.46	6.65	9.9	9.34	8.93
ENSDARG00000055787	dzank1	double zinc ribbon and ankyrin repeat domains 1 [Source:ZFIN;Acc:ZDB-GENE-051113-296]	22	25049563	25063998		0.97	0.49	0.48	0.85	0.86	1.02
ENSDARG00000055791	zgc:158423	zgc:158423 [Source:ZFIN;Acc:ZDB-GENE-061215-58]	7	20535869	20542545		21.43	26.51	27.45	20.28	22.09	21.05
ENSDARG00000055792	foxo4	forkhead box O4 [Source:ZFIN;Acc:ZDB-GENE-150311-1]	14	30910114	30917928		12.89	8.57	7.27	20.38	20.39	21.1
ENSDARG00000055797	cnpy4	canopy FGF signaling regulator 4 [Source:NCBI gene;Acc:568776]	7	20459671	20464468		4.49	6.66	5.63	6.16	6.17	6.09
ENSDARG00000055798	heyl	hes related family bHLH transcription factor with YRPW motif like [Source:NCBI gene;Acc:335134]	19	32553874	32560591		0.94	0.53	0.93	0.83	0.98	1.09
ENSDARG00000055809	vtg2	vitellogenin 2 [Source:ZFIN;Acc:ZDB-GENE-001201-2]	22	24869690	24880824		0	0	0.01	0.01	0.03	0.01
ENSDARG00000055813	si:dkey-225f5.5	si:dkey-225f5.5 [Source:ZFIN;Acc:ZDB-GENE-141216-18]	3	23471180	23474416		1.41	1.47	1.17	0.81	1.02	0.9
ENSDARG00000055825	celsr3	cadherin, EGF LAG seven-pass G-type receptor 3 [Source:ZFIN;Acc:ZDB-GENE-070122-3]	8	26125218	26212523		3.96	0.76	1	8.27	7.86	8.7
ENSDARG00000055831	si:dkey-182g1.6	si:dkey-182g1.6 [Source:ZFIN;Acc:ZDB-GENE-100922-82]	22	8799855	8809496		0.02	0	0.01	0.09	0.03	0.01
ENSDARG00000055833	lectin	lectin [Source:ZFIN;Acc:ZDB-GENE-070912-438]	9	33236844	33238249		0	0	0	0.03	0.2	0.1
ENSDARG00000055835	slc26a6	solute carrier family 26 member 6 [Source:ZFIN;Acc:ZDB-GENE-081105-83]	8	26268100	26288506		3.61	5.9	3.38	3	2.97	2.82
ENSDARG00000055837	arhgef1b	Rho guanine nucleotide exchange factor (GEF) 1b [Source:ZFIN;Acc:ZDB-GENE-060616-136]	16	26140895	26232411		11.04	9.13	8.38	22.79	23.37	22.74
ENSDARG00000055838	mettl16	methyltransferase like 16 [Source:NCBI gene;Acc:445217]	15	25214171	25269028		2.77	2.28	1.98	1.44	1.76	1.78
ENSDARG00000055839	march4l	membrane-associated ring finger (C3HC4) 4, like [Source:NCBI gene;Acc:100000640]	1	29954690	30039332		1.78	0.51	0.65	2.69	3.07	3.1
ENSDARG00000055841	CABZ01046427.1		22	8843870	8876699		0.12	0.21	0.19	0.24	0.24	0.23
ENSDARG00000055843	cdh10a	cadherin 10, type 2a (T2-cadherin) [Source:ZFIN;Acc:ZDB-GENE-060614-1]	2	28889936	28934284		5.18	1.37	2.01	4.59	4.89	5.14
ENSDARG00000055852	mtnr1ab	melatonin receptor 1A b [Source:ZFIN;Acc:ZDB-GENE-080107-4]	14	30105611	30141162		0.93	0.33	0.35	0.67	0.82	0.69
ENSDARG00000055854	nr4a3	nuclear receptor subfamily 4, group A, member 3 [Source:ZFIN;Acc:ZDB-GENE-070824-4]	16	27345383	27369782		1.83	1.02	0.99	2.96	3.53	3.69
ENSDARG00000055855	kcnc3a	potassium voltage-gated channel, Shaw-related subfamily, member 3a [Source:ZFIN;Acc:ZDB-GENE-100901-1]	3	31953145	32053929		9.66	3.32	4.1	15.28	14.57	15.11
ENSDARG00000055856	tor1l1	torsin family 1 like 1 [Source:ZFIN;Acc:ZDB-GENE-080723-36]	23	37530718	37536127		0.07	0.04	0	0.07	0.05	0.02
ENSDARG00000055857	dop1b	DOP1 leucine zipper like protein B [Source:ZFIN;Acc:ZDB-GENE-040426-1195]	9	33154841	33195586		9.26	5.14	4.4	10.04	9.79	10.41
ENSDARG00000055868	rsl1d1	ribosomal L1 domain containing 1 [Source:ZFIN;Acc:ZDB-GENE-130603-67]	12	19384615	19393968		53.98	70.05	67.88	31.75	33.06	33.01
ENSDARG00000055874	cpe	carboxypeptidase E [Source:ZFIN;Acc:ZDB-GENE-090313-68]	1	20839482	20911297		58.83	17.89	25.82	76.33	83.93	82.07

ENSDARG00000055875	pag1	phosphoprotein membrane anchor with glycosphingolipid microdomains 1 [Source:ZFIN;Acc:ZDB-GENE-060503-586]	19	32048379	32150214		9.44	6.63	5.61	10.22	10.91	11.22
ENSDARG00000055876	msmo1	methylsterol monooxygenase 1 [Source:NCBI gene;Acc:406662]	1	20916551	20928772		14.75	24.27	19.21	3.54	4.42	3.9
ENSDARG00000055883	dmc1	DNA meiotic recombinase 1 [Source:ZFIN;Acc:ZDB-GENE-060331-93]	12	19356623	19362186		0	0	0.04	0.02	0	0.07
ENSDARG00000055885	tex10	testis expressed 10 [Source:NCBI gene;Acc:327196]	16	27508381	27543092		10.67	8.76	8.62	9.92	9.93	9.56
ENSDARG00000055889	zgc:153215	zgc:153215 [Source:ZFIN;Acc:ZDB-GENE-061103-58]	16	27561114	27566552		2.51	3.22	2.51	1.27	1.43	1.64
ENSDARG00000055893	nmnat1-rbp7a	nmnat1-rbp7a readthrough [Source:ZFIN;Acc:ZDB-GENE-160617-1]	23	29554591	29569827		0	0	0	0	0	0
ENSDARG00000055897	zgc:154093	zgc:154093 [Source:ZFIN;Acc:ZDB-GENE-061103-517]	15	23693514	23699737		7.76	4.04	3	9.45	10.27	10.62
ENSDARG00000055899	lzic	leucine zipper and CTNNBIP1 domain containing [Source:NCBI gene;Acc:436871]	23	29569893	29575691		4.95	5.41	4.68	4.1	4.31	3.99
ENSDARG00000055900	zswim5	zinc finger, SWIM-type containing 5 [Source:ZFIN;Acc:ZDB-GENE-030925-30]	2	27491066	27543942		13.15	6.35	5.28	27.42	24.87	26.68
ENSDARG00000055901	steap4	STEAP family member 4 [Source:ZFIN;Acc:ZDB-GENE-070424-244]	16	27734872	27749172		5.19	6.57	5.86	10.27	10.53	10.73
ENSDARG00000055903	luc7l	LUC7-like (S. cerevisiae) [Source:ZFIN;Acc:ZDB-GENE-040625-111]	24	39638555	39654132		33.64	19.01	26.14	32.97	31.63	31.18
ENSDARG00000055912	nipa2	NIPA magnesium transporter 2 [Source:ZFIN;Acc:ZDB-GENE-040426-2134]	6	37733608	37745508		9.64	6.23	6.36	6.92	7.97	6.62
ENSDARG00000055917	nipa1	NIPA magnesium transporter 1 [Source:ZFIN;Acc:ZDB-GENE-041010-164]	6	37745719	37749711		2.76	3.93	3.46	1.82	2.34	2.29
ENSDARG00000055920	olfcq18	olfactory receptor C family, q18 [Source:ZFIN;Acc:ZDB-GENE-060503-454]	18	33685119	33689274		0.01	0.01	0.01	0.01	0	0
ENSDARG00000055926	foxi3a	forkhead box I3a [Source:ZFIN;Acc:ZDB-GENE-031126-3]	21	30351256	30352951		12.78	12.73	11.48	6.32	5.86	6.51
ENSDARG00000055930	zc3h7bb	zinc finger CCCH-type containing 7Bb [Source:ZFIN;Acc:ZDB-GENE-080208-7]	12	19158832	19185865		20.11	15.31	13.3	27.07	27.11	25.83
ENSDARG00000055934	si:ch1073-416j23.1	si:ch1073-416j23.1 [Source:ZFIN;Acc:ZDB-GENE-100922-35]	9	10781687	10805231		0.02	0.01	0.01	0.04	0.02	0.08
ENSDARG00000055938	tspan14	tetraspanin 14 [Source:ZFIN;Acc:ZDB-GENE-060825-329]	13	21638454	21650404		17.47	19.37	17.52	22.91	24.18	23.06
ENSDARG00000055945	asph	aspartate beta-hydroxylase [Source:ZFIN;Acc:ZDB-GENE-031112-5]	2	26655744	26682182		23.29	19.81	16.84	27.9	27.31	26.86
ENSDARG00000055952	olfcx2	olfactory receptor C family, x2 [Source:ZFIN;Acc:ZDB-GENE-050419-39]	18	33237127	33240503		0.11	0.09	0.06	0.1	0.06	0.21
ENSDARG00000055955	lcp2a	lymphocyte cytosolic protein 2a [Source:ZFIN;Acc:ZDB-GENE-041114-97]	21	30306369	30347592		0.79	0.78	0.66	0.86	0.82	1.05
ENSDARG00000055965	klc3	kinesin light chain 3 [Source:ZFIN;Acc:ZDB-GENE-060929-772]	15	23657051	23677446		2.43	0.96	1.03	1.34	1.28	1.7
ENSDARG00000055966	cflara	CASP8 and FADD-like apoptosis regulator a [Source:ZFIN;Acc:ZDB-GENE-030826-3]	11	30332959	30341431		3.43	3.34	2.71	4.98	4.93	5.34
ENSDARG00000055969	rfc3	replication factor C (activator 1) 3 [Source:ZFIN;Acc:ZDB-GENE-020809-3]	10	34021368	34033680		3.52	3.65	4.8	2.29	2.02	2.09
ENSDARG00000055970	zgc:136971	zgc:136971 [Source:ZFIN;Acc:ZDB-GENE-060421-3674]	8	26432639	26449831		1.5	0.73	0.82	1.78	1.74	1.82
ENSDARG00000055973	zar1l	zygote arrest 1-like [Source:ZFIN;Acc:ZDB-GENE-060810-119]	10	33999869	34002185		0.02	0.02	0.03	0	0.06	0.05
ENSDARG00000055974	tpmt.1	thiopurine S-methyltransferase, tandem duplicate 1 [Source:NCBI gene;Acc:436842]	19	47290287	47298657		7.64	12.41	10.61	6.45	5.42	5.82
ENSDARG00000055976	pecr	peroxisomal trans-2-enoyl-CoA reductase [Source:ZFIN;Acc:ZDB-GENE-050417-232]	11	30355442	30364847		6.63	9.62	7.82	4.71	5.34	4.88
ENSDARG00000055986	gzm3.2	granzyme 3, tandem duplicate 2 [Source:ZFIN;Acc:ZDB-GENE-070912-133]	9	32657695	32663496		0.18	0.21	0.08	0.06	0.07	0.16
ENSDARG00000055989	tmem161b	transmembrane protein 161B [Source:NCBI gene;Acc:406680]	5	48027981	48070779		3.7	2.46	2.18	4.3	4.74	4.86
ENSDARG00000055991	hmbsb	hydroxymethylbilane synthase, b [Source:ZFIN;Acc:ZDB-GENE-050522-491]	15	23511407	23529945		5.41	5.48	5.12	7.96	8.34	7.87
ENSDARG00000055994	olfcq9	olfactory receptor C family, q9 [Source:ZFIN;Acc:ZDB-GENE-050419-131]	18	33490315	33493613		0	0	0	0.01	0	0.01
ENSDARG00000055996	rps8a	ribosomal protein S8a [Source:NCBI gene;Acc:407690]	2	26498446	26501868		733.57	981.46	1092.85	525.75	582.58	588.32
ENSDARG00000055999	BRD8	si:ch211-59d17.3 [Source:ZFIN;Acc:ZDB-GENE-091113-54]	21	30194904	30236796	BRD8	3.94	1.09	1.33	3.49	3.45	3.41
ENSDARG00000056000	olfcq10	olfactory receptor C family, q10 [Source:ZFIN;Acc:ZDB-GENE-050419-265]	18	33521609	33526012		0.02	0	0	0.02	0	0.01
ENSDARG00000056001	fryb	furry homolog b (Drosophila) [Source:ZFIN;Acc:ZDB-GENE-080215-5]	10	33895315	33999781		7.1	2.92	2.58	11.68	11.02	11.63
ENSDARG00000056004	sprn	shadow of prion protein [Source:NCBI gene;Acc:386702]	13	21682770	21688176		11.77	4.67	6.57	11.23	12.05	11.31
ENSDARG00000056005	dnajc18	DnaJ (Hsp40) homolog, subfamily C, member 18 [Source:ZFIN;Acc:ZDB-GENE-030131-8019]	21	30239549	30254185		13.32	9.63	8.36	11.32	10.23	10.67
ENSDARG00000056006	olfcq12	olfactory receptor C family, q12 [Source:ZFIN;Acc:ZDB-GENE-060503-735]	18	33557125	33561739		0.14	0.05	0.16	0.05	0.17	0.06
ENSDARG00000056007	cyb5d1	cytochrome b5 domain containing 1 [Source:NCBI gene;Acc:795592]	7	20771754	20778584		0.5	0.32	0.37	0.41	0.43	0.46
ENSDARG00000056008	atp5mc1	ATP synthase membrane subunit c locus 1 [Source:ZFIN;Acc:ZDB-GENE-040426-1966]	3	23599290	23604396		292.98	388.56	400.08	213.55	210.07	207.37
ENSDARG00000056009	olfcq8	olfactory receptor C family, q8 [Source:ZFIN;Acc:ZDB-GENE-050419-176]	18	33464345	33473581		0	0	0	0	0.01	0
ENSDARG00000056012	eve1	even-skipped-like1 [Source:ZFIN;Acc:ZDB-GENE-980526-69]	3	23641935	23643830		0.79	0.8	0.83	0.89	0.87	0.82
ENSDARG00000056014	olfcq16	olfactory receptor C family, q16 [Source:ZFIN;Acc:ZDB-GENE-060503-264]	18	33652976	33656425		0	0	0	0	0	0
ENSDARG00000056015	hoxb13a	homeobox B13a [Source:NCBI gene;Acc:559921]	3	23654233	23655834		4.47	5.43	6.17	3.74	4.44	4.31
ENSDARG00000056018	itga6l	integrin, alpha 6, like [Source:ZFIN;Acc:ZDB-GENE-040914-57]	14	41528635	41600040		0.51	0.55	0.5	0.15	0.19	0.19
ENSDARG00000056019	ttbk1a	tau tubulin kinase 1a [Source:ZFIN;Acc:ZDB-GENE-100609-4]	11	30663300	30728241		6.71	1.41	1.94	7.36	7.78	7.46
ENSDARG00000056021	sostdc1b	sclerostin domain containing 1b [Source:ZFIN;Acc:ZDB-GENE-060503-653]	19	31061718	31063020		2.36	2.66	2.17	1.83	2.33	2.32
ENSDARG00000056023	hoxb9a	homeobox B9a [Source:NCBI gene;Acc:30344]	3	23677351	23682436		3.19	4.81	4.08	10.85	10.75	10.39
ENSDARG00000056024	eva1c	eva-1 homolog C (C. elegans) [Source:ZFIN;Acc:ZDB-GENE-091204-329]	10	33580285	33588310		0.51	0.36	0.36	0.31	0.47	0.31
ENSDARG00000056026	tprg1	tumor protein p63 regulated 1 [Source:ZFIN;Acc:ZDB-GENE-070410-113]	6	28752943	28790319		10.03	11.25	9.04	7.79	6.84	7.2
ENSDARG00000056027	hoxb8a	homeobox B8a [Source:NCBI gene;Acc:30343]	3	23687909	23690481		9.45	8.1	7.95	17.33	17.87	18.71
ENSDARG00000056028	slc22a7a	solute carrier family 22 member 7a [Source:ZFIN;Acc:ZDB-GENE-050506-52]	11	30729745	30742966		13.22	18.18	20.58	16.61	17.51	15.3
ENSDARG00000056029	cyp26c1	cytochrome P450, family 26, subfamily C, polypeptide 1 [Source:ZFIN;Acc:ZDB-GENE-050714-2]	17	19522856	19535328		2.78	2.17	1.61	4.87	3.8	4.15
ENSDARG00000056030	hoxb7a	homeobox B7a [Source:NCBI gene;Acc:58044]	3	23691847	23696208		3.44	5.79	4.12	6.05	6.18	6.2
ENSDARG00000056034	pip4p2	phosphatidylinositol-4,5-bisphosphate 4-phosphatase 2 [Source:ZFIN;Acc:ZDB-GENE-060503-854]	19	31404686	31431027		9.43	3.79	5.68	11.21	9.85	10.04
ENSDARG00000056036	stxbp1b	syntaxin binding protein 1b [Source:ZFIN;Acc:ZDB-GENE-060531-166]	5	28972935	29002322		23.3	4.18	3.11	22.57	21.49	22.89
ENSDARG00000056037	itih6	inter-alpha-trypsin inhibitor heavy chain family member 6 [Source:ZFIN;Acc:ZDB-GENE-050603-1]	23	26039524	26056072		2.17	2.07	1.68	6.88	7.68	6.94
ENSDARG00000056045	si:ch73-139e5.4	si:ch73-139e5.4 [Source:ZFIN;Acc:ZDB-GENE-130531-31]	12	19065679	19091214		3.05	4.43	3.61	1.54	1.97	1.75
ENSDARG00000056047	noxa1	NADPH oxidase activator 1 [Source:ZFIN;Acc:ZDB-GENE-050913-32]	5	29142797	29152464		0.77	0.56	0.38	1.16	1.29	0.96
ENSDARG00000056050	kctd17	potassium channel tetramerization domain containing 17 [Source:ZFIN;Acc:ZDB-GENE-130531-80]	12	19036380	19065298		8.99	3.17	3.54	9.08	9.23	8.62
ENSDARG00000056057	grem2a	gremlin 2, DAN family BMP antagonist a [Source:ZFIN;Acc:ZDB-GENE-131127-498]	17	19730001	19749179		0.52	0.32	0.3	0.37	0.41	0.19
ENSDARG00000056059	rangap1b	Ran GTPase activating protein 1b [Source:ZFIN;Acc:ZDB-GENE-040426-1921]	12	19007926	19028068		9.63	8.47	8.78	7.53	6.73	6.71
ENSDARG00000056062	adgre14	adhesion G protein-coupled receptor E14 [Source:ZFIN;Acc:ZDB-GENE-131120-49]	3	52145511	52166846		0.01	0.05	0.03	0.06	0.06	0.11
ENSDARG00000056065	mov10b.2	Moloney leukemia virus 10b, tandem duplicate 2 [Source:NCBI gene;Acc:555950]	8	27630692	27656765		0.24	0.11	0.11	0.09	0.17	0.2
ENSDARG00000056075	rca2.1	regulator of complement activation group 2 gene 1 [Source:ZFIN;Acc:ZDB-GENE-050320-60]	23	26094481	26120281		22.23	22.11	17.76	26.9	28.09	28.35
ENSDARG00000056077	dcst1	DC-STAMP domain containing 1 [Source:ZFIN;Acc:ZDB-GENE-070809-5]	16	28711556	28727763		0.22	0.03	0.06	0.12	0.2	0.2
ENSDARG00000056078	rftn2	raftlin family member 2 [Source:ZFIN;Acc:ZDB-GENE-040426-2760]	9	32312920	32343673		7.48	6.28	5.32	9.48	9.16	8.92
ENSDARG00000056079	l3mbtl2	L3MBTL histone methyl-lysine binding protein 2 [Source:ZFIN;Acc:ZDB-GENE-030131-8713]	12	18970790	18991178		3.7	2.88	3.19	3.42	3.51	3.33
ENSDARG00000056080	si:dkey-191g9.5	si:dkey-191g9.5 [Source:ZFIN;Acc:ZDB-GENE-090313-217]	13	21712284	21739142		16.38	3.18	3.83	9.24	10.55	9.69
ENSDARG00000056084	igsf21b	immunoglobin superfamily, member 21b [Source:ZFIN;Acc:ZDB-GENE-070424-59]	23	21638258	21661916		9.97	1.18	1.98	8.6	7.7	8.64
ENSDARG00000056085	mob4	MOB family member 4, phocein [Source:ZFIN;Acc:ZDB-GENE-040801-174]	9	32306862	32312862		72.16	65.27	73.78	64.63	66.17	65.39
ENSDARG00000056087	ecrg4a	ECRG4 augurin precursor a [Source:NCBI gene;Acc:791762]	9	6371919	6380653		19.56	21.57	21.72	17.61	16.59	15.47
ENSDARG00000056089	nrbp1	nuclear receptor binding protein 1 [Source:ZFIN;Acc:ZDB-GENE-041001-146]	20	26002534	26024924		10.58	7.84	7.22	12.86	11.98	12.52
ENSDARG00000056090	capza1b	capping actin protein of muscle Z-line subunit alpha 1b [Source:ZFIN;Acc:ZDB-GENE-031002-24]	8	27807266	27827660		106.4	124.43	126.76	88.23	83.77	84.54
ENSDARG00000056091	cttnbp2nlb	CTTNBP2 N-terminal like b [Source:ZFIN;Acc:ZDB-GENE-040724-65]	8	27823012	27858465		9.66	6.2	6.59	9.4	8.31	8.81
ENSDARG00000056092	si:dkey-12h9.6	si:dkey-12h9.6 [Source:ZFIN;Acc:ZDB-GENE-041001-145]	20	26026518	26042070		7.39	1.73	3.9	8.3	8.1	8.9
ENSDARG00000056099	gtf2h5	general transcription factor IIH, polypeptide 5 [Source:ZFIN;Acc:ZDB-GENE-041001-144]	20	26045253	26047787		15.43	18.78	15.9	13.87	14.74	14.5
ENSDARG00000056101	kcnd3	potassium voltage-gated channel, Shal-related subfamily, member 3 [Source:ZFIN;Acc:ZDB-GENE-030131-5626]	8	28065803	28232817		2.67	0.67	0.66	5.08	4.82	4.93
ENSDARG00000056102	uxs1	UDP-glucuronate decarboxylase 1 [Source:NCBI gene;Acc:192315]	9	6255682	6370693		11.15	9.78	9.51	13.24	12.31	12.74
ENSDARG00000056105	syt11b	synaptotagmin XIb [Source:ZFIN;Acc:ZDB-GENE-090601-7]	16	28842414	28856112		15.19	4.54	8.09	17.06	17.88	17.44
ENSDARG00000056108	ndufa4	NADH:ubiquinone oxidoreductase subunit A4 [Source:NCBI gene;Acc:406298]	12	9880493	9884326		145.2	206.52	188.76	186.3	196.8	182.82
ENSDARG00000056111	lmod1a	leiomodin 1a (smooth muscle) [Source:ZFIN;Acc:ZDB-GENE-081104-348]	8	28297066	28304534		1.33	1.74	1.41	2.04	2.45	2.26
ENSDARG00000056112	pikfyve	phosphoinositide kinase, FYVE finger containing [Source:ZFIN;Acc:ZDB-GENE-030131-9636]	9	32073606	32106034		7.49	4.06	4.31	9.5	9.36	10.03
ENSDARG00000056119	eef1g	eukaryotic translation elongation factor 1 gamma [Source:NCBI gene;Acc:195822]	14	26719691	26729289		972.82	1164.95	1311.54	815.06	844.61	845.63
ENSDARG00000056121	serac1	serine active site containing 1 [Source:NCBI gene;Acc:568022]	20	26048304	26060154		5.62	4.46	4.73	5.5	5.74	6.25
ENSDARG00000056122	gdi1	GDP dissociation inhibitor 1 [Source:ZFIN;Acc:ZDB-GENE-050522-504]	23	26063149	26077038		59.77	28.46	34.26	56.4	58.65	58.24
ENSDARG00000056125	myct1a	myc target 1a [Source:NCBI gene;Acc:796556]	20	26062519	26066020		3.48	3.56	3.55	3.75	3.96	3.93
ENSDARG00000056127	polr2gl	polymerase (RNA) II (DNA directed) polypeptide G-like [Source:NCBI gene;Acc:324088]	14	26715626	26719053		19.7	21.99	20.61	16.31	16.12	15.69
ENSDARG00000056128	fastkd1	FAST kinase domains 1 [Source:ZFIN;Acc:ZDB-GENE-040426-1833]	9	48232571	48267646		3.16	2.64	2.32	1.72	1.91	1.91
ENSDARG00000056130	neurog1	neurogenin 1 [Source:NCBI gene;Acc:30239]	14	26703331	26704829		2.93	1.21	1.93	3.61	4.04	4.59
ENSDARG00000056133	kitb	KIT proto-oncogene, receptor tyrosine kinase b [Source:ZFIN;Acc:ZDB-GENE-050916-2]	1	19473028	19502378		2.72	1.87	1.95	2.56	2.55	2.94
ENSDARG00000056134	c1qtnf5	C1q and TNF related 5 [Source:ZFIN;Acc:ZDB-GENE-050706-101]	15	23369020	23376541		5.85	9.02	10.84	6.83	6.9	7.27
ENSDARG00000056138	sf3b1	splicing factor 3b, subunit 1 [Source:ZFIN;Acc:ZDB-GENE-040827-3]	9	32161623	32177117		69.07	34.21	40.07	84.33	87.67	88.52
ENSDARG00000056141	coq10b	coenzyme Q10B [Source:NCBI gene;Acc:550442]	9	32178050	32188445		13.94	18.37	17.44	18.05	17.38	16.02
ENSDARG00000056144	zgc:123244	zgc:123244 [Source:NCBI gene;Acc:556933]	3	24220354	24226028		1.89	3.08	2.25	0.35	0.63	0.43
ENSDARG00000056145	si:dkey-159a18.1	si:dkey-159a18.1 [Source:ZFIN;Acc:ZDB-GENE-041210-204]	4	20110921	20118468		1.49	1.17	1.2	0.67	0.63	0.78
ENSDARG00000056150	rbms2b	RNA binding motif, single stranded interacting protein 2b [Source:ZFIN;Acc:ZDB-GENE-040801-51]	23	25708787	25768765		8.56	7.13	8.54	15.39	16.43	15.41
ENSDARG00000056151	tyrp1b	tyrosinase-related protein 1b [Source:ZFIN;Acc:ZDB-GENE-040718-249]	1	19332837	19346048		56.01	64.14	73.28	71.39	68.91	68.38
ENSDARG00000056152	fam3c	family with sequence similarity 3 member C [Source:NCBI gene;Acc:334205]	4	20101992	20108833		8.72	9.12	7.52	8.99	9.05	8.91
ENSDARG00000056154	olfcx3	olfactory receptor C family, x3 [Source:ZFIN;Acc:ZDB-GENE-050419-129]	18	33248996	33254039		0.01	0.02	0.02	0.01	0.02	0
ENSDARG00000056156	npdc1b	neural proliferation, differentiation and control, 1b [Source:ZFIN;Acc:ZDB-GENE-140106-58]	5	28870372	28915130		9.69	3.51	4.71	13.08	13.57	14.26
ENSDARG00000056160	hspd1	heat shock 60 protein 1 [Source:ZFIN;Acc:ZDB-GENE-021206-1]	9	32286605	32300783		80.22	92.52	78.86	59.5	60.06	57.13
ENSDARG00000056162	nr2c2ap	nuclear receptor 2C2-associated protein [Source:NCBI gene;Acc:724051]	22	18156000	18160269		3.71	4.4	4.58	3.81	3.83	3.55
ENSDARG00000056163	slc25a51a	solute carrier family 25 member 51a [Source:ZFIN;Acc:ZDB-GENE-040426-1722]	1	18811679	18816806		3.72	1	2.07	4.28	3.29	3.67
ENSDARG00000056165	si:ch211-215a10.4	si:ch211-215a10.4 [Source:ZFIN;Acc:ZDB-GENE-030131-5518]	19	30450125	30468234		7.75	6.42	5.77	7.57	7.78	6.95
ENSDARG00000056166	jac2	jacalin 2 [Source:ZFIN;Acc:ZDB-GENE-160114-69]	7	8598995	8602864		0.07	0.05	0.02	0.23	0.1	0.12
ENSDARG00000056167	hspe1	heat shock 10 protein 1 [Source:ZFIN;Acc:ZDB-GENE-000906-2]	9	32301017	32305007		48.49	67.37	62.7	34.59	35.47	34.3
ENSDARG00000056175	scrt2	scratch family zinc finger 2 [Source:ZFIN;Acc:ZDB-GENE-030131-4097]	8	28527776	28534245		9.93	2.41	3.57	14.04	12.71	13.25
ENSDARG00000056178	dpt	dermatopontin [Source:ZFIN;Acc:ZDB-GENE-060312-9]	6	29184137	29195642		1.53	1.99	2.41	0.63	0.7	0.69
ENSDARG00000056180	olfck1	olfactory receptor C family, k1 [Source:ZFIN;Acc:ZDB-GENE-050419-130]	18	33284202	33294131		0	0	0.02	0.04	0.01	0
ENSDARG00000056181	ncam1a	neural cell adhesion molecule 1a [Source:ZFIN;Acc:ZDB-GENE-990415-31]	21	23338059	23746947		16.98	5.44	5.24	24.41	23.57	26.35
ENSDARG00000056184	dvl2	dishevelled segment polarity protein 2 [Source:ZFIN;Acc:ZDB-GENE-041118-20]	7	20260172	20295432		4.82	4.97	3.87	8.36	9.36	8.83
ENSDARG00000056186	eif5a2	eukaryotic translation initiation factor 5A2 [Source:ZFIN;Acc:ZDB-GENE-040426-2405]	2	25839193	25845337		46.56	54.41	46.67	64.93	63.69	58.89
ENSDARG00000056191	si:ch211-168k14.2	si:ch211-168k14.2 [Source:ZFIN;Acc:ZDB-GENE-070912-149]	9	31534601	31586956		0.32	0.22	0.24	0.38	0.28	0.54
ENSDARG00000056193	nme7	NME/NM23 family member 7 [Source:ZFIN;Acc:ZDB-GENE-000210-35]	6	29227489	29288155		6.51	5.47	6.25	5.51	5.08	5.22
ENSDARG00000056196	slc2a2	solute carrier family 2 member 2 [Source:ZFIN;Acc:ZDB-GENE-030131-1213]	2	25816843	25837986		11.7	17.48	14.17	6.9	7.79	7.35
ENSDARG00000056200	abcb9	ATP-binding cassette, sub-family B (MDR/TAP), member 9 [Source:ZFIN;Acc:ZDB-GENE-050517-12]	5	28018246	28029558		3.29	3.15	3.21	2.42	2.69	3.14
ENSDARG00000056203	si:ch211-201h21.5	si:ch211-201h21.5 [Source:ZFIN;Acc:ZDB-GENE-030131-9599]	6	29305190	29312330		9.76	18.69	14.34	4.25	5.89	4.81
ENSDARG00000056204	zgc:113436	zgc:113436 [Source:ZFIN;Acc:ZDB-GENE-050220-6]	5	28030954	28052883		2.26	1.56	1.77	1.92	1.86	2.04
ENSDARG00000056206	camk2g2	calcium/calmodulin-dependent protein kinase (CaM kinase) II gamma 2 [Source:ZFIN;Acc:ZDB-GENE-070323-2]	13	22119798	22243311		20.41	8.88	10.5	25.93	26.54	26.81
ENSDARG00000056209	myoz1a	myozenin 1a [Source:ZFIN;Acc:ZDB-GENE-040426-1875]	13	22264914	22278415		48.21	76.09	89.49	59	55.96	56.39
ENSDARG00000056210	hspa1b	heat shock protein family A (Hsp70) member 1B [Source:ZFIN;Acc:ZDB-GENE-060503-867]	8	24968098	24970790		1.06	1.34	1.51	1.76	1.76	2.08
ENSDARG00000056211	ogfod2	2-oxoglutarate and iron-dependent oxygenase domain containing 2 [Source:NCBI gene;Acc:790923]	5	28041967	28047353		2.18	1.65	1.61	1.16	1.41	1.64
ENSDARG00000056213	si:ch73-130a3.4	si:ch73-130a3.4 [Source:ZFIN;Acc:ZDB-GENE-141219-41]	19	29854223	29860334		3.2	2.42	2.45	2.62	2.03	2.48
ENSDARG00000056214	gnrh3	gonadotropin-releasing hormone 3 [Source:ZFIN;Acc:ZDB-GENE-030724-4]	17	20204122	20207376		2.11	2.26	2.87	3.03	3.49	4.05
ENSDARG00000056218	tnika	TRAF2 and NCK interacting kinase a [Source:ZFIN;Acc:ZDB-GENE-030131-3767]	2	25657958	25817227		5.23	1.66	2.05	7.26	7.13	7.63
ENSDARG00000056226	creb3l3a	cAMP responsive element binding protein 3-like 3a [Source:NCBI gene;Acc:553700]	22	20112715	20126230		3.17	4.89	3.06	2.35	2.63	2.34
ENSDARG00000056228	pld1a	phospholipase D1a [Source:ZFIN;Acc:ZDB-GENE-031002-2]	2	25560556	25632090		5.12	6.76	5.43	4.06	4.37	4.34
ENSDARG00000056230	ghsra	growth hormone secretagogue receptor a [Source:ZFIN;Acc:ZDB-GENE-070912-238]	2	25551457	25553632		0.06	0.02	0.13	0.08	0.45	0.13
ENSDARG00000056233	zgc:110591	zgc:110591 [Source:NCBI gene;Acc:402849]	7	19909774	19923249		6.35	8.05	7.43	6.25	6.74	7.29
ENSDARG00000056235	smndc1	survival motor neuron domain containing 1 [Source:NCBI gene;Acc:572679]	17	20237727	20243962		28.66	28.76	27.88	25.8	27.81	30.5
ENSDARG00000056239	tmem45b	transmembrane protein 45B [Source:NCBI gene;Acc:402977]	21	25236297	25247052		22.07	23.53	21.25	13.41	12.41	11.57
ENSDARG00000056244	zgc:171740	zgc:171740 [Source:ZFIN;Acc:ZDB-GENE-071004-29]	21	25054420	25062590		12.08	4.34	5.96	10.12	9.29	8.59
ENSDARG00000056245	zgc:162952	zgc:162952 [Source:ZFIN;Acc:ZDB-GENE-070424-98]	5	28526569	28553754		1.61	1.15	1.27	2.42	2.51	2.17
ENSDARG00000056247	bricd5	BRICHOS domain containing 5 [Source:ZFIN;Acc:ZDB-GENE-050208-85]	12	18551597	18560500		5.05	5.79	7.9	2.79	3.28	2.93
ENSDARG00000056248	si:dkey-183i3.5	si:dkey-183i3.5 [Source:ZFIN;Acc:ZDB-GENE-030131-8568]	21	25187210	25197103		954.48	1185.62	1167.95	80.23	49.95	56.91
ENSDARG00000056250	add3b	adducin 3 (gamma) b [Source:ZFIN;Acc:ZDB-GENE-080718-6]	17	20248177	20287588		5.83	3.27	3.99	4.05	4.36	4.17
ENSDARG00000056252	sort1b	sortilin 1b [Source:ZFIN;Acc:ZDB-GENE-030131-7447]	8	25041713	25074319		10.54	5.83	5.05	8.75	8.28	8.31
ENSDARG00000056258	cdc27	cell division cycle 27 [Source:ZFIN;Acc:ZDB-GENE-990706-2]	3	22035863	22057600		8.15	5.84	5.58	6.81	6.71	6.51
ENSDARG00000056259	tmem131	transmembrane protein 131 [Source:ZFIN;Acc:ZDB-GENE-130530-662]	6	29313220	29377092		14.73	9.96	7.04	17.9	17.86	17.79
ENSDARG00000056262	slc35g2a	solute carrier family 35 member G2a [Source:ZFIN;Acc:ZDB-GENE-041114-202]	2	24990398	24996441		6.59	2.64	3.33	9.74	10.68	10.29
ENSDARG00000056267	dixdc1b	DIX domain containing 1b [Source:ZFIN;Acc:ZDB-GENE-041008-148]	21	25068215	25119942		3.03	2.02	2.24	1.89	1.96	2.03
ENSDARG00000056268	atxn7l2b	ataxin 7-like 2b [Source:ZFIN;Acc:ZDB-GENE-040718-403]	8	25091407	25106682		5.06	2.88	3.06	7.16	6.91	6.61
ENSDARG00000056270	atpaf2	ATP synthase mitochondrial F1 complex assembly factor 2 [Source:ZFIN;Acc:ZDB-GENE-050411-18]	12	18410608	18414539		7.17	6.38	6.24	6.27	5.95	6.67
ENSDARG00000056277	or132-5	odorant receptor, family H, subfamily 132, member 5 [Source:ZFIN;Acc:ZDB-GENE-070806-21]	21	25375172	25376738		0.34	0.26	0.35	0.21	0.54	0.26
ENSDARG00000056279	ccdc157	coiled-coil domain containing 157 [Source:ZFIN;Acc:ZDB-GENE-030616-155]	8	36541949	36554732		0.49	0.42	0.56	0.45	0.4	0.49
ENSDARG00000056281	cyb561a3a	cytochrome b561 family, member A3a [Source:NCBI gene;Acc:100002371]	5	27421025	27429216		3.61	4.13	4.19	4.62	4.1	3.78
ENSDARG00000056284	drd7	dopamine receptor D7 [Source:ZFIN;Acc:ZDB-GENE-060526-372]	5	27437481	27438812		0.55	0.26	0.16	0.58	0.41	0.5
ENSDARG00000056292	vsx1	visual system homeobox 1 homolog, chx10-like [Source:NCBI gene;Acc:30598]	17	21063403	21066105		42.83	5.63	5.9	54.58	46.07	60.77
ENSDARG00000056294	SBK1	si:ch211-183d21.3 [Source:ZFIN;Acc:ZDB-GENE-091118-114]	24	37361089	37375262	SBK1	6.94	1.58	2.22	8.98	7.98	9.18
ENSDARG00000056307	znf706	zinc finger protein 706 [Source:ZFIN;Acc:ZDB-GENE-040718-454]	19	12206158	12212796		50.46	54.73	52.91	50.18	52.02	49.78
ENSDARG00000056314	a2ml	alpha-2-macroglobulin-like [Source:ZFIN;Acc:ZDB-GENE-090212-1]	15	21113965	21132480		48.67	66.94	63.13	42.46	44.49	41.75
ENSDARG00000056318	kansl2	KAT8 regulatory NSL complex subunit 2 [Source:ZFIN;Acc:ZDB-GENE-041010-67]	23	24598910	24609494		0.04	0.02	0.03	0.12	0.08	0.06
ENSDARG00000056322	ldb3a	LIM domain binding 3a [Source:ZFIN;Acc:ZDB-GENE-040121-6]	13	22476742	22552687		12.16	17.89	16.13	18.55	16.49	16.4
ENSDARG00000056324	zgc:123295	zgc:123295 [Source:ZFIN;Acc:ZDB-GENE-051127-11]	3	21189766	21198744		2.28	2.02	2.43	1.96	1.74	2.03
ENSDARG00000056328	rhot2	ras homolog family member T2 [Source:NCBI gene;Acc:557574]	24	37533728	37559496		6.18	5.67	5.9	6.85	7.77	6.99
ENSDARG00000056330	mhc2dbb	major histocompatibility complex class II DBB gene [Source:ZFIN;Acc:ZDB-GENE-010112-2]	18	29922939	29925717		0.66	0.64	0.52	0.27	0.46	0.68
ENSDARG00000056331	ahcyl1	adenosylhomocysteinase-like 1 [Source:ZFIN;Acc:ZDB-GENE-021206-7]	8	24971890	25011157		30.13	21.85	19.12	24.16	25.59	23.78
ENSDARG00000056334	mlh3	mutL homolog 3 (E. coli) [Source:ZFIN;Acc:ZDB-GENE-060810-45]	23	24589633	24598877		1.55	1.25	0.95	1.19	1.38	1.39
ENSDARG00000056338	szrd1	SUZ RNA binding domain containing 1 [Source:NCBI gene;Acc:554231]	23	24501918	24508821		18.18	16.72	15.85	15.92	16.24	15.74
ENSDARG00000056339	stk31	serine/threonine kinase 31 [Source:ZFIN;Acc:ZDB-GENE-140106-59]	16	21491891	21540077		0.1	0.09	0.11	0.23	0.15	0.21
ENSDARG00000056346	acap1	ArfGAP with coiled-coil, ankyrin repeat and PH domains 1 [Source:ZFIN;Acc:ZDB-GENE-070112-1242]	7	20058857	20158380		2.16	1.91	1.93	2.39	2.32	2.28
ENSDARG00000056347	rab3aa	RAB3A, member RAS oncogene family, a [Source:ZFIN;Acc:ZDB-GENE-040801-2]	2	24867534	24878813		12.43	5.45	5.23	12.86	12.79	13.18
ENSDARG00000056367	mpv17l2	MPV17 mitochondrial membrane protein-like 2 [Source:NCBI gene;Acc:436840]	2	24770435	24778912		5.72	7.05	6.32	5.67	5.01	5.42
ENSDARG00000056369	ufsp2	ufm1-specific peptidase 2 [Source:NCBI gene;Acc:336782]	1	17699471	17711676		13.79	16.39	16.54	10.02	9.88	11.4
ENSDARG00000056374	noxo1b	NADPH oxidase organizer 1b [Source:ZFIN;Acc:ZDB-GENE-101203-1]	12	18445604	18454422		0.5	0.28	0.34	1.31	1.66	1.42
ENSDARG00000056376	ankrd37	ankyrin repeat domain 37 [Source:ZFIN;Acc:ZDB-GENE-070308-1]	1	17695426	17698773		3.58	2.22	1.48	31.01	35.14	31.63
ENSDARG00000056378	ifi30	IFI30 lysosomal thiol reductase [Source:NCBI gene;Acc:336503]	2	24762567	24767470		31.5	41.77	42.63	21.66	21.9	22.48
ENSDARG00000056379	si:ch73-86n18.1	si:ch73-86n18.1 [Source:ZFIN;Acc:ZDB-GENE-141216-19]	16	21779920	21785261		8.59	13.47	13.68	30.2	30.95	30.8
ENSDARG00000056381	cfap97	cilia and flagella associated protein 97 [Source:ZFIN;Acc:ZDB-GENE-030131-9405]	1	17681044	17693273		3.48	3.03	3.06	5.87	6.29	5.65
ENSDARG00000056386	TMC1	transmembrane channel-like 1 [Source:NCBI gene;Acc:567237]	5	25652727	25668787	TMC1	0.55	0.54	0.57	0.63	0.54	0.5
ENSDARG00000056387	rnf17	ring finger protein 17 [Source:ZFIN;Acc:ZDB-GENE-030131-6451]	9	29548195	29571228		0.35	0.4	0.32	0.43	0.42	0.47
ENSDARG00000056394	TOM1L2	target of myb1 like 2 membrane trafficking protein [Source:NCBI gene;Acc:100535615]	3	62091298	62125701	TOM1L2	5.05	3.15	2.61	5.67	5.9	5.64
ENSDARG00000056395	onecut3a	one cut homeobox 3a [Source:ZFIN;Acc:ZDB-GENE-050208-744]	22	20461488	20473567		4.92	3.88	4.63	6.9	5.59	7.09
ENSDARG00000056396	dusp22a	dual specificity phosphatase 22a [Source:NCBI gene;Acc:436787]	18	30511230	30564688		5.55	6.61	6.71	7.72	7.32	7.96
ENSDARG00000056400	helt	helt bHLH transcription factor [Source:NCBI gene;Acc:404275]	1	17593392	17595045		2.64	1.6	2.68	2.42	2.36	3.44
ENSDARG00000056407	irf8	interferon regulatory factor 8 [Source:ZFIN;Acc:ZDB-GENE-040718-367]	18	30567945	30573849		1.34	1.69	1.61	2.54	2.35	2.26
ENSDARG00000056410	fdx1	ferredoxin 1 [Source:ZFIN;Acc:ZDB-GENE-071015-2]	9	29520696	29535453		23.04	31.83	28.42	17.47	17.87	17.18
ENSDARG00000056414	usp1	ubiquitin specific peptidase 1 [Source:ZFIN;Acc:ZDB-GENE-030131-726]	6	32394502	32411727		9.99	8	7.87	7.28	7.6	6.99
ENSDARG00000056427	auts2a	activator of transcription and developmental regulator AUTS2 a [Source:ZFIN;Acc:ZDB-GENE-030616-571]	10	27182704	27741793		6.39	3.06	3.12	13.95	13.2	14.01
ENSDARG00000056438	her9	hairy-related 9 [Source:ZFIN;Acc:ZDB-GENE-011213-1]	23	23399520	23401305		23.71	20.06	22.43	21	22.08	19.36
ENSDARG00000056439	4-Mar	membrane-associated ring finger (C3HC4) 4 [Source:ZFIN;Acc:ZDB-GENE-041210-304]	9	52492639	52539704		0.77	0.24	0.28	0.66	0.92	0.84

ENSDARG00000056443	zgc:152753	zgc:152753 [Source:ZFIN;Acc:ZDB-GENE-060818-8]	3	1931815	1938665		0.01	0.01	0	0.02	0.01	0
ENSDARG00000056450	MANEAL	si:ch211-30b16.2 [Source:NCBI gene;Acc:566679]	19	17259912	17277208	MANEAL	6.18	2.53	3.77	6.66	6.51	6.86
ENSDARG00000056453	afap1l1b	actin filament associated protein 1-like 1b [Source:ZFIN;Acc:ZDB-GENE-061013-428]	21	30418034	30467905		1.88	2.15	2.02	2.32	1.9	2.29
ENSDARG00000056458	lhfpl5b	LHFPL tetraspan subfamily member 5b [Source:ZFIN;Acc:ZDB-GENE-080220-51]	8	23876928	23885016		0.17	0.26	0.23	0.11	0.07	0.16
ENSDARG00000056462	crp2	C-reactive protein 2 [Source:ZFIN;Acc:ZDB-GENE-030131-5826]	24	38082471	38085789		0.81	1.07	0.5	0.93	0.33	0.95
ENSDARG00000056464	fitm1l	fat storage-inducing transmembrane protein 1 [Source:NCBI gene;Acc:503747]	2	24304854	24308682		10.7	16.66	17.49	9.21	8.56	7.74
ENSDARG00000056467	tmub2	transmembrane and ubiquitin-like domain containing 2 [Source:ZFIN;Acc:ZDB-GENE-030616-254]	3	20012891	20021596		15.45	11.7	13.32	15.07	16.18	17.28
ENSDARG00000056468	asb16	ankyrin repeat and SOCS box containing 16 [Source:ZFIN;Acc:ZDB-GENE-031031-3]	3	20001608	20011807		2.28	2.89	3.11	1.97	1.27	1.57
ENSDARG00000056473	chaf1b	chromatin assembly factor 1, subunit B [Source:ZFIN;Acc:ZDB-GENE-040426-1947]	9	30543139	30555725		6.47	6.19	4.96	4.23	3.87	4.07
ENSDARG00000056475	trnau1apb	tRNA selenocysteine 1 associated protein 1b [Source:NCBI gene;Acc:791478]	2	24310635	24317436		24.24	12.81	15.24	27.11	25.54	26.01
ENSDARG00000056477	ccdc125	coiled-coil domain containing 125 [Source:NCBI gene;Acc:565477]	5	26171564	26187097		5.91	4.88	5.19	3.44	4.66	3.92
ENSDARG00000056478	ACAP2	si:ch211-168b3.1 [Source:ZFIN;Acc:ZDB-GENE-081104-34]	2	5887626	5924301	ACAP2	6.49	1.9	3.14	8.32	8.18	8.53
ENSDARG00000056479	rab1bb	RAB1B, member RAS oncogene family b [Source:ZFIN;Acc:ZDB-GENE-040625-133]	10	26926654	26933708		18.09	13.76	15.33	16.29	15.1	15.89
ENSDARG00000056480	cpvl	carboxypeptidase vitellogenic like [Source:ZFIN;Acc:ZDB-GENE-031219-2]	16	20496691	20515806		10.93	11	11.17	8.29	8.56	9.18
ENSDARG00000056481	vat1	vesicle amine transport 1 [Source:NCBI gene;Acc:368752]	3	19914332	19971774		39.91	28.26	19.71	58.29	57.43	61.22
ENSDARG00000056483	ssbp4	single stranded DNA binding protein 4 [Source:ZFIN;Acc:ZDB-GENE-030131-469]	22	20951211	21046843		11.39	6.09	5.92	20.08	20.14	20.44
ENSDARG00000056490	SSBP4	zgc:110158 [Source:ZFIN;Acc:ZDB-GENE-050522-195]	2	41577507	41723487	SSBP4	9.64	4	3.67	22.62	21.92	22.67
ENSDARG00000056491	ikzf5	IKAROS family zinc finger 5 [Source:NCBI gene;Acc:402893]	17	21760032	21822840		3.86	1.73	1.87	4.85	4	4.82
ENSDARG00000056498	crp2	C-reactive protein 2 [Source:ZFIN;Acc:ZDB-GENE-030131-5826]	24	38109799	38113208		46.58	76.15	62.71	16.93	7.75	14.94
ENSDARG00000056499	ca6	carbonic anhydrase VI [Source:ZFIN;Acc:ZDB-GENE-030131-7091]	23	22634442	22650992		43.24	49.5	41.06	48.03	49.15	45.73
ENSDARG00000056501	si:ch211-208g24.8	si:ch211-208g24.8 [Source:ZFIN;Acc:ZDB-GENE-131127-183]	17	21826825	21832190		0	0.01	0	0	0	0
ENSDARG00000056502	si:ch73-334d15.4	si:ch73-334d15.4 [Source:ZFIN;Acc:ZDB-GENE-091204-184]	24	36636208	36638807		13.49	15.95	16.43	14.55	13.34	14.05
ENSDARG00000056504	frg1	FSHD region gene 1 [Source:ZFIN;Acc:ZDB-GENE-050417-324]	1	16673841	16689023		6.43	6.17	6.28	5.01	5.45	5.29
ENSDARG00000056506	si:ch73-113g13.2	si:ch73-113g13.2 [Source:ZFIN;Acc:ZDB-GENE-091204-438]	18	22712638	22713621		0	0.02	0.08	0	0.11	0.03
ENSDARG00000056509	pdik1l	PDLIM1 interacting kinase 1 like [Source:NCBI gene;Acc:569665]	19	15485287	15518874		1.48	1.66	1.47	1.64	1.68	1.5
ENSDARG00000056510	gstk1	glutathione S-transferase kappa 1 [Source:ZFIN;Acc:ZDB-GENE-040718-298]	16	32009203	32013929		21.89	31.68	29.35	14.85	15.56	15.82
ENSDARG00000056511	arr3a	arrestin 3a, retinal (X-arrestin) [Source:ZFIN;Acc:ZDB-GENE-040718-102]	10	26800213	26811397		163.58	45.15	36.83	114.42	123.45	120.46
ENSDARG00000056515	spsb1	splA/ryanodine receptor domain and SOCS box containing 1 [Source:ZFIN;Acc:ZDB-GENE-030131-6122]	23	22496314	22523303		4.46	3.34	3.38	5.49	5.77	5.43
ENSDARG00000056517	thoc3	THO complex 3 [Source:ZFIN;Acc:ZDB-GENE-040808-14]	14	25505468	25510955		14.01	12.43	12.98	11.2	10.7	9.91
ENSDARG00000056519	si:dkey-280e21.3	si:dkey-280e21.3 [Source:ZFIN;Acc:ZDB-GENE-140106-61]	14	25464681	25502537		3.28	1.17	1.41	3.5	3.44	3.25
ENSDARG00000056525	bcar1	BCAR1 scaffold protein, Cas family member [Source:ZFIN;Acc:ZDB-GENE-030131-2774]	18	22504200	22688073		7.01	6.45	4.92	12.44	11.63	12.36
ENSDARG00000056530	cpamd8	C3 and PZP like alpha-2-macroglobulin domain containing 8 [Source:ZFIN;Acc:ZDB-GENE-050208-428]	22	21252790	21304247		5.74	5.52	4.05	7.43	6.13	5.9
ENSDARG00000056531	nutf2	nuclear transport factor 2 [Source:ZFIN;Acc:ZDB-GENE-041010-77]	18	21913685	21929451		36.69	42.51	43.57	41.36	36.36	37.15
ENSDARG00000056532	serinc2	serine incorporator 2 [Source:ZFIN;Acc:ZDB-GENE-041111-296]	19	15408363	15420718		18.05	18.83	15.89	15.85	13.39	13.95
ENSDARG00000056535	pla2g4f.1	phospholipase A2, group IVF, tandem duplicate 1 [Source:ZFIN;Acc:ZDB-GENE-131121-408]	17	21452258	21467430		3.66	3.1	2.63	1.6	1.39	1.61
ENSDARG00000056541	nr0b1	nuclear receptor subfamily 0, group B, member 1 [Source:ZFIN;Acc:ZDB-GENE-070130-1]	11	29682909	29685853		2.32	2.05	1.86	2.29	1.86	2.77
ENSDARG00000056549	vps26a	VPS26, retromer complex component A [Source:NCBI gene;Acc:393881]	13	23058268	23067381		17.97	15.54	15.27	17.94	17.29	17.4
ENSDARG00000056550	tango2	transport and golgi organization 2 homolog (Drosophila) [Source:ZFIN;Acc:ZDB-GENE-040808-42]	5	26686279	26726220		2.65	3.15	2.39	2.87	3.08	2.81
ENSDARG00000056551	slc22a13a	solute carrier family 22 member 13a [Source:ZFIN;Acc:ZDB-GENE-070912-473]	2	23731194	23745290		0.05	0.04	0.04	0.04	0.03	0.03
ENSDARG00000056554	rap1gap	RAP1 GTPase activating protein [Source:ZFIN;Acc:ZDB-GENE-080402-9]	23	22267374	22425257		5.74	2.32	2.44	5.23	5.37	5.39
ENSDARG00000056557	copb1	coatomer protein complex, subunit beta 1 [Source:NCBI gene;Acc:338138]	7	27802480	27820976		36.34	33.82	32.84	34.57	36.68	34.46
ENSDARG00000056559	ccz1	CCZ1 homolog, vacuolar protein trafficking and biogenesis associated [Source:NCBI gene;Acc:393103]	12	17657224	17686404		11.72	12.28	11.35	11.58	11.26	11.86
ENSDARG00000056561	asb11	ankyrin repeat and SOCS box containing 11 [Source:ZFIN;Acc:ZDB-GENE-070112-532]	9	30364667	30371247		0.08	0.29	0.37	0.18	0.27	0.22
ENSDARG00000056566	necab1	N-terminal EF-hand calcium binding protein 1 [Source:ZFIN;Acc:ZDB-GENE-050417-395]	19	31437689	31522677		8.74	4.09	5.87	6.17	6.7	5.84
ENSDARG00000056572	nrsn1l	neurensin 1-like [Source:ZFIN;Acc:ZDB-GENE-060503-68]	19	28356753	28367413		10.52	3.96	5.89	8.87	10.52	9.06
ENSDARG00000056582	arf2b	ADP-ribosylation factor 2b [Source:ZFIN;Acc:ZDB-GENE-030131-5234]	24	36373588	36400458		1	0.93	0.86	1.25	1.13	1.13
ENSDARG00000056583	ndufs6	NADH:ubiquinone oxidoreductase subunit S6 [Source:ZFIN;Acc:ZDB-GENE-040912-86]	19	28281201	28283877		73.1	91.24	82.55	43.05	48.46	51.27
ENSDARG00000056587	cyp2r1	cytochrome P450, family 2, subfamily R, polypeptide 1 [Source:ZFIN;Acc:ZDB-GENE-110114-1]	7	27455321	27697234		1.66	2.68	2.32	0.88	0.84	0.8
ENSDARG00000056590	calca	calcitonin/calcitonin-related polypeptide, alpha [Source:ZFIN;Acc:ZDB-GENE-040718-173]	7	27680556	27696958		4.79	4.73	4.23	8.17	9.06	8.8
ENSDARG00000056591	gucy1b2	guanylate cyclase 1, soluble, beta 2 [Source:ZFIN;Acc:ZDB-GENE-130530-664]	11	29739348	29755142		0.14	0.07	0.08	0.08	0.07	0.11
ENSDARG00000056594	irx1b	iroquois homeobox 1b [Source:ZFIN;Acc:ZDB-GENE-010716-1]	19	28127654	28130658		5.4	3.04	3.41	7.45	6.89	7.26
ENSDARG00000056598	pimr47	Pim proto-oncogene, serine/threonine kinase, related 47 [Source:ZFIN;Acc:ZDB-GENE-090312-19]	13	13660564	13662624		0.02	0	0.01	0.05	0	0
ENSDARG00000056600	papss2b	3'-phosphoadenosine 5'-phosphosulfate synthase 2b [Source:ZFIN;Acc:ZDB-GENE-010323-5]	12	17458949	17479078		16.04	15.04	13.65	20.08	20.73	20.41
ENSDARG00000056601	plekha3	pleckstrin homology domain containing, family A (phosphoinositide binding specific) member 3 [Source:ZFIN;Acc:ZDB-GENE-04042	11	29756860	29768054		5.52	5.03	5.04	6.3	7.15	6.77
ENSDARG00000056603	mcf2b	MCF.2 cell line derived transforming sequence b [Source:ZFIN;Acc:ZDB-GENE-091118-4]	10	26771047	26793448		0.8	0.26	0.3	0.9	0.76	0.85
ENSDARG00000056605	wbp2	WW domain binding protein 2 [Source:ZFIN;Acc:ZDB-GENE-050522-137]	12	45200744	45218478		7.74	2.34	2.2	7.74	7.67	7.81
ENSDARG00000056609	atad1b	ATPase family AAA domain containing 1b [Source:NCBI gene;Acc:368412]	12	17436904	17456083		15.57	14.03	12.87	11.9	12.22	11.74
ENSDARG00000056615	cybb	cytochrome b-245, beta polypeptide (chronic granulomatous disease) [Source:ZFIN;Acc:ZDB-GENE-040426-1380]	11	29795155	29818866		1.17	1.12	1.12	2.15	2.45	2.34
ENSDARG00000056616	pimr48	Pim proto-oncogene, serine/threonine kinase, related 48 [Source:ZFIN;Acc:ZDB-GENE-090312-25]	13	13668991	13671097		0.03	0	0	0	0	0
ENSDARG00000056617	rpgra	retinitis pigmentosa GTPase regulator a [Source:ZFIN;Acc:ZDB-GENE-070912-423]	9	30274637	30288446		1.79	0.37	0.22	0.8	0.81	0.81
ENSDARG00000056618	xk	X-linked Kx blood group (McLeod syndrome) [Source:ZFIN;Acc:ZDB-GENE-080908-1]	11	29818389	29833698		2.78	0.69	1.98	3.61	3.9	3.65
ENSDARG00000056619	arhgap42a	Rho GTPase activating protein 42a [Source:NCBI gene;Acc:566275]	21	22738939	22796064		4.08	2.96	2.89	6.96	7.53	6.98
ENSDARG00000056620	grpr	gastrin-releasing peptide receptor [Source:ZFIN;Acc:ZDB-GENE-081015-4]	11	29856032	29877090		0.96	0.37	0.51	1.13	0.81	0.73
ENSDARG00000056621	ctcf	CCCTC-binding factor (zinc finger protein) [Source:ZFIN;Acc:ZDB-GENE-040624-5]	18	22285992	22295064		18.86	12.01	11.07	19.54	18.75	18.93
ENSDARG00000056623	ptenb	phosphatase and tensin homolog B [Source:ZFIN;Acc:ZDB-GENE-030616-47]	12	17399852	17424174		20.94	12.16	9.89	24.29	24.74	25.28
ENSDARG00000056624	vegfd	vascular endothelial growth factor D [Source:ZFIN;Acc:ZDB-GENE-070810-6]	11	29889828	29916607		0.99	1.01	0.92	1.68	1.28	1.25
ENSDARG00000056625	trpc6a	transient receptor potential cation channel, subfamily C, member 6a [Source:ZFIN;Acc:ZDB-GENE-040724-114]	21	22801257	22812449		0.68	1.19	0.71	2.18	2.5	2.67
ENSDARG00000056627	cxcl14	chemokine (C-X-C motif) ligand 14 [Source:ZFIN;Acc:ZDB-GENE-000619-1]	14	24400799	24410673		25.06	28.53	24.28	26.3	26.89	25.92
ENSDARG00000056628	tmem170a	transmembrane protein 170A [Source:NCBI gene;Acc:393662]	18	22405707	22414565		4.36	3.07	2.95	3.97	3.77	4.07
ENSDARG00000056630	angptl5	angiopoietin-like 5 [Source:ZFIN;Acc:ZDB-GENE-030131-5054]	21	22820970	22831388		3.86	4.73	4.94	4.67	5.79	5.42
ENSDARG00000056633	fgf13b	fibroblast growth factor 13b [Source:ZFIN;Acc:ZDB-GENE-040426-1793]	10	26716362	26744166		5.55	2.45	3.53	8.07	8.86	8.53
ENSDARG00000056638	pir	pirin [Source:ZFIN;Acc:ZDB-GENE-040718-288]	11	29917613	29936755		6.39	7.28	7.04	4.53	4.97	4.91
ENSDARG00000056639	carmil2	capping protein regulator and myosin 1 linker 2 [Source:ZFIN;Acc:ZDB-GENE-050419-197]	18	22302635	22396371		4.4	1.96	1.78	8.05	8.06	8.78
ENSDARG00000056640	rpia	ribose 5-phosphate isomerase A (ribose 5-phosphate epimerase) [Source:ZFIN;Acc:ZDB-GENE-041114-24]	13	13930263	13944288		15.39	17.87	17.85	17.56	17.64	16.88
ENSDARG00000056642	hdac9b	histone deacetylase 9b [Source:NCBI gene;Acc:393789]	16	19840146	20006270		5.08	1.46	1.49	4.72	4.8	4.9
ENSDARG00000056643	slc22a7b.1	solute carrier family 22 member 7b, tandem duplicate 1 [Source:ZFIN;Acc:ZDB-GENE-040426-1311]	17	22002857	22010668		2.19	3.83	2.87	1.18	1.63	1.84
ENSDARG00000056647	klhl3	kelch-like family member 3 [Source:NCBI gene;Acc:568556]	14	24283915	24305086		4.61	1.52	2.26	5.41	5.4	5.64
ENSDARG00000056648	ext2	exostosin glycosyltransferase 2 [Source:ZFIN;Acc:ZDB-GENE-041124-3]	7	26844261	26892613		12.13	9.37	9.71	14.53	14.9	15.08
ENSDARG00000056649	htatsf1	HIV-1 Tat specific factor 1 [Source:ZFIN;Acc:ZDB-GENE-040426-1578]	10	26652859	26667243		26.76	23.16	25.1	18.29	18.26	19.7
ENSDARG00000056650	zgc:113276	zgc:113276 [Source:ZFIN;Acc:ZDB-GENE-050522-7]	11	29936743	29946927		3.98	5.91	5.6	2.26	2.27	2.19
ENSDARG00000056651	gfra4a	GDNF family receptor alpha 4a [Source:ZFIN;Acc:ZDB-GENE-080402-10]	13	14191293	14487524		2.63	0.72	1.02	2.86	2.88	2.89
ENSDARG00000056652	zmp:0000001102	zmp:0000001102 [Source:ZFIN;Acc:ZDB-GENE-140106-62]	17	22014345	22021213		0.69	1.37	1.11	0.2	0.25	0.19
ENSDARG00000056653	fhl1b	four and a half LIM domains 1b [Source:ZFIN;Acc:ZDB-GENE-031219-1]	10	26597990	26621189		4.28	5.83	5.93	7.6	7.59	7.79
ENSDARG00000056654	gna15.3	guanine nucleotide binding protein (G protein), alpha 15 (Gq class), tandem duplicate 3 [Source:ZFIN;Acc:ZDB-GENE-050208-700]	22	22004082	22014961		0.13	0.11	0.15	0.52	0.55	0.32
ENSDARG00000056656	tspan18a	tetraspanin 18a [Source:ZFIN;Acc:ZDB-GENE-040718-232]	7	26762717	26837282		9.95	7.48	7.84	10.93	12.76	11.95
ENSDARG00000056659	ttbk1b	tau tubulin kinase 1b [Source:ZFIN;Acc:ZDB-GENE-100609-5]	17	22032296	22067451		6.28	1.95	2.31	6.05	6.38	5.98
ENSDARG00000056664	arfip2b	ADP-ribosylation factor interacting protein 2b [Source:ZFIN;Acc:ZDB-GENE-050417-131]	10	26205779	26225678		0.04	0	0.04	0.03	0.04	0.05
ENSDARG00000056665	nsun2	NOP2/Sun RNA methyltransferase 2 [Source:ZFIN;Acc:ZDB-GENE-030131-4017]	19	27858866	27902509		7.18	7.38	5.36	5.33	6.16	5.66
ENSDARG00000056666	sp8b	sp8 transcription factor b [Source:NCBI gene;Acc:324931]	16	19536614	19538776		4.15	3.39	2.78	6.34	6.53	6.87
ENSDARG00000056672	abcb8	ATP-binding cassette, sub-family B (MDR/TAP), member 8 [Source:NCBI gene;Acc:548340]	24	33781485	33801668		9.4	8.54	7.89	6.53	6.89	6.77
ENSDARG00000056677	BX530018.2	OCIA domain-containing protein 1-like [Source:NCBI gene;Acc:553528]	1	14417785	14426804		2.74	3.38	2.54	1.71	1.87	1.54
ENSDARG00000056678	trim47	tripartite motif containing 47 [Source:ZFIN;Acc:ZDB-GENE-040426-1000]	10	26118122	26128179		8.17	5.72	5.64	6.65	6.97	6.5
ENSDARG00000056679	morc3a	MORC family CW-type zinc finger 3a [Source:ZFIN;Acc:ZDB-GENE-030131-1502]	9	30558508	30576522		4.95	3.36	3.72	4.02	4.23	4.05
ENSDARG00000056680	stc2a	stanniocalcin 2a [Source:ZFIN;Acc:ZDB-GENE-041008-14]	14	24215046	24221965		6.98	5.47	6.52	19.82	20.88	20.52
ENSDARG00000056683	cdk5	cyclin-dependent kinase 5 [Source:ZFIN;Acc:ZDB-GENE-010131-2]	24	33767771	33780387		6.77	3.39	3.96	8.76	9.31	8.4
ENSDARG00000056690	mtmr1a	myotubularin related protein 1a [Source:ZFIN;Acc:ZDB-GENE-050522-518]	7	25858380	25878560		10.91	2.32	3.63	11.62	12.15	12.51
ENSDARG00000056691	cpeb4a	cytoplasmic polyadenylation element binding protein 4a [Source:NCBI gene;Acc:393956]	14	24096588	24111292		7.91	3.39	3.52	8.68	7.69	7.92
ENSDARG00000056695	phc2a	polyhomeotic homolog 2a (Drosophila) [Source:NCBI gene;Acc:171465]	23	22063484	22130778		8.9	7	5.96	10.63	10.37	10.61
ENSDARG00000056704	RAMP1	zgc:91818 [Source:ZFIN;Acc:ZDB-GENE-040718-446]	9	28778649	28867908	RAMP1	3.3	2.75	3.09	3.99	3.7	3.81
ENSDARG00000056713	pskh1	protein serine kinase H1 [Source:NCBI gene;Acc:554840]	18	21728194	21748966		1.41	1.43	1.11	2.05	2.1	2.27
ENSDARG00000056717	vars2	valyl-tRNA synthetase 2, mitochondrial [Source:ZFIN;Acc:ZDB-GENE-060503-575]	19	27399168	27448606		4.68	3.83	3.99	3.41	4.04	3.39
ENSDARG00000056719	slc6a19b	solute carrier family 6 member 19b [Source:ZFIN;Acc:ZDB-GENE-030131-4755]	16	18974064	18998549		5.33	7.51	4.58	2.82	2.84	2.72
ENSDARG00000056720	utp3	UTP3 small subunit processome component [Source:ZFIN;Acc:ZDB-GENE-040912-163]	11	26370381	26375575		11.36	14.29	10.55	8.53	7.71	7.57
ENSDARG00000056722	cd99l2	CD99 molecule-like 2 [Source:NCBI gene;Acc:323266]	7	25878553	25895472		44.77	20.85	24.99	46.72	42.71	44.66
ENSDARG00000056723	fhad1	forkhead-associated (FHA) phosphopeptide binding domain 1 [Source:ZFIN;Acc:ZDB-GENE-041210-354]	8	21383347	21393457		1.52	1.65	1.85	1.06	0.82	0.91
ENSDARG00000056725	hmgb3a	high mobility group box 3a [Source:ZFIN;Acc:ZDB-GENE-050428-1]	7	25913225	25918281		139.76	66.6	87.13	156.96	154.41	161.47
ENSDARG00000056726	mettl7a	methyltransferase like 7A [Source:ZFIN;Acc:ZDB-GENE-061027-239]	6	39493864	39497974		4.9	4.01	3.1	2.88	3.08	3.28
ENSDARG00000056729	her4.2	hairy-related 4, tandem duplicate 2 [Source:ZFIN;Acc:ZDB-GENE-060815-1]	23	21469203	21471022		2.54	1.72	2.09	1.53	1.5	1
ENSDARG00000056732	her4.1	hairy-related 4, tandem duplicate 1 [Source:ZFIN;Acc:ZDB-GENE-980526-521]	23	21452557	21453614		5.1	4.16	3.37	4.73	3.93	4.37
ENSDARG00000056739	tas1r1	taste receptor, type 1, member 1 [Source:ZFIN;Acc:ZDB-GENE-060208-2]	23	21424747	21440054		0.26	0.07	0.17	0.36	0.26	0.27
ENSDARG00000056740	tmeff1b	transmembrane protein with EGF-like and two follistatin-like domains 1b [Source:ZFIN;Acc:ZDB-GENE-050706-143]	24	33708804	33756003		42.88	18.66	25.66	54.29	55.96	53.98
ENSDARG00000056741	senp3b	SUMO specific peptidase 3b [Source:ZFIN;Acc:ZDB-GENE-030131-6714]	7	26518843	26532089		11.11	8.97	10.15	8.56	9.83	9.41
ENSDARG00000056742	crmp1	collapsin response mediator protein 1 [Source:ZFIN;Acc:ZDB-GENE-050720-1]	1	14348399	14364710		24.06	5.09	6.6	25.25	24.1	24.56
ENSDARG00000056743	cavin4b	caveolae associated protein 4b [Source:ZFIN;Acc:ZDB-GENE-041212-87]	24	33697130	33703504		24.96	34.16	31.81	26.58	25.05	25.82
ENSDARG00000056744	ela2	elastase 2 [Source:ZFIN;Acc:ZDB-GENE-041117-1]	8	21376290	21381734		606.15	897.73	726.89	11.22	8.61	11.34
ENSDARG00000056745	necab2	N-terminal EF-hand calcium binding protein 2 [Source:ZFIN;Acc:ZDB-GENE-050706-167]	18	21408794	21620816		24	7.05	10.24	27.62	28.78	28.01
ENSDARG00000056750	si:ch211-254p10.2	si:ch211-254p10.2 [Source:ZFIN;Acc:ZDB-GENE-100922-17]	16	19013018	19023116		0.07	0.04	0.06	0.36	0.38	0.32
ENSDARG00000056753	dctn1b	dynactin 1b [Source:ZFIN;Acc:ZDB-GENE-050419-28]	13	11581942	11667661		9.02	2.18	2.63	13.17	14.5	15.27
ENSDARG00000056757	slc39a8	solute carrier family 39 member 8 [Source:ZFIN;Acc:ZDB-GENE-050419-124]	13	11690277	11699530		1.55	2.02	1.79	1.35	1.51	1.46
ENSDARG00000056762	zgc:172079	zgc:172079 [Source:ZFIN;Acc:ZDB-GENE-080204-103]	7	26446020	26457812		0.59	0.22	0.34	0.54	0.65	0.46
ENSDARG00000056764	hydin	HYDIN axonemal central pair apparatus protein [Source:ZFIN;Acc:ZDB-GENE-050419-219]	18	21273749	21401401		0.69	0.28	0.27	0.64	0.67	0.69
ENSDARG00000056765	ela2l	elastase 2 like [Source:ZFIN;Acc:ZDB-GENE-040511-1]	8	21366771	21372485		387.37	592.18	547.4	13.79	11.16	14.31
ENSDARG00000056767	itgb3a	integrin beta 3a [Source:ZFIN;Acc:ZDB-GENE-071207-1]	3	19621034	19649535		13	12.37	7.93	10.75	7.66	6.98
ENSDARG00000056768	rprml	reprimo-like [Source:NCBI gene;Acc:100073335]	3	19609638	19610990		4.38	1.82	2.94	3.84	3.58	4.06
ENSDARG00000056773	zgc:163079	zgc:163079 [Source:ZFIN;Acc:ZDB-GENE-030131-7428]	3	19557604	19561058		0	0.04	0.01	0	0.02	0
ENSDARG00000056774	gpr37l1b	G protein-coupled receptor 37 like 1b [Source:ZFIN;Acc:ZDB-GENE-081104-324]	8	21265263	21268303		11.91	3.34	6.11	13.37	12.45	13.54
ENSDARG00000056780	ssh1a	slingshot protein phosphatase 1a [Source:ZFIN;Acc:ZDB-GENE-040724-201]	5	20255568	20289292		2.13	1.05	1.22	2.31	2.56	2.12
ENSDARG00000056781	zanl	zonadhesin, like [Source:ZFIN;Acc:ZDB-GENE-060131-2]	7	26326462	26365420		7.55	11.12	4.9	2.52	2.52	2.62
ENSDARG00000056783	raraa	retinoic acid receptor, alpha a [Source:NCBI gene;Acc:30680]	12	10952794	11157237		7.9	6.04	5.71	10.49	9.91	10.42
ENSDARG00000056784	aire	autoimmune regulator [Source:ZFIN;Acc:ZDB-GENE-071008-4]	2	22808946	22846553		0.09	0.12	0.02	0.07	0.11	0.07
ENSDARG00000056791	pde6ga	phosphodiesterase 6G, cGMP-specific, rod, gamma, paralog a [Source:ZFIN;Acc:ZDB-GENE-050522-144]	3	19460991	19466015		8.39	3.13	2.42	10.57	11.18	11.95
ENSDARG00000056793	jazf1b	JAZF zinc finger 1b [Source:ZFIN;Acc:ZDB-GENE-041212-56]	16	20738740	20800498		8.19	4.58	5.51	9.24	8.37	7.9
ENSDARG00000056794	arl3a	ADP ribosylation factor like GTPase 3a [Source:ZFIN;Acc:ZDB-GENE-050417-368]	13	11963789	11971161		1.86	1.39	1.31	2.95	2.72	3.27
ENSDARG00000056795	serpine1	serpin peptidase inhibitor, clade E (nexin, plasminogen activator inhibitor type 1), member 1 [Source:ZFIN;Acc:ZDB-GENE-070912-6	7	26265584	26270014		3.86	4.4	5.17	19.53	19.14	17.75
ENSDARG00000056797	ppp2r2ca	protein phosphatase 2, regulatory subunit B, gamma a [Source:ZFIN;Acc:ZDB-GENE-051120-168]	1	14283692	14306838		10.8	2.48	4.23	14.11	13.64	14.27
ENSDARG00000056798	zgc:123297	zgc:123297 [Source:ZFIN;Acc:ZDB-GENE-051127-9]	3	19446997	19457773		0.17	0.35	0.16	0.09	0.07	0.1
ENSDARG00000056801	sufu	suppressor of fused homolog (Drosophila) [Source:ZFIN;Acc:ZDB-GENE-030131-6223]	13	11828516	11872143		5.9	5.12	5	7.14	7.18	7.16
ENSDARG00000056803	ap1s1	adaptor related protein complex 1 subunit sigma 1 [Source:ZFIN;Acc:ZDB-GENE-040426-1119]	7	26245061	26263206		21.29	21.94	18.06	21.24	19.69	18.24
ENSDARG00000056804	si:dkey-40g16.6	si:dkey-40g16.6 [Source:ZFIN;Acc:ZDB-GENE-030131-9110]	15	21694388	21702317		0.07	0.11	0.11	0.18	0.14	0.18
ENSDARG00000056805	plch2b	phospholipase C, eta 2b [Source:ZFIN;Acc:ZDB-GENE-101112-1]	11	25752198	25777318		1.39	0.28	0.2	1.58	1.54	1.57
ENSDARG00000056806	pde5aa	phosphodiesterase 5A, cGMP-specific, a [Source:ZFIN;Acc:ZDB-GENE-100414-2]	1	14257158	14258409		3.23	0.61	0.94	2.89	2.57	2.56
ENSDARG00000056819	hoxa9b	homeobox A9b [Source:NCBI gene;Acc:58048]	16	20915319	20917584		2.77	4.25	4.55	8.63	8.23	8.49
ENSDARG00000056829	zmp:0000000662	zmp:0000000662 [Source:ZFIN;Acc:ZDB-GENE-130530-665]	13	11767603	11800281		7.08	6.17	5.09	6.32	6.34	6.41
ENSDARG00000056830	si:ch211-107m4.1	si:ch211-107m4.1 [Source:ZFIN;Acc:ZDB-GENE-081107-3]	14	22481833	22495604		2.14	1.48	1.45	2.57	2.38	2.28
ENSDARG00000056831	gng2	guanine nucleotide binding protein (G protein), gamma 2 [Source:ZFIN;Acc:ZDB-GENE-050417-59]	13	12045475	12088835		29.49	16.45	19.29	31.59	33	34.13
ENSDARG00000056832	exo1	exonuclease 1 [Source:NCBI gene;Acc:406778]	17	22563836	22573402		0.87	0.77	0.81	0.61	0.49	0.42
ENSDARG00000056833	svopa	SV2 related protein a [Source:ZFIN;Acc:ZDB-GENE-060526-336]	5	20147830	20165688		5.25	1.1	2.19	6.36	6.65	6.08
ENSDARG00000056835	laynb	layilin b [Source:ZFIN;Acc:ZDB-GENE-071012-6]	15	21842638	21852991		1.92	2.1	2.17	1.92	2.27	2.26
ENSDARG00000056836	si:ch211-125o16.4	si:ch211-125o16.4 [Source:ZFIN;Acc:ZDB-GENE-131120-100]	17	22544648	22552678		45.39	35.94	25.24	24.46	22.7	20.16
ENSDARG00000056838	pimr93	Pim proto-oncogene, serine/threonine kinase, related 93 [Source:ZFIN;Acc:ZDB-GENE-141216-82]	11	11797026	11804720		0.08	0.02	0.03	0.03	0.01	0.01
ENSDARG00000056840	otud5b	OTU deubiquitinase 5b [Source:ZFIN;Acc:ZDB-GENE-040801-50]	11	25681335	25689873		19.43	14.42	14.05	17.46	18.84	19.14
ENSDARG00000056842	usp30	ubiquitin specific peptidase 30 [Source:NCBI gene;Acc:559099]	5	20125934	20135679		1.81	1.82	1.76	1.82	1.76	1.54
ENSDARG00000056847	si:dkey-27c15.3	si:dkey-27c15.3 [Source:ZFIN;Acc:ZDB-GENE-060503-728]	19	26681848	26723369		2.63	1.59	1.53	2.54	2.56	2.68
ENSDARG00000056855	gatc	glutamyl-tRNA amidotransferase subunit C [Source:NCBI gene;Acc:558898]	5	20103085	20108469		15.08	16.88	18.75	10.37	10.05	10.9
ENSDARG00000056856	tax1bp1b	Tax1 (human T-cell leukemia virus type I) binding protein 1b [Source:NCBI gene;Acc:324152]	16	20805322	20932896		48.81	42.9	40.84	45.94	50.22	50.92
ENSDARG00000056859	spx	spexin hormone [Source:NCBI gene;Acc:101882535]	4	16794418	16797485		1.75	1.17	1.57	2.56	2.06	3.34
ENSDARG00000056862	cep63	centrosomal protein 63 [Source:NCBI gene;Acc:393574]	6	27896657	27925470		6.44	5.1	5.04	5.03	4.74	5.46
ENSDARG00000056871	ap4m1	adaptor related protein complex 4 subunit mu 1 [Source:ZFIN;Acc:ZDB-GENE-040718-421]	10	22854803	22862078		3.55	2.75	3.29	3.03	2.84	2.69
ENSDARG00000056873	and3	actinodin3 [Source:ZFIN;Acc:ZDB-GENE-040724-185]	10	22848556	22854758		139.07	174.54	147.84	92.14	91.99	86.09
ENSDARG00000056874	lygl1	lysozyme g-like 1 [Source:ZFIN;Acc:ZDB-GENE-040718-461]	14	20893065	20898499		10.7	17.27	16.25	15.57	14	13.25
ENSDARG00000056877	vamp2	vesicle-associated membrane protein 2 [Source:ZFIN;Acc:ZDB-GENE-030131-8225]	10	22838364	22845485		42.15	9.95	11.15	86.8	91.49	94.3
ENSDARG00000056885	per1a	period circadian clock 1a [Source:ZFIN;Acc:ZDB-GENE-011220-1]	10	22809715	22831611		4.77	2.14	1.71	4.42	4.23	4.28
ENSDARG00000056886	tbccd1	TBCC domain containing 1 [Source:NCBI gene;Acc:100037389]	9	27740968	27749936		1.73	1.23	1.58	2.06	2.21	1.69
ENSDARG00000056888	dnah5l	dynein, axonemal, heavy chain 5 like [Source:ZFIN;Acc:ZDB-GENE-091204-287]	24	33589667	33697002		0.62	0.49	0.37	1.33	1.49	1.32
ENSDARG00000056892	mpp6a	membrane protein, palmitoylated 6a (MAGUK p55 subfamily member 6) [Source:ZFIN;Acc:ZDB-GENE-050506-35]	16	21442362	21489641		5.57	4.82	3.38	4.72	4.65	4.88
ENSDARG00000056896	ttc12	tetratricopeptide repeat domain 12 [Source:ZFIN;Acc:ZDB-GENE-071016-4]	15	21947117	21981546		0.48	0.24	0.44	0.94	1	0.77
ENSDARG00000056903	mrpl13	mitochondrial ribosomal protein L13 [Source:ZFIN;Acc:ZDB-GENE-050522-167]	16	15052513	15097513		29.36	34.66	34.21	20.27	19.3	19.11
ENSDARG00000056907	si:rp71-45g20.10	si:rp71-45g20.10 [Source:ZFIN;Acc:ZDB-GENE-070912-707]	9	27804027	27805832		0.07	0.01	0.06	0.01	0	0.03
ENSDARG00000056909	LO018309.1	epidermal growth factor receptor-like [Source:NCBI gene;Acc:571431]	24	33552198	33578392		8.56	10.89	7.05	3.03	3.72	2.5
ENSDARG00000056910	CU234171.1		23	17899226	17907404		2.06	1.61	1.47	2.6	2.64	2.96
ENSDARG00000056911	or133-1	odorant receptor, family H, subfamily 133, member 1 [Source:ZFIN;Acc:ZDB-GENE-070806-34]	21	21599225	21600640		0.37	0.21	0.22	0.07	0.26	0.24
ENSDARG00000056913	pcolcea	procollagen C-endopeptidase enhancer a [Source:ZFIN;Acc:ZDB-GENE-041014-370]	10	22789319	22803854		16.76	24.85	28.07	16.78	16.74	15.67
ENSDARG00000056914	emg1	EMG1 N1-specific pseudouridine methyltransferase [Source:ZFIN;Acc:ZDB-GENE-060421-2909]	16	17254489	17258111		8.59	9.4	8.26	6.94	5.87	5.54
ENSDARG00000056915	si:ch211-237l4.6	si:ch211-237l4.6 [Source:ZFIN;Acc:ZDB-GENE-040724-42]	10	22782522	22786450		6.14	1.93	1.6	2.09	2.16	2.71
ENSDARG00000056917	si:rp71-45g20.4	si:rp71-45g20.4 [Source:ZFIN;Acc:ZDB-GENE-070912-709]	9	27855684	27857413		0.06	0.03	0.01	0.08	0.05	0.07
ENSDARG00000056920	tmem88a	transmembrane protein 88 a [Source:ZFIN;Acc:ZDB-GENE-040426-1808]	10	22771176	22780157		1.68	1.82	1.83	2.16	2.25	2.08
ENSDARG00000056921	ankk1	ankyrin repeat and kinase domain containing 1 [Source:ZFIN;Acc:ZDB-GENE-071015-4]	15	22014029	22024954		2.33	1.95	1.32	1.79	1.4	1.36

ENSDARG00000056922	ltbp1	latent transforming growth factor beta binding protein 1 [Source:ZFIN;Acc:ZDB-GENE-091202-8]	17	22956427	23116523		6.48	5.62	4.84	10.46	10.47	10.13
ENSDARG00000056923	dbr1	debranching RNA lariats 1 [Source:NCBI gene;Acc:323746]	9	27860332	27868267		12.65	10.06	8.87	10.13	8.75	9.92
ENSDARG00000056924	sap130a	Sin3A-associated protein a [Source:ZFIN;Acc:ZDB-GENE-070615-5]	6	28018390	28049817		4.56	3.21	2.61	6.79	6.08	6.52
ENSDARG00000056926	drd2a	dopamine receptor D2a [Source:ZFIN;Acc:ZDB-GENE-021119-2]	15	22046557	22074315		1.19	0.36	0.56	1.26	1.37	1.38
ENSDARG00000056929	kdm6bb	lysine (K)-specific demethylase 6B, b [Source:ZFIN;Acc:ZDB-GENE-040724-166]	10	22724059	22760659		24.05	11.95	10.48	38.24	37.09	39.51
ENSDARG00000056932	tfip11	tuftelin interacting protein 11 [Source:NCBI gene;Acc:436994]	23	17847040	17866100		8.34	5.58	6	8.37	7.79	8.49
ENSDARG00000056934	hepacama	hepatic and glial cell adhesion molecule a [Source:ZFIN;Acc:ZDB-GENE-050522-198]	15	22311803	22325973		6.86	3.16	4.29	13.55	12.49	13.37
ENSDARG00000056938	kera	keratocan [Source:NCBI gene;Acc:567017]	4	16337156	16345227		10.96	10.25	8.14	5.47	4.18	4.08
ENSDARG00000056940	or125-8	odorant receptor, family E, subfamily 125, member 8 [Source:ZFIN;Acc:ZDB-GENE-070806-98]	21	21655755	21657308		0.15	0.01	0.04	0.12	0.12	0.22
ENSDARG00000056949	htr7a	5-hydroxytryptamine (serotonin) receptor 7a [Source:ZFIN;Acc:ZDB-GENE-130530-666]	17	23542115	23545716		0.52	0.09	0.37	0.51	0.32	0.52
ENSDARG00000056950	epyc	epiphycan [Source:ZFIN;Acc:ZDB-GENE-041008-9]	4	16317296	16334362		15.58	17.98	15.35	20.32	20.3	20.58
ENSDARG00000056954	si:dkeyp-38g8.5	si:dkeyp-38g8.5 [Source:ZFIN;Acc:ZDB-GENE-081104-62]	4	16315692	16319762		9.4	8.12	8.05	6.19	5.39	6.17
ENSDARG00000056961	ccdc30	coiled-coil domain containing 30 [Source:ZFIN;Acc:ZDB-GENE-130530-667]	23	20705849	20730155		2.57	1.75	1.47	2.48	2.49	2.44
ENSDARG00000056964	ilk	integrin-linked kinase [Source:ZFIN;Acc:ZDB-GENE-040426-1435]	10	24722580	24759616		7.14	7.3	6.83	11.4	11.19	11.04
ENSDARG00000056966	nek7	NIMA-related kinase 7 [Source:ZFIN;Acc:ZDB-GENE-040801-136]	22	23105059	23228669		10.2	11.63	9.97	11.33	12.27	11.42
ENSDARG00000056976	ifit16	interferon-induced protein with tetratricopeptide repeats 16 [Source:ZFIN;Acc:ZDB-GENE-131120-86]	17	23598205	23603263		0.02	0.05	0.04	0.02	0.07	0.02
ENSDARG00000056978	letm1	leucine zipper-EF-hand containing transmembrane protein 1 [Source:NCBI gene;Acc:570745]	13	13033424	13099800		5.65	3.46	3.05	5.92	5.67	6.16
ENSDARG00000056979	lhx9	LIM homeobox 9 [Source:NCBI gene;Acc:550405]	22	23238790	23253481		7.15	2.17	4	7.48	7.61	7.92
ENSDARG00000056984	rad9a	RAD9 checkpoint clamp component A [Source:ZFIN;Acc:ZDB-GENE-040426-935]	14	21923741	21932403		1.94	1.89	1.53	1.66	1.44	1.46
ENSDARG00000056985	tpte	transmembrane phosphatase with tensin homology [Source:ZFIN;Acc:ZDB-GENE-030131-5503]	10	24690534	24707956		10.4	14.59	14.12	18.1	17.53	18.72
ENSDARG00000056986	dact2	dishevelled-binding antagonist of beta-catenin 2 [Source:NCBI gene;Acc:337795]	13	43400443	43407199		3.53	3.85	2.99	3.87	4.16	3.93
ENSDARG00000056995	snai1a	snail family zinc finger 1a [Source:ZFIN;Acc:ZDB-GENE-990415-255]	11	25251495	25257595		3.62	4.38	4.02	5.86	6.12	5.82
ENSDARG00000056998	kirrel1b	kirre like nephrin family adhesion molecule 1b [Source:ZFIN;Acc:ZDB-GENE-070912-173]	2	43961626	44038776		5.66	1.81	2.32	8.02	7.87	8.35
ENSDARG00000057000	camkvl	CaM kinase-like vesicle-associated, like [Source:ZFIN;Acc:ZDB-GENE-090311-58]	23	20563779	20636740		9.26	1.89	2.46	7.09	7.23	7.74
ENSDARG00000057004	si:ch211-287a12.9	si:ch211-287a12.9 [Source:ZFIN;Acc:ZDB-GENE-130530-668]	10	24682307	24689725		0.76	1.29	1.16	1.21	1.33	1.22
ENSDARG00000057006	fam241a	family with sequence similarity 241 member A [Source:ZFIN;Acc:ZDB-GENE-050522-524]	13	12664866	12667220		8.01	7.04	5.78	6.35	5.93	5.8
ENSDARG00000057007	ctbp1	C-terminal binding protein 1 [Source:ZFIN;Acc:ZDB-GENE-010130-1]	14	21820034	21846181		32.73	18.18	16.43	44.27	40.89	44.34
ENSDARG00000057010	si:dkey-154b15.1	si:dkey-154b15.1 [Source:ZFIN;Acc:ZDB-GENE-060503-52]	19	24750466	24758109		0.91	0.76	0.65	0.64	0.7	0.83
ENSDARG00000057011	dpm1	dolichyl-phosphate mannosyltransferase subunit 1, catalytic [Source:ZFIN;Acc:ZDB-GENE-040801-115]	23	20513104	20517692		11.63	13.57	11.42	10.71	11.14	10.96
ENSDARG00000057013	cadm3	cell adhesion molecule 3 [Source:ZFIN;Acc:ZDB-GENE-060512-196]	2	44049081	44183613		14.96	3.63	4.7	20.63	21.3	22.69
ENSDARG00000057016	cdc14ab	cell division cycle 14Ab [Source:NCBI gene;Acc:565969]	22	16190231	16275236		3.95	2.41	1.76	5.2	4.82	5.31
ENSDARG00000057017	slc6a4b	solute carrier family 6 member 4b [Source:ZFIN;Acc:ZDB-GENE-060314-2]	5	19969794	19988772		0.86	0.26	0.34	0.71	0.54	0.82
ENSDARG00000057021	vps36	vacuolar protein sorting 36 homolog [Source:NCBI gene;Acc:335810]	10	24643079	24680425		3.77	3.17	2.84	2.7	2.91	3.06
ENSDARG00000057025	chrna2b	cholinergic receptor, nicotinic, alpha 2b (neuronal) [Source:ZFIN;Acc:ZDB-GENE-041001-99]	20	19793620	19824515		0.62	0.33	0.76	1.5	1.4	1.74
ENSDARG00000057026	ran	RAN, member RAS oncogene family [Source:NCBI gene;Acc:30572]	14	21685537	21693914		305.11	325.89	355.75	216.55	220.72	224.56
ENSDARG00000057027	rtn2b	reticulon 2b [Source:ZFIN;Acc:ZDB-GENE-060331-95]	21	21374277	21386120		12.23	19.59	19.37	12.3	10.71	9.95
ENSDARG00000057029	htr2aa	5-hydroxytryptamine (serotonin) receptor 2A, genome duplicate a [Source:ZFIN;Acc:ZDB-GENE-070912-500]	9	25198249	25255490		0.82	0.23	0.44	1.23	1.32	1.47
ENSDARG00000057032	ppm1nb	protein phosphatase, Mg2+/Mn2+ dependent, 1Nb (putative) [Source:ZFIN;Acc:ZDB-GENE-071004-34]	21	21367914	21373242		16.44	4.49	4.84	10.69	11.19	12.06
ENSDARG00000057035	stoml3b	stomatin (EPB72)-like 3b [Source:ZFIN;Acc:ZDB-GENE-050417-366]	10	24641229	24648231		45.52	81.21	65.26	18.77	21.77	19.66
ENSDARG00000057037	kcnk12l	potassium channel, subfamily K, member 12 like [Source:ZFIN;Acc:ZDB-GENE-091118-74]	21	21357309	21361287		0.07	0	0.02	0.13	0.28	0.11
ENSDARG00000057039	si:ch211-191j22.8	si:ch211-191j22.8 [Source:ZFIN;Acc:ZDB-GENE-091204-10]	21	21309164	21315773		1.38	1.23	1.06	1.83	0.75	1.59
ENSDARG00000057042	tm2d1	TM2 domain containing 1 [Source:NCBI gene;Acc:100093710]	22	16759010	16775517		10.57	8.69	10.73	9.91	8.19	9.39
ENSDARG00000057049	kcnk18	potassium channel, subfamily K, member 18 [Source:ZFIN;Acc:ZDB-GENE-100405-2]	17	23770848	23779316		1.6	1.56	1.51	1.86	1.37	1.29
ENSDARG00000057053	SGMS1	zgc:91976 [Source:ZFIN;Acc:ZDB-GENE-041114-201]	17	23729717	23765068	SGMS1	6.71	5.14	4.52	7.93	7.69	7.85
ENSDARG00000057054	lrit3b	leucine-rich repeat, immunoglobulin-like and transmembrane domains 3b [Source:ZFIN;Acc:ZDB-GENE-070424-130]	13	12591508	12602920		0.33	0.14	0.09	0.27	0.38	0.75
ENSDARG00000057055	ndufs8b	NADH:ubiquinone oxidoreductase core subunit S8b [Source:ZFIN;Acc:ZDB-GENE-050522-131]	14	21671107	21678917		0.39	0.14	0.08	0.02	0.08	0.05
ENSDARG00000057058	minpp1a	multiple inositol-polyphosphate phosphatase 1a [Source:ZFIN;Acc:ZDB-GENE-060929-1194]	17	23710659	23727978		9.27	8.14	7.78	7.8	8.2	8.79
ENSDARG00000057060	tvp23b	trans-golgi network vesicle protein 23 homolog B (S. cerevisiae) [Source:ZFIN;Acc:ZDB-GENE-050522-404]	12	46683114	46695839		13.08	14.32	13.71	13.97	13.11	12.8
ENSDARG00000057062	si:dkey-157g16.6	si:dkey-157g16.6 [Source:ZFIN;Acc:ZDB-GENE-090313-189]	23	27134942	27235654		7.83	6.29	5.07	10.21	9.18	9.74
ENSDARG00000057064	enpep	glutamyl aminopeptidase [Source:ZFIN;Acc:ZDB-GENE-050309-218]	13	12543634	12581388		10.95	12.14	9.01	5.04	5.41	5.64
ENSDARG00000057074	rpgrb	retinitis pigmentosa GTPase regulator b [Source:ZFIN;Acc:ZDB-GENE-081015-2]	11	29770966	29790222		5.42	1.39	2.07	5.85	6.32	6.64
ENSDARG00000057075	lamtor3	late endosomal/lysosomal adaptor, MAPK and MTOR activator 3 [Source:NCBI gene;Acc:553597]	13	12479264	12486076		8.73	8.92	10.57	7.7	8.03	8.36
ENSDARG00000057089	ftsj1	FtsJ RNA 2'-O-methyltransferase 1 [Source:ZFIN;Acc:ZDB-GENE-041114-83]	21	21167837	21178751		2.96	3.41	3.53	2.14	1.99	2.33
ENSDARG00000057090	zmp:0000001103	zmp:0000001103 [Source:ZFIN;Acc:ZDB-GENE-140106-63]	10	23548674	23550956		0.02	0	0.01	0.02	0	0.02
ENSDARG00000057093	kdm5ba	lysine (K)-specific demethylase 5Ba [Source:ZFIN;Acc:ZDB-GENE-030131-5379]	11	24819324	24851464		20.33	12.08	9.34	34.6	36.12	36.14
ENSDARG00000057096	ndr1	nodal-related 1 [Source:ZFIN;Acc:ZDB-GENE-990415-256]	21	21010786	21013085		0	0.04	0	0.01	0.02	0.02
ENSDARG00000057098	htr1ab	5-hydroxytryptamine (serotonin) receptor 1A b [Source:ZFIN;Acc:ZDB-GENE-090409-2]	21	20949976	20951413		1.23	0.8	1	1.29	1.22	1.25
ENSDARG00000057099	papss1	3'-phosphoadenosine 5'-phosphosulfate synthase 1 [Source:ZFIN;Acc:ZDB-GENE-080721-5]	1	50487586	50527964		11	5.72	6.35	10.74	9.48	8.76
ENSDARG00000057100	zwilch	zwilch kinetochore protein [Source:NCBI gene;Acc:336439]	18	19456648	19472646		2.68	2.88	2.71	1.82	1.7	1.8
ENSDARG00000057101	rnf180	ring finger protein 180 [Source:ZFIN;Acc:ZDB-GENE-040426-1620]	21	20940022	20945658		2.28	1.99	1.96	1.59	2.07	1.96
ENSDARG00000057105	nle1	notchless homolog 1 (Drosophila) [Source:ZFIN;Acc:ZDB-GENE-050522-321]	10	23070741	23099869		7.12	7.73	6.98	5.73	5.7	5.41
ENSDARG00000057107	si:ch73-383g2.1	si:ch73-383g2.1 [Source:ZFIN;Acc:ZDB-GENE-131121-54]	24	29622345	29729799		2.07	0.95	1.02	2.2	2.04	2.22
ENSDARG00000057108	serpine3	serpin peptidase inhibitor, clade E (nexin, plasminogen activator inhibitor type 1), member 3 [Source:NCBI gene;Acc:556612]	9	23842884	23850978		0.47	0.53	0.72	0.74	1.04	0.75
ENSDARG00000057110	slc25a1a	solute carrier family 25 member 1a [Source:NCBI gene;Acc:393579]	10	23060391	23069323		8.08	3.34	4.66	6.07	5.61	5.57
ENSDARG00000057112	gpr25	G protein-coupled receptor 25 [Source:ZFIN;Acc:ZDB-GENE-141216-12]	11	24703108	24704437		0.07	0.02	0.01	0	0	0
ENSDARG00000057113	c6	complement component 6 [Source:ZFIN;Acc:ZDB-GENE-040426-1358]	21	20914888	20932603		3.09	3.46	2.87	22.43	23.32	23.67
ENSDARG00000057114	hpf1	histone PARylation factor 1 [Source:NCBI gene;Acc:100002184]	1	14020445	14026780		14.43	14.23	12.98	10.77	10.95	10.72
ENSDARG00000057117	ghsrb	growth hormone secretagogue receptor b [Source:ZFIN;Acc:ZDB-GENE-090327-1]	24	26742226	26746179		0.42	0.17	0.4	0.58	0.69	0.49
ENSDARG00000057121	c7b	complement component 7b [Source:ZFIN;Acc:ZDB-GENE-021120-1]	21	20901505	20915291		10	10.55	9.48	4.75	4.91	5.3
ENSDARG00000057125	pld1b	phospholipase D1b [Source:ZFIN;Acc:ZDB-GENE-070510-3]	24	26659010	26715174		2.52	2.27	1.85	1.65	1.59	1.72
ENSDARG00000057128	hadhaa	hydroxyacyl-CoA dehydrogenase trifunctional multienzyme complex subunit alpha a [Source:ZFIN;Acc:ZDB-GENE-031222-5]	20	47953047	47984895		14.35	17.89	15.86	12.59	12.06	12.2
ENSDARG00000057138	zgc:174164	zgc:174164 [Source:ZFIN;Acc:ZDB-GENE-030131-4971]	12	11315849	11349899		3.33	2.66	2.23	2.39	1.76	1.85
ENSDARG00000057141	zgc:110045	zgc:110045 [Source:ZFIN;Acc:ZDB-GENE-050706-149]	13	16230556	16257848		8.26	2.08	1.54	11.86	10.76	11.31
ENSDARG00000057142	zgc:194252	zgc:194252 [Source:ZFIN;Acc:ZDB-GENE-081022-86]	7	18989241	18990075		0.14	0.06	0.07	0.08	0.19	0.06
ENSDARG00000057143	nradd	neurotrophin receptor associated death domain [Source:ZFIN;Acc:ZDB-GENE-030131-2537]	16	16318028	16341027		5.49	7.65	6.23	7.76	8.07	7.06
ENSDARG00000057148	mocos	molybdenum cofactor sulfurase [Source:NCBI gene;Acc:100331902]	13	16261245	16278920		1.65	1.84	1.93	1.16	1.36	1.32
ENSDARG00000057151	prep	prolyl endopeptidase [Source:ZFIN;Acc:ZDB-GENE-050522-14]	20	48037019	48061351		7.53	5.69	5.57	9.21	8.28	8.04
ENSDARG00000057159	ankrd29	ankyrin repeat domain 29 [Source:NCBI gene;Acc:553798]	22	16418688	16431139		2.6	1.51	2.22	2.45	2.56	2.55
ENSDARG00000057166	lrmda	leucine rich melanocyte differentiation associated [Source:ZFIN;Acc:ZDB-GENE-050522-285]	13	16922705	17464654		0.6	0.58	0.36	0.5	0.59	0.39
ENSDARG00000057167	eif4g2b	eukaryotic translation initiation factor 4, gamma 2b [Source:ZFIN;Acc:ZDB-GENE-030131-6550]	18	16715864	16726206		46.33	39.94	34.2	59.92	61.98	62.74
ENSDARG00000057169	abca4a	ATP-binding cassette, sub-family A (ABC1), member 4a [Source:ZFIN;Acc:ZDB-GENE-050517-3]	24	28561038	28645334		2.71	0.54	0.33	1.76	1.93	1.75
ENSDARG00000057173	ifit8	interferon-induced protein with tetratricopeptide repeats 8 [Source:ZFIN;Acc:ZDB-GENE-051127-17]	13	17694845	17697158		0	0.01	0.03	0.01	0	0.01
ENSDARG00000057180	il17c	interleukin 17c [Source:ZFIN;Acc:ZDB-GENE-061031-5]	18	17075985	17077596		0	0.03	0.03	0.03	0.04	0
ENSDARG00000057184	C2H7orf25	zgc:55781 [Source:NCBI gene;Acc:325979]	2	21620177	21625493	C2H7orf25	7.55	6.31	5.66	6.55	5.83	6.09
ENSDARG00000057206	nmt1b	N-myristoyltransferase 1b [Source:ZFIN;Acc:ZDB-GENE-081222-3]	12	15666949	15676285		3.57	4.55	4.49	6.26	8.07	6.16
ENSDARG00000057209	plppr5a	phospholipid phosphatase related 5a [Source:ZFIN;Acc:ZDB-GENE-070705-281]	2	20262479	20323901		2.54	0.51	0.52	2.52	2.79	2.7
ENSDARG00000057210	has3	hyaluronan synthase 3 [Source:ZFIN;Acc:ZDB-GENE-021118-1]	18	17027874	17030870		0.61	0.3	0.55	1.79	1.91	1.95
ENSDARG00000057218	tbc1d15	TBC1 domain family, member 15 [Source:ZFIN;Acc:ZDB-GENE-041111-251]	18	17002164	17020231		10.06	8.93	7.71	9.59	9.34	9.8
ENSDARG00000057227	si:dkey-222b8.4	si:dkey-222b8.4 [Source:ZFIN;Acc:ZDB-GENE-060503-438]	19	23875382	23895839		1.1	0.29	0.28	0.67	0.99	0.82
ENSDARG00000057231	rorca	RAR-related orphan receptor C a [Source:ZFIN;Acc:ZDB-GENE-990415-250]	8	18885108	18899427		15.1	7.58	6.51	12.03	11.61	10.79
ENSDARG00000057234	chtopa	chromatin target of PRMT1a [Source:ZFIN;Acc:ZDB-GENE-030131-210]	19	23897666	23918136		26.58	17.72	19.17	32.69	33.32	32.74
ENSDARG00000057238	si:dkey-30k6.5	si:dkey-30k6.5 [Source:ZFIN;Acc:ZDB-GENE-091204-299]	21	20386865	20396541		2.27	2.45	2.15	2.12	2.11	1.97
ENSDARG00000057239	tph2	tryptophan hydroxylase 2 (tryptophan 5-monooxygenase) [Source:ZFIN;Acc:ZDB-GENE-040624-4]	18	16989764	17001056		2.2	1.96	1.86	2.38	2.91	2.6
ENSDARG00000057241	tnfsf10	TNF superfamily member 10 [Source:ZFIN;Acc:ZDB-GENE-040718-335]	24	26932472	26937526		17.6	19.74	19.19	13.72	14.48	14.16
ENSDARG00000057245	im:7138535	im:7138535 [Source:ZFIN;Acc:ZDB-GENE-041111-49]	5	61788819	61797220		3.61	3.79	3.83	2.08	2.15	2.7
ENSDARG00000057248	sclt1	sodium channel and clathrin linker 1 [Source:ZFIN;Acc:ZDB-GENE-030131-3278]	1	12364456	12397258		5.12	3.06	4.03	4.44	4.39	4.32
ENSDARG00000057249	zmynd11	zinc finger, MYND-type containing 11 [Source:ZFIN;Acc:ZDB-GENE-050522-301]	24	27223952	27256673		10.73	5.42	5.87	14.02	14.39	14.58
ENSDARG00000057253	pimr141	Pim proto-oncogene, serine/threonine kinase, related 141 [Source:ZFIN;Acc:ZDB-GENE-070912-462]	2	19505263	19508136		0.62	0.38	0.63	0.44	0.4	0.39
ENSDARG00000057255	emc1	ER membrane protein complex subunit 1 [Source:ZFIN;Acc:ZDB-GENE-060810-98]	23	21261313	21277522		11.93	7.95	6.02	14.65	13.97	14.38
ENSDARG00000057262	si:dkey-91i10.3	si:dkey-91i10.3 [Source:ZFIN;Acc:ZDB-GENE-081105-124]	9	23009608	23023288		7.73	11.06	8.11	5.74	5.86	5.49
ENSDARG00000057263	zgc:173729	zgc:173729 [Source:ZFIN;Acc:ZDB-GENE-071004-64]	16	51281959	51288178		0.11	0.09	0.07	0.09	0.16	0.21
ENSDARG00000057265	pimr52	Pim proto-oncogene, serine/threonine kinase, related 52 [Source:ZFIN;Acc:ZDB-GENE-070912-460]	2	19518520	19520690		0	0	0	0.02	0	0
ENSDARG00000057272	slc30a9	solute carrier family 30 member 9 [Source:NCBI gene;Acc:497184]	14	44804326	44826422		9.11	7.43	6.84	8.39	7.86	8.18
ENSDARG00000057273	alox5a	arachidonate 5-lipoxygenase a [Source:ZFIN;Acc:ZDB-GENE-090311-47]	13	18128275	18174864		1.47	1.54	1.34	1.16	1.6	1.1
ENSDARG00000057276	iqca1	IQ motif containing with AAA domain 1 [Source:ZFIN;Acc:ZDB-GENE-050208-768]	6	15762647	15893049		0.83	0.58	0.85	0.93	1.07	1.12
ENSDARG00000057284	pimr54	Pim proto-oncogene, serine/threonine kinase, related 54 [Source:ZFIN;Acc:ZDB-GENE-070912-425]	2	19633493	19635654		0	0	0.01	0	0.02	0.02
ENSDARG00000057286	swap70b	switching B cell complex subunit SWAP70b [Source:ZFIN;Acc:ZDB-GENE-030131-3587]	18	16876833	16916840		7.09	6.67	6.76	9.01	9.05	9.1
ENSDARG00000057287	slc25a16	solute carrier family 25 member 16 [Source:ZFIN;Acc:ZDB-GENE-030131-3299]	13	18179096	18195942		11.45	13.4	13.08	9.73	9.53	8.72
ENSDARG00000057296	cbln1	cerebellin 1 precursor [Source:ZFIN;Acc:ZDB-GENE-040718-226]	18	18104235	18110032		20.88	7.35	12.86	18.84	18.28	20.29
ENSDARG00000057299	pbdc1	polysaccharide biosynthesis domain containing 1 [Source:ZFIN;Acc:ZDB-GENE-040718-459]	10	20103067	20106012		31.62	35.74	35.61	20.21	22.32	21.85
ENSDARG00000057303	galnt7	UDP-N-acetyl-alpha-D-galactosamine: polypeptide N-acetylgalactosaminyltransferase 7 [Source:ZFIN;Acc:ZDB-GENE-050522-284	1	38142354	38170906		9.81	6.83	5.4	8.47	7.78	7.7
ENSDARG00000057315	rwdd3	RWD domain containing 3 [Source:NCBI gene;Acc:393729]	2	19640952	19649341		3.06	2.48	2.68	1.62	2.31	2.07
ENSDARG00000057317	nexn	nexilin (F actin binding protein) [Source:ZFIN;Acc:ZDB-GENE-041114-92]	8	18515711	18537882		14.18	13.27	10.65	14.12	13.4	14
ENSDARG00000057321	tut1	terminal uridylyl transferase 1, U6 snRNA-specific [Source:NCBI gene;Acc:564388]	12	10337674	10352001		3.47	3.28	3.43	2.57	2.83	2.55
ENSDARG00000057322	tmem47	transmembrane protein 47 [Source:NCBI gene;Acc:406640]	8	18565904	18582922		11.4	7.28	7.5	14.66	14.99	14.44
ENSDARG00000057323	e2f8	E2F transcription factor 8 [Source:NCBI gene;Acc:565952]	7	16576246	16598212		3.66	3.07	2.27	3.66	3.47	3.38
ENSDARG00000057328	cth1	cysteine three histidine 1 [Source:ZFIN;Acc:ZDB-GENE-990806-20]	7	17947217	17950315		0	0	0	0	0.02	0.06
ENSDARG00000057331	kcnk7	potassium channel, subfamily K, member 7 [Source:ZFIN;Acc:ZDB-GENE-110304-2]	7	17973097	17980221		0.19	0.24	0.28	0.16	0.21	0.15
ENSDARG00000057334	cmasa	cytidine monophosphate N-acetylneuraminic acid synthetase a [Source:NCBI gene;Acc:561018]	4	14727018	14737102		19.55	19.96	19.41	17.7	17.32	17.68
ENSDARG00000057352	slc25a29	solute carrier family 25 member 29 [Source:ZFIN;Acc:ZDB-GENE-050913-70]	20	53600638	53624664		3.2	2.83	2.88	4.51	4.21	4.22
ENSDARG00000057353	ehbp1l1a	EH domain binding protein 1-like 1a [Source:ZFIN;Acc:ZDB-GENE-080917-27]	7	17992455	18074041		1.66	2.02	1.35	1.77	1.26	1.33
ENSDARG00000057365	elovl8b	ELOVL fatty acid elongase 8b [Source:ZFIN;Acc:ZDB-GENE-050522-453]	8	18200331	18203342		1.22	1.54	1.42	2.09	2.17	1.91
ENSDARG00000057368	rps8b	ribosomal protein S8b [Source:ZFIN;Acc:ZDB-GENE-050522-202]	8	18193116	18200003		8.4	8.3	7.89	6.55	6.79	6.6
ENSDARG00000057374	sfxn3	sideroflexin 3 [Source:ZFIN;Acc:ZDB-GENE-070112-2092]	13	18677428	18695289		9.56	5.82	5.72	11.57	11.19	10.17
ENSDARG00000057378	selenou1a	Danio rerio selenoprotein U 1a (selenou1a), transcript variant 2, mRNA. [Source:RefSeq mRNA;Acc:NM_001354552]	13	18659325	18669575		39.08	44.92	41.9	34.99	36.04	35.66
ENSDARG00000057392	lrrc31	leucine rich repeat containing 31 [Source:ZFIN;Acc:ZDB-GENE-091204-286]	24	26354768	26369185		2.07	1.43	1.02	1.07	1.16	1.3
ENSDARG00000057394	rab33a	RAB33A, member RAS oncogene family [Source:ZFIN;Acc:ZDB-GENE-060825-283]	14	17593284	17599452		3.49	1.48	1.64	4.14	4.1	3.7
ENSDARG00000057395	si:ch211-130m23.3	si:ch211-130m23.3 [Source:ZFIN;Acc:ZDB-GENE-060531-14]	5	46207017	46273938		2.37	1.37	1.55	4.19	4.45	4.43
ENSDARG00000057403	CR847953.1		23	18869416	18898522		9.03	4.93	4.36	8.13	8.61	9.45
ENSDARG00000057408	CABZ01067232.1	otolin-1-like [Source:NCBI gene;Acc:101886389]	15	45668851	45674213		48.54	37.46	32.6	10.88	12.24	9.95
ENSDARG00000057410	rxfp3	relaxin family peptide receptor 3 [Source:ZFIN;Acc:ZDB-GENE-080723-67]	21	19487707	19489087		1.28	0.47	0.59	1	1.02	1.18
ENSDARG00000057414	phb	prohibitin [Source:ZFIN;Acc:ZDB-GENE-030131-6577]	3	15736811	15746999		84.07	98.97	94.08	73.35	74.25	71.39
ENSDARG00000057417	nudt13	nudix (nucleoside diphosphate linked moiety X)-type motif 13 [Source:ZFIN;Acc:ZDB-GENE-040724-60]	12	9519250	9538578		2.47	2.42	2.65	1.37	1.19	1.14
ENSDARG00000057419	slc44a5b	solute carrier family 44 member 5b [Source:ZFIN;Acc:ZDB-GENE-081105-4]	8	17838924	17917668		3.47	2.92	2.76	4.86	5.39	5.41
ENSDARG00000057426	oard1	O-acyl-ADP-ribose deacylase 1 [Source:ZFIN;Acc:ZDB-GENE-050522-480]	23	19220381	19225709		1.52	1.16	1.22	1.45	1.25	1.45
ENSDARG00000057427	sv2ba	synaptic vesicle glycoprotein 2Ba [Source:ZFIN;Acc:ZDB-GENE-070209-134]	7	15266093	15306015		22.17	5.11	3.75	19.78	21.32	20.63
ENSDARG00000057429	arg1	arginase 1 [Source:ZFIN;Acc:ZDB-GENE-040724-181]	12	9597151	9617770		1.64	1.61	0.88	1.67	2	2.34
ENSDARG00000057433	st6galnac5b	ST6 (alpha-N-acetyl-neuraminyl-2,3-beta-galactosyl-1,3)-N-acetylgalactosaminide alpha-2,6-sialyltransferase 5b [Source:ZFIN;Acc:	8	17800380	17822259		3.22	2.86	3.12	2.52	2.66	2.55
ENSDARG00000057435	amacr	alpha-methylacyl-CoA racemase [Source:ZFIN;Acc:ZDB-GENE-050522-484]	21	19418563	19441617		4.35	6.39	5.59	2.54	2.61	2.44
ENSDARG00000057437	apodb	apolipoprotein Db [Source:ZFIN;Acc:ZDB-GENE-051023-8]	24	26306362	26328721		34.68	43.04	27.18	25.96	27.8	27.82
ENSDARG00000057439	phf1	PHD finger protein 1 [Source:ZFIN;Acc:ZDB-GENE-100922-277]	16	45424907	45443805		1.86	0.32	0.76	1.92	1.86	1.74
ENSDARG00000057440	si:dkey-228d14.5	si:dkey-228d14.5 [Source:ZFIN;Acc:ZDB-GENE-090313-252]	13	18332685	18345854		2.4	1.63	1.4	1.32	1.72	1.17
ENSDARG00000057452	ston2	stonin 2 [Source:ZFIN;Acc:ZDB-GENE-050913-137]	17	16755287	16808413		5.63	4.91	4.32	6.88	6.46	6.65
ENSDARG00000057456	ppp3ccb	protein phosphatase 3, catalytic subunit, gamma isozyme, b [Source:ZFIN;Acc:ZDB-GENE-070112-1102]	10	20180163	20234383		18.36	12.54	9.97	22.2	21.64	20.25
ENSDARG00000057460	crygm2d13	crystallin, gamma M2d13 [Source:ZFIN;Acc:ZDB-GENE-040718-105]	9	22091161	22092089		53.87	94.64	147.88	214.71	202.29	245.77
ENSDARG00000057465	gfpt1	glutamine--fructose-6-phosphate transaminase 1 [Source:ZFIN;Acc:ZDB-GENE-070423-1]	8	49975160	50037559		13.76	12.49	10.58	16.06	17.1	16.64
ENSDARG00000057468	kcnc2	potassium voltage-gated channel, Shaw-related subfamily, member 2 [Source:ZFIN;Acc:ZDB-GENE-070410-118]	4	2060524	2196928		2.82	0.63	0.76	3.25	3.39	3.23
ENSDARG00000057477	lrrc28	leucine rich repeat containing 28 [Source:ZFIN;Acc:ZDB-GENE-061128-1]	7	15618127	15653635		3.01	2.52	2.29	3.25	2.64	3.23
ENSDARG00000057479	hrh2b	histamine receptor H2b [Source:ZFIN;Acc:ZDB-GENE-070928-20]	10	21899753	21909696		0.14	0.22	0.17	0.13	0.08	0.16
ENSDARG00000057481	RNF157	si:dkey-49c17.3 [Source:ZFIN;Acc:ZDB-GENE-130531-49]	12	31729075	31765822	RNF157	11.44	2.48	4.01	12.49	12.86	13.88
ENSDARG00000057484	srsf2a	serine and arginine rich splicing factor 2a [Source:ZFIN;Acc:ZDB-GENE-040426-2706]	12	31720673	31726748		52.48	28.54	32.68	58.51	54.4	53.42
ENSDARG00000057491	n4bp1	nedd4 binding protein 1 [Source:NCBI gene;Acc:327327]	18	18405992	18422543		3.37	2.31	1.99	4.23	4.39	4.34
ENSDARG00000057497	stk35l	serine/threonine kinase 35, like [Source:ZFIN;Acc:ZDB-GENE-041212-52]	6	38845676	38861349		5.3	3.05	2.84	9.5	9.62	9.12
ENSDARG00000057498	habp2	hyaluronan binding protein 2 [Source:ZFIN;Acc:ZDB-GENE-030131-4721]	12	31713239	31720066		11.27	16.13	15.39	4.07	3.95	4.29
ENSDARG00000057504	si:dkey-202g17.3	si:dkey-202g17.3 [Source:ZFIN;Acc:ZDB-GENE-131127-371]	15	9072821	9089507		0.02	0.12	0.21	3.81	3.31	3.88
ENSDARG00000057513	mdm4	MDM4 regulator of p53 [Source:NCBI gene;Acc:334932]	11	22890997	22916641		18.28	10.6	10.69	23.18	22.57	23.09
ENSDARG00000057519	pcdh1g9	protocadherin 1 gamma 9 [Source:ZFIN;Acc:ZDB-GENE-050609-5]	10	21701551	21820133		2.12	0.48	0.67	2.76	2.79	2.98
ENSDARG00000057527	mef2ab	myocyte enhancer factor 2ab [Source:ZFIN;Acc:ZDB-GENE-120614-2]	7	15659280	15712421		5.91	1.18	1.07	4.08	4.28	3.91
ENSDARG00000057529	itpa	inosine triphosphatase (nucleoside triphosphate pyrophosphatase) [Source:NCBI gene;Acc:557834]	20	14941264	14949443		10.66	11.29	11.72	8.84	9.22	8.35
ENSDARG00000057531	pgap3	post-GPI attachment to proteins phospholipase 3 [Source:NCBI gene;Acc:100136873]	12	9449665	9468618		2.85	1.32	1.97	4.99	5.07	4.27
ENSDARG00000057536	crygm2a	crystallin, gamma M2a [Source:ZFIN;Acc:ZDB-GENE-050516-9]	9	22075528	22076368		0	0.04	0	0	0	0
ENSDARG00000057556	rpl17	ribosomal protein L17 [Source:ZFIN;Acc:ZDB-GENE-030131-8585]	21	18997503	19067517		466.01	554.52	630.06	303.05	313.38	314.45
ENSDARG00000057562	polr3c	polymerase (RNA) III (DNA directed) polypeptide C [Source:NCBI gene;Acc:393201]	16	14249546	14280600		4.67	4.34	4.4	3.48	3.86	3.63
ENSDARG00000057568	nefla	neurofilament, light polypeptide a [Source:ZFIN;Acc:ZDB-GENE-091117-1]	21	19008168	19011040		9.65	7.89	6.41	5.02	5.4	5
ENSDARG00000057571	pgam2	phosphoglycerate mutase 2 (muscle) [Source:ZFIN;Acc:ZDB-GENE-040116-6]	21	19001669	19006641		158.05	238.83	301.8	110.86	100.26	104.88
ENSDARG00000057575	pnp4a	purine nucleoside phosphorylase 4a [Source:ZFIN;Acc:ZDB-GENE-040625-83]	11	24251141	24299538		5.79	8.53	7.61	4.94	5.07	4.88
ENSDARG00000057577	mbtps2	membrane-bound transcription factor peptidase, site 2 [Source:ZFIN;Acc:ZDB-GENE-030131-6598]	24	25447911	25461267		6.25	4.62	4.81	6.49	6.5	6.38
ENSDARG00000057583	prrc2c	proline-rich coiled-coil 2C [Source:ZFIN;Acc:ZDB-GENE-081104-156]	20	15023263	15062606		7.13	5.88	4.68	12.69	11.89	12.18
ENSDARG00000057586	sall3b	spalt-like transcription factor 3b [Source:ZFIN;Acc:ZDB-GENE-030131-9140]	19	22062202	22074081		2.49	0.82	0.84	5.41	4.78	4.85
ENSDARG00000057590	si:ch1073-396h14.1	si:ch1073-396h14.1 [Source:ZFIN;Acc:ZDB-GENE-070705-6]	22	15643174	15693085		3.24	3.78	3.23	3.17	2.91	2.58
ENSDARG00000057598	s100b	S100 calcium binding protein, beta (neural) [Source:ZFIN;Acc:ZDB-GENE-040718-290]	22	13846130	13857729		3.81	4.07	3.35	4.1	4.46	5.11
ENSDARG00000057606	arl4ca	ADP-ribosylation factor-like 4Ca [Source:ZFIN;Acc:ZDB-GENE-050417-265]	22	13977772	13979393		13.14	8.34	7.52	12.53	12.49	12.56
ENSDARG00000057610	cdkn2c	cyclin-dependent kinase inhibitor 2C (p18, inhibits CDK4) [Source:ZFIN;Acc:ZDB-GENE-081104-145]	8	16407884	16414886		0.68	0.58	0.59	0.58	0.56	0.61
ENSDARG00000057620	nitr3b	novel immune-type receptor 3b [Source:ZFIN;Acc:ZDB-GENE-001106-16]	7	17412992	17457872		0.01	0.04	0.06	0.06	0.03	0.04
ENSDARG00000057624	cops5	COP9 signalosome subunit 5 [Source:NCBI gene;Acc:393698]	24	23716918	23725371		26.13	23.75	26.83	19.65	20.27	20.14
ENSDARG00000057625	dusp14	dual specificity phosphatase 14 [Source:ZFIN;Acc:ZDB-GENE-041010-162]	15	15856178	15877832		4.17	1.56	2.16	6.4	7	6.65
ENSDARG00000057626	josd2	Josephin domain containing 2 [Source:ZFIN;Acc:ZDB-GENE-040426-850]	16	13683807	13706770		12.86	9.81	10.04	13.66	12.59	14.35
ENSDARG00000057630	galm	galactose mutarotase [Source:ZFIN;Acc:ZDB-GENE-040718-66]	13	8306399	8318216		16.6	22.7	21.42	9.64	9.58	8.5
ENSDARG00000057632	ppp1r42	protein phosphatase 1, regulatory subunit 42 [Source:NCBI gene;Acc:431749]	24	23730061	23740899		0.58	0.65	0.84	0.47	0.59	0.52
ENSDARG00000057633	cxcr4a	chemokine (C-X-C motif) receptor 4a [Source:ZFIN;Acc:ZDB-GENE-020102-1]	6	12922002	12923727		2.26	2.91	2.03	3.34	3.84	3.17
ENSDARG00000057635	wdr34	WD repeat domain 34 [Source:ZFIN;Acc:ZDB-GENE-070928-9]	21	13217896	13225402		0.96	0.6	0.74	0.99	0.93	0.96
ENSDARG00000057644	adam8b	ADAM metallopeptidase domain 8b [Source:ZFIN;Acc:ZDB-GENE-070808-1]	12	9096963	9132682		3.45	2.51	2.19	4.06	3.97	3.64
ENSDARG00000057648	dnttip2	deoxynucleotidyltransferase, terminal, interacting protein 2 [Source:ZFIN;Acc:ZDB-GENE-080513-5]	8	15277874	15286309		14.66	16.2	14.51	11.33	11.33	11.37

ENSDARG00000057649	satb1a	SATB homeobox 1a [Source:ZFIN;Acc:ZDB-GENE-031010-36]	19	20453831	20482261		5.8	1.95	2.36	5.7	5.75	5.7
ENSDARG00000057652	dbpb	D site albumin promoter binding protein b [Source:ZFIN;Acc:ZDB-GENE-100922-6]	16	13599693	13613475		19.34	9.73	6.87	17.12	16.57	17.45
ENSDARG00000057660	dhx57	DEAH (Asp-Glu-Ala-Asp/His) box polypeptide 57 [Source:ZFIN;Acc:ZDB-GENE-030131-4078]	13	8320455	8353560		4.16	3.05	2.66	4.25	4.23	4.16
ENSDARG00000057661	aldoca	aldolase C, fructose-bisphosphate, a [Source:NCBI gene;Acc:792692]	15	16079586	16098531		10.3	8.15	10.18	4.85	6.02	5.95
ENSDARG00000057665	sprn2	shadow of prion protein 2 [Source:ZFIN;Acc:ZDB-GENE-041221-1]	10	20635960	20637098		6.45	2.02	2.9	5.8	6.18	6.37
ENSDARG00000057671	epas1b	endothelial PAS domain protein 1b [Source:ZFIN;Acc:ZDB-GENE-060607-11]	13	8375612	8446341		6.64	5.7	4.08	5.41	5.19	5.28
ENSDARG00000057672	plpp5	phospholipid phosphatase 5 [Source:ZFIN;Acc:ZDB-GENE-040704-54]	10	20527427	20537062		12.15	9.33	10.89	12.07	11.85	11.62
ENSDARG00000057676	golga7	golgin A7 [Source:ZFIN;Acc:ZDB-GENE-040625-142]	10	20364009	20372269		15.14	13.55	14.89	16.96	17.09	18.19
ENSDARG00000057677	neu4	sialidase 4 [Source:ZFIN;Acc:ZDB-GENE-050522-125]	15	2490204	2497568		0.07	0.11	0.1	0.08	0.15	0.1
ENSDARG00000057678	sfrp1b	secreted frizzled-related protein 1b [Source:ZFIN;Acc:ZDB-GENE-061103-1]	10	20351413	20357013		5.63	7.19	6.46	5.26	5.58	4.98
ENSDARG00000057679	sh2b1	SH2B adaptor protein 1 [Source:ZFIN;Acc:ZDB-GENE-070209-188]	3	15358459	15392466		10.05	7.08	5.52	16.02	14.82	15.86
ENSDARG00000057680	foxj2	forkhead box J2 [Source:ZFIN;Acc:ZDB-GENE-100922-240]	16	12758494	12787029		3.21	3.69	2.56	4.82	4.32	4.77
ENSDARG00000057681	si:ch211-266o15.1	si:ch211-266o15.1 [Source:ZFIN;Acc:ZDB-GENE-131127-116]	17	14731315	14780578		8.45	5.51	4.57	10.18	9.97	10.76
ENSDARG00000057683	mcm6	minichromosome maintenance complex component 6 [Source:ZFIN;Acc:ZDB-GENE-030909-6]	6	12968101	12985390		33.82	28.93	25.61	16.6	15.95	14.78
ENSDARG00000057687	sbk3	SH3 domain binding kinase family, member 3 [Source:ZFIN;Acc:ZDB-GENE-080204-39]	16	12710904	12723738		1.96	2.11	1.94	0.91	0.91	0.97
ENSDARG00000057688	ical1	islet cell autoantigen 1-like [Source:ZFIN;Acc:ZDB-GENE-030910-4]	6	12870744	12912606		6.6	3.96	4.07	7.78	8.39	8.11
ENSDARG00000057691	srsf1a	serine and arginine rich splicing factor 1a [Source:NCBI gene;Acc:406288]	15	16072080	16076518		50.92	39.34	42.42	58.93	58.33	58.42
ENSDARG00000057698	ctsd	cathepsin D [Source:ZFIN;Acc:ZDB-GENE-010131-8]	18	16133595	16146081		110.17	136.8	144.56	112.56	118.85	115.6
ENSDARG00000057699	styx	serine/threonine/tyrosine interacting protein [Source:ZFIN;Acc:ZDB-GENE-050522-45]	17	15220042	15229787		6.93	5.9	5.47	7.54	7.74	6.76
ENSDARG00000057706	si:ch211-137i24.10	si:ch211-137i24.10 [Source:ZFIN;Acc:ZDB-GENE-060526-34]	5	24165141	24174956		22.3	24.12	20.71	14.68	16.33	15.41
ENSDARG00000057707	zgc:66443	zgc:66443 [Source:ZFIN;Acc:ZDB-GENE-040426-2945]	3	15669952	15679107		4.26	3.52	3.78	3.22	3.41	3.42
ENSDARG00000057708	phtf1	putative homeodomain transcription factor 1 [Source:ZFIN;Acc:ZDB-GENE-080204-123]	11	18799186	18819178		4.51	4.61	4.05	3.85	3.77	3.96
ENSDARG00000057714	cmah	cytidine monophospho-N-acetylneuraminic acid hydroxylase [Source:NCBI gene;Acc:431739]	4	2228754	2250264		18.47	18.7	15.85	7.64	6.22	7.31
ENSDARG00000057719	atl2	atlastin GTPase 2 [Source:ZFIN;Acc:ZDB-GENE-030131-6505]	13	8281223	8304605		13.77	18.99	16.49	10.61	10.57	9.97
ENSDARG00000057728	slc17a8	solute carrier family 17 member 8 [Source:NCBI gene;Acc:563467]	18	15616167	15635407		2.2	2.06	2.21	1.63	1.57	1.86
ENSDARG00000057729	ephb6	eph receptor B6 [Source:ZFIN;Acc:ZDB-GENE-100922-51]	16	12335274	12470034		2.28	0.59	0.55	3.35	3.14	3.18
ENSDARG00000057736	ano10a	anoctamin 10a [Source:NCBI gene;Acc:566425]	16	7985886	8041348		15.53	18.19	17.4	14.28	14.99	15
ENSDARG00000057737	rxraa	retinoid X receptor, alpha a [Source:NCBI gene;Acc:555578]	21	17768174	17944808		7.32	5.03	4.07	7.77	7.68	7.5
ENSDARG00000057738	hells	helicase, lymphoid specific [Source:ZFIN;Acc:ZDB-GENE-030131-9923]	13	8255106	8270718		12.89	12.49	11.41	6.03	5.93	5.1
ENSDARG00000057741	nr1h4	nuclear receptor subfamily 1, group H, member 4 [Source:ZFIN;Acc:ZDB-GENE-040718-313]	18	15644559	15666942		4.27	4.91	4.05	1.85	2	2.19
ENSDARG00000057743	si:dkey-10f21.4	si:dkey-10f21.4 [Source:ZFIN;Acc:ZDB-GENE-070912-350]	2	15035090	15041846		1.59	1.32	1.59	1.42	1.08	1.45
ENSDARG00000057751	zbtb47a	zinc finger and BTB domain containing 47a [Source:ZFIN;Acc:ZDB-GENE-081104-121]	2	21309272	21321749		1.22	0.69	0.84	0.76	0.81	0.81
ENSDARG00000057767	nudt4a	nudix (nucleoside diphosphate linked moiety X)-type motif 4a [Source:ZFIN;Acc:ZDB-GENE-031010-33]	18	15795566	15837342		17.76	20.33	18.81	21.9	21.7	22.15
ENSDARG00000057769	pimr55	Pim proto-oncogene, serine/threonine kinase, related 55 [Source:ZFIN;Acc:ZDB-GENE-070912-349]	2	14992879	14995050		0	0	0	0	0.02	0
ENSDARG00000057772	7-Mar	membrane-associated ring finger (C3HC4) 7 [Source:ZFIN;Acc:ZDB-GENE-080204-121]	6	11781231	11812224		3.01	2.38	2.03	4.21	3.99	4.54
ENSDARG00000057782	baz2ba	bromodomain adjacent to zinc finger domain, 2Ba [Source:ZFIN;Acc:ZDB-GENE-070615-37]	6	11829867	12002935		12.51	6.74	6.38	27.04	27.36	29.89
ENSDARG00000057787	itgae.2	integrin, alpha E, tandem duplicate 2 [Source:ZFIN;Acc:ZDB-GENE-130530-671]	15	17177635	17190540		0.8	0.68	0.6	1.4	1.94	1.52
ENSDARG00000057789	lyz	lysozyme [Source:ZFIN;Acc:ZDB-GENE-020515-2]	24	24035863	24038800		0.88	0.92	0.46	1.68	1.95	1.84
ENSDARG00000057790	ankrd6a	ankyrin repeat domain 6a [Source:ZFIN;Acc:ZDB-GENE-120525-1]	17	15756828	15777350		5.67	0.38	0.51	3.32	2.79	3.66
ENSDARG00000057792	cx52.7	connexin 52.7 [Source:ZFIN;Acc:ZDB-GENE-050616-15]	17	15744090	15747296		1.17	0.1	0.13	0.61	0.58	0.45
ENSDARG00000057805	mrpl28	mitochondrial ribosomal protein L28 [Source:ZFIN;Acc:ZDB-GENE-050522-113]	15	17128282	17138640		27.1	29.53	28.42	20.33	21.12	20.69
ENSDARG00000057826	si:ch73-61d6.3	si:ch73-61d6.3 [Source:ZFIN;Acc:ZDB-GENE-091204-109]	8	13190314	13211527		8.15	8.71	5.96	11.04	10.96	11.96
ENSDARG00000057830	dnmt3bb.2	DNA (cytosine-5-)-methyltransferase 3 beta, duplicate b.2 [Source:ZFIN;Acc:ZDB-GENE-990712-11]	23	16981929	17004783		1.38	1.22	1.08	1.02	0.91	0.88
ENSDARG00000057833	gulp1b	GULP PTB domain containing engulfment adaptor 1b [Source:ZFIN;Acc:ZDB-GENE-120228-2]	6	11497646	11614339		2.11	1.23	1.45	1.64	1.74	2
ENSDARG00000057844	zgc:171470	zgc:171470 [Source:ZFIN;Acc:ZDB-GENE-080204-4]	1	57497959	57501299		1.36	1.45	1.43	0.44	0.5	0.75
ENSDARG00000057853	atp6v0ca	ATPase H+ transporting V0 subunit ca [Source:ZFIN;Acc:ZDB-GENE-020419-23]	3	16044680	16055463		219.59	182.52	194.67	202.96	210.91	201.08
ENSDARG00000057854	sap18	Sin3A-associated protein [Source:ZFIN;Acc:ZDB-GENE-040426-1679]	9	21266684	21268576		33.1	35.46	36.28	28.85	25.24	28.51
ENSDARG00000057855	mrps31	mitochondrial ribosomal protein S31 [Source:ZFIN;Acc:ZDB-GENE-030131-3715]	5	24138446	24154437		23.32	27.64	24.77	14.52	14.64	15.21
ENSDARG00000057857	arfrp1	ADP-ribosylation factor related protein 1 [Source:ZFIN;Acc:ZDB-GENE-050227-14]	6	58522557	58531664		8.27	7.84	6.23	8.59	8.1	9.46
ENSDARG00000057859	hoxd10a	homeobox D10a [Source:NCBI gene;Acc:30404]	9	1967707	1970071		2.88	5.24	5.08	5.01	4.49	5.13
ENSDARG00000057863	dnmt3bb.3	DNA (cytosine-5-)-methyltransferase beta, duplicate b.3 [Source:ZFIN;Acc:ZDB-GENE-050314-2]	23	16961269	16981985		1.75	1.05	1.18	1.34	1.19	1.23
ENSDARG00000057867	lasp1	LIM and SH3 protein 1 [Source:ZFIN;Acc:ZDB-GENE-030131-1936]	3	16072966	16110351		61.29	62.24	59.03	67.12	68.53	68.87
ENSDARG00000057869	cdc42l2	cell division cycle 42 like 2 [Source:ZFIN;Acc:ZDB-GENE-060312-21]	23	16956185	16960972		2.03	2.23	1.99	1.4	1.59	1.7
ENSDARG00000057879	p3h3	prolyl 3-hydroxylase 3 [Source:ZFIN;Acc:ZDB-GENE-060503-231]	16	11992498	12006828		7.04	7.91	7.65	9.26	9.28	8.86
ENSDARG00000057881	si:dkey-33c12.3	si:dkey-33c12.3 [Source:ZFIN;Acc:ZDB-GENE-030131-7320]	13	9313777	9318891		25.5	22.75	19.98	19.24	20.71	20.06
ENSDARG00000057882	arpc3	actin related protein 2/3 complex, subunit 3 [Source:ZFIN;Acc:ZDB-GENE-040625-107]	21	17024002	17029464		113.88	130.12	124.32	101.07	102.04	100.11
ENSDARG00000057884	zgc:114174	zgc:114174 [Source:ZFIN;Acc:ZDB-GENE-050626-103]	23	16864130	16884098		2.09	2.84	2.71	2.92	2.8	3.18
ENSDARG00000057886	BX901922.1	butyrophilin-like protein 2 [Source:NCBI gene;Acc:108191960]	13	9333317	9335891		0.34	0.2	0.15	0.15	0.42	0.12
ENSDARG00000057887	nts	neurotensin [Source:ZFIN;Acc:ZDB-GENE-091204-433]	18	16330025	16339902		1.52	1.25	1.18	2.2	2.88	3.19
ENSDARG00000057890	si:dkey-33c12.4	si:dkey-33c12.4 [Source:ZFIN;Acc:ZDB-GENE-030131-2527]	13	9339919	9368535		14.03	13.3	11.35	10.6	9.38	9.09
ENSDARG00000057899	zgc:114081	zgc:114081 [Source:ZFIN;Acc:ZDB-GENE-050913-78]	23	16807209	16811727		0.74	0.87	0.91	0.82	0.47	0.73
ENSDARG00000057903	si:ch211-266g18.10	si:ch211-266g18.10 [Source:ZFIN;Acc:ZDB-GENE-131127-188]	17	15449401	15498275		7.8	11.99	8.79	18.9	14.28	14.79
ENSDARG00000057907	si:ch73-181d5.4	si:ch73-181d5.4 [Source:ZFIN;Acc:ZDB-GENE-100922-118]	23	457569	478201		3.59	1.86	1.58	3.89	3.65	4.08
ENSDARG00000057910	mrps34	mitochondrial ribosomal protein S34 [Source:ZFIN;Acc:ZDB-GENE-041114-71]	3	16003122	16010968		46.54	42.8	40.23	31.32	28.9	30.44
ENSDARG00000057911	ACTC1	zgc:86709 [Source:ZFIN;Acc:ZDB-GENE-040625-35]	20	9970786	9980318	ACTC1	31.67	39.24	37.26	21.98	22.94	21.02
ENSDARG00000057912	eif1axb	eukaryotic translation initiation factor 1A X-linked b [Source:ZFIN;Acc:ZDB-GENE-030131-1319]	24	24164227	24168533		64.59	76.95	71.73	50.19	50.12	53.94
ENSDARG00000057913	syt11a	synaptotagmin XIa [Source:ZFIN;Acc:ZDB-GENE-040426-2815]	19	23982466	24034710		21.16	5.67	8.16	30.81	31.28	33.09
ENSDARG00000057918	tsc1b	TSC complex subunit 1b [Source:ZFIN;Acc:ZDB-GENE-030131-3404]	21	17301790	17339832		4.88	3.83	3.38	8.88	9.15	9.52
ENSDARG00000057921	ccdc136b	coiled-coil domain containing 136b [Source:ZFIN;Acc:ZDB-GENE-081104-244]	4	10017049	10049186		15.02	4.97	6.77	12.46	13.64	13.94
ENSDARG00000057927	rps6ka3b	ribosomal protein S6 kinase, polypeptide 3b [Source:ZFIN;Acc:ZDB-GENE-070209-74]	24	24183752	24271629		2.74	1.72	1.61	3.76	3.84	3.53
ENSDARG00000057928	mzt2b	mitotic spindle organizing protein 2B [Source:NCBI gene;Acc:445174]	21	16079124	16087880		8.72	5.98	6.45	9.72	9.4	9.3
ENSDARG00000057929	naf1	nuclear assembly factor 1 homolog (S. cerevisiae) [Source:ZFIN;Acc:ZDB-GENE-030131-4546]	4	10003523	10012303		6.26	5.77	5.27	3.03	3.13	2.98
ENSDARG00000057936	lhx5	LIM homeobox 5 [Source:NCBI gene;Acc:30465]	21	15913128	15929041		10.18	3.27	4.63	14.38	14.27	14.65
ENSDARG00000057939	ythdf3	YTH N(6)-methyladenosine RNA binding protein 3 [Source:ZFIN;Acc:ZDB-GENE-030131-6123]	24	24285751	24300350		5.25	4.21	3.34	6.46	6.1	6.85
ENSDARG00000057940	dido1	death inducer-obliterator 1 [Source:ZFIN;Acc:ZDB-GENE-030131-6117]	11	18531984	18557929		9.07	6.07	5.17	10.32	9.81	10.08
ENSDARG00000057942	glt8d2	glycosyltransferase 8 domain containing 2 [Source:ZFIN;Acc:ZDB-GENE-041210-98]	4	9843923	9852318		0.67	0.9	0.83	0.52	0.51	0.59
ENSDARG00000057949	slc43a3b	solute carrier family 43 member 3b [Source:ZFIN;Acc:ZDB-GENE-060312-28]	14	17125428	17162558		3.5	4.43	3.79	4.34	4.71	4.49
ENSDARG00000057973	dph1	diphthamide biosynthesis 1 [Source:NCBI gene;Acc:550559]	15	25910501	26089064		5.58	6.25	5.74	3.91	3.89	3.5
ENSDARG00000057974	parp12b	poly (ADP-ribose) polymerase family, member 12b [Source:ZFIN;Acc:ZDB-GENE-030131-2617]	4	9813753	9822519		6.39	3.91	4.78	5.97	6.02	5.76
ENSDARG00000057975	plcd4a	phospholipase C, delta 4a [Source:ZFIN;Acc:ZDB-GENE-050208-654]	22	12729329	12746539		0.53	0.37	0.47	1.05	0.98	1.06
ENSDARG00000057983	svopl	SVOP-like [Source:NCBI gene;Acc:492766]	4	9769050	9781132		4.24	4.06	4.62	3.82	3.19	2.81
ENSDARG00000057984	eps8l1a	eps8 like 1a [Source:ZFIN;Acc:ZDB-GENE-050306-14]	3	16513219	16537441		9.98	8.75	7.58	4.66	4.26	4.67
ENSDARG00000057986	ints8	integrator complex subunit 8 [Source:ZFIN;Acc:ZDB-GENE-050522-556]	19	17834769	17996336		3.41	2.49	2.83	3.23	3.27	3.12
ENSDARG00000057987	uba3	ubiquitin-like modifier activating enzyme 3 [Source:NCBI gene;Acc:406776]	11	17785182	17803137		15.04	12.99	14.1	14.43	14.45	14.21
ENSDARG00000057992	fstb	follistatin b [Source:ZFIN;Acc:ZDB-GENE-031118-139]	10	8968203	8976823		7.91	9.05	7.95	12.75	14.4	13.58
ENSDARG00000057995	arl15b	ADP-ribosylation factor-like 15b [Source:ZFIN;Acc:ZDB-GENE-080813-2]	10	9006574	9089636		14.6	12.79	13.79	14.85	14.33	13.84
ENSDARG00000057997	tmf1	TATA element modulatory factor 1 [Source:ZFIN;Acc:ZDB-GENE-040801-25]	11	17757342	17782930		12.74	9.18	8.59	10.47	11.17	11.26
ENSDARG00000058000	cep350	centrosomal protein 350 [Source:ZFIN;Acc:ZDB-GENE-030131-6975]	8	14559378	14609284		5.68	3.69	3.07	7.49	6.87	7.07
ENSDARG00000058003	wfdc1	WAP four-disulfide core domain 1 [Source:ZFIN;Acc:ZDB-GENE-070112-352]	18	14595546	14617707		10.84	12.05	9.87	10.36	9.48	8.62
ENSDARG00000058004	six2a	SIX homeobox 2a [Source:ZFIN;Acc:ZDB-GENE-010412-1]	13	10232695	10235565		13.66	13.3	12.21	11.59	12.74	13.08
ENSDARG00000058005	hgd	homogentisate 1,2-dioxygenase [Source:ZFIN;Acc:ZDB-GENE-020802-5]	24	23959107	23975132		10.29	14.26	16.39	6.58	7.04	6.8
ENSDARG00000058008	six3a	SIX homeobox 3a [Source:ZFIN;Acc:ZDB-GENE-990415-127]	13	10258007	10261383		18.31	3.97	4.18	17.36	17.78	17.65
ENSDARG00000058011	arxa	aristaless related homeobox a [Source:NCBI gene;Acc:30657]	24	23936829	23942722		7.66	5.17	4.37	7.66	7.11	7.84
ENSDARG00000058015	ano3	anoctamin 3 [Source:ZFIN;Acc:ZDB-GENE-111031-3]	25	35375848	35468555		2.48	0.52	0.6	3.58	3.48	3.84
ENSDARG00000058020	fbxw9	F-box and WD repeat domain containing 9 [Source:ZFIN;Acc:ZDB-GENE-091204-198]	3	13445772	13461361		2.47	1.69	1.88	2.77	3.04	2.36
ENSDARG00000058022	ormdl2	ORMDL sphingolipid biosynthesis regulator 2 [Source:ZFIN;Acc:ZDB-GENE-060331-113]	23	31962383	31969786		7.32	7.71	6.29	7.47	7.21	7.07
ENSDARG00000058023	si:dkey-11o15.7	si:dkey-11o15.7 [Source:ZFIN;Acc:ZDB-GENE-091204-328]	21	15295685	15306081		0.12	0.23	0.19	0.22	0.12	0.16
ENSDARG00000058030	hspa14	heat shock protein 14 [Source:NCBI gene;Acc:565232]	4	9592486	9601691		15.12	16.55	16.93	12.36	11.37	10.59
ENSDARG00000058032	tpk2	thiamin pyrophosphokinase 2 [Source:ZFIN;Acc:ZDB-GENE-060503-173]	8	14158021	14170576		9.92	12.7	13.77	8.74	7.81	7.25
ENSDARG00000058038	eml3	EMAP like 3 [Source:ZFIN;Acc:ZDB-GENE-110304-3]	7	17816006	17905528		2.89	3.15	2.28	2.6	3.14	2.91
ENSDARG00000058039	bhlhe22	basic helix-loop-helix family, member e22 [Source:ZFIN;Acc:ZDB-GENE-040426-1411]	24	24461341	24463346		24.47	6.19	8.04	25.26	22.58	27.46
ENSDARG00000058041	ndufa8	NADH:ubiquinone oxidoreductase subunit A8 [Source:ZFIN;Acc:ZDB-GENE-040426-1688]	10	9410304	9419994		140.8	185.63	195.4	94.91	94.53	97.68
ENSDARG00000058042	kitlgb	kit ligand b [Source:ZFIN;Acc:ZDB-GENE-070424-2]	4	9508505	9515398		2.65	2.09	1.27	1.49	0.78	1.08
ENSDARG00000058044	rab1ba	zRAB1B, member RAS oncogene family a [Source:ZFIN;Acc:ZDB-GENE-040426-873]	7	18100996	18116392		45.15	34.87	32.94	51.55	52.73	51.82
ENSDARG00000058045	tlr21	toll-like receptor 21 [Source:ZFIN;Acc:ZDB-GENE-040220-4]	16	11785967	11789108		0.23	0.4	0.26	0.47	0.43	0.46
ENSDARG00000058047	pafah1b3	platelet-activating factor acetylhydrolase, isoform Ib, gamma subunit [Source:ZFIN;Acc:ZDB-GENE-040426-1026]	16	11773395	11779661		11.2	9.82	9.09	12.26	11.08	11.6
ENSDARG00000058048	ugt5e1	UDP glucuronosyltransferase 5 family, polypeptide E1 [Source:ZFIN;Acc:ZDB-GENE-081105-106]	8	14058646	14060244		3.43	4.27	3.93	1.76	2.45	2
ENSDARG00000058050	smyd3	SET and MYND domain containing 3 [Source:ZFIN;Acc:ZDB-GENE-051120-138]	17	11728920	12058171		3.16	3.54	3.37	2.21	2.46	2.03
ENSDARG00000058053	serping1	serpin peptidase inhibitor, clade G (C1 inhibitor), member 1 [Source:ZFIN;Acc:ZDB-GENE-030131-1262]	8	13960655	13972626		5.51	8.24	6.45	6.75	7.44	8.07
ENSDARG00000058058	igf3	insulin-like growth factor 3 [Source:ZFIN;Acc:ZDB-GENE-080611-1]	8	13978486	13985032		0.13	0.1	0.15	0.07	0.15	0.22
ENSDARG00000058062	magt1	magnesium transporter 1 [Source:NCBI gene;Acc:324944]	14	12370375	12391506		20.22	26.43	24.82	21.65	22.23	20.97
ENSDARG00000058064	cx31.7	connexin 31.7 [Source:ZFIN;Acc:ZDB-GENE-050616-3]	14	12316606	12334053		0.13	0.03	0.04	0.1	0.04	0.04
ENSDARG00000058068	tnfsf10l4	tumor necrosis factor (ligand) superfamily, member 10 like 4 [Source:ZFIN;Acc:ZDB-GENE-050227-6]	5	22579975	22583542		1.47	1.4	1.36	1.64	1.57	1.09
ENSDARG00000058076	snf8	SNF8 subunit of ESCRT-II [Source:NCBI gene;Acc:492771]	12	20652957	20658285		12.35	12.97	11.75	10.46	8.91	10.21
ENSDARG00000058079	txndc17	thioredoxin domain containing 17 [Source:NCBI gene;Acc:445062]	15	16328450	16333015		26.9	36.81	33.98	18.6	17.97	20.13
ENSDARG00000058082	birc7	baculoviral IAP repeat containing 7 [Source:ZFIN;Acc:ZDB-GENE-070615-35]	23	14217508	14230506		2.25	2.03	2.77	3.85	4.78	5.21
ENSDARG00000058088	aifm1	apoptosis inducing factor mitochondrion associated 1 [Source:ZFIN;Acc:ZDB-GENE-030826-11]	5	22550614	22573673		34.54	36.96	32.5	28.72	29.27	26.95
ENSDARG00000058089	rprd2a	regulation of nuclear pre-mRNA domain containing 2a [Source:ZFIN;Acc:ZDB-GENE-030131-6641]	19	8893952	8926575		6.42	3.81	3.33	8.87	8.05	8.62
ENSDARG00000058090	cfap57	cilia and flagella associated protein 57 [Source:ZFIN;Acc:ZDB-GENE-110411-266]	2	13462305	13673494		0.63	0.65	0.65	0.88	1.16	1
ENSDARG00000058094	ciarta	circadian associated repressor of transcription a [Source:ZFIN;Acc:ZDB-GENE-030131-7016]	19	8928434	8940068		24.81	16.89	17.5	31.22	32.17	31.1
ENSDARG00000058100	b3gnt3.4	UDP-GlcNAc:betaGal beta-1,3-N-acetylglucosaminyltransferase 3, tandem duplicate 4 [Source:ZFIN;Acc:ZDB-GENE-030131-8875]	8	13630492	13641022		2.08	3.06	2.23	2.88	2.78	3.19
ENSDARG00000058102	sardh	sarcosine dehydrogenase [Source:ZFIN;Acc:ZDB-GENE-040426-996]	10	10386435	10564968		9.42	13.31	11.37	12.59	12.43	12.92
ENSDARG00000058103	glra4b	glycine receptor, alpha 4b [Source:ZFIN;Acc:ZDB-GENE-020402-2]	5	22515347	22544151		2.45	0.56	0.41	2.29	2.27	2.36
ENSDARG00000058105	rpl36a	ribosomal protein L36A [Source:NCBI gene;Acc:195819]	5	22510639	22515186		762.36	1017.99	1065.85	579.37	600.88	615.8
ENSDARG00000058108	eps8l2	EPS8 like 2 [Source:ZFIN;Acc:ZDB-GENE-131121-340]	25	3994391	4129303		6.36	6.73	4.58	5.96	6	6.43
ENSDARG00000058114	impad1	inositol monophosphatase domain containing 1 [Source:NCBI gene;Acc:641570]	2	11633253	11648731		14.66	13.28	9.22	14.39	15.77	15.46
ENSDARG00000058115	fgfr2	fibroblast growth factor receptor 2 [Source:NCBI gene;Acc:352940]	13	46298560	46429433		20.7	18.04	12.44	29.49	28.55	27.41
ENSDARG00000058117	snap25b	synaptosome associated protein 25b [Source:NCBI gene;Acc:30711]	17	12297984	12389259		101.96	25.36	26.79	90.21	91.53	94.58
ENSDARG00000058120	GPR83	si:dkey-27p18.2 [Source:ZFIN;Acc:ZDB-GENE-060526-286]	5	22363015	22369916	GPR83	0.81	0.17	0.41	0.76	0.79	0.56
ENSDARG00000058127	hcfc2	host cell factor C2 [Source:ZFIN;Acc:ZDB-GENE-050417-203]	4	9184481	9196291		3.82	2.32	2	3.04	2.77	2.99
ENSDARG00000058128	msna	moesin a [Source:ZFIN;Acc:ZDB-GENE-021211-2]	5	22133153	22174037		42.38	45.29	44.14	45.21	47.36	47.06
ENSDARG00000058133	foxd2	forkhead box D2 [Source:ZFIN;Acc:ZDB-GENE-980605-6]	8	19674369	19676640		7.29	5.76	6.52	7.7	8.54	8.54
ENSDARG00000058140	ttc25	tetratricopeptide repeat domain 25 [Source:NCBI gene;Acc:393286]	3	17910569	17928233		1.18	1.2	1.47	1.49	1.74	1.34
ENSDARG00000058145	atp7b	ATPase copper transporting beta [Source:ZFIN;Acc:ZDB-GENE-061220-5]	6	10094061	10132102		0.88	0.97	0.66	0.86	0.93	0.98
ENSDARG00000058147	dnajc5b	DnaJ (Hsp40) homolog, subfamily C, member 5 beta [Source:ZFIN;Acc:ZDB-GENE-050413-1]	24	24808955	24824899		2.88	3.81	3.73	0.59	1.06	0.77
ENSDARG00000058148	dnajc7	DnaJ (Hsp40) homolog, subfamily C, member 7 [Source:ZFIN;Acc:ZDB-GENE-040426-2483]	3	17878124	17910375		21.94	17.69	18.41	20.68	20.19	19.67
ENSDARG00000058154	znf385c	zinc finger protein 385C [Source:ZFIN;Acc:ZDB-GENE-091112-1]	3	17744339	17860047		2.85	0.97	0.96	7.83	8.33	8.55
ENSDARG00000058158	trim55b	tripartite motif containing 55b [Source:ZFIN;Acc:ZDB-GENE-060312-37]	24	24831727	24846603		3.54	5.69	6.8	8.21	7.96	8.42
ENSDARG00000058159	ipo8	importin 8 [Source:ZFIN;Acc:ZDB-GENE-060503-3]	4	9360532	9389359		11.67	5.15	7.25	13.72	12.64	12.82
ENSDARG00000058160	tnfaip2b	tumor necrosis factor, alpha-induced protein 2b [Source:ZFIN;Acc:ZDB-GENE-081107-56]	13	7442023	7453702		1.41	2.52	2.37	2.88	3.1	3.71
ENSDARG00000058162	pcyt1ba	phosphate cytidylyltransferase 1, choline, beta a [Source:ZFIN;Acc:ZDB-GENE-061220-4]	24	24923166	24935849		5.04	1.35	2.1	4.47	4.96	4.45
ENSDARG00000058165	htr2ab	5-hydroxytryptamine (serotonin) receptor 2A, genome duplicate b [Source:ZFIN;Acc:ZDB-GENE-120215-60]	6	10077756	10088463		0.82	0.18	0.35	0.42	0.54	0.78
ENSDARG00000058178	zdhhc20b	zinc finger DHHC-type containing 20b [Source:ZFIN;Acc:ZDB-GENE-091117-30]	24	24989145	25004611		6.3	3.24	4.12	7.65	6.88	7.31
ENSDARG00000058179	ackr3b	atypical chemokine receptor 3b [Source:ZFIN;Acc:ZDB-GENE-031116-61]	6	15751982	15757867		22.51	26.07	25.51	28.91	29.23	29.37
ENSDARG00000058180	exoc3l4	exocyst complex component 3-like 4 [Source:ZFIN;Acc:ZDB-GENE-081028-71]	13	7387822	7414096		3.14	3.83	3.32	2.04	2.45	2.43
ENSDARG00000058200	plcxd2	phosphatidylinositol-specific phospholipase C, X domain containing 2 [Source:ZFIN;Acc:ZDB-GENE-091204-217]	24	25173470	25186895		2.08	0.57	0.43	1.41	1.12	1.47
ENSDARG00000058202	pex16	peroxisomal biogenesis factor 16 [Source:NCBI gene;Acc:573990]	18	44913422	44935174		7.45	5.79	5.25	6.12	6.54	6.41
ENSDARG00000058203	smc1al	structural maintenance of chromosomes 1A, like [Source:ZFIN;Acc:ZDB-GENE-040426-57]	23	12840080	12897815		34.51	26.43	26.46	29.53	29.95	30.29
ENSDARG00000058206	si:ch211-153b23.5	si:ch211-153b23.5 [Source:ZFIN;Acc:ZDB-GENE-030131-9744]	14	11457500	11461002		19.7	22.23	23.11	29.03	26.03	22.89
ENSDARG00000058207	abi2a	abl-interactor 2a [Source:ZFIN;Acc:ZDB-GENE-060929-192]	9	13733468	13799692		8.99	7.12	7.06	9.08	8.48	9.07
ENSDARG00000058208	slc25a26	solute carrier family 25 member 26 [Source:NCBI gene;Acc:560478]	11	16216909	16324575		10.73	13.32	13.42	8.66	8.55	8.8
ENSDARG00000058212	ndnl2	necdin-like 2 [Source:ZFIN;Acc:ZDB-GENE-031107-3]	23	12897878	12913131		2.28	3.27	2.18	1.67	1.52	1.53
ENSDARG00000058218	fam151a	family with sequence similarity 151 member A [Source:NCBI gene;Acc:100005645]	2	11069055	11082914		3	5.2	3.01	1.1	1.18	1.41
ENSDARG00000058219	tubd1	tubulin, delta 1 [Source:ZFIN;Acc:ZDB-GENE-040625-73]	15	17397321	17406056		1.65	1.81	1.59	1.25	1.25	1.39
ENSDARG00000058220	tada3l	transcriptional adaptor 3 (NGG1 homolog, yeast)-like [Source:NCBI gene;Acc:334785]	11	16152316	16166705		11.03	7.25	8.33	9.65	9.64	10.1
ENSDARG00000058221	ugcg	UDP-glucose ceramide glucosyltransferase [Source:ZFIN;Acc:ZDB-GENE-030131-9885]	10	11038192	11094127		3.64	2.47	2.6	4.5	4.82	4.72
ENSDARG00000058222	mpp4a	membrane protein, palmitoylated 4a (MAGUK p55 subfamily member 4) [Source:ZFIN;Acc:ZDB-GENE-070912-523]	9	13682133	13711991		7.59	0.7	0.83	6.42	5.53	5.23
ENSDARG00000058225	arpc4l	actin related protein 2/3 complex, subunit 4, like [Source:ZFIN;Acc:ZDB-GENE-040808-18]	11	16139897	16152400		12.45	13.25	12.75	12.5	12.99	12.73
ENSDARG00000058226	ak3	adenylate kinase 3 [Source:ZFIN;Acc:ZDB-GENE-040426-2142]	10	11000894	11012000		17.74	25.52	19.63	30.06	33.65	30.4
ENSDARG00000058229	acot11a	acyl-CoA thioesterase 11a [Source:ZFIN;Acc:ZDB-GENE-050522-538]	2	11028923	11063878		3.62	3.88	2.62	3.77	4.37	3.91
ENSDARG00000058230	rps6kb1b	ribosomal protein S6 kinase b, polypeptide 1b [Source:ZFIN;Acc:ZDB-GENE-040426-2038]	15	17406920	17424590		27.76	25.58	23.93	23.68	24.7	23.57
ENSDARG00000058231	nt5c2b	5'-nucleotidase, cytosolic IIb [Source:ZFIN;Acc:ZDB-GENE-061013-522]	1	31809173	31856676		14.7	9.48	10.63	19.17	20.04	19.53
ENSDARG00000058232	fbrsl1	fibrosin-like 1 [Source:ZFIN;Acc:ZDB-GENE-070705-487]	5	17624463	17753961		6.81	3.16	3.45	13.95	12.32	13.16
ENSDARG00000058237	ssbp3a	single stranded DNA binding protein 3a [Source:ZFIN;Acc:ZDB-GENE-070705-484]	2	10934479	11027410		3.59	2.63	2.1	6.1	5.74	5.88
ENSDARG00000058243	phactr3a	phosphatase and actin regulator 3a [Source:ZFIN;Acc:ZDB-GENE-080220-4]	23	12232773	12345764		7.67	2.26	3.15	6.98	6.52	7.58
ENSDARG00000058244	il17ra1a	interleukin 17 receptor A1a [Source:ZFIN;Acc:ZDB-GENE-070705-242]	4	12317461	12323228		1.79	1.29	1.03	1.49	1.28	1.5
ENSDARG00000058246	cysltr1	cysteinyl leukotriene receptor 1 [Source:ZFIN;Acc:ZDB-GENE-050522-250]	14	10450216	10462758		0.44	0.75	0.41	1.07	0.84	0.98
ENSDARG00000058248	si:dkeyp-77h1.4	si:dkeyp-77h1.4 [Source:ZFIN;Acc:ZDB-GENE-050208-18]	16	10346277	10369298		8.49	2.15	2.6	9.11	8.83	8.14
ENSDARG00000058252	adamts15b	ADAM metallopeptidase with thrombospondin type 1 motif, 15b [Source:ZFIN;Acc:ZDB-GENE-070814-5]	15	17621375	17652807		0.95	1.23	0.82	0.73	0.79	0.86
ENSDARG00000058254	si:ch211-213d14.1	si:ch211-213d14.1 [Source:ZFIN;Acc:ZDB-GENE-131127-437]	15	17722054	17748319		0.08	0.04	0.09	0.17	0.09	0.14
ENSDARG00000058256	draxin	dorsal inhibitory axon guidance protein [Source:NCBI gene;Acc:619245]	11	15931188	16013114		1.91	0.64	0.84	1.93	1.83	1.98
ENSDARG00000058257	slc39a1	solute carrier family 39 member 1 [Source:NCBI gene;Acc:321324]	7	13896769	13906409		2.53	2.98	2.7	3.69	3.93	4.18
ENSDARG00000058259	celsr1b	cadherin EGF LAG seven-pass G-type receptor 1b [Source:ZFIN;Acc:ZDB-GENE-050823-4]	25	19753890	19869495		1.72	0.73	0.72	2.6	2.43	2.51
ENSDARG00000058260	cip2a	cell proliferation regulating inhibitor of protein phosphatase 2A [Source:ZFIN;Acc:ZDB-GENE-030131-6252]	24	25210015	25233751		2.55	2.29	1.94	2.3	2.32	2.4
ENSDARG00000058267	glmna	glomulin, FKBP associated protein a [Source:ZFIN;Acc:ZDB-GENE-041114-120]	2	10821127	10857898		5.26	4.29	4.46	5.23	5.35	5.47
ENSDARG00000058270	COLGALT1	si:ch211-114l13.7 [Source:NCBI gene;Acc:567859]	1	58013076	58036509	COLGALT1	8.83	7.11	4.84	12.49	11.8	11.5
ENSDARG00000058280	si:dkey-208b23.5	si:dkey-208b23.5 [Source:ZFIN;Acc:ZDB-GENE-081104-362]	8	13036221	13064330		0.44	0.21	0.34	0.41	0.51	0.54
ENSDARG00000058284	CT990561.1	EPH receptor A5 [Source:NCBI gene;Acc:797595]	10	12517059	12730451		0.89	0.43	0.4	1.34	1.41	1.59
ENSDARG00000058285	cpt1b	carnitine palmitoyltransferase 1B (muscle) [Source:ZFIN;Acc:ZDB-GENE-041010-9]	18	8340886	8367566		4.74	5.27	5.11	4.75	3.82	3.81
ENSDARG00000058287	gpalpp1	GPALPP motifs containing 1 [Source:ZFIN;Acc:ZDB-GENE-040801-212]	1	34685405	34694365		11.39	7.18	7.58	9.69	9.1	11.03
ENSDARG00000058292	sephs1	selenophosphate synthetase 1 [Source:NCBI gene;Acc:324947]	18	8367602	8380120		17.62	14.86	15.73	13.42	12.12	12.34
ENSDARG00000058297	timm13	translocase of inner mitochondrial membrane 13 homolog (yeast) [Source:NCBI gene;Acc:436738]	22	10771115	10774735		41.37	52.87	53.41	31.49	32.1	33.18
ENSDARG00000058299	zgc:173737	zgc:173737 [Source:ZFIN;Acc:ZDB-GENE-071004-109]	8	3334708	3336273		0.22	0.27	0.26	0.27	0.19	0.25

ENSDARG00000058302	sh3bgrl	SH3 domain binding glutamate-rich protein like [Source:ZFIN;Acc:ZDB-GENE-040426-1376]	14	9405099	9420911	12.04	12.49	10.42	14.26	12.75	12.5
ENSDARG00000058306	prpf18	PRP18 pre-mRNA processing factor 18 homolog (yeast) [Source:NCBI gene;Acc:431751]	18	8402129	8408518	14.22	13.17	13.63	12.06	12.56	12.6
ENSDARG00000058318	c1qtnf9	C1q and TNF related 9 [Source:ZFIN;Acc:ZDB-GENE-080303-33]	24	21905717	21914276	0.79	1.28	0.7	1.99	1.97	1.81
ENSDARG00000058319	cep76	centrosomal protein 76 [Source:NCBI gene;Acc:564926]	16	10025896	10053049	1.21	0.68	1.04	1.74	1.47	1.33
ENSDARG00000058322	enc3	ectodermal-neural cortex 3 [Source:ZFIN;Acc:ZDB-GENE-030131-5742]	22	10781894	10795953	7.91	6.6	6.04	6.86	7.12	7.02
ENSDARG00000058323	tmbim1a	transmembrane BAX inhibitor motif containing 1a [Source:ZFIN;Acc:ZDB-GENE-041010-69]	6	12996038	13022166	9.56	7.96	6.95	10.15	9.91	9.79
ENSDARG00000058325	casp8	caspase 8, apoptosis-related cysteine peptidase [Source:ZFIN;Acc:ZDB-GENE-000713-1]	6	13045885	13058979	1.6	1.6	1.35	1.88	1.89	1.7
ENSDARG00000058327	neu3.3	sialidase 3 (membrane sialidase), tandem duplicate 3 [Source:ZFIN;Acc:ZDB-GENE-061103-511]	21	21803363	21809107	2.28	3.07	2.64	0.35	0.34	0.35
ENSDARG00000058328	lsm7	LSM7 homolog, U6 small nuclear RNA and mRNA degradation associated [Source:ZFIN;Acc:ZDB-GENE-030131-8284]	22	10752511	10756047	41.24	46.19	40.7	27.6	28.77	30.13
ENSDARG00000058329	btr21	bloodthirsty-related gene family, member 21 [Source:ZFIN;Acc:ZDB-GENE-081031-80]	15	18212190	18218227	0.24	0.23	0.18	0.1	0.03	0.01
ENSDARG00000058332	krt18a.2	keratin 18a, tandem duplicate 2 [Source:ZFIN;Acc:ZDB-GENE-090312-205]	23	10352921	10355552	9.71	13.97	10.02	4	4.18	4.16
ENSDARG00000058336	slc25a22b	solute carrier family 25 member 22b [Source:ZFIN;Acc:ZDB-GENE-110408-66]	7	12976173	12999913	1.22	0.65	0.8	0.47	0.49	0.35
ENSDARG00000058342	grhl2a	grainyhead-like transcription factor 2a [Source:ZFIN;Acc:ZDB-GENE-050913-123]	16	9807263	9830451	3.73	4.12	3.8	2.6	2.68	2.29
ENSDARG00000058345	si:dkey-5i3.5	si:dkey-5i3.5 [Source:ZFIN;Acc:ZDB-GENE-070424-162]	18	8901846	8909935	6.13	5.84	5.71	4.58	4.52	4.21
ENSDARG00000058346	sass6	SAS-6 centriolar assembly protein [Source:NCBI gene;Acc:406747]	22	10735909	10752471	1.29	0.85	0.87	0.8	0.77	0.72
ENSDARG00000058348	scinlb	scinderin like b [Source:ZFIN;Acc:ZDB-GENE-040426-2068]	2	10396579	10450784	13.71	10.4	9.55	13.77	13.96	13.67
ENSDARG00000058349	polr2l	polymerase (RNA) II (DNA directed) polypeptide L [Source:ZFIN;Acc:ZDB-GENE-050522-181]	7	13363898	13371484	49.46	61.37	61.62	35.95	33.63	36.04
ENSDARG00000058351	tapbpl	TAP binding protein like [Source:ZFIN;Acc:ZDB-GENE-121003-1]	16	9787383	9802998	0.3	0.2	0.22	0.23	0.14	0.22
ENSDARG00000058352	acot9.1	acyl-CoA thioesterase 9, tandem duplicate 1 [Source:ZFIN;Acc:ZDB-GENE-040724-139]	24	21959708	21973365	6.26	5.47	5.21	5.9	6.39	6.2
ENSDARG00000058354	selenot1a	selenoprotein T, 1a [Source:NCBI gene;Acc:352919]	2	9684305	9696283	51.2	43.26	42	38.32	42.05	41.5
ENSDARG00000058357	ankrd33ba	ankyrin repeat domain 33ba [Source:ZFIN;Acc:ZDB-GENE-040724-39]	24	22039964	22059801	5.3	1.63	1.16	3.22	4.11	3.92
ENSDARG00000058358	krt8	keratin 8 [Source:NCBI gene;Acc:797433]	23	10249626	10254288	348.55	412.4	455.38	320.53	358.43	358.21
ENSDARG00000058365	hspb8	heat shock protein b8 [Source:ZFIN;Acc:ZDB-GENE-030131-2480]	5	15819651	15840485	6.77	10.26	6.89	9.09	9.4	8.46
ENSDARG00000058366	si:dkey-222f8.3	si:dkey-222f8.3 [Source:ZFIN;Acc:ZDB-GENE-030131-3658]	4	12033873	12040345	14.88	18.33	15.35	12.55	12.91	11.92
ENSDARG00000058367	abhd14a	abhydrolase domain containing 14A [Source:NCBI gene;Acc:404598]	22	10698549	10706873	3.88	2.15	2.02	5.75	5.38	5.79
ENSDARG00000058369	mex3b	mex-3 RNA binding family member B [Source:ZFIN;Acc:ZDB-GENE-030131-2628]	7	12061787	12065668	19.81	9.04	9.31	31.44	29.85	32.52
ENSDARG00000058370	ropn1l	rhophilin associated tail protein 1-like [Source:NCBI gene;Acc:798354]	24	22021675	22031079	1.66	1.64	1.99	0.97	1.27	1.2
ENSDARG00000058371	krt5	keratin 5 [Source:ZFIN;Acc:ZDB-GENE-991110-23]	23	10173196	10177458	2367.79	2636.03	2232.87	647.65	570.47	541.43
ENSDARG00000058372	mcph1	microcephalin 1 [Source:ZFIN;Acc:ZDB-GENE-060421-6122]	13	6286594	6324011	0.6	0.44	0.48	0.41	0.45	0.39
ENSDARG00000058379	otpb	orthopedia homeobox b [Source:NCBI gene;Acc:30316]	5	51615025	51619742	5.86	1.24	2.79	7.76	7.05	7.58
ENSDARG00000058381	zgc:171775	zgc:171775 [Source:ZFIN;Acc:ZDB-GENE-030131-4309]	23	10146542	10165033	11.51	14.18	11.67	11.32	11.48	10.88
ENSDARG00000058386	pimr57	Pim proto-oncogene, serine/threonine kinase, related 57 [Source:ZFIN;Acc:ZDB-GENE-141211-43]	14	8725216	8727352	0.03	0	0	0	0.02	0.04
ENSDARG00000058389	ccl19a.1	chemokine (C-C motif) ligand 19a, tandem duplicate 1 [Source:ZFIN;Acc:ZDB-GENE-060526-181]	5	13353866	13392962	1.21	1.7	1.33	0.94	1.2	1.15
ENSDARG00000058390	acot9.2	acyl-CoA thioesterase 9, tandem duplicate 2 [Source:ZFIN;Acc:ZDB-GENE-051113-136]	24	21938741	21952164	1.91	1.71	2.05	1.27	1.1	1.42
ENSDARG00000058392	adcy2a	adenylate cyclase 2a [Source:ZFIN;Acc:ZDB-GENE-061109-1]	16	9275689	9383629	2.73	0.64	1	2.82	3.22	3
ENSDARG00000058394	tagln3b	transgelin 3b [Source:ZFIN;Acc:ZDB-GENE-030131-4199]	24	21917012	21934571	3.98	4.79	4.65	3.57	3.45	3.68
ENSDARG00000058409	hce2l1	high choriolytic enzyme 2-like 1 [Source:ZFIN;Acc:ZDB-GENE-070912-147]	9	12894578	12903567	0.17	0.07	0.05	0.29	0.11	0.28
ENSDARG00000058410	CR956623.1	acyl-CoA binding domain containing 5b [Source:NCBI gene;Acc:100004736]	2	9918678	9941405	2.38	0.56	0.59	2.02	2.28	2.12
ENSDARG00000058413	rnaset2l	ribonuclease T2, like [Source:ZFIN;Acc:ZDB-GENE-090819-2]	15	20395309	20400438	0.85	1.57	1.52	0.72	0.91	0.56
ENSDARG00000058414	bglap	bone gamma-carboxyglutamate (gla) protein [Source:NCBI gene;Acc:792433]	3	1405504	1408487	0	0.06	0	0.19	0.07	0
ENSDARG00000058416	si:dkey-11m19.5	si:dkey-11m19.5 [Source:ZFIN;Acc:ZDB-GENE-120709-97]	11	12879635	12955738	0.06	0.05	0.05	0.13	0.13	0.12
ENSDARG00000058419	gcn1	GCN1 activator of EIF2AK4 [Source:ZFIN;Acc:ZDB-GENE-040724-264]	8	3530761	3643374	18.66	18.27	15.25	24.69	24.24	23.64
ENSDARG00000058421	lgi1b	leucine-rich, glioma inactivated 1b [Source:ZFIN;Acc:ZDB-GENE-060217-1]	12	5189776	5204629	5.02	1.81	3.32	5.28	5.06	5.34
ENSDARG00000058425	rab35b	RAB35, member RAS oncogene family b [Source:ZFIN;Acc:ZDB-GENE-040801-62]	8	3649036	3674915	13.02	12.45	11.49	11.09	10.94	10.57
ENSDARG00000058444	snx6	sorting nexin 6 [Source:ZFIN;Acc:ZDB-GENE-050913-152]	17	9963436	10000339	0.45	0.31	0.18	0.58	0.54	0.54
ENSDARG00000058445	zgc:136472	zgc:136472 [Source:ZFIN;Acc:ZDB-GENE-060421-2552]	1	9234928	9249943	8.19	16.6	12.13	2.47	3.06	2.63
ENSDARG00000058448	zrsr2	zinc finger (CCCH type), RNA-binding motif and serine/arginine rich 2 [Source:ZFIN;Acc:ZDB-GENE-030131-1495]	11	12732247	12744082	3.67	1.76	2.2	2.12	2.3	2.44
ENSDARG00000058451	rpl6	ribosomal protein L6 [Source:ZFIN;Acc:ZDB-GENE-030131-8671]	8	2493794	2506384	2295.29	2714.39	3339.09	1818.31	1902.82	1883.21
ENSDARG00000058452	abhd17c	abhydrolase domain containing 17C [Source:NCBI gene;Acc:393126]	7	10911396	11005919	8.19	5.23	5.47	10.67	10.78	9.67
ENSDARG00000058454	dynll1	dynein, light chain, LC8-type 1 [Source:ZFIN;Acc:ZDB-GENE-040426-1961]	8	2487250	2491471	159.2	179.27	189.18	140.38	142.23	140.01
ENSDARG00000058459	plekhg2	pleckstrin homology domain containing, family G (with RhoGef domain) member 2 [Source:ZFIN;Acc:ZDB-GENE-090819-1]	15	20281305	20352045	2.66	2.64	1.95	4.14	4.14	4.29
ENSDARG00000058460	cacng3a	calcium channel, voltage-dependent, gamma subunit 3a [Source:ZFIN;Acc:ZDB-GENE-090312-124]	1	9082417	9091935	0.58	0.08	0.01	0.49	0.52	0.54
ENSDARG00000058461	ice1	KIAA0947-like (H. sapiens) [Source:ZFIN;Acc:ZDB-GENE-030131-5566]	16	9400661	9420358	3.3	2.24	2.17	3.13	3.24	3.17
ENSDARG00000058462	zgc:158846	zgc:158846 [Source:ZFIN;Acc:ZDB-GENE-070209-50]	6	39336731	39344259	147.67	250.04	191.56	44.28	45.38	44.78
ENSDARG00000058463	ndufab1a	NADH:ubiquinone oxidoreductase subunit AB1a [Source:ZFIN;Acc:ZDB-GENE-040801-1]	1	9118100	9123465	78.69	101.35	106.34	56.15	50.34	50.54
ENSDARG00000058464	rasd3	RASD family member 3 [Source:ZFIN;Acc:ZDB-GENE-060818-9]	1	9144958	9145896	1.74	0.86	1.29	1.58	1.5	1.18
ENSDARG00000058467	prpf40a	PRP40 pre-mRNA processing factor 40 homolog A [Source:ZFIN;Acc:ZDB-GENE-030131-274]	9	4675193	4721993	18.6	13.59	10.25	18.93	19.05	18.85
ENSDARG00000058470	mapk13	mitogen-activated protein kinase 13 [Source:ZFIN;Acc:ZDB-GENE-041111-17]	8	10823069	10857042	5.33	4.11	4.6	7.05	6.42	6.15
ENSDARG00000058471	plk1	polo-like kinase 1 (Drosophila) [Source:ZFIN;Acc:ZDB-GENE-021115-7]	1	9153141	9161843	14.31	11.04	11.48	13.14	13.21	13.58
ENSDARG00000058473	st6galnac3	ST6 (alpha-N-acetyl-neuraminyl-2,3-beta-galactosyl-1,3)-N-acetylgalactosaminide alpha-2,6-sialyltransferase 3 [Source:ZFIN;Acc:Z	2	9330146	9489611	4.55	1.82	2.27	5.22	4.95	5.22
ENSDARG00000058475	ern2	endoplasmic reticulum to nucleus signaling 2 [Source:ZFIN;Acc:ZDB-GENE-090312-15]	1	9169807	9195760	2.52	2.36	1.81	2.73	2.69	2.52
ENSDARG00000058476	stc1l	stanniocalcin 1, like [Source:ZFIN;Acc:ZDB-GENE-040426-1521]	10	42677891	42685512	1.57	2.42	1.55	13.53	14.84	12.56
ENSDARG00000058477	pimr72	Pim proto-oncogene, serine/threonine kinase, related 72 [Source:ZFIN;Acc:ZDB-GENE-091118-122]	21	18970857	18972206	0	0	0	0.06	0.03	0.09
ENSDARG00000058479	zgc:110425	zgc:110425 [Source:ZFIN;Acc:ZDB-GENE-050522-103]	7	6469133	6470431	0.55	0.83	0.7	0.92	1.02	0.45
ENSDARG00000058480	chtf18	CTF18, chromosome transmission fidelity factor 18 homolog (S. cerevisiae) [Source:ZFIN;Acc:ZDB-GENE-050522-508]	1	9199031	9227204	3.13	2.43	2.24	1.99	2.07	1.83
ENSDARG00000058486	caps2	calcyphosine 2 [Source:ZFIN;Acc:ZDB-GENE-091204-449]	18	7317545	7343389	0.82	0.32	0.54	0.86	0.76	0.87
ENSDARG00000058488	adora2ab	adenosine A2a receptor b [Source:ZFIN;Acc:ZDB-GENE-081022-41]	21	13301798	13311387	0.63	0.93	0.65	1.54	1.79	1.61
ENSDARG00000058492	si:dkey-30c15.17	si:dkey-30c15.17 [Source:ZFIN;Acc:ZDB-GENE-060503-450]	18	7363242	7374783	1.28	2.57	1.79	1.14	1.38	1.37
ENSDARG00000058494	dnaja3a	DnaJ heat shock protein family (Hsp40) member A3a [Source:ZFIN;Acc:ZDB-GENE-030131-7837]	3	11926030	11947672	14.38	14.77	12.34	11.92	12.53	12.68
ENSDARG00000058498	slc9a3.1	solute carrier family 9 member A3, tandem duplicate 1 [Source:ZFIN;Acc:ZDB-GENE-060503-545]	19	10692166	10730488	0.17	0.37	0.15	2.92	4.07	3.48
ENSDARG00000058502	olah	oleoyl-ACP hydrolase [Source:ZFIN;Acc:ZDB-GENE-060312-17]	19	10649424	10656796	3.05	3.72	3.94	2.53	2.3	2.44
ENSDARG00000058503	flt3	fms-related tyrosine kinase 3 [Source:ZFIN;Acc:ZDB-GENE-050114-4]	24	21691939	21713883	0.06	0.06	0.14	0.13	0.16	0.13
ENSDARG00000058504	ap1s2	adaptor related protein complex 1 subunit sigma 2 [Source:ZFIN;Acc:ZDB-GENE-030131-5448]	11	12719944	12728353	28.48	33.2	33.64	30.84	33.22	30.9
ENSDARG00000058508	cfap70	cilia and flagella associated protein 70 [Source:ZFIN;Acc:ZDB-GENE-091204-50]	3	11977271	12026741	0.77	0.42	0.57	0.66	0.91	0.92
ENSDARG00000058522	shq1	SHQ1, H/ACA ribonucleoprotein assembly factor [Source:NCBI gene;Acc:567601]	6	44089189	44161262	2.5	2.51	2.63	1.71	1.83	1.76
ENSDARG00000058528	cnot10	CCR4-NOT transcription complex, subunit 10 [Source:NCBI gene;Acc:449918]	16	7703534	7730836	7.84	4.76	5.68	6.14	6.08	6.06
ENSDARG00000058532	pimr209	Pim proto-oncogene, serine/threonine kinase, related 209 [Source:ZFIN;Acc:ZDB-GENE-110411-216]	16	7688368	7692568	0.1	0.05	0.11	0.19	0.3	0.2
ENSDARG00000058533	pole	polymerase (DNA directed), epsilon [Source:ZFIN;Acc:ZDB-GENE-070705-557]	5	12743640	12804906	3.75	3.22	2.74	2.6	2.55	2.29
ENSDARG00000058535	si:dkey-211g8.7	si:dkey-211g8.7 [Source:ZFIN;Acc:ZDB-GENE-060503-907]	19	10624644	10627752	0	0	0.01	0	0.01	0
ENSDARG00000058537	si:dkey-102c8.2	si:dkey-102c8.2 [Source:ZFIN;Acc:ZDB-GENE-050208-329]	22	10085572	10086446	3.16	3.27	3.13	2.86	2.73	2.69
ENSDARG00000058538	alcamb	activated leukocyte cell adhesion molecule b [Source:ZFIN;Acc:ZDB-GENE-030131-1768]	15	19838458	19878987	25.38	22.01	19.98	27.62	26.73	26.51
ENSDARG00000058543	lama5	laminin, alpha 5 [Source:ZFIN;Acc:ZDB-GENE-030131-9823]	23	9567334	9768700	13.81	10.51	6.25	17.48	14.8	15.15
ENSDARG00000058546	si:dkey-30c15.12	si:dkey-30c15.12 [Source:ZFIN;Acc:ZDB-GENE-060503-920]	18	7400929	7422787	0.06	0.05	0.04	0.1	0.16	0.16
ENSDARG00000058547	si:ch73-160i9.3	si:ch73-160i9.3 [Source:ZFIN;Acc:ZDB-GENE-141212-252]	19	10527228	10528344	0	0	0.08	0.14	0.31	0.19
ENSDARG00000058548	bves	blood vessel epicardial substance [Source:NCBI gene;Acc:415107]	16	7662609	7681144	4.57	6.23	4.76	5.54	5.01	5.3
ENSDARG00000058551	popdc3	popeye domain containing 3 [Source:ZFIN;Acc:ZDB-GENE-040624-10]	16	7653905	7658707	1.59	2.61	1.97	2.43	1.95	2
ENSDARG00000058553	sprb	sepiapterin reductase b [Source:ZFIN;Acc:ZDB-GENE-070719-1]	8	1065458	1070636	2.39	3	3.08	3.39	2.94	2.24
ENSDARG00000058556	muc5.2	mucin 5.2 [Source:ZFIN;Acc:ZDB-GENE-121108-1]	25	8407892	8445400	1.52	1.28	0.53	3.07	2.77	2.65
ENSDARG00000058557	il11b	interleukin 11b [Source:ZFIN;Acc:ZDB-GENE-051019-2]	19	10429810	10432134	0.04	0.04	0	1.22	1.31	1.75
ENSDARG00000058558	lzts3b	leucine zipper, putative tumor suppressor family member 3b [Source:ZFIN;Acc:ZDB-GENE-110411-249]	8	1082100	1110352	2.74	0.55	0.86	4.92	4.64	5.13
ENSDARG00000058560	adrm1	adhesion regulating molecule 1 [Source:NCBI gene;Acc:796450]	23	9560797	9567306	33.37	35.98	33.73	29.85	30.56	29.46
ENSDARG00000058561	fbxo30a	F-box protein 30a [Source:ZFIN;Acc:ZDB-GENE-040426-1215]	17	7620220	7628458	3.25	2.24	2.52	3.34	3.17	3.08
ENSDARG00000058562	znf991	zinc finger protein 991 [Source:ZFIN;Acc:ZDB-GENE-050208-409]	22	9898028	9901788	3.77	3.02	2.82	3.78	3.26	3.39
ENSDARG00000058563	ubox5	U-box domain containing 5 [Source:ZFIN;Acc:ZDB-GENE-110411-166]	8	1118328	1133945	4.21	3.4	4.23	4.39	4.66	4.03
ENSDARG00000058564	si:ch73-368j24.17	si:ch73-368j24.17 [Source:ZFIN;Acc:ZDB-GENE-160113-31]	7	6385664	6394666	0	0	0.01	0	0	0
ENSDARG00000058565	socs6a	suppressor of cytokine signaling 6a [Source:ZFIN;Acc:ZDB-GENE-080722-35]	24	15277216	15278775	3.44	2.01	2.37	5.57	4.86	4.53
ENSDARG00000058566	si:dkey-253d23.5	si:dkey-253d23.5 [Source:ZFIN;Acc:ZDB-GENE-050208-729]	22	9886903	9890386	1.68	1.18	1.48	1.73	1.66	1.6
ENSDARG00000058567	avp	arginine vasopressin [Source:ZFIN;Acc:ZDB-GENE-030407-2]	8	1148876	1157380	1.56	1.07	1.19	1.82	1.97	2.38
ENSDARG00000058570	ccl27a	chemokine (C-C motif) ligand 27a [Source:ZFIN;Acc:ZDB-GENE-000208-28]	8	1170015	1183948	2.78	3.42	3.7	0.51	0.93	0.63
ENSDARG00000058571	pdp2	putative pyruvate dehydrogenase phosphatase isoenzyme 2 [Source:ZFIN;Acc:ZDB-GENE-000921-2]	18	7452809	7456378	2.77	1.8	1.91	2.98	3.08	2.66
ENSDARG00000058574	abcg2c	ATP-binding cassette, sub-family G (WHITE), member 2c [Source:ZFIN;Acc:ZDB-GENE-050517-37]	13	5031197	5065539	6.19	5.61	4.93	7.9	7.24	7.56
ENSDARG00000058578	ccdc106b	coiled-coil domain containing 106b [Source:ZFIN;Acc:ZDB-GENE-050306-6]	19	10325360	10330831	0.66	0.56	0.5	0.78	0.77	0.52
ENSDARG00000058579	si:ch211-274f20.2	si:ch211-274f20.2 [Source:ZFIN;Acc:ZDB-GENE-091204-91]	14	9009600	9028955	3.24	5.58	4.06	2.53	2.66	2.98
ENSDARG00000058585	chst2b	carbohydrate (N-acetylglucosamine-6-O) sulfotransferase 2b [Source:ZFIN;Acc:ZDB-GENE-060720-33]	24	5784264	5786759	6.14	4.77	5.62	3.71	4.19	4.25
ENSDARG00000058587	terb1	telomere repeat binding bouquet formation protein 1 [Source:NCBI gene;Acc:559140]	18	7456597	7478152	0.06	0.07	0.01	0.05	0.03	0.05
ENSDARG00000058593	sri	sorcin [Source:ZFIN;Acc:ZDB-GENE-040426-1356]	16	7242610	7260345	39.85	56.23	44.07	46.77	47.41	45.76
ENSDARG00000058597	nt5c3a	5'-nucleotidase, cytosolic IIIA [Source:NCBI gene;Acc:321720]	16	7214005	7239508	12.69	16.18	17.33	19.26	19.46	19.11
ENSDARG00000058598	sox18	SRY-box transcription factor 18 [Source:ZFIN;Acc:ZDB-GENE-080725-1]	23	8797143	8799880	4.41	3.56	2.6	6.16	6.83	6.37
ENSDARG00000058601	gdap1	ganglioside induced differentiation associated protein 1 [Source:ZFIN;Acc:ZDB-GENE-050522-424]	24	14713776	14725763	8.98	4.24	5.69	9.02	10.34	9.66
ENSDARG00000058603	jph1a	junctophilin 1a [Source:ZFIN;Acc:ZDB-GENE-040724-233]	24	14623831	14712427	5.88	8.16	5.93	10.42	9.22	9.05
ENSDARG00000058605	vsig10	V-set and immunoglobulin domain containing 10 [Source:NCBI gene;Acc:100005564]	5	12562188	12587053	3.86	3.84	2.77	3.39	2.79	2.39
ENSDARG00000058606	sik1	salt-inducible kinase 1 [Source:ZFIN;Acc:ZDB-GENE-030131-9446]	9	9112159	9122562	9.26	4.75	3.53	19.5	18.56	19.31
ENSDARG00000058608	wsb2	WD repeat and SOCS box containing 2 [Source:ZFIN;Acc:ZDB-GENE-081104-26]	5	12549551	12560569	2.28	1.51	0.99	1.65	1.63	1.65
ENSDARG00000058630	zgc:162472	zgc:162472 [Source:ZFIN;Acc:ZDB-GENE-050809-88]	21	11330234	11367271	6.74	3.59	3.59	8.43	8.25	9
ENSDARG00000058631	CABZ01030107.1		12	4533921	4540564	2.37	3.48	3.33	2.52	2.31	2.67
ENSDARG00000058638	si:ch211-71m22.1	si:ch211-71m22.1 [Source:ZFIN;Acc:ZDB-GENE-070705-193]	2	8586415	8611675	8.57	13.46	10.38	5.51	5.79	4.51
ENSDARG00000058644	dnajb2	DnaJ heat shock protein family (Hsp40) member B2 [Source:ZFIN;Acc:ZDB-GENE-061013-537]	9	7621622	7655301	3.43	2.62	2.42	4.9	4.14	4.11
ENSDARG00000058646	ptprna	protein tyrosine phosphatase receptor type Na [Source:ZFIN;Acc:ZDB-GENE-030131-3511]	9	7548533	7619622	13.88	4.72	3.98	22.57	24.2	25.58
ENSDARG00000058647	hck	HCK proto-oncogene, Src family tyrosine kinase [Source:ZFIN;Acc:ZDB-GENE-090313-72]	23	7505943	7545960	0.44	0.48	0.45	0.95	1.12	0.81
ENSDARG00000058649	trim46b	tripartite motif containing 46b [Source:ZFIN;Acc:ZDB-GENE-060503-466]	19	8582685	8604429	6.06	1.44	2.33	8.81	9.52	8.74
ENSDARG00000058650	dpm3	dolichyl-phosphate mannosyltransferase subunit 3, regulatory [Source:ZFIN;Acc:ZDB-GENE-040426-1782]	19	8533989	8536474	14.31	14.52	15.48	10.44	10.03	10.35
ENSDARG00000058656	desma	desmin a [Source:ZFIN;Acc:ZDB-GENE-980526-221]	9	7515864	7539326	109.01	136.12	144.23	172.89	169.84	171.27
ENSDARG00000058658	si:dkey-178e17.3	si:dkey-178e17.3 [Source:ZFIN;Acc:ZDB-GENE-081104-335]	8	1873345	1909840	6.94	7.08	8.31	9.07	9.49	9.19
ENSDARG00000058660	ilf3a	interleukin enhancer binding factor 3a [Source:ZFIN;Acc:ZDB-GENE-030131-3151]	6	8363629	8392200	13.24	6.8	7.06	14.57	14.54	14.59
ENSDARG00000058663	pimr94	Pim proto-oncogene, serine/threonine kinase, related 94 [Source:ZFIN;Acc:ZDB-GENE-141216-85]	11	11842724	11849995	0.03	0.02	0.01	0.02	0.01	0.02
ENSDARG00000058666	dennd2da	DENN/MADD domain containing 2Da [Source:ZFIN;Acc:ZDB-GENE-061013-542]	8	12989372	13029297	8.36	9.91	8.18	5.72	6.06	5.54
ENSDARG00000058672	nuggc.3	nuclear GTPase, germinal center associated, tandem duplicate 3 [Source:ZFIN;Acc:ZDB-GENE-061207-67]	19	7895086	7923910	0	0.01	0	0.01	0.02	0.02
ENSDARG00000058673	nkl.1	NK-lysin tandem duplicate 1 [Source:ZFIN;Acc:ZDB-GENE-131121-525]	17	8896383	8907682	0.02	0.07	0.02	0.02	0.1	0.02
ENSDARG00000058678	aqp10b	aquaporin 10b [Source:ZFIN;Acc:ZDB-GENE-060503-57]	19	7810028	7816886	1.67	2.53	1.67	0.22	0.24	0.23
ENSDARG00000058679	zgc:114104	zgc:114104 [Source:ZFIN;Acc:ZDB-GENE-050913-63]	21	11521163	11529370	4.22	3.89	2.88	3.26	3.42	3.42
ENSDARG00000058682	cd8b	cd8 beta [Source:ZFIN;Acc:ZDB-GENE-091204-31]	21	11535304	11544499	0.01	0.02	0.02	0.03	0	0.02
ENSDARG00000058683	pimr204	Pim proto-oncogene, serine/threonine kinase, related 204 [Source:ZFIN;Acc:ZDB-GENE-141216-237]	11	16137302	16141536	0	0	0	0	0	0.01
ENSDARG00000058685	si:dkey-204a24.11	si:dkey-204a24.11 [Source:ZFIN;Acc:ZDB-GENE-120215-129]	19	7694487	7706926	0.01	0	0	0.01	0.02	0.02
ENSDARG00000058690	cgnb	cingulin b [Source:NCBI gene;Acc:100005752]	19	7636941	7680056	5.26	3.45	3.37	4.03	3.88	4.05
ENSDARG00000058692	fzd3b	frizzled class receptor 3b [Source:ZFIN;Acc:ZDB-GENE-070122-1]	17	8498782	8570367	3.38	1.61	1.41	4.82	4.72	3.98
ENSDARG00000058693	cast	calpastatin [Source:ZFIN;Acc:ZDB-GENE-050810-2]	21	11565888	11657043	58.63	54.85	42.06	45.7	41.75	41.7
ENSDARG00000058695	ddr2b	discoidin domain receptor tyrosine kinase 2b [Source:ZFIN;Acc:ZDB-GENE-060503-361]	2	6963296	6996486	3.12	2.43	2.8	5.19	5.21	5.31
ENSDARG00000058696	tbl1xr1a	transducin (beta)-like 1 X-linked receptor 1a [Source:ZFIN;Acc:ZDB-GENE-050522-314]	11	8565323	8682447	12.89	7.86	7.43	15.33	16.24	16.24
ENSDARG00000058699	tmeff2b	transmembrane protein with EGF-like and two follistatin-like domains 2b [Source:ZFIN;Acc:ZDB-GENE-101001-4]	6	8756177	8865543	2.45	1.36	1.67	3.69	3.73	3.58
ENSDARG00000058700	si:dkey-73p2.2	si:dkey-73p2.2 [Source:ZFIN;Acc:ZDB-GENE-131119-50]	3	4911207	4911989	1.65	1.62	1.23	0.4	0.62	0.9
ENSDARG00000058701	nos1apb	nitric oxide synthase 1 (neuronal) adaptor protein b [Source:ZFIN;Acc:ZDB-GENE-100824-1]	2	6926100	6952754	3.73	0.81	1.42	4.43	5.27	4.91
ENSDARG00000058702	vax2	ventral anterior homeobox 2 [Source:NCBI gene;Acc:373869]	7	8844285	8881514	1.51	0.33	0.31	2.05	1.76	1.88
ENSDARG00000058710	terf1	telomeric repeat binding factor (NIMA-interacting) 1 [Source:ZFIN;Acc:ZDB-GENE-090612-2]	24	13336327	13349802	1.16	1.45	1.05	0.65	0.6	0.69
ENSDARG00000058716	cept1a	choline/ethanolamine phosphotransferase 1a [Source:ZFIN;Acc:ZDB-GENE-081105-47]	8	12951155	12969237	0.36	0.18	0.15	0.58	0.58	0.71
ENSDARG00000058719	si:dkey-119f1.1	si:dkey-119f1.1 [Source:ZFIN;Acc:ZDB-GENE-070705-287]	2	6321623	6373829	2.66	2.36	2.11	1.32	1.71	1.13
ENSDARG00000058724	or126-7	odorant receptor, family E, subfamily 126, member 7 [Source:ZFIN;Acc:ZDB-GENE-070806-104]	15	5162274	5165013	0	0	0	0	0	0
ENSDARG00000058725	rfesd	Rieske (Fe-S) domain containing [Source:ZFIN;Acc:ZDB-GENE-050417-254]	21	11829836	11834452	0.91	1.48	1.02	1.54	2.19	1.53
ENSDARG00000058728	nudt2	nudix (nucleoside diphosphate linked moiety X)-type motif 2 [Source:ZFIN;Acc:ZDB-GENE-040718-7]	21	11834880	11840092	8.69	8.35	6.89	6.77	6.59	6.42
ENSDARG00000058729	akirin2	akirin 2 [Source:ZFIN;Acc:ZDB-GENE-040426-2944]	20	2715949	2725930	16.61	13.38	13.24	18.84	18.8	18.48
ENSDARG00000058730	rdh10a	retinol dehydrogenase 10a [Source:NCBI gene;Acc:562840]	24	13251480	13272843	16.94	15.12	14.37	24.57	24.76	25.96
ENSDARG00000058731	slc2a6	solute carrier family 2 member 6 [Source:ZFIN;Acc:ZDB-GENE-081104-301]	8	12854105	12867434	0.36	0.49	0.33	0.7	0.63	0.67
ENSDARG00000058732	scgn	secretagogin, EF-hand calcium binding protein [Source:NCBI gene;Acc:573010]	16	6356460	6377672	2.3	2.37	2.21	1.55	1.38	1.96
ENSDARG00000058733	ihha	Indian hedgehog signaling molecule a [Source:ZFIN;Acc:ZDB-GENE-051010-1]	9	7358749	7367278	4.19	4.6	3.37	5.71	6.21	6.77
ENSDARG00000058734	prdx1	peroxiredoxin 1 [Source:ZFIN;Acc:ZDB-GENE-050320-35]	2	6104378	6115688	7.28	12.45	9.96	6.38	6.09	5.86
ENSDARG00000058736	gabra6b	gamma-aminobutyric acid (GABA) A receptor, alpha 6b [Source:ZFIN;Acc:ZDB-GENE-080815-1]	21	42156949	42202792	1.84	0.22	0.24	1.23	1.27	1.25
ENSDARG00000058738	si:ch211-117c9.2	si:ch211-117c9.2 [Source:ZFIN;Acc:ZDB-GENE-110408-11]	23	6882410	6920107	0.1	0.11	0.1	0.38	0.45	0.53
ENSDARG00000058740	ube2r2	ubiquitin-conjugating enzyme E2R 2 [Source:ZFIN;Acc:ZDB-GENE-040718-344]	21	11845241	11856143	9.8	6.8	6.97	9.68	9.36	10.09
ENSDARG00000058746	ubap1	ubiquitin associated protein 1 [Source:ZFIN;Acc:ZDB-GENE-040426-950]	21	11865972	11884517	5.43	4.34	3.61	7.05	6.96	7.03
ENSDARG00000058753	si:ch73-213k20.5	si:ch73-213k20.5 [Source:ZFIN;Acc:ZDB-GENE-120727-11]	22	6952107	6960943	0.25	0.25	0.2	0.35	0.37	0.33
ENSDARG00000058762	or128-3	odorant receptor, family E, subfamily 128, member 3 [Source:ZFIN;Acc:ZDB-GENE-070806-108]	15	5228580	5230423	0.04	0.03	0.07	0.03	0.02	0.02
ENSDARG00000058765	or128-2	odorant receptor, family E, subfamily 128, member 2 [Source:ZFIN;Acc:ZDB-GENE-070806-107]	15	5237596	5239536	0	0	0	0	0	0.01
ENSDARG00000058771	nav1b	neuron navigator 1b [Source:ZFIN;Acc:ZDB-GENE-120215-84]	6	54888493	55022544	4.53	2	1.98	9.87	9.75	9.87
ENSDARG00000058774	CABZ01053221.1	chemokine (C motif) receptor 1b, duplicate 2 [Source:NCBI gene;Acc:101883597]	2	50706439	50707494	0.07	0.13	0.19	0.13	0.17	0.09
ENSDARG00000058775	2-Oct	organic cation transporter 2 [Source:ZFIN;Acc:ZDB-GENE-080204-92]	17	6331994	6349044	0.76	0.85	0.85	0.89	1.16	1.04
ENSDARG00000058780	slc9a2	solute carrier family 9 member 2 [Source:ZFIN;Acc:ZDB-GENE-070912-541]	9	6963039	6991650	1.68	1.44	1.26	1.12	1.25	1.12
ENSDARG00000058782	si:ch73-242m19.1	si:ch73-242m19.1 [Source:ZFIN;Acc:ZDB-GENE-131121-474]	17	6828550	6932061	0.17	0.06	0.09	0.49	0.48	0.47
ENSDARG00000058791	zgc:171887	zgc:171887 [Source:ZFIN;Acc:ZDB-GENE-080220-39]	22	6096303	6099424	0	0.06	0	0.02	0.02	0
ENSDARG00000058794	si:dkey-19a16.2	si:dkey-19a16.2 [Source:ZFIN;Acc:ZDB-GENE-050208-493]	22	6073628	6082447	1.14	1.21	0.82	1.56	1.47	1.65
ENSDARG00000058799	mybpha	myosin binding protein Ha [Source:ZFIN;Acc:ZDB-GENE-040426-1531]	23	6077503	6126003	10.53	15.51	12.11	6.16	5.64	5.5
ENSDARG00000058800	rab25a	RAB25, member RAS oncogene family a [Source:ZFIN;Acc:ZDB-GENE-041212-69]	19	7413942	7420867	5.65	5.69	4.5	3.82	3.59	2.94
ENSDARG00000058801	rev3l	REV3 like, DNA directed polymerase zeta catalytic subunit [Source:ZFIN;Acc:ZDB-GENE-050302-55]	20	3346242	3403033	6.86	4.07	3.66	6.5	6.24	6.5
ENSDARG00000058803	grk1a	G protein-coupled receptor kinase 1 a [Source:ZFIN;Acc:ZDB-GENE-050823-1]	1	245076	258138	10.4	1.95	1.3	6.56	7.36	7.58
ENSDARG00000058806	naxe	NAD(P)HX epimerase [Source:NCBI gene;Acc:436891]	19	7408235	7412647	13.86	14.43	16.91	8.75	9.7	9.87
ENSDARG00000058815	ihhb	Indian hedgehog signaling molecule b [Source:NCBI gene;Acc:30299]	6	7059999	7082108	0.05	0.03	0.07	1.29	1.12	1.72
ENSDARG00000058818	rbm38	RNA binding motif protein 38 [Source:ZFIN;Acc:ZDB-GENE-030131-5923]	23	6586467	6632106	6.43	4.62	3.55	7.94	7.66	6.71
ENSDARG00000058819	nog1	noggin 1 [Source:NCBI gene;Acc:30174]	3	10310090	10312232	3.2	3.64	2.9	3.17	4.14	4.02
ENSDARG00000058820	bin1b	bridging integrator 1b [Source:ZFIN;Acc:ZDB-GENE-030425-1]	6	6999487	7052603	8.97	8.29	7.68	10.24	11.08	9.79

ENSDARG00000058821	sema5a	sema domain, seven thrombospondin repeats (type 1 and type 1-like), transmembrane domain (TM) and short cytoplasmic domain,	24	16393302	16706981		5.9	4.45	3.59	10.14	10.37	11.09
ENSDARG00000058822	wnt3a	wingless-type MMTV integration site family, member 3A [Source:ZFIN;Acc:ZDB-GENE-001106-1]	2	3106237	3158919		1.97	1.53	1.2	2.44	1.66	1.82
ENSDARG00000058824	mrpl4	mitochondrial ribosomal protein L4 [Source:NCBI gene;Acc:553771]	11	11319141	11331146		35.05	41.81	44.67	24.74	22	24.06
ENSDARG00000058828	ccdc51	coiled-coil domain containing 51 [Source:ZFIN;Acc:ZDB-GENE-060825-200]	22	5655756	5663354		4.59	5.25	5.45	3.37	3.73	3.21
ENSDARG00000058829	zgc:171424	zgc:171424 [Source:ZFIN;Acc:ZDB-GENE-080204-26]	7	1980829	2004159		0.02	0.03	0	0.02	0.06	0.14
ENSDARG00000058830	zdhhc3b	zinc finger DHHC-type containing 3b [Source:ZFIN;Acc:ZDB-GENE-061110-19]	19	1003332	1013213		4.72	3.74	2.44	5.77	5.54	5.63
ENSDARG00000058835	si:ch73-212j7.3	si:ch73-212j7.3 [Source:ZFIN;Acc:ZDB-GENE-081104-259]	20	3145497	3160687		3.38	1.7	1.59	2.77	3.25	2.71
ENSDARG00000058839	susd1	sushi domain containing 1 [Source:ZFIN;Acc:ZDB-GENE-060526-169]	5	9246018	9278521		0.4	0.5	0.31	0.61	0.73	0.49
ENSDARG00000058848	mcoln1b	mucolipin 1b [Source:ZFIN;Acc:ZDB-GENE-130514-1]	3	5991765	6035766		3.34	1.04	1.53	4.16	4.14	4.96
ENSDARG00000058853	sash1b	SAM and SH3 domain containing 1b [Source:ZFIN;Acc:ZDB-GENE-110228-1]	17	6985428	7060477		2.32	1.75	1.77	2.49	2.69	2.94
ENSDARG00000058857	C7H19orf12	zgc:112052 [Source:NCBI gene;Acc:550358]	18	13123723	13133268	C7H19orf12	7.33	7.54	7.86	7.17	7.02	7.2
ENSDARG00000058861	ncln	nicalin [Source:NCBI gene;Acc:336844]	22	5233396	5252012		15.61	12.87	9.6	19.31	19.39	19.35
ENSDARG00000058865	endog	endonuclease G [Source:ZFIN;Acc:ZDB-GENE-050522-402]	8	12118097	12122228		10.08	11.77	11.87	7.82	7.02	6.24
ENSDARG00000058868	apc	APC regulator of WNT signaling pathway [Source:ZFIN;Acc:ZDB-GENE-031112-7]	10	1849874	1893095		27.66	8.93	8.98	31.37	31.04	32.38
ENSDARG00000058869	CABZ01040556.1	NIMA related kinase 3 [Source:NCBI gene;Acc:100536894]	15	3674765	3689119		2.13	1.39	1.33	1.57	1.26	1.13
ENSDARG00000058871	zgc:171857	zgc:171857 [Source:ZFIN;Acc:ZDB-GENE-080305-14]	23	4392147	4409668		1.97	2.27	1.82	2.33	2.34	2.31
ENSDARG00000058873	ptpdc1b	protein tyrosine phosphatase domain containing 1b [Source:ZFIN;Acc:ZDB-GENE-090312-138]	23	4373336	4390318		4.32	0.66	0.48	2.25	2.05	1.96
ENSDARG00000058874	zgc:113227	zgc:113227 [Source:ZFIN;Acc:ZDB-GENE-050327-32]	6	6662479	6666426		2.56	1.7	2	2.01	2.22	1.91
ENSDARG00000058876	mmp16b	matrix metallopeptidase 16b (membrane-inserted) [Source:ZFIN;Acc:ZDB-GENE-061009-22]	2	3630307	3678029		3.63	0.75	0.98	4.59	4.44	4.4
ENSDARG00000058880	cldnj	claudin j [Source:ZFIN;Acc:ZDB-GENE-010328-10]	15	2631421	2632891		1.71	1.56	2.19	2.06	2.06	2.01
ENSDARG00000058885	ttc29	tetratricopeptide repeat domain 29 [Source:ZFIN;Acc:ZDB-GENE-100922-181]	16	4497302	4512795		0.15	0.08	0.23	0.6	0.38	0.34
ENSDARG00000058892	cep170ab	centrosomal protein 170Ab [Source:ZFIN;Acc:ZDB-GENE-050309-106]	6	6445065	6487876		1.82	0.53	0.54	2.36	2.23	2.46
ENSDARG00000058893	nhej1	nonhomologous end-joining factor 1 [Source:NCBI gene;Acc:406593]	6	7085889	7109063		3.8	3.63	3.92	2.6	2.87	2.71
ENSDARG00000058903	GPR161	si:ch211-237c6.4 [Source:ZFIN;Acc:ZDB-GENE-120215-39]	6	7123980	7133531	GPR161	4.89	4.41	4.52	5.35	5.07	5.04
ENSDARG00000058906	si:ch211-181l5.2	si:ch211-181l5.2 [Source:ZFIN;Acc:ZDB-GENE-120215-133]	6	7240403	7245216		0.9	0.34	0.76	1.99	1.66	1.72
ENSDARG00000058917	nnr	nanor [Source:ZFIN;Acc:ZDB-GENE-030128-1]	15	1589813	1590858		0.02	0	0	0	0.03	0
ENSDARG00000058924	pou1f1	POU class 1 homeobox 1 [Source:ZFIN;Acc:ZDB-GENE-040630-7]	9	19132885	19161982		0.32	0.02	0.1	0.24	0.07	0.17
ENSDARG00000058930	si:busm1-52i16.2	si:busm1-52i16.2 [Source:ZFIN;Acc:ZDB-GENE-030616-509]	6	7287102	7292221		0.22	0.07	0.1	0.2	0.03	0.05
ENSDARG00000058937	cldn10a	claudin 10a [Source:ZFIN;Acc:ZDB-GENE-030616-510]	6	7299194	7306056		0.05	0.05	0.27	0.24	0.38	0.47
ENSDARG00000058939	b4galnt3b	beta-1,4-N-acetyl-galactosaminyl transferase 3b [Source:ZFIN;Acc:ZDB-GENE-030131-8615]	18	6558146	6619842		2.67	1.33	0.93	2.02	2.08	1.74
ENSDARG00000058940	si:ch211-248e11.2	si:ch211-248e11.2 [Source:NCBI gene;Acc:557793]	8	11542852	11550273		1.09	1.18	1	2.5	2.56	2.94
ENSDARG00000058943	cdcp1a	CUB domain containing protein 1a [Source:ZFIN;Acc:ZDB-GENE-050327-55]	16	2847565	2870522		1.51	1.44	1.26	2.19	1.77	1.67
ENSDARG00000058944	si:dkey-8e10.3	si:dkey-8e10.3 [Source:ZFIN;Acc:ZDB-GENE-081104-442]	8	10033326	10048059		3.86	4.29	3.84	4.66	4.56	4.59
ENSDARG00000058945	acot22	acyl-CoA thioesterase 22 [Source:ZFIN;Acc:ZDB-GENE-110407-17]	16	2814233	2818170		0.01	0	0	0.04	0.03	0.01
ENSDARG00000058946	CU856539.2	myosin-IIIb [Source:NCBI gene;Acc:556989]	10	2600830	2667208		10.07	9.98	7.99	9.25	10.05	9.66
ENSDARG00000058949	spina	spindlin a [Source:ZFIN;Acc:ZDB-GENE-161230-18]	10	2568793	2575387		23.66	9.53	14.61	24.1	23.79	25.81
ENSDARG00000058953	abcc4	ATP-binding cassette, sub-family C (CFTR/MRP), member 4 [Source:ZFIN;Acc:ZDB-GENE-030616-511]	6	7322587	7390458		6.36	4.73	3.67	5.17	4.79	4.61
ENSDARG00000058958	trim35-7	tripartite motif containing 35-7 [Source:ZFIN;Acc:ZDB-GENE-050522-333]	3	5344999	5362347		0.29	0.18	0.21	0.2	0.19	0.2
ENSDARG00000058960	otol1b	otolin 1b [Source:ZFIN;Acc:ZDB-GENE-080416-3]	15	1705167	1711472		1.33	0.43	0.66	1.84	1.88	2.23
ENSDARG00000058961	ngfa	nerve growth factor a (beta polypeptide) [Source:NCBI gene;Acc:30270]	23	4929442	4975517		1.7	1.51	1.45	0.91	0.94	1.28
ENSDARG00000058962	txnl4b	thioredoxin-like 4B [Source:ZFIN;Acc:ZDB-GENE-051030-63]	1	513986	518076		3.83	3.68	3.8	2.51	2.67	2.94
ENSDARG00000058964	themis2	thymocyte selection associated family member 2 [Source:ZFIN;Acc:ZDB-GENE-110411-89]	16	4725829	4746492		0.36	0.25	0.33	0.44	0.26	0.45
ENSDARG00000058966	zgc:112332	zgc:112332 [Source:ZFIN;Acc:ZDB-GENE-050522-387]	24	18448108	18467064		2	1.19	2.07	4.98	4.22	5.38
ENSDARG00000058968	haus1	HAUS augmin-like complex, subunit 1 [Source:ZFIN;Acc:ZDB-GENE-091204-387]	21	5066557	5077715		1.8	2.03	2.14	1.41	1.23	1.31
ENSDARG00000058969	cntnap2a	contactin associated protein like 2a [Source:ZFIN;Acc:ZDB-GENE-120328-3]	24	17417150	18179535		4.14	1.3	1.66	5.98	5.92	5.94
ENSDARG00000058978	zgc:113423	zgc:113423 [Source:ZFIN;Acc:ZDB-GENE-050913-14]	23	1042624	1056808		4.19	1.64	2.22	2.78	2.66	2.53
ENSDARG00000058984	rad23ab	RAD23 homolog A, nucleotide excision repair protein b [Source:ZFIN;Acc:ZDB-GENE-051113-160]	22	4745105	4760187		5.26	5.25	4.64	4.67	4.77	4.78
ENSDARG00000058985	KCNJ6	potassium inwardly rectifying channel subfamily J member 6 [Source:NCBI gene;Acc:569498]	10	124995	135432	KCNJ6	4.63	1.23	2.34	4.94	4.15	4.51
ENSDARG00000058988	KCNJ15	potassium inwardly rectifying channel subfamily J member 15 [Source:NCBI gene;Acc:572476]	10	158590	159597	KCNJ15	6.4	5.07	3.93	3.68	4.22	4.52
ENSDARG00000058990	slc35a2	solute carrier family 35 member 2 [Source:ZFIN;Acc:ZDB-GENE-030616-62]	8	8947623	8957679		5.1	3.77	3.64	8.07	8.04	8.32
ENSDARG00000058992	cers2b	ceramide synthase 2b [Source:ZFIN;Acc:ZDB-GENE-110408-40]	16	1362250	1399328		6.15	7.27	5.22	10.72	10.7	10.13
ENSDARG00000058993	rock1	Rho-associated, coiled-coil containing protein kinase 1 [Source:ZFIN;Acc:ZDB-GENE-100712-2]	2	2818645	2900825		14.31	11	9.47	14.43	14.67	14.3
ENSDARG00000058994	stx1a	syntaxin 1A (brain) [Source:ZFIN;Acc:ZDB-GENE-100802-1]	15	1734444	1746963		1.42	1.9	1.6	0.65	0.58	0.52
ENSDARG00000058995	jmjd4	jumonji domain containing 4 [Source:NCBI gene;Acc:767690]	2	3403264	3414068		4.13	3.56	3.76	4.29	3.63	4.05
ENSDARG00000058996	jam2a	junctional adhesion molecule 2a [Source:ZFIN;Acc:ZDB-GENE-031204-3]	1	594584	608247		6.1	6.94	8.13	5.89	6.29	6.09
ENSDARG00000059001	pim2	Pim-2 proto-oncogene, serine/threonine kinase [Source:NCBI gene;Acc:58054]	8	8927870	8934069		4.37	4.82	4.75	7.37	8.08	6.79
ENSDARG00000059003	strip2	striatin interacting protein 2 [Source:ZFIN;Acc:ZDB-GENE-120328-1]	4	4971042	5019113		3.61	1.44	1.62	3.77	3.5	3.88
ENSDARG00000059006	otud5a	OTU deubiquitinase 5a [Source:NCBI gene;Acc:555459]	8	8860902	8922631		15.36	11.63	10.77	18.26	17.27	19.38
ENSDARG00000059015	lim2.2	lens intrinsic membrane protein 2.2 [Source:ZFIN;Acc:ZDB-GENE-060124-1]	22	3223489	3232634		0.83	0.9	1.06	1.68	1.57	1.62
ENSDARG00000059020	thap1	THAP domain containing, apoptosis associated protein 1 [Source:NCBI gene;Acc:692315]	5	431994	441090		18.14	16.61	18.31	12.22	11.79	12.22
ENSDARG00000059026	zgc:123217	zgc:123217 [Source:ZFIN;Acc:ZDB-GENE-051113-188]	12	4036409	4044132		5.7	4.68	3.77	2.82	3.76	2.68
ENSDARG00000059028	srrd	SRR1 domain containing [Source:ZFIN;Acc:ZDB-GENE-060825-269]	8	4434422	4444573		4.65	4.56	5.03	3.51	2.94	3.02
ENSDARG00000059029	mmp28	matrix metallopeptidase 28 [Source:ZFIN;Acc:ZDB-GENE-100226-1]	15	1778773	1822548		3.6	3.55	2.77	2.89	2.66	3.19
ENSDARG00000059035	porb	P450 (cytochrome) oxidoreductase b [Source:ZFIN;Acc:ZDB-GENE-030131-5767]	15	1847135	1885247		27.2	32.8	19.16	27.3	29.69	28.33
ENSDARG00000059039	FO904903.1	major histocompatibility complex class I ULA [Source:NCBI gene;Acc:796419]	22	3914318	3929075		0.13	0.08	0.16	2.6	3.08	2.29
ENSDARG00000059043	ntf3	neurotrophin 3 [Source:ZFIN;Acc:ZDB-GENE-031118-66]	4	4397503	4431975		3.14	1.25	1.21	4.39	5	4.09
ENSDARG00000059044	epm2a	EPM2A glucan phosphatase, laforin [Source:ZFIN;Acc:ZDB-GENE-100922-143]	23	1526708	1557195		0.79	1.19	1.2	1.57	1.71	1.53
ENSDARG00000059046	psmd5	proteasome 26S subunit, non-ATPase 5 [Source:ZFIN;Acc:ZDB-GENE-050417-28]	21	5368130	5393125		8.7	7.34	8.82	6.24	6.14	6.02
ENSDARG00000059048	mpzl1l	myelin protein zero-like 1 like [Source:ZFIN;Acc:ZDB-GENE-050417-455]	10	213878	223885		22.25	17.01	18.88	24.18	23.54	24.4
ENSDARG00000059049	zgc:174904	zgc:174904 [Source:ZFIN;Acc:ZDB-GENE-080204-76]	19	5690933	5699703		9.07	14.1	12.15	8.84	10.28	9.87
ENSDARG00000059053	slc13a4	solute carrier family 13 member 4 [Source:ZFIN;Acc:ZDB-GENE-070424-71]	4	4803698	4832149		12.04	19.9	20.32	7.43	7.11	6.79
ENSDARG00000059054	pdk2b	pyruvate dehydrogenase kinase, isozyme 2b [Source:ZFIN;Acc:ZDB-GENE-040426-939]	19	5674907	5687743		9.79	11.87	11.29	31.28	37.8	40.55
ENSDARG00000059056	ft1	alpha(1,3)fucosyltransferase gene 1 [Source:ZFIN;Acc:ZDB-GENE-990715-17]	19	5651325	5655001		0.64	0.76	0.41	0.36	0.62	0.25
ENSDARG00000059057	atpv0e2	ATPase H+ transporting V0 subunit e2 [Source:ZFIN;Acc:ZDB-GENE-050522-135]	22	34701848	34707460		66.52	48.29	43.03	49.83	49.2	54.63
ENSDARG00000059058	vipr1b	vasoactive intestinal peptide receptor 1b [Source:ZFIN;Acc:ZDB-GENE-050311-6]	24	20708174	20808283		1.85	0.45	0.7	1.73	1.66	1.53
ENSDARG00000059059	emsy	EMSY transcriptional repressor, BRCA2 interacting [Source:NCBI gene;Acc:335889]	10	284303	302827		7.35	4.54	4.63	11.11	10.53	10.03
ENSDARG00000059060	lgalsla	lectin, galactoside-binding-like a [Source:NCBI gene;Acc:723995]	1	55080797	55089987		1.1	0.69	0.88	1.63	1.63	1.48
ENSDARG00000059064	rassf3	Ras association domain family member 3 [Source:ZFIN;Acc:ZDB-GENE-100422-9]	4	4540223	4592287		4.87	4.7	3.4	5.57	6.18	5.8
ENSDARG00000059067	jupb	junction plakoglobin b [Source:ZFIN;Acc:ZDB-GENE-110407-10]	19	5480327	5590012		5.93	1.75	1.97	5.5	5.11	4.67
ENSDARG00000059070	gars1	glycyl-tRNA synthetase 1 [Source:ZFIN;Acc:ZDB-GENE-030131-9174]	24	20536056	20554969		59.1	69.49	69.84	66.49	66.06	66.84
ENSDARG00000059073	gsc	goosecoid [Source:NCBI gene;Acc:30212]	17	19343015	19345521		5.93	6.13	6.34	6.15	5.53	5.71
ENSDARG00000059075	nip7	NIP7 nucleolar pre-rRNA processing protein [Source:NCBI gene;Acc:553616]	18	5843583	5850683		9.88	12.93	12.63	6.79	6.01	5.57
ENSDARG00000059079	vps25	vacuolar protein sorting 25 homolog [Source:NCBI gene;Acc:407684]	12	3714076	3721957		18.36	21.8	20.34	18.29	17.83	17.03
ENSDARG00000059081	PRSS35	inactive serine protease 35-like [Source:NCBI gene;Acc:100002503]	23	2446978	2448234	PRSS35	0.09	0.15	0.05	0.13	0.02	0.06
ENSDARG00000059083	ppp2r5ca	protein phosphatase 2, regulatory subunit B', gamma a [Source:ZFIN;Acc:ZDB-GENE-120406-9]	17	956821	981781		4.24	2.48	2.6	6.29	6.16	5.95
ENSDARG00000059090	sstr2a	somatostatin receptor 2a [Source:ZFIN;Acc:ZDB-GENE-140130-1]	12	1556147	1557286		6.16	6.26	6.73	6.86	7.39	6.91
ENSDARG00000059093	ank1a	ankyrin 1, erythrocytic a [Source:ZFIN;Acc:ZDB-GENE-041010-44]	5	68899473	69041102		6.48	3.31	3.77	6.86	6.74	7.3
ENSDARG00000059097	nktr	natural killer cell triggering receptor [Source:ZFIN;Acc:ZDB-GENE-030131-1725]	24	20643658	20658668		18.93	7.35	10.44	21.32	22.89	23.73
ENSDARG00000059103	si:ch73-126o18.1	si:ch73-126o18.1 [Source:ZFIN;Acc:ZDB-GENE-160113-97]	7	1812480	1833922		0	0	0	0.03	0	0
ENSDARG00000059109	si:dkeyp-113d7.1	si:dkeyp-113d7.1 [Source:ZFIN;Acc:ZDB-GENE-030131-755]	19	5315987	5320330		14.65	8.93	8.87	18.27	19.4	18.6
ENSDARG00000059116	heatr5b	HEAT repeat containing 5B [Source:ZFIN;Acc:ZDB-GENE-130530-676]	13	450002	479130		3.76	2.26	1.76	5.77	5.78	5.6
ENSDARG00000059121	EFEMP1	EGF containing fibulin extracellular matrix protein 1 [Source:HGNC Symbol;Acc:HGNC:3218]	13	432790	438705	EFEMP1	0.47	0.3	0.33	0.56	0.68	0.47
ENSDARG00000059123	plcd1a	phospholipase C, delta 1a [Source:ZFIN;Acc:ZDB-GENE-071004-78]	24	20372497	20419254		7.81	8.18	7.11	6.44	6.39	5.97
ENSDARG00000059125	prkacbb	protein kinase, cAMP-dependent, catalytic, beta b [Source:ZFIN;Acc:ZDB-GENE-050904-4]	2	1594344	1622641		10.54	7.29	6.81	13.11	12.9	12.01
ENSDARG00000059128	uqcrh	ubiquinol-cytochrome c reductase hinge protein [Source:ZFIN;Acc:ZDB-GENE-051030-93]	6	3509036	3514753		201.66	266.77	296.35	145.97	142.41	147.22
ENSDARG00000059130	gata1b	GATA binding protein 1b [Source:ZFIN;Acc:ZDB-GENE-081104-43]	8	7463911	7475917		0.11	0.15	0.1	0.17	0.38	0.32
ENSDARG00000059131	spdya	speedy/RINGO cell cycle regulator family member A [Source:ZFIN;Acc:ZDB-GENE-041010-194]	17	24809221	24815220		0.2	0.17	0.16	0.22	0.21	0.13
ENSDARG00000059139	wfikkn2b	WAP, follistatin/kazal, immunoglobulin, kunitz and netrin domain containing 2b [Source:ZFIN;Acc:ZDB-GENE-110414-1]	12	804314	808443		0.2	0.2	0.1	0.13	0.1	0.16
ENSDARG00000059150	tmem107	transmembrane protein 107 [Source:NCBI gene;Acc:406492]	14	247456	251089		5.47	2.9	4.18	7.68	7.74	8.45
ENSDARG00000059163	rbp3	retinol binding protein 3 [Source:ZFIN;Acc:ZDB-GENE-990415-132]	12	2665081	2670156		115.03	30.8	17.15	63.72	73.34	68.86
ENSDARG00000059166	slc22a13b	solute carrier family 22 member 13b [Source:ZFIN;Acc:ZDB-GENE-061013-532]	24	20006711	20016817		0.76	0.82	0.76	1.43	1.42	1.62
ENSDARG00000059173	gdf2	growth differentiation factor 2 [Source:NCBI gene;Acc:563287]	12	2648043	2651276		0.28	0.1	0.2	0.14	0.11	0.15
ENSDARG00000059175	cep19	centrosomal protein 19 [Source:NCBI gene;Acc:619252]	22	2937485	2942954		3.11	1.99	2.32	2.97	2.97	3.17
ENSDARG00000059177	tax1bp3	Tax1 (human T-cell leukemia virus type I) binding protein 3 [Source:ZFIN;Acc:ZDB-GENE-040426-2830]	11	6136220	6145158		14.73	19.97	17.2	14.31	14.85	14.93
ENSDARG00000059196	CU467633.1		4	1958240	1974894		0.22	0.14	0.25	0.09	0.22	0.04
ENSDARG00000059202	tspan2b	tetraspanin 2b [Source:ZFIN;Acc:ZDB-GENE-100922-60]	23	423351	452365		5.75	4.45	4.26	11.44	12.58	12.78
ENSDARG00000059209	abhd13	abhydrolase domain containing 13 [Source:NCBI gene;Acc:561333]	9	5345236	5351017		3.41	2.69	2.33	4.27	4.01	3.18
ENSDARG00000059212	ciao1	cytosolic iron-sulfur assembly component 1 [Source:NCBI gene;Acc:795104]	8	4779744	4789034		18.96	22.14	23.33	15.59	15.99	14.89
ENSDARG00000059226	zbtb8a	zinc finger and BTB domain containing 8A [Source:ZFIN;Acc:ZDB-GENE-060421-5153]	17	24869371	24879003		2.04	1.38	1.5	1.82	1.63	1.58
ENSDARG00000059227	fabp1b.1	fatty acid binding protein 1b, tandem duplicate 1 [Source:NCBI gene;Acc:554095]	8	999421	1006174		768.02	1512.88	1121.7	155.14	143.72	166.71
ENSDARG00000059231	hephl1a	hephaestin-like 1a [Source:ZFIN;Acc:ZDB-GENE-070801-4]	15	2421432	2472933		1.15	1.36	1.27	2.36	2.44	2.09
ENSDARG00000059233	runx2b	RUNX family transcription factor 2b [Source:ZFIN;Acc:ZDB-GENE-040629-4]	20	43994848	44090660		0.69	0.71	0.42	1.43	1.52	1.54
ENSDARG00000059234	mrps27	mitochondrial ribosomal protein S27 [Source:ZFIN;Acc:ZDB-GENE-040808-39]	8	926673	944055		18.77	20.98	21.48	13.44	12.64	12.73
ENSDARG00000059235	zgc:113274	zgc:113274 [Source:ZFIN;Acc:ZDB-GENE-050508-2]	10	5135842	5147582		3.1	2.49	3.07	2.24	1.85	2.03
ENSDARG00000059236	ptger4a	prostaglandin E receptor 4 (subtype EP4) a [Source:ZFIN;Acc:ZDB-GENE-060222-1]	8	902750	912821		0.44	0.34	0.63	0.42	0.17	0.25
ENSDARG00000059244	arfip2a	ADP-ribosylation factor interacting protein 2a [Source:ZFIN;Acc:ZDB-GENE-041010-221]	15	32249062	32267153		14.48	6.37	8.43	15.02	14.16	14.52
ENSDARG00000059246	hnrnpd	heterogeneous nuclear ribonucleoprotein D [Source:ZFIN;Acc:ZDB-GENE-070424-97]	10	5000623	5020378		52.08	37.62	39.04	43.43	41.27	42.6
ENSDARG00000059247	tmem54a	transmembrane protein 54a [Source:ZFIN;Acc:ZDB-GENE-040426-1304]	17	25326054	25342274		10.24	9.83	8.93	7.82	7.23	8.01
ENSDARG00000059252	zpcx	zona pellucida protein C [Source:ZFIN;Acc:ZDB-GENE-040824-1]	17	25328351	25331447		0.01	0	0	0	0	0
ENSDARG00000059254	illr1	immune-related, lectin-like receptor 1 [Source:ZFIN;Acc:ZDB-GENE-050311-2]	19	3247555	3255725		0.11	0.09	0.06	0.13	0.02	0.08
ENSDARG00000059255	evx2	even-skipped homeobox 2 [Source:ZFIN;Acc:ZDB-GENE-980526-215]	9	2002701	2007106		1.56	0.58	0.7	4.54	4.31	4.46
ENSDARG00000059256	hoxd13a	homeobox D13a [Source:NCBI gene;Acc:30407]	9	1986816	1990323		1.16	1.23	1.33	1.6	1.89	1.75
ENSDARG00000059259	pabpc4	poly(A) binding protein, cytoplasmic 4 (inducible form) [Source:ZFIN;Acc:ZDB-GENE-030131-9663]	17	25303671	25326685		72.35	94.99	93.24	107.57	108.94	99.17
ENSDARG00000059263	hoxd12a	homeobox D12a [Source:NCBI gene;Acc:100006598]	9	1981882	1986014		2.11	2.43	2.14	2.98	3.08	3.96
ENSDARG00000059267	hoxd11a	homeobox D11a [Source:NCBI gene;Acc:30405]	9	1975818	1978090		3.24	3.97	4.19	4.9	4.96	4.73
ENSDARG00000059274	hoxd9a	homeobox D9a [Source:NCBI gene;Acc:30350]	9	1964005	1965727		4.08	6.97	6.66	6.93	7.88	8.03
ENSDARG00000059276	hoxd4a	homeobox D4a [Source:NCBI gene;Acc:30329]	9	1948465	1951144		1.63	1.15	1.33	3.42	2.99	2.68
ENSDARG00000059279	tfap2a	transcription factor AP-2 alpha [Source:ZFIN;Acc:ZDB-GENE-011212-6]	24	8721676	8732519		45.53	24.97	21.48	48.81	44.99	48.22
ENSDARG00000059280	hoxd3a	homeobox D3a [Source:NCBI gene;Acc:30349]	9	1937049	1959917		3.76	1.77	2.08	8.65	8.18	8.34
ENSDARG00000059287	mak	male germ cell-associated kinase [Source:ZFIN;Acc:ZDB-GENE-030131-7279]	24	8742279	8777781		12.68	7.94	8.26	11.23	11.89	11.62
ENSDARG00000059294	marco	macrophage receptor with collagenous structure [Source:ZFIN;Acc:ZDB-GENE-120514-2]	9	795609	813693		1.37	1.57	1.83	2.19	2.94	2.8
ENSDARG00000059298	spcs1	signal peptidase complex subunit 1 [Source:ZFIN;Acc:ZDB-GENE-050809-130]	11	3989403	3993738		48.33	64.36	60.93	44.86	44.4	44.04
ENSDARG00000059303	hnrnpl2	heterogeneous nuclear ribonucleoprotein L2 [Source:ZFIN;Acc:ZDB-GENE-040426-2707]	15	5784212	5799183		35.32	25.73	27.17	40.27	38.23	39.4
ENSDARG00000059304	chchd2	coiled-coil-helix-coiled-coil-helix domain containing 2 [Source:ZFIN;Acc:ZDB-GENE-040426-1737]	15	5773119	5780648		55.1	58.99	50.4	55.72	54.3	54.93
ENSDARG00000059305	tmem37	transmembrane protein 37 [Source:ZFIN;Acc:ZDB-GENE-040426-2208]	9	917060	923251		1.32	1.82	1.27	2.63	2.67	2.8
ENSDARG00000059308	golt1bb	golgi transport 1Bb [Source:ZFIN;Acc:ZDB-GENE-050913-94]	25	3341774	3347418		6.2	6.38	6.76	9.93	9.26	8.38
ENSDARG00000059311	dpysl5b	dihydropyrimidinase like 5b [Source:ZFIN;Acc:ZDB-GENE-050720-4]	4	798696	815871		29.67	6.57	7.24	46.62	48.29	52.42
ENSDARG00000059315	ora3	olfactory receptor class A related 3 [Source:ZFIN;Acc:ZDB-GENE-100721-3]	22	381143	382955		0.58	0.23	0.6	0.33	0.18	0.25
ENSDARG00000059322	topbp1	DNA topoisomerase II binding protein 1 [Source:ZFIN;Acc:ZDB-GENE-060626-1]	24	9693951	9737230		2.29	1.57	1.63	2.35	2.12	2.12
ENSDARG00000059323	rpn1	ribophorin I [Source:ZFIN;Acc:ZDB-GENE-030131-4286]	11	3892386	3907937		36.33	33.09	21.44	49.11	46.79	47.24
ENSDARG00000059327	gata2a	GATA binding protein 2a [Source:ZFIN;Acc:ZDB-GENE-980526-260]	11	3847442	3865472		4.21	3.71	3.55	7.73	6.47	7.36
ENSDARG00000059333	si:ch211-285f17.1	si:ch211-285f17.1 [Source:ZFIN;Acc:ZDB-GENE-000607-77]	24	9372292	9598456		3.3	3.08	2.29	5.59	5.44	5.57
ENSDARG00000059337	tgif1	TGFB-induced factor homeobox 1 [Source:ZFIN;Acc:ZDB-GENE-030131-475]	24	9287409	9300160		12.23	9.13	7.42	16.23	15.26	15.59
ENSDARG00000059340	dlgap2a	discs, large (Drosophila) homolog-associated protein 2a [Source:ZFIN;Acc:ZDB-GENE-101215-1]	17	24936059	25175560		0.63	0.15	0.31	1	0.97	1.07
ENSDARG00000059342	avil	advillin [Source:ZFIN;Acc:ZDB-GENE-060421-6683]	11	169394	183328		1.08	0.37	0.5	1.53	1.62	1.76
ENSDARG00000059347	calml4b	calmodulin-like 4b [Source:ZFIN;Acc:ZDB-GENE-071009-6]	25	1266945	1323928		0.17	0.02	0.01	0.05	0.03	0.02
ENSDARG00000059348	rxfp3.3b	relaxin family peptide receptor 3.3b [Source:ZFIN;Acc:ZDB-GENE-121030-6]	25	1304173	1305384		3.25	0.84	1.25	2.33	2.65	2.9
ENSDARG00000059349	homer2	homer scaffold protein 2 [Source:ZFIN;Acc:ZDB-GENE-050522-417]	18	1615	2758		66.2	60.04	64.8	44.63	43.51	40.51
ENSDARG00000059351	hnrnpa3	heterogeneous nuclear ribonucleoprotein A3 [Source:ZFIN;Acc:ZDB-GENE-060224-1]	9	1692926	1703801		16.42	4.73	5.56	14.85	14.71	15.85
ENSDARG00000059354	glmp	glycosylated lysosomal membrane protein [Source:NCBI gene;Acc:447841]	19	798589	808265		9.56	8.9	9.77	9.3	9.31	9.67
ENSDARG00000059357	sarnp	SAP domain containing ribonucleoprotein [Source:ZFIN;Acc:ZDB-GENE-050522-161]	11	3308656	3318747		24.37	28.99	25.98	20.94	21.67	24.1
ENSDARG00000059360	srsf3b	serine and arginine rich splicing factor 3b [Source:ZFIN;Acc:ZDB-GENE-071005-2]	22	613265	622967		53.7	27.96	35.83	60.81	56.76	57.94
ENSDARG00000059361	slc39a4	solute carrier family 39 member 4 [Source:ZFIN;Acc:ZDB-GENE-060315-8]	19	1119416	1127722		1.29	2.11	1.46	0.43	0.55	0.36
ENSDARG00000059362	cavin1b	caveolae associated protein 1b [Source:ZFIN;Acc:ZDB-GENE-030131-5509]	24	7631797	7668158		25.22	32.85	28.71	23.1	25.52	23.79
ENSDARG00000059363	tgfbr2a	transforming growth factor beta receptor 2a [Source:ZFIN;Acc:ZDB-GENE-120404-1]	19	917852	932264		1.78	1.23	1.49	7.42	7.47	7.51
ENSDARG00000059365	nipal4	NIPA like domain containing 4 [Source:ZFIN;Acc:ZDB-GENE-040718-412]	21	26097732	26114886		4.43	4.36	3.14	2.84	3.42	3.29
ENSDARG00000059367	mfap2	microfibril associated protein 2 [Source:ZFIN;Acc:ZDB-GENE-030131-6710]	22	22888	24275		42.69	55.25	60.85	41.9	41.79	44.52
ENSDARG00000059368	gria4b	glutamate receptor, ionotropic, AMPA 4b [Source:ZFIN;Acc:ZDB-GENE-030131-8013]	21	21894067	22082510		15.81	2.86	7.07	17.12	16.78	17.63
ENSDARG00000059369	si:ch1073-15f12.3	si:ch1073-15f12.3 [Source:ZFIN;Acc:ZDB-GENE-081104-69]	22	1073413	1079773		0.15	0.08	0.11	0.74	0.65	1.1
ENSDARG00000059370	nr1d4b	nuclear receptor subfamily 1, group D, member 4b [Source:ZFIN;Acc:ZDB-GENE-080403-6]	11	1944729	1967438		8.77	3.46	3.4	8.04	8.84	9.97
ENSDARG00000059373	dnajc8	DnaJ (Hsp40) homolog, subfamily C, member 8 [Source:ZFIN;Acc:ZDB-GENE-030131-66]	19	69238	72398		59.74	46.86	53.07	48.86	46.82	48.15
ENSDARG00000059387	fgf7	fibroblast growth factor 7 [Source:ZFIN;Acc:ZDB-GENE-080122-1]	18	5428472	5439775		1.04	0.94	1.05	0.92	0.71	0.67
ENSDARG00000059388	bdh1	3-hydroxybutyrate dehydrogenase, type 1 [Source:ZFIN;Acc:ZDB-GENE-070410-130]	24	3328354	3336400		13.61	12.65	13.37	16.46	21.17	17.59
ENSDARG00000059391	gyg1b	glycogenin 1b [Source:ZFIN;Acc:ZDB-GENE-040625-30]	24	3307857	3326369		40.48	56.68	66.8	14.6	15.05	15.08
ENSDARG00000059397	tas1r2.2	taste receptor, type 1, member 2, tandem duplicate 2 [Source:ZFIN;Acc:ZDB-GENE-070917-2]	8	46610020	46619620		0	0.02	0.02	0	0	0
ENSDARG00000059398	myef2	myelin expression factor 2 [Source:ZFIN;Acc:ZDB-GENE-051120-114]	18	5233354	5248404		55.61	36.61	43.52	51.65	51.15	50.07
ENSDARG00000059399	si:ch211-160o17.6	si:ch211-160o17.6 [Source:ZFIN;Acc:ZDB-GENE-141215-39]	11	2691243	2700397		0.41	0.11	0.24	2.1	2.17	2.25
ENSDARG00000059400	tmem167b	transmembrane protein 167B [Source:NCBI gene;Acc:445304]	11	2699951	2707028		10.48	10.2	9.25	9.73	9.48	10.01
ENSDARG00000059404	rab43	RAB43, member RAS oncogene family [Source:ZFIN;Acc:ZDB-GENE-060825-25]	11	440305	450099		2.57	3.17	2.1	3.27	3.51	3.33
ENSDARG00000059406	raf1b	Raf-1 proto-oncogene, serine/threonine kinase b [Source:ZFIN;Acc:ZDB-GENE-090826-2]	11	506465	540252		10.02	8.21	7.91	12.67	13.14	13.4
ENSDARG00000059409	abcd2	ATP-binding cassette, sub-family D (ALD), member 2 [Source:ZFIN;Acc:ZDB-GENE-050517-28]	4	75292781	75308985		0.12	0.04	0.06	0.18	0.17	0.2
ENSDARG00000059410	ccr12b.1	chemokine (C-C motif) receptor 12b, tandem duplicate 1 [Source:ZFIN;Acc:ZDB-GENE-090420-5]	24	260902	261906		0.05	0	0.08	0.07	0.03	0
ENSDARG00000059411	impa2	inositol(myo)-1(or 4)-monophosphatase 2 [Source:ZFIN;Acc:ZDB-GENE-050522-349]	24	792429	799026		7.26	3.89	4.35	7.41	8.82	7.76
ENSDARG00000059412	zgc:111976	zgc:111976 [Source:ZFIN;Acc:ZDB-GENE-050522-332]	24	1023839	1036384		0.13	0.06	0.01	0.06	0.13	0.08
ENSDARG00000059423	prune2	prune homolog 2 with BCH domain [Source:ZFIN;Acc:ZDB-GENE-050309-222]	5	55371248	55395964		10.55	5.31	5.87	10.63	11.24	10.96
ENSDARG00000059424	rnf215	ring finger protein 215 [Source:ZFIN;Acc:ZDB-GENE-060526-65]	5	18040054	18046053		3.26	2.52	2.46	4.88	4.62	4.27
ENSDARG00000059432	fsd1l	fibronectin type III and SPRY domain containing 1-like [Source:ZFIN;Acc:ZDB-GENE-070501-9]	5	69808763	69851412		6.55	3.58	3.95	5.72	5.78	5.49
ENSDARG00000059437	fktn	fukutin [Source:ZFIN;Acc:ZDB-GENE-070410-96]	5	69907222	69924642		5.53	3.52	4.31	6.01	5.42	5.69
ENSDARG00000059438	galnt18b	UDP-N-acetyl-alpha-D-galactosamine:polypeptide N-acetylgalactosaminyltransferase 18b [Source:ZFIN;Acc:ZDB-GENE-091014-1]	7	66259046	66452827		4.71	2.65	2.78	3.98	4.11	3.81
ENSDARG00000059442	smtnb	smoothelin b [Source:ZFIN;Acc:ZDB-GENE-070912-2]	5	69289990	69316206		8.75	5.05	3.46	8.06	8.38	7.4
ENSDARG00000059455	dqx1	DEAQ box RNA-dependent ATPase 1 [Source:ZFIN;Acc:ZDB-GENE-150224-1]	5	72066835	72075456		7.43	4.53	4.76	11	10.41	9.84
ENSDARG00000059459	C5H2orf68	si:ch211-283h6.4 [Source:NCBI gene;Acc:100001127]	5	68817034	68826177	C5H2orf68	14.15	10.18	10.93	8.97	8.38	10.19
ENSDARG00000059460	sbf2	SET binding factor 2 [Source:ZFIN;Acc:ZDB-GENE-030911-10]	7	66884291	67171468		4.67	2.87	2.28	6.46	6.6	6.93

ENSDARG00000059461	mepce	methylphosphate capping enzyme [Source:NCBI gene;Acc:561384]	5	68765883	68782641		4.57	2.83	2.91	4.38	4.57	4.44
ENSDARG00000059466	wasf3a	WASP family member 3a [Source:ZFIN;Acc:ZDB-GENE-060531-55]	5	68007172	68022631		5.84	0.88	0.53	5.77	6.19	5.16
ENSDARG00000059472	arhgap31	Rho GTPase activating protein 31 [Source:ZFIN;Acc:ZDB-GENE-100922-256]	5	67671207	67721368		3.38	3.68	3.18	5.07	5.5	6
ENSDARG00000059473	kank4	KN motif and ankyrin repeat domains 4 [Source:ZFIN;Acc:ZDB-GENE-120423-1]	6	32410848	32507298		5.84	3.62	3.64	7.56	7.73	7.91
ENSDARG00000059474	mlxip	MLX interacting protein [Source:ZFIN;Acc:ZDB-GENE-060526-8]	5	67315816	67349916		3.05	2.96	2.08	5.5	5.39	5.42
ENSDARG00000059475	camsap3	calmodulin regulated spectrin-associated protein family, member 3 [Source:ZFIN;Acc:ZDB-GENE-060503-811]	3	53352018	53449437		4.24	2.64	1.96	6.62	6.45	6.6
ENSDARG00000059483	tead1b	TEA domain family member 1b [Source:ZFIN;Acc:ZDB-GENE-091013-5]	7	65813301	65924821		3.67	2.39	2.31	6.54	6.58	6.06
ENSDARG00000059485	emb	embigin [Source:ZFIN;Acc:ZDB-GENE-070705-158]	5	66353589	66362023		1.94	1.48	1.68	0.93	0.98	1.18
ENSDARG00000059486	cplx4b	complexin 4b [Source:ZFIN;Acc:ZDB-GENE-070705-157]	5	66347006	66351700		0.06	0	0	0	0.07	0
ENSDARG00000059498	arl16	ADP-ribosylation factor-like 16 [Source:ZFIN;Acc:ZDB-GENE-070410-114]	3	50271962	50277959		7.15	3.25	4.21	6.8	7.47	7.8
ENSDARG00000059503	ACSF3	acyl-CoA synthetase family member 3 [Source:NCBI gene;Acc:562292]	7	55950229	56038023	ACSF3	3.67	4.29	2.32	2.31	2.76	2.26
ENSDARG00000059507		si:dkey-163m14.2 [Source:ZFIN;Acc:ZDB-GENE-120215-47]	21	8076666	8085635		0.81	0.75	0.88	2.67	2.34	2.8
ENSDARG00000059510	coq2	coenzyme Q2 4-hydroxybenzoate polyprenyltransferase [Source:ZFIN;Acc:ZDB-GENE-070410-84]	5	66203154	66215928		5.45	6.57	6.72	4.7	4.41	4.44
ENSDARG00000059523	rims4	regulating synaptic membrane exocytosis 4 [Source:ZFIN;Acc:ZDB-GENE-110103-1]	6	52081752	52156427		2.3	0.58	0.84	2.55	2.68	2.6
ENSDARG00000059529		uncharacterized protein KIAA0895-like [Source:NCBI gene;Acc:562097]	7	56735195	56746338		3.45	2.49	2.88	3.22	3.23	3.65
ENSDARG00000059534	JPH3	si:ch211-146m13.3 [Source:ZFIN;Acc:ZDB-GENE-110408-64]	7	56777668	56793739	JPH3	6.8	1.66	2.75	8.23	8.05	8.12
ENSDARG00000059549	akap10	A kinase (PRKA) anchor protein 10 [Source:ZFIN;Acc:ZDB-GENE-030131-2535]	5	62009765	62043299		7.06	3.87	3.88	8.42	8.51	7.81
ENSDARG00000059553	sympk	symplekin [Source:ZFIN;Acc:ZDB-GENE-060526-361]	5	61862199	61892917		7.36	4.95	4.79	6.74	6.84	7.06
ENSDARG00000059556	recql5	RecQ helicase-like 5 [Source:ZFIN;Acc:ZDB-GENE-071214-1]	3	59986120	60027308		7.22	4.54	5	5.61	5.56	5.39
ENSDARG00000059557	xylt2	xylosyltransferase II [Source:ZFIN;Acc:ZDB-GENE-081022-125]	3	60177774	60226027		1.83	1.11	1.2	2.15	2.11	1.96
ENSDARG00000059558	tecta	tectorin alpha [Source:ZFIN;Acc:ZDB-GENE-110411-120]	5	63177355	63233216		0.92	0.32	0.31	1.37	1.05	1.35
ENSDARG00000059567	si:ch73-281f12.4	si:ch73-281f12.4 [Source:ZFIN;Acc:ZDB-GENE-130530-682]	3	58714741	58798815		2.79	1.49	1.23	3.82	3.75	4.44
ENSDARG00000059574	fscn2a	fascin actin-bundling protein 2a, retinal [Source:ZFIN;Acc:ZDB-GENE-110901-1]	3	57234093	57257075		7.92	1.51	1.27	4.53	4.82	4.65
ENSDARG00000059575	uhmk1	U2AF homology motif (UHM) kinase 1 [Source:ZFIN;Acc:ZDB-GENE-060929-1014]	6	35052721	35061467		6.11	4.93	4.68	6.4	6.31	6.56
ENSDARG00000059581	mbd5	methyl-CpG binding domain protein 5 [Source:ZFIN;Acc:ZDB-GENE-070209-125]	6	48862	60202		4.47	2.9	2.61	8.54	7.09	8.16
ENSDARG00000059582	sowahcb	sosondowah ankyrin repeat domain family Cb [Source:ZFIN;Acc:ZDB-GENE-061215-96]	6	68220	69904		5.1	1.98	2.9	5.88	4.84	5.52
ENSDARG00000059583	rcc1l	RCC1 like [Source:ZFIN;Acc:ZDB-GENE-060526-370]	5	61404497	61431809		9.5	11.4	9.99	10.86	10.79	10.52
ENSDARG00000059587	ABR	ABR activator of RhoGEF and GTPase [Source:HGNC Symbol;Acc:HGNC:81]	5	62374092	62545913	ABR	9.57	5.68	4.87	10.45	11.14	11.12
ENSDARG00000059596	clip2	CAP-GLY domain containing linker protein 2 [Source:ZFIN;Acc:ZDB-GENE-100727-3]	5	59392183	59480161		8.59	3.86	4.77	8.83	8.93	8.47
ENSDARG00000059600	rabep1	rabaptin, RAB GTPase binding effector protein 1 [Source:ZFIN;Acc:ZDB-GENE-060526-165]	5	59030096	59083298		6.41	3.74	3.2	7.45	7.35	6.9
ENSDARG00000059601	map1aa	microtubule-associated protein 1Aa [Source:ZFIN;Acc:ZDB-GENE-100426-2]	7	52830254	52876683		19.14	5.25	7.51	19.93	20.27	20.72
ENSDARG00000059604	mtss1	MTSS I-BAR domain containing 1 [Source:ZFIN;Acc:ZDB-GENE-081105-141]	6	50580940	50685862		7.5	6.89	5.98	9.45	9.57	9.48
ENSDARG00000059606	ccdc84	coiled-coil domain containing 84 [Source:ZFIN;Acc:ZDB-GENE-060929-1174]	5	58687541	58696264		10.1	4.22	5.24	8.17	7.56	8.1
ENSDARG00000059610	gpr146	G protein-coupled receptor 146 [Source:ZFIN;Acc:ZDB-GENE-050823-3]	3	45687266	45717575		12.1	8.94	7.87	15	15.58	15.32
ENSDARG00000059613	nprl2	NPR2 like, GATOR1 complex subunit [Source:ZFIN;Acc:ZDB-GENE-060825-93]	6	22352223	22369125		7.99	7.77	6.95	8.19	7.28	7.81
ENSDARG00000059616	hs3st2	heparan sulfate (glucosamine) 3-O-sulfotransferase 2 [Source:ZFIN;Acc:ZDB-GENE-070202-7]	3	44947355	45000645		2.57	0.82	0.87	3.86	3.31	2.63
ENSDARG00000059629	asphd2	aspartate beta-hydroxylase domain containing 2 [Source:NCBI gene;Acc:569819]	8	39965117	39984593		7.97	3.87	4.22	5.81	5.84	5.96
ENSDARG00000059630	mlec	malectin [Source:NCBI gene;Acc:569613]	8	39870714	39884359		7.58	6.38	4.69	8.55	9.11	7.93
ENSDARG00000059634	thumpd3	THUMP domain containing 3 [Source:ZFIN;Acc:ZDB-GENE-030131-9745]	6	21992820	22022636		8.23	9.18	7.83	6.28	7.45	6.07
ENSDARG00000059636	C5H2orf42	si:ch211-127d4.3 [Source:ZFIN;Acc:ZDB-GENE-060526-22]	5	56927703	56948058	C5H2orf42	2.15	1.06	1.12	1.62	2.11	1.94
ENSDARG00000059642	mtmr14	myotubularin related protein 14 [Source:ZFIN;Acc:ZDB-GENE-030131-2649]	6	21697128	21726758		5.55	4.47	3.69	5.52	5.6	5.66
ENSDARG00000059646	nt5dc2	5'-nucleotidase domain containing 2 [Source:ZFIN;Acc:ZDB-GENE-060810-17]	8	23639124	23656730		22.15	21.98	17.79	67.3	70.6	72.88
ENSDARG00000059651	cidec	cell death inducing DFFA like effector c [Source:ZFIN;Acc:ZDB-GENE-030131-4591]	6	21202639	21212367		2.29	4.34	2.87	1.87	2.21	2.01
ENSDARG00000059653	kdm2aa	lysine (K)-specific demethylase 2Aa [Source:ZFIN;Acc:ZDB-GENE-061215-144]	1	44838706	44869972		14.03	9.66	8.55	14.67	14.53	14.65
ENSDARG00000059654	eif3ba	eukaryotic translation initiation factor 3, subunit Ba [Source:ZFIN;Acc:ZDB-GENE-030131-2748]	3	41714966	41724932		220.95	232.53	221.5	179.02	178.43	179.94
ENSDARG00000059657	mks1	MKS transition zone complex subunit 1 [Source:ZFIN;Acc:ZDB-GENE-030131-3813]	5	56090851	56119028		2.6	1.71	1.97	2.64	3.17	3.18
ENSDARG00000059658	mrm1	mitochondrial rRNA methyltransferase 1 homolog (S. cerevisiae) [Source:ZFIN;Acc:ZDB-GENE-060526-229]	5	56119975	56125769		1.11	0.99	1.37	1.32	1.22	0.99
ENSDARG00000059672	arhgap36	Rho GTPase activating protein 36 [Source:ZFIN;Acc:ZDB-GENE-060616-298]	7	51364290	51461664		2.64	1.8	1.43	2.59	2.92	3.1
ENSDARG00000059680	fscn1a	fascin actin-bundling protein 1a [Source:ZFIN;Acc:ZDB-GENE-031009-1]	3	40576447	40589489		64.45	33.32	42.57	68.35	69.5	71.51
ENSDARG00000059682	slc43a3a	solute carrier family 43 member 3a [Source:ZFIN;Acc:ZDB-GENE-110208-8]	1	44141758	44161417		3.61	1.25	1.24	3.64	3.17	3.9
ENSDARG00000059689	mblac2	metallo-beta-lactamase domain containing 2 [Source:ZFIN;Acc:ZDB-GENE-081104-313]	5	48659747	48664522		2.42	1.67	1.83	3.66	4.6	3.83
ENSDARG00000059690	slc29a4	Danio rerio solute carrier family 29 member 4 (slc29a4), transcript variant 1, mRNA. [Source:RefSeq mRNA;Acc:NM_001362610]	3	40315410	40349733		6.18	2.14	3.24	7.04	6.98	7.51
ENSDARG00000059693	adam19a	ADAM metallopeptidase domain 19a [Source:ZFIN;Acc:ZDB-GENE-070813-1]	7	61480296	61533934		3.38	3.13	2.22	3.55	3.93	3.97
ENSDARG00000059695	dusp28	dual specificity phosphatase 28 [Source:ZFIN;Acc:ZDB-GENE-131127-257]	2	5446087	5450198		0.98	0.68	0.78	1.41	1.35	1.25
ENSDARG00000059699	dnajc16	DnaJ (Hsp40) homolog, subfamily C, member 16 [Source:ZFIN;Acc:ZDB-GENE-130530-683]	23	37493532	37514493		3.93	1.01	2.05	3.55	4.2	4.21
ENSDARG00000059700	agmat	agmatine ureohydrolase (agmatinase) [Source:ZFIN;Acc:ZDB-GENE-060929-696]	23	37482727	37490249		9.02	11.83	7.34	5.08	4.77	5.14
ENSDARG00000059701	flii	FLII actin remodeling protein [Source:ZFIN;Acc:ZDB-GENE-071212-3]	3	40206031	40232615		9.45	8.14	7.05	11.13	11.44	10.85
ENSDARG00000059705	vwa5b1	von Willebrand factor A domain containing 5B1 [Source:ZFIN;Acc:ZDB-GENE-090313-299]	23	37185247	37198890		1.12	0.24	0.16	1.7	1.75	1.2
ENSDARG00000059707		si:ch211-216l23.1 [Source:NCBI gene;Acc:566696]	18	17786548	17997474		5.56	2.81	2.75	8.27	8.19	8.64
ENSDARG00000059711	nol6	nucleolar protein 6 (RNA-associated) [Source:ZFIN;Acc:ZDB-GENE-030131-6294]	5	50869091	50899688		4.65	4.47	4.05	4.33	4.38	4.06
ENSDARG00000059712	slc19a2	solute carrier family 19 member 2 [Source:ZFIN;Acc:ZDB-GENE-110411-262]	6	16436572	16456169		1.19	1.39	1.28	1.94	2.2	1.9
ENSDARG00000059714	arsk	arylsulfatase family, member K [Source:NCBI gene;Acc:562412]	5	50898849	50911256		1.91	1.81	1.88	2.79	3.18	3.16
ENSDARG00000059719	fam169aa	family with sequence similarity 169 member Aa [Source:ZFIN;Acc:ZDB-GENE-060825-117]	5	51079504	51096579		9.02	5.74	2.4	4.64	4.17	4.11
ENSDARG00000059722	ubac1	UBA domain containing 1 [Source:ZFIN;Acc:ZDB-GENE-030131-9372]	5	51226846	51243925		10.06	9.12	7.3	9.12	7.62	7.48
ENSDARG00000059725	hypk	huntingtin interacting protein K [Source:ZFIN;Acc:ZDB-GENE-080515-6]	7	50407575	50410524		109.73	138.38	142.39	78.65	78.92	75.62
ENSDARG00000059729	sec14l8	SEC14-like lipid binding 8 [Source:ZFIN;Acc:ZDB-GENE-060526-180]	5	13427826	13469307		8.97	12.9	8.69	4.81	4.81	4.89
ENSDARG00000059738	ptprsa	protein tyrosine phosphatase receptor type Sa [Source:ZFIN;Acc:ZDB-GENE-020107-3]	22	3389347	3595439		5.96	2.7	2.85	13.26	13.39	13.94
ENSDARG00000059743	tmem98	transmembrane protein 98 [Source:ZFIN;Acc:ZDB-GENE-070112-1092]	3	39099716	39110228		2.51	3.35	2.45	2.54	2.75	2.6
ENSDARG00000059746	plod1a	procollagen-lysine, 2-oxoglutarate 5-dioxygenase 1a [Source:ZFIN;Acc:ZDB-GENE-030131-3165]	8	47850073	47895817		8.46	8.82	6.8	30.5	32.33	33.04
ENSDARG00000059747	kcnj19a	potassium inwardly rectifying channel subfamily J member 19a [Source:ZFIN;Acc:ZDB-GENE-060503-706]	3	38793499	38859305		2.55	0.43	0.67	2.17	1.95	2.2
ENSDARG00000059751	abtb2a	ankyrin repeat and BTB (POZ) domain containing 2a [Source:ZFIN;Acc:ZDB-GENE-100422-13]	7	49272702	49320952		0.52	0.24	0.28	2.17	1.91	2.22
ENSDARG00000059760	wdtc1	WD and tetratricopeptide repeats 1 [Source:ZFIN;Acc:ZDB-GENE-030131-3224]	16	54947768	54978981		7.38	4.63	4.53	7.31	6.78	7.67
ENSDARG00000059763	gabrd	gamma-aminobutyric acid (GABA) A receptor, delta [Source:ZFIN;Acc:ZDB-GENE-081105-170]	8	53185079	53198154		9.92	2.21	4.16	10.16	10.54	10.54
ENSDARG00000059768	gpatch8	G patch domain containing 8 [Source:ZFIN;Acc:ZDB-GENE-030131-6966]	3	37662444	37699992		24.18	8.03	10.49	27.59	26.32	27.75
ENSDARG00000059770	cpt1aa	carnitine palmitoyltransferase 1Aa (liver) [Source:ZFIN;Acc:ZDB-GENE-060503-925]	7	48805534	48830121		9.67	7.03	5.91	9.03	8.81	9.16
ENSDARG00000059773	SRCIN1	si:ch211-278a6.1 [Source:ZFIN;Acc:ZDB-GENE-060503-699]	3	37235924	37476475	SRCIN1	3.63	1	1.11	6.19	5.85	6.13
ENSDARG00000059774	dub	duboraya [Source:ZFIN;Acc:ZDB-GENE-060825-81]	21	22917221	22951646		6.48	7.34	7.13	3.95	3.75	3.62
ENSDARG00000059775	slc32a1	solute carrier family 32 member 1 [Source:ZFIN;Acc:ZDB-GENE-061201-1]	6	60142619	60147517		32.1	6.89	6.9	42.34	42.84	43.03
ENSDARG00000059781	atcayb	ATCAY kinesin light chain interacting caytaxin b [Source:ZFIN;Acc:ZDB-GENE-070912-220]	2	55914699	55970970		1.69	0.38	0.63	2.66	2.64	2.81
ENSDARG00000059789	parp8	poly (ADP-ribose) polymerase family, member 8 [Source:ZFIN;Acc:ZDB-GENE-060929-432]	5	40844968	40910880		4.9	1.51	2.02	6.98	6.69	6.73
ENSDARG00000059792	trpm5	transient receptor potential cation channel, subfamily M, member 5 [Source:ZFIN;Acc:ZDB-GENE-060503-736]	7	48667081	48702051		0.77	0.3	0.36	1.01	1.06	1.08
ENSDARG00000059794	kdm6al	lysine (K)-specific demethylase 6A, like [Source:ZFIN;Acc:ZDB-GENE-070112-2002]	6	59854224	59895080		4.65	3.09	3.26	7.89	7.62	7.92
ENSDARG00000059798	kcnq1.1	potassium voltage-gated channel, KQT-like subfamily, member 1.1 [Source:ZFIN;Acc:ZDB-GENE-061214-5]	7	48555400	48658172		0.82	0.59	0.4	0.57	0.63	0.57
ENSDARG00000059801	pdzd2	PDZ domain containing 2 [Source:ZFIN;Acc:ZDB-GENE-060526-297]	5	40931658	41049690		3.95	1.62	1.34	4.89	4.72	5.27
ENSDARG00000059804	retreg3	reticulophagy regulator family member 3 [Source:ZFIN;Acc:ZDB-GENE-080520-1]	3	37083765	37107873		3.58	1.96	1.74	3.64	3.57	3.68
ENSDARG00000059805	slc25a38a	solute carrier family 25 member 38a [Source:NCBI gene;Acc:767662]	3	4644031	4663602		1.2	0.89	1.43	8.33	10.71	9.78
ENSDARG00000059806	sacm1lb	SAC1 like phosphatidylinositide phosphatase b [Source:NCBI gene;Acc:791142]	16	53603945	53633569		7.06	5.58	5.11	8.25	8.23	8.33
ENSDARG00000059809	caska	calcium/calmodulin-dependent serine protein kinase a [Source:ZFIN;Acc:ZDB-GENE-020802-4]	9	33366683	33608427		13.02	10.49	9.94	17.01	18.47	19.18
ENSDARG00000059811	smpd5	sphingomyelin phosphodiesterase 5 [Source:NCBI gene;Acc:567592]	16	53519048	53542242		7.64	3.33	4.09	6.35	6.59	6.63
ENSDARG00000059812	sin3ab	SIN3 transcription regulator family member Ab [Source:ZFIN;Acc:ZDB-GENE-030131-2066]	7	48345747	48396195		5.48	3.4	2.9	7.3	7.1	7.08
ENSDARG00000059815	oaz2b	ornithine decarboxylase antizyme 2b [Source:ZFIN;Acc:ZDB-GENE-070313-1]	7	48288762	48296227		5.55	2.83	3.25	10.07	10.98	11.47
ENSDARG00000059816	zgc:136864	zgc:136864 [Source:NCBI gene;Acc:677749]	1	55007272	55008882		0	0	0	0	0	0
ENSDARG00000059817	mtmr12	myotubularin related protein 12 [Source:NCBI gene;Acc:569130]	5	41105602	41124241		3.68	2.45	2.55	3.56	3.27	2.72
ENSDARG00000059818	kif5ab	kinesin family member 5A, b [Source:ZFIN;Acc:ZDB-GENE-110510-3]	6	58772209	58828398		4.28	0.99	1.73	4.19	4.86	4.66
ENSDARG00000059824	soat2	sterol O-acyltransferase 2 [Source:ZFIN;Acc:ZDB-GENE-061013-742]	6	58737168	58764672		3.17	4.81	5.23	7.02	9.16	9.15
ENSDARG00000059826	crtac1a	cartilage acidic protein 1a [Source:ZFIN;Acc:ZDB-GENE-030131-9704]	1	54934522	54947592		2.95	0.95	1.7	2.52	1.88	2.36
ENSDARG00000059832	adgrb3	adhesion G protein-coupled receptor B3 [Source:ZFIN;Acc:ZDB-GENE-070424-99]	13	38302665	38593701		10.38	2.01	3.19	11.55	11.24	11.65
ENSDARG00000059835	zfyve27	zinc finger, FYVE domain containing 27 [Source:ZFIN;Acc:ZDB-GENE-061013-502]	1	54889733	54907823		9.27	6.58	6.1	9.41	8.95	8.7
ENSDARG00000059836	ddit3	DNA-damage-inducible transcript 3 [Source:ZFIN;Acc:ZDB-GENE-070410-90]	6	58915889	58919086		39.89	20.39	25.37	44.01	44	49.88
ENSDARG00000059841	exosc1	exosome component 1 [Source:ZFIN;Acc:ZDB-GENE-080926-3]	1	54700657	54706039		48.19	46.5	37.6	29.6	32.21	32.37
ENSDARG00000059843	fam135a	family with sequence similarity 135 member A [Source:ZFIN;Acc:ZDB-GENE-080130-1]	13	39182099	39228382		7.55	4.22	3.99	7.98	8.07	7.88
ENSDARG00000059846	epg5	ectopic P-granules autophagy protein 5 homolog (C. elegans) [Source:NCBI gene;Acc:569543]	5	41582643	41638039		3.93	2.95	2.44	4.11	4.08	4.29
ENSDARG00000059850	slc35f3b	solute carrier family 35 member F3b [Source:ZFIN;Acc:ZDB-GENE-090312-118]	13	39346298	39399967		0.95	0.38	0.37	1.31	1.38	1.09
ENSDARG00000059853	manbal	mannosidase beta like [Source:ZFIN;Acc:ZDB-GENE-161222-1]	6	55212928	55218839		12.97	13.2	13.35	10.01	10.3	10.24
ENSDARG00000059854	CABZ01041604.1	E3 ubiquitin-protein ligase RNF182-like [Source:NCBI gene;Acc:797048]	6	55277419	55278162		5.24	1.05	1.92	4.47	4.96	4.2
ENSDARG00000059855	wdr66	WD repeat domain 66 [Source:ZFIN;Acc:ZDB-GENE-060526-328]	5	41774366	41792672		0.62	0.34	0.55	0.63	0.81	0.67
ENSDARG00000059856	alkbh2	alkB homolog 2, alpha-ketoglutarate-dependent dioxygenase [Source:ZFIN;Acc:ZDB-GENE-060526-327]	5	41800082	41804398		4.01	3.82	3.76	3.6	3.97	4.04
ENSDARG00000059857	blvra	biliverdin reductase A [Source:ZFIN;Acc:ZDB-GENE-060929-312]	2	49844630	49860723		2.52	1.99	2.14	2.05	2.13	1.26
ENSDARG00000059858	pappab	pregnancy-associated plasma protein A, pappalysin 1b [Source:ZFIN;Acc:ZDB-GENE-060526-71]	5	63913310	64104012		0.46	0.34	0.42	1.46	1.36	1.39
ENSDARG00000059866	cactin	cactin [Source:NCBI gene;Acc:100126813]	2	49608796	49627103		13.31	10.56	9.59	10.55	11.25	11.17
ENSDARG00000059870	clk2b	CDC-like kinase 2b [Source:ZFIN;Acc:ZDB-GENE-060929-1126]	16	54474621	54498109		7.05	5.01	5.37	6.76	6.69	6.8
ENSDARG00000059871	shrprbck1r	sharpin and rbck1 related [Source:NCBI gene;Acc:569903]	7	40058757	40082053		4.58	3.41	2.88	5.59	5.51	5.68
ENSDARG00000059883	trpv1	transient receptor potential cation channel, subfamily V, member 1 [Source:ZFIN;Acc:ZDB-GENE-030912-8]	5	42136359	42158556		1.53	1	1.15	2.18	2.42	2.31
ENSDARG00000059885	frmd3	FERM domain containing 3 [Source:ZFIN;Acc:ZDB-GENE-070523-1]	8	49617946	49705058		3.04	0.9	1.21	4.02	4.51	4.46
ENSDARG00000059886	fam222ba	family with sequence similarity 222 member Ba [Source:ZFIN;Acc:ZDB-GENE-060526-355]	5	42164079	42180205		8.74	3.9	4.03	13.51	13.46	12.08
ENSDARG00000059887	eral1	Era-like 12S mitochondrial rRNA chaperone 1 [Source:NCBI gene;Acc:569087]	5	42258994	42270105		7.63	6.29	5.76	5.84	6.08	6.3
ENSDARG00000059888	ago3a	argonaute RISC catalytic component 3a [Source:ZFIN;Acc:ZDB-GENE-051107-16]	16	51001957	51072406		2.32	2.13	1.92	4.91	4.61	4.55
ENSDARG00000059891	AL929217.1	gamma-aminobutyric acid receptor subunit pi-like [Source:NCBI gene;Acc:562831]	13	51140464	51247529		2.06	0.19	0.41	2.47	2.25	2.51
ENSDARG00000059897	ntrk2a	neurotrophic tyrosine kinase, receptor, type 2a [Source:ZFIN;Acc:ZDB-GENE-010126-1]	8	49778486	49865375		9.01	3.07	4.44	12.05	13.07	11.9
ENSDARG00000059899	CR847944.2	adenosine receptor A1-like [Source:NCBI gene;Acc:108179103]	6	54459413	54465377		0.25	0.27	0.2	0.3	0.36	0.22
ENSDARG00000059900	tbc1d9	TBC1 domain family, member 9 (with GRAM domain) [Source:ZFIN;Acc:ZDB-GENE-060810-33]	1	53321878	53361108		5.87	2.97	2.69	8.08	7.89	8.53
ENSDARG00000059902	vps16	vacuolar protein sorting 16 homolog [Source:ZFIN;Acc:ZDB-GENE-050809-9]	6	9107063	9130843		15.27	10.07	9.3	12.47	12.29	13.53
ENSDARG00000059903	hs3st3l	heparan sulfate (glucosamine) 3-O-sulfotransferase 3-like [Source:ZFIN;Acc:ZDB-GENE-070202-6]	12	415711	431249		6.67	1.44	1.16	6.37	6.51	7.42
ENSDARG00000059906	sdc4	syndecan 4 [Source:ZFIN;Acc:ZDB-GENE-061111-1]	6	52663097	52675630		38.04	39.15	33.56	83.18	86.21	85.91
ENSDARG00000059908	si:ch211-217k17.7	si:ch211-217k17.7 [Source:ZFIN;Acc:ZDB-GENE-061009-11]	1	52452010	52461322		36.6	53.59	47.18	20.9	20.66	20.59
ENSDARG00000059911	bbs7	Bardet-Biedl syndrome 7 [Source:ZFIN;Acc:ZDB-GENE-030219-90]	14	46375241	46395659		5.11	2.4	2.86	4.47	4.08	4.74
ENSDARG00000059914	sdr42e2	short chain dehydrogenase/reductase family 42E, member 2 [Source:ZFIN;Acc:ZDB-GENE-101203-3]	3	58379450	58416968		6.23	7.79	9.19	4.57	3.88	4.03
ENSDARG00000059916	hyal1	hyaluronidase 1 [Source:ZFIN;Acc:ZDB-GENE-060312-42]	6	54063032	54078623		3.73	2.72	2.88	2.42	2.42	2.39
ENSDARG00000059917	ccnj	cyclin J [Source:ZFIN;Acc:ZDB-GENE-100721-4]	13	50450190	50463938		0.31	0.21	0.21	0.29	0.23	0.31
ENSDARG00000059919	zgc:162613	zgc:162613 [Source:ZFIN;Acc:ZDB-GENE-070410-89]	3	32862730	32870055		4.6	5.05	4.94	3.62	3.54	3.68
ENSDARG00000059923	slc25a47a	solute carrier family 25 member 47a [Source:NCBI gene;Acc:724009]	20	53628534	53641863		1.11	1.76	1.04	1.23	1.2	0.97
ENSDARG00000059925	usp24	ubiquitin specific peptidase 24 [Source:ZFIN;Acc:ZDB-GENE-030131-4356]	20	7443224	7583486		7.06	7.09	5.35	9.57	8.75	8.21
ENSDARG00000059931	hyal2a	hyaluronidase 2a [Source:ZFIN;Acc:ZDB-GENE-131121-41]	6	54093057	54111928		1.41	1.27	1.08	1.77	1.67	1.9
ENSDARG00000059933	plpp3	phospholipid phosphatase 3 [Source:ZFIN;Acc:ZDB-GENE-060526-241]	20	7964356	8024582		14.04	14.36	12.77	23.86	25.15	25.57
ENSDARG00000059936	herc4	HECT and RLD domain containing E3 ubiquitin protein ligase 4 [Source:ZFIN;Acc:ZDB-GENE-070801-1]	13	50387365	50446872		5.05	3.64	3.58	5.24	5.29	5.1
ENSDARG00000059939	dab1a	DAB adaptor protein 1a [Source:ZFIN;Acc:ZDB-GENE-060528-1]	20	8122693	8443425		4.91	1.93	1.57	7.39	7.13	7.32
ENSDARG00000059944	c1qtnf2	C1q and TNF related 2 [Source:ZFIN;Acc:ZDB-GENE-030131-5133]	14	46020282	46027226		0.81	0.79	0.85	0.53	0.44	0.51
ENSDARG00000059945	sv2a	synaptic vesicle glycoprotein 2A [Source:ZFIN;Acc:ZDB-GENE-131001-1]	16	46111849	46202705		31.29	6.2	6.22	48.99	47.2	48.84
ENSDARG00000059948	si:dkey-121b10.7	si:dkey-121b10.7 [Source:ZFIN;Acc:ZDB-GENE-090312-176]	1	52264131	52292235		0.98	0.71	0.77	0.77	0.43	0.85
ENSDARG00000059950	plxdc2	plexin domain containing 2 [Source:ZFIN;Acc:ZDB-GENE-140106-69]	7	41517173	41627956		14.28	16.92	14.79	16.73	18.01	16.67
ENSDARG00000059951	pld6	phospholipase D family, member 6 [Source:NCBI gene;Acc:567338]	1	52203009	52210950		0.11	0.11	0.15	0.14	0.14	0.33
ENSDARG00000059956	ptk6b	PTK6 protein tyrosine kinase 6b [Source:ZFIN;Acc:ZDB-GENE-060929-1150]	23	17387325	17404011		4.82	7.62	5.32	2.08	1.44	1.76
ENSDARG00000059960	plch2a	phospholipase C, eta 2a [Source:ZFIN;Acc:ZDB-GENE-030131-6367]	8	47342586	47622532		10.36	2.22	2.48	9.88	9.37	10.03
ENSDARG00000059961	chpf2	chondroitin polymerizing factor 2 [Source:ZFIN;Acc:ZDB-GENE-030131-7797]	7	41421998	41433786		6.54	5.84	5.76	8.08	8.3	7.36
ENSDARG00000059963	polk	polymerase (DNA directed) kappa [Source:ZFIN;Acc:ZDB-GENE-060526-137]	5	45895613	45914457		2.08	1.33	1.27	1.64	1.86	1.66
ENSDARG00000059965	camsap2b	calmodulin regulated spectrin-associated protein family, member 2b [Source:ZFIN;Acc:ZDB-GENE-070912-80]	2	45300512	45376408		3.03	1.87	1.86	3.27	3.34	3.23
ENSDARG00000059972	raly	RALY heterogeneous nuclear ribonucleoprotein [Source:ZFIN;Acc:ZDB-GENE-041010-127]	6	50021410	50204262		6.36	3.27	3.96	5.71	5.75	5.79
ENSDARG00000059978	cplx4a	complexin 4a [Source:ZFIN;Acc:ZDB-GENE-060526-116]	21	10739846	10749542		35.97	6.37	4.38	29.98	31.06	31.28
ENSDARG00000059982	poc5	POC5 centriolar protein homolog (Chlamydomonas) [Source:ZFIN;Acc:ZDB-GENE-060526-135]	5	45938316	45958838		1.12	1.09	0.78	1.04	1.01	1.1
ENSDARG00000059983	zufsp	zinc finger containing ubiquitin peptidase 1 [Source:ZFIN;Acc:ZDB-GENE-070209-211]	16	32184485	32196771		2.66	1.7	2.11	1.78	1.73	2.13
ENSDARG00000059987	dnah12	dynein, axonemal, heavy chain 12 [Source:ZFIN;Acc:ZDB-GENE-090311-9]	23	19506023	19558556		0.42	0.35	0.28	0.66	0.58	0.57
ENSDARG00000059993	trpm4a	transient receptor potential cation channel, subfamily M, member 4a [Source:ZFIN;Acc:ZDB-GENE-090302-3]	3	32492467	32518947		8.63	7.58	5.7	9.22	9.01	8.96
ENSDARG00000059997	sv2ca	synaptic vesicle glycoprotein 2Ca [Source:ZFIN;Acc:ZDB-GENE-060526-233]	5	45976130	46066558		2.59	0.86	1.37	4.74	4.02	4.92
ENSDARG00000060001	mettl9	methyltransferase like 9 [Source:ZFIN;Acc:ZDB-GENE-061013-752]	12	992442	999800		6.43	5.49	4.7	6.08	5.48	5.74
ENSDARG00000060002	ogg1	8-oxoguanine DNA glycosylase [Source:ZFIN;Acc:ZDB-GENE-070112-932]	8	46386601	46406382		1.57	1.22	1.16	0.94	1.04	1.16
ENSDARG00000060004	fzd7a	frizzled class receptor 7a [Source:ZFIN;Acc:ZDB-GENE-990415-223]	9	13352308	13355071		28.47	26.54	29.3	38.9	40.96	39.68
ENSDARG00000060005	mtrf1l	mitochondrial translational release factor 1-like [Source:ZFIN;Acc:ZDB-GENE-030131-8198]	13	46927350	46937057		2.87	2.07	2.76	2.75	2.68	2.6
ENSDARG00000060006	nedd4l	NEDD4 like E3 ubiquitin protein ligase [Source:ZFIN;Acc:ZDB-GENE-051118-2]	21	10890258	11054876		6.33	4.52	3.68	10.68	10.38	11.09
ENSDARG00000060007	n4bp3	NEDD4 binding protein 3 [Source:ZFIN;Acc:ZDB-GENE-141216-2]	14	49264184	49286773		1.3	1.07	1.12	1.34	1.42	0.99
ENSDARG00000060008	hhipl2	HHIP-like 2 [Source:ZFIN;Acc:ZDB-GENE-131120-145]	17	45501378	45525936		3.99	4.48	3.21	2.45	1.9	2.01
ENSDARG00000060010	iqgap2	IQ motif containing GTPase activating protein 2 [Source:ZFIN;Acc:ZDB-GENE-030131-2878]	5	46077710	46196708		4.63	5.7	4.37	3.86	4	3.78
ENSDARG00000060012	f2r	coagulation factor II (thrombin) receptor [Source:ZFIN;Acc:ZDB-GENE-060526-30]	5	46196147	46198034		0.95	1.41	1.49	3.52	3.81	3.72
ENSDARG00000060016	nsd1a	nuclear receptor binding SET domain protein 1a [Source:ZFIN;Acc:ZDB-GENE-080519-3]	14	49152341	49214607		4.89	2.59	2.42	6.97	6.53	6.53
ENSDARG00000060018	si:ch1073-44g3.1	si:ch1073-44g3.1 [Source:ZFIN;Acc:ZDB-GENE-141216-124]	14	49135264	49137035		14.66	4.76	6.34	24.08	22.73	23.21
ENSDARG00000060021	vcpkmt	valosin containing protein lysine (K) methyltransferase [Source:ZFIN;Acc:ZDB-GENE-060825-19]	17	45639247	45647054		4.37	3.51	3.88	4.34	4.19	4.17
ENSDARG00000060023	ncapg2	non-SMC condensin II complex, subunit G2 [Source:ZFIN;Acc:ZDB-GENE-070410-100]	7	40205394	40227602		2.97	2.56	2.6	2.59	2.29	2.42
ENSDARG00000060025	nsmfa	NMDA receptor synaptonuclear signaling and neuronal migration factor a [Source:ZFIN;Acc:ZDB-GENE-091204-32]	21	13455308	13535601		1.31	0.36	0.6	2.95	3.93	3.69
ENSDARG00000060027	nom1	nucleolar protein with MIF4G domain 1 [Source:ZFIN;Acc:ZDB-GENE-060503-321]	7	40644769	40657831		5.66	6.31	6.24	4	3.86	4.44
ENSDARG00000060029	fam160b2	family with sequence similarity 160 member B2 [Source:NCBI gene;Acc:777718]	8	45961617	46005359		5.86	5.1	5.67	5.74	5.93	6.08
ENSDARG00000060030	setd1a	SET domain containing 1A, histone lysine methyltransferase [Source:ZFIN;Acc:ZDB-GENE-080521-4]	3	32714157	32742578		4.73	3.53	2.72	7.77	7.38	7.84
ENSDARG00000060034	tmem151ba	transmembrane protein 151Ba [Source:NCBI gene;Acc:100006371]	20	51312883	51334529		2.13	0.69	1.21	2.89	2.19	2.46
ENSDARG00000060035	rnf32	ring finger protein 32 [Source:ZFIN;Acc:ZDB-GENE-061013-39]	7	40715756	40738774		1.22	1.18	0.89	0.98	1.36	1.01
ENSDARG00000060036	aimp1a	aminoacyl tRNA synthetase complex interacting multifunctional protein 1a [Source:ZFIN;Acc:ZDB-GENE-060825-144]	1	50293938	50319654		51.83	57.78	59.09	43.92	42.93	46.32
ENSDARG00000060041	lig1	ligase I, DNA, ATP-dependent [Source:ZFIN;Acc:ZDB-GENE-110404-2]	2	44301276	44344321		21.06	19.54	17.79	9.58	9.12	8.95
ENSDARG00000060049	zgc:195075	zgc:195075 [Source:ZFIN;Acc:ZDB-GENE-080723-70]	7	5155968	5162292		0.35	0.35	0.29	0.13	0.29	0.36
ENSDARG00000060051	slc47a2.1	solute carrier family 47 member 2.1 [Source:NCBI gene;Acc:560024]	21	39992151	40015530		1.62	2.53	1.33	2.13	2.04	2.1
ENSDARG00000060054	epc1b	enhancer of polycomb homolog 1 (Drosophila) b [Source:ZFIN;Acc:ZDB-GENE-090609-1]	2	43585571	43605556		8.66	5.08	4.55	12.99	12.62	13.52
ENSDARG00000060058	cylda	cylindromatosis (turban tumor syndrome), a [Source:ZFIN;Acc:ZDB-GENE-061108-2]	7	37514780	37555208		7.39	4.09	3.67	7.8	7.74	8.02
ENSDARG00000060062	lrrc74a	leucine rich repeat containing 74A [Source:ZFIN;Acc:ZDB-GENE-131127-263]	17	52600506	52609728		0.19	0.2	0.27	0.21	0.29	0.3
ENSDARG00000060065	ubap2b	ubiquitin associated protein 2b [Source:ZFIN;Acc:ZDB-GENE-070209-122]	8	45293743	45328652		43.64	55	50.26	70.06	65.69	64.45
ENSDARG00000060068	fam98b	family with sequence similarity 98 member B [Source:ZFIN;Acc:ZDB-GENE-061201-63]	17	52632094	52636937		7.37	5.42	4.84	7.13	7.21	7.64
ENSDARG00000060069	isoc1	isochorismatase domain containing 1 [Source:NCBI gene;Acc:559897]	10	16547259	16563953		6.88	3.76	4.5	8.94	8.38	8.89
ENSDARG00000060070	adcy7	adenylate cyclase 7 [Source:ZFIN;Acc:ZDB-GENE-040713-1]	7	37761524	37858666		1.02	0.67	0.59	1.43	1.38	1.36

**ENSDARG00000060071**	tent4b	terminal nucleotidyltransferase 4B [Source:ZFIN;Acc:ZDB-GENE-041021-2]	7	37861247	37895771		8.31	5.27	4.27	8.59	8.77	8.64
**ENSDARG00000060072**	abi3a	ABI family, member 3a [Source:ZFIN;Acc:ZDB-GENE-060929-958]	3	33395942	33417826		1.83	0.85	0.62	3.72	3.71	3.69
**ENSDARG00000060081**	crb2b	crumbs cell polarity complex component 2b [Source:ZFIN;Acc:ZDB-GENE-060610-1]	8	2878756	2906214		1.61	1.29	1.07	3.68	3.97	3.84
**ENSDARG00000060085**	kcnq3	potassium voltage-gated channel, KQT-like subfamily, member 3 [Source:ZFIN;Acc:ZDB-GENE-070912-301]	2	43001512	43083755		2.42	0.61	0.92	4.39	4.88	5.12
**ENSDARG00000060089**	btaf1	BTAF1 RNA polymerase II, B-TFIID transcription factor-associated [Source:ZFIN;Acc:ZDB-GENE-021025-1]	13	42480852	42536960		9.29	5.12	4.87	9.96	9.88	10.15
**ENSDARG00000060092**	zgc:165481	zgc:165481 [Source:ZFIN;Acc:ZDB-GENE-070615-21]	7	38456426	38477235		3.93	2.27	2.66	2.45	2.81	3.42
**ENSDARG00000060093**	dapk1	death-associated protein kinase 1 [Source:ZFIN;Acc:ZDB-GENE-060526-177]	5	44654535	44795906		4.67	3.05	2.27	5.17	5.2	4.99
**ENSDARG00000060094**	ptgis	prostaglandin I2 (prostacyclin) synthase [Source:NCBI gene;Acc:559148]	6	46568119	46589726		4.17	4.03	4.09	2.7	2.49	2.36
**ENSDARG00000060095**	kcnb1	potassium voltage-gated channel, Shab-related subfamily, member 1 [Source:ZFIN;Acc:ZDB-GENE-090831-3]	6	46507609	46565466		1.2	0.27	0.42	1.88	1.72	1.81
**ENSDARG00000060096**	tbc1d10ab	TBC1 domain family, member 10Ab [Source:ZFIN;Acc:ZDB-GENE-081105-145]	10	8534750	8576218		2.31	1.09	1.24	2.23	2.1	2.68
**ENSDARG00000060101**	trappc12	trafficking protein particle complex 12 [Source:ZFIN;Acc:ZDB-GENE-070112-1282]	17	51231210	51262430		6.81	4.07	3.51	7.47	7.07	7.06
**ENSDARG00000060102**	kank1a	KN motif and ankyrin repeat domains 1a [Source:ZFIN;Acc:ZDB-GENE-060526-215]	5	44846280	44942760		8.45	8.34	6.63	8.08	8.09	7.74
**ENSDARG00000060103**	cpeb3	cytoplasmic polyadenylation element binding protein 3 [Source:ZFIN;Acc:ZDB-GENE-090312-68]	13	42406883	42472060		3.44	2.64	2.7	5.12	4.96	4.68
**ENSDARG00000060106**	crb2a	crumbs cell polarity complex component 2a [Source:ZFIN;Acc:ZDB-GENE-060612-1]	21	8478774	8513192		2.72	1.06	0.93	3.67	3.9	3.94
**ENSDARG00000060109**	aggf1	angiogenic factor with G patch and FHA domains 1 [Source:ZFIN;Acc:ZDB-GENE-060929-836]	21	7741781	7781697		4.5	3.34	3.65	4.12	4.38	4.45
**ENSDARG00000060112**	sybu	syntabulin (syntaxin-interacting) [Source:ZFIN;Acc:ZDB-GENE-060929-1202]	16	39146696	39168840		4.1	0.88	1	2.9	3.07	3.02
**ENSDARG00000060113**	znf395a	zinc finger protein 395a [Source:ZFIN;Acc:ZDB-GENE-061215-52]	17	16090160	16108384		20.25	15.23	12.94	52.72	60.22	61.13
**ENSDARG00000060115**	lrrn1	leucine rich repeat neuronal 1 [Source:ZFIN;Acc:ZDB-GENE-071126-1]	6	43076516	43092175		21.06	8.2	10.28	22.25	22.45	24.03
**ENSDARG00000060116**	camk1a	calcium/calmodulin-dependent protein kinase Ia [Source:ZFIN;Acc:ZDB-GENE-131120-147]	6	46353910	46403475		4.03	1.08	1.49	3.91	3.18	3.38
**ENSDARG00000060120**	glyctk	glycerate kinase [Source:NCBI gene;Acc:570678]	6	43019642	43047774		9.7	13.83	11.81	5.87	6.37	6.71
**ENSDARG00000060123**	ca16b	carbonic anhydrase XVI b [Source:ZFIN;Acc:ZDB-GENE-080818-1]	6	45951893	46162585		6.21	2.1	2.57	6.92	7.41	7.02
**ENSDARG00000060124**	timm17b	translocase of inner mitochondrial membrane 17 homolog B (yeast) [Source:ZFIN;Acc:ZDB-GENE-081104-144]	8	44921123	44926077		7.4	4.71	4.48	5.21	6.67	6.21
**ENSDARG00000060126**	acyp1	acylphosphatase 1, erythrocyte (common) type [Source:ZFIN;Acc:ZDB-GENE-050309-125]	17	50244443	50248023		5.7	4.44	4.68	5.38	5.83	5.9
**ENSDARG00000060127**	adamts3	ADAM metallopeptidase with thrombospondin type 1 motif, 3 [Source:ZFIN;Acc:ZDB-GENE-110223-1]	5	45322734	45532848		1.92	1.92	1.73	5.49	6.21	5.89
**ENSDARG00000060130**	dusp3b	dual specificity phosphatase 3b [Source:ZFIN;Acc:ZDB-GENE-030616-38]	3	34683096	34688277		0.46	1.03	0.8	0.98	0.72	0.84
**ENSDARG00000060148**	sh3pxd2aa	SH3 and PX domains 2Aa [Source:NCBI gene;Acc:562018]	1	47585700	47686888		1.69	1.04	0.96	3.41	3.23	3.78
**ENSDARG00000060149**	ablim1a	actin binding LIM protein 1a [Source:ZFIN;Acc:ZDB-GENE-080219-41]	13	43507383	43599939		4.78	1.39	2.33	5.1	5	5.57
**ENSDARG00000060150**	psmd11a	proteasome 26S subunit, non-ATPase 11a [Source:NCBI gene;Acc:503934]	3	34801146	34816979		8.92	7.83	7.62	8.1	6.74	7.18
**ENSDARG00000060152**	fam155b	family with sequence similarity 155 member B [Source:NCBI gene;Acc:791161]	5	36415978	36422162		7.36	3.11	3.93	5.92	6.16	6.98
**ENSDARG00000060153**	h6pd	hexose-6-phosphate dehydrogenase (glucose 1-dehydrogenase) [Source:ZFIN;Acc:ZDB-GENE-110408-60]	6	45918981	45929465		7.86	9.82	6.97	7.91	7.96	7.82
**ENSDARG00000060165**	dnah7	dynein, axonemal, heavy chain 7 [Source:ZFIN;Acc:ZDB-GENE-070912-282]	9	24951129	25055722		0.32	0.23	0.19	0.52	0.56	0.52
**ENSDARG00000060168**	fam160b1	family with sequence similarity 160 member B1 [Source:ZFIN;Acc:ZDB-GENE-070424-63]	13	43610350	43637371		3.47	2.76	2.67	5.87	5.54	6.13
**ENSDARG00000060169**	mns1	meiosis-specific nuclear structural 1 [Source:NCBI gene;Acc:573382]	7	33120167	33130552		4.56	3.24	3.57	4.05	4.14	4.07
**ENSDARG00000060176**	trmt10b	tRNA methyltransferase 10B [Source:NCBI gene;Acc:768135]	7	7783113	7797684		1.87	2.04	2.4	1.76	1.3	1.01
**ENSDARG00000060177**	vcpip1	valosin containing protein (p97)/p47 complex interacting protein 1 [Source:ZFIN;Acc:ZDB-GENE-070912-613]	2	42072231	42083513		2.94	1.83	1.69	3.13	3.04	3
**ENSDARG00000060184**	pkn1a	protein kinase N1a [Source:ZFIN;Acc:ZDB-GENE-061207-17]	1	45754868	45836167		5.74	4.41	3.65	7.73	7.53	7.38
**ENSDARG00000060187**	si:ch211-214c7.5	si:ch211-214c7.5 [Source:NCBI gene;Acc:100038798]	1	45744118	45752261		0.05	0.02	0.02	0.1	0.04	0.08
**ENSDARG00000060192**	rpusd1	RNA pseudouridine synthase domain containing 1 [Source:NCBI gene;Acc:767786]	3	35542067	35549478		3.27	2.4	2.12	2.88	2.8	2.84
**ENSDARG00000060196**	lhpp	phospholysine phosphohistidine inorganic pyrophosphate phosphatase [Source:NCBI gene;Acc:100073345]	12	46950584	46959990		11.92	15.48	16.04	18.46	19.22	19.15
**ENSDARG00000060197**	atp2c1	ATPase secretory pathway Ca2+ transporting 1 [Source:ZFIN;Acc:ZDB-GENE-060503-615]	16	41585645	41667101		7.72	3.91	3.45	6.48	6.51	5.96
**ENSDARG00000060199**	mrpl18	mitochondrial ribosomal protein L18 [Source:ZFIN;Acc:ZDB-GENE-061013-632]	14	35499678	35505537		36.09	49.77	52.49	22.63	22.53	23.67
**ENSDARG00000060200**	CU019662.1	regulator of G protein signaling 14b [Source:NCBI gene;Acc:100003648]	21	33093331	33126697		0.09	0.15	0.14	0.17	0.13	0.25
**ENSDARG00000060207**	traf7	TNF receptor-associated factor 7 [Source:ZFIN;Acc:ZDB-GENE-070112-2212]	3	35608385	35634727		8.09	6.31	6.28	10.67	10.29	10.48
**ENSDARG00000060210**	d2hgdh	D-2-hydroxyglutarate dehydrogenase [Source:NCBI gene;Acc:565889]	2	41546271	41562868		2.02	1.51	1.88	2.11	2.02	1.8
**ENSDARG00000060211**	ankef1b	ankyrin repeat and EF-hand domain containing 1b [Source:ZFIN;Acc:ZDB-GENE-060810-68]	17	12397033	12408109		0	0	0.01	0.03	0.01	0.02
**ENSDARG00000060215**	cep85	centrosomal protein 85 [Source:ZFIN;Acc:ZDB-GENE-030131-1998]	16	41685001	41717063		3.13	2.24	2.44	1.99	2.11	2.12
**ENSDARG00000060222**	scn1ba	sodium channel, voltage-gated, type I, beta a [Source:ZFIN;Acc:ZDB-GENE-060503-604]	16	41873708	41897745		13.97	5.36	8.74	16.34	15.59	18.53
**ENSDARG00000060235**	mst1ra	macrophage stimulating 1 receptor a [Source:ZFIN;Acc:ZDB-GENE-071218-2]	6	42475730	42505007		2.28	2.18	1.71	2.14	2.44	2.31
**ENSDARG00000060236**	dok7	docking protein 7 [Source:ZFIN;Acc:ZDB-GENE-090312-103]	1	41142881	41192206		1.76	2.26	1.62	2.08	2.05	1.94
**ENSDARG00000060238**	uacab	uveal autoantigen with coiled-coil domains and ankyrin repeats b [Source:ZFIN;Acc:ZDB-GENE-030521-32]	7	33798131	33869011		13.71	14.96	12.77	11.03	10.3	9.86
**ENSDARG00000060243**	enox1	ecto-NOX disulfide-thiol exchanger 1 [Source:ZFIN;Acc:ZDB-GENE-081104-328]	9	18310228	18568927		6.24	1.55	3.13	8.33	9.09	9.74
**ENSDARG00000060246**	slc16a6b	solute carrier family 16 member 6b [Source:ZFIN;Acc:ZDB-GENE-110208-3]	3	36313532	36328672		3.51	2.75	1.95	12.57	12.52	12.53
**ENSDARG00000060248**	fgd4b	FYVE, RhoGEF and PH domain containing 4b [Source:ZFIN;Acc:ZDB-GENE-110421-6]	4	3312852	3338940		0.87	0.61	0.75	0.96	1.02	1.15
**ENSDARG00000060249**	wdr44	WD repeat domain 44 [Source:ZFIN;Acc:ZDB-GENE-030131-9549]	5	37316683	37341044		17.08	14.59	12.21	18.42	19.79	20.75
**ENSDARG00000060252**	mogs	mannosyl-oligosaccharide glucosidase [Source:ZFIN;Acc:ZDB-GENE-070112-412]	1	41609254	41620873		4.25	3.67	2.97	4.12	3.78	3.63
**ENSDARG00000060253**	si:ch211-216b21.2	si:ch211-216b21.2 [Source:ZFIN;Acc:ZDB-GENE-130603-96]	12	36669128	36740781		0.89	0.59	0.56	0.82	0.77	0.69
**ENSDARG00000060256**	alkbh8	alkB homolog 8, tRNA methyltransferase [Source:ZFIN;Acc:ZDB-GENE-100922-251]	16	42156064	42175617		3.38	2.77	2.59	2.51	2.57	2.64
**ENSDARG00000060257**	zranb1a	zinc finger, RAN-binding domain containing 1a [Source:ZFIN;Acc:ZDB-GENE-100212-3]	12	47044707	47072007		16.41	9.7	10.76	20.96	22.39	22.25
**ENSDARG00000060259**	zgc:162160	zgc:162160 [Source:ZFIN;Acc:ZDB-GENE-070424-22]	16	42193163	42238120		1.55	0.7	0.85	1.64	1.83	2.25
**ENSDARG00000060263**	pecam1	platelet/endothelial cell adhesion molecule 1 [Source:ZFIN;Acc:ZDB-GENE-130530-689]	3	59784632	59822428		1.96	2.05	2.32	2.95	3.27	3.65
**ENSDARG00000060264**	si:dkey-37g12.1	si:dkey-37g12.1 [Source:ZFIN;Acc:ZDB-GENE-090312-145]	1	41886830	41924484		0.02	0.04	0.04	0.05	0.03	0.05
**ENSDARG00000060280**	pde9a	phosphodiesterase 9A [Source:ZFIN;Acc:ZDB-GENE-130920-1]	9	19421841	19477248		2.03	1.1	1.87	1.48	1.64	1.95
**ENSDARG00000060282**	rnf121	ring finger protein 121 [Source:ZFIN;Acc:ZDB-GENE-050809-3]	21	44773237	44784723		13.32	9.17	7.67	15.33	14.78	14.66
**ENSDARG00000060288**	plpbp	pyridoxal phosphate binding protein [Source:ZFIN;Acc:ZDB-GENE-030131-1378]	23	33963619	33976116		27.51	25.64	25.68	23.01	24.32	25.65
**ENSDARG00000060297**	arhgap35b	Rho GTPase activating protein 35b [Source:ZFIN;Acc:ZDB-GENE-060526-44]	5	37864705	37881345		6.15	2.71	2.29	9.59	8.46	9.2
**ENSDARG00000060298**	nin	ninein (GSK3B interacting protein) [Source:ZFIN;Acc:ZDB-GENE-070725-1]	13	36800334	36844945		1.49	0.77	0.79	1.35	1.21	1.31
**ENSDARG00000060303**	slc4a10b	solute carrier family 4 member 10b [Source:ZFIN;Acc:ZDB-GENE-081105-62]	9	51289904	51370293		6.28	1.09	1.64	8.13	8.29	8.32
**ENSDARG00000060306**	tfcp2	transcription factor CP2 [Source:ZFIN;Acc:ZDB-GENE-030131-2523]	23	33654231	33680265		6.9	3.85	4.11	7.21	7.44	7.37
**ENSDARG00000060308**	urb1	URB1 ribosome biogenesis homolog [Source:ZFIN;Acc:ZDB-GENE-030131-5181]	15	5627877	5720666		2.04	1.87	1.5	1.78	1.78	1.66
**ENSDARG00000060309**	srgap3	SLIT-ROBO Rho GTPase activating protein 3 [Source:ZFIN;Acc:ZDB-GENE-060524-4]	6	41554794	41757181		4.53	1.92	1.89	6.84	6.87	7.26
**ENSDARG00000060312**	wdr62	WD repeat domain 62 [Source:ZFIN;Acc:ZDB-GENE-060503-291]	15	5565641	5624456		2.21	1.67	1.61	2.48	2.1	2.23
**ENSDARG00000060315**	rabgap1l2	RAB GTPase activating protein 1-like 2 [Source:ZFIN;Acc:ZDB-GENE-070912-414]	2	36898982	36905195		18.51	17.31	14.18	4.79	3.99	4.74
**ENSDARG00000060316**	cish	cytokine inducible SH2-containing protein [Source:ZFIN;Acc:ZDB-GENE-050907-1]	6	41503854	41506749		9.12	8.24	8.19	3.68	3.15	3.36
**ENSDARG00000060319**	scn4bb	sodium channel, voltage-gated, type IV, beta b [Source:ZFIN;Acc:ZDB-GENE-031116-71]	5	37918810	37935621		1.15	1.1	1.11	1.06	1.24	1.44
**ENSDARG00000060320**	dcaf5	ddb1 and cul4 associated factor 5 [Source:ZFIN;Acc:ZDB-GENE-060929-808]	13	36406274	36439061		2.88	2.36	1.92	2.84	2.9	2.86
**ENSDARG00000060322**	zgc:153654	zgc:153654 [Source:ZFIN;Acc:ZDB-GENE-060929-1102]	2	36914408	36918709		0.14	0.24	0.11	0.27	0.27	0.3
**ENSDARG00000060323**	exoc5	exocyst complex component 5 [Source:ZFIN;Acc:ZDB-GENE-080204-126]	17	44413770	44440832		5.83	4.27	4.87	6.33	7.15	7.45
**ENSDARG00000060325**	fam20cl	family with sequence similarity 20 member C, like [Source:ZFIN;Acc:ZDB-GENE-070705-319]	22	24954103	24967475		0	0.04	0.05	0.01	0.03	0.01
**ENSDARG00000060326**	map1sb	microtubule-associated protein 1Sb [Source:ZFIN;Acc:ZDB-GENE-070912-412]	2	36917613	36932253		3.05	0.87	1.23	3.58	4.06	4.23
**ENSDARG00000060330**	si:ch211-284e13.4	si:ch211-284e13.4 [Source:ZFIN;Acc:ZDB-GENE-060526-161]	5	38088594	38094130		2.92	0.55	0.96	3.82	3.66	3.8
**ENSDARG00000060338**	trmt12	tRNA methyltransferase 12 homolog [Source:NCBI gene;Acc:564925]	14	35414632	35422777		2.57	2.36	2.82	1.88	1.83	1.95
**ENSDARG00000060340**	si:dkey-157l19.2	si:dkey-157l19.2 [Source:ZFIN;Acc:ZDB-GENE-090313-190]	13	35995238	36034582		5.94	8.47	6.69	6.17	6.8	7.02
**ENSDARG00000060345**	apoda.1	apolipoprotein Da, duplicate 1 [Source:ZFIN;Acc:ZDB-GENE-060929-148]	2	37207799	37210397		28.42	47.47	44.89	20.11	22.16	23.17
**ENSDARG00000060348**	map3k3	mitogen-activated protein kinase kinase kinase 3 [Source:ZFIN;Acc:ZDB-GENE-070910-1]	3	31815184	31845816		2.9	2.16	1.77	3.82	4.3	4.03
**ENSDARG00000060349**	wasf1	WASP family member 1 [Source:ZFIN;Acc:ZDB-GENE-070705-555]	20	53300588	53366137		6.16	2.74	3.06	9.44	9.43	10.07
**ENSDARG00000060350**	apoda.2	apolipoprotein Da, duplicate 2 [Source:ZFIN;Acc:ZDB-GENE-070912-551]	2	37207461	37215989		151.52	213.64	185.61	13.35	9.64	11.54
**ENSDARG00000060354**	samd7	sterile alpha motif domain containing 7 [Source:ZFIN;Acc:ZDB-GENE-070912-549]	2	37227011	37242537		4.29	1.26	1.23	4.31	4.14	4.93
**ENSDARG00000060361**	cep104	centrosomal protein 104 [Source:ZFIN;Acc:ZDB-GENE-060503-195]	8	22297813	22326744		4.13	2.52	2.36	4.06	3.95	3.33
**ENSDARG00000060362**	nadkb	NAD kinase b [Source:ZFIN;Acc:ZDB-GENE-061103-433]	2	37267241	37281748		8.62	7.61	5.83	9.39	8.9	9.29
**ENSDARG00000060366**	slc12a9	solute carrier family 12 member 9 [Source:NCBI gene;Acc:793341]	5	38215924	38248462		2.68	2.31	1.79	3.72	3.76	3.73
**ENSDARG00000060368**	syn1	synapsin I [Source:ZFIN;Acc:ZDB-GENE-081105-93]	8	31119548	31148319		13.98	3.82	4.51	23.31	23.32	24.15
**ENSDARG00000060372**	plxna2	plexin A2 [Source:ZFIN;Acc:ZDB-GENE-090311-6]	23	32579495	33038423		6.61	1.65	1.83	11.67	10.96	12.2
**ENSDARG00000060374**	ttll11	tubulin tyrosine ligase-like family, member 11 [Source:ZFIN;Acc:ZDB-GENE-061013-747]	5	33611951	33668960		2.41	1.08	0.97	2.57	2.61	2.23
**ENSDARG00000060380**	coq6	coenzyme Q6 monooxygenase [Source:NCBI gene;Acc:751691]	17	45009868	45030025		5.35	4.95	5.48	4.62	4.35	4.49
**ENSDARG00000060383**	mmaa	metabolism of cobalamin associated A [Source:ZFIN;Acc:ZDB-GENE-070928-15]	1	35956435	35970259		8.13	8.17	6.11	7.19	6.58	6.69
**ENSDARG00000060390**	stk26	serine/threonine protein kinase 26 [Source:ZFIN;Acc:ZDB-GENE-080516-5]	21	43420304	43457554		5.4	4.79	3.94	7.85	8.25	7.47
**ENSDARG00000060392**	ash2l	ash2 like, histone lysine methyltransferase complex subunit [Source:ZFIN;Acc:ZDB-GENE-030131-494]	10	2759226	2788792		22.45	15.72	19.95	20.88	19.74	19.27
**ENSDARG00000060393**	fibcd1	fibrinogen C domain containing 1 [Source:ZFIN;Acc:ZDB-GENE-070424-245]	5	34066591	34185497		3.91	0.99	1.25	5.48	5.4	5.49
**ENSDARG00000060394**	mtfr2	mitochondrial fission regulator 2 [Source:NCBI gene;Acc:768141]	23	31901350	31913289		2.87	2.69	2.81	2.79	3.11	3.03
**ENSDARG00000060395**	si:dkey-57k2.7	si:dkey-57k2.7 [Source:ZFIN;Acc:ZDB-GENE-070912-559]	2	37449294	37458527		5.57	4.72	3.89	5.56	5.15	6.05
**ENSDARG00000060397**	hhip	hedgehog interacting protein [Source:ZFIN;Acc:ZDB-GENE-030131-4827]	1	35790082	35835863		10.63	9.2	8.66	16.23	16.75	16.64
**ENSDARG00000060398**	rft1	RFT1 homolog [Source:ZFIN;Acc:ZDB-GENE-110411-126]	6	40843786	40854458		3.62	2.54	2.47	3.65	3.19	3.36
**ENSDARG00000060400**	gpr185a	G protein-coupled receptor 185 a [Source:ZFIN;Acc:ZDB-GENE-120215-99]	21	43559123	43562630		0.98	0.11	0.31	1.29	0.98	1.57
**ENSDARG00000060410**	thbs2a	thrombospondin 2a [Source:ZFIN;Acc:ZDB-GENE-020708-2]	13	35635672	35677353		1.24	1.5	0.96	1.71	1.75	1.81
**ENSDARG00000060411**	ddx28	DEAD (Asp-Glu-Ala-Asp) box polypeptide 28 [Source:ZFIN;Acc:ZDB-GENE-061013-64]	7	34963748	34965654		4.86	4.9	4.09	4.11	3.99	3.96
**ENSDARG00000060413**	si:ch211-157b11.8	si:ch211-157b11.8 [Source:ZFIN;Acc:ZDB-GENE-131127-436]	6	40775800	40783641		0.89	1.01	0.74	0.39	0.62	0.46
**ENSDARG00000060415**	arhgef28a	Rho guanine nucleotide exchange factor (GEF) 28a [Source:ZFIN;Acc:ZDB-GENE-060531-28]	5	34827614	34964830		1.6	1.58	1.45	4.14	3.92	4.21
**ENSDARG00000060430**	zfyve28	zinc finger, FYVE domain containing 28 [Source:NCBI gene;Acc:777615]	17	43565628	43594864		4.01	2.33	2.03	4.41	4.6	4.67
**ENSDARG00000060434**	map1b	microtubule-associated protein 1B [Source:ZFIN;Acc:ZDB-GENE-130530-690]	5	35270859	35301800		8.16	2.64	2.97	8.03	7.64	7.83
**ENSDARG00000060435**	bnipl	BCL2 interacting protein like [Source:ZFIN;Acc:ZDB-GENE-060503-81]	16	38277339	38299644		8.66	7.57	6.49	5.33	5.24	4.79
**ENSDARG00000060438**	tbl1x	transducin beta like 1 X-linked [Source:ZFIN;Acc:ZDB-GENE-070209-131]	9	55093078	55128839		2.69	1.99	1.73	3.88	3.9	3.77
**ENSDARG00000060439**	clcn2c	chloride channel 2c [Source:ZFIN;Acc:ZDB-GENE-050506-73]	15	46736794	46779934		10.55	10.14	9.54	3.9	5	4.06
**ENSDARG00000060442**	tmem259	transmembrane protein 259 [Source:ZFIN;Acc:ZDB-GENE-080225-14]	11	14071689	14102158		4.08	3.11	2.97	5.12	4.99	5.16
**ENSDARG00000060445**	elp6	elongator acetyltransferase complex subunit 6 [Source:NCBI gene;Acc:751679]	16	38350704	38359637		1.53	1.5	1.62	1.29	1.28	1.4
**ENSDARG00000060452**	sdk2b	sidekick cell adhesion molecule 2b [Source:ZFIN;Acc:ZDB-GENE-030131-8203]	12	37463513	37734973		2.86	1.51	1.09	6.07	6.08	6.49
**ENSDARG00000060457**	pmp22b	peripheral myelin protein 22b [Source:ZFIN;Acc:ZDB-GENE-060421-4337]	12	37275336	37299671		41.75	56.15	45.1	65.39	61.48	58.44
**ENSDARG00000060469**	pik3r4	phosphoinositide-3-kinase, regulatory subunit 4 [Source:ZFIN;Acc:ZDB-GENE-050309-84]	16	36721230	36748374		8.24	5.7	5.11	9	8.76	9.34
**ENSDARG00000060471**	gcnt3	glucosaminyl (N-acetyl) transferase 3, mucin type [Source:ZFIN;Acc:ZDB-GENE-110411-253]	25	34274691	34284144		0.66	0.65	0.58	0.53	0.53	0.33
**ENSDARG00000060477**	vps8	VPS8 subunit of CORVET complex [Source:ZFIN;Acc:ZDB-GENE-060503-348]	1	30083578	30310689		7.04	4.62	5.18	6.42	6.93	6.86
**ENSDARG00000060480**	ubr1	ubiquitin protein ligase E3 component n-recognin 1 [Source:ZFIN;Acc:ZDB-GENE-091110-2]	17	39790388	39817543		8.7	5.72	4.3	6.85	6.99	7.27
**ENSDARG00000060481**	atl1	atlastin GTPase 1 [Source:ZFIN;Acc:ZDB-GENE-030131-8003]	13	36764715	36784400		17.88	5.08	7.22	18.99	18.77	21.6
**ENSDARG00000060482**	mid1	midline 1 [Source:ZFIN;Acc:ZDB-GENE-110411-205]	9	53909566	54001502		2.92	0.46	0.46	2.95	2.7	2.73
**ENSDARG00000060484**	selenoi	selenoprotein I [Source:ZFIN;Acc:ZDB-GENE-091204-137]	23	31000243	31022418		5.3	4.66	3.42	5.25	5.58	4.55
**ENSDARG00000060489**	mrpl32	mitochondrial ribosomal protein L32 [Source:ZFIN;Acc:ZDB-GENE-060825-188]	16	43344475	43346916		17.61	25.9	22.78	14.05	14.79	15.5
**ENSDARG00000060494**	eprs1	glutamyl-prolyl-tRNA synthetase 1 [Source:ZFIN;Acc:ZDB-GENE-030131-638]	17	39697621	39739994		29.44	27.47	21.13	41.65	41.18	40.2
**ENSDARG00000060496**	cacna1ha	calcium channel, voltage-dependent, T type, alpha 1H subunit a [Source:ZFIN;Acc:ZDB-GENE-130103-1]	3	39759130	39952441		2.89	0.99	0.87	3.34	3.01	3.49
**ENSDARG00000060498**	tnfrsf9a	tumor necrosis factor receptor superfamily, member 9a [Source:ZFIN;Acc:ZDB-GENE-060421-8213]	23	30006206	30010042		1.49	1.34	1.38	4.64	3.46	3.63
**ENSDARG00000060500**	l2hgdh	L-2-hydroxyglutarate dehydrogenase [Source:ZFIN;Acc:ZDB-GENE-090319-6]	13	36666662	36680531		10.9	15.06	12.68	6.23	6.37	6.18
**ENSDARG00000060501**	dpcd	deleted in primary ciliary dyskinesia homolog (mouse) [Source:NCBI gene;Acc:751660]	1	31654203	31657854		4.01	4.66	4.57	3.13	3.22	3.93
**ENSDARG00000060504**	pfkla	phosphofructokinase, liver a [Source:ZFIN;Acc:ZDB-GENE-121207-1]	9	52628899	52678995		10.5	7.51	5.84	11.65	11.32	11.84
**ENSDARG00000060506**	sos2	son of sevenless homolog 2 (Drosophila) [Source:ZFIN;Acc:ZDB-GENE-090319-5]	13	36638955	36663358		6.5	4.78	4.29	7.9	7.68	8.06
**ENSDARG00000060510**	cul4b	cullin 4B [Source:ZFIN;Acc:ZDB-GENE-041008-208]	14	33486179	33505825		16.59	12.33	12.36	15.91	16.58	16.93
**ENSDARG00000060512**	kbtbd3	kelch repeat and BTB (POZ) domain containing 3 [Source:ZFIN;Acc:ZDB-GENE-131016-5]	15	47828637	47841259		1.3	0.35	0.6	1.16	1.24	1.09
**ENSDARG00000060515**	zdhhc9	zinc finger DHHC-type containing 9 [Source:ZFIN;Acc:ZDB-GENE-071004-8]	14	33525196	33567334		14.31	6.72	5.5	17.41	16.48	15.97
**ENSDARG00000060518**	pcsk5b	proprotein convertase subtilisin/kexin type 5b [Source:ZFIN;Acc:ZDB-GENE-070822-7]	8	38649989	38810233		12.27	13.82	12.16	12.38	11.81	12.01
**ENSDARG00000060521**	rbm27	RNA binding motif protein 27 [Source:ZFIN;Acc:ZDB-GENE-110411-31]	9	54237100	54280035		3.01	1.86	1.82	4.82	4.73	4.83
**ENSDARG00000060522**	rfk	riboflavin kinase [Source:ZFIN;Acc:ZDB-GENE-081104-321]	8	38564942	38573105		5.87	7.38	5.84	5.3	5.99	5.3
**ENSDARG00000060526**	bmp3	bone morphogenetic protein 3 [Source:ZFIN;Acc:ZDB-GENE-030131-7192]	5	39667272	39673477		7.17	7.13	6.01	10.6	10.64	11.41
**ENSDARG00000060529**	nkx6.3	NK6 homeobox 3 [Source:ZFIN;Acc:ZDB-GENE-070810-7]	8	38415374	38419647		0.58	0.27	0.34	0.74	0.84	0.7
**ENSDARG00000060532**	adam22	ADAM metallopeptidase domain 22 [Source:ZFIN;Acc:ZDB-GENE-070810-1]	16	43152727	43284939		8.96	2.17	3.5	11.05	11.81	12.04
**ENSDARG00000060534**	sgpp1	sphingosine-1-phosphate phosphatase 1 [Source:ZFIN;Acc:ZDB-GENE-090319-2]	13	37491804	37519817		2.77	2.76	2.03	3.09	3.61	3.05
**ENSDARG00000060537**	atad2b	ATPase family AAA domain containing 2B [Source:ZFIN;Acc:ZDB-GENE-110411-210]	20	44582318	44792557		7.52	5.27	4.38	9.95	9.34	9.98
**ENSDARG00000060539**	shank1	SH3 and multiple ankyrin repeat domains 1 [Source:ZFIN;Acc:ZDB-GENE-130530-691]	3	30714111	30861177		4.42	1.02	1.78	6.62	6.69	6.14
**ENSDARG00000060542**	lgr4	Danio rerio leucine-rich repeat containing G protein-coupled receptor 4 (lgr4), mRNA. [Source:RefSeq mRNA;Acc:NM_001353862]	7	32369026	32460223		6.57	5.35	3.68	10.47	9.91	9.97
**ENSDARG00000060549**	ecel1	endothelin converting enzyme-like 1 [Source:ZFIN;Acc:ZDB-GENE-130530-692]	15	47008307	47075260		2.3	1.36	1.41	3.62	3.57	3.61
**ENSDARG00000060551**	rps6ka5	ribosomal protein S6 kinase, polypeptide 5 [Source:ZFIN;Acc:ZDB-GENE-130530-693]	17	38393189	38442362		3.94	1.83	1.99	4.81	4.62	4.87
**ENSDARG00000060554**	mut	methylmalonyl CoA mutase [Source:ZFIN;Acc:ZDB-GENE-010430-3]	20	44372745	44460789		44.09	49.41	45.28	30.3	30.76	31.04
**ENSDARG00000060559**	dis3	DIS3 exosome endoribonuclease and 3'-5' exoribonuclease [Source:ZFIN;Acc:ZDB-GENE-080213-3]	1	30736449	30762264		5.24	4.7	4.61	3.47	3.49	3.86
**ENSDARG00000060564**	slc25a21	solute carrier family 25 member 21 [Source:ZFIN;Acc:ZDB-GENE-060929-664]	17	38030327	38233021		3.36	4.46	3.03	3.57	3.48	2.76
**ENSDARG00000060566**	cluhb	clustered mitochondria (cluA/CLU1) homolog b [Source:ZFIN;Acc:ZDB-GENE-091113-48]	21	39361040	39403012		2.29	0.86	1.02	1.86	1.91	1.94
**ENSDARG00000060569**	tmem229b	transmembrane protein 229B [Source:NCBI gene;Acc:767735]	17	37624704	37649828		14.05	7.4	7.35	11.68	11.38	10.91
**ENSDARG00000060578**	si:dkey-226m8.10	si:dkey-226m8.10 [Source:ZFIN;Acc:ZDB-GENE-060526-258]	5	33199998	33215389		11.36	4.53	6.68	7.41	5.86	7.03
**ENSDARG00000060580**	tasp1	taspase, threonine aspartase, 1 [Source:ZFIN;Acc:ZDB-GENE-070424-242]	13	34689663	34750299		2.46	1.78	1.54	2.11	2.06	2.18
**ENSDARG00000060584**	kank1b	KN motif and ankyrin repeat domains 1b [Source:ZFIN;Acc:ZDB-GENE-060526-260]	5	33222124	33236637		0.49	0.31	0.34	0.43	0.44	0.39
**ENSDARG00000060586**	ctdspl2b	CTD (carboxy-terminal domain, RNA polymerase II, polypeptide A) small phosphatase like 2b [Source:NCBI gene;Acc:323089]	7	31374383	31395511		4.47	2.59	2.72	5.17	5.63	4.82
**ENSDARG00000060594**	hadhab	hydroxyacyl-CoA dehydrogenase trifunctional multienzyme complex subunit alpha b [Source:ZFIN;Acc:ZDB-GENE-041111-204]	17	37227936	37252810		48.59	58.04	47.34	37.89	37.69	35.73
**ENSDARG00000060596**	prkaa1	protein kinase, AMP-activated, alpha 1 catalytic subunit [Source:ZFIN;Acc:ZDB-GENE-030325-1]	5	33238545	33255759		12.17	9.92	8.26	13.12	13.13	12.96
**ENSDARG00000060597**	zgc:158659	zgc:158659 [Source:ZFIN;Acc:ZDB-GENE-070112-492]	23	44190852	44233410		2.99	0.78	1.19	5.14	4.37	5.03
**ENSDARG00000060600**	capn15	calpain 15 [Source:ZFIN;Acc:ZDB-GENE-140106-70]	24	39027481	39084453		3.84	2.09	2.07	6.5	6.83	6.34
**ENSDARG00000060601**	rgs7bpa	regulator of G protein signaling 7 binding protein a [Source:NCBI gene;Acc:767669]	8	31901318	31919624		5.33	2.19	3.62	5.65	5.62	5.71
**ENSDARG00000060609**	cd109	CD109 molecule [Source:ZFIN;Acc:ZDB-GENE-120215-253]	20	49119633	49175503		5.46	3.58	3.49	2.16	2.18	1.9
**ENSDARG00000060610**	pcdh7b	protocadherin 7b [Source:ZFIN;Acc:ZDB-GENE-081104-299]	7	63324888	63570206		7.25	3.02	2.87	10.95	10.27	11.18
**ENSDARG00000060618**	ipo13	importin 13 [Source:ZFIN;Acc:ZDB-GENE-070706-1]	20	48292634	48407944		6.67	4.38	3.06	11.89	11.63	11.94
**ENSDARG00000060620**	lig4	ligase IV, DNA, ATP-dependent [Source:ZFIN;Acc:ZDB-GENE-070820-10]	1	29267841	29271473		2.44	2.78	2.48	2.15	1.64	1.71
**ENSDARG00000060622**	si:ch73-14h1.2	si:ch73-14h1.2 [Source:ZFIN;Acc:ZDB-GENE-070912-263]	2	20539402	20555743		0.99	1.32	0.83	3.24	2.85	2.53
**ENSDARG00000060626**	dgkaa	diacylglycerol kinase, alpha a [Source:ZFIN;Acc:ZDB-GENE-060616-305]	23	32238337	32304754		10.25	10.64	9.99	12.64	12.39	11.94
**ENSDARG00000060627**	hip1ra	huntingtin interacting protein 1 related a [Source:ZFIN;Acc:ZDB-GENE-170110-1]	10	44473851	44482911		3.46	2.02	1.83	2.38	1.95	1.84
**ENSDARG00000060631**	GARNL3	si:ch211-182d3.1 [Source:NCBI gene;Acc:560594]	5	33356425	33460959	GARNL3	6.53	3.16	2.7	9.67	10.05	10.74
**ENSDARG00000060633**	usp16	ubiquitin specific peptidase 16 [Source:NCBI gene;Acc:569700]	15	41290948	41311295		0.05	0.01	0.01	0.08	0.06	0.12
**ENSDARG00000060637**	clstn2	calsyntenin 2 [Source:ZFIN;Acc:ZDB-GENE-070911-2]	2	39108339	39555667		1.47	0.41	0.49	3.87	4.1	3.85
**ENSDARG00000060638**	CLSTN2	si:ch211-276c2.2 [Source:ZFIN;Acc:ZDB-GENE-121214-77]	15	41555593	41664198	CLSTN2	2.76	0.34	0.49	2.74	2.43	2.64
**ENSDARG00000060639**	apba2b	amyloid beta (A4) precursor protein-binding, family A, member 2b [Source:ZFIN;Acc:ZDB-GENE-030131-5371]	7	30787903	30915965		15.06	4.01	4.57	28.76	29.05	31.51
**ENSDARG00000060641**	prkaa2	protein kinase, AMP-activated, alpha 2 catalytic subunit [Source:ZFIN;Acc:ZDB-GENE-081120-5]	20	8028613	8067973		3.35	2.47	2.23	3.28	2.97	3.06
**ENSDARG00000060645**	sirt7	sirtuin 7 [Source:ZFIN;Acc:ZDB-GENE-050208-612]	22	38935060	38946971		2.76	1.12	1.53	3.58	3.01	3.5
**ENSDARG00000060646**	senp5	SUMO specific peptidase 5 [Source:ZFIN;Acc:ZDB-GENE-050208-772]	22	38914983	38929371		2.73	1.85	1.81	2.84	2.45	2.69
**ENSDARG00000060655**	BX005421.2	interferon-induced very large GTPase 1-like [Source:NCBI gene;Acc:101883028]	3	3992590	4007814		0.02	0.03	0.01	0.02	0.02	0.01
**ENSDARG00000060656**	si:ch211-10a23.2	si:ch211-10a23.2 [Source:ZFIN;Acc:ZDB-GENE-090313-9]	13	33117528	33123240		9.74	6.23	6.91	7.95	7.78	8.17

ENSDARG00000060661	klf8	Kruppel-like factor 8 [Source:ZFIN;Acc:ZDB-GENE-070424-152]	21	38033226	38111218		9.97	6.53	5.78	13.71	12.93	14
ENSDARG00000060662	prf1.9	perforin 1.9 [Source:ZFIN;Acc:ZDB-GENE-131127-344]	3	30968176	30975502		0.09	0.11	0.1	0.16	0.23	0.17
ENSDARG00000060668	tm4sf18	transmembrane 4 L six family member 18 [Source:ZFIN;Acc:ZDB-GENE-050208-690]	22	38159823	38168571		2.09	1.91	2.03	5.44	6	6.59
ENSDARG00000060671	lct	lactase [Source:ZFIN;Acc:ZDB-GENE-081104-434]	9	46369932	46399611		4.84	9.27	5.67	0.38	0.37	0.37
ENSDARG00000060678	ndst2b	N-deacetylase/N-sulfotransferase (heparan glucosaminyl) 2b [Source:ZFIN;Acc:ZDB-GENE-100430-2]	12	35203091	35339564		3.59	1.37	1.44	4.18	4.25	4.52
ENSDARG00000060679	kdm1a	lysine (K)-specific demethylase 1a [Source:ZFIN;Acc:ZDB-GENE-030131-7828]	17	28082441	28101989		24.76	13.27	14.04	29.4	29.54	30.92
ENSDARG00000060680	si:ch73-127m5.1	si:ch73-127m5.1 [Source:ZFIN;Acc:ZDB-GENE-130531-25]	12	35119762	35194800		10.25	2.43	3.73	17.94	17.7	17.74
ENSDARG00000060682	agr1	anterior gradient 1 [Source:ZFIN;Acc:ZDB-GENE-030131-7249]	9	45591239	45603009		61.56	64.09	57.11	49.7	42.94	47.65
ENSDARG00000060683	lrsam1	leucine rich repeat and sterile alpha motif containing 1 [Source:ZFIN;Acc:ZDB-GENE-060526-97]	5	32865364	32882162		8.19	5.46	5.76	10.09	11.11	11.51
ENSDARG00000060687	chd2	chromodomain helicase DNA binding protein 2 [Source:ZFIN;Acc:ZDB-GENE-050419-256]	18	24933473	24989580		13.29	8.9	7.21	21.14	23.53	23.46
ENSDARG00000060688	trim69	tripartite motif containing 69 [Source:ZFIN;Acc:ZDB-GENE-030131-857]	10	40836378	40851677		2.44	2.68	2.3	3.6	3.4	3.43
ENSDARG00000060695	znf346	zinc finger protein 346 [Source:NCBI gene;Acc:560756]	21	37090585	37111264		7.7	5.61	5.27	7.81	7.76	7.55
ENSDARG00000060697	kmt2bb	lysine (K)-specific methyltransferase 2Bb [Source:ZFIN;Acc:ZDB-GENE-080521-1]	15	37457009	37543839		4.41	2.65	2.05	7.43	7.13	7.12
ENSDARG00000060702	npffr2b	neuropeptide FF receptor 2b [Source:ZFIN;Acc:ZDB-GENE-070705-159]	5	33067283	33104366		0.37	0.02	0.09	0.49	0.34	0.3
ENSDARG00000060705	nsd1b	nuclear receptor binding SET domain protein 1b [Source:ZFIN;Acc:ZDB-GENE-080519-2]	21	37355716	37398134		3.07	1.44	1.24	2.67	2.75	2.89
ENSDARG00000060707	pex11a	peroxisomal biogenesis factor 11 alpha [Source:ZFIN;Acc:ZDB-GENE-050419-121]	18	25546227	25550960		4.7	5.9	4.89	4.5	3.62	3.52
ENSDARG00000060711	sv2bb	synaptic vesicle glycoprotein 2Bb [Source:ZFIN;Acc:ZDB-GENE-030131-2789]	25	11034750	11088839		13.87	3.63	4.57	14.78	14.9	15.18
ENSDARG00000060716	prkx	protein kinase X-linked [Source:ZFIN;Acc:ZDB-GENE-090313-408]	1	33217911	33283689		2.66	1.55	1.49	3.09	3.09	2.7
ENSDARG00000060723	stim1a	stromal interaction molecule 1a [Source:ZFIN;Acc:ZDB-GENE-060503-914]	15	38221038	38300598		5.38	3.39	2.82	4.67	4.49	3.98
ENSDARG00000060725	dnajc16l	DnaJ (Hsp40) homolog, subfamily C, member 16 like [Source:ZFIN;Acc:ZDB-GENE-130530-696]	11	3593127	3613558		5.78	3.14	3.73	6.15	6.54	6.35
ENSDARG00000060729	trim8b	tripartite motif containing 8b [Source:ZFIN;Acc:ZDB-GENE-030131-4325]	12	33916939	33935522		12.4	7.6	6.23	16.4	16.81	16.88
ENSDARG00000060734	hesx1	HESX homeobox 1 [Source:ZFIN;Acc:ZDB-GENE-990415-130]	11	42600731	42635440		5.28	2.97	3.02	5.65	5.41	5.82
ENSDARG00000060741	syt17	synaptotagmin XVII [Source:ZFIN;Acc:ZDB-GENE-090527-1]	3	31201542	31226841		0.93	0.62	1.42	1.39	1.17
ENSDARG00000060745	pgm5	phosphoglucomutase 5 [Source:ZFIN;Acc:ZDB-GENE-060503-838]	8	30456161	30494617		1.28	1.31	1.13	1.9	2.22	2.27
ENSDARG00000060748	tsfm	Ts translation elongation factor, mitochondrial [Source:NCBI gene;Acc:567785]	6	39897543	39903431		8.62	12.25	11.83	7.86	7.41	7.03
ENSDARG00000060751	taok3a	TAO kinase 3a [Source:ZFIN;Acc:ZDB-GENE-100921-50]	5	15359995	15494297		4.74	2.97	2.86	5.94	6.36	6.25
ENSDARG00000060753	pear1	platelet endothelial aggregation receptor 1 [Source:ZFIN;Acc:ZDB-GENE-091230-1]	16	30438041	30520157		2.55	2.88	2.47	2.68	3.09	3.08
ENSDARG00000060756	abhd6a	abhydrolase domain containing 6a [Source:ZFIN;Acc:ZDB-GENE-090925-1]	11	42062067	42099645		1.48	1.8	1.34	0.92	1	0.95
ENSDARG00000060758	nphs1	NPHS1 adhesion molecule, nephrin [Source:ZFIN;Acc:ZDB-GENE-051102-1]	15	36553823	36939655		0.27	0.13	0.1	0.64	0.78	0.72
ENSDARG00000060766	pxnb	paxillin b [Source:ZFIN;Acc:ZDB-GENE-130530-697]	8	3496204	3519152		4.07	4.39	3.3	9.83	8.3	8.05
ENSDARG00000060767	smg7	SMG7 nonsense mediated mRNA decay factor [Source:ZFIN;Acc:ZDB-GENE-030131-9122]	2	36046126	36079907		11.77	6.94	5.99	20.54	20.93	21.77
ENSDARG00000060768	ankib1a	ankyrin repeat and IBR domain containing 1a [Source:NCBI gene;Acc:566524]	19	43905671	43960753		1.94	1.5	1.28	2.81	3.12	2.86
ENSDARG00000060771	map7d3	MAP7 domain containing 3 [Source:ZFIN;Acc:ZDB-GENE-121129-3]	14	31668510	31715412		12.1	7.77	7.2	11.75	11.54	10.98
ENSDARG00000060783	acap2	ArfGAP with coiled-coil, ankyrin repeat and PH domains 2 [Source:ZFIN;Acc:ZDB-GENE-050208-640]	22	37733107	37797695		6.32	6.15	5.51	5.63	6.01	5.66
ENSDARG00000060785	pcnx4	pecanex 4 [Source:NCBI gene;Acc:563650]	13	31520537	31535508		3.65	1.77	1.68	5.22	5.22	4.96
ENSDARG00000060788	lrrc14b	leucine rich repeat containing 14B [Source:NCBI gene;Acc:566643]	19	44039849	44047176		1.5	2.03	1.99	2.17	2.29	2.26
ENSDARG00000060796	slc20a2	solute carrier family 20 member 2 [Source:ZFIN;Acc:ZDB-GENE-060929-828]	8	29770098	29851706		9.78	8.02	5.77	19.74	20.12	20.36
ENSDARG00000060797	pfkmb	phosphofructokinase, muscle b [Source:ZFIN;Acc:ZDB-GENE-081114-1]	6	39737408	39765546		7.37	10.8	10.53	15.41	12.1	11.93
ENSDARG00000060805	map1sa	microtubule-associated protein 1Sa [Source:ZFIN;Acc:ZDB-GENE-030131-3982]	22	36794438	36829025		4.29	3.73	2.54	5.69	5.56	5.16
ENSDARG00000060808	mecom	MDS1 and EVI1 complex locus [Source:ZFIN;Acc:ZDB-GENE-050208-123]	15	35372907	35410860		2.19	1.33	1.24	7.37	7	7.95
ENSDARG00000060813	plekha7a	pleckstrin homology domain containing, family A member 7a [Source:NCBI gene;Acc:571486]	18	27101096	27307129		2.07	2.07	1.62	2.75	2.62	2.44
ENSDARG00000060829	car15	carbonic anhydrase 15 [Source:ZFIN;Acc:ZDB-GENE-091204-152]	10	42027285	42039169		0.48	0.41	0.53	0.67	0.95	0.6
ENSDARG00000060830	fam83hb	family with sequence similarity 83 member Hb [Source:NCBI gene;Acc:565918]	19	43612179	43639331		9.57	8.86	5.77	7.69	7.02	7.43
ENSDARG00000060835	tecpr2	tectonin beta-propeller repeat containing 2 [Source:ZFIN;Acc:ZDB-GENE-060503-358]	17	29282863	29312506		4.83	2.42	2.38	4.54	4.46	4.83
ENSDARG00000060837	trpc5b	transient receptor potential cation channel, subfamily C, member 5b [Source:ZFIN;Acc:ZDB-GENE-091112-24]	21	34132747	34211658		1.07	0.15	0.39	0.93	1.03	1.09
ENSDARG00000060838	trim35-33	tripartite motif containing 35-33 [Source:ZFIN;Acc:ZDB-GENE-081031-1]	22	36692681	36716767		0.05	0.01	0.01	0.02	0.03	0
ENSDARG00000060841	pik3c2a	phosphatidylinositol-4-phosphate 3-kinase, catalytic subunit type 2 alpha [Source:ZFIN;Acc:ZDB-GENE-030328-39]	18	27000850	27078219		5.39	5.49	4.19	7.29	7.35	7.17
ENSDARG00000060845	antxr1d	ANTXR cell adhesion molecule 1d [Source:ZFIN;Acc:ZDB-GENE-090313-28]	13	31297016	31335769		1.05	0.99	0.71	1.67	1.63	1.69
ENSDARG00000060846	cabp7b	calcium binding protein 7b [Source:ZFIN;Acc:ZDB-GENE-060526-366]	5	32086448	32092856		11.84	4.38	8.68	9.89	11.63	9.91
ENSDARG00000060847	setd1ba	SET domain containing 1B, histone lysine methyltransferase a [Source:NCBI gene;Acc:567970]	10	41986685	42022566		3.45	1.98	1.7	4.58	4.3	4.34
ENSDARG00000060849	dlgap4a	discs, large (Drosophila) homolog-associated protein 4a [Source:ZFIN;Acc:ZDB-GENE-080219-51]	23	42836125	42883522		7.35	5.34	5.08	9.15	8.94	8.87
ENSDARG00000060853	nckap1	NCK-associated protein 1 [Source:NCBI gene;Acc:561894]	9	44721808	44825419		37.43	20.87	21.32	42.72	42.83	42.15
ENSDARG00000060854	kctd3	potassium channel tetramerization domain containing 3 [Source:ZFIN;Acc:ZDB-GENE-060503-169]	17	29345606	29370314		4.31	2.51	2.36	8.27	9.01	9.28
ENSDARG00000060860	pstpip1a	proline-serine-threonine phosphatase interacting protein 1a [Source:ZFIN;Acc:ZDB-GENE-050419-251]	18	26930956	26963473		0.38	0.33	0.65	1.02	0.83	0.99
ENSDARG00000060861	dars2	aspartyl-tRNA synthetase 2, mitochondrial [Source:ZFIN;Acc:ZDB-GENE-070912-443]	2	34138376	34173489		3.7	3.16	2.29	3.52	3.61	3.39
ENSDARG00000060862	atxn1b	ataxin 1b [Source:ZFIN;Acc:ZDB-GENE-061218-2]	16	25555309	25568512		6.21	1.93	2.33	10.89	11.07	11.55
ENSDARG00000060865	scai	suppressor of cancer cell invasion [Source:ZFIN;Acc:ZDB-GENE-060526-57]	5	31995683	32046297		3.24	0.58	1.18	3.85	4.14	4.46
ENSDARG00000060868	mbtd1	mbt domain containing 1 [Source:ZFIN;Acc:ZDB-GENE-060810-138]	12	33541600	33558879		12.28	6.45	6.62	12.72	13.33	13.06
ENSDARG00000060871	mctp1b	multiple C2 domains, transmembrane 1b [Source:ZFIN;Acc:ZDB-GENE-090313-382]	10	43862964	43928937		1.59	1.35	0.9	1.32	1.21	0.86
ENSDARG00000060875	pde1a	phosphodiesterase 1A, calmodulin-dependent [Source:ZFIN;Acc:ZDB-GENE-090831-2]	9	44498182	44642108		3.68	1.64	1.84	3.72	4.32	4.1
ENSDARG00000060877	edil3b	EGF-like repeats and discoidin I-like domains 3b [Source:ZFIN;Acc:ZDB-GENE-090312-42]	10	43355895	43404027		1.84	0.24	0.63	2.11	2.12	2.36
ENSDARG00000060879	slc28a1	solute carrier family 28 member 1 [Source:ZFIN;Acc:ZDB-GENE-050419-117]	18	26829086	26850069		7.15	14.15	9.55	1.58	1.52	1.7
ENSDARG00000060884	nmur1a	neuromedin U receptor 1a [Source:ZFIN;Acc:ZDB-GENE-081104-220]	22	35485100	35489371		0.09	0.04	0.11	0.04	0.24	0.09
ENSDARG00000060885	znf592	zinc finger protein 592 [Source:ZFIN;Acc:ZDB-GENE-030131-9613]	18	26800061	26811959		6.45	4.28	4.33	6.13	6.05	6.69
ENSDARG00000060889	mettl7a	methyltransferase like 7A [Source:ZFIN;Acc:ZDB-GENE-061027-239]	6	39506043	39508375		8.08	11.84	9.16	1.79	1.8	1.85
ENSDARG00000060893	col8a2	collagen, type VIII, alpha 2 [Source:ZFIN;Acc:ZDB-GENE-060503-488]	19	35496282	35596207		8.26	7.97	6.08	29.88	28.93	30.57
ENSDARG00000060900	znf362a	zinc finger protein 362a [Source:ZFIN;Acc:ZDB-GENE-061103-547]	11	40175161	40204621		7.23	4.39	4.31	7.36	7.46	7.75
ENSDARG00000060901	trim62.1	tripartite motif containing 62, tandem duplicate 1 [Source:ZFIN;Acc:ZDB-GENE-070112-502]	11	40331791	40418975		1.83	0.99	0.83	2.87	2.79	2.48
ENSDARG00000060911	si:ch211-126i22.5	si:ch211-126i22.5 [Source:ZFIN;Acc:ZDB-GENE-091204-183]	10	11282883	11312342		5.31	2.76	3.3	5.27	5.21	5.29
ENSDARG00000060915	fbxl16	F-box and leucine-rich repeat protein 16 [Source:ZFIN;Acc:ZDB-GENE-120215-180]	3	28634467	28665291		10.24	2.78	2.96	13.3	12.89	12.75
ENSDARG00000060917	anln	anillin, actin binding protein [Source:ZFIN;Acc:ZDB-GENE-030131-4486]	19	35433155	35450857		4.54	3.22	2.49	5.12	4.83	4.64
ENSDARG00000060921	pla2g6	phospholipase A2, group VI (cytosolic, calcium-independent) [Source:ZFIN;Acc:ZDB-GENE-040426-2079]	22	29087308	29130549		4.73	3.03	2.61	4.78	4.84	5
ENSDARG00000060922	ess2	ess-2 splicing factor homolog [Source:ZFIN;Acc:ZDB-GENE-030131-2843]	10	42089267	42108137		10.69	8	7.95	10.76	10.92	10.89
ENSDARG00000060925	jarid2a	jumonji, AT rich interactive domain 2a [Source:ZFIN;Acc:ZDB-GENE-061218-1]	16	25407021	25500729		5.93	3.06	2.93	10.52	9.7	9.71
ENSDARG00000060926	rnf19b	ring finger protein 19B [Source:NCBI gene;Acc:562396]	19	35379899	35400819		4.09	1.76	1.61	7.16	7.54	7.83
ENSDARG00000060927	si:ch211-136a13.1	si:ch211-136a13.1 [Source:ZFIN;Acc:ZDB-GENE-050419-214]	18	49225552	49247479		2.6	2.04	1.94	16.77	18.34	17.35
ENSDARG00000060929	rnf220a	ring finger protein 220a [Source:ZFIN;Acc:ZDB-GENE-060929-1006]	2	33051730	33216740		5.92	3.53	3.72	5.65	5.8	6.19
ENSDARG00000060933	baiap2l2b	BAR/IMD domain containing adaptor protein 2 like 2b [Source:NCBI gene;Acc:564798]	22	29135846	29166693		2.29	1.44	1.8	1.01	1.31	1.02
ENSDARG00000060937	adnp2a	ADNP homeobox 2a [Source:ZFIN;Acc:ZDB-GENE-040914-55]	16	25371653	25380969		2.49	1.43	1.29	2.81	2.6	2.62
ENSDARG00000060941	ANKFN1	si:ch211-277e21.2 [Source:ZFIN;Acc:ZDB-GENE-130603-21]	12	32292564	32374156	ANKFN1	0.95	0.38	0.34	1.05	1.15	1.27
ENSDARG00000060944	rnf217	ring finger protein 217 [Source:ZFIN;Acc:ZDB-GENE-060503-400]	20	39749448	39789846		1.54	1.12	0.96	1.3	1.38	1.21
ENSDARG00000060946	pip5kl1	phosphatidylinositol-4-phosphate 5-kinase-like 1 [Source:ZFIN;Acc:ZDB-GENE-060526-248]	5	31693316	31712399		0.98	0.59	0.65	2.06	2.31	2.35
ENSDARG00000060948	dync1i1	dynein, cytoplasmic 1, intermediate chain 1 [Source:ZFIN;Acc:ZDB-GENE-061215-62]	16	25316973	25337834		12.27	4.09	7.52	11.42	10.62	11.64
ENSDARG00000060951	polg	polymerase (DNA directed), gamma [Source:ZFIN;Acc:ZDB-GENE-060303-1]	25	8477843	8513255		1.94	1.49	1.37	2.08	2.09	1.83
ENSDARG00000060952	si:ch1073-214b20.2	si:ch1073-214b20.2 [Source:ZFIN;Acc:ZDB-GENE-131121-231]	22	34615591	34626033		0.01	0.01	0.03	0.02	0.11	0.06
ENSDARG00000060954	tmcc1b	transmembrane and coiled-coil domain family 1b [Source:ZFIN;Acc:ZDB-GENE-061013-154]	11	18205766	18244561		10.05	6.41	6.19	10.35	9.77	9.73
ENSDARG00000060962	btr04	bloodthirsty-related gene family, member 4 [Source:ZFIN;Acc:ZDB-GENE-060526-109]	7	16185376	16195144		6.16	6.31	5.38	4.63	4.57	4.18
ENSDARG00000060977	zgc:153018	zgc:153018 [Source:ZFIN;Acc:ZDB-GENE-060929-752]	14	45339986	45346558		10.1	8.69	7.78	14.59	15.49	15.11
ENSDARG00000060978	atp2a3	ATPase sarcoplasmic/endoplasmic reticulum Ca2+ transporting 3 [Source:ZFIN;Acc:ZDB-GENE-060531-103]	5	31168096	31269354		7.89	4.87	3.47	9.83	8.73	8.14
ENSDARG00000060980	atp8b1	ATPase phospholipid transporting 8B1 [Source:ZFIN;Acc:ZDB-GENE-091116-14]	21	3618519	3700334		6.95	4.61	3.25	4.98	4.59	4.23
ENSDARG00000060983	smyd4	SET and MYND domain containing 4 [Source:ZFIN;Acc:ZDB-GENE-060929-248]	10	36092564	36106146		0.99	0.71	0.79	0.78	0.65	0.78
ENSDARG00000060987	C25H12orf4	zgc:154077 [Source:ZFIN;Acc:ZDB-GENE-061110-34]	25	16853386	16877107	C25H12orf4	6.69	4.93	5.1	5.46	4.93	5.68
ENSDARG00000060991	mief1	mitochondrial elongation factor 1 [Source:NCBI gene;Acc:564985]	3	25136513	25148047		6.86	4.88	3.95	6.81	6.54	7.48
ENSDARG00000060992	rpusd2	RNA pseudouridine synthase domain containing 2 [Source:ZFIN;Acc:ZDB-GENE-120411-43]	17	31205064	31212420		3.67	3.08	2.8	2.76	2.37	2.73
ENSDARG00000060994	fbxw7	F-box and WD repeat domain containing 7 [Source:ZFIN;Acc:ZDB-GENE-090313-79]	1	24870661	25144439		7.56	4.84	4.22	9.28	8.58	9.08
ENSDARG00000061000	klhdc8a	kelch domain containing 8A [Source:ZFIN;Acc:ZDB-GENE-120810-3]	11	38033115	38083473		2.33	0.59	1.21	3.9	3.94	3.59
ENSDARG00000061004	cox11	cytochrome c oxidase assembly homolog 11 (yeast) [Source:ZFIN;Acc:ZDB-GENE-050506-82]	12	32068294	32073619		8.76	11.55	9.95	7.81	7.11	7.35
ENSDARG00000061006	cyb5d2	cytochrome b5 domain containing 2 [Source:NCBI gene;Acc:795950]	5	31032149	31035198		9.05	7.1	7.4	6.09	6.65	6.2
ENSDARG00000061011	hlfb	HLF transcription factor, PAR bZIP family member b [Source:ZFIN;Acc:ZDB-GENE-110420-3]	12	32159272	32165227		10.93	6.55	5.26	8.34	8.58	7.7
ENSDARG00000061013	ankfy1	ankyrin repeat and FYVE domain containing 1 [Source:ZFIN;Acc:ZDB-GENE-041222-1]	5	30996322	31030840		10.58	7.57	7.22	12.98	12.98	11.94
ENSDARG00000061014	kcnj5	potassium inwardly rectifying channel subfamily J member 5 [Source:ZFIN;Acc:ZDB-GENE-120215-97]	18	48576527	48643857		0.73	0.24	0.29	1.04	1.1	1.19
ENSDARG00000061017	spns2	spinster homolog 2 (Drosophila) [Source:NCBI gene;Acc:568117]	5	30802798	30924453		2.93	1.83	1.72	4.95	5.28	5.1
ENSDARG00000061020	ccdc186	si:ch211-225b10.4 [Source:ZFIN;Acc:ZDB-GENE-100318-3]	12	30367079	30422456		5.88	2.96	2.62	4.56	4.66	4.93
ENSDARG00000061021	fam160a1a	family with sequence similarity 160 member A1a [Source:ZFIN;Acc:ZDB-GENE-090312-132]	1	24469313	24514673		2.64	1.85	1.23	2	2	1.63
ENSDARG00000061023	si:dkey-72l14.4	si:dkey-72l14.4 [Source:ZFIN;Acc:ZDB-GENE-060531-151]	8	25959940	25971552		7.69	4.92	4.32	9.66	9.77	9.57
ENSDARG00000061024	rabggta	Rab geranylgeranyltransferase subunit alpha [Source:ZFIN;Acc:ZDB-GENE-060929-1042]	23	1702624	1726560		8.22	5.23	4.76	9.07	9.45	8.89
ENSDARG00000061030	vezf1b	vascular endothelial zinc finger 1b [Source:ZFIN;Acc:ZDB-GENE-060825-121]	15	31244025	31265375		6.78	3.49	3.11	7.78	7.61	7.72
ENSDARG00000061031	dner	delta/notch-like EGF repeat containing [Source:ZFIN;Acc:ZDB-GENE-130502-1]	18	46699731	46763170		13.08	2.62	3.8	17.93	18.41	19.72
ENSDARG00000061039	atp10a	ATPase phospholipid transporting 10A [Source:ZFIN;Acc:ZDB-GENE-101102-1]	6	38626684	38730530		0.93	1	0.71	2.04	1.85	1.78
ENSDARG00000061040	pum2	pumilio RNA-binding family member 2 [Source:ZFIN;Acc:ZDB-GENE-081031-76]	20	6089507	6131824		5.34	3.09	2.56	8.9	9.26	8.92
ENSDARG00000061042	gabbr2	gamma-aminobutyric acid (GABA) B receptor, 2 [Source:ZFIN;Acc:ZDB-GENE-060503-620]	19	34311374	34679738		3.1	0.79	1.01	3.9	4.4	4.43
ENSDARG00000061044	si:dkey-72l14.3	si:dkey-72l14.3 [Source:ZFIN;Acc:ZDB-GENE-060526-332]	8	25973151	25980694		1.03	0.46	0.25	1.17	1.17	1.43
ENSDARG00000061047	abcg4a	ATP-binding cassette, sub-family G (WHITE), member 4a [Source:ZFIN;Acc:ZDB-GENE-050517-39]	5	30624183	30672482		3.09	0.95	1.34	4.59	4.64	4.95
ENSDARG00000061048	ranbp9	RAN binding protein 9 [Source:NCBI gene;Acc:558976]	2	31806602	31827960		14.74	10.3	8.94	15.12	16.09	15.61
ENSDARG00000061049	rnf182	ring finger protein 182 [Source:ZFIN;Acc:ZDB-GENE-060825-49]	2	31804582	31805604		0.56	0.2	0.47	0.76	1.12	1.47
ENSDARG00000061051	bmp3a.2	bone morphogenetic protein/retinoic acid inducible neural-specific 3a, tandem duplicate 2 [Source:ZFIN;Acc:ZDB-GENE-131119-53]	6	36127505	36134080		1.17	0.36	0.55	1.26	1.26	1.43
ENSDARG00000061052	nipblb	NIPBL cohesin loading factor b [Source:NCBI gene;Acc:794108]	10	36475860	36547128		11.34	6.25	5.35	12.22	11.77	11.68
ENSDARG00000061057	cyhr1	cysteine/histidine-rich 1 [Source:NCBI gene;Acc:767773]	19	42919279	42928979		19.77	16.22	17.46	20.82	21.34	22.82
ENSDARG00000061060	stpg2		5	6954162	7005117		0.48	0.27	0.22	0.49	0.6	0.6
ENSDARG00000061061	dpagt1	dolichyl-phosphate (UDP-N-acetylglucosamine) N-acetylglucosaminephosphotransferase 1 (GlcNAc-1-P transferase) [Source:ZFIN;]	5	30588856	30595372		11.26	12.77	11.32	12.79	12.65	13.25
ENSDARG00000061062	nemp2	nuclear envelope integral membrane protein 2 [Source:ZFIN;Acc:ZDB-GENE-081104-413]	9	41459759	41481650		3.27	2.14	2.06	3.25	3.3	3.45
ENSDARG00000061066	c2cd2l	c2cd2 like [Source:ZFIN;Acc:ZDB-GENE-041019-1]	5	30537640	30588549		8.09	2.68	2.67	7.59	7.45	7.11
ENSDARG00000061070	chst3a	carbohydrate (chondroitin 6) sulfotransferase 3a [Source:ZFIN;Acc:ZDB-GENE-090312-180]	13	29510023	29520852		0.62	0.7	0.51	1.53	1.56	1.52
ENSDARG00000061081	arpp21	cAMP-regulated phosphoprotein, 21 [Source:ZFIN;Acc:ZDB-GENE-081104-296]	19	42886413	42917226		4.16	2.86	2.58	13.31	12.81	13.52
ENSDARG00000061083	igl4v8	immunoglobulin light 4 variable 8 [Source:ZFIN;Acc:ZDB-GENE-051013-6]	25	37313713	37314700		0.26	0	0.02	0.03	0.03	0
ENSDARG00000061091	gbpbp6	GTP binding protein 6 (putative) [Source:ZFIN;Acc:ZDB-GENE-031118-62]	6	37441789	37454910		2.78	2.07	2.23	1.85	1.66	1.83
ENSDARG00000061093	plekhb1a	pleckstrin homology-like domain, family B, member 1a [Source:ZFIN;Acc:ZDB-GENE-060526-272]	5	30427198	30491172		4.09	3.91	3.55	6.21	6.61	6.91
ENSDARG00000061095	stk36	serine/threonine kinase 36 (fused homolog, Drosophila) [Source:ZFIN;Acc:ZDB-GENE-030912-6]	5	30392148	30411165		0.83	0.61	0.75	1.22	1.54	1.68
ENSDARG00000061096	dapk2a	death-associated protein kinase 2a [Source:ZFIN;Acc:ZDB-GENE-080402-8]	7	29278580	29292206		2.05	2.5	2.47	2.77	2.51	2.44
ENSDARG00000061099	nfasca	neurofascin homolog (chicken) a [Source:ZFIN;Acc:ZDB-GENE-080229-6]	11	23704410	23884805		7.31	2.28	2.35	9.08	8.9	9.49
ENSDARG00000061100	nars1	asparaginyl-tRNA synthetase 1 [Source:ZFIN;Acc:ZDB-GENE-030131-1016]	21	944715	965598		36.37	36.7	32.9	54.41	58.7	57.32
ENSDARG00000061101	snx19a	sorting nexin 19a [Source:ZFIN;Acc:ZDB-GENE-060526-204]	5	30351233	30380593		3.84	0.89	0.79	2.35	2.67	2.94
ENSDARG00000061103	slc19a3b	solute carrier family 19 member 3b [Source:ZFIN;Acc:ZDB-GENE-050419-166]	18	46370385	46380537		0.2	0.47	0.28	0.12	0.08	0.14
ENSDARG00000061108	ep300b	E1A binding protein p300 b [Source:ZFIN;Acc:ZDB-GENE-080403-15]	3	24996930	25055795		9.54	5.06	4.4	15.35	14.51	15.06
ENSDARG00000061110	zbtb44	zinc finger and BTB domain containing 44 [Source:ZFIN;Acc:ZDB-GENE-060526-201]	5	30131275	30151815		0.91	0.4	0.33	1.39	1.25	1.38
[truncated: 3,111,543 more chars]
