# Supplementary material for: Succinate supplementation ameliorates musculoskeletal defects caused by PLOD3 mutations in a BCARD syndrome model
Source: Genome Med. 2026 Mar 13;18:29. doi: 10.1186/s13073-026-01608-y (PMC12994257; doi:10.1186/s13073-026-01608-y)
Supplement: Supplementary file 6 — Additional file 6: Table S11. Enrichr Reactome downregulated. [file 13073_2026_1608_MOESM6_ESM.pdf]

Table:S11 Enrichr Reactome Down

Term	Overlap	P-value	Adjusted P-value	Old P-value	Old Adjusted P-value	Odds Ratio	Combined Score	Genes
Metabolism	276/2181	3.43E-86	4.03E-83	0	0	5.643571908	1110.597497	PRSS1;RPL32;RPL31;SLC23A1;ECI1;ECI2;RPL7;GCSH;RPS14;NSDHL;RPS17;TDO2;SCP2;RPL36;LRAT;CHAC2;RPS11;MCCC2;GSTK1;TSTD1;CTRB2;RPL22;CTRB1;OMD;ENTPD8;ACSL5;HOGA1;SLC51A;SLC5A8;RBP4
Aerobic Respiration and Respiratory Electron Transport	60/260	1.83E-31	1.08E-28	0	0	8.645619335	611.8802291	NDUFA11;NDUFA12;NDUFA10;ETFB;COX6A1;COX7C;COX6A2;MPC2;ME1;COA5;NDUFC2;SDHC;COX6B2;NDUFS8;NDUFS7;NDUFS6;NDUFS4;TMEM126A;SUCLG2;SUCLG1;TMEM126B;L2HGDH;SLC25A4;NDUFB9;ND
Metabolism of Amino Acids and Derivatives	61/358	2.17E-24	8.49E-22	0	0	5.897813231	321.3623932	RPL32;RPL31;RPLP1;TAT;HNMT;FTCD;RPL7;GCSH;RPS14;RPS17;TDO2;IYD;RPL36;RPS11;MCCC2;RPS9;TSTD1;HGD;RPS8;RPL22;AMT;PIPOX;RPL13A;RPS3A;HOGA1;PSMA6;ALDH7A1;HPD;IDO1;ALDH9A1;GCDH;GLS
Respiratory Electron Transport	39/158	5.76E-22	1.69E-19	0	0	9.193265007	449.6059879	NDUFB9;NDUFB8;NDUFB7;NDUFA11;NDUFB10;NDUFB6;NDUFA12;NDUFB11;NDUFA10;COX7A2;ETFB;UQCR10;COX6A1;COX6A2;COX7C;COX5A;UQCRH;COX14;NDUFV3;NDUFV2;NDUFV1;COA5;NDUFA9;NDUFA8;ND
Complex I Biogenesis	25/68	3.52E-19	8.28E-17	0	0	16.04467652	681.7427474	NDUFB9;NDUFB8;NDUFB7;NDUFA11;NDUFB10;NDUFB6;NDUFA12;NDUFB11;NDUFA10;NDUFV3;NDUFV2;NDUFV1;NDUFA9;NDUFA8;NDUFA7;COA1;NDUFA5;NDUFC2;NDUFS8;NDUFS7;NDUFS6;NDUFS4;NDUFAB1;TM
Metabolism of Lipids	81/758	1.33E-18	2.61E-16	0	0	3.471959203	142.9085534	ACAA2;MOGAT3;MOGAT2;ECI1;HEXA;ECI2;AKR1B1;DBI;MSMO1;AHR;CYP46A1;FADS2;NSDHL;SCP2;ACADL;CYP2R1;ME1;ANKRD1;CD36;ACADM;GGT1;ACAA1;HADH;ACADS;ENPP7;CBR1;PCYT1B;DGAT2;GPX1;DGA
DNA Strand Elongation	17/32	8.51E-17	1.43E-14	0	0	30.96652482	1145.854785	GINS1;RFC5;PRIM2;GINS2;FEN1;PCNA;LIG1;RFC2;PRIM1;GINS3;GINS4;RPA2;CDC45;RPA3;MCM3;MCM5;MCM2
Fatty Acid Metabolism	33/176	5.5E-15	8.08E-13	0	0	6.408953891	210.4322126	PON3;ACAA2;SLC22A5;ECI1;ECI2;PON1;DBI;FADS2;SCP2;ACADL;LTA4H;ACADM;ACAA1;GGT1;HADH;ACADS;CBR1;GPX1;GPX4;ELOVL2;FAAH2;ACOT13;ACSL5;PTGR1;ACSF2;HADHB;NUDT7;AMACR;PRXL2B;EHHAD
rRNA Processing in the Nucleus and Cytosol	34/199	3.74E-14	4.88E-12	0	0	5.724471459	176.9877598	RPL32;RPL31;RPLP1;NIP7;RPL11;RPL36A;DDX21;SNU13;RPL7;RPS14;FBL;RPS17;EXOSC5;RPL36;RPS11;RPS27A;EXOSC2;RPL17;RPS9;RPS8;RPL22;RPL13A;RPL35A;RPS3A;GNL3;BYSL;RPS25;DKC1;TSR1;RPS20;FA
rRNA Processing	36/237	2.68E-13	2.94E-11	0	0	4.980723206	144.1868961	RPL32;RPL31;RPLP1;NIP7;RPL11;RPL36A;DDX21;SNU13;HSD17B10;RPL7;RPS14;FBL;RPS17;EXOSC5;RPL36;RPS11;RPS27A;EXOSC2;RPL17;RPS9;RPS8;RPL22;MTERF4;RPL13A;RPL35A;RPS3A;GNL3;BYSL;RPS25;D
Major Pathway of rRNA Processing in the Nucleolus and Cytosol	32/189	2.75E-13	2.94E-11	0	0	5.648223022	163.3561846	RPL32;RPL31;RPLP1;NIP7;RPL11;RPL36A;DDX21;SNU13;RPL7;FBL;RPS14;RPS17;EXOSC5;RPL36;RPS11;RPS27A;EXOSC2;RPL17;RPS9;RPS8;RPL22;RPL13A;RPL35A;RPS3A;GNL3;BYSL;RPS25;TSR1;RPS20;FAU;NO
Eukaryotic Translation Termination	23/98	5.39E-13	5.27E-11	0	0	8.424778255	237.9993679	RPS9;RPL32;RPL31;RPS8;RPLP1;RPL22;RPL11;RPL36A;RPL13A;RPL35A;RPS3A;RPL7;RPS14;RPS25;RPS17;RPL36;RPS20;ETF1;FAU;RPS11;RPS27A;RPL17;RPS24
Eukaryotic Translation Elongation	23/99	6.78E-13	6.13E-11	0	0	8.31349296	232.9406781	RPS9;RPL32;RPL31;RPS8;RPLP1;RPL22;RPL11;RPL36A;RPL13A;RPL35A;RPS3A;RPL7;RPS14;RPS25;RPS17;EEF1D;RPL36;RPS20;FAU;RPS11;RPS27A;RPL17;RPS24
Nonsense Mediated Decay (NMD) Independent of the Exon Junction Complex (EJC)	23/100	8.51E-13	7.14E-11	0	0	8.205098192	228.0383668	RPS9;RPL32;RPL31;RPS8;RPLP1;RPL22;RPL11;RPL36A;RPL13A;RPL35A;RPS3A;RPL7;RPS14;RPS25;RPS17;RPL36;RPS20;ETF1;FAU;RPS11;RPS27A;RPL17;RPS24
Selenoamino Acid Metabolism	25/122	1.4E-12	1.09E-10	0	0	7.092620805	193.6059899	RPL32;RPL31;RPLP1;RPL11;RPL36A;HNMT;RPL7;RPS14;RPS17;CBS;RPL36;RPS11;RPS27A;RPL17;RPS9;RPS8;RPL22;GSR;RPL13A;RPL35A;RPS3A;RPS25;RPS20;FAU;RPS24
Peptide Chain Elongation	22/94	1.83E-12	1.35E-10	0	0	8.383571429	226.5659957	RPS9;RPL32;RPL31;RPS8;RPLP1;RPL22;RPL11;RPL36A;RPL13A;RPL35A;RPS3A;RPL7;RPS14;RPS25;RPS17;RPL36;RPS20;FAU;RPS11;RPS27A;RPL17;RPS24
Formation of a Pool of Free 40S Subunits	23/106	3.14E-12	2.05E-10	0	0	7.609579951	201.5636505	RPS9;RPL32;RPL31;RPS8;RPLP1;RPL22;RPL11;RPL36A;RPL13A;RPL35A;RPS3A;RPL7;RPS14;RPS25;RPS17;EIF3M;RPL36;RPS20;FAU;RPS11;RPS27A;RPL17;RPS24
Response of EIF2AK4 (GCN2) to Amino Acid Deficiency	23/106	3.14E-12	2.05E-10	0	0	7.609579951	201.5636505	RPS9;RPL32;RPL31;RPS8;RPLP1;RPL22;RPL11;RPL36A;RPL13A;RPL35A;RPS3A;RPL7;RPS14;RPS25;RPS17;IMPACT;RPL36;RPS20;FAU;RPS11;RPS27A;RPL17;RPS24
Selenocysteine Synthesis	22/98	4.5E-12	2.79E-10	0	0	7.940676692	207.4574689	RPS9;RPL32;RPL31;RPS8;RPLP1;RPL22;RPL11;RPL36A;RPL13A;RPL35A;RPS3A;RPL7;RPS14;RPS25;RPS17;RPL36;RPS20;FAU;RPS11;RPS27A;RPL17;RPS24
Translation	39/302	5.18E-12	3.04E-10	0	0	4.128425811	107.2861087	MRPS17;RPL32;RPL31;MRPL18;RPLP1;RPL11;MRPL16;RPL36A;MRPL14;MRPL36;MRPL34;MRPL54;RPL7;MRPL4;RPS14;MRPL42;RPS17;RPL36;RPS11;RPS27A;RPL17;CHCHD1;RPS9;MRPS24;RPS8;RPL22;MRPL27;F
Viral mRNA Translation	22/102	1.06E-11	5.91E-10	0	0	7.542071429	190.6151812	RPS9;RPL32;RPL31;RPS8;RPLP1;RPL22;RPL11;RPL36A;RPL13A;RPL35A;RPS3A;RPL7;RPS14;RPS25;RPS17;RPL36;RPS20;FAU;RPS11;RPS27A;RPL17;RPS24
Formation of ATP by Chemiosmotic Coupling	11/20	1.57E-11	8.39E-10	0	0	33.12376934	824.0136779	ATP5PF;ATP5PD;ATP5PB;ATP5PO;ATP5MC3;ATP5MK;ATP5MJ;ATP5F1C;ATP5F1E;ATP5MC1;ATP5ME
L13a-mediated Translational Silencing of Ceruloplasmin Expression	23/116	2.25E-11	1.15E-09	0	0	6.787807467	166.4124596	RPS9;RPL32;RPL31;RPS8;RPLP1;RPL22;RPL11;RPL36A;RPL13A;RPL35A;RPS3A;RPL7;RPS14;RPS25;RPS17;EIF3M;RPL36;RPS20;FAU;RPS11;RPS27A;RPL17;RPS24
GTP Hydrolysis and Joining of the 60S Ribosomal Subunit	23/117	2.71E-11	1.33E-09	0	0	6.715246705	163.3922614	RPS9;RPL32;RPL31;RPS8;RPLP1;RPL22;RPL11;RPL36A;RPL13A;RPL35A;RPS3A;RPL7;RPS14;RPS25;RPS17;EIF3M;RPL36;RPS20;FAU;RPS11;RPS27A;RPL17;RPS24
Nonsense Mediated Decay (NMD) Enhanced by the Exon Junction Complex (EJC)	23/120	4.66E-11	2.11E-09	0	0	6.506541009	154.7858721	RPS9;RPL32;RPL31;RPS8;RPLP1;RPL22;RPL11;RPL36A;RPL13A;RPL35A;RPS3A;RPL7;RPS14;RPS25;RPS17;RPL36;RPS20;ETF1;FAU;RPS11;RPS27A;RPL17;RPS24
Nonsense-Mediated Decay (NMD)	23/120	4.66E-11	2.11E-09	0	0	6.506541009	154.7858721	RPS9;RPL32;RPL31;RPS8;RPLP1;RPL22;RPL11;RPL36A;RPL13A;RPL35A;RPS3A;RPL7;RPS14;RPS25;RPS17;RPL36;RPS20;ETF1;FAU;RPS11;RPS27A;RPL17;RPS24
Cap-dependent Translation Initiation	23/124	9.35E-11	3.92E-09	0	0	6.247553081	144.2778153	RPS9;RPL32;RPL31;RPS8;RPLP1;RPL22;RPL11;RPL36A;RPL13A;RPL35A;RPS3A;RPL7;RPS14;RPS25;RPS17;EIF3M;RPL36;RPS20;FAU;RPS11;RPS27A;RPL17;RPS24
Eukaryotic Translation Initiation	23/124	9.35E-11	3.92E-09	0	0	6.247553081	144.2778153	RPS9;RPL32;RPL31;RPS8;RPLP1;RPL22;RPL11;RPL36A;RPL13A;RPL35A;RPS3A;RPL7;RPS14;RPS25;RPS17;EIF3M;RPL36;RPS20;FAU;RPS11;RPS27A;RPL17;RPS24
Biological Oxidations	31/222	1.11E-10	4.5E-09	0	0	4.483198339	102.7592136	SLC26A1;SLC35D1;TPMT;MGST3;GSTP1;MGST1;GSTT2;AHR;CYP46A1;CYP7A1;CNDP2;AKR7A3;ALDH2;MAT2A;CYP4V2;ESD;CYP2R1;CHAC2;GGT1;GSTK1;AOC1;GSTO2;GSTO1;EPHX1;CYP51A1;AKR1A1;NR1H4;CYF
SRP-dependent Cotranslational Protein Targeting to Membrane	22/119	2.56E-10	1E-08	0	0	6.214756996	137.266604	RPS9;RPL32;RPL31;RPS8;RPLP1;RPL22;RPL11;RPL36A;RPL13A;RPL35A;RPS3A;RPL7;RPS14;RPS25;RPS17;RPL36;RPS20;FAU;RPS11;RPS27A;RPL17;RPS24
Cristae Formation	12/33	7.91E-10	3E-08	0	0	15.49859155	324.8227726	APOO;ATP5PF;ATP5PD;ATP5PB;ATP5PO;ATP5MC3;ATP5MK;ATP5MJ;ATP5F1C;ATP5F1E;ATP5MC1;ATP5ME
Unwinding of DNA	8/12	1.21E-09	4.44E-08	0	0	53.98879552	1108.596842	GINS1;GINS2;CDC45;GINS3;GINS4;MCM3;MCM5;MCM2
Transport of Small Molecules	60/724	1.86E-09	6.64E-08	0	0	2.540767299	51.07126963	ABCD3;AQP8;AQP10;SLC2A2;PCSK5;BEST4;SLC22A16;FTH1;SLC22A18;CA4;SLC39A4;SLC13A1;SLC34A2;ABCG8;SLC15A1;SLC6A19;ABCG5;SLC13A4;SLC30A8;ABCC2;AQP12A;SLC34A1;APOA1;APOA4;PSMA6;SLC
Glutathione Conjugation	12/37	3.61E-09	1.25E-07	0	0	13.01611268	253.0118046	GSTK1;GSTO2;GSTO1;GSTP1;MGST3;ESD;MGST1;AKR1A1;GSTT2;CHAC2;GGT1;CNDP2
Influenza Viral RNA Transcription and Replication	23/149	4.01E-09	1.35E-07	0	0	5.001430615	96.69659595	RPS9;RPL32;RPL31;RPS8;RPLP1;RPL22;RPL11;RPL36A;RPL13A;RPL35A;RPS3A;RPL7;RPS14;RPS25;RPS17;RPL36;POLR2E;RPS20;FAU;RPS11;RPS27A;RPL17;RPS24
Regulation of Expression of SLITs and ROBOs	24/164	5.51E-09	1.8E-07	0	0	4.700286533	89.38584025	RPS9;RPL32;RPL31;RPS8;RPLP1;RPL22;RPL11;RPL36A;RPL13A;RPL35A;RPS3A;RPL7;RPS14;RPS25;PSMA6;RPS17;RPL36;RPS20;ETF1;FAU;RPS11;RPS27A;RPL17;RPS24
Synthesis of DNA	19/108	1E-08	3.18E-07	0	0	5.827209232	107.3324273	RFC5;GINS1;PRIM2;GINS2;FEN1;PCNA;LIG1;RFC2;PRIM1;GINS3;GINS4;RPA2;PSMA6;CDC45;RPA3;MCM3;MCM5;RPS27A;MCM2
Lagging Strand Synthesis	9/20	1.16E-08	3.6E-07	0	0	22.10926941	403.9020476	RFC5;PRIM2;FEN1;PCNA;LIG1;RFC2;PRIM1;RPA3;RPA2
Cellular Response to Starvation	23/161	1.82E-08	5.49E-07	0	0	4.563662375	81.32351623	RPS9;RPL32;RPL31;RPS8;RPLP1;RPL22;RPL11;RPL36A;RPL13A;RPL35A;RPS3A;RPL7;RPS14;RPS25;RPS17;IMPACT;RPL36;RPS20;FAU;RPS11;RPS27A;RPL17;RPS24
Protein Localization	23/164	2.6E-08	7.64E-07	0	0	4.465863087	77.99805744	TIMM8B;GSTK1;CYB5A;NDUFB8;ABCD3;CMC4;IDH1;ECI2;PIPOX;CHCHD10;ATP5MC1;DHRS4;NUDT7;AMACR;DAO;SCP2;EHHADH;CHCHD7;ACAA1;RPS27A;SLC27A2;SLC25A4;STX1A
Metabolism of Steroids	22/155	4.18E-08	1.2E-06	0	0	4.52406015	76.86319794	HSD3B7;SRD5A2;CYP51A1;NR1H4;AKR1B1;MSMO1;SLC51A;CYP46A1;ABCB11;HSD17B7;LSS;CYP7A1;SQLE;NSDHL;EBP;AMACR;SCP2;CYP2R1;SC5D;MVD;GC;SLC27A2
Influenza Infection	23/169	4.6E-08	1.29E-06	0	0	4.311795716	72.84475593	RPS9;RPL32;RPL31;RPS8;RPLP1;RPL22;RPL11;RPL36A;RPL13A;RPL35A;RPS3A;RPL7;RPS14;RPS25;RPS17;RPL36;POLR2E;RPS20;FAU;RPS11;RPS27A;RPL17;RPS24
Mitochondrial Fatty Acid Beta-Oxidation	11/38	6.21E-08	1.7E-06	0	0	11.03094233	183.0511418	HADHB;ACAA2;ACADL;ECI1;NDUFAB1;ACOT13;DBI;ACADM;HADH;ACADS;ACSF2
S Phase	21/150	1.08E-07	2.88E-06	0	0	4.446903095	71.34611511	RFC5;GINS1;PRIM2;GINS2;CDKN1A;FEN1;PCNA;LIG1;RFC2;PRIM1;GINS3;GINS4;RPA2;PTK6;PSMA6;CDC45;RPA3;MCM3;MCM5;RPS27A;MCM2
SARS-CoV-1 Modulates Host Translation Machinery	11/41	1.48E-07	3.86E-06	0	0	9.926300985	156.1195855	RPS14;RPS25;RPS17;RPS9;RPS8;RPS3A;RPS20;FAU;RPS11;RPS27A;RPS24
Signaling by ROBO Receptors	25/210	1.74E-07	4.46E-06	0	0	3.701772073	57.60489659	RPL32;RPL31;RPLP1;RPL11;RPL36A;RPL7;RPS14;RPS17;RPL36;RPS11;RPS27A;RPL17;RPS9;RPS8;RPL22;RPL13A;RPL35A;RPS3A;RPS25;PSMA6;RPS20;ETF1;FAU;PFN1;RPS24
Extension of Telomeres	12/51	1.95E-07	4.87E-06	0	0	8.3375948	128.8296975	RFC5;PRIM2;FEN1;PCNA;LIG1;RFC2;DKC1;DSCC1;PRIM1;RPA3;RPA2;NOP10
Telomere C-strand (Lagging Strand) Synthesis	10/34	2.11E-07	5.17E-06	0	0	11.26755618	173.1951318	RFC5;PRIM2;FEN1;PCNA;LIG1;RFC2;DSCC1;PRIM1;RPA3;RPA2
Metabolism of Vitamins and Cofactors	24/198	2.19E-07	5.25E-06	0	0	3.775121035	57.88953301	MCCC2;CYB5A;PRSS1;GSTO2;GSTO1;SLC23A1;CTRB2;IDH1;CTRB1;APOA1;MTHFS;APOA4;DHFR;RBP4;SLC5A8;TTR;MTHFD1;RBP2;TCN2;APOC2;SLC19A3;LRAT;APOB;BCO1
Cholesterol Biosynthesis	9/27	2.59E-07	6.08E-06	0	0	13.50631136	204.8504532	SQLE;EBP;NSDHL;CYP51A1;SC5D;MSMO1;MVD;HSD17B7;LSS
Formation of the Ternary Complex, and Subsequently, the 43S Complex	12/53	3.06E-07	7.06E-06	0	0	7.930058399	118.9412288	RPS14;RPS25;RPS17;RPS9;EIF3M;RPS8;RPS3A;RPS20;FAU;RPS11;RPS27A;RPS24
Peroxisomal Protein Import	13/63	3.14E-07	7.1E-06	0	0	7.051170663	105.5795691	GSTK1;IDH1;ECI2;PIPOX;DHRS4;NUDT7;AMACR;DAO;SCP2;EHHADH;ACAA1;RPS27A;SLC27A2
Striated Muscle Contraction	10/36	3.83E-07	8.44E-06	0	0	10.39974071	153.6525715	MYBPC2;DES;TMOD4;TNNT1;TNNC1;TPM2;MYL2;TNNC2;TNNT3;TNNI2
Processive Synthesis on the Lagging Strand	7/15	3.88E-07	8.44E-06	0	0	23.58216783	348.1404462	PRIM2;FEN1;PCNA;LIG1;PRIM1;RPA3;RPA2
SARS-CoV-2 Modulates Host Translation Machinery	12/55	4.71E-07	1E-05	0	0	7.560432362	110.136716	RPS14;RPS25;RPS17;RPS9;RPS8;RPS3A;SNRPE;RPS20;FAU;RPS11;RPS27A;RPS24
Phase II - Conjugation of Compounds	17/111	4.77E-07	1E-05	0	0	4.921201147	71.63561416	GSTK1;SLC26A1;TPMT;SLC35D1;GSTO2;GSTO1;MGST3;GSTP1;MGST1;AKR1A1;GSTT2;CNDP2;MAT2A;ESD;CHAC2;GGT1;SULT6B1
Phenylalanine and Tyrosine Metabolism	6/11	8.58E-07	1.77E-05	0	0	32.30111732	451.2164392	HGD;PAH;TAT;KYAT1;FAH;HPD
DNA Replication	19/143	9.79E-07	1.98E-05	0	0	4.174803836	57.76590789	RFC5;GINS1;PRIM2;GINS2;FEN1;PCNA;LIG1;RFC2;PRIM1;GINS3;GINS4;RPA2;PSMA6;CDC45;RPA3;MCM3;MCM5;RPS27A;MCM2
Cellular Response to Chemical Stress	23/202	1.19E-06	2.36E-05	0	0	3.510817529	47.90645027	NQO1;CDKN1A;GPX1;AQP8;GSTP1;IDH1;GSR;PTK6;COX7A2;COX6A1;COX6B2;SOD2;COX7C;COX5A;COX6A2;ATOX1;SOD1;PRDX3;FABP1;PRDX2;PSMA6;ME1;RPS27A
Ribosomal Scanning and Start Codon Recognition	12/60	1.27E-06	2.45E-05	0	0	6.771126761	91.90425317	RPS14;RPS25;RPS17;RPS9;EIF3M;RPS8;RPS3A;RPS20;FAU;RPS11;RPS27A;RPS24
Translation Initiation Complex Formation	12/60	1.27E-06	2.45E-05	0	0	6.771126761	91.90425317	RPS14;RPS25;RPS17;RPS9;EIF3M;RPS8;RPS3A;RPS20;FAU;RPS11;RPS27A;RPS24
mRNA Activation Upon Binding of the Cap-Binding Complex and eIFs, and Subsequent Binding to 43S	12/61	1.54E-06	2.91E-05	0	0	6.632595573	88.79026485	RPS14;RPS25;RPS17;RPS9;EIF3M;RPS8;RPS3A;RPS20;FAU;RPS11;RPS27A;RPS24
Disorders of Transmembrane Transporters	20/164	2.01E-06	3.75E-05	0	0	3.785612536	49.65310115	ABCG8;SLC34A2;ABCG5;SLC6A19;GCKR;SLC22A5;ABCC2;SLC34A1;ABCB4;SLC2A2;APOA1;SLC5A1;ABCB11;GCK;PSMA6;SLC22A18;SLC17A5;RPS27A;SLC26A3;SLC39A4
Digestion and Absorption	8/26	2.43E-06	4.47E-05	0	0	11.98879552	154.972552	GUCY2C;AMY2A;SLC2A2;ALPI;SLC5A1;CEL;SLC2A5;LCT
Detoxification of Reactive Oxygen Species	9/36	3.88E-06	7.02E-05	0	0	9	112.1267291	PRDX3;PRDX2;GPX1;AQP8;GSTP1;GSR;SOD2;ATOX1;SOD1
Mitochondrial Translation Elongation	14/91	4.59E-06	7.93E-05	0	0	4.930919363	60.61155849	MRPS17;MRPS24;MRPL18;MRPL16;MRPL27;MRPL36;MRPL14;MRPL48;MRPL34;MRPL24;MRPL54;MRPL4;MRPL42;CHCHD1
Mitochondrial Translation Initiation	14/91	4.59E-06	7.93E-05	0	0	4.930919363	60.61155849	MRPS17;MRPS24;MRPL18;MRPL16;MRPL27;MRPL36;MRPL14;MRPL48;MRPL34;MRPL24;MRPL54;MRPL4;MRPL42;CHCHD1
Mitochondrial Translation Termination	14/91	4.59E-06	7.93E-05	0	0	4.930919363	60.61155849	MRPS17;MRPS24;MRPL18;MRPL16;MRPL27;MRPL36;MRPL14;MRPL48;MRPL34;MRPL24;MRPL54;MRPL4;MRPL42;CHCHD1
Removal of the Flap Intermediate	6/14	5.08E-06	8.53E-05	0	0	20.18505587	246.0641609	PRIM2;FEN1;PCNA;PRIM1;RPA3;RPA2
Tryptophan Catabolism	6/14	5.08E-06	8.53E-05	0	0	20.18505587	246.0641609	TDO2;HAAO;KYAT3;KYAT1;ACMSD;IDO1
PCNA-Dependent Long Patch Base Excision Repair	7/21	5.8E-06	9.59E-05	0	0	13.47132867	162.4420759	RFC5;FEN1;PCNA;LIG1;RFC2;RPA3;RPA2
Triglyceride Metabolism	9/38	6.3E-06	1.03E-04	0	0	8.378439812	100.3250804	FABP1;FABP2;FABP3;DGAT2;MOGAT3;DGAT1;MOGAT2;AGMO;FABP7
Metabolism of Proteins	112/2067	7.15E-06	1.15E-04	0	0	1.626913756	19.27727398	SPON2;RPL32;RPL31;ECI1;PARK7;RPL7;GCSH;RPS14;RPS17;RAB42;ANPEP;RPL36;RPS11;HADH;CHCHD1;SLC34A2;RPS9;SLC30A8;CISH;IGFBP5;RPS8;FBXO2;SLC34A1;RPL22;IGFBP2;ACE2;PSME1;SUCLG2;STX1A
Arachidonate Metabolism	11/59	7.24E-06	1.15E-04	0	0	6.198136428	73.35879303	CBR1;PON3;GPX1;GPX4;PRXL2B;PON1;FAAH2;CYP1A1;LTA4H;GGT1;PTGR1
Formation of Xylulose-5-Phosphate	4/5	8.18E-06	1.26E-04	0	0	107.3927577	1257.960944	DCXR;AKR1A1;SORD;CRYL1
Tyrosine Catabolism	4/5	8.18E-06	1.26E-04	0	0	107.3927577	1257.960944	HGD;TAT;FAH;HPD
Mitochondrial Biogenesis	14/97	9.84E-06	1.46E-04	0	0	4.573037914	52.72378421	ATP5PF;ATP5PD;ATP5PB;ATP5MC3;ATP5F1C;SOD2;PRKAG3;ATP5F1E;ATP5MC1;APOO;ATP5PO;ATP5MK;ATP5MJ;ATP5ME
Mitochondrial Protein Degradation	14/97	9.84E-06	1.46E-04	0	0	4.573037914	52.72378421	ATP5PF;ATP5PD;NDUFB6;ECI1;ATP5F1C;COX5A;HSD17B10;SMDT1;ALDH2;ATP5PO;SUCLG2;NDUFV3;HADH;NDUFV1
Mitochondrial Translation	14/97	9.84E-06	1.46E-04	0	0	4.573037914	52.72378421	MRPS17;MRPS24;MRPL18;MRPL16;MRPL27;MRPL14;MRPL36;MRPL48;MRPL34;MRPL24;MRPL54;MRPL4;MRPL42;CHCHD1
Cellular Responses to Stress	53/787	1.02E-05	1.5E-04	0	0	2.001506987	23.00697848	CDKN1A;RPL32;RPL31;RPLP1;AQP8;COX6A1;COX7C;COX6A2;RPL7;RPS14;RPS17;RPL36;ME1;RPS11;RPS9;GPX1;RPS8;RPL22;RPL13A;APOA1;RPS3A;COX6B2;PSMA6;IMPACT;MRPL18;GSTP1;RPL11;RPL36A;COX7A
Synthesis of Bile Acids and Bile Salts via 7Alpha-Hydroxycholesterol	7/23	1.15E-05	1.66E-04	0	0	11.78618881	134.0747019	HSD3B7;AMACR;SCP2;NR1H4;ABCB11;CYP7A1;SLC27A2
Translesion Synthesis by REV1	6/16	1.27E-05	1.82E-04	0	0	16.14636872	181.9951715	RFC5;PCNA;RFC2;RPA3;RPA2;RPS27A
SLC-mediated Transmembrane Transport	24/249	1.29E-05	1.83E-04	0	0	2.911633238	32.77646216	SLC13A1;SLC34A2;SLC6A19;SLC15A1;SLC22A5;SLC30A8;SLC26A1;SLC13A4;SLC35D1;SLC34A1;SLC43A1;SLC2A2;SLC5A1;SLC5A8;SLC22A16;LCN15;SLC22A18;SLC17A5;APOD;RHBG;SLC28A1;SLC26A3;SLC25A4;
Chylomicron Assembly	5/10	1.31E-05	1.83E-04	0	0	26.88005579	302.2058753	APOC2;MTTP;APOA1;APOA4;APOB
Activation of the Pre-Replicative Complex	8/33	1.73E-05	2.36E-04	0	0	8.628795518	94.62144062	PRIM2;CDC45;PRIM1;RPA3;MCM3;RPA2;MCM5;MCM2
Synthesis of Bile Acids and Bile Salts	8/33	1.73E-05	2.36E-04	0	0	8.628795518	94.62144062	HSD3B7;AMACR;SCP2;NR1H4;ABCB11;CYP46A1;CYP7A1;SLC27A2
Cellular Responses to Stimuli	57/887	1.85E-05	2.5E-04	0	0	1.905129088	20.7600675	CDKN1A;RPL32;RPL31;RPLP1;AQP8;COX6A1;COX7C;COX6A2;RPL7;RPS14;RPS17;CAPNS1;CSRP1;RPL36;ME1;RPS11;RPS9;GPX1;ANXA2;RPS8;RPL22;RPL13A;APOA1;RPS3A;COX6B2;PSMA6;IMPACT;MRPL18;GSTF
Translesion Synthesis by POLI	6/17	1.91E-05	2.52E-04	0	0	14.67775521	159.5066592	RFC5;PCNA;RFC2;RPA3;RPA2;RPS27A
Translesion Synthesis by POLK	6/17	1.91E-05	2.52E-04	0	0	14.67775521	159.5066592	RFC5;PCNA;RFC2;RPA3;RPA2;RPS27A
Gap-filling DNA Repair Synthesis and Ligation in GG-NER	7/25	2.11E-05	2.73E-04	0	0	10.47552448	112.7720228	RFC5;PCNA;LIG1;RFC2;RPA3;RPA2;RPS27A
Resolution of AP Sites via the Multiple-Nucleotide Patch Replacement Pathway	7/25	2.11E-05	2.73E-04	0	0	10.47552448	112.7720228	RFC5;FEN1;PCNA;LIG1;RFC2;RPA3;RPA2
Bile Acid and Bile Salt Metabolism	9/44	2.26E-05	2.85E-04	0	0	6.93997195	74.24390212	HSD3B7;AMACR;SCP2;NR1H4;SLC51A;ABCB11;CYP46A1;CYP7A1;SLC27A2
Retinoid Metabolism and Transport	9/44	2.26E-05	2.85E-04	0	0	6.93997195	74.24390212	RBP4;TTR;RBP2;APOC2;APOA1;LRAT;APOA4;APOB;BCO1
Mitochondrial Fatty Acid Beta-Oxidation of Saturated Fatty Acids	5/11	2.33E-05	2.91E-04	0	0	22.39888424	238.9248767	HADHB;ACADL;ACADM;HADH;ACADS
Mitochondrial Fatty Acid Beta-Oxidation of Unsaturated Fatty Acids	4/6	2.38E-05	2.95E-04	0	0	53.69359331	571.521892	HADHB;ACADL;ECI1;ACADM
Telomere Maintenance	13/92	2.56E-05	3.14E-04	0	0	4.456035422	47.11020074	RFC5;PRIM2;FEN1;PCNA;LIG1;RFC2;DSCC1;PRIM1;RPA2;DKC1;RPA3;POLR2E;NOP10
G1 S Transition	15/121	3.09E-05	3.74E-04	0	0	3.837367564	39.85049369	PRIM2;CDKN1A;PCNA;PRIM1;RPA2;PTK6;TYMS;DHFR;PSMA6;CDC45;RPA3;MCM3;MCM5;RPS27A;MCM2
Glyoxylate Metabolism and Glycine Degradation	6/19	3.93E-05	4.62E-04	0	0	12.41834981	125.9737725	GCSH;GRHPR;DAO;GOT2;AMT;HOGA1
Plasma Lipoprotein Assembly	6/19	3.93E-05	4.62E-04	0	0	12.41834981	125.9737725	APOC2;MTTP;APOC1;APOA1;APOA4;APOB
Translesion Synthesis by POLH	6/19	3.93E-05	4.62E-04	0	0	12.41834981	125.9737725	RFC5;PCNA;RFC2;RPA3;RPA2;RPS27A
Activation of ATR in Response to Replication Stress	8/37	4.23E-05	4.92E-04	0	0	7.43707138	74.89671612	RFC5;CDC45;RFC2;RPA3;MCM3;RPA2;MCM5;MCM2
Complex IV Assembly	9/48	4.7E-05	5.35E-04	0	0	6.226885317	62.05639111	COA1;COX7A2;COX14;COX6A1;COX6B2;COX5A;COX6A2;COX7C;COA5
Metabolism of Fat-Soluble Vitamins	9/48	4.7E-05	5.35E-04	0	0	6.226885317	62.05639111	RBP4;TTR;RBP2;APOC2;APOA1;LRAT;APOA4;APOB;BCO1
Peroxisomal Lipid Metabolism	7/28	4.73E-05	5.35E-04	0	0	8.977622378	89.40184127	NUDT7;AMACR;SCP2;EHHADH;ECI2;ACAA1;SLC27A2
Plasma Lipoprotein Assembly, Remodeling, and Clearance	11/75	7.51E-05	8.4E-04	0	0	4.644734529	44.11112873	NCEH1;APOC2;MTTP;APOC1;ANGPTL3;APOA1;APOA4;APOB;RPS27A;PCSK5;CES3
Leading Strand Synthesis	5/14	9.23E-05	0.001003722	0	0	14.93026499	138.7160777	RFC5;PRIM2;PCNA;RFC2;PRIM1
Polymerase Switching	5/14	9.23E-05	0.001003722	0	0	14.93026499	138.7160777	RFC5;PRIM2;PCNA;RFC2;PRIM1
Triglyceride Biosynthesis	5/14	9.23E-05	0.001003722	0	0	14.93026499	138.7160777	DGAT2;DGAT1;MOGAT3;MOGAT2;AGMO
The Canonical Retinoid Cycle in Rods (Twilight Vision)	6/22	9.85E-05	0.001062053	0	0	10.08833799	93.06720984	RBP4;TTR;DHRS9;CYP4V2;LRAT;STRA6
Chromosome Maintenance	14/119	1E-04	0.001070898	0	0	3.610734463	33.24692401	RFC5;PRIM2;CENPU;FEN1;PCNA;LIG1;RFC2;DSCC1;PRIM1;RPA2;DKC1;RPA3;POLR2E;NOP10
Phase I - Functionalization of Compounds	13/105	1.04E-04	0.001101295	0	0	3.823787331	35.05802404	AOC1;EPHX1;CYP51A1;NR1H4;AHR;CYP46A1;CYP7A1;ALDH2;CYP4V2;CYP2R1;CYP1A1;CES3;CES2
Vitamin C (Ascorbate) Metabolism	4/8	1.05E-04	0.001101295	0	0	26.84401114	245.9392694	CYB5A;GSTO2;GSTO1;SLC23A1
Metabolism of RNA	48/761	1.33E-04	0.001377878	0	0	1.85432889	16.55701806	SF3B5;POP5;RPL32;TP53RK;RPL31;RPLP1;NIP7;RPL11;RPL36A;ZBTB8OS;DDX21;SNU13;HSD17B10;OSGEP;RPL7;RPS14;FBL;EXOSC5;RPS17;RPL36;POLR2E;RPS27A;RPS11;RPL17;EXOSC2;PPIL1;RPS9;APOBEC2;F
Mitotic G1 Phase and G1 S Transition	15/139	1.54E-04	0.001582659	0	0	3.277250536	28.77909034	PRIM2;CDKN1A;PCNA;PRIM1;RPA2;PTK6;TYMS;DHFR;PSMA6;CDC45;RPA3;MCM3;MCM5;RPS27A;MCM2
Plasma Lipoprotein Remodeling	7/34	1.78E-04	0.001819902	0	0	6.98041958	60.26240783	APOC2;MTTP;ANGPTL3;APOA1;APOA4;APOB;PCSK5
Atorvastatin ADME	4/9	1.84E-04	0.001843377	0	0	21.47409471	184.7417597	PON3;ABCB1;ABCC2;PON1
Synthesis of 5-Eicosatetraenoic Acids	4/9	1.84E-04	0.001843377	0	0	21.47409471	184.7417597	PON3;GPX1;GPX4;PON1
Metabolism of Water-Soluble Vitamins and Cofactors	14/127	2.02E-04	0.00200679	0	0	3.353707315	28.53856628	MCCC2;CYB5A;PRSS1;GSTO2;GSTO1;SLC23A1;CTRB2;CTRB1;MTHFS;DHFR;SLC5A8;MTHFD1;TCN2;SLC19A3
Metabolism of Nucleotides	12/98	2.12E-04	0.002091683	0	0	3.771765477	31.90795206	DUT;GDA;DPYS;GSR;NME2;ENTPD8;GLRX;TYMS;DNPH1;XDH;ADA;TYMP
SLC Transporter Disorders	12/99	2.33E-04	0.002265413	0	0	3.728217581	31.17994579	SLC34A2;SLC6A19;GCKR;SLC22A5;SLC34A1;SLC22A18;SLC17A5;SLC2A2;SLC5A1;SLC26A3;SLC39A4;GCK
Visual Phototransduction	12/99	2.33E-04	0.002265413	0	0	3.728217581	31.17994579	RBP4;TTR;RBP2;DHRS9;CYP4V2;APOC2;APOA1;LRAT;APOA4;APOB;BCO1;STRA6
Endogenous Sterols	6/26	2.69E-04	0.002567357	0	0	8.068994413	66.34099055	CYP4V2;CYP51A1;NR1H4;AHR;CYP46A1;CYP7A1
Polymerase Switching on the C-strand of the Telomere	6/26	2.69E-04	0.002567357	0	0	8.068994413	66.34099055	RFC5;PRIM2;PCNA;RFC2;DSCC1;PRIM1
Chylomicron Remodeling	4/10	2.97E-04	0.0028161	0	0	17.89415042	145.3209783	APOC2;APOA1;APOA4;APOB
Neutrophil Degranulation	33/478	3.06E-04	0.002880715	0	0	2.027002169	16.39931216	CFD;SERPINA1;GSTP1;FGL2;MGST1;MOSPD2;SLC2A5;PLAC8;PGRMC1;TTR;PLAU;ANPEP;FTH1;LTA4H;CD36;ACAA1;GYG1;S100A11;SERPINB1;AOC1;GSN;ANXA2;DGAT1;AHSG;IDH1;TMT1A;NME2;NDUFC2;MIF;CEA
Resolution of Abasic Sites (AP Sites)	7/38	3.69E-04	0.003436971	0	0	6.078457027	48.0542079	RFC5;FEN1;PCNA;LIG1;RFC2;RPA3;RPA2
Metabolism of Carbohydrates	23/290	3.71E-04	0.003436971	0	0	2.342849335	18.50382619	GCKR;SLC26A1;PKLR;GLYCTK;DCXR;RPE;OMD;HEXA;PGAM2;AKR1A1;SORD;AKR1B1;GCK;GYS2;GNPDA1;PPP1R3C;HKDC1;CRYL1;RPS27A;GYG1;FBP1;PFKM;RBKS
Beta Oxidation of hexanoyl-CoA to butanoyl-CoA	3/5	4.44E-04	0.003949196	0	0	40.21418637	310.4722121	HADHB;HADH;ACADS
Beta Oxidation of lauroyl-CoA to decanoyl-CoA-CoA	3/5	4.44E-04	0.003949196	0	0	40.21418637	310.4722121	HADHB;ACADL;HADH
Beta Oxidation of octanoyl-CoA to hexanoyl-CoA	3/5	4.44E-04	0.003949196	0	0	40.21418637	310.4722121	HADHB;ACADM;HADH
Intestinal Absorption	3/5	4.44E-04	0.003949196	0	0	40.21418637	310.4722121	SLC2A2;SLC5A1;SLC2A5
VLDL Assembly	3/5	4.44E-04	0.003949196	0	0	40.21418637	310.4722121	APOC1;MTTP;APOB
Beta-oxidation of Very Long Chain Fatty Acids	4/11	4.54E-04	0.004008199	0	0	15.33704735	118.0659352	EHHADH;ECI2;ACAA1;SLC27A2
Processive Synthesis on the C-strand of the Telomere	5/19	4.61E-04	0.004043319	0	0	9.59553696	73.71169561	FEN1;PCNA;LIG1;RPA3;RPA2
Drug ADME	12/109	5.7E-04	0.004962994	0	0	3.34212284	24.96396865	PON3;ABCB1;ABCC2;TPMT;GSTP1;PON1;NME2;GGT1;XDH;CES2;ADA;CNDP2
Dual Incision in GG-NER	7/41	5.98E-04	0.005166025	0	0	5.541258741	41.12733499	RFC5;PCNA;RFC2;RPA3;RPA2;RPS27A;DDB2
Recognition of DNA Damage by PCNA-containing Replication Complex	6/30	6.13E-04	0.0052572	0	0	6.722765363	49.72963527	RFC5;PCNA;RFC2;RPA3;RPA2;RPS27A
SARS-CoV-1-host Interactions	12/112	7.29E-04	0.006204827	0	0	3.241352113	23.41616778	RPS14;RPS25;RPS17;RPS9;PKLR;RPS8;RPS20;RPS3A;FAU;RPS11;RPS27A;RPS24
Digestion	5/21	7.6E-04	0.006424747	0	0	8.395223152	60.29573077	GUCY2C;AMY2A;ALPI;CEL;LCT
Beta Oxidation of decanoyl-CoA to octanoyl-CoA-CoA	3/6	8.63E-04	0.007195597	0	0	26.80806676	189.1188333	HADHB;ACADM;HADH
Scavenging by Class B Receptors	3/6	8.63E-04	0.007195597	0	0	26.80806676	189.1188333	APOA1;CD36;APOB
Termination of Translesion DNA Synthesis	6/32	8.8E-04	0.007280399	0	0	6.204984959	43.65682192	RFC5;PCNA;RFC2;RPA3;RPA2;RPS27A
Alpha-Linolenic (Omega3) and Linoleic (Omega6) Acid Metabolism	4/13	9.28E-04	0.007366337	0	0	11.9275766	83.28604343	FADS2;SCP2;ELOVL2;ACAA1
Alpha-Linolenic Acid (ALA) Metabolism	4/13	9.28E-04	0.007366337	0	0	11.9275766	83.28604343	FADS2;SCP2;ELOVL2;ACAA1
Diseases Associated With Visual Transduction	4/13	9.28E-04	0.007366337	0	0	11.9275766	83.28604343	RBP4;TTR;LRAT;STRA6
Diseases of the Neuronal System	4/13	9.28E-04	0.007366337	0	0	11.9275766	83.28604343	RBP4;TTR;LRAT;STRA6
Retinoid Cycle Disease Events	4/13	9.28E-04	0.007366337	0	0	11.9275766	83.28604343	RBP4;TTR;LRAT;STRA6
Synthesis of Bile Acids and Bile Salts via 24-Hydroxycholesterol	4/13	9.28E-04	0.007366337	0	0	11.9275766	83.28604343	HSD3B7;AMACR;CYP46A1;SLC27A2
Peptide Hormone Metabolism	10/86	0.001053	0.008254246	0	0	3.548565937	24.32689476	DPP4;ACE2;GH1;ENPEP;SLC30A8;CPB1;ANPEP;STX1A;GIP;GNG13
TP53 Regulates Metabolic Genes	10/86	0.001053	0.008254246	0	0	3.548565937	24.32689476	PRDX2;GLS2;GSR;COX7A2;COX6A1;COX6B2;COX6A2;COX7C;COX5A;PRKAG3
Cytoprotection by HMOX1	8/59	0.001208	0.00940719	0	0	4.224089636	28.37753808	FABP1;COX7A2;PTK6;COX6A1;COX6B2;COX6A2;COX7C;COX5A

Diseases of Carbohydrate Metabolism	6/34	0.0012282	0.009494691	0	0	5.761173184	38.61233551	GYS2;PPP1R3C;DCXR;RPS27A;LCT;GYG1
Glycogen Synthesis	4/14	0.001262	0.009567395	0	0	10.73426184	71.65111814	GYS2;PPP1R3C;RPS27A;GYG1
Mismatch Repair (MMR) Directed by MSH2 MSH3 (MutSbeta)	4/14	0.001262	0.009567395	0	0	10.73426184	71.65111814	PCNA;LIG1;RPA3;RPA2
Mismatch Repair (MMR) Directed by MSH2 MSH6 (MutSalpha)	4/14	0.001262	0.009567395	0	0	10.73426184	71.65111814	PCNA;LIG1;RPA3;RPA2
Nucleotide Catabolism	6/35	0.0014377	0.010829036	0	0	5.562223078	36.40303485	GDA;DPYS;ENTPD8;DNPH1;XDH;TYMP
Fructose Metabolism	3/7	0.0014705	0.01100574	0	0	20.10500695	131.1271867	GLYCTK;SORD;AKR1B1
ABC-family Proteins Mediated Transport	10/90	0.0014969	0.011132297	0	0	3.370435393	21.92243455	ABCG8;PSMA6;ABCG5;ABCD3;ABCC2;ABCB1;ABCB4;ABCB5;APOA1;RPS27A
Diseases of Metabolism	20/264	0.0015241	0.011263088	0	0	2.222455747	14.41561174	MCCC2;SPON2;SLC34A2;SLC35D1;TPMT;IDH1;DCXR;OMD;HEXA;PPM1K;GYS2;PPP1R3C;TCN2;PAH;CYP2R1;RPS27A;GGT1;LCT;GYG1;ADA
Mismatch Repair	4/15	0.0016721	0.012238531	0	0	9.757913396	62.38838518	PCNA;LIG1;RPA3;RPA2
Gap-filling DNA Repair Synthesis and Ligation in TC-NER	8/62	0.0016769	0.012238531	0	0	3.988795518	25.49153495	RFC5;PCNA;LIG1;RFC2;RPA3;POLR2E;RPA2;RPS27A
SARS-CoV-1 Infection	14/157	0.0016902	0.012259725	0	0	2.64598396	16.8889528	RPS9;PKLR;RPS8;RPS3A;RPS14;ACE2;RPS25;RPS17;CHMP4C;RPS20;FAU;RPS11;RPS27A;RPS24
Plasma Lipoprotein Clearance	6/37	0.0019371	0.013964273	0	0	5.202829339	32.49961072	NCEH1;APOC1;APOA1;APOB;RPS27A;CES3
Glycogen Storage Diseases	4/16	0.0021664	0.015521567	0	0	8.944289694	54.87037142	GYS2;PPP1R3C;RPS27A;GYG1
ABC Transporter Disorders	8/65	0.0022777	0.016111618	0	0	3.778269202	22.9891125	ABCG8;PSMA6;ABCG5;ABCC2;ABCB4;APOA1;ABCB11;RPS27A
Cytochrome P450 - Arranged by Substrate Type	8/65	0.0022777	0.016111618	0	0	3.778269202	22.9891125	CYP4V2;CYP2R1;CYP51A1;NR1H4;CYP1A1;AHR;CYP46A1;CYP7A1
Histidine Catabolism	3/8	0.0022899	0.016111618	0	0	16.08317107	97.77352597	AMDHD1;HNMT;FTCD
Platelet Degranulation	12/128	0.0023249	0.016260682	0	0	2.791937834	16.93049163	CFD;CYB5R1;TF;SCCPDH;SERPINA1;AHSG;SERPINF2;ANXA5;APOA1;CD36;PFN1;SOD1
Translesion Synthesis by Y Family DNA Polymerases Bypasses Lesions on DNA Template	6/39	0.0025577	0.017783396	0	0	4.886998476	29.16861135	RFC5;PCNA;RFC2;RPA3;RPA2;RPS27A
Metabolism of Angiotensinogen to Angiotensins	4/17	0.0027528	0.018915668	0	0	8.255838869	48.66919854	ACE2;ENPEP;CPB1;ANPEP
Removal of the Flap Intermediate From the C-strand	4/17	0.0027528	0.018915668	0	0	8.255838869	48.66919854	FEN1;PCNA;RPA3;RPA2
Response to Elevated Platelet Cytosolic Ca2+	12/133	0.0031957	0.021831448	0	0	2.675870097	15.37536951	CFD;CYB5R1;TF;SCCPDH;SERPINA1;AHSG;SERPINF2;ANXA5;APOA1;CD36;PFN1;SOD1
Transport of Bile Salts and Organic Acids, Metal Ions and Amine Compounds	9/84	0.0032609	0.022071874	0	0	3.231921459	18.50509782	SLC13A1;SLC6A19;SLC13A4;SLC30A8;SLC22A5;SLC22A16;SLC22A18;RHBG;SLC39A4
Transcriptional Regulation by TP53	24/362	0.0032685	0.022071874	0	0	1.926722165	11.02743881	RFC5;CDKN1A;PCNA;TP53RK;RFC2;GLS2;GSR;RPA2;COX7A2;NOC2L;COX6A1;COX6B2;COX7C;COX5A;COX6A2;PRKAG3;DDB2;PRDX2;ING5;AIFM2;RPA3;SMYD2;POLR2E;RPS27A
Myoclonic Epilepsy of Lafora	3/9	0.003343	0.022446449	0	0	13.40194715	76.40262827	PPP1R3C;RPS27A;GYG1
PPARA Activates Gene Expression	11/117	0.0034028	0.022717823	0	0	2.798237932	15.90279173	FABP1;ABCB4;CYP1A1;ANKRD1;ME1;NR1H4;APOA1;AHR;ACADM;CD36;CYP7A1
ABC Transporters in Lipid Homeostasis	4/18	0.0034393	0.022831703	0	0	7.665738162	43.48373806	ABCG8;ABCG5;ABCD3;APOA1
Regulation of Lipid Metabolism by PPARalpha	11/119	0.0038768	0.025591609	0	0	2.746132208	15.24853238	FABP1;ABCB4;CYP1A1;ANKRD1;ME1;NR1H4;APOA1;AHR;ACADM;CD36;CYP7A1
Interconversion of Nucleotide Di- and Triphosphates	5/30	0.0040709	0.026722757	0	0	5.370432357	29.55820119	DUT;GSR;NME2;GLRX;TYMS
Aflatoxin Activation and Detoxification	4/19	0.0042335	0.027331813	0	0	7.154317549	39.09634709	AKR7A3;MGST3;MGST1;GGT1
Assembly of Active LPL and LIPC Lipase Complexes	4/19	0.0042335	0.027331813	0	0	7.154317549	39.09634709	APOC2;ANGPTL3;APOA4;PCSK5
Biosynthesis of Specialized Proresolving Mediators (SPMs)	4/19	0.0042335	0.027331813	0	0	7.154317549	39.09634709	GPX4;CYP1A1;LTA4H;PTGR1
Organic Cation Transport	3/10	0.0046484	0.029524126	0	0	11.4867872	61.69800507	SLC22A5;SLC22A16;SLC22A18
Thyroxine Biosynthesis	3/10	0.0046484	0.029524126	0	0	11.4867872	61.69800507	DUOX1;IYD;DUOX2
Uptake of Dietary Cobalamins Into Enterocytes	3/10	0.0046484	0.029524126	0	0	11.4867872	61.69800507	PRSS1;CTRB2;CTRB1
Transcription-Coupled Nucleotide Excision Repair (TC-NER)	8/76	0.0060264	0.038070318	0	0	3.165266106	16.1795651	RFC5;PCNA;LIG1;RFC2;RPA3;POLR2E;RPA2;RPS27A
Glycerophospholipid Biosynthesis	11/128	0.0066999	0.042098647	0	0	2.533701179	12.68283269	HADHB;DUSP23;PCYT1B;DGAT2;DGAT1;PLA2G1B;GPD1;ALPI;CHPT1;PLA1A;ETNPPL
DNA Damage Bypass	6/48	0.007320	0.045388498	0	0	3.837988827	18.87190478	RFC5;PCNA;RFC2;RPA3;RPA2;RPS27A
Cobalamin (Cbl, Vitamin B12) Transport and Metabolism	4/22	0.0073338	0.045388498	0	0	5.961002786	29.29986098	PRSS1;TCN2;CTRB2;CTRB1
Muscle Contraction	15/204	0.0073394	0.045388498	0	0	2.142857143	10.53106264	ANXA1;MYBPC2;ANXA2;TMOD4;TNNC1;TPM2;TNNC2;ATP1A1;DES;ALDH2;TNNT1;MYL2;TNNT3;TNNI2;CASQ1
Dual Incision in TC-NER	7/63	0.0074373	0.045753584	0	0	3.36048951	16.47054933	RFC5;PCNA;RFC2;RPA3;POLR2E;RPA2;RPS27A
Erythrocytes Take up Carbon Dioxide and Release Oxygen	3/12	0.0080749	0.048907819	0	0	8.933240612	43.04913697	CYB5R2;CYB5R1;CA4
Lysine Catabolism	3/12	0.0080749	0.048907819	0	0	8.933240612	43.04913697	GCDH;PIPOX;ALDH7A1
O2 CO2 Exchange in Erythrocytes	3/12	0.0080749	0.048907819	0	0	8.933240612	43.04913697	CYB5R2;CYB5R1;CA4
Mitochondrial Protein Import	7/65	0.008800	0.053025735	0	0	3.244272968	15.35514977	TIMM8B;NDUFB8;CMC4;CHCHD7;CHCHD10;SLC25A4;ATP5MC1
Triglyceride Catabolism	4/24	0.0100639	0.060037473	0	0	5.364345404	24.66950265	FABP1;FABP2;FABP3;FABP7
Gene and Protein Expression by JAK-STAT Signaling After Interleukin-12 Stimulation	5/37	0.0101256	0.060037473	0	0	4.194124826	19.26230019	ANXA2;GSTO1;MIF;SOD2;SOD1
Dissolution of Fibrin Clot	3/13	0.0102191	0.060037473	0	0	8.039499305	36.84898417	ANXA2;PLAU;SERPINF2
Glutathione Synthesis and Recycling	3/13	0.0102191	0.060037473	0	0	8.039499305	36.84898417	CHAC2;GGT1;CNDP2
Passive Transport by Aquaporins	3/13	0.0102191	0.060037473	0	0	8.039499305	36.84898417	AQP12A;AQP10;AQP8
DNA Replication Pre-Initiation	10/118	0.0104244	0.060938894	0	0	2.492977528	11.37695768	PSMA6;PRIM2;CDC45;PRIM1;RPA3;MCM3;RPA2;MCM5;RPS27A;MCM2
Global Genome Nucleotide Excision Repair (GG-NER)	8/84	0.0108561	0.063148125	0	0	2.830900781	12.80424306	RFC5;PCNA;LIG1;RFC2;RPA3;RPA2;RPS27A;DDB2
Glucose Metabolism	8/85	0.0116169	0.066748743	0	0	2.793990323	12.44804492	GCKR;GNPDA1;PKLR;HKDC1;PGAM2;FBP1;PFKM;GCK
Defective Intrinsic Pathway for Apoptosis	4/25	0.0116455	0.066748743	0	0	5.108635097	22.74790025	PRDX2;CAPNS1;C1QBP;SOD2
Glycogen Metabolism	4/25	0.0116455	0.066748743	0	0	5.108635097	22.74790025	GYS2;PPP1R3C;RPS27A;GYG1
Beta Oxidation of butanoyl-CoA to acetyl-CoA	2/5	0.0121024	0.068040119	0	0	17.84722222	78.78383078	HADH;ACADS
Biosynthesis of E-series 18(S)-resolvins	2/5	0.0121024	0.068040119	0	0	17.84722222	78.78383078	GPX4;LTA4H
Sodium-coupled Phosphate Cotransporters	2/5	0.0121024	0.068040119	0	0	17.84722222	78.78383078	SLC34A2;SLC34A1
Sodium-coupled Sulphate, Di- and Tri-Carboxylate Transporters	2/5	0.0121024	0.068040119	0	0	17.84722222	78.78383078	SLC13A1;SLC13A4
Glutamate and Glutamine Metabolism	3/14	0.0126619	0.070178463	0	0	7.308256417	31.93087559	GLS2;GOT2;KYAT1
Methylation	3/14	0.0126619	0.070178463	0	0	7.308256417	31.93087559	TPMT;MAT2A;GSTO1
Synthesis of Bile Acids and Bile Salts via 27-Hydroxycholesterol	3/14	0.0126619	0.070178463	0	0	7.308256417	31.93087559	HSD3B7;NR1H4;CYP7A1
NR1H2 and NR1H3-mediated Signaling	6/54	0.0128548	0.07091274	0	0	3.357192737	14.6173395	ABCG8;ABCG5;APOC2;APOC1;ANGPTL3;APOD
Disease	96/2131	0.0130315	0.071551608	0	0	1.299407336	5.63992644	SPON2;SH3GL3;CDKN1A;FEN1;RPL32;TPMT;RPL31;RPLP1;TAT;HEXA;SLC2A2;RPL7;GYS2;RPS14;RPS17;CAPNS1;SLC22A18;C1QBP;CYP2R1;RPL36;LRAT;AP1S3;CD36;RPS11;GGT1;GYG1;SLC39A4;MBL2;SH3GL1;RF
Base Excision Repair	7/71	0.0139834	0.076421103	0	0	2.939204545	12.55005602	RFC5;FEN1;PCNA;LIG1;RFC2;RPA3;RPA2
Regulation of IGF Transport and Uptake by Insulin-like Growth Factor Binding Proteins (IGFBPs)	10/124	0.014430	0.078497033	0	0	2.361028977	10.00707841	TF;SERPINA1;IGFBP5;SERPIND1;AHSG;IGFBP2;APOA1;IGFBP6;MELTF;APOB
Interleukin-12 Family Signaling	6/56	0.015217	0.082396654	0	0	3.222569832	13.48753921	ANXA2;GSTO1;EBI3;MIF;SOD2;SOD1
Transport of Inorganic Cations Anions and Amino Acids Oligopeptides	9/107	0.0153716	0.08267623	0	0	2.470446804	10.31468619	SLC34A2;SLC15A1;SLC6A19;SLC5A8;SLC26A1;SLC34A1;SLC43A1;SLC17A5;SLC26A3
Organic Cation Anion Zwitterion Transport	3/15	0.0154094	0.08267623	0	0	6.698887344	27.95294855	SLC22A5;SLC22A16;SLC22A18
Nucleotide Excision Repair	9/108	0.0162513	0.086797212	0	0	2.445365294	10.07386979	RFC5;PCNA;LIG1;RFC2;RPA3;POLR2E;RPA2;RPS27A;DDB2
Glycolysis	7/74	0.0172631	0.091783689	0	0	2.807160004	11.39477168	GCKR;GNPDA1;PKLR;HKDC1;PGAM2;PFKM;GCK
Biosynthesis of EPA-derived SPMs	2/6	0.0177219	0.092138538	0	0	13.38472222	53.97992105	GPX4;LTA4H
LDL Remodeling	2/6	0.0177219	0.092138538	0	0	13.38472222	53.97992105	MTTP;APOB
Phenylalanine Metabolism	2/6	0.0177219	0.092138538	0	0	13.38472222	53.97992105	PAH;KYAT1
Synthesis of 15-Eicosatetraenoic Acid Derivatives	2/6	0.0177219	0.092138538	0	0	13.38472222	53.97992105	GPX1;GPX4
VLDL Clearance	2/6	0.0177219	0.092138538	0	0	13.38472222	53.97992105	APOC1;APOB
Regulation of TP53 Activity Through Phosphorylation	8/92	0.0180816	0.093594505	0	0	2.56022409	10.2738132	RFC5;TP53RK;RFC2;RPA3;RPA2;NOC2L;RPS27A;PRKAG3
InlB-mediated Entry of Listeria Monocytogenes Into Host Cell	3/16	0.0184655	0.095162285	0	0	6.183267359	24.682672	SH3GL3;RPS27A;SH3GL1
Activation of Gene Expression by SREBF (SREBP)	5/43	0.0187508	0.095792441	0	0	3.530793511	14.04025552	SQLE;CYP51A1;SC5D;MVD;LSS
Transport of Vitamins, Nucleosides, and Related Molecules	5/43	0.0187508	0.095792441	0	0	3.530793511	14.04025552	SLC35D1;LCN15;APOD;SLC25A4;SLC28A1
G1 S-Specific Transcription	4/29	0.0195252	0.099316811	0	0	4.290362117	16.88706326	DHFR;PCNA;CDC45;TYMS
NR1H3 & NR1H2 Regulate Gene Expression Linked to Cholesterol Transport and Efflux	5/44	0.0205454	0.104055424	0	0	3.440081536	13.36512152	ABCG8;ABCG5;APOC2;APOC1;APOD
Biosynthesis of DHA-derived SPMs	3/17	0.0218325	0.109050219	0	0	5.741307371	21.95678859	GPX4;CYP1A1;LTA4H
Metabolism of Folate and Pterines	3/17	0.0218325	0.109050219	0	0	5.741307371	21.95678859	DHFR;MTHFD1;MTHFS
Purine Catabolism	3/17	0.0218325	0.109050219	0	0	5.741307371	21.95678859	GDA;DNPH1;XDH
Paracetamol ADME	4/30	0.0219028	0.109050219	0	0	4.125133919	15.76270764	ABCC2;GSTP1;GGT1;CNDP2
Acyl Chain Remodeling of DAG and TAG	2/7	0.0242225	0.118097486	0	0	10.70722222	39.83591338	DGAT2;DGAT1
Glycine Degradation	2/7	0.0242225	0.118097486	0	0	10.70722222	39.83591338	GCSH;AMT
Mitochondrial Uncoupling	2/7	0.0242225	0.118097486	0	0	10.70722222	39.83591338	PM20D1;SLC25A4
Synthesis of 12-Eicosatetraenoic Acid Derivatives	2/7	0.0242225	0.118097486	0	0	10.70722222	39.83591338	GPX1;GPX4
TYSND1 Cleaves Peroxisomal Proteins	2/7	0.0242225	0.118097486	0	0	10.70722222	39.83591338	SCP2;ACAA1
Interleukin-12 Signaling	5/46	0.0244621	0.118773037	0	0	3.27193251	12.14091967	ANXA2;GSTO1;MIF;SOD2;SOD1
Metabolism of Amine-Derived Hormones	3/18	0.0255111	0.122851056	0	0	5.358275382	19.65757152	DUOX1;IYD;DUOX2
Recycling of Bile Acids and Salts	3/18	0.0255111	0.122851056	0	0	5.358275382	19.65757152	NR1H4;SLC51A;ABCB11
rRNA Modification in the Nucleus and Cytosol	6/63	0.0258214	0.123837512	0	0	2.825786533	10.33263024	FBL;RPS14;RPS9;DKC1;SNU13;NOP10
G2 M Checkpoints	10/137	0.0268990	0.128481373	0	0	2.117911174	7.657653558	RFC5;PSMA6;CDC45;RFC2;RPA3;MCM3;RPA2;MCM5;RPS27A;MCM2
LDL Clearance	3/19	0.0295007	0.139771791	0	0	5.023122392	17.69816267	NCEH1;APOB;CES3
Scavenging by Class A Receptors	3/19	0.0295007	0.139771791	0	0	5.023122392	17.69816267	FTH1;APOA1;APOB
Pyruvate Metabolism	5/49	0.0311852	0.145873225	0	0	3.048370737	10.57116713	FAHD1;PKLR;MPC2;ME1;RPS27A
DNA Replication Initiation	2/8	0.0315334	0.145873225	0	0	8.922222222	30.84150371	PRIM2;PRIM1
Linoleic Acid (LA) Metabolism	2/8	0.0315334	0.145873225	0	0	8.922222222	30.84150371	FADS2;ELOVL2
Metabolism of Ingested SeMet, Sec, MeSec Into H2Se	2/8	0.0315334	0.145873225	0	0	8.922222222	30.84150371	CBS;HNMT
POLB-Dependent Long Patch Base Excision Repair	2/8	0.0315334	0.145873225	0	0	8.922222222	30.84150371	FEN1;LIG1
Transport of Fatty Acids	2/8	0.0315334	0.145873225	0	0	8.922222222	30.84150371	LCN15;APOD
Metabolic Disorders of Biological Oxidation Enzymes	4/34	0.0331259	0.152639125	0	0	3.574373259	12.17945772	SLC35D1;TPMT;CYP2R1;GGT1
Synthesis of Leukotrienes (LT) and Eoxins (EX)	3/20	0.0337992	0.155133289	0	0	4.727399166	16.0131976	LTA4H;PTGR1;GGT1
Impaired BRCA2 Binding to RAD51	4/35	0.0363681	0.166274614	0	0	3.458891185	11.46297948	RFC5;RFC2;RPA3;RPA2
HDR Through Homologous Recombination (HRR)	6/69	0.0381049	0.172889203	0	0	2.555865922	8.351062056	RFC5;PCNA;RFC2;RPA3;RPA2;RPS27A
Branched-chain Amino Acid Catabolism	3/21	0.0384034	0.172889203	0	0	4.464534075	14.5526281	MCCC2;PPM1K;HSD17B10
Negative Regulation of MET Activity	3/21	0.0384034	0.172889203	0	0	4.464534075	14.5526281	SH3GL3;RPS27A;SH3GL1
Regulation of Gene Expression in Beta Cells	3/21	0.0384034	0.172889203	0	0	4.464534075	14.5526281	PKLR;SLC2A2;GCK
Beta-oxidation of pristanoyl-CoA	2/9	0.0395878	0.174871144	0	0	7.647222222	24.69466248	AMACR;SCP2
Digestion of Dietary Carbohydrate	2/9	0.0395878	0.174871144	0	0	7.647222222	24.69466248	AMY2A;LCT
Malate-aspartate Shuttle	2/9	0.0395878	0.174871144	0	0	7.647222222	24.69466248	MDH1;GOT2
Multifunctional Anion Exchangers	2/9	0.0395878	0.174871144	0	0	7.647222222	24.69466248	SLC26A1;SLC26A3
PTK6 Regulates RTKs and Their Effectors AKT1 and DOK1	2/9	0.0395878	0.174871144	0	0	7.647222222	24.69466248	PTK6;RPS27A
Post-translational Protein Phosphorylation	8/107	0.0398827	0.175514174	0	0	2.1706137	6.993305459	TF;SERPINA1;IGFBP5;SERPIND1;AHSG;APOA1;MELTF;APOB
Deregulated CDK5 Triggers Multiple Neurodegenerative Pathways in Alzheimer's Disease Models	3/22	0.0433092	0.187404235	0	0	4.229338994	13.27754399	PRDX2;CAPNS1;SOD2
Listeria Monocytogenes Entry Into Host Cells	3/22	0.0433092	0.187404235	0	0	4.229338994	13.27754399	SH3GL3;RPS27A;SH3GL1
Neurodegenerative Diseases	3/22	0.0433092	0.187404235	0	0	4.229338994	13.27754399	PRDX2;CAPNS1;SOD2
RA Biosynthesis Pathway	3/22	0.0433092	0.187404235	0	0	4.229338994	13.27754399	SDR16C5;DHRS9;DHRS4
HDR Through Single Strand Annealing (SSA)	4/37	0.043382	0.187404235	0	0	3.248923778	10.19417631	RFC5;RFC2;RPA3;RPA2
Creatine Metabolism	2/10	0.0483227	0.206469755	0	0	6.690972222	20.27266675	CKMT2;GATM
HDL Remodeling	2/10	0.0483227	0.206469755	0	0	6.690972222	20.27266675	APOC2;APOA1
Protein Lipoylation	2/10	0.0483227	0.206469755	0	0	6.690972222	20.27266675	GCSH;NDUFAB1
Azathioprine ADME	3/23	0.0485113	0.206524634	0	0	4.017663421	12.15728134	TPMT;NME2;XDH
Regulation of Cholesterol Biosynthesis by SREBP (SREBF)	5/56	0.0510187	0.215987452	0	0	2.629009763	7.822783512	SQLE;CYP51A1;SC5D;MVD;LSS
Glycosphingolipid Catabolism	4/39	0.0511017	0.215987452	0	0	3.062952646	9.109029059	NEU3;ASAH2;HEXA;ENPP7
SARS-CoV-2-host Interactions	13/218	0.0533468	0.224668534	0	0	1.705934157	4.999992007	RPS9;RPS8;RPS3A;RPS14;RPS25;RPS17;RPS20;SNRPE;FAU;RPS11;RPS27A;RPS24;MBL2
Incretin Synthesis, Secretion, and Inactivation	3/24	0.0540038	0.226623088	0	0	3.826147427	11.16737982	DPP4;GIP;GNG13
Presynaptic Phase of Homologous DNA Pairing and Strand Exchange	4/40	0.0552246	0.230921684	0	0	2.977715877	8.624493728	RFC5;RFC2;RPA3;RPA2
Cell Cycle, Mitotic	26/516	0.0554744	0.231143493	0	0	1.432348112	4.142111446	PRIM2;CDKN1A;FEN1;PCNA;PRIM1;TYMS;CDC45;RPS27A;RFC5;GINS1;CENPU;GINS2;LIG1;RFC2;GINS3;GINS4;RPA2;PTK6;DHFR;PSMA6;RPA3;MCM3;CHMP4C;MCM5;MAPRE1;MCM2
Ribavirin ADME	2/11	0.0576785	0.238208706	0	0	5.947222222	16.96665238	NME2;ADA
Glycosphingolipid Metabolism	5/58	0.0577782	0.238208706	0	0	2.529538696	7.21207483	NEU3;ASAH2;B3GNT5;HEXA;ENPP7
Orc1 Removal From Chromatin	5/58	0.0577782	0.238208706	0	0	2.529538696	7.21207483	PSMA6;MCM3;MCM5;RPS27A;MCM2
Defective Homologous Recombination Repair (HRR) Due to BRCA2 Loss of Function	4/41	0.0595215	0.24368598	0	0	2.897086502	8.173886389	RFC5;RFC2;RPA3;RPA2
Diseases of DNA Double-Strand Break Repair	4/41	0.0595215	0.24368598	0	0	2.897086502	8.173886389	RFC5;RFC2;RPA3;RPA2
Diseases of Programmed Cell Death	6/78	0.0625762	0.255302342	0	0	2.235335196	6.194940293	PRIM2;PRDX2;CAPNS1;C1QBP;PRIM1;SOD2
Regulation of Beta-Cell Development	4/42	0.0639909	0.260170899	0	0	2.820700777	7.754143952	NR5A2;PKLR;SLC2A2;GCK
Regulation of TP53 Activity	10/160	0.0649730	0.263252963	0	0	1.791011236	4.896234955	RFC5;ING5;TP53RK;RFC2;RPA3;SMYD2;RPA2;NOC2L;RPS27A;PRKAG3
Butyrophilin (BTN) Family Interactions	2/12	0.0675992	0.269251304	0	0	5.352222222	14.41973333	PPL;XDH
Organic Anion Transporters	2/12	0.0675992	0.269251304	0	0	5.352222222	14.41973333	SLC5A8;SLC17A5
Pyrimidine Catabolism	2/12	0.0675992	0.269251304	0	0	5.352222222	14.41973333	DPYS;TYMP
Transport of Nucleosides and Free Purine and Pyrimidine Bases Across the Plasma Membrane	2/12	0.0675992	0.269251304	0	0	5.352222222	14.41973333	SLC25A4;SLC28A1
Vitamin D (Calciferol) Metabolism	2/12	0.0675992	0.269251304	0	0	5.352222222	14.41973333	CYP2R1;GC
Formation of Incision Complex in GG-NER	4/43	0.0686309	0.271519891	0	0	2.748232269	7.362544936	RPA3;RPA2;RPS27A;DDB2
Homologous DNA Pairing and Strand Exchange	4/43	0.0686309	0.271519891	0	0	2.748232269	7.362544936	RFC5;RFC2;RPA3;RPA2
Amyloid Fiber Formation	6/80	0.0690309	0.272185843	0	0	2.174694247	5.813392965	APCS;TTR;GSN;APOA1;APOA4;RPS27A
Organelle Biogenesis and Maintenance	16/297	0.0732304	0.287638224	0	0	1.532124243	4.00519409	ARF4;ATP5PF;ATP5PD;ATP5PB;ATP5MC3;ATP5F1C;SOD2;PRKAG3;ATP5F1E;ATP5MC1;APOO;ATP5PO;ATP5MK;ATP5MJ;MAPRE1;ATP5ME
Smooth Muscle Contraction	4/44	0.0734395	0.287638224	0	0	2.679387187	6.996664216	ANXA1;ALDH2;ANXA2;TPM2
Diseases of Branched-Chain Amino Acid Catabolism	2/13	0.078032	0.301604044	0	0	4.86540404	12.4098745	MCCC2;PPM1K
Inhibition of Replication Initiation of Damaged DNA by RB1 E2F1	2/13	0.078032	0.301604044	0	0	4.86540404	12.4098745	PRIM2;PRIM1
Synthesis, Secretion, and Inactivation of Glucose-dependent Insulinotropic Polypeptide (GIP)	2/13	0.078032	0.301604044	0	0	4.86540404	12.4098745	DPP4;GIP
Telomere C-strand Synthesis Initiation	2/13	0.078032	0.301604044	0	0	4.86540404	12.4098745	PRIM2;PRIM1
Sulfur Amino Acid Metabolism	3/28	0.0787337	0.302327497	0	0	3.213296245	8.16717963	TSTD1;CBS;GOT2
TGFBR3 Expression	3/28	0.0787337	0.302327497	0	0	3.213296245	8.16717963	HELLS;MYF6;MYF5
Synthesis of Substrates in N-glycan Biosynthesis	5/64	0.0809693	0.309898825	0	0	2.271588303	5.71005764	NEU3;PGM3;SLC17A5;MVD;NANS
Phospholipid Metabolism	12/211	0.0813472	0.310334654	0	0	1.620411919	4.065658355	HADHB;DUSP23;PCYT1B;DGAT2;DGAT1;PLA2G1B;GPD1;GDPD3;ALPI;CHPT1;PLA1A;ETNPPL

Nervous System Development	27/567	0.088044	0.334797008	0	0	1.348057554	3.275662612	RPL32;RPL31;RPLP1;RPL11;RPL36A;RPL7;RPS14;RPS17;RPL36;MBP;RPS27A;RPS11;RPL17;RPS9;RPS8;RPL22;CYP51A1;RPL13A;RPL35A;RPS3A;PSMA6;RPS25;ETF1;RPS20;FAU;PFN1;RPS24
Stimuli-sensing Channels	7/107	0.092481	0.350003404	0	0	1.877566434	4.470010765	STOML3;BEST4;TRPV6;CLCA1;CASQ1;RPS27A;CLCN2
HSF1 Activation	3/30	0.092639	0.350003404	0	0	2.974965229	7.077569948	MRPL18;RPA3;RPA2
Nuclear Events Mediated by NFE2L2	6/87	0.094525	0.35598536	0	0	1.98603352	4.684826489	PSMA6;NQO1;IDH1;GSR;ME1;RPS27A
EGFR Downregulation	3/31	0.099945	0.3680594	0	0	2.868567455	6.606692249	SH3GL3;RPS27A;SH3GL1
FOXO-mediated Transcription of Oxidative Stress, Metabolic and Neuronal Genes	3/31	0.099945	0.3680594	0	0	2.868567455	6.606692249	TRIM63;SOD2;GCK
Cell Cycle	30/649	0.100042	0.3680594	0	0	1.306811284	3.008482914	PRIM2;CDKN1A;FEN1;PCNA;PRIM1;DSCC1;TYMS;CDC45;POLR2E;RPS27A;RFC5;GINS1;CENPU;GINS2;LIG1;RFC2;GINS3;GINS4;RPA2;PTK6;DHFR;PSMA6;DKC1;RPA3;MCM3;CHMP4C;MCM5;MAPRE1;NOP10;MCM2
Zinc Transporters	2/15	0.100237	0.3680594	0	0	4.116452991	9.468720209	SLC30A8;SLC39A4
Degradation of Cysteine and Homocysteine	2/15	0.100237	0.3680594	0	0	4.116452991	9.468720209	TSTD1;GOT2
Keratan Sulfate Degradation	2/15	0.100237	0.3680594	0	0	4.116452991	9.468720209	HEXA;OMD
Pentose Phosphate Pathway	2/15	0.100237	0.3680594	0	0	4.116452991	9.468720209	RPE;RBKS
Synthesis of Prostaglandins (PG) and Thromboxanes (TX)	2/15	0.100237	0.3680594	0	0	4.116452991	9.468720209	CBR1;PRXL2B
SCF(Skp2)-mediated Degradation of P27 P21	4/50	0.105680	0.386838115	0	0	2.329175245	5.234428393	PSMA6;CDKN1A;PTK6;RPS27A
Diseases of DNA Repair	4/51	0.111589	0.404630832	0	0	2.279499793	4.99877323	RFC5;RFC2;RPA3;RPA2
mRNA Decay by 3' to 5' Exoribonuclease	2/16	0.111919	0.404630832	0	0	3.822222222	8.370584097	EXOSC5;EXOSC2
Heme Degradation	2/16	0.111919	0.404630832	0	0	3.822222222	8.370584097	FABP1;ABCC2
TGF-beta Receptor Signaling in EMT (Epithelial to Mesenchymal Transition)	2/16	0.111919	0.404630832	0	0	3.822222222	8.370584097	F11R;RPS27A
Activation of Matrix Metalloproteinases	3/33	0.115215	0.414000165	0	0	2.67705146	5.784980143	PRSS1;CTRB2;CTRB1
Sialic Acid Metabolism	3/33	0.115215	0.414000165	0	0	2.67705146	5.784980143	NEU3;SLC17A5;NANS
Aquaporin-mediated Transport	4/52	0.117642	0.421431272	0	0	2.23189415	4.776495393	AQP12A;AQP10;AQP8;GNG13
Citric Acid Cycle (TCA Cycle)	3/34	0.123159	0.435983835	0	0	2.590560366	5.425345692	SUCLG2;SDHC;SUCLG1
Late Endosomal Microautophagy	3/34	0.123159	0.435983835	0	0	2.590560366	5.425345692	CHMP4C;PARK7;RPS27A
Butyrate Response Factor 1 (BRF1) Binds and Destabilizes mRNA	2/17	0.123930	0.435983835	0	0	3.567222222	7.448476096	EXOSC5;EXOSC2
KSRP (KHSRP) Binds and Destabilizes mRNA	2/17	0.123930	0.435983835	0	0	3.567222222	7.448476096	EXOSC5;EXOSC2
Regulation of MITF-M-dependent Genes Involved in Cell Cycle and Proliferation	2/17	0.123930	0.435983835	0	0	3.567222222	7.448476096	CDKN1A;HINT1
Tristetraprolin (TTP, ZFP36) Binds and Destabilizes mRNA	2/17	0.123930	0.435983835	0	0	3.567222222	7.448476096	EXOSC5;EXOSC2
Axon Guidance	25/541	0.124579	0.436956431	0	0	1.304177371	2.716359781	RPL32;RPL31;RPLP1;RPL11;RPL36A;RPL7;RPS14;RPS17;RPL36;RPS27A;RPS11;RPL17;RPS9;RPS8;RPL22;RPL13A;RPL35A;RPS3A;PSMA6;RPS25;ETF1;RPS20;FAU;PFN1;RPS24
Mitochondrial Unfolded Protein Response (UPRmt)	2/18	0.136233	0.474931387	0	0	3.344097222	6.66608112	HSPE1;SOD2
RNA Polymerase III Chain Elongation	2/18	0.136233	0.474931387	0	0	3.344097222	6.66608112	POLR3GL;POLR2E
Sensory Processing of Sound by Outer Hair Cells of the Cochlea	4/55	0.136618	0.474931387	0	0	2.100278552	4.18073565	CLIC5;GSN;USH1C;PLS1
KEAP1-NFE2L2 Pathway	7/119	0.139343	0.48297698	0	0	1.67534965	3.301795184	PSMA6;NQO1;CDKN1A;IDH1;GSR;ME1;RPS27A
Sensory Processing of Sound	5/77	0.145293	0.502117373	0	0	1.860181311	3.588288233	CLIC5;GSN;USH1C;STX1A;PLS1
Fatty acyl-CoA Biosynthesis	3/37	0.148106	0.509703123	0	0	2.361613352	4.510266556	ELOVL2;ACSL5;SLC27A2
NFE2L2 Regulating Anti-Oxidant Detoxification Enzymes	2/19	0.148789	0.509703123	0	0	3.147222222	5.996150137	NQO1;GSR
Regulation of TP53 Activity Through Methylation	2/19	0.148789	0.509703123	0	0	3.147222222	5.996150137	SMYD2;RPS27A
Cell Cycle Checkpoints	13/261	0.151692	0.518135134	0	0	1.406968015	2.653402334	RFC5;CENPU;CDKN1A;RFC2;RPA2;PSMA6;CDC45;RPA3;MCM3;MCM5;MAPRE1;RPS27A;MCM2
Homology Directed Repair	7/122	0.152581	0.519662115	0	0	1.63138948	3.067102711	RFC5;FEN1;PCNA;RFC2;RPA3;RPA2;RPS27A
Platelet Activation, Signaling and Aggregation	13/262	0.154707	0.525380767	0	0	1.401243904	2.615024719	CFD;SERPINA1;AHSG;SERPINF2;ANXA5;APOA1;GNG13;SOD1;CYB5R1;TF;SCCPDH;CD36;PFN1
Biosynthesis of the N-glycan Precursor (Dolichol LLO) and Transfer to a Nascent Protein	5/79	0.156715	0.527771898	0	0	1.809717667	3.353986023	NEU3;PGM3;SLC17A5;MVD;NANS
Switching of Origins to a Post-Replicative State	5/79	0.156715	0.527771898	0	0	1.809717667	3.353986023	PSMA6;MCM3;MCM5;RPS27A;MCM2
Fanconi Anemia Pathway	3/38	0.156759	0.527771898	0	0	2.294019471	4.250915817	RPA3;RPA2;RPS27A
Processing of DNA Double-Strand Break Ends	5/80	0.162561	0.530471855	0	0	1.785495119	3.243705145	RFC5;RFC2;RPA3;RPA2;RPS27A
SARS-CoV-2 Infection	15/313	0.162975	0.530471855	0	0	1.351300039	2.451467584	RPS9;RPS8;RPS3A;RPS14;ACE2;RPS25;RPS17;CHMP4C;RPS20;SNRPE;FAU;RPS11;RPS27A;RPS24;MBL2
Formation of Fibrin Clot (Clotting Cascade)	3/39	0.165564	0.530471855	0	0	2.230180807	4.010747438	TF;SERPIND1;C1QBP
NGF-stimulated Transcription	3/39	0.165564	0.530471855	0	0	2.230180807	4.010747438	SH3GL3;TF;ASCL1
Synthesis of PA	3/39	0.165564	0.530471855	0	0	2.230180807	4.010747438	PLA2G1B;GPD1;ALPI
Zinc Efflux and Compartmentalization by the SLC30 Family	1/5	0.167945	0.530471855	0	0	6.683079057	11.92340129	SLC30A8
Abacavir Transmembrane Transport	1/5	0.167945	0.530471855	0	0	6.683079057	11.92340129	ABCB1
Alternative Complement Activation	1/5	0.167945	0.530471855	0	0	6.683079057	11.92340129	CFD
Chylomicron Clearance	1/5	0.167945	0.530471855	0	0	6.683079057	11.92340129	APOB
Extrinsic Pathway of Fibrin Clot Formation	1/5	0.167945	0.530471855	0	0	6.683079057	11.92340129	TF
Formation of the Active Cofactor, UDP-glucuronate	1/5	0.167945	0.530471855	0	0	6.683079057	11.92340129	SLC35D1
Fructose Catabolism	1/5	0.167945	0.530471855	0	0	6.683079057	11.92340129	GLYCTK
H139Hfs13* PPM1K Causes a Mild Variant of MSUD	1/5	0.167945	0.530471855	0	0	6.683079057	11.92340129	PPM1K
HDL Clearance	1/5	0.167945	0.530471855	0	0	6.683079057	11.92340129	APOA1
Maple Syrup Urine Disease	1/5	0.167945	0.530471855	0	0	6.683079057	11.92340129	PPM1K
Maturation of Protein E 9683683	1/5	0.167945	0.530471855	0	0	6.683079057	11.92340129	RPS27A
Maturation of Protein E 9694493	1/5	0.167945	0.530471855	0	0	6.683079057	11.92340129	RPS27A
PTK6 Expression	1/5	0.167945	0.530471855	0	0	6.683079057	11.92340129	PTK6
PTK6 Regulates Proteins Involved in RNA Processing	1/5	0.167945	0.530471855	0	0	6.683079057	11.92340129	PTK6
Signaling by Membrane-Tethered Fusions of PDGFRA or PDGFRB	1/5	0.167945	0.530471855	0	0	6.683079057	11.92340129	BIN2
TFAP2 (AP-2) Family Regulates Transcription of Cell Cycle Factors	1/5	0.167945	0.530471855	0	0	6.683079057	11.92340129	CDKN1A
Threonine Catabolism	1/5	0.167945	0.530471855	0	0	6.683079057	11.92340129	SDSL
Vitamin B1 (Thiamin) Metabolism	1/5	0.167945	0.530471855	0	0	6.683079057	11.92340129	SLC19A3
Synthesis, Secretion, and Inactivation of Glucagon-like Peptide-1 (GLP-1)	2/21	0.174531	0.549796785	0	0	2.815643275	4.915132071	DPP4;GNG13
Signaling by Retinoic Acid	3/41	0.183588	0.576783161	0	0	2.112583266	3.580948032	SDR16C5;DHRS9;DHRS4
Acyl Chain Remodelling of PS	2/22	0.187653	0.577206265	0	0	2.674722222	4.475233995	PLA2G1B;PLA1A
Cellular Hexose Transport	2/22	0.187653	0.577206265	0	0	2.674722222	4.475233995	SLC2A2;SLC5A1
Chaperone Mediated Autophagy	2/22	0.187653	0.577206265	0	0	2.674722222	4.475233995	PARK7;RPS27A
Defects in Vitamin and Cofactor Metabolism	2/22	0.187653	0.577206265	0	0	2.674722222	4.475233995	MCCC2;TCN2
E2F Mediated Regulation of DNA Replication	2/22	0.187653	0.577206265	0	0	2.674722222	4.475233995	PRIM2;PRIM1
Early Phase of HIV Life Cycle	2/22	0.187653	0.577206265	0	0	2.674722222	4.475233995	FEN1;LIG1
Insertion of Tail-Anchored Proteins Into the Endoplasmic Reticulum Membrane	2/22	0.187653	0.577206265	0	0	2.674722222	4.475233995	CYB5A;STX1A
Regulation of TLR by Endogenous Ligand	2/22	0.187653	0.577206265	0	0	2.674722222	4.475233995	CD36;APOB
Integration of Energy Metabolism	6/108	0.195661	0.590451762	0	0	1.575418994	2.570088034	PKLR;ADIPOQ;SLC2A2;CD36;STX1A;GNG13
Acyl Chain Remodeling of CL	1/6	0.197989	0.590451762	0	0	5.346185853	8.658361054	HADHB
Alpha-oxidation of Phytanate	1/6	0.197989	0.590451762	0	0	5.346185853	8.658361054	SLC27A2
Biosynthesis of Lipoxins (LX)	1/6	0.197989	0.590451762	0	0	5.346185853	8.658361054	PTGR1
Choline Catabolism	1/6	0.197989	0.590451762	0	0	5.346185853	8.658361054	ALDH7A1
Fibronectin Matrix Formation	1/6	0.197989	0.590451762	0	0	5.346185853	8.658361054	CEACAM1
LTC4-CYSLTR Mediated IL4 Production	1/6	0.197989	0.590451762	0	0	5.346185853	8.658361054	GGT1
PTK6 Regulates Cell Cycle	1/6	0.197989	0.590451762	0	0	5.346185853	8.658361054	PTK6
Sulfide Oxidation to Sulfate	1/6	0.197989	0.590451762	0	0	5.346185853	8.658361054	TSTD1
Synthesis of Dolichyl-phosphate	1/6	0.197989	0.590451762	0	0	5.346185853	8.658361054	MVD
Transport and Synthesis of PAPS	1/6	0.197989	0.590451762	0	0	5.346185853	8.658361054	SLC26A1
Vitamins	1/6	0.197989	0.590451762	0	0	5.346185853	8.658361054	CYP2R1
Intrinsic Pathway of Fibrin Clot Formation	2/23	0.200905	0.593124281	0	0	2.547222222	4.088094876	SERPIND1;C1QBP
Nucleotide Salvage	2/23	0.200905	0.593124281	0	0	2.547222222	4.088094876	ADA;TYMP
RNA Polymerase III Transcription Termination	2/23	0.200905	0.593124281	0	0	2.547222222	4.088094876	POLR3GL;POLR2E
Telomere Extension By Telomerase	2/23	0.200905	0.593124281	0	0	2.547222222	4.088094876	DKC1;NOP10
tRNA Modification in the Nucleus and Cytosol	3/43	0.202100	0.595158179	0	0	2.00674548	3.208766098	TP53RK;TPRKB;OSGEP
The Role of GTSE1 in G2 M Progression After G2 Checkpoint	4/65	0.207410	0.609269683	0	0	1.755057309	2.760798445	PSMA6;CDKN1A;MAPRE1;RPS27A
Xenobiotics	2/24	0.214259	0.620086687	0	0	2.431313131	3.745598979	CYP1A1;AHR
Cytosolic Sulfonation of Small Molecules	2/24	0.214259	0.620086687	0	0	2.431313131	3.745598979	SLC26A1;SULT6B1
Glutamate Neurotransmitter Release Cycle	2/24	0.214259	0.620086687	0	0	2.431313131	3.745598979	GLS2;STX1A
Growth Hormone Receptor Signaling	2/24	0.214259	0.620086687	0	0	2.431313131	3.745598979	GH1;CISH
Metal Ion SLC Transporters	2/24	0.214259	0.620086687	0	0	2.431313131	3.745598979	SLC30A8;SLC39A4
Synthesis of Very Long-Chain Fatty acyl-CoAs	2/24	0.214259	0.620086687	0	0	2.431313131	3.745598979	ELOVL2;ACSL5
FOXO-mediated Transcription	4/67	0.222680	0.640045464	0	0	1.699164345	2.552172369	CDKN1A;TRIM63;SOD2;GCK
Aryl Hydrocarbon Receptor Signalling	1/7	0.226951	0.640045464	0	0	4.454923717	6.606746076	AHR
Defective HLCS Causes Multiple Carboxylase Deficiency	1/7	0.226951	0.640045464	0	0	4.454923717	6.606746076	MCCC2
Digestion of Dietary Lipid	1/7	0.226951	0.640045464	0	0	4.454923717	6.606746076	CEL
FLT3 Signaling by CBL Mutants	1/7	0.226951	0.640045464	0	0	4.454923717	6.606746076	RPS27A
Glycerophospholipid Catabolism	1/7	0.226951	0.640045464	0	0	4.454923717	6.606746076	GDPD3
PTK6 Promotes HIF1A Stabilization	1/7	0.226951	0.640045464	0	0	4.454923717	6.606746076	PTK6
RUNX3 Regulates CDKN1A Transcription	1/7	0.226951	0.640045464	0	0	4.454923717	6.606746076	CDKN1A
Scavenging by Class F Receptors	1/7	0.226951	0.640045464	0	0	4.454923717	6.606746076	APOB
Complex III Assembly	2/25	0.227692	0.640045464	0	0	2.325483092	3.441152354	UQCR10;UQCRH
Insulin Processing	2/25	0.227692	0.640045464	0	0	2.325483092	3.441152354	SLC30A8;STX1A
Metabolism of Cofactors	2/25	0.227692	0.640045464	0	0	2.325483092	3.441152354	DHFR;IDH1
Sensory Processing of Sound by Inner Hair Cells of the Cochlea	4/69	0.238231	0.668072592	0	0	1.646710949	2.362224884	CLIC5;USH1C;STX1A;PLS1
Cyclin D Associated Events in G1	3/47	0.240278	0.670610572	0	0	1.823934758	2.600853034	CDKN1A;PTK6;RPS27A
G1 Phase	3/47	0.240278	0.670610572	0	0	1.823934758	2.600853034	CDKN1A;PTK6;RPS27A
Gluconeogenesis	2/26	0.241181	0.671535162	0	0	2.228472222	3.169348824	PGAM2;FBP1
HDR Through Homologous Recombination (HRR) or Single Strand Annealing (SSA)	6/116	0.241949	0.672083189	0	0	1.460233621	2.072107138	RFC5;PCNA;RFC2;RPA3;RPA2;RPS27A
RNA Polymerase III Transcription Initiation From Type 2 Promoter	2/27	0.254703	0.67296554	0	0	2.139222222	2.92571929	POLR3GL;POLR2E
AMPK Inhibits chREBP Transcriptional Activation Activity	1/8	0.254867	0.67296554	0	0	3.818307906	5.219666233	ADIPOQ
mRNA Editing C to U Conversion	1/8	0.254867	0.67296554	0	0	3.818307906	5.219666233	APOBEC2
rRNA Modification in the Mitochondrion	1/8	0.254867	0.67296554	0	0	3.818307906	5.219666233	MTERF4
ChREBP Activates Metabolic Gene Expression	1/8	0.254867	0.67296554	0	0	3.818307906	5.219666233	PKLR
Cross-presentation of Particulate Exogenous Antigens (Phagosomes)	1/8	0.254867	0.67296554	0	0	3.818307906	5.219666233	CD36
Defective B4GALT1 Causes B4GALT1-CDG (CDG-2d)	1/8	0.254867	0.67296554	0	0	3.818307906	5.219666233	OMD
Defective CHST6 Causes MCDC1	1/8	0.254867	0.67296554	0	0	3.818307906	5.219666233	OMD
Defective ST3GAL3 Causes MCT12 and EIEE15	1/8	0.254867	0.67296554	0	0	3.818307906	5.219666233	OMD
Defects in Biotin (Btn) Metabolism	1/8	0.254867	0.67296554	0	0	3.818307906	5.219666233	MCCC2
Erythrocytes Take up Oxygen and Release Carbon Dioxide	1/8	0.254867	0.67296554	0	0	3.818307906	5.219666233	CA4
Formation of the Editosome	1/8	0.254867	0.67296554	0	0	3.818307906	5.219666233	APOBEC2
HDL Assembly	1/8	0.254867	0.67296554	0	0	3.818307906	5.219666233	APOA1
Lectin Pathway of Complement Activation	1/8	0.254867	0.67296554	0	0	3.818307906	5.219666233	MBL2
Phosphate Bond Hydrolysis by NTPDase Proteins	1/8	0.254867	0.67296554	0	0	3.818307906	5.219666233	ENTPD8
Regulation of Gene Expression in Early Pancreatic Precursor Cells	1/8	0.254867	0.67296554	0	0	3.818307906	5.219666233	NR5A2
Synthesis of PG	1/8	0.254867	0.67296554	0	0	3.818307906	5.219666233	DUSP23
Synthesis of UDP-N-acetyl-glucosamine	1/8	0.254867	0.67296554	0	0	3.818307906	5.219666233	PGM3
Synthesis of diphthamide-EEF2	1/8	0.254867	0.67296554	0	0	3.818307906	5.219666233	DPH6
Synthesis of Epoxy (EET) and Dihydroxyeicosatrienoic Acids (DHET)	1/8	0.254867	0.67296554	0	0	3.818307906	5.219666233	CYP1A1
Terminal Pathway of Complement	1/8	0.254867	0.67296554	0	0	3.818307906	5.219666233	C7
Transport of RCbl Within the Body	1/8	0.254867	0.67296554	0	0	3.818307906	5.219666233	TCN2
Signaling by TGFBR3	3/49	0.259798	0.684445932	0	0	1.744451835	2.351259583	HELLS;MYF6;MYF5
ATF4 Activates Genes in Response to Endoplasmic Reticulum Stress	2/28	0.268239	0.697304022	0	0	2.056837607	2.706541234	EXOSC5;EXOSC2
FLT3 Signaling in Disease	2/28	0.268239	0.697304022	0	0	2.056837607	2.706541234	CDKN1A;RPS27A
Metabolism of Porphyrins	2/28	0.268239	0.697304022	0	0	2.056837607	2.706541234	FABP1;ABCC2
RNA Polymerase III Transcription Initiation From Type 1 Promoter	2/28	0.268239	0.697304022	0	0	2.056837607	2.706541234	POLR3GL;POLR2E
RNA Polymerase III Transcription Initiation From Type 3 Promoter	2/28	0.268239	0.697304022	0	0	2.056837607	2.706541234	POLR3GL;POLR2E
Synthesis of PC	2/28	0.268239	0.697304022	0	0	2.056837607	2.706541234	PCYT1B;CHPT1
Cyclin E Associated Events During G1 S Transition	4/73	0.270027	0.700401245	0	0	1.550926487	2.030524451	PSMA6;CDKN1A;PTK6;RPS27A
Signaling by Nuclear Receptors	12/270	0.271276	0.702091877	0	0	1.245987553	1.625536834	ABCG8;SDR16C5;ABCG5;NR5A2;DHRS9;APOC2;APOC1;ANGPTL3;POLR2E;APOD;GNG13;DHRS4
EGR2 and SOX10-mediated Initiation of Schwann Cell Myelination	2/29	0.281770	0.712019166	0	0	1.980555556	2.508692323	CYP51A1;MBP
Myogenesis	2/29	0.281770	0.712019166	0	0	1.980555556	2.508692323	MYF6;MYF5
Abacavir ADME	1/9	0.281777	0.712019166	0	0	3.340846047	4.231637445	ABCB1
tRNA Modification in the Mitochondrion	1/9	0.281777	0.712019166	0	0	3.340846047	4.231637445	HSD17B10
Downregulation of ERBB4 Signaling	1/9	0.281777	0.712019166	0	0	3.340846047	4.231637445	RPS27A
Formyl Peptide Receptors Bind Formyl Peptides and Many Other Ligands	1/9	0.281777	0.712019166	0	0	3.340846047	4.231637445	ANXA1
Lipophagy	1/9	0.281777	0.712019166	0	0	3.340846047	4.231637445	PRKAG3
NR1H2 & NR1H3 Regulate Gene Expression Linked to Lipogenesis	1/9	0.281777	0.712019166	0	0	3.340846047	4.231637445	ANGPTL3
Regulation of PTEN Localization	1/9	0.281777	0.712019166	0	0	3.340846047	4.231637445	RPS27A
Synthesis of (16-20)-Hydroxyeicosatetraenoic Acids (HETE)	1/9	0.281777	0.712019166	0	0	3.340846047	4.231637445	CYP1A1
Transport of Nucleotide Sugars	1/9	0.281777	0.712019166	0	0	3.340846047	4.231637445	SLC35D1

Cyclin A Cdk2-associated Events at S Phase Entry	4/75	0.2861972	0.721634705	0	0	1.507081486	1.885470477	PSMA6;CDKN1A;PTK6;RPS27A
Apoptotic Execution Phase	3/52	0.2894238	0.726651668	0	0	1.637393205	2.030143551	GSN;STK24;HMGB2
Proteasome Assembly	3/52	0.2894238	0.726651668	0	0	1.637393205	2.030143551	PSMA6;PSME1;PSMA8
Regulation of mRNA Stability by Proteins That Bind AU-rich Elements	4/76	0.2943347	0.731417838	0	0	1.486072423	1.817522231	PSMA6;EXOSC5;RPS27A;EXOSC2
SUMOylation of Intracellular Receptors	2/30	0.2952803	0.731417838	0	0	1.909722222	2.329536748	NR5A2;NR1H4
Surfactant Metabolism	2/30	0.2952803	0.731417838	0	0	1.909722222	2.329536748	SLC34A2;SLC34A1
mRNA Splicing - Minor Pathway	3/53	0.2993592	0.731417838	0	0	1.604561892	1.935279798	SF3B5;POLR2E;SNRPE
p53-Dependent G1 DNA Damage Response	3/53	0.2993592	0.731417838	0	0	1.604561892	1.935279798	PSMA6;CDKN1A;RPS27A
p53-Dependent G1 S DNA Damage Checkpoint	3/53	0.2993592	0.731417838	0	0	1.604561892	1.935279798	PSMA6;CDKN1A;RPS27A
Nuclear Receptor Transcription Pathway	3/53	0.2993592	0.731417838	0	0	1.604561892	1.935279798	NR5A2;NR1H4;NR0B2
Signaling by EGFR	3/53	0.2993592	0.731417838	0	0	1.604561892	1.935279798	SH3GL3;RPS27A;SH3GL1
Assembly of the Pre-Replicative Complex	5/101	0.3009187	0.731417838	0	0	1.393392608	1.673346036	PSMA6;MCM3;MCM5;RPS27A;MCM2
G2 M DNA Damage Checkpoint	4/77	0.3025014	0.731417838	0	0	1.465638951	1.752419498	RFC5;RFC2;RPA3;RPA2
Host Interactions of HIV Factors	6/126	0.3036634	0.731417838	0	0	1.337849162	1.594495742	PSMA6;RANBP1;TAT;AP1S3;RPS27A;SLC25A4
HIV Infection	10/227	0.3052763	0.731417838	0	0	1.233689743	1.463819676	PSMA6;RANBP1;FEN1;LIG1;TAT;CHMP4C;POLR2E;AP1S3;RPS27A;SLC25A4
Zinc Influx Into Cells by the SLC39 Gene Family	1/10	0.3077172	0.731417838	0	0	2.969486824	3.499759747	SLC39A4
mRNA Editing	1/10	0.3077172	0.731417838	0	0	2.969486824	3.499759747	APOBEC2
Activation of PPARGC1A (PGC-1alpha) by Phosphorylation	1/10	0.3077172	0.731417838	0	0	2.969486824	3.499759747	PRKAG3
Caspase Activation via Dependence Receptors in the Absence of Ligand	1/10	0.3077172	0.731417838	0	0	2.969486824	3.499759747	DAPK2
Diseases Associated With Surfactant Metabolism	1/10	0.3077172	0.731417838	0	0	2.969486824	3.499759747	SLC34A2
IRAK2 Mediated Activation of TAK1 Complex	1/10	0.3077172	0.731417838	0	0	2.969486824	3.499759747	RPS27A
InlA-mediated Entry of Listeria Monocytogenes Into Host Cells	1/10	0.3077172	0.731417838	0	0	2.969486824	3.499759747	RPS27A
Neurotransmitter Clearance	1/10	0.3077172	0.731417838	0	0	2.969486824	3.499759747	ALDH2
Prednisone ADME	1/10	0.3077172	0.731417838	0	0	2.969486824	3.499759747	ABCB1
SLBP Independent Processing of Histone Pre-mRNAs	1/10	0.3077172	0.731417838	0	0	2.969486824	3.499759747	SNRPE
STAT5 Activation Downstream of FLT3 ITD Mutants	1/10	0.3077172	0.731417838	0	0	2.969486824	3.499759747	CDKN1A
Synthesis of Pyrophosphates in the Cytosol	1/10	0.3077172	0.731417838	0	0	2.969486824	3.499759747	ITPK1
Tetrahydrobiopterin (BH4) Synthesis, Recycling, Salvage and Regulation	1/10	0.3077172	0.731417838	0	0	2.969486824	3.499759747	DHFR
Endosomal Sorting Complex Required For Transport (ESCRT)	2/31	0.3087517	0.731417838	0	0	1.843773946	2.166836117	CHMP4C;RPS27A
Tight Junction Interactions	2/31	0.3087517	0.731417838	0	0	1.843773946	2.166836117	CLDN15;F11R
Turbulent Flow Shear Stress Activates Signaling by PIEZO1 and Integrins in Endothelial Cells	2/31	0.3087517	0.731417838	0	0	1.843773946	2.166836117	CAPNS1;ANXA2
DNA Double-Strand Break Repair	7/152	0.3101055	0.733060553	0	0	1.291835061	1.51253557	RFC5;FEN1;PCNA;RFC2;RPA3;RPA2;RPS27A
Regulation of Insulin Secretion	4/78	0.3106928	0.733060553	0	0	1.445757735	1.690018968	SLC2A2;CD36;STX1A;GNG13
G1 S DNA Damage Checkpoints	3/55	0.3192751	0.750930507	0	0	1.542687493	1.761289361	PSMA6;CDKN1A;RPS27A
Defective TPR May Confer Susceptibility Towards Thyroid Papillary Carcinoma (TPC)	2/32	0.3221702	0.750930507	0	0	1.782222222	2.018678997	GCKR;GCK
Pausing and Recovery of Tat-mediated HIV Elongation	2/32	0.3221702	0.750930507	0	0	1.782222222	2.018678997	TAT;POLR2E
Regulation of Glucokinase by Glucokinase Regulatory Protein	2/32	0.3221702	0.750930507	0	0	1.782222222	2.018678997	GCKR;GCK
Tat-mediated HIV Elongation Arrest and Recovery	2/32	0.3221702	0.750930507	0	0	1.782222222	2.018678997	TAT;POLR2E
Formation of the Cornified Envelope	6/129	0.3227377	0.750930507	0	0	1.305014307	1.475860518	KRT18;CAPNS1;KRT15;KRT5;EVPL;PPL
Cargo Recognition for Clathrin-Mediated Endocytosis	5/105	0.3294027	0.750930507	0	0	1.337377964	1.485123415	SH3GL3;TF;APOB;RPS27A;SH3GL1
Alpha-protein Kinase 1 Signaling Pathway	1/11	0.3327211	0.750930507	0	0	2.672399445	2.940843591	RPS27A
Androgen Biosynthesis	1/11	0.3327211	0.750930507	0	0	2.672399445	2.940843591	SRD5A2
Attachment and Entry 9678110	1/11	0.3327211	0.750930507	0	0	2.672399445	2.940843591	ACE2
Biotin Transport and Metabolism	1/11	0.3327211	0.750930507	0	0	2.672399445	2.940843591	MCCC2
Free Fatty Acids Regulate Insulin Secretion	1/11	0.3327211	0.750930507	0	0	2.672399445	2.940843591	CD36
Interaction With Cumulus Cells And The Zona Pellucida	1/11	0.3327211	0.750930507	0	0	2.672399445	2.940843591	SPAM1
Interleukin-27 Signaling	1/11	0.3327211	0.750930507	0	0	2.672399445	2.940843591	EBI3
Modulation by Mtb of Host Immune System	1/11	0.3327211	0.750930507	0	0	2.672399445	2.940843591	RPS27A
Nef Mediated Downregulation of MHC Class I Complex Cell Surface Expression	1/11	0.3327211	0.750930507	0	0	2.672399445	2.940843591	AP1S3
Pexophagy	1/11	0.3327211	0.750930507	0	0	2.672399445	2.940843591	RPS27A
Pyrimidine Salvage	1/11	0.3327211	0.750930507	0	0	2.672399445	2.940843591	TYMP
Regulation of TBK1, IKK?-mediated Activation of IRF3, IRF7 Upon TLR3 Ligation	1/11	0.3327211	0.750930507	0	0	2.672399445	2.940843591	RPS27A
SLBP Dependent Processing of Replication-Dependent Histone Pre-mRNAs	1/11	0.3327211	0.750930507	0	0	2.672399445	2.940843591	SNRPE
TICAM1,TRAF6-dependent Induction of TAK1 Complex	1/11	0.3327211	0.750930507	0	0	2.672399445	2.940843591	RPS27A
Type I Hemidesmosome Assembly	1/11	0.3327211	0.750930507	0	0	2.672399445	2.940843591	KRT5
Amino Acid Transport Across the Plasma Membrane	2/33	0.3355221	0.750930507	0	0	1.724641577	1.88342472	SLC6A19;SLC43A1
Glucagon-type Ligand Receptors	2/33	0.3355221	0.750930507	0	0	1.724641577	1.88342472	GIP;GNG13
PERK Regulates Gene Expression	2/33	0.3355221	0.750930507	0	0	1.724641577	1.88342472	EXOSC5;EXOSC2
RNA Polymerase I Transcription Termination	2/33	0.3355221	0.750930507	0	0	1.724641577	1.88342472	POLR2E;POLR1E
Regulation of Pyruvate Metabolism	2/33	0.3355221	0.750930507	0	0	1.724641577	1.88342472	ME1;RPS27A
E3 Ubiquitin Ligases Ubiquitinate Target Proteins	3/57	0.3392043	0.757728363	0	0	1.485396384	1.605940061	PCNA;RPS27A;RNF152
Sphingolipid Metabolism	5/107	0.3437474	0.766419801	0	0	1.311018131	1.399968213	NEU3;ASAH2;B3GNT5;HEXA;ENPP7
HIV Life Cycle	7/158	0.3451280	0.768040744	0	0	1.240114852	1.319283336	RANBP1;FEN1;LIG1;TAT;CHMP4C;POLR2E;RPS27A
Ion Channel Transport	8/184	0.3468958	0.770515409	0	0	1.216068245	1.287488688	STOML3;BEST4;TRPV6;CLCA1;CASQ1;ATP1A1;RPS27A;CLCN2
Aspartate and Asparagine Metabolism	1/12	0.3568231	0.779306974	0	0	2.429327954	2.503459177	GOT2
Caspase-mediated Cleavage of Cytoskeletal Proteins	1/12	0.3568231	0.779306974	0	0	2.429327954	2.503459177	GSN
Ethanol Oxidation	1/12	0.3568231	0.779306974	0	0	2.429327954	2.503459177	ALDH2
HDR Through MMEJ (alt-NHEJ)	1/12	0.3568231	0.779306974	0	0	2.429327954	2.503459177	FEN1
Hyaluronan Uptake and Degradation	1/12	0.3568231	0.779306974	0	0	2.429327954	2.503459177	HEXA
Interleukin-35 Signalling	1/12	0.3568231	0.779306974	0	0	2.429327954	2.503459177	EBI3
Josephin Domain DUBs	1/12	0.3568231	0.779306974	0	0	2.429327954	2.503459177	RPS27A
Pregnenolone Biosynthesis	1/12	0.3568231	0.779306974	0	0	2.429327954	2.503459177	AKR1B1
Reversible Hydration of Carbon Dioxide	1/12	0.3568231	0.779306974	0	0	2.429327954	2.503459177	CA4
B-WICH Complex Positively Regulates rRNA Expression	3/59	0.3590939	0.78136182	0	0	1.432197497	1.466815494	POLR2E;POLR1E;DDX21
Iron Uptake and Transport	3/59	0.3590939	0.78136182	0	0	1.432197497	1.466815494	TF;FTH1;RPS27A
Lysosome Vesicle Biogenesis	2/35	0.3619765	0.786178242	0	0	1.619949495	1.646153619	AP4S1;AP1S3
Budding and Maturation of HIV Virion	2/36	0.3750566	0.804622416	0	0	1.572222222	1.54184423	CHMP4C;RPS27A
Keratan Sulfate Keratin Metabolism	2/36	0.3750566	0.804622416	0	0	1.572222222	1.54184423	HEXA;OMD
Metabolism of Steroid Hormones	2/36	0.3750566	0.804622416	0	0	1.572222222	1.54184423	SRD5A2;AKR1B1
RNA Polymerase III Transcription Initiation	2/36	0.3750566	0.804622416	0	0	1.572222222	1.54184423	POLR3GL;POLR2E
Nuclear Events (Kinase and Transcription Factor Activation)	3/61	0.3788949	0.804622416	0	0	1.382667498	1.341873542	SH3GL3;TF;ASCL1
p75NTR Recruits Signalling Complexes	1/13	0.3800556	0.804622416	0	0	2.226768377	2.154259172	RPS27A
Apoptosis Induced DNA Fragmentation	1/13	0.3800556	0.804622416	0	0	2.226768377	2.154259172	HMGB2
Downregulation of ERBB2 ERBB3 Signaling	1/13	0.3800556	0.804622416	0	0	2.226768377	2.154259172	RPS27A
ERBB2 Activates PTK6 Signaling	1/13	0.3800556	0.804622416	0	0	2.226768377	2.154259172	PTK6
NF-kB Is Activated and Signals Survival	1/13	0.3800556	0.804622416	0	0	2.226768377	2.154259172	RPS27A
Purine Salvage	1/13	0.3800556	0.804622416	0	0	2.226768377	2.154259172	ADA
Synthesis of PE	1/13	0.3800556	0.804622416	0	0	2.226768377	2.154259172	ETNPPL
TICAM1-dependent Activation of IRF3 IRF7	1/13	0.3800556	0.804622416	0	0	2.226768377	2.154259172	RPS27A
TRAF6 Mediated IRF7 Activation in TLR7 8 or 9 Signaling	1/13	0.3800556	0.804622416	0	0	2.226768377	2.154259172	RPS27A
Molecules Associated With Elastic Fibres	2/37	0.3880251	0.818545056	0	0	1.527222222	1.445798406	MFAP5;VTN
Regulation of Activated PAK-2p34 by Proteasome Mediated Degradation	2/37	0.3880251	0.818545056	0	0	1.527222222	1.445798406	PSMA6;RPS27A
rRNA Processing in the Mitochondrion	2/38	0.4008732	0.825268736	0	0	1.484722222	1.357199302	MTERF4;HSD17B10
Apoptotic Cleavage of Cellular Proteins	2/38	0.4008732	0.825268736	0	0	1.484722222	1.357199302	GSN;STK24
DNA Damage Recognition in GG-NER	2/38	0.4008732	0.825268736	0	0	1.484722222	1.357199302	RPS27A;DDB2
Regulation of Ornithine Decarboxylase (ODC)	2/38	0.4008732	0.825268736	0	0	1.484722222	1.357199302	NQO1;PSMA6
AKT Phosphorylates Targets in the Cytosol	1/14	0.4024502	0.825268736	0	0	2.055371813	1.870766363	CDKN1A
CS DS Degradation	1/14	0.4024502	0.825268736	0	0	2.055371813	1.870766363	HEXA
Carnitine Shuttle	1/14	0.4024502	0.825268736	0	0	2.055371813	1.870766363	SLC22A5
Defects in Cobalamin (B12) Metabolism	1/14	0.4024502	0.825268736	0	0	2.055371813	1.870766363	TCN2
Glycogen Breakdown (Glycogenolysis)	1/14	0.4024502	0.825268736	0	0	2.055371813	1.870766363	GYG1
IRAK1 Recruits IKK Complex	1/14	0.4024502	0.825268736	0	0	2.055371813	1.870766363	RPS27A
IRAK1 Recruits IKK Complex Upon TLR7 8 or 9 Stimulation	1/14	0.4024502	0.825268736	0	0	2.055371813	1.870766363	RPS27A
LGI-ADAM Interactions	1/14	0.4024502	0.825268736	0	0	2.055371813	1.870766363	STX1A
PTK6 Regulates RHO GTPases, RAS GTPase and MAP Kinases	1/14	0.4024502	0.825268736	0	0	2.055371813	1.870766363	PTK6
Regulation of TBK1, IKK? (IKBKE)-mediated Activation of IRF3, IRF7	1/14	0.4024502	0.825268736	0	0	2.055371813	1.870766363	RPS27A
Response to Metal Ions	1/14	0.4024502	0.825268736	0	0	2.055371813	1.870766363	CSRP1
TP53 Regulates Transcription of Genes Involved in G1 Cell Cycle Arrest	1/14	0.4024502	0.825268736	0	0	2.055371813	1.870766363	CDKN1A
Apoptosis	7/168	0.4042971	0.827611744	0	0	1.162480997	1.052748805	PSMA6;GSN;STK24;DAPK2;C1QBP;HMGB2;RPS27A
Cytosolic Sensors of Pathogen-Associated DNA	3/64	0.4083330	0.834419698	0	0	1.314462254	1.177327239	POLR3GL;POLR2E;RPS27A
p53-Independent DNA Damage Response	2/39	0.4135926	0.837881722	0	0	1.44451952	1.275328228	PSMA6;RPS27A
p53-Independent G1 S DNA Damage Checkpoint	2/39	0.4135926	0.837881722	0	0	1.44451952	1.275328228	PSMA6;RPS27A
Autodegradation of the E3 Ubiquitin Ligase COP1	2/39	0.4135926	0.837881722	0	0	1.44451952	1.275328228	PSMA6;RPS27A
Ubiquitin Mediated Degradation of Phosphorylated Cdc25A	2/39	0.4135926	0.837881722	0	0	1.44451952	1.275328228	PSMA6;RPS27A
Ubiquitin-dependent Degradation of Cyclin D	2/39	0.4135926	0.837881722	0	0	1.44451952	1.275328228	PSMA6;RPS27A
Constitutive Signaling by NOTCH1 HD Domain Mutants	1/15	0.4240368	0.8473041	0	0	1.908460472	1.637335015	RPS27A
IRAK2 Mediated Activation of TAK1 Complex Upon TLR7 8 or 9 Stimulation	1/15	0.4240368	0.8473041	0	0	1.908460472	1.637335015	RPS27A
Membrane Binding and Targetting of GAG Proteins	1/15	0.4240368	0.8473041	0	0	1.908460472	1.637335015	RPS27A
Negative Regulation of FLT3	1/15	0.4240368	0.8473041	0	0	1.908460472	1.637335015	RPS27A
Prolactin Receptor Signaling	1/15	0.4240368	0.8473041	0	0	1.908460472	1.637335015	GH1
Regulation of Innate Immune Responses to Cytosolic DNA	1/15	0.4240368	0.8473041	0	0	1.908460472	1.637335015	RPS27A
Signaling by NOTCH1 HD Domain Mutants in Cancer	1/15	0.4240368	0.8473041	0	0	1.908460472	1.637335015	RPS27A
Synthesis And Processing Of GAG, GAGPOL Polyproteins	1/15	0.4240368	0.8473041	0	0	1.908460472	1.637335015	RPS27A
Innate Immune System	43/1149	0.4254445	0.8473041	0	0	1.040510058	0.889241296	CFD;SERPINA1;PGLYRP2;GSTP1;FGL2;MGST1;MOSPD2;SLC2A5;PLAC8;VTN;PGRMC1;TTR;C7;PLAU;ANPEP;FTH1;POLR2E;LTA4H;CD36;RPS27A;APOB;ACAA1;GYG1;S100A11;MBL2;SERPINB1;AOC1;GSN;ANXA2;DG
Regulation of Apoptosis	2/40	0.4261759	0.8473041	0	0	1.406432749	1.199550747	PSMA6;RPS27A
Vpu Mediated Degradation of CD4	2/40	0.4261759	0.8473041	0	0	1.406432749	1.199550747	PSMA6;RPS27A
tRNA Processing	6/146	0.4324149	0.858255929	0	0	1.145530726	0.960378285	POP5;TP53RK;TPRKB;ZBTB8OS;HSD17B10;OSGEP
Defective Pyroptosis	2/41	0.4386162	0.861107859	0	0	1.370299145	1.129305039	PRIM2;PRIM1
Diseases Associated With Glycosaminoglycan Metabolism	2/41	0.4386162	0.861107859	0	0	1.370299145	1.129305039	HEXA;OMD
RNA Polymerase III Abortive And Retractive Initiation	2/41	0.4386162	0.861107859	0	0	1.370299145	1.129305039	POLR3GL;POLR2E
RNA Polymerase III Transcription	2/41	0.4386162	0.861107859	0	0	1.370299145	1.129305039	POLR3GL;POLR2E
MITF-M-dependent Gene Expression	4/94	0.4419599	0.861107859	0	0	1.187743733	0.969835494	CDKN1A;HINT1;CEACAM1;LIG1
p75NTR Signals via NF-kB	1/16	0.4448446	0.861107859	0	0	1.781137309	1.442774915	RPS27A
Elevation of Cytosolic Ca2+ Levels	1/16	0.4448446	0.861107859	0	0	1.781137309	1.442774915	P2RX4
MAP3K8 (TPL2)-dependent MAPK1 3 Activation	1/16	0.4448446	0.861107859	0	0	1.781137309	1.442774915	RPS27A
NRIF Signals Cell Death From the Nucleus	1/16	0.4448446	0.861107859	0	0	1.781137309	1.442774915	RPS27A
Processing of SMDT1	1/16	0.4448446	0.861107859	0	0	1.781137309	1.442774915	SMDT1
Regulation of BACH1 Activity	1/16	0.4448446	0.861107859	0	0	1.781137309	1.442774915	RPS27A
Signaling by FLT3 ITD and TKD Mutants	1/16	0.4448446	0.861107859	0	0	1.781137309	1.442774915	CDKN1A
Spry Regulation of FGF Signaling	1/16	0.4448446	0.861107859	0	0	1.781137309	1.442774915	RPS27A
TRAF6-mediated Induction of TAK1 Complex Within TLR4 Complex	1/16	0.4448446	0.861107859	0	0	1.781137309	1.442774915	RPS27A
VLDLR Internalisation and Degradation	1/16	0.4448446	0.861107859	0	0	1.781137309	1.442774915	RPS27A
Programmed Cell Death	8/202	0.4457191	0.861381495	0	0	1.10219758	0.890648758	PSMA6;GSN;STK24;DAPK2;C1QBP;CHMP4C;HMGB2;RPS27A
AUF1 (hnRNP D0) Binds and Destabilizes mRNA	2/42	0.4509076	0.86289334	0	0	1.335972222	1.064092104	PSMA6;RPS27A
Degradation of AXIN	2/42	0.4509076	0.86289334	0	0	1.335972222	1.064092104	PSMA6;RPS27A
FBXL7 Down-Regulates AURKA During Mitotic Entry and in Early Mitosis	2/42	0.4509076	0.86289334	0	0	1.335972222	1.064092104	PSMA6;RPS27A
GSK3B and BTRC CUL1-mediated-degradation of NFE2L2	2/42	0.4509076	0.86289334	0	0	1.335972222	1.064092104	PSMA6;RPS27A
SCF-beta-TrCP Mediated Degradation of Emi1	2/42	0.4509076	0.86289334	0	0	1.335972222	1.064092104	PSMA6;RPS27A
Vif-mediated Degradation of APOBEC3G	2/42	0.4509076	0.86289334	0	0	1.335972222	1.064092104	PSMA6;RPS27A
Aggrephagy	2/43	0.4630445	0.869840093	0	0	1.303319783	1.003467485	PARK7;RPS27A
Aspirin ADME	2/43	0.4630445	0.869840093	0	0	1.303319783	1.003467485	ABCC2;CES2
Hh Mutants Are Degraded by ERAD	2/43	0.4630445	0.869840093	0	0	1.303319783	1.003467485	PSMA6;RPS27A
Aberrant Regulation of Mitotic G1 S Transition in Cancer Due to RB1 Defects	1/17	0.4649017	0.869840093	0	0	1.669729542	1.278894522	CDKN1A
Acetylcholine Neurotransmitter Release Cycle	1/17	0.4649017	0.869840093	0	0	1.669729542	1.278894522	STX1A
Acyl Chain Remodelling of PI	1/17	0.4649017	0.869840093	0	0	1.669729542	1.278894522	PLA2G1B
Defective Binding of RB1 Mutants to E2F1,(E2F2, E2F3)	1/17	0.4649017	0.869840093	0	0	1.669729542	1.278894522	CDKN1A
FOXO-mediated Transcription of Cell Cycle Genes	1/17	0.4649017	0.869840093	0	0	1.669729542	1.278894522	CDKN1A

Formation of Senescence-Associated Heterochromatin Foci (SAHF)	1/17	0.4649017	0.869840093	0	0	1.669729542	1.278894522	CDKN1A
Hyaluronan Metabolism	1/17	0.4649017	0.869840093	0	0	1.669729542	1.278894522	HEXA
Platelet Sensitization by LDL	1/17	0.4649017	0.869840093	0	0	1.669729542	1.278894522	APOB
Protein Methylation	1/17	0.4649017	0.869840093	0	0	1.669729542	1.278894522	ETFB
TNF Receptor Superfamily (TNFSF) Members Mediating Non-Canonical NF-kB Pathway	1/17	0.4649017	0.869840093	0	0	1.669729542	1.278894522	TNFSF14
Translation of Replicase and Assembly of the Replication Transcription Complex 9694676	1/17	0.4649017	0.869840093	0	0	1.669729542	1.278894522	CHMP4C
Degradation of DVL	2/44	0.4750222	0.877596055	0	0	1.272222222	0.947034244	PSMA6;RPS27A
Elastic Fibre Formation	2/44	0.4750222	0.877596055	0	0	1.272222222	0.947034244	MFAP5;VTN
Formation of HIV-1 Elongation Complex Containing HIV-1 Tat	2/44	0.4750222	0.877596055	0	0	1.272222222	0.947034244	TAT;POLR2E
HIV Transcription Elongation	2/44	0.4750222	0.877596055	0	0	1.272222222	0.947034244	TAT;POLR2E
Negative Regulation of NOTCH4 Signaling	2/44	0.4750222	0.877596055	0	0	1.272222222	0.947034244	PSMA6;RPS27A
Stabilization of P53	2/44	0.4750222	0.877596055	0	0	1.272222222	0.947034244	PSMA6;RPS27A
Tat-mediated Elongation of the HIV-1 Transcript	2/44	0.4750222	0.877596055	0	0	1.272222222	0.947034244	TAT;POLR2E
Uptake and Actions of Bacterial Toxins	2/44	0.4750222	0.877596055	0	0	1.272222222	0.947034244	GUCY2C;STX1A
Activation of IRF3, IRF7 Mediated by TBK1, IKK? (IKBKE)	1/18	0.4842352	0.87969338	0	0	1.571428571	1.139575683	RPS27A
Acyl Chain Remodelling of PG	1/18	0.4842352	0.87969338	0	0	1.571428571	1.139575683	PLA2G1B
Formation of the Nephric Duct	1/18	0.4842352	0.87969338	0	0	1.571428571	1.139575683	PLAC8
G Beta Gamma Signalling Through BTK	1/18	0.4842352	0.87969338	0	0	1.571428571	1.139575683	GNG13
Norepinephrine Neurotransmitter Release Cycle	1/18	0.4842352	0.87969338	0	0	1.571428571	1.139575683	STX1A
Prevention of Phagosomal-Lysosomal Fusion	1/18	0.4842352	0.87969338	0	0	1.571428571	1.139575683	RPS27A
Regulation of NF-kappa B Signaling	1/18	0.4842352	0.87969338	0	0	1.571428571	1.139575683	RPS27A
Serotonin Neurotransmitter Release Cycle	1/18	0.4842352	0.87969338	0	0	1.571428571	1.139575683	STX1A
TP53 Regulates Transcription of Genes Involved in G2 Cell Cycle Arrest	1/18	0.4842352	0.87969338	0	0	1.571428571	1.139575683	PCNA
Translation of Replicase and Assembly of the Replication Transcription Complex 9679504	1/18	0.4842352	0.87969338	0	0	1.571428571	1.139575683	CHMP4C
trans-Golgi Network Vesicle Budding	3/72	0.4843928	0.87969338	0	0	1.16157707	0.841979589	FTH1;AP4S1;AP1S3
Regulation of RUNX3 Expression and Activity	2/45	0.4868361	0.882766197	0	0	1.242571059	0.894436976	PSMA6;RPS27A
Early SARS-CoV-2 Infection Events	2/46	0.4984826	0.885867286	0	0	1.214267677	0.845356691	ACE2;CHMP4C
Hh Mutants Abrogate Ligand Secretion	2/46	0.4984826	0.885867286	0	0	1.214267677	0.845356691	PSMA6;RPS27A
Metabolism of Polyamines	2/46	0.4984826	0.885867286	0	0	1.214267677	0.845356691	NQO1;PSMA6
NIK-->noncanonical NF-kB Signaling	2/46	0.4984826	0.885867286	0	0	1.214267677	0.845356691	PSMA6;RPS27A
PKMTs Methylate Histone Lysines	2/46	0.4984826	0.885867286	0	0	1.214267677	0.845356691	SETD6;SMYD2
Constitutive Signaling by Ligand-Responsive EGFR Cancer Variants	1/19	0.5028710	0.885867286	0	0	1.484049931	1.020167844	RPS27A
GABA Synthesis, Release, Reuptake and Degradation	1/19	0.5028710	0.885867286	0	0	1.484049931	1.020167844	STX1A
Glycosphingolipid Biosynthesis	1/19	0.5028710	0.885867286	0	0	1.484049931	1.020167844	B3GNT5
MyD88 Deficiency (TLR2 4)	1/19	0.5028710	0.885867286	0	0	1.484049931	1.020167844	CD36
Na+ Cl- Dependent Neurotransmitter Transporters	1/19	0.5028710	0.885867286	0	0	1.484049931	1.020167844	SLC6A19
Nicotinamide Salvaging	1/19	0.5028710	0.885867286	0	0	1.484049931	1.020167844	SLC5A8
Prostacyclin Signalling Through Prostacyclin Receptor	1/19	0.5028710	0.885867286	0	0	1.484049931	1.020167844	GNG13
Regulation of MITF-M-dependent Genes Involved in Apoptosis	1/19	0.5028710	0.885867286	0	0	1.484049931	1.020167844	HINT1
Signaling by FGFR2 IIIa TM	1/19	0.5028710	0.885867286	0	0	1.484049931	1.020167844	POLR2E
Signaling by FLT3 Fusion Proteins	1/19	0.5028710	0.885867286	0	0	1.484049931	1.020167844	CDKN1A
Signaling by Ligand-Responsive EGFR Variants in Cancer	1/19	0.5028710	0.885867286	0	0	1.484049931	1.020167844	RPS27A
Synthesis, Secretion, and Deacylation of Ghrelin	1/19	0.5028710	0.885867286	0	0	1.484049931	1.020167844	GH1
TICAM1, RIP1-mediated IKK Complex Recruitment	1/19	0.5028710	0.885867286	0	0	1.484049931	1.020167844	RPS27A
Transcription of E2F Targets Under Negative Control by DREAM Complex	1/19	0.5028710	0.885867286	0	0	1.484049931	1.020167844	PCNA
Degradation of GLI1 by the Proteasome	2/47	0.5099584	0.892997233	0	0	1.187222222	0.799506415	PSMA6;RPS27A
Degradation of GLI2 by the Proteasome	2/47	0.5099584	0.892997233	0	0	1.187222222	0.799506415	PSMA6;RPS27A
GLI3 Is Processed to GLI3R by the Proteasome	2/47	0.5099584	0.892997233	0	0	1.187222222	0.799506415	PSMA6;RPS27A
Heme Signaling	2/47	0.5099584	0.892997233	0	0	1.187222222	0.799506415	APOA1;APOB
Positive Epigenetic Regulation of rRNA Expression	3/75	0.5117053	0.894722911	0	0	1.113004172	0.745719797	POLR2E;POLR1E;DDX21
Apoptotic Factor-Mediated Response	1/20	0.5208344	0.895440243	0	0	1.405869042	0.917080837	C1QBP
Class I Peroxisomal Membrane Protein Import	1/20	0.5208344	0.895440243	0	0	1.405869042	0.917080837	ABCD3
G Beta Gamma Signalling Through CDC42	1/20	0.5208344	0.895440243	0	0	1.405869042	0.917080837	GNG13
G Beta Gamma Signalling Through PLC Beta	1/20	0.5208344	0.895440243	0	0	1.405869042	0.917080837	GNG13
IRAK4 Deficiency (TLR2 4)	1/20	0.5208344	0.895440243	0	0	1.405869042	0.917080837	CD36
Induction of Cell-Cell Fusion	1/20	0.5208344	0.895440243	0	0	1.405869042	0.917080837	ACE2
Maturation of TCA Enzymes and Regulation of TCA Cycle	1/20	0.5208344	0.895440243	0	0	1.405869042	0.917080837	SDHC
Protein Hydroxylation	1/20	0.5208344	0.895440243	0	0	1.405869042	0.917080837	ETF1
Signaling by PDGFR in Disease	1/20	0.5208344	0.895440243	0	0	1.405869042	0.917080837	BIN2
TP53 Regulates Transcription of Genes Involved in Cytochrome C Release	1/20	0.5208344	0.895440243	0	0	1.405869042	0.917080837	AIFM2
Defective CFTR Causes Cystic Fibrosis	2/48	0.5212605	0.895440243	0	0	1.161352657	0.75662741	PSMA6;RPS27A
RNA Polymerase I Transcription Initiation	2/48	0.5212605	0.895440243	0	0	1.161352657	0.75662741	POLR2E;POLR1E
Protein Ubiquitination	3/77	0.5294817	0.908235166	0	0	1.082810209	0.688511941	PCNA;RPS27A;RNF152
Dectin-1 Mediated Noncanonical NF-kB Signaling	2/49	0.5323865	0.908655403	0	0	1.136583924	0.716485904	PSMA6;RPS27A
Signaling by NOTCH3	2/49	0.5323865	0.908655403	0	0	1.136583924	0.716485904	FABP7;RPS27A
TP53 Regulates Transcription of Cell Cycle Genes	2/49	0.5323865	0.908655403	0	0	1.136583924	0.716485904	CDKN1A;PCNA
Viral Infection Pathways	37/1029	0.5347918	0.908655403	0	0	0.99567636	0.623171682	FEN1;RPL32;RPL31;RPLP1;TAT;RPL11;RPL36A;ATP1A1;RPL7;RPS14;RPS17;RPL36;POLR2E;AP1S3;RPS27A;RPS11;RPL17;MBL2;RANBP1;RPS9;DUT;PKLR;LIG1;RPS8;RPL22;RPL13A;RPL35A;RPS3A;ACE2;PSMA6;RPS
Transcriptional Regulation by RUNX2	4/106	0.5354709	0.908655403	0	0	1.04735376	0.654186228	PSMA6;CDKN1A;UCMA;RPS27A
ER Quality Control Compartment (ERQC)	1/21	0.5381495	0.908655403	0	0	1.335506241	0.827504691	RPS27A
Neurotoxicity of Clostridium Toxins	1/21	0.5381495	0.908655403	0	0	1.335506241	0.827504691	STX1A
Presynaptic Function of Kainate Receptors	1/21	0.5381495	0.908655403	0	0	1.335506241	0.827504691	GNG13
Regulation of FZD by Ubiquitination	1/21	0.5381495	0.908655403	0	0	1.335506241	0.827504691	RPS27A
VxPx Cargo-Targeting to Cilium	1/21	0.5381495	0.908655403	0	0	1.335506241	0.827504691	ARF4
Interferon Alpha Beta Signaling	3/78	0.5382333	0.908655403	0	0	1.068317107	0.661783046	IFI27;RPS27A;IFIT2
Signaling by ERBB2	2/50	0.5433345	0.91595134	0	0	1.112847222	0.67887025	PTK6;RPS27A
PCP CE Pathway	3/79	0.5468910	0.919308939	0	0	1.054205402	0.636218999	PSMA6;PFN1;RPS27A
Signaling by MET	3/79	0.5468910	0.919308939	0	0	1.054205402	0.636218999	SH3GL3;RPS27A;SH3GL1
Asparagine N-linked Glycosylation	11/306	0.5488978	0.920532111	0	0	0.995556509	0.597177453	NEU3;ARF4;SEC16B;SERPINA1;SLC17A5;PGM3;MVD;NANS;RPS27A;ASGR1;ASGR2
Asymmetric Localization of PCP Proteins	2/51	0.5541027	0.920532111	0	0	1.090079365	0.643588468	PSMA6;RPS27A
Formation of TC-NER Pre-Incision Complex	2/51	0.5541027	0.920532111	0	0	1.090079365	0.643588468	POLR2E;RPS27A
Neurotransmitter Release Cycle	2/51	0.5541027	0.920532111	0	0	1.090079365	0.643588468	GLS2;STX1A
ADP Signalling Through P2Y Purinoceptor 12	1/22	0.5548398	0.920532111	0	0	1.27184466	0.749212851	GNG13
Common Pathway of Fibrin Clot Formation	1/22	0.5548398	0.920532111	0	0	1.27184466	0.749212851	SERPIND1
JNK (c-Jun Kinases) Phosphorylation and Activation Mediated by Activated Human TAK1	1/22	0.5548398	0.920532111	0	0	1.27184466	0.749212851	RPS27A
Nef-mediates Down Modulation of Cell Surface Receptors by Recruiting Them to Clathrin Adapters	1/22	0.5548398	0.920532111	0	0	1.27184466	0.749212851	AP1S3
Regulation of Signaling by CBL	1/22	0.5548398	0.920532111	0	0	1.27184466	0.749212851	RPS27A
ER-Phagosome Pathway	3/80	0.5554529	0.920532111	0	0	1.040460235	0.611760752	PSMA6;CD36;RPS27A
Hedgehog Ligand Biogenesis	2/52	0.5646898	0.934522012	0	0	1.068222222	0.610466091	PSMA6;RPS27A
Activated TAK1 Mediates P38 MAPK Activation	1/23	0.5709277	0.936290416	0	0	1.213970496	0.680421404	RPS27A
Abortive Elongation of HIV-1 Transcript in the Absence of Tat	1/23	0.5709277	0.936290416	0	0	1.213970496	0.680421404	POLR2E
Dopamine Neurotransmitter Release Cycle	1/23	0.5709277	0.936290416	0	0	1.213970496	0.680421404	STX1A
IKK Complex Recruitment Mediated by RIP1	1/23	0.5709277	0.936290416	0	0	1.213970496	0.680421404	RPS27A
Mitochondrial Calcium Ion Transport	1/23	0.5709277	0.936290416	0	0	1.213970496	0.680421404	SMDT1
Suppression of Phagosomal Maturation	1/23	0.5709277	0.936290416	0	0	1.213970496	0.680421404	RPS27A
Hemostasis	25/707	0.5719611	0.936290416	0	0	0.978007969	0.546397681	CFD;SERPINA1;F11R;CYB5R1;PLAU;C1QBP;CD36;APOB;CD74;ANXA2;SERPIND1;AHSG;SERPINF2;ANXA5;ITPK1;APOA1;MIF;GNG13;SOD1;TF;CEACAM1;SCCPDH;P2RX4;KIF16B;PFN1
Negative Epigenetic Regulation of rRNA Expression	3/82	0.5722831	0.936290416	0	0	1.014013838	0.565942893	POLR2E;POLR1E;RRP8
Degradation of the Extracellular Matrix	5/140	0.5729300	0.936290416	0	0	0.988842399	0.550776956	PRSS1;CAPN9;CAPNS1;CTRB2;CTRB1
Oxygen-dependent Proline Hydroxylation of Hypoxia-inducible Factor Alpha	2/53	0.5750950	0.937221417	0	0	1.047222222	0.579344288	PSMA6;RPS27A
RHOD GTPase Cycle	2/53	0.5750950	0.937221417	0	0	1.047222222	0.579344288	MOSPD2;FILIP1
Regulation of HSF1-mediated Heat Shock Response	3/83	0.5805482	0.941340474	0	0	1.001286509	0.54448189	MRPL18;RPA3;RPA2
Activation of NF-kappaB in B Cells	2/54	0.5853174	0.941340474	0	0	1.027029915	0.550078215	PSMA6;RPS27A
Autodegradation of Cdh1 by Cdh1 APC C	2/54	0.5853174	0.941340474	0	0	1.027029915	0.550078215	PSMA6;RPS27A
Ion Homeostasis	2/54	0.5853174	0.941340474	0	0	1.027029915	0.550078215	CASQ1;ATP1A1
APC C Cdc20 Mediated Degradation of Cyclin B	1/24	0.5864350	0.941340474	0	0	1.161128867	0.619686692	RPS27A
Assembly Of The HIV Virion	1/24	0.5864350	0.941340474	0	0	1.161128867	0.619686692	RPS27A
G-protein Activation	1/24	0.5864350	0.941340474	0	0	1.161128867	0.619686692	GNG13
Other Interleukin Signaling	1/24	0.5864350	0.941340474	0	0	1.161128867	0.619686692	STX1A
RAS Processing	1/24	0.5864350	0.941340474	0	0	1.161128867	0.619686692	RPS27A
RUNX2 Regulates Osteoblast Differentiation	1/24	0.5864350	0.941340474	0	0	1.161128867	0.619686692	UCMA
Thromboxane Signalling Through TP Receptor	1/24	0.5864350	0.941340474	0	0	1.161128867	0.619686692	GNG13
Bacterial Infection Pathways	5/143	0.5918339	0.946904283	0	0	0.967194227	0.507321543	GUCY2C;SH3GL3;RPS27A;STX1A;SH3GL1
APC C Cdc20 Mediated Degradation of Securin	2/55	0.5953567	0.946904283	0	0	1.007599581	0.52253557	PSMA6;RPS27A
Regulation of RAS by GAPs	2/55	0.5953567	0.946904283	0	0	1.007599581	0.52253557	PSMA6;RPS27A
Signaling by Non-Receptor Tyrosine Kinases	2/55	0.5953567	0.946904283	0	0	1.007599581	0.52253557	PTK6;RPS27A
Signaling by PTK6	2/55	0.5953567	0.946904283	0	0	1.007599581	0.52253557	PTK6;RPS27A
ADP Signalling Through P2Y Purinoceptor 1	1/25	0.6013826	0.946904283	0	0	1.112690707	0.56582973	GNG13
G Beta Gamma Signalling Through PI3Kgamma	1/25	0.6013826	0.946904283	0	0	1.112690707	0.56582973	GNG13
Inactivation of CSF3 (G-CSF) Signaling	1/25	0.6013826	0.946904283	0	0	1.112690707	0.56582973	RPS27A
Interleukin-7 Signaling	1/25	0.6013826	0.946904283	0	0	1.112690707	0.56582973	CISH
NOTCH2 Activation and Transmission of Signal to the Nucleus	1/25	0.6013826	0.946904283	0	0	1.112690707	0.56582973	RPS27A
NOTCH3 Activation and Transmission of Signal to the Nucleus	1/25	0.6013826	0.946904283	0	0	1.112690707	0.56582973	RPS27A
NOTCH3 Intracellular Domain Regulates Transcription	1/25	0.6013826	0.946904283	0	0	1.112690707	0.56582973	FABP7
Signaling by EGFR in Cancer	1/25	0.6013826	0.946904283	0	0	1.112690707	0.56582973	RPS27A
Clathrin-mediated Endocytosis	5/145	0.6041765	0.946904283	0	0	0.953277545	0.480345966	SH3GL3;TF;APOB;RPS27A;SH3GL1
Late Phase of HIV Life Cycle	5/145	0.6041765	0.946904283	0	0	0.953277545	0.480345966	RANBP1;TAT;CHMP4C;POLR2E;RPS27A
Transcriptional Regulation by RUNX3	3/86	0.6047294	0.946904283	0	0	0.964944619	0.485342177	PSMA6;CDKN1A;RPS27A
Deadenylation-dependent mRNA Decay	2/56	0.6052128	0.946904283	0	0	0.988888889	0.496595317	EXOSC5;EXOSC2
Golgi Associated Vesicle Biogenesis	2/56	0.6052128	0.946904283	0	0	0.988888889	0.496595317	FTH1;AP1S3
Regulation of PTEN Stability and Activity	2/56	0.6052128	0.946904283	0	0	0.988888889	0.496595317	PSMA6;RPS27A
Neddylation	8/234	0.6121518	0.947060339	0	0	0.944547731	0.463560296	PSMA6;CDKN1A;CISH;ASB10;FBXO2;RPS27A;ASB18;DDB2
Platelet Homeostasis	3/87	0.6125816	0.947060339	0	0	0.95340751	0.46723926	P2RX4;APOB;GNG13
Chemokine Receptors Bind Chemokines	2/57	0.6148858	0.947060339	0	0	0.970858586	0.472146555	CXCR3;CCL27
Meiotic Recombination	2/57	0.6148858	0.947060339	0	0	0.970858586	0.472146555	RPA3;RPA2
APC-Cdc20 Mediated Degradation of Nek2A	1/26	0.6157907	0.947060339	0	0	1.068127601	0.517879648	RPS27A
Attachment and Entry 9694614	1/26	0.6157907	0.947060339	0	0	1.068127601	0.517879648	ACE2
Calnexin Calreticulin Cycle	1/26	0.6157907	0.947060339	0	0	1.068127601	0.517879648	RPS27A
Constitutive Signaling by AKT1 E17K in Cancer	1/26	0.6157907	0.947060339	0	0	1.068127601	0.517879648	CDKN1A
Downregulation of TGF-beta Receptor Signaling	1/26	0.6157907	0.947060339	0	0	1.068127601	0.517879648	RPS27A
Fertilization	1/26	0.6157907	0.947060339	0	0	1.068127601	0.517879648	SPAM1
Glucuronidation	1/26	0.6157907	0.947060339	0	0	1.068127601	0.517879648	SLC35D1
MicroRNA (miRNA) Biogenesis	1/26	0.6157907	0.947060339	0	0	1.068127601	0.517879648	POLR2E
Synthesis of IP3 and IP4 in the Cytosol	1/26	0.6157907	0.947060339	0	0	1.068127601	0.517879648	ITPK1
TNFR2 Non-Canonical NF-kB Pathway	3/88	0.6203284	0.951377224	0	0	0.942141864	0.449878492	PSMA6;TNFSF14;RPS27A
Signaling by ERBB4	2/58	0.6243761	0.951377224	0	0	0.953472222	0.449087528	STMN1;RPS27A
Selective Autophagy	3/89	0.6279692	0.951377224	0	0	0.931138209	0.433225184	PARK7;RPS27A;PRKAG3
SARS-CoV Infections	17/498	0.6284291	0.951377224	0	0	0.942330547	0.437742702	RPS9;PKLR;RPS8;RPS3A;ATP1A1;ACE2;RPS14;RPS25;RPS17;CHMP4C;SNRPE;RPS20;FAU;RPS27A;RPS11;RPS24;MBL2
Acyl Chain Remodelling of PC	1/27	0.6296786	0.951377224	0	0	1.026992425	0.47503084	PLA2G1B
Attenuation Phase	1/27	0.6296786	0.951377224	0	0	1.026992425	0.47503084	MRPL18
Cell Recruitment (Pro-Inflammatory Response)	1/27	0.6296786	0.951377224	0	0	1.026992425	0.47503084	P2RX4
G0 and Early G1	1/27	0.6296786	0.951377224	0	0	1.026992425	0.47503084	PCNA
Purinergic Signaling in Leishmaniasis Infection	1/27	0.6296786	0.951377224	0	0	1.026992425	0.47503084	P2RX4
Pyroptosis	1/27	0.6296786	0.951377224	0	0	1.026992425	0.47503084	CHMP4C
RNA Pol II CTD Phosphorylation and Interaction With CE	1/27	0.6296786	0.951377224	0	0	1.026992425	0.47503084	POLR2E
RNA Pol II CTD Phosphorylation and Interaction With CE During HIV Infection	1/27	0.6296786	0.951377224	0	0	1.026992425	0.47503084	POLR2E
Syndecan Interactions	1/27	0.6296786	0.951377224	0	0	1.026992425	0.47503084	VTN
tRNA Processing in the Nucleus	2/59	0.6336843	0.951377224	0	0	0.936695906	0.427324737	POP5;ZBTB8OS
Non-integrin membrane-ECM Interactions	2/59	0.6336843	0.951377224	0	0	0.936695906	0.427324737	VTN;TTR

RNA Polymerase I Promoter Escape	2/59	0.6336843	0.951377224	0	0	0.936695906	0.427324737	POLR2E;POLR1E
Regulation of RUNX2 Expression and Activity	2/59	0.6336843	0.951377224	0	0	0.936695906	0.427324737	PSMA6;RPS27A
Developmental Biology	48/1385	0.6389733	0.951377224	0	0	0.955644973	0.428026196	CDKN1A;RPL32;RPL31;RPLP1;RPL11;RPL36A;SLC2A2;PPL;EVPL;RPL7;PLAC8;RPS14;RPS17;HINT1;CAPNS1;RPL36;POLR2E;MBP;CD36;RPS27A;RPS11;RPL17;RPS9;PKLR;LIG1;RPS8;RPL22;CYP51A1;ADIPOQ;RPL13
CDK-mediated Phosphorylation and Removal of Cdc6	2/60	0.6428110	0.951377224	0	0	0.920498084	0.406772154	PSMA6;RPS27A
Cdc20 Phospho-APC C Mediated Degradation of Cyclin A	2/60	0.6428110	0.951377224	0	0	0.920498084	0.406772154	PSMA6;RPS27A
Activation of AMPK Downstream of NMDARs	1/28	0.6430653	0.951377224	0	0	0.9889043	0.436610137	PRKAG3
Adrenaline,noradrenaline Inhibits Insulin Secretion	1/28	0.6430653	0.951377224	0	0	0.9889043	0.436610137	GNG13
Caspase Activation via Extrinsic Apoptotic Signalling Pathway	1/28	0.6430653	0.951377224	0	0	0.9889043	0.436610137	DAPK2
FGFR2 Alternative Splicing	1/28	0.6430653	0.951377224	0	0	0.9889043	0.436610137	POLR2E
Keratan Sulfate Biosynthesis	1/28	0.6430653	0.951377224	0	0	0.9889043	0.436610137	OMD
Platelet Calcium Homeostasis	1/28	0.6430653	0.951377224	0	0	0.9889043	0.436610137	P2RX4
TRP Channels	1/28	0.6430653	0.951377224	0	0	0.9889043	0.436610137	TRPV6
Extracellular Matrix Organization	10/300	0.6460715	0.951377224	0	0	0.919604804	0.401724825	MFAP5;VTN;PRSS1;CEACAM1;CAPN9;TTR;CAPNS1;CTRB2;CTRB1;F11R
Binding and Uptake of Ligands by Scavenger Receptors	4/122	0.6465213	0.951377224	0	0	0.904584297	0.394533529	FTH1;APOA1;CD36;APOB
APC C Cdh1 Mediated Degradation of Cdc20 and Other APC C Cdh1 Targets in Late Mitosis Early G1	2/61	0.6517573	0.951377224	0	0	0.904849341	0.387350525	PSMA6;RPS27A
APC Cdc20 Mediated Degradation of Cell Cycle Proteins Prior to Satisfation of the Checkpoint	2/61	0.6517573	0.951377224	0	0	0.904849341	0.387350525	PSMA6;RPS27A
Gamma Carboxylation, Hypusinylation, Hydroxylation, and Arylsulfatase Activation	2/61	0.6517573	0.951377224	0	0	0.904849341	0.387350525	ETF1;DPH6
Regulated Necrosis	2/61	0.6517573	0.951377224	0	0	0.904849341	0.387350525	CHMP4C;RPS27A
mRNA Capping	1/29	0.6559686	0.951377224	0	0	0.953536755	0.402051383	POLR2E
Activation of G Protein Gated Potassium Channels	1/29	0.6559686	0.951377224	0	0	0.953536755	0.402051383	GNG13
Acyl Chain Remodelling of PE	1/29	0.6559686	0.951377224	0	0	0.953536755	0.402051383	PLA2G1B
Downregulation of ERBB2 Signaling	1/29	0.6559686	0.951377224	0	0	0.953536755	0.402051383	RPS27A
Energy Dependent Regulation of mTOR by LKB1-AMPK	1/29	0.6559686	0.951377224	0	0	0.953536755	0.402051383	PRKAG3
G Protein Gated Potassium Channels	1/29	0.6559686	0.951377224	0	0	0.953536755	0.402051383	GNG13
Inhibition of Voltage Gated Ca2+ Channels via Gbeta Gamma Subunits	1/29	0.6559686	0.951377224	0	0	0.953536755	0.402051383	GNG13
Negative Regulation of FGFR3 Signaling	1/29	0.6559686	0.951377224	0	0	0.953536755	0.402051383	RPS27A
PIWI-interacting RNA (piRNA) Biogenesis	1/29	0.6559686	0.951377224	0	0	0.953536755	0.402051383	POLR2E
Processing of Capped Intronless Pre-mRNA	1/29	0.6559686	0.951377224	0	0	0.953536755	0.402051383	SNRPE
TNFs Bind Their Physiological Receptors	1/29	0.6559686	0.951377224	0	0	0.953536755	0.402051383	TNFSF14
The Role of Nef in HIV-1 Replication and Disease Pathogenesis	1/29	0.6559686	0.951377224	0	0	0.953536755	0.402051383	AP1S3
Cellular Responses to Mechanical Stimuli	3/93	0.6574623	0.951377224	0	0	0.889568846	0.37305646	CAPNS1;ANXA2;GNG13
Post-translational Modification Synthesis of GPI-anchored Proteins	3/93	0.6574623	0.951377224	0	0	0.889568846	0.37305646	XPNPEP2;ALPI;MELTF
Response of Endothelial Cells to Shear Stress	3/93	0.6574623	0.951377224	0	0	0.889568846	0.37305646	CAPNS1;ANXA2;GNG13
Cellular Response to Hypoxia	2/62	0.6605243	0.953459602	0	0	0.889722222	0.368986742	PSMA6;RPS27A
TP53 Regulates Transcription of DNA Repair Genes	2/62	0.6605243	0.953459602	0	0	0.889722222	0.368986742	POLR2E;DDB2
Activation of Kainate Receptors Upon Glutamate Binding	1/30	0.6684061	0.959961028	0	0	0.92060835	0.370875566	GNG13
Regulation of TP53 Activity Through Acetylation	1/30	0.6684061	0.959961028	0	0	0.92060835	0.370875566	ING5
Signaling by CSF3 (G-CSF)	1/30	0.6684061	0.959961028	0	0	0.92060835	0.370875566	RPS27A
Synthesis of Active Ubiquitin Roles of E1 and E2 Enzymes	1/30	0.6684061	0.959961028	0	0	0.92060835	0.370875566	RPS27A
APC C Cdc20 Mediated Degradation of Mitotic Proteins	2/63	0.6691132	0.959961028	0	0	0.875091075	0.351613286	PSMA6;RPS27A
Activation of APC C and APC C Cdc20 Mediated Degradation of Mitotic Proteins	2/64	0.6775255	0.963209306	0	0	0.8609319	0.335167729	PSMA6;RPS27A
Antigen processing-Cross Presentation	3/96	0.6784526	0.963209306	0	0	0.860738481	0.333915451	PSMA6;CD36;RPS27A
Activated NOTCH1 Transmits Signal to the Nucleus	1/31	0.6803946	0.963209306	0	0	0.889875173	0.342675151	RPS27A
Downregulation of SMAD2 3 SMAD4 Transcriptional Activity	1/31	0.6803946	0.963209306	0	0	0.889875173	0.342675151	RPS27A
Metalloprotease DUBs	1/31	0.6803946	0.963209306	0	0	0.889875173	0.342675151	RPS27A
Negative Regulation of FGFR4 Signaling	1/31	0.6803946	0.963209306	0	0	0.889875173	0.342675151	RPS27A
Nicotinate Metabolism	1/31	0.6803946	0.963209306	0	0	0.889875173	0.342675151	SLC5A8
RUNX2 Regulates Bone Development	1/31	0.6803946	0.963209306	0	0	0.889875173	0.342675151	UCMA
Sealing of the Nuclear Envelope (NE) by ESCRT-III	1/31	0.6803946	0.963209306	0	0	0.889875173	0.342675151	CHMP4C
Signaling by CSF1 (M-CSF) in Myeloid Cells	1/31	0.6803946	0.963209306	0	0	0.889875173	0.342675151	RPS27A
Transferrin Endocytosis and Recycling	1/31	0.6803946	0.963209306	0	0	0.889875173	0.342675151	TF
O-linked Glycosylation of Mucins	2/65	0.6857626	0.969640354	0	0	0.847222222	0.319592282	B3GNT5;GCNT4
G-protein Beta Gamma Signalling	1/32	0.6919502	0.972537765	0	0	0.861124782	0.31710161	GNG13
Nuclear Signaling by ERBB4	1/32	0.6919502	0.972537765	0	0	0.861124782	0.31710161	STMN1
PINK1-PRKN Mediated Mitophagy	1/32	0.6919502	0.972537765	0	0	0.861124782	0.31710161	RPS27A
Regulation of Necroptotic Cell Death	1/32	0.6919502	0.972537765	0	0	0.861124782	0.31710161	RPS27A
Thrombin Signalling Through Proteinase Activated Receptors (PARs)	1/32	0.6919502	0.972537765	0	0	0.861124782	0.31710161	GNG13
DNA Repair	10/312	0.6934912	0.973539101	0	0	0.882506139	0.323011937	RFC5;FEN1;PCNA;LIG1;RFC2;RPA3;POLR2E;RPA2;RPS27A;DDB2
Signaling by TGFB Family Members	5/161	0.69478	0.974184368	0	0	0.854790259	0.311280446	HELLS;MYF6;F11R;RPS27A;MYF5
Cargo Concentration in the ER	1/33	0.7030886	0.974208906	0	0	0.83417129	0.293855444	SERPINA1
FGFR2 Mutant Receptor Activation	1/33	0.7030886	0.974208906	0	0	0.83417129	0.293855444	POLR2E
Formation of the Early Elongation Complex	1/33	0.7030886	0.974208906	0	0	0.83417129	0.293855444	POLR2E
Formation of the HIV-1 Early Elongation Complex	1/33	0.7030886	0.974208906	0	0	0.83417129	0.293855444	POLR2E
Glucagon Signaling in Metabolic Regulation	1/33	0.7030886	0.974208906	0	0	0.83417129	0.293855444	GNG13
HIV Elongation Arrest and Recovery	1/33	0.7030886	0.974208906	0	0	0.83417129	0.293855444	POLR2E
Negative Regulation of FGFR1 Signaling	1/33	0.7030886	0.974208906	0	0	0.83417129	0.293855444	RPS27A
Pausing and Recovery of HIV Elongation	1/33	0.7030886	0.974208906	0	0	0.83417129	0.293855444	POLR2E
Signal Amplification	1/33	0.7030886	0.974208906	0	0	0.83417129	0.293855444	GNG13
TNFR1-induced NF-kappa-B Signaling Pathway	1/33	0.7030886	0.974208906	0	0	0.83417129	0.293855444	RPS27A
Downstream Signaling Events of B Cell Receptor (BCR)	2/68	0.7094406	0.980303679	0	0	0.808585859	0.277570144	PSMA6;RPS27A
Regulation of APC C Activators Between G1 S and Early Anaphase	2/68	0.7094406	0.980303679	0	0	0.808585859	0.277570144	PSMA6;RPS27A
Cellular Response to Heat Stress	3/101	0.7112892	0.980303679	0	0	0.816610372	0.278199653	MRPL18;RPA3;RPA2
Negative Regulation of FGFR2 Signaling	1/34	0.7138248	0.980303679	0	0	0.808851343	0.272678119	RPS27A
RIPK1-mediated Regulated Necrosis	1/34	0.7138248	0.980303679	0	0	0.808851343	0.272678119	RPS27A
Infectious Disease	44/1311	0.7165044	0.980303679	0	0	0.922537851	0.307547242	GUCY2C;SH3GL3;FEN1;RPL32;RPL31;RPLP1;TAT;RPL11;RPL36A;ATP1A1;RPL7;RPS14;RPS17;RPL36;POLR2E;AP1S3;RPS27A;RPS11;GGT1;RPL17;MBL2;SH3GL1;RANBP1;RPS9;DUT;PKLR;LIG1;RPS8;RPL22;RPL13A;F
COPII-mediated Vesicle Transport	2/69	0.7169949	0.980303679	0	0	0.796475954	0.264976774	SEC16B;SERPINA1
High Laminar Flow Shear Stress Activates Signaling by PIEZO1 and PECAM1 CDH5 KDR in Endothelial	2/69	0.7169949	0.980303679	0	0	0.796475954	0.264976774	CAPNS1;GNG13
Beta-catenin Independent WNT Signaling	4/134	0.7173682	0.980303679	0	0	0.820569959	0.272565408	PSMA6;PFN1;RPS27A;GNG13
Signaling by NTRKs	4/134	0.7173682	0.980303679	0	0	0.820569959	0.272565408	SH3GL3;TF;ASCL1;PCSK5
Epigenetic Regulation of Adipogenesis Genes by MLL3 and MLL4 Complexes	3/103	0.7236800	0.980303679	0	0	0.800194715	0.25878775	DGAT2;ADIPOQ;CD36
Epigenetic Regulation of Gene Expression by MLL3 and MLL4 Complexes	3/103	0.7236800	0.980303679	0	0	0.800194715	0.25878775	DGAT2;ADIPOQ;CD36
MLL4 and MLL3 Complexes Regulate Expression of PPARG Targets in Adipogenesis and Hepatic Steat	3/103	0.7236800	0.980303679	0	0	0.800194715	0.25878775	DGAT2;ADIPOQ;CD36
Diseases Associated With the TLR Signaling Cascade	1/35	0.7241732	0.980303679	0	0	0.785020804	0.253345518	CD36
Diseases of Immune System	1/35	0.7241732	0.980303679	0	0	0.785020804	0.253345518	CD36
Inwardly Rectifying K+ Channels	1/35	0.7241732	0.980303679	0	0	0.785020804	0.253345518	GNG13
N-glycan Trimming in the ER and Calnexin Calreticulin Cycle	1/35	0.7241732	0.980303679	0	0	0.785020804	0.253345518	RPS27A
Negative Regulators of DDX58 IFIH1 Signaling	1/35	0.7241732	0.980303679	0	0	0.785020804	0.253345518	RPS27A
Oncogene Induced Senescence	1/35	0.7241732	0.980303679	0	0	0.785020804	0.253345518	RPS27A
Translation of Structural Proteins 9683701	1/35	0.7241732	0.980303679	0	0	0.785020804	0.253345518	RPS27A
MITF-M-regulated Melanocyte Development	4/136	0.7280957	0.98447935	0	0	0.808052672	0.256413421	CDKN1A;HINT1;CEACAM1;LIG1
Transcription of the HIV Genome	2/71	0.7316083	0.984730507	0	0	0.773309179	0.24166684	TAT;POLR2E
Aberrant Regulation of Mitotic Cell Cycle Due to RB1 Defects	1/36	0.7341480	0.984730507	0	0	0.762552011	0.235662588	CDKN1A
Regulation of TP53 Degradation	1/36	0.7341480	0.984730507	0	0	0.762552011	0.235662588	RPS27A
Rev-mediated Nuclear Export of HIV RNA	1/36	0.7341480	0.984730507	0	0	0.762552011	0.235662588	RANBP1
SMAD2 SMAD3 SMAD4 Heterotrimer Regulates Transcription	1/36	0.7341480	0.984730507	0	0	0.762552011	0.235662588	RPS27A
Signaling by NOTCH2	1/36	0.7341480	0.984730507	0	0	0.762552011	0.235662588	RPS27A
Transcriptional Regulation by the AP-2 (TFAP2) Family of Transcription Factors	1/36	0.7341480	0.984730507	0	0	0.762552011	0.235662588	CDKN1A
Degradation of Beta-Catenin by the Destruction Complex	2/72	0.7386710	0.988541169	0	0	0.762222222	0.2308785	PSMA6;RPS27A
Signaling by NOTCH4	2/72	0.7386710	0.988541169	0	0	0.762222222	0.2308785	PSMA6;RPS27A
Complement Cascade	4/139	0.7436124	0.988598386	0	0	0.789972145	0.234017605	CFD;VTN;C7;MBL2
Cross-presentation of Soluble Exogenous Antigens (Endosomes)	1/37	0.7437625	0.988598386	0	0	0.741331484	0.219458935	PSMA6
Evasion by RSV of Host Interferon Responses	1/37	0.7437625	0.988598386	0	0	0.741331484	0.219458935	RPS27A
HSF1-dependent Transactivation	1/37	0.7437625	0.988598386	0	0	0.741331484	0.219458935	MRPL18
NOD1 2 Signaling Pathway	1/37	0.7437625	0.988598386	0	0	0.741331484	0.219458935	RPS27A
Regulation of TP53 Expression and Degradation	1/37	0.7437625	0.988598386	0	0	0.741331484	0.219458935	RPS27A
Hedgehog 'On' State	2/73	0.7455755	0.989888502	0	0	0.751447574	0.220624067	PSMA6;RPS27A
RHOC GTPase Cycle	2/74	0.7523225	0.991939472	0	0	0.740972222	0.21087338	ABCD3;C1QBP
RHOG GTPase Cycle	2/74	0.7523225	0.991939472	0	0	0.740972222	0.21087338	NDUFA5;HSPE1
Cooperation of PDCL (PhLP1) and TRiC CCT in G-protein Beta Folding	1/38	0.7530297	0.991939472	0	0	0.721258013	0.204585184	GNG13
Defective B3GALTL Causes PpS	1/38	0.7530297	0.991939472	0	0	0.721258013	0.204585184	SPON2
Diseases of Mitotic Cell Cycle	1/38	0.7530297	0.991939472	0	0	0.721258013	0.204585184	CDKN1A
FLT3 Signaling	1/38	0.7530297	0.991939472	0	0	0.721258013	0.204585184	RPS27A
Interactions of Rev With Host Cellular Proteins	1/38	0.7530297	0.991939472	0	0	0.721258013	0.204585184	RANBP1
APC C-mediated Degradation of Cell Cycle Proteins	2/75	0.7589147	0.994784163	0	0	0.730783866	0.201598258	PSMA6;RPS27A
Regulation of Mitotic Cell Cycle	2/75	0.7589147	0.994784163	0	0	0.730783866	0.201598258	PSMA6;RPS27A
Signaling by FGFR2	2/75	0.7589147	0.994784163	0	0	0.730783866	0.201598258	POLR2E;RPS27A
Mitophagy	1/39	0.7619623	0.994784163	0	0	0.702241039	0.19090995	RPS27A
O-glycosylation of TSR Domain-Containing Proteins	1/39	0.7619623	0.994784163	0	0	0.702241039	0.19090995	SPON2
Ovarian Tumor Domain Proteases	1/39	0.7619623	0.994784163	0	0	0.702241039	0.19090995	RPS27A
Response of Mtb to Phagocytosis	1/39	0.7619623	0.994784163	0	0	0.702241039	0.19090995	RPS27A
SIRT1 Negatively Regulates rRNA Expression	1/39	0.7619623	0.994784163	0	0	0.702241039	0.19090995	RRP8
Macroautophagy	4/143	0.7632410	0.995347609	0	0	0.767078816	0.207250441	CHMP4C;PARK7;RPS27A;PRKAG3
ISG15 Antiviral Mechanism	2/76	0.7653546	0.995893413	0	0	0.720870871	0.192772344	FLNB;RPS27A
NoRC Negatively Regulates rRNA Expression	2/76	0.7653546	0.995893413	0	0	0.720870871	0.192772344	POLR2E;POLR1E
Class C 3 (Metabotropic Glutamate Pheromone Receptors)	1/40	0.7705722	0.999996012	0	0	0.684199296	0.178317307	GPRC6A
Signaling by FGFR3	1/40	0.7705722	0.999996012	0	0	0.684199296	0.178317307	RPS27A
Interleukin-4 and Interleukin-13 Signaling	3/112	0.7743386	0.999996012	0	0	0.733779076	0.187661053	CDKN1A;ANXA1;CD36
MTOR Signalling	1/41	0.7788711	0.999996012	0	0	0.667059639	0.166704669	PRKAG3
Sensory Perception of Sweet, Bitter, and Umami (Glutamate) Taste	1/41	0.7788711	0.999996012	0	0	0.667059639	0.166704669	GNG13
Signaling by FGFR4	1/41	0.7788711	0.999996012	0	0	0.667059639	0.166704669	RPS27A
RNA Polymerase I Promoter Clearance	2/79	0.7837843	0.999996012	0	0	0.692676768	0.1687509	POLR2E;POLR1E
O-linked Glycosylation	3/114	0.7845018	0.999996012	0	0	0.720482652	0.174865712	SPON2;B3GNT5;GCNT4
tRNA Processing in the Mitochondrion	1/42	0.7868702	0.999996012	0	0	0.650756064	0.155980994	HSD17B10
Deactivation of the Beta-Catenin Transactivating Complex	1/42	0.7868702	0.999996012	0	0	0.650756064	0.155980994	RPS27A
Glucagon-like Peptide-1 (GLP1) Regulates Insulin Secretion	1/42	0.7868702	0.999996012	0	0	0.650756064	0.155980994	GNG13
RHOF GTPase Cycle	1/42	0.7868702	0.999996012	0	0	0.650756064	0.155980994	BAIAP2L2
Signaling by NTRK1 (TRKA)	3/115	0.7894399	0.999996012	0	0	0.714012517	0.168815089	SH3GL3;TF;ASCL1
Late SARS-CoV-2 Infection Events	2/80	0.7896389	0.999996012	0	0	0.683760684	0.16149024	ACE2;RPS27A
MAPK6 MAPK4 Signaling	2/80	0.7896389	0.999996012	0	0	0.683760684	0.16149024	PSMA6;RPS27A
ADORA2B Mediated Anti-Inflammatory Cytokines Production	1/43	0.7945803	0.999996012	0	0	0.635228849	0.146065274	GNG13
Activation of GABAB Receptors	1/43	0.7945803	0.999996012	0	0	0.635228849	0.146065274	GNG13
GABA B Receptor Activation	1/43	0.7945803	0.999996012	0	0	0.635228849	0.146065274	GNG13
Interactions of Vpr With Host Cellular Proteins	1/43	0.7945803	0.999996012	0	0	0.635228849	0.146065274	SLC25A4
Negative Regulation of MAPK Pathway	1/43	0.7945803	0.999996012	0	0	0.635228849	0.146065274	RPS27A
Signaling by FGFR2 in Disease	1/43	0.7945803	0.999996012	0	0	0.635228849	0.146065274	POLR2E
Vasopressin Regulates Renal Water Homeostasis via Aquaporins	1/43	0.7945803	0.999996012	0	0	0.635228849	0.146065274	GNG13
Voltage Gated Potassium Channels	1/43	0.7945803	0.999996012	0	0	0.635228849	0.146065274	KCNA7
RNA Polymerase I Transcription	2/81	0.7953533	0.999996012	0	0	0.675070323	0.15457	POLR2E;POLR1E
Senescence-Associated Secretory Phenotype (SASP)	2/81	0.7953533	0.999996012	0	0	0.675070323	0.15457	CDKN1A;RPS27A
Keratinization	6/217	0.7996020	0.999996012	0	0	0.757248008	0.169351743	KRT18;CAPNS1;KRT15;KRT5;EVPL;PPL
UCH Proteinases	2/82	0.8009301	0.999996012	0	0	0.666597222	0.147972281	PSMA6;RPS27A
SUMOylation of Transcription Cofactors	1/44	0.8020119	0.999996012	0	0	0.62042383	0.136885242	PARK7
TP53 Regulates Transcription of Cell Death Genes	1/44	0.8020119	0.999996012	0	0	0.62042383	0.136885242	AIFM2
Cell Junction Organization	3/119	0.8082598	0.999996012	0	0	0.689247518	0.146721294	CLDN15;KRT5;F11R
Formation of HIV Elongation Complex in the Absence of HIV Tat	1/45	0.8091749	0.999996012	0	0	0.606291766	0.128376277	POLR2E
GPER1 Signaling	1/45	0.8091749	0.999996012	0	0	0.606291766	0.128376277	GNG13
Somitogenesis	1/45	0.8091749	0.999996012	0	0	0.606291766	0.128376277	PSMA6

TAK1-dependent IKK and NF-kappa-B Activation	1/45	0.8091749	0.999996012	0	0	0.606291766	0.128376277	RPS27A
Transcriptional Regulation of White Adipocyte Differentiation	2/84	0.8116804	0.999996012	0	0	0.650271003	0.135678117	ADIPOQ;CD36
Kidney Development	1/46	0.8160792	0.999996012	0	0	0.592787795	0.120480458	PLAC8
SUMOylation of DNA Replication Proteins	1/46	0.8160792	0.999996012	0	0	0.592787795	0.120480458	PCNA
Integrin Cell Surface Interactions	2/85	0.8168589	0.999996012	0	0	0.642402945	0.129950967	VTN;F11R
RUNX1 Regulates Transcription of Genes Involved in Differentiation of HSCs	2/85	0.8168589	0.999996012	0	0	0.642402945	0.129950967	PSMA6;RPS27A
HIV Transcription Initiation	1/47	0.8227340	0.999996012	0	0	0.579870952	0.113145758	POLR2E
Inhibition of DNA Recombination at Telomere	1/47	0.8227340	0.999996012	0	0	0.579870952	0.113145758	POLR2E
RNA Polymerase II HIV Promoter Escape	1/47	0.8227340	0.999996012	0	0	0.579870952	0.113145758	POLR2E
RNA Polymerase II Promoter Escape	1/47	0.8227340	0.999996012	0	0	0.579870952	0.113145758	POLR2E
RNA Polymerase II Transcription Initiation	1/47	0.8227340	0.999996012	0	0	0.579870952	0.113145758	POLR2E
RNA Polymerase II Transcription Initiation And Promoter Clearance	1/47	0.8227340	0.999996012	0	0	0.579870952	0.113145758	POLR2E
RNA Polymerase II Transcription Pre-Initiation And Promoter Opening	1/47	0.8227340	0.999996012	0	0	0.579870952	0.113145758	POLR2E
Sensory Perception of Taste	1/47	0.8227340	0.999996012	0	0	0.579870952	0.113145758	GNG13
TGF-beta Receptor Signaling Activates SMADs	1/47	0.8227340	0.999996012	0	0	0.579870952	0.113145758	RPS27A
Autophagy	4/158	0.8265319	0.999996012	0	0	0.691820714	0.131803384	CHMP4C;PARK7;RPS27A;PRKAG3
CLEC7A (Dectin-1) Signaling	2/87	0.8268349	0.999996012	0	0	0.627222222	0.119266402	PSMA6;RPS27A
Regulation of PLK1 Activity at G2 M Transition	2/87	0.8268349	0.999996012	0	0	0.627222222	0.119266402	MAPRE1;RPS27A
G Alpha (Z) Signalling Events	1/48	0.8291483	0.999996012	0	0	0.567503763	0.106325348	GNG13
Inositol Phosphate Metabolism	1/48	0.8291483	0.999996012	0	0	0.567503763	0.106325348	ITPK1
Interleukin-3, Interleukin-5 and GM-CSF Signaling	1/48	0.8291483	0.999996012	0	0	0.567503763	0.106325348	RPS27A
NOTCH1 Intracellular Domain Regulates Transcription	1/48	0.8291483	0.999996012	0	0	0.567503763	0.106325348	RPS27A
Signaling by Interleukins	13/452	0.8348291	0.999996012	0	0	0.786847271	0.142048082	CDKN1A;ANXA1;ANXA2;CISH;GSTO1;EBI3;MIF;SOD2;SOD1;PSMA6;CD36;RPS27A;STX1A
Viral Messenger RNA Synthesis	1/49	0.8353308	0.999996012	0	0	0.555651872	0.09997699	POLR2E
Meiosis	2/89	0.8363194	0.999996012	0	0	0.612739464	0.109523878	RPA3;RPA2
MHC Class II Antigen Presentation	3/126	0.8377774	0.999996012	0	0	0.649784592	0.115013666	CD74;AP1S3;IFI30
Chondroitin Sulfate Dermatan Sulfate Metabolism	1/50	0.8412899	0.999996012	0	0	0.544283733	0.094062515	HEXA
Regulation of TNFR1 Signaling	1/50	0.8412899	0.999996012	0	0	0.544283733	0.094062515	RPS27A
Glycosaminoglycan Metabolism	3/127	0.8416578	0.999996012	0	0	0.644510745	0.111101885	SLC26A1;OMD;HEXA
Reproduction	3/127	0.8416578	0.999996012	0	0	0.644510745	0.111101885	RPA3;RPA2;SPAM1
Downstream TCR Signaling	2/91	0.8453318	0.999996012	0	0	0.598907615	0.100632075	PSMA6;RPS27A
Signaling by FGFR	2/91	0.8453318	0.999996012	0	0	0.598907615	0.100632075	POLR2E;RPS27A
Cargo Trafficking to the Periciliary Membrane	1/51	0.8470330	0.999996012	0	0	0.533370319	0.088547371	ARF4
Transcriptional Activity of SMAD2 SMAD3 SMAD4 Heterotrimer	1/51	0.8470330	0.999996012	0	0	0.533370319	0.088547371	RPS27A
Transcriptional and Post-Translational Regulation of MITF-M Expression and Activity	1/51	0.8470330	0.999996012	0	0	0.533370319	0.088547371	HINT1
Cell-cell Junction Organization	2/92	0.8496669	0.999996012	0	0	0.592222222	0.096479407	CLDN15;F11R
Signaling by FGFR1	1/52	0.8525698	0.999996012	0	0	0.522884882	0.083400222	RPS27A
Signaling by ALK Fusions and Activated Point Mutants	2/93	0.8538912	0.999996012	0	0	0.585683761	0.092509607	CDKN1A;RPS27A
Signaling by ALK in Cancer	2/93	0.8538912	0.999996012	0	0	0.585683761	0.092509607	CDKN1A;RPS27A
Deposition of New CENPA-containing Nucleosomes at the Centromere	1/53	0.8579058	0.999996012	0	0	0.512802731	0.078592604	CENPU
Nucleosome Assembly	1/53	0.8579058	0.999996012	0	0	0.512802731	0.078592604	CENPU
Amplification of Signal From Unattached Kinetochores via a MAD2 Inhibitory Signal	2/94	0.8580069	0.999996012	0	0	0.57928744	0.088713853	CENPU;MAPRE1
Amplification of Signal From the Kinetochores	2/94	0.8580069	0.999996012	0	0	0.57928744	0.088713853	CENPU;MAPRE1
Class B 2 (Secretin Family Receptors)	2/94	0.8580069	0.999996012	0	0	0.57928744	0.088713853	GIP;GNG13
Signaling by TGF-beta Receptor Complex	2/94	0.8580069	0.999996012	0	0	0.57928744	0.088713853	F11R;RPS27A
Antimicrobial Peptides	2/95	0.8620164	0.999996012	0	0	0.573028674	0.085083801	PGLYRP2;ATOX1
Unfolded Protein Response (UPR)	2/95	0.8620164	0.999996012	0	0	0.573028674	0.085083801	EXOSC5;EXOSC2
snRNP Assembly	1/54	0.8630490	0.999996012	0	0	0.503101039	0.074098617	SNRPE
Metabolism of Non-Coding RNA	1/54	0.8630490	0.999996012	0	0	0.503101039	0.074098617	SNRPE
SARS-CoV-1 Activates Modulates Innate Immune Responses	1/54	0.8630490	0.999996012	0	0	0.503101039	0.074098617	RPS27A
mRNA Splicing - Major Pathway	5/204	0.8638162	0.999996012	0	0	0.668579999	0.097876887	SF3B5;PPIL1;POLR2E;SNU13;SNRPE
SUMO E3 Ligases SUMOylate Target Proteins	4/170	0.8664672	0.999996012	0	0	0.641406853	0.091933441	NR5A2;PCNA;NR1H4;PARK7
Intrinsic Pathway for Apoptosis	1/55	0.8680062	0.999996012	0	0	0.493758669	0.06989466	C1QBP
Ion Transport by P-type ATPases	1/55	0.8680062	0.999996012	0	0	0.493758669	0.06989466	ATP1A1
Epigenetic Regulation by WDR5-containing Histone Modifying Complexes	3/135	0.8699010	0.999996012	0	0	0.605196611	0.084349351	DGAT2;ADIPOQ;CD36
Transcriptional Activation of Mitochondrial Biogenesis	1/56	0.8727843	0.999996012	0	0	0.484756021	0.065959196	SOD2
Hedgehog 'Off' State	2/99	0.8770378	0.999996012	0	0	0.549284078	0.07206886	PSMA6;RPS27A
Neurexins and Neuroligins	1/57	0.8773896	0.999996012	0	0	0.476074896	0.06227254	STX1A
Constitutive Signaling by NOTCH1 HD+PEST Domain Mutants	1/58	0.8818284	0.999996012	0	0	0.467698372	0.05881668	RPS27A
Constitutive Signaling by NOTCH1 PEST Domain Mutants	1/58	0.8818284	0.999996012	0	0	0.467698372	0.05881668	RPS27A
Formation of RNA Pol II Elongation Complex	1/58	0.8818284	0.999996012	0	0	0.467698372	0.05881668	POLR2E
RNA Polymerase II Transcription Elongation	1/58	0.8818284	0.999996012	0	0	0.467698372	0.05881668	POLR2E
Signaling by NOTCH1 HD+PEST Domain Mutants in Cancer	1/58	0.8818284	0.999996012	0	0	0.467698372	0.05881668	RPS27A
Signaling by NOTCH1 PEST Domain Mutants in Cancer	1/58	0.8818284	0.999996012	0	0	0.467698372	0.05881668	RPS27A
Signaling by NOTCH1 in Cancer	1/58	0.8818284	0.999996012	0	0	0.467698372	0.05881668	RPS27A
Transcriptional Regulation of Granulopoiesis	1/58	0.8818284	0.999996012	0	0	0.467698372	0.05881668	CDKN1A
RHO GTPases Activate Formins	3/139	0.8822822	0.999996012	0	0	0.587273992	0.073552102	CENPU;PFN1;MAPRE1
SUMOylation	4/176	0.8832947	0.999996012	0	0	0.618837857	0.076795514	NR5A2;PCNA;NR1H4;PARK7
COPI-dependent Golgi-to-ER Retrograde Traffic	2/101	0.8839695	0.999996012	0	0	0.538131313	0.066369146	ARF4;KIF16B
Separation of Sister Chromatids	4/177	0.8859125	0.999996012	0	0	0.615228557	0.074526933	PSMA6;CENPU;MAPRE1;RPS27A
Formation of Paraxial Mesoderm	1/59	0.8861068	0.999996012	0	0	0.459610694	0.055575108	PSMA6
TNF Signaling	1/59	0.8861068	0.999996012	0	0	0.459610694	0.055575108	RPS27A
Interleukin-1 Signaling	2/102	0.8872982	0.999996012	0	0	0.532722222	0.063699801	PSMA6;RPS27A
Sensory Perception	18/640	0.8896660	0.999996012	0	0	0.766881029	0.089654856	CLIC5;GSN;USH1C;APOA1;APOA4;GNG13;RBP4;TTR;RBP2;DHRS9;APOC2;CYP4V2;LRAT;BCO1;APOB;STX1A;PLS1;STRA6
DNA Damage Telomere Stress Induced Senescence	1/60	0.8902304	0.999996012	0	0	0.451797174	0.052532679	CDKN1A
GABA Receptor Activation	1/60	0.8902304	0.999996012	0	0	0.451797174	0.052532679	GNG13
Recruitment and ATM-med Phosphorylation of Repair and Signaling Proteins at DNA Double Strand Bre	1/60	0.8902304	0.999996012	0	0	0.451797174	0.052532679	RPS27A
Potassium Channels	2/103	0.8905381	0.999996012	0	0	0.527420242	0.061143462	KCNA7;GNG13
mRNA Splicing	5/215	0.8915639	0.999996012	0	0	0.633193863	0.072676803	SF3B5;PPIL1;POLR2E;SNU13;SNRPE
Initial Triggering of Complement	2/104	0.8936915	0.999996012	0	0	0.522222222	0.058694976	CFD;MBL2
DNA Double Strand Break Response	1/61	0.8942049	0.999996012	0	0	0.444244105	0.049675481	RPS27A
Nucleotide-binding Domain, Leucine Rich Repeat Containing Receptor (NLR) Signaling Pathways	1/61	0.8942049	0.999996012	0	0	0.444244105	0.049675481	RPS27A
Kinesins	1/62	0.8980358	0.999996012	0	0	0.436938678	0.046990719	KIF16B
Diseases of Glycosylation	3/146	0.9014421	0.999996012	0	0	0.558322067	0.057931152	SPON2;OMD;HEXA
Ca2+ Pathway	1/63	0.9017280	0.999996012	0	0	0.42986891	0.04446661	GNG13
MAP Kinase Activation	1/63	0.9017280	0.999996012	0	0	0.42986891	0.04446661	RPS27A
Signaling by FGFR in Disease	1/63	0.9017280	0.999996012	0	0	0.42986891	0.04446661	POLR2E
Translation of Structural Proteins 9694635	1/63	0.9017280	0.999996012	0	0	0.42986891	0.04446661	RPS27A
G2 M Transition	4/184	0.9028428	0.999996012	0	0	0.591086351	0.060413019	PSMA6;CDKN1A;MAPRE1;RPS27A
Epigenetic Regulation of Gene Expression	7/291	0.9044673	0.999996012	0	0	0.654771989	0.06574503	DGAT2;ADIPOQ;POLR2E;POLR1E;DDX21;CD36;RRP8
Mitotic Anaphase	5/221	0.9045406	0.999996012	0	0	0.615411437	0.061742987	PSMA6;CENPU;CHMP4C;MAPRE1;RPS27A
Mitotic Metaphase and Anaphase	5/222	0.9065667	0.999996012	0	0	0.612543303	0.060084728	PSMA6;CENPU;CHMP4C;MAPRE1;RPS27A
Mitotic G2-G2 M Phases	4/186	0.9072542	0.999996012	0	0	0.584529676	0.056893781	PSMA6;CDKN1A;MAPRE1;RPS27A
RAB Geranylgeranylation	1/65	0.9087169	0.999996012	0	0	0.416392164	0.039857755	RAB42
Adipogenesis	2/110	0.9109031	0.999996012	0	0	0.493055556	0.046011296	ADIPOQ;CD36
Antigen Processing Ubiquitination & Proteasome Degradation	7/296	0.9131431	0.999996012	0	0	0.643274373	0.058449605	PSMA6;ZNRF2;TRIM63;ASB10;FBXO2;RPS27A;ASB18
Mitotic Spindle Checkpoint	2/111	0.9135048	0.999996012	0	0	0.488506626	0.044193538	CENPU;MAPRE1
RNA Polymerase II Transcription Termination	1/67	0.9152093	0.999996012	0	0	0.40373219	0.035771674	SNRPE
Immune System	67/2150	0.9153058	0.999996012	0	0	0.844396477	0.074726575	CDKN1A;SERPINA1;PGLYRP2;TAT;IFI30;SLC2A5;IFIT2;PLAU;ANPEP;FTH1;AP1S3;CD36;ACAA1;GYG1;MBL2;SERPINB1;ANXA1;TRIM63;ANXA2;DGAT1;CISH;AHSG;GSTO1;FBXO2;TMT1A;NME2;NDUFC2;MIF;PSMA6;CEA
TCR Signaling	2/113	0.9184957	0.999996012	0	0	0.479654655	0.040779268	PSMA6;RPS27A
Loss of Nlp From Mitotic Centrosomes	1/69	0.9212405	0.999996012	0	0	0.391816921	0.032142348	MAPRE1
Loss of Proteins Required for Interphase Microtubule Organization From the Centrosome	1/69	0.9212405	0.999996012	0	0	0.391816921	0.032142348	MAPRE1
ER to Golgi Anterograde Transport	3/155	0.9219280	0.999996012	0	0	0.52501647	0.042677576	SEC16B;ARF4;SERPINA1
MyD88 MAL(TIRAP) Cascade Initiated on Plasma Membrane	2/115	0.9232150	0.999996012	0	0	0.471116028	0.037638923	CD36;RPS27A
Toll Like Receptor TLR6 TLR2 Cascade	2/115	0.9232150	0.999996012	0	0	0.471116028	0.037638923	CD36;RPS27A
Circadian Clock	1/70	0.9240935	0.999996012	0	0	0.386118314	0.030480947	RPS27A
EML4 and NUDC in Mitotic Spindle Formation	2/116	0.9254769	0.999996012	0	0	0.466959064	0.036164125	CENPU;MAPRE1
Cell-Cell Communication	3/157	0.9259168	0.999996012	0	0	0.518143887	0.039881959	CLDN15;KRT5;F11R
Cell Surface Interactions at the Vascular Wall	5/233	0.9264868	0.999996012	0	0	0.582654335	0.044488794	CD74;CEACAM1;MIF;F11R;APOB
Diseases Associated With O-glycosylation of Proteins	1/71	0.9268433	0.999996012	0	0	0.380582524	0.028913143	SPON2
Translocation of SLC2A4 (GLUT4) to the Plasma Membrane	1/71	0.9268433	0.999996012	0	0	0.380582524	0.028913143	PRKAG3
Antiviral Mechanism by IFN-stimulated Genes	3/158	0.9278404	0.999996012	0	0	0.514774104	0.038554262	TAT;FLNB;RPS27A
AURKA Activation by TPX2	1/72	0.9294936	0.999996012	0	0	0.375202672	0.027433077	MAPRE1
Interleukin-17 Signaling	1/72	0.9294936	0.999996012	0	0	0.375202672	0.027433077	RPS27A
Toll Like Receptor 2 (TLR2) Cascade	2/118	0.9298136	0.999996012	0	0	0.458860153	0.033391776	CD36;RPS27A
Toll Like Receptor TLR1 TLR2 Cascade	2/118	0.9298136	0.999996012	0	0	0.458860153	0.033391776	CD36;RPS27A
Post-translational Protein Modification	43/1457	0.9329688	0.999996012	0	0	0.800069992	0.055511656	ARF4;SPON2;CDKN1A;SERPINA1;PCNA;PARK7;ETFB;ASB18;ASGR1;ASGR2;PSMA8;GCSH;NEU3;RAB42;ASB10;SLC17A5;PGM3;GCNT4;DPH6;RPS27A;APOB;NANS;RNF152;SEC16B;SERPIND1;CISH;IGFBP5;AHSG;FE
Signaling by NOTCH1	1/74	0.9345099	0.999996012	0	0	0.364885148	0.024714754	RPS27A
Estrogen-dependent Gene Expression	2/121	0.9358737	0.999996012	0	0	0.447222222	0.029639512	NR5A2;POLR2E
Extra-nuclear Estrogen Signaling	1/75	0.9368828	0.999996012	0	0	0.359935525	0.023466716	GNG13
Nuclear Envelope (NE) Reassembly	1/75	0.9368828	0.999996012	0	0	0.359935525	0.023466716	CHMP4C
Transcriptional Regulation by Small RNAs	1/75	0.9368828	0.999996012	0	0	0.359935525	0.023466716	POLR2E
Cell Death Signalling via NRAGE, NRIF and NADE	1/76	0.9391699	0.999996012	0	0	0.355117892	0.022286797	RPS27A
ECM Proteoglycans	1/76	0.9391699	0.999996012	0	0	0.355117892	0.022286797	VTN
Interferon Signaling	6/280	0.9414444	0.999996012	0	0	0.581209477	0.035070122	IFI27;TAT;FLNB;IFI30;RPS27A;IFIT2
Resolution of Sister Chromatid Cohesion	2/126	0.9448858	0.999996012	0	0	0.429077061	0.024324881	CENPU;MAPRE1
G Alpha (12 13) Signalling Events	1/80	0.9475197	0.999996012	0	0	0.337067013	0.018170428	GNG13
PKR-mediated Signaling	1/80	0.9475197	0.999996012	0	0	0.337067013	0.018170428	TAT
Post NMDA Receptor Activation Events	1/80	0.9475197	0.999996012	0	0	0.337067013	0.018170428	PRKAG3
Regulation of Complement Cascade	2/128	0.9481419	0.999996012	0	0	0.422222222	0.022483798	VTN;C7
Centrosome Maturation	1/81	0.9494218	0.999996012	0	0	0.332836338	0.017274892	MAPRE1
RNA Polymerase II Pre-transcription Events	1/81	0.9494218	0.999996012	0	0	0.332836338	0.017274892	POLR2E
RNA Polymerase II Transcribes snRNA Genes	1/81	0.9494218	0.999996012	0	0	0.332836338	0.017274892	POLR2E
Recruitment of Mitotic Centrosome Proteins and Complexes	1/81	0.9494218	0.999996012	0	0	0.332836338	0.017274892	MAPRE1
Cardiac Conduction	2/130	0.9512141	0.999996012	0	0	0.415581597	0.020785765	CASQ1;ATP1A1
Infection With Mycobacterium Tuberculosis	1/82	0.9512550	0.999996012	0	0	0.328710125	0.016426645	RPS27A
C-type Lectin Receptors (CLRs)	2/131	0.9526843	0.999996012	0	0	0.412338501	0.019986738	PSMA6;RPS27A
Toll-like Receptor Cascades	3/174	0.9529810	0.999996012	0	0	0.4662177	0.022453182	CD36;APOB;RPS27A
PI Metabolism	1/84	0.9547248	0.999996012	0	0	0.320755978	0.014861305	GDPD3
DDX58 IFIH1-mediated Induction of Interferon-Alpha Beta	1/86	0.9579479	0.999996012	0	0	0.313176144	0.013454638	RPS27A
Golgi-to-ER Retrograde Transport	2/135	0.9581525	0.999996012	0	0	0.399853801	0.017093041	ARF4;KIF16B
Cytokine Signaling in Immune System	20/776	0.9583059	0.999996012	0	0	0.698005698	0.029726768	CDKN1A;ANXA1;ANXA2;CISH;TNFSF14;GSTO1;TAT;EBI3;MIF;IFI30;SOD2;IFIT2;SOD1;PSMA6;GH1;IFI27;FLNB;CD36;RPS27A;STX1A
Signaling by Hedgehog	2/136	0.9594220	0.999996012	0	0	0.396849088	0.016439169	PSMA6;RPS27A
Protein-protein Interactions at Synapses	1/88	0.9609418	0.999996012	0	0	0.305944808	0.012189275	STX1A
Class I MHC Mediated Antigen Processing & Presentation	8/373	0.9616771	0.999996012	0	0	0.58057634	0.022686885	PSMA6;ZNRF2;TRIM63;ASB10;FBXO2;CD36;RPS27A;ASB18
SARS-CoV-2 Activates Modulates Innate and Adaptive Immune Responses	2/138	0.9618510	0.999996012	0	0	0.390972222	0.015207131	RPS27A;MBL2
Opioid Signalling	1/90	0.9637228	0.999996012	0	0	0.299038477	0.011049932	GNG13
Interleukin-1 Family Signaling	2/140	0.9641402	0.999996012	0	0	0.3852657	0.014069347	PSMA6;RPS27A
Activation of HOX Genes During Differentiation	1/91	0.9650382	0.999996012	0	0	0.295700416	0.010523241	POLR2E
Activation of Anterior HOX Genes in Hindbrain Development During Early Embryogenesis	1/91	0.9650382	0.999996012	0	0	0.295700416	0.010523241	POLR2E
Chaperonin-mediated Protein Folding	1/91	0.9650382	0.999996012	0	0	0.295700416	0.010523241	GNG13
Transport to the Golgi and Subsequent Modification	3/186	0.9661835	0.999996012	0	0	0.435372444	0.014977461	SEC16B;ARF4;SERPINA1
Scavenging of Heme From Plasma	1/92	0.9663060	0.999996012	0	0	0.29243572	0.010023128	APOA1
Activation of NMDA Receptors and Postsynaptic Events	1/93	0.9675279	0.999996012	0	0	0.289241995	0.00954815	PRKAG3
Transmission Across Chemical Synapses	5/269	0.9679055	0.999996012	0	0	0.502250539	0.016383815	ALDH2;GLS2;STX1A;PRKAG3;GNG13
Oxidative Stress Induced Senescence	1/94	0.9687056	0.999996012	0	0	0.286116952	0.009096952	RPS27A
Recruitment of NuMA to Mitotic Centrosomes	1/94	0.9687056	0.999996012	0	0	0.286116952	0.009096952	MAPRE1

PTEN Regulation	2/145	0.969300	0.999996012	0	0	0.371697747	0.011589906	PSMA6;RPS27A
Ub-specific Processing Proteases	3/190	0.969745	0.999996012	0	0	0.425970413	0.01308638	PSMA6;RPS27A;DDB2
Creation of C4 and C2 Activators	1/96	0.970934	0.999996012	0	0	0.280064238	0.008260863	MBL2
FCERI Mediated NF-kB Activation	2/147	0.971157	0.999996012	0	0	0.366532567	0.010727309	PSMA6;RPS27A
ESR-mediated Signaling	3/192	0.971390	0.999996012	0	0	0.421418637	0.012232204	NR5A2;POLR2E;GNG13
Anchoring of the Basal Body to the Plasma Membrane	1/97	0.971988	0.999996012	0	0	0.277132455	0.007873638	MAPRE1
Protein Folding	1/97	0.971988	0.999996012	0	0	0.277132455	0.007873638	GNG13
Toll Like Receptor 4 (TLR4) Cascade	2/148	0.972044	0.999996012	0	0	0.364003044	0.010320813	CD36;RPS27A
RHOA GTPase Cycle	2/149	0.972905	0.999996012	0	0	0.361507937	0.009929981	ABCD3;C1QBP
P75 NTR Receptor-Mediated Signalling	1/98	0.973004	0.999996012	0	0	0.274261121	0.007505516	RPS27A
MyD88 Cascade Initiated on Plasma Membrane	1/98	0.973004	0.999996012	0	0	0.274261121	0.007505516	RPS27A
Toll Like Receptor 10 (TLR10) Cascade	1/98	0.973004	0.999996012	0	0	0.274261121	0.007505516	RPS27A
Toll Like Receptor 5 (TLR5) Cascade	1/98	0.973004	0.999996012	0	0	0.274261121	0.007505516	RPS27A
Interferon Gamma Signaling	1/99	0.973983	0.999996012	0	0	0.271448385	0.00715549	IFI30
Peptide Ligand-Binding Receptors	3/198	0.975832	0.999996012	0	0	0.408323526	0.009989156	ANXA1;CXCR3;CCL27
COPI-mediated Anterograde Transport	1/101	0.975837	0.999996012	0	0	0.265991678	0.006505991	ARF4
TRAF6 Mediated Induction of NFkB and MAP Kinases Upon TLR7 8 or 9 Activation	1/102	0.976713	0.999996012	0	0	0.263344365	0.006204778	RPS27A
MyD88 Dependent Cascade Initiated on Endosome	1/103	0.977558	0.999996012	0	0	0.26074896	0.005918178	RPS27A
Processing of Capped Intron-Containing Pre-mRNA	5/285	0.978213	0.999996012	0	0	0.473152022	0.010422392	SF3B5;PPIL1;POLR2E;SNU13;SNRPE
Factors Involved in Megakaryocyte Development and Platelet Production	2/158	0.979585	0.999996012	0	0	0.340491453	0.007023045	KIF16B;ITPK1
G Alpha (S) Signalling Events	2/158	0.979585	0.999996012	0	0	0.340491453	0.007023045	GNG13;GIP
Toll Like Receptor 3 (TLR3) Cascade	1/106	0.979914	0.999996012	0	0	0.253259362	0.005138739	RPS27A
Anti-inflammatory Response Favouring Leishmania Parasite Infection	2/159	0.980220	0.999996012	0	0	0.338305025	0.006758634	GGT1;GNG13
Leishmania Parasite Growth and Survival	2/159	0.980220	0.999996012	0	0	0.338305025	0.006758634	GGT1;GNG13
Toll Like Receptor 7 8 (TLR7 8) Cascade	1/107	0.980642	0.999996012	0	0	0.250857038	0.004903472	RPS27A
HATs Acetylate Histones	1/108	0.981345	0.999996012	0	0	0.248499618	0.004679448	ING5
Signaling by the B Cell Receptor (BCR)	2/162	0.982013	0.999996012	0	0	0.331909722	0.006024349	PSMA6;RPS27A
RSV-host Interactions	1/109	0.982022	0.999996012	0	0	0.246185853	0.004466101	RPS27A
Signaling by NOTCH	3/209	0.982328	0.999996012	0	0	0.386297041	0.00688757	PSMA6;FABP7;RPS27A
MyD88-independent TLR4 Cascade	1/110	0.982674	0.999996012	0	0	0.243914543	0.004262893	RPS27A
PI3K AKT Signaling in Cancer	1/110	0.982674	0.999996012	0	0	0.243914543	0.004262893	CDKN1A
TRIF (TICAM1)-mediated TLR4 Signaling	1/110	0.982674	0.999996012	0	0	0.243914543	0.004262893	RPS27A
Toll Like Receptor 9 (TLR9) Cascade	1/110	0.982674	0.999996012	0	0	0.243914543	0.004262893	RPS27A
Generic Transcription Pathway	32/1230	0.982773	0.999996012	0	0	0.699910479	0.012162109	CDKN1A;PCNA;TP53RK;UCMA;GLS2;COX7A2;NOC2L;COX6A1;COX7C;COX5A;COX6A2;PRKAG3;PRDX2;ING5;AIFM2;POLR2E;SMYD2;RPS27A;RFC5;TRIM63;RFC2;GSR;NR1H4;RPA2;COX6B2;SOD2;NR0B2;GCK;DDB2
Potential Therapeutics for SARS	2/164	0.983119	0.999996012	0	0	0.327777778	0.005580216	ACE2;ATP1A1
Membrane Trafficking	14/633	0.984135	0.999996012	0	0	0.596063452	0.009532352	ARF4;SEC16B;SH3GL3;SERPINA1;PRKAG3;TF;KIF16B;FTH1;CHMP4C;AP4S1;AP1S3;RPS27A;APOB;SH3GL1
Cellular Senescence	2/167	0.984656	0.999996012	0	0	0.321767677	0.004975293	CDKN1A;RPS27A
G Alpha (Q) Signalling Events	3/219	0.986755	0.999996012	0	0	0.36821975	0.004909344	GPRC6A;ANXA1;GNG13
Gastrulation	1/120	0.988030	0.999996012	0	0	0.223300971	0.002689002	PSMA6
Chromatin Modifying Enzymes	3/238	0.992412	0.999996012	0	0	0.338111443	0.002575157	ING5;SETD6;SMYD2
Chromatin Organization	3/238	0.992412	0.999996012	0	0	0.338111443	0.002575157	ING5;SETD6;SMYD2
TCF Dependent Signaling in Response to WNT	2/189	0.992436	0.999996012	0	0	0.283585859	0.002153036	PSMA6;RPS27A
Respiratory Syncytial Virus Infection Pathway	1/133	0.992600	0.999996012	0	0	0.201172614	0.001494177	RPS27A
Gene Silencing by RNA	1/134	0.992869	0.999996012	0	0	0.199649609	0.001428802	POLR2E
Signaling by WNT	4/287	0.993119	0.999996012	0	0	0.373928364	0.002581867	PSMA6;PFN1;RPS27A;GNG13
Vesicle-mediated Transport	16/752	0.993425	0.999996012	0	0	0.570944698	0.003765802	SEC16B;ARF4;SH3GL3;SERPINA1;APOA1;PRKAG3;TF;KIF16B;FTH1;CHMP4C;AP4S1;AP1S3;CD36;RPS27A;APOB;SH3GL1
Leishmania Infection	3/245	0.993837	0.999996012	0	0	0.328210669	0.002028898	P2RX4;GGT1;GNG13
Parasitic Infection Pathways	3/245	0.993837	0.999996012	0	0	0.328210669	0.002028898	P2RX4;GGT1;GNG13
Fc Epsilon Receptor (FCERI) Signaling	2/197	0.994168	0.999996012	0	0	0.271837607	0.001589775	PSMA6;RPS27A
Cilium Assembly	2/200	0.994712	0.999996012	0	0	0.267676768	0.001419065	ARF4;MAPRE1
Transcriptional Regulation by RUNX1	2/202	0.995047	0.999996012	0	0	0.264972222	0.001315619	PSMA6;RPS27A
Mitotic Prometaphase	2/203	0.995206	0.999996012	0	0	0.263640133	0.001266773	CENPU;MAPRE1
Intra-Golgi and Retrograde Golgi-to-ER Traffic	2/204	0.995360	0.999996012	0	0	0.262321232	0.001219749	ARF4;KIF16B
Neurotransmitter Receptors and Postsynaptic Signal Transmission	2/204	0.995360	0.999996012	0	0	0.262321232	0.001219749	PRKAG3;GNG13
RNA Polymerase II Transcription	33/1360	0.995666	0.999996012	0	0	0.647906657	0.002813887	CDKN1A;PCNA;TP53RK;UCMA;GLS2;COX7A2;NOC2L;COX6A1;COX7C;COX5A;COX6A2;PRKAG3;PRDX2;ING5;AIFM2;POLR2E;SMYD2;RPS27A;RFC5;TRIM63;RFC2;GSR;NR1H4;RPA2;COX6B2;SOD2;NR0B2;GCK;DDB2
Deubiquitination	3/268	0.996916	0.999996012	0	0	0.299362322	9.24E-04	PSMA6;RPS27A;DDB2
Gene Expression (Transcription)	40/1615	0.996930	0.999996012	0	0	0.659237537	0.002026595	CDKN1A;PCNA;TP53RK;UCMA;GLS2;DDX21;COX7A2;NOC2L;COX6A1;COX7C;COX5A;COX6A2;RRP8;PRKAG3;PRDX2;ING5;AIFM2;POLR2E;SMYD2;CD36;RPS27A;RFC5;DGAT2;TRIM63;POLR3GL;RFC2;GSR;ADIPOQ;N
G Alpha (I) Signalling Events	4/316	0.996955	0.999996012	0	0	0.338654382	0.001032491	ANXA1;CXCR3;CCL27;GNG13
Death Receptor Signaling	1/157	0.996956	0.999996012	0	0	0.170009602	5.18E-04	RPS27A
RHO GTPase Cycle	7/450	0.997218	0.999996012	0	0	0.416249665	0.001159463	BAIAP2L2;ABCD3;NDUFA5;C1QBP;MOSPD2;FILIP1;HSPE1
Neuronal System	6/411	0.997435	0.999996012	0	0	0.390502793	0.001002856	ALDH2;GLS2;KCNA7;PRKAG3;STX1A;GNG13
Signaling by Receptor Tyrosine Kinases	9/536	0.997507	0.999996012	0	0	0.449124553	0.001120631	SH3GL3;TF;STMN1;POLR2E;PTK6;ASCL1;RPS27A;PCSK5;SH3GL1
M Phase	5/372	0.997737	0.999996012	0	0	0.3593348	8.14E-04	PSMA6;CENPU;CHMP4C;MAPRE1;RPS27A
PIP3 Activates AKT Signaling	3/281	0.997927	0.999996012	0	0	0.285168249	5.92E-04	PSMA6;CDKN1A;RPS27A
HCMV Late Events	1/171	0.998187	0.999996012	0	0	0.155894591	2.83E-04	CHMP4C
RHO GTPase Effectors	3/291	0.998476	0.999996012	0	0	0.275121697	4.19E-04	CENPU;PFN1;MAPRE1
HCMV Infection	2/259	0.999252	0.999996012	0	0	0.205587981	1.54E-04	DUT;CHMP4C
RAF MAP Kinase Cascade	2/265	0.999389	0.999996012	0	0	0.20083439	1.23E-04	PSMA6;RPS27A
Intracellular Signaling by Second Messengers	3/321	0.999401	0.999996012	0	0	0.248773191	1.49E-04	PSMA6;CDKN1A;RPS27A
GPCR Ligand Binding	6/467	0.999422	0.999996012	0	0	0.342048498	1.98E-04	GPRC6A;ANXA1;CXCR3;CCL27;GNG13;GIP
HCMV Early Events	1/205	0.999484	0.999996012	0	0	0.129680999	6.68E-05	DUT
MAPK1 MAPK3 Signaling	2/271	0.999500	0.999996012	0	0	0.196292854	9.8E-05	PSMA6;RPS27A
Class A 1 (Rhodopsin-like Receptors)	3/333	0.999589	0.999996012	0	0	0.239575168	9.84E-05	ANXA1;CXCR3;CCL27
Signaling by Rho GTPases	10/672	0.999744	0.999996012	0	0	0.394955701	1.01E-04	CENPU;BAIAP2L2;ABCD3;NDUFA5;C1QBP;MOSPD2;FILIP1;PFN1;MAPRE1;HSPE1
Diseases of Signal Transduction by Growth Factor Receptors and Second Messengers	5/451	0.999754	0.999996012	0	0	0.294450594	7.24E-05	PSMA6;CDKN1A;BIN2;POLR2E;RPS27A
Signaling by Rho GTPases, Miro GTPases and RHOBTB3	10/688	0.999827	0.999996012	0	0	0.385303769	6.65E-05	CENPU;BAIAP2L2;ABCD3;NDUFA5;C1QBP;MOSPD2;FILIP1;PFN1;MAPRE1;HSPE1
MAPK Family Signaling Cascades	2/314	0.999882	0.999996012	0	0	0.168856838	1.99E-05	PSMA6;RPS27A
Adaptive Immune System	13/854	0.999934	0.999996012	0	0	0.401967904	2.65E-05	CD74;TRIM63;FBXO2;IFI30;PPL;ASB18;PSMA6;ZNRF2;ASB10;AP1S3;CD36;RPS27A;XDH
GPCR Downstream Signalling	6/633	0.999990	0.999996012	0	0	0.249271605	2.27E-06	GPRC6A;ANXA1;CXCR3;CCL27;GNG13;GIP
Signal Transduction	43/2613	0.999993	0.999996012	0	0	0.411708681	2.65E-06	SH3GL3;CDKN1A;ABCD3;MOSPD2;ASCL1;F11R;PCSK5;PRKAG3;GPRC6A;SDR16C5;BAIAP2L2;C1QBP;CXCR3;STMN1;POLR2E;APOD;RPS27A;SH3GL1;CENPU;HELLS;ABCG8;ABCG5;ANXA1;NDUFA5;PTK6;HSPE1;DH
Signaling by GPCR	6/706	0.999994	0.999996012	0	0	0.222402235	1.12E-06	GPRC6A;ANXA1;CXCR3;CCL27;GNG13;GIP
Olfactory Signaling Pathway	1/422	0.999996	0.999996012	0	0	0.062123403	2.48E-07	GNG13
